# Frequency and patterns of early recanalization after vasectomy

**DOI:** 10.1186/1471-2490-6-25

**Published:** 2006-09-19

**Authors:** Michel Labrecque, Melissa Hays, Mario Chen-Mok, Mark A Barone, David Sokal

**Affiliations:** 1 Evaluation Research Unit, D1-724, Centre de recherche du Centre Hospitalier Universitaire de Québec (CHUQ), Hôpital Saint-François d'Assise, 10, rue de l'Espinay, Québec, (Qc), G1L 3L5, Canada; 2 Family Health International, 2224 East NC Highway 54, Durham, NC 27713, USA; 3 EngenderHealth, 440 Ninth Ave. New York, NY 10001, USA

## Abstract

**Background:**

Our understanding of early post-vasectomy recanalization is limited to histopathological studies. The objective of this study was to estimate the frequency and to describe semen analysis patterns of early recanalization after vasectomy.

**Methods:**

Charts displaying serial post-vasectomy semen analyses were created using the semen analysis results from 826 and 389 men participating in a randomized trial of fascial interposition (FI) and an observational study of cautery, respectively. In the FI trial, participants were randomly allocated to vas occlusion by ligation and excision with or without FI. In the cautery study, sites used their usual cautery occlusion technique, two with and two without FI. Presumed early recanalization was based on the assessment of individual semen analysis charts by three independent reviewers. Discrepancies were resolved by consensus.

**Results:**

Presumed early recanalization was characterized by a very low sperm concentration within two weeks after vasectomy followed by return to large numbers of sperm over the next few weeks. The overall proportion of men with presumed early recanalization was 13% (95% CI 12%–15%). The risk was highest with ligation and excision without FI (25%) and lowest for thermal cautery with FI (0%). The highest proportion of presumed early recanalization was observed among men classified as vasectomy failures.

**Conclusion:**

Early recanalization, occurring within the first weeks after vasectomy, is more common than generally recognized. Its frequency depends on the occlusion technique performed.

## Background

Vasectomy success is usually assumed when one or two post-vasectomy semen analyses show azoospermia or when only very rare non-motile sperm are observed, otherwise failure of adequate vas occlusion is implied[1]. Failure can be attributed to surgical errors such as cutting a structure other than the vas, repeating the vasectomy twice on the same vas, or, very rarely, by overlooking a congenital duplication of the vas. However, most vasectomy failures are presumed to result from recanalization of the severed vas.

Our understanding of recanalization is limited to histopathological studies that have been conducted on specimens collected from men undergoing repeat vasectomy or vaso-vasostomy [2-7]. Recanalization results from the proliferation of epithelial microtubules through granulomatous tissue between the severed ends of the vas, producing a fistula that allows the passage of sperm.

Data from two studies – a randomized clinical trial of the effectiveness of fascial interposition (FI)[8] and an observational study of vasectomy using cautery[9] – in which sequential semen analyses were performed early after vasectomy, provided an opportunity to enhance our clinical understanding of post-vasectomy recanalization. The objectives of this secondary analysis were to estimate the frequency of post-vasectomy early recanalization and to describe semen analysis patterns associated with presumed early recanalization.

## Methods

### Vasectomy studies

The methods of the FI and cautery studies have been previously described[8,9] and are summarized in Additional file 1. Briefly, the FI trial[8] involved eight sites in seven countries. It was a randomized clinical trial comparing two occlusion techniques: ligation and excision with versus without FI. All surgeons used the no-scalpel vasectomy (NSV) approach to the vas and a standardized occlusion technique. The study was halted following a planned interim analysis that demonstrated a clear benefit from the use of FI[10]. Of the 841 men who were randomized in the FI study, 826 were included in the analysis reported here; 410 had FI and 416 did not. Fifteen men were excluded because they did not return for any semen analyses after vasectomy.

The cautery study[9] involved four sites in four countries. It was a prospective observational study designed to estimate the effectiveness of cautery as currently performed at each site and to describe trends in sperm counts after vas occlusion by cautery. Each surgeon used his or her customary cautery occlusion technique, which differed among the sites: two sites performed electrocautery alone and two sites used thermal cautery combined with FI. A small vas segment was excised in one site using electrocautery and in one site using thermal cautery. The other two sites did not remove any vas tissue. Three sites used the NSV approach to the vas. Of 400 men enrolled, 389 were included in the analysis reported here. Eleven men were excluded because they did not provide any semen samples after vasectomy.

Both studies conducted frequent semen analyses, beginning at two weeks after vasectomy. The FI trial conducted subsequent semen analyses every four weeks until a man had provided two consecutive azoospermic specimens, was declared a vasectomy failure, or reached the end of study follow-up at 34 weeks. After the first sample at two weeks, the cautery study conducted subsequent semen analyses at weeks 5, 8, 12, 16, 20 and 24 regardless of semen analysis findings.

Semen analysis methods for both studies were based on World Health Organization recommendations, but differed somewhat between the two studies. Freshly collected semen was examined in the FI trial and data were obtained on sperm concentration, motility, and viability. For the cautery study, two of the four sites did not routinely collect fresh specimens, so semen analysis data from those two sites were limited to sperm concentrations. In addition, specimens showing azoospermia or very low sperm concentrations were centrifuged in the FI trial but not in the cautery study. During both studies, the laboratories conducted periodic quality control tests.

Definitions of success and failure were different in each study. In the FI trial, success was defined as two consecutive azoospermic specimens at least two weeks apart. Failure was defined as 5 million or more motile sperm/mL at 14+ weeks or 100,000 sperm or more/mL with any motility at 26+ weeks. Men who did not meet the criteria for success or failure were classified as indeterminate. In the cautery study, success was defined as less than 100,000 sperm/mL in two consecutive specimens taken at least two weeks apart, and failure as not meeting the definition of success by 24 weeks or having more than 10 million sperm/mL at 12+ weeks. Men who had less then 12 weeks of follow-up without meeting the criteria for success or having been declared a failure by a study site clinician were classified as indeterminate.

The data collection forms, study monitoring, and laboratory quality control procedures were similar for both studies. One research site was common to both studies. Both studies were organized and managed by researchers and staff at FHI and EngenderHealth and received approval from FHI's Institutional Review Board (IRB) and by local IRB, when present.

### Assessment of early recanalization

Semen analysis results over time were used as a surrogate marker to determine early recanalization. Charts presenting the log of the sperm concentration at each follow-up time point were created for each participant. Participant identifiers, study sites, and vasectomy technique assignments were not included and charts were presented in random order. The percentage of motile sperm was included in the charts for the FI trial, but not for the cautery study. Data on cumulative number of ejaculations at each time point were included in the cautery study charts as was the participant's age, because age seemed to be associated with longer times to azoospermia[8]. The FI trial charts indicated participants' final vasectomy outcome (success, failure, indeterminate) because participants were discontinued from the FI trial when failure or success was determined. However, the cautery study charts did not include final vasectomy outcome as sperm concentration data were collected throughout the entire follow-up period. All charts from both studies are available in Additional files 2 (FI trial) and 3 (cautery study).

Since there are no established criteria for determining early recanalization based on semen analysis data, we used an adjudication process. Three experts in vasectomy research (ML, DS, and MB) independently reviewed the charts to determine if early recanalization had taken place according to their own criteria, which were set prior to reviewing the FI trial charts. These criteria (Table 1) were collected after review of the FI trial charts and were not discussed until the adjudication process for the cautery study was completed.

**Table 1:** Individual and consensus criteria used by the reviewers to assess the presence of early recanalization.

Reviewers	Criteria used to assess early recanalization
1	Absence or very rare sperm at 2 weeks followed by an increasing number of sperm in any subsequent semen analyses. If motility was available, reappearance of motility after complete disappearance was considered as recanalization. Persistence of high numbers of motile sperm with no evidence of an initial decrease in sperm numbers was not considered as recanalization.
2	A severe drop in sperm counts immediately or soon after the vasectomy, down to about 1 million/mL or less, followed by a subsequent rise to above about 10 million, with motility increasing the probability of recanalization. Vasectomy success, included for the FI trial, was considered as evidence against recanalization.
3	Azoospermia or count(s) of less than 1 million/mL and then subsequent count(s) over 1 million/mL or reappearance of motile sperm. No motility for several samples followed by reappearance of motile sperm. No recanalization if steady decline to azoospermia or to low sperm numbers (less than1 million/mL) even if azoospermia was not reached.
Consensus	1) Azoospermia or low sperm count (less than 1 million/mL) within two to six weeks after the vasectomy and then at least one subsequent count of over 1 million/mL. The probability of recanalization was assumed to increase if the sperm count was higher.
	2) When motility was available, azoospermia or low sperm count with complete or near-complete loss of motility followed by the appearance of increasing numbers of motile sperm. Persistence of numerous motile sperm with no evidence of an early and significant decrease in sperm count was considered as a technical failure and not a recanalization.
	3) When motility was not available, a slow decline to azoospermia or low sperm numbers (less than 100,000/mL) was not considered as a recanalization, even if azoospermia was not reached.
	4) If recanalization could not be agreed upon due to missing or ambiguous data, then it was assumed that no recanalization had occurred.

Classification of participants was done first for the FI trial and three months later for the cautery study as part of the procedures of each study. In the FI trial, reviewers were asked to provide their assessment on whether early recanalization had occurred by selecting either yes or no for each chart. In the cautery study an indeterminate category was included as well for those cases where the reviewers felt they could not classify a participant due to insufficient information because of missing data or loss to follow-up. After the adjudication process of the two studies was completed, discrepancies were discussed among the three reviewers and resolved by consensus. In the final consensus assessment, the indeterminate classification was not allowed, and such cases were reclassified as not indicative of recanalization by default. Consensus definitions agreed upon at the time of the final adjudication are presented in Table 1.

**Figure 1:**
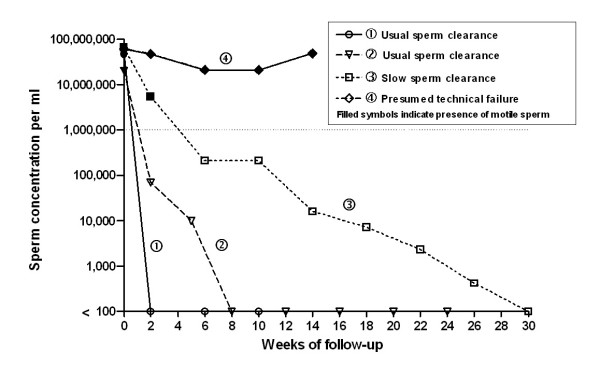
**Semen analysis charts of four men without presumed early recanalization**. Sperm concentration is illustrated on a log scale. Since a logarithmic scale has no true zero, we used <100 on the graph to indicate azoospermia. The dotted line indicates low sperm cut-off (1,000,000 sperm/mL) according to reviewers' consensus (see Table 1). For case no 2, pre-vasectomy sperm concentrations were not available. We assumed a count of 20,000,000 sperm/mL with presence of motile sperm.

**Figure 2:**
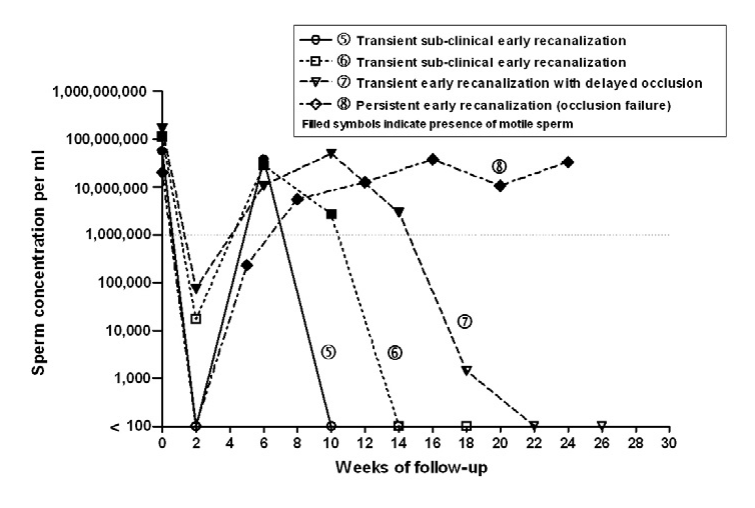
**Semen analysis charts of four men with presumed early recanalization**. Sperm concentration is illustrated on a log scale. Since a logarithmic scale has no true zero, we used <100 on the graph to indicate azoospermia. The dotted line indicates low sperm cut-off (1,000,000 sperm/mL) according to reviewers' consensus (see Table 1). For case no 8, pre-vasectomy sperm concentrations were not available. We assumed a count of 20,000,000 sperm/mL with presence of motile sperm.

Charts best illustrating patterns of sperm clearance after vasectomy (success or failure, with and without early recanalization) were selected by consensus of the reviewers and are presented in Figures 1 and 2.

### Statistical analysis

We computed crude agreement, kappa statistic, and its 95% confidence interval to study the level of agreement among the three reviewers based on their initial independent review. The frequency of early recanalization after reviewers' consensus was calculated. We produced a cross-sectional tabulation to examine the relationship between early recanalization, vasectomy occlusion technique, and final vasectomy outcome. Association between the risks of early recanalization and study center and vasectomy related adverse events in the FI trial were evaluated with the Cochran-Mantel-Haenszel Chi-square test. The probabilities of semen analysis showing any motile sperm, when data available, or 1 million sperm/mL or more at each follow-up time point (weeks) were calculated by early recanalization status and vasectomy occlusion technique.

## Results

A total of 826 charts from the FI trial and 389 from the cautery study were analyzed. Table 2 shows the agreement of the reviewers after independent assessment. According to kappa coefficients, their agreement was moderate to substantial in the FI trial, where they had to classify the charts as either recanalization or not, and was fair to moderate in the cautery study, where in addition they could classify a chart as indeterminate[11]. Discordance between the three reviewers was observed in 104 (12.6%) of the FI trial charts (77 of which were classified as early recanalization by consensus) and in 54 (13.9%) of the cautery study charts (13 of which were classified as early recanalization by consensus).

**Table 2:** Agreement of reviewers on early recanalization in the fascial interposition trial and the cautery study.

Reviewers	Crude agreement	Kappa coefficient	95% CI
FI trial			
1 vs. 2	0.89	0.60	0.53 – 0.68
1 vs. 3	0.95	0.84	0.79 – 0.89
2 vs. 3	0.90	0.58	0.49 – 0.66
All three	0.87	0.68	0.64 – 0.72
Cautery study			
1 vs. 2	0.93	0.40	0.22 – 0.58
1 vs. 3	0.88	0.35	0.21 – 0.49
2 vs. 3	0.91	0.42	0.27 – 0.57
All three	0.86	0.38	0.34 – 0.43

FI = fascial interposition

Note: agreement was assessed on two categories (recanalization, no recanalization) in the FI trial and on three categories (recanalization, no recanalization, indeterminate) in the cautery study.

Based on final consensus, the overall estimate of presumed early recanalization was 13% (95% CI 12% – 15%). The frequency of presumed early recanalization according to the vasectomy occlusion technique and outcomes as defined in each of the two studies is presented in Table 3. The proportion of men with presumed early recanalization was 25% (95% CI 21%–30%) with ligation and excision without FI, 10% (95% CI 7%–13%) with ligation and excision with FI, 9% (95% CI 5%–13%) with electrocautery without FI, and 0% (95% CI 0%–2%) with thermal cautery with FI.

**Table 3:** Frequency of presumed early recanalization following the consensus process, according to vasectomy occlusion technique and outcome in the fascial interposition trial and the cautery study.

Vasectomy technique	Early recanalization	Vasectomy outcome			Total
		Failure^a ^n (%)	Success^b ^n (%)	Indeterminate^c ^n (%)	
FI trial					
LE					
	Yes	46 (87)	49 (15)	10 (31)	105 (25)
	No	7 (13)	282 (85)	22 (69)	311 (75)
	Total	53 (100)	331 (100)	32 (100)	416 (100)
LE and FI					
	Yes	20 (83)	20 (6)	1 (2)	41 (10)
	No	4 (17)	323 (94)	42 (98)	369 (90)
	Total	24 (100)	343 (100)	43 (100)	410 (100)
Cautery study					
EC					
	Yes	2 (100)	13 (7)	2 (40)	17 (9)
	No	0 (0)	177 (93)	3 (60)	180 (91)
	Total	2 (100)	190 (100)	5 (100)	197 (100)
TC and FI					
	Yes	0 (0)	0 (0)	0 (0)	0 (0)
	No	1 (100)	188 (100)	3 (100)	192 (100)
	Total	1 (100)	188 (100)	3 (100)	192 (100)

FI = fascial interposition, LE = ligation and excision, EC = electrocautery, TC = thermal cautery

^a ^FI trial definition: 5 million or more motile sperm/mL at 14+ weeks or 100,000 sperm or more/mL with any motility at 26+ weeks. Cautery study definition: Not meeting success definition by 24 weeks or having more than 10 million sperm/mL at 12+ weeks

^b ^FI trial definition: Two consecutive azoospermic specimens taken at least two weeks apart. Cautery study definition: Less than 100,000 sperm/mL in two consecutive specimens taken at least two weeks apart.

^c ^Neither a success nor a failure.

In both studies most vasectomy failures, 85% overall, were classified as early recanalizations (Table 3). However, there were about twice as many recanalizations as failures in both groups in the FI trial. The risk of early recanalization was similar whether or not the men experienced a vasectomy related adverse event (without FI 32% vs 24%, p = 0.15; with FI 10% vs 11%, p = 0.83). There was no association between the risk of early recanalization and study centers (p = 0.1).

In the cautery study, all presumed early recanalizations were encountered in the two centers where electrocautery alone was performed. The early recanalization risk was similar in both of these centers (8.2% and 9%). There were over eight times more recanalizations than failures. In the two centers combining thermal cautery with FI, no early recanalization was observed; there was, however, one failure, classified as a surgical error[9].

Table 4 presents the probabilities of semen analysis showing any motile sperm and 1 million sperm/mL or more according to the vasectomy occlusion technique, the early recanalization status, and the number of weeks after vasectomy. In men with early recanalization, the trends regarding motility or sperm count were similar in all three occlusion techniques in which recanalization was encountered. Within each technique, the proportions of men with motile sperm or with 1 million sperm/mL or more were much lower before five weeks after vasectomy than between five and 10 weeks. All these proportions then decreased after 12 weeks or more to a similar or lower level than observed at the time of the first post-vasectomy semen analysis.

**Table 4:** Probabilities of semen analysis showing any motile sperm or 1 million sperm/mL or more by vasectomy occlusion technique, early recanalization status, and number of weeks after vasectomy.

Week	Semen analysis with any motile sperm				Semen analysis with 1 × 10^6 ^sperm/mL or more			
	Vasectomy Technique				Vasectomy Technique			
	LE	LE and FI	EC^a^	TC and FI^a^	LE	LE and FI	EC^b^	TC and FI^b^
	n/N (%)^c^	n/N (%)	n/N (%)	n/N (%)	n/N (%)	n/N (%)	n/N (%)	n/N (%)

**Recanalization**								
<5	45/105 (43)	21/41(51)	2/7 (29)	0	24/105 (23)	11/40 (28)	4/15 (27)	0
5–6	76/90 (84)	26/37 (70)	8/8 (100)	0	66/90 (73)	22/37 (59)	8/13 (62)	0
8–10	76/96 (79)	29/38 (76)	7/9 (78)	0	61/96 (64)	24/38 (63)	8/17 (47)	0
12–14	55/91 (60)	19/38 (50)	3/7 (43)	0	41/91 (45)	16/38 (42)	1/11 (9)	0
16–18	36/75 (48)	12/27 (44)	2/6 (33)	0	21/75 (28)	8/27 (30)	2/12 (17)	0
20–22	17/52 (33)	8/20 (40)	2/8 (25)	0	13/52 (25)	5/20 (25)	2/11 (18)	0
24–26	11/30 (37)	6/12 (50)	2/7 (28)	0	6/30 (20)	3/12 (25)	2/12 (17)	0
**No recanalization**								
<5	87/300 (29)	121/349 (35)	29/85 (34)	14/93 (15)	75/300 (25)	87/349 (25)	46/170 (27)	28/177 (16)
5–6	39/245 (16)	17/298 (6)	13/79 (16)	2/86 (2)	19/245 (8)	14/298 (5)	15/153 (10)	7/163 (4)
8–10	12/236 (5)	4/279 (1)	3/82 (4)	0/80 (0)	9/236 (4)	7/279 (3)	3/154 (2)	1/162 (1)
12–14	8/170 (5)	6/162 (4)	0/81 (0)	0/83 (0)	8/170 (5)	4/162 (2)	0/155 (0)	0/160 (0)
16–18	5/94 (5)	2/104 (2)	0/89 (0)	0/90 (0)	4/94 (4)	2/104 (2)	0/154 (0)	0/159 (0)
20–22	3/57 (5)	0/62 (0)	0/91 (0)	0/83 (0)	4/57 (7)	0/62 (0)	0/151 (0)	0/160 (0)
24–26	4/41 (10)	1/46 (2)	0/91 (0)	0/62 (0)	4/41 (10)	1/46 (2)	0/127 (0)	0/128 (0)

FI = fascial interposition, LE = ligation and excision, EC = electrocautery, TC = thermal cautery

^a ^restricted to the two centers in the cautery study that had information about motility.

^b ^includes all centers in the cautery study.

^c ^n/N (%) = the number of men with the outcome of interest (semen analysis with any motile sperm or with 1 × 10^6 ^sperm/mL or more) divided by the total number of men who provided a semen sample for a given vasectomy technique and early recanalization status for that time period.

In men with no early recanalization, about one fourth to one third of men had either motile sperm or 1 million sperm/mL or more at the time of the first semen analysis (< five weeks), except when thermal cautery with fascial interposition was performed in which case these proportions were about 15%. Motility and high sperm count decreased rapidly with all occlusion techniques over the following weeks with no motility observed with thermal cautery combined with fascial interposition six weeks after vasectomy. The proportions in the latter weeks in the ligation and excision techniques appear spuriously high due to the fact that men in the FI trial were discontinued from the study after two azoospermic semen analyses whereas men in the cautery study provided semen samples throughout the duration of the study, independent of semen analysis results.

Figures 1 and 2 illustrate selected semen analysis charts that are typical of sperm clearance associated with the different outcomes. Figure 1 shows the sperm clearance patterns of four men without presumed early recanalization. Lines no 1 and 2 show examples of the usual patterns of sperm clearance after vasectomy observed in both studies where sperm clearance is rapid, reaching azoospermia within two to eight weeks. Line no 3 illustrates slow sperm clearance characterized as low numbers of residual, non-motile sperm persisting for many weeks. Line no 4 illustrates a presumed technical failure with persistence of numerous motile sperm with no decrease in sperm concentration.

Figure 2 shows the sperm clearance patterns of four men with presumed early recanalization, characterized as a very low sperm concentration within two weeks after vasectomy followed by return to large numbers of sperm over the next few weeks. In lines no 5 and 6, the recanalization closed off with sperm concentrations falling again before 12–14 weeks and resulting in a successful vasectomy. These were categorized as sub-clinical transient recanalization, since the recanalization would not have been identified at the time of a routine first post-vasectomy semen analysis at 12 weeks or later. In line no 7, a transient early recanalization also occurred but the closing off process occurred later, between 14 and 22 weeks. This pattern has been described as a delayed success[12]. In line no 8, early recanalization persisted until the 24-week study endpoint resulting in an occlusion failure and need for a repeat vasectomy.

## Discussion

This is the first large study describing the semen patterns of early recanalization after vasectomy. Our findings suggest that early recanalization is far more frequent than commonly believed. As expected, almost all vasectomy failures are explained by presumed early recanalization. However, the number of early recanalizations far exceeds the number of failures, indicating that many are transient, eventually closing off, and resulting in a successful vasectomy.

Based on visual analyses of semen analysis charts, we observed that the vast majority of early recanalizations occur within a few weeks after vasectomy, most usually somewhere between two to six weeks. Similar observations based on one[13] and eight[14] cases were reported earlier. Our results imply that in clinical practice where the first post-vasectomy semen analysis is usually requested eight to 14 weeks after the procedure, many men assumed to have a successful vasectomy could in fact have had unnoticed sub-clinical transient recanalization that scarred down and spontaneously occluded before the first semen analysis. The partners of these men would be at higher risk of post-vasectomy pregnancy than the partners of men without recanalization if not using another contraception method until sterility is confirmed, as generally recommended. Recent evidence showing delayed vasectomy success in more than 50% of men who have motile sperm at the time of the first post-vasectomy semen analysis further supports the common occurrence of transient early recanalization[12].

In our blinded review of the semen analysis data, we observed large differences in early recanalization risk, suggesting that early recanalization is associated with occlusion technique. The highest risk of recanalization was seen with ligation and excision alone; one fourth of the men in this group were classified as having had an early recanalization. The risk was lower (about 10%) when ligation and excision with fascial interposition or electrocautery alone (with or without excision) was used. The lowest risk was observed in men whose vasectomy was performed with thermal cautery combined with fascial interposition.

When thermal cautery combined with fascial interposition was performed, no cases were classified as early recanalizations and motile sperm were cleared in all cases by six weeks post-vasectomy. These findings suggest that the current 12-week interval after the procedure before testing for sterility[15,16] could be shortened with this technique.

In the ligation and excision groups, a final outcome of early recanalization was equally as likely to be a success as a failure. However, when electrocautery without fascial interposition was used, the final outcome of most early recanalizations was vasectomy success. This suggests that early recanalizations occurring with cautery are less likely to result in vasectomy failure compared to those after ligation and excision.

Association between presumed early recanalization and vas occlusion technique is consistent with the primary and secondary analyses of the FI trial and cautery studies[8,9,17]. These analyses showed that most failures occurred in the ligation and excision alone group and the least in men who had a vasectomy performed with cautery. These findings are also in line with the results of a systematic review of comparative studies showing that the failure risk of vasectomy varies widely according to occlusion technique[18]. In that review, ligation and excision was associated with the highest risk of failure whereas cautery combined with FI appeared to have the lowest risk.

High risk of early recanalization with ligation and excision without FI may partly explain the unacceptably high contraceptive failure rate following vasectomy observed in Asian countries where this occlusion technique is still the most commonly performed[19]. A study involving 1052 men in Nepal showed that within 3 years after vasectomy 4.2% had an unplanned pregnancy[20]. A similar failure rate (4.1%) was also found in Vietnam after more than 5 years of follow-up[21]. In a study conducted in China, among 1,555 couples using vasectomy as a contraceptive method, the risk of an unplanned pregnancy was 9.5% after 5 years[22].

### Strengths and limitations

The major strength of our study is that it was based on results from prospective studies with standardized and excellent follow-up, including early and frequent post-vasectomy semen analyses. However, the primary purpose of this secondary analysis was not to compare the studies and techniques but to generate hypotheses based on observation. Thus, a number of limitations must be emphasized.

As study design of the two studies was different, comparisons between the four occlusion techniques are based on non randomized groups, apart from the two groups in the FI trial. There were also slight methodological differences between the two studies, such as sample size, timing of semen analyses, and definitions of success and failure. In addition, although all participating sites in both studies followed standardized methods for performing semen analysis, semen samples showing azoospermia or very low sperm counts were centrifuged in the FI trial but not in the cautery study. Non-centrifuged semen samples categorized as azoospermic commonly have some sperm if examined after centrifugation, but the numbers would be too low to have interfered with chart adjudication relative to whether or not there was a recanalization[23]. In laboratories that do not centrifuge specimens, sperm concentrations below 100,000/mL are likely to be read as azoospermic (D Sokal, manuscript in preparation).

The level of expertise of participating surgeons in the two studies may explain differences between the results observed with the four occlusion techniques. Some of the surgeons involved in the FI trial were not well-experienced with the FI technique and different methods of FI were used in the cautery study. Despite some training provided before starting the study, technical errors may partly explain the high failure and recanalization risk encountered with this technique. In fact, FI may be difficult to adequately master[19]. In the cautery study, excellent results obtained with thermal cautery and FI combined may be due to the extensive expertise of the two participating surgeons with this technique. Differences in the level of expertise of participating surgeons would not however influence the findings and conclusions related to the failure/recanalization ratio within each individual technique.

Independent review of charts by three experienced vasectomy clinicians/researchers with consensus on discordant cases was used to estimate the risk of presumed early recanalization after vasectomy. Although this method minimizes the risk of bias, it does not exclude it. Recanalization cannot be proven on clinical grounds and no histology was done to confirm recanalization.

Since reviewers knew the outcome of the cases while reviewing the charts from the FI trial, the proportion of failures explained by early recanalization may have been overestimated. Furthermore, knowing the outcome may have led to underestimating the numbers of transient early recanalizations that are followed by subsequent azoospermia and vasectomy success. However, failure or success was included as a criterion to identify early recanalization by only one reviewer and not retained as a consensus criterion (Table 1).

Semen analysis data from two of the four cautery study sites were limited to sperm concentrations, so we could not consider sperm motility when adjudicating charts from the cautery study. The risk of early recanalization may have been underestimated without this information. Using the results from the two sites where motility was assessed and the consensus criteria that include motility (see Table 1), early recanalization was presumed in 1% (1/99) and 18% (18/97) of cases with cautery with and without FI, respectively, whereas according to the classification obtained without motility, these figures were 0% (0/99) and 9% (9/97). Some argue that motility found as early as three weeks after vasectomy is either due to a surgical error or to early recanalization[13,24-26].

This possible underestimation does not change our primary results showing that cautery combined with FI is associated with the lowest risk of early recanalization and simple ligation and excision with the highest. The risk associated with cautery alone and ligation and excision with FI is lying in between.

Other factors could also have led to a slight underestimation of early recanalization risk. In the few instances where sperm counts never fell to sub-fertile levels, it was assumed that failure was due to a surgical error. However, it is possible that early recanalization could have occurred within the first two weeks after the procedure, before any semen analysis was performed. In addition, in cases where lack of data meant a participant could not be classified definitively as a presumed early recanalization or not, he was considered as not having had a recanalization.

Adjudication of the charts from the cautery study was done about three months after adjudication of the FI trial charts. Although individual criteria were collected after adjudication of the FI trial charts and not discussed until after completing the adjudication of the cautery study charts, the criteria and interpretation of charts may have changed in time from "learning" about recanalization. This potential bias was minimized using multiple independent reviewers and consensus was needed in only a small and similar proportion of charts in both studies (13% and 14%).

We were unable to asses the risk of late recanalization in this study. Late recanalization is defined as the reappearance of motile sperm after the vasectomy was declared a success, often only discovered by an unexpected pregnancy. This situation suggests that recanalization can occur at any time after vasectomy and not only a few weeks after vasectomy as observed in our study. However, late recanalization is believed to be a very rare phenomenon occurring in only about one out of 2000 to 3000 men [26-28].

## Conclusion

Patterns and criteria of presumed early recanalization after vasectomy were identified in this study. As the occlusive effectiveness of vasectomy has probably been overestimated in most vasectomy research, further studies on occlusion techniques should take into account the occurrence of presumed early recanalization when setting effectiveness end-points. Our consensus criteria may be useful to this end and they should be further validated for that specific purpose.

From a clinical point of view, our results reinforce the recent recommendation to avoid ligation and excision as the sole method for occluding the vas[27,29,30]. They also support the use of cautery combined with FI as probably the most effective vas occlusion technique. If early recanalization could be reliably prevented, the currently recommended 12-week waiting period before performing the post-vasectomy semen analysis[15,16] could probably be shortened.

## Competing interests

The author(s) declare that they have no competing interests.

## Authors' contributions

All authors participated in the conception, design, data collection, and analysis of the study. ML drafted the manuscript, and all authors reviewed and approved the final manuscript.

## Pre-publication history

The pre-publication history for this paper can be accessed here:



## Supplementary Material

Additional file 1 Characteristics of vasectomies studies. This table provides a comparative summary of the characteristics of the two studies. Click here for file

Additional file 2 Semen analysis charts from the Fascial Interposition trial. This file provides all semen analysis charts from the Fascial Interposition trial. Click here for file

Additional file 3 Semen analysis charts from the Cautery study. This file provides all semen analysis charts from the Cautery study. Click here for file
