# Supplementary material for: Frequency and patterns of early recanalization after vasectomy
Source: BMC Urol. 2006 Sep 19;6:25. doi: 10.1186/1471-2490-6-25 (PMC1586021; doi:10.1186/1471-2490-6-25)
Supplement: Additional file 1 — Characteristics of vasectomies studies. This table provides a comparative summary of the characteristics of the two studies. [file 1471-2490-6-25-S1.doc]

## Additional file 1 – Characteristics of vasectomies studies

| **Characteristics** | **Fascial Interposition Trial**[8] | **Cautery Study**[9] |
| --- | --- | --- |
| **Study Sites** | Eight sites in seven countries | Four sites in four countries |
| **Study Design** | Randomized controlled trial comparing ligation and excision with versus without fascial interposition | Prospective observational study to estimate the effectiveness of cautery occlusion and describe trends in sperm counts after cautery occlusion |
| **Vasectomy technique**   - Approach to the vas - Occlusion method |  |  |
|  | No-scalpel vasectomy | Three sites used no-scalpel vasectomy |
|  | Standardized occlusion technique   - Vas occluded with two silk sutures - A segment of vas was excised (approximately 1 cm) - For FI, a suture was used to contain the testicular end of the vas inside the fascial sheath | Customary cautery occlusion techniques used at each site   - Two sites used electrocautery alone - Two sites used thermal cautery with fascial interposition - A small vas segment was excised in one site using electrocautery and in one site using thermal cautery |
| **Timing of semen analysis**   - Began at two weeks after vasectomy in both studies | - Subsequent semen analyses every 4 weeks until a man had provided two consecutive azoospermic specimens, was declared a vasectomy failure, or reached the end of study follow-up at 34 weeks. | - Subsequent semen analyses at weeks 5, 8, 12, 16, 20 and 24 regardless of semen analysis findings |
| **Semen analysis procedures**   - Based on World Health Organization recommendations - Laboratories conducted periodic quality control tests | - Freshly collected semen specimens were examined - Data obtained on sperm concentration, motility and viability - Specimens showing azoospermia or very low sperm concentrations were centrifuged | - All sites did not examine fresh specimens - Data limited to sperm concentration at 2 sites - No centrifugation of specimens |
| **Adverse Events** | - Vasectomy related adverse events were collected | Vasectomy related adverse events were not collected |
| **Definition of success and failure**   - Success - Failure - Indeterminate |  |  |
|  | - Two consecutive azoospermic specimens at least two weeks apart | - Less than 100,000 sperm/mL in two consecutive specimens taken at least two weeks |
|  | - 5 million or more motile sperm/mL at 14+ weeks or 100,000 sperm or more/mL with any motility at 26+ weeks | - Not meeting success definition by 24 weeks or having more than 10 millions sperm/mL at 12+ weeks |
|  | - Neither success nor failure | - Men who had less then 12 weeks of follow-up without having been declared a failure by a study site clinician where classified as indeterminate |
