# Supplementary material for: Frequency and patterns of early recanalization after vasectomy
Source: BMC Urol. 2006 Sep 19;6:25. doi: 10.1186/1471-2490-6-25 (PMC1586021; doi:10.1186/1471-2490-6-25)

id= 1 outcome= Success

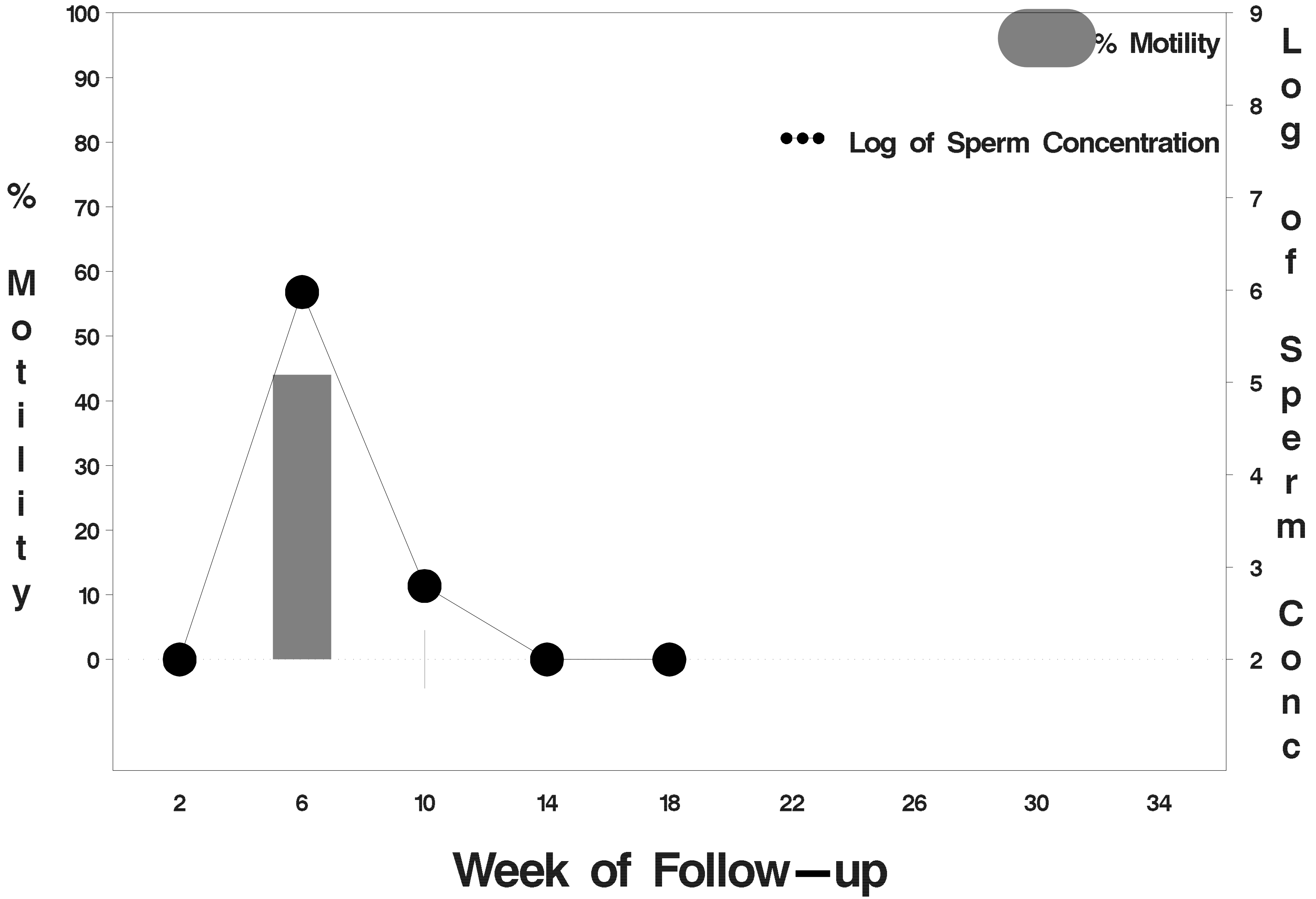

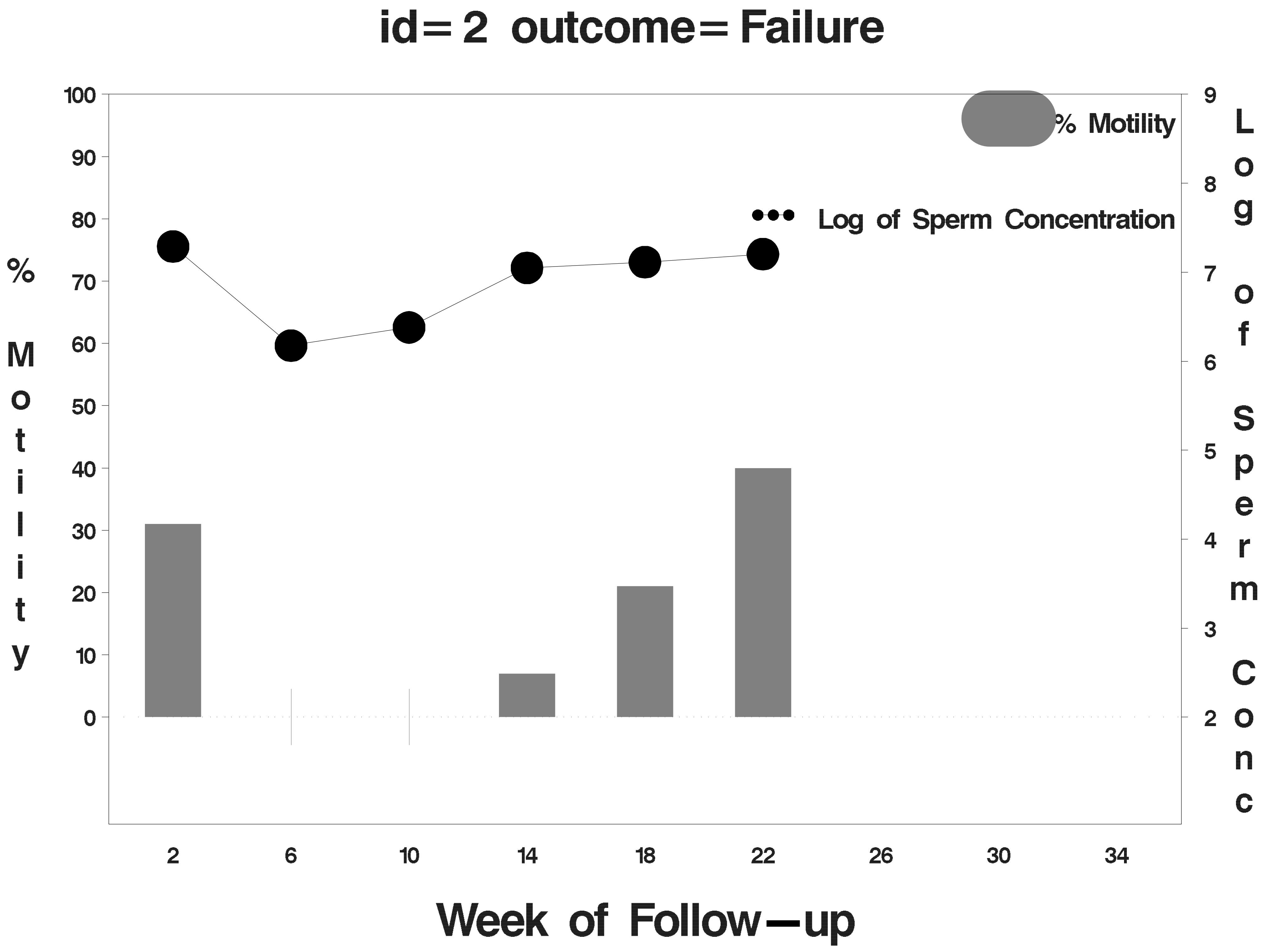
id= 2 outcome= Failure
% Motility
Log of Sperm Concentration
% Motility
Log of Sperm Conc
Week of Follow−up
100
90
80
70
60
50
40
30
20
10
0
9
8
7
6
5
4
3
2
2
6
10
14
18
22
26
30
34

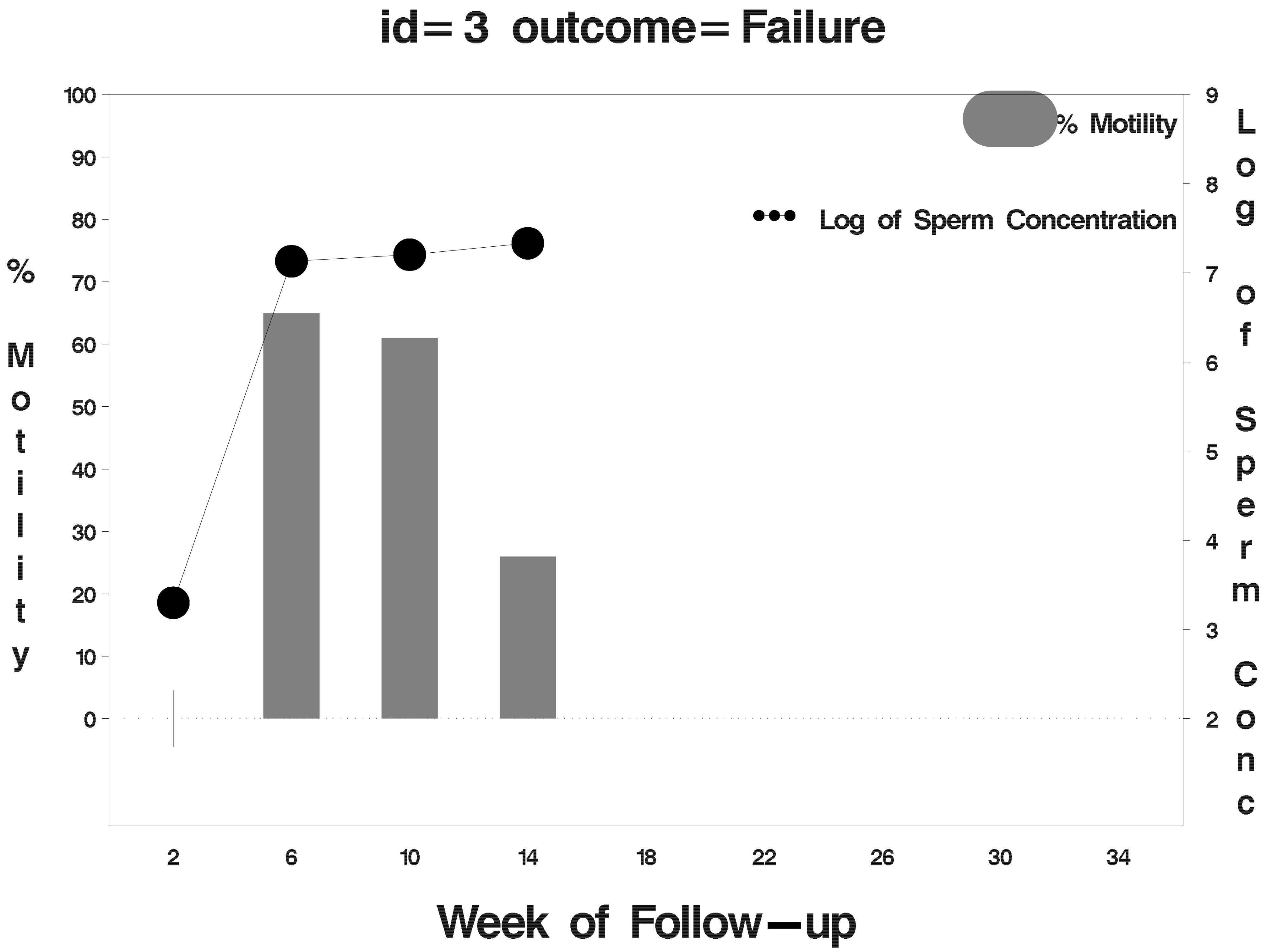

id= 3 outcome= Failure
% Motility
Log of Sperm Concentration
100
90
80
70
60
50
40
30
20
10
0
9
8
7
6
5
4
3
2
2
6
10
14
18
22
26
30
34
Week of Follow−up
Log of Sperm Conc

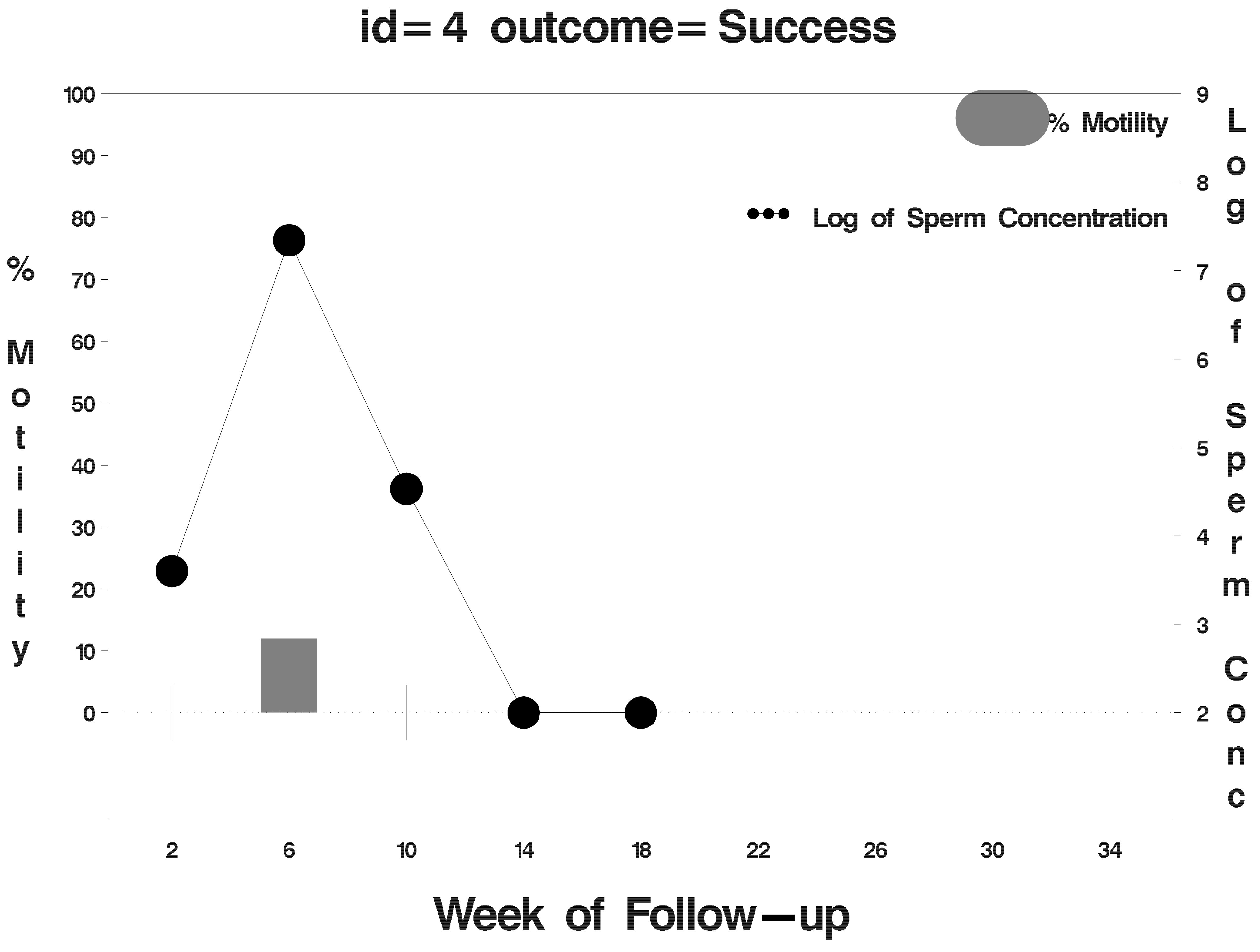
id= 4 outcome= Success
% Motility
Log of Sperm Concentration
100
90
80
70
60
50
40
30
20
10
0
9
8
7
6
5
4
3
2
2
6
10
14
18
22
26
30
34
Week of Follow−up
Log of Sperm Conc

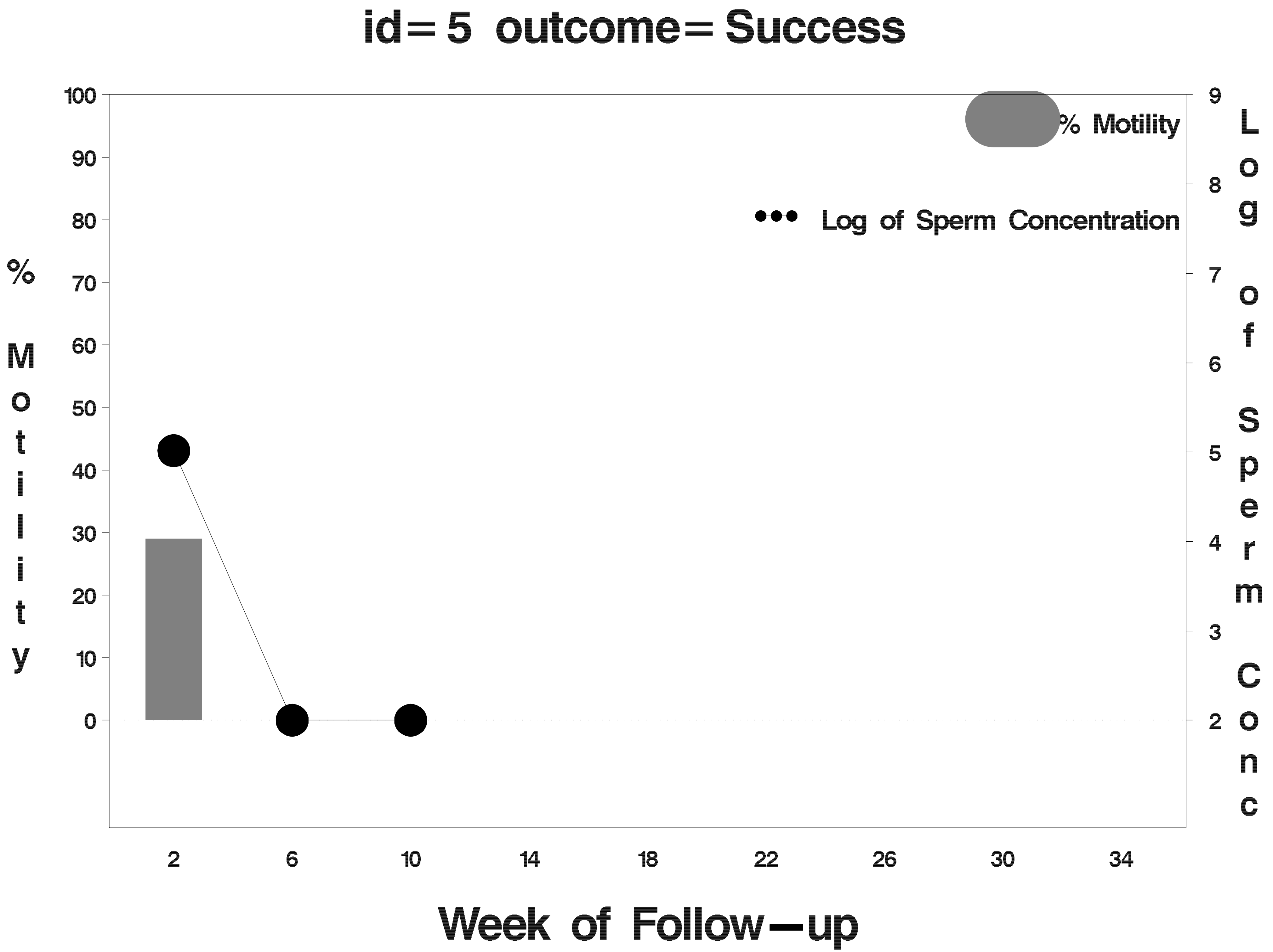

id= 5 outcome= Success
% Motility
Log of Sperm Concentration
100
90
80
70
60
50
40
30
20
10
0
9
8
7
6
5
4
3
2
2
6
10
14
18
22
26
30
34
Week of Follow-up
% Motility
Log of Sperm Conc

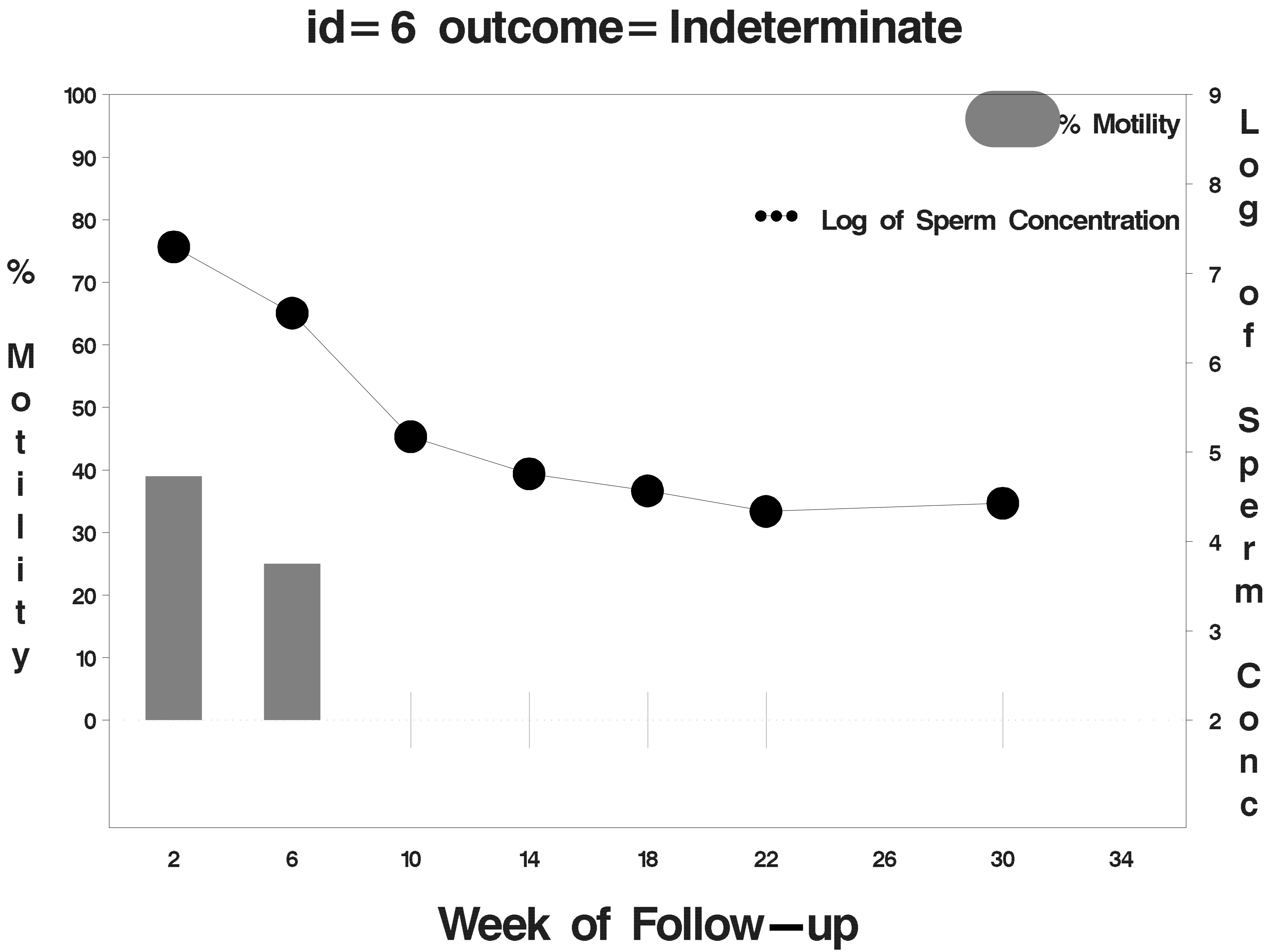

id= 6 outcome= Indeterminate
% Motility
Log of Sperm Concentration
100
90
80
70
60
50
40
30
20
10
0
9
8
7
6
5
4
3
2
2
6
10
14
18
22
26
30
34
Week of Follow—up
Log of Sperm Conc

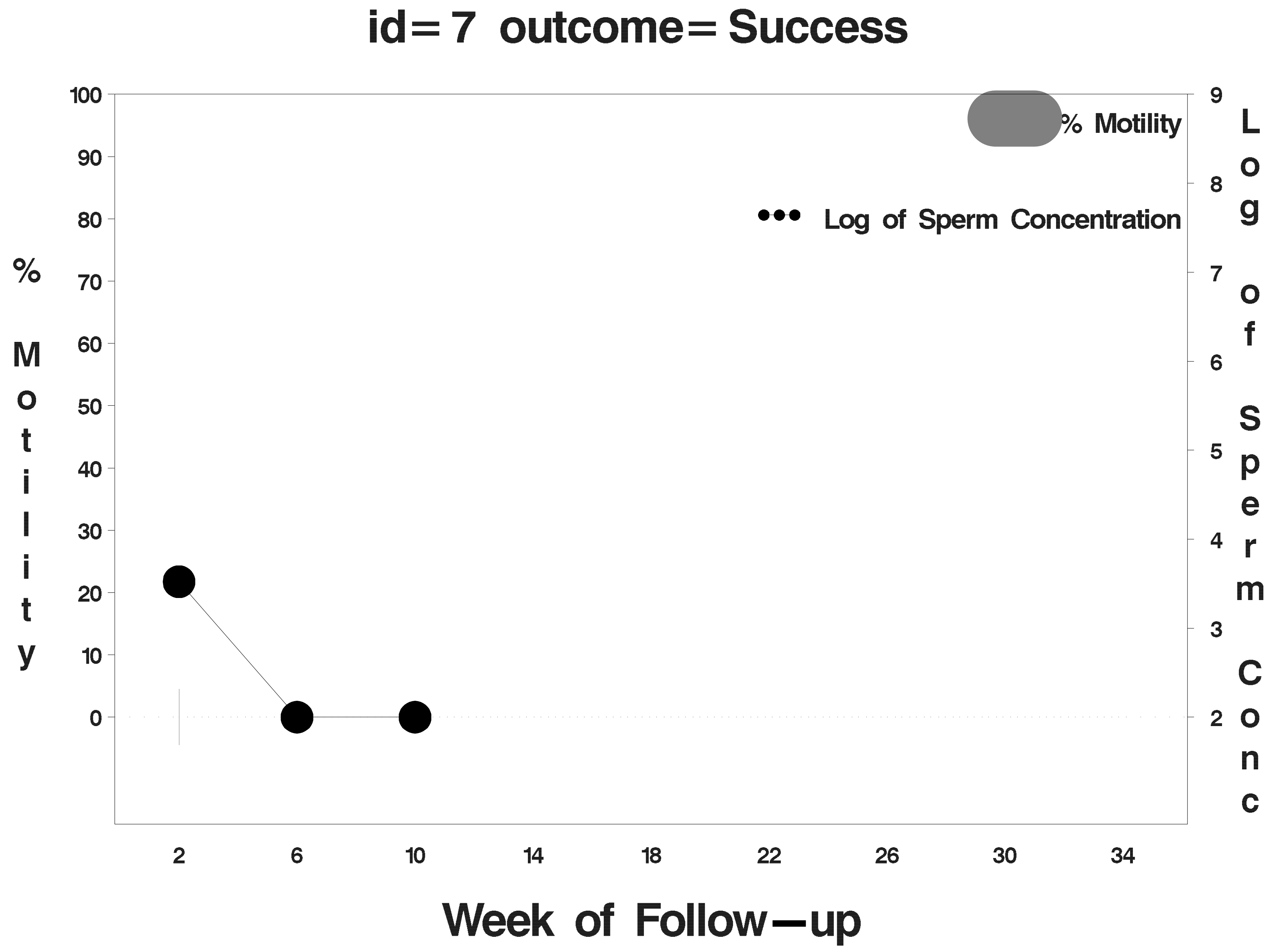

id= 7 outcome= Success
% Motility
Log of Sperm Concentration
100
90
80
70
60
50
40
30
20
10
0
9
8
7
6
5
4
3
2
2
6
10
14
18
22
26
30
34
Week of Follow—up
% Motility
Log of Sperm Conc

id= 8 outcome= Success

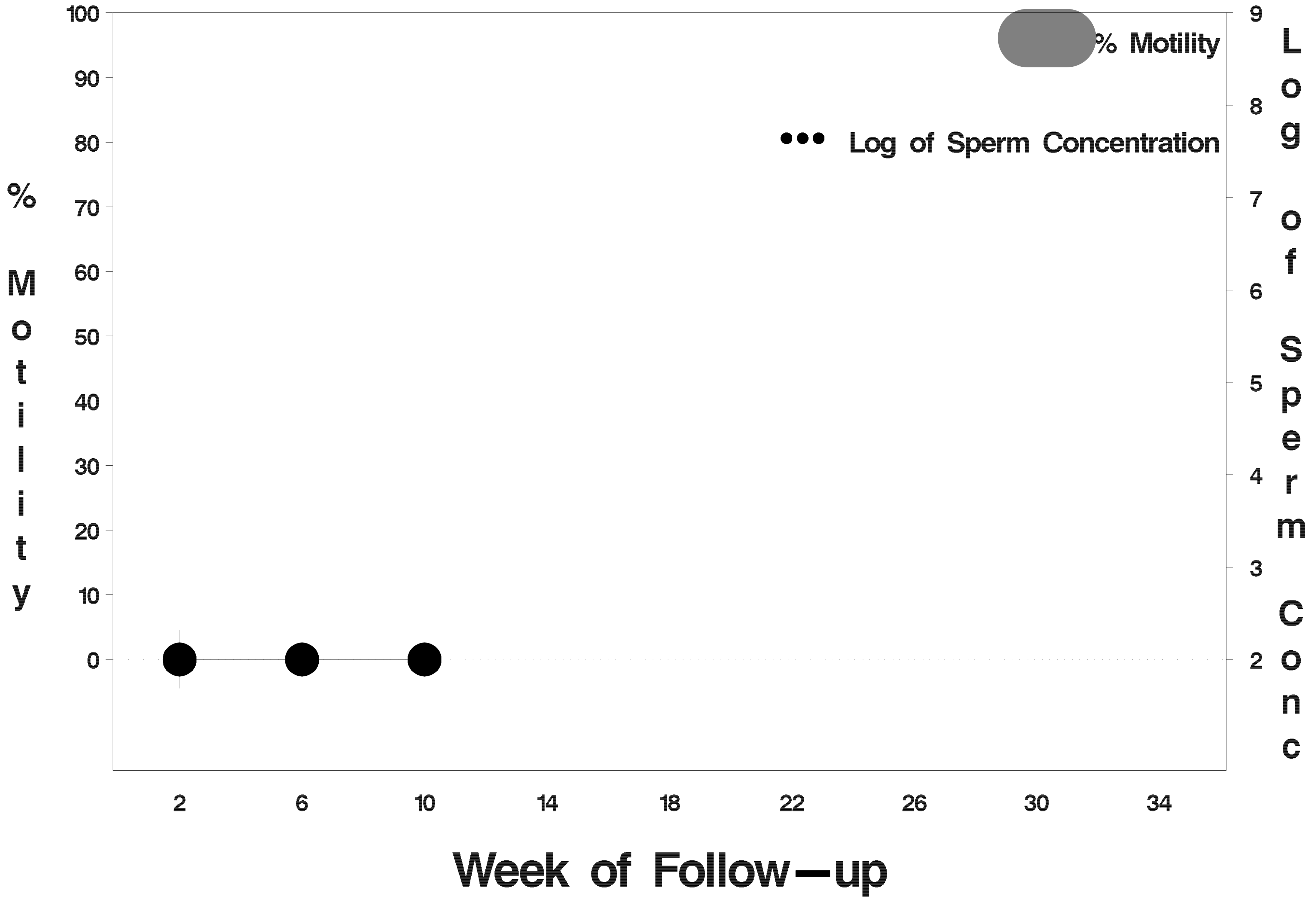

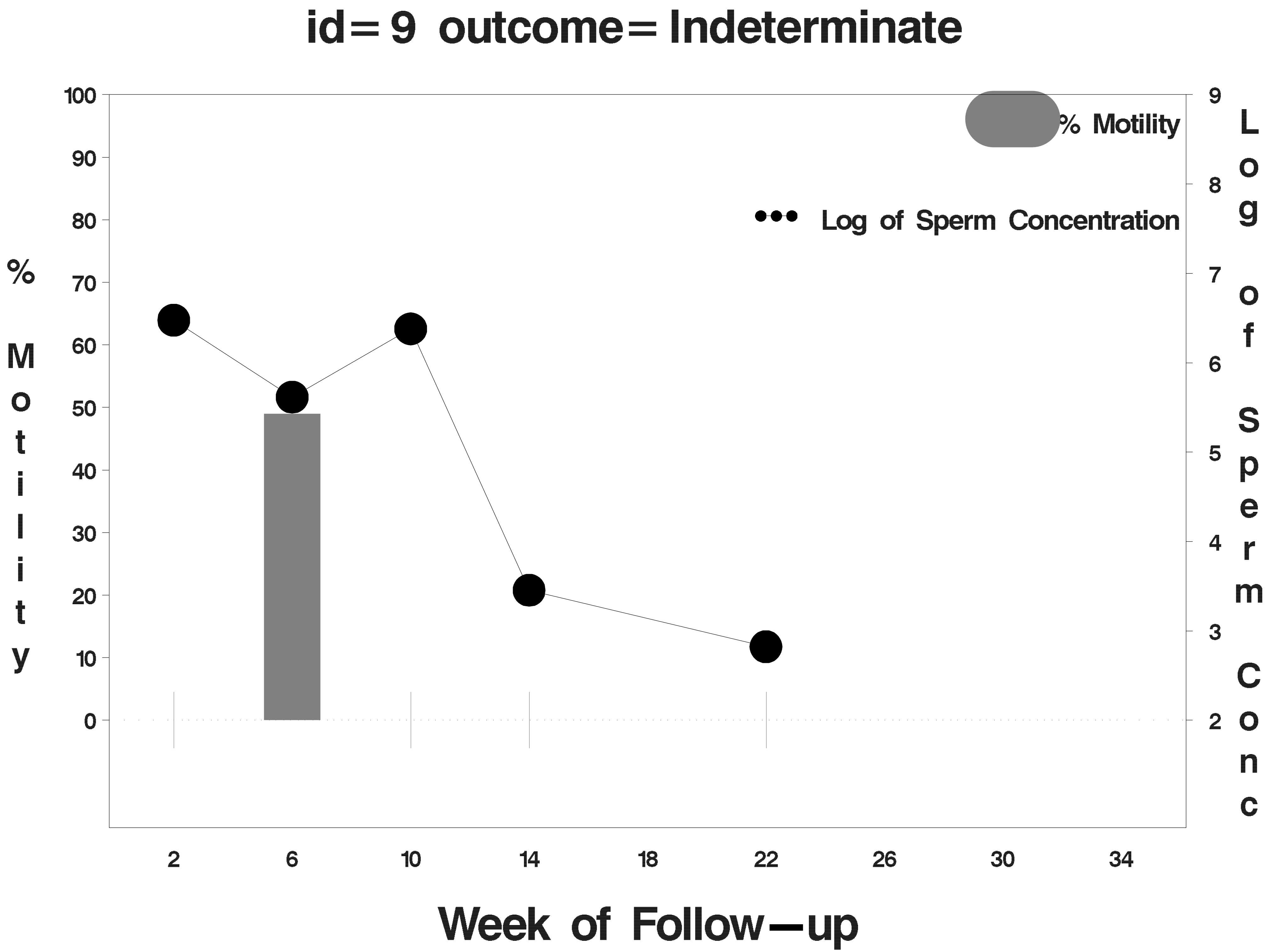

id= 9 outcome= Indeterminate
% Motility
Log of Sperm Concentration
100
90
80
70
60
50
40
30
20
10
0
9
8
7
6
5
4
3
2
2
6
10
14
18
22
26
30
34
Week of Follow—up
Log of Sperm Conc

id= 10 outcome= Success

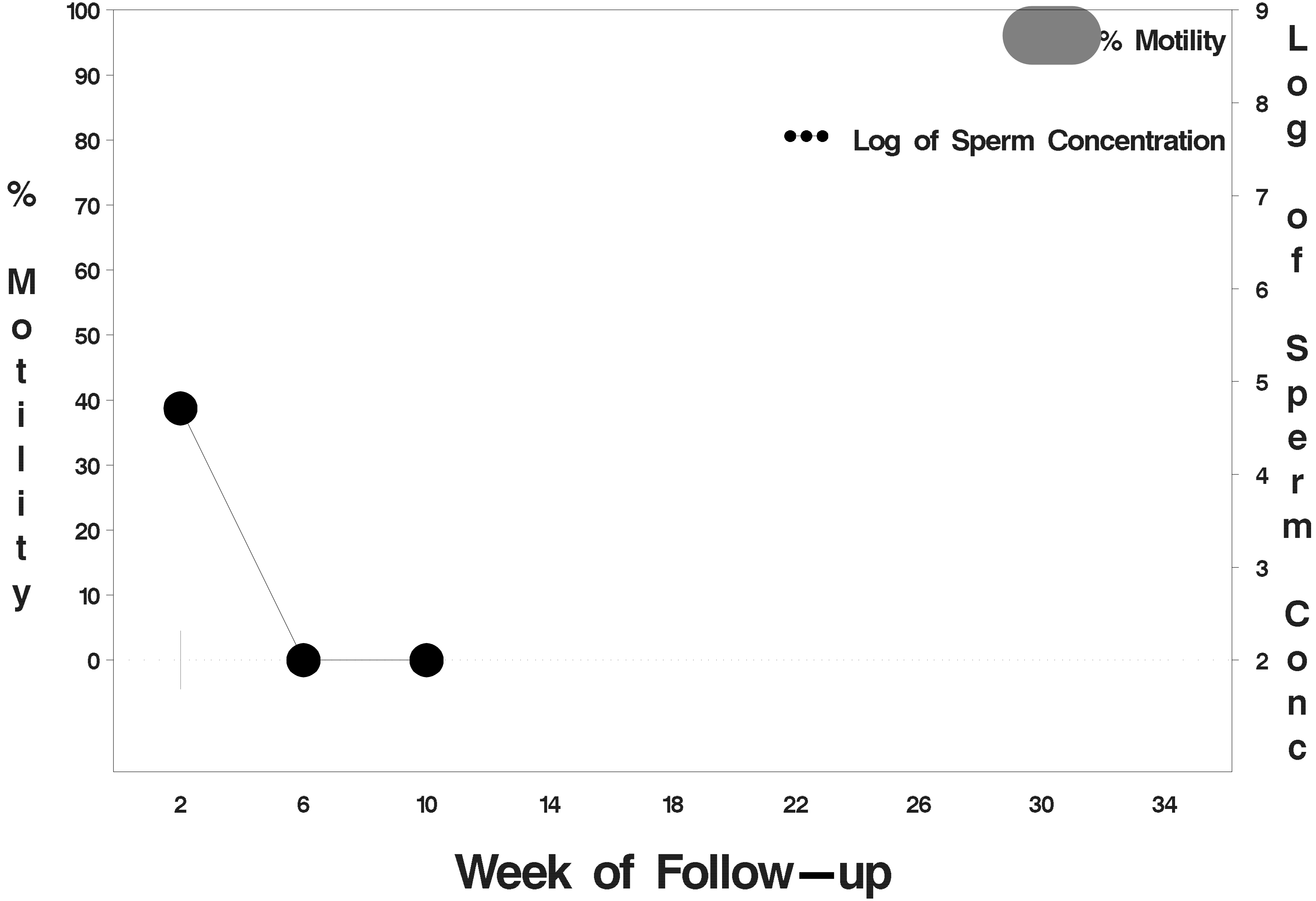

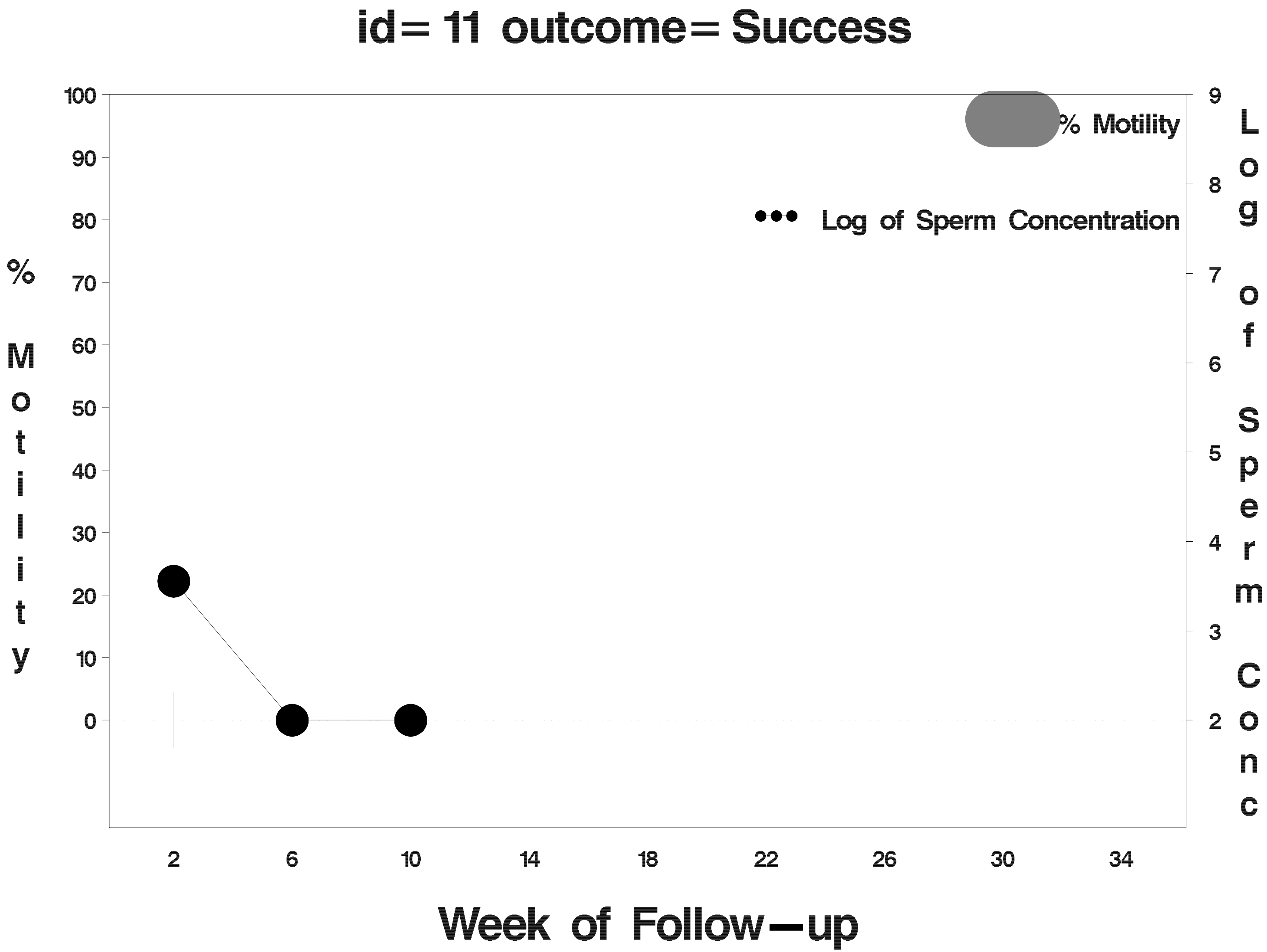
id= 11 outcome= Success
% Motility
Log of Sperm Concentration
100
90
80
70
60
50
40
30
20
10
0
9
8
7
6
5
4
3
2
2
6
10
14
18
22
26
30
34
Week of Follow−up
% Motility
Log of Sperm Conc

id= 12 outcome= Indeterminate

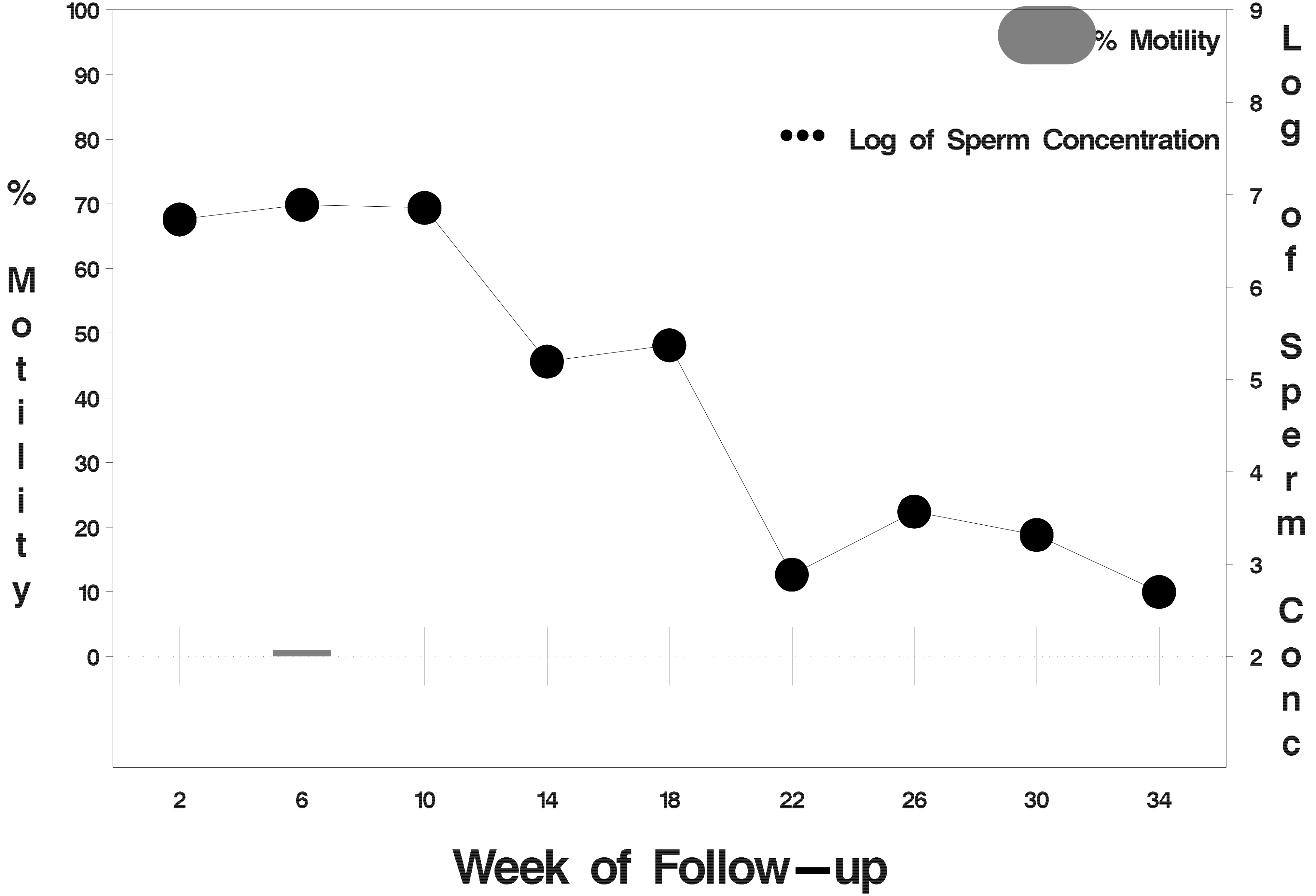

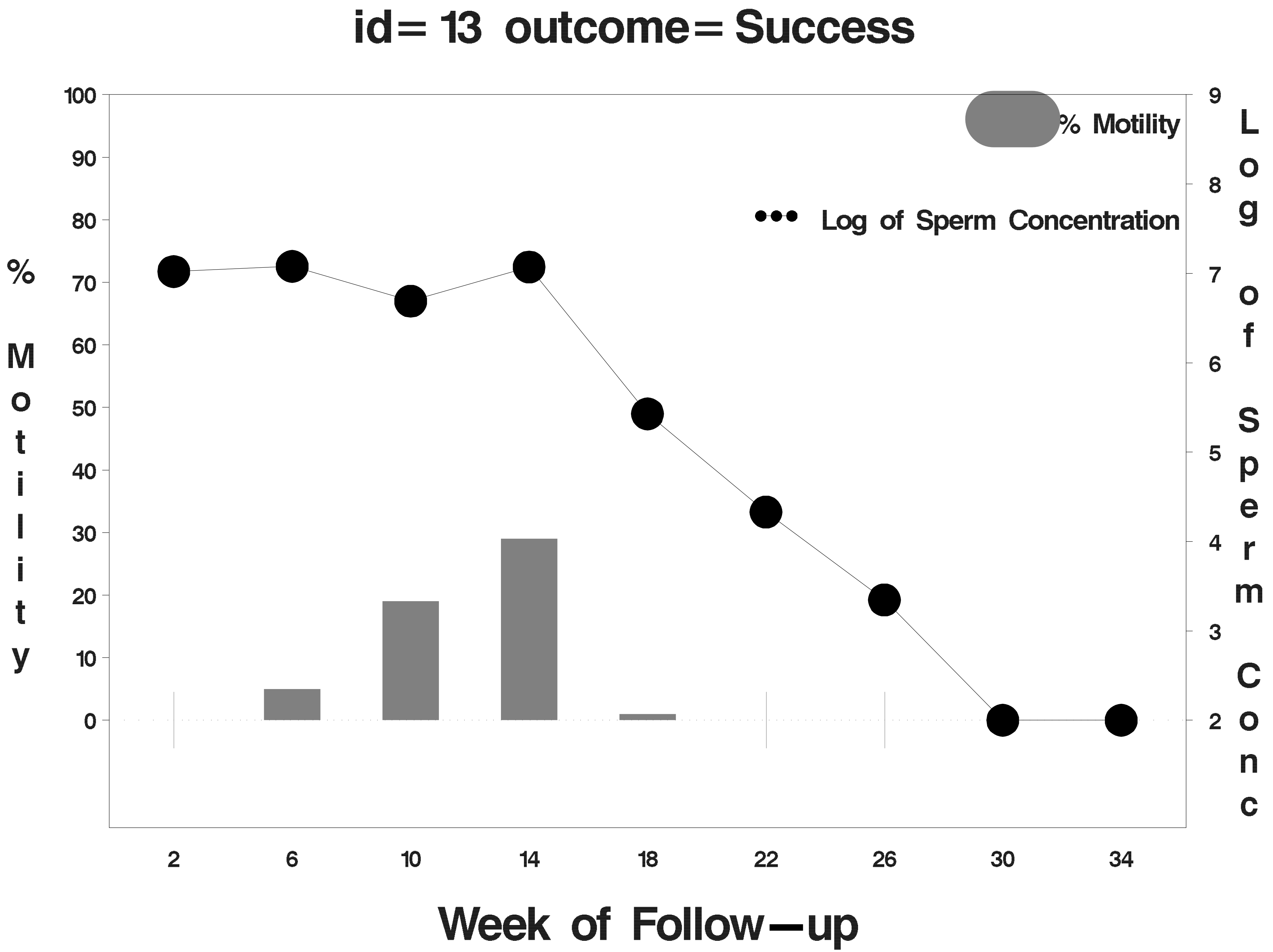

id= 13 outcome= Success
% Motility
Log of Sperm Concentration
% Motility
Log of Sperm Conc
Week of Follow−up
100
90
80
70
60
50
40
30
20
10
0
9
8
7
6
5
4
3
2
2
6
10
14
18
22
26
30
34

id= 14 outcome= Success

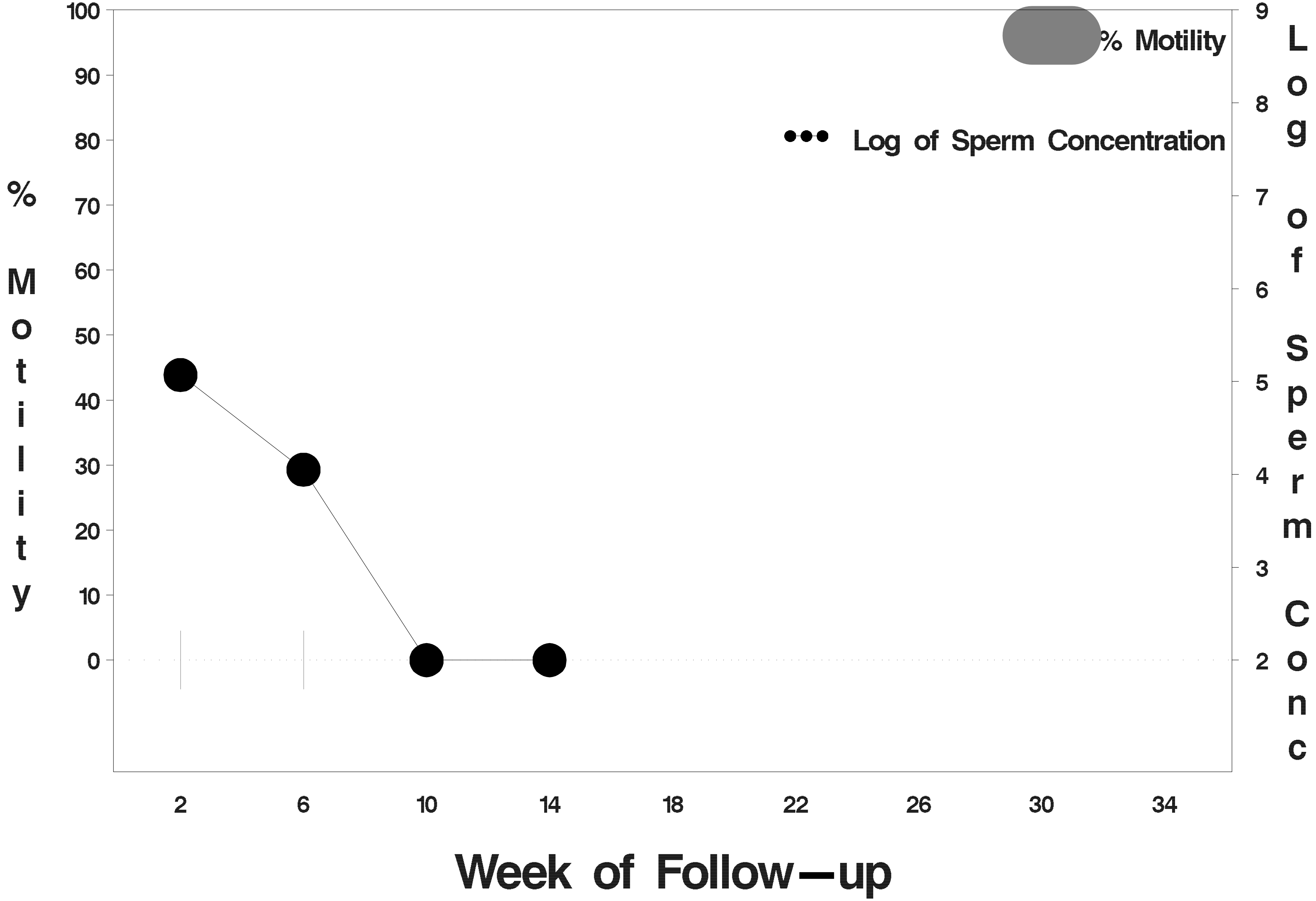

id= 15 outcome= Success

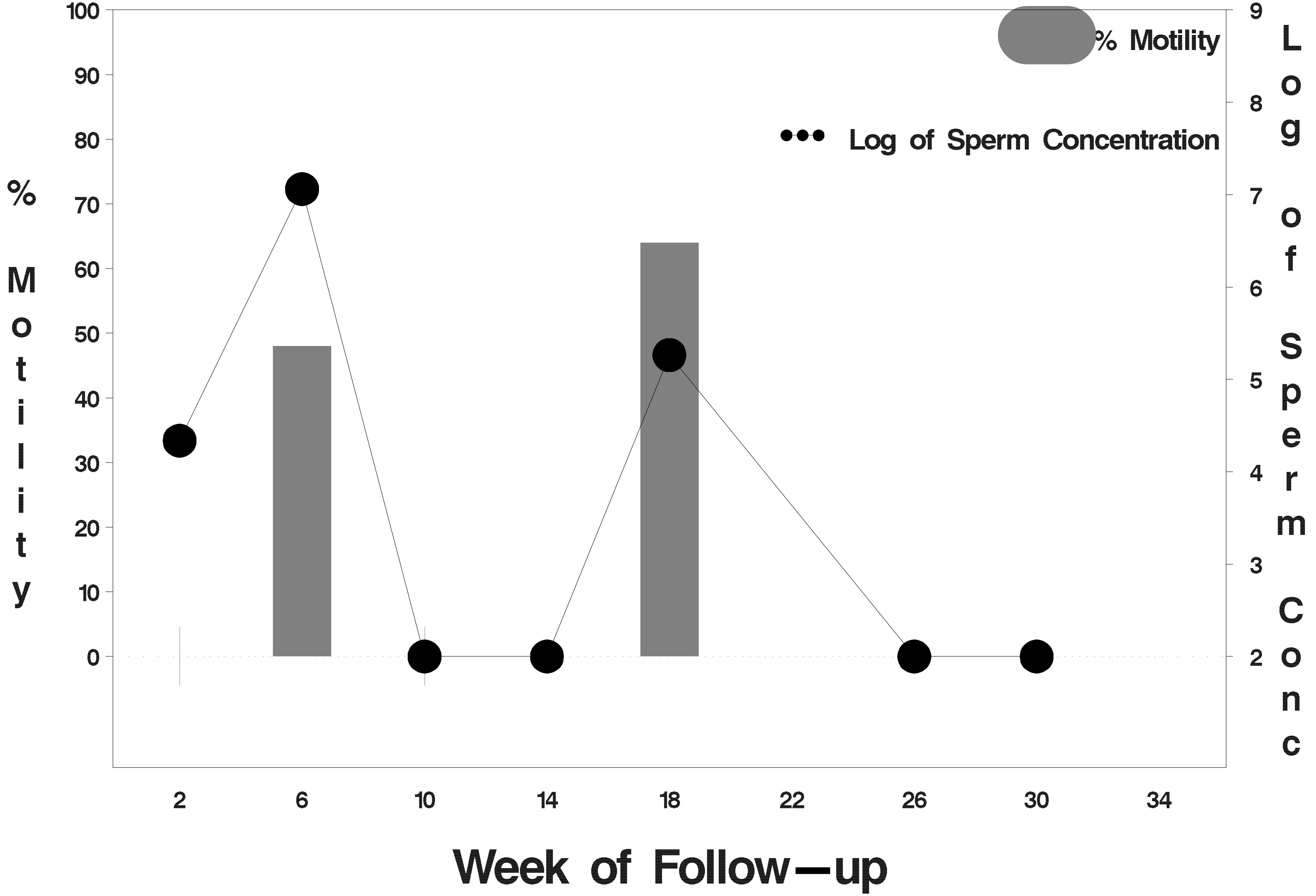

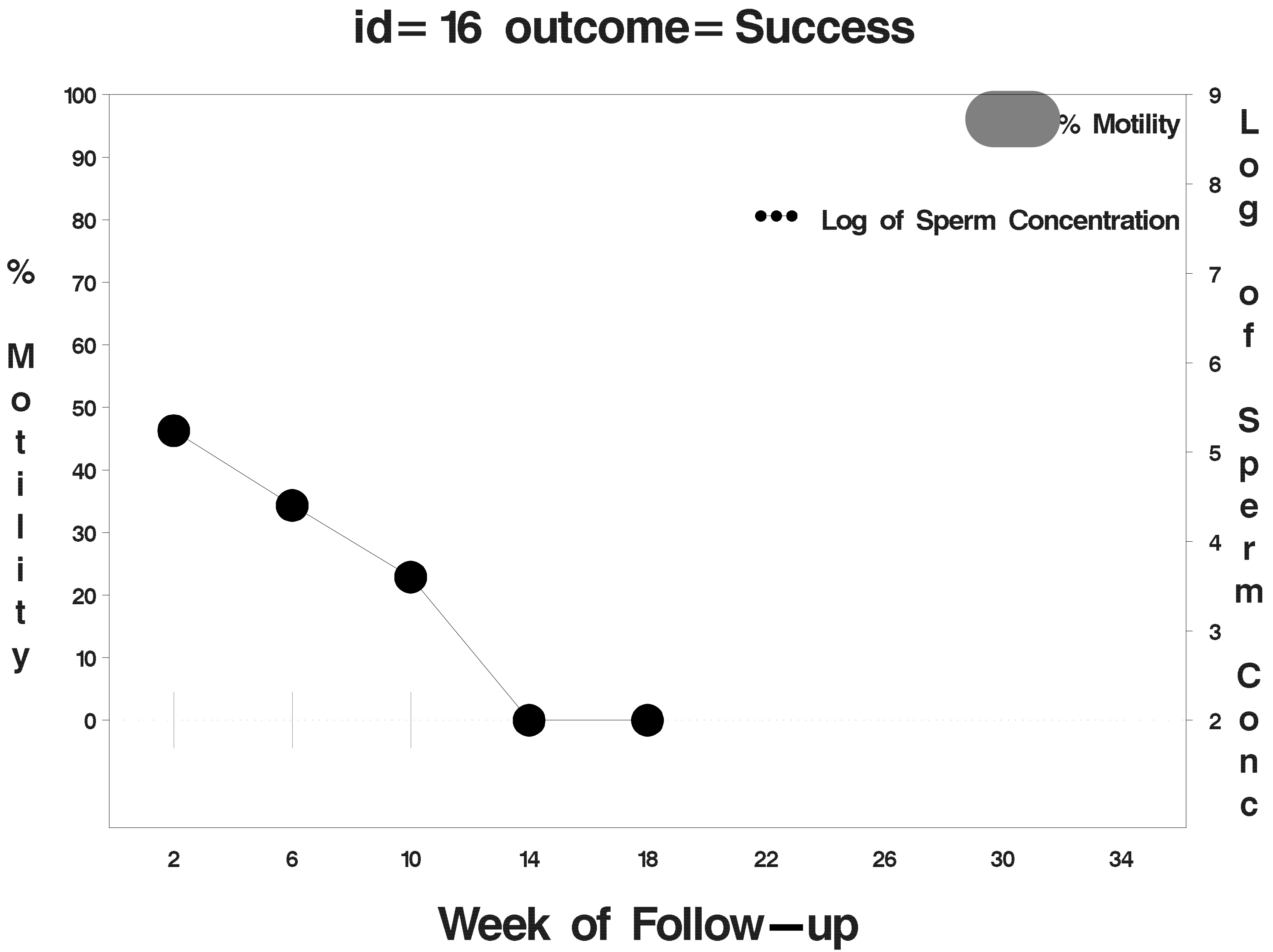

id= 16 outcome= Success
% Motility
Log of Sperm Concentration
100
90
80
70
60
50
40
30
20
10
0
9
8
7
6
5
4
3
2
2
6
10
14
18
22
26
30
34
Week of Follow−up
% Motility
Log of Sperm Conc

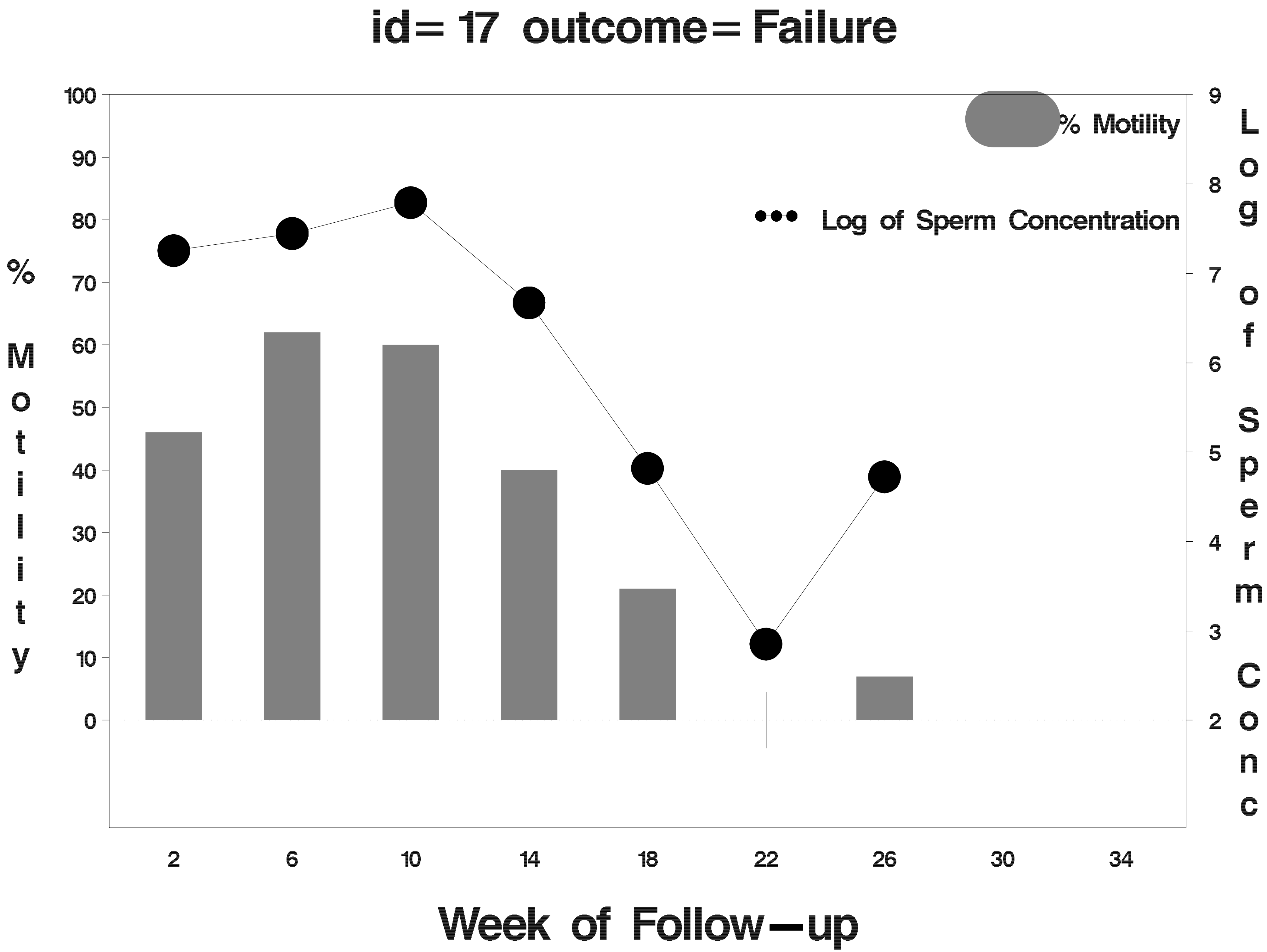
id= 17 outcome= Failure
% Motility
Log of Sperm Concentration
% Motility
Log of Sperm Conc
Week of Follow−up
100
90
80
70
60
50
40
30
20
10
0
9
8
7
6
5
4
3
2
2
6
10
14
18
22
26
30
34

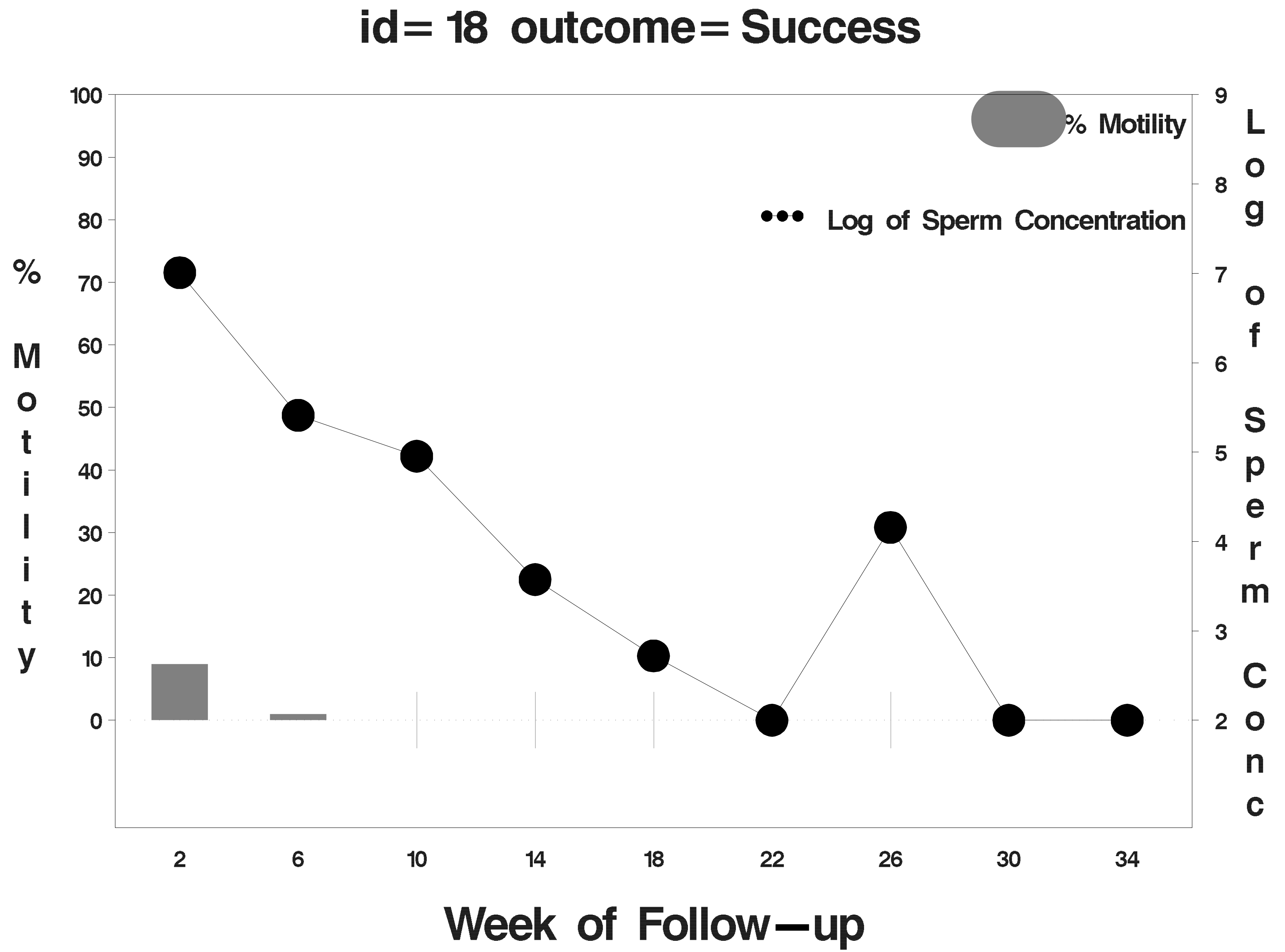
id= 18 outcome= Success
% Motility
Log of Sperm Concentration
100
90
80
70
60
50
40
30
20
10
0
9
8
7
6
5
4
3
2
2
6
10
14
18
22
26
30
34
Week of Follow—up
Log of Sperm Conc

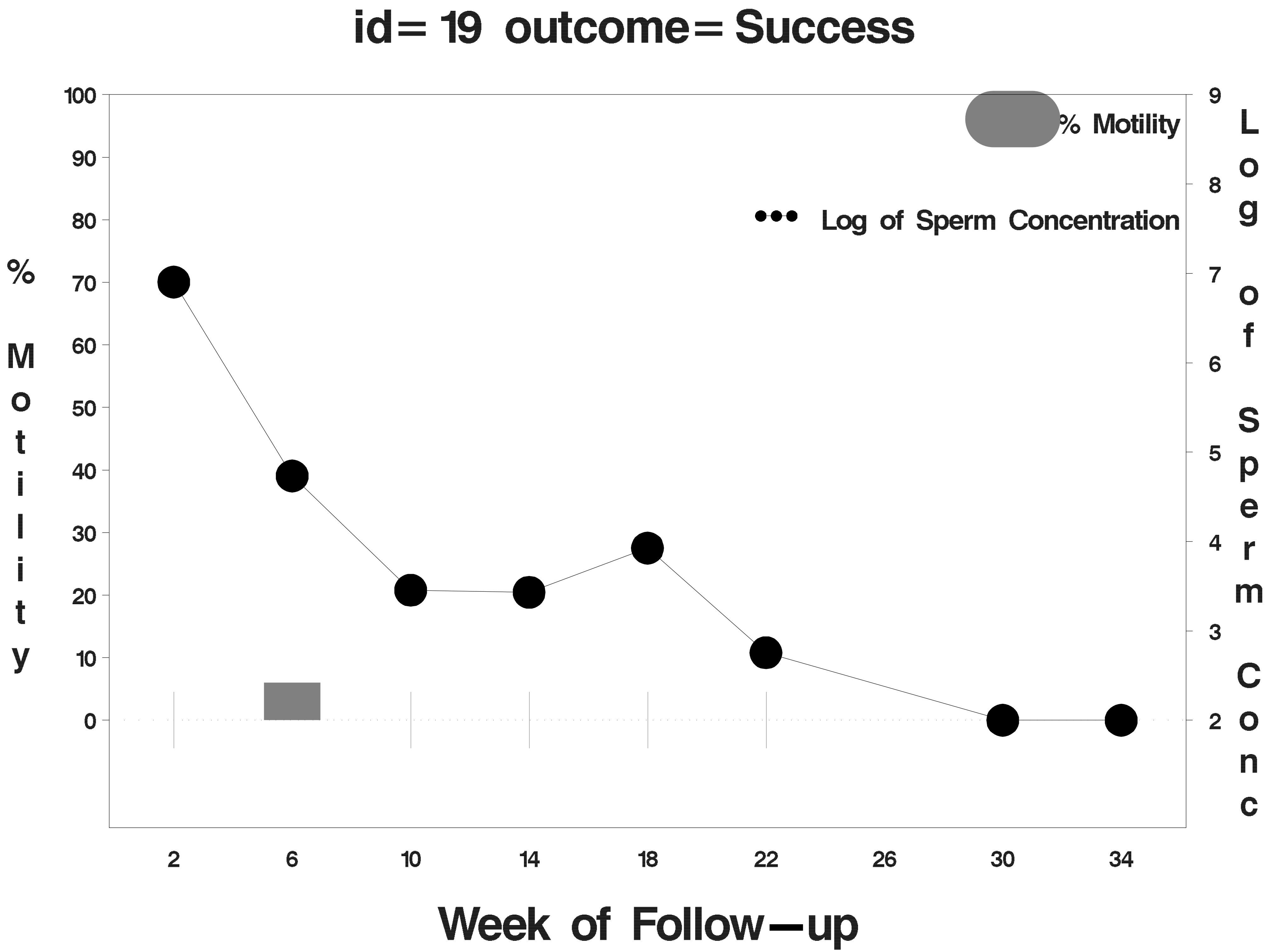
id= 19 outcome= Success
% Motility
Log of Sperm Concentration
% Motility
Log of Sperm Conc
100
90
80
70
60
50
40
30
20
10
0
9
8
7
6
5
4
3
2
2
6
10
14
18
22
26
30
34
Week of Follow−up

id= 20 outcome= Success

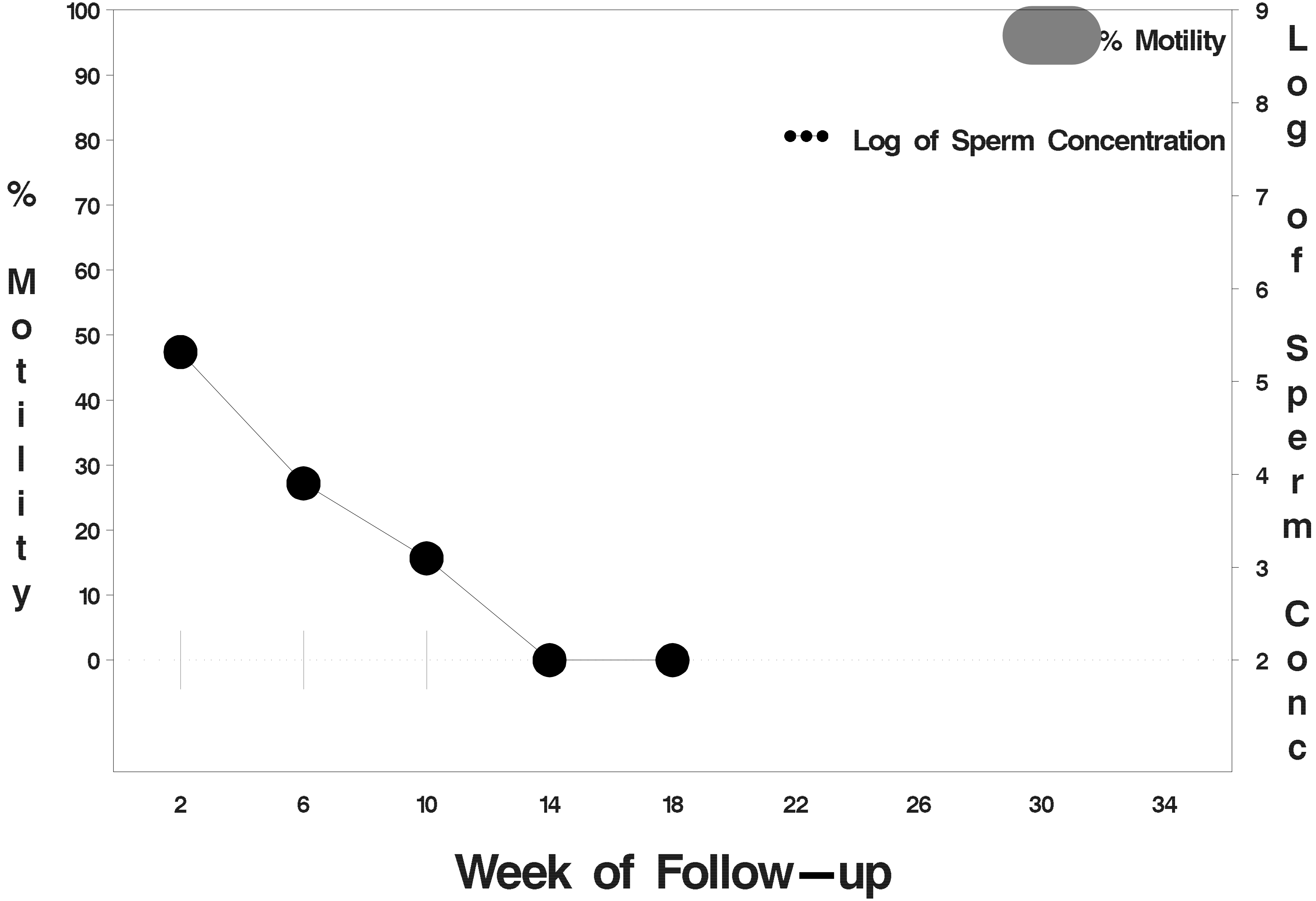

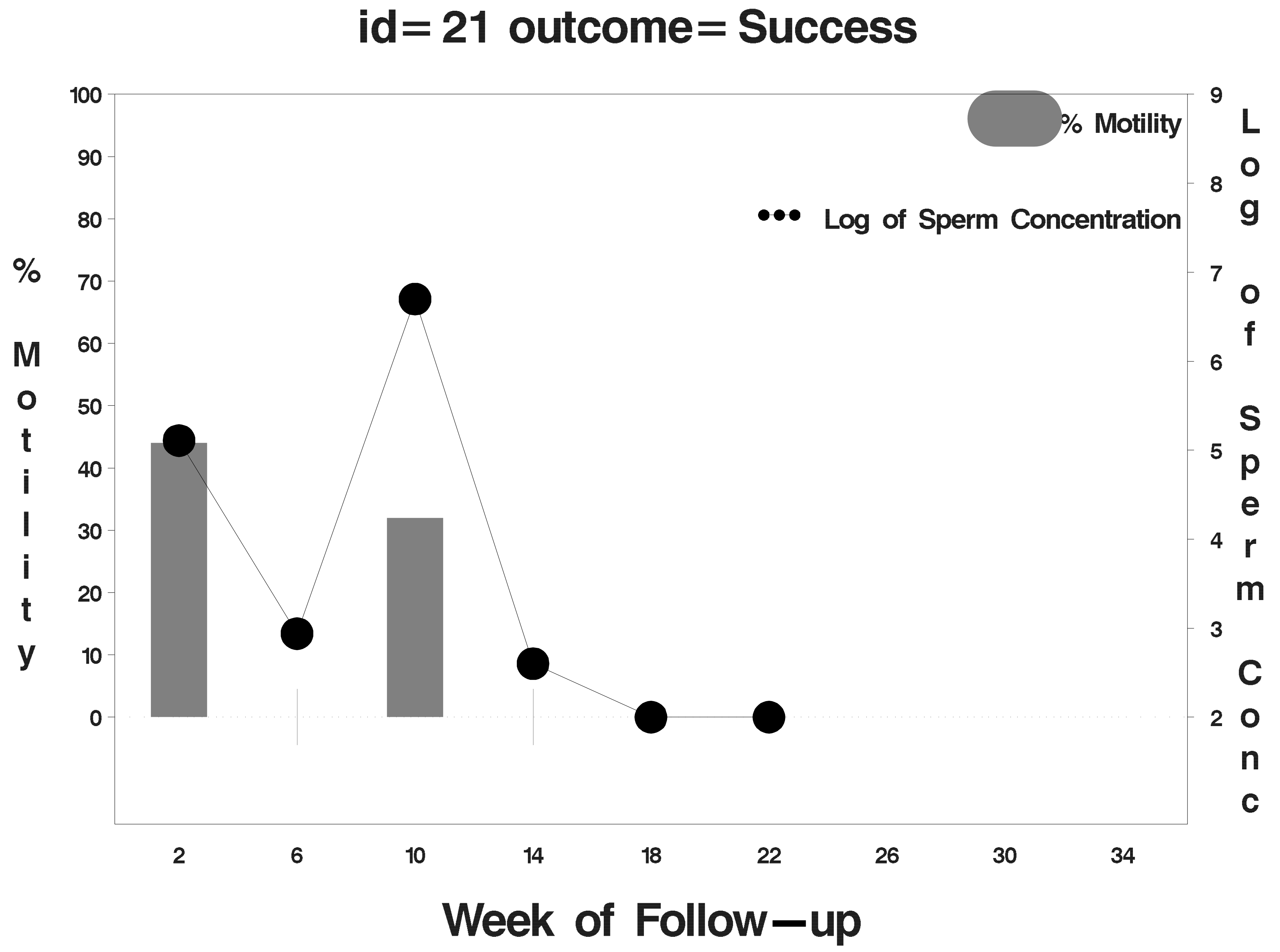

id= 21 outcome= Success
% Motility
Log of Sperm Concentration
100
90
80
70
60
50
40
30
20
10
0
9
8
7
6
5
4
3
2
2
6
10
14
18
22
26
30
34
Week of Follow-up
Log of Sperm Conc

id= 22 outcome= Success

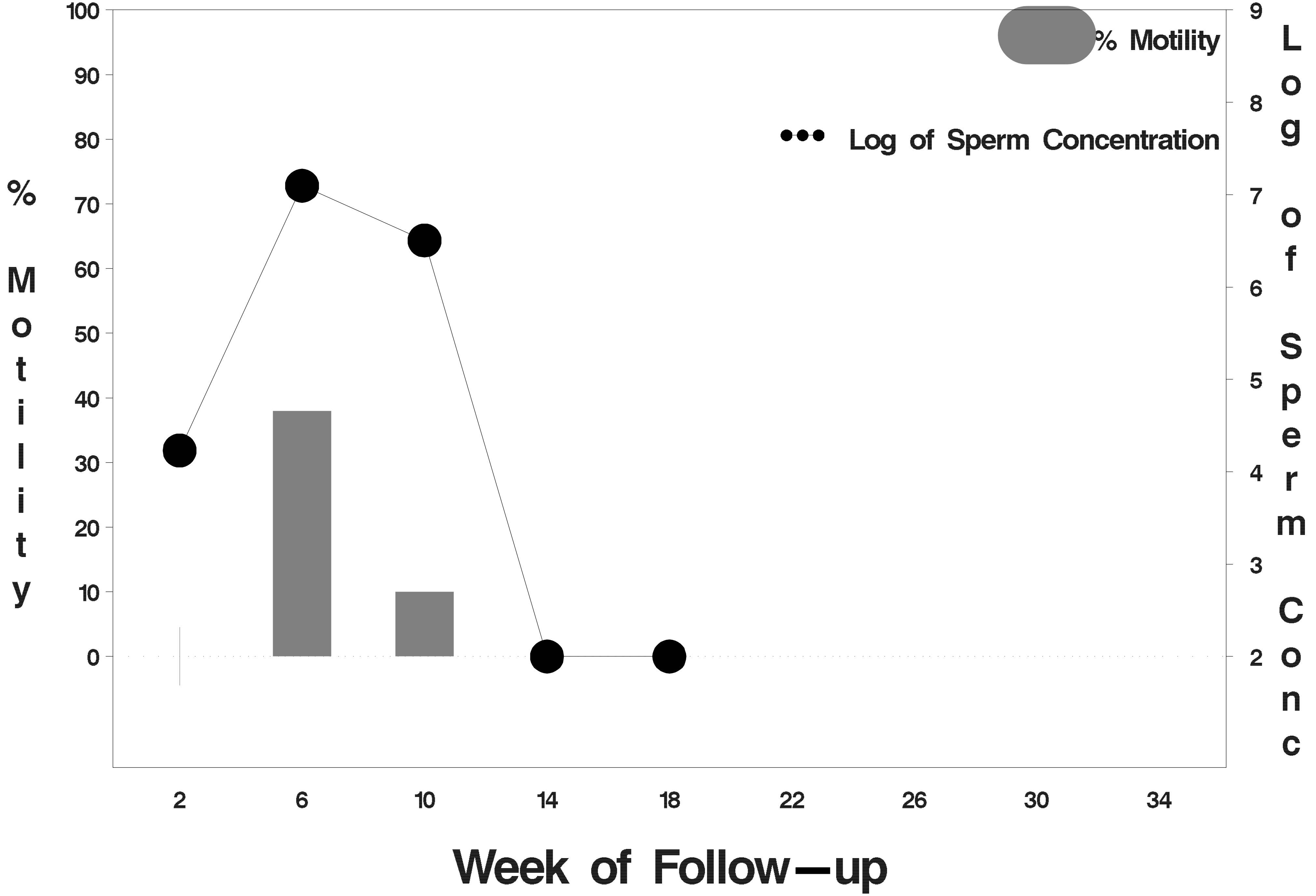

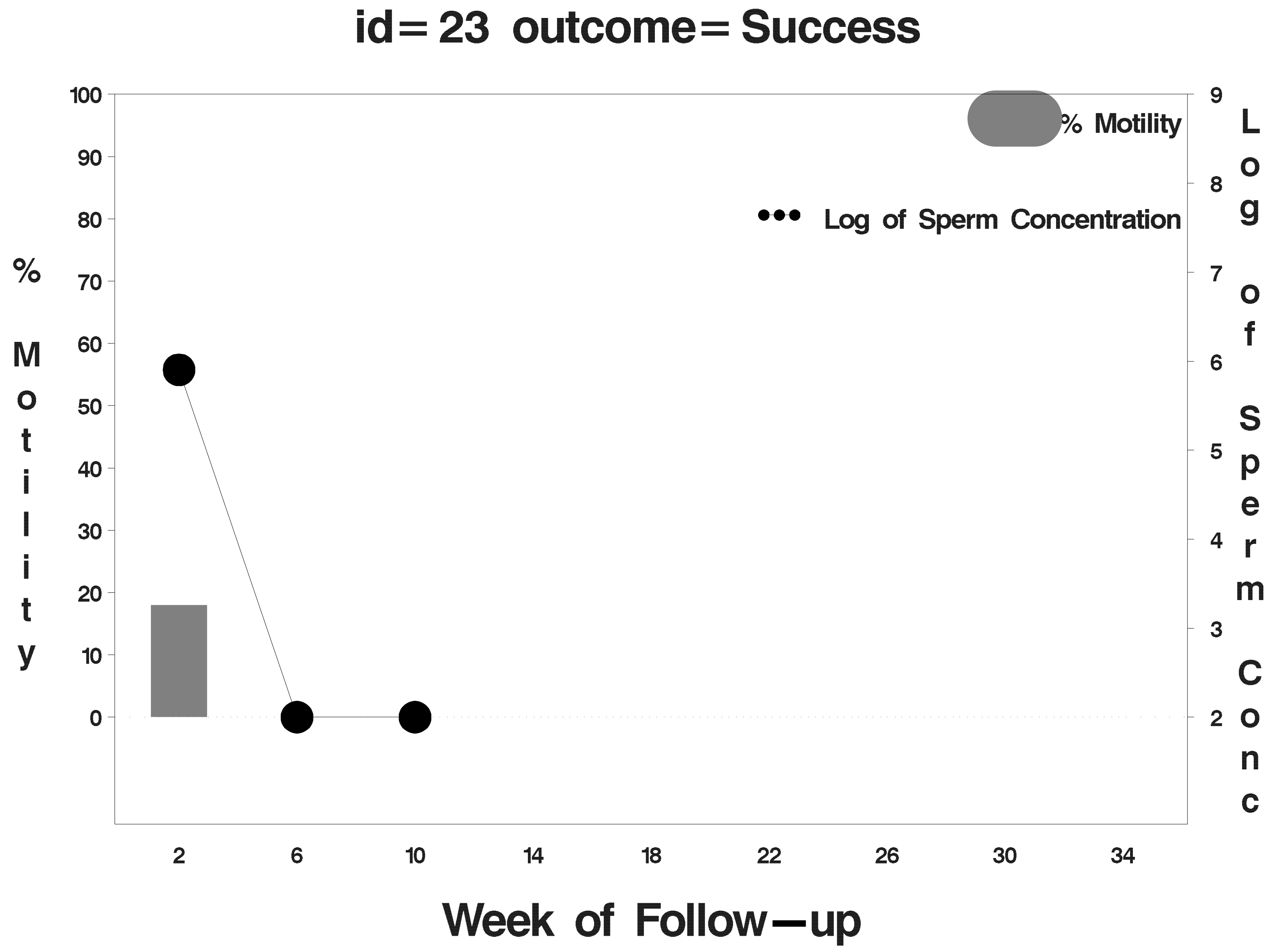
id= 23 outcome= Success
% Motility
Log of Sperm Concentration
100
90
80
70
60
50
40
30
20
10
0
9
8
7
6
5
4
3
2
% Motility
Log of Sperm Conc
2
6
10
14
18
22
26
30
34
Week of Follow—up

id= 24 outcome= Success

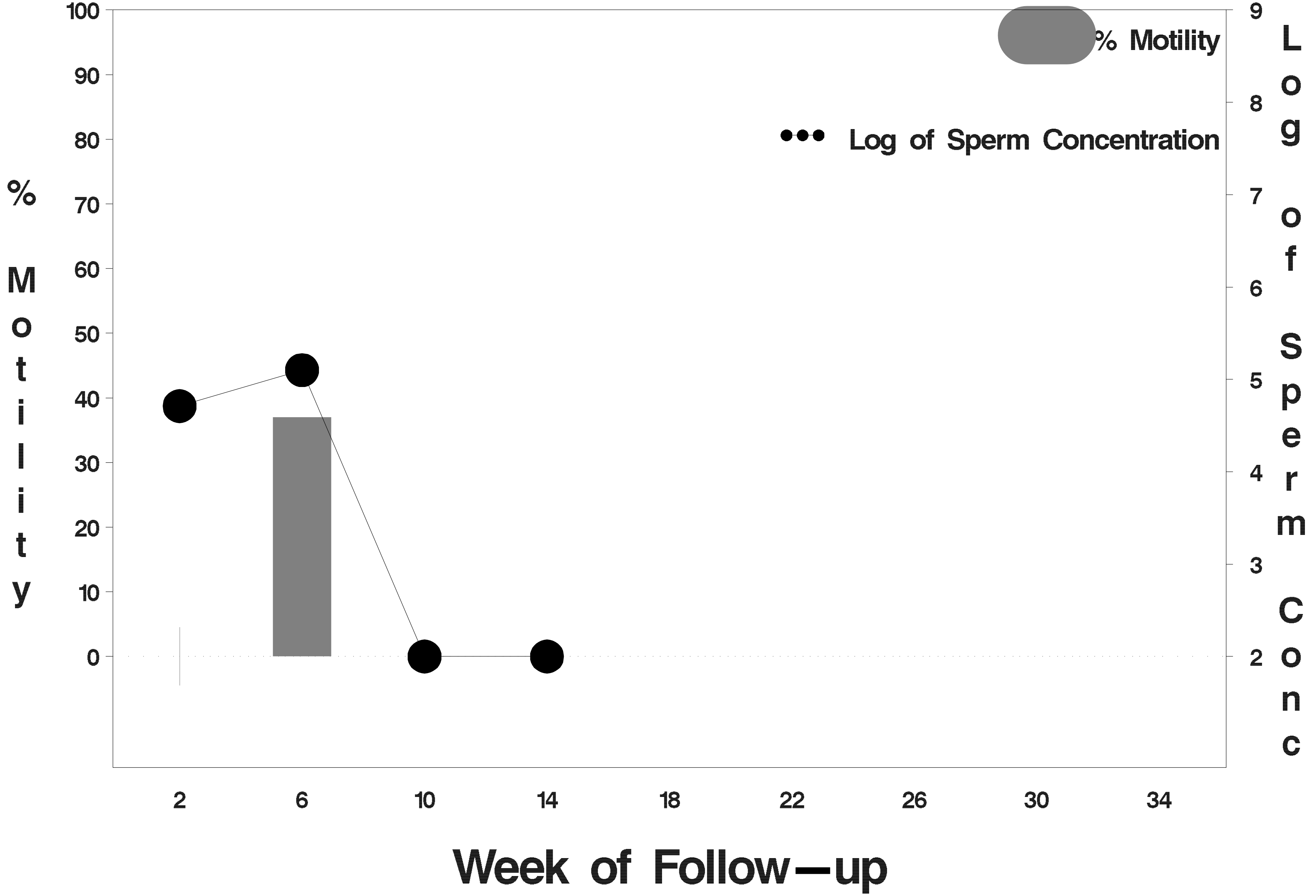

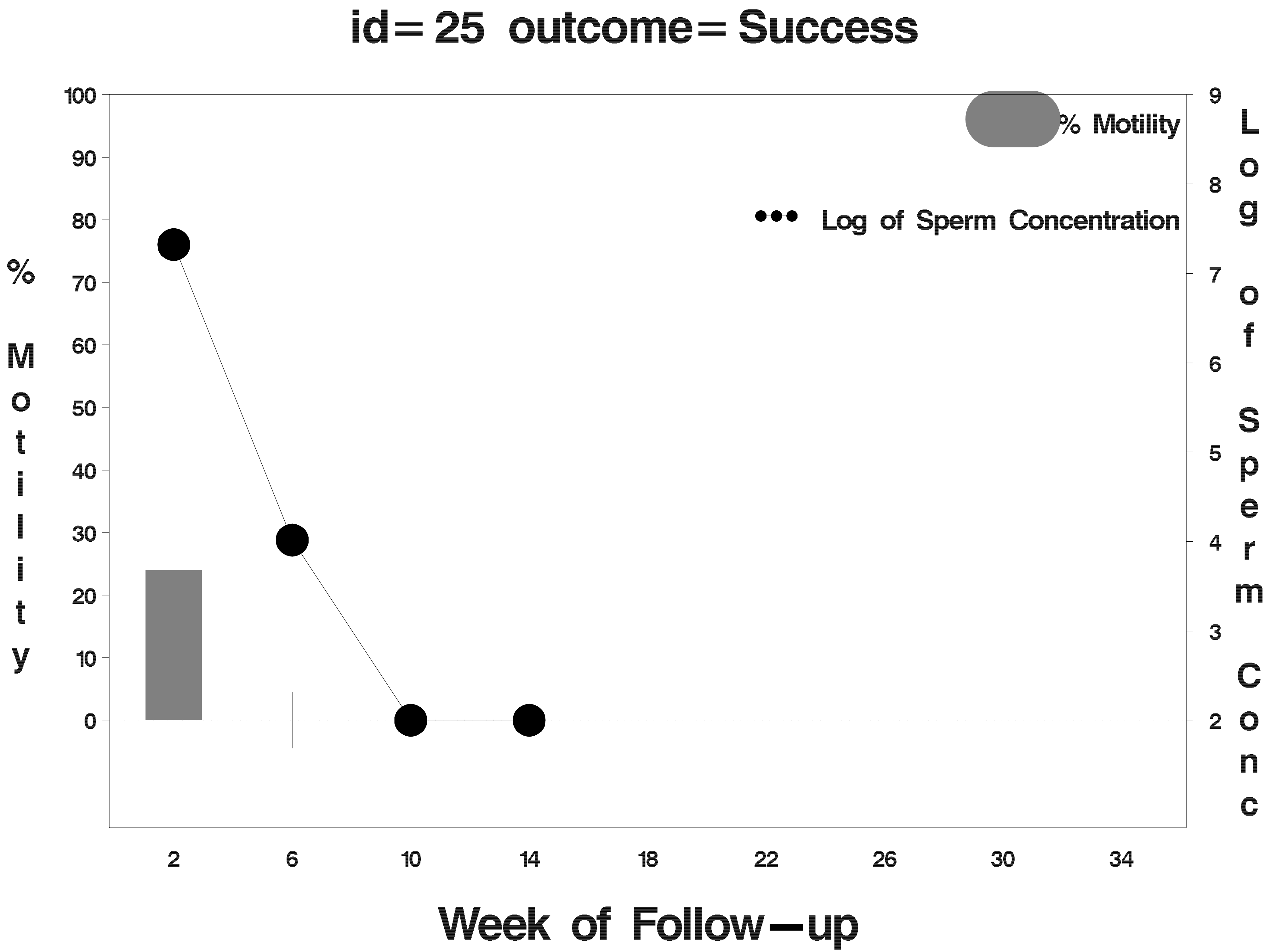

id= 25 outcome= Success
% Motility
Log of Sperm Concentration
100
90
80
70
60
50
40
30
20
10
0
9
8
7
6
5
4
3
2
2
6
10
14
18
22
26
30
34
Week of Follow—up
% Motility
Log of Sperm Conc

id= 26 outcome= Success

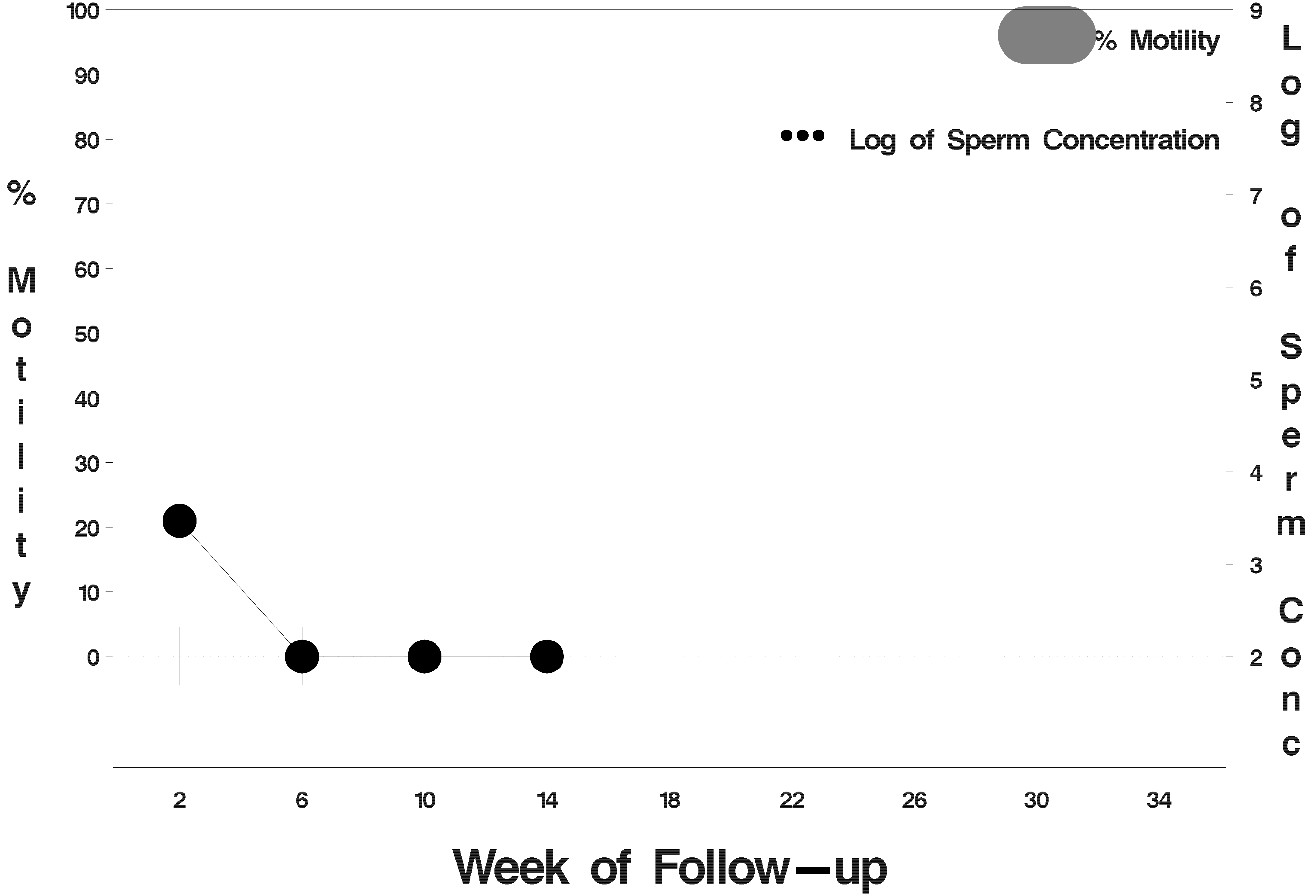

id= 27 outcome= Success

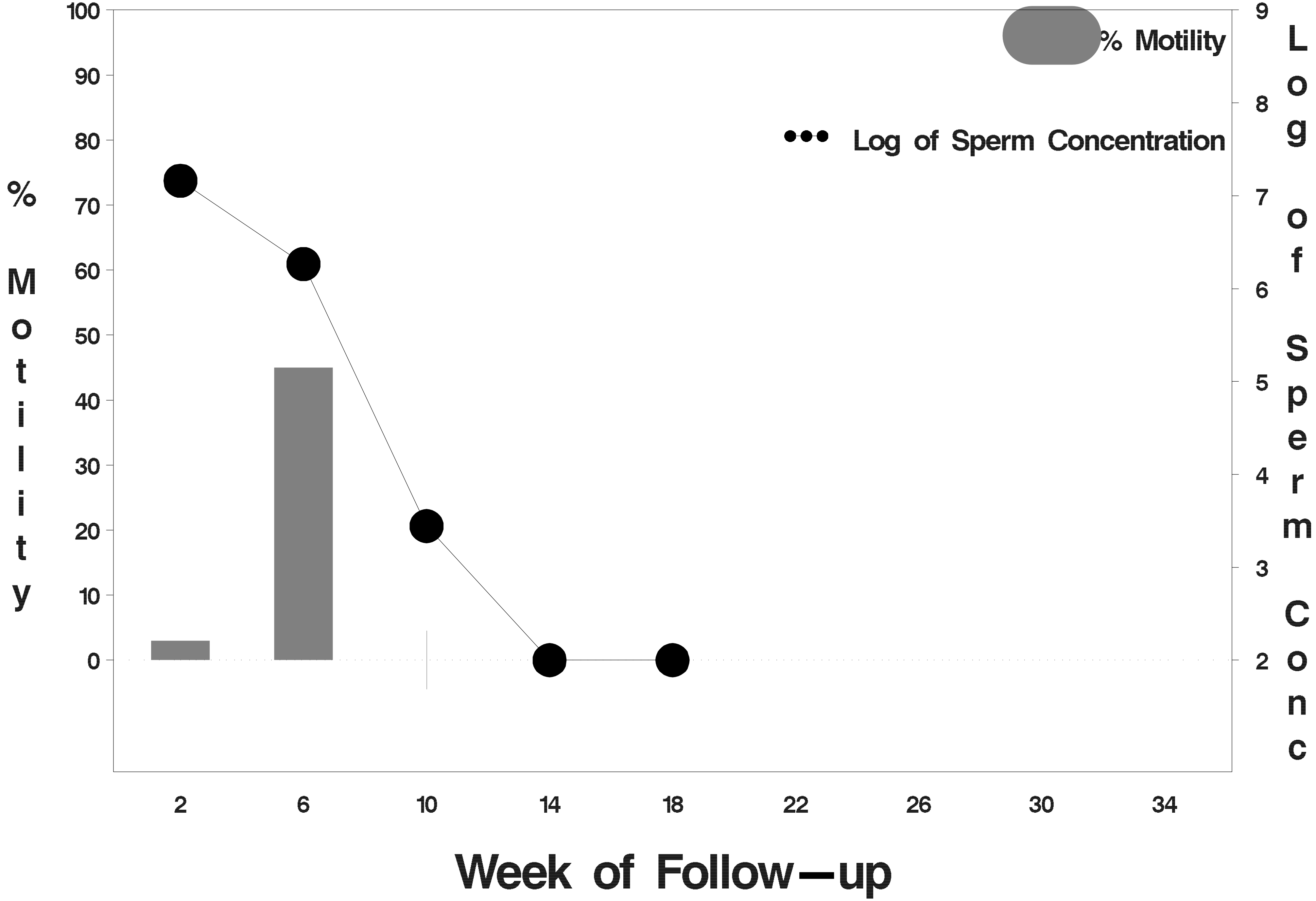

id= 28 outcome= Success

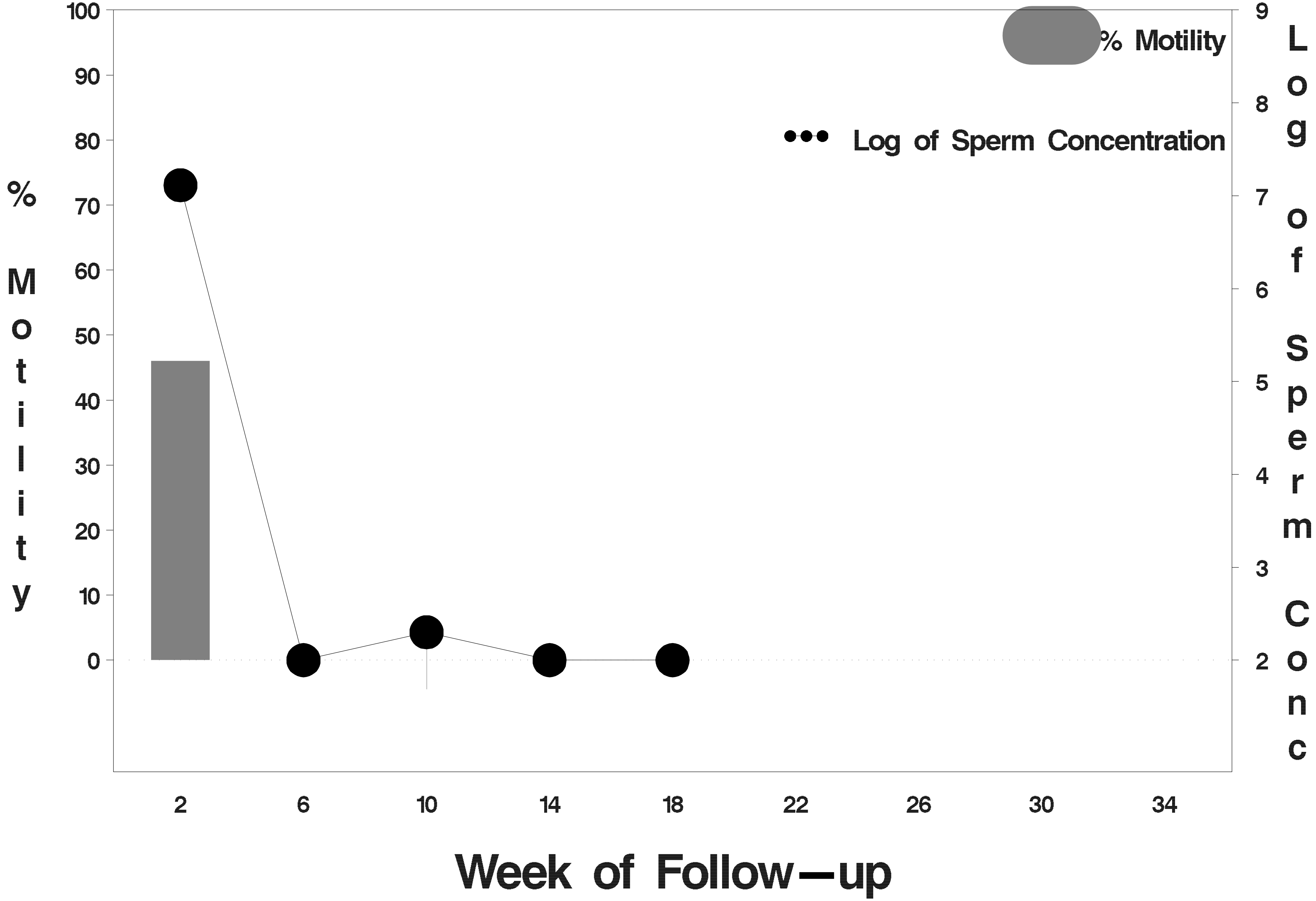

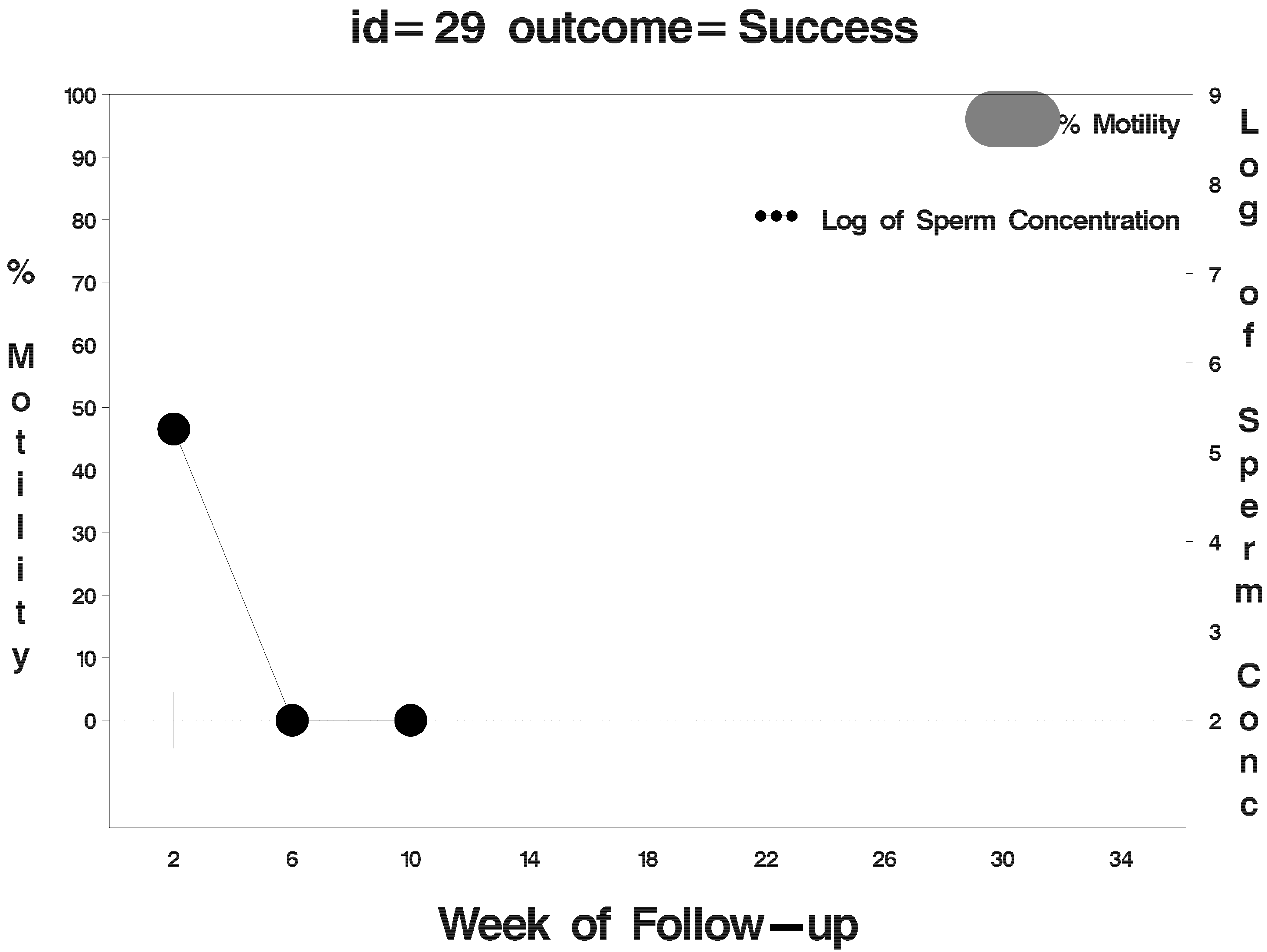
id= 29 outcome= Success
% Motility
Log of Sperm Concentration
100
90
80
70
60
50
40
30
20
10
0
9
8
7
6
5
4
3
2
2
6
10
14
18
22
26
30
34
Week of Follow−up
% Motility
Log of Sperm Conc

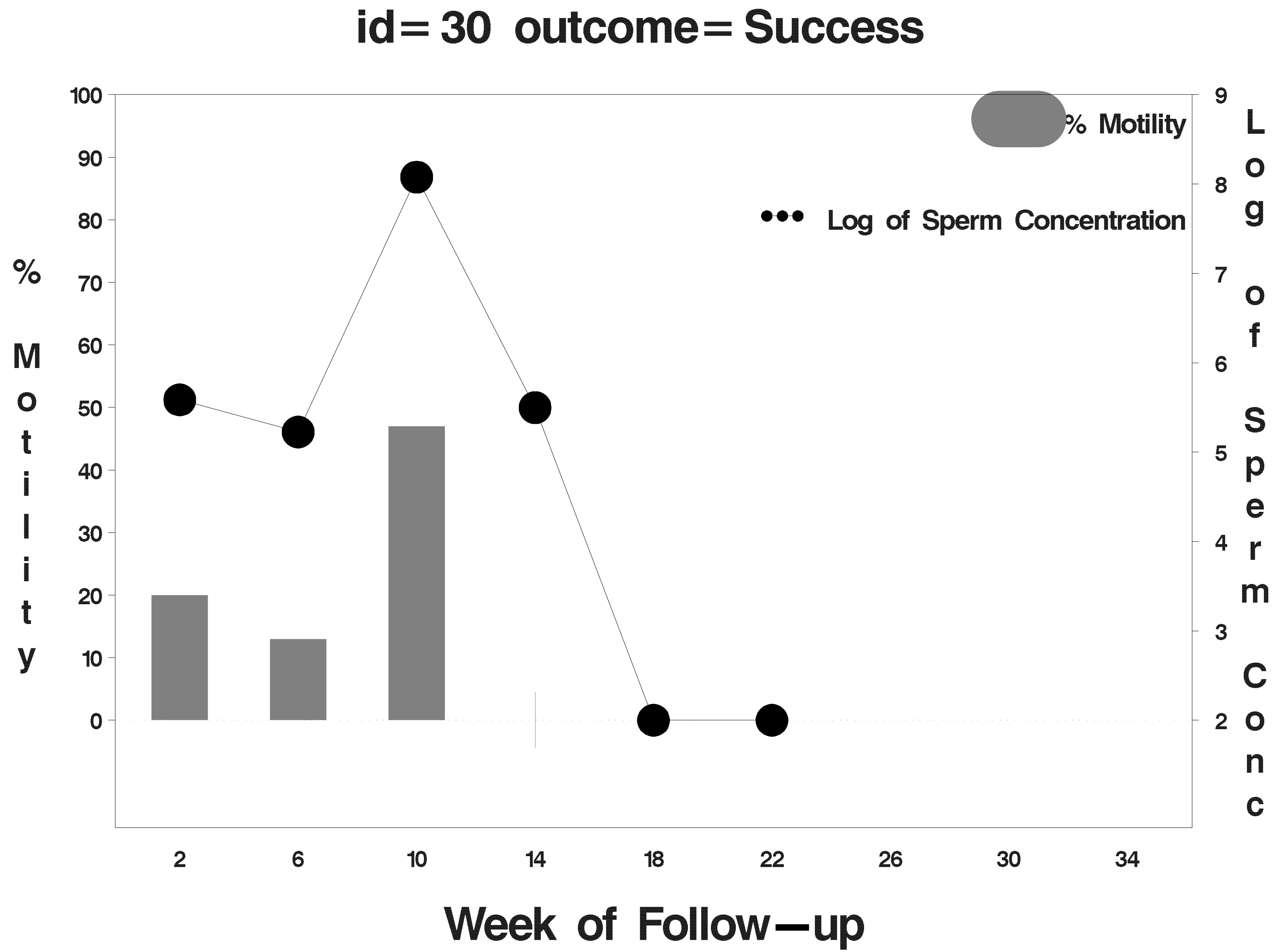
id= 30 outcome= Success
% Motility
Log of Sperm Concentration
% Motility
Log of Sperm Conc
Week of Follow−up
100
90
80
70
60
50
40
30
20
10
0
9
8
7
6
5
4
3
2
2
6
10
14
18
22
26
30
34

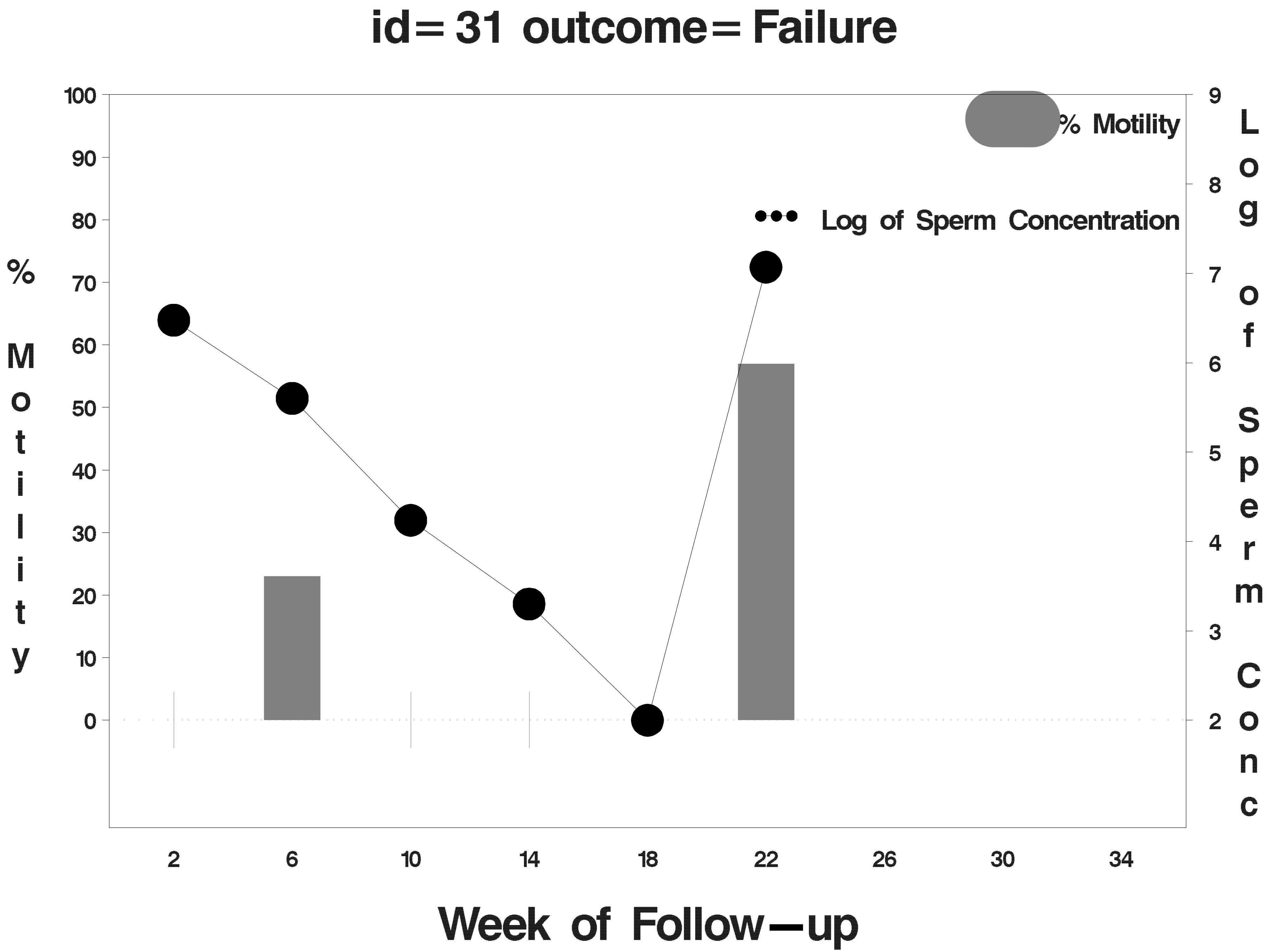

id= 31 outcome= Failure
% Motility
Log of Sperm Concentration
% Motility
Log of Sperm Conc
Week of Follow-up
100
90
80
70
60
50
40
30
20
10
0
9
8
7
6
5
4
3
2
2
6
10
14
18
22
26
30
34

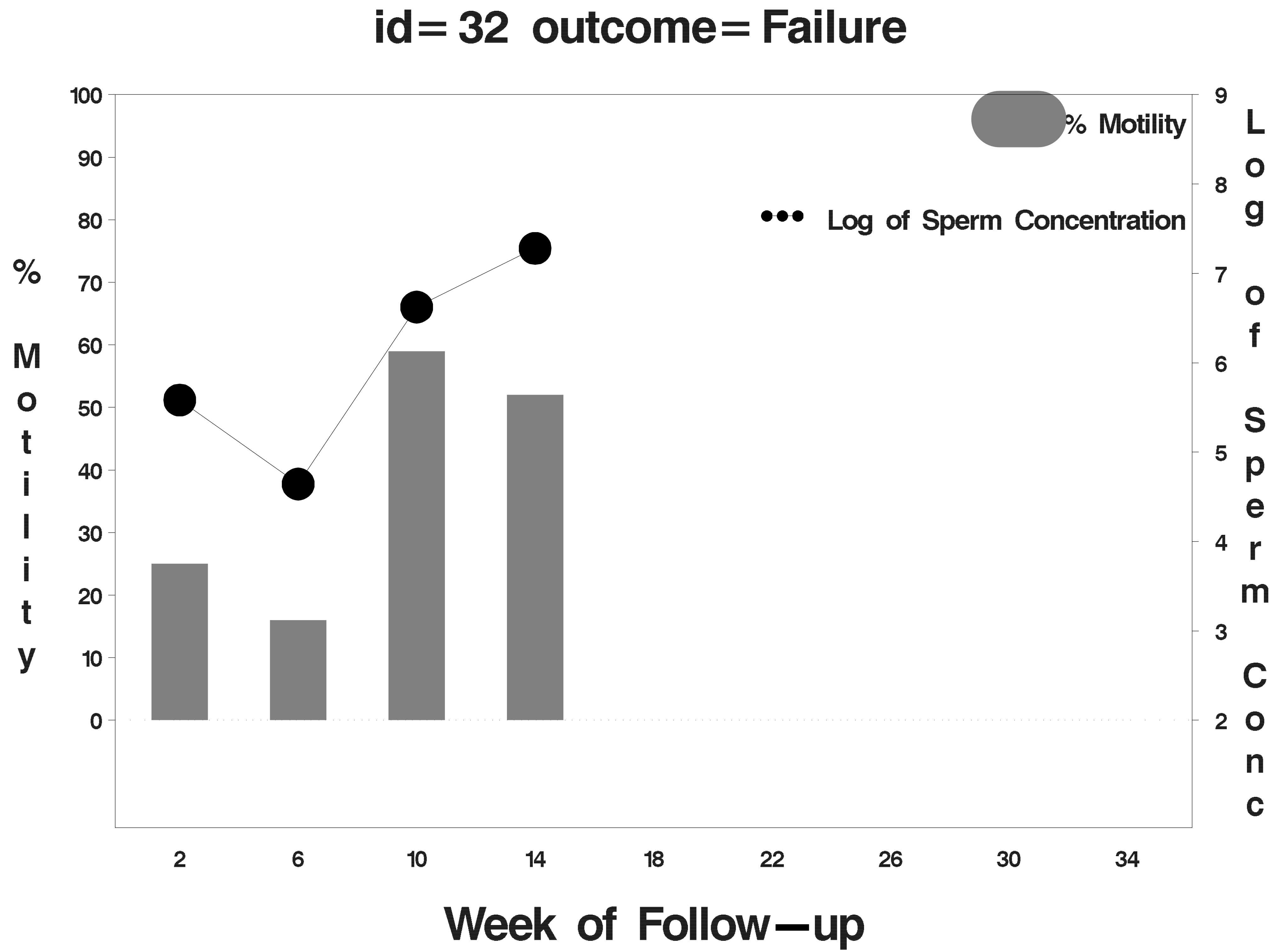

id= 32 outcome= Failure
% Motility
Log of Sperm Concentration
% Motility
Log of Sperm Conc
Week of Follow-up
100
90
80
70
60
50
40
30
20
10
0
9
8
7
6
5
4
3
2
2
6
10
14
18
22
26
30
34

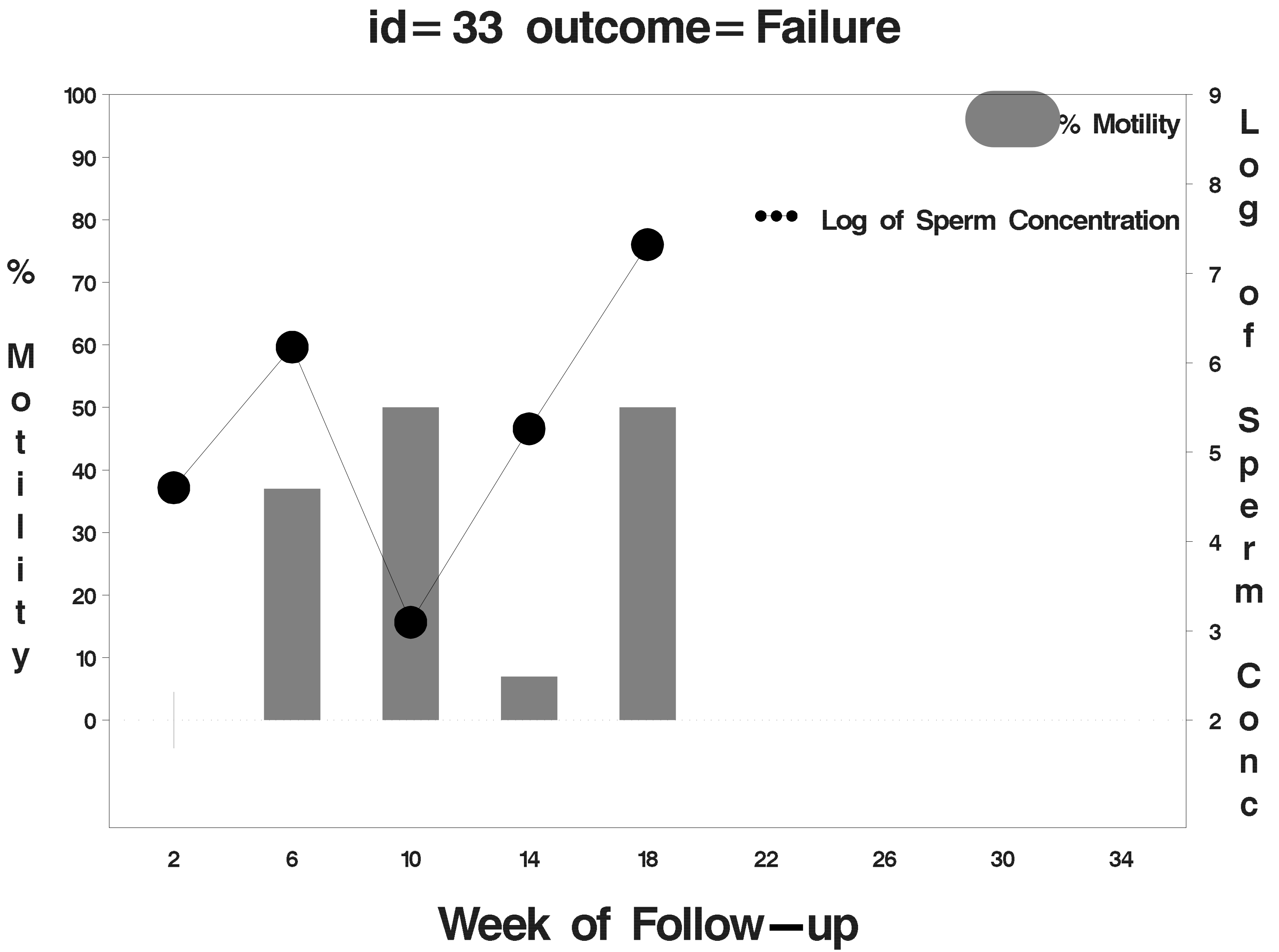
id= 33 outcome= Failure
% Motility
Log of Sperm Concentration
% Motility
Log of Sperm Conc
Week of Follow-up
100
90
80
70
60
50
40
30
20
10
0
9
8
7
6
5
4
3
2
2
6
10
14
18
22
26
30
34

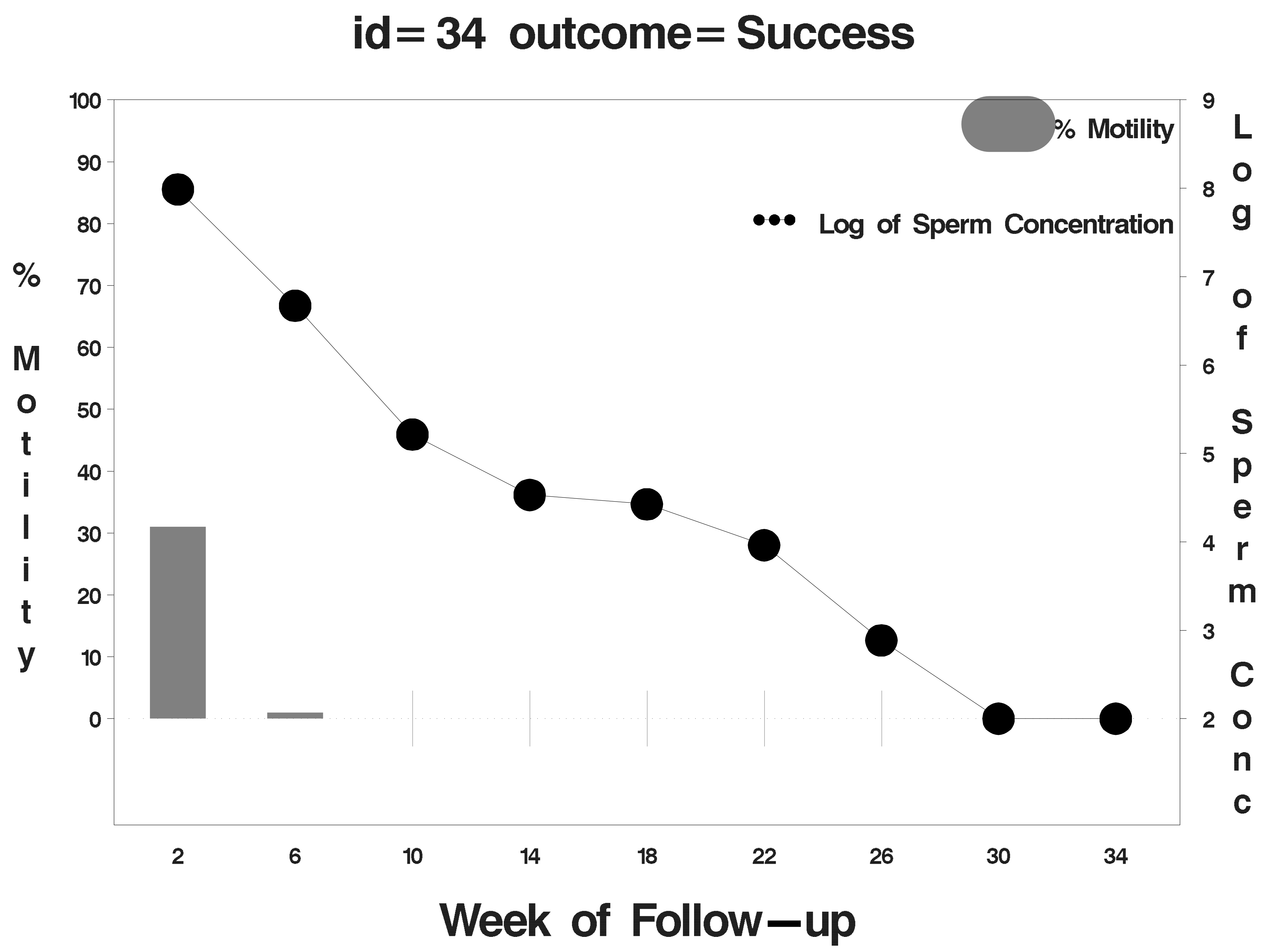
id= 34 outcome= Success
% Motility
Log of Sperm Concentration
100
90
80
70
60
50
40
30
20
10
0
9
8
7
6
5
4
3
2
2
6
10
14
18
22
26
30
34
Week of Follow—up
Log of Sperm Conc

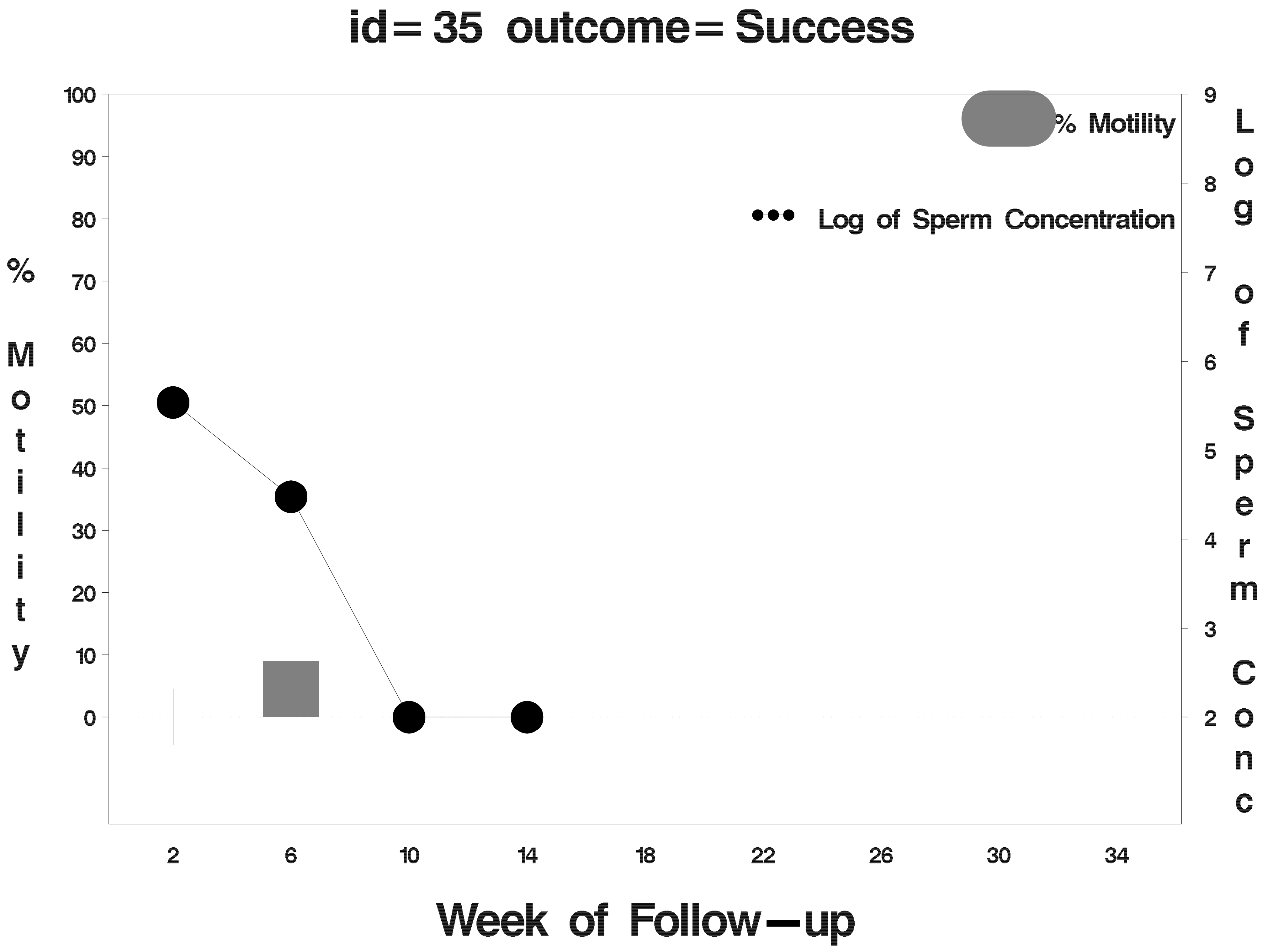

id= 35 outcome= Success
% Motility
Log of Sperm Concentration
100
90
80
70
60
50
40
30
20
10
0
9
8
7
6
5
4
3
2
2
6
10
14
18
22
26
30
34
Week of Follow-up

id= 36 outcome= Success

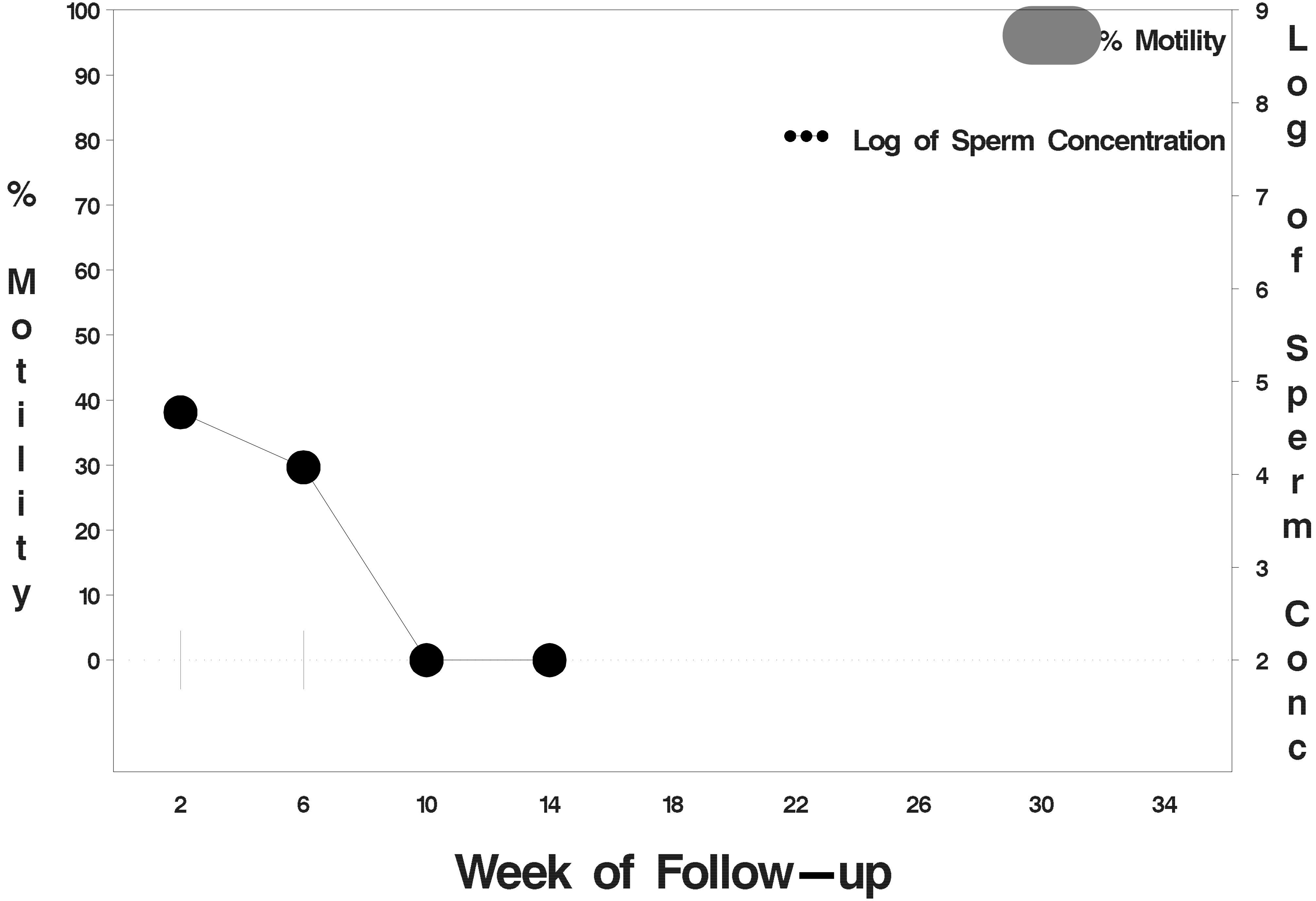

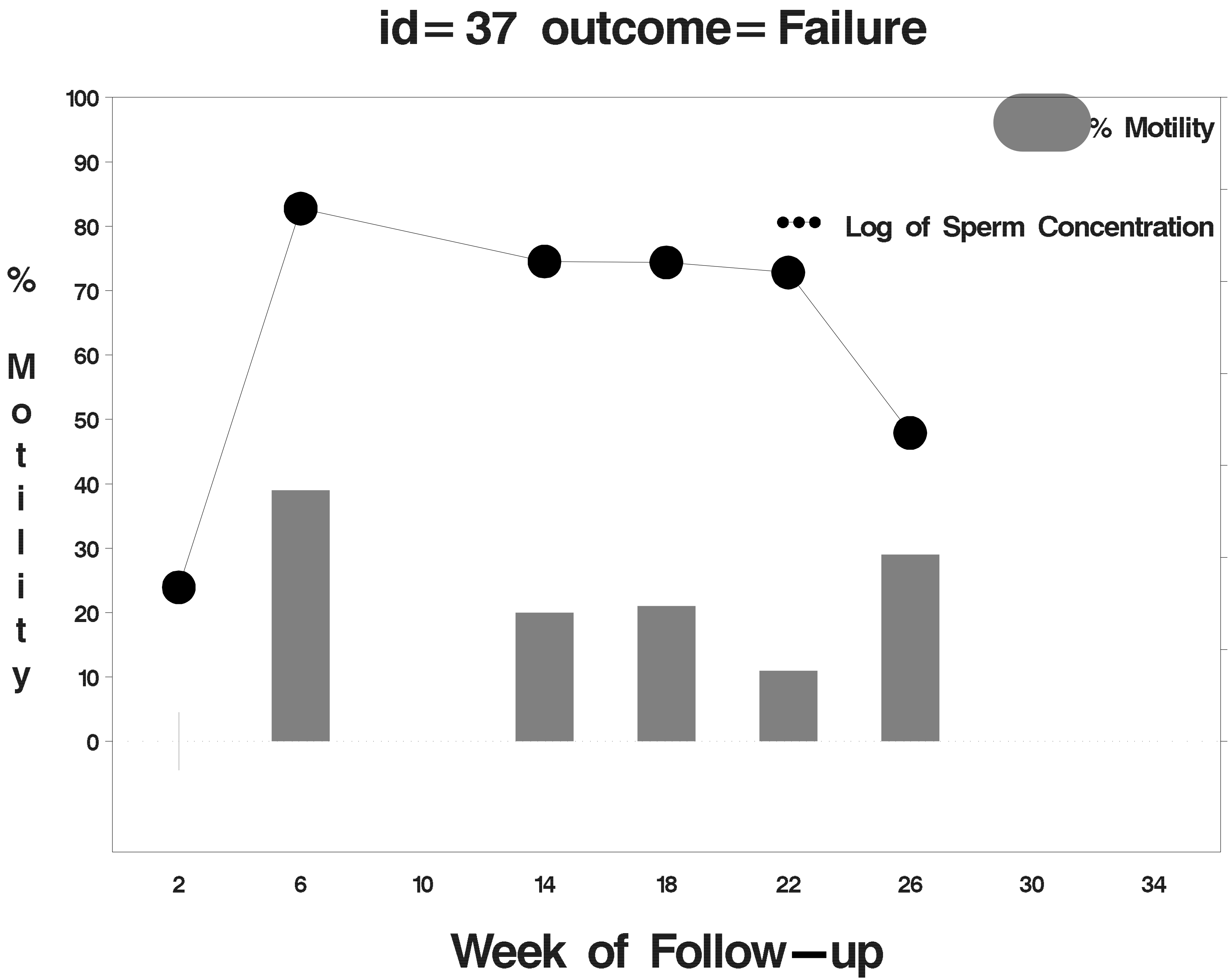

id= 37 outcome= Failure
% Motility
Log of Sperm Concentration
100
90
80
70
60
50
40
30
20
10
0
9
8
7
6
5
4
3
2
2
6
10
14
18
22
26
30
34
Week of Follow−up
Log of Sperm Conc

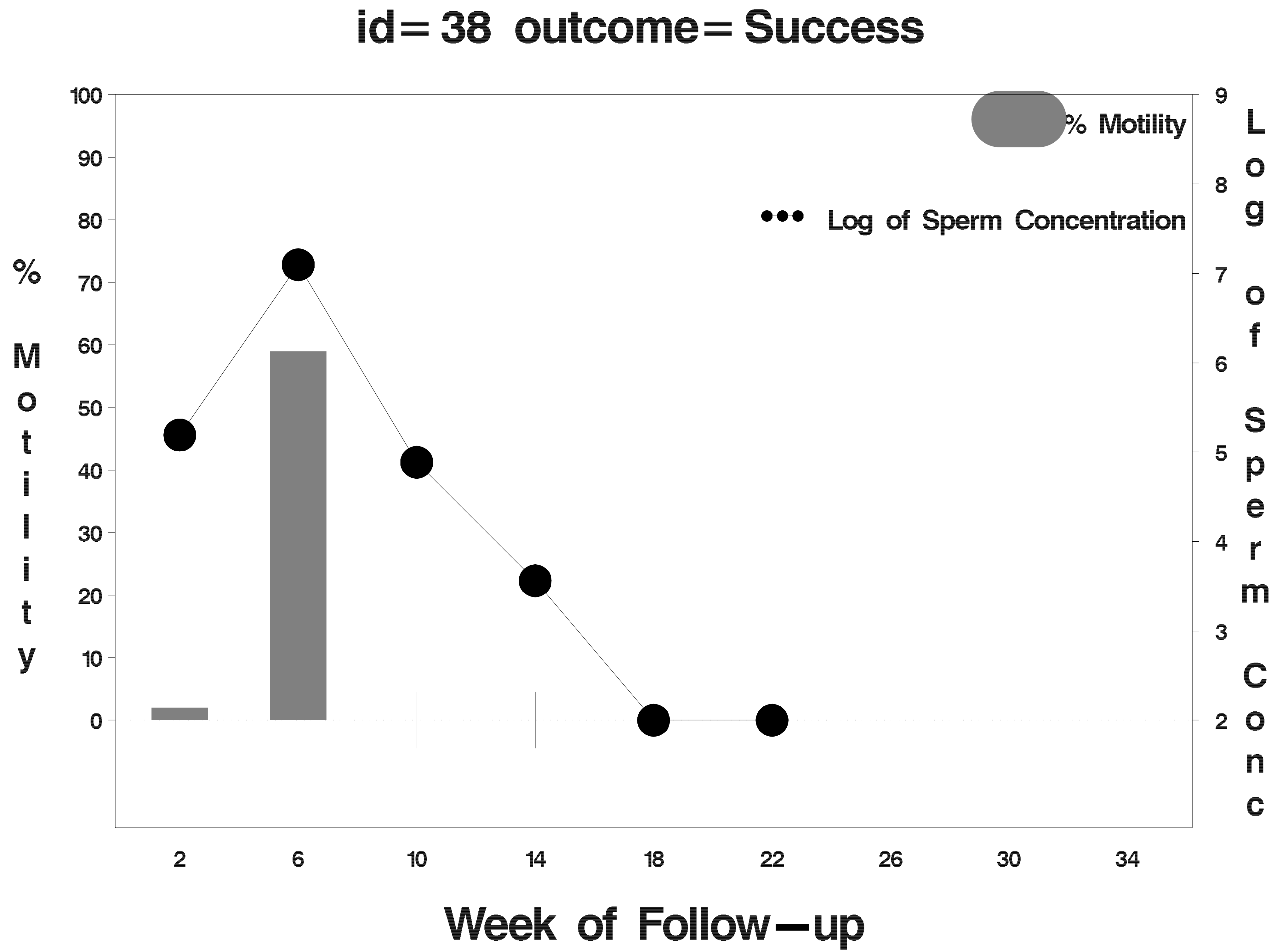
id= 38 outcome= Success
% Motility
Log of Sperm Concentration
100
90
80
70
60
50
40
30
20
10
0
9
8
7
6
5
4
3
2
% Motility
Log of Sperm Conc
2
6
10
14
18
22
26
30
34
Week of Follow−up

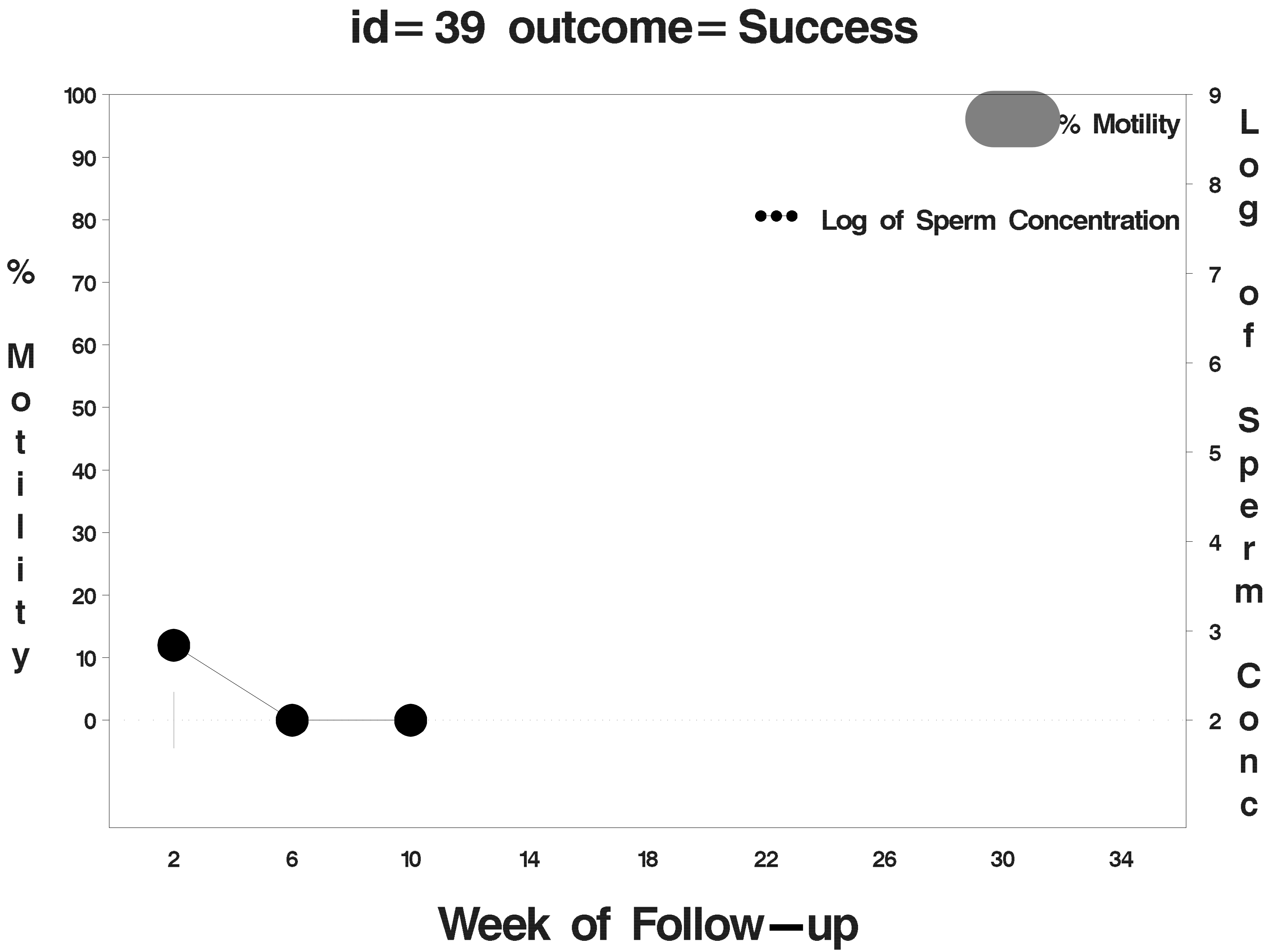

id= 39 outcome= Success
% Motility
Log of Sperm Concentration
% Motility
Log of Sperm Conc
Week of Follow—up
100
90
80
70
60
50
40
30
20
10
0
9
8
7
6
5
4
3
2
2
6
10
14
18
22
26
30
34

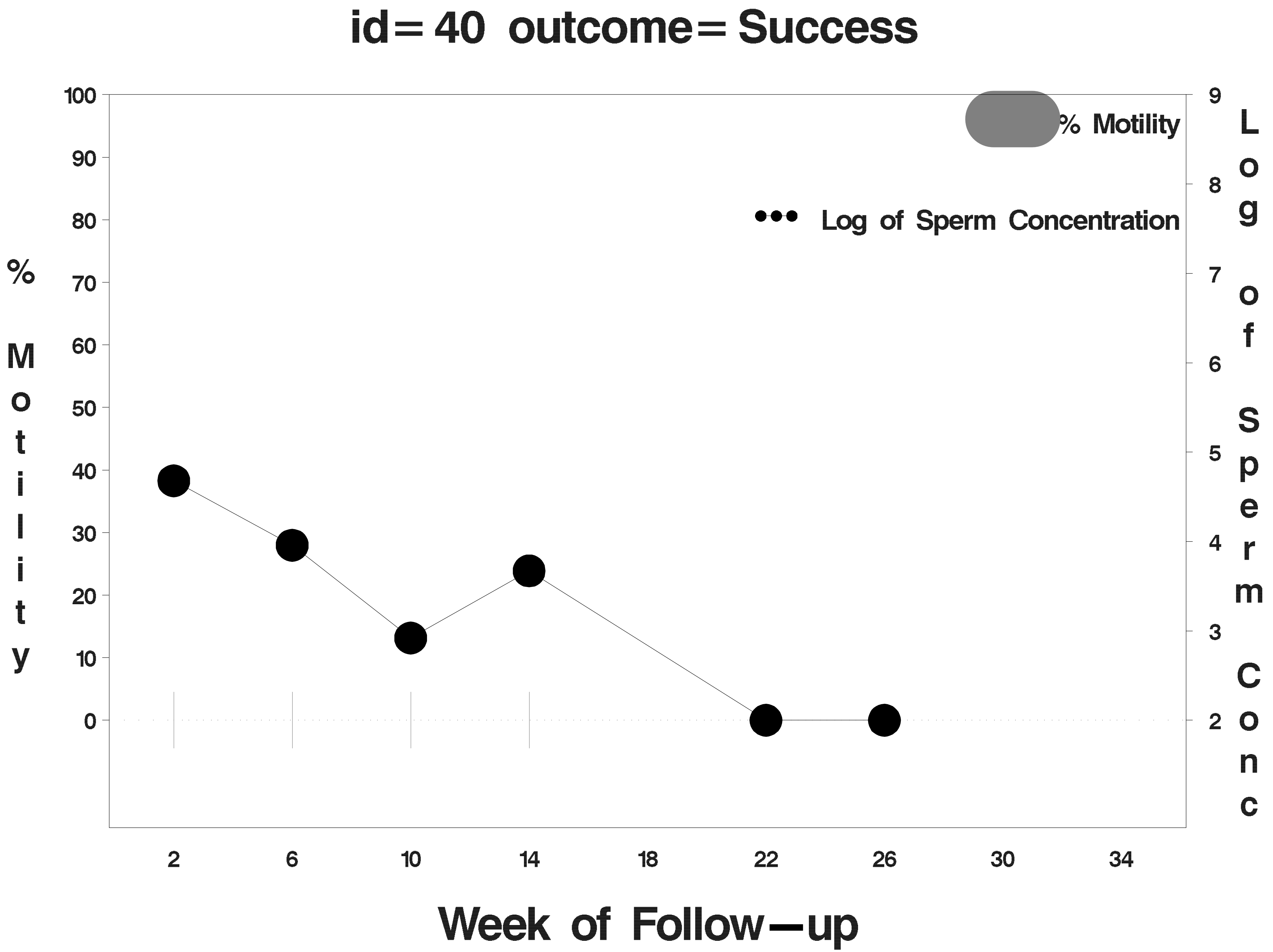
id= 40 outcome= Success
% Motility
Log of Sperm Concentration
% Motility
Log of Sperm Conc
Week of Follow-up
100
90
80
70
60
50
40
30
20
10
0
9
8
7
6
5
4
3
2
2
6
10
14
18
22
26
30
34

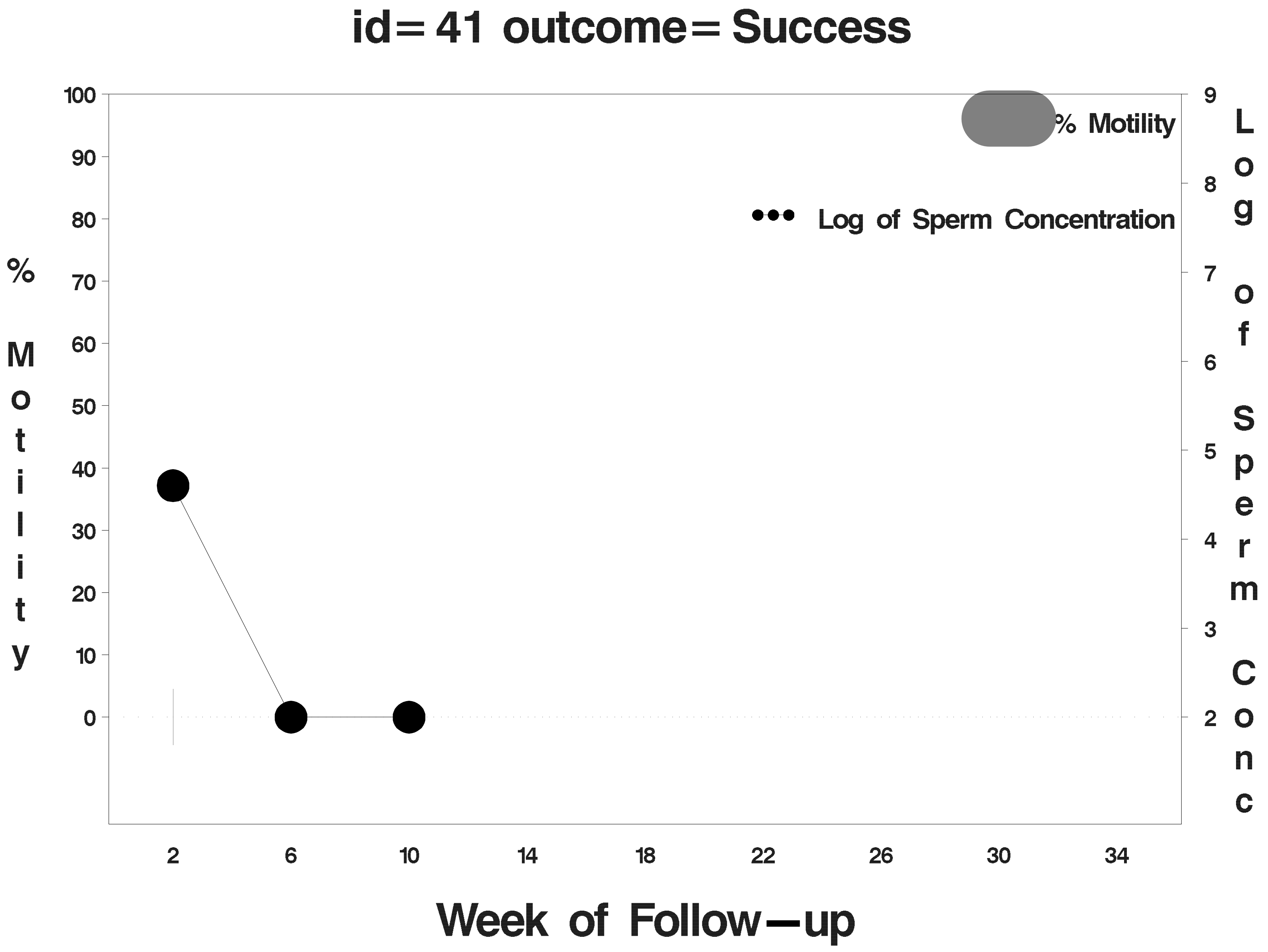

id= 41 outcome= Success
% Motility
Log of Sperm Concentration
100
90
80
70
60
50
40
30
20
10
0
9
8
7
6
5
4
3
2
2
6
10
14
18
22
26
30
34
Week of Follow—up
% Motility
Log of Sperm Conc

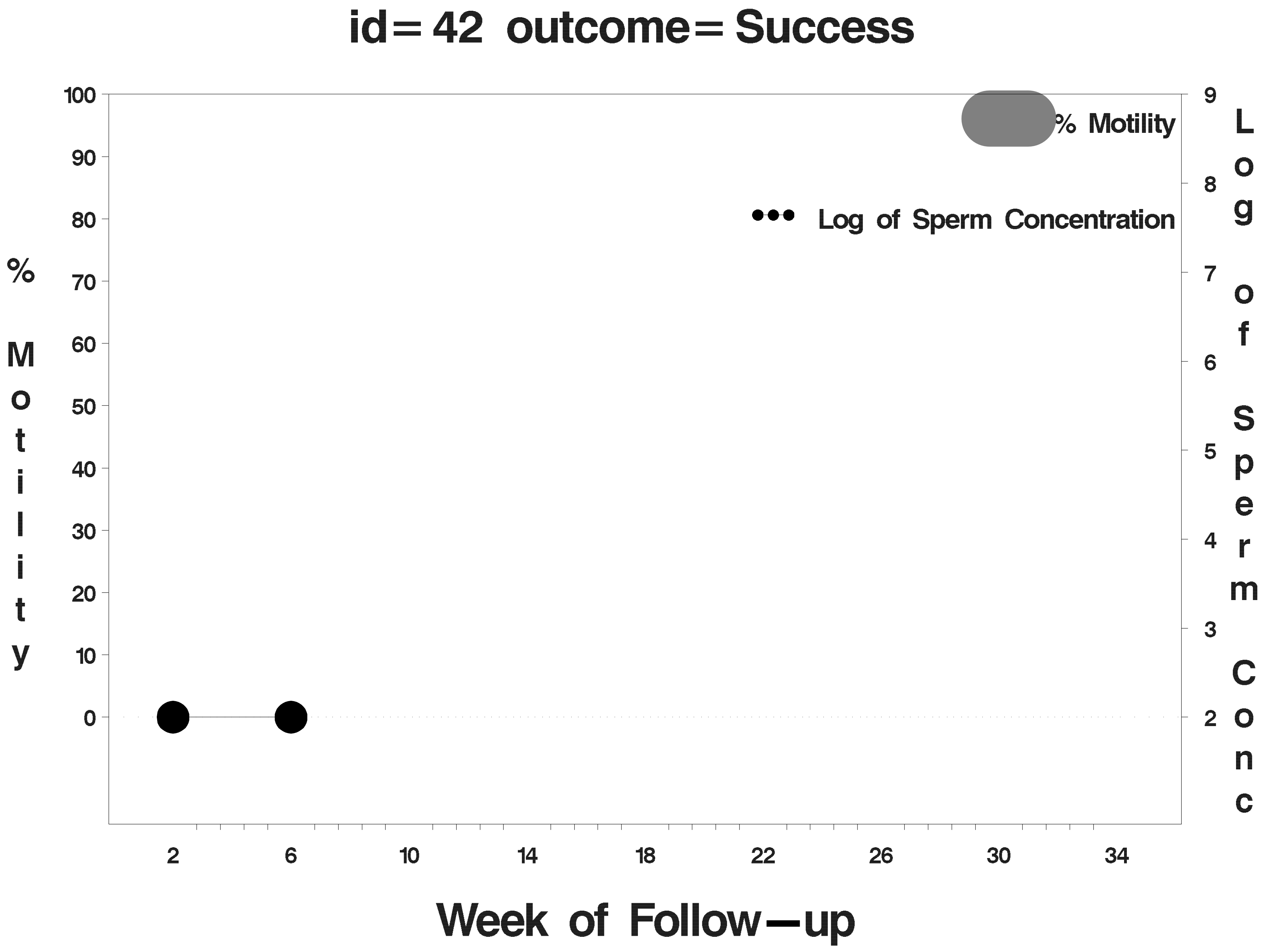

id= 42 outcome= Success
% Motility
Log of Sperm Concentration
100
90
80
70
60
50
40
30
20
10
0
9
8
7
6
5
4
3
2
2
6
10
14
18
22
26
30
34
Week of Follow−up
Log of Sperm Conc

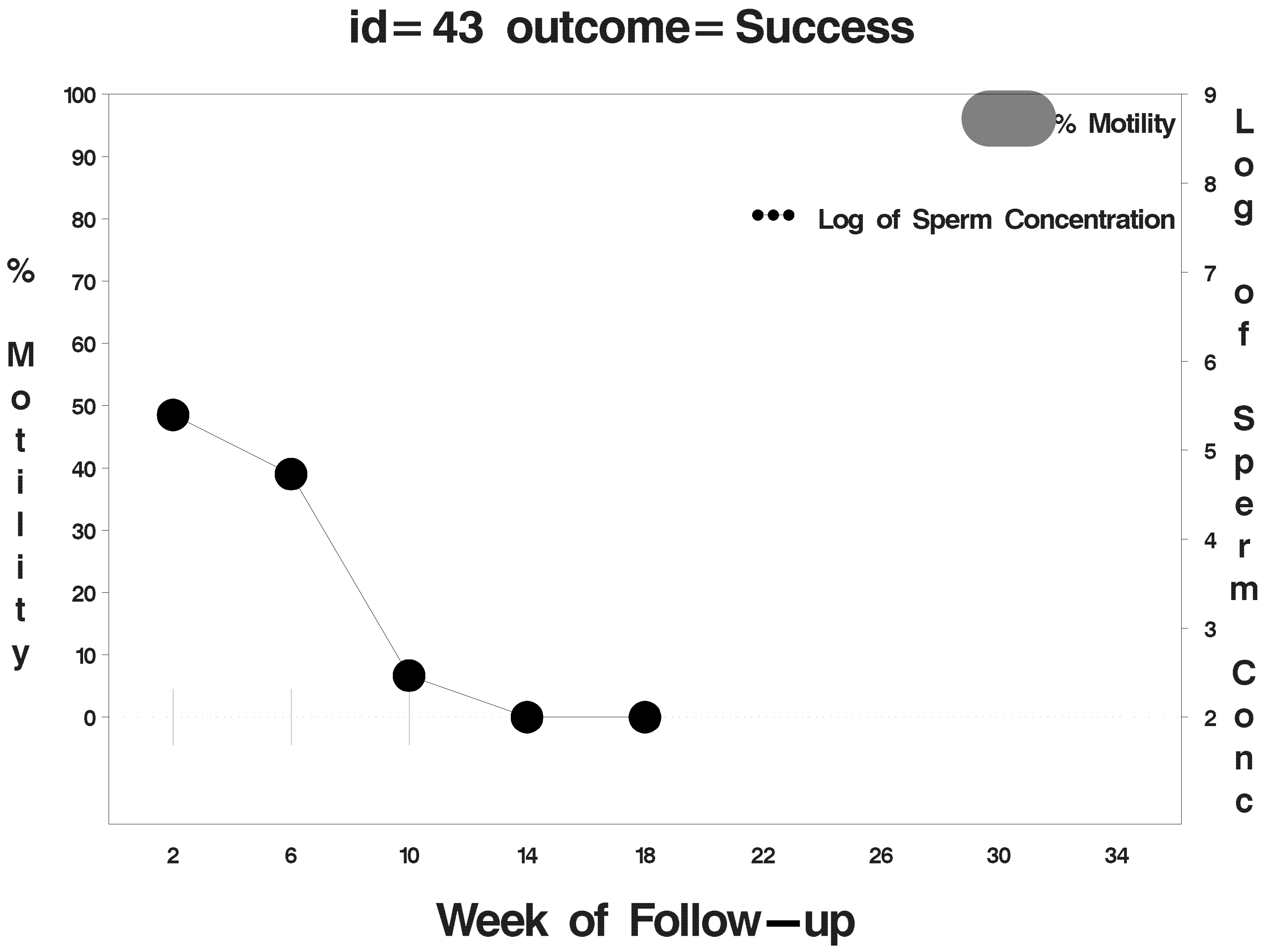
id= 43 outcome= Success
% Motility
Log of Sperm Concentration
100
90
80
70
60
50
40
30
20
10
0
9
8
7
6
5
4
3
2
2
6
10
14
18
22
26
30
34
Week of Follow—up
% Motility
Log of Sperm Conc

id= 44 outcome= Success

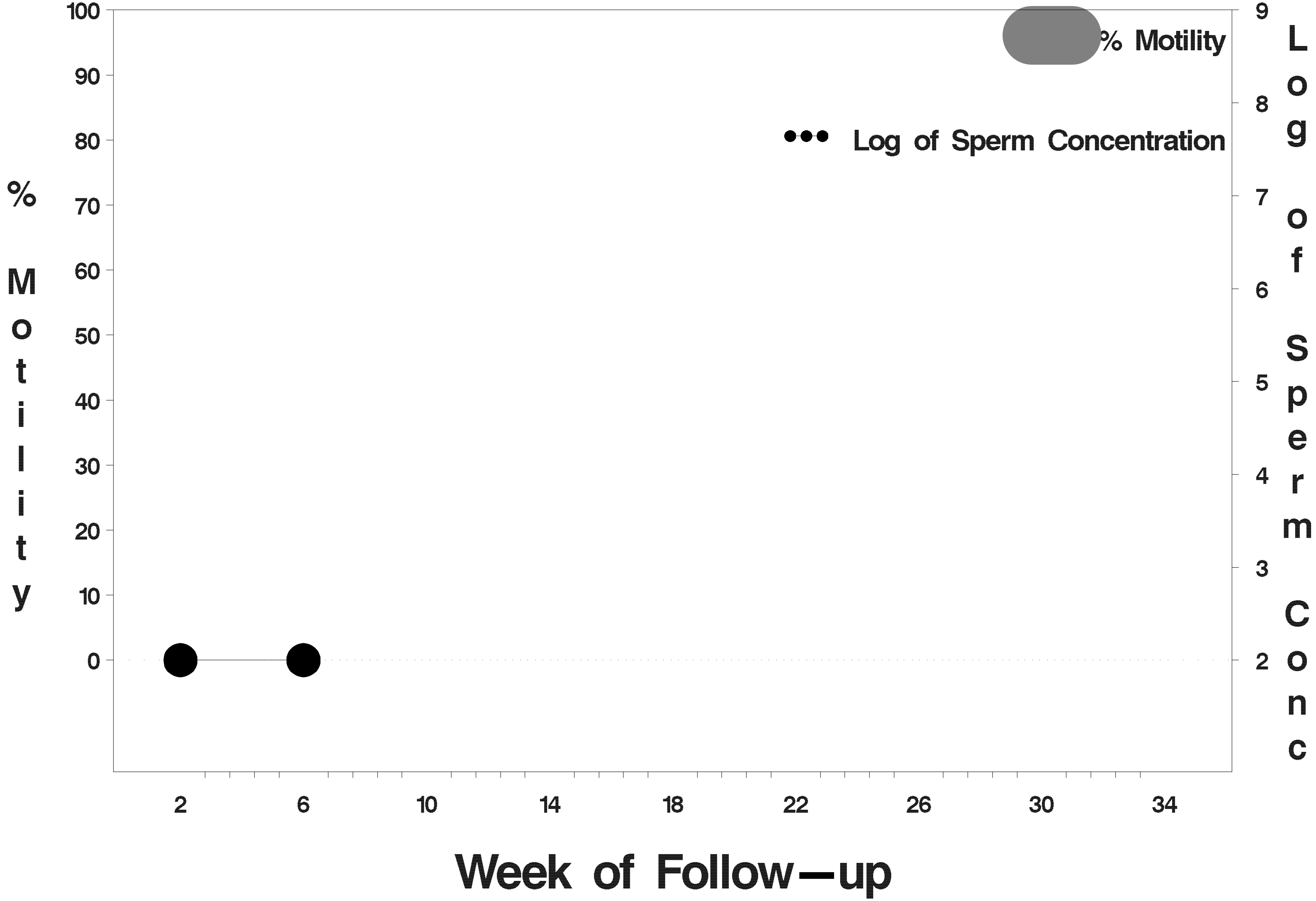

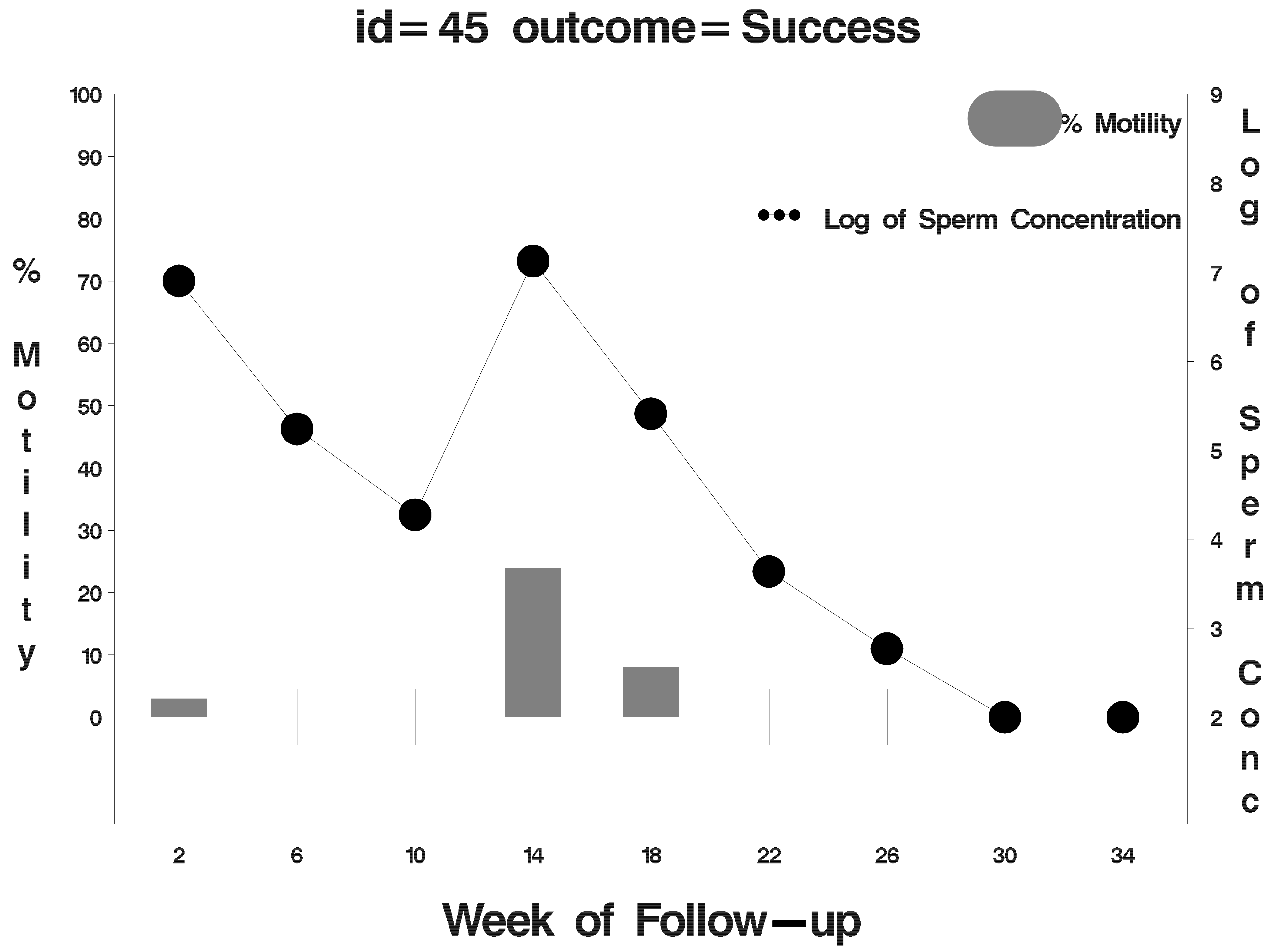

id= 45 outcome= Success
% Motility
Log of Sperm Concentration
100
90
80
70
60
50
40
30
20
10
0
9
8
7
6
5
4
3
2
2
6
10
14
18
22
26
30
34
Week of Follow-up
Log of Sperm Conc

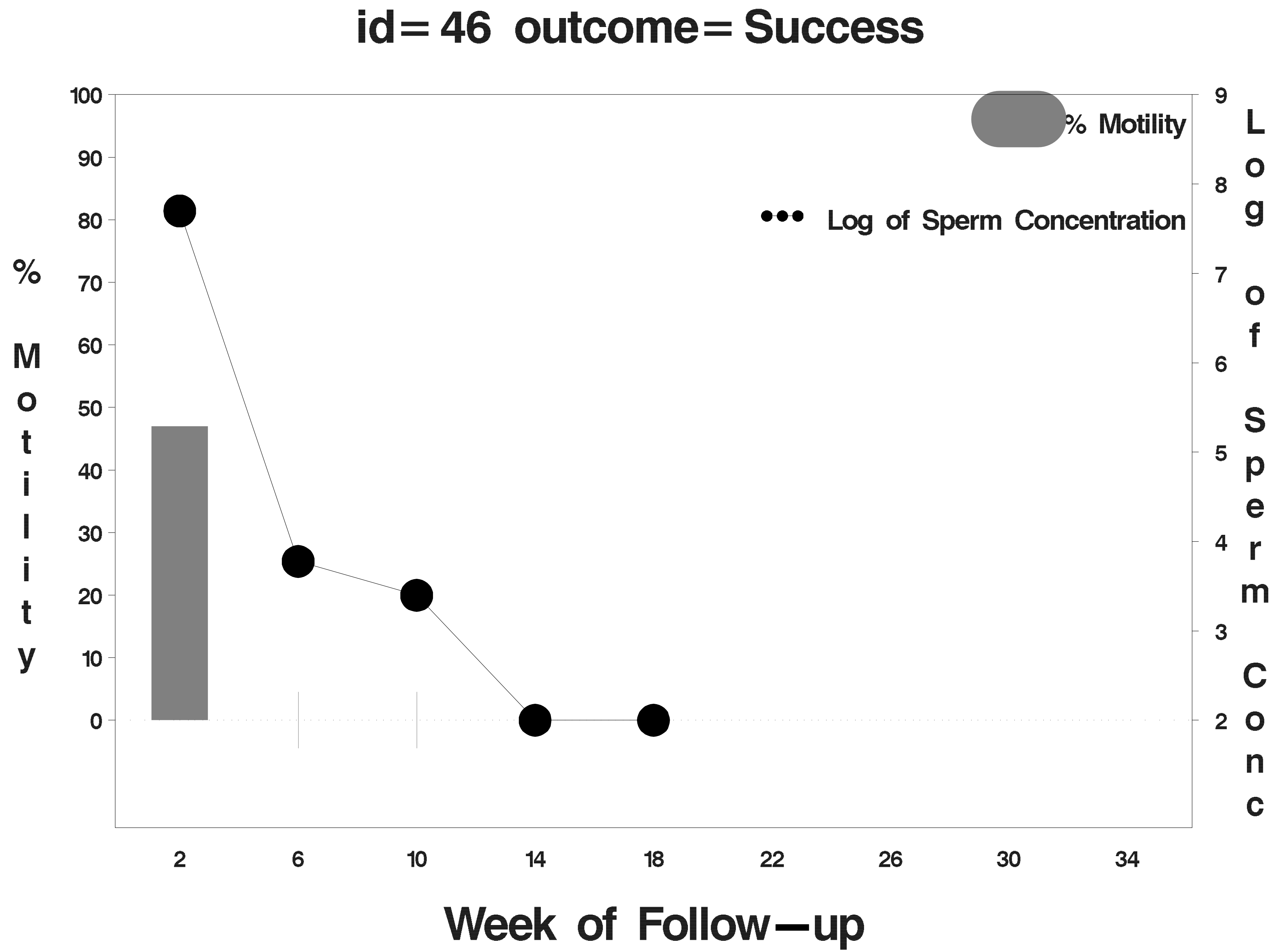

id= 46 outcome= Success
% Motility
Log of Sperm Concentration
100
90
80
70
60
50
40
30
20
10
0
9
8
7
6
5
4
3
2
2
6
10
14
18
22
26
30
34
Week of Follow—up
Log of Sperm Conc

id= 47 outcome= Success

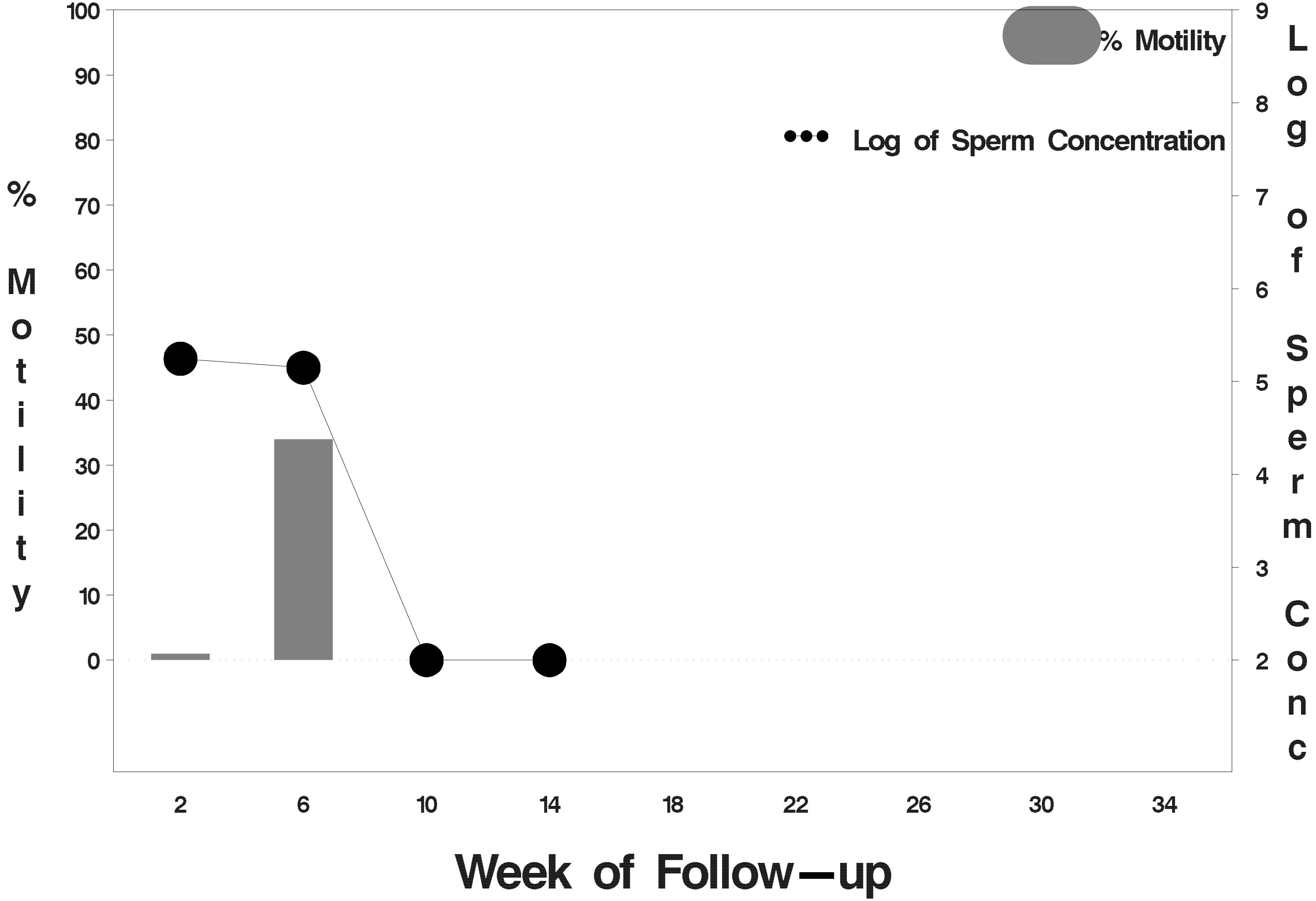

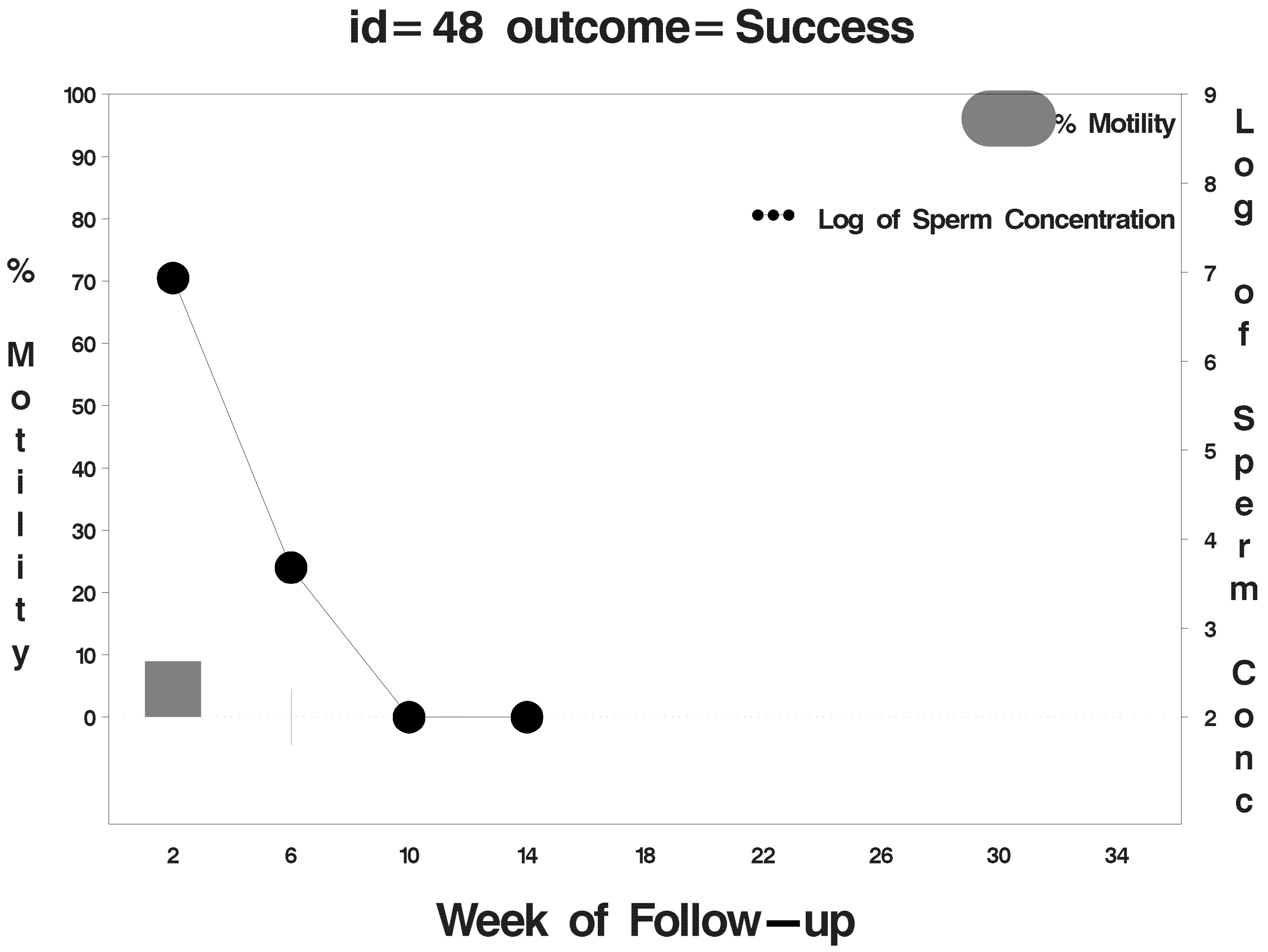

id= 48 outcome= Success
% Motility
Log of Sperm Concentration
100
90
80
70
60
50
40
30
20
10
0
9
8
7
6
5
4
3
2
2
6
10
14
18
22
26
30
34
Week of Follow—up
Log of Sperm Conc

id= 49 outcome= Success

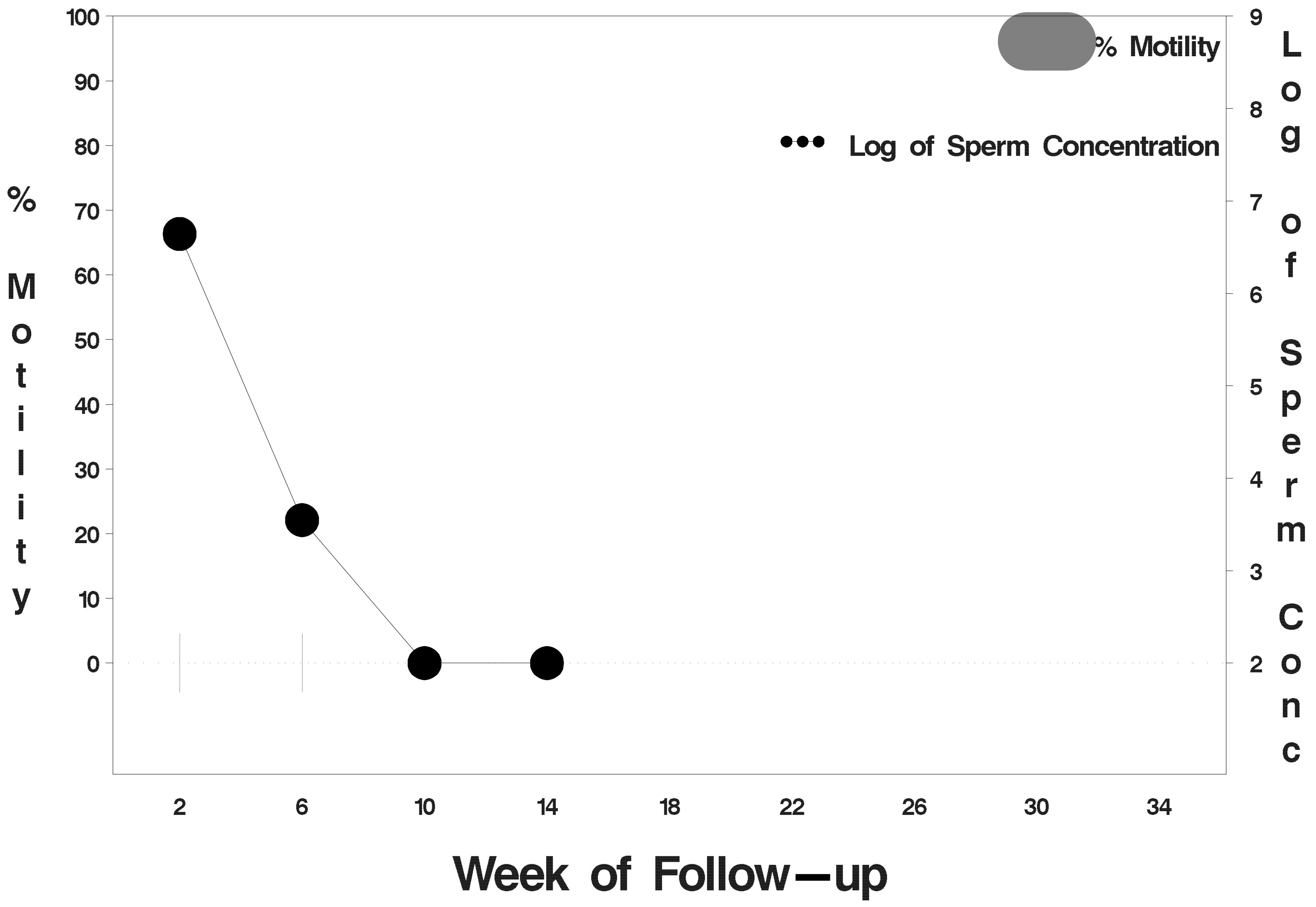

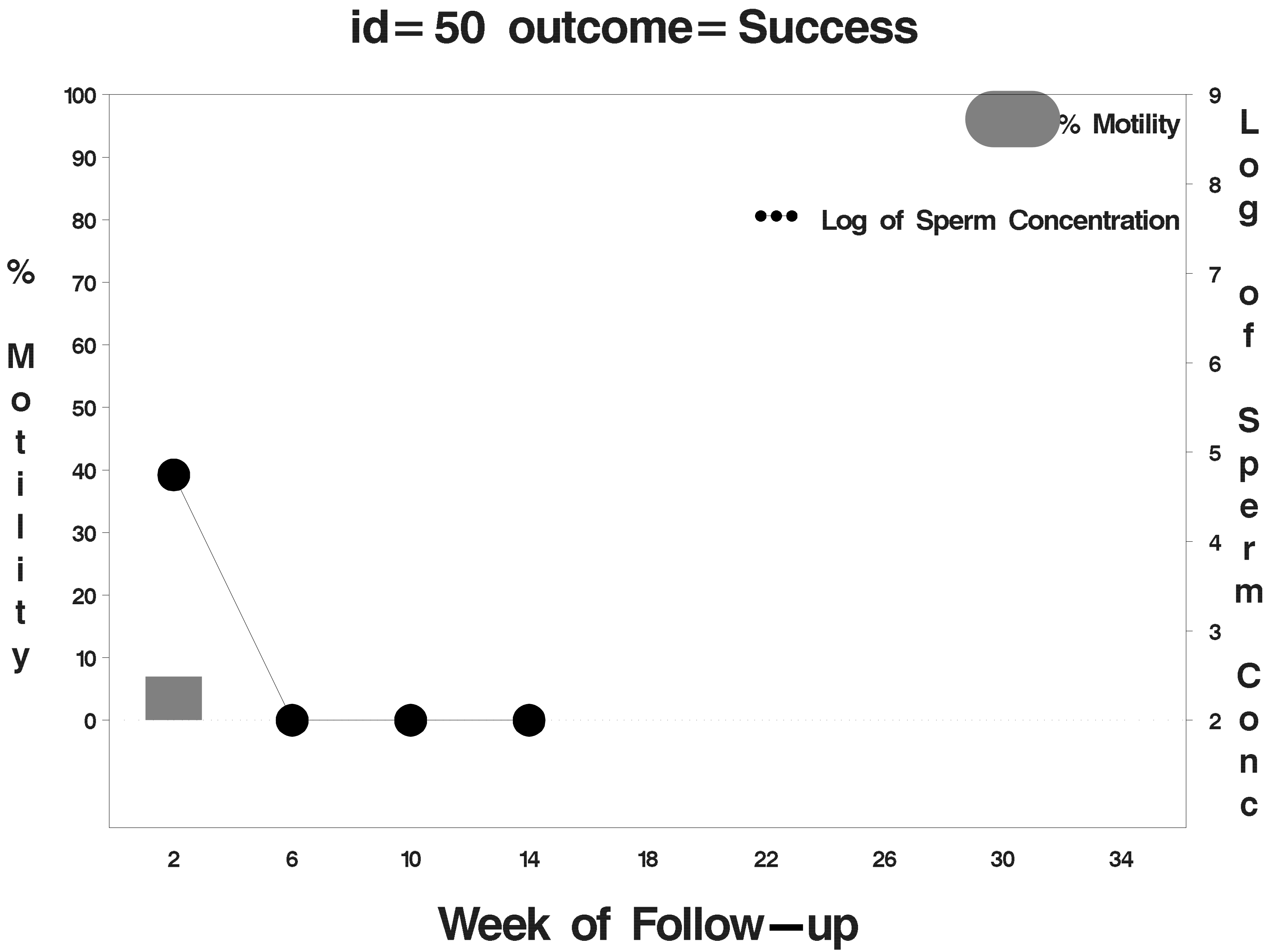

id= 50 outcome= Success
% Motility
Log of Sperm Concentration
100
90
80
70
60
50
40
30
20
10
0
9
8
7
6
5
4
3
2
2
6
10
14
18
22
26
30
34
Week of Follow−up
% Motility
Log of Sperm Conc

id= 51 outcome= Success

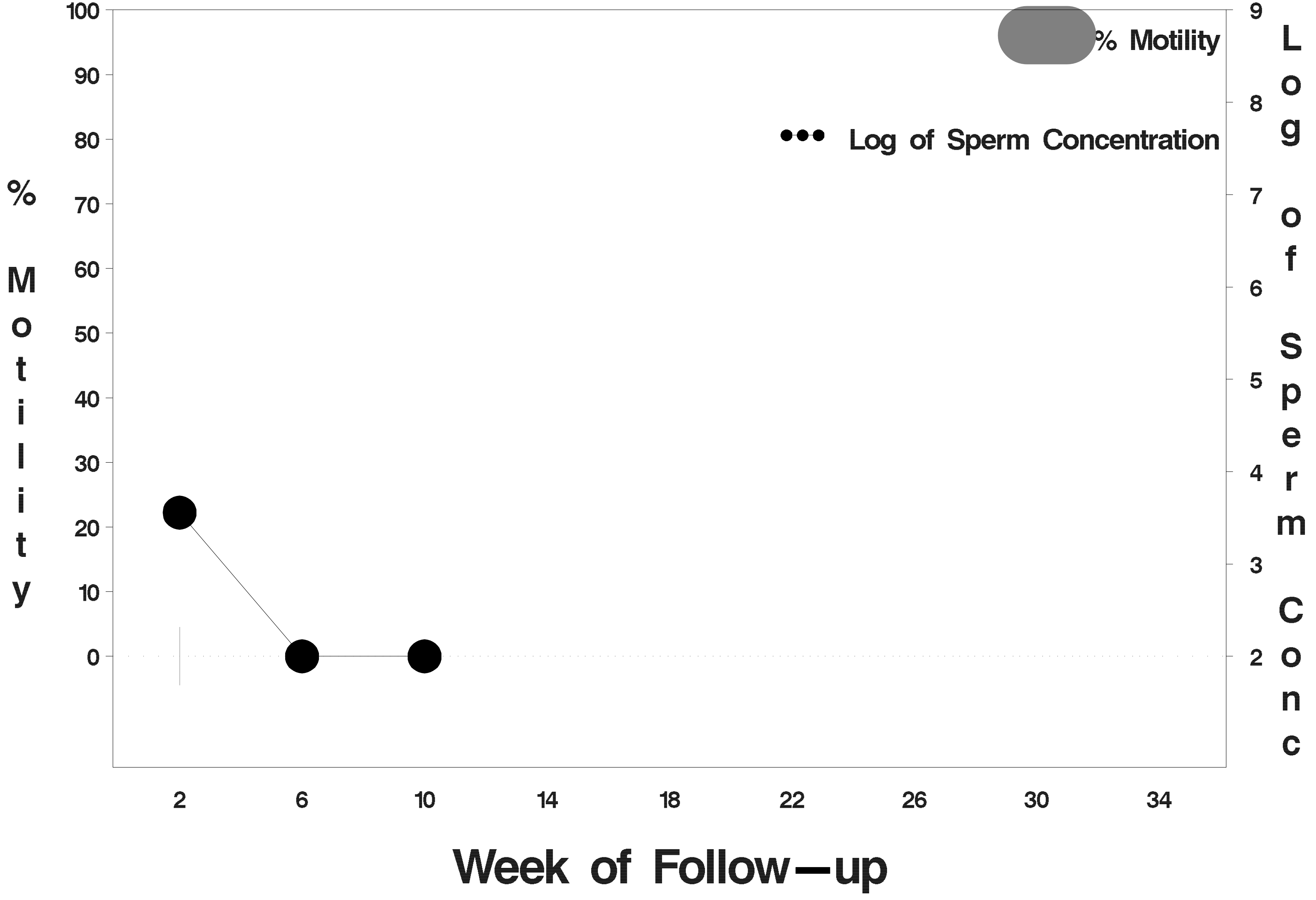

id= 52 outcome= Success

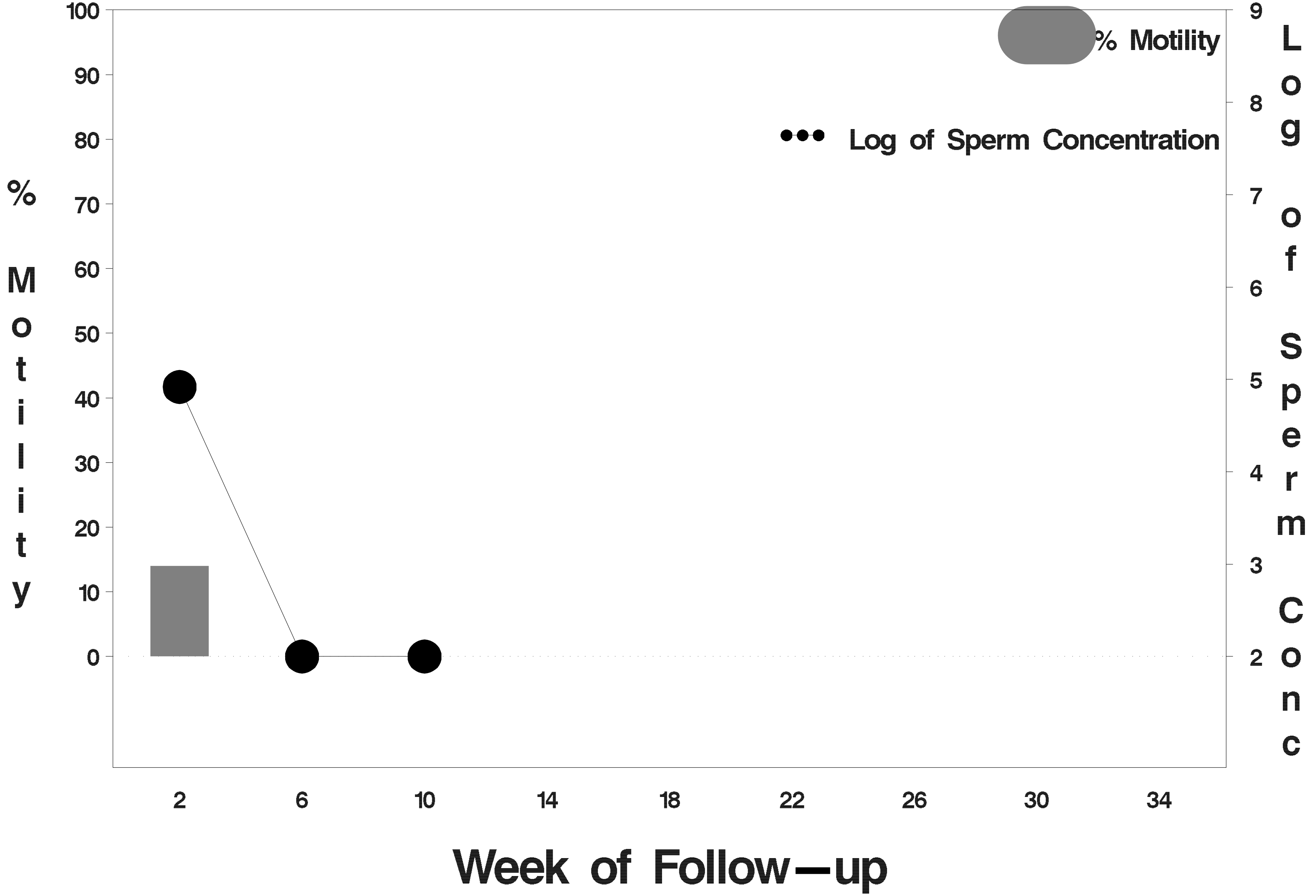

id= 53 outcome= Success

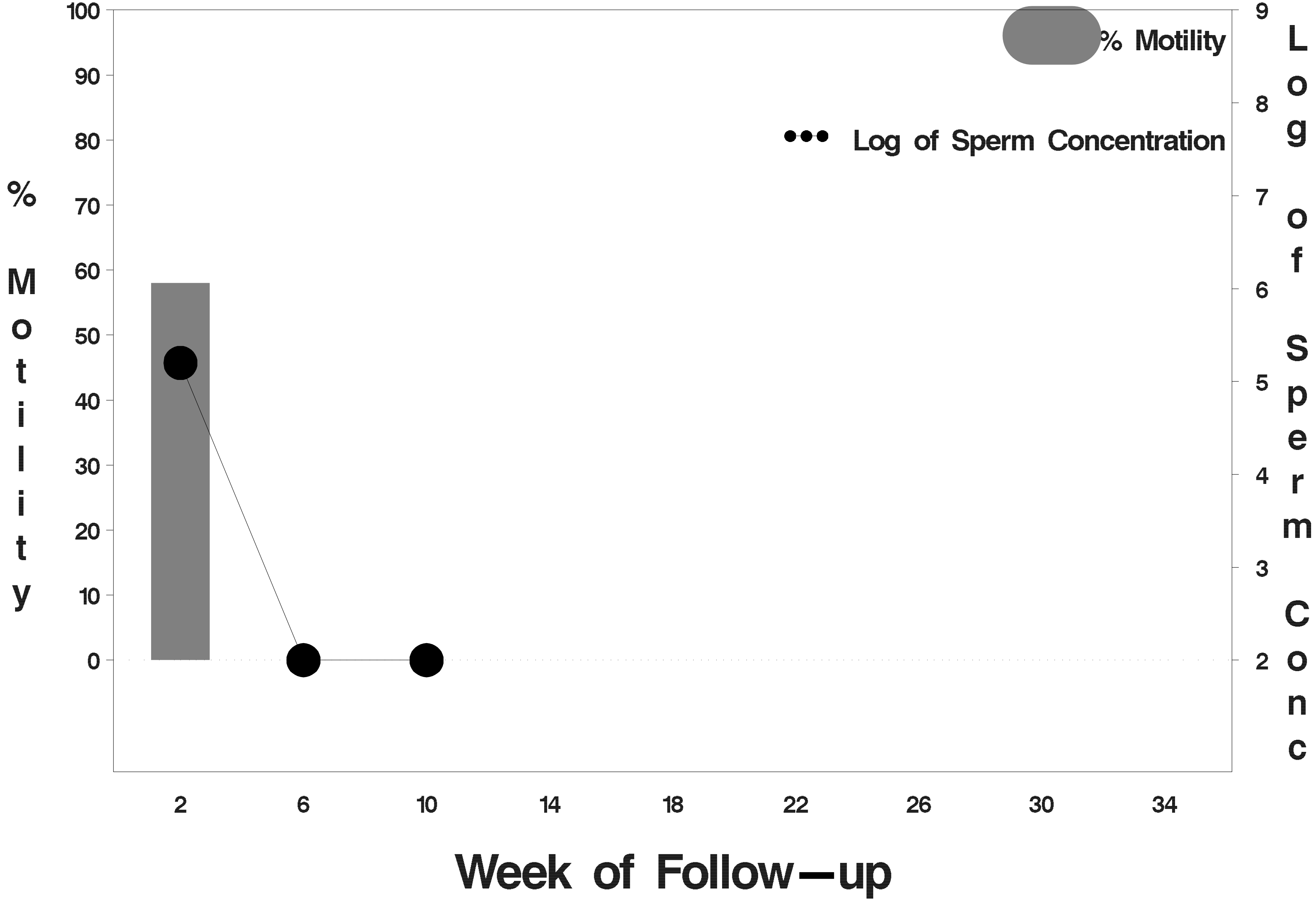

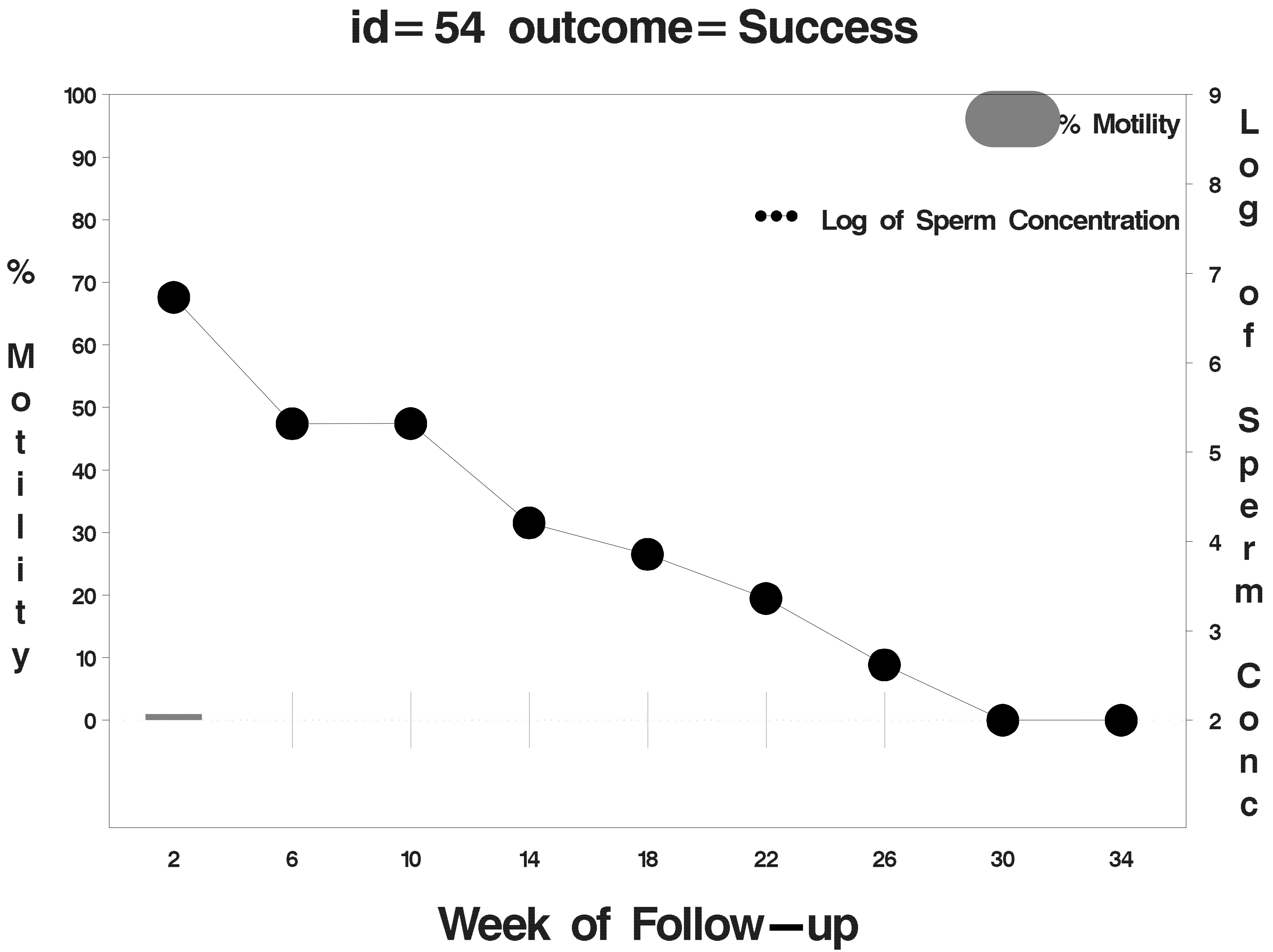

id= 54 outcome= Success
% Motility
Log of Sperm Concentration
100
90
80
70
60
50
40
30
20
10
0
9
8
7
6
5
4
3
2
2
6
10
14
18
22
26
30
34
Week of Follow—up
% Motility
Log of Sperm Conc

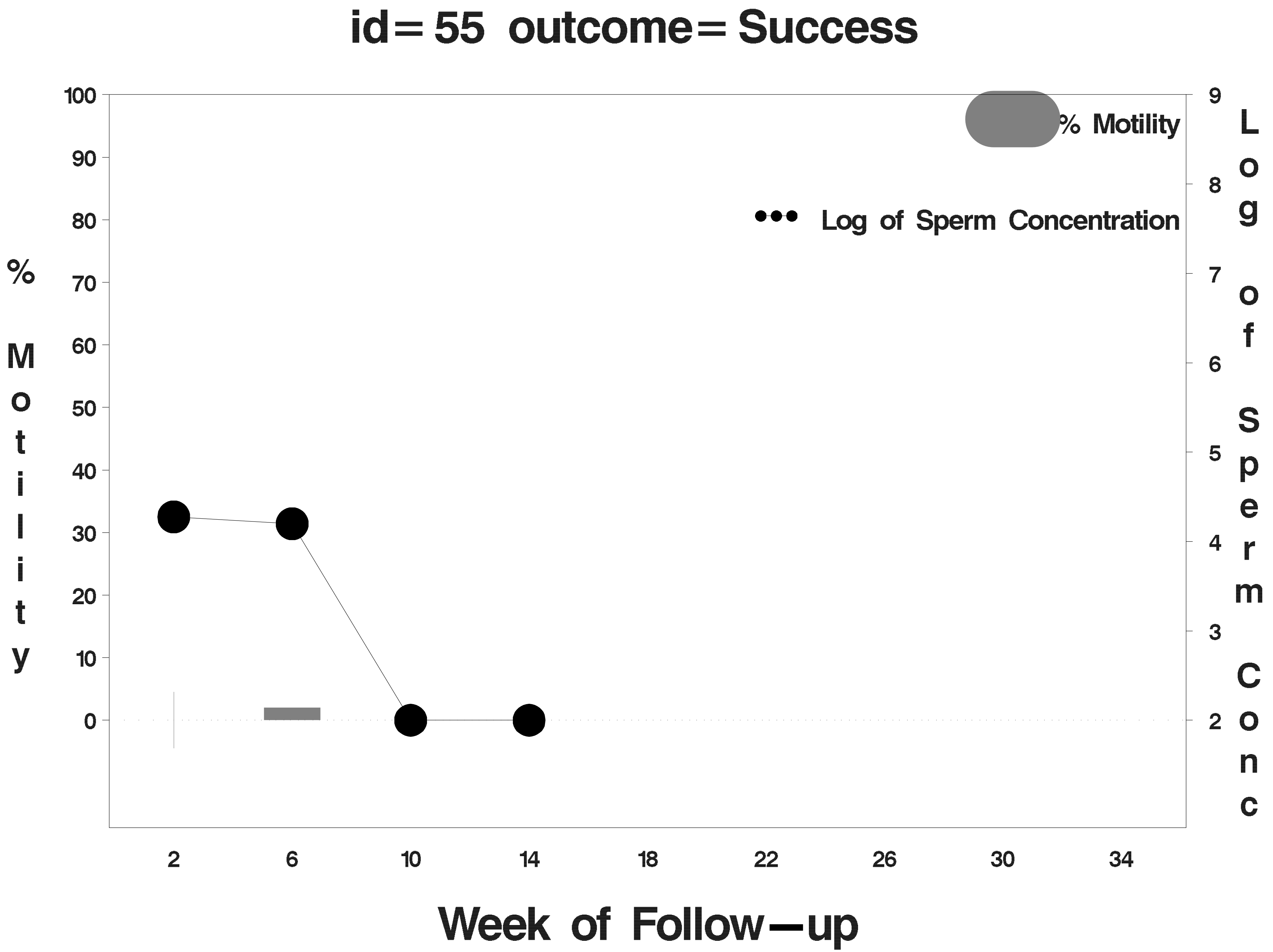

id= 55 outcome= Success
% Motility
Log of Sperm Concentration
100
90
80
70
60
50
40
30
20
10
0
9
8
7
6
5
4
3
2
2
6
10
14
18
22
26
30
34
Week of Follow—up

id= 56 outcome= Success

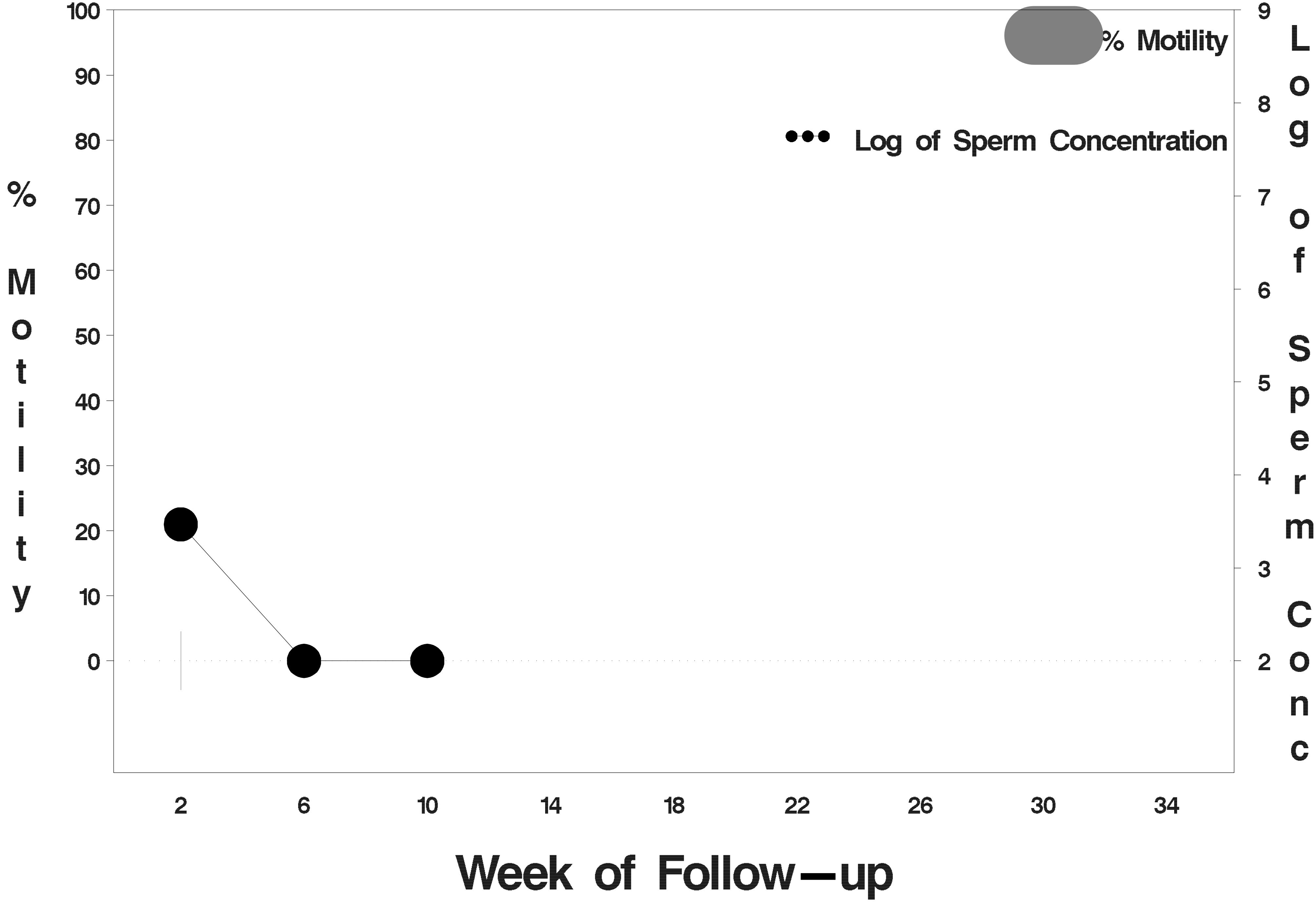

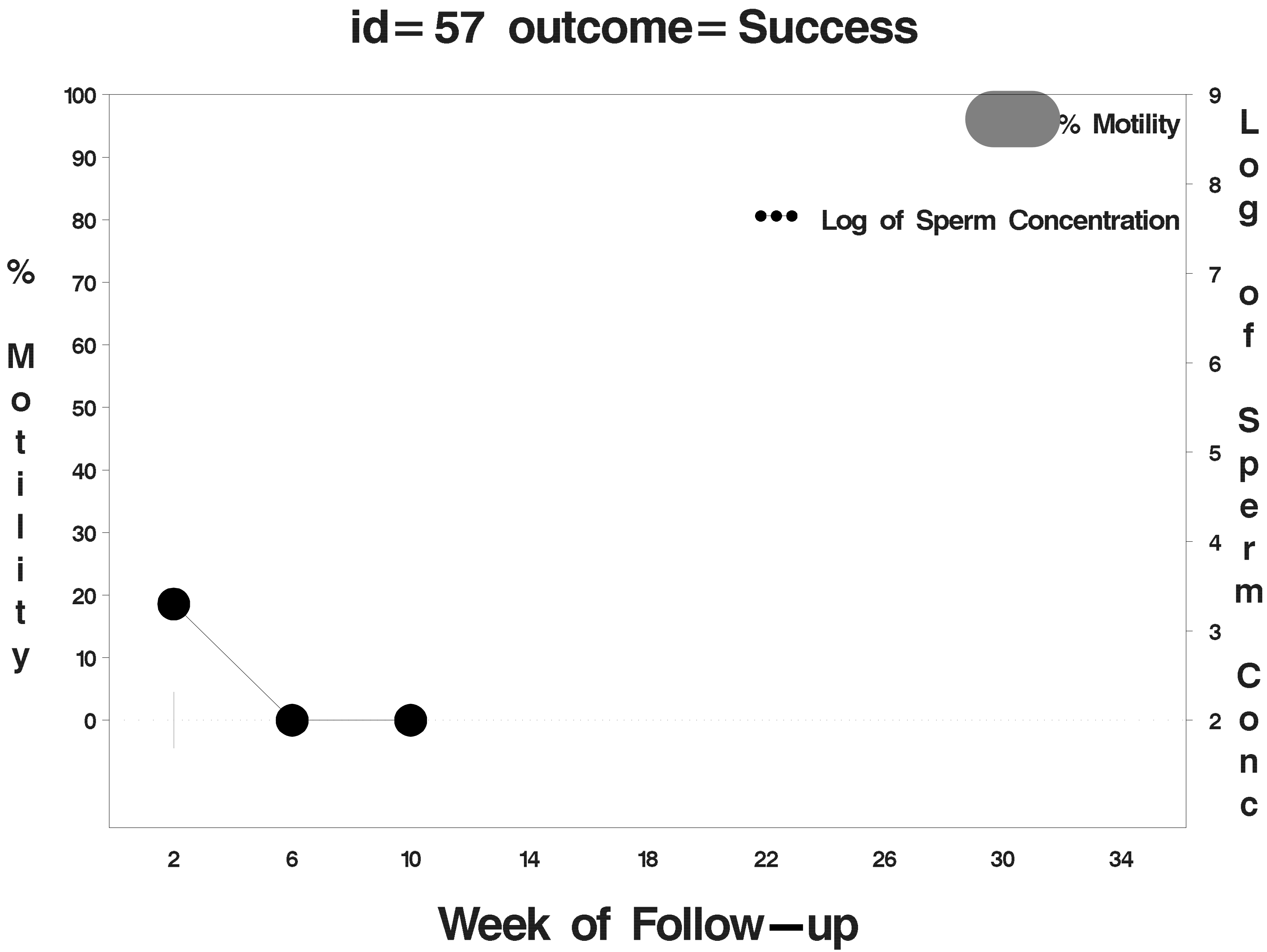

id= 57 outcome= Success
% Motility
Log of Sperm Concentration
100
90
80
70
60
50
40
30
20
10
0
9
8
7
6
5
4
3
2
2
6
10
14
18
22
26
30
34
Week of Follow−up
Log of Sperm Conc

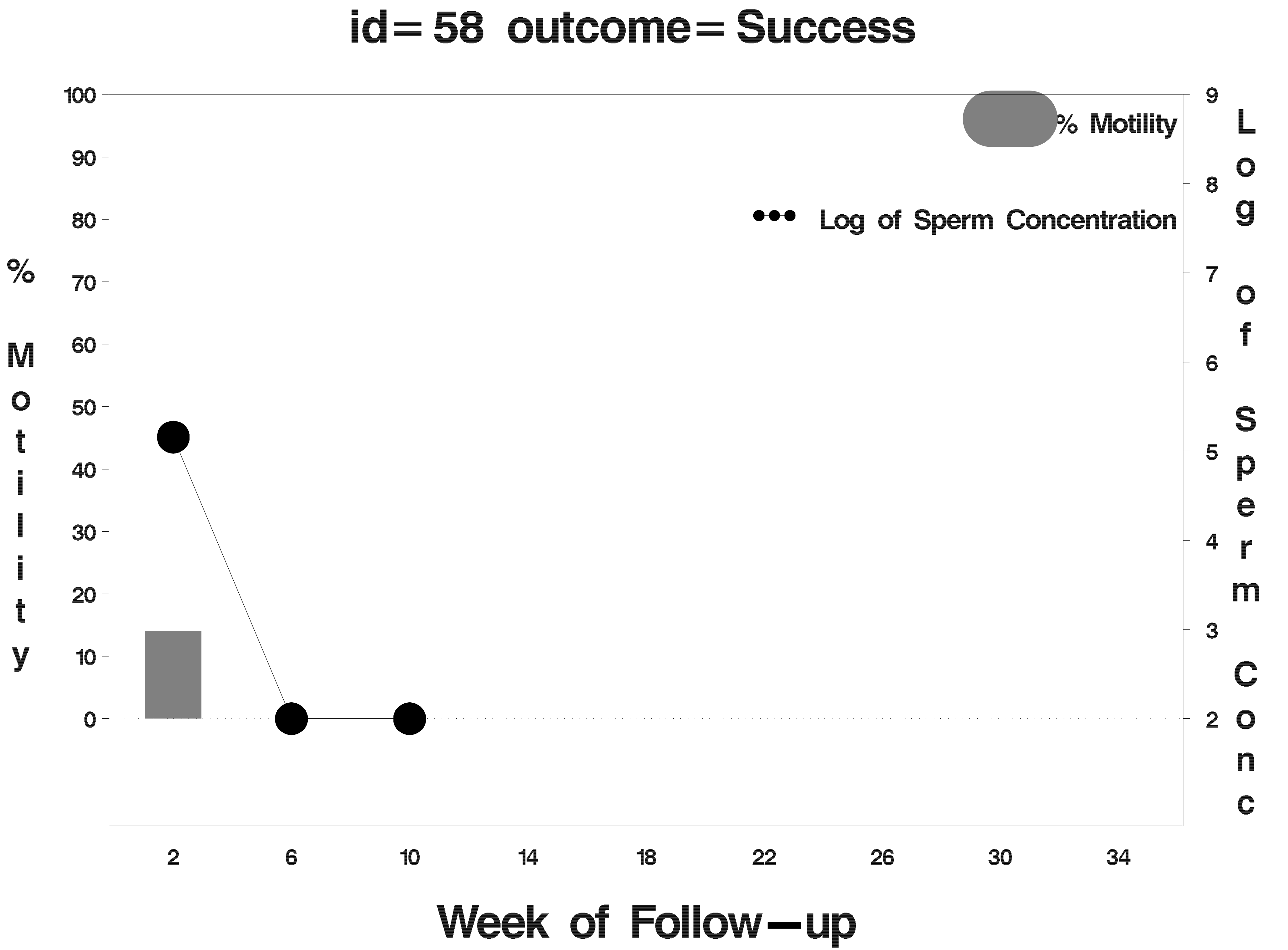

id= 58 outcome= Success
% Motility
Log of Sperm Concentration
100
90
80
70
60
50
40
30
20
10
0
9
8
7
6
5
4
3
2
2
6
10
14
18
22
26
30
34
Week of Follow—up
% Motility
Log of Sperm Conc

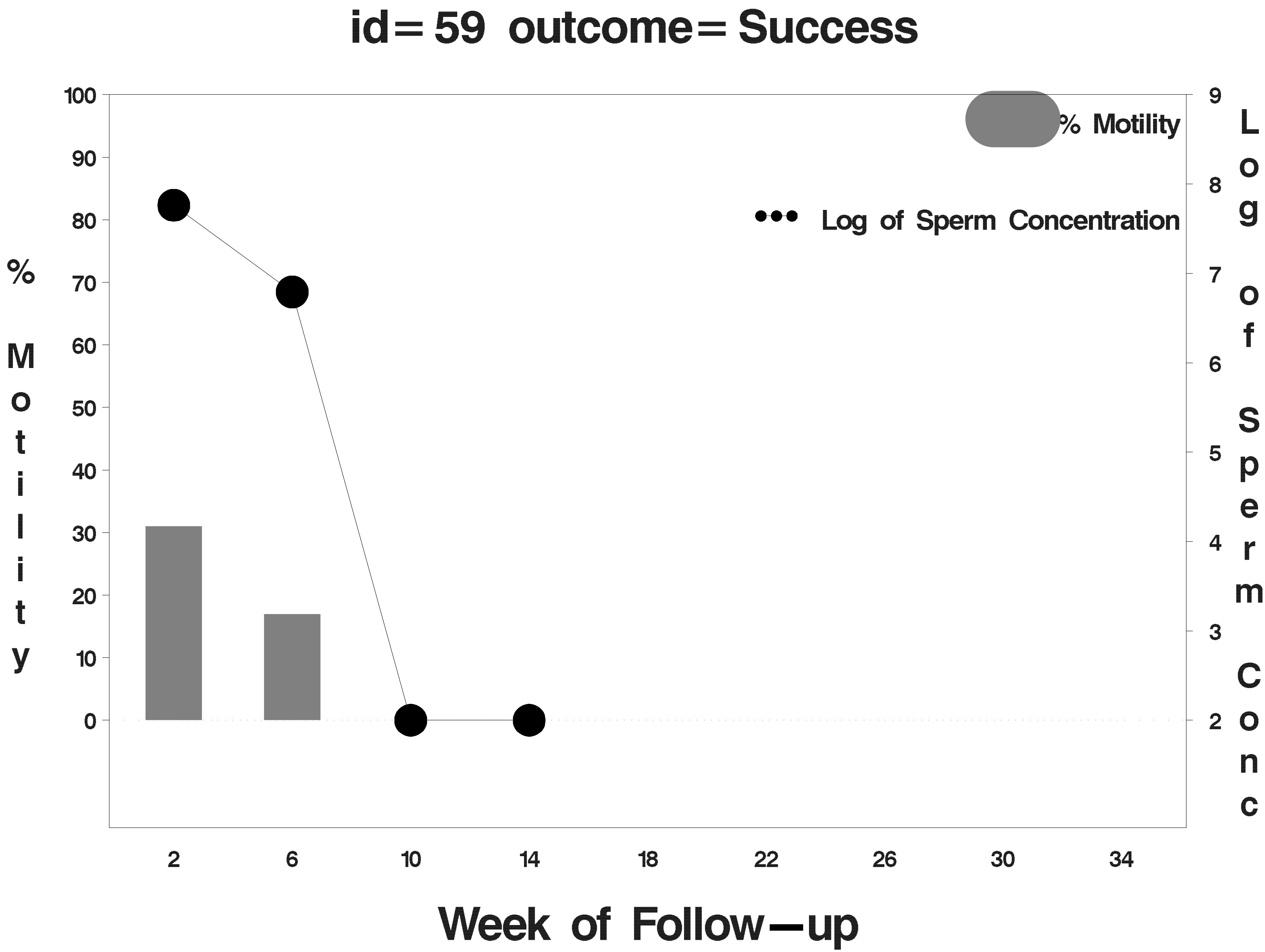

id= 59 outcome= Success
% Motility
Log of Sperm Concentration
100
90
80
70
60
50
40
30
20
10
0
9
8
7
6
5
4
3
2
2
6
10
14
18
22
26
30
34
Week of Follow—up
Log of Sperm Conc

id= 60 outcome= Success

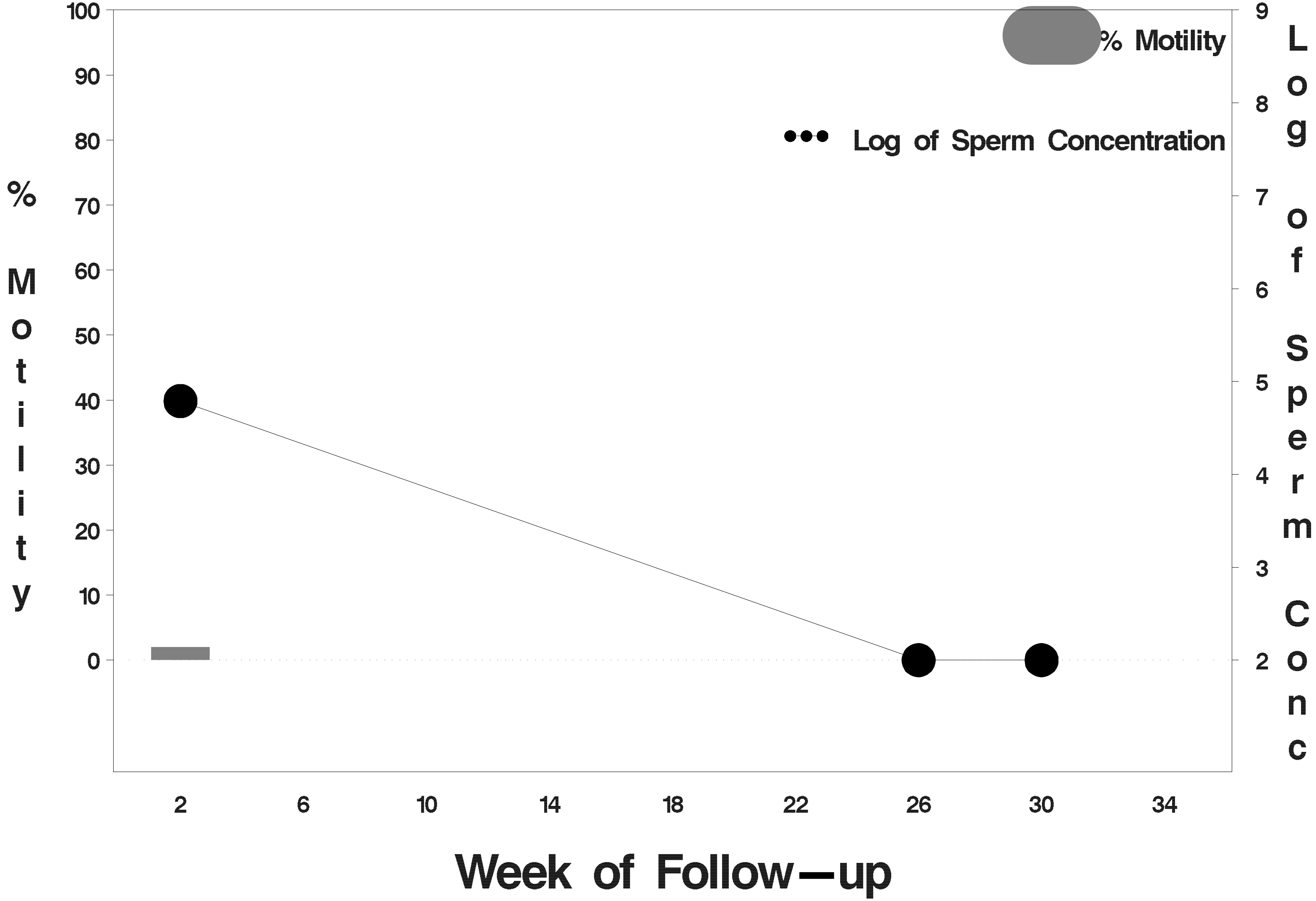

id= 61 outcome= Success

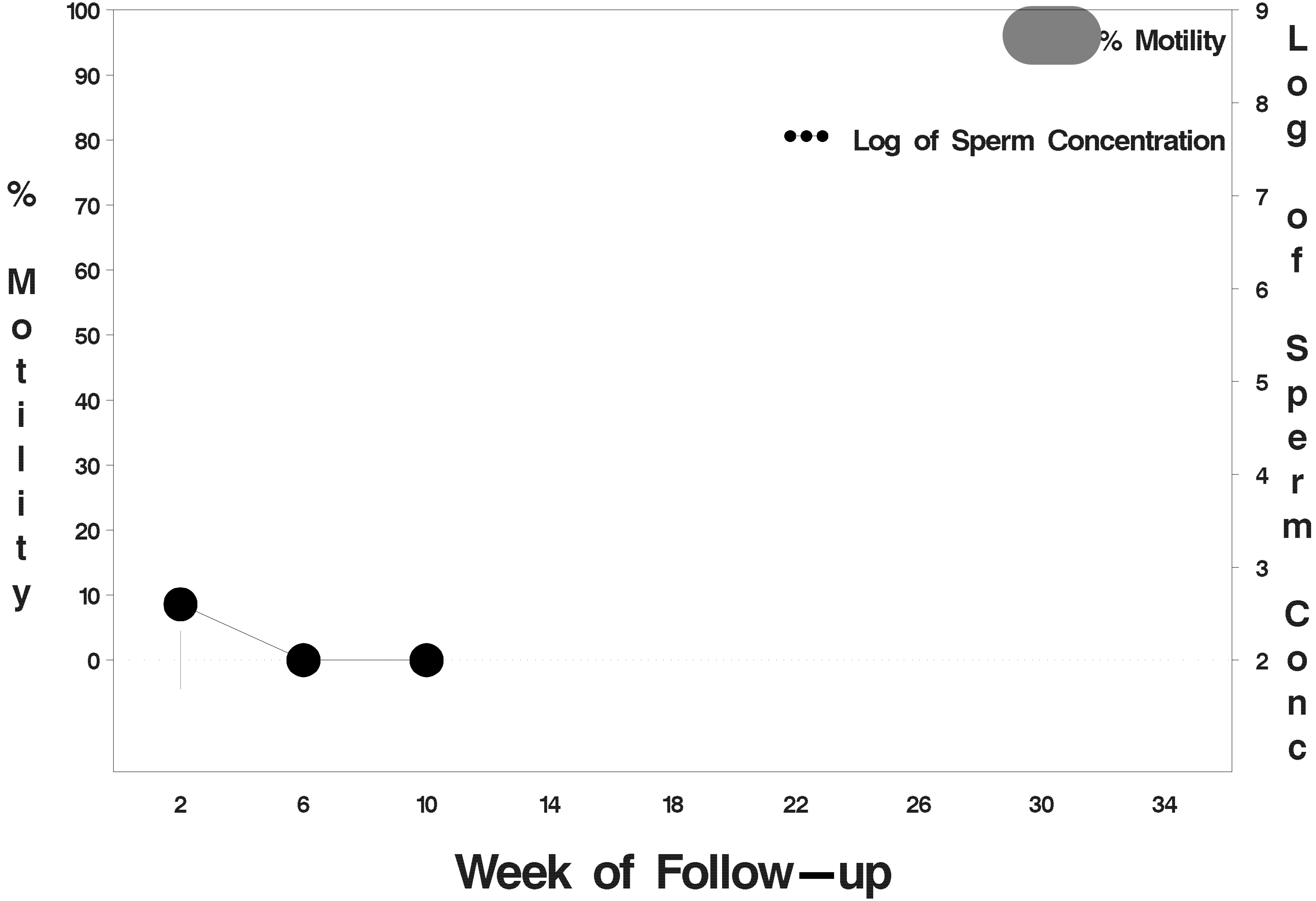

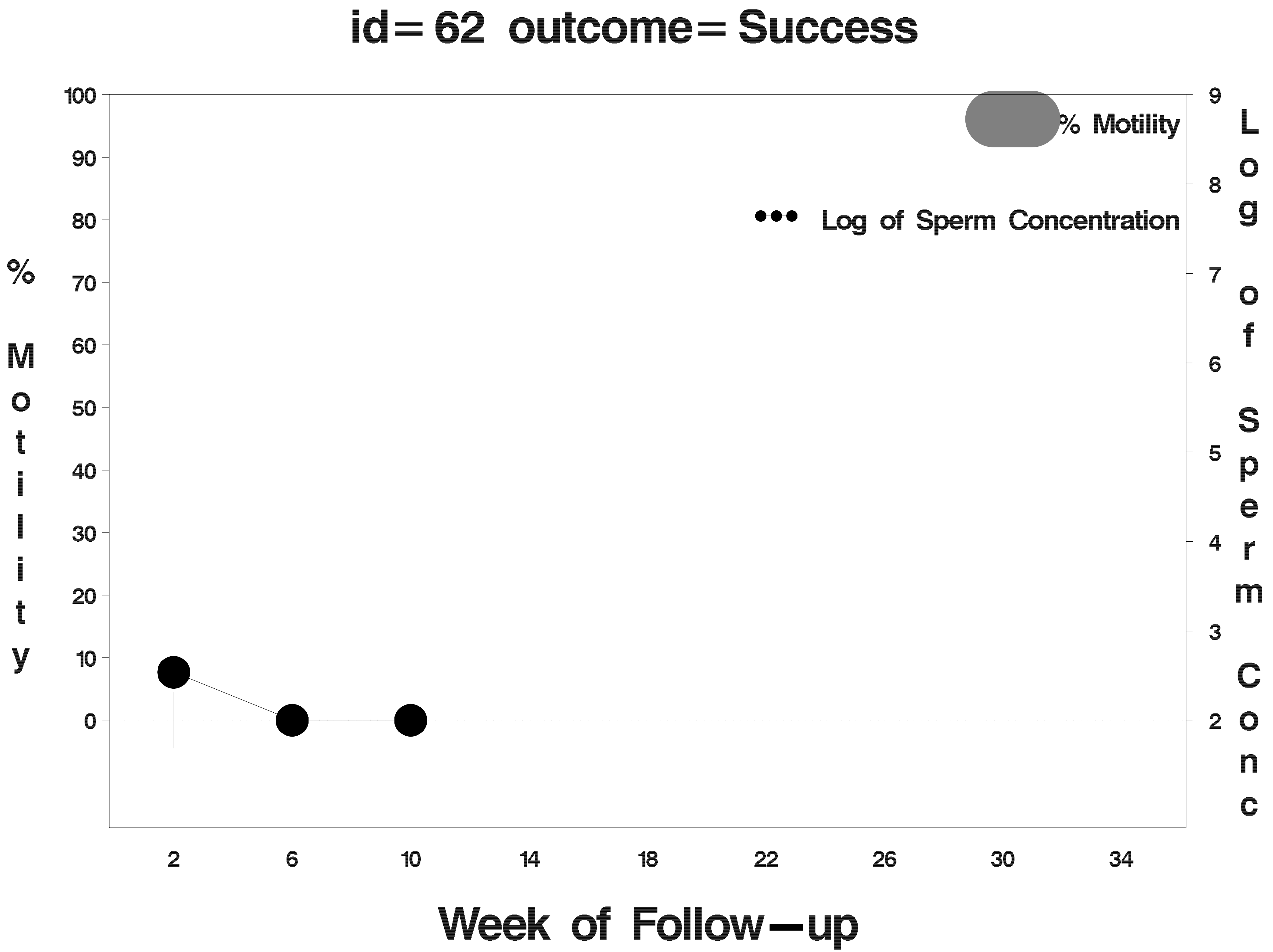
id= 62 outcome= Success
% Motility
Log of Sperm Concentration
100
90
80
70
60
50
40
30
20
10
0
9
8
7
6
5
4
3
2
2
6
10
14
18
22
26
30
34
Week of Follow—up
Log of Sperm Conc

id= 63 outcome= Success

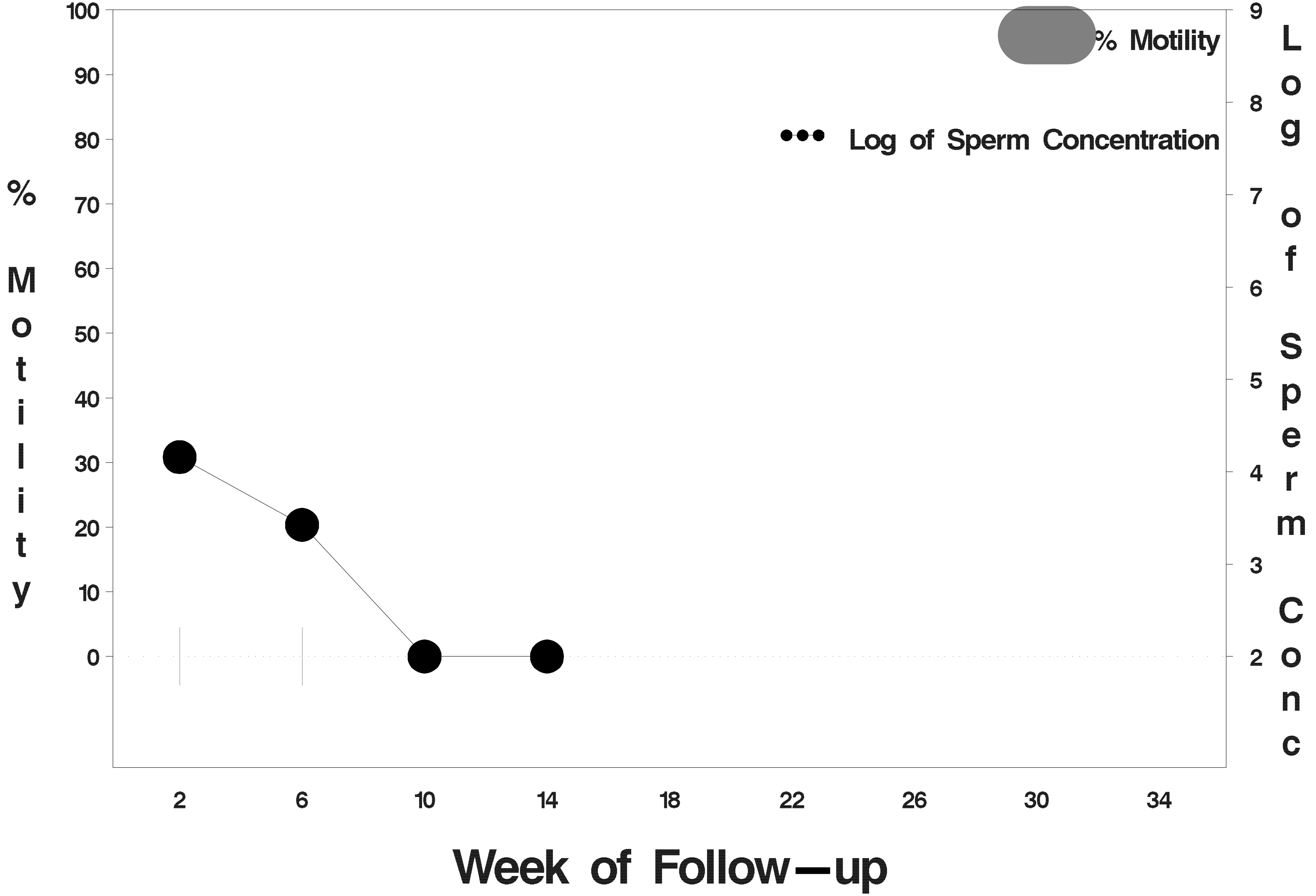

id= 64 outcome= Success

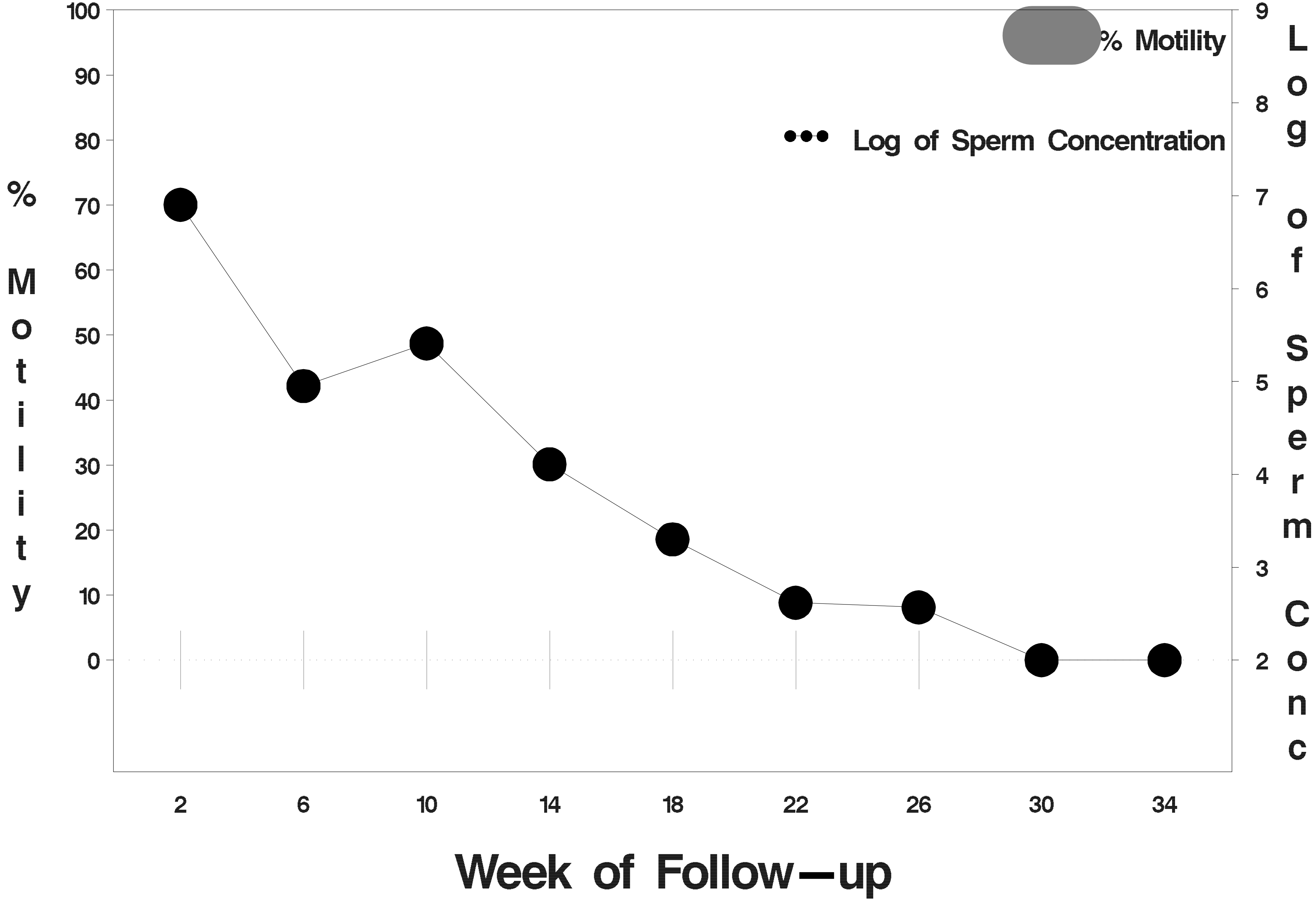

id= 65 outcome= Success

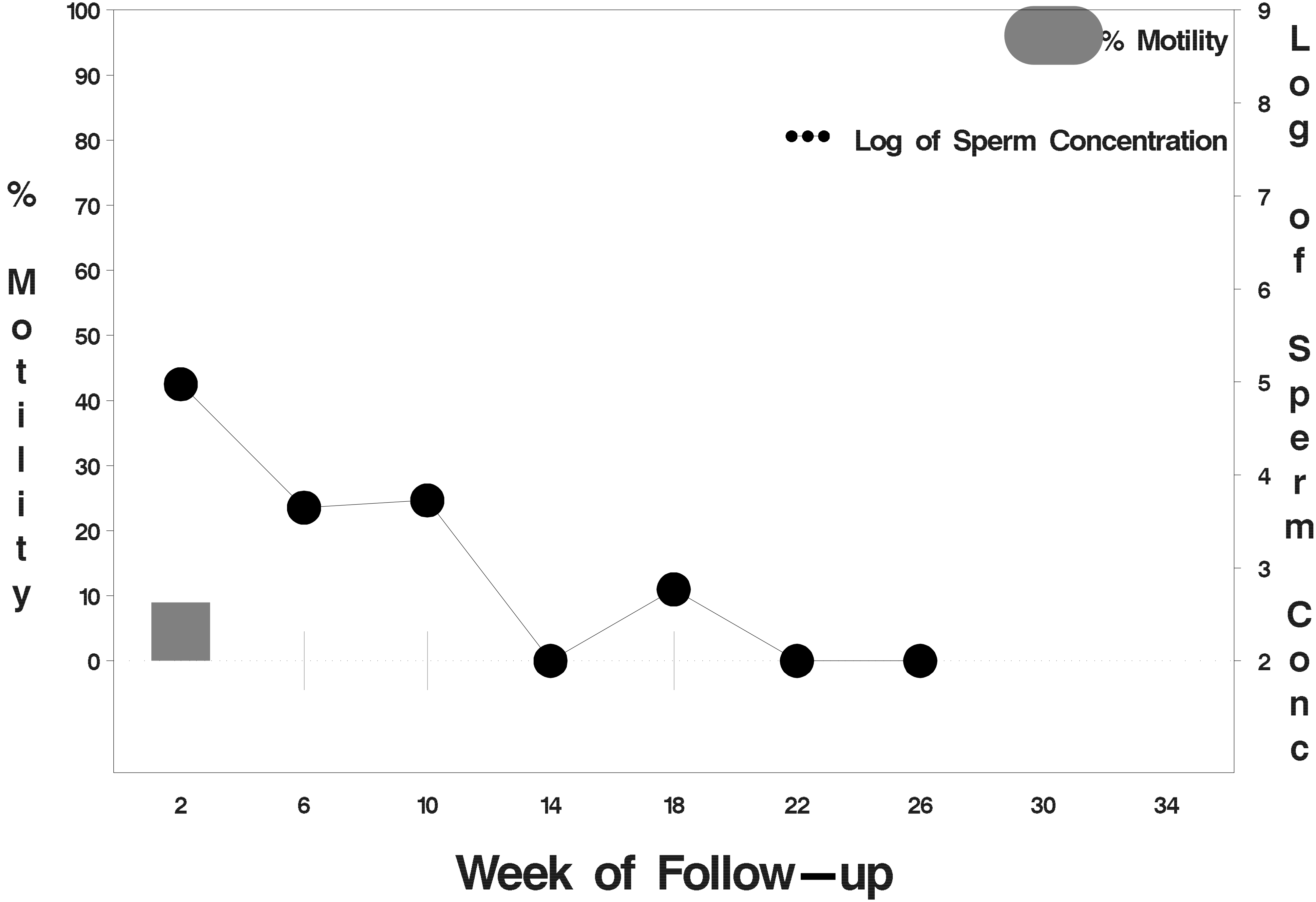

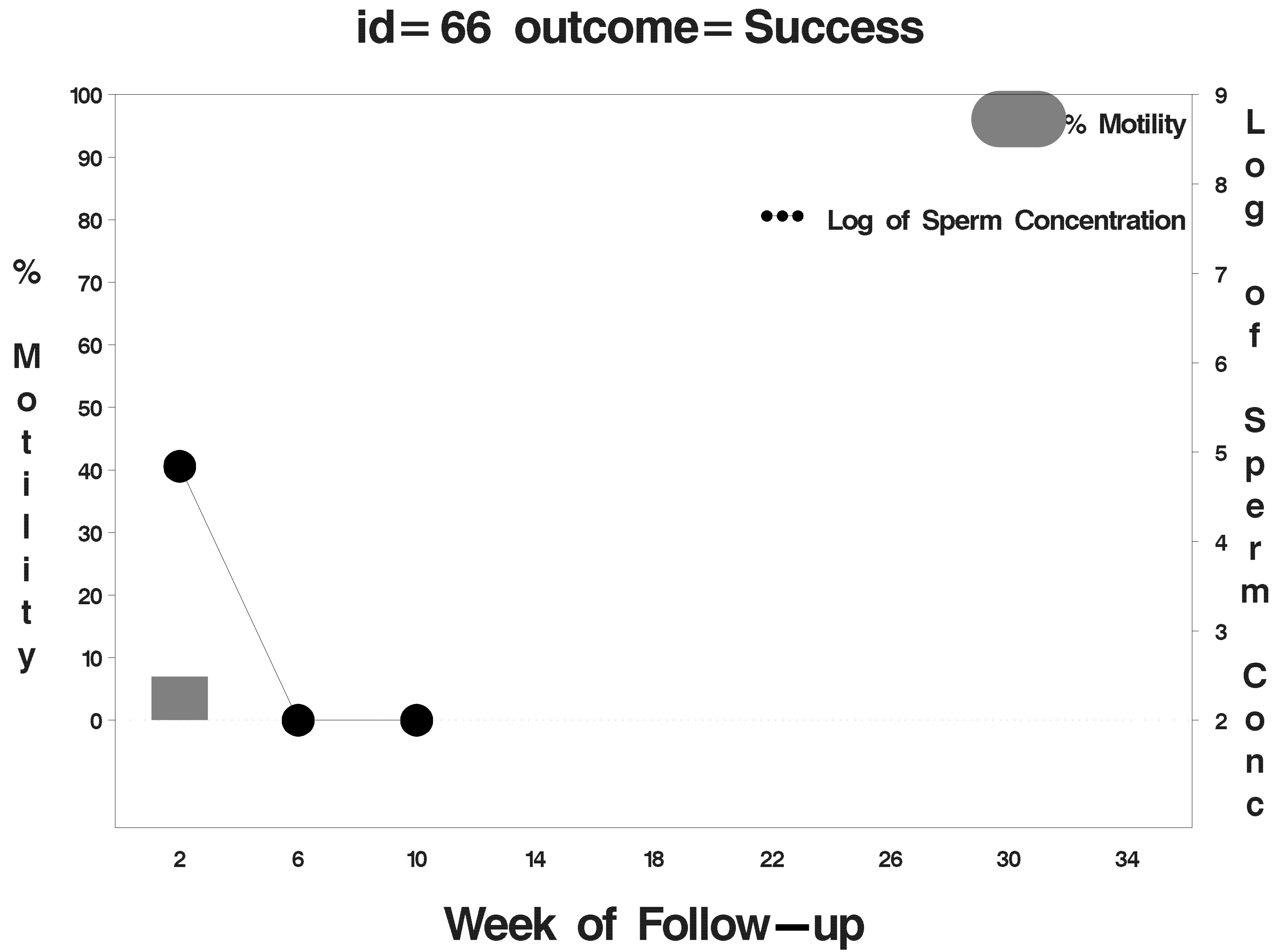

id= 66 outcome= Success
% Motility
Log of Sperm Concentration
100
90
80
70
60
50
40
30
20
10
0
9
8
7
6
5
4
3
2
% Motility
Log of Sperm Conc
2
6
10
14
18
22
26
30
34
Week of Follow—up

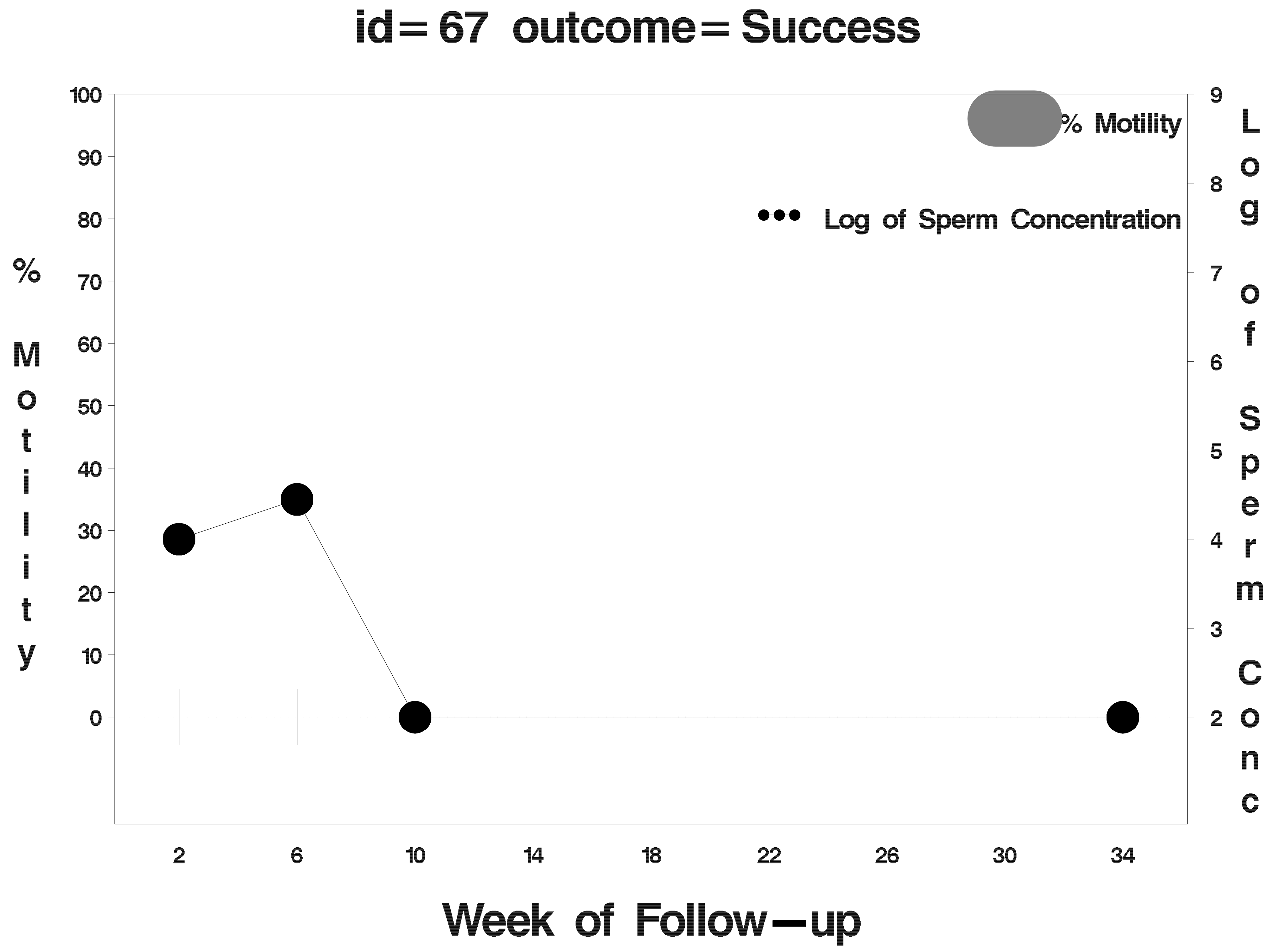
id= 67 outcome= Success
% Motility
Log of Sperm Concentration
100
90
80
70
60
50
40
30
20
10
0
9
8
7
6
5
4
3
2
2
6
10
14
18
22
26
30
34
Week of Follow—up
Log of Sperm Conc

id= 68 outcome= Success

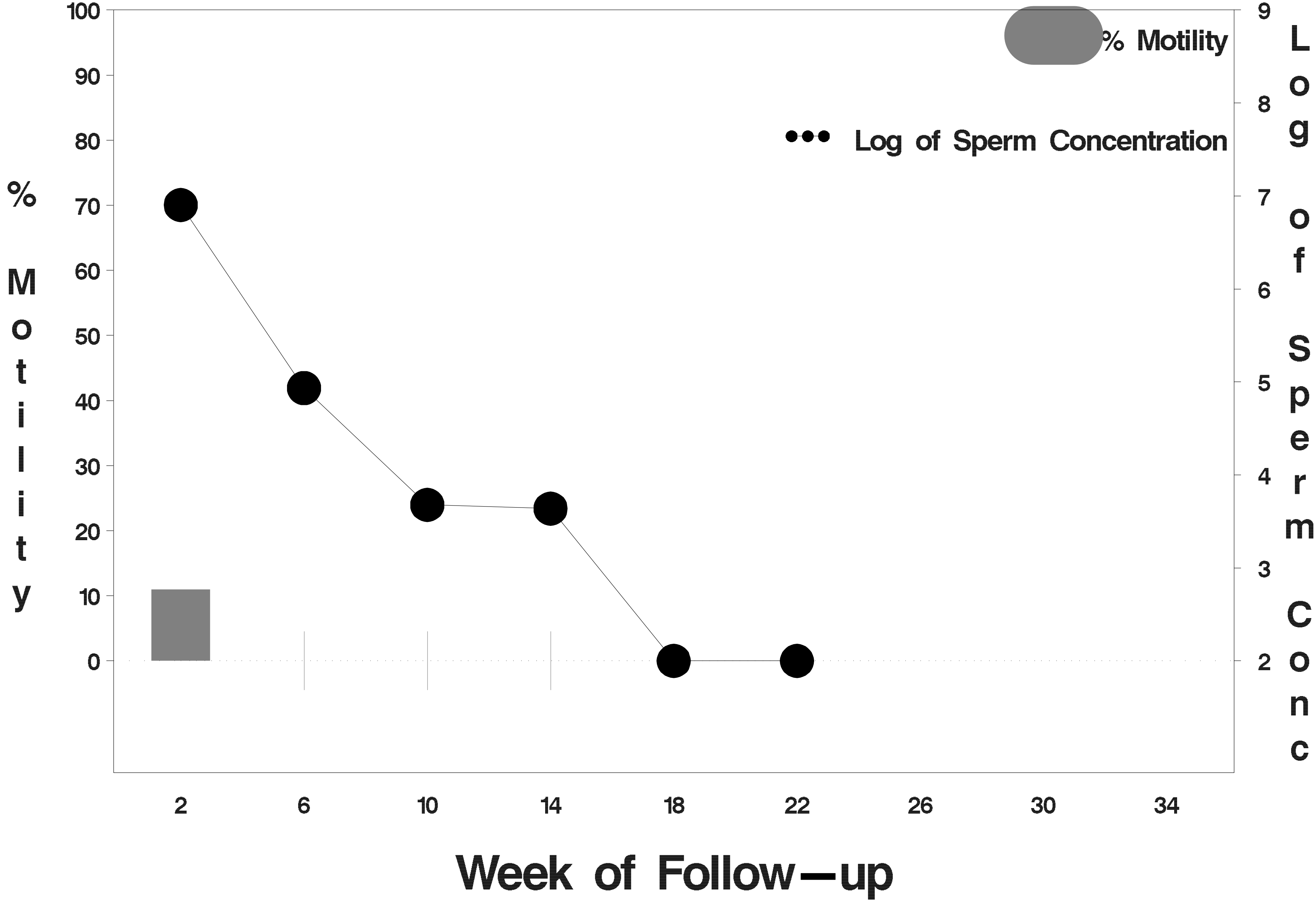

id= 69 outcome= Indeterminate

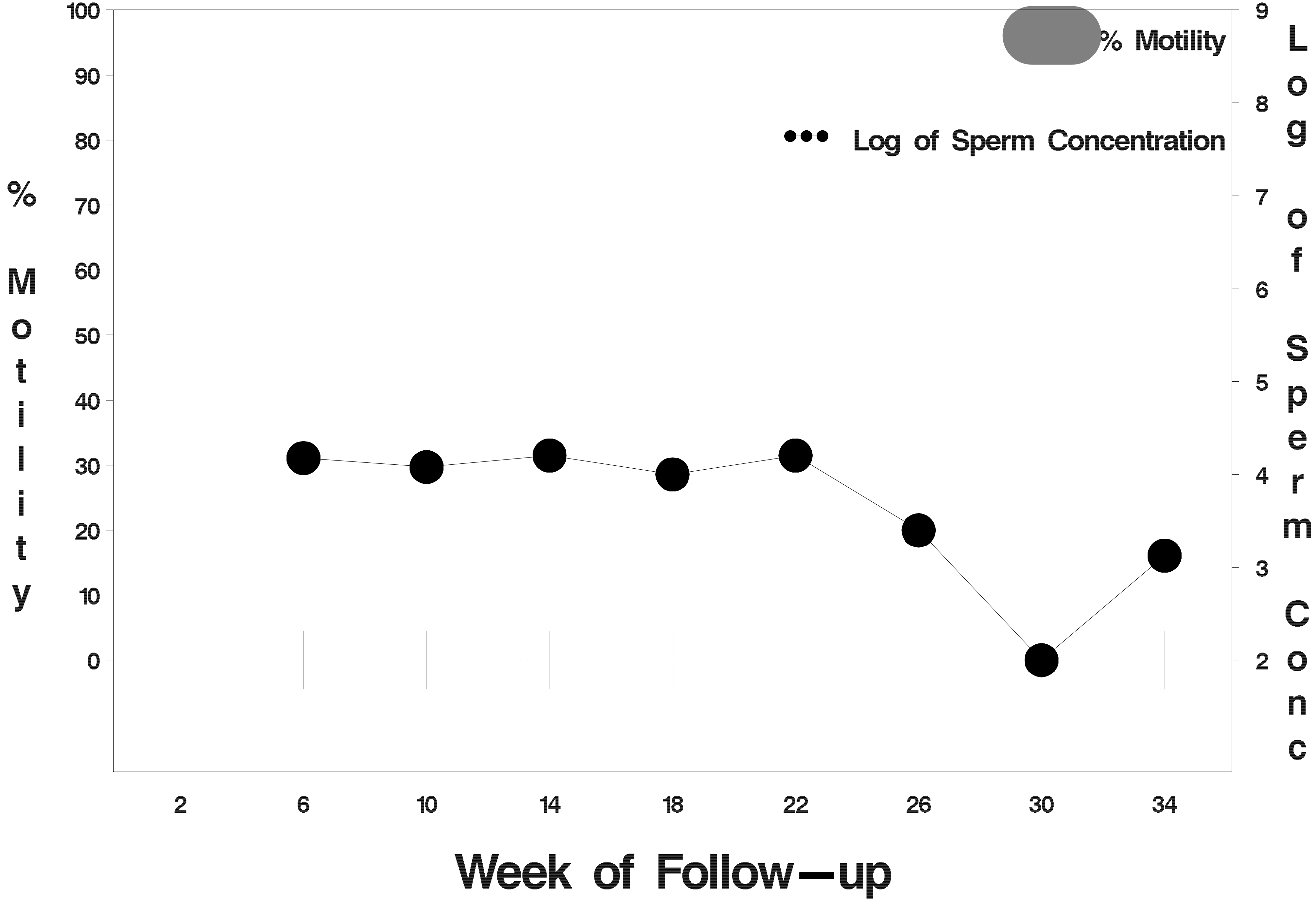

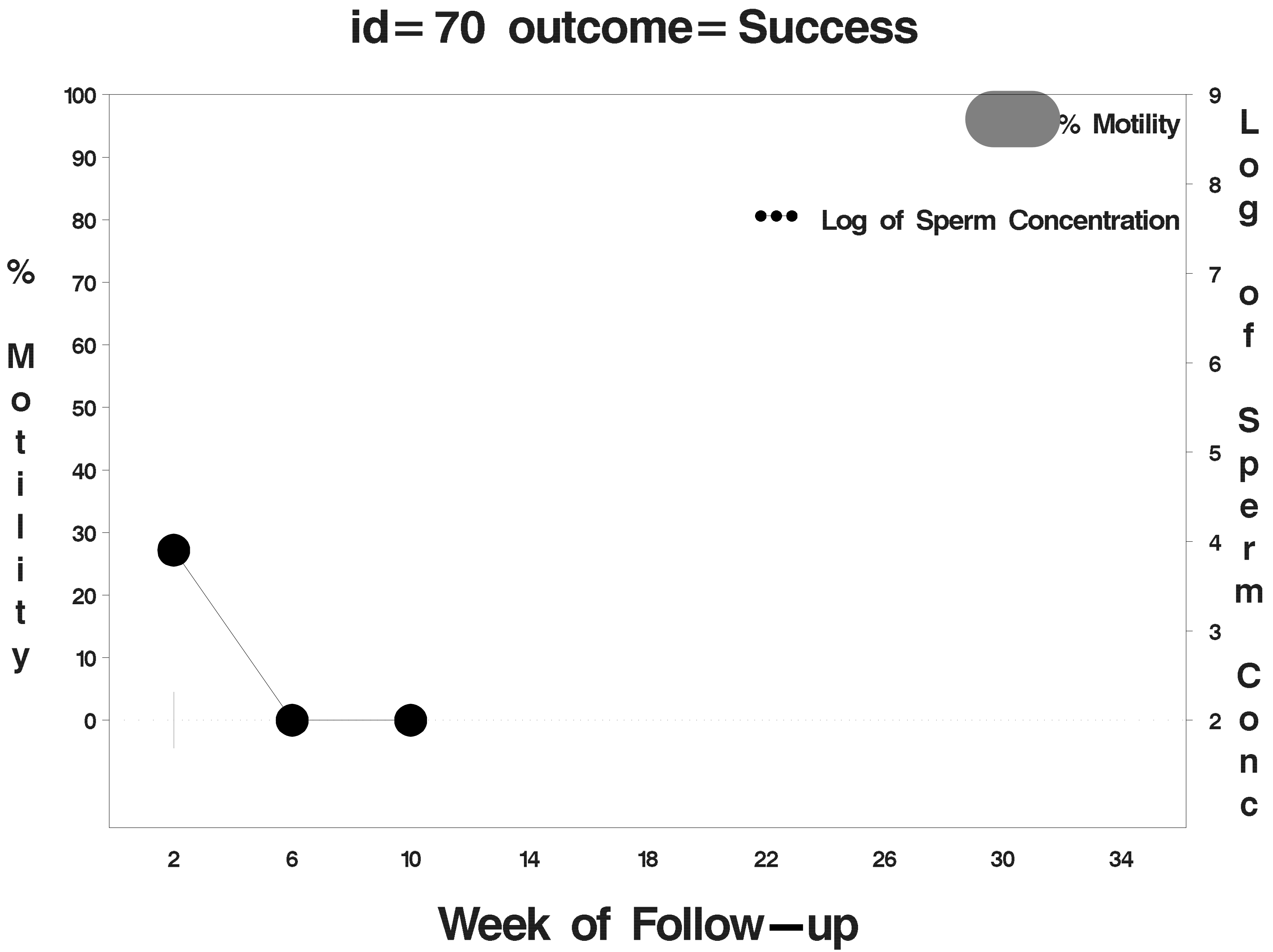

id= 70 outcome= Success
% Motility
Log of Sperm Concentration
% Motility
Log of Sperm Conc
100
90
80
70
60
50
40
30
20
10
0
9
8
7
6
5
4
3
2
2
6
10
14
18
22
26
30
34
Week of Follow—up

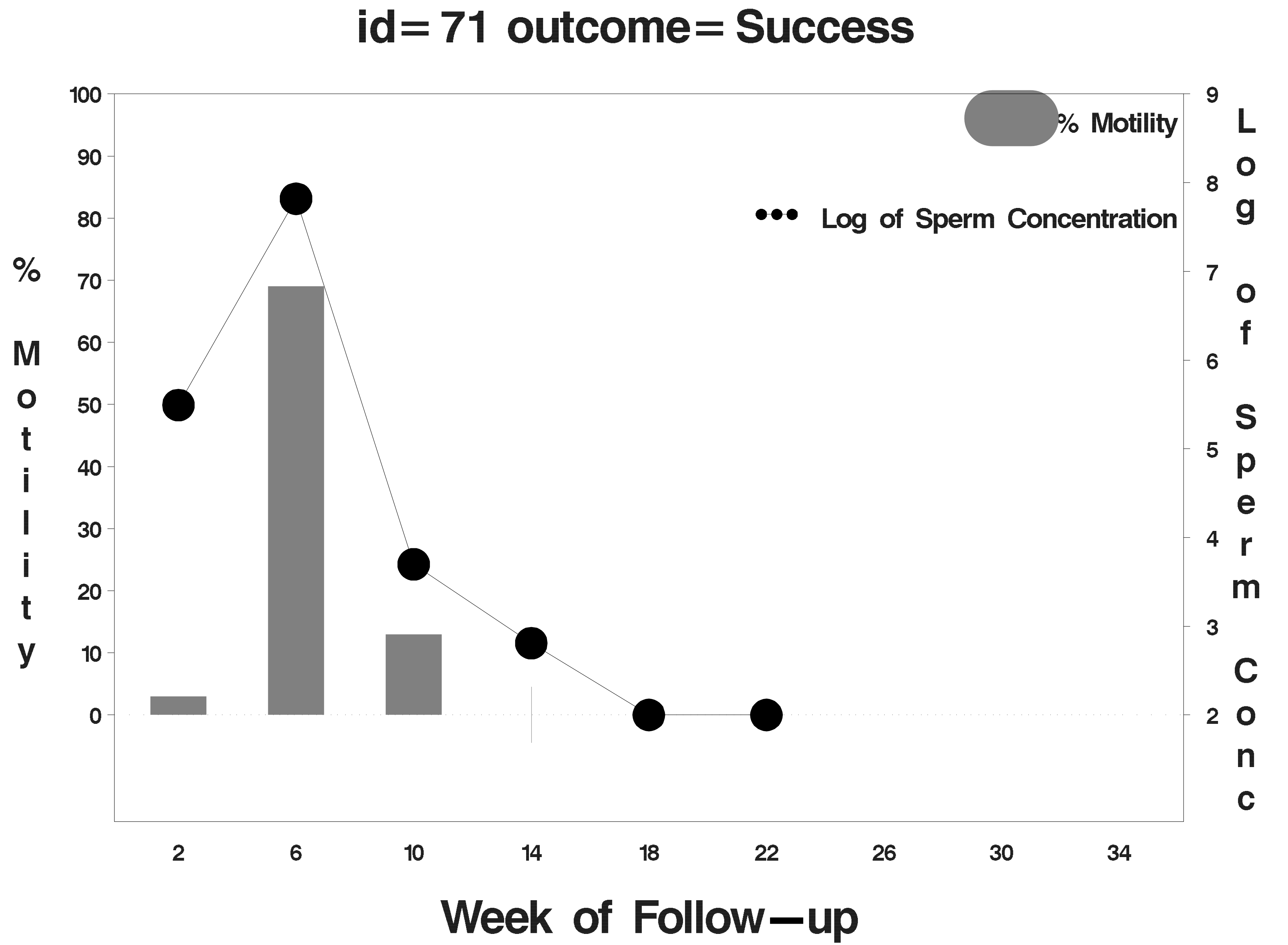

id= 71 outcome= Success
% Motility
Log of Sperm Concentration
100
90
80
70
60
50
40
30
20
10
0
9
8
7
6
5
4
3
2
2
6
10
14
18
22
26
30
34
Week of Follow−up
Log of Sperm Conc

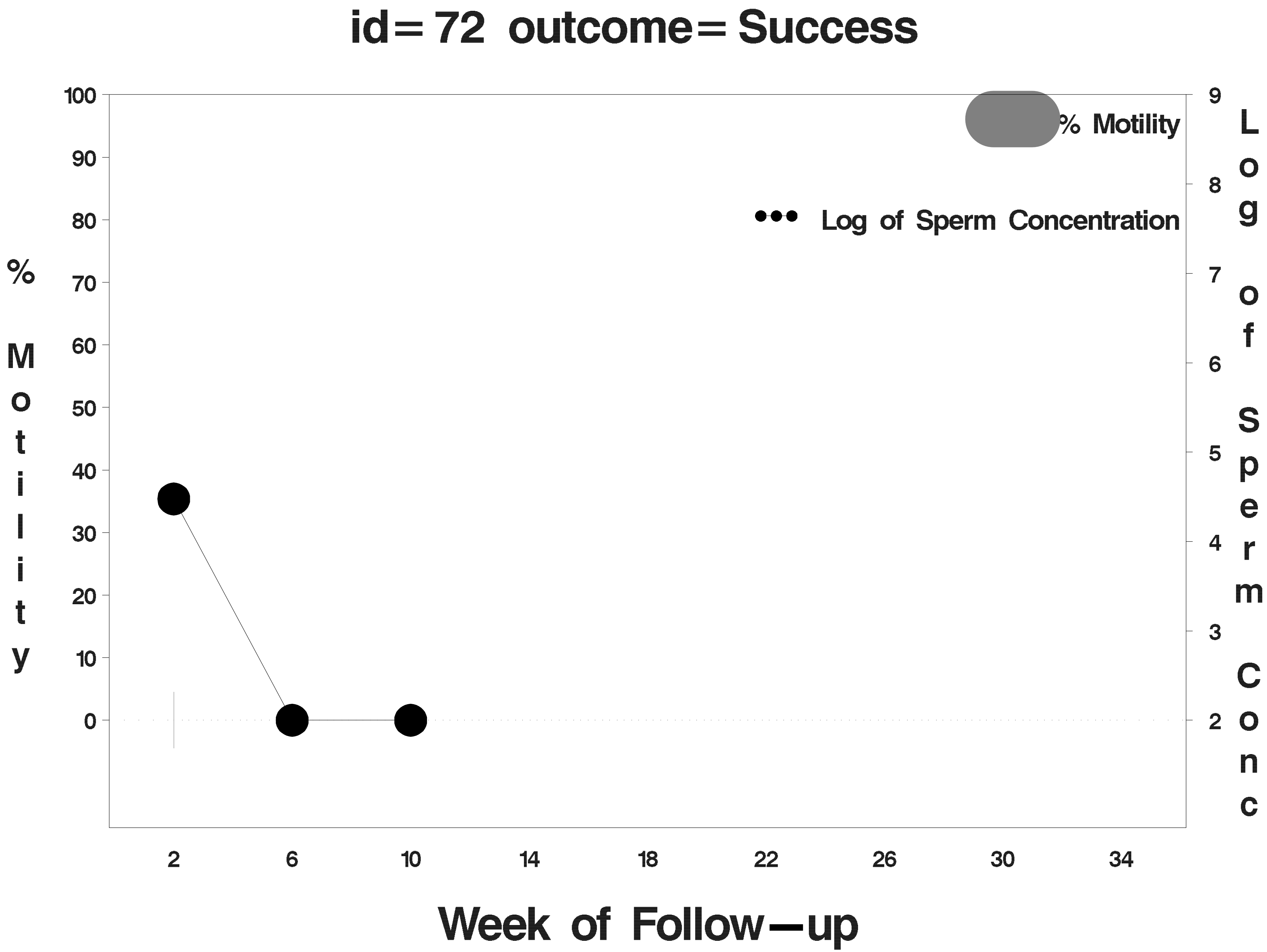
id= 72 outcome= Success
% Motility
Log of Sperm Concentration
% Motility
Log of Sperm Conc
Week of Follow−up
100
90
80
70
60
50
40
30
20
10
0
9
8
7
6
5
4
3
2
2
6
10
14
18
22
26
30
34

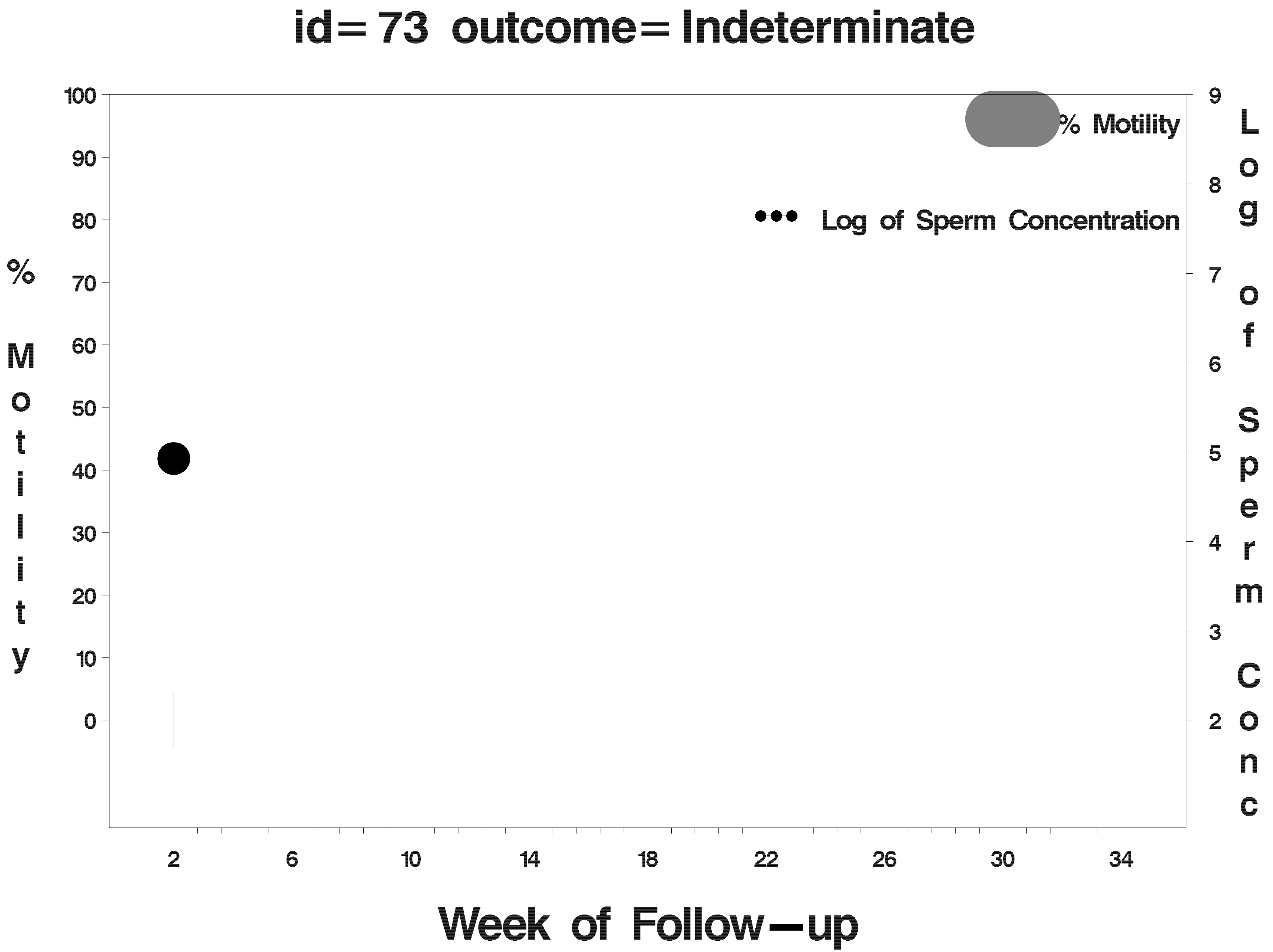

id= 73 outcome= Indeterminate
% Motility
Log of Sperm Concentration
% Motility
Log of Sperm Conc
Week of Follow-up
100
90
80
70
60
50
40
30
20
10
0
9
8
7
6
5
4
3
2
2
6
10
14
18
22
26
30
34

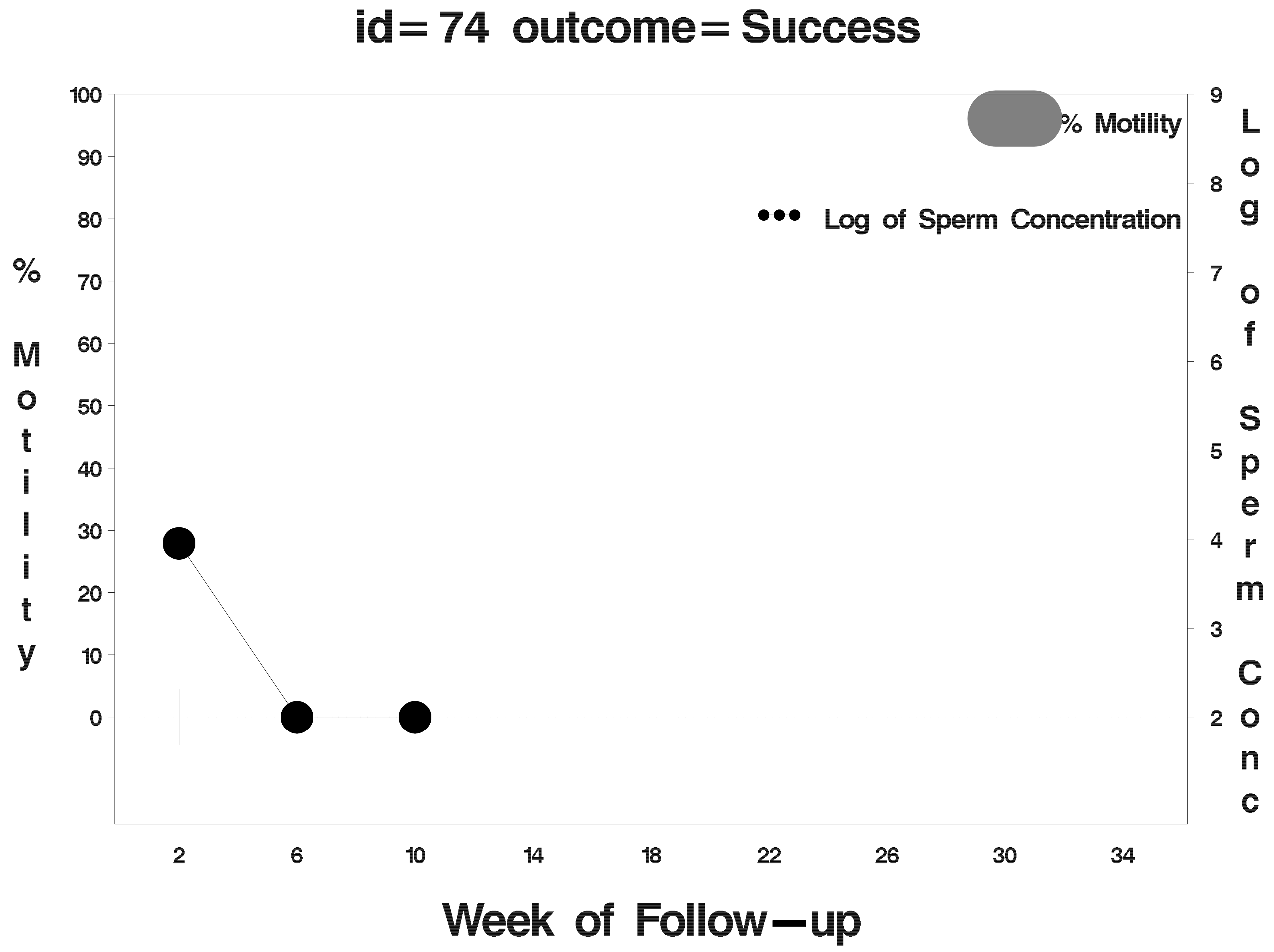
id= 74 outcome= Success
% Motility
Log of Sperm Concentration
100
90
80
70
60
50
40
30
20
10
0
9
8
7
6
5
4
3
2
Log of Sperm Conc
2
6
10
14
18
22
26
30
34
Week of Follow—up

id= 75 outcome= Success

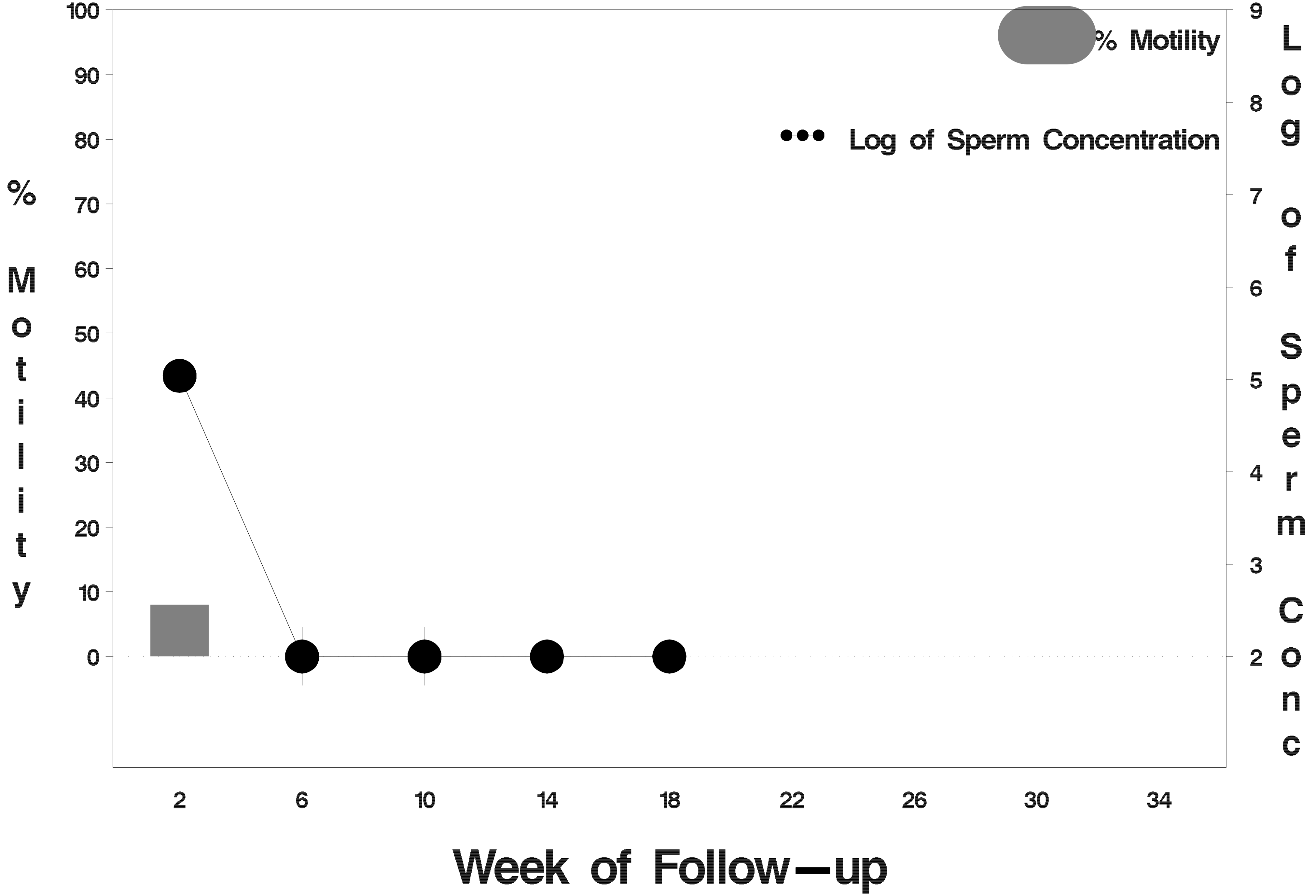

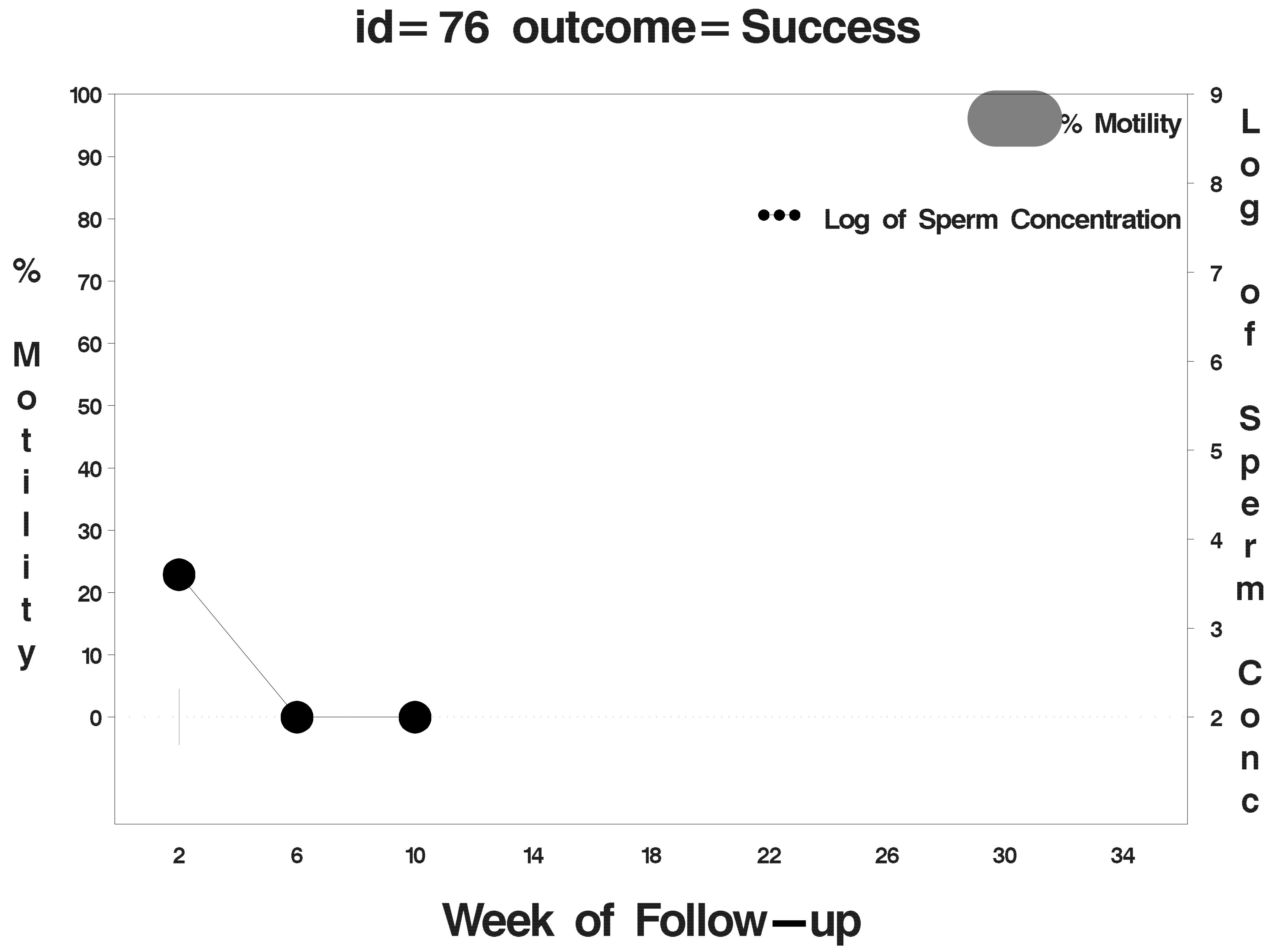

id= 76 outcome= Success
% Motility
Log of Sperm Concentration
% Motility
Log of Sperm Conc
Week of Follow−up
100
90
80
70
60
50
40
30
20
10
0
9
8
7
6
5
4
3
2
2
6
10
14
18
22
26
30
34

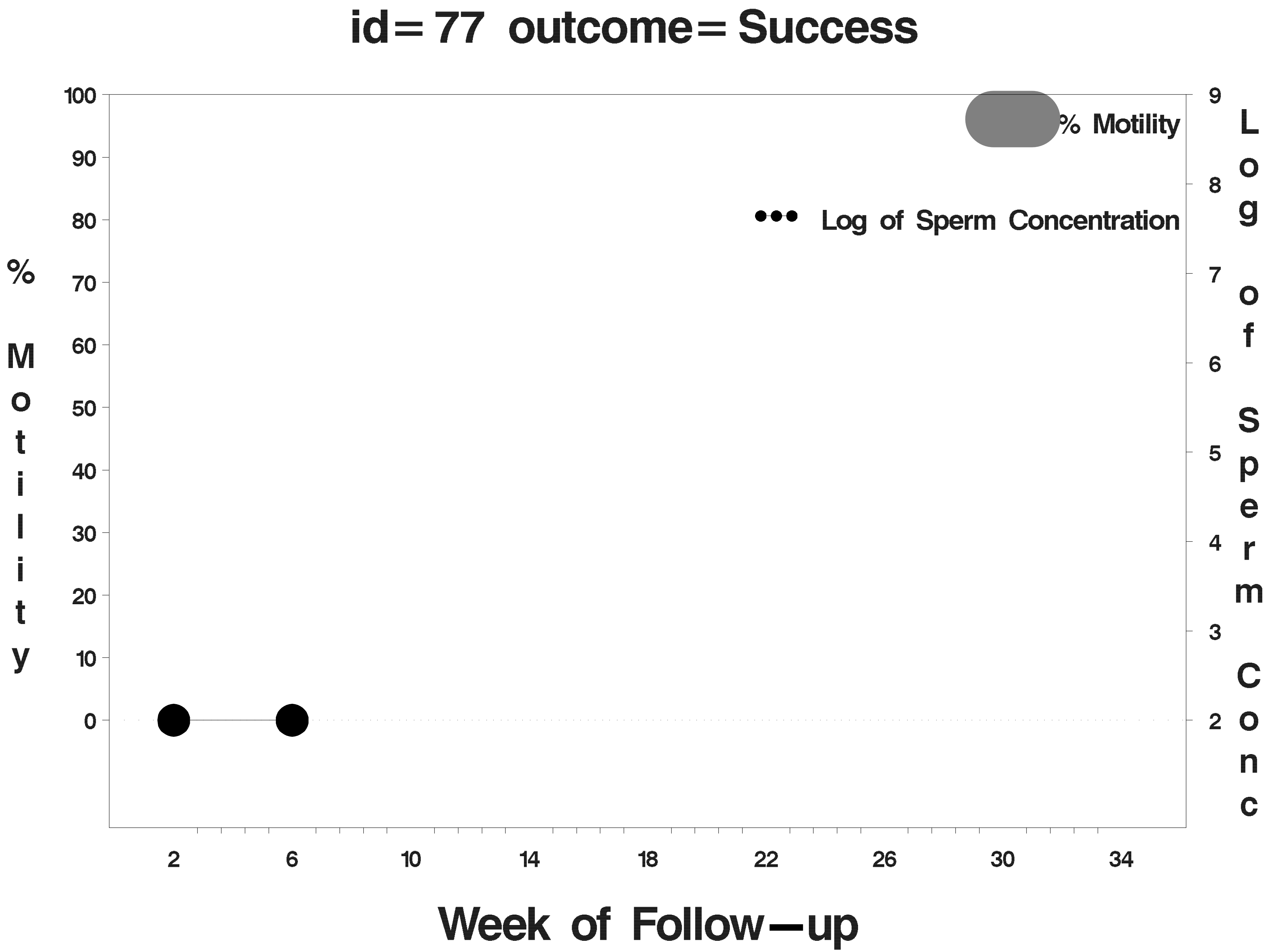

id= 77 outcome= Success
% Motility
Log of Sperm Concentration
100
90
80
70
60
50
40
30
20
10
0
9
8
7
6
5
4
3
2
2
6
10
14
18
22
26
30
34
Week of Follow-up
Log of Sperm Conc

id= 78 outcome= Success

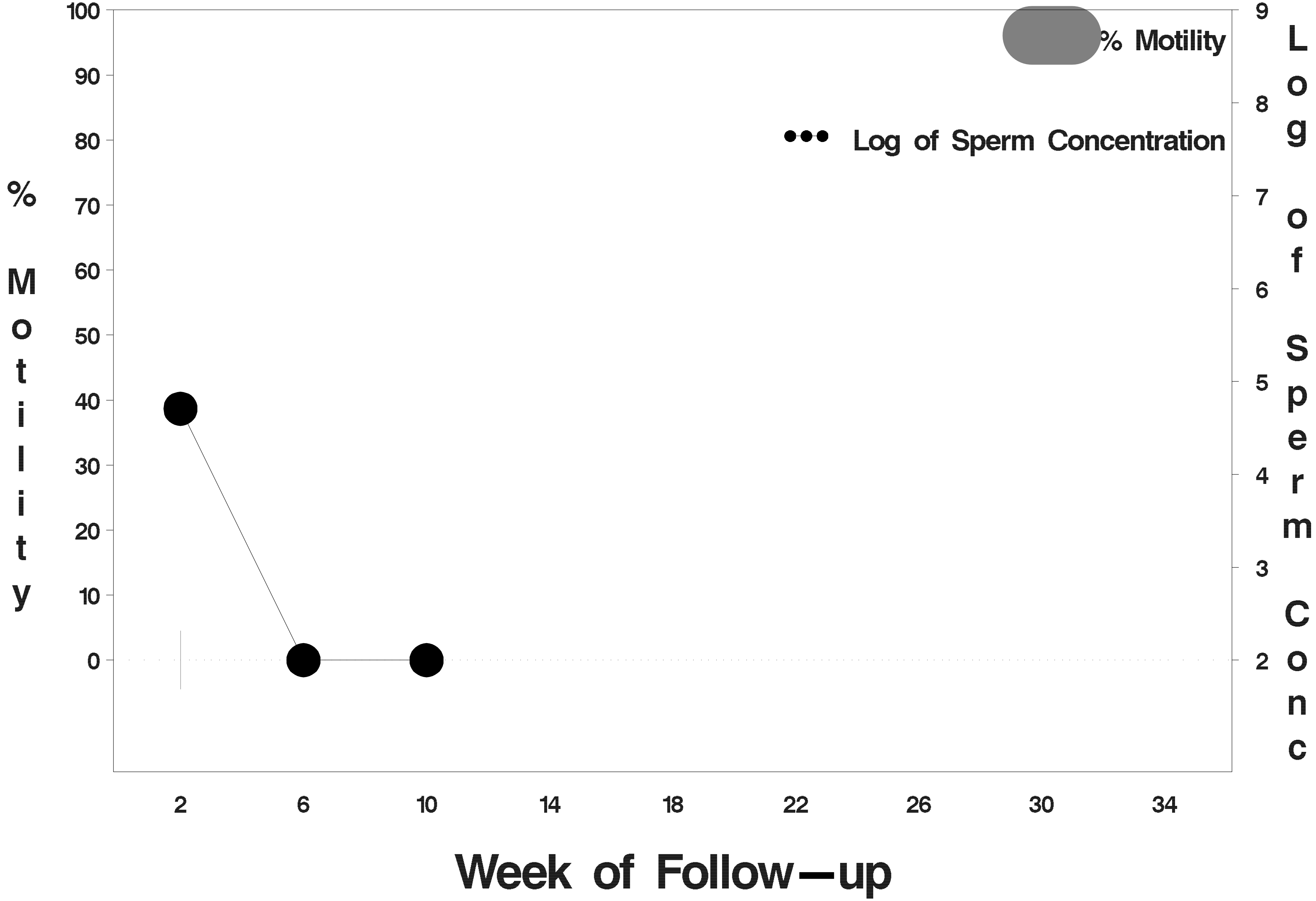

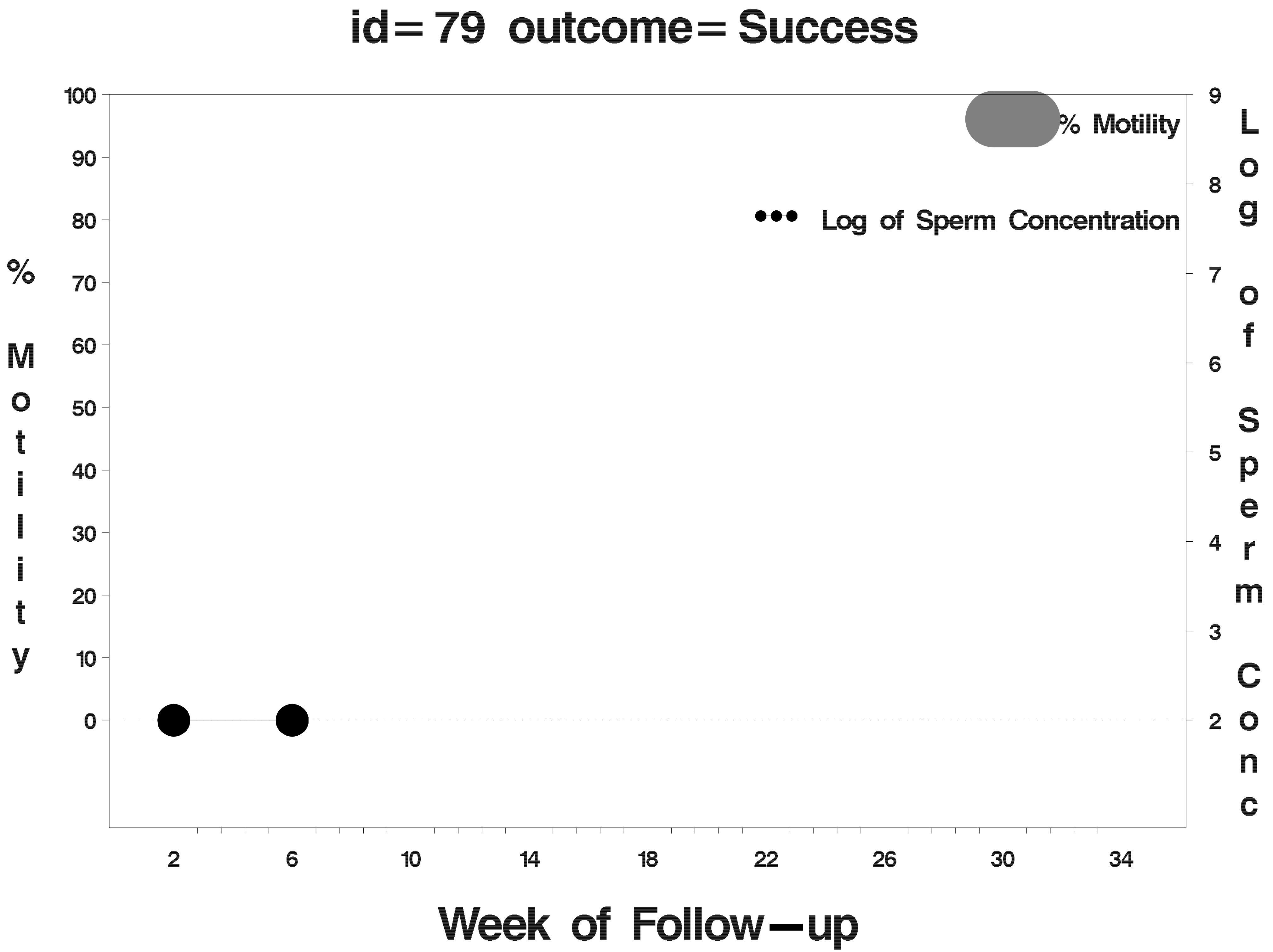

id= 79 outcome= Success
% Motility
Log of Sperm Concentration
% Motility
Log of Sperm Conc
Week of Follow-up
100
90
80
70
60
50
40
30
20
10
0
9
8
7
6
5
4
3
2
2
6
10
14
18
22
26
30
34

id= 80 outcome= Success

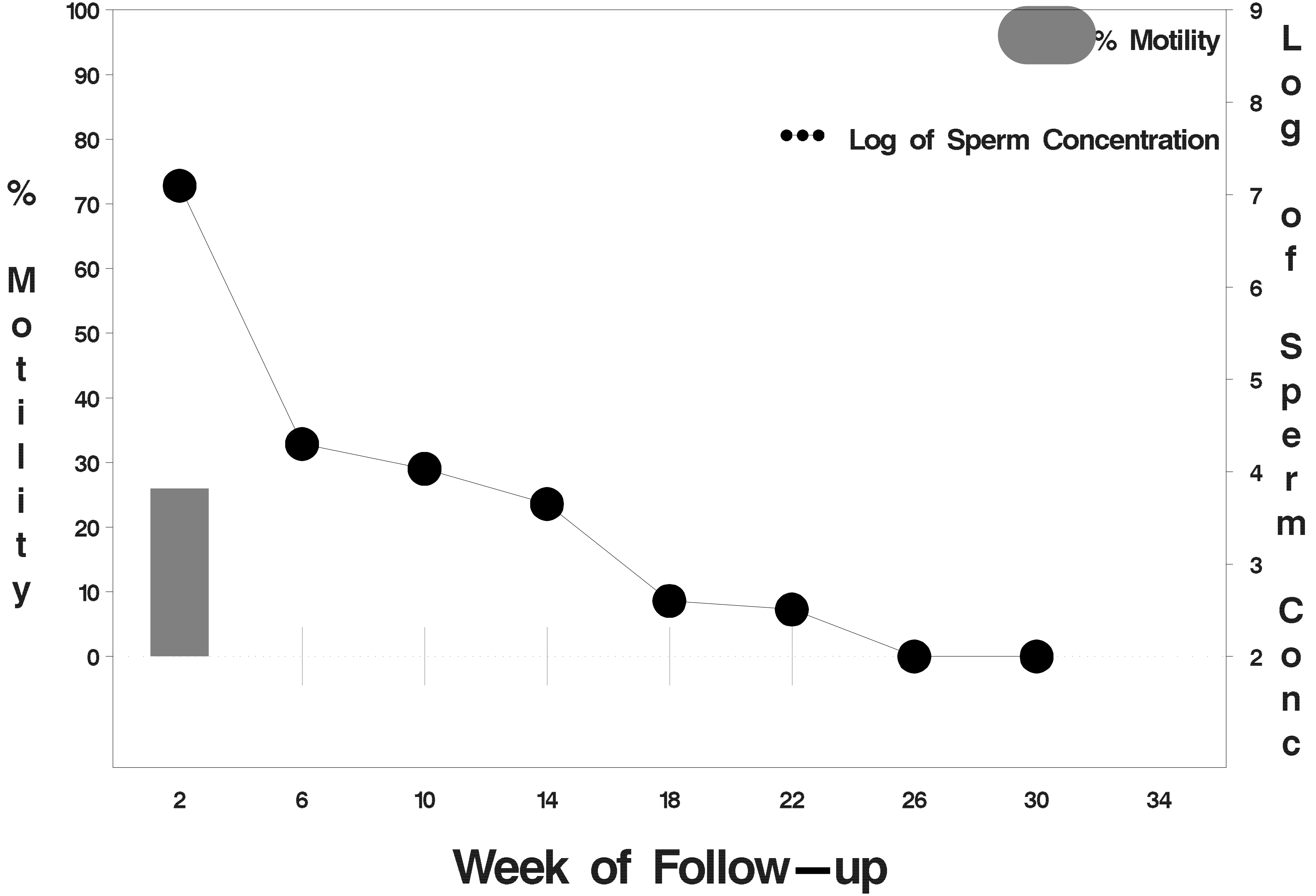

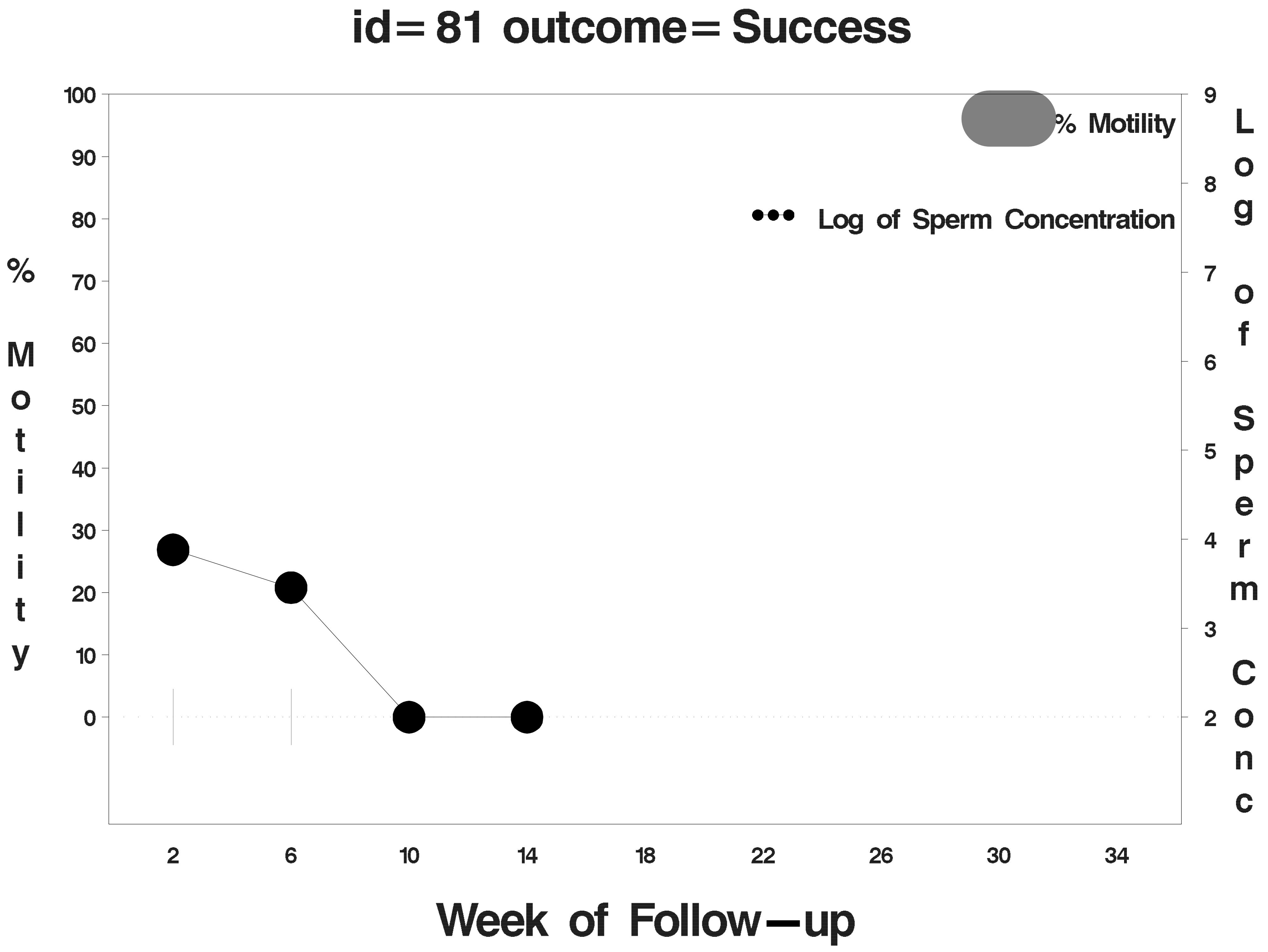

id= 81 outcome= Success
% Motility
Log of Sperm Concentration
100
90
80
70
60
50
40
30
20
10
0
9
8
7
6
5
4
3
2
2
6
10
14
18
22
26
30
34
Week of Follow—up
Log of Sperm Conc

id= 82 outcome= Success

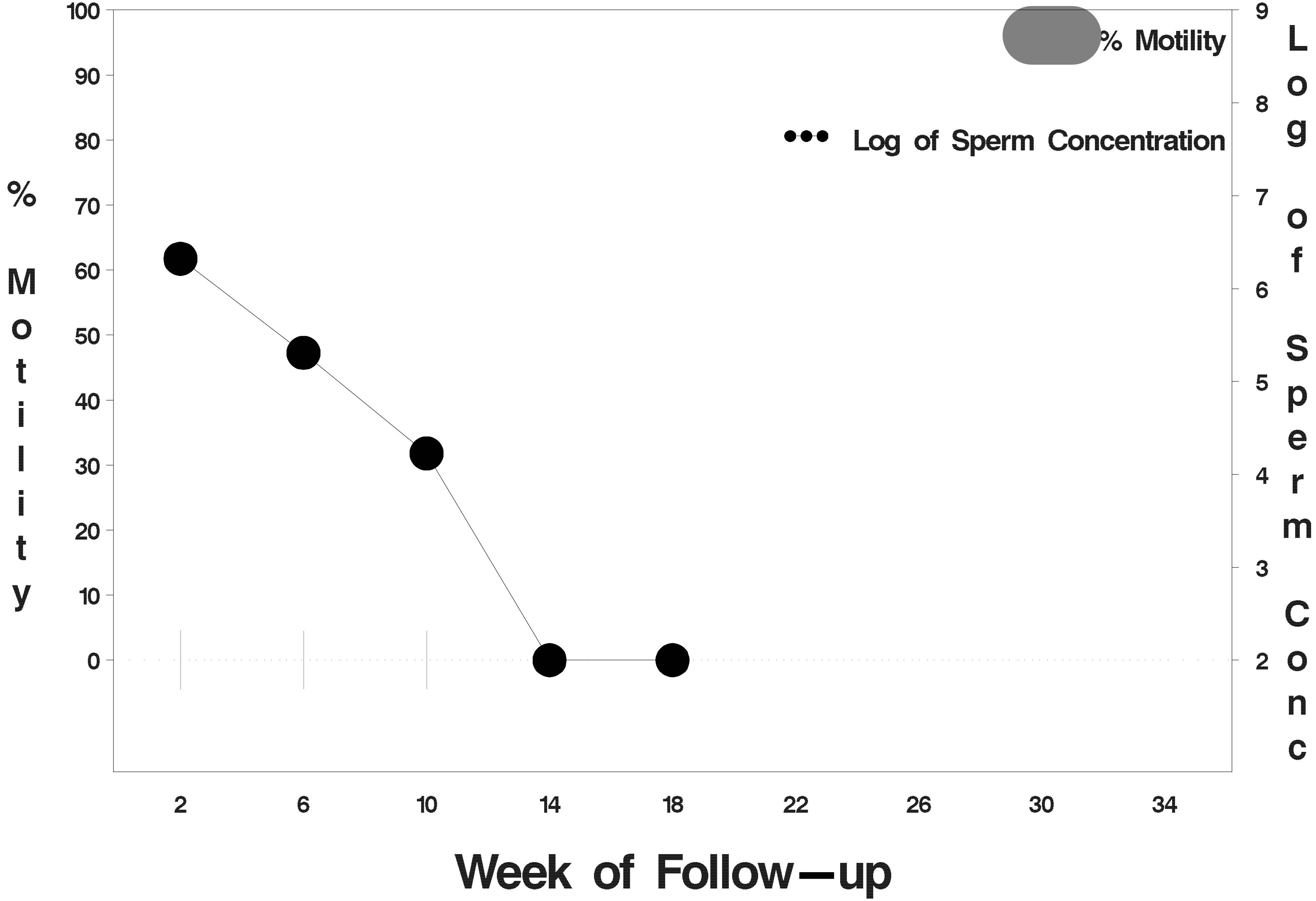

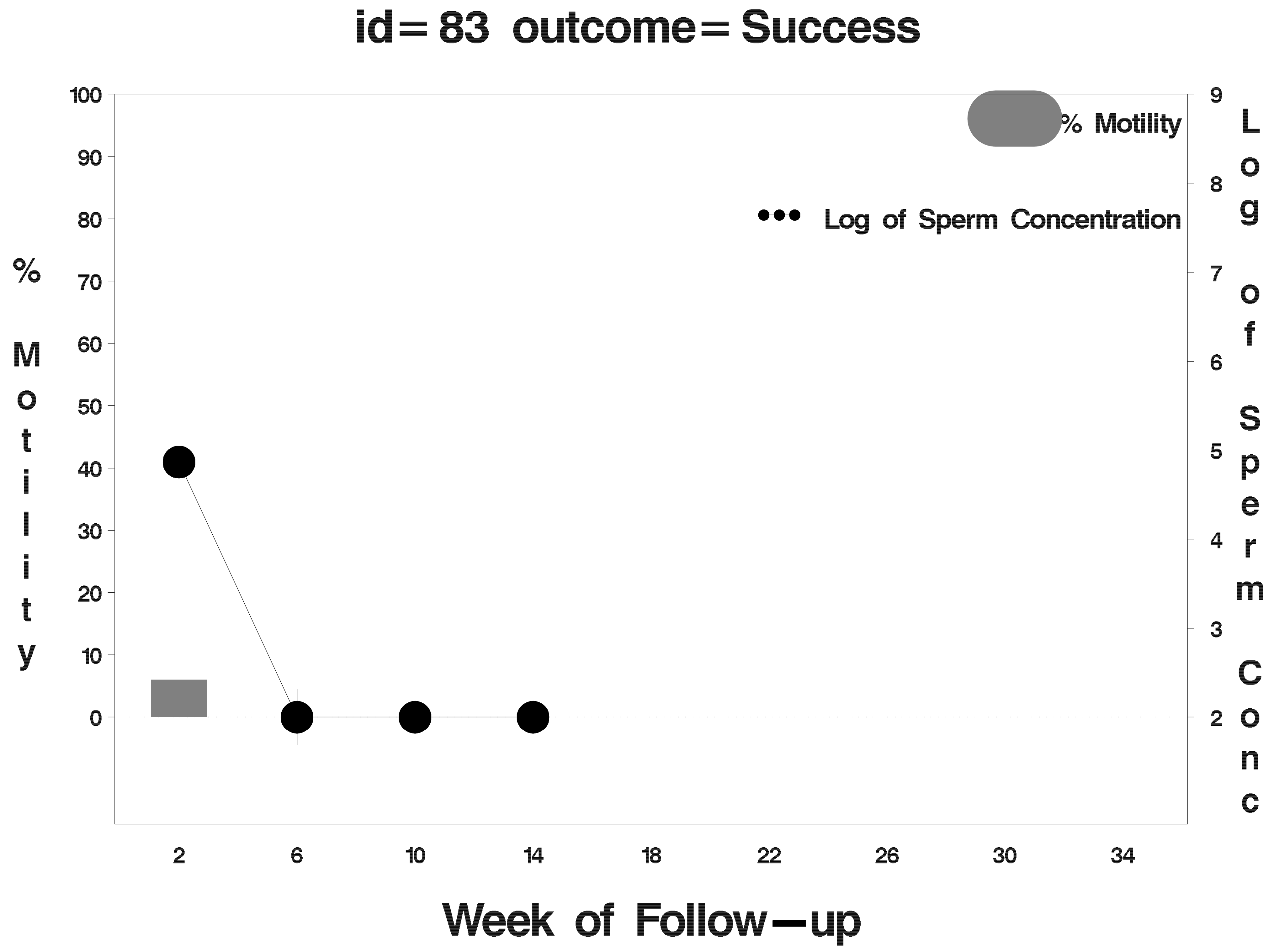

id= 83 outcome= Success
% Motility
Log of Sperm Concentration
% Motility
Log of Sperm Conc
Week of Follow−up
100
90
80
70
60
50
40
30
20
10
0
9
8
7
6
5
4
3
2
2
6
10
14
18
22
26
30
34

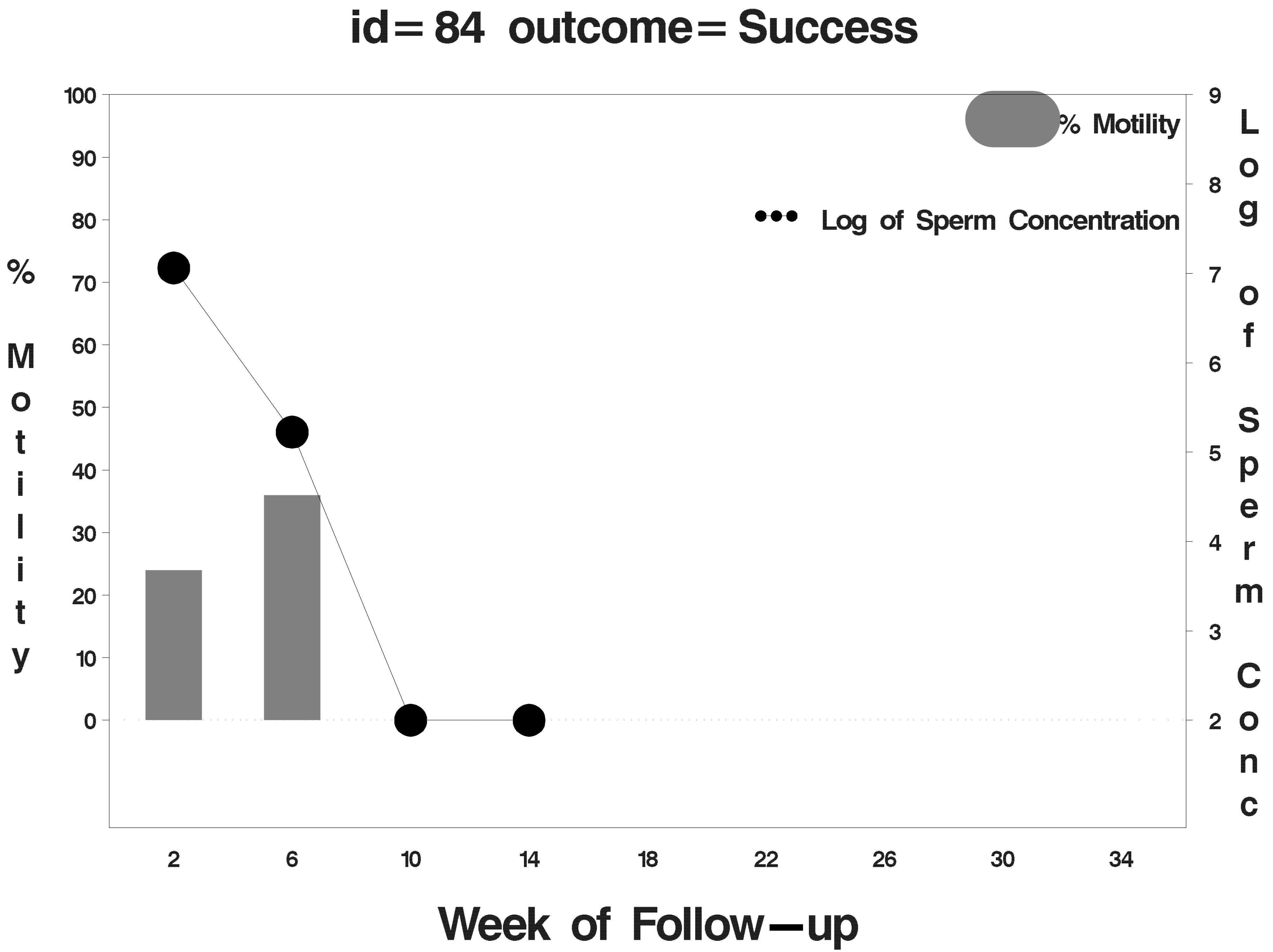

id= 84 outcome= Success
% Motility
Log of Sperm Concentration
% Motility
Log of Sperm Conc
Week of Follow-up
100
90
80
70
60
50
40
30
20
10
0
9
8
7
6
5
4
3
2
2
6
10
14
18
22
26
30
34

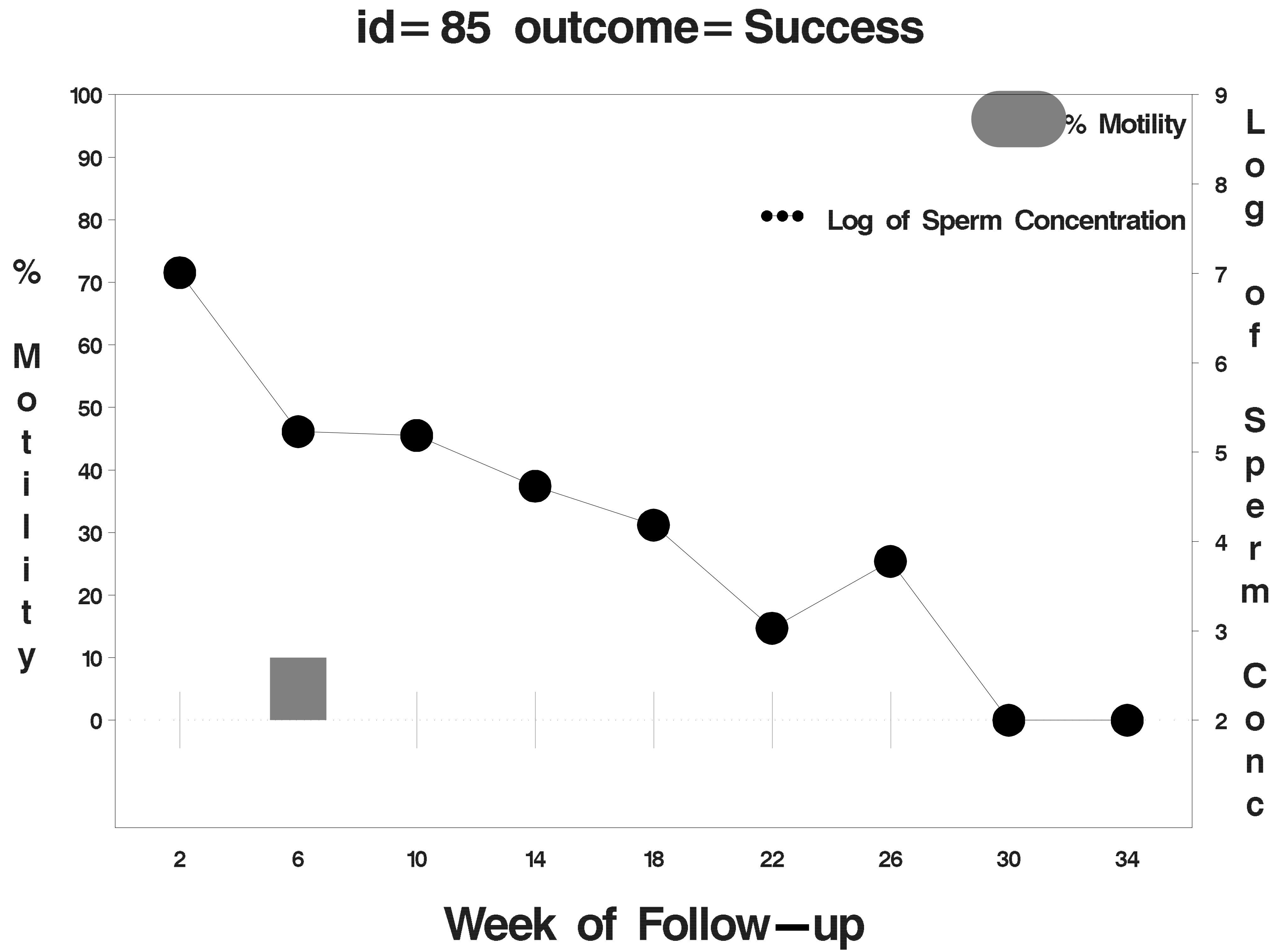

id= 85 outcome= Success
% Motility
Log of Sperm Concentration
% Motility
Log of Sperm Conc
Week of Follow—up
100
90
80
70
60
50
40
30
20
10
0
9
8
7
6
5
4
3
2
2
6
10
14
18
22
26
30
34

id= 86 outcome= Success

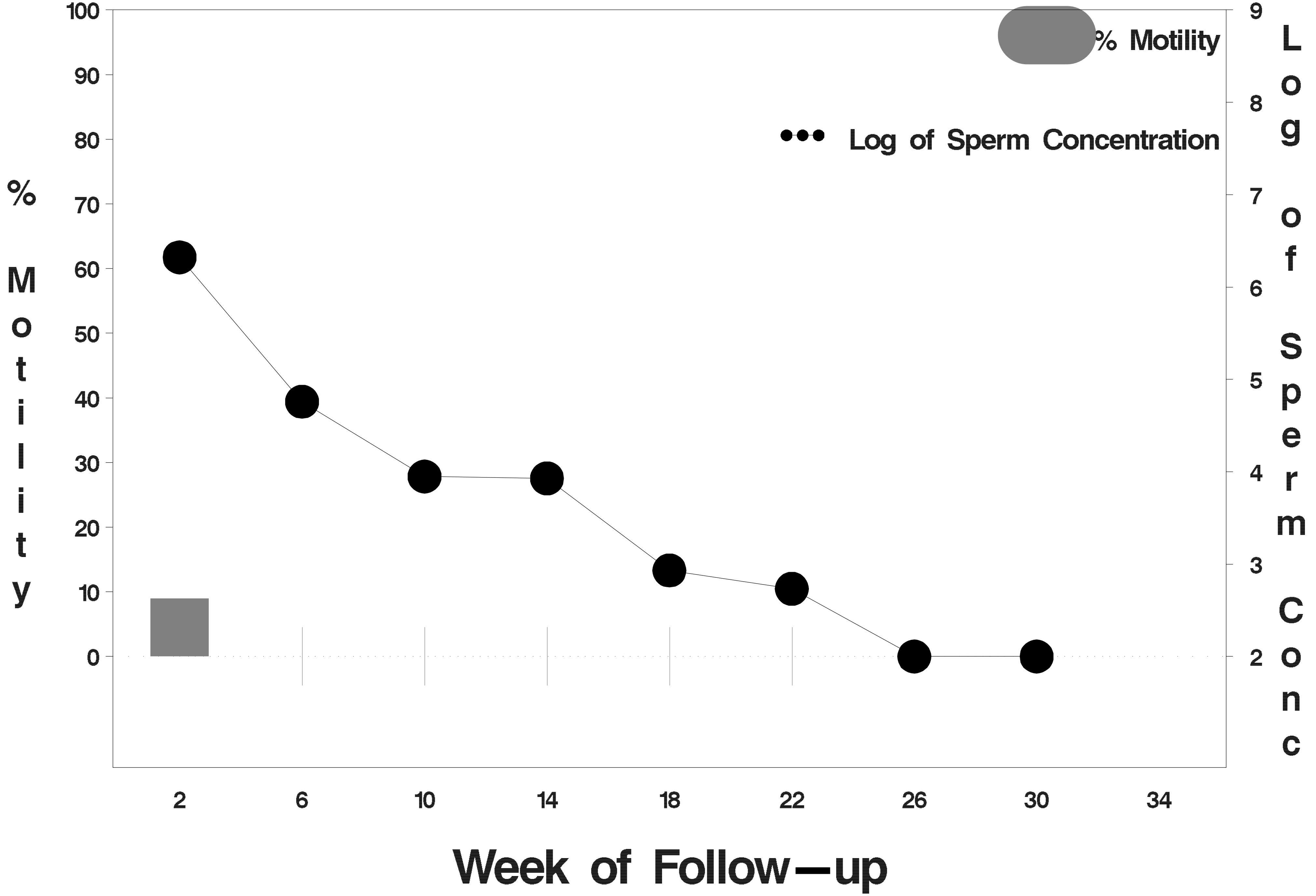

id= 87 outcome= Success

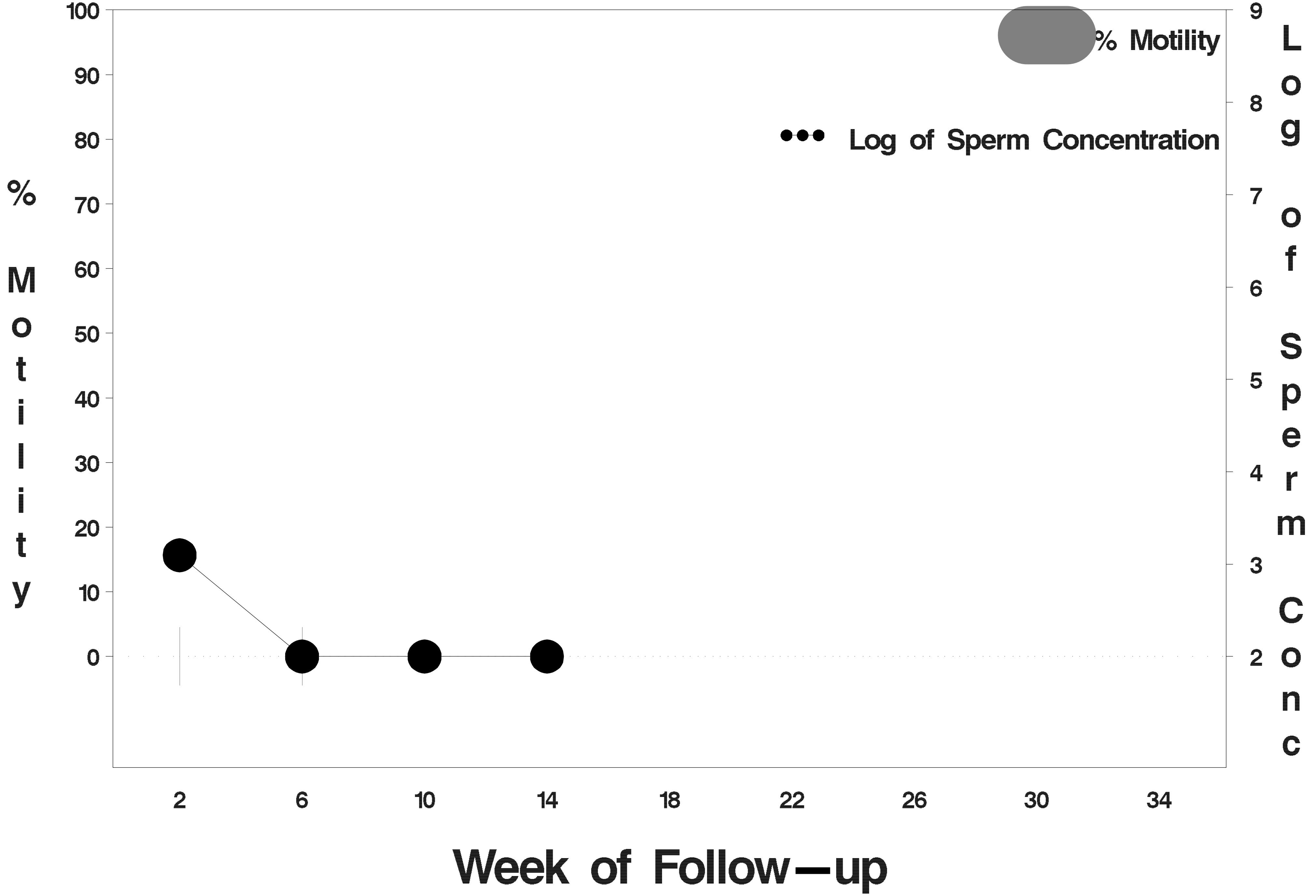

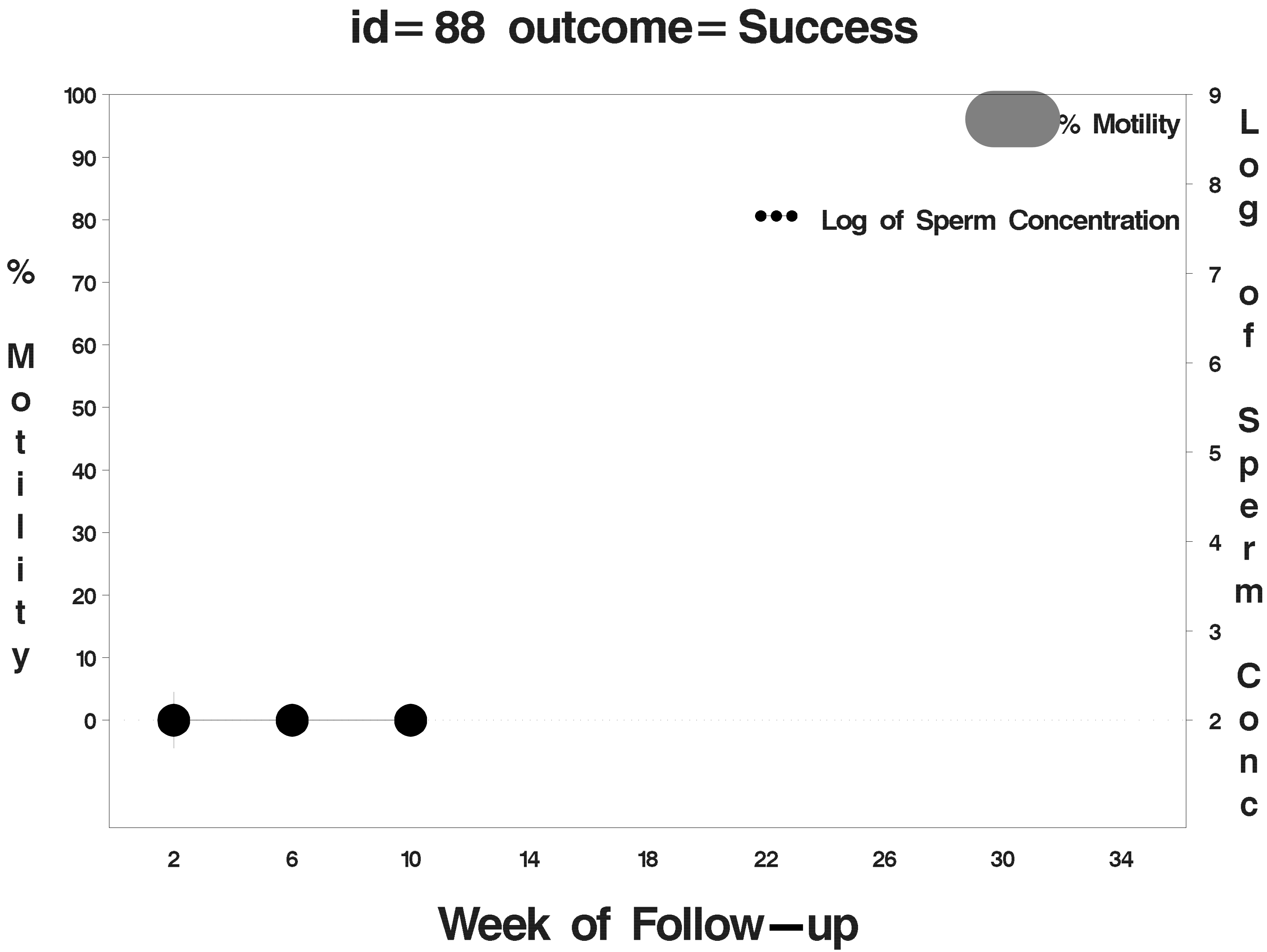
id= 88 outcome= Success
% Motility
Log of Sperm Concentration
100
90
80
70
60
50
40
30
20
10
0
9
8
7
6
5
4
3
2
2
6
10
14
18
22
26
30
34
Week of Follow—up
Log of Sperm Conc

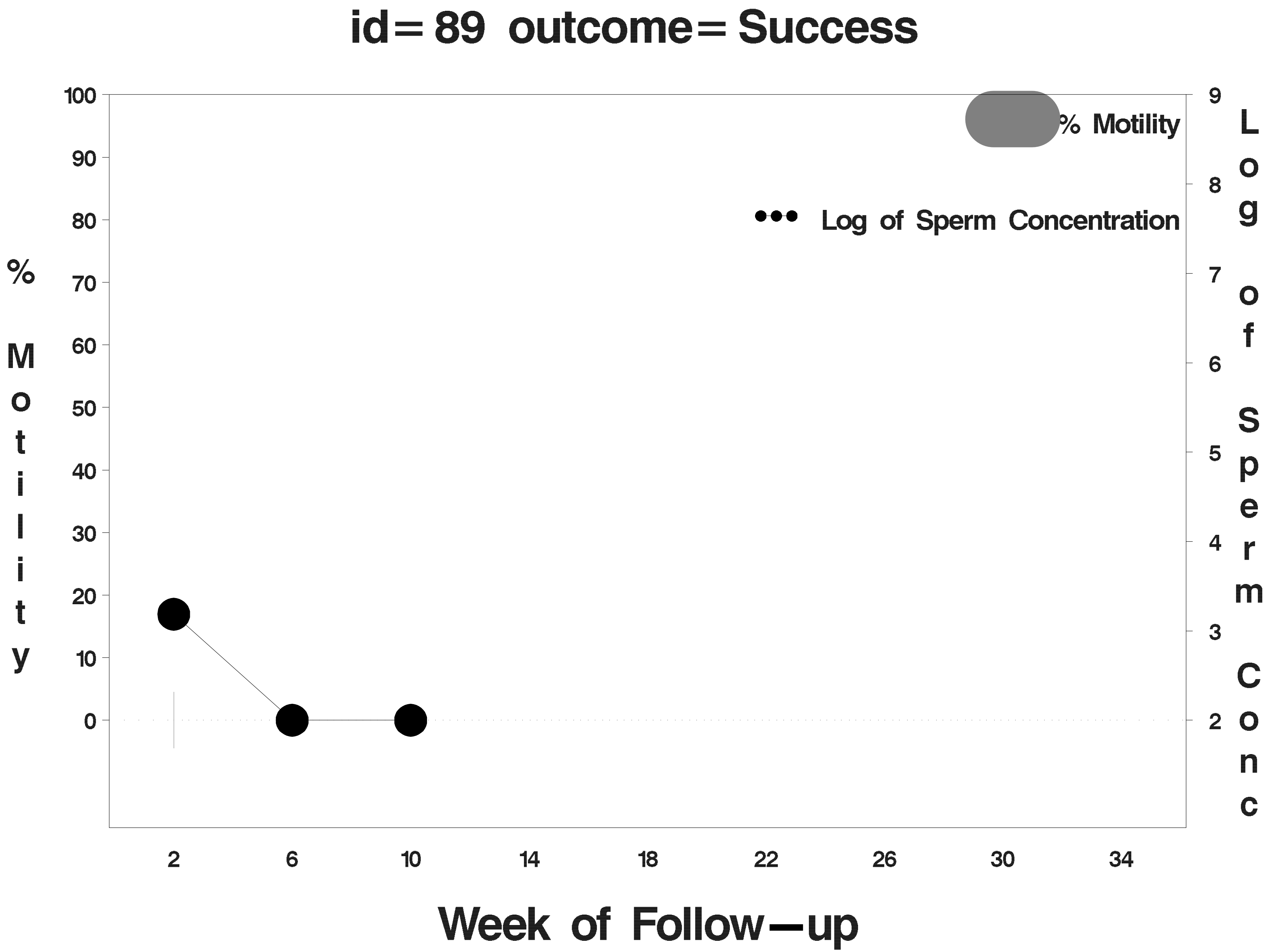

id= 89 outcome= Success
% Motility
Log of Sperm Concentration
100
90
80
70
60
50
40
30
20
10
0
9
8
7
6
5
4
3
2
2
6
10
14
18
22
26
30
34
Week of Follow—up
Log of Sperm Conc

id= 90 outcome= Success

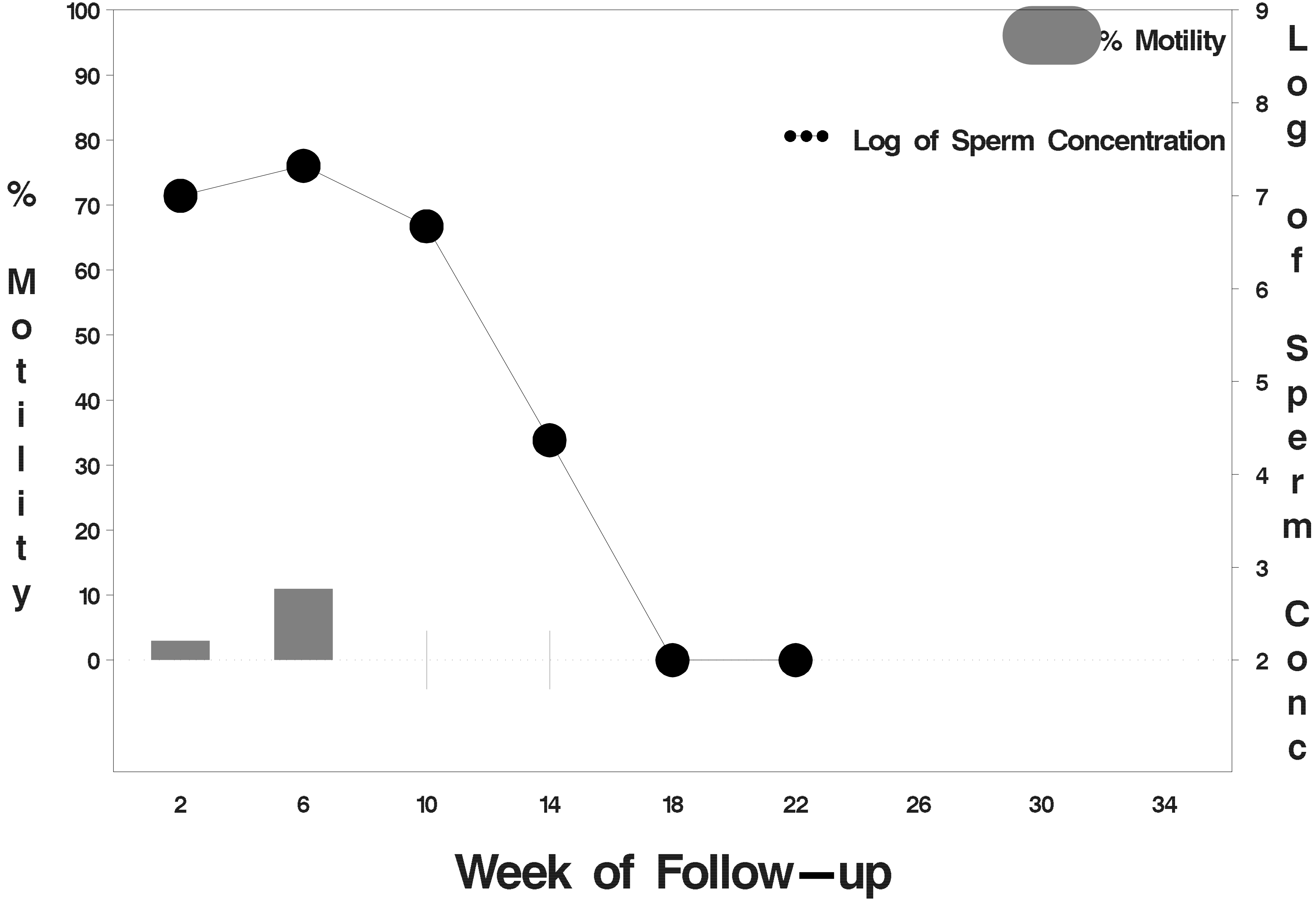

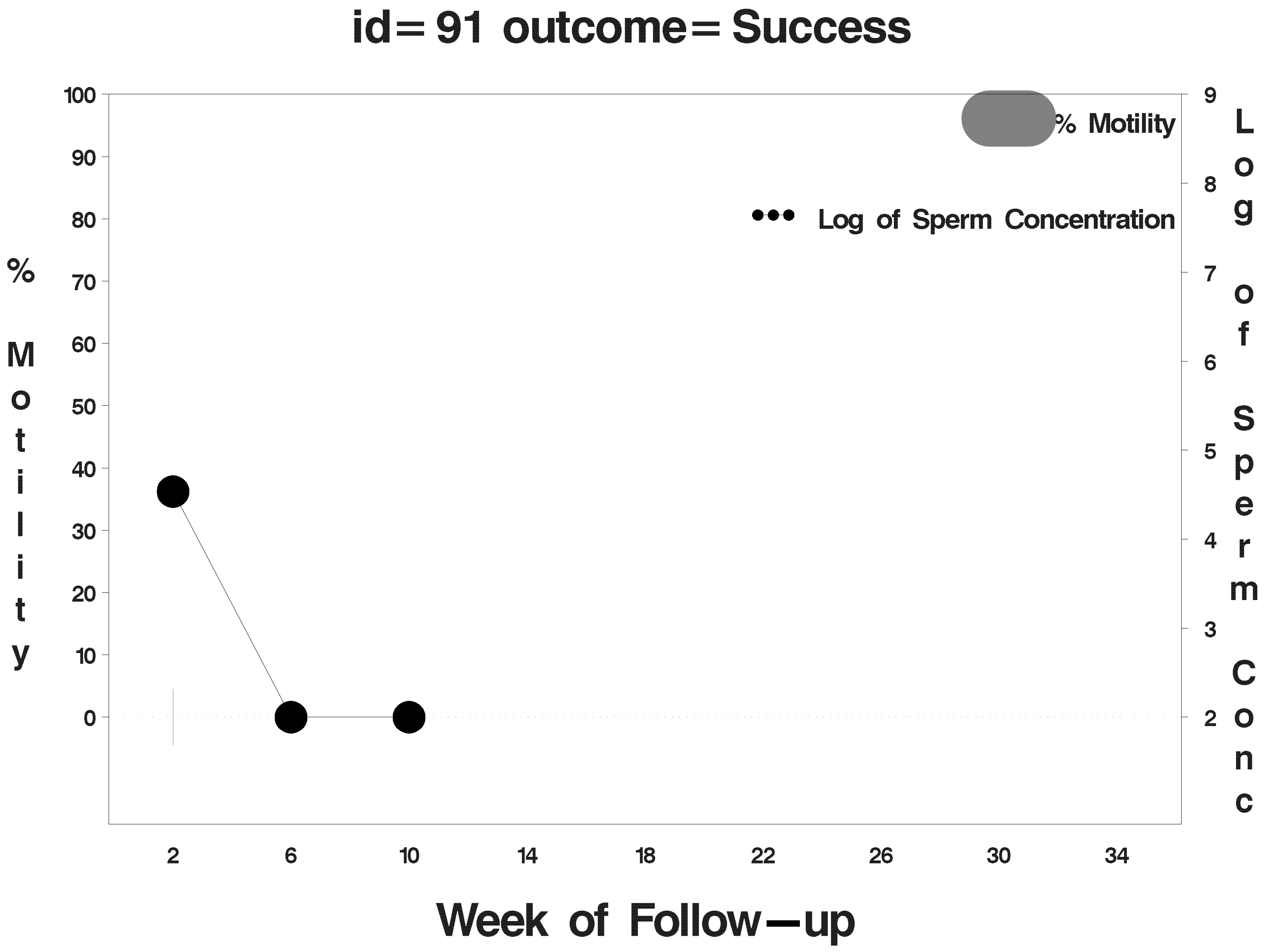

id= 91 outcome= Success
% Motility
Log of Sperm Concentration
100
90
80
70
60
50
40
30
20
10
0
9
8
7
6
5
4
3
2
2
6
10
14
18
22
26
30
34
Week of Follow—up
% Motility
Log of Sperm Conc

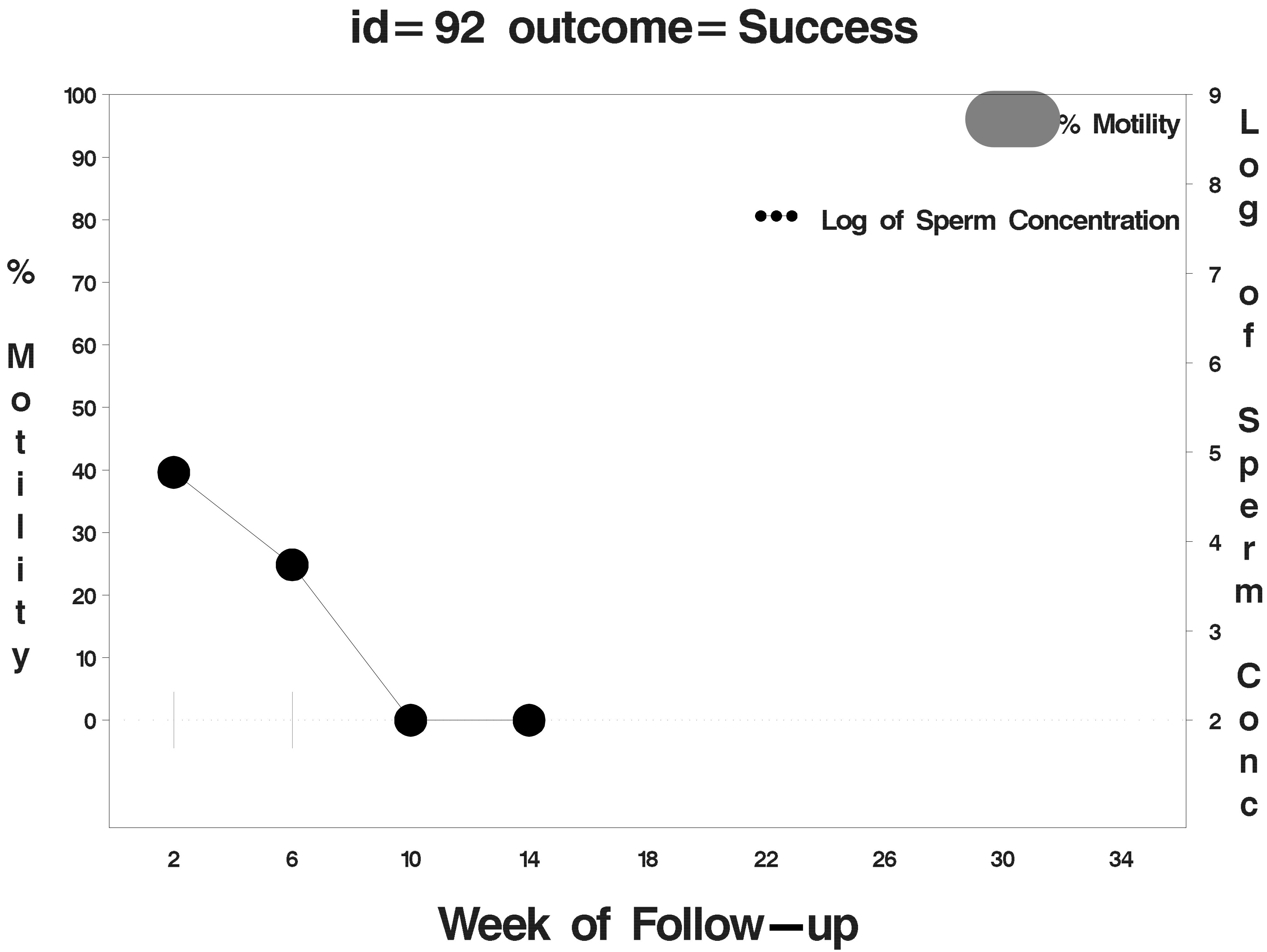
id= 92 outcome= Success
% Motility
Log of Sperm Concentration
100
90
80
70
60
50
40
30
20
10
0
9
8
7
6
5
4
3
2
2
6
10
14
18
22
26
30
34
% Motility
Log of Sperm Conc
Week of Follow-up

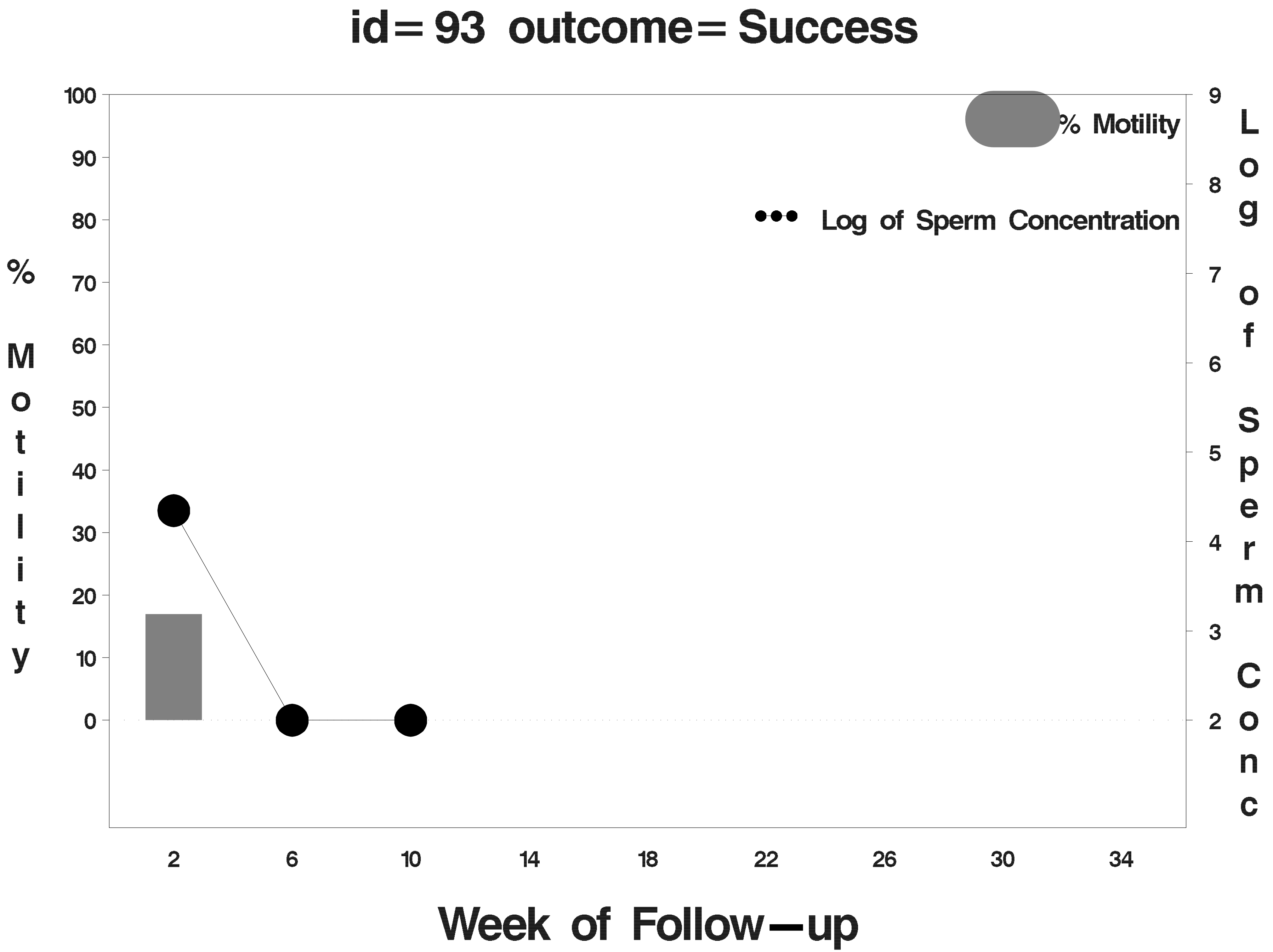
id= 93 outcome= Success
% Motility
Log of Sperm Concentration
100
90
80
70
60
50
40
30
20
10
0
9
8
7
6
5
4
3
2
2
6
10
14
18
22
26
30
34
Week of Follow—up
Log of Sperm Conc

id= 94 outcome= Success

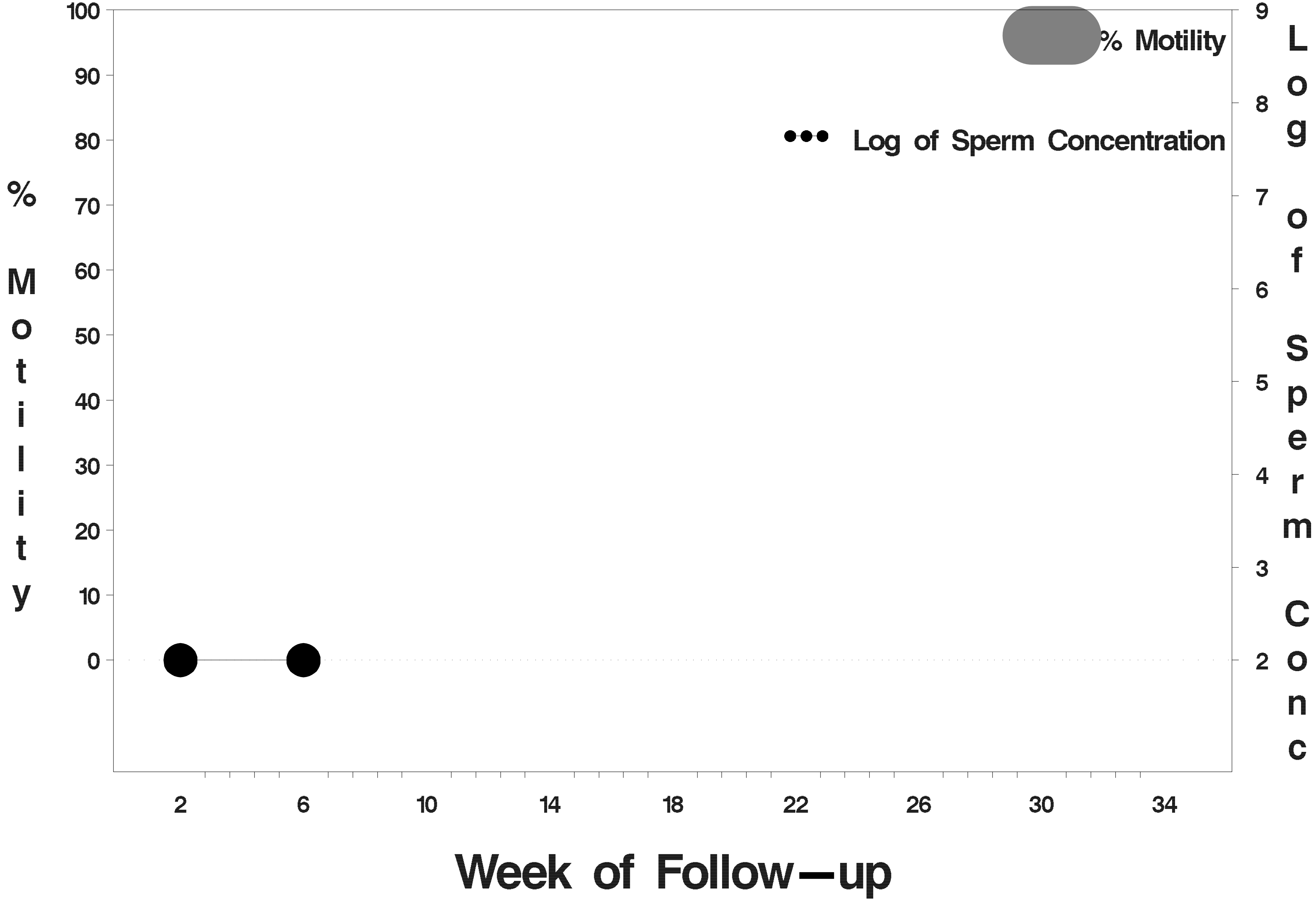

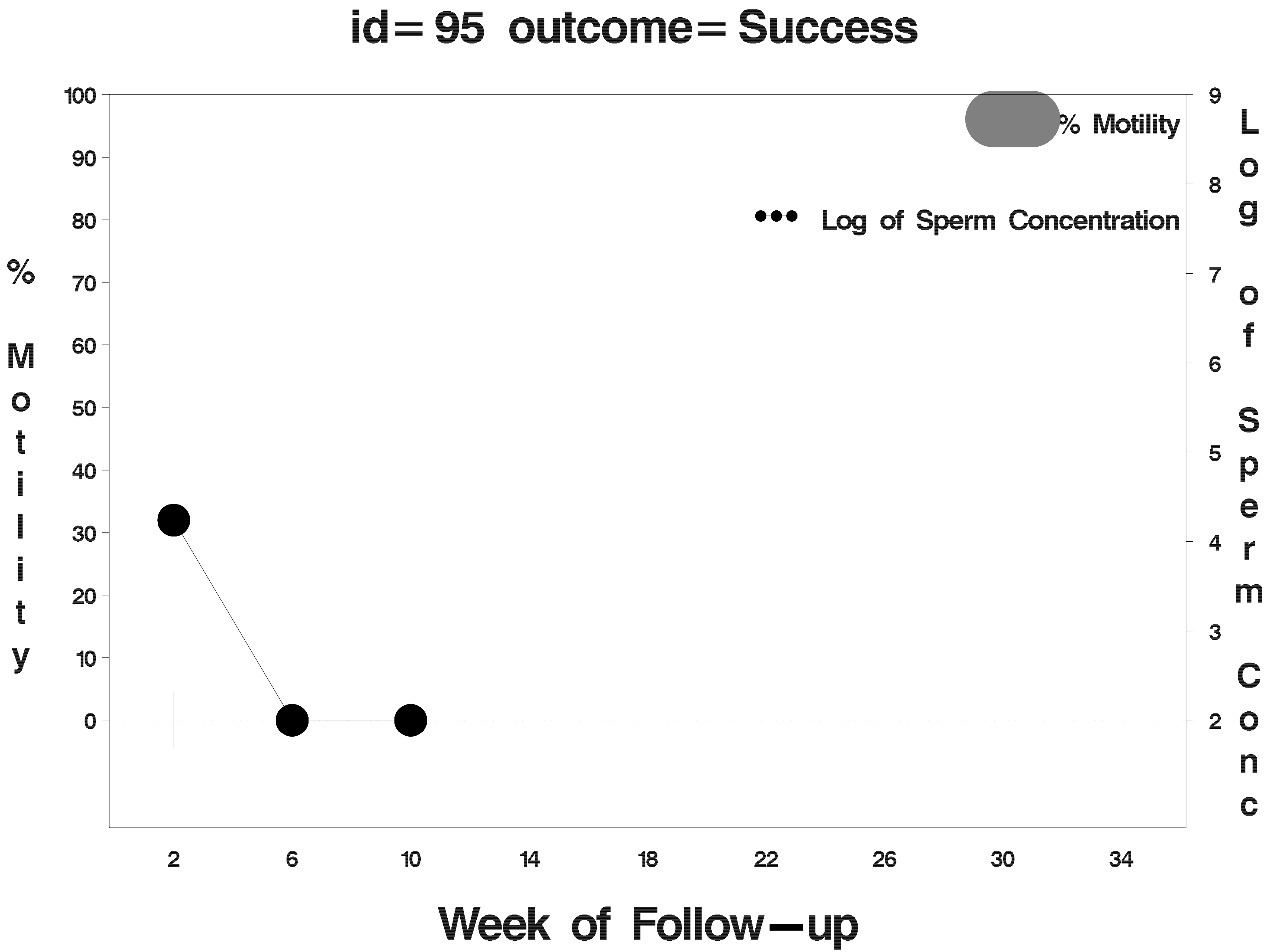

id= 95 outcome= Success
% Motility
Log of Sperm Concentration
100
90
80
70
60
50
40
30
20
10
0
9
8
7
6
5
4
3
2
L o g o f S p e r m C o n c
2
6
10
14
18
22
26
30
34
Week of Follow—up

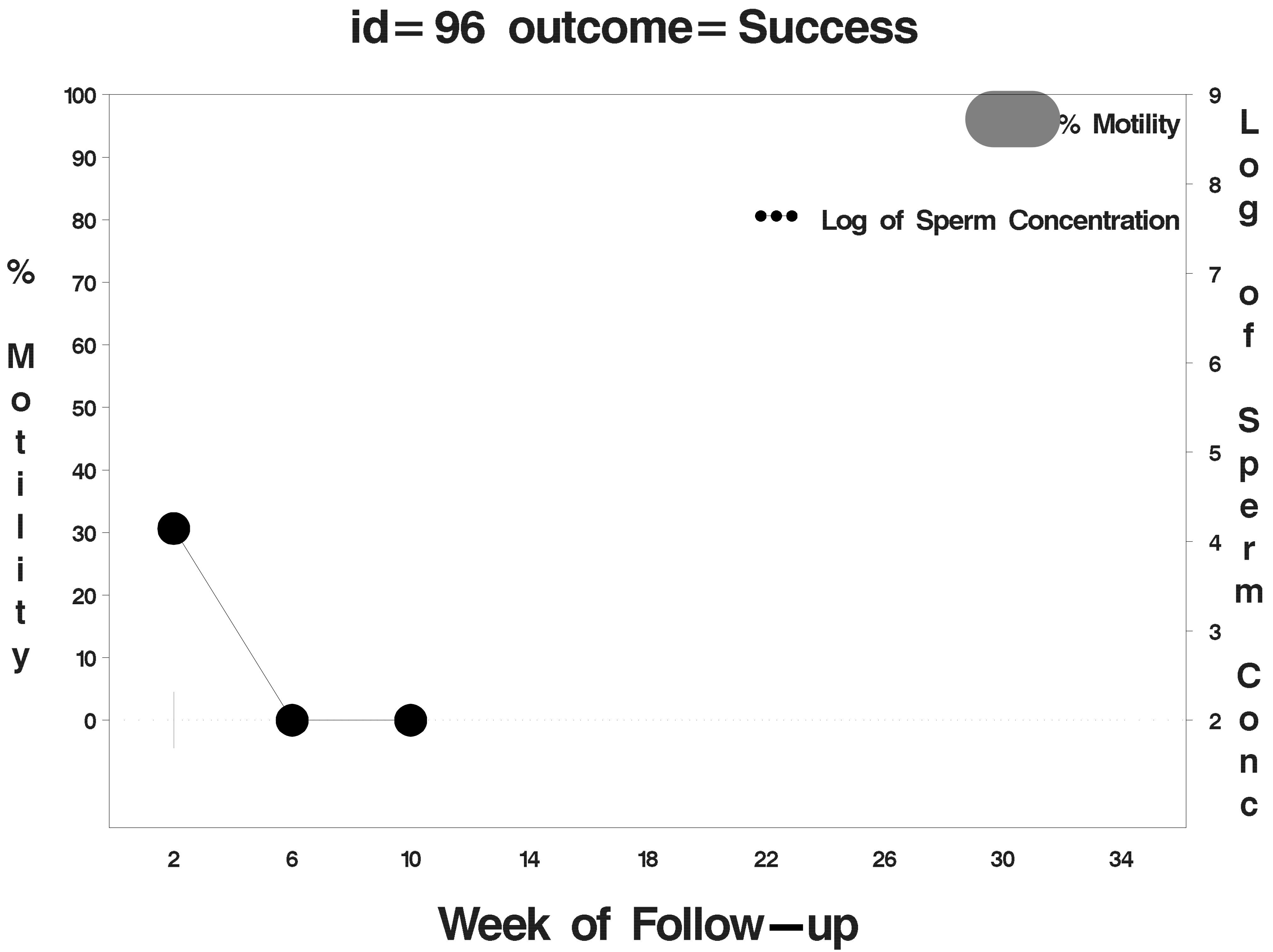
id= 96 outcome= Success
% Motility
Log of Sperm Concentration
100
90
80
70
60
50
40
30
20
10
0
9
8
7
6
5
4
3
2
2
6
10
14
18
22
26
30
34
Week of Follow—up
% Motility
Log of Sperm Conc

id= 97 outcome= Success

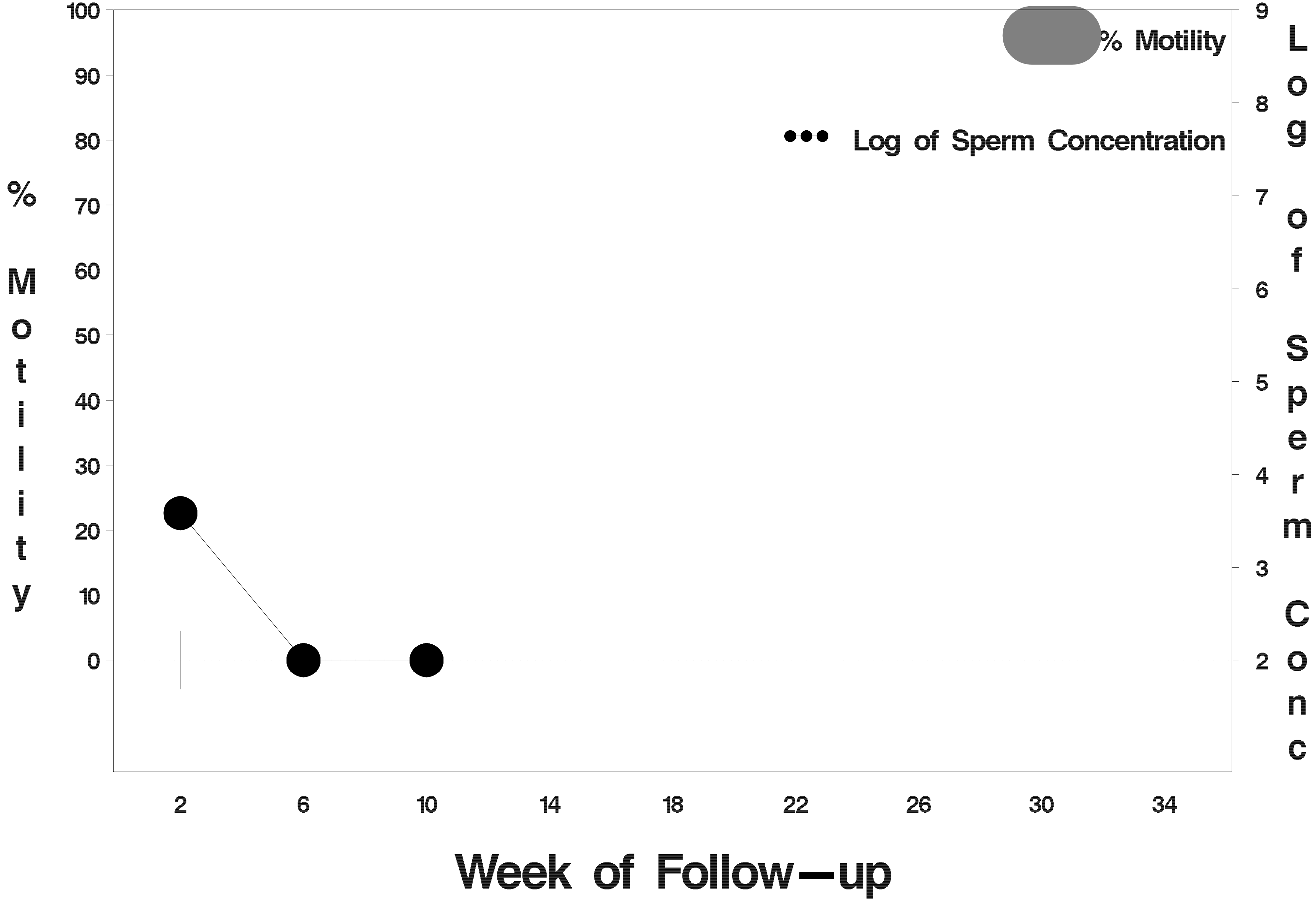

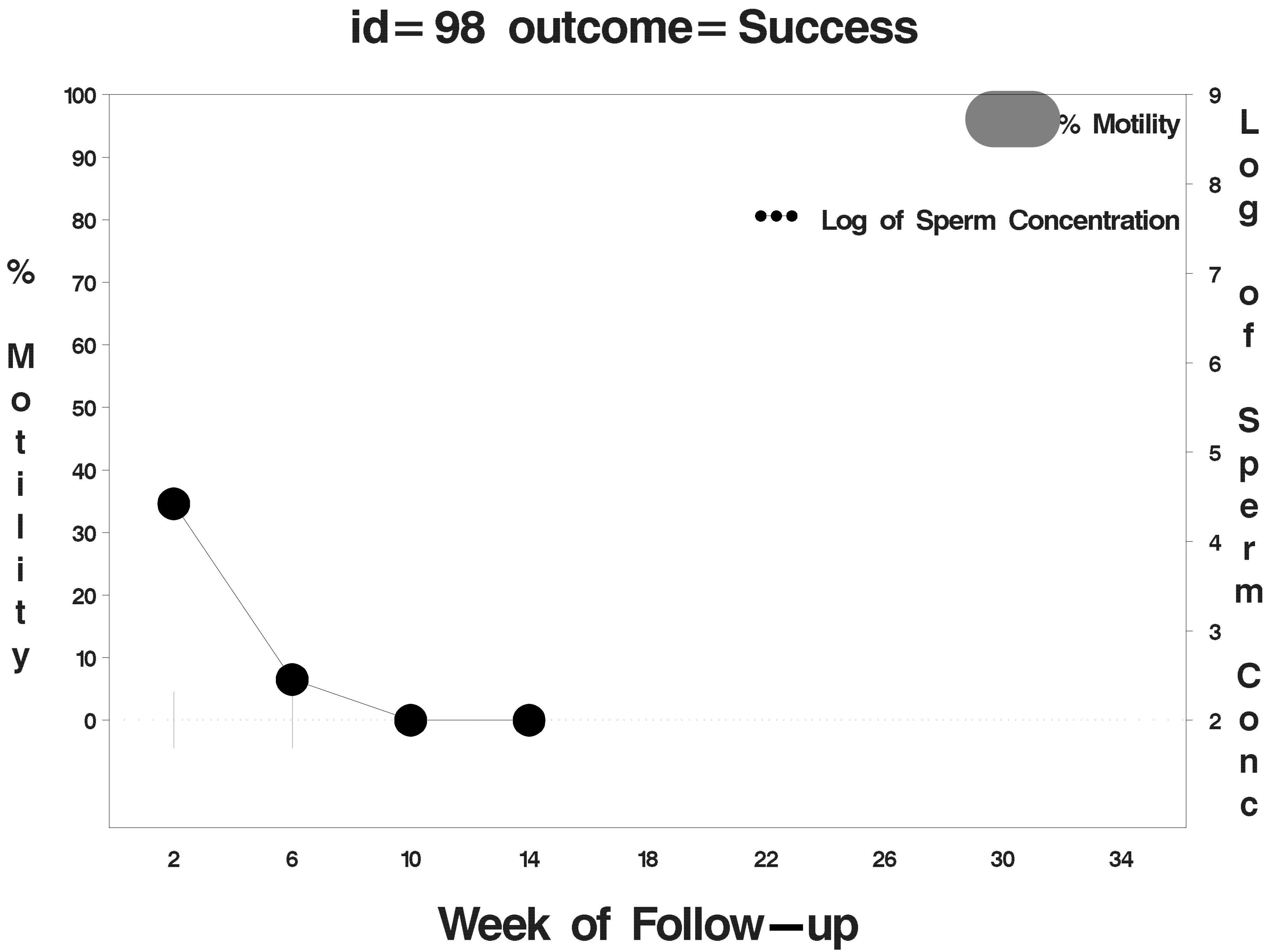

id= 98 outcome= Success
% Motility
Log of Sperm Concentration
% Motility
Log of Sperm Conc
Week of Follow—up
100
90
80
70
60
50
40
30
20
10
0
9
8
7
6
5
4
3
2
2
6
10
14
18
22
26
30
34

id= 99 outcome= Success

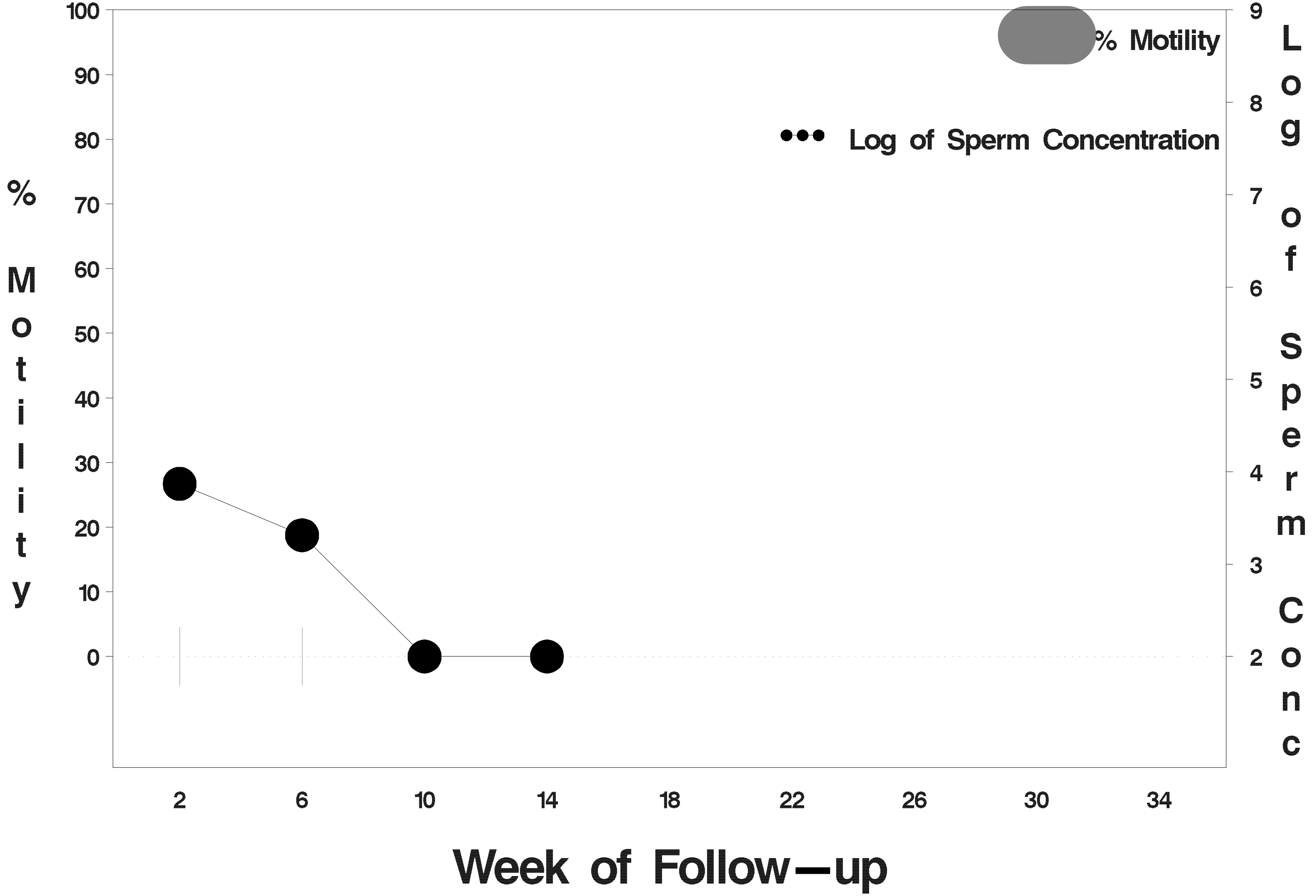

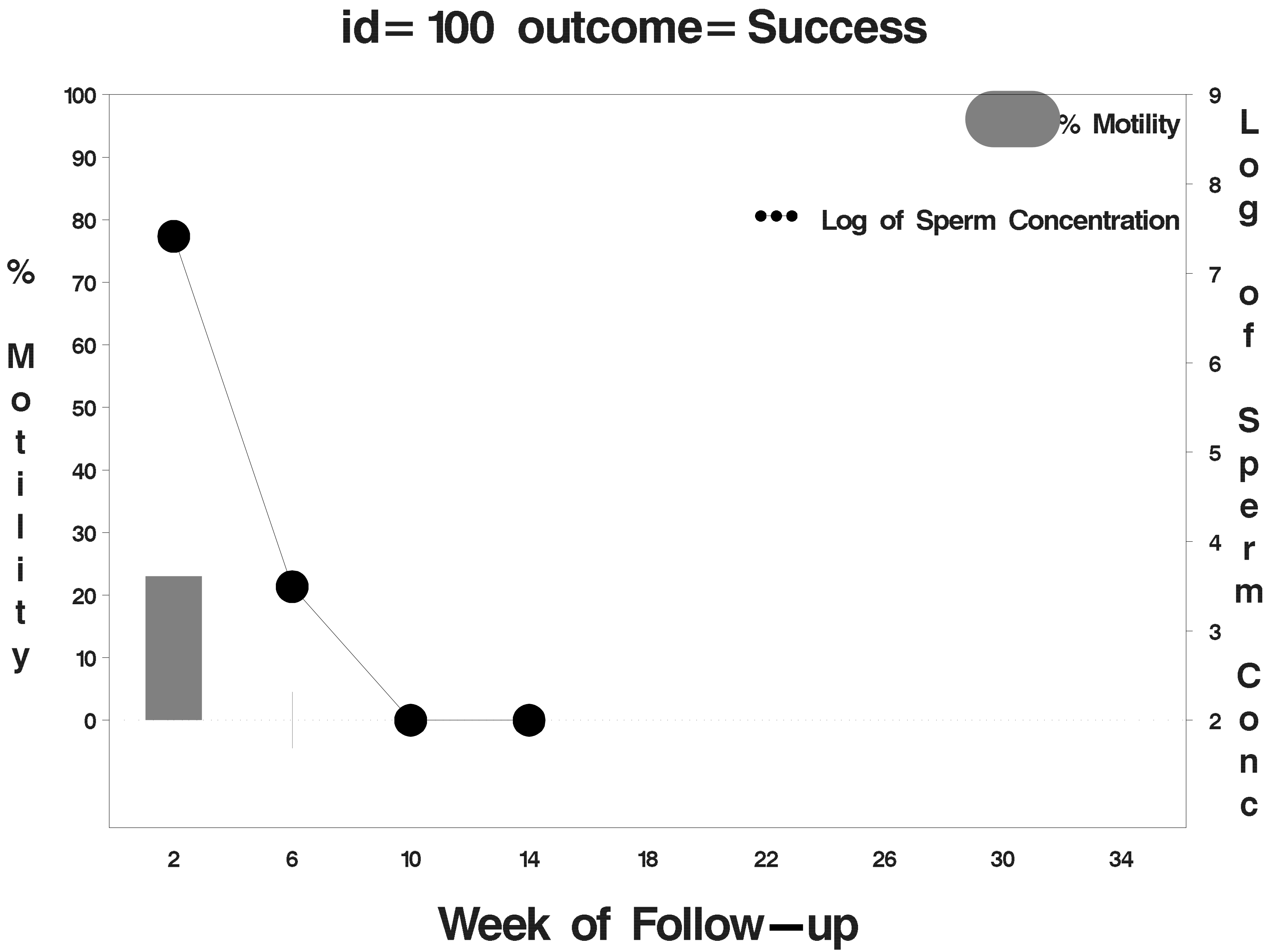

id= 100 outcome= Success
% Motility
Log of Sperm Concentration
% Motility
Log of Sperm Conc
Week of Follow−up
100
90
80
70
60
50
40
30
20
10
0
9
8
7
6
5
4
3
2
2
6
10
14
18
22
26
30
34

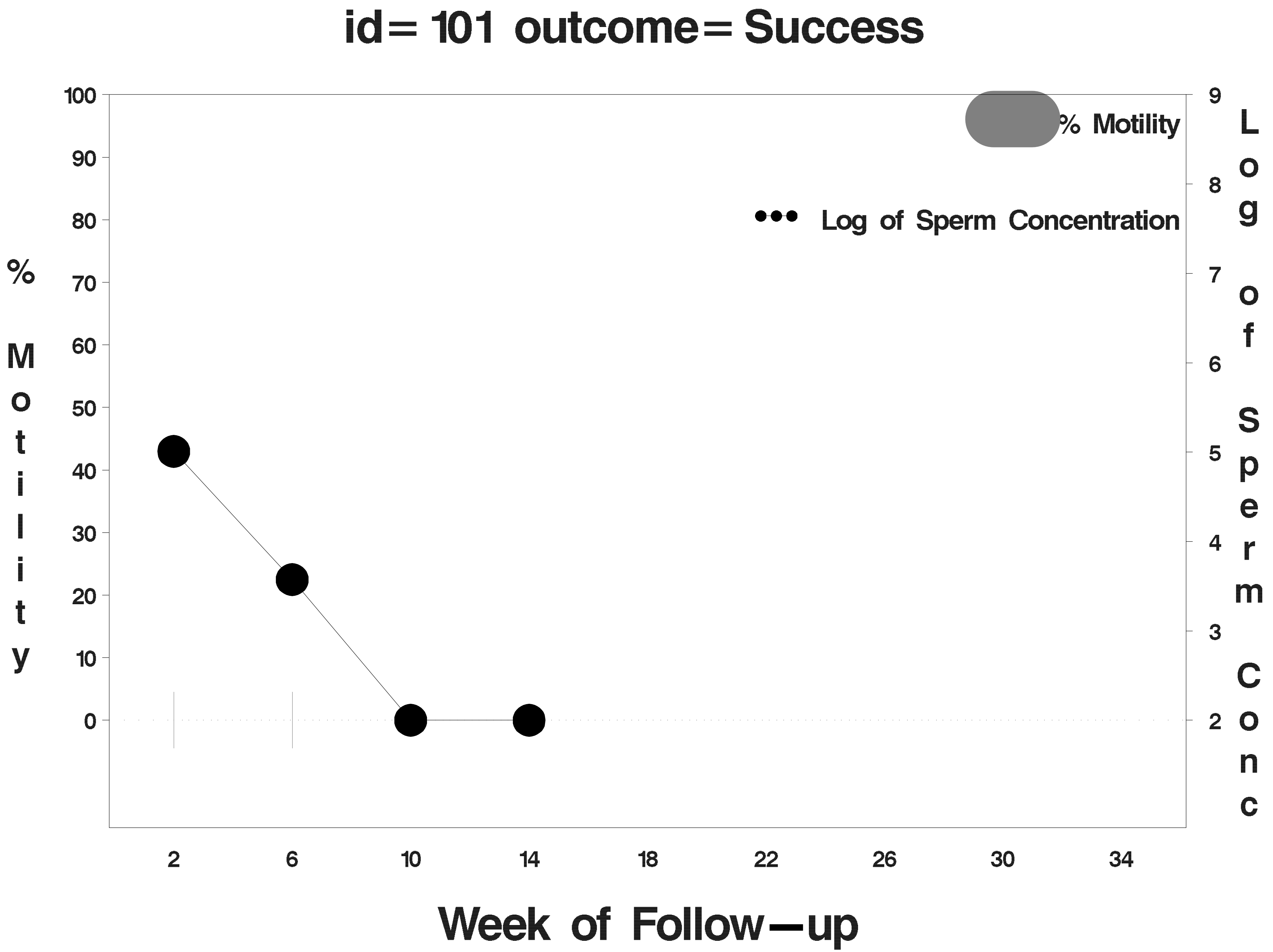
id= 101 outcome= Success
% Motility
Log of Sperm Concentration
100
90
80
70
60
50
40
30
20
10
0
9
8
7
6
5
4
3
2
2
6
10
14
18
22
26
30
34
Week of Follow-up
Log of Sperm Conc

id= 102 outcome= Success

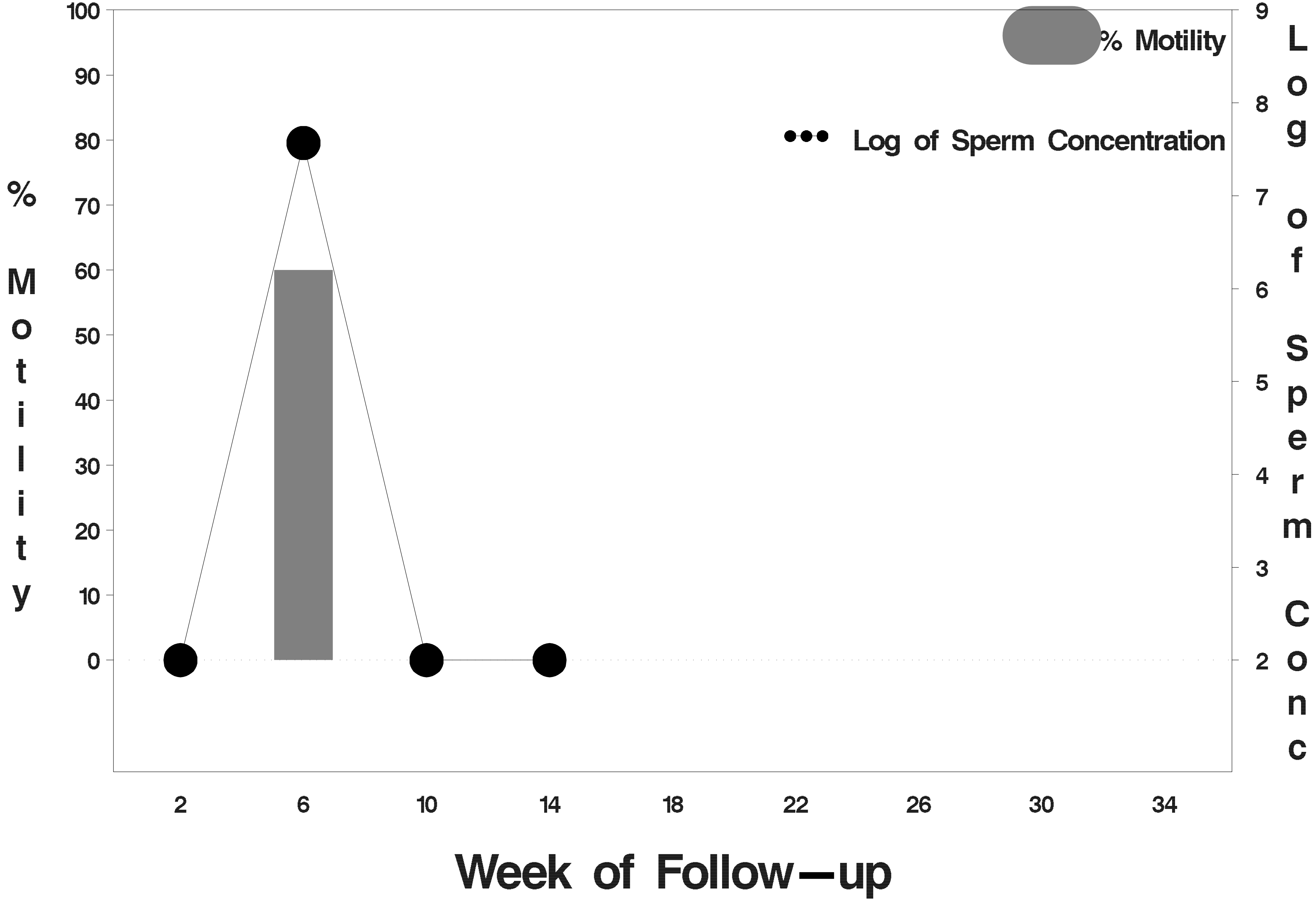

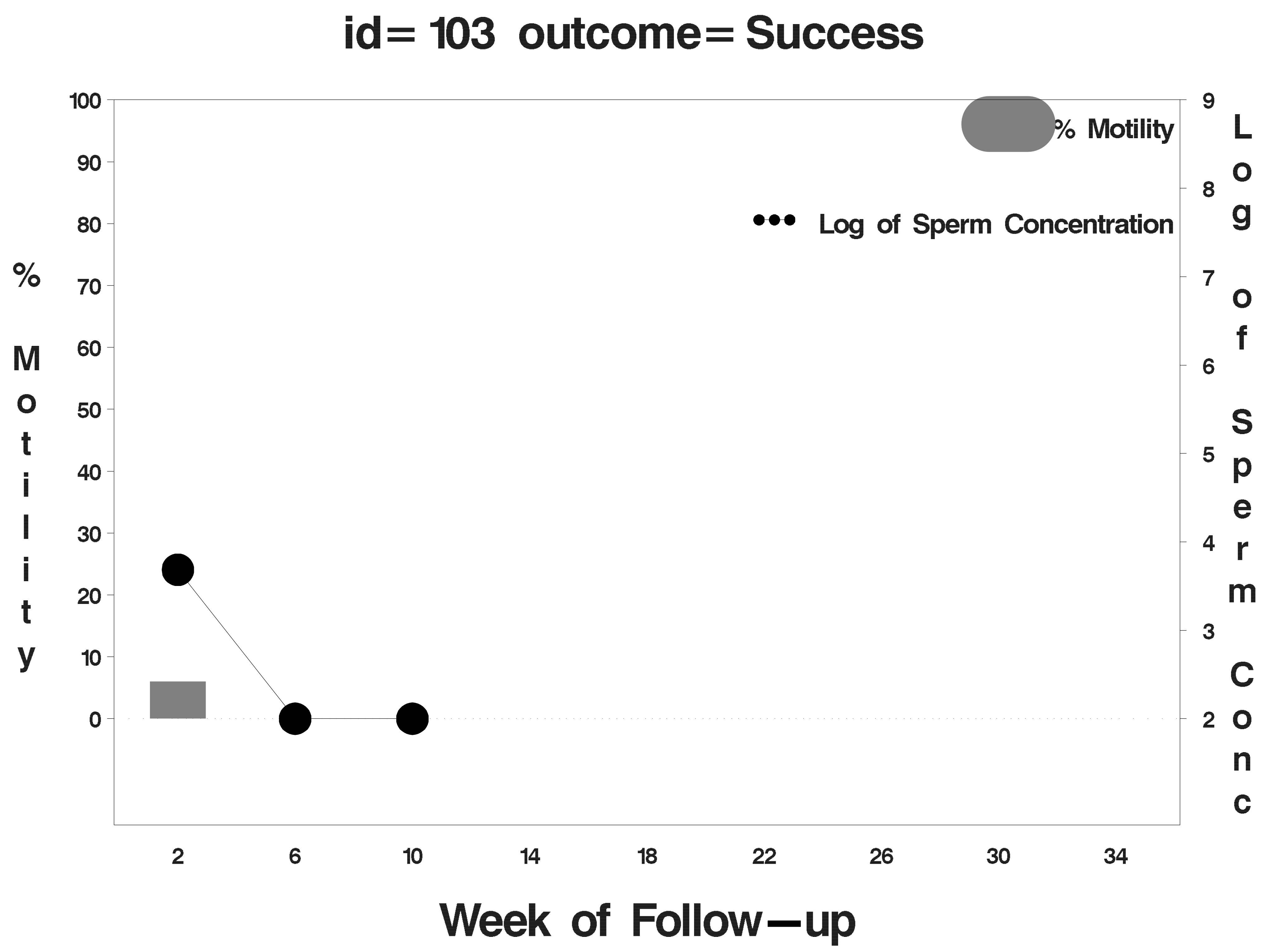

id= 103 outcome= Success
% Motility
Log of Sperm Concentration
% Motility
Log of Sperm Conc
Week of Follow-up
100
90
80
70
60
50
40
30
20
10
0
9
8
7
6
5
4
3
2
2
6
10
14
18
22
26
30
34

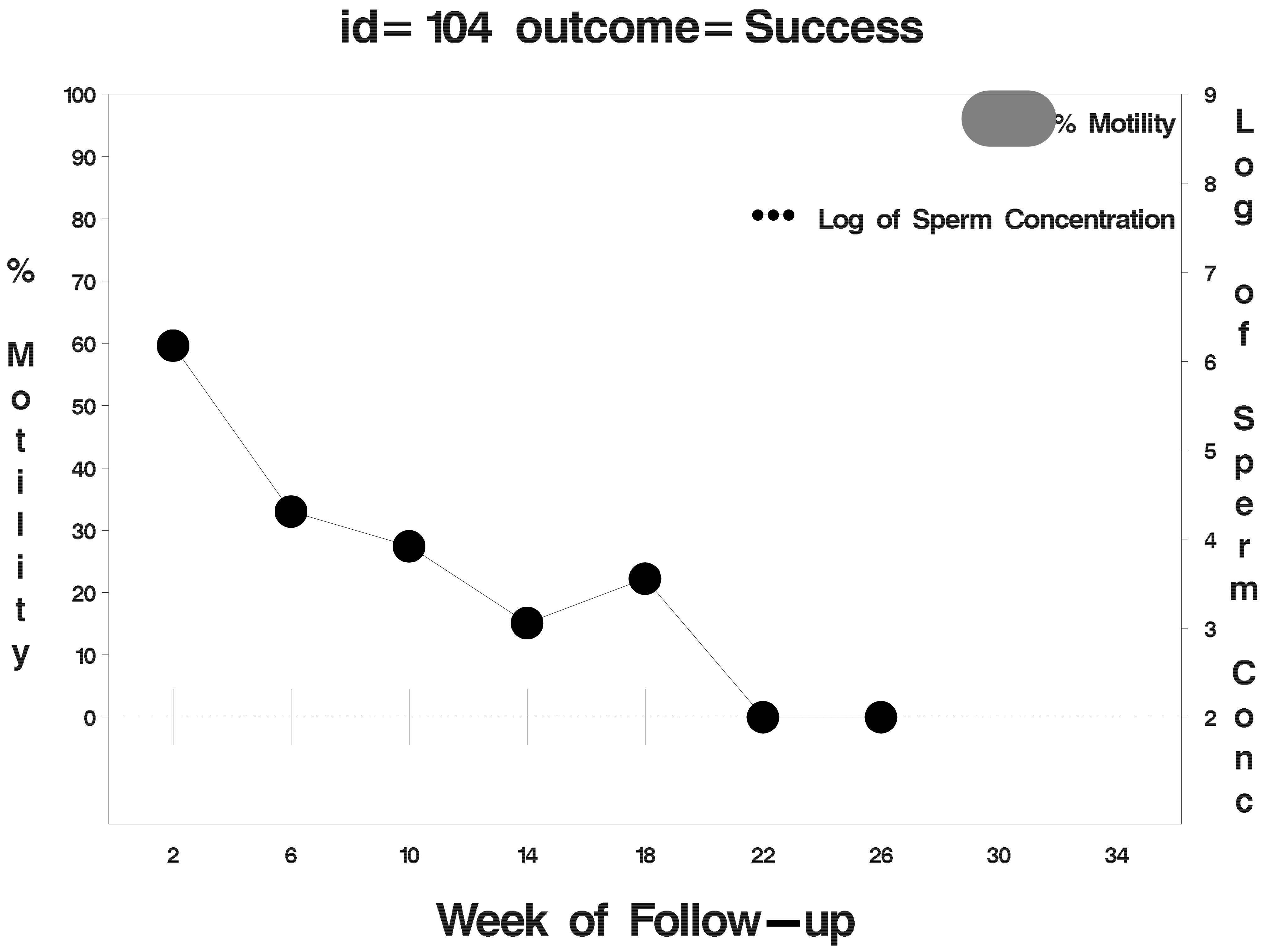

id= 104 outcome= Success
% Motility
Log of Sperm Concentration
100
90
80
70
60
50
40
30
20
10
0
9
8
7
6
5
4
3
2
2
6
10
14
18
22
26
30
34
Week of Follow—up
Log of Sperm Conc

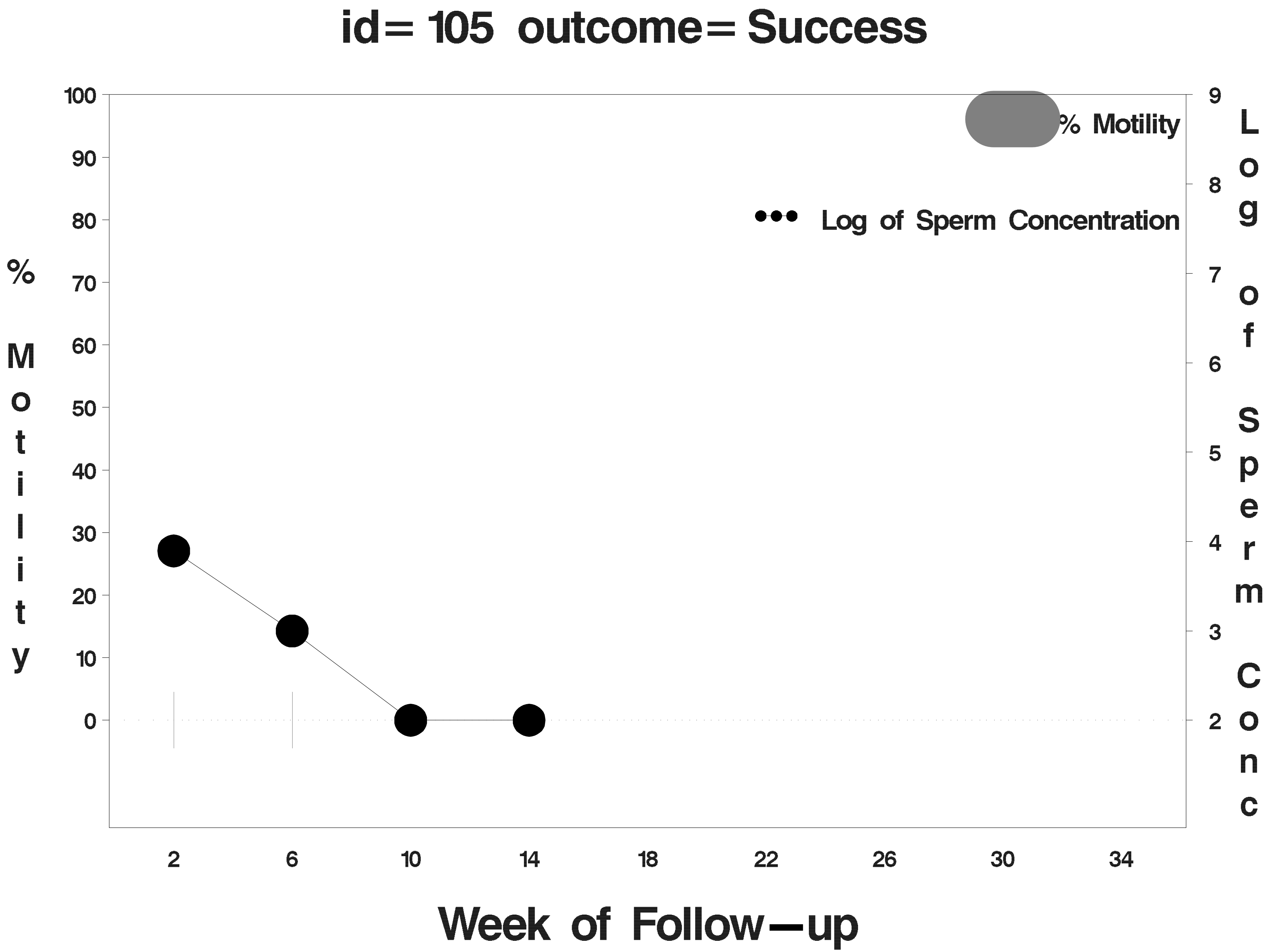

id= 105 outcome= Success
% Motility
Log of Sperm Concentration
100
90
80
70
60
50
40
30
20
10
0
9
8
7
6
5
4
3
2
2
6
10
14
18
22
26
30
34
Week of Follow−up
Log of Sperm Conc

id= 106 outcome= Success

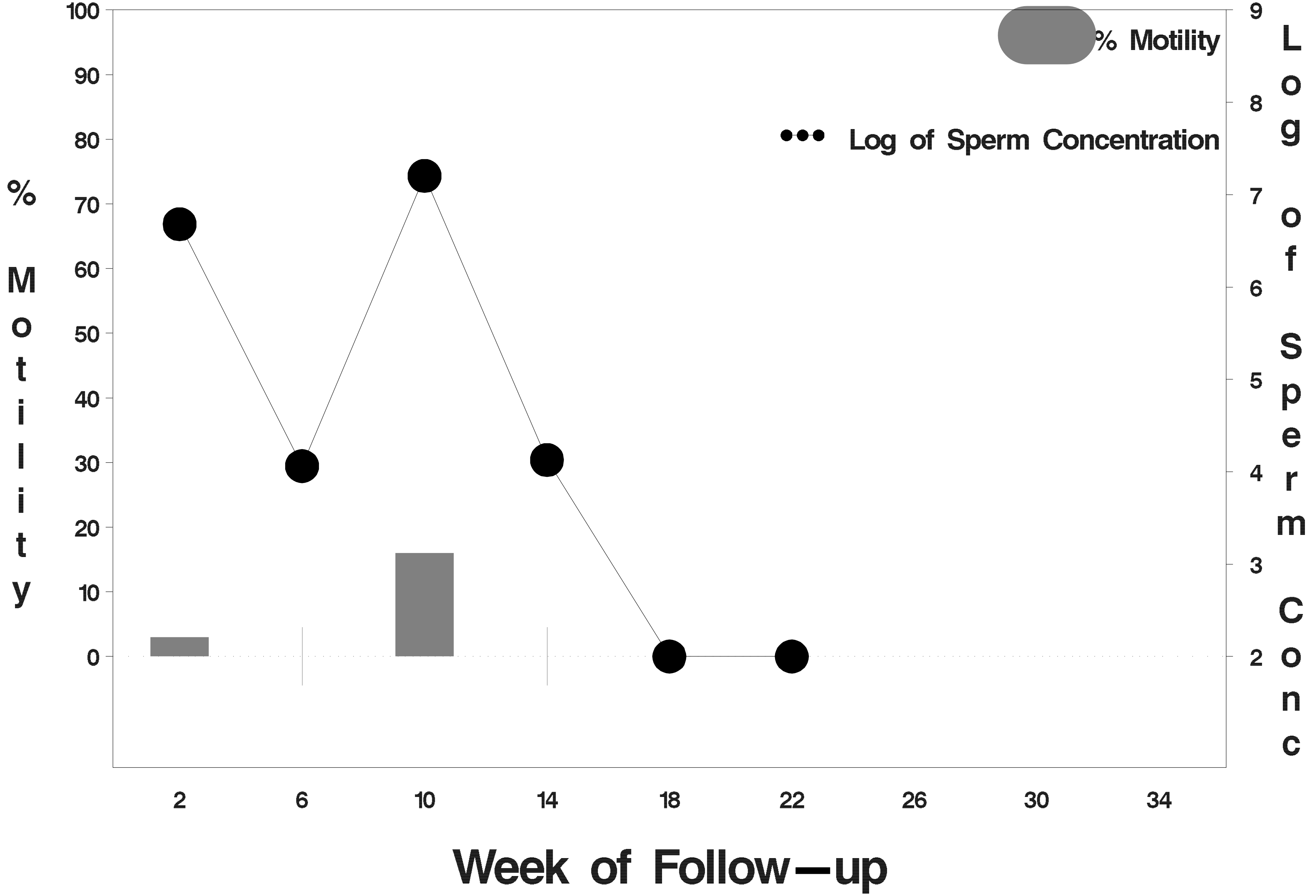

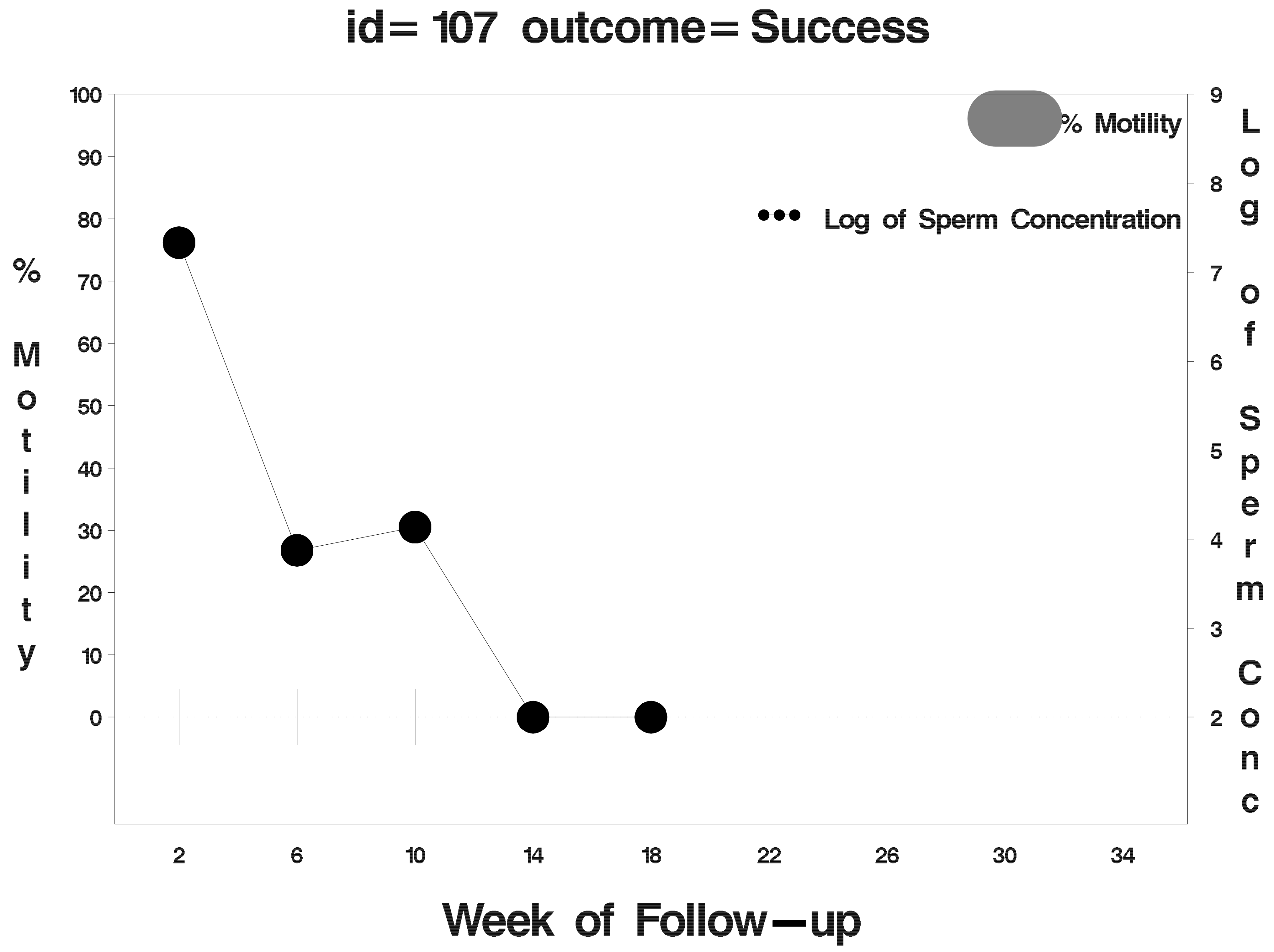

id= 107 outcome= Success
% Motility
Log of Sperm Concentration
100
90
80
70
60
50
40
30
20
10
0
9
8
7
6
5
4
3
2
2
6
10
14
18
22
26
30
34
Week of Follow−up
Log of Sperm Conc

id= 108 outcome= Success

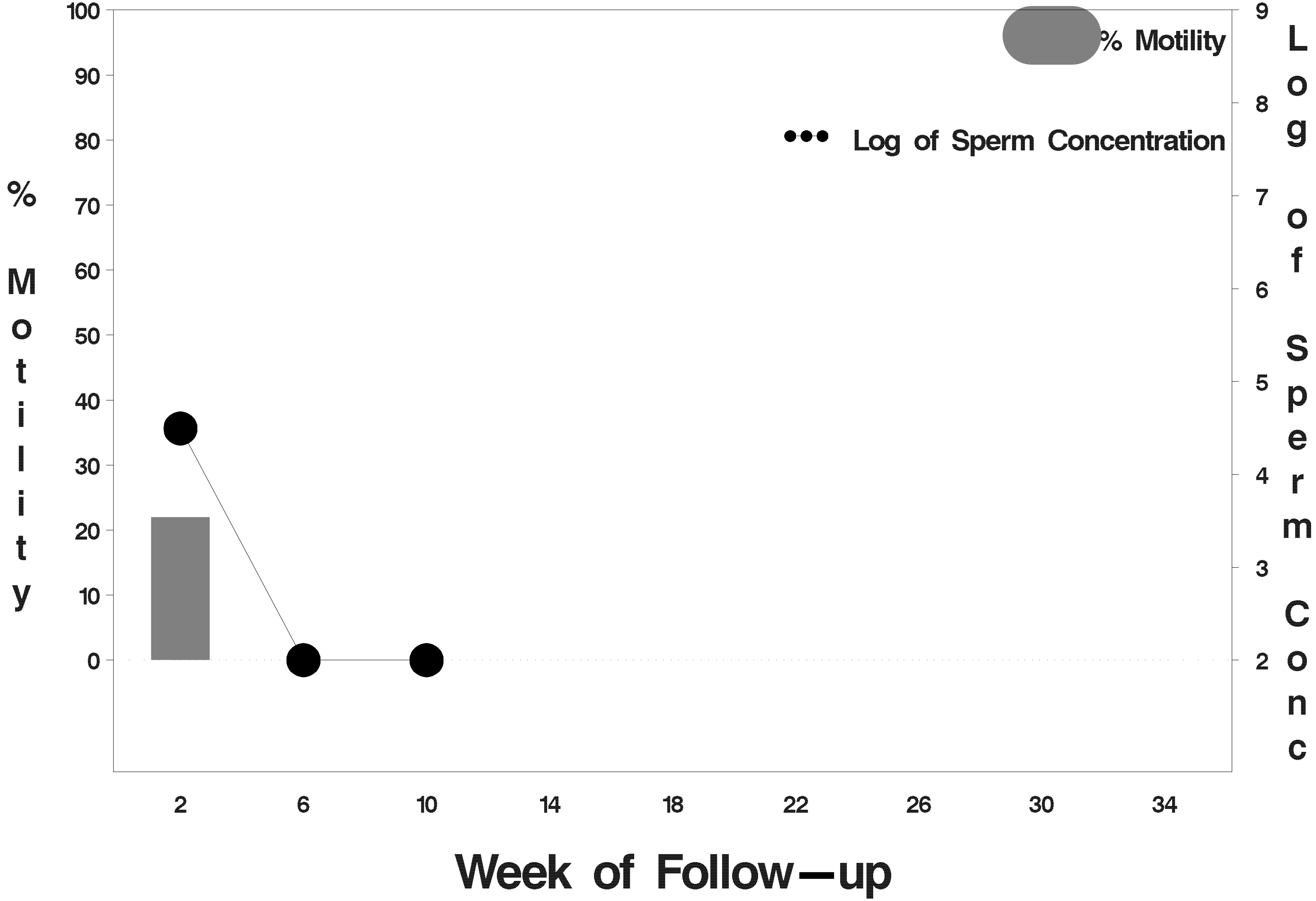

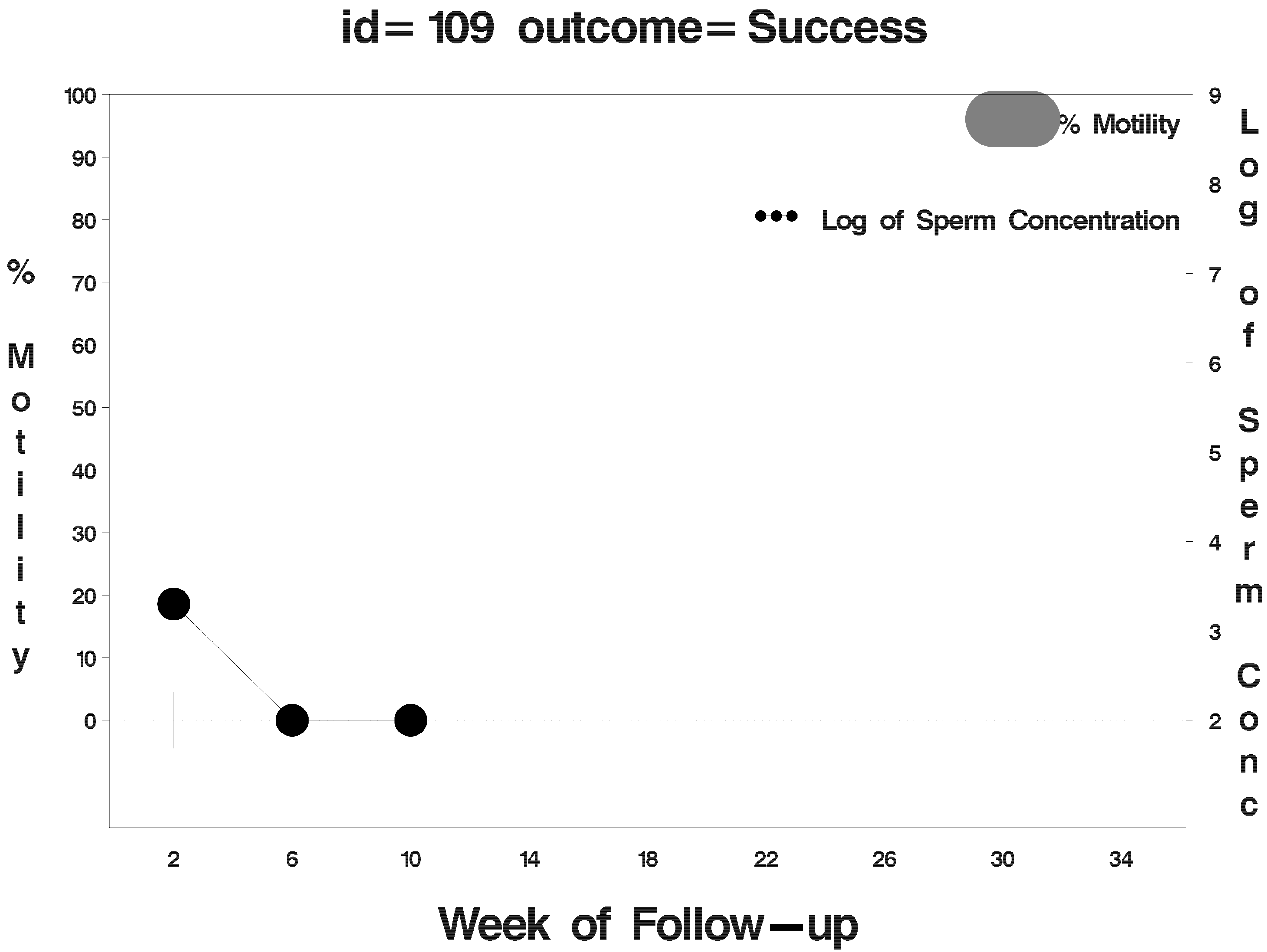
id= 109 outcome= Success
% Motility
Log of Sperm Concentration
100
90
80
70
60
50
40
30
20
10
0
9
8
7
6
5
4
3
2
2
6
10
14
18
22
26
30
34
Week of Follow—up
Log of Sperm Conc

id= 110 outcome= Success

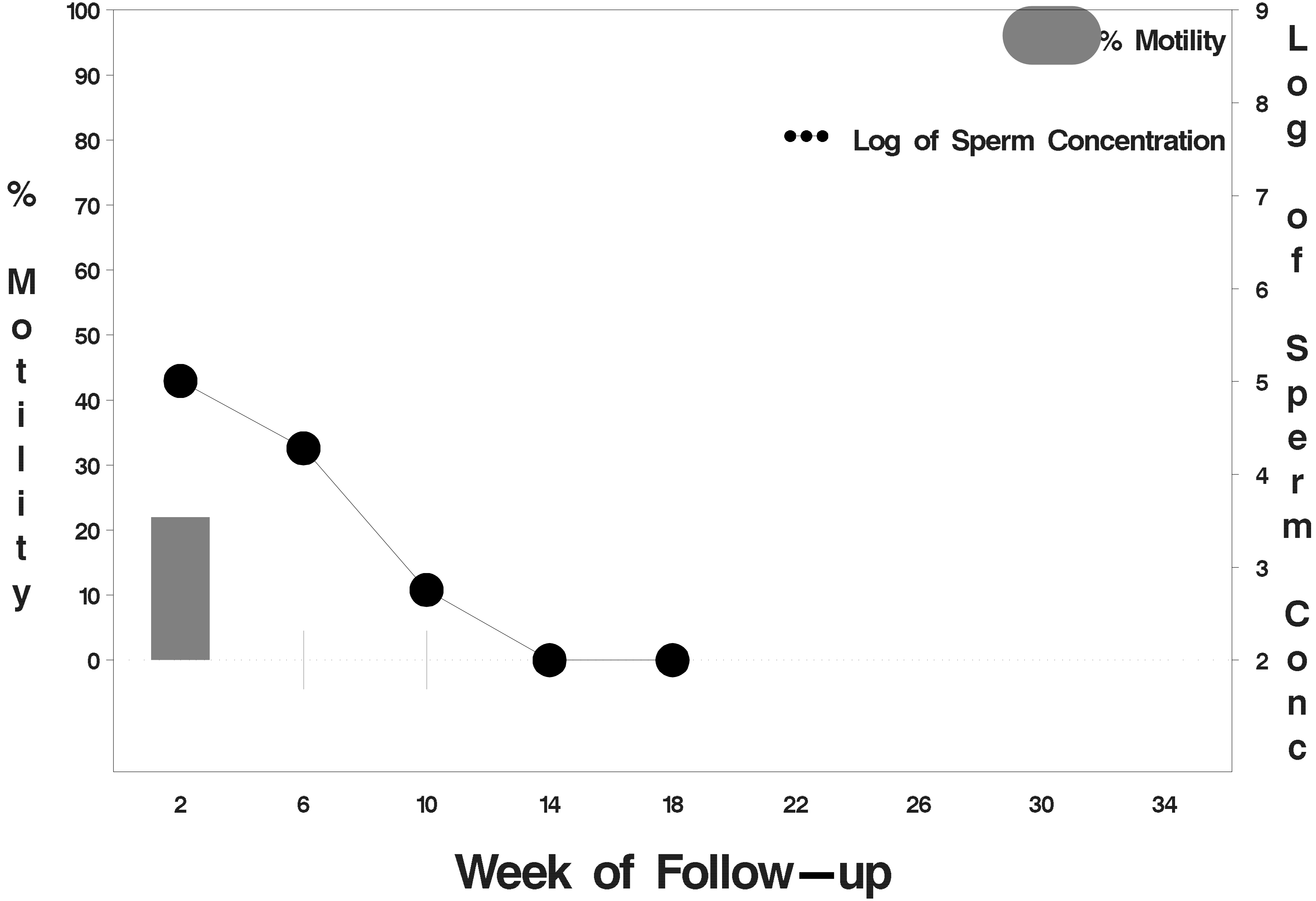

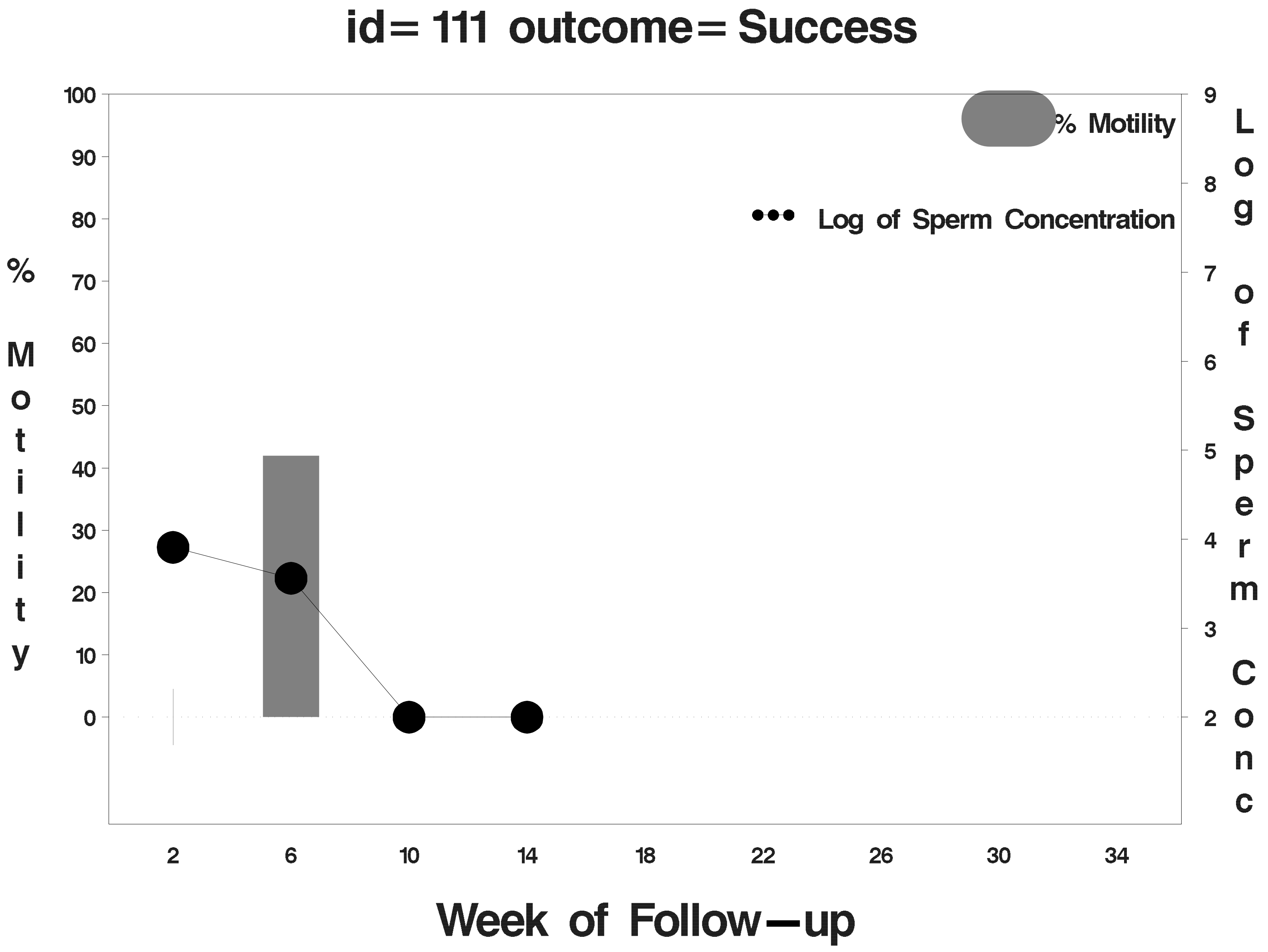

id= 111 outcome= Success
% Motility
Log of Sperm Concentration
100
90
80
70
60
50
40
30
20
10
0
9
8
7
6
5
4
3
2
2
6
10
14
18
22
26
30
34
Week of Follow−up
Log of Sperm Conc

id= 112 outcome= Success

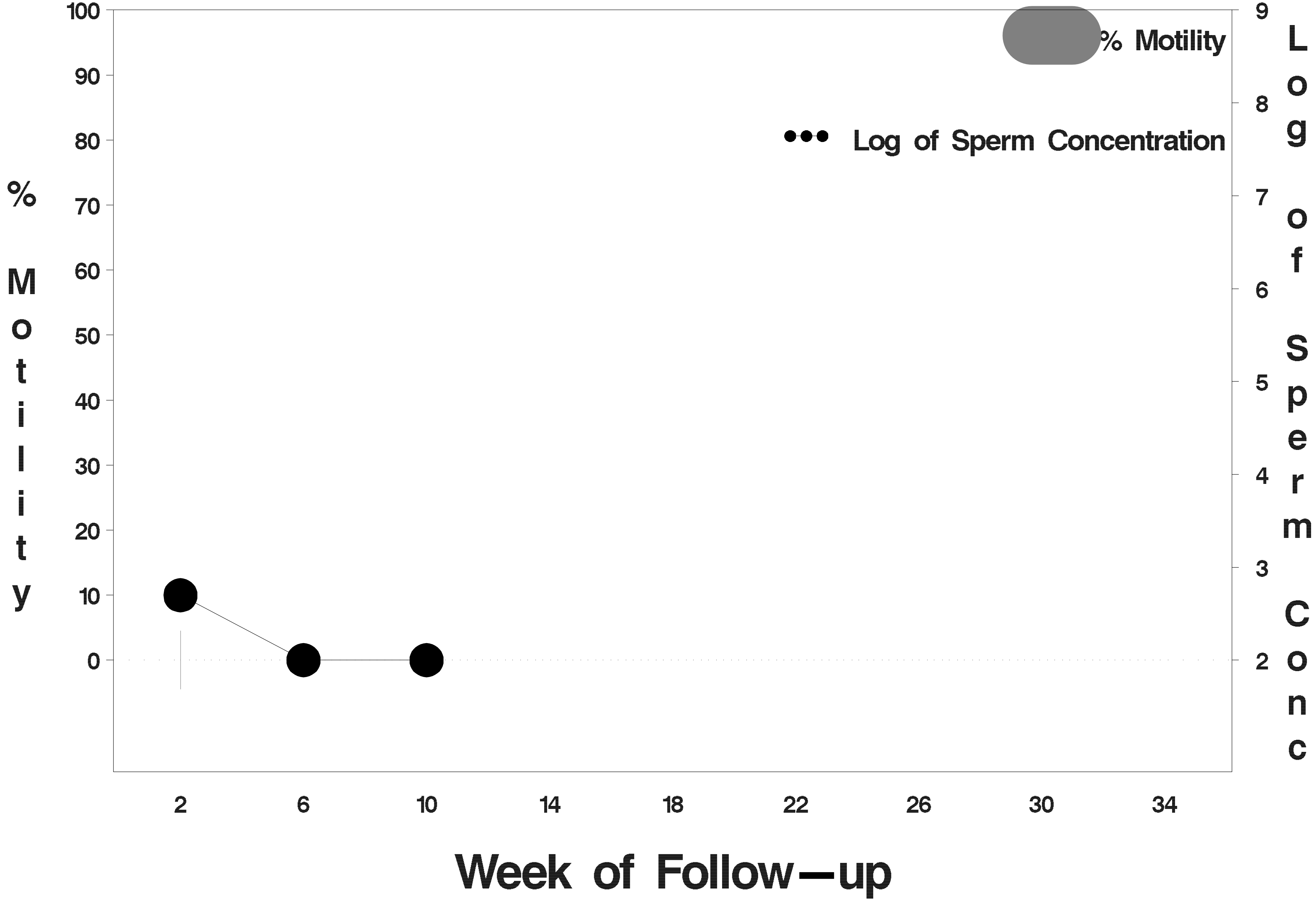

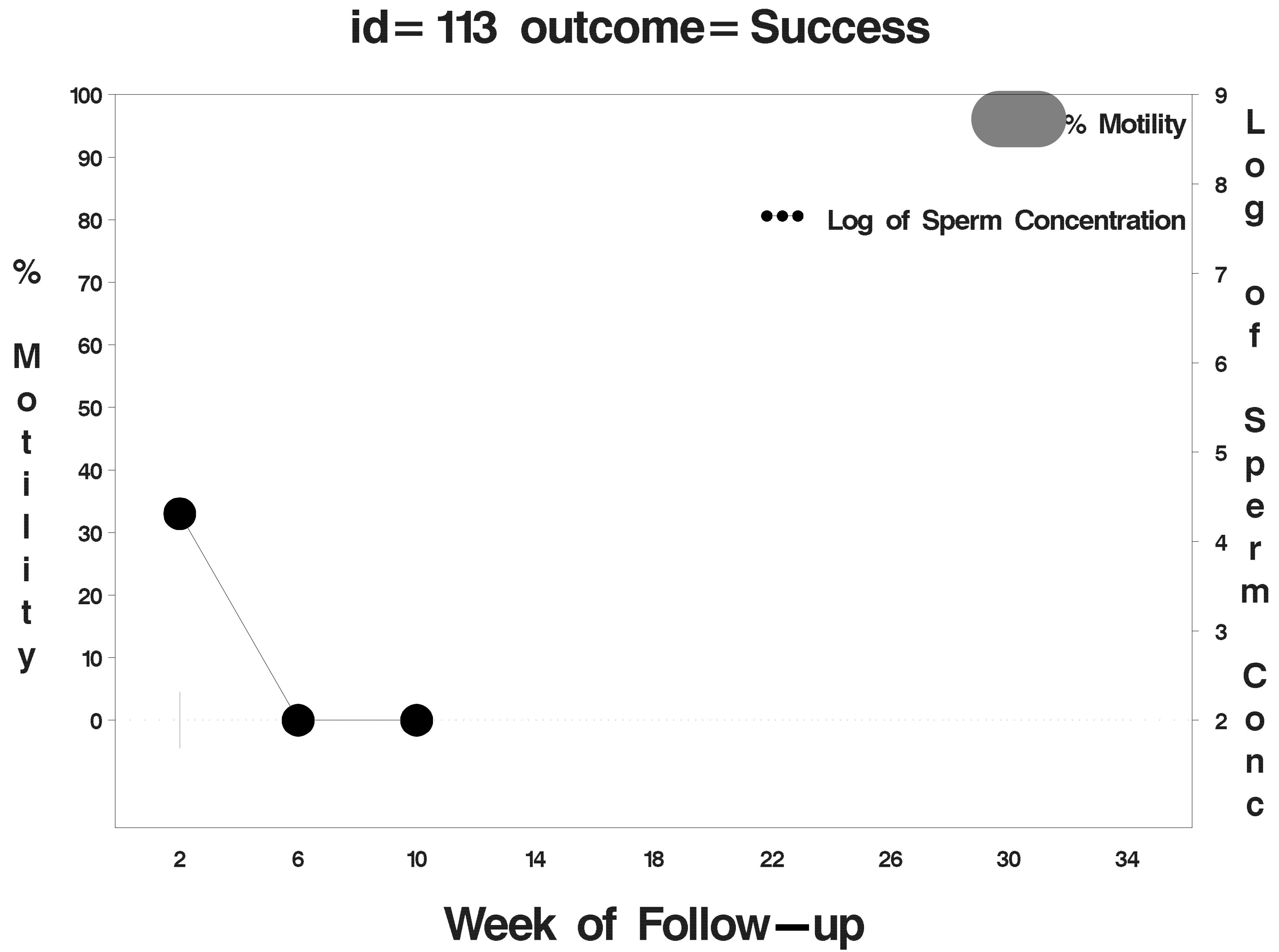

id= 113 outcome= Success
% Motility
Log of Sperm Concentration
100
90
80
70
60
50
40
30
20
10
0
9
8
7
6
5
4
3
2
2
6
10
14
18
22
26
30
34
Week of Follow−up
Log of Sperm Conc

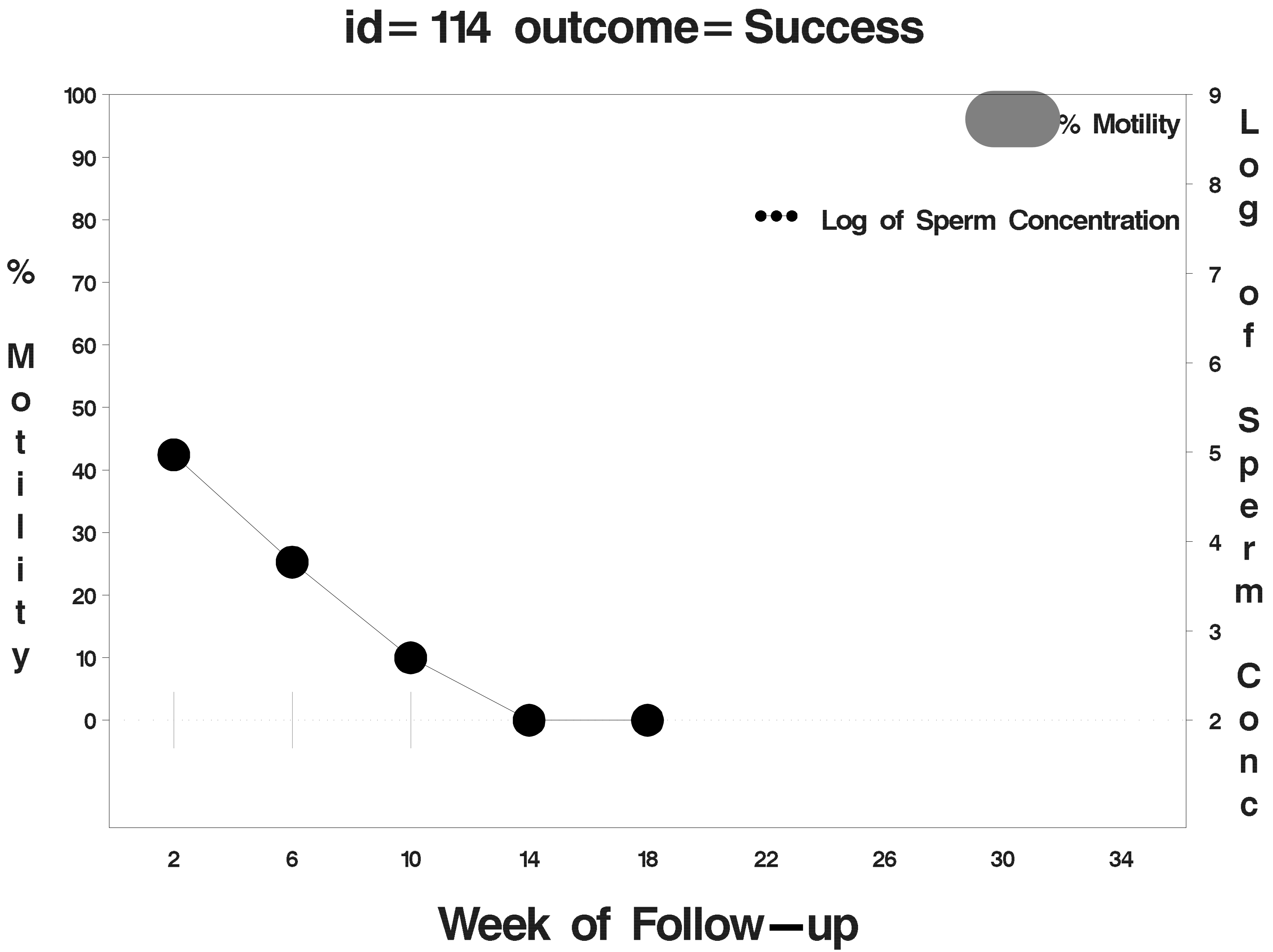

id= 114 outcome= Success
% Motility
Log of Sperm Concentration
% Motility
Log of Sperm Conc
Week of Follow−up
100
90
80
70
60
50
40
30
20
10
0
9
8
7
6
5
4
3
2
2
6
10
14
18
22
26
30
34

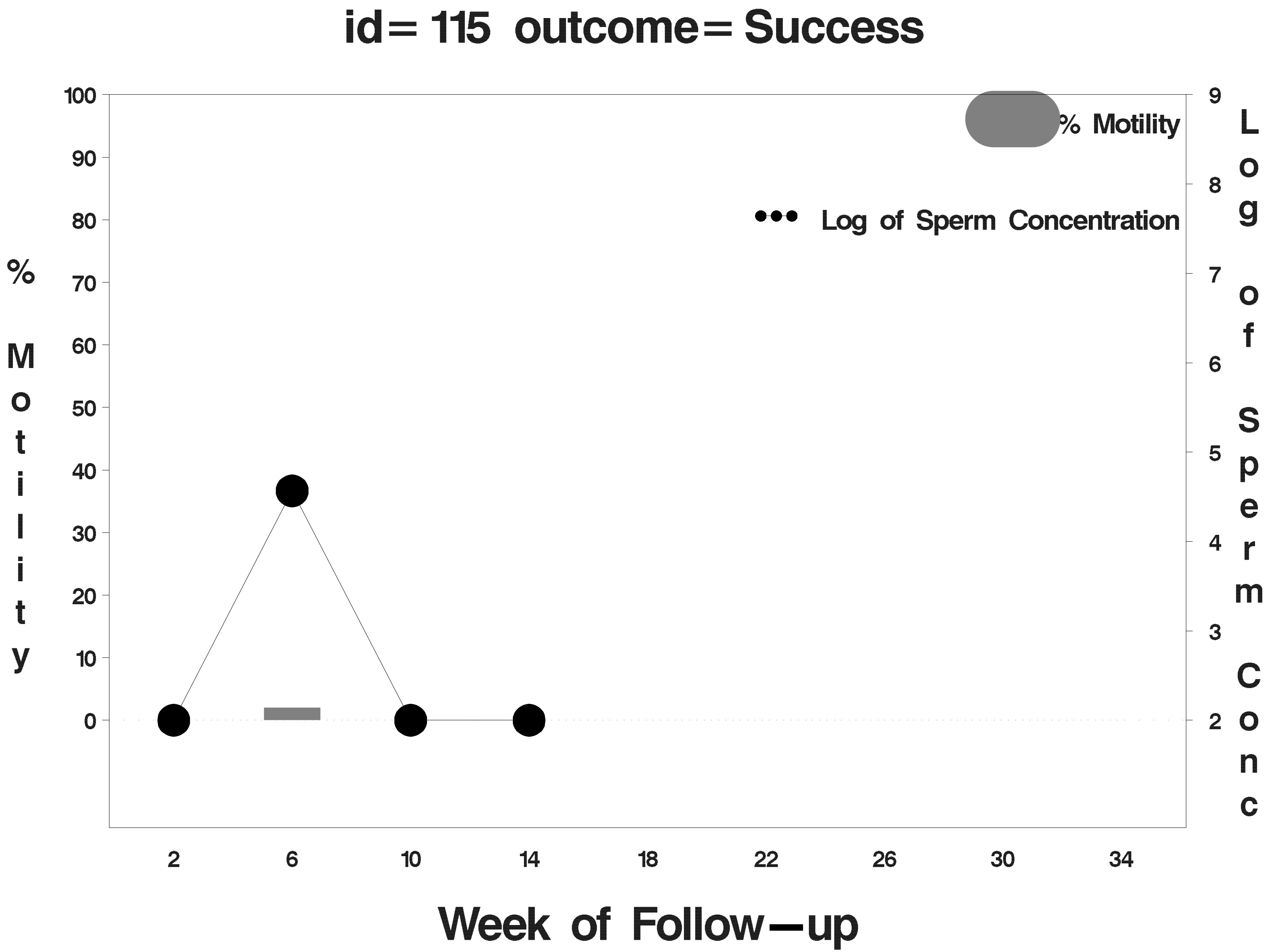
id= 115 outcome= Success
% Motility
Log of Sperm Concentration
100
90
80
70
60
50
40
30
20
10
0
9
8
7
6
5
4
3
2
% Motility
Log of Sperm Conc
2
6
10
14
18
22
26
30
34
Week of Follow—up

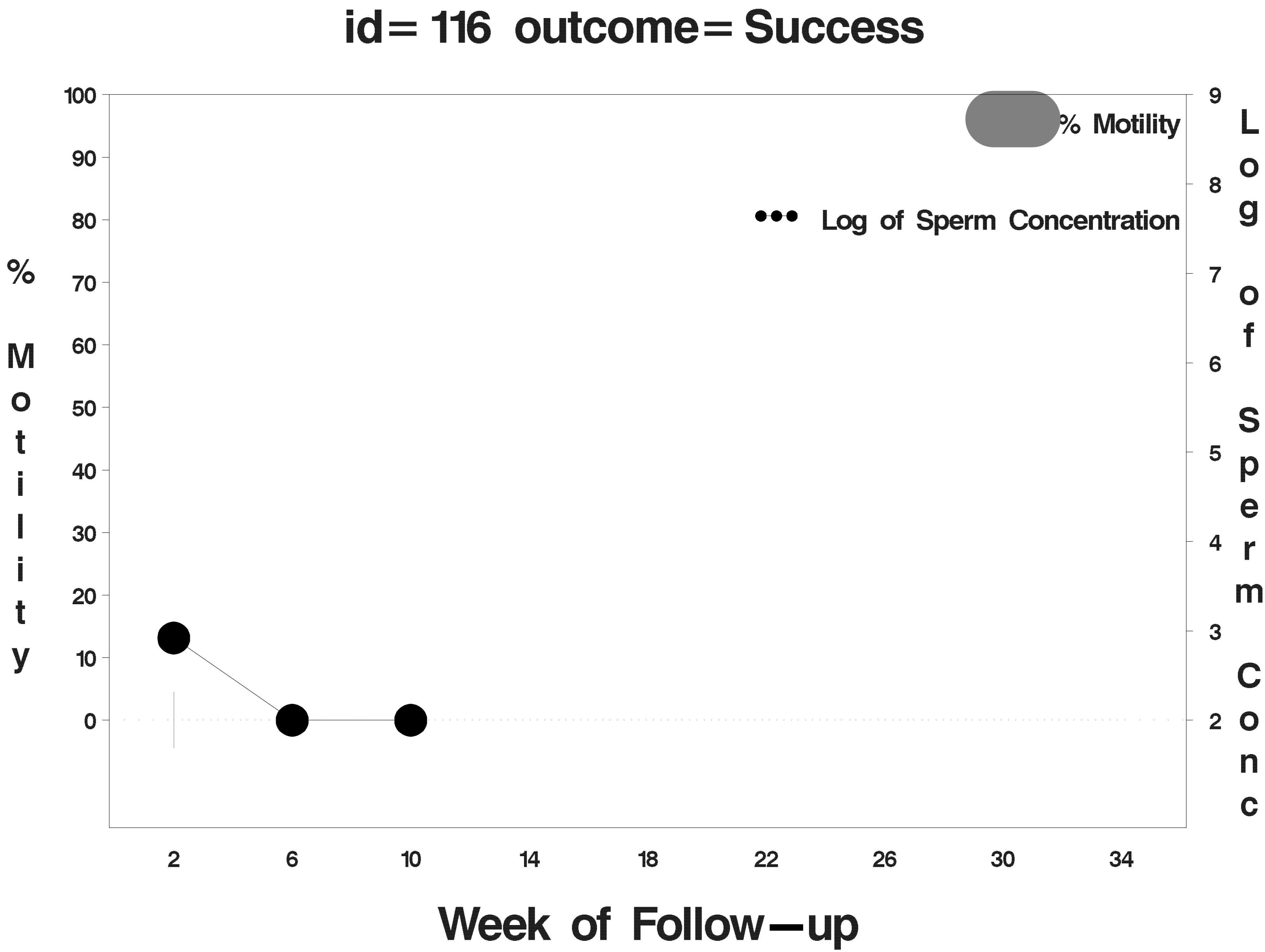

id= 116 outcome= Success
% Motility
Log of Sperm Concentration
100
90
80
70
60
50
40
30
20
10
0
9
8
7
6
5
4
3
2
2
6
10
14
18
22
26
30
34
Week of Follow—up
% Motility
Log of Sperm Conc

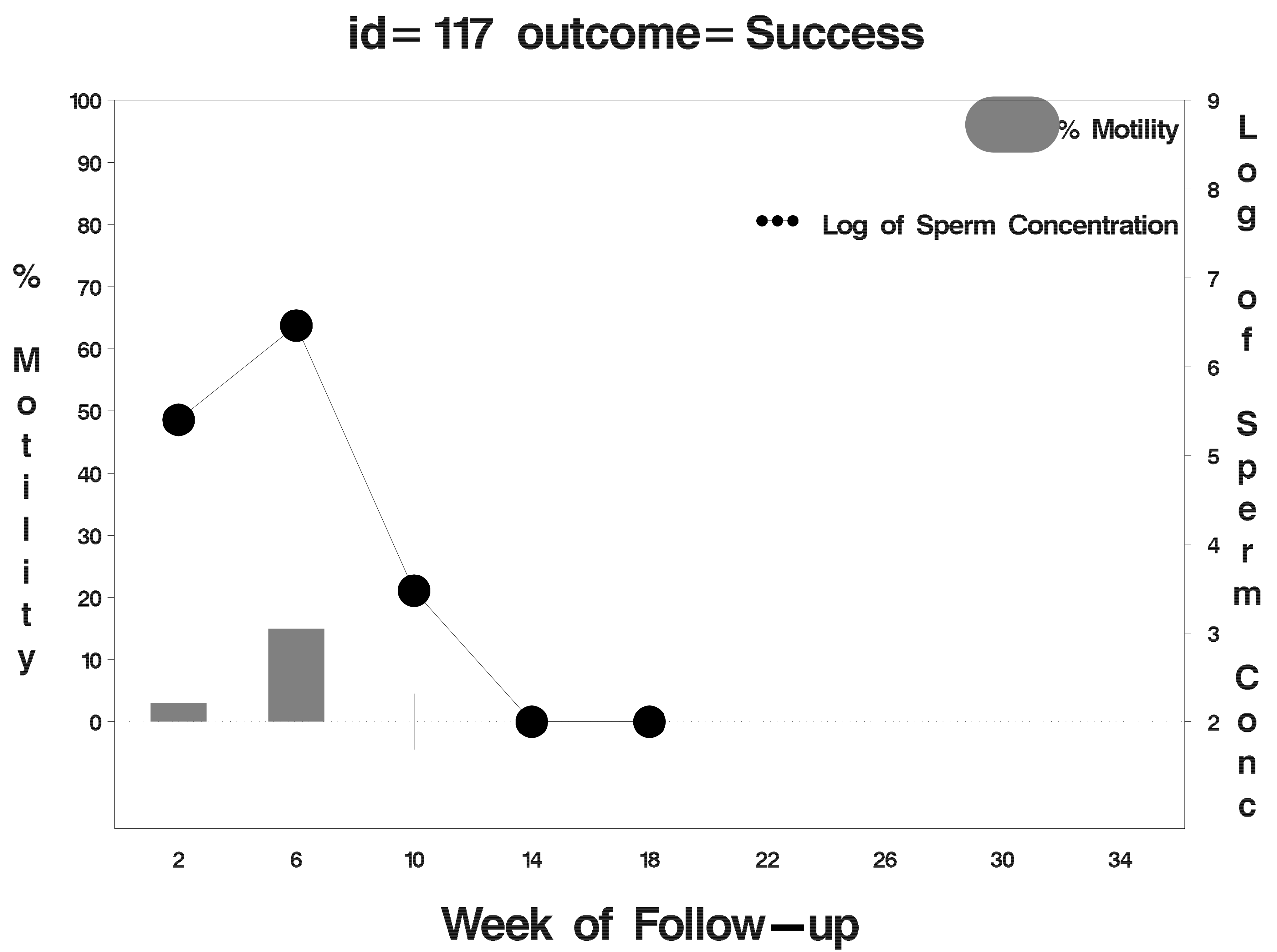
id= 117 outcome= Success
% Motility
Log of Sperm Concentration
100
90
80
70
60
50
40
30
20
10
0
9
8
7
6
5
4
3
2
2
6
10
14
18
22
26
30
34
% Motility
Log of Sperm Conc
Week of Follow—up

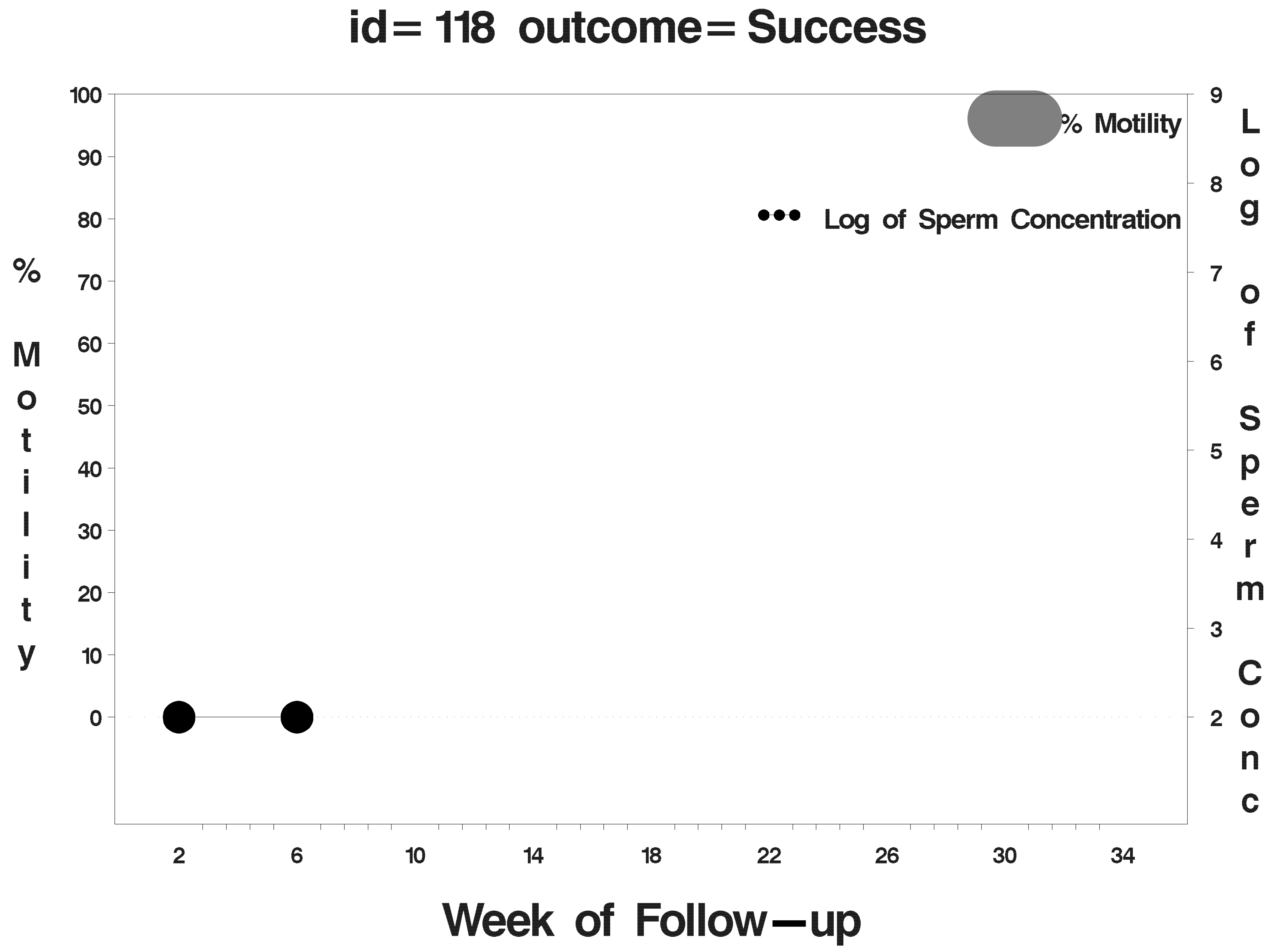
id= 118 outcome= Success
% Motility
Log of Sperm Concentration
% Motility
Log of Sperm Conc
Week of Follow−up
100
90
80
70
60
50
40
30
20
10
0
9
8
7
6
5
4
3
2
2
6
10
14
18
22
26
30
34

id= 119 outcome= Success

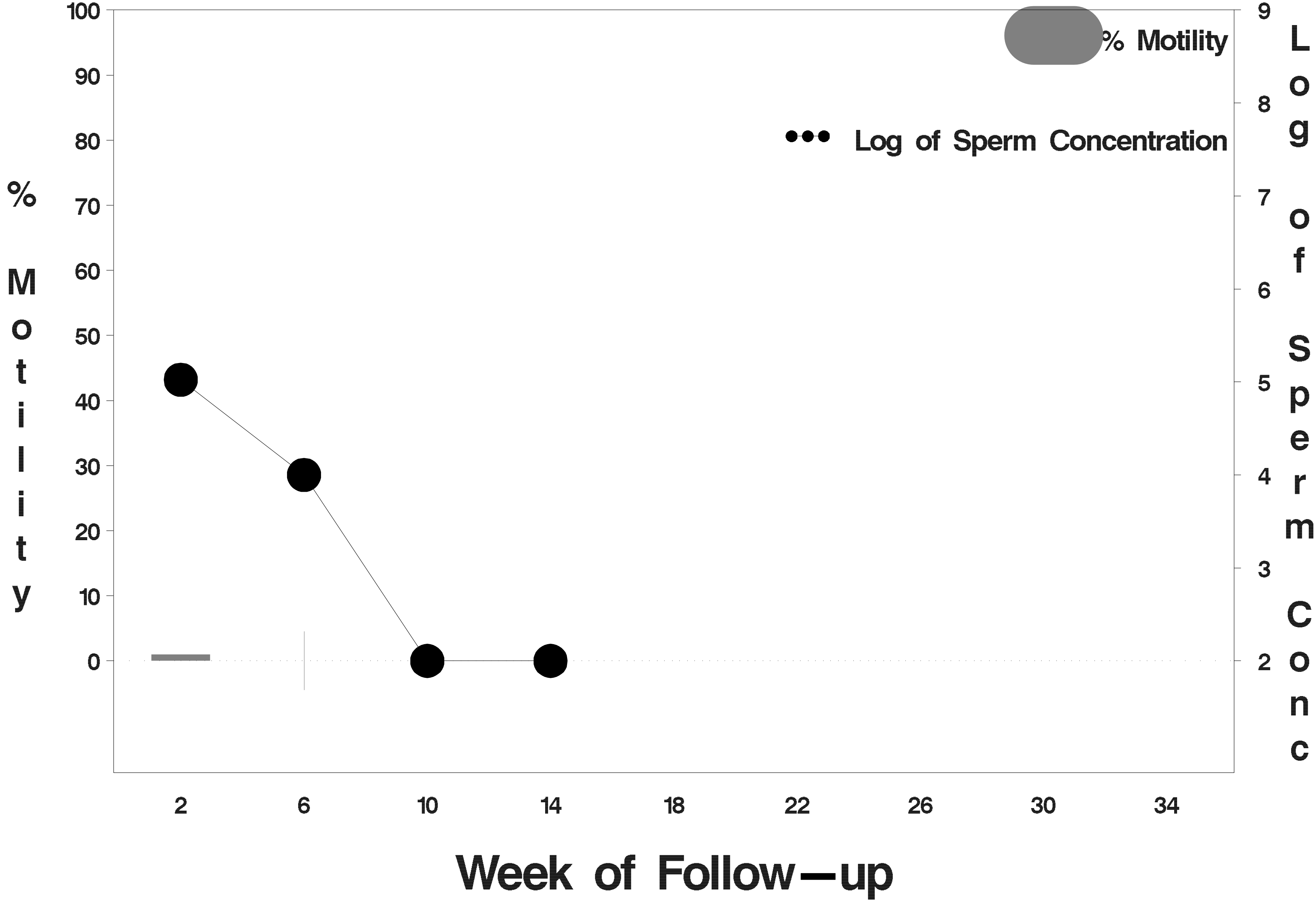

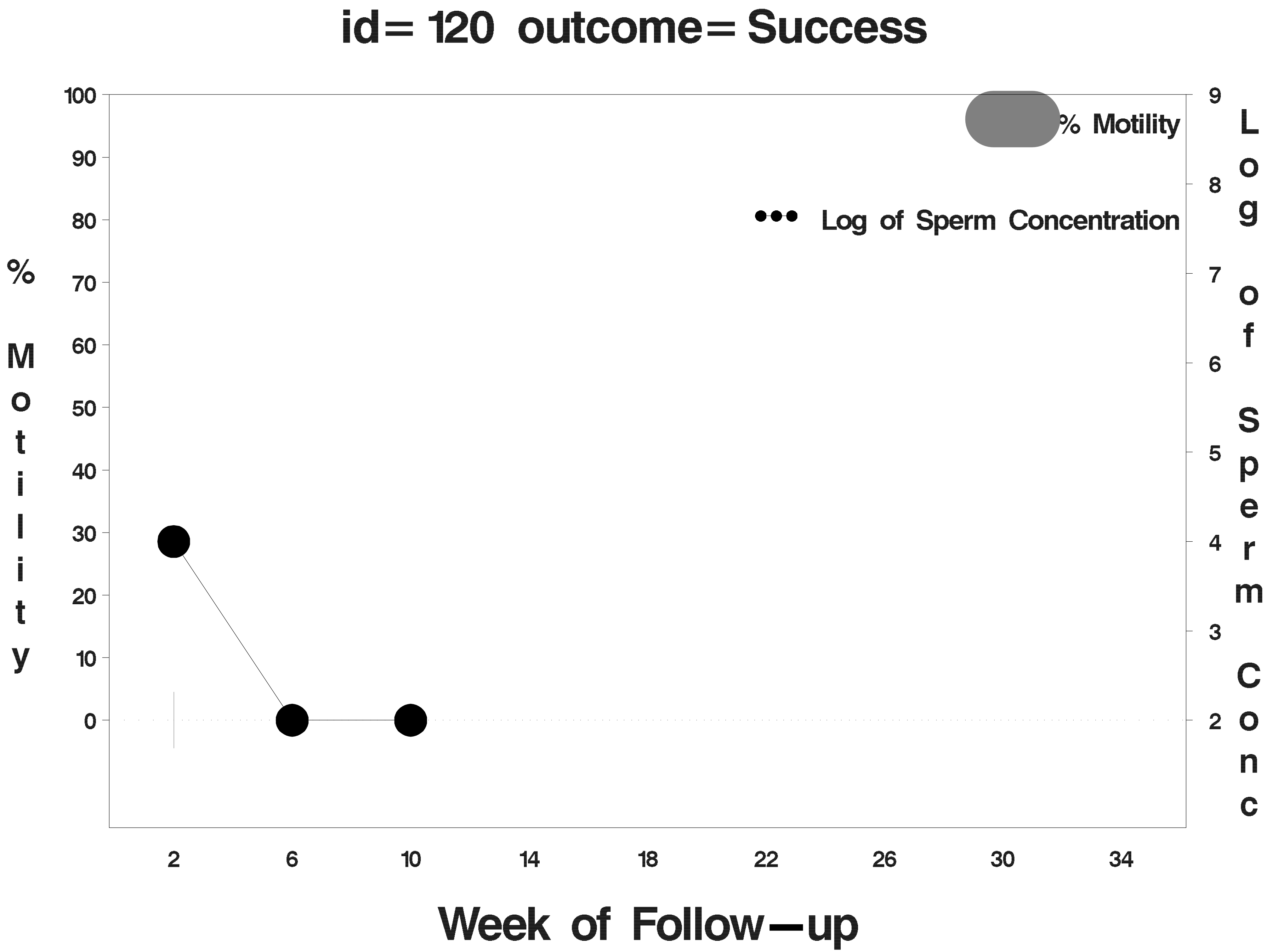

id= 120 outcome= Success
% Motility
Log of Sperm Concentration
% Motility
Log of Sperm Conc
100
90
80
70
60
50
40
30
20
10
0
9
8
7
6
5
4
3
2
2
6
10
14
18
22
26
30
34
Week of Follow-up

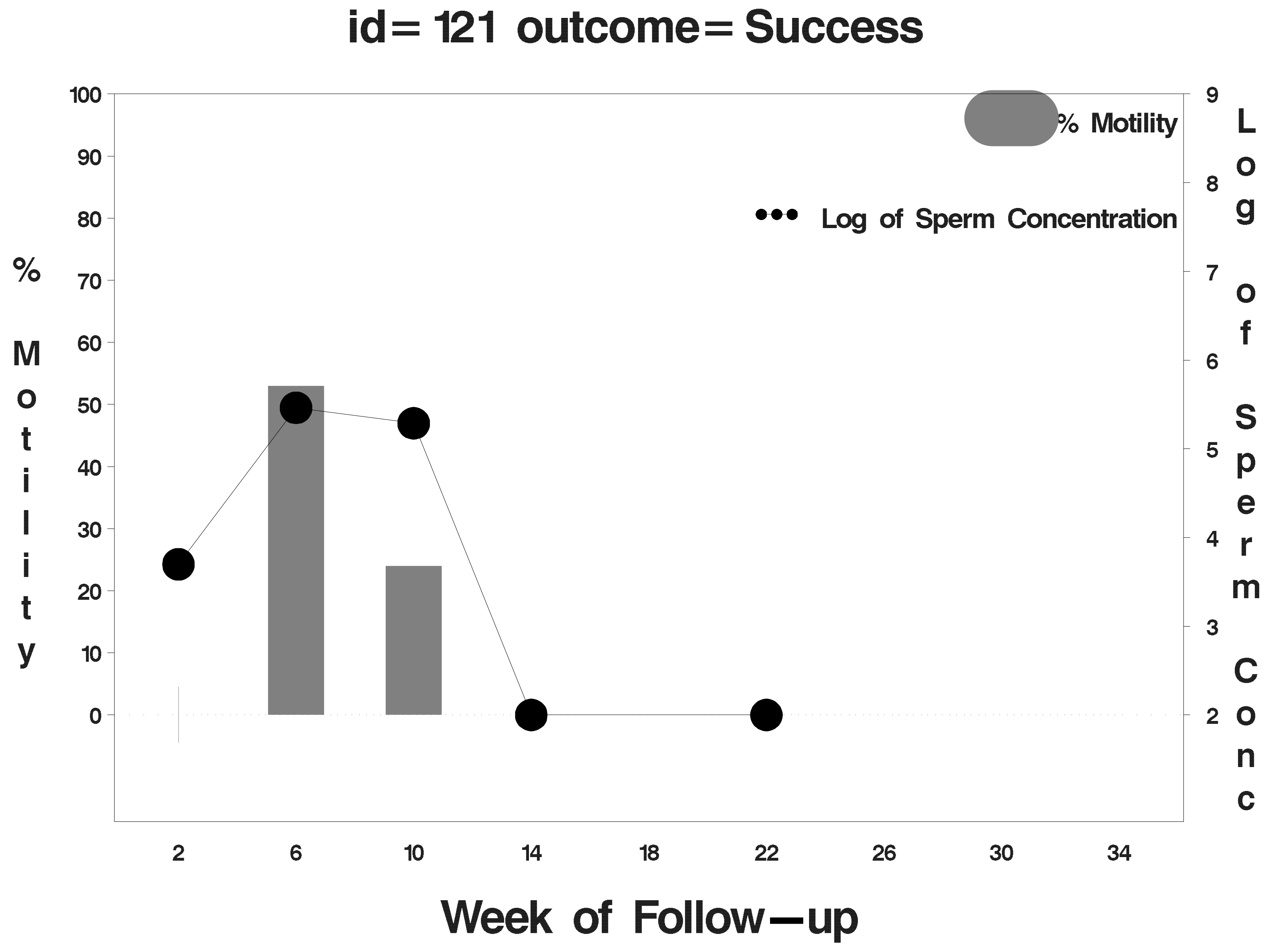

id= 121 outcome= Success
% Motility
Log of Sperm Concentration
100
90
80
70
60
50
40
30
20
10
0
9
8
7
6
5
4
3
2
2
6
10
14
18
22
26
30
34
Week of Follow−up
Log of Sperm Conc

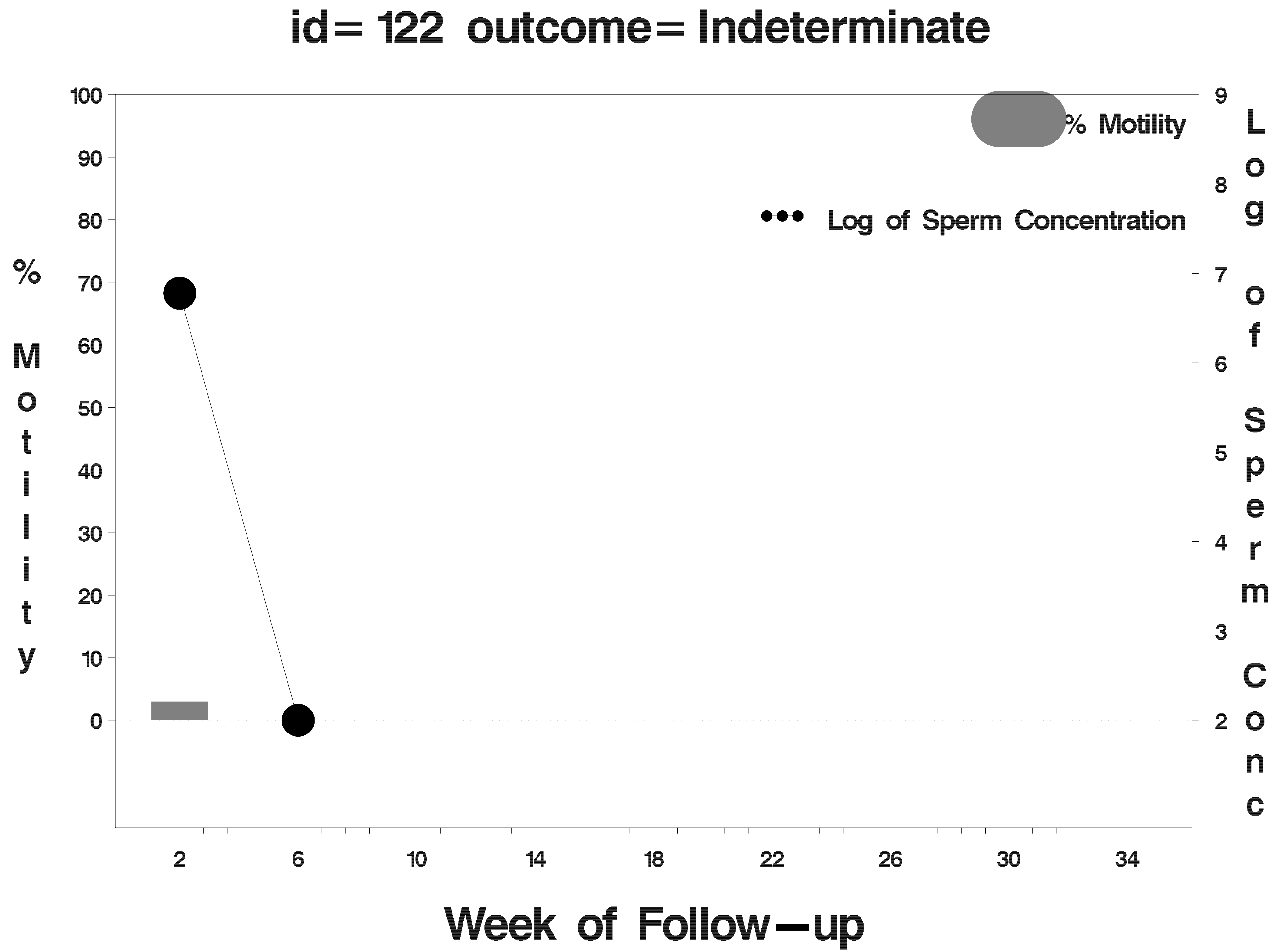

id= 122 outcome= Indeterminate
% Motility
Log of Sperm Concentration
100
90
80
70
60
50
40
30
20
10
0
9
8
7
6
5
4
3
2
Log of Sperm Conc
2
6
10
14
18
22
26
30
34
Week of Follow-up

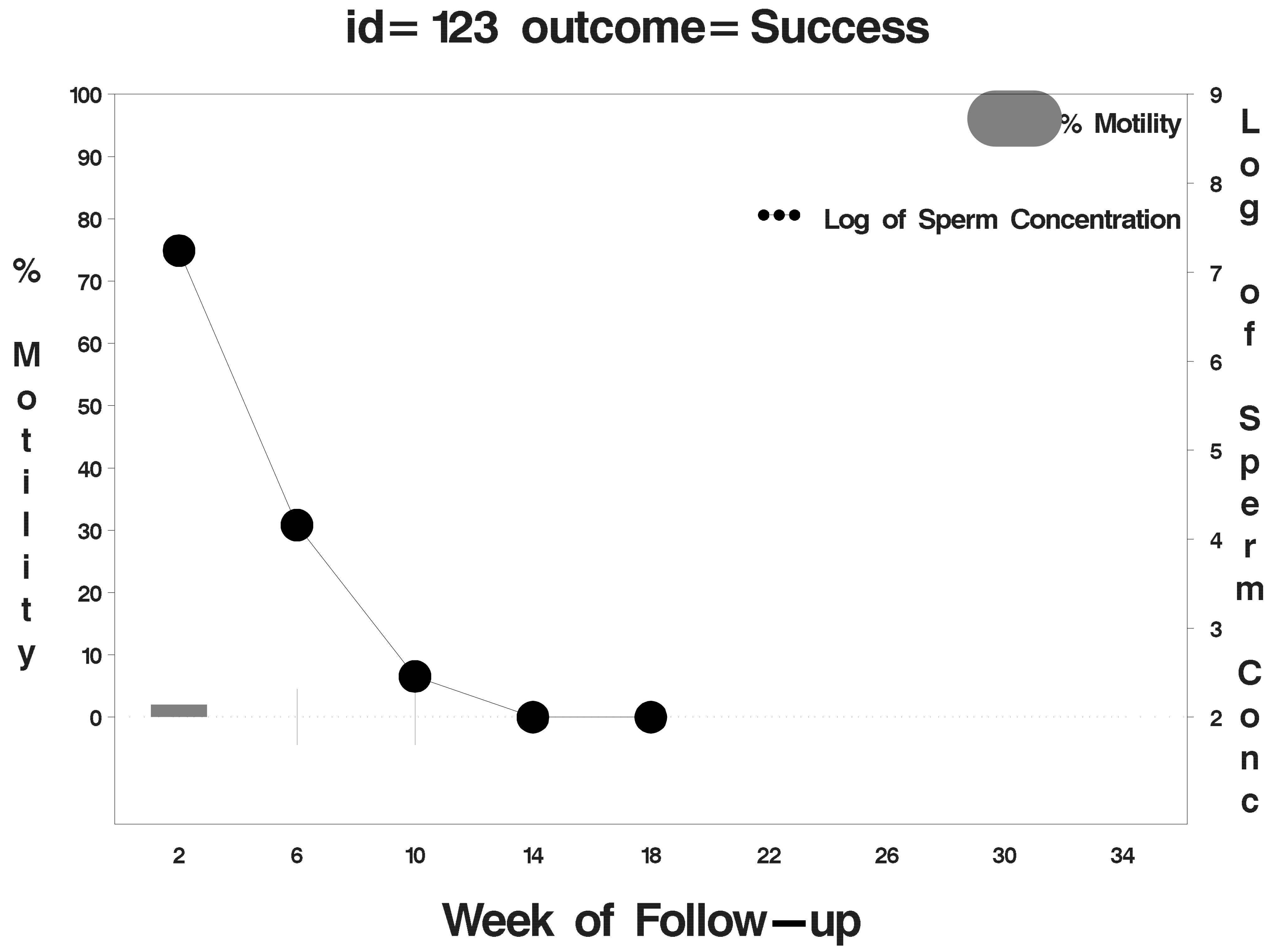
id= 123 outcome= Success
% Motility
Log of Sperm Concentration
100
90
80
70
60
50
40
30
20
10
0
9
8
7
6
5
4
3
2
2
6
10
14
18
22
26
30
34
% Motility
Log of Sperm Conc
Week of Follow-up

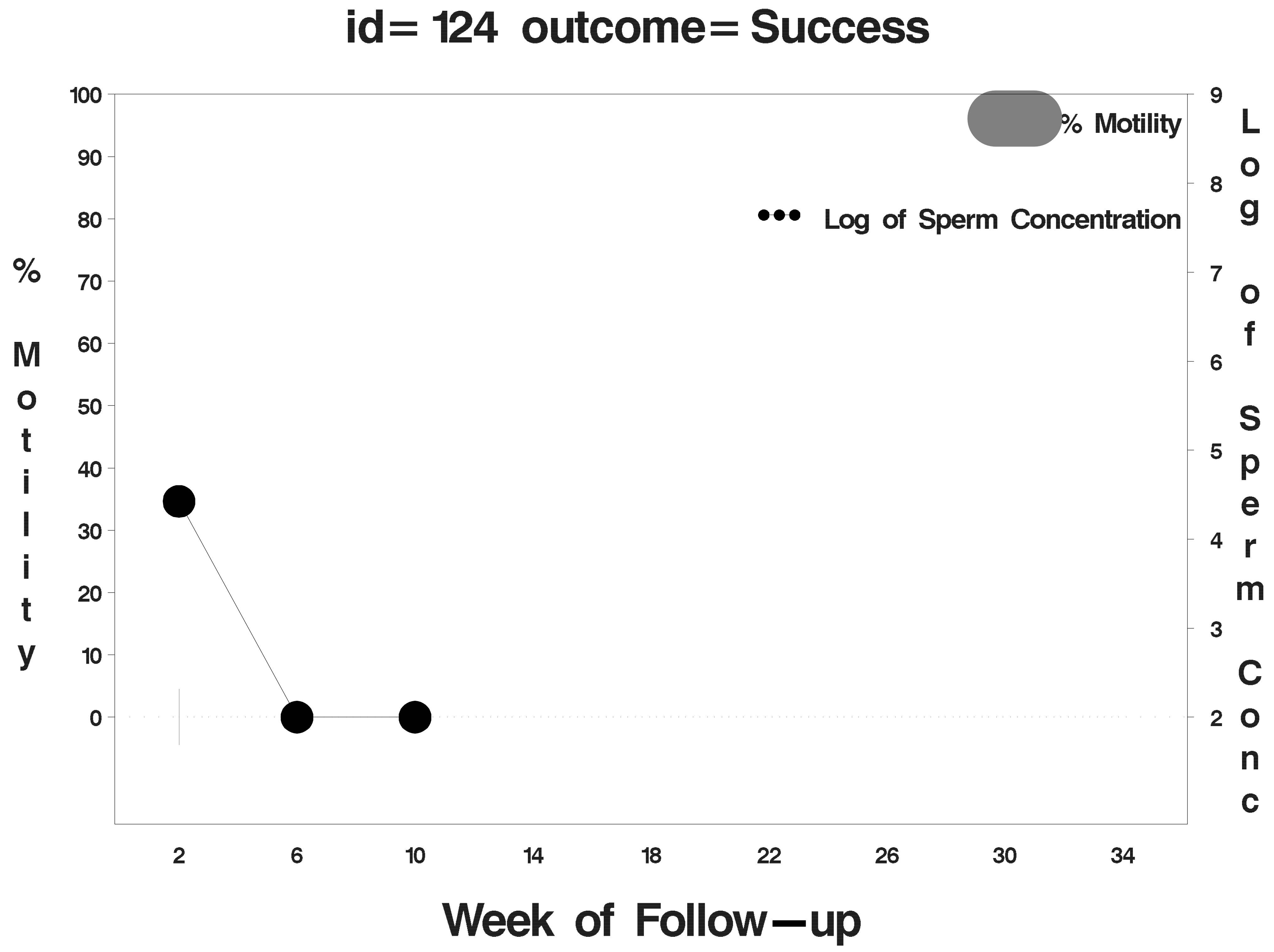

id= 124 outcome= Success
% Motility
Log of Sperm Concentration
100
90
80
70
60
50
40
30
20
10
0
9
8
7
6
5
4
3
2
2
6
10
14
18
22
26
30
34
% Motility
Log of Sperm Conc
Week of Follow—up

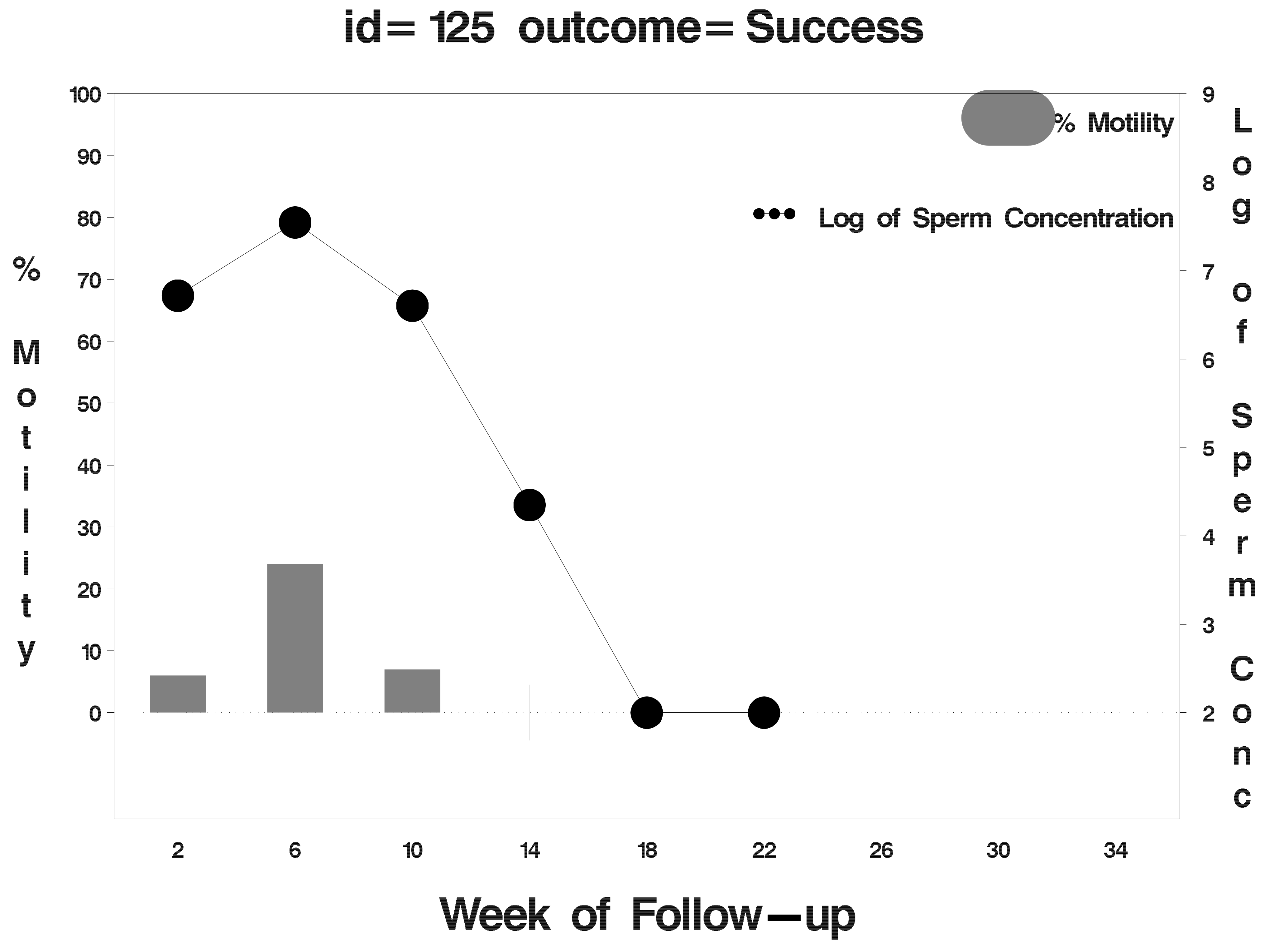

id= 125 outcome= Success
% Motility
Log of Sperm Concentration
% Motility
Log of Sperm Conc
Week of Follow−up
100
90
80
70
60
50
40
30
20
10
0
9
8
7
6
5
4
3
2
2
6
10
14
18
22
26
30
34

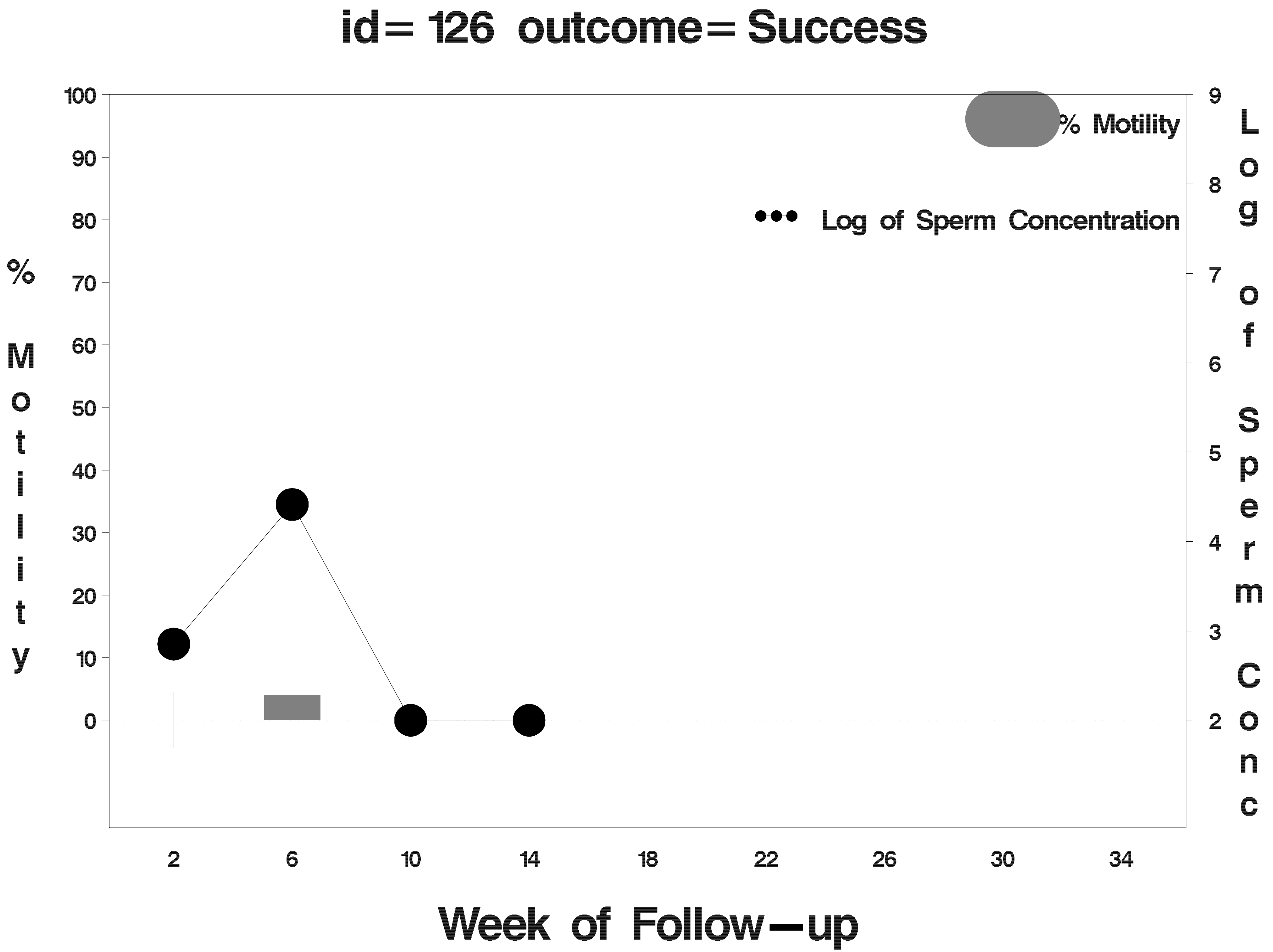

id= 126 outcome= Success
% Motility
Log of Sperm Concentration
100
90
80
70
60
50
40
30
20
10
0
9
8
7
6
5
4
3
2
Log of Sperm Conc
2
6
10
14
18
22
26
30
34
Week of Follow—up

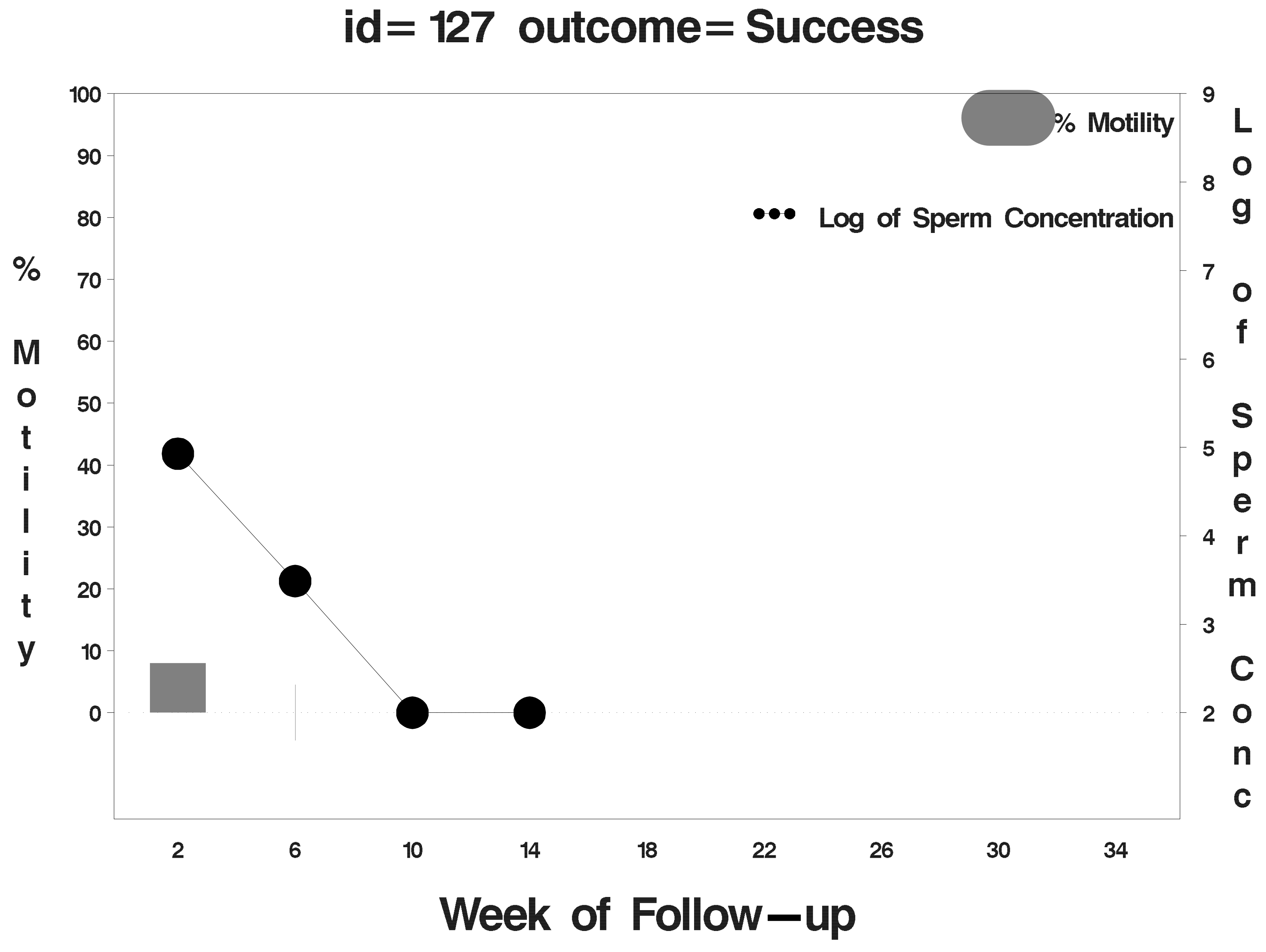
id= 127 outcome= Success
% Motility
Log of Sperm Concentration
100
90
80
70
60
50
40
30
20
10
0
9
8
7
6
5
4
3
2
2
6
10
14
18
22
26
30
34
Week of Follow—up
Log of Sperm Conc

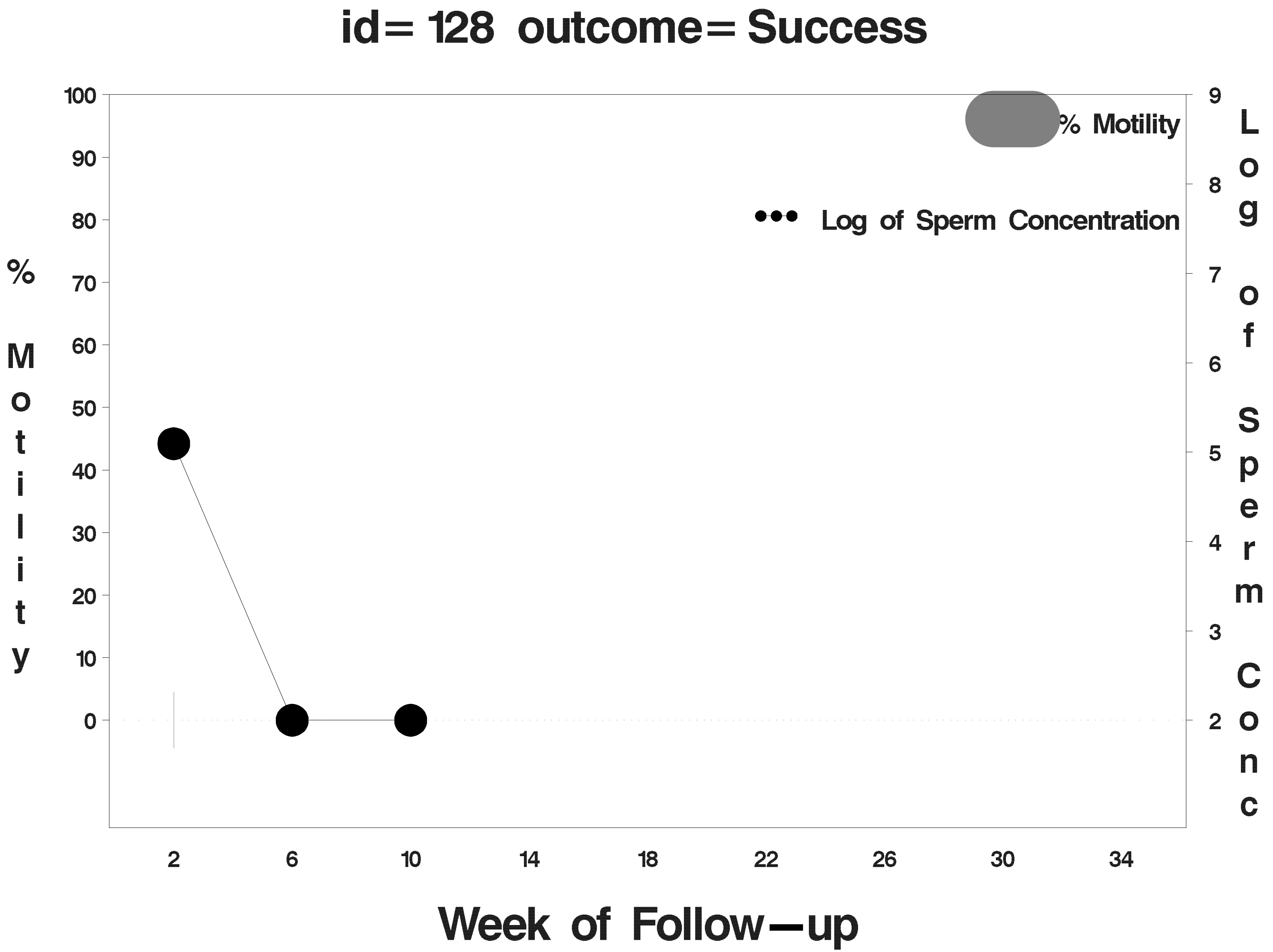

id= 128 outcome= Success
% Motility
Log of Sperm Concentration
100
90
80
70
60
50
40
30
20
10
0
9
8
7
6
5
4
3
2
2
6
10
14
18
22
26
30
34
Week of Follow—up
% Motility
Log of Sperm Conc

id= 129 outcome= Success

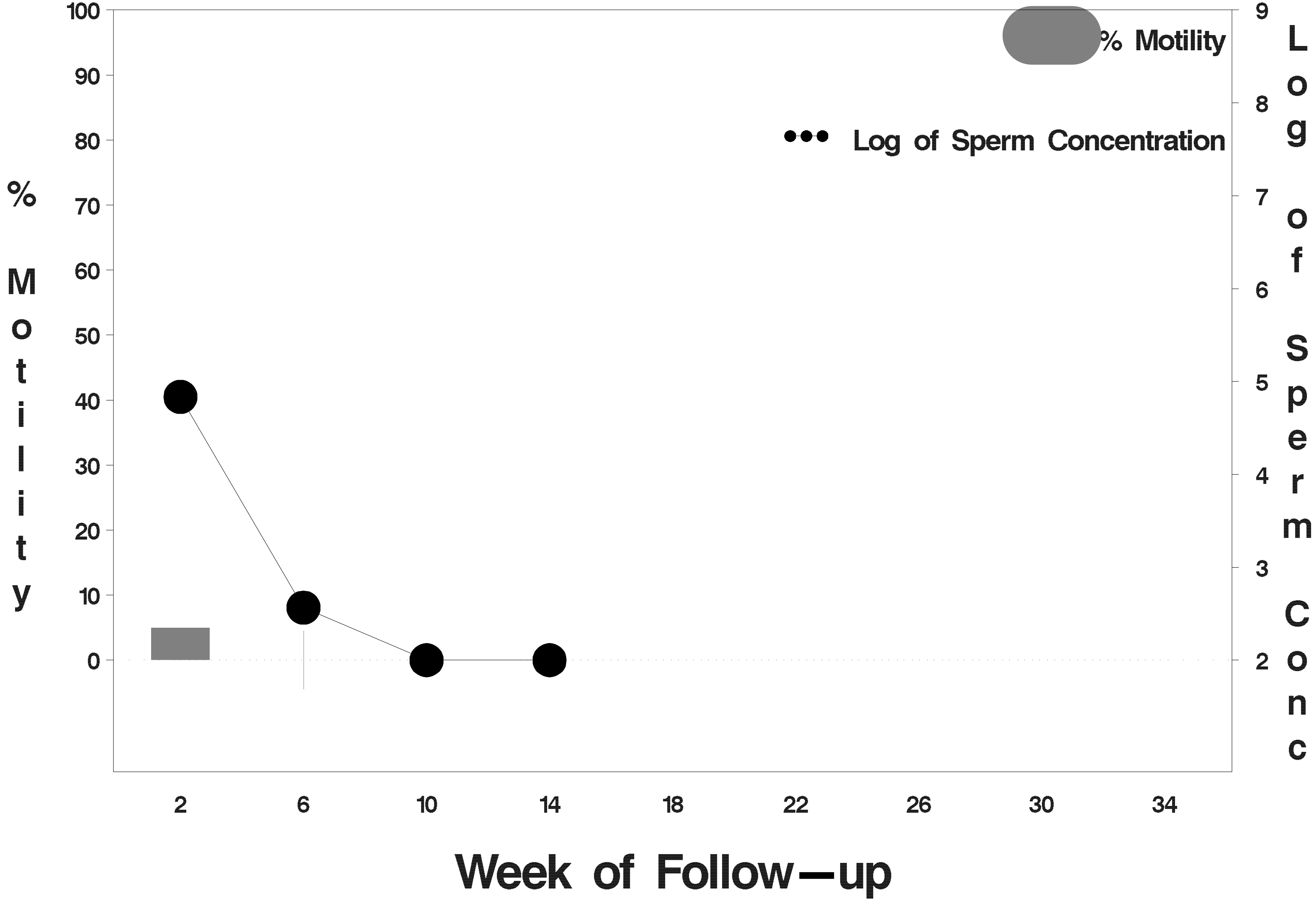

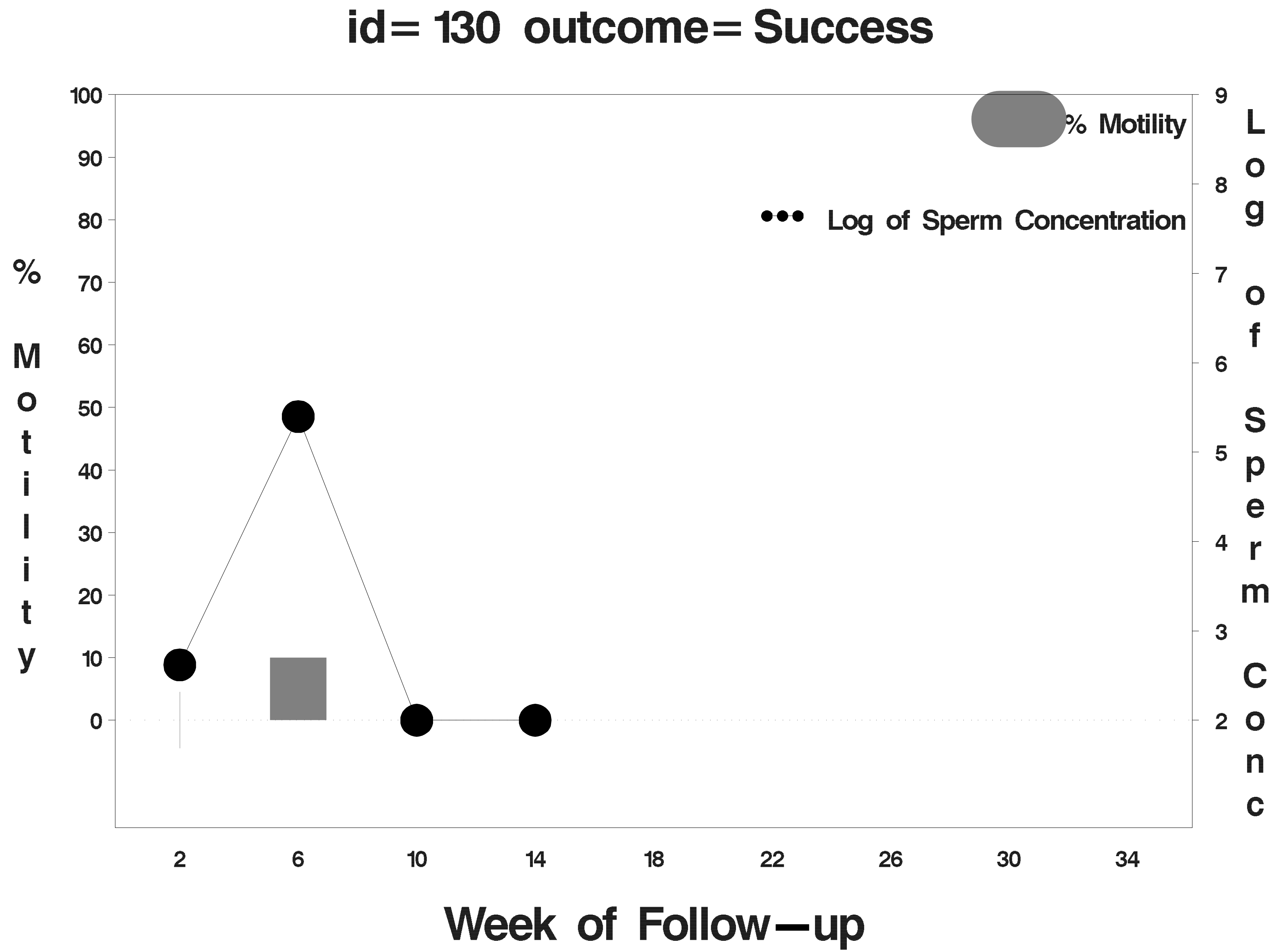

id= 130 outcome= Success
% Motility
Log of Sperm Concentration
100
90
80
70
60
50
40
30
20
10
0
9
8
7
6
5
4
3
2
% Motility
Log of Sperm Conc
2
6
10
14
18
22
26
30
34
Week of Follow—up

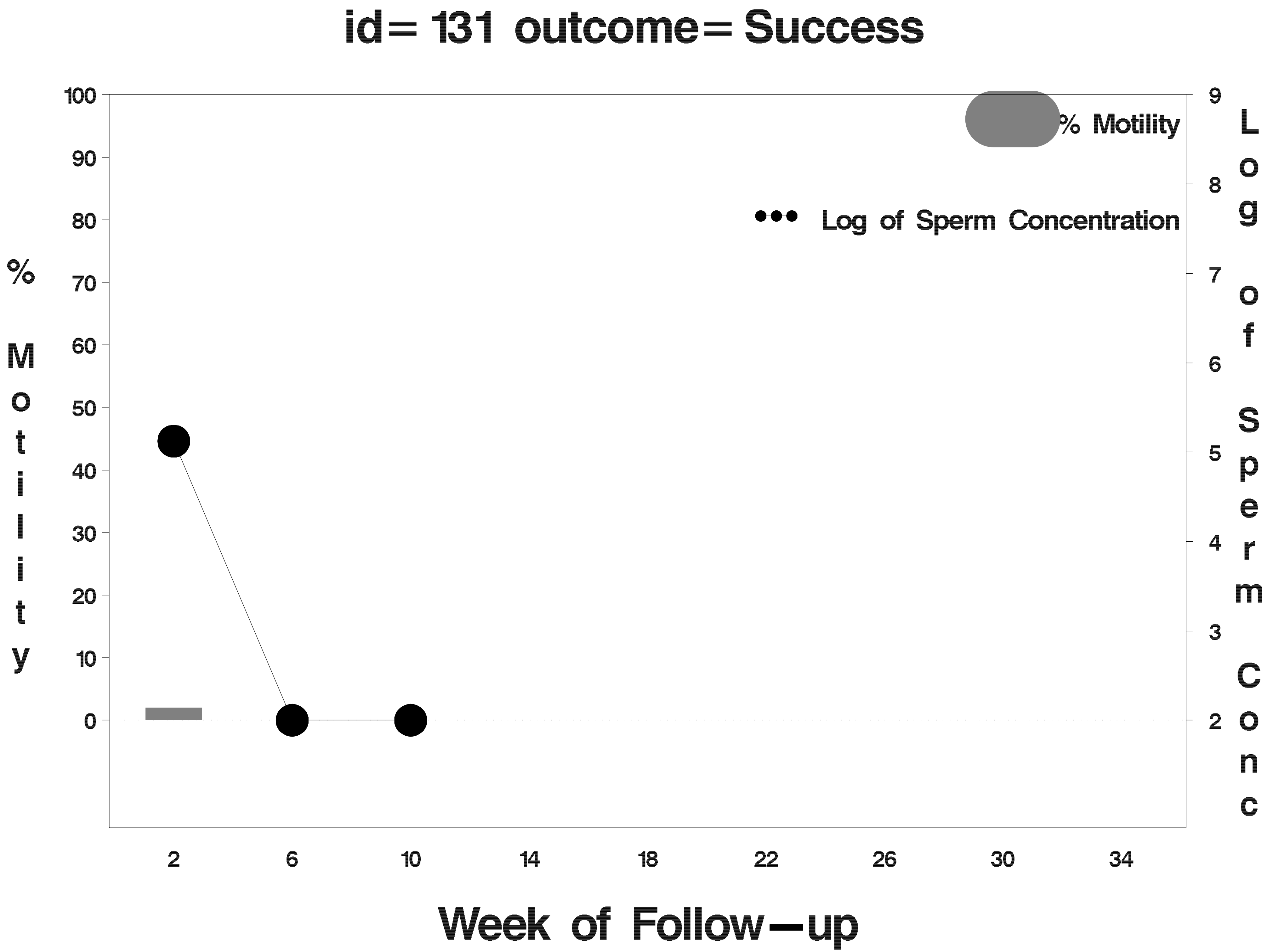

id= 131 outcome= Success
% Motility
Log of Sperm Concentration
% Motility
Log of Sperm Conc
Week of Follow−up
100
90
80
70
60
50
40
30
20
10
0
9
8
7
6
5
4
3
2
2
6
10
14
18
22
26
30
34

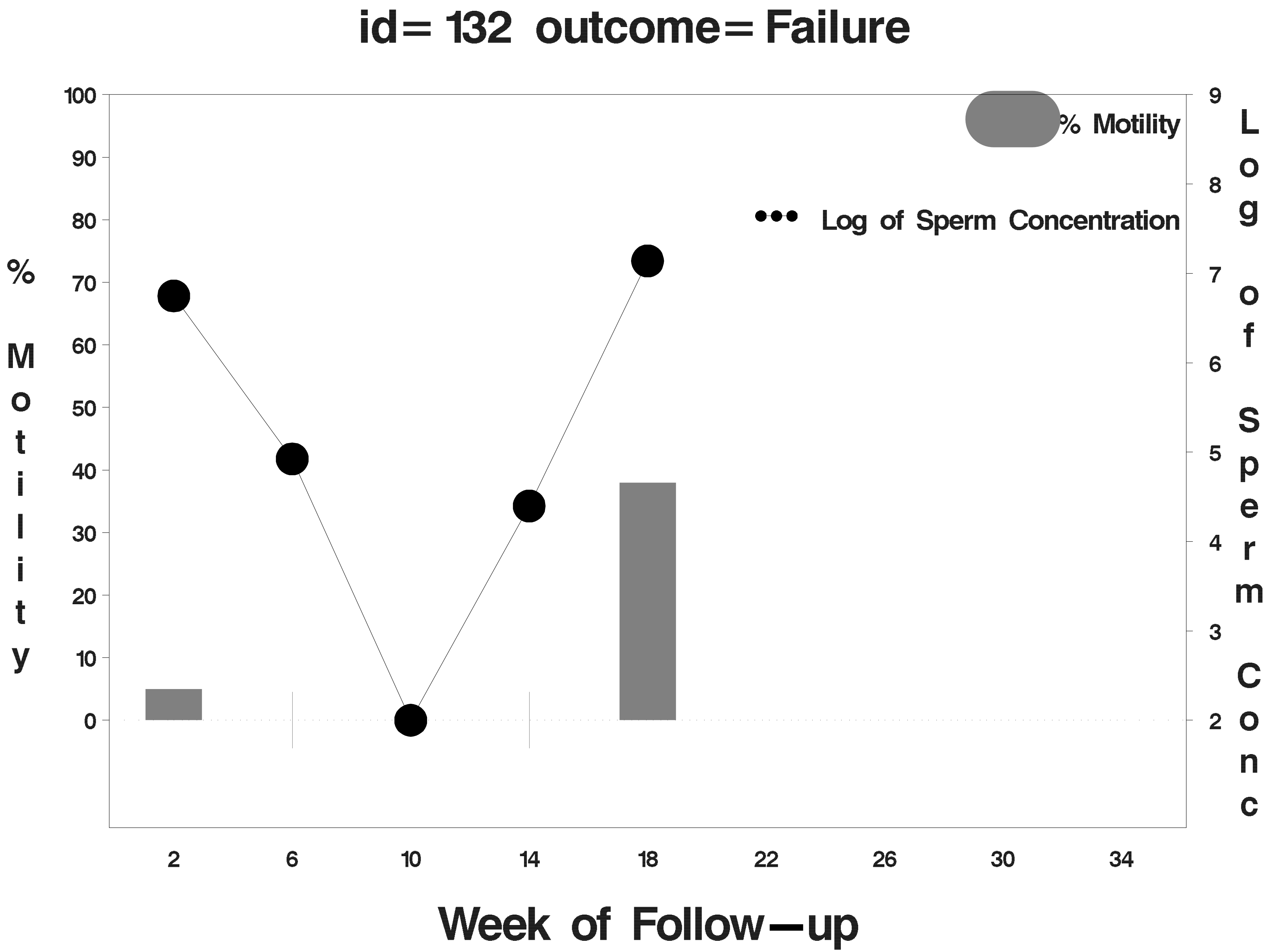

id= 132 outcome= Failure
% Motility
Log of Sperm Concentration
100
90
80
70
60
50
40
30
20
10
0
9
8
7
6
5
4
3
2
2
6
10
14
18
22
26
30
34
Week of Follow—up
Log of Sperm Conc

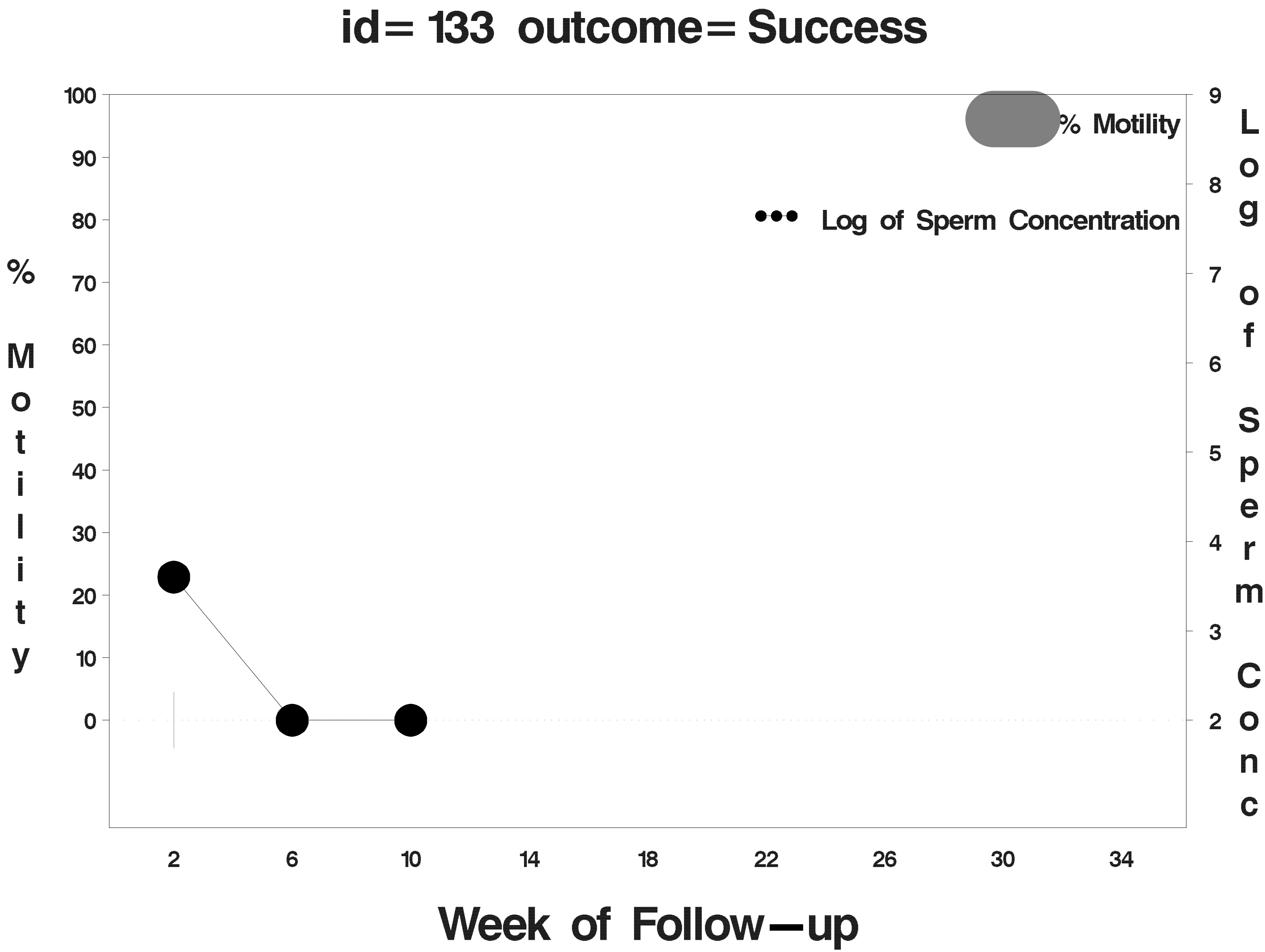

id= 133 outcome= Success
% Motility
Log of Sperm Concentration
100
90
80
70
60
50
40
30
20
10
0
9
8
7
6
5
4
3
2
2
6
10
14
18
22
26
30
34
Week of Follow−up
Log of Sperm Conc

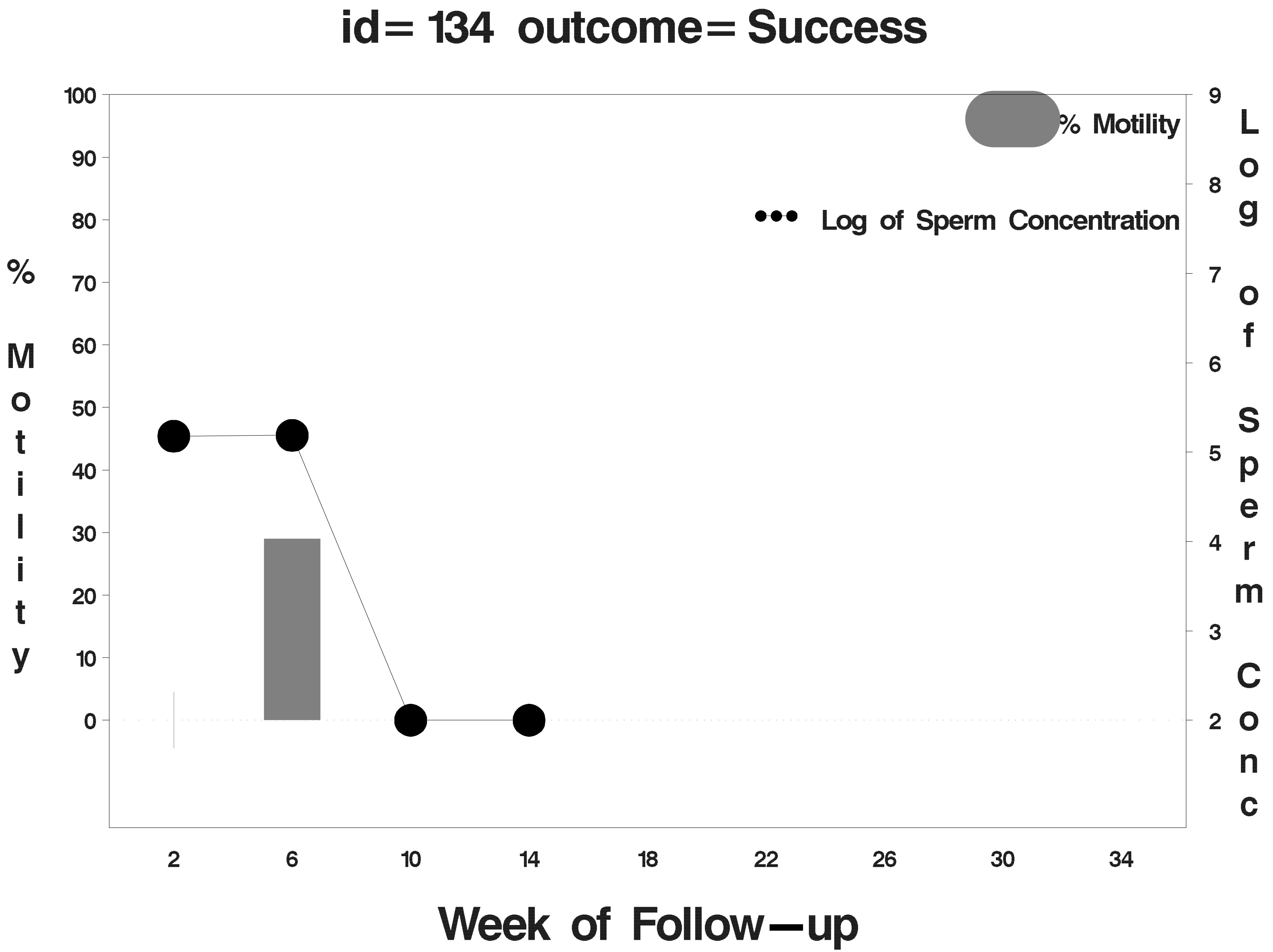

id= 134 outcome= Success
% Motility
Log of Sperm Concentration
100
90
80
70
60
50
40
30
20
10
0
9
8
7
6
5
4
3
2
2
6
10
14
18
22
26
30
34
Week of Follow—up
Log of Sperm Conc

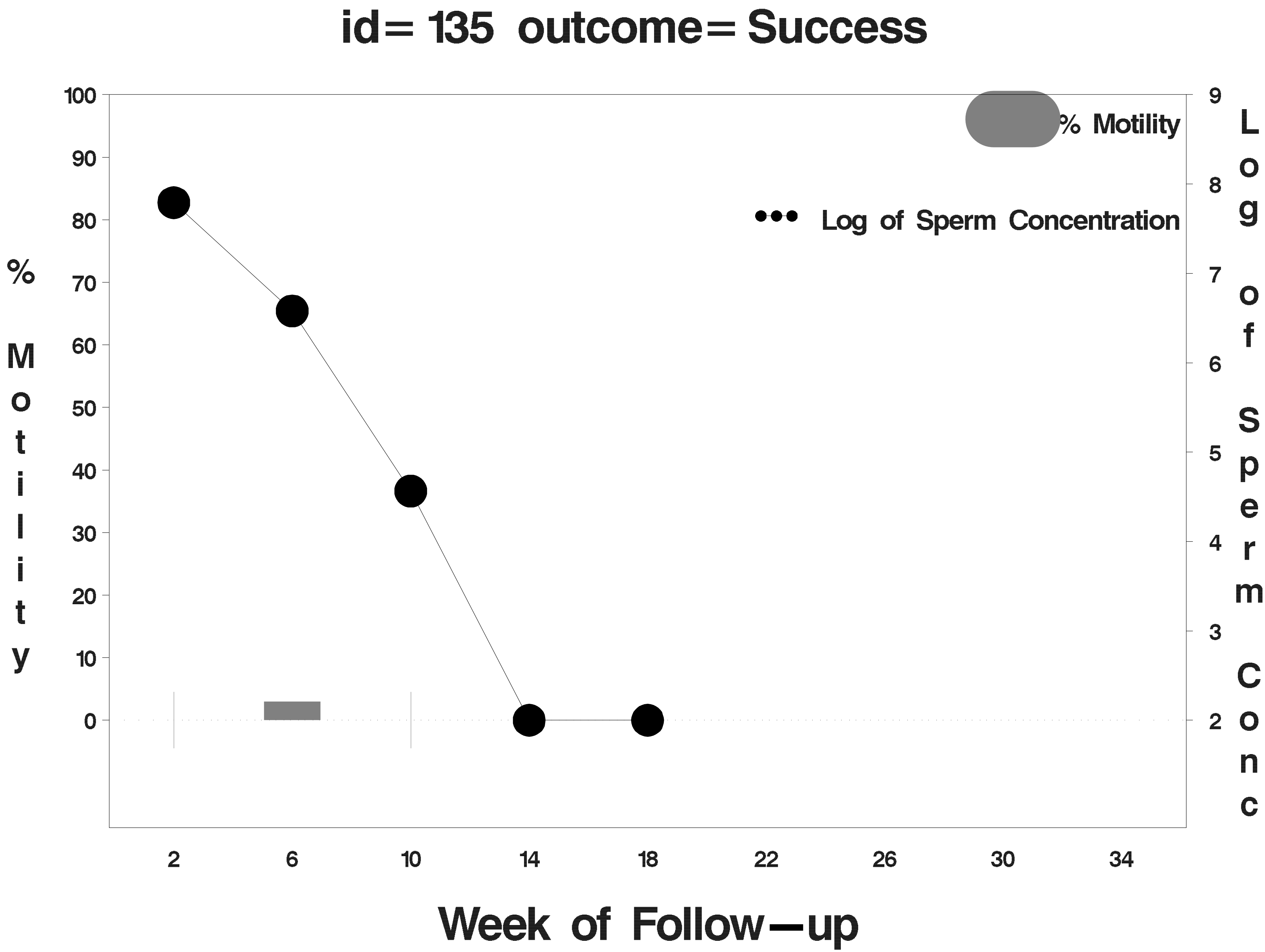
id= 135 outcome= Success
% Motility
Log of Sperm Concentration
100
90
80
70
60
50
40
30
20
10
0
9
8
7
6
5
4
3
2
% Motility
Log of Sperm Conc
2
6
10
14
18
22
26
30
34
Week of Follow—up

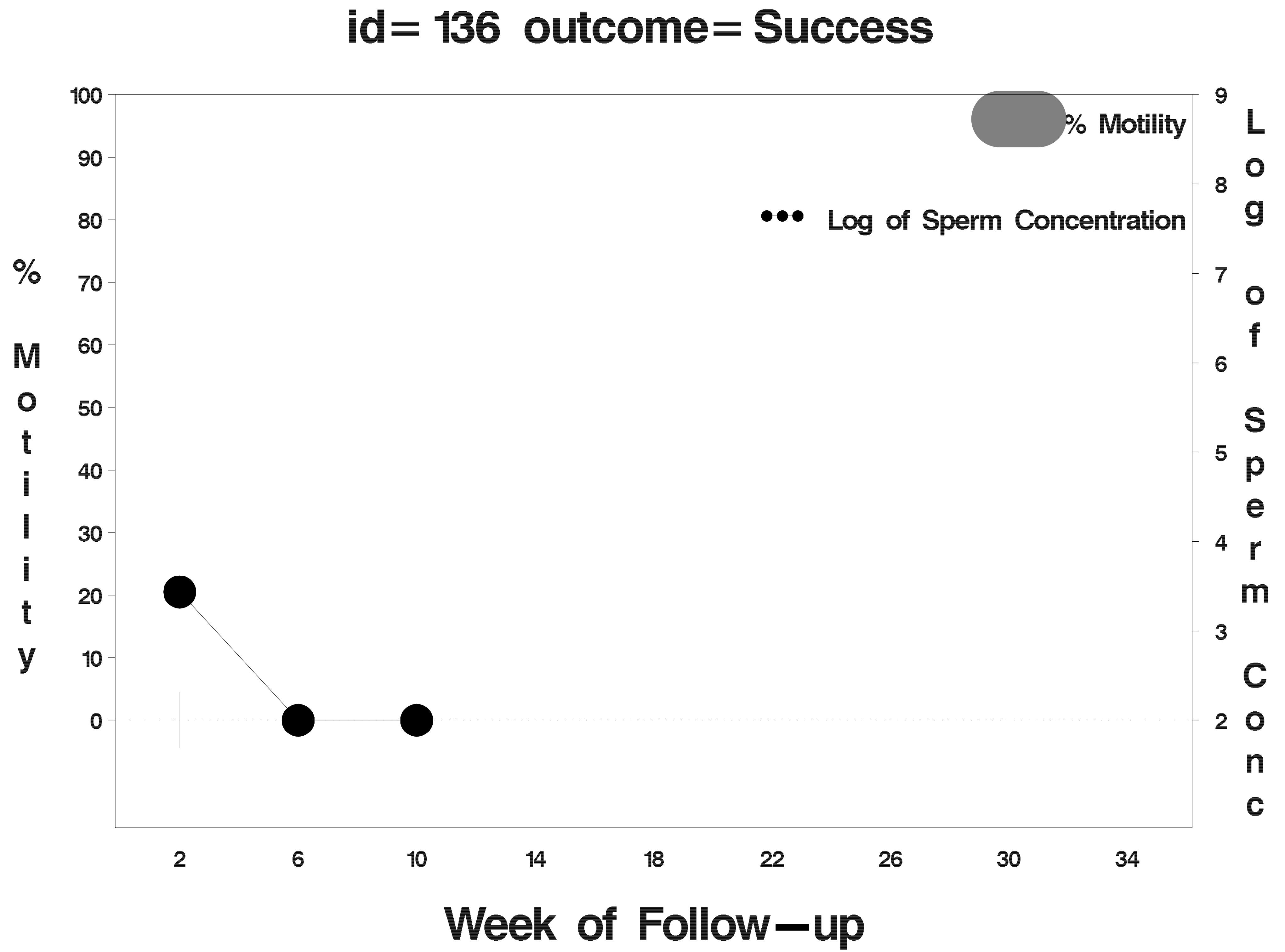
id= 136 outcome= Success
% Motility
Log of Sperm Concentration
100
90
80
70
60
50
40
30
20
10
0
9
8
7
6
5
4
3
2
2
6
10
14
18
22
26
30
34
Week of Follow—up
Log of Sperm Conc

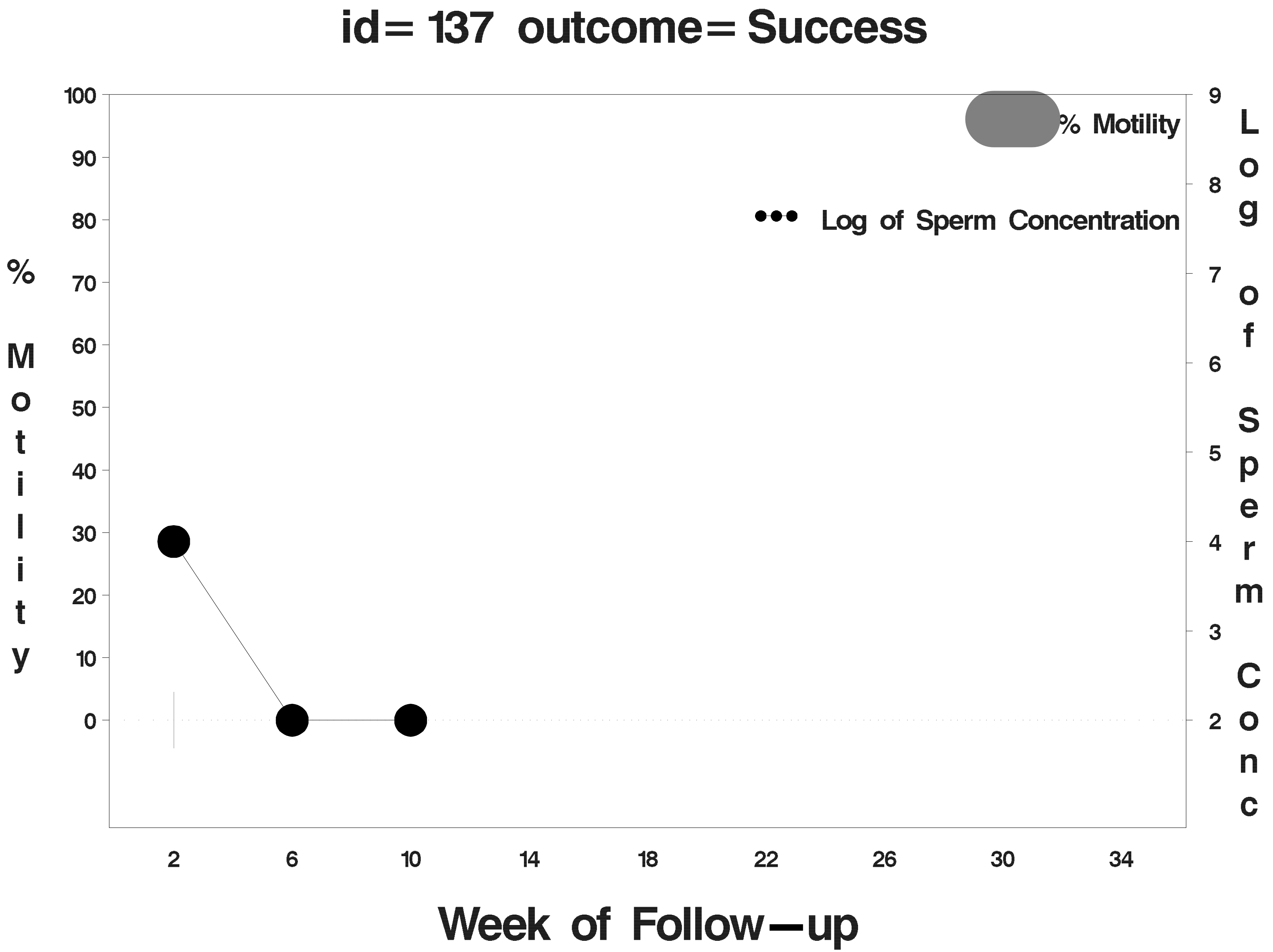

id= 137 outcome= Success
% Motility
Log of Sperm Concentration
100
90
80
70
60
50
40
30
20
10
0
9
8
7
6
5
4
3
2
% Motility
Log of Sperm Conc
2
6
10
14
18
22
26
30
34
Week of Follow—up

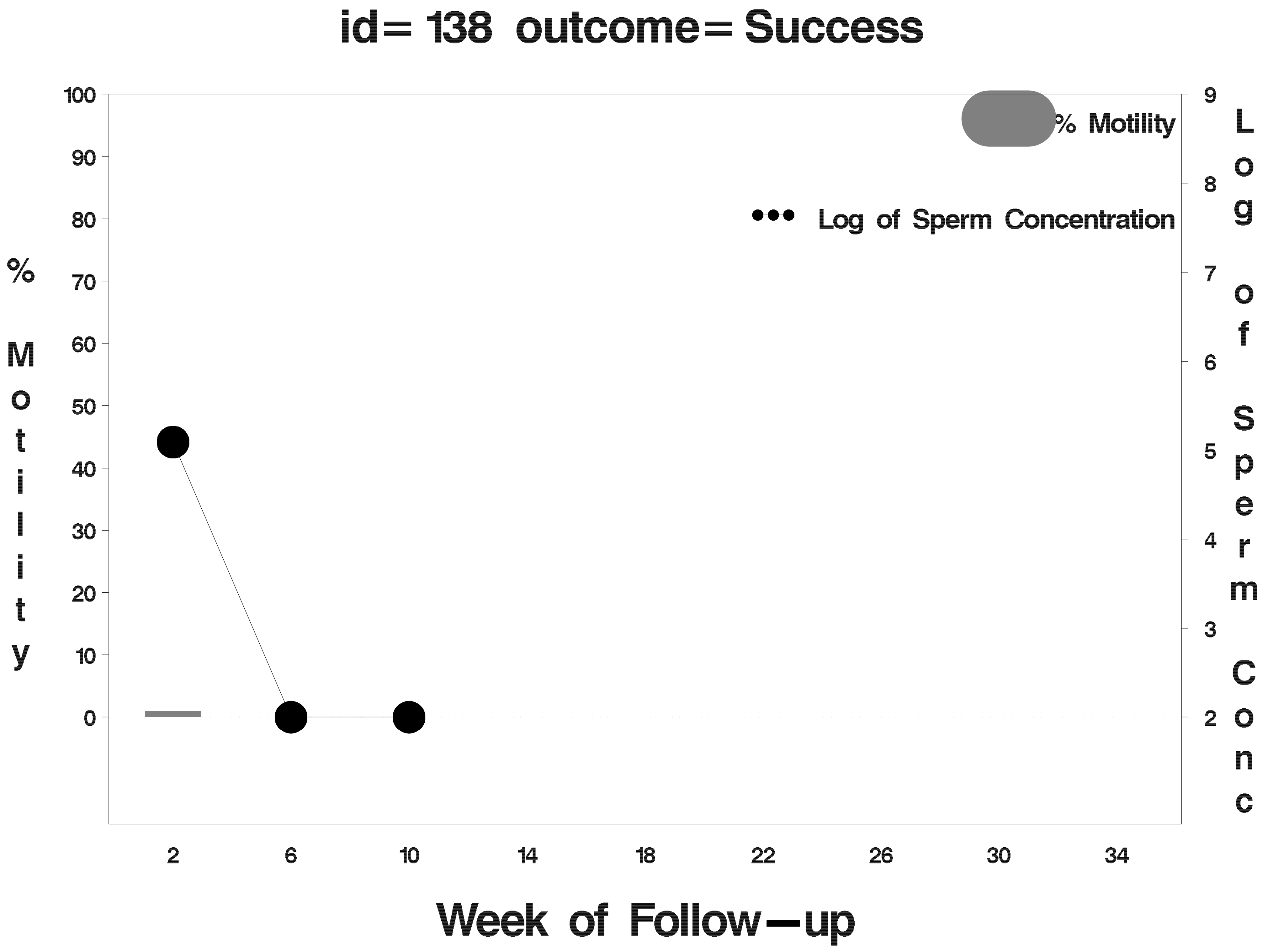

id= 138 outcome= Success
% Motility
Log of Sperm Concentration
% Motility
Log of Sperm Conc
Week of Follow—up
100
90
80
70
60
50
40
30
20
10
0
9
8
7
6
5
4
3
2
2
6
10
14
18
22
26
30
34

id= 139 outcome= Indeterminate

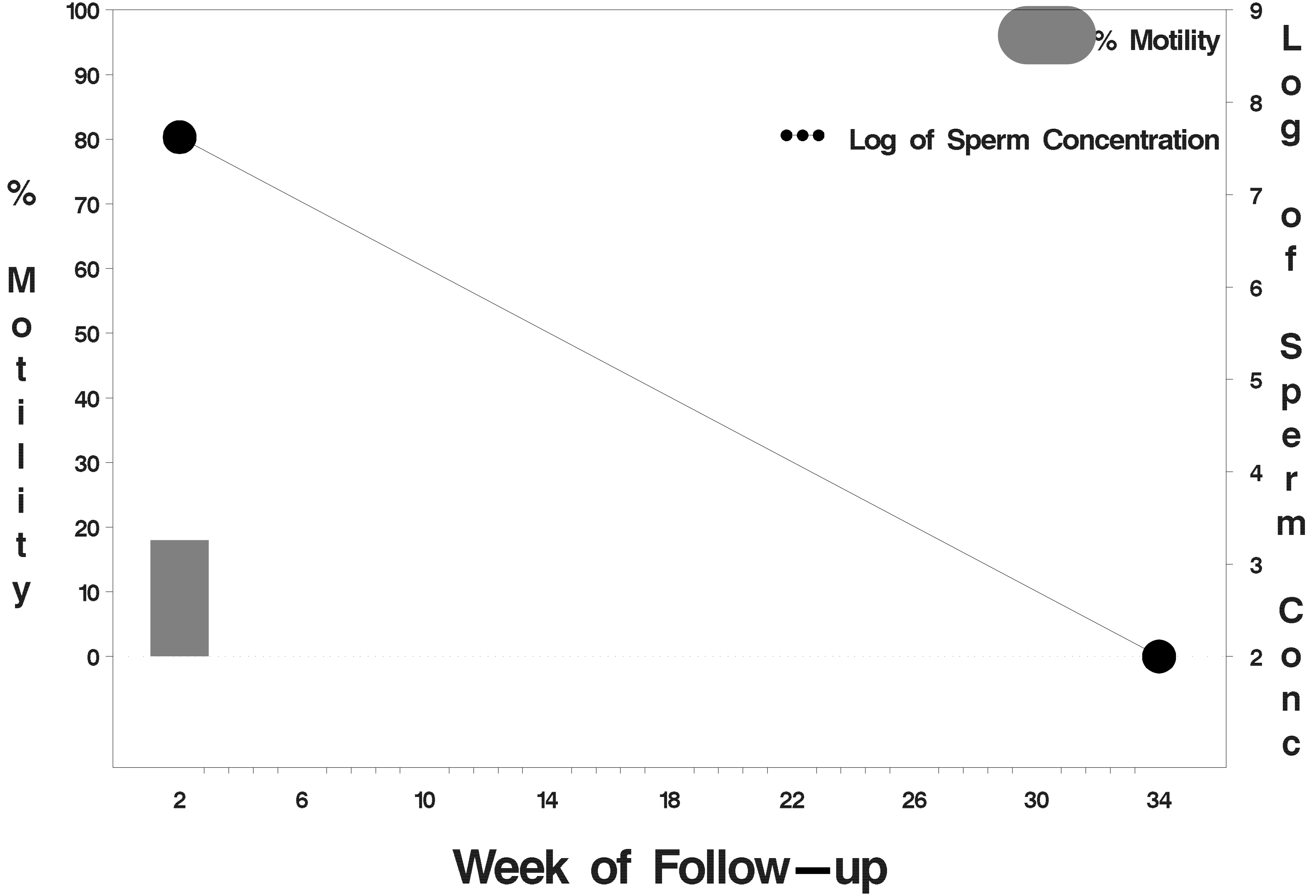

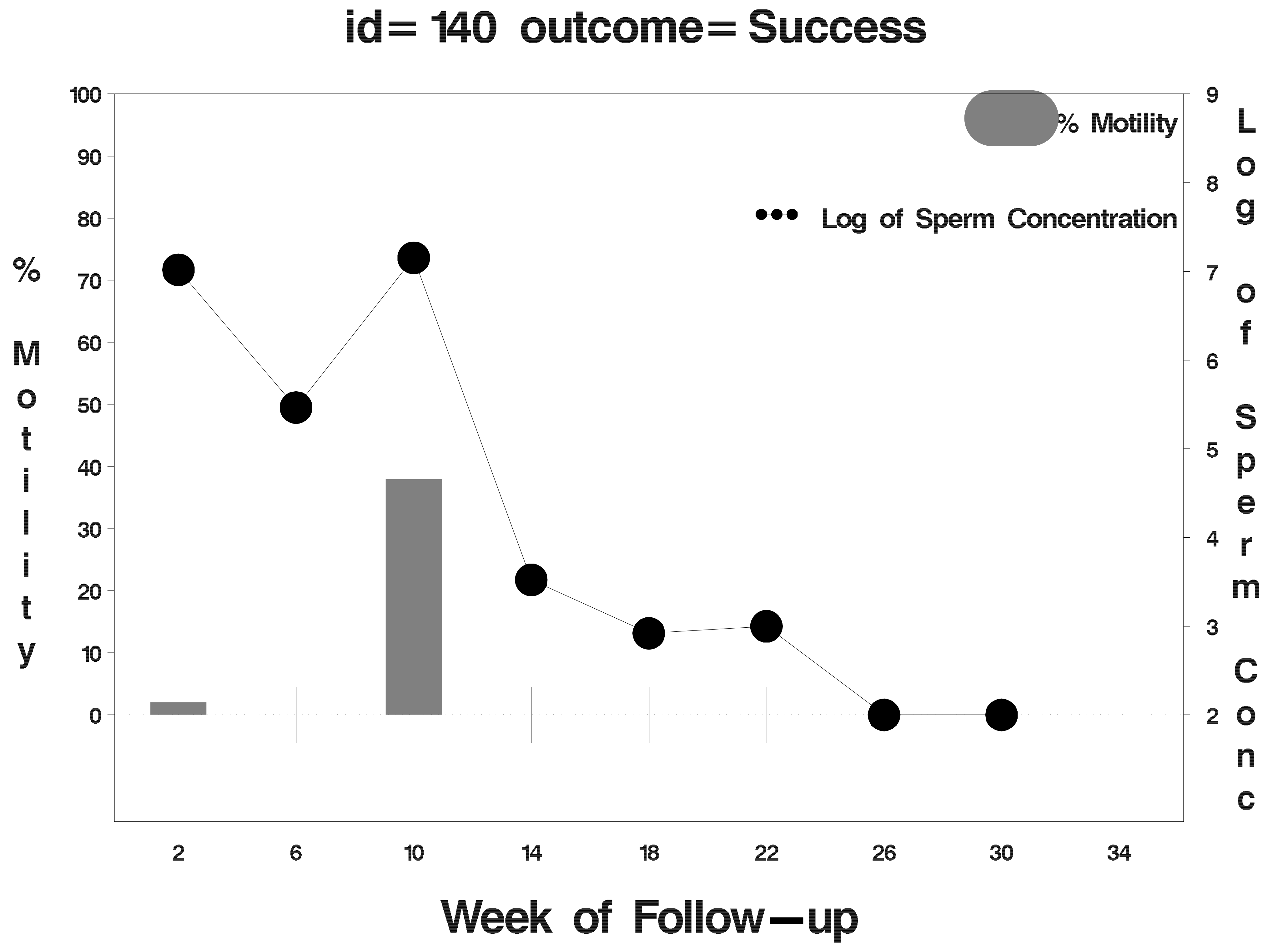
id= 140 outcome= Success
% Motility
Log of Sperm Concentration
100
90
80
70
60
50
40
30
20
10
0
9
8
7
6
5
4
3
2
% Motility
Log of Sperm Conc
2
6
10
14
18
22
26
30
34
Week of Follow−up

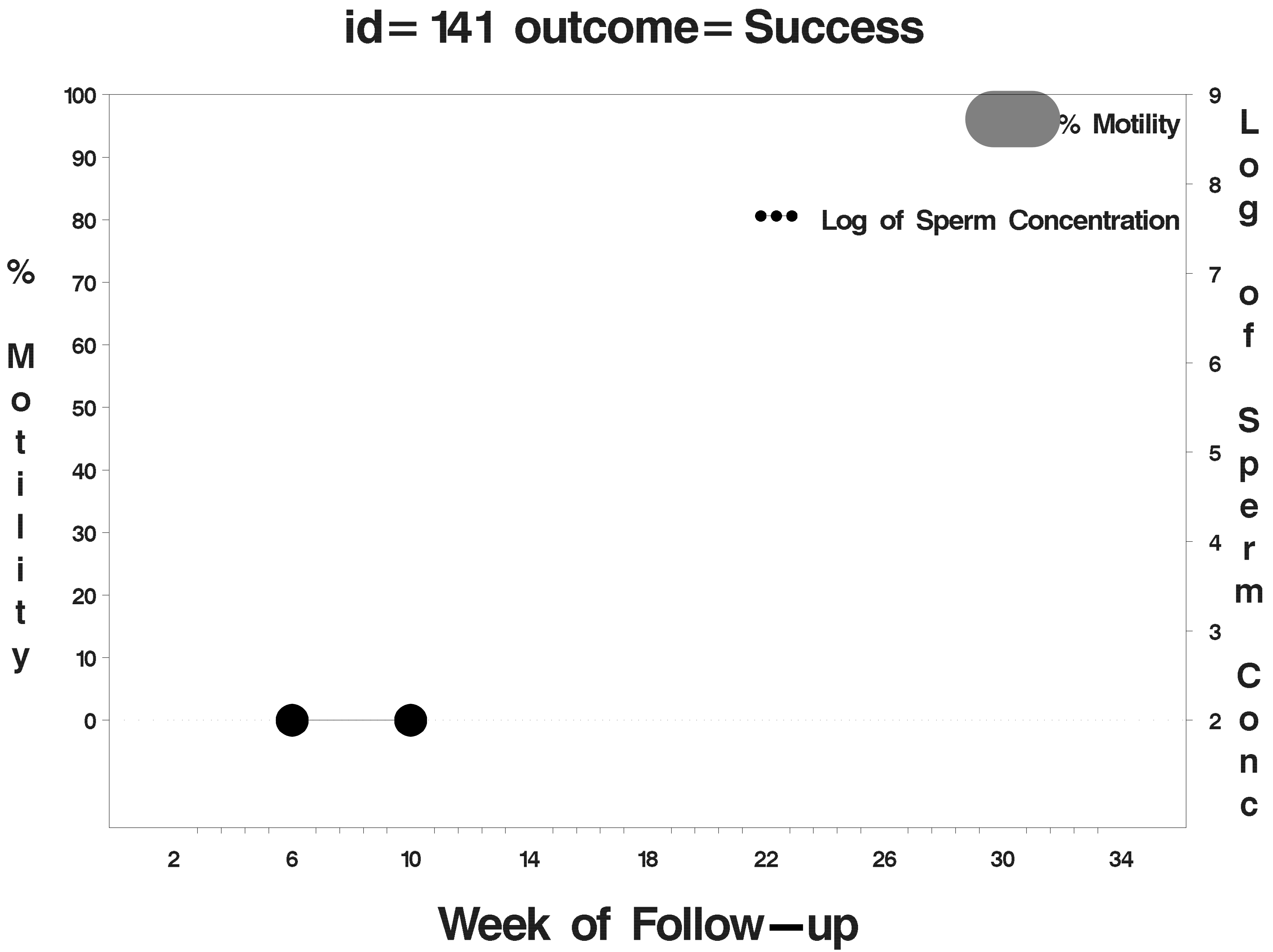
id= 141 outcome= Success
% Motility
Log of Sperm Concentration
% Motility
Log of Sperm Conc
Week of Follow-up
0
10
20
30
40
50
60
70
80
90
100
2
3
4
5
6
7
8
9
2
6
10
14
18
22
26
30
34

id= 142 outcome= Success

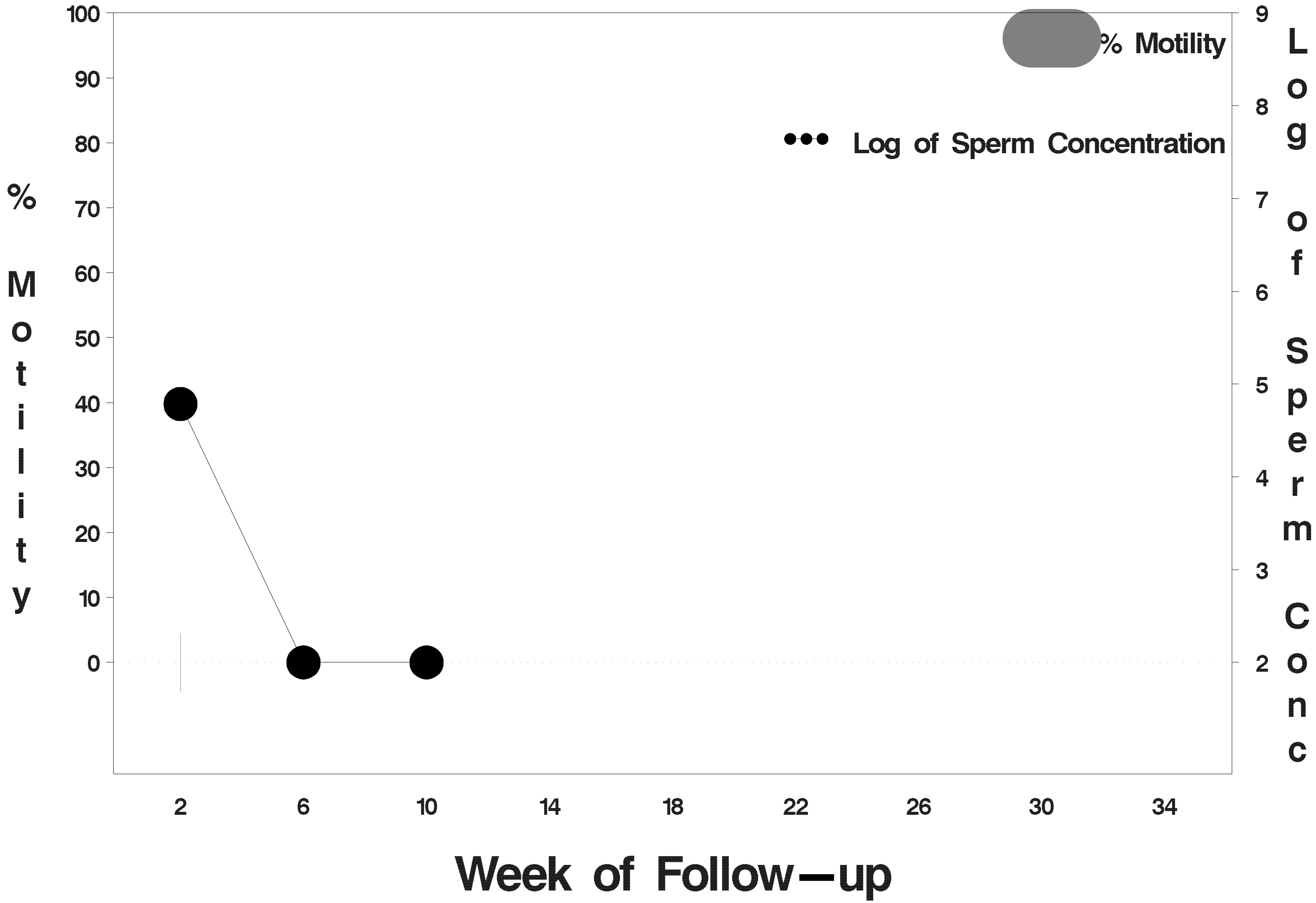

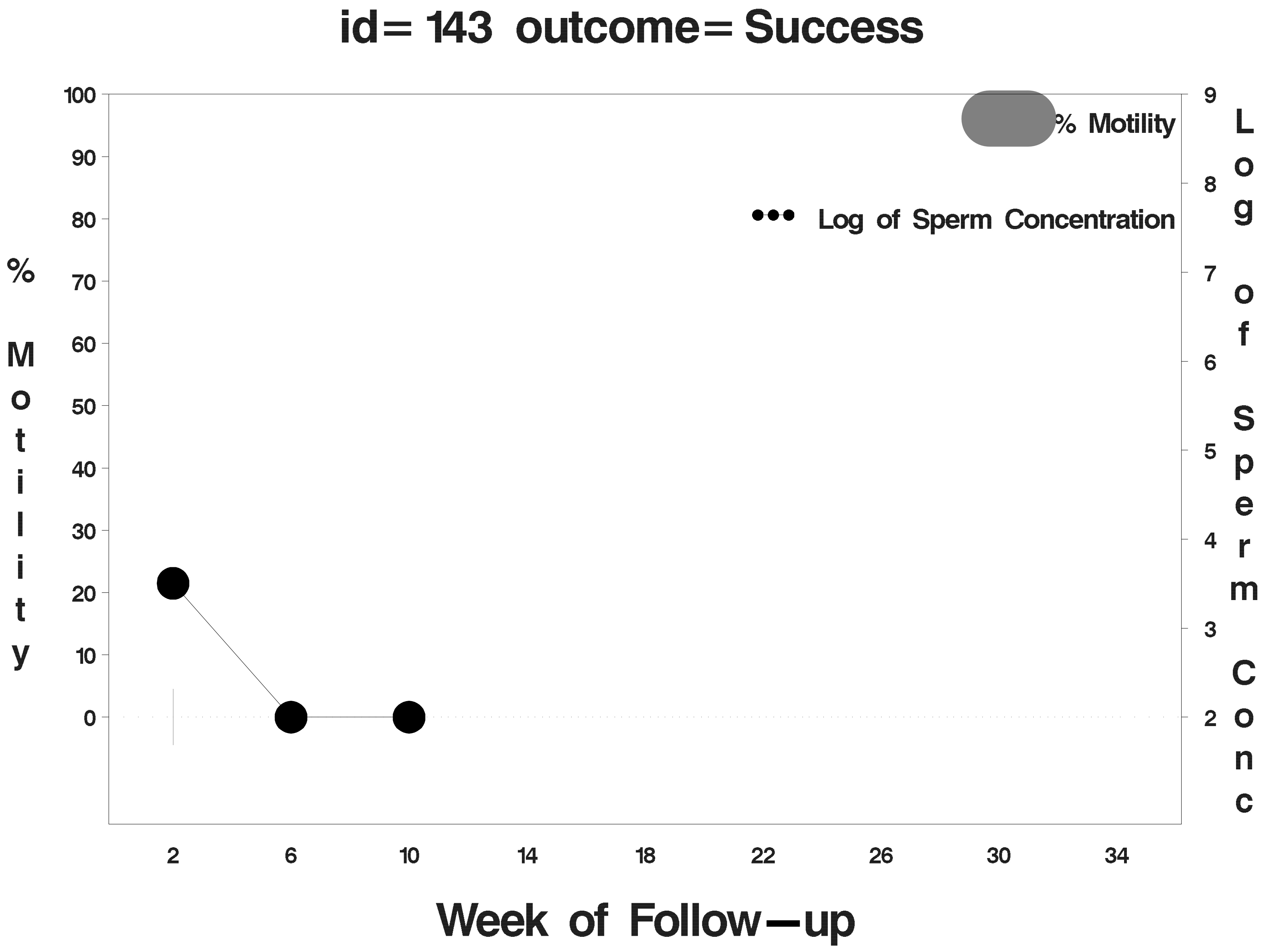
id= 143 outcome= Success
% Motility
Log of Sperm Concentration
100
90
80
70
60
50
40
30
20
10
0
9
8
7
6
5
4
3
2
2
6
10
14
18
22
26
30
34
Week of Follow—up
% Motility
Log of Sperm Conc

id= 144 outcome= Success

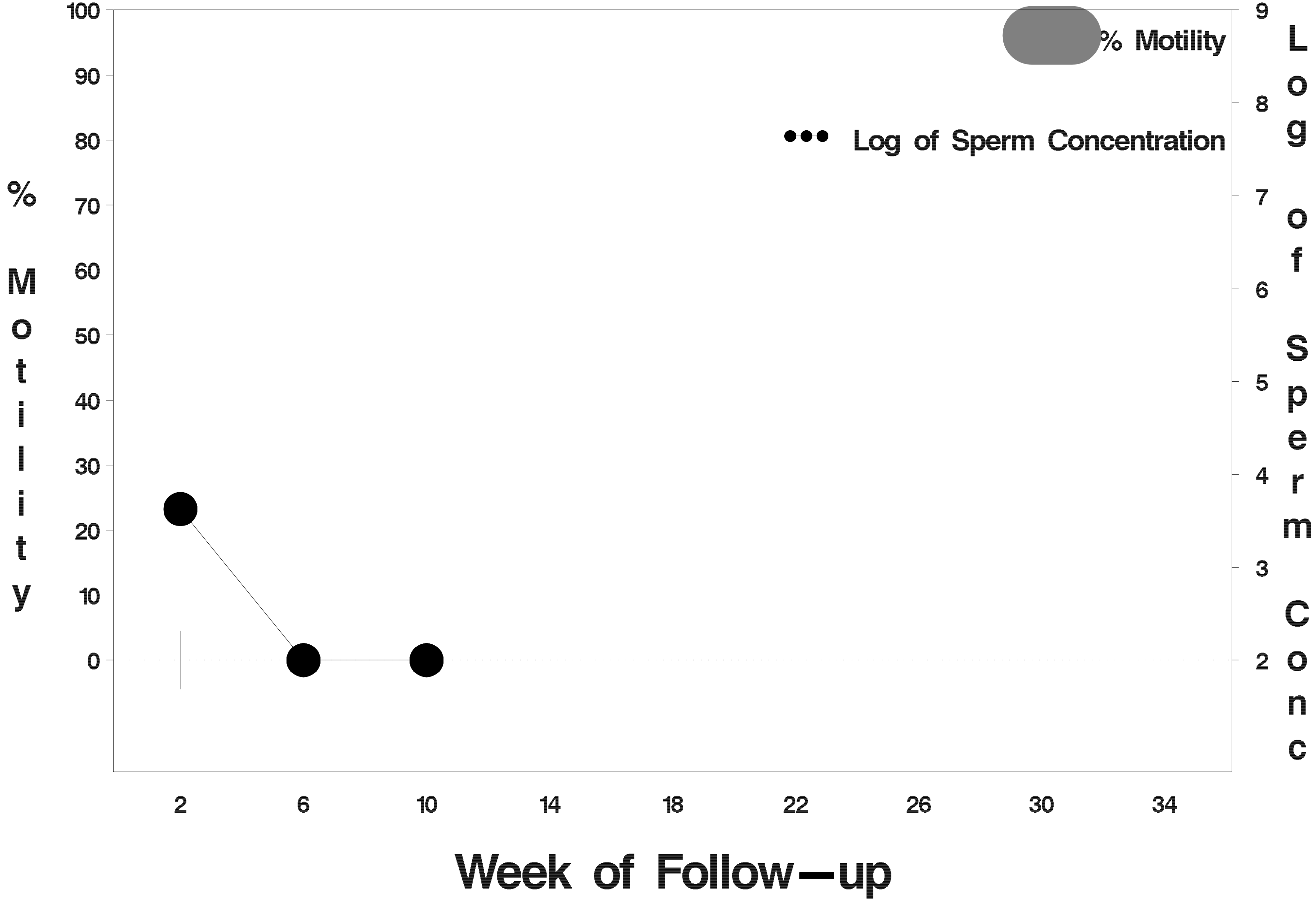

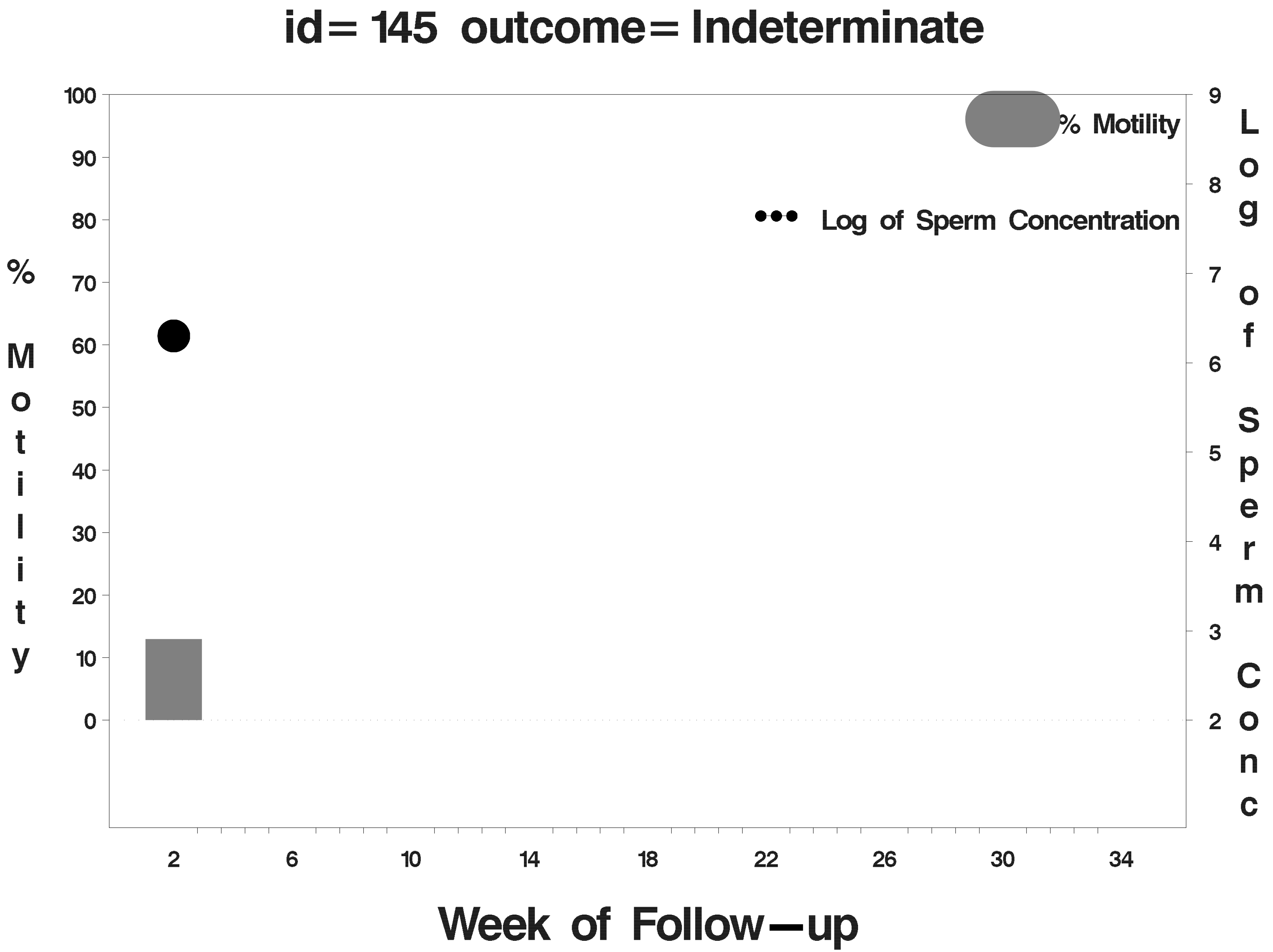
id= 145 outcome= Indeterminate
% Motility
Log of Sperm Concentration
100
90
80
70
60
50
40
30
20
10
0
9
8
7
6
5
4
3
2
2
6
10
14
18
22
26
30
34
Week of Follow—up
Log of Sperm Conc

id= 146 outcome= Indeterminate

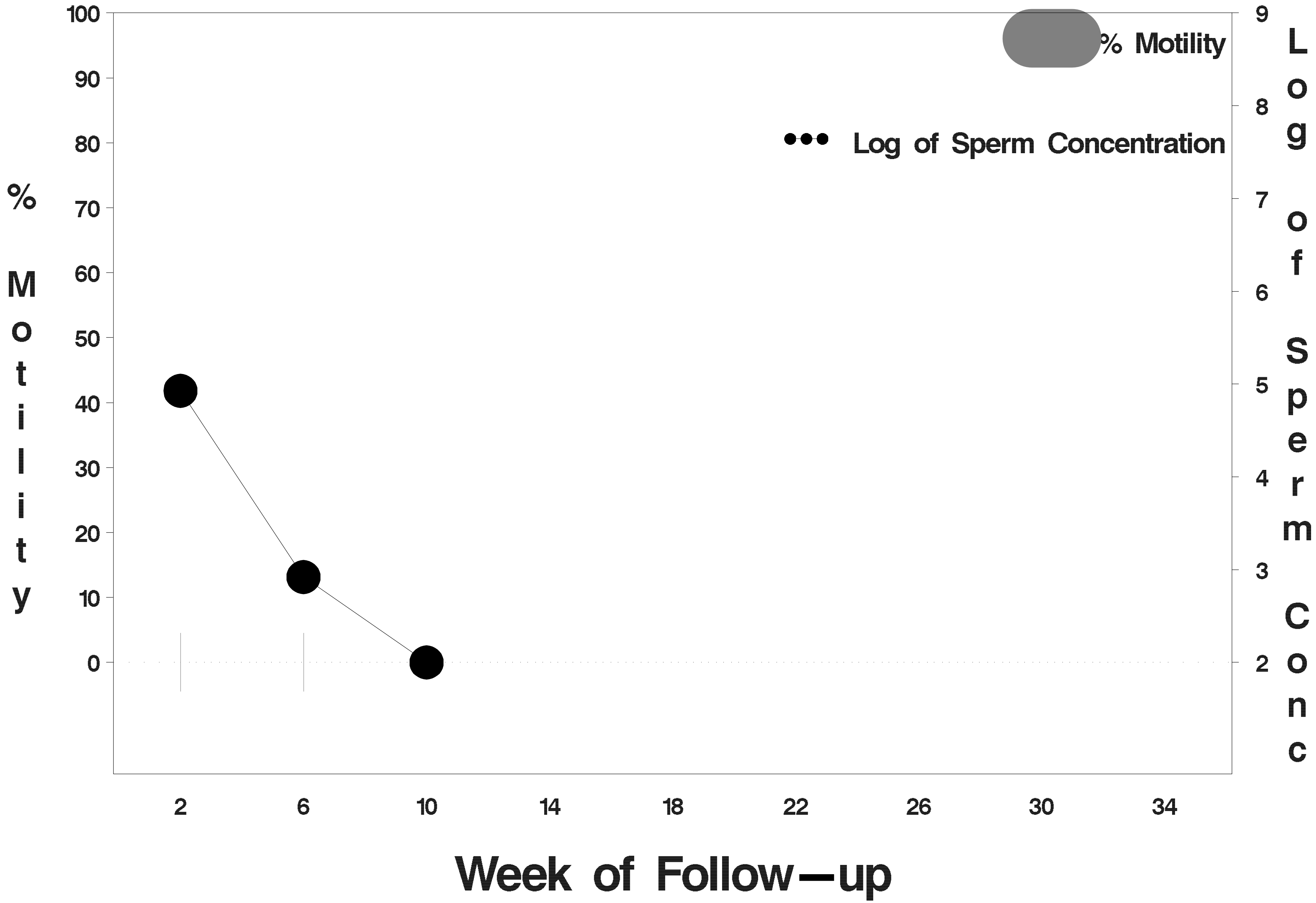

id= 147 outcome= Success

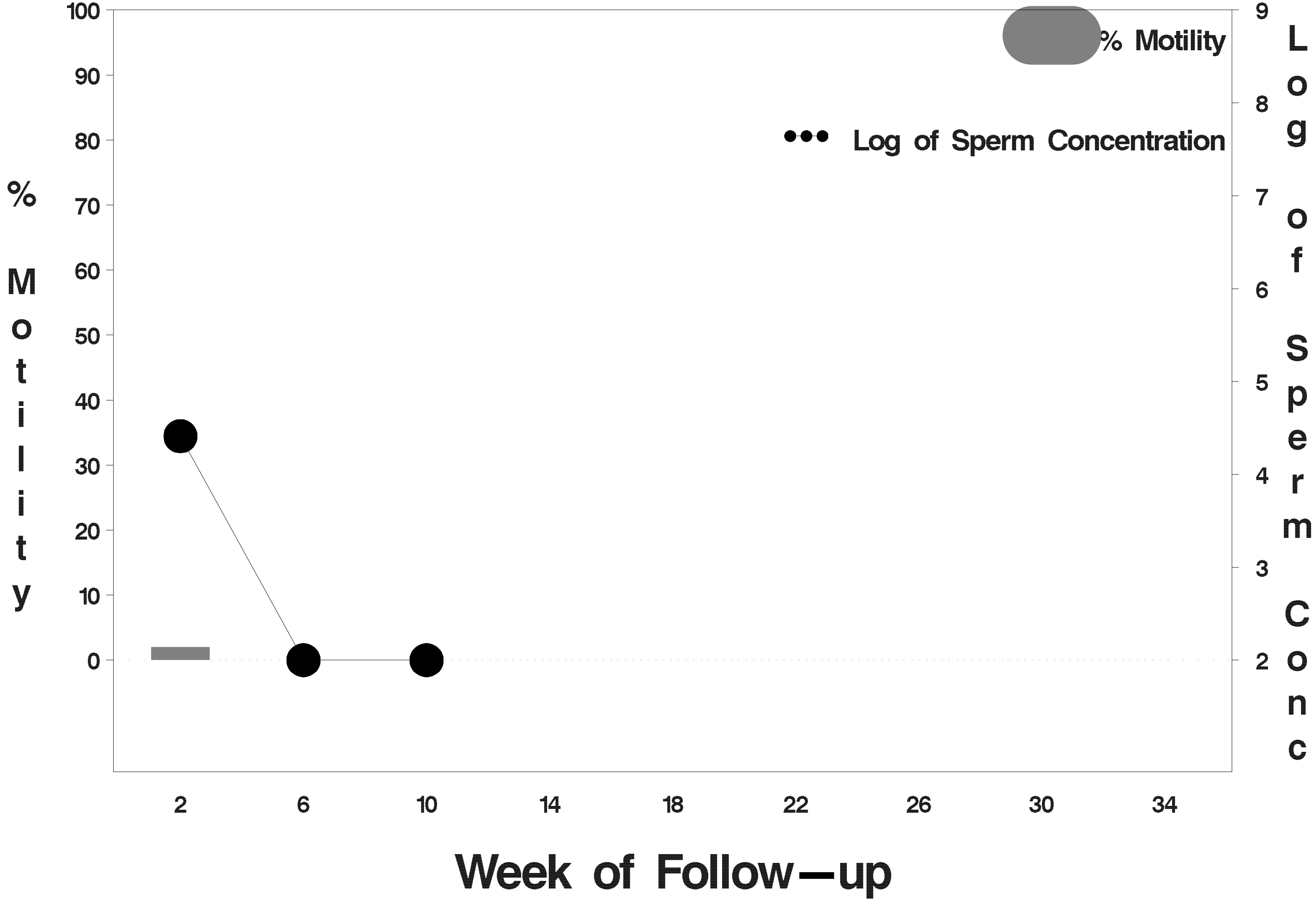

id= 148 outcome= Success

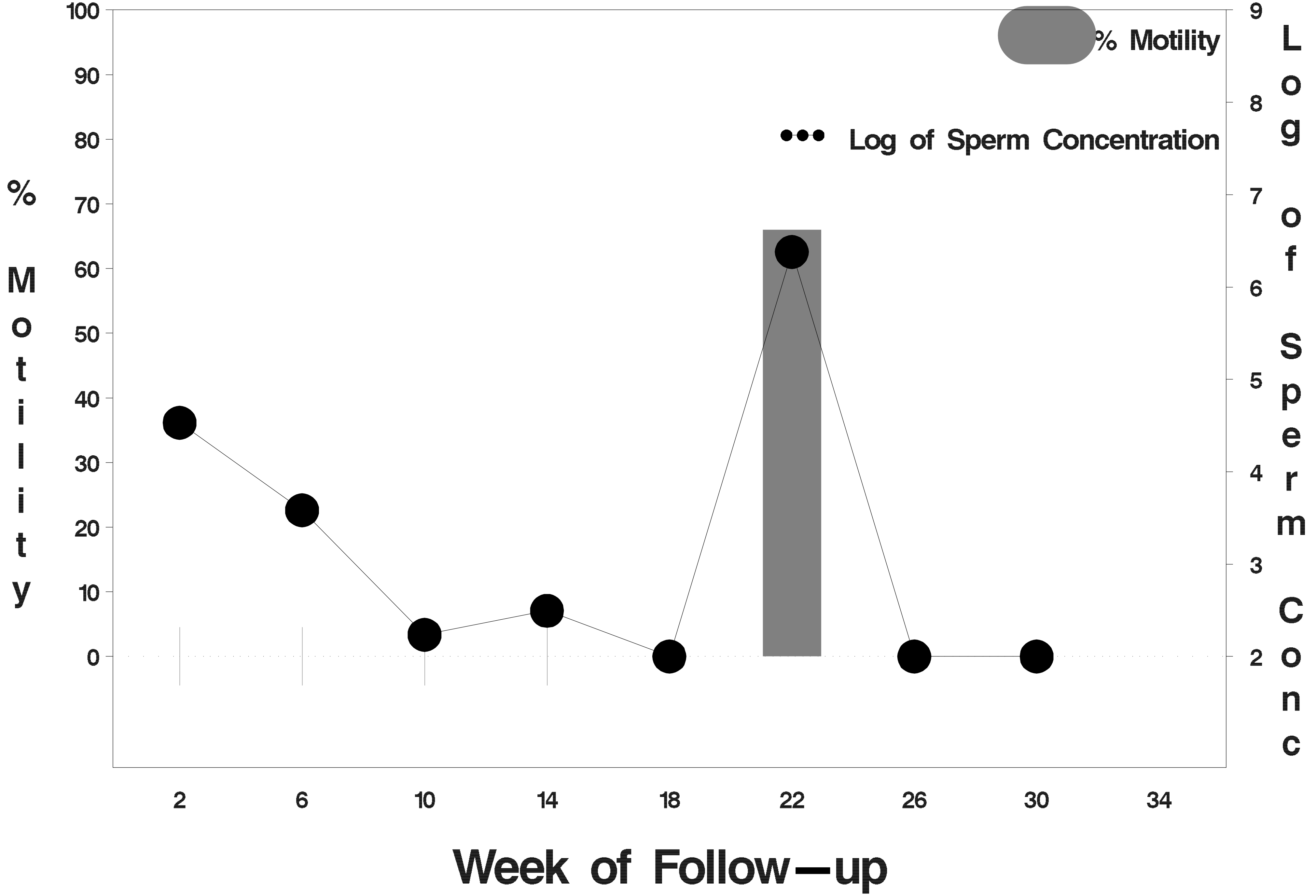

id= 149 outcome= Success

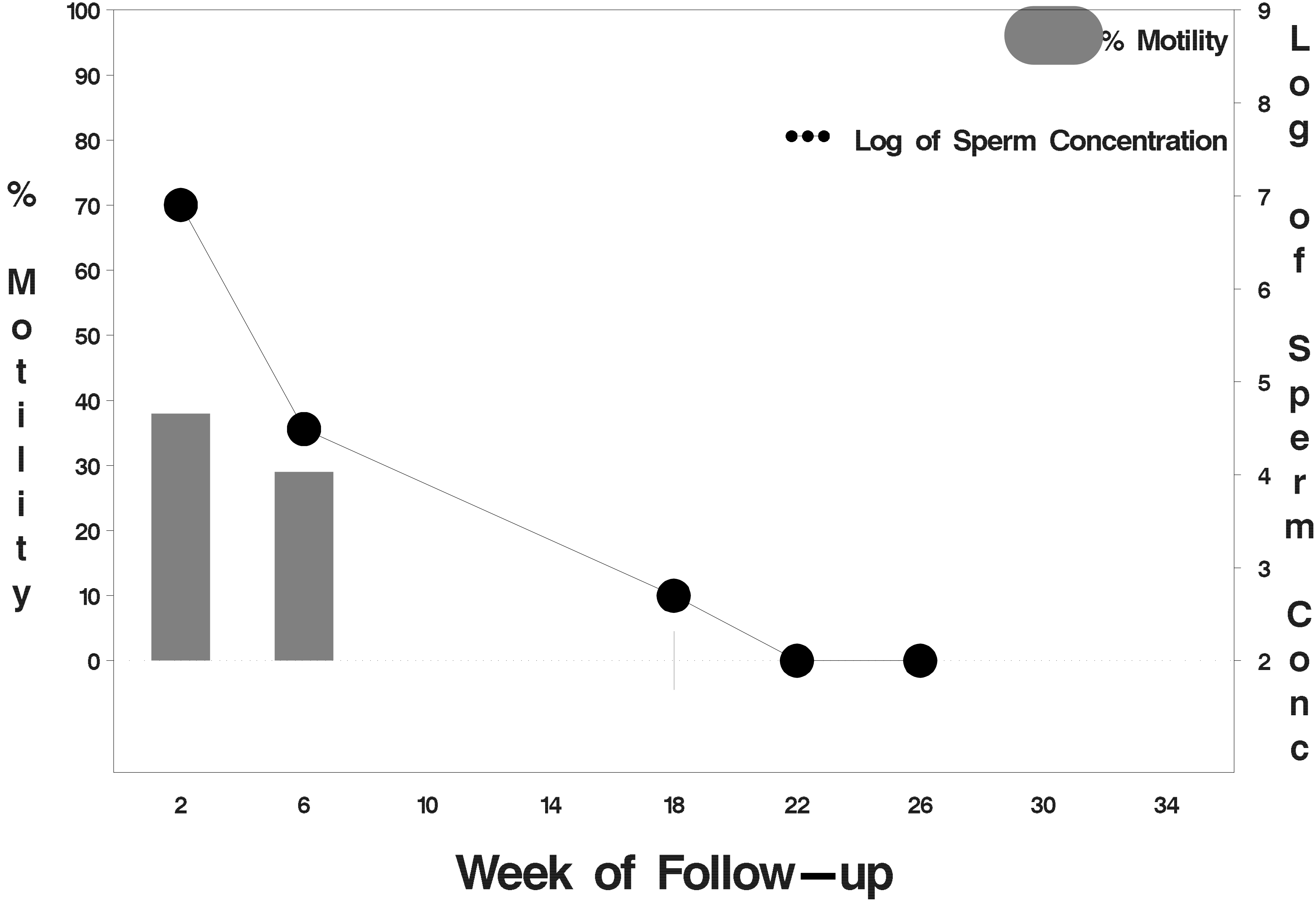

id= 150 outcome= Success

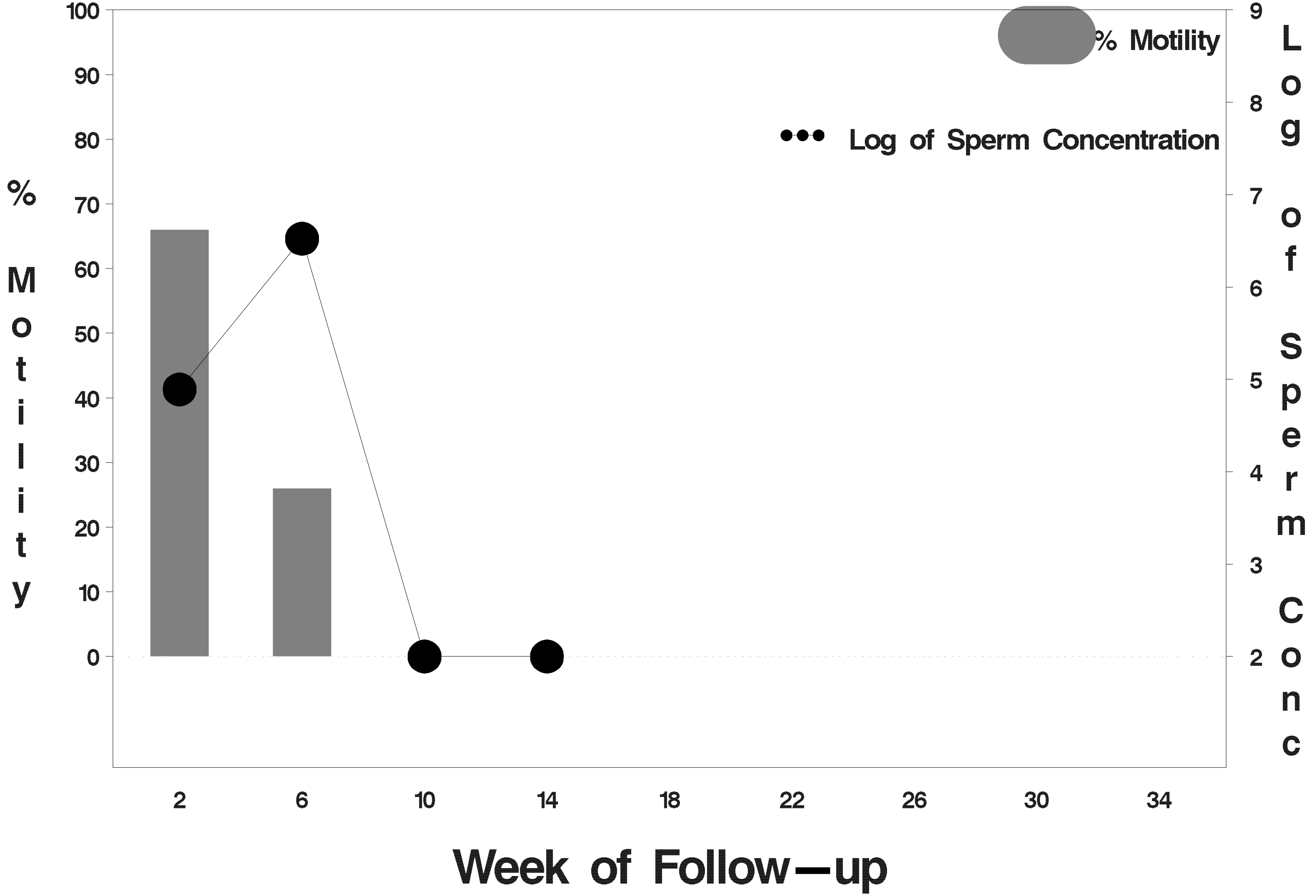

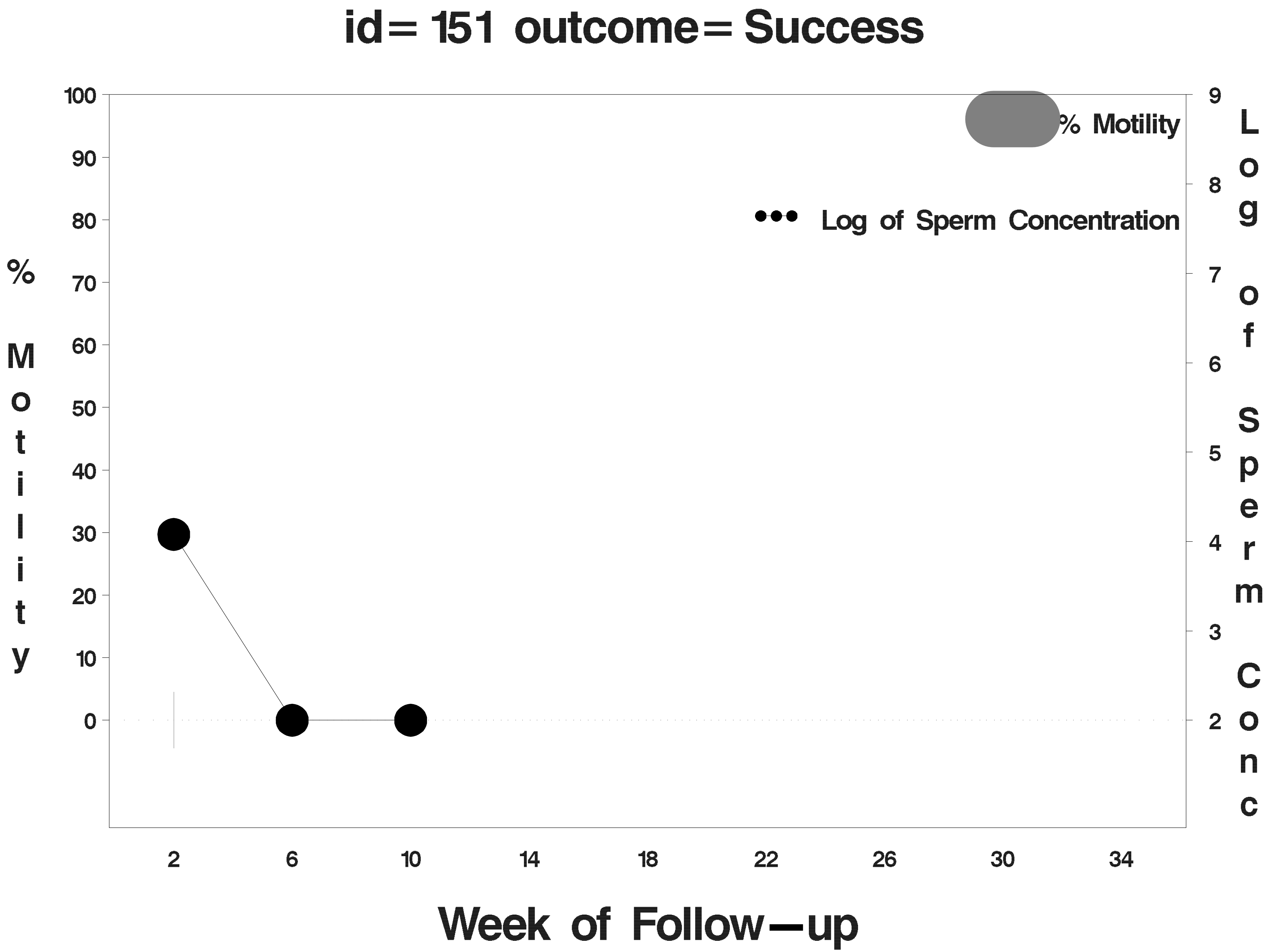
id= 151 outcome= Success
% Motility
Log of Sperm Concentration
100
90
80
70
60
50
40
30
20
10
0
9
8
7
6
5
4
3
2
2
6
10
14
18
22
26
30
34
Week of Follow—up
Log of Sperm Conc

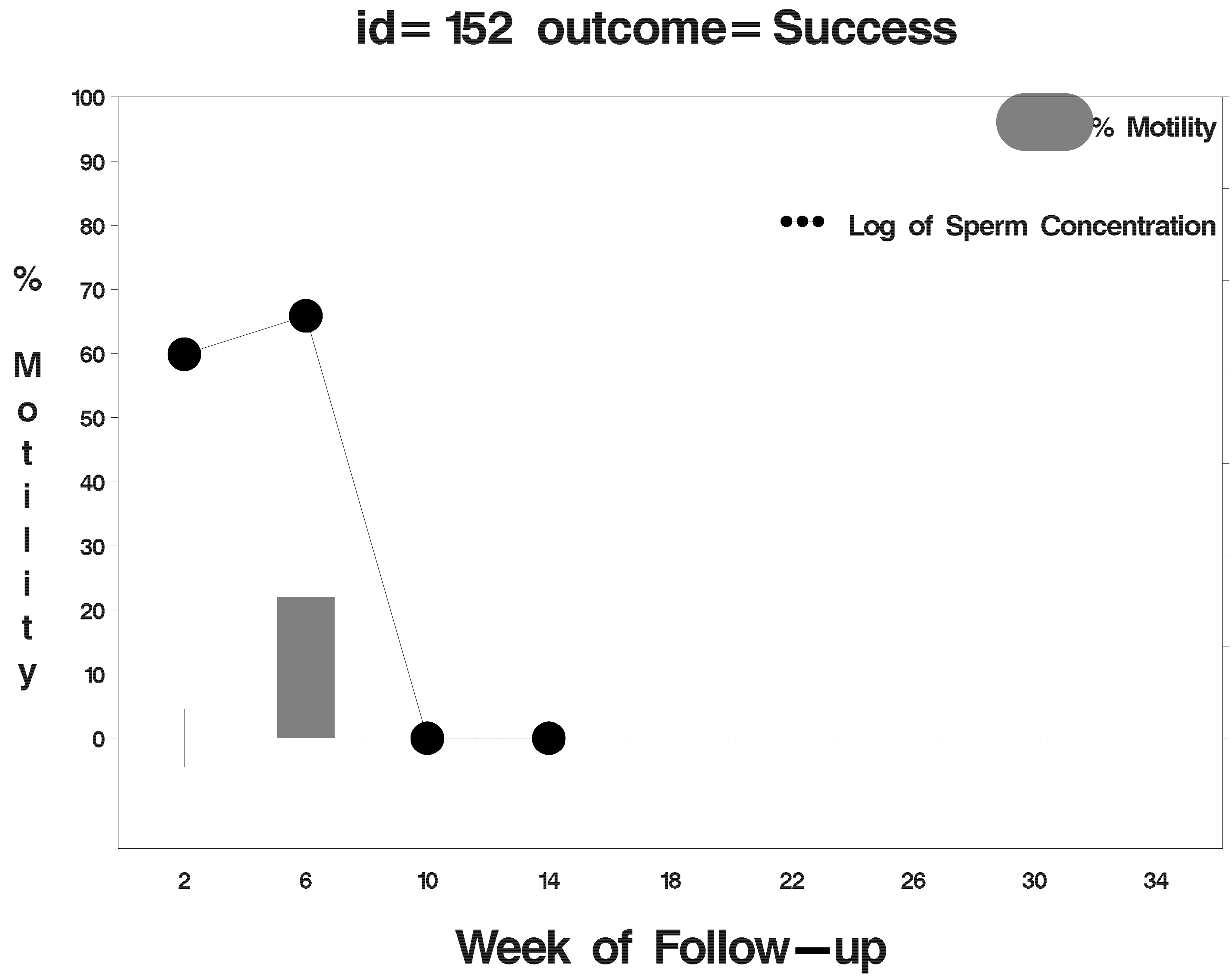

id= 152 outcome= Success
% Motility
Log of Sperm Concentration
100
90
80
70
60
50
40
30
20
10
0
9
8
7
6
5
4
3
2
2
6
10
14
18
22
26
30
34
Week of Follow−up
Log of Sperm Conc

id= 153 outcome= Success

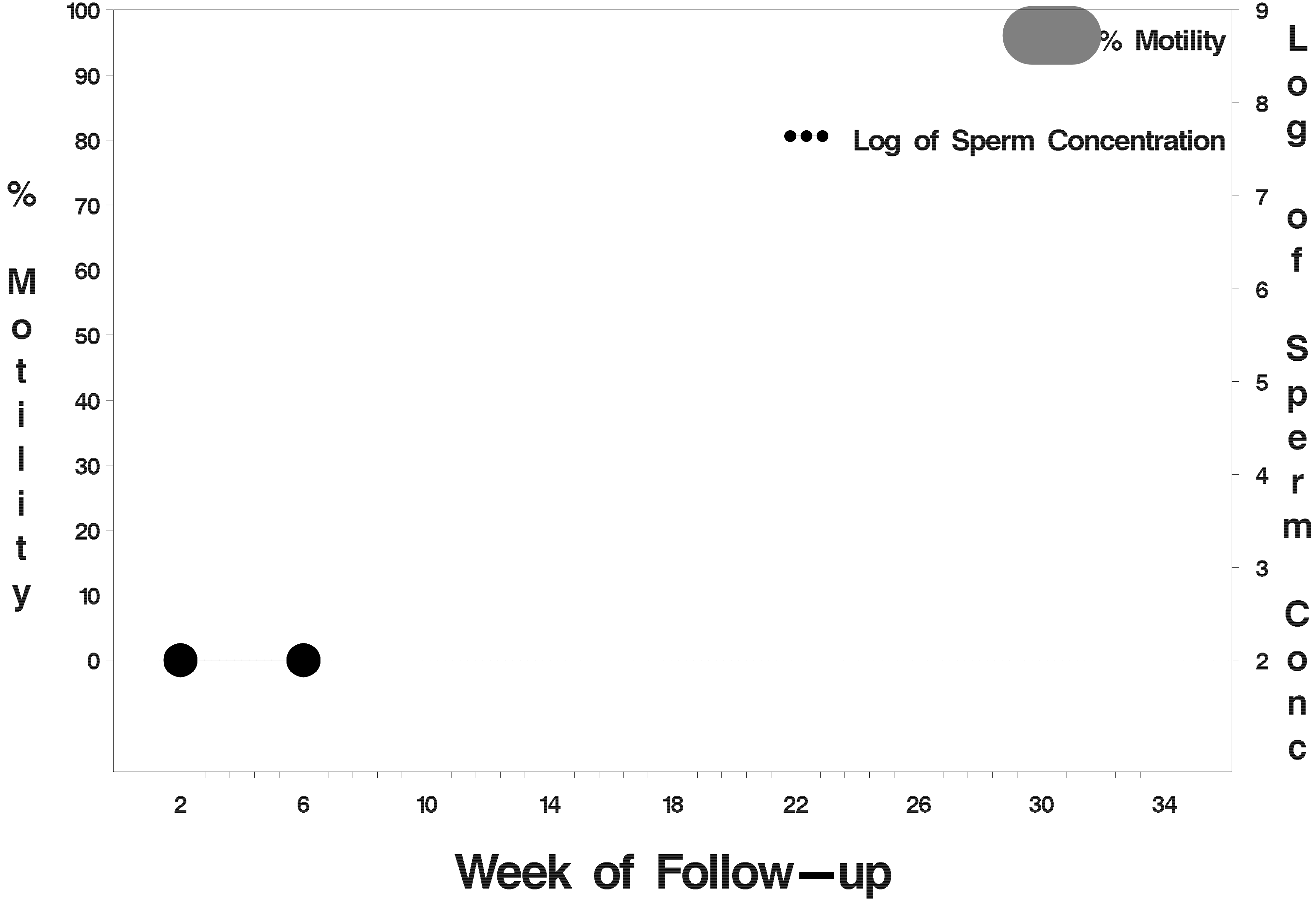

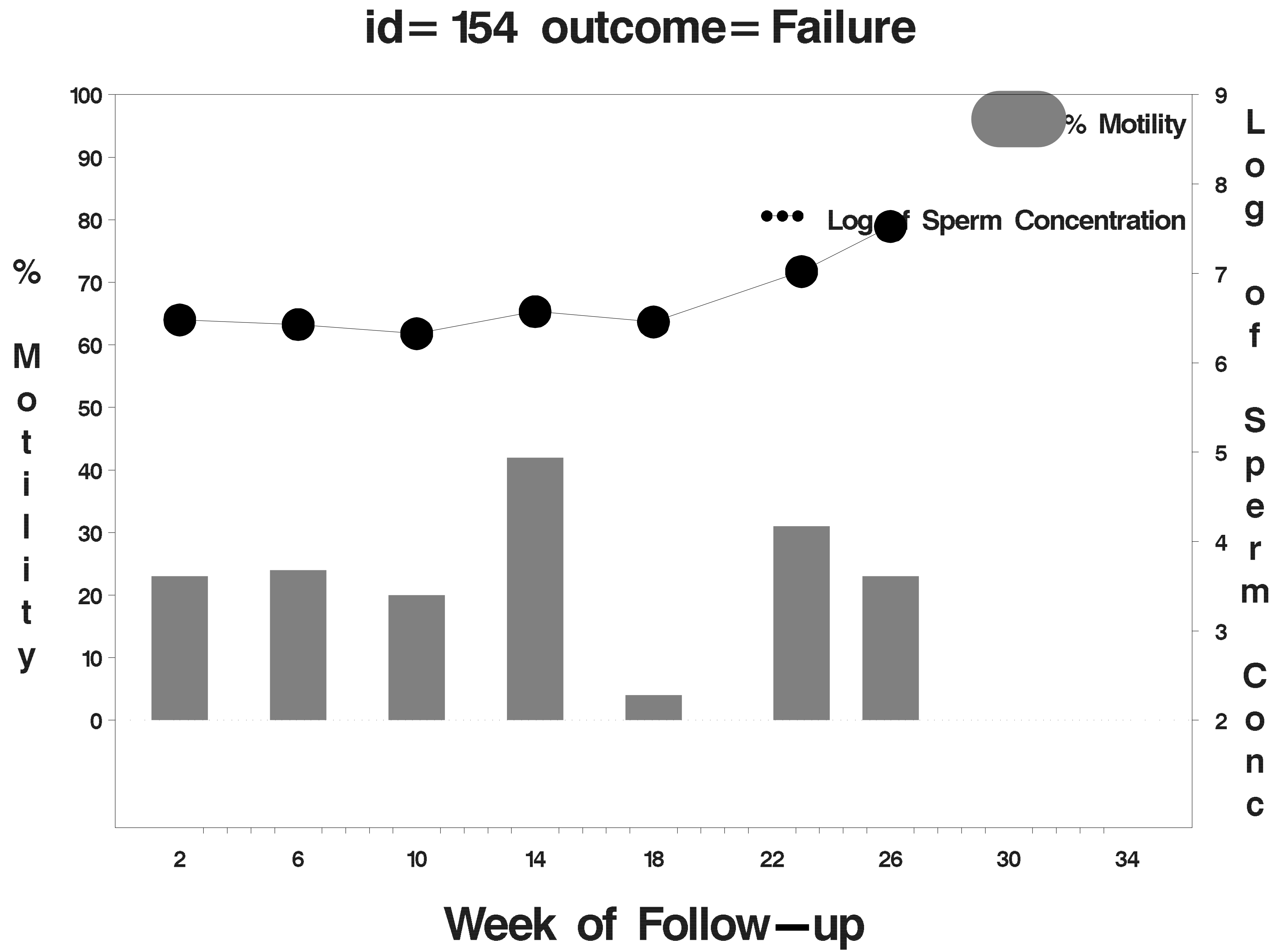

id= 154 outcome= Failure
% Motility
Log of Sperm Concentration
100
90
80
70
60
50
40
30
20
10
0
9
8
7
6
5
4
3
2
L o g o f S p e r m C o n c
2
6
10
14
18
22
26
30
34
Week of Follow—up

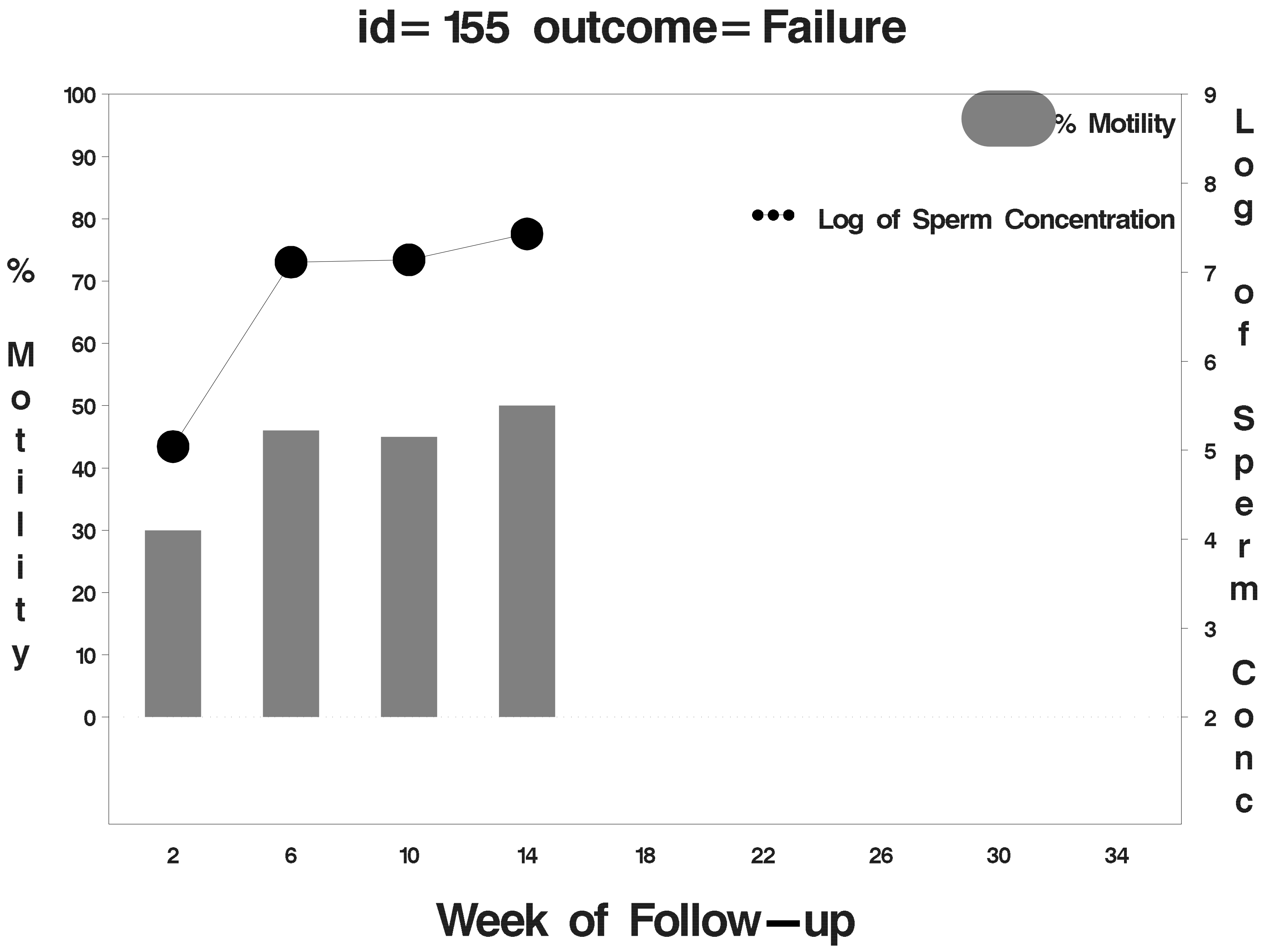
id= 155 outcome= Failure
% Motility
Log of Sperm Concentration
% Motility
Log of Sperm Conc
Week of Follow−up
100
90
80
70
60
50
40
30
20
10
0
9
8
7
6
5
4
3
2
2
6
10
14
18
22
26
30
34

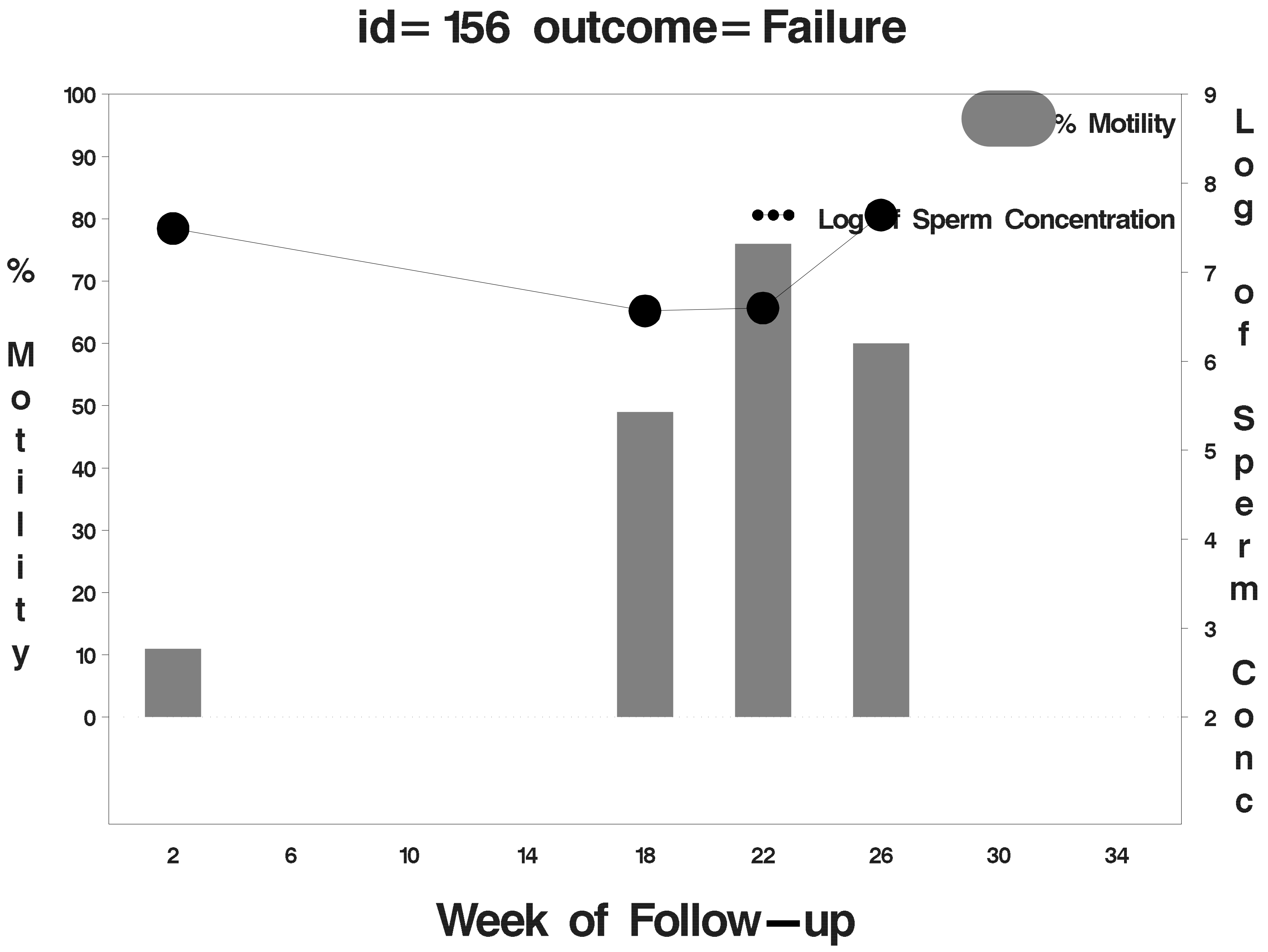

id= 156 outcome= Failure
% Motility
Log of Sperm Concentration
100
90
80
70
60
50
40
30
20
10
0
9
8
7
6
5
4
3
2
2
6
10
14
18
22
26
30
34
Week of Follow—up
% Motility
Log of Sperm Conc

id= 157 outcome= Success

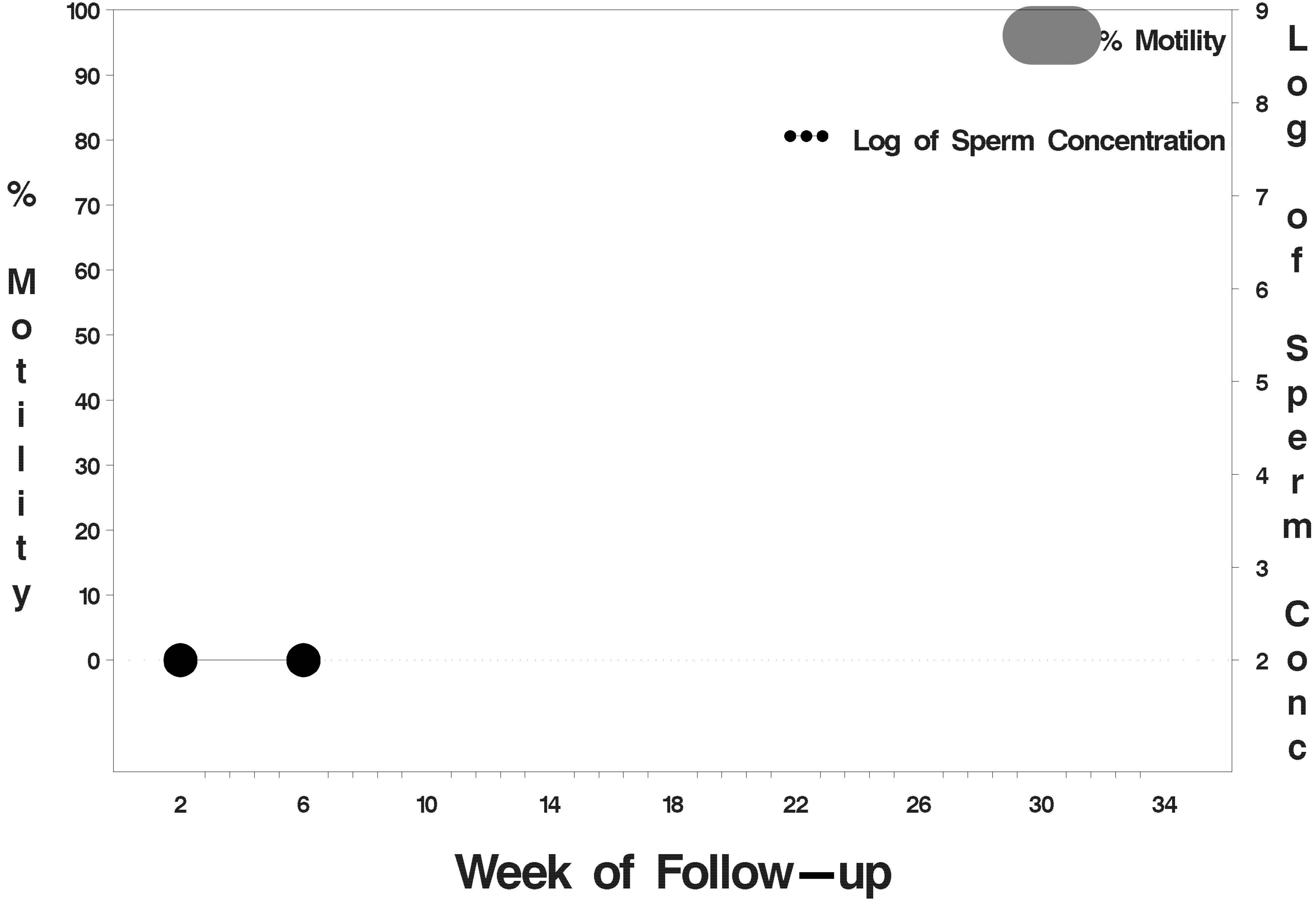

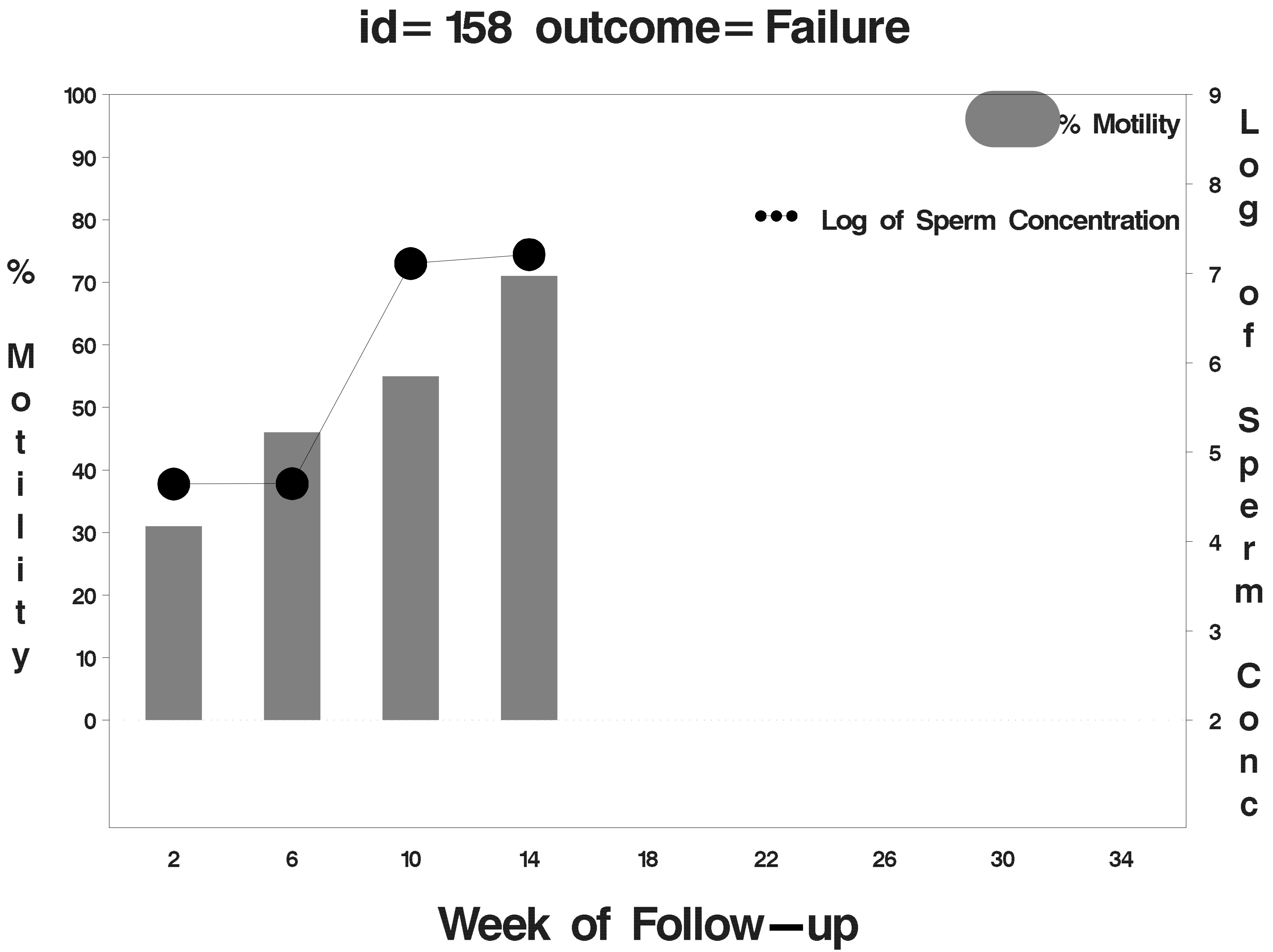

id= 158 outcome= Failure
% Motility
Log of Sperm Concentration
100
90
80
70
60
50
40
30
20
10
0
9
8
7
6
5
4
3
2
2
6
10
14
18
22
26
30
34
Week of Follow−up
Log of Sperm Conc

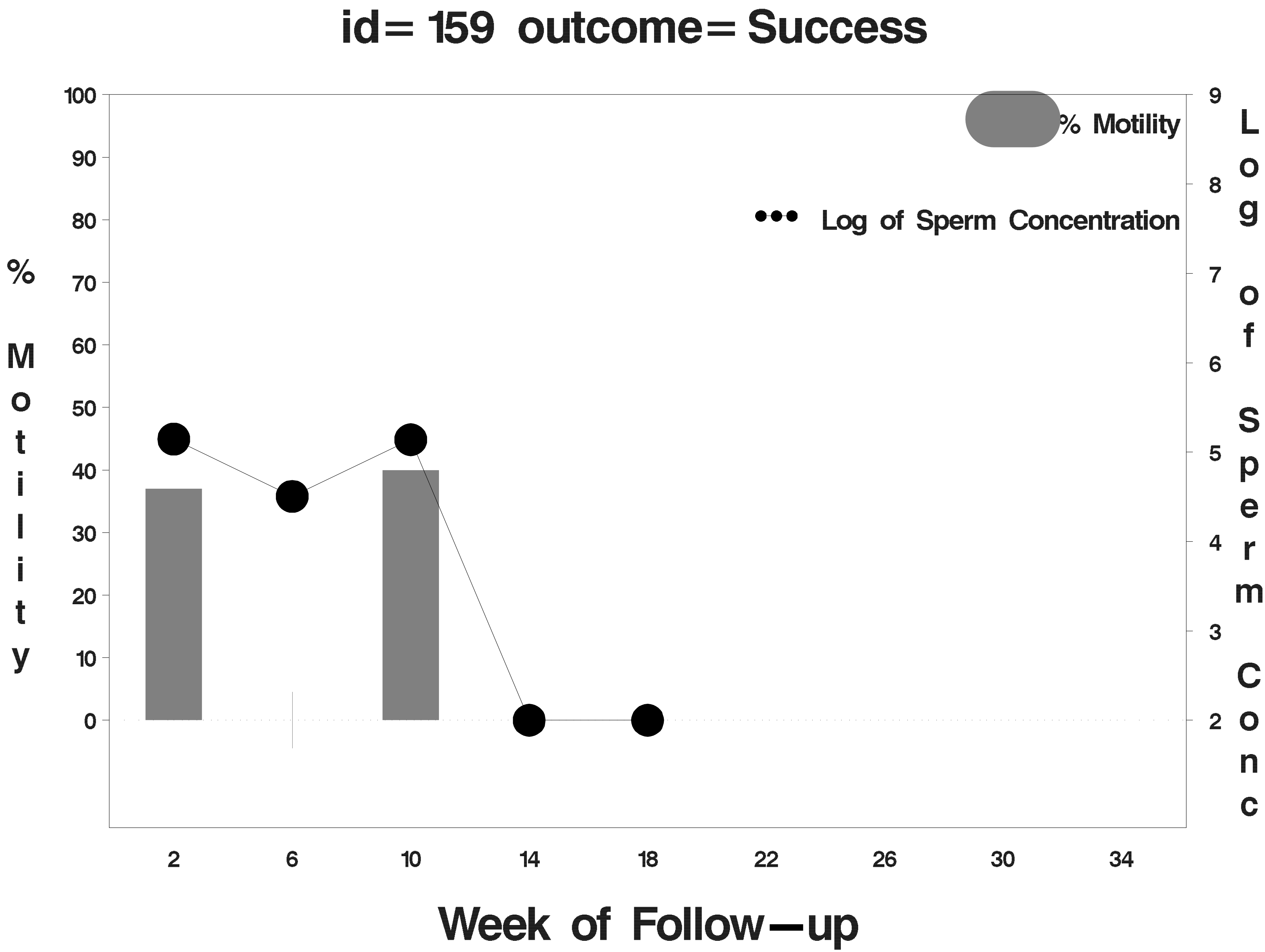

id= 159 outcome= Success
% Motility
Log of Sperm Concentration
100
90
80
70
60
50
40
30
20
10
0
9
8
7
6
5
4
3
2
Log of Sperm Conc
2
6
10
14
18
22
26
30
34
Week of Follow−up

id= 160 outcome= Success

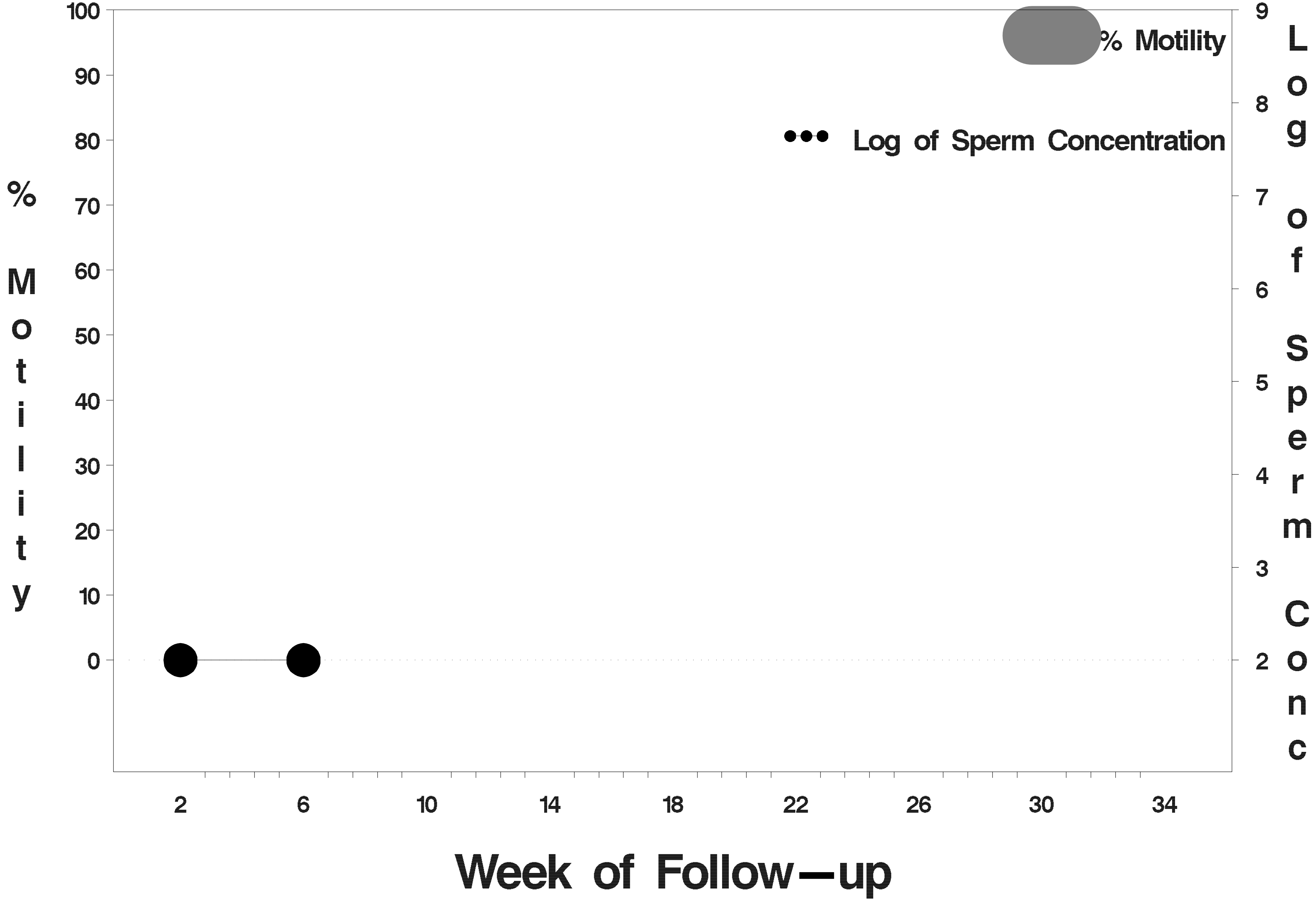

id= 161 outcome= Success

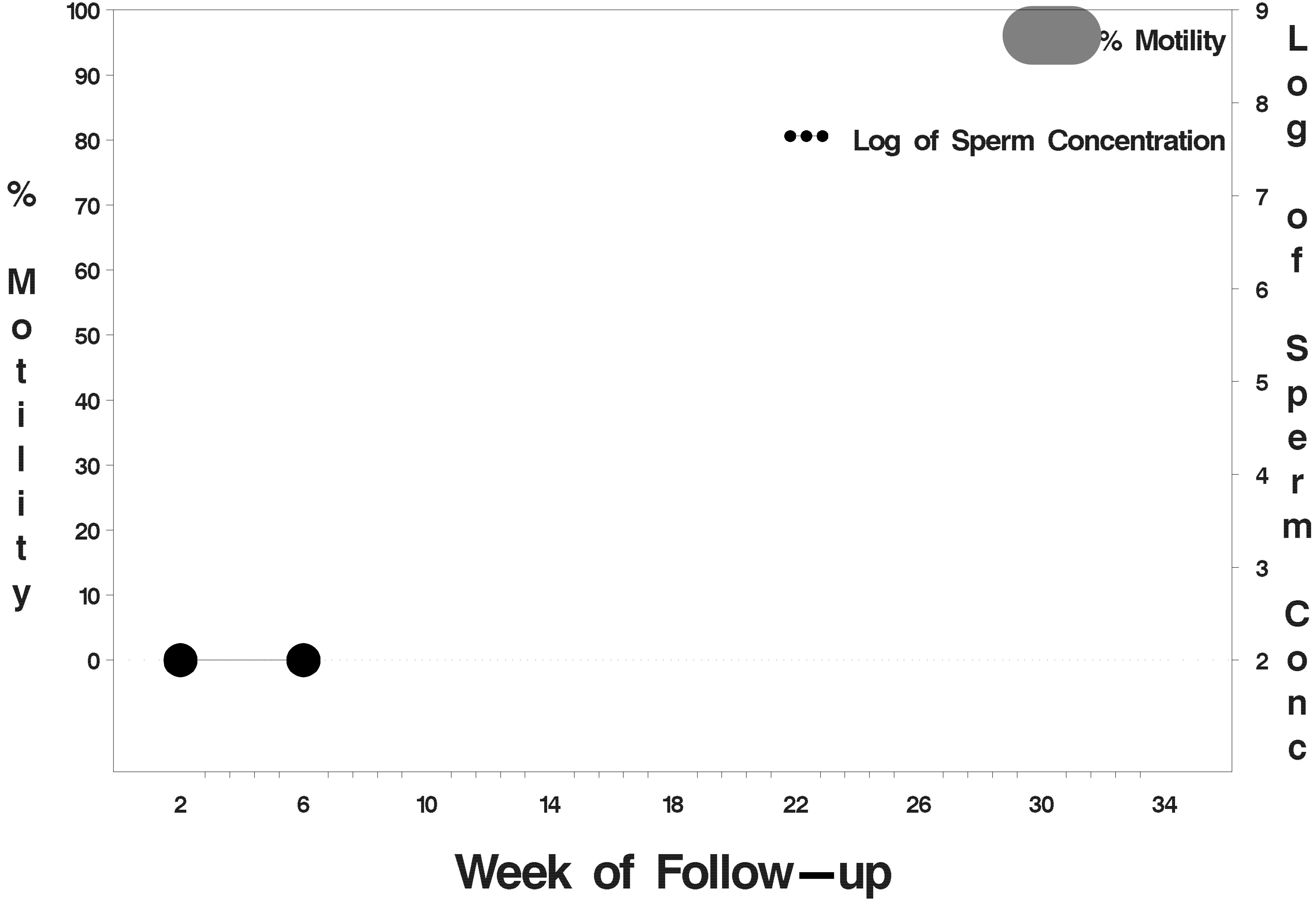

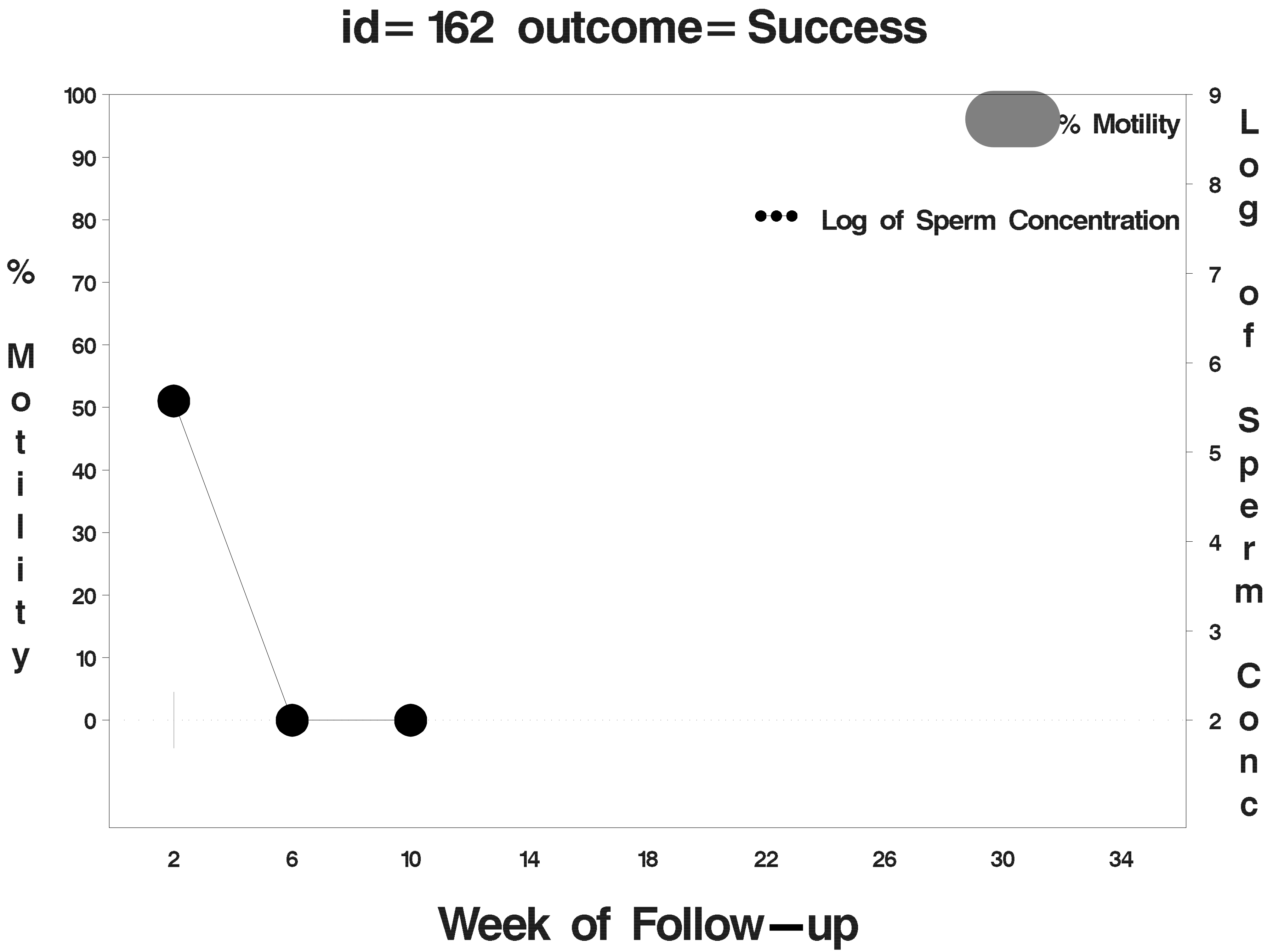

id= 162 outcome= Success
% Motility
Log of Sperm Concentration
% Motility
Log of Sperm Conc
Week of Follow-up
100
90
80
70
60
50
40
30
20
10
0
9
8
7
6
5
4
3
2
2
6
10
14
18
22
26
30
34

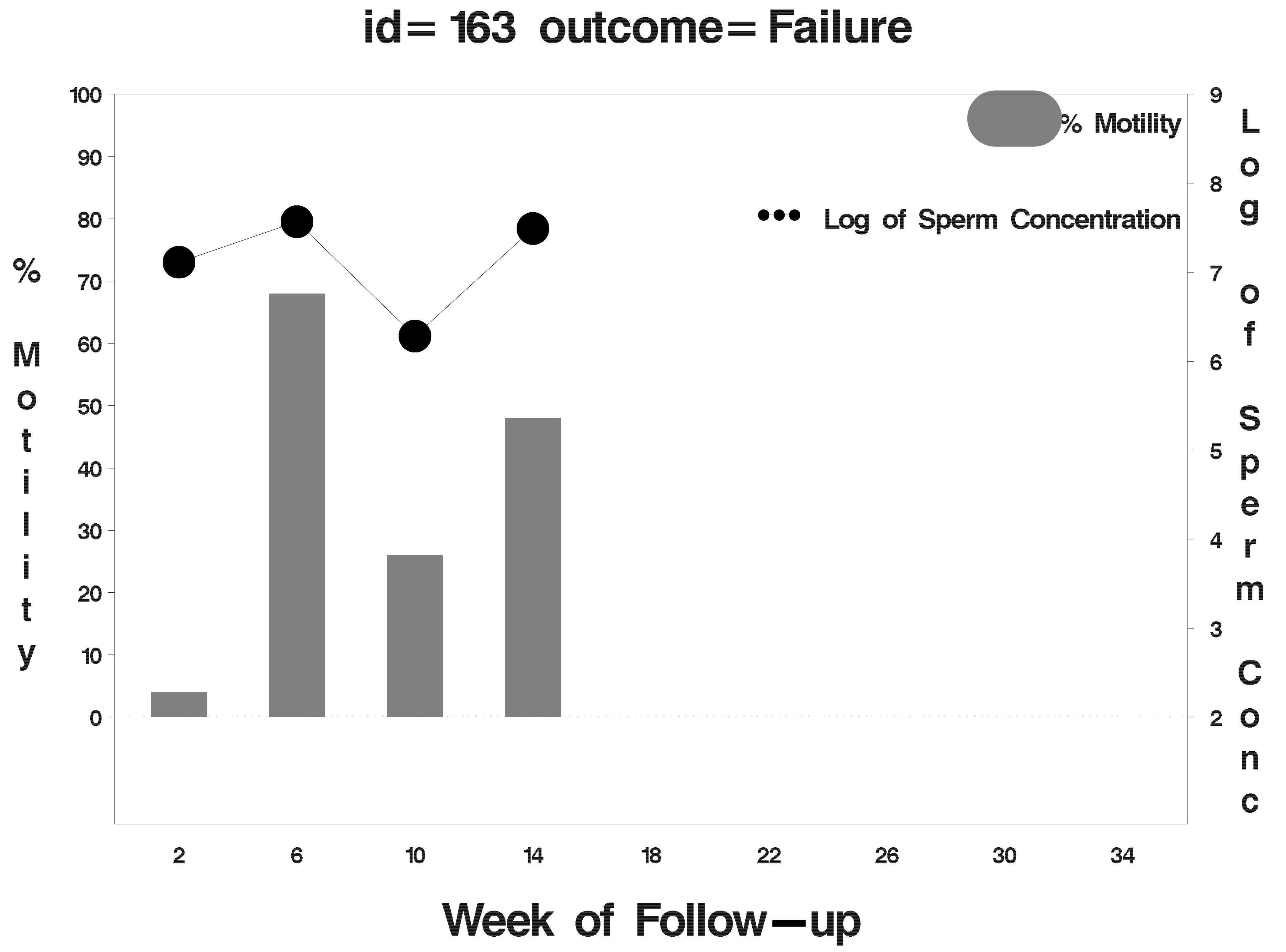

id= 163 outcome= Failure
% Motility
Log of Sperm Concentration
% Motility
Log of Sperm Conc
Week of Follow−up
100
90
80
70
60
50
40
30
20
10
0
9
8
7
6
5
4
3
2
2
6
10
14
18
22
26
30
34

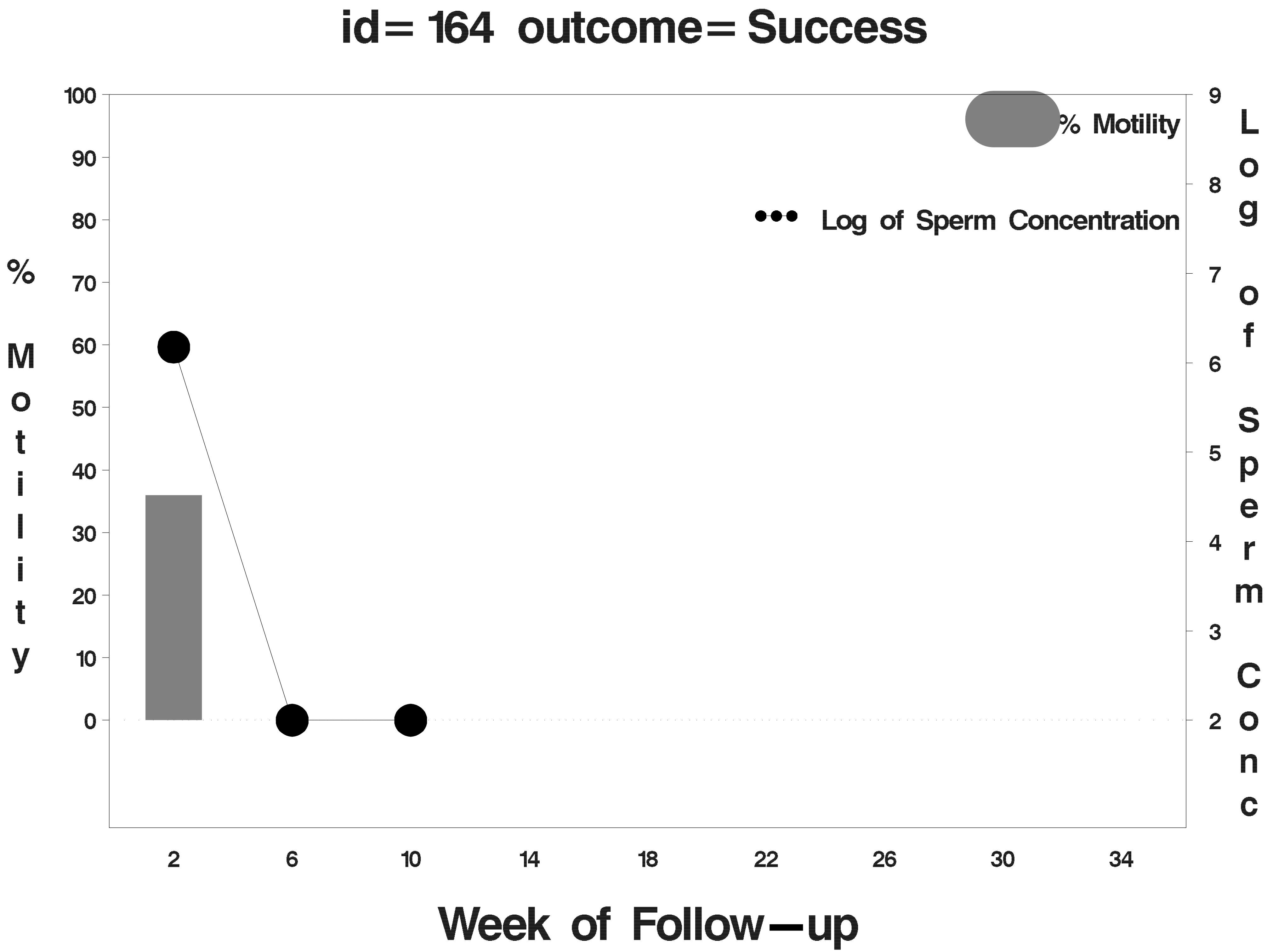

id= 164 outcome= Success
% Motility
Log of Sperm Concentration
% Motility
Log of Sperm Conc
Week of Follow-up
100
90
80
70
60
50
40
30
20
10
0
9
8
7
6
5
4
3
2
2
6
10
14
18
22
26
30
34

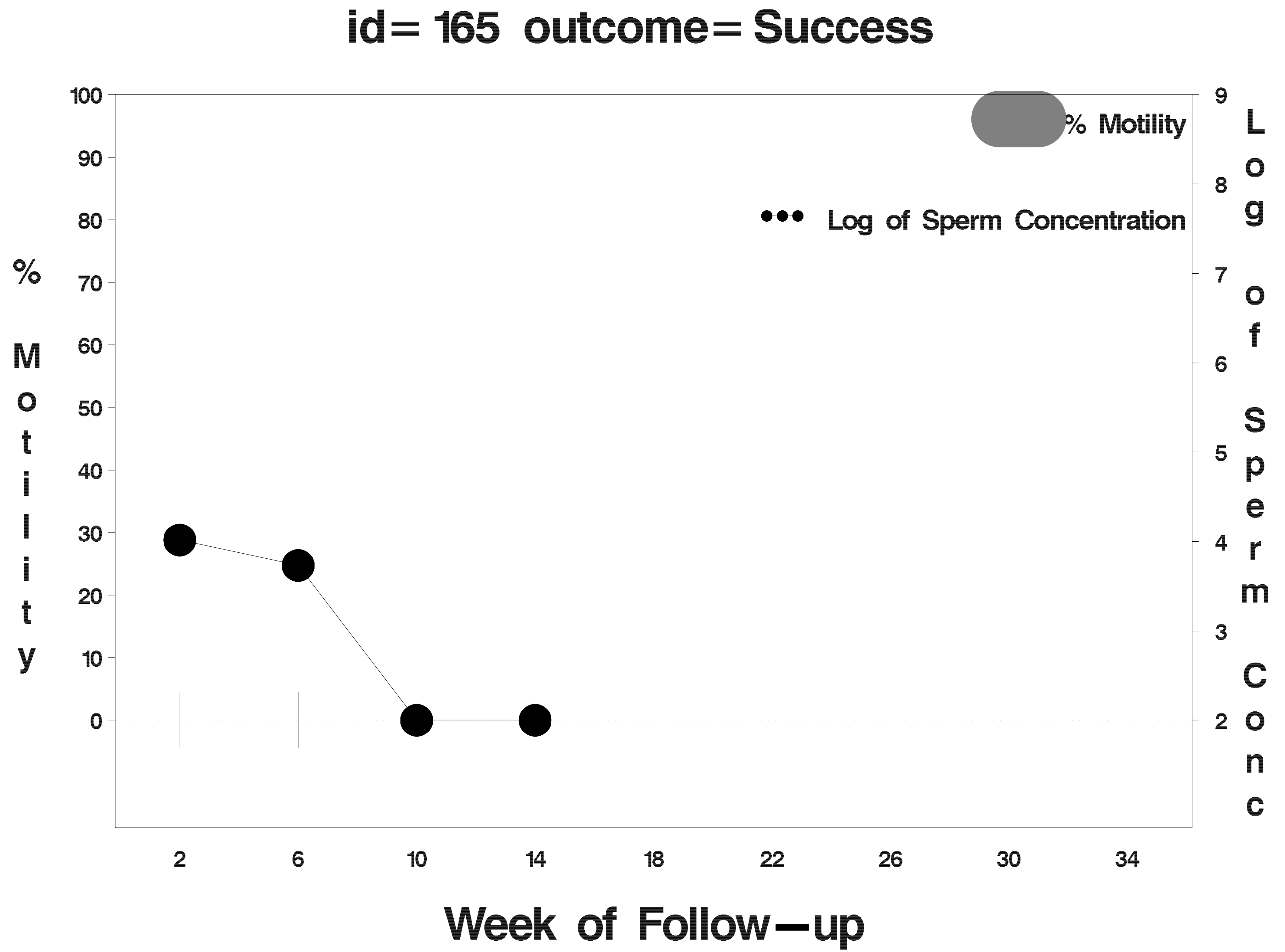
id= 165 outcome= Success
% Motility
Log of Sperm Concentration
% Motility
Log of Sperm Conc
Week of Follow-up
100
90
80
70
60
50
40
30
20
10
0
9
8
7
6
5
4
3
2
2
6
10
14
18
22
26
30
34

id= 166 outcome= Success

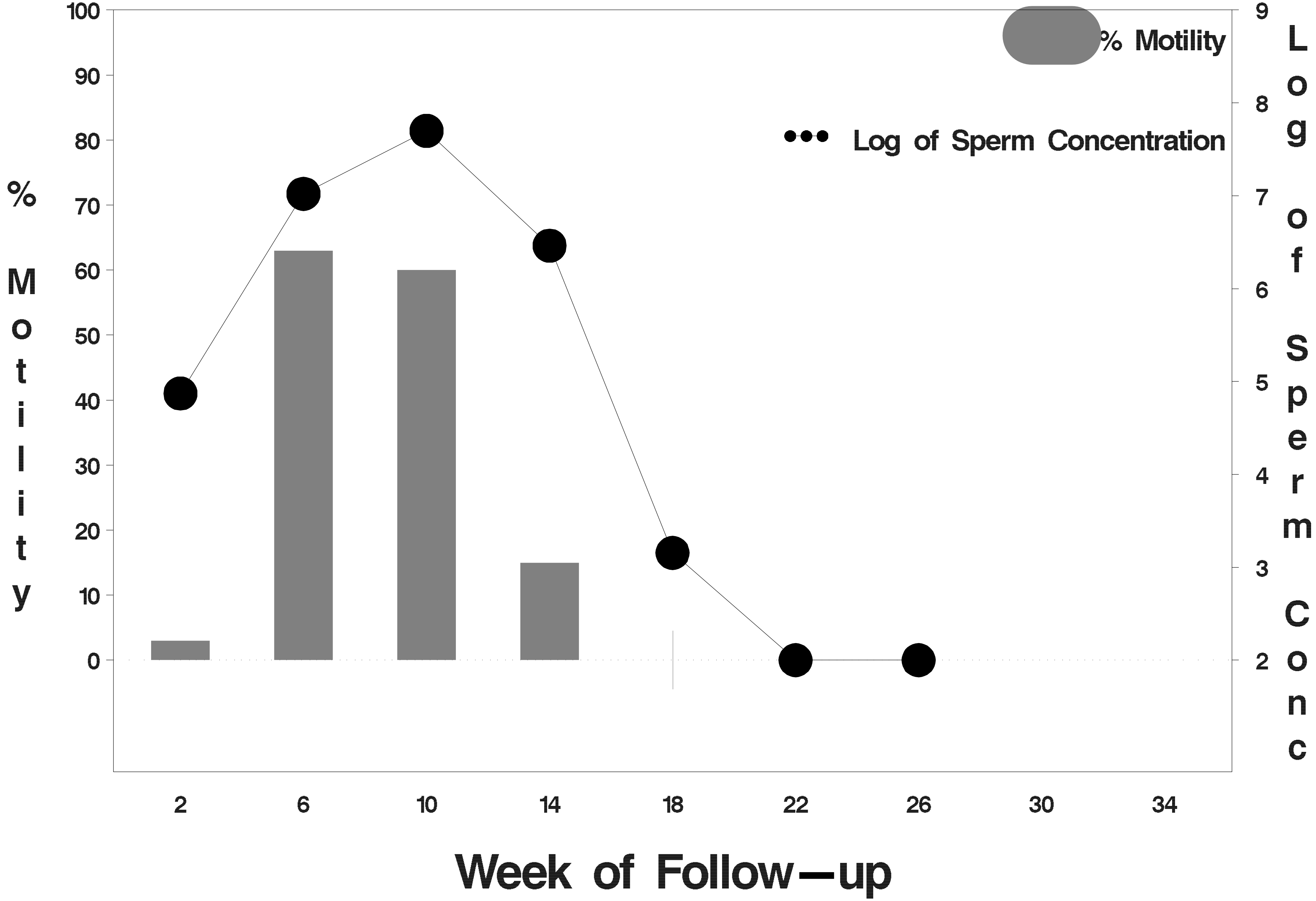

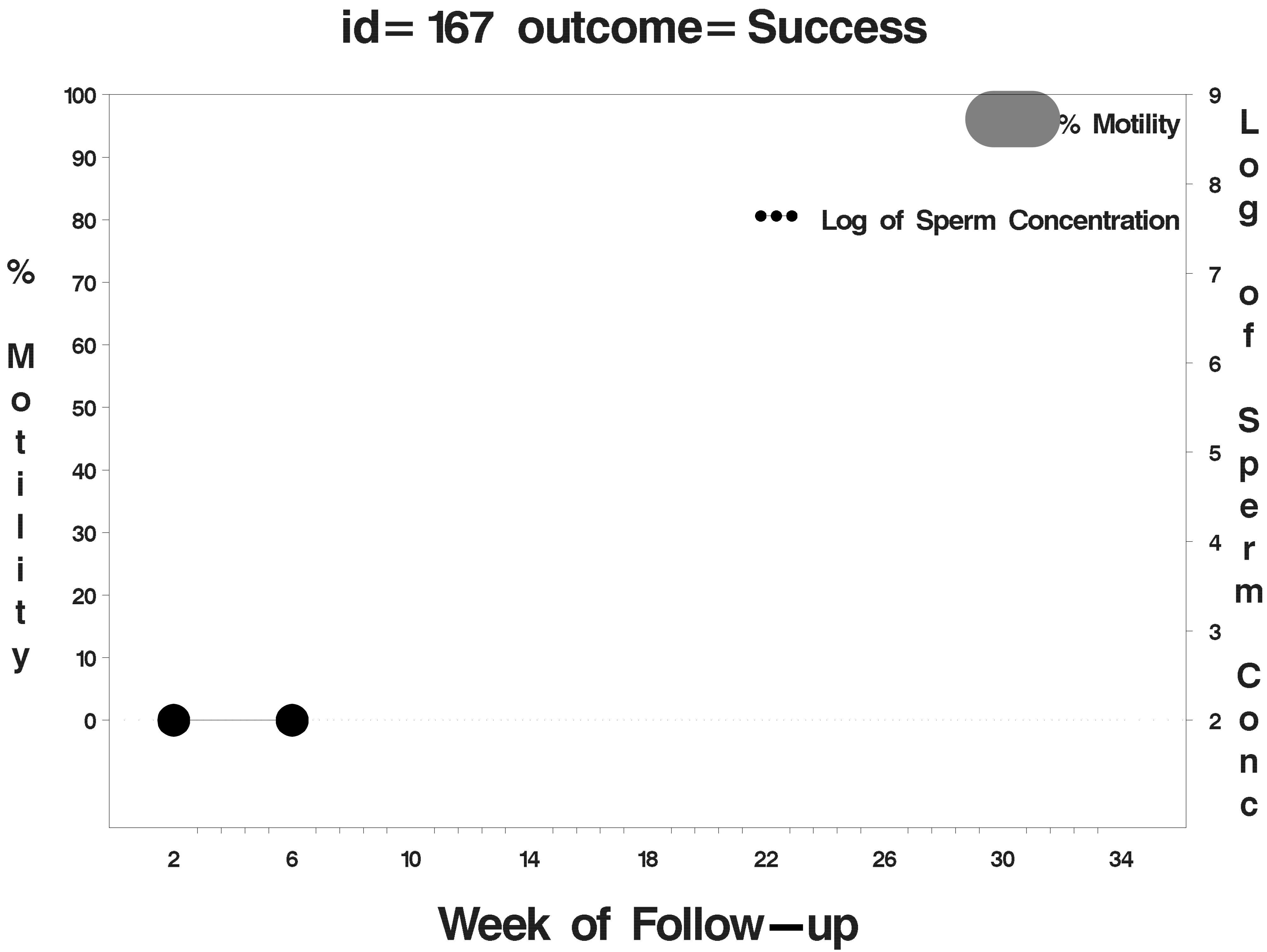
id= 167 outcome= Success
% Motility
Log of Sperm Concentration
100
90
80
70
60
50
40
30
20
10
0
9
8
7
6
5
4
3
2
2
6
10
14
18
22
26
30
34
Week of Follow-up
Log of Sperm Conc

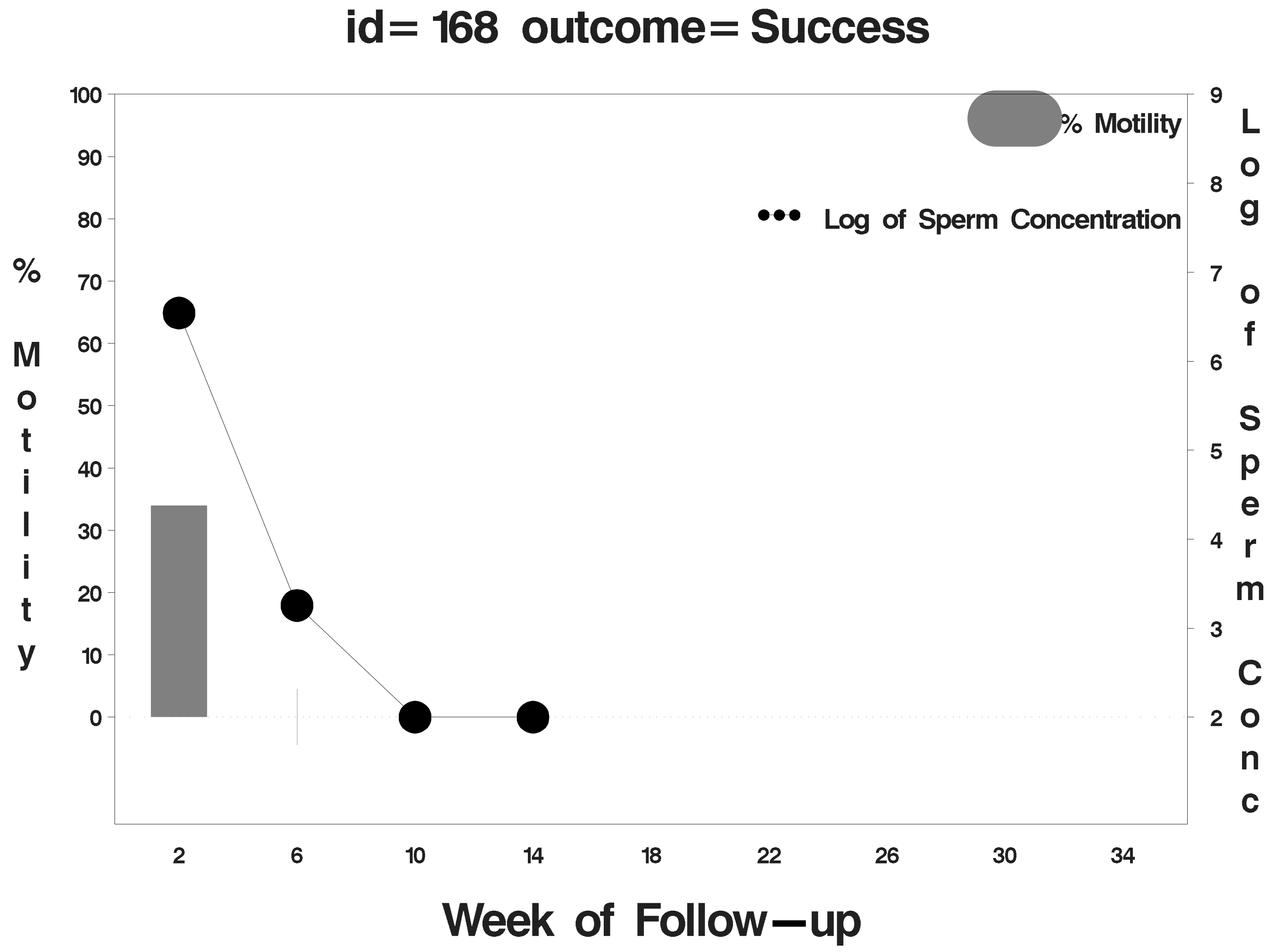
id= 168 outcome= Success
% Motility
Log of Sperm Concentration
% Motility
Log of Sperm Conc
Week of Follow-up
100
90
80
70
60
50
40
30
20
10
0
9
8
7
6
5
4
3
2
2
6
10
14
18
22
26
30
34

id= 169 outcome= Success

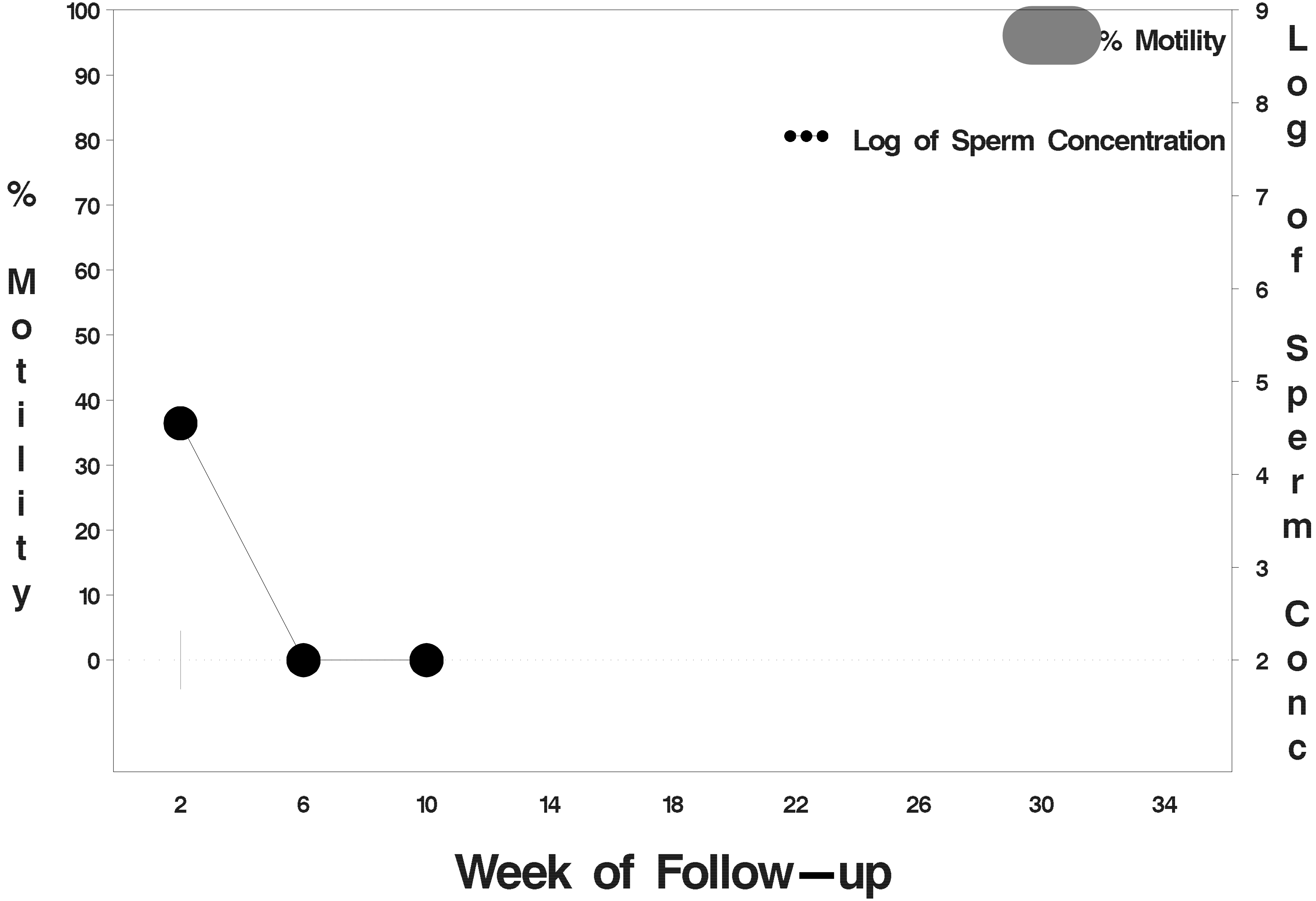

id= 170 outcome= Success

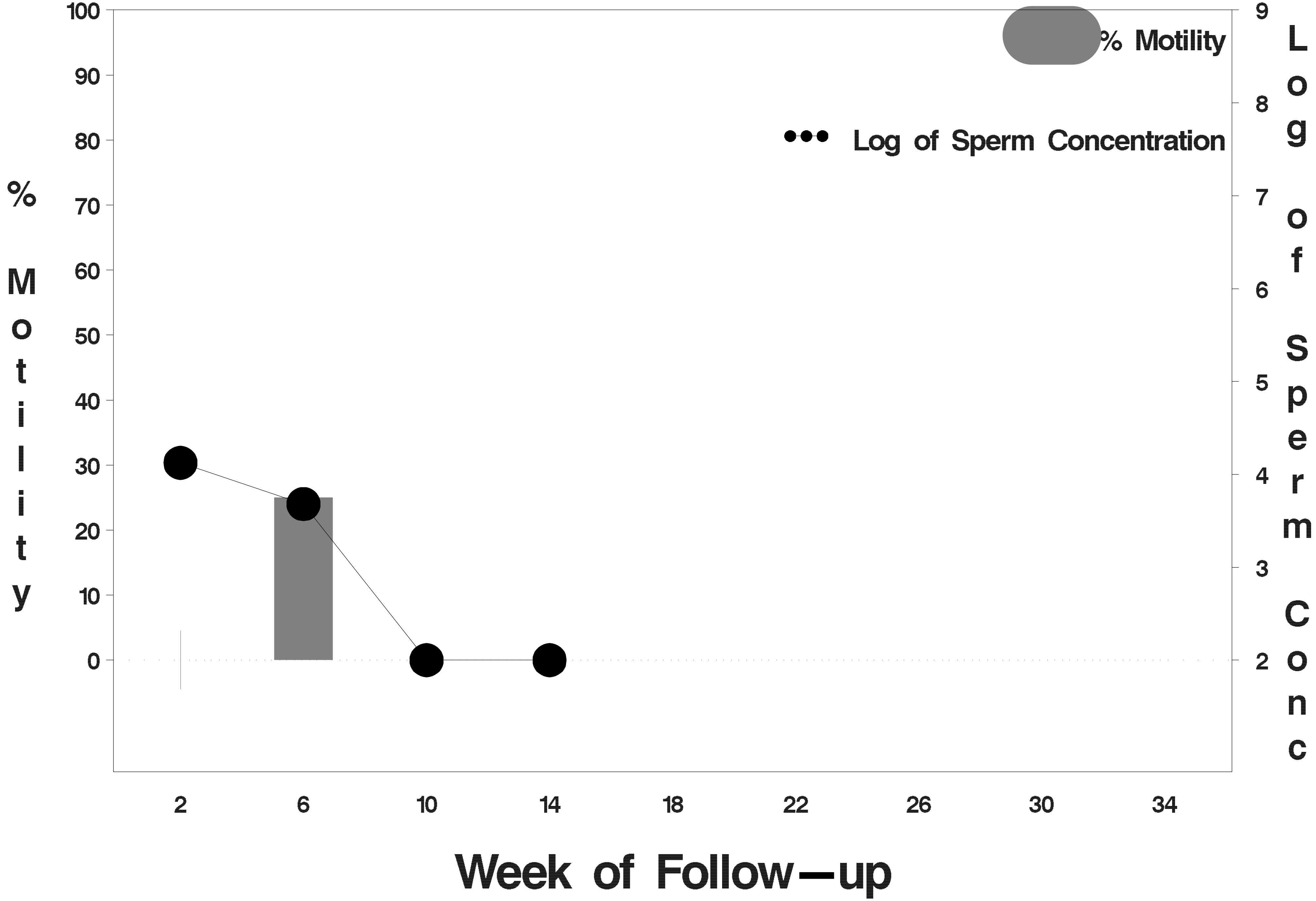

id= 171 outcome= Success

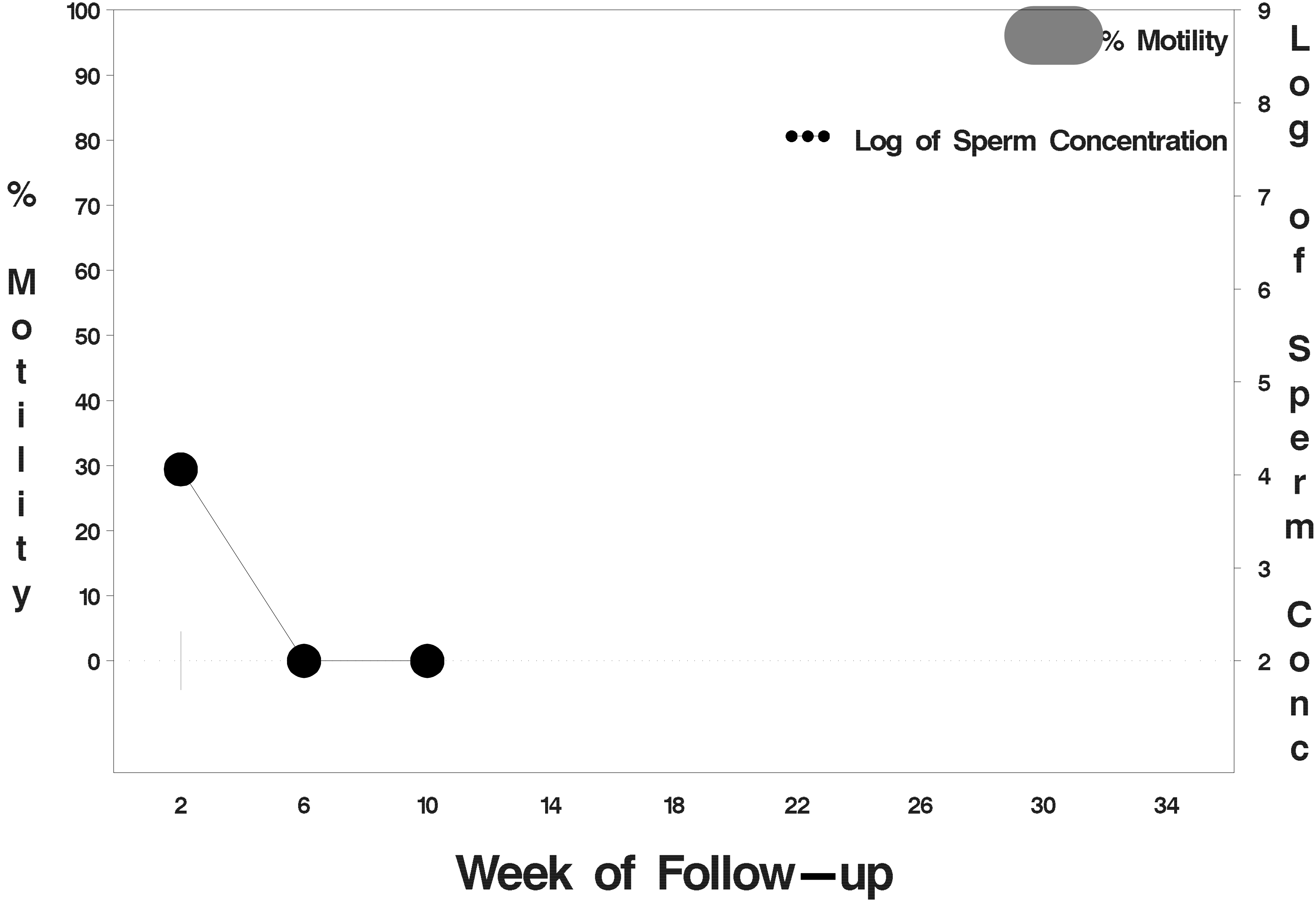

id= 172 outcome= Success

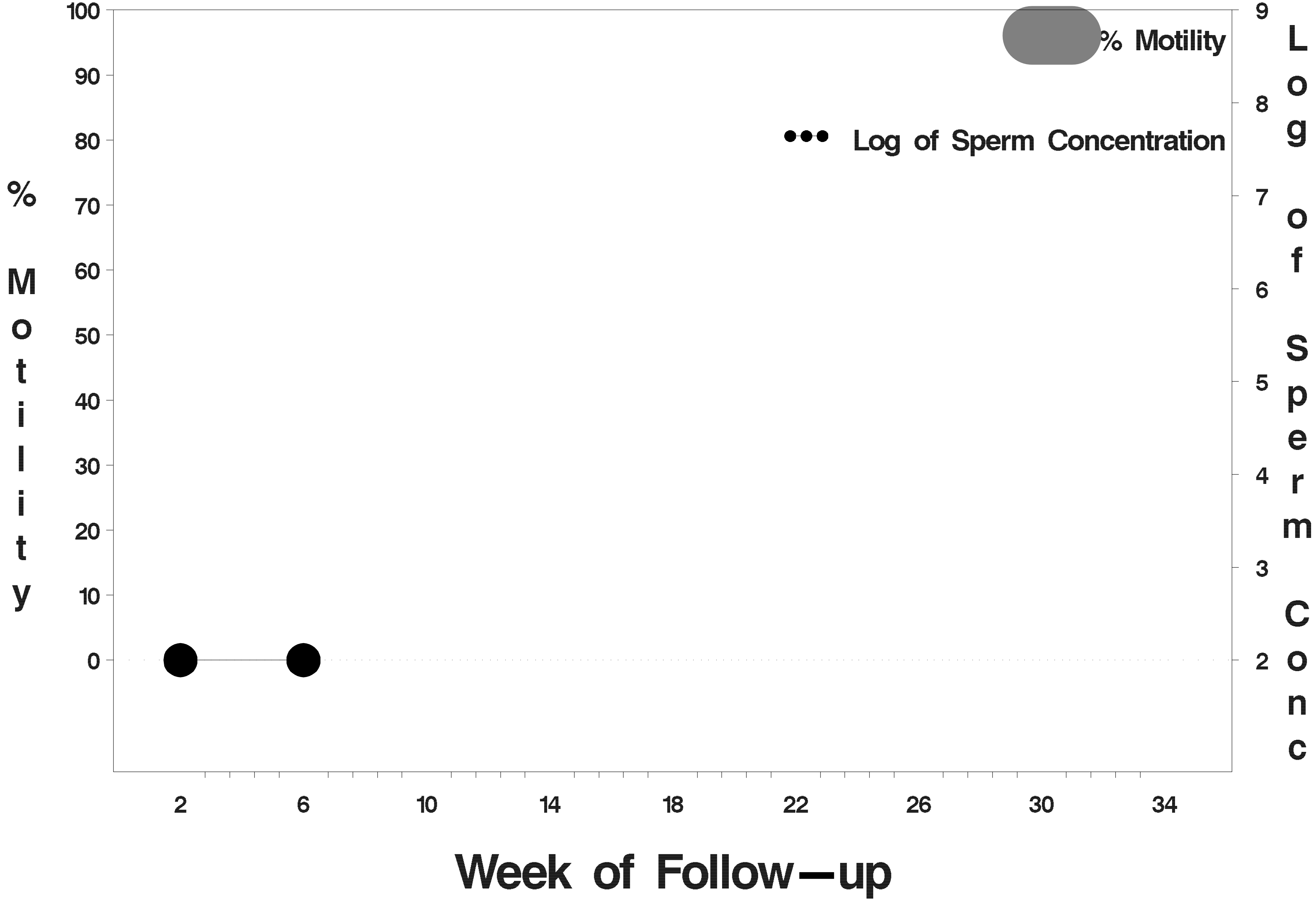

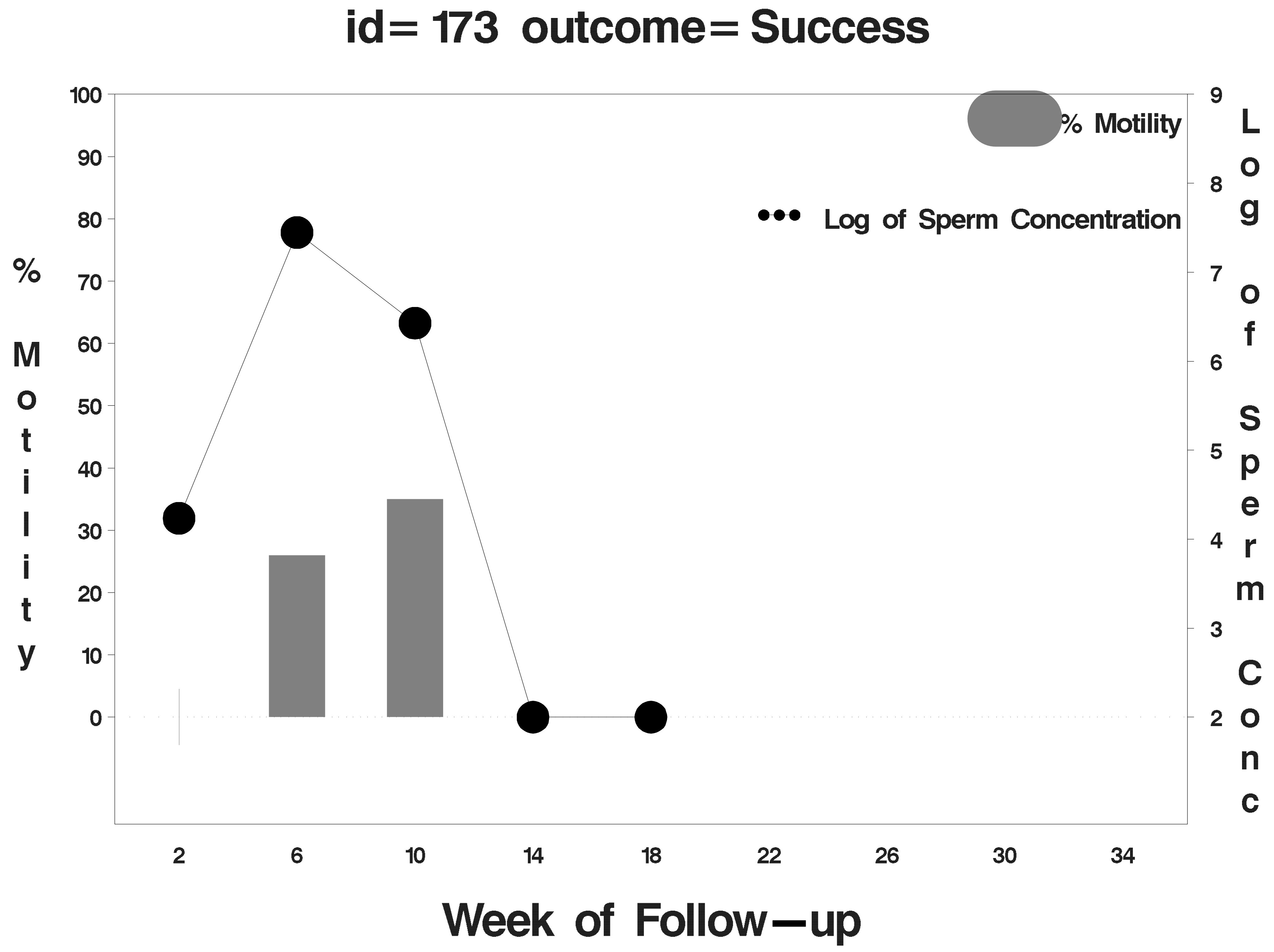

id= 173 outcome= Success
% Motility
Log of Sperm Concentration
100
90
80
70
60
50
40
30
20
10
0
9
8
7
6
5
4
3
2
% Motility
Log of Sperm Conc
2
6
10
14
18
22
26
30
34
Week of Follow−up

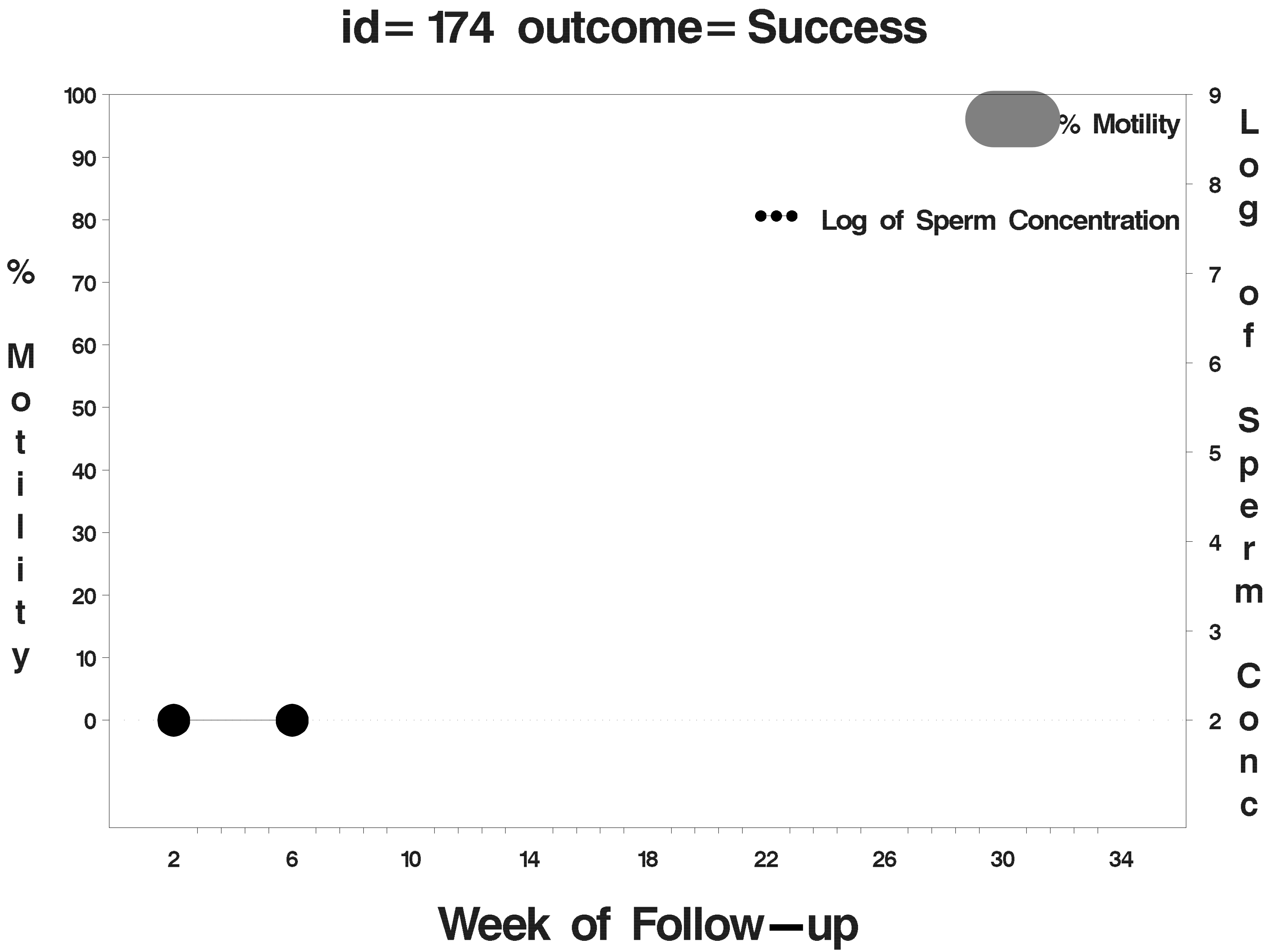
id= 174 outcome= Success
% Motility
Log of Sperm Concentration
% Motility
Log of Sperm Conc
Week of Follow−up
100
90
80
70
60
50
40
30
20
10
0
9
8
7
6
5
4
3
2
2
6
10
14
18
22
26
30
34

id= 175 outcome= Success

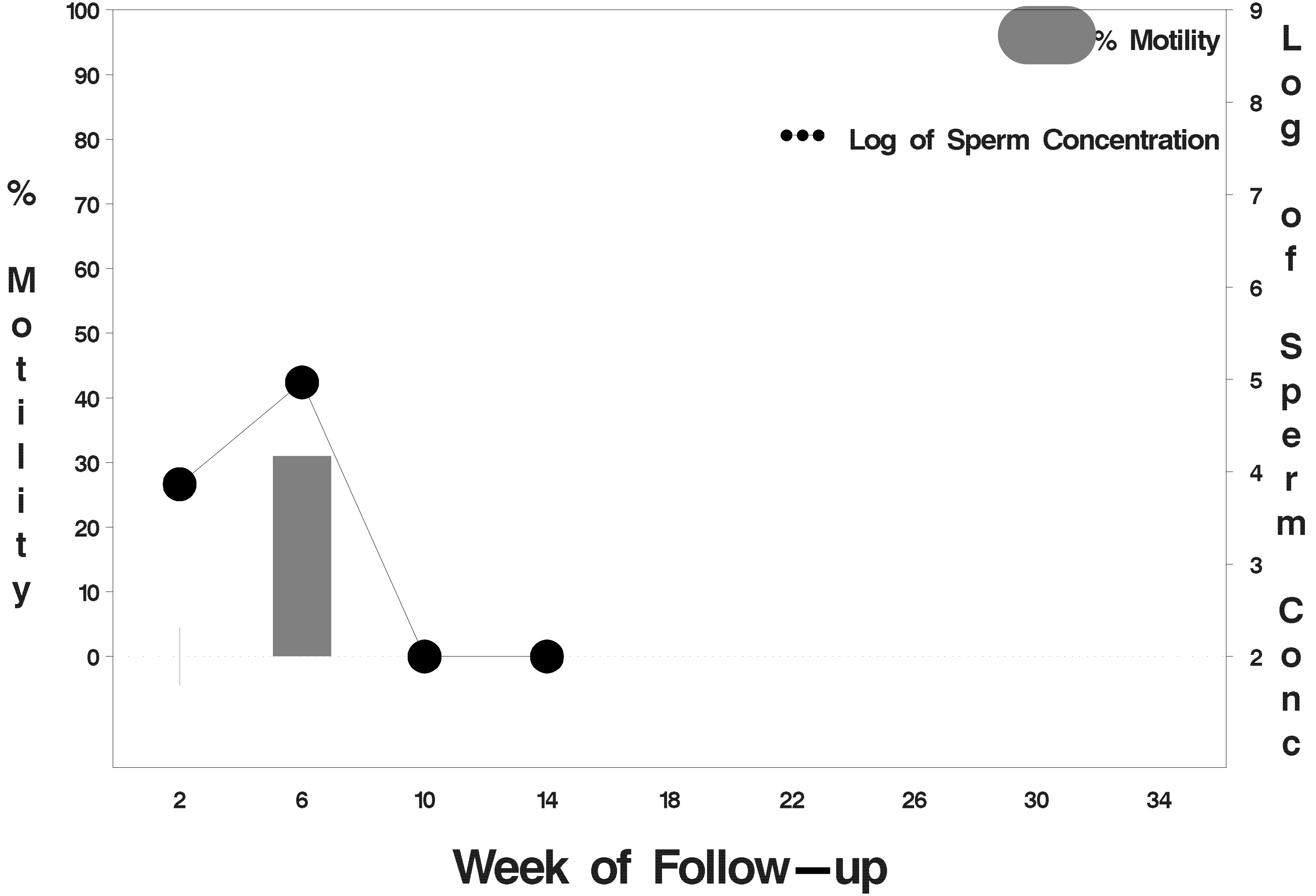

id= 176 outcome= Success

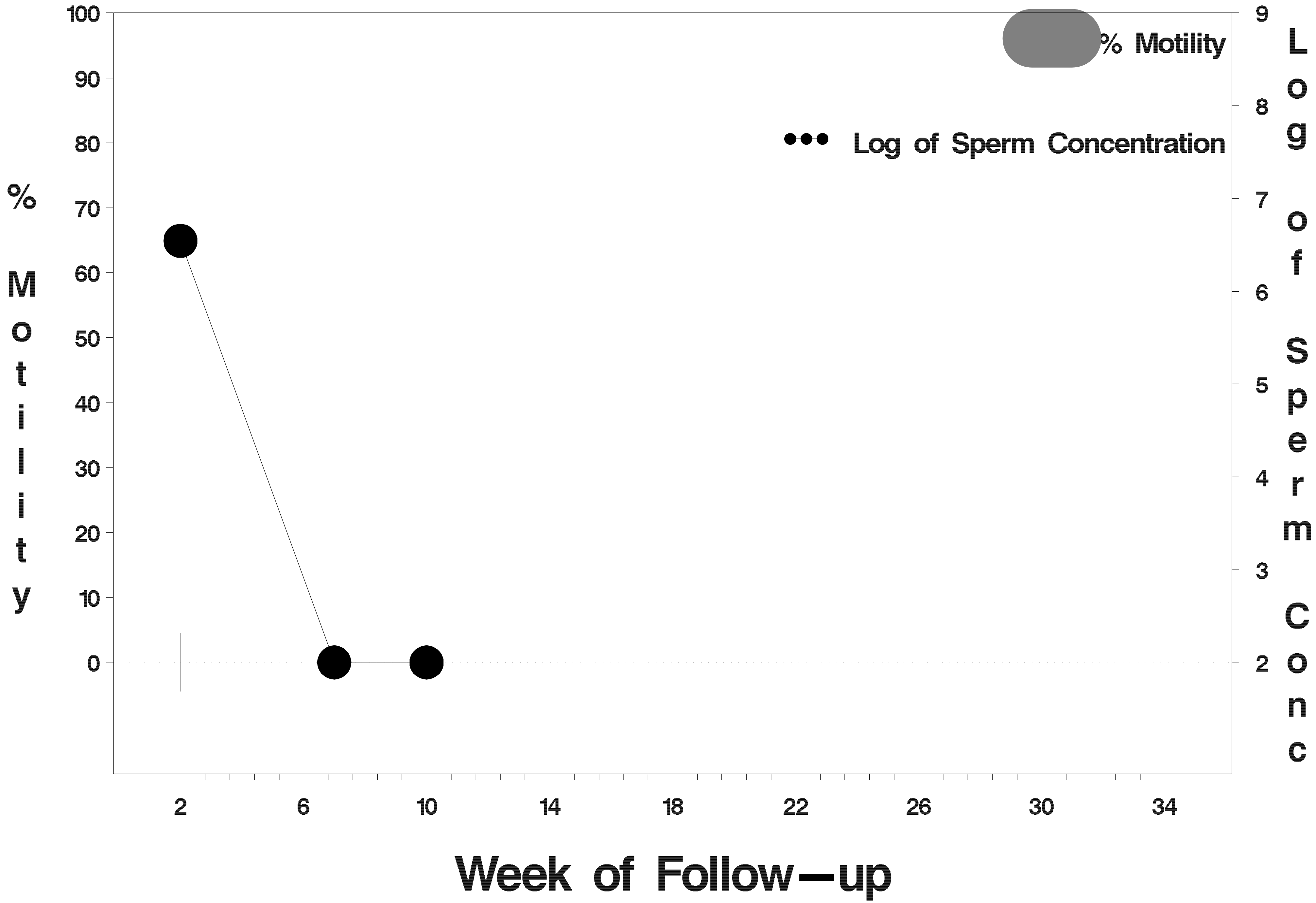

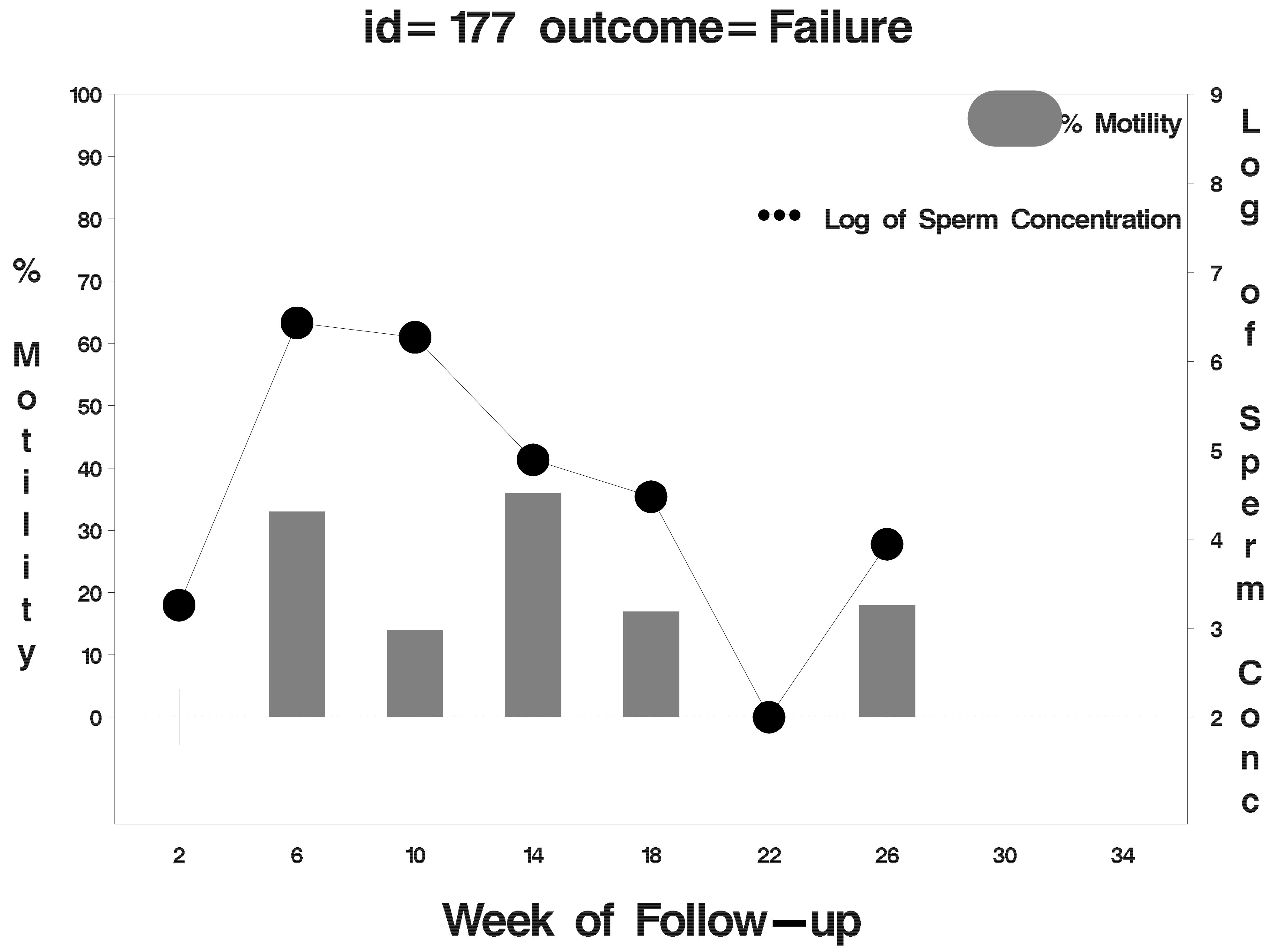

id= 177 outcome= Failure
% Motility
Log of Sperm Concentration
100
90
80
70
60
50
40
30
20
10
0
9
8
7
6
5
4
3
2
2
6
10
14
18
22
26
30
34
Week of Follow−up
Log of Sperm Conc

id= 178 outcome= Success

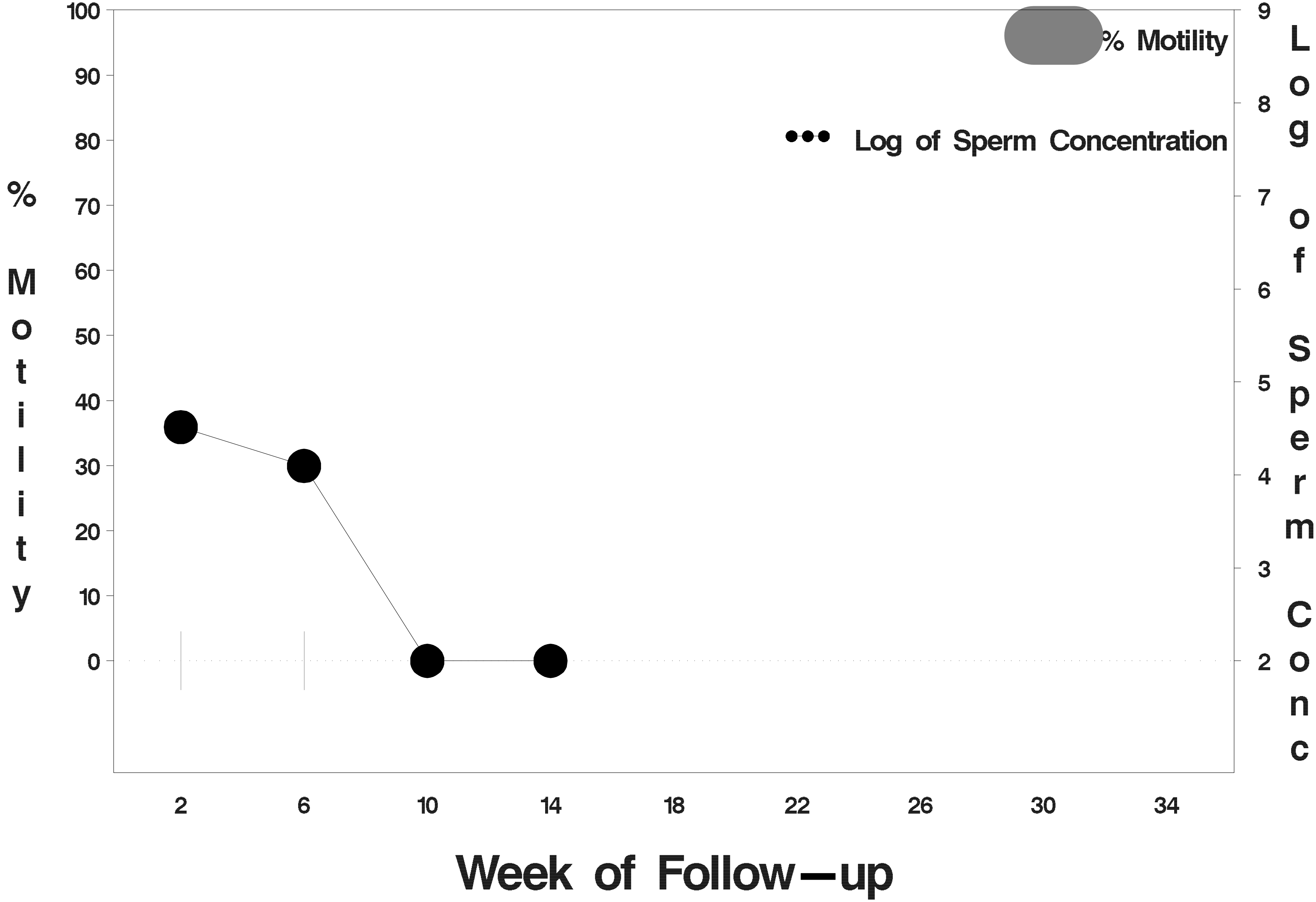

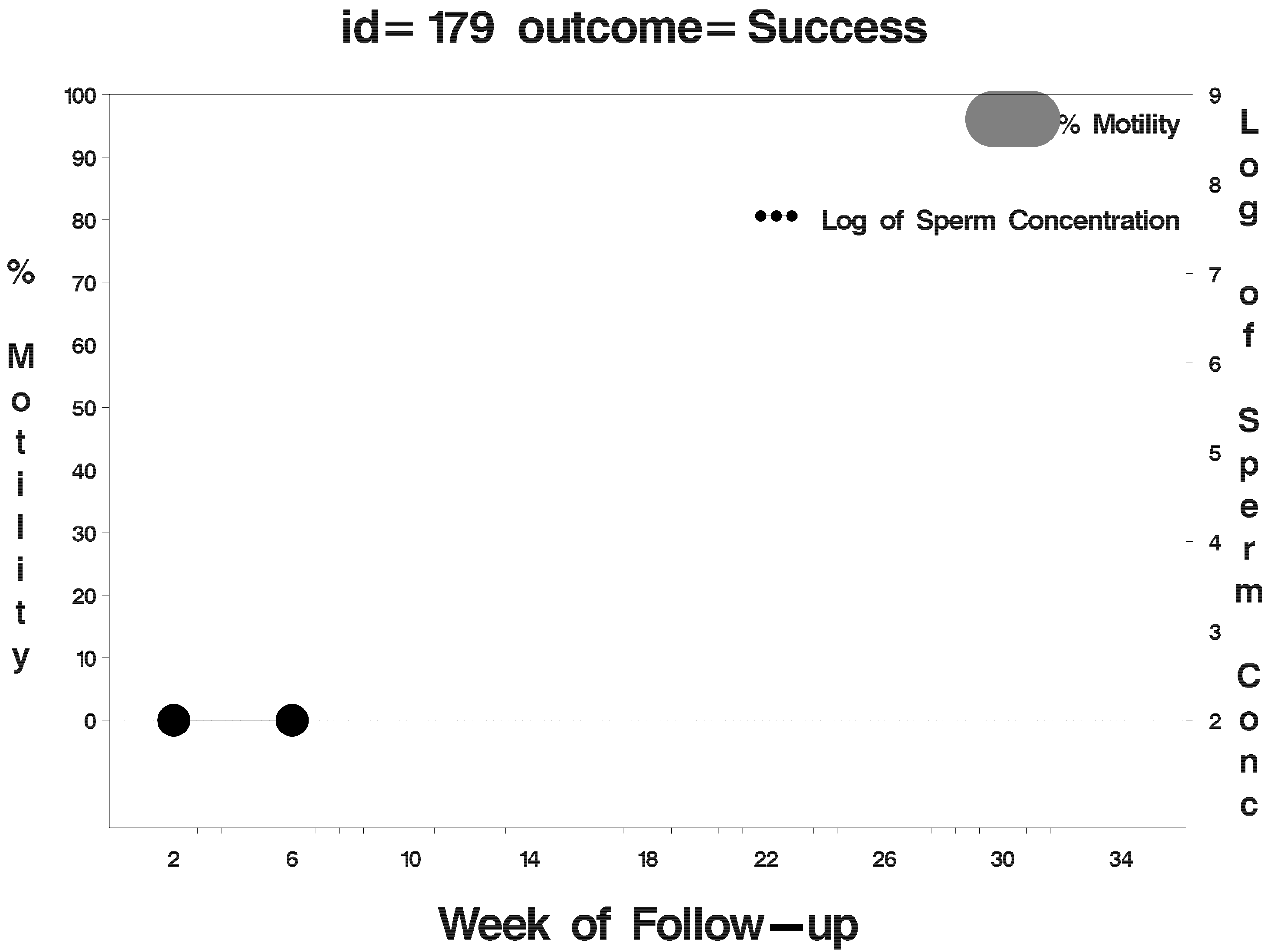

id= 179 outcome= Success
% Motility
Log of Sperm Concentration
100
90
80
70
60
50
40
30
20
10
0
9
8
7
6
5
4
3
2
2
6
10
14
18
22
26
30
34
Week of Follow-up
Log of Sperm Conc

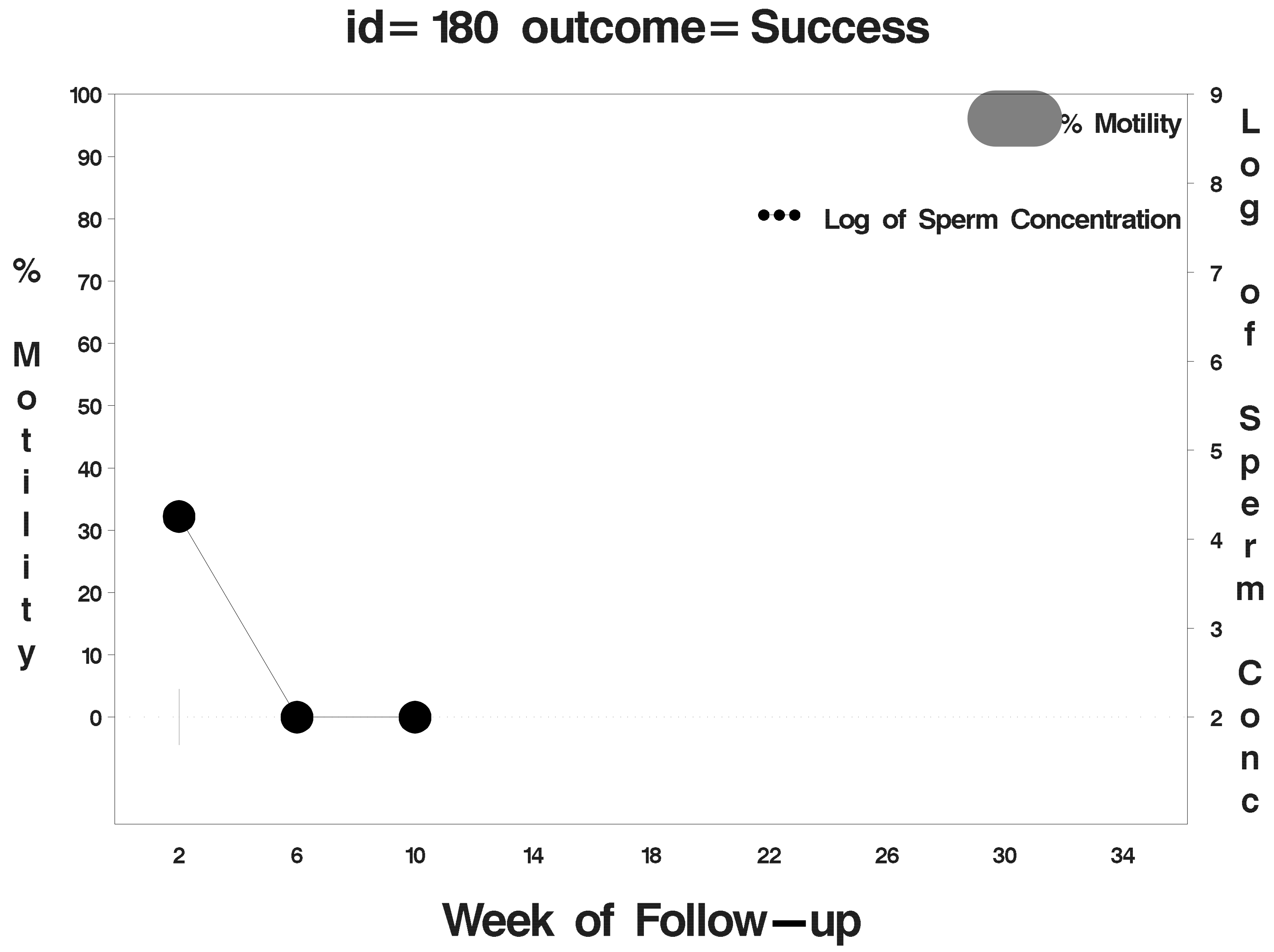
id= 180 outcome= Success
% Motility
Log of Sperm Concentration
100
90
80
70
60
50
40
30
20
10
0
9
8
7
6
5
4
3
2
% Motility
Log of Sperm Conc
2
6
10
14
18
22
26
30
34
Week of Follow—up

id= 181 outcome= Success

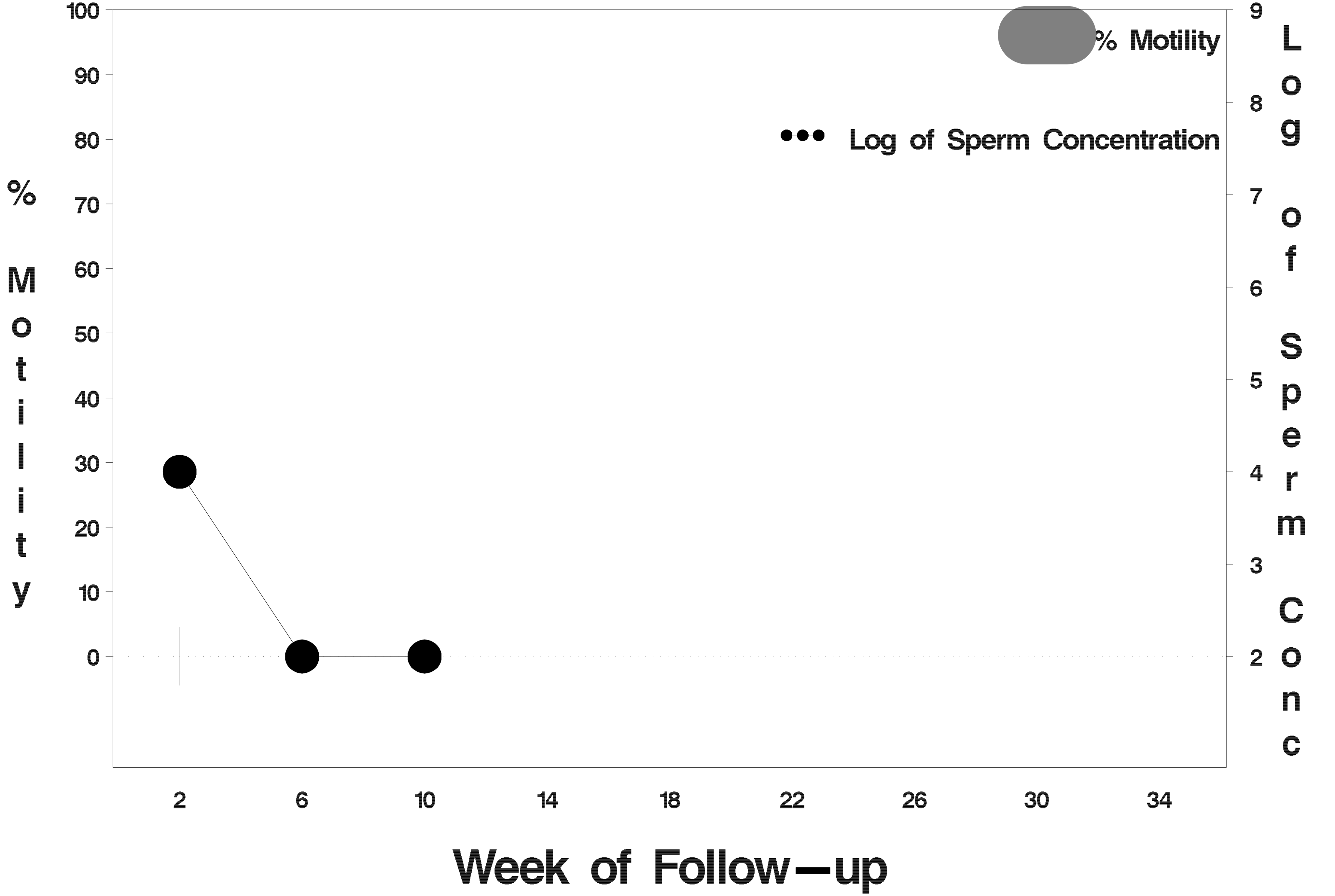

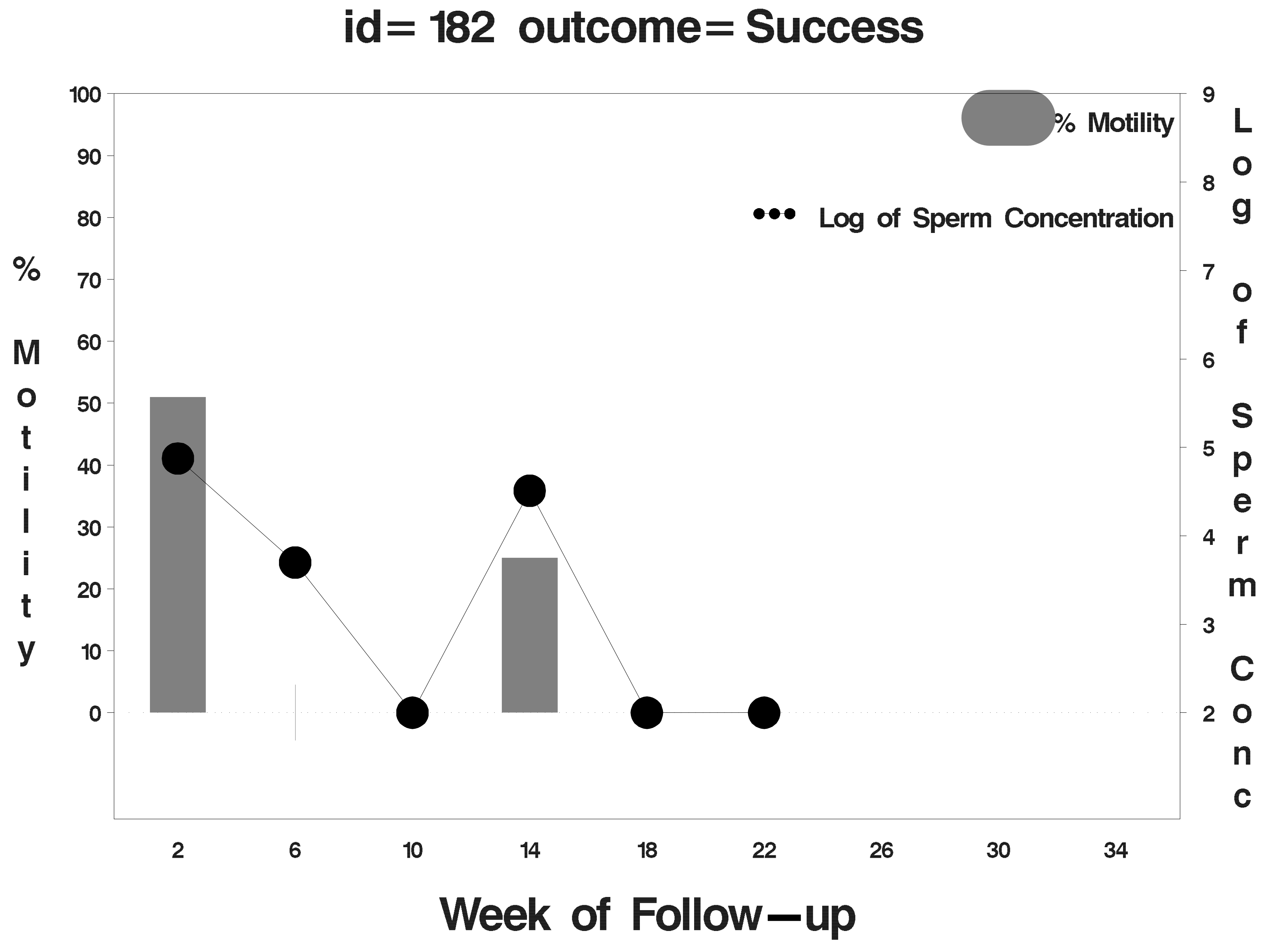
id= 182 outcome= Success
% Motility
Log of Sperm Concentration
100
90
80
70
60
50
40
30
20
10
0
9
8
7
6
5
4
3
2
2
6
10
14
18
22
26
30
34
Week of Follow−up
% Motility
Log of Sperm Conc

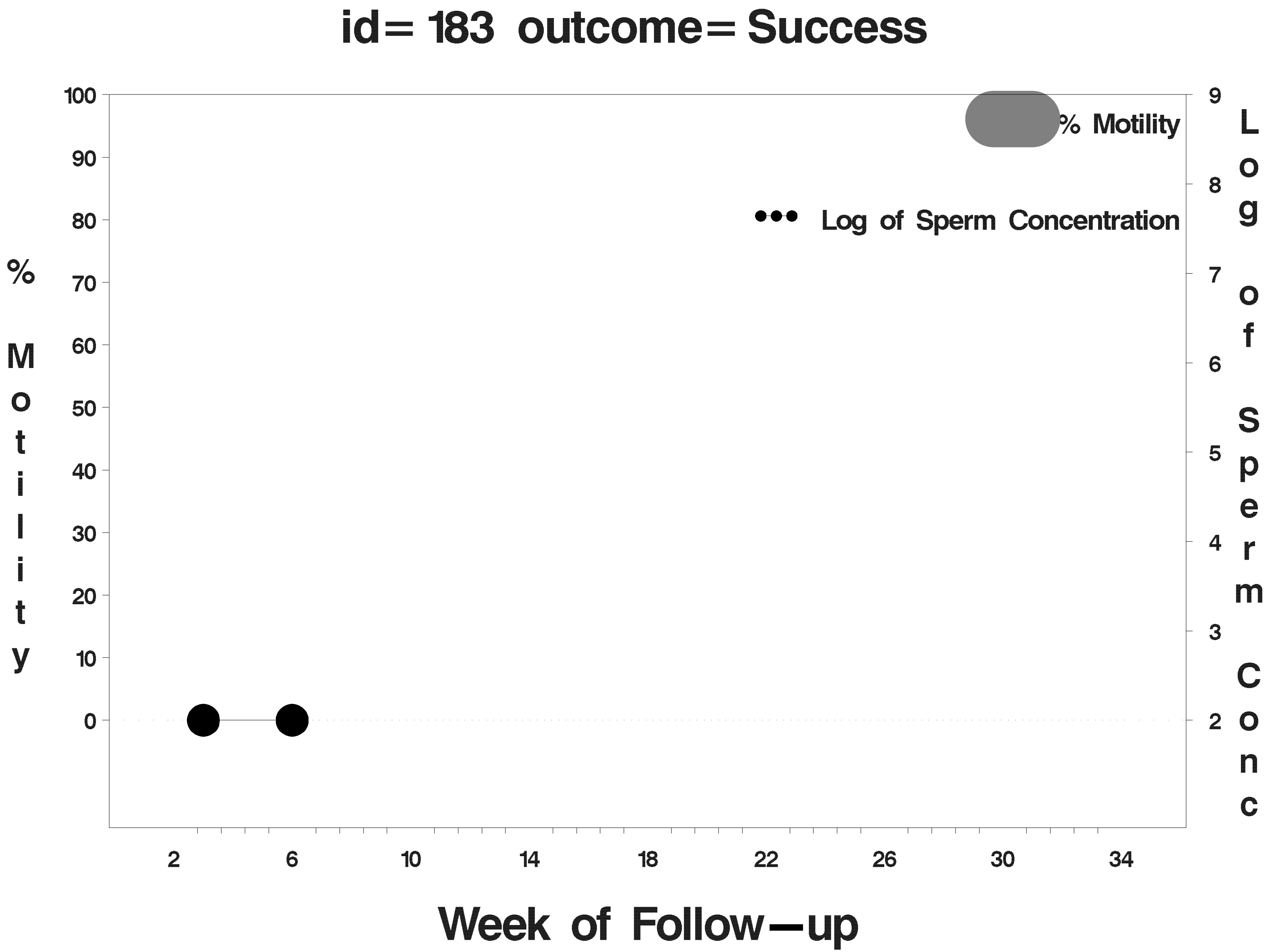
id= 183 outcome= Success
% Motility
Log of Sperm Concentration
100
90
80
70
60
50
40
30
20
10
0
9
8
7
6
5
4
3
2
2
6
10
14
18
22
26
30
34
Week of Follow—up
Log of Sperm Conc

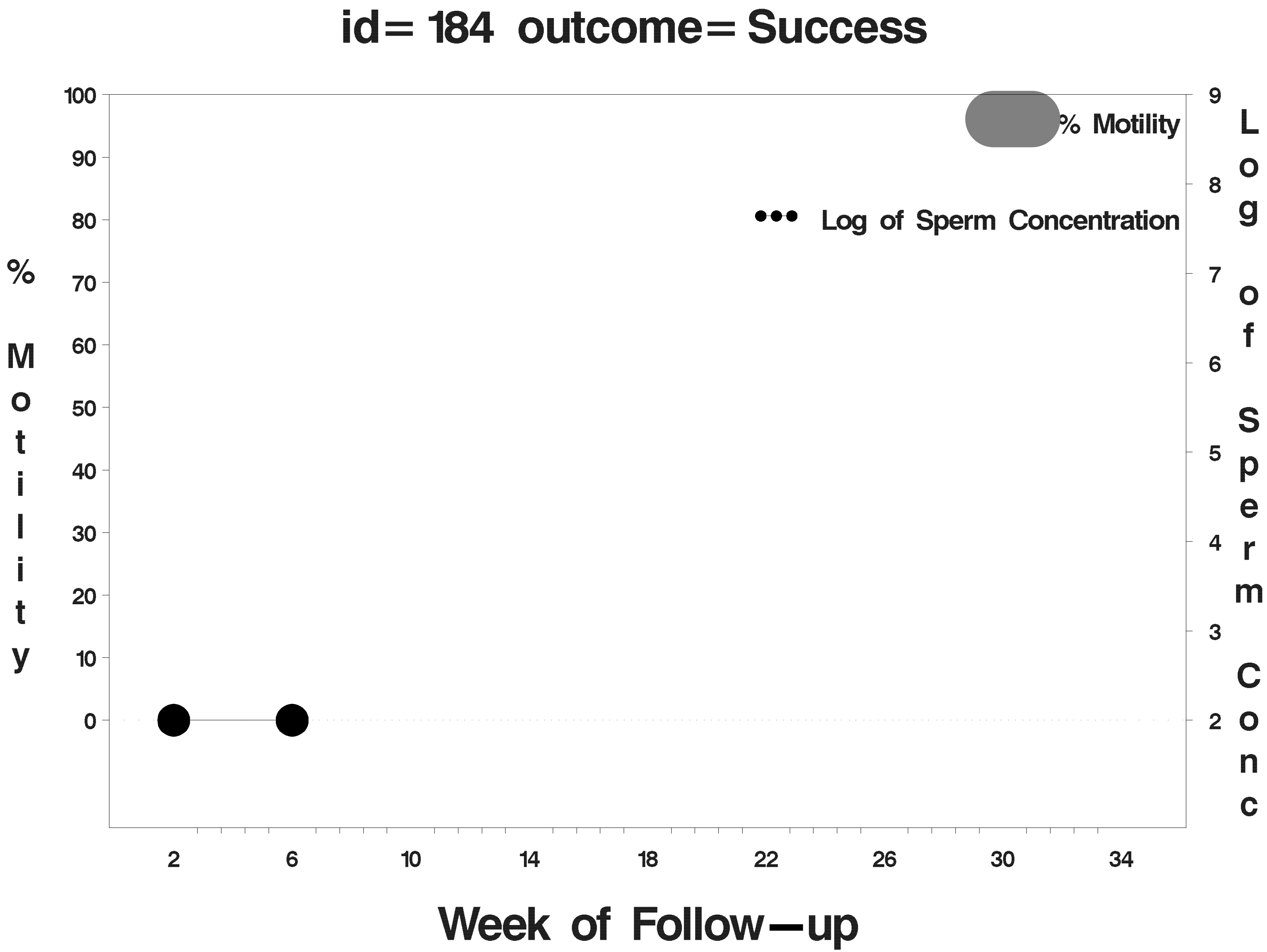
id= 184 outcome= Success
% Motility
Log of Sperm Concentration
100
90
80
70
60
50
40
30
20
10
0
9
8
7
6
5
4
3
2
2
6
10
14
18
22
26
30
34
Week of Follow−up
Log of Sperm Conc

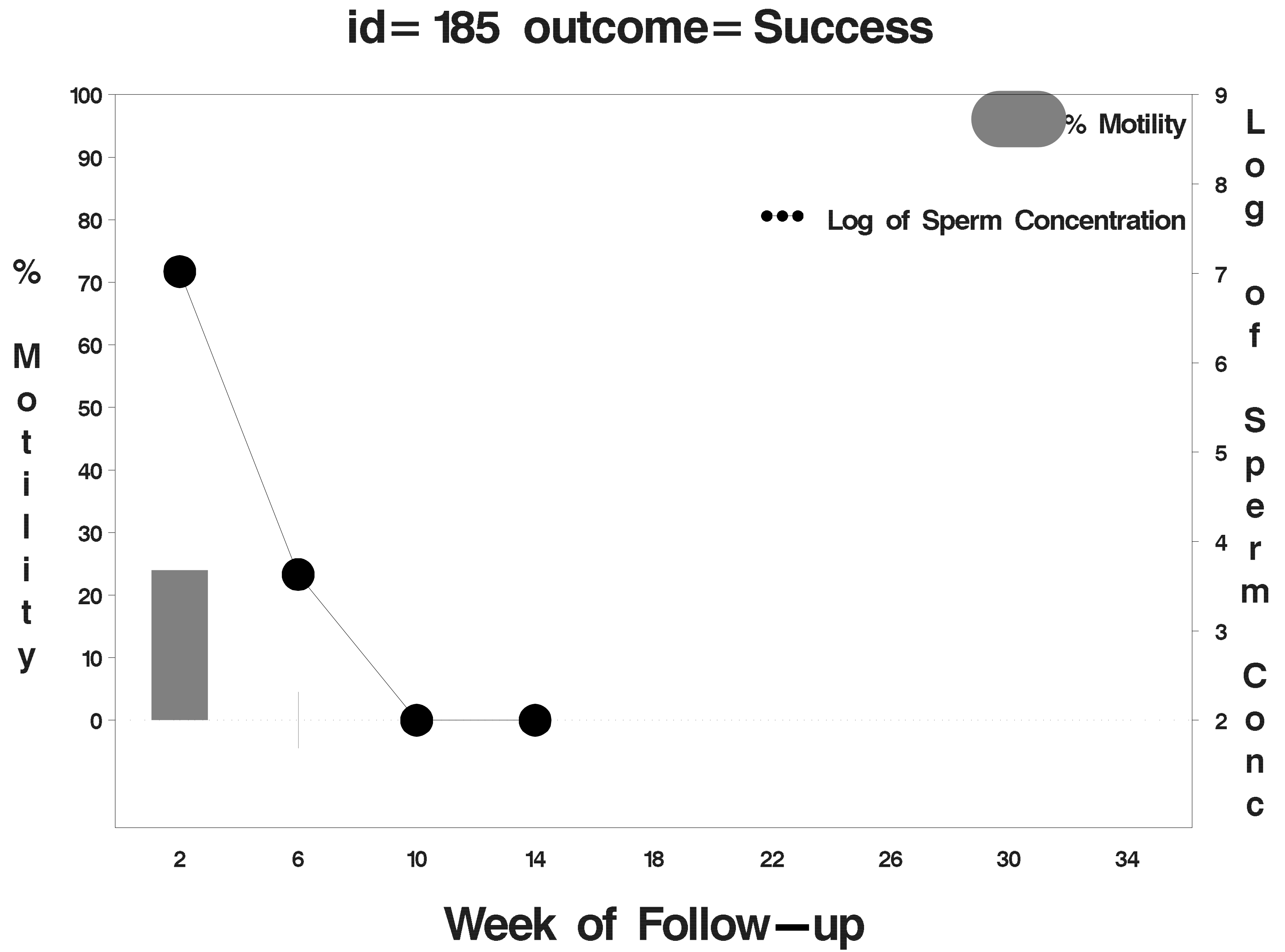

id= 185 outcome= Success
% Motility
Log of Sperm Concentration
% Motility
Log of Sperm Conc
Week of Follow—up
100
90
80
70
60
50
40
30
20
10
0
9
8
7
6
5
4
3
2
2
6
10
14
18
22
26
30
34

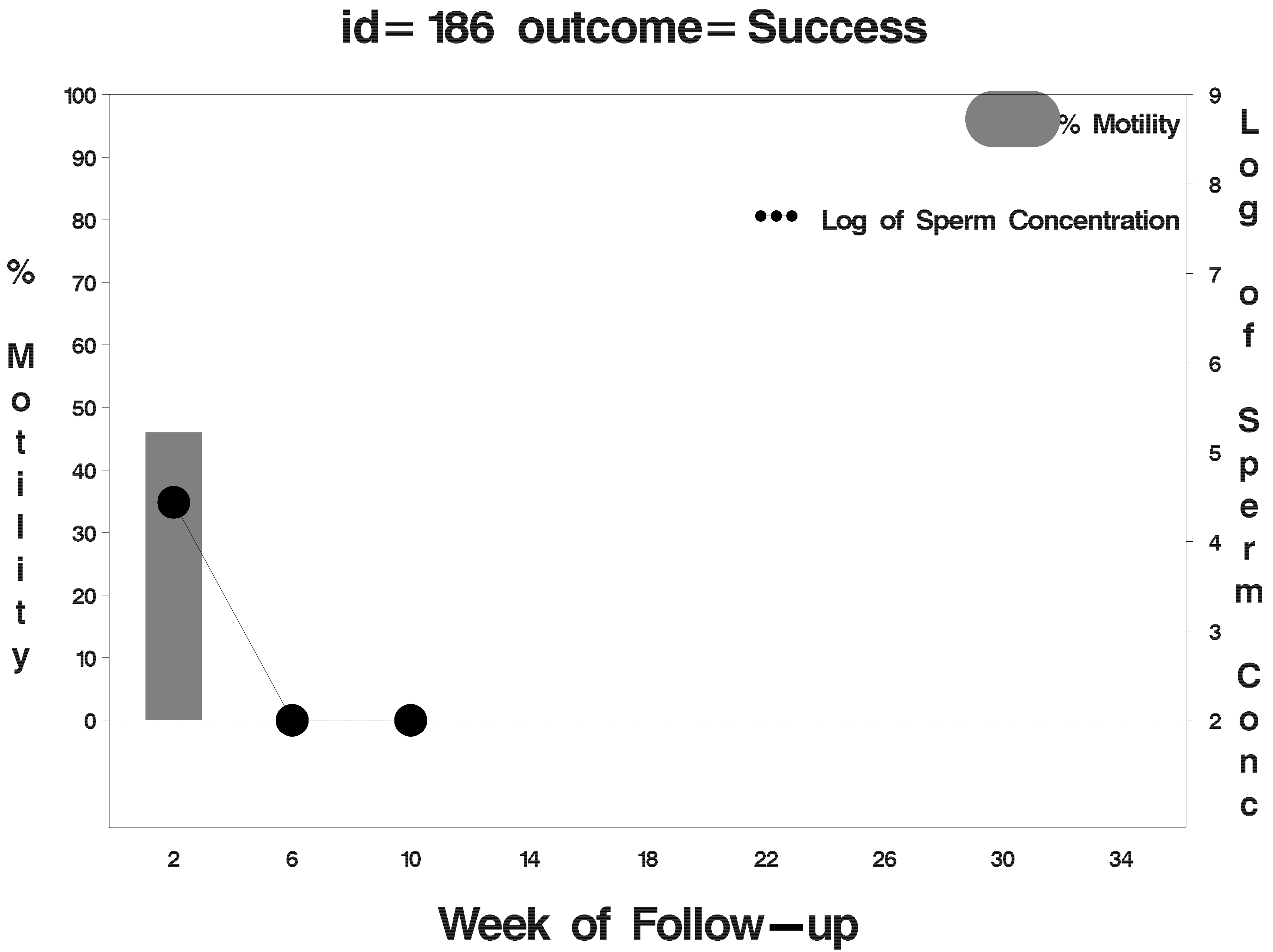

id= 186 outcome= Success
% Motility
Log of Sperm Concentration
% Motility
Log of Sperm Conc
Week of Follow-up
100
90
80
70
60
50
40
30
20
10
0
9
8
7
6
5
4
3
2
2
6
10
14
18
22
26
30
34

id= 187 outcome= Success

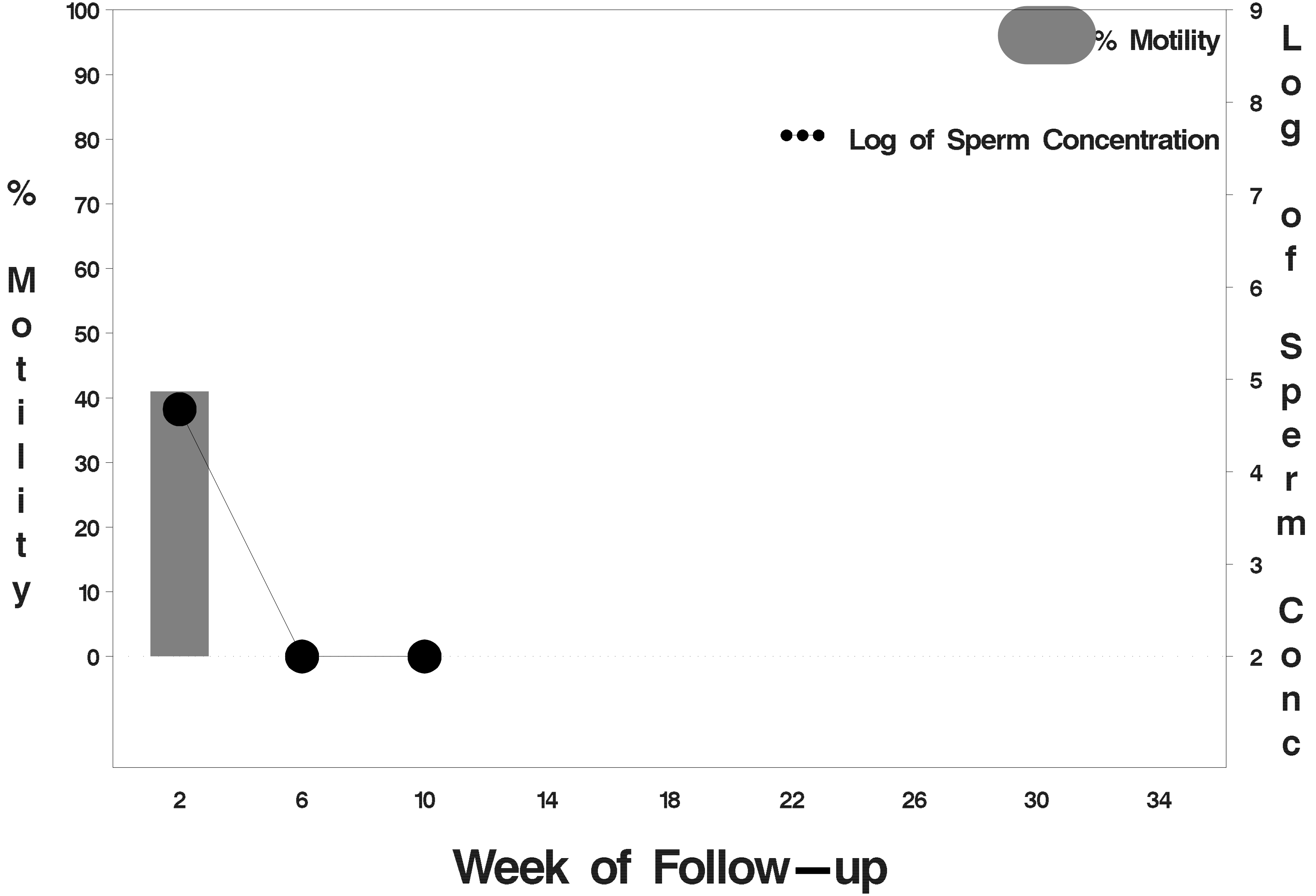

id= 188 outcome= Success

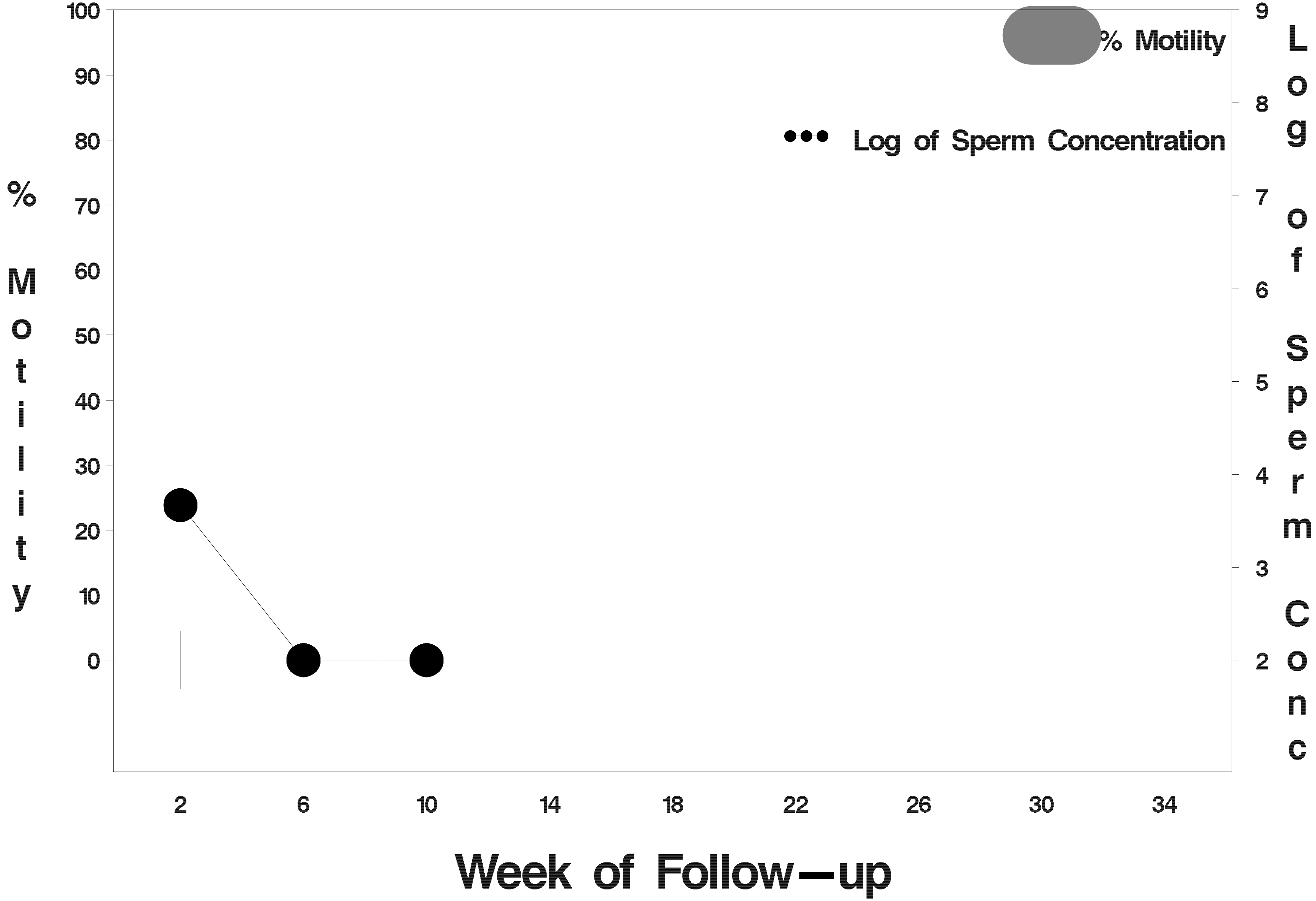

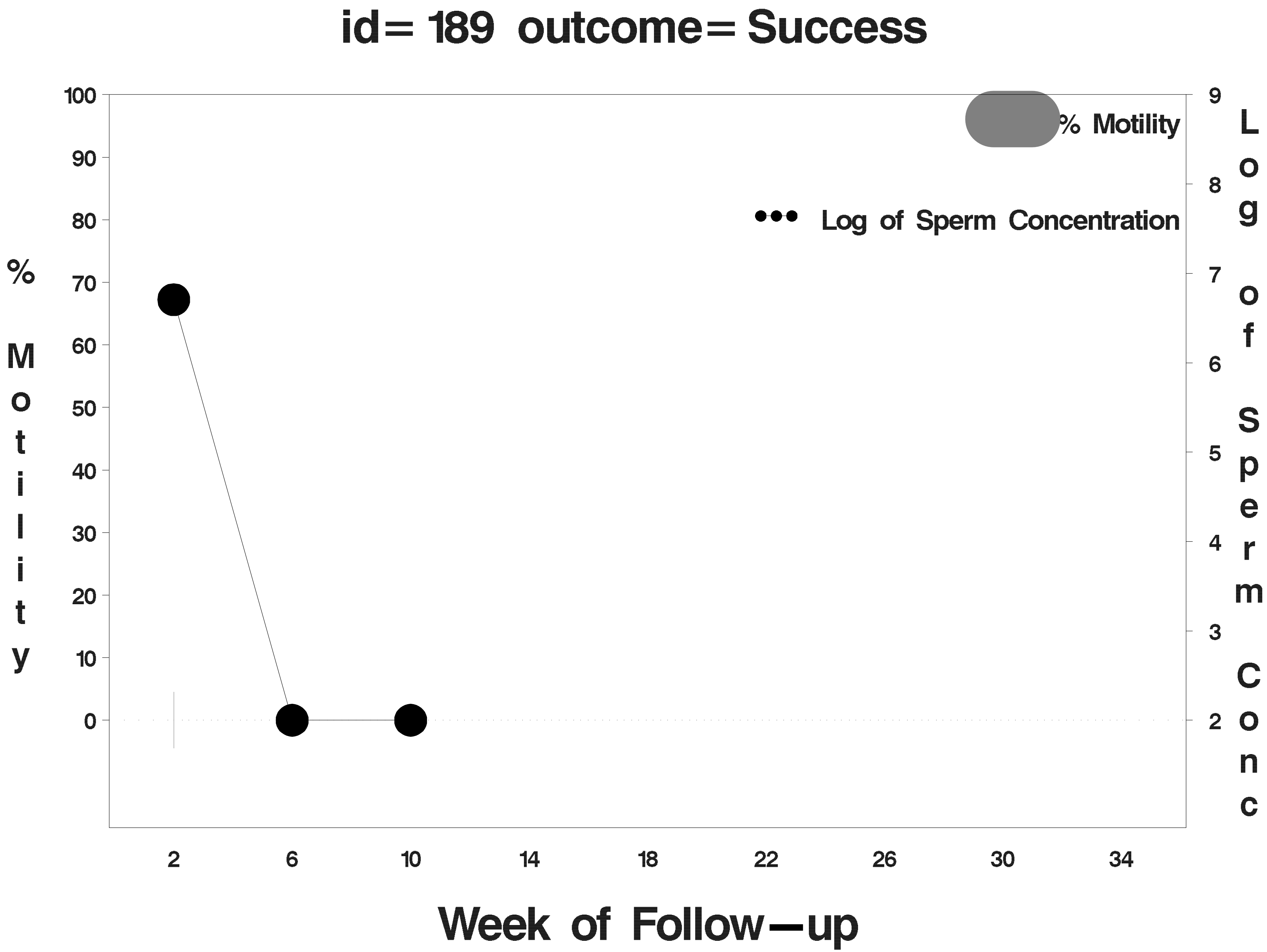

id= 189 outcome= Success
% Motility
Log of Sperm Concentration
100
90
80
70
60
50
40
30
20
10
0
9
8
7
6
5
4
3
2
2
6
10
14
18
22
26
30
34
Week of Follow—up
% Motility
Log of Sperm Conc

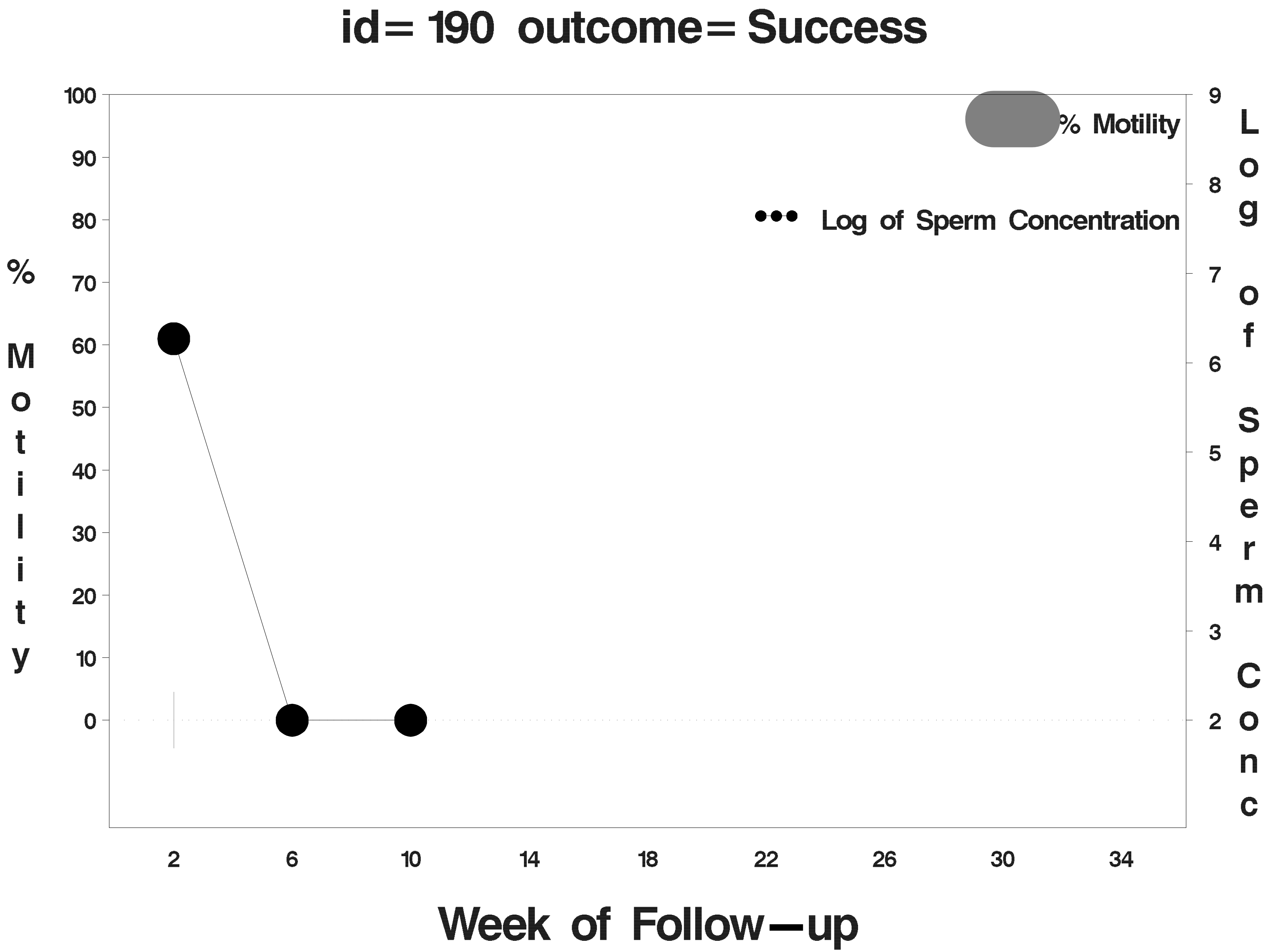
id= 190 outcome= Success
% Motility
Log of Sperm Concentration
100
90
80
70
60
50
40
30
20
10
0
9
8
7
6
5
4
3
2
% Motility
Log of Sperm Conc
2
6
10
14
18
22
26
30
34
Week of Follow—up

id= 191 outcome= Success

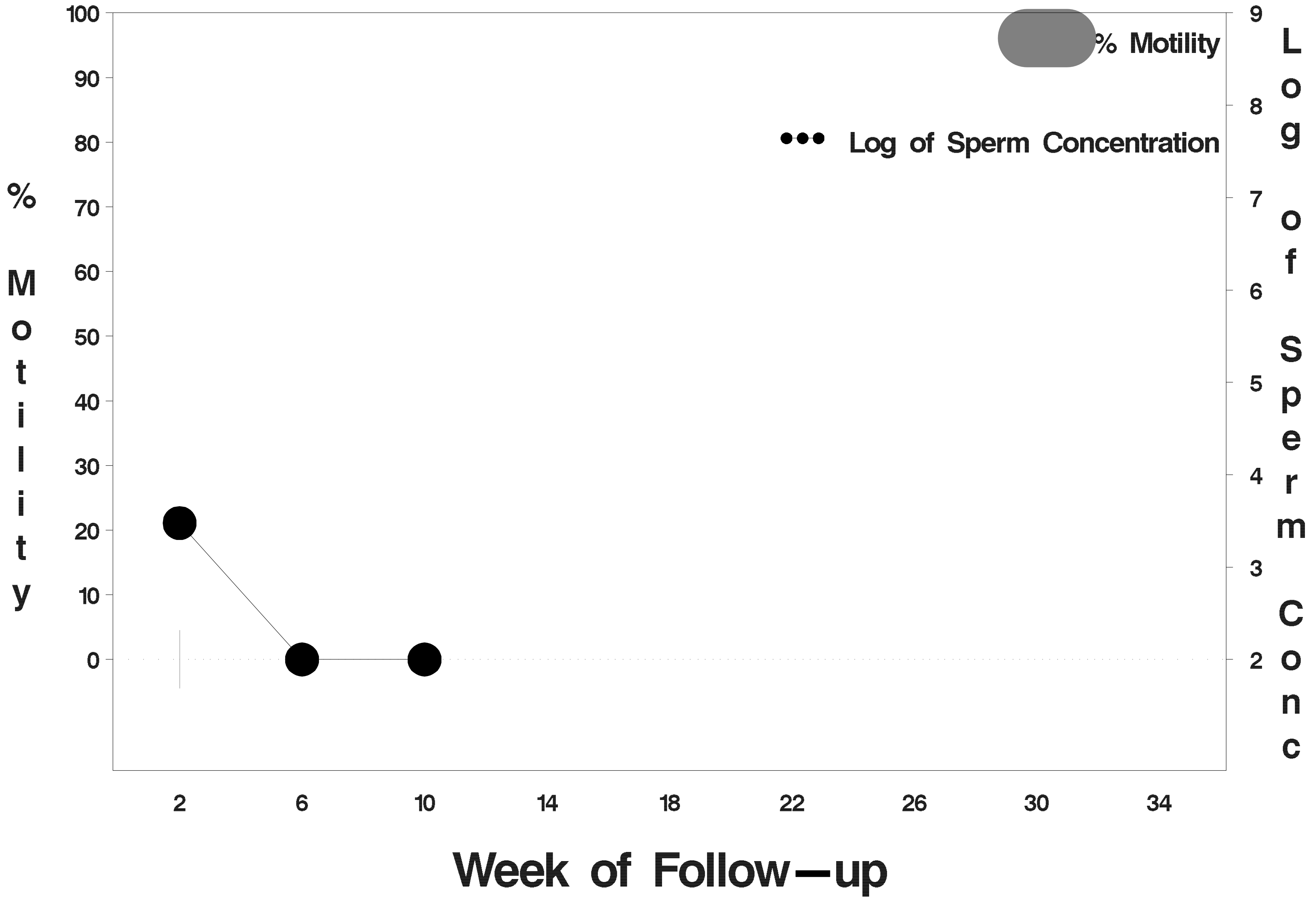

id= 192 outcome= Failure

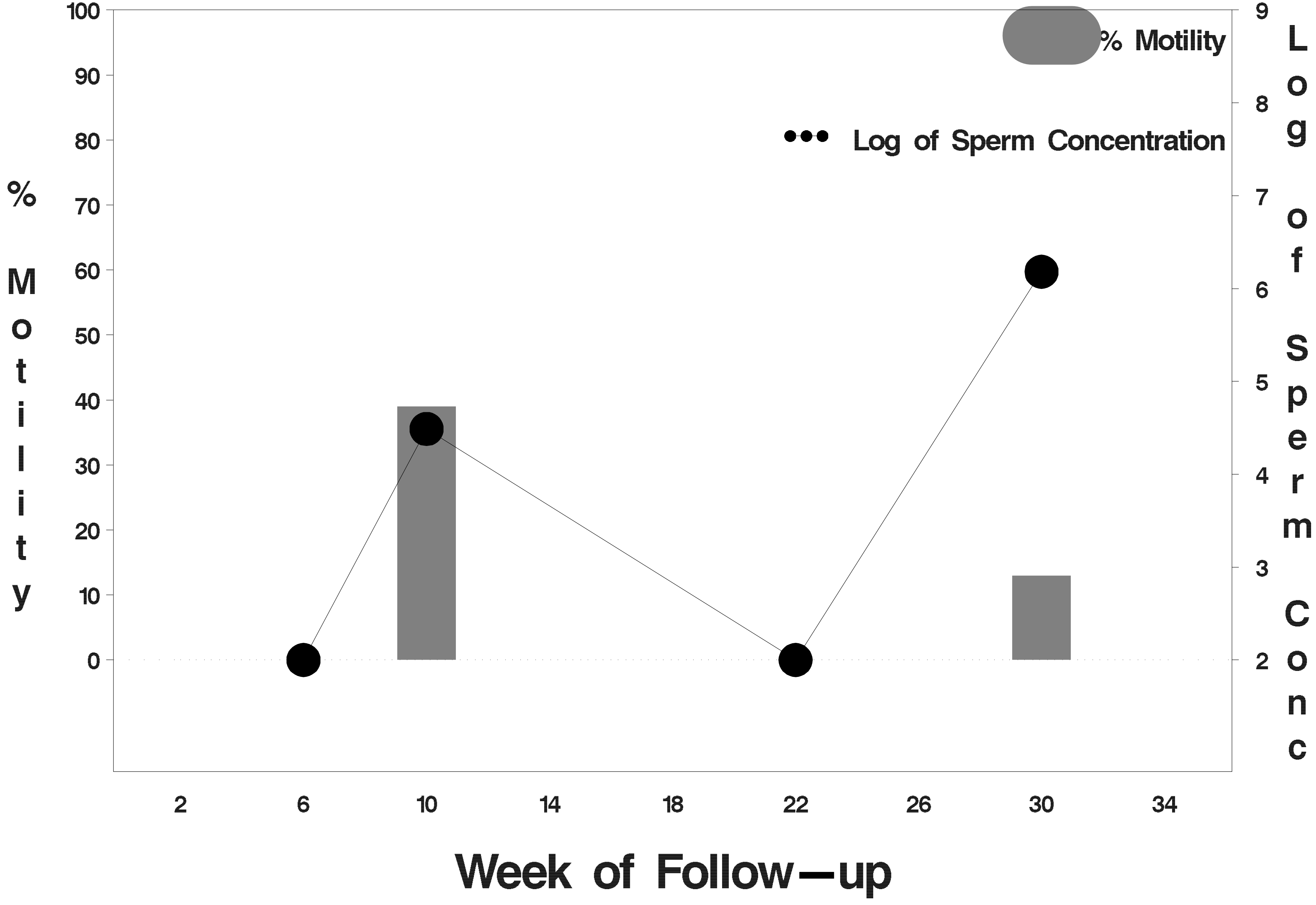

id= 193 outcome= Success

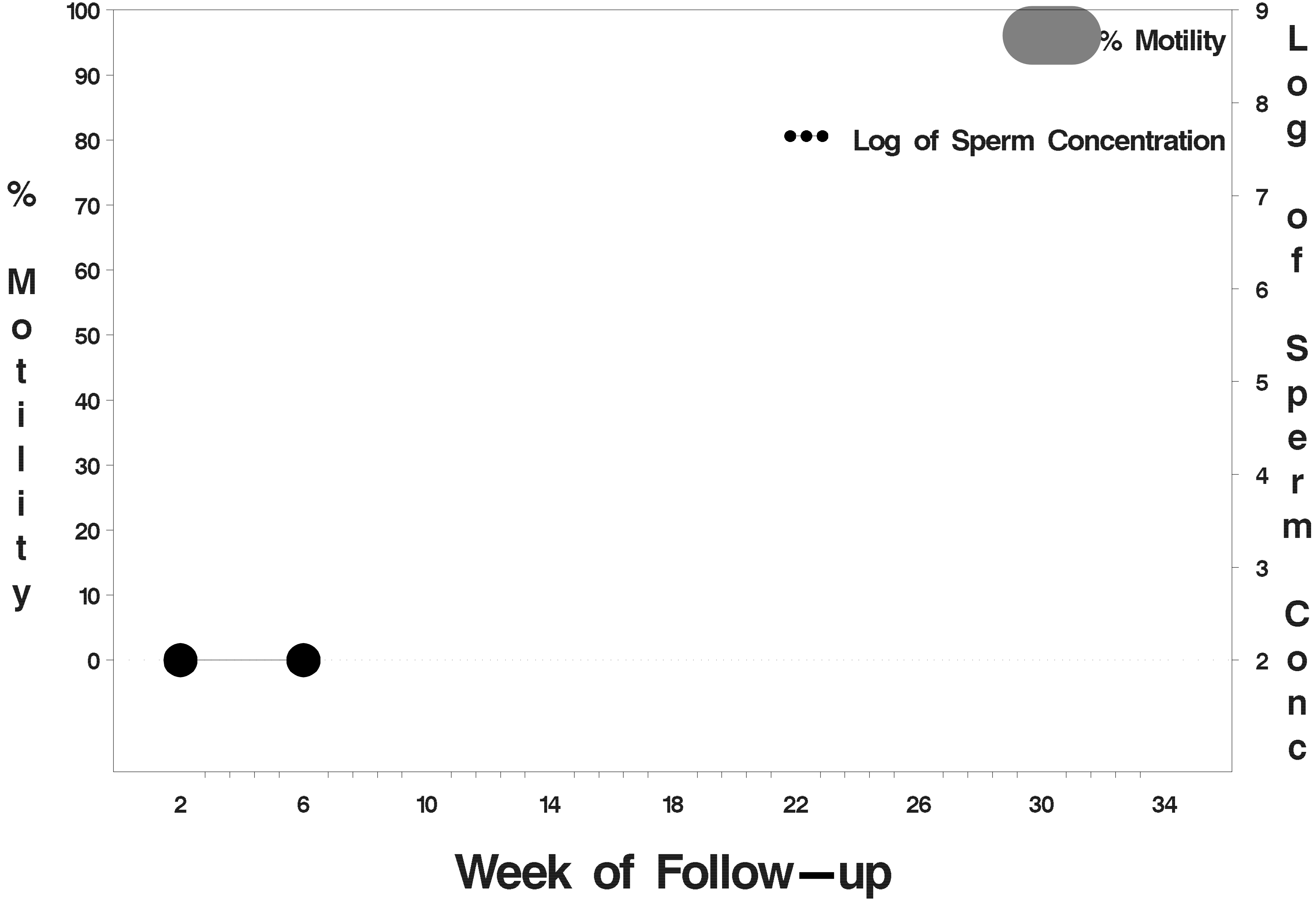

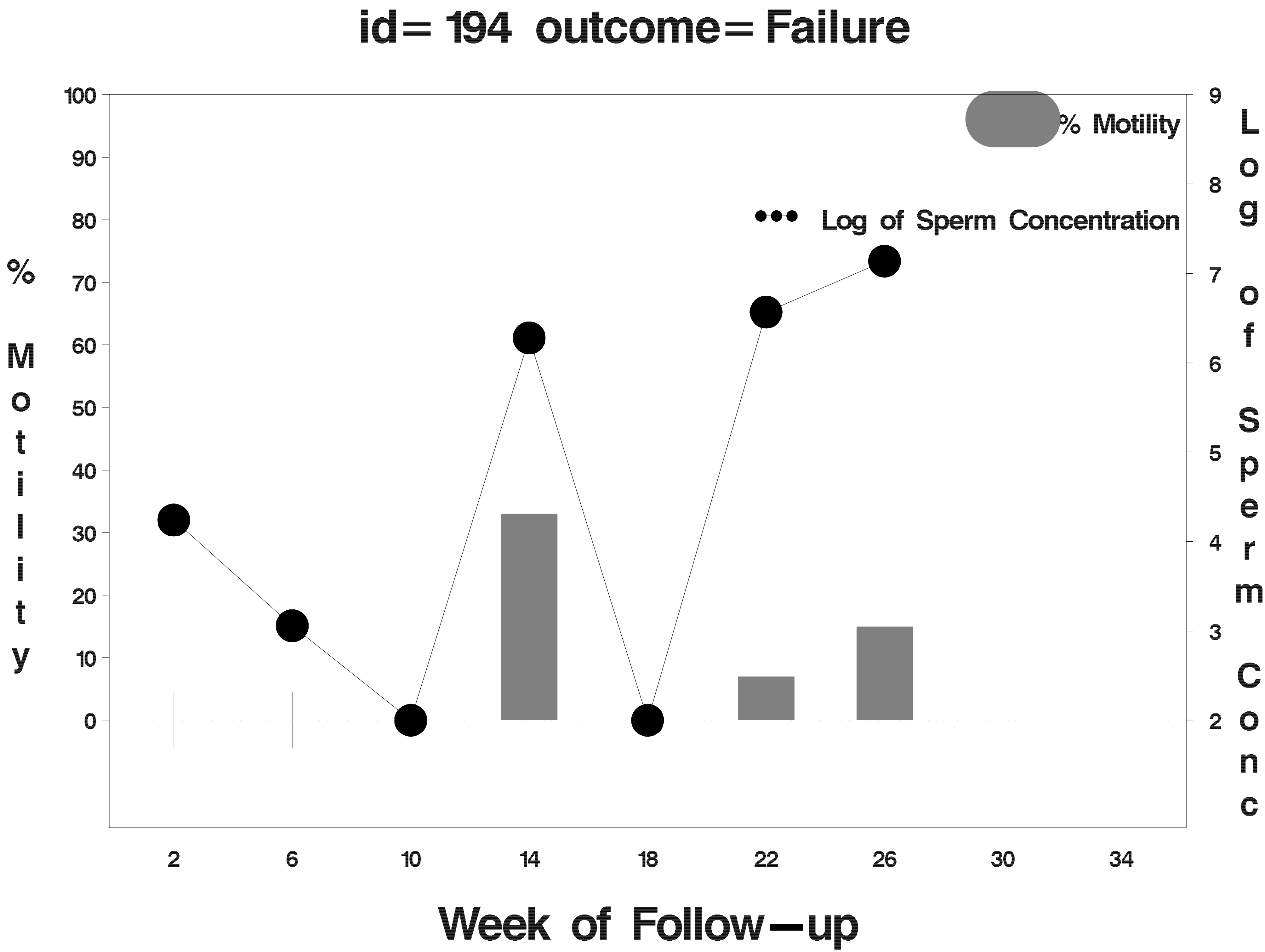
id= 194 outcome= Failure
% Motility
Log of Sperm Concentration
% Motility
Log of Sperm Conc
Week of Follow-up
0
10
20
30
40
50
60
70
80
90
100
2
3
4
5
6
7
8
9
2
6
10
14
18
22
26
30
34

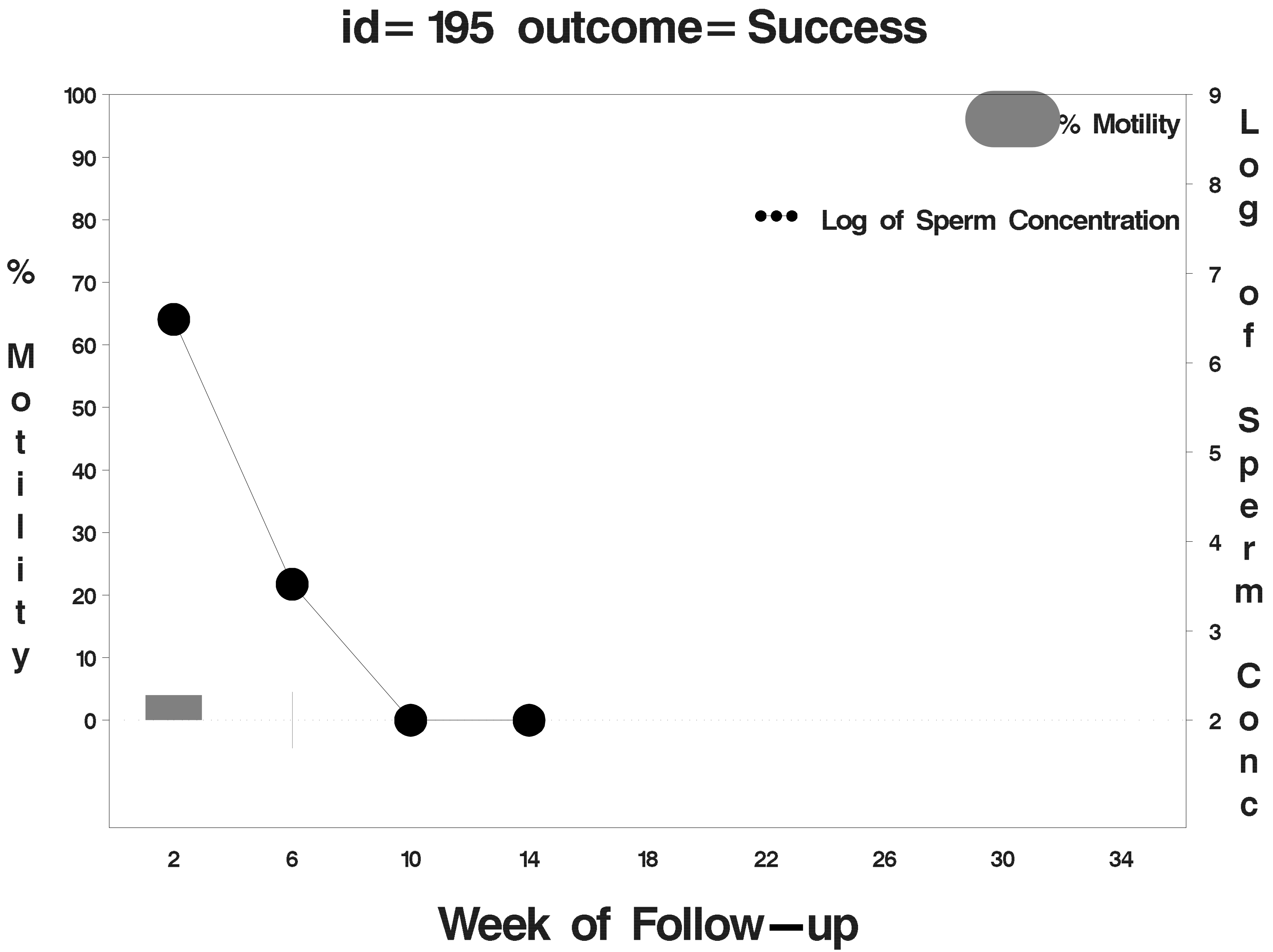
id= 195 outcome= Success
% Motility
Log of Sperm Concentration
100
90
80
70
60
50
40
30
20
10
0
9
8
7
6
5
4
3
2
2
6
10
14
18
22
26
30
34
Week of Follow—up
Log of Sperm Conc

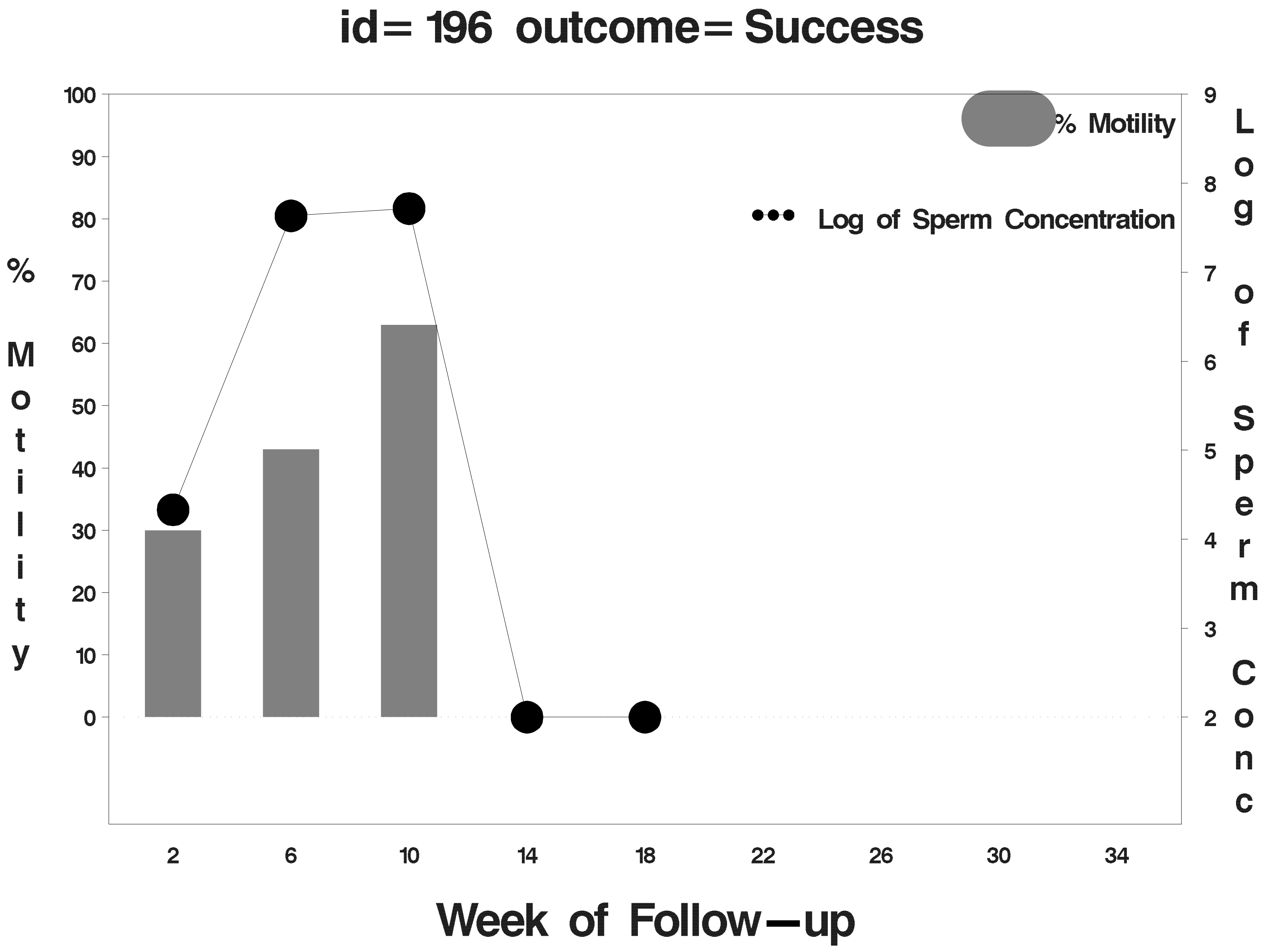
id= 196 outcome= Success
% Motility
Log of Sperm Concentration
100
90
80
70
60
50
40
30
20
10
0
9
8
7
6
5
4
3
2
2
6
10
14
18
22
26
30
34
Week of Follow—up
Log of Sperm Conc

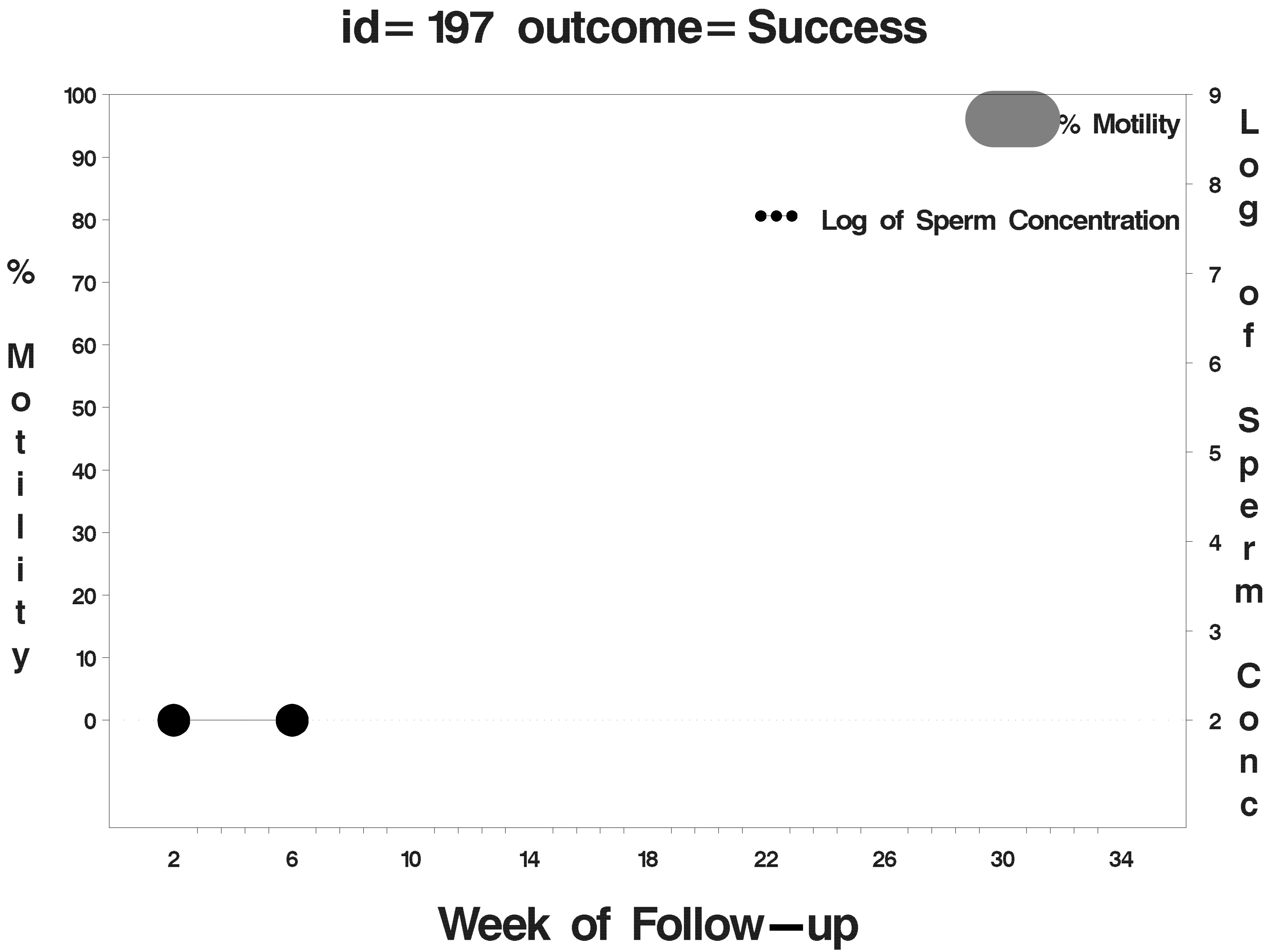
id= 197 outcome= Success
% Motility
Log of Sperm Concentration
100
90
80
70
60
50
40
30
20
10
0
9
8
7
6
5
4
3
2
2
6
10
14
18
22
26
30
34
Week of Follow−up
% Motility
Log of Sperm Conc

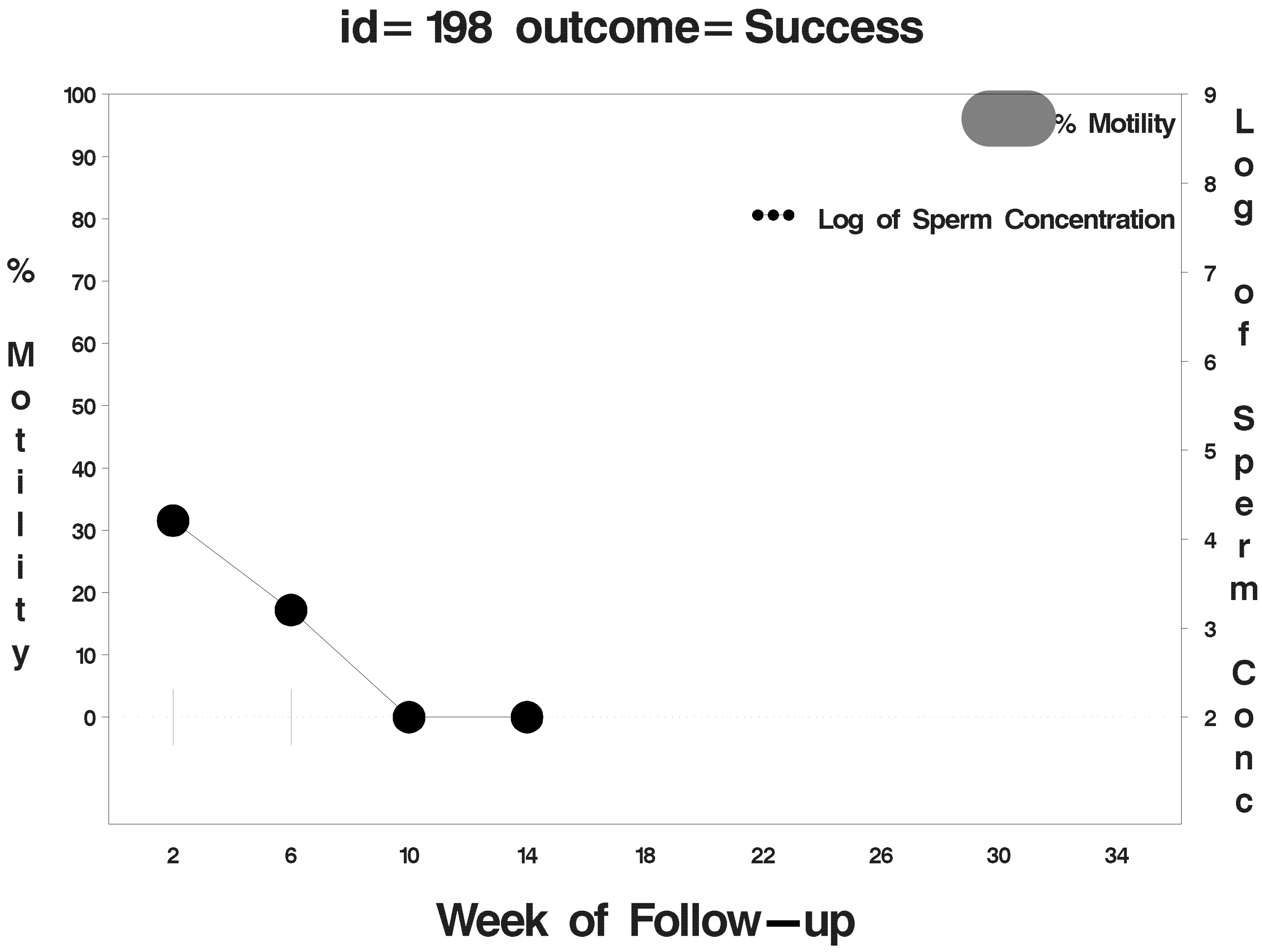

id= 198 outcome= Success
% Motility
Log of Sperm Concentration
100
90
80
70
60
50
40
30
20
10
0
9
8
7
6
5
4
3
2
2
6
10
14
18
22
26
30
34
Week of Follow−up

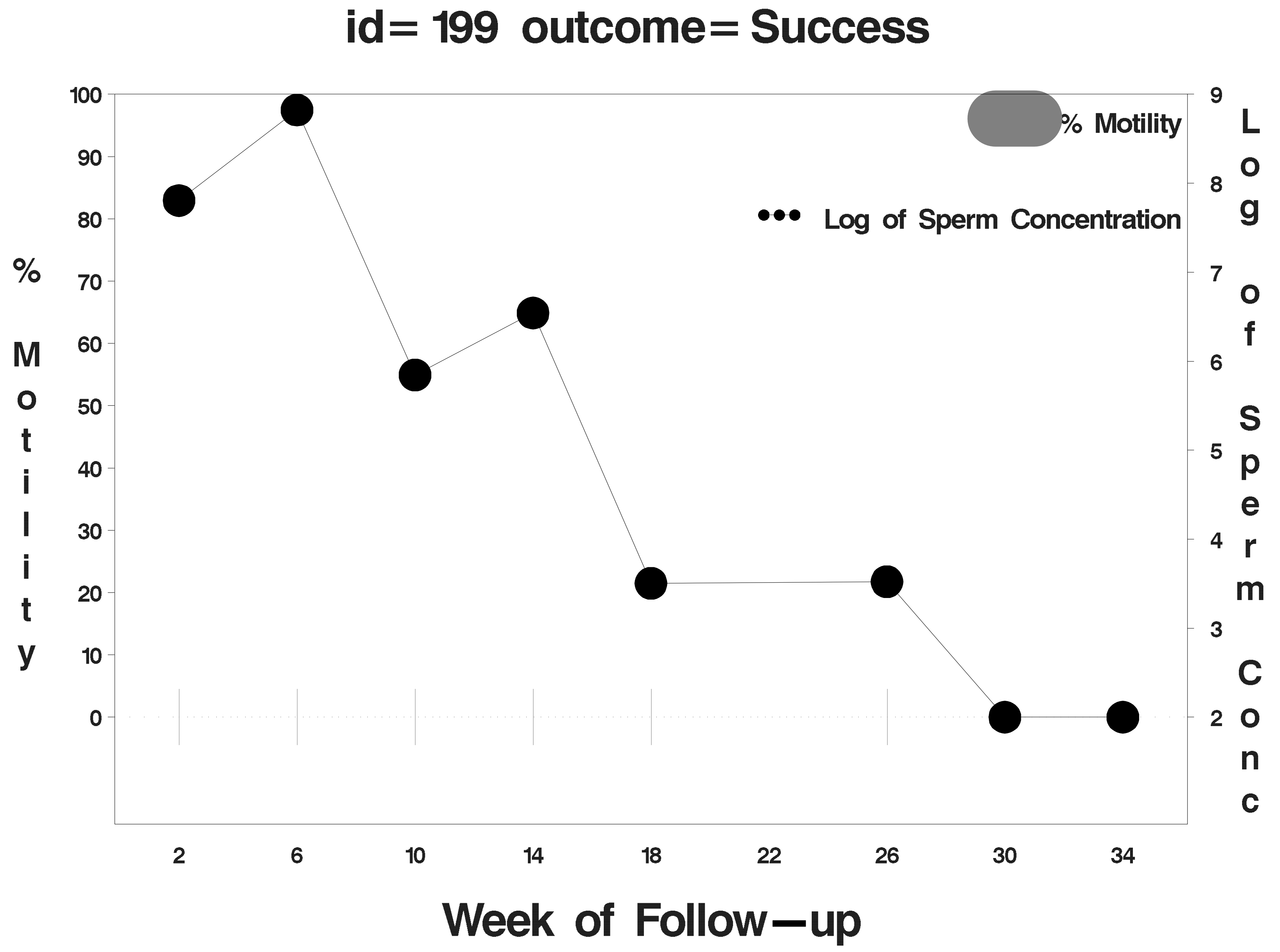

id= 199 outcome= Success
% Motility
Log of Sperm Concentration
% Motility
Log of Sperm Conc
Week of Follow-up
100
90
80
70
60
50
40
30
20
10
0
9
8
7
6
5
4
3
2
2
6
10
14
18
22
26
30
34

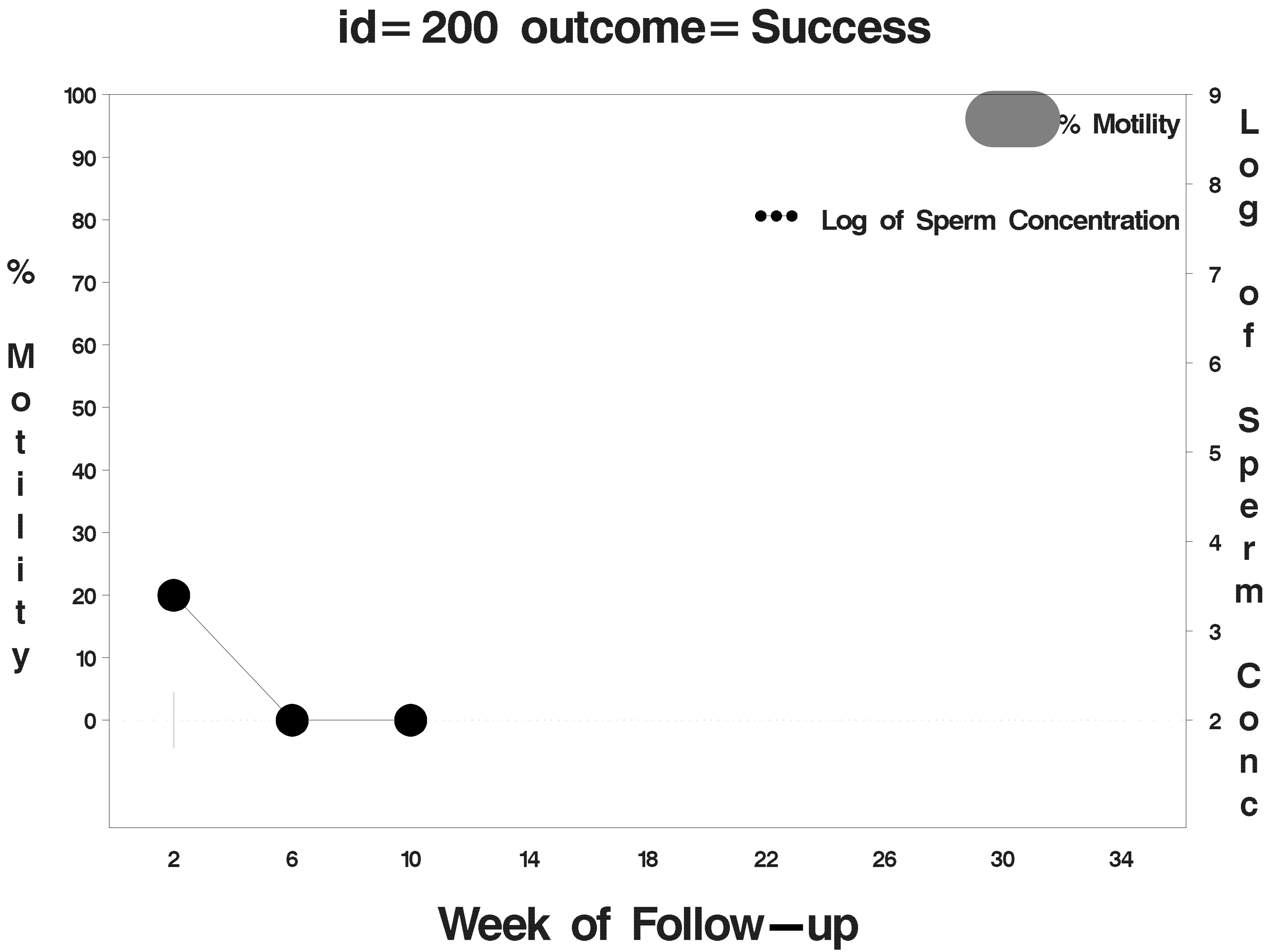

id= 200 outcome= Success
% Motility
Log of Sperm Concentration
100
90
80
70
60
50
40
30
20
10
0
9
8
7
6
5
4
3
2
2
6
10
14
18
22
26
30
34
Week of Follow—up

id= 201 outcome= Success

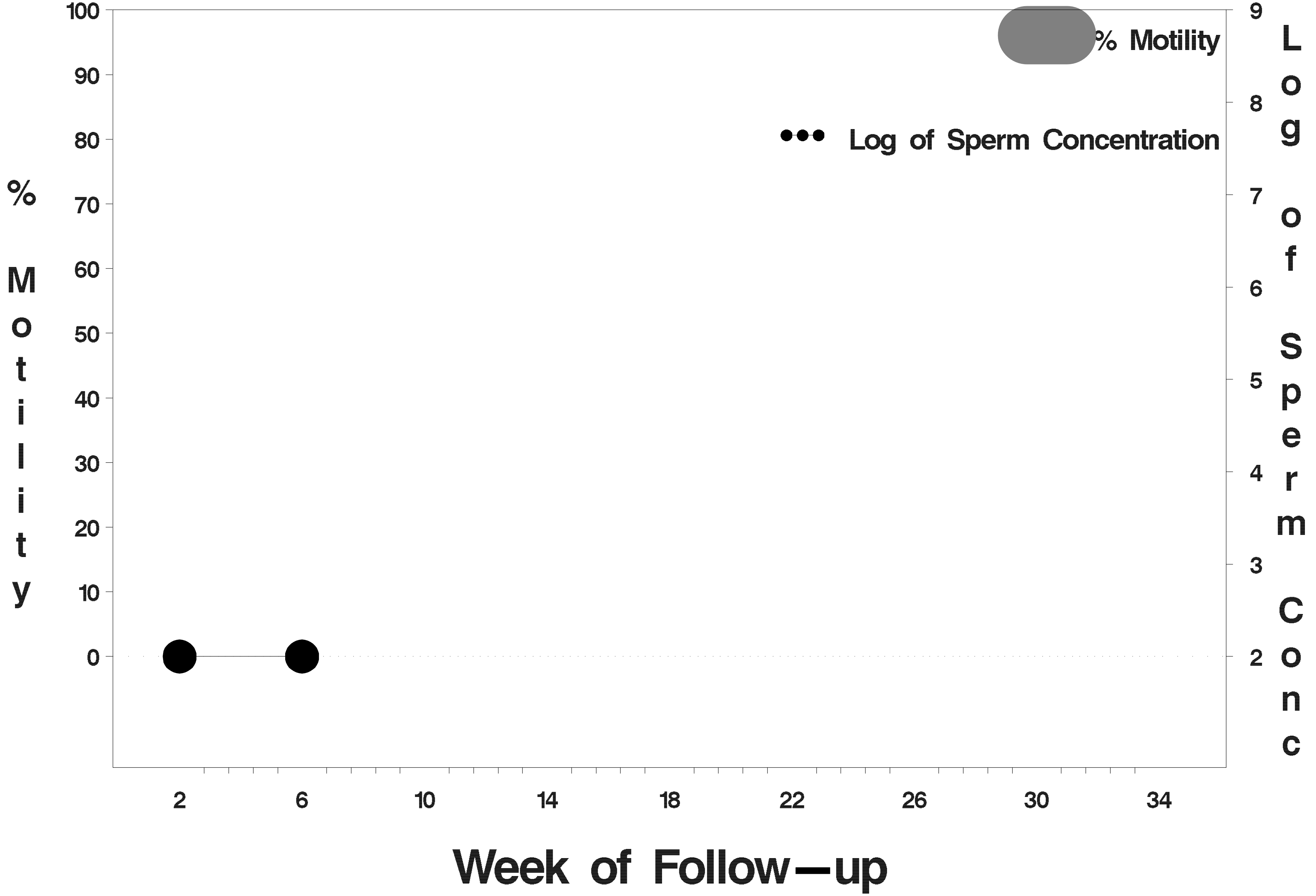

id= 202 outcome= Success

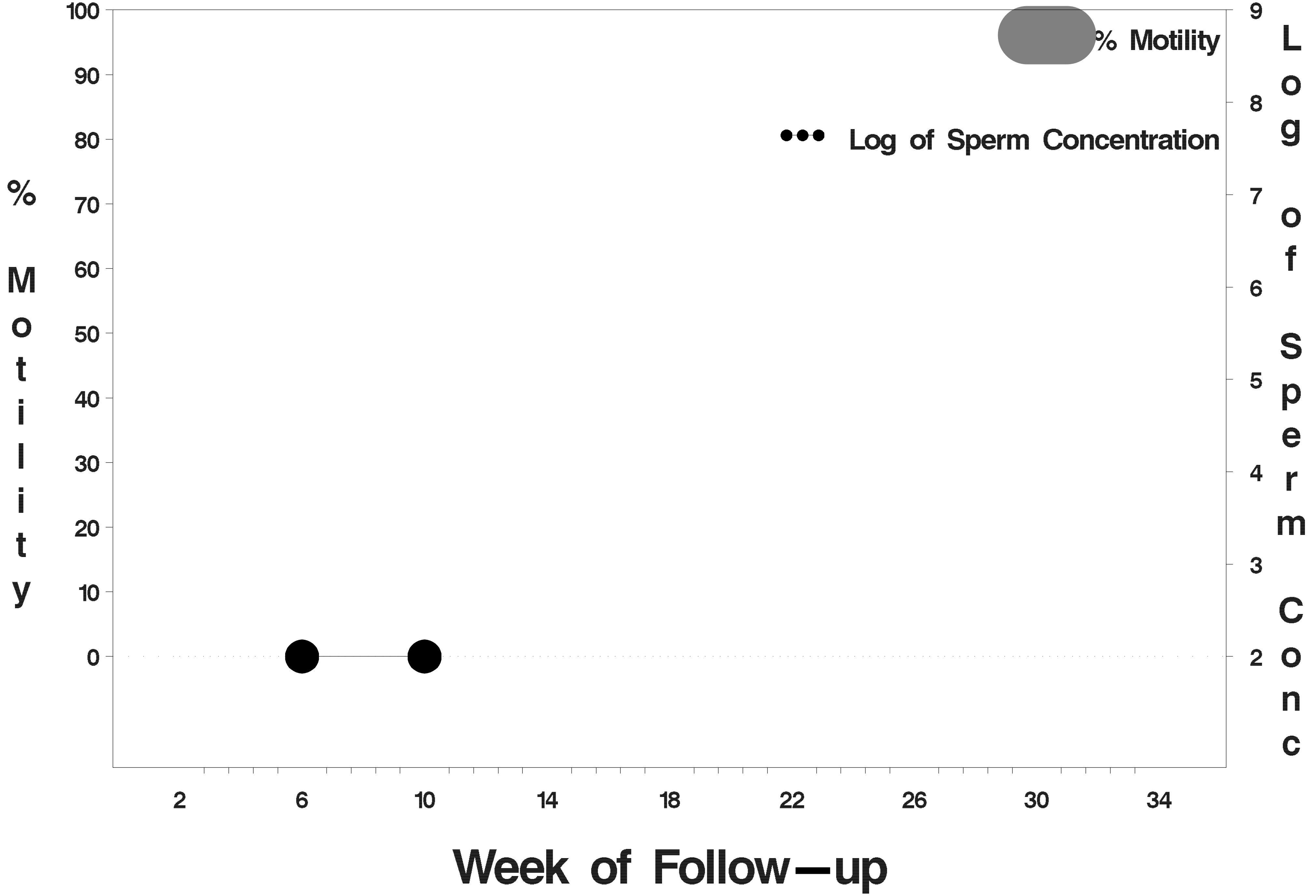

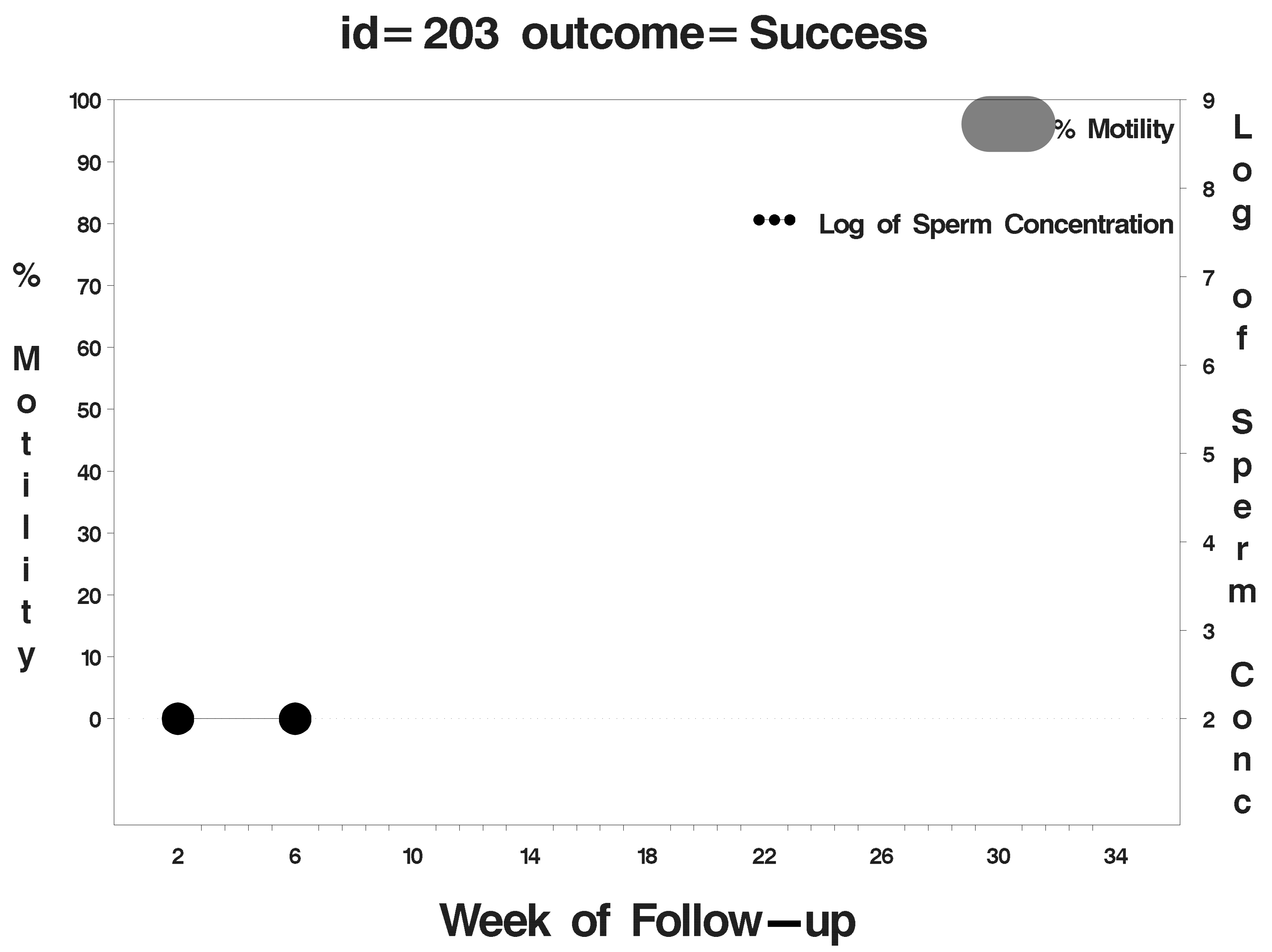

id= 203 outcome= Success
% Motility
Log of Sperm Concentration
100
90
80
70
60
50
40
30
20
10
0
9
8
7
6
5
4
3
2
2
6
10
14
18
22
26
30
34
Week of Follow—up

id= 204 outcome= Success

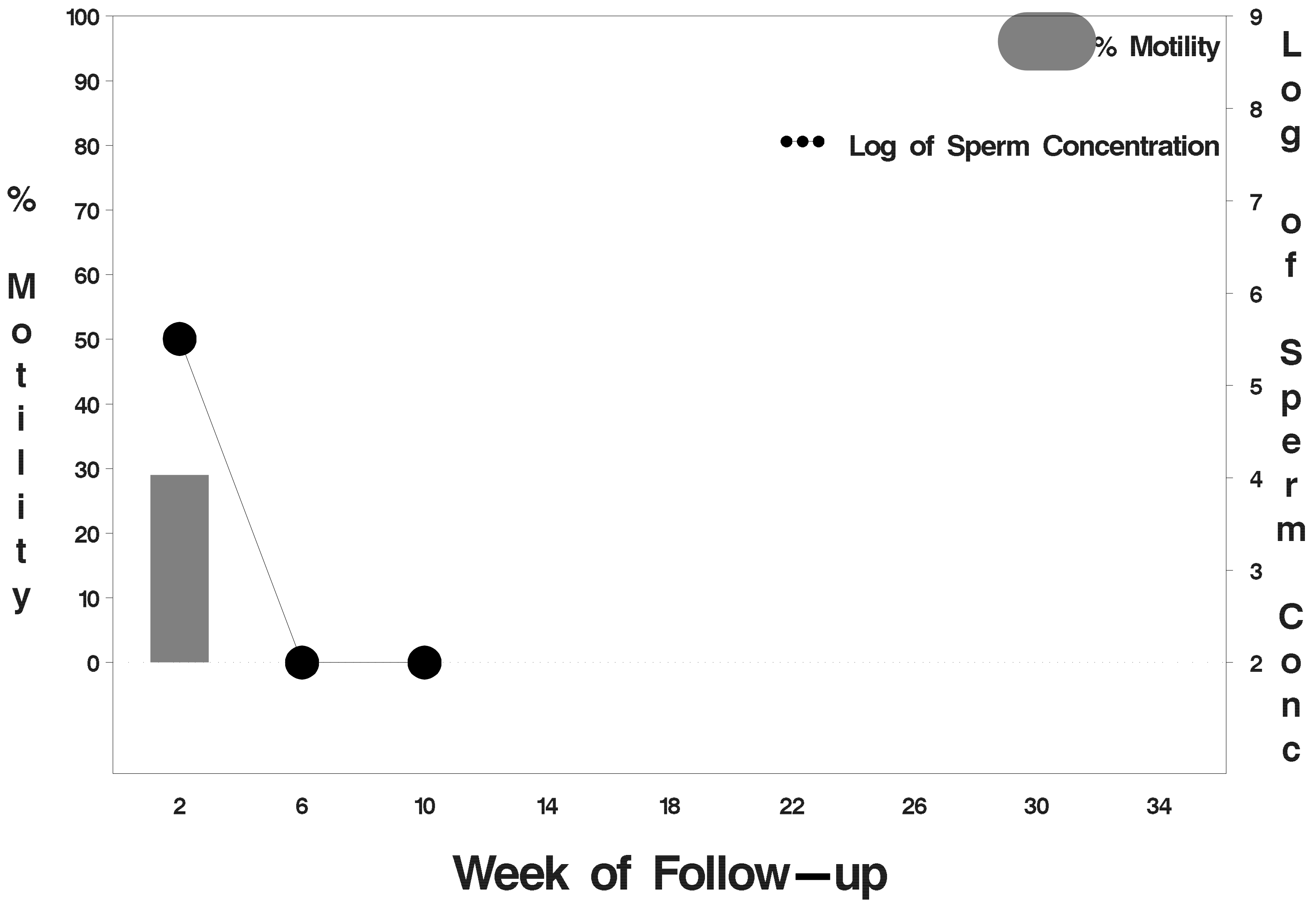

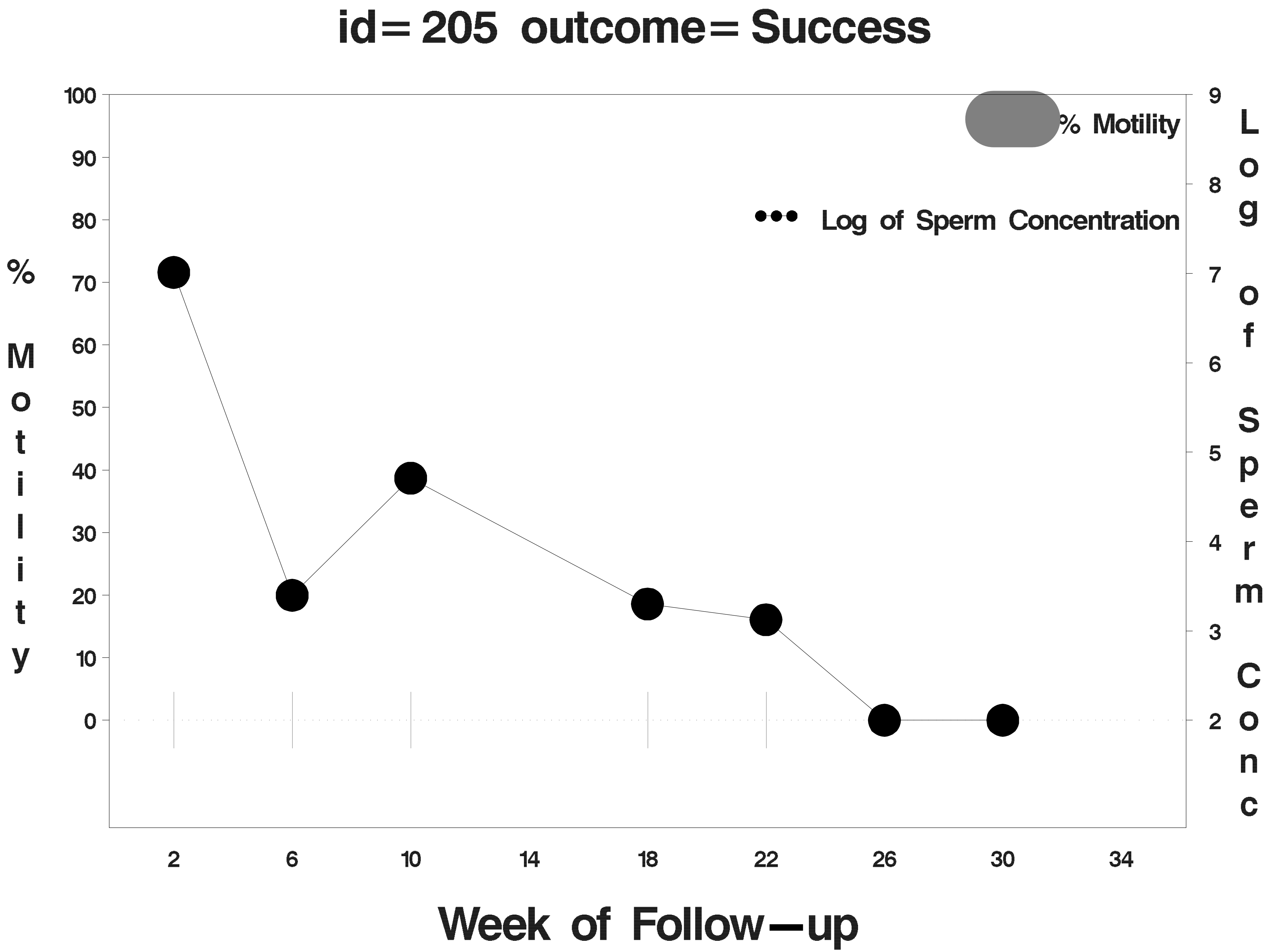
id= 205 outcome= Success
% Motility
Log of Sperm Concentration
% Motility
Log of Sperm Conc
Week of Follow-up
100
90
80
70
60
50
40
30
20
10
0
9
8
7
6
5
4
3
2
2
6
10
14
18
22
26
30
34

id= 206 outcome= Success

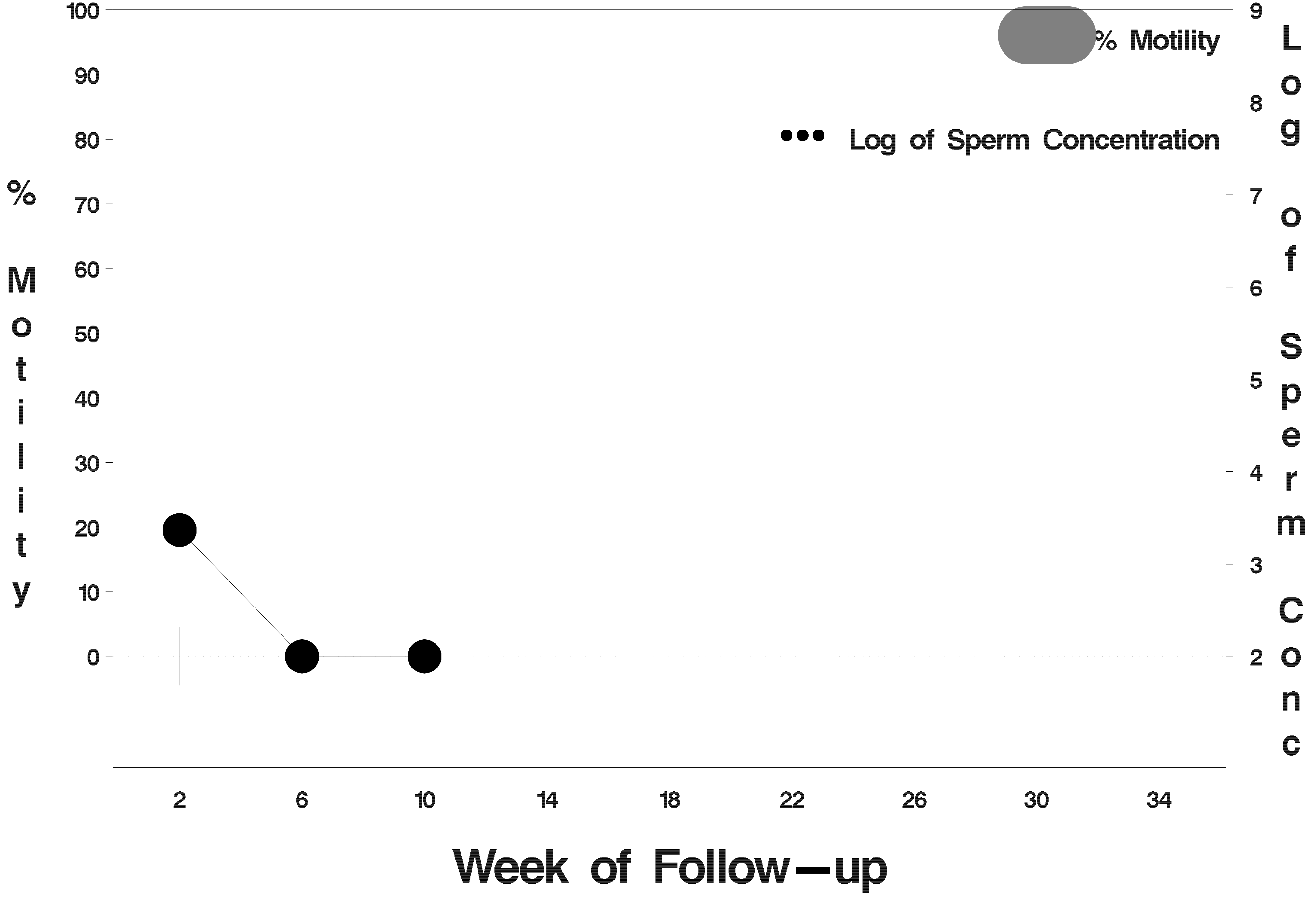

id= 207 outcome= Indeterminate

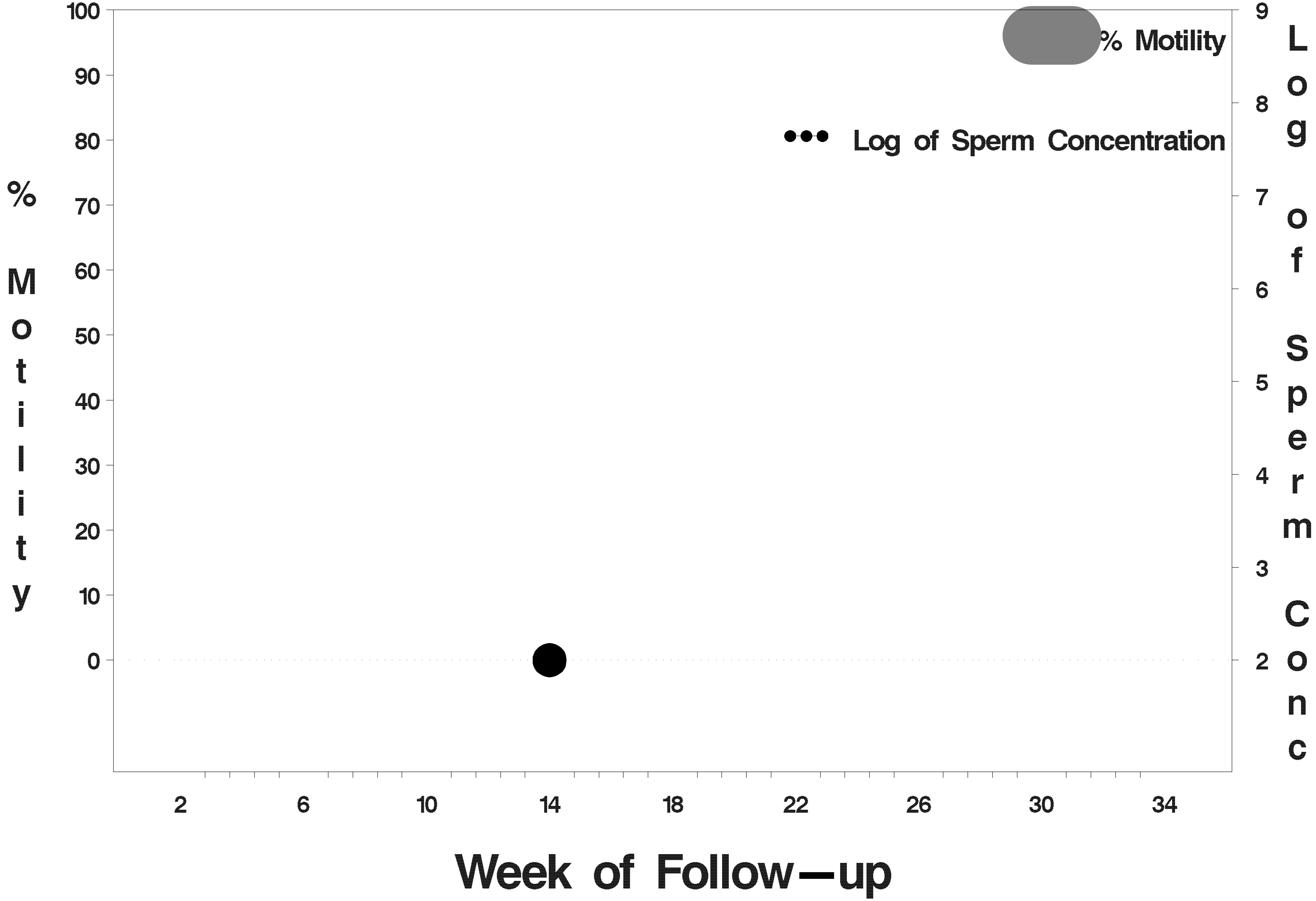

id= 208 outcome= Success

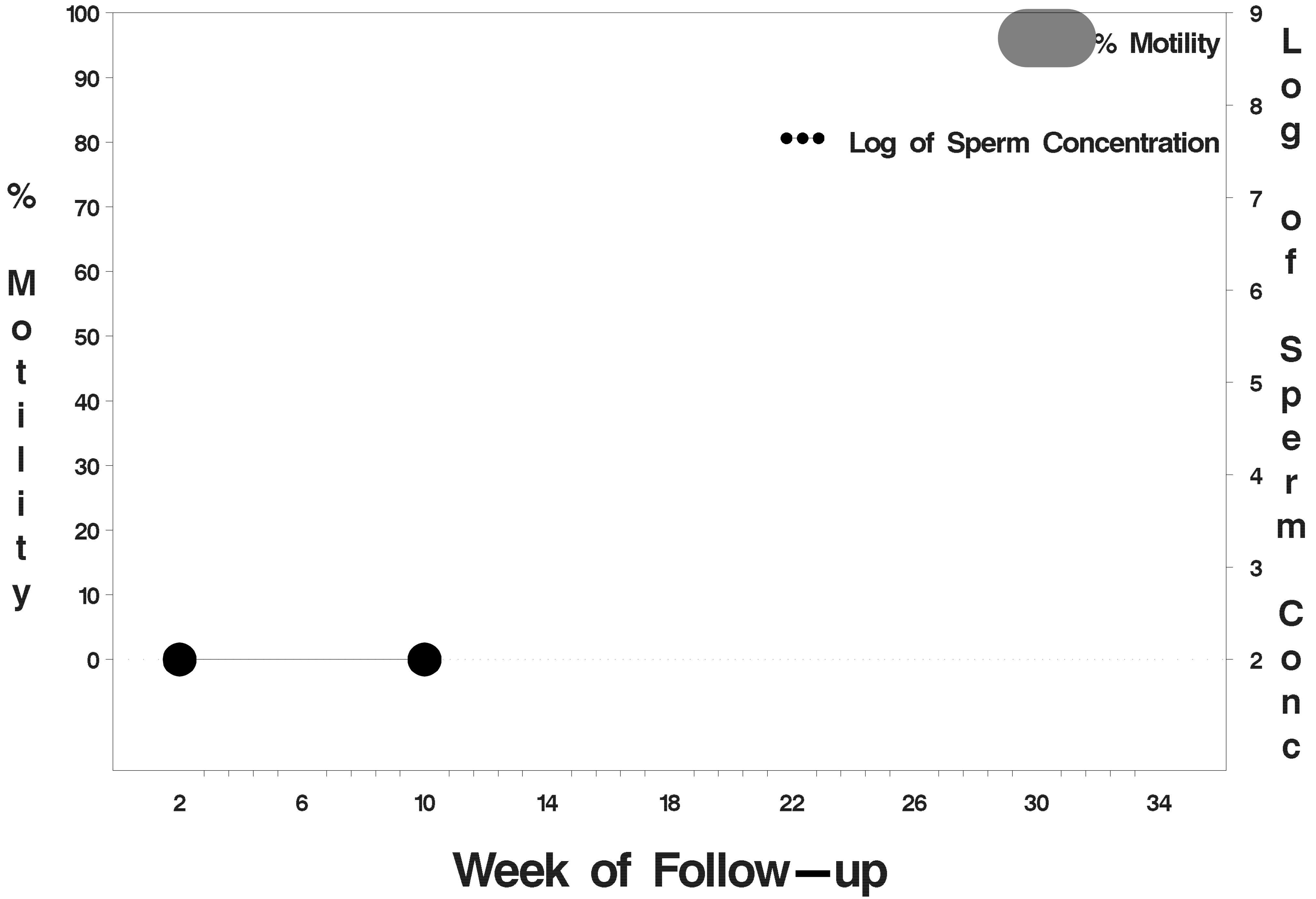

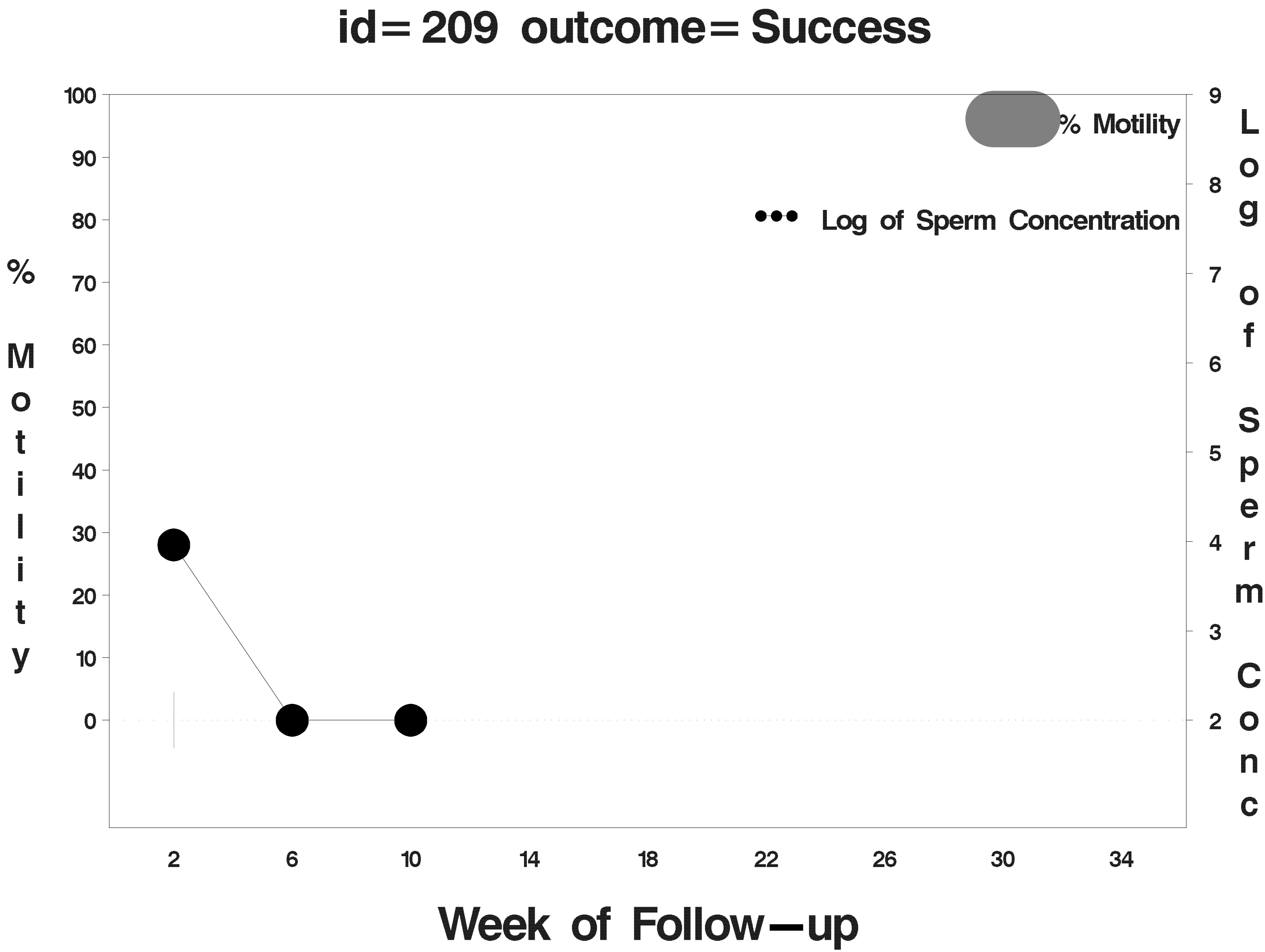

id= 209 outcome= Success
% Motility
Log of Sperm Concentration
% Motility
Log of Sperm Conc
Week of Follow-up
100
90
80
70
60
50
40
30
20
10
0
9
8
7
6
5
4
3
2
2
6
10
14
18
22
26
30
34

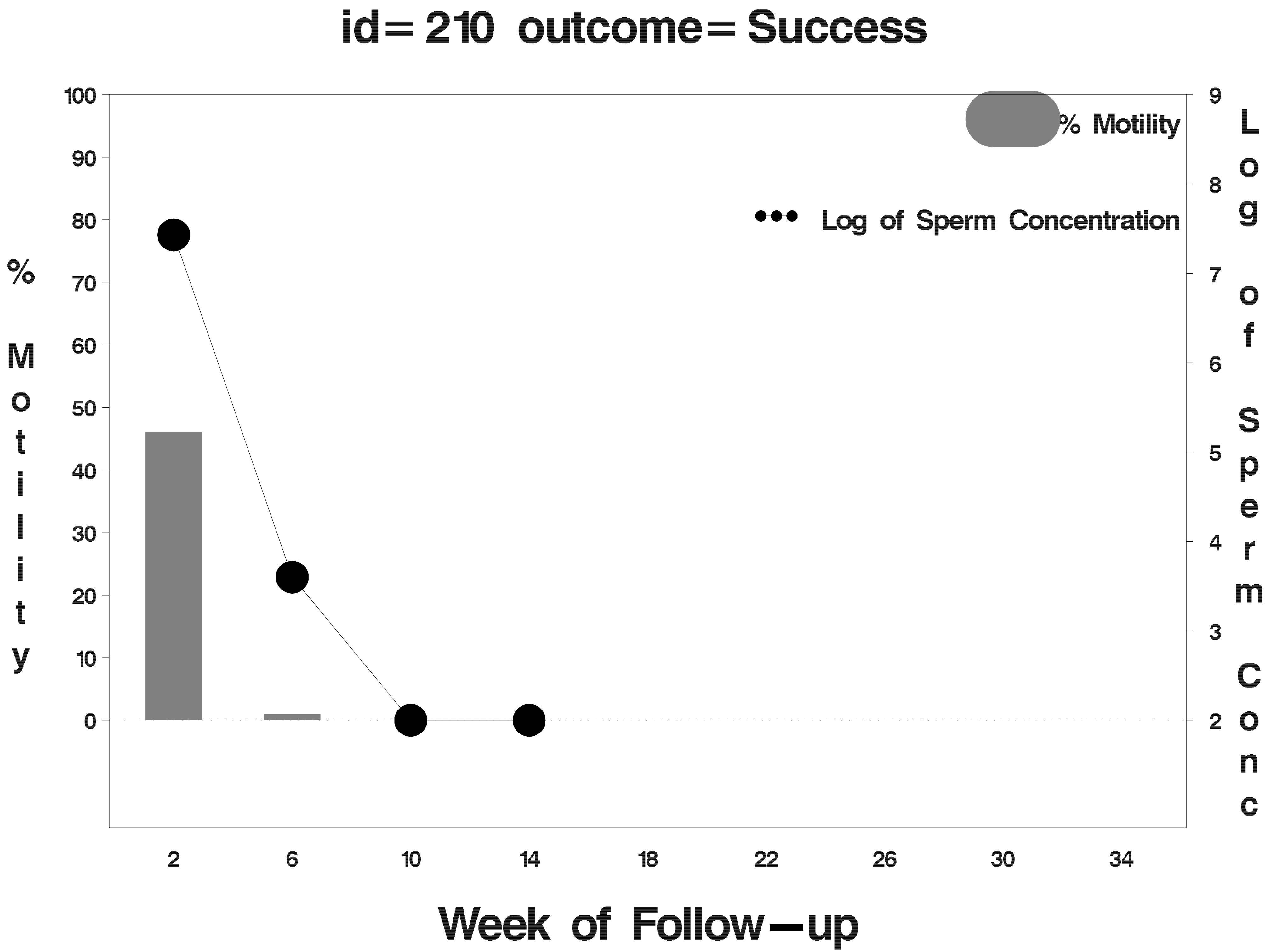
id= 210 outcome= Success
% Motility
Log of Sperm Concentration
% Motility
Log of Sperm Conc
Week of Follow—up
100
90
80
70
60
50
40
30
20
10
0
9
8
7
6
5
4
3
2
2
6
10
14
18
22
26
30
34

id= 211 outcome= Success

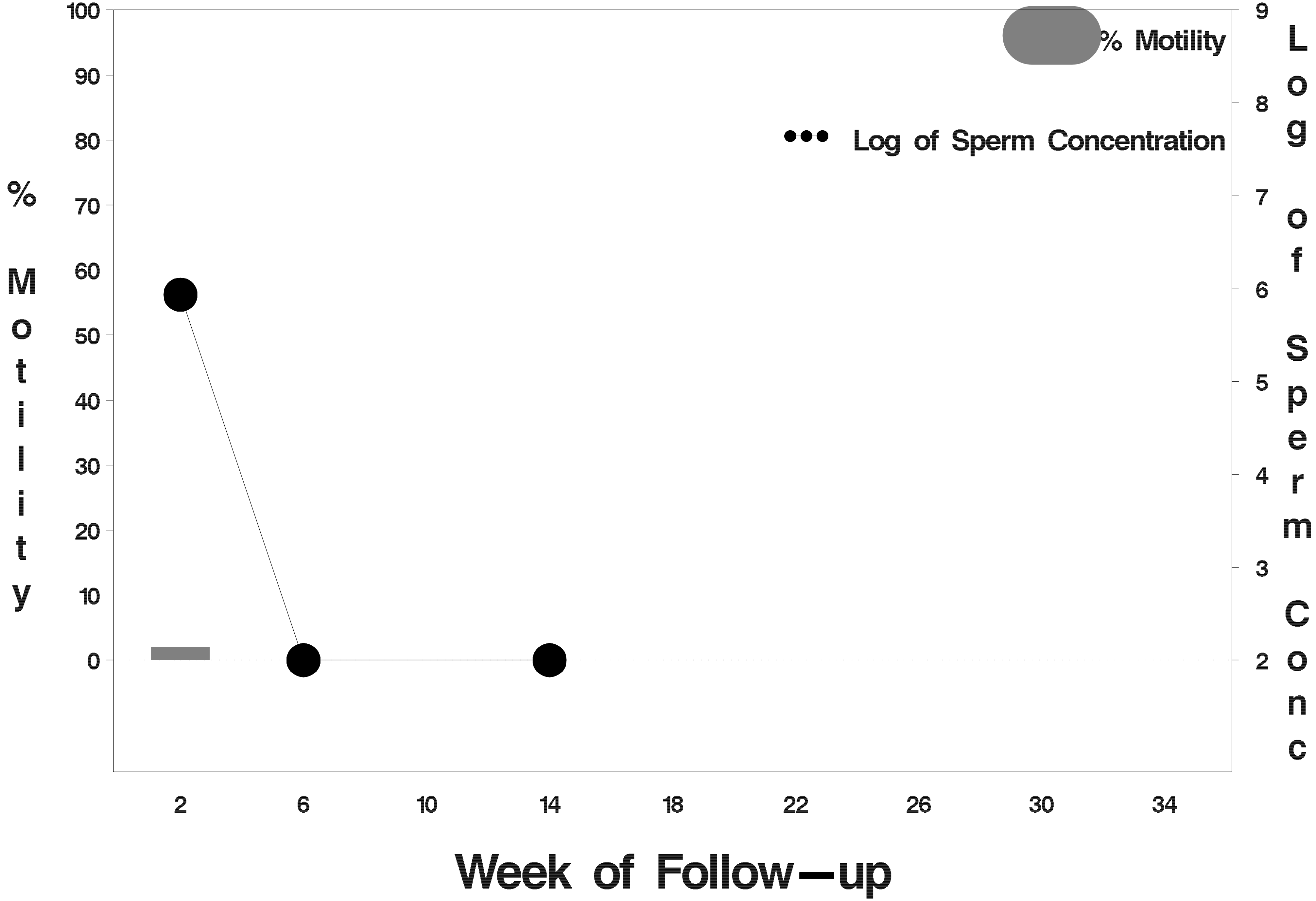

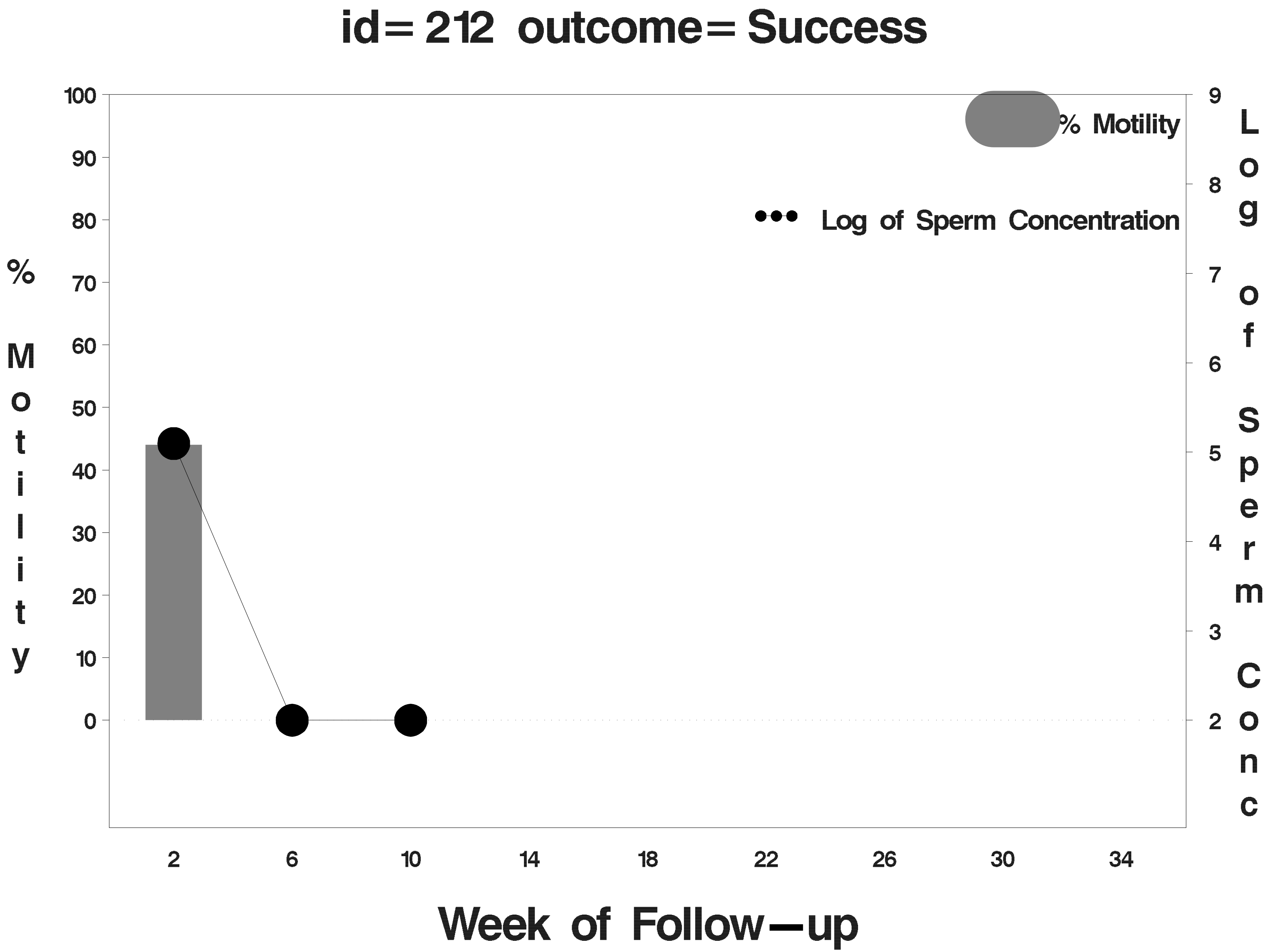
id= 212 outcome= Success
% Motility
Log of Sperm Concentration
100
90
80
70
60
50
40
30
20
10
0
9
8
7
6
5
4
3
2
Log of Sperm Conc
2
6
10
14
18
22
26
30
34
Week of Follow−up

id= 213 outcome= Indeterminate

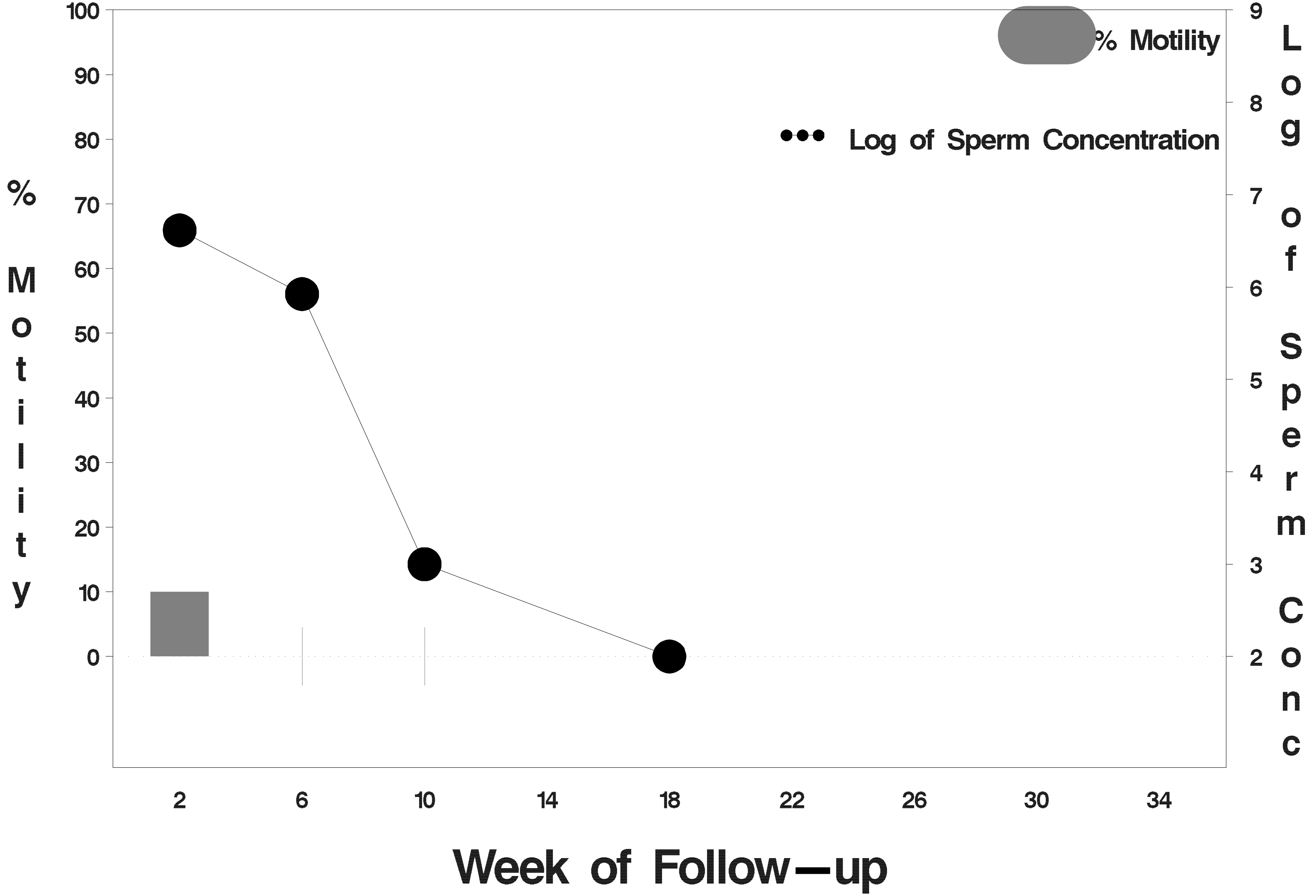

id= 214 outcome= Success

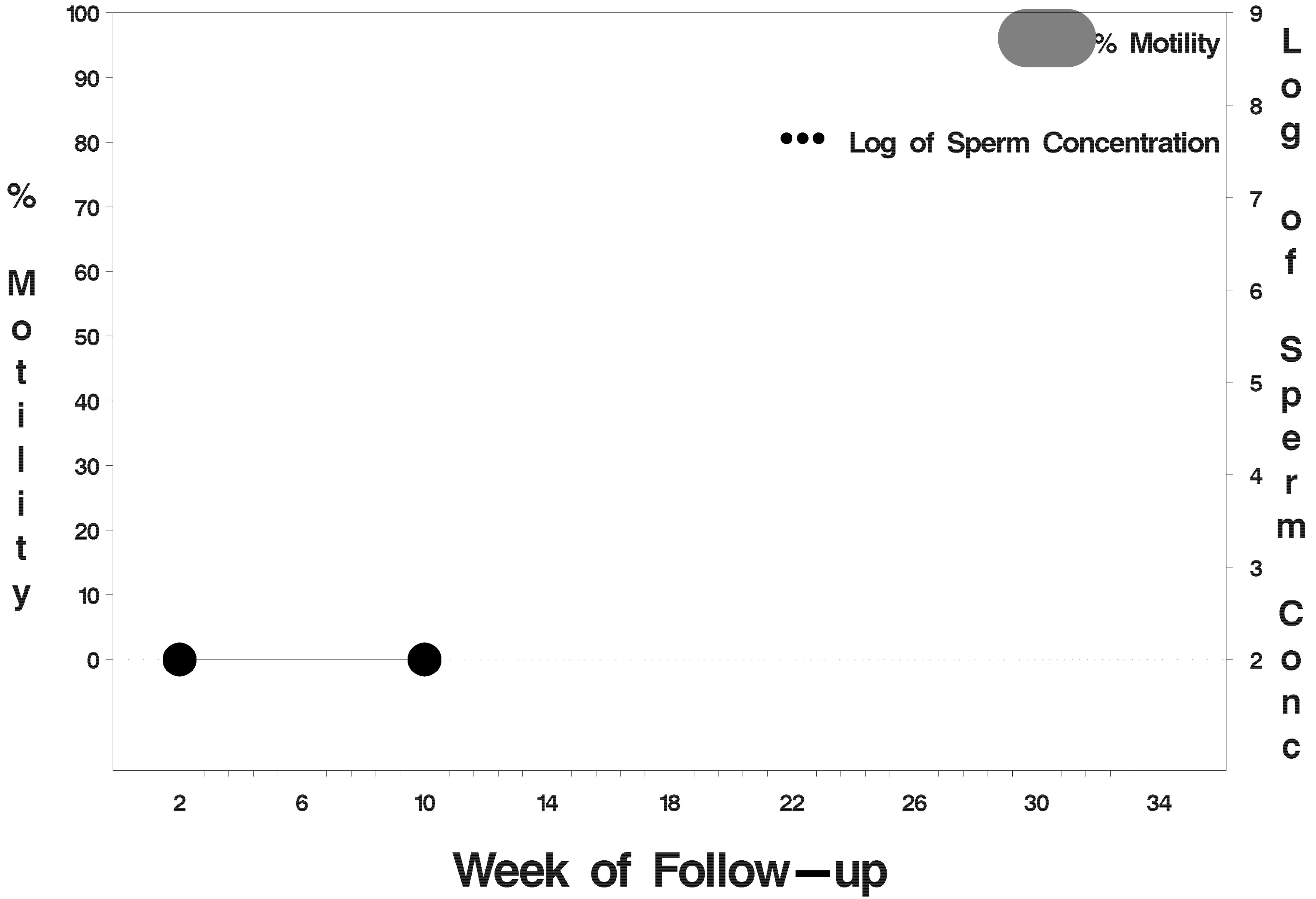

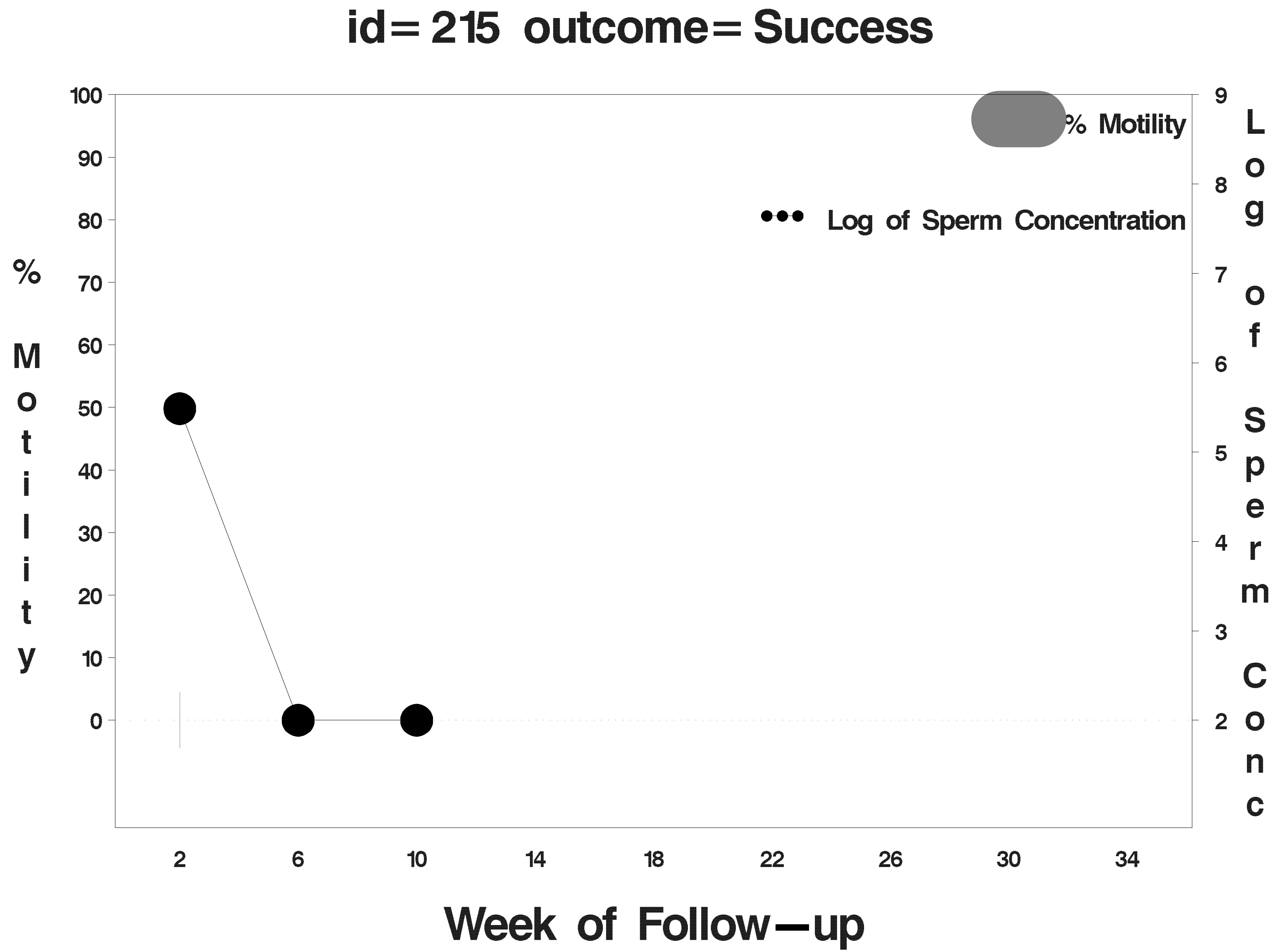

id= 215 outcome= Success
% Motility
Log of Sperm Concentration
100
90
80
70
60
50
40
30
20
10
0
9
8
7
6
5
4
3
2
2
6
10
14
18
22
26
30
34
Week of Follow−up
Log of Sperm Conc

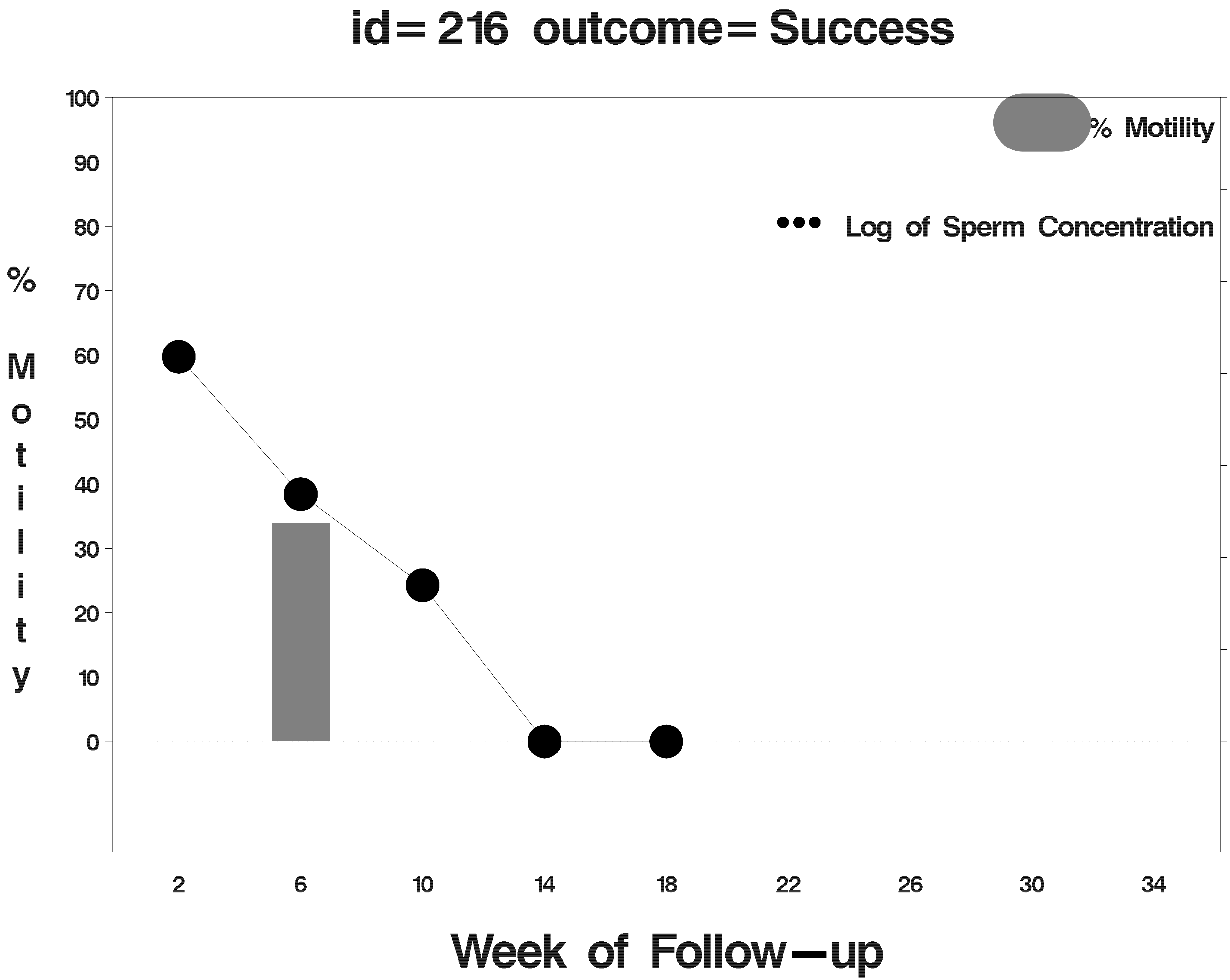

id= 216 outcome= Success
% Motility
Log of Sperm Concentration
100
90
80
70
60
50
40
30
20
10
0
9
8
7
6
5
4
3
2
2
6
10
14
18
22
26
30
34
Week of Follow−up
Log of Sperm Conc

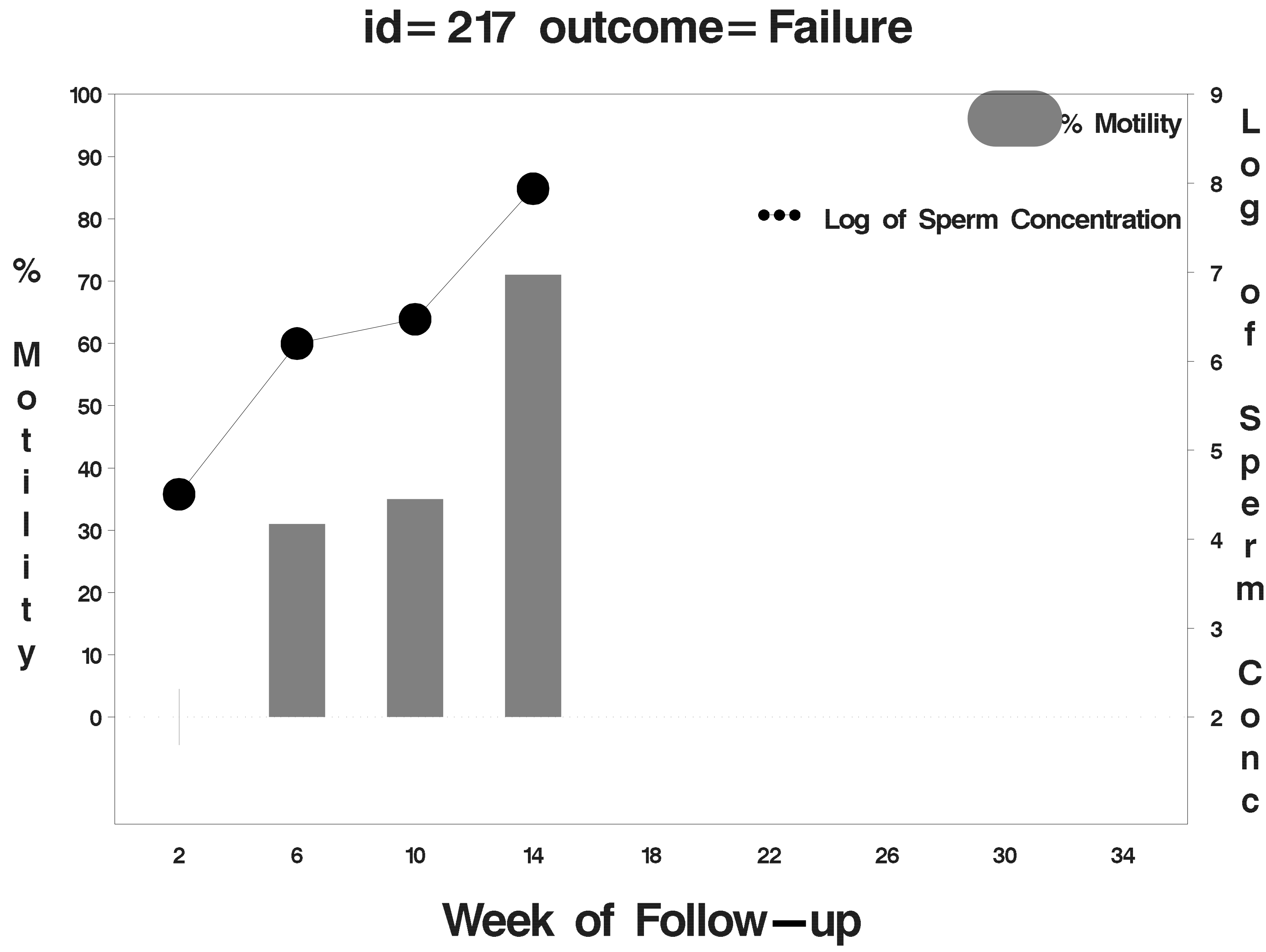

id= 217 outcome= Failure
% Motility
Log of Sperm Concentration
% Motility
Log of Sperm Conc
Week of Follow−up
100
90
80
70
60
50
40
30
20
10
0
9
8
7
6
5
4
3
2
2
6
10
14
18
22
26
30
34

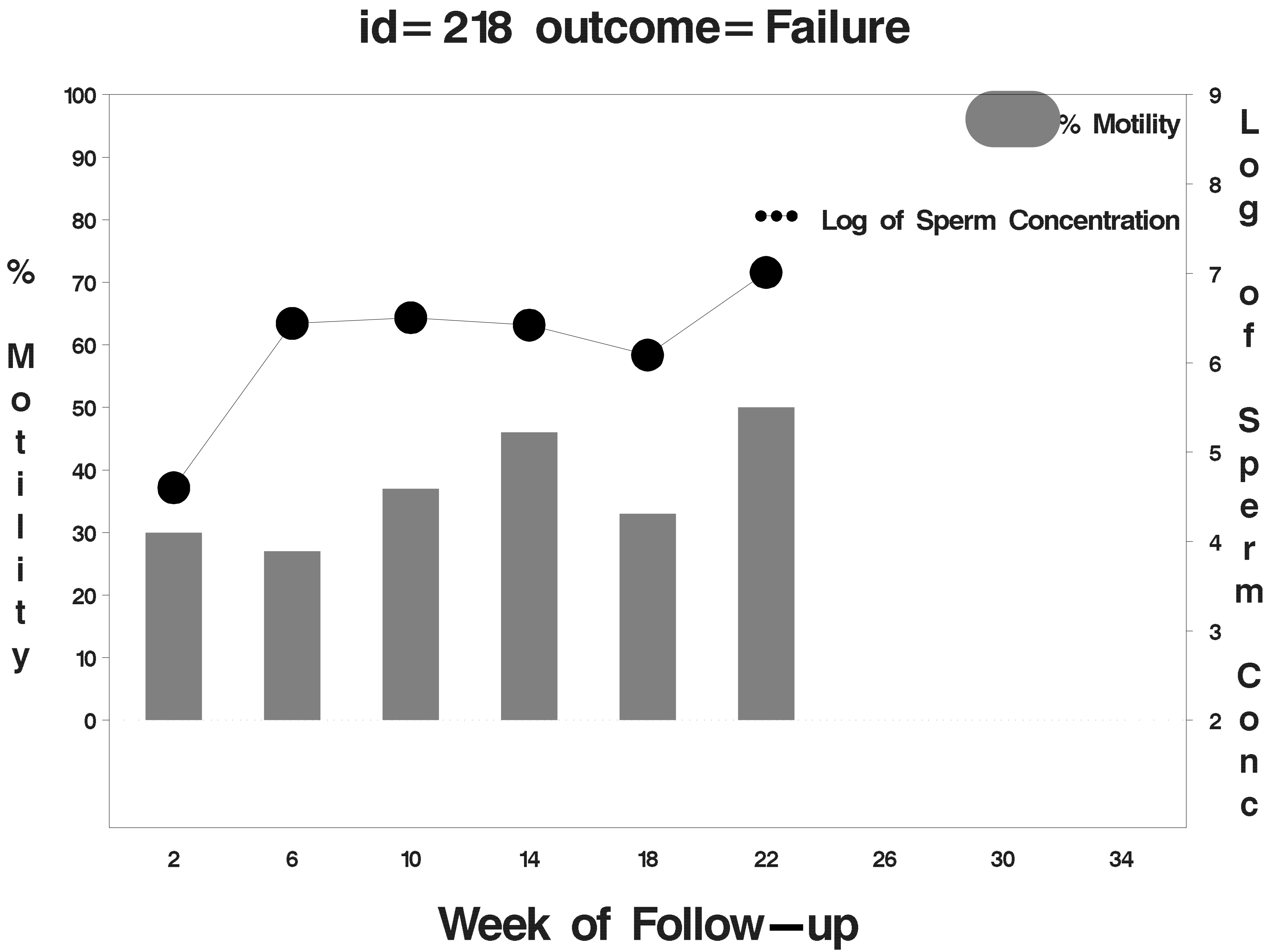
id= 218 outcome= Failure
% Motility
Log of Sperm Concentration
% Motility
Log of Sperm Conc
Week of Follow-up
100
90
80
70
60
50
40
30
20
10
0
9
8
7
6
5
4
3
2
2
6
10
14
18
22
26
30
34

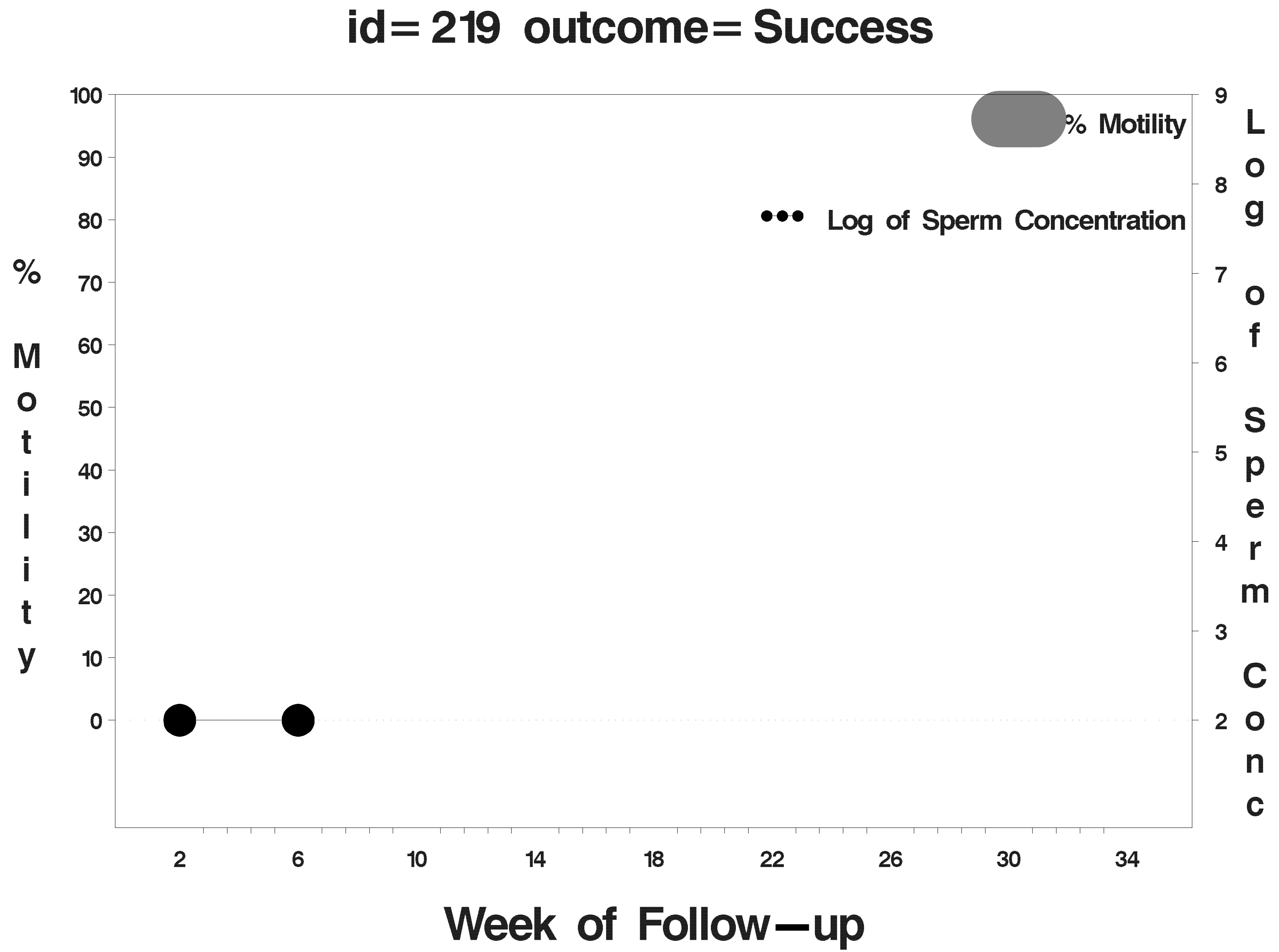

id= 219 outcome= Success
% Motility
Log of Sperm Concentration
% Motility
Log of Sperm Conc
100
90
80
70
60
50
40
30
20
10
0
9
8
7
6
5
4
3
2
2
6
10
14
18
22
26
30
34
Week of Follow—up

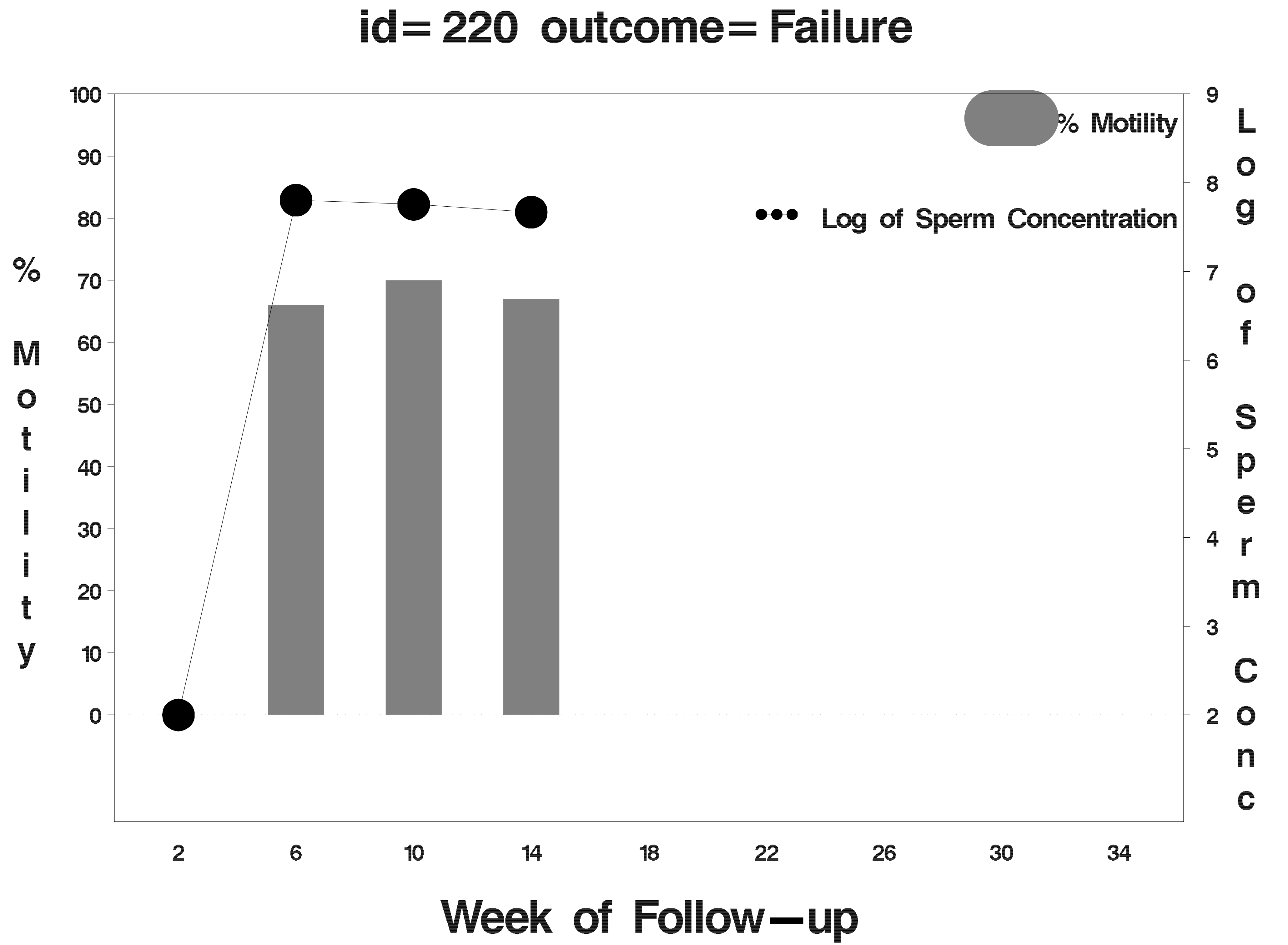

id= 220 outcome= Failure
% Motility
Log of Sperm Concentration
100
90
80
70
60
50
40
30
20
10
0
9
8
7
6
5
4
3
2
2
6
10
14
18
22
26
30
34
Week of Follow−up
% Motility
Log of Sperm Conc

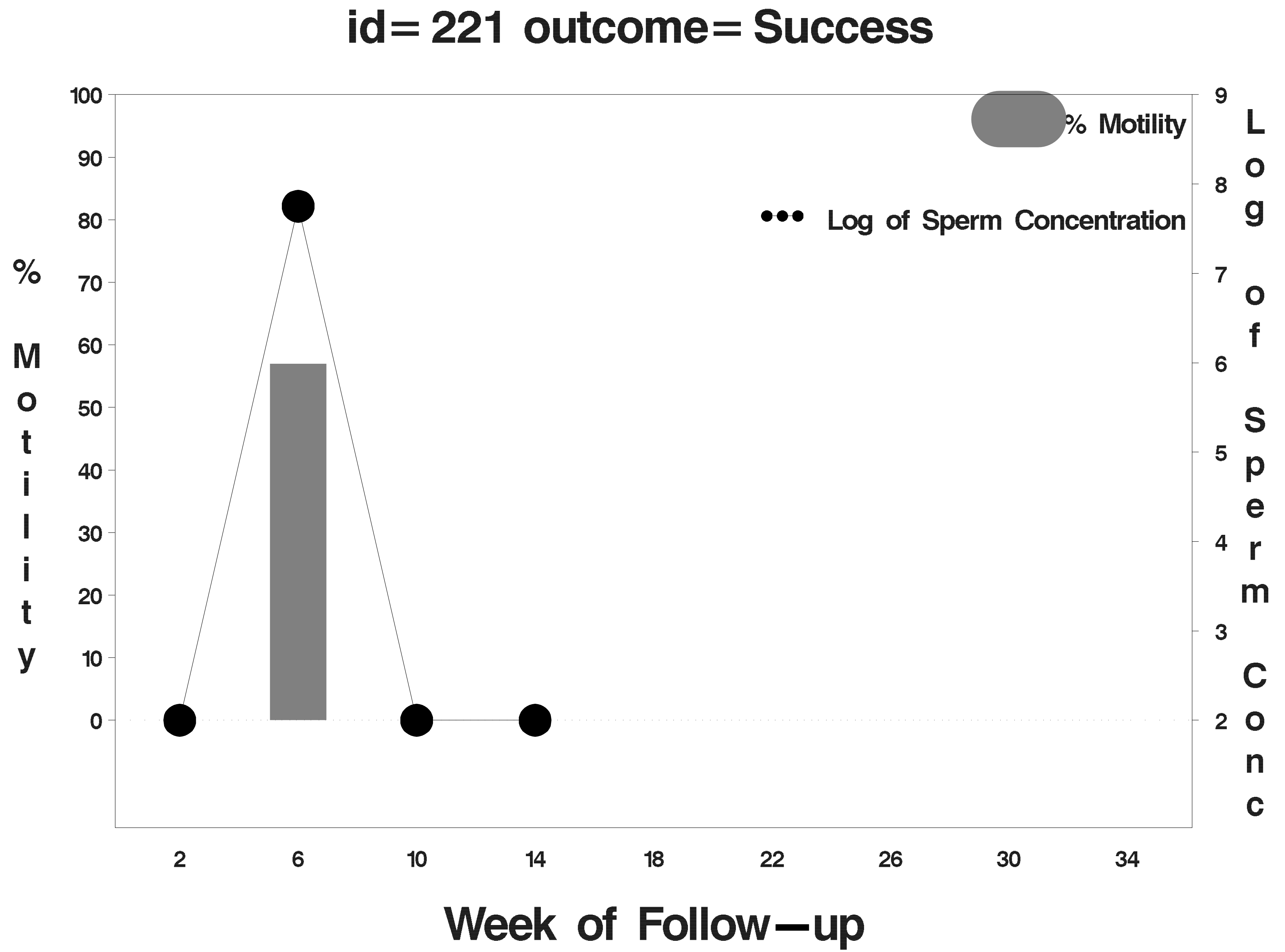

id= 221 outcome= Success
% Motility
Log of Sperm Concentration
100
90
80
70
60
50
40
30
20
10
0
9
8
7
6
5
4
3
2
2
6
10
14
18
22
26
30
34
Week of Follow−up
Log of Sperm Conc

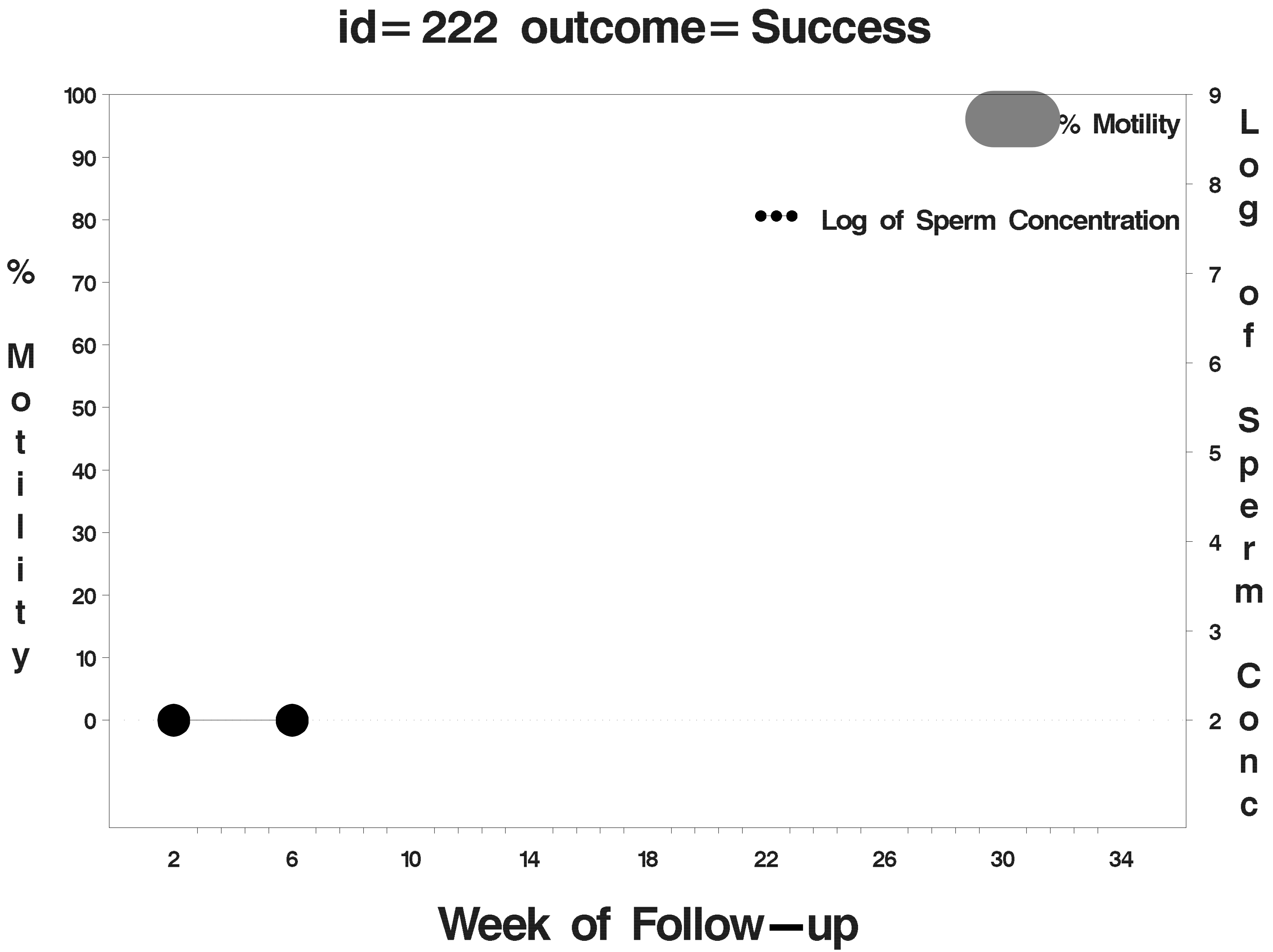

id= 222 outcome= Success
% Motility
Log of Sperm Concentration
100
90
80
70
60
50
40
30
20
10
0
9
8
7
6
5
4
3
2
2
6
10
14
18
22
26
30
34
Week of Follow—up
Log of Sperm Conc

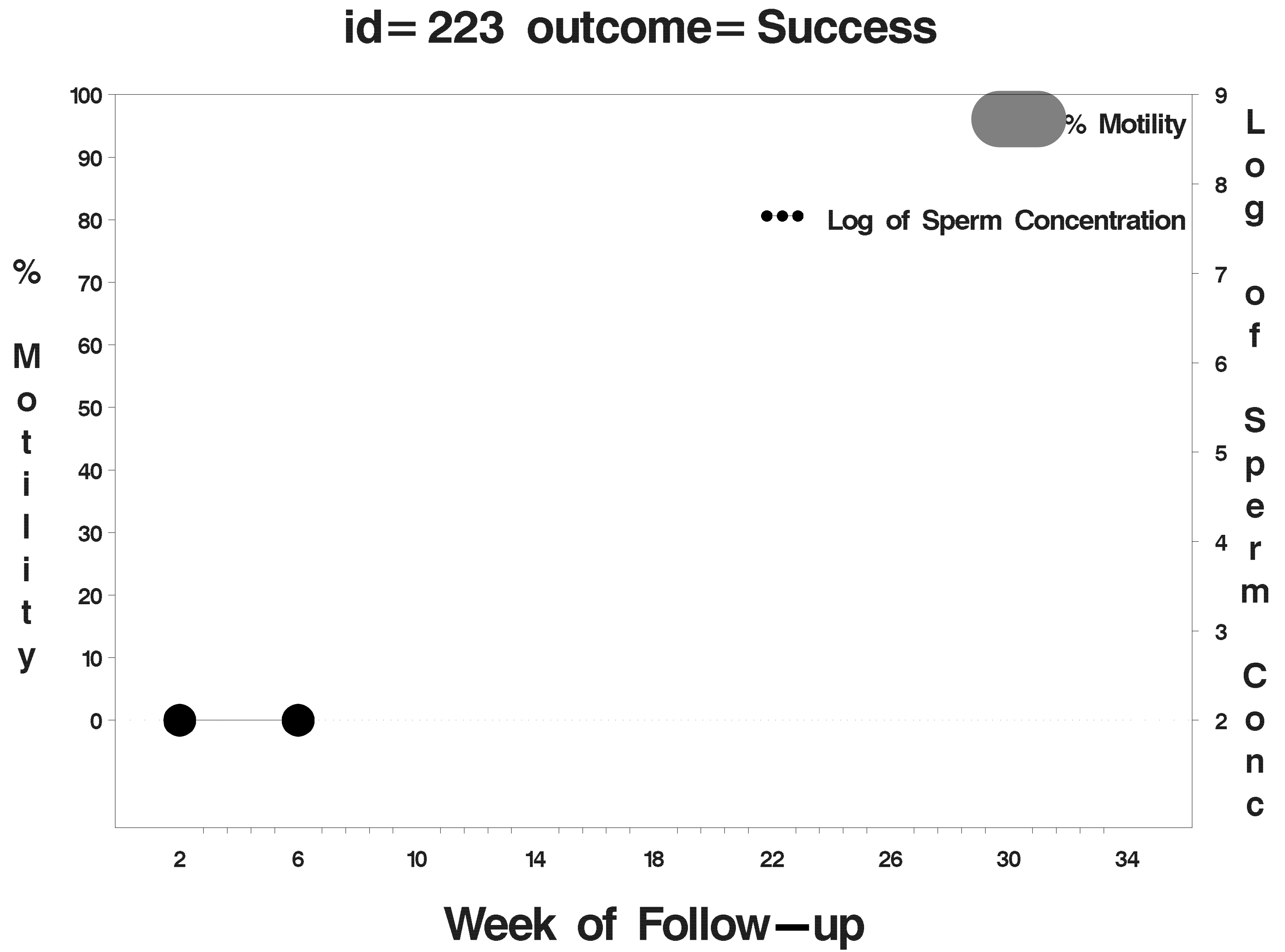
id= 223 outcome= Success
% Motility
Log of Sperm Concentration
100
90
80
70
60
50
40
30
20
10
0
9
8
7
6
5
4
3
2
2
6
10
14
18
22
26
30
34
Week of Follow—up
% Motility
Log of Sperm Conc

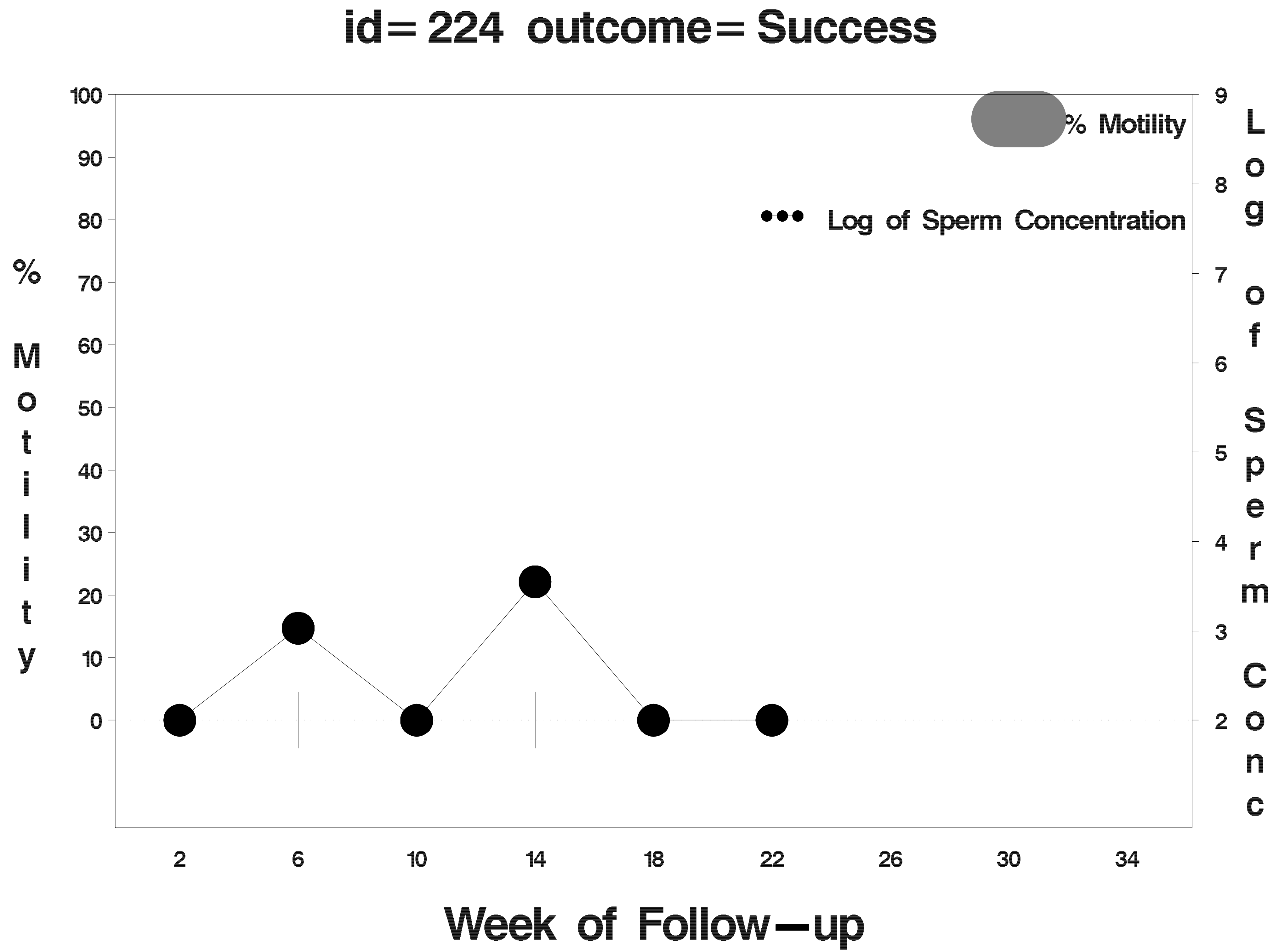

id= 224 outcome= Success
% Motility
Log of Sperm Concentration
100
90
80
70
60
50
40
30
20
10
0
9
8
7
6
5
4
3
2
2
6
10
14
18
22
26
30
34
Week of Follow−up

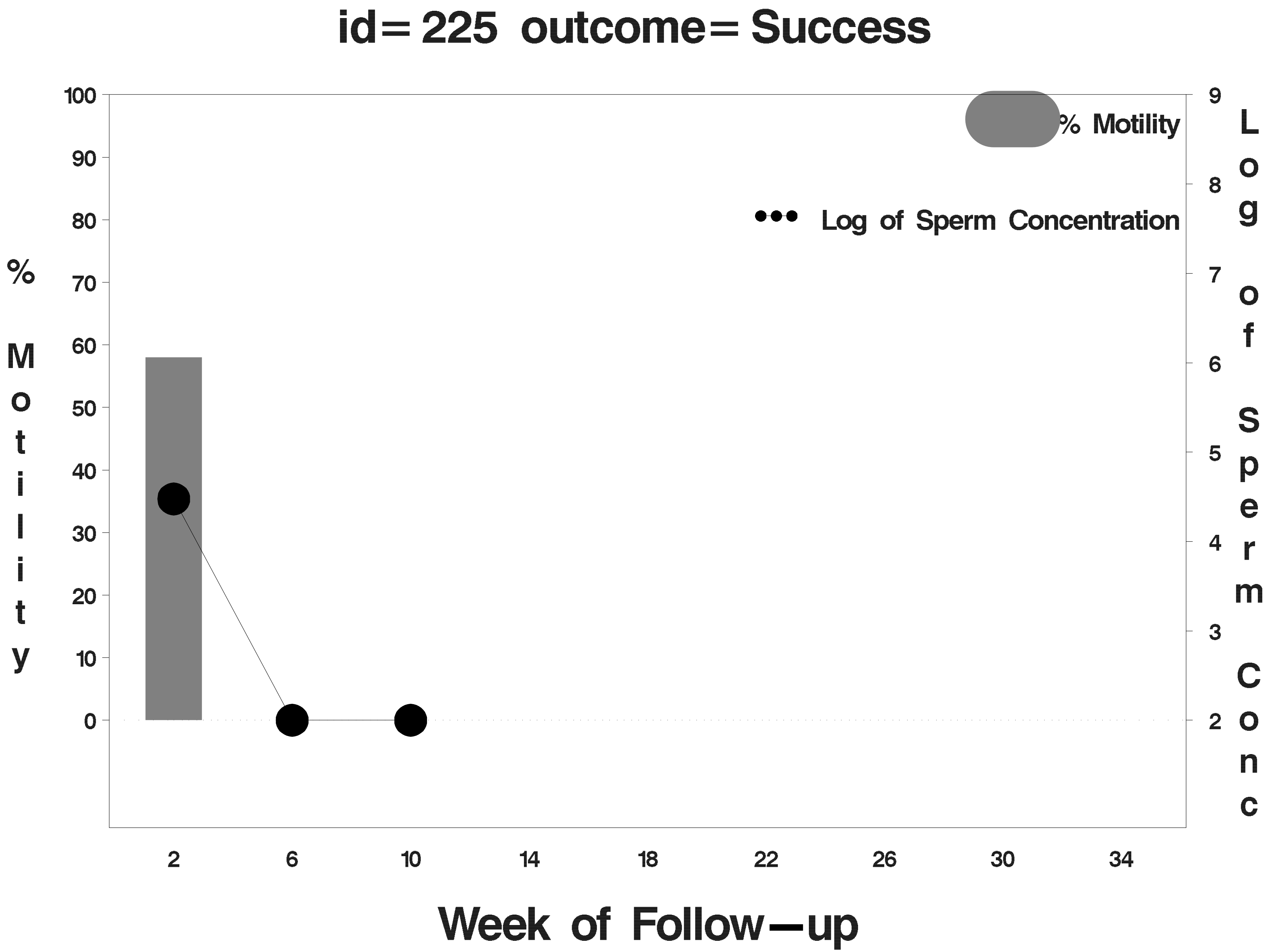

id= 225 outcome= Success
% Motility
Log of Sperm Concentration
100
90
80
70
60
50
40
30
20
10
0
9
8
7
6
5
4
3
2
2
6
10
14
18
22
26
30
34
Week of Follow—up
Log of Sperm Conc

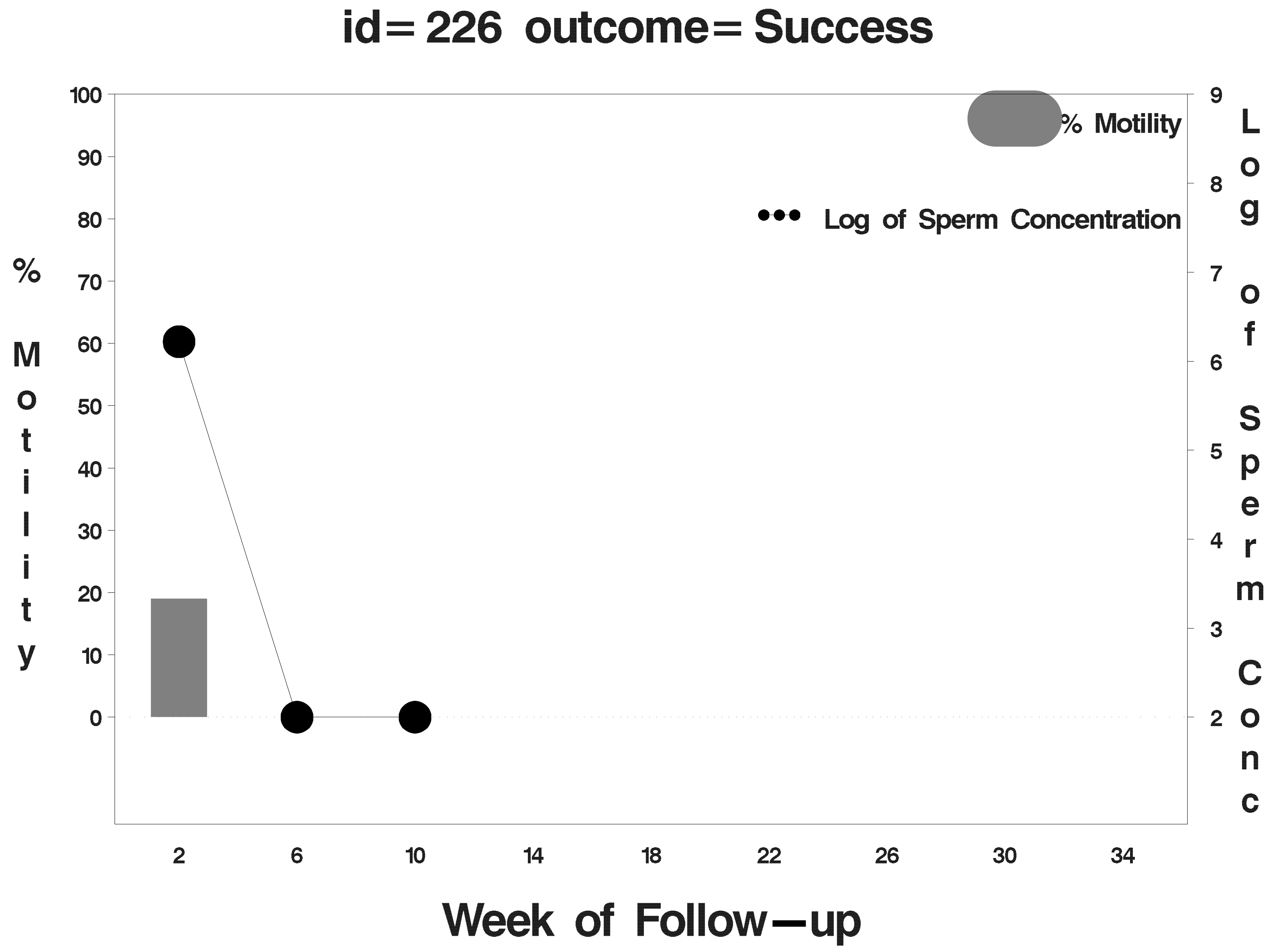

id= 226 outcome= Success
% Motility
Log of Sperm Concentration
100
90
80
70
60
50
40
30
20
10
0
9
8
7
6
5
4
3
2
2
6
10
14
18
22
26
30
34
Week of Follow—up
Log of Sperm Conc

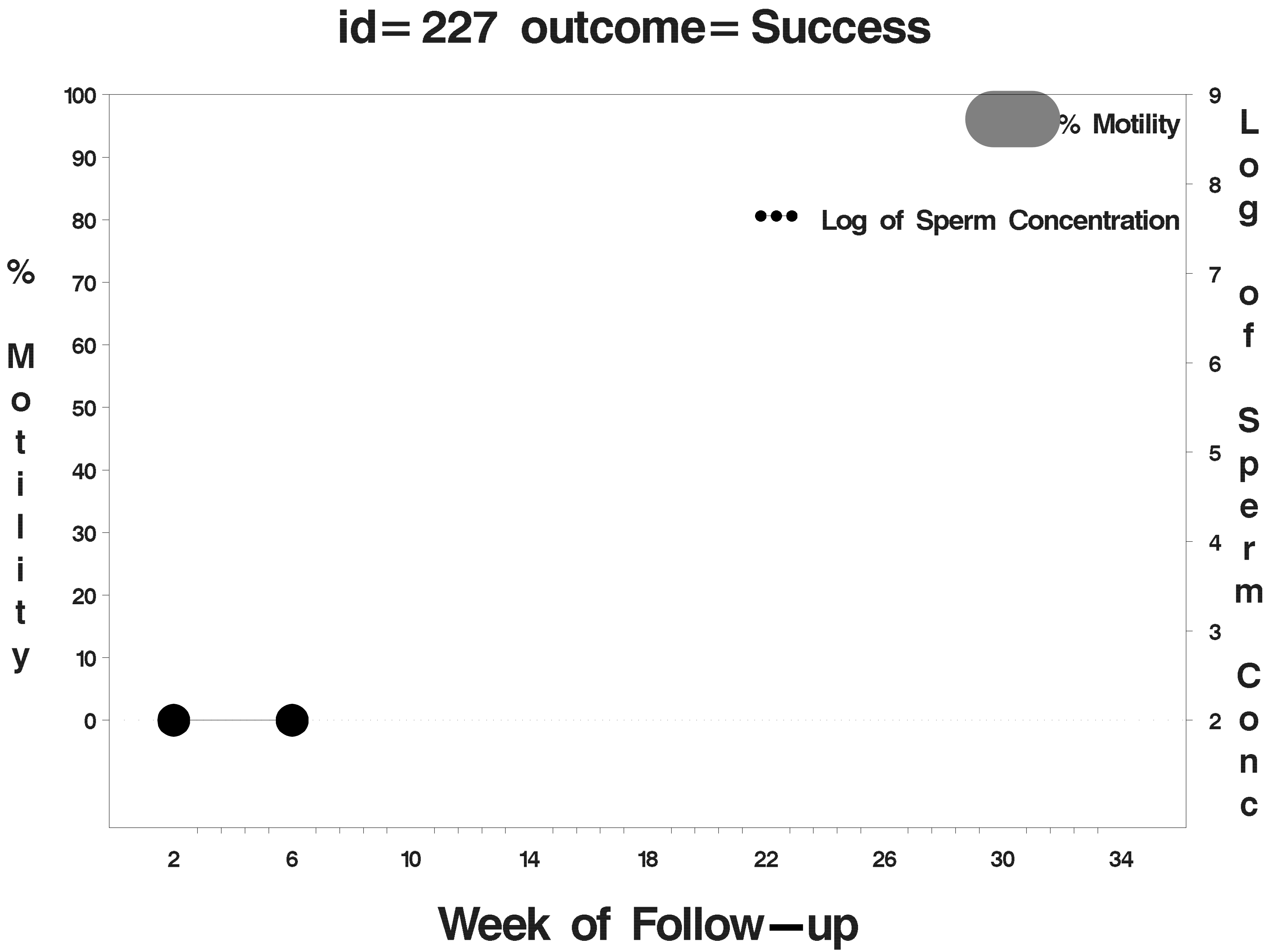

id= 227 outcome= Success
% Motility
Log of Sperm Concentration
100
90
80
70
60
50
40
30
20
10
0
9
8
7
6
5
4
3
2
2
6
10
14
18
22
26
30
34
Week of Follow—up
Log of Sperm Conc

id= 228 outcome= Success

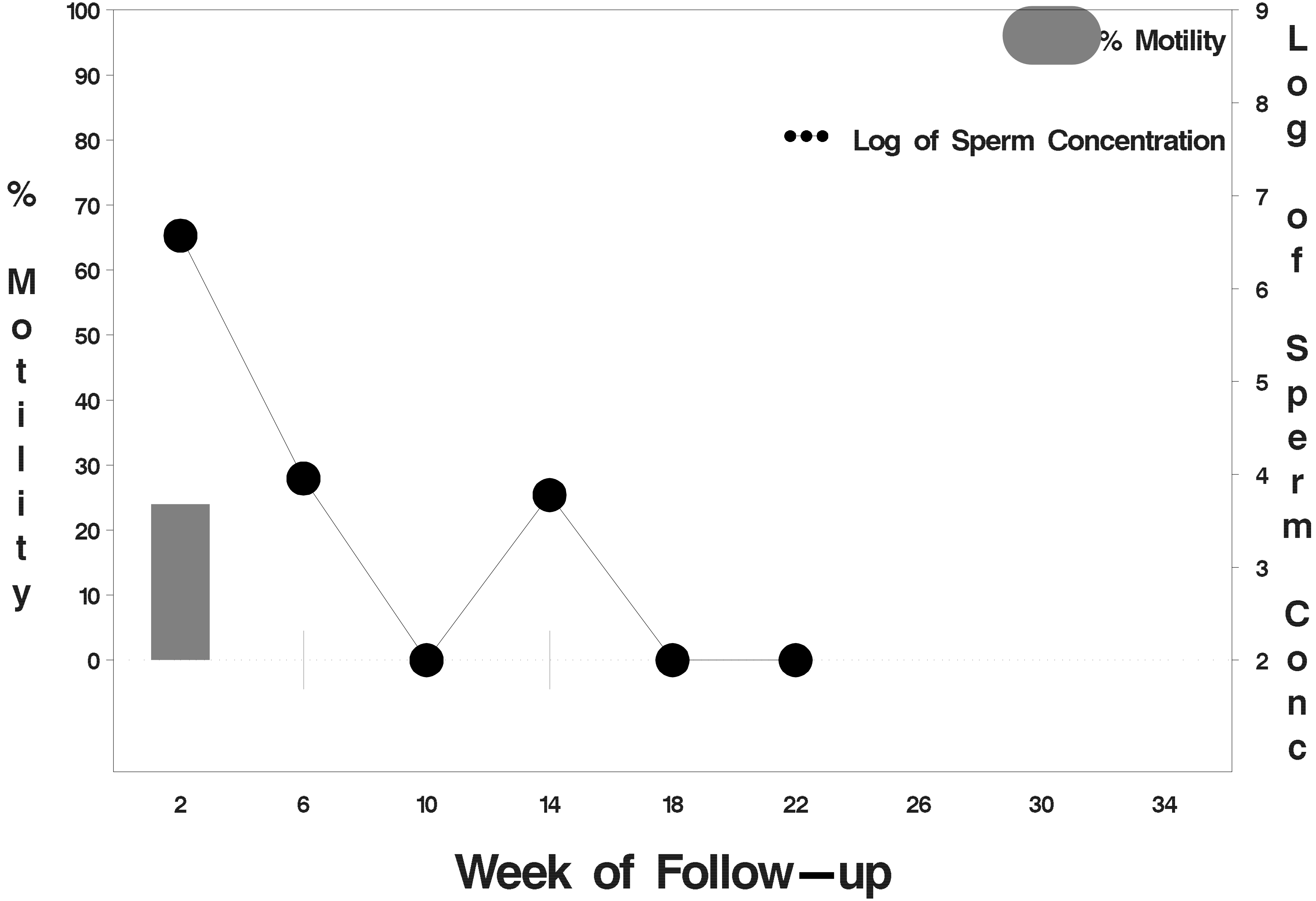

id= 229 outcome= Failure

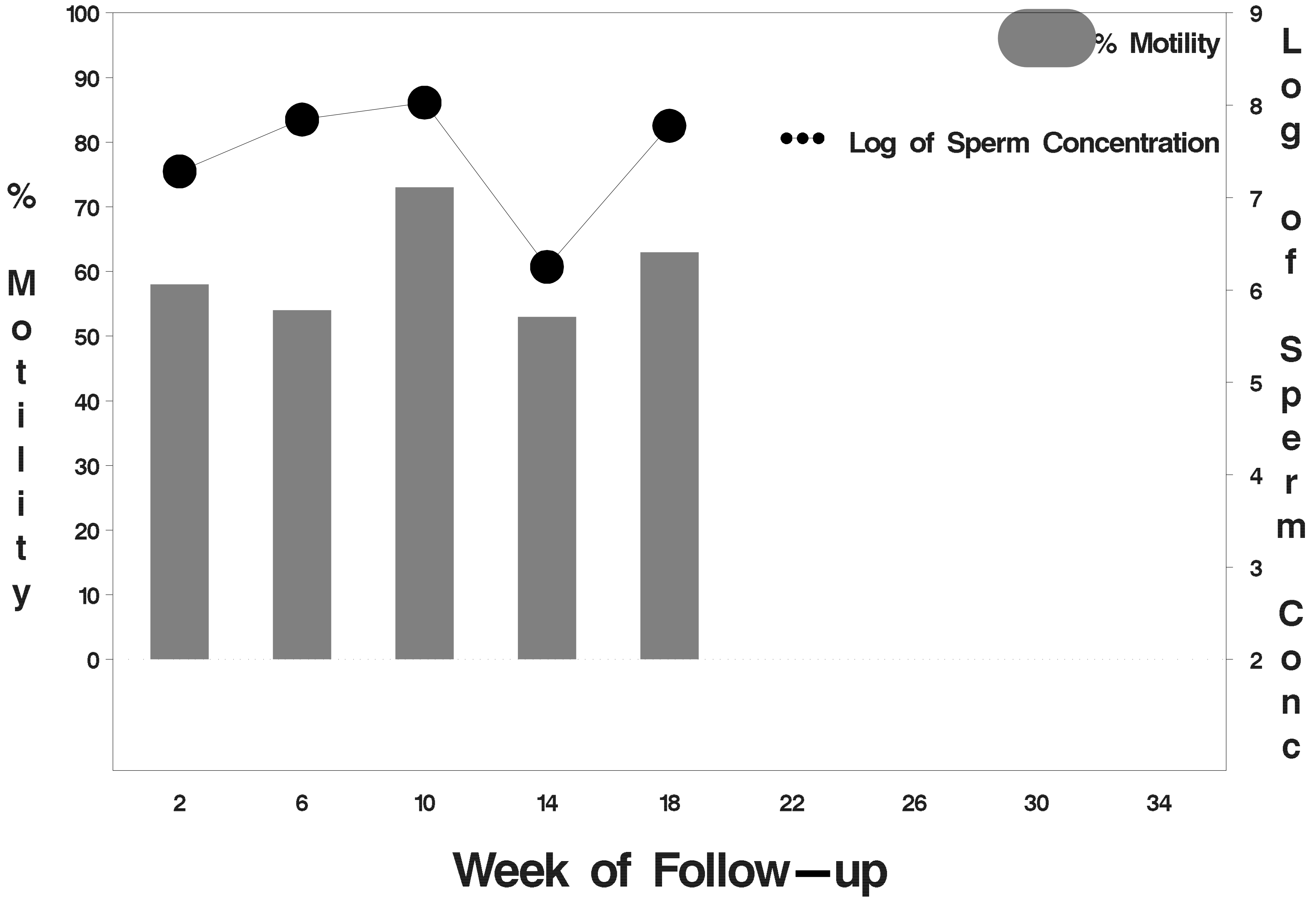

id= 230 outcome= Success

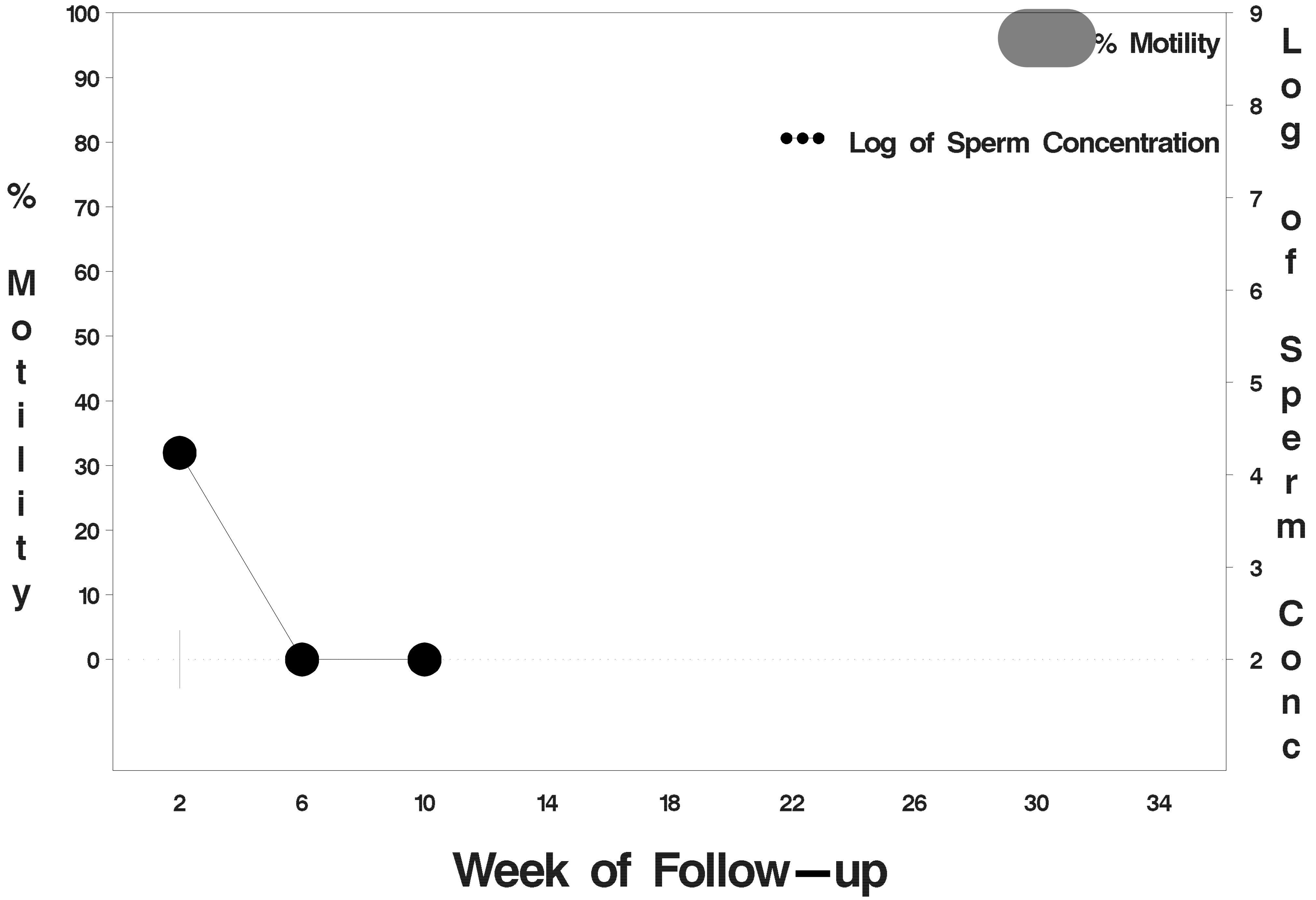

id= 231 outcome= Indeterminate

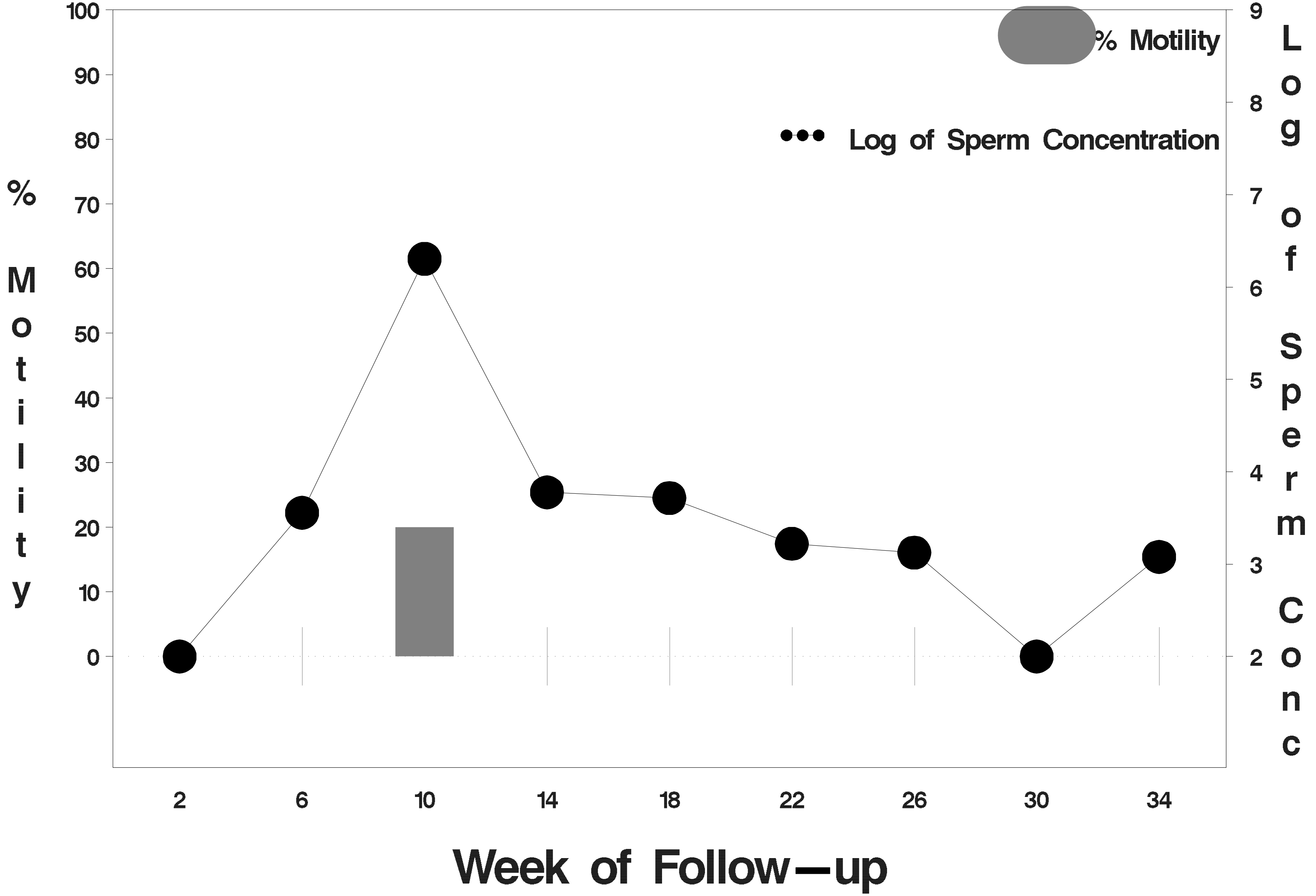

id= 232 outcome= Failure

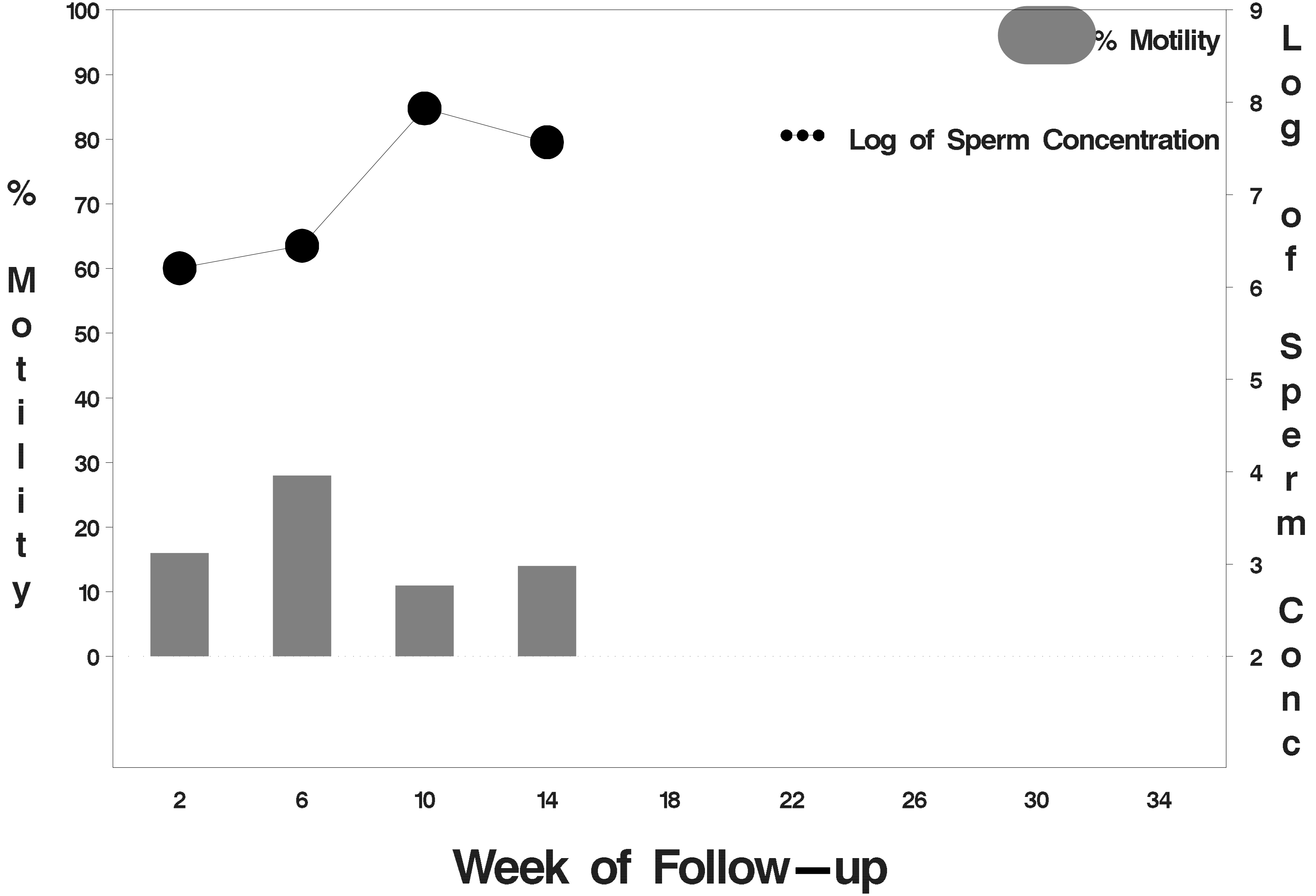

id= 233 outcome= Success

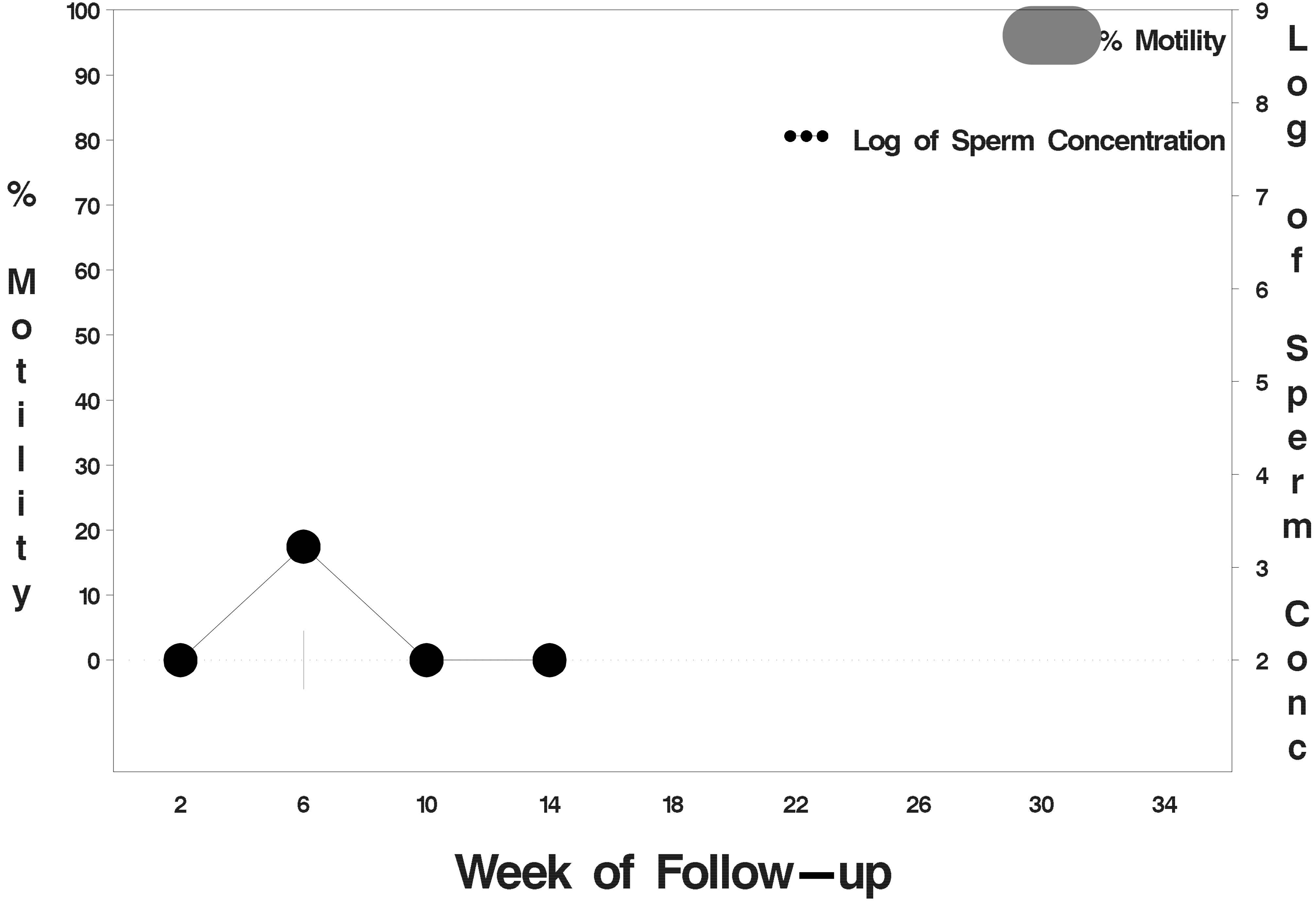

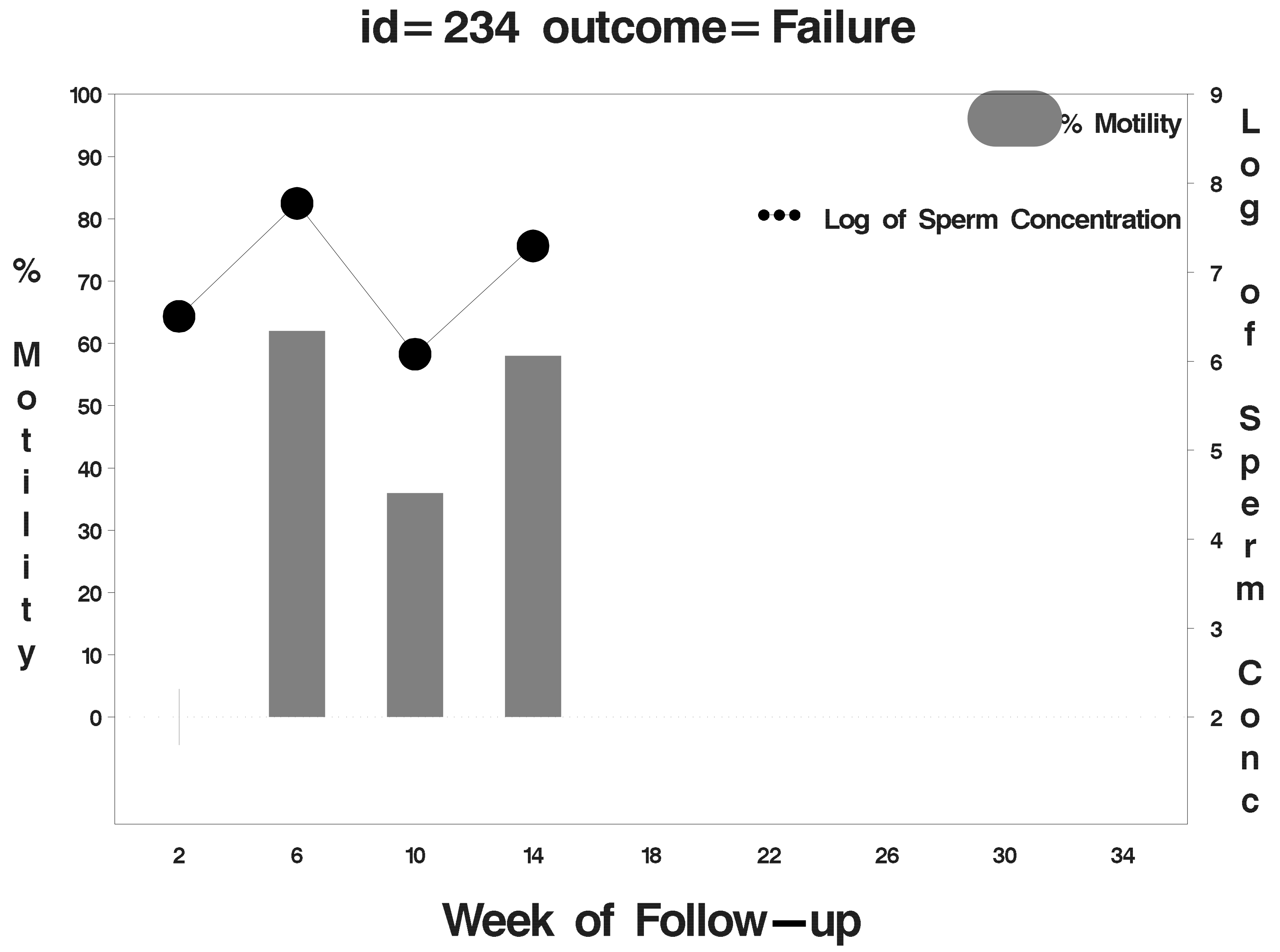
id= 234 outcome= Failure
% Motility
Log of Sperm Concentration
100
90
80
70
60
50
40
30
20
10
0
9
8
7
6
5
4
3
2
2
6
10
14
18
22
26
30
34
Week of Follow−up
Log of Sperm Conc

id= 235 outcome= Success

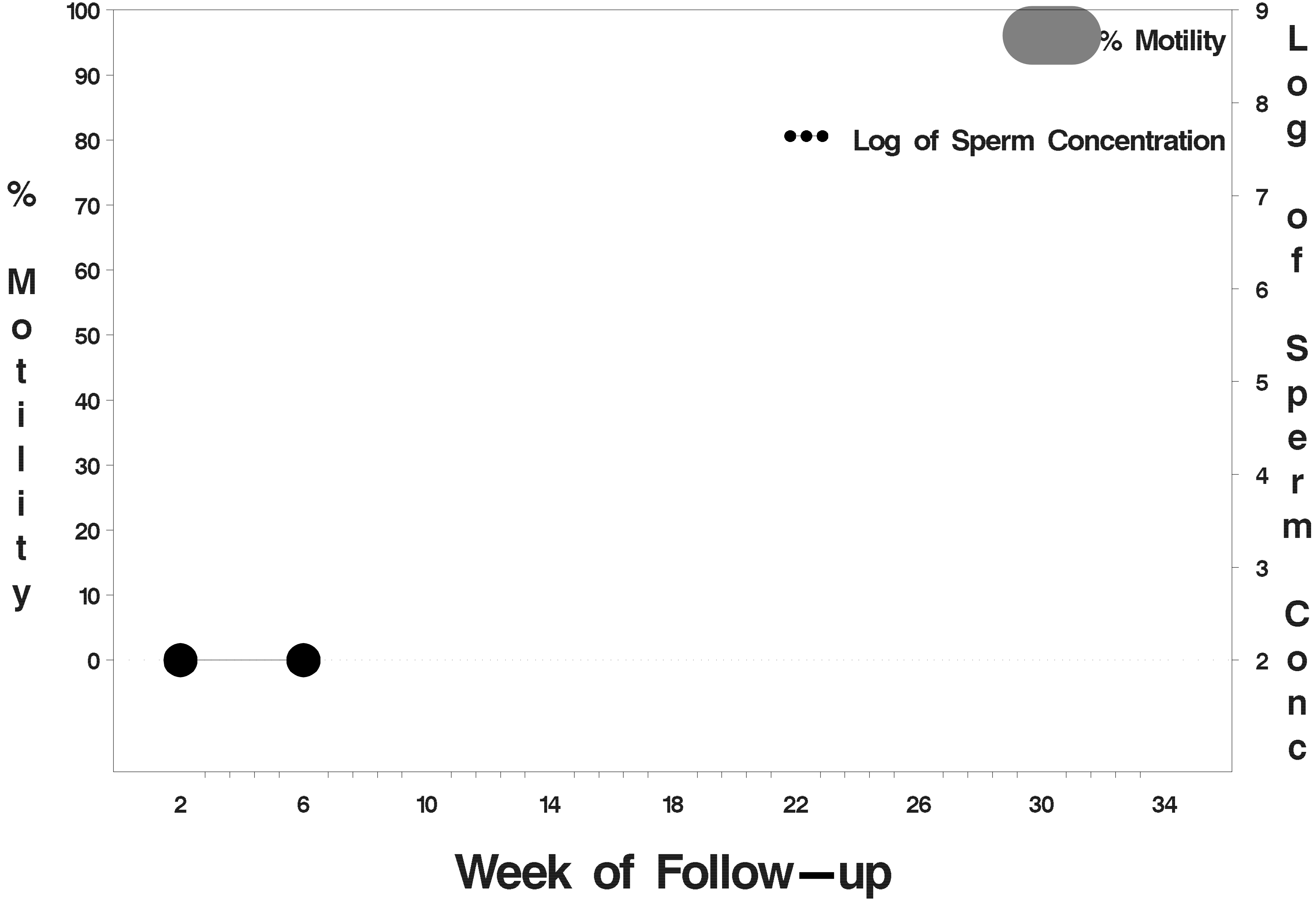

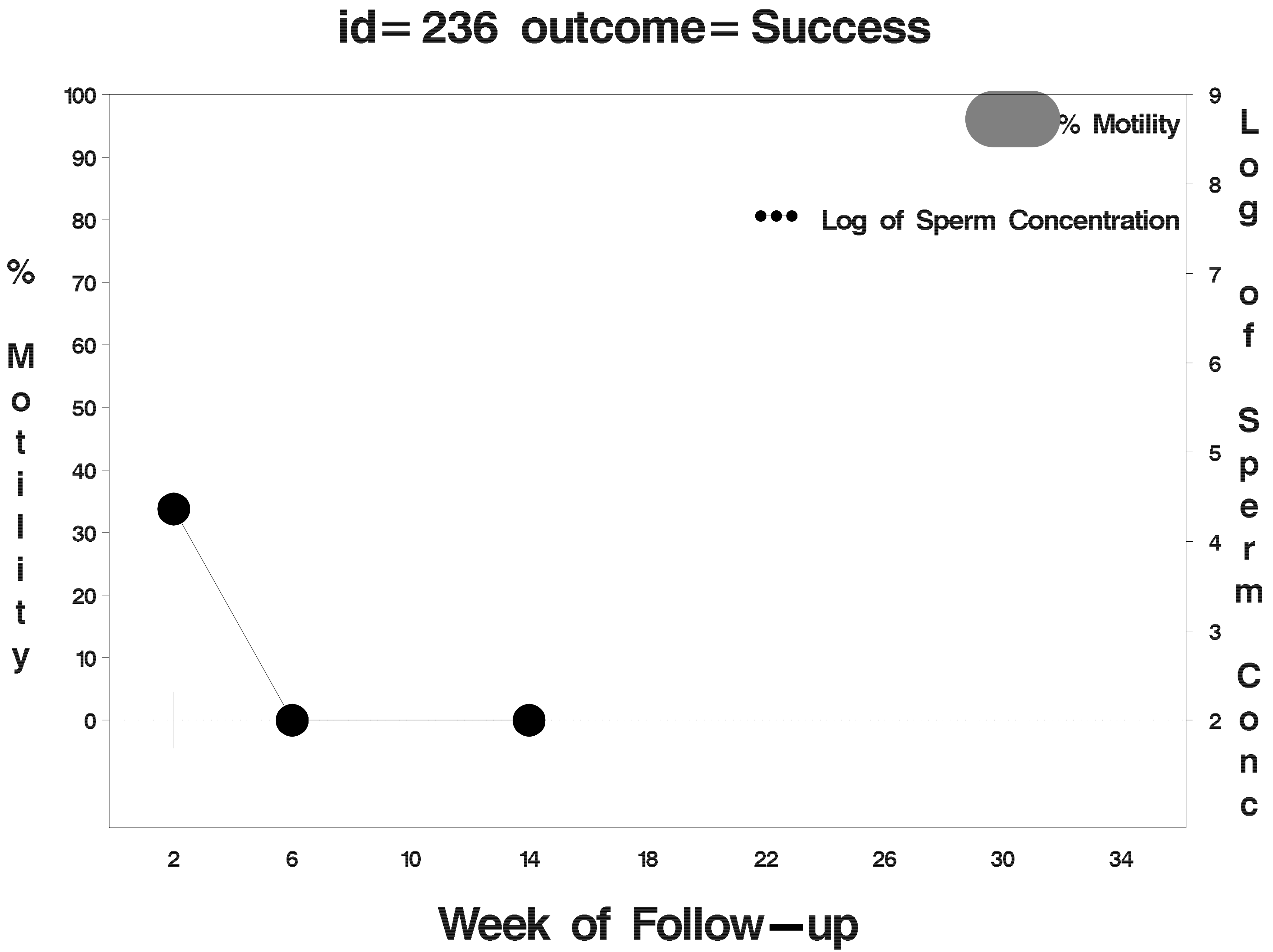
id= 236 outcome= Success
% Motility
Log of Sperm Concentration
100
90
80
70
60
50
40
30
20
10
0
9
8
7
6
5
4
3
2
Log of Sperm Conc
2
6
10
14
18
22
26
30
34
Week of Follow—up

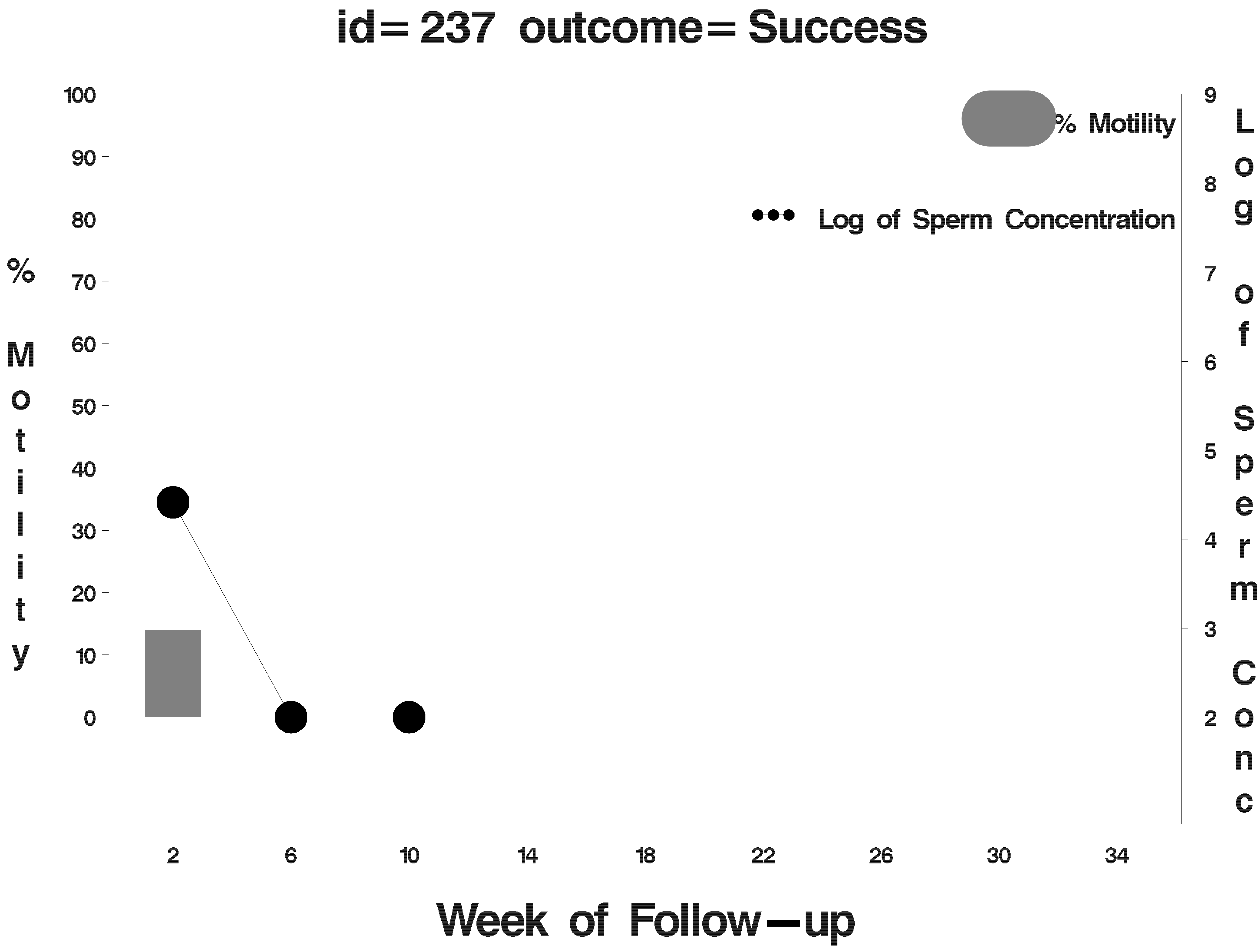

id= 237 outcome= Success
% Motility
Log of Sperm Concentration
% Motility
Log of Sperm Conc
Week of Follow—up
100
90
80
70
60
50
40
30
20
10
0
9
8
7
6
5
4
3
2
2
6
10
14
18
22
26
30
34

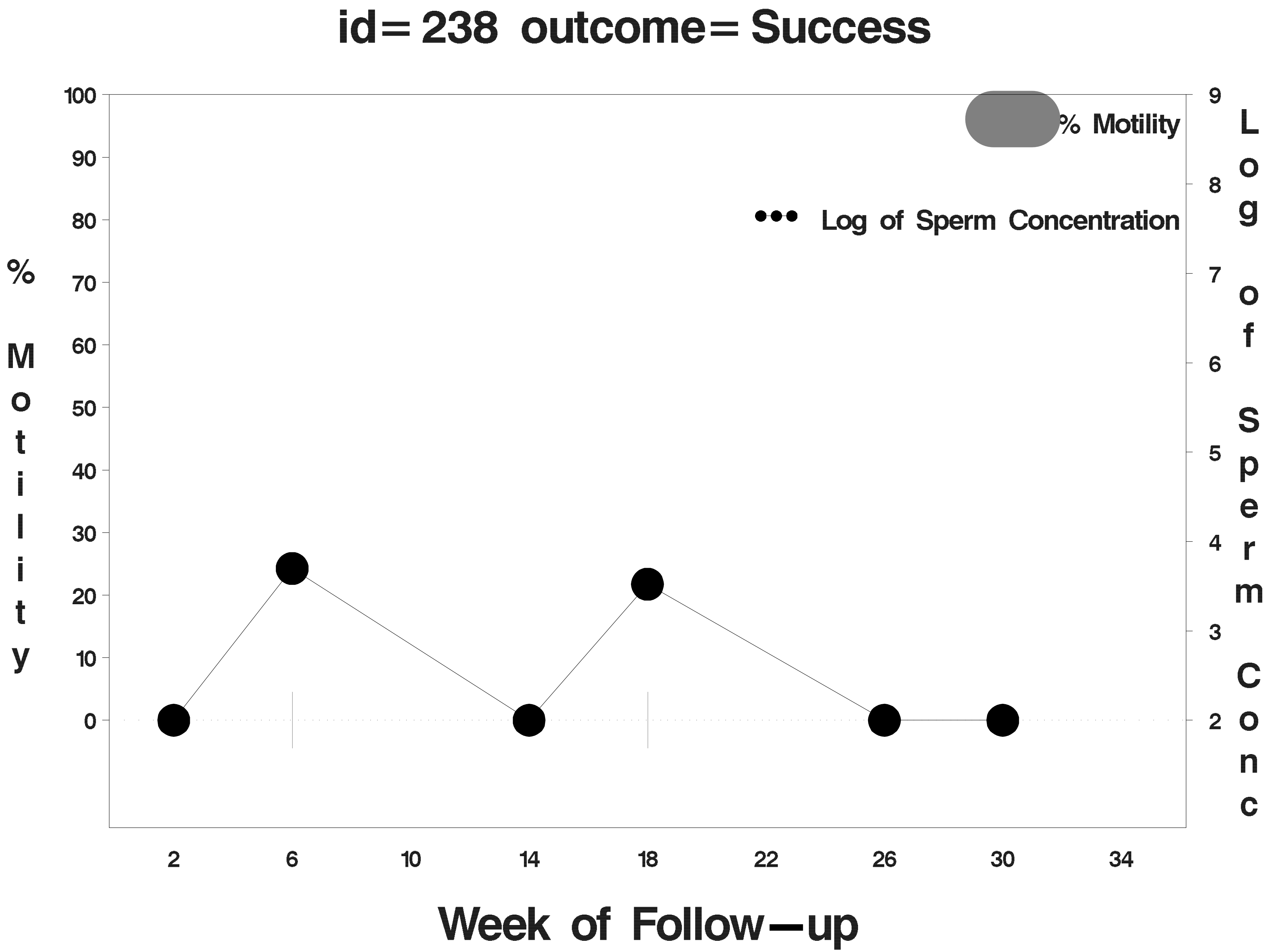

id= 238 outcome= Success
% Motility
Log of Sperm Concentration
% Motility
Log of Sperm Conc
Week of Follow—up
100
90
80
70
60
50
40
30
20
10
0
9
8
7
6
5
4
3
2
2
6
10
14
18
22
26
30
34

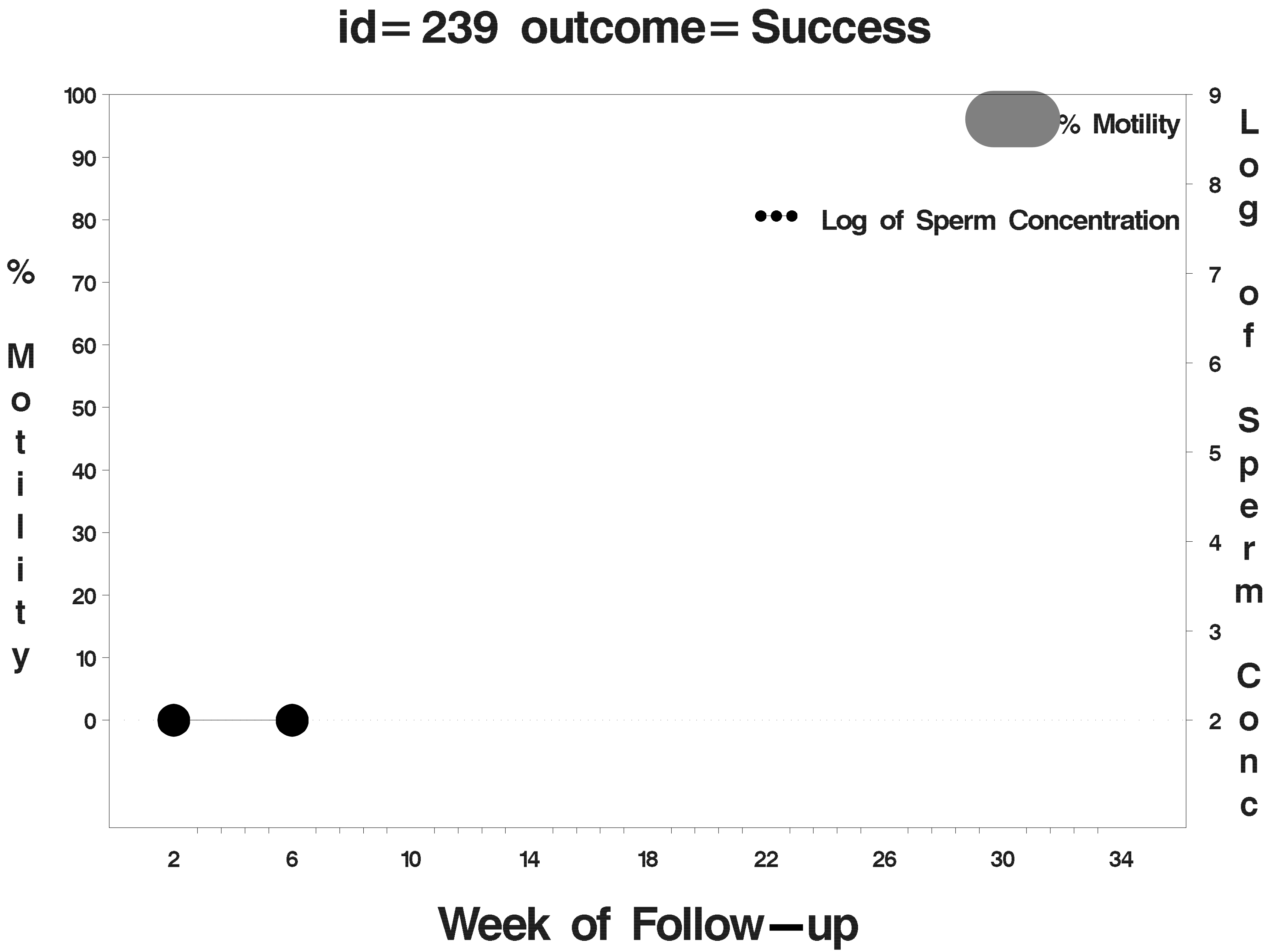
id= 239 outcome= Success
% Motility
Log of Sperm Concentration
% Motility
Log of Sperm Conc
Week of Follow-up
100
90
80
70
60
50
40
30
20
10
0
9
8
7
6
5
4
3
2
2
6
10
14
18
22
26
30
34

id= 240 outcome= Success

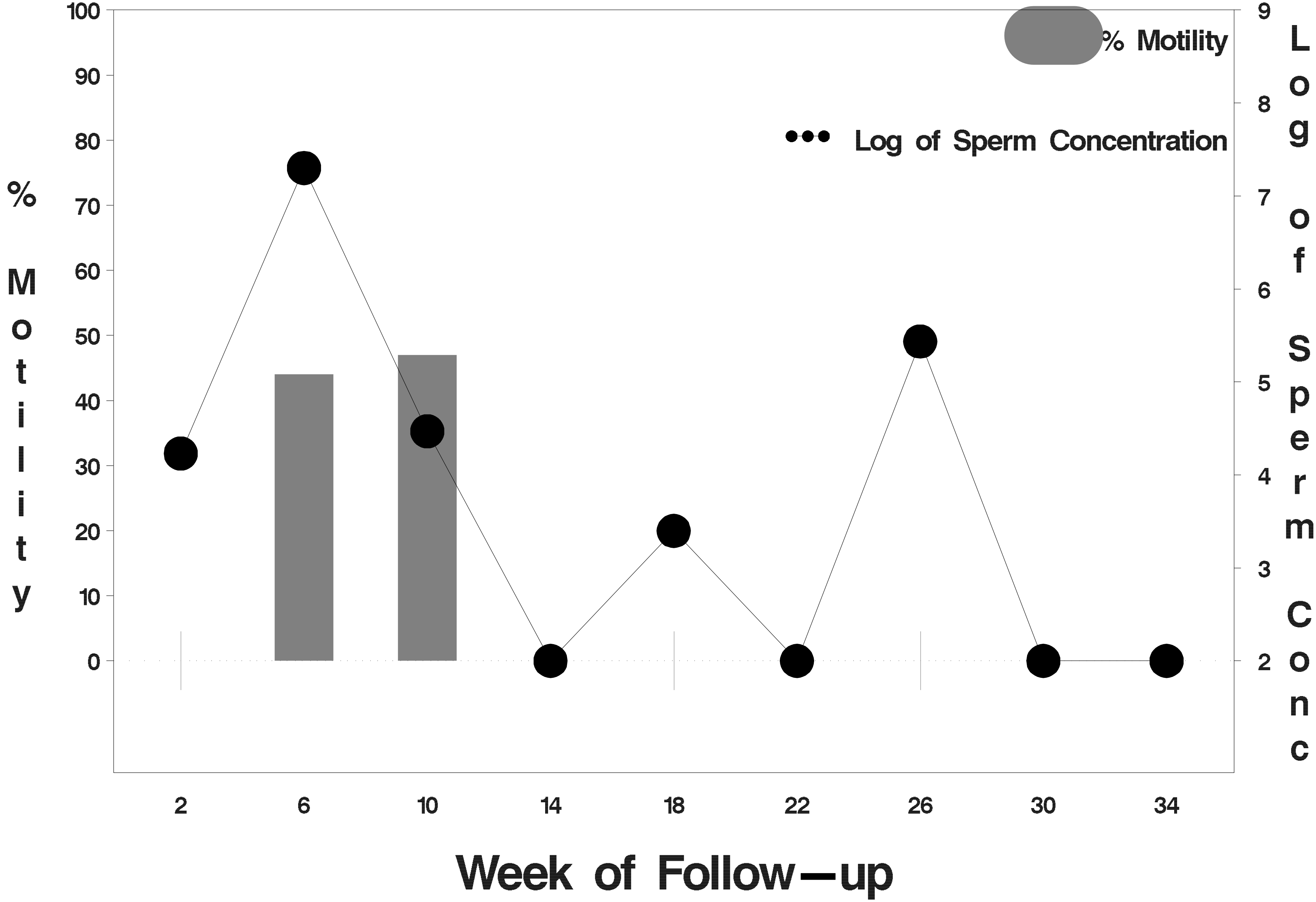

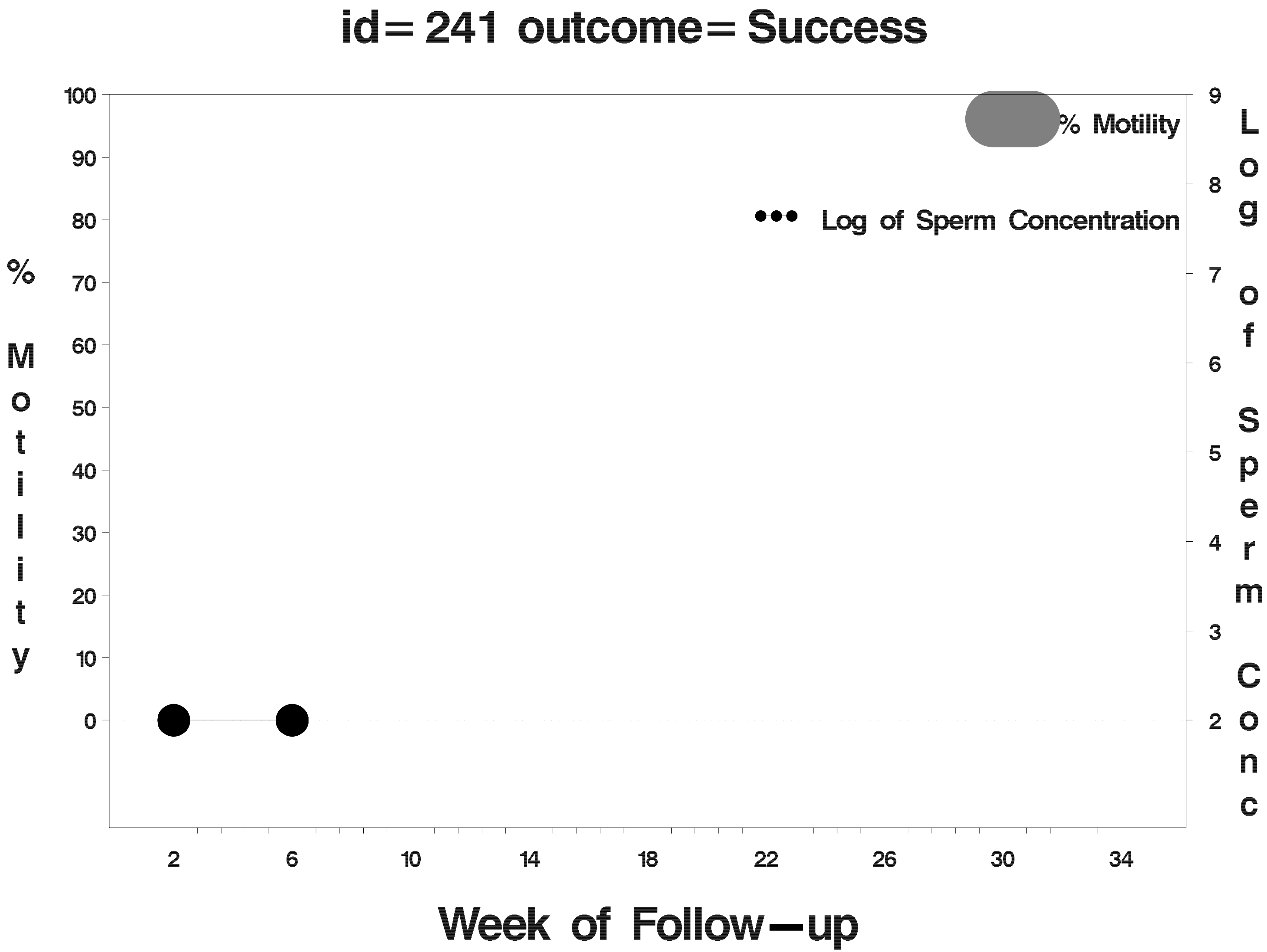
id= 241 outcome= Success
% Motility
Log of Sperm Concentration
% Motility
Log of Sperm Conc
Week of Follow−up
100
90
80
70
60
50
40
30
20
10
0
9
8
7
6
5
4
3
2
2
6
10
14
18
22
26
30
34

id= 242 outcome= Success

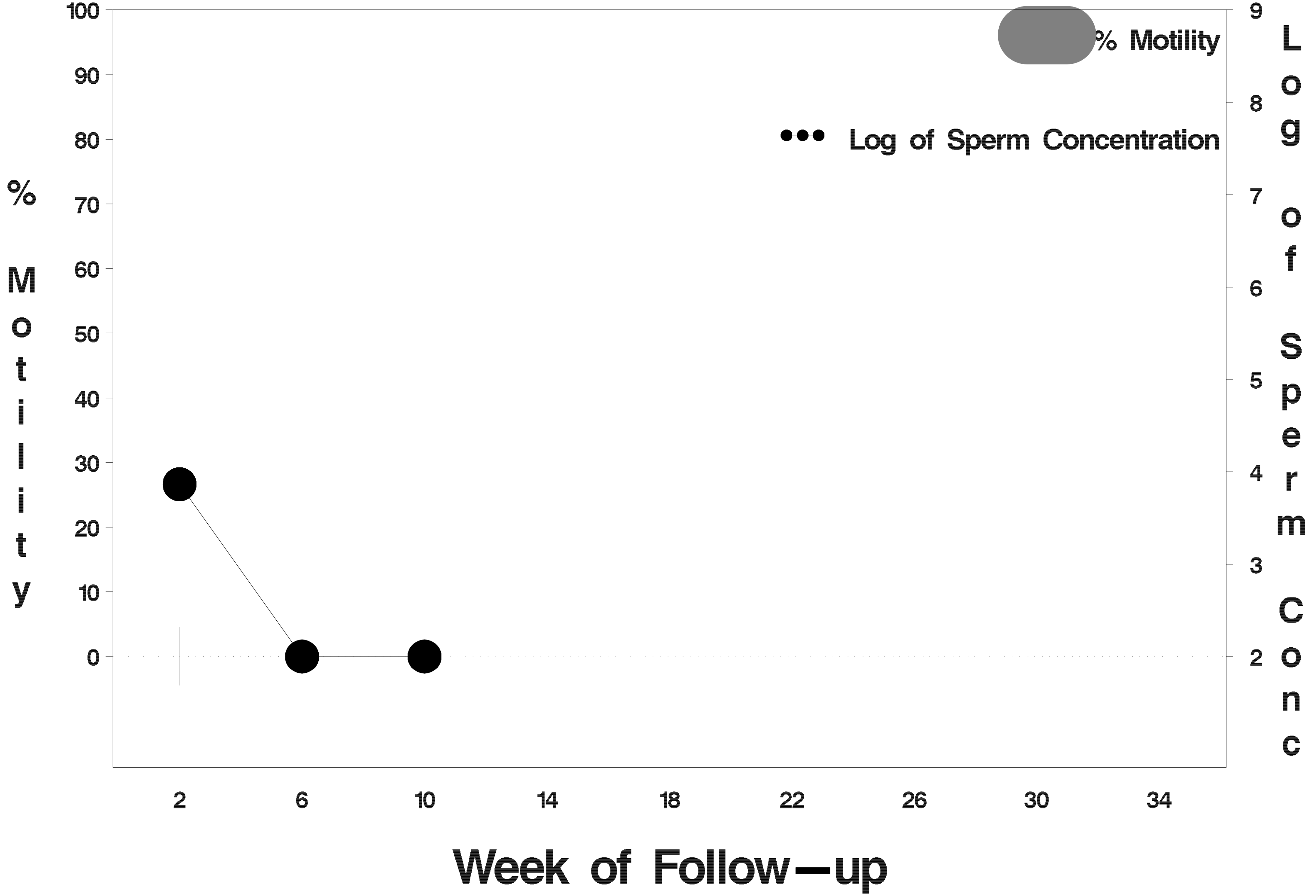

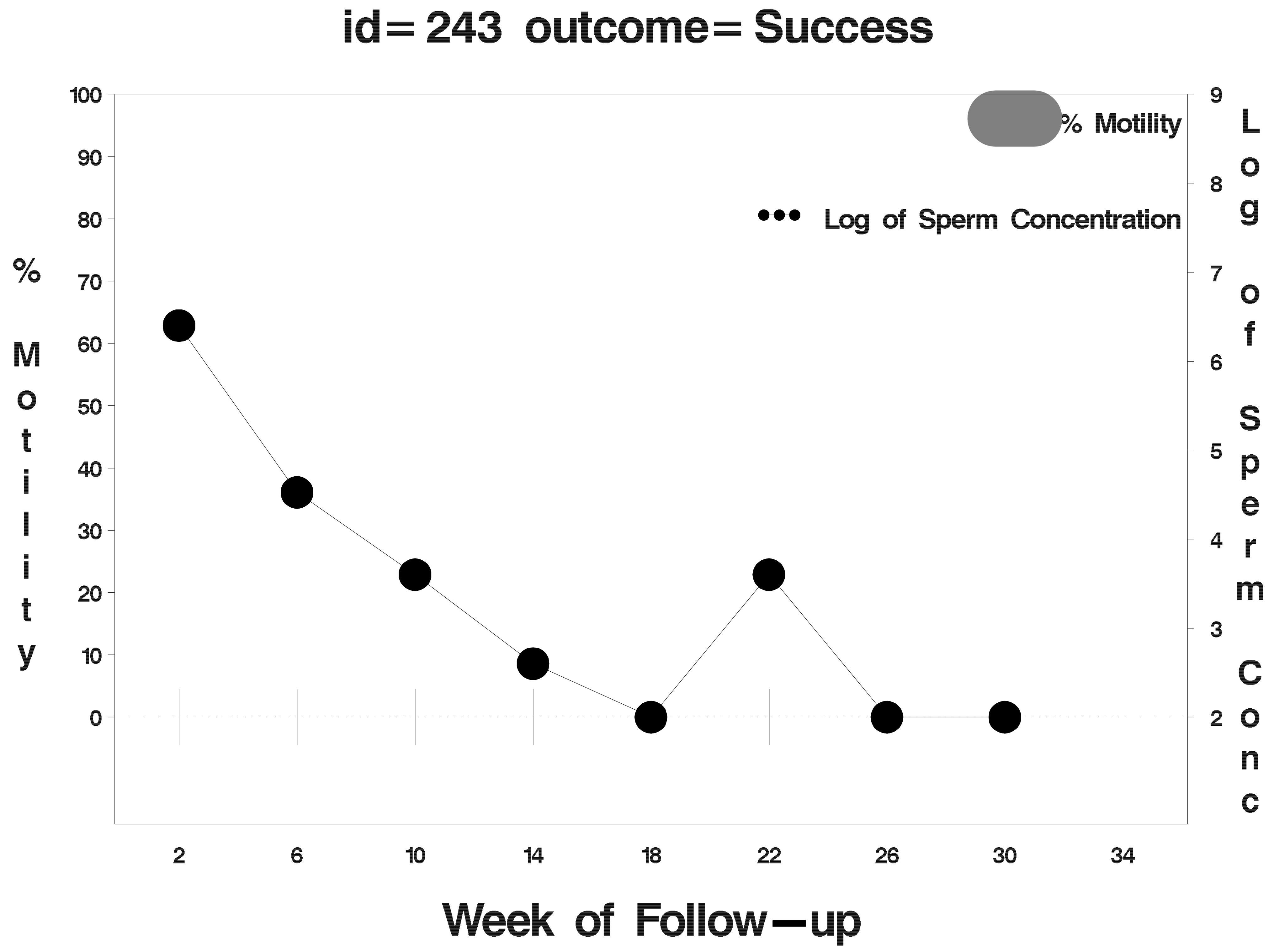

id= 243 outcome= Success
% Motility
Log of Sperm Concentration
100
90
80
70
60
50
40
30
20
10
0
9
8
7
6
5
4
3
2
2
6
10
14
18
22
26
30
34
Week of Follow—up
% Motility
Log of Sperm Conc

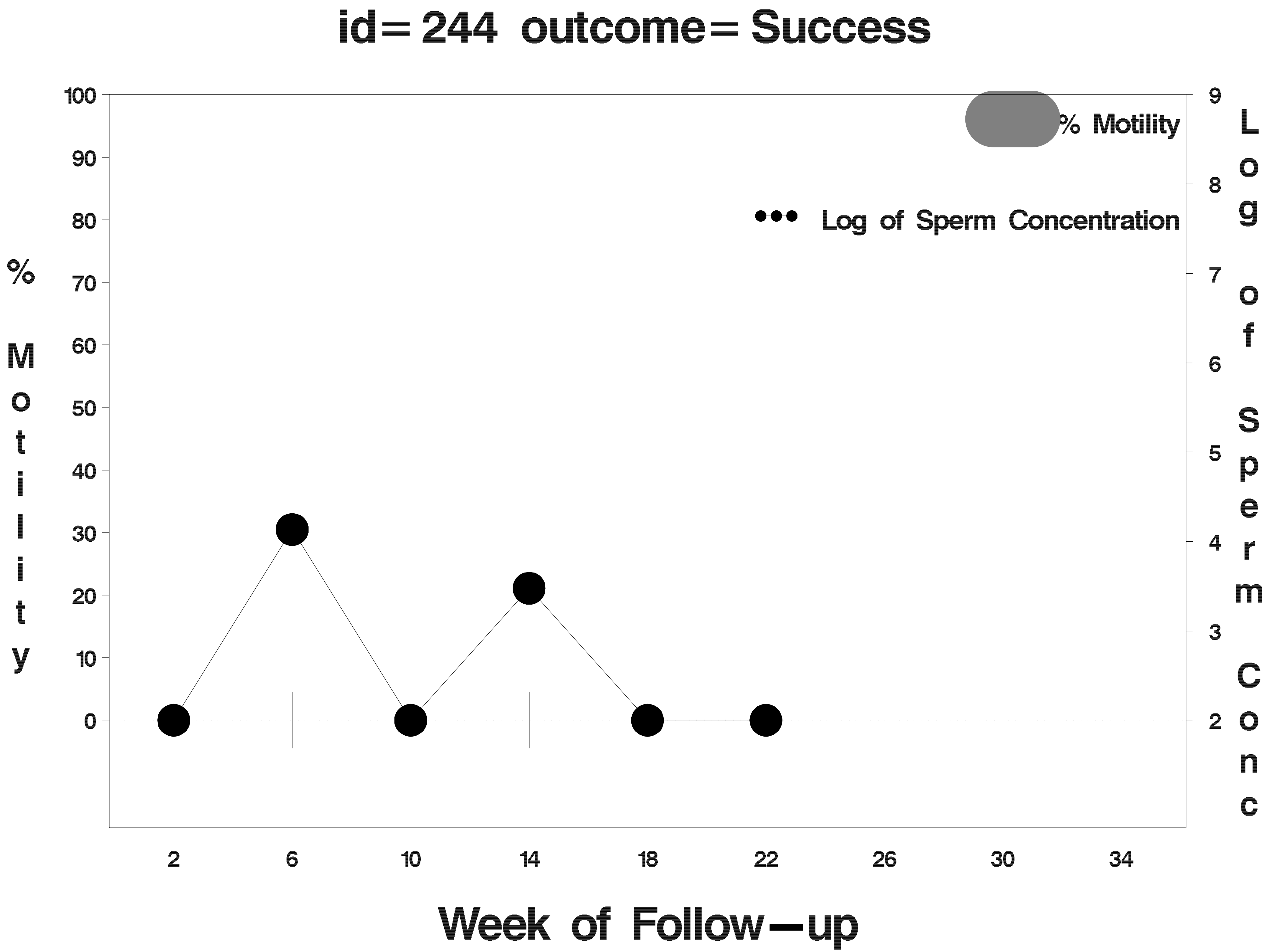

id= 244 outcome= Success
% Motility
Log of Sperm Concentration
100
90
80
70
60
50
40
30
20
10
0
9
8
7
6
5
4
3
2
2
6
10
14
18
22
26
30
34
Week of Follow−up

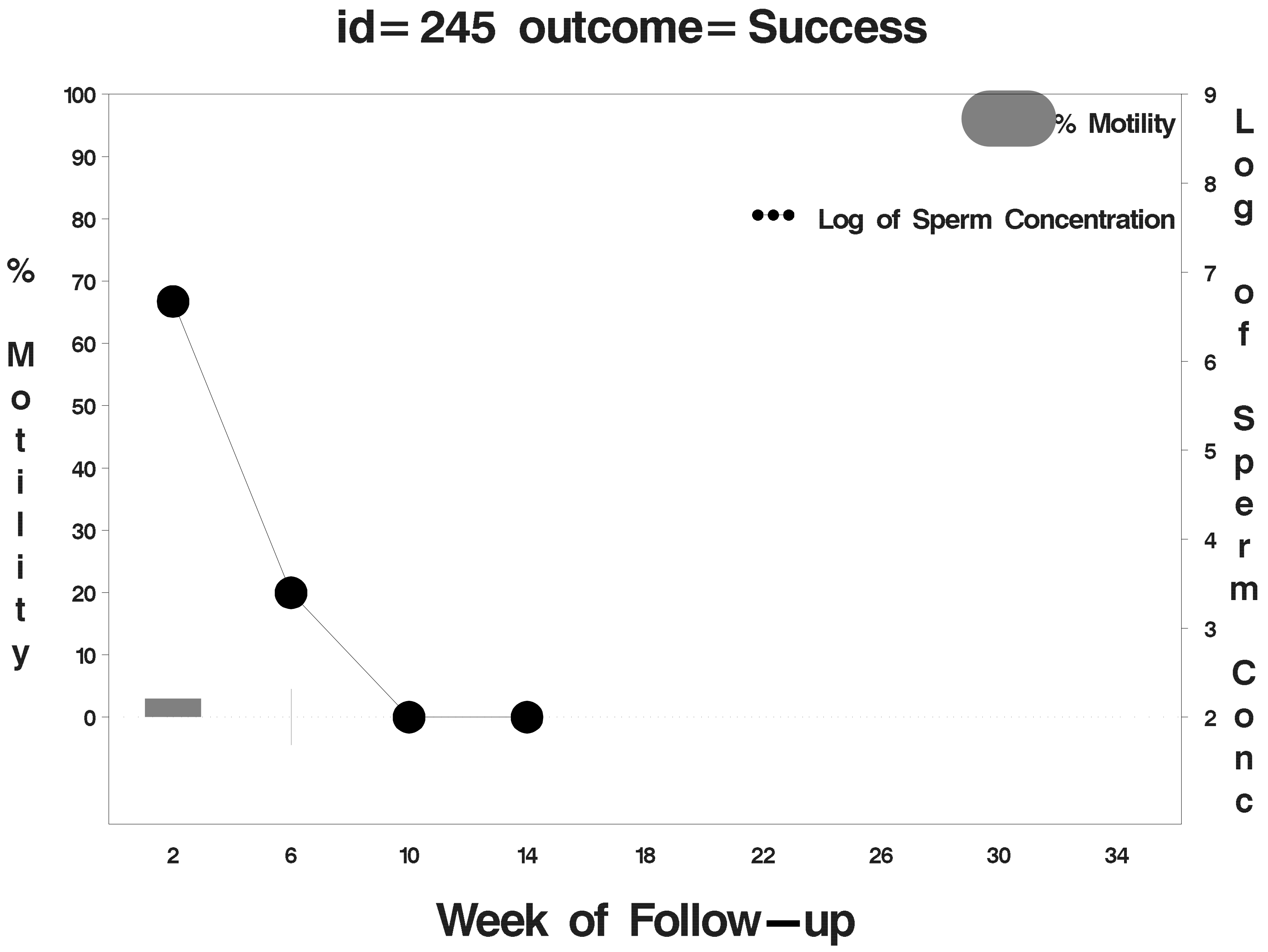
id= 245 outcome= Success
% Motility
Log of Sperm Concentration
100
90
80
70
60
50
40
30
20
10
0
9
8
7
6
5
4
3
2
2
6
10
14
18
22
26
30
34
Week of Follow—up
Log of Sperm Conc

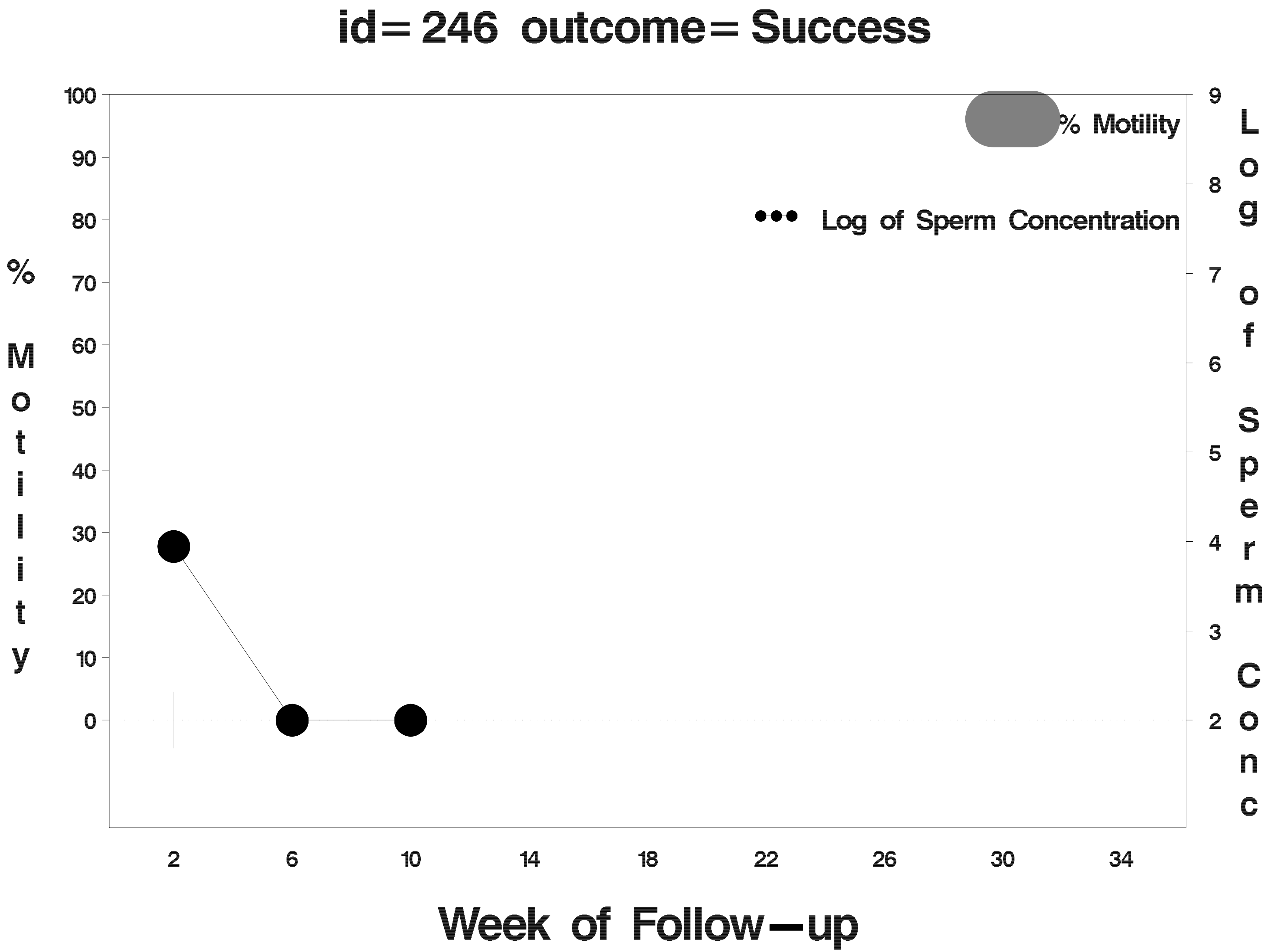

id= 246 outcome= Success
% Motility
Log of Sperm Concentration
% Motility
Log of Sperm Conc
Week of Follow−up
100
90
80
70
60
50
40
30
20
10
0
9
8
7
6
5
4
3
2
2
6
10
14
18
22
26
30
34

id= 247 outcome= Success

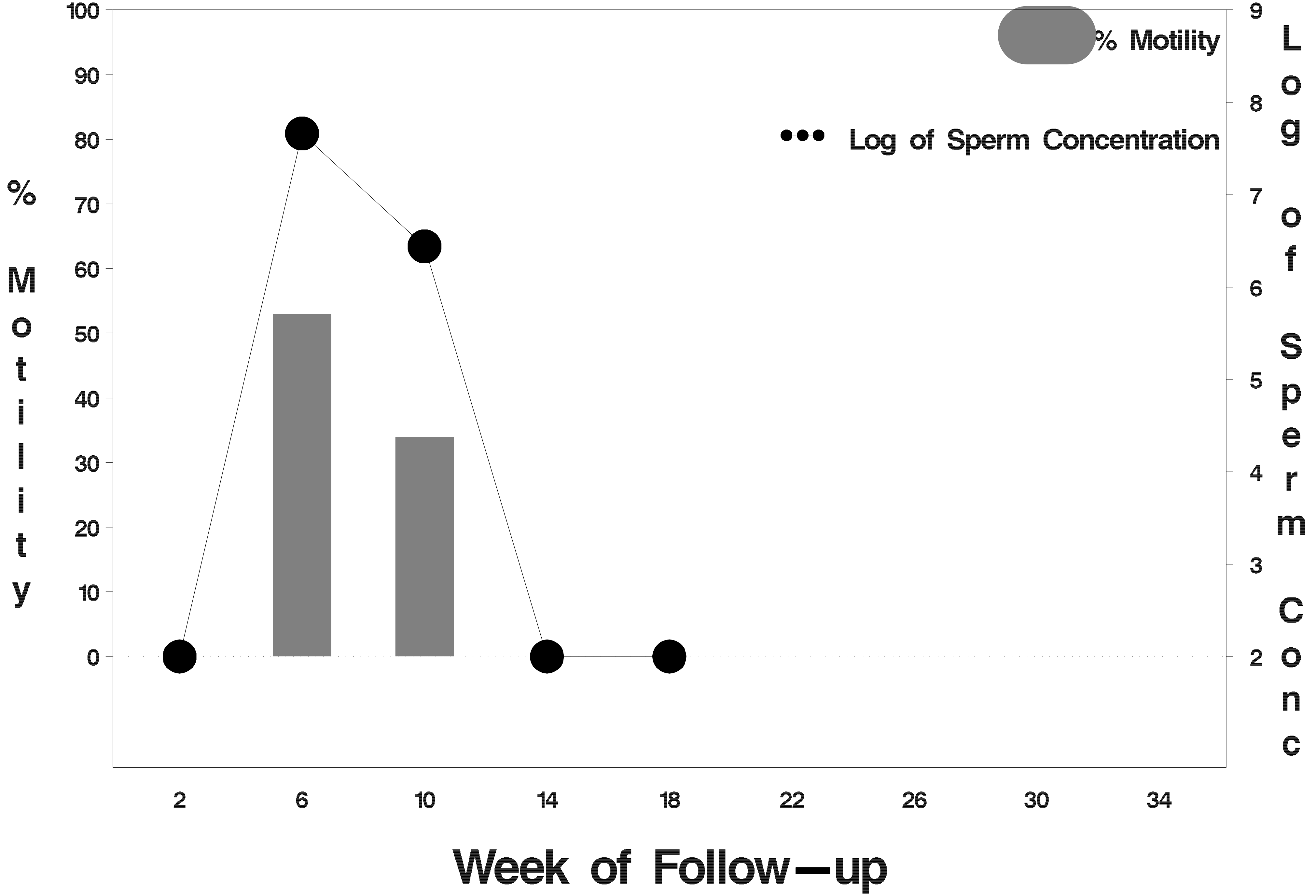

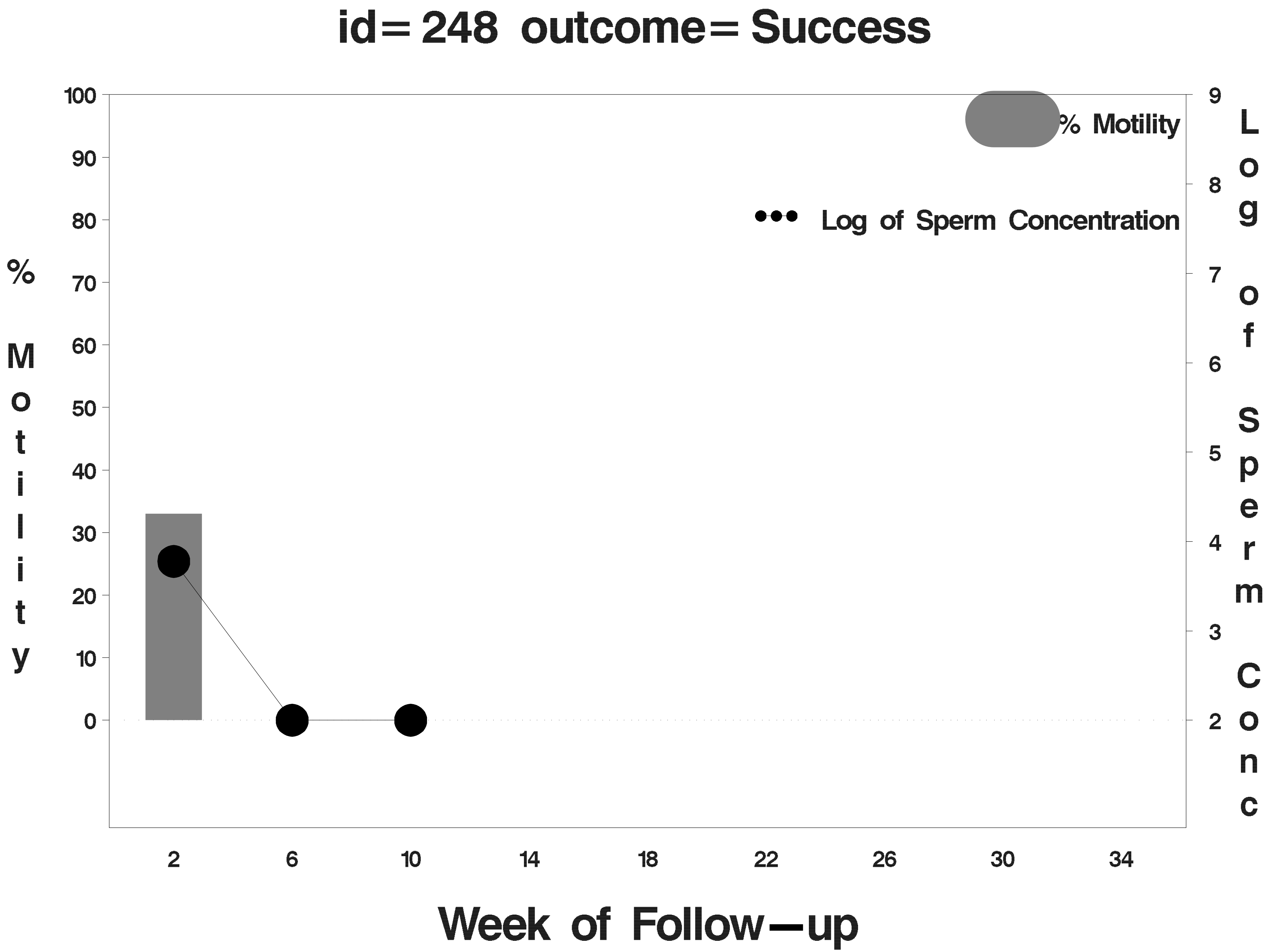

id= 248 outcome= Success
% Motility
Log of Sperm Concentration
100
90
80
70
60
50
40
30
20
10
0
9
8
7
6
5
4
3
2
2
6
10
14
18
22
26
30
34
Week of Follow—up
Log of Sperm Conc

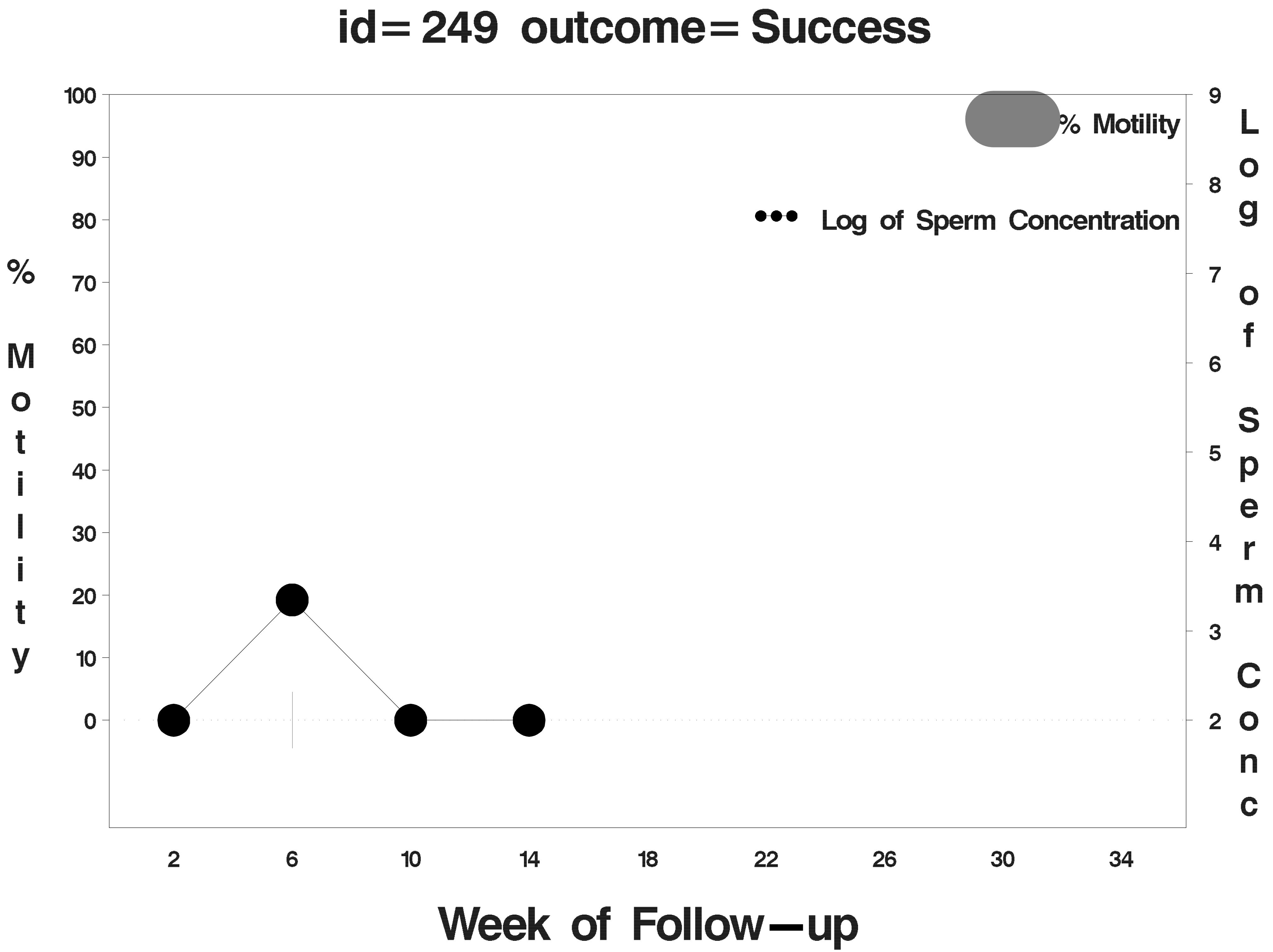
id= 249 outcome= Success
% Motility
Log of Sperm Concentration
100
90
80
70
60
50
40
30
20
10
0
9
8
7
6
5
4
3
2
2
6
10
14
18
22
26
30
34
Week of Follow—up
Log of Sperm Conc

id= 250 outcome= Success

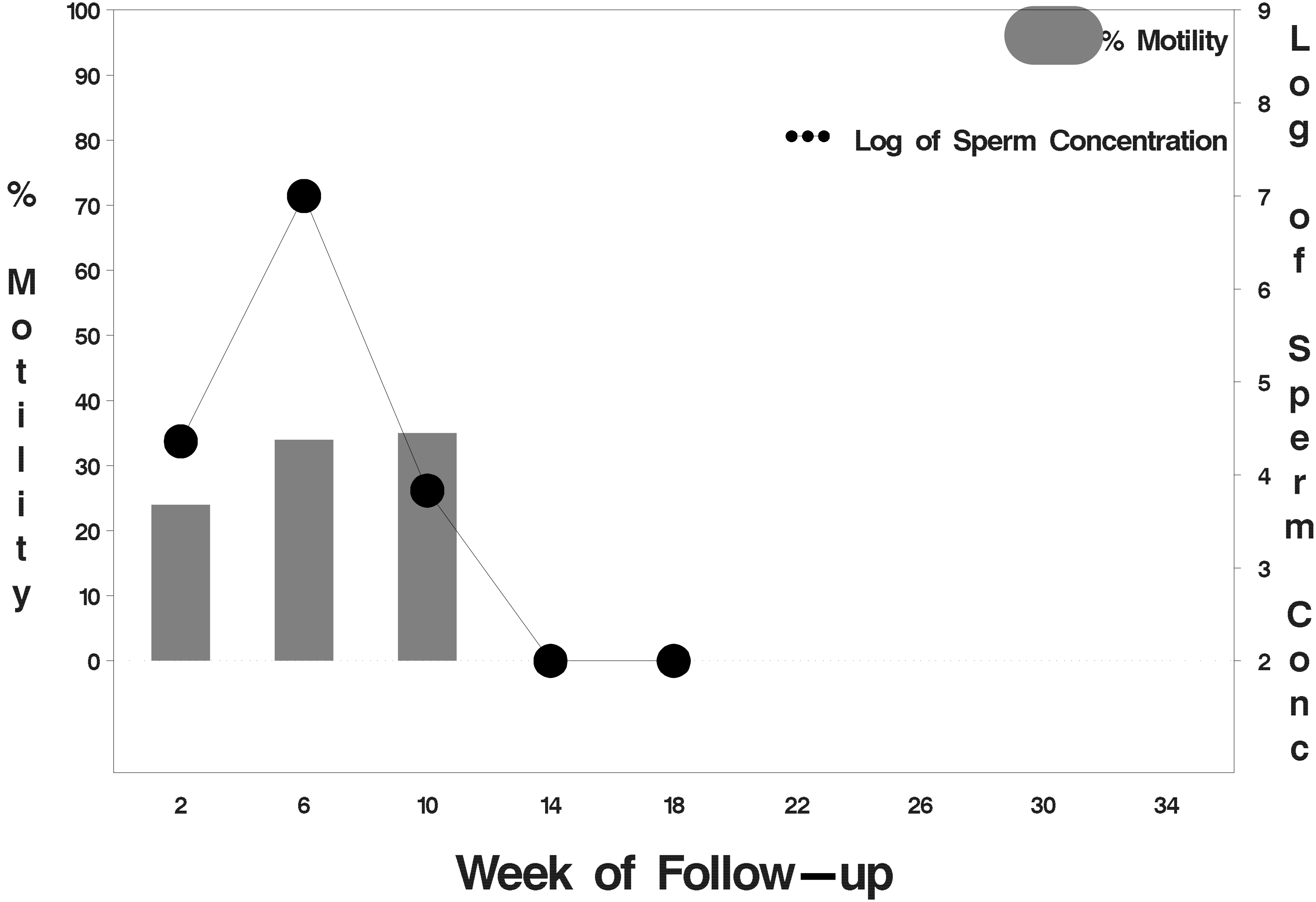

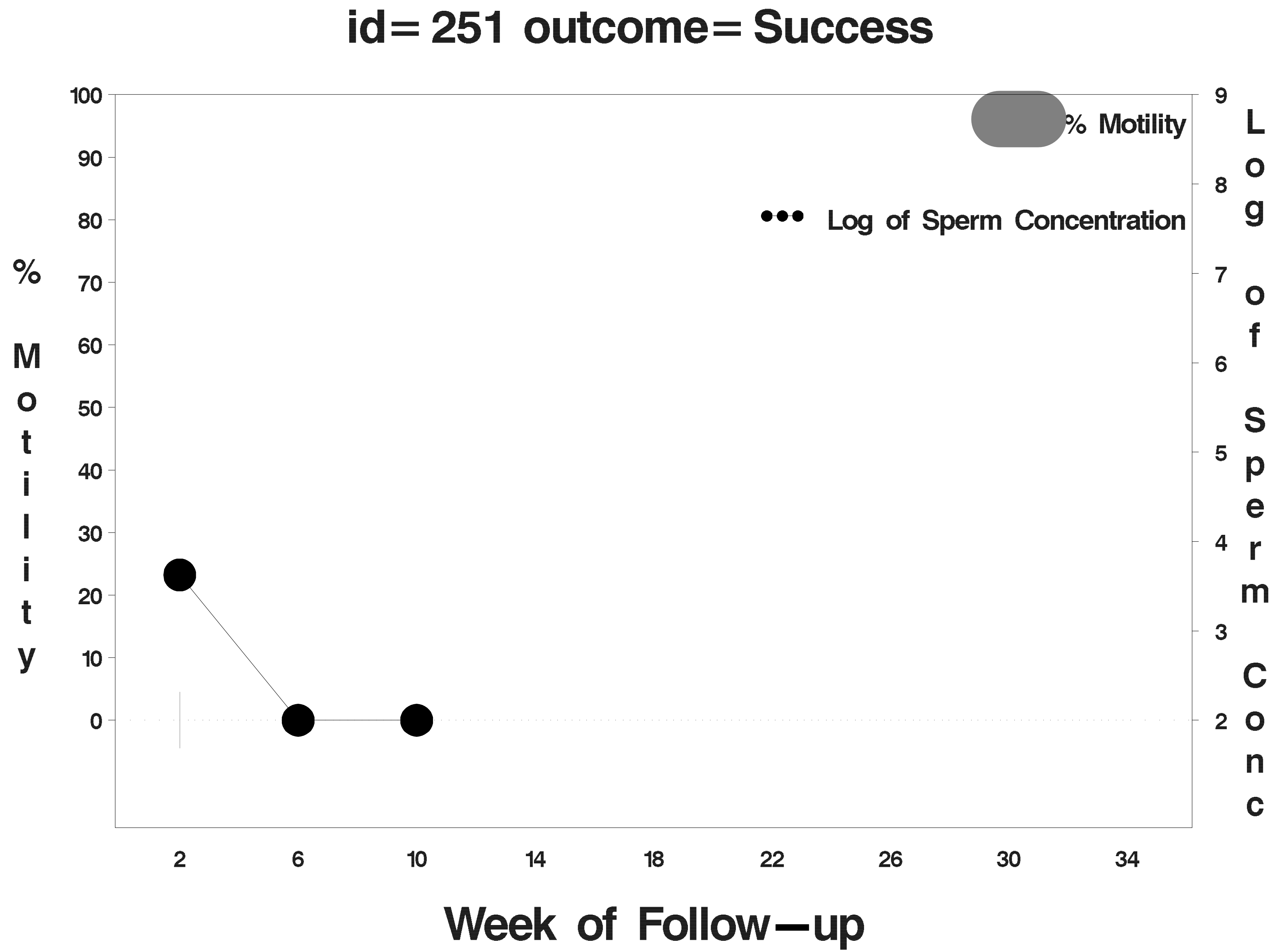

id= 251 outcome= Success
% Motility
Log of Sperm Concentration
100
90
80
70
60
50
40
30
20
10
0
9
8
7
6
5
4
3
2
2
6
10
14
18
22
26
30
34
Week of Follow—up
Log of Sperm Conc

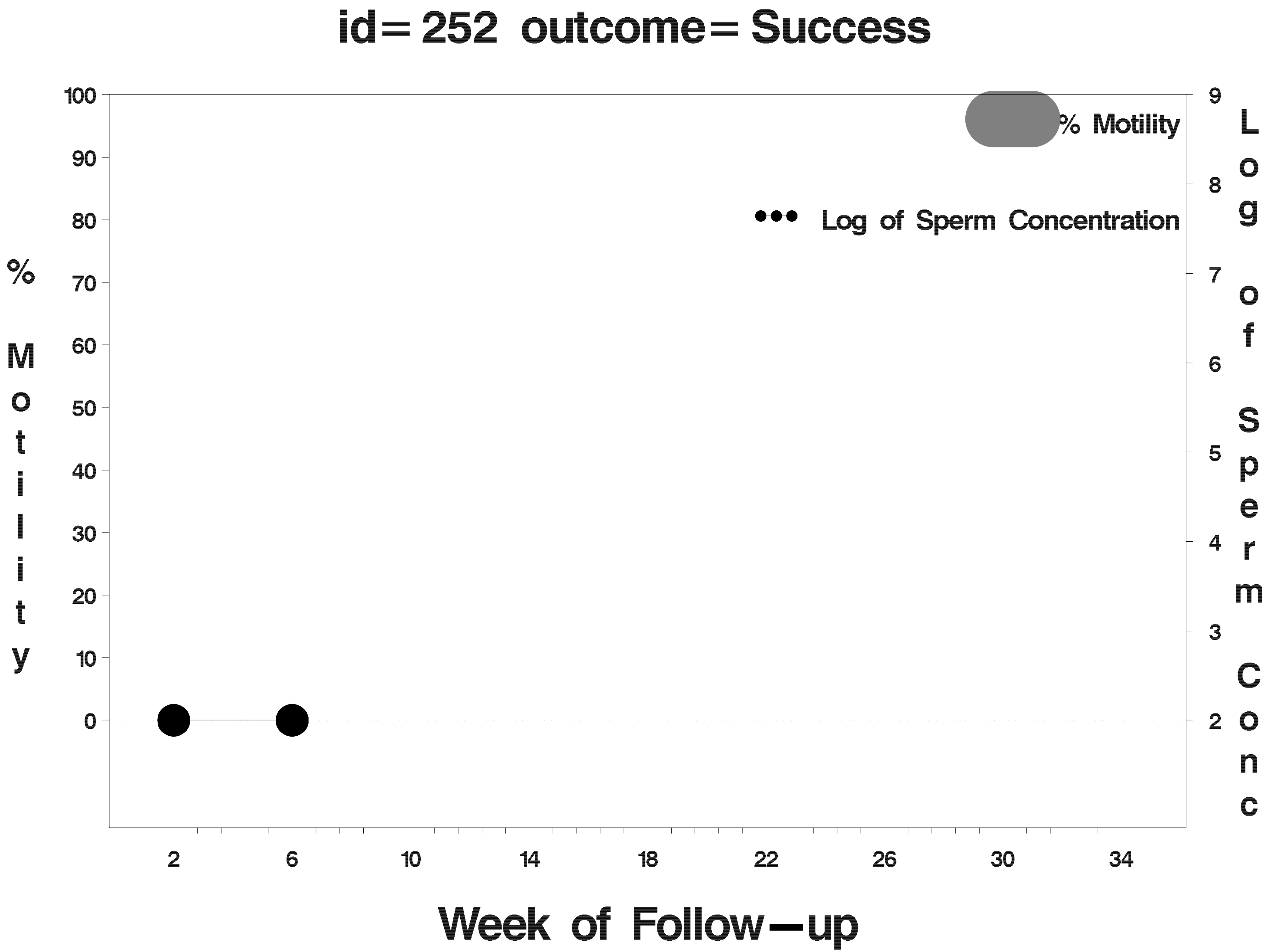

id= 252 outcome= Success
% Motility
Log of Sperm Concentration
100
90
80
70
60
50
40
30
20
10
0
9
8
7
6
5
4
3
2
2
6
10
14
18
22
26
30
34
Week of Follow-up
Log of Sperm Conc

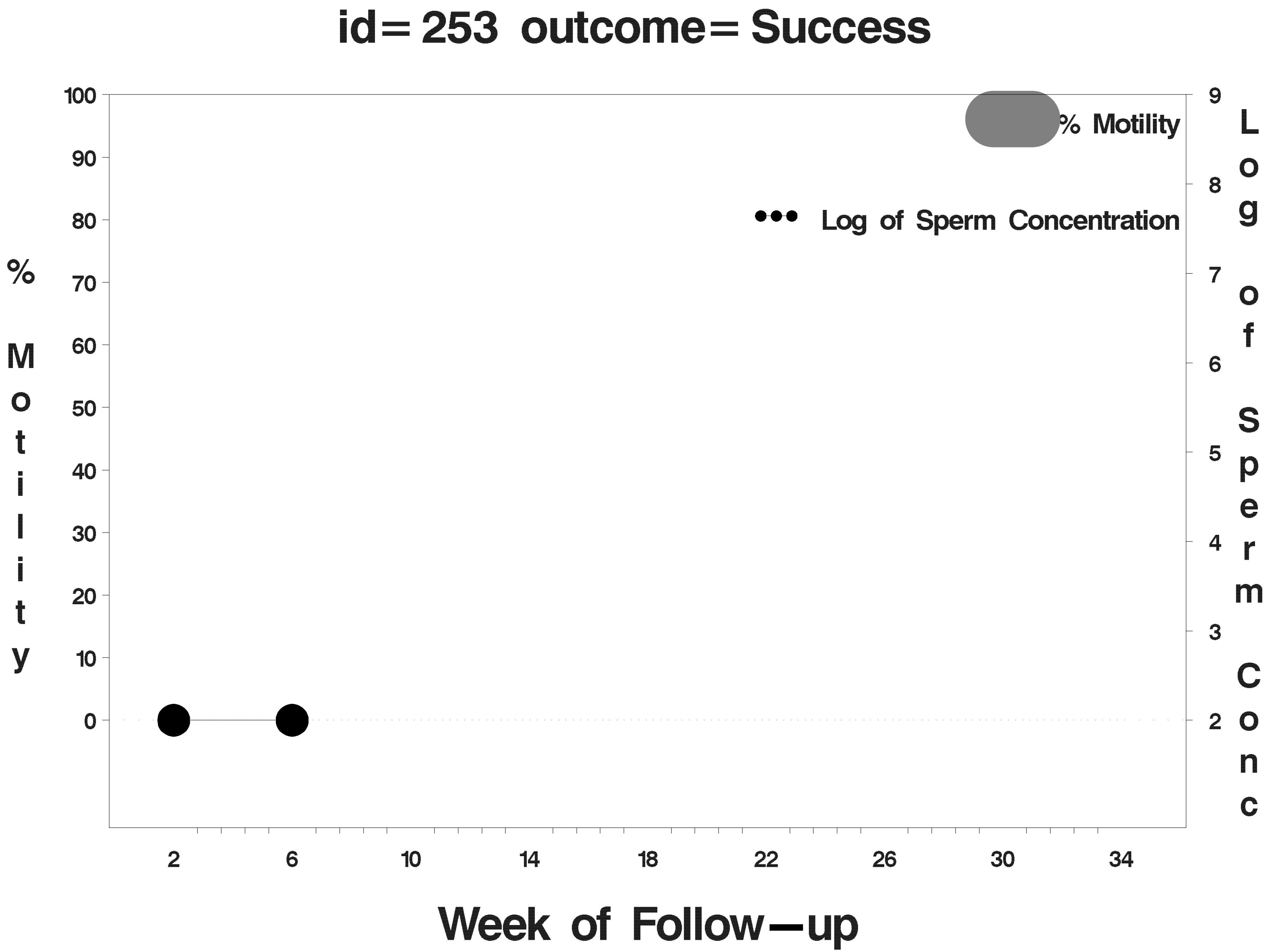

id= 253 outcome= Success
% Motility
Log of Sperm Concentration
100
90
80
70
60
50
40
30
20
10
0
9
8
7
6
5
4
3
2
2
6
10
14
18
22
26
30
34
Week of Follow-up

id= 254 outcome= Success

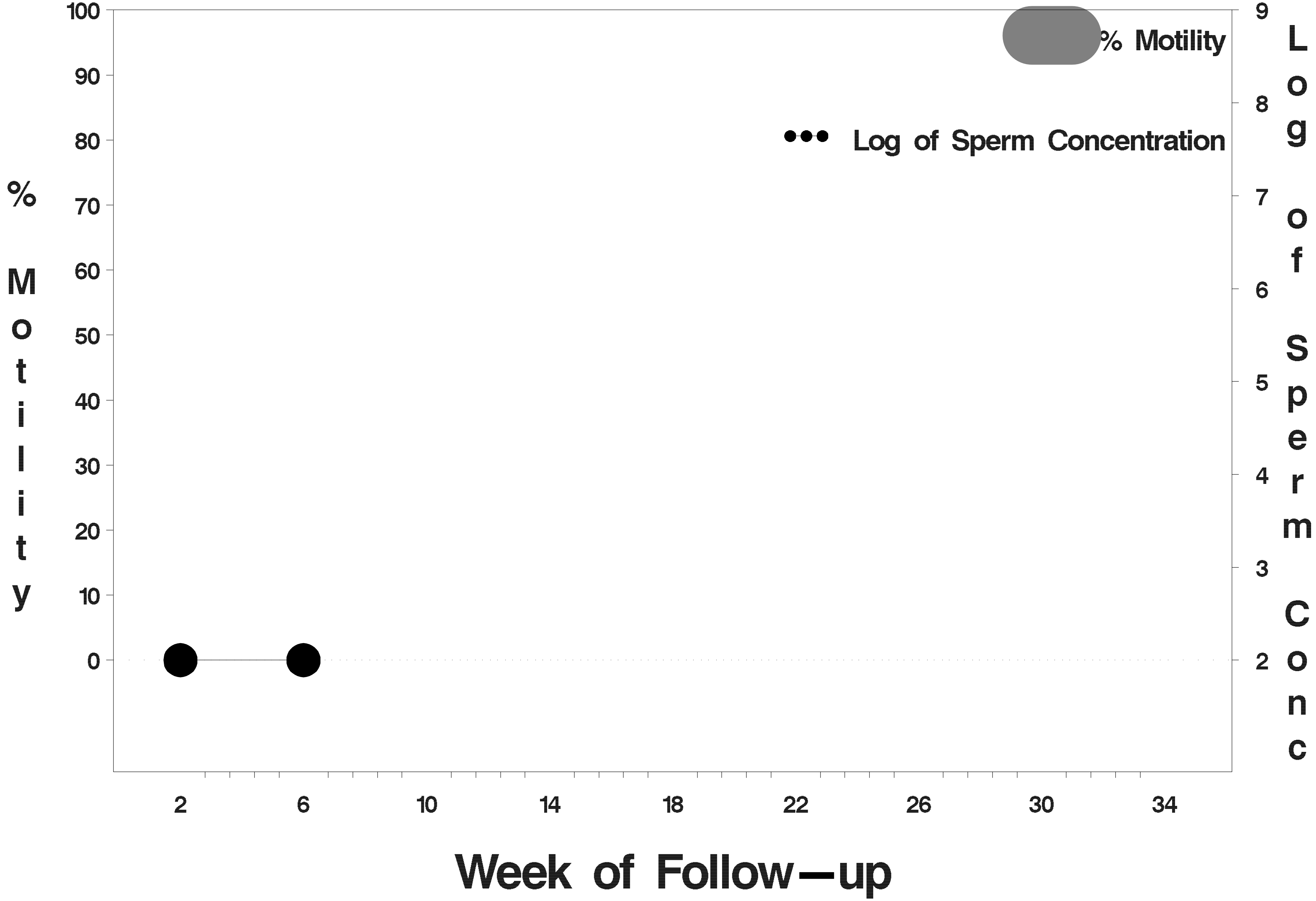

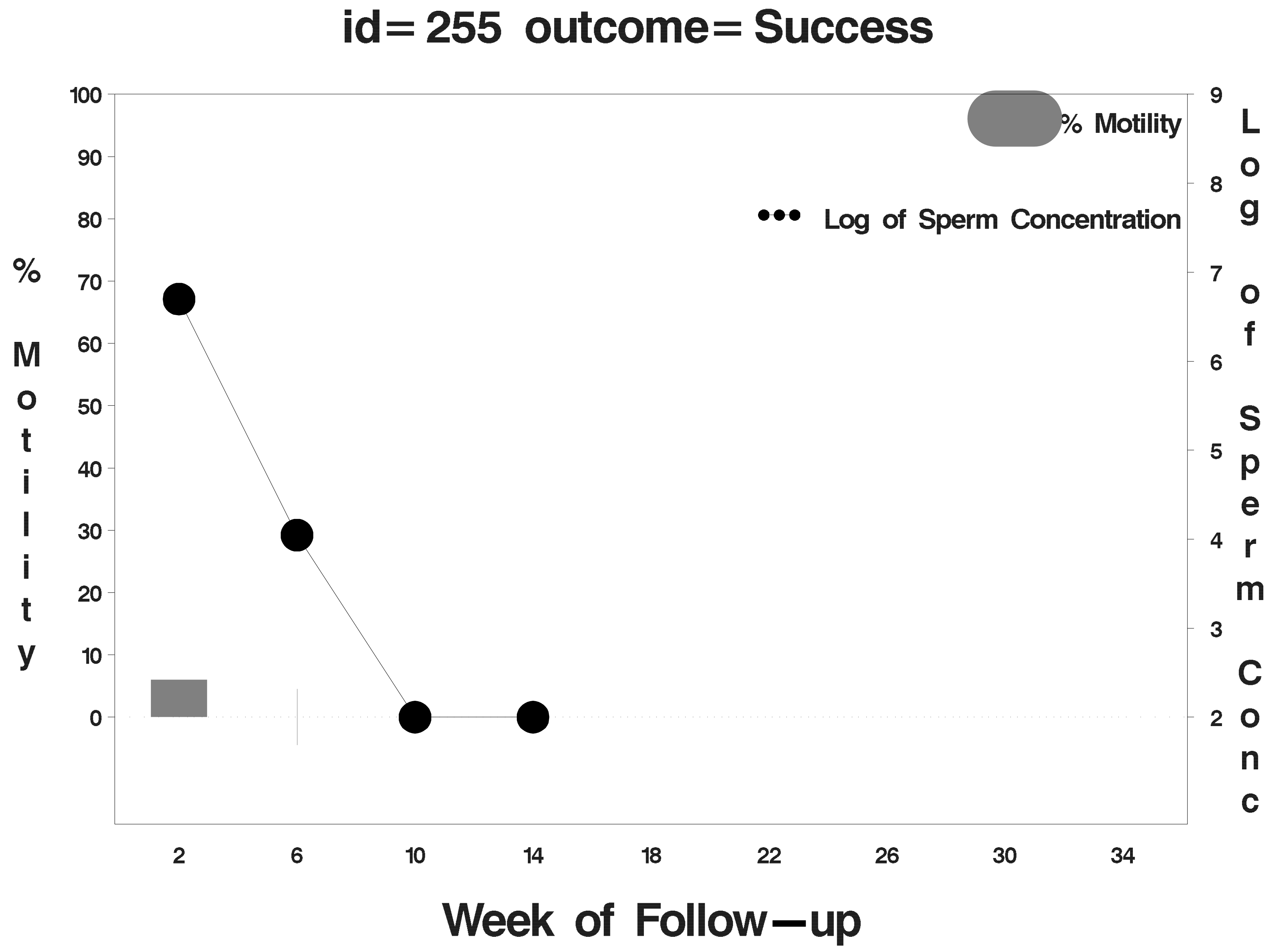
id= 255 outcome= Success
% Motility
Log of Sperm Concentration
100
90
80
70
60
50
40
30
20
10
0
9
8
7
6
5
4
3
2
Log of Sperm Conc
2
6
10
14
18
22
26
30
34
Week of Follow−up

id= 256 outcome= Success

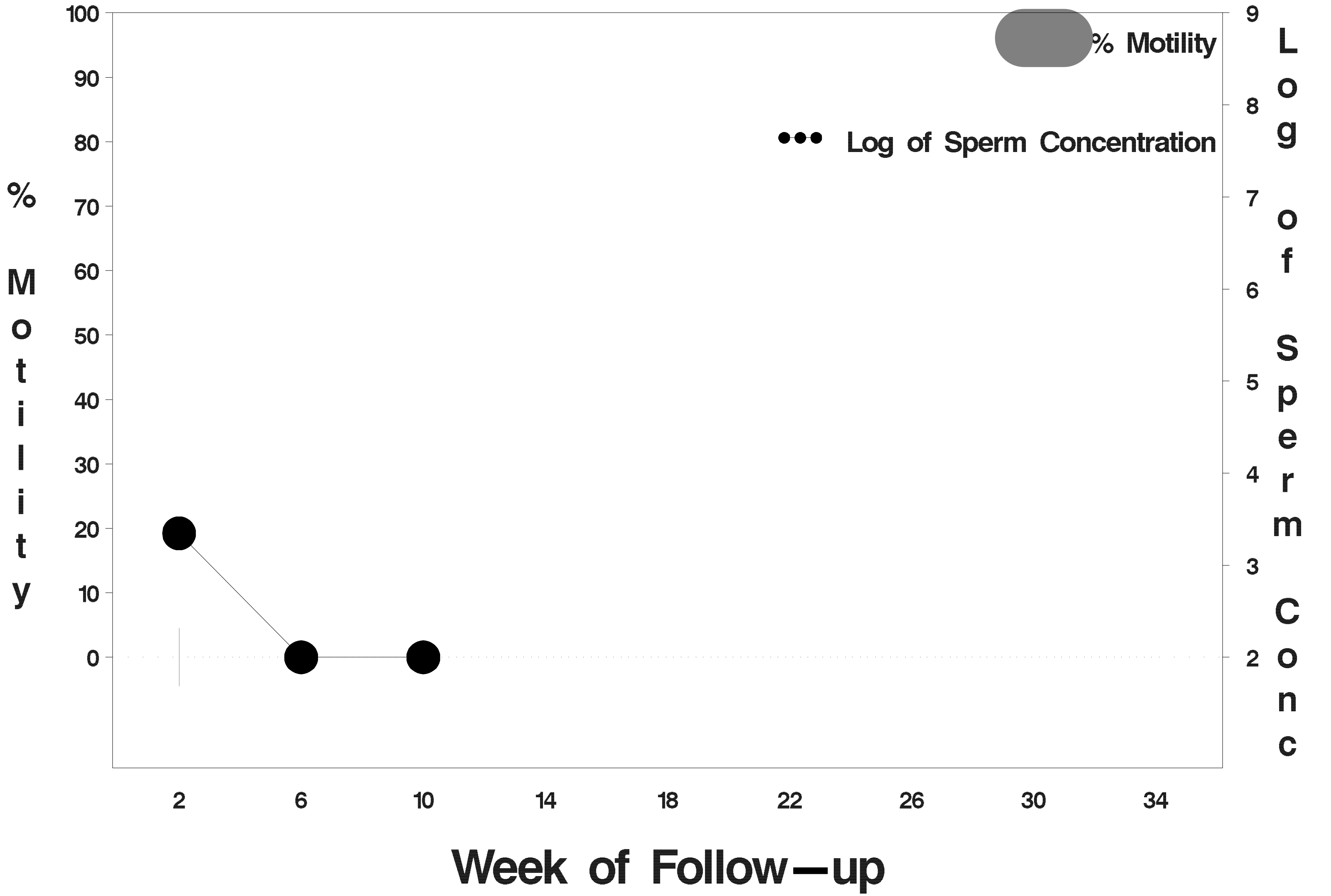

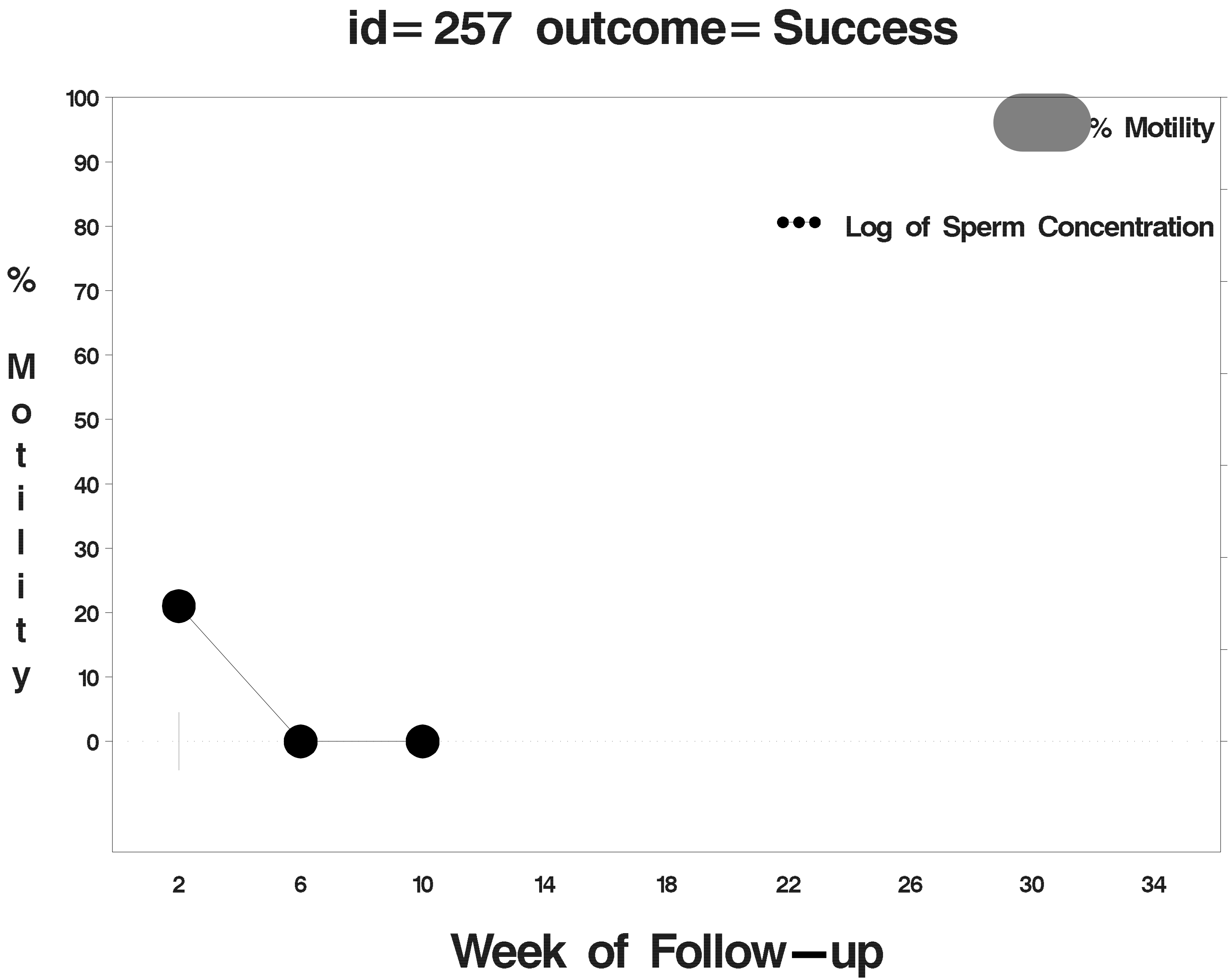
id= 257 outcome= Success
% Motility
Log of Sperm Concentration
100
90
80
70
60
50
40
30
20
10
0
9
8
7
6
5
4
3
2
2
6
10
14
18
22
26
30
34
Week of Follow—up
% Motility
Log of Sperm Conc

id= 258 outcome= Success

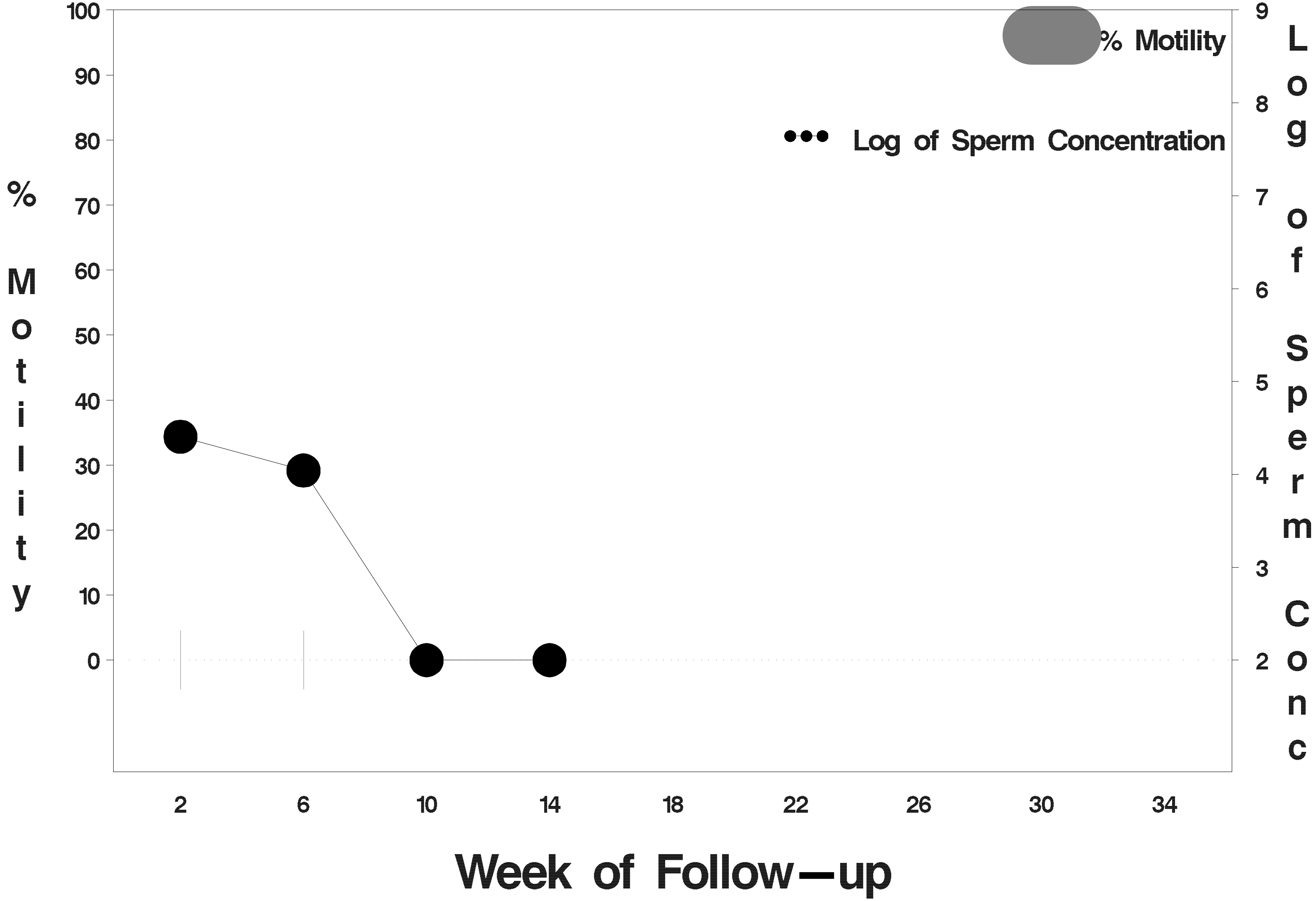

id= 259 outcome= Success

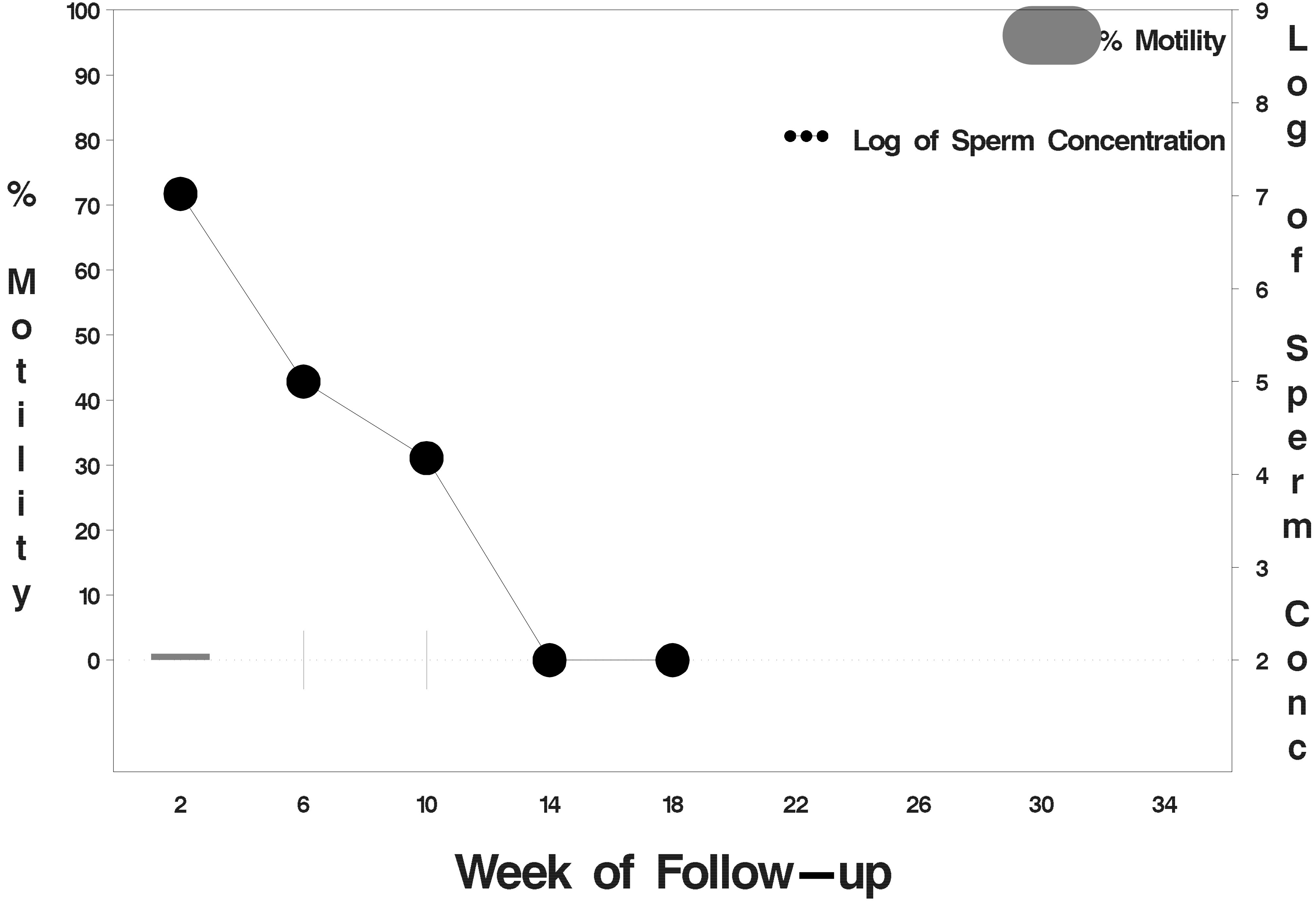

id= 260 outcome= Success

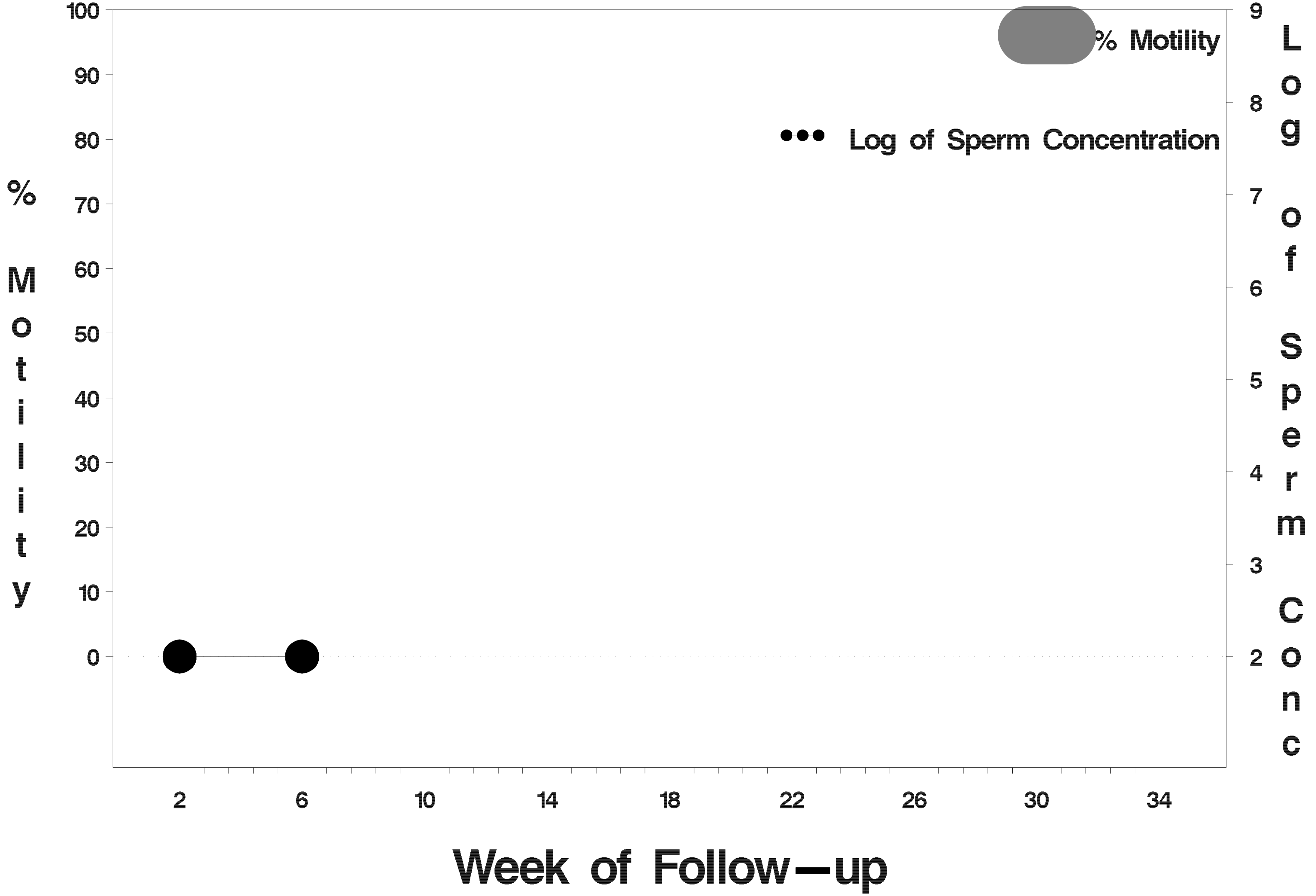

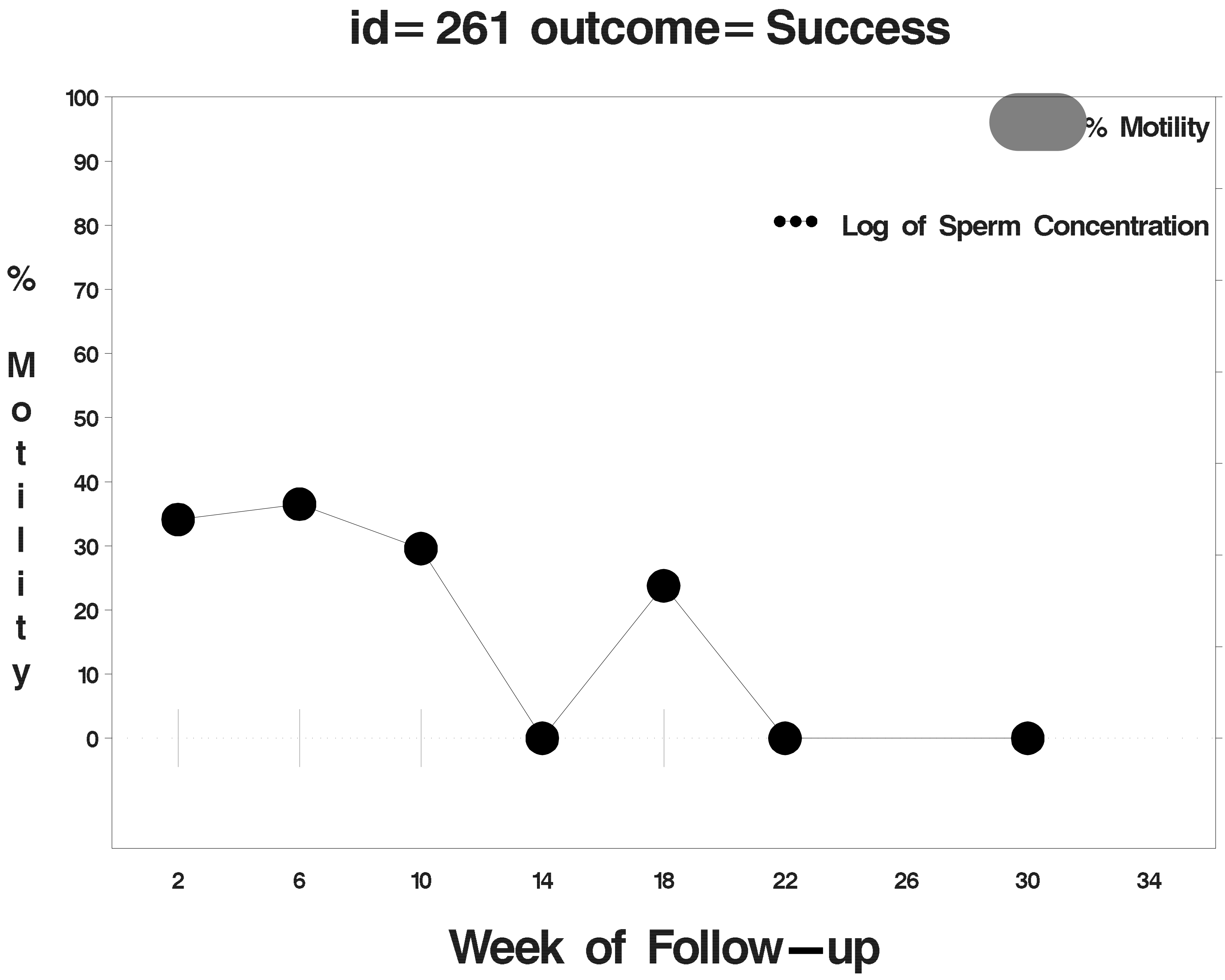

id= 261 outcome= Success
% Motility
Log of Sperm Concentration
100
90
80
70
60
50
40
30
20
10
0
9
8
7
6
5
4
3
2
2
6
10
14
18
22
26
30
34
Week of Follow—up
Log of Sperm Conc

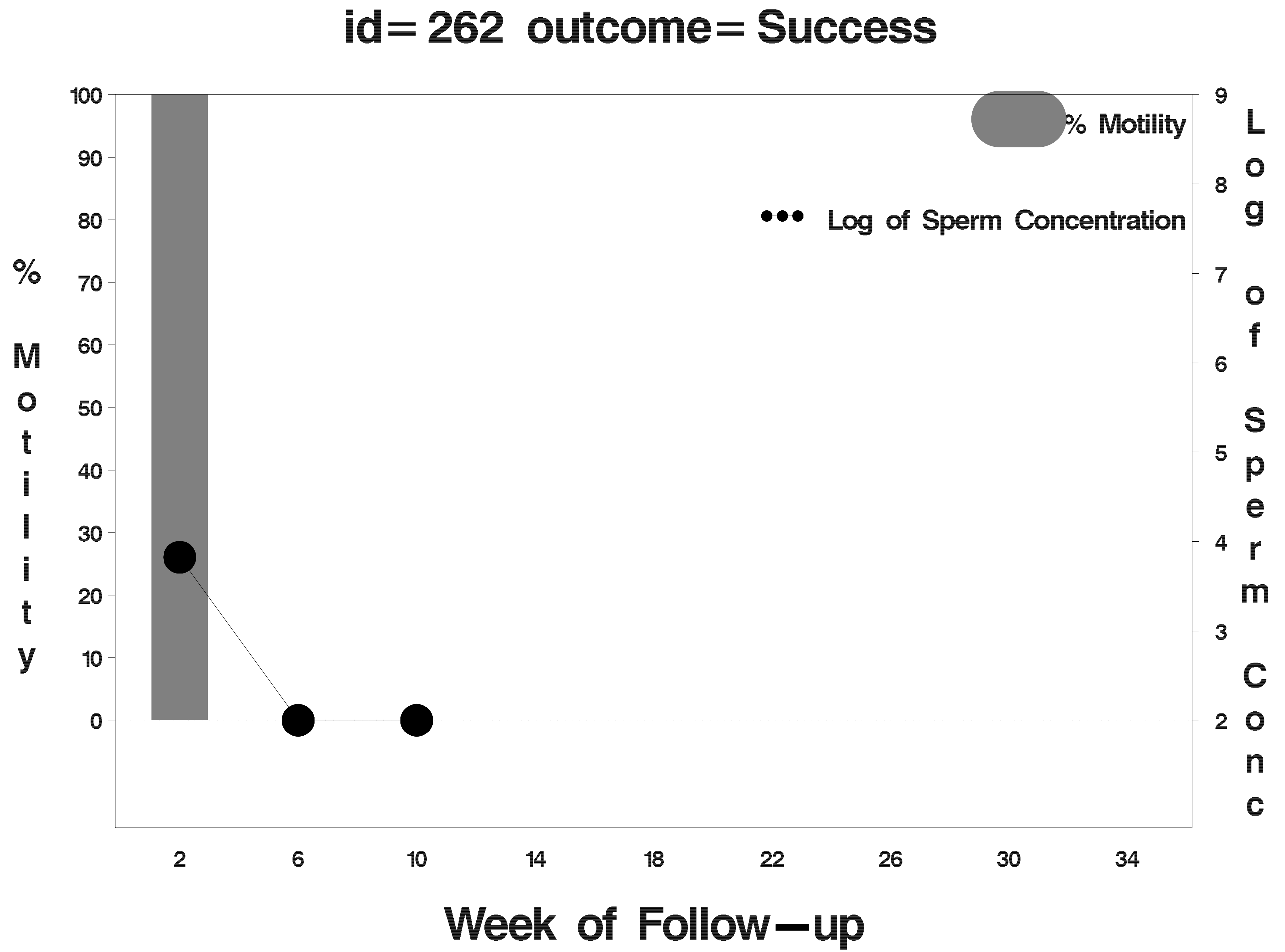

id= 262 outcome= Success
% Motility
Log of Sperm Concentration
% Motility
Log of Sperm Conc
100
90
80
70
60
50
40
30
20
10
0
9
8
7
6
5
4
3
2
2
6
10
14
18
22
26
30
34
Week of Follow-up

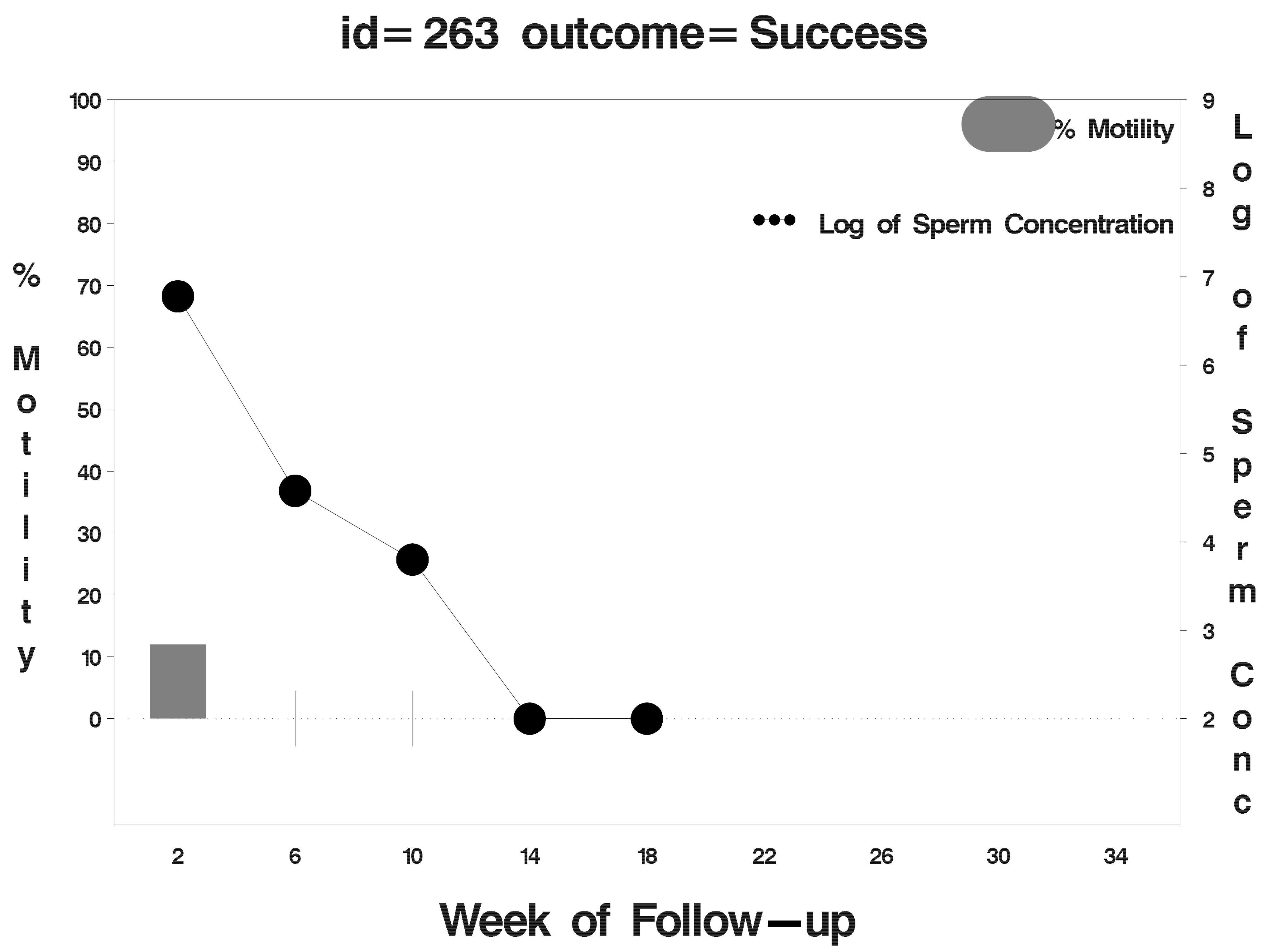

id= 263 outcome= Success
% Motility
Log of Sperm Concentration
% Motility
Log of Sperm Conc
Week of Follow—up
100
90
80
70
60
50
40
30
20
10
0
9
8
7
6
5
4
3
2
2
6
10
14
18
22
26
30
34

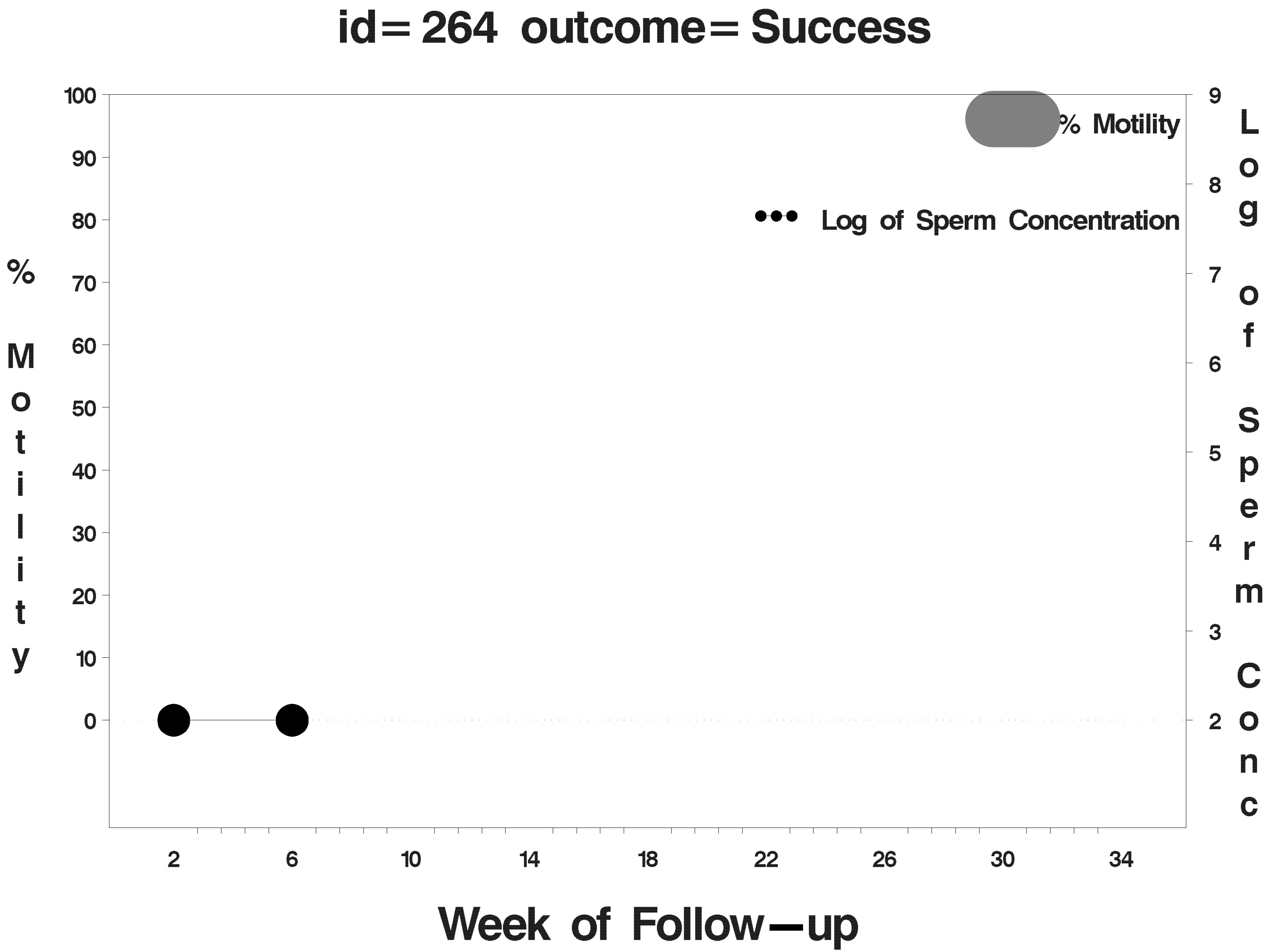

id= 264 outcome= Success
% Motility
Log of Sperm Concentration
% Motility
Log of Sperm Conc
Week of Follow—up
100
90
80
70
60
50
40
30
20
10
0
9
8
7
6
5
4
3
2
2
6
10
14
18
22
26
30
34

id= 265 outcome= Success

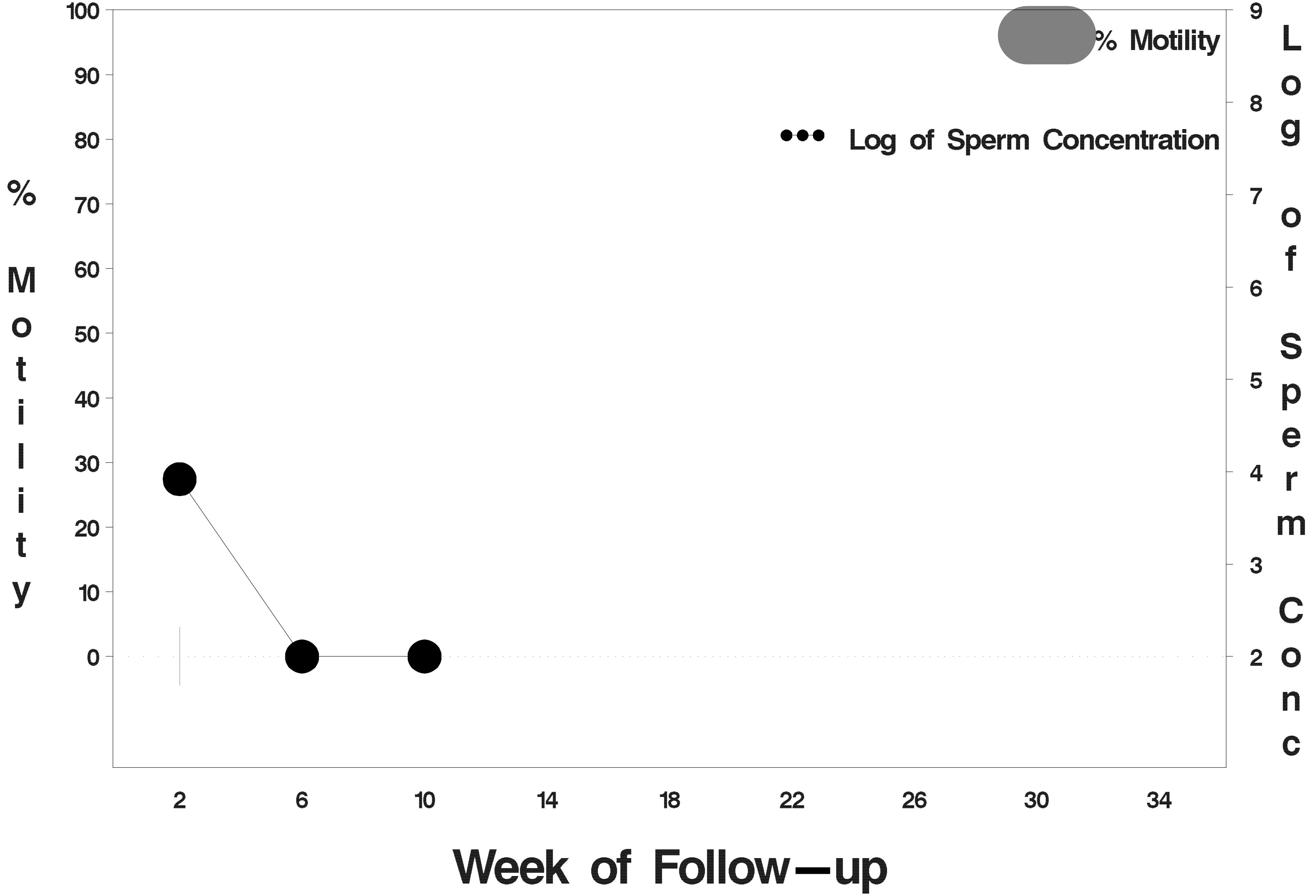

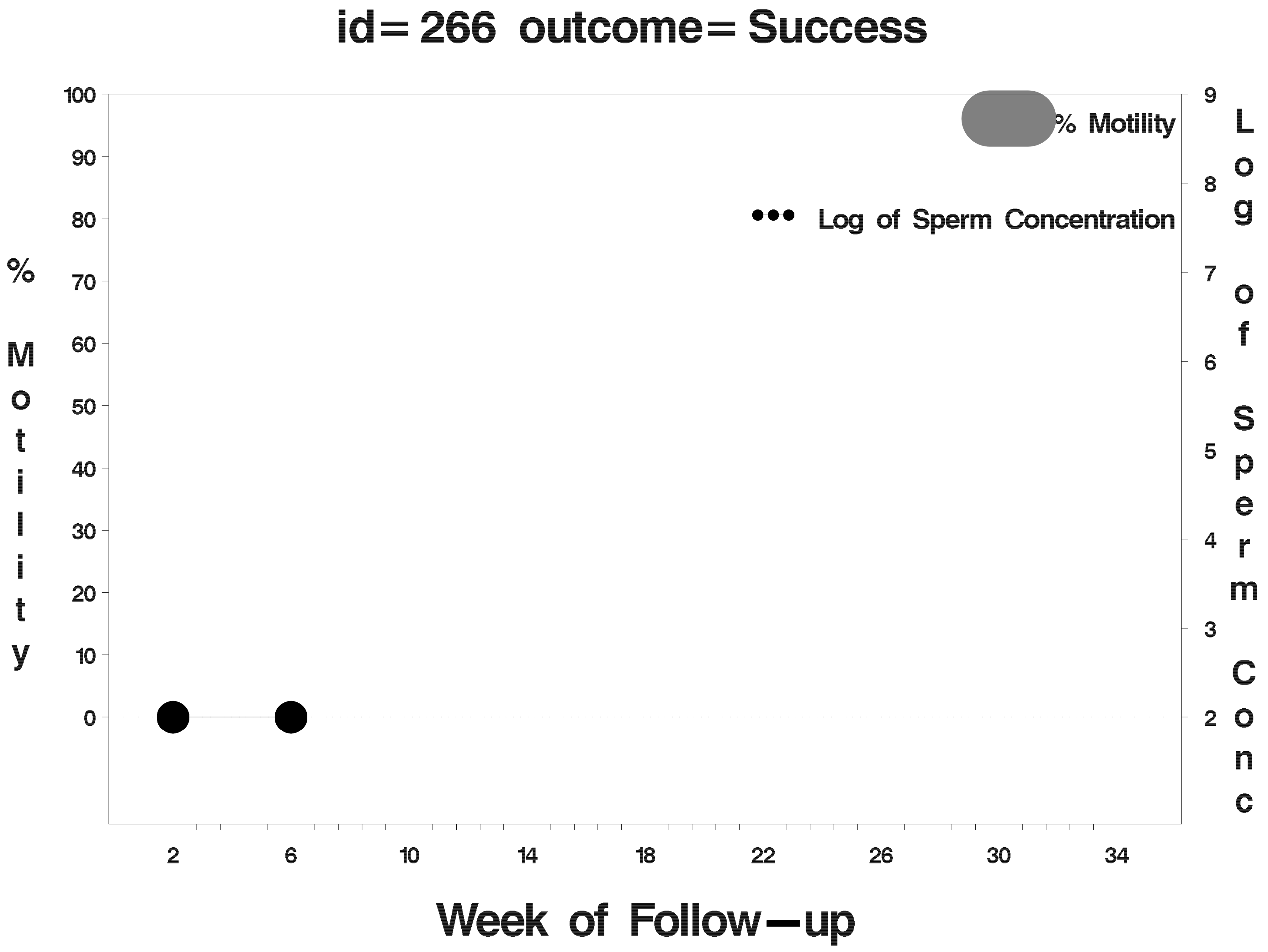

id= 266 outcome= Success
% Motility
Log of Sperm Concentration
% Motility
Log of Sperm Conc
Week of Follow-up
100
90
80
70
60
50
40
30
20
10
0
9
8
7
6
5
4
3
2
2
6
10
14
18
22
26
30
34

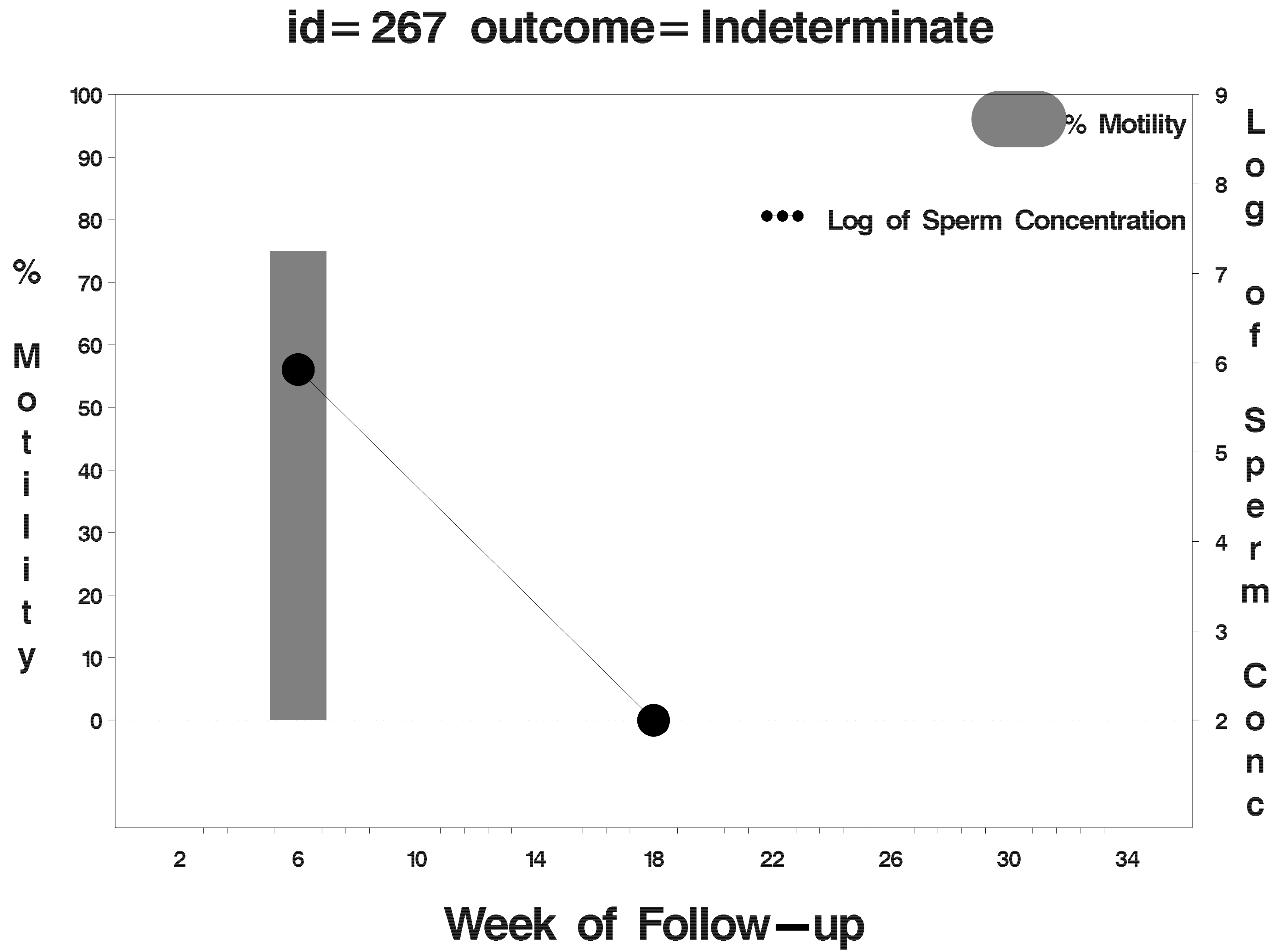

id= 267 outcome= Indeterminate
% Motility
Log of Sperm Concentration
% Motility
Log of Sperm Conc
Week of Follow−up
100
90
80
70
60
50
40
30
20
10
0
9
8
7
6
5
4
3
2
2
6
10
14
18
22
26
30
34

id= 268 outcome= Success

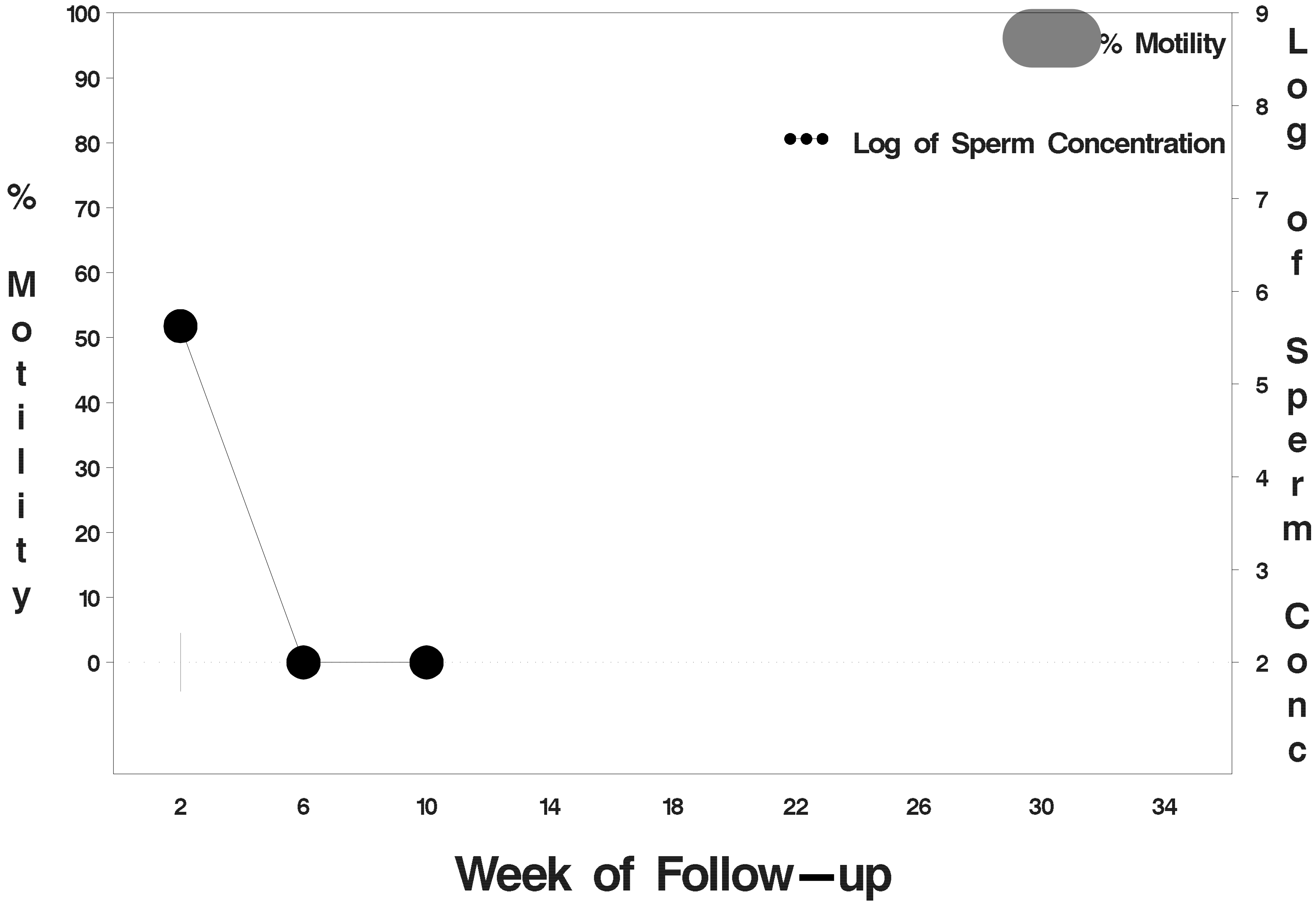

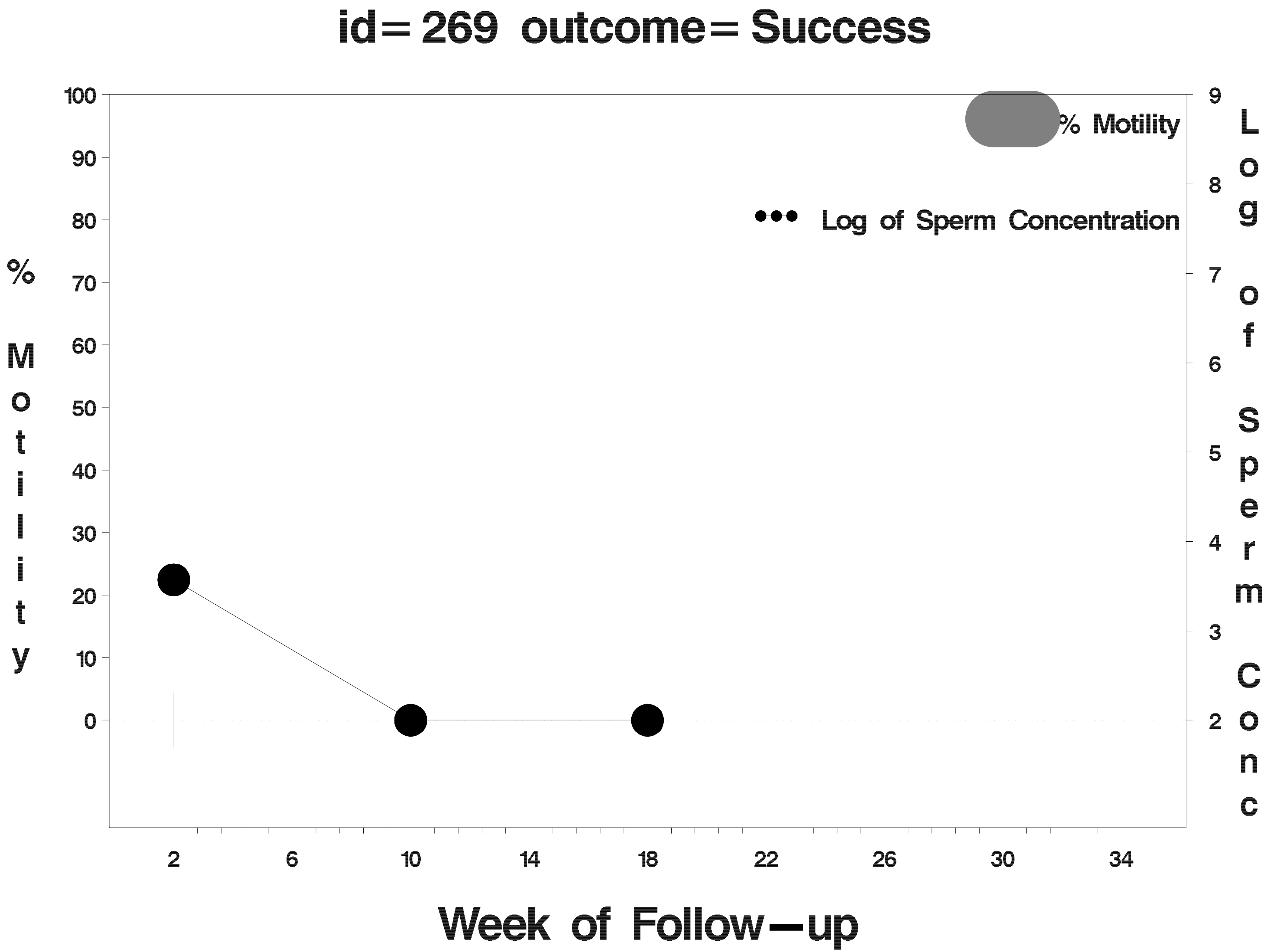

id= 269 outcome= Success
% Motility
Log of Sperm Concentration
% Motility
Log of Sperm Conc
Week of Follow−up
100
90
80
70
60
50
40
30
20
10
0
9
8
7
6
5
4
3
2
2
6
10
14
18
22
26
30
34

id= 270 outcome= Success

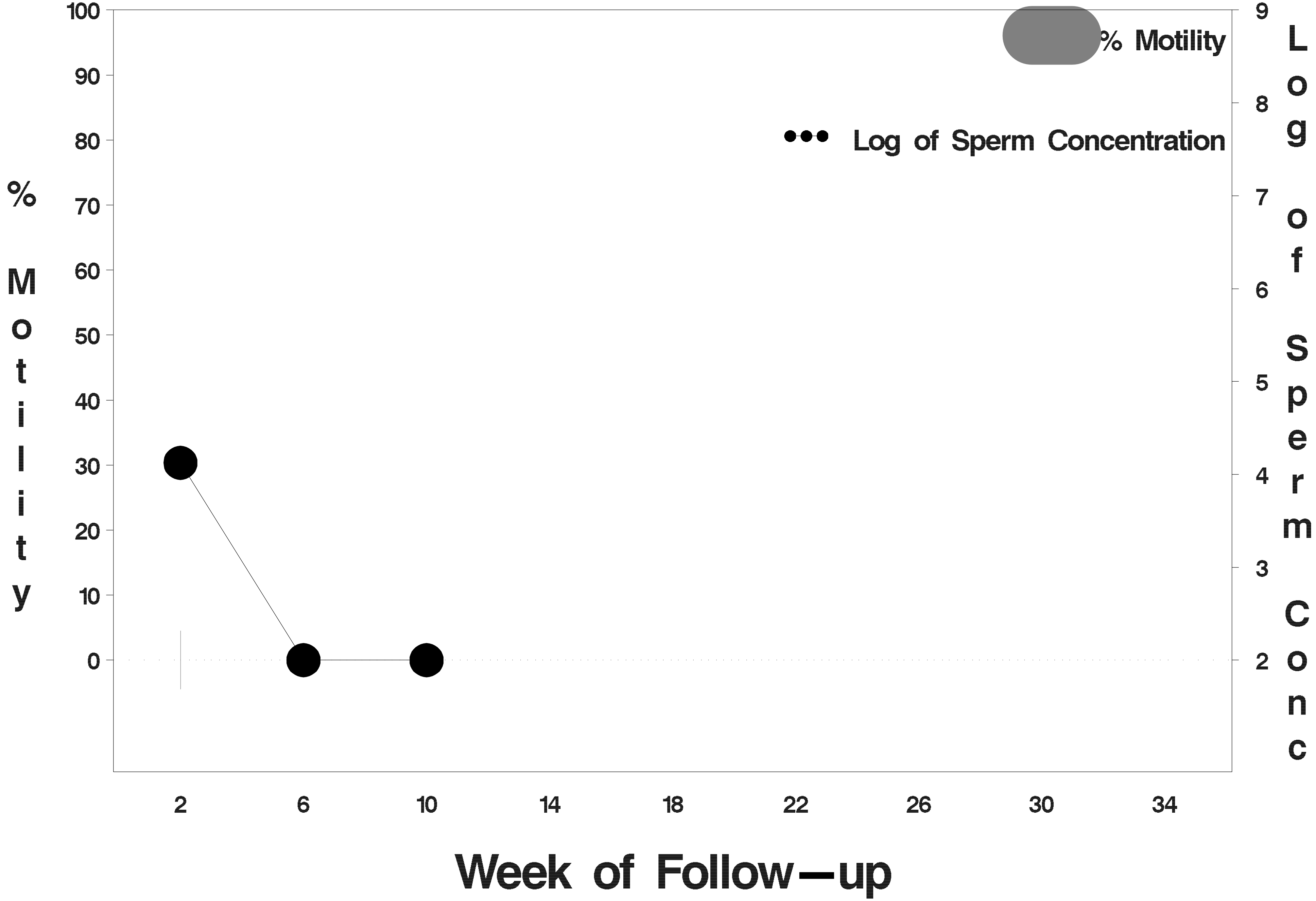

id= 271 outcome= Success

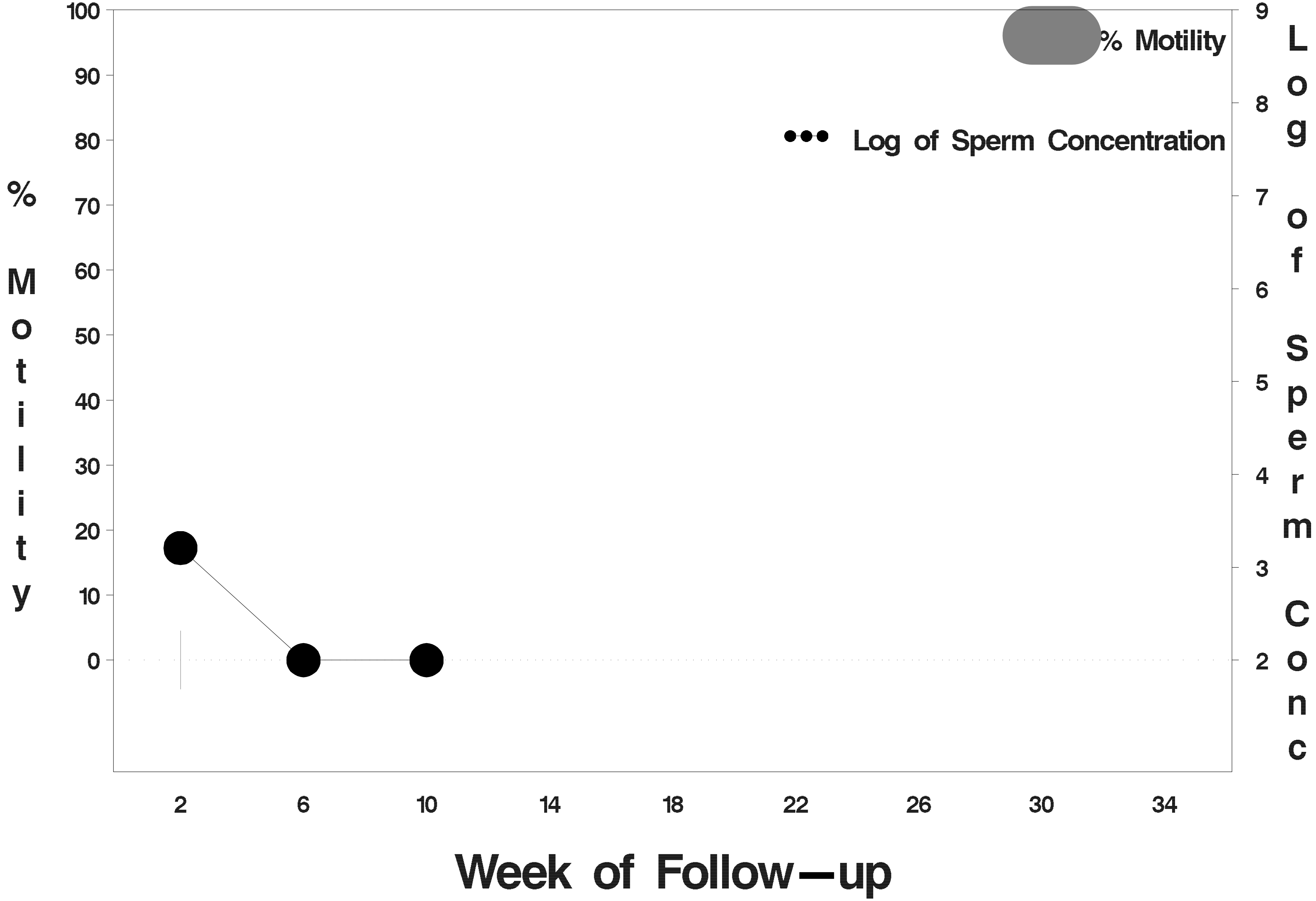

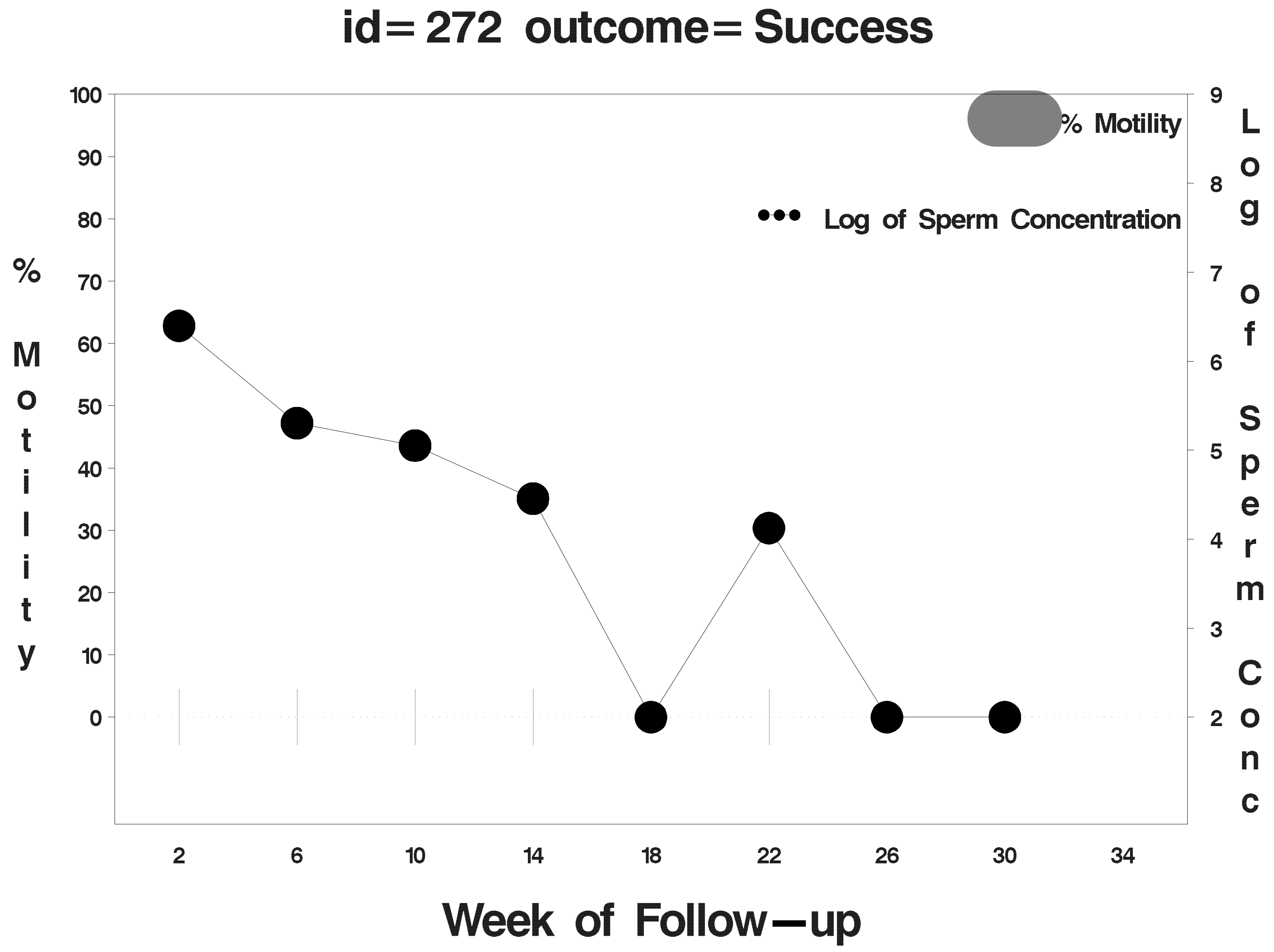

id= 272 outcome= Success
% Motility
Log of Sperm Concentration
% Motility
Log of Sperm Conc
Week of Follow−up
100
90
80
70
60
50
40
30
20
10
0
9
8
7
6
5
4
3
2
2
6
10
14
18
22
26
30
34

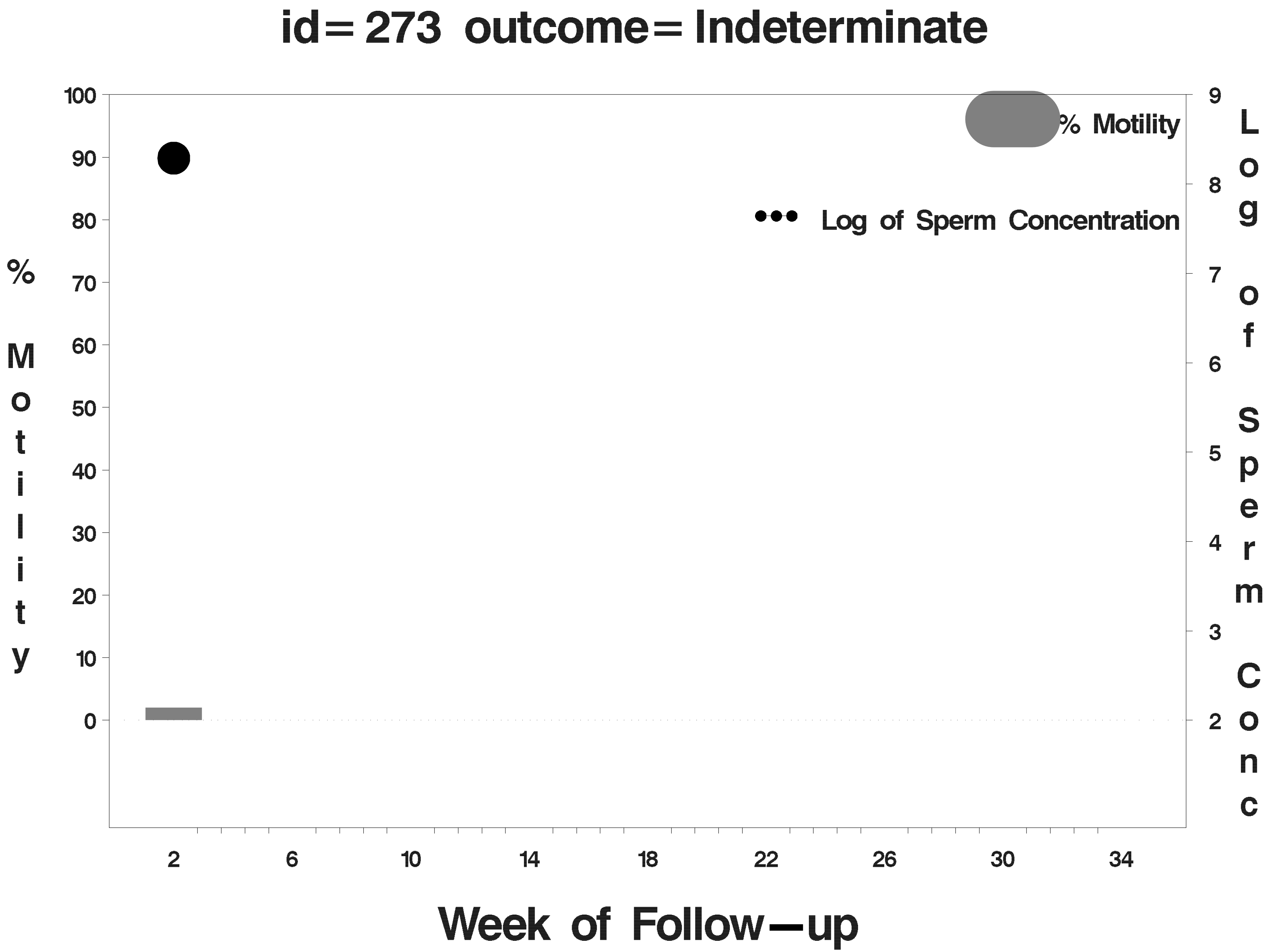

id= 273 outcome= Indeterminate
% Motility
Log of Sperm Concentration
% Motility
Log of Sperm Conc
Week of Follow-up
100
90
80
70
60
50
40
30
20
10
0
9
8
7
6
5
4
3
2
2
6
10
14
18
22
26
30
34

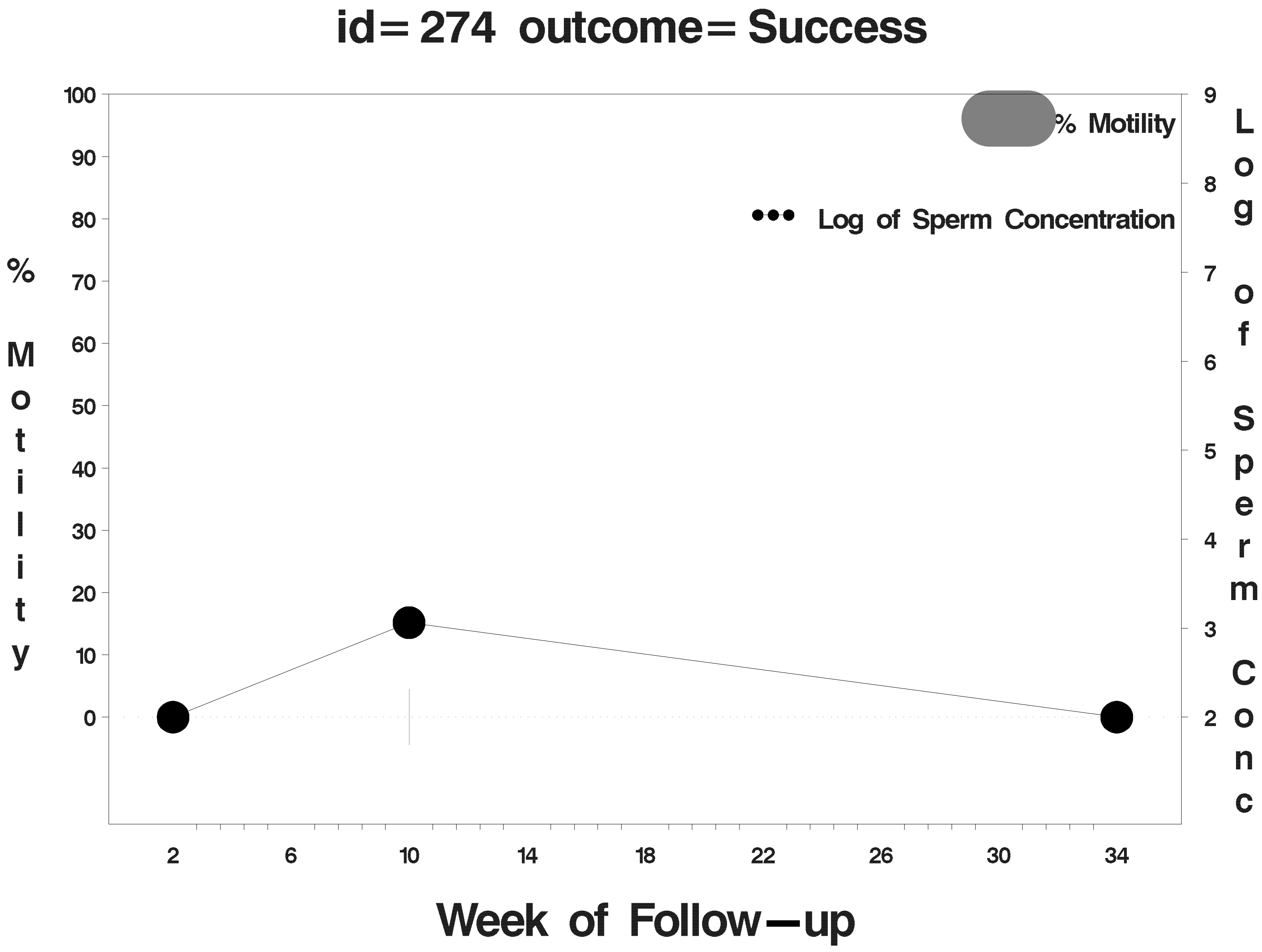

id= 274 outcome= Success
% Motility
Log of Sperm Concentration
% Motility
Log of Sperm Conc
Week of Follow-up
100
90
80
70
60
50
40
30
20
10
0
9
8
7
6
5
4
3
2
2
6
10
14
18
22
26
30
34

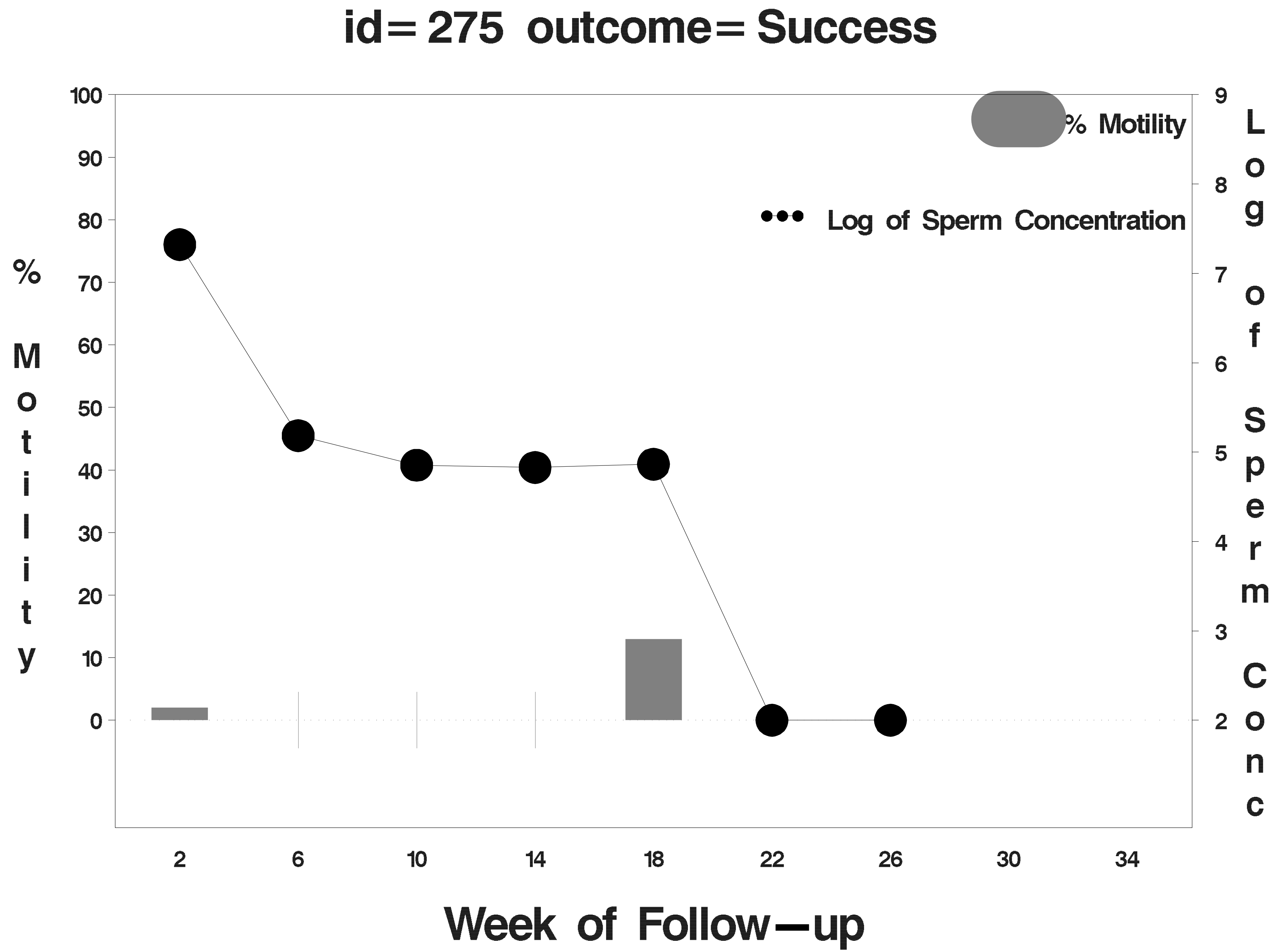

id= 275 outcome= Success
% Motility
Log of Sperm Concentration
100
90
80
70
60
50
40
30
20
10
0
9
8
7
6
5
4
3
2
2
6
10
14
18
22
26
30
34
Week of Follow-up
Log of Sperm Conc

id= 276 outcome= Success

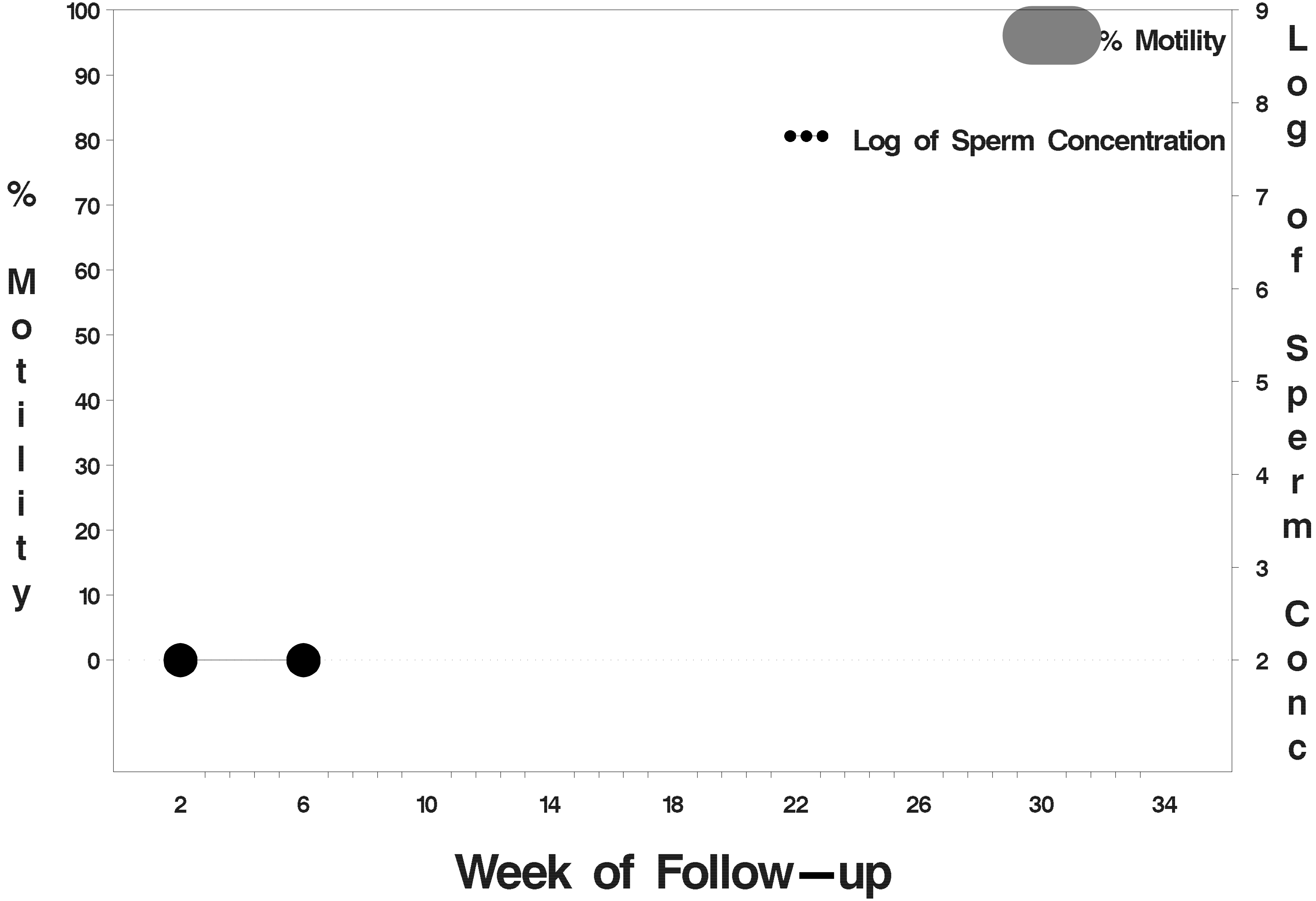

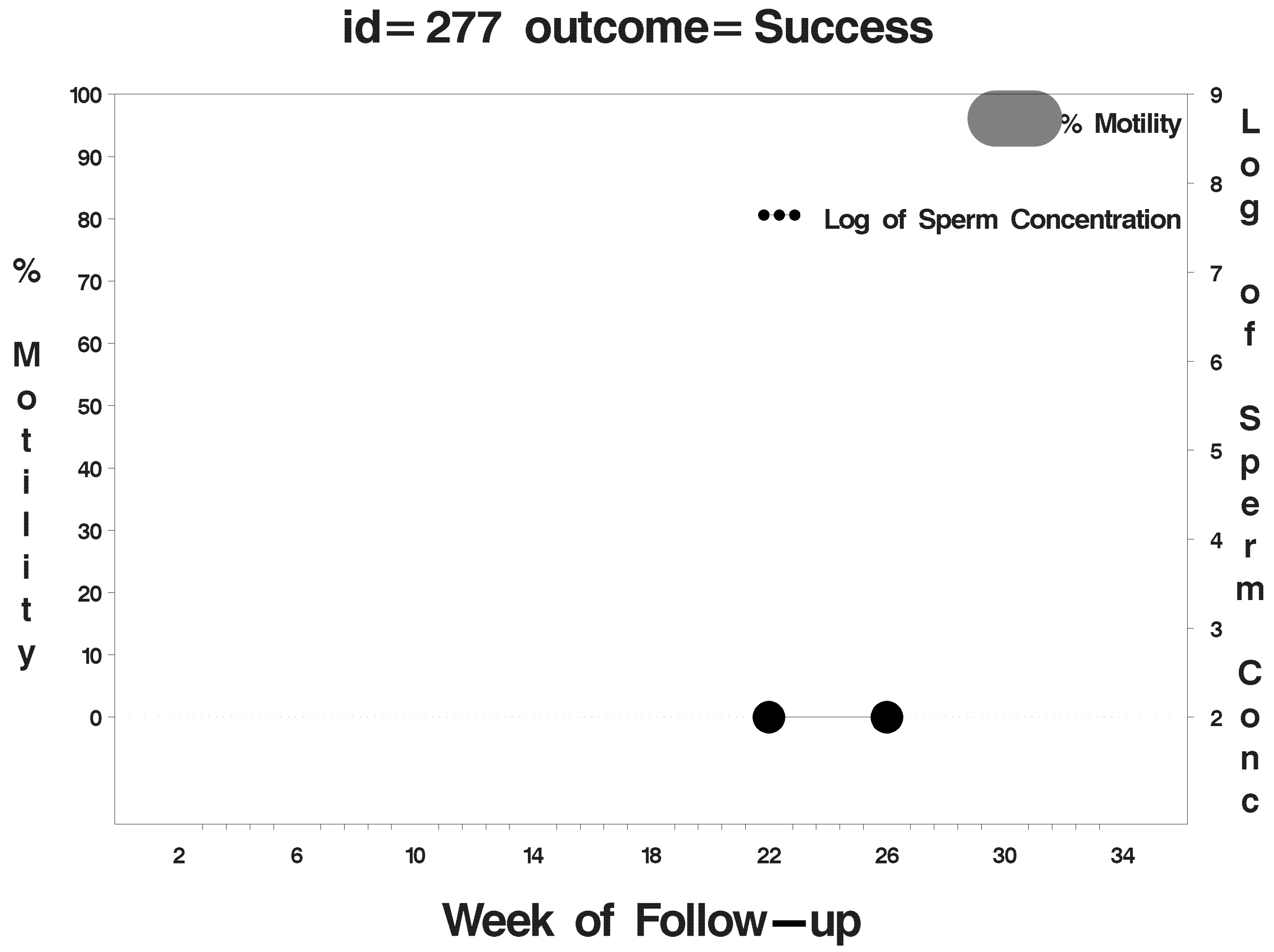

id= 277 outcome= Success
% Motility
Log of Sperm Concentration
100
90
80
70
60
50
40
30
20
10
0
9
8
7
6
5
4
3
2
2
6
10
14
18
22
26
30
34
Week of Follow—up
% Motility
Log of Sperm Conc

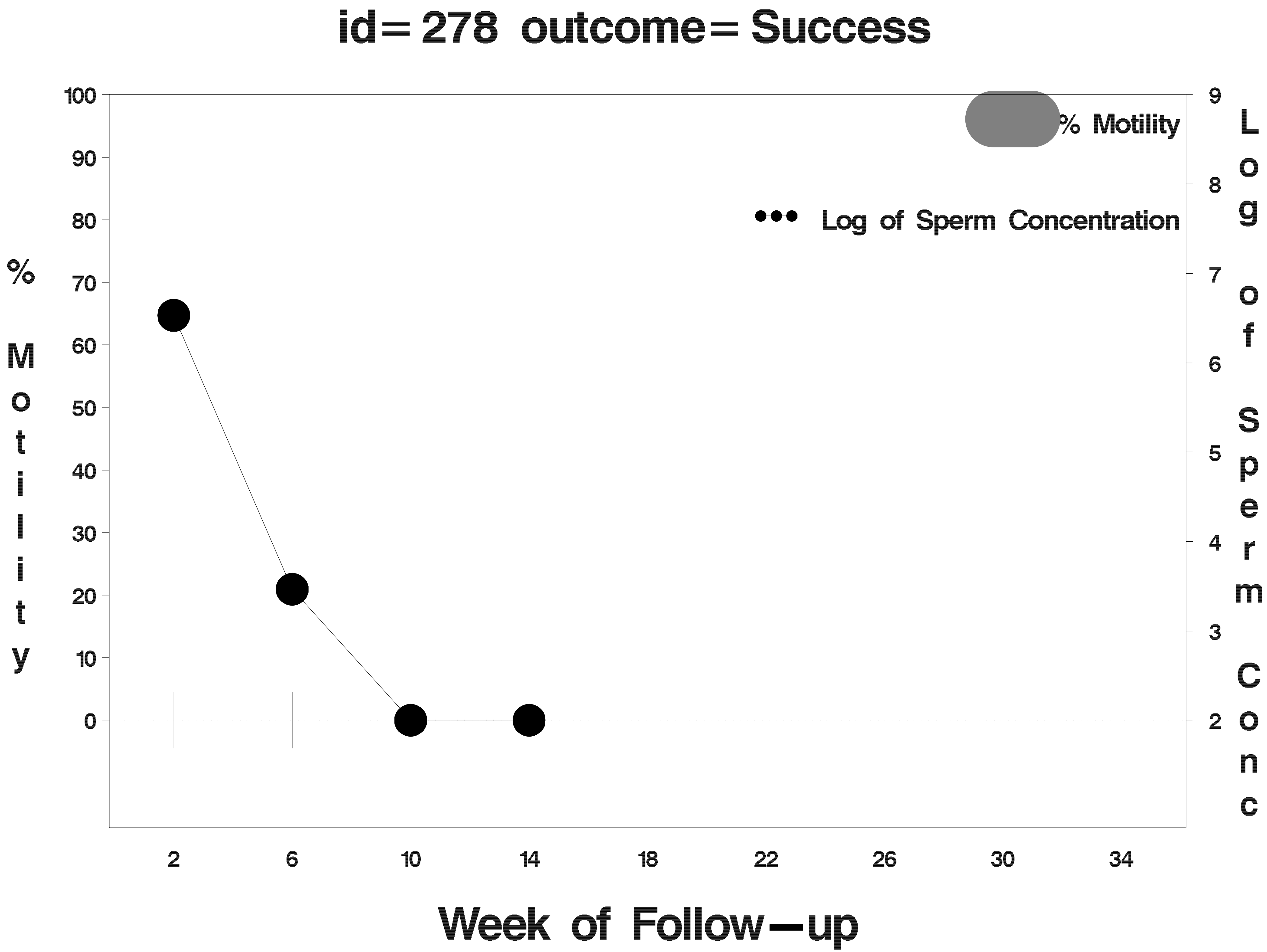

id= 278 outcome= Success
% Motility
Log of Sperm Concentration
% Motility
Log of Sperm Conc
Week of Follow—up
100
90
80
70
60
50
40
30
20
10
0
9
8
7
6
5
4
3
2
2
6
10
14
18
22
26
30
34

id= 279 outcome= Success

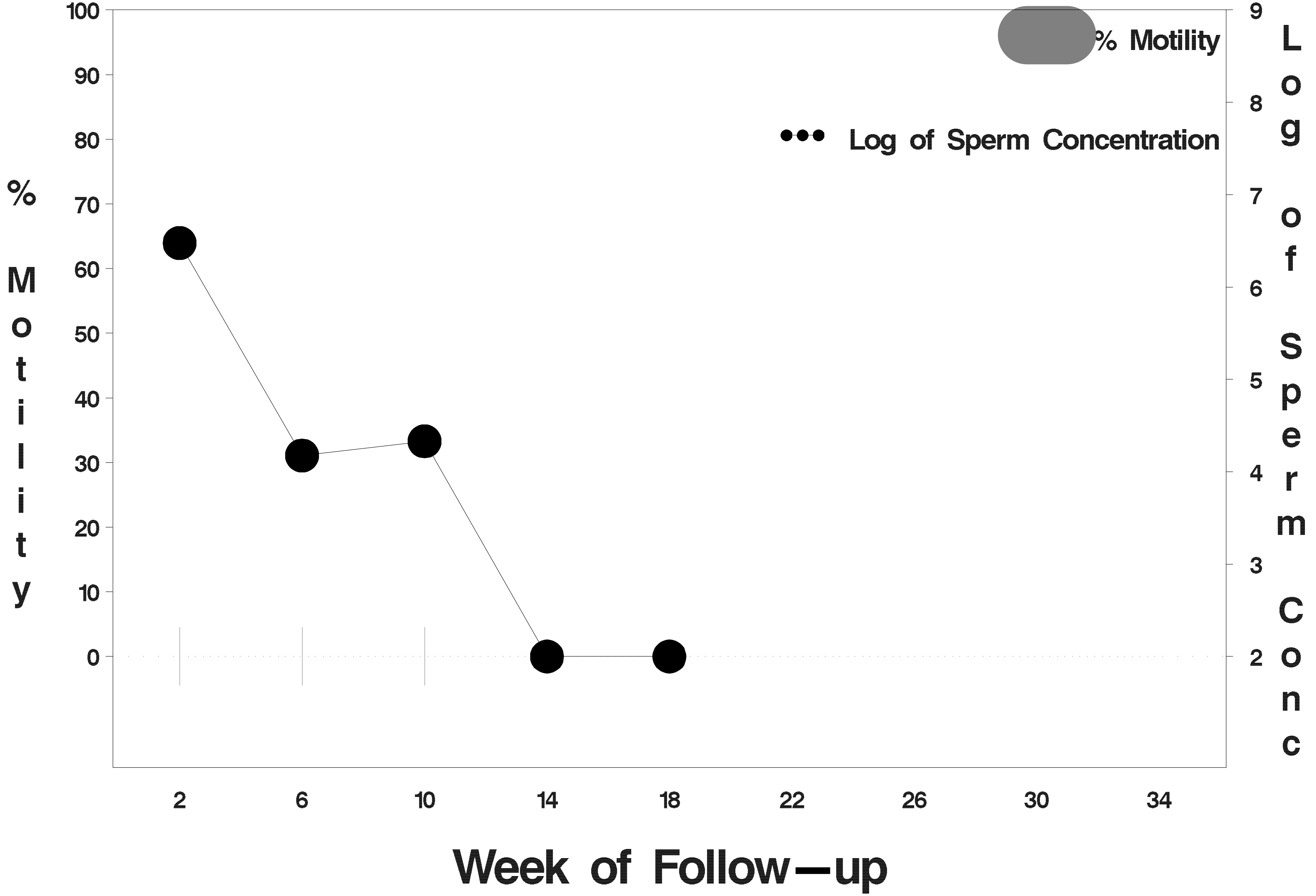

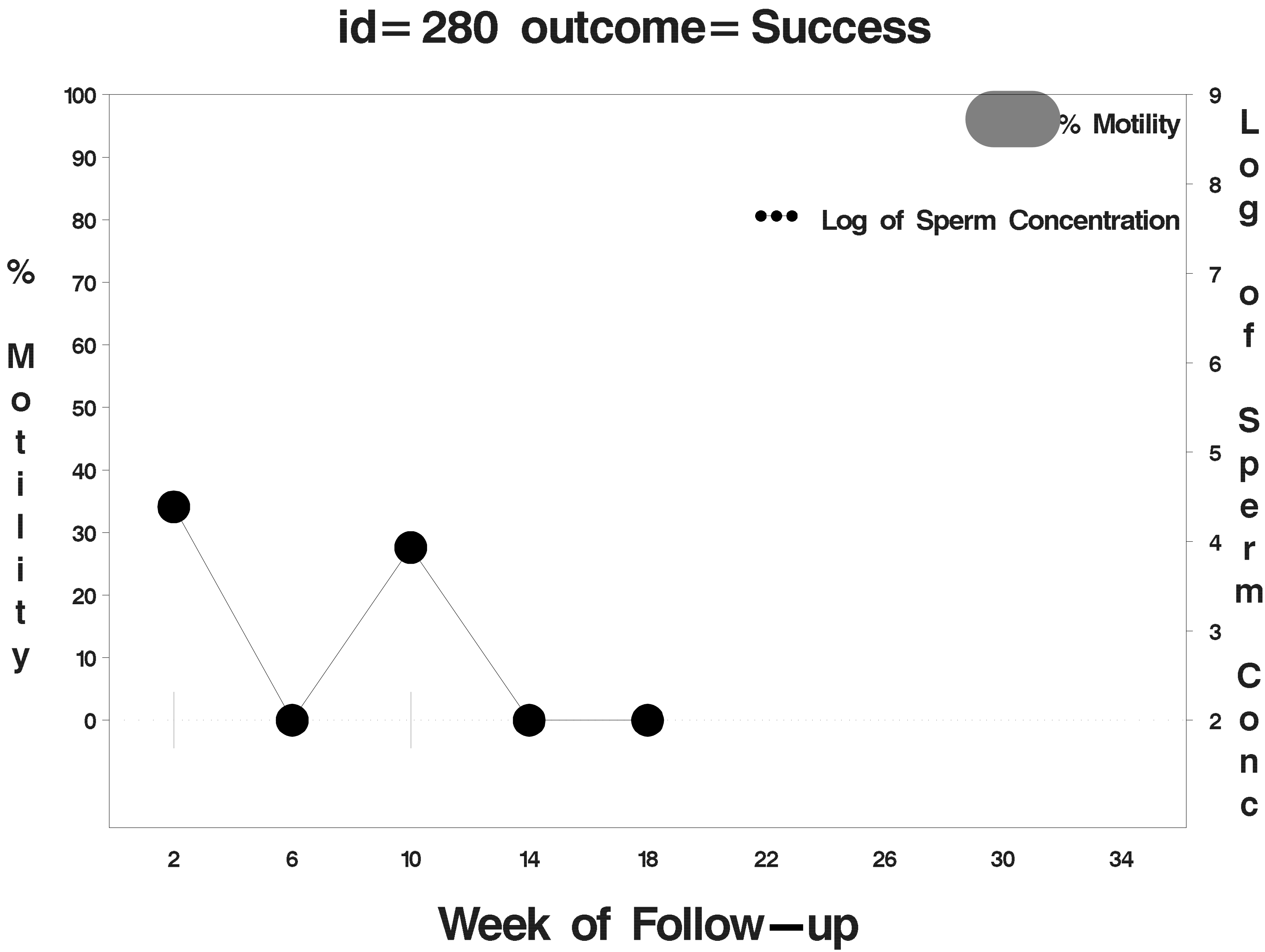

id= 280 outcome= Success
% Motility
Log of Sperm Concentration
% Motility
Log of Sperm Conc
Week of Follow—up
100
90
80
70
60
50
40
30
20
10
0
9
8
7
6
5
4
3
2
2
6
10
14
18
22
26
30
34

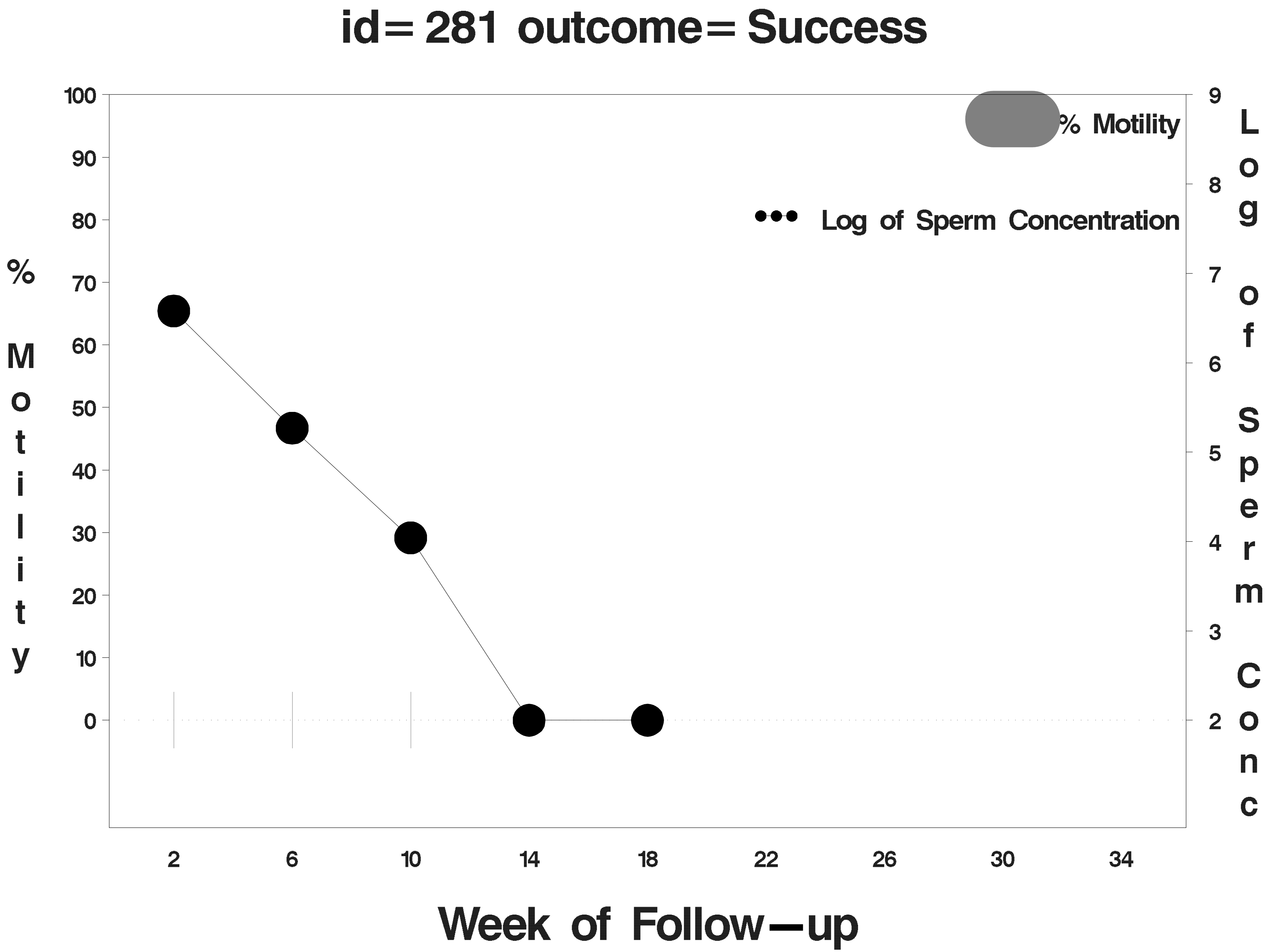
id= 281 outcome= Success
% Motility
Log of Sperm Concentration
100
90
80
70
60
50
40
30
20
10
0
9
8
7
6
5
4
3
2
2
6
10
14
18
22
26
30
34
Week of Follow−up
Log of Sperm Conc

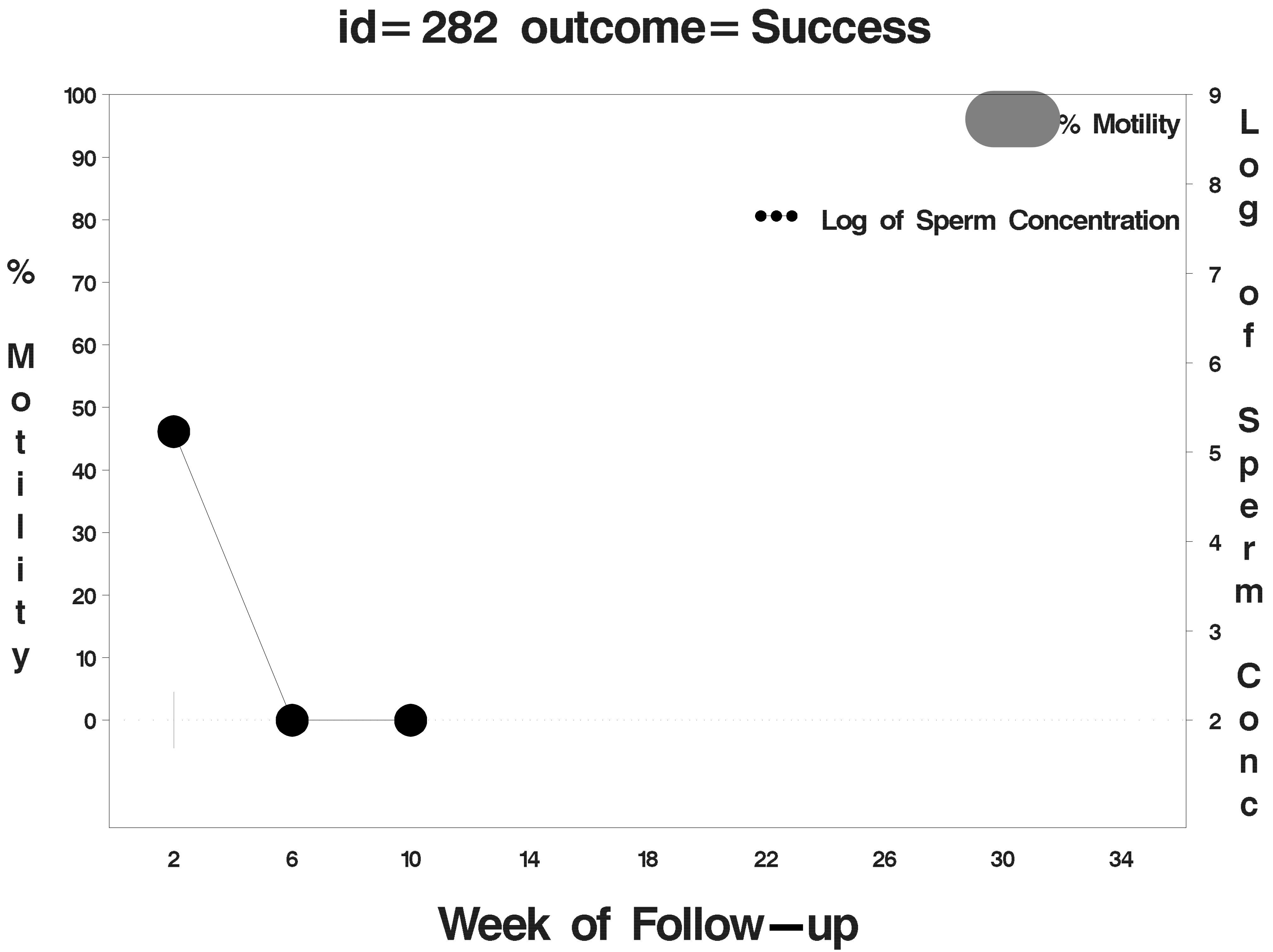

id= 282 outcome= Success
% Motility
Log of Sperm Concentration
% Motility
Log of Sperm Conc
Week of Follow−up
100
90
80
70
60
50
40
30
20
10
0
9
8
7
6
5
4
3
2
2
6
10
14
18
22
26
30
34

id= 283 outcome= Success

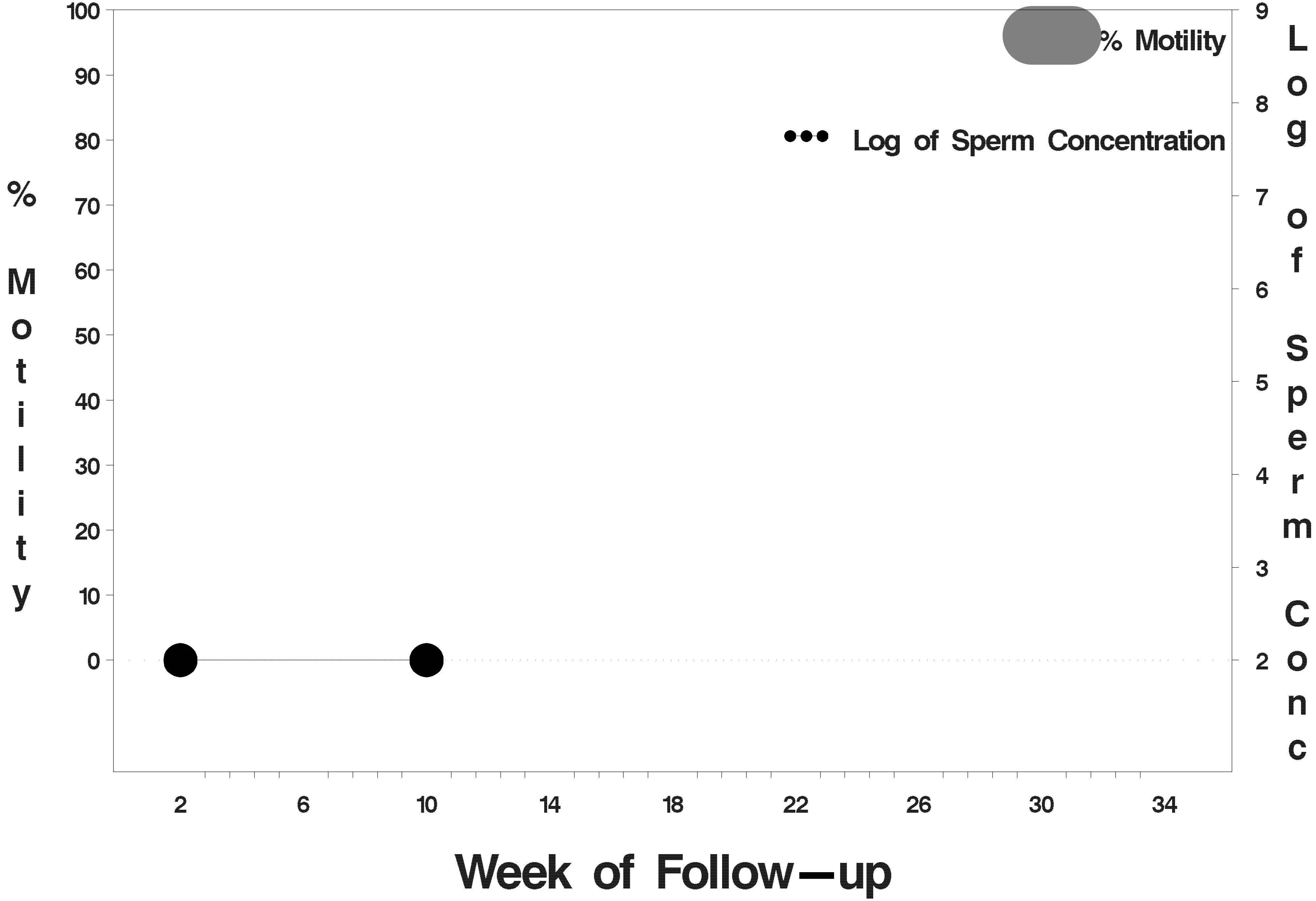

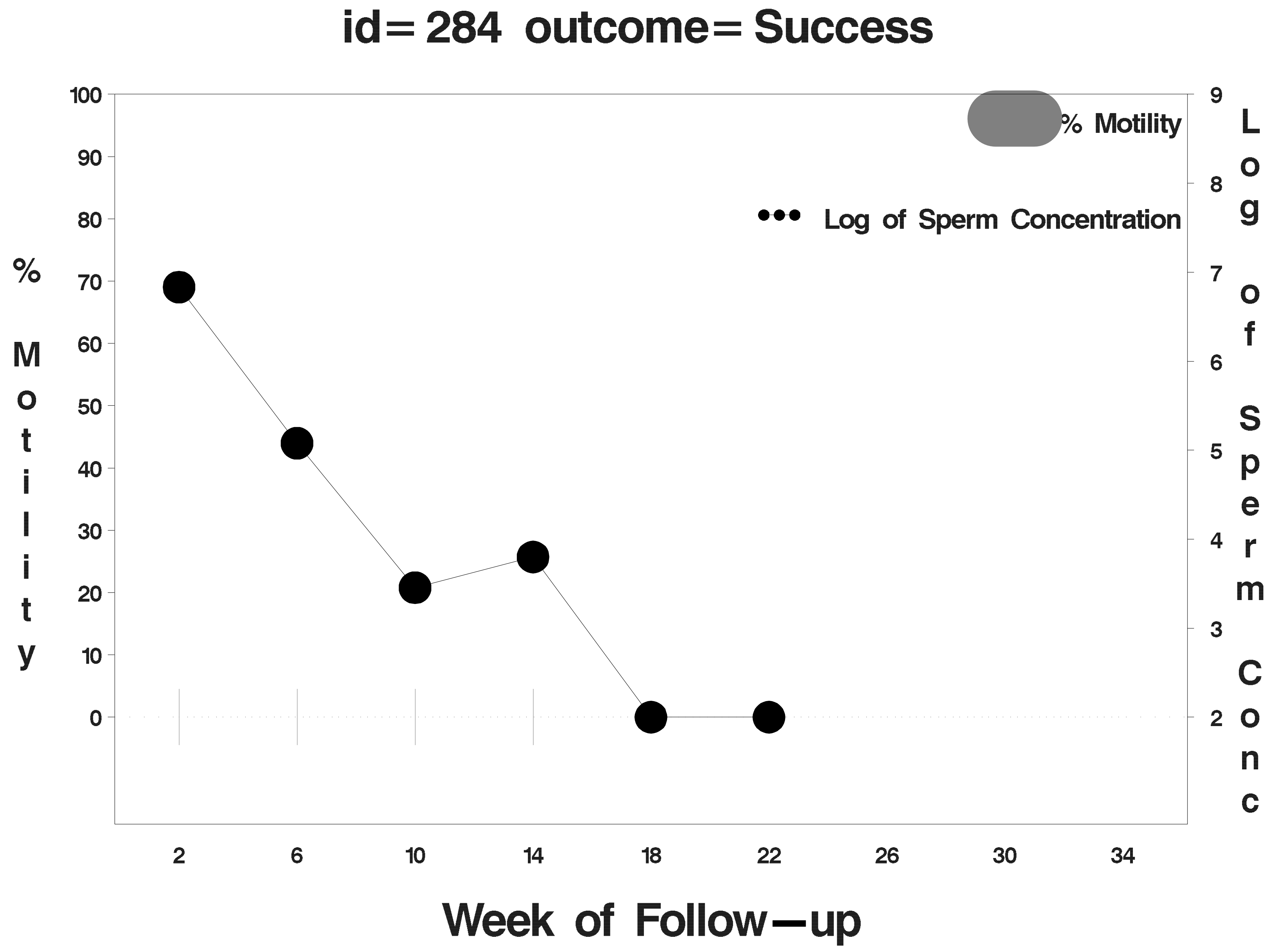
id= 284 outcome= Success
% Motility
Log of Sperm Concentration
100
90
80
70
60
50
40
30
20
10
0
9
8
7
6
5
4
3
2
2
6
10
14
18
22
26
30
34
Week of Follow-up
Log of Sperm Conc

id= 285 outcome= Success

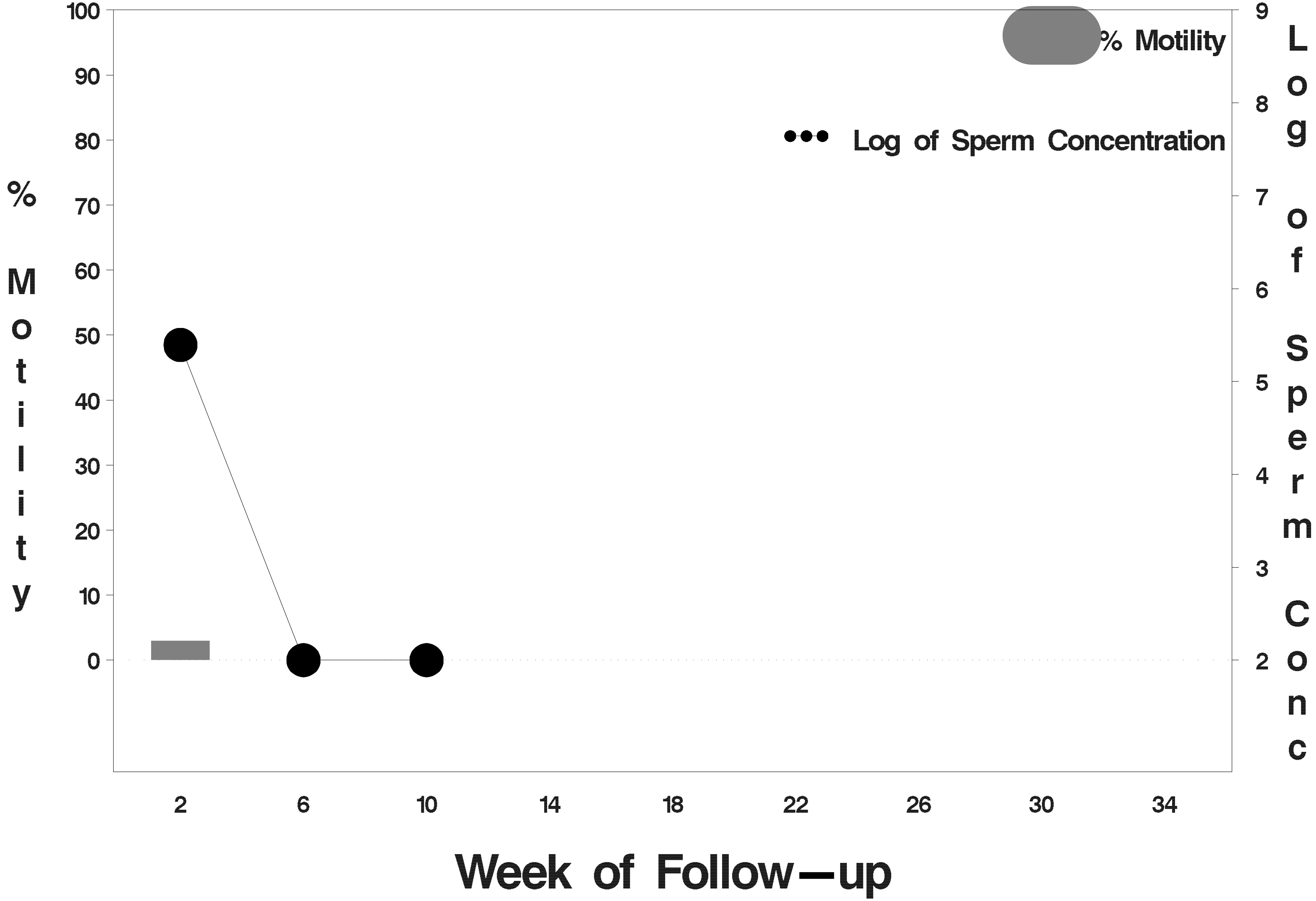

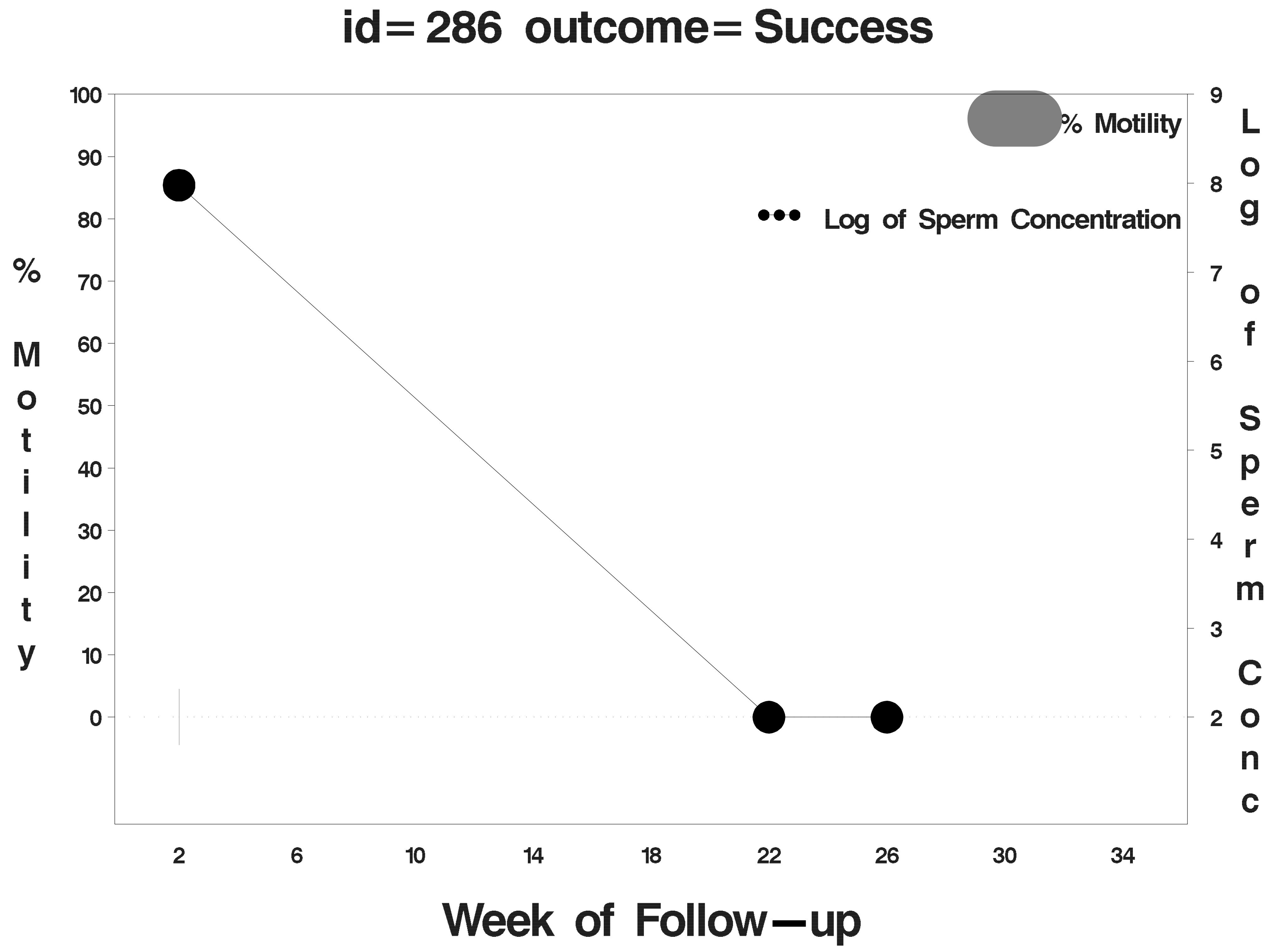

id= 286 outcome= Success
% Motility
Log of Sperm Concentration
100
90
80
70
60
50
40
30
20
10
0
9
8
7
6
5
4
3
2
2
6
10
14
18
22
26
30
34
Week of Follow−up
% Motility
Log of Sperm Conc

id= 287 outcome= Success

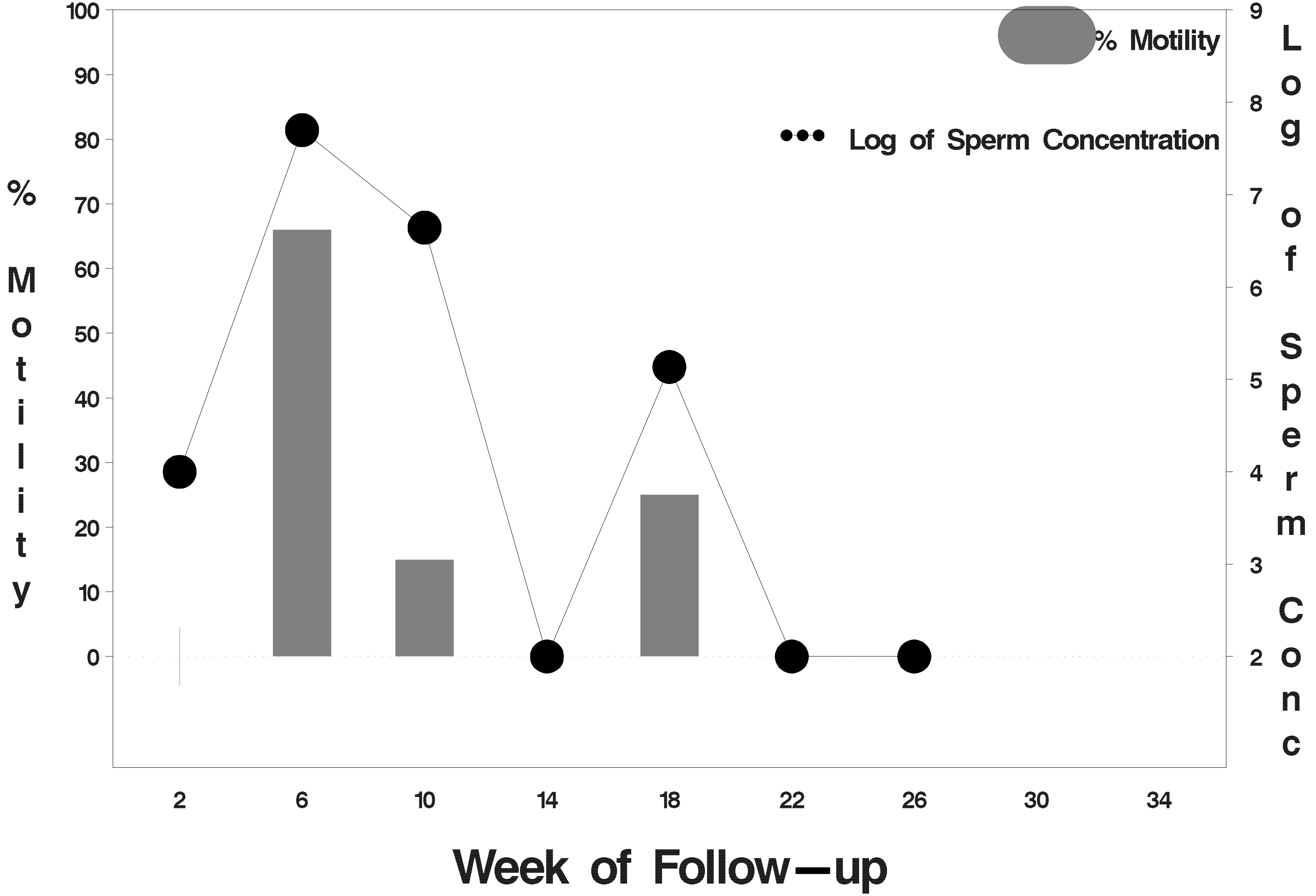

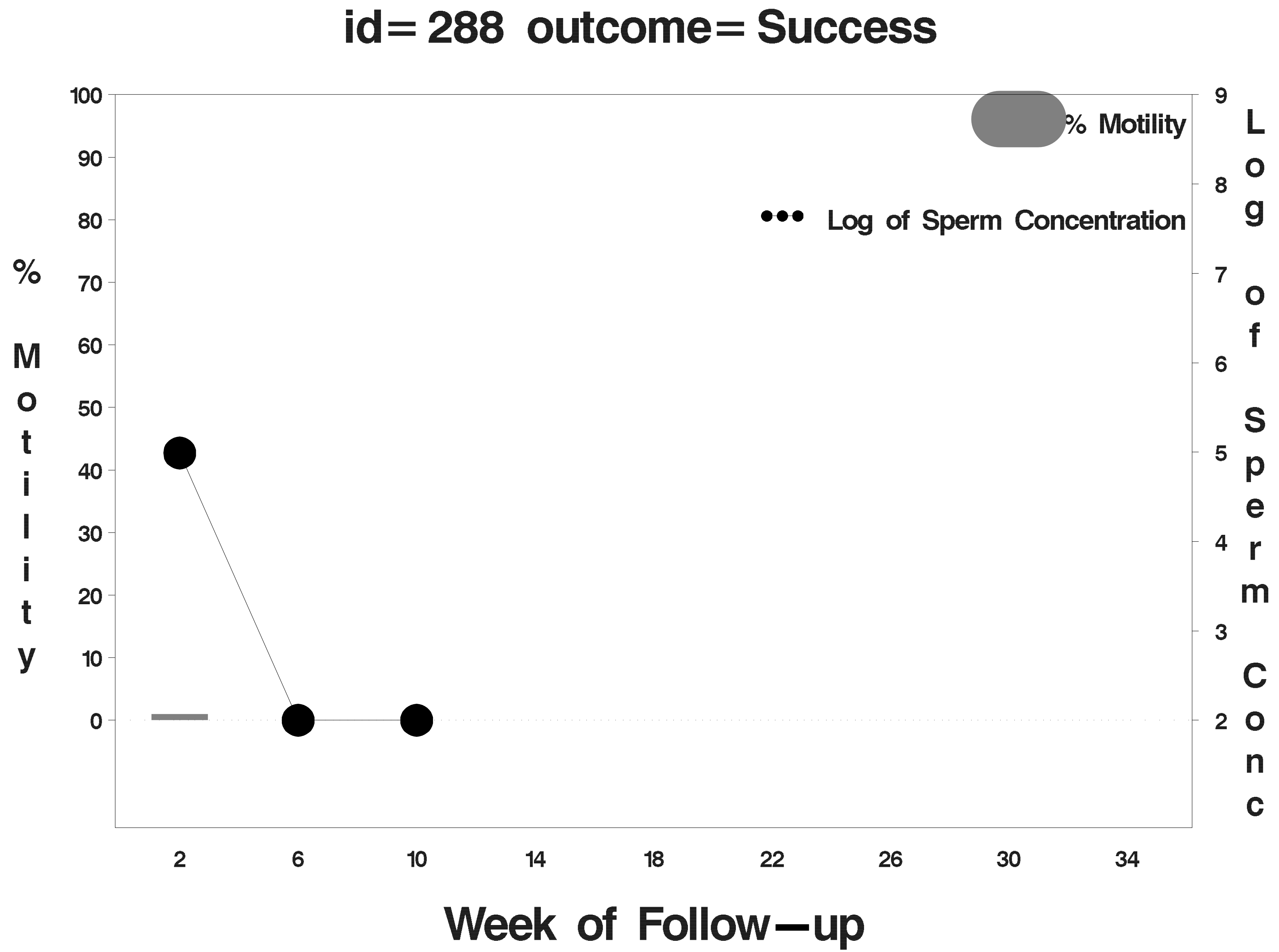

id= 288 outcome= Success
% Motility
Log of Sperm Concentration
100
90
80
70
60
50
40
30
20
10
0
9
8
7
6
5
4
3
2
2
6
10
14
18
22
26
30
34
Week of Follow—up
Log of Sperm Conc

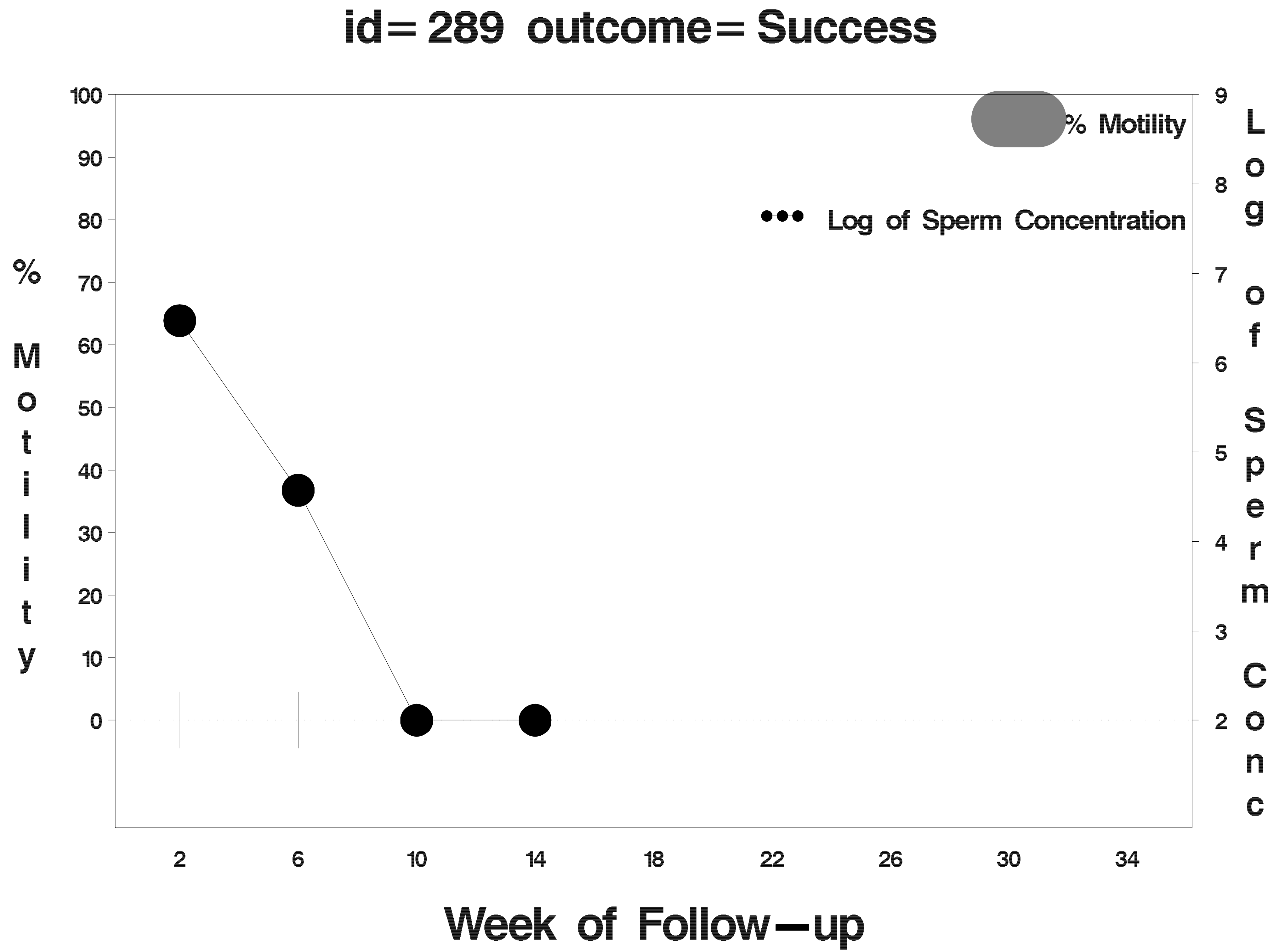
id= 289 outcome= Success
% Motility
Log of Sperm Concentration
100
90
80
70
60
50
40
30
20
10
0
9
8
7
6
5
4
3
2
2
6
10
14
18
22
26
30
34
Week of Follow−up
% Motility
Log of Sperm Conc

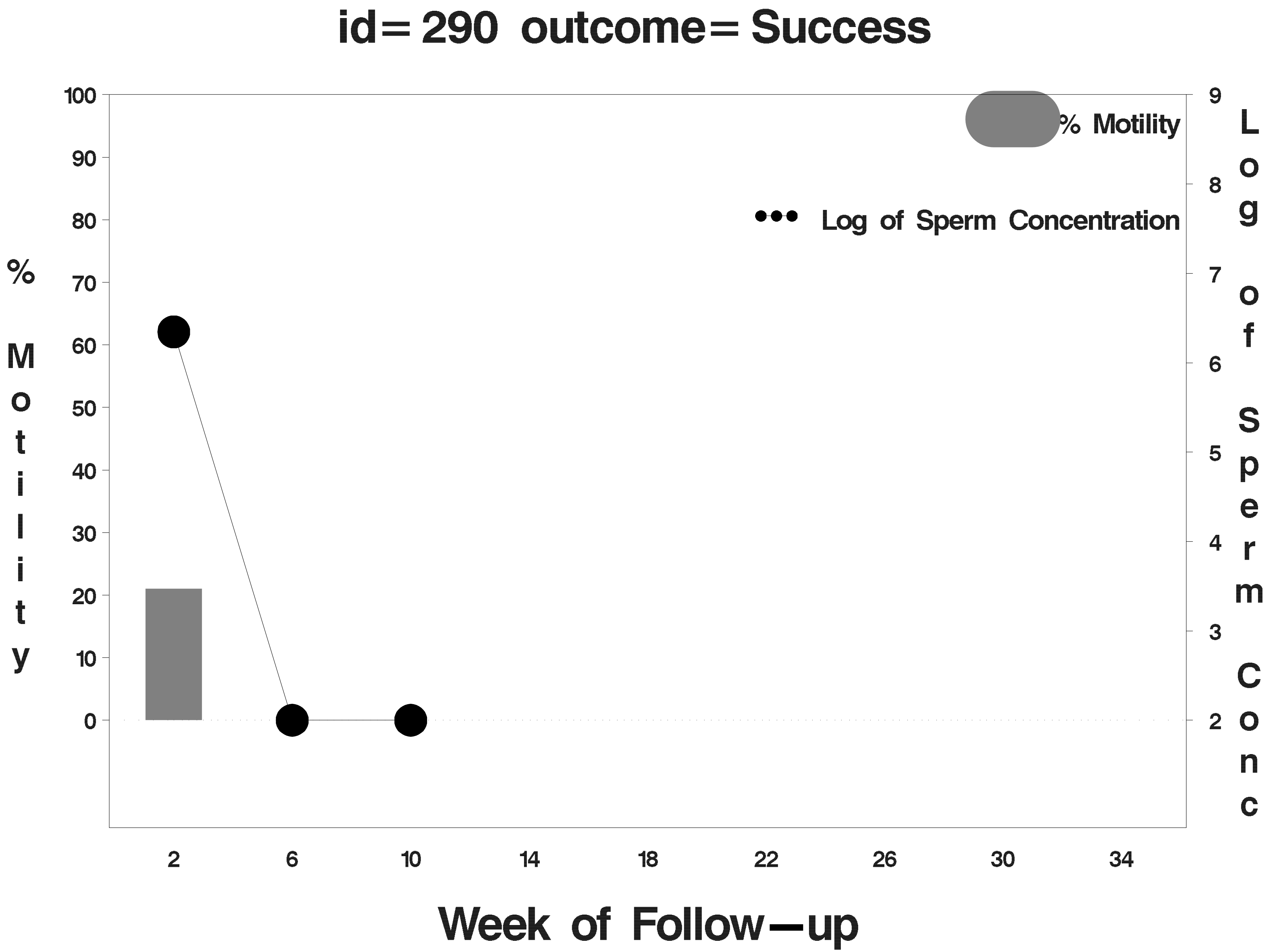

id= 290 outcome= Success
% Motility
Log of Sperm Concentration
% Motility
Log of Sperm Conc
Week of Follow-up
100
90
80
70
60
50
40
30
20
10
0
9
8
7
6
5
4
3
2
2
6
10
14
18
22
26
30
34

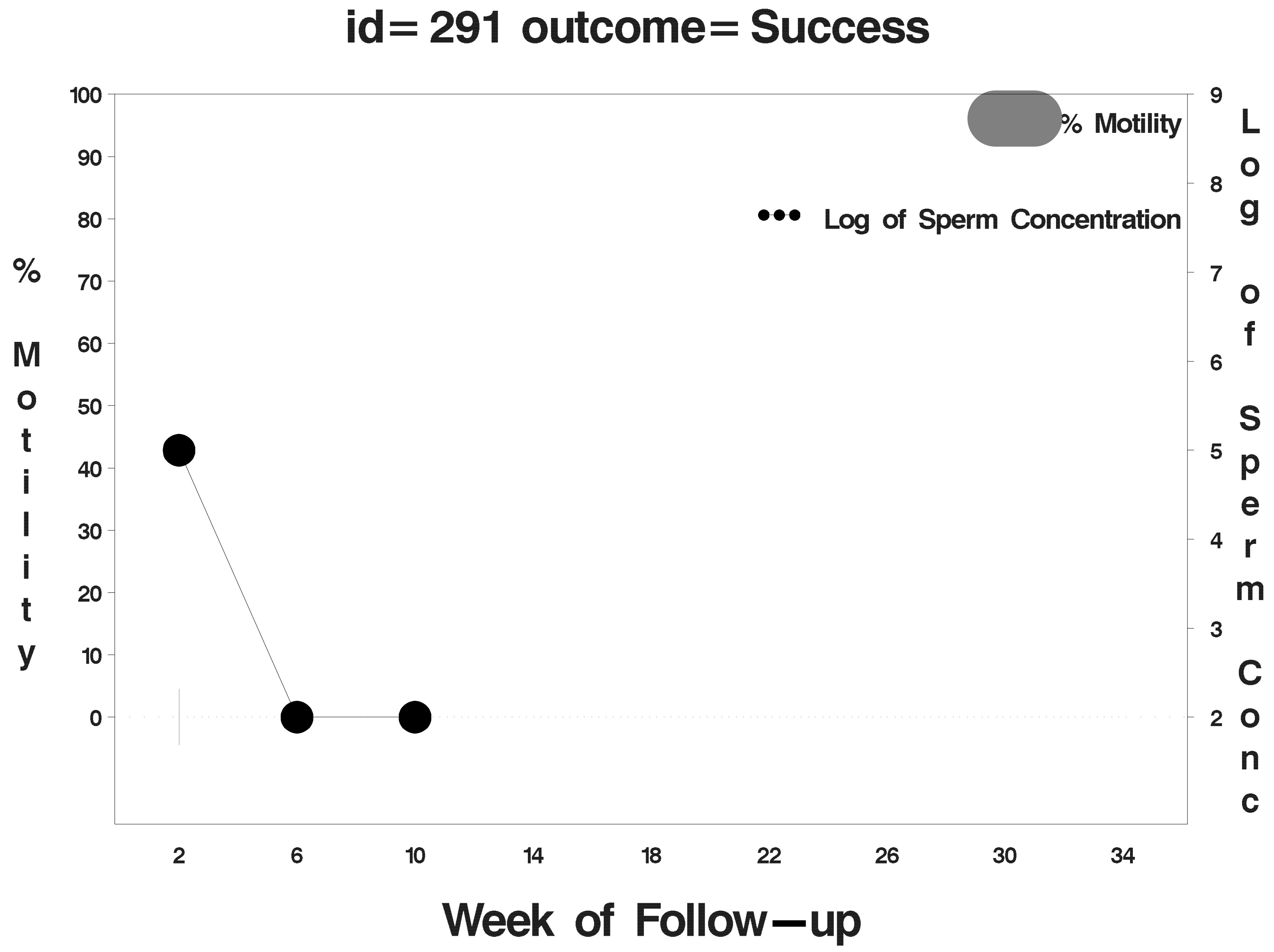
id= 291 outcome= Success
% Motility
Log of Sperm Concentration
100
90
80
70
60
50
40
30
20
10
0
9
8
7
6
5
4
3
2
2
6
10
14
18
22
26
30
34
% Motility
Log of Sperm Conc
Week of Follow—up

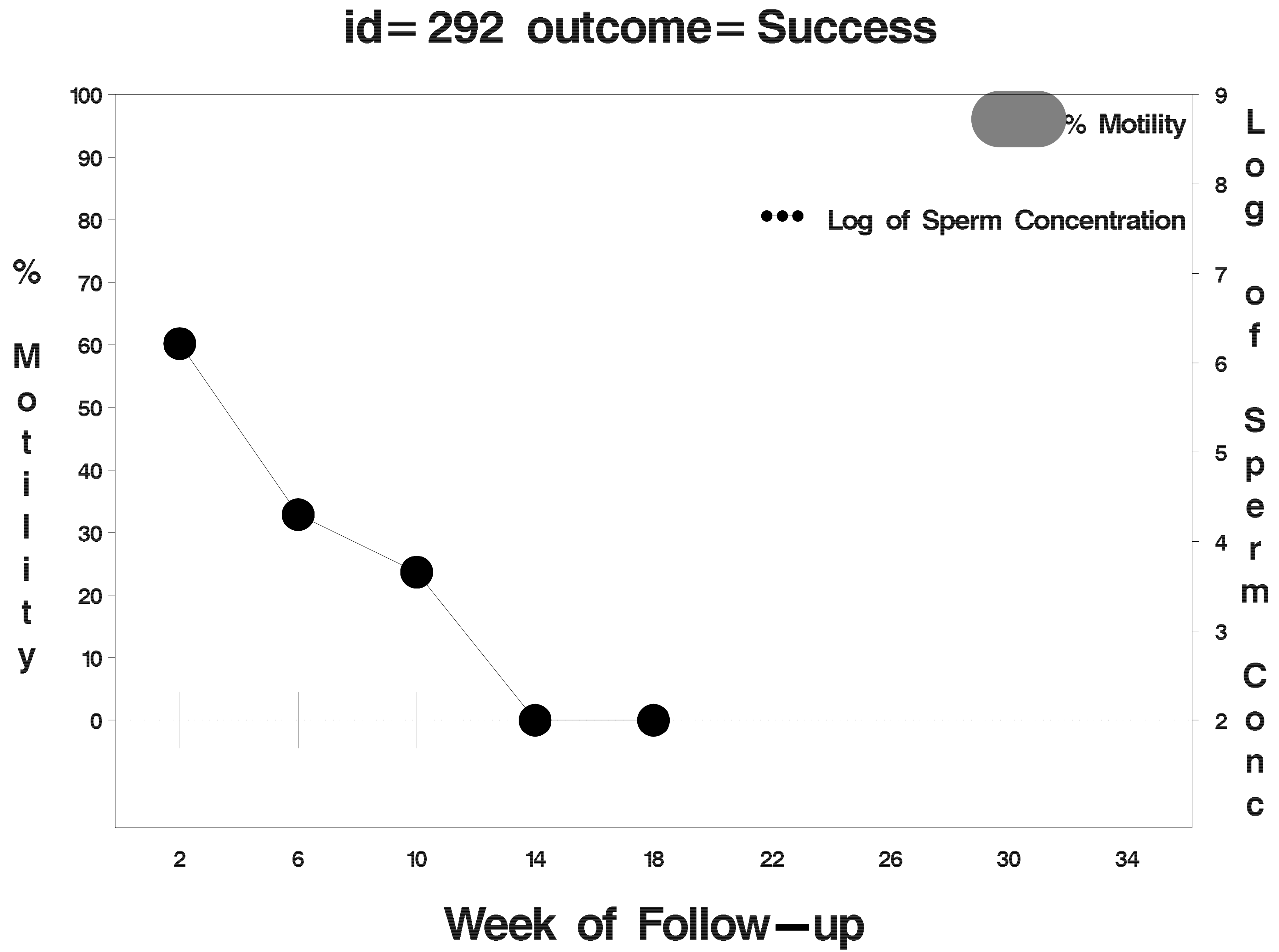

id= 292 outcome= Success
% Motility
Log of Sperm Concentration
% Motility
Log of Sperm Conc
Week of Follow−up
100
90
80
70
60
50
40
30
20
10
0
9
8
7
6
5
4
3
2
2
6
10
14
18
22
26
30
34

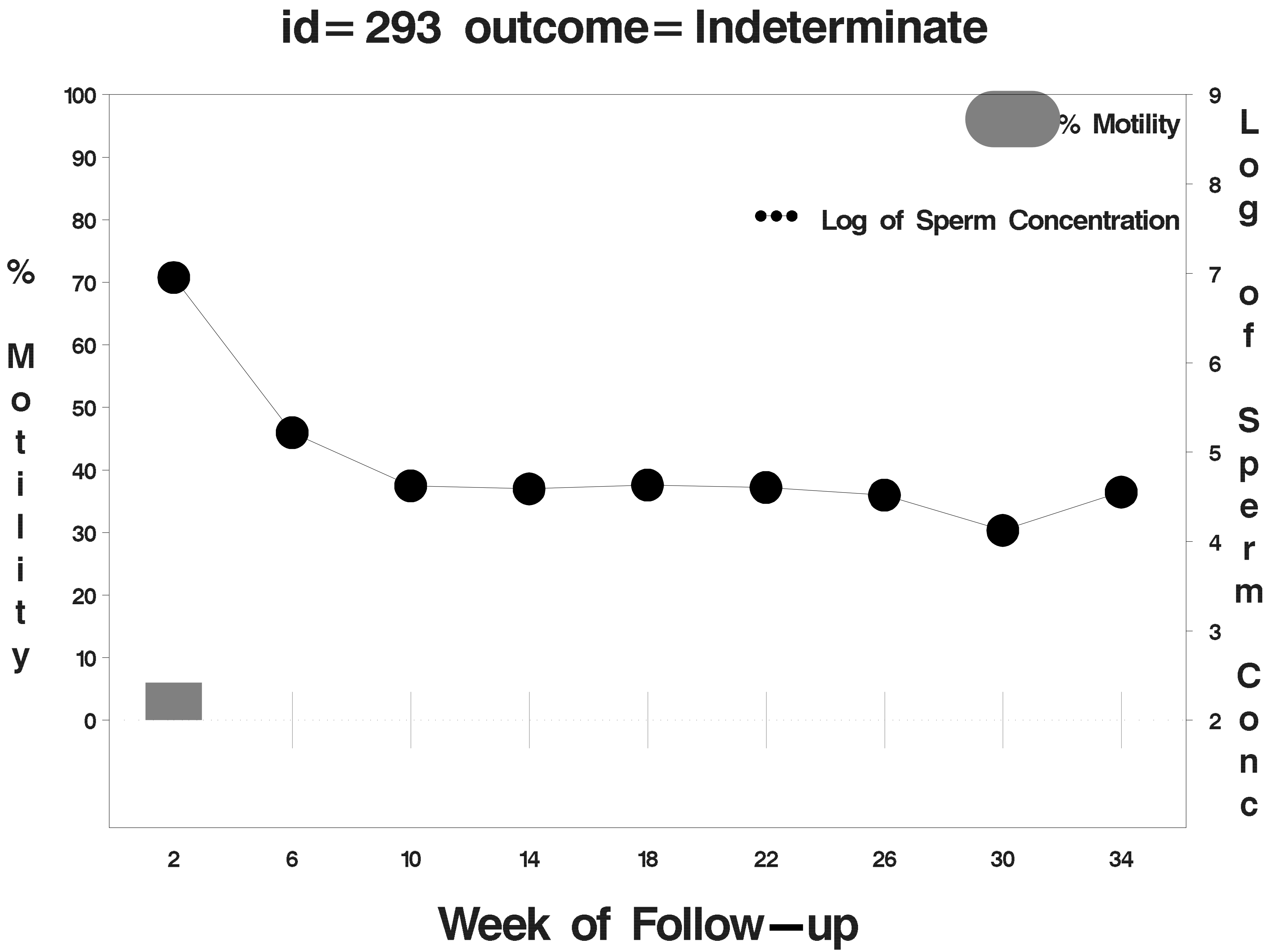
id= 293 outcome= Indeterminate
% Motility
Log of Sperm Concentration
% Motility
Log of Sperm Conc
Week of Follow—up
100
90
80
70
60
50
40
30
20
10
0
9
8
7
6
5
4
3
2
2
6
10
14
18
22
26
30
34

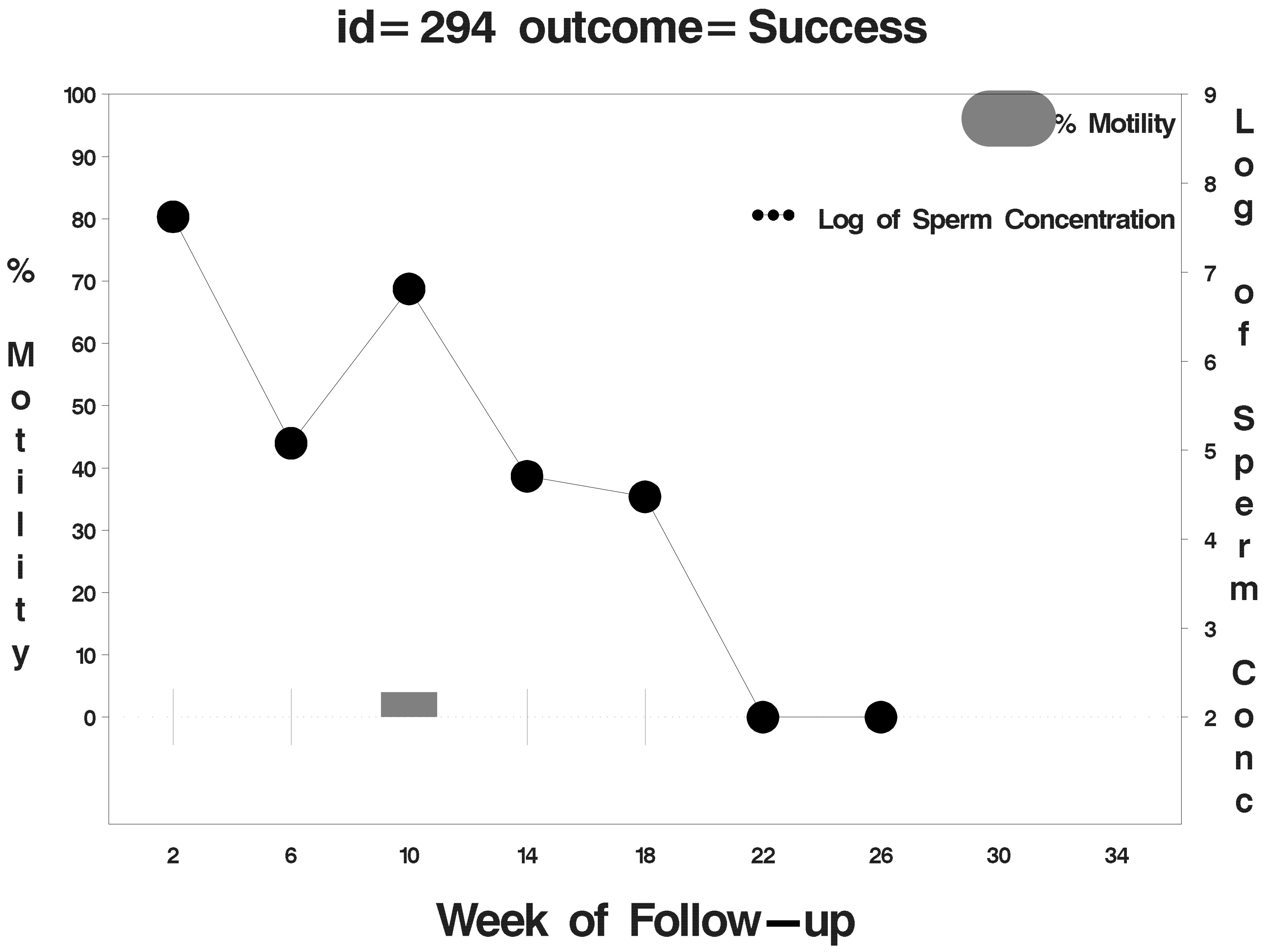

id= 294 outcome= Success
% Motility
Log of Sperm Concentration
% Motility
Log of Sperm Conc
Week of Follow—up
100
90
80
70
60
50
40
30
20
10
0
9
8
7
6
5
4
3
2
2
6
10
14
18
22
26
30
34

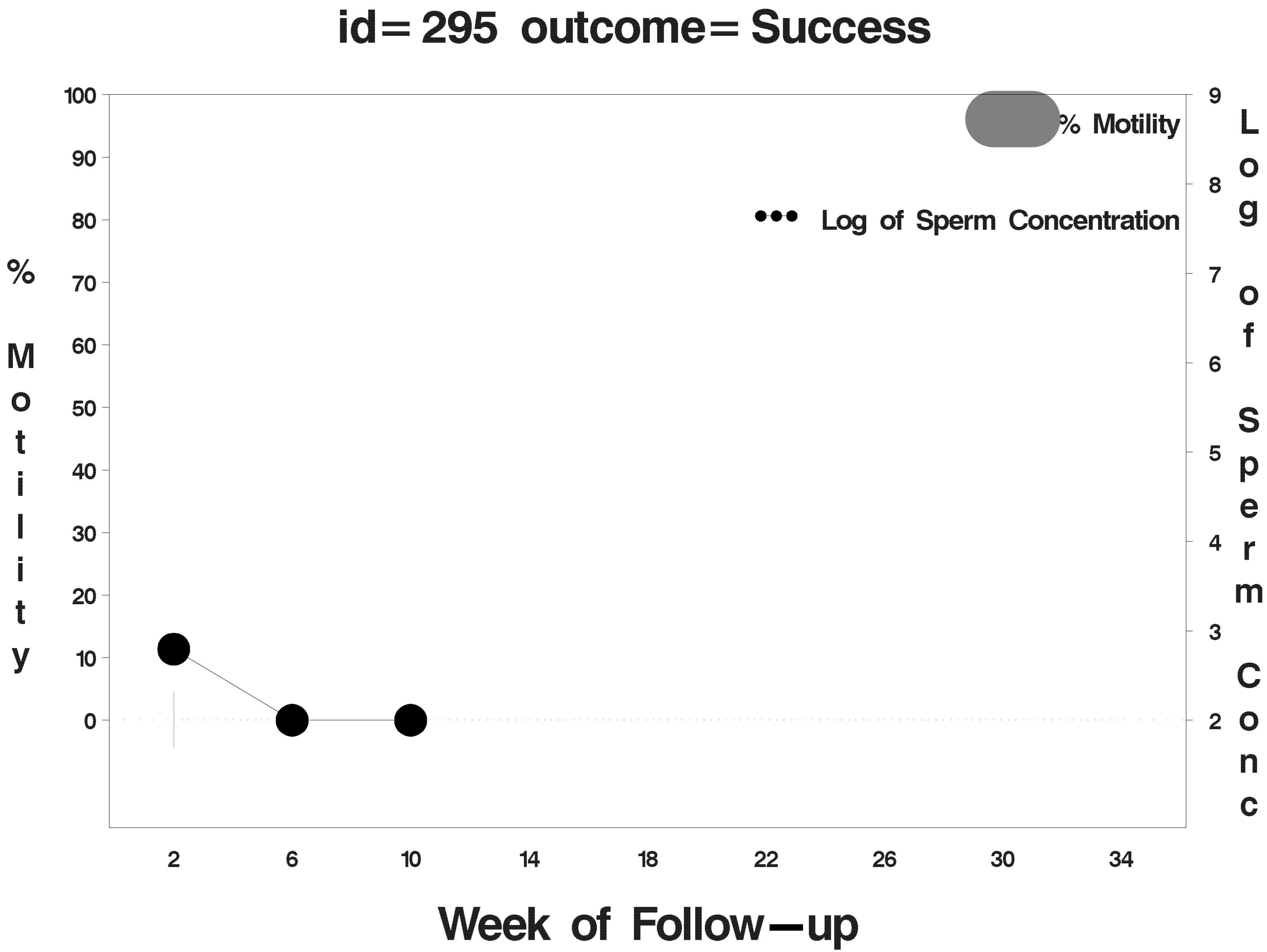

id= 295 outcome= Success
% Motility
Log of Sperm Concentration
% Motility
Log of Sperm Conc
Week of Follow—up
100
90
80
70
60
50
40
30
20
10
0
9
8
7
6
5
4
3
2
2
6
10
14
18
22
26
30
34

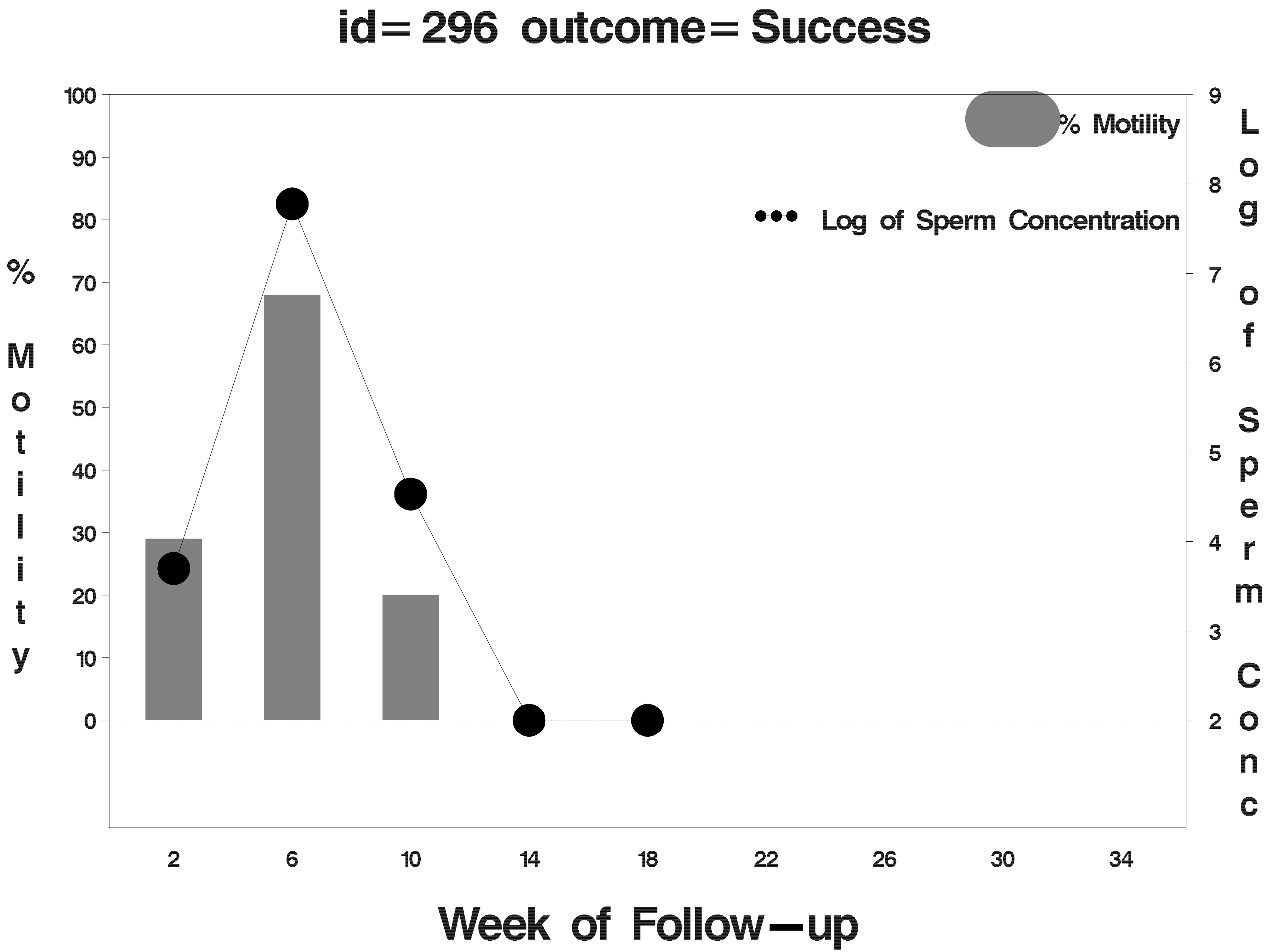

id= 296 outcome= Success
% Motility
Log of Sperm Concentration
100
90
80
70
60
50
40
30
20
10
0
9
8
7
6
5
4
3
2
Log of Sperm Conc
2
6
10
14
18
22
26
30
34
Week of Follow−up

id= 297 outcome= Success

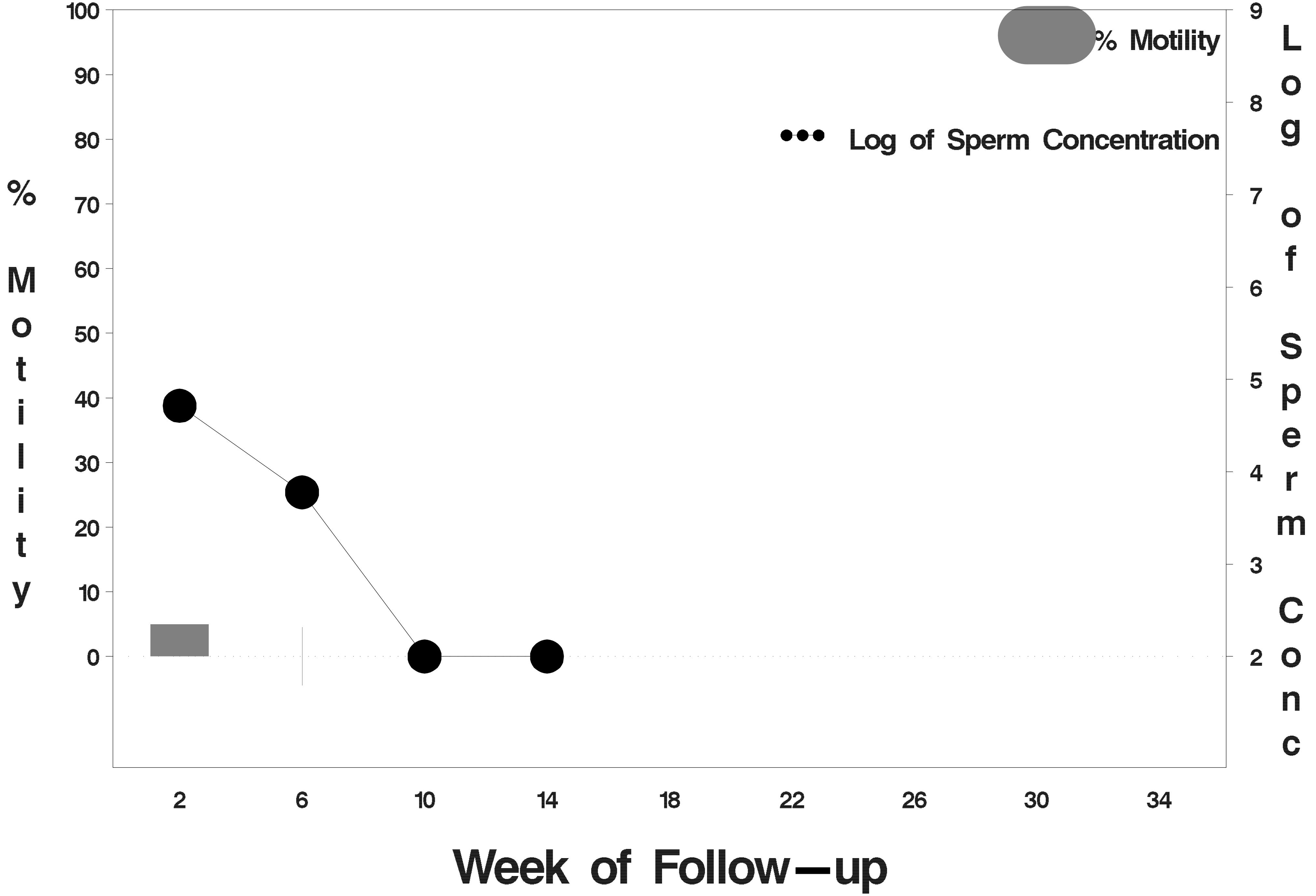

id= 298 outcome= Indeterminate

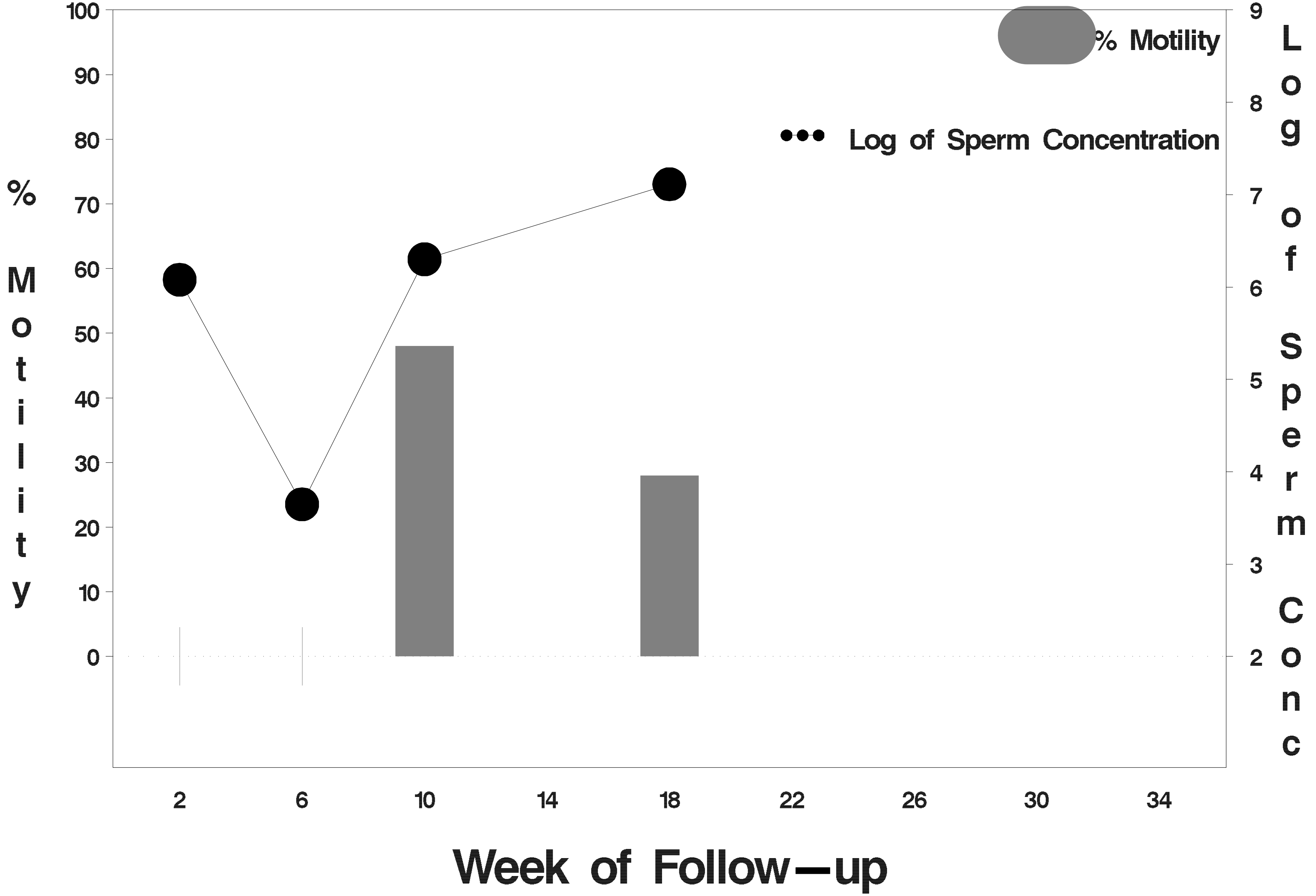

id= 299 outcome= Success

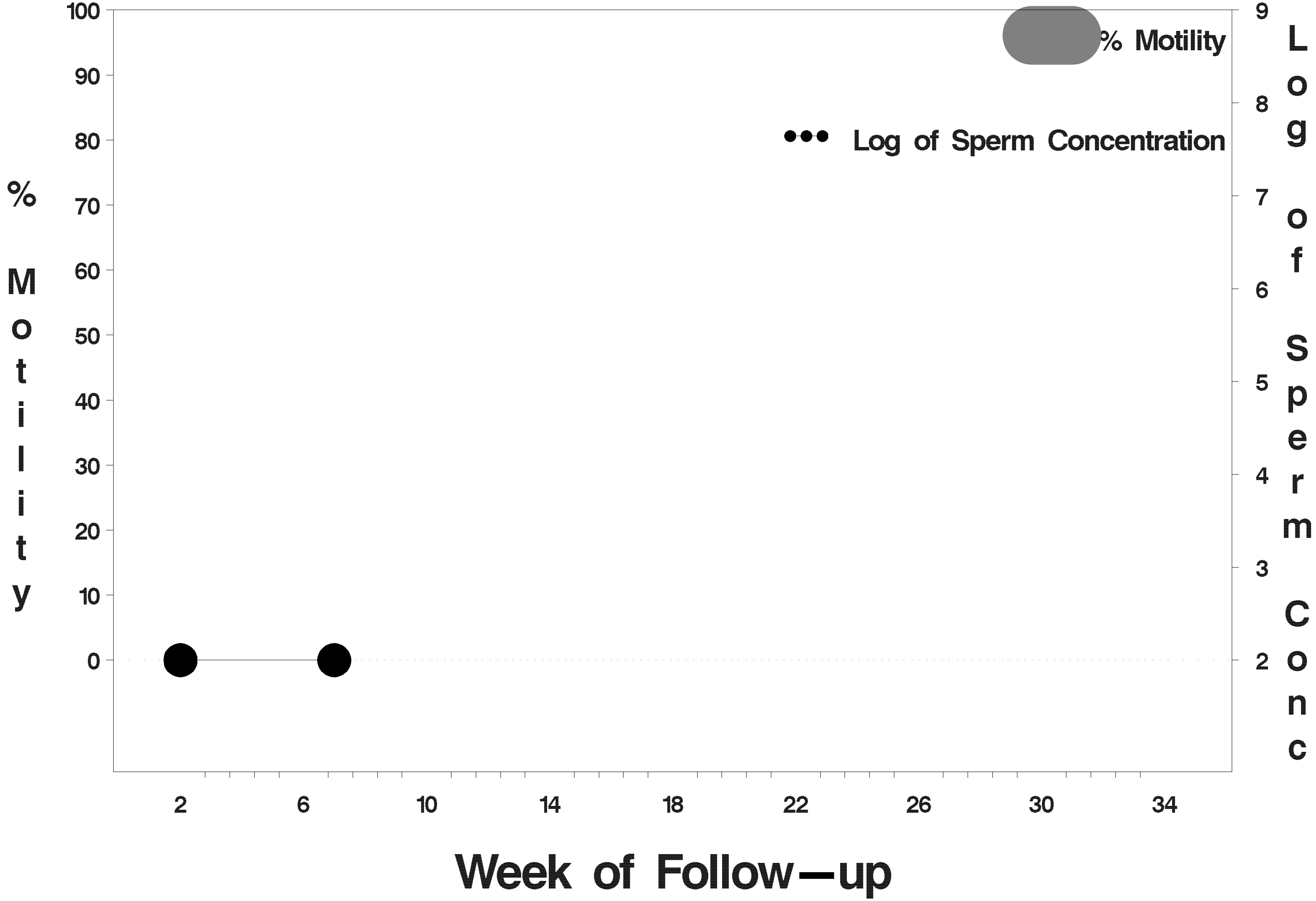

id= 300 outcome= Success

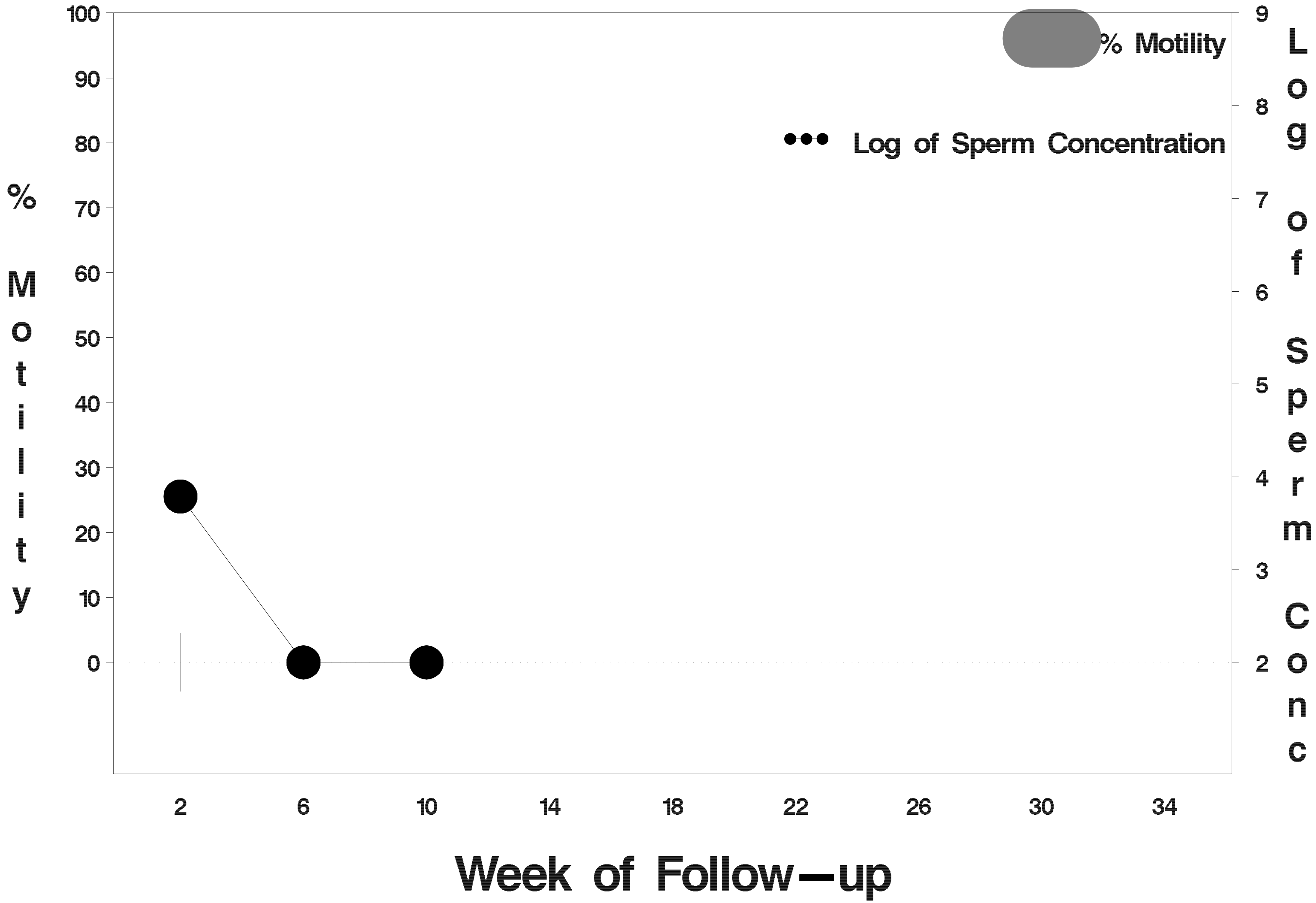

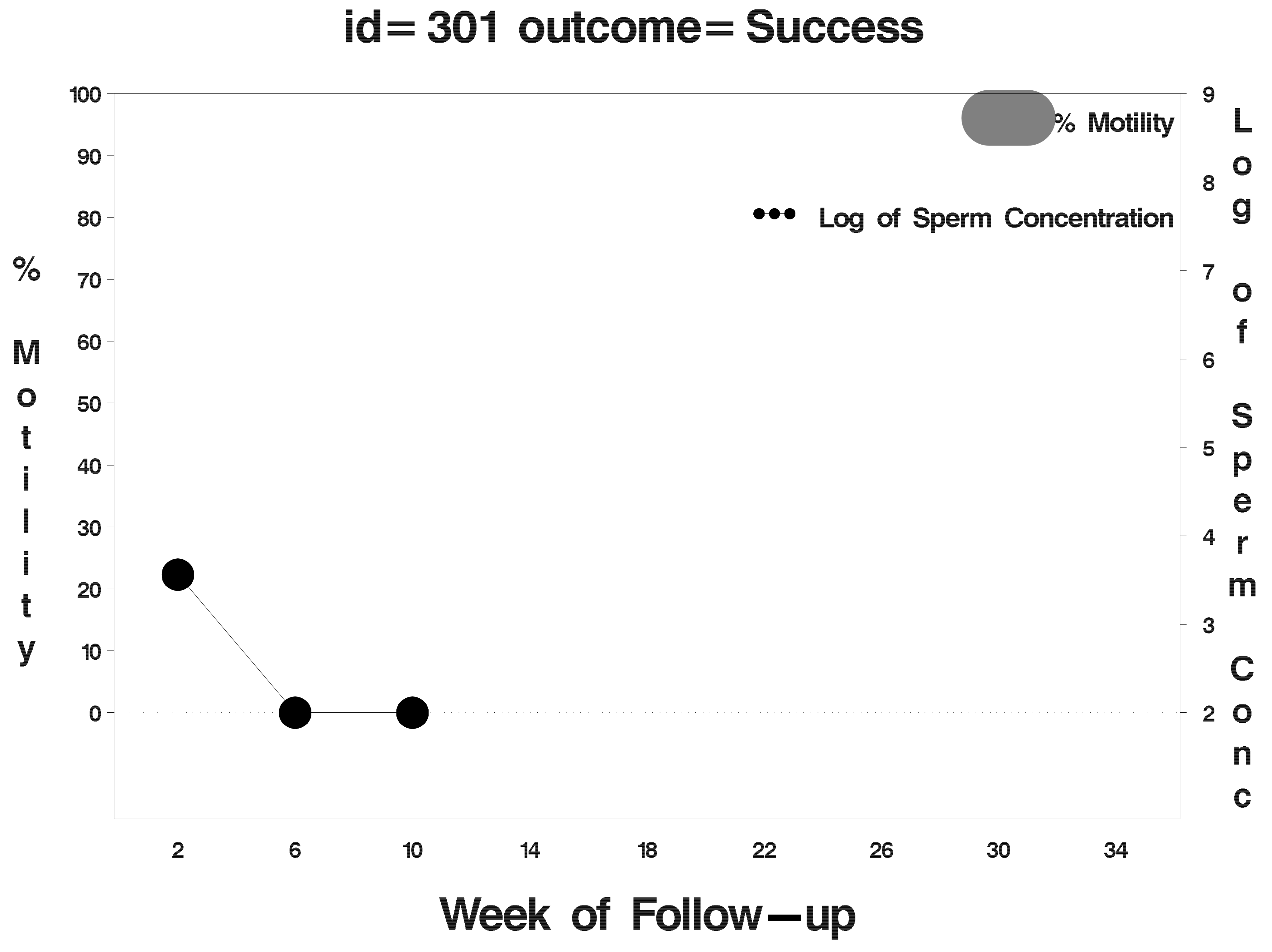
id= 301 outcome= Success
% Motility
Log of Sperm Concentration
100
90
80
70
60
50
40
30
20
10
0
9
8
7
6
5
4
3
2
2
6
10
14
18
22
26
30
34
% Motility
Log of Sperm Conc
Week of Follow−up

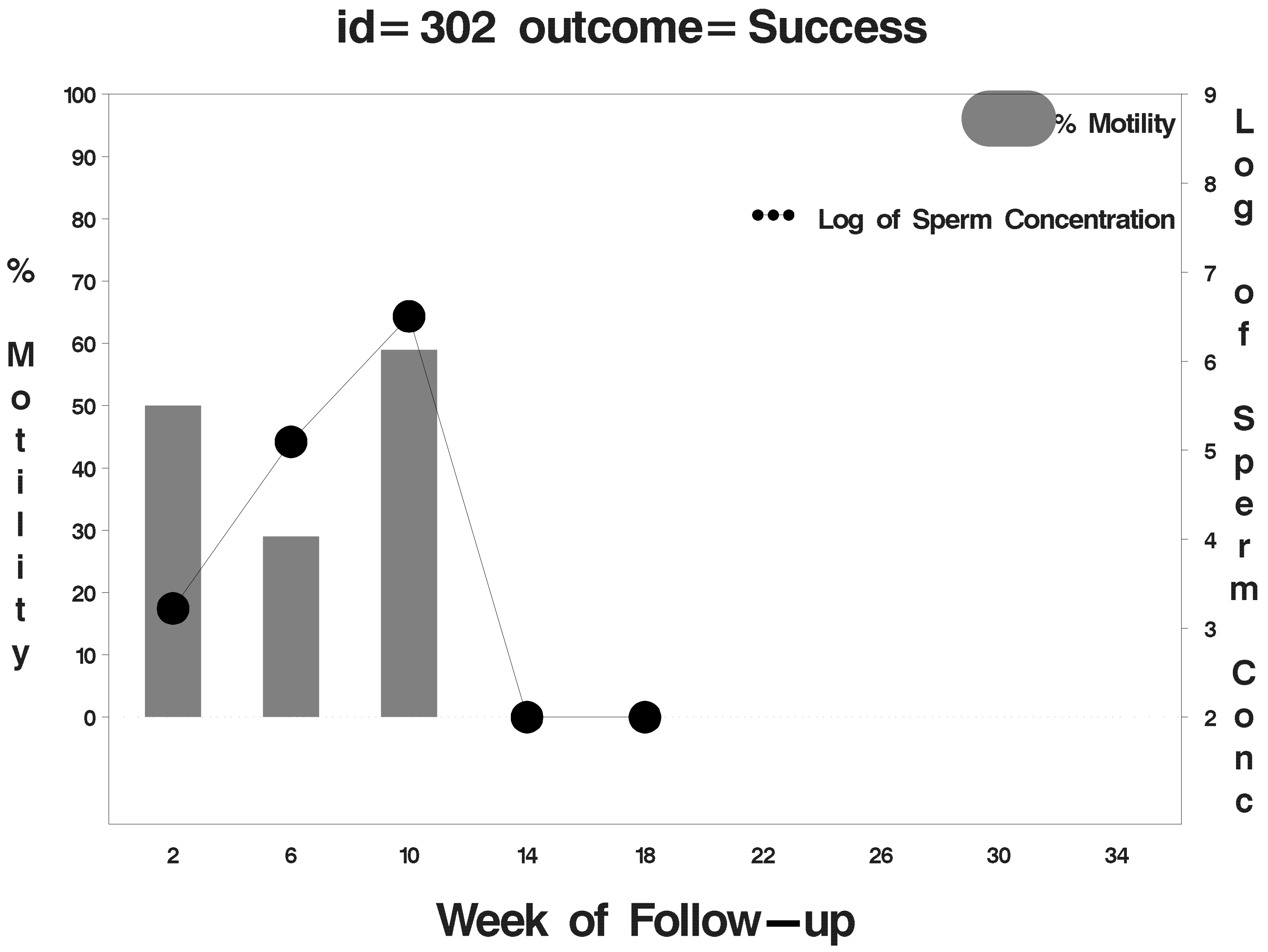
id= 302 outcome= Success
% Motility
Log of Sperm Concentration
100
90
80
70
60
50
40
30
20
10
0
9
8
7
6
5
4
3
2
2
6
10
14
18
22
26
30
34
Week of Follow—up
Log of Sperm Conc

id= 303 outcome= Success

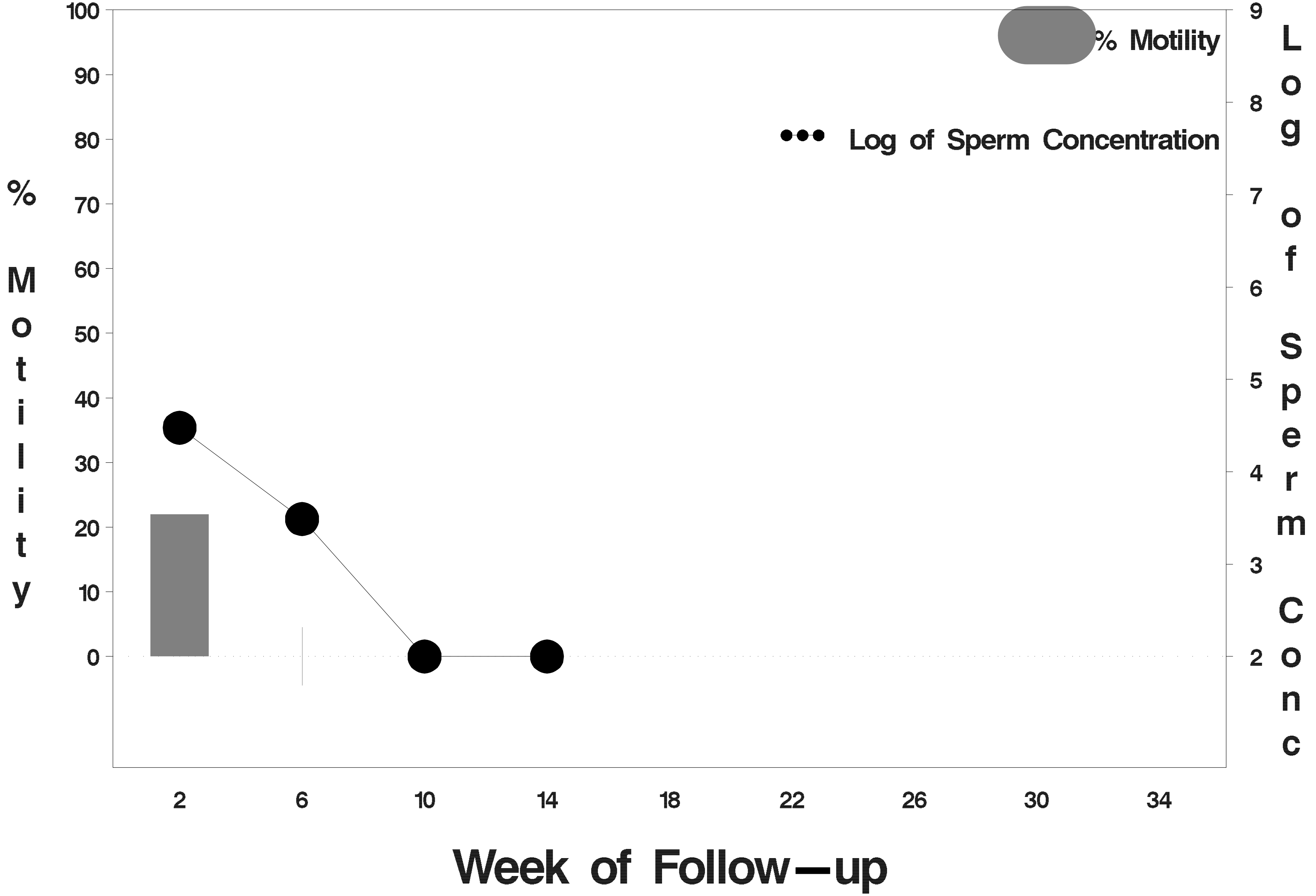

id= 304 outcome= Failure

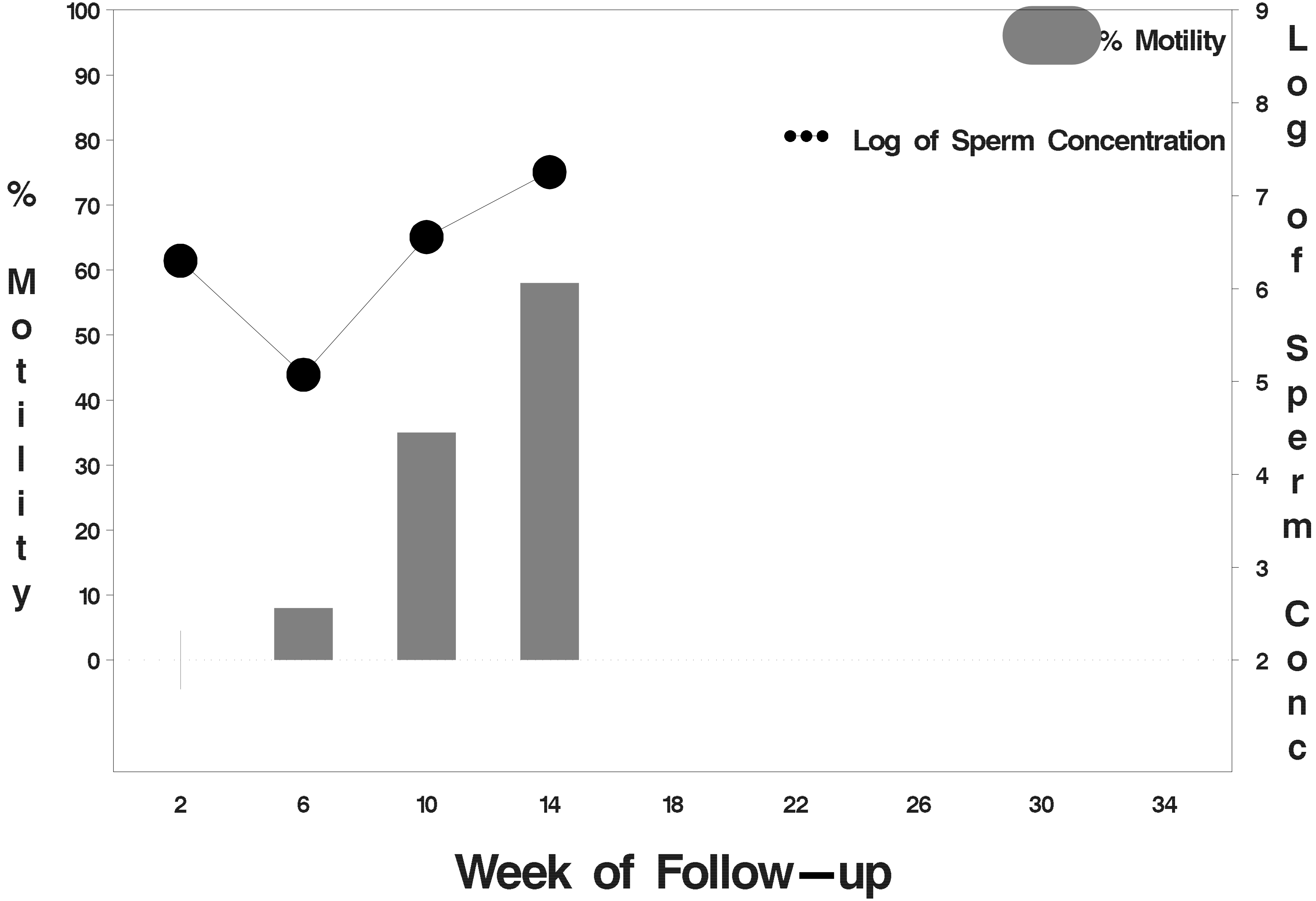

id= 305 outcome= Success

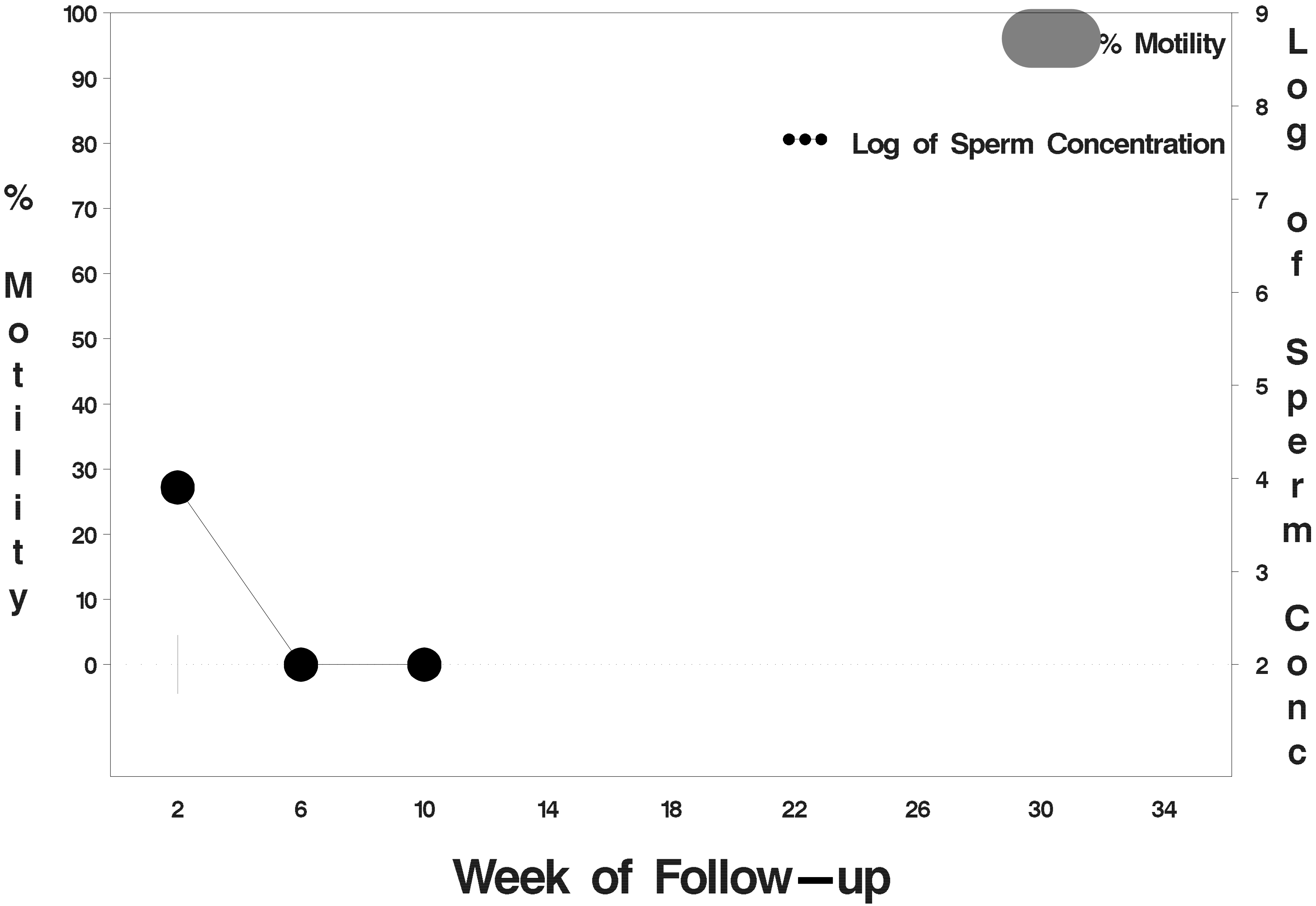

id= 306 outcome= Success

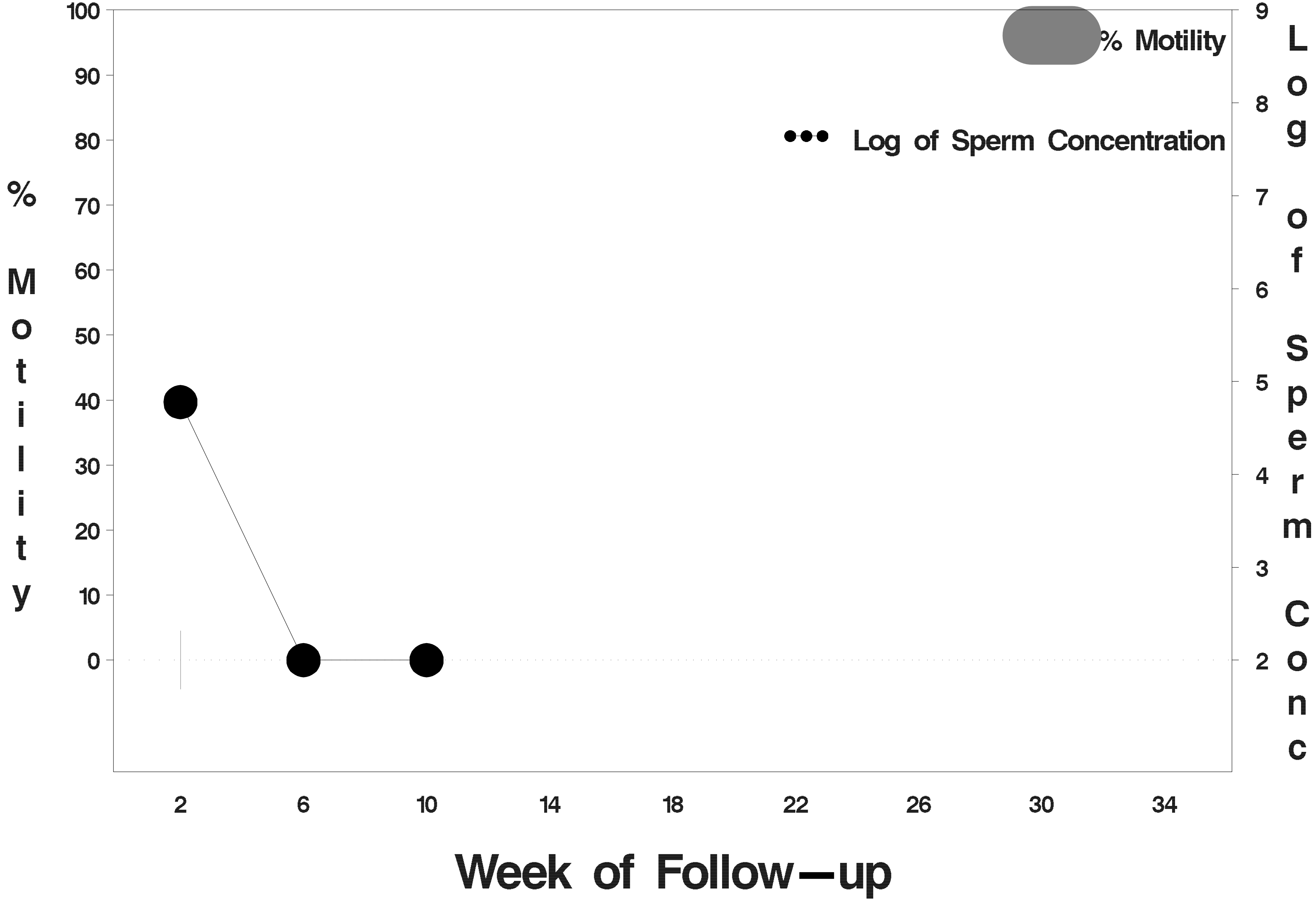

id= 307 outcome= Success

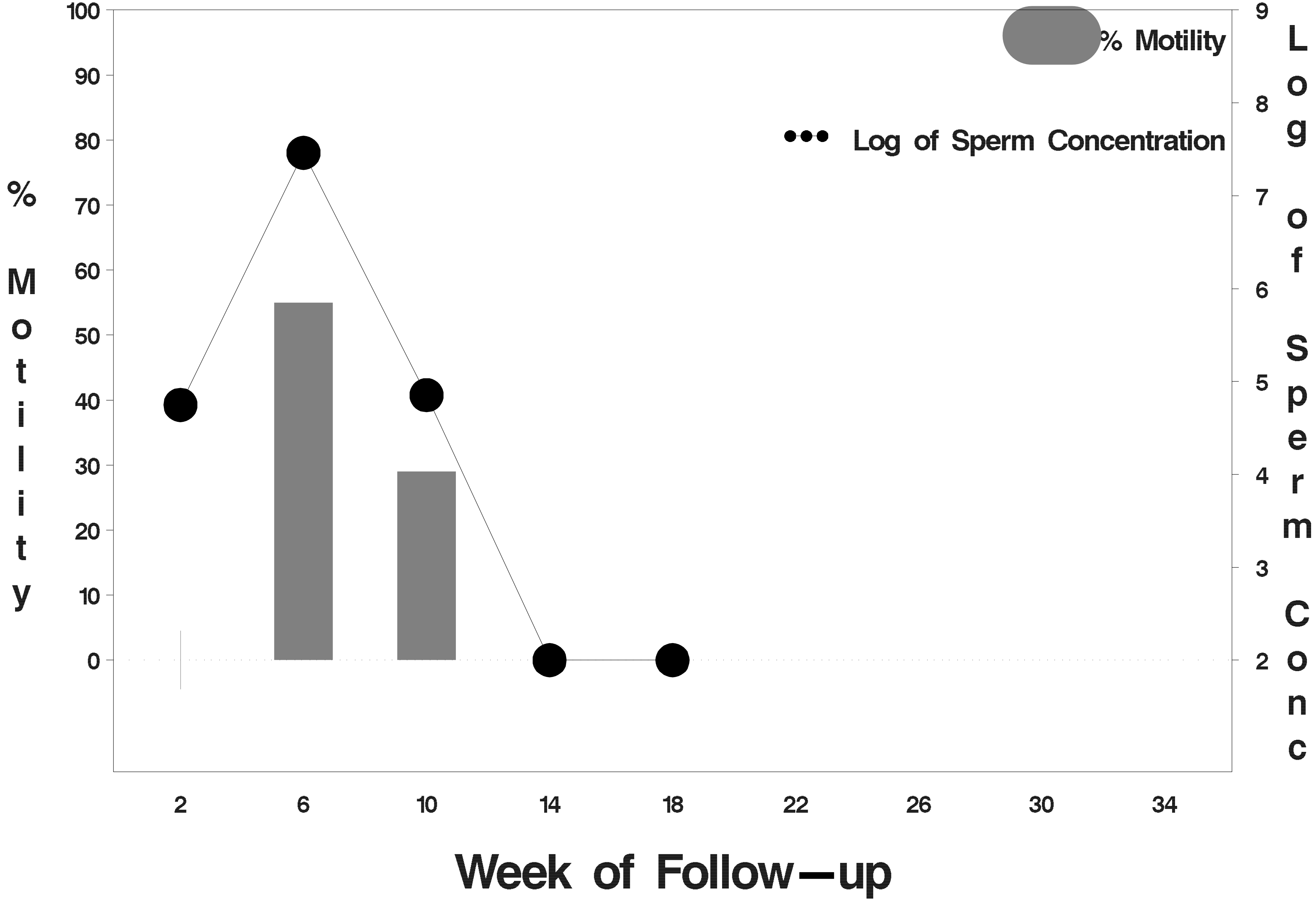

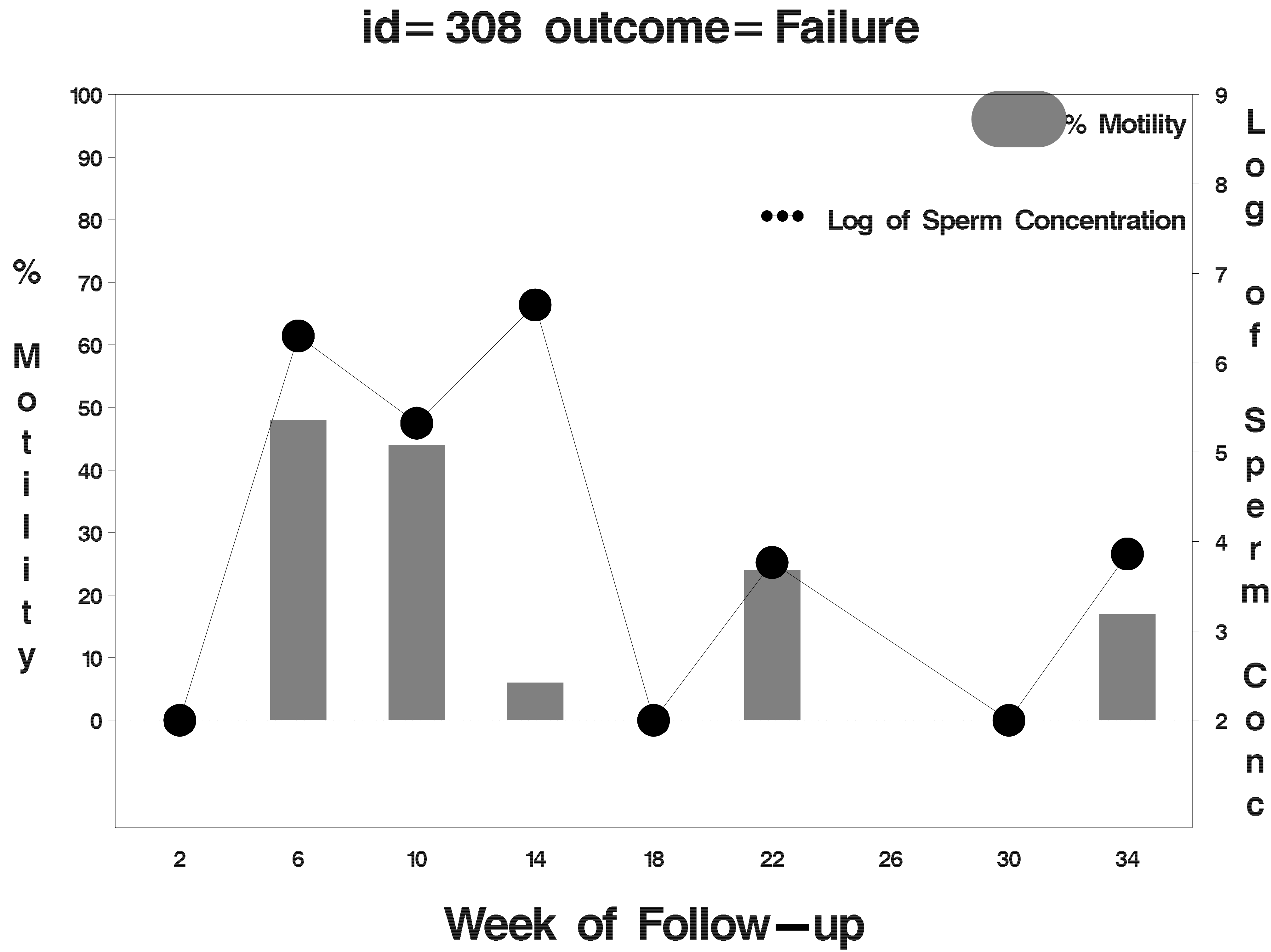

id= 308 outcome= Failure
% Motility
Log of Sperm Concentration
% Motility
Log of Sperm Conc
Week of Follow-up
100
90
80
70
60
50
40
30
20
10
0
9
8
7
6
5
4
3
2
2
6
10
14
18
22
26
30
34

id= 309 outcome= Success

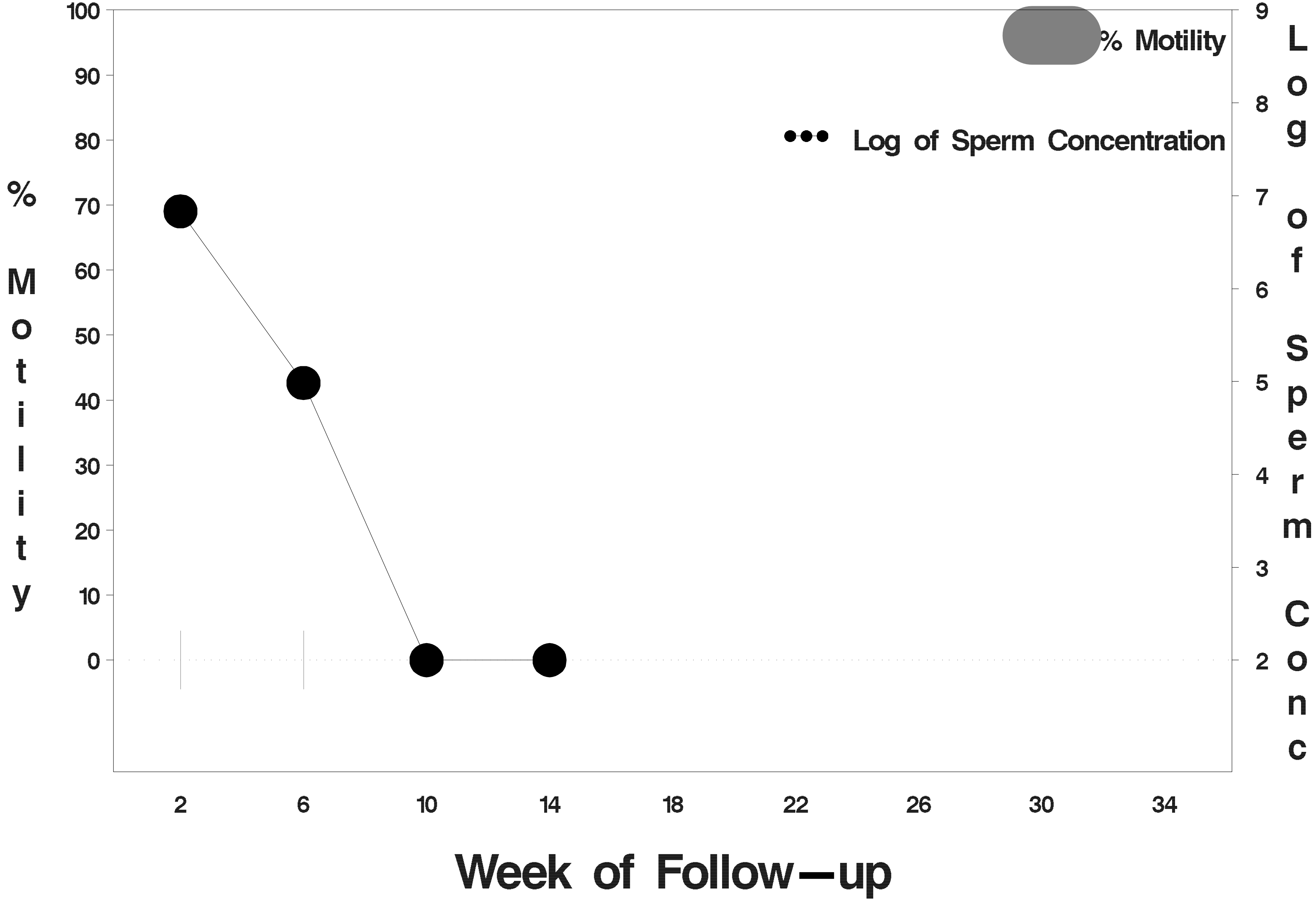

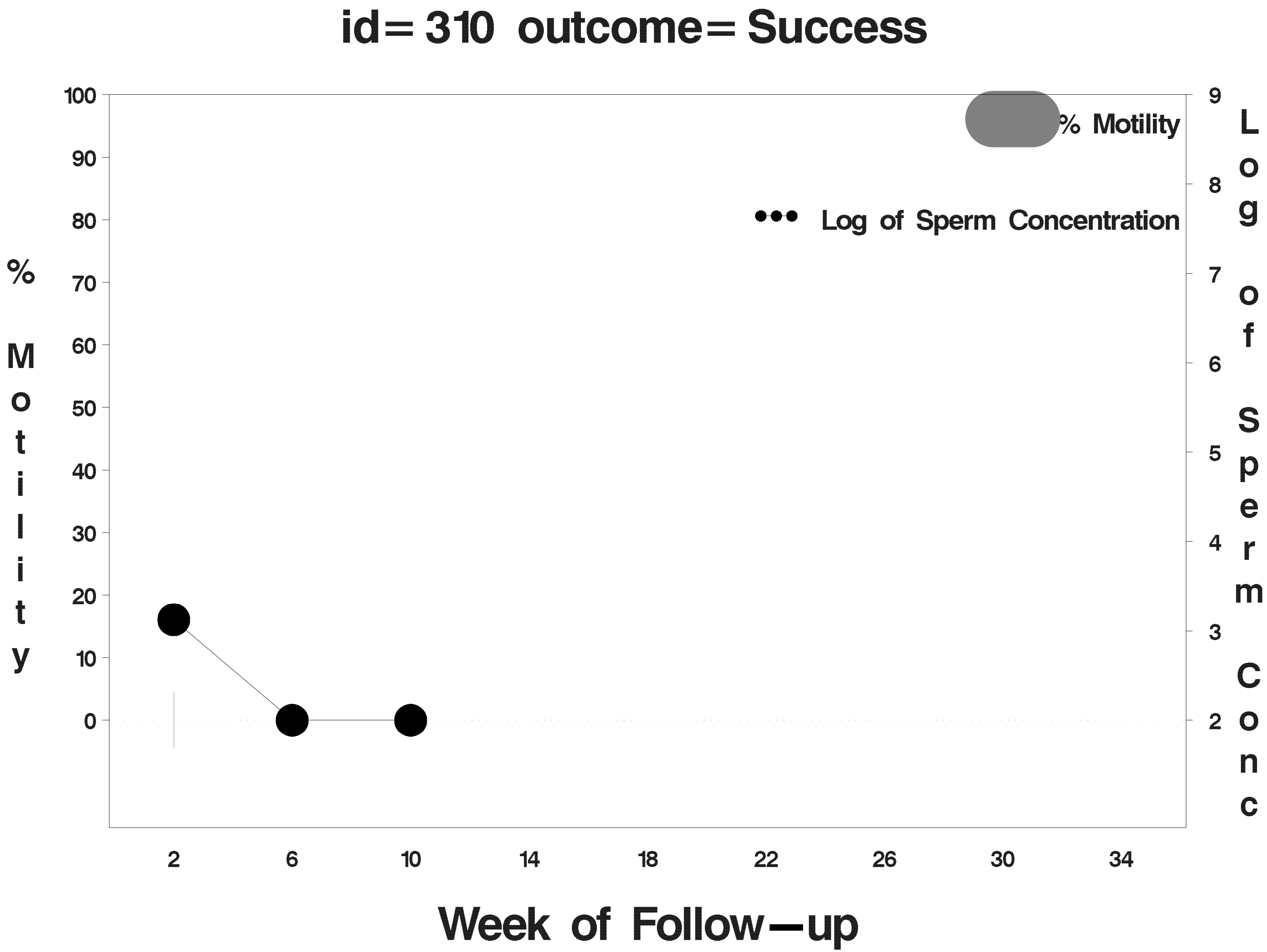

id= 310 outcome= Success
% Motility
Log of Sperm Concentration
% Motility
Log of Sperm Conc
Week of Follow—up
100
90
80
70
60
50
40
30
20
10
0
9
8
7
6
5
4
3
2
2
6
10
14
18
22
26
30
34

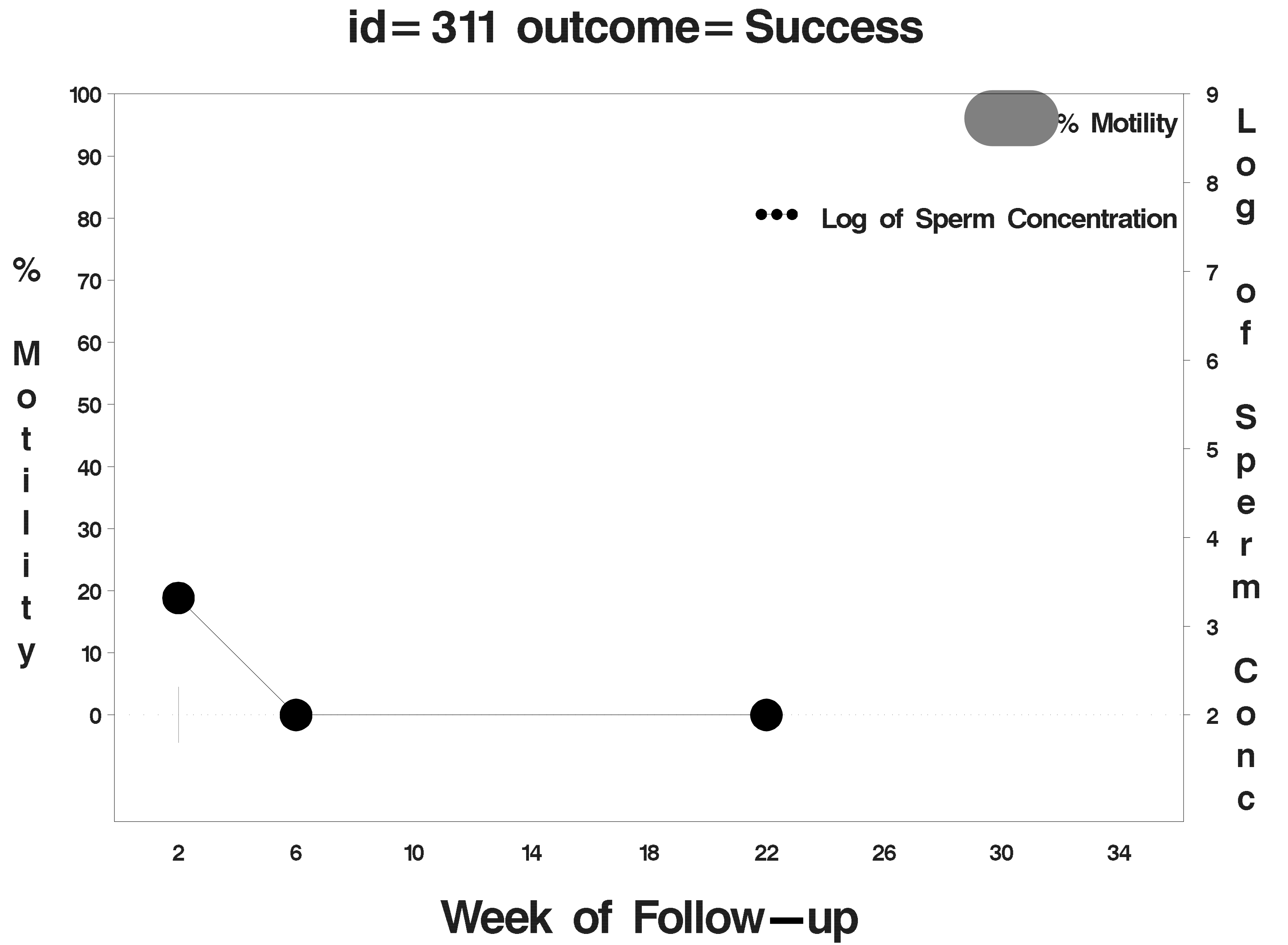
id= 311 outcome= Success
% Motility
Log of Sperm Concentration
% Motility
Log of Sperm Conc
Week of Follow-up
100
90
80
70
60
50
40
30
20
10
0
9
8
7
6
5
4
3
2
2
6
10
14
18
22
26
30
34

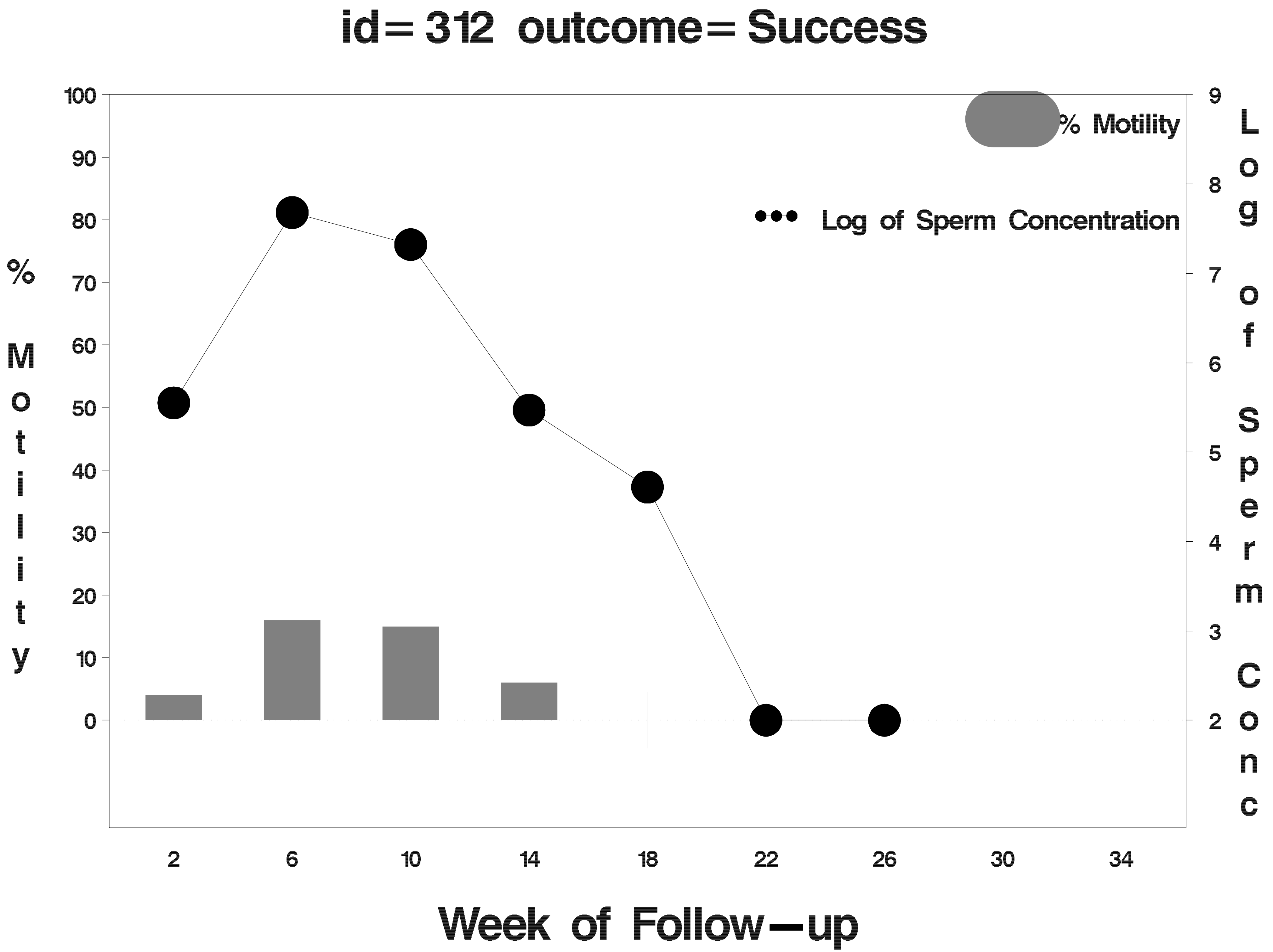
id= 312 outcome= Success
% Motility
Log of Sperm Concentration
100
90
80
70
60
50
40
30
20
10
0
9
8
7
6
5
4
3
2
2
6
10
14
18
22
26
30
34
Week of Follow−up
Log of Sperm Conc

id= 313 outcome= Success

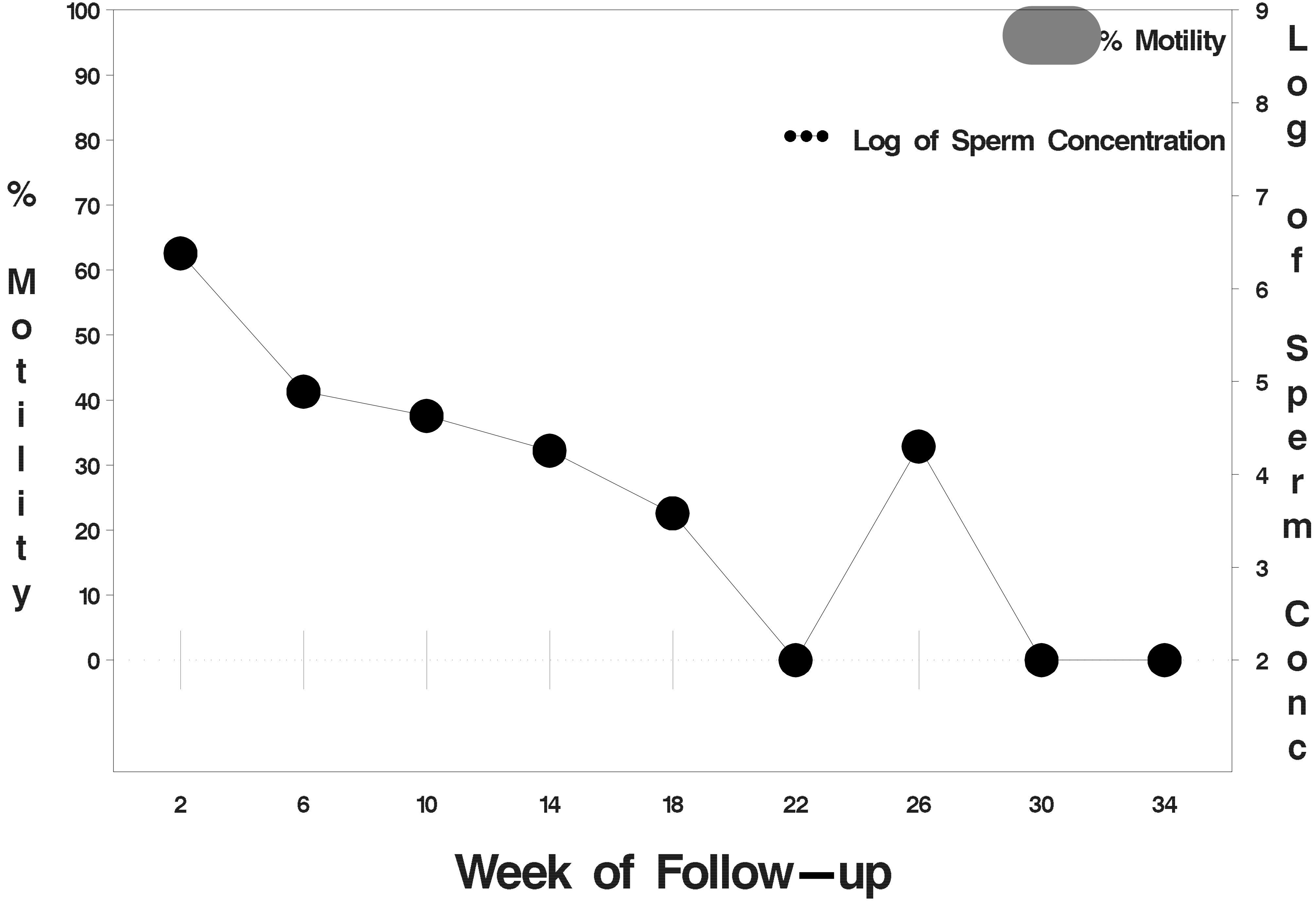

id= 314 outcome= Success

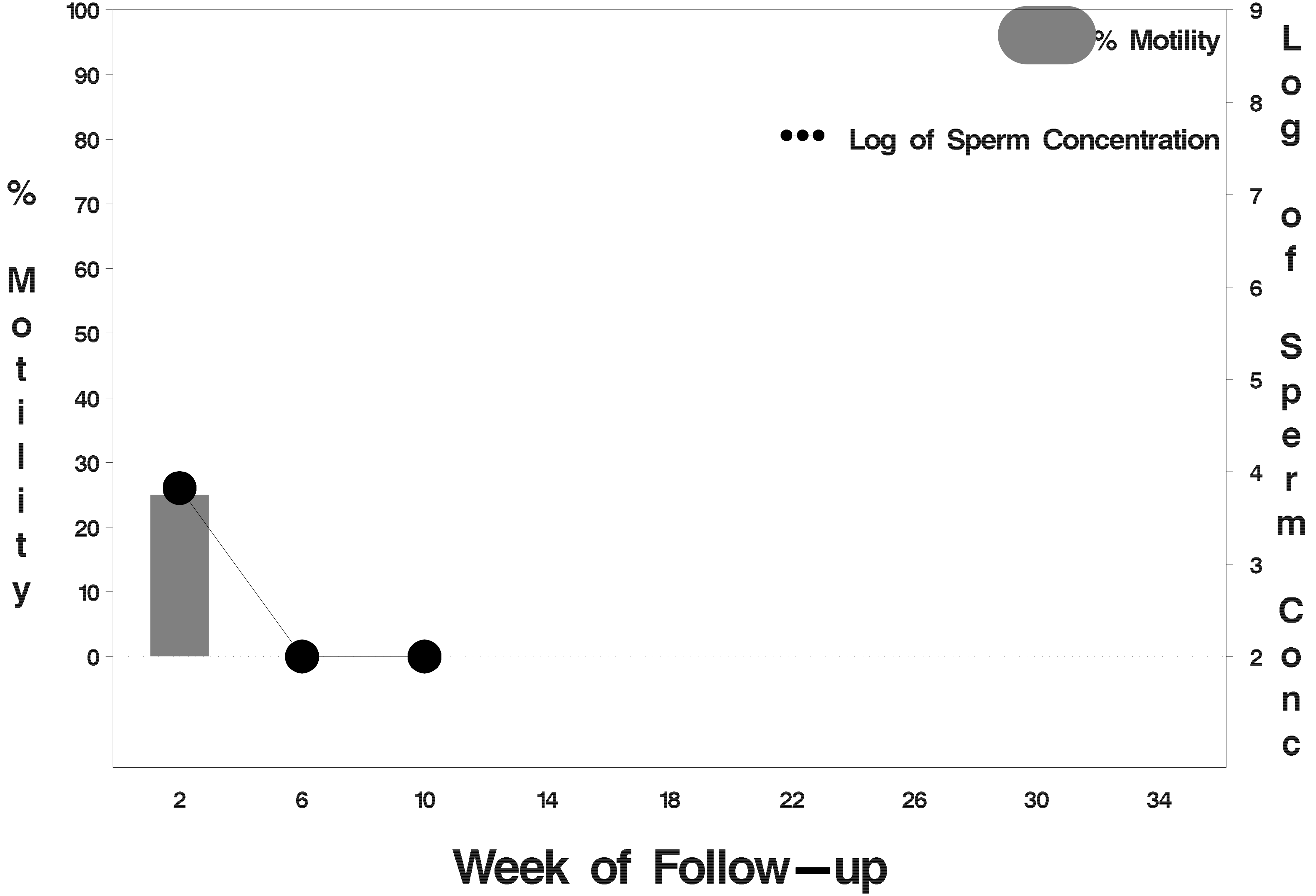

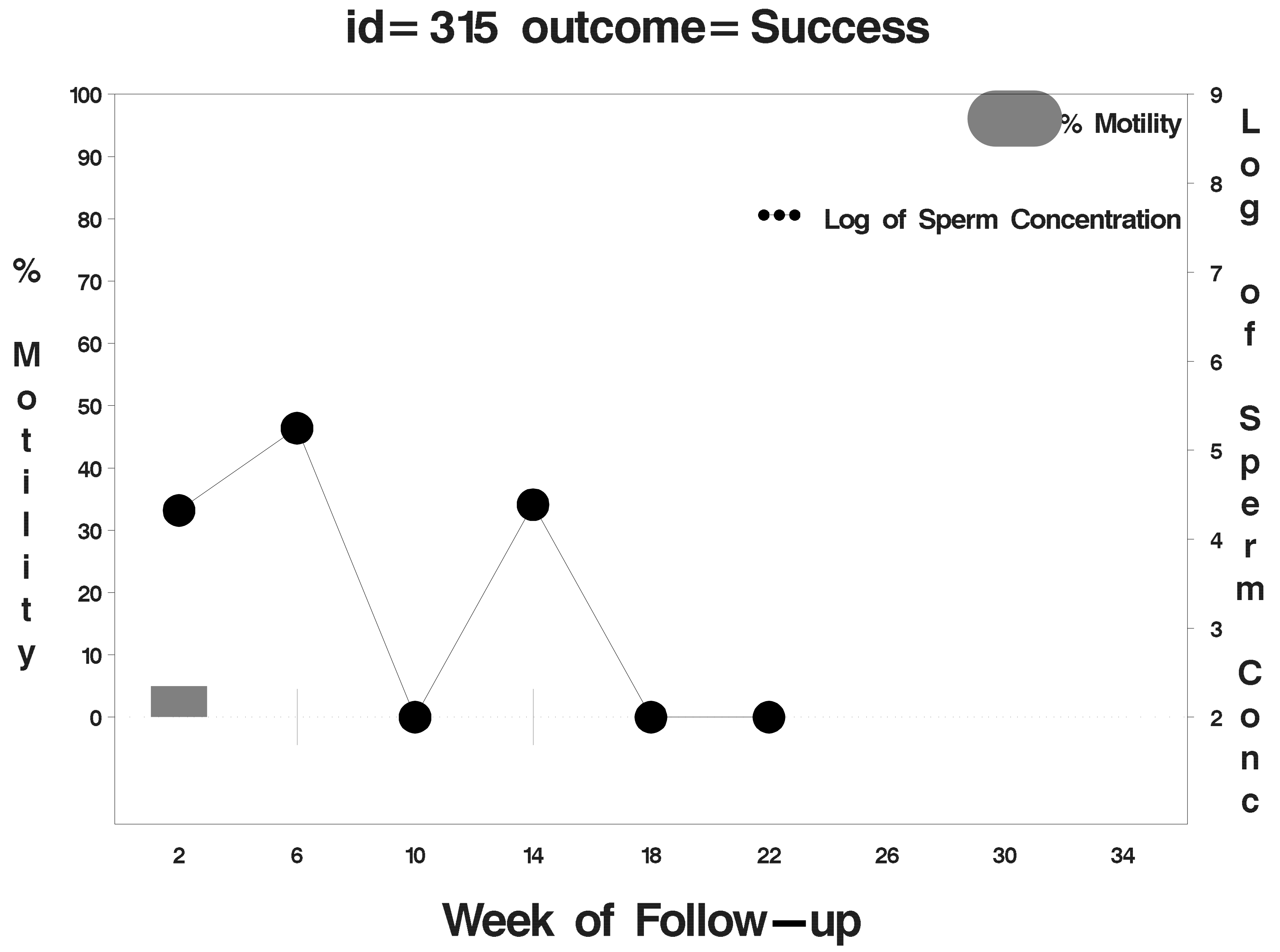

id= 315 outcome= Success
% Motility
Log of Sperm Concentration
100
90
80
70
60
50
40
30
20
10
0
9
8
7
6
5
4
3
2
2
6
10
14
18
22
26
30
34
Week of Follow−up
% Motility
Log of Sperm Conc

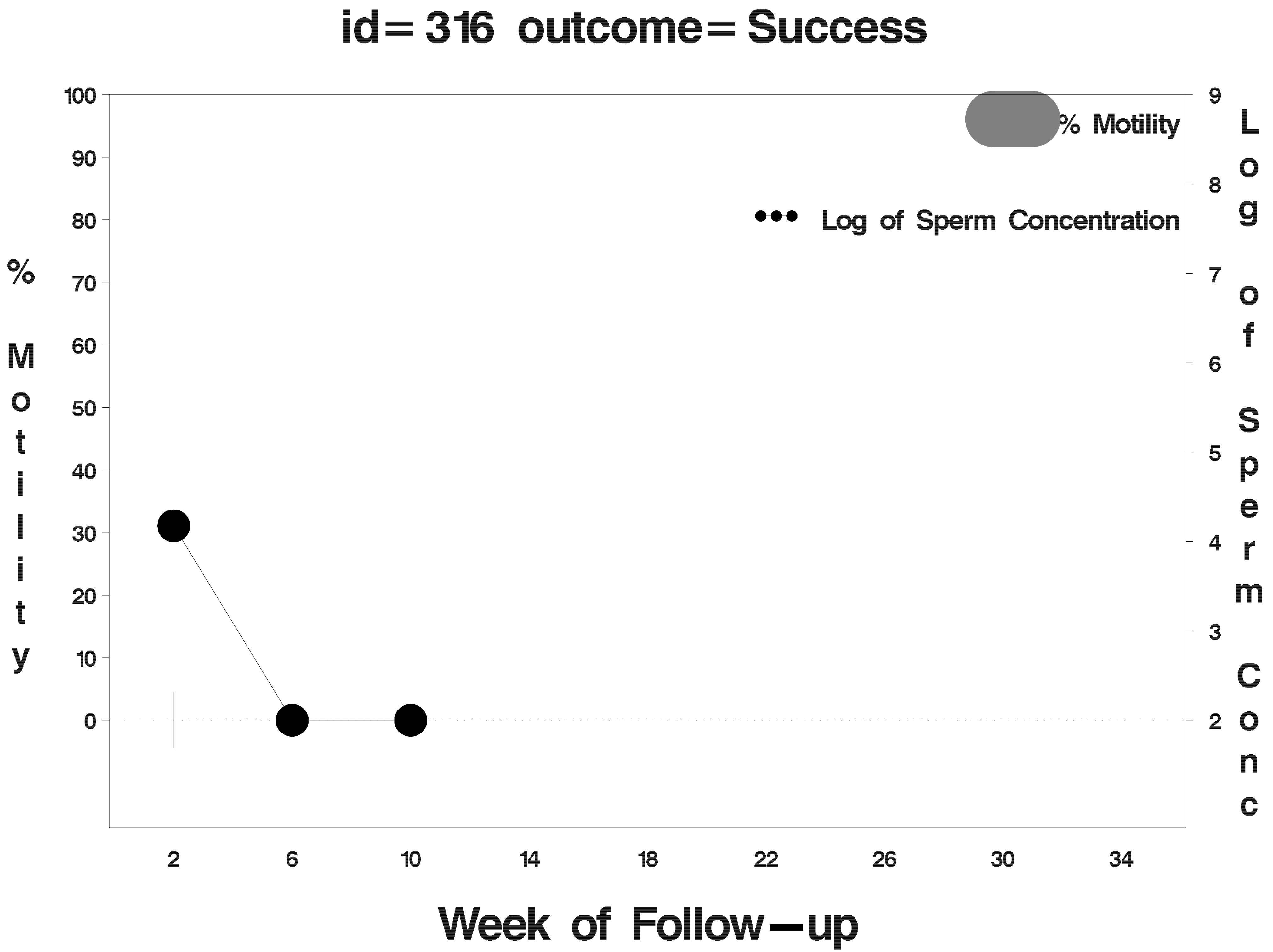

id= 316 outcome= Success
% Motility
Log of Sperm Concentration
100
90
80
70
60
50
40
30
20
10
0
9
8
7
6
5
4
3
2
2
6
10
14
18
22
26
30
34
Week of Follow−up
Log of Sperm Conc

id= 317 outcome= Success

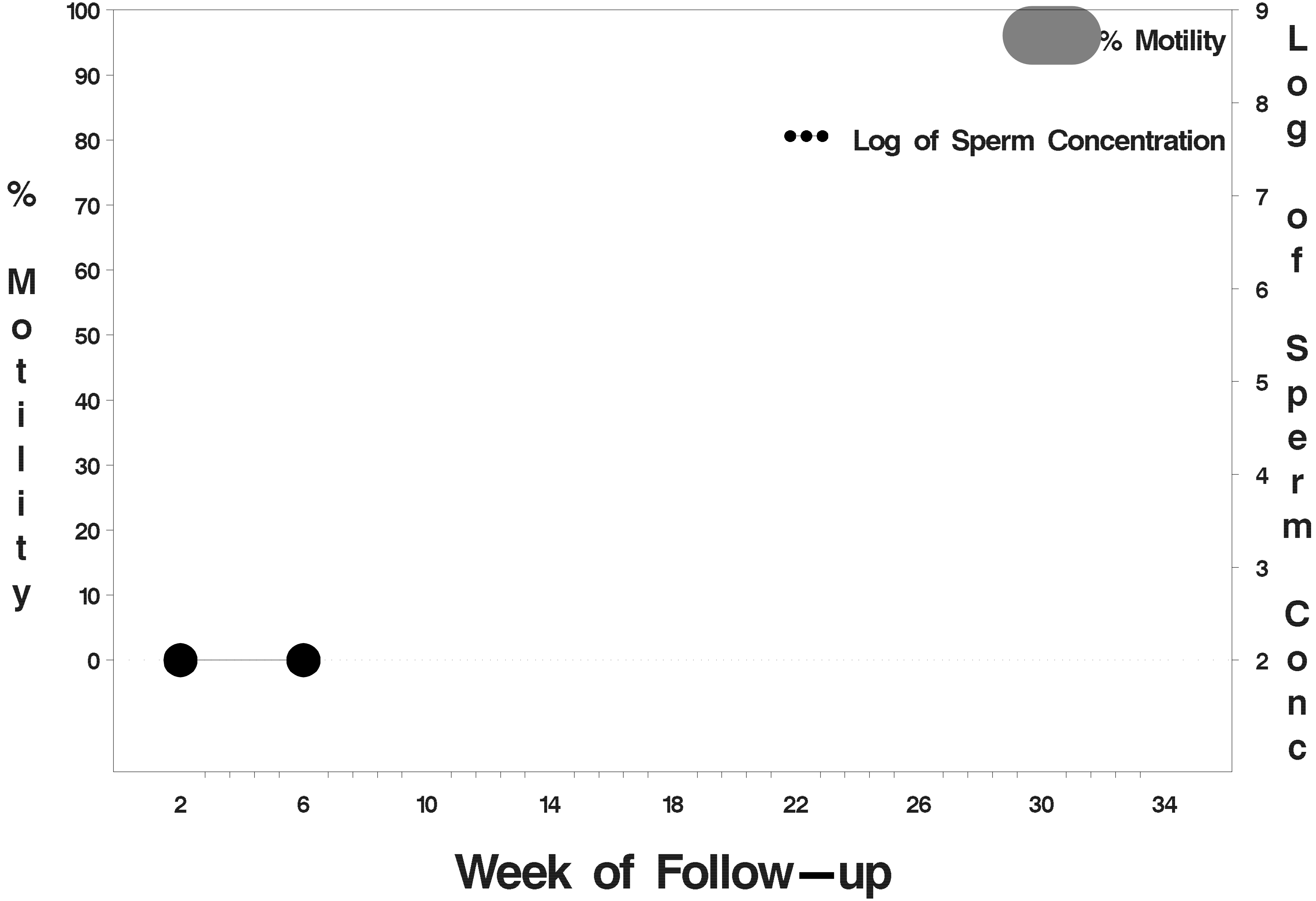

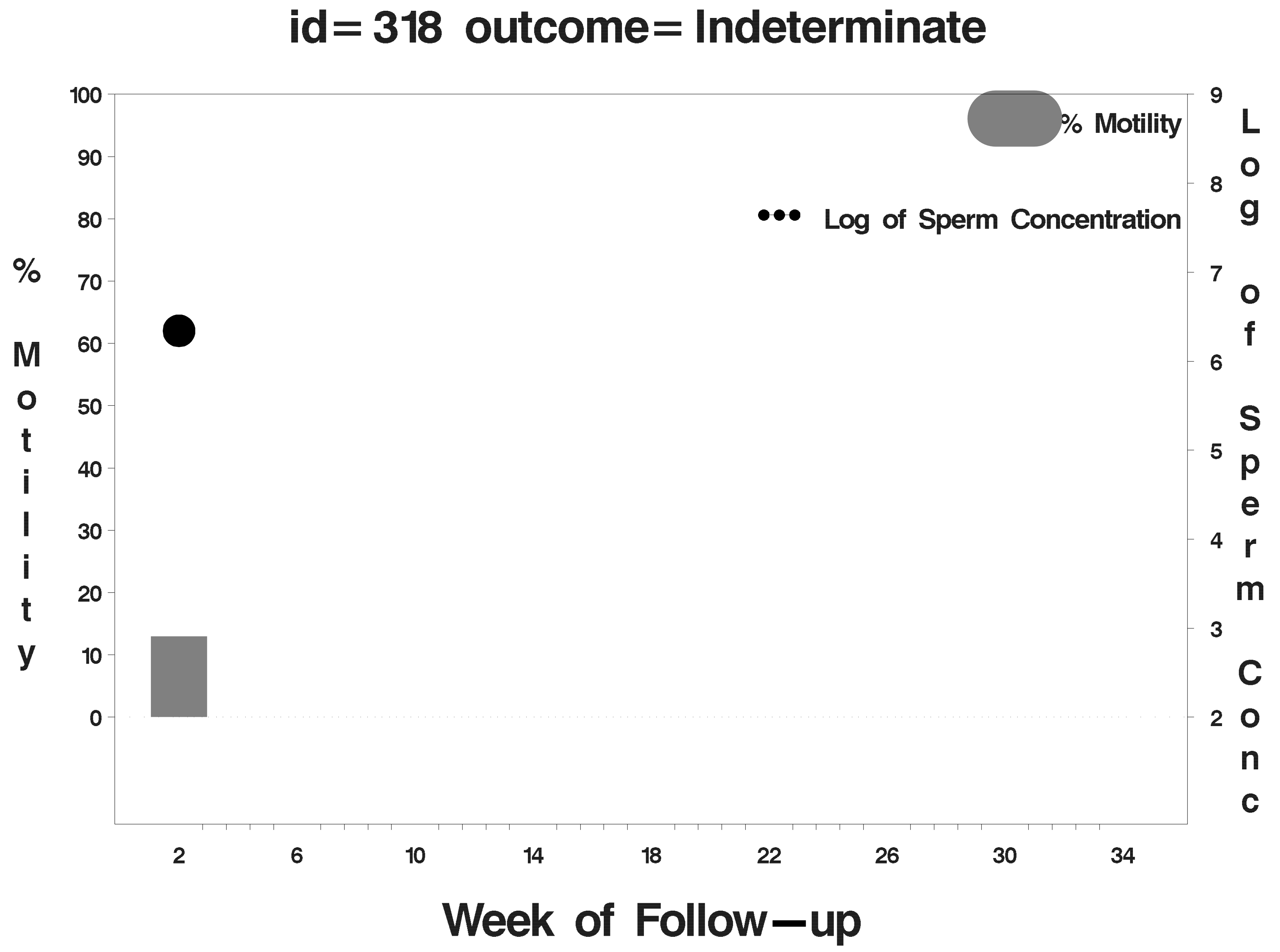
id= 318 outcome= Indeterminate
% Motility
Log of Sperm Concentration
100
90
80
70
60
50
40
30
20
10
0
9
8
7
6
5
4
3
2
2
6
10
14
18
22
26
30
34
Week of Follow−up
% Motility
Log of Sperm Conc

id= 319 outcome= Indeterminate

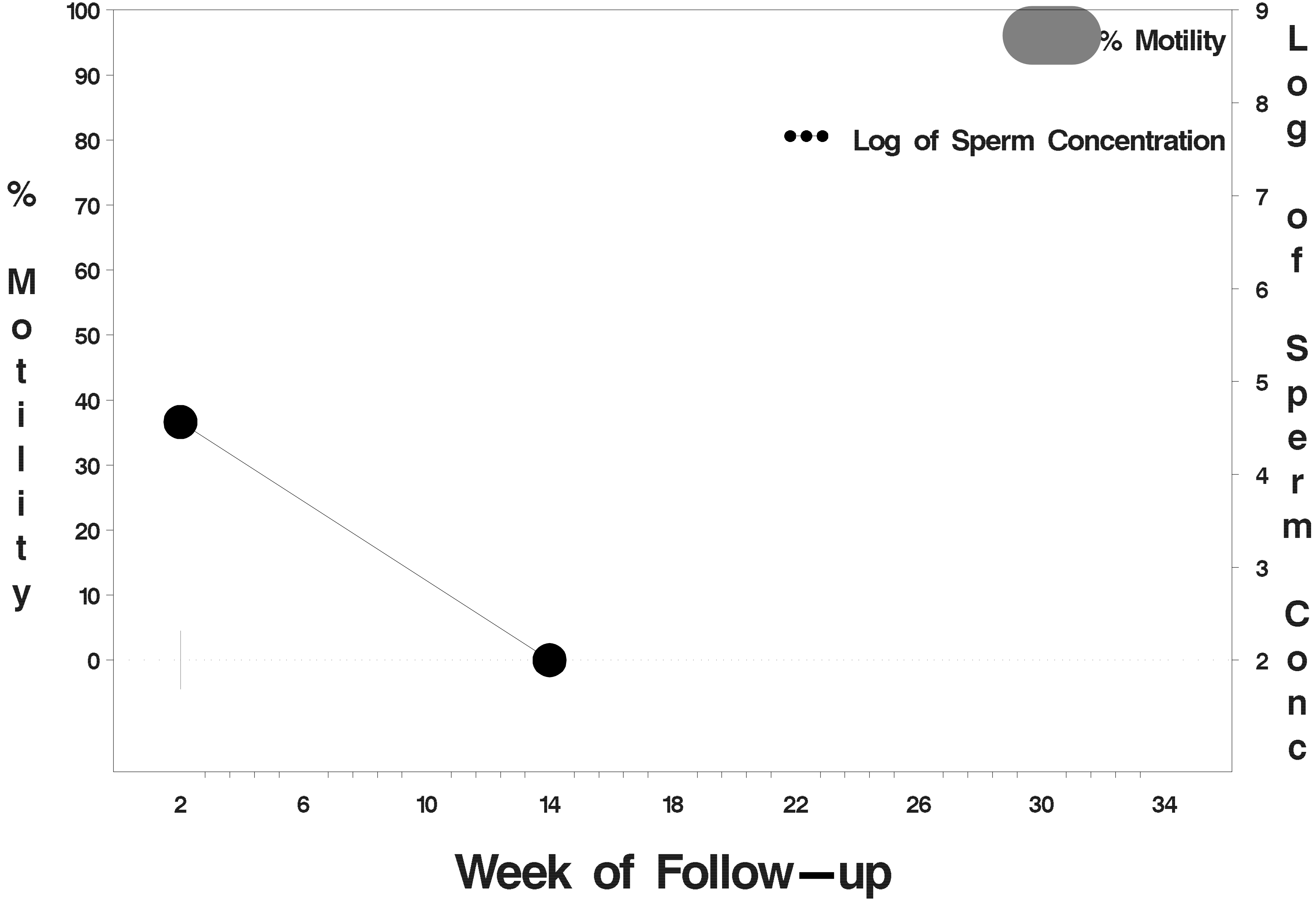

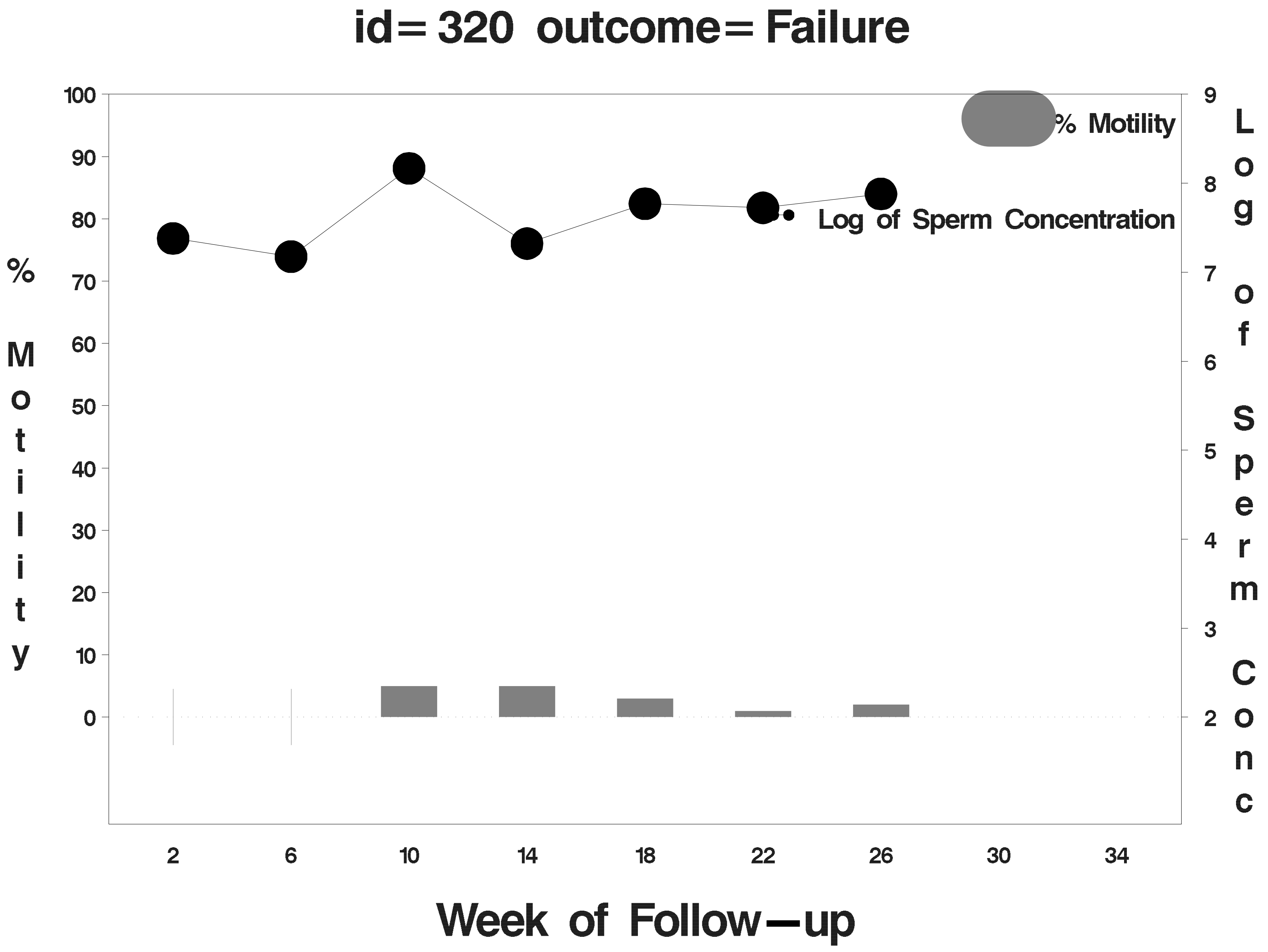
id= 320 outcome= Failure
% Motility
Log of Sperm Concentration
100
90
80
70
60
50
40
30
20
10
0
9
8
7
6
5
4
3
2
2
6
10
14
18
22
26
30
34
Week of Follow-up
% Motility
Log of Sperm Conc

id= 321 outcome= Success

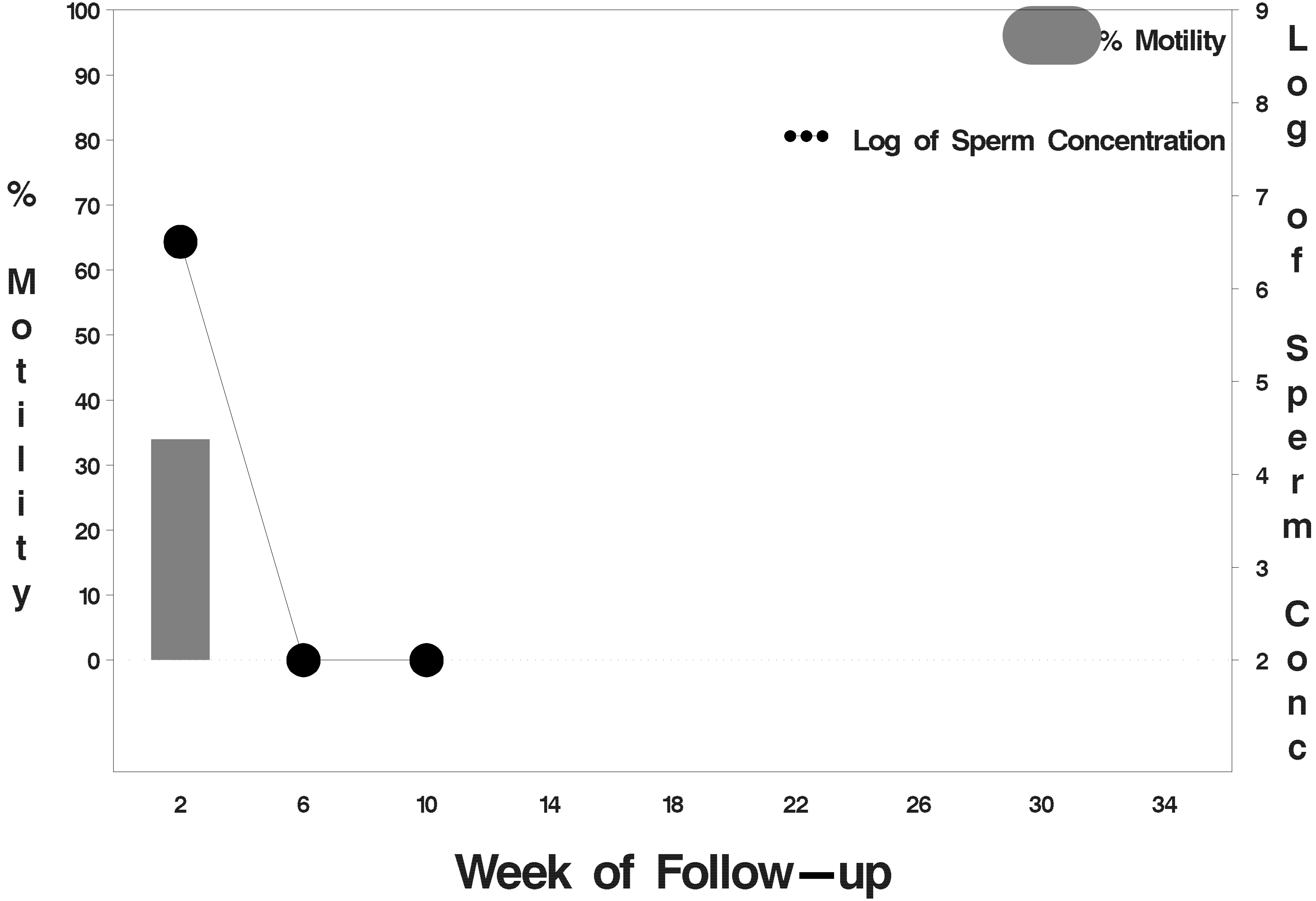

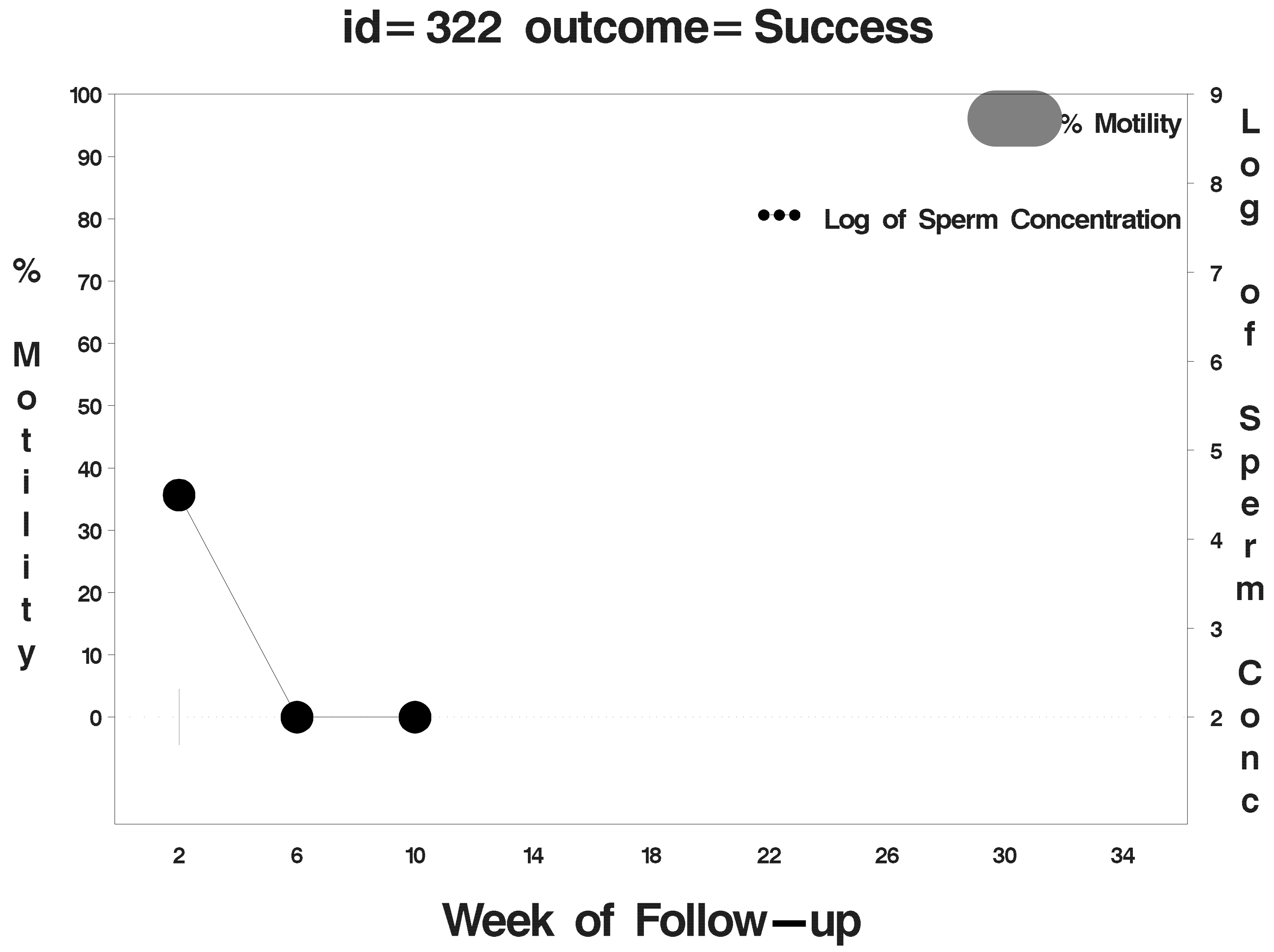
id= 322 outcome= Success
% Motility
Log of Sperm Concentration
% Motility
Log of Sperm Conc
Week of Follow—up
100
90
80
70
60
50
40
30
20
10
0
9
8
7
6
5
4
3
2
2
6
10
14
18
22
26
30
34

id= 323 outcome= Success

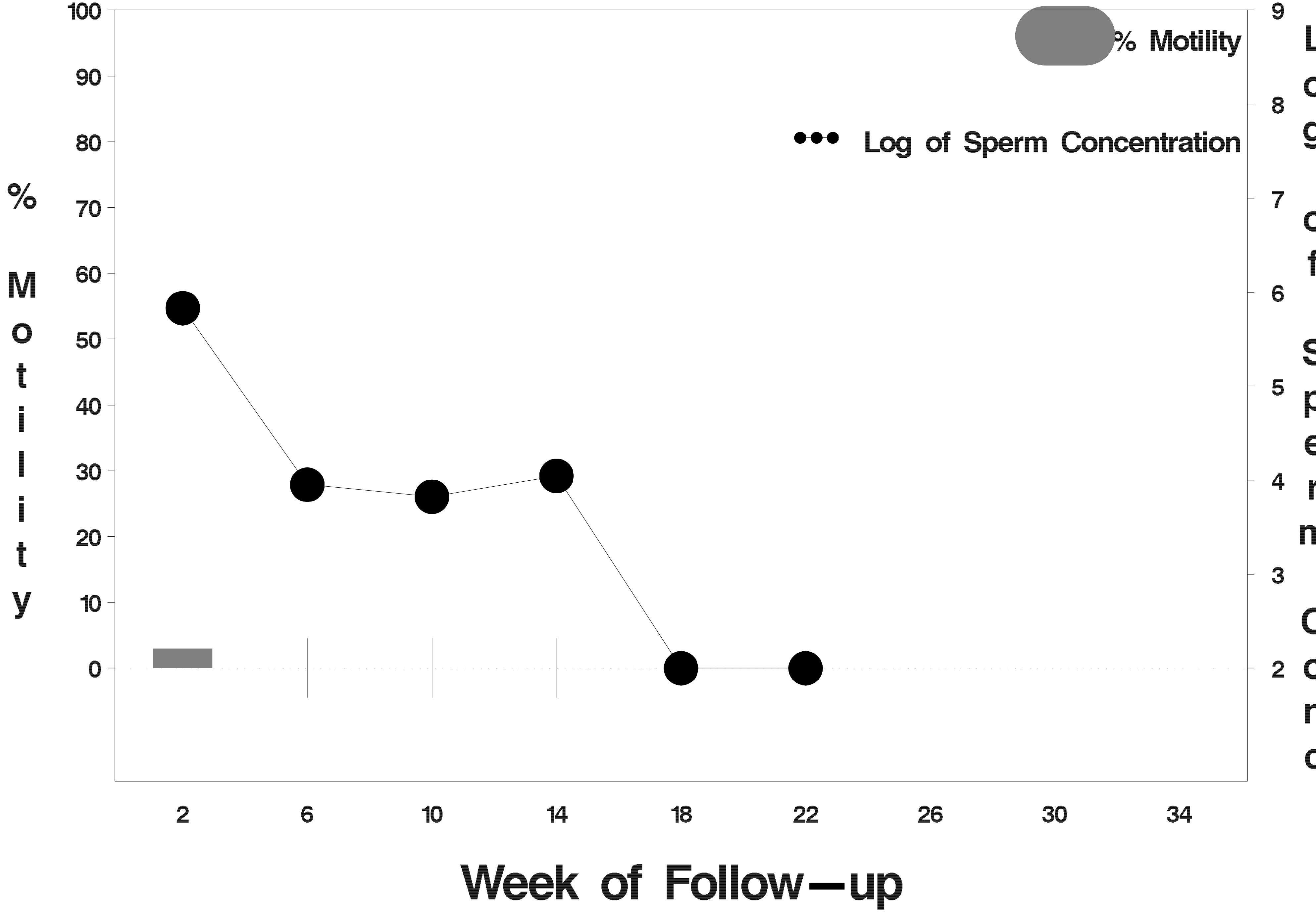

id= 324 outcome= Success

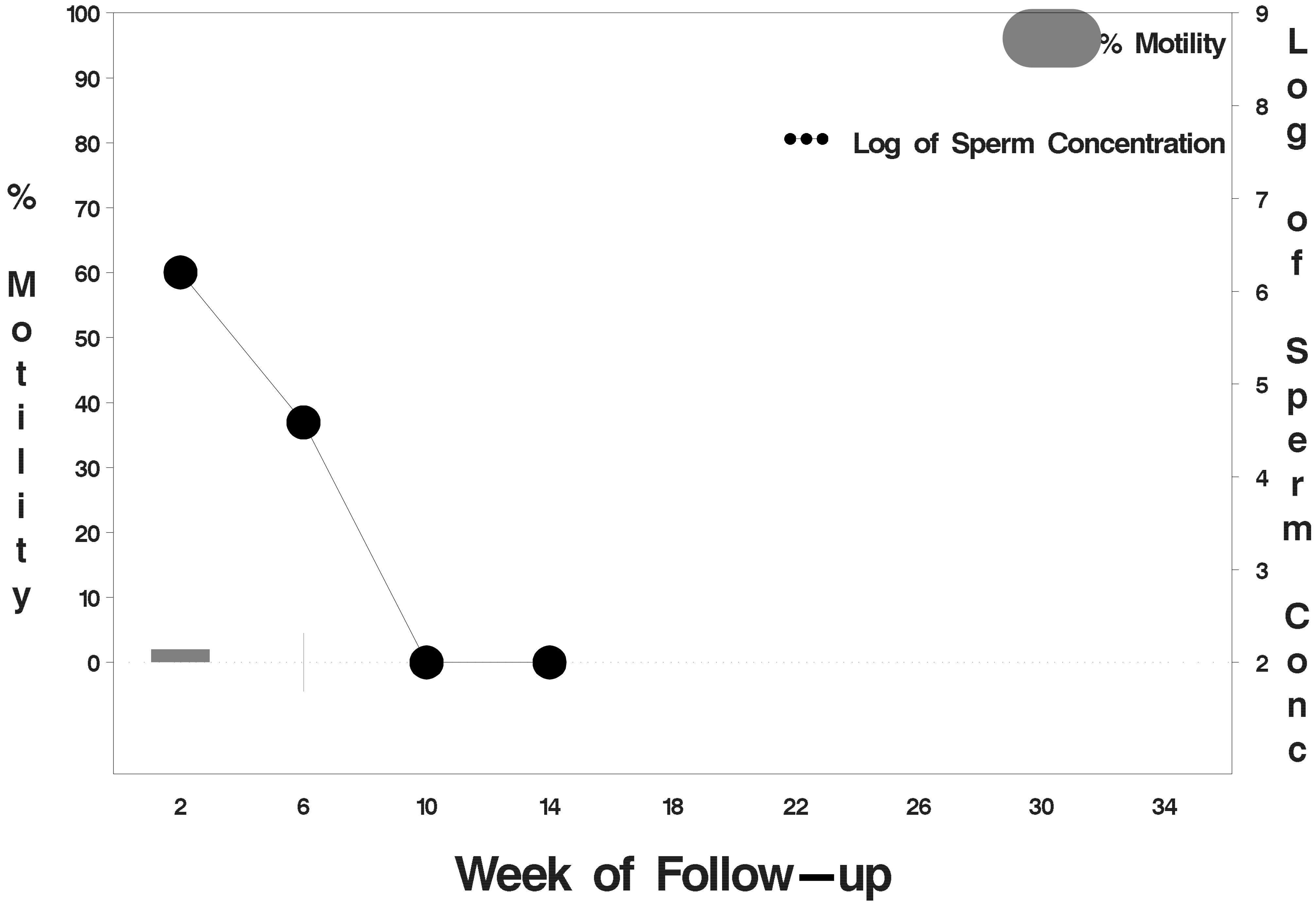

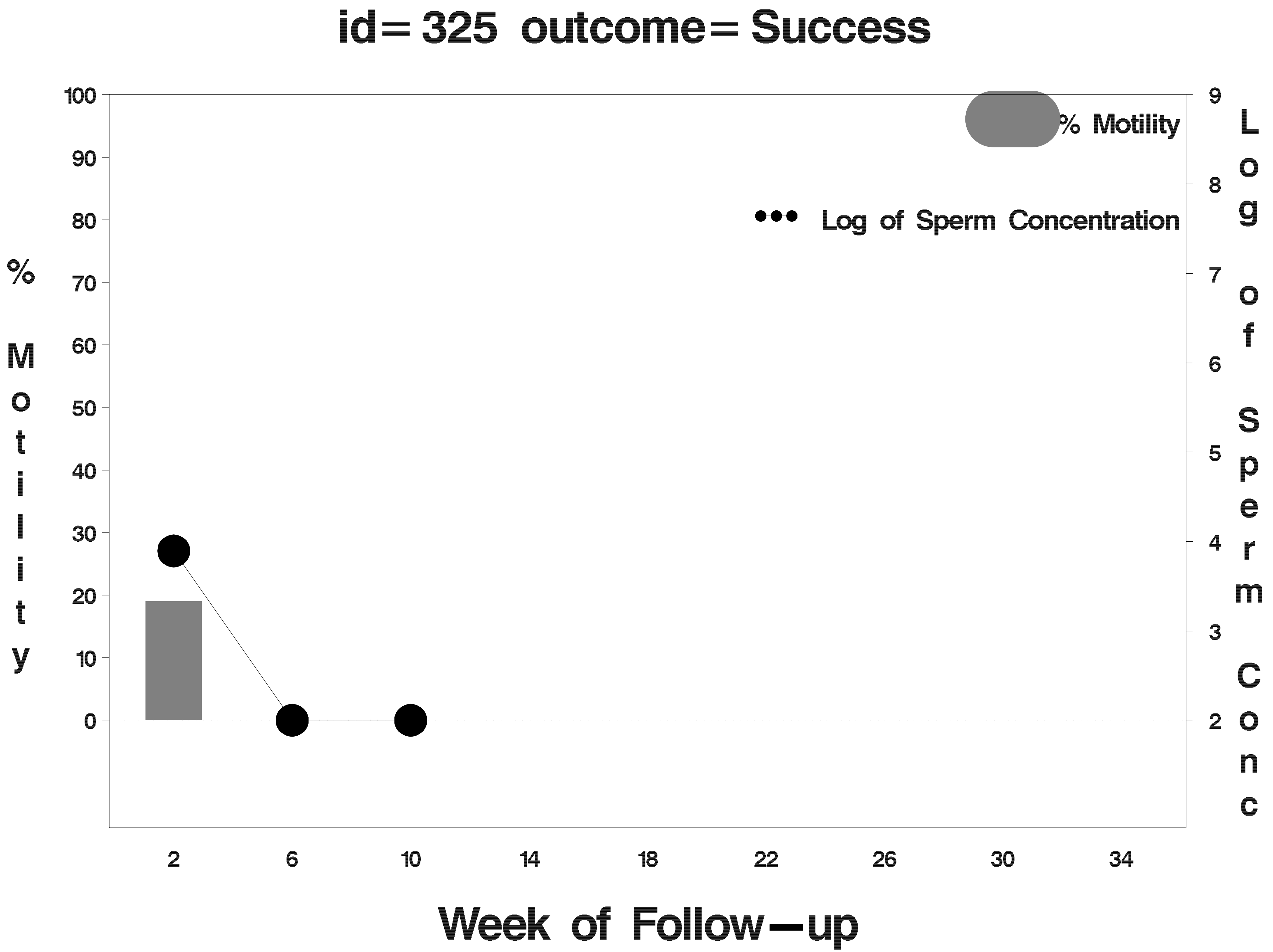
id= 325 outcome= Success
% Motility
Log of Sperm Concentration
100
90
80
70
60
50
40
30
20
10
0
9
8
7
6
5
4
3
2
2
6
10
14
18
22
26
30
34
Week of Follow—up
% Motility
Log of Sperm Conc

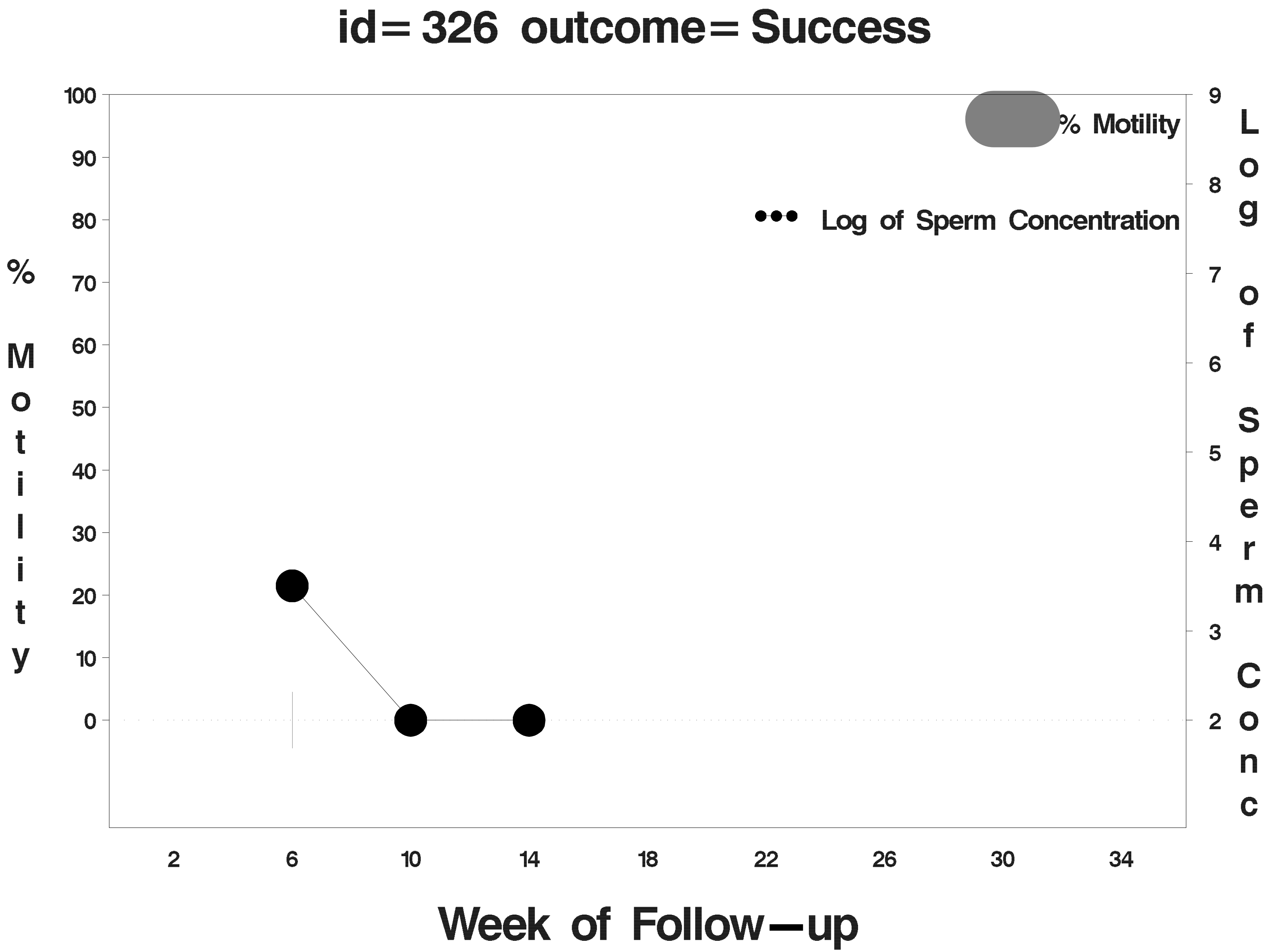

id= 326 outcome= Success
% Motility
Log of Sperm Concentration
100
90
80
70
60
50
40
30
20
10
0
9
8
7
6
5
4
3
2
2
6
10
14
18
22
26
30
34
Week of Follow−up
Log of Sperm Conc

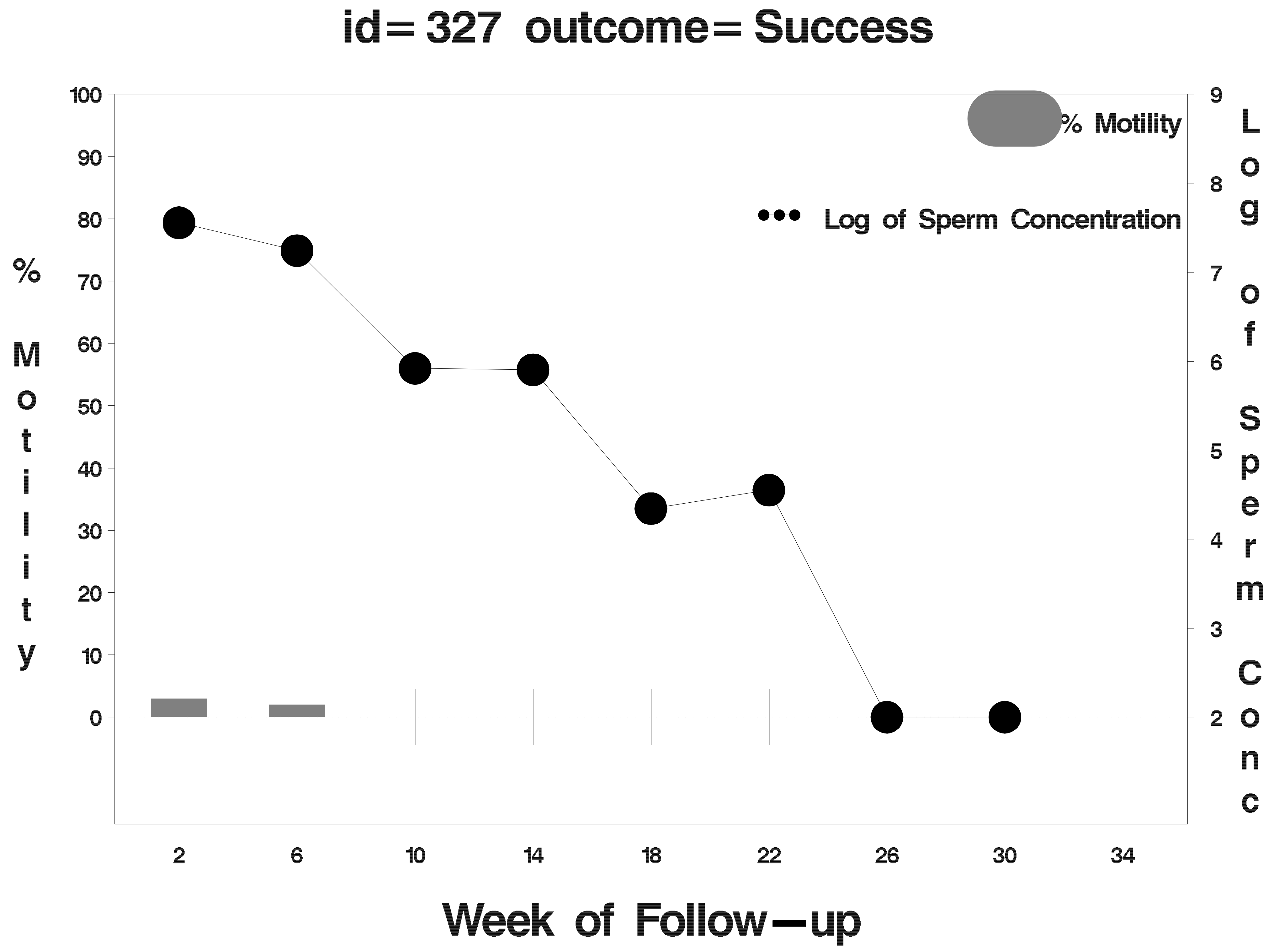

id= 327 outcome= Success
% Motility
Log of Sperm Concentration
% Motility
Log of Sperm Conc
Week of Follow−up
100
90
80
70
60
50
40
30
20
10
0
9
8
7
6
5
4
3
2
2
6
10
14
18
22
26
30
34

id= 328 outcome= Success

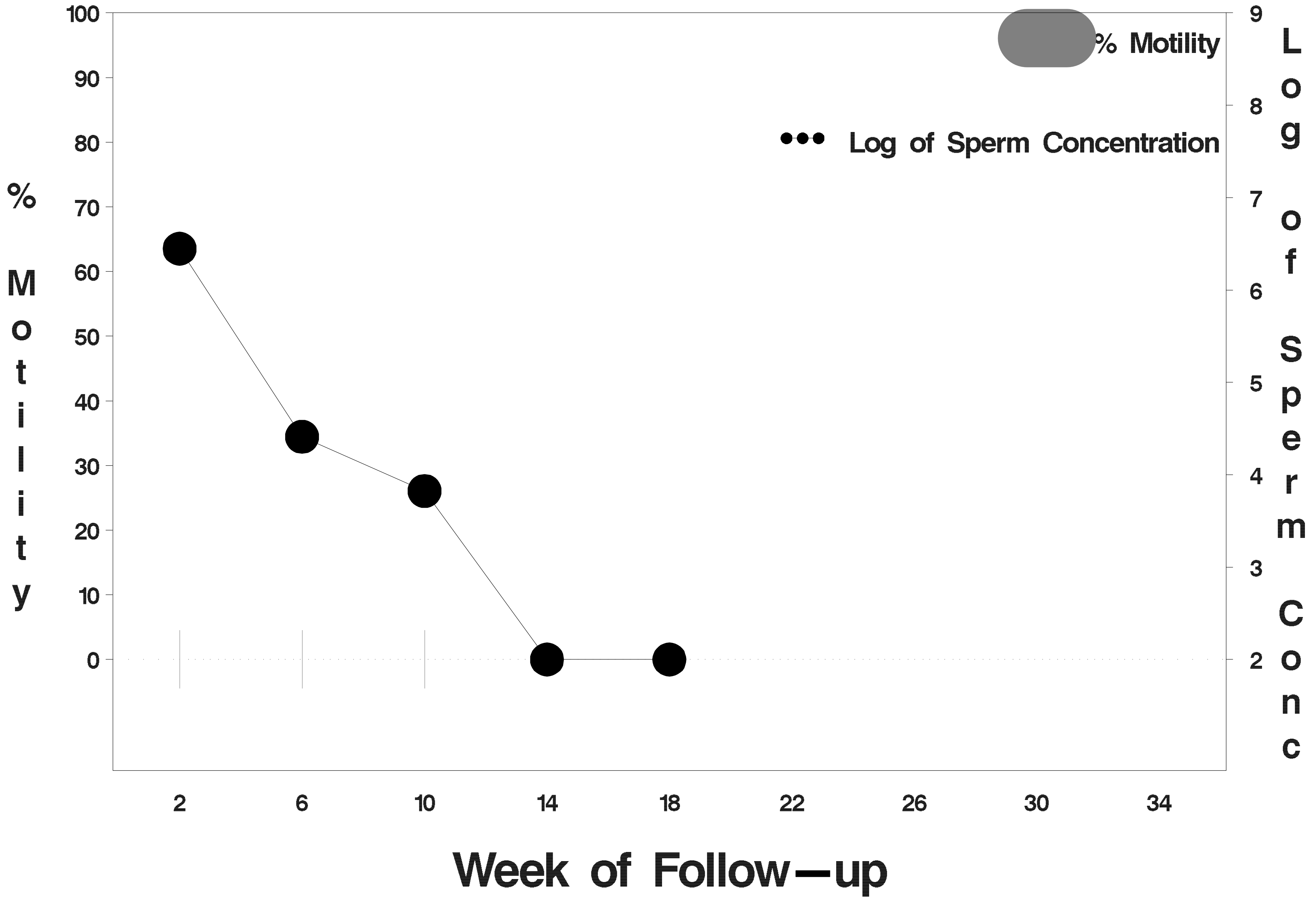

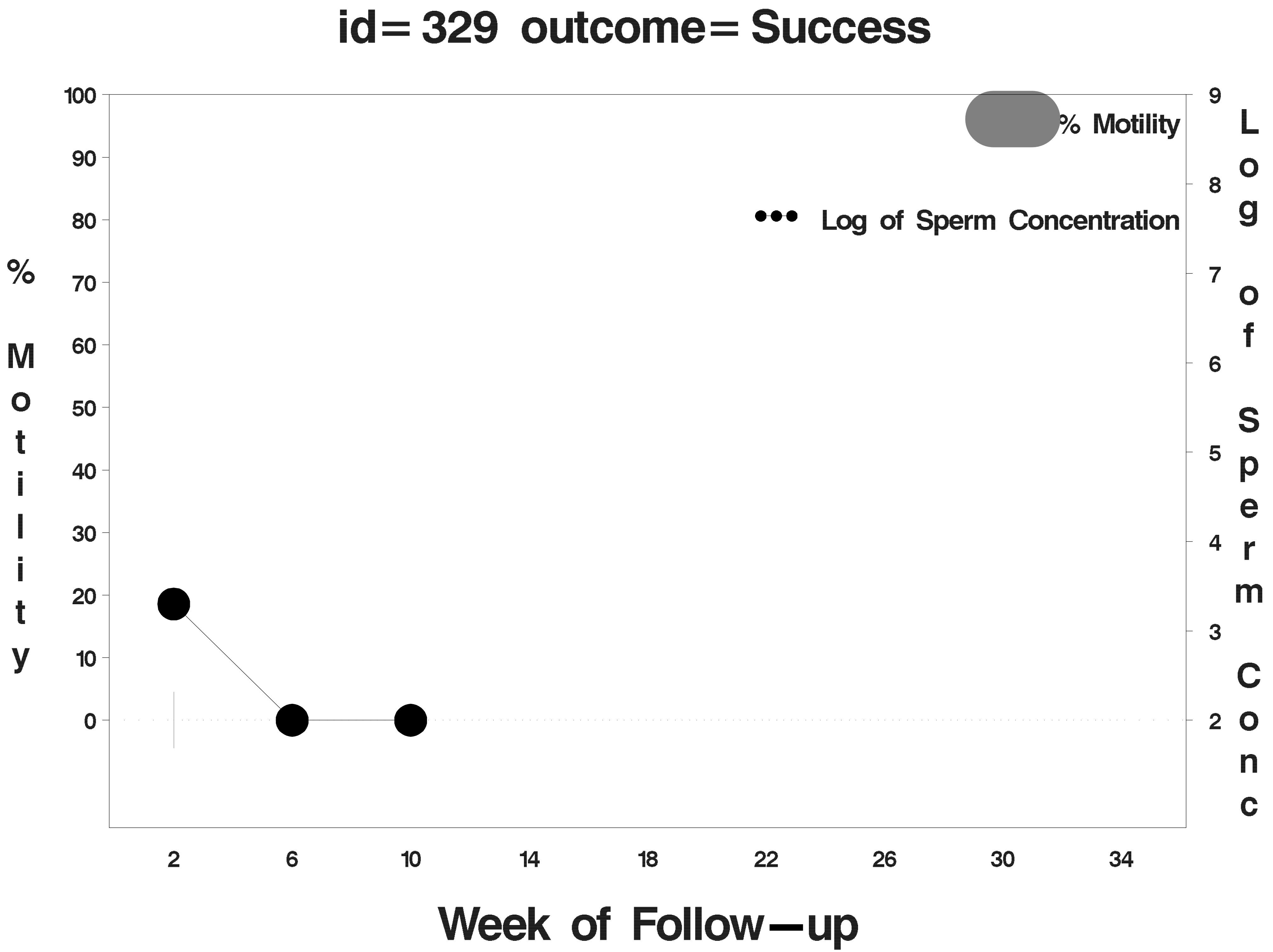

id= 329 outcome= Success
% Motility
Log of Sperm Concentration
100
90
80
70
60
50
40
30
20
10
0
9
8
7
6
5
4
3
2
2
6
10
14
18
22
26
30
34
Week of Follow-up
% Motility
Log of Sperm Conc

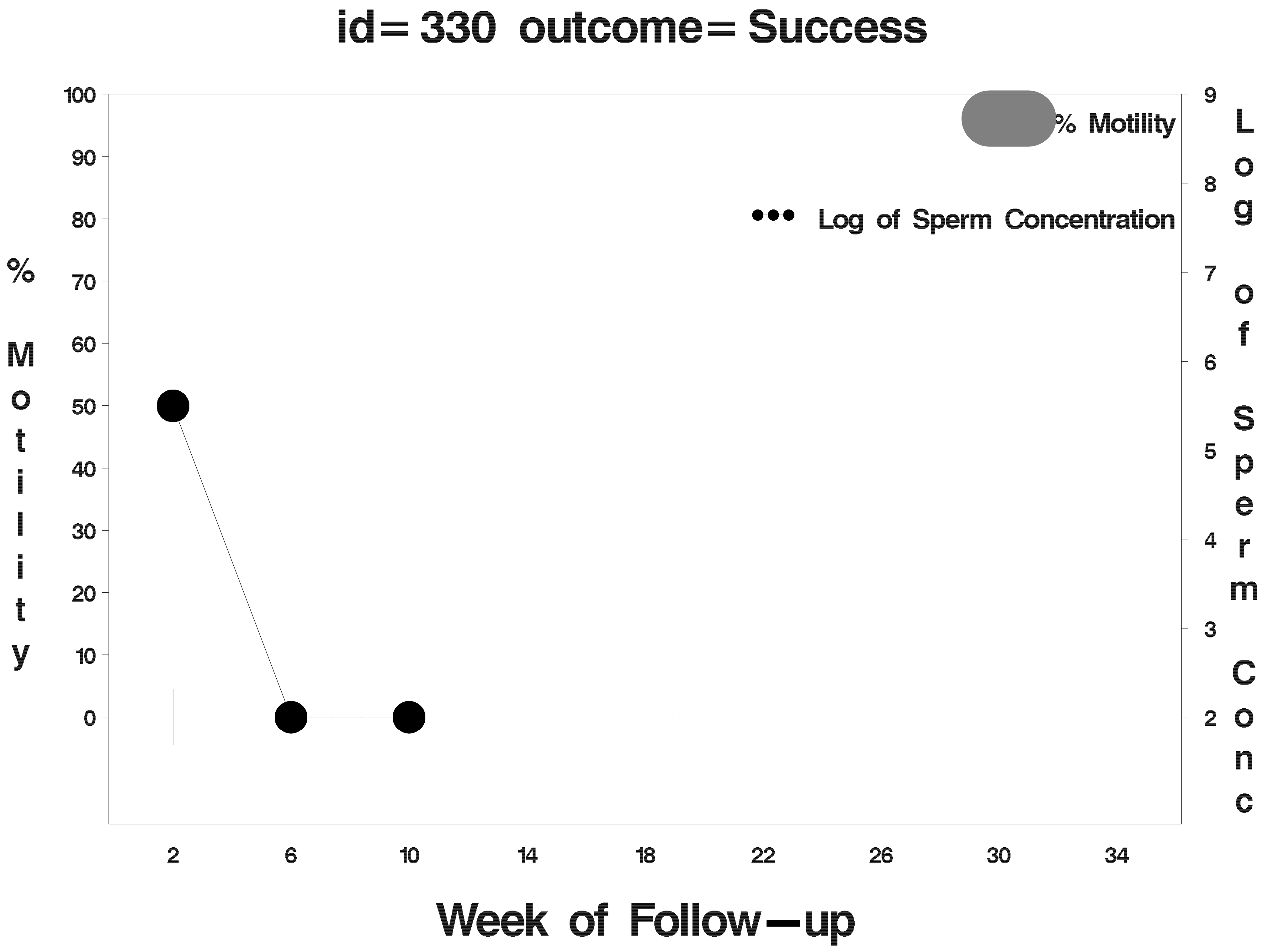

id= 330 outcome= Success
% Motility
Log of Sperm Concentration
100
90
80
70
60
50
40
30
20
10
0
9
8
7
6
5
4
3
2
2
6
10
14
18
22
26
30
34
Week of Follow−up
Log of Sperm Conc

id= 331 outcome= Success

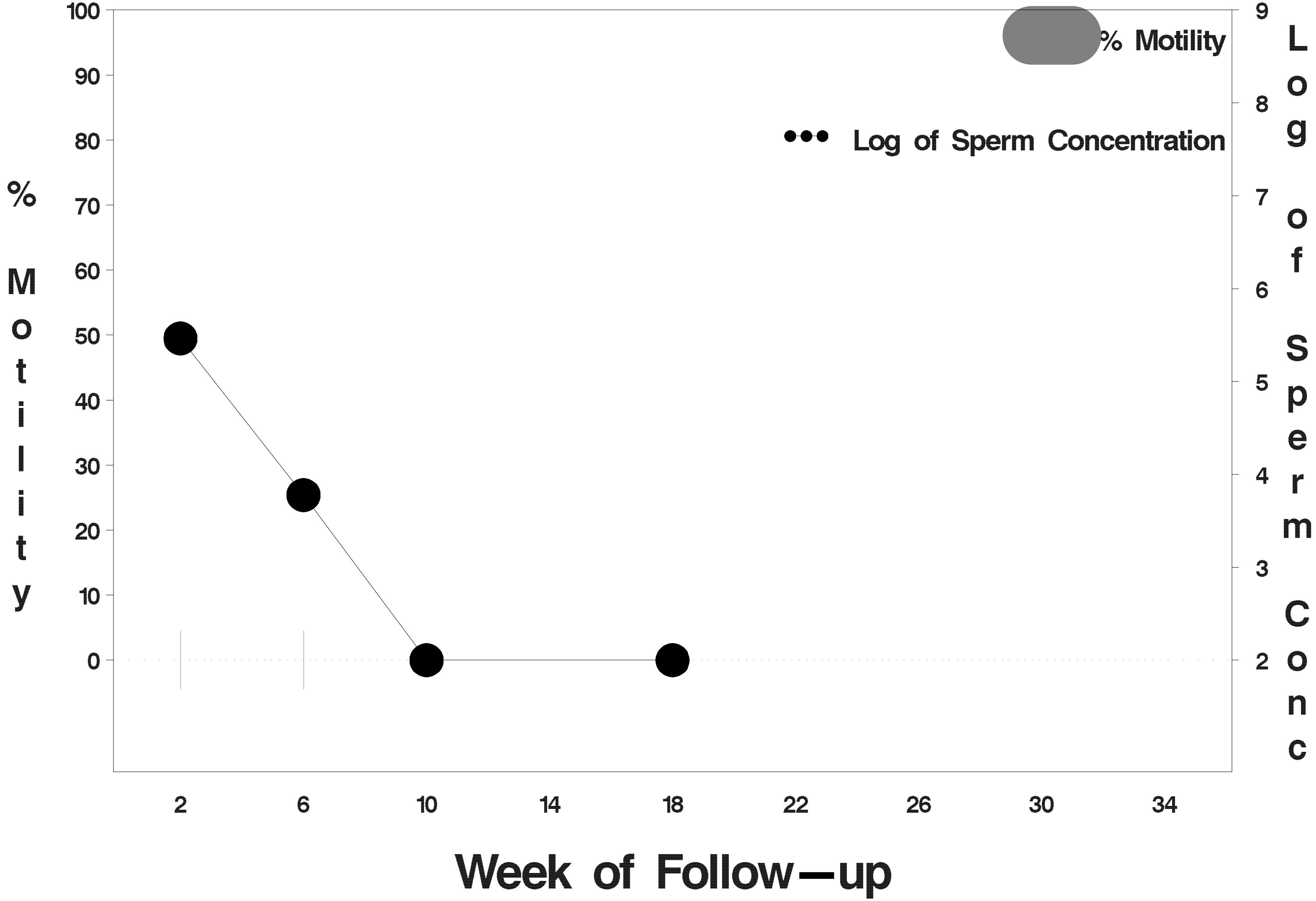

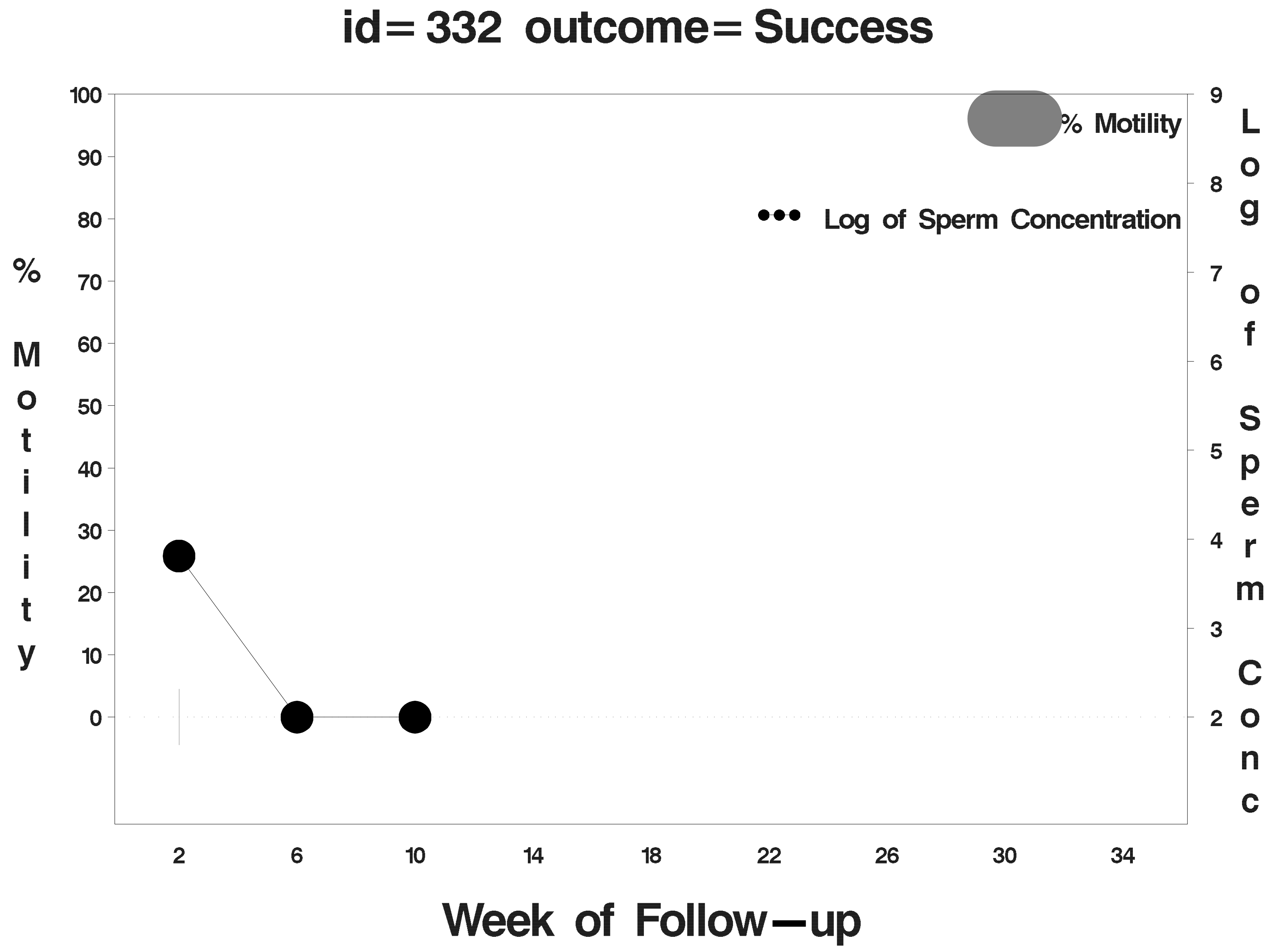

id= 332 outcome= Success
% Motility
Log of Sperm Concentration
100
90
80
70
60
50
40
30
20
10
0
9
8
7
6
5
4
3
2
2
6
10
14
18
22
26
30
34
Week of Follow−up
% Motility
Log of Sperm Conc

id= 333 outcome= Indeterminate

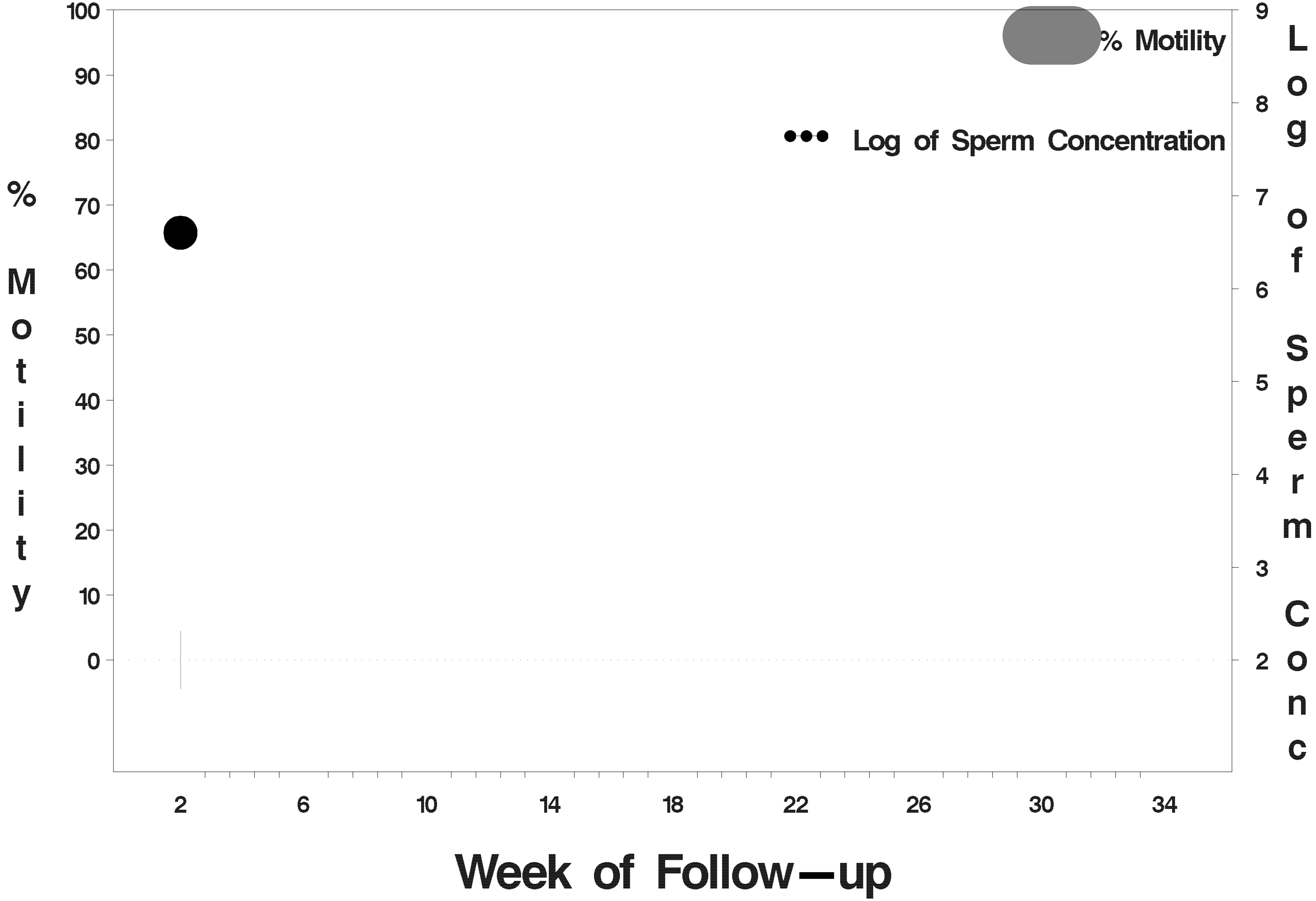

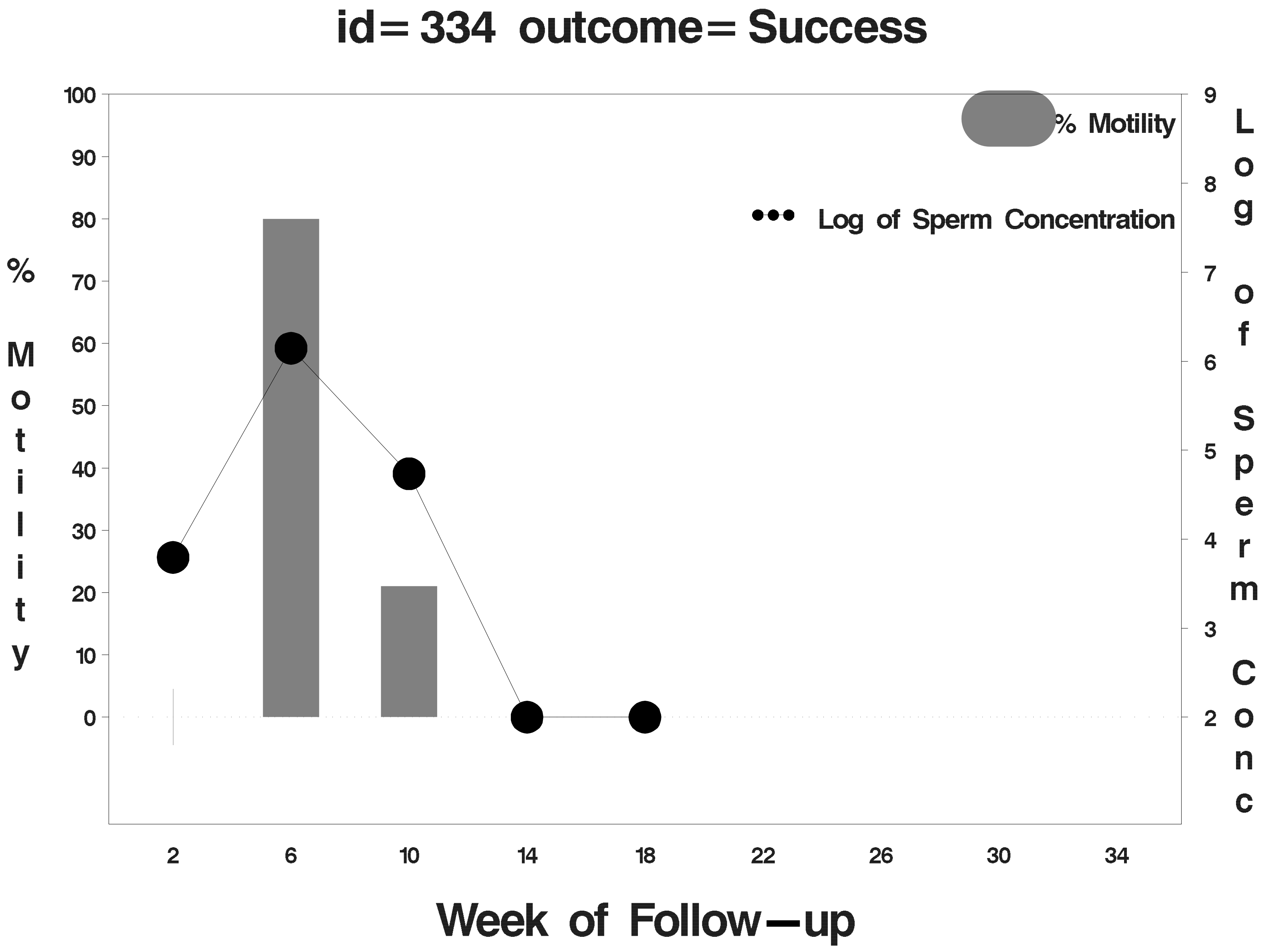

id= 334 outcome= Success
% Motility
Log of Sperm Concentration
100
90
80
70
60
50
40
30
20
10
0
9
8
7
6
5
4
3
2
% Motility
Log of Sperm Conc
2
6
10
14
18
22
26
30
34
Week of Follow-up

id= 335 outcome= Success

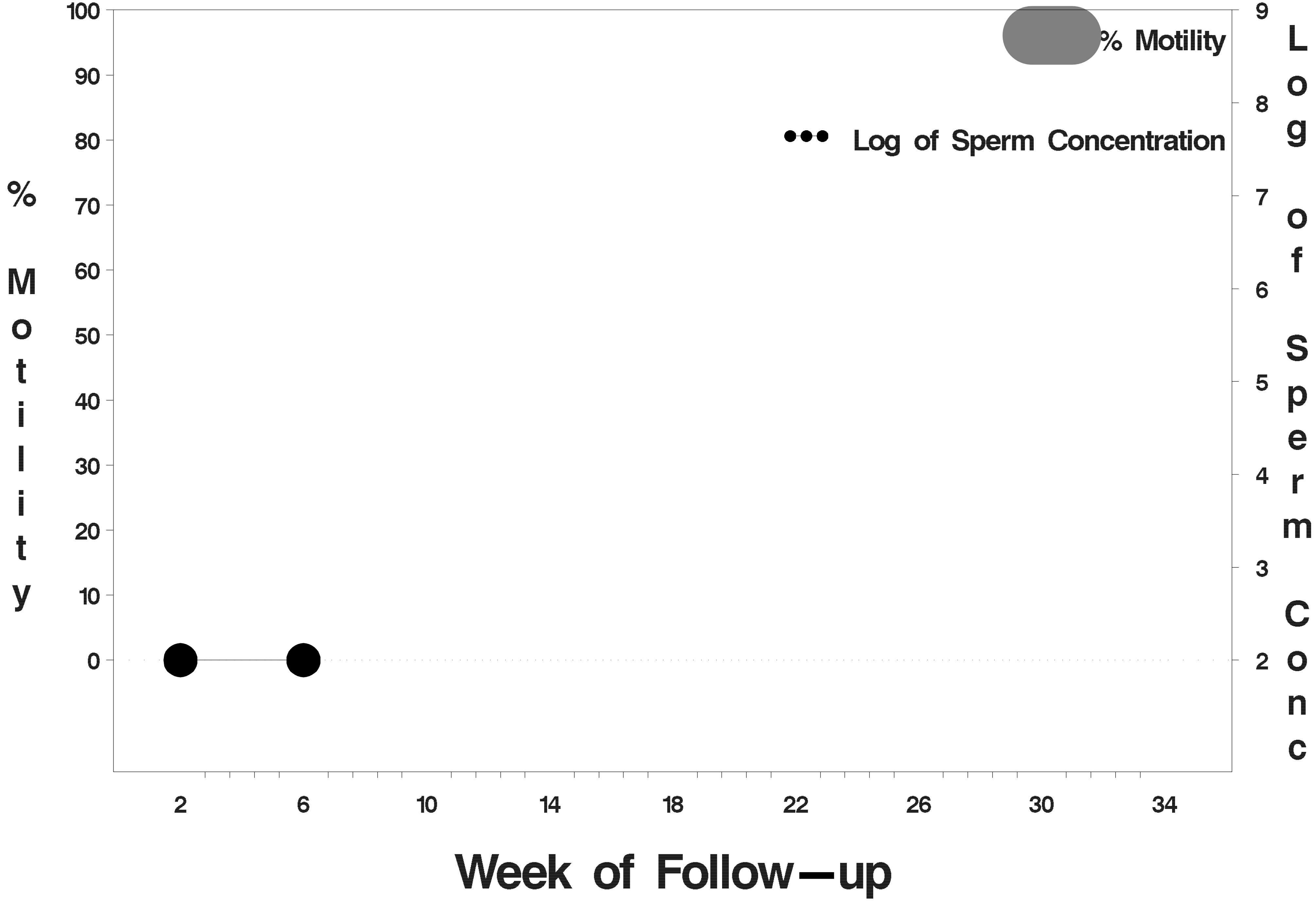

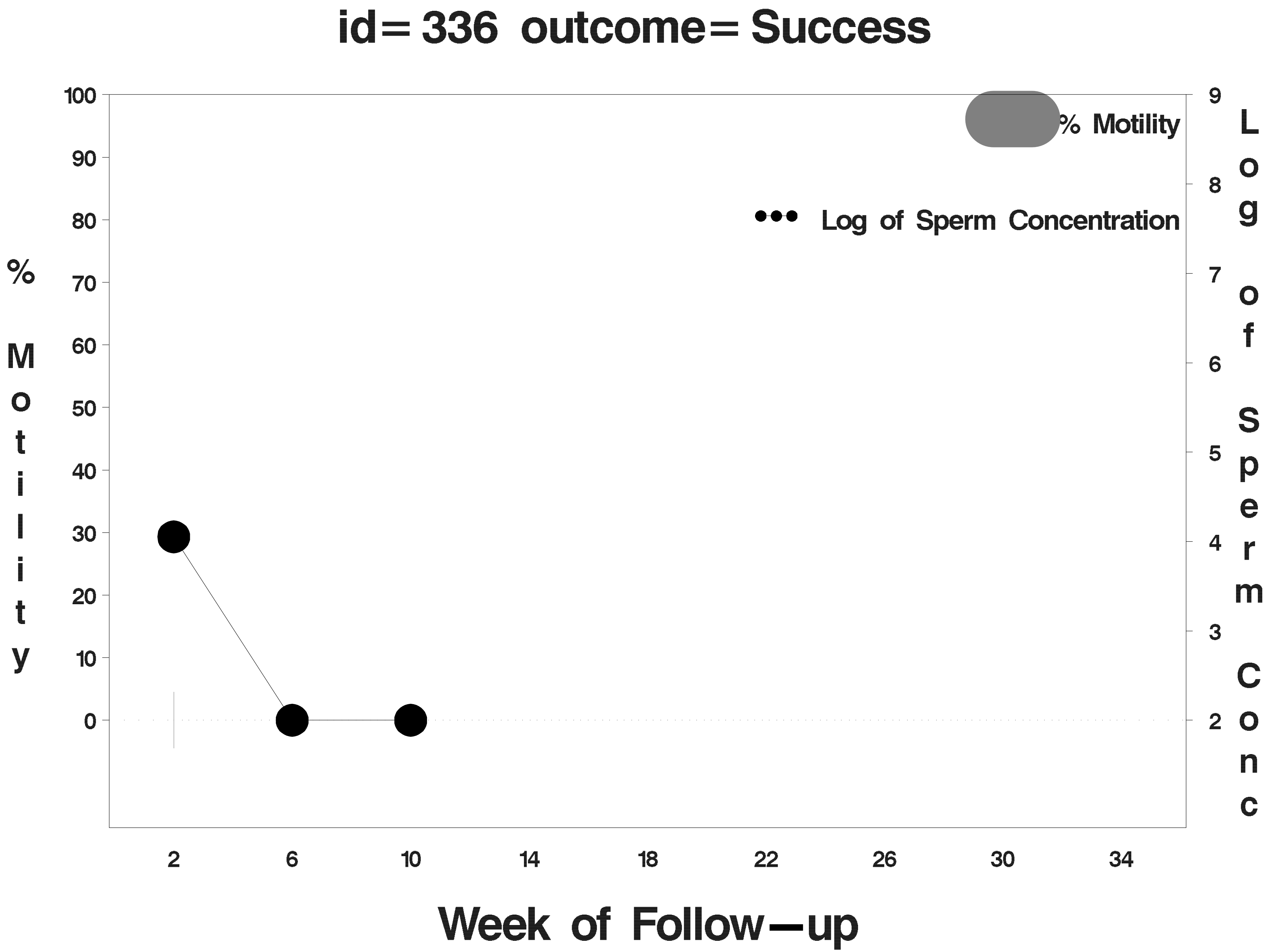

id= 336 outcome= Success
% Motility
Log of Sperm Concentration
100
90
80
70
60
50
40
30
20
10
0
9
8
7
6
5
4
3
2
% Motility
Log of Sperm Conc
2
6
10
14
18
22
26
30
34
Week of Follow—up

id= 337 outcome= Success

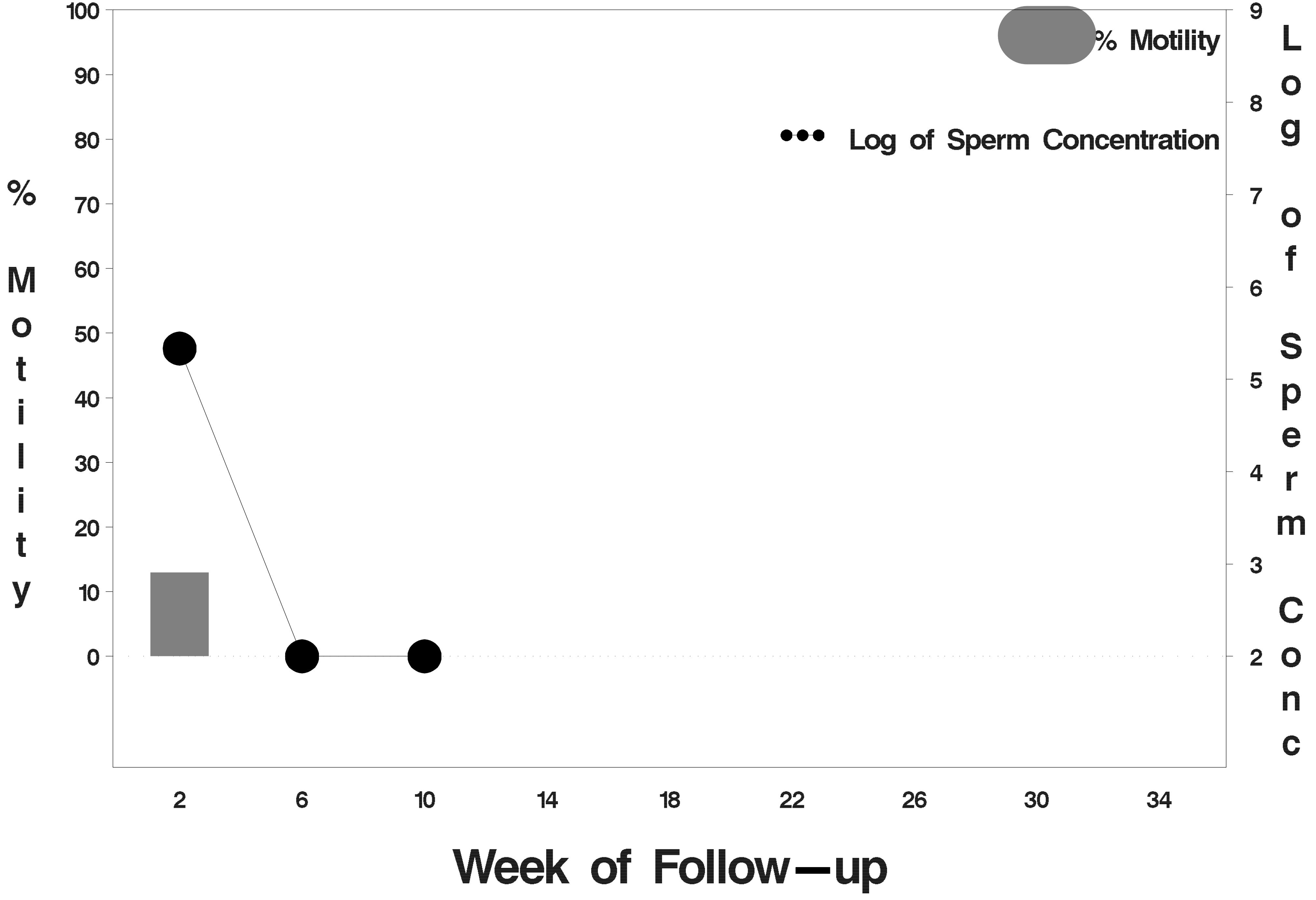

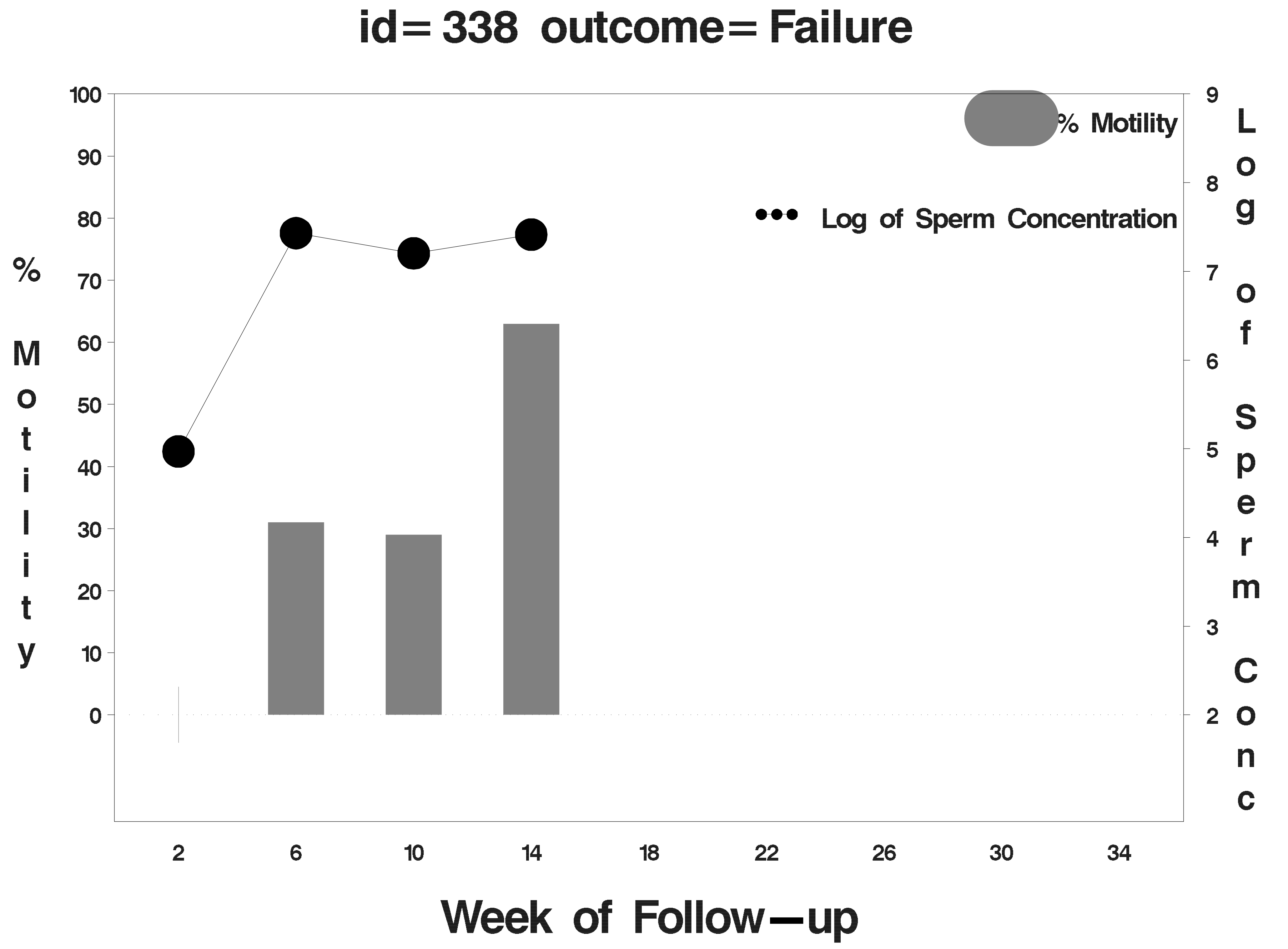

id= 338 outcome= Failure
% Motility
Log of Sperm Concentration
% Motility
Log of Sperm Conc
Week of Follow-up
100
90
80
70
60
50
40
30
20
10
0
9
8
7
6
5
4
3
2
2
6
10
14
18
22
26
30
34

id= 339 outcome= Success

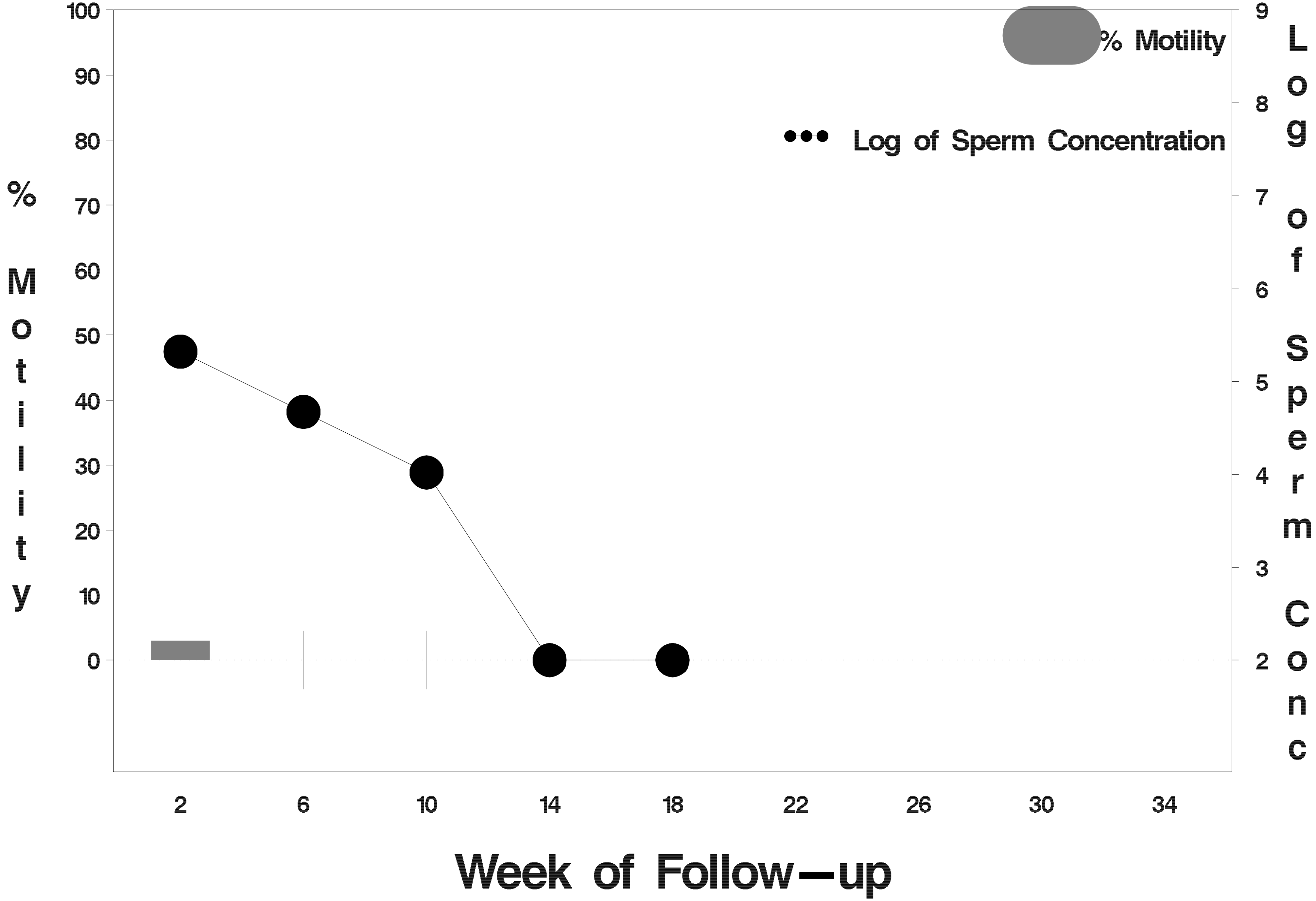

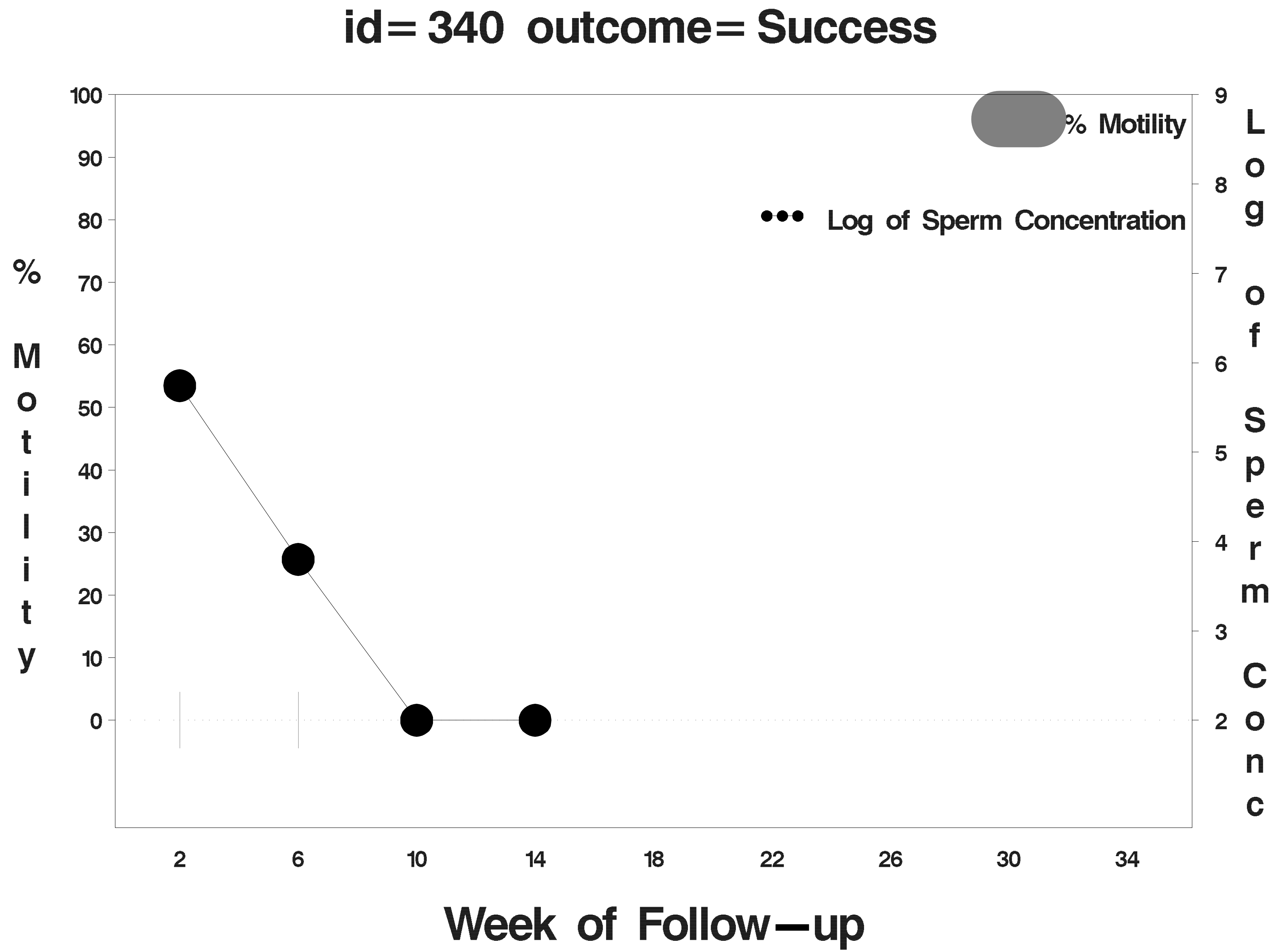

id= 340 outcome= Success
% Motility
Log of Sperm Concentration
% Motility
Log of Sperm Conc
Week of Follow−up
100
90
80
70
60
50
40
30
20
10
0
9
8
7
6
5
4
3
2
2
6
10
14
18
22
26
30
34

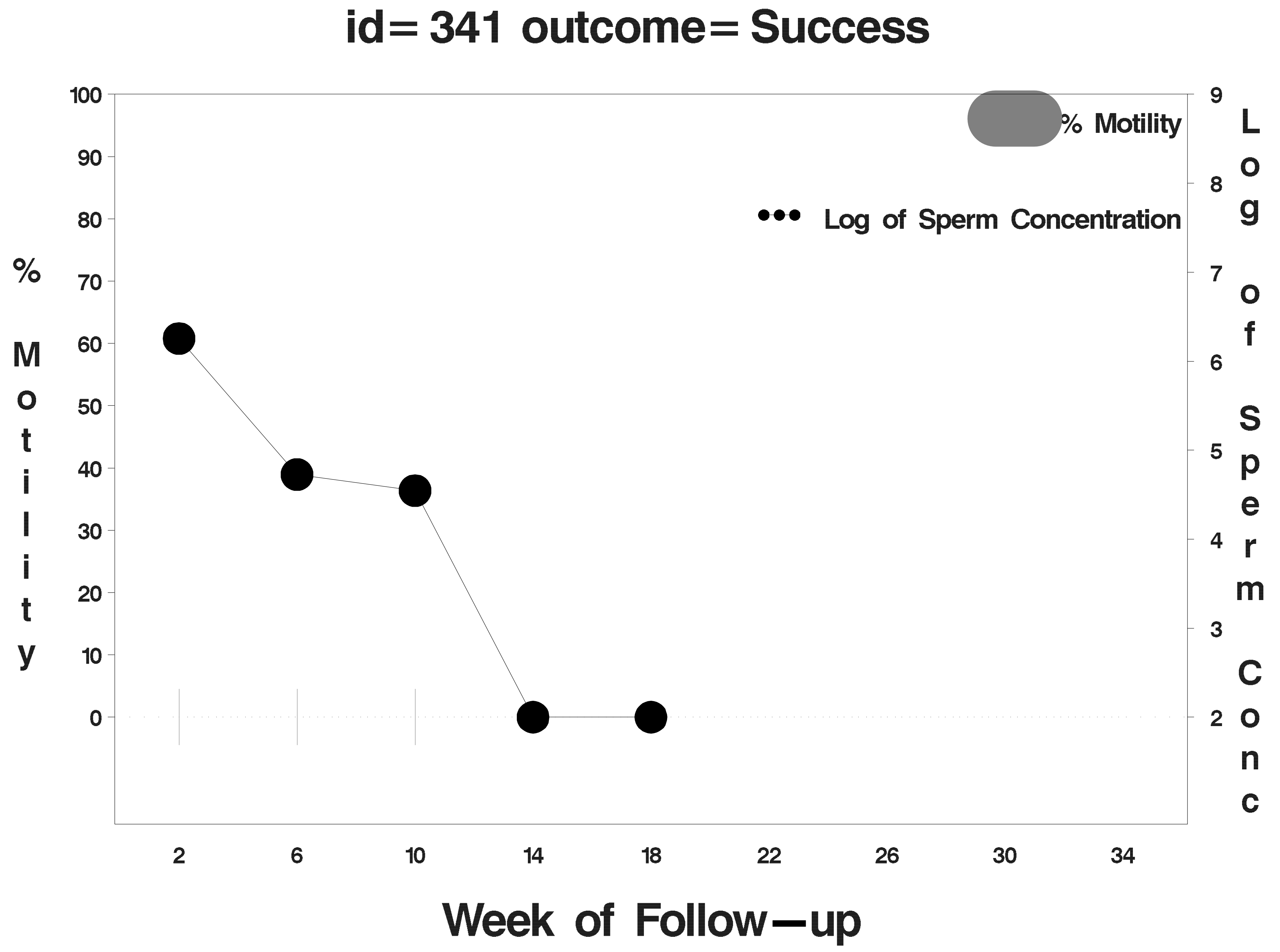
id= 341 outcome= Success
% Motility
Log of Sperm Concentration
% Motility
Log of Sperm Conc
Week of Follow−up
100
90
80
70
60
50
40
30
20
10
0
9
8
7
6
5
4
3
2
2
6
10
14
18
22
26
30
34

id= 342 outcome= Success

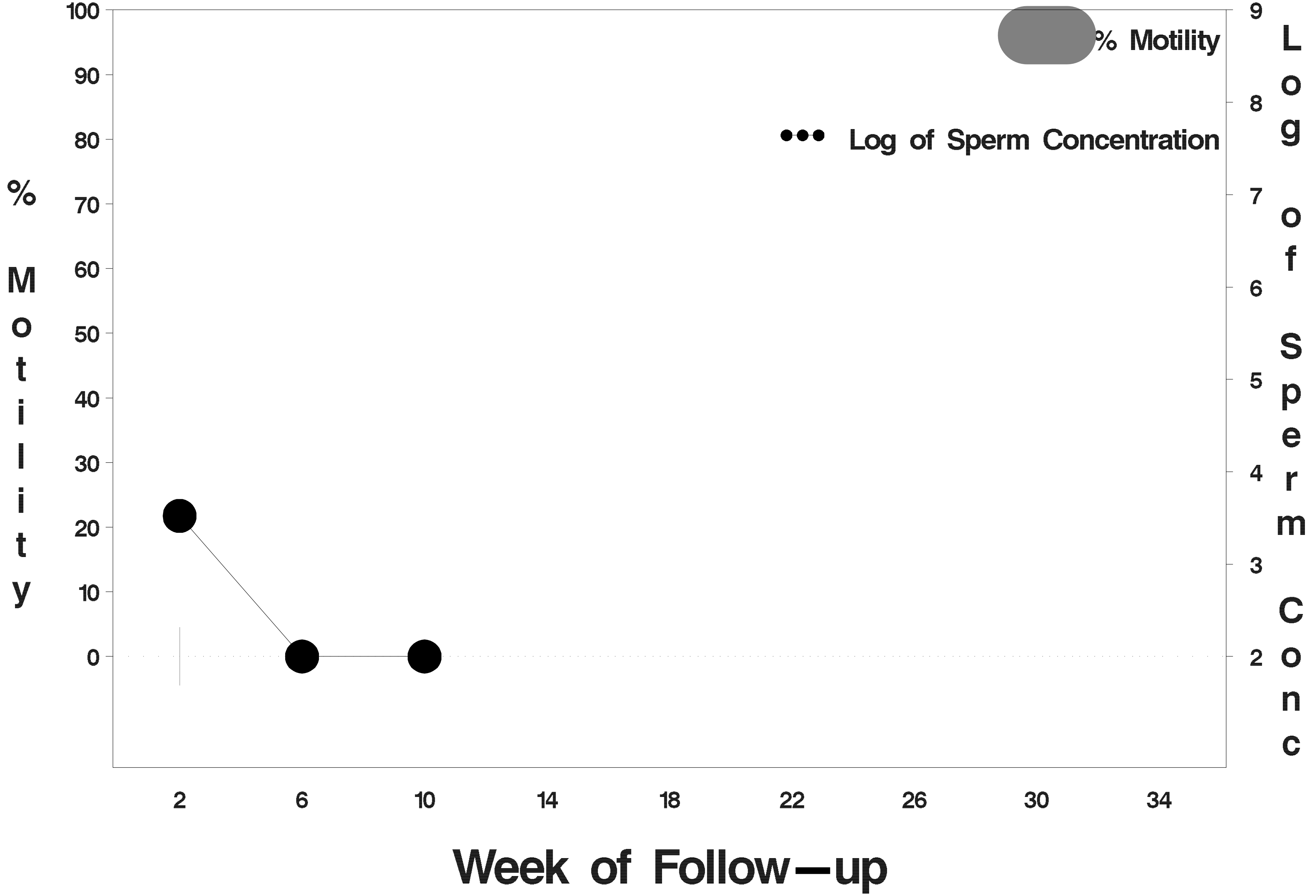

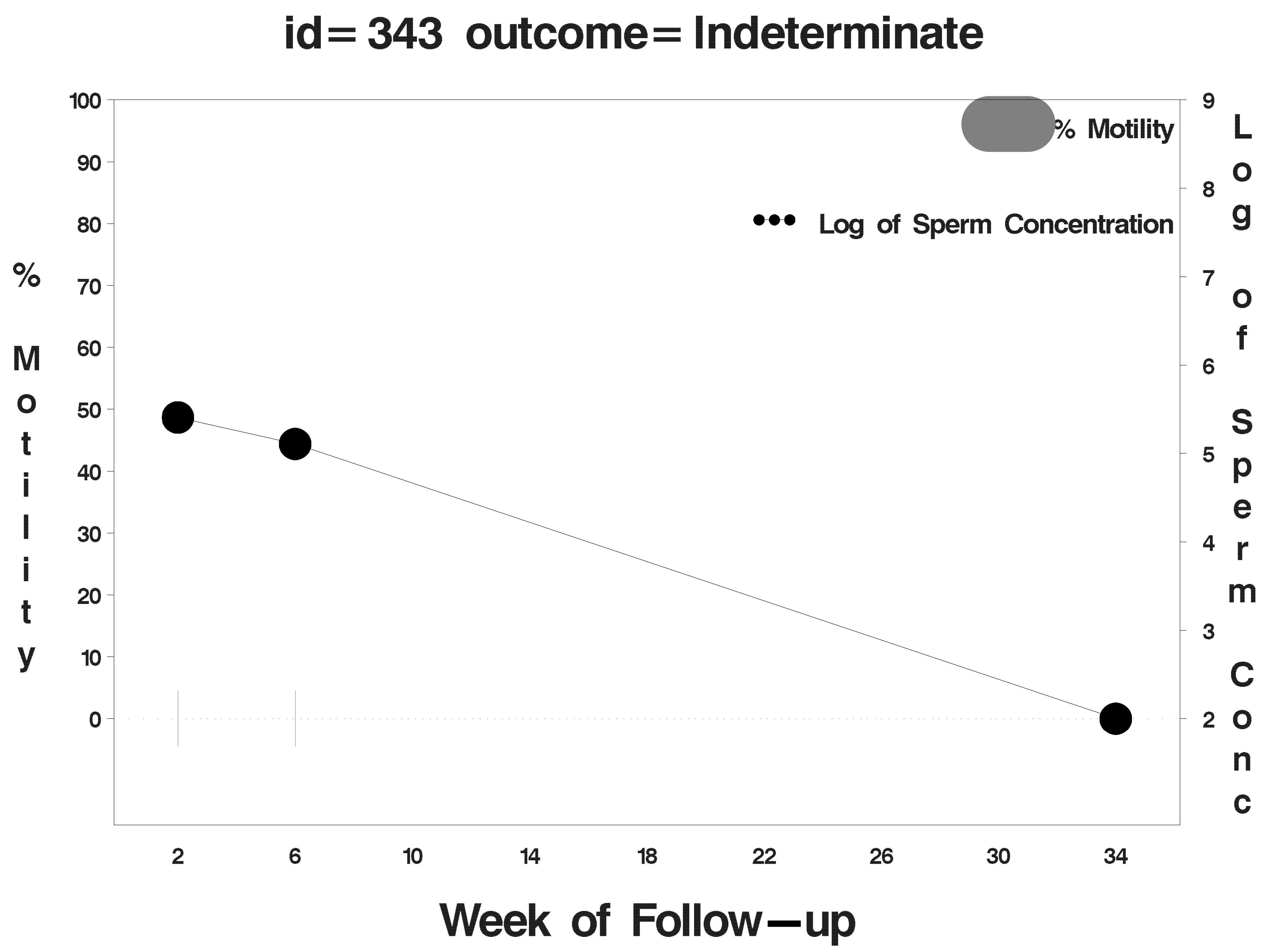

id= 343 outcome= Indeterminate
% Motility
Log of Sperm Concentration
100
90
80
70
60
50
40
30
20
10
0
9
8
7
6
5
4
3
2
2
6
10
14
18
22
26
30
34
Week of Follow—up
Log of Sperm Conc

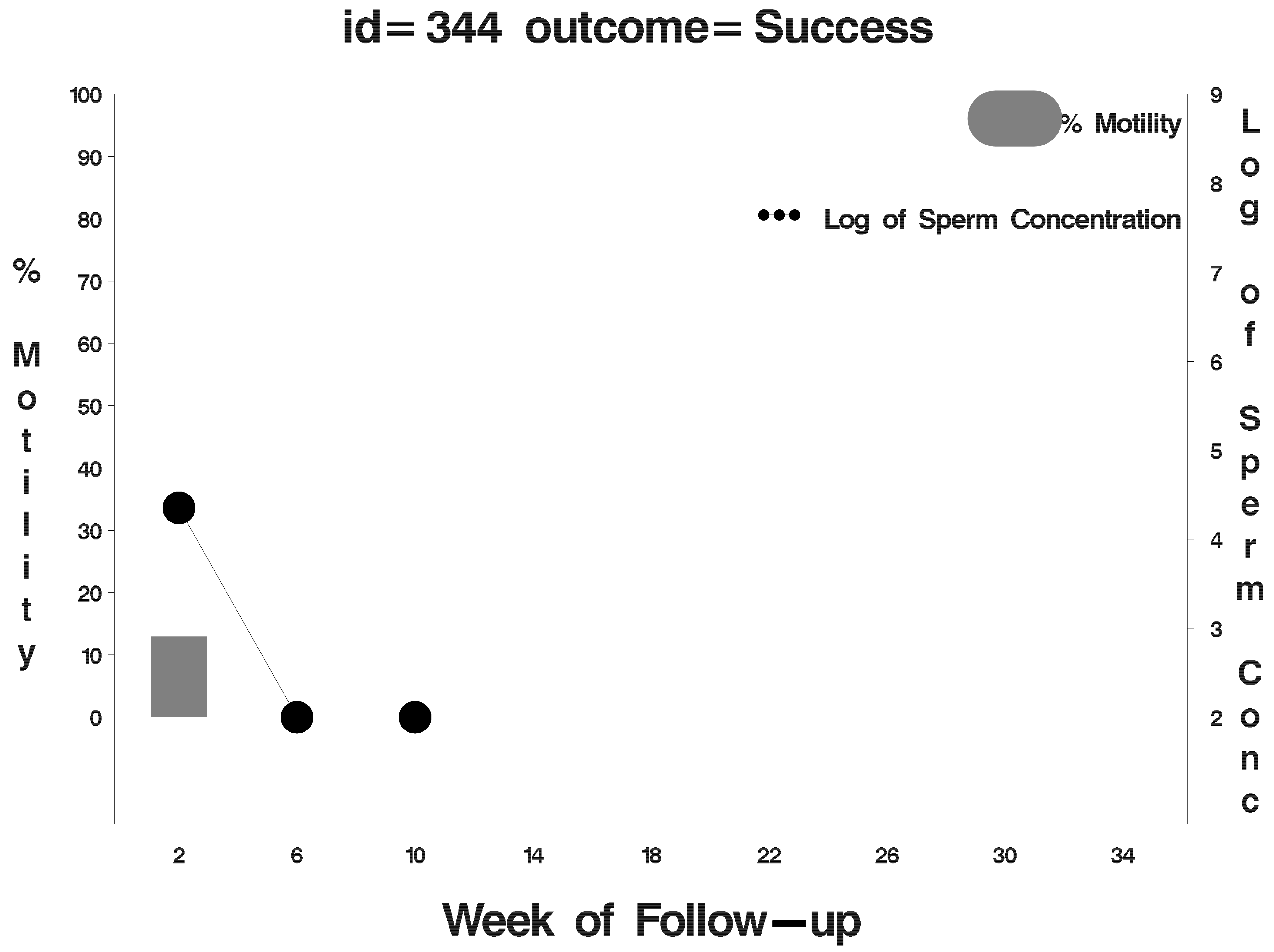
id= 344 outcome= Success
% Motility
Log of Sperm Concentration
100
90
80
70
60
50
40
30
20
10
0
9
8
7
6
5
4
3
2
2
6
10
14
18
22
26
30
34
Week of Follow—up
% Motility
Log of Sperm Conc

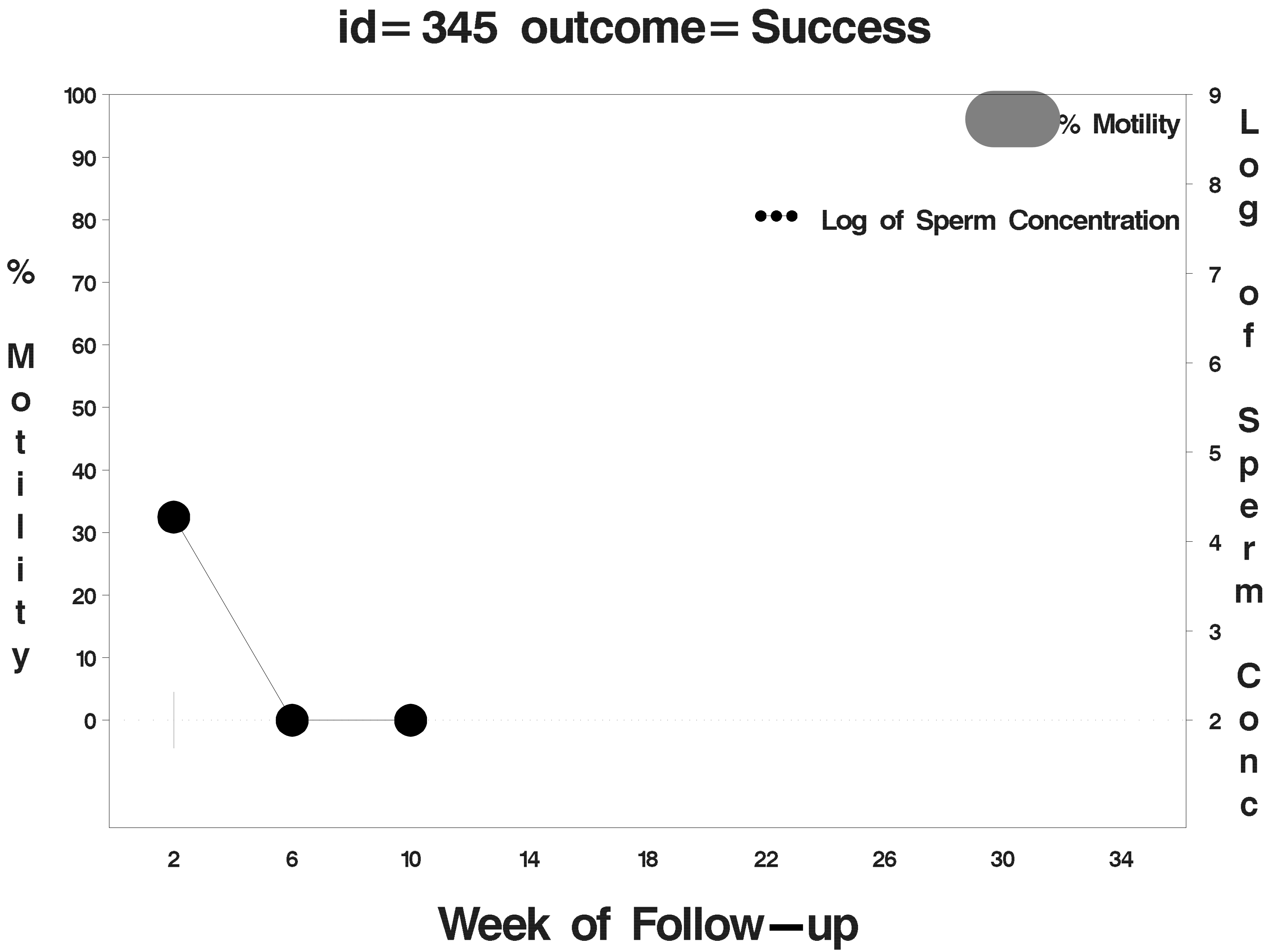
id= 345 outcome= Success
% Motility
Log of Sperm Concentration
100
90
80
70
60
50
40
30
20
10
0
9
8
7
6
5
4
3
2
2
6
10
14
18
22
26
30
34
Week of Follow-up
Log of Sperm Conc

id= 346 outcome= Indeterminate

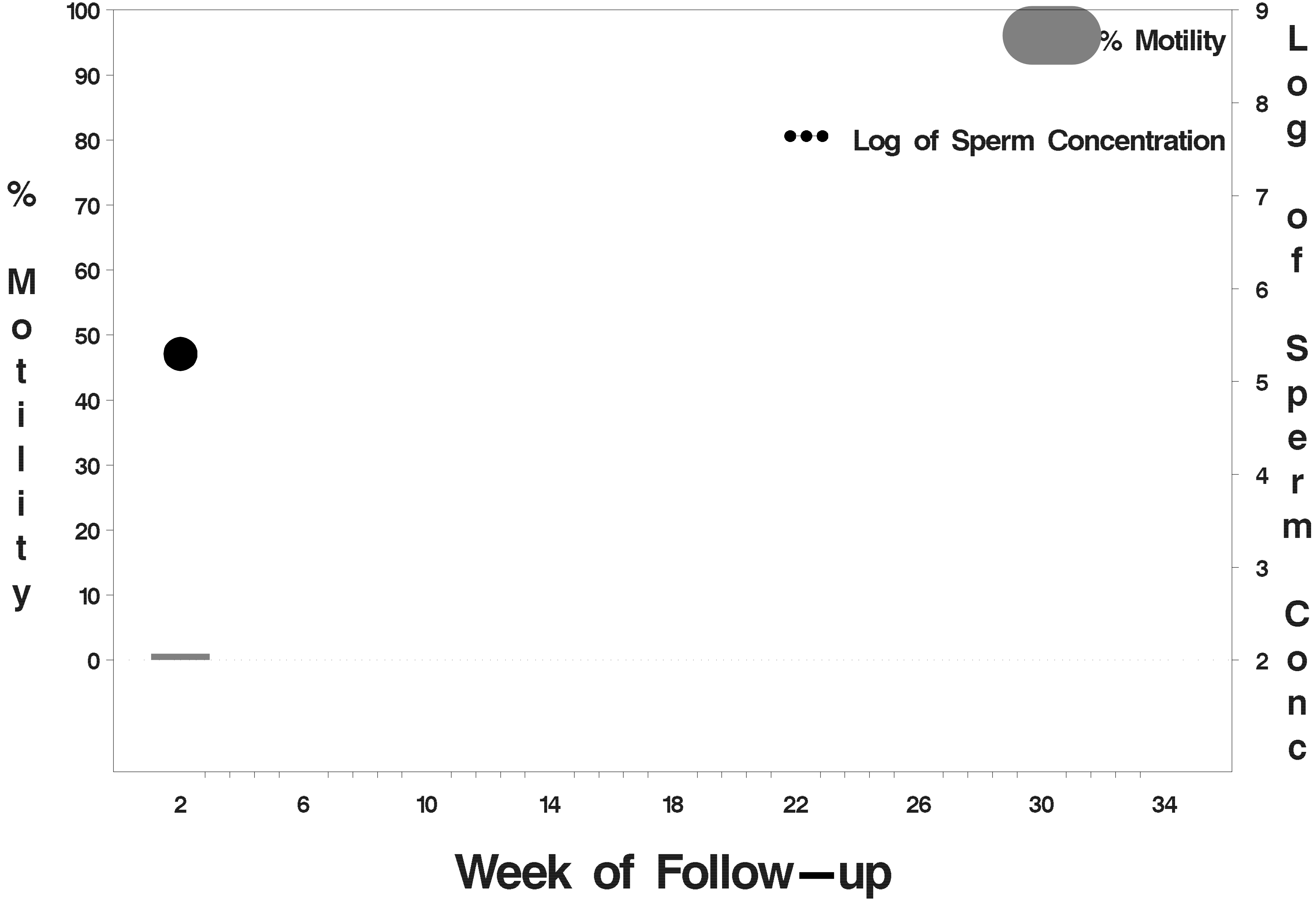

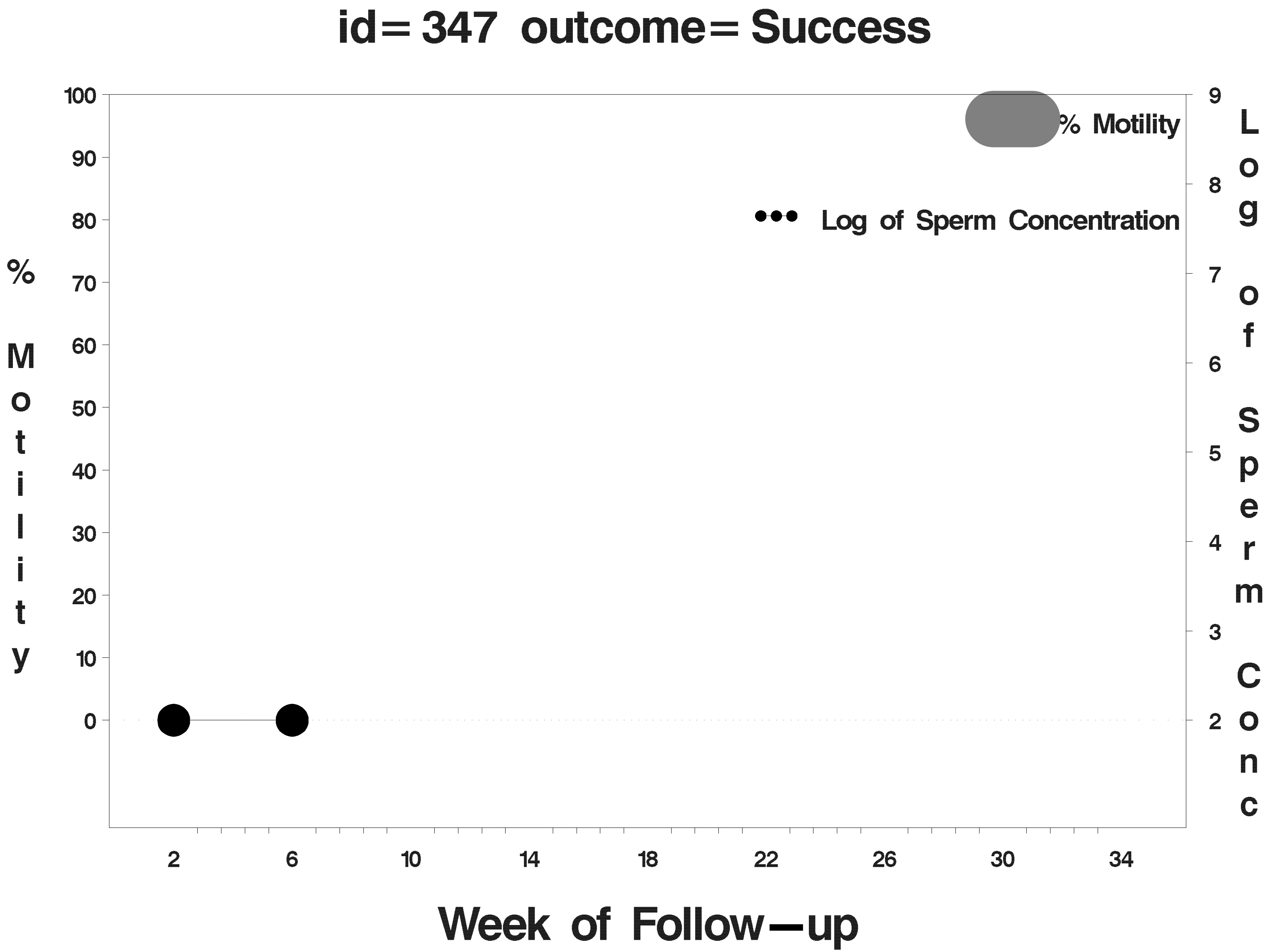

id= 347 outcome= Success
% Motility
Log of Sperm Concentration
% Motility
Log of Sperm Conc
Week of Follow—up
100
90
80
70
60
50
40
30
20
10
0
9
8
7
6
5
4
3
2
2
6
10
14
18
22
26
30
34

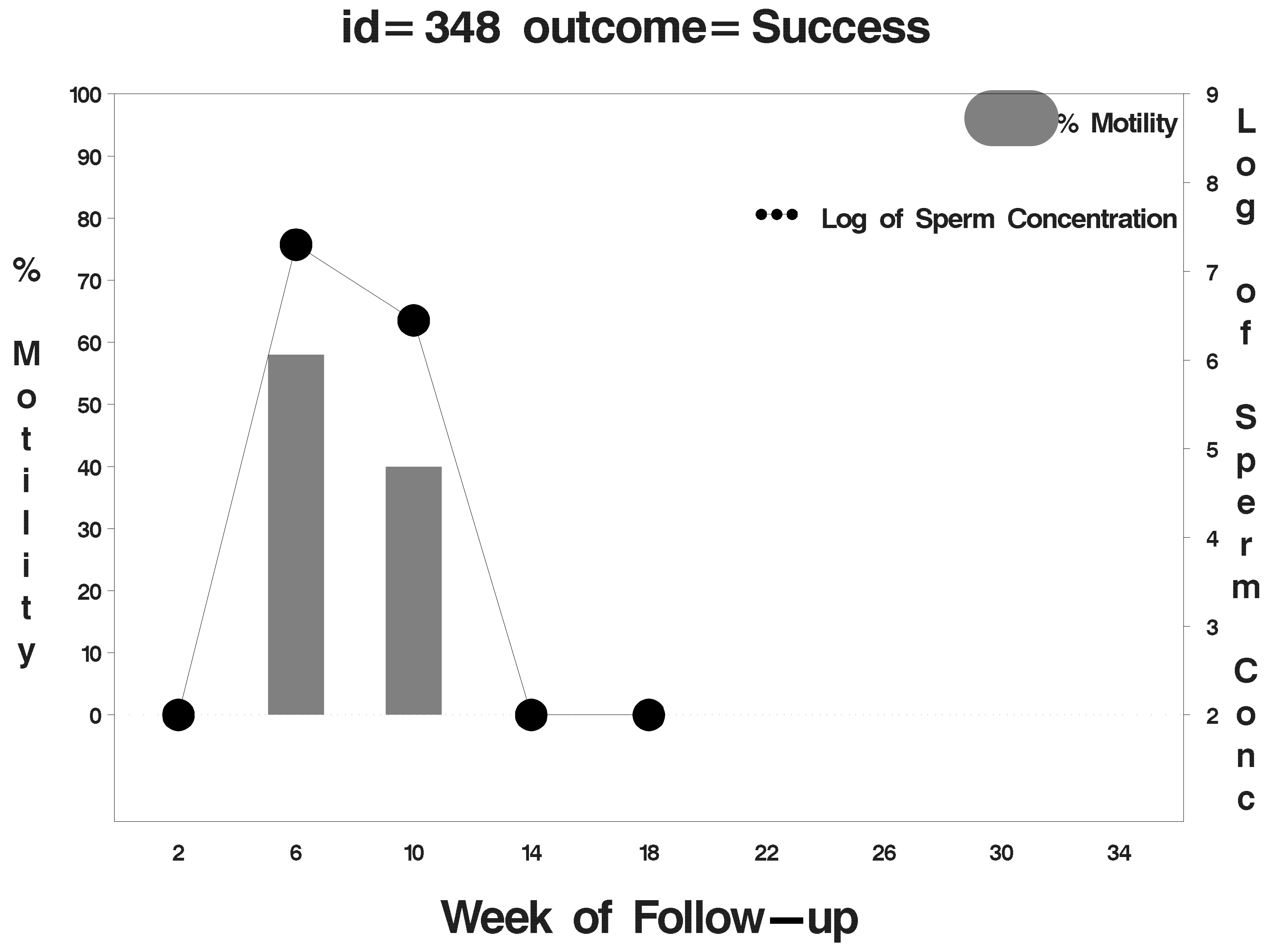

id= 348 outcome= Success
% Motility
Log of Sperm Concentration
100
90
80
70
60
50
40
30
20
10
0
9
8
7
6
5
4
3
2
2
6
10
14
18
22
26
30
34
Week of Follow−up
Log of Sperm Conc

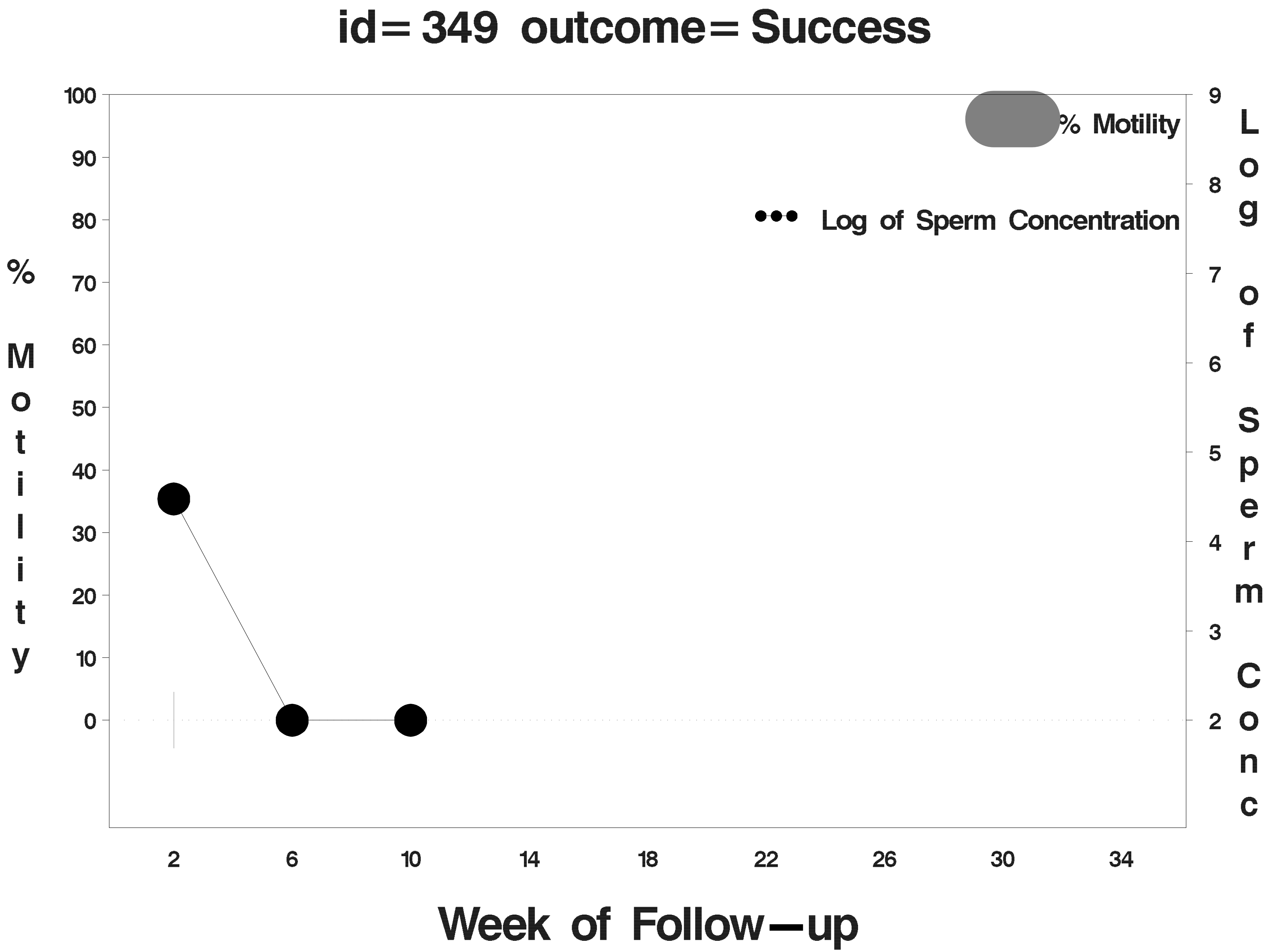
id= 349 outcome= Success
% Motility
Log of Sperm Concentration
100
90
80
70
60
50
40
30
20
10
0
9
8
7
6
5
4
3
2
2
6
10
14
18
22
26
30
34
% Motility
Log of Sperm Conc
Week of Follow—up

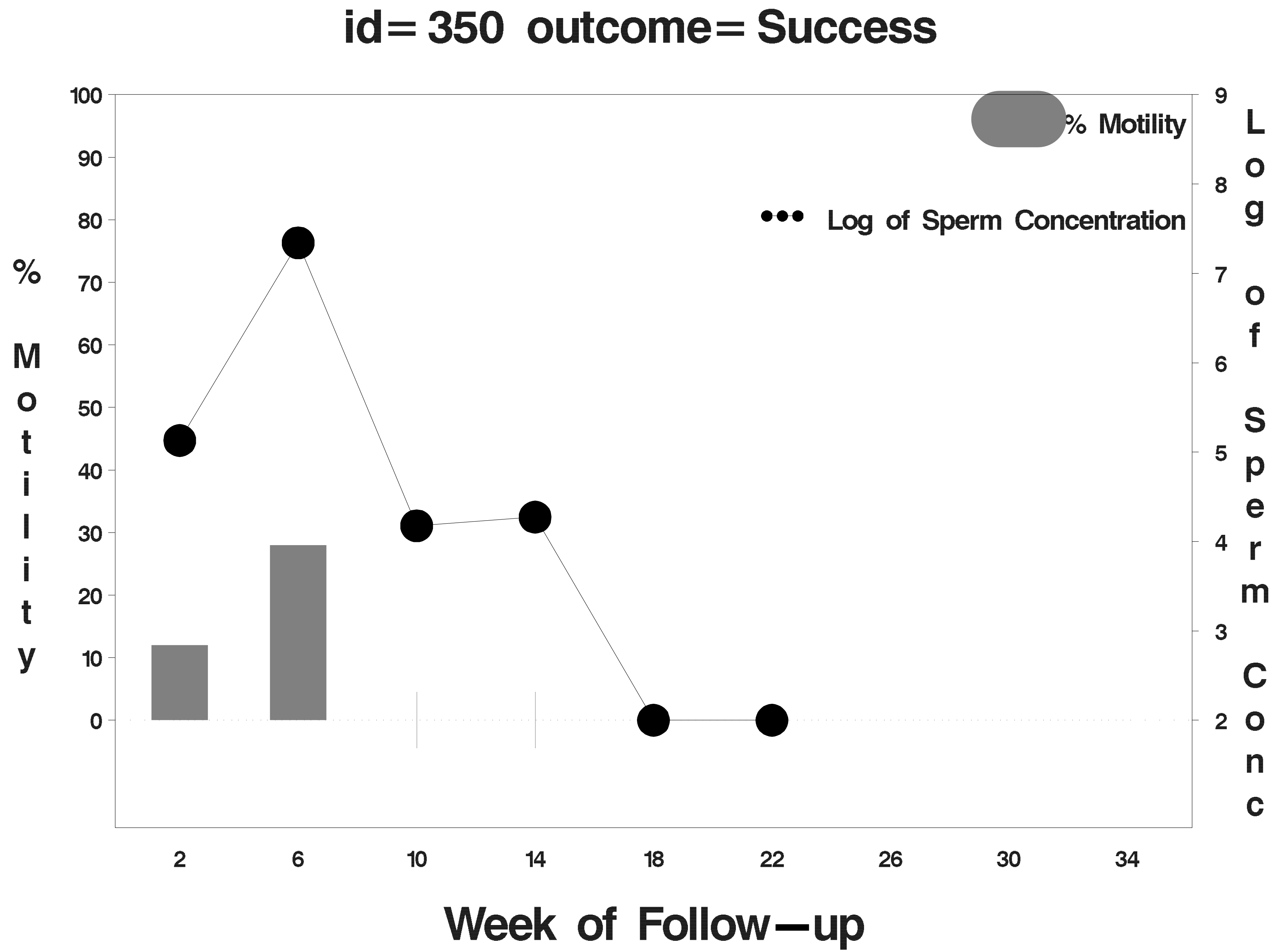
id= 350 outcome= Success
% Motility
Log of Sperm Concentration
100
90
80
70
60
50
40
30
20
10
0
9
8
7
6
5
4
3
2
2
6
10
14
18
22
26
30
34
Week of Follow−up
% Motility
Log of Sperm Conc

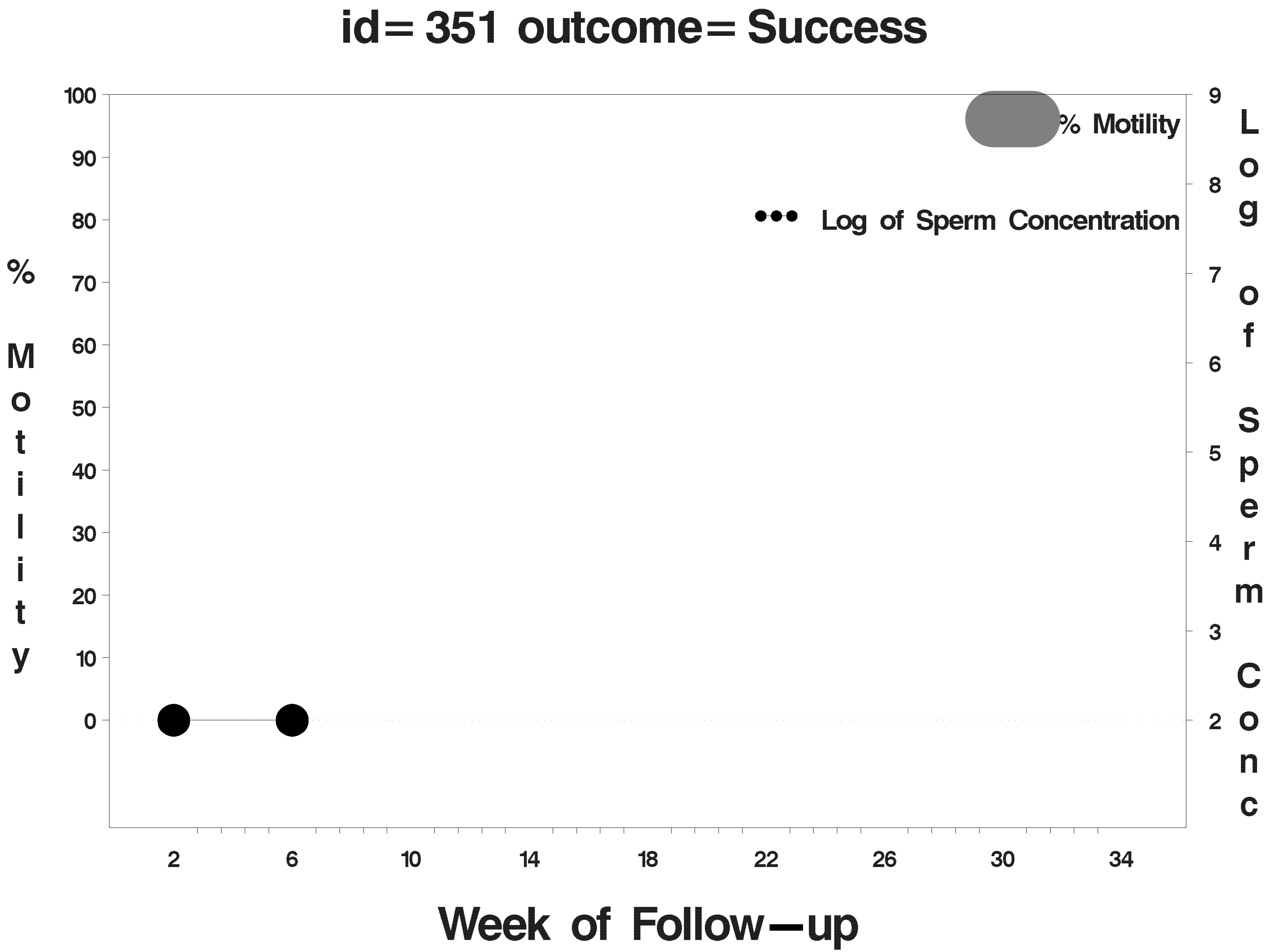

id= 351 outcome= Success
% Motility
Log of Sperm Concentration
% Motility
Log of Sperm Conc
Week of Follow−up
100
90
80
70
60
50
40
30
20
10
0
9
8
7
6
5
4
3
2
2
6
10
14
18
22
26
30
34

id= 352 outcome= Failure

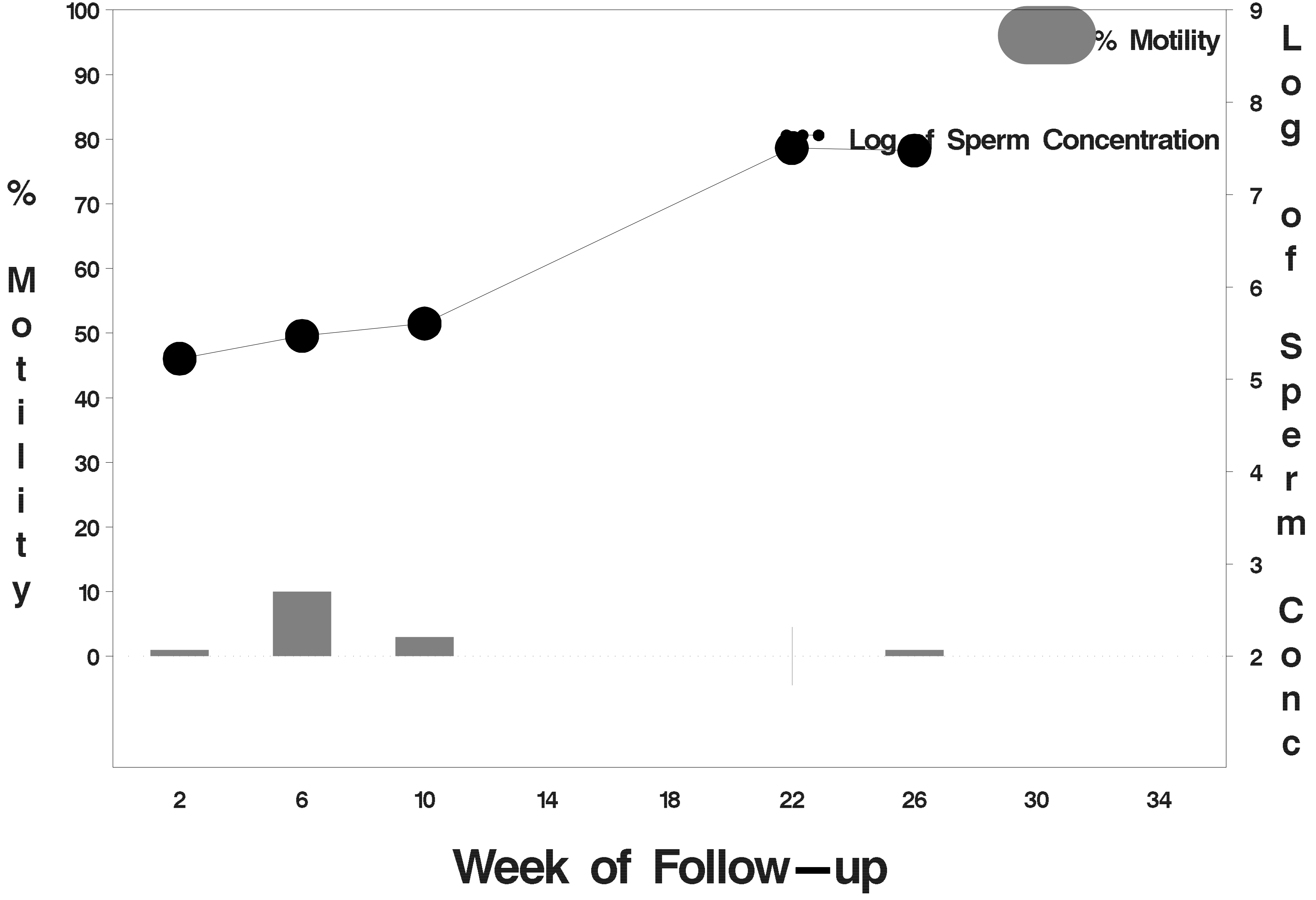

id= 353 outcome= Success

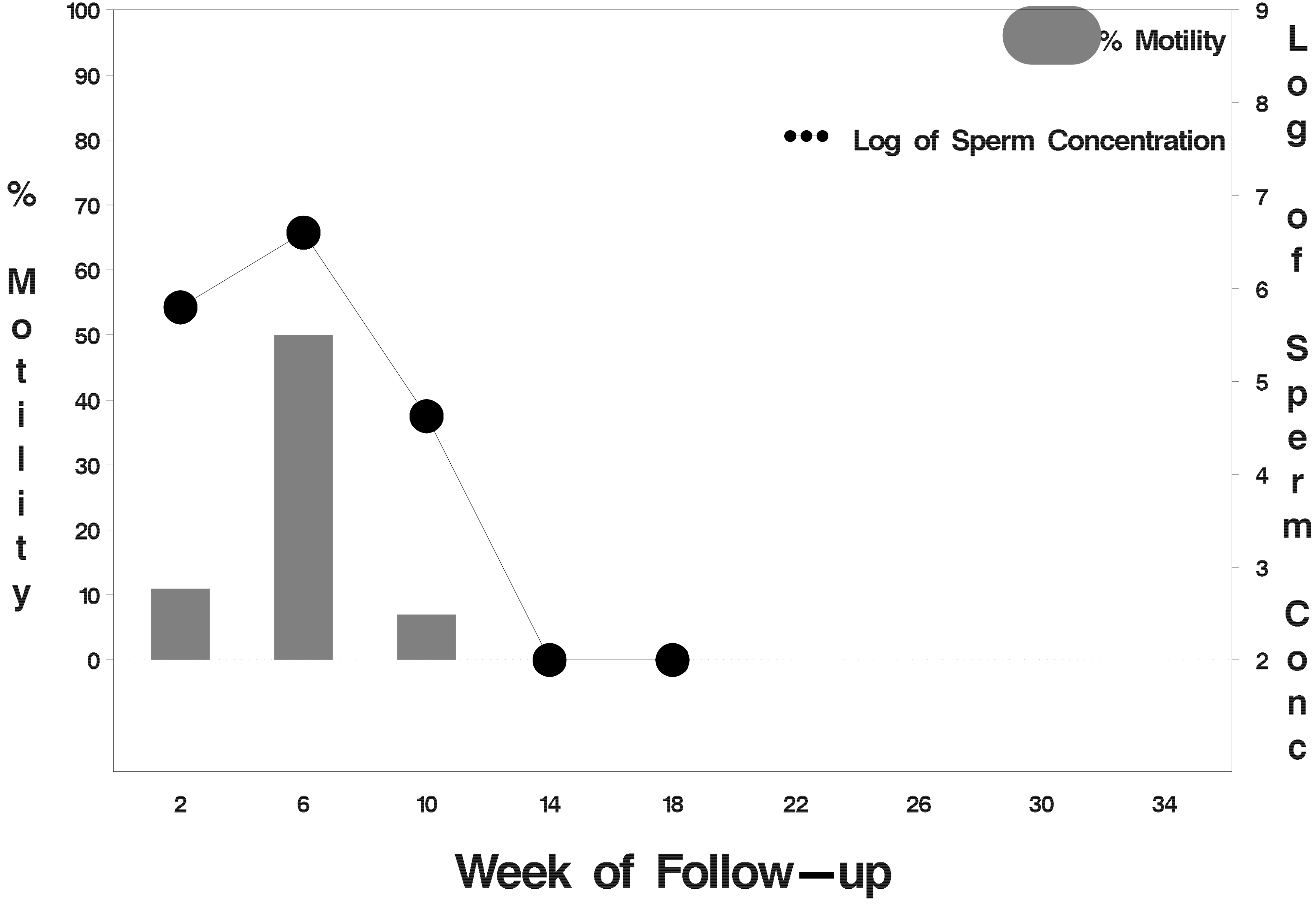

id= 354 outcome= Success

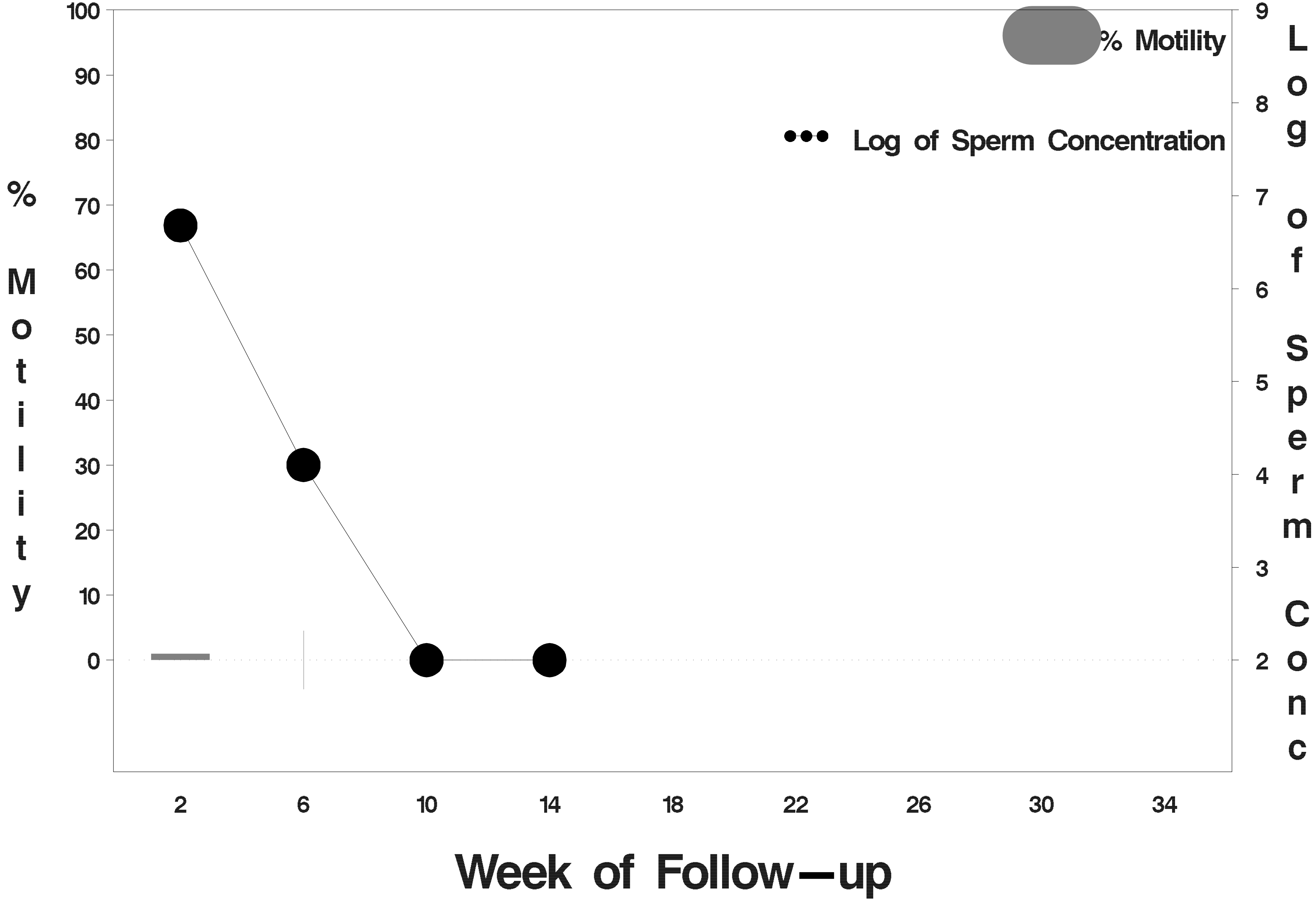

id= 355 outcome= Success

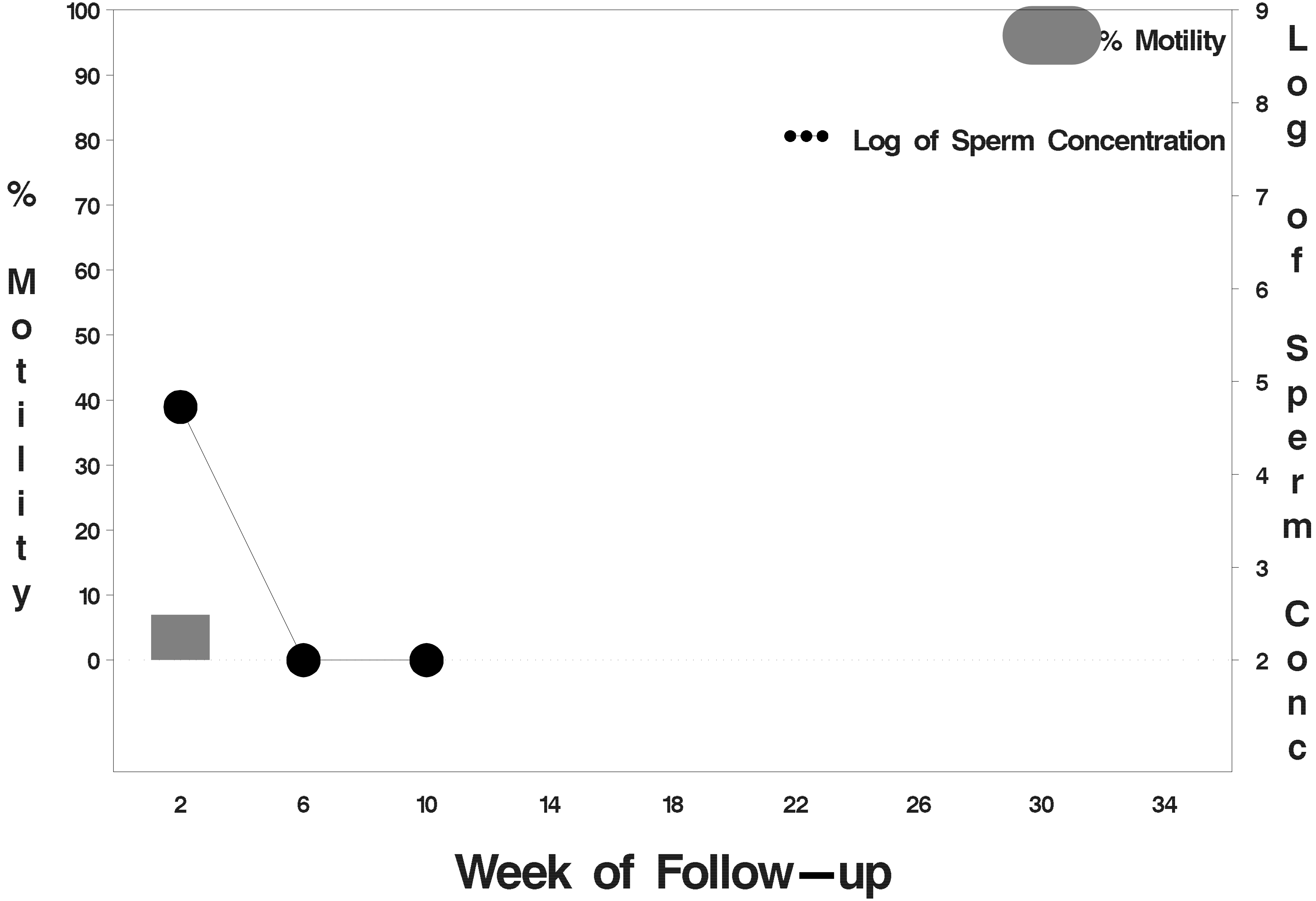

id= 356 outcome= Indeterminate

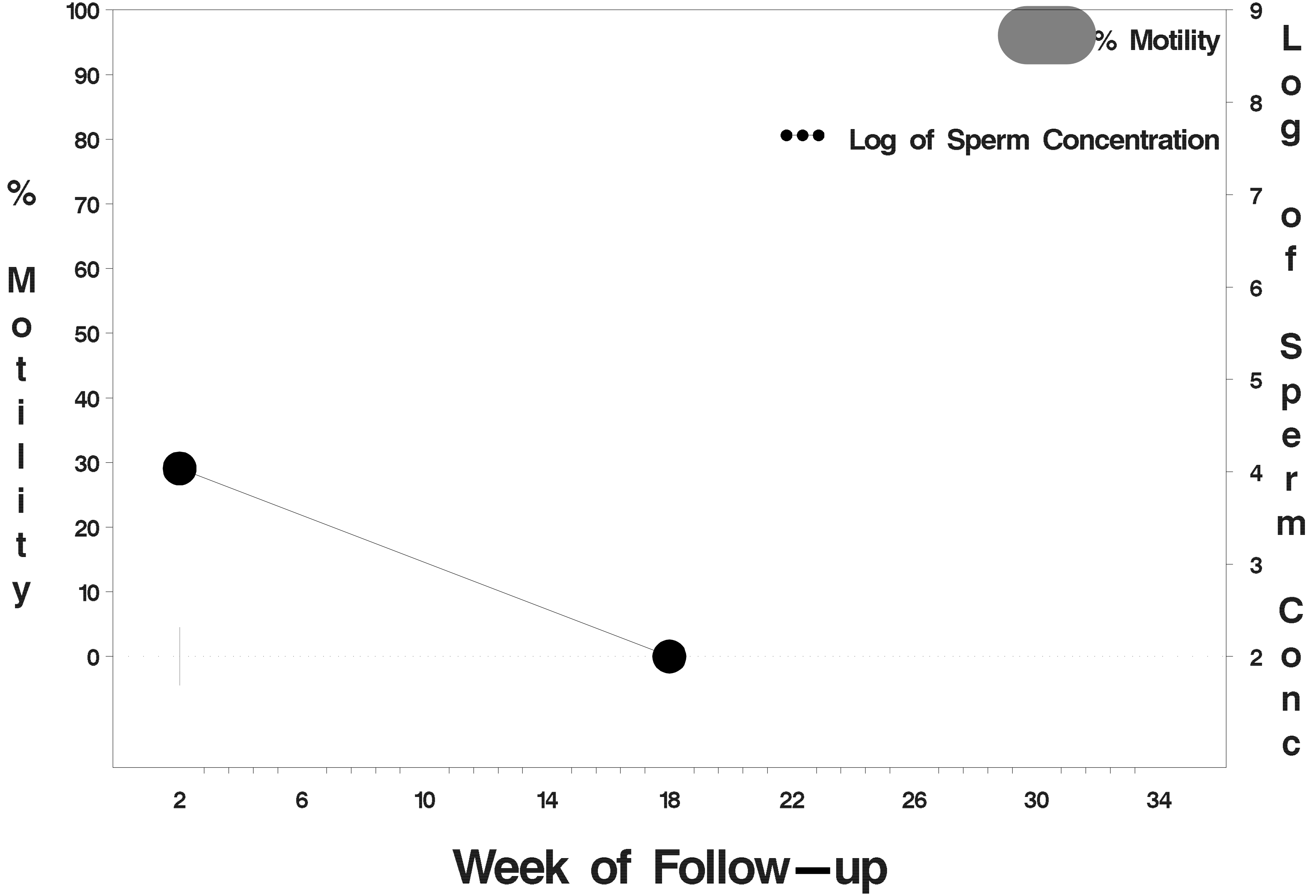

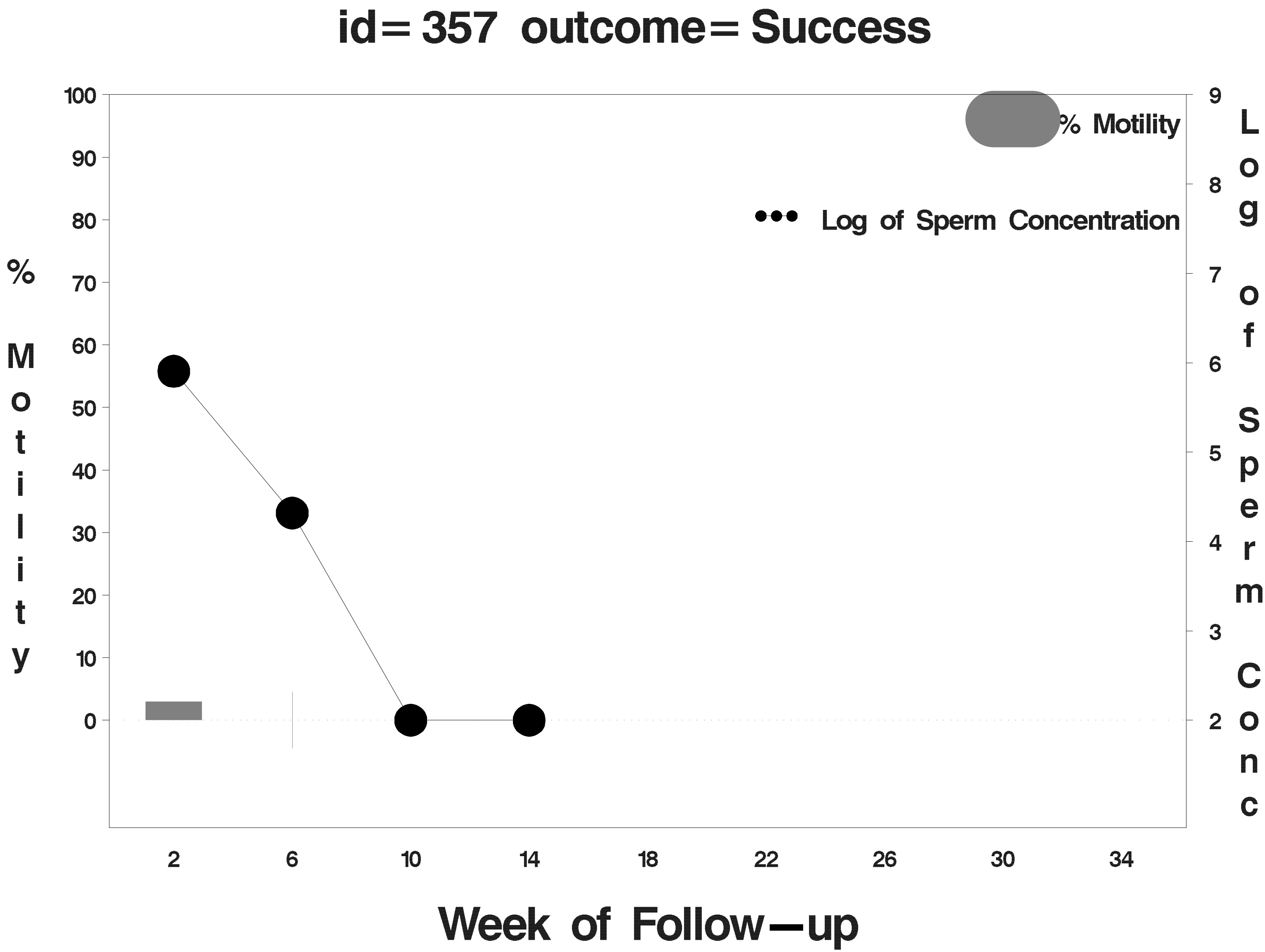

id= 357 outcome= Success
% Motility
Log of Sperm Concentration
100
90
80
70
60
50
40
30
20
10
0
9
8
7
6
5
4
3
2
2
6
10
14
18
22
26
30
34
Week of Follow−up

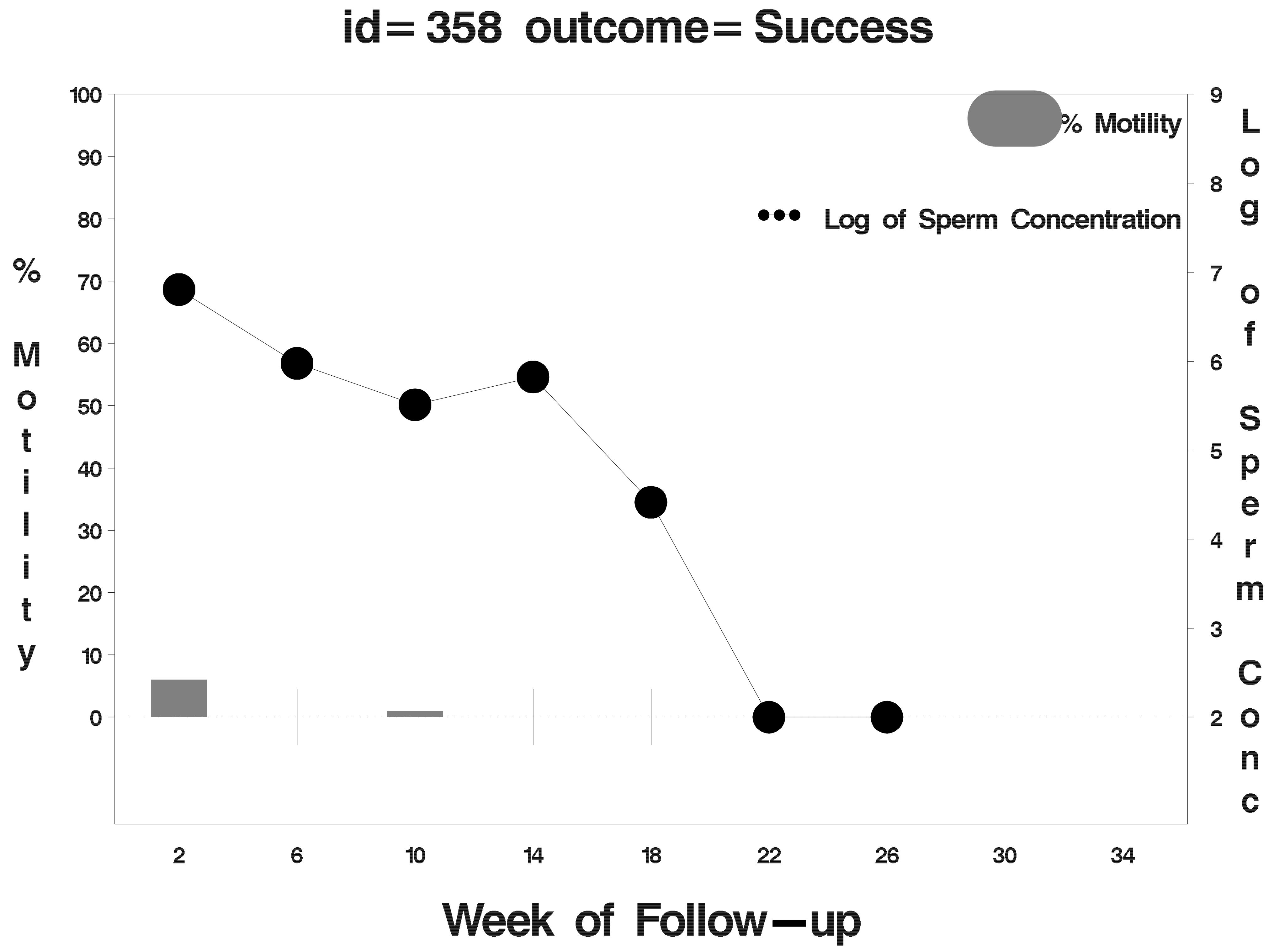

id= 358 outcome= Success
% Motility
Log of Sperm Concentration
% Motility
Log of Sperm Conc
100
90
80
70
60
50
40
30
20
10
0
9
8
7
6
5
4
3
2
2
6
10
14
18
22
26
30
34
Week of Follow−up

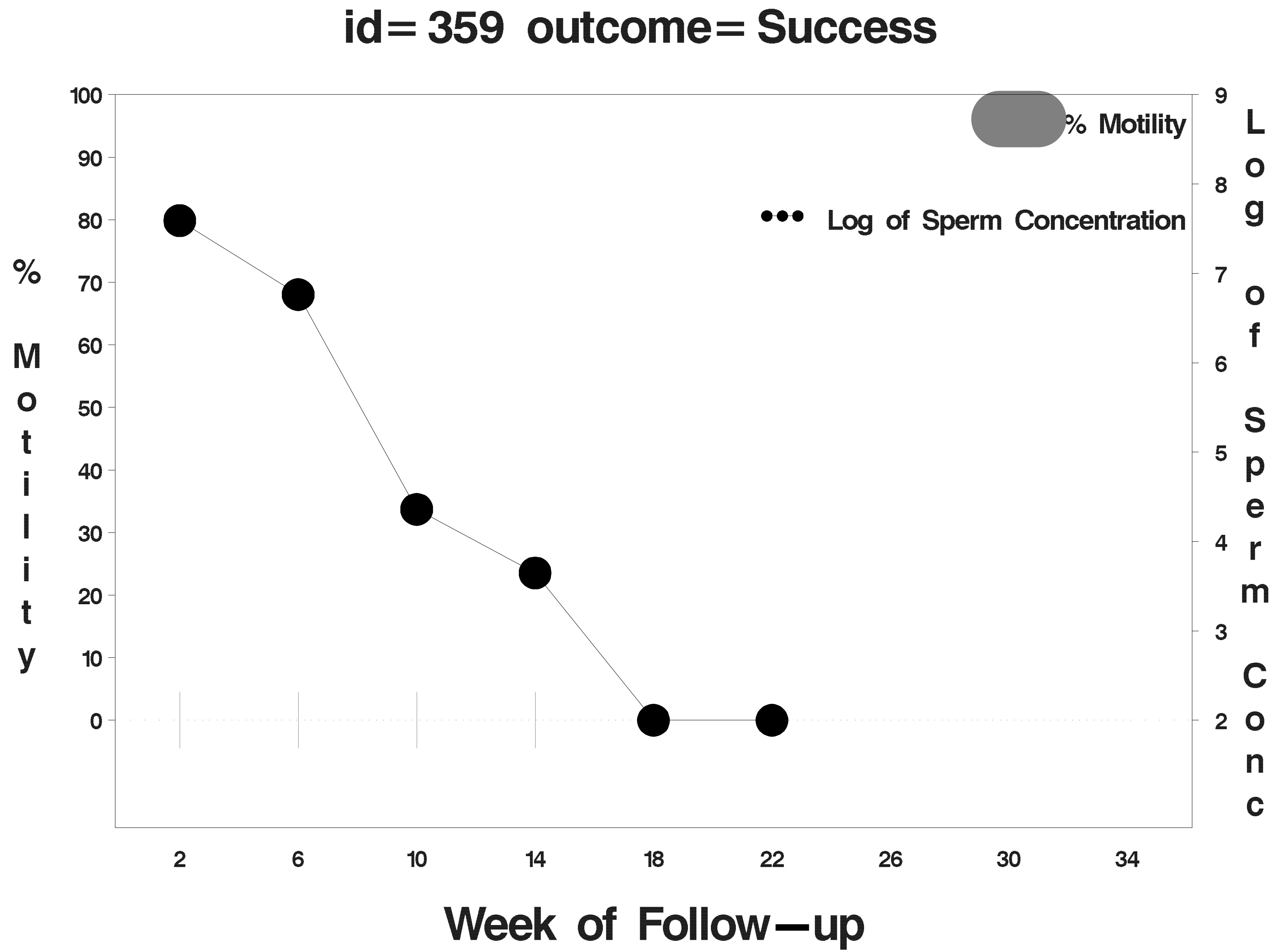
id= 359 outcome= Success
% Motility
Log of Sperm Concentration
% Motility
Log of Sperm Conc
Week of Follow−up
0
10
20
30
40
50
60
70
80
90
100
2
3
4
5
6
7
8
9
2
6
10
14
18
22
26
30
34

id= 360 outcome= Success

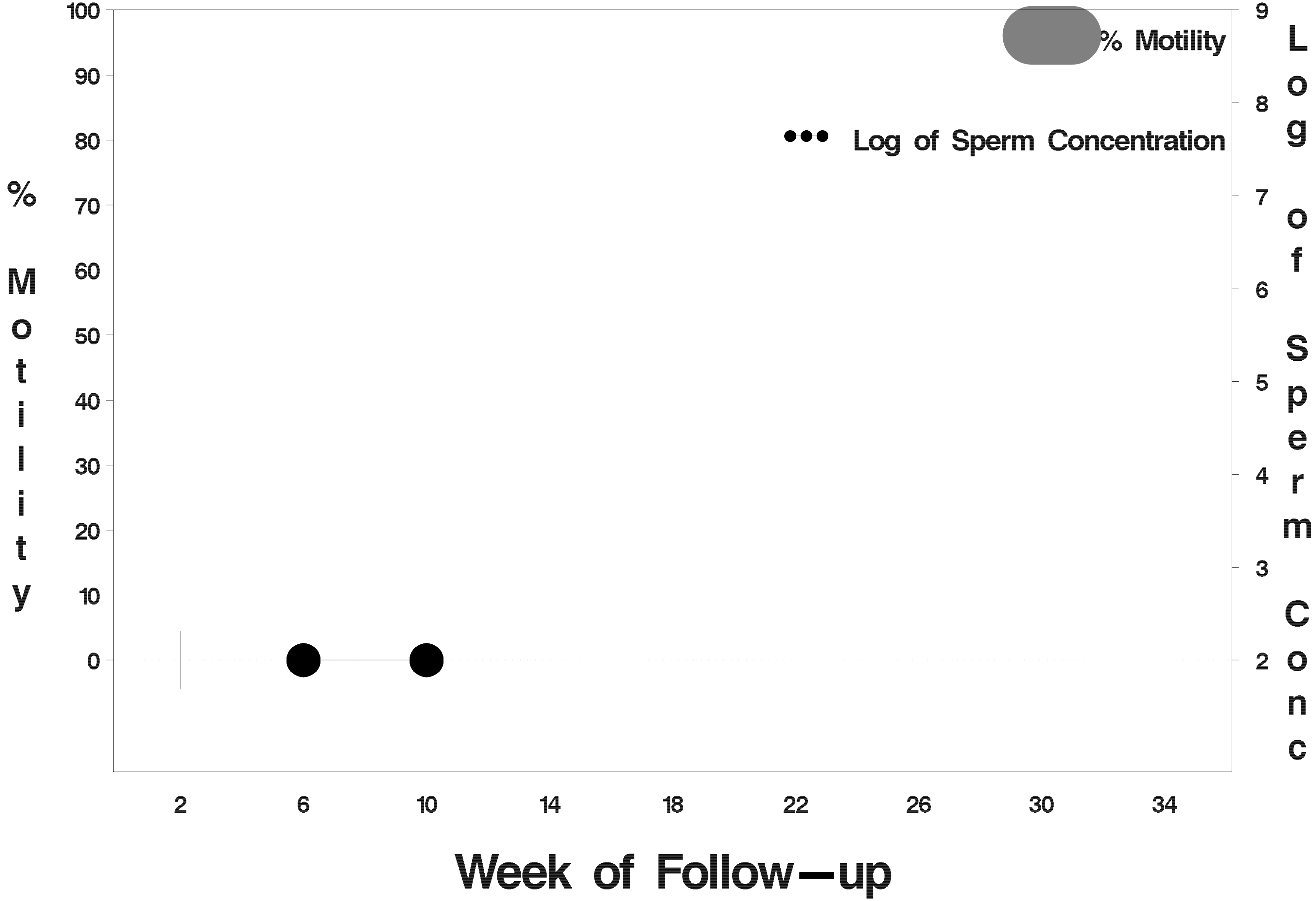

id= 361 outcome= Success

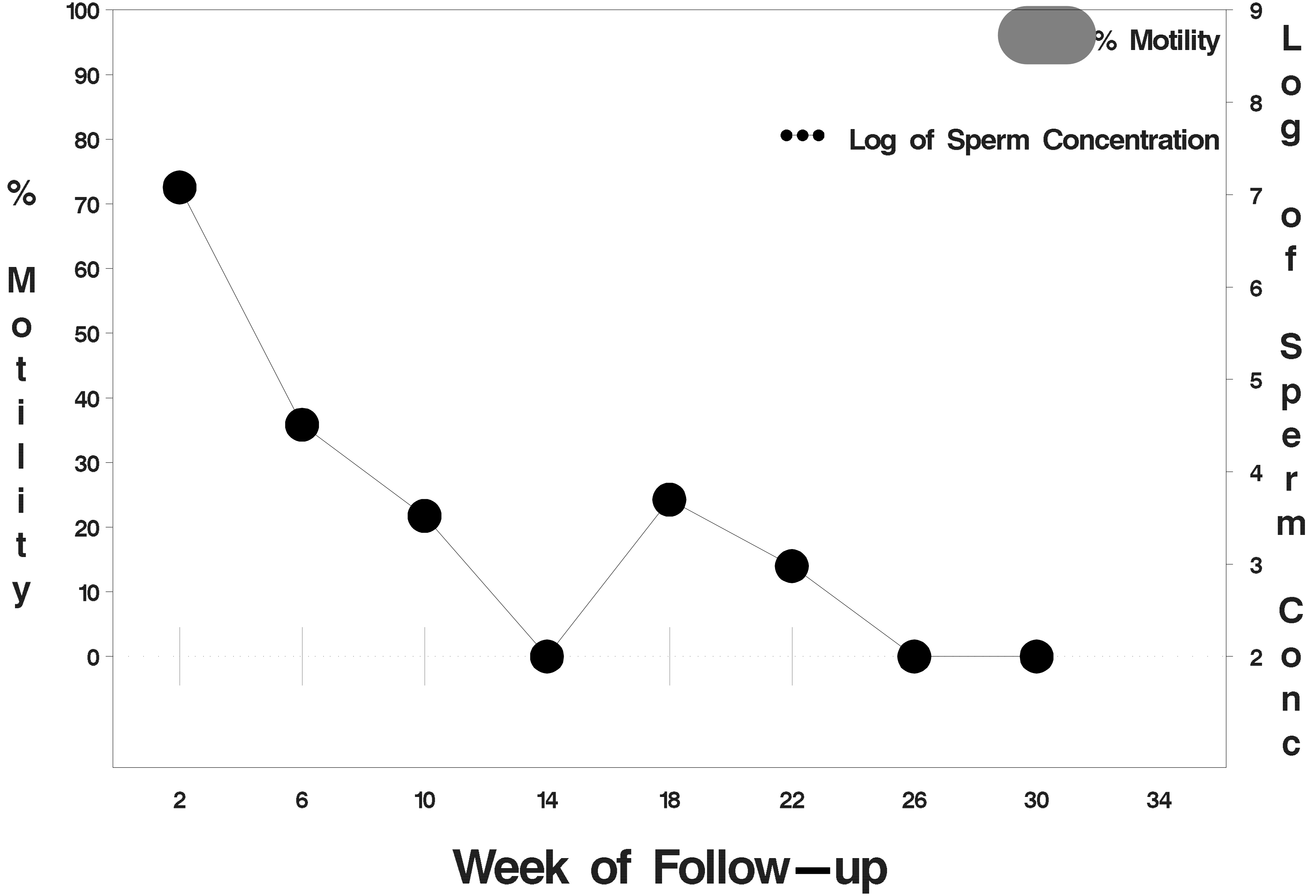

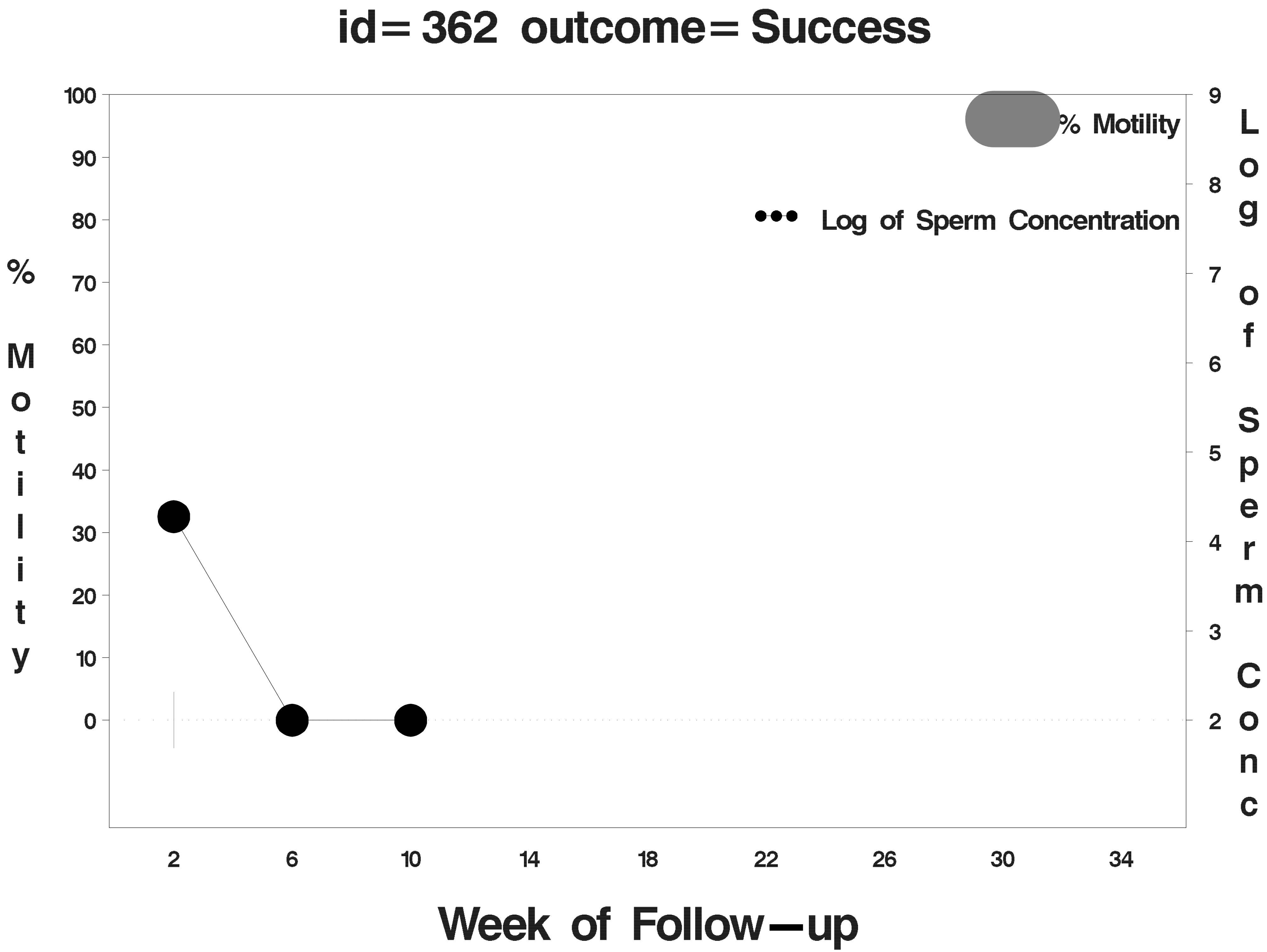

id= 362 outcome= Success
% Motility
Log of Sperm Concentration
100
90
80
70
60
50
40
30
20
10
0
9
8
7
6
5
4
3
2
2
6
10
14
18
22
26
30
34
Week of Follow-up
% Motility
Log of Sperm Conc

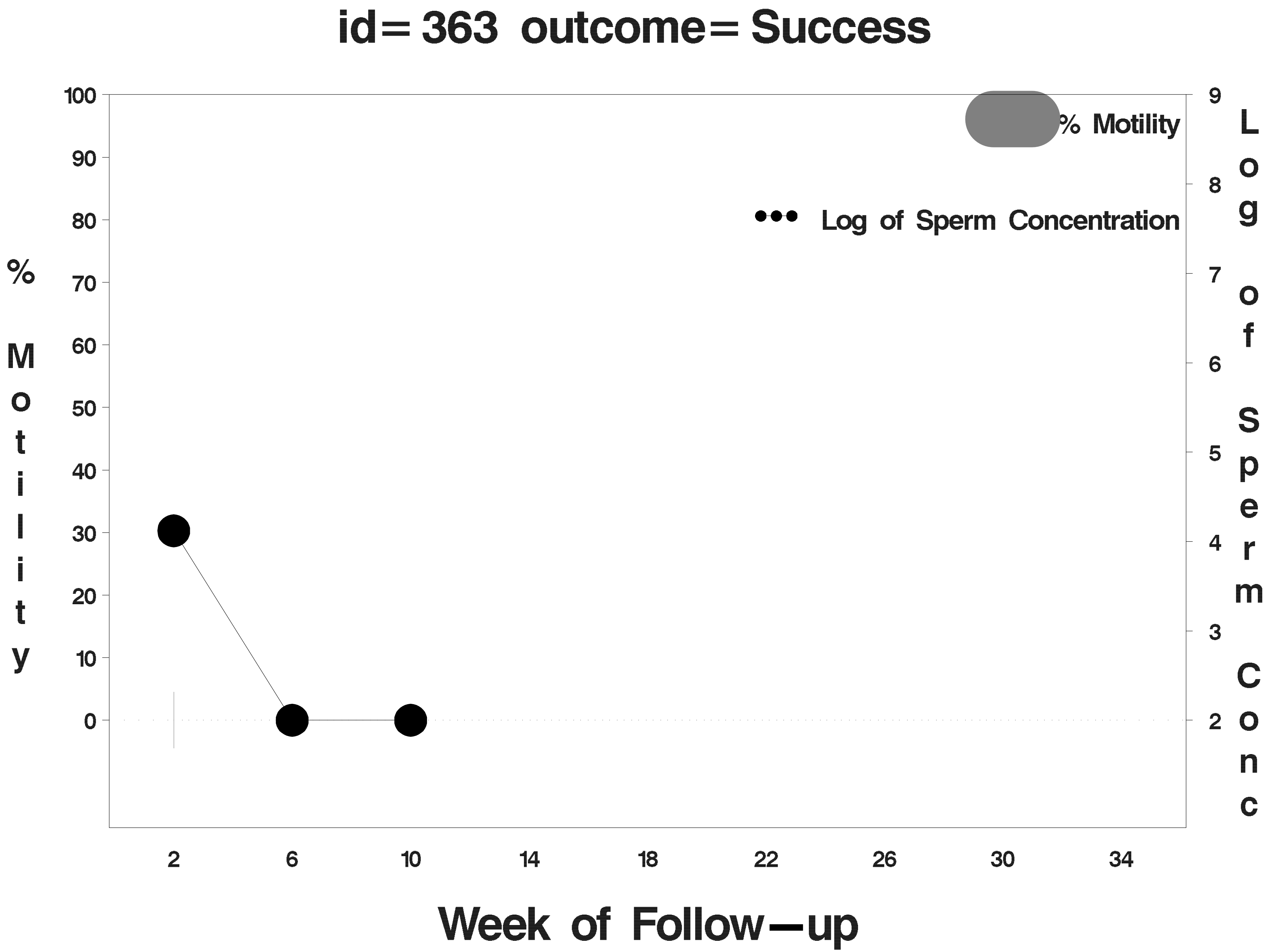

id= 363 outcome= Success
% Motility
Log of Sperm Concentration
100
90
80
70
60
50
40
30
20
10
0
9
8
7
6
5
4
3
2
2
6
10
14
18
22
26
30
34
Week of Follow—up
Log of Sperm Conc

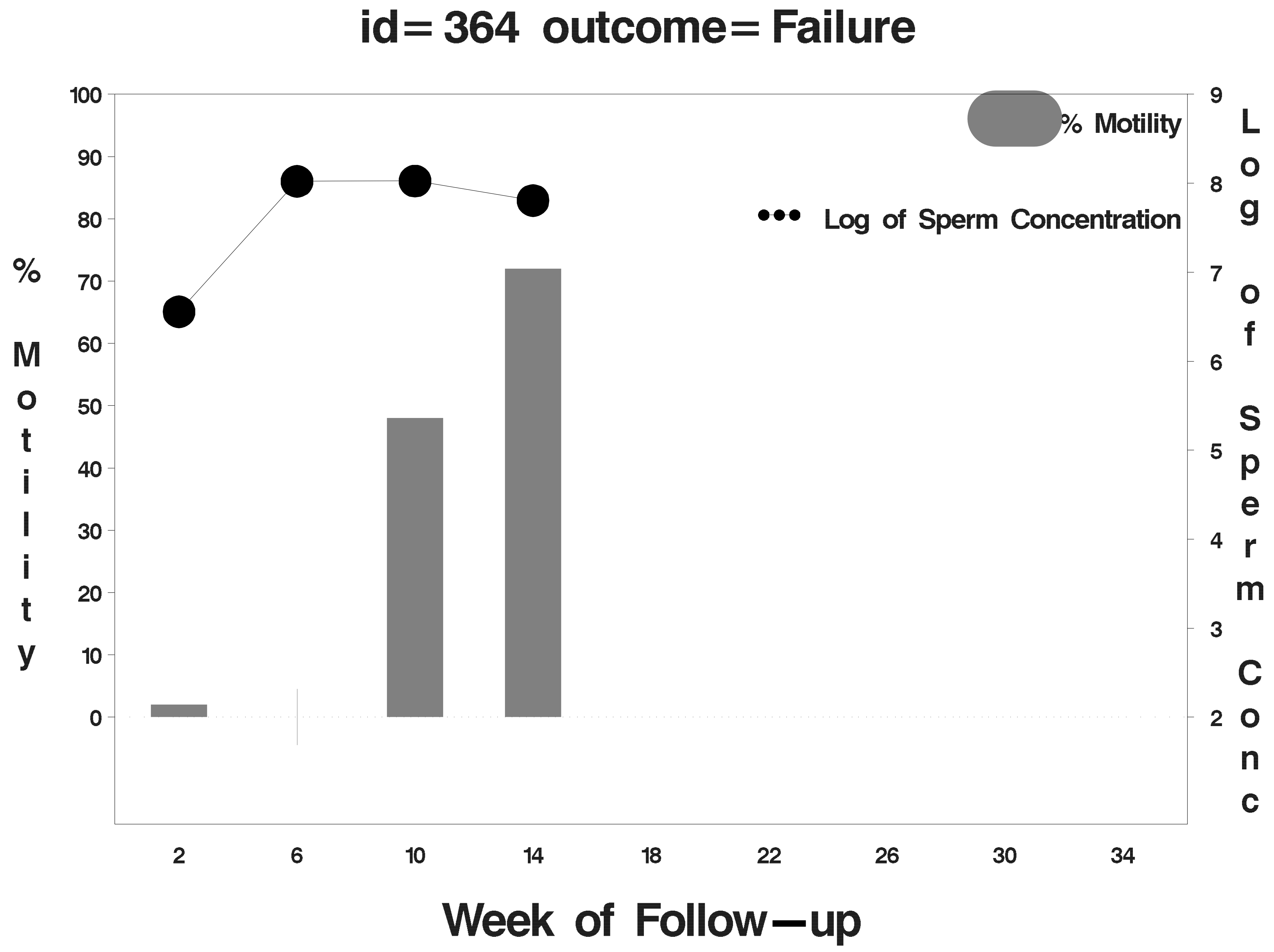
id= 364 outcome= Failure
% Motility
Log of Sperm Concentration
100
90
80
70
60
50
40
30
20
10
0
9
8
7
6
5
4
3
2
2
6
10
14
18
22
26
30
34
Week of Follow−up
Log of Sperm Conc

id= 365 outcome= Success

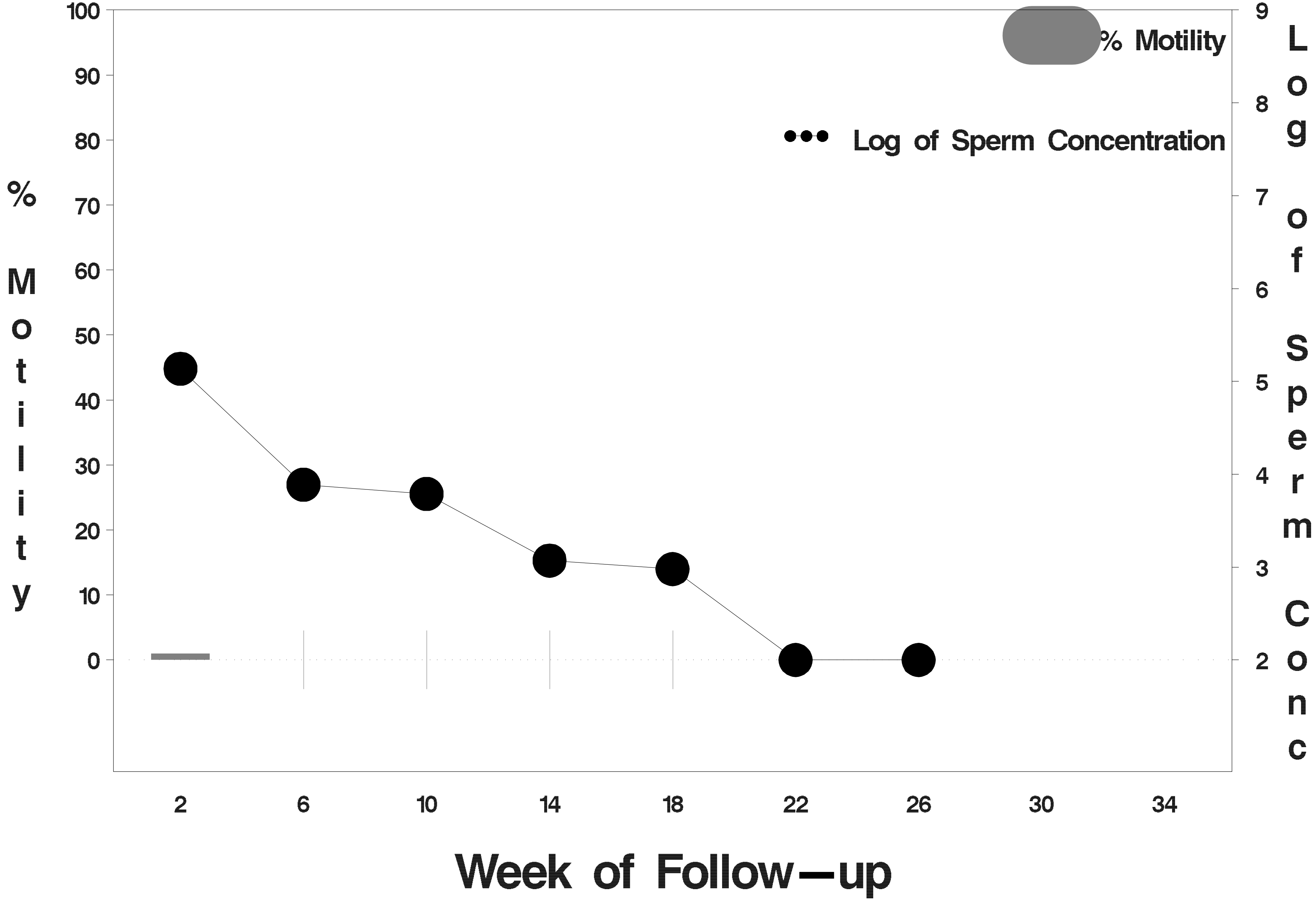

id= 366 outcome= Success

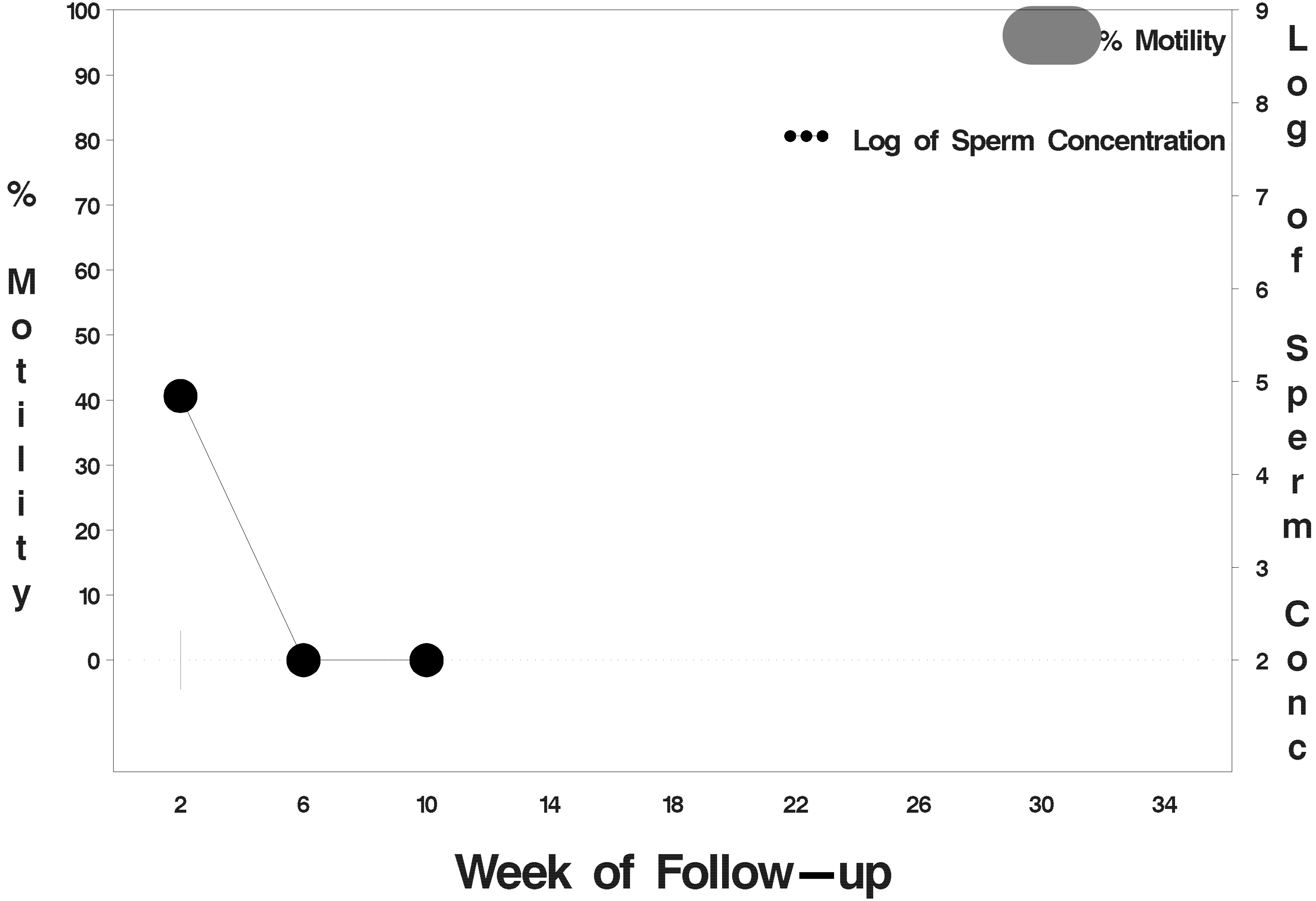

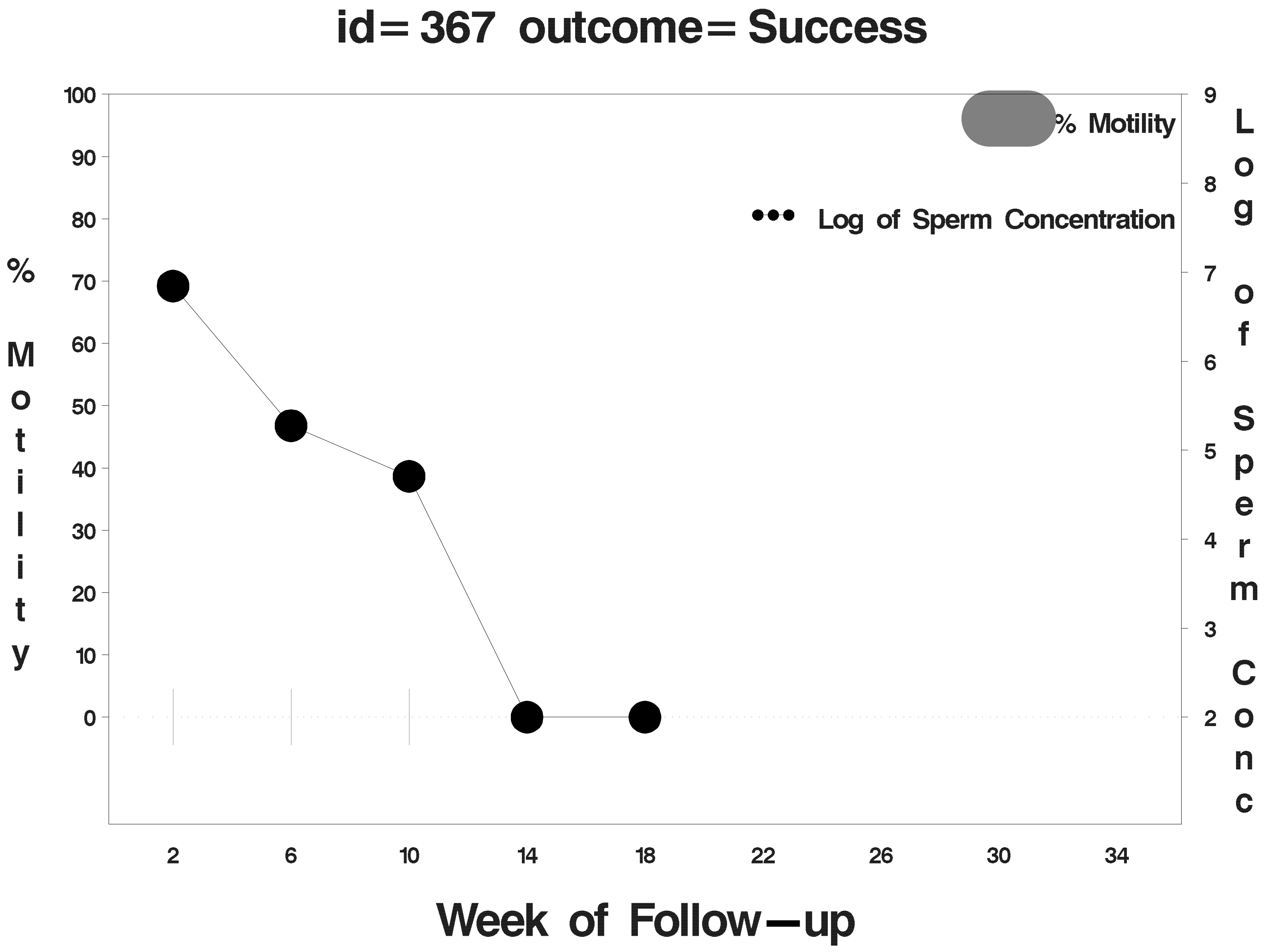
id= 367 outcome= Success
% Motility
Log of Sperm Concentration
100
90
80
70
60
50
40
30
20
10
0
9
8
7
6
5
4
3
2
% Motility
Log of Sperm Conc
2
6
10
14
18
22
26
30
34
Week of Follow—up

id= 368 outcome= Success

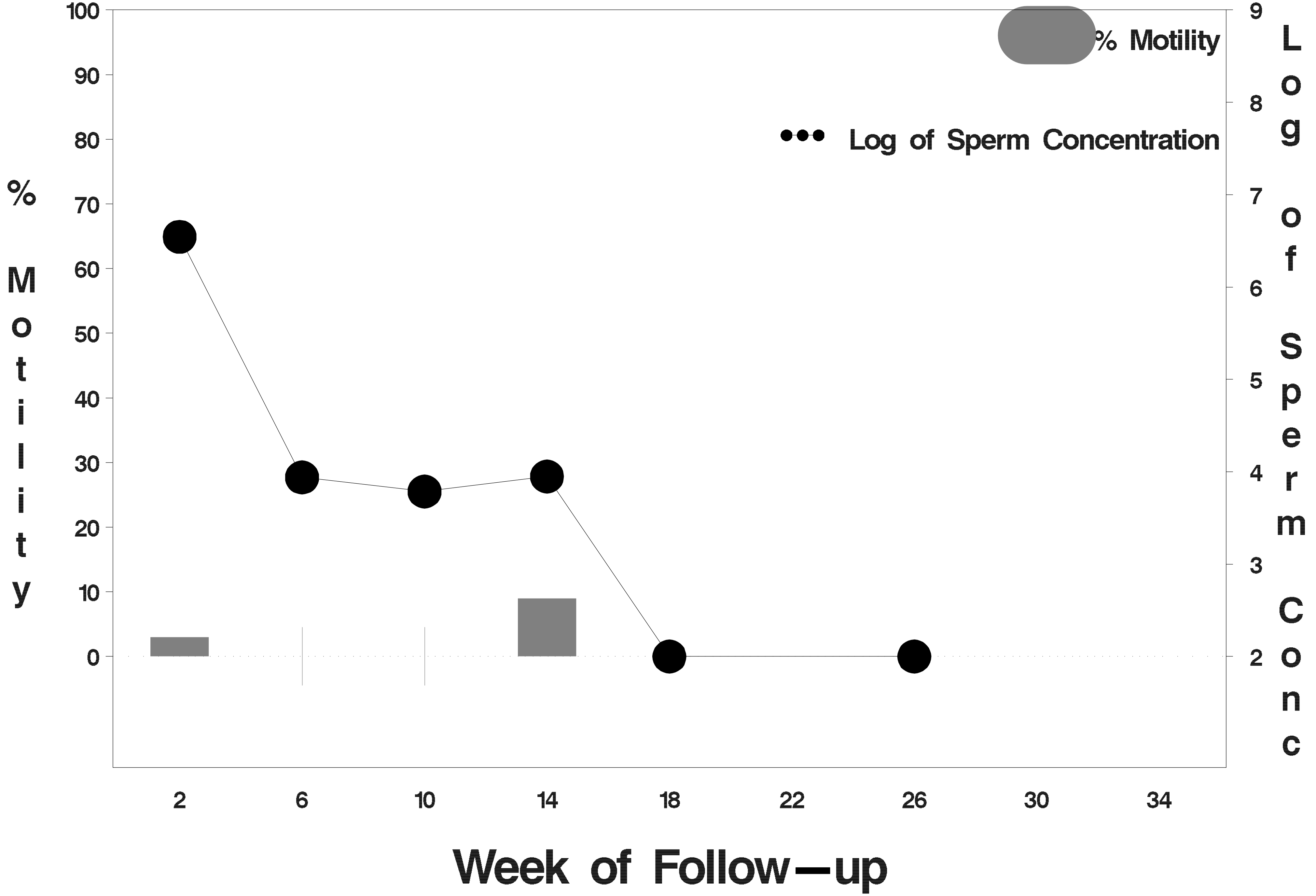

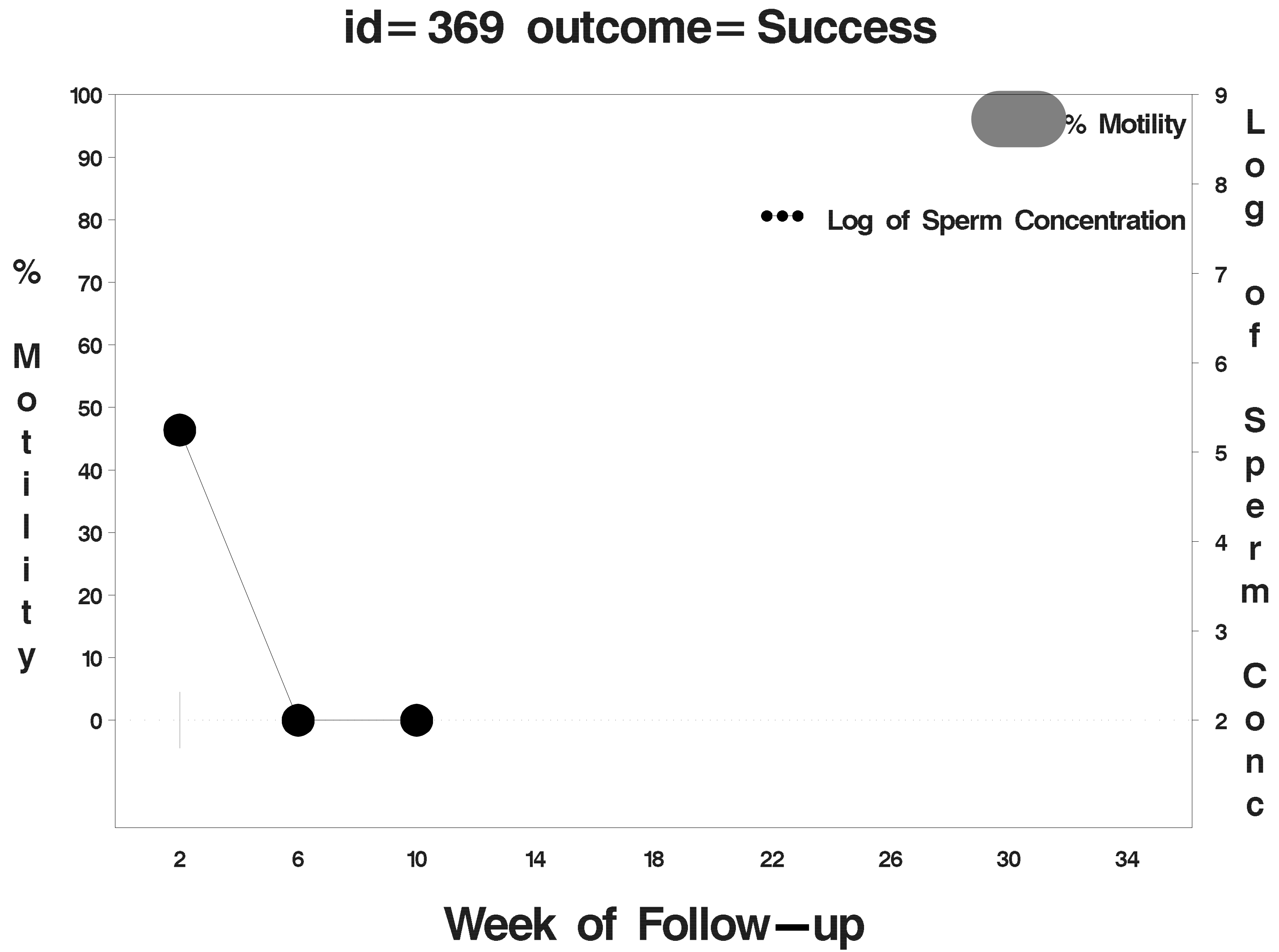

id= 369 outcome= Success
% Motility
Log of Sperm Concentration
100
90
80
70
60
50
40
30
20
10
0
9
8
7
6
5
4
3
2
2
6
10
14
18
22
26
30
34
Week of Follow−up
Log of Sperm Conc

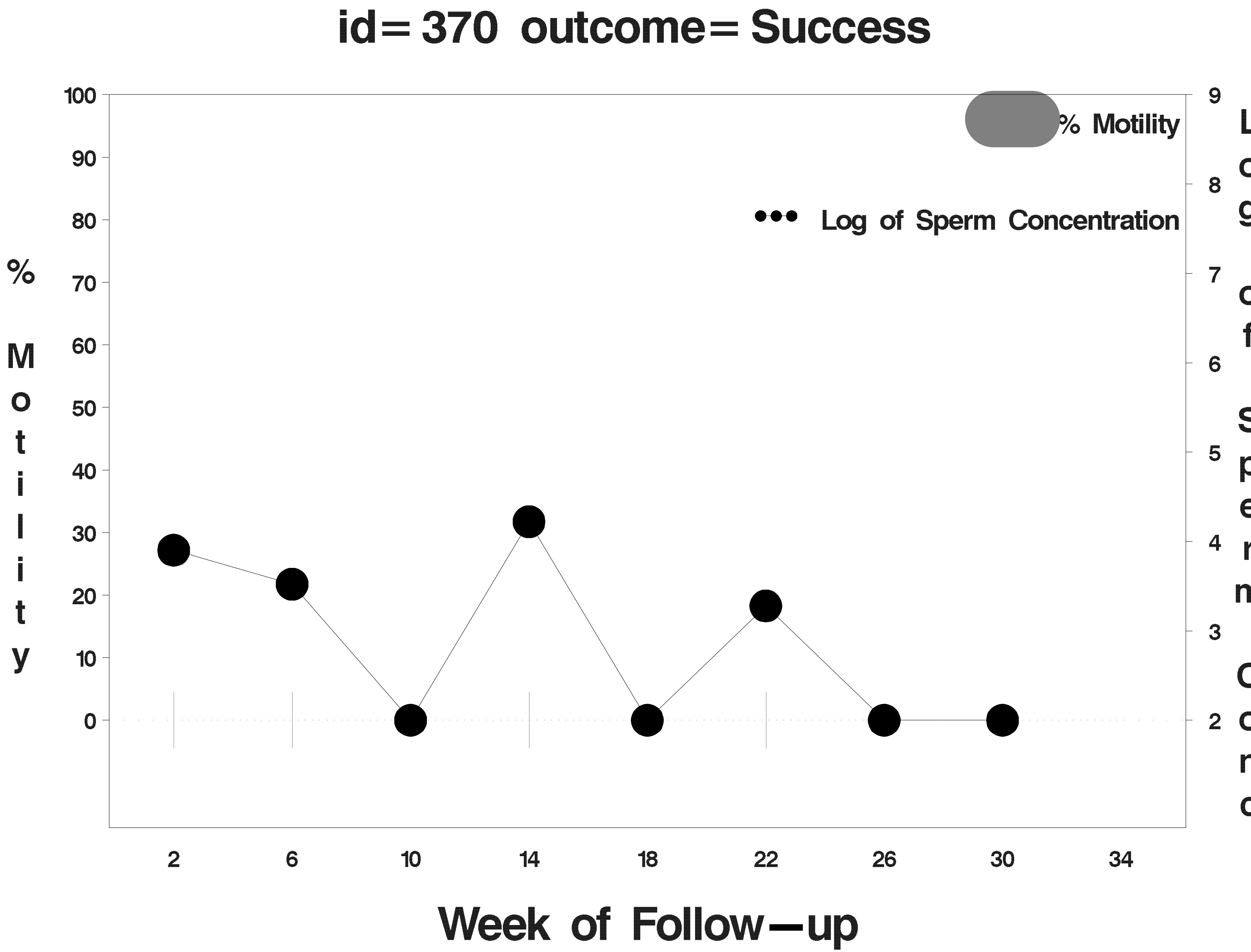
id= 370 outcome= Success
% Motility
Log of Sperm Concentration
100
90
80
70
60
50
40
30
20
10
0
9
8
7
6
5
4
3
2
2
6
10
14
18
22
26
30
34
Week of Follow—up
% Motility
Log of Sperm Conc

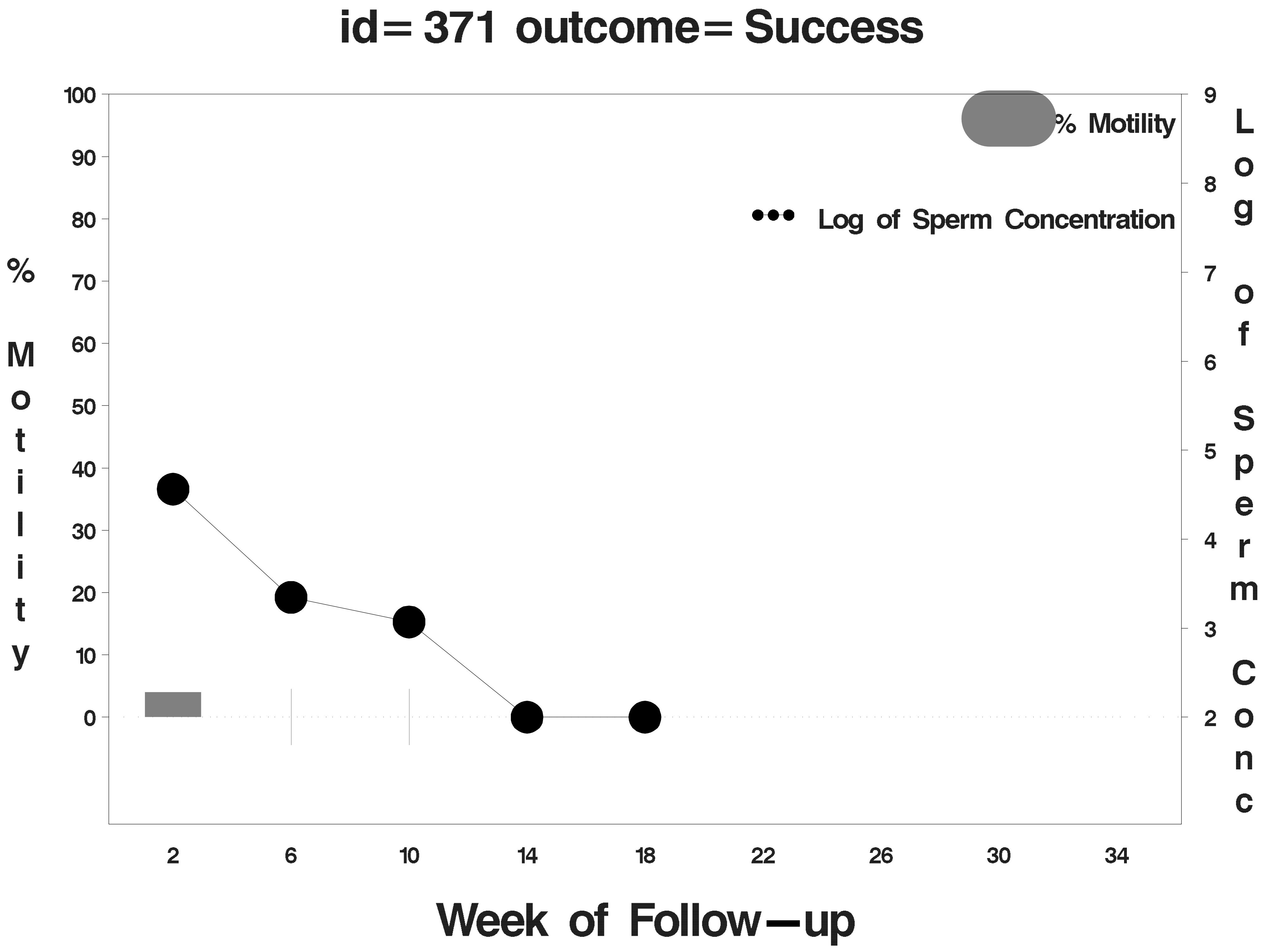

id= 371 outcome= Success
% Motility
Log of Sperm Concentration
100
90
80
70
60
50
40
30
20
10
0
9
8
7
6
5
4
3
2
2
6
10
14
18
22
26
30
34
Week of Follow—up
% Motility
Log of Sperm Conc

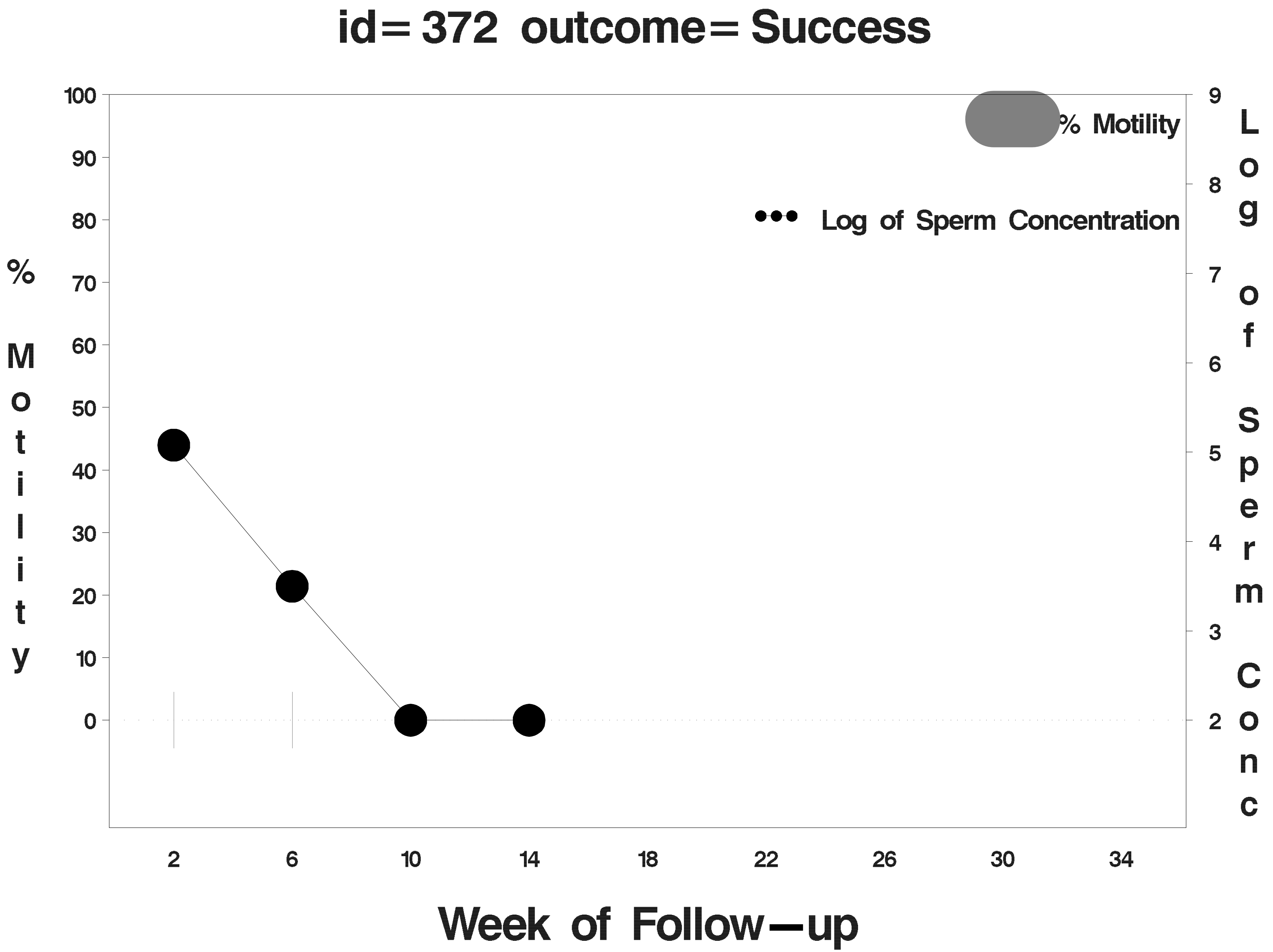

id= 372 outcome= Success
% Motility
Log of Sperm Concentration
100
90
80
70
60
50
40
30
20
10
0
9
8
7
6
5
4
3
2
2
6
10
14
18
22
26
30
34
Week of Follow−up
% Motility
Log of Sperm Conc

id= 373 outcome= Success

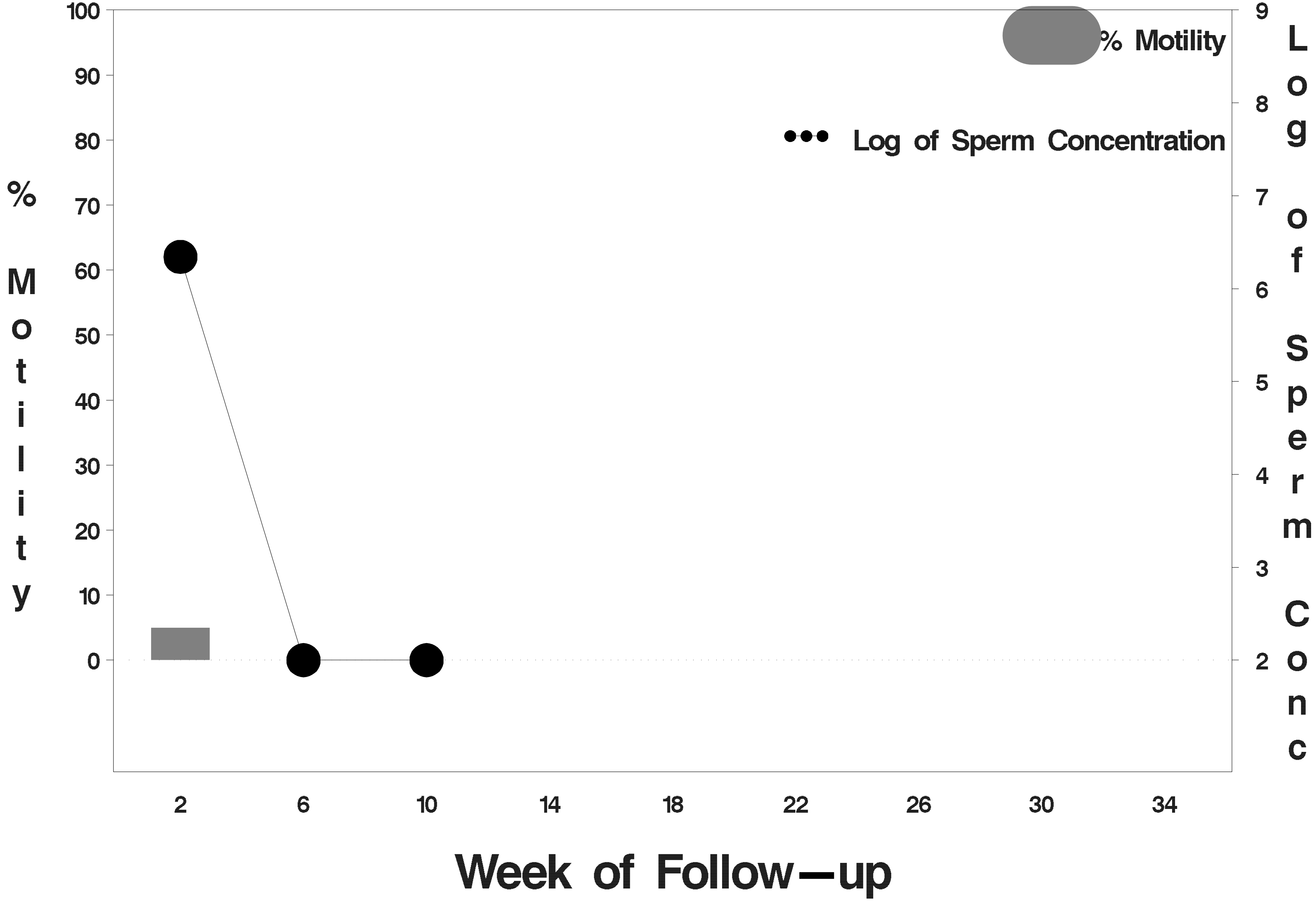

id= 374 outcome= Failure

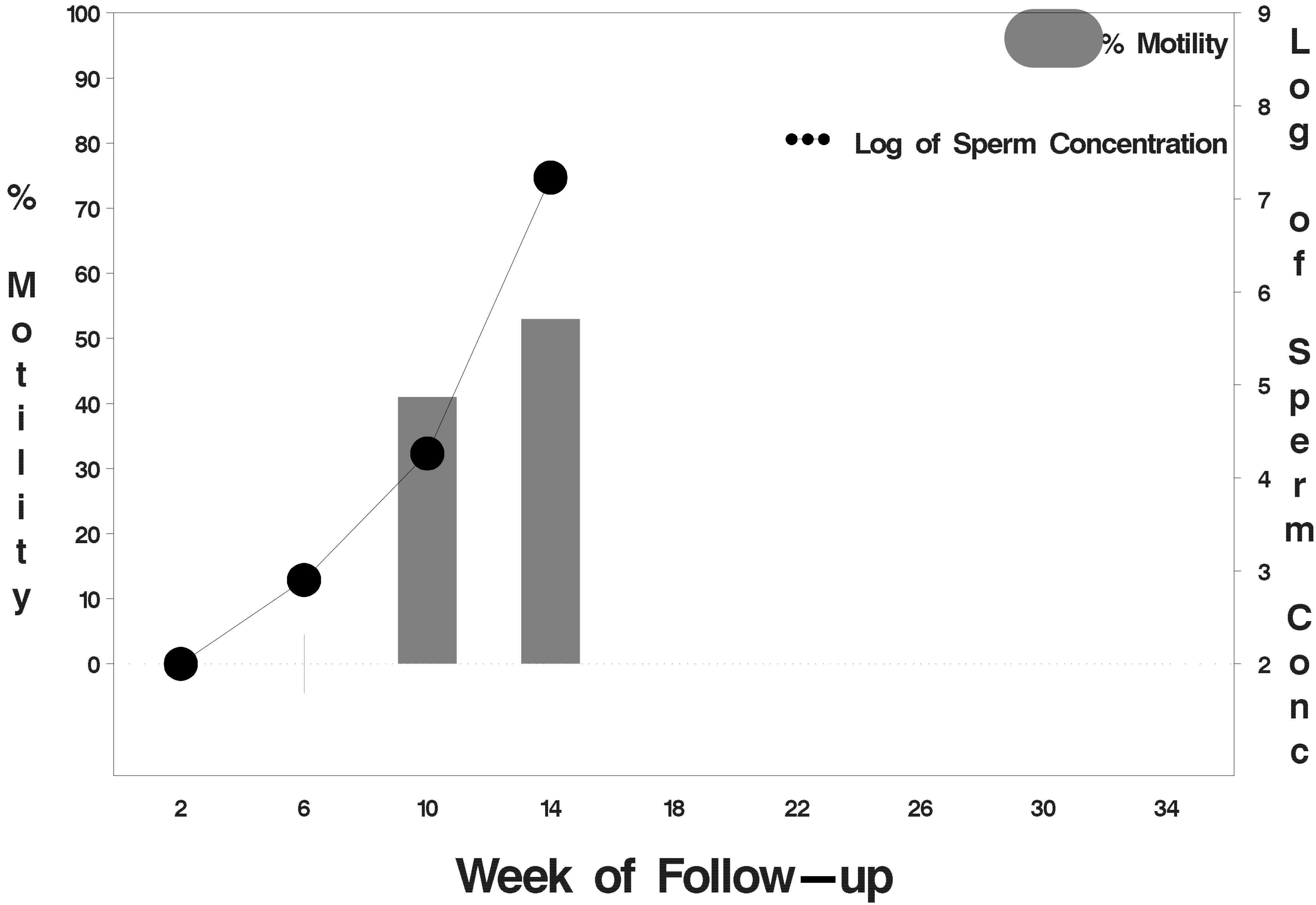

id= 375 outcome= Success

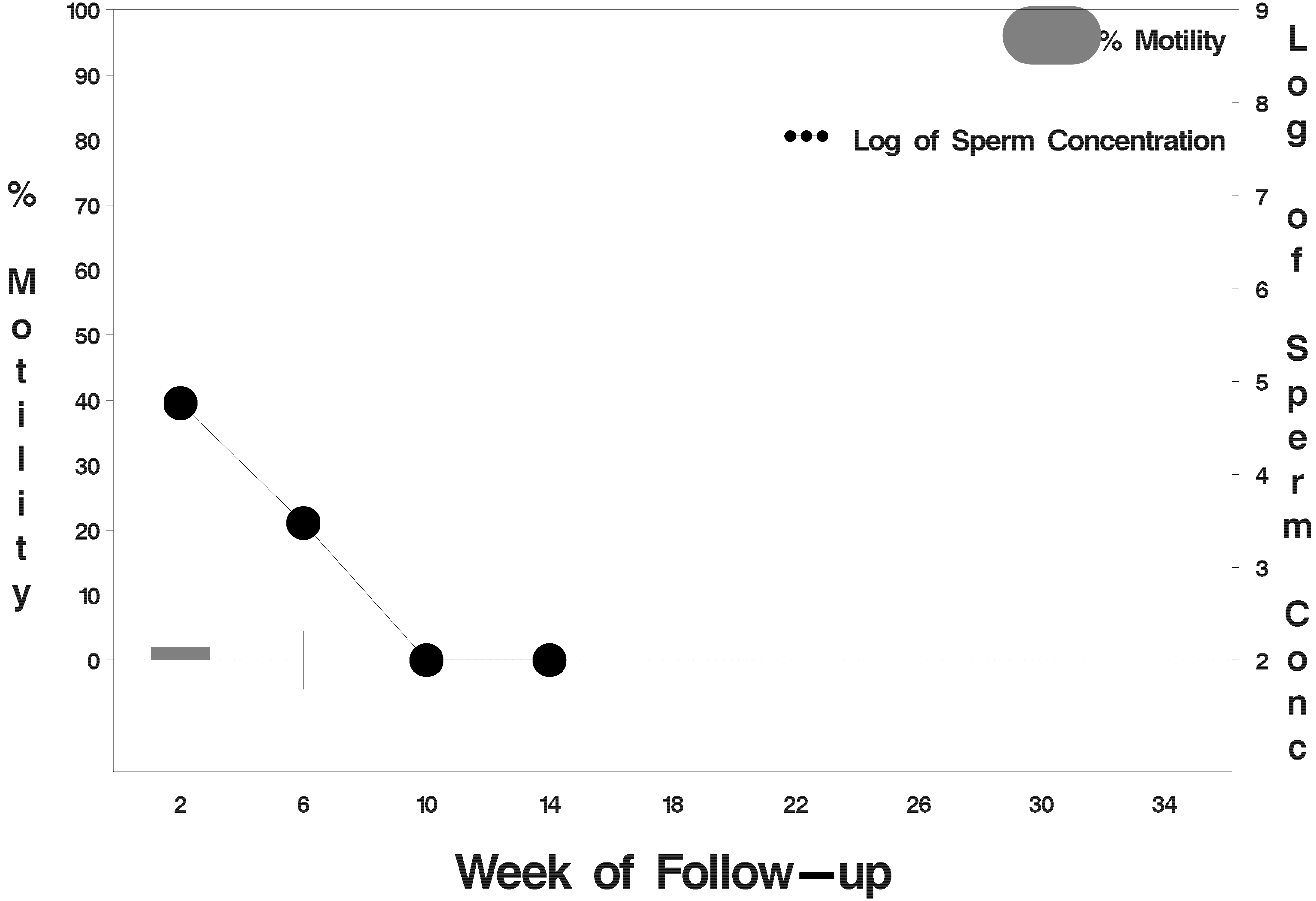

id= 376 outcome= Success

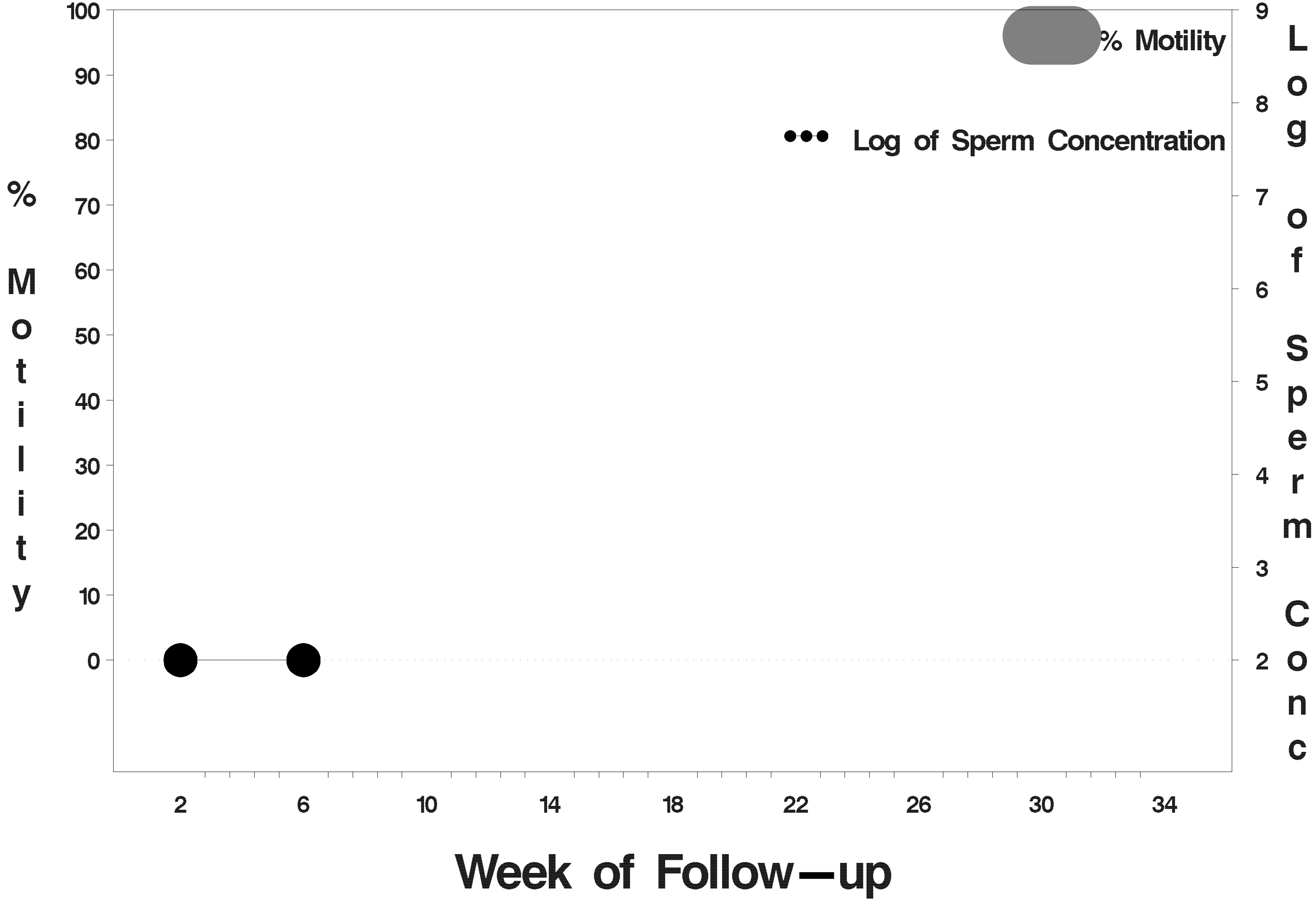

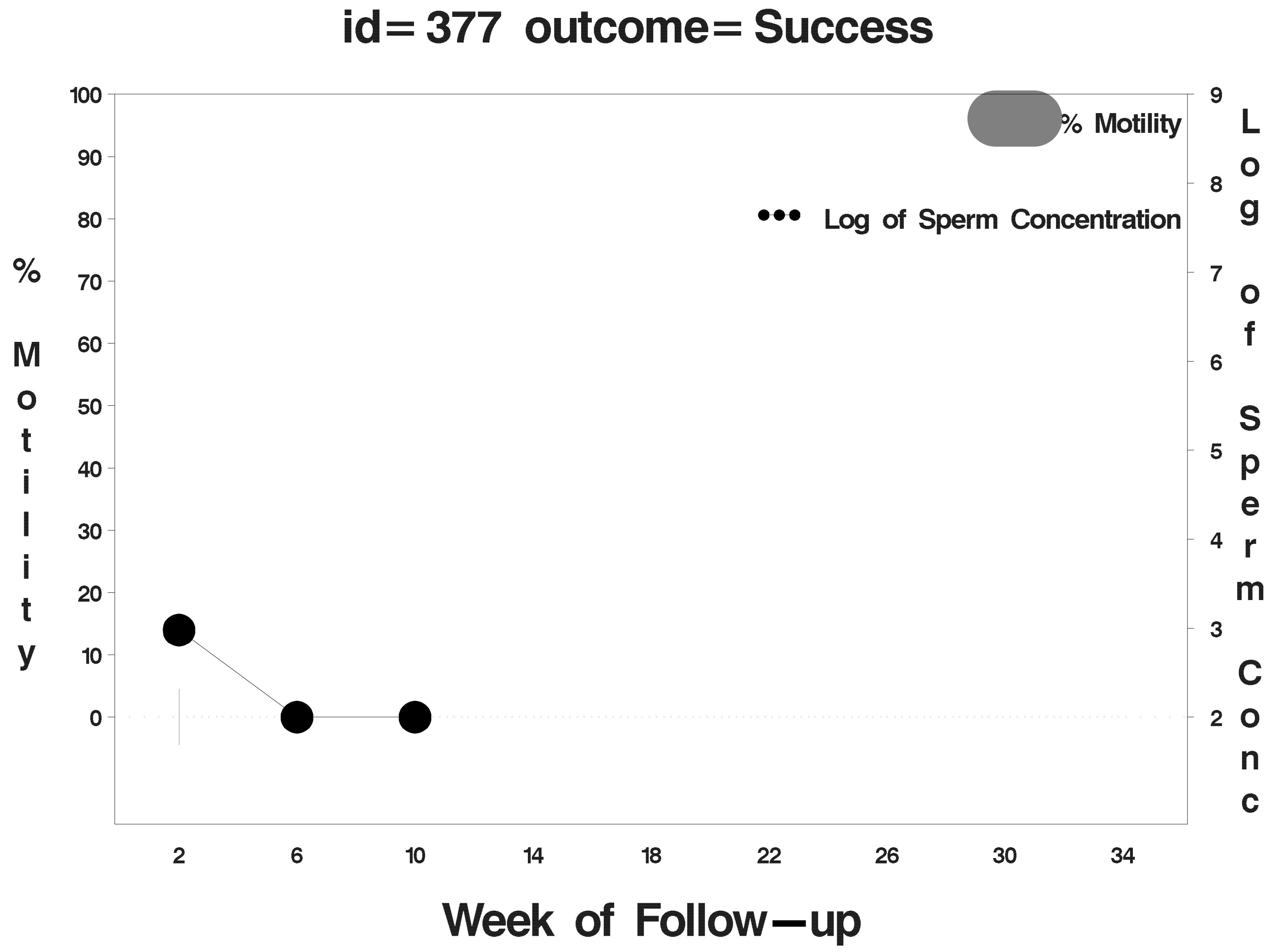
id= 377 outcome= Success
% Motility
Log of Sperm Concentration
100
90
80
70
60
50
40
30
20
10
0
9
8
7
6
5
4
3
2
2
6
10
14
18
22
26
30
34
Week of Follow−up
% Motility
Log of Sperm Conc

id= 378 outcome= Success

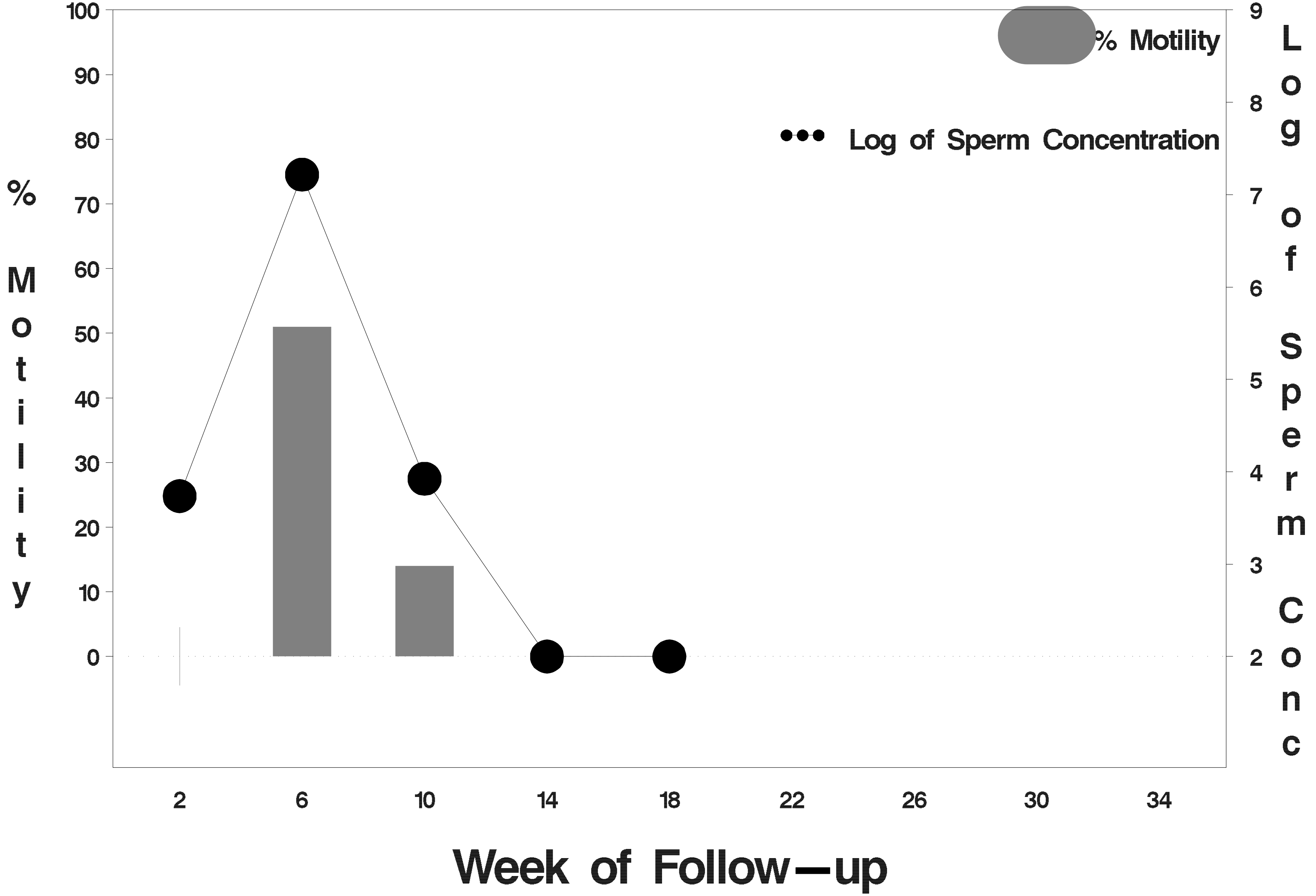

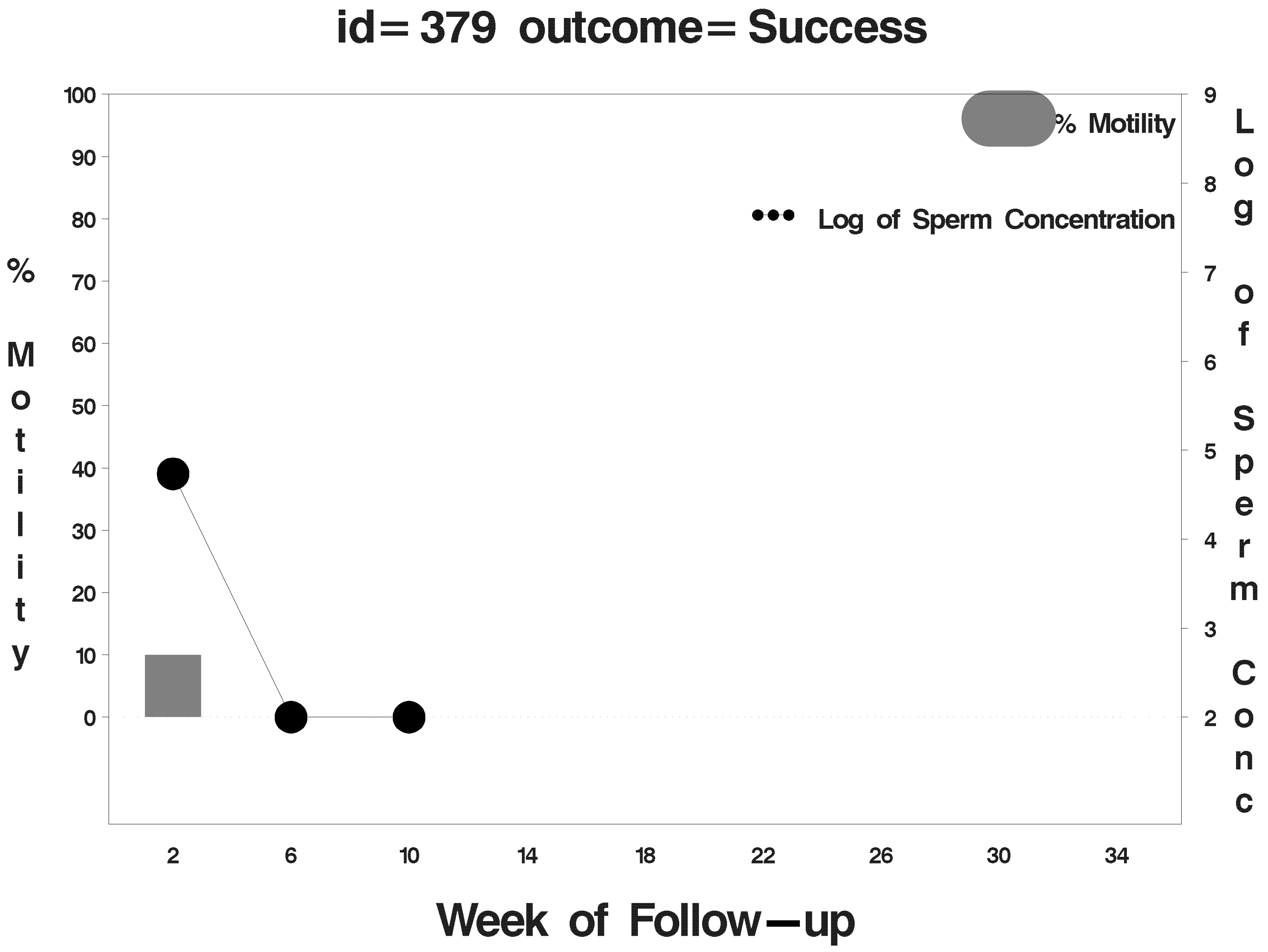

id= 379 outcome= Success
% Motility
Log of Sperm Concentration
100
90
80
70
60
50
40
30
20
10
0
9
8
7
6
5
4
3
2
2
6
10
14
18
22
26
30
34
Week of Follow—up
% Motility
Log of Sperm Conc

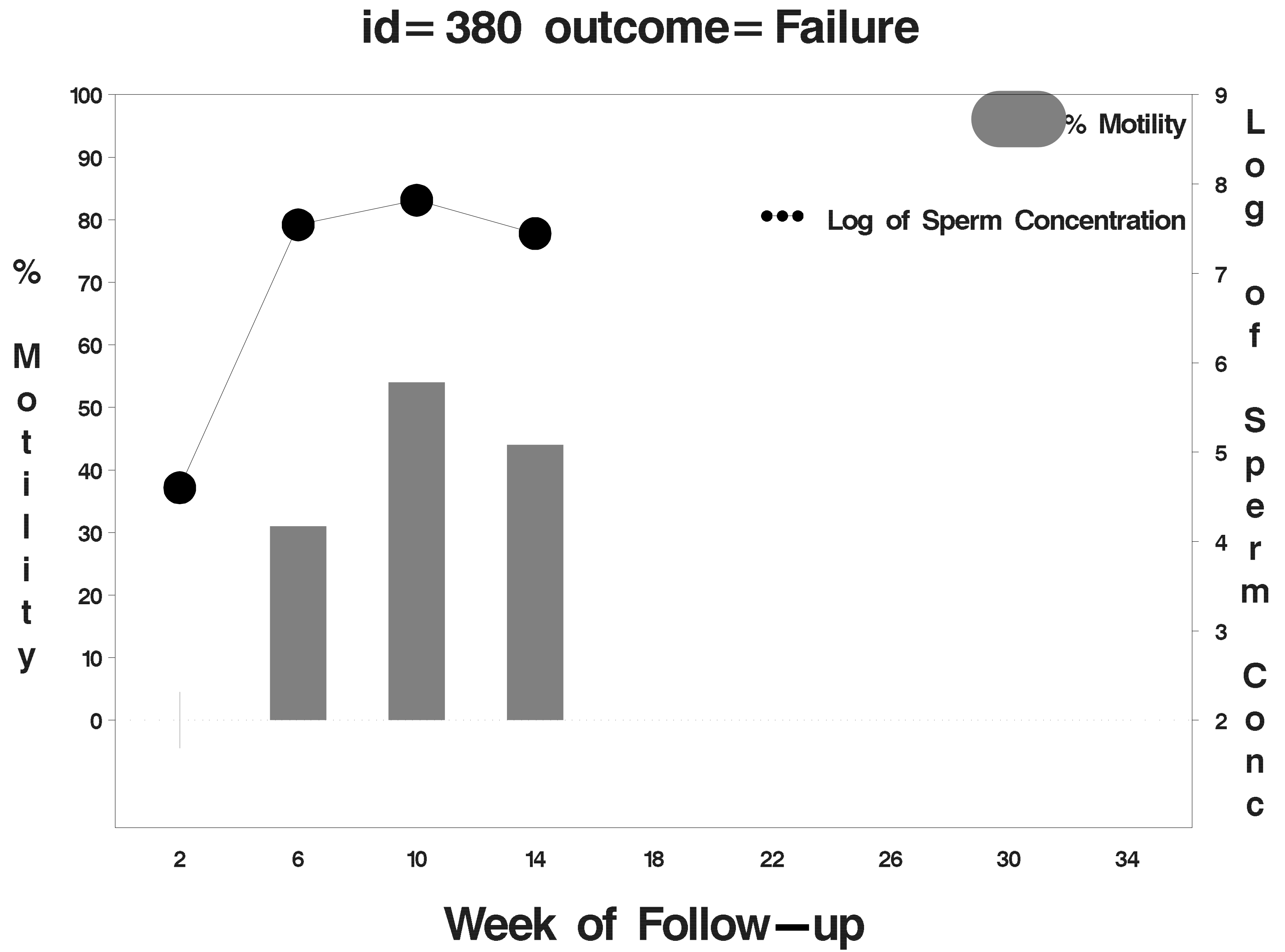

id= 380 outcome= Failure
% Motility
Log of Sperm Concentration
100
90
80
70
60
50
40
30
20
10
0
9
8
7
6
5
4
3
2
2
6
10
14
18
22
26
30
34
Week of Follow—up
Log of Sperm Conc

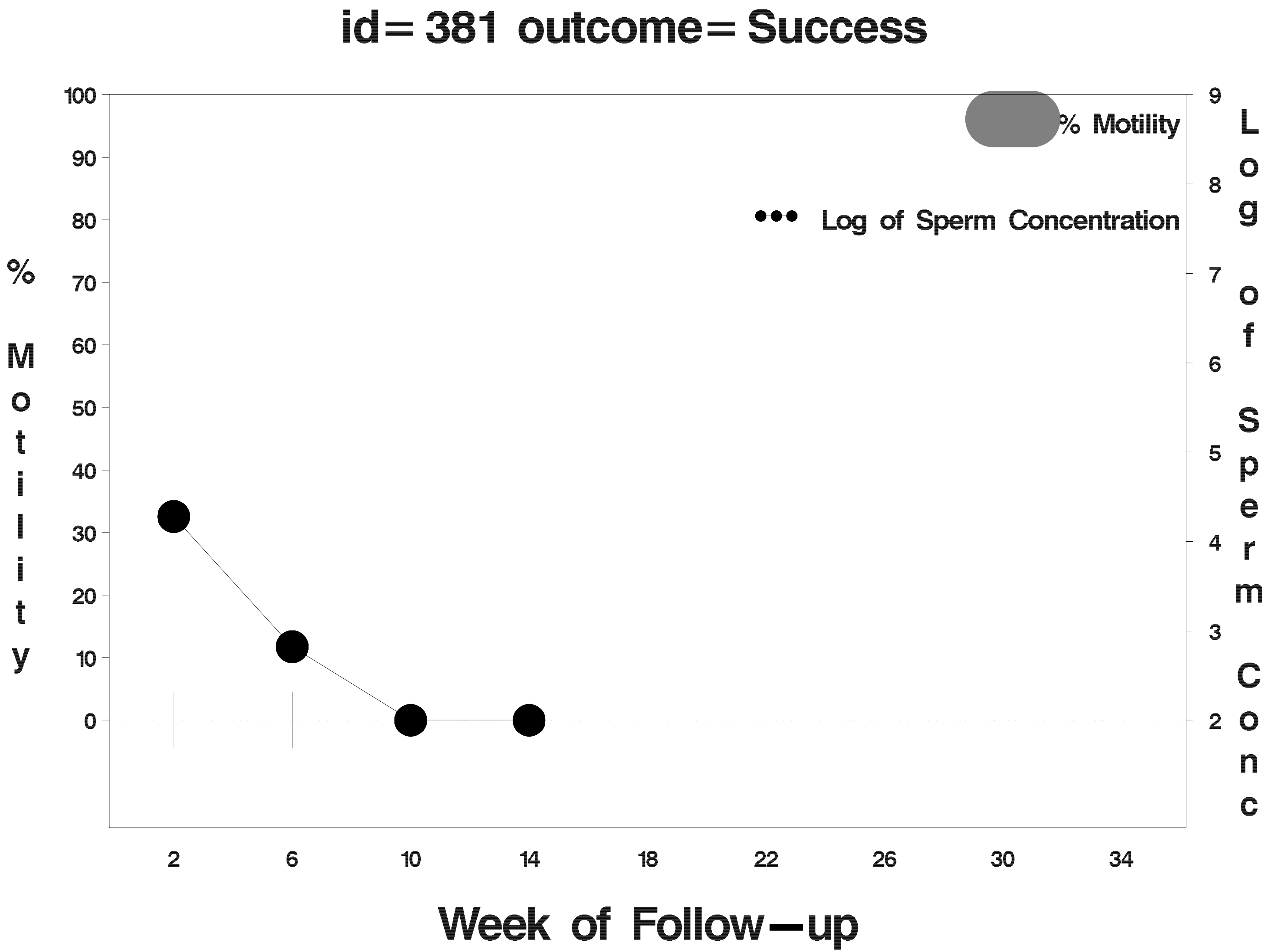
id= 381 outcome= Success
% Motility
Log of Sperm Concentration
% Motility
Log of Sperm Conc
Week of Follow−up
100
90
80
70
60
50
40
30
20
10
0
9
8
7
6
5
4
3
2
2
6
10
14
18
22
26
30
34

id= 382 outcome= Success

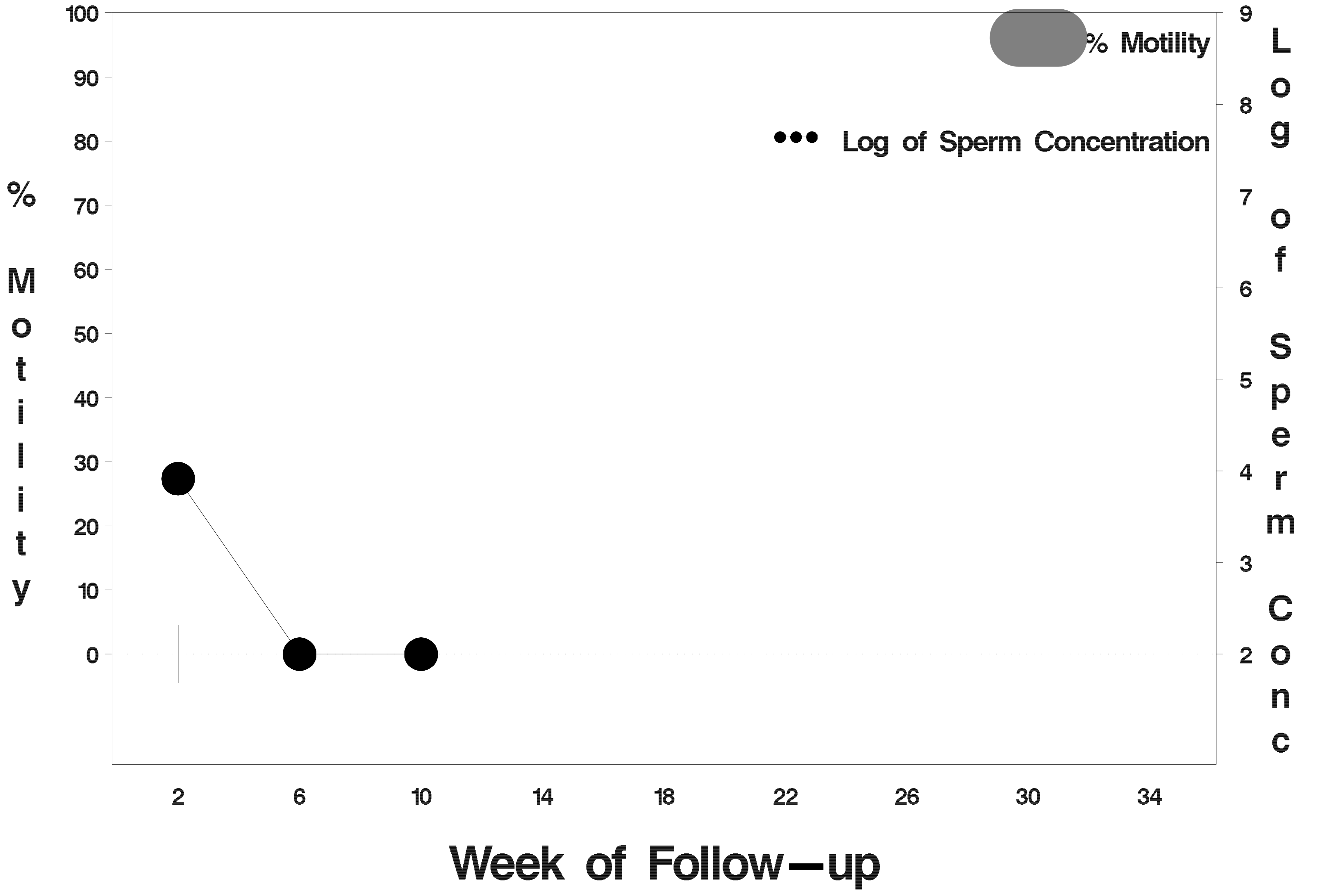

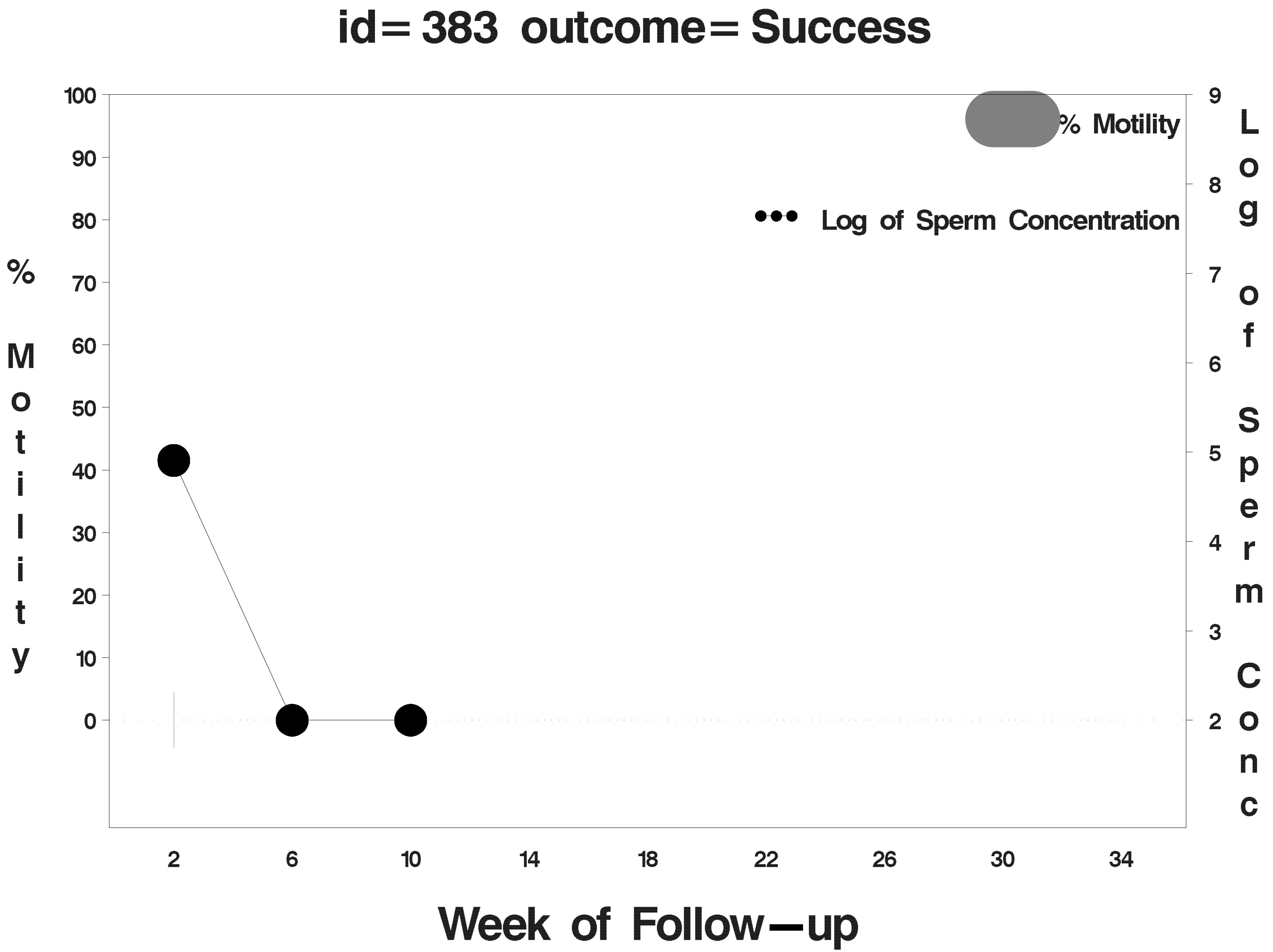

id= 383 outcome= Success
% Motility
Log of Sperm Concentration
100
90
80
70
60
50
40
30
20
10
0
9
8
7
6
5
4
3
2
2
6
10
14
18
22
26
30
34
Week of Follow—up

id= 384 outcome= Success

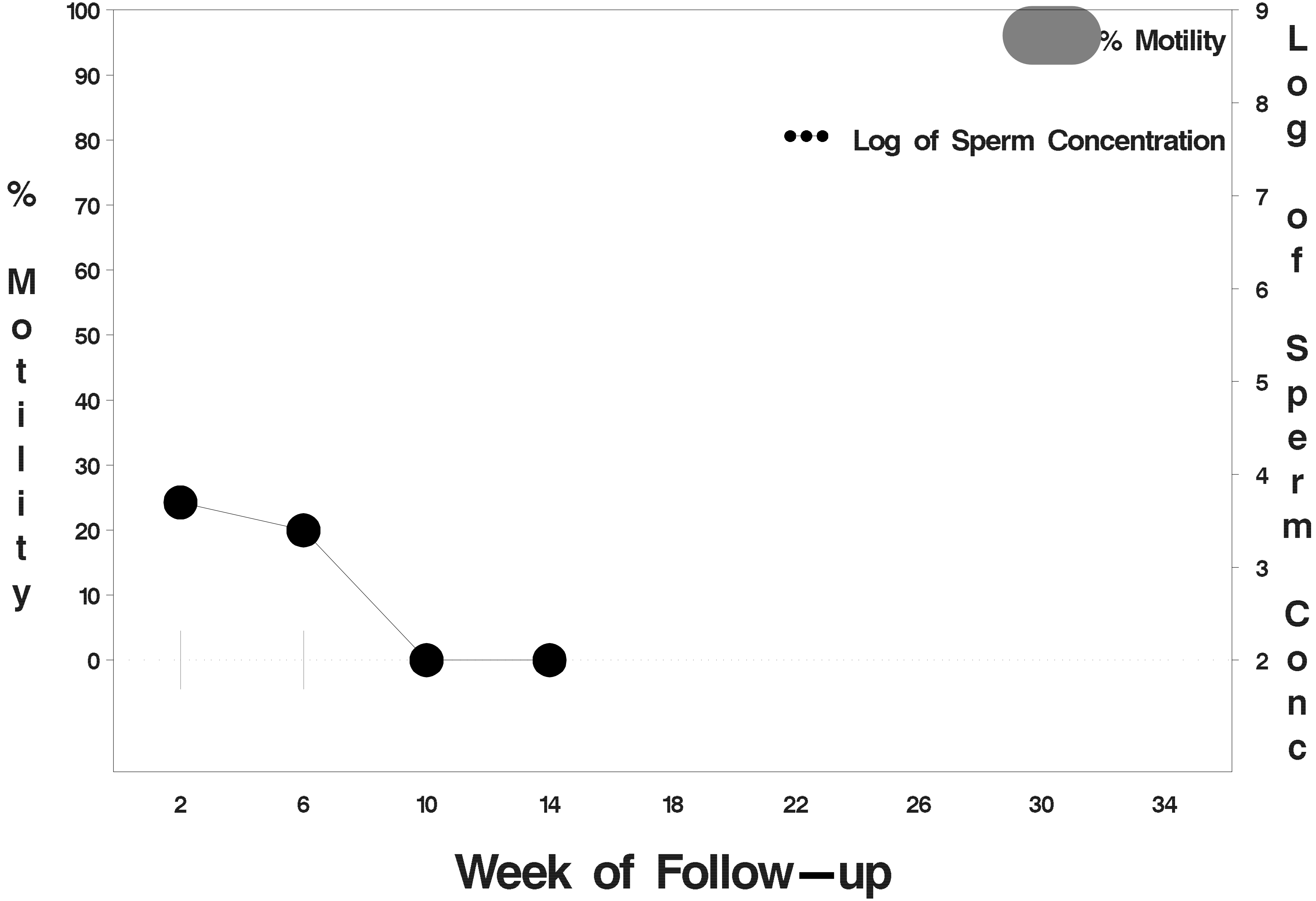

id= 385 outcome= Success

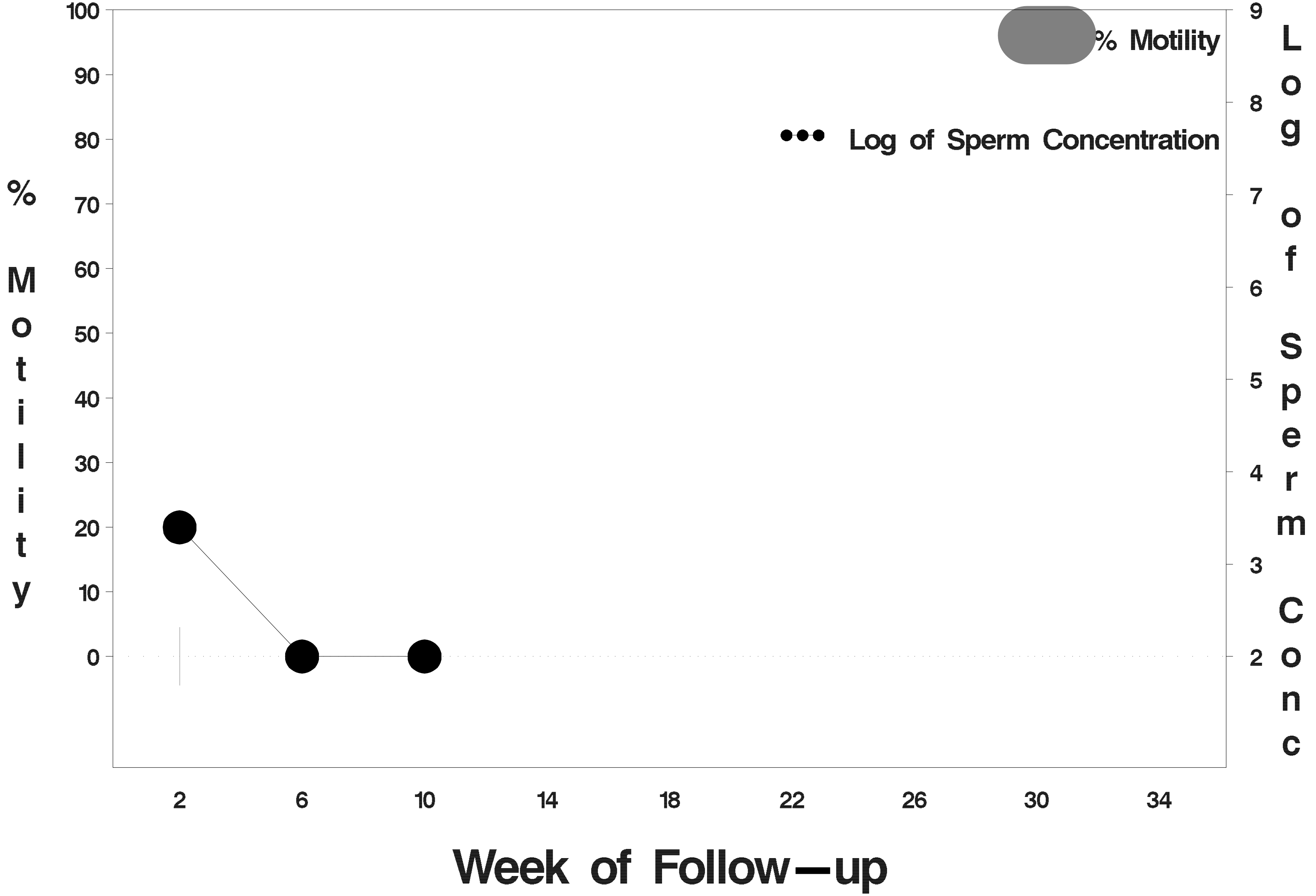

id= 386 outcome= Success

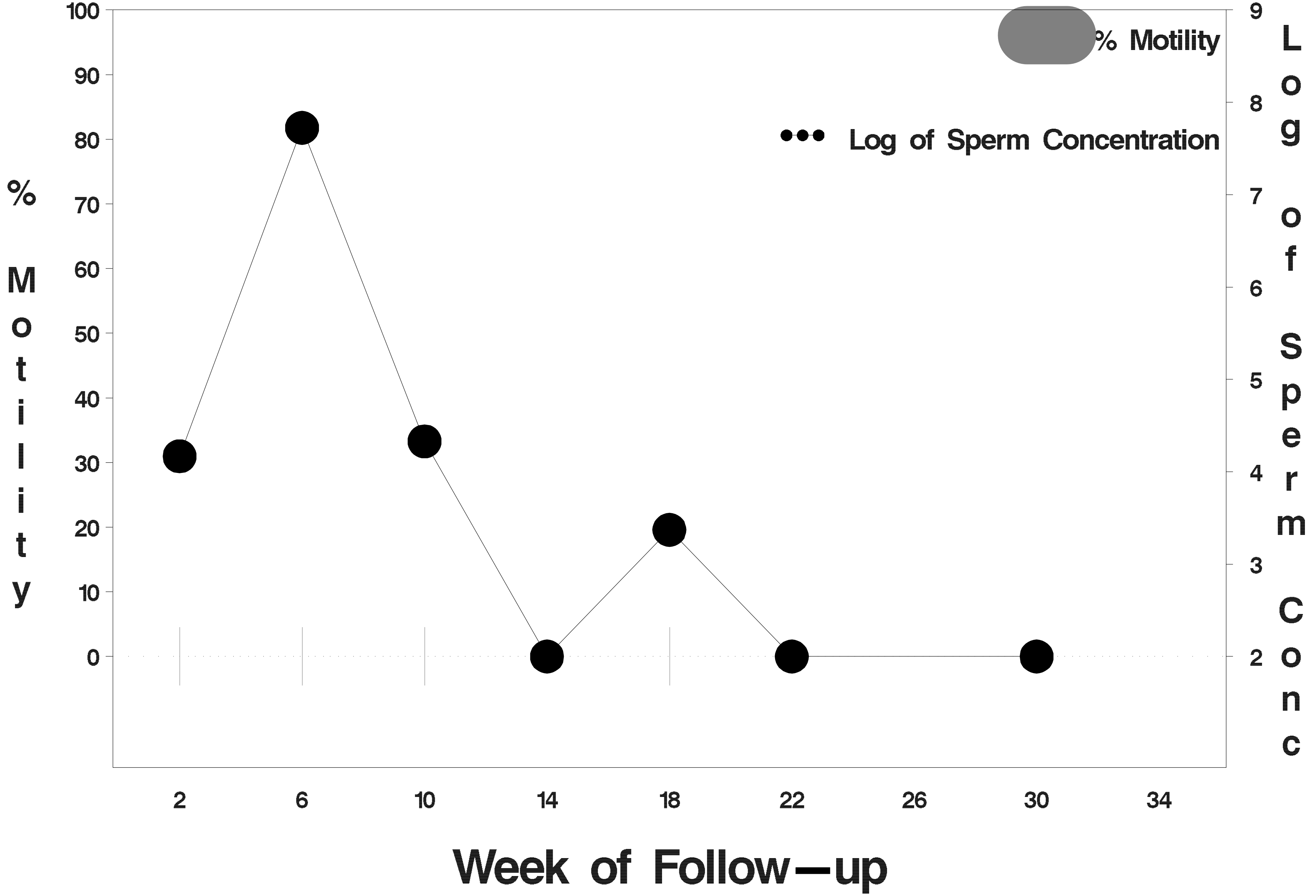

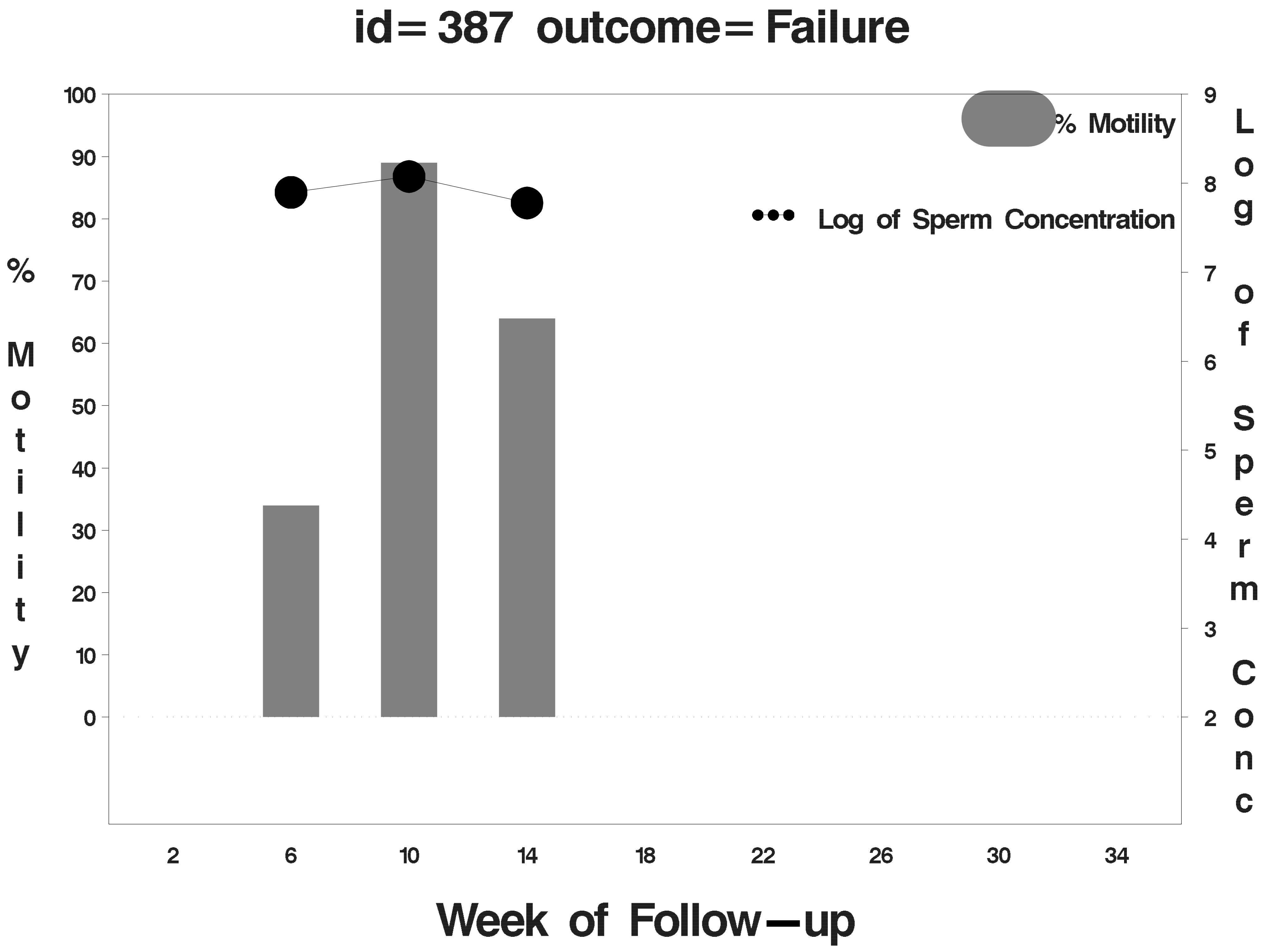
id= 387 outcome= Failure
% Motility
Log of Sperm Concentration
% Motility
Log of Sperm Conc
Week of Follow—up
100
90
80
70
60
50
40
30
20
10
0
9
8
7
6
5
4
3
2
2
6
10
14
18
22
26
30
34

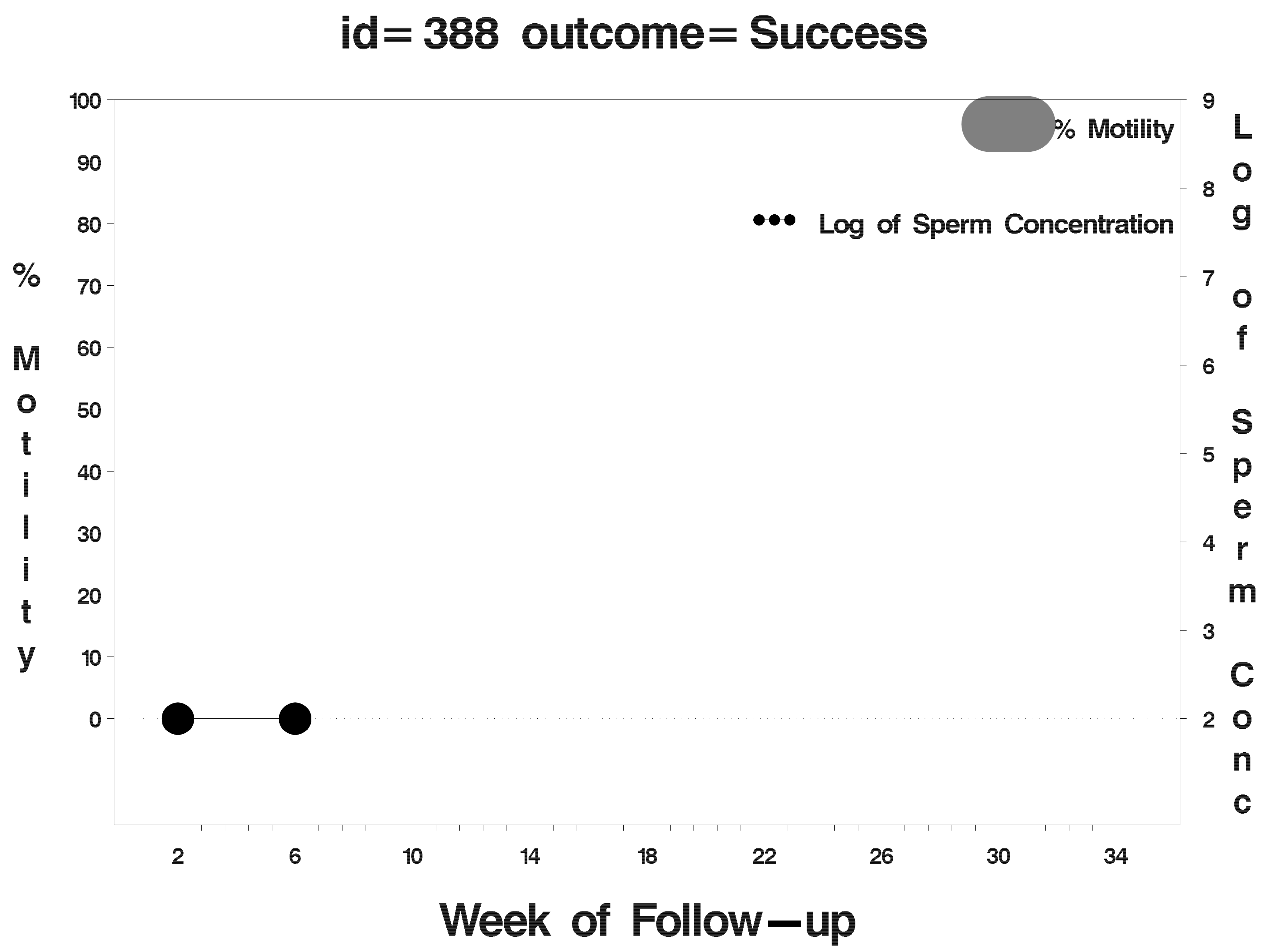

id= 388 outcome= Success
% Motility
Log of Sperm Concentration
100
90
80
70
60
50
40
30
20
10
0
9
8
7
6
5
4
3
2
2
6
10
14
18
22
26
30
34
Week of Follow—up
Log of Sperm Conc

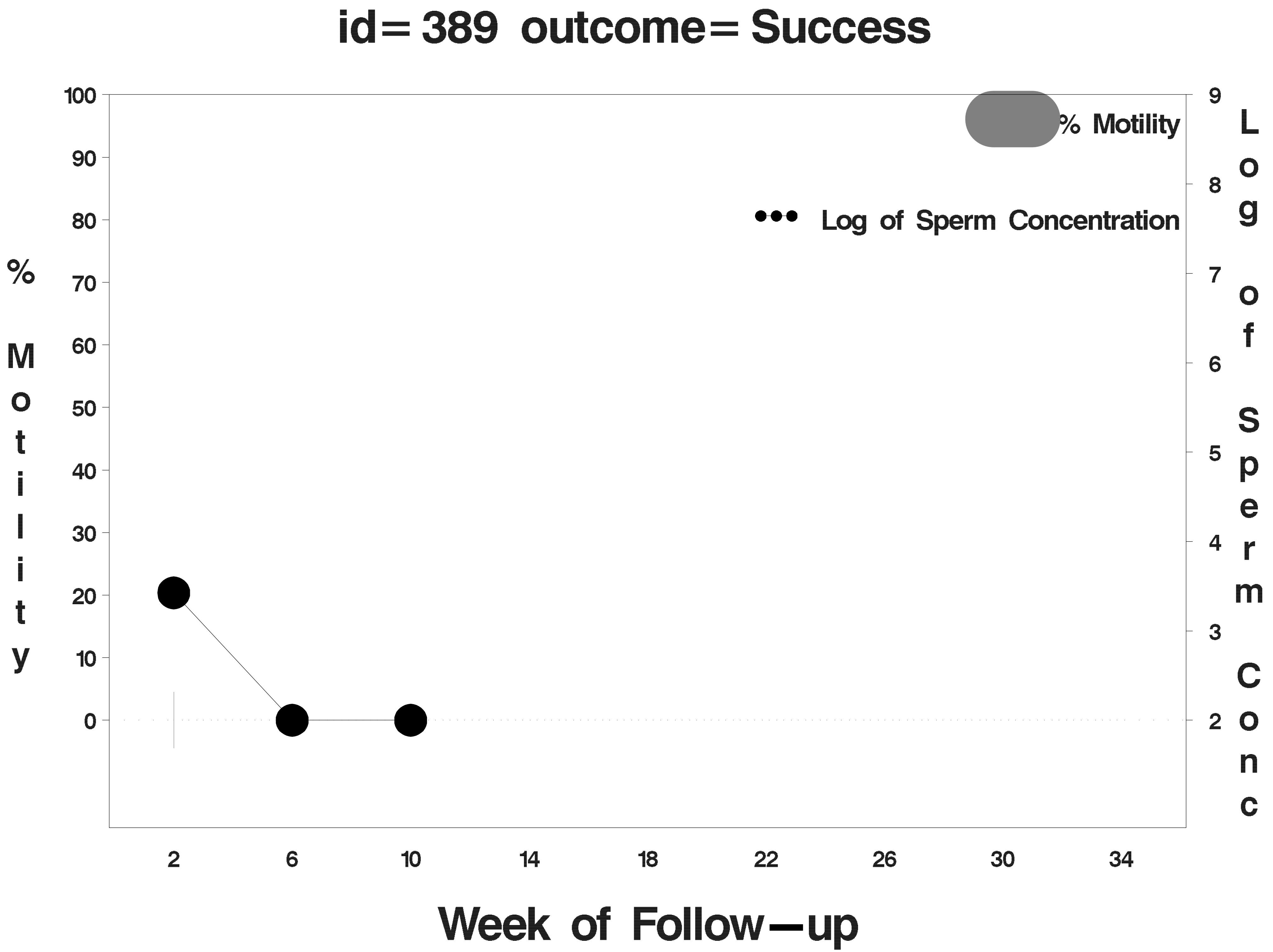
id= 389 outcome= Success
% Motility
Log of Sperm Concentration
100
90
80
70
60
50
40
30
20
10
0
9
8
7
6
5
4
3
2
2
6
10
14
18
22
26
30
34
Week of Follow—up
% Motility
Log of Sperm Conc

id= 390 outcome= Success

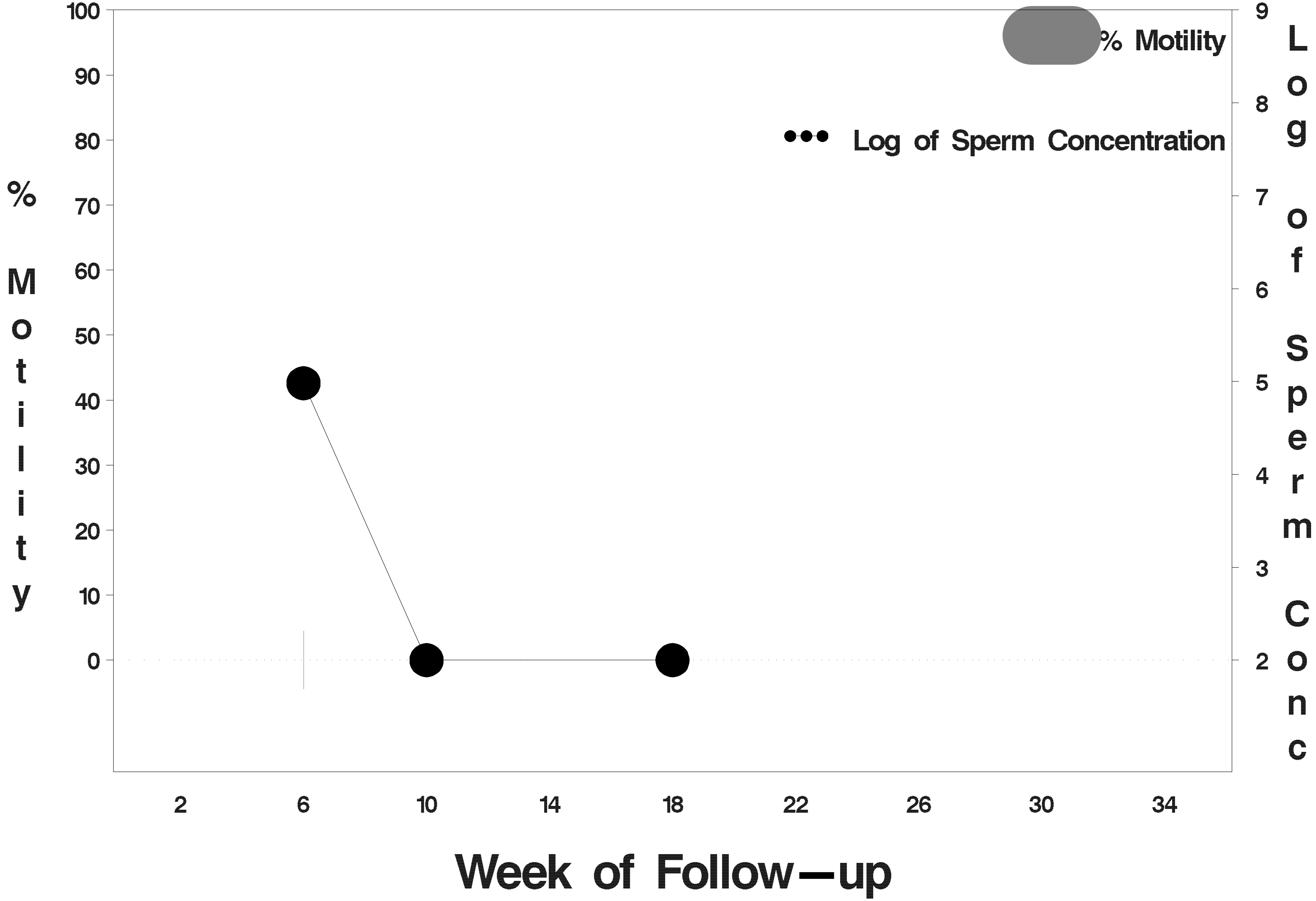

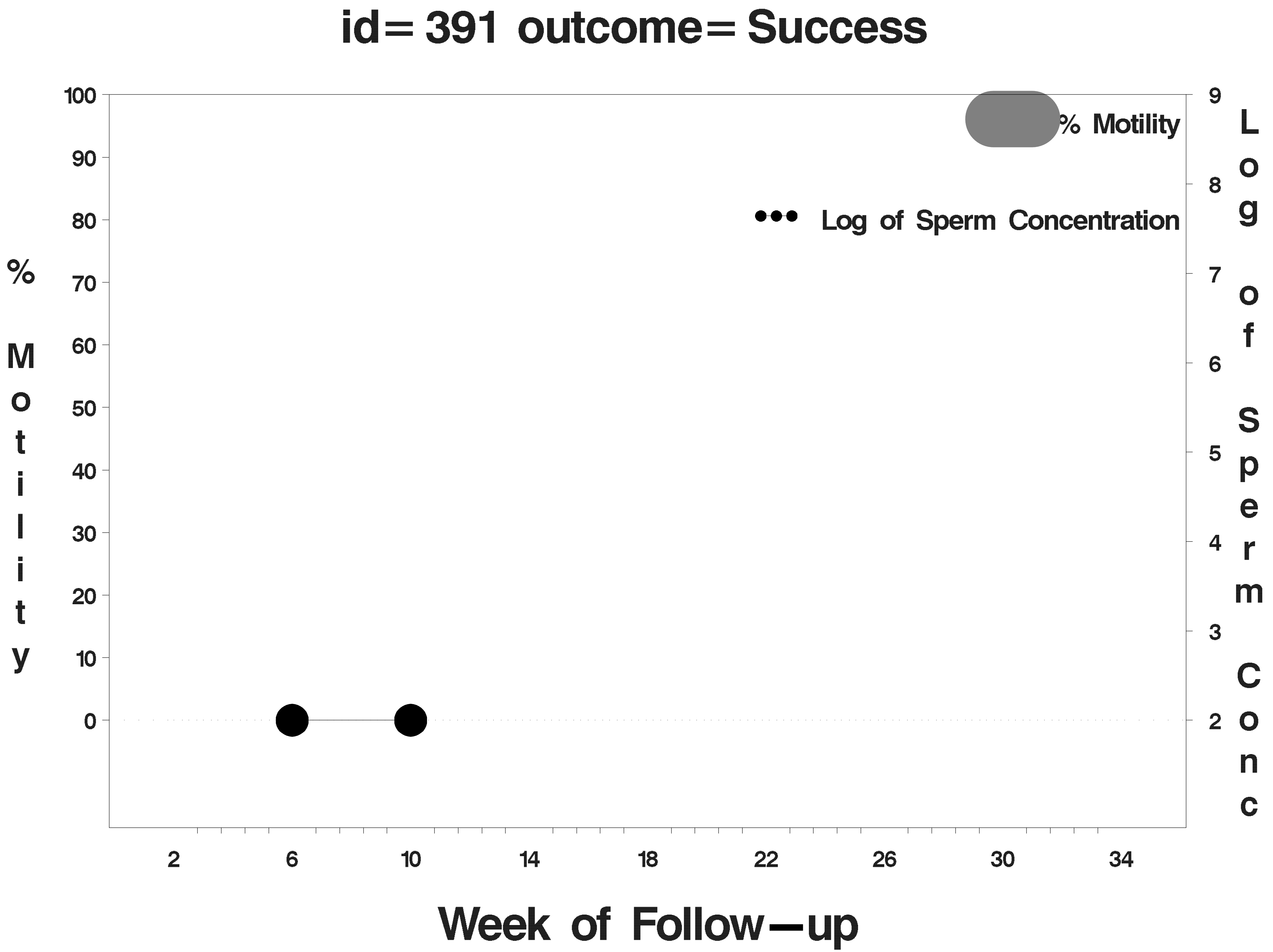

id= 391 outcome= Success
% Motility
Log of Sperm Concentration
% Motility
Log of Sperm Conc
Week of Follow-up
100
90
80
70
60
50
40
30
20
10
0
9
8
7
6
5
4
3
2
2
6
10
14
18
22
26
30
34

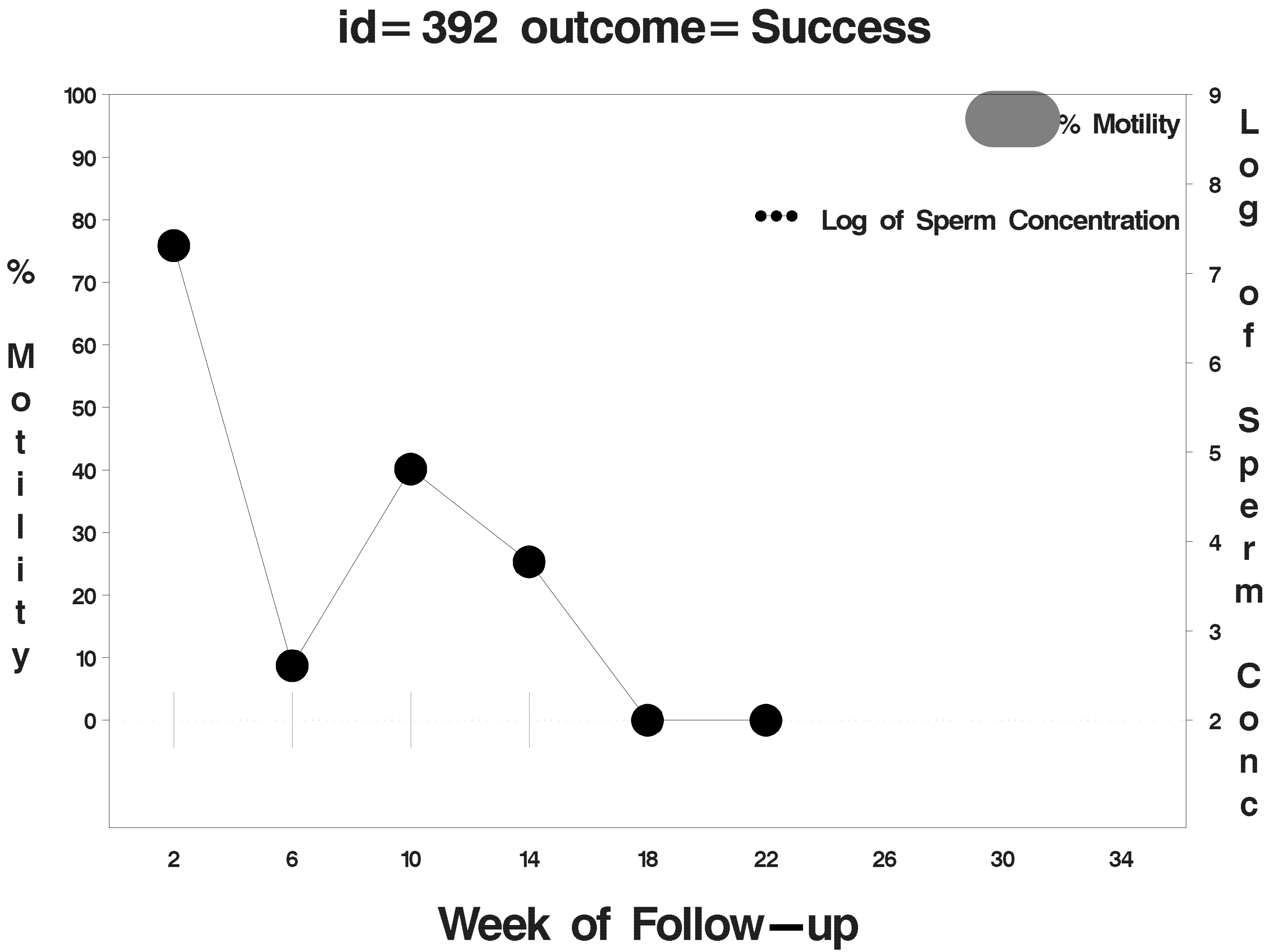

id= 392 outcome= Success
% Motility
Log of Sperm Concentration
100
90
80
70
60
50
40
30
20
10
0
9
8
7
6
5
4
3
2
2
6
10
14
18
22
26
30
34
Week of Follow—up
% Motility
Log of Sperm Conc

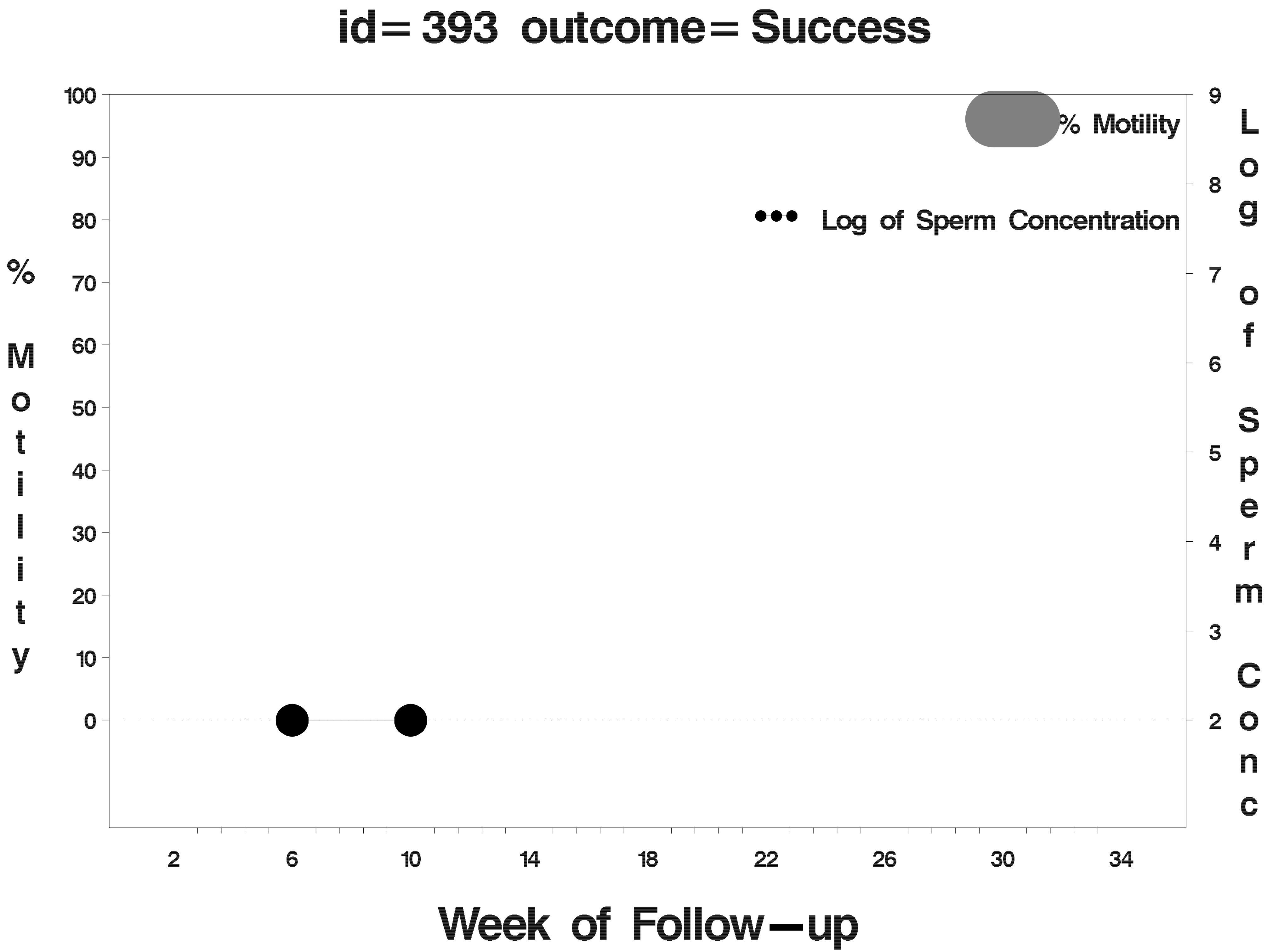

id= 393 outcome= Success
% Motility
Log of Sperm Concentration
100
90
80
70
60
50
40
30
20
10
0
9
8
7
6
5
4
3
2
2
6
10
14
18
22
26
30
34
Week of Follow—up
Log of Sperm Conc

id= 394 outcome= Success

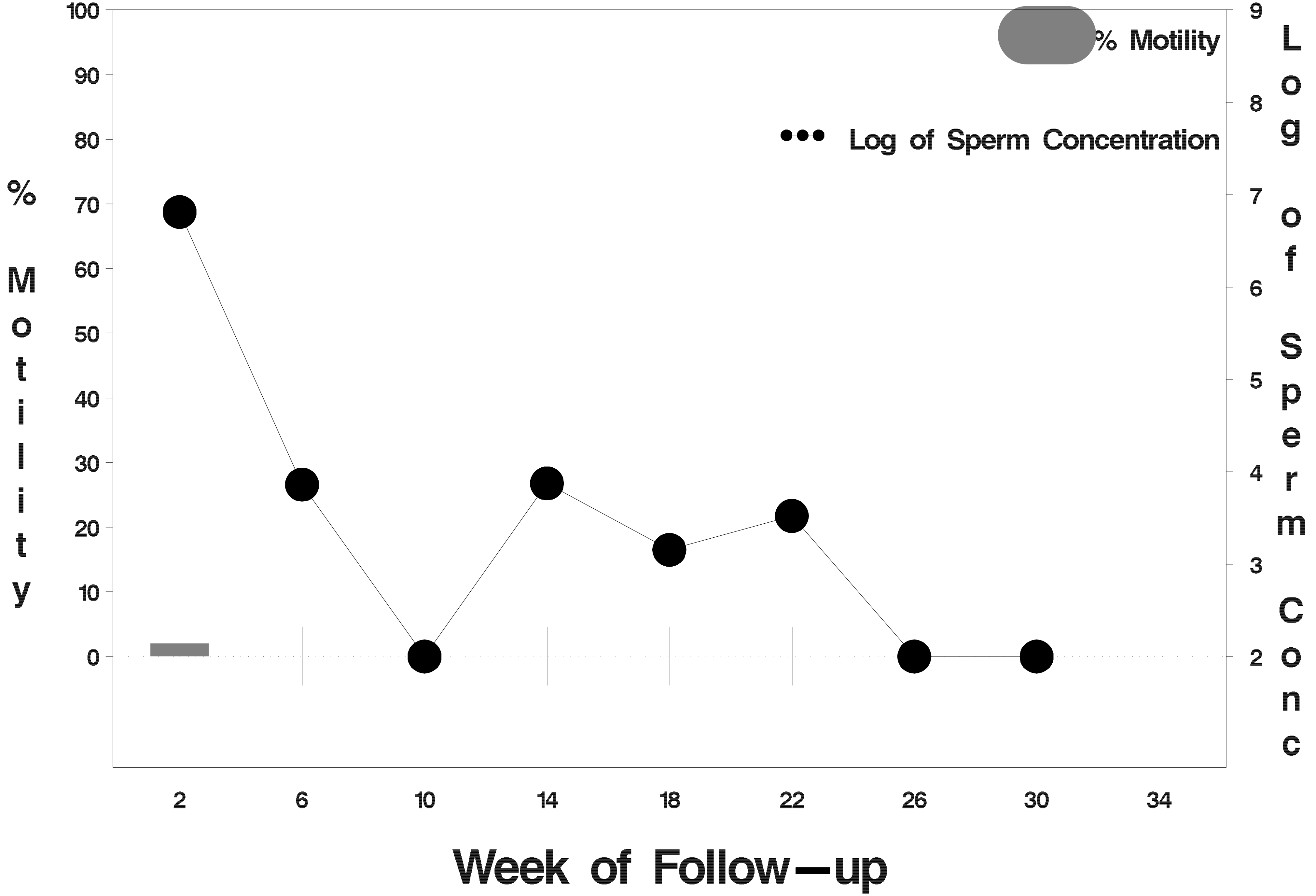

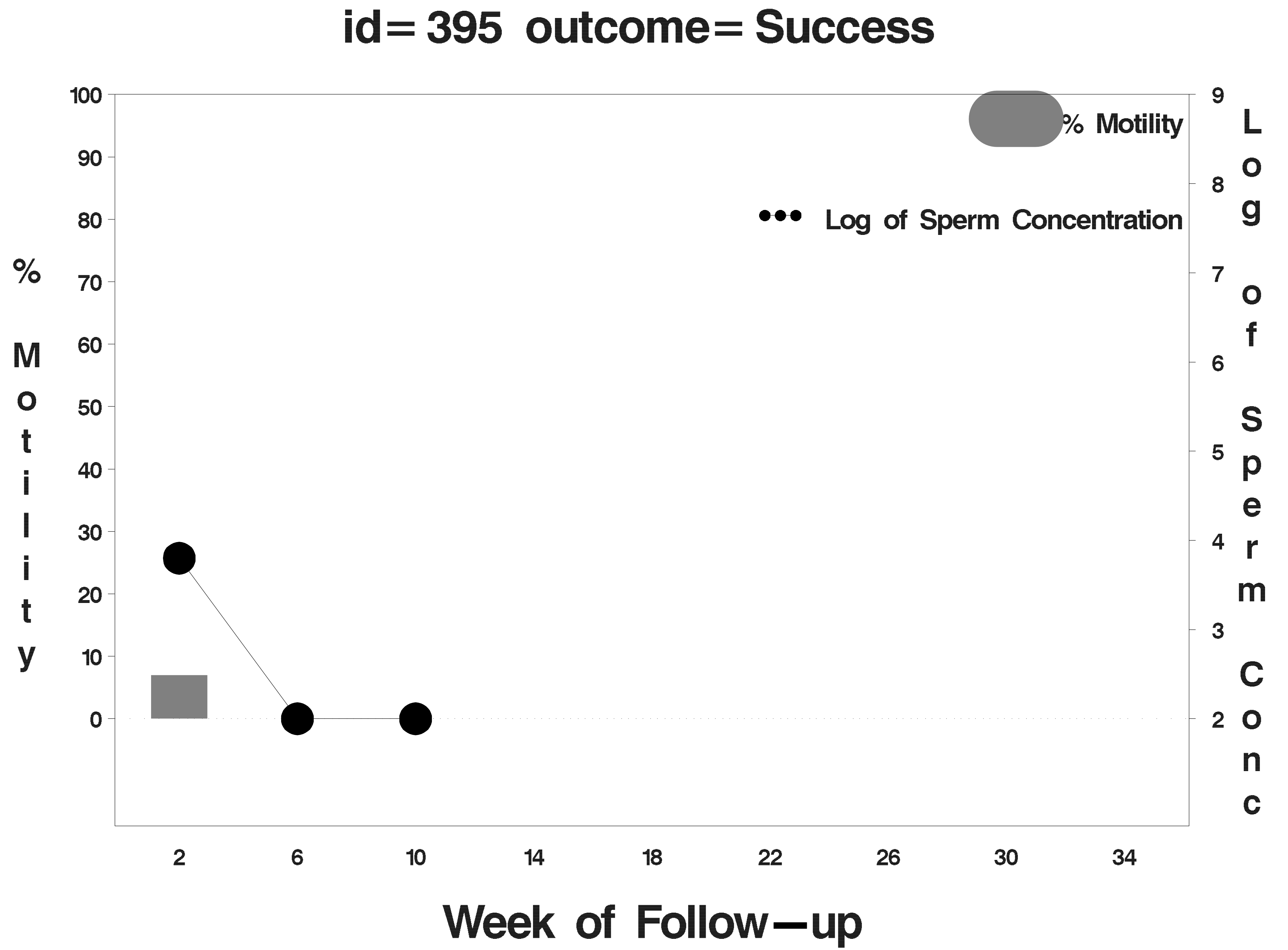

id= 395 outcome= Success
% Motility
Log of Sperm Concentration
100
90
80
70
60
50
40
30
20
10
0
9
8
7
6
5
4
3
2
2
6
10
14
18
22
26
30
34
Week of Follow—up
Log of Sperm Conc

id= 396 outcome= Success

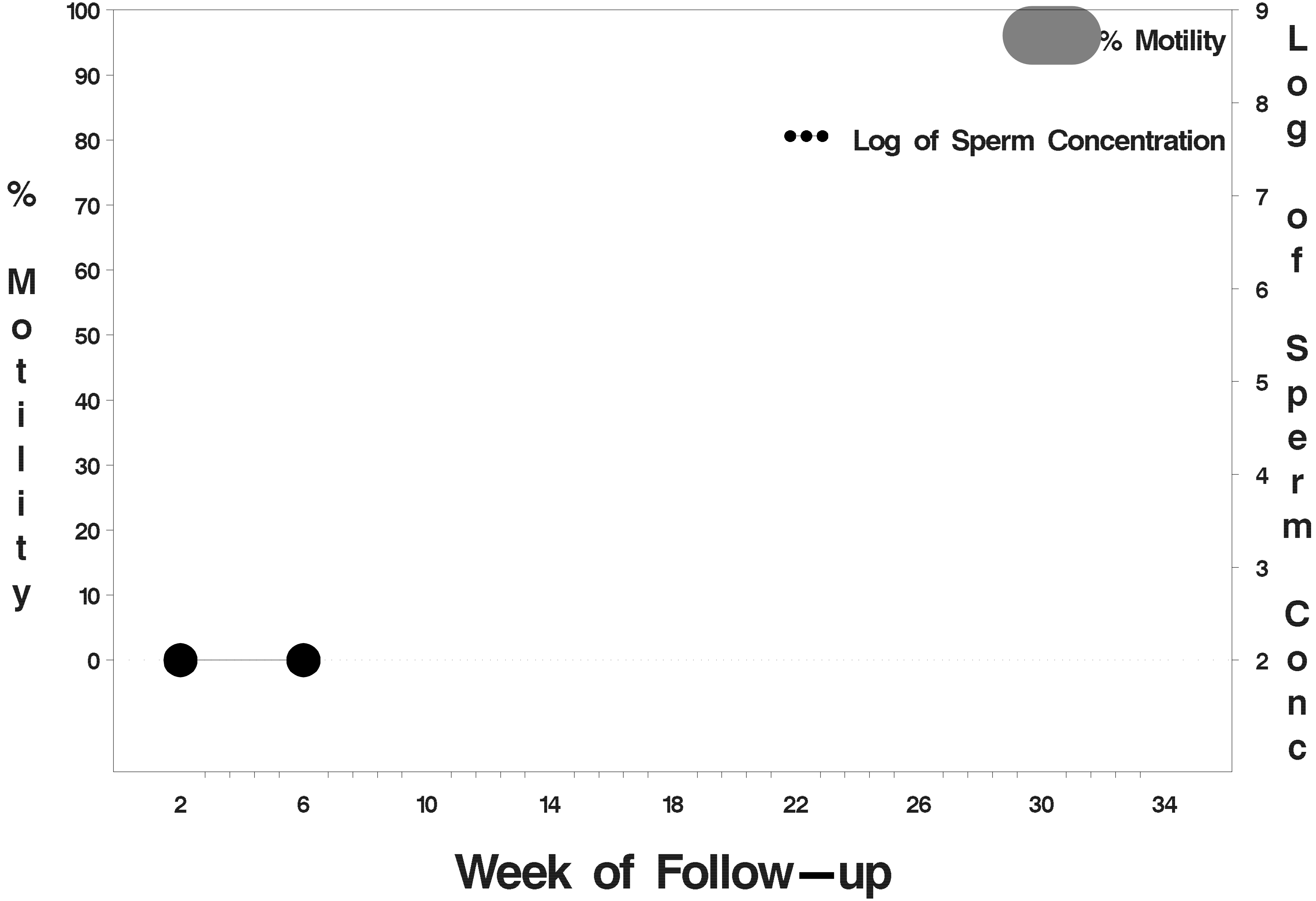

id= 397 outcome= Failure

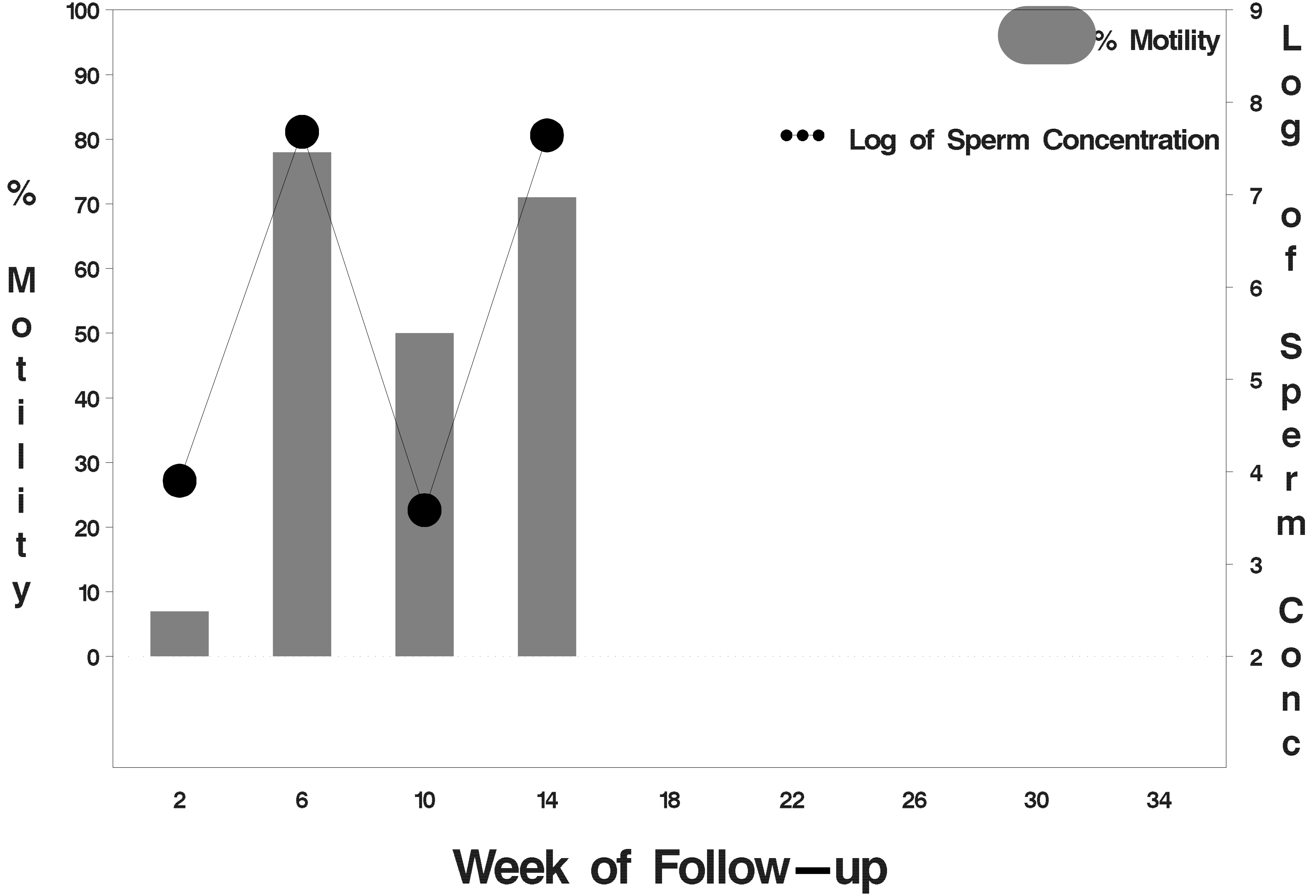

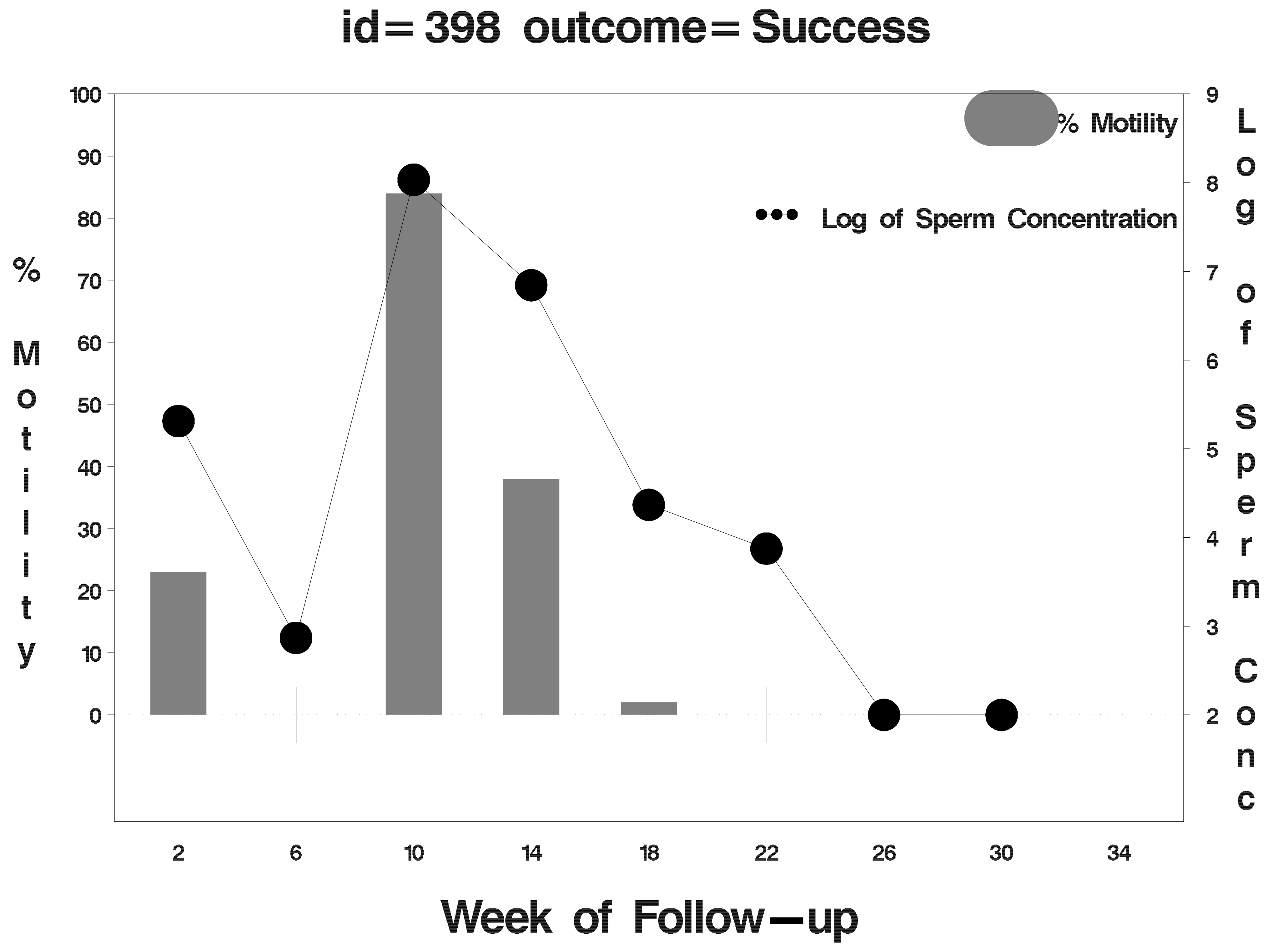

id= 398 outcome= Success
% Motility
Log of Sperm Concentration
% Motility
Log of Sperm Conc
Week of Follow-up
100
90
80
70
60
50
40
30
20
10
0
9
8
7
6
5
4
3
2
2
6
10
14
18
22
26
30
34

id= 399 outcome= Success

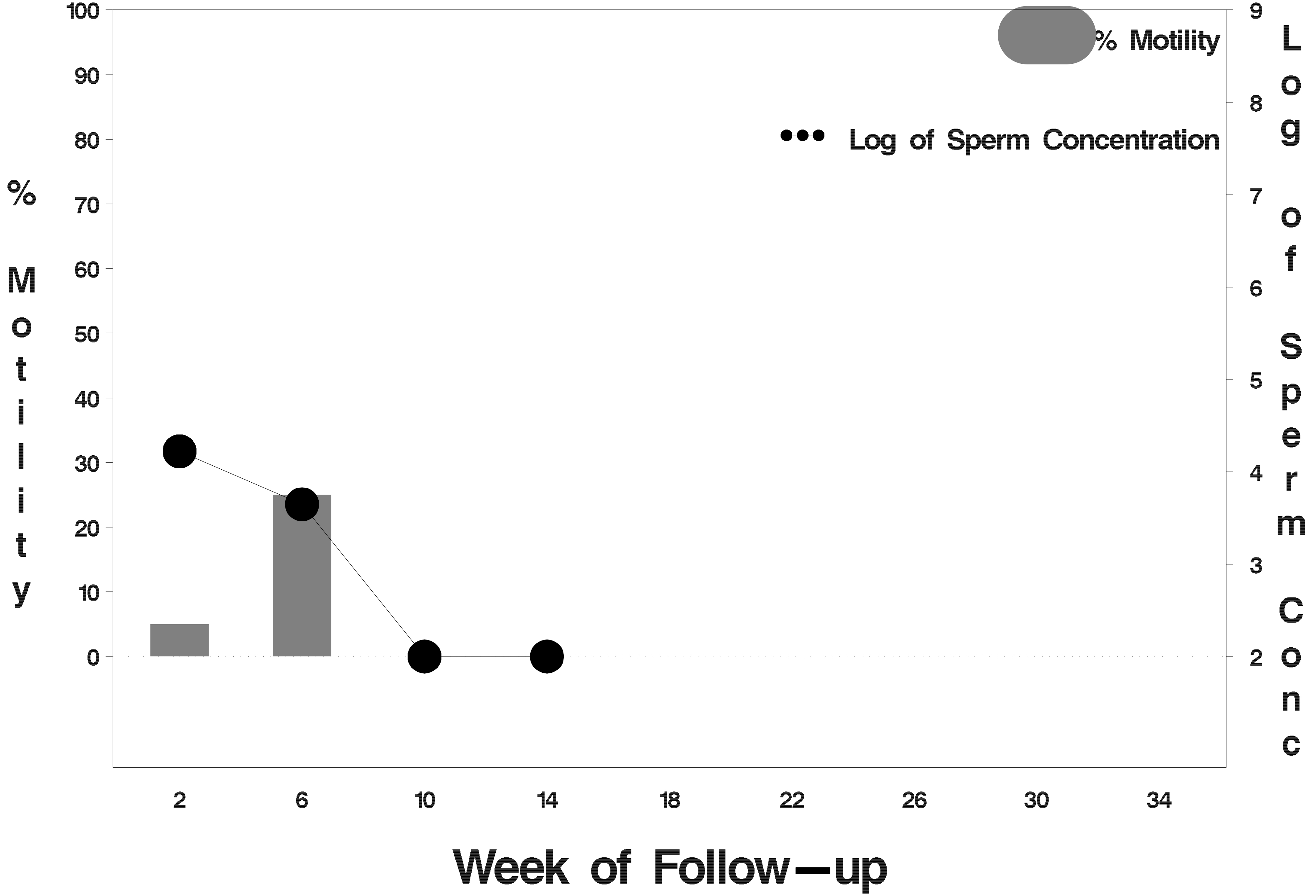

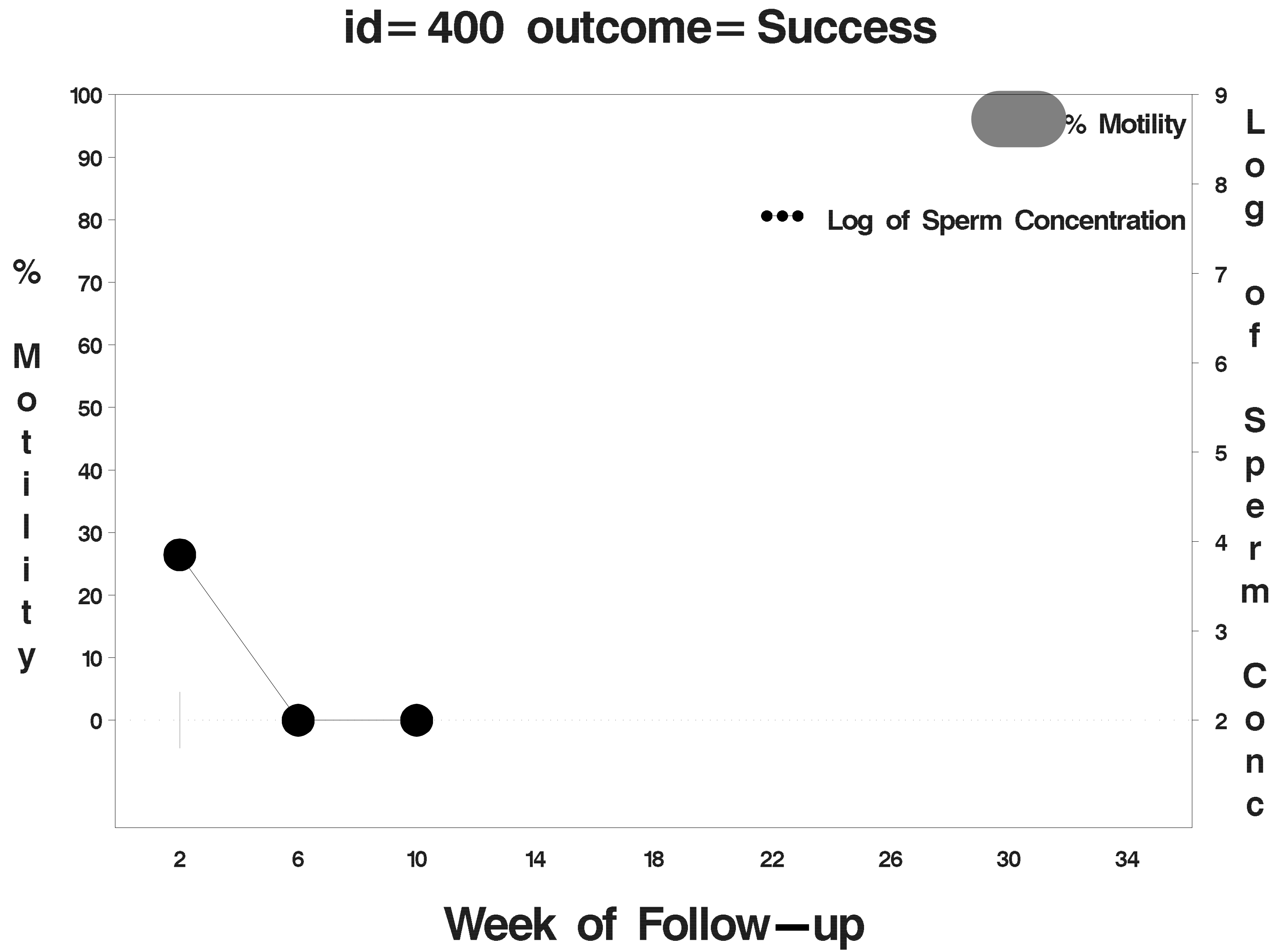

id= 400 outcome= Success
% Motility
Log of Sperm Concentration
100
90
80
70
60
50
40
30
20
10
0
9
8
7
6
5
4
3
2
2
6
10
14
18
22
26
30
34
Week of Follow−up
Log of Sperm Conc

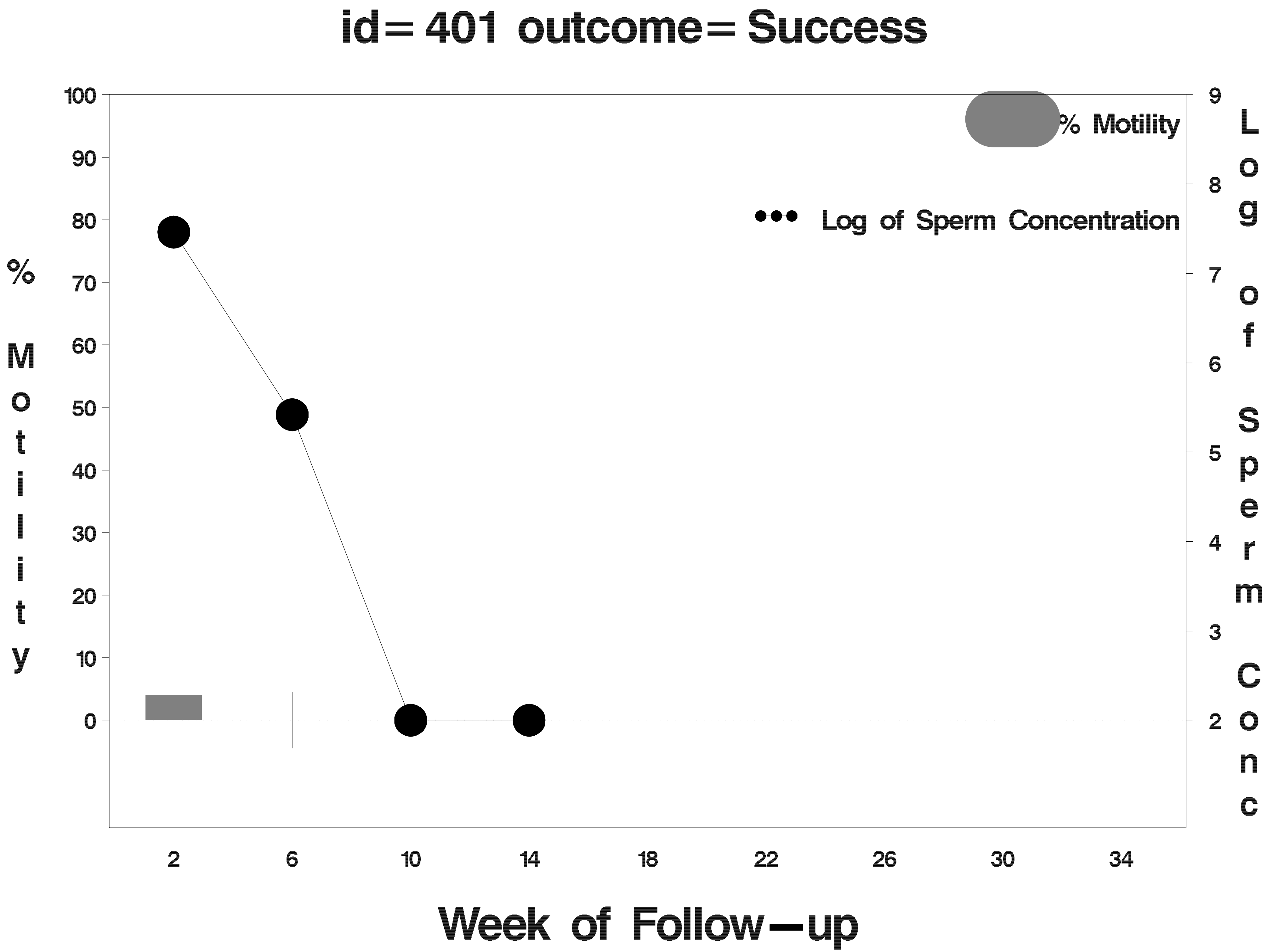

id= 401 outcome= Success
% Motility
Log of Sperm Concentration
100
90
80
70
60
50
40
30
20
10
0
9
8
7
6
5
4
3
2
2
6
10
14
18
22
26
30
34
Week of Follow—up
Log of Sperm Conc

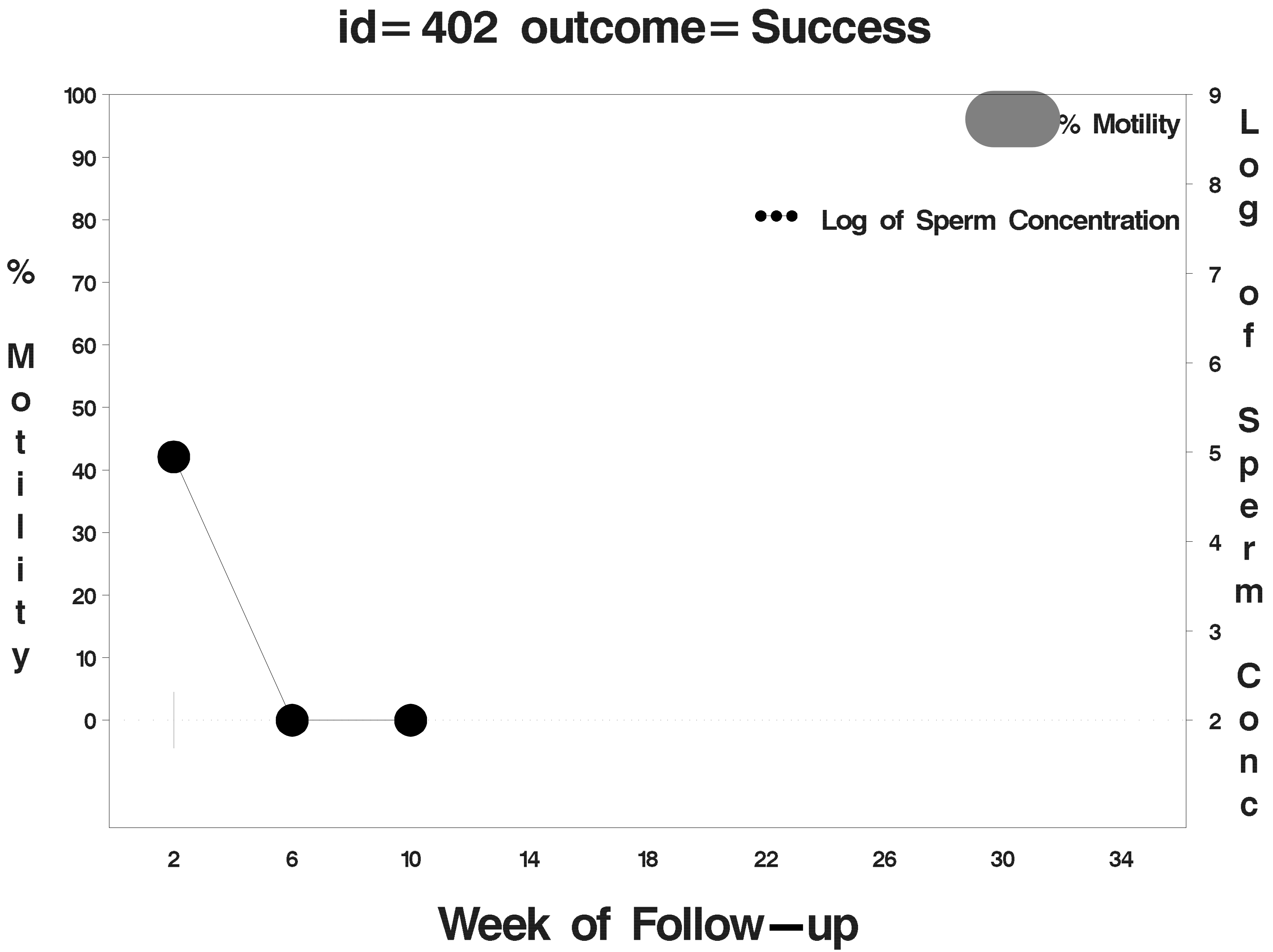
id= 402 outcome= Success
% Motility
Log of Sperm Concentration
% Motility
Log of Sperm Conc
Week of Follow−up
100
90
80
70
60
50
40
30
20
10
0
9
8
7
6
5
4
3
2
2
6
10
14
18
22
26
30
34

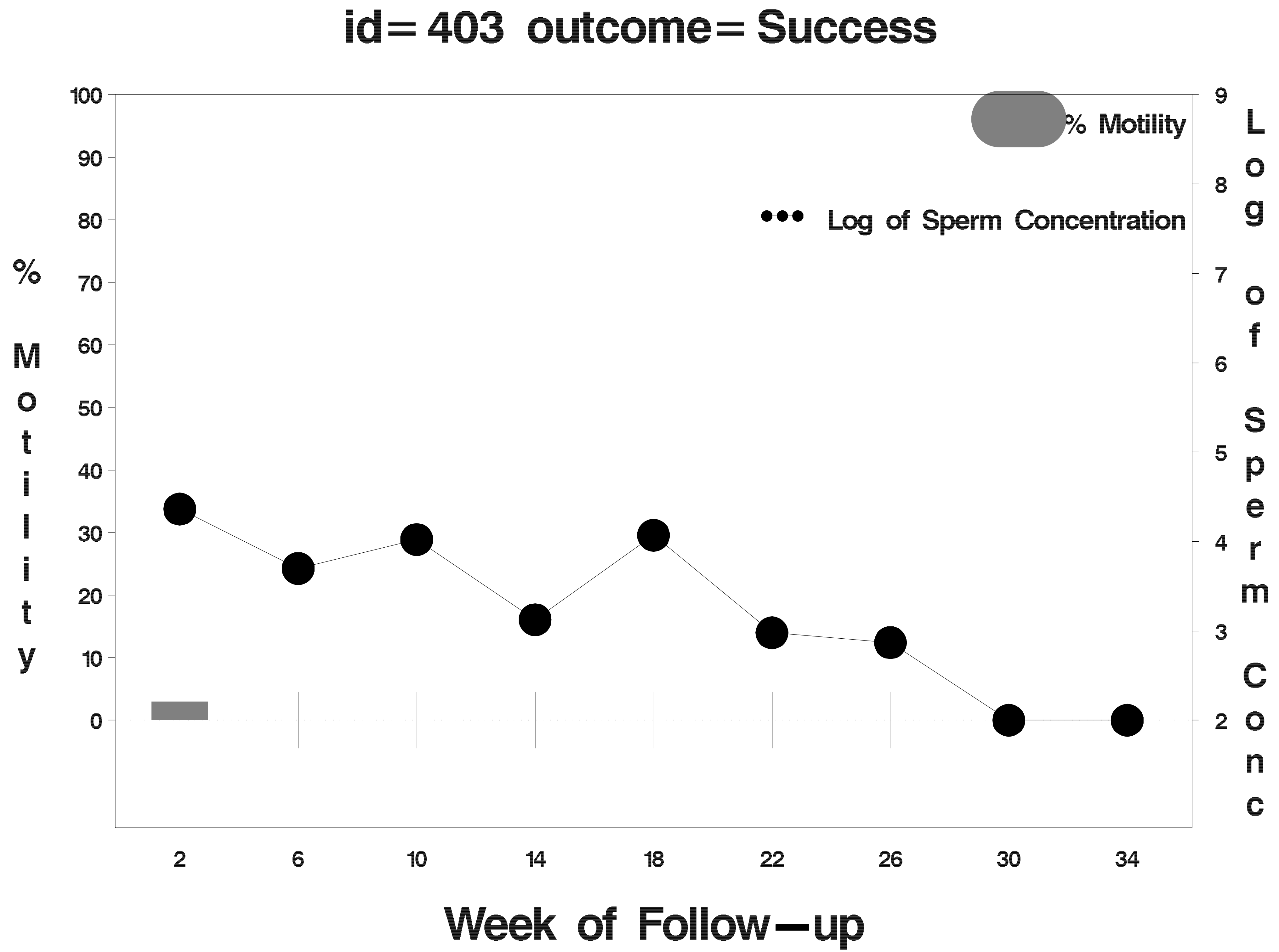

id= 403 outcome= Success
% Motility
Log of Sperm Concentration
% Motility
Log of Sperm Conc
100
90
80
70
60
50
40
30
20
10
0
9
8
7
6
5
4
3
2
2
6
10
14
18
22
26
30
34
Week of Follow−up

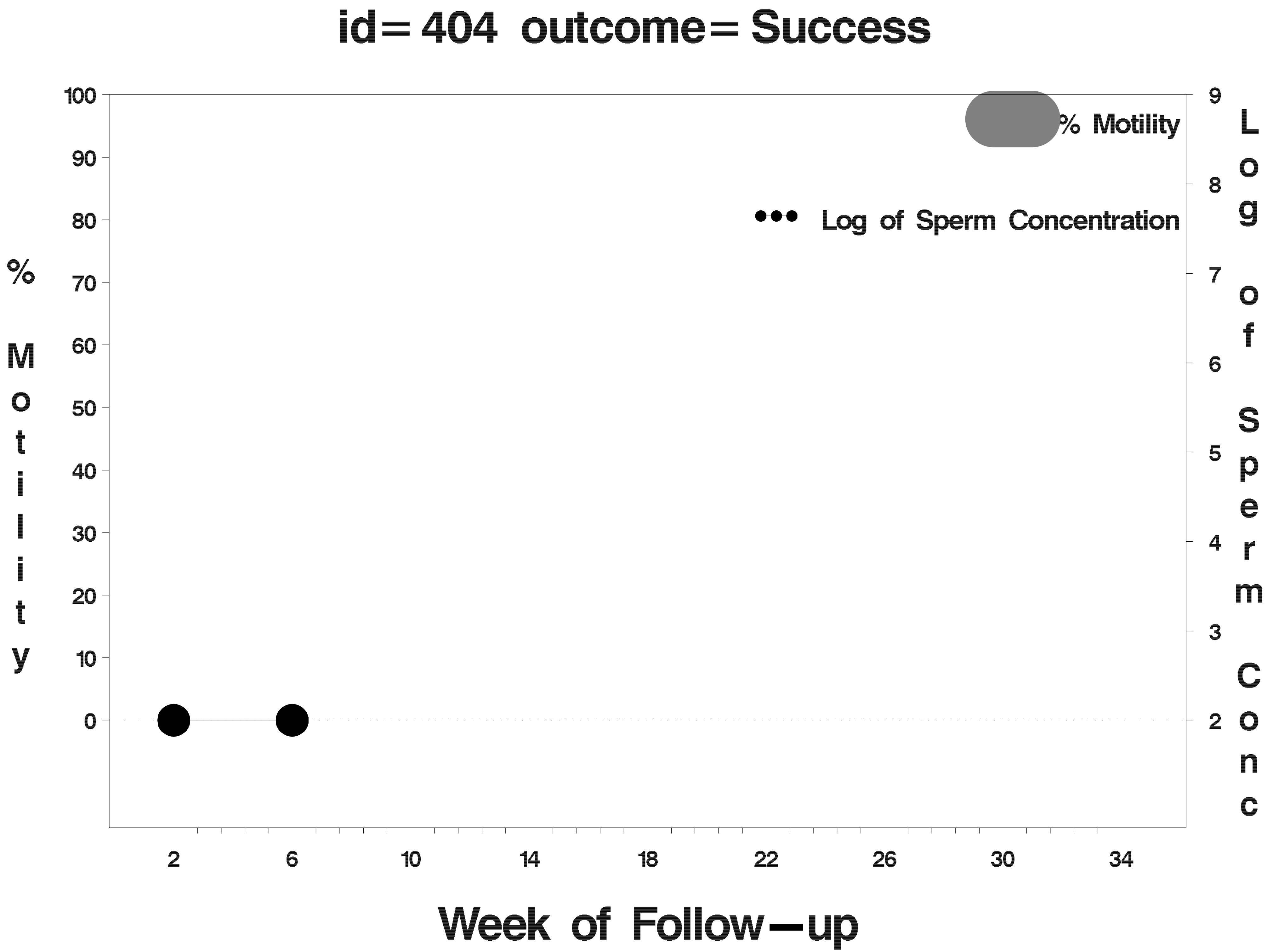

id= 404 outcome= Success
% Motility
Log of Sperm Concentration
% Motility
Log of Sperm Conc
Week of Follow−up
100
90
80
70
60
50
40
30
20
10
0
9
8
7
6
5
4
3
2
2
6
10
14
18
22
26
30
34

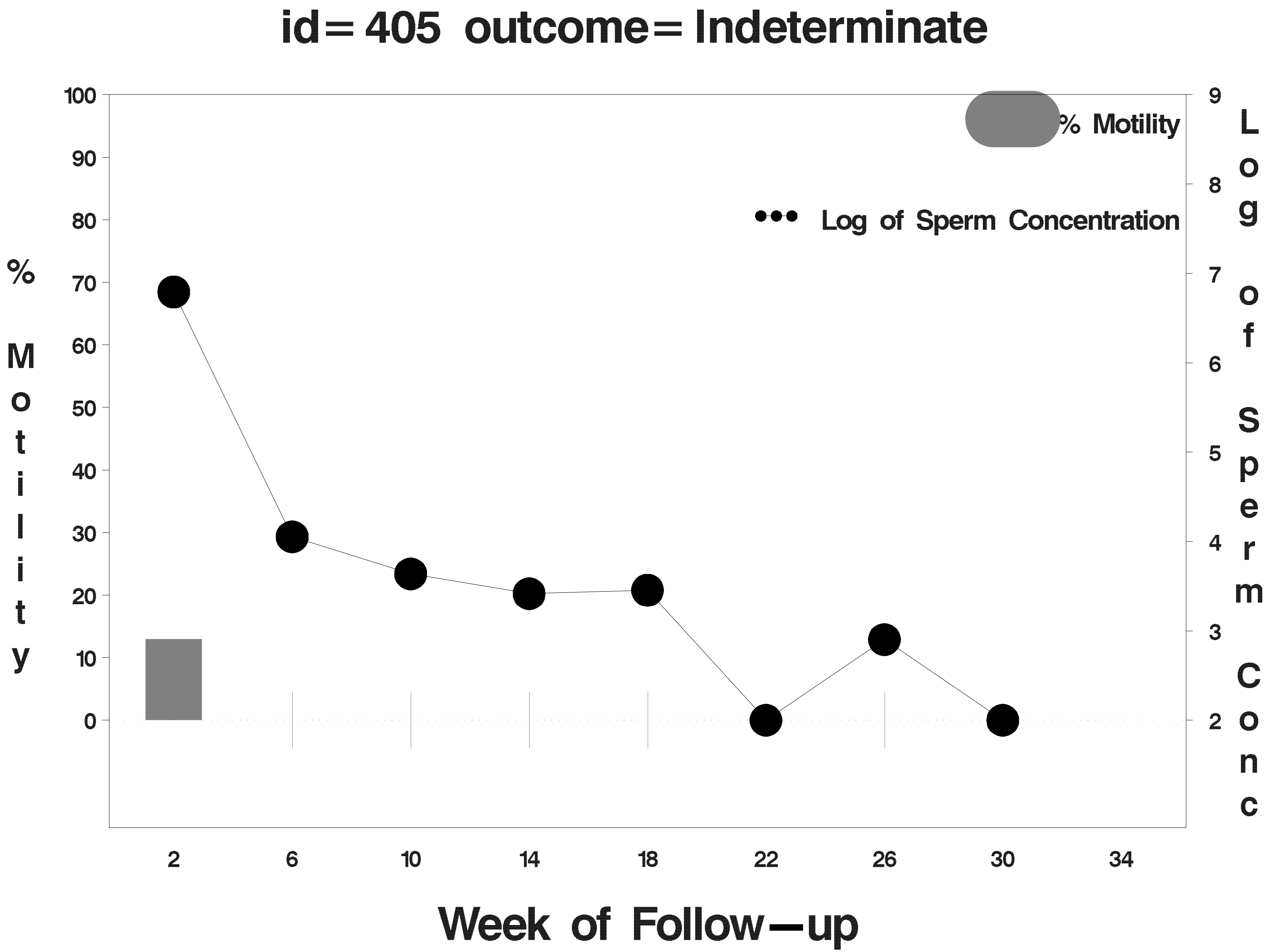

id= 405 outcome= Indeterminate
% Motility
Log of Sperm Concentration
% Motility
Log of Sperm Conc
100
90
80
70
60
50
40
30
20
10
0
9
8
7
6
5
4
3
2
2
6
10
14
18
22
26
30
34
Week of Follow—up

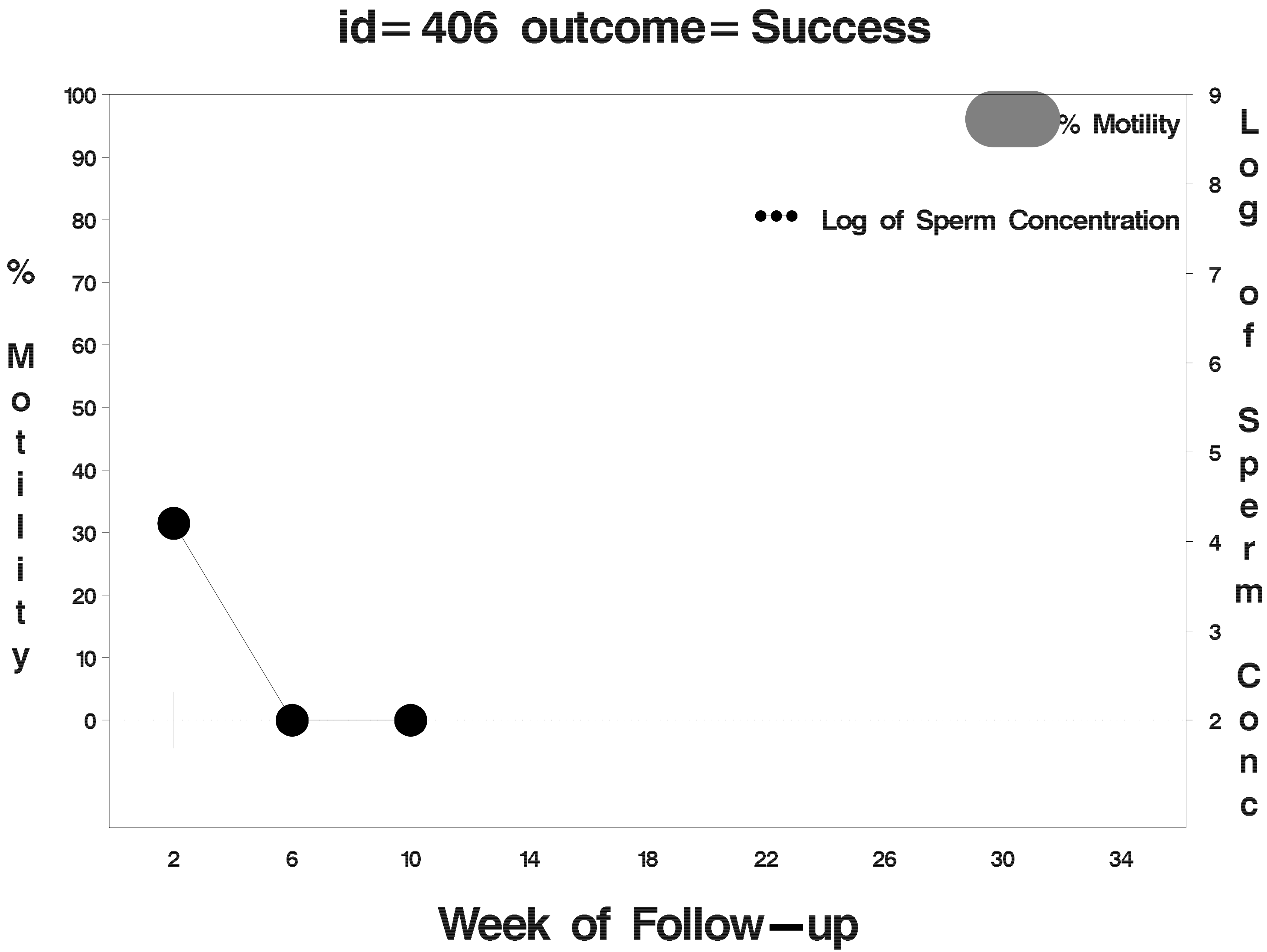
id= 406 outcome= Success
% Motility
Log of Sperm Concentration
100
90
80
70
60
50
40
30
20
10
0
9
8
7
6
5
4
3
2
2
6
10
14
18
22
26
30
34
Week of Follow-up
Log of Sperm Conc

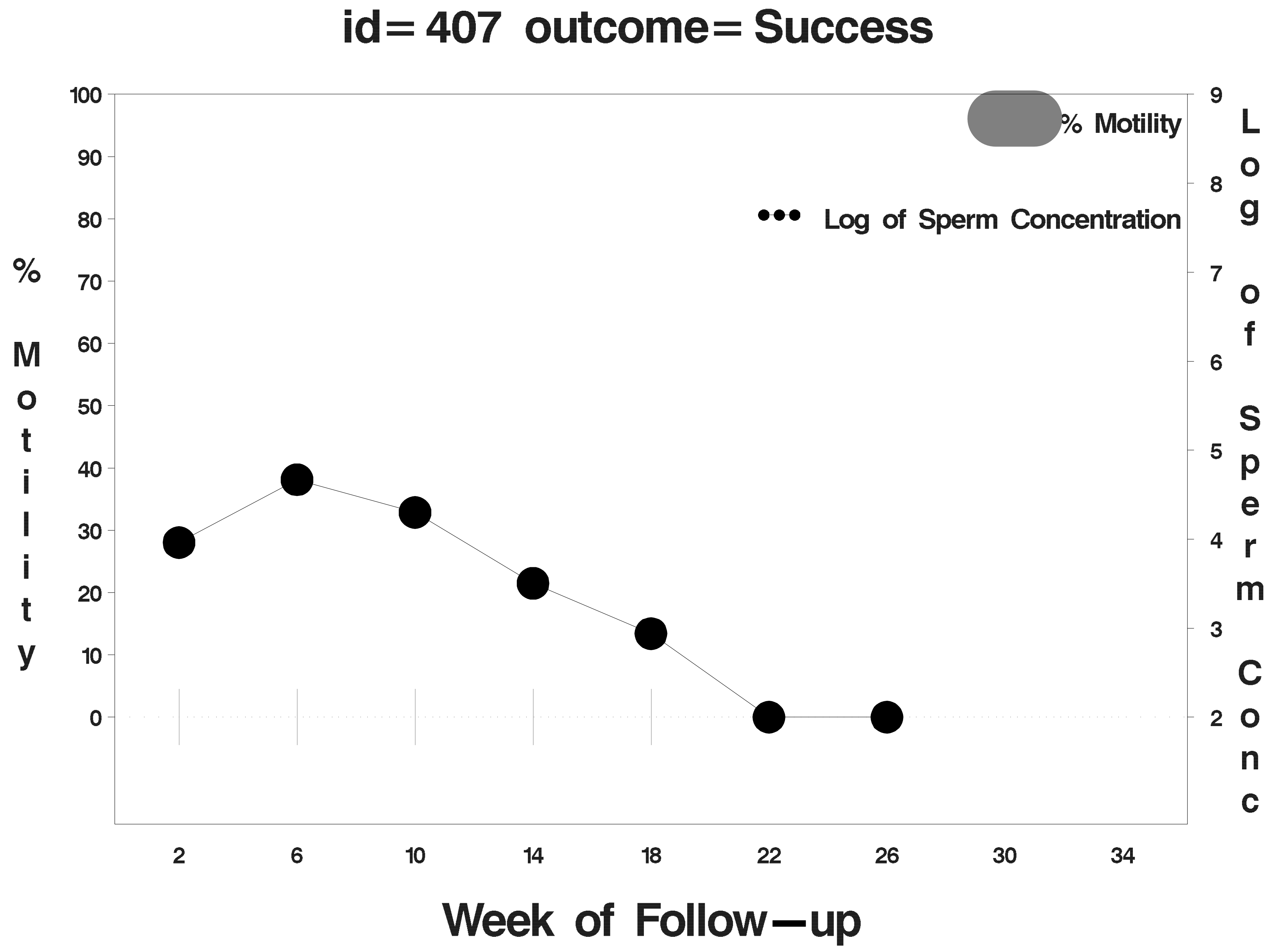
id= 407 outcome= Success
% Motility
Log of Sperm Concentration
% Motility
Log of Sperm Conc
Week of Follow−up
100
90
80
70
60
50
40
30
20
10
0
9
8
7
6
5
4
3
2
2
6
10
14
18
22
26
30
34

id= 408 outcome= Success

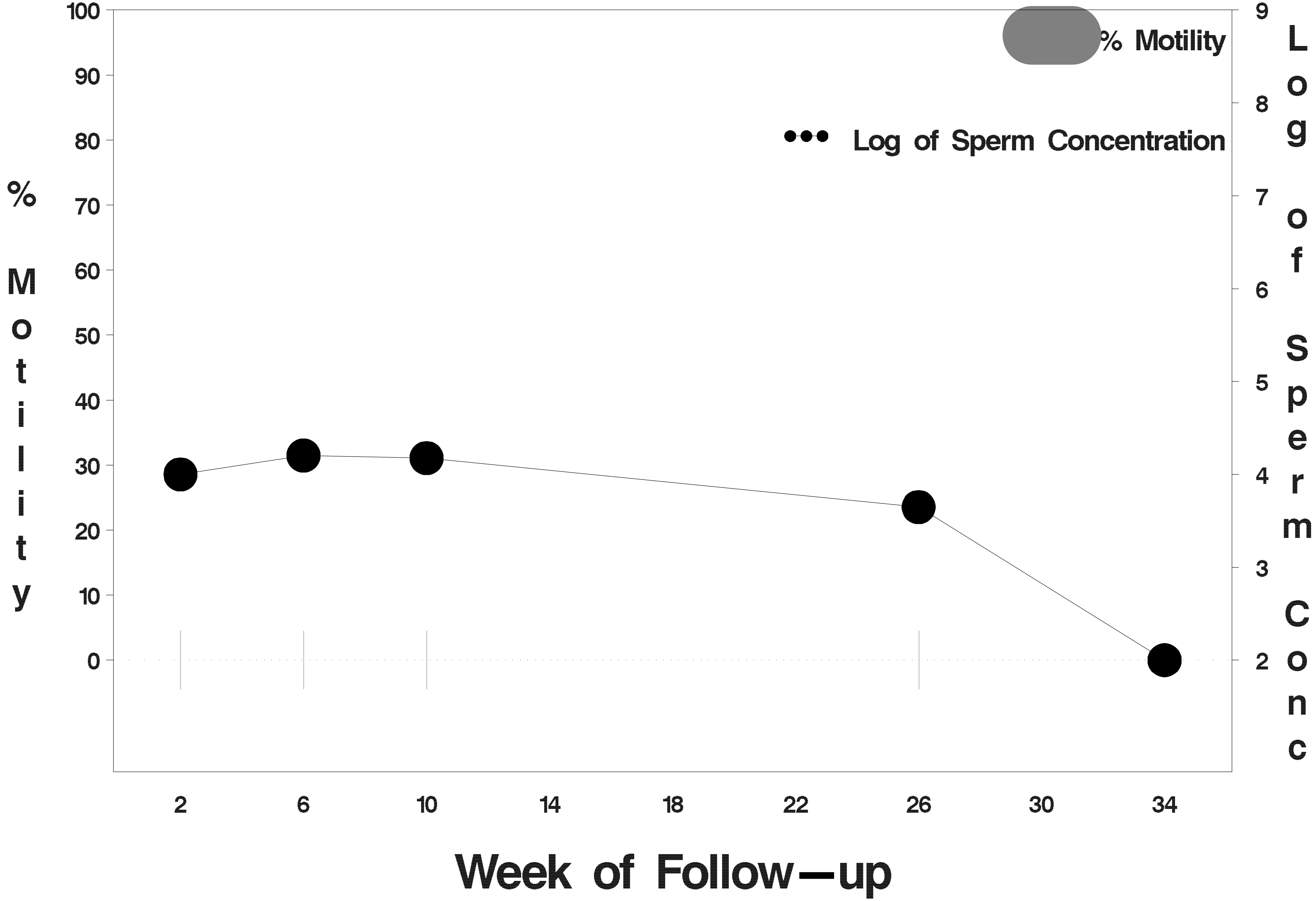

id= 409 outcome= Success

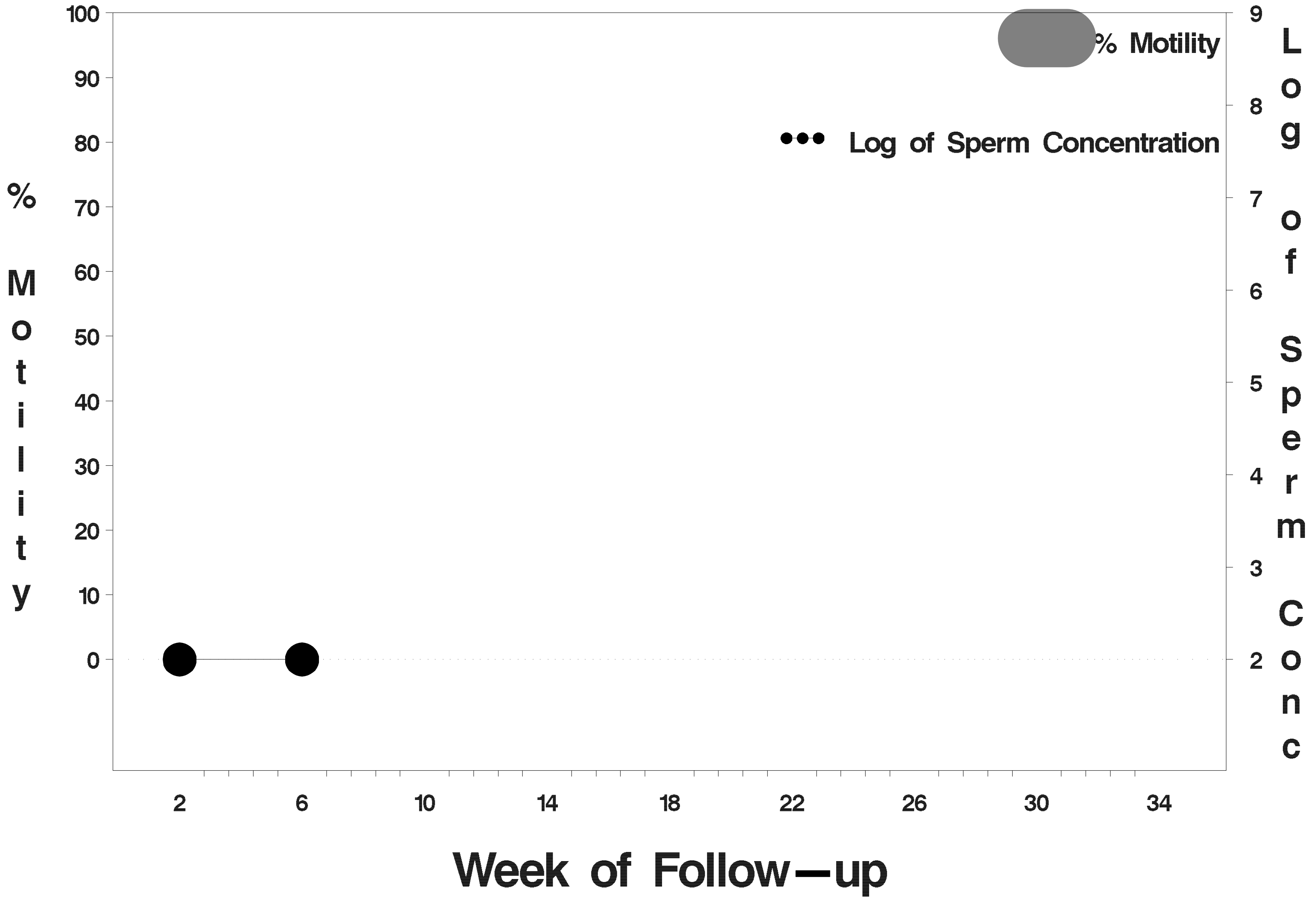

id= 410 outcome= Success

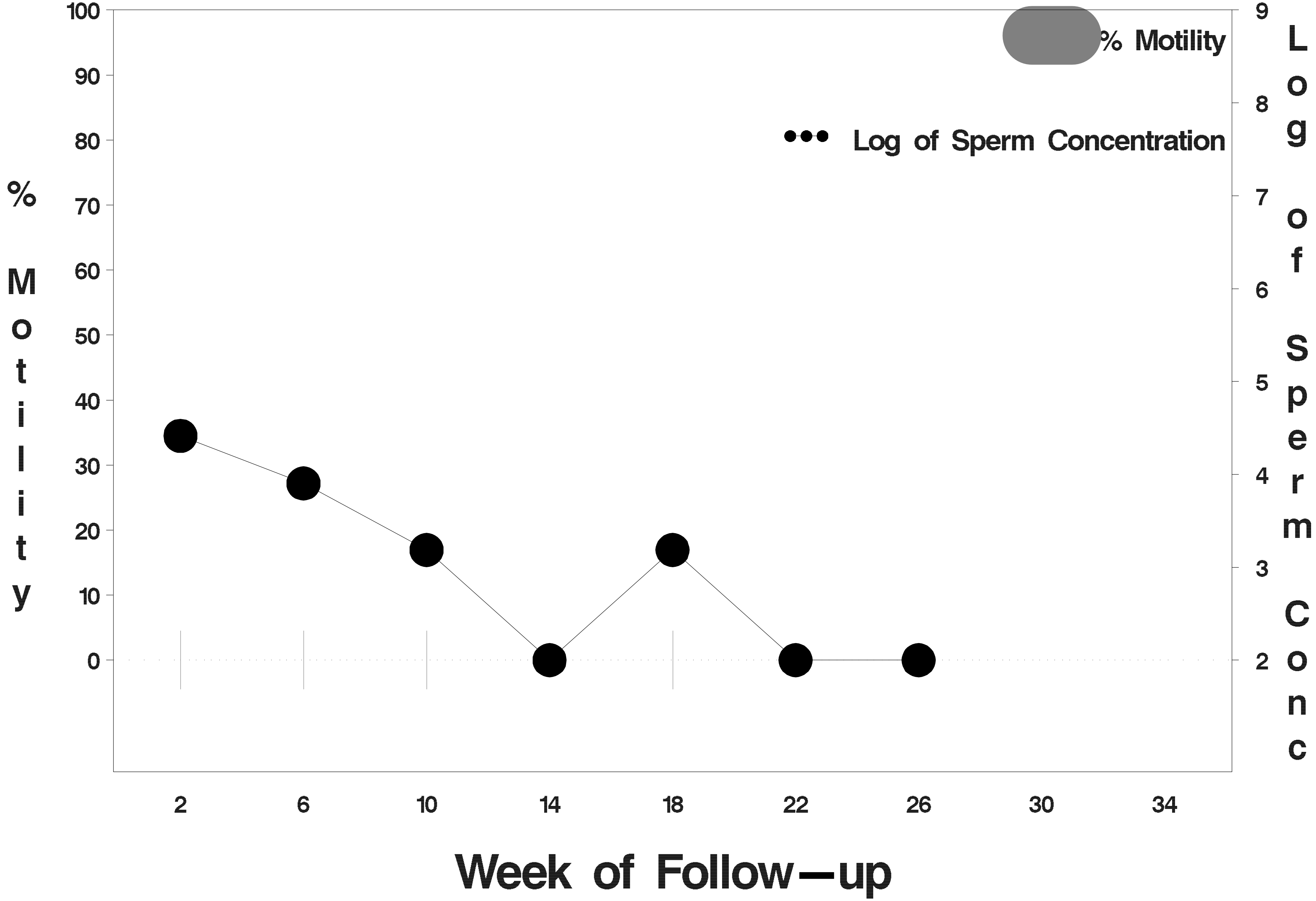

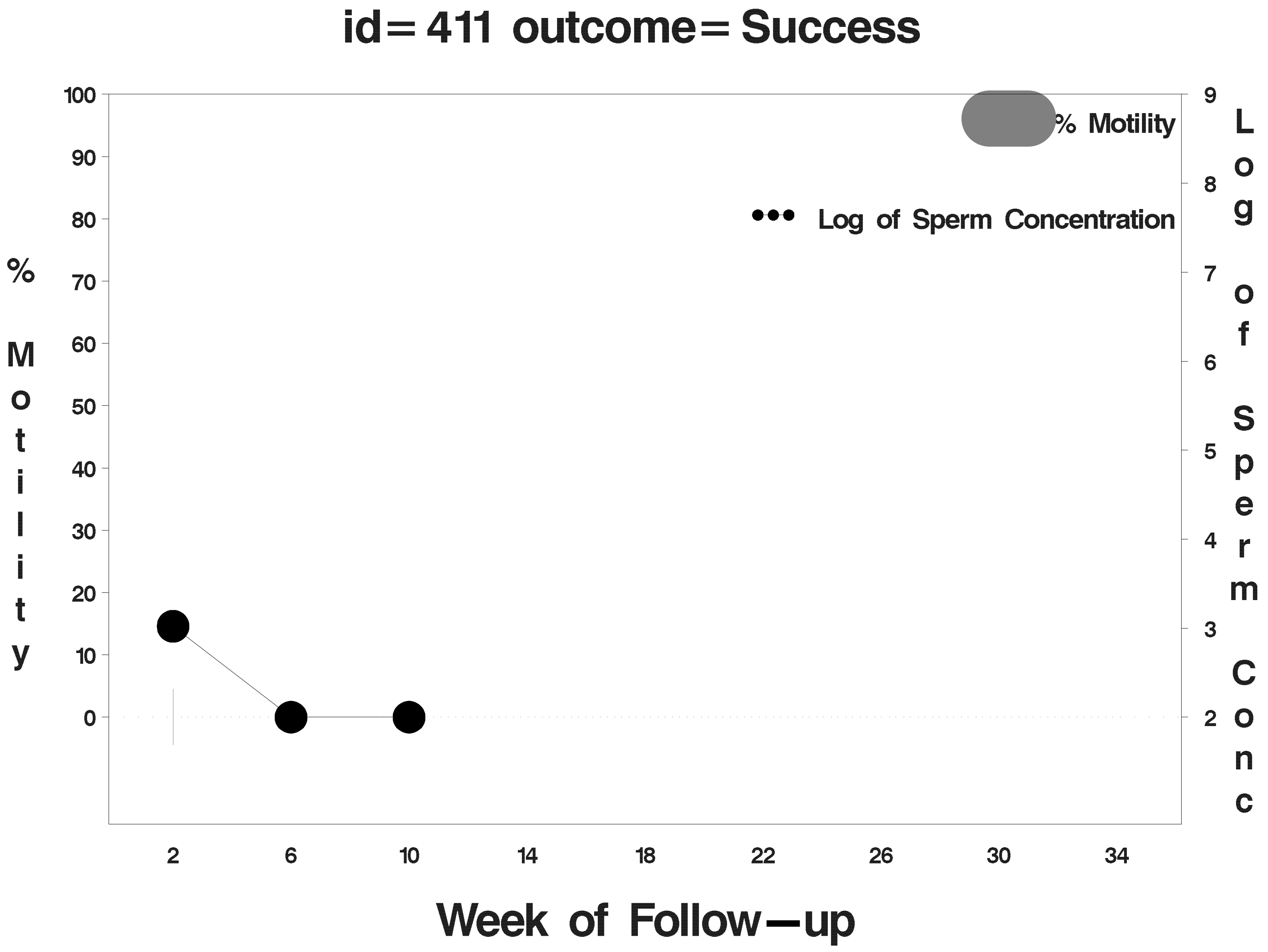
id= 411 outcome= Success
% Motility
Log of Sperm Concentration
100
90
80
70
60
50
40
30
20
10
0
9
8
7
6
5
4
3
2
2
6
10
14
18
22
26
30
34
Week of Follow−up
Log of Sperm Conc

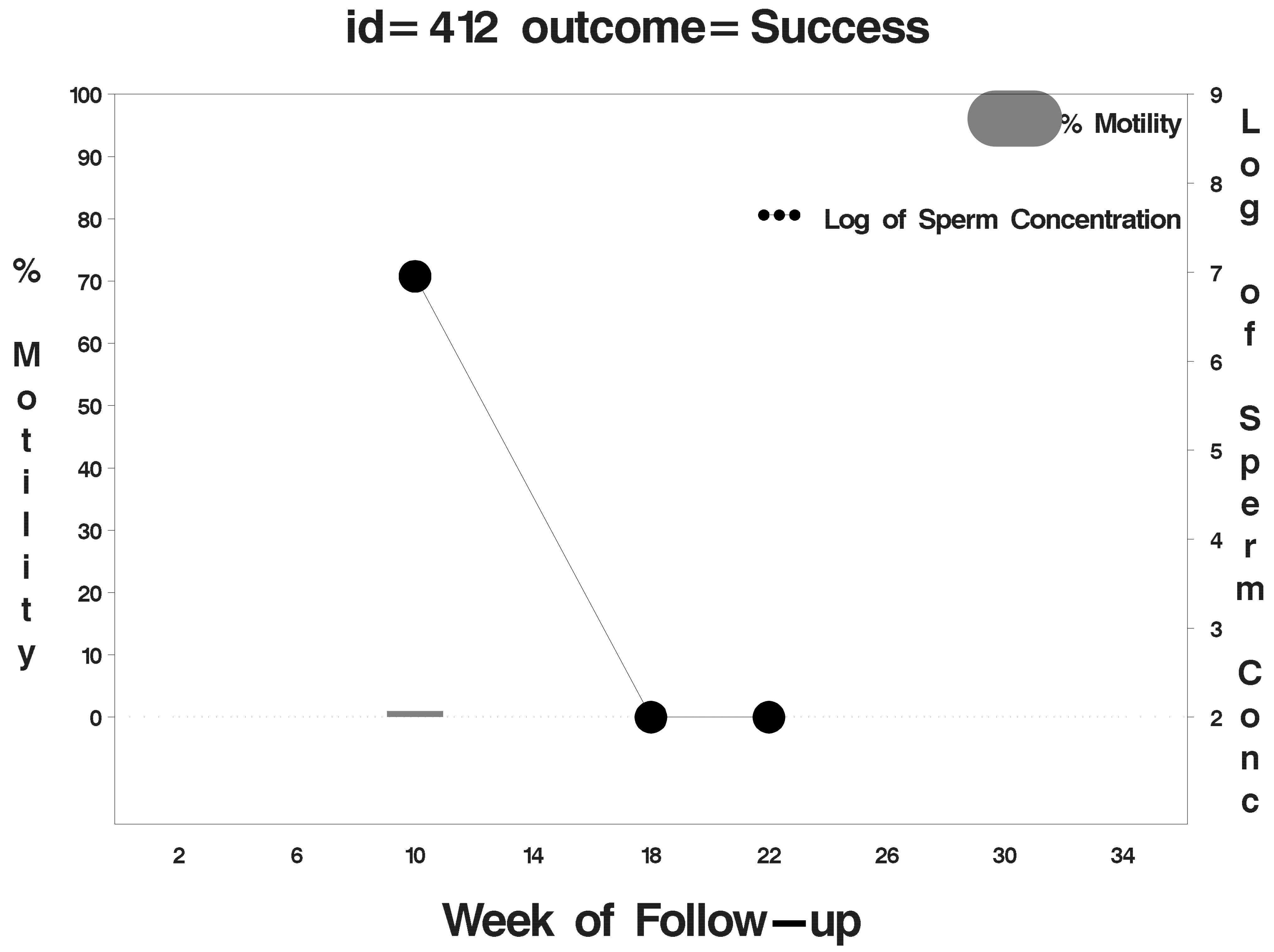
id= 412 outcome= Success
% Motility
Log of Sperm Concentration
100
90
80
70
60
50
40
30
20
10
0
9
8
7
6
5
4
3
2
2
6
10
14
18
22
26
30
34
Week of Follow−up
% Motility
Log of Sperm Conc

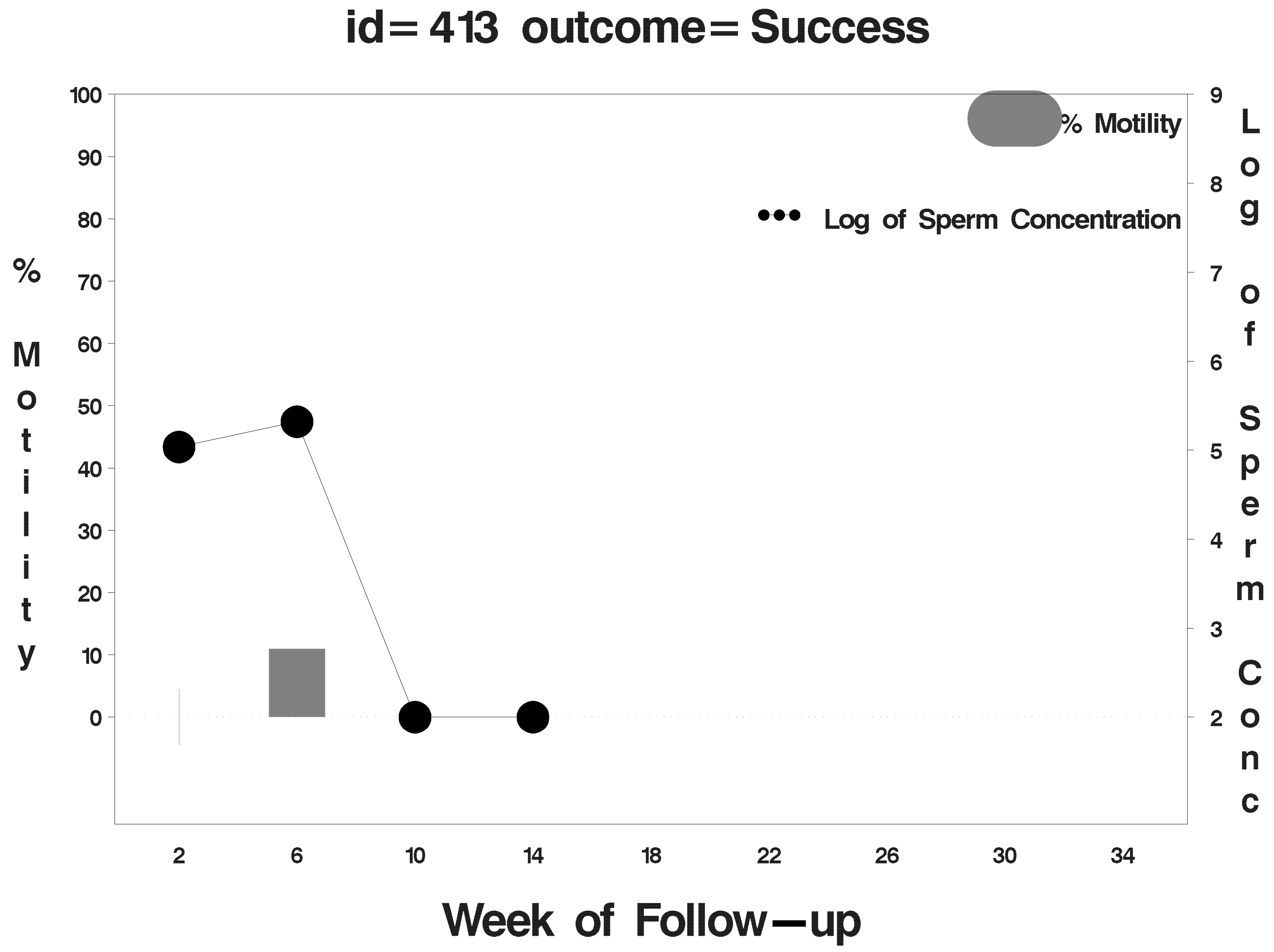

id= 413 outcome= Success
% Motility
Log of Sperm Concentration
100
90
80
70
60
50
40
30
20
10
0
9
8
7
6
5
4
3
2
2
6
10
14
18
22
26
30
34
Week of Follow-up
Log of Sperm Conc

id= 414 outcome= Success

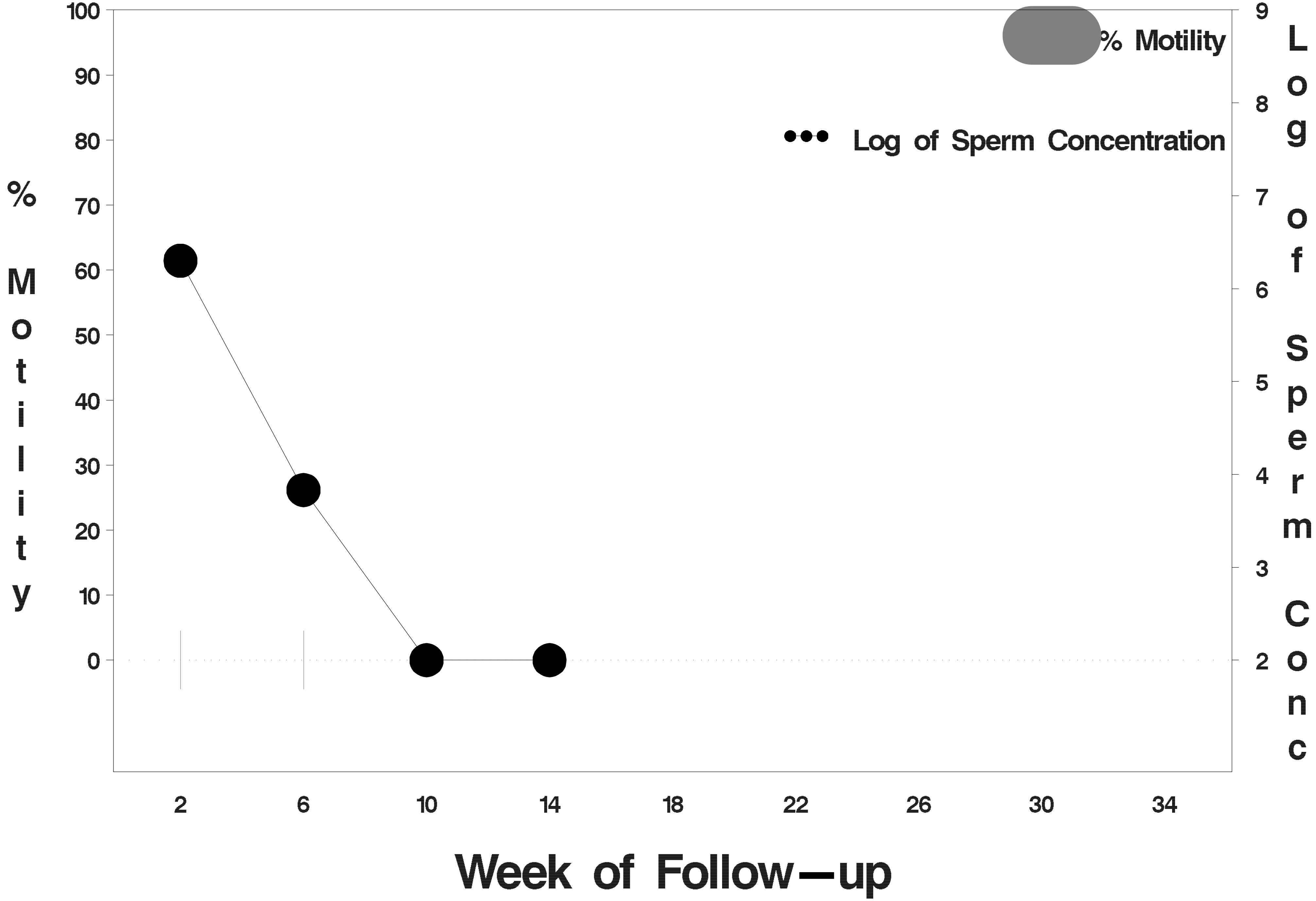

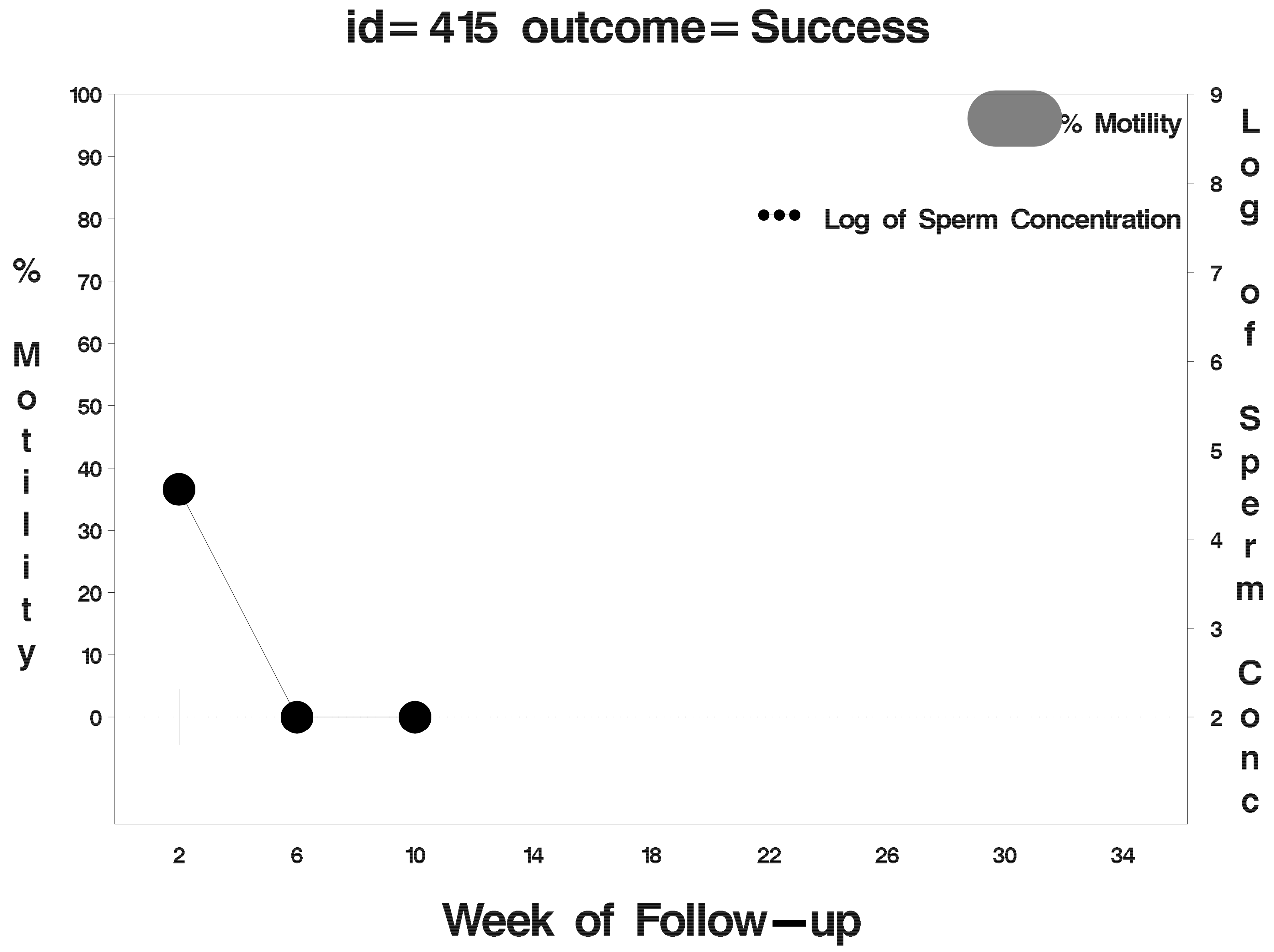

id= 415 outcome= Success
% Motility
Log of Sperm Concentration
% Motility
Log of Sperm Conc
100
90
80
70
60
50
40
30
20
10
0
9
8
7
6
5
4
3
2
2
6
10
14
18
22
26
30
34
Week of Follow-up

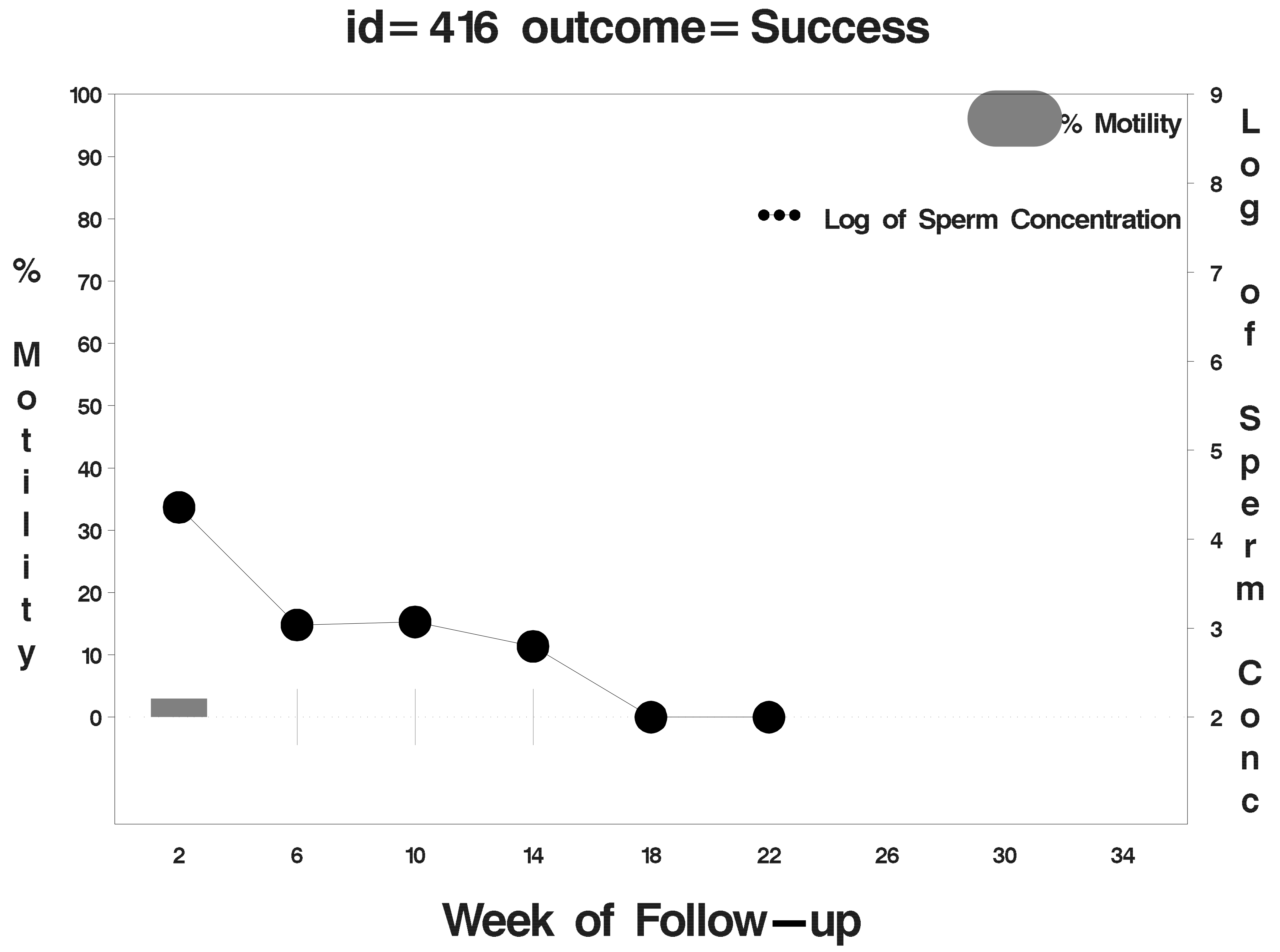

id= 416 outcome= Success
% Motility
Log of Sperm Concentration
% Motility
Log of Sperm Conc
100
90
80
70
60
50
40
30
20
10
0
9
8
7
6
5
4
3
2
2
6
10
14
18
22
26
30
34
Week of Follow-up

id= 417 outcome= Success

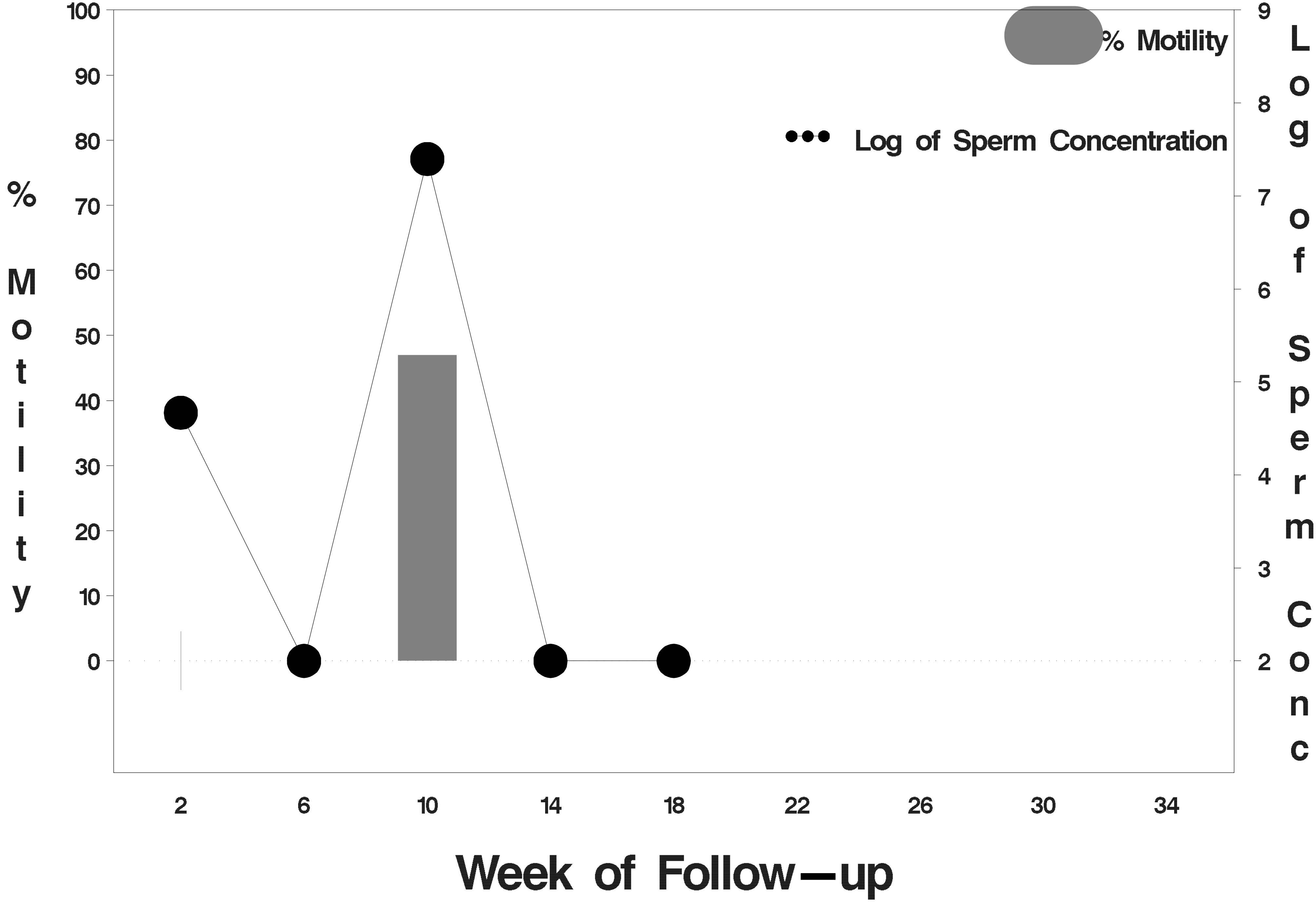

id= 418 outcome= Success

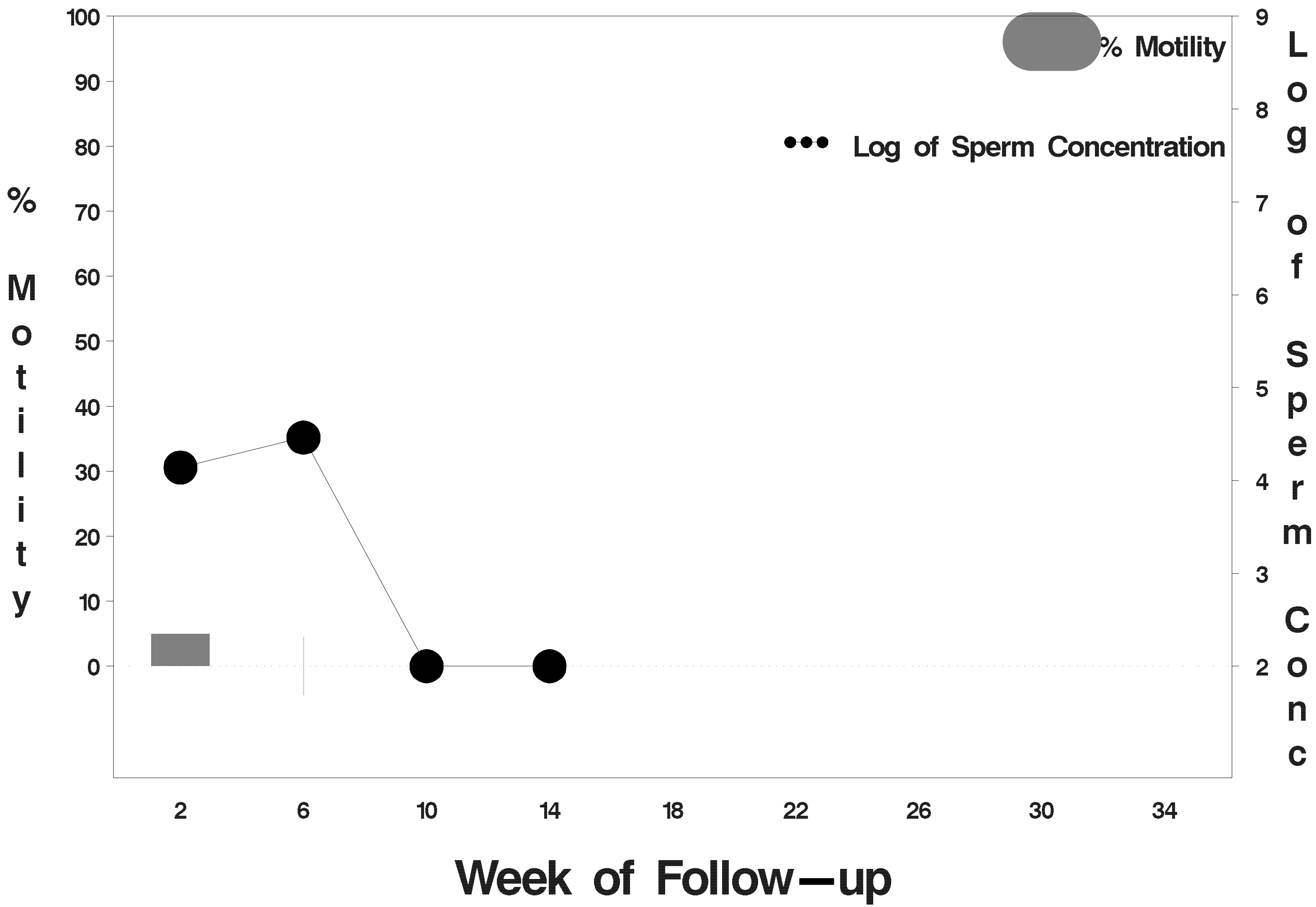

id= 419 outcome= Success

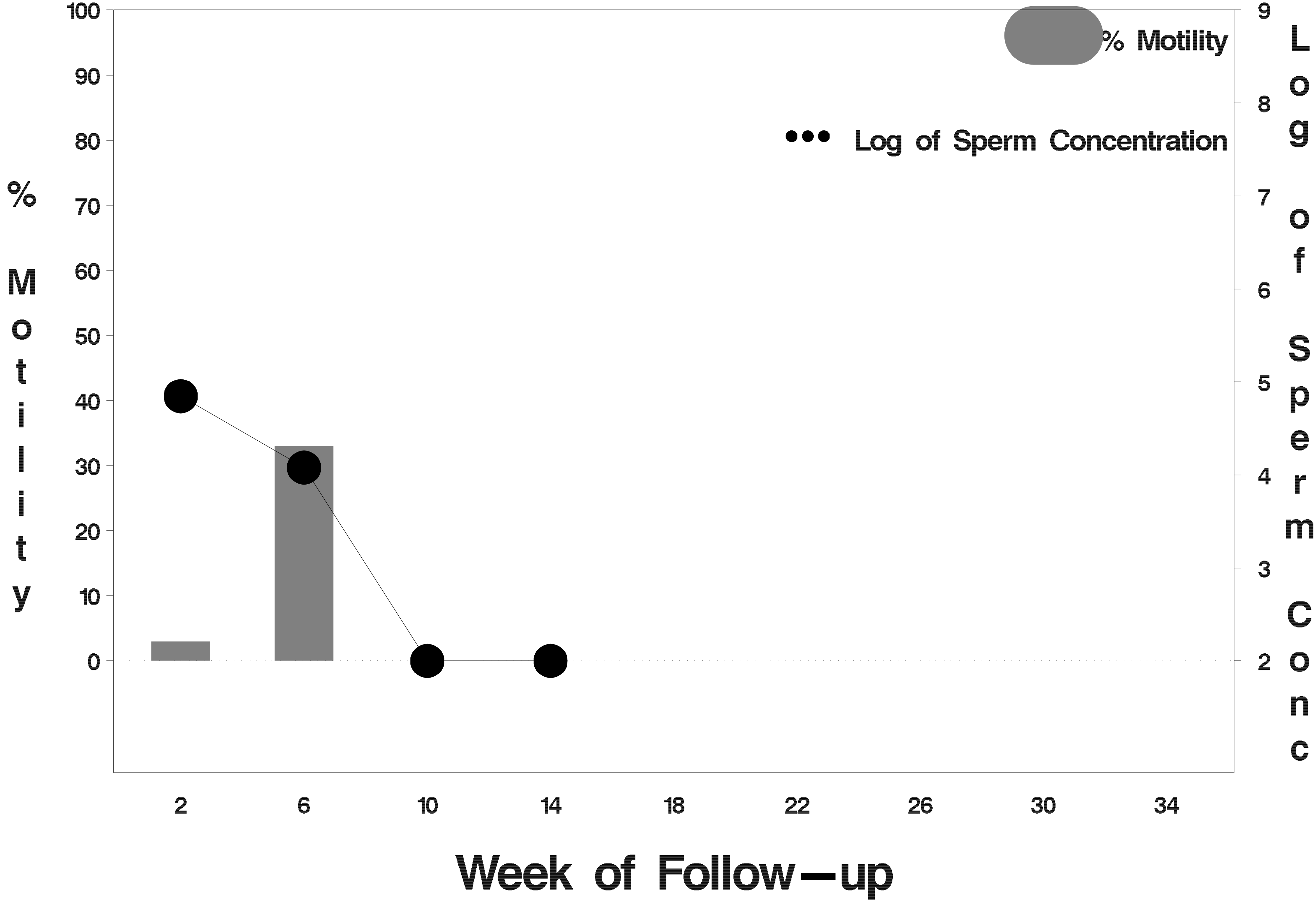

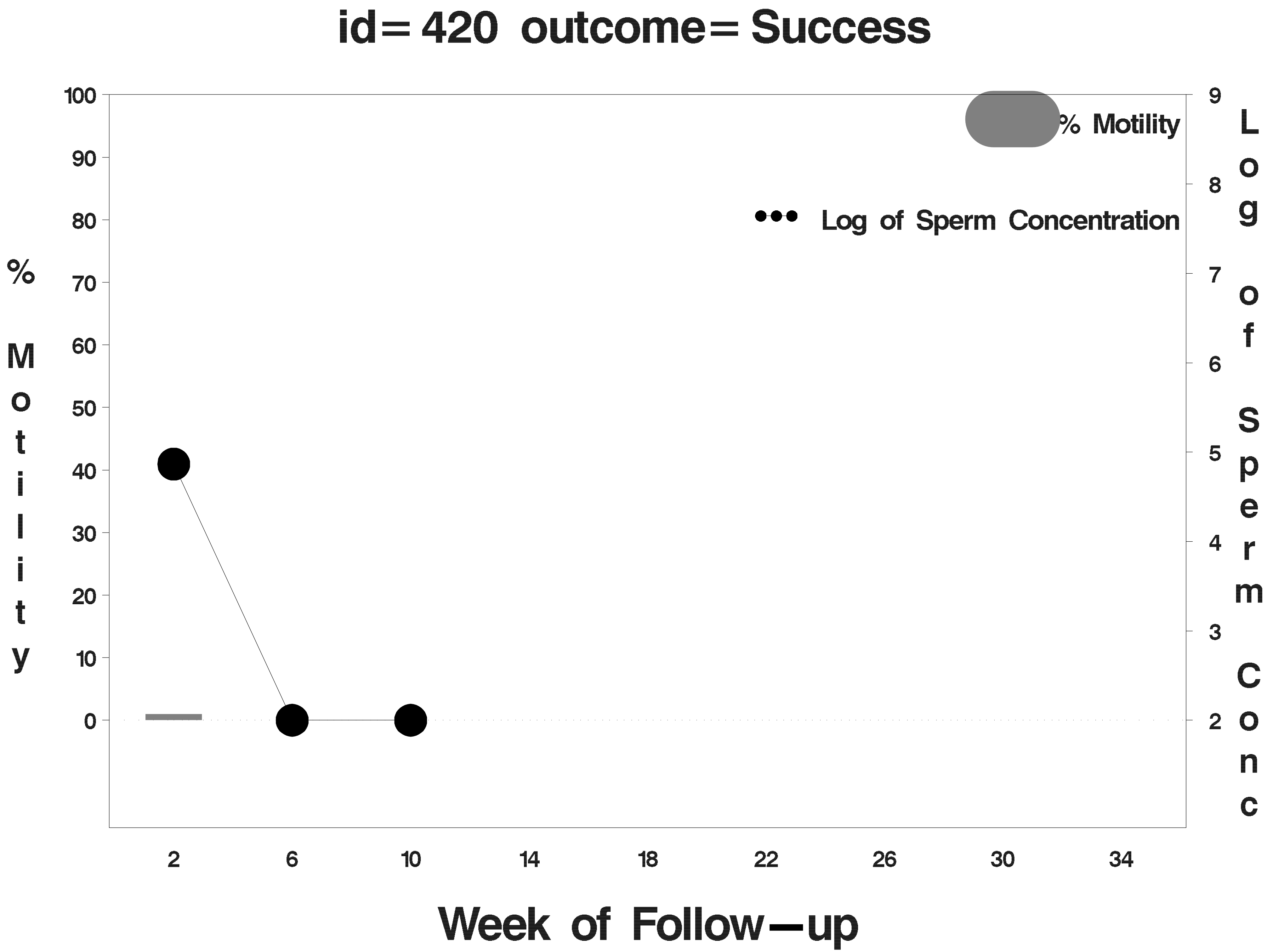

id= 420 outcome= Success
% Motility
Log of Sperm Concentration
100
90
80
70
60
50
40
30
20
10
0
9
8
7
6
5
4
3
2
2
6
10
14
18
22
26
30
34
Week of Follow—up
% Motility
Log of Sperm Conc

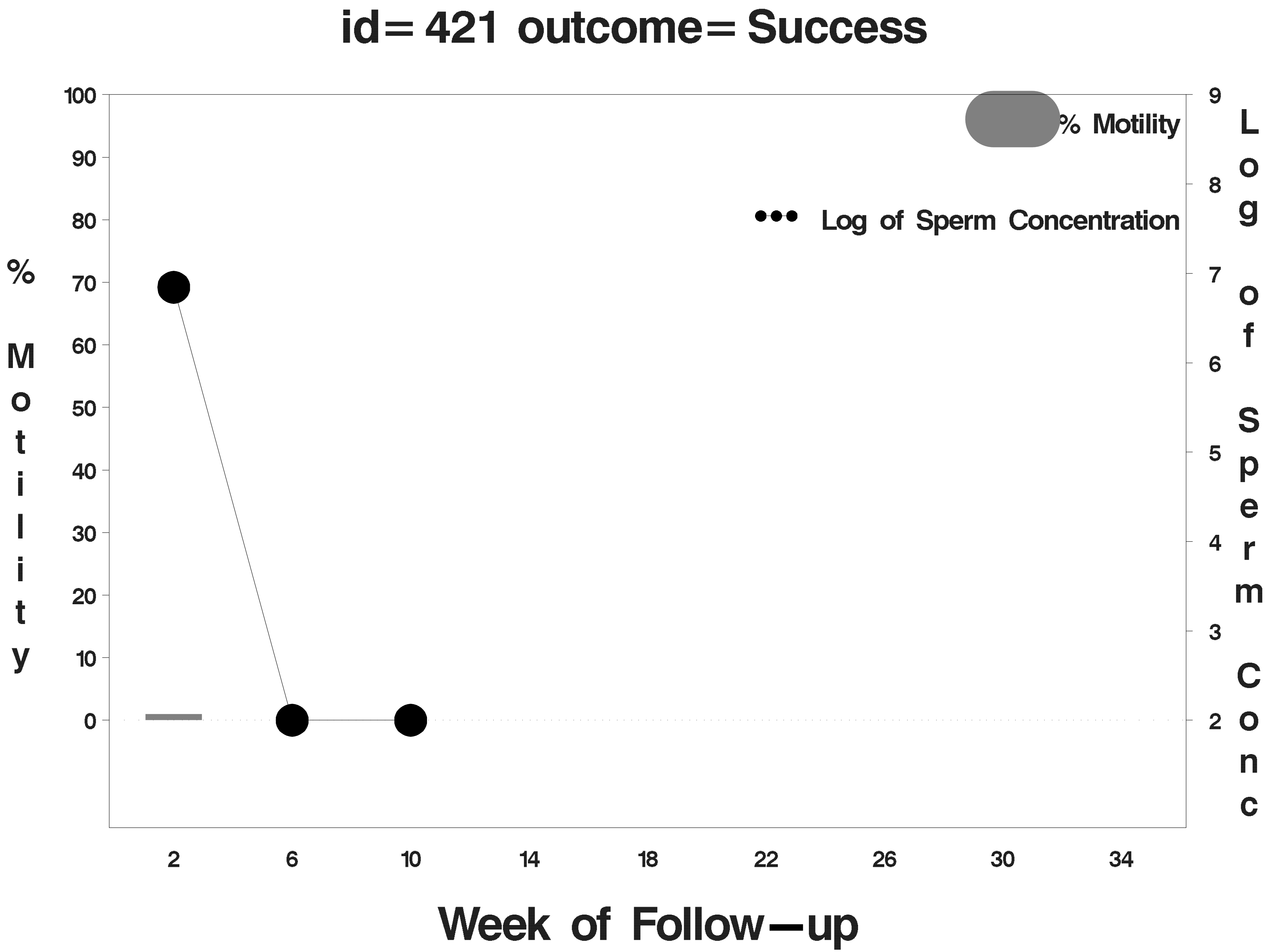
id= 421 outcome= Success
% Motility
Log of Sperm Concentration
100
90
80
70
60
50
40
30
20
10
0
9
8
7
6
5
4
3
2
% Motility
Log of Sperm Conc
2
6
10
14
18
22
26
30
34
Week of Follow−up

id= 422 outcome= Success

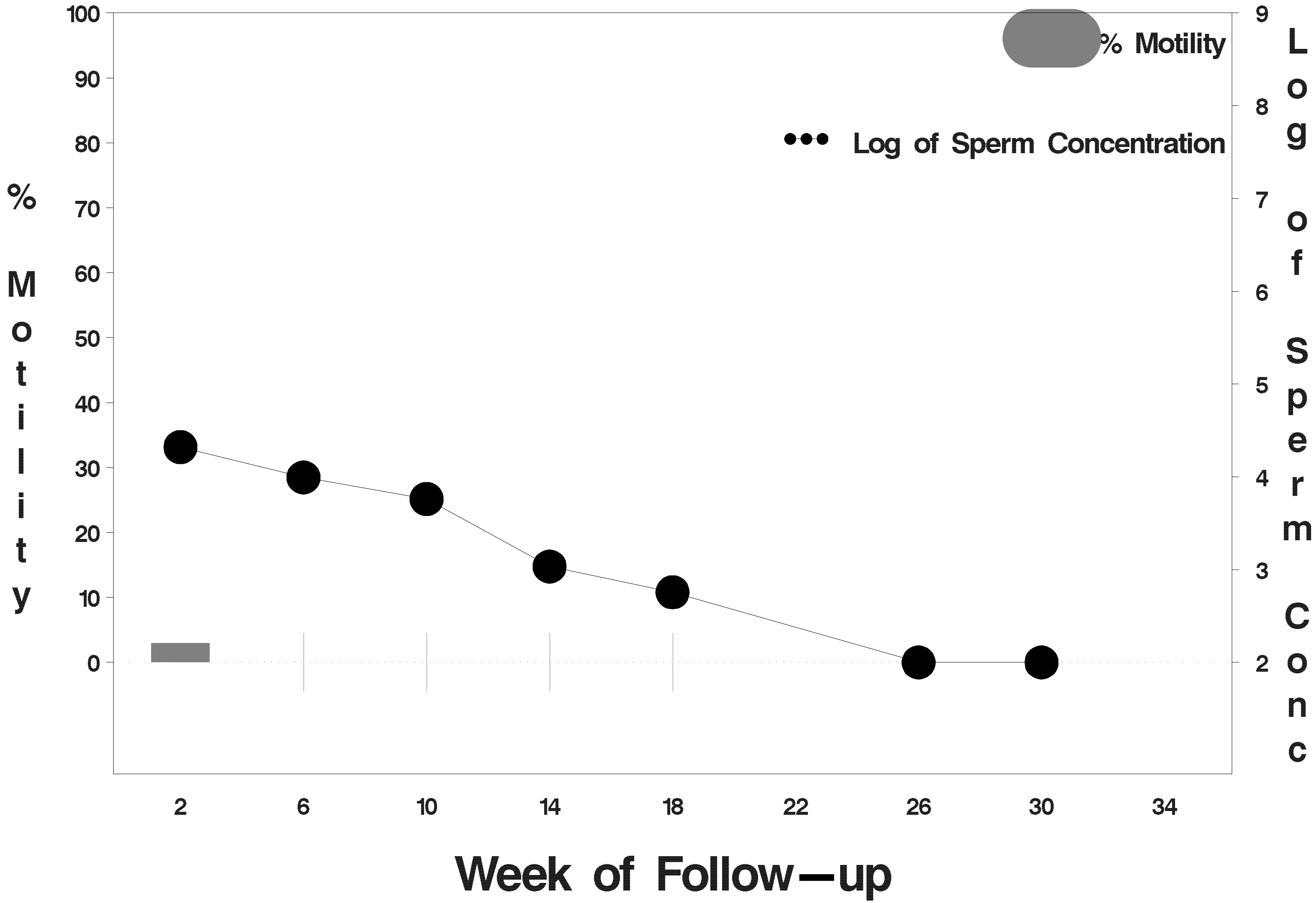

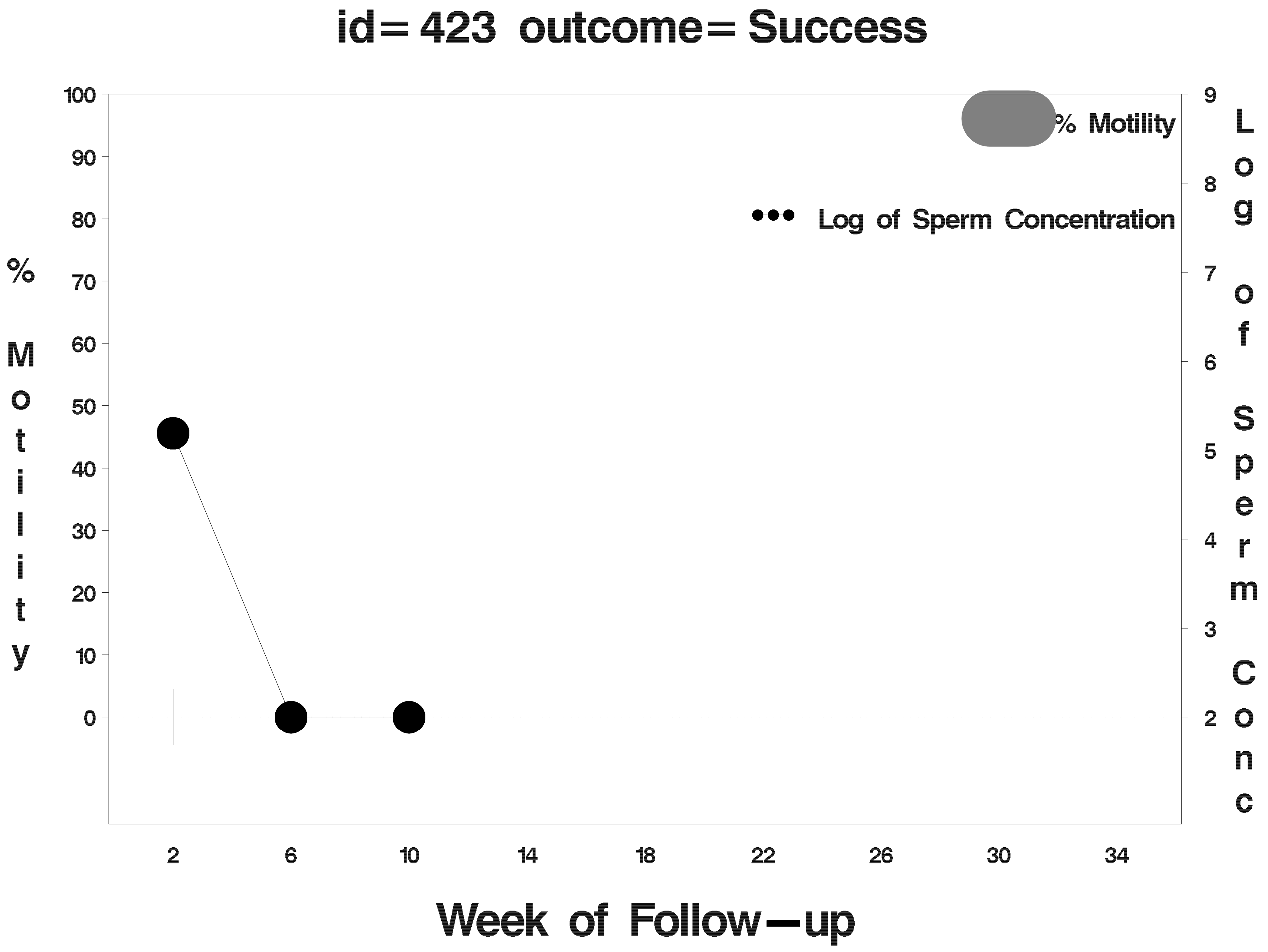

id= 423 outcome= Success
% Motility
Log of Sperm Concentration
100
90
80
70
60
50
40
30
20
10
0
9
8
7
6
5
4
3
2
2
6
10
14
18
22
26
30
34
Week of Follow—up

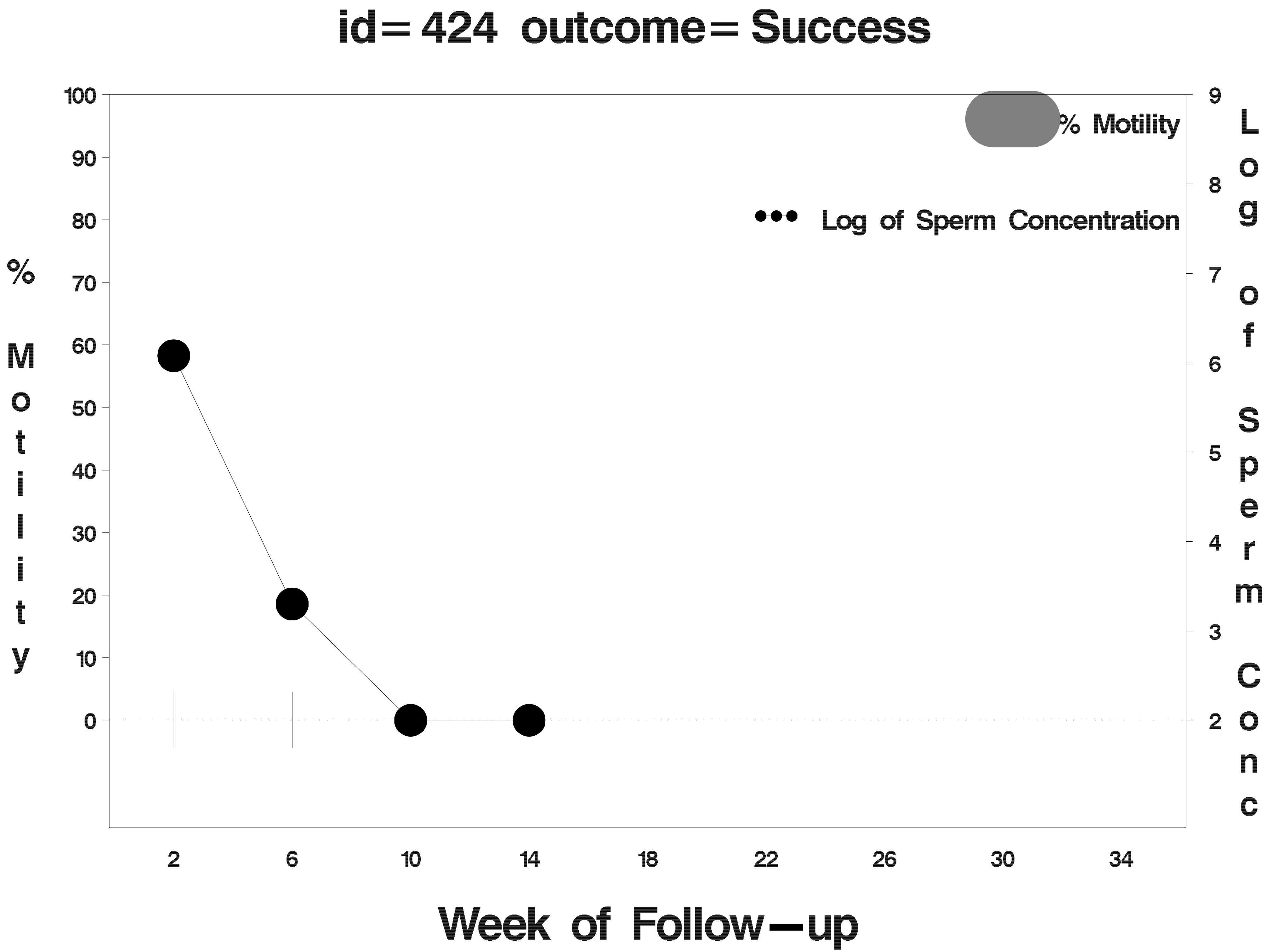

id= 424 outcome= Success
% Motility
Log of Sperm Concentration
100
90
80
70
60
50
40
30
20
10
0
9
8
7
6
5
4
3
2
2
6
10
14
18
22
26
30
34
Week of Follow−up

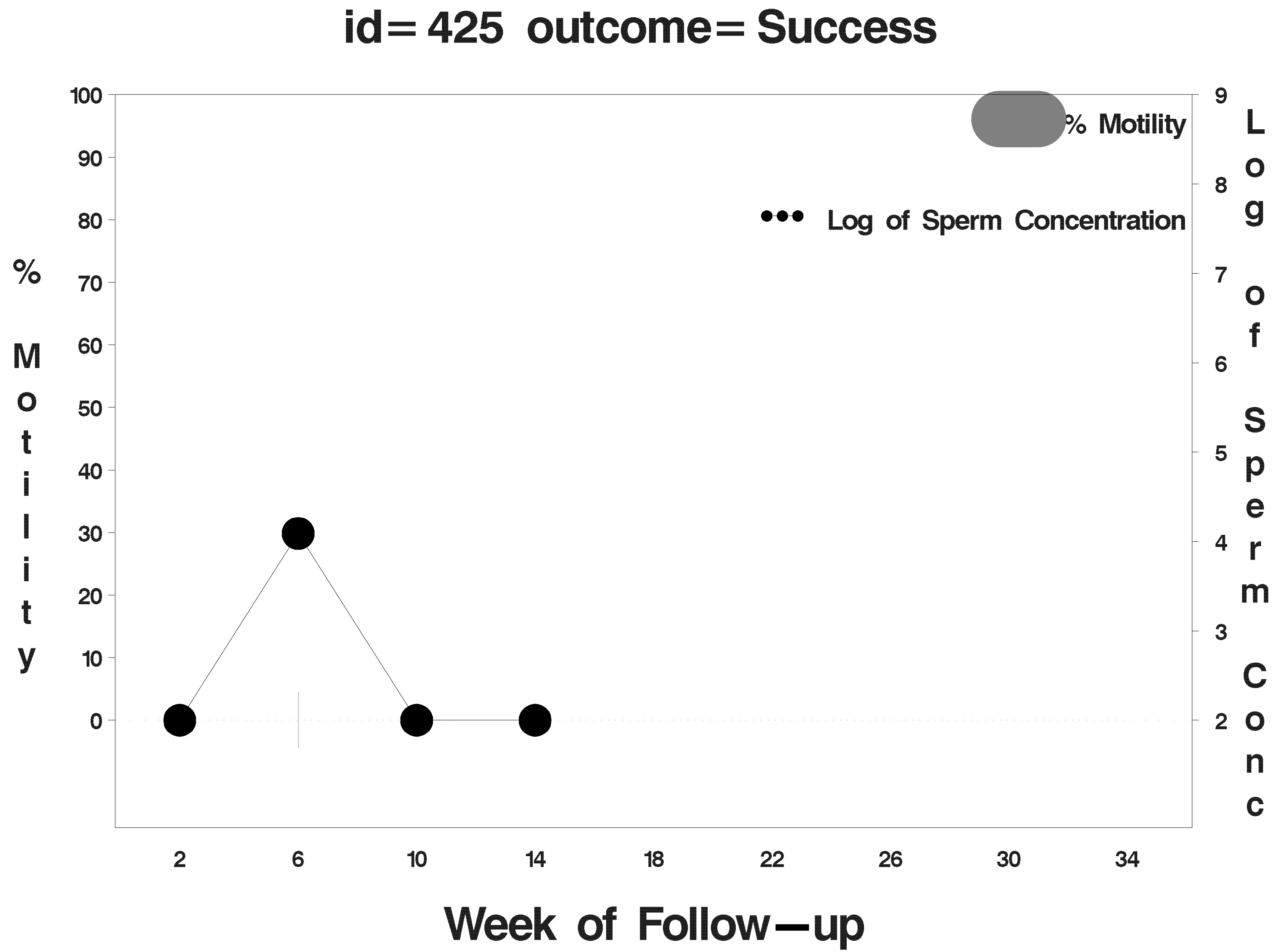
id= 425 outcome= Success
% Motility
Log of Sperm Concentration
100
90
80
70
60
50
40
30
20
10
0
9
8
7
6
5
4
3
2
2
6
10
14
18
22
26
30
34
Week of Follow—up
% Motility
Log of Sperm Conc

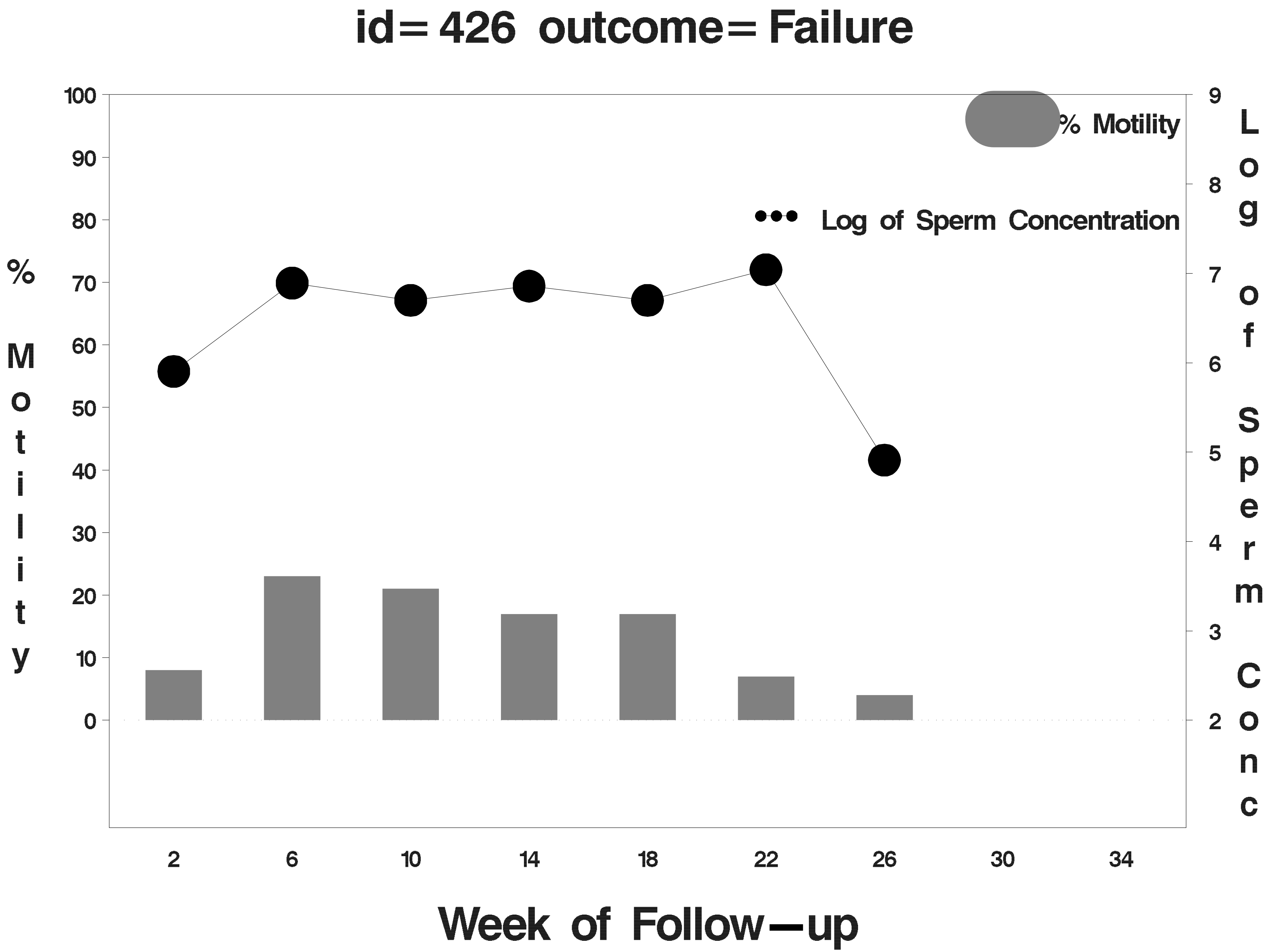

id= 426 outcome= Failure
% Motility
Log of Sperm Concentration
100
90
80
70
60
50
40
30
20
10
0
9
8
7
6
5
4
3
2
% Motility
Log of Sperm Conc
2
6
10
14
18
22
26
30
34
Week of Follow-up

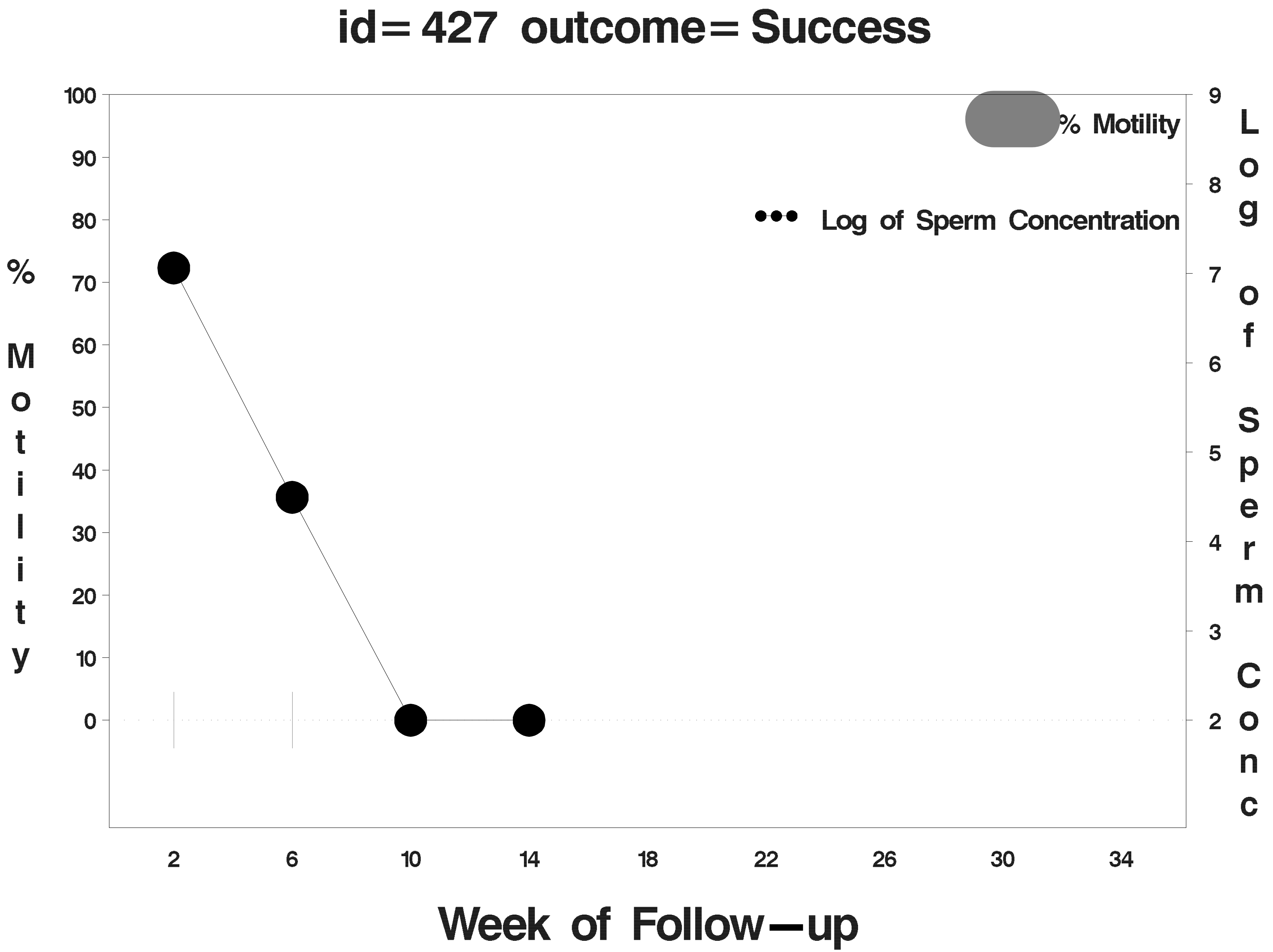

id= 427 outcome= Success
% Motility
Log of Sperm Concentration
% Motility
Log of Sperm Conc
Week of Follow—up
100
90
80
70
60
50
40
30
20
10
0
9
8
7
6
5
4
3
2
2
6
10
14
18
22
26
30
34

id= 428 outcome= Indeterminate

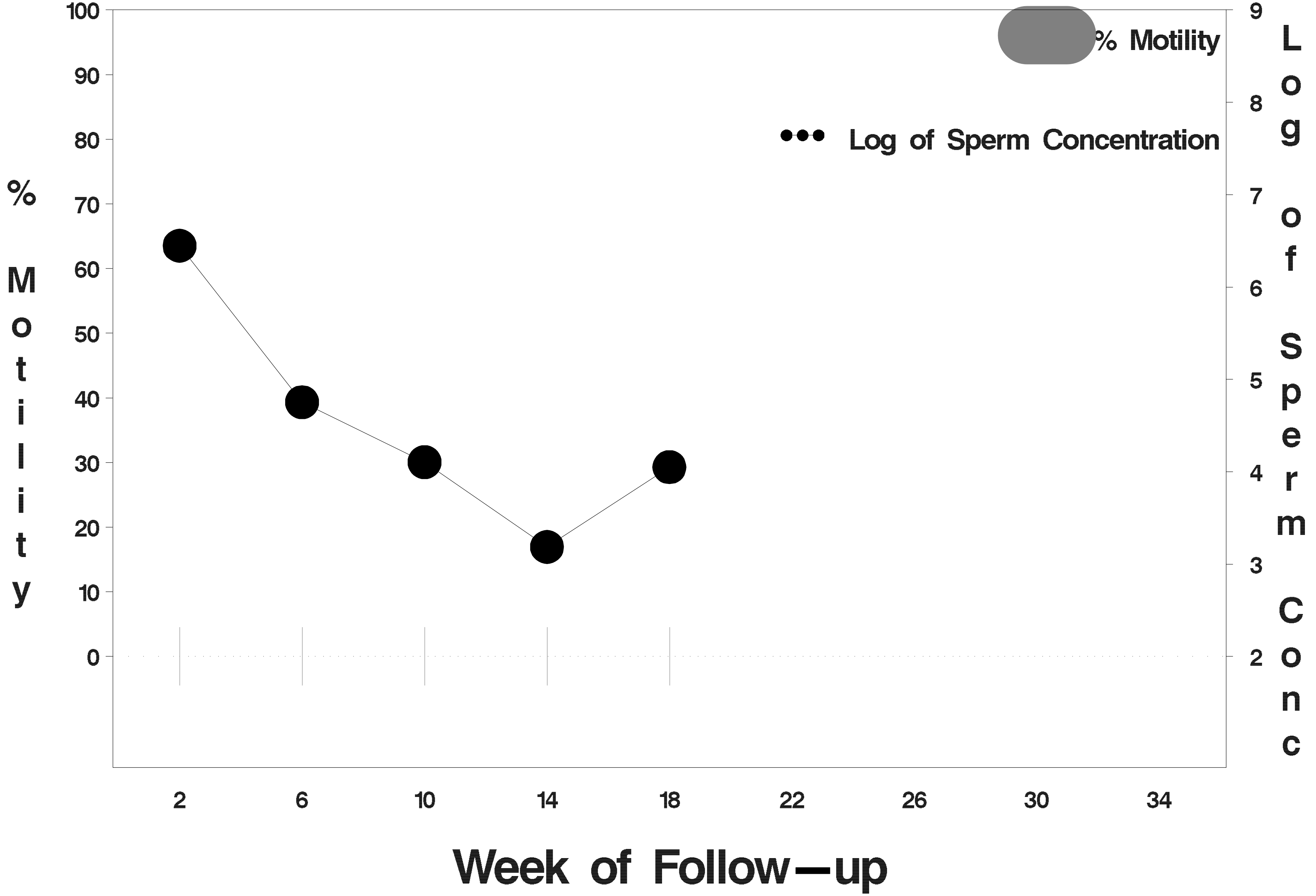

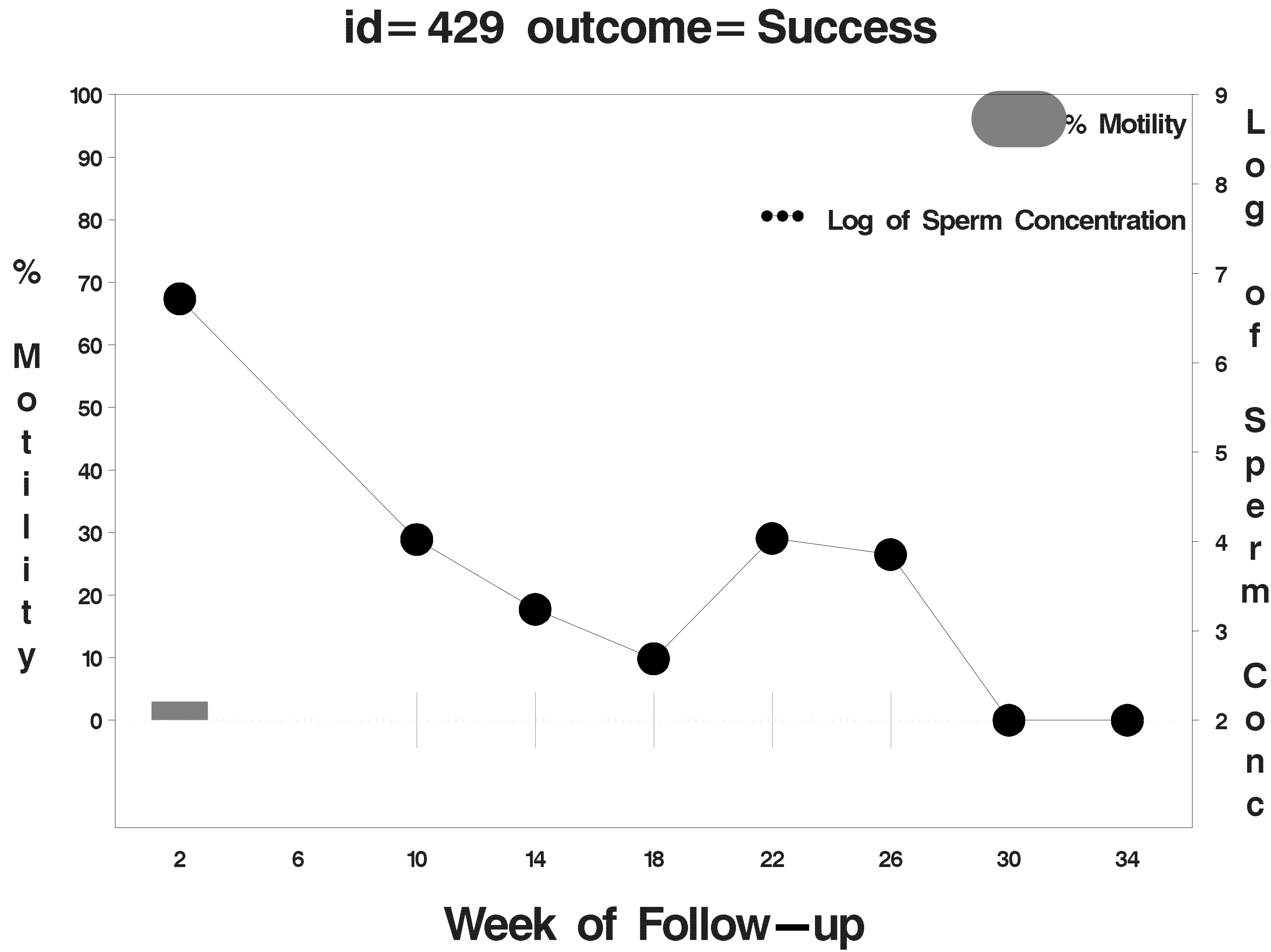

id= 429 outcome= Success
% Motility
Log of Sperm Concentration
100
90
80
70
60
50
40
30
20
10
0
9
8
7
6
5
4
3
2
Log of Sperm Conc
2
6
10
14
18
22
26
30
34
Week of Follow−up

id= 430 outcome= Success

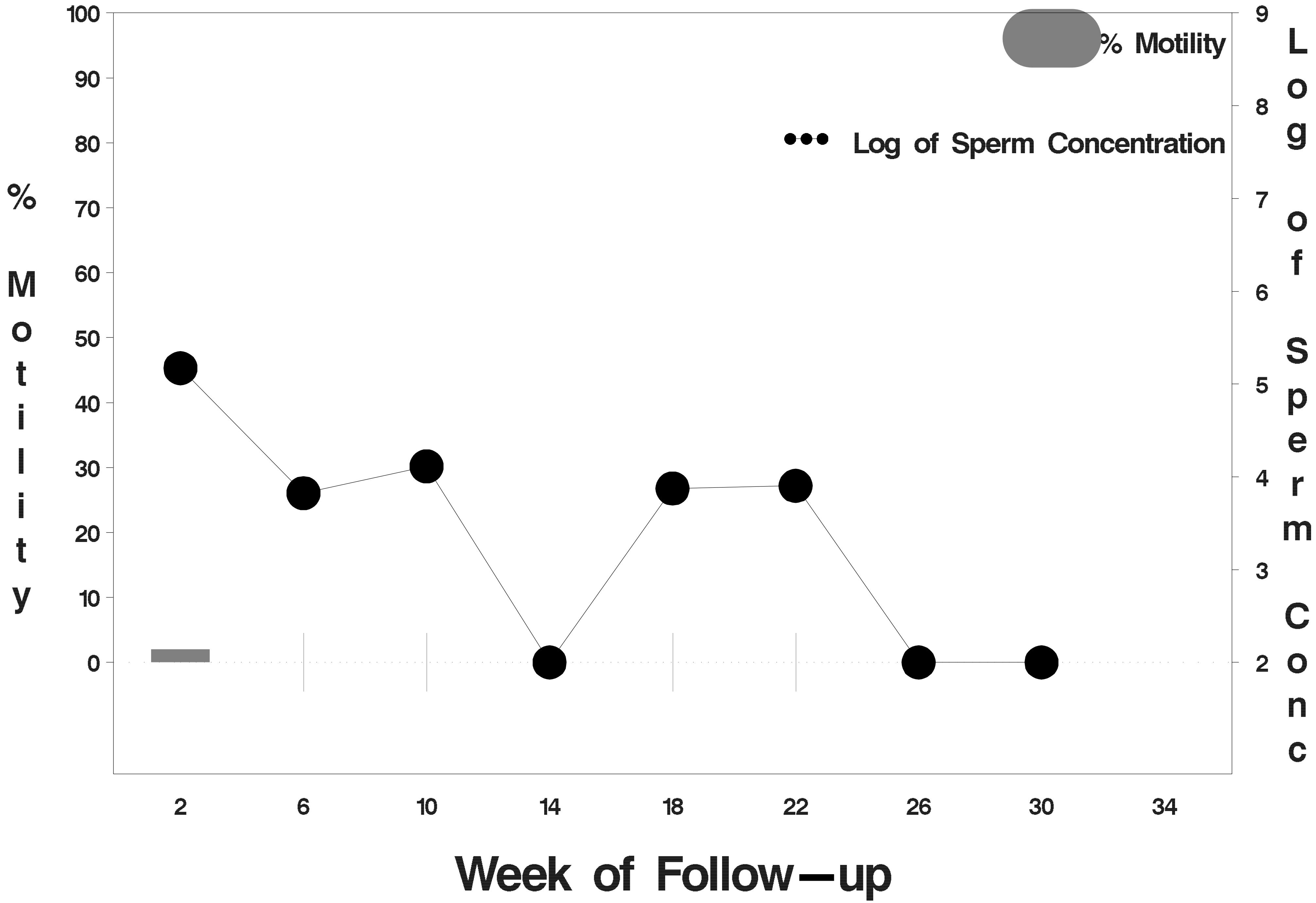

id= 431 outcome= Failure

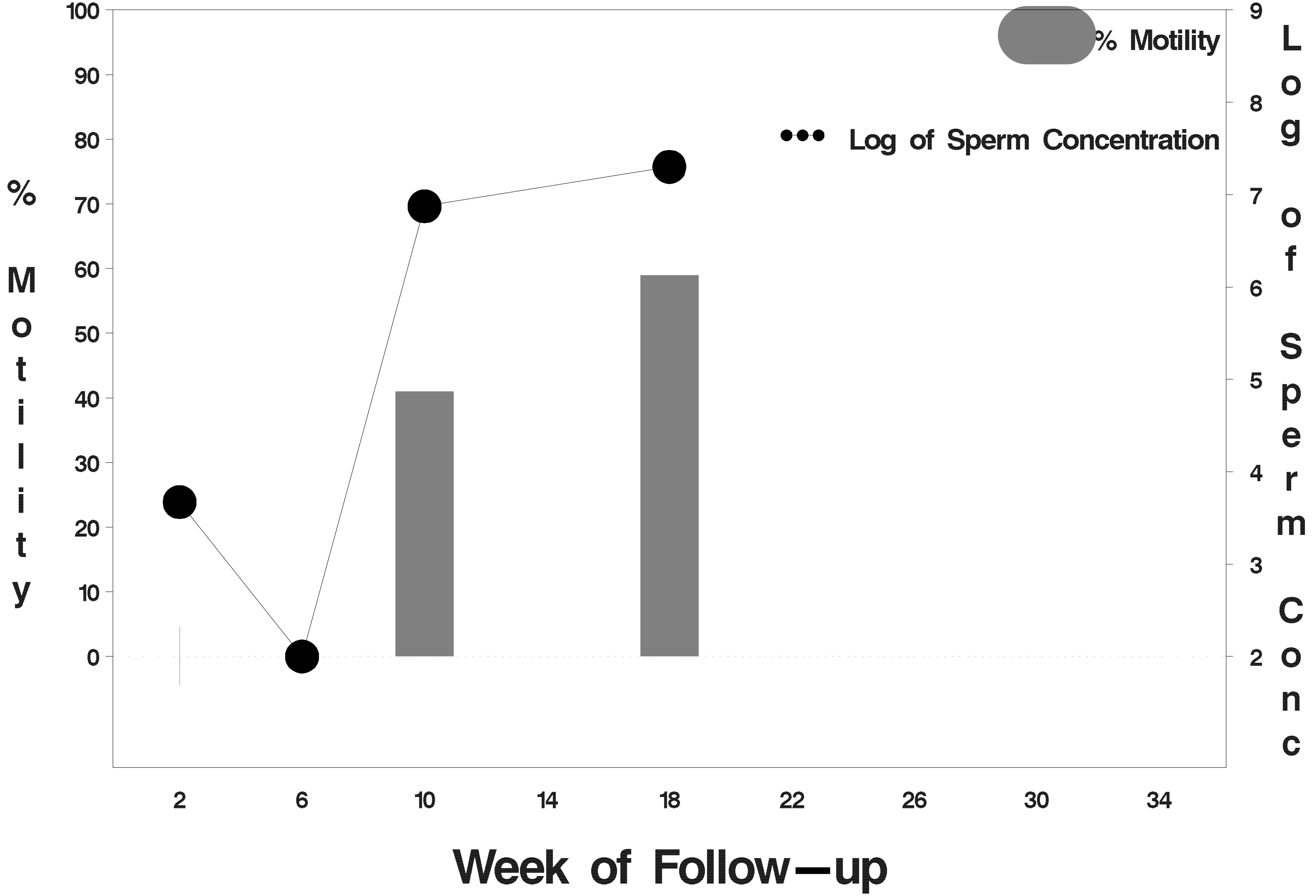

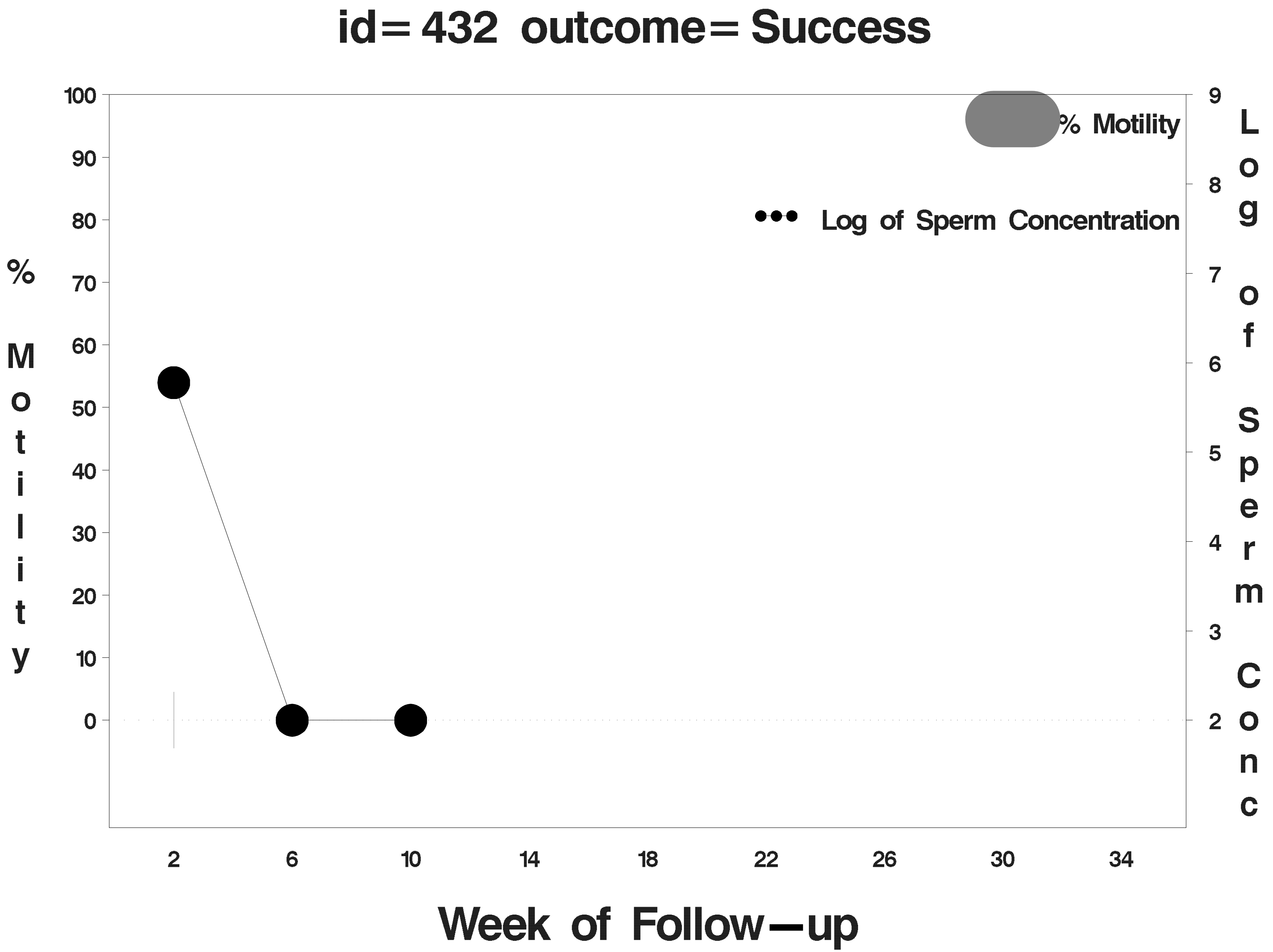

id= 432 outcome= Success
% Motility
Log of Sperm Concentration
100
90
80
70
60
50
40
30
20
10
0
9
8
7
6
5
4
3
2
% Motility
Log of Sperm Conc
2
6
10
14
18
22
26
30
34
Week of Follow—up

id= 433 outcome= Success

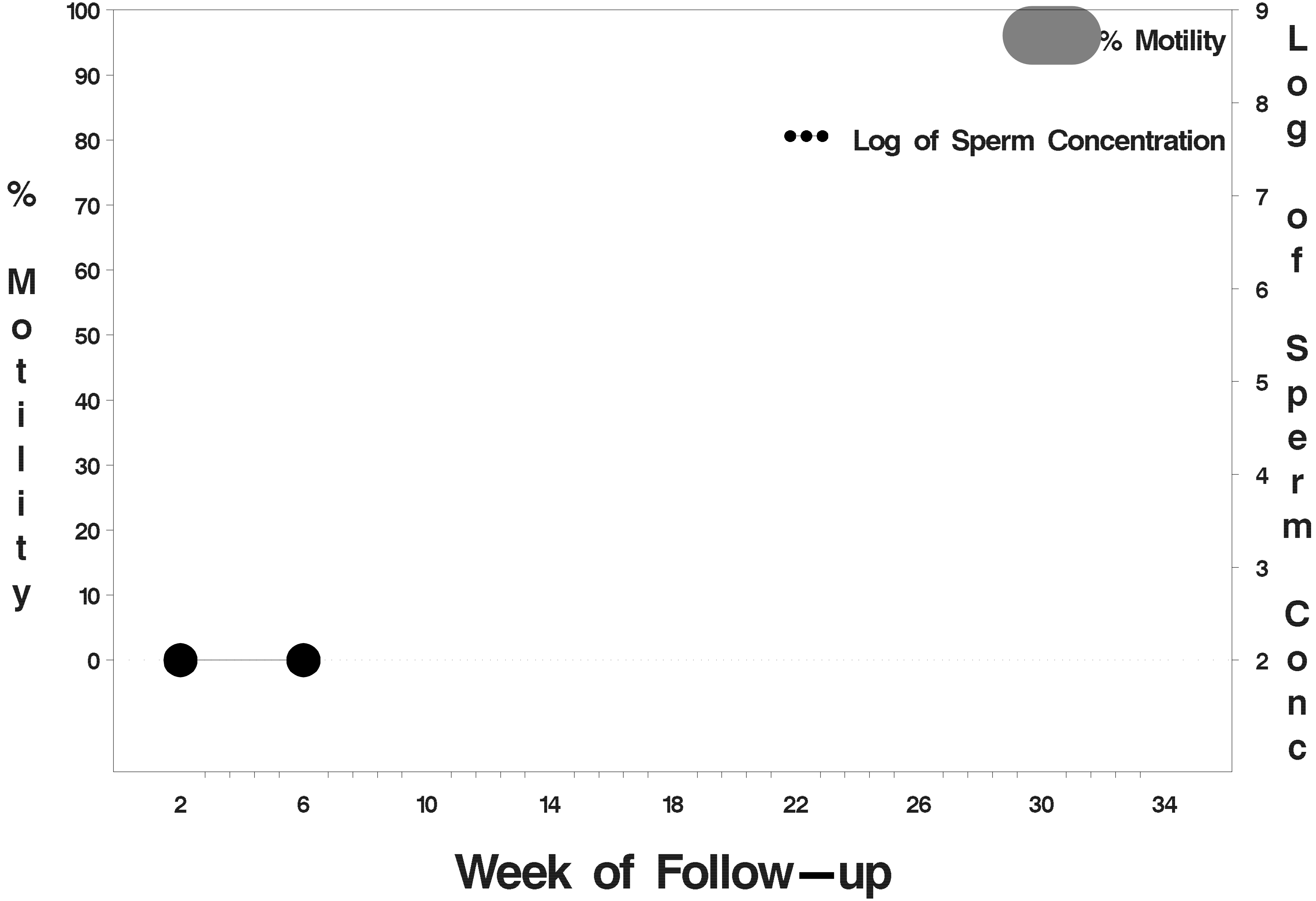

id= 434 outcome= Success

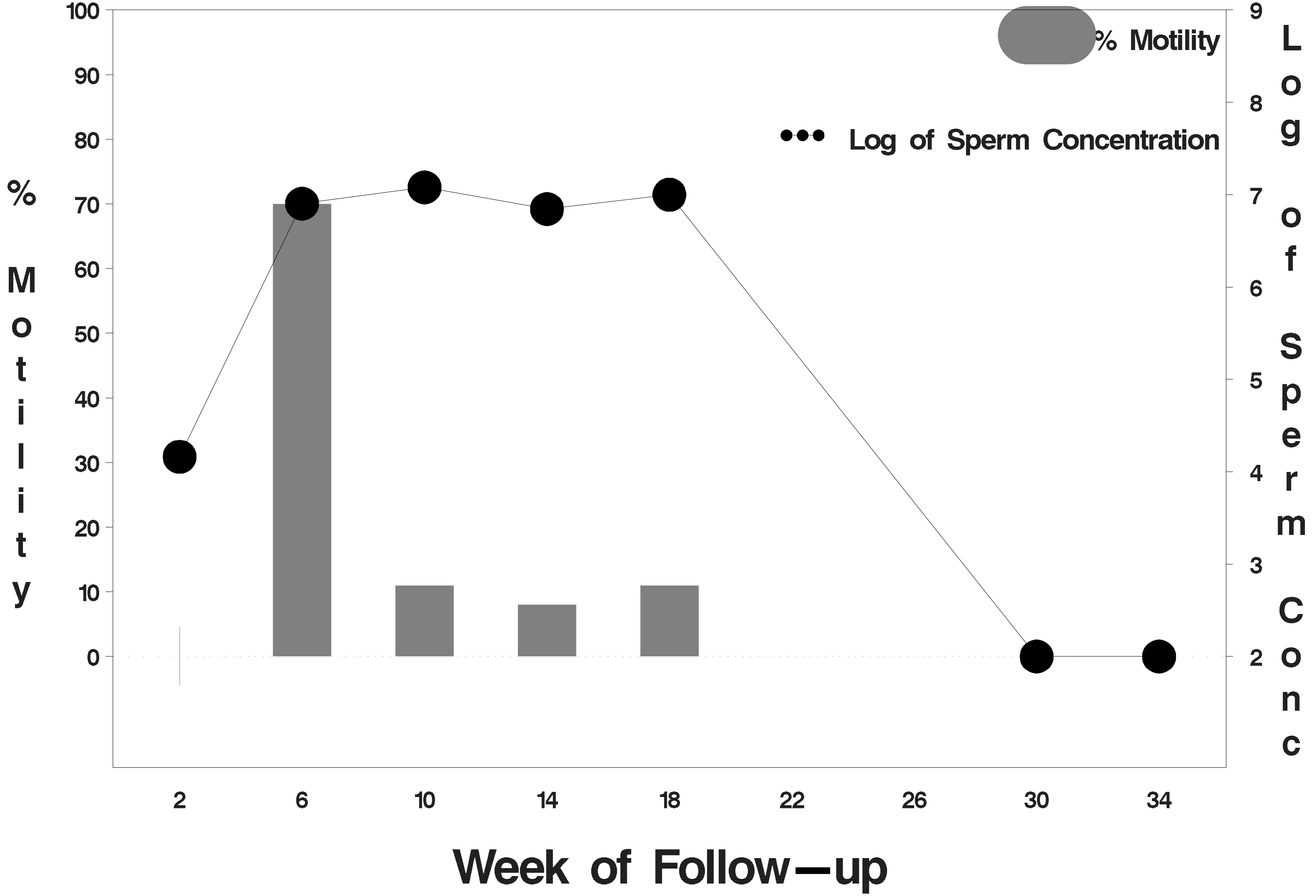

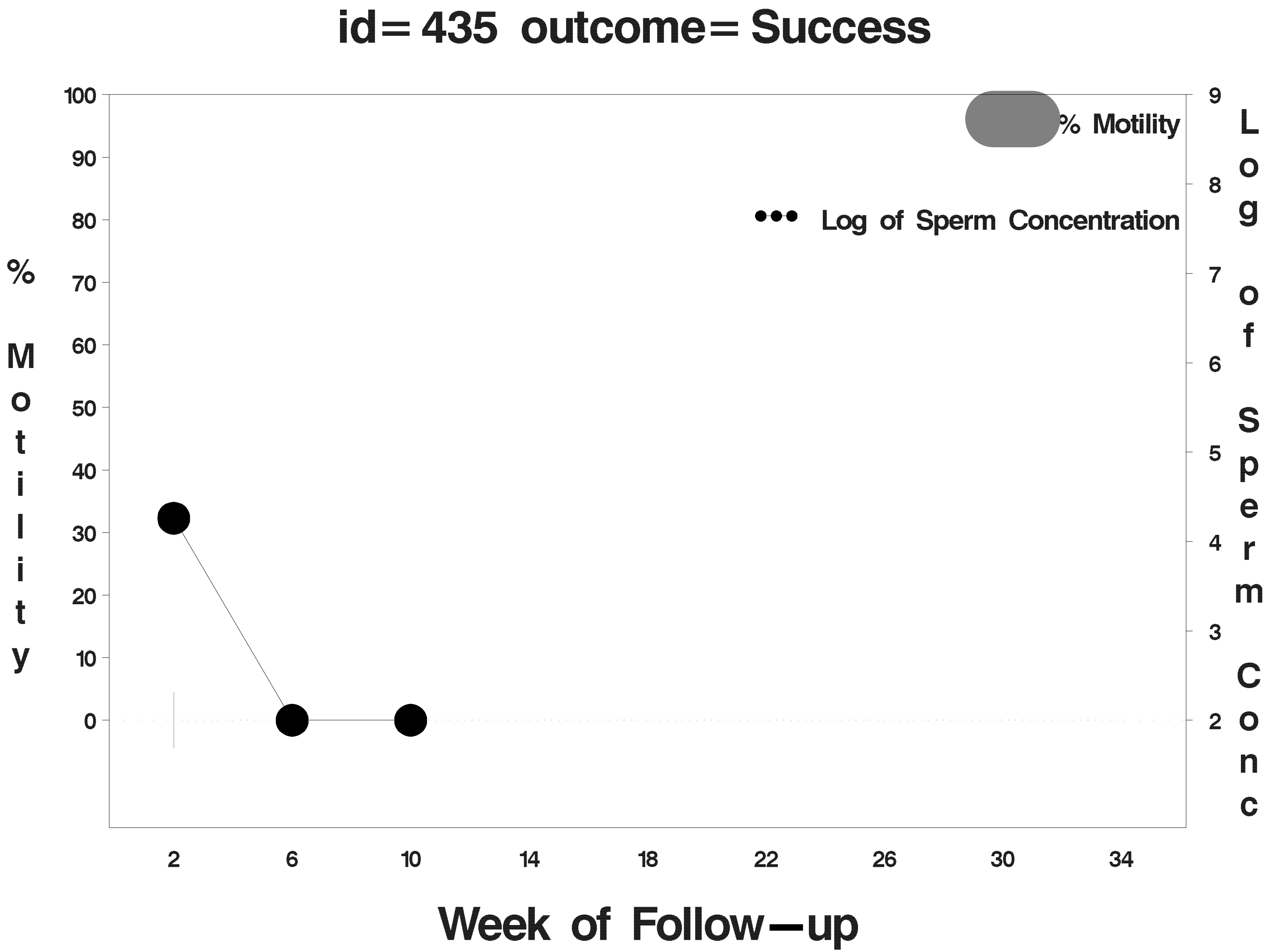
id= 435 outcome= Success
% Motility
Log of Sperm Concentration
100
90
80
70
60
50
40
30
20
10
0
9
8
7
6
5
4
3
2
Log of Sperm Conc
2
6
10
14
18
22
26
30
34
Week of Follow—up

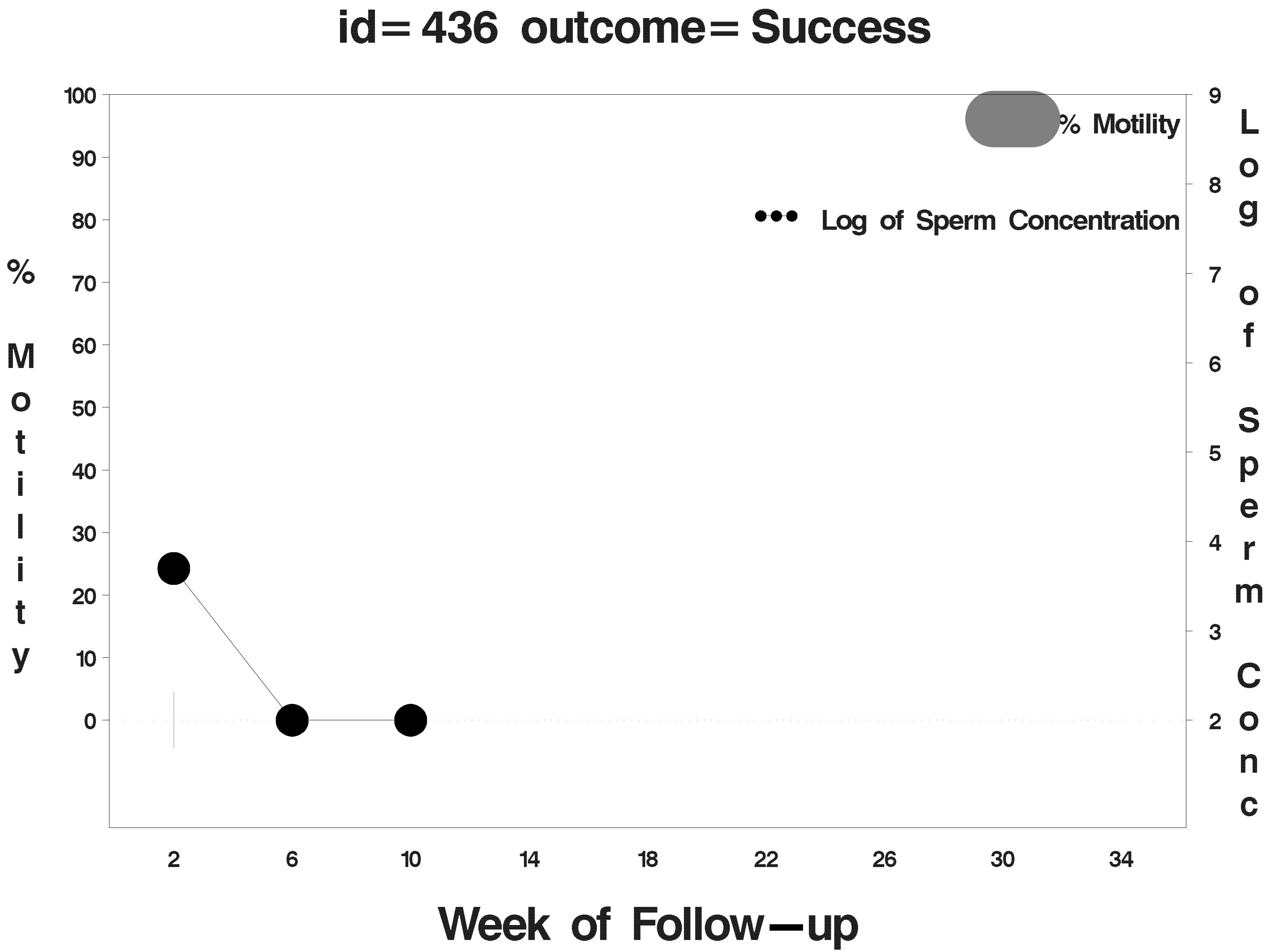

id= 436 outcome= Success
% Motility
Log of Sperm Concentration
100
90
80
70
60
50
40
30
20
10
0
9
8
7
6
5
4
3
2
2
6
10
14
18
22
26
30
34
Week of Follow—up
Log of Sperm Conc

id= 437 outcome= Success

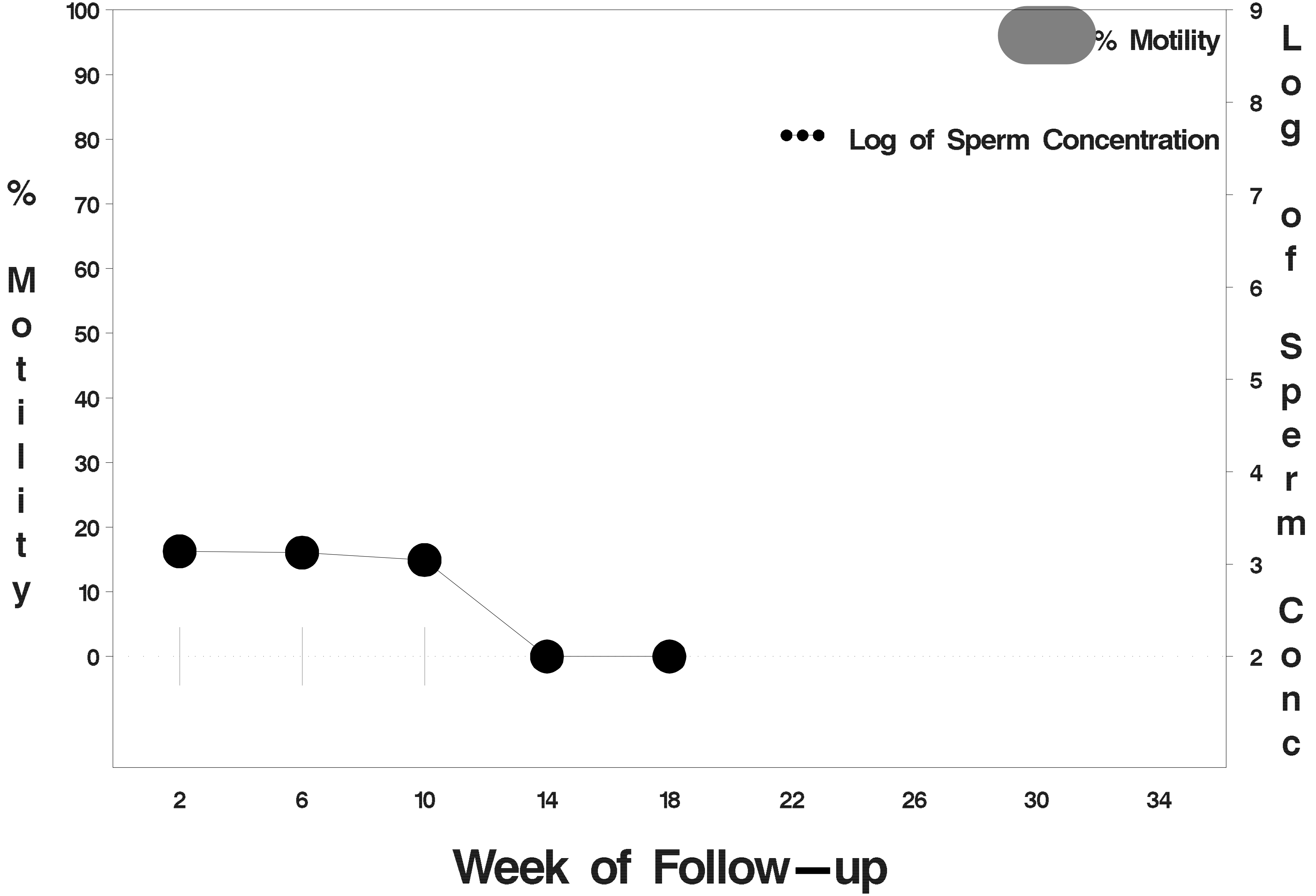

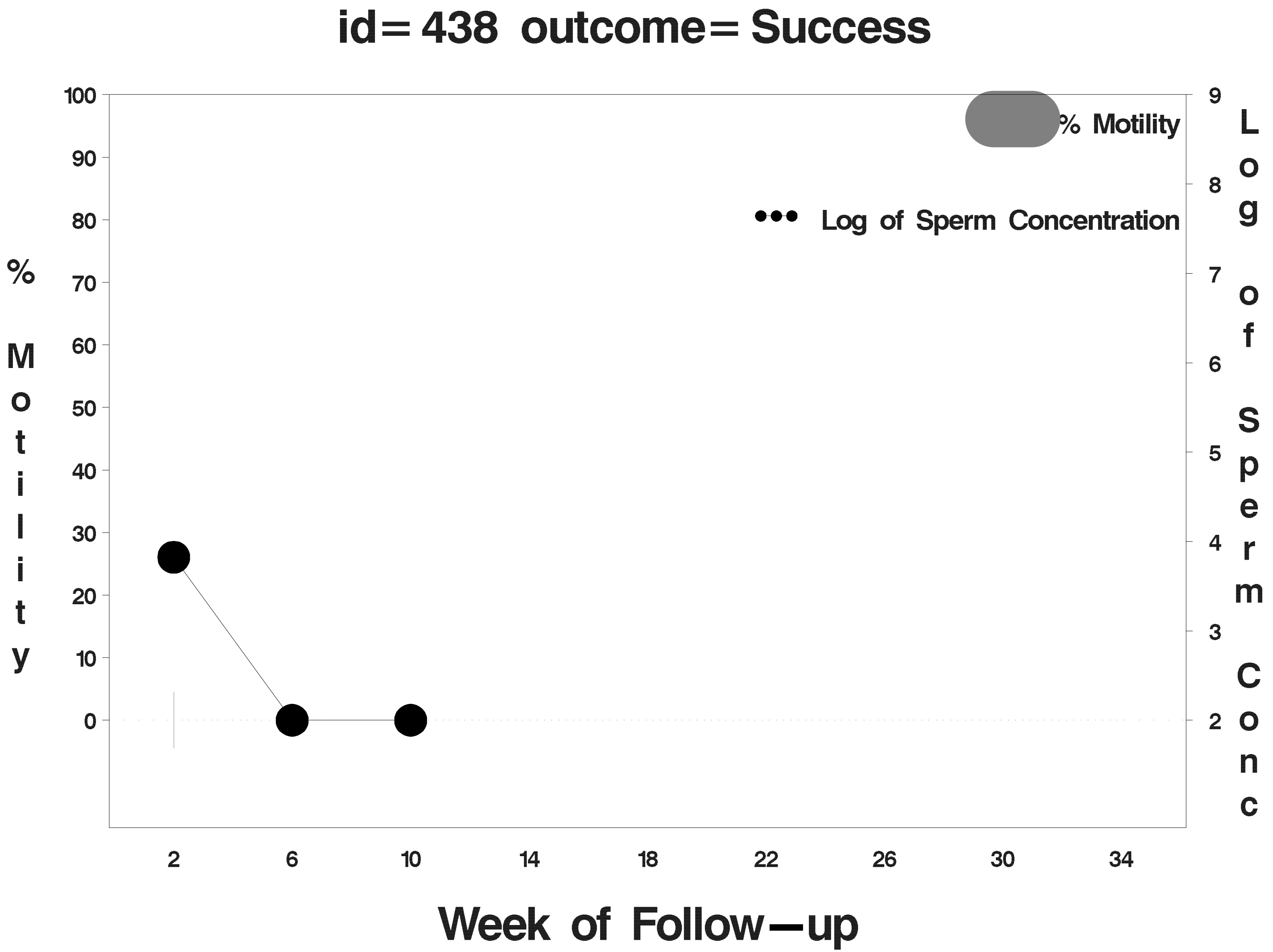

id= 438 outcome= Success
% Motility
Log of Sperm Concentration
100
90
80
70
60
50
40
30
20
10
0
9
8
7
6
5
4
3
2
2
6
10
14
18
22
26
30
34
Week of Follow—up

id= 439 outcome= Success

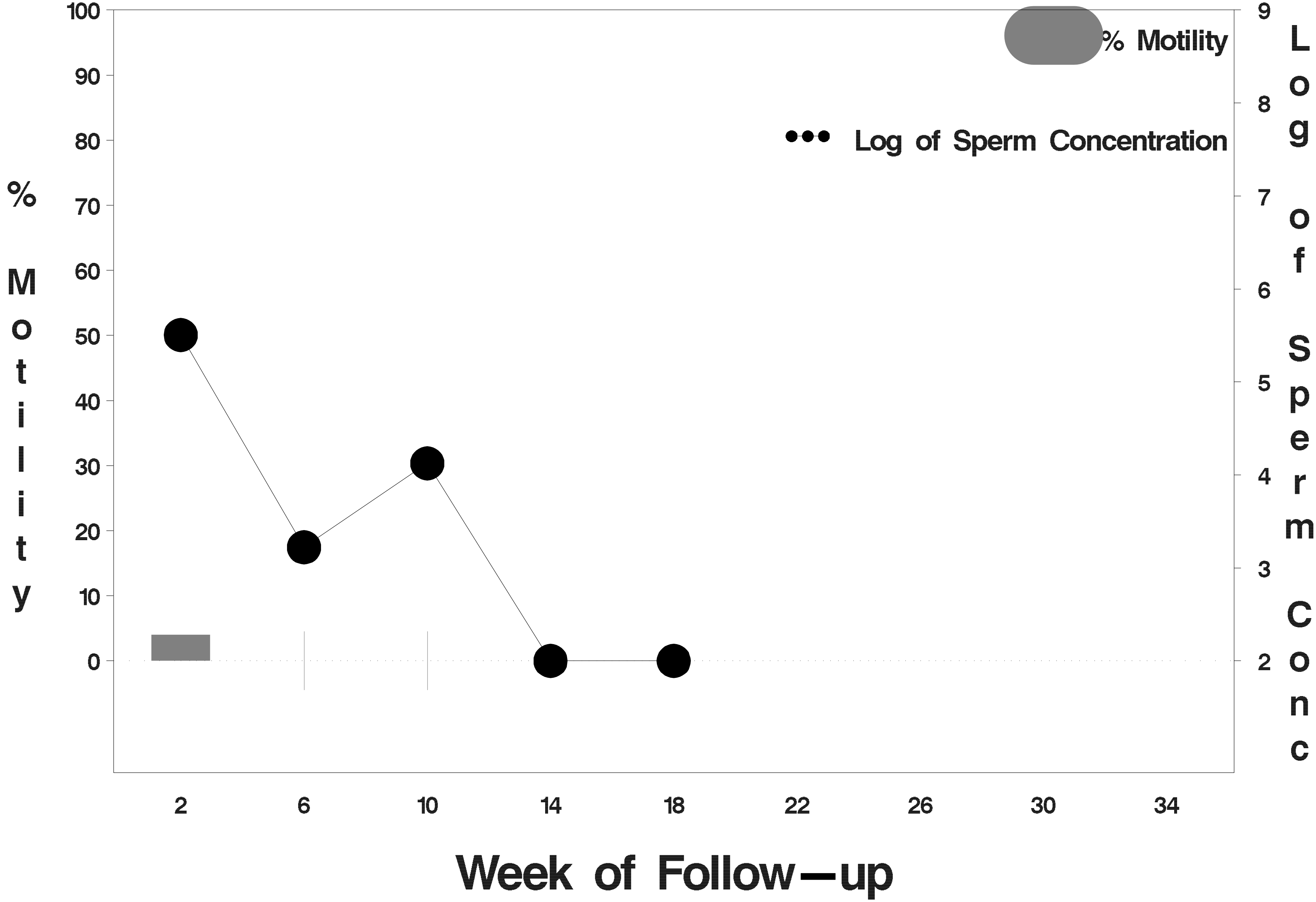

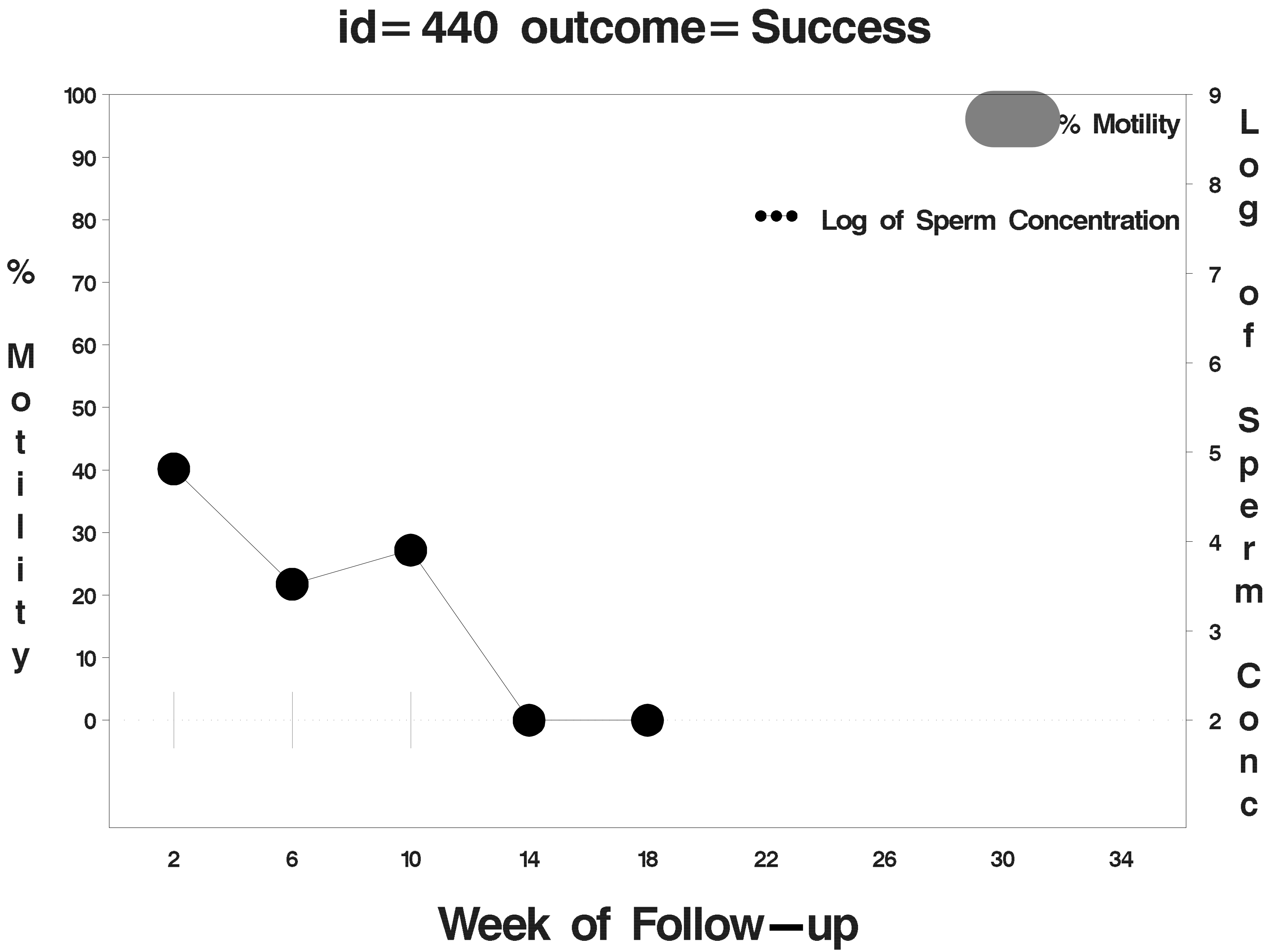

id= 440 outcome= Success
% Motility
Log of Sperm Concentration
100
90
80
70
60
50
40
30
20
10
0
9
8
7
6
5
4
3
2
2
6
10
14
18
22
26
30
34
Week of Follow−up
Log of Sperm Conc

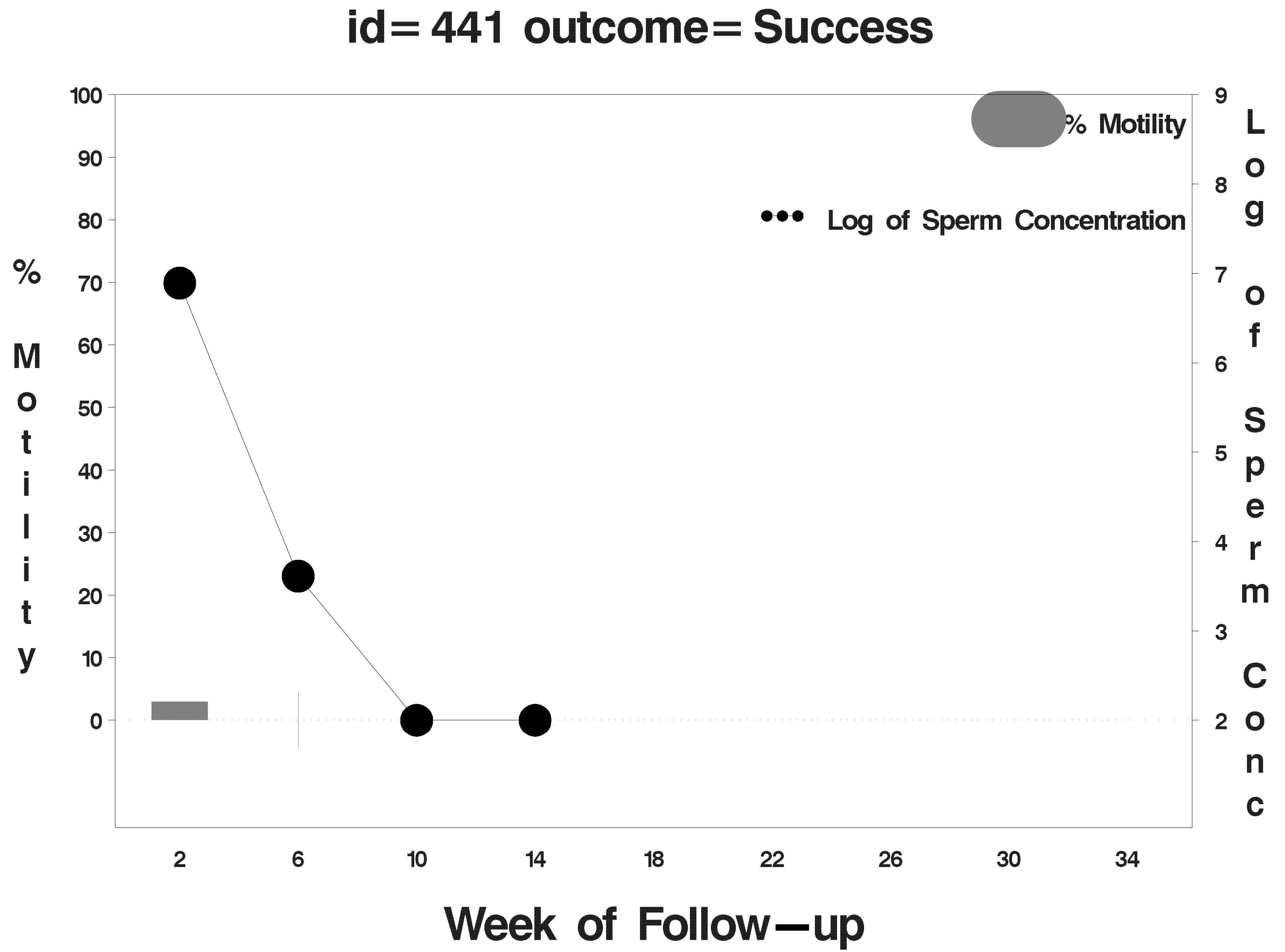
id= 441 outcome= Success
% Motility
Log of Sperm Concentration
100
90
80
70
60
50
40
30
20
10
0
9
8
7
6
5
4
3
2
2
6
10
14
18
22
26
30
34
Week of Follow-up
Log of Sperm Conc

id= 442 outcome= Success

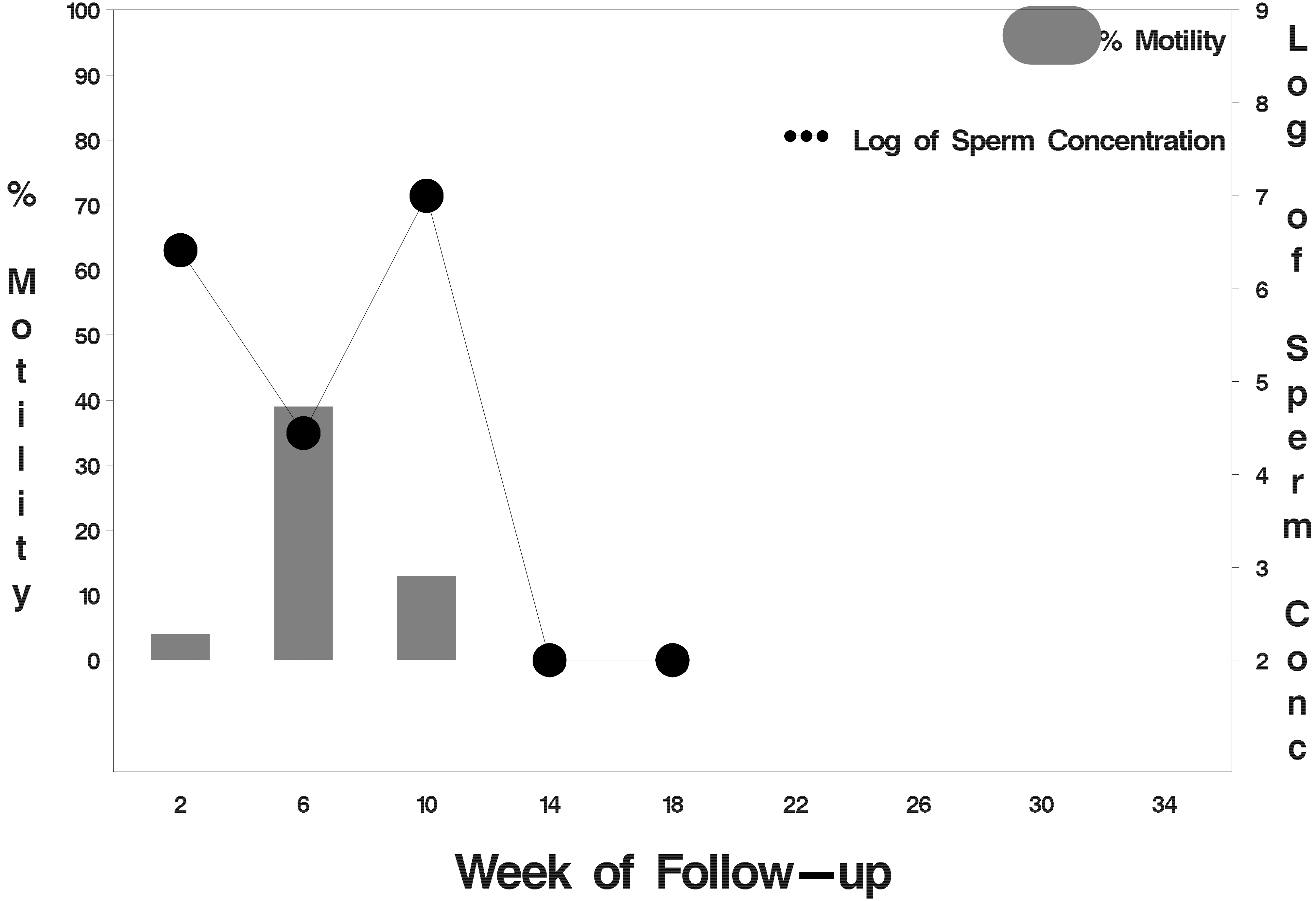

id= 443 outcome= Success

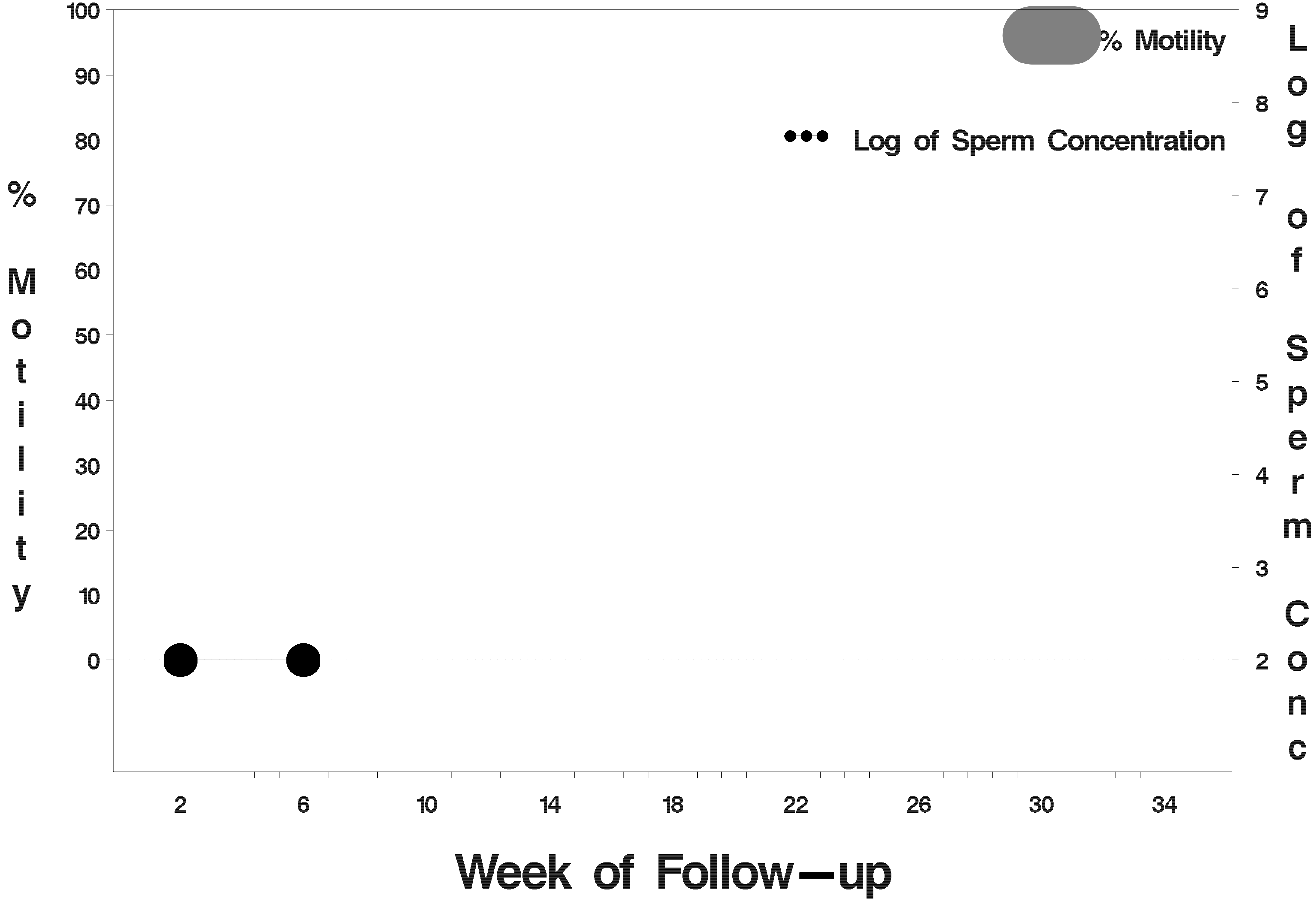

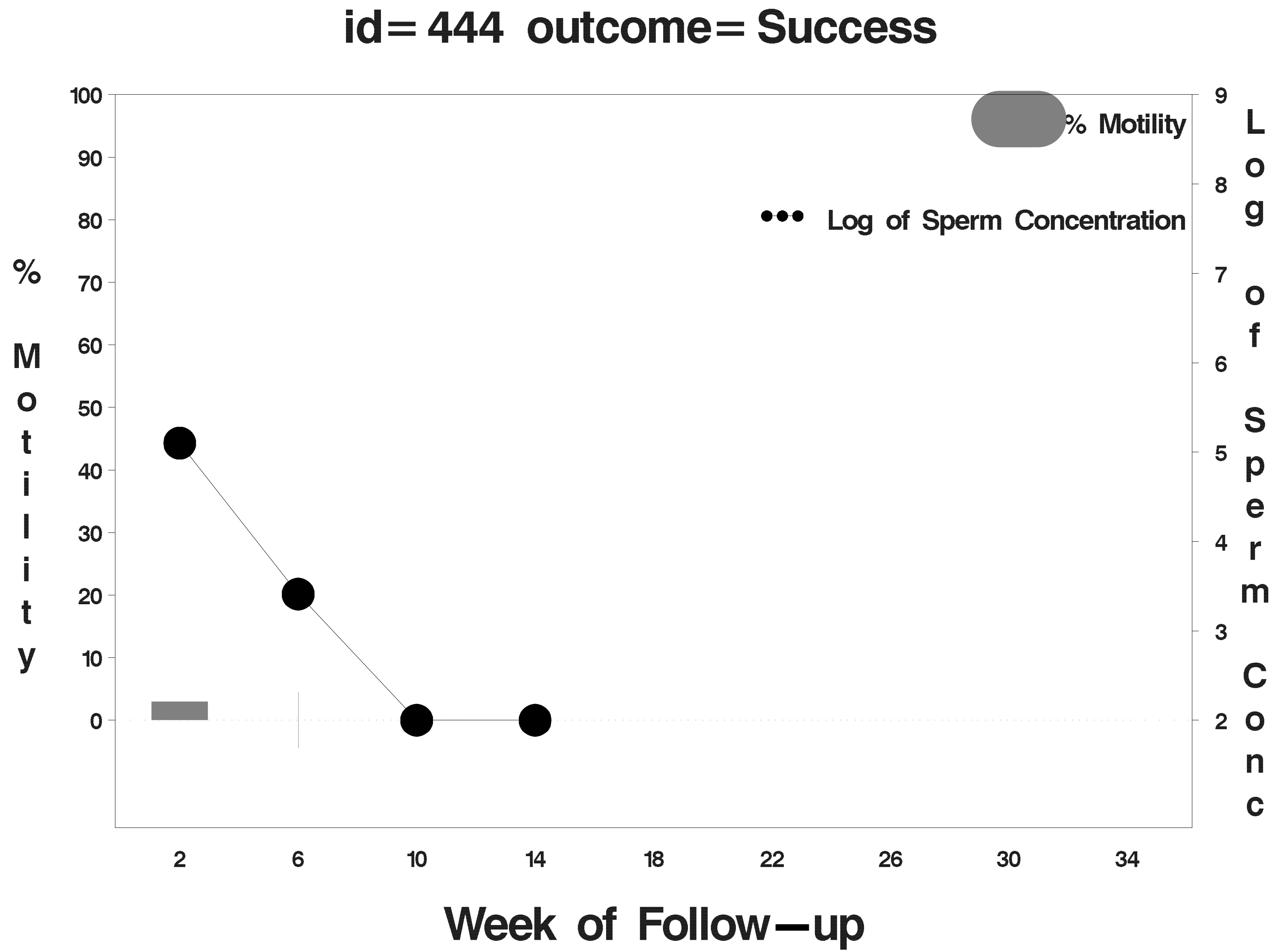

id= 444 outcome= Success
% Motility
Log of Sperm Concentration
100
90
80
70
60
50
40
30
20
10
0
9
8
7
6
5
4
3
2
2
6
10
14
18
22
26
30
34
Week of Follow—up
Log of Sperm Conc

id= 445 outcome= Success

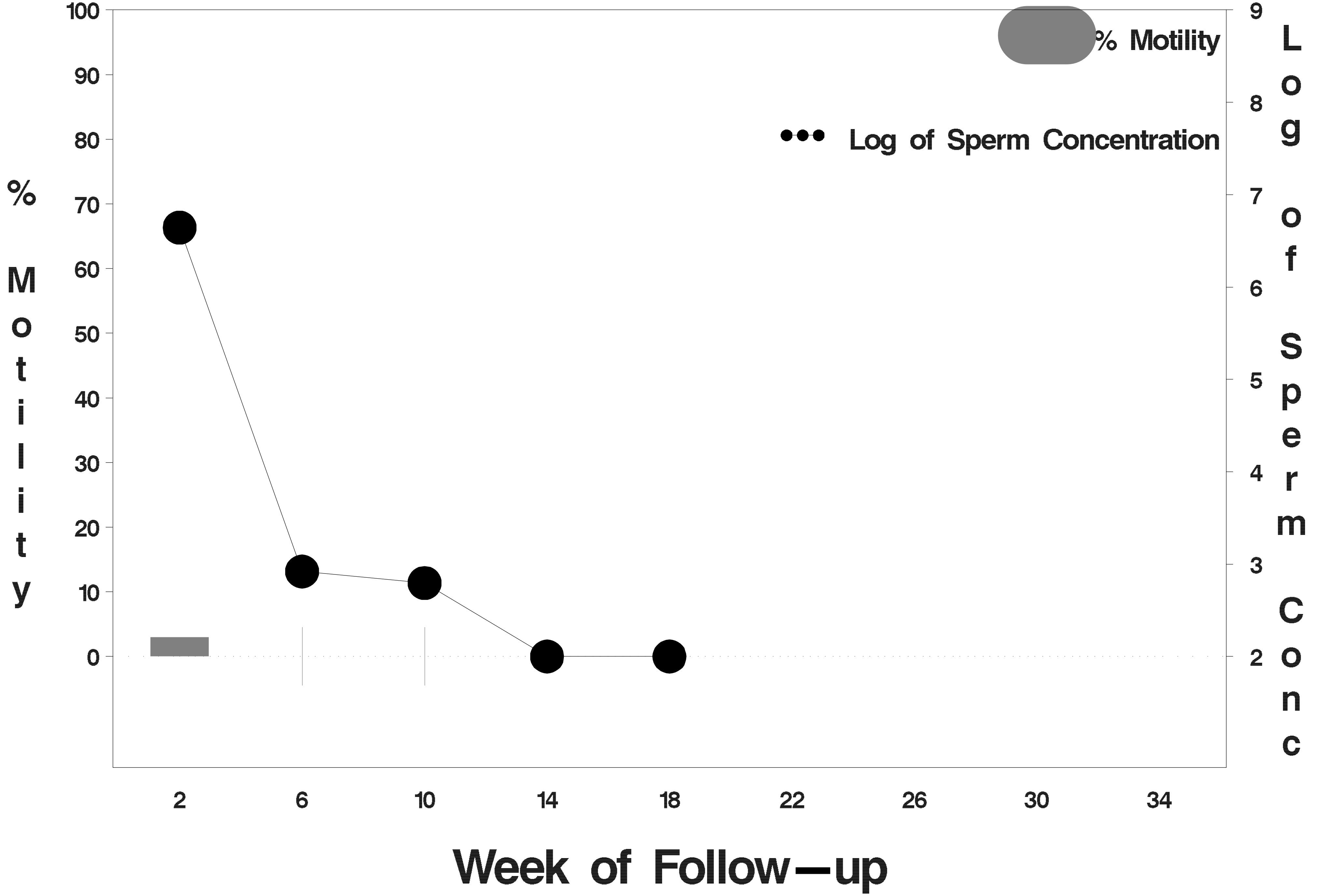

id= 446 outcome= Success

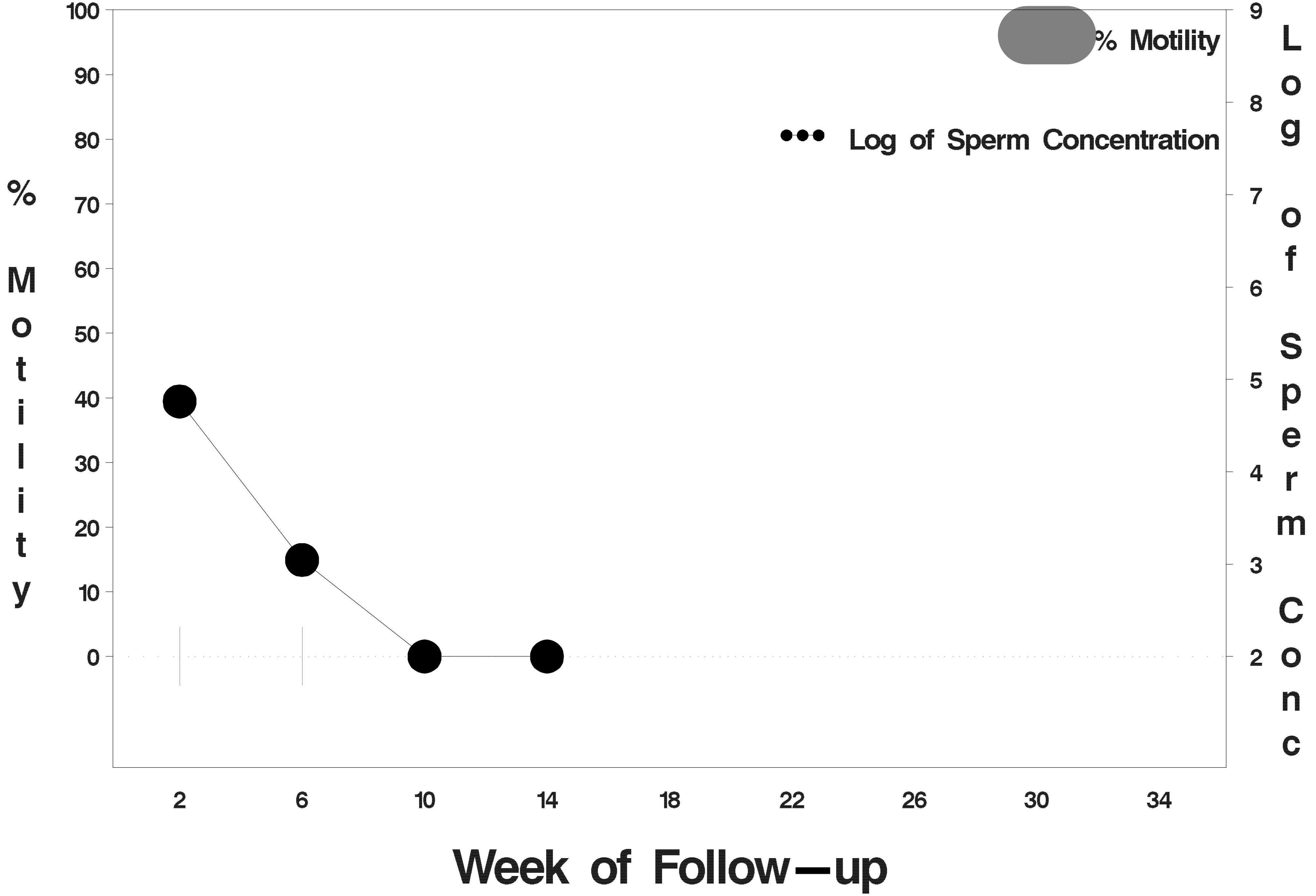

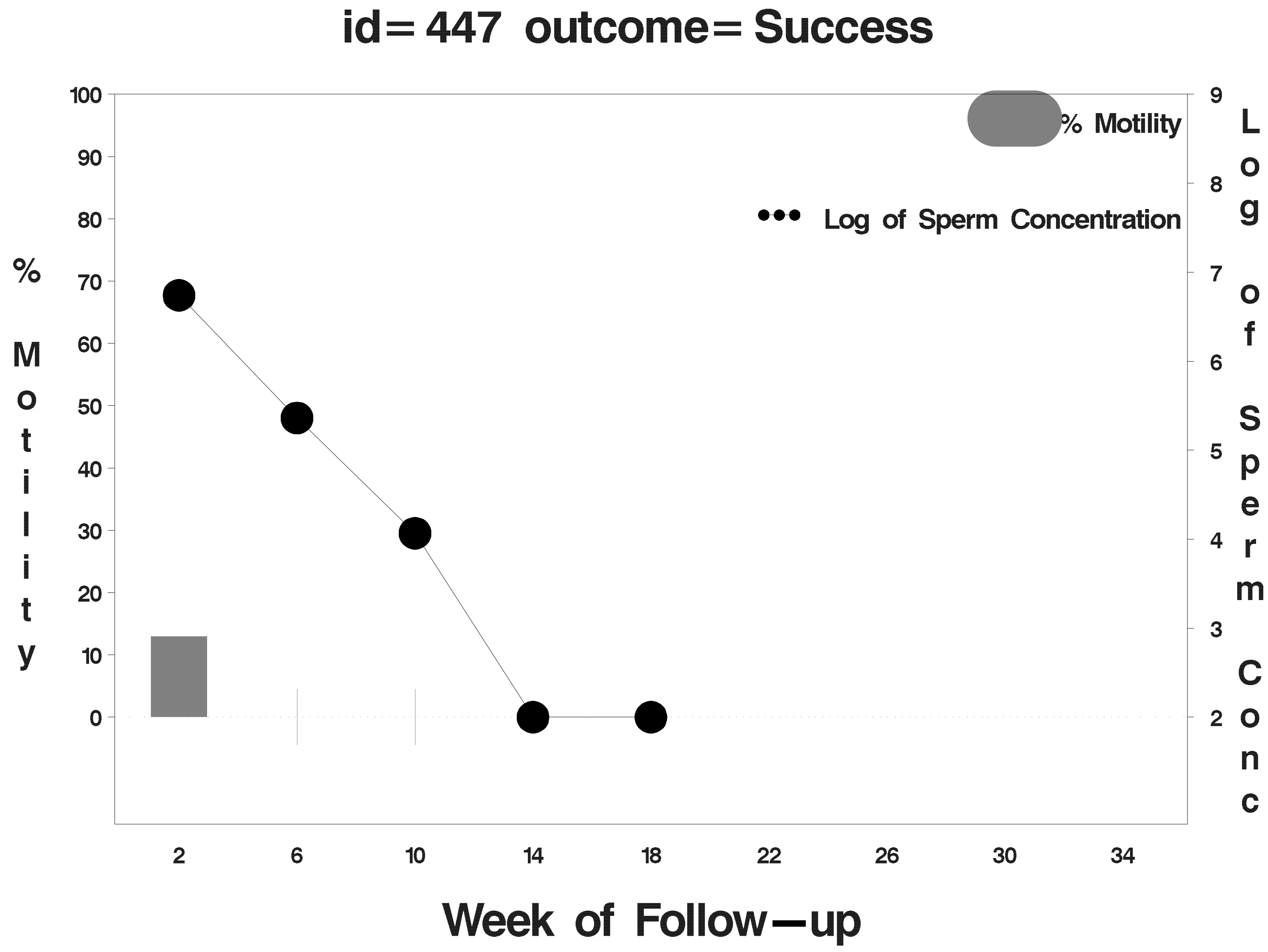

id= 447 outcome= Success
% Motility
Log of Sperm Concentration
100
90
80
70
60
50
40
30
20
10
0
9
8
7
6
5
4
3
2
2
6
10
14
18
22
26
30
34
Week of Follow−up
Log of Sperm Conc

id= 448 outcome= Success

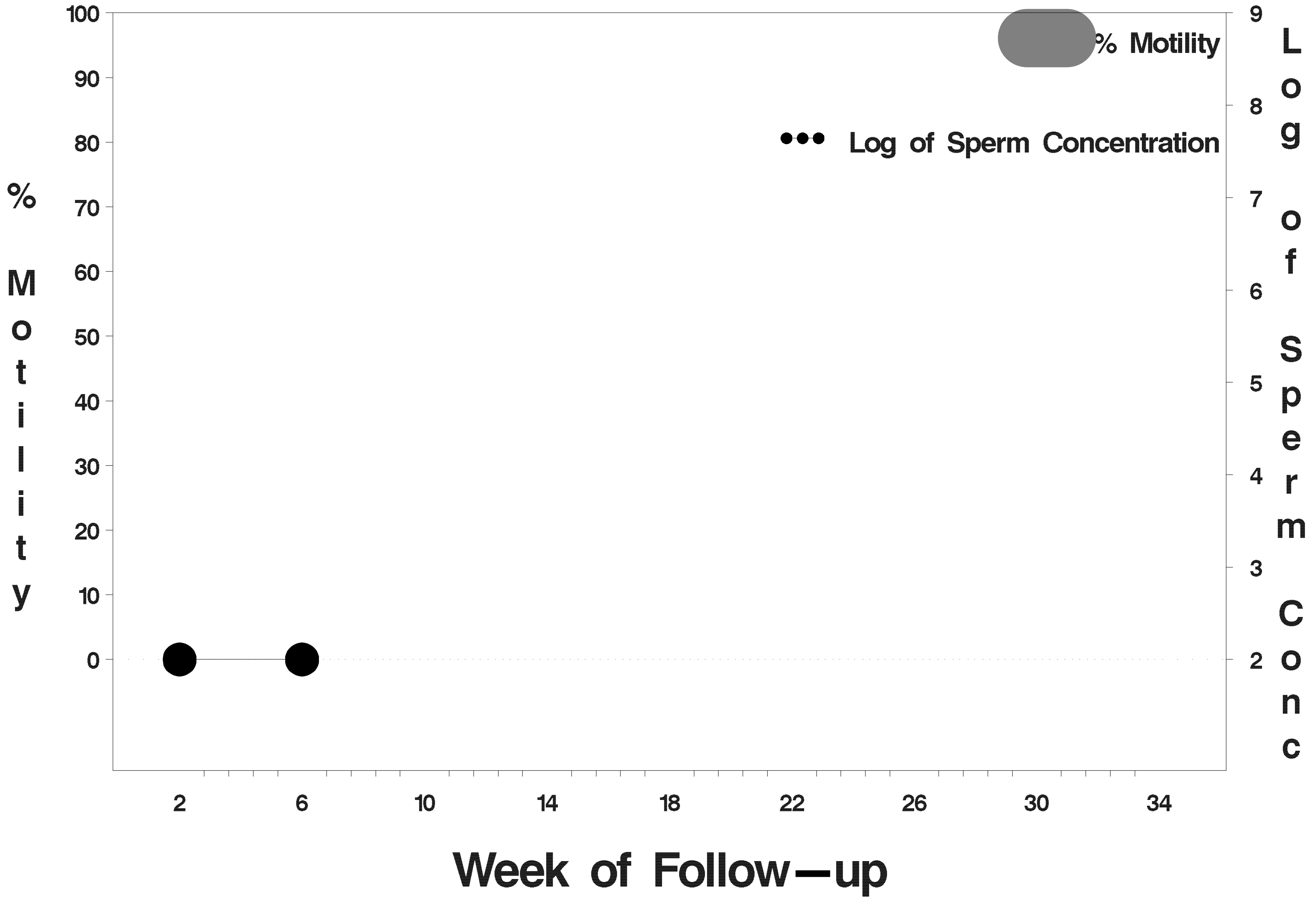

id= 449 outcome= Success

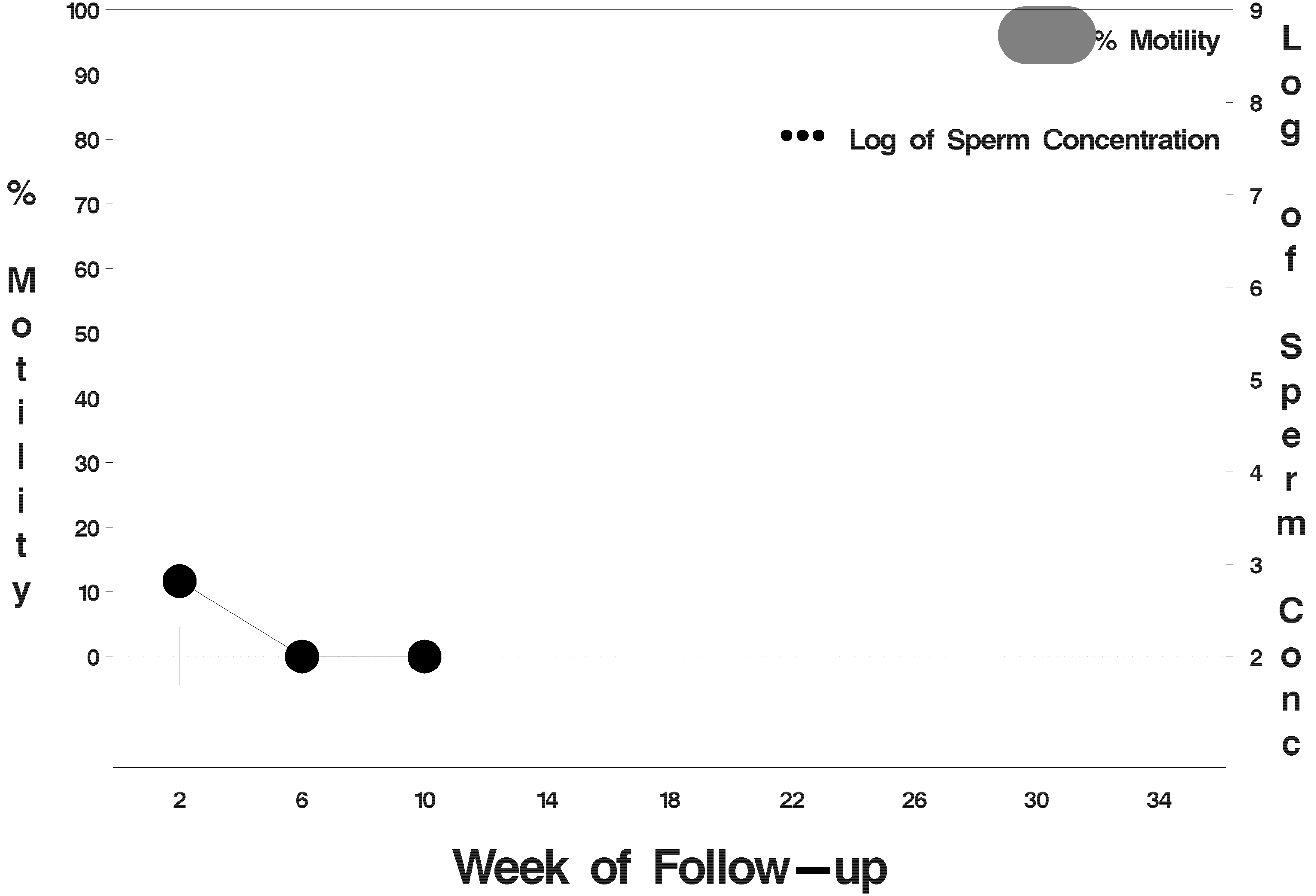

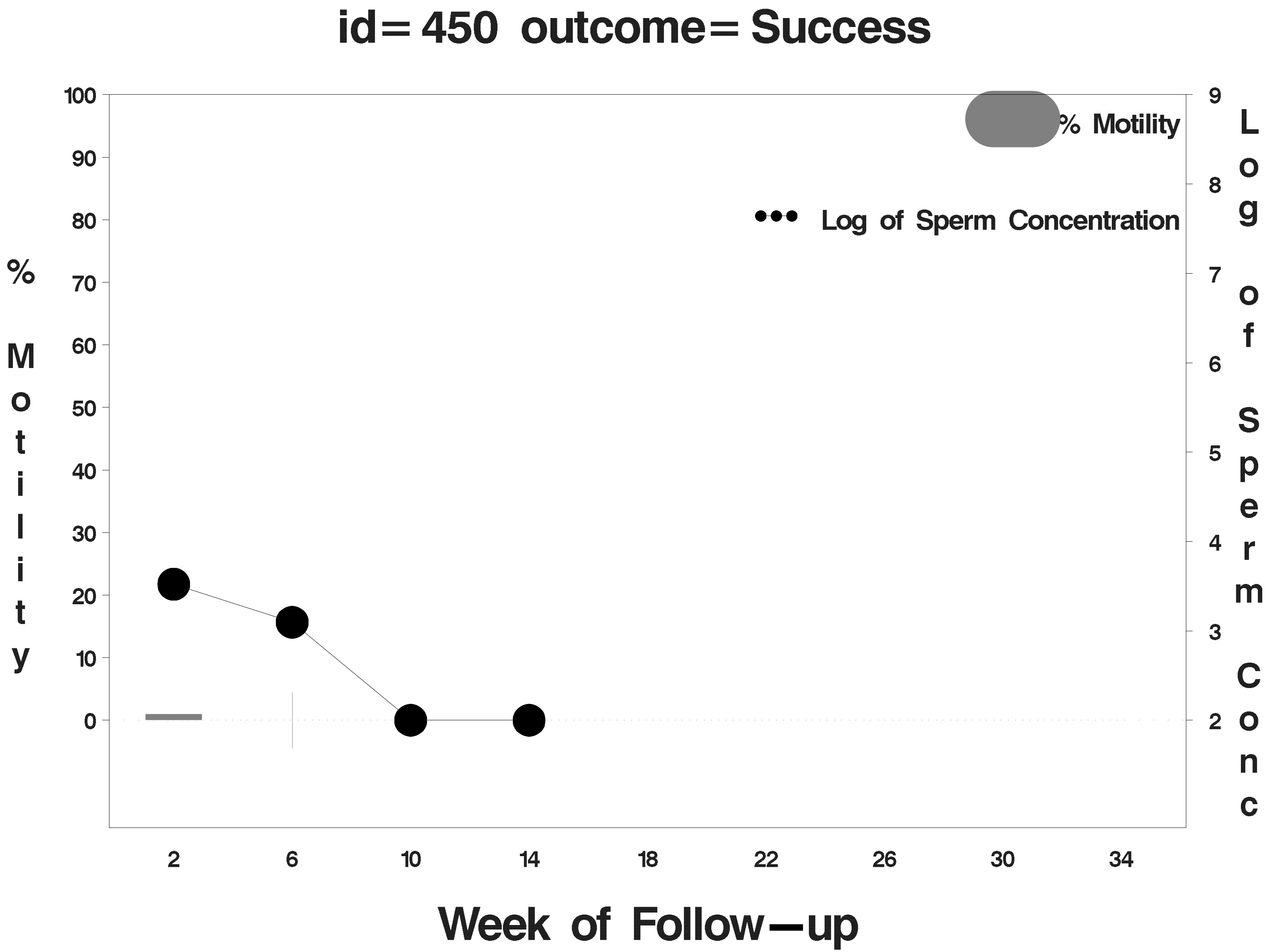
id= 450 outcome= Success
% Motility
Log of Sperm Concentration
100
90
80
70
60
50
40
30
20
10
0
9
8
7
6
5
4
3
2
2
6
10
14
18
22
26
30
34
Week of Follow—up
Log of Sperm Conc

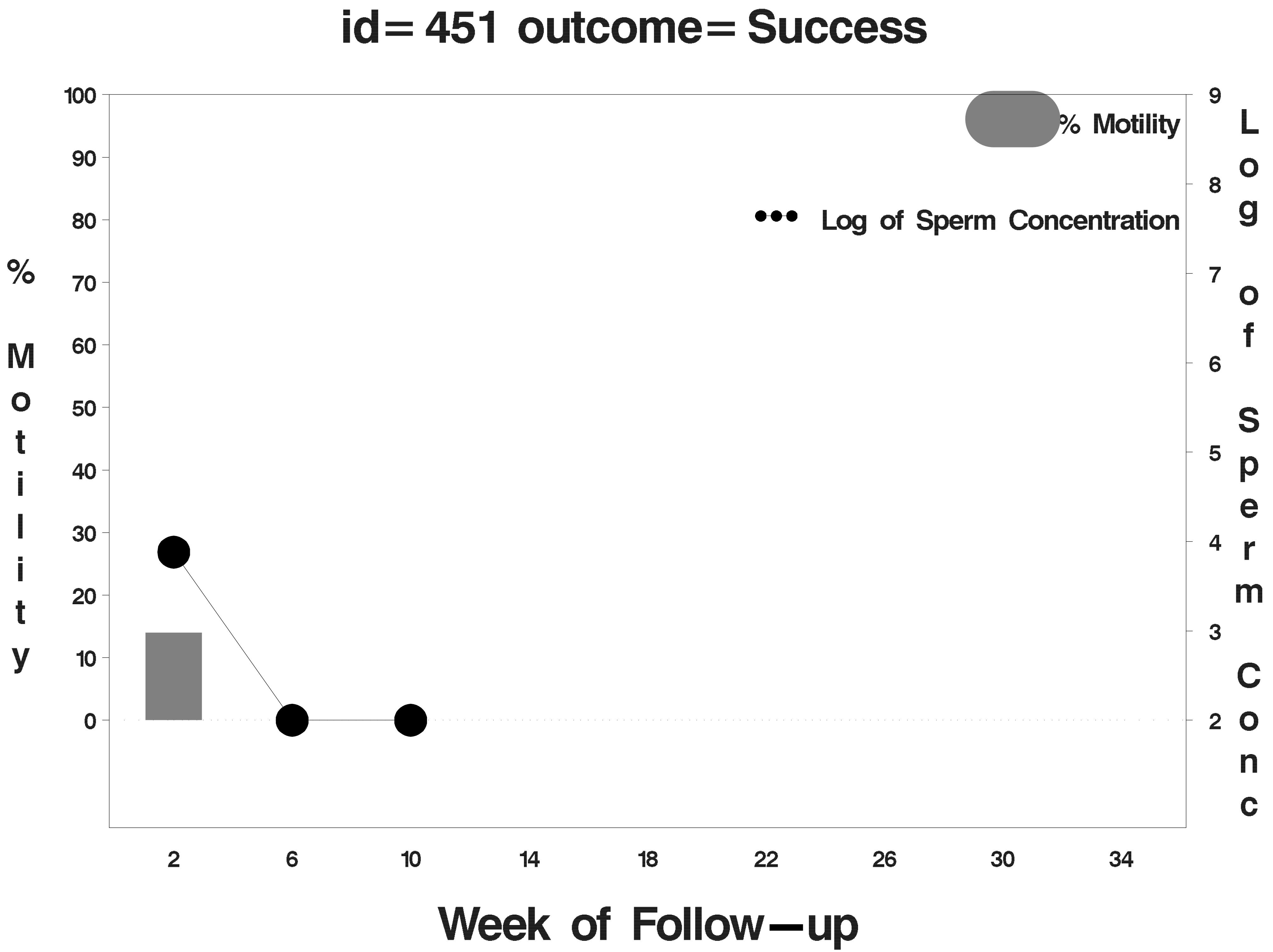

id= 451 outcome= Success
% Motility
Log of Sperm Concentration
100
90
80
70
60
50
40
30
20
10
0
9
8
7
6
5
4
3
2
2
6
10
14
18
22
26
30
34
Week of Follow-up

id= 452 outcome= Success

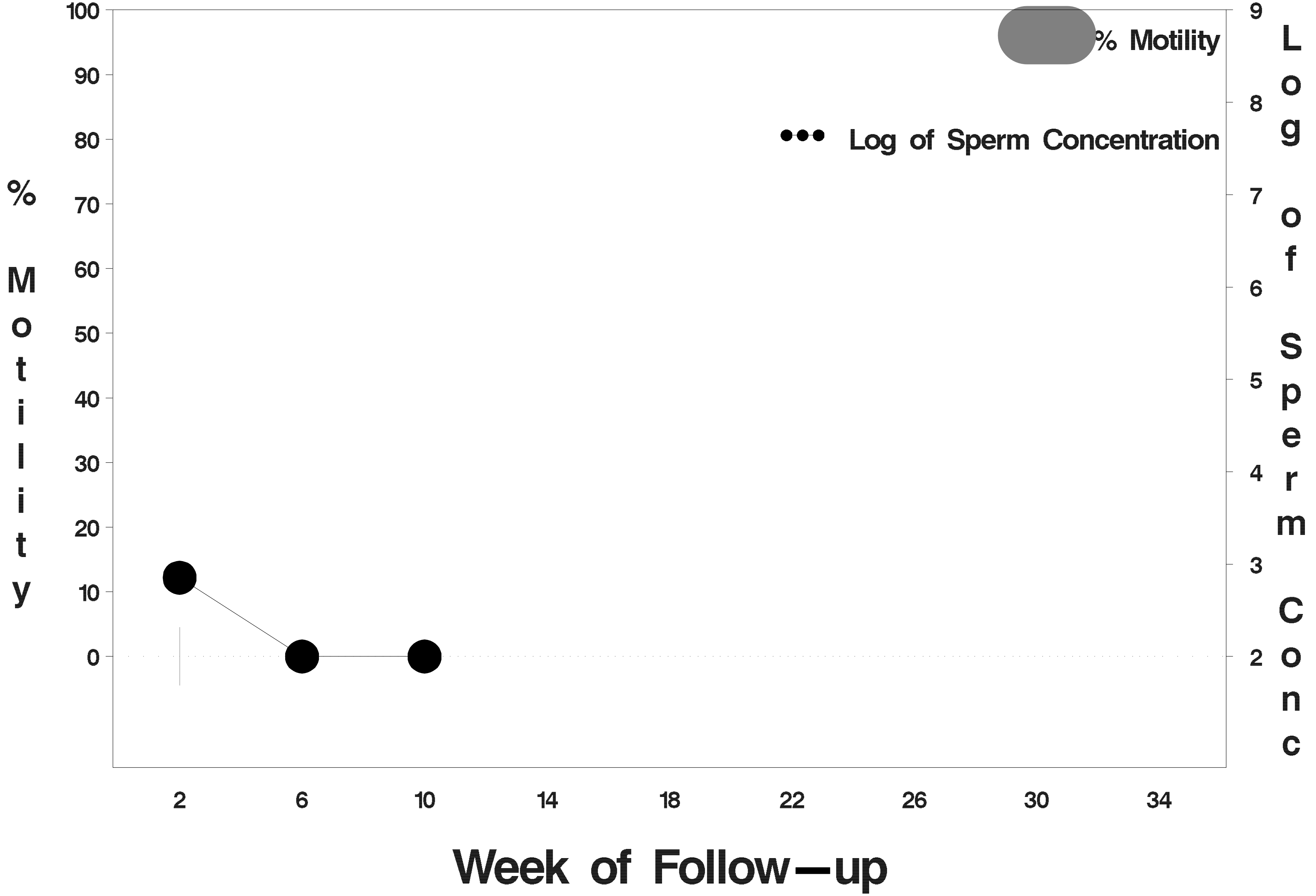

id= 453 outcome= Failure

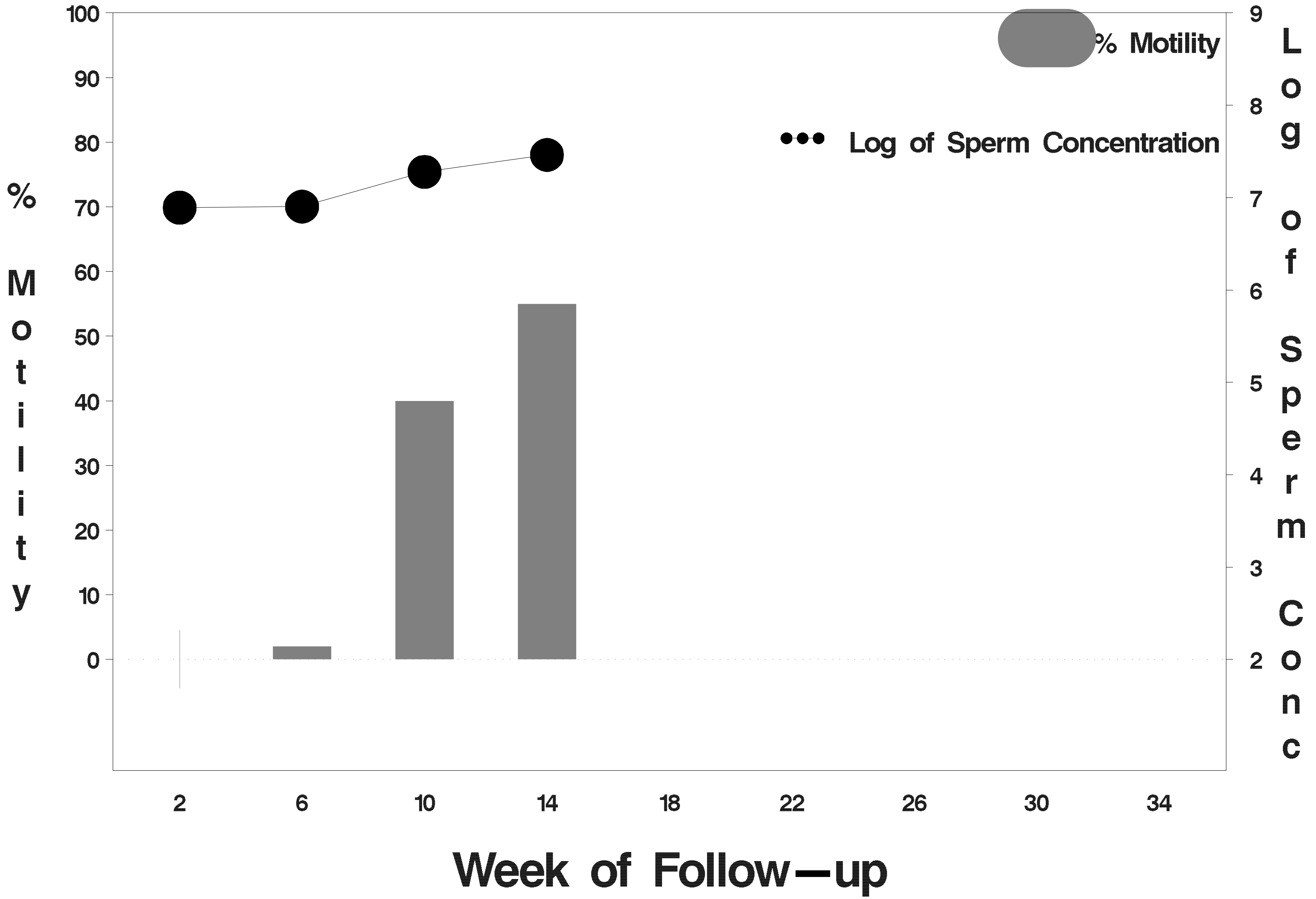

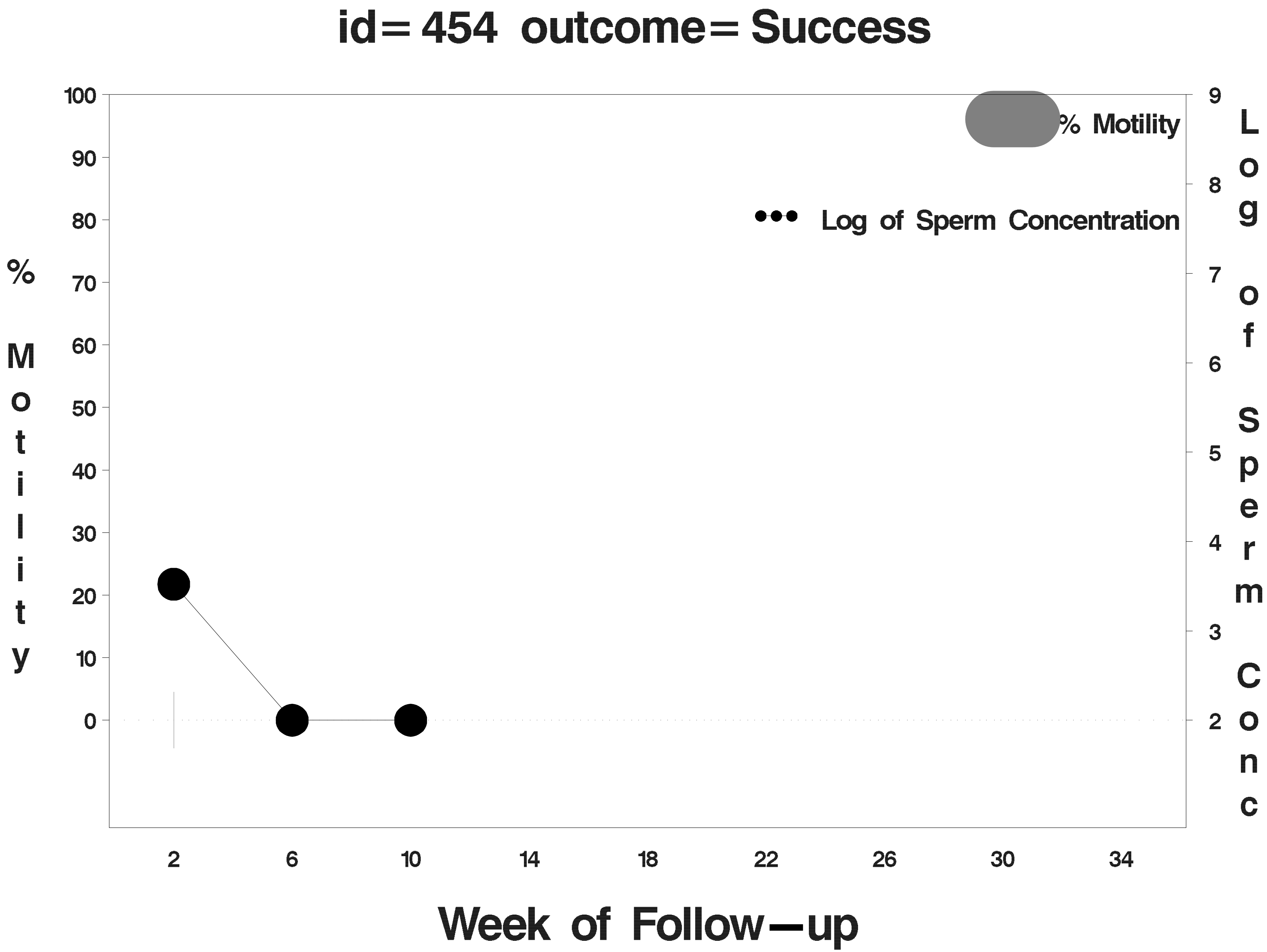
id= 454 outcome= Success
% Motility
Log of Sperm Concentration
100
90
80
70
60
50
40
30
20
10
0
9
8
7
6
5
4
3
2
2
6
10
14
18
22
26
30
34
Week of Follow—up
Log of Sperm Conc

id= 455 outcome= Success

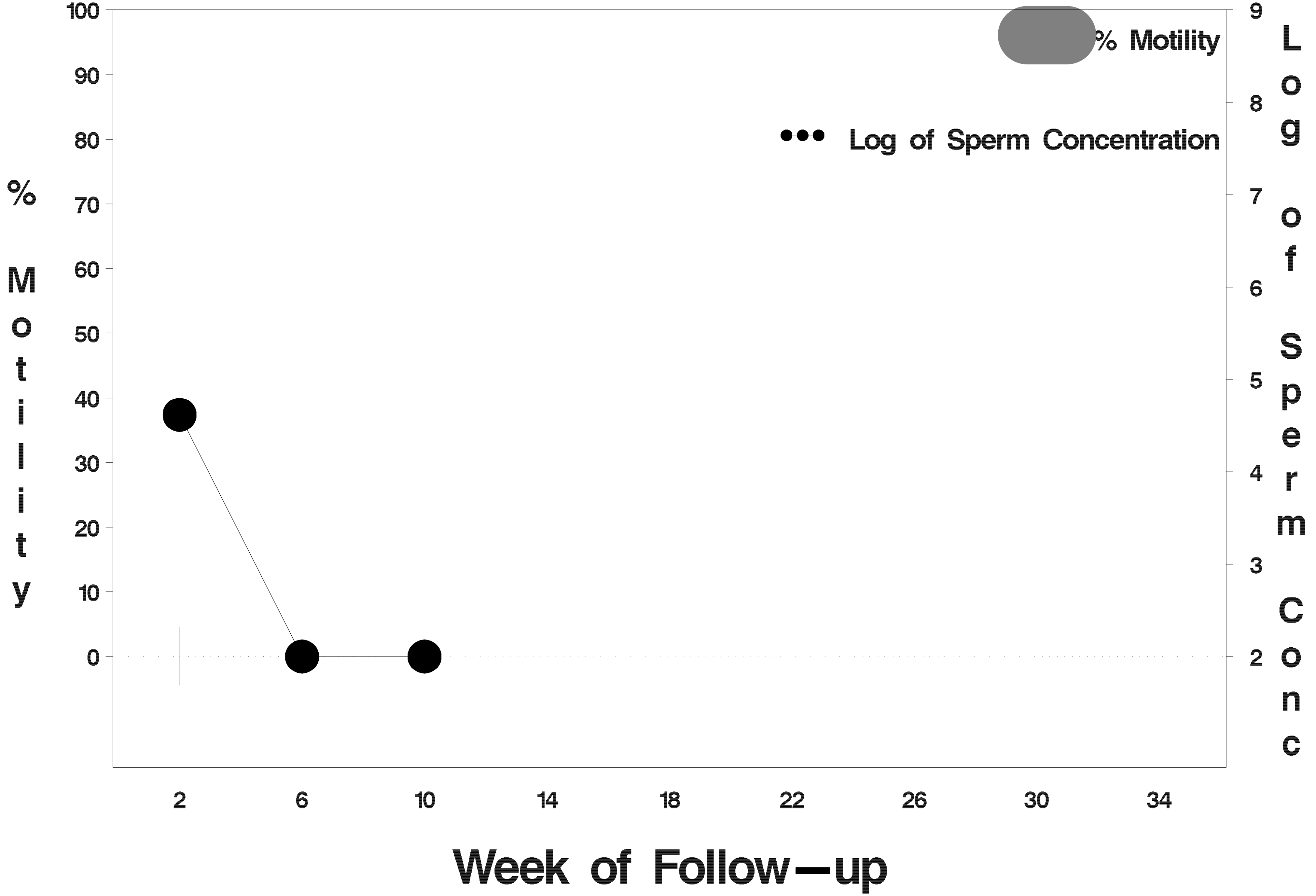

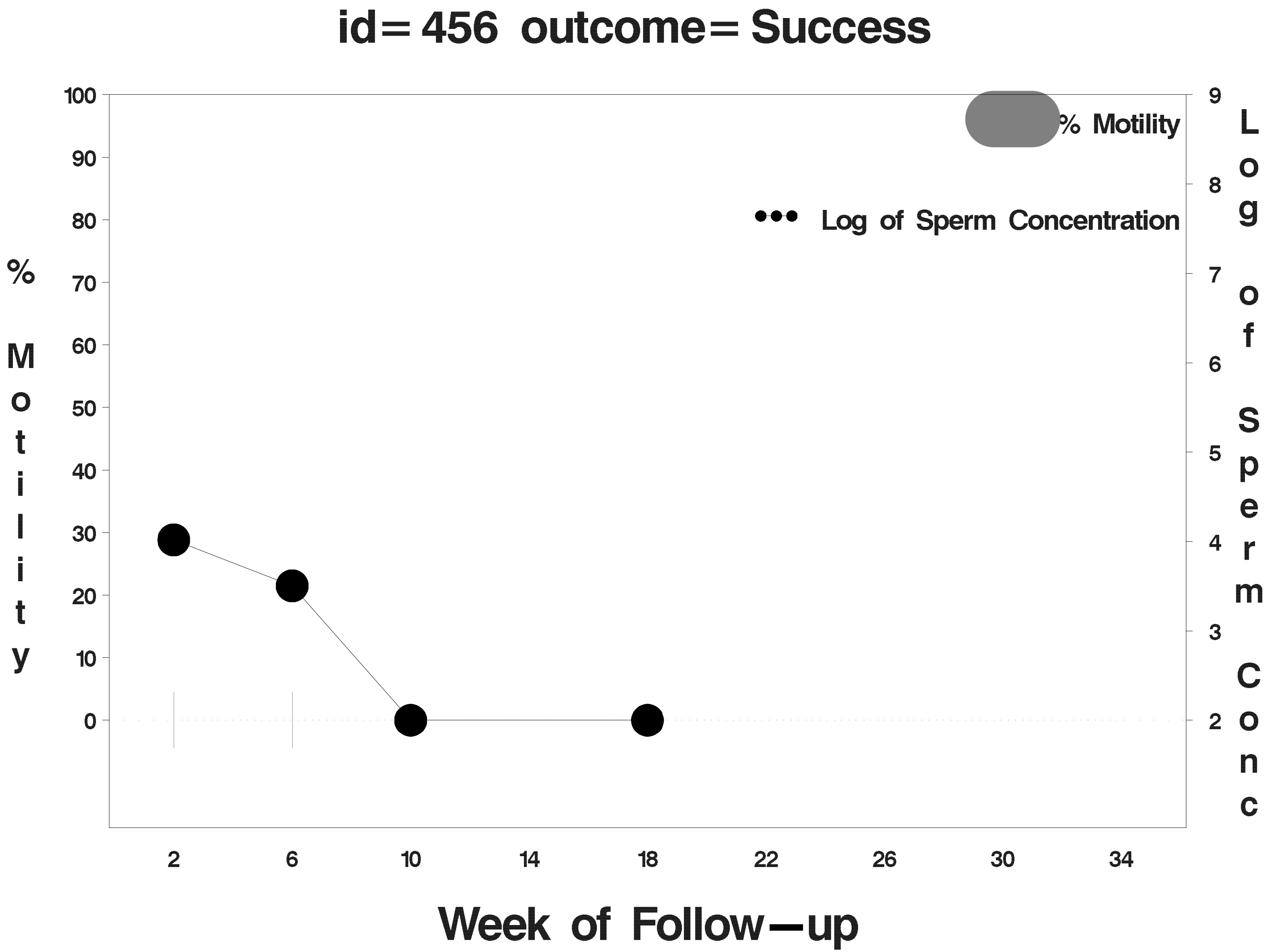

id= 456 outcome= Success
% Motility
Log of Sperm Concentration
100
90
80
70
60
50
40
30
20
10
0
9
8
7
6
5
4
3
2
2
6
10
14
18
22
26
30
34
Week of Follow—up
Log of Sperm Conc

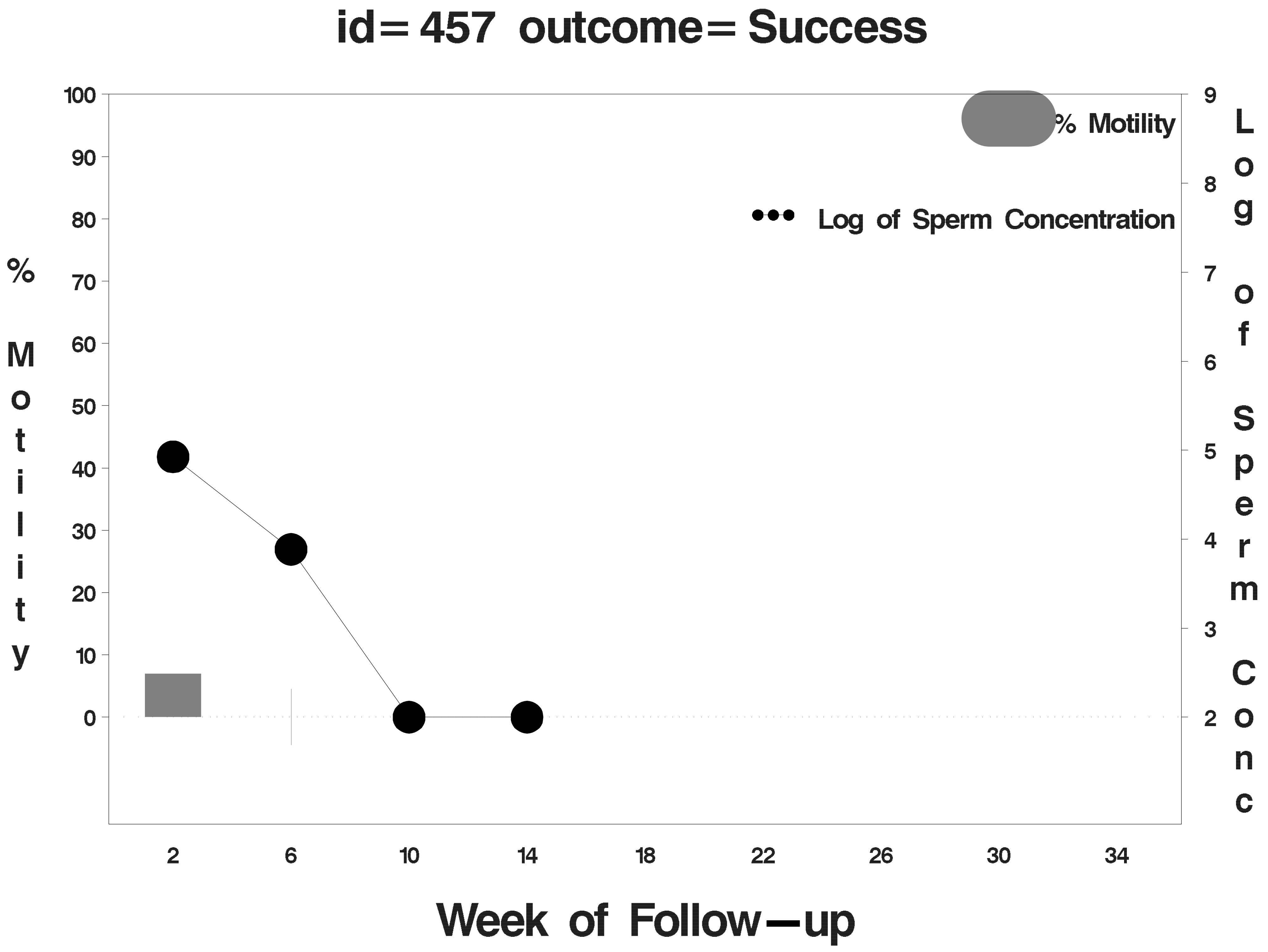

id= 457 outcome= Success
% Motility
Log of Sperm Concentration
100
90
80
70
60
50
40
30
20
10
0
9
8
7
6
5
4
3
2
2
6
10
14
18
22
26
30
34
Week of Follow-up

id= 458 outcome= Failure

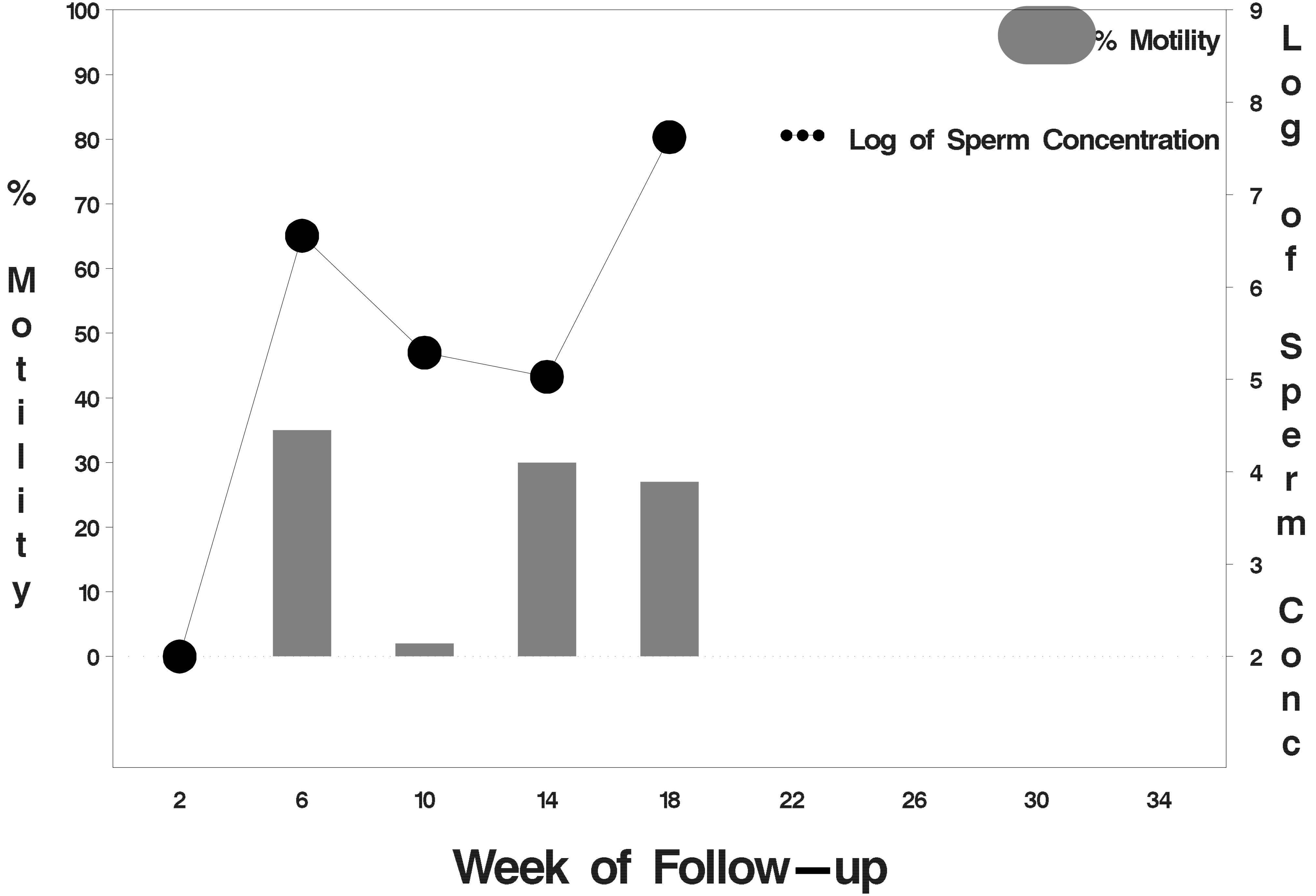

id= 459 outcome= Indeterminate

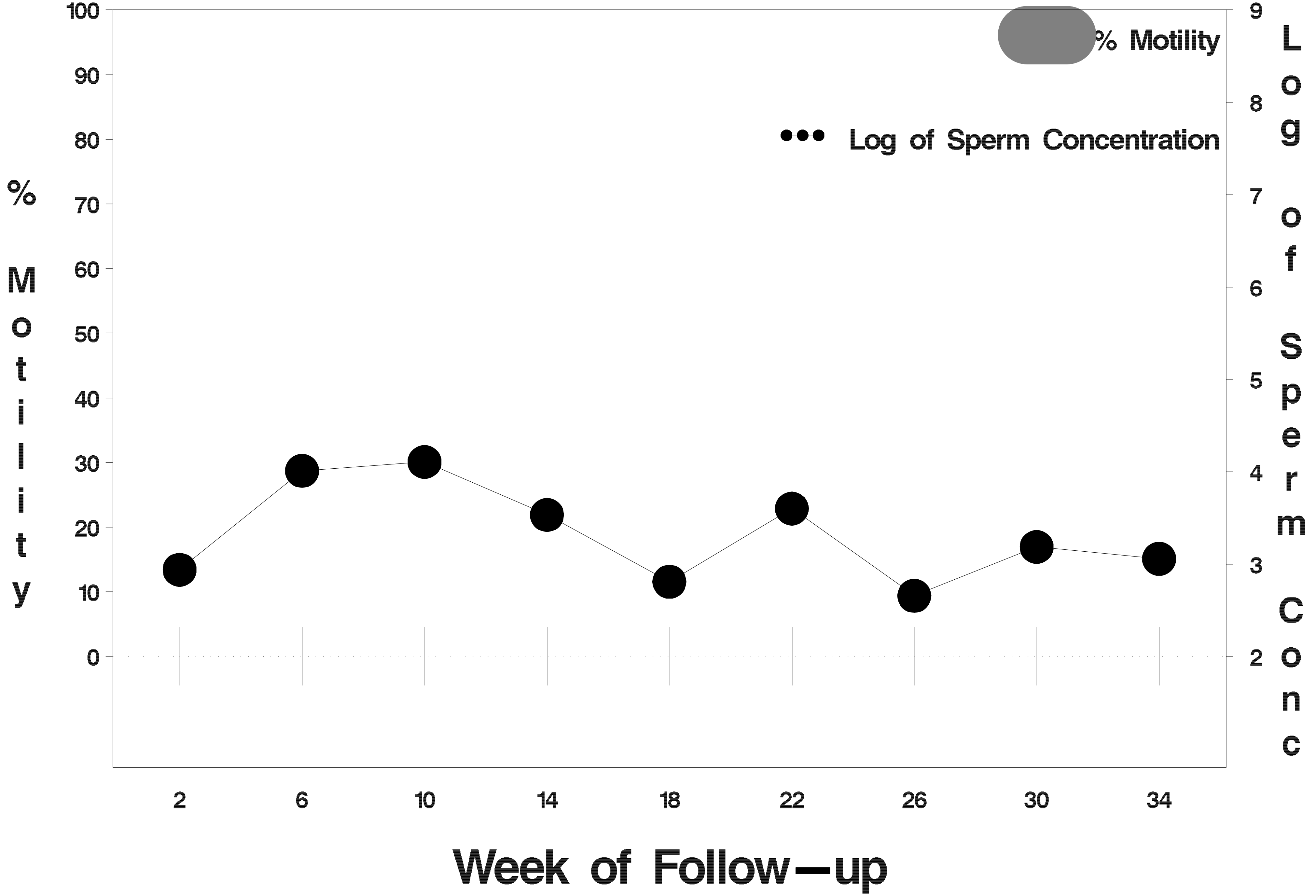

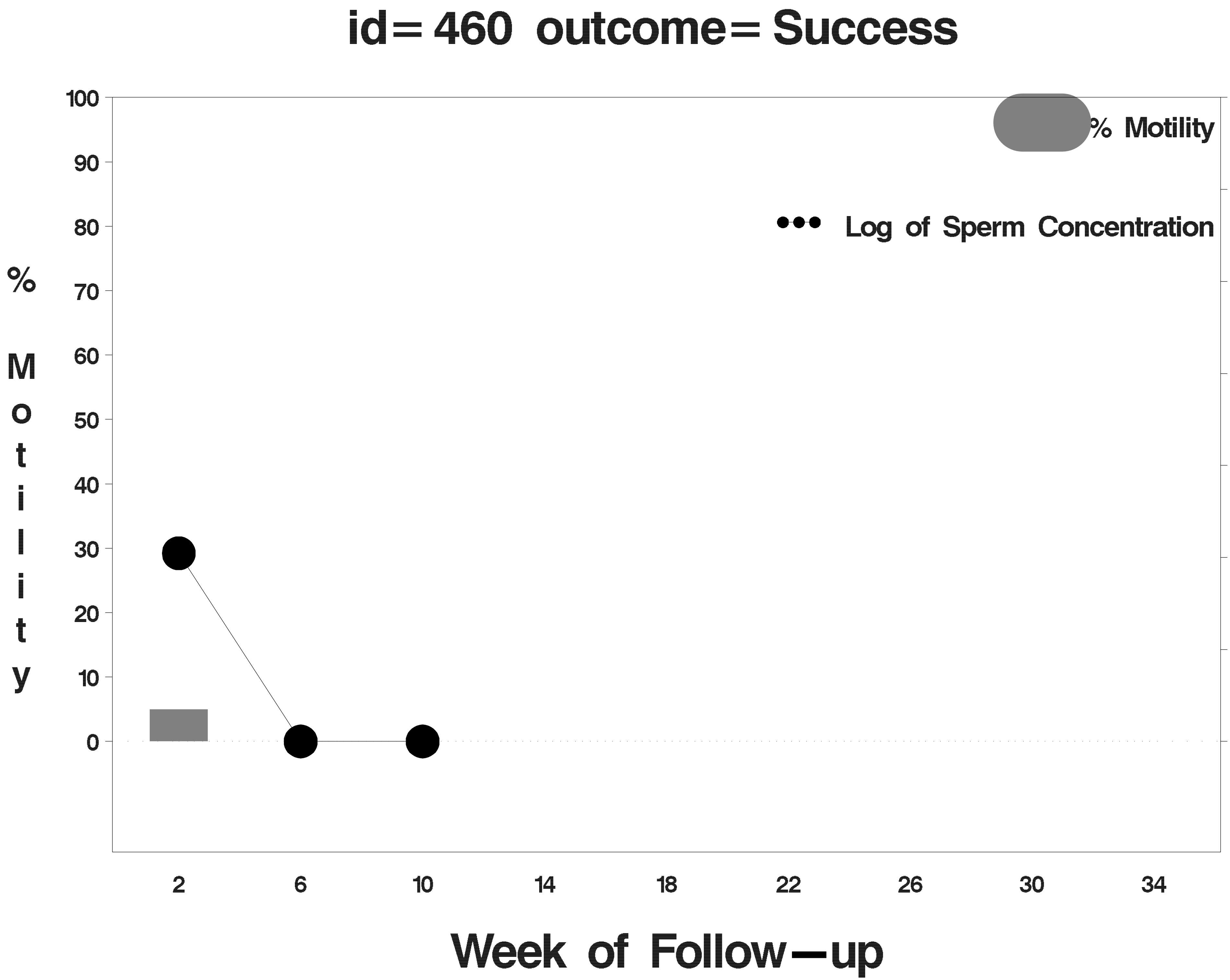

id= 460 outcome= Success
% Motility
Log of Sperm Concentration
100
90
80
70
60
50
40
30
20
10
0
9
8
7
6
5
4
3
2
2
6
10
14
18
22
26
30
34
Week of Follow—up
Log of Sperm Conc

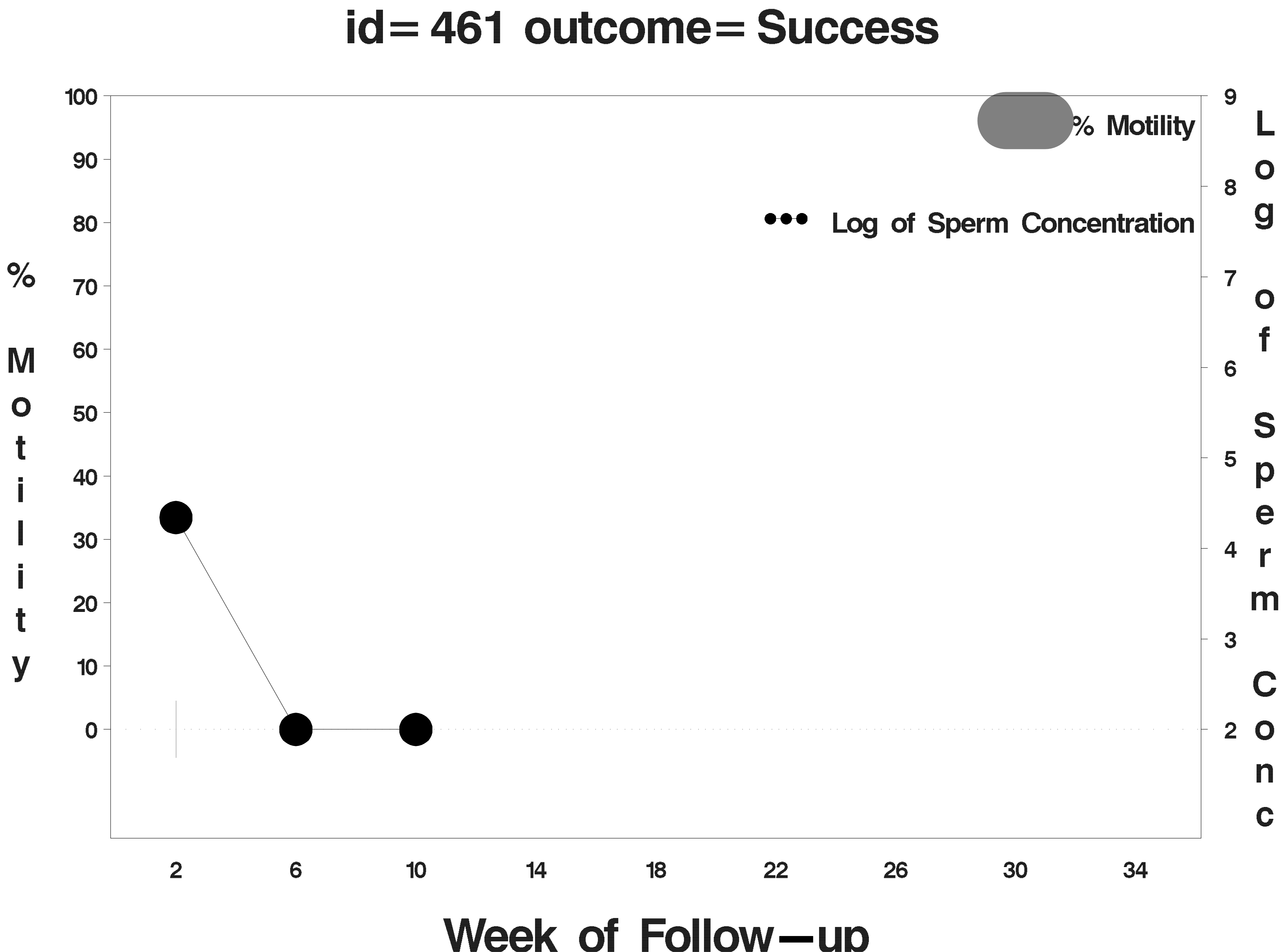

id= 461 outcome= Success
% Motility
Log of Sperm Concentration
100
90
80
70
60
50
40
30
20
10
0
9
8
7
6
5
4
3
2
2
6
10
14
18
22
26
30
34
Week of Follow−up

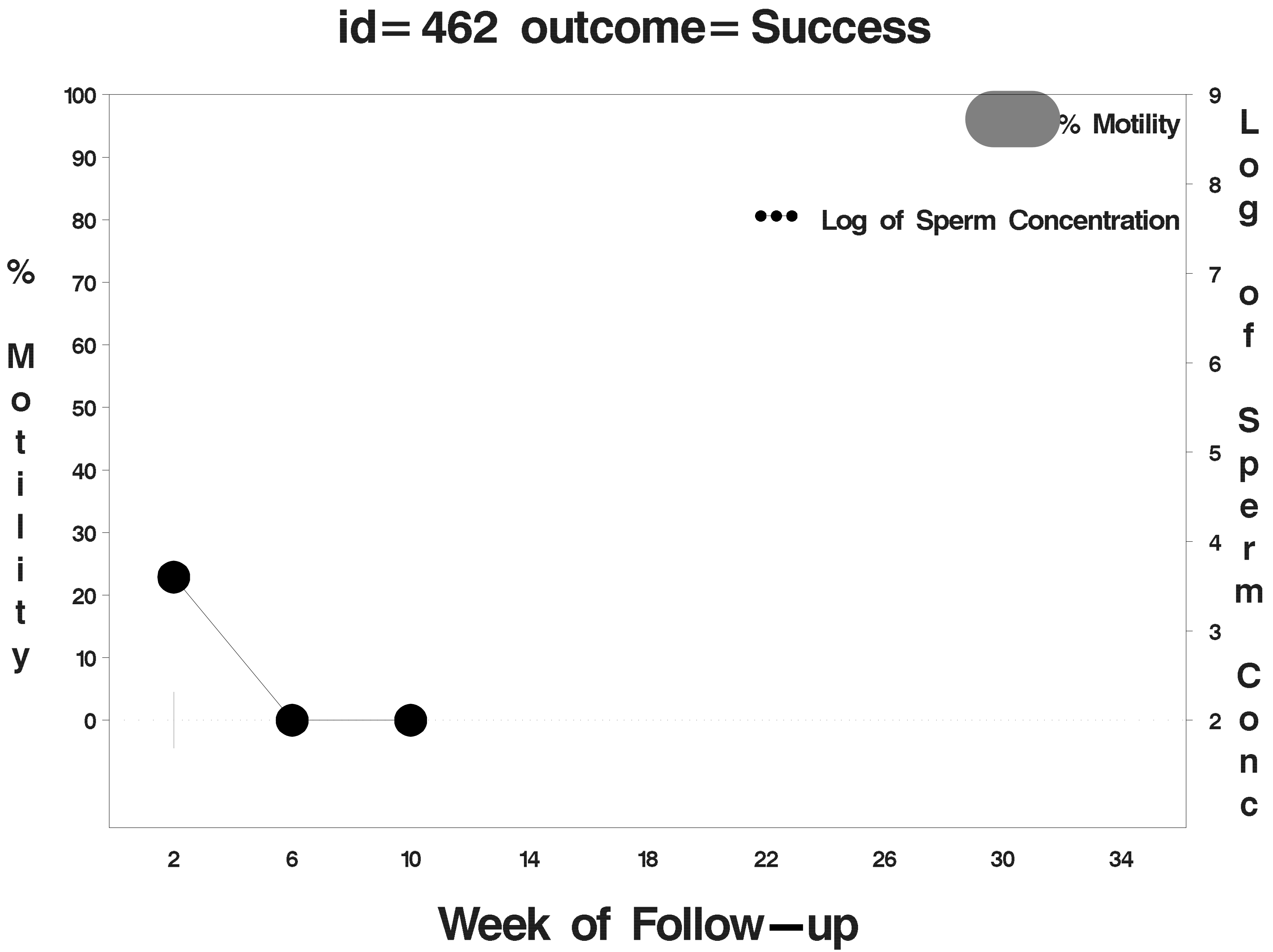
id= 462 outcome= Success
% Motility
Log of Sperm Concentration
100
90
80
70
60
50
40
30
20
10
0
9
8
7
6
5
4
3
2
2
6
10
14
18
22
26
30
34
Week of Follow—up
% Motility
Log of Sperm Conc

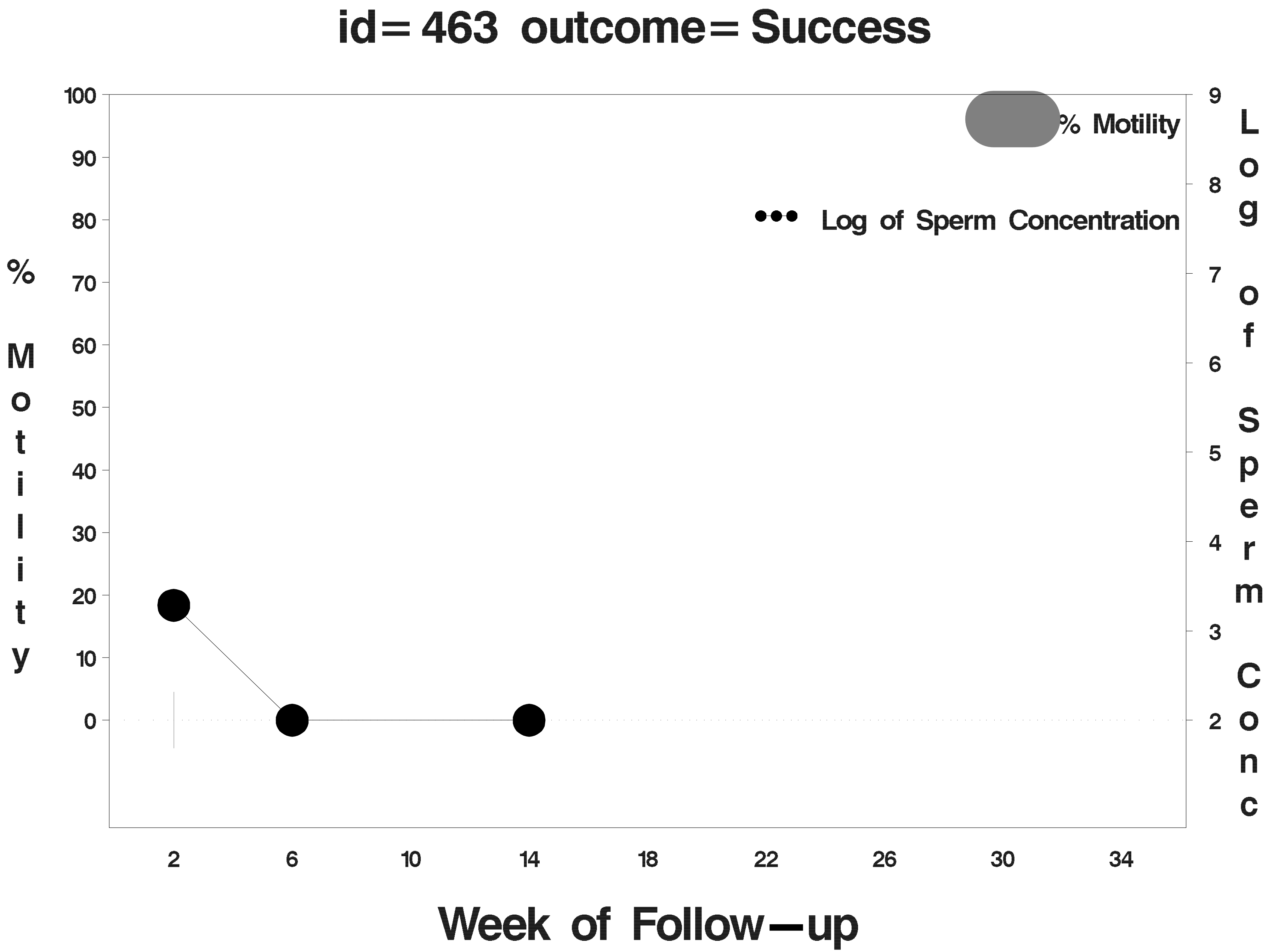
id= 463 outcome= Success
% Motility
Log of Sperm Concentration
100
90
80
70
60
50
40
30
20
10
0
9
8
7
6
5
4
3
2
2
6
10
14
18
22
26
30
34
Week of Follow—up
Log of Sperm Conc

id= 464 outcome= Success

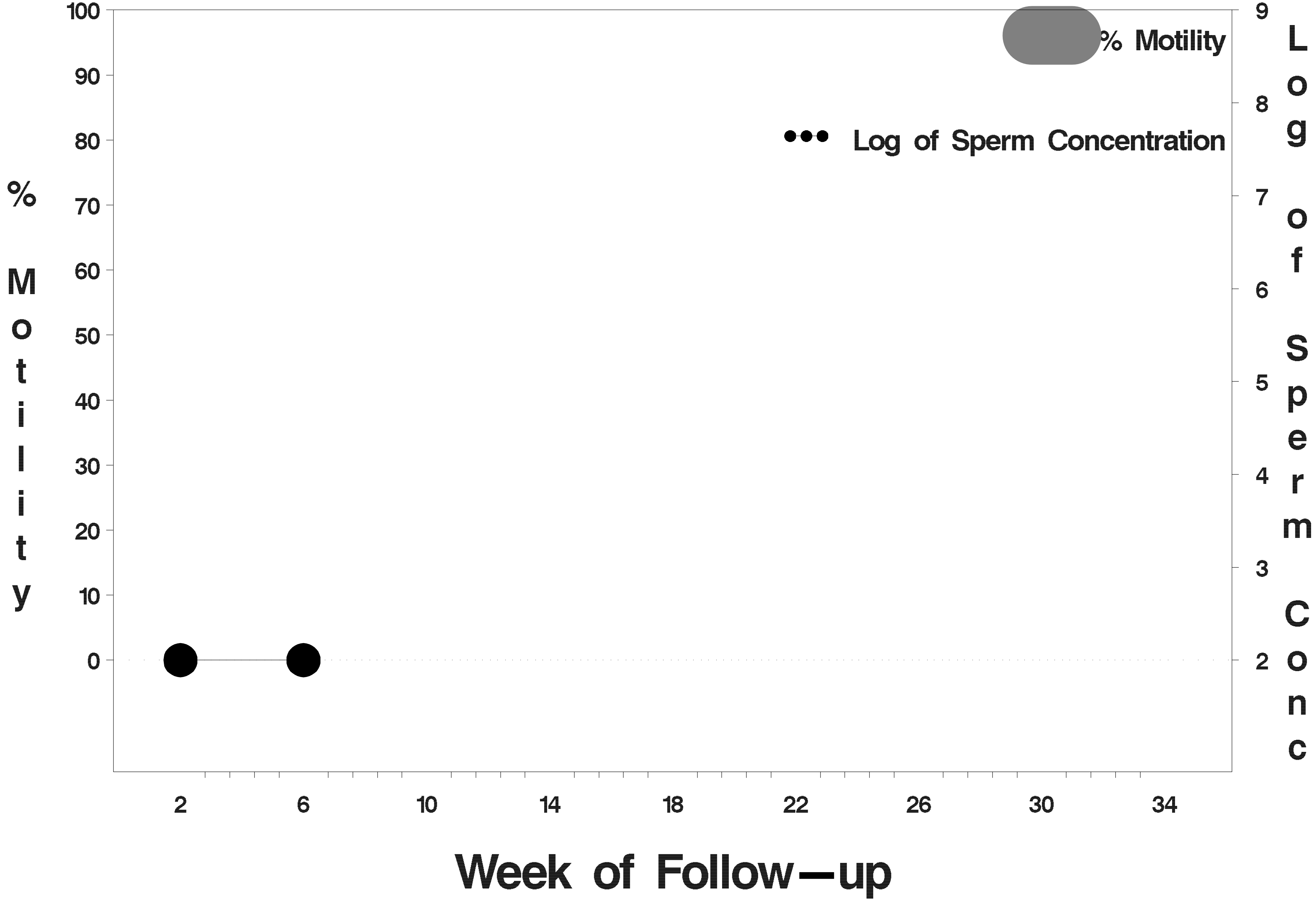

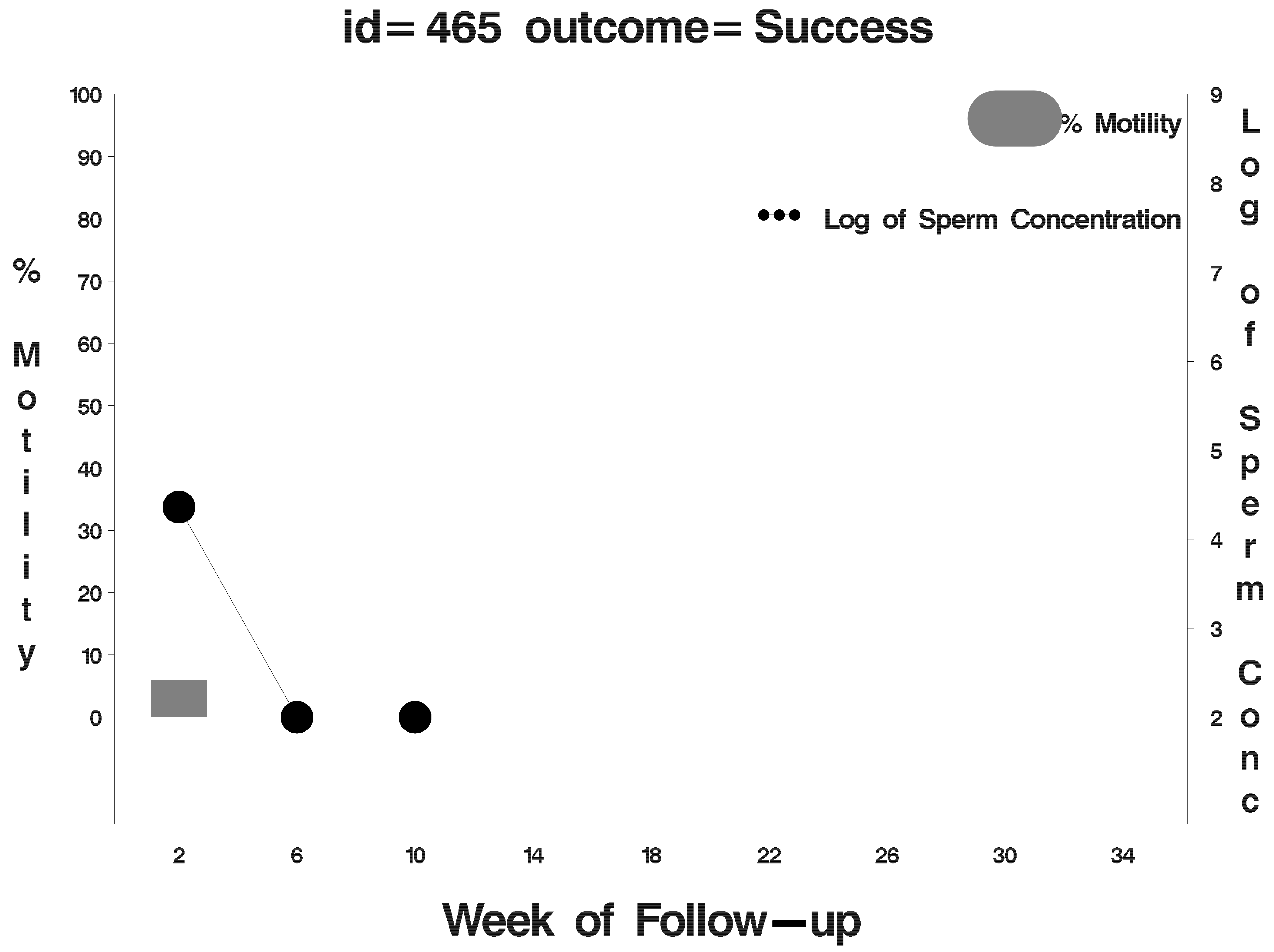
id= 465 outcome= Success
% Motility
Log of Sperm Concentration
% Motility
Log of Sperm Conc
Week of Follow−up
100
90
80
70
60
50
40
30
20
10
0
9
8
7
6
5
4
3
2
2
6
10
14
18
22
26
30
34

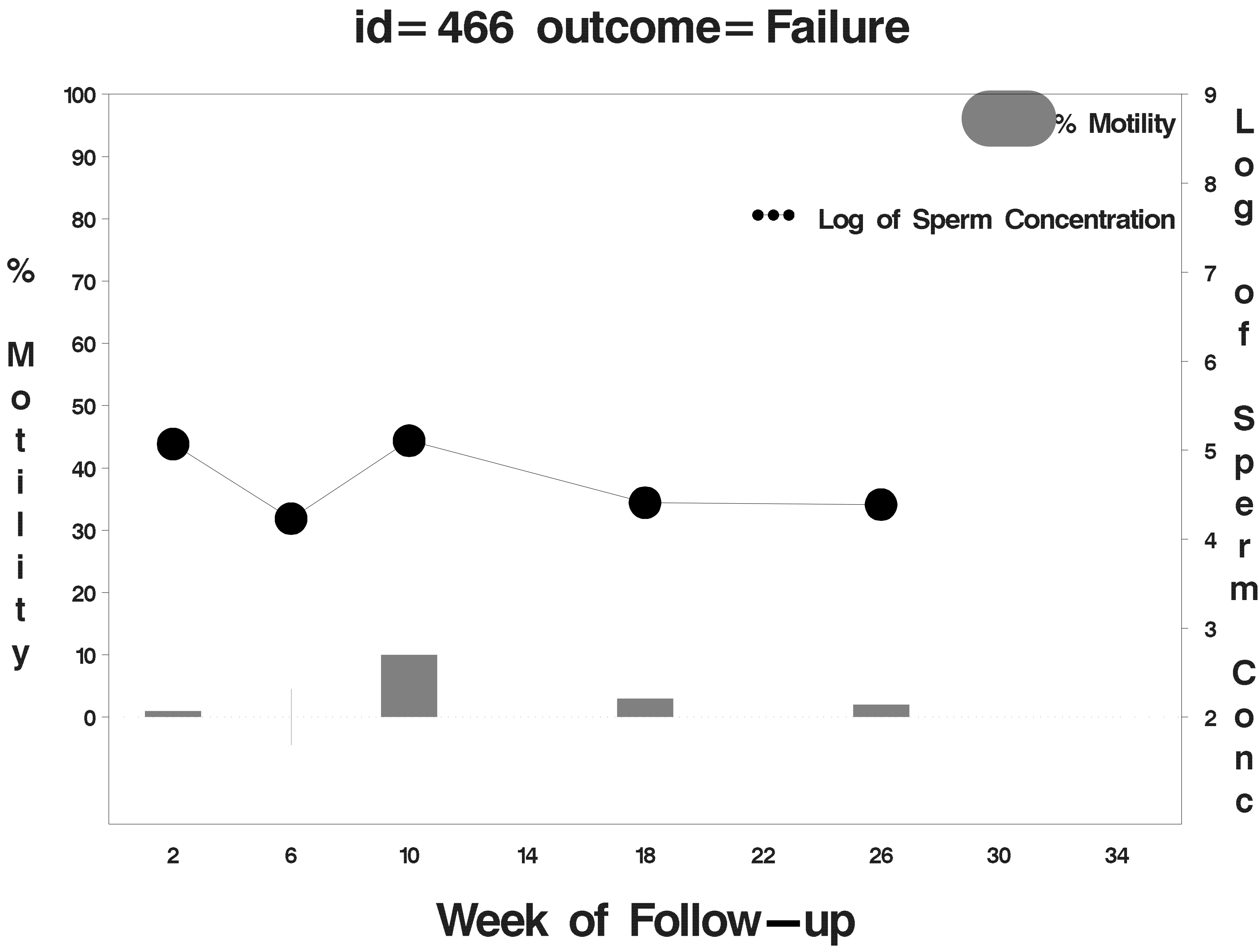

id= 466 outcome= Failure
% Motility
Log of Sperm Concentration
Week of Follow−up
Log of Sperm Conc
100
90
80
70
60
50
40
30
20
10
0
9
8
7
6
5
4
3
2
2
6
10
14
18
22
26
30
34

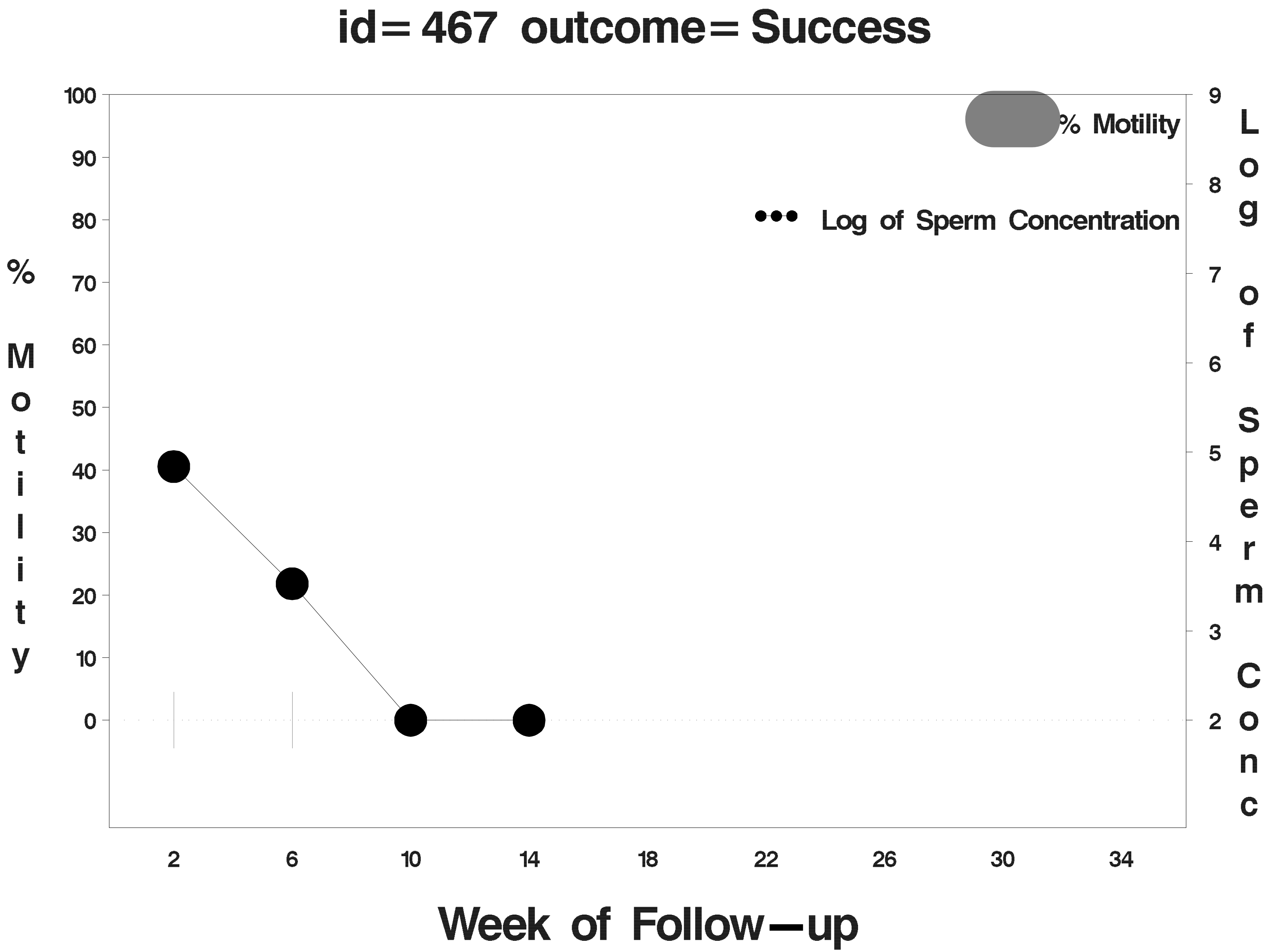

id= 467 outcome= Success
% Motility
Log of Sperm Concentration
% Motility
Log of Sperm Conc
Week of Follow-up
100
90
80
70
60
50
40
30
20
10
0
9
8
7
6
5
4
3
2
2
6
10
14
18
22
26
30
34

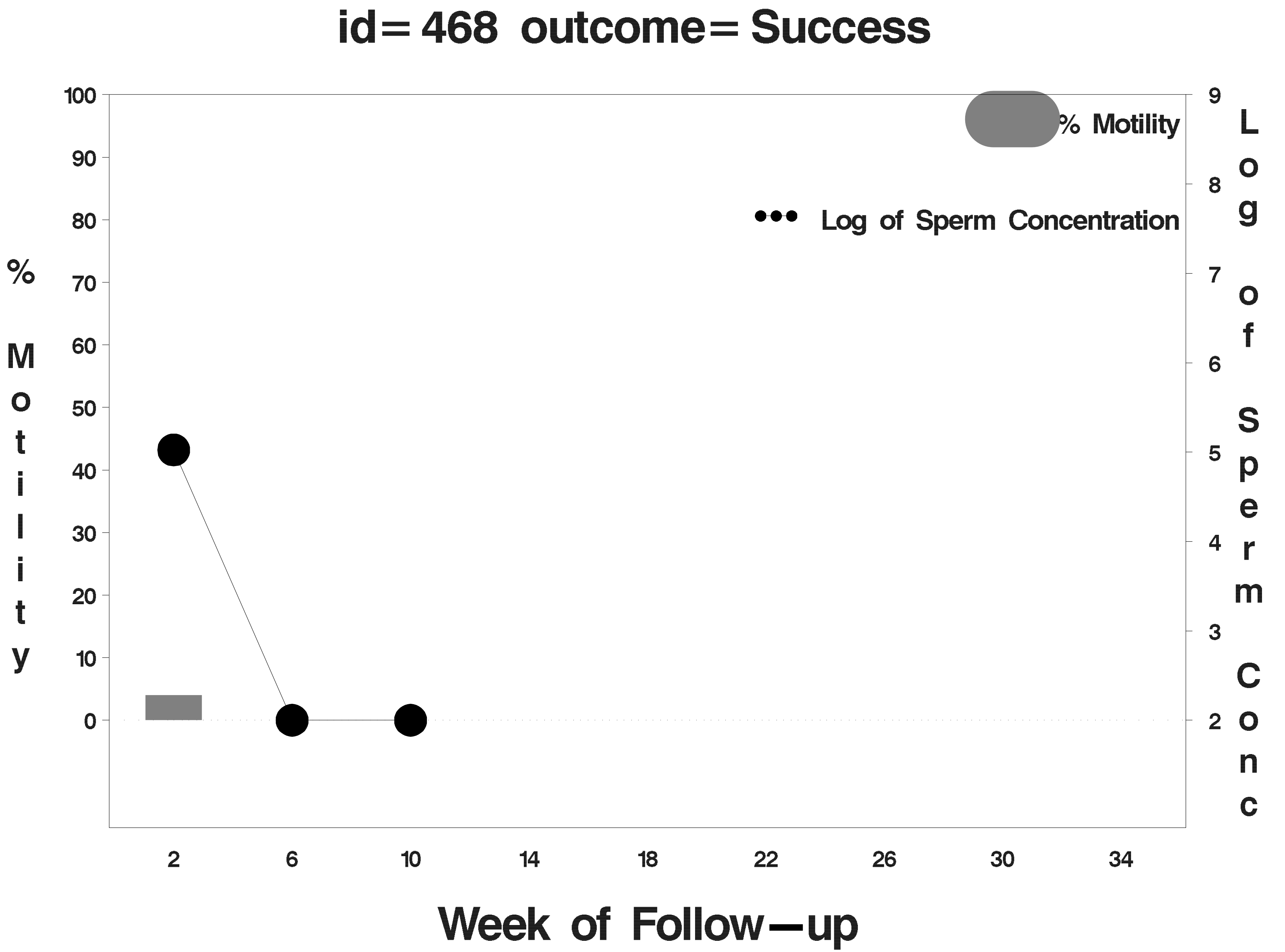
id= 468 outcome= Success
% Motility
Log of Sperm Concentration
100
90
80
70
60
50
40
30
20
10
0
9
8
7
6
5
4
3
2
2
6
10
14
18
22
26
30
34
Week of Follow-up
% Motility
Log of Sperm Conc

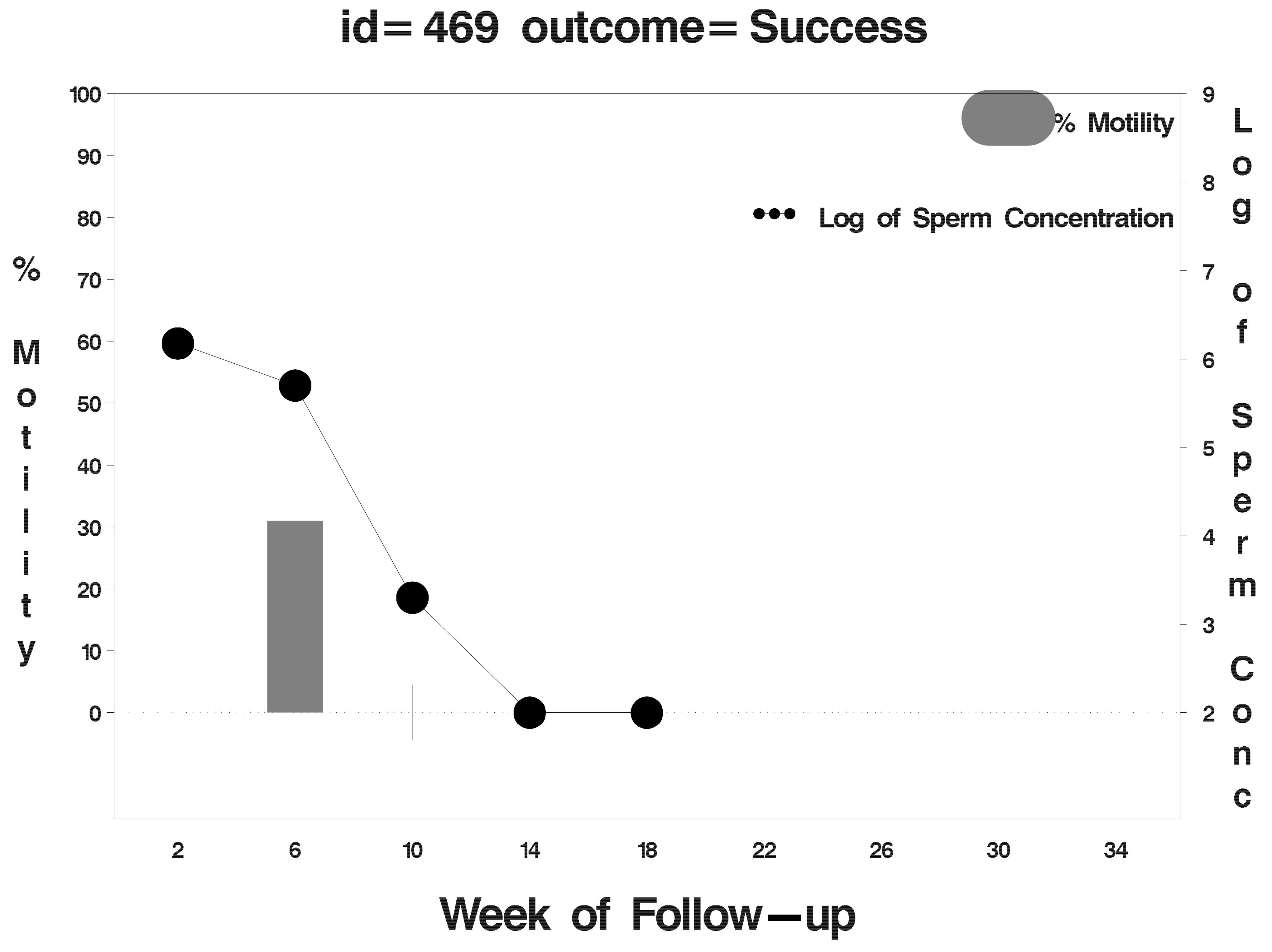

id= 469 outcome= Success
% Motility
Log of Sperm Concentration
100
90
80
70
60
50
40
30
20
10
0
9
8
7
6
5
4
3
2
2
6
10
14
18
22
26
30
34
Week of Follow−up
Log of Sperm Conc

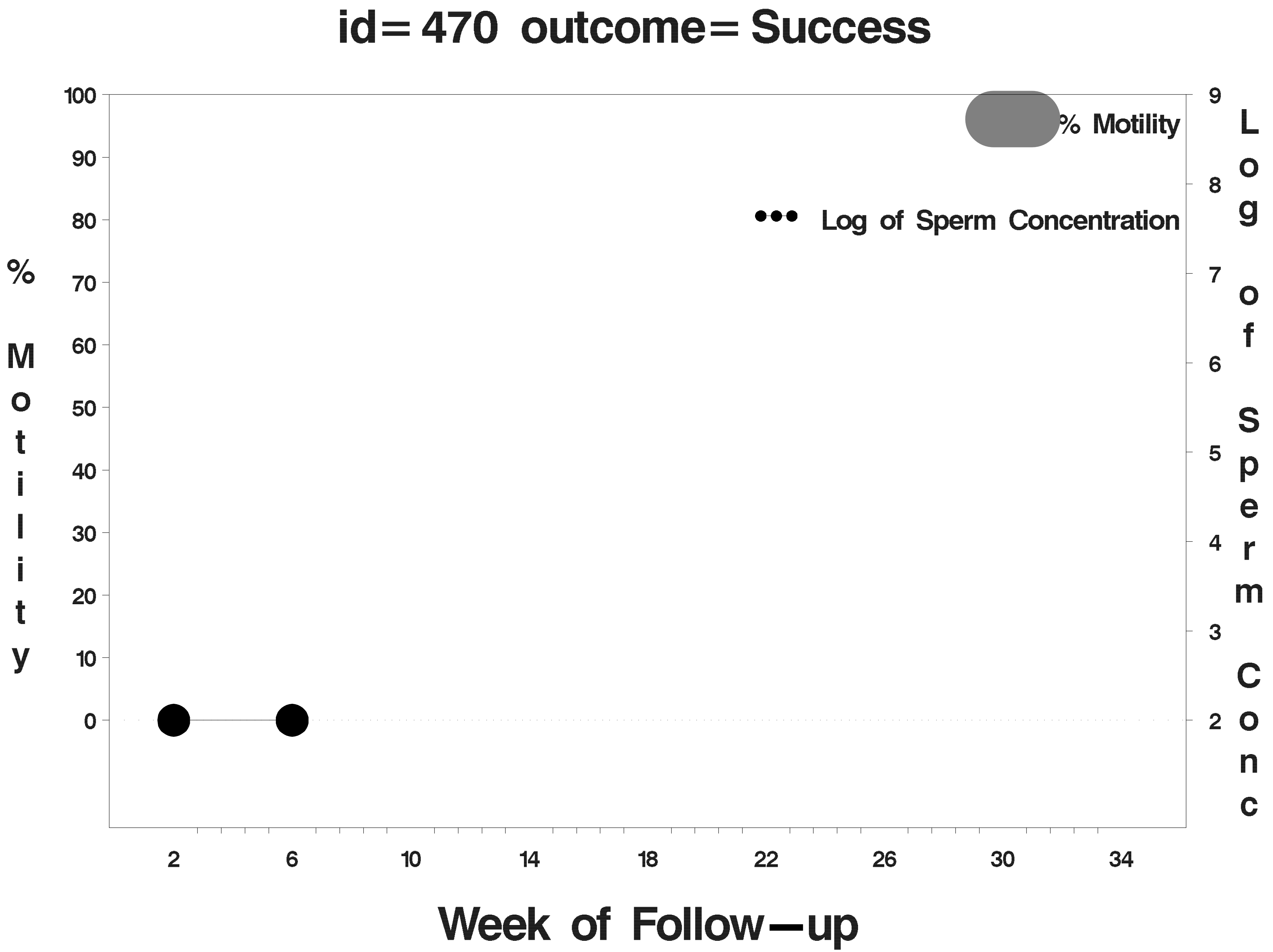

id= 470 outcome= Success
% Motility
Log of Sperm Concentration
% Motility
Log of Sperm Conc
Week of Follow−up
100
90
80
70
60
50
40
30
20
10
0
9
8
7
6
5
4
3
2
2
6
10
14
18
22
26
30
34

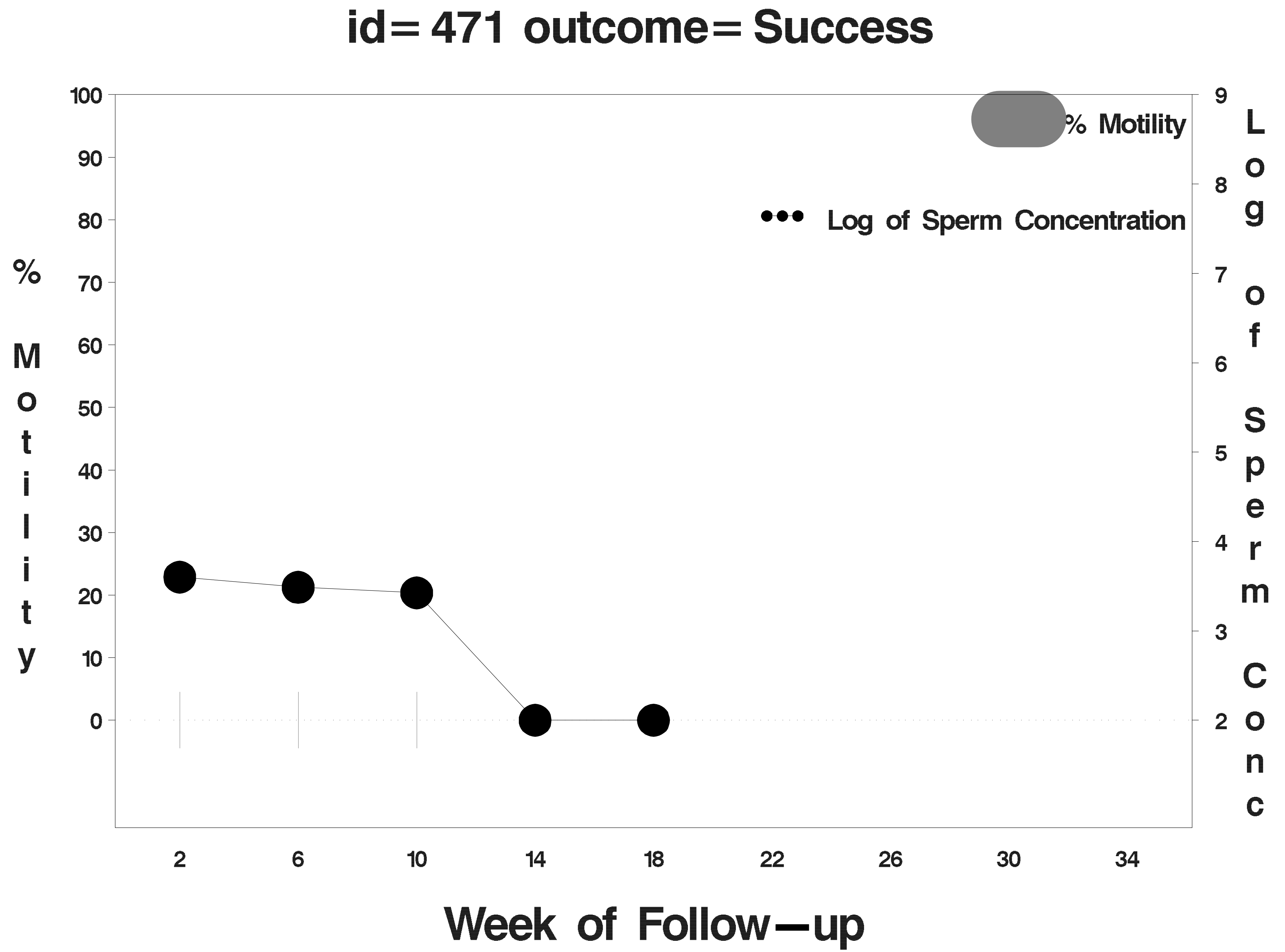

id= 471 outcome= Success
% Motility
Log of Sperm Concentration
100
90
80
70
60
50
40
30
20
10
0
9
8
7
6
5
4
3
2
2
6
10
14
18
22
26
30
34
Week of Follow−up
Log of Sperm Conc

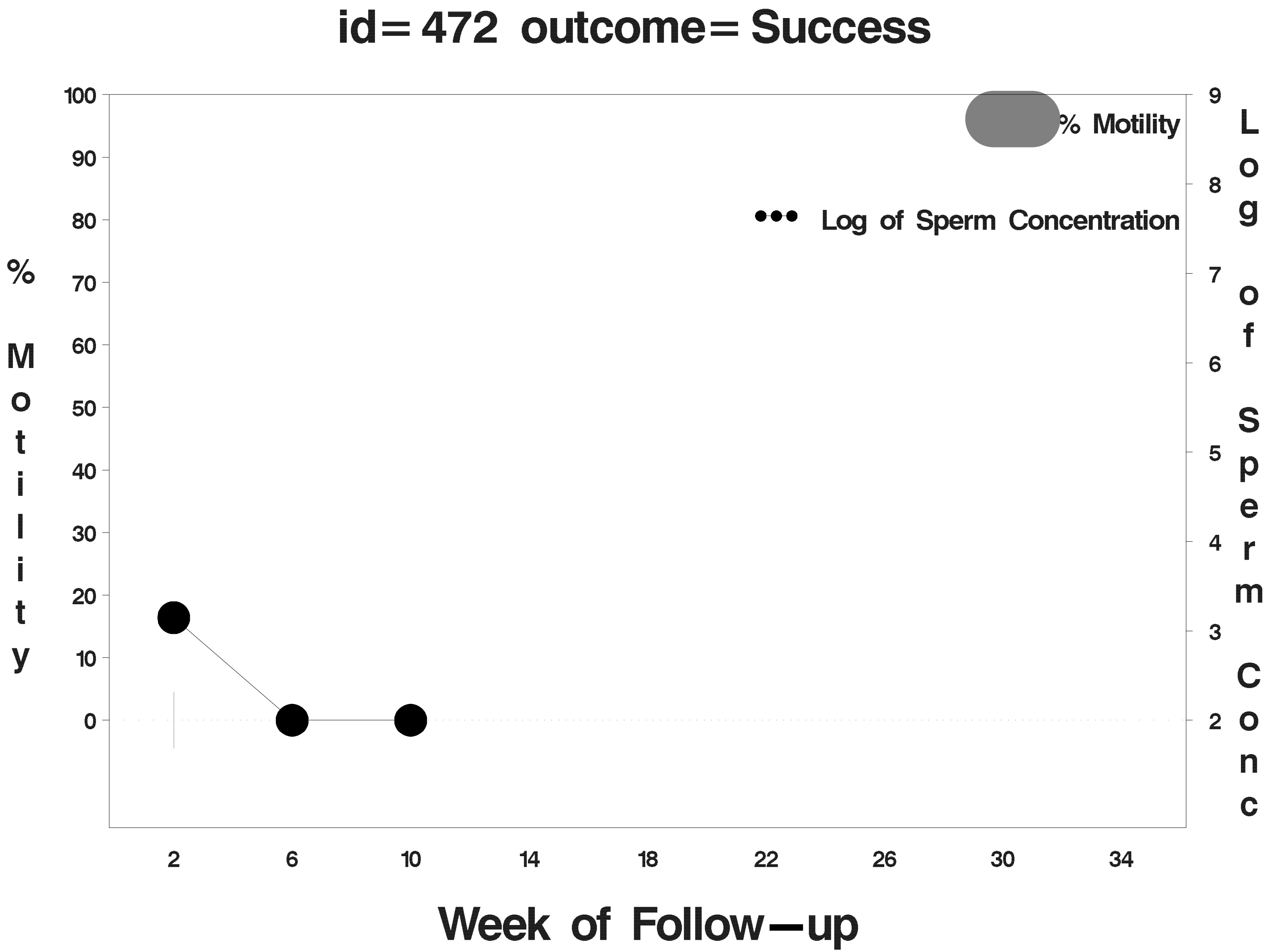
id= 472 outcome= Success
% Motility
Log of Sperm Concentration
% Motility
Log of Sperm Conc
Week of Follow-up
100
90
80
70
60
50
40
30
20
10
0
9
8
7
6
5
4
3
2
2
6
10
14
18
22
26
30
34

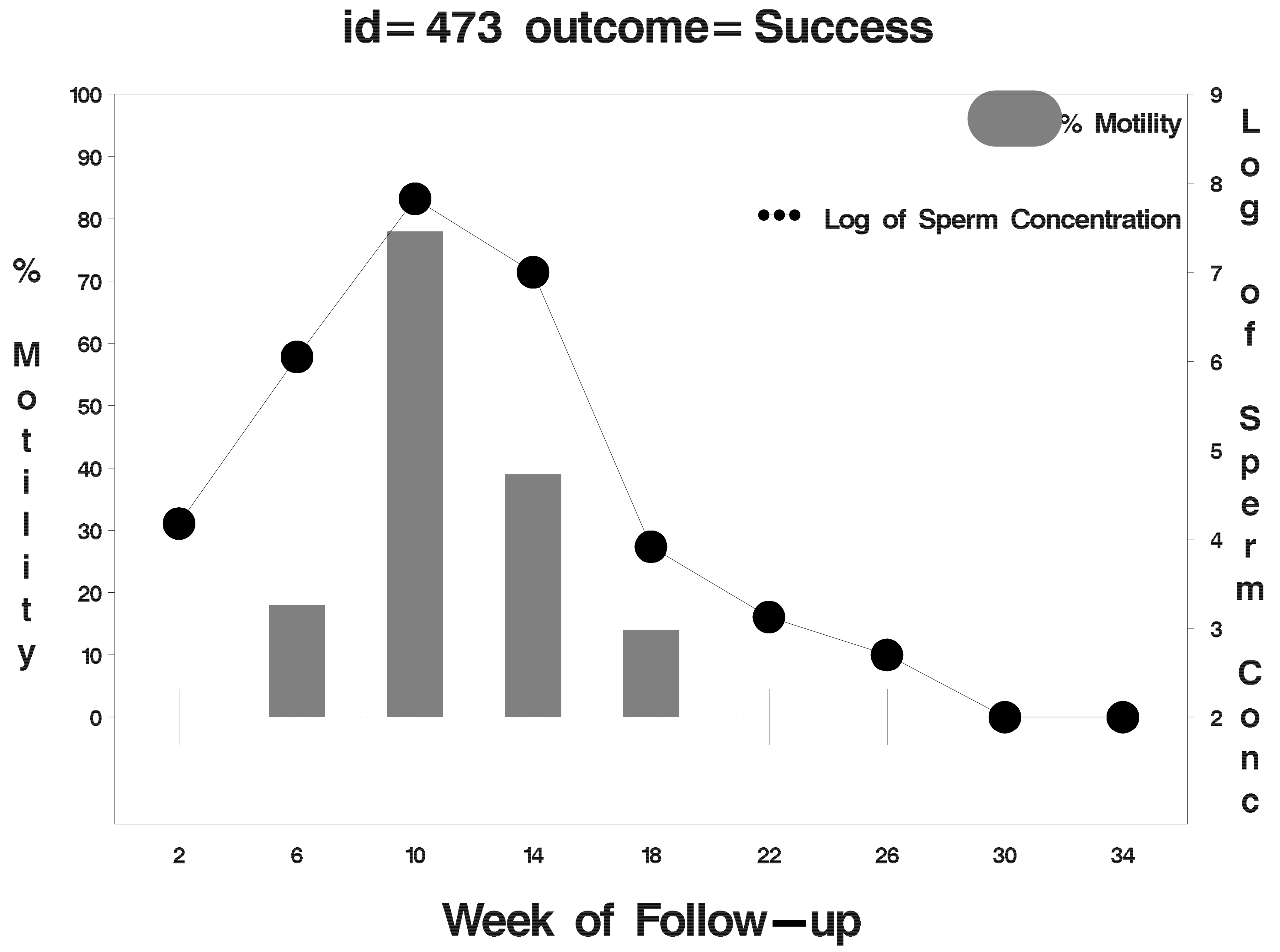

id= 473 outcome= Success
% Motility
Log of Sperm Concentration
100
90
80
70
60
50
40
30
20
10
0
9
8
7
6
5
4
3
2
2
6
10
14
18
22
26
30
34
Week of Follow—up
Log of Sperm Conc

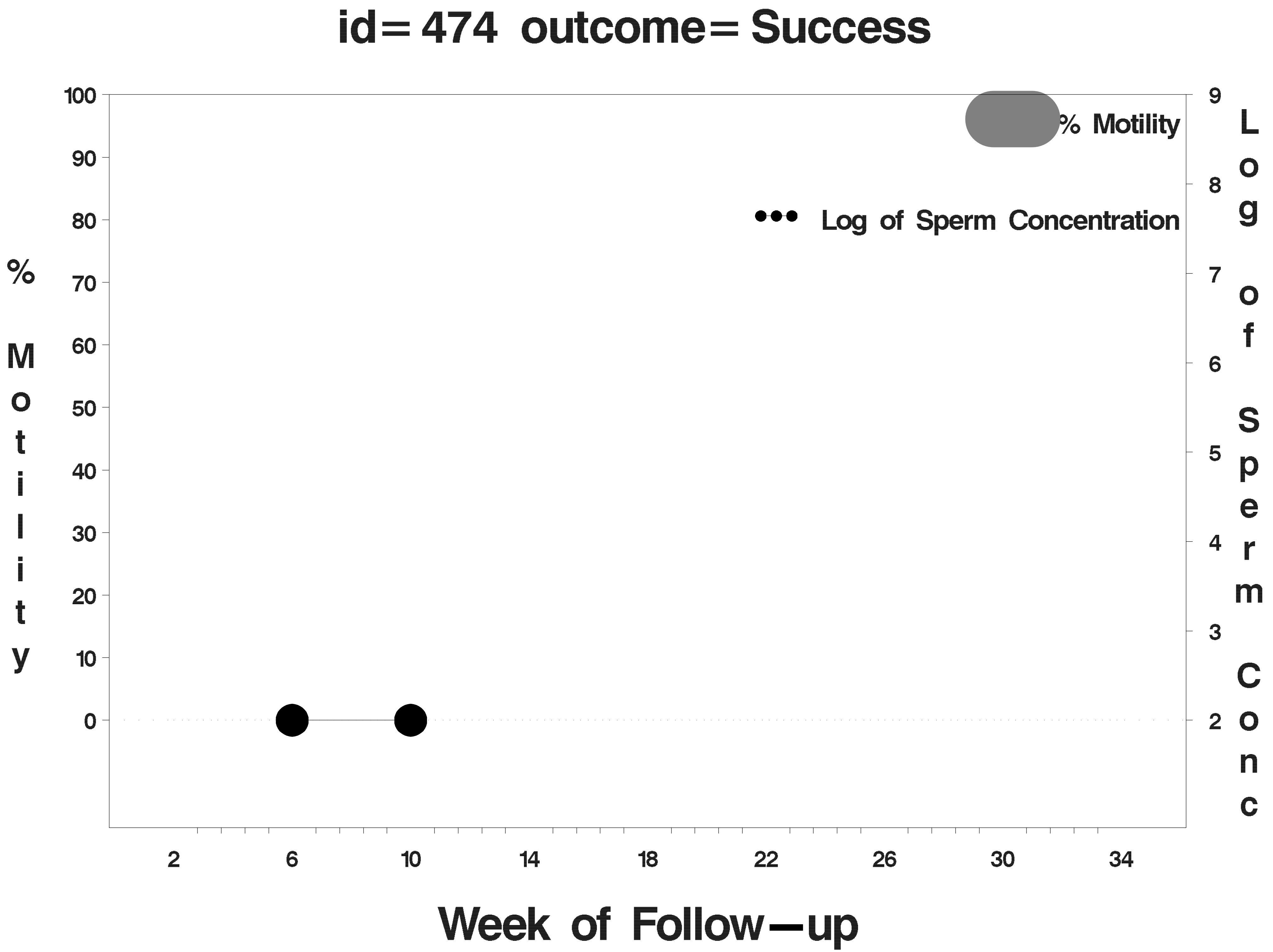
id= 474 outcome= Success
% Motility
Log of Sperm Concentration
100
90
80
70
60
50
40
30
20
10
0
9
8
7
6
5
4
3
2
2
6
10
14
18
22
26
30
34
% Motility
Log of Sperm Conc
Week of Follow—up

id= 475 outcome= Failure

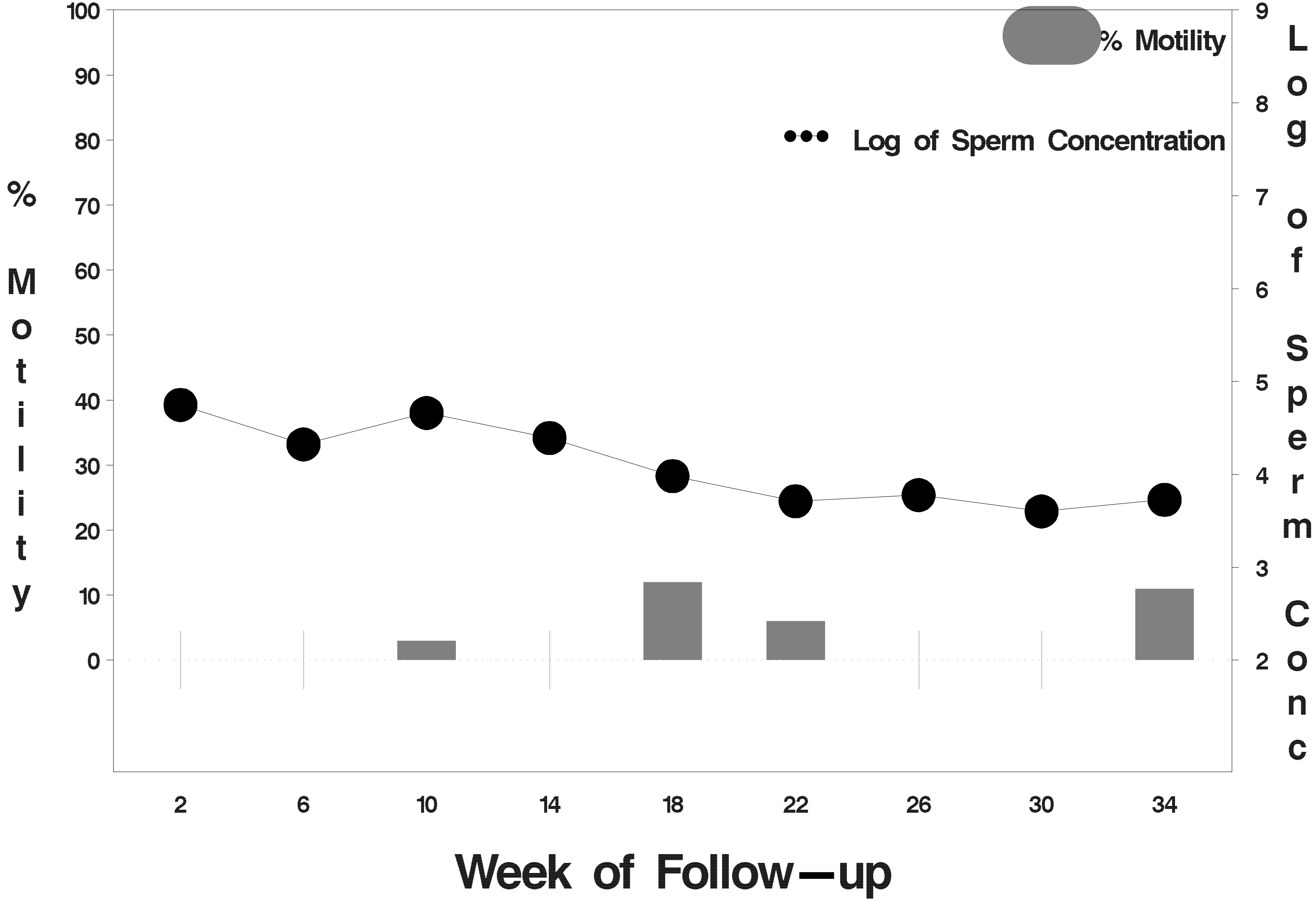

id= 476 outcome= Success

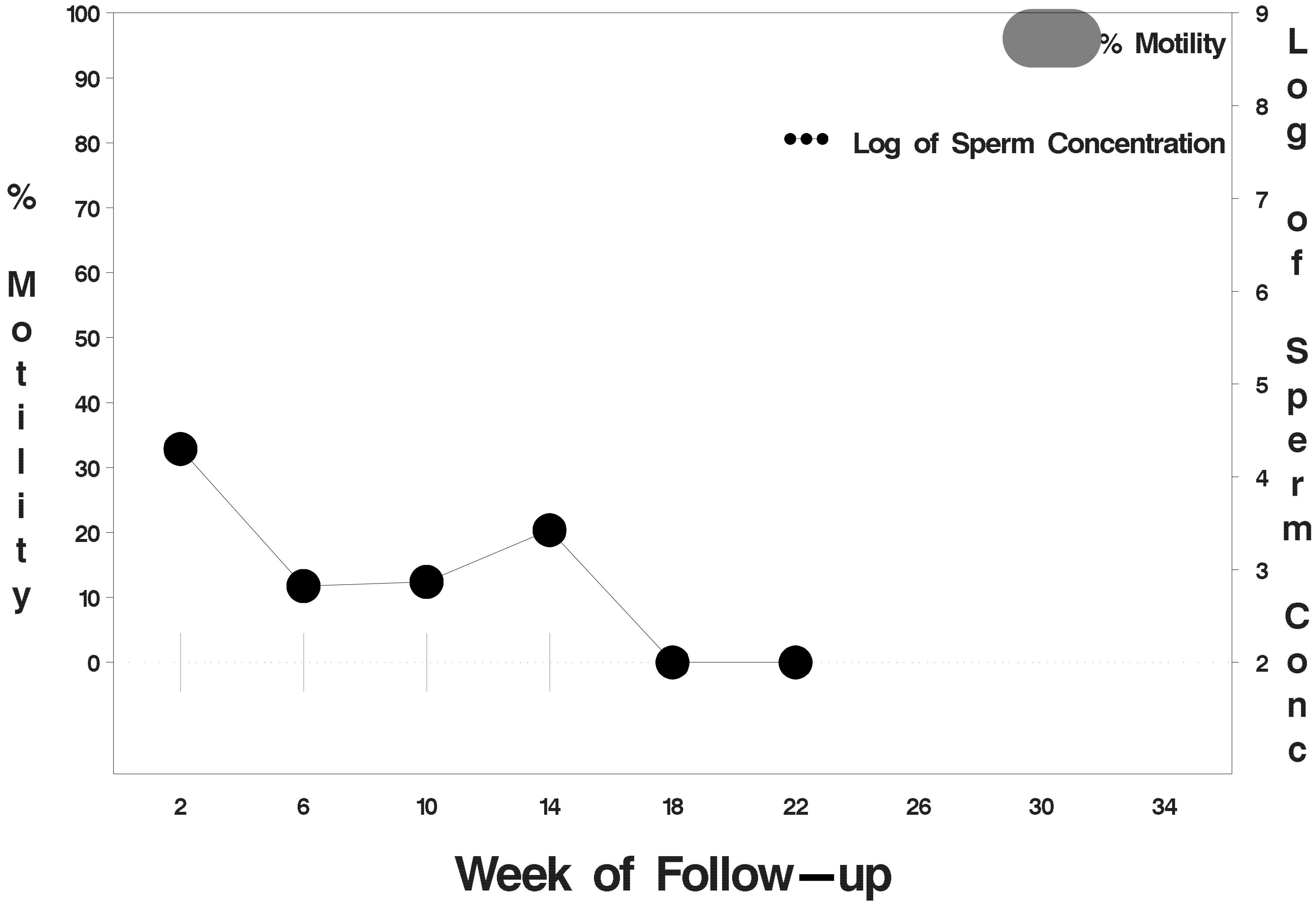

id= 477 outcome= Success

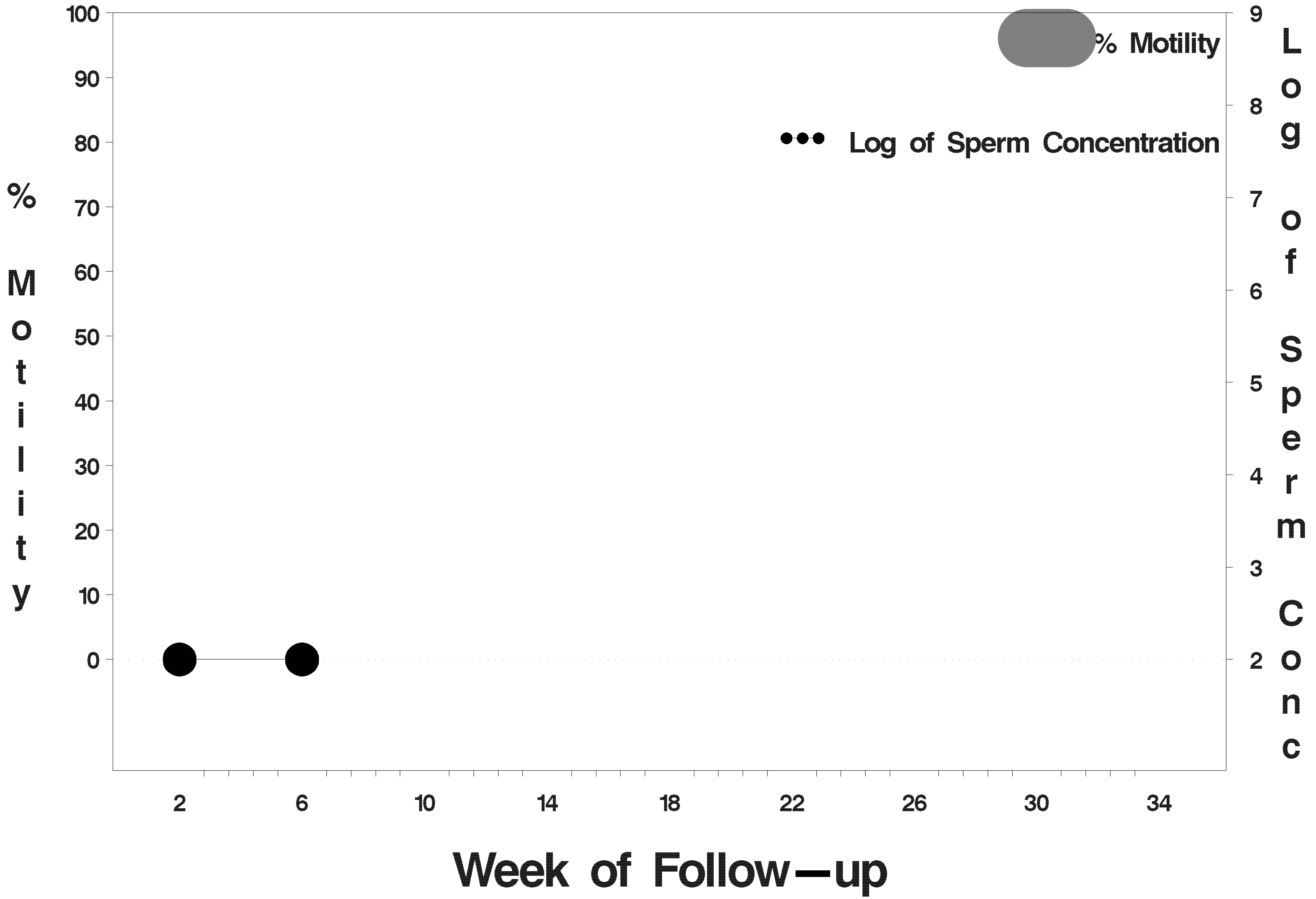

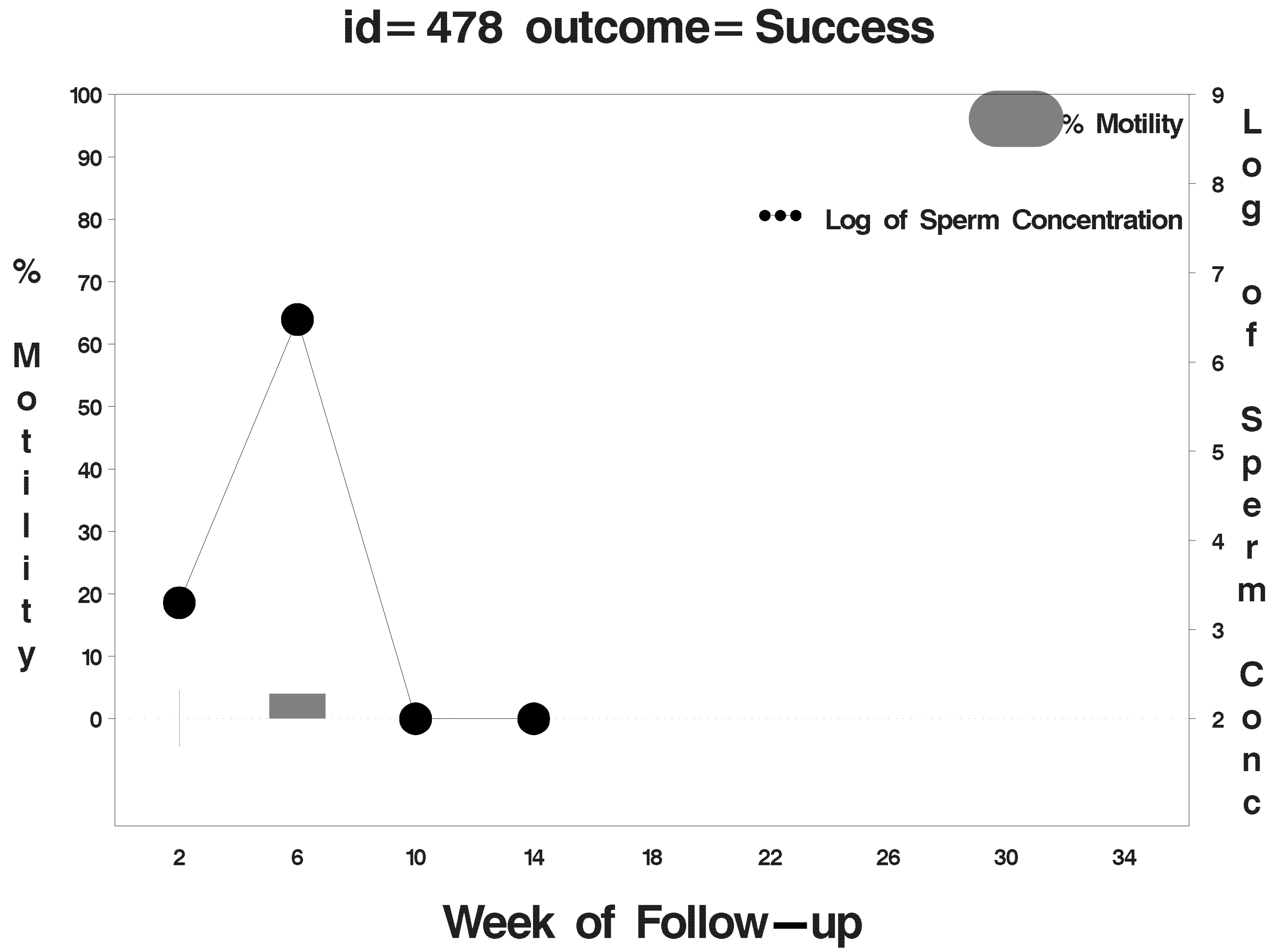

id= 478 outcome= Success
% Motility
Log of Sperm Concentration
100
90
80
70
60
50
40
30
20
10
0
9
8
7
6
5
4
3
2
2
6
10
14
18
22
26
30
34
Week of Follow−up
Log of Sperm Conc

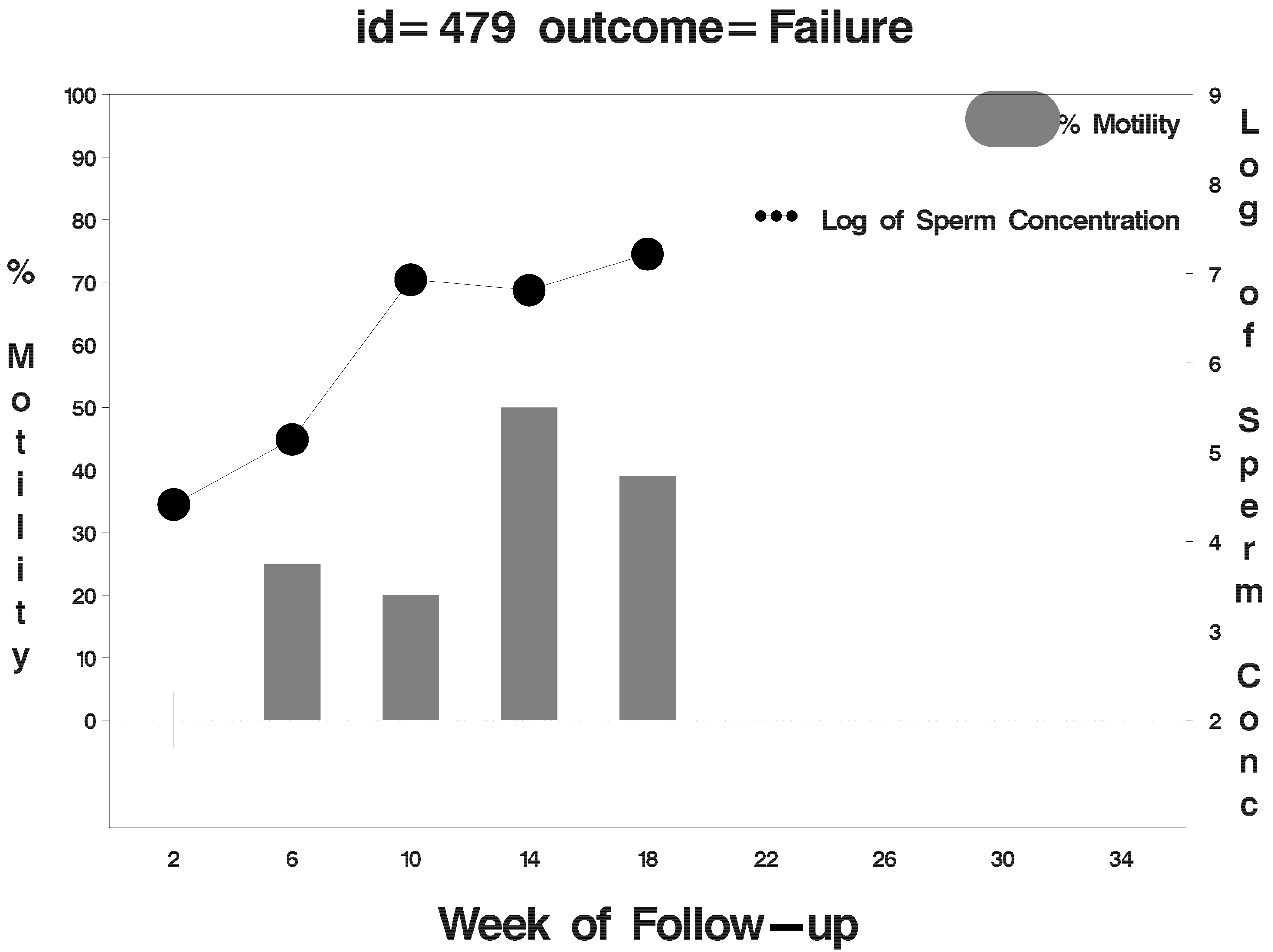

id= 479 outcome= Failure
% Motility
Log of Sperm Concentration
100
90
80
70
60
50
40
30
20
10
0
9
8
7
6
5
4
3
2
2
6
10
14
18
22
26
30
34
Week of Follow—up
Log of Sperm Conc

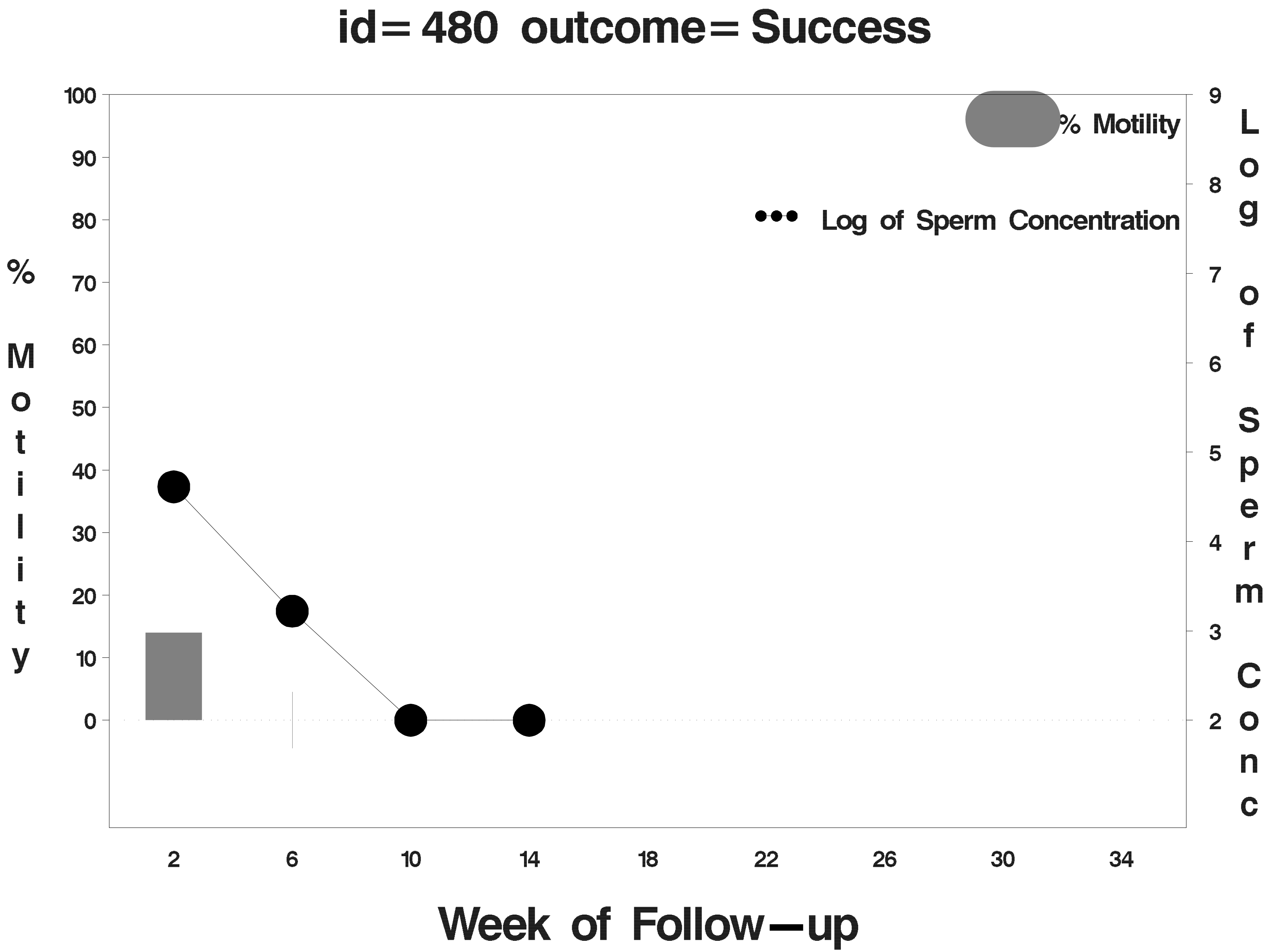

id= 480 outcome= Success
% Motility
Log of Sperm Concentration
100
90
80
70
60
50
40
30
20
10
0
9
8
7
6
5
4
3
2
2
6
10
14
18
22
26
30
34
Week of Follow—up
Log of Sperm Conc

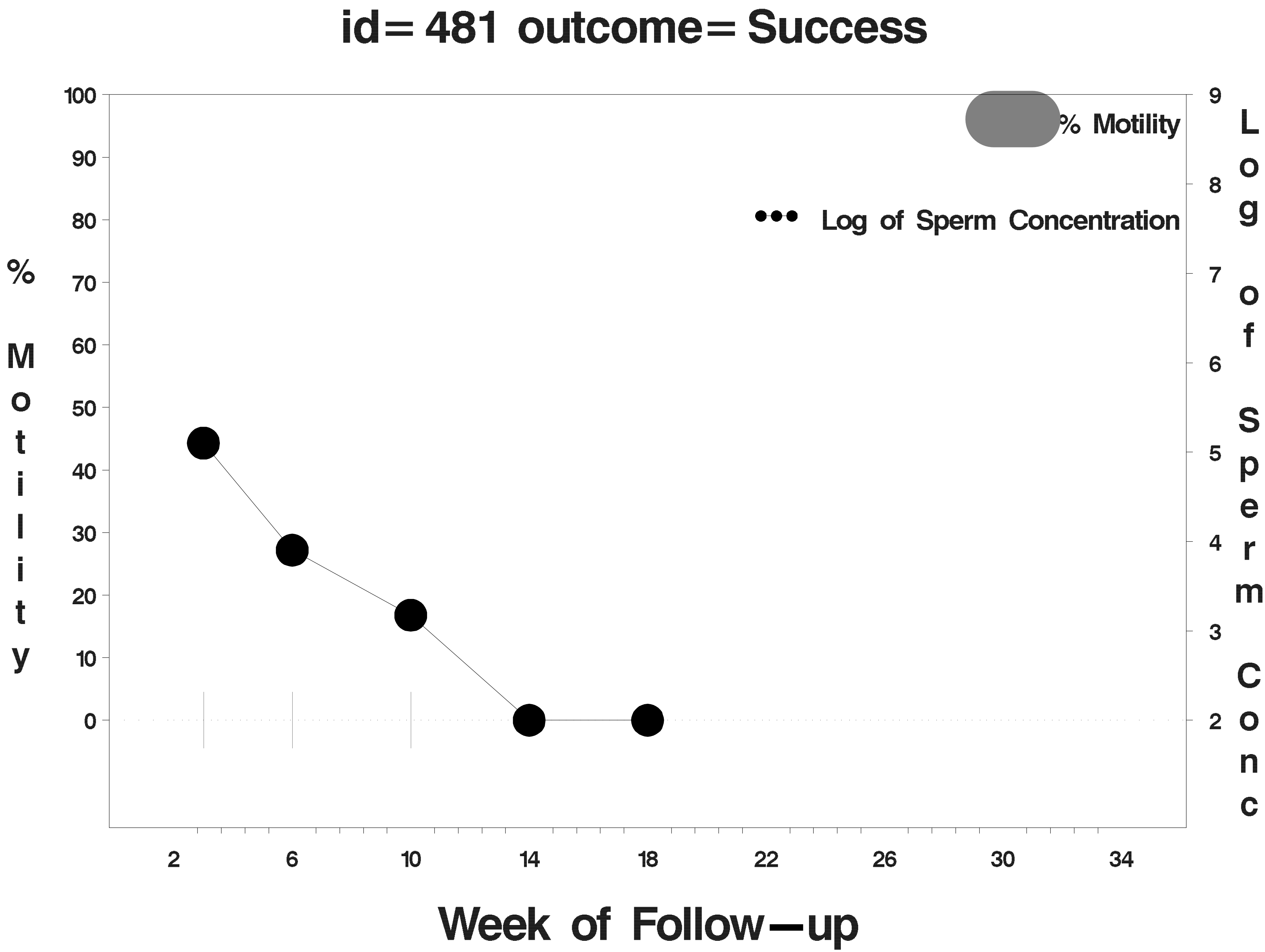
id= 481 outcome= Success
% Motility
Log of Sperm Concentration
100
90
80
70
60
50
40
30
20
10
0
9
8
7
6
5
4
3
2
2
6
10
14
18
22
26
30
34
Week of Follow−up
% Motility
Log of Sperm Conc

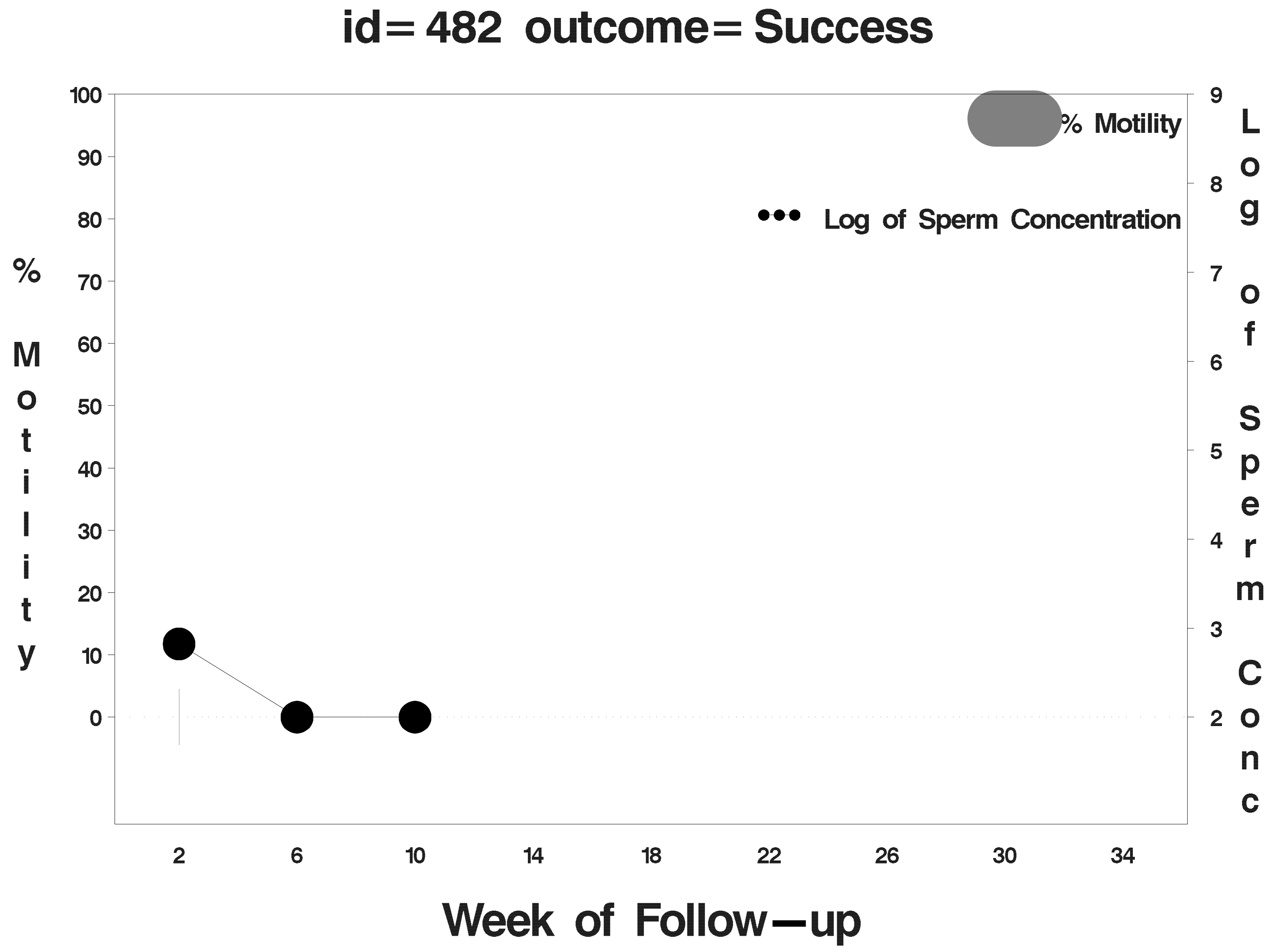

id= 482 outcome= Success
% Motility
Log of Sperm Concentration
100
90
80
70
60
50
40
30
20
10
0
9
8
7
6
5
4
3
2
2
6
10
14
18
22
26
30
34
Week of Follow−up
Log of Sperm Conc

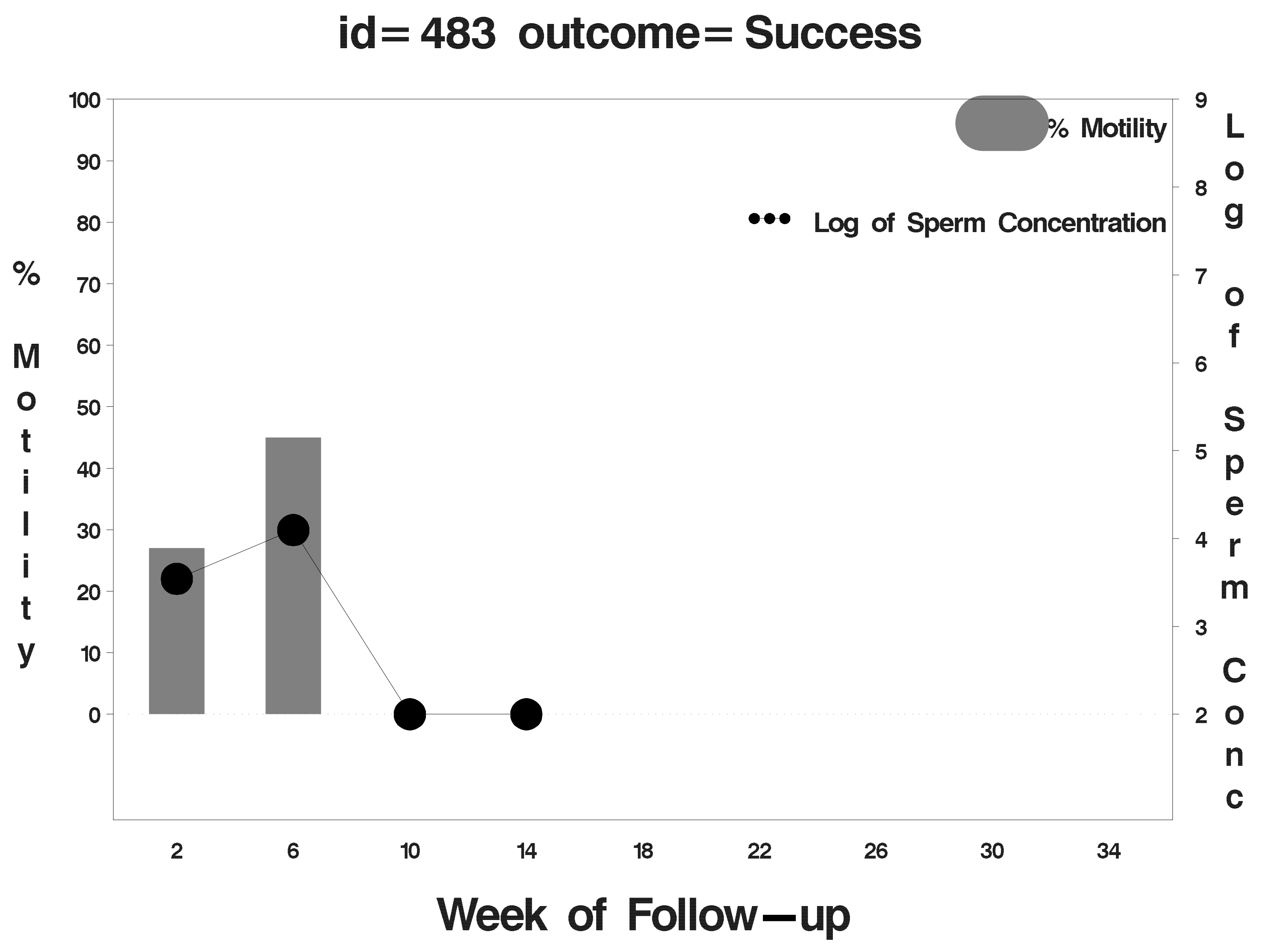
id= 483 outcome= Success
% Motility
Log of Sperm Concentration
100
90
80
70
60
50
40
30
20
10
0
9
8
7
6
5
4
3
2
2
6
10
14
18
22
26
30
34
Week of Follow-up
Log of Sperm Conc

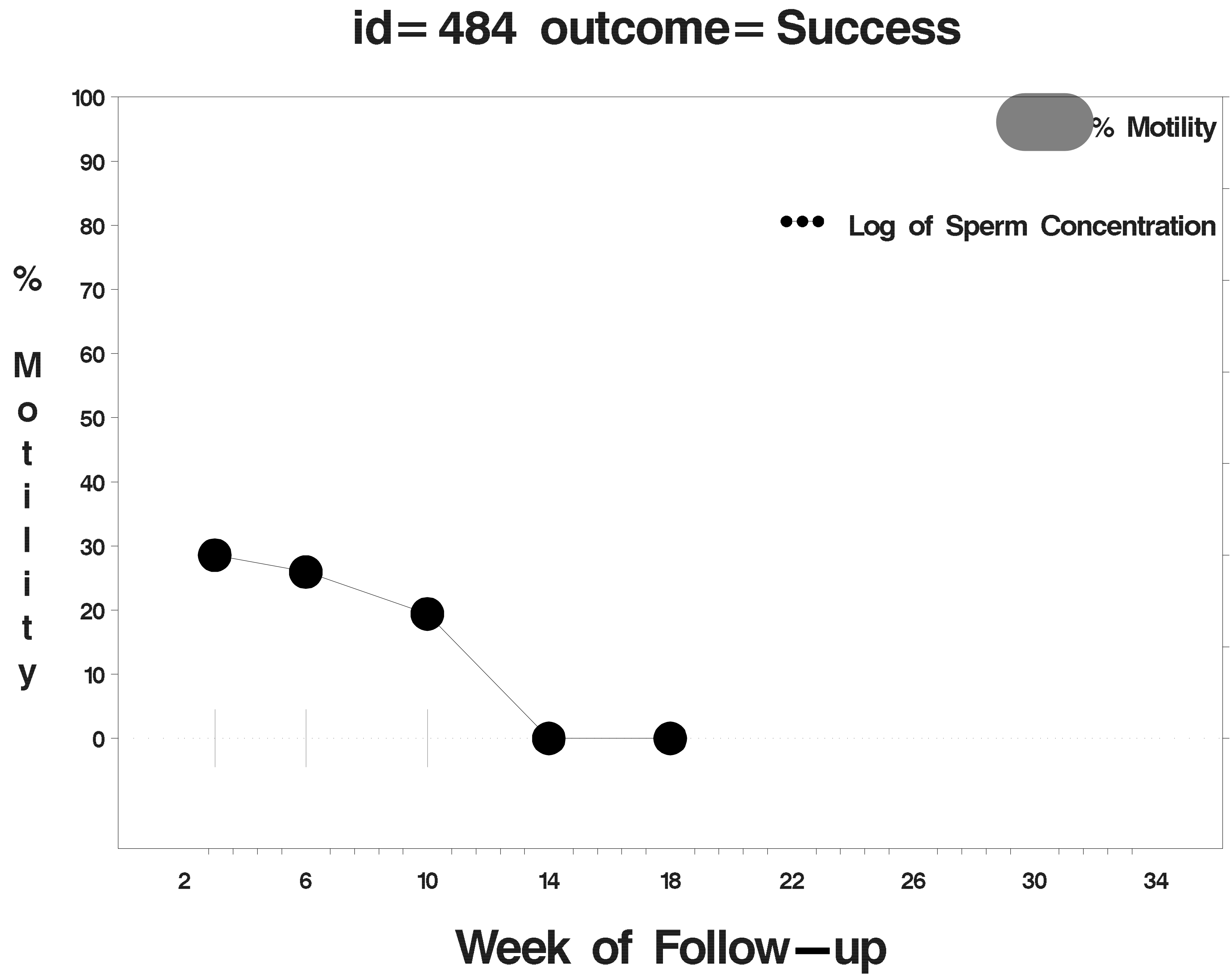

id= 484 outcome= Success
% Motility
Log of Sperm Concentration
100
90
80
70
60
50
40
30
20
10
0
9
8
7
6
5
4
3
2
2
6
10
14
18
22
26
30
34
Week of Follow−up
Log of Sperm Conc

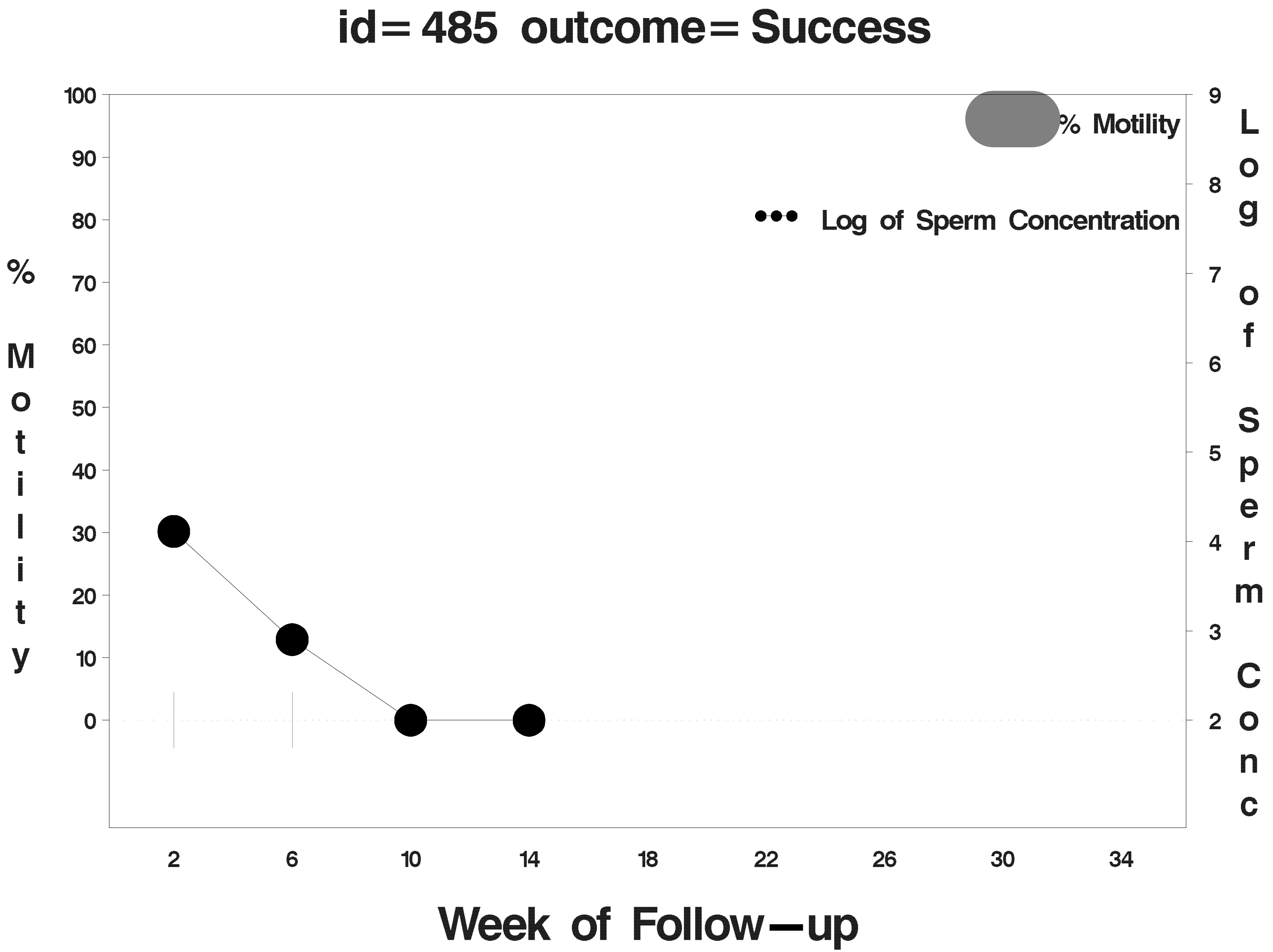

id= 485 outcome= Success
% Motility
Log of Sperm Concentration
100
90
80
70
60
50
40
30
20
10
0
9
8
7
6
5
4
3
2
2
6
10
14
18
22
26
30
34
Week of Follow−up
% Motility
Log of Sperm Conc

id= 486 outcome= Success

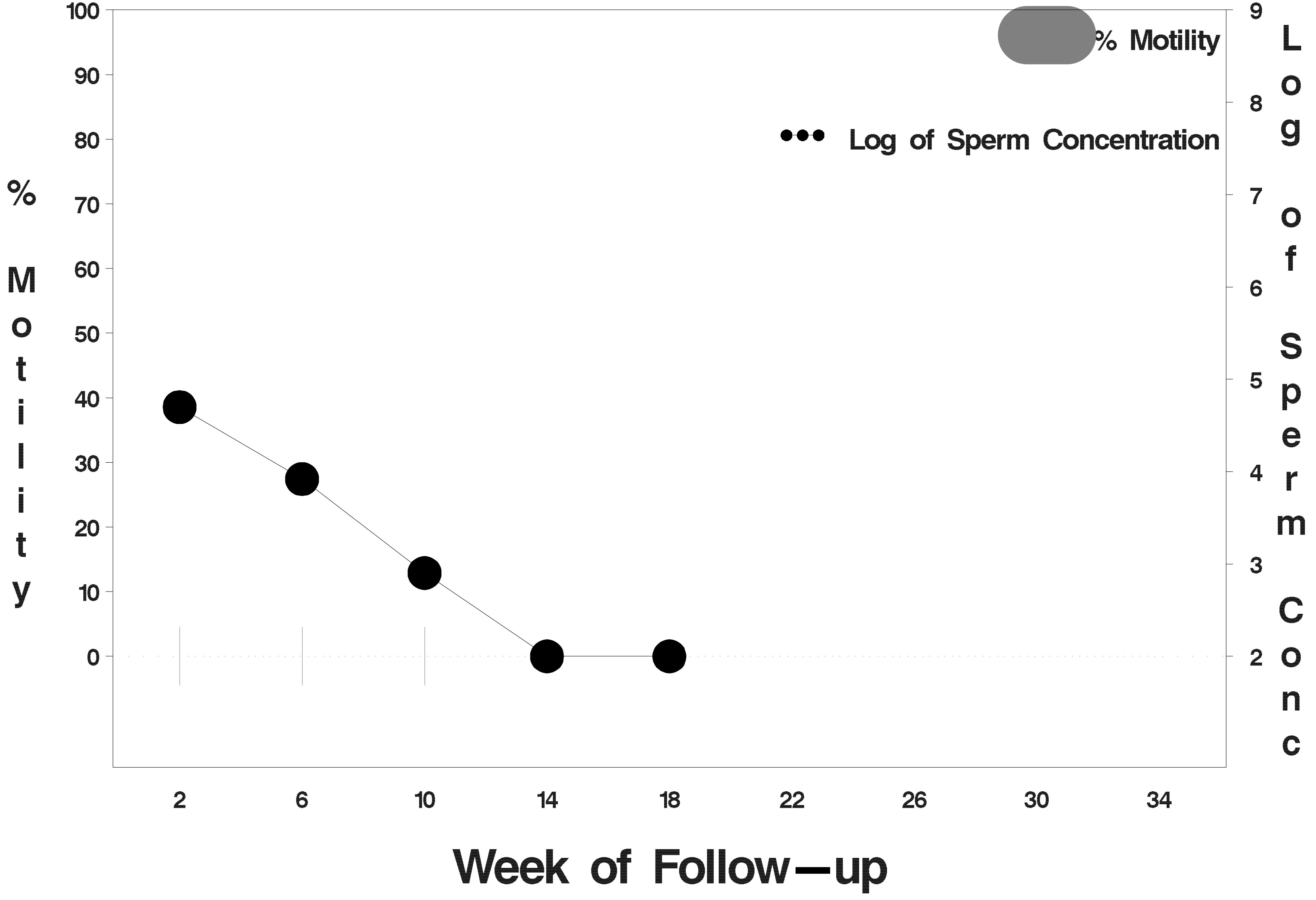

id= 487 outcome= Success

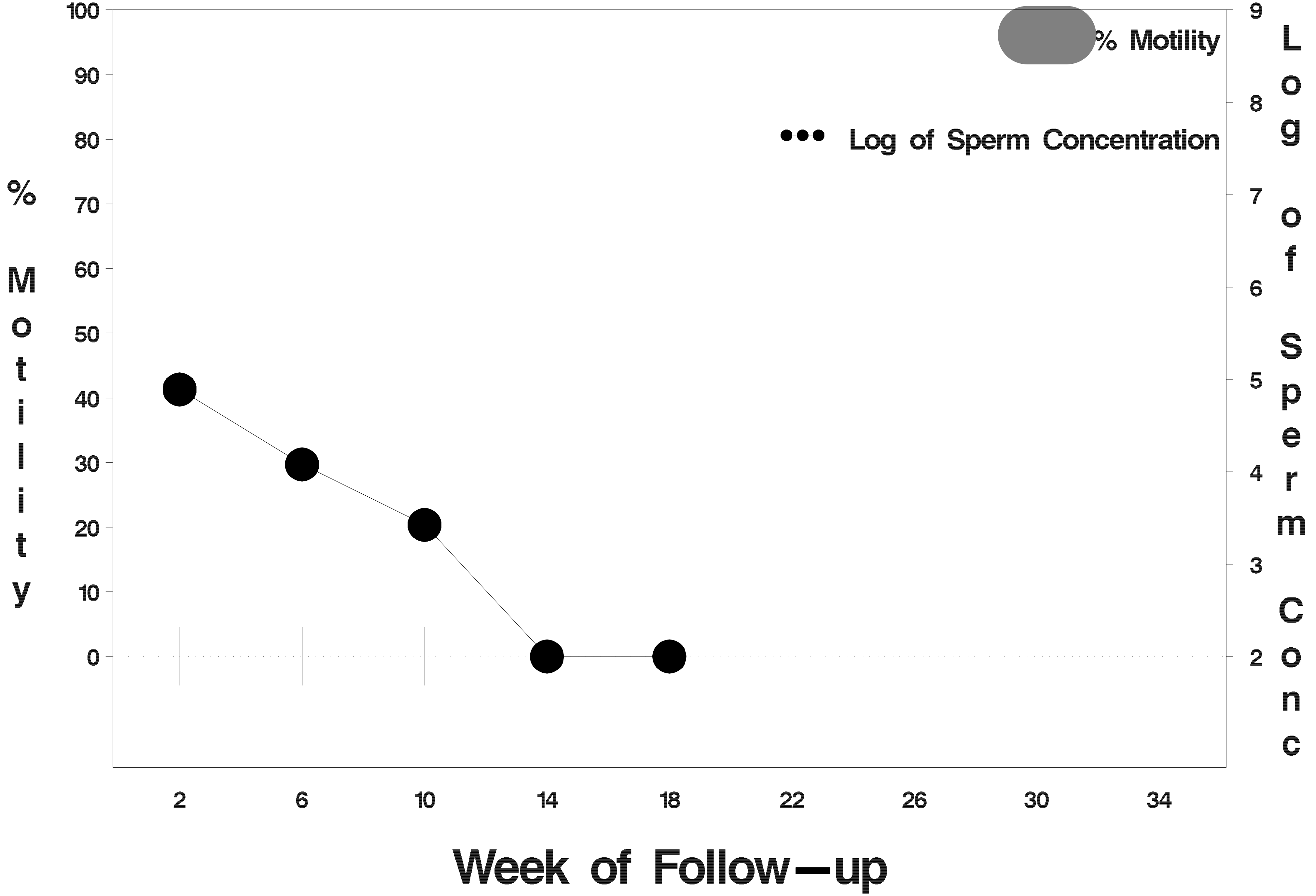

id= 488 outcome= Success

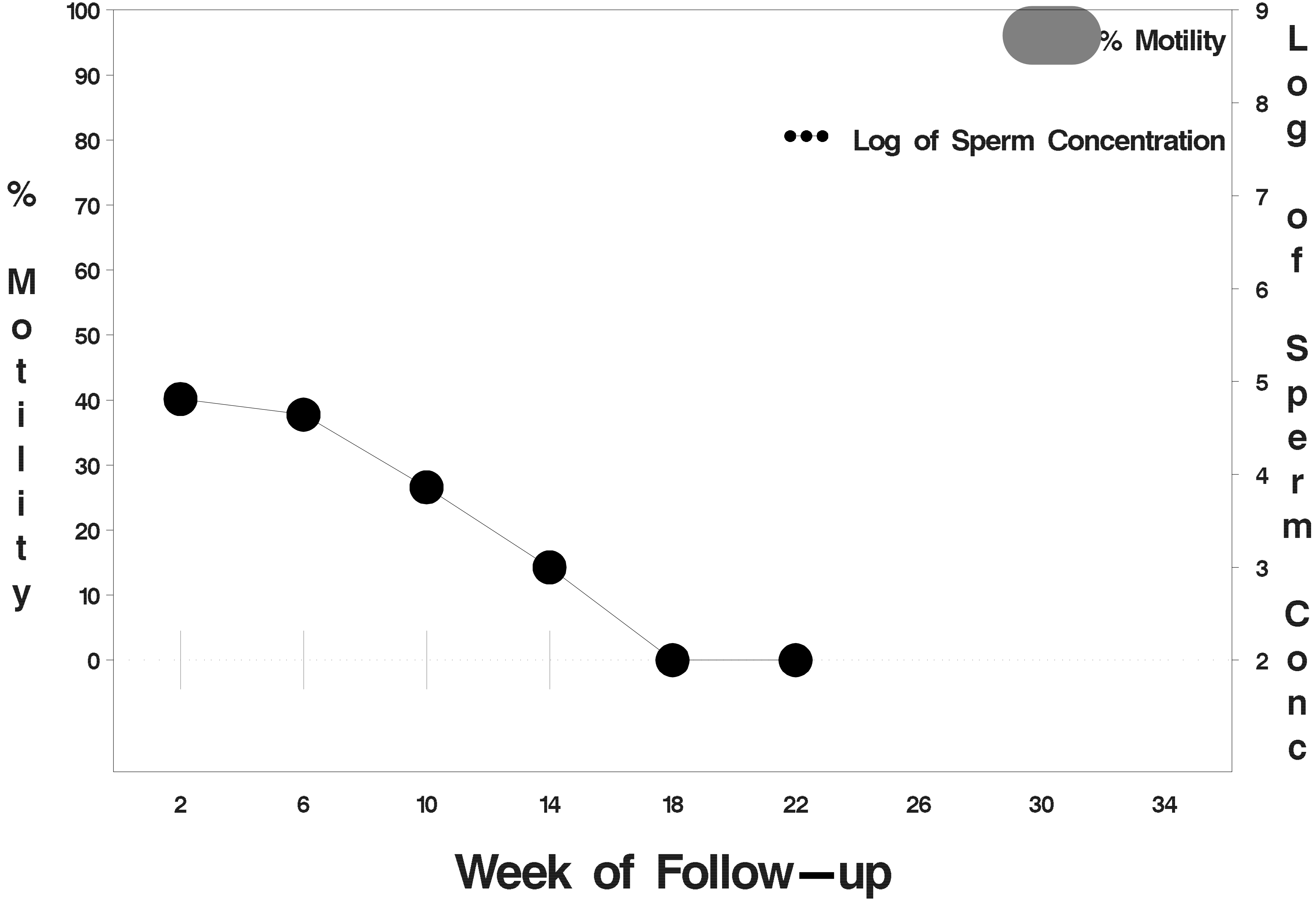

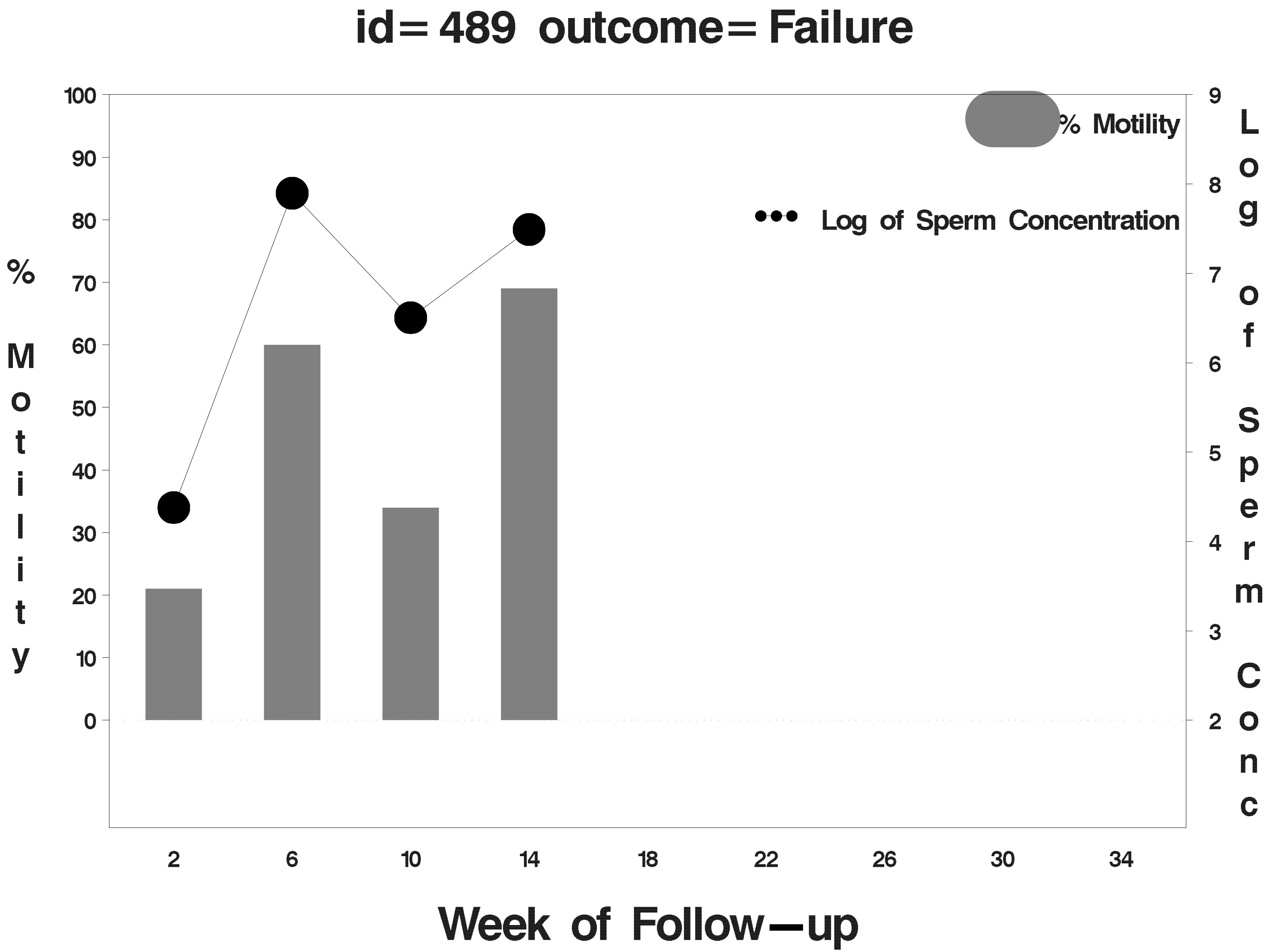

id= 489 outcome= Failure
% Motility
Log of Sperm Concentration
100
90
80
70
60
50
40
30
20
10
0
9
8
7
6
5
4
3
2
2
6
10
14
18
22
26
30
34
Week of Follow−up

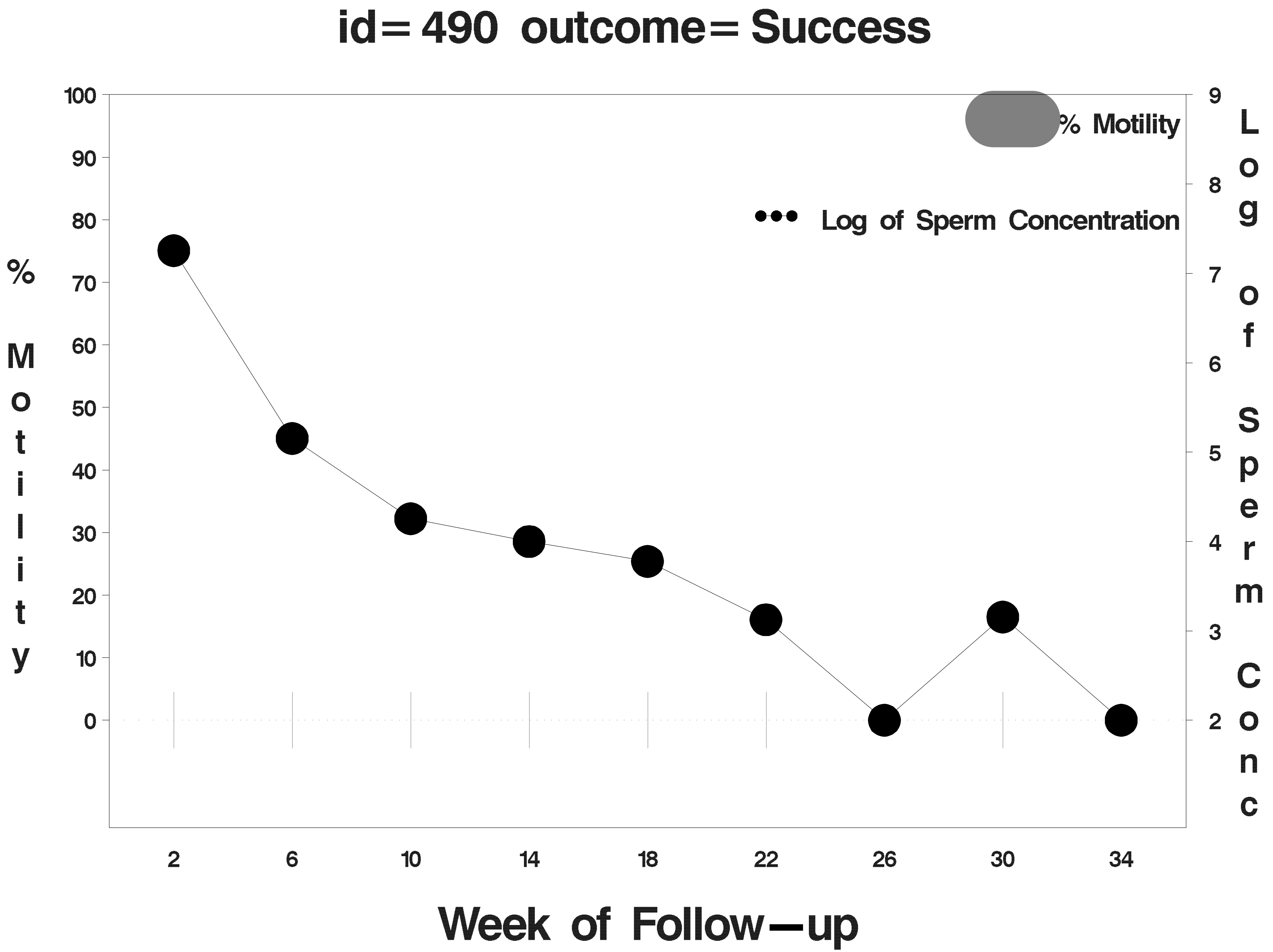
id= 490 outcome= Success
% Motility
Log of Sperm Concentration
100
90
80
70
60
50
40
30
20
10
0
9
8
7
6
5
4
3
2
2
6
10
14
18
22
26
30
34
Week of Follow—up
Log of Sperm Conc

id= 491 outcome= Failure

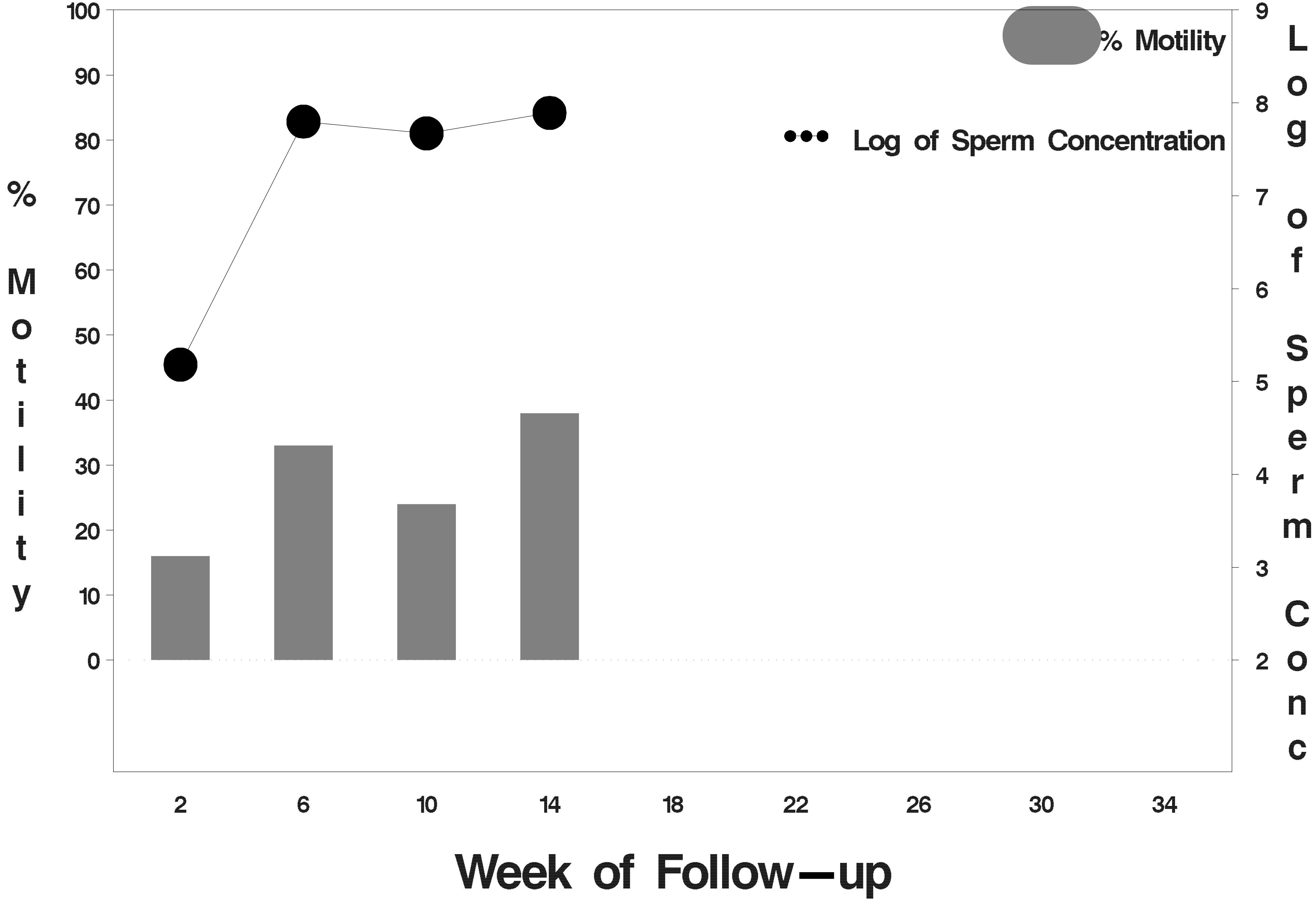

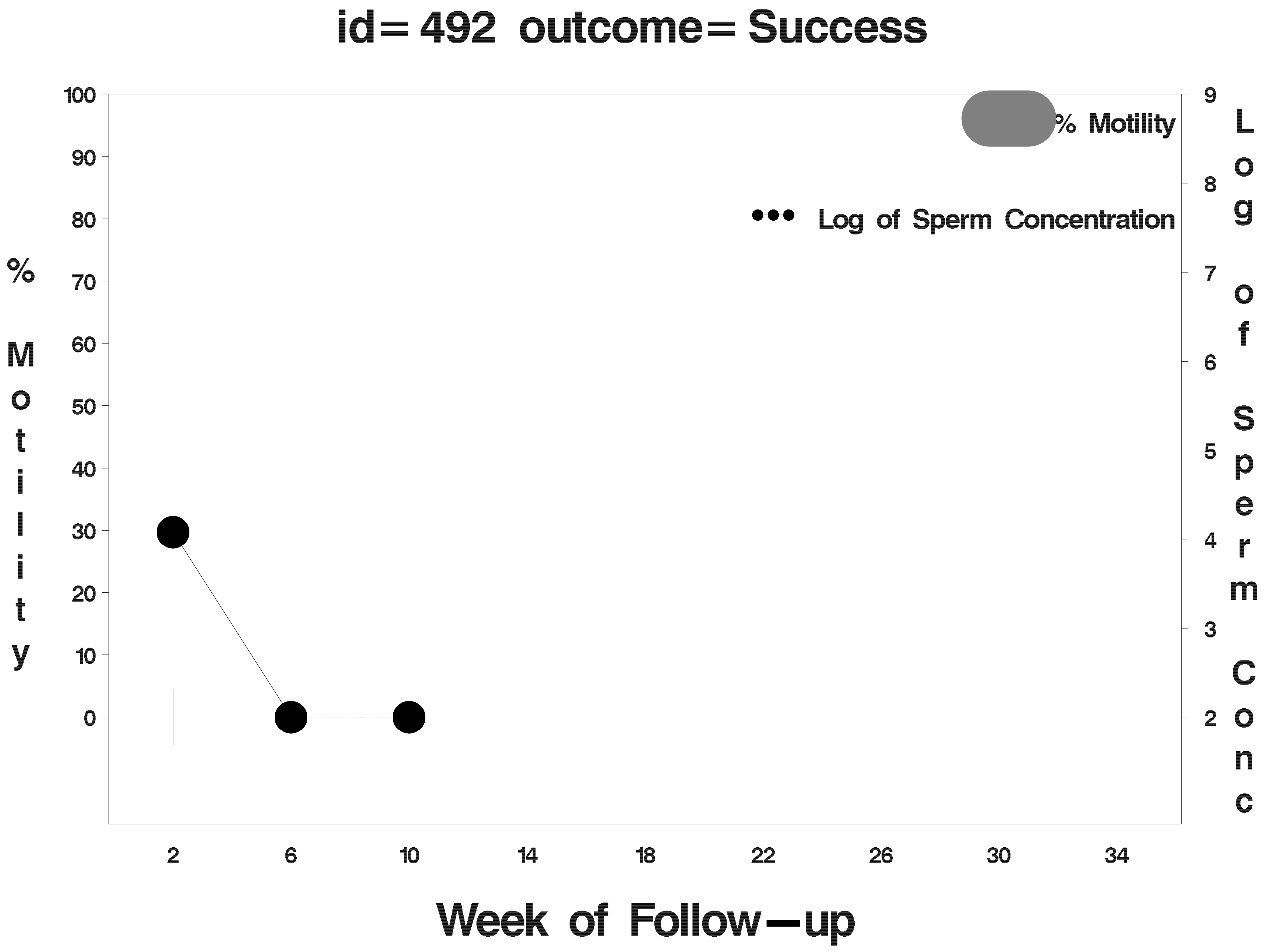

id= 492 outcome= Success
% Motility
Log of Sperm Concentration
100
90
80
70
60
50
40
30
20
10
0
9
8
7
6
5
4
3
2
2
6
10
14
18
22
26
30
34
Week of Follow—up

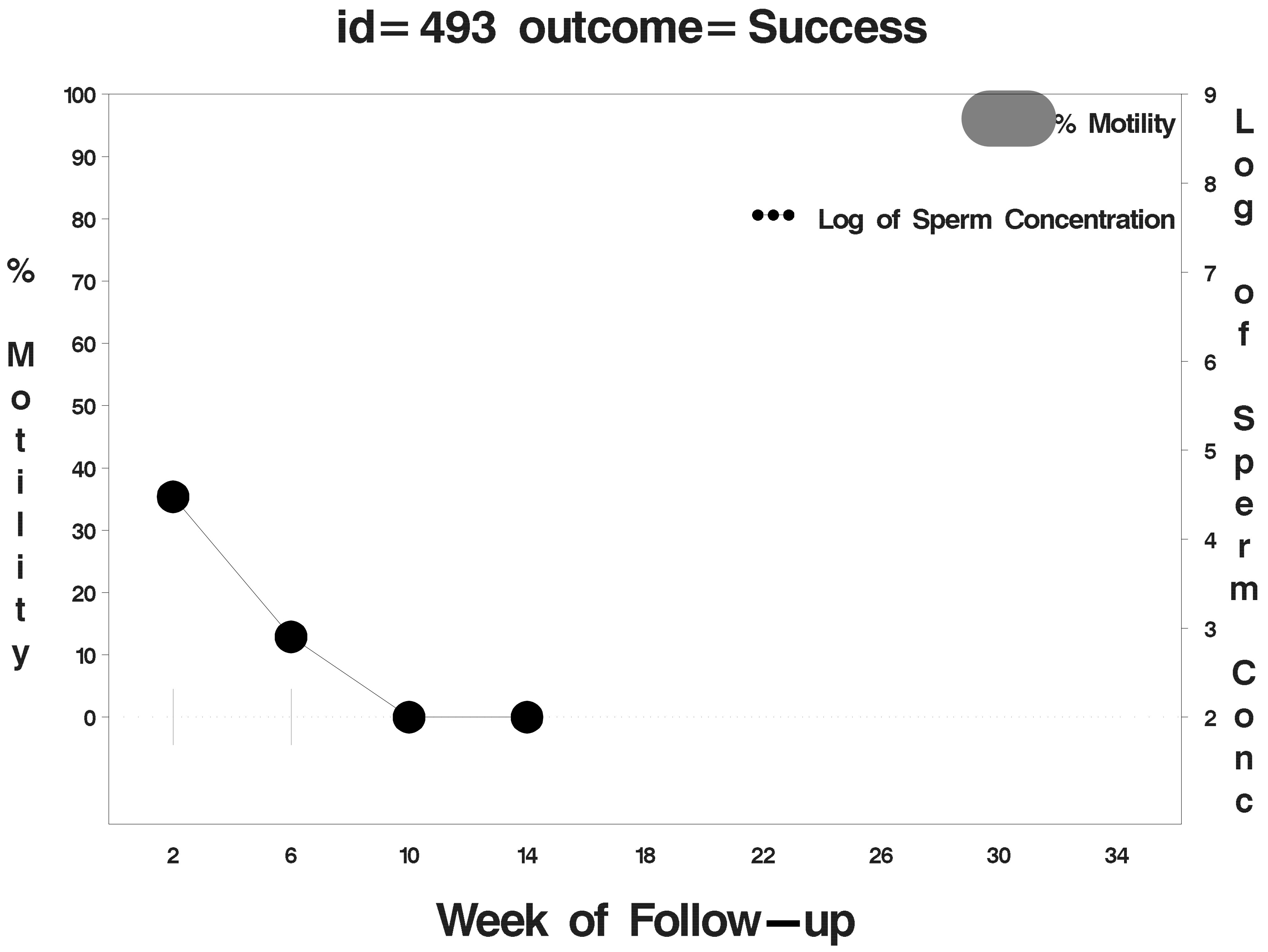

id= 493 outcome= Success
% Motility
Log of Sperm Concentration
% Motility
Log of Sperm Conc
Week of Follow−up
100
90
80
70
60
50
40
30
20
10
0
9
8
7
6
5
4
3
2
2
6
10
14
18
22
26
30
34

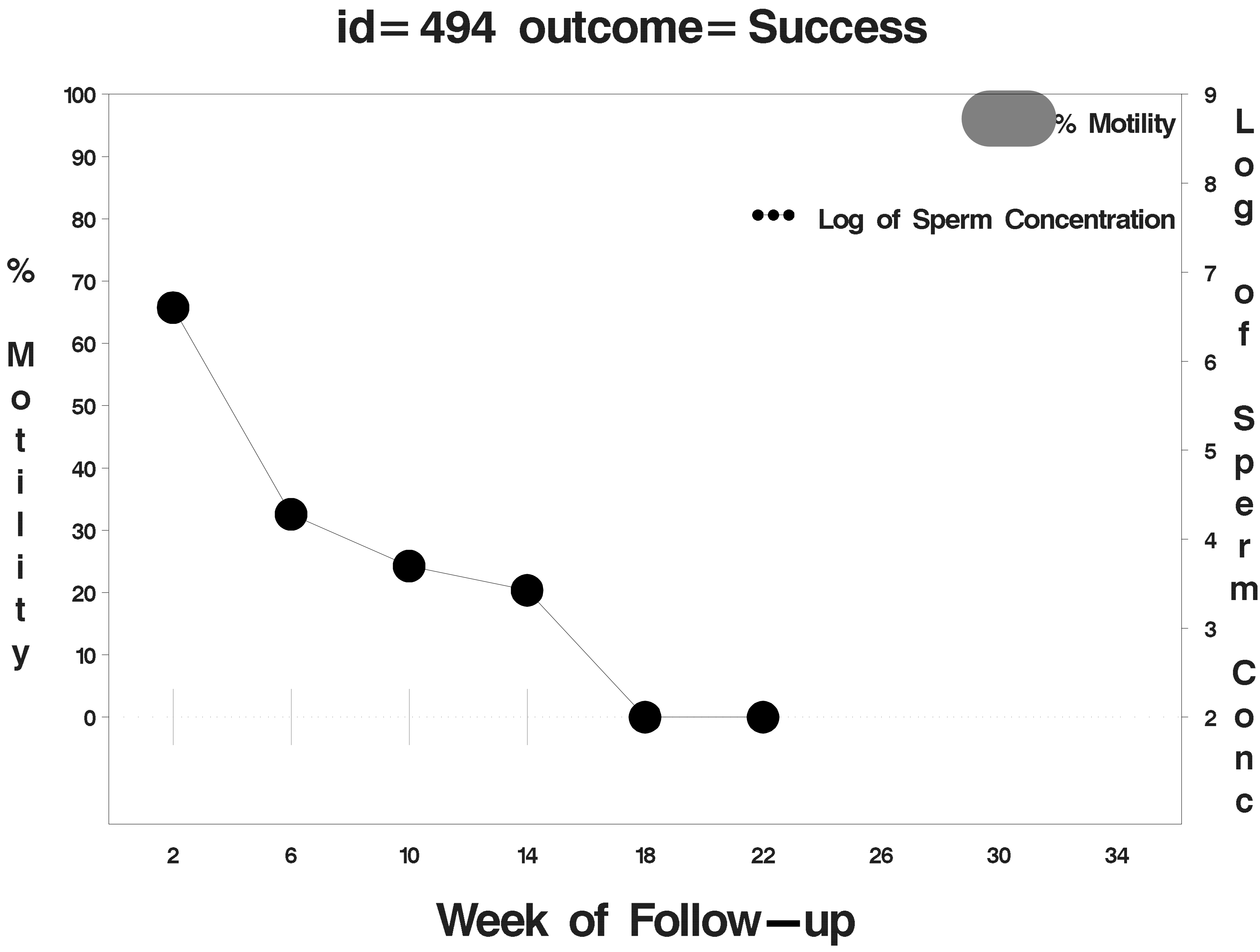

id= 494 outcome= Success
% Motility
Log of Sperm Concentration
% Motility
Log of Sperm Conc
Week of Follow−up
100
90
80
70
60
50
40
30
20
10
0
9
8
7
6
5
4
3
2
2
6
10
14
18
22
26
30
34

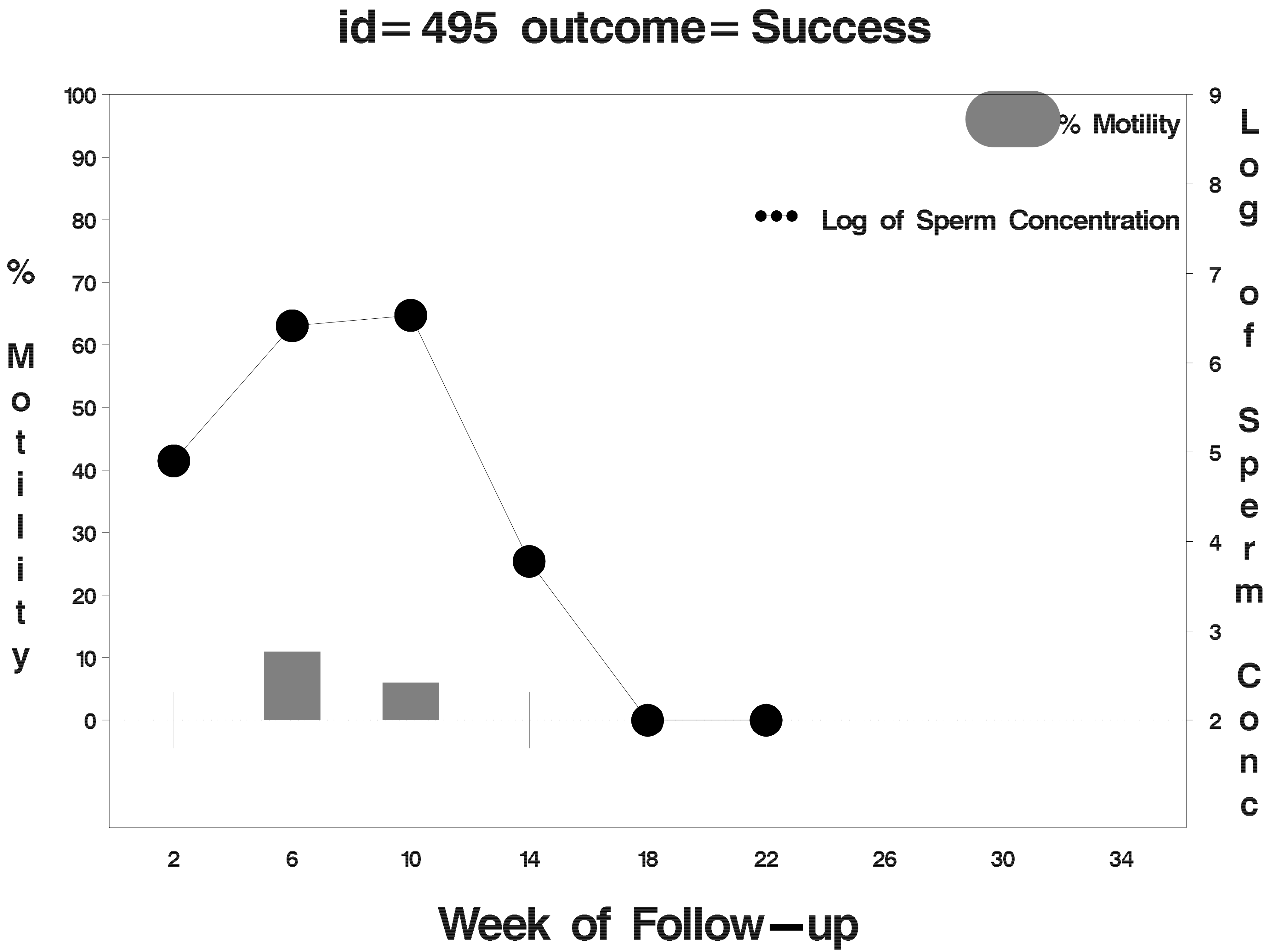

id= 495 outcome= Success
% Motility
Log of Sperm Concentration
% Motility
Log of Sperm Conc
Week of Follow−up
100
90
80
70
60
50
40
30
20
10
0
9
8
7
6
5
4
3
2
2
6
10
14
18
22
26
30
34

id= 496 outcome= Success

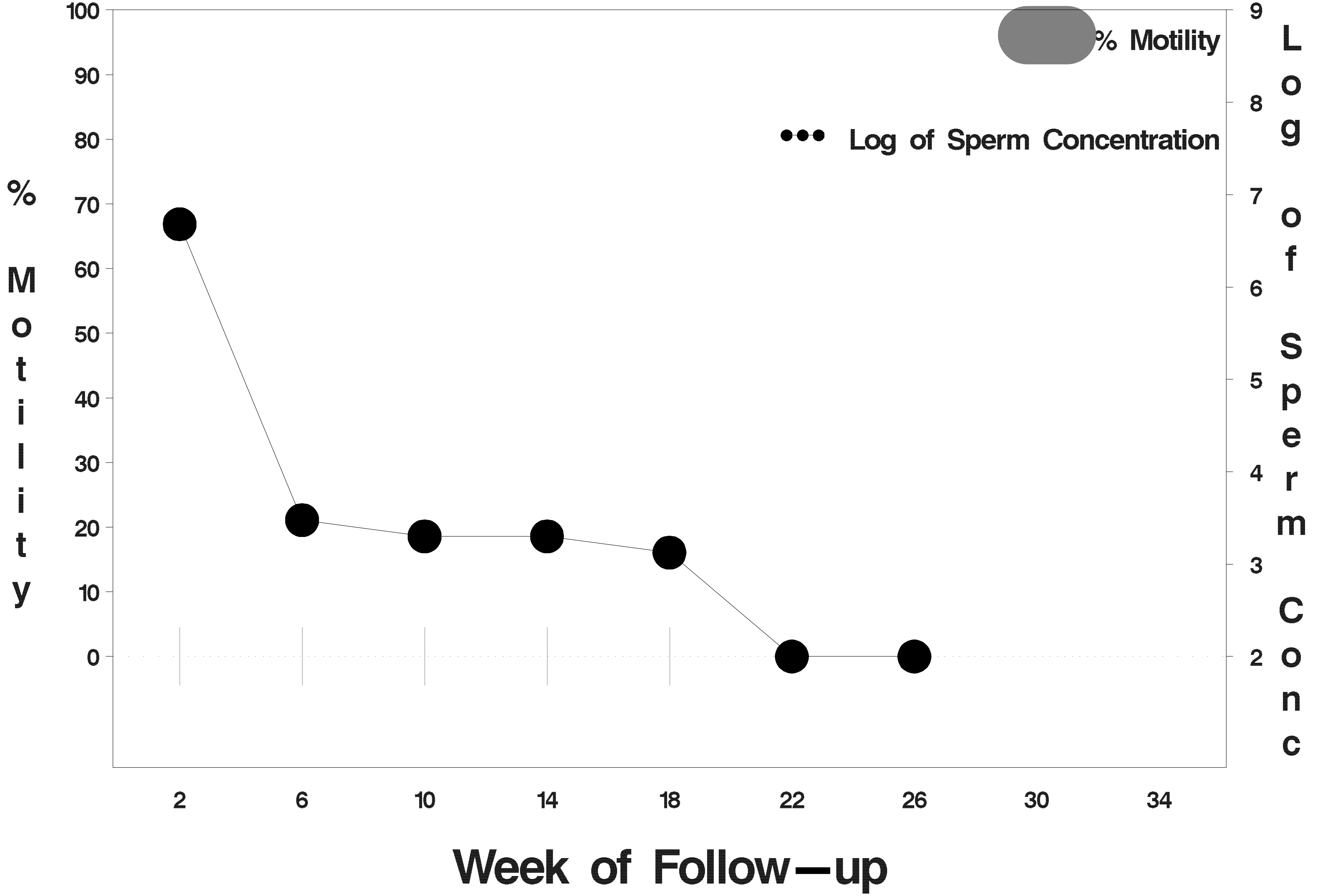

id= 497 outcome= Failure

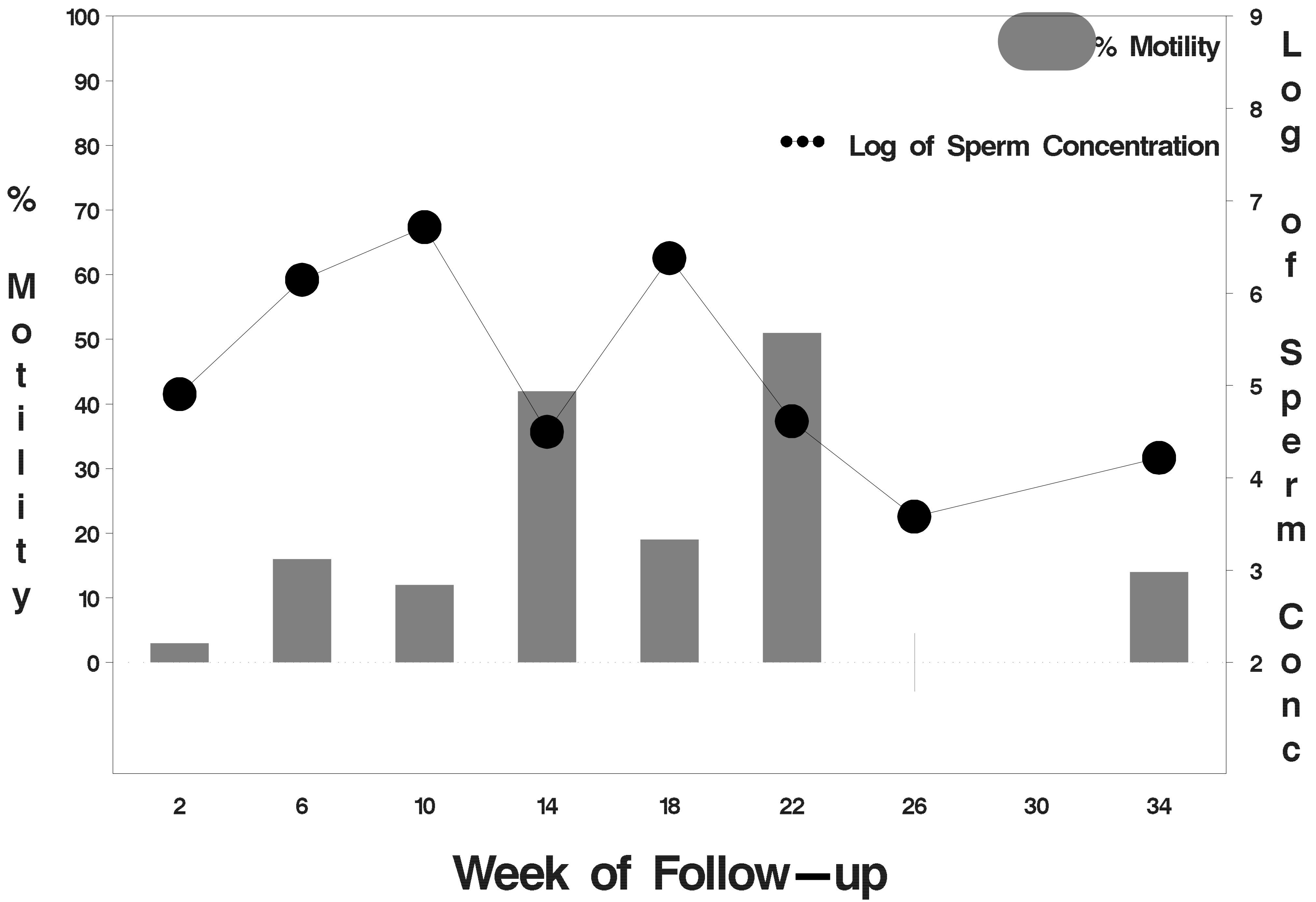

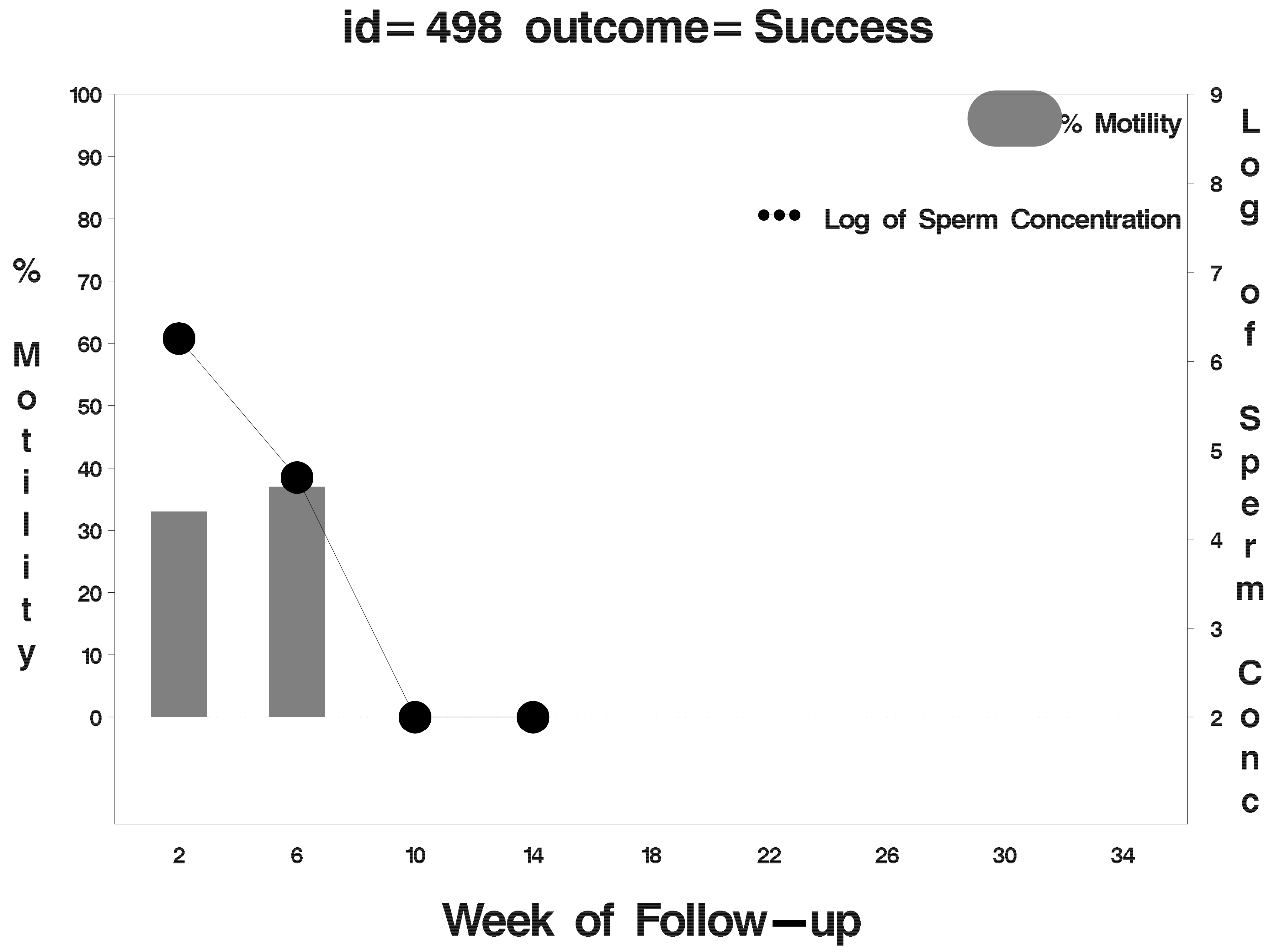
id= 498 outcome= Success
% Motility
Log of Sperm Concentration
% Motility
Log of Sperm Conc
Week of Follow—up
100
90
80
70
60
50
40
30
20
10
0
9
8
7
6
5
4
3
2
2
6
10
14
18
22
26
30
34

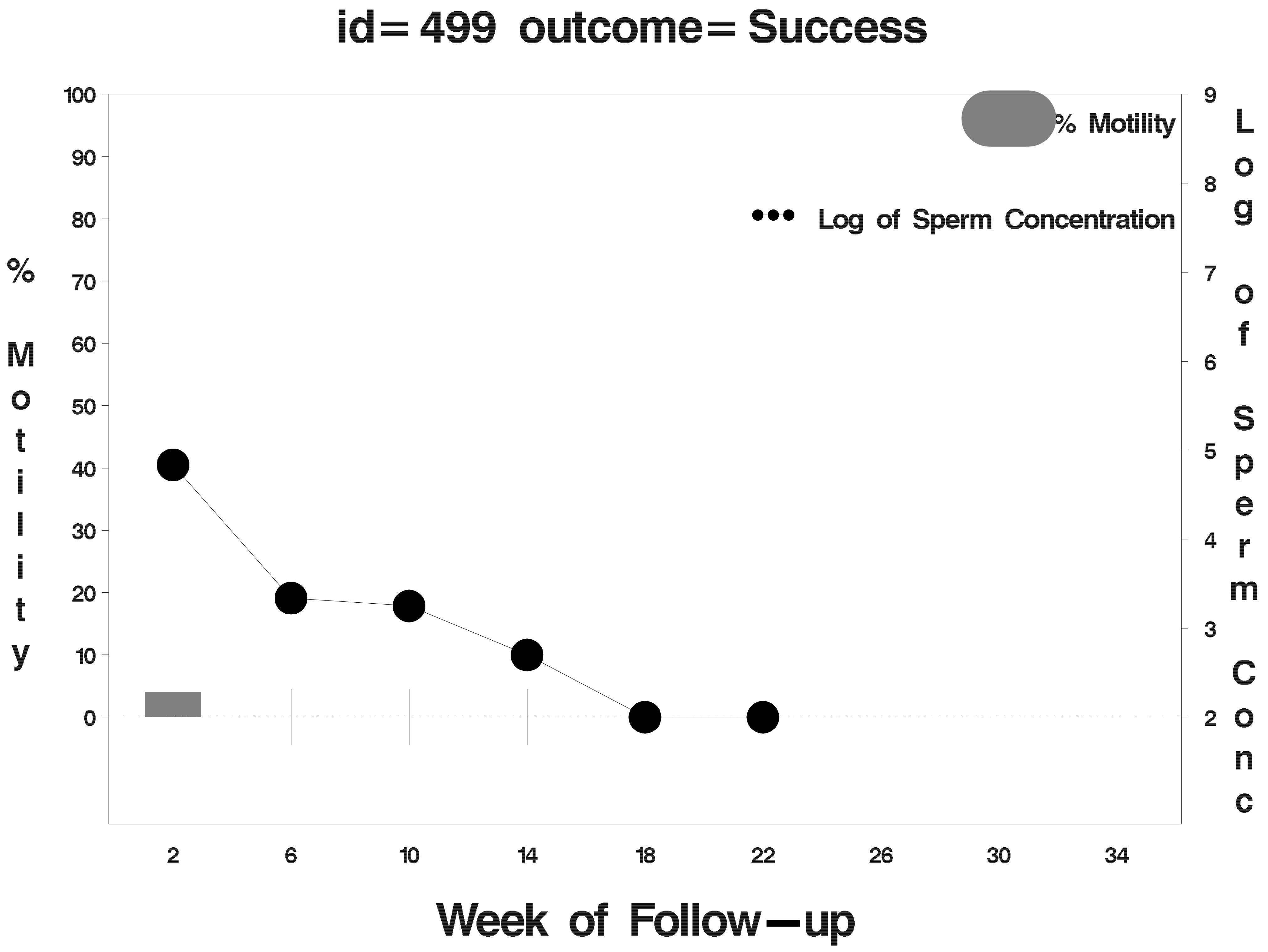

id= 499 outcome= Success
% Motility
Log of Sperm Concentration
100
90
80
70
60
50
40
30
20
10
0
9
8
7
6
5
4
3
2
2
6
10
14
18
22
26
30
34
Week of Follow—up
Log of Sperm Conc

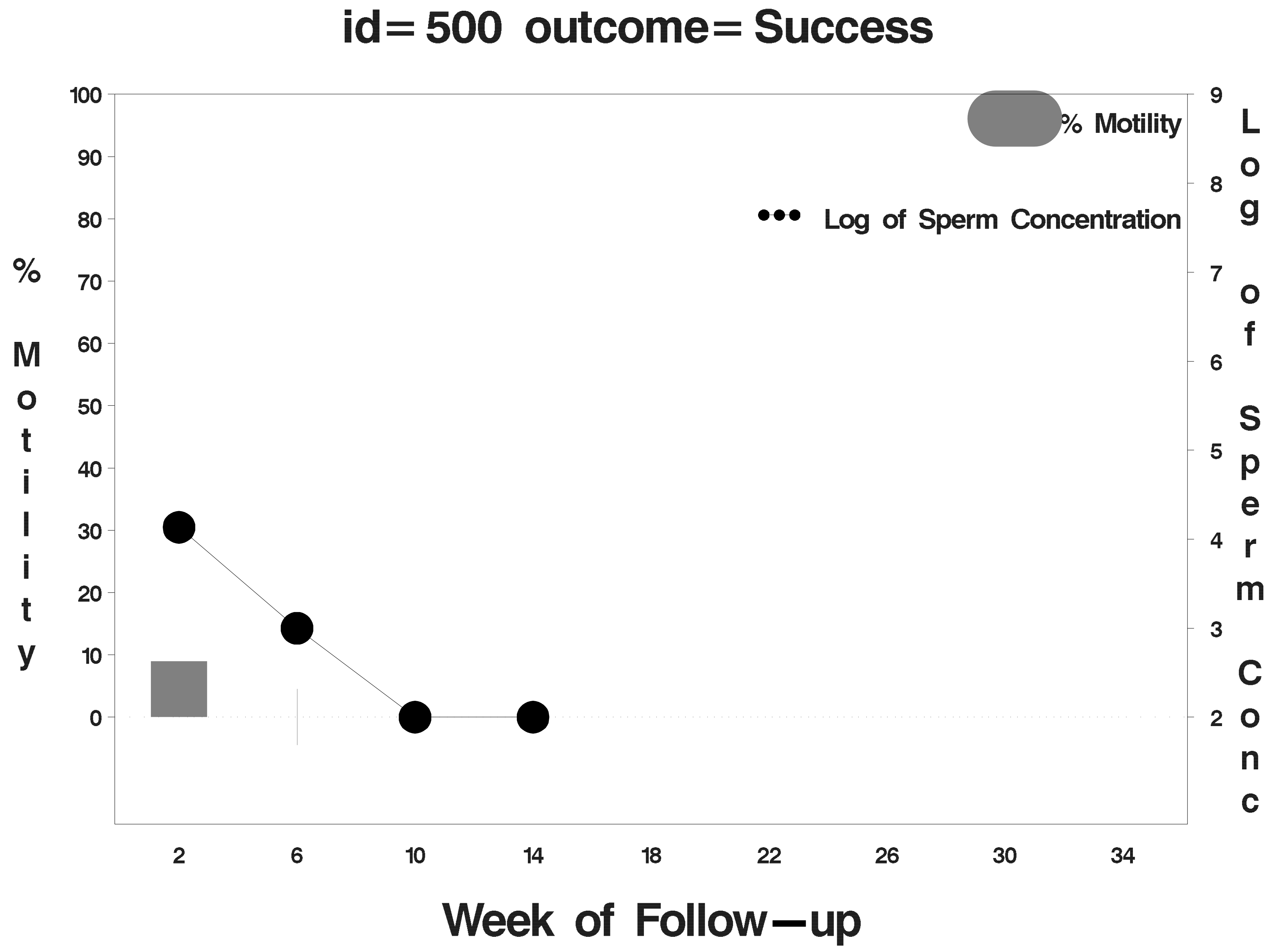

id= 500 outcome= Success
% Motility
Log of Sperm Concentration
% Motility
Log of Sperm Conc
Week of Follow−up
100
90
80
70
60
50
40
30
20
10
0
9
8
7
6
5
4
3
2
2
6
10
14
18
22
26
30
34

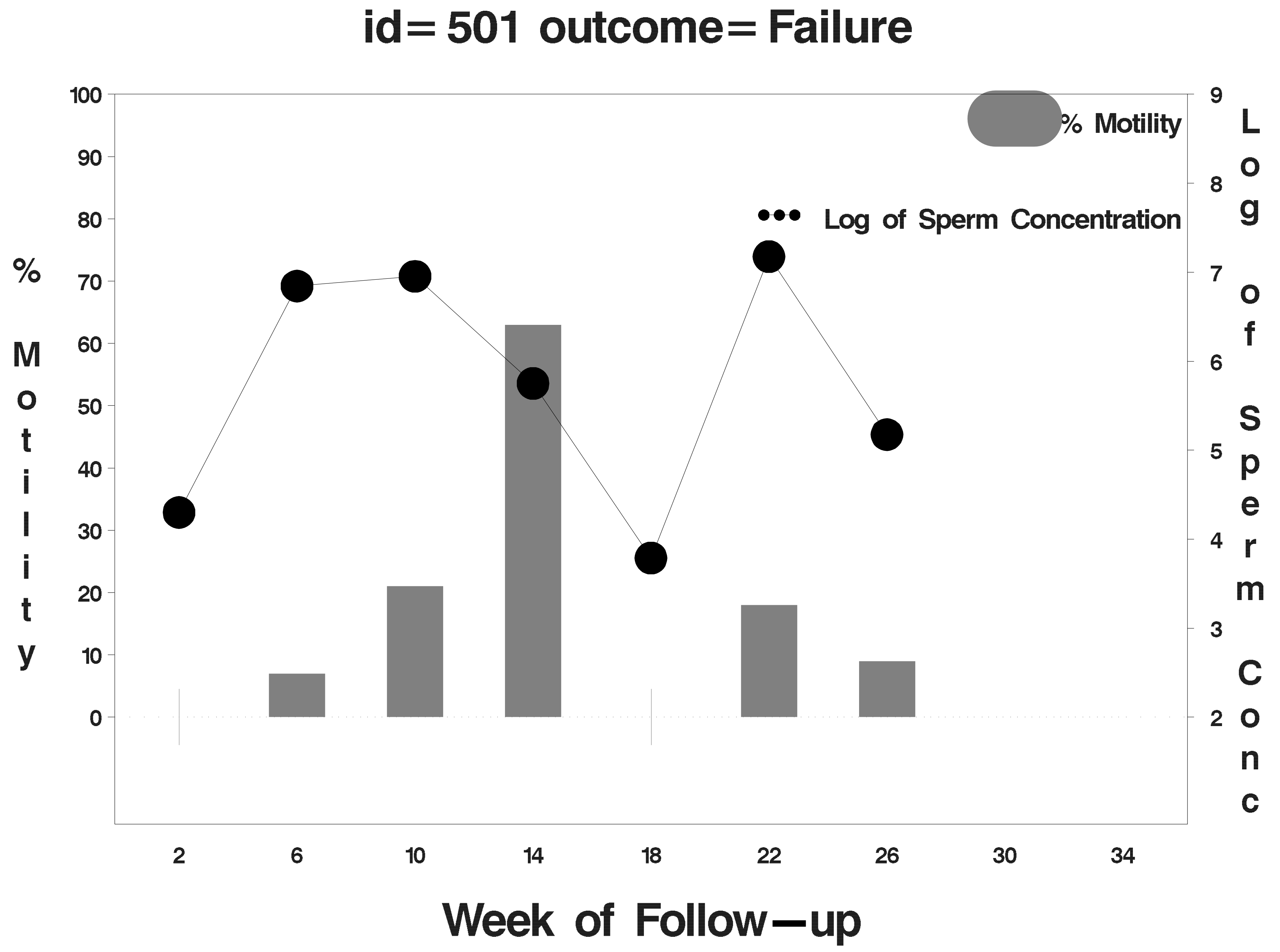
id= 501 outcome= Failure
% Motility
Log of Sperm Concentration
100
90
80
70
60
50
40
30
20
10
0
9
8
7
6
5
4
3
2
2
6
10
14
18
22
26
30
34
Week of Follow−up
Log of Sperm Conc

id= 502 outcome= Success

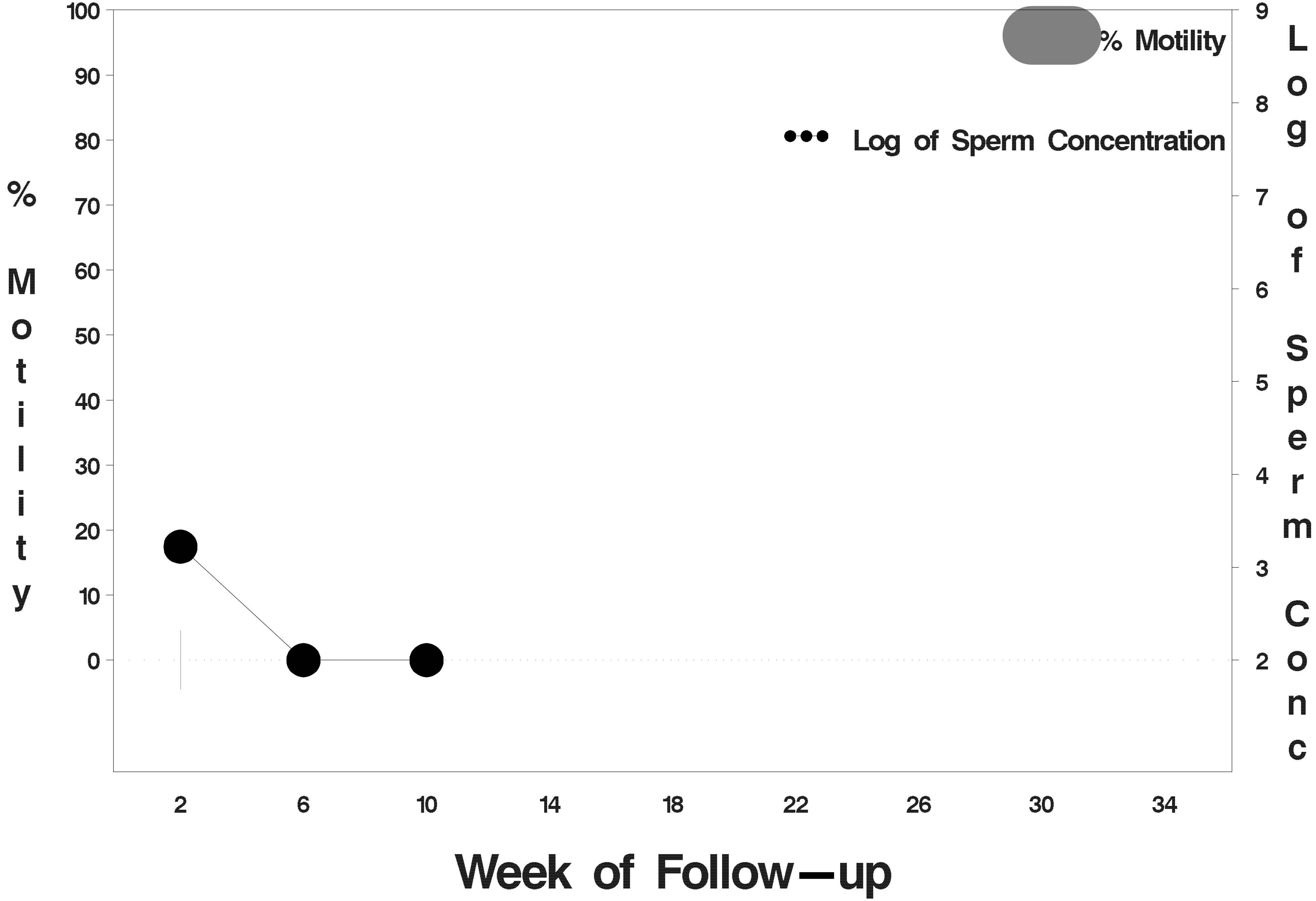

id= 503 outcome= Indeterminate

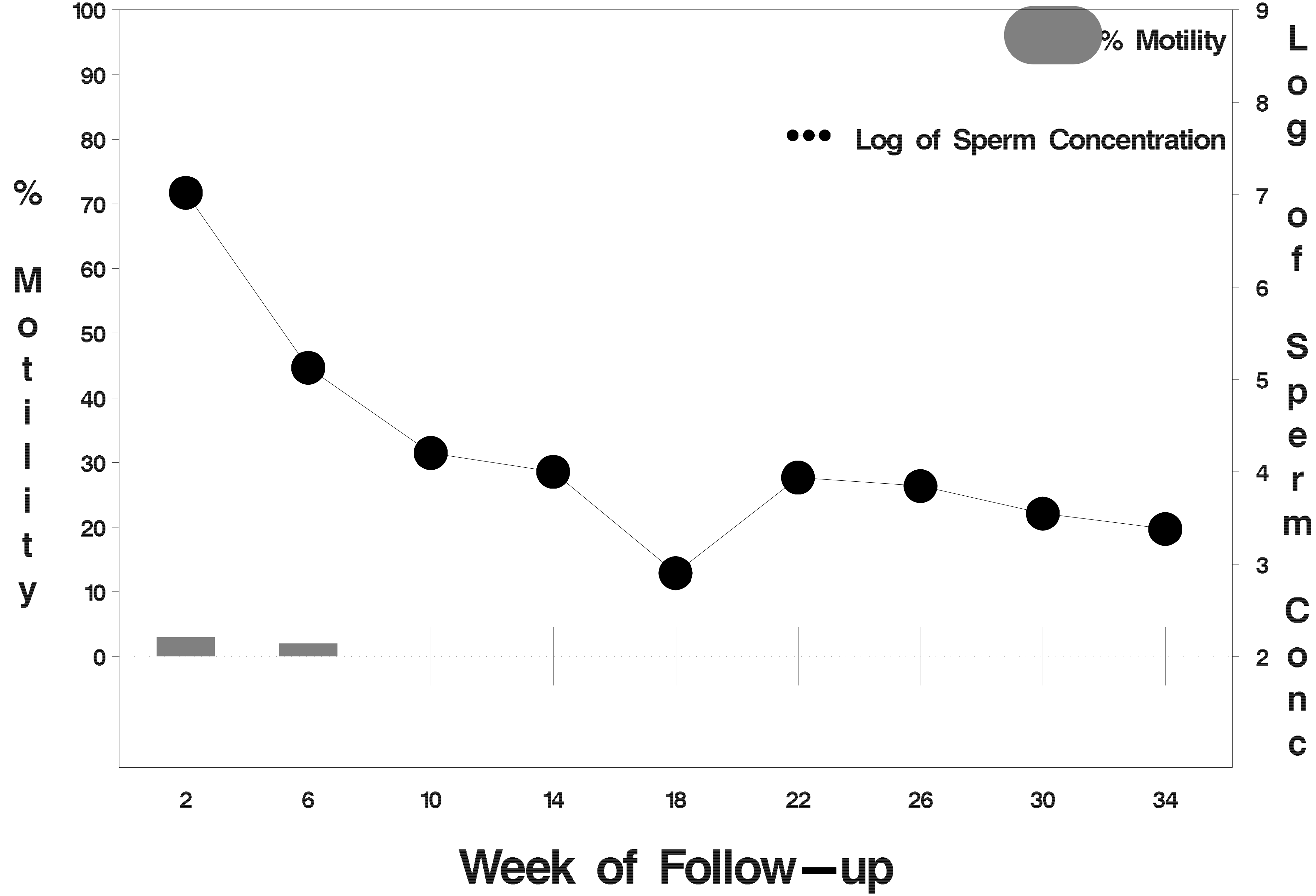

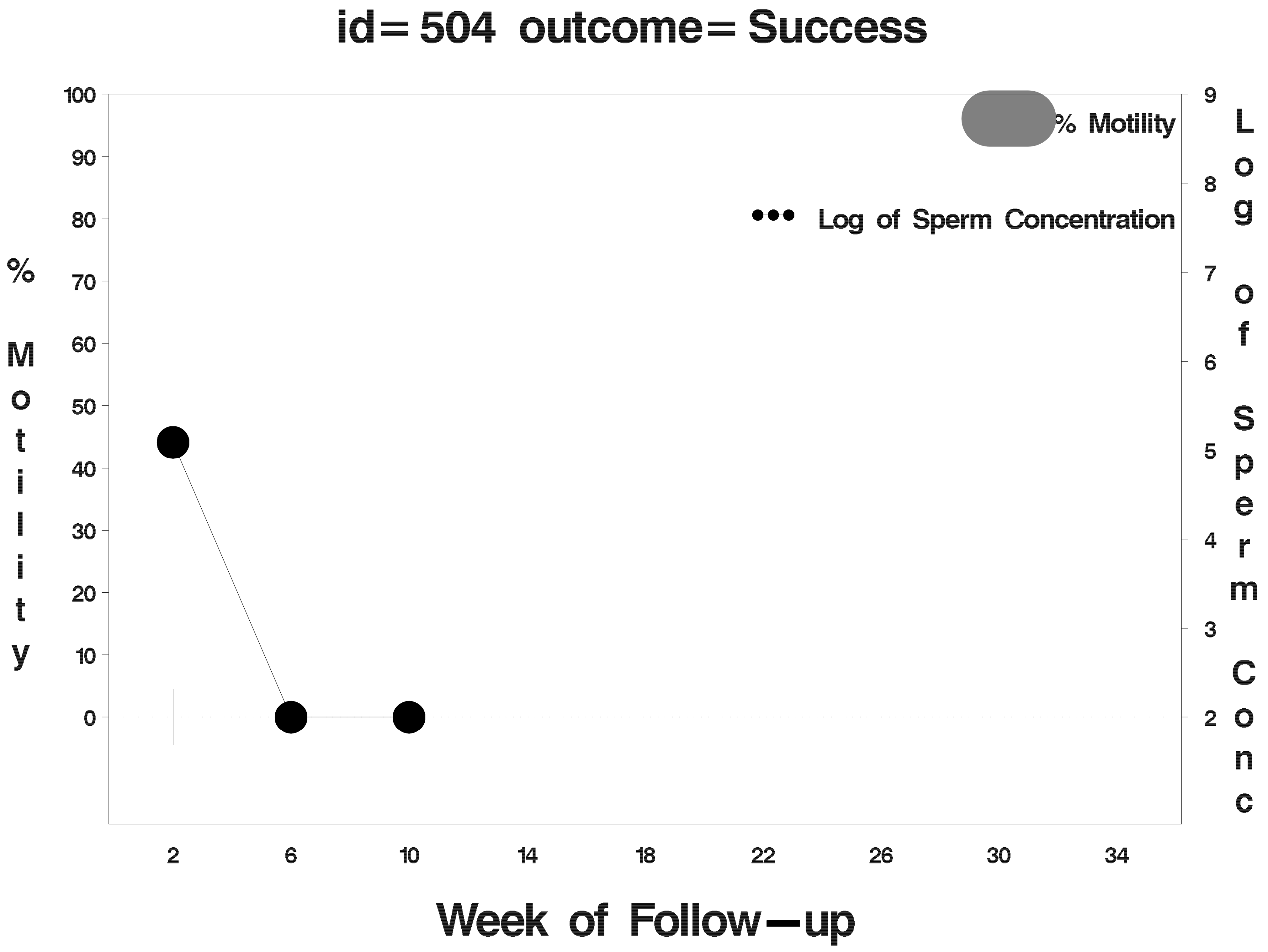
id= 504 outcome= Success
% Motility
Log of Sperm Concentration
% Motility
Log of Sperm Conc
Week of Follow−up
100
90
80
70
60
50
40
30
20
10
0
9
8
7
6
5
4
3
2
2
6
10
14
18
22
26
30
34

id= 505 outcome= Success

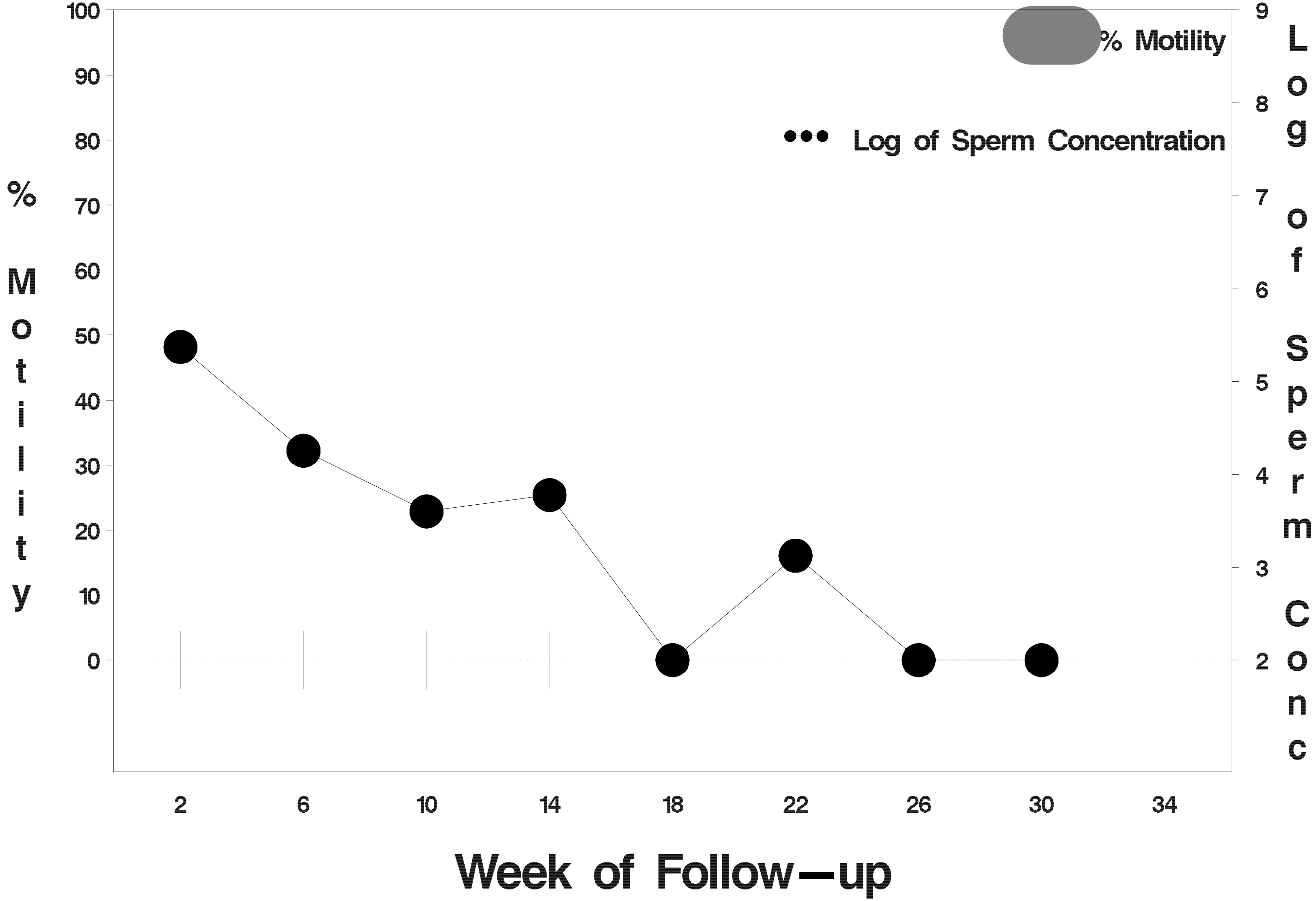

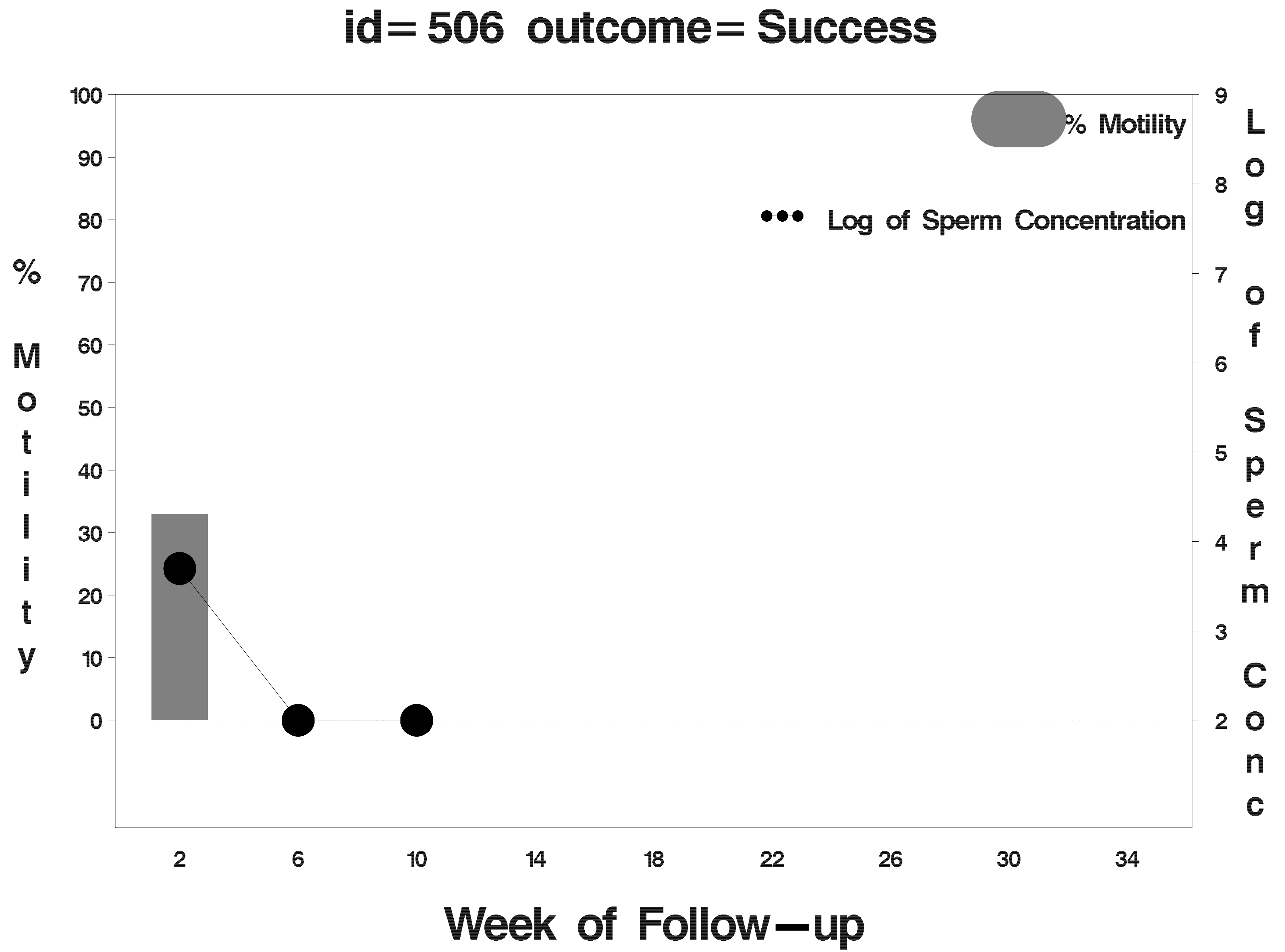
id= 506 outcome= Success
% Motility
Log of Sperm Concentration
100
90
80
70
60
50
40
30
20
10
0
9
8
7
6
5
4
3
2
2
6
10
14
18
22
26
30
34
Week of Follow−up
% Motility
Log of Sperm Conc

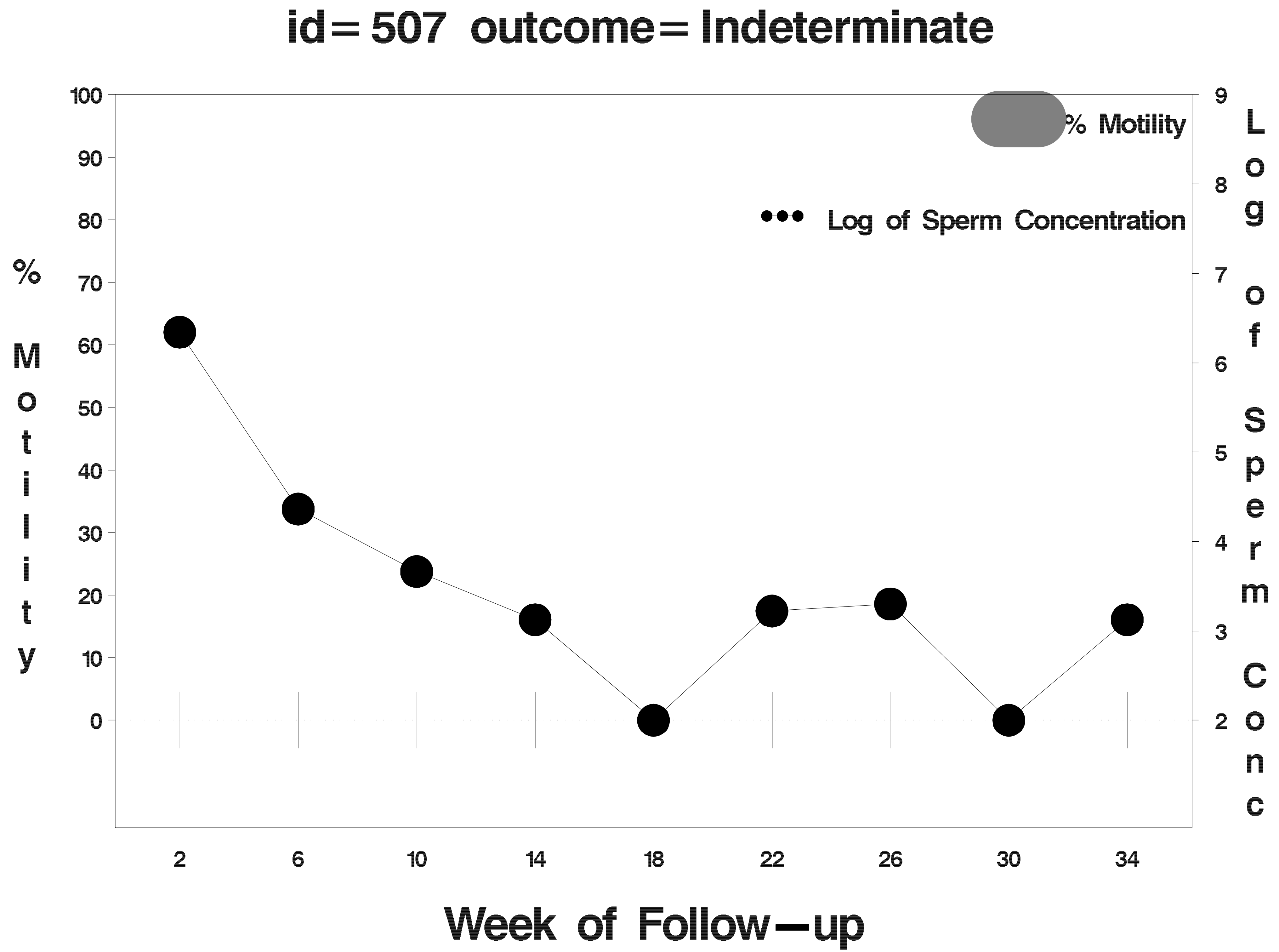

id= 507 outcome= Indeterminate
% Motility
Log of Sperm Concentration
% Motility
Log of Sperm Conc
Week of Follow−up
100
90
80
70
60
50
40
30
20
10
0
9
8
7
6
5
4
3
2
2
6
10
14
18
22
26
30
34

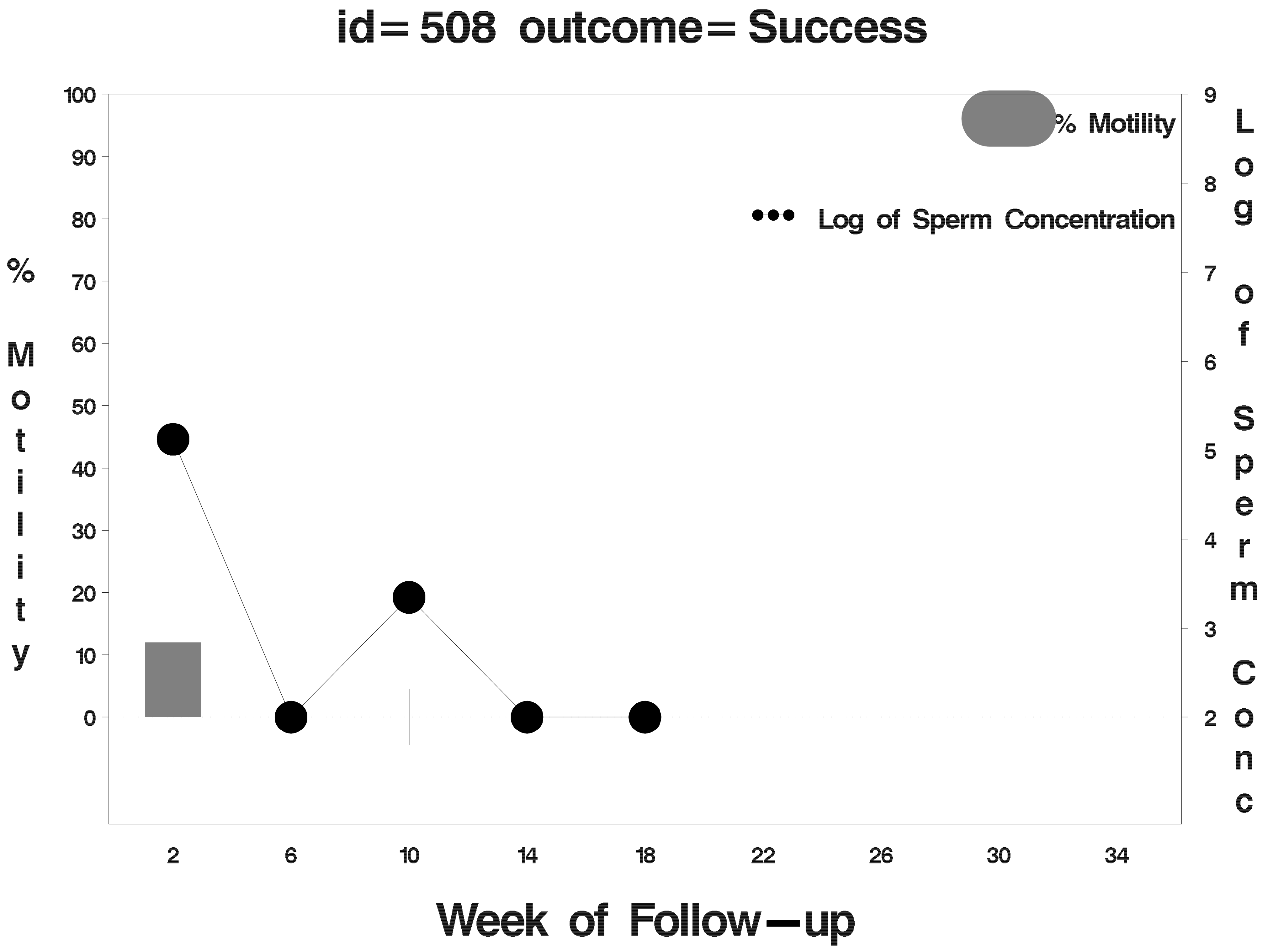
id= 508 outcome= Success
% Motility
Log of Sperm Concentration
100
90
80
70
60
50
40
30
20
10
0
9
8
7
6
5
4
3
2
2
6
10
14
18
22
26
30
34
Week of Follow−up
Log of Sperm Conc

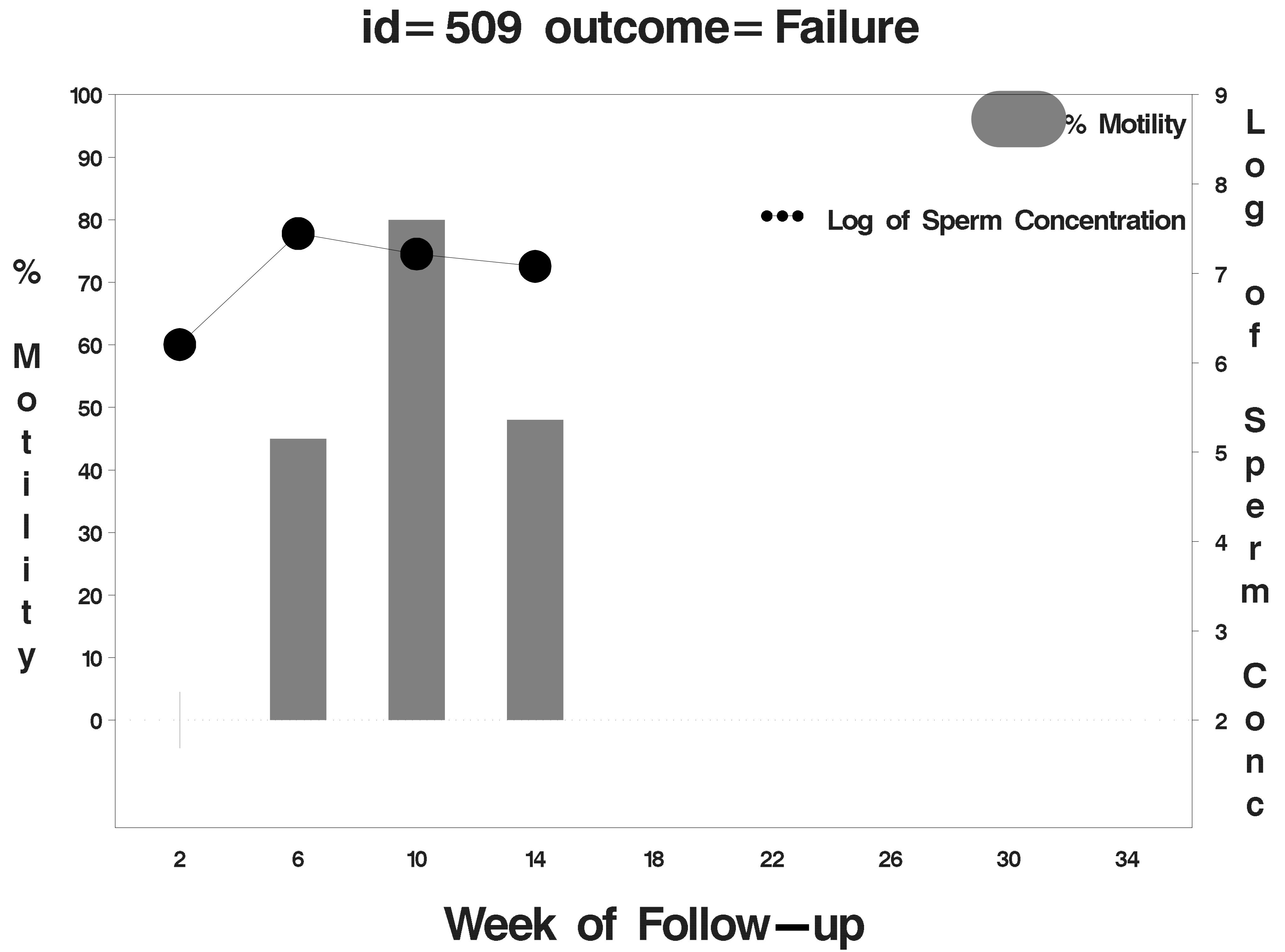

id= 509 outcome= Failure
% Motility
Log of Sperm Concentration
100
90
80
70
60
50
40
30
20
10
0
9
8
7
6
5
4
3
2
2
6
10
14
18
22
26
30
34
Week of Follow—up
Log of Sperm Conc

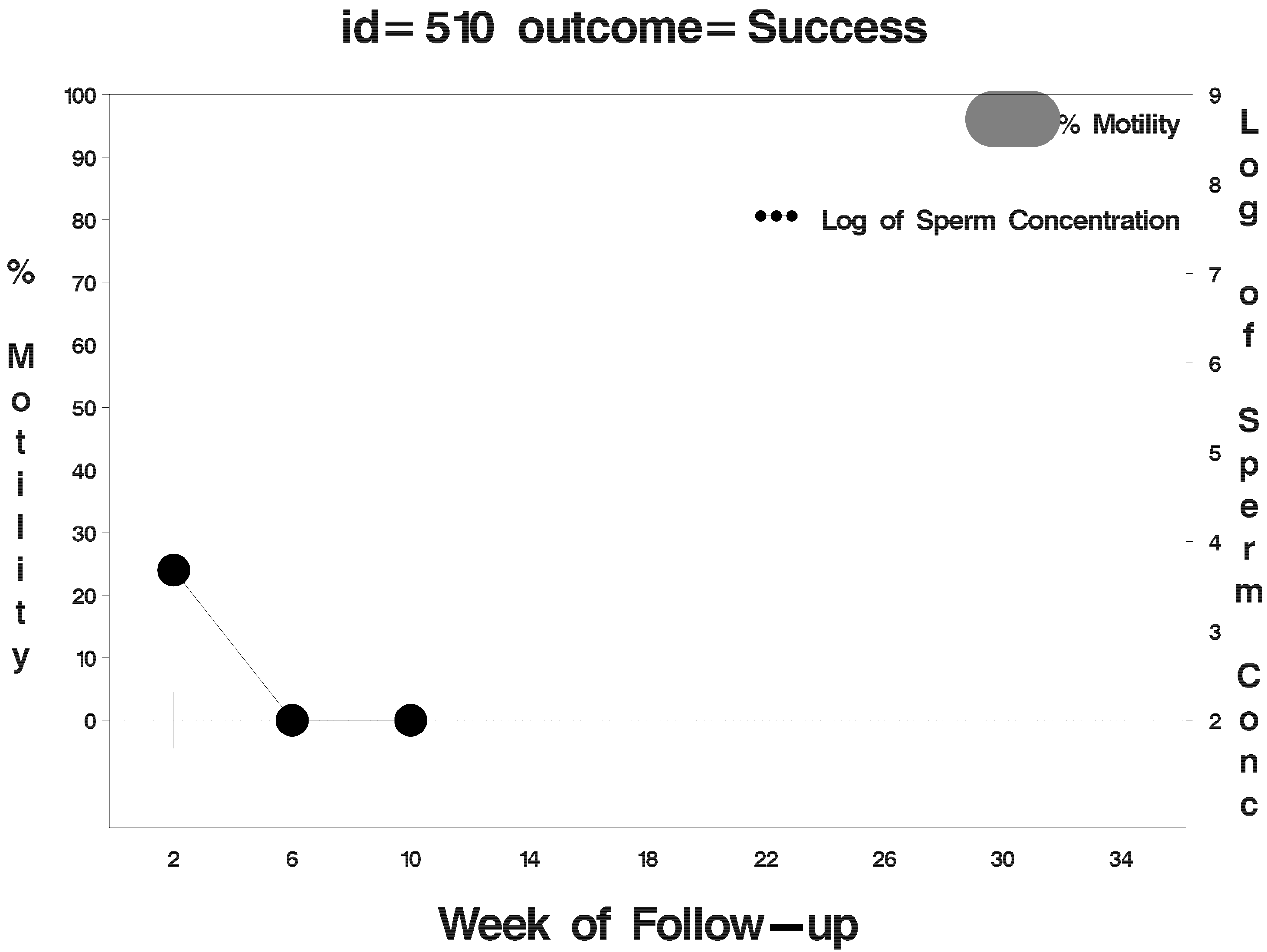

id= 511 outcome= Success

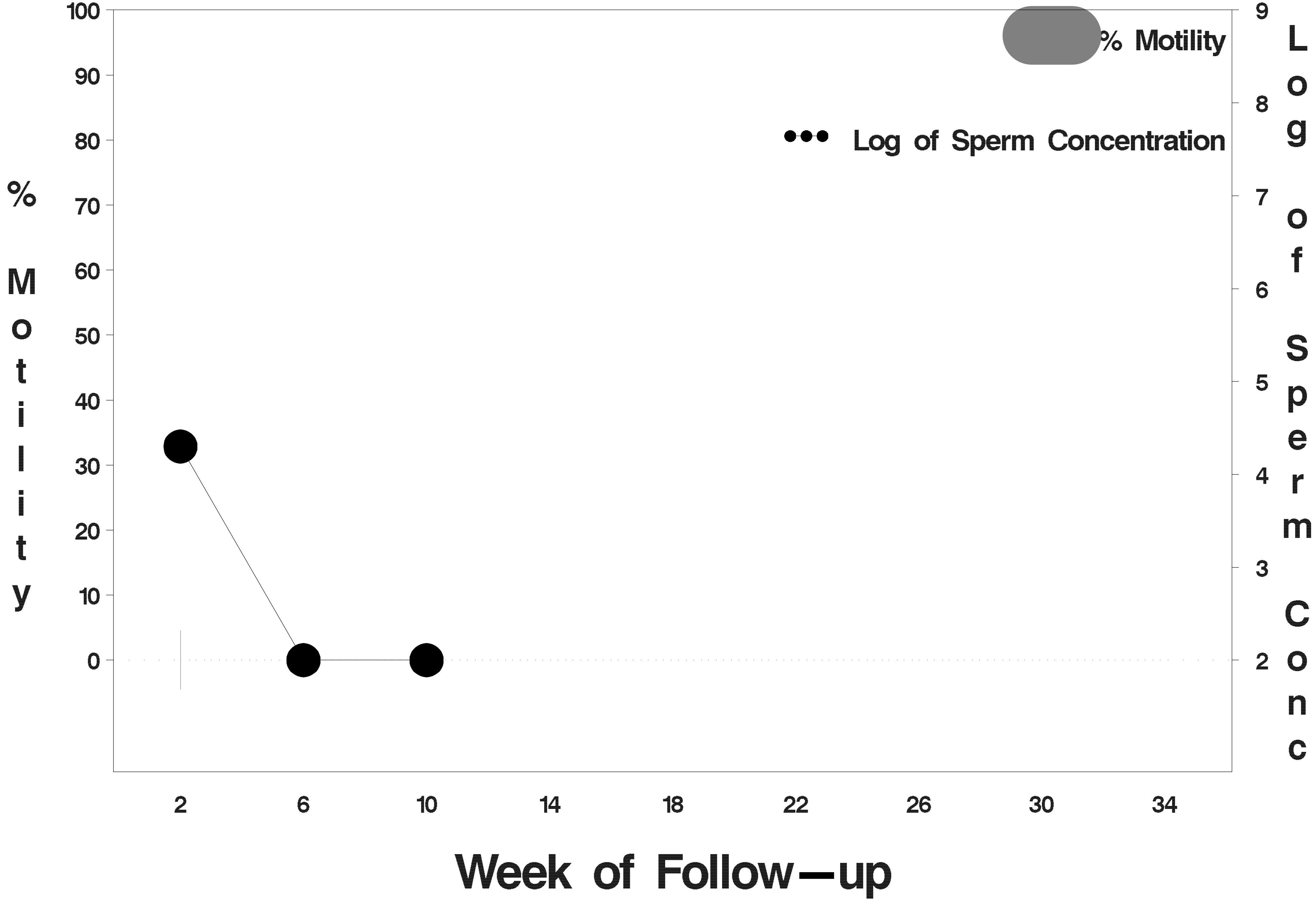

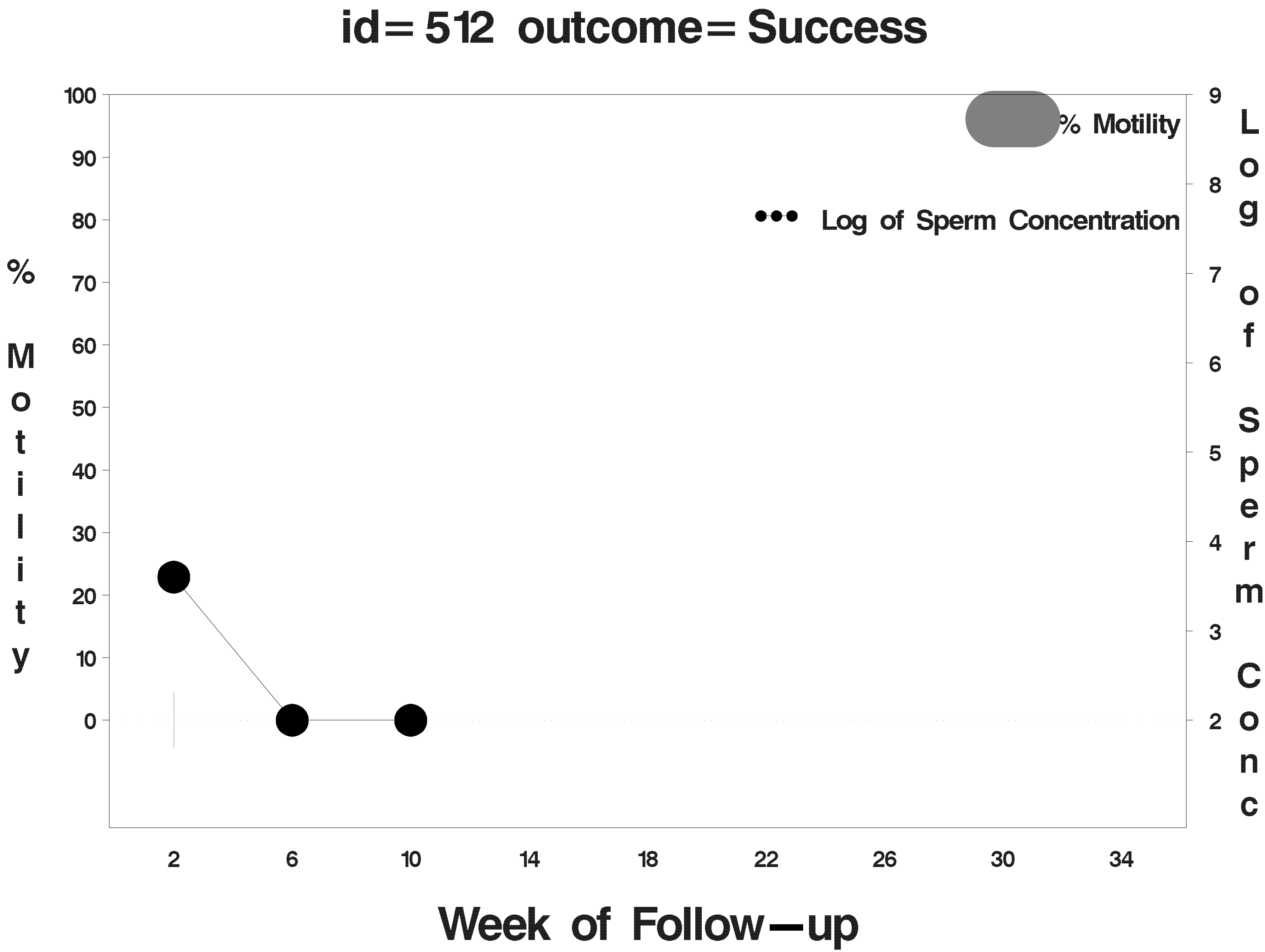

id= 512 outcome= Success
% Motility
Log of Sperm Concentration
100
90
80
70
60
50
40
30
20
10
0
9
8
7
6
5
4
3
2
2
6
10
14
18
22
26
30
34
Week of Follow−up
Log of Sperm Conc

id= 513 outcome= Failure

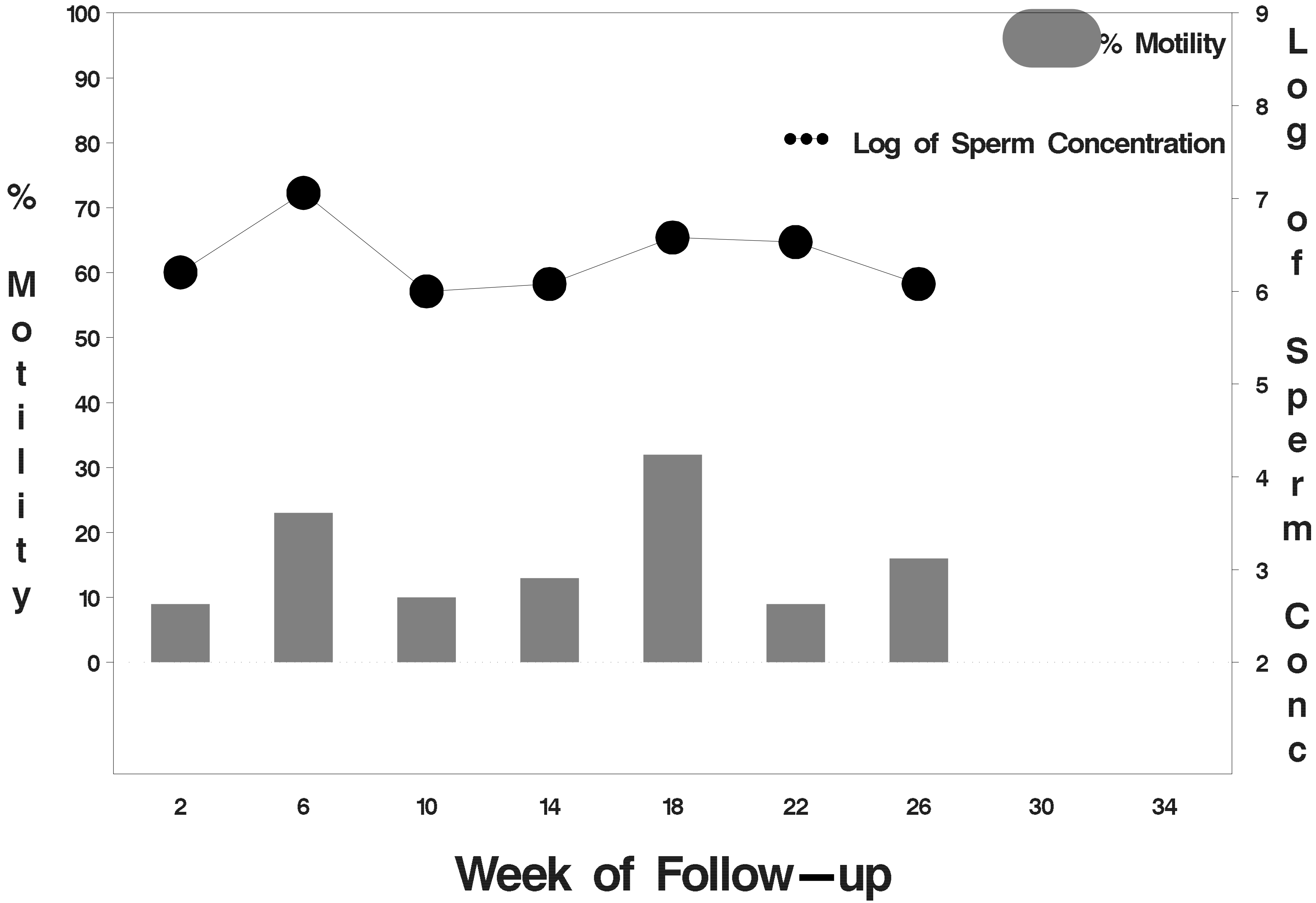

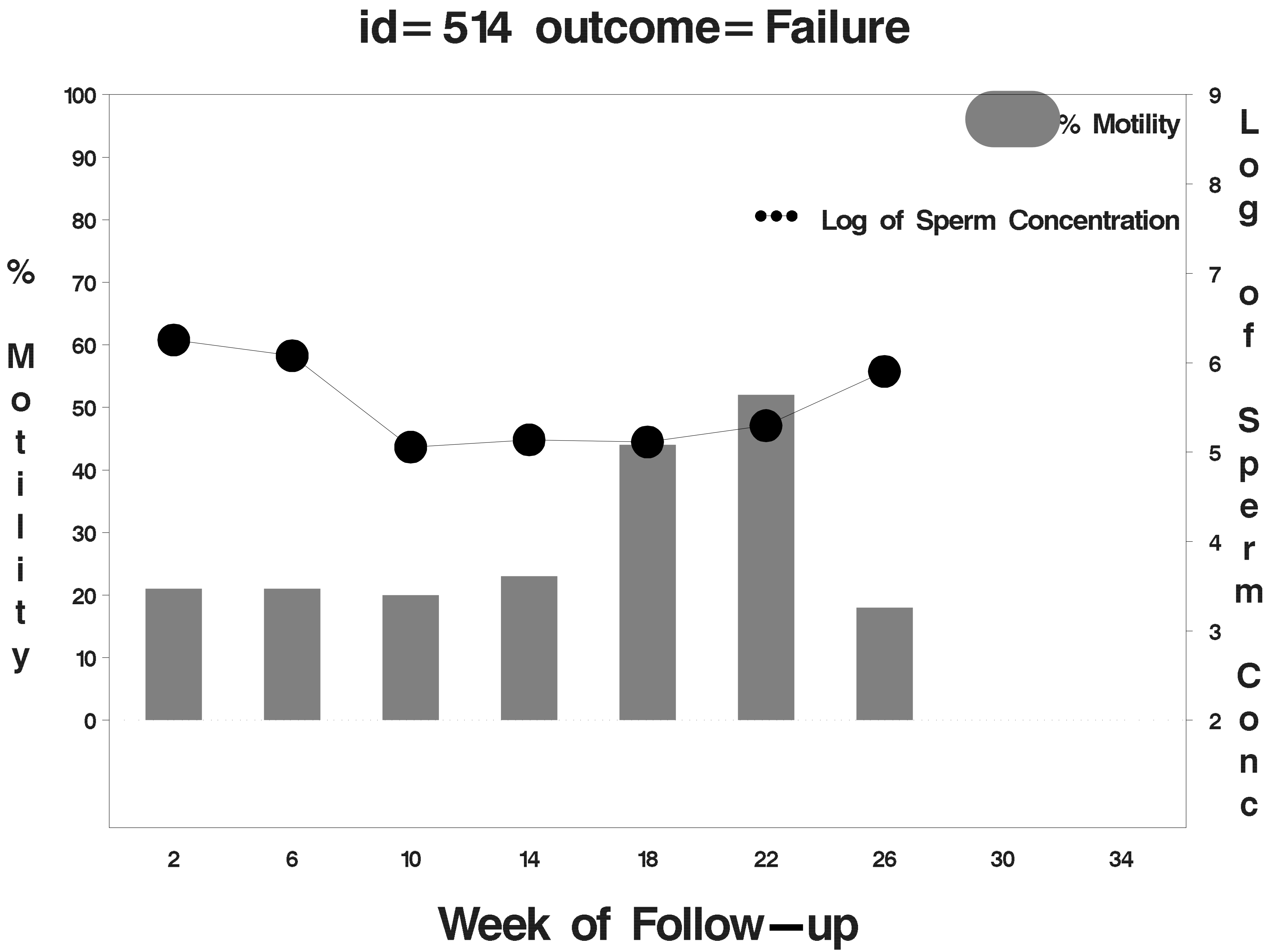

id= 514 outcome= Failure
% Motility
Log of Sperm Concentration
100
90
80
70
60
50
40
30
20
10
0
9
8
7
6
5
4
3
2
2
6
10
14
18
22
26
30
34
Week of Follow—up
% Motility
Log of Sperm Conc

id= 515 outcome= Success

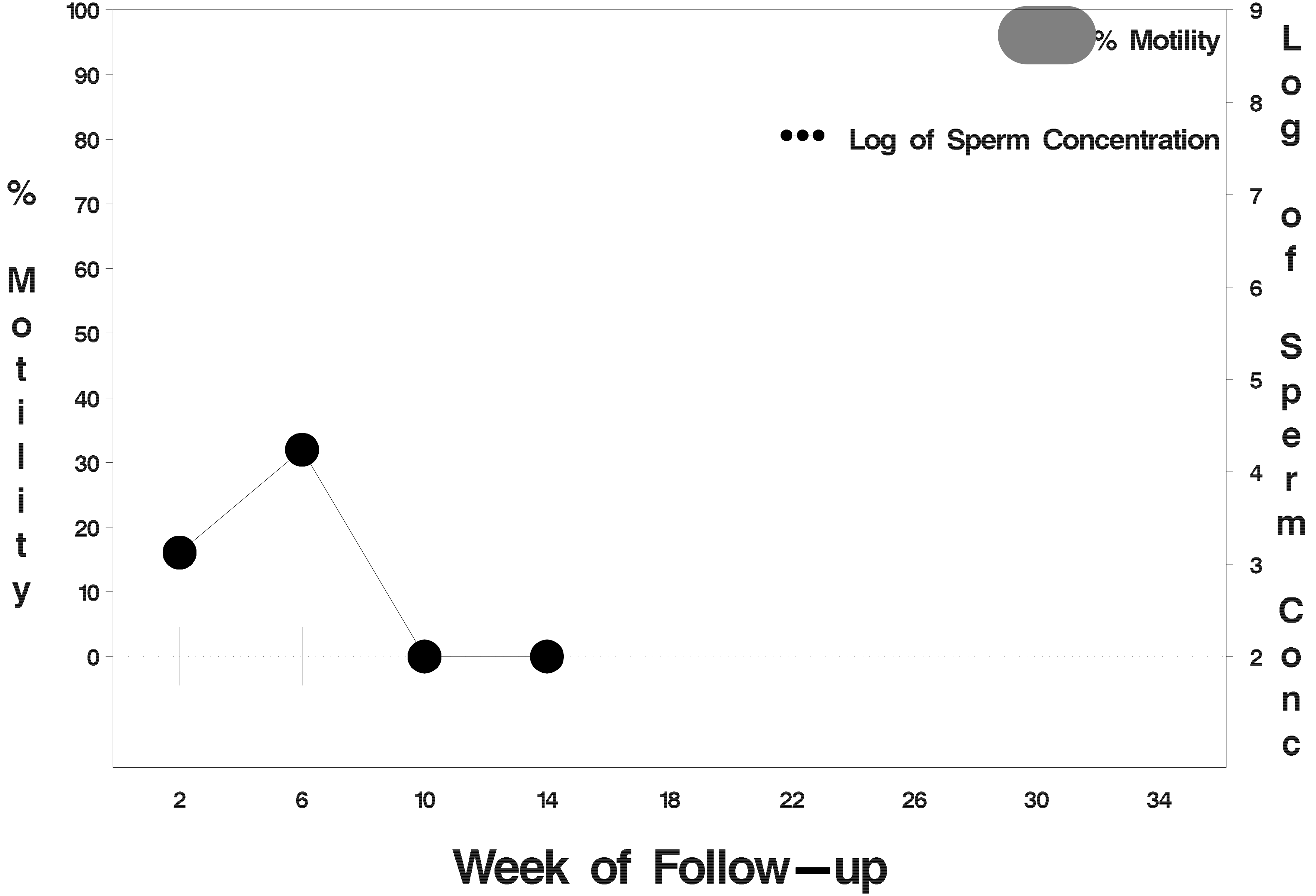

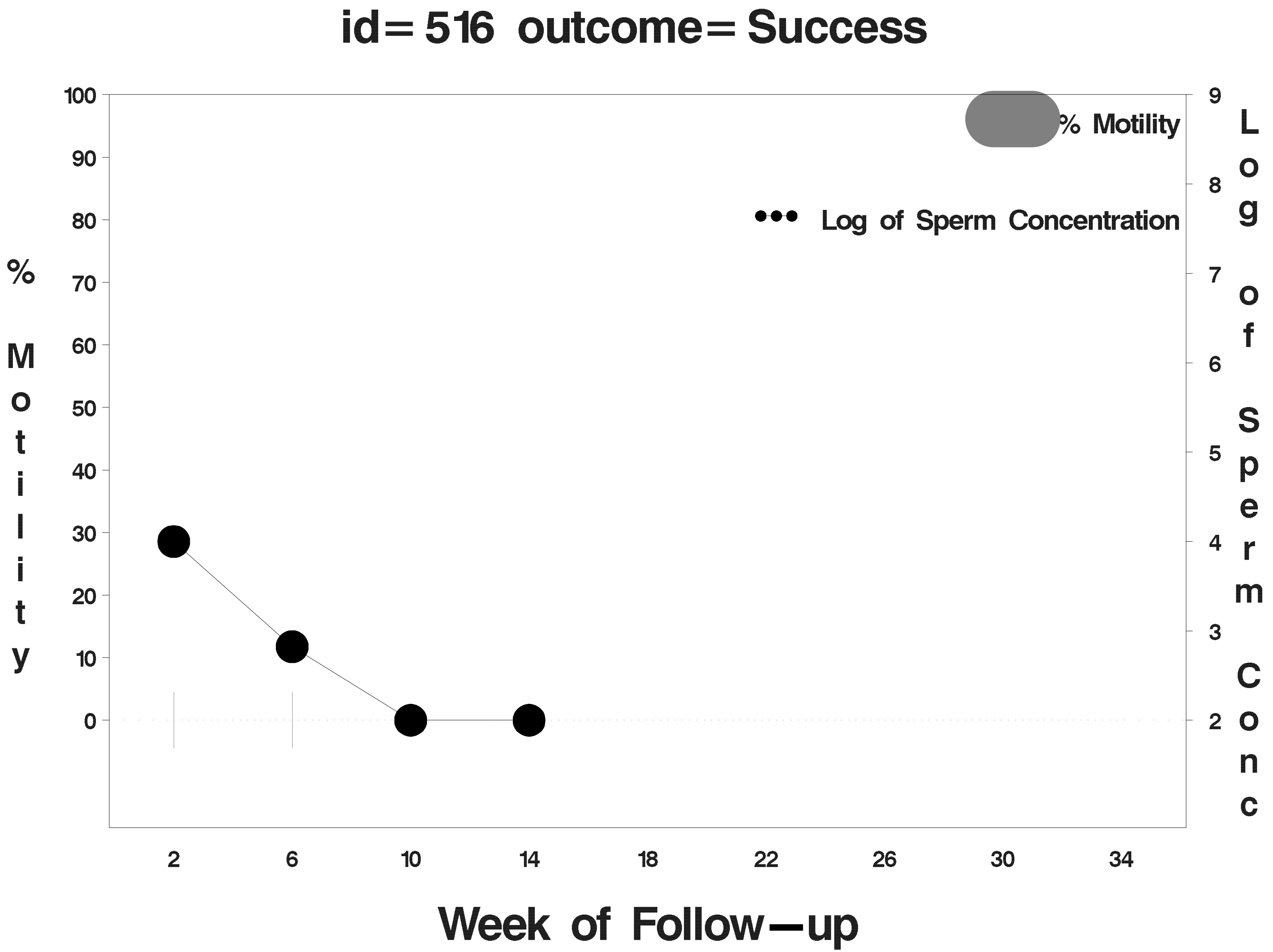
id= 516 outcome= Success
% Motility
Log of Sperm Concentration
100
90
80
70
60
50
40
30
20
10
0
9
8
7
6
5
4
3
2
2
6
10
14
18
22
26
30
34
Week of Follow—up
Log of Sperm Conc

id= 517 outcome= Success

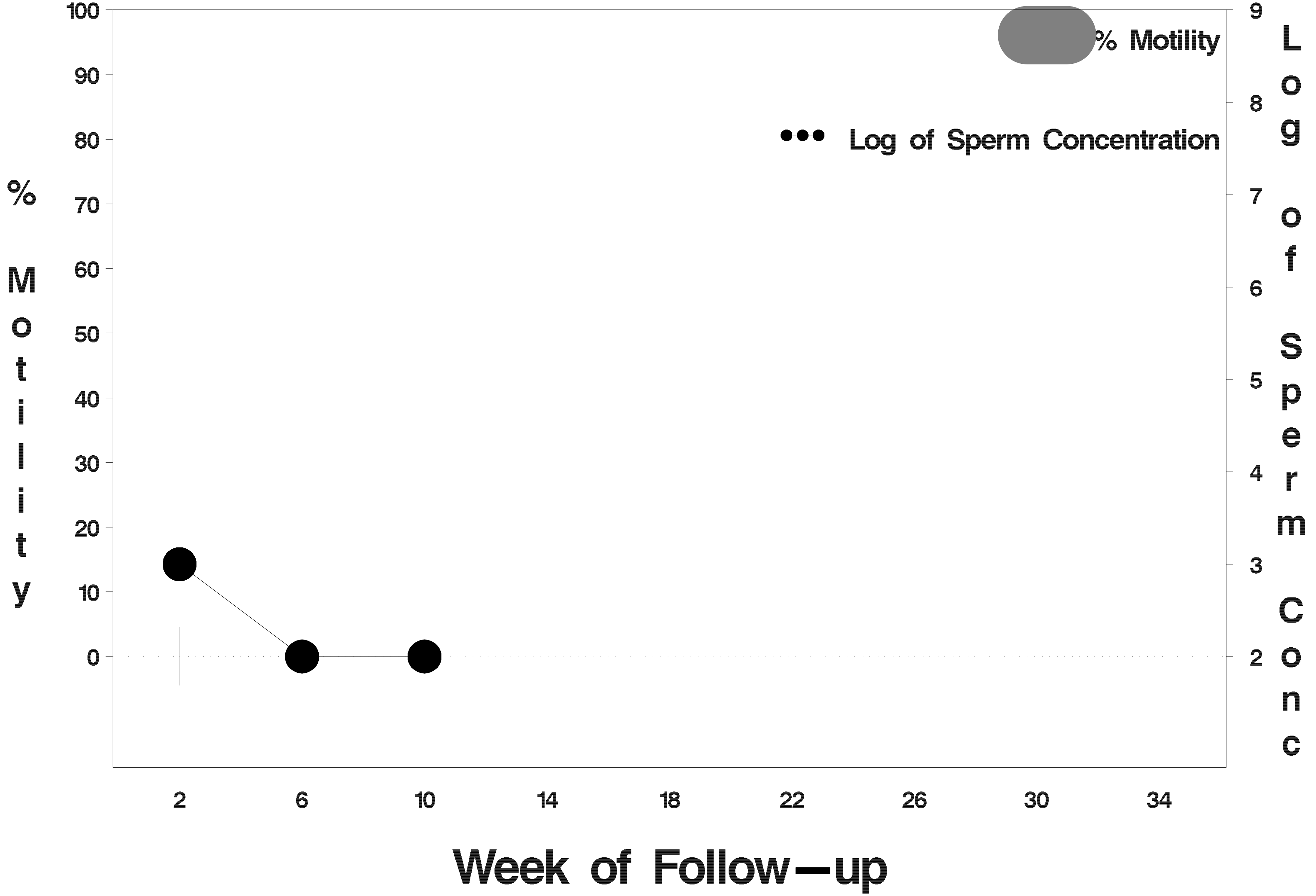

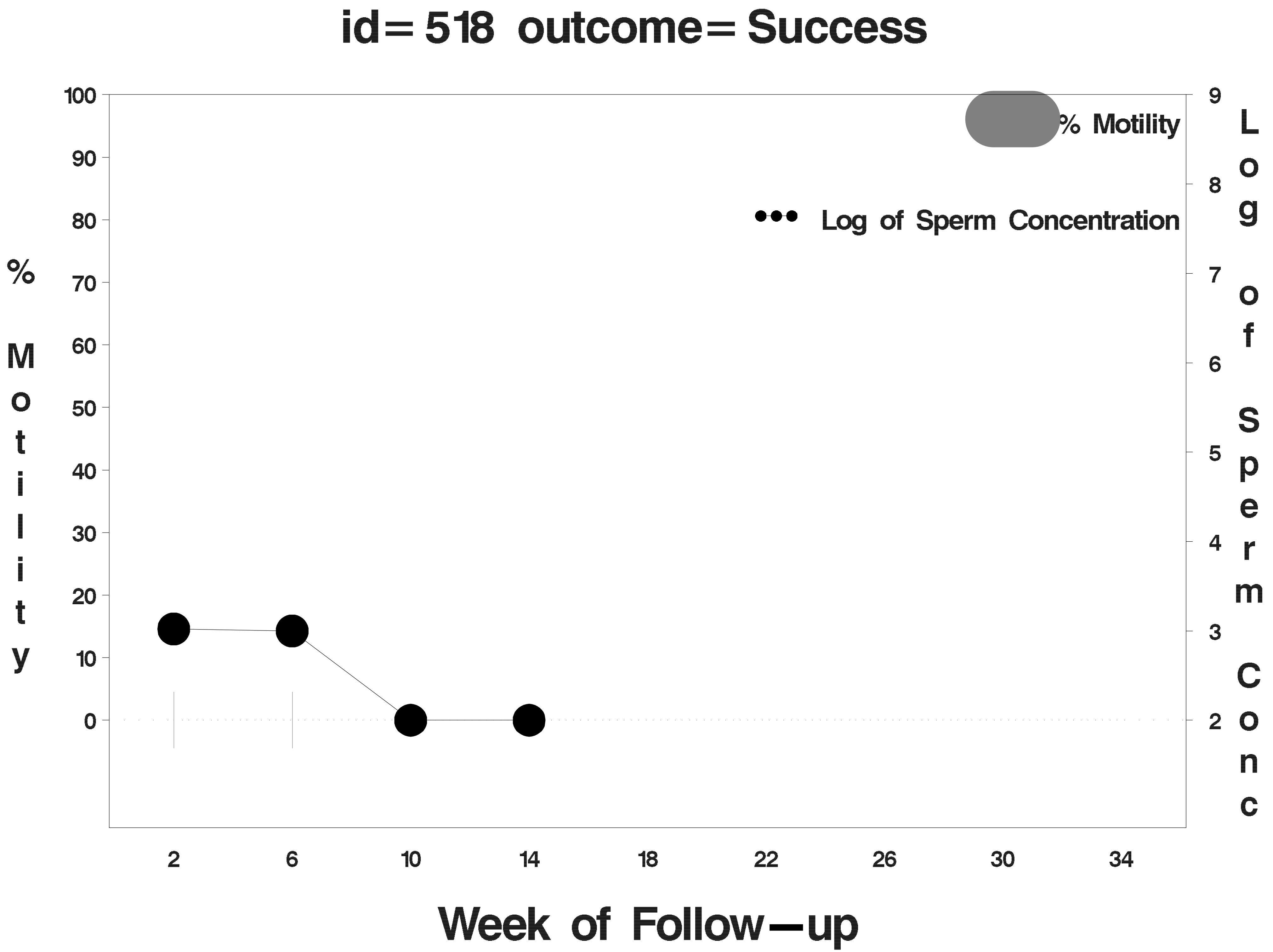

id= 518 outcome= Success
% Motility
Log of Sperm Concentration
% Motility
Log of Sperm Conc
Week of Follow-up
100
90
80
70
60
50
40
30
20
10
0
9
8
7
6
5
4
3
2
2
6
10
14
18
22
26
30
34

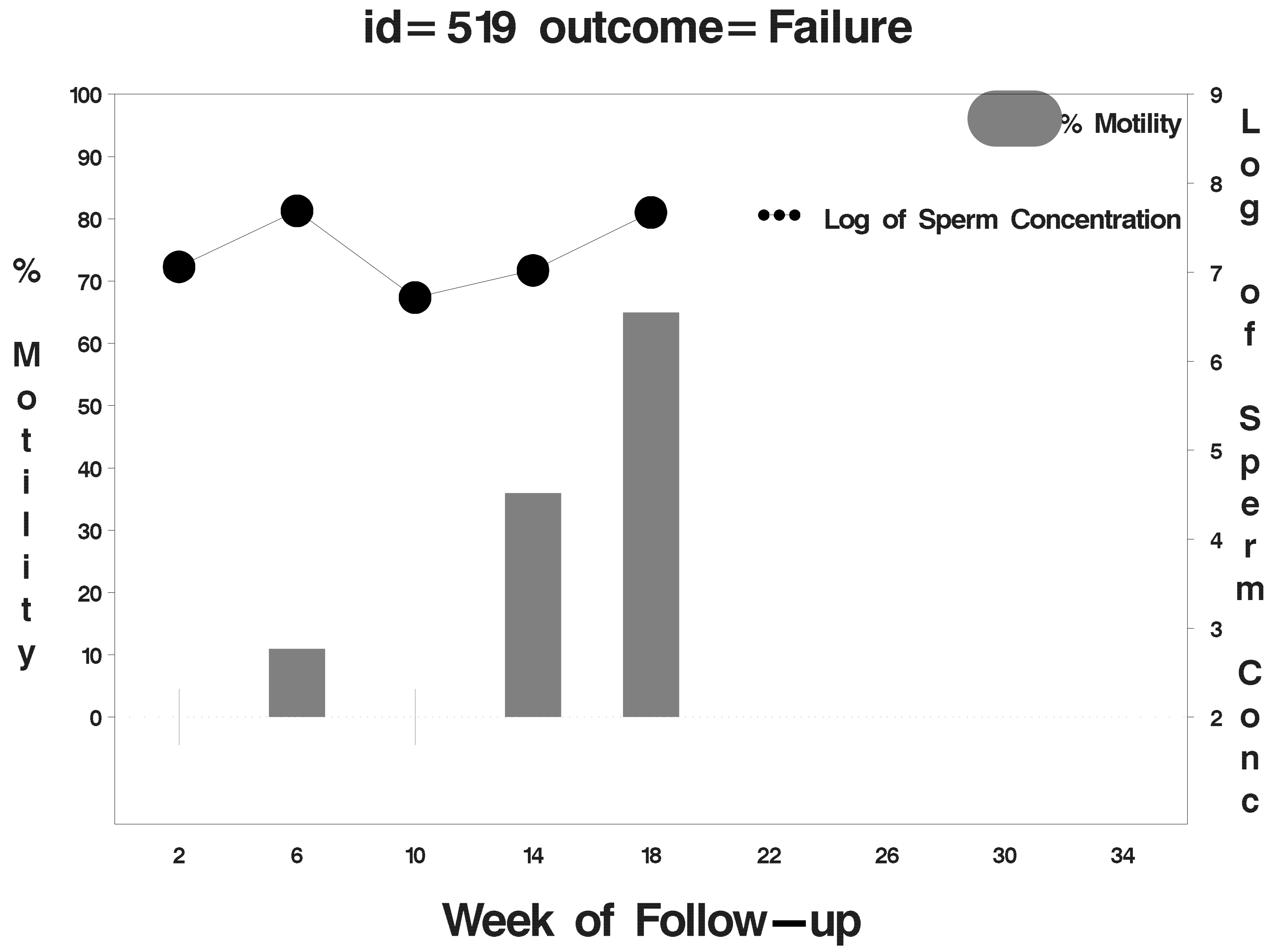
id= 519 outcome= Failure
% Motility
Log of Sperm Concentration
100
90
80
70
60
50
40
30
20
10
0
9
8
7
6
5
4
3
2
2
6
10
14
18
22
26
30
34
Week of Follow−up
% Motility
Log of Sperm Conc

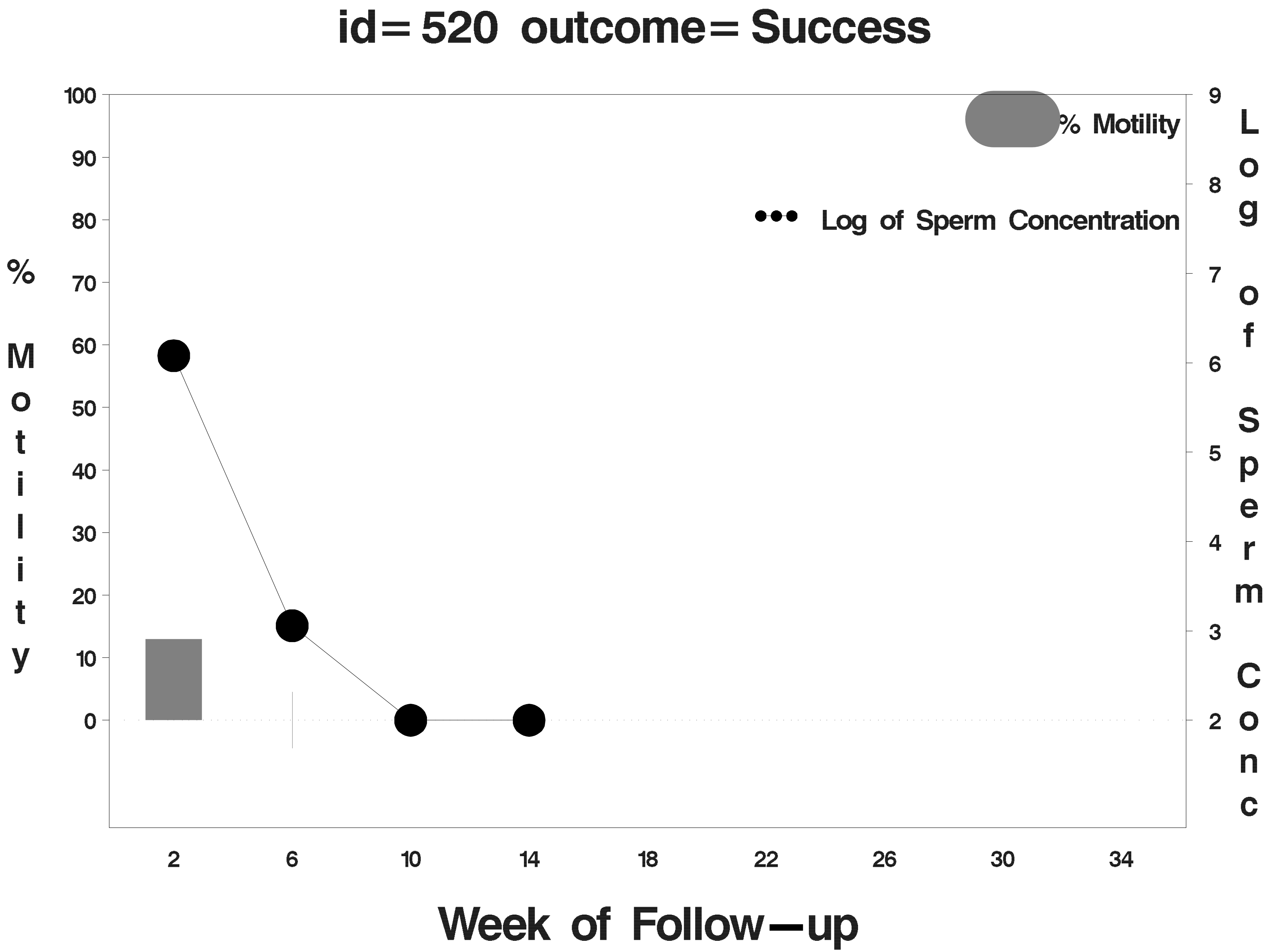

id= 520 outcome= Success
% Motility
Log of Sperm Concentration
100
90
80
70
60
50
40
30
20
10
0
9
8
7
6
5
4
3
2
2
6
10
14
18
22
26
30
34
Week of Follow-up
Log of Sperm Conc

id= 521 outcome= Success

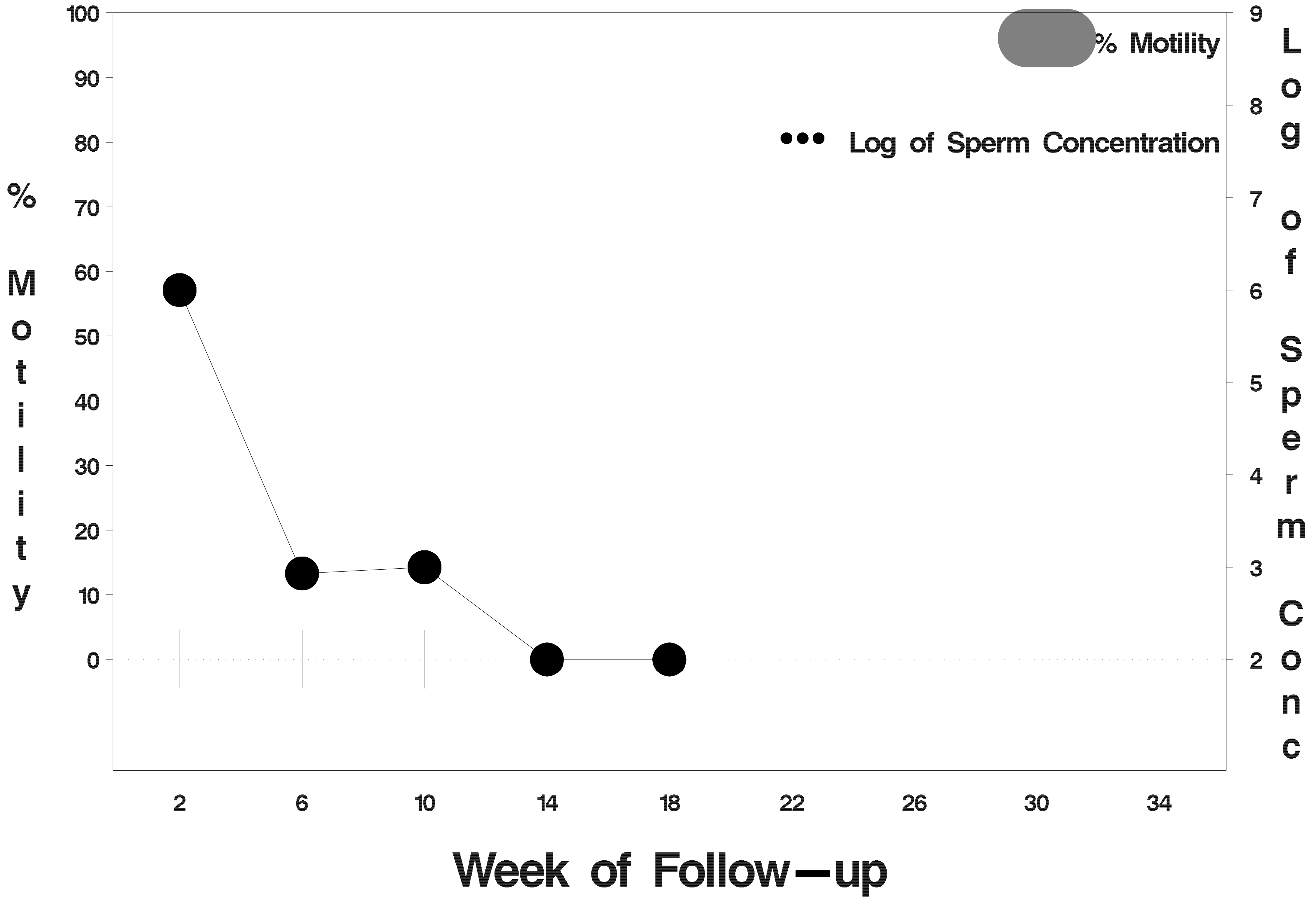

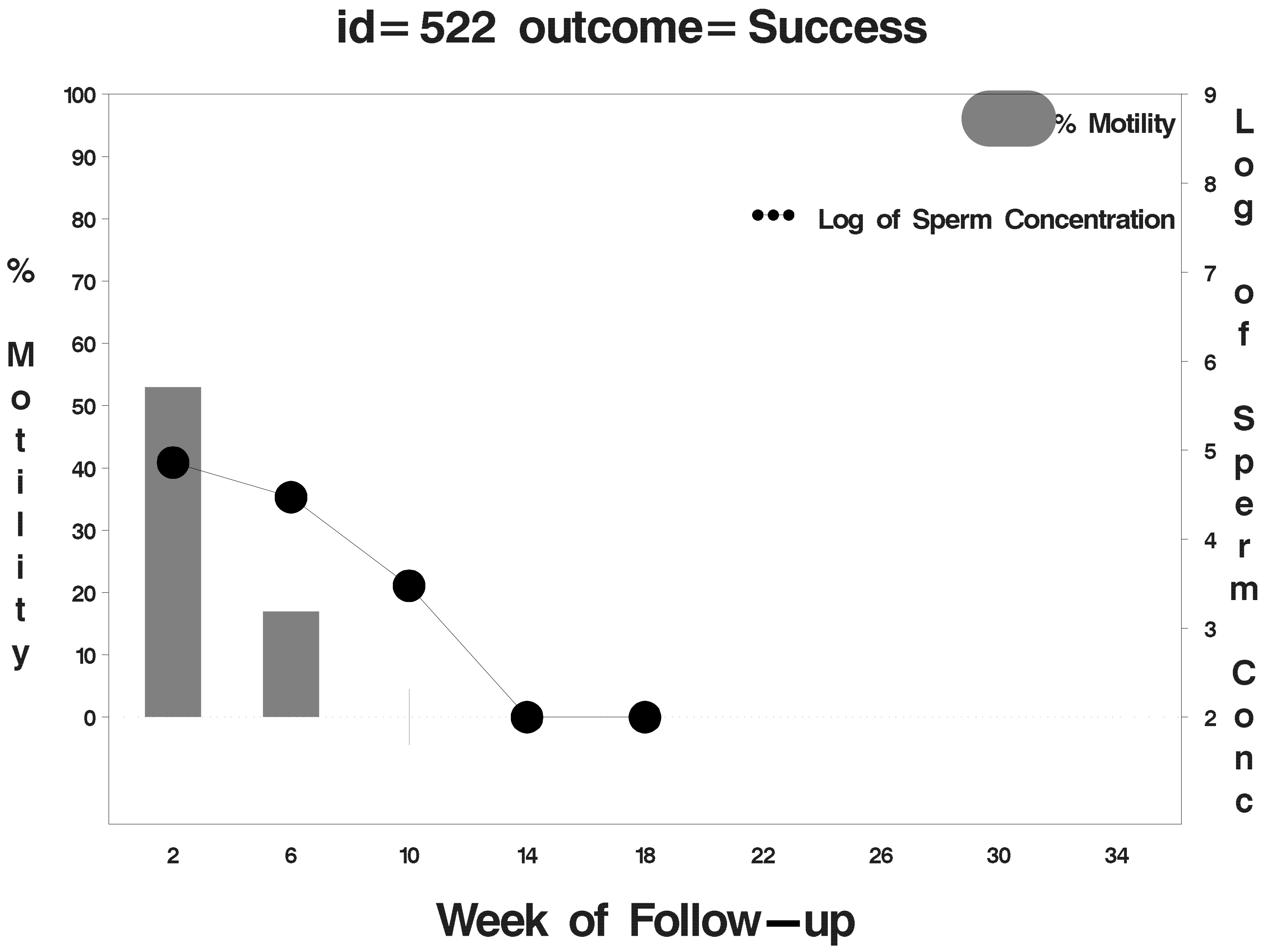

id= 522 outcome= Success
% Motility
Log of Sperm Concentration
100
90
80
70
60
50
40
30
20
10
0
9
8
7
6
5
4
3
2
2
6
10
14
18
22
26
30
34
% Motility
Log of Sperm Conc
Week of Follow−up

id= 523 outcome= Success

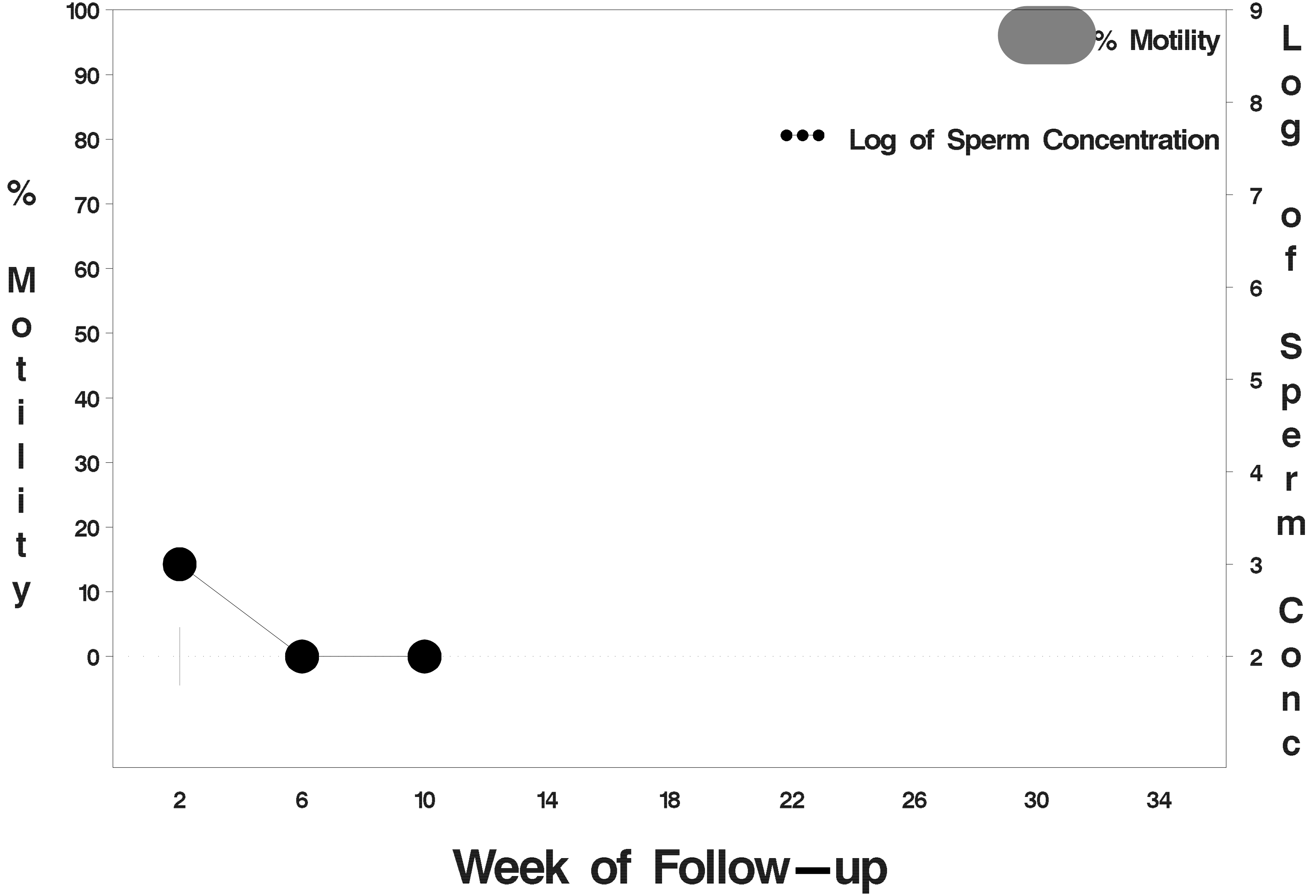

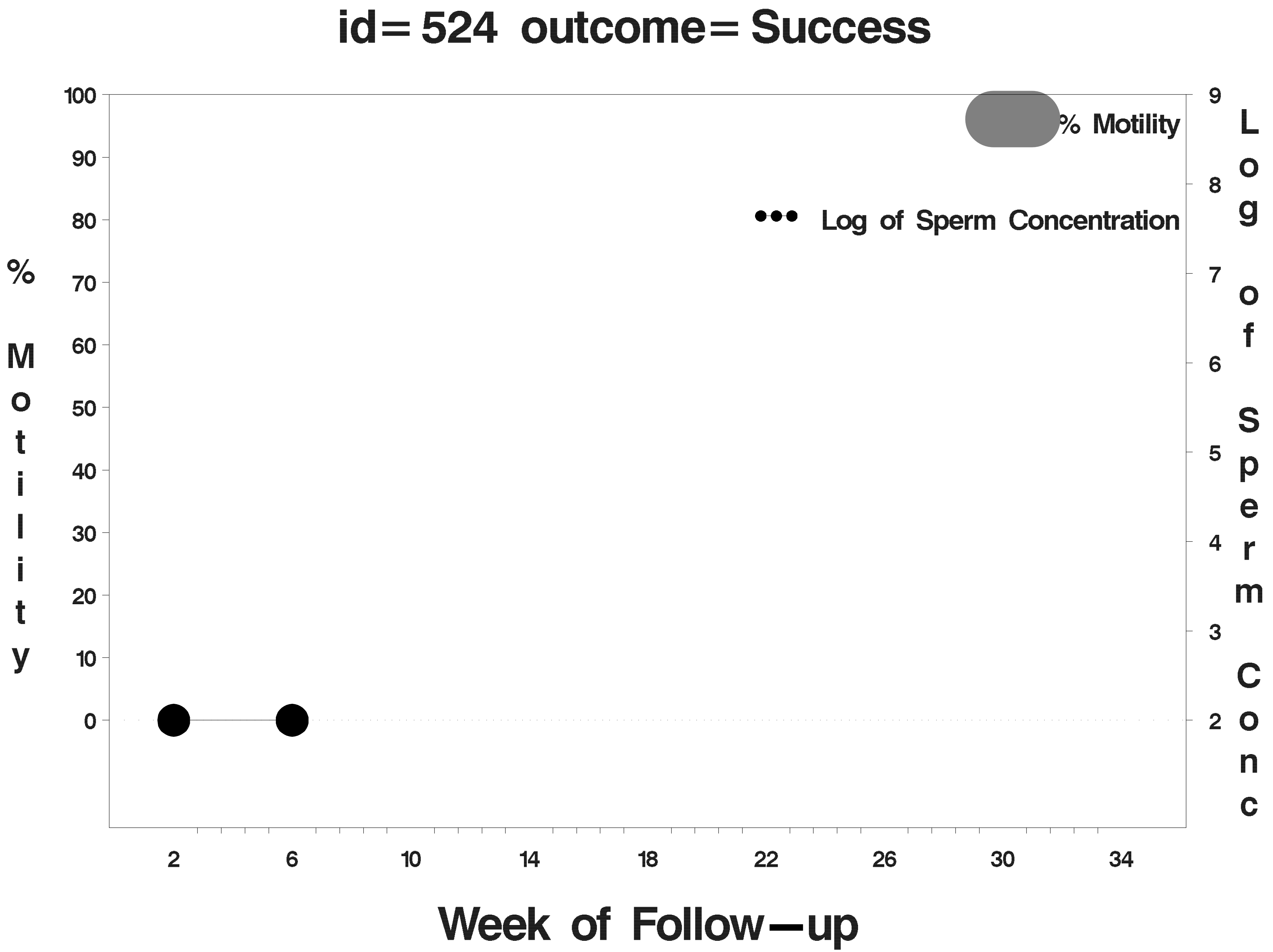
id= 524 outcome= Success
% Motility
Log of Sperm Concentration
100
90
80
70
60
50
40
30
20
10
0
9
8
7
6
5
4
3
2
2
6
10
14
18
22
26
30
34
Week of Follow-up
Log of Sperm Conc

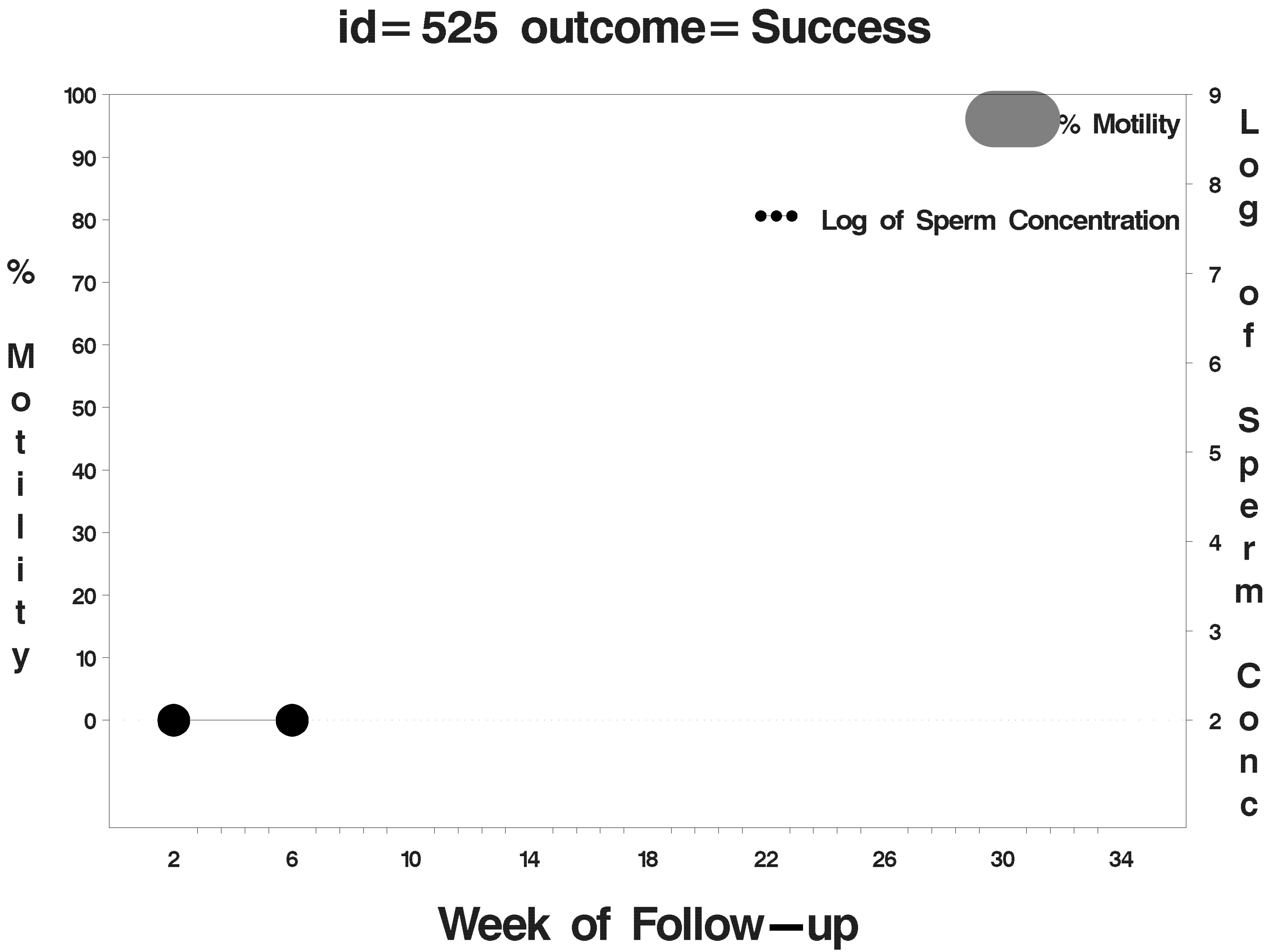

id= 525 outcome= Success
% Motility
Log of Sperm Concentration
% Motility
Log of Sperm Conc
Week of Follow-up
100
90
80
70
60
50
40
30
20
10
0
9
8
7
6
5
4
3
2
2
6
10
14
18
22
26
30
34

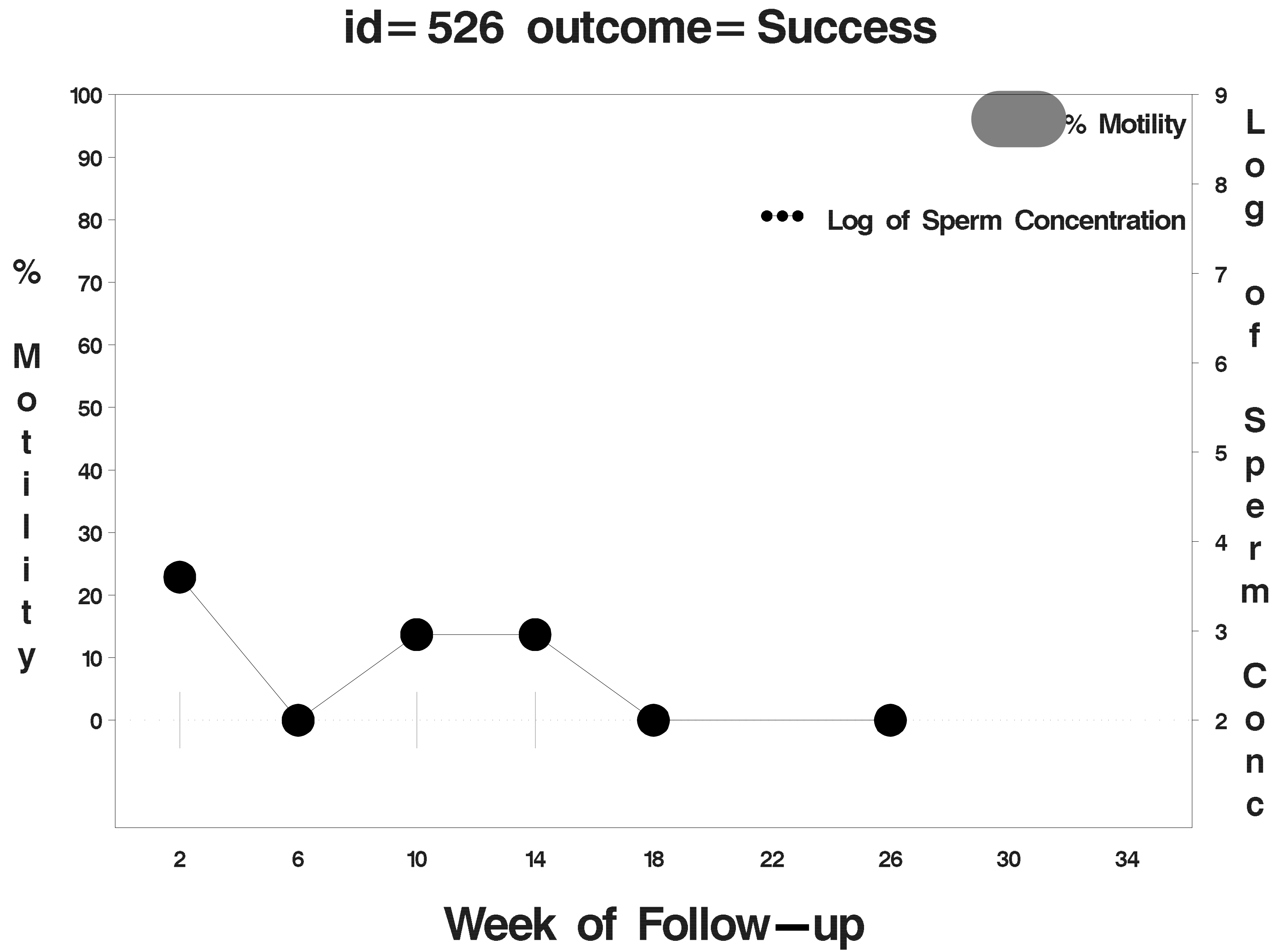

id= 526 outcome= Success
% Motility
Log of Sperm Concentration
100
90
80
70
60
50
40
30
20
10
0
9
8
7
6
5
4
3
2
2
6
10
14
18
22
26
30
34
Week of Follow—up

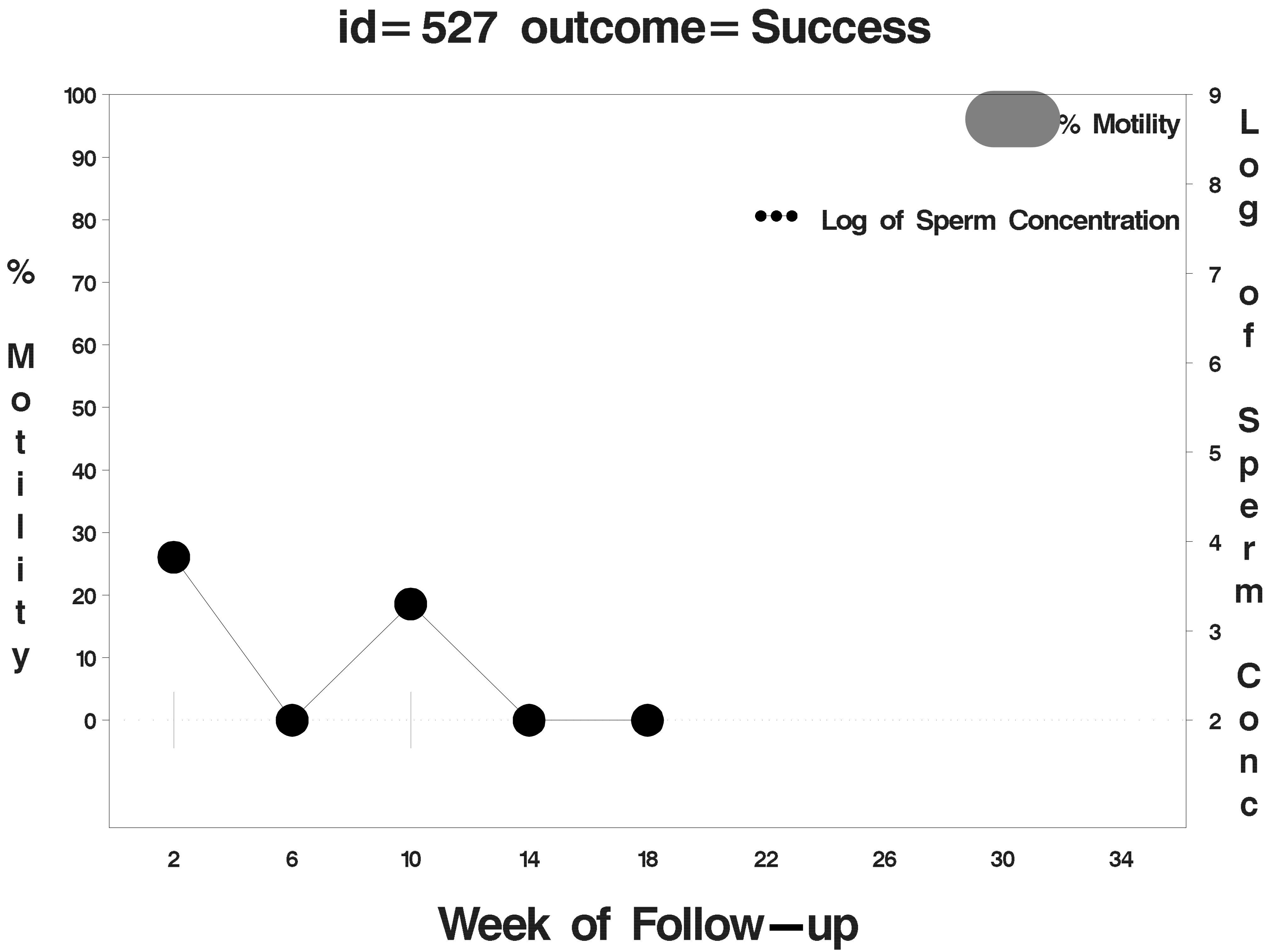

id= 527 outcome= Success
% Motility
Log of Sperm Concentration
100
90
80
70
60
50
40
30
20
10
0
9
8
7
6
5
4
3
2
2
6
10
14
18
22
26
30
34
Week of Follow−up
Log of Sperm Conc

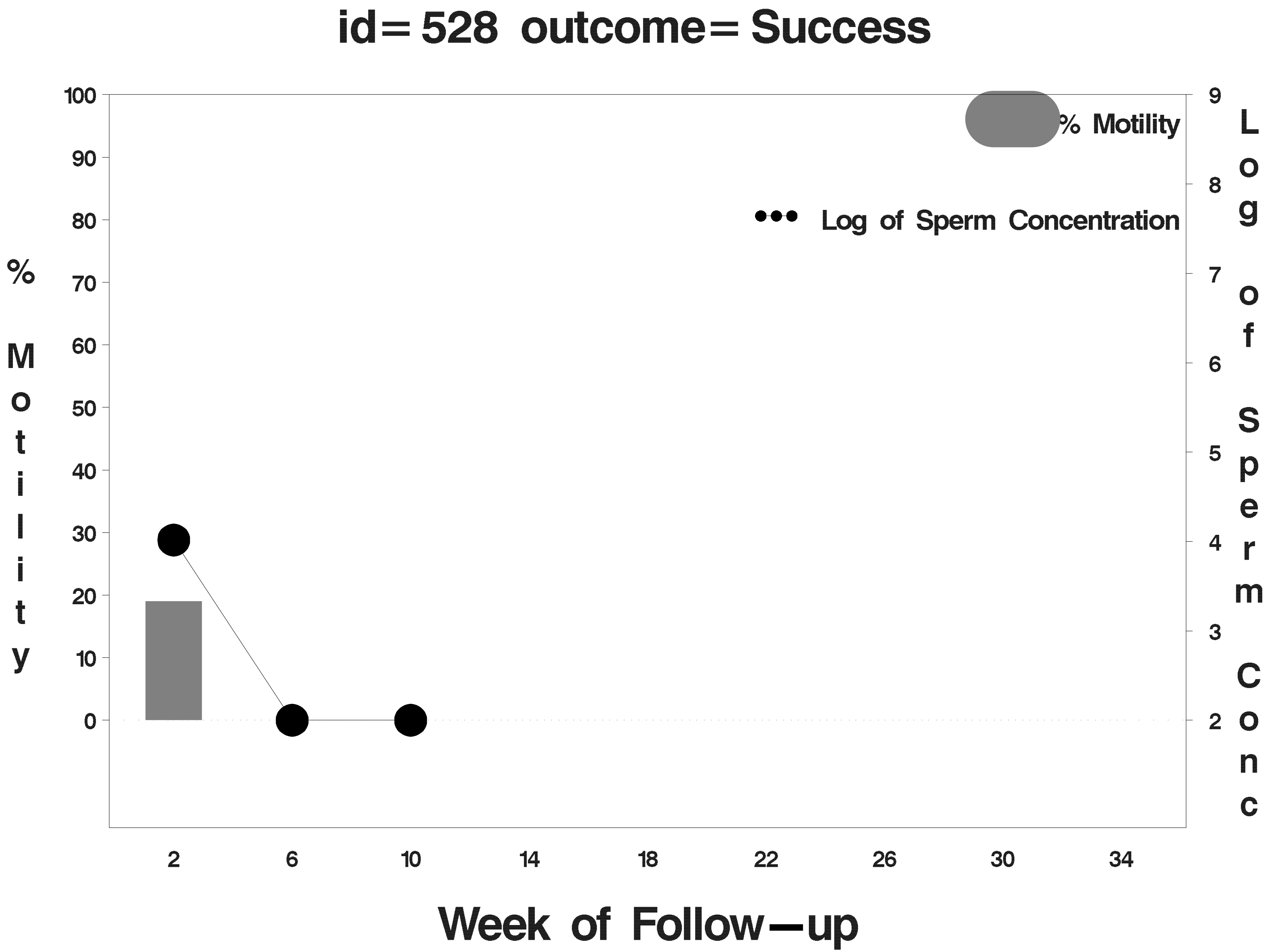

id= 528 outcome= Success
% Motility
Log of Sperm Concentration
% Motility
Log of Sperm Conc
Week of Follow—up
100
90
80
70
60
50
40
30
20
10
0
9
8
7
6
5
4
3
2
2
6
10
14
18
22
26
30
34

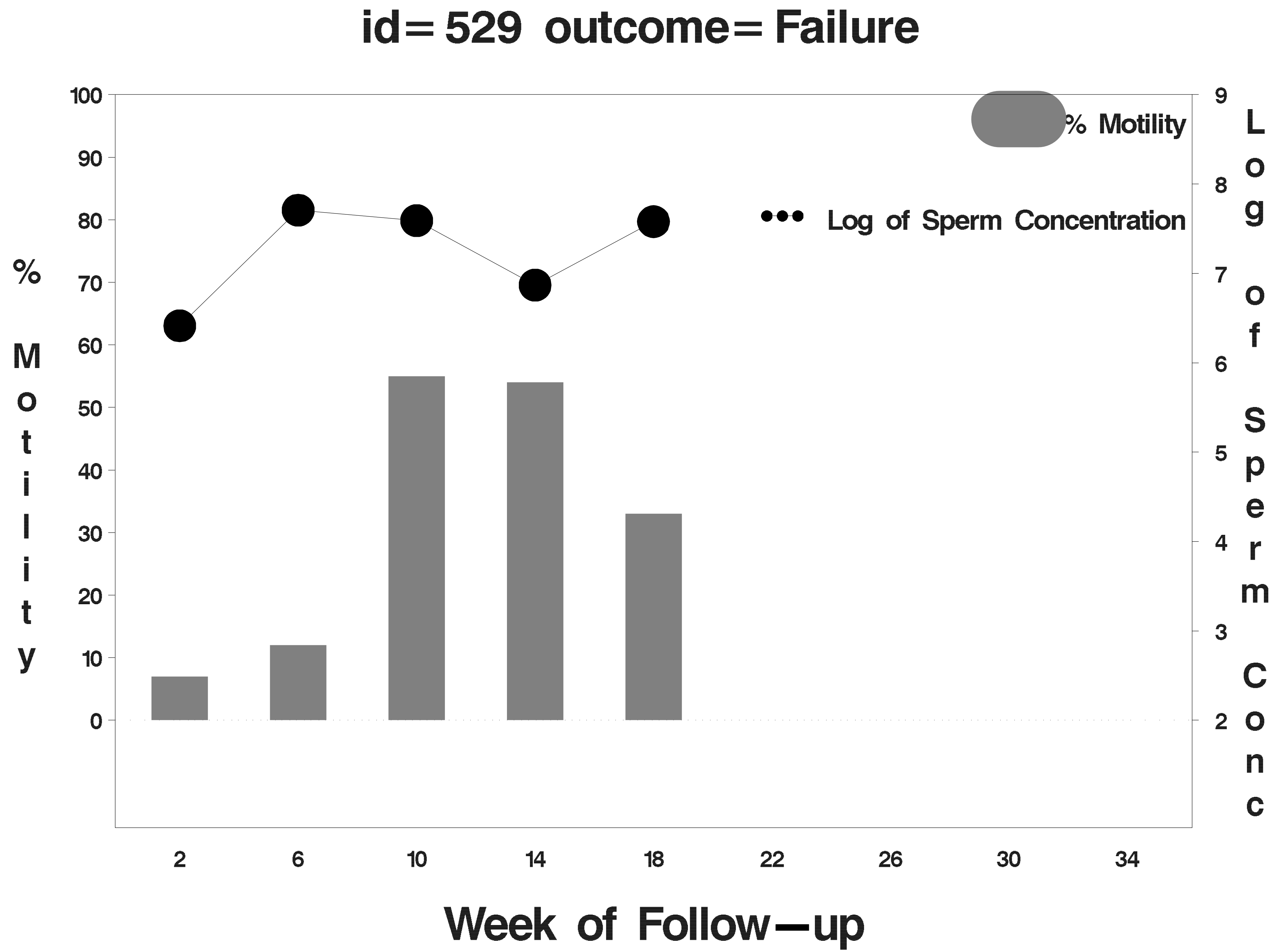
id= 529 outcome= Failure
% Motility
Log of Sperm Concentration
% Motility
Log of Sperm Conc
Week of Follow-up
100
90
80
70
60
50
40
30
20
10
0
9
8
7
6
5
4
3
2
2
6
10
14
18
22
26
30
34

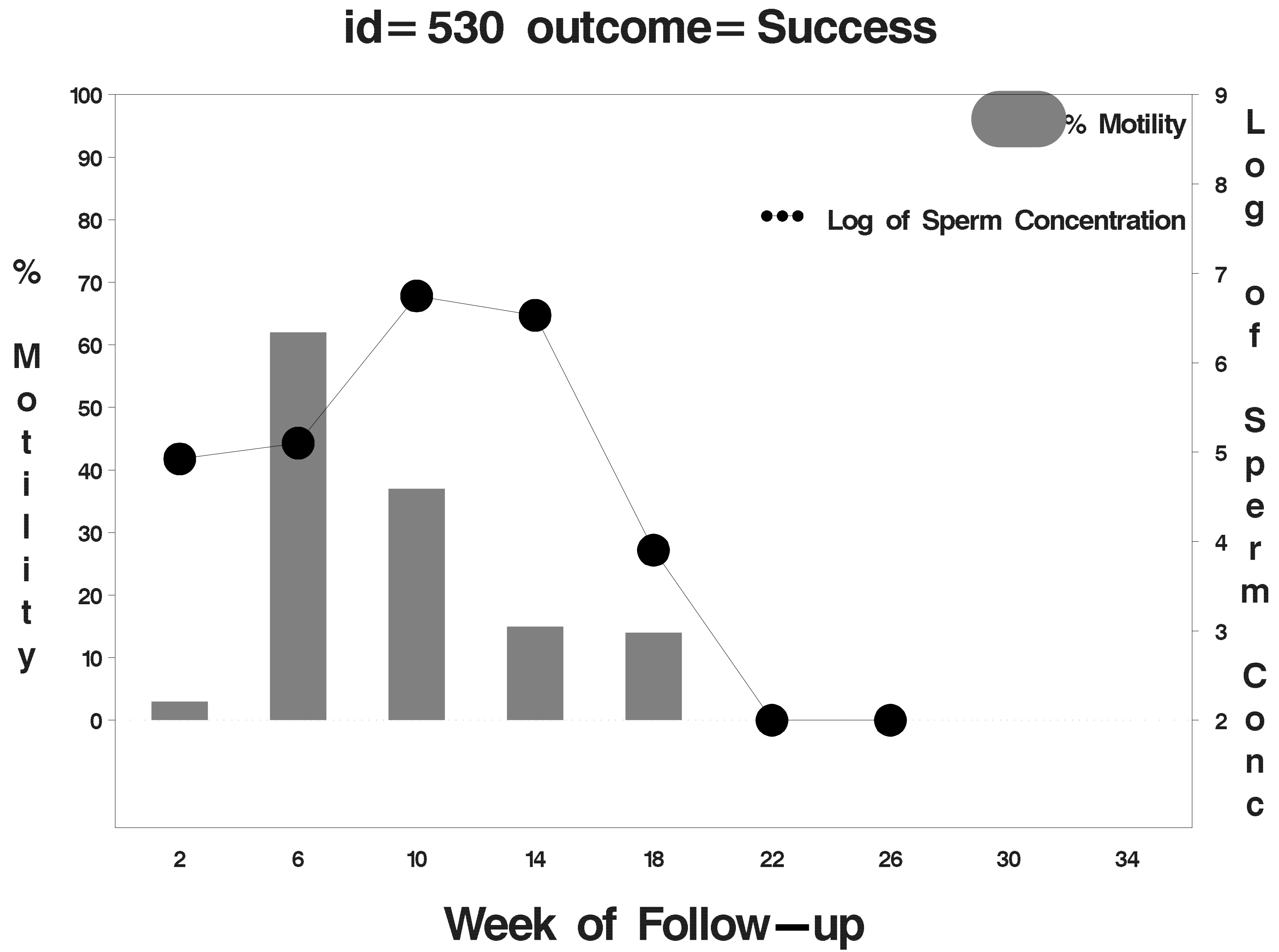
id= 530 outcome= Success
% Motility
Log of Sperm Concentration
100
90
80
70
60
50
40
30
20
10
0
9
8
7
6
5
4
3
2
Log of Sperm Conc
2
6
10
14
18
22
26
30
34
Week of Follow-up

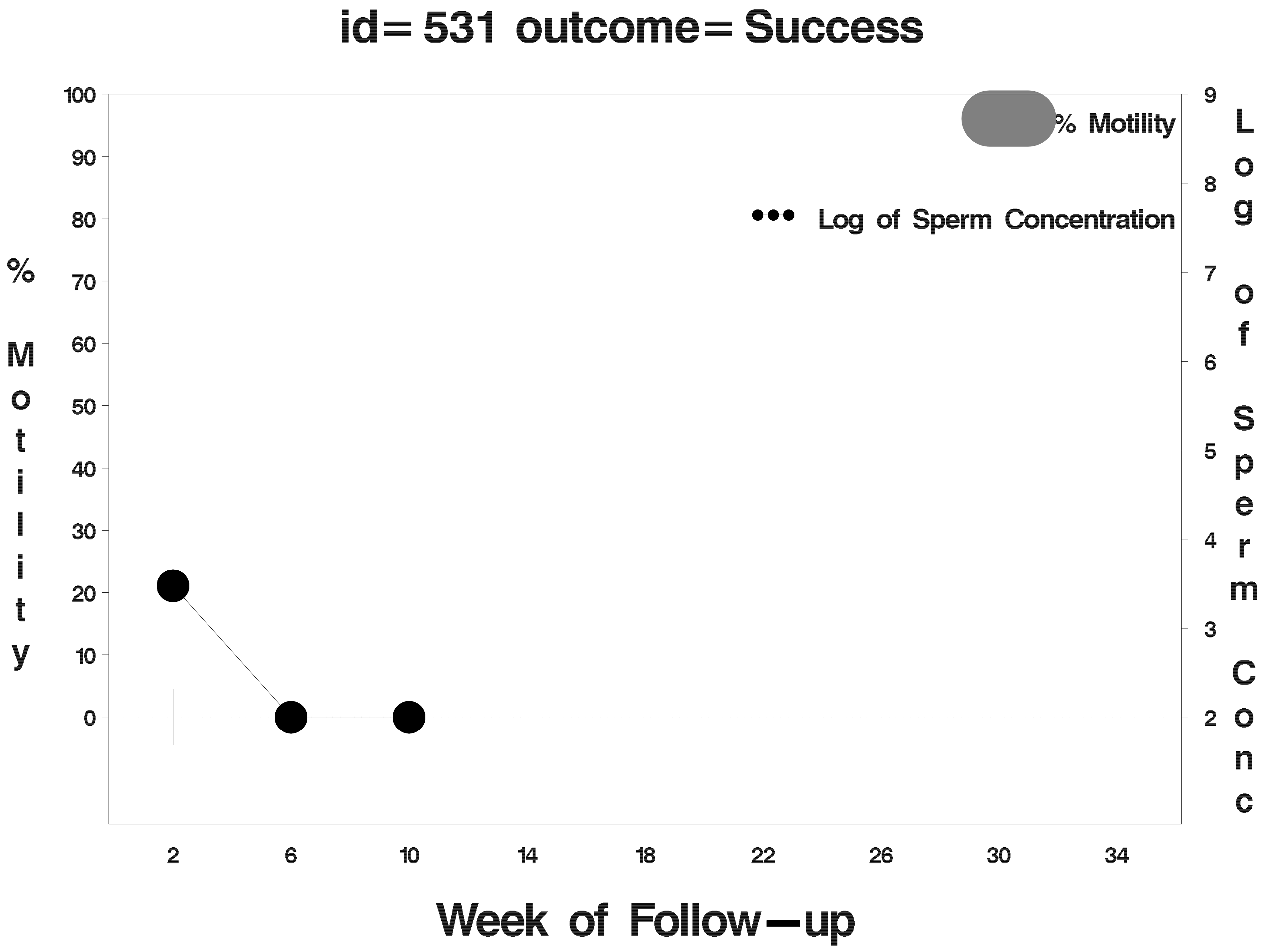

id= 531 outcome= Success
% Motility
Log of Sperm Concentration
% Motility
Log of Sperm Conc
Week of Follow—up
100
90
80
70
60
50
40
30
20
10
0
9
8
7
6
5
4
3
2
2
6
10
14
18
22
26
30
34

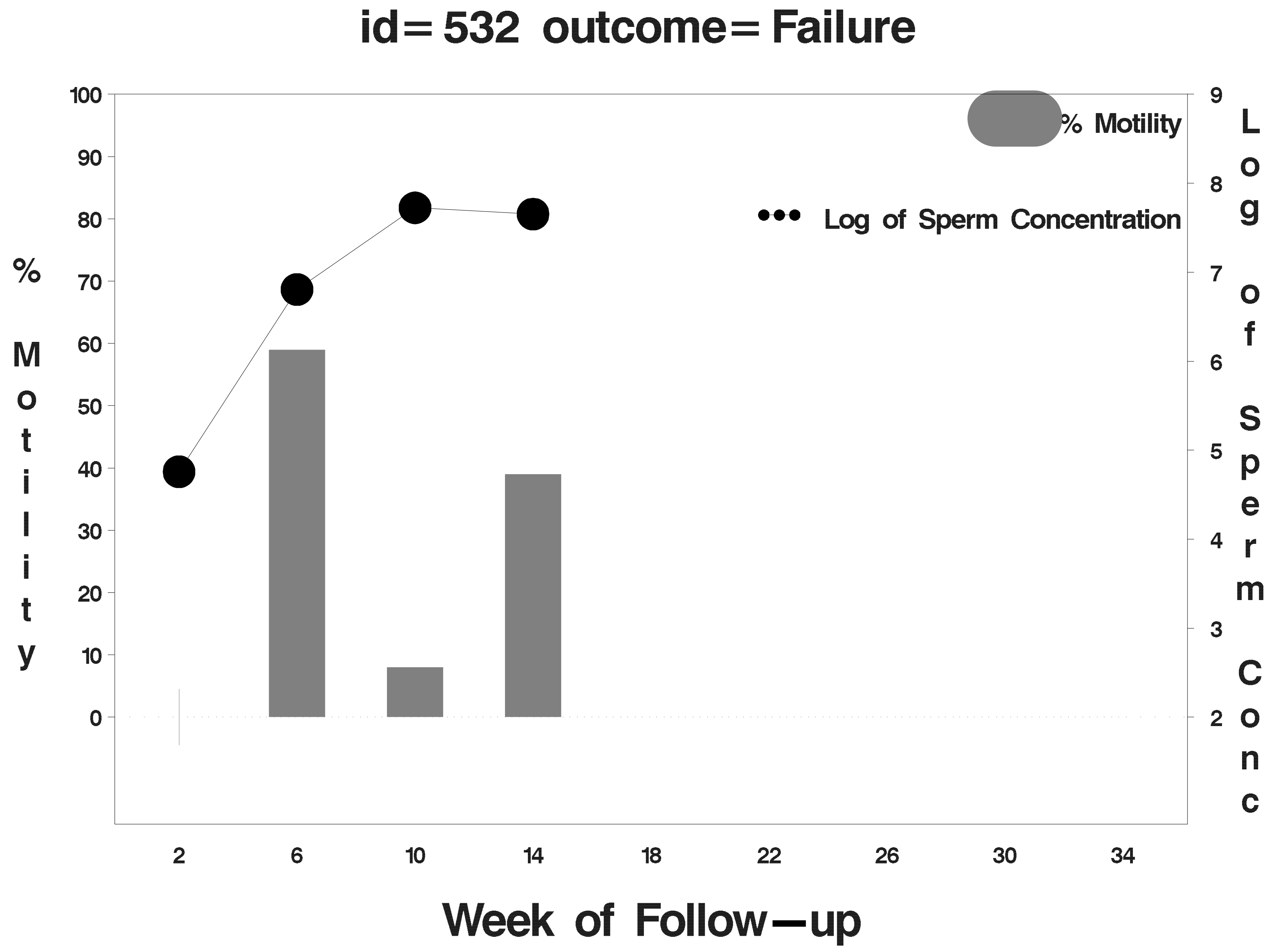
id= 532 outcome= Failure
% Motility
Log of Sperm Concentration
100
90
80
70
60
50
40
30
20
10
0
9
8
7
6
5
4
3
2
% Motility
Log of Sperm Conc
2
6
10
14
18
22
26
30
34
Week of Follow−up

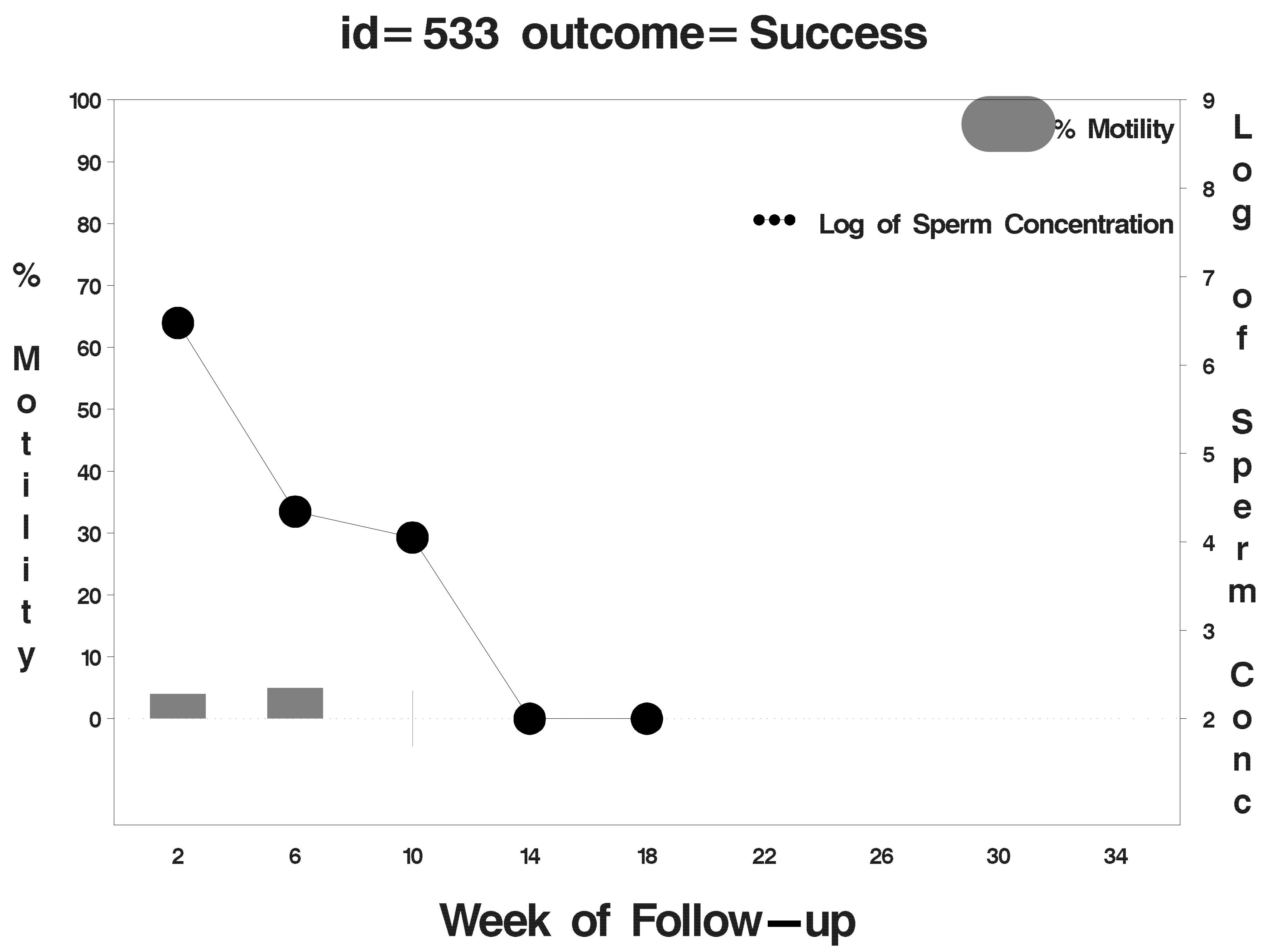

id= 533 outcome= Success
% Motility
Log of Sperm Concentration
% Motility
Log of Sperm Conc
Week of Follow—up
100
90
80
70
60
50
40
30
20
10
0
9
8
7
6
5
4
3
2
2
6
10
14
18
22
26
30
34

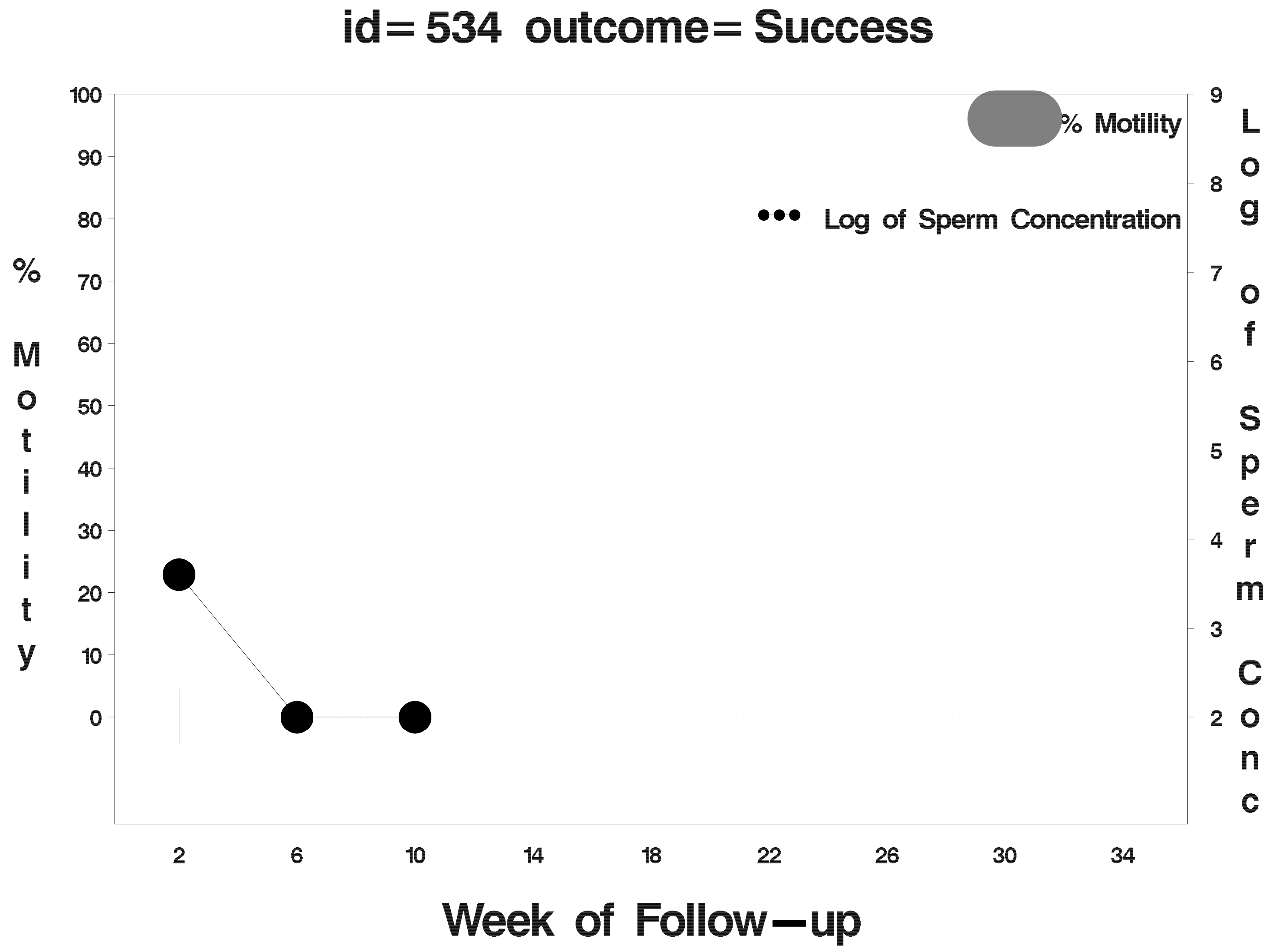
id= 534 outcome= Success
% Motility
Log of Sperm Concentration
% Motility
Log of Sperm Conc
Week of Follow—up
100
90
80
70
60
50
40
30
20
10
0
9
8
7
6
5
4
3
2
2
6
10
14
18
22
26
30
34

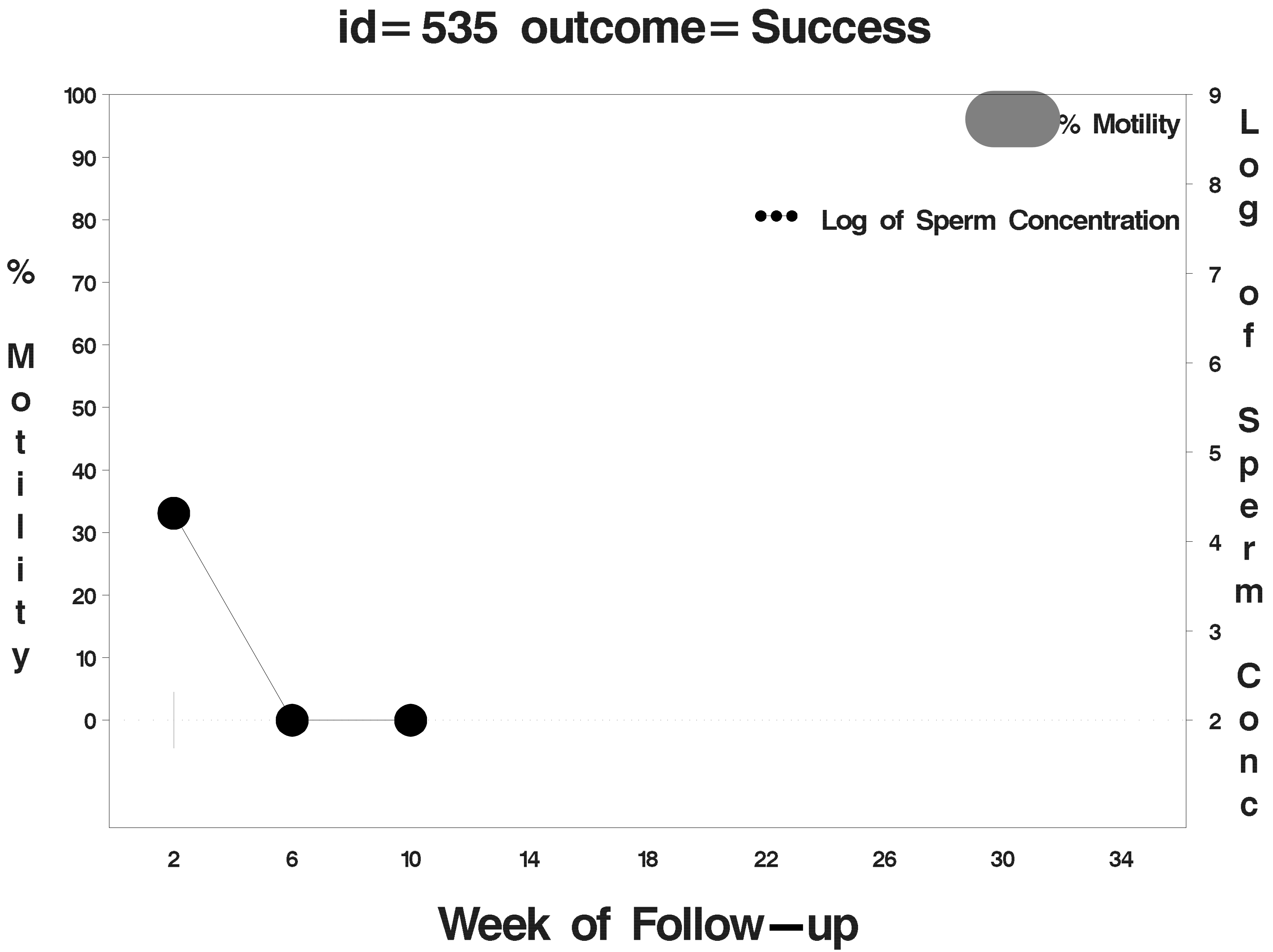
id= 535 outcome= Success
% Motility
Log of Sperm Concentration
100
90
80
70
60
50
40
30
20
10
0
9
8
7
6
5
4
3
2
2
6
10
14
18
22
26
30
34
% Motility
Log of Sperm Conc
Week of Follow−up

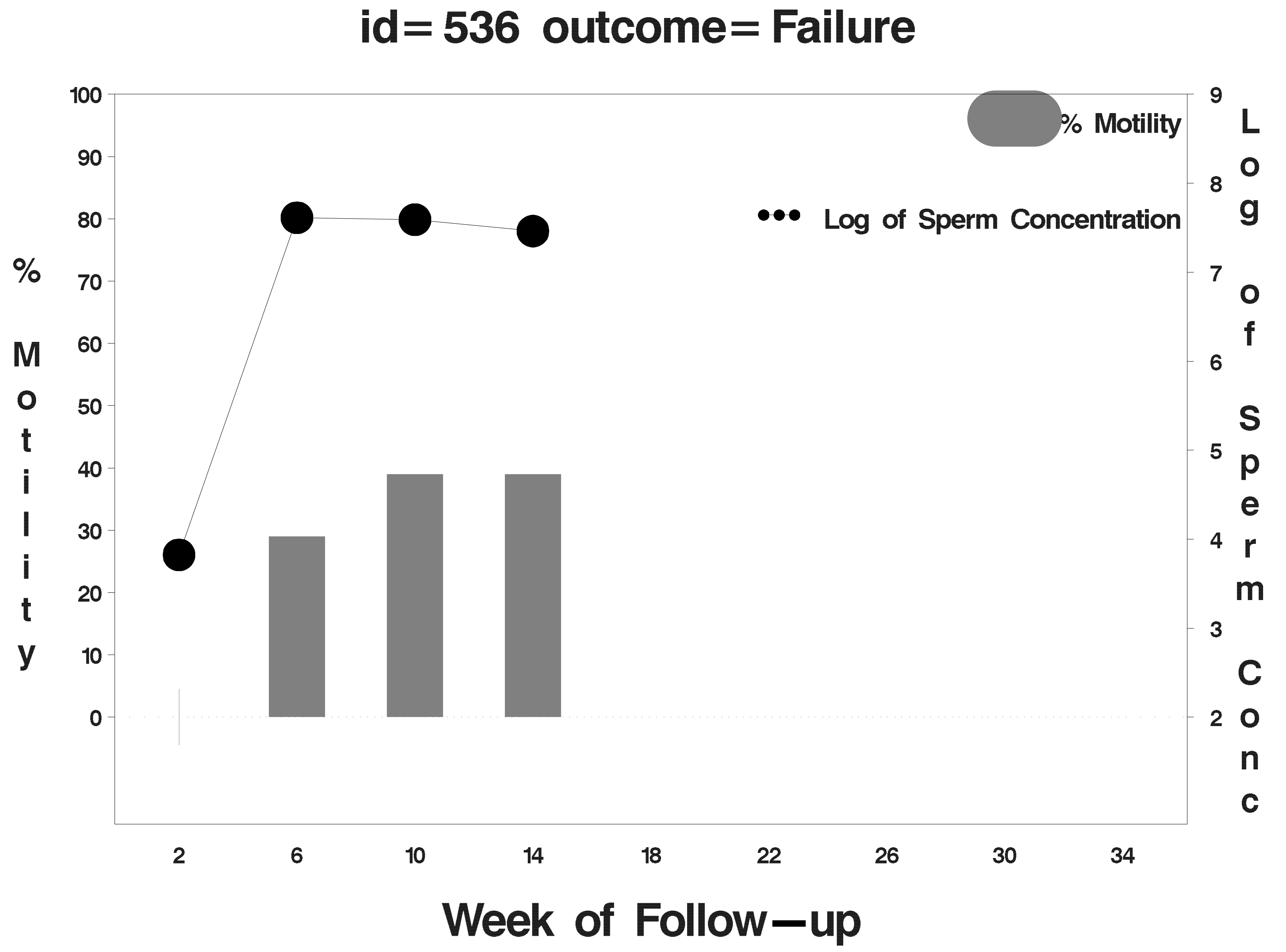
id= 536 outcome= Failure
% Motility
Log of Sperm Concentration
% Motility
Log of Sperm Conc
Week of Follow-up
100
90
80
70
60
50
40
30
20
10
0
9
8
7
6
5
4
3
2
2
6
10
14
18
22
26
30
34

id= 537 outcome= Success

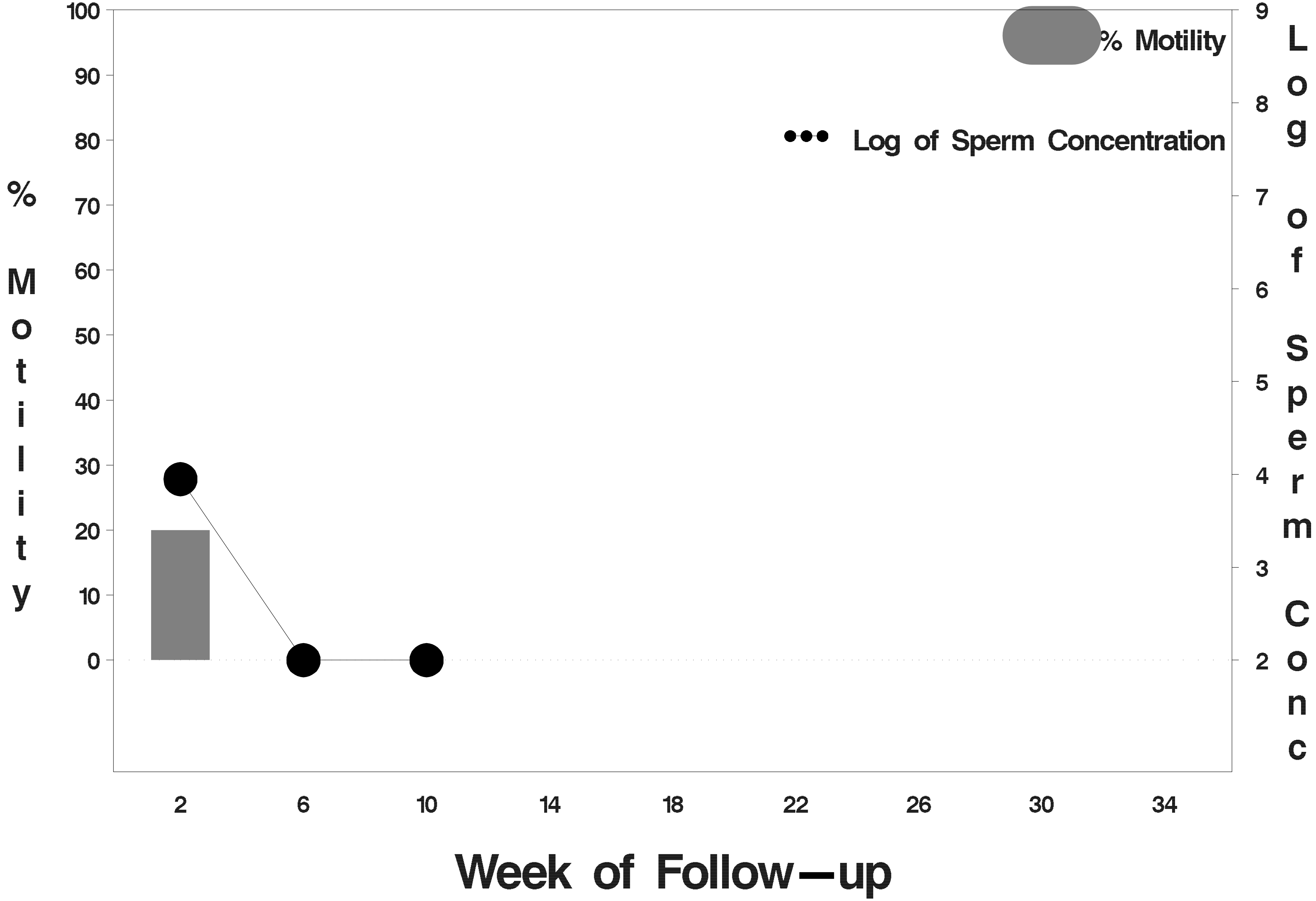

id= 538 outcome= Success

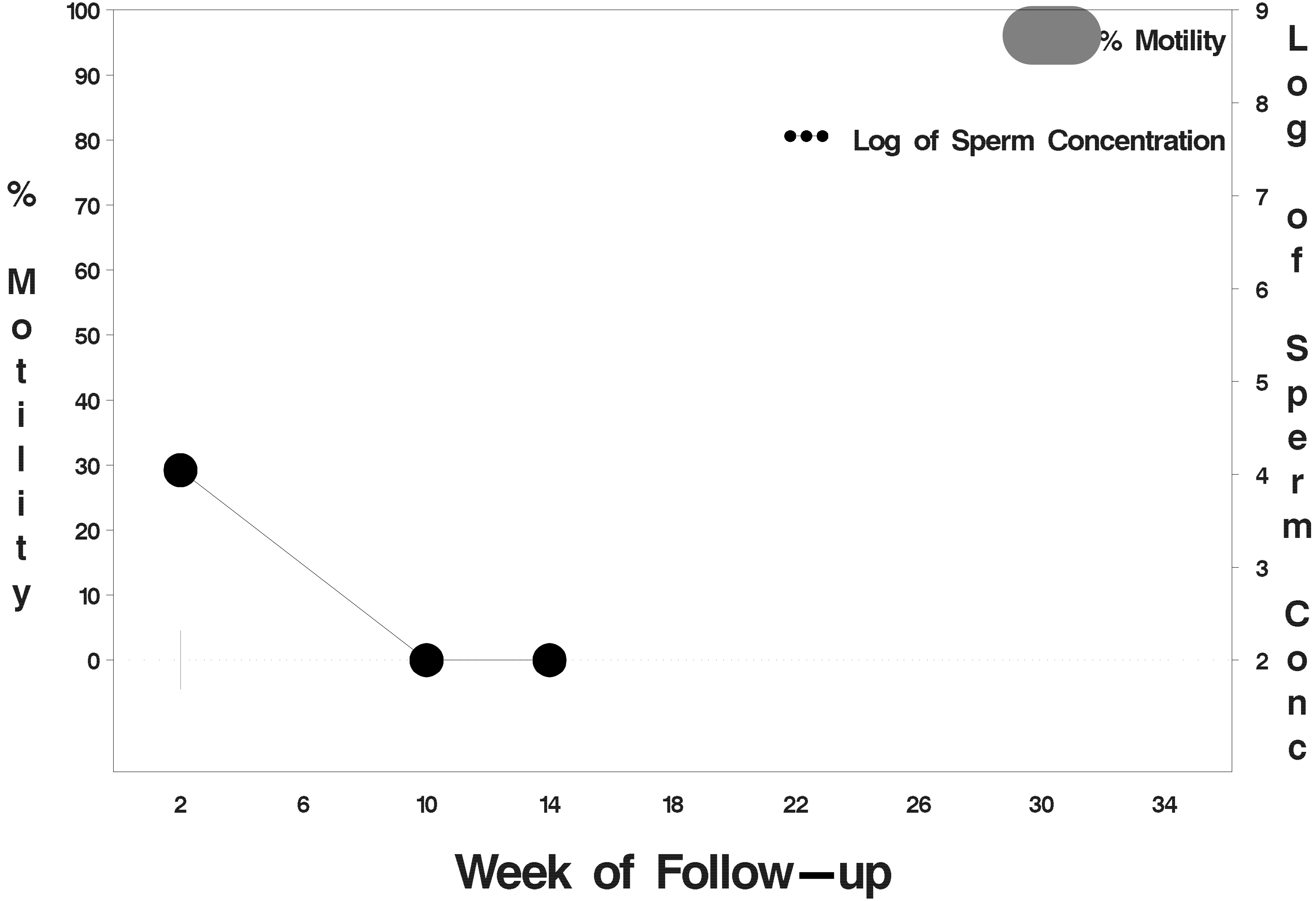

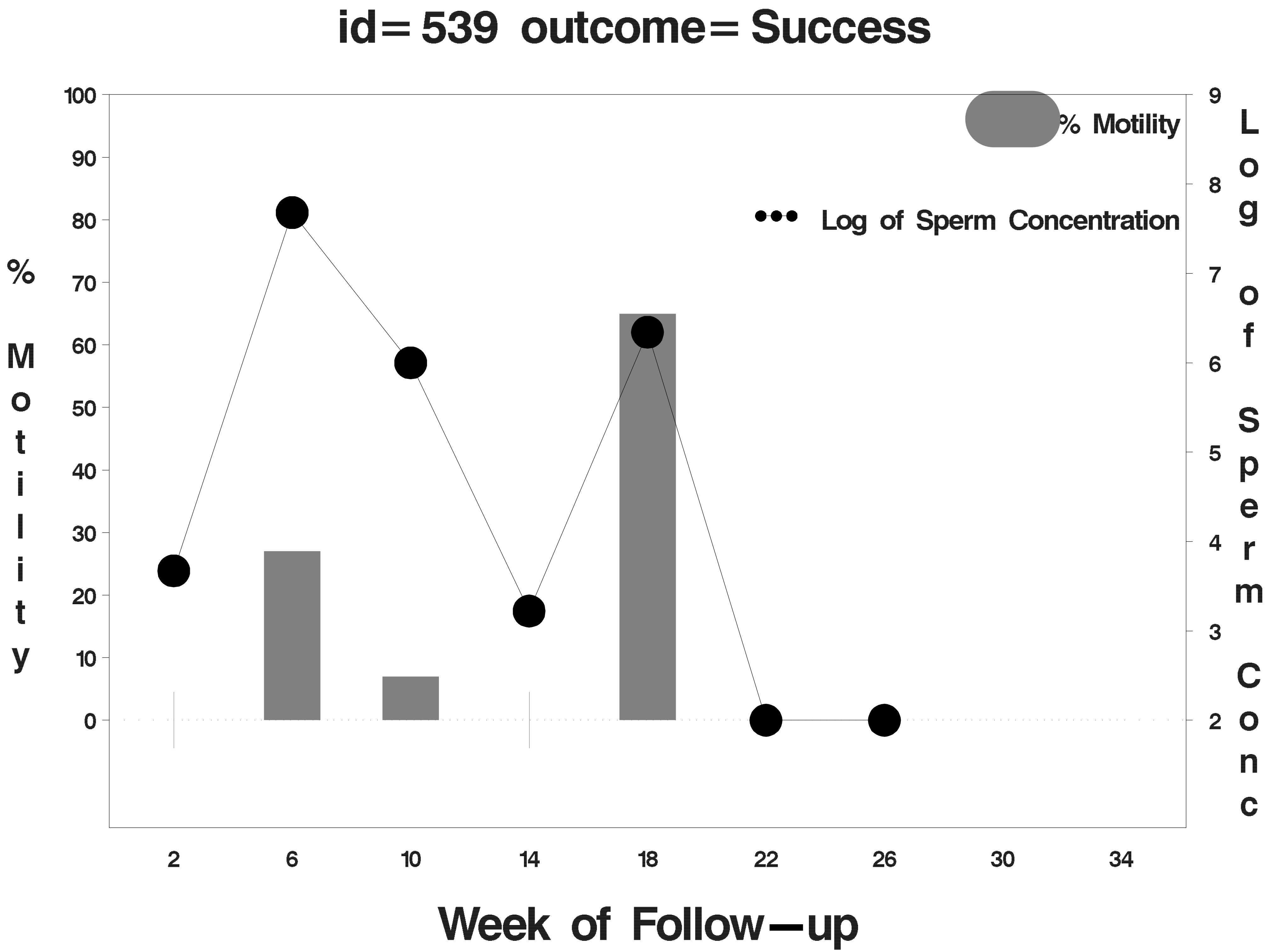
id= 539 outcome= Success
% Motility
Log of Sperm Concentration
100
90
80
70
60
50
40
30
20
10
0
9
8
7
6
5
4
3
2
% Motility
Log of Sperm Conc
2
6
10
14
18
22
26
30
34
Week of Follow−up

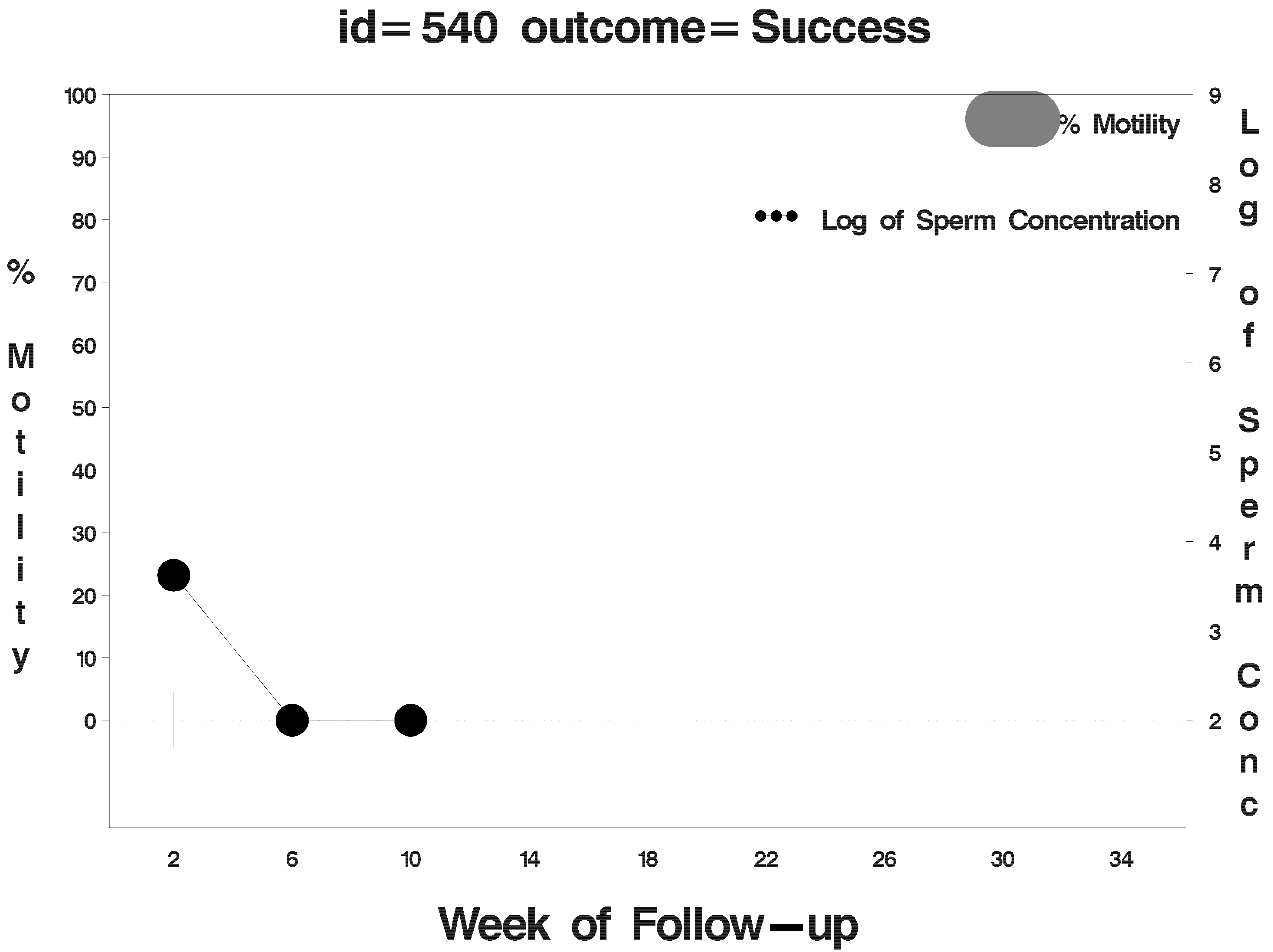

id= 540 outcome= Success
% Motility
Log of Sperm Concentration
% Motility
Log of Sperm Conc
Week of Follow-up
100
90
80
70
60
50
40
30
20
10
0
9
8
7
6
5
4
3
2
2
6
10
14
18
22
26
30
34

id= 541 outcome= Failure

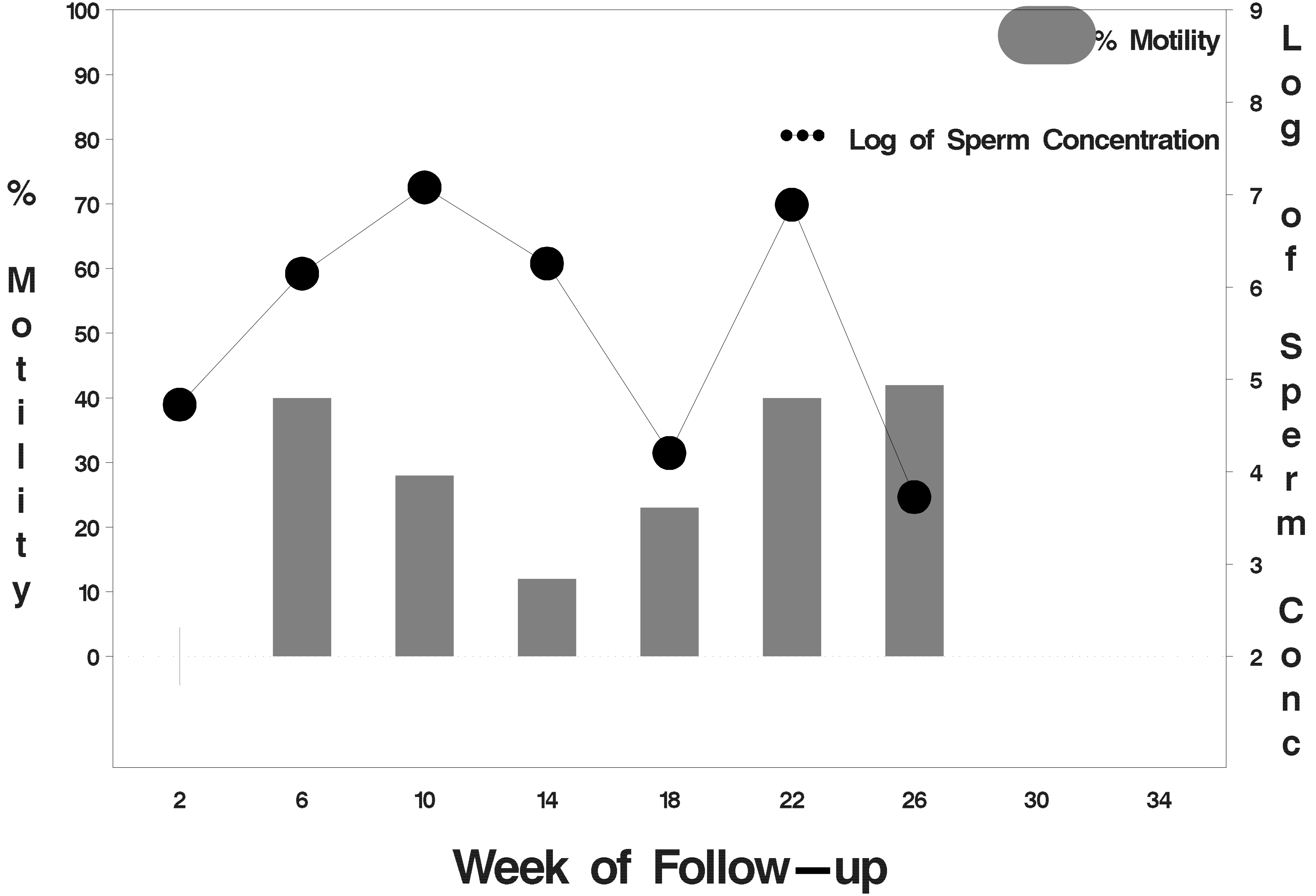

id= 542 outcome= Failure

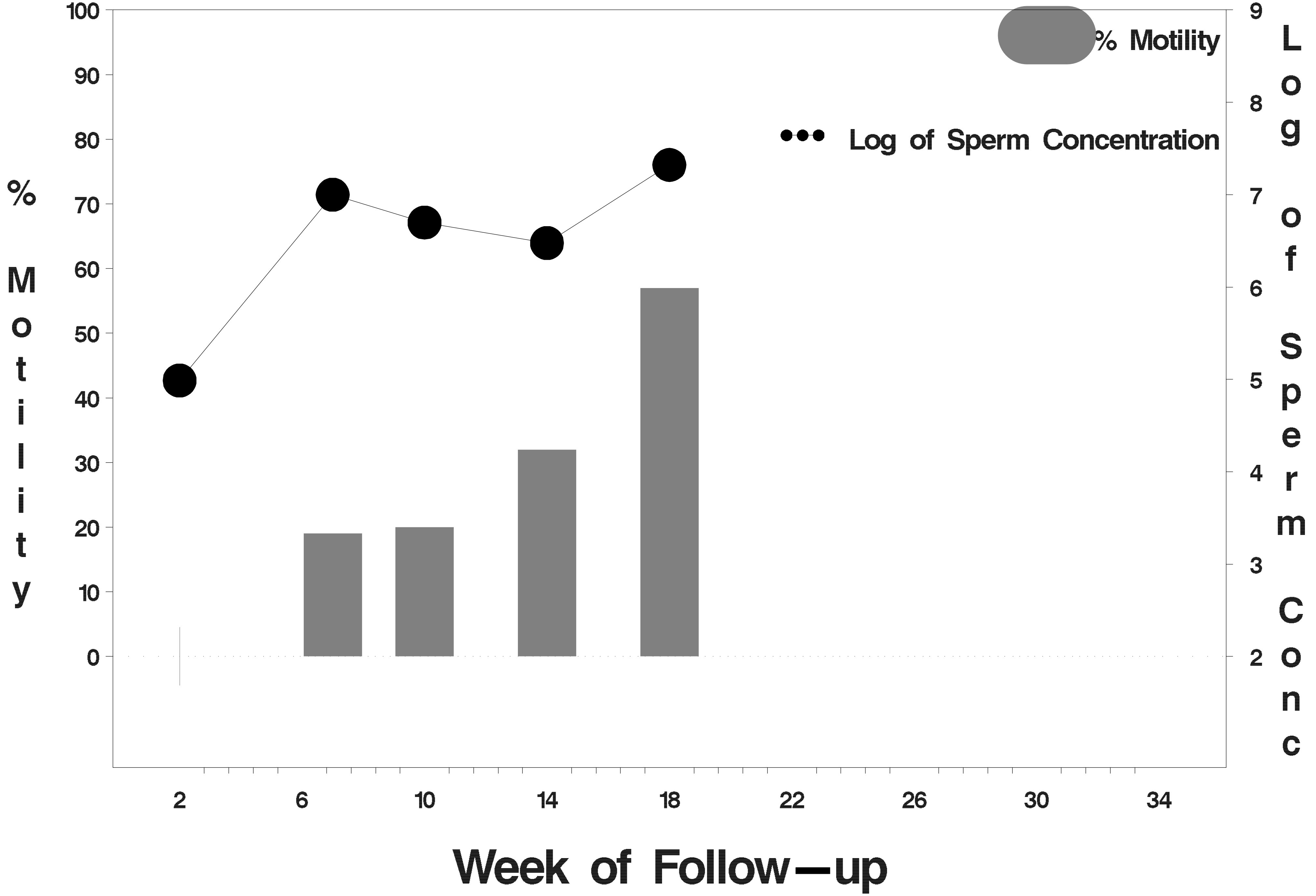

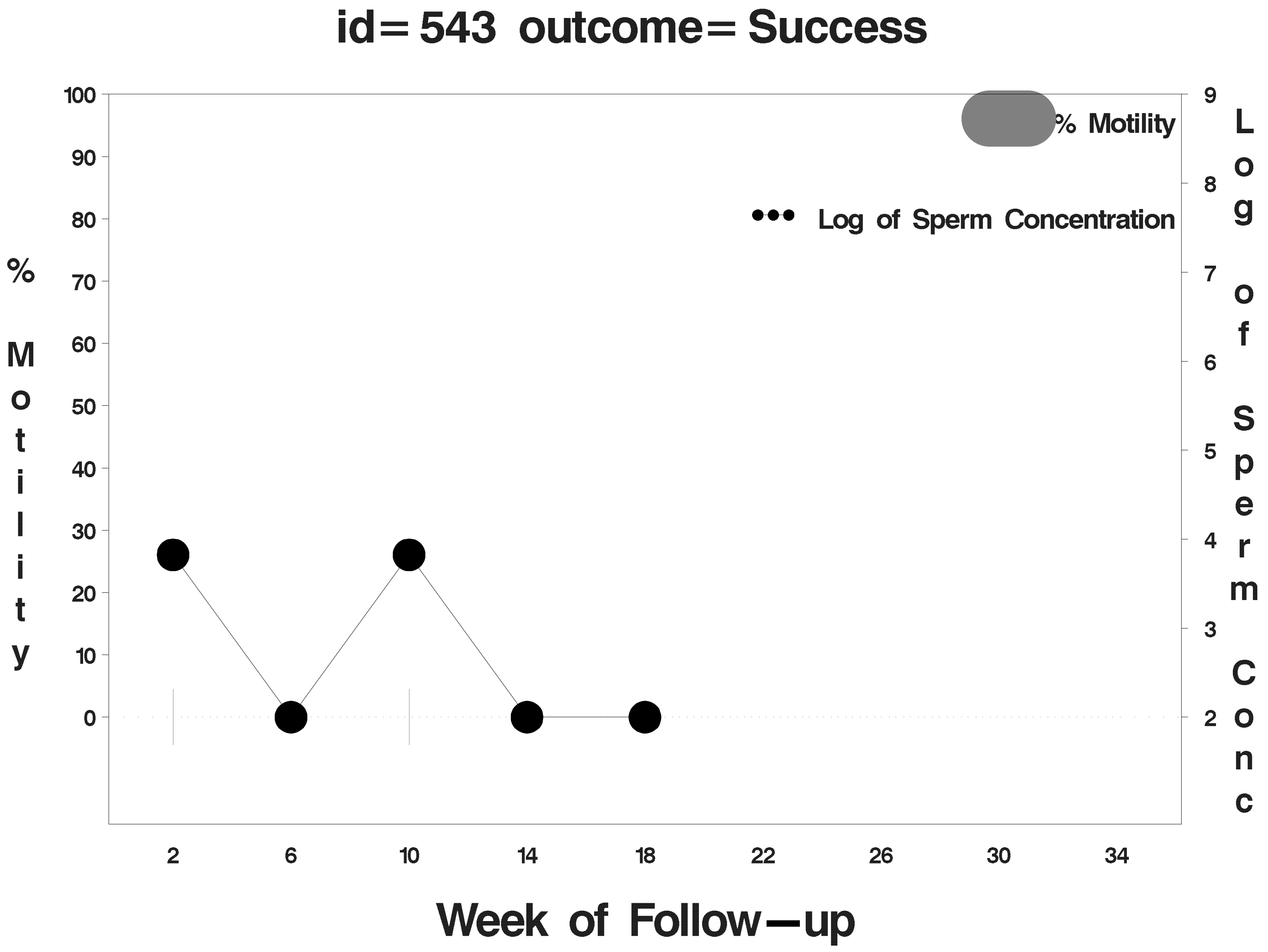

id= 543 outcome= Success
% Motility
Log of Sperm Concentration
100
90
80
70
60
50
40
30
20
10
0
9
8
7
6
5
4
3
2
2
6
10
14
18
22
26
30
34
Week of Follow—up
Log of Sperm Conc

id= 544 outcome= Success

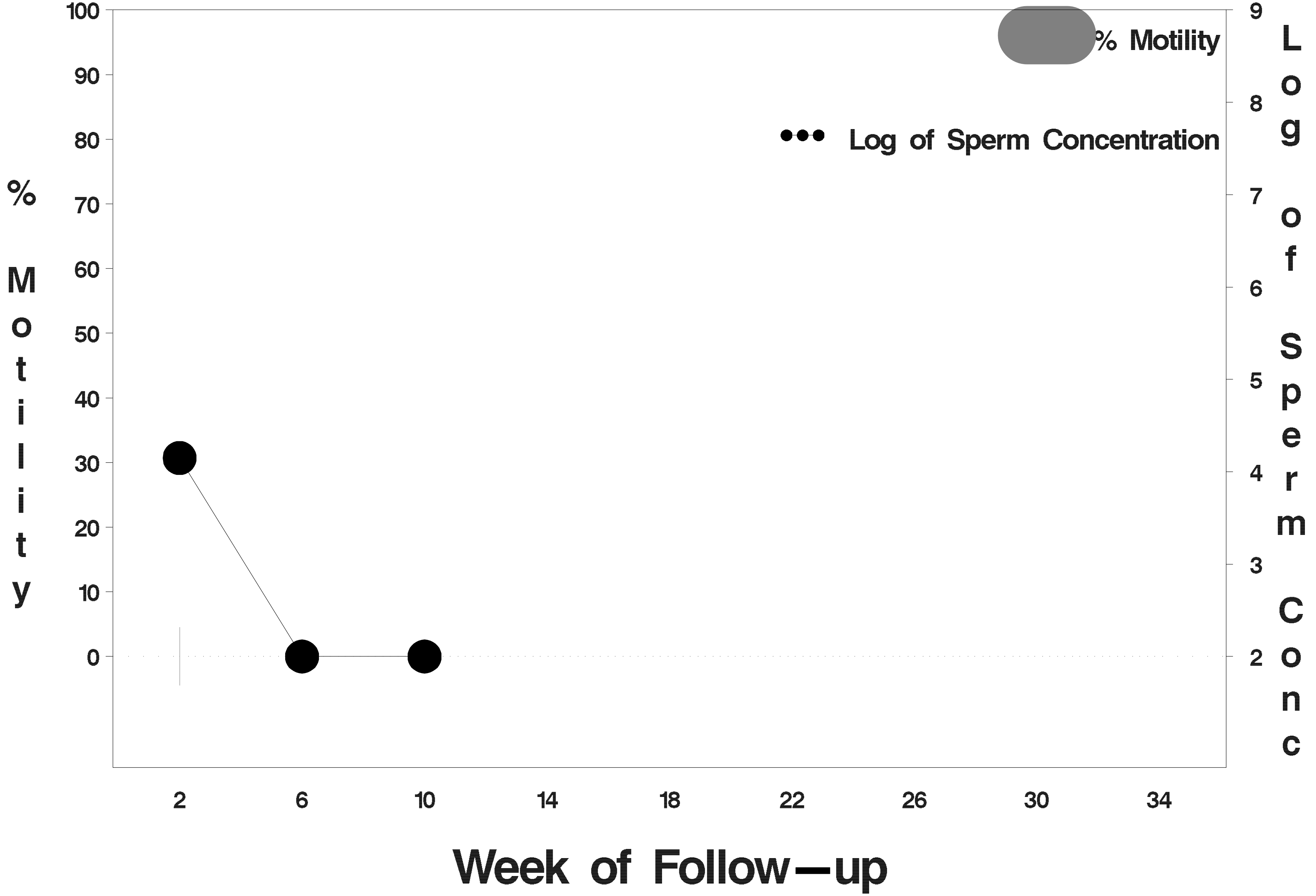

id= 545 outcome= Success

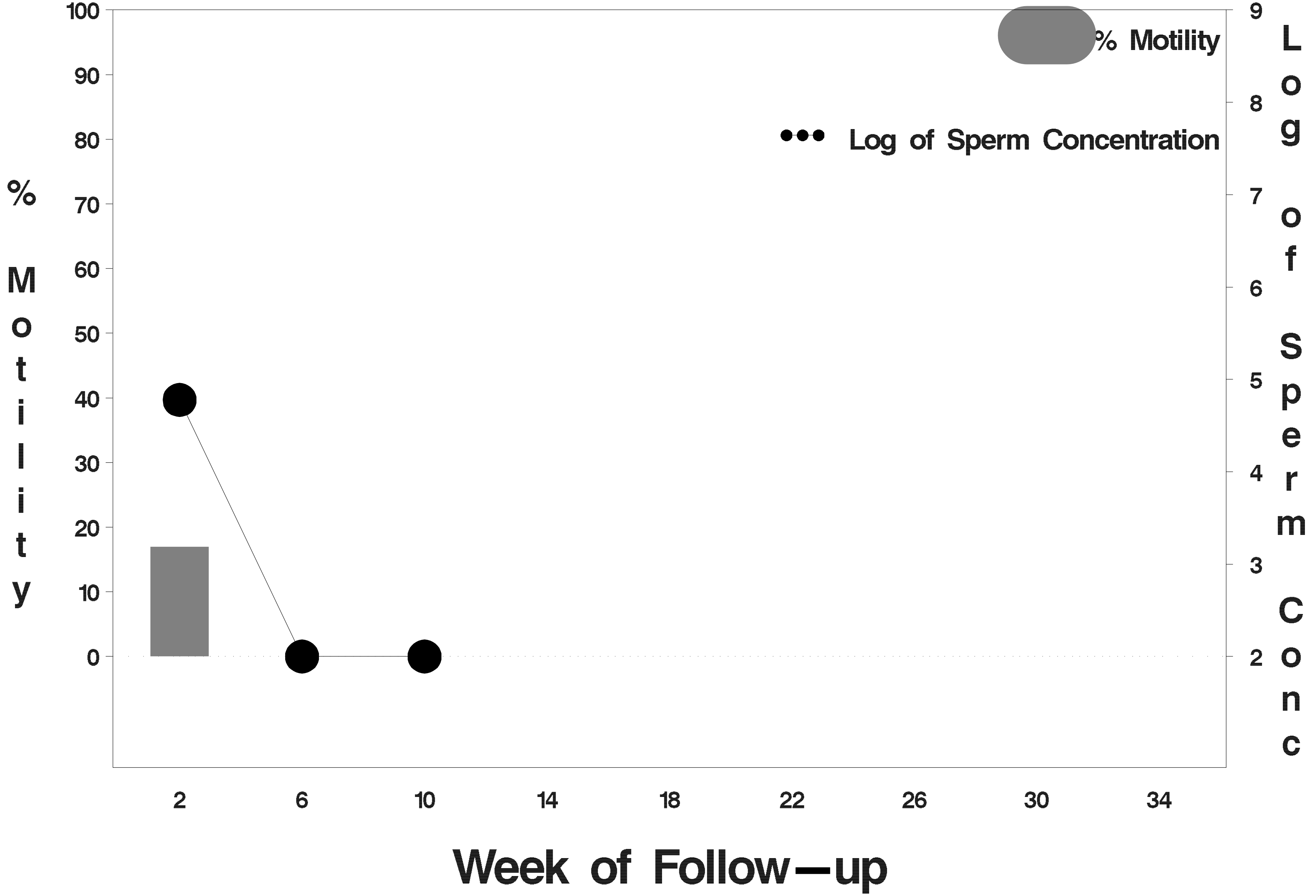

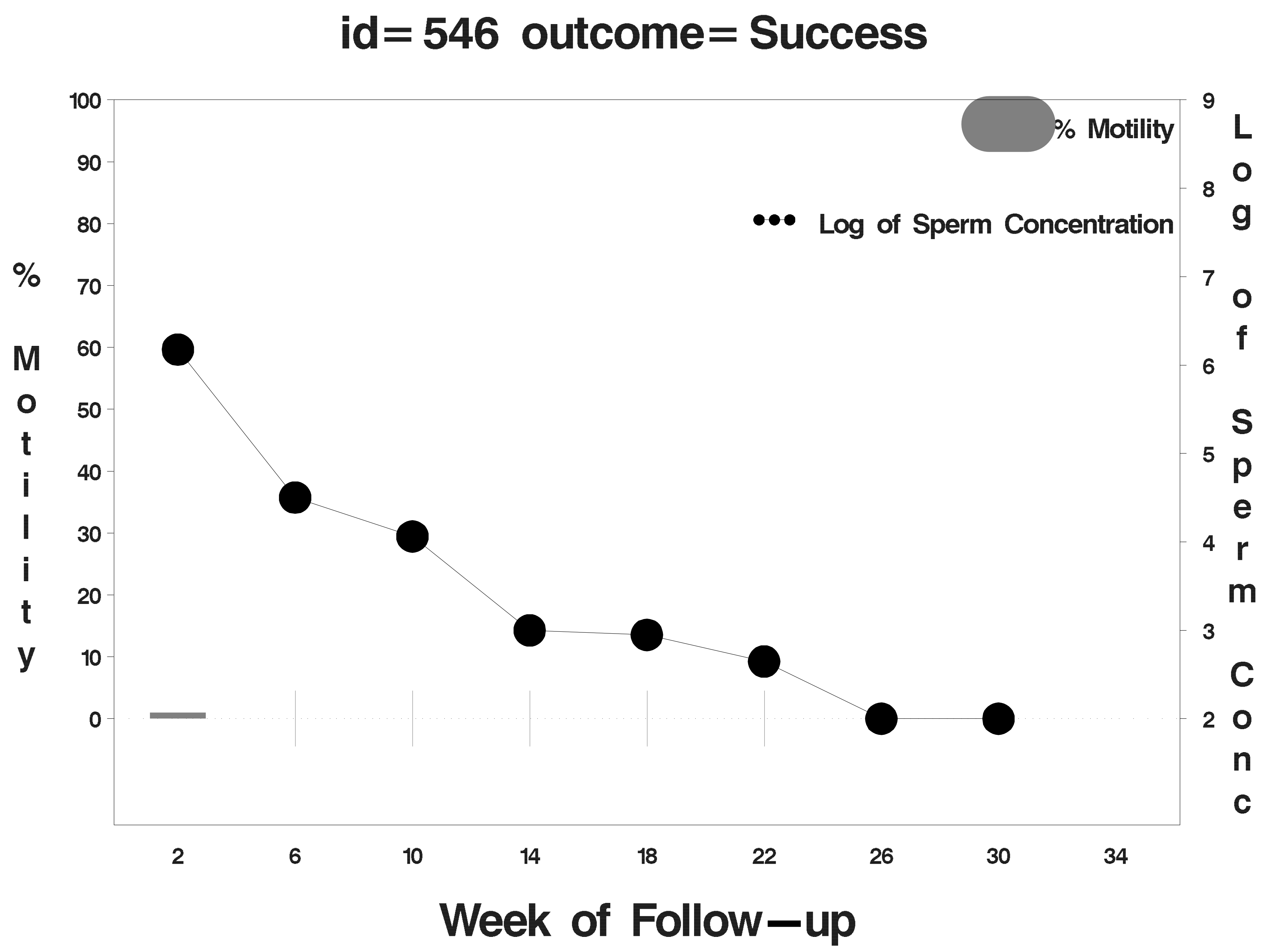
id= 546 outcome= Success
% Motility
Log of Sperm Concentration
100
90
80
70
60
50
40
30
20
10
0
9
8
7
6
5
4
3
2
2
6
10
14
18
22
26
30
34
% Motility
Log of Sperm Conc
Week of Follow—up

id= 547 outcome= Success

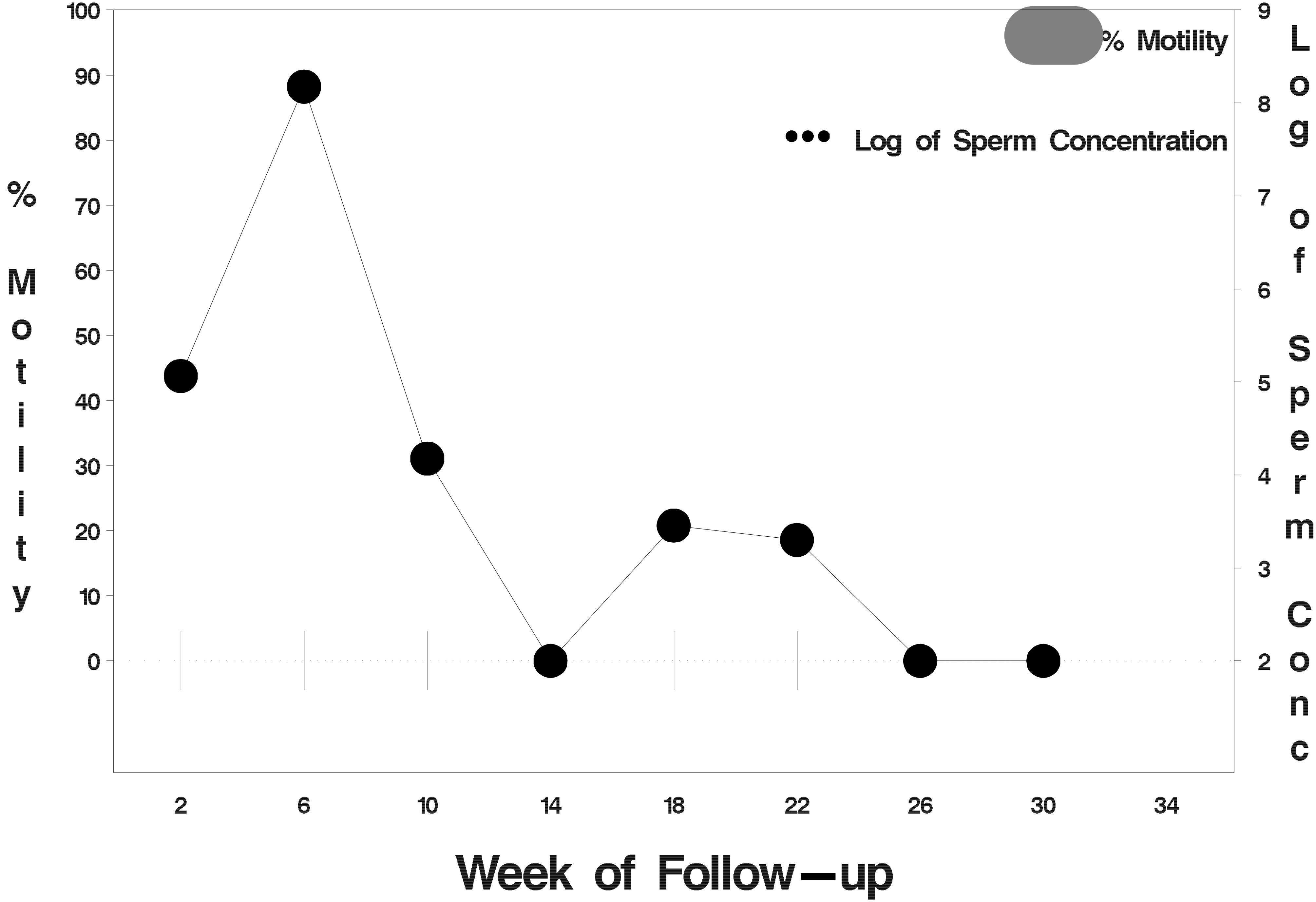

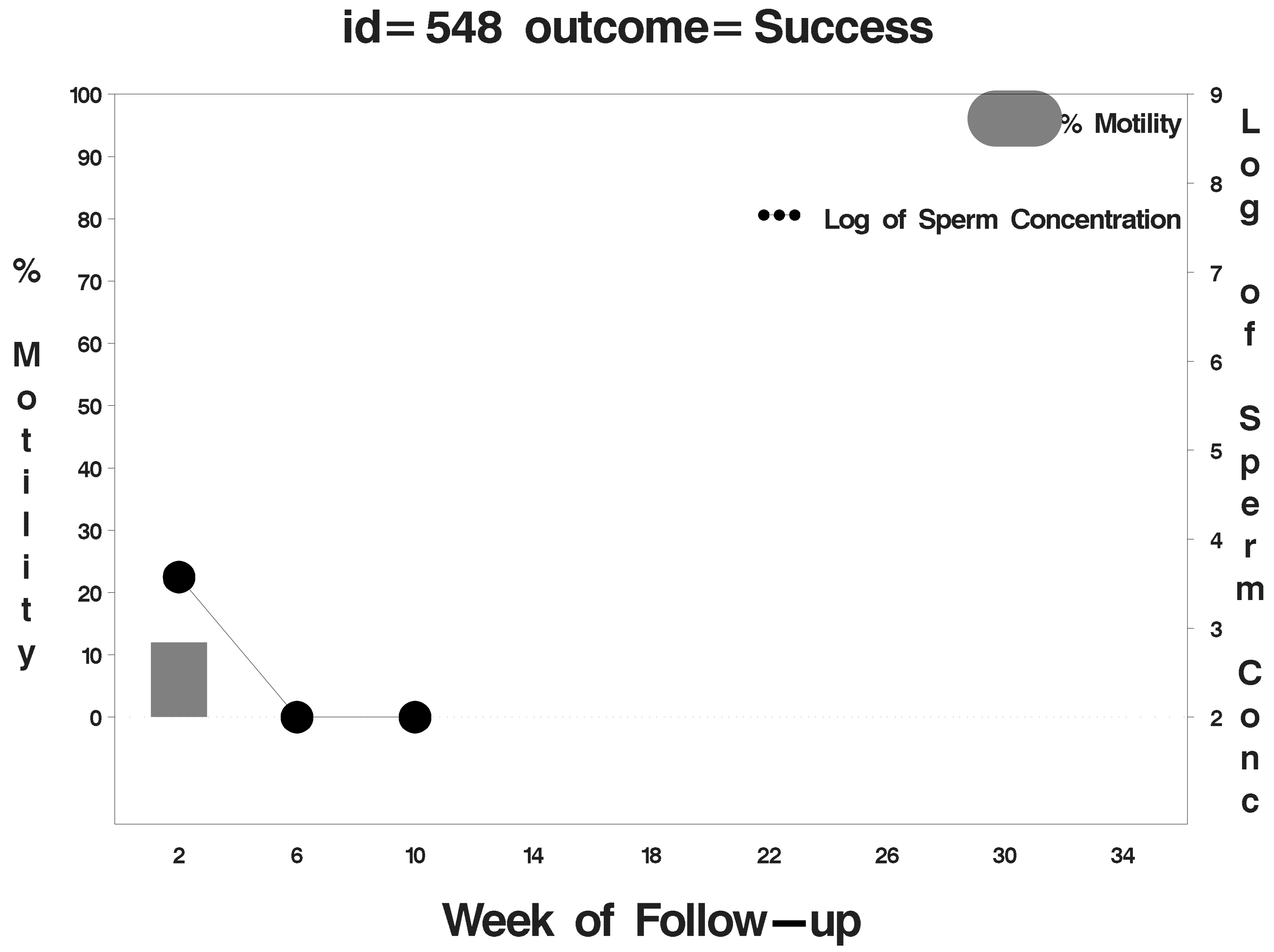

id= 548 outcome= Success
% Motility
Log of Sperm Concentration
100
90
80
70
60
50
40
30
20
10
0
9
8
7
6
5
4
3
2
2
6
10
14
18
22
26
30
34
Week of Follow-up
% Motility
Log of Sperm Conc

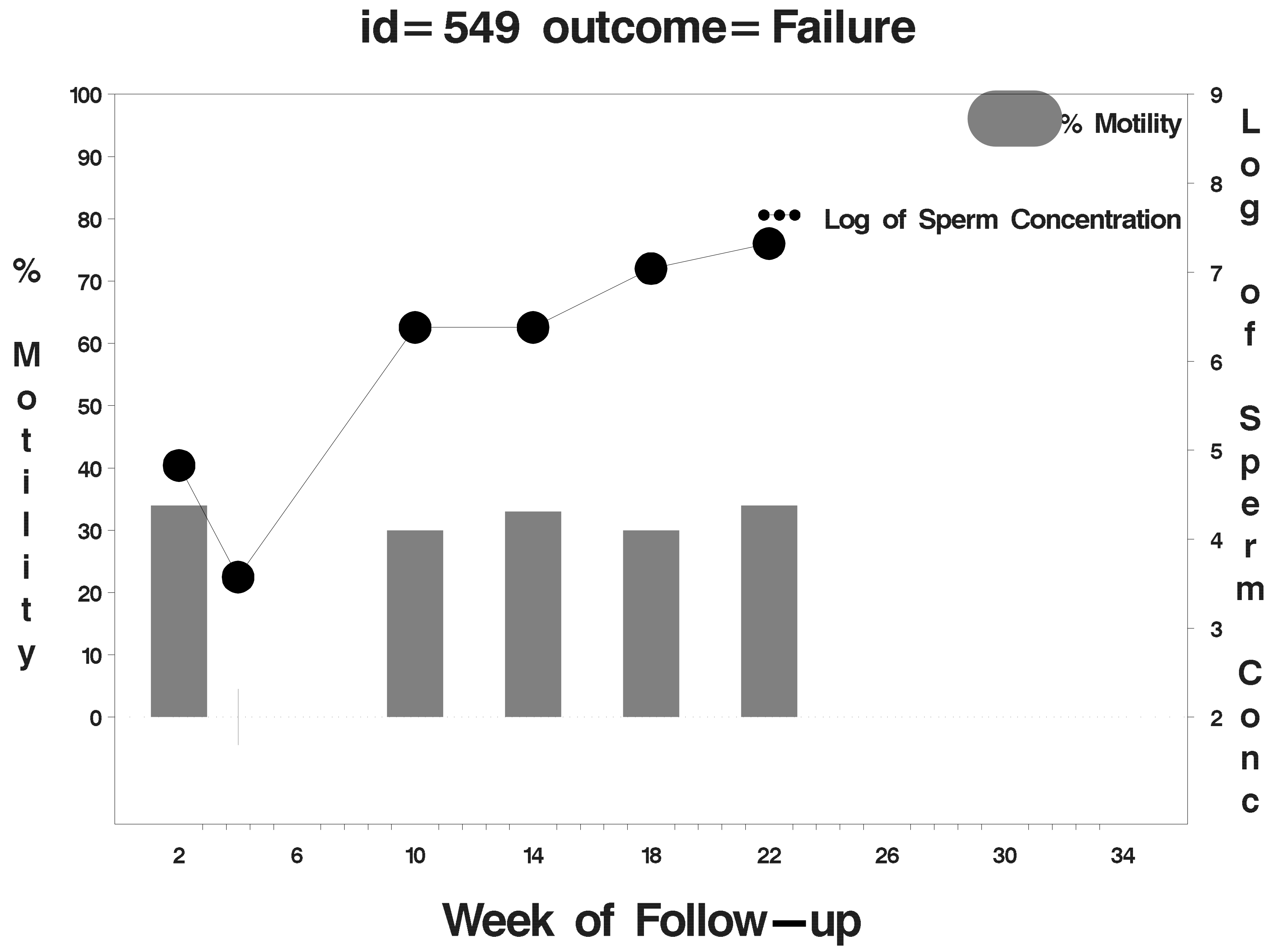

id= 549 outcome= Failure
% Motility
Log of Sperm Concentration
100
90
80
70
60
50
40
30
20
10
0
9
8
7
6
5
4
3
2
2
6
10
14
18
22
26
30
34
Week of Follow-up
Log of Sperm Conc

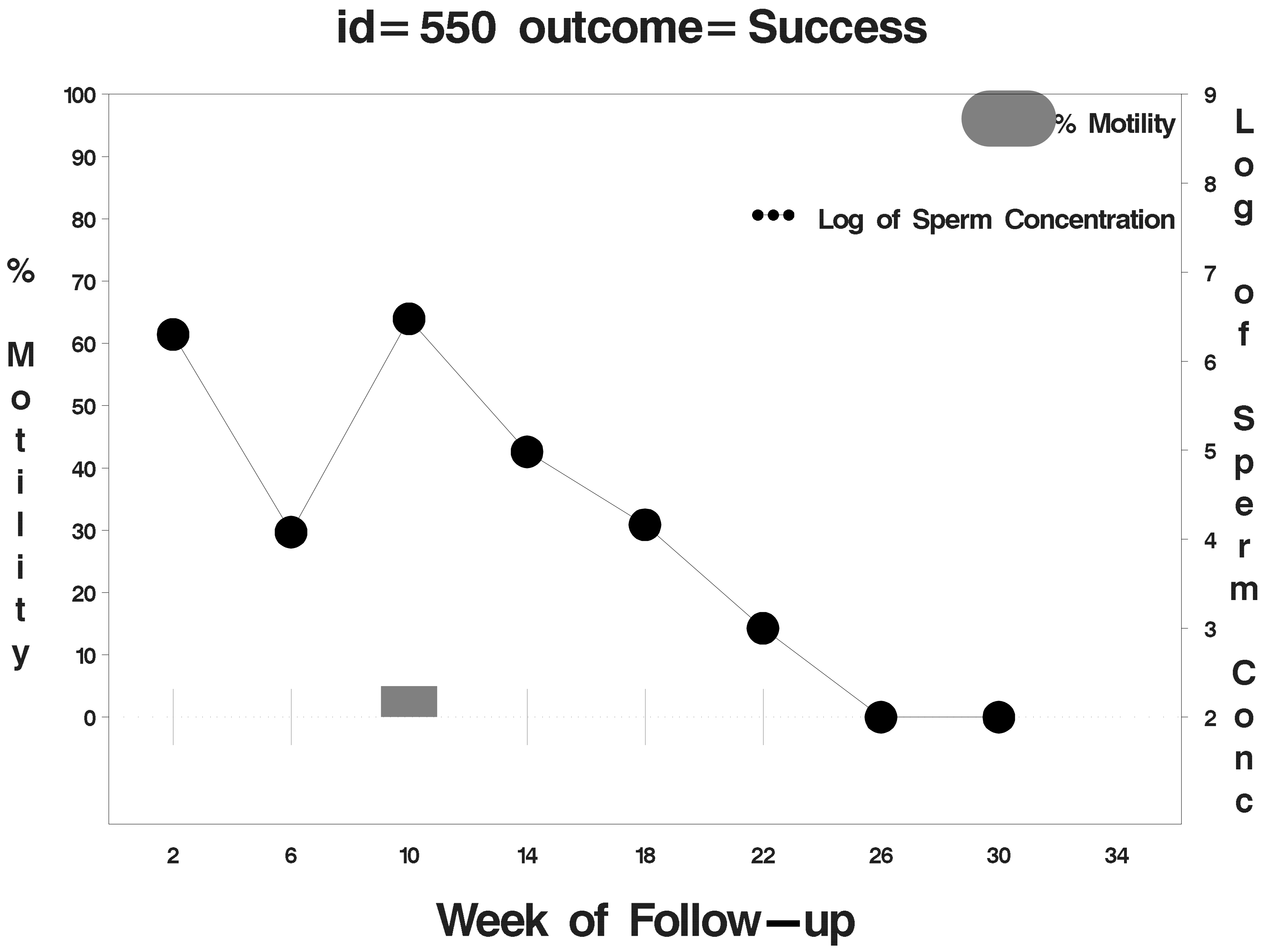
id= 550 outcome= Success
% Motility
Log of Sperm Concentration
100
90
80
70
60
50
40
30
20
10
0
9
8
7
6
5
4
3
2
2
6
10
14
18
22
26
30
34
Week of Follow—up
% Motility
Log of Sperm Conc

id= 551 outcome= Success

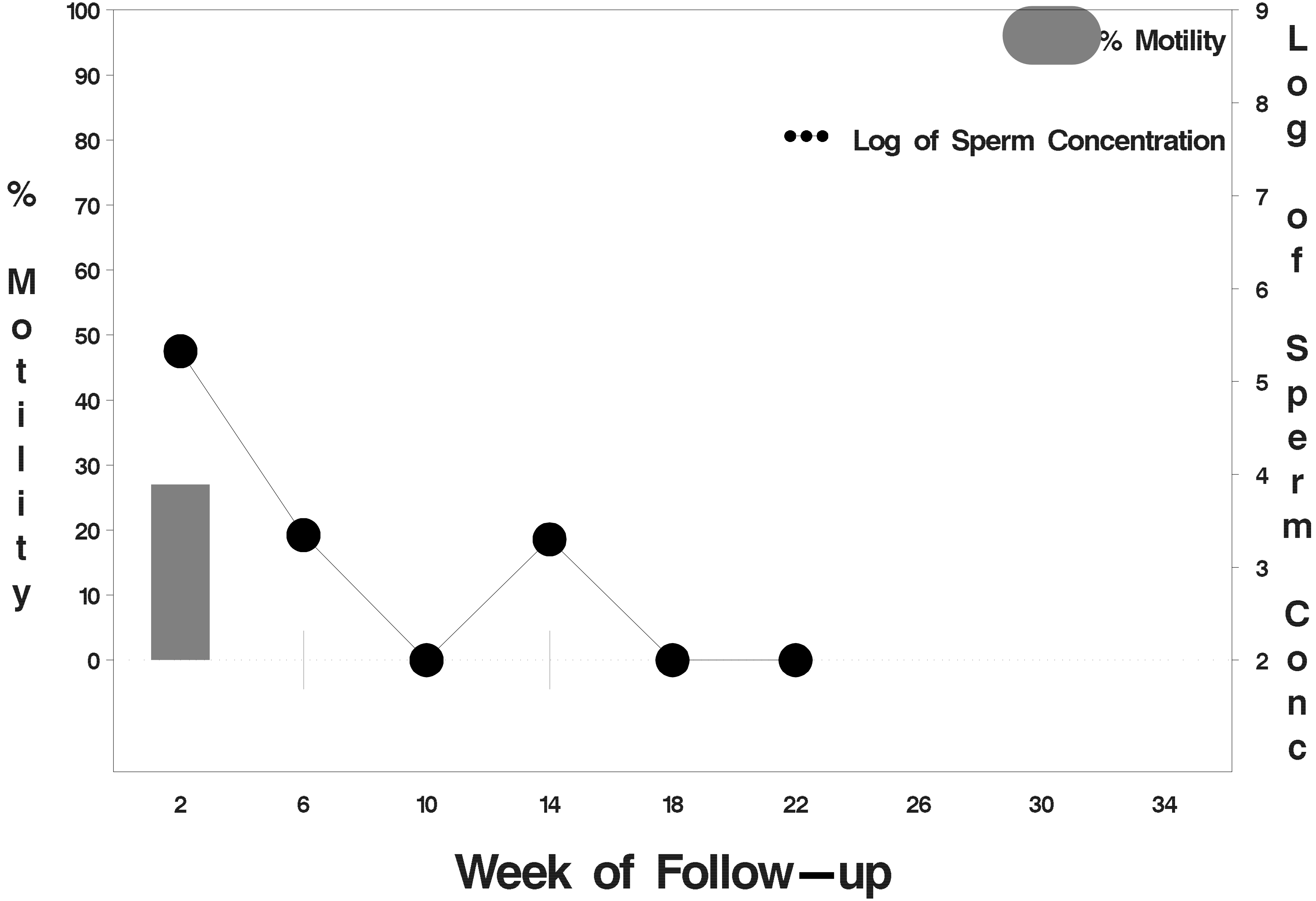

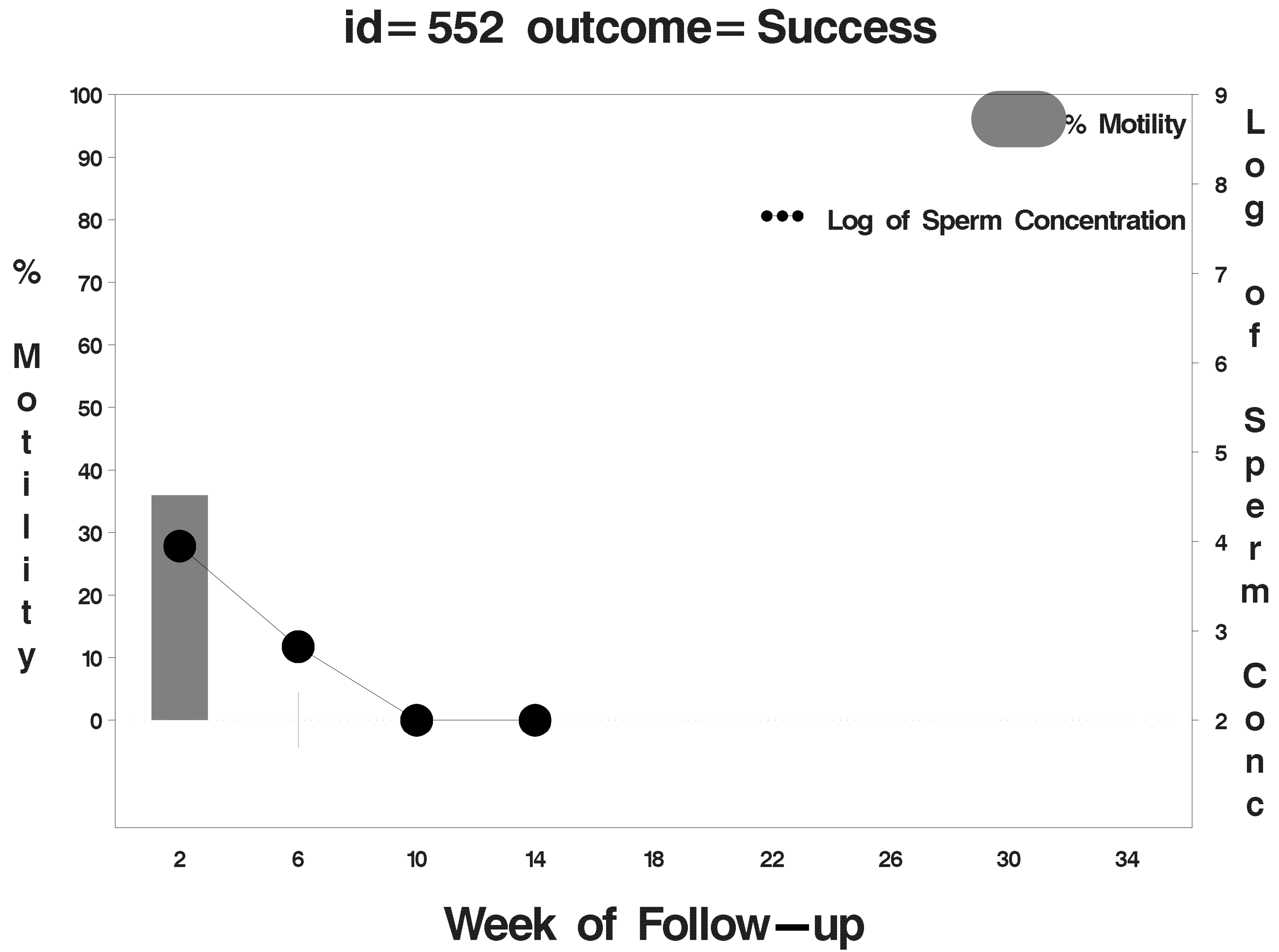

id= 552 outcome= Success
% Motility
Log of Sperm Concentration
100
90
80
70
60
50
40
30
20
10
0
9
8
7
6
5
4
3
2
2
6
10
14
18
22
26
30
34
Week of Follow−up
Log of Sperm Conc

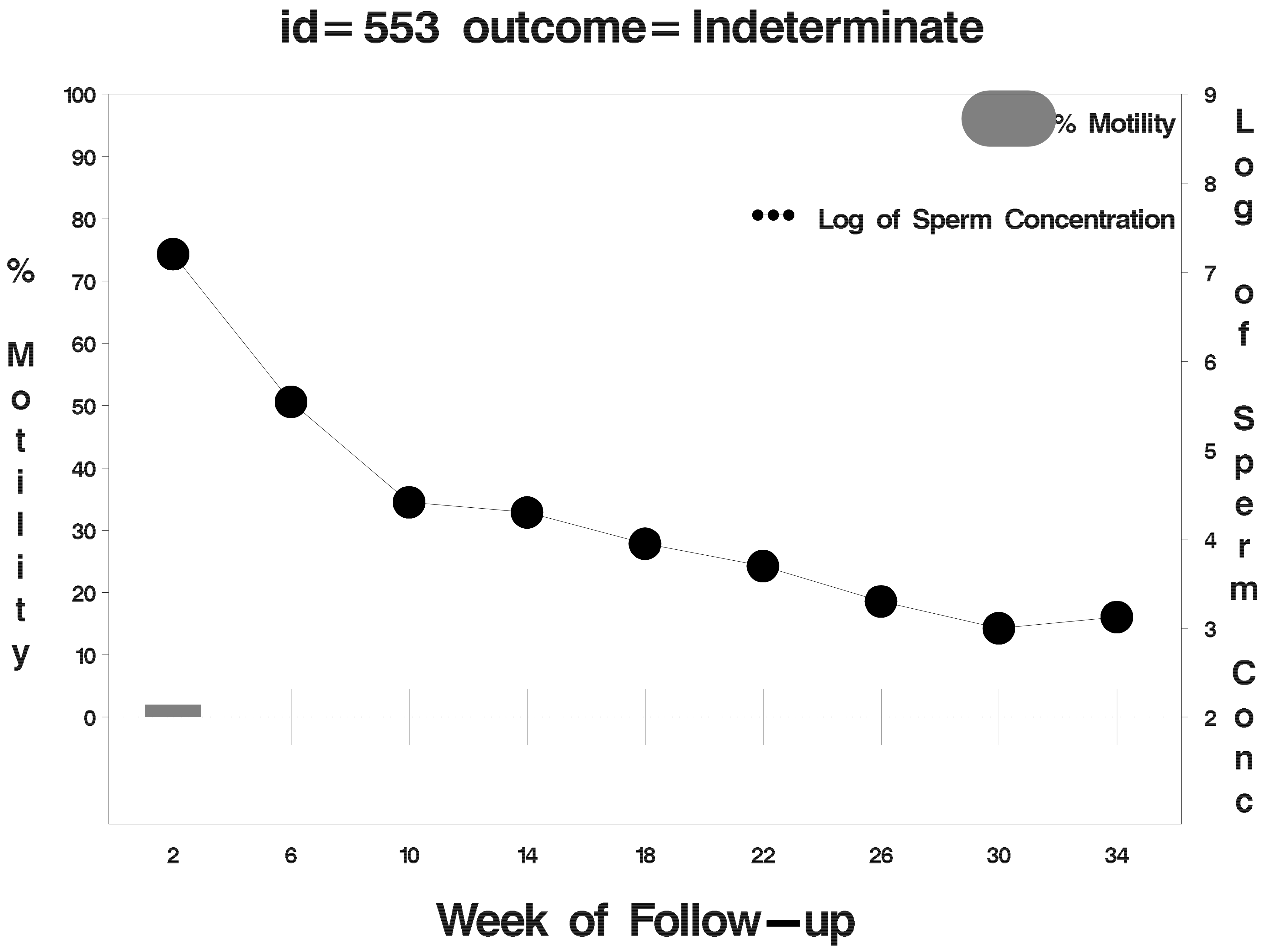

id= 553 outcome= Indeterminate
% Motility
Log of Sperm Concentration
% Motility
Log of Sperm Conc
Week of Follow−up
100
90
80
70
60
50
40
30
20
10
0
9
8
7
6
5
4
3
2
2
6
10
14
18
22
26
30
34

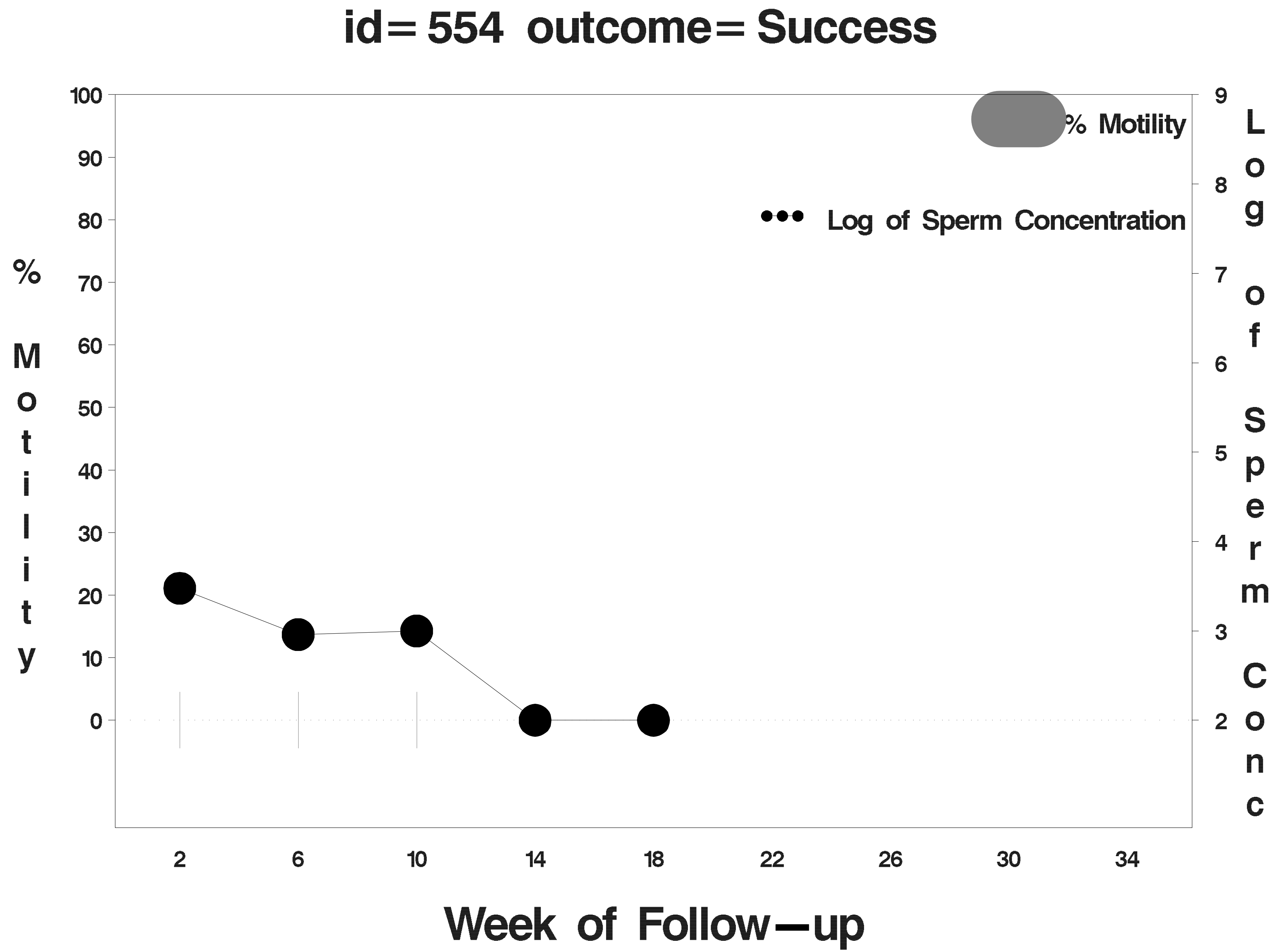

id= 554 outcome= Success
% Motility
Log of Sperm Concentration
100
90
80
70
60
50
40
30
20
10
0
9
8
7
6
5
4
3
2
2
6
10
14
18
22
26
30
34
Week of Follow—up
Log of Sperm Conc

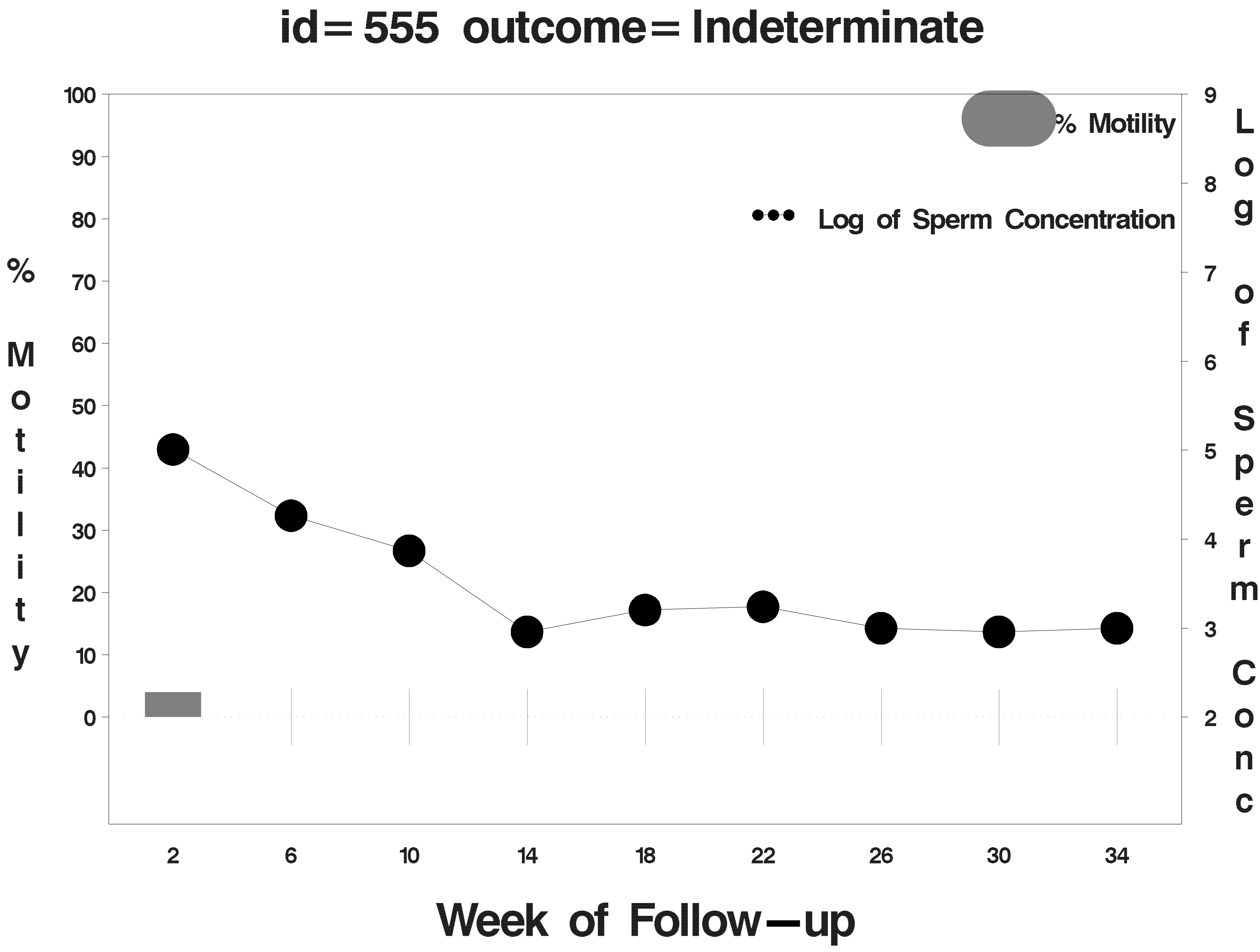

id=555 outcome=Indeterminate
% Motility
Log of Sperm Concentration
% Motility
Log of Sperm Conc
Week of Follow-up
100
90
80
70
60
50
40
30
20
10
0
9
8
7
6
5
4
3
2
2
6
10
14
18
22
26
30
34

id= 556 outcome= Failure

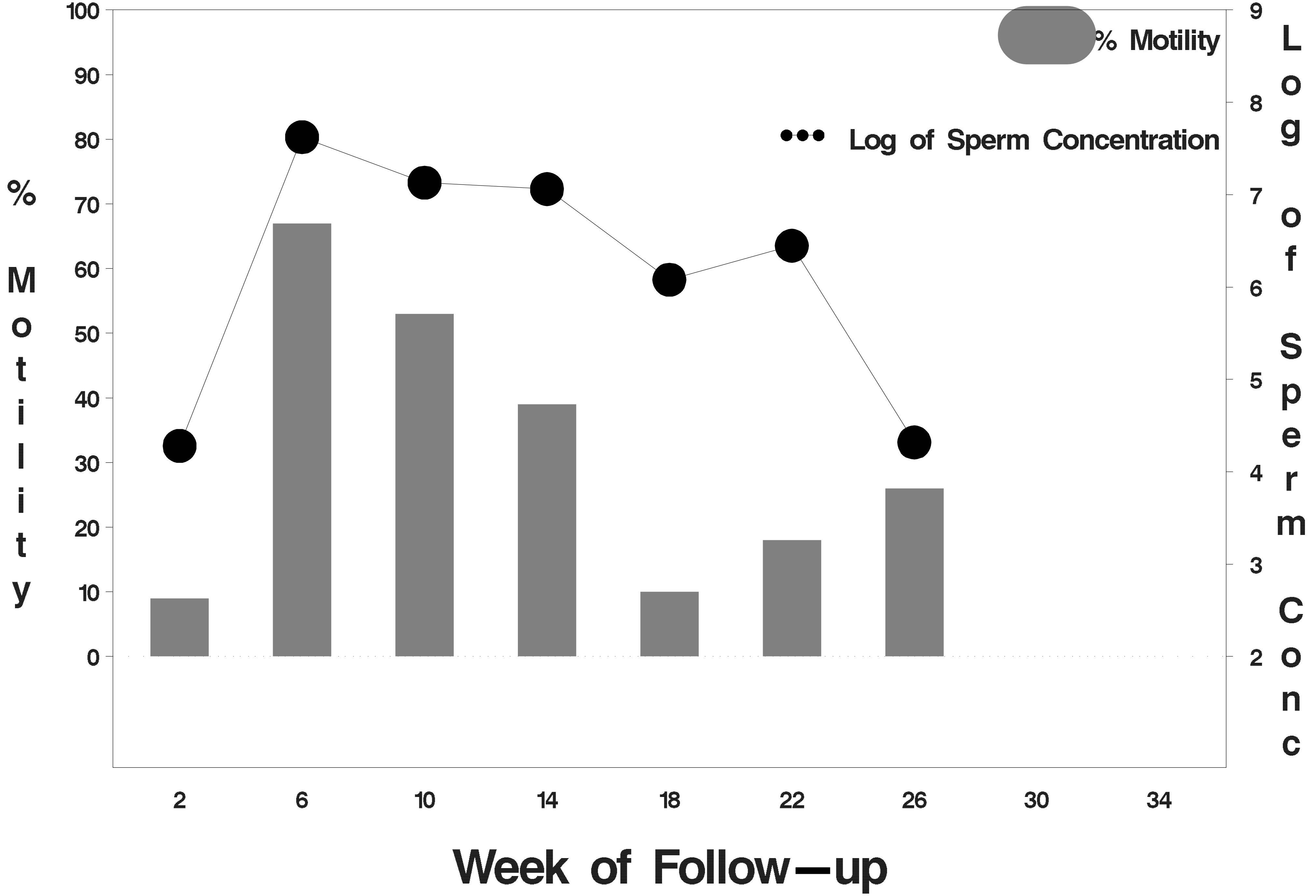

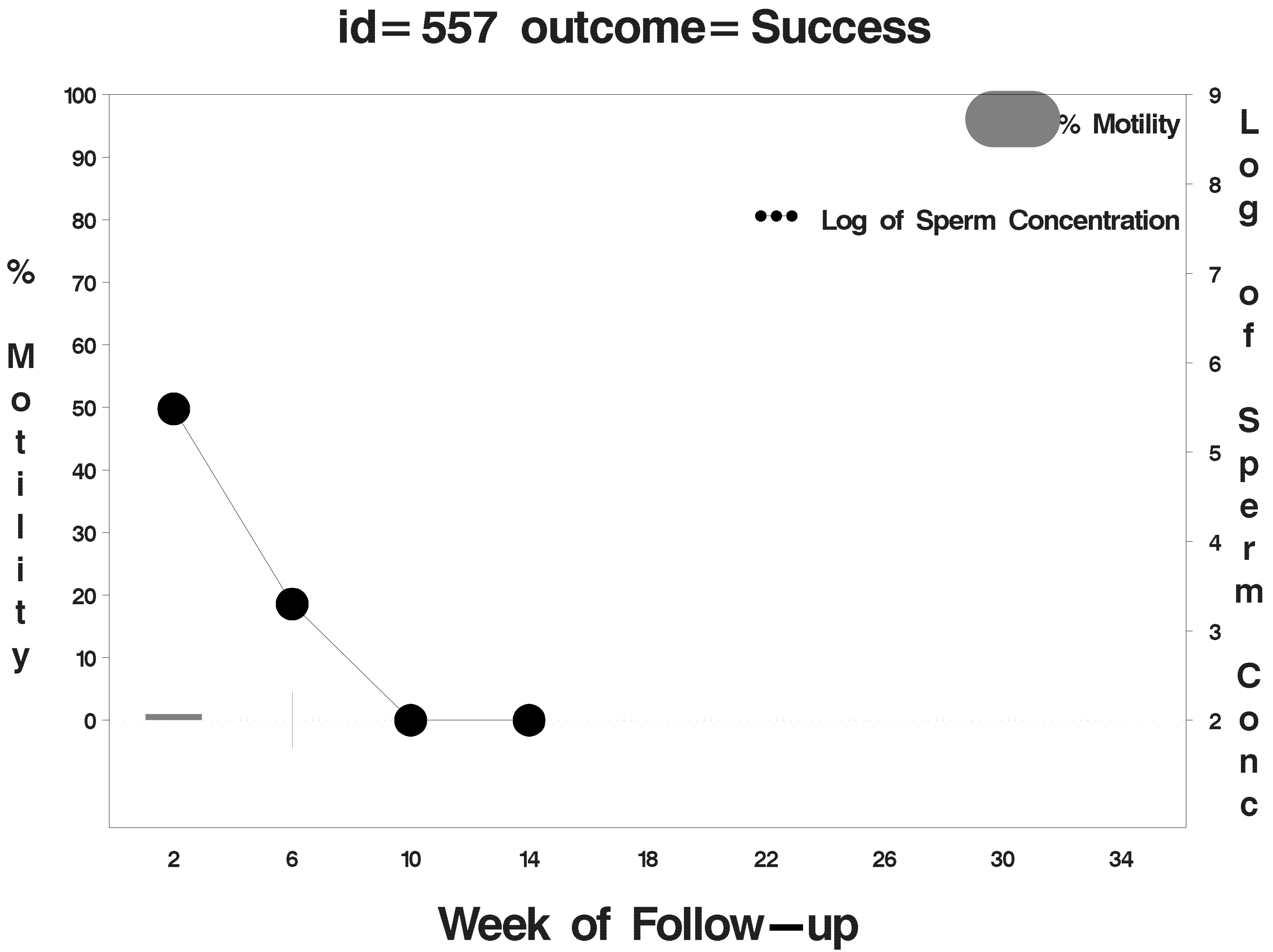
id= 557 outcome= Success
% Motility
Log of Sperm Concentration
100
90
80
70
60
50
40
30
20
10
0
9
8
7
6
5
4
3
2
2
6
10
14
18
22
26
30
34
Week of Follow-up
% Motility
Log of Sperm Conc

id= 558 outcome= Success

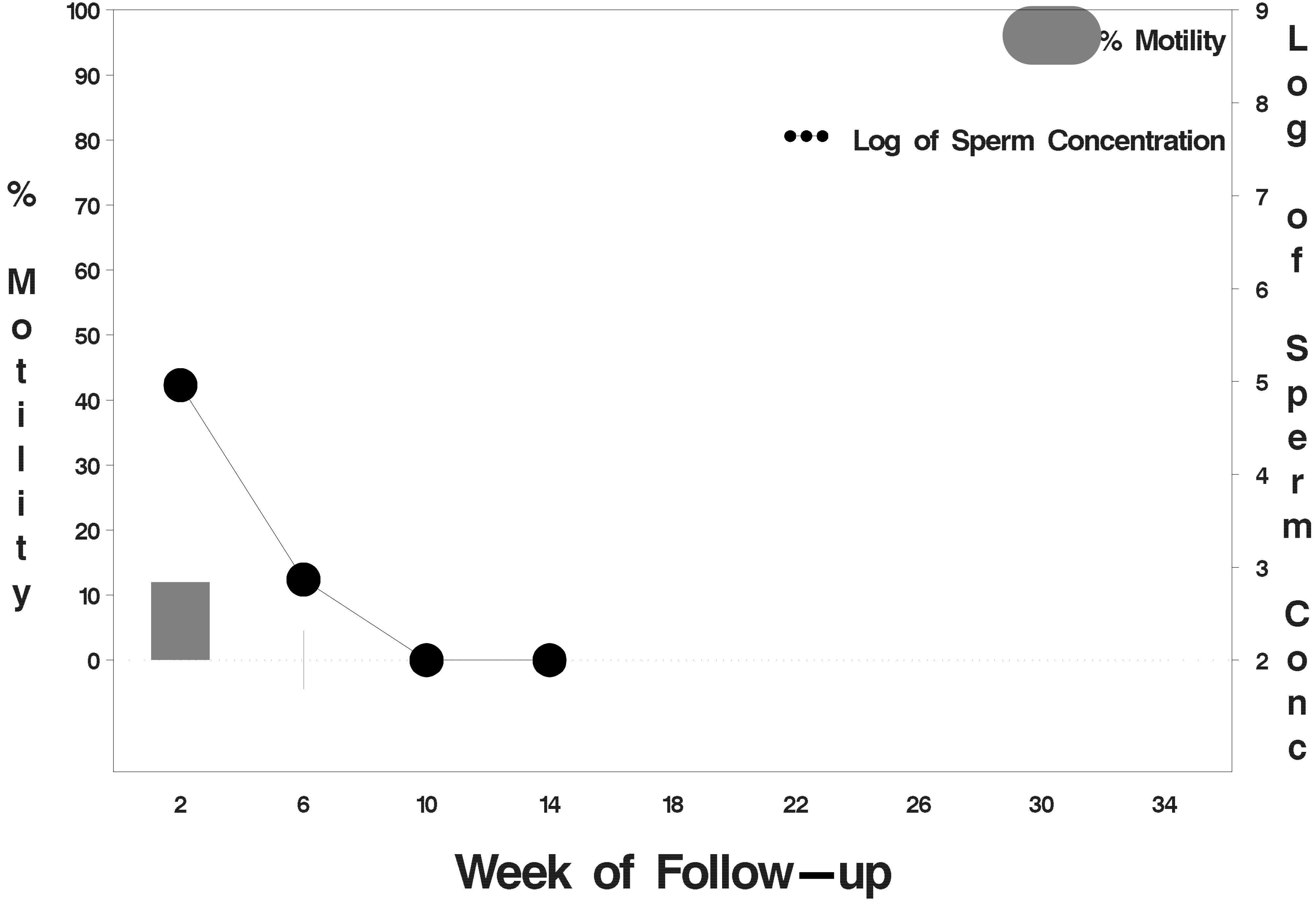

id= 559 outcome= Success

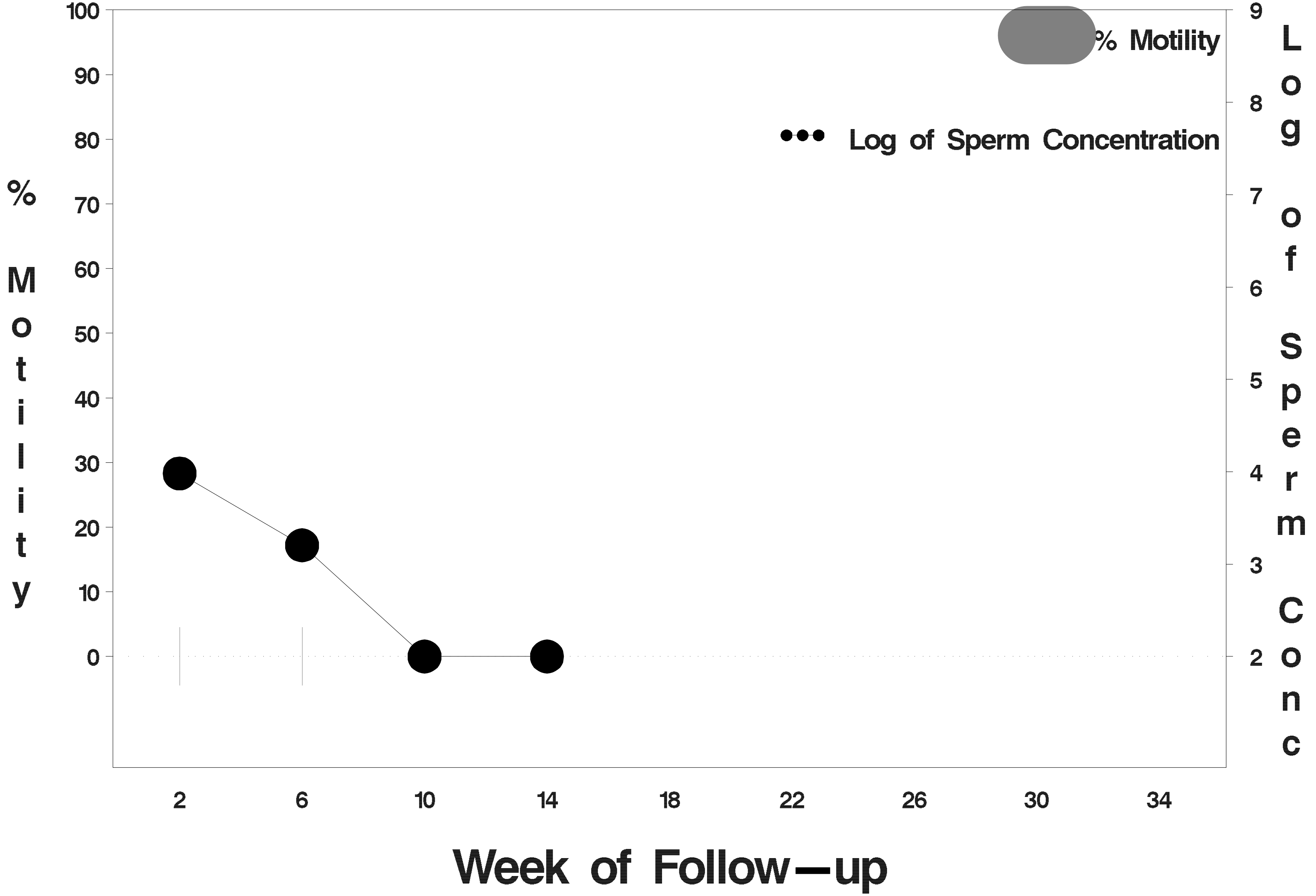

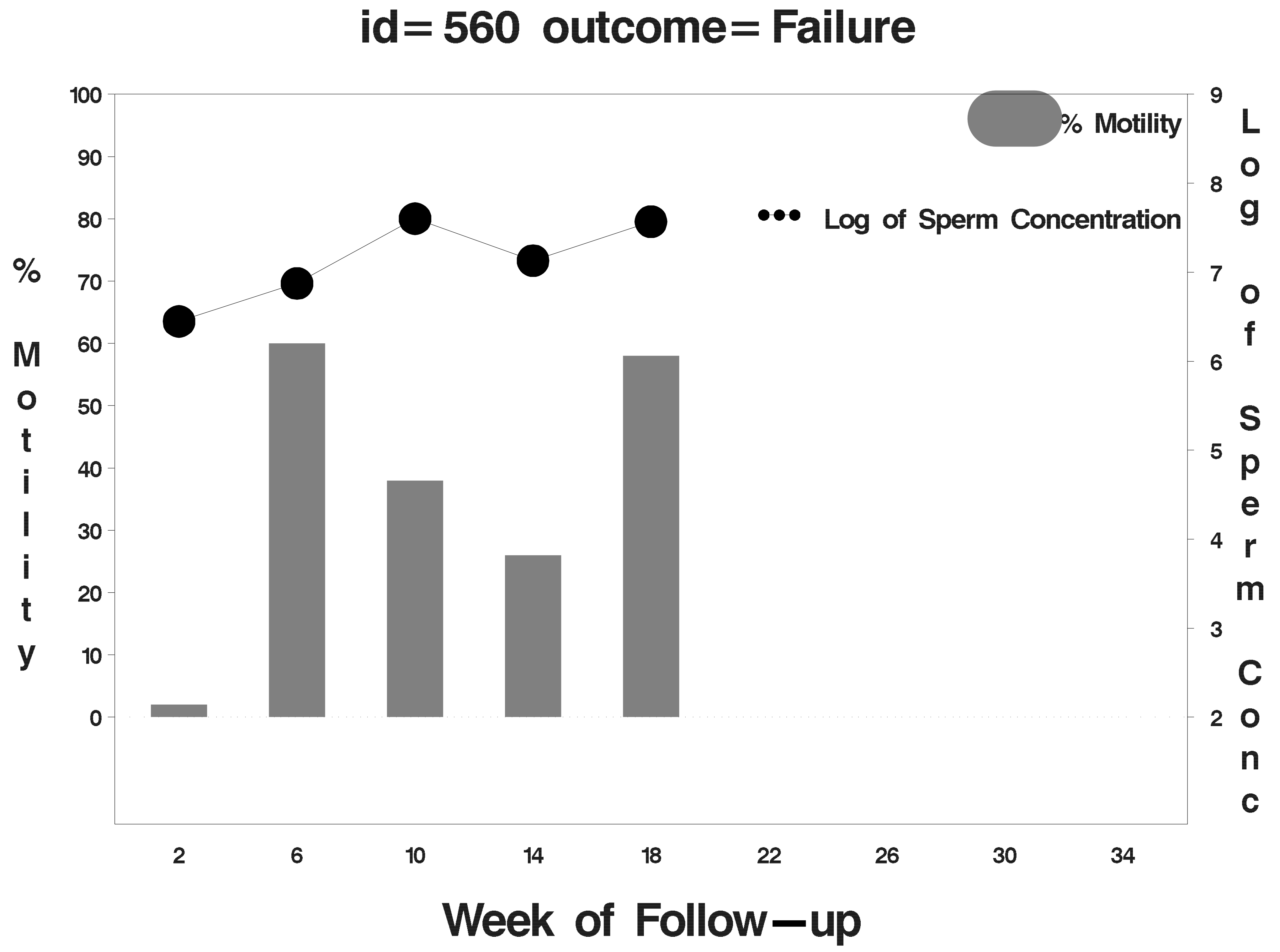
id= 560 outcome= Failure
% Motility
Log of Sperm Concentration
100
90
80
70
60
50
40
30
20
10
0
9
8
7
6
5
4
3
2
2
6
10
14
18
22
26
30
34
Week of Follow−up
% Motility
Log of Sperm Conc

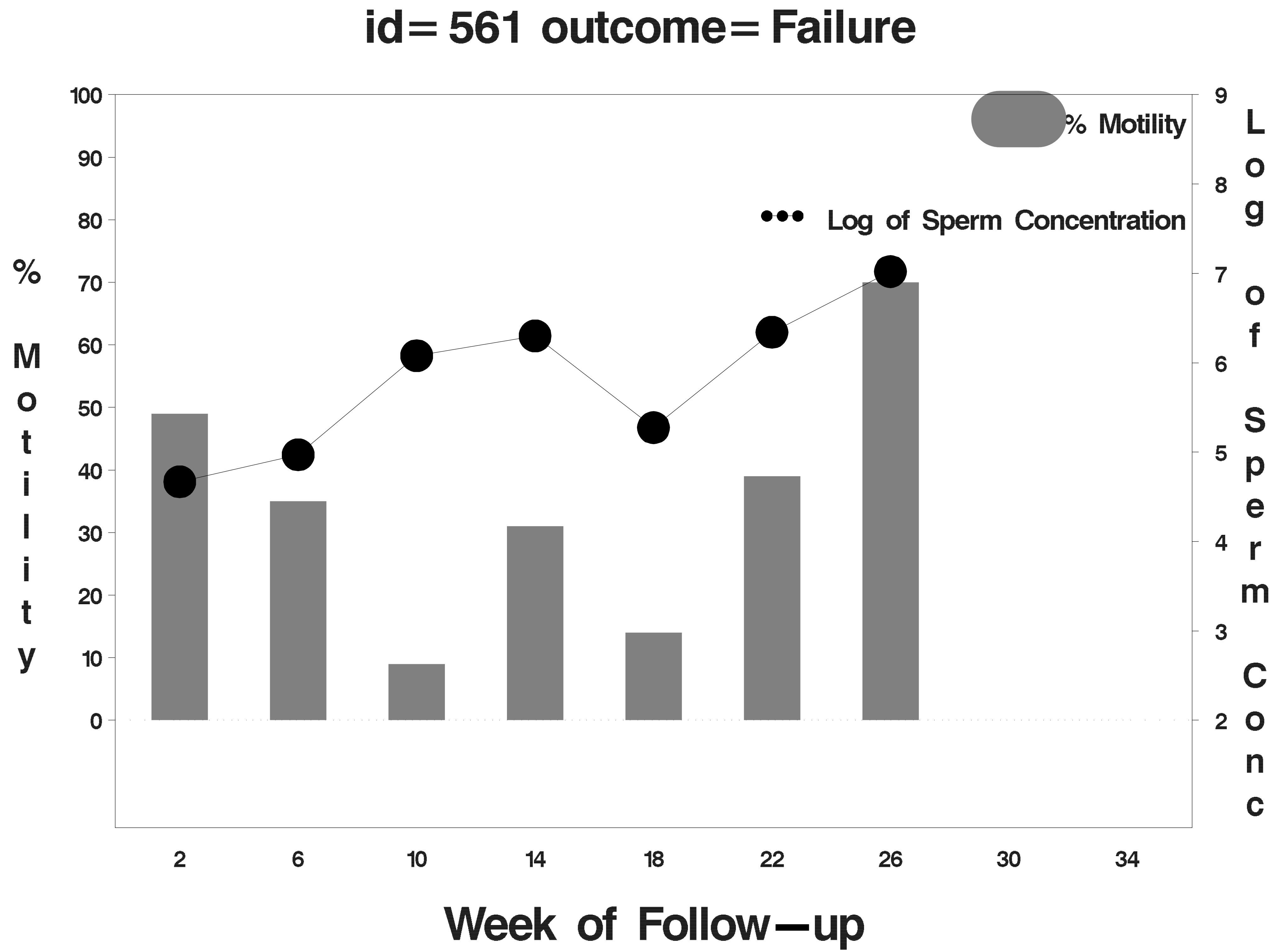

id= 561 outcome= Failure
% Motility
Log of Sperm Concentration
100
90
80
70
60
50
40
30
20
10
0
9
8
7
6
5
4
3
2
% Motility
Log of Sperm Conc
2
6
10
14
18
22
26
30
34
Week of Follow—up

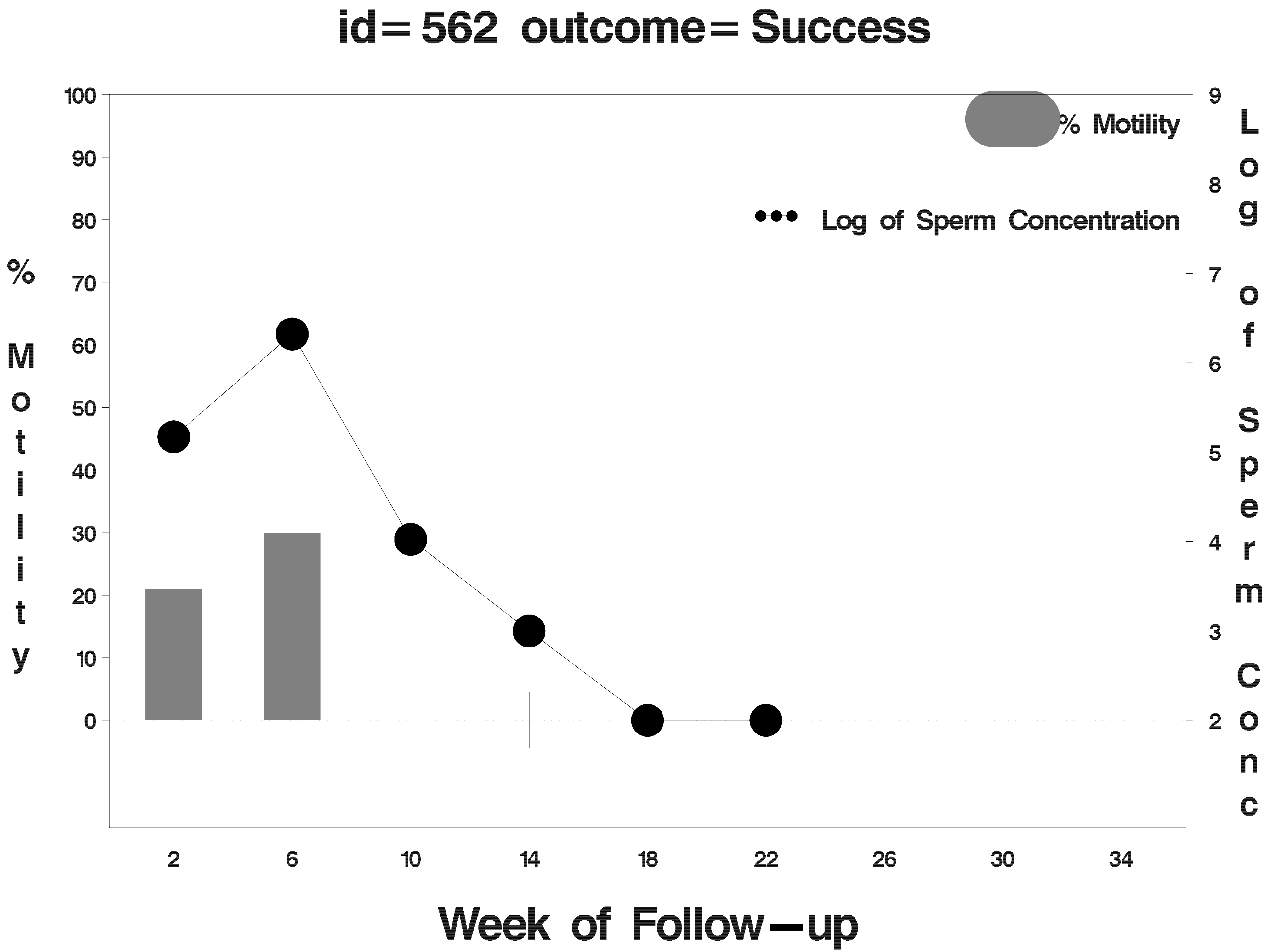
id= 562 outcome= Success
% Motility
Log of Sperm Concentration
100
90
80
70
60
50
40
30
20
10
0
9
8
7
6
5
4
3
2
2
6
10
14
18
22
26
30
34
Week of Follow−up
% Motility
Log of Sperm Conc

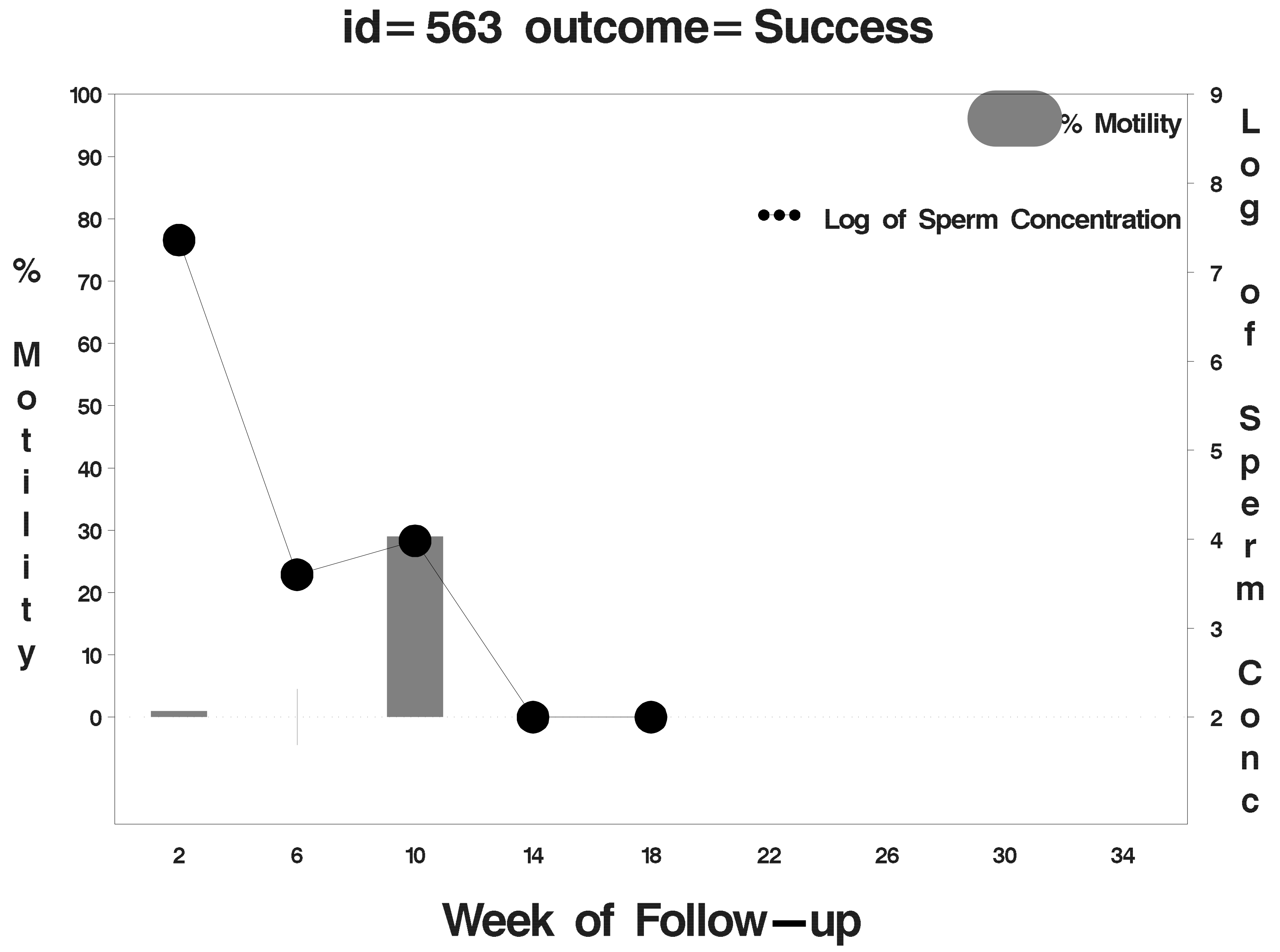
id= 563 outcome= Success
% Motility
Log of Sperm Concentration
100
90
80
70
60
50
40
30
20
10
0
9
8
7
6
5
4
3
2
2
6
10
14
18
22
26
30
34
Week of Follow−up
Log of Sperm Conc

id= 564 outcome= Indeterminate

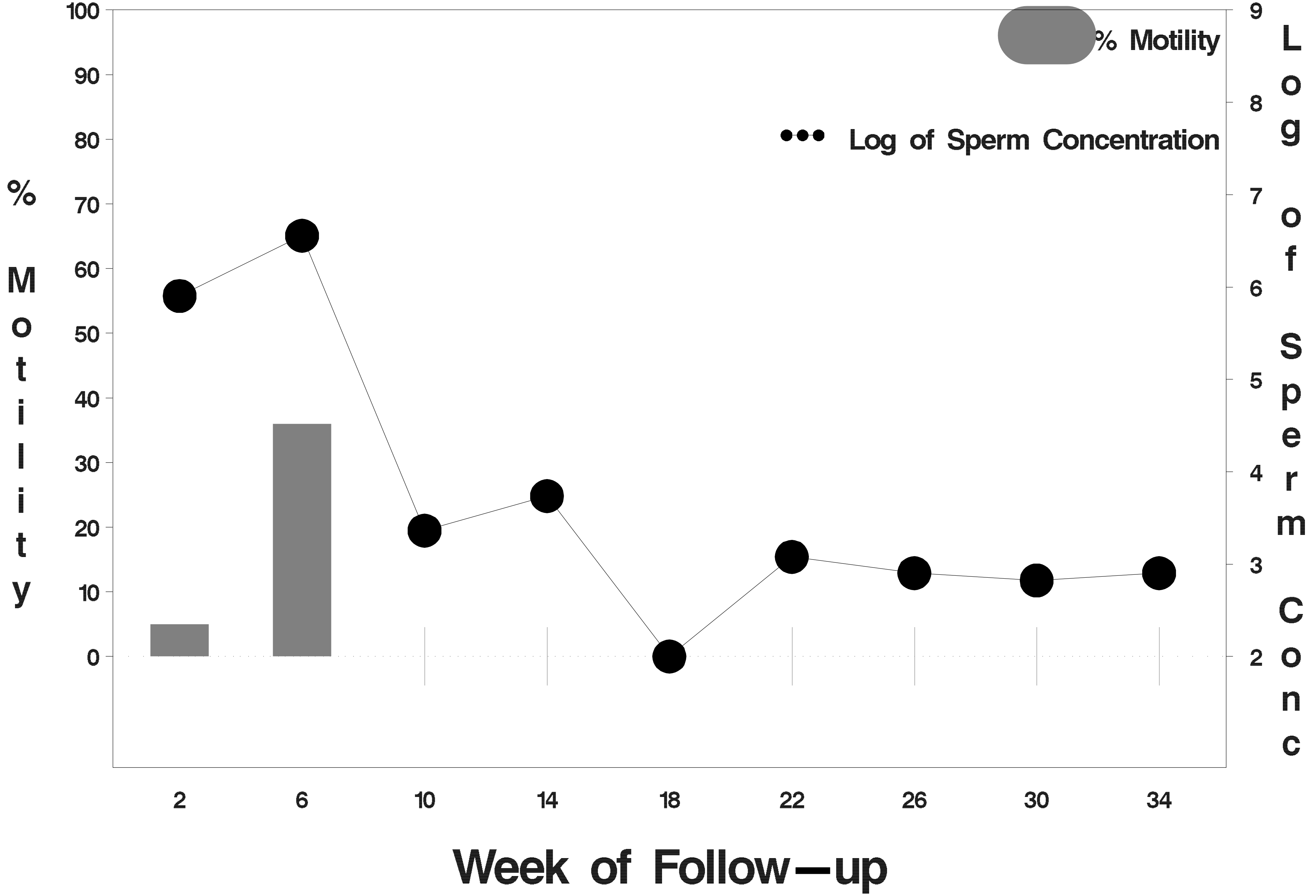

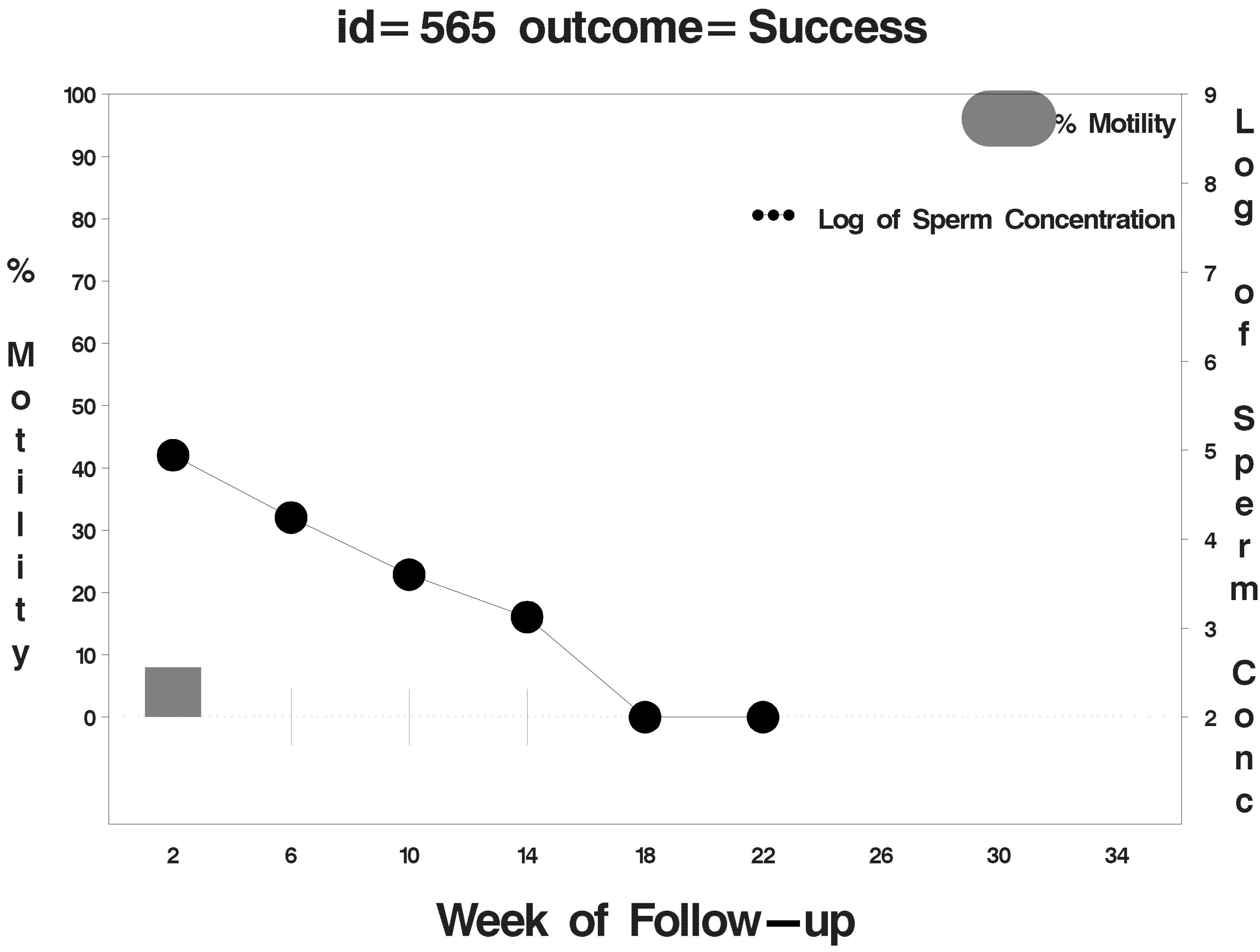
id= 565 outcome= Success
% Motility
Log of Sperm Concentration
100
90
80
70
60
50
40
30
20
10
0
9
8
7
6
5
4
3
2
2
6
10
14
18
22
26
30
34
Week of Follow—up
Log of Sperm Conc

id= 566 outcome= Failure

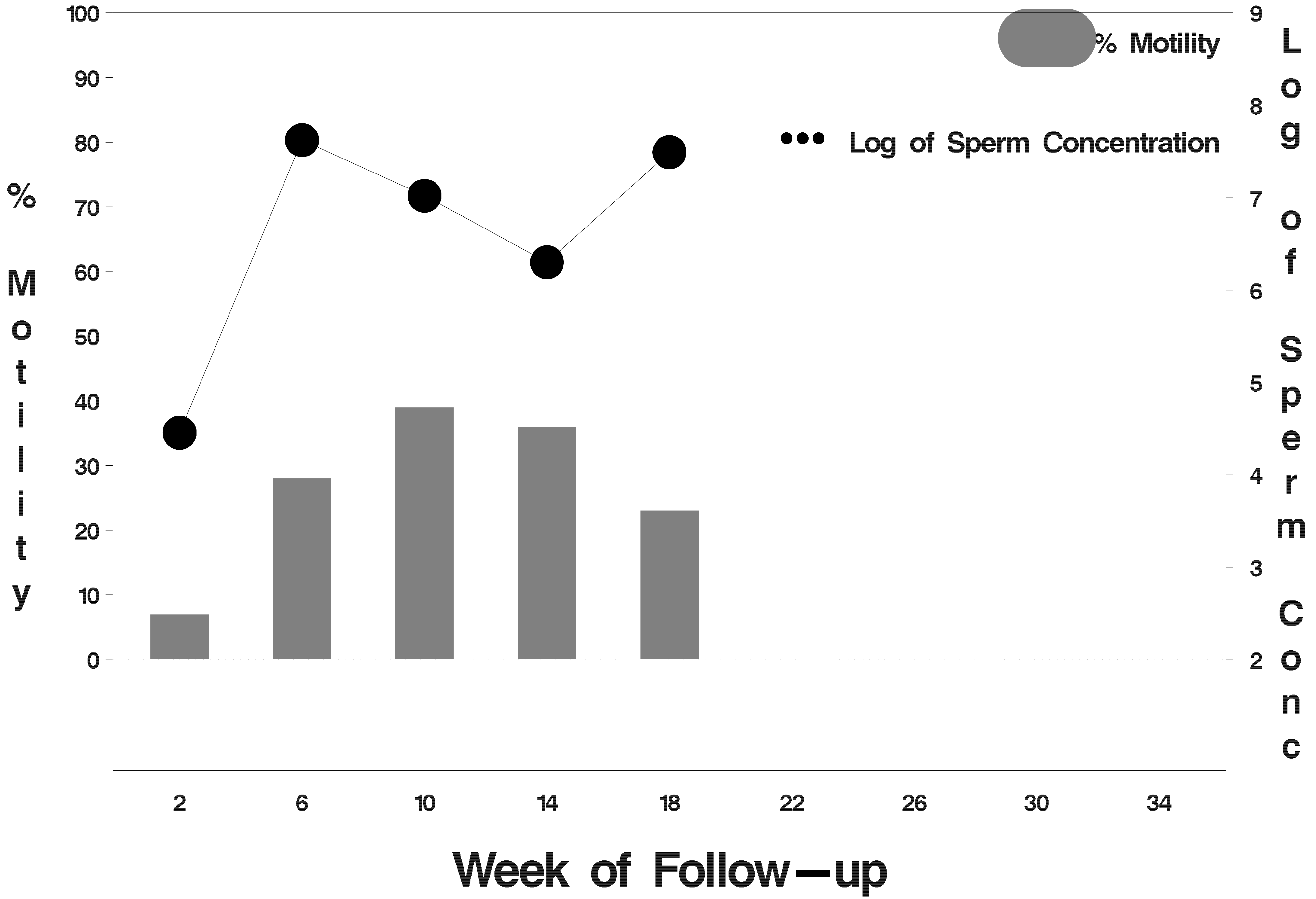

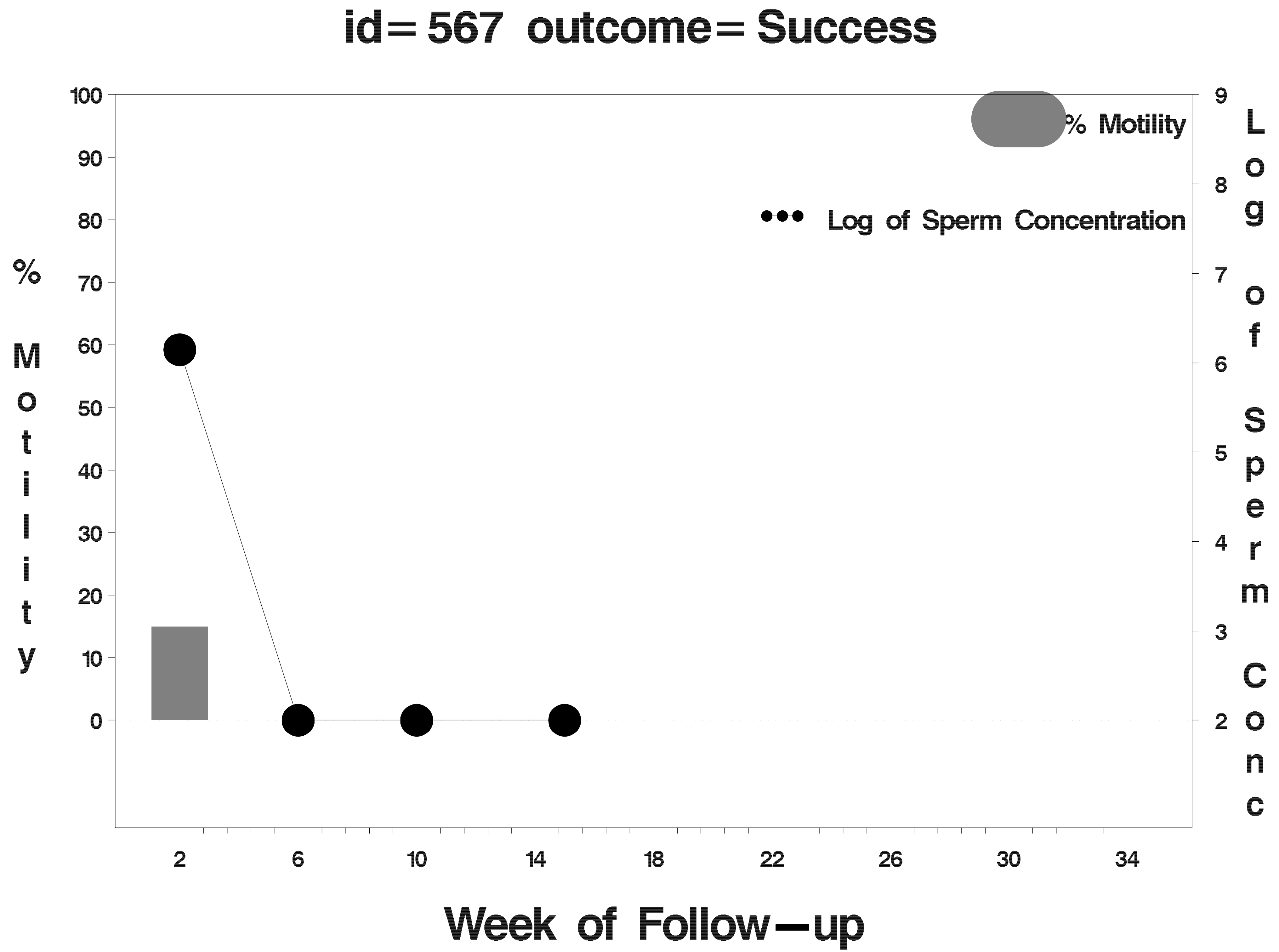

id= 567 outcome= Success
% Motility
Log of Sperm Concentration
100
90
80
70
60
50
40
30
20
10
0
9
8
7
6
5
4
3
2
2
6
10
14
18
22
26
30
34
Week of Follow−up
% Motility
Log of Sperm Conc

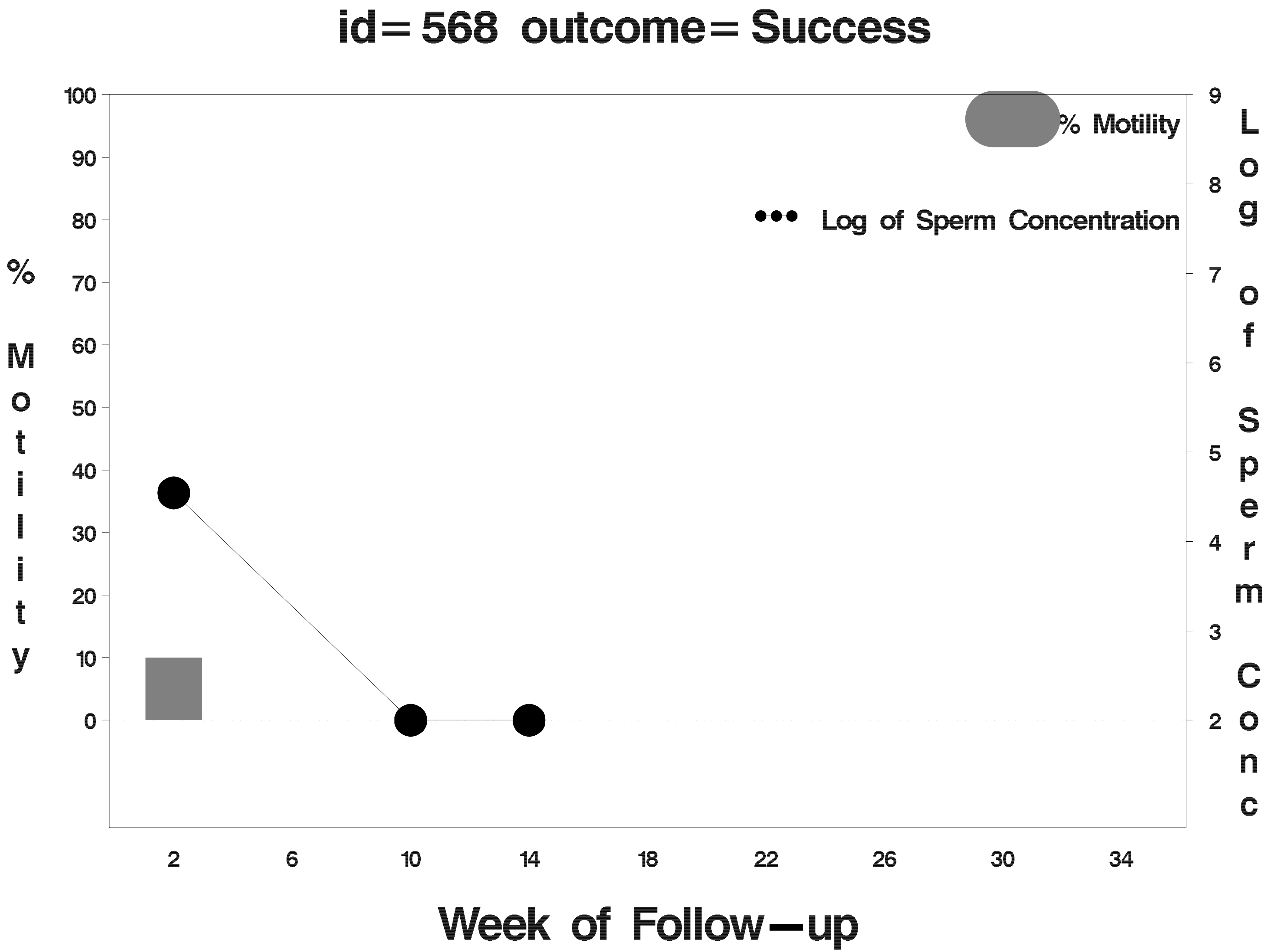

id= 568 outcome= Success
% Motility
Log of Sperm Concentration
100
90
80
70
60
50
40
30
20
10
0
9
8
7
6
5
4
3
2
% Motility
Log of Sperm Conc
2
6
10
14
18
22
26
30
34
Week of Follow—up

id= 569 outcome= Indeterminate

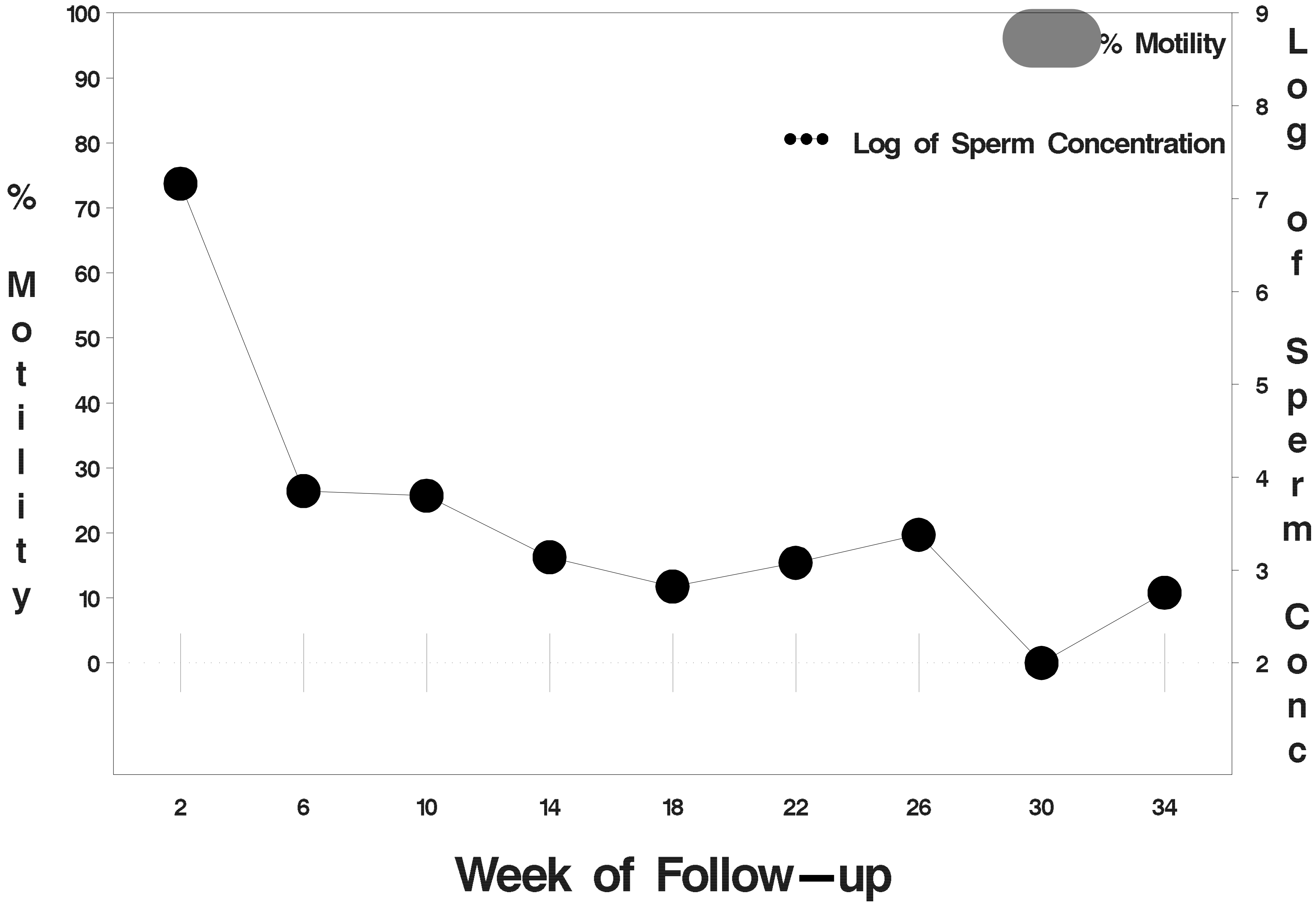

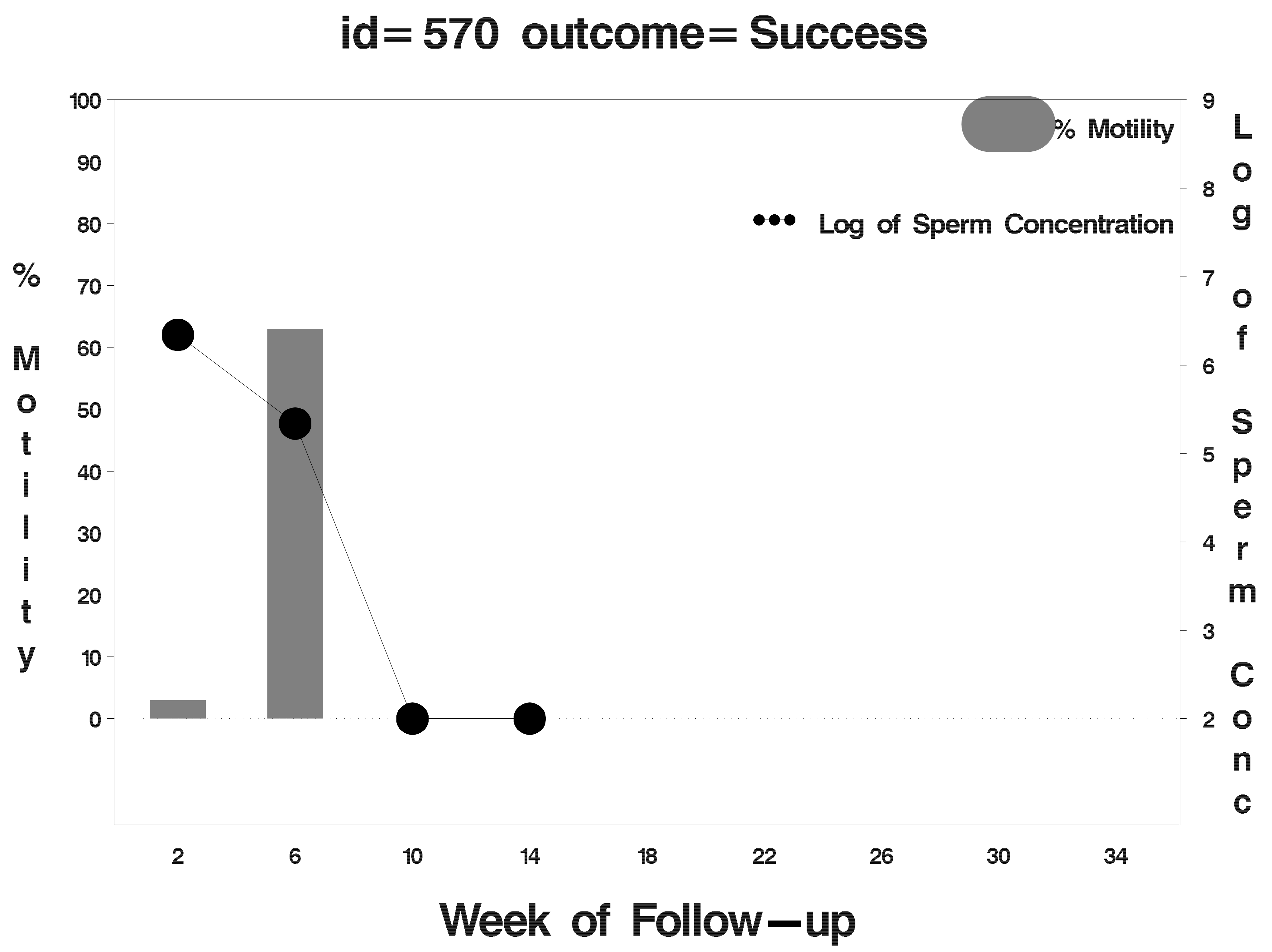

id= 570 outcome= Success
% Motility
Log of Sperm Concentration
100
90
80
70
60
50
40
30
20
10
0
9
8
7
6
5
4
3
2
2
6
10
14
18
22
26
30
34
Week of Follow—up
% Motility
Log of Sperm Conc

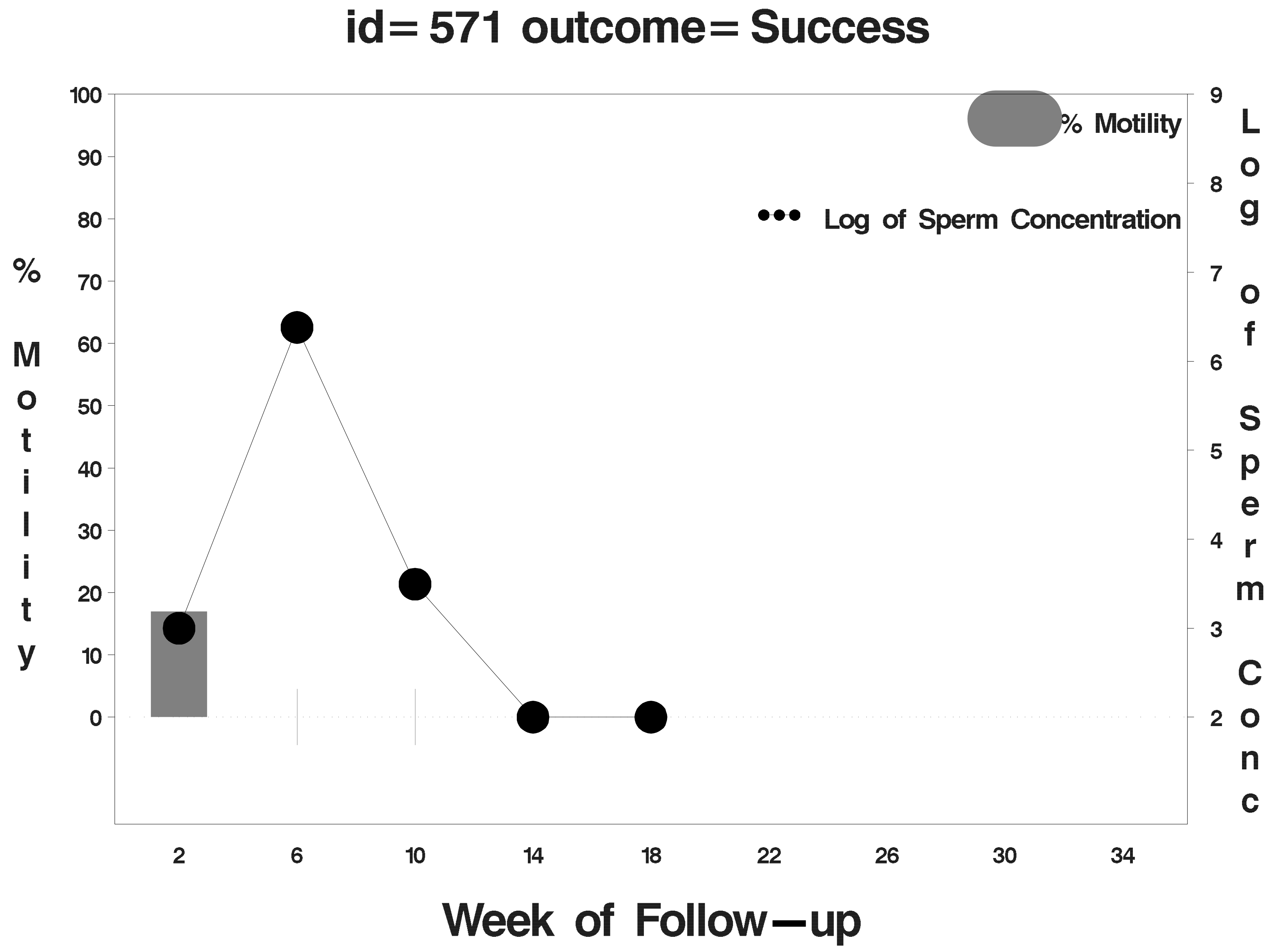
id= 571 outcome= Success
% Motility
Log of Sperm Concentration
100
90
80
70
60
50
40
30
20
10
0
9
8
7
6
5
4
3
2
2
6
10
14
18
22
26
30
34
Week of Follow−up
Log of Sperm Conc

id= 572 outcome= Success

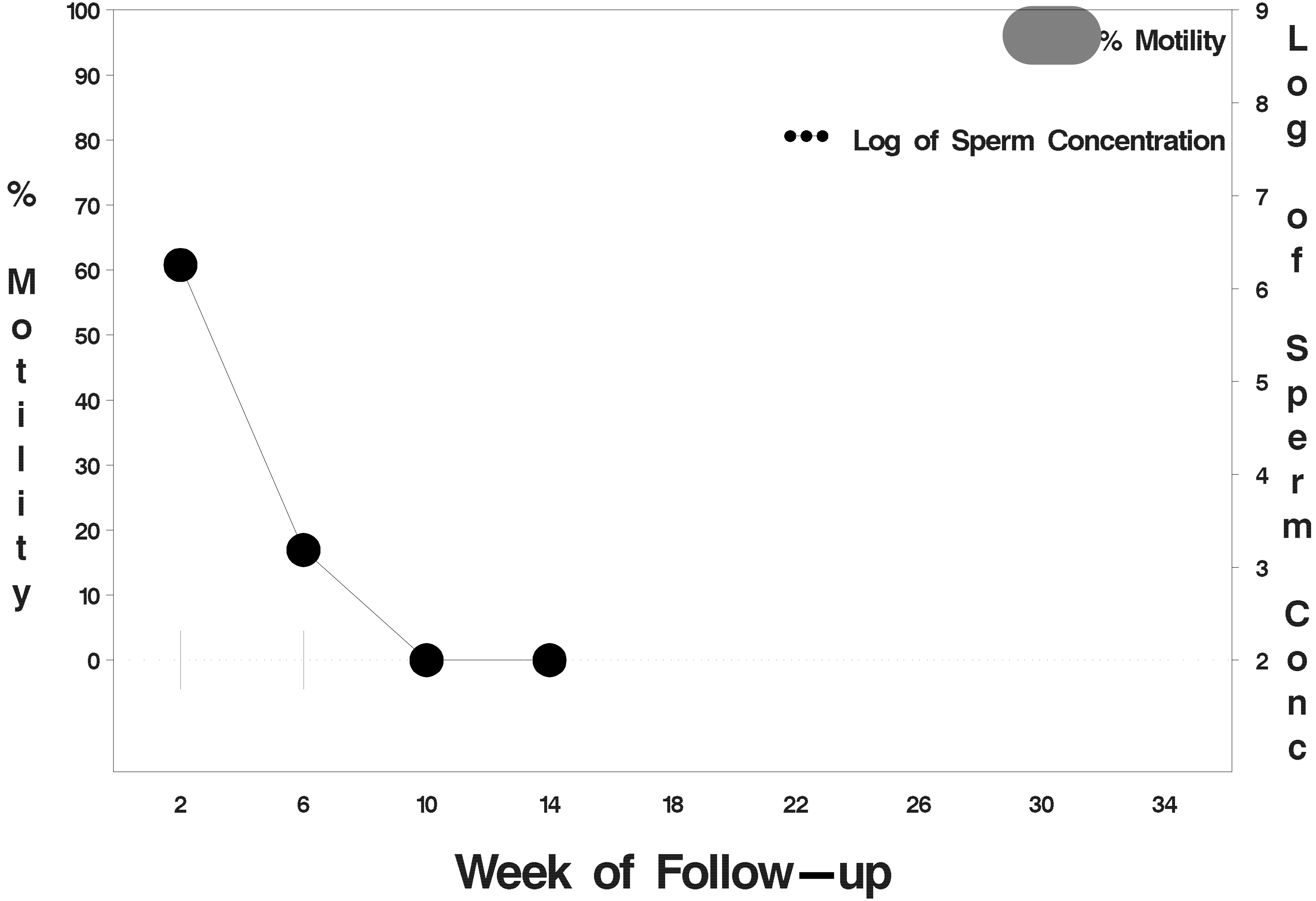

id= 573 outcome= Success

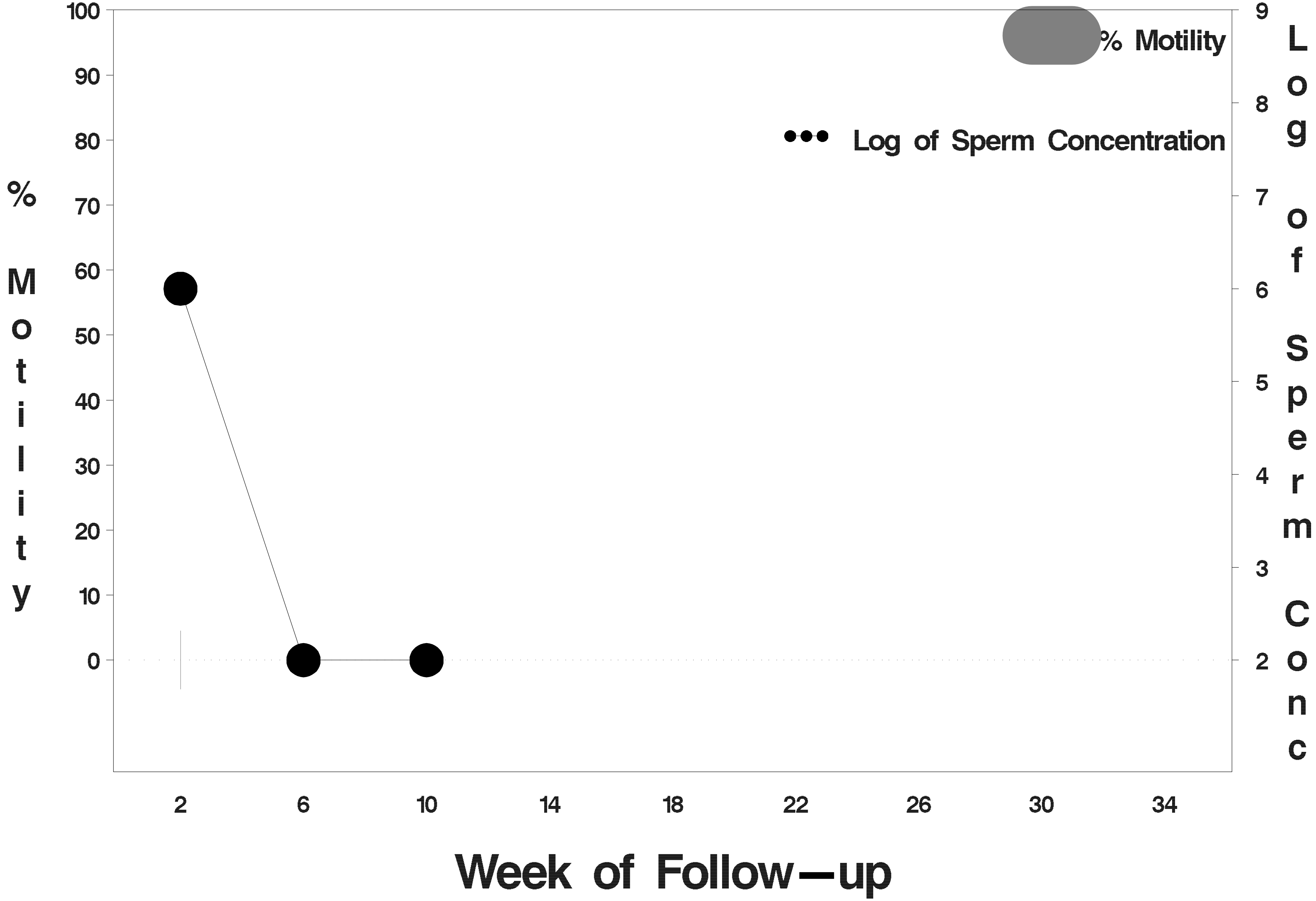

id= 574 outcome= Success

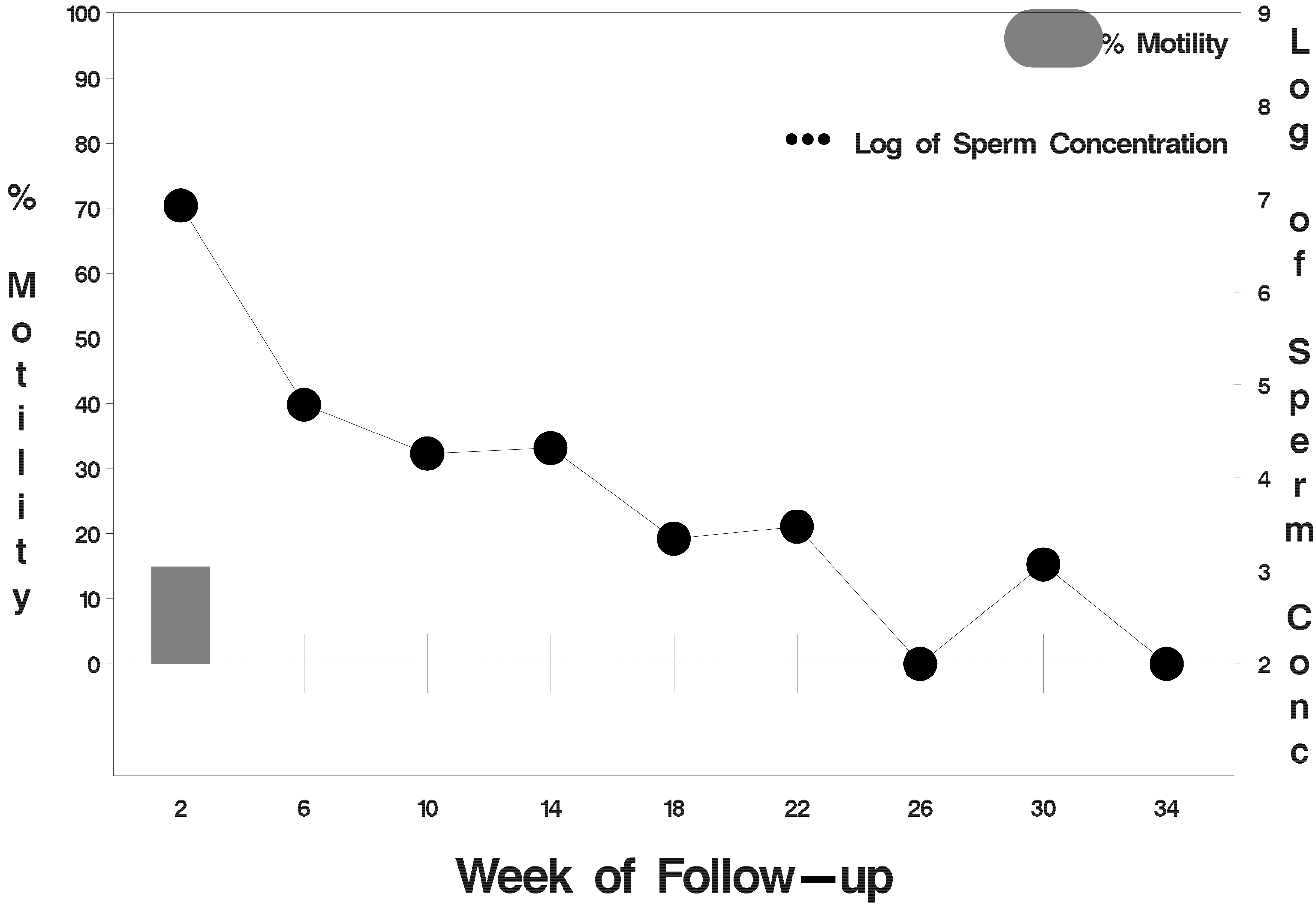

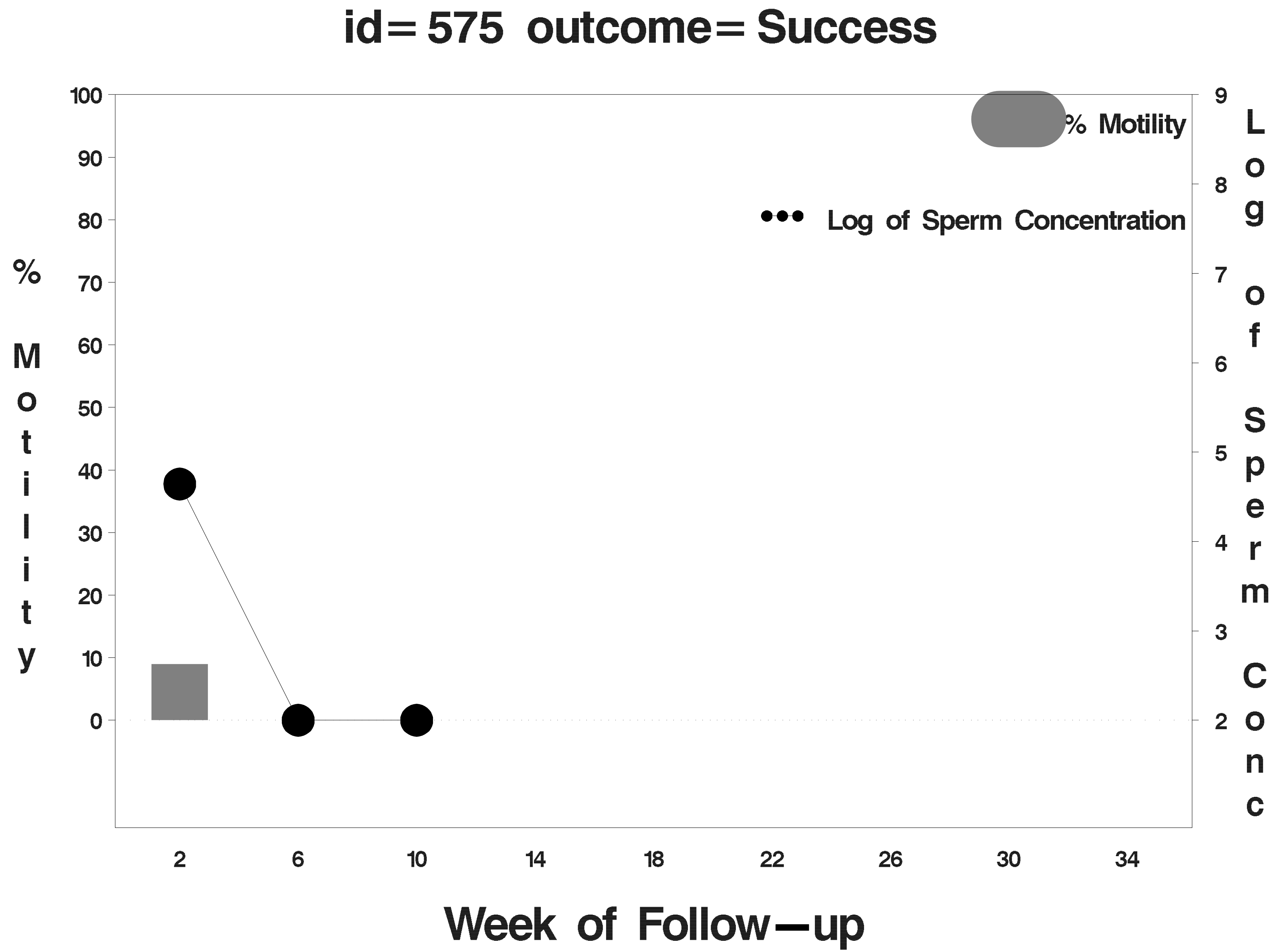

id= 575 outcome= Success
% Motility
Log of Sperm Concentration
100
90
80
70
60
50
40
30
20
10
0
9
8
7
6
5
4
3
2
% Motility
Log of Sperm Conc
2
6
10
14
18
22
26
30
34
Week of Follow-up

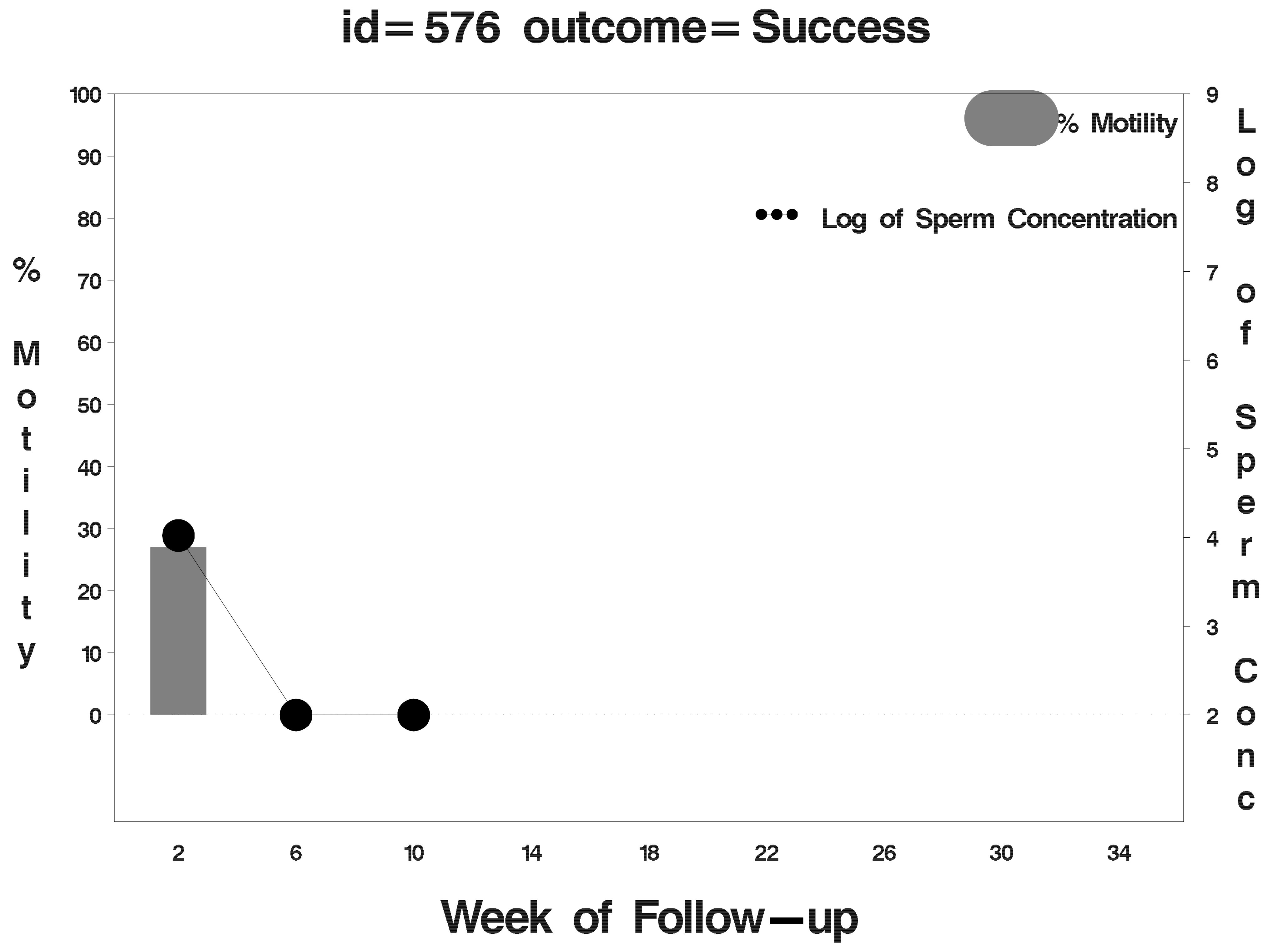
id= 576 outcome= Success
% Motility
Log of Sperm Concentration
% Motility
Log of Sperm Conc
Week of Follow−up
100
90
80
70
60
50
40
30
20
10
0
9
8
7
6
5
4
3
2
2
6
10
14
18
22
26
30
34

id= 577 outcome= Success

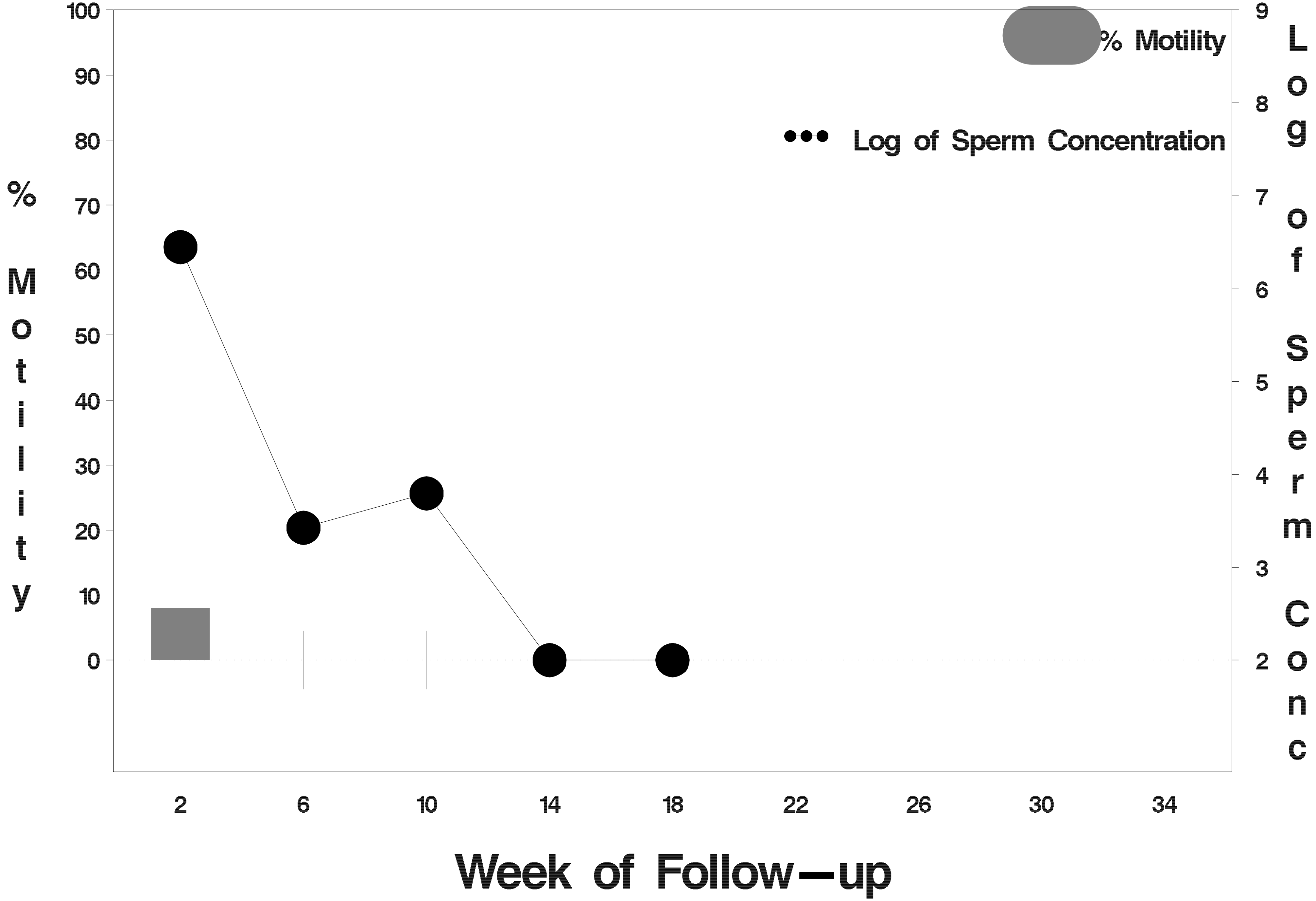

id= 578 outcome= Failure

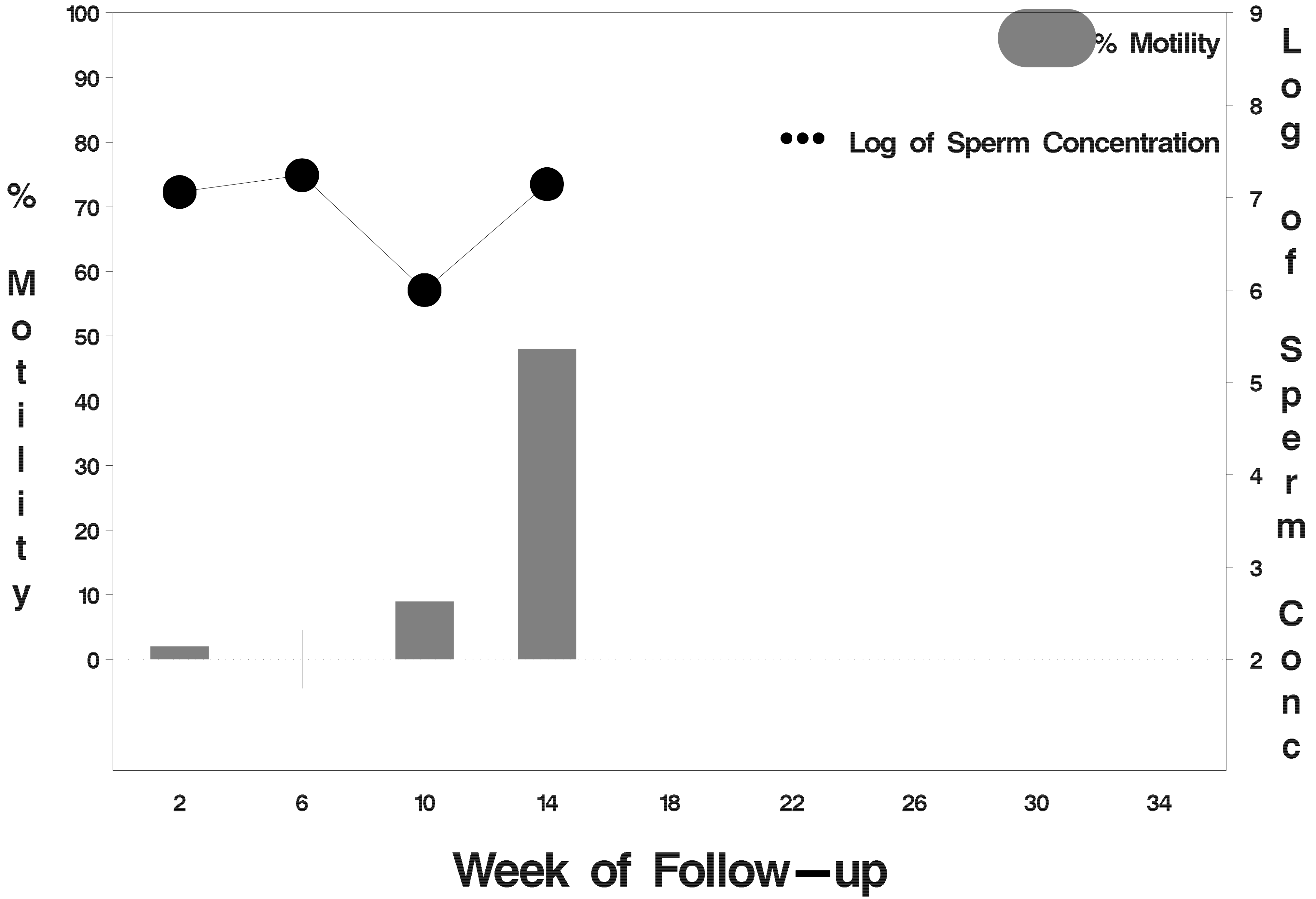

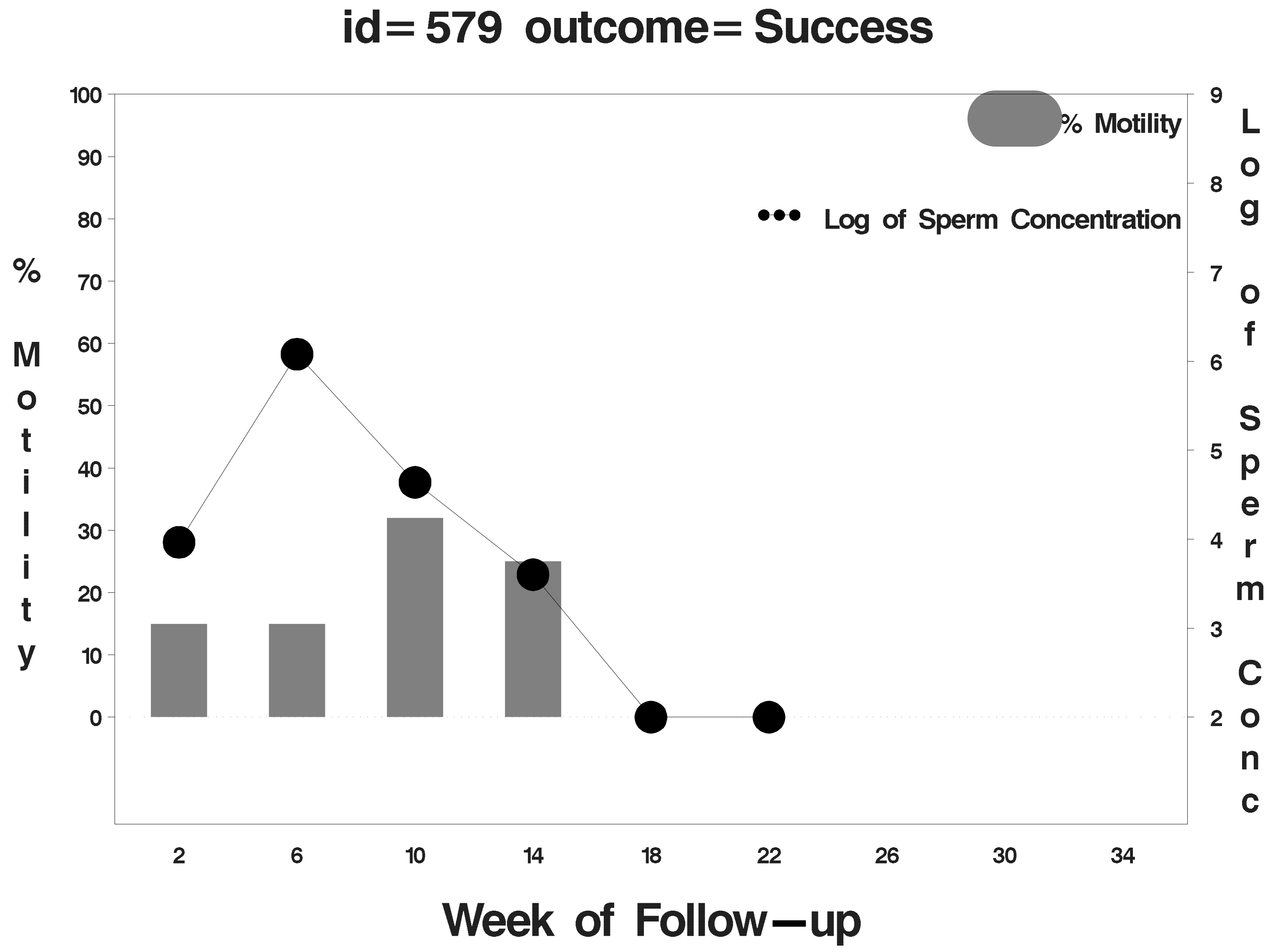
id= 579 outcome= Success
% Motility
Log of Sperm Concentration
% Motility
Log of Sperm Conc
Week of Follow-up
100
90
80
70
60
50
40
30
20
10
0
9
8
7
6
5
4
3
2
2
6
10
14
18
22
26
30
34

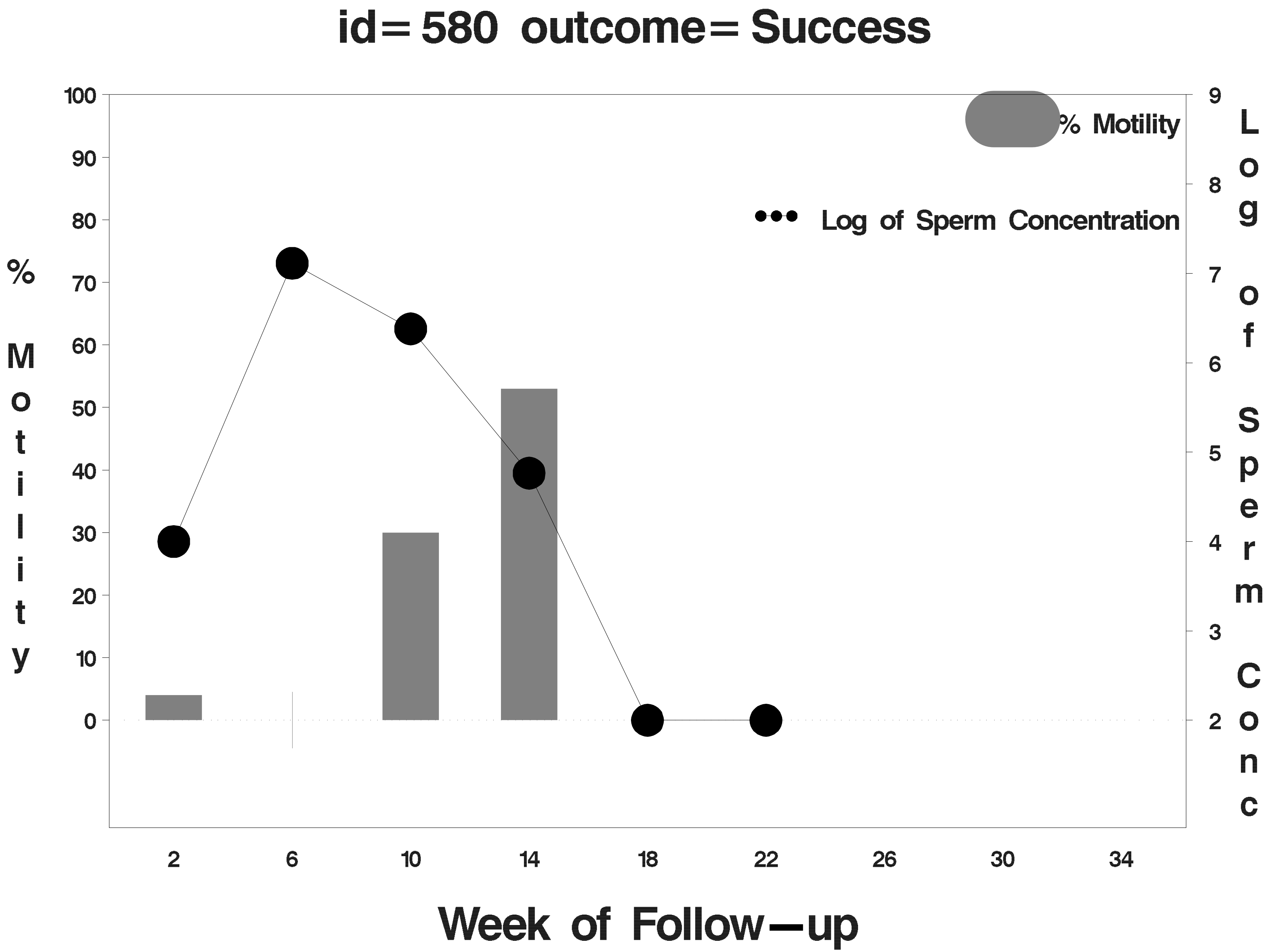
id= 580 outcome= Success
% Motility
Log of Sperm Concentration
100
90
80
70
60
50
40
30
20
10
0
9
8
7
6
5
4
3
2
2
6
10
14
18
22
26
30
34
Week of Follow−up
Log of Sperm Conc

id= 581 outcome= Success

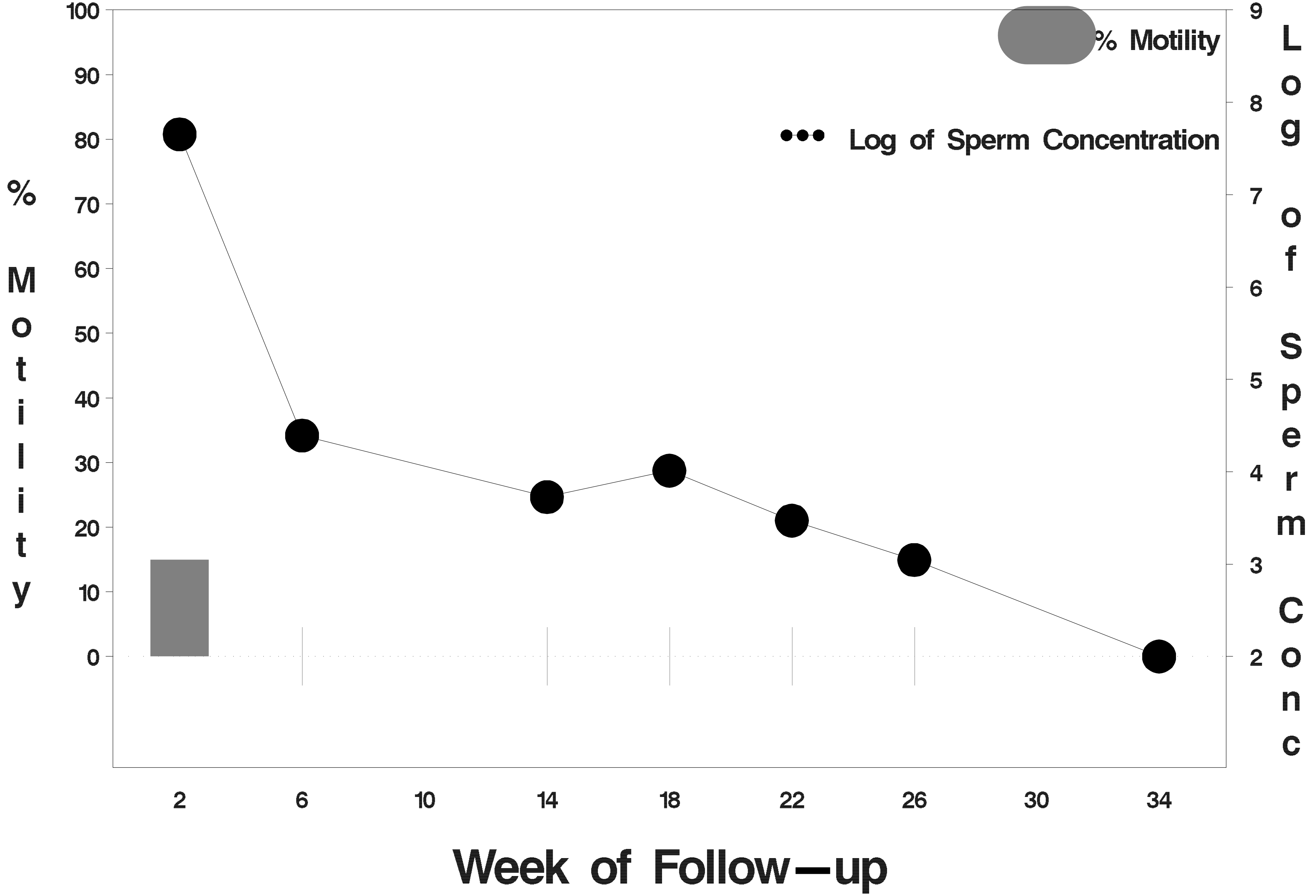

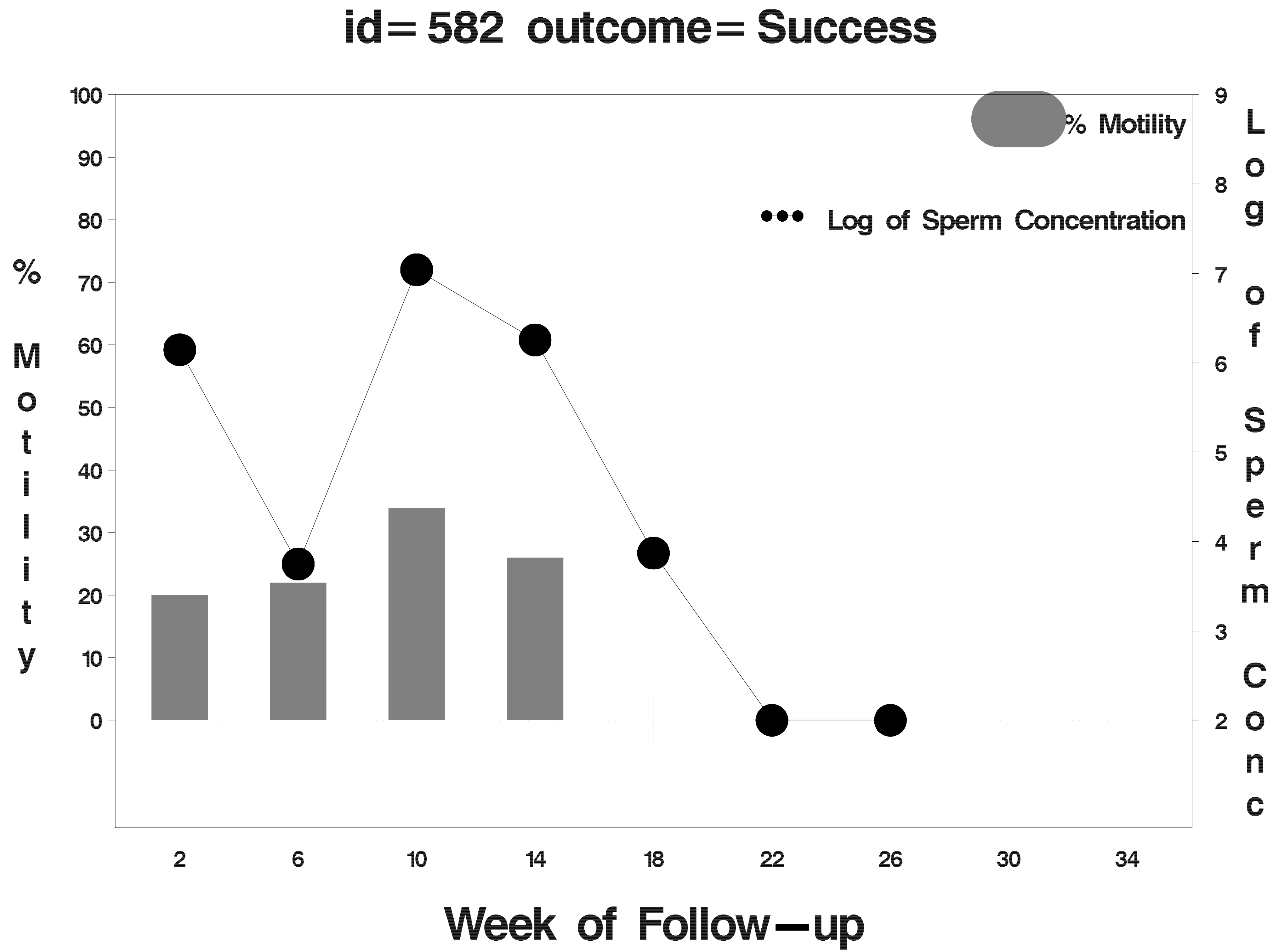

id= 582 outcome= Success
% Motility
Log of Sperm Concentration
100
90
80
70
60
50
40
30
20
10
0
9
8
7
6
5
4
3
2
2
6
10
14
18
22
26
30
34
Week of Follow−up
Log of Sperm Conc

id= 583 outcome= Success

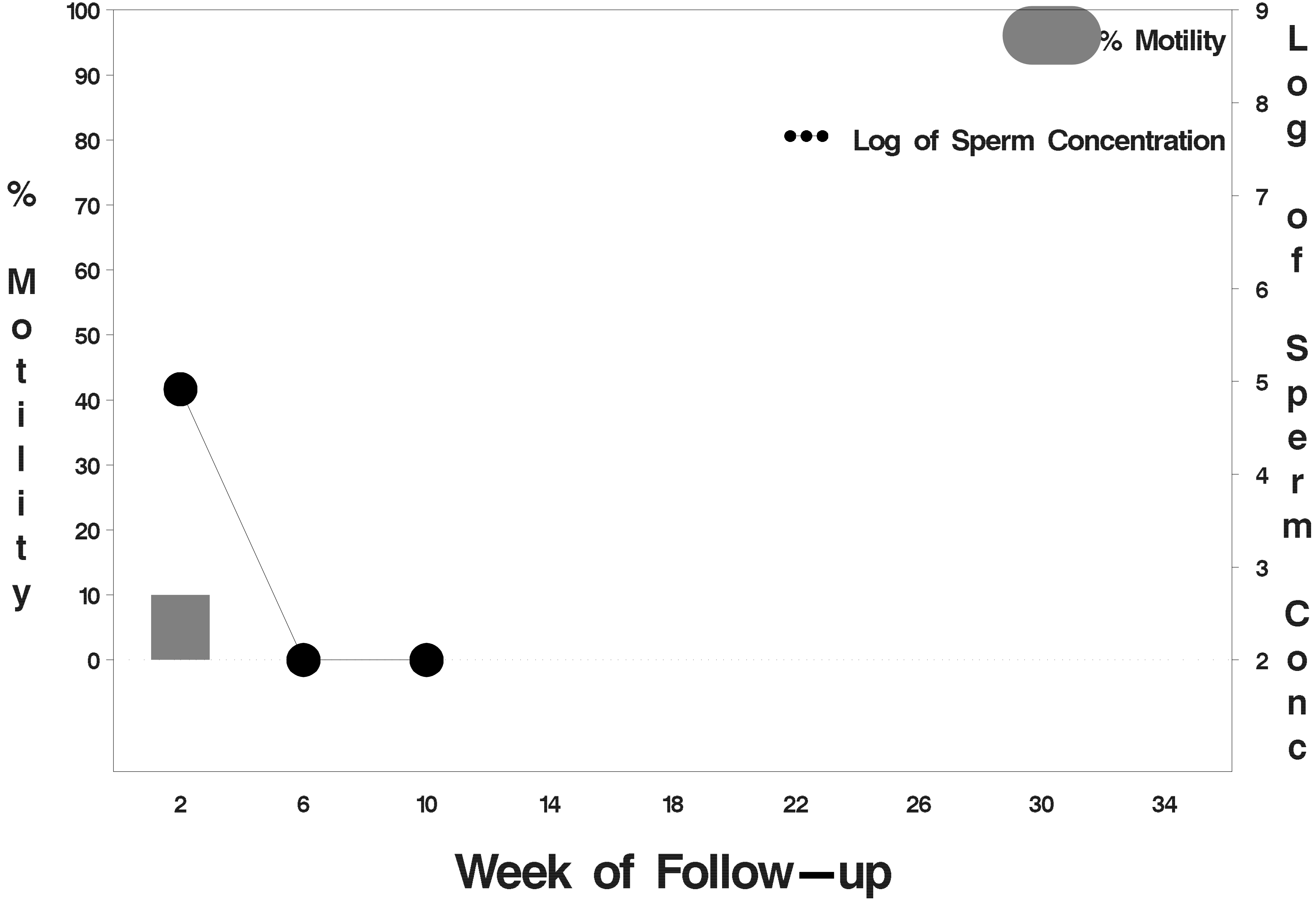

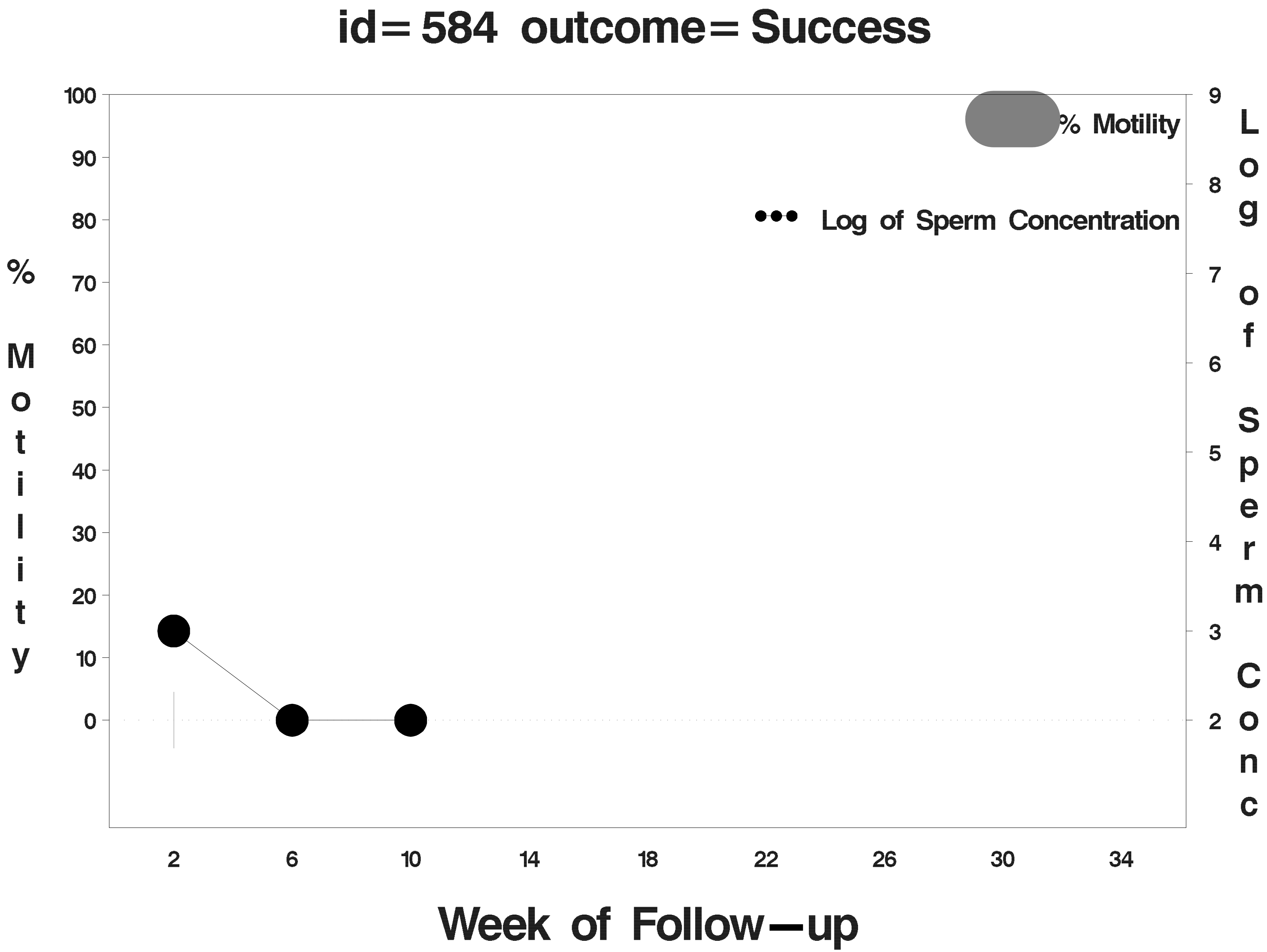

id= 584 outcome= Success
% Motility
Log of Sperm Concentration
% Motility
Log of Sperm Conc
Week of Follow—up
100
90
80
70
60
50
40
30
20
10
0
9
8
7
6
5
4
3
2
2
6
10
14
18
22
26
30
34

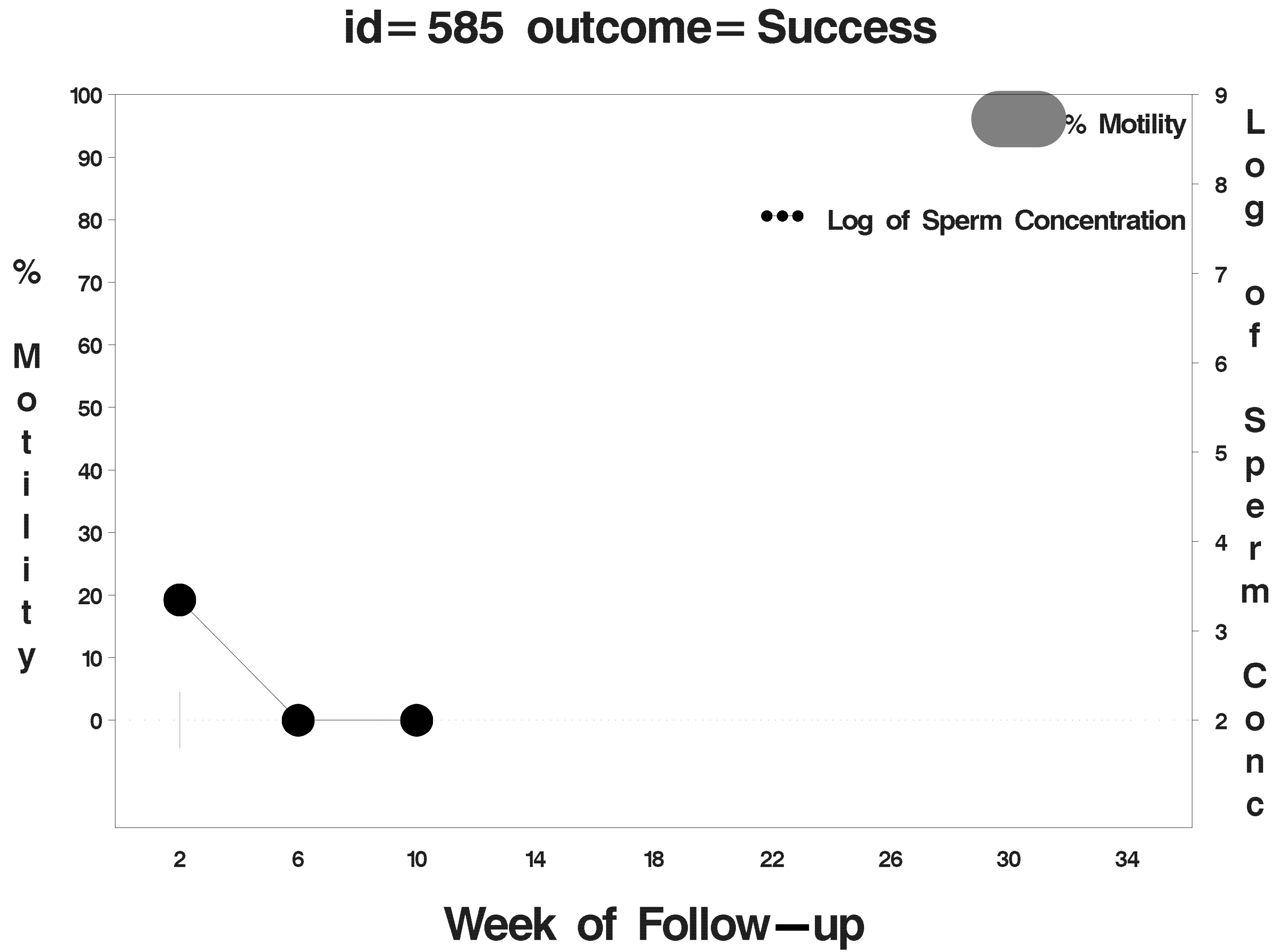

id= 585 outcome= Success
% Motility
Log of Sperm Concentration
100
90
80
70
60
50
40
30
20
10
0
9
8
7
6
5
4
3
2
2
6
10
14
18
22
26
30
34
Week of Follow−up

id= 586 outcome= Success

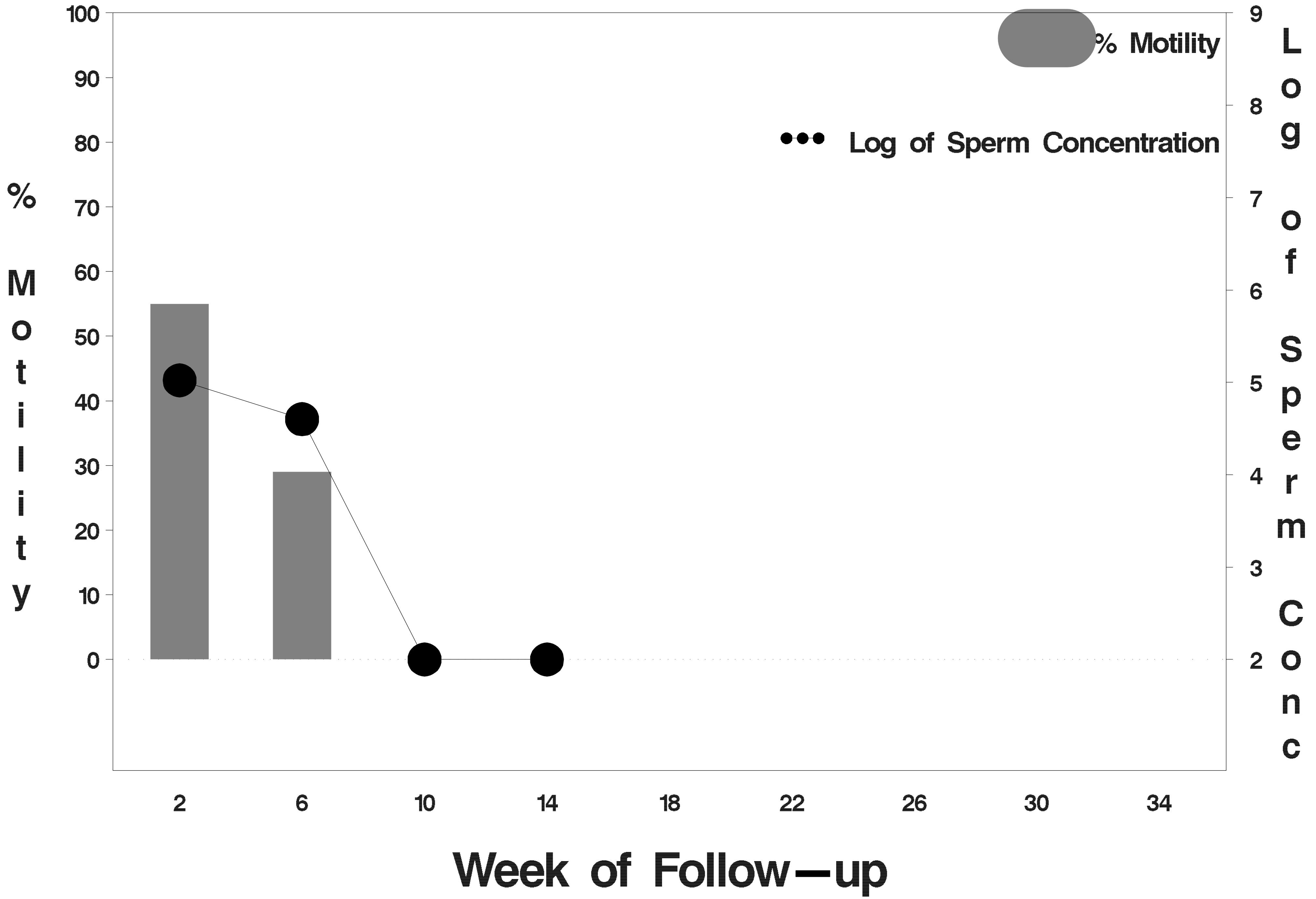

id= 587 outcome= Success

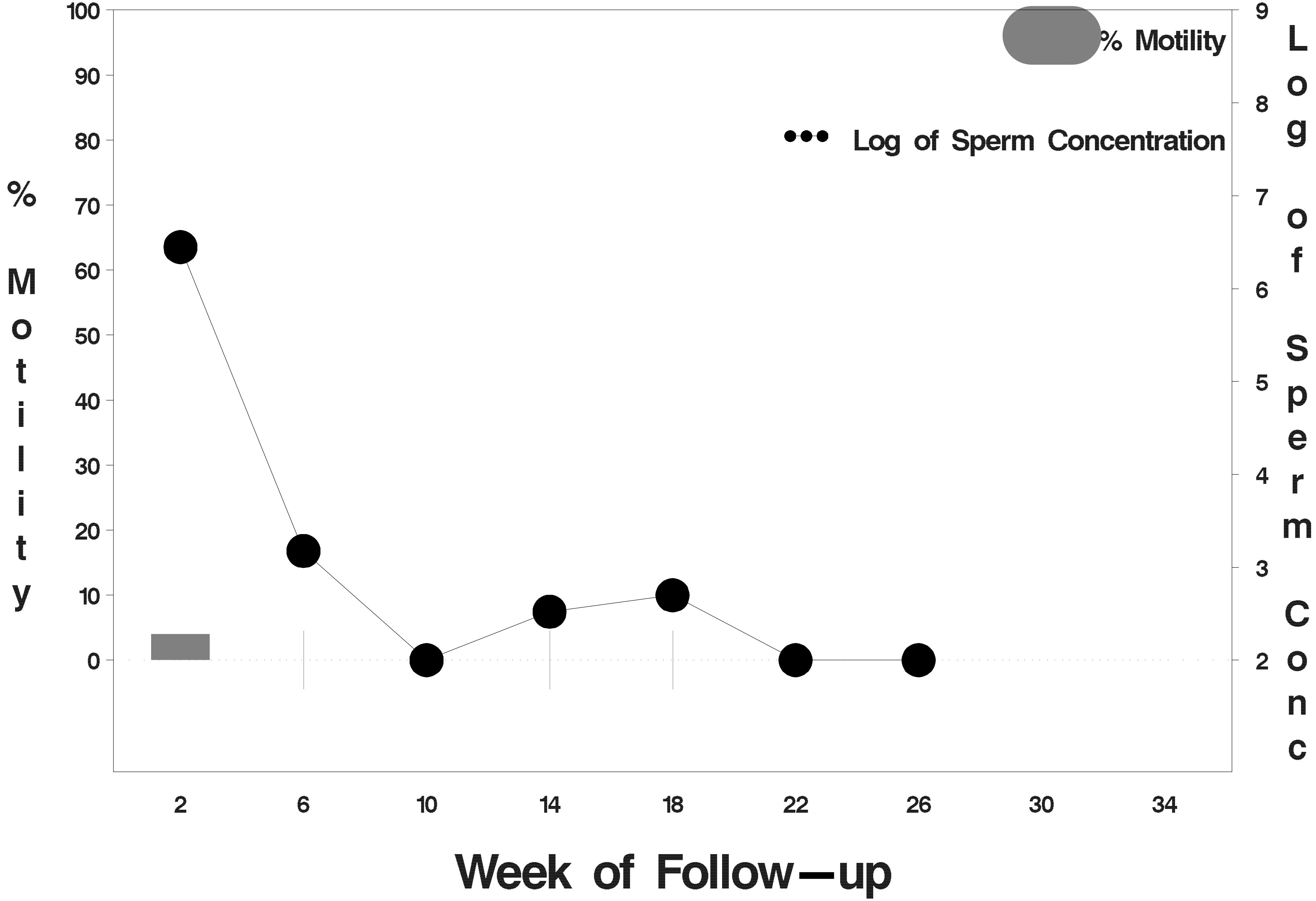

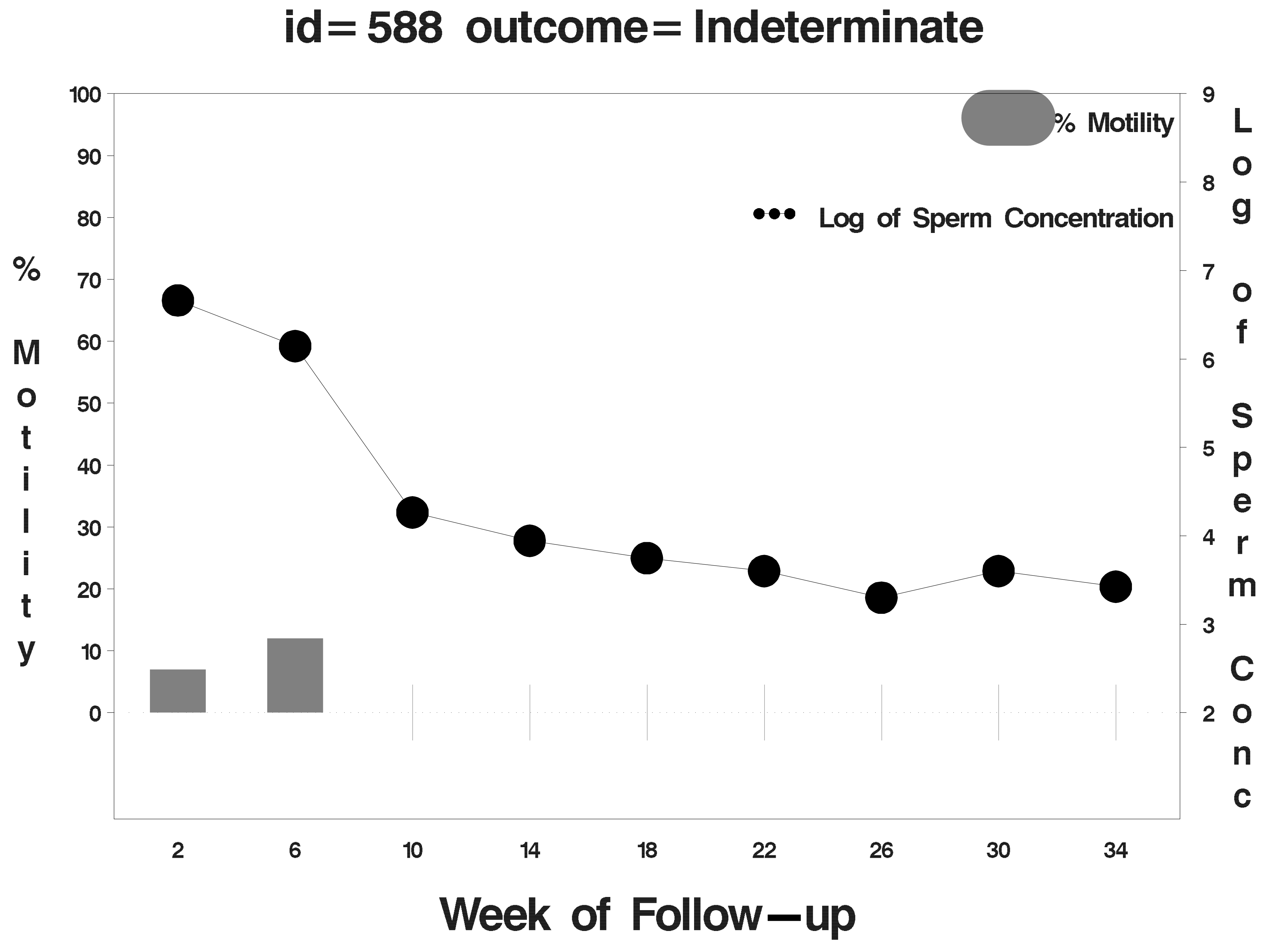

id= 588 outcome= Indeterminate
% Motility
Log of Sperm Concentration
100
90
80
70
60
50
40
30
20
10
0
9
8
7
6
5
4
3
2
2
6
10
14
18
22
26
30
34
Week of Follow-up
Log of Sperm Conc

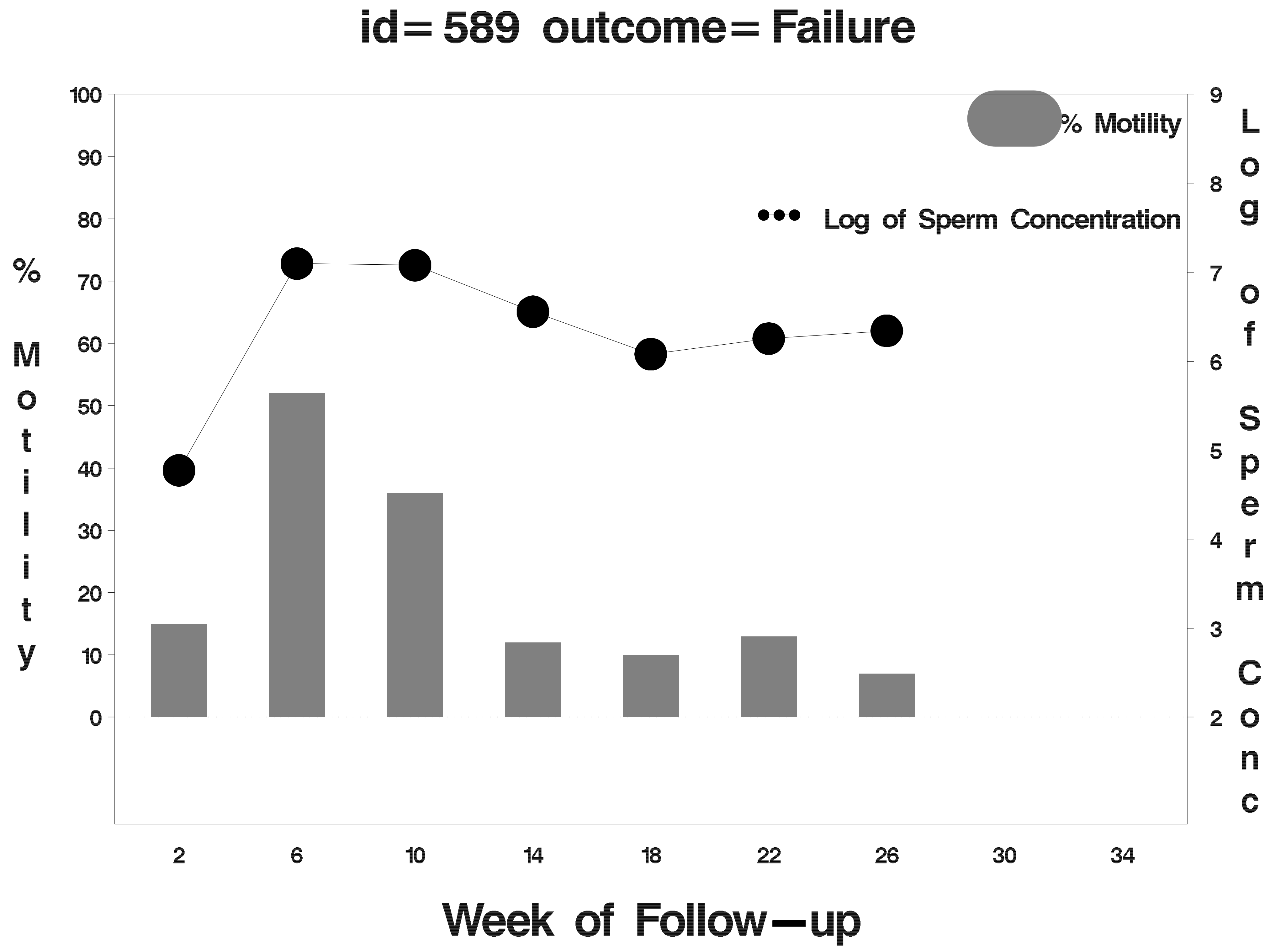

id= 589 outcome= Failure
% Motility
Log of Sperm Concentration
% Motility
Log of Sperm Conc
100
90
80
70
60
50
40
30
20
10
0
9
8
7
6
5
4
3
2
2
6
10
14
18
22
26
30
34
Week of Follow-up

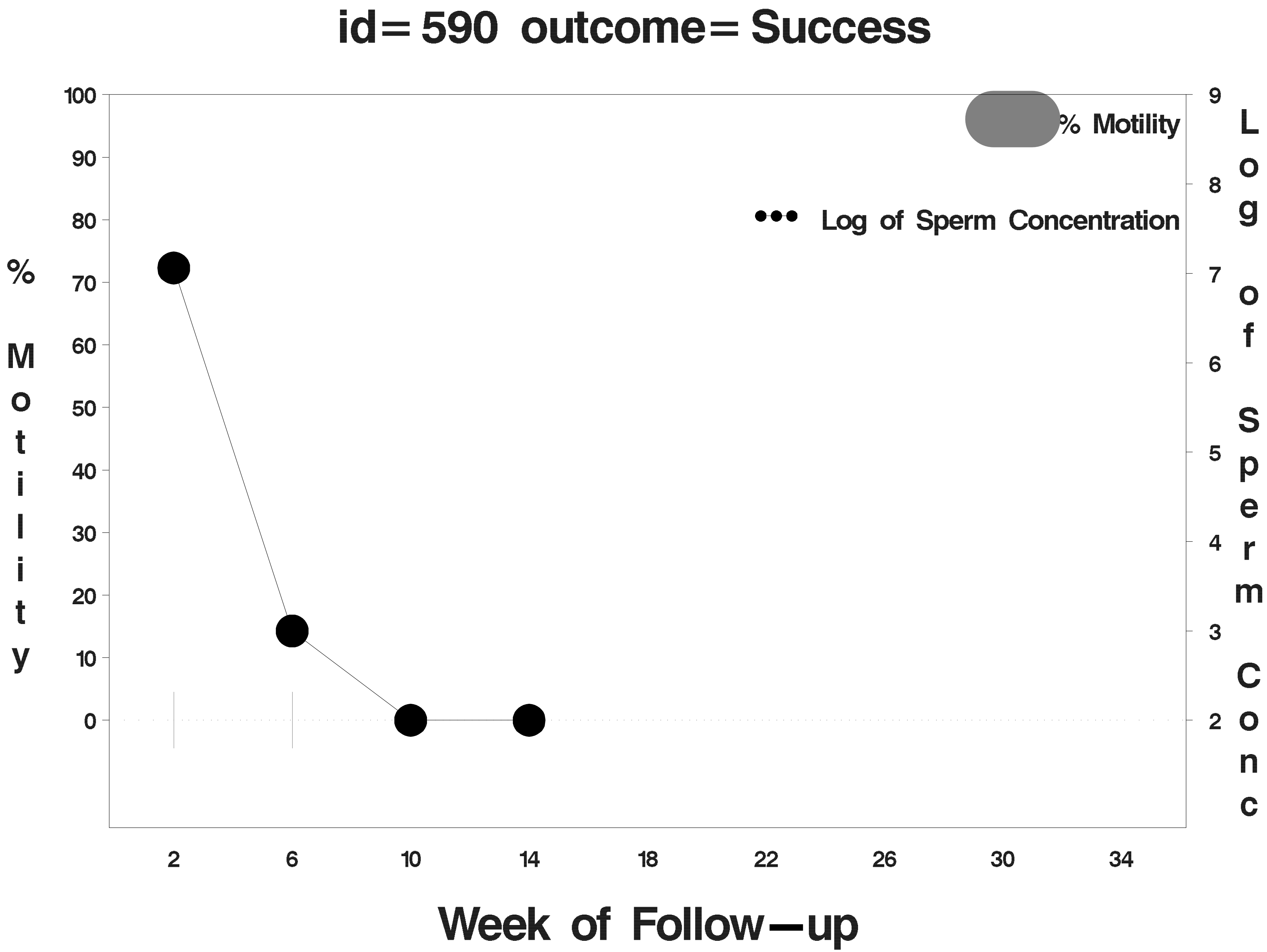
id= 590 outcome= Success
100
90
80
70
60
50
40
30
20
10
0
% Motility
% Motility
Log of Sperm Concentration
9
8
7
6
5
4
3
2
Log of Sperm Conc
2
6
10
14
18
22
26
30
34
Week of Follow−up

id= 591 outcome= Success

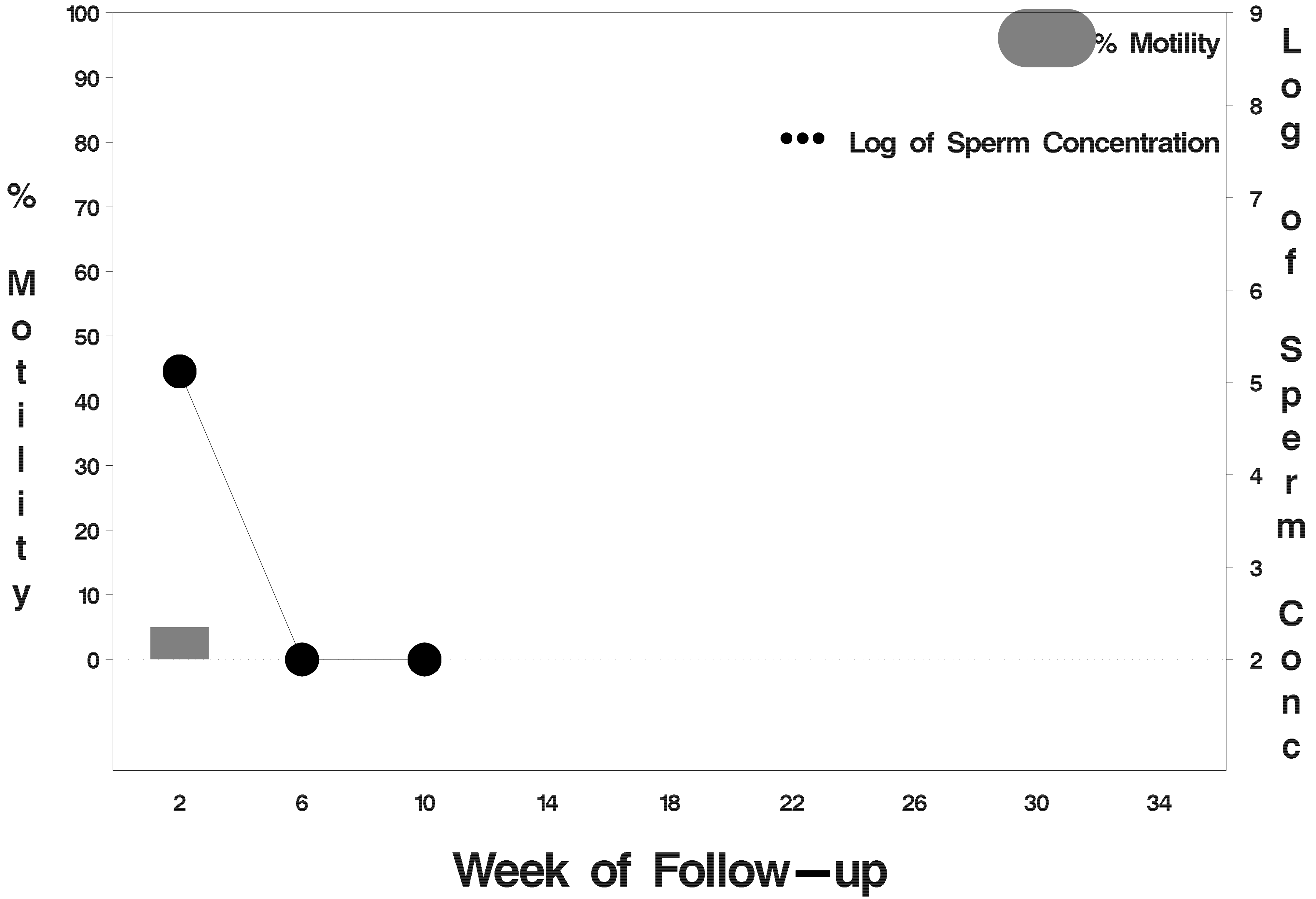

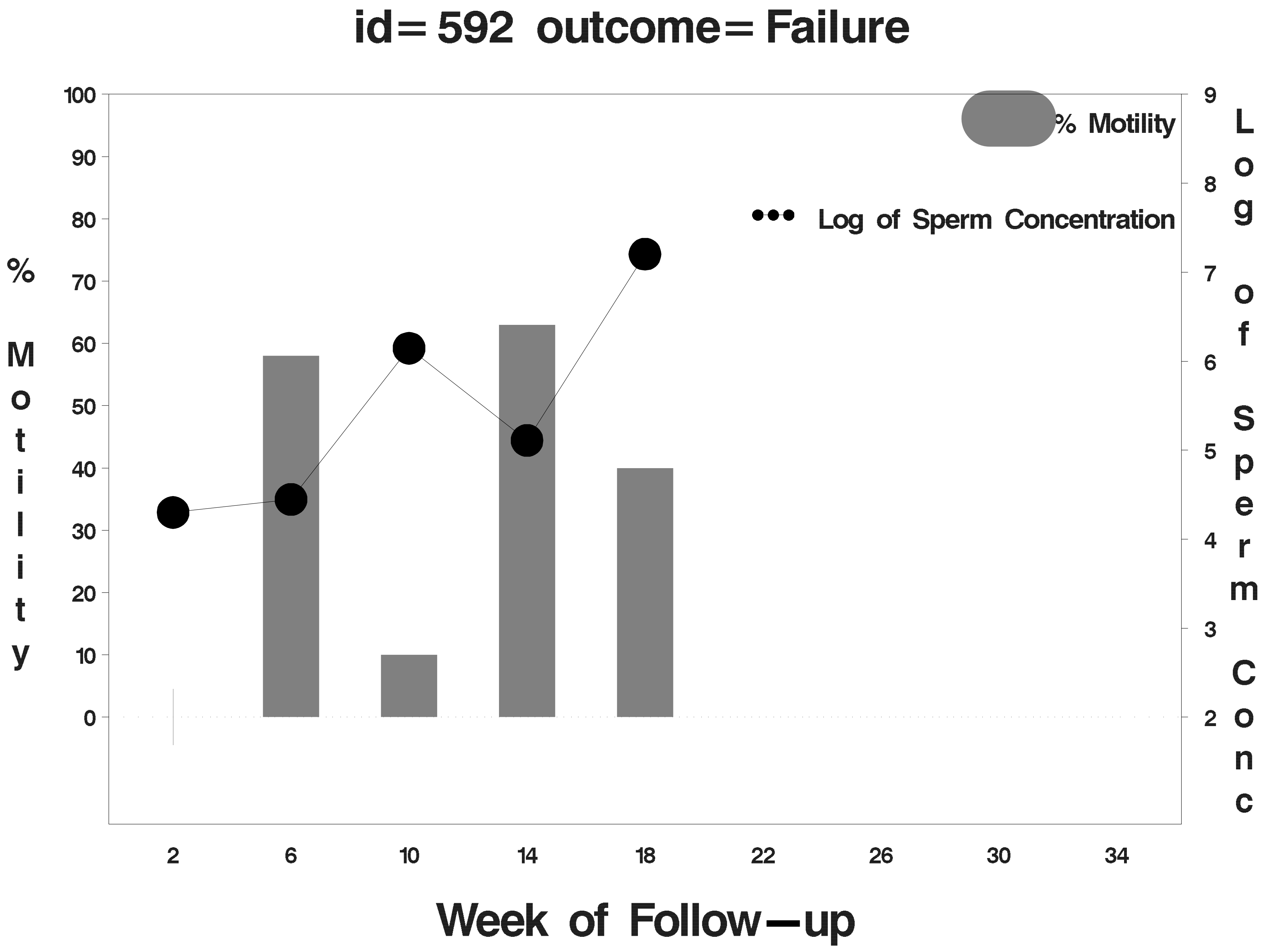
id= 592 outcome= Failure
% Motility
Log of Sperm Concentration
100
90
80
70
60
50
40
30
20
10
0
9
8
7
6
5
4
3
2
2
6
10
14
18
22
26
30
34
Week of Follow−up
Log of Sperm Conc

id= 593 outcome= Success

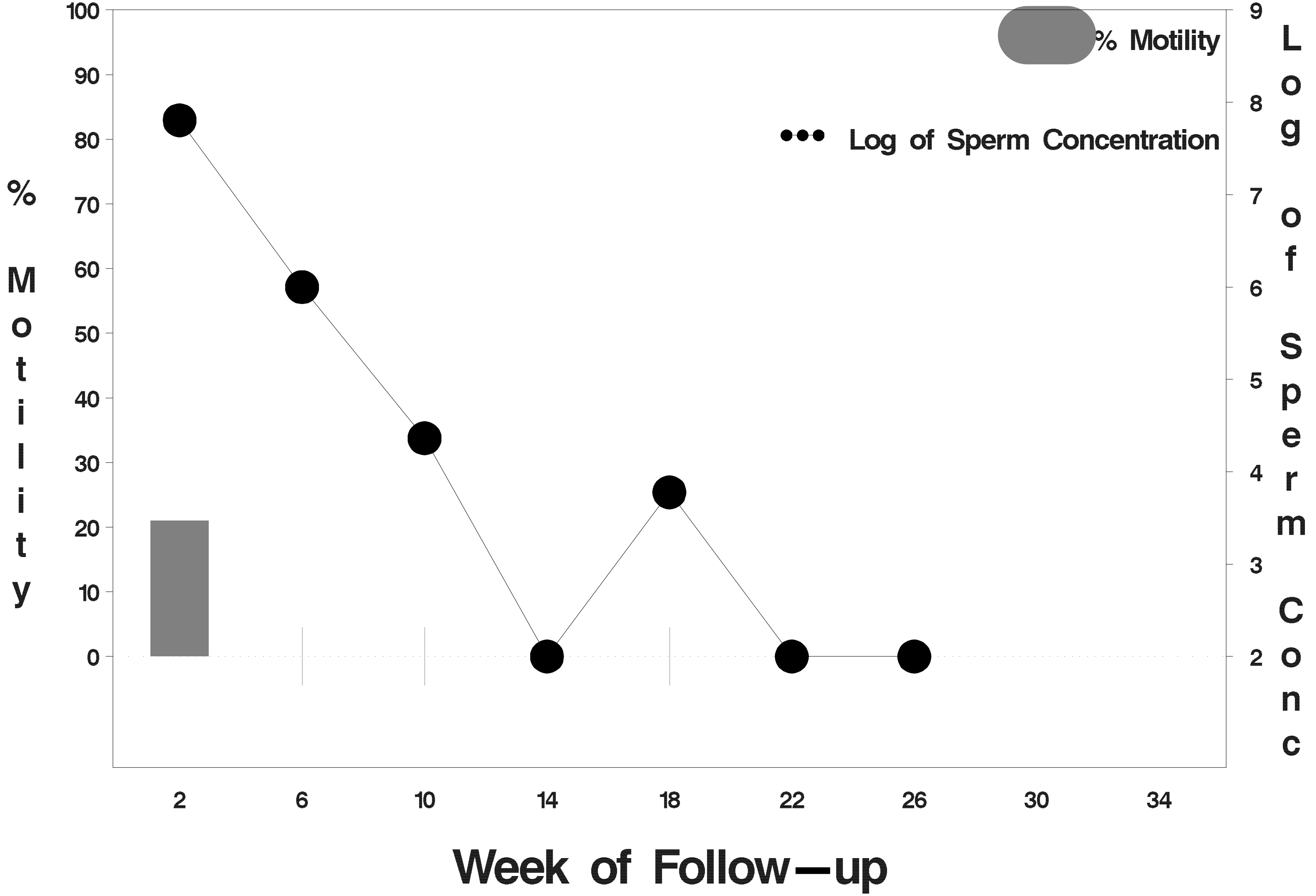

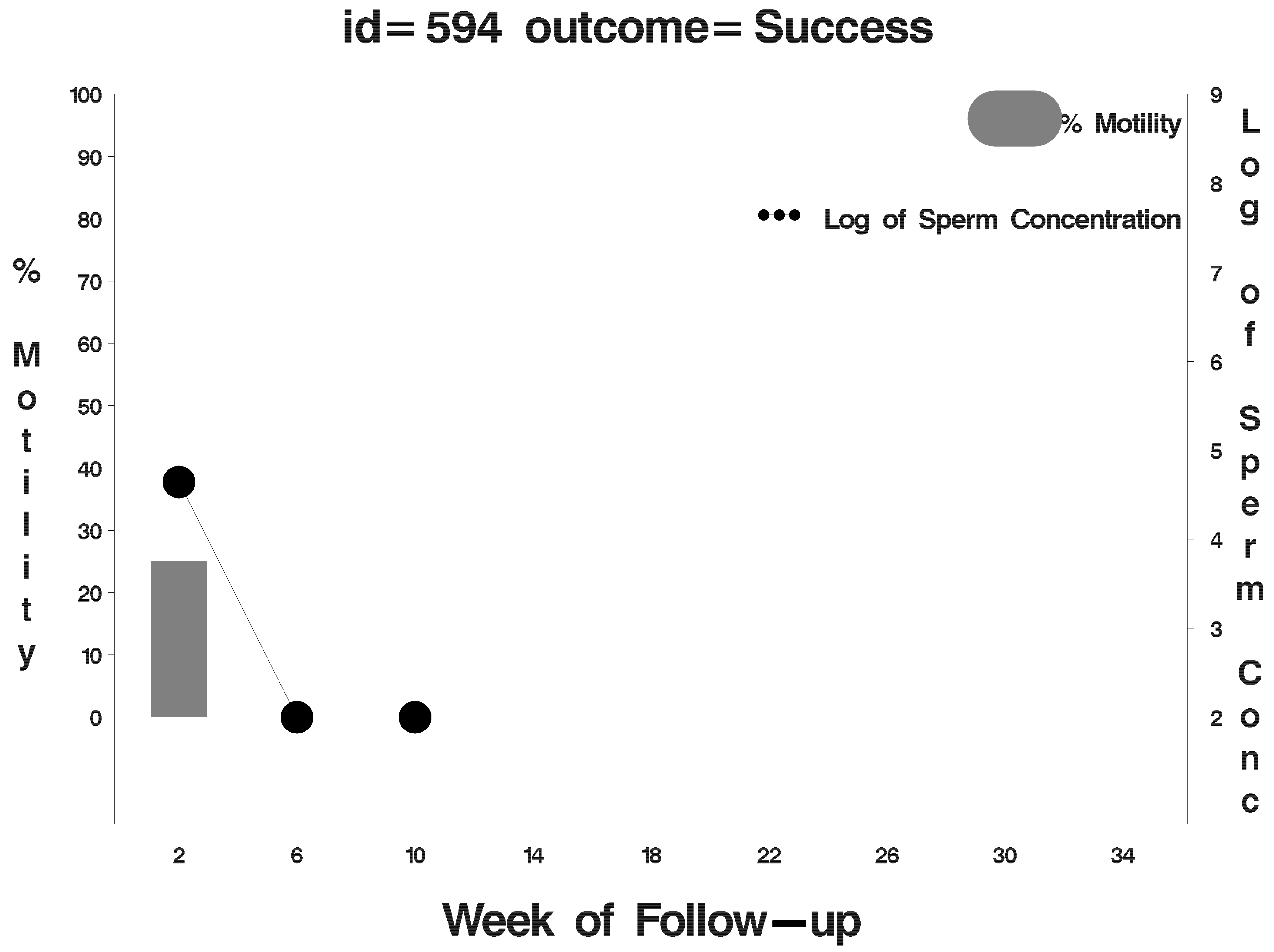
id= 594 outcome= Success
% Motility
Log of Sperm Concentration
100
90
80
70
60
50
40
30
20
10
0
9
8
7
6
5
4
3
2
2
6
10
14
18
22
26
30
34
Week of Follow-up
Log of Sperm Conc

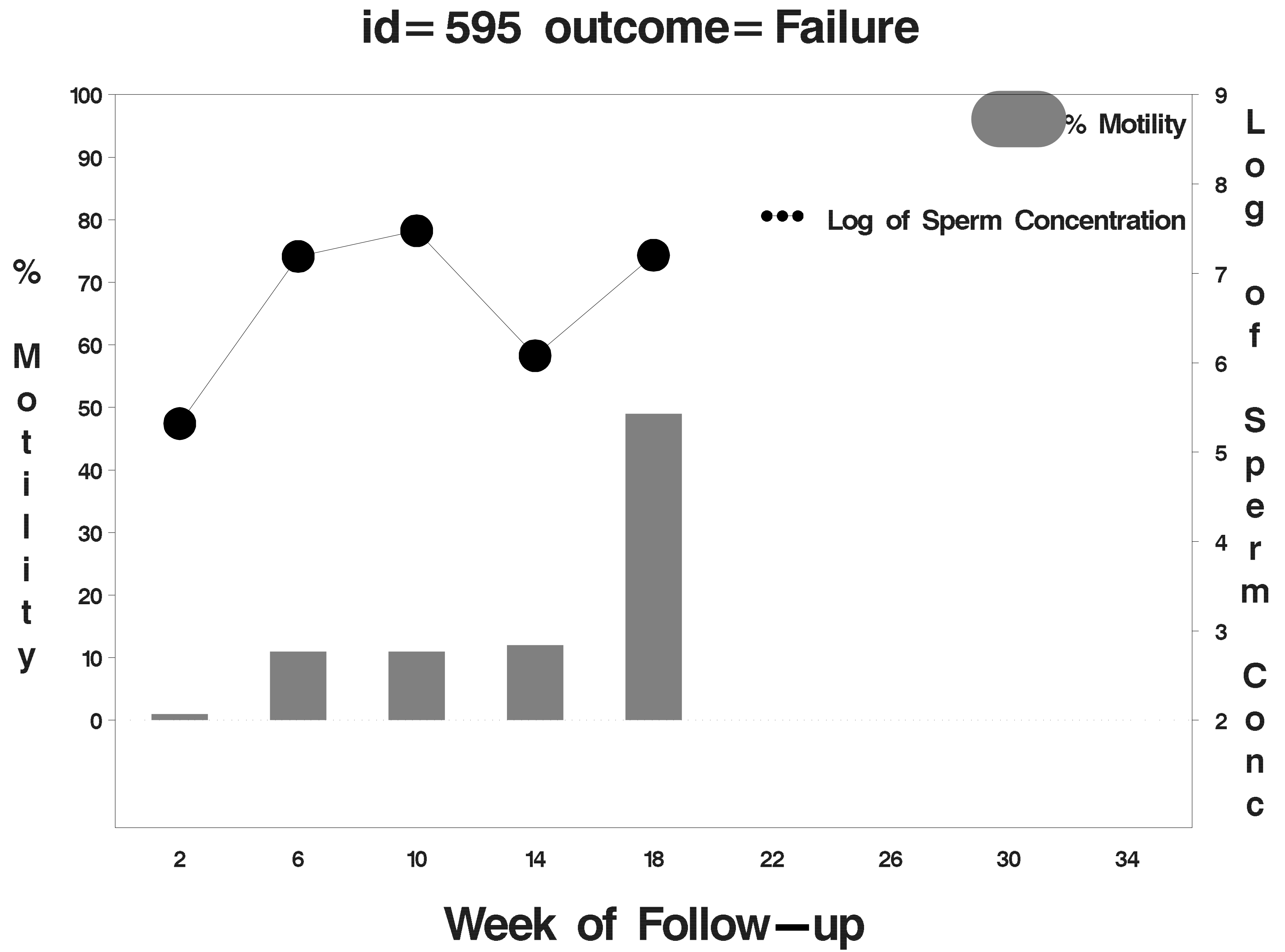
id= 595 outcome= Failure
% Motility
Log of Sperm Concentration
100
90
80
70
60
50
40
30
20
10
0
9
8
7
6
5
4
3
2
2
6
10
14
18
22
26
30
34
Week of Follow-up
Log of Sperm Conc

id= 596 outcome= Success

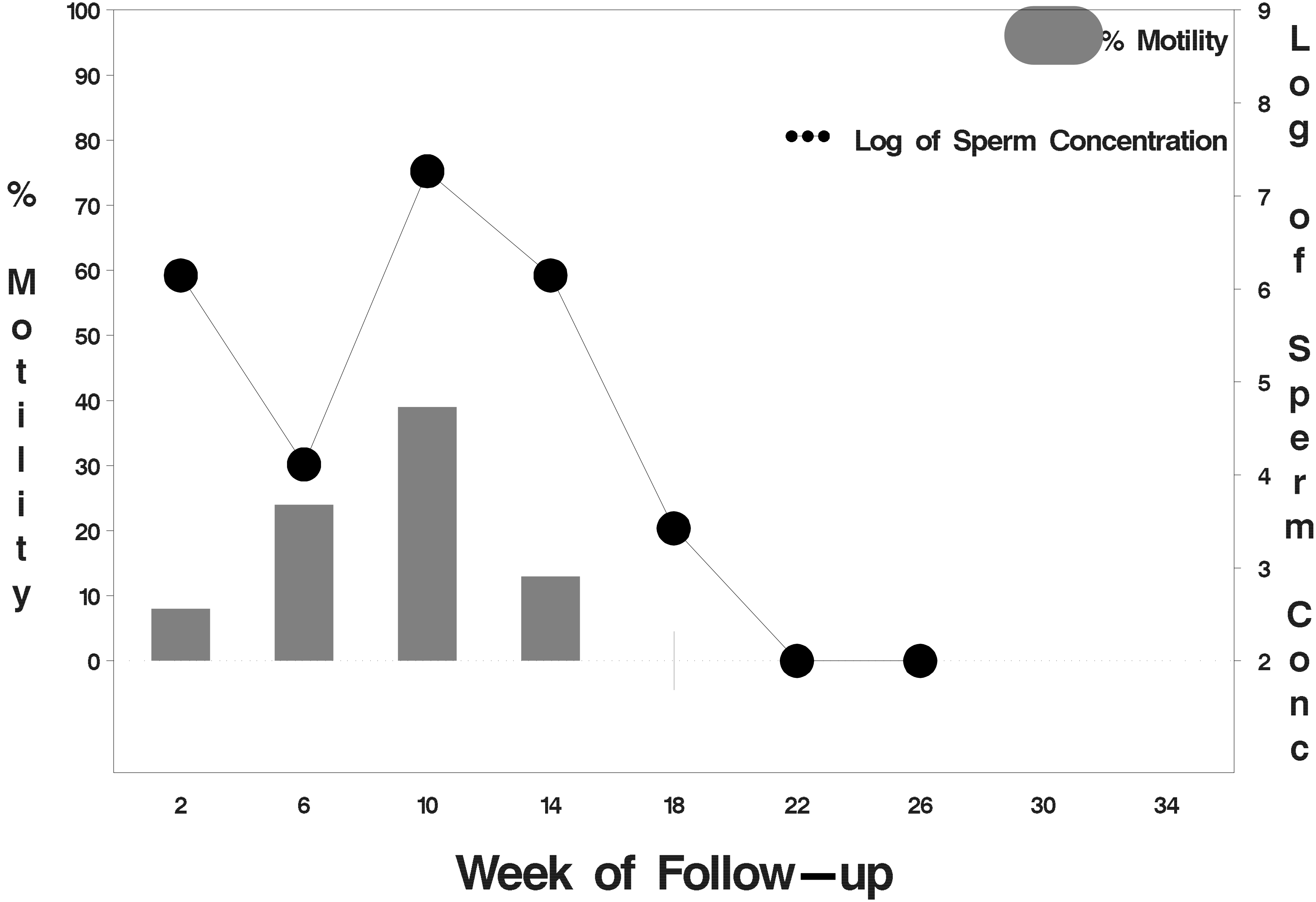

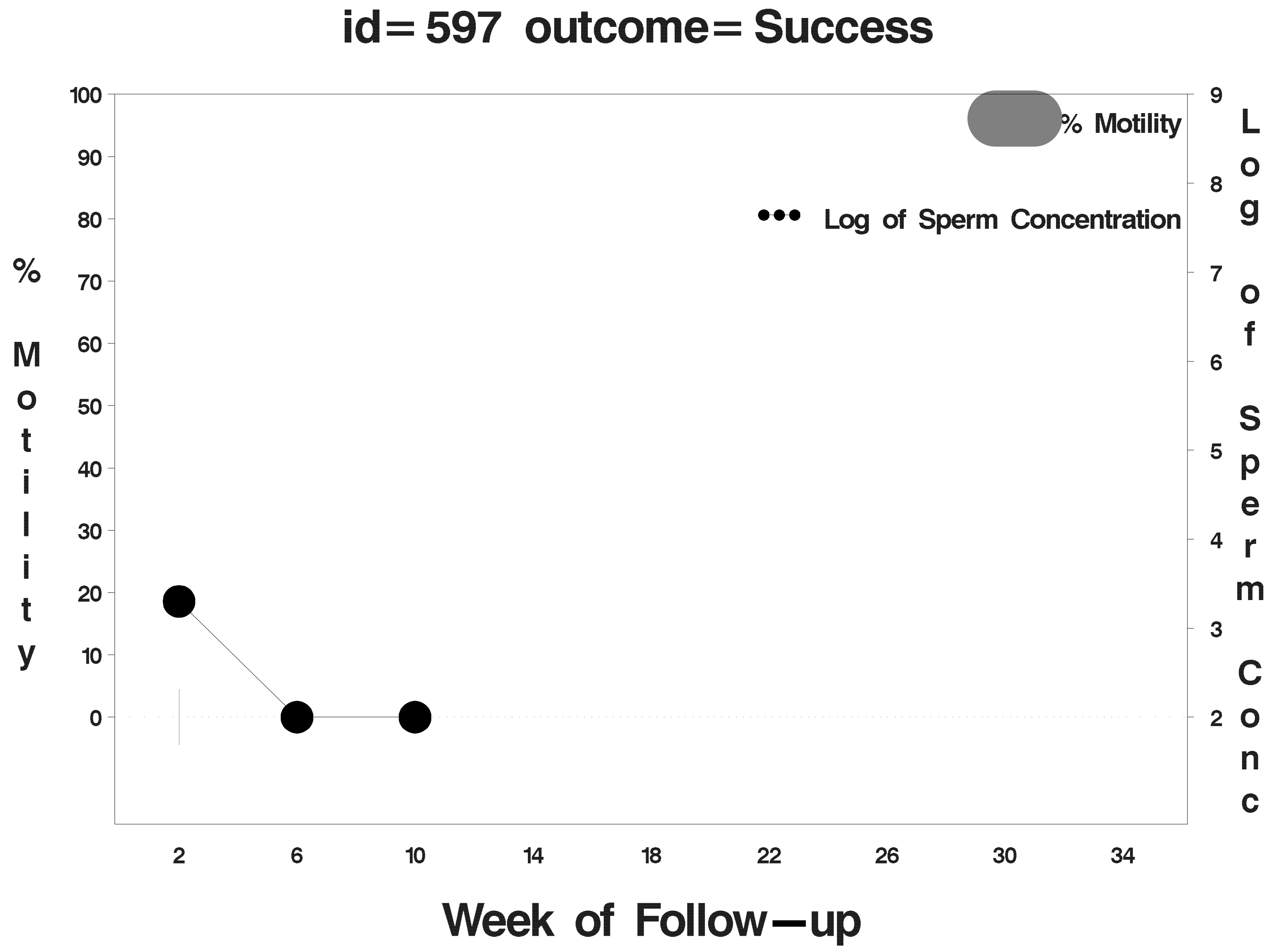
id= 597 outcome= Success
% Motility
Log of Sperm Concentration
100
90
80
70
60
50
40
30
20
10
0
9
8
7
6
5
4
3
2
2
6
10
14
18
22
26
30
34
% Motility
Log of Sperm Conc
Week of Follow−up

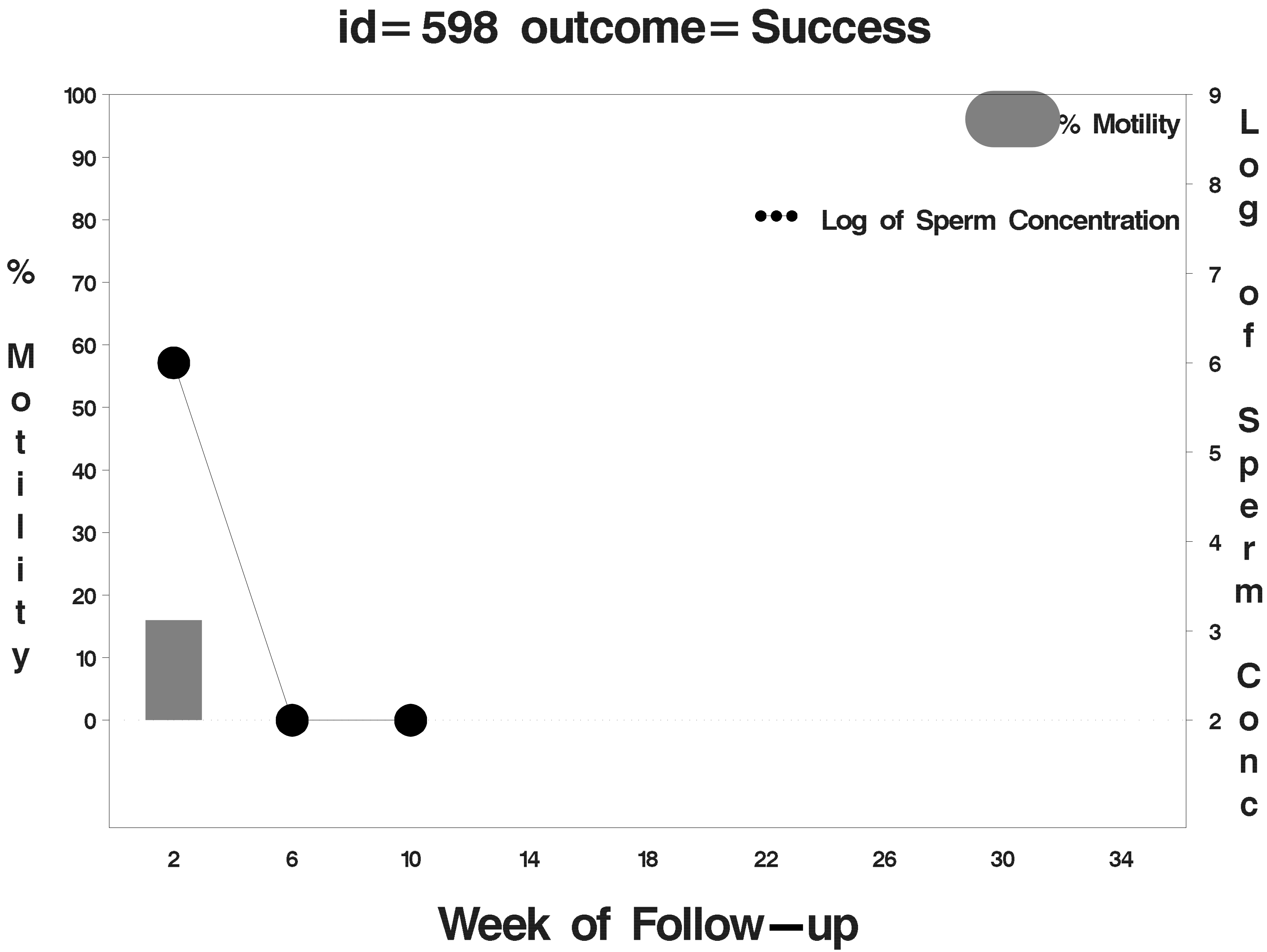

id= 598 outcome= Success
% Motility
Log of Sperm Concentration
% Motility
Log of Sperm Conc
Week of Follow-up
100
90
80
70
60
50
40
30
20
10
0
9
8
7
6
5
4
3
2
2
6
10
14
18
22
26
30
34

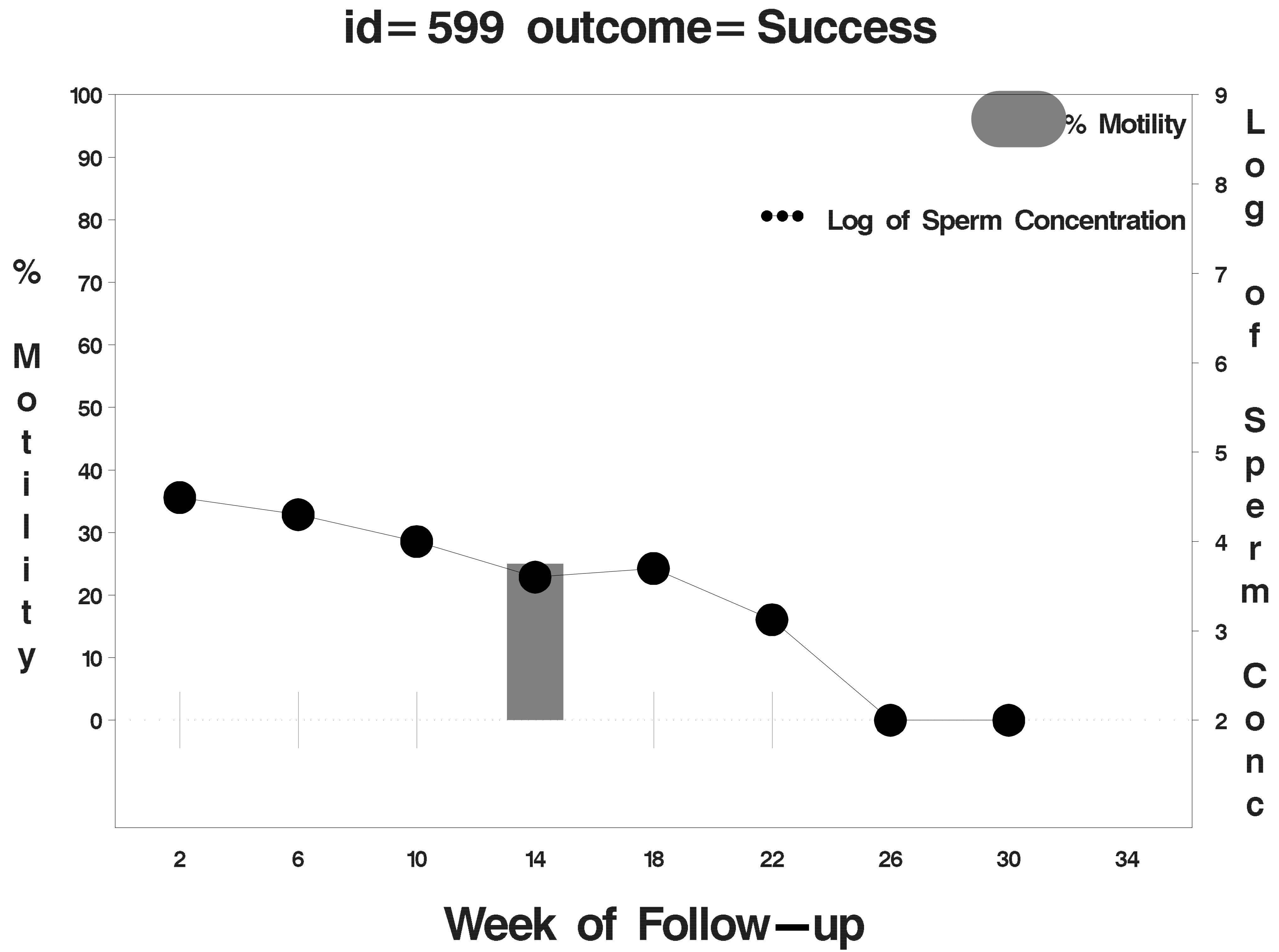
id= 599 outcome= Success
% Motility
Log of Sperm Concentration
100
90
80
70
60
50
40
30
20
10
0
9
8
7
6
5
4
3
2
2
6
10
14
18
22
26
30
34
Week of Follow−up
% Motility
Log of Sperm Conc

id= 600 outcome= Success

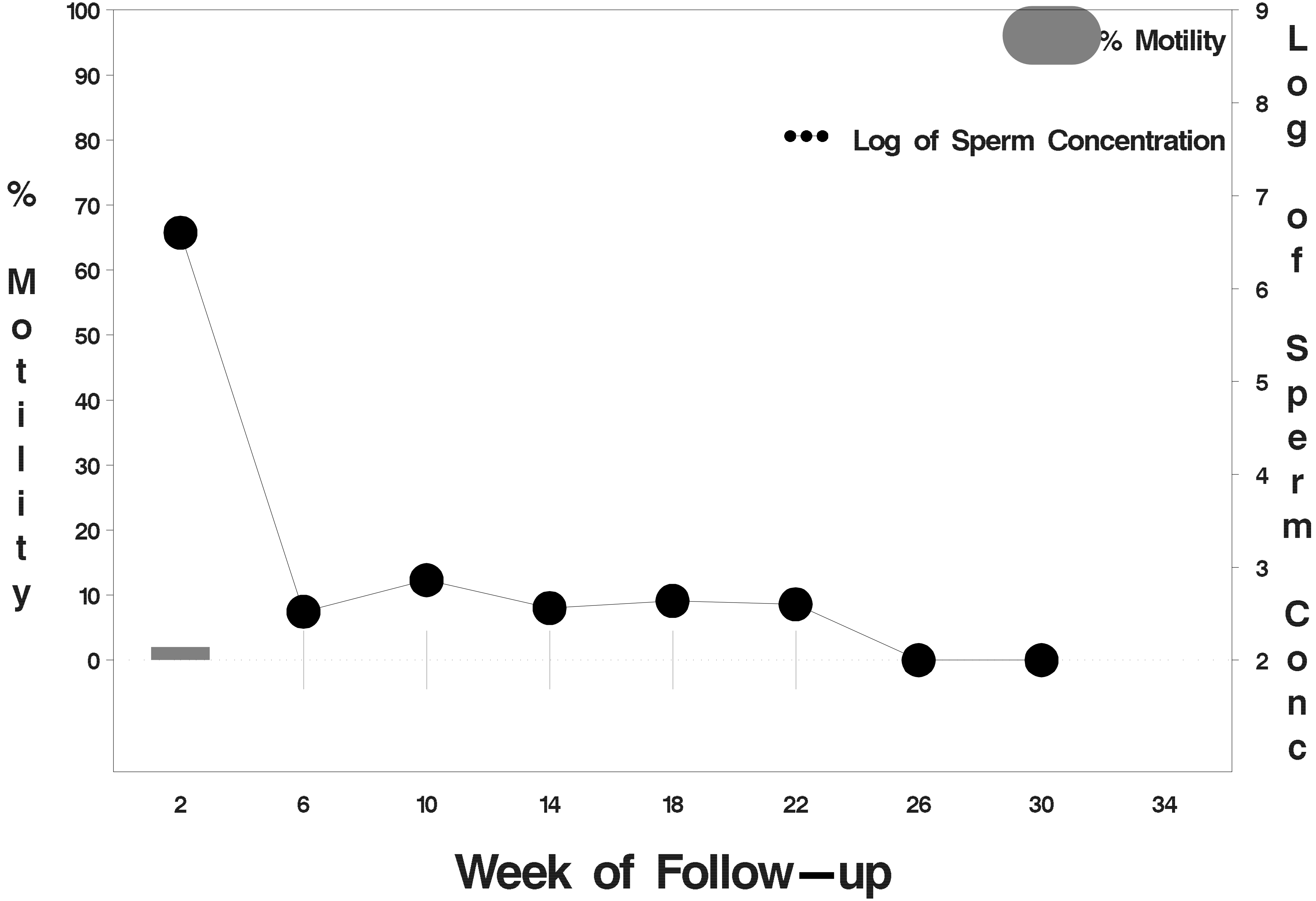

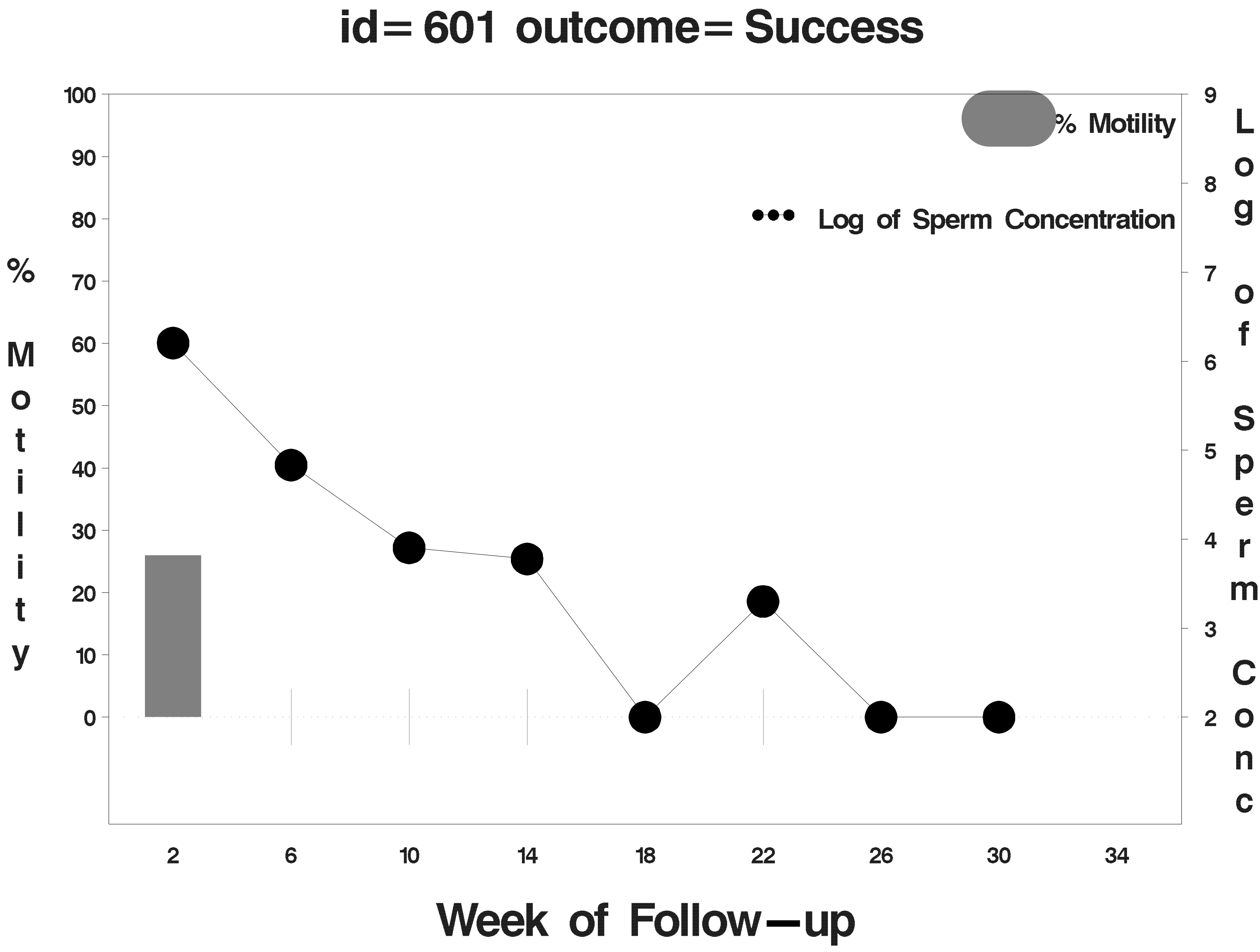

id= 601 outcome= Success
% Motility
Log of Sperm Concentration
100
90
80
70
60
50
40
30
20
10
0
9
8
7
6
5
4
3
2
2
6
10
14
18
22
26
30
34
% Motility
Log of Sperm Conc
Week of Follow−up

id= 602 outcome= Success

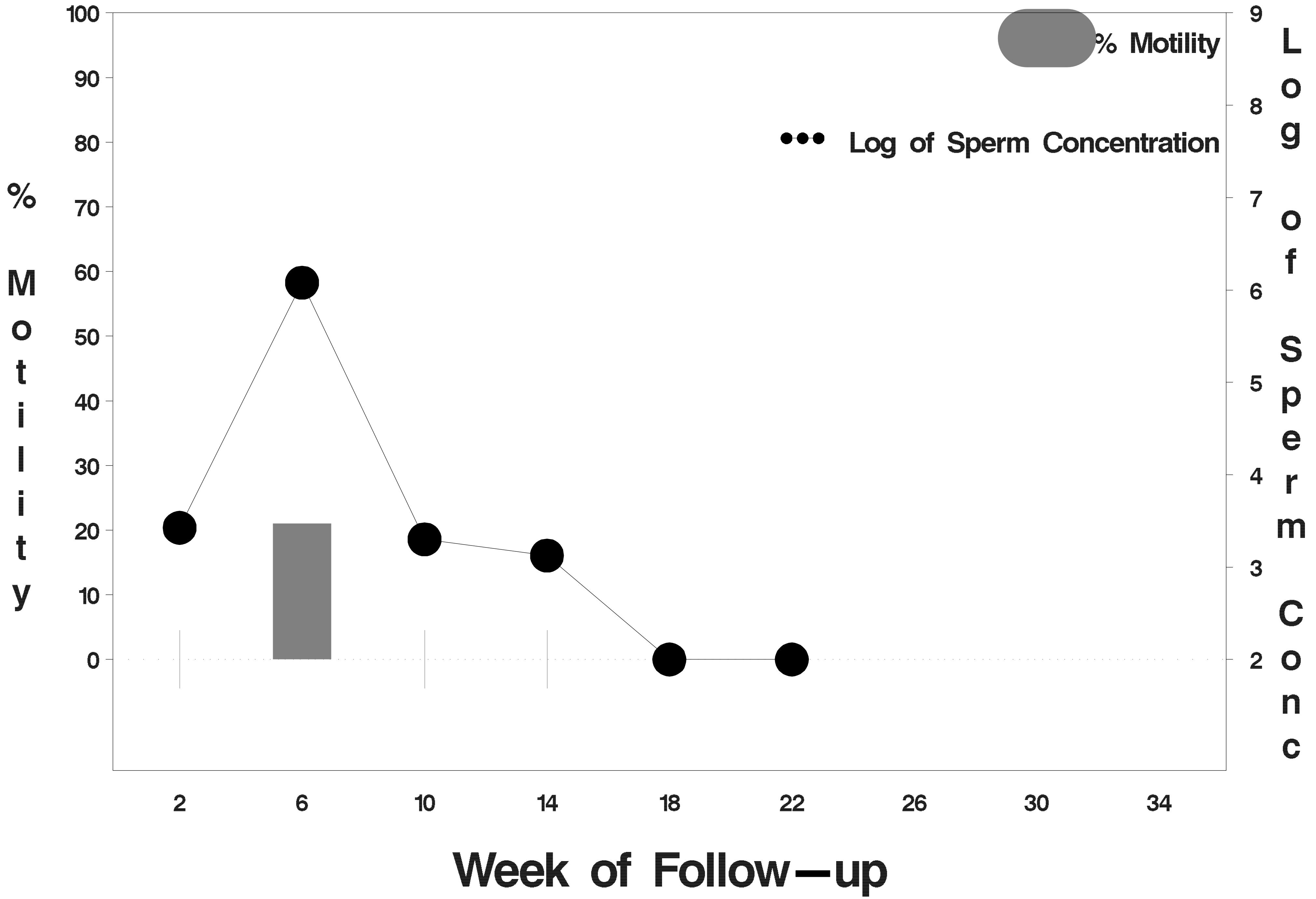

id= 603 outcome= Indeterminate

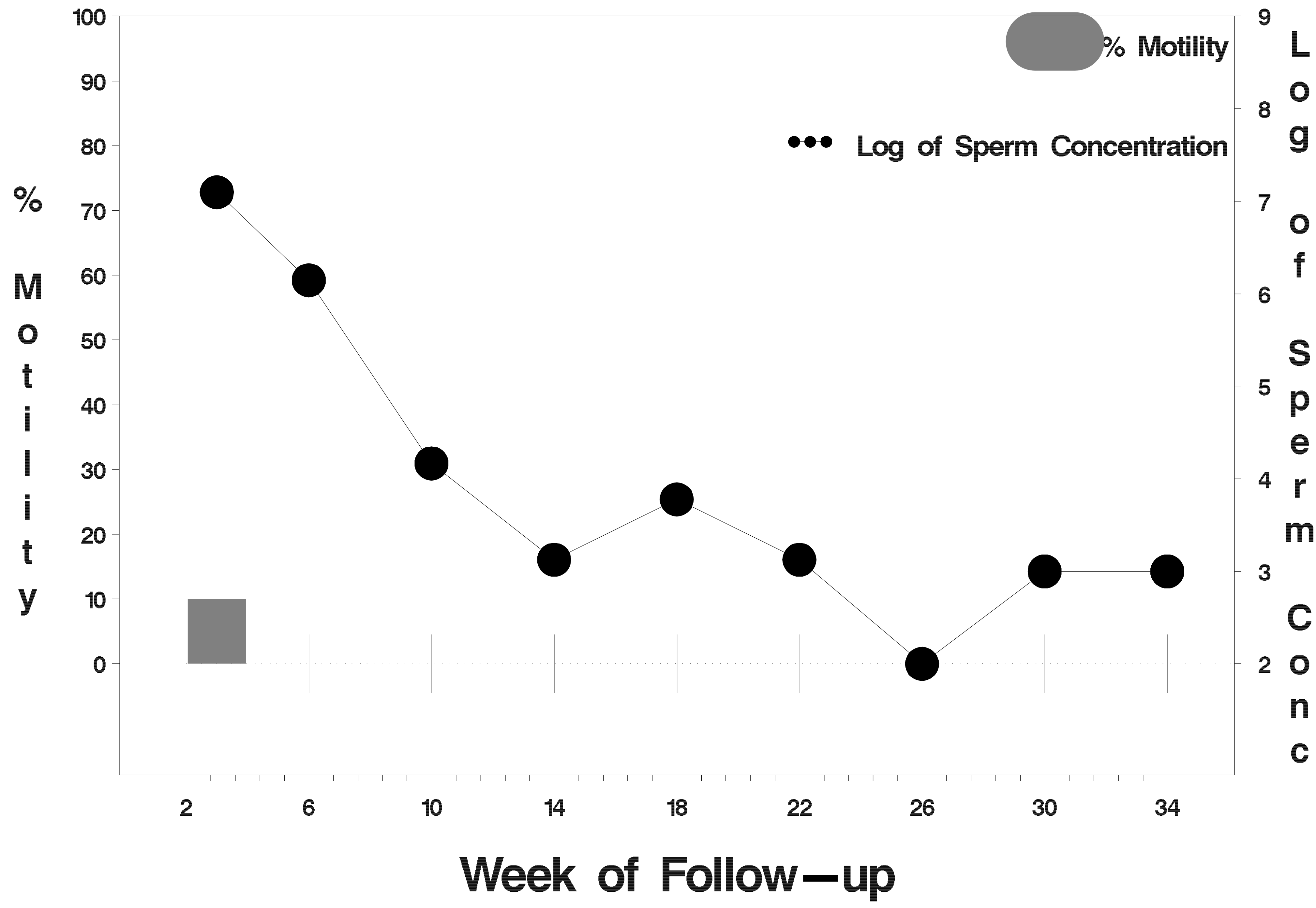

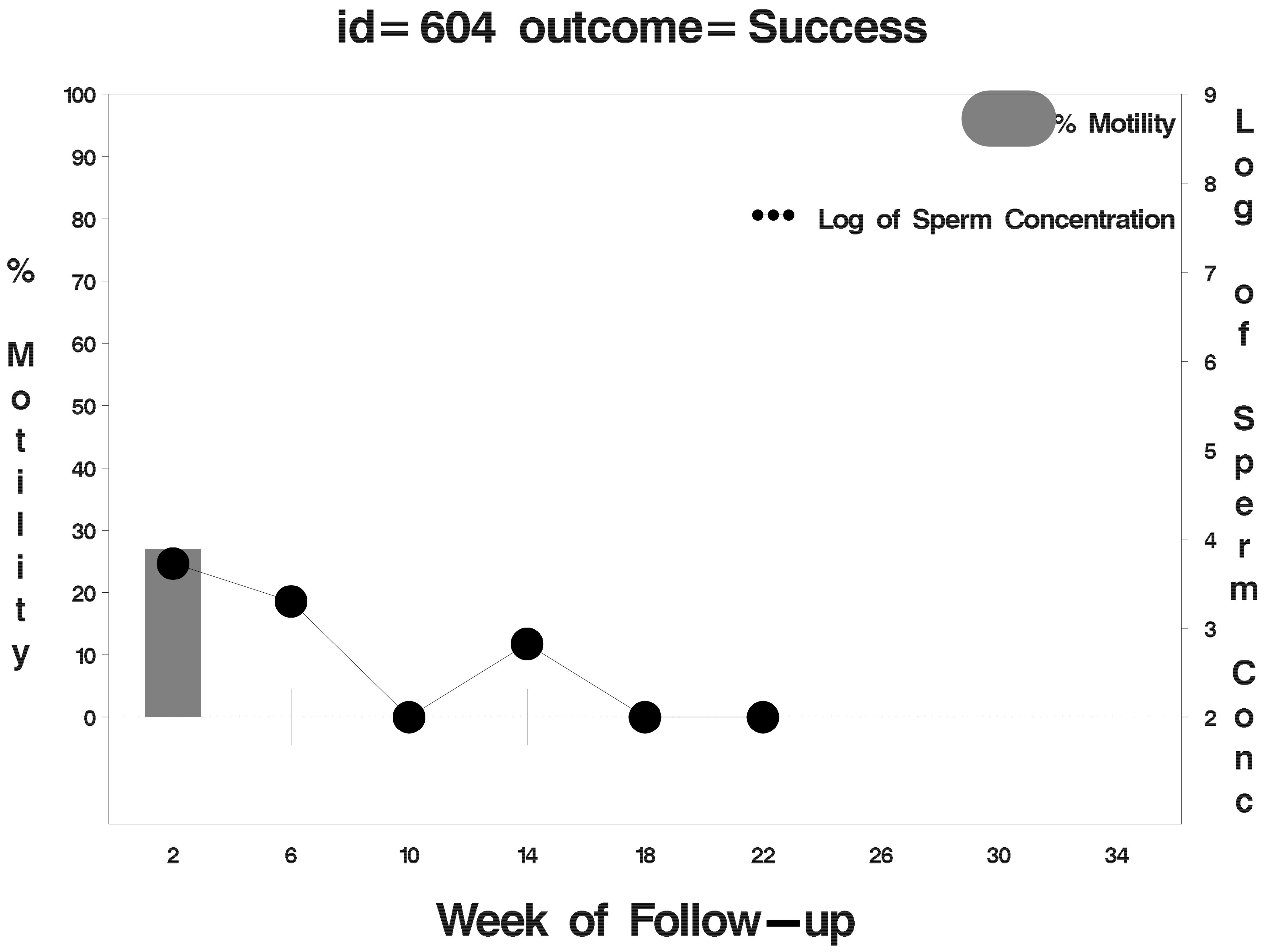
id= 604 outcome= Success
% Motility
Log of Sperm Concentration
100
90
80
70
60
50
40
30
20
10
0
9
8
7
6
5
4
3
2
2
6
10
14
18
22
26
30
34
Week of Follow—up
% Motility
Log of Sperm Conc

id= 605 outcome= Success

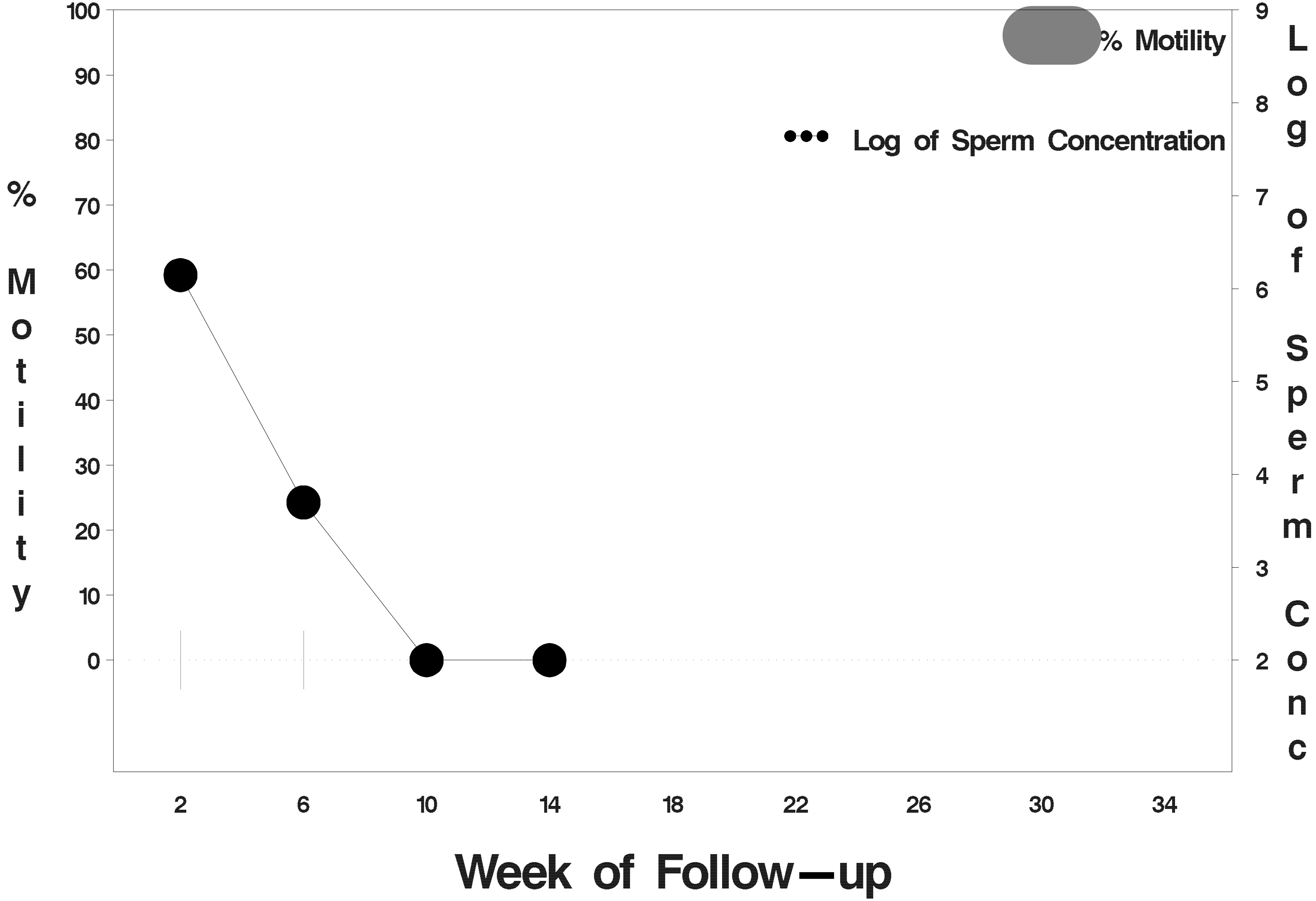

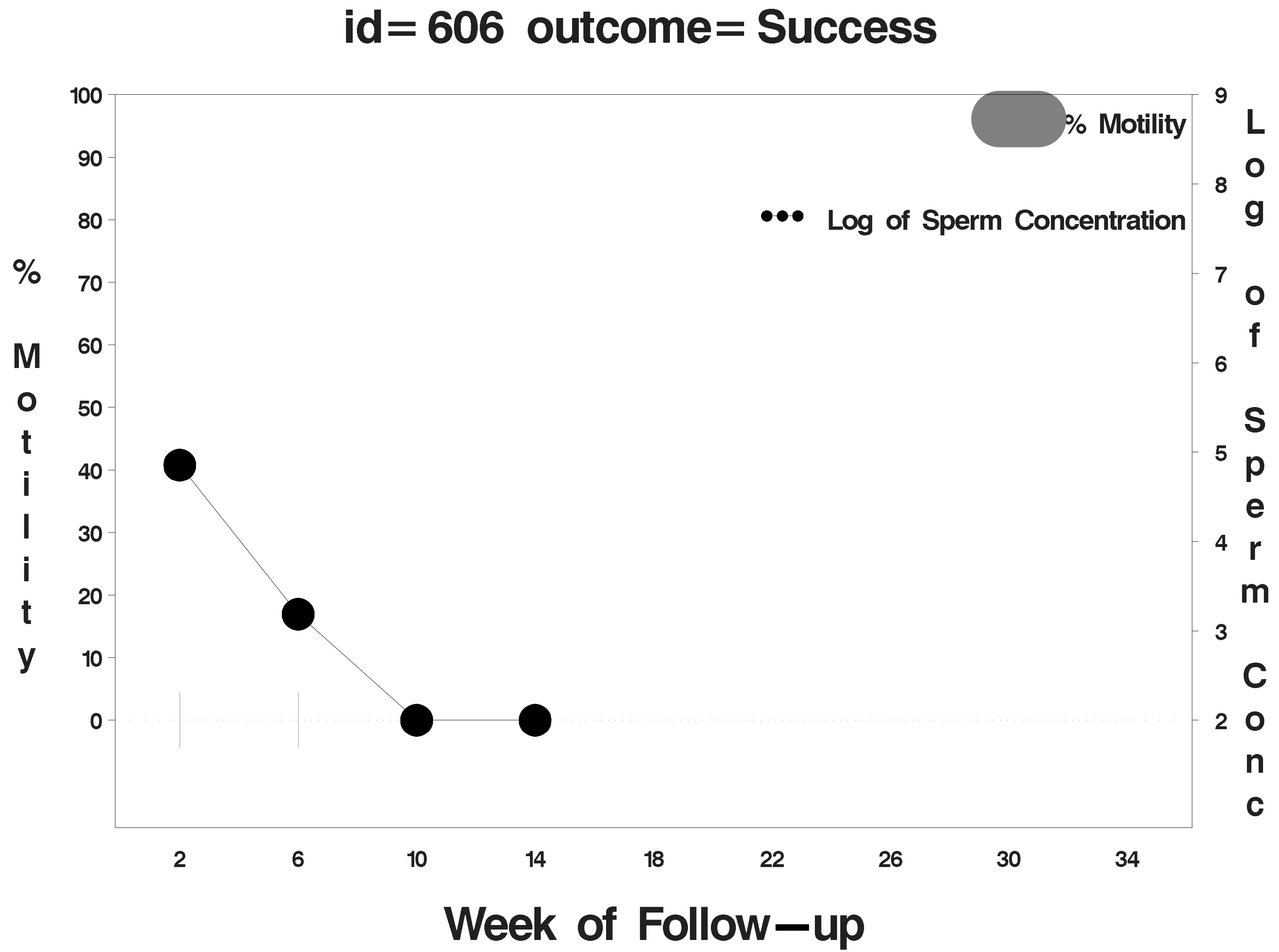

id= 606 outcome= Success
% Motility
Log of Sperm Concentration
100
90
80
70
60
50
40
30
20
10
0
9
8
7
6
5
4
3
2
2
6
10
14
18
22
26
30
34
Week of Follow—up
Log of Sperm Conc

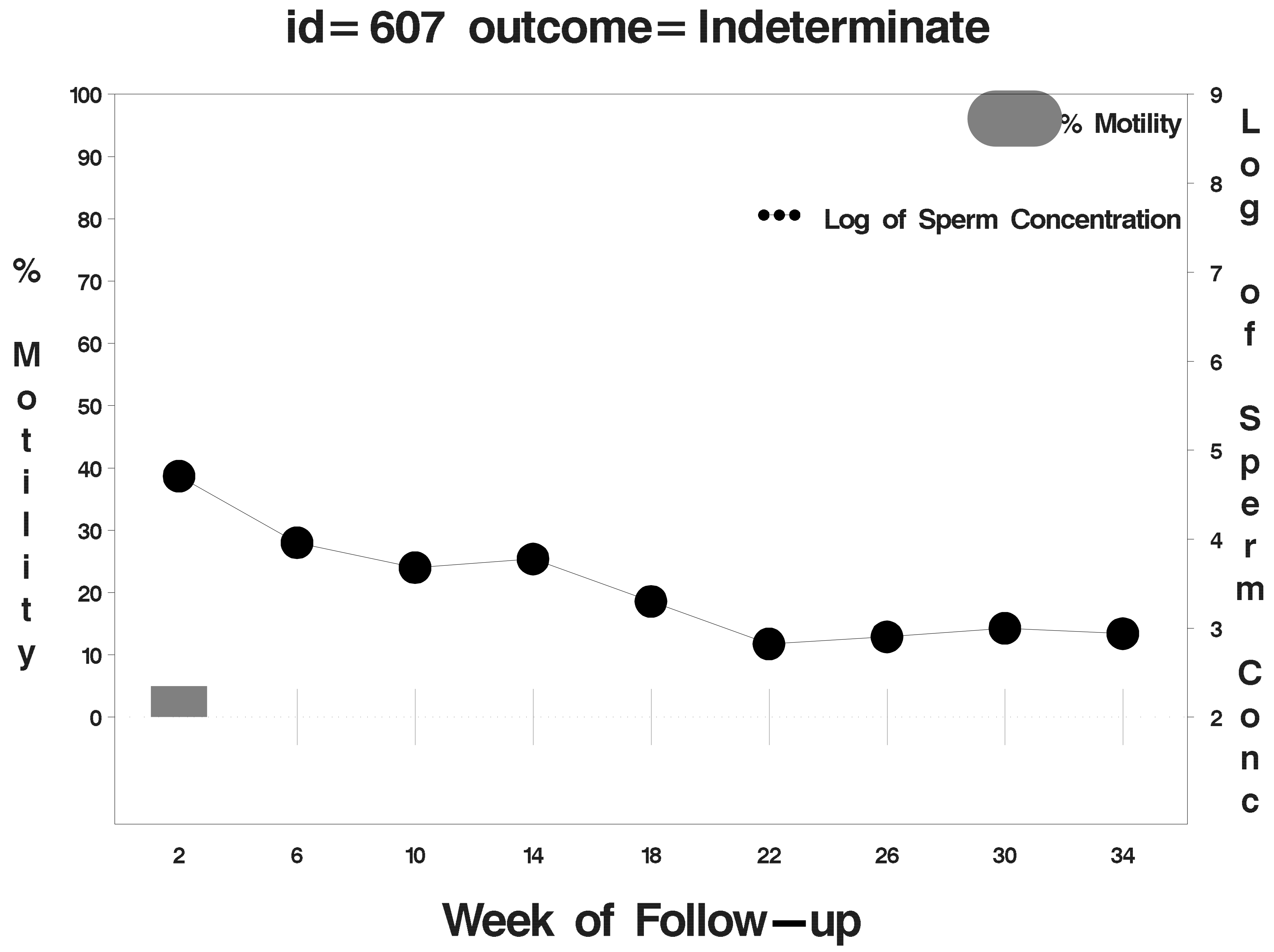

id= 607 outcome= Indeterminate
% Motility
Log of Sperm Concentration
100
90
80
70
60
50
40
30
20
10
0
9
8
7
6
5
4
3
2
2
6
10
14
18
22
26
30
34
Week of Follow—up

id= 608 outcome= Indeterminate

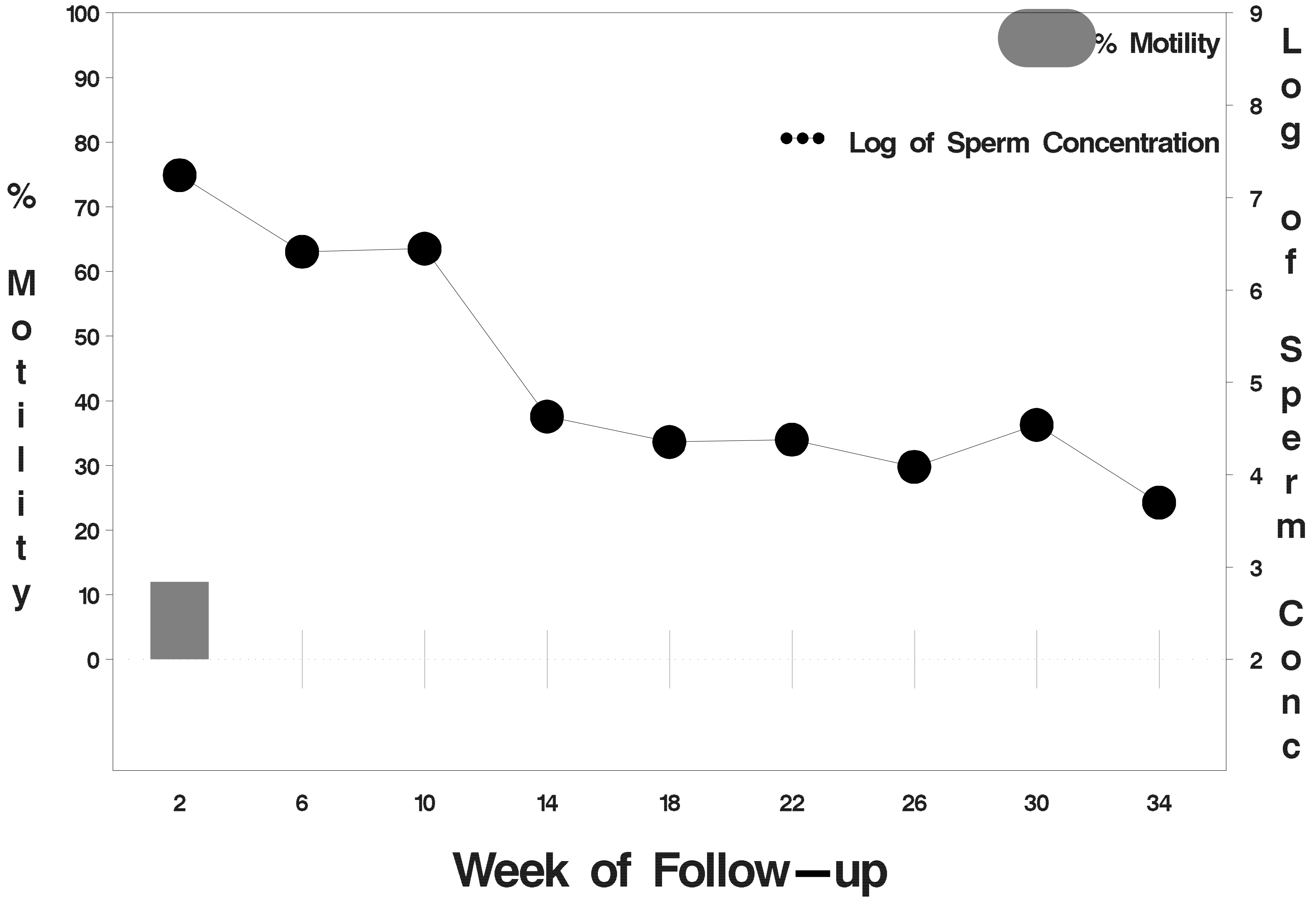

id= 609 outcome= Indeterminate

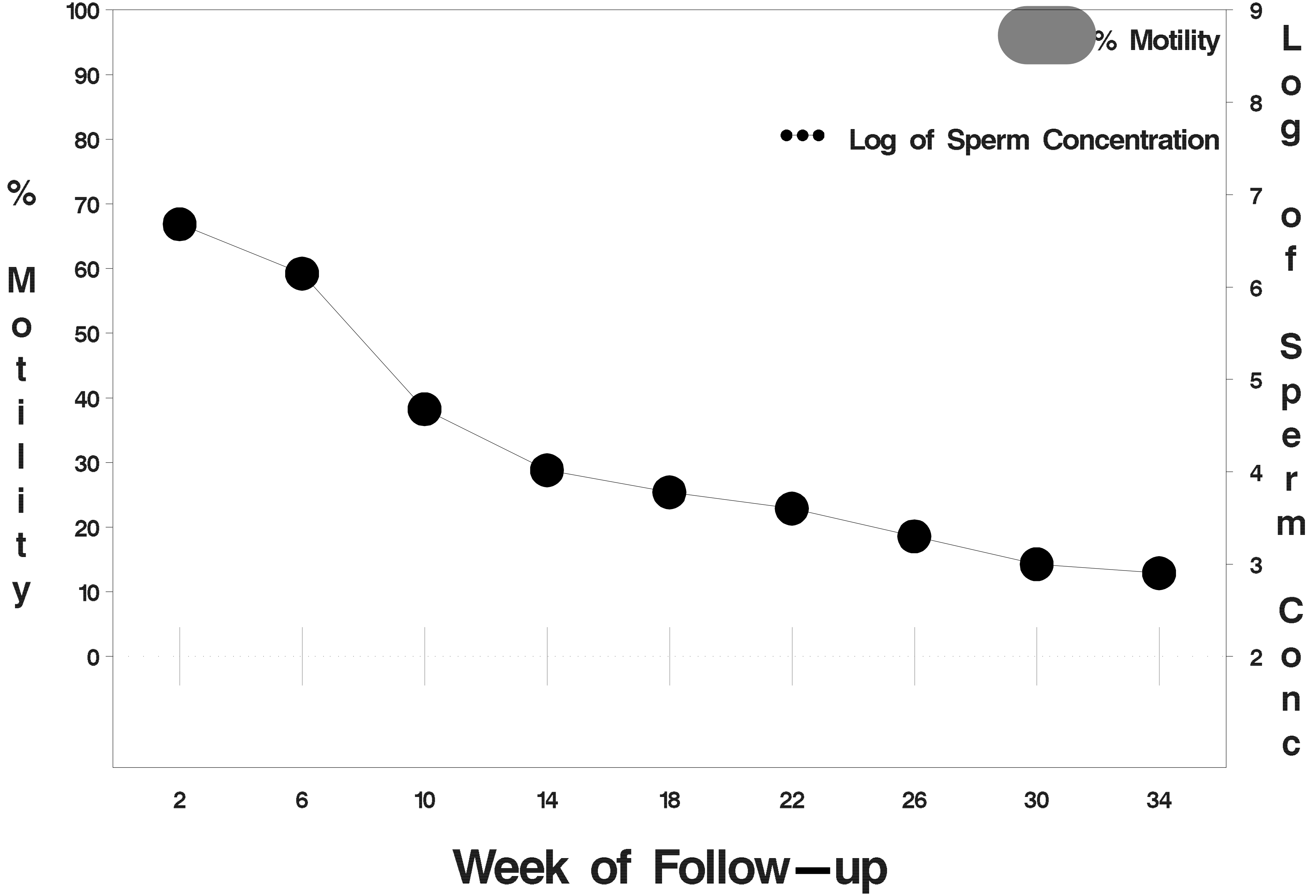

id= 610 outcome= Success

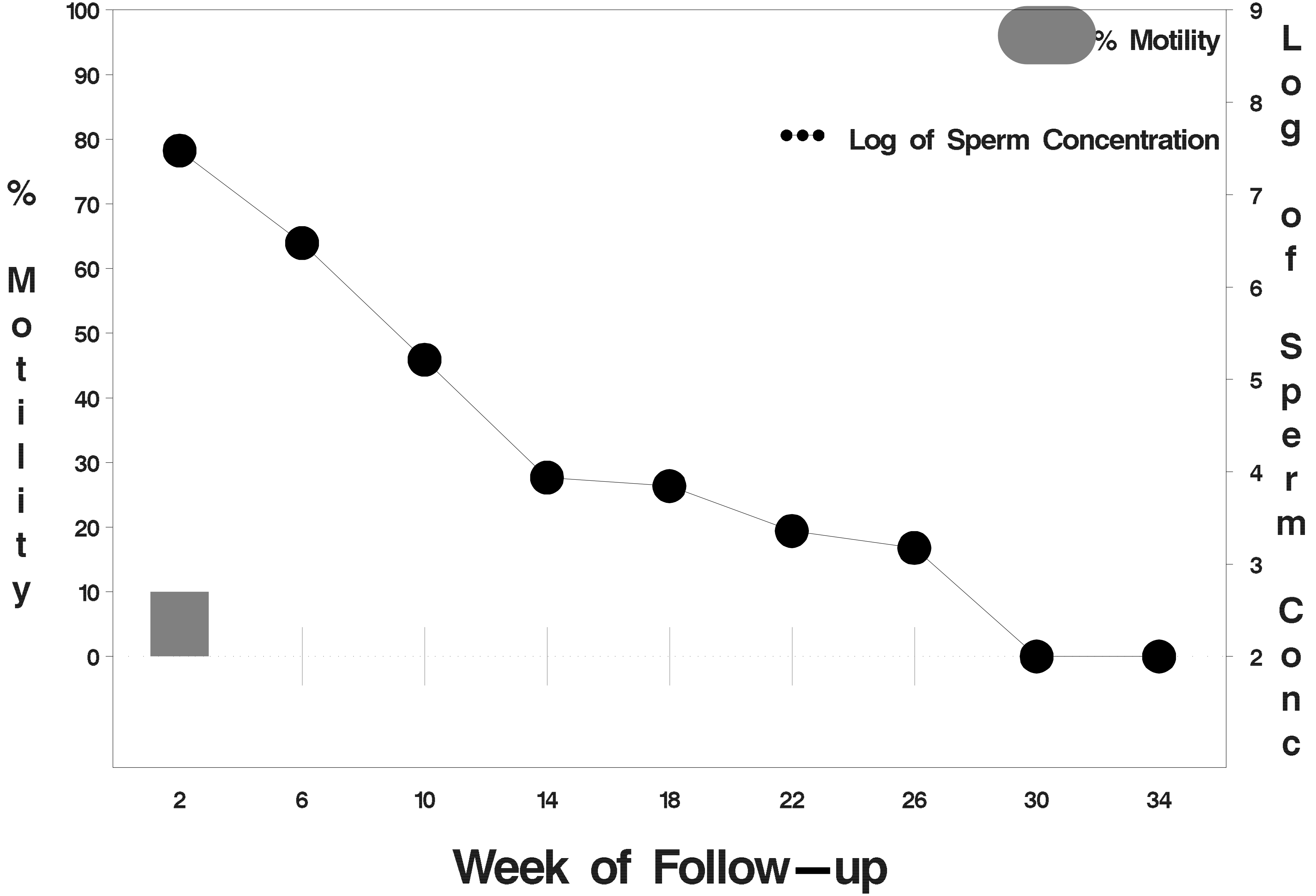

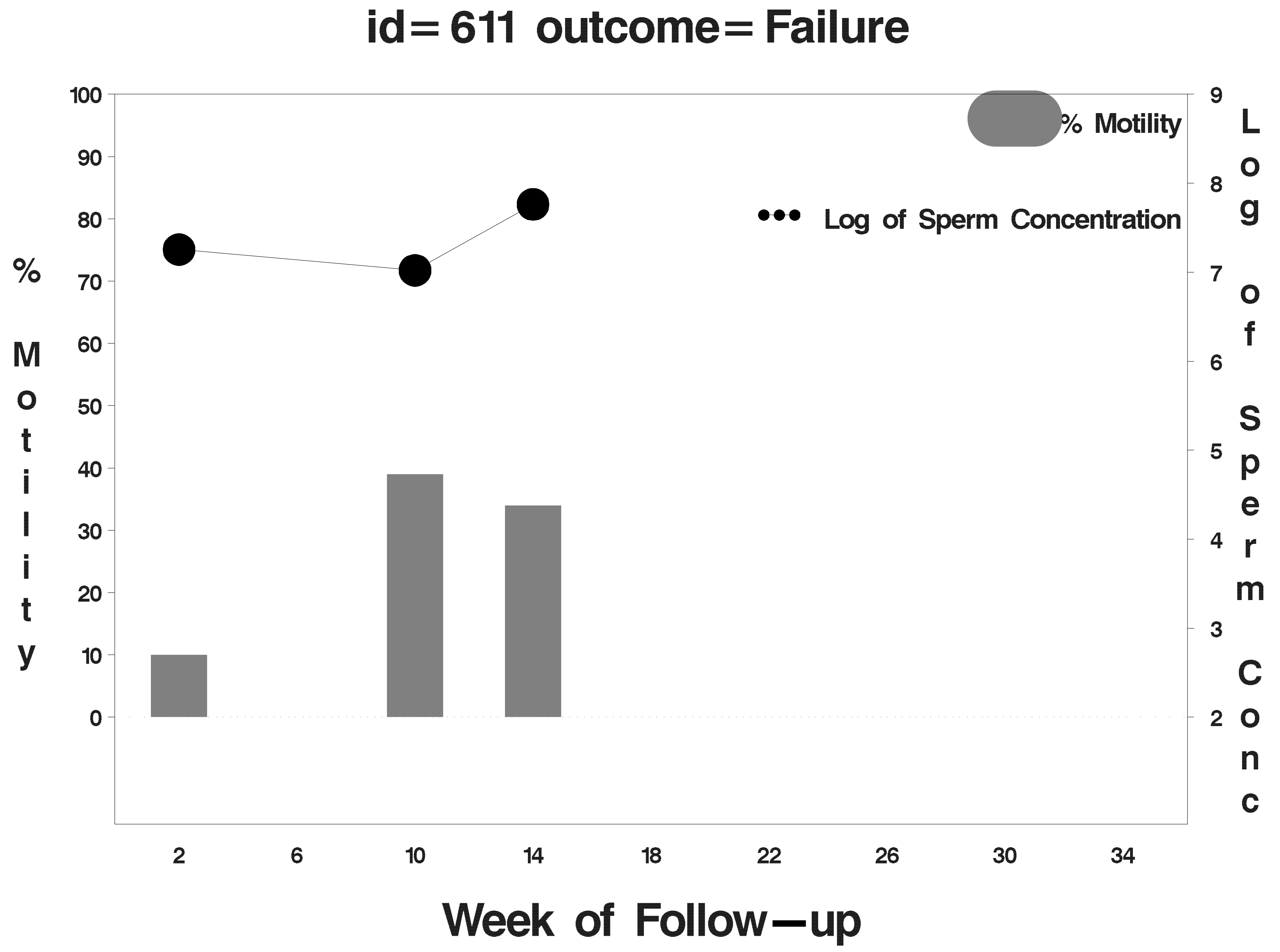

id= 611 outcome= Failure
% Motility
Log of Sperm Concentration
100
90
80
70
60
50
40
30
20
10
0
9
8
7
6
5
4
3
2
2
6
10
14
18
22
26
30
34
Week of Follow-up
Log of Sperm Conc

id= 612 outcome= Success

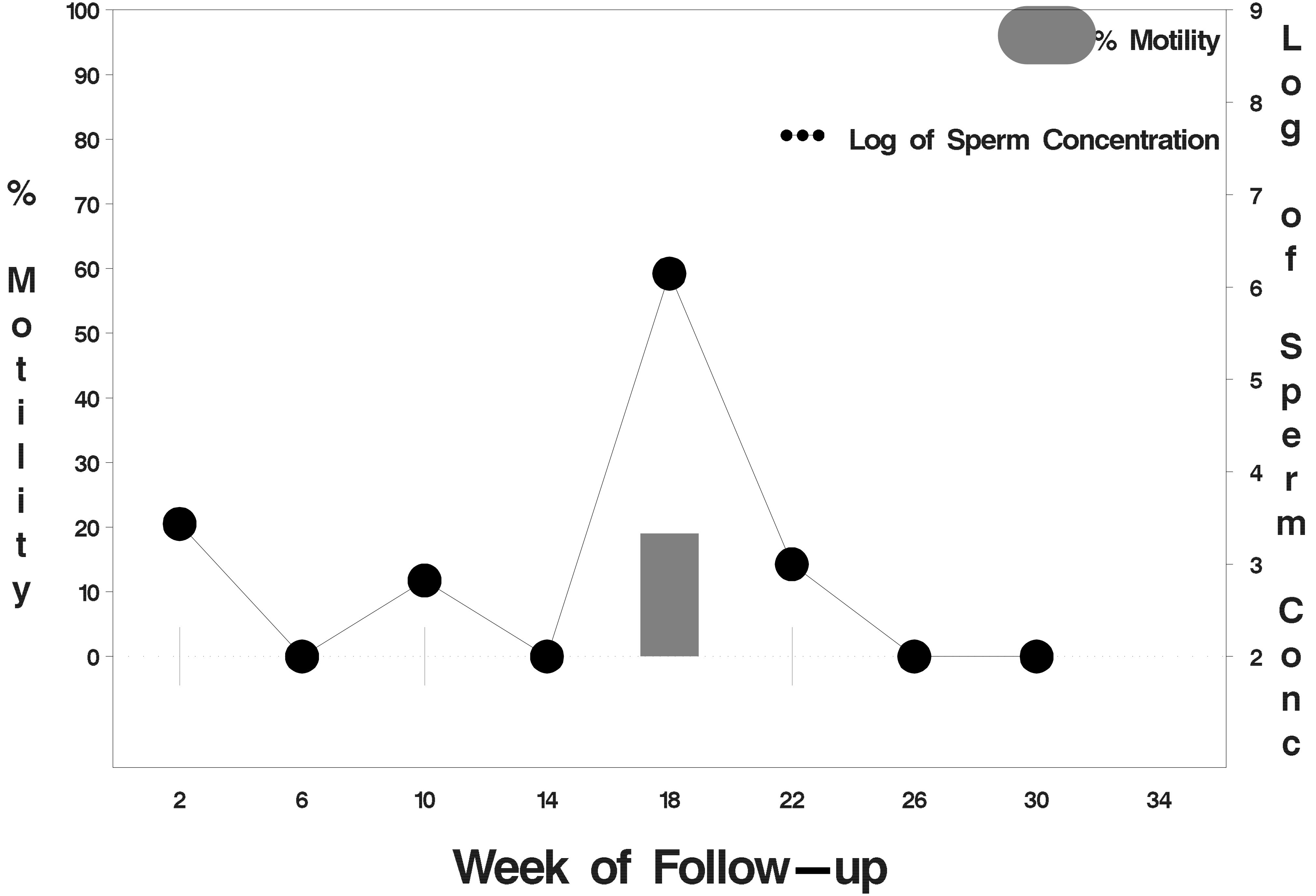

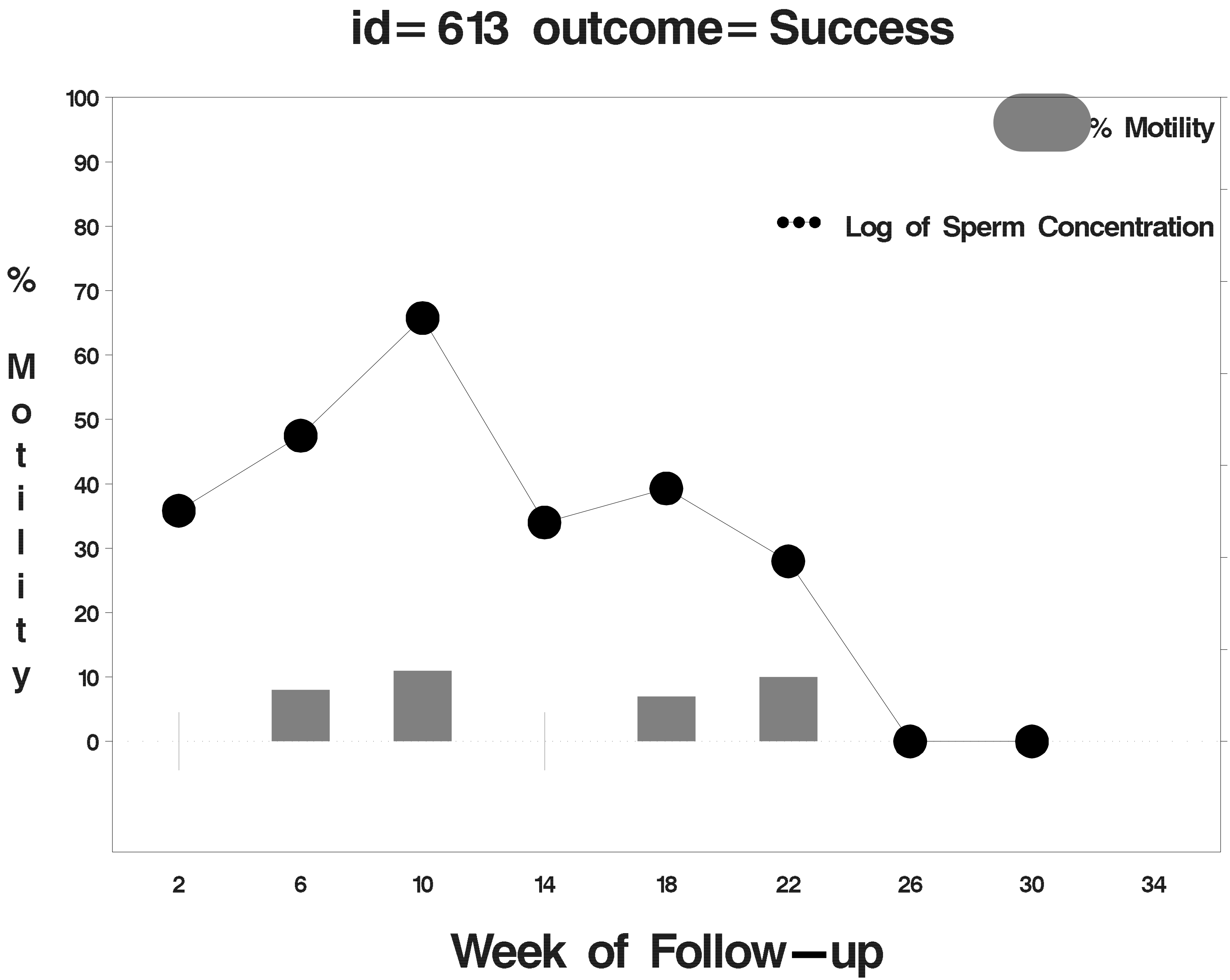

id= 613 outcome= Success
% Motility
Log of Sperm Concentration
100
90
80
70
60
50
40
30
20
10
0
9
8
7
6
5
4
3
2
2
6
10
14
18
22
26
30
34
Week of Follow—up
Log of Sperm Conc

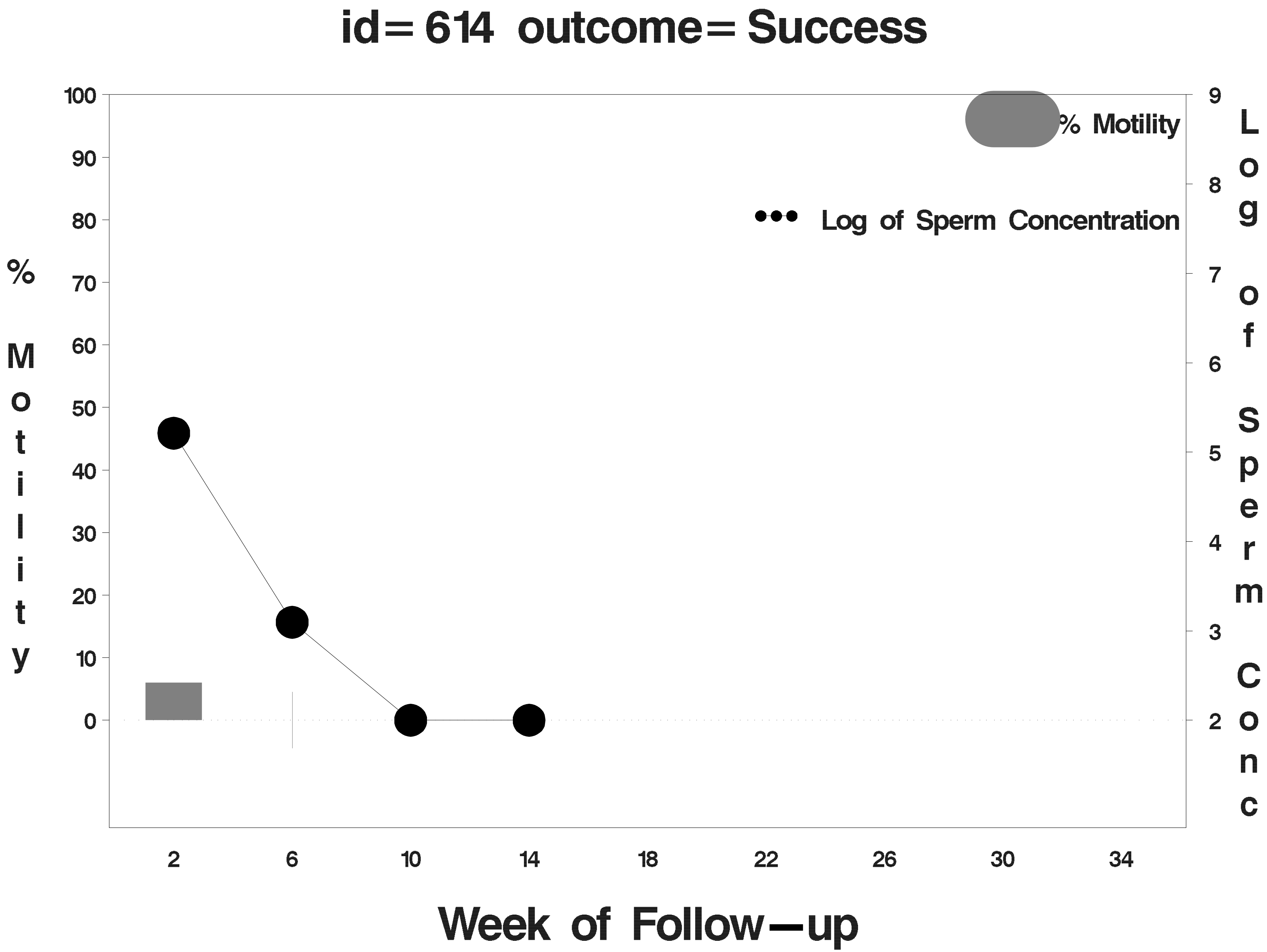

id= 614 outcome= Success
% Motility
Log of Sperm Concentration
100
90
80
70
60
50
40
30
20
10
0
9
8
7
6
5
4
3
2
2
6
10
14
18
22
26
30
34
Week of Follow−up
% Motility
Log of Sperm Conc

id= 615 outcome= Failure

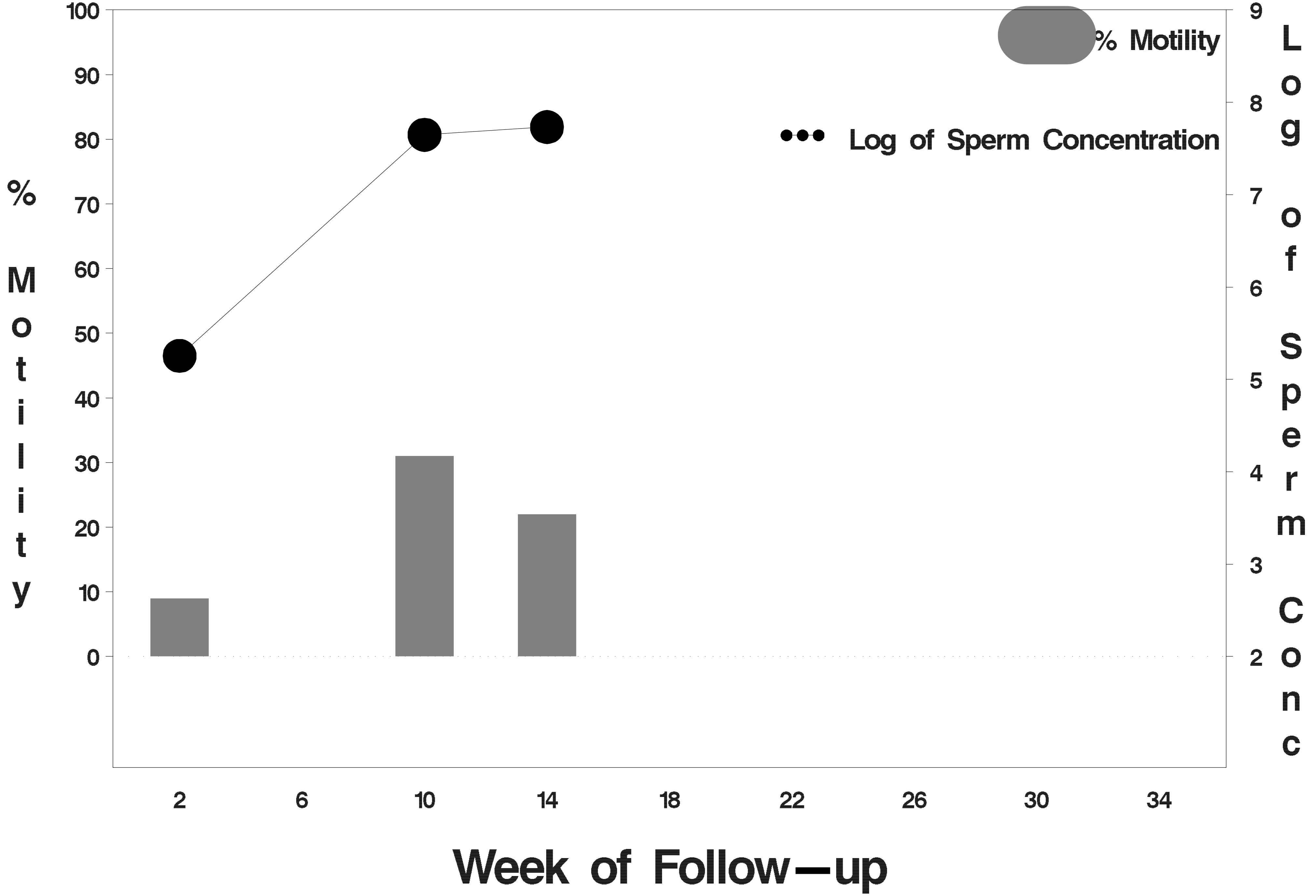

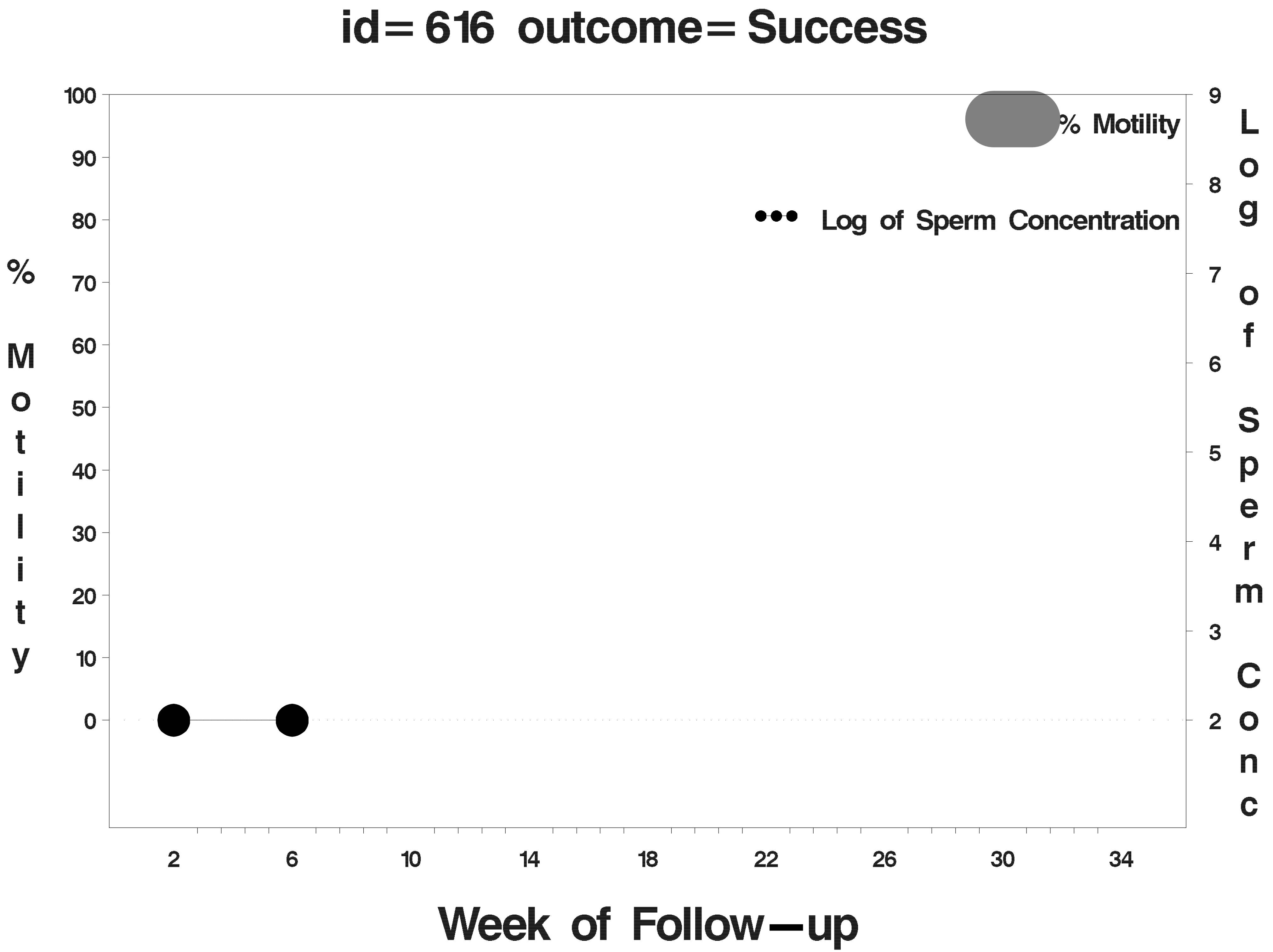

id= 616 outcome= Success
% Motility
Log of Sperm Concentration
% Motility
Log of Sperm Conc
Week of Follow−up
100
90
80
70
60
50
40
30
20
10
0
9
8
7
6
5
4
3
2
2
6
10
14
18
22
26
30
34

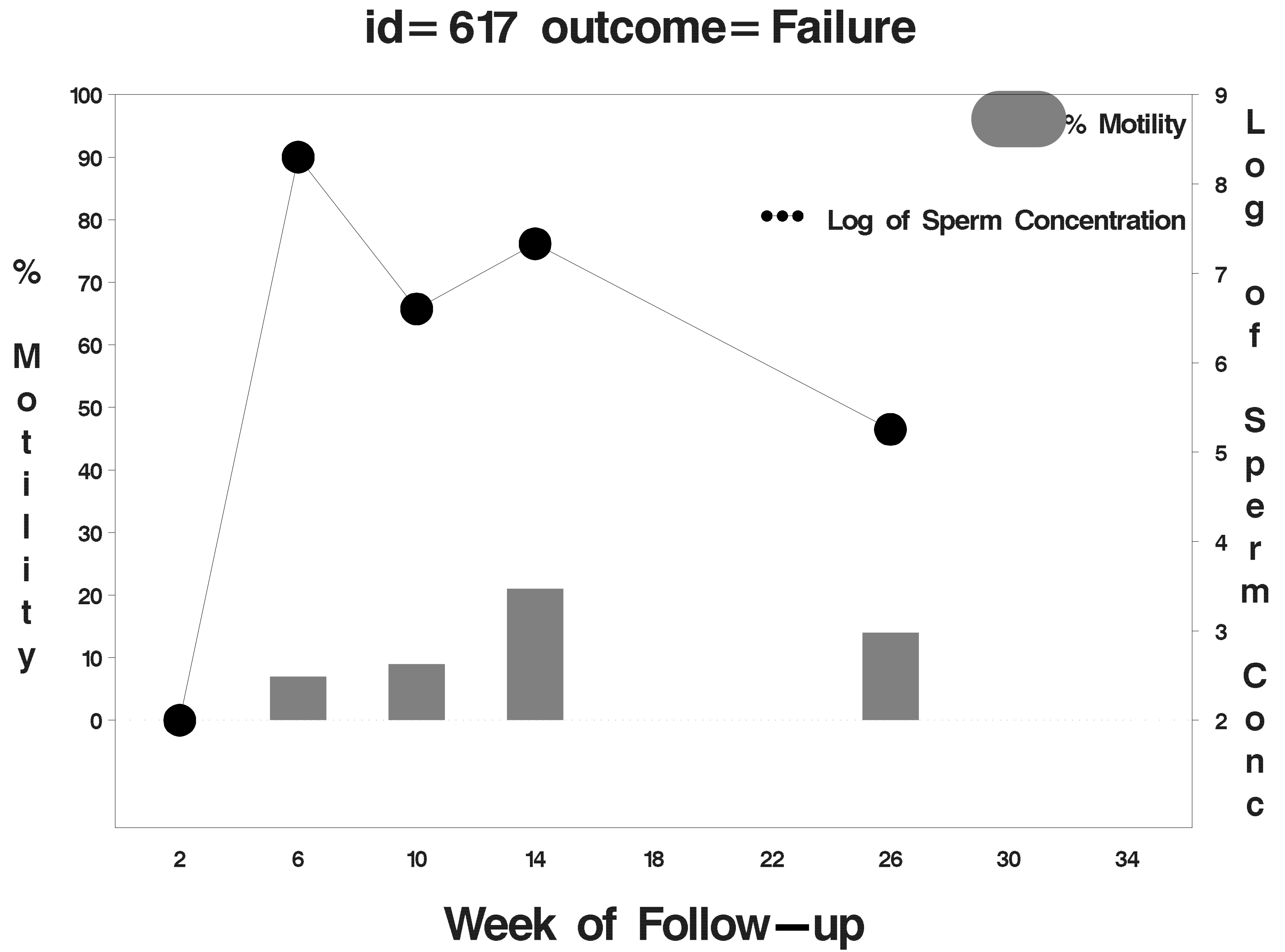
id= 617 outcome= Failure
% Motility
Log of Sperm Concentration
% Motility
Log of Sperm Conc
Week of Follow-up
100
90
80
70
60
50
40
30
20
10
0
9
8
7
6
5
4
3
2
2
6
10
14
18
22
26
30
34

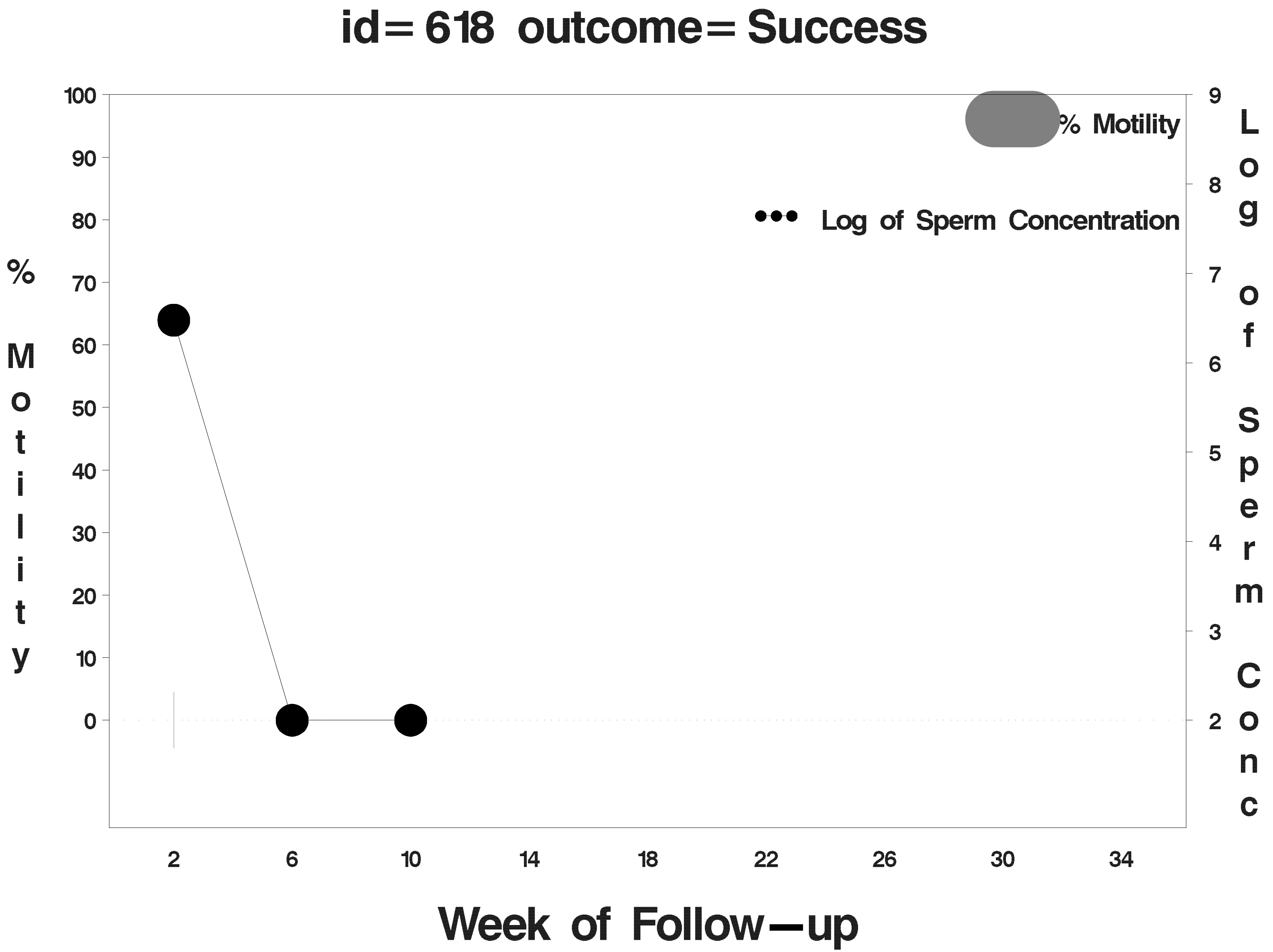
id= 618 outcome= Success
% Motility
Log of Sperm Concentration
% Motility
Log of Sperm Conc
Week of Follow—up
100
90
80
70
60
50
40
30
20
10
0
9
8
7
6
5
4
3
2
2
6
10
14
18
22
26
30
34

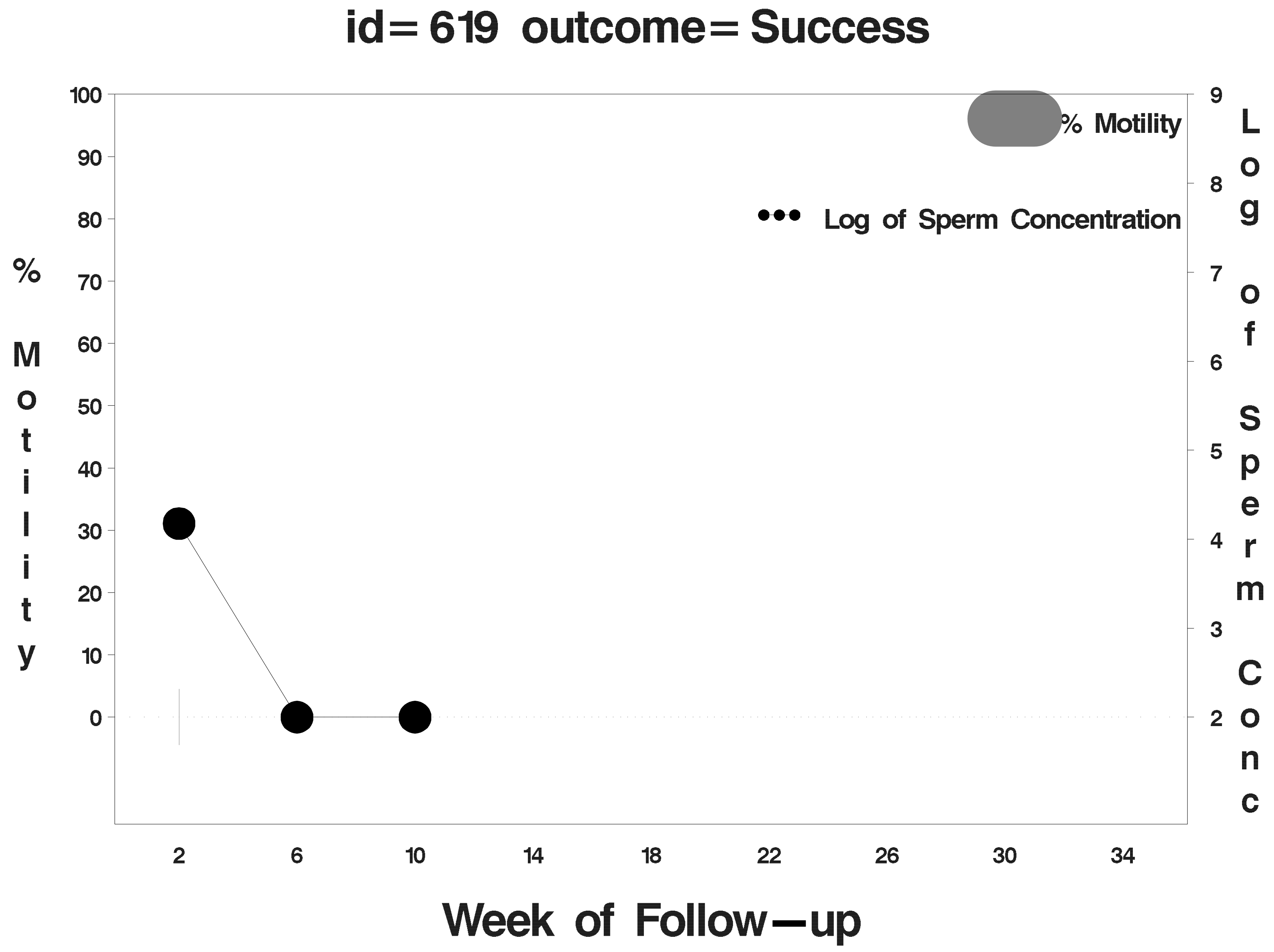

id= 619 outcome= Success
% Motility
Log of Sperm Concentration
100
90
80
70
60
50
40
30
20
10
0
9
8
7
6
5
4
3
2
2
6
10
14
18
22
26
30
34
Week of Follow—up
Log of Sperm Conc

id= 620 outcome= Indeterminate

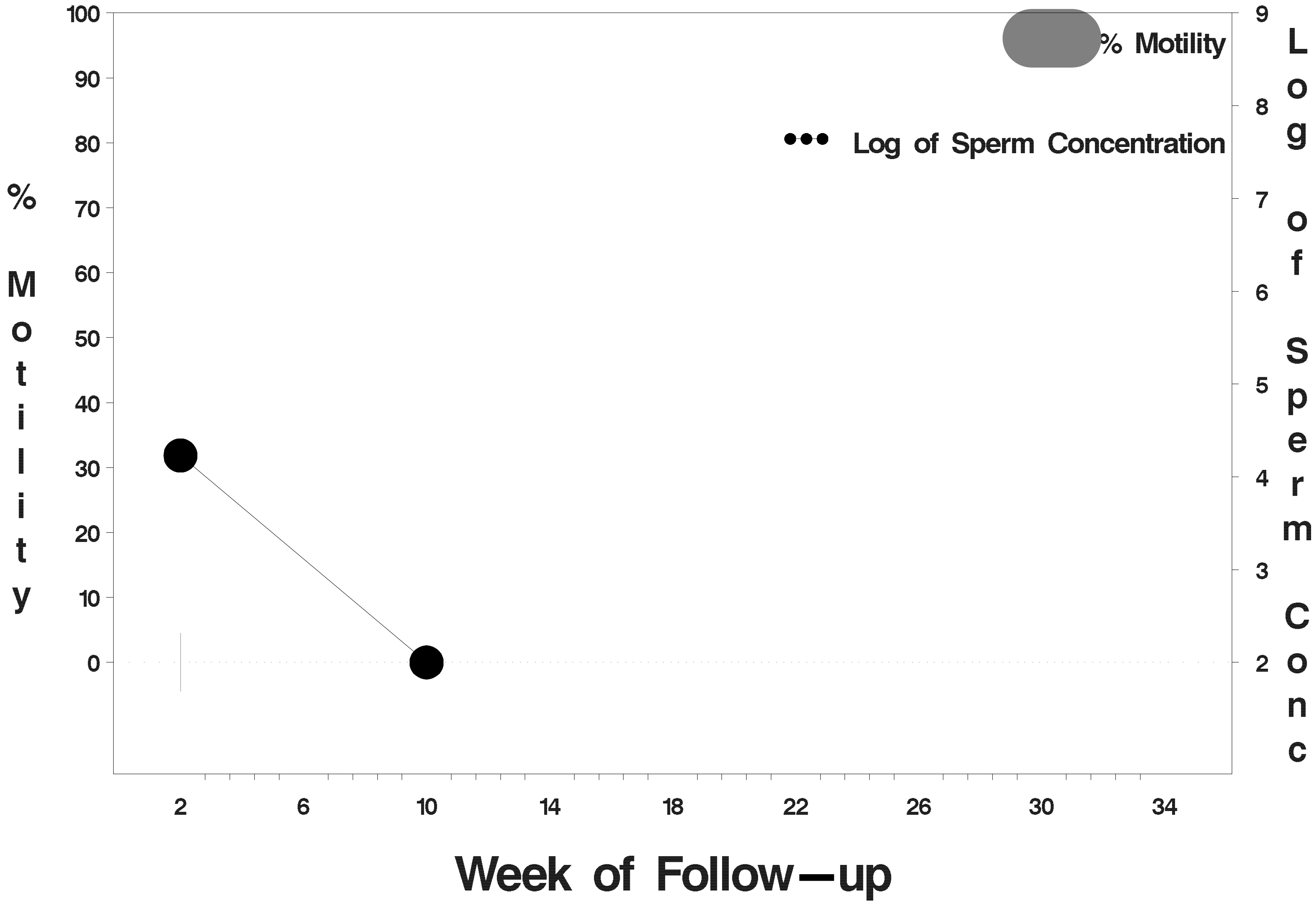

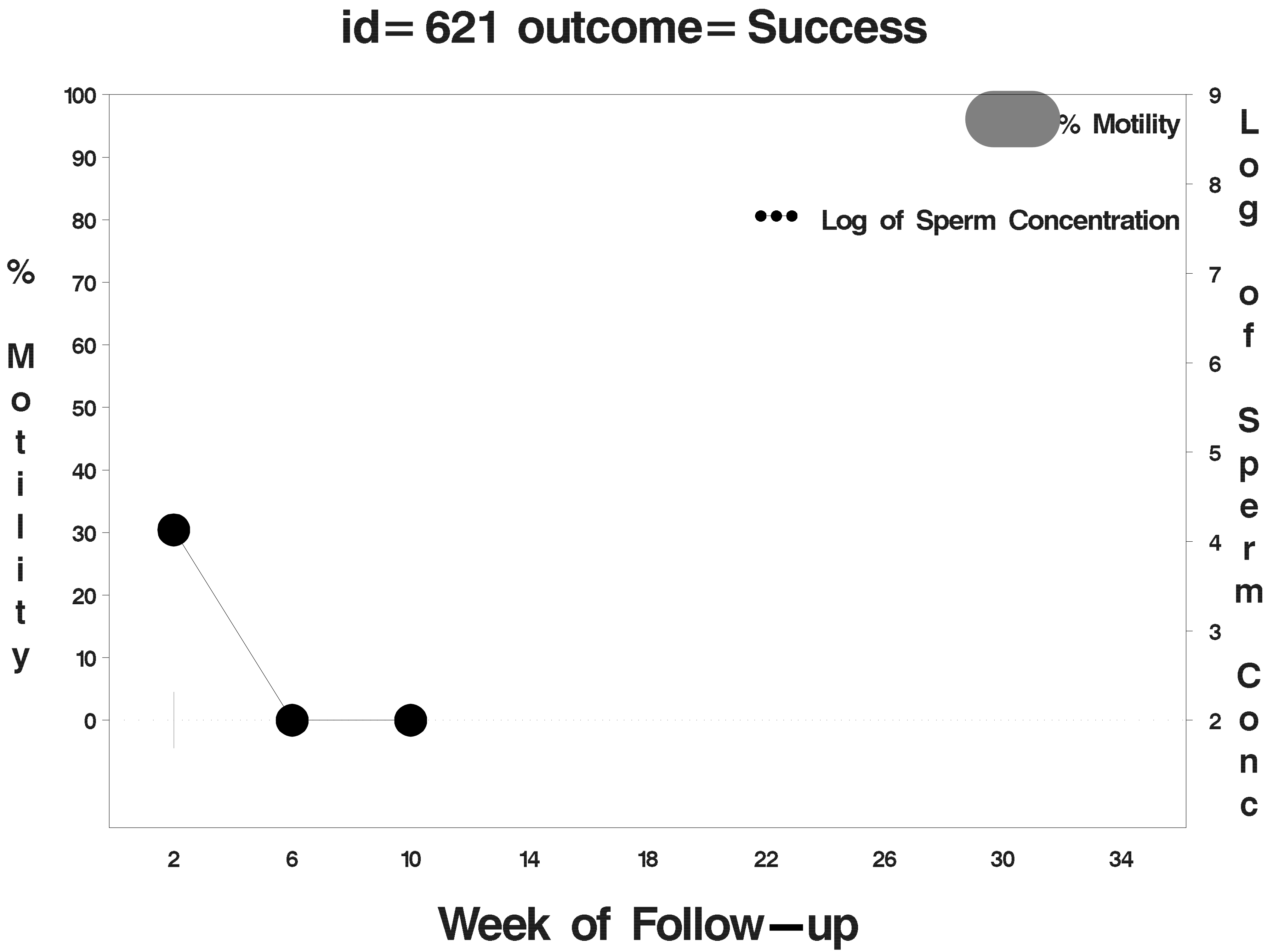
id= 621 outcome= Success
% Motility
Log of Sperm Concentration
100
90
80
70
60
50
40
30
20
10
0
9
8
7
6
5
4
3
2
2
6
10
14
18
22
26
30
34
% Motility
Log of Sperm Conc
Week of Follow-up

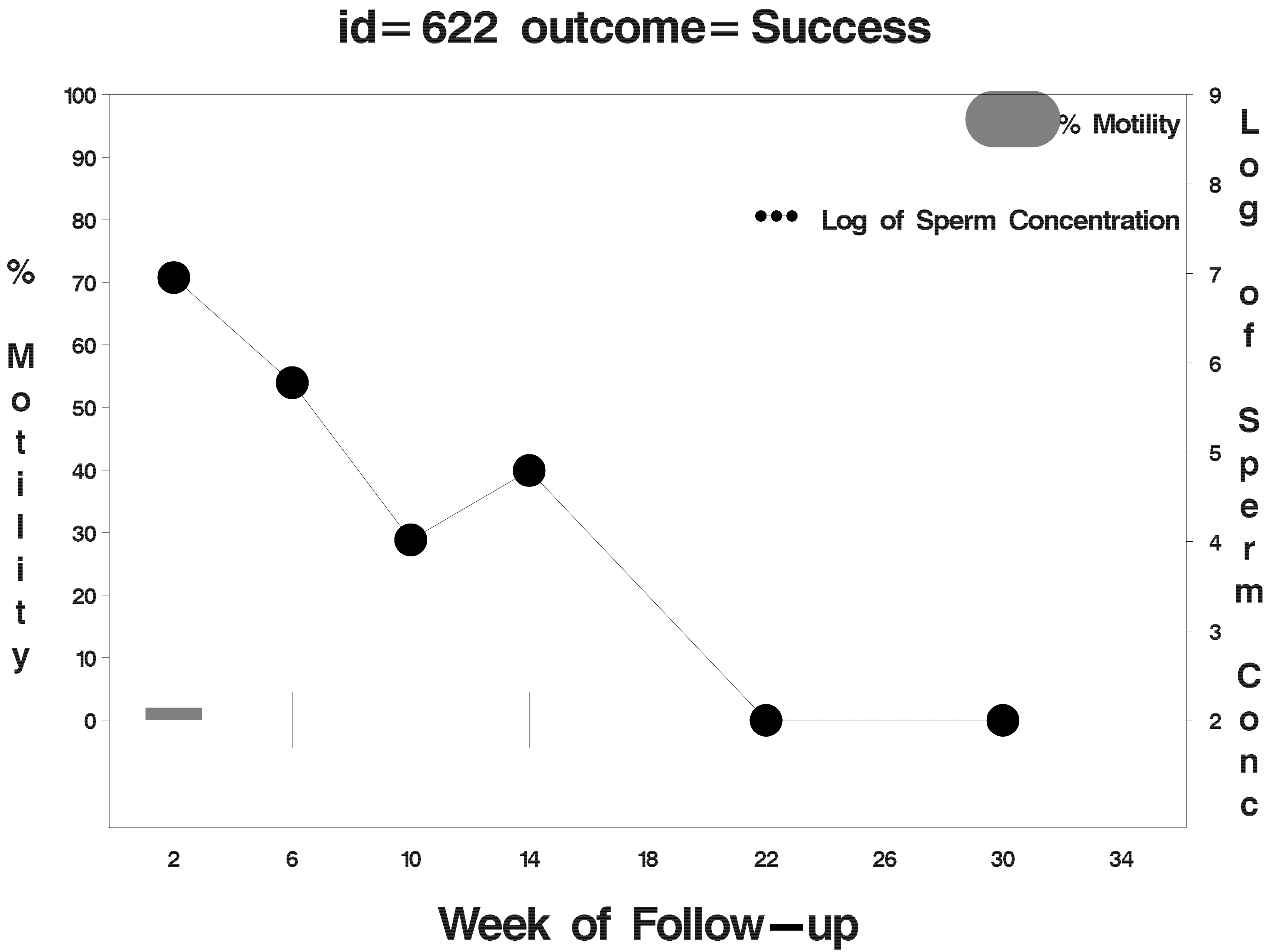

id= 622 outcome= Success
% Motility
Log of Sperm Concentration
% Motility
Log of Sperm Conc
Week of Follow−up
100
90
80
70
60
50
40
30
20
10
0
9
8
7
6
5
4
3
2
2
6
10
14
18
22
26
30
34

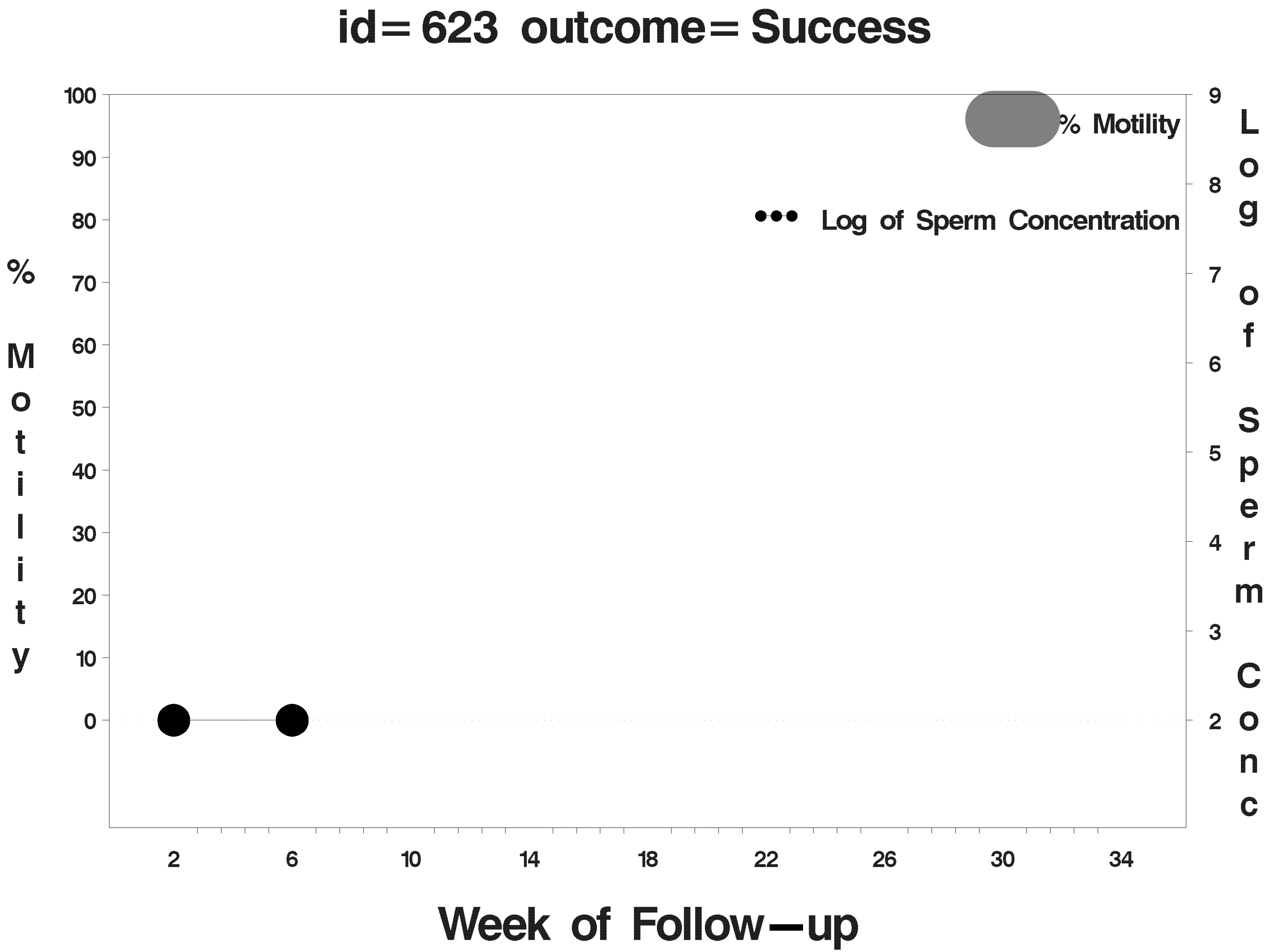

id= 623 outcome= Success
% Motility
Log of Sperm Concentration
% Motility
Log of Sperm Conc
Week of Follow—up
100
90
80
70
60
50
40
30
20
10
0
9
8
7
6
5
4
3
2
2
6
10
14
18
22
26
30
34

id= 624 outcome= Success

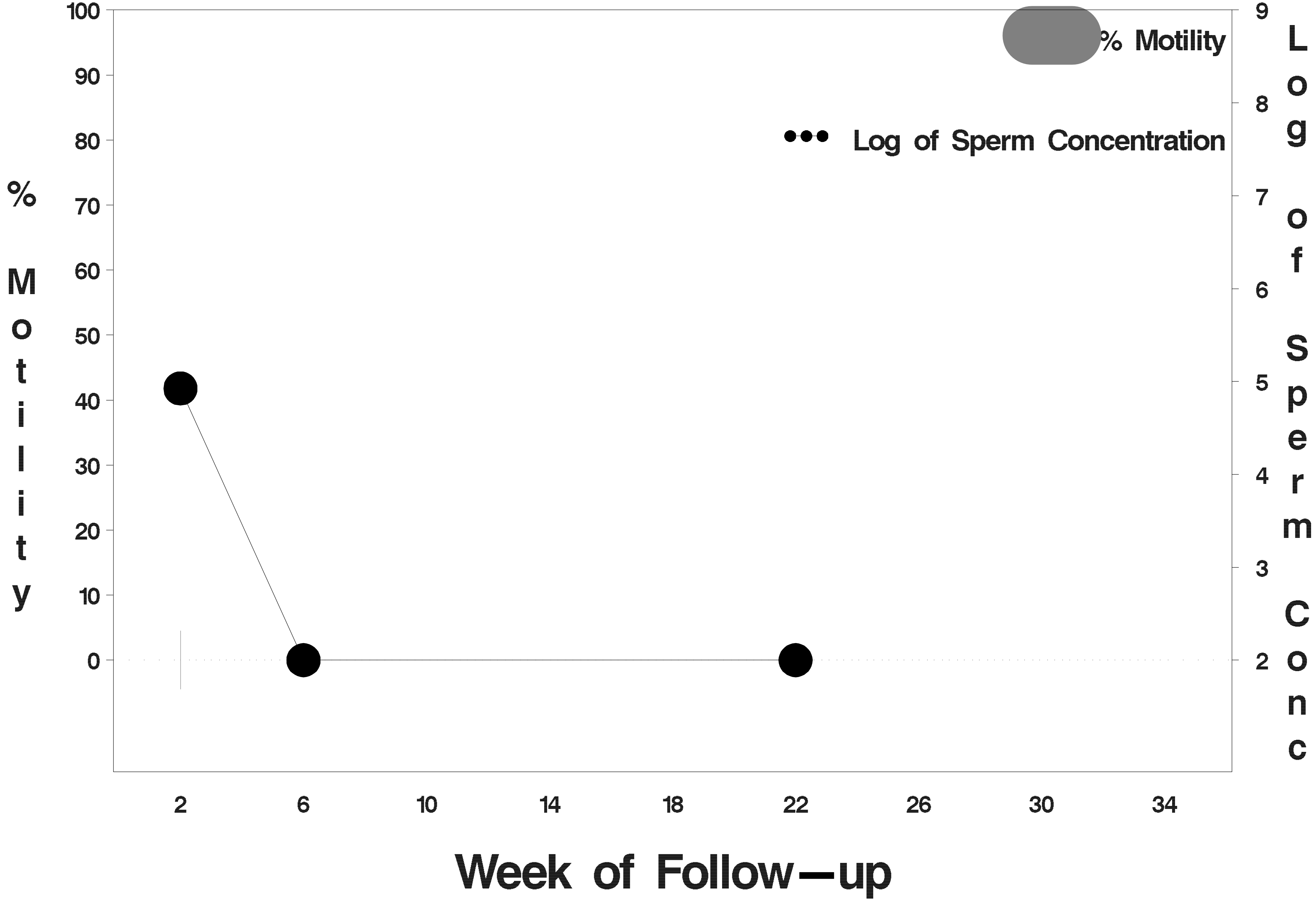

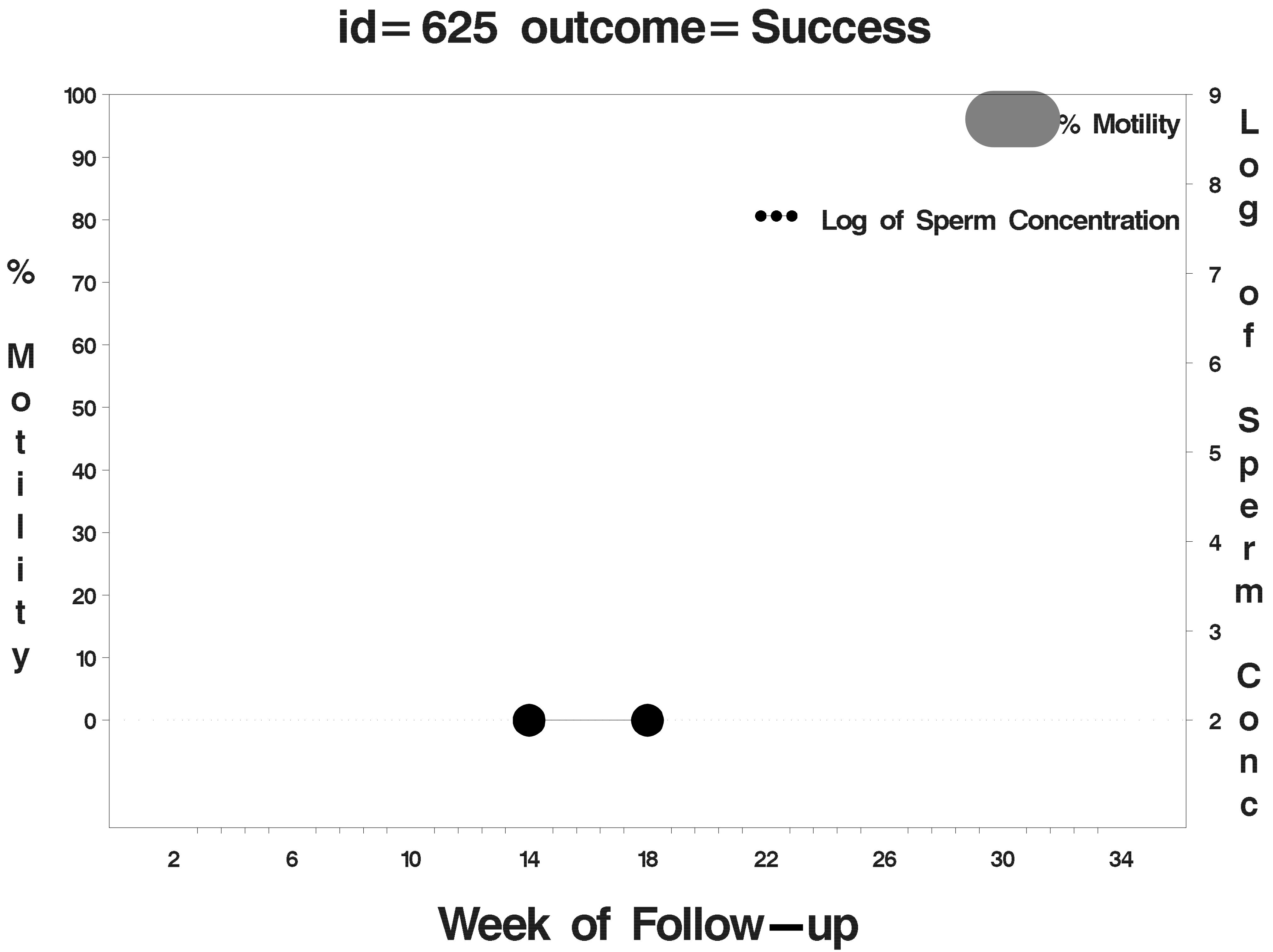
id= 625 outcome= Success
% Motility
Log of Sperm Concentration
% Motility
Log of Sperm Conc
Week of Follow-up
100
90
80
70
60
50
40
30
20
10
0
9
8
7
6
5
4
3
2
2
6
10
14
18
22
26
30
34

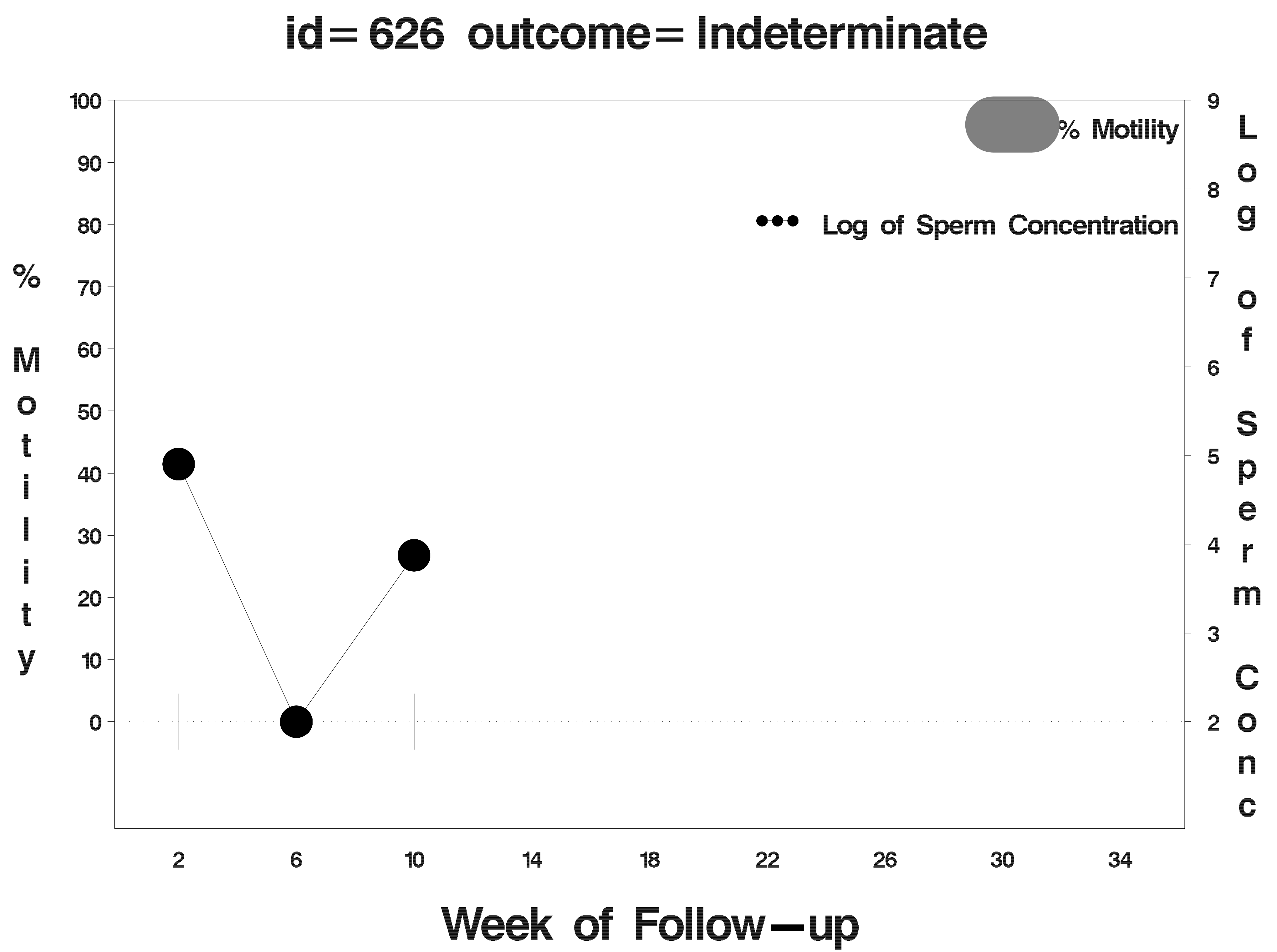
id= 626 outcome= Indeterminate
% Motility
Log of Sperm Concentration
100
90
80
70
60
50
40
30
20
10
0
9
8
7
6
5
4
3
2
Log of Sperm Conc
2
6
10
14
18
22
26
30
34
Week of Follow−up

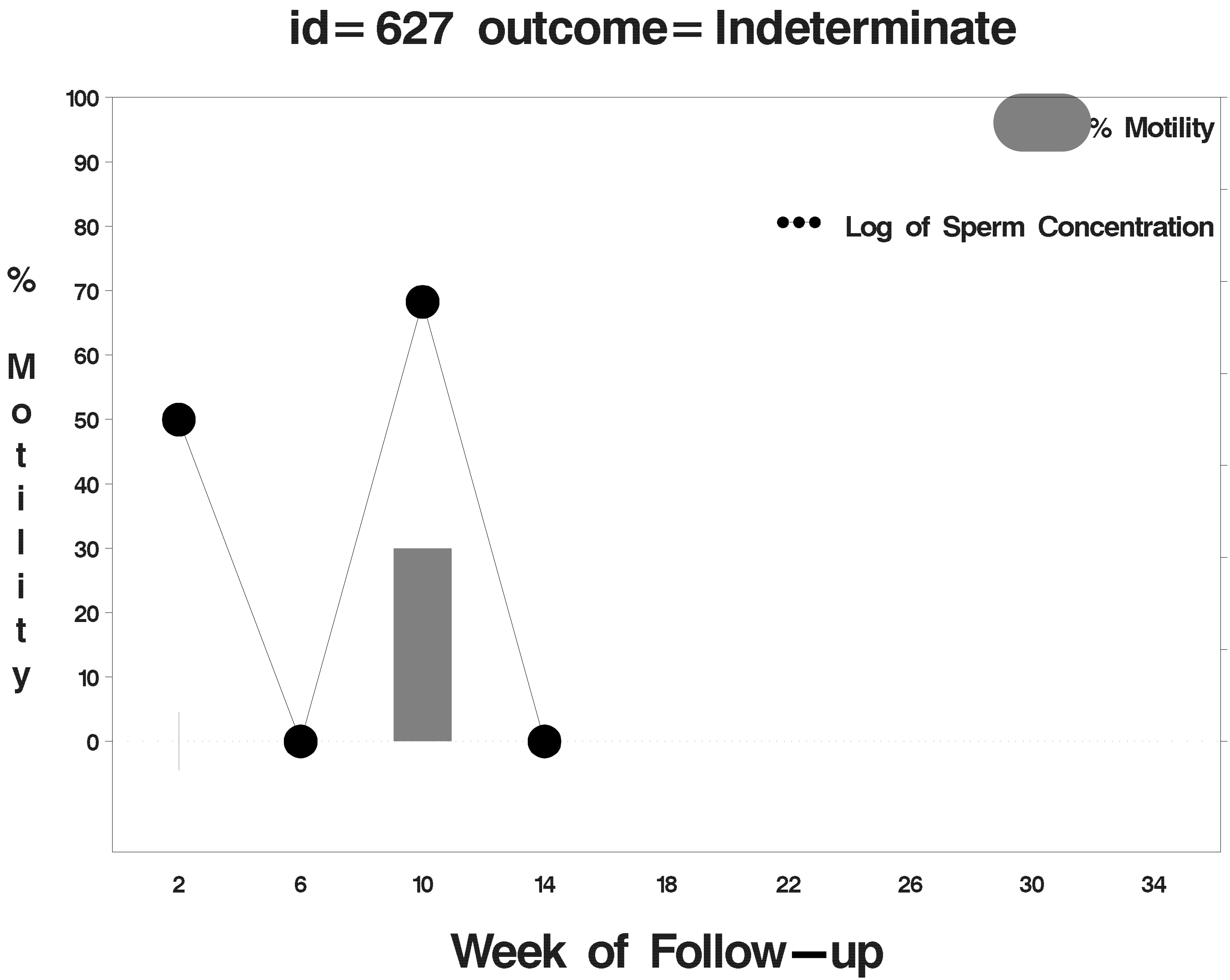

id= 627 outcome= Indeterminate
% Motility
Log of Sperm Concentration
100
90
80
70
60
50
40
30
20
10
0
9
8
7
6
5
4
3
2
2
6
10
14
18
22
26
30
34
Week of Follow-up
Log of Sperm Conc

id= 628 outcome= Success

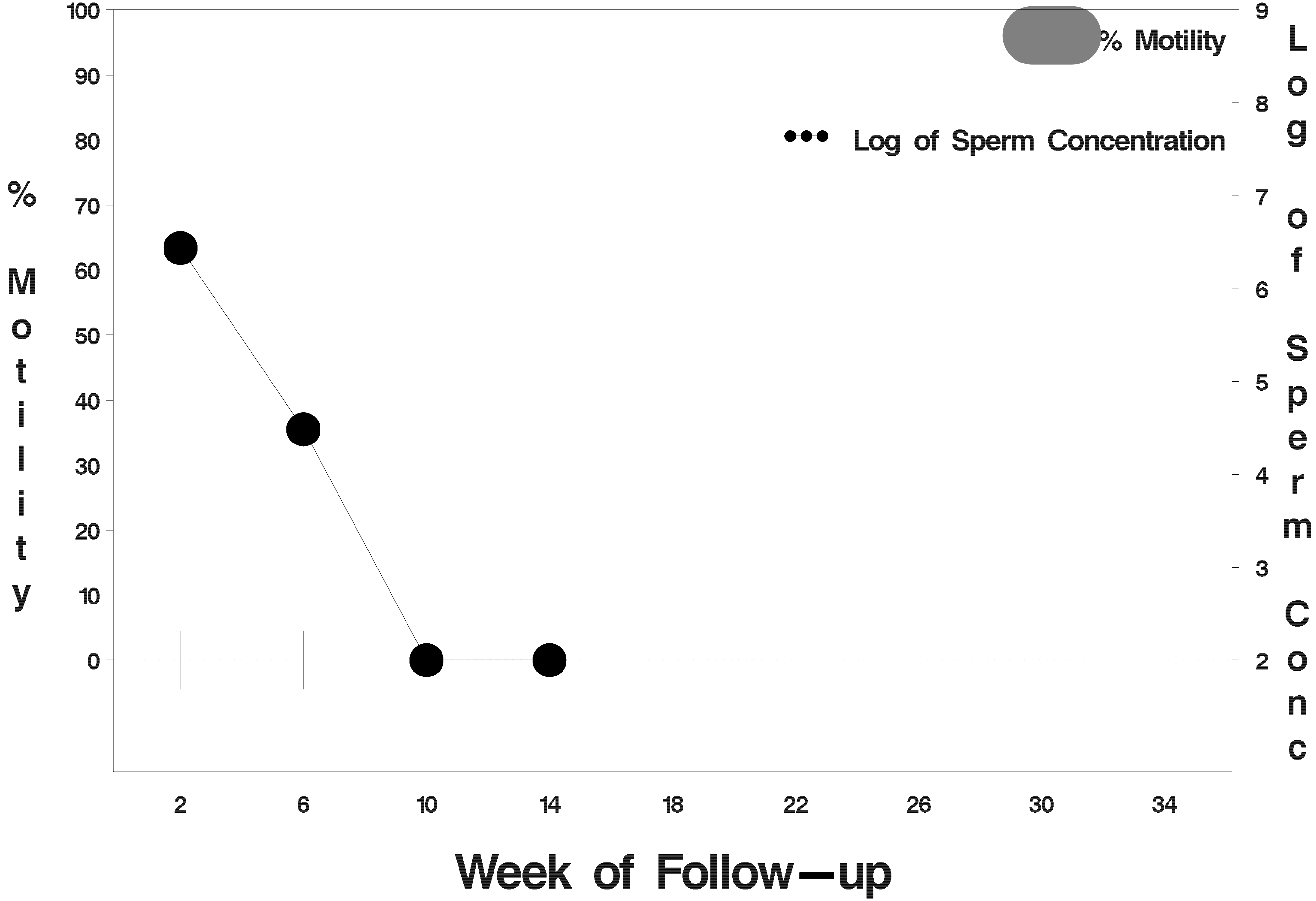

id= 629 outcome= Indeterminate

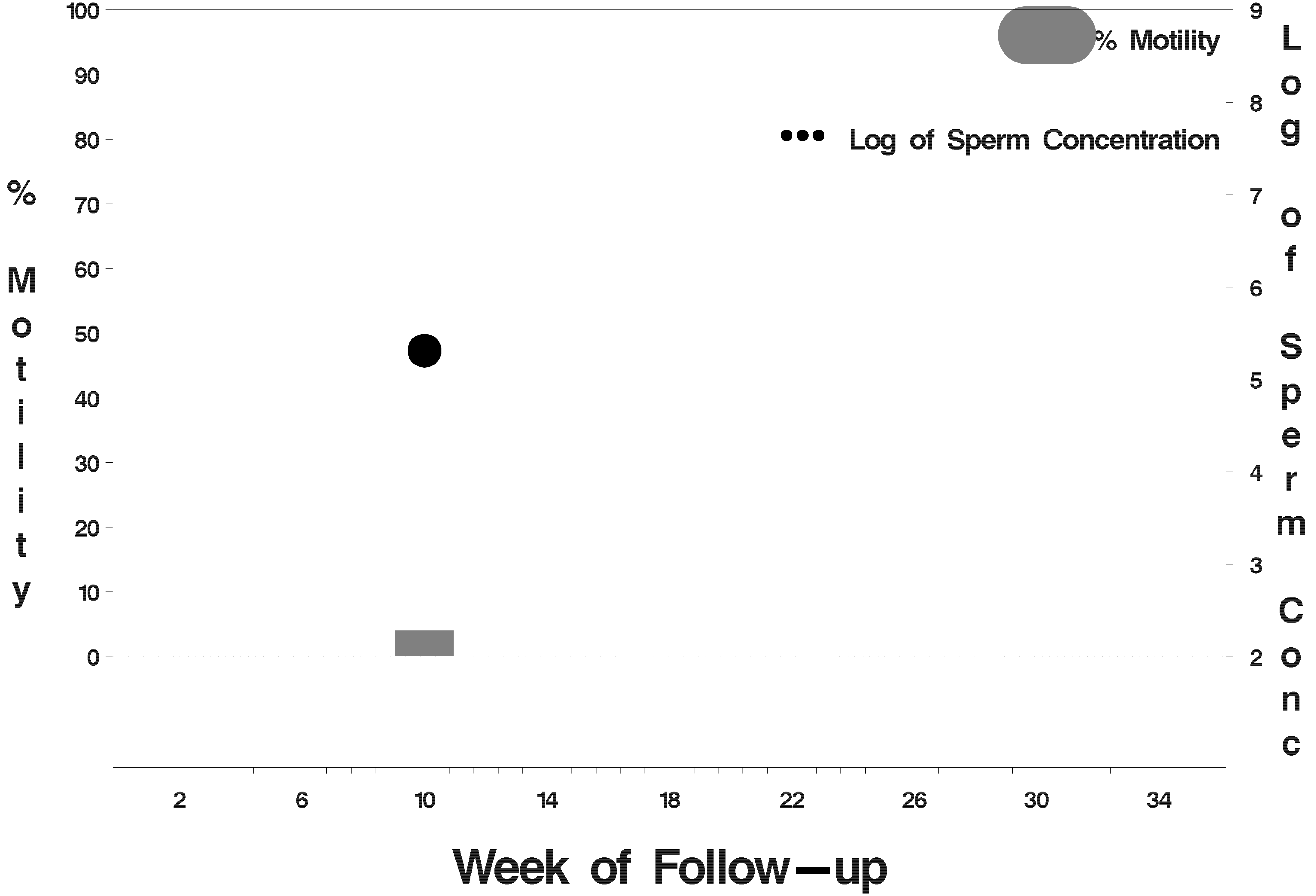

id= 630 outcome= Failure

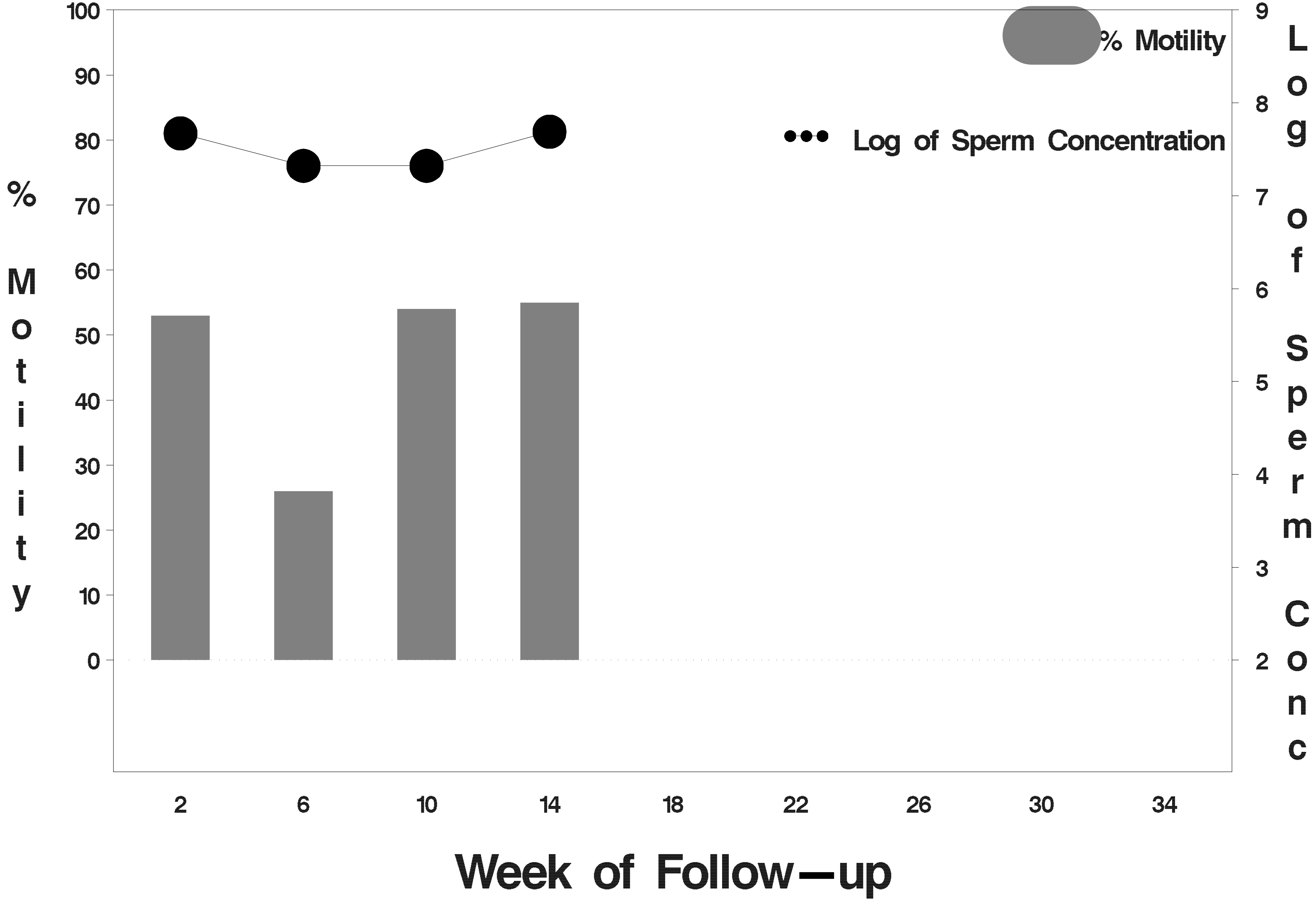

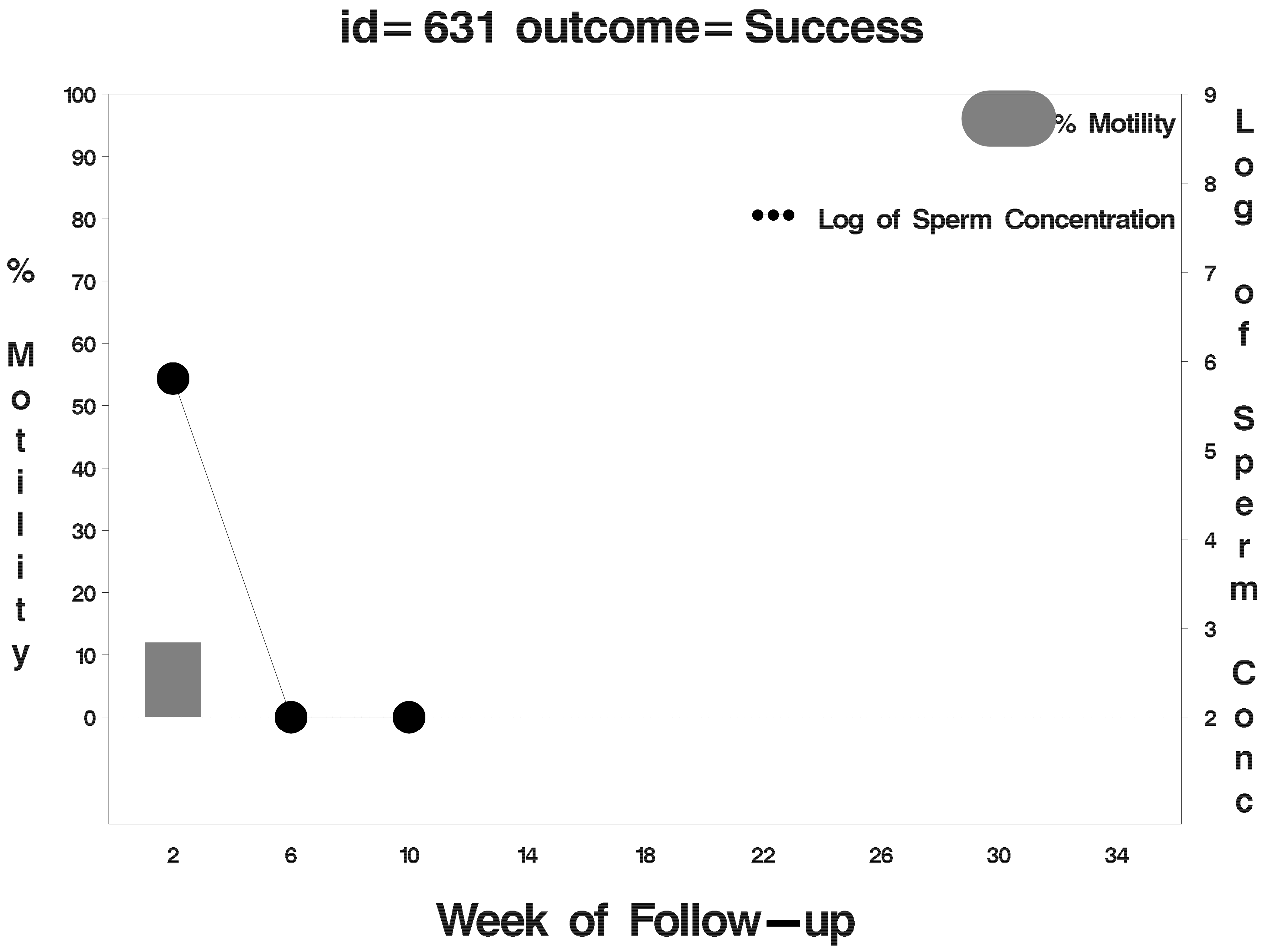
id= 631 outcome= Success
% Motility
Log of Sperm Concentration
100
90
80
70
60
50
40
30
20
10
0
9
8
7
6
5
4
3
2
2
6
10
14
18
22
26
30
34
Week of Follow—up
Log of Sperm Conc

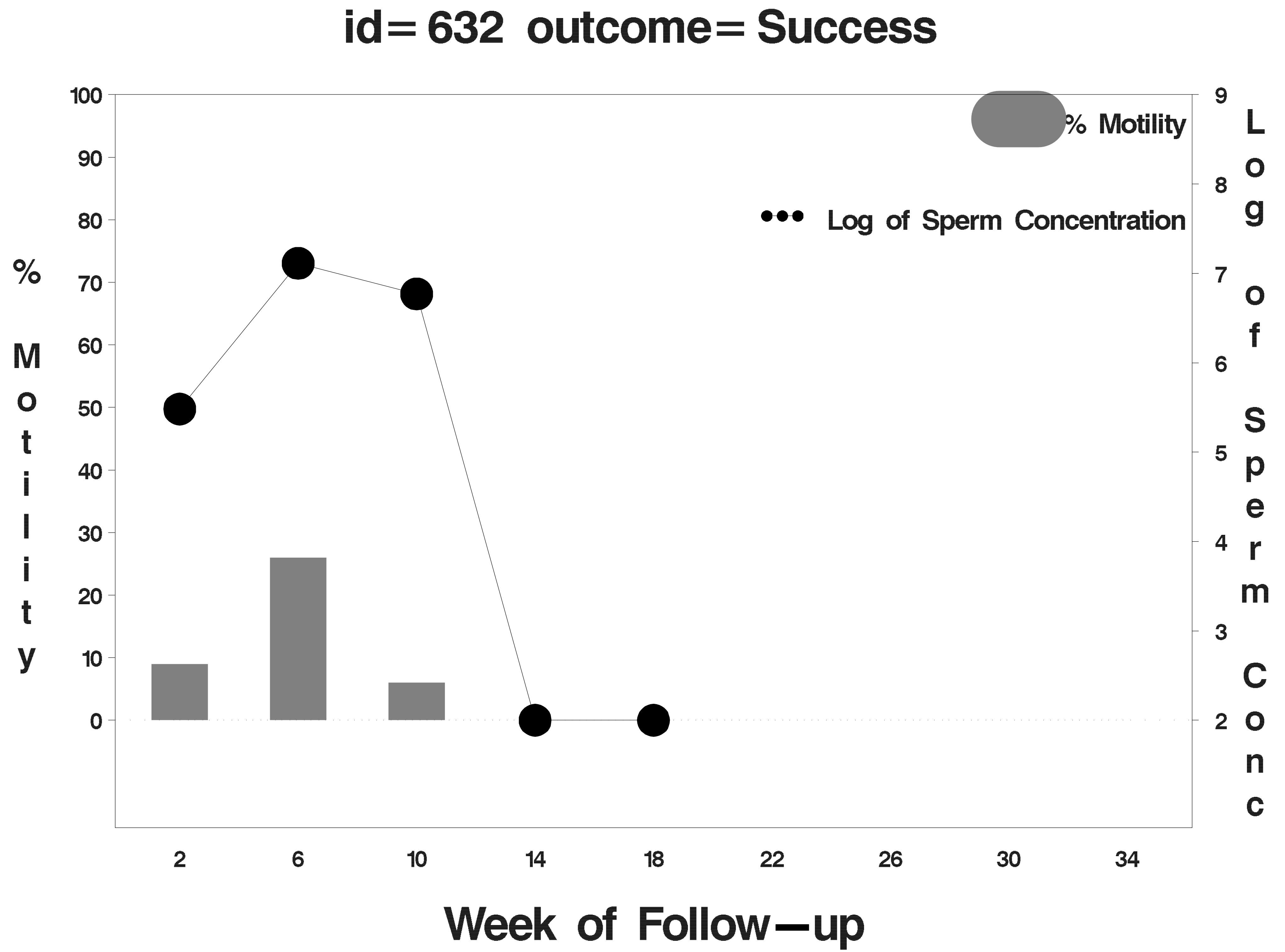
id= 632 outcome= Success
% Motility
Log of Sperm Concentration
% Motility
Log of Sperm Conc
Week of Follow−up
100
90
80
70
60
50
40
30
20
10
0
9
8
7
6
5
4
3
2
2
6
10
14
18
22
26
30
34

id= 633 outcome= Success

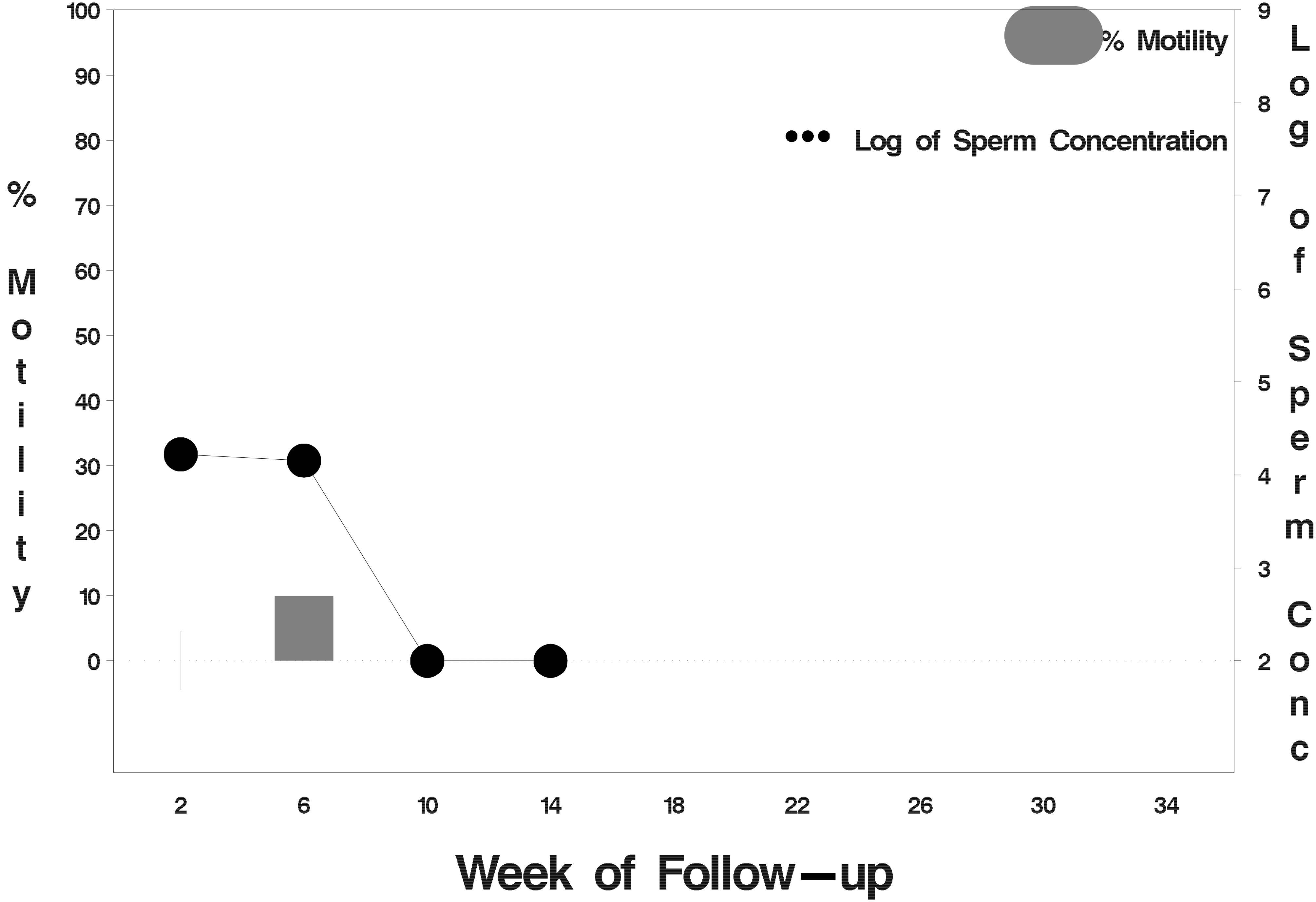

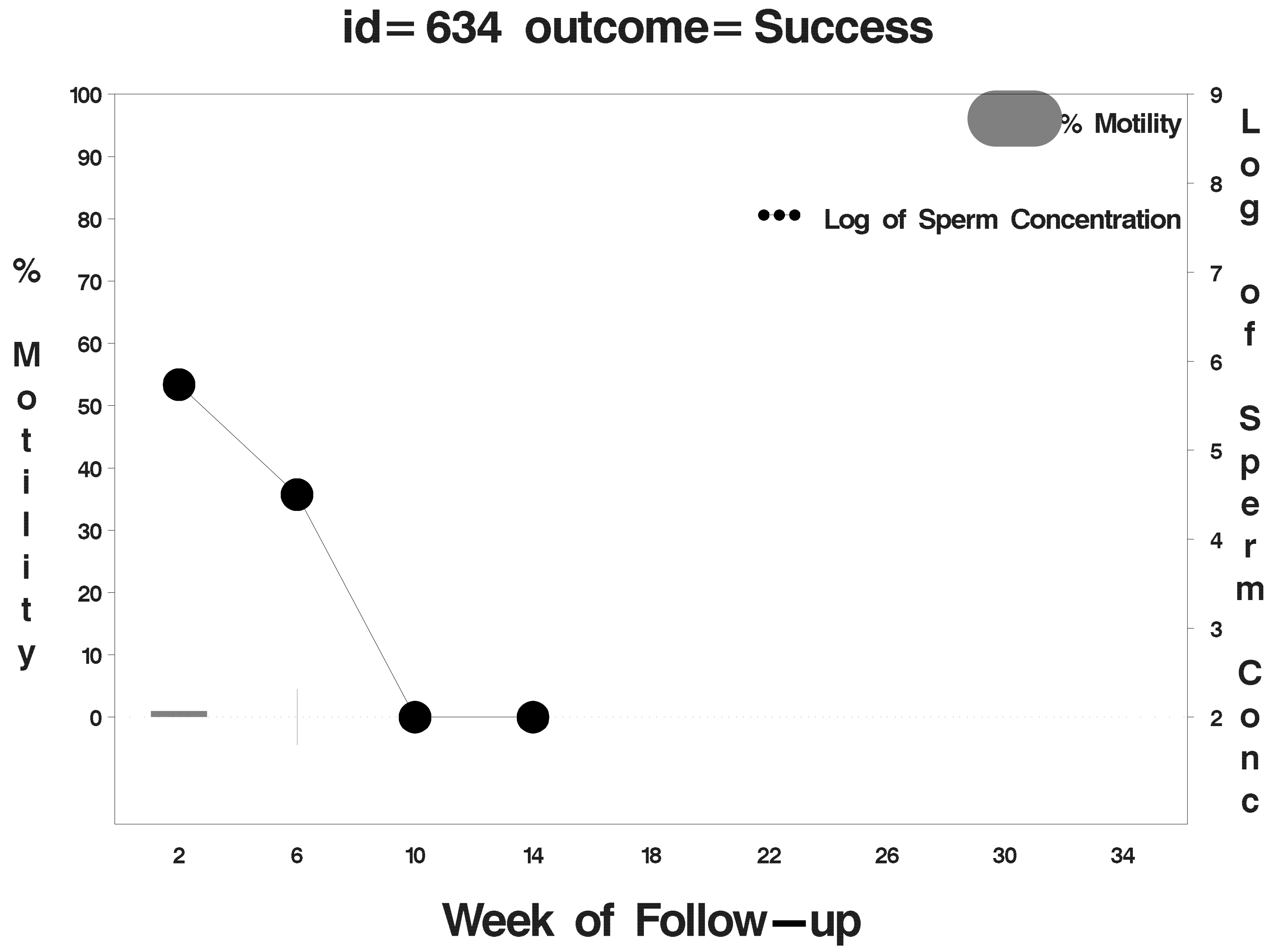

id= 634 outcome= Success
% Motility
Log of Sperm Concentration
% Motility
Log of Sperm Conc
Week of Follow-up
100
90
80
70
60
50
40
30
20
10
0
9
8
7
6
5
4
3
2
2
6
10
14
18
22
26
30
34

id= 635 outcome= Indeterminate

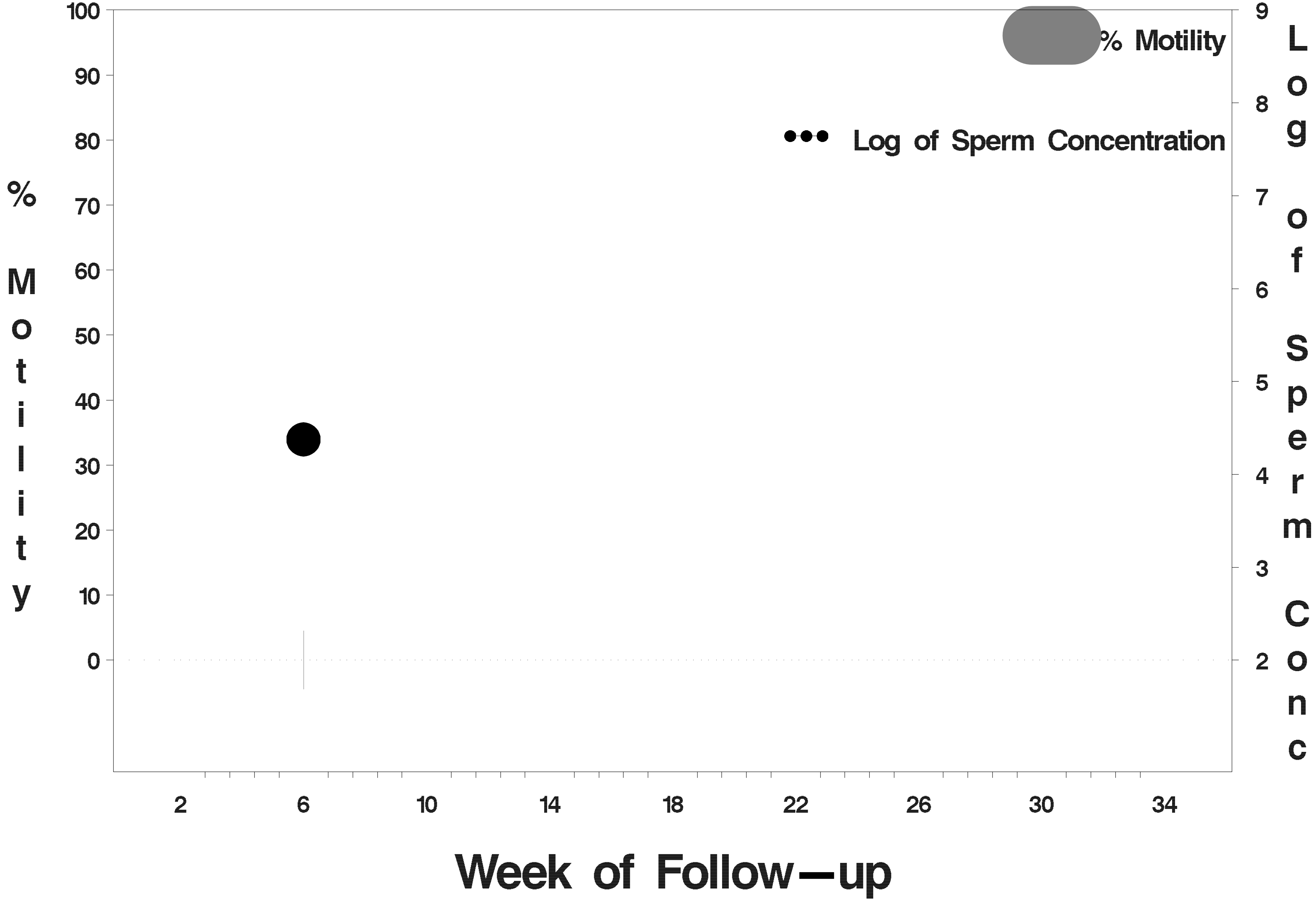

id= 636 outcome= Success

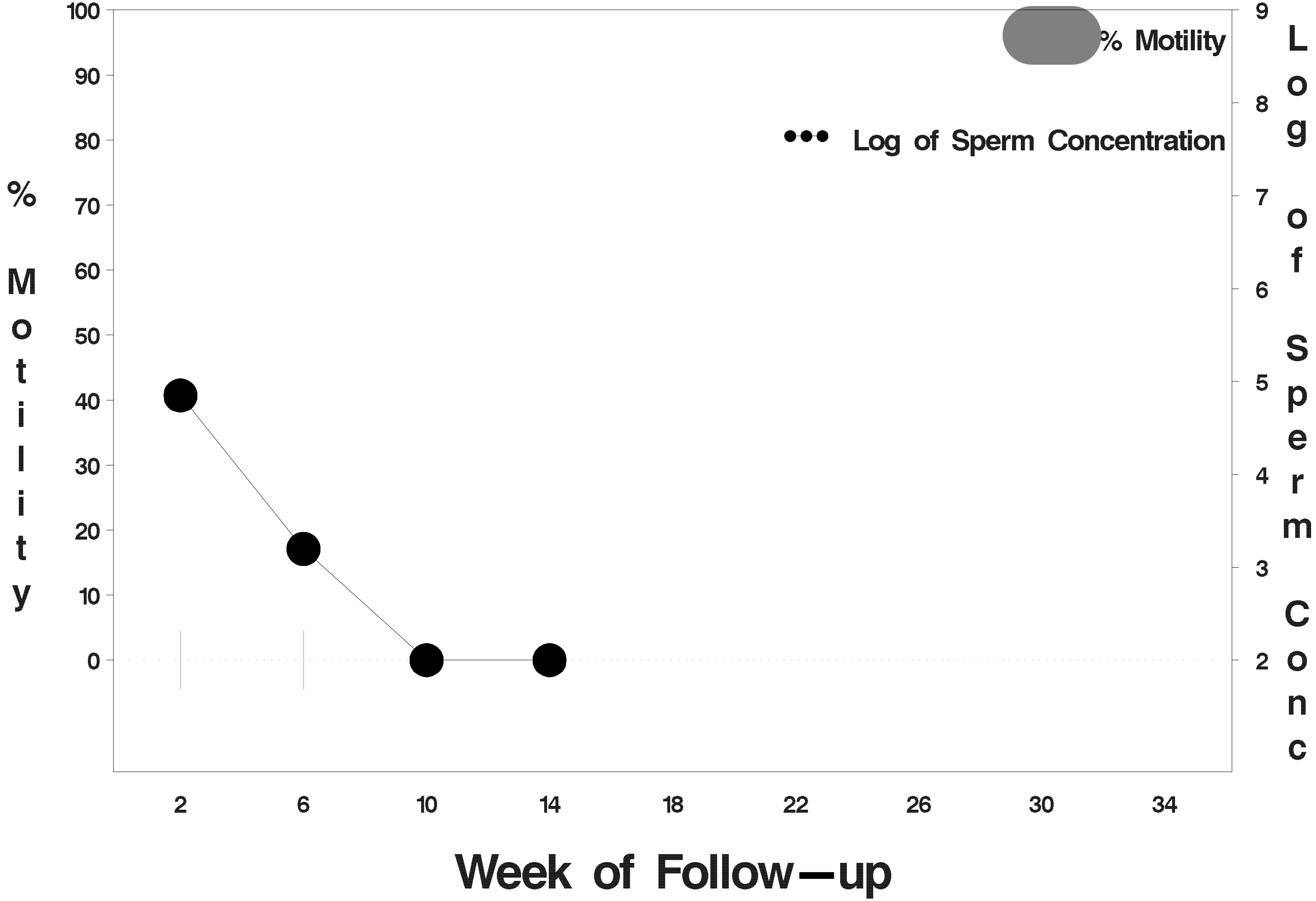

id= 637 outcome= Success

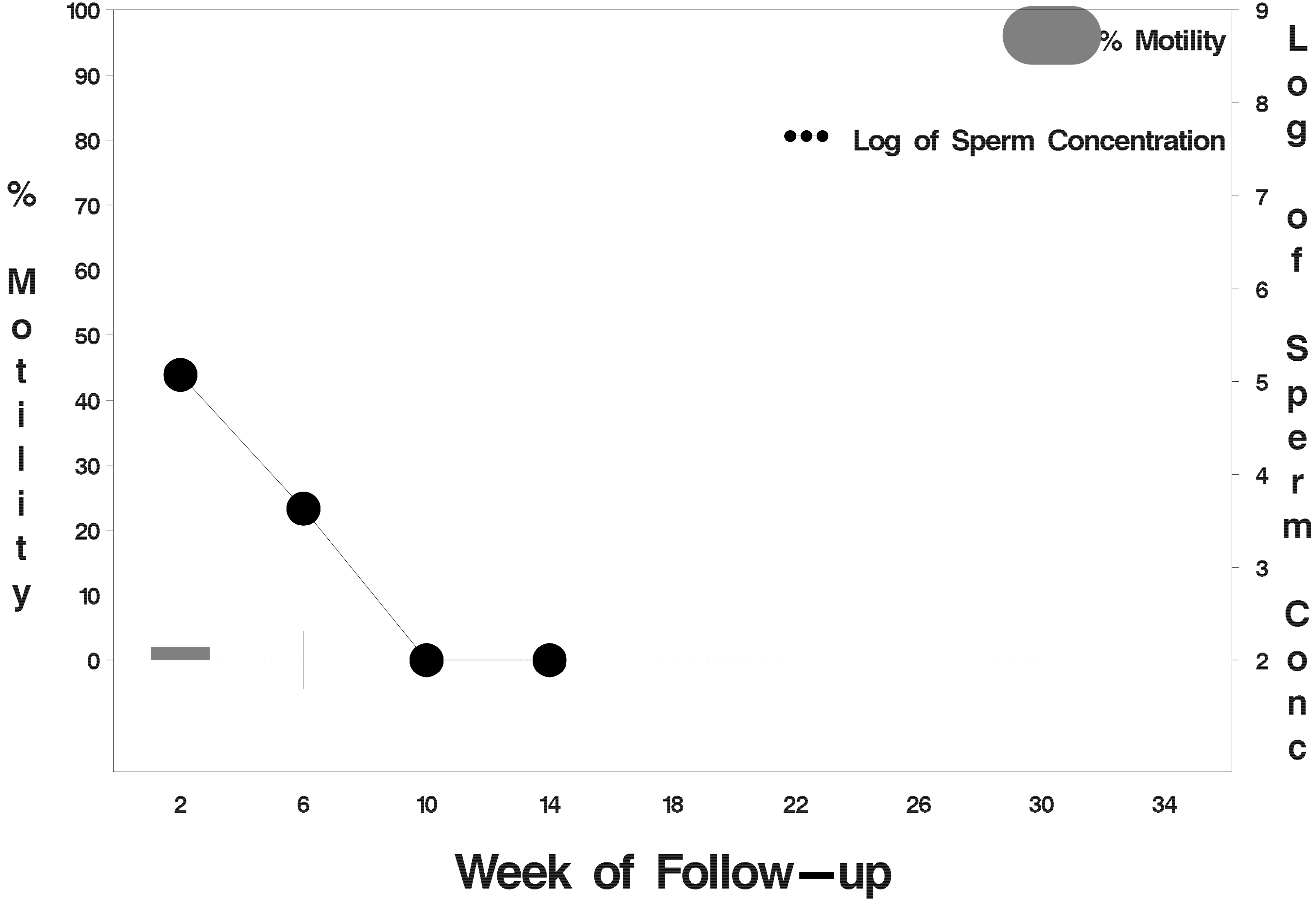

id= 638 outcome= Success

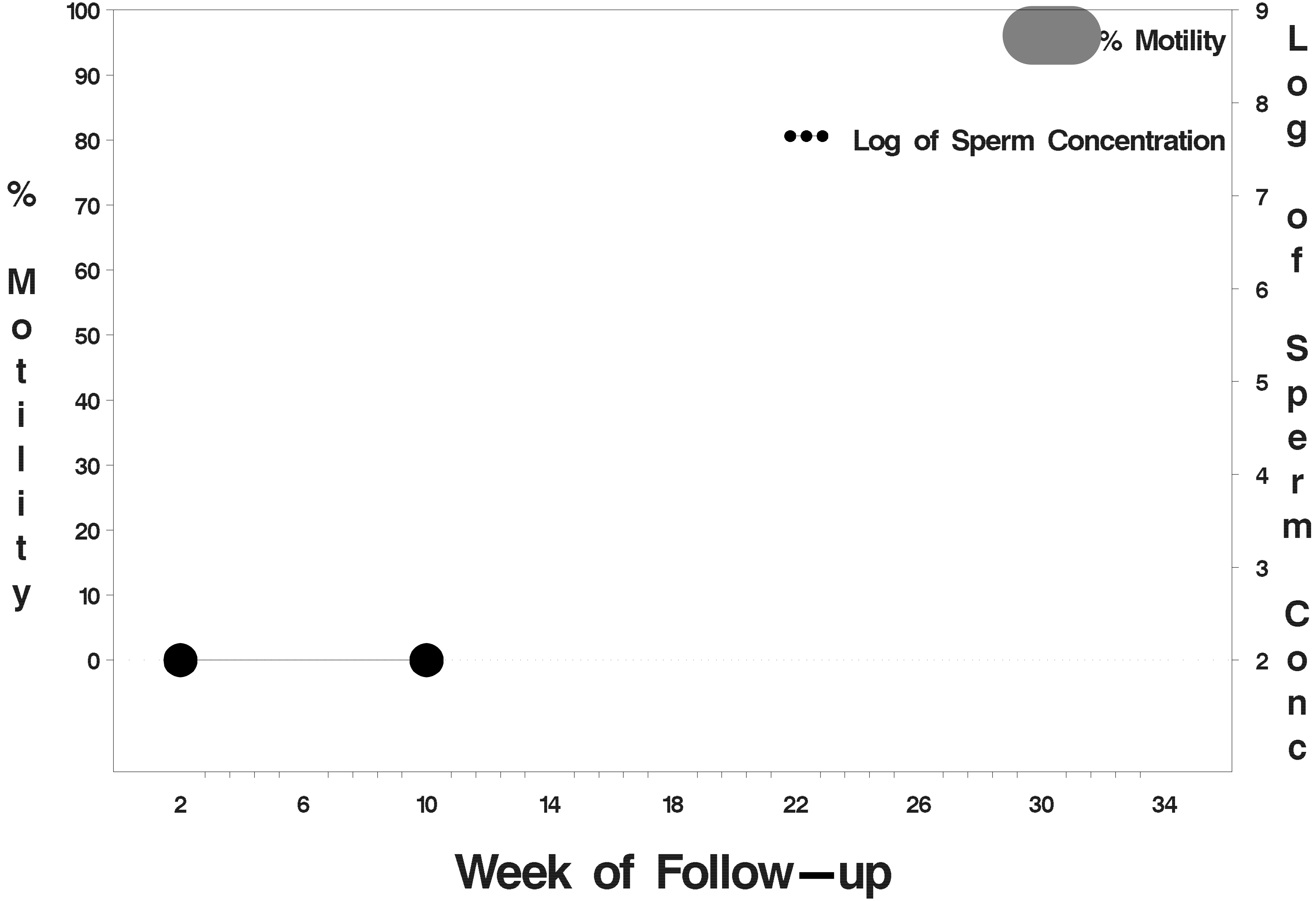

id= 639 outcome= Success

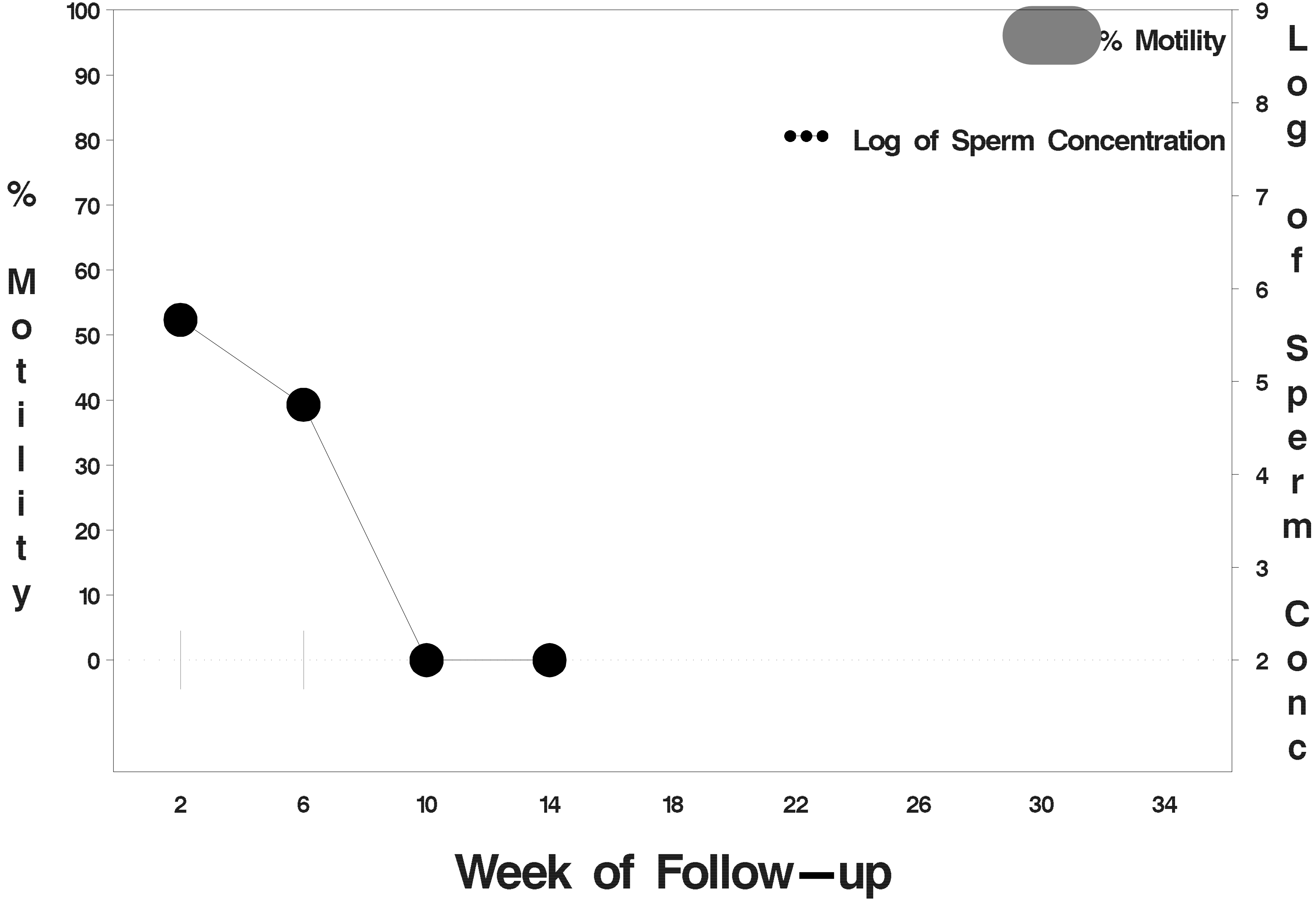

id= 640 outcome= Success

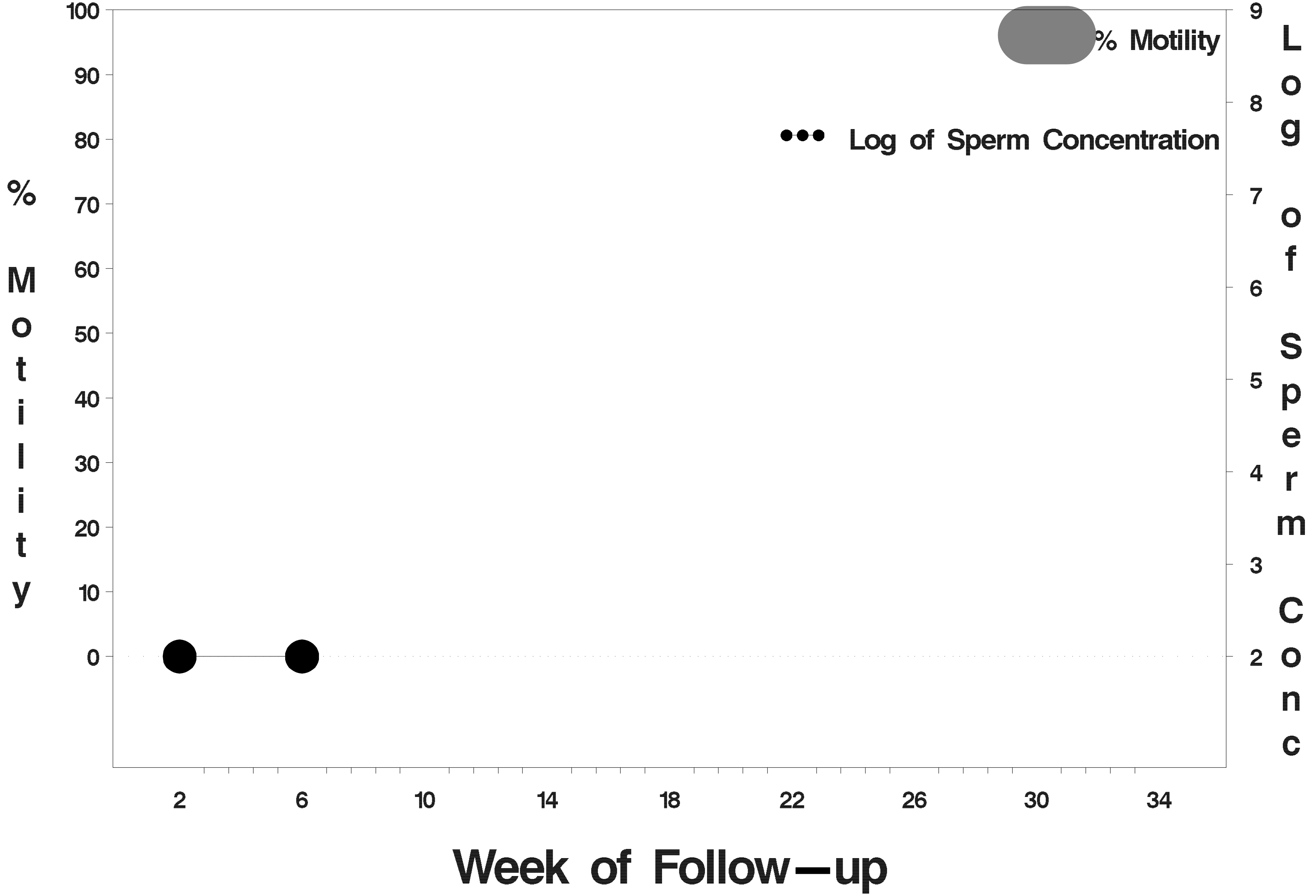

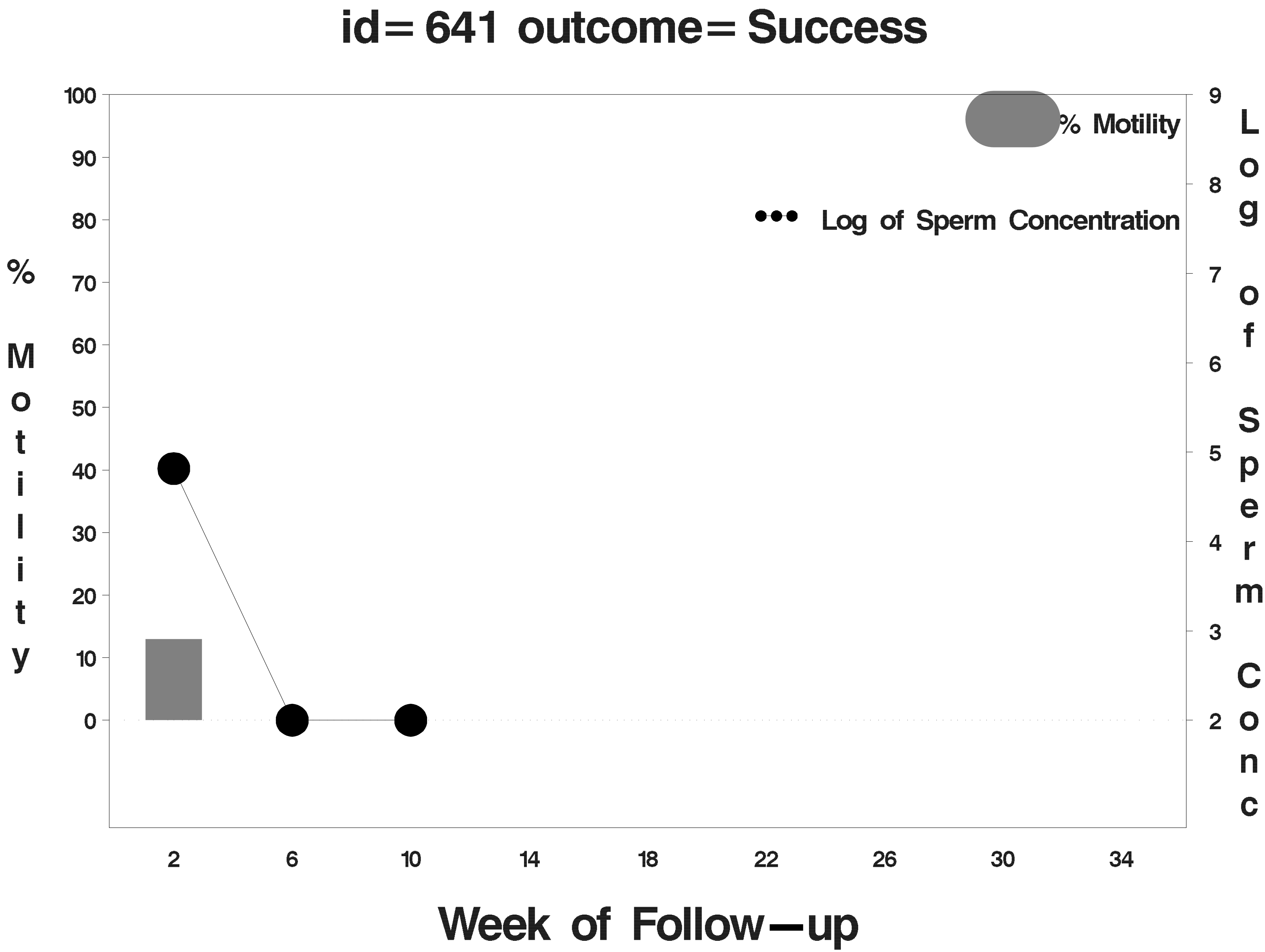

id= 641 outcome= Success
% Motility
Log of Sperm Concentration
100
90
80
70
60
50
40
30
20
10
0
9
8
7
6
5
4
3
2
2
6
10
14
18
22
26
30
34
Week of Follow—up
% Motility
Log of Sperm Conc

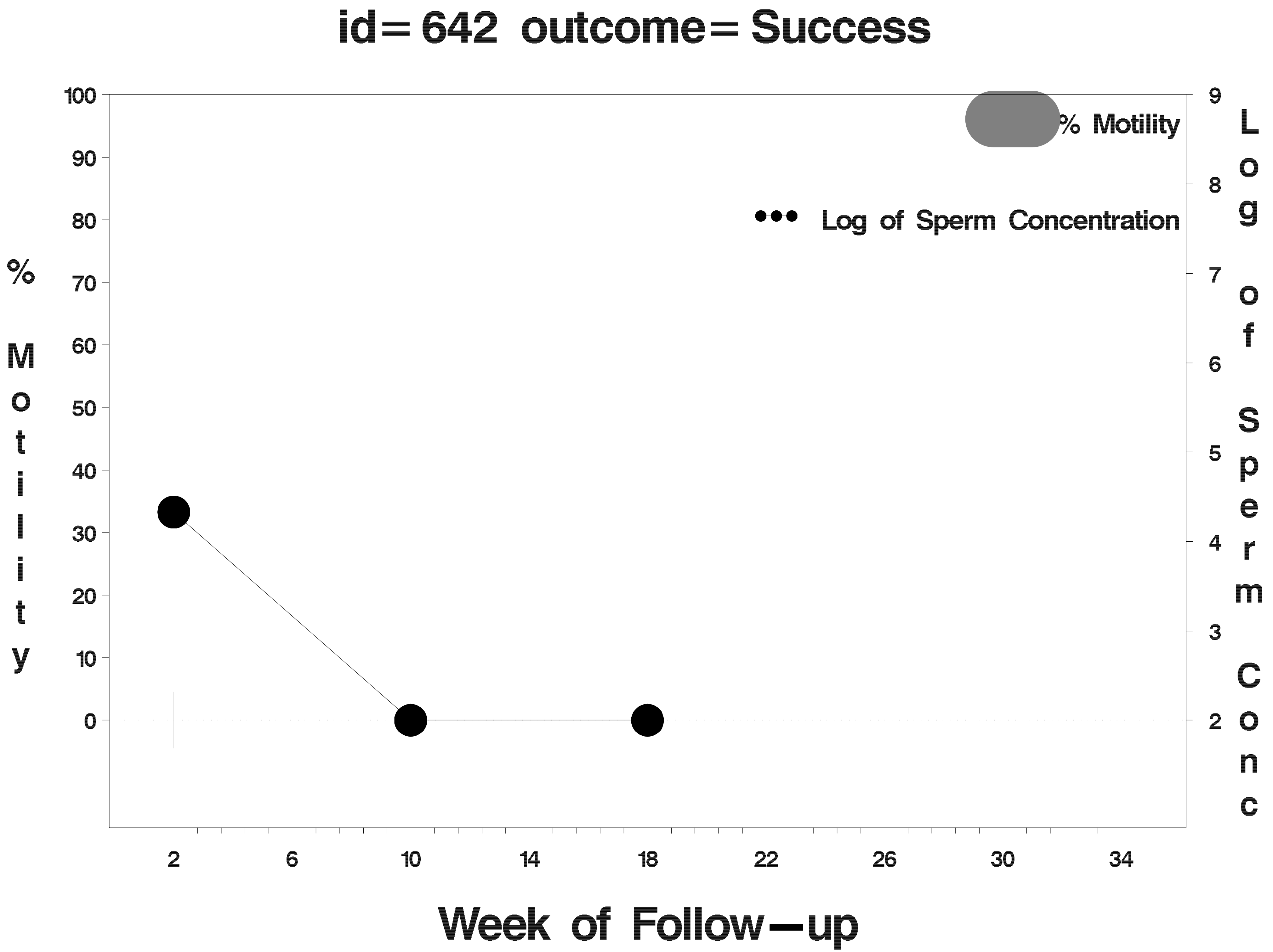

id= 642 outcome= Success
% Motility
Log of Sperm Concentration
100
90
80
70
60
50
40
30
20
10
0
9
8
7
6
5
4
3
2
2
6
10
14
18
22
26
30
34
Week of Follow−up

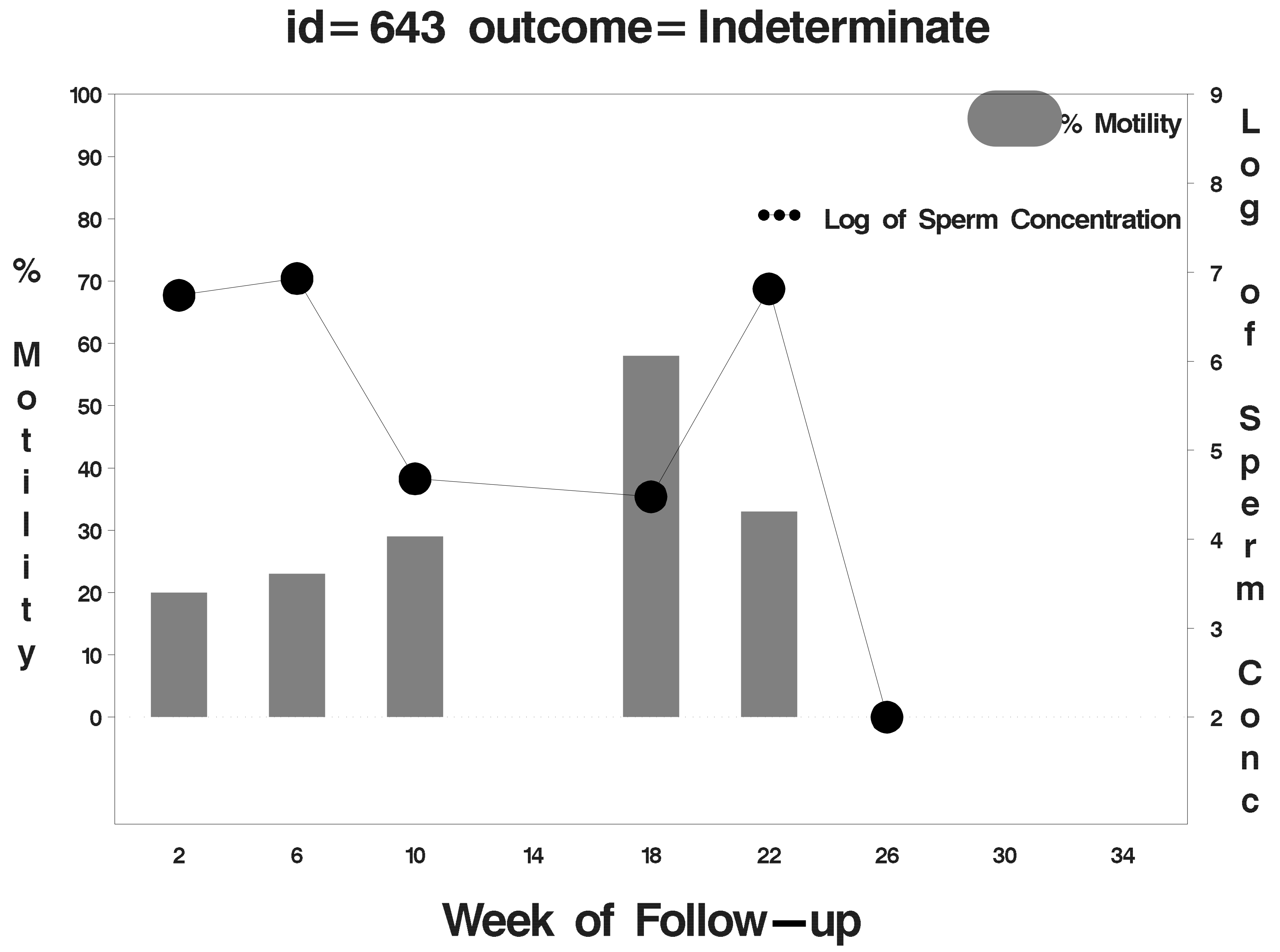

id= 643 outcome= Indeterminate
% Motility
Log of Sperm Concentration
100
90
80
70
60
50
40
30
20
10
0
9
8
7
6
5
4
3
2
2
6
10
14
18
22
26
30
34
Week of Follow−up
Log of Sperm Conc

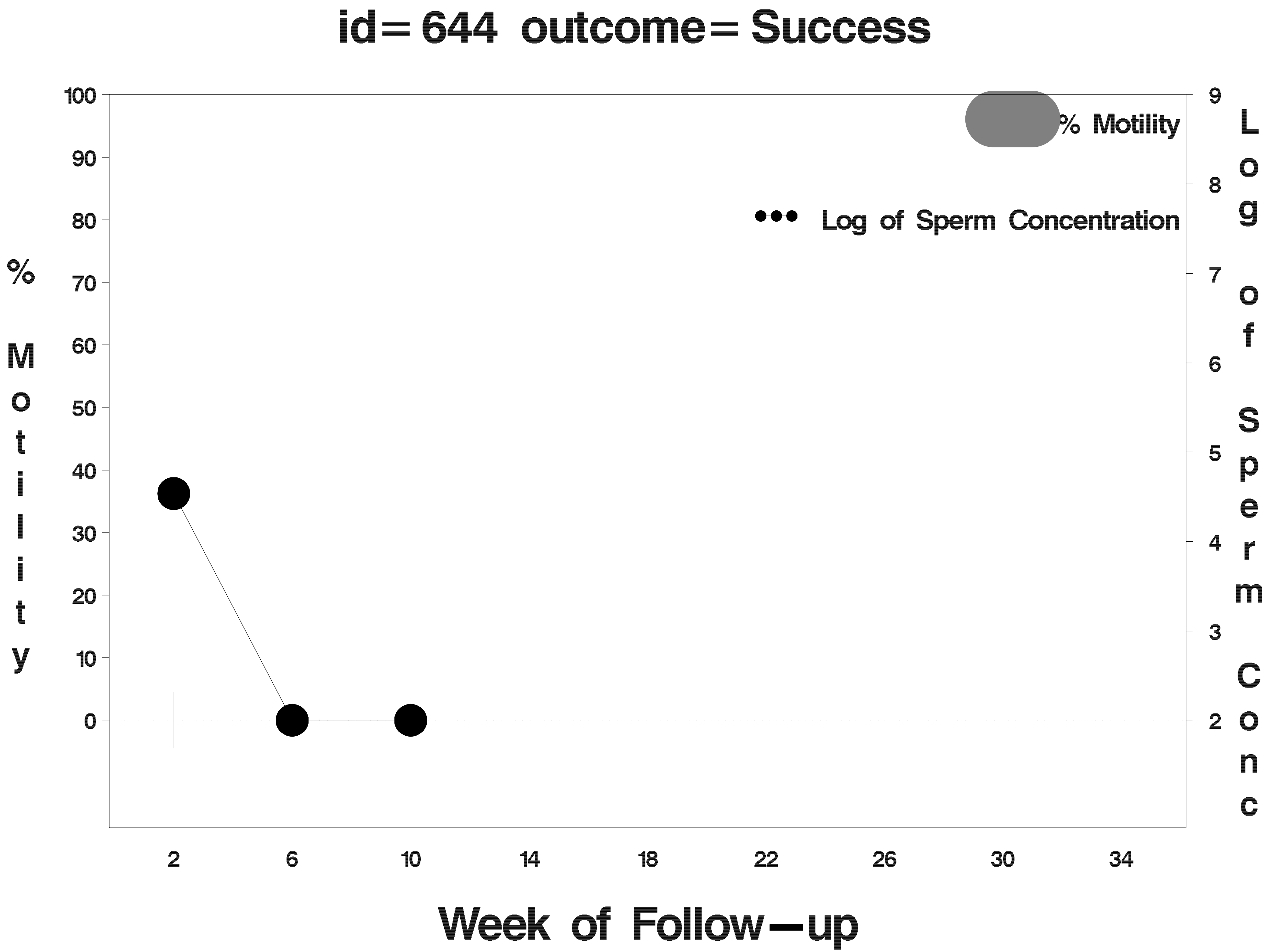

id= 644 outcome= Success
% Motility
Log of Sperm Concentration
100
90
80
70
60
50
40
30
20
10
0
9
8
7
6
5
4
3
2
2
6
10
14
18
22
26
30
34
Week of Follow-up
Log of Sperm Conc

id= 645 outcome= Indeterminate

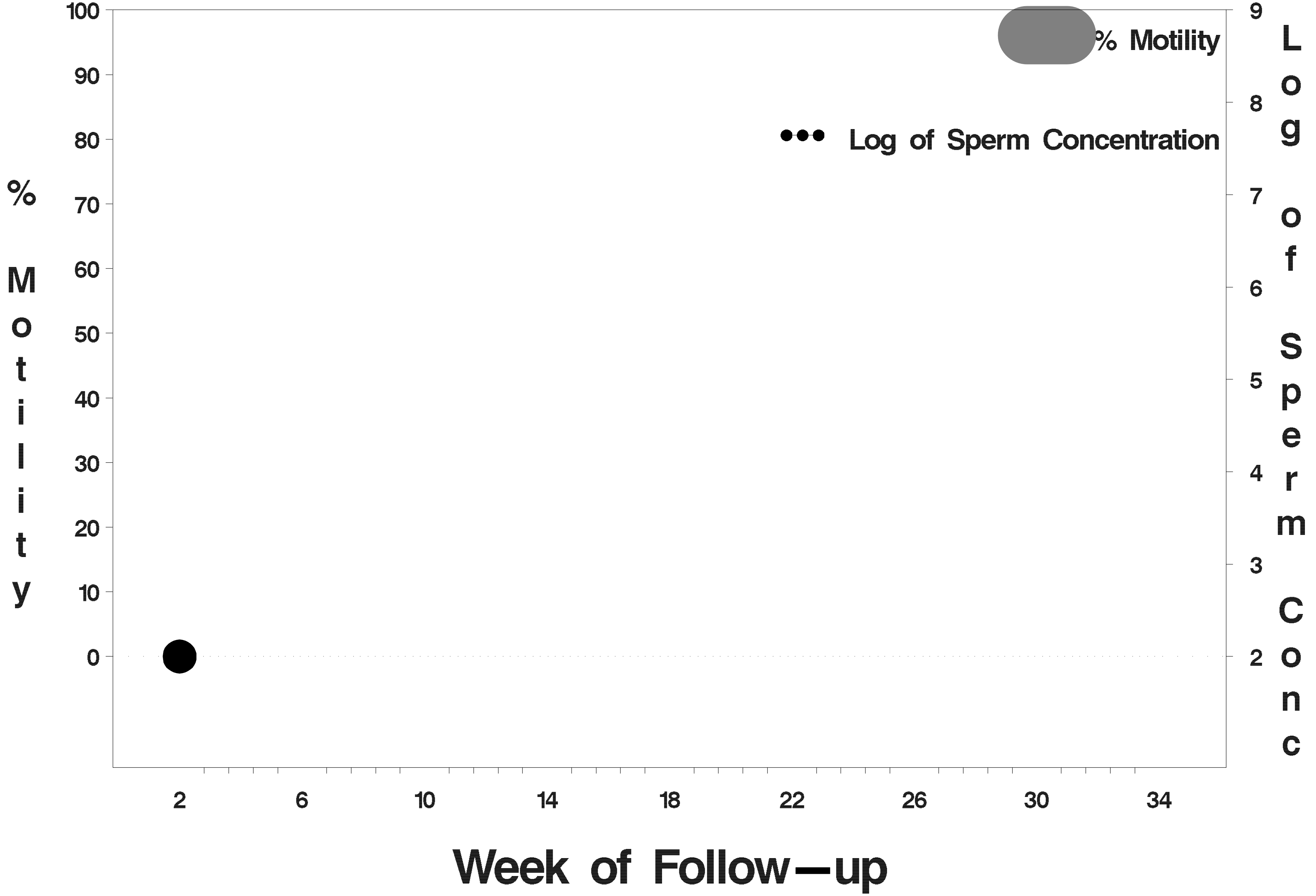

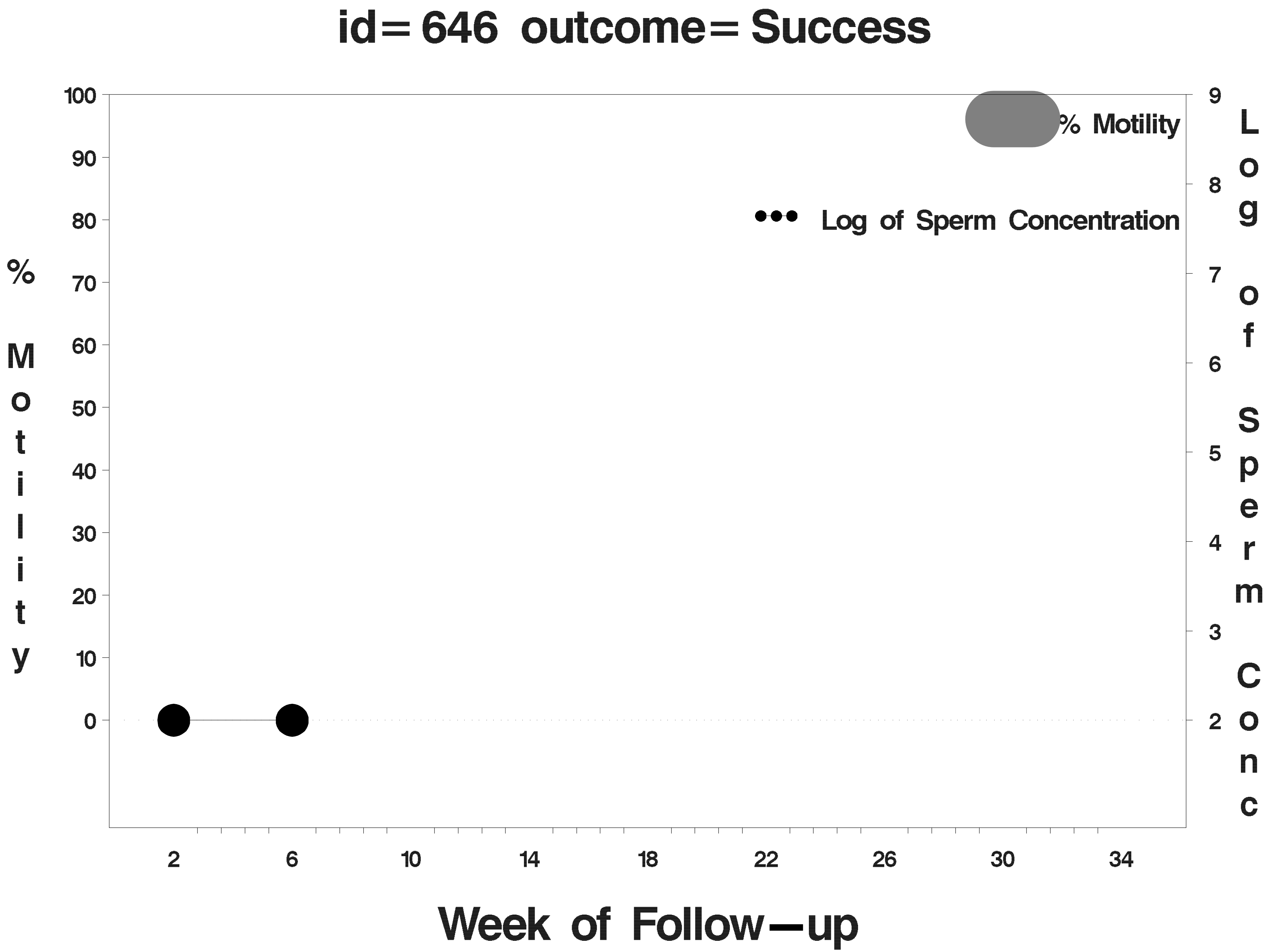
id= 646 outcome= Success
% Motility
Log of Sperm Concentration
100
90
80
70
60
50
40
30
20
10
0
9
8
7
6
5
4
3
2
2
6
10
14
18
22
26
30
34
Week of Follow—up
Log of Sperm Conc

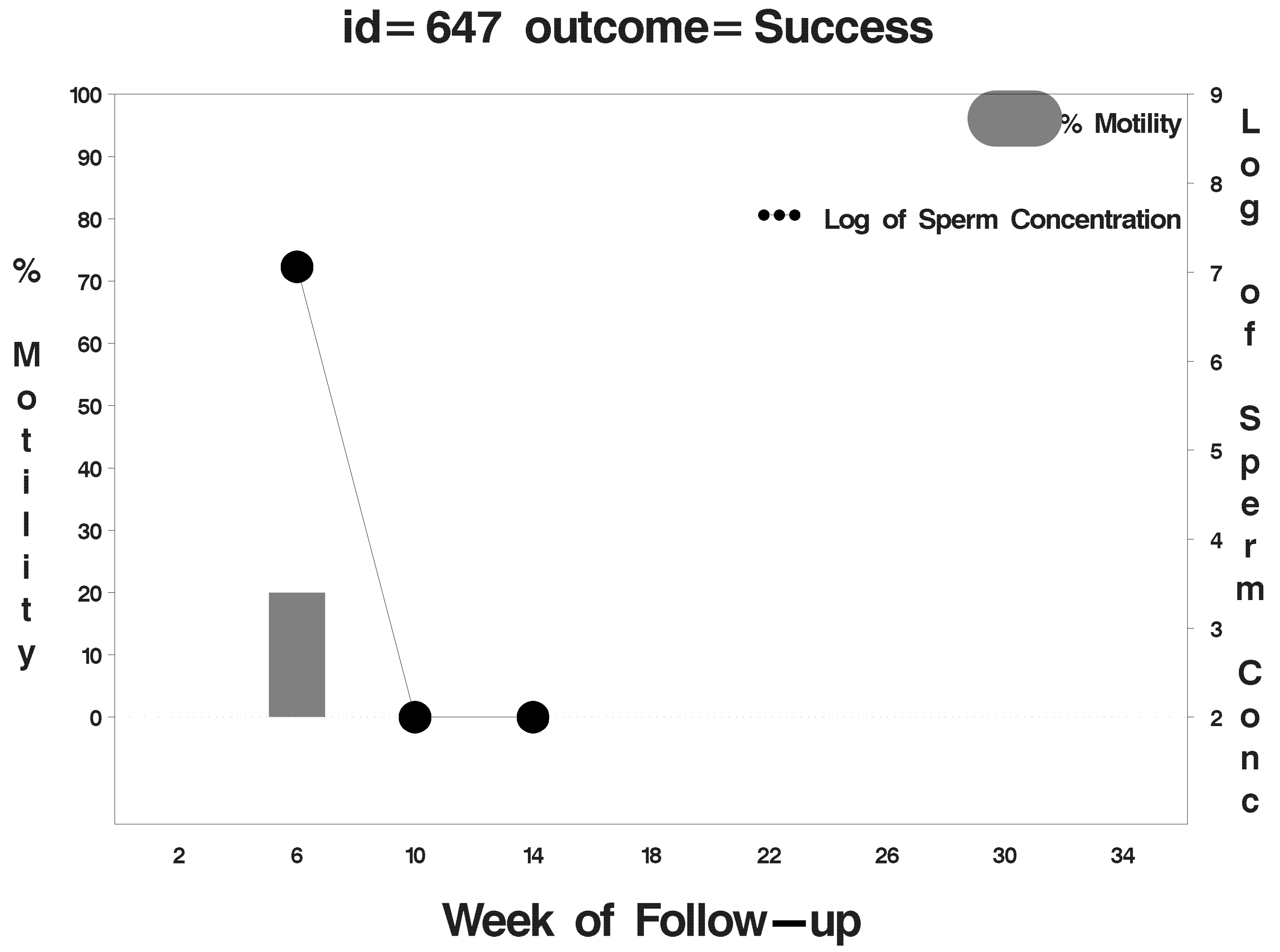

id= 647 outcome= Success
% Motility
Log of Sperm Concentration
% Motility
Log of Sperm Conc
100
90
80
70
60
50
40
30
20
10
0
9
8
7
6
5
4
3
2
2
6
10
14
18
22
26
30
34
Week of Follow−up

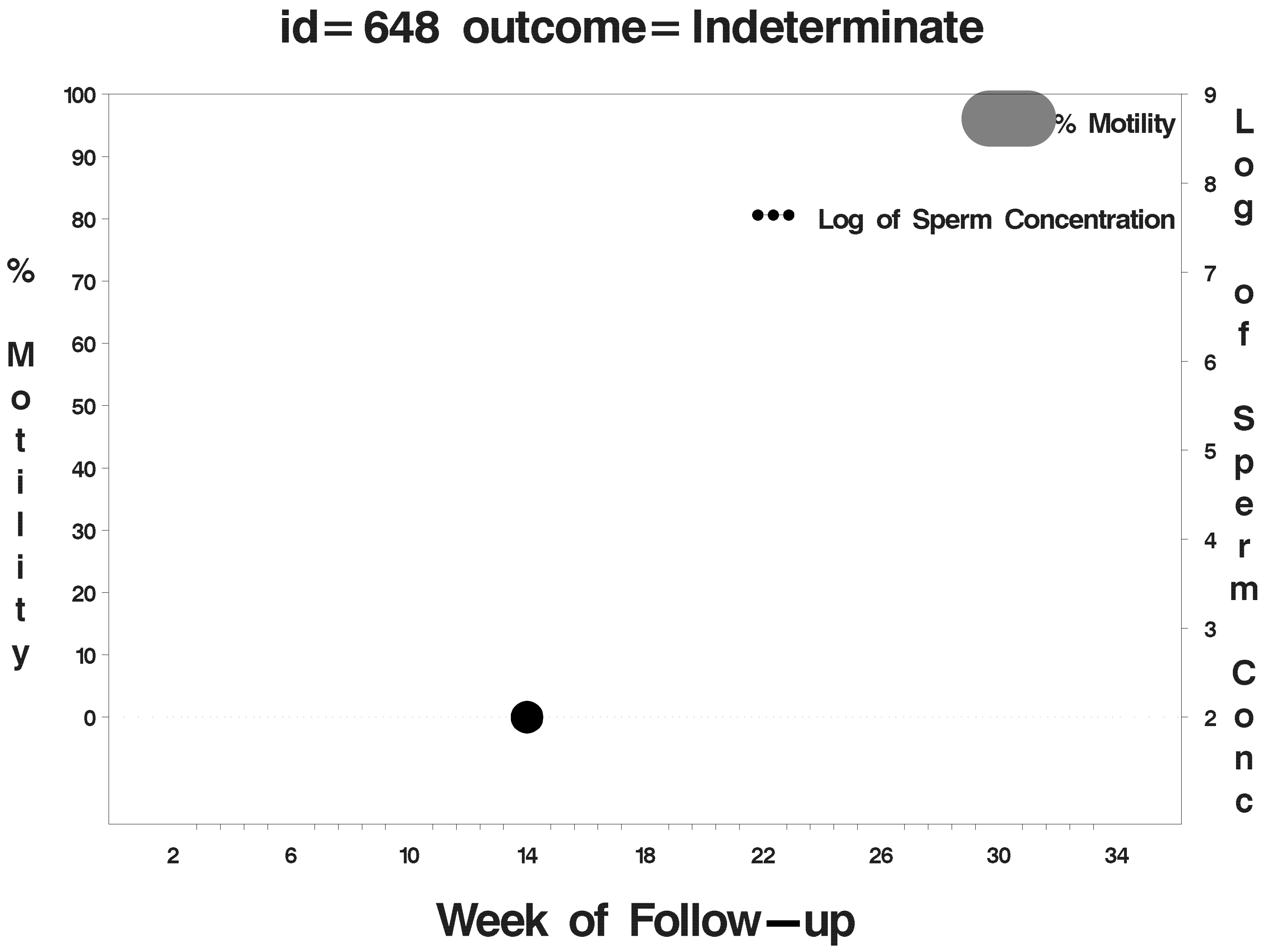

id= 648 outcome= Indeterminate
% Motility
Log of Sperm Concentration
100
90
80
70
60
50
40
30
20
10
0
9
8
7
6
5
4
3
2
2
6
10
14
18
22
26
30
34
Week of Follow-up
Log of Sperm Conc

id= 649 outcome= Success

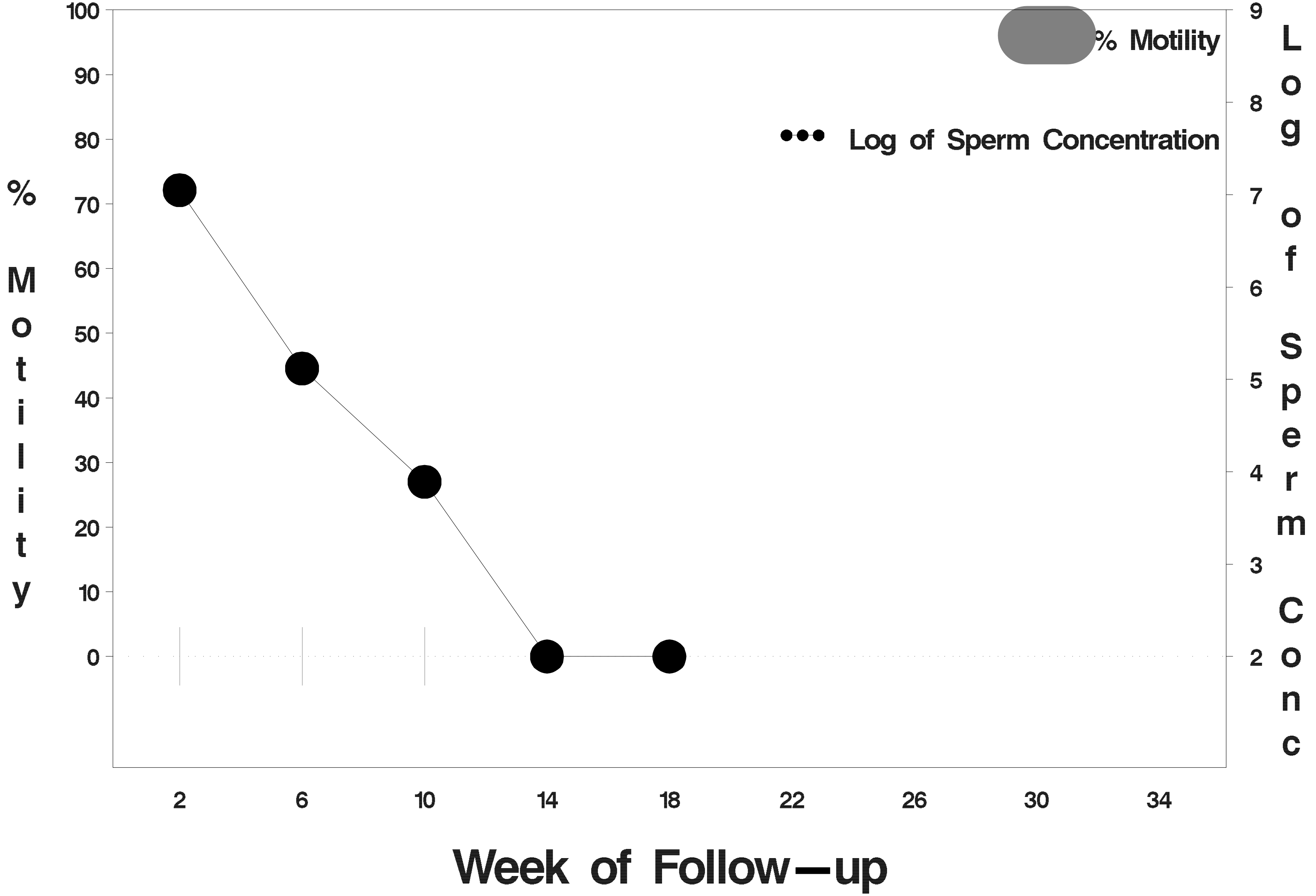

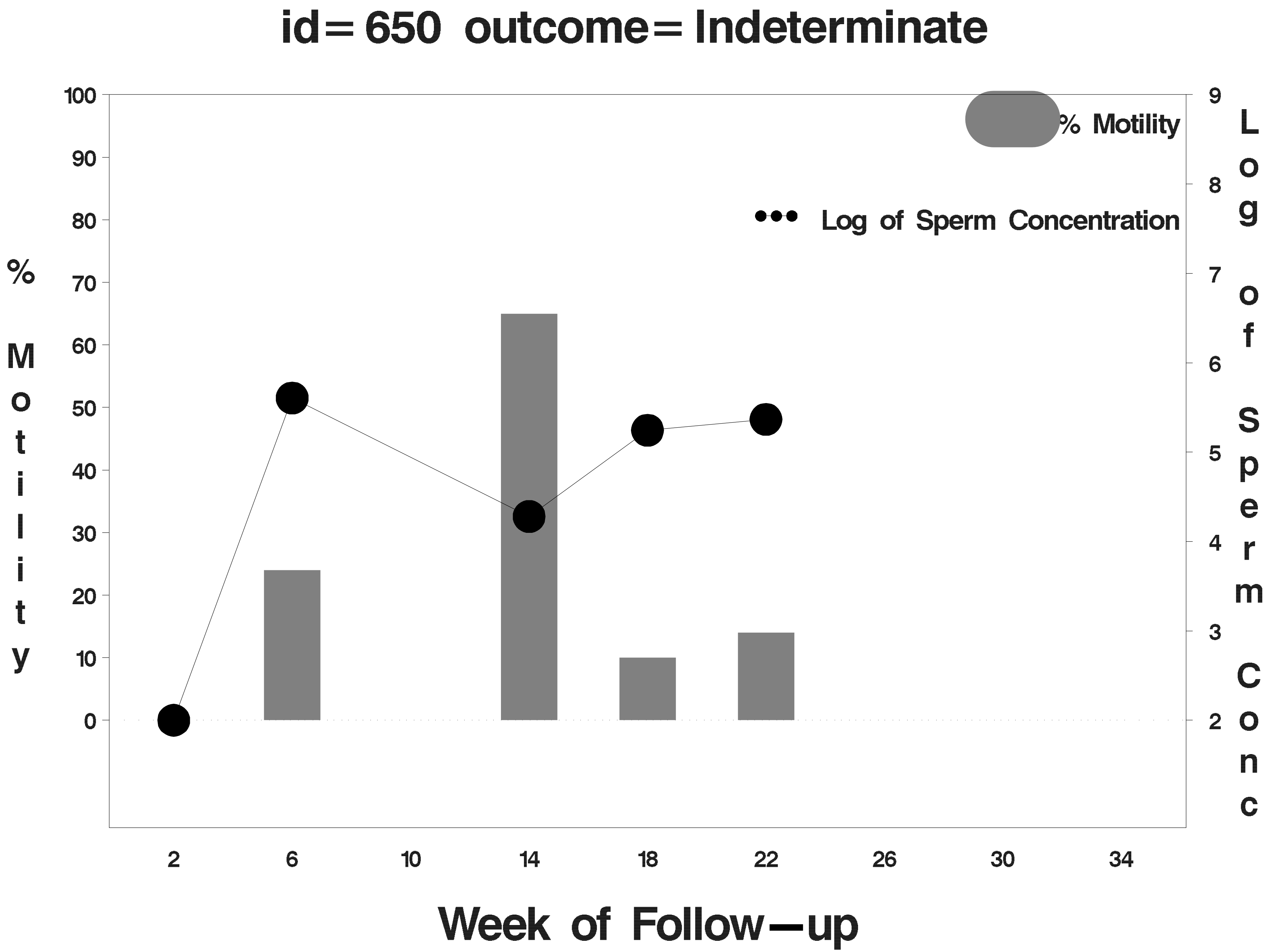

id= 650 outcome= Indeterminate
% Motility
Log of Sperm Concentration
% Motility
Log of Sperm Conc
Week of Follow—up
100
90
80
70
60
50
40
30
20
10
0
9
8
7
6
5
4
3
2
2
6
10
14
18
22
26
30
34

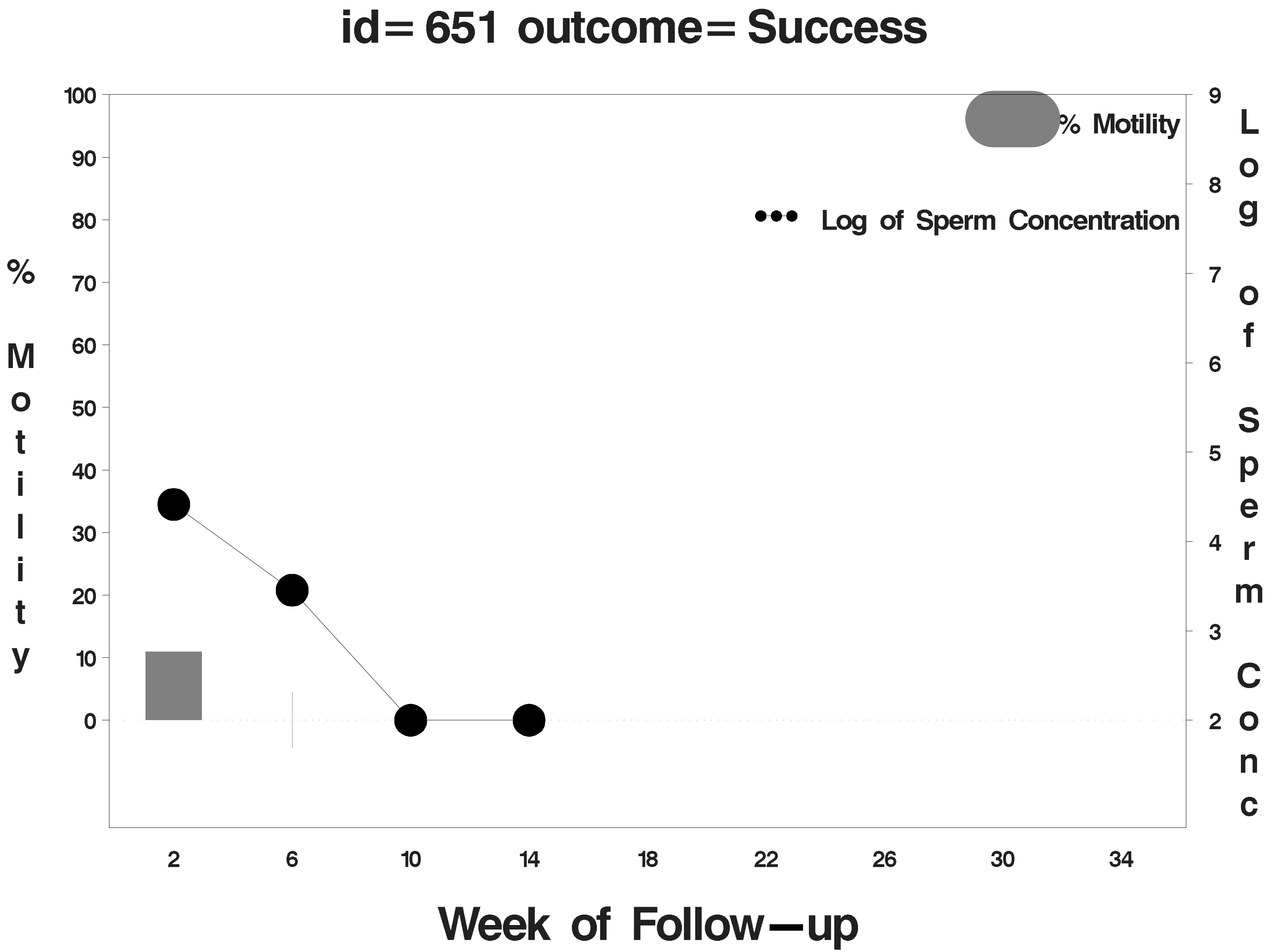

id= 651 outcome= Success
% Motility
Log of Sperm Concentration
100
90
80
70
60
50
40
30
20
10
0
9
8
7
6
5
4
3
2
2
6
10
14
18
22
26
30
34
Week of Follow—up
Log of Sperm Conc

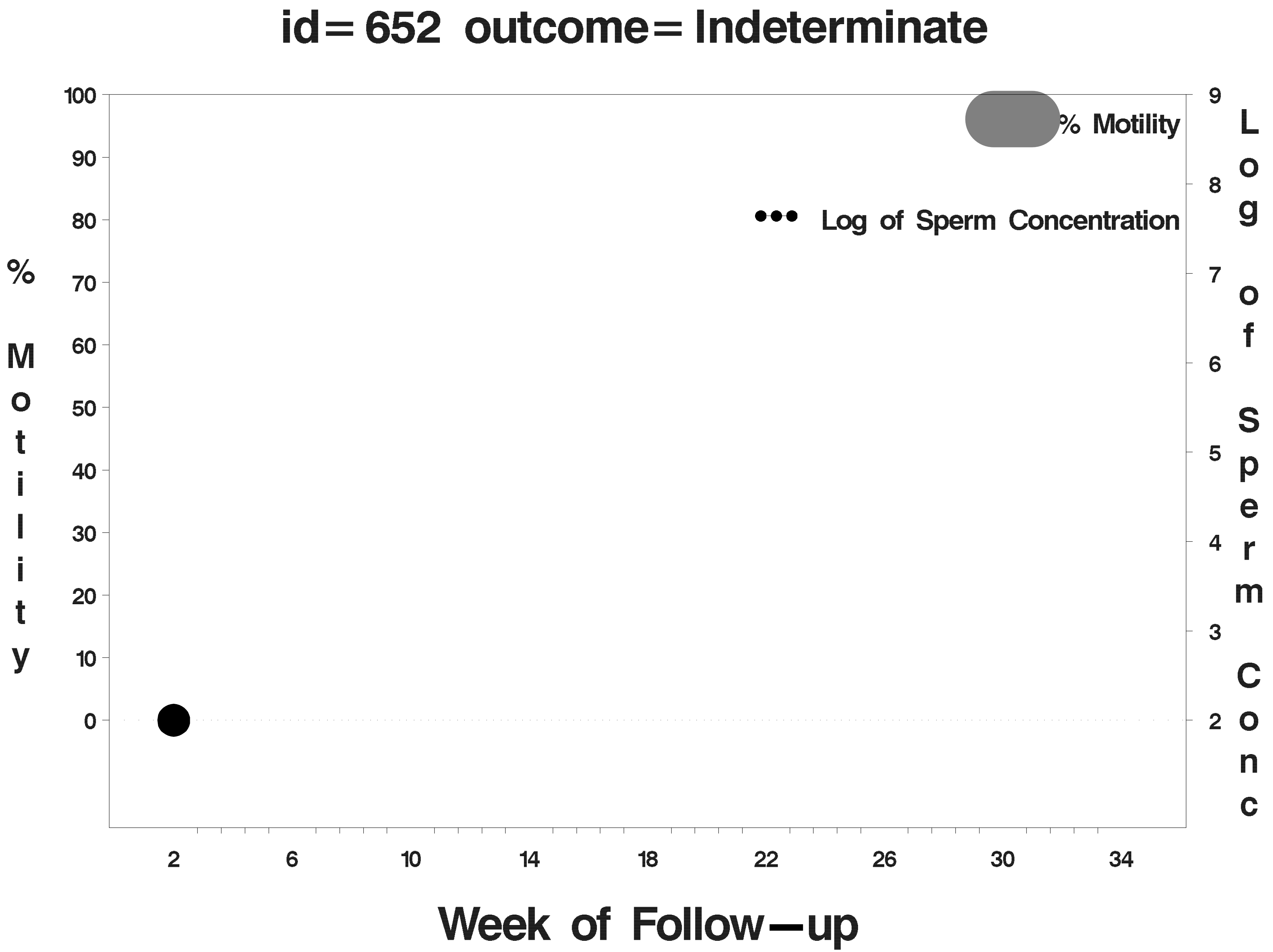

id= 652 outcome= Indeterminate
% Motility
Log of Sperm Concentration
% Motility
100
90
80
70
60
50
40
30
20
10
0
Log of Sperm Conc
9
8
7
6
5
4
3
2
Week of Follow-up
2
6
10
14
18
22
26
30
34

id= 653 outcome= Failure

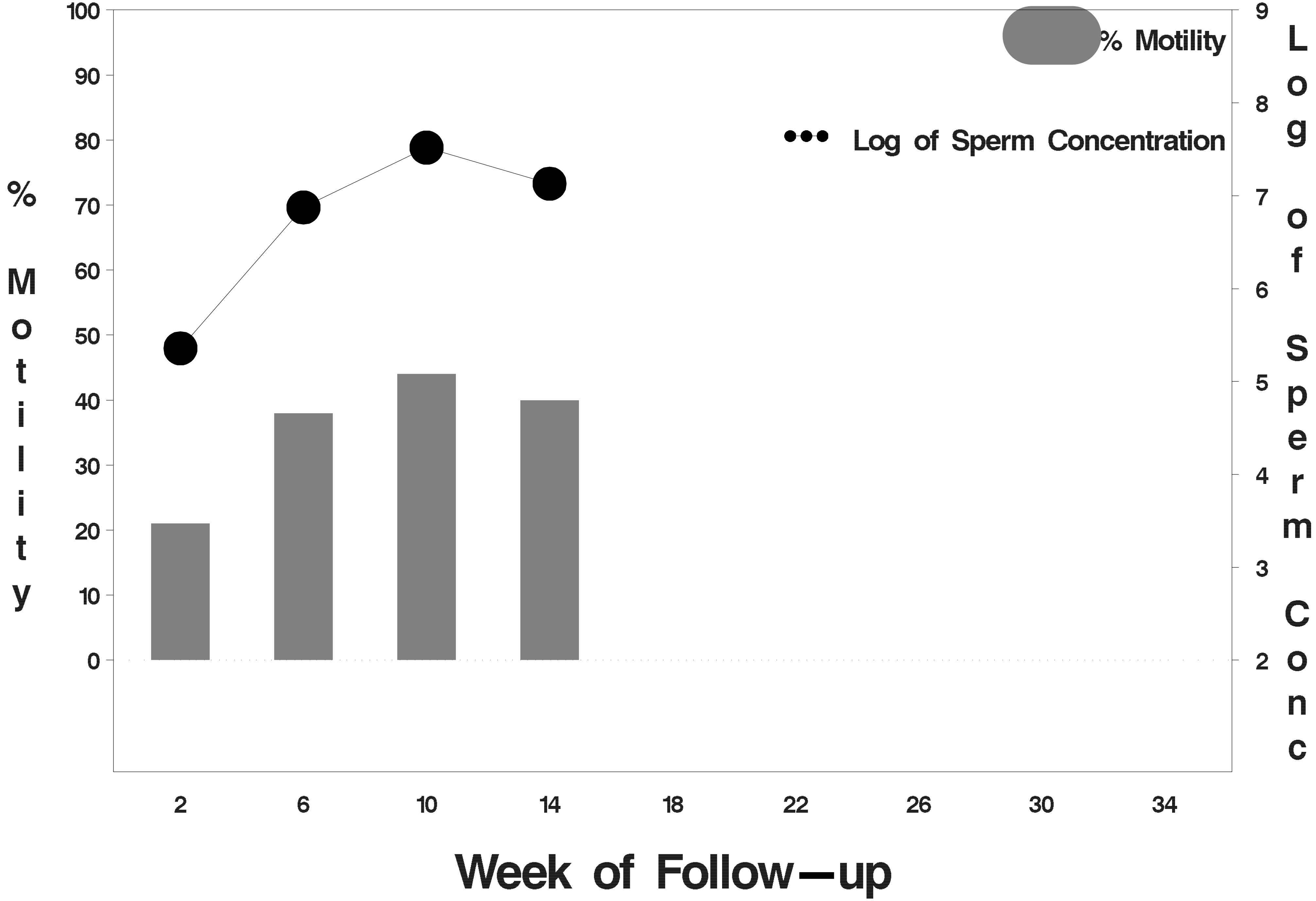

id= 654 outcome= Success

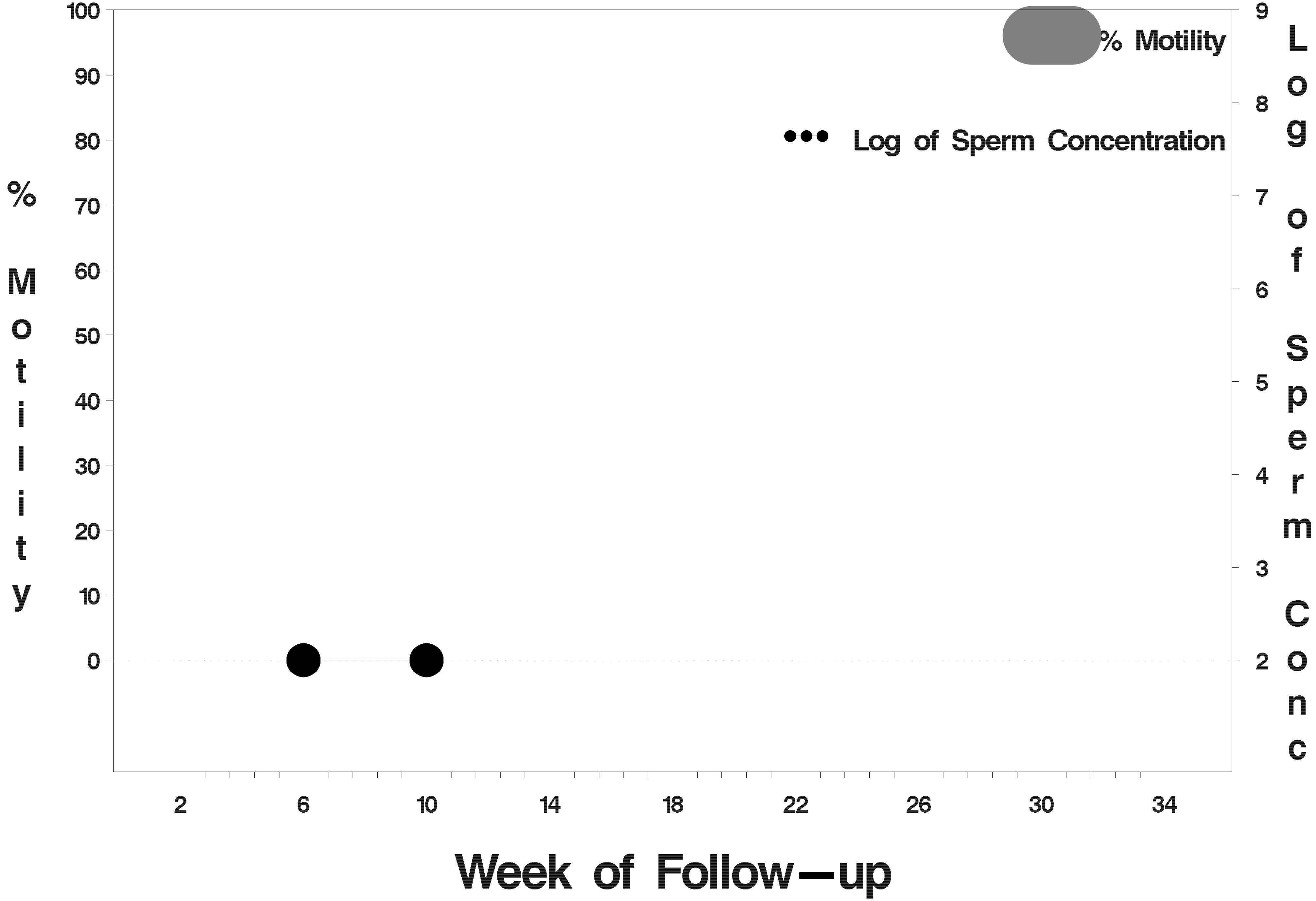

id= 655 outcome= Failure

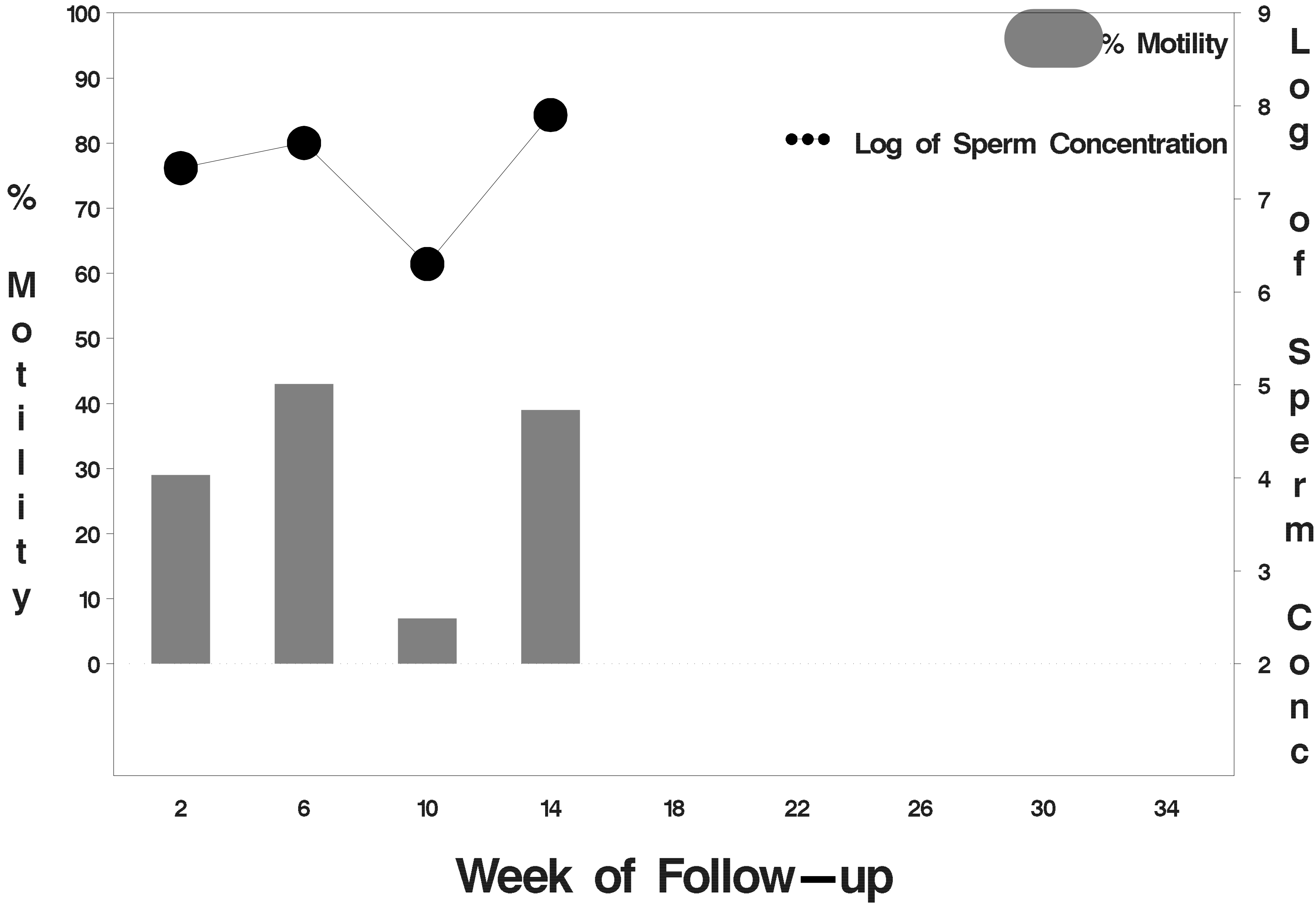

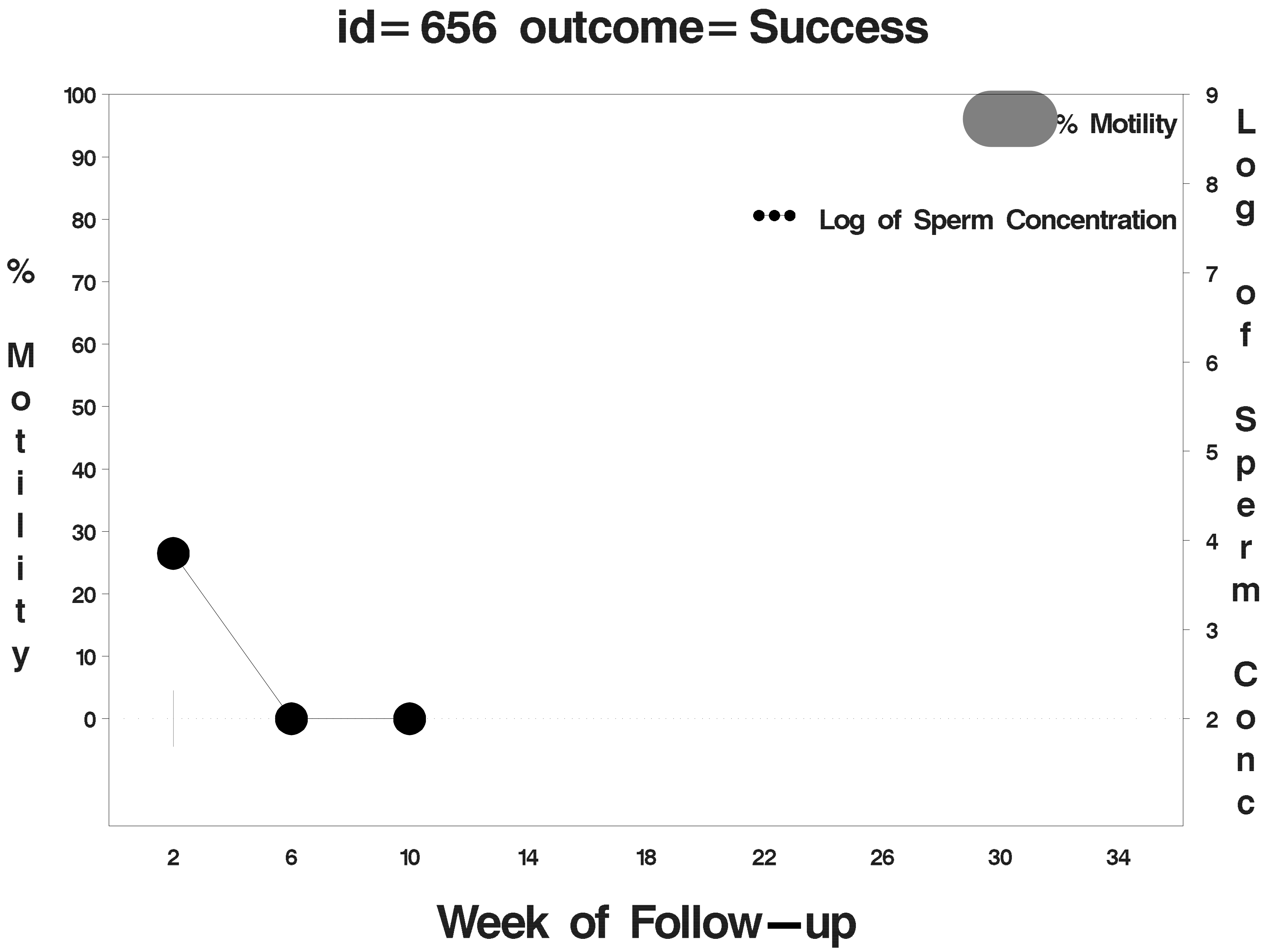

id= 656 outcome= Success
% Motility
Log of Sperm Concentration
100
90
80
70
60
50
40
30
20
10
0
9
8
7
6
5
4
3
2
% Motility
Log of Sperm Conc
2
6
10
14
18
22
26
30
34
Week of Follow—up

id= 657 outcome= Success

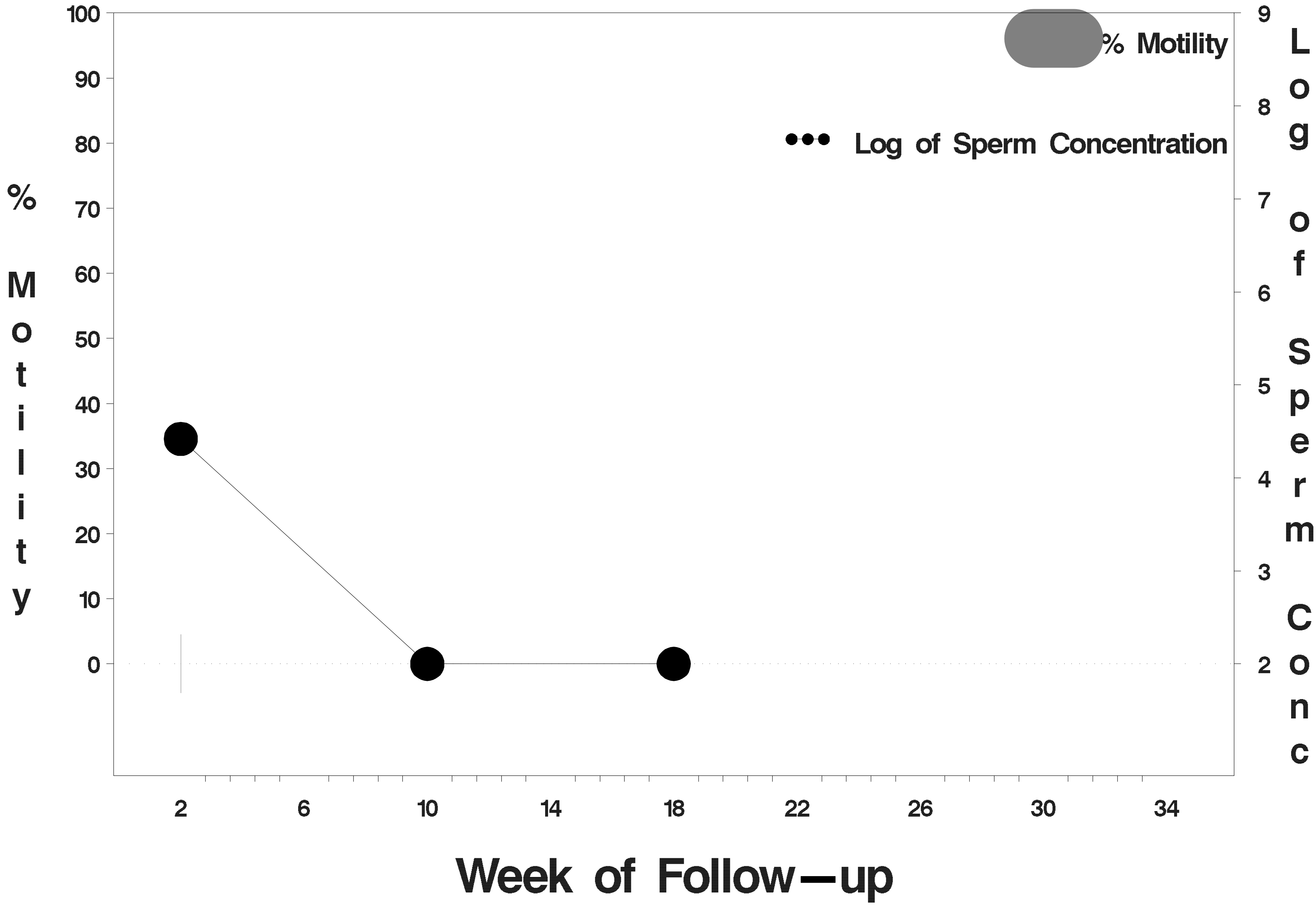

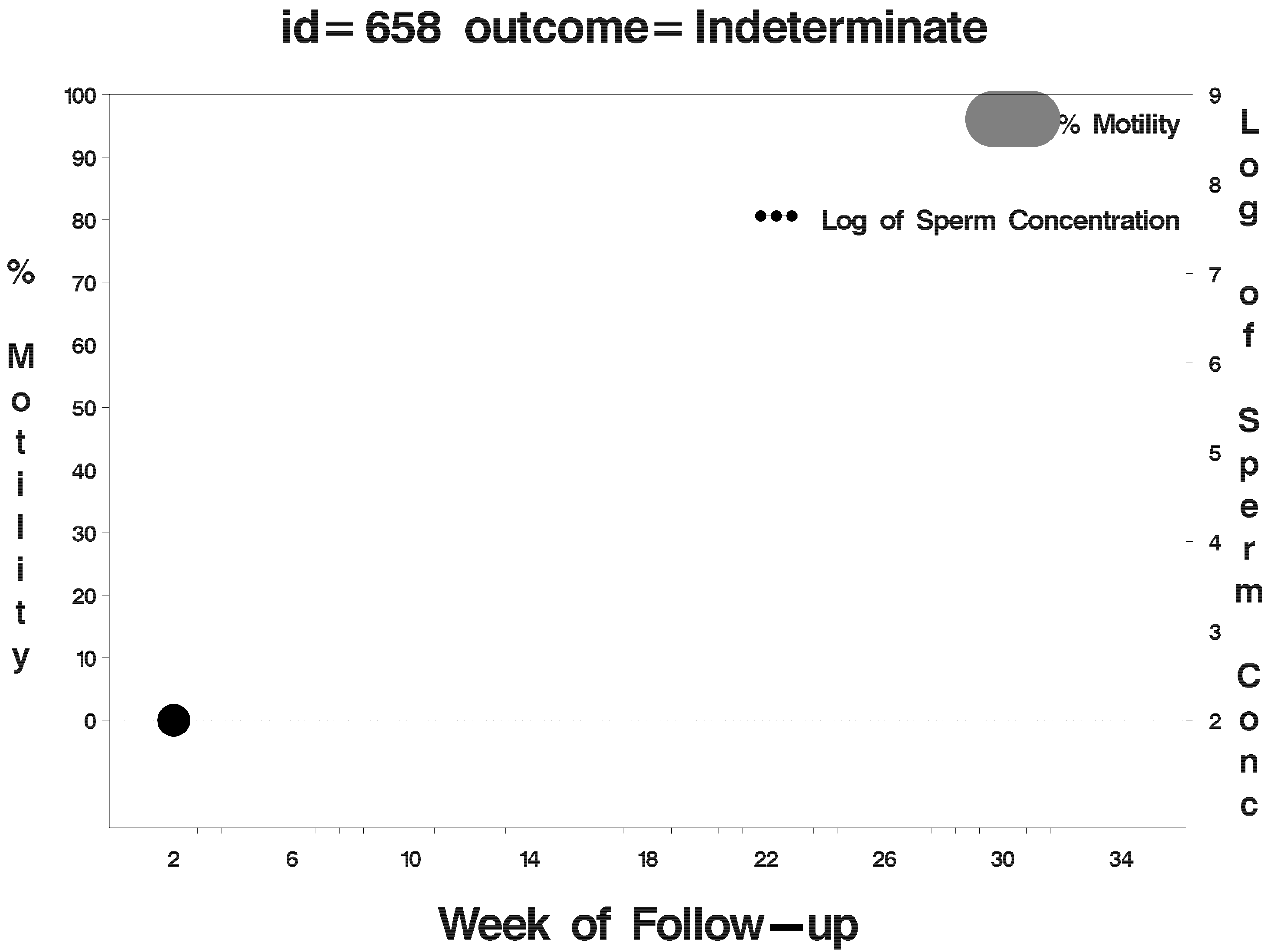

id= 658 outcome= Indeterminate
% Motility
Log of Sperm Concentration
100
90
80
70
60
50
40
30
20
10
0
9
8
7
6
5
4
3
2
2
6
10
14
18
22
26
30
34
Week of Follow-up
Log of Sperm Conc

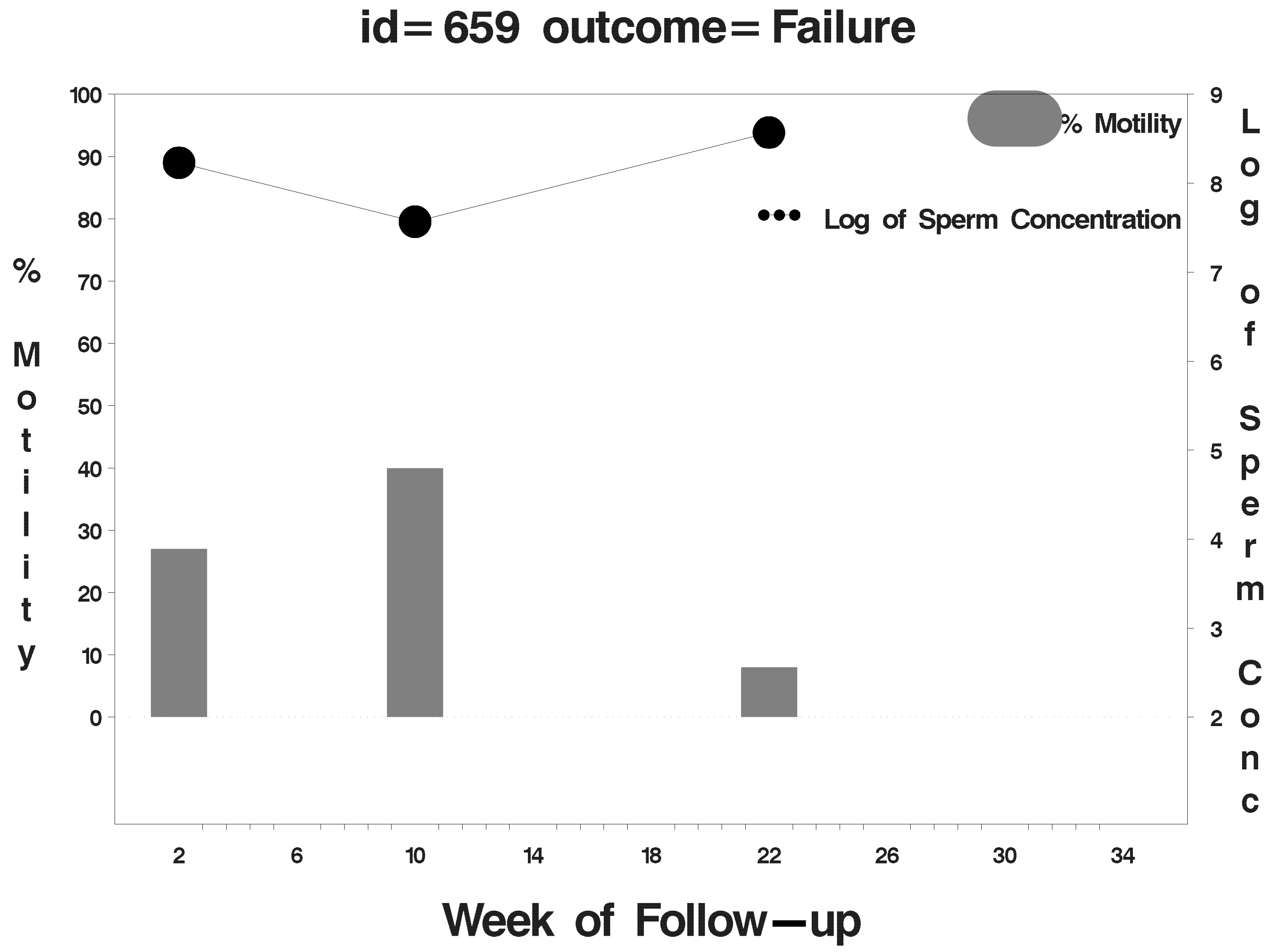

id= 659 outcome= Failure
% Motility
Log of Sperm Concentration
% Motility
Log of Sperm Conc
Week of Follow—up
100
90
80
70
60
50
40
30
20
10
0
9
8
7
6
5
4
3
2
2
6
10
14
18
22
26
30
34

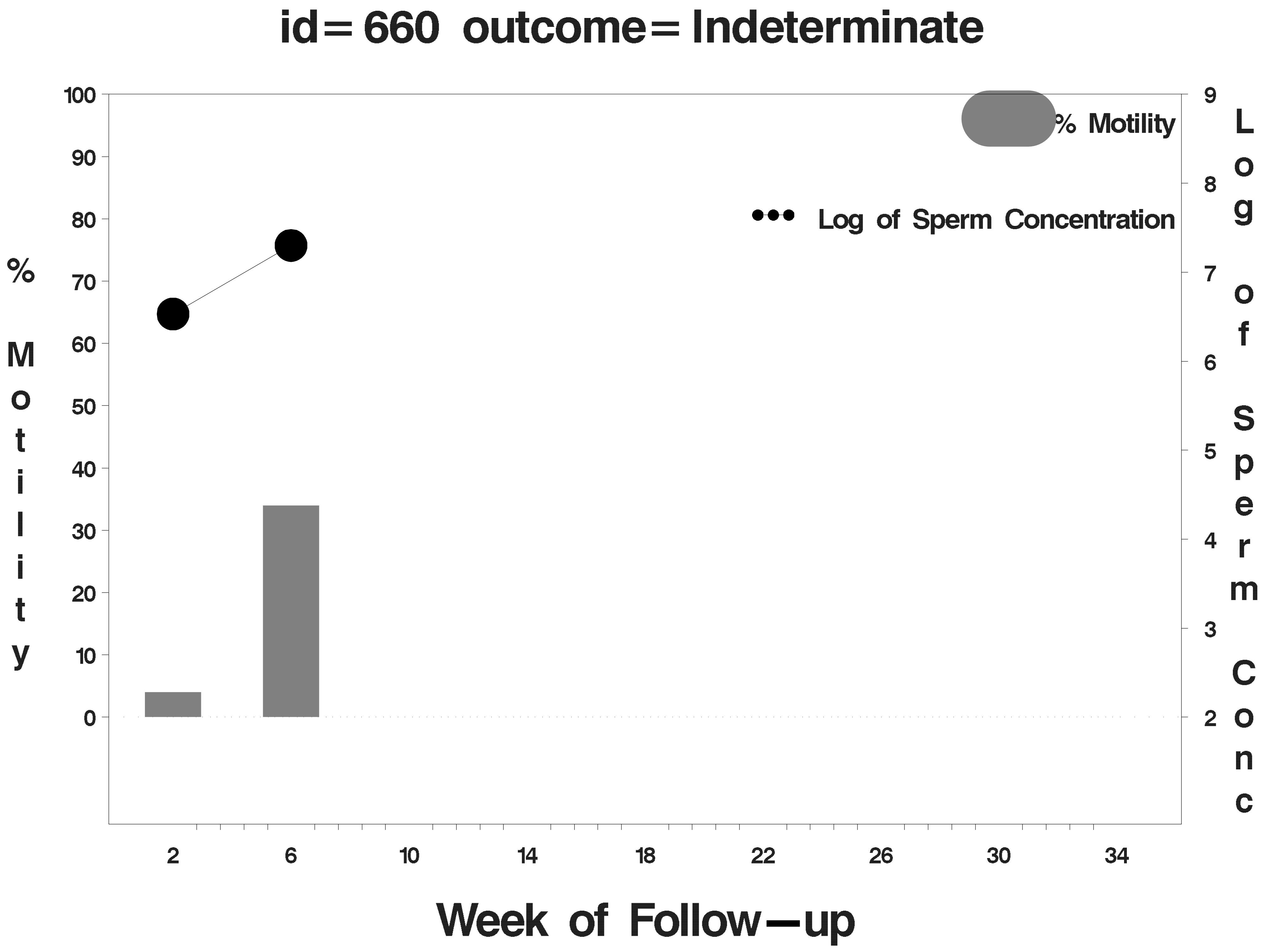
id= 660 outcome= Indeterminate
% Motility
Log of Sperm Concentration
100
90
80
70
60
50
40
30
20
10
0
9
8
7
6
5
4
3
2
2
6
10
14
18
22
26
30
34
Week of Follow−up
Log of Sperm Conc

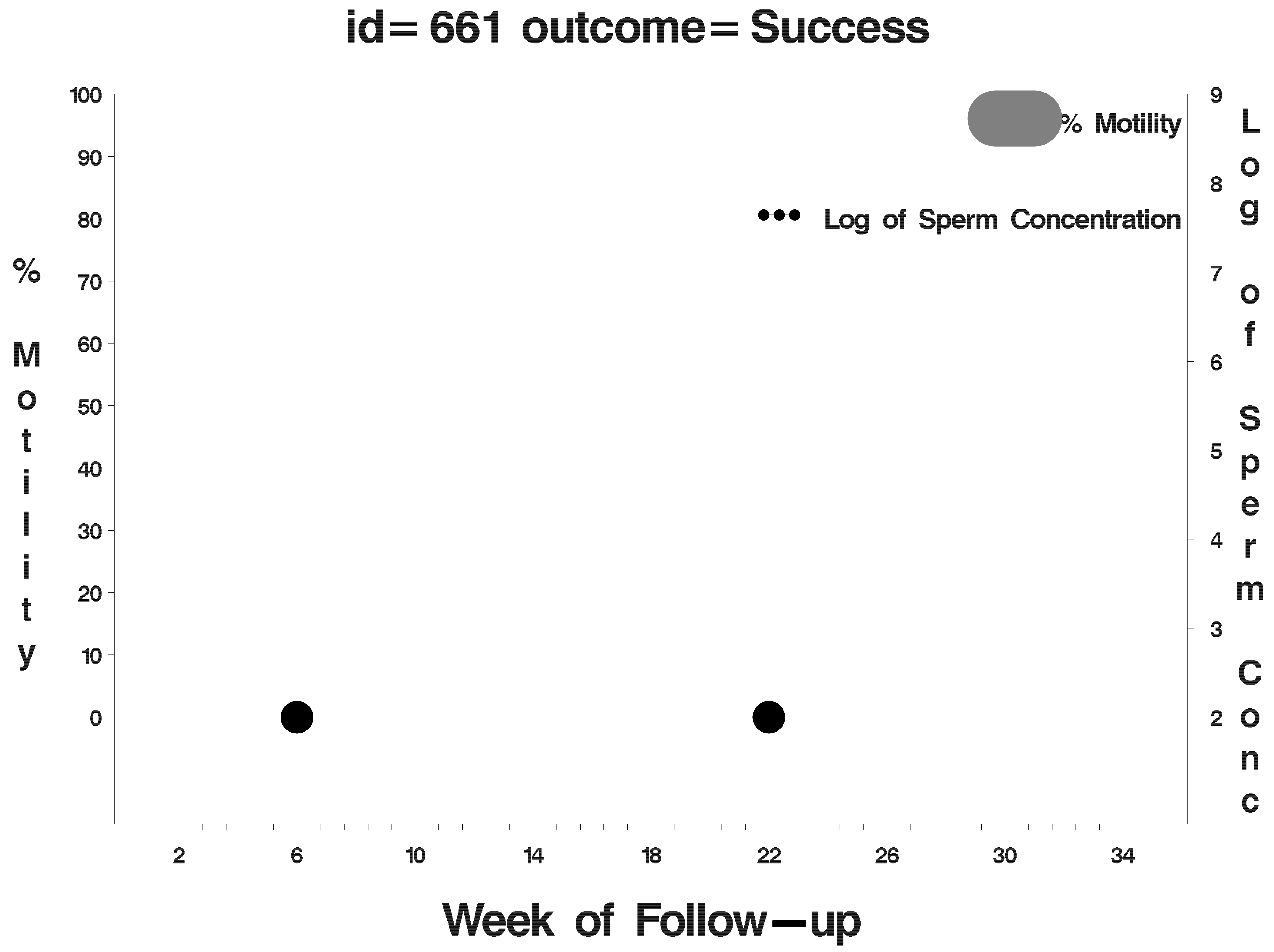

id= 661 outcome= Success
% Motility
Log of Sperm Concentration
100
90
80
70
60
50
40
30
20
10
0
9
8
7
6
5
4
3
2
2
6
10
14
18
22
26
30
34
Week of Follow−up
Log of Sperm Conc

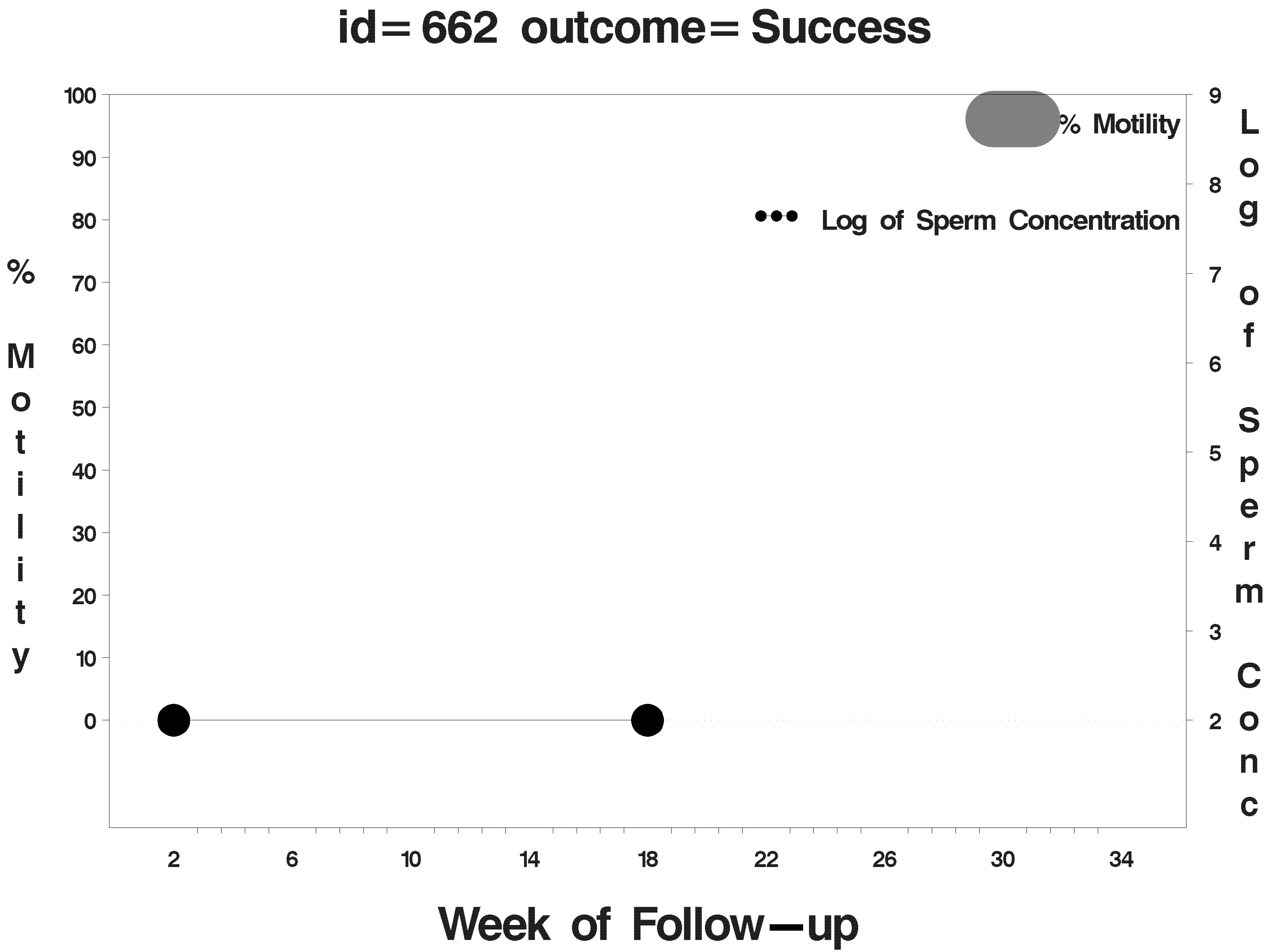

id= 662 outcome= Success
% Motility
Log of Sperm Concentration
100
90
80
70
60
50
40
30
20
10
0
9
8
7
6
5
4
3
2
2
6
10
14
18
22
26
30
34
Week of Follow−up

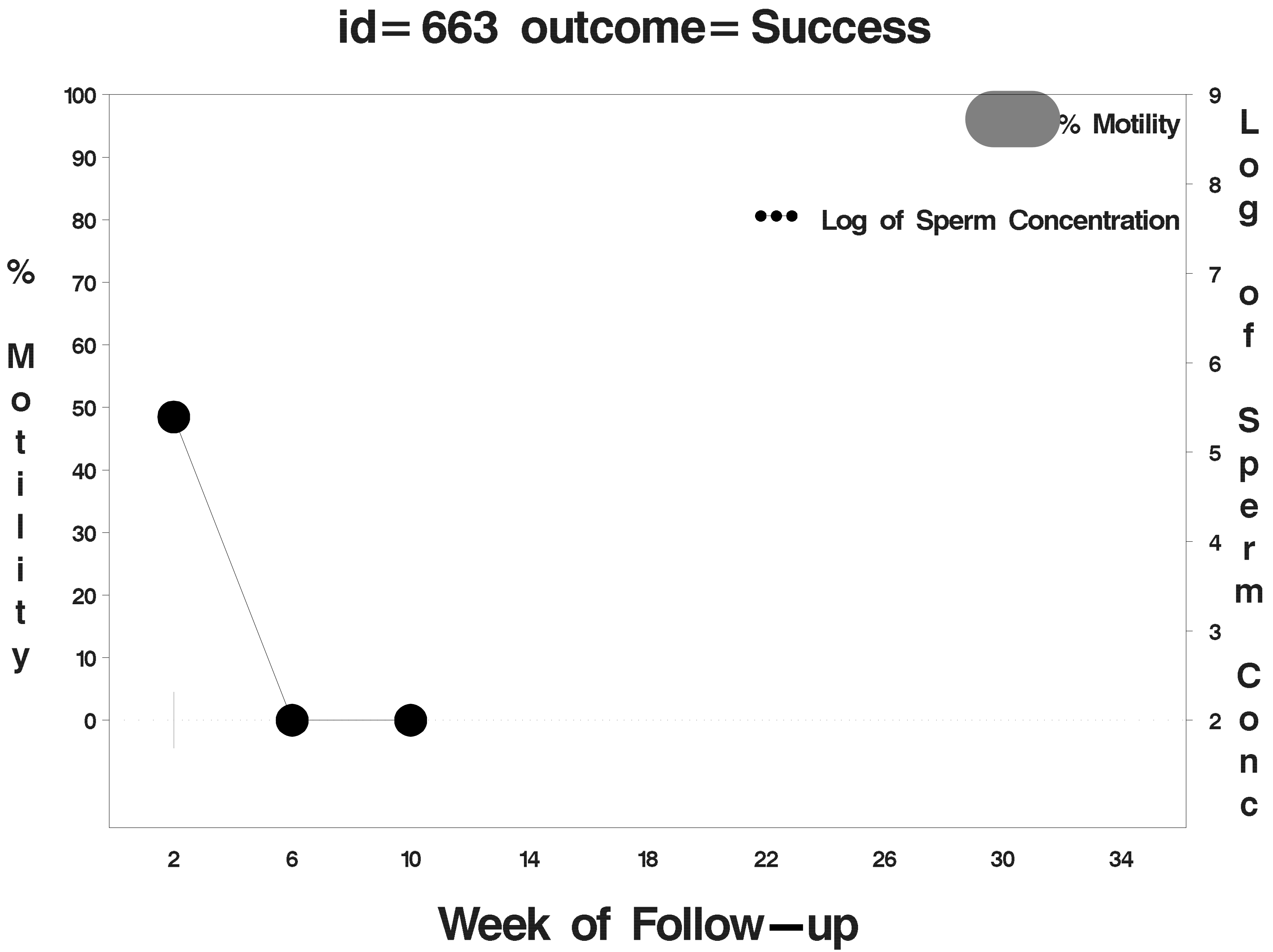

id= 663 outcome= Success
% Motility
Log of Sperm Concentration
100
90
80
70
60
50
40
30
20
10
0
9
8
7
6
5
4
3
2
2
6
10
14
18
22
26
30
34
Week of Follow—up
Log of Sperm Conc

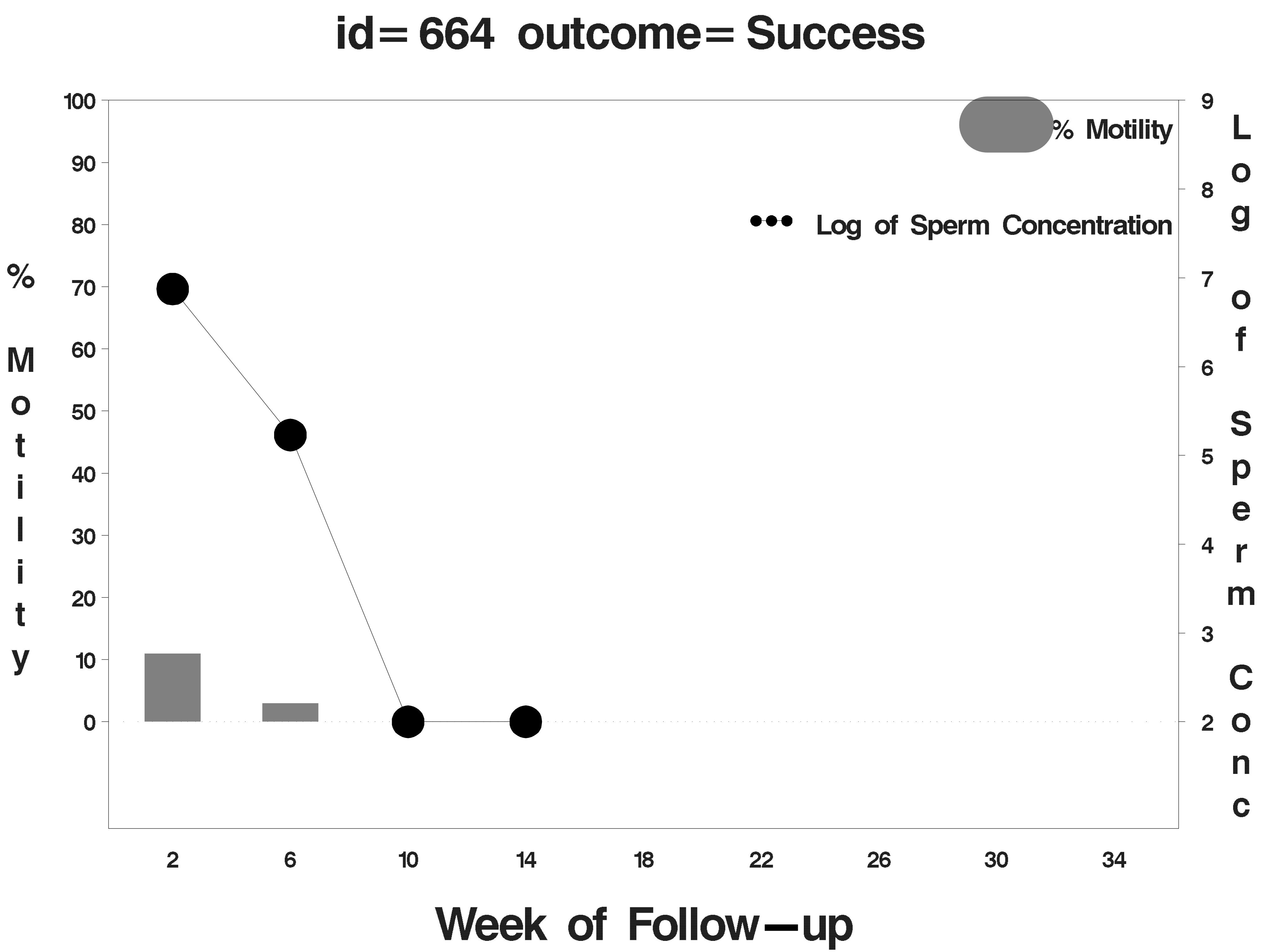

id= 664 outcome= Success
% Motility
Log of Sperm Concentration
% Motility
Log of Sperm Conc
Week of Follow—up
100
90
80
70
60
50
40
30
20
10
0
9
8
7
6
5
4
3
2
2
6
10
14
18
22
26
30
34

id= 665 outcome= Indeterminate

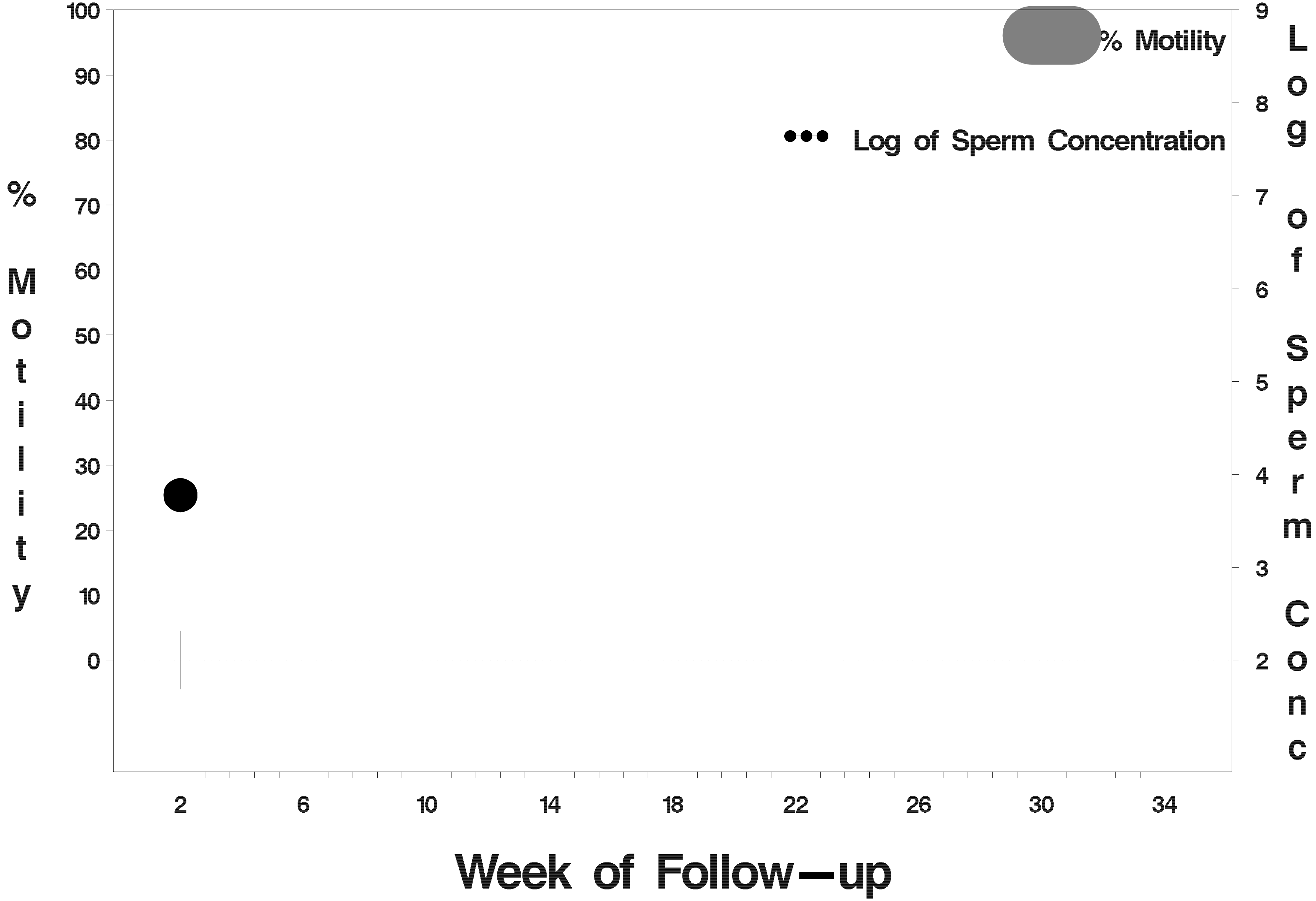

id= 666 outcome= Success

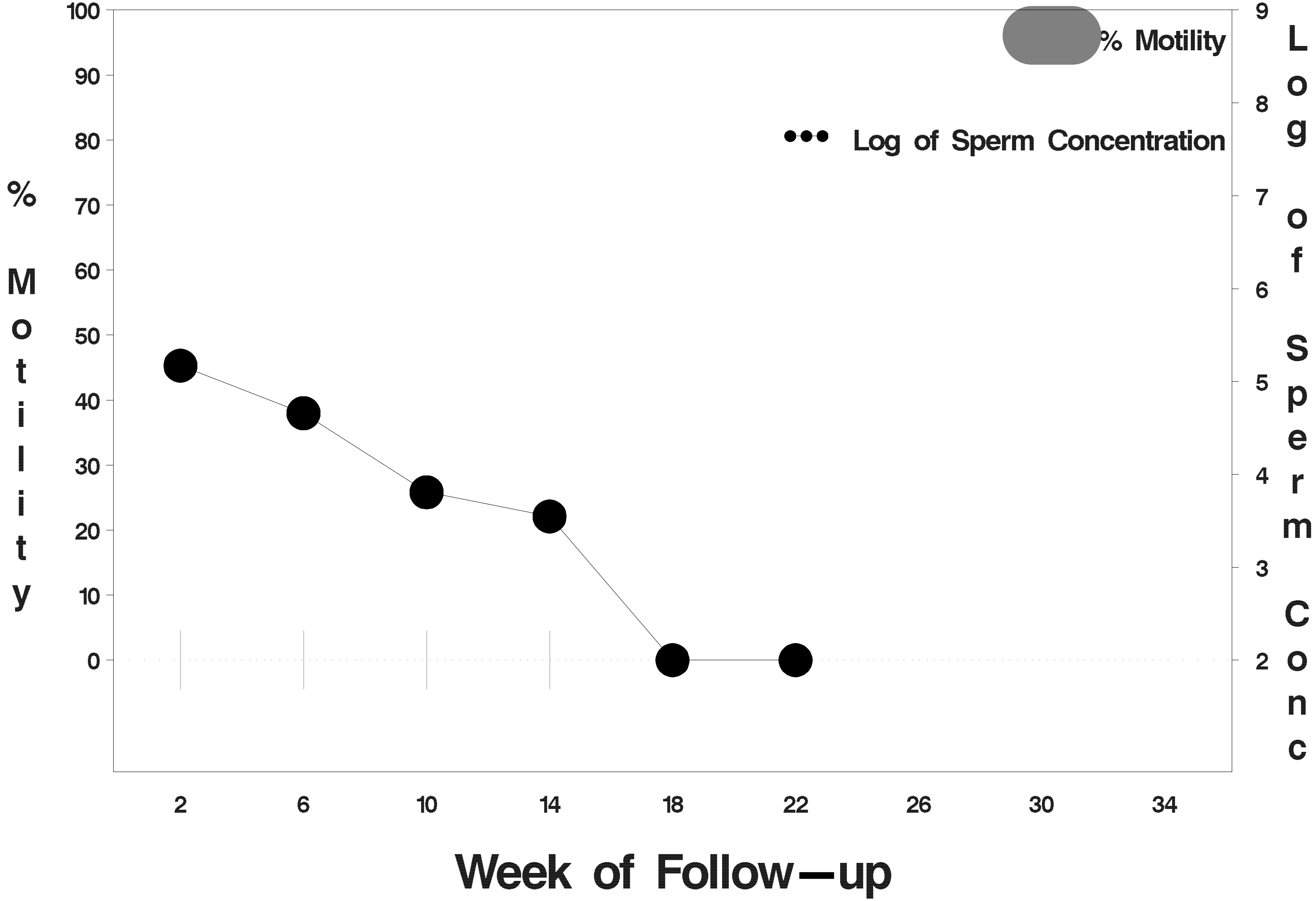

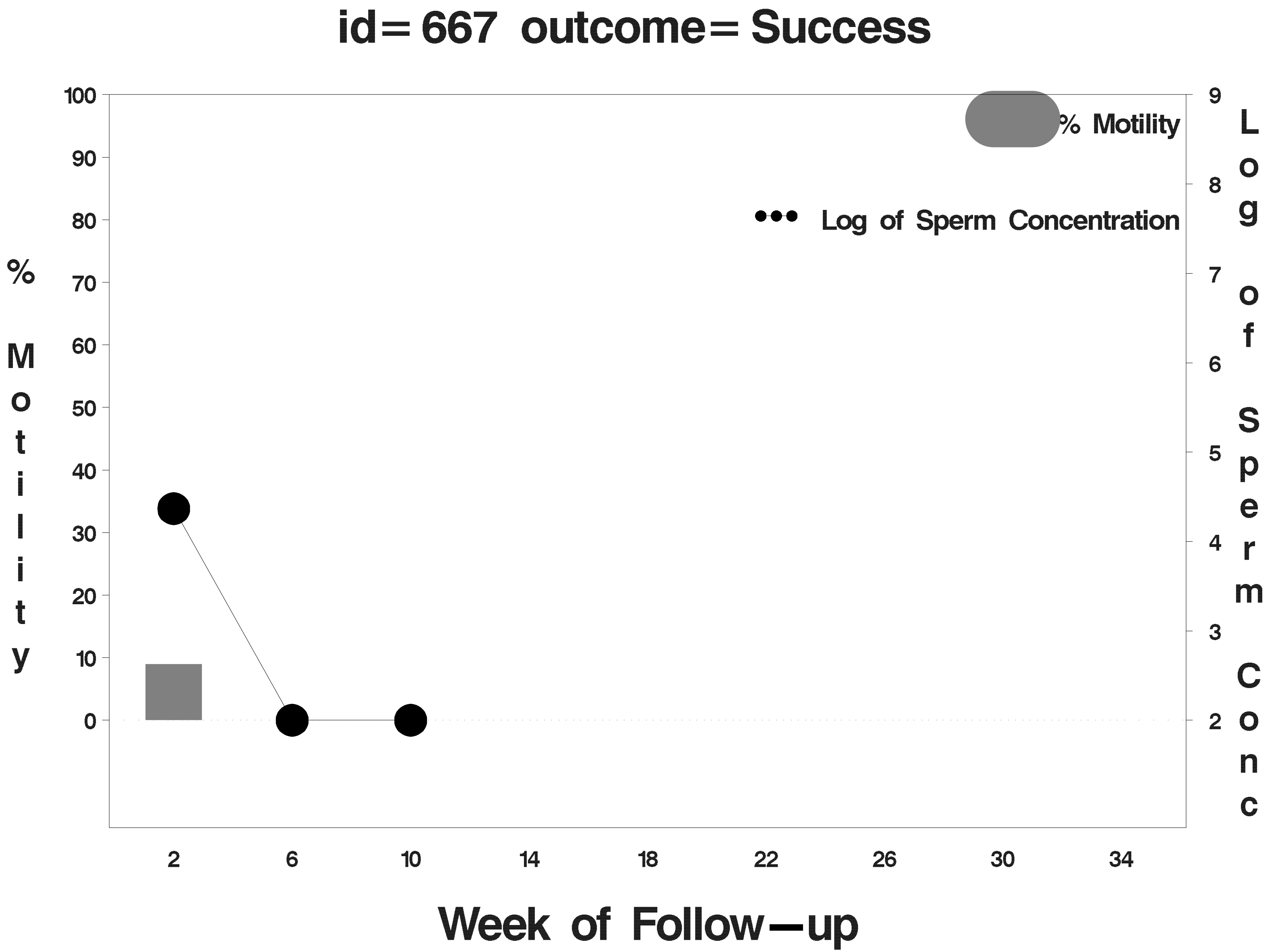

id= 667 outcome= Success
% Motility
Log of Sperm Concentration
% Motility
Log of Sperm Conc
Week of Follow-up
100
90
80
70
60
50
40
30
20
10
0
9
8
7
6
5
4
3
2
2
6
10
14
18
22
26
30
34

id= 668 outcome= Success

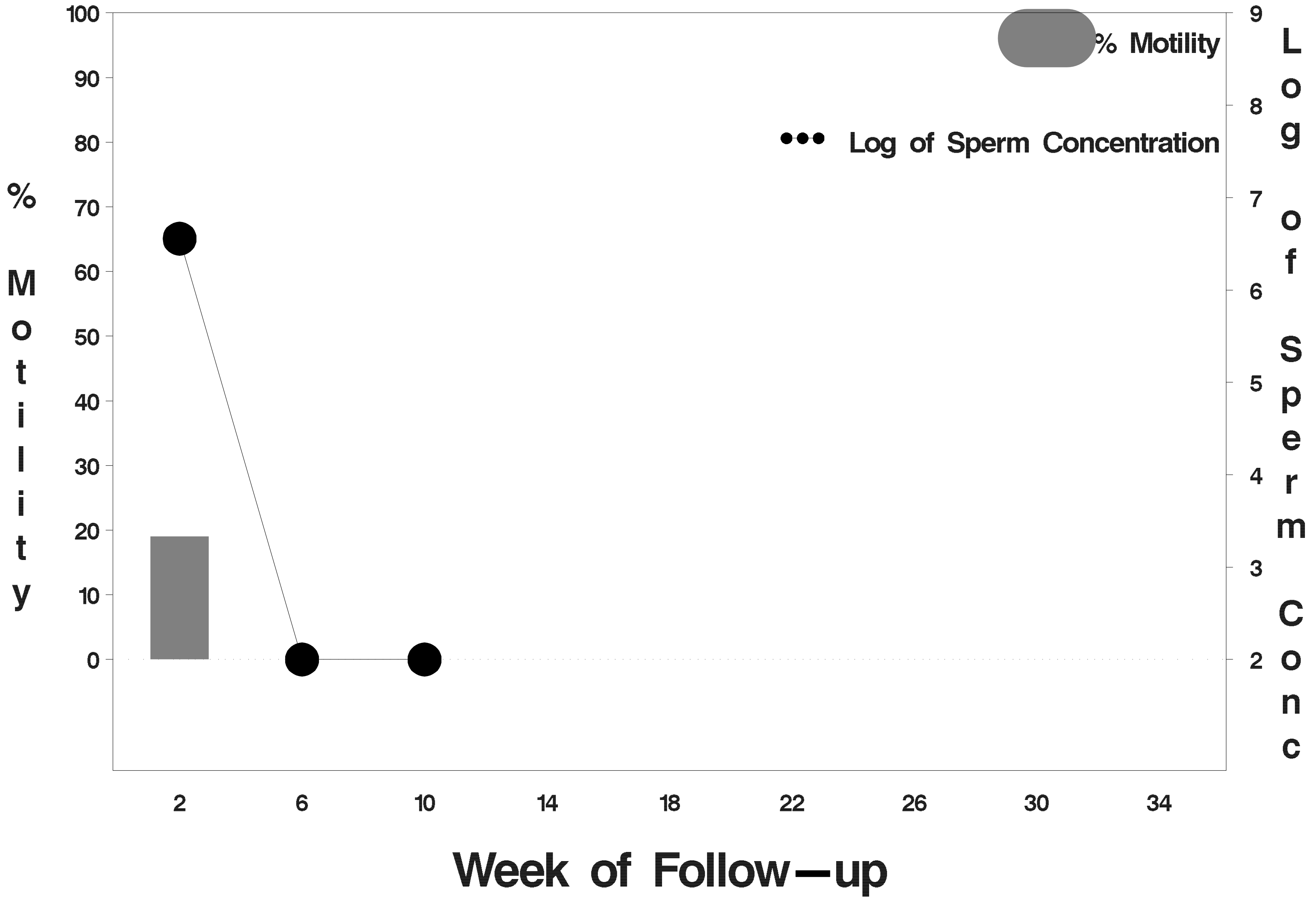

id= 669 outcome= Success

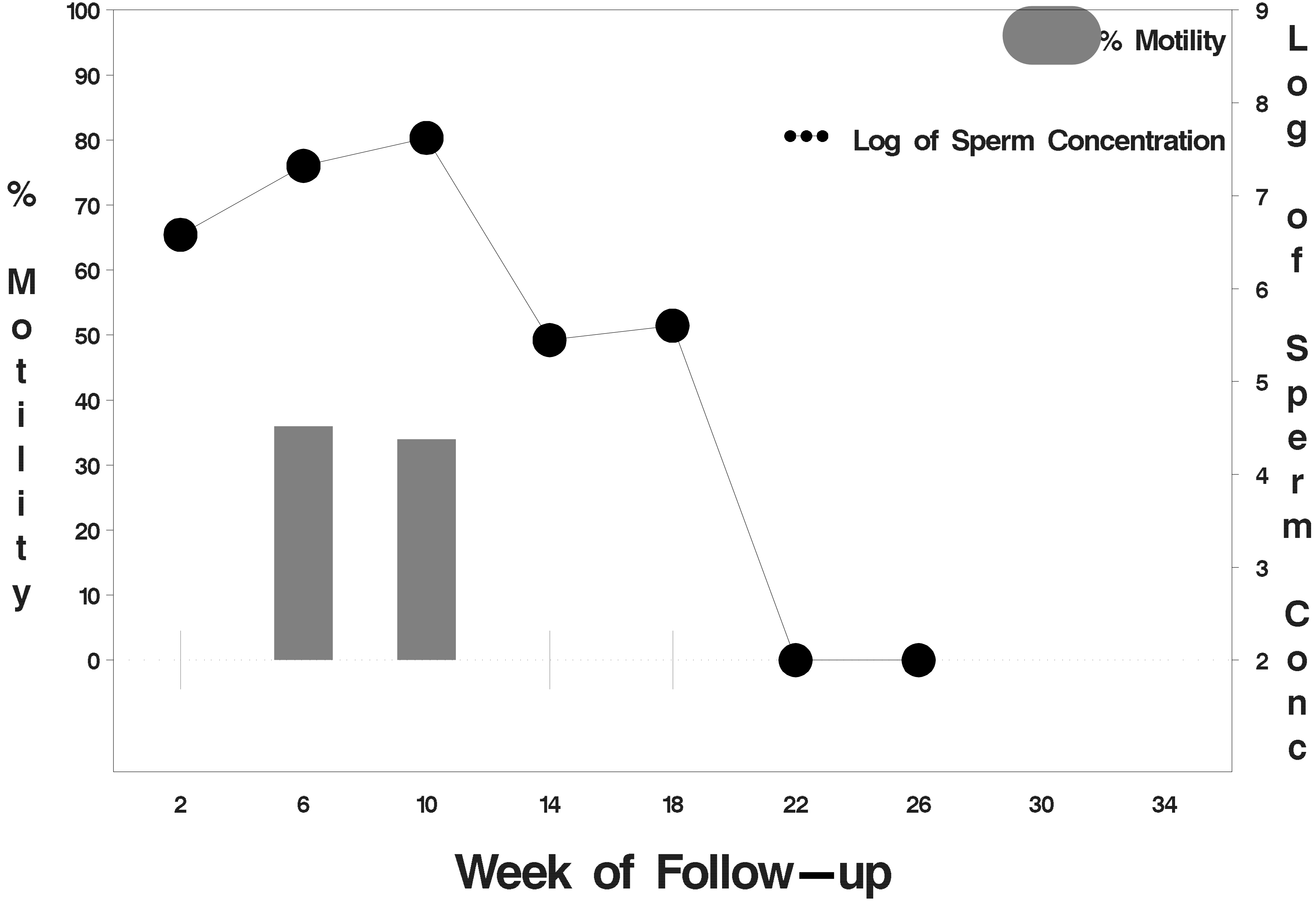

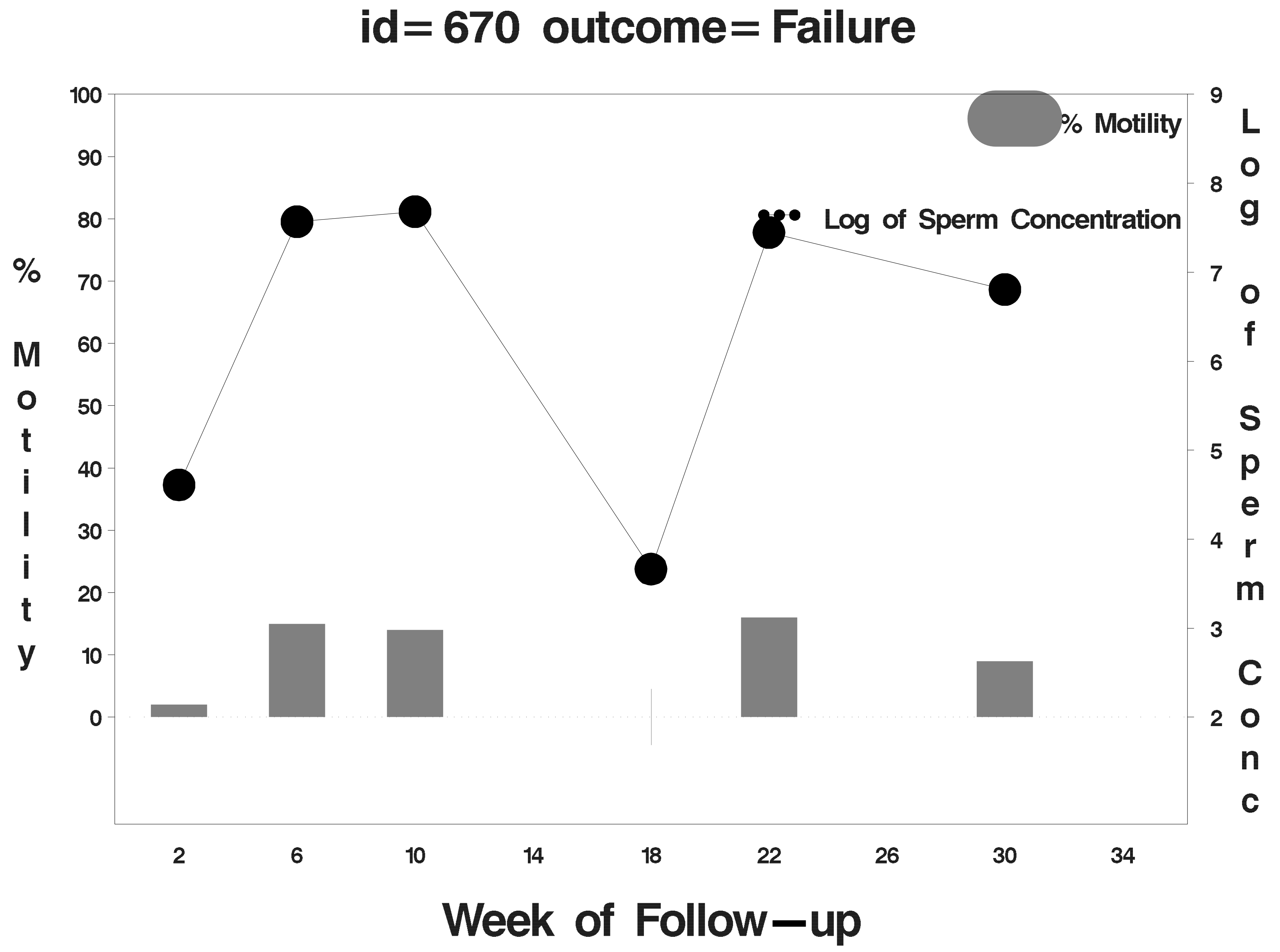

id= 670 outcome= Failure
% Motility
Log of Sperm Concentration
100
90
80
70
60
50
40
30
20
10
0
9
8
7
6
5
4
3
2
2
6
10
14
18
22
26
30
34
Week of Follow−up
% Motility
Log of Sperm Conc

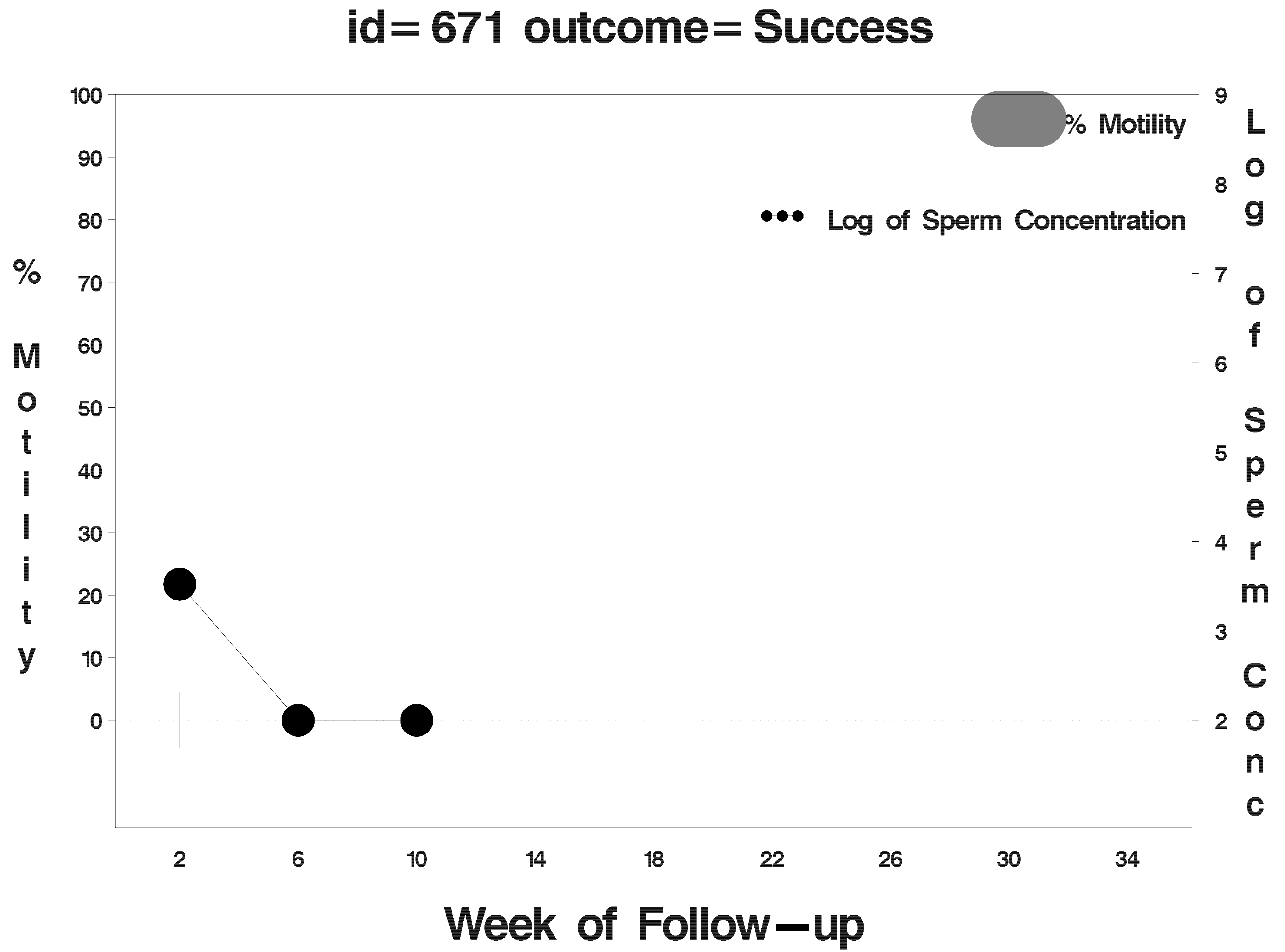
id= 671 outcome= Success
% Motility
Log of Sperm Concentration
% Motility
Log of Sperm Conc
Week of Follow—up
100
90
80
70
60
50
40
30
20
10
0
9
8
7
6
5
4
3
2
2
6
10
14
18
22
26
30
34

id= 672 outcome= Success

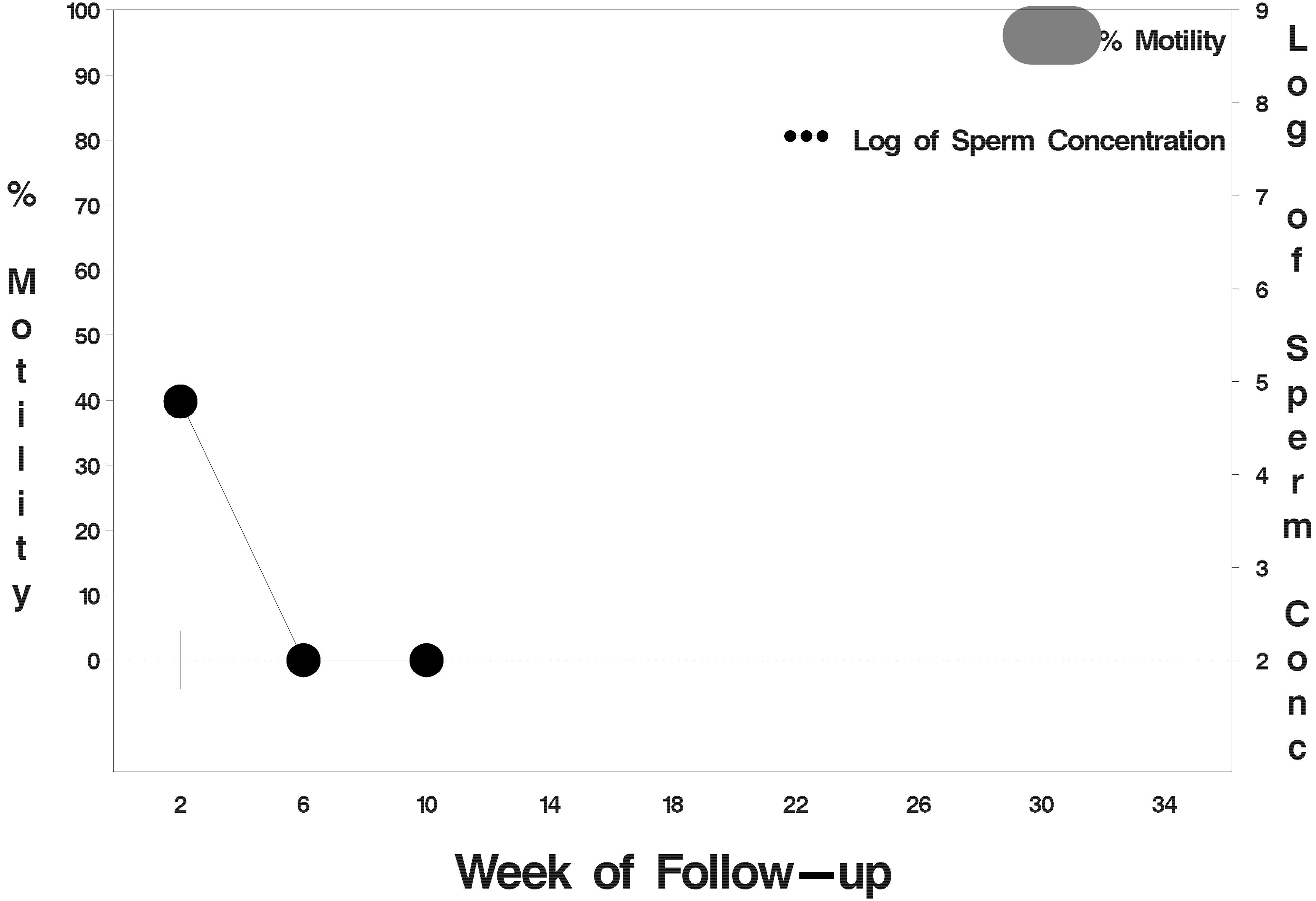

id= 673 outcome= Success

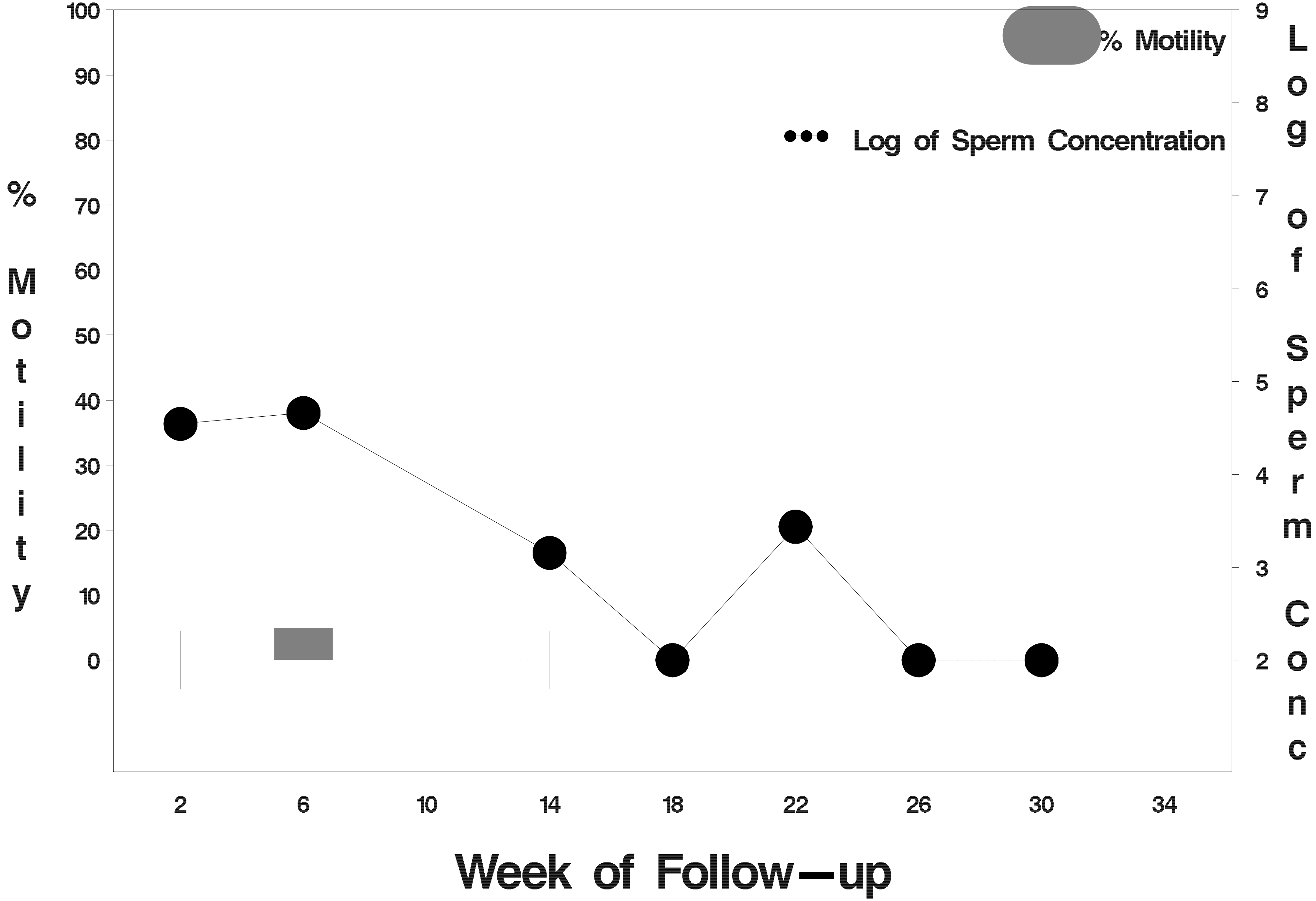

id= 674 outcome= Success

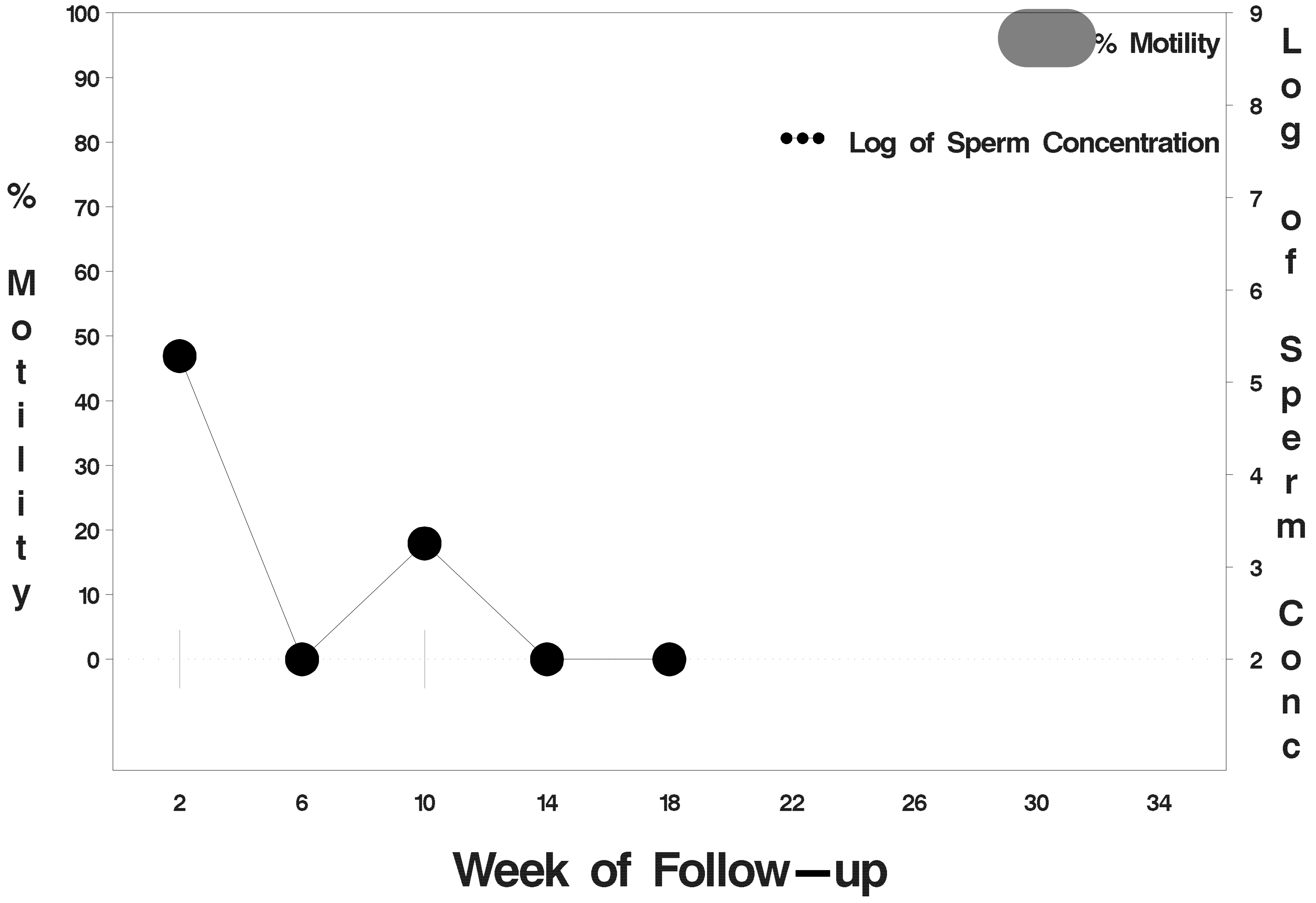

id= 675 outcome= Success

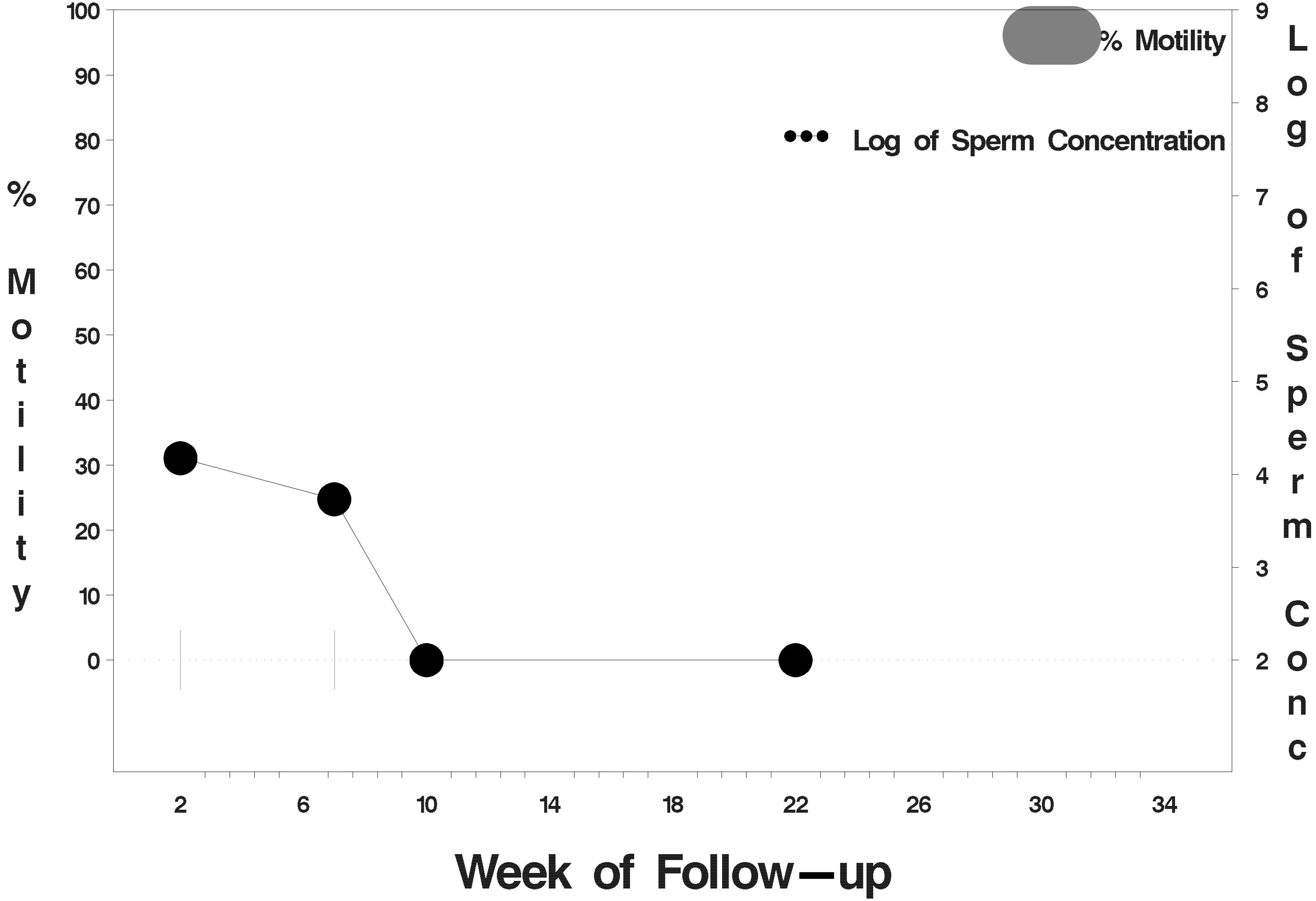

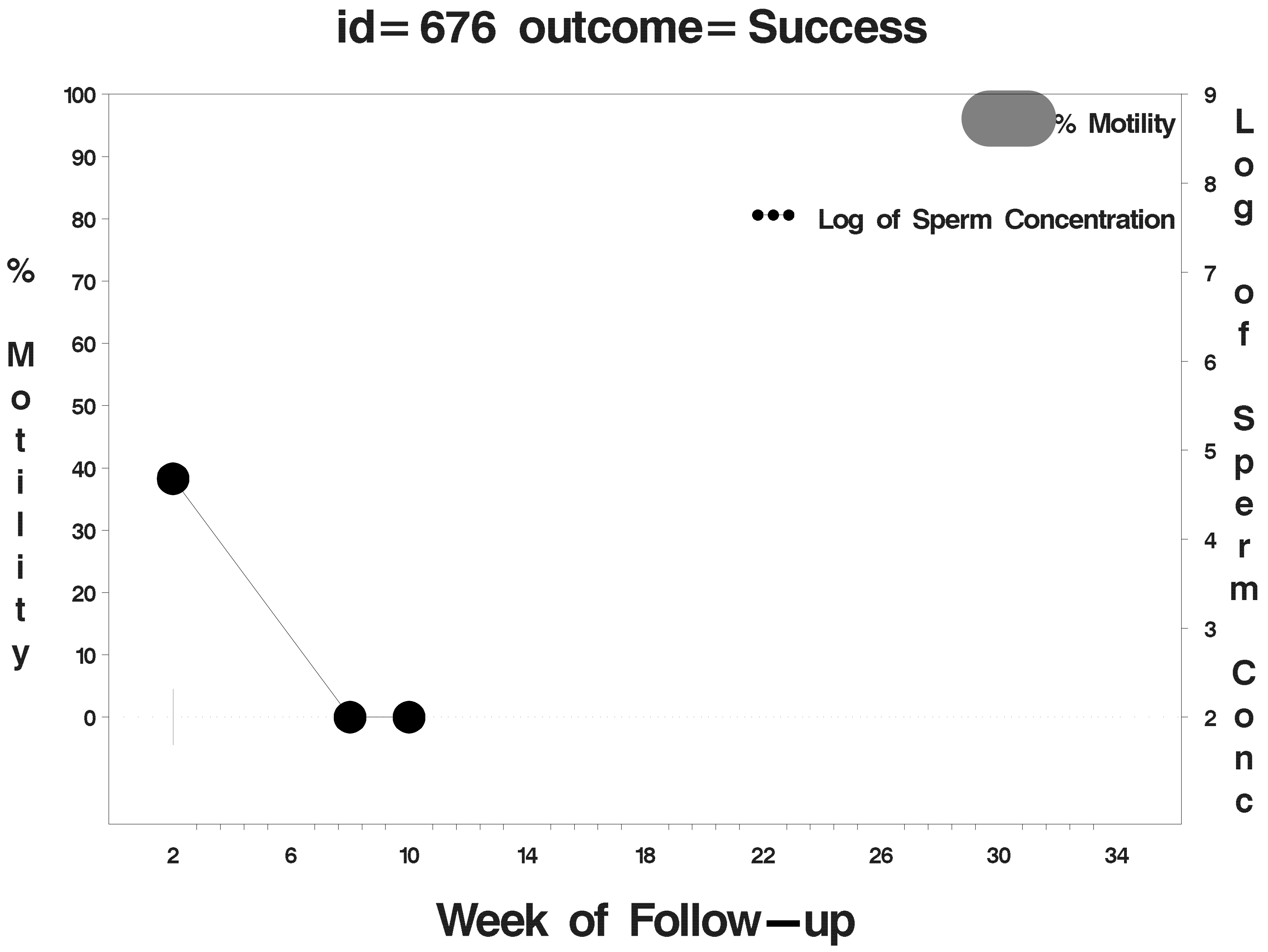
id= 676 outcome= Success
% Motility
Log of Sperm Concentration
100
90
80
70
60
50
40
30
20
10
0
9
8
7
6
5
4
3
2
2
6
10
14
18
22
26
30
34
% Motility
Log of Sperm Conc
Week of Follow—up

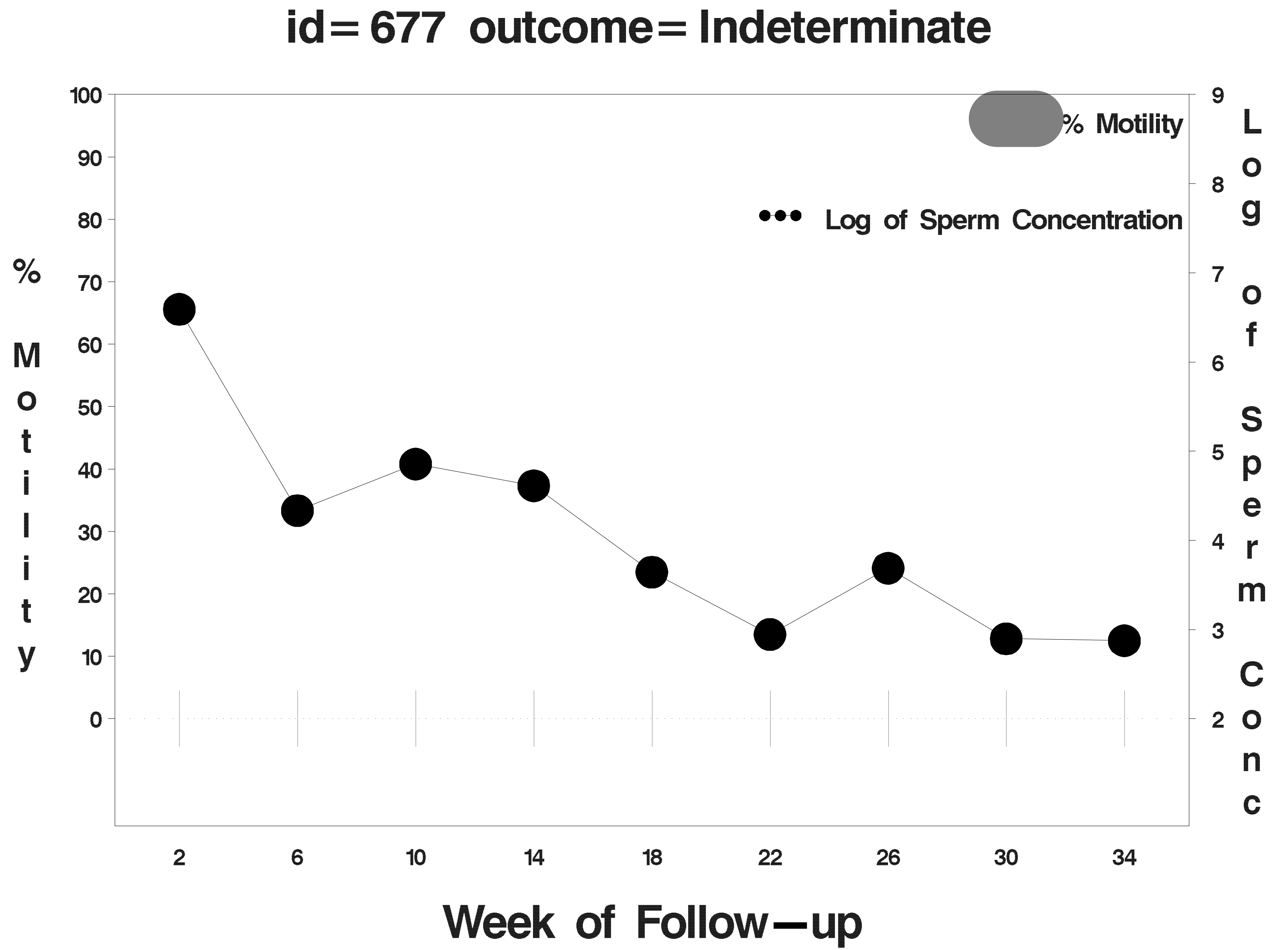

id= 677 outcome= Indeterminate
% Motility
Log of Sperm Concentration
100
90
80
70
60
50
40
30
20
10
0
9
8
7
6
5
4
3
2
% Motility
Log of Sperm Conc
2
6
10
14
18
22
26
30
34
Week of Follow—up

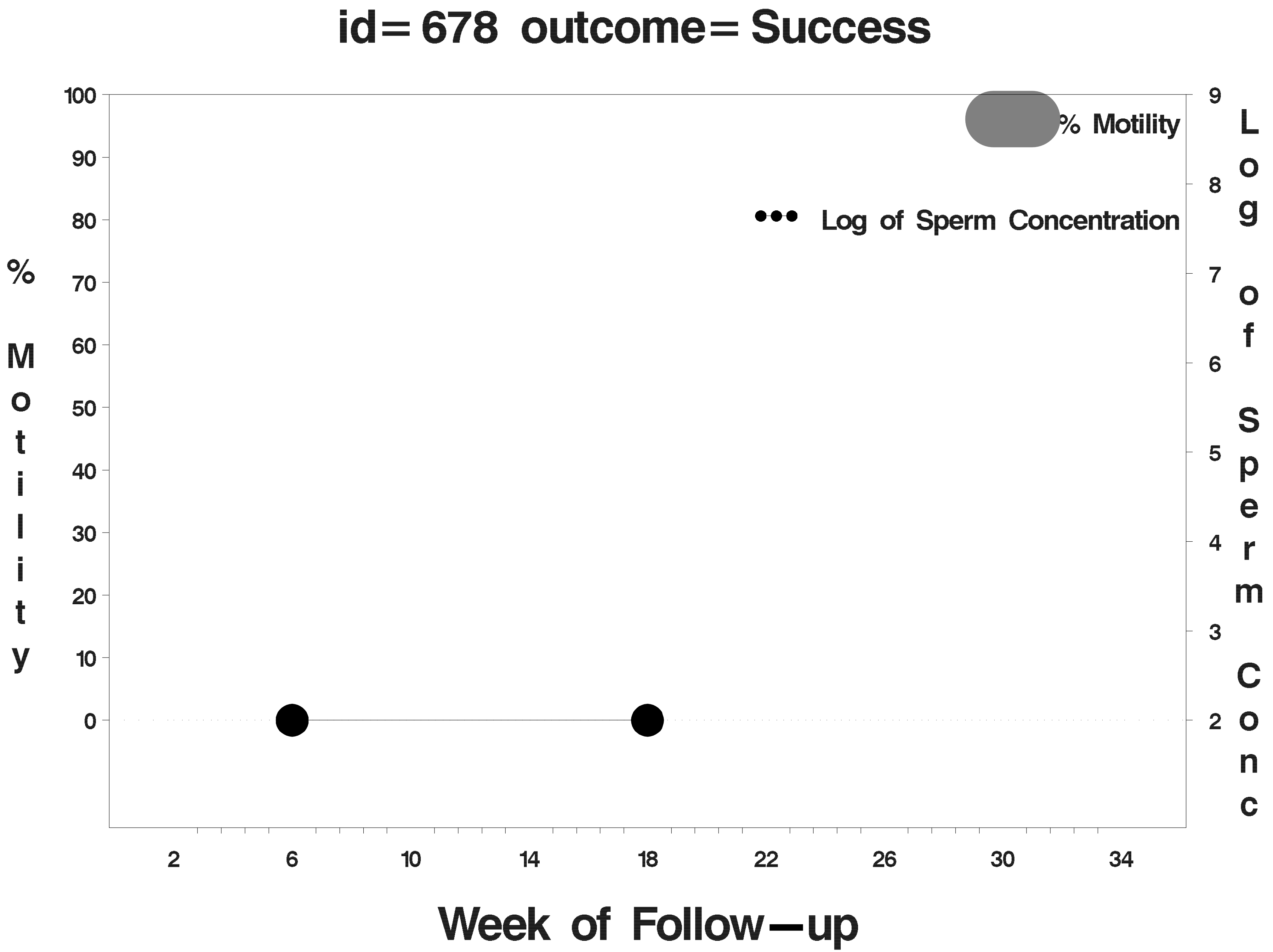
id= 678 outcome= Success
% Motility
Log of Sperm Concentration
% Motility
Log of Sperm Conc
Week of Follow-up
100
90
80
70
60
50
40
30
20
10
0
9
8
7
6
5
4
3
2
2
6
10
14
18
22
26
30
34

id= 679 outcome= Success

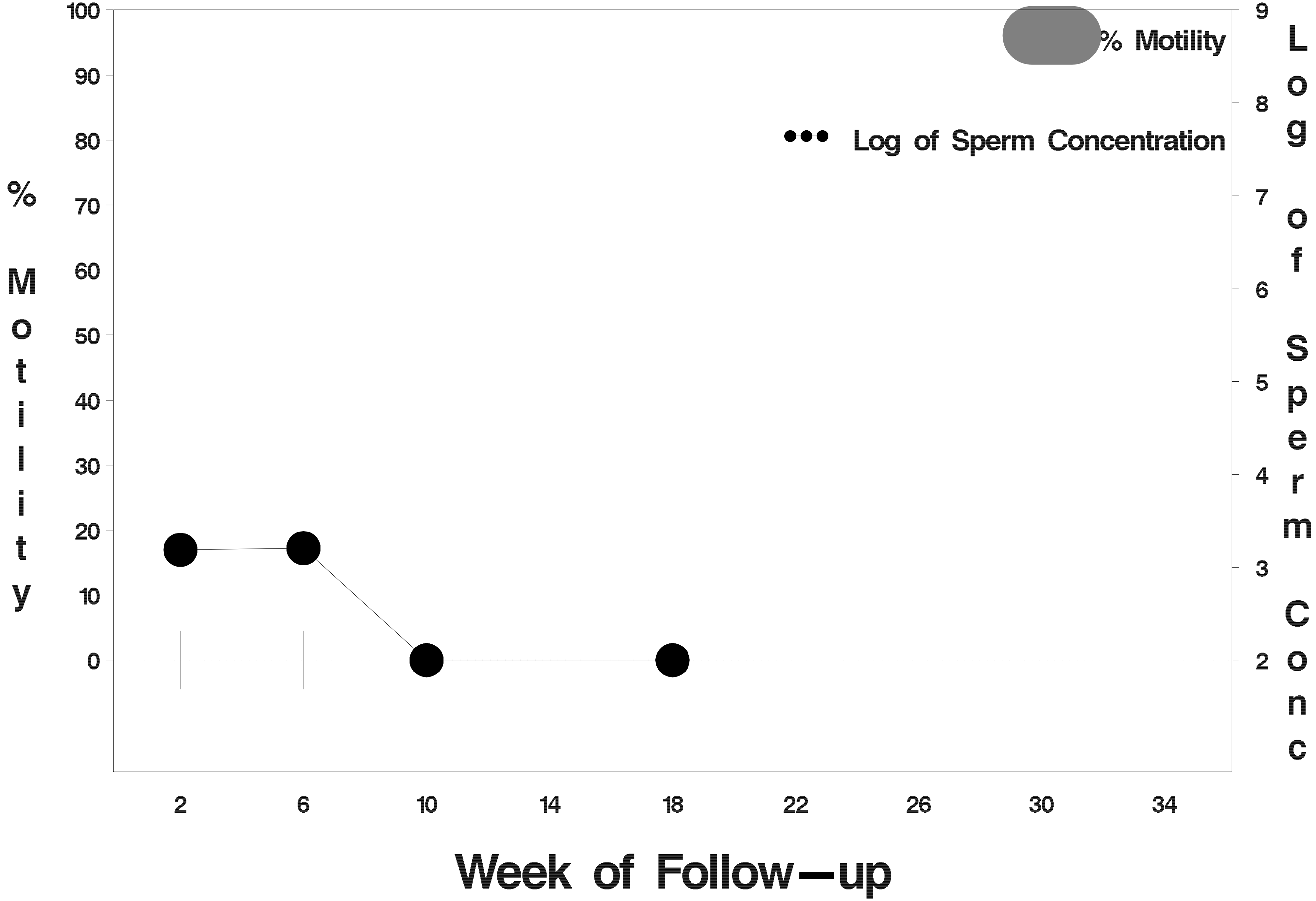

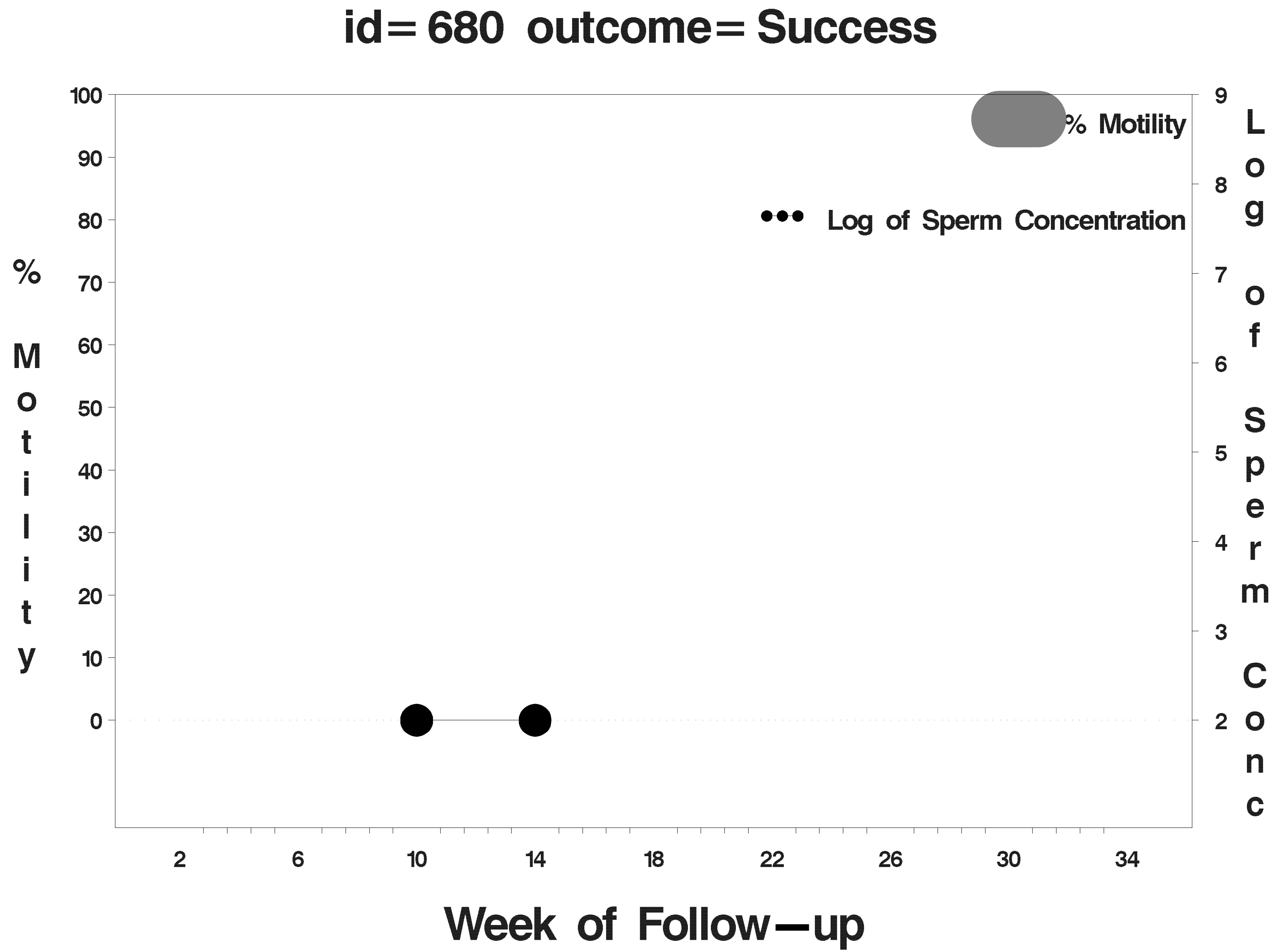

id= 680 outcome= Success
% Motility
Log of Sperm Concentration
100
90
80
70
60
50
40
30
20
10
0
9
8
7
6
5
4
3
2
2
6
10
14
18
22
26
30
34
Week of Follow—up
% Motility
Log of Sperm Conc

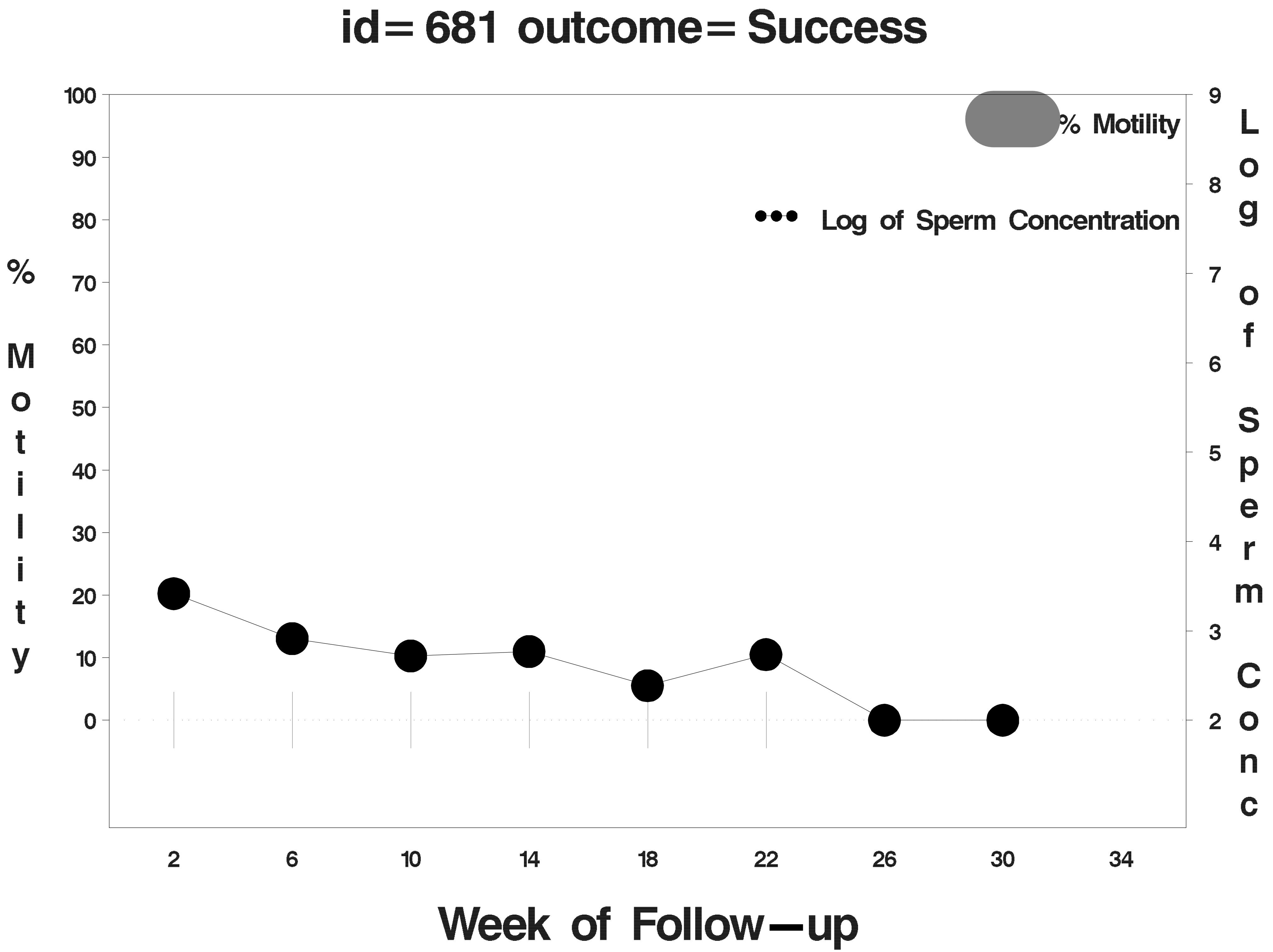

id= 681 outcome= Success
% Motility
Log of Sperm Concentration
100
90
80
70
60
50
40
30
20
10
0
9
8
7
6
5
4
3
2
2
6
10
14
18
22
26
30
34
Week of Follow—up
Log of Sperm Conc

id= 682 outcome= Success

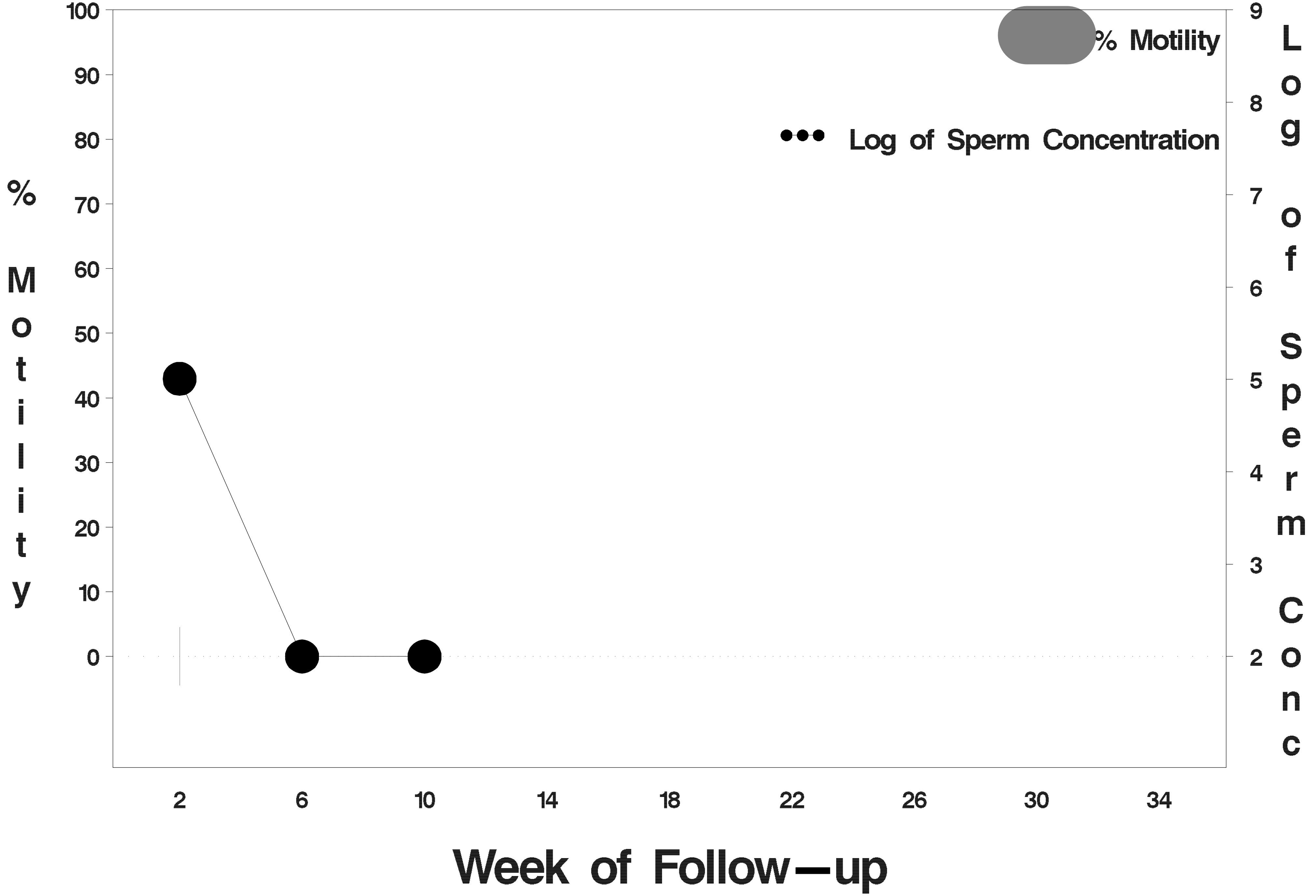

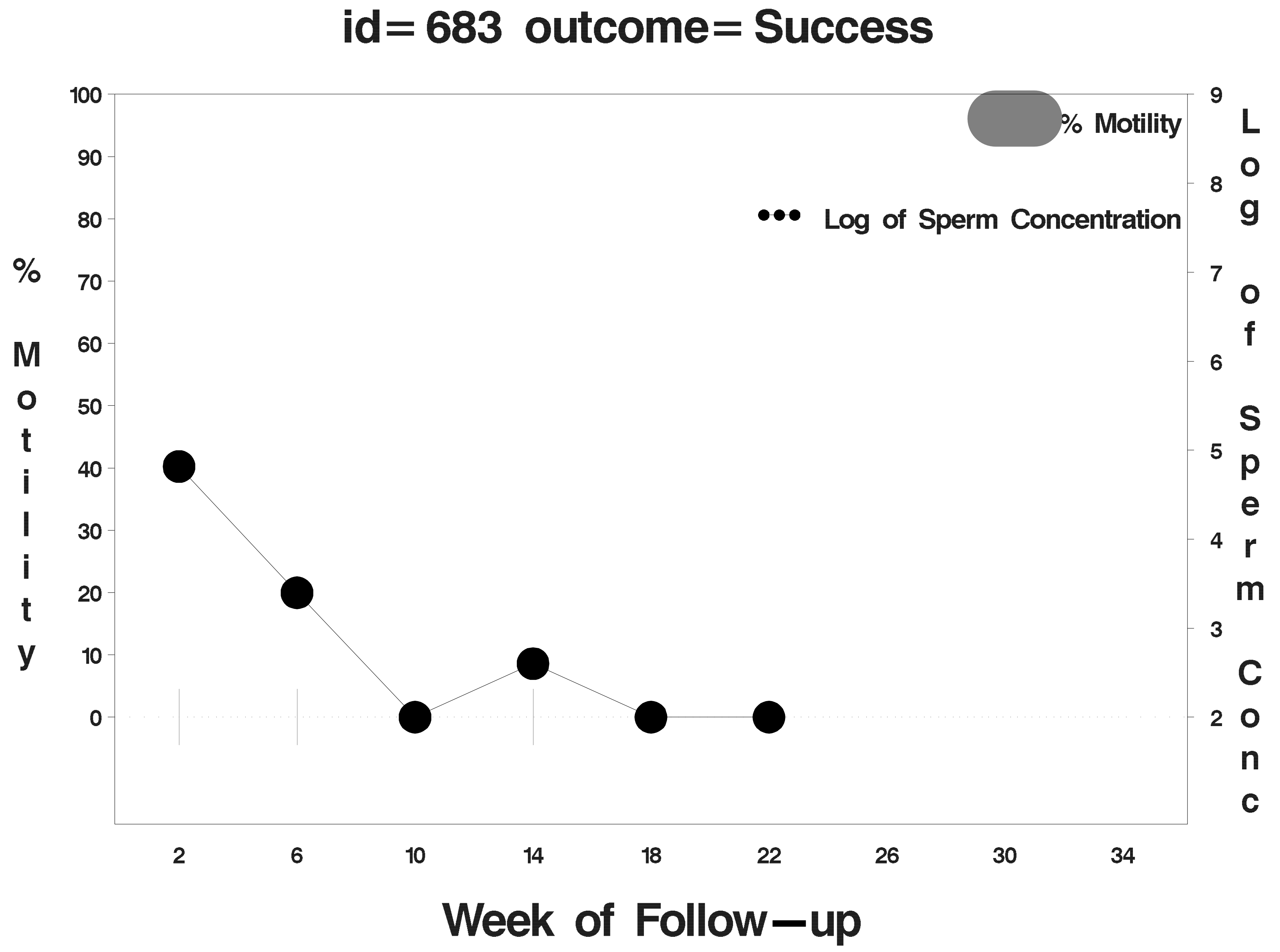
id= 683 outcome= Success
% Motility
Log of Sperm Concentration
% Motility
Log of Sperm Conc
Week of Follow−up
100
90
80
70
60
50
40
30
20
10
0
9
8
7
6
5
4
3
2
2
6
10
14
18
22
26
30
34

id= 684 outcome= Indeterminate

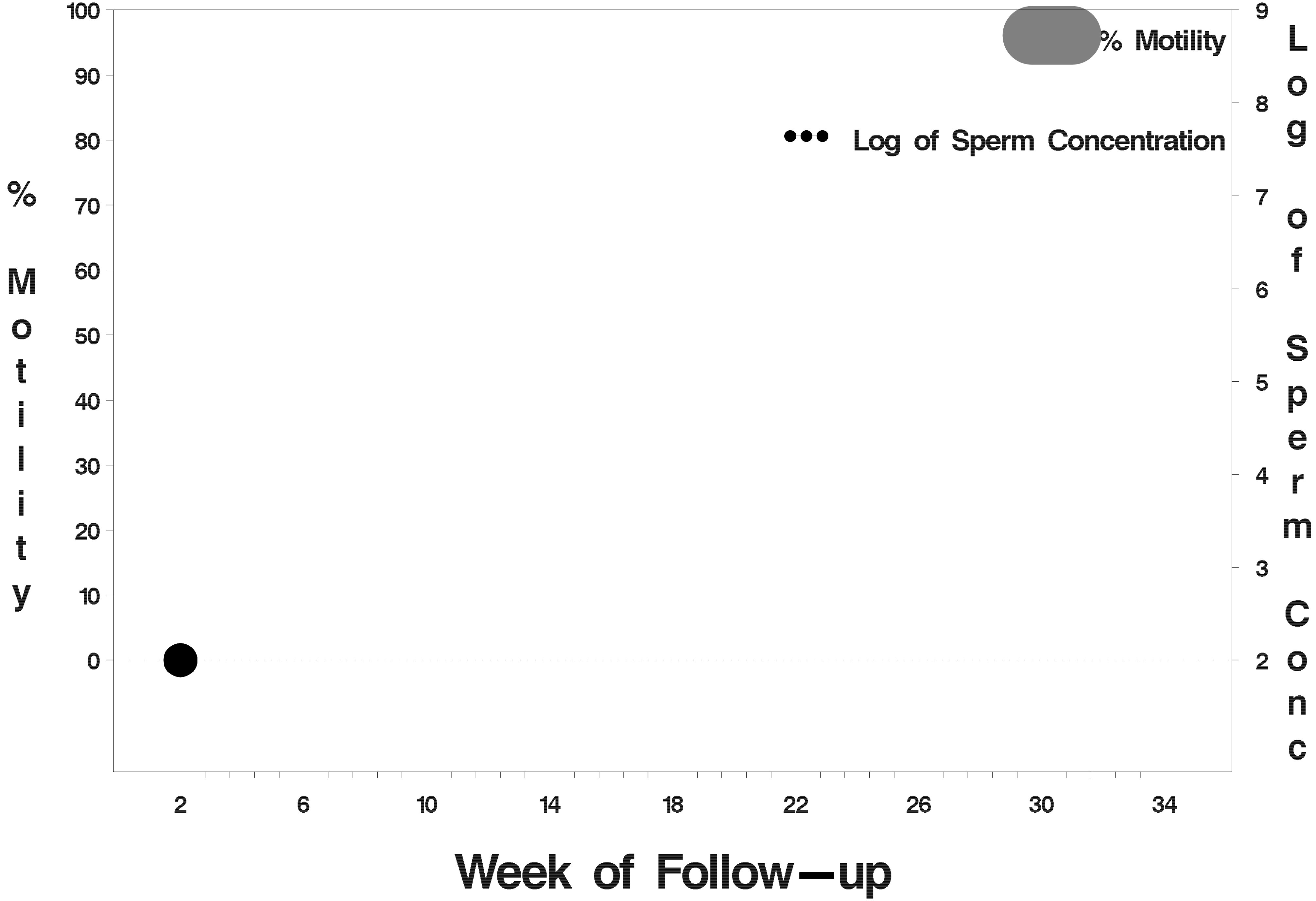

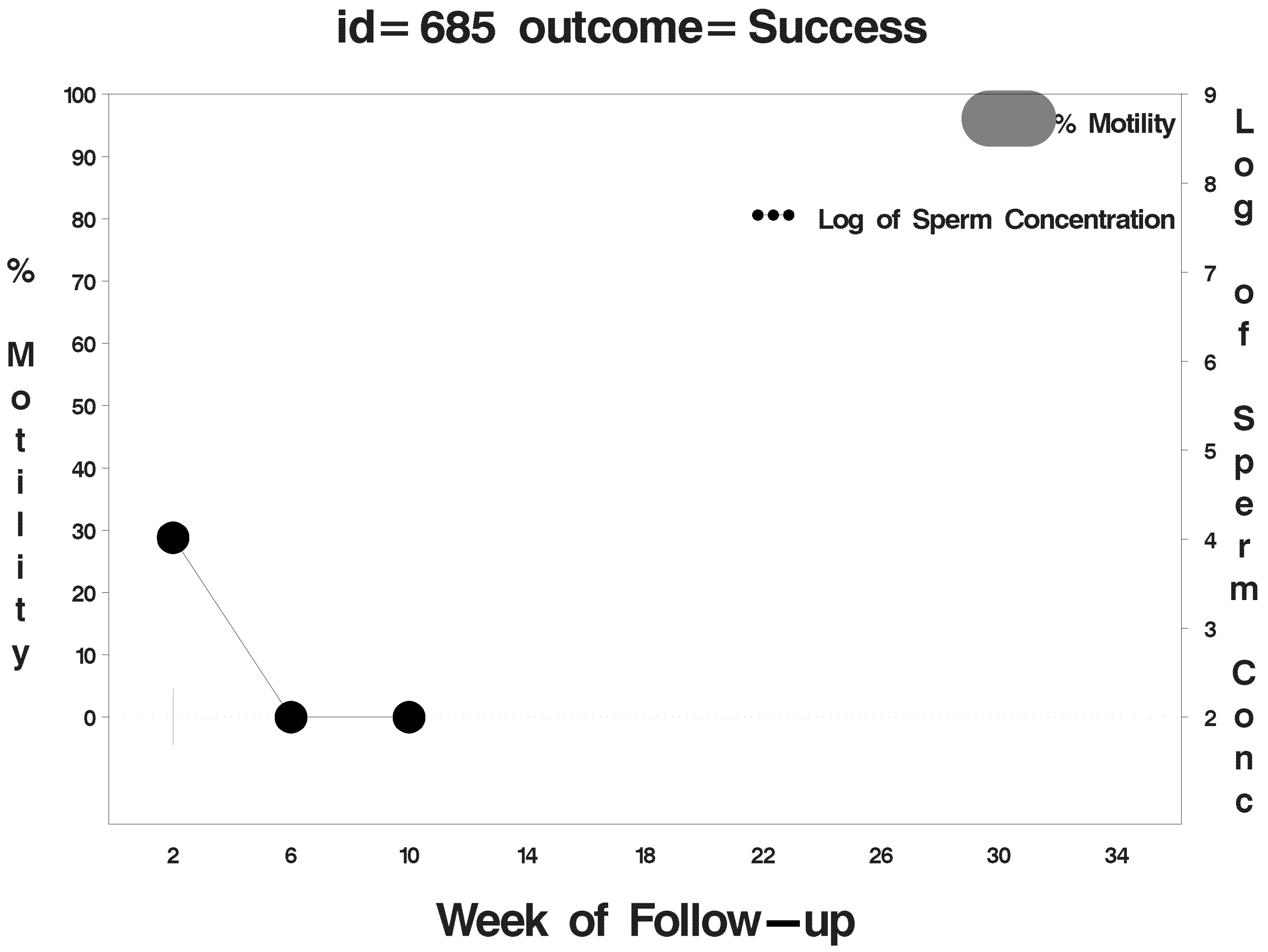
id= 685 outcome= Success
% Motility
Log of Sperm Concentration
100
90
80
70
60
50
40
30
20
10
0
9
8
7
6
5
4
3
2
2
6
10
14
18
22
26
30
34
Week of Follow-up
Log of Sperm Conc

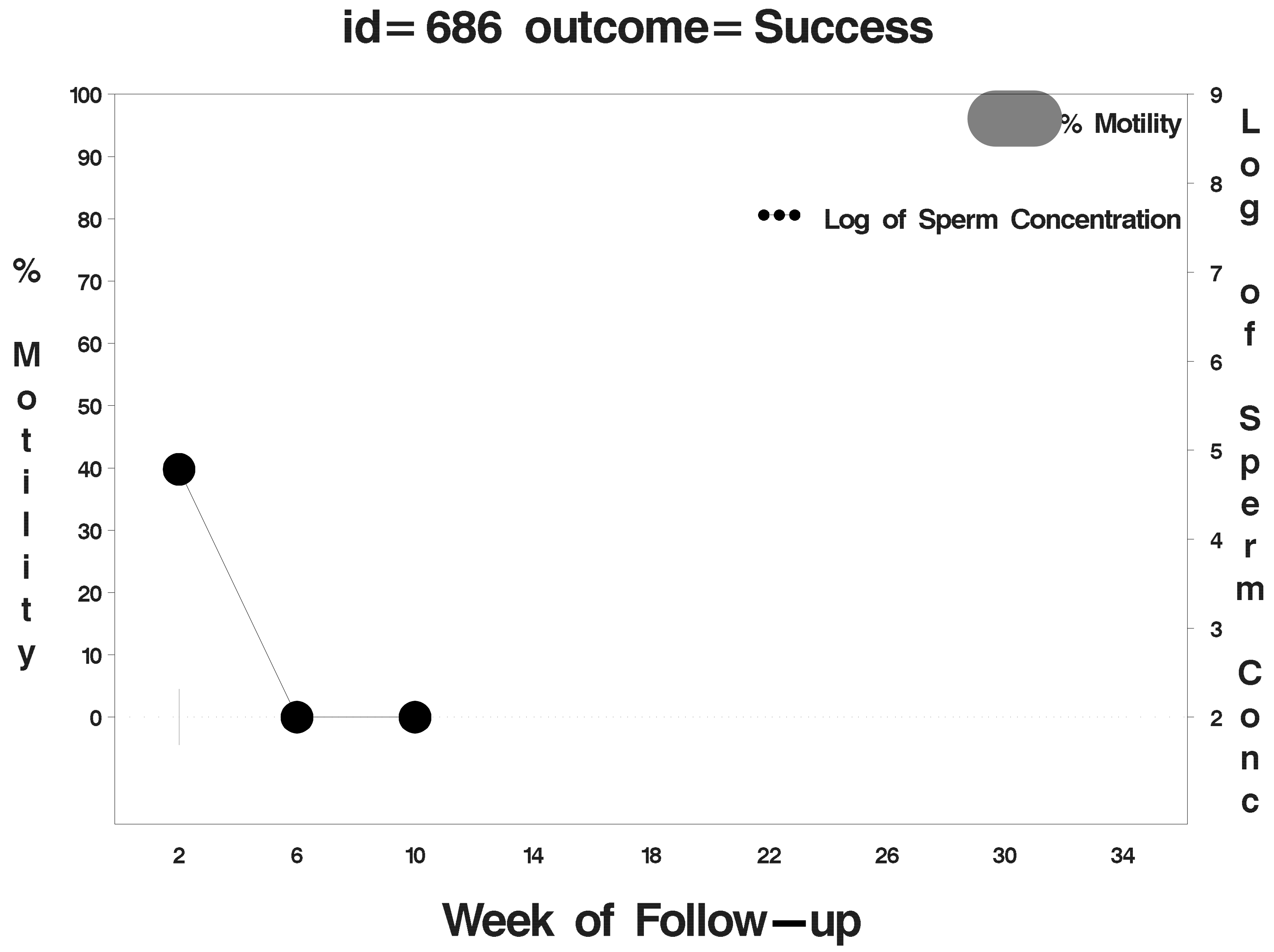

id= 686 outcome= Success
% Motility
Log of Sperm Concentration
100
90
80
70
60
50
40
30
20
10
0
9
8
7
6
5
4
3
2
% Motility
Log of Sperm Conc
2
6
10
14
18
22
26
30
34
Week of Follow−up

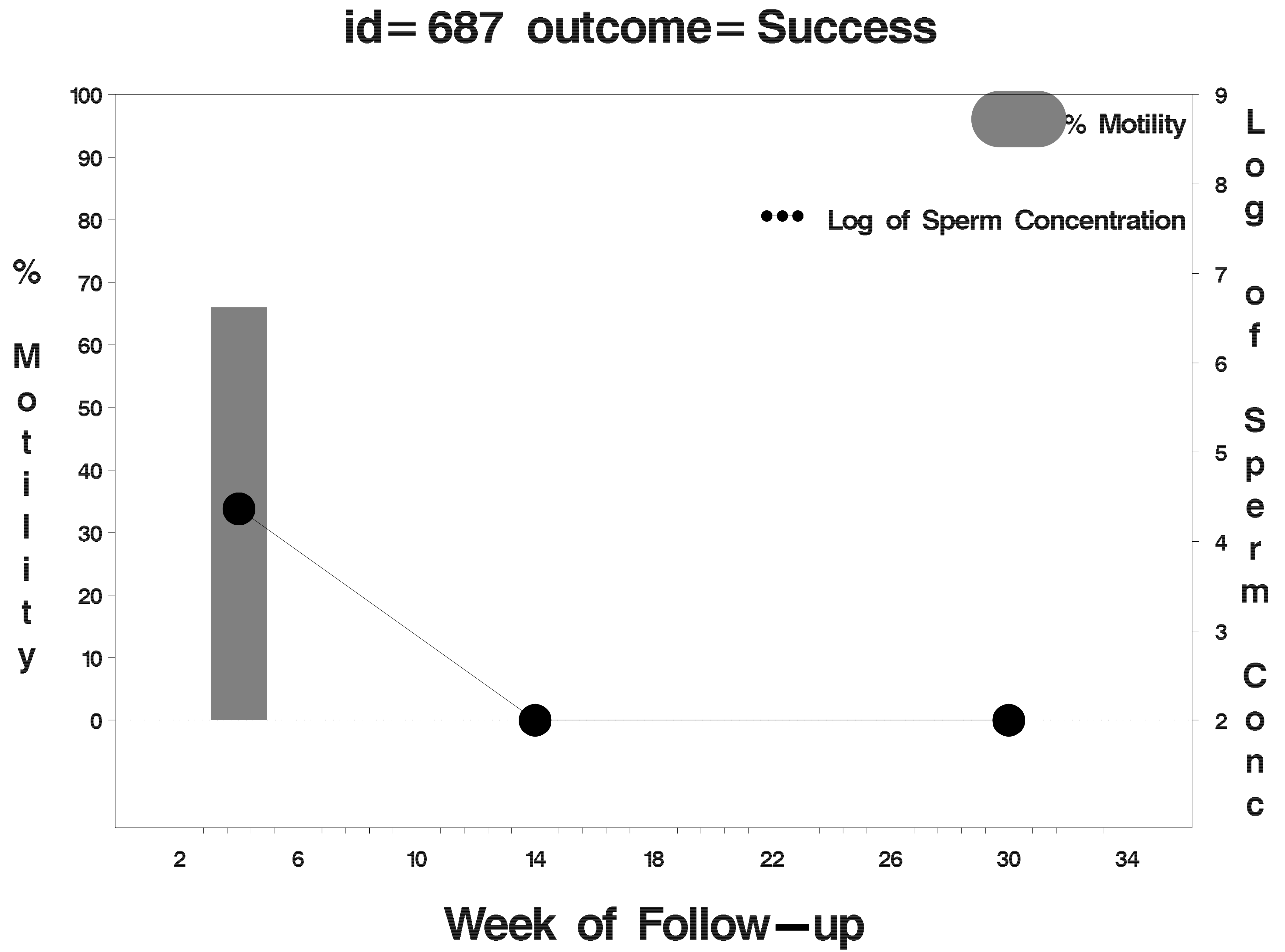
id= 687 outcome= Success
% Motility
Log of Sperm Concentration
100
90
80
70
60
50
40
30
20
10
0
9
8
7
6
5
4
3
2
2
6
10
14
18
22
26
30
34
% Motility
Log of Sperm Conc
Week of Follow—up

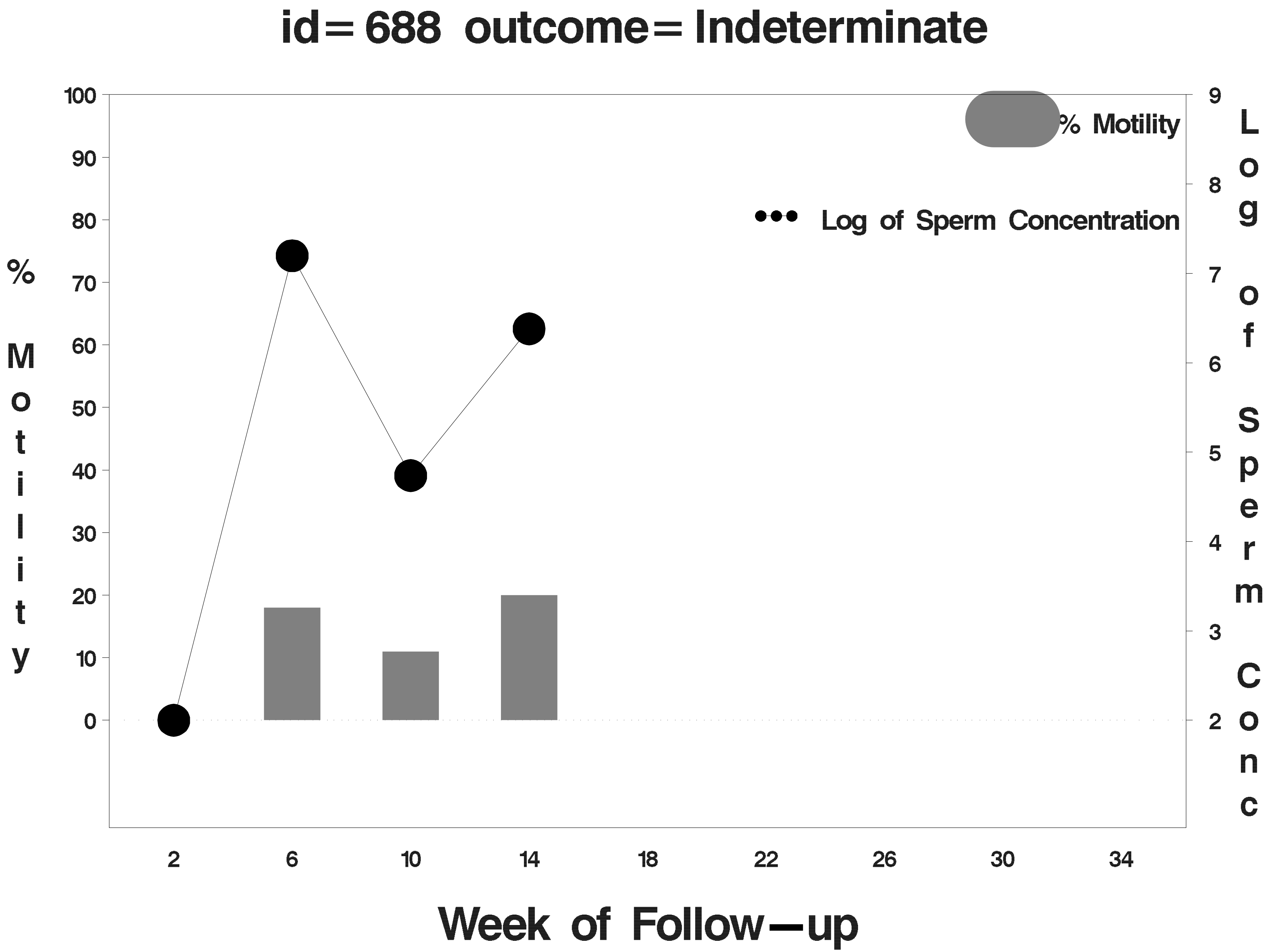
id= 688 outcome= Indeterminate
% Motility
Log of Sperm Concentration
100
90
80
70
60
50
40
30
20
10
0
9
8
7
6
5
4
3
2
2
6
10
14
18
22
26
30
34
% Motility
Log of Sperm Conc
Week of Follow−up

id= 689 outcome= Success

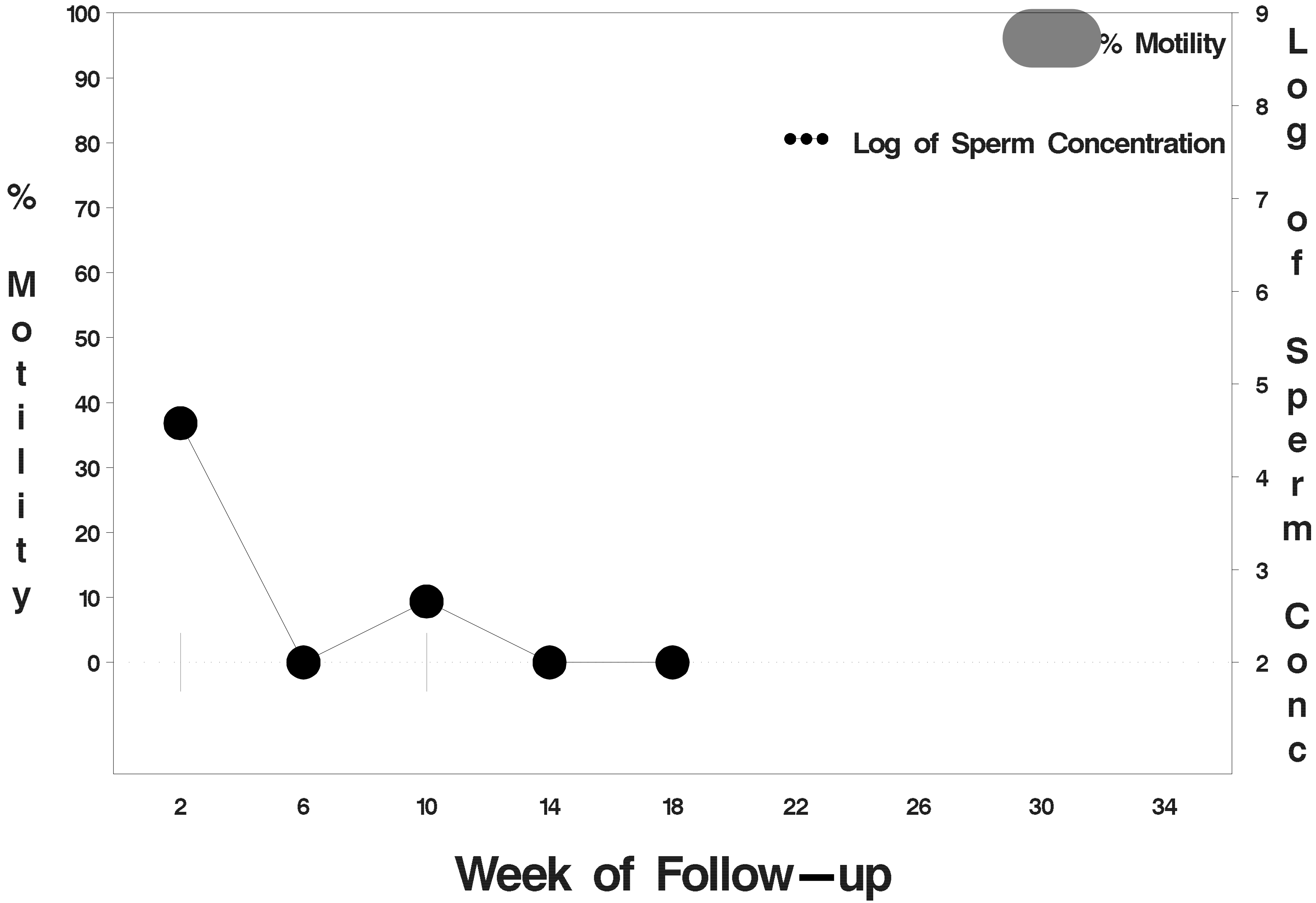

id= 690 outcome= Success

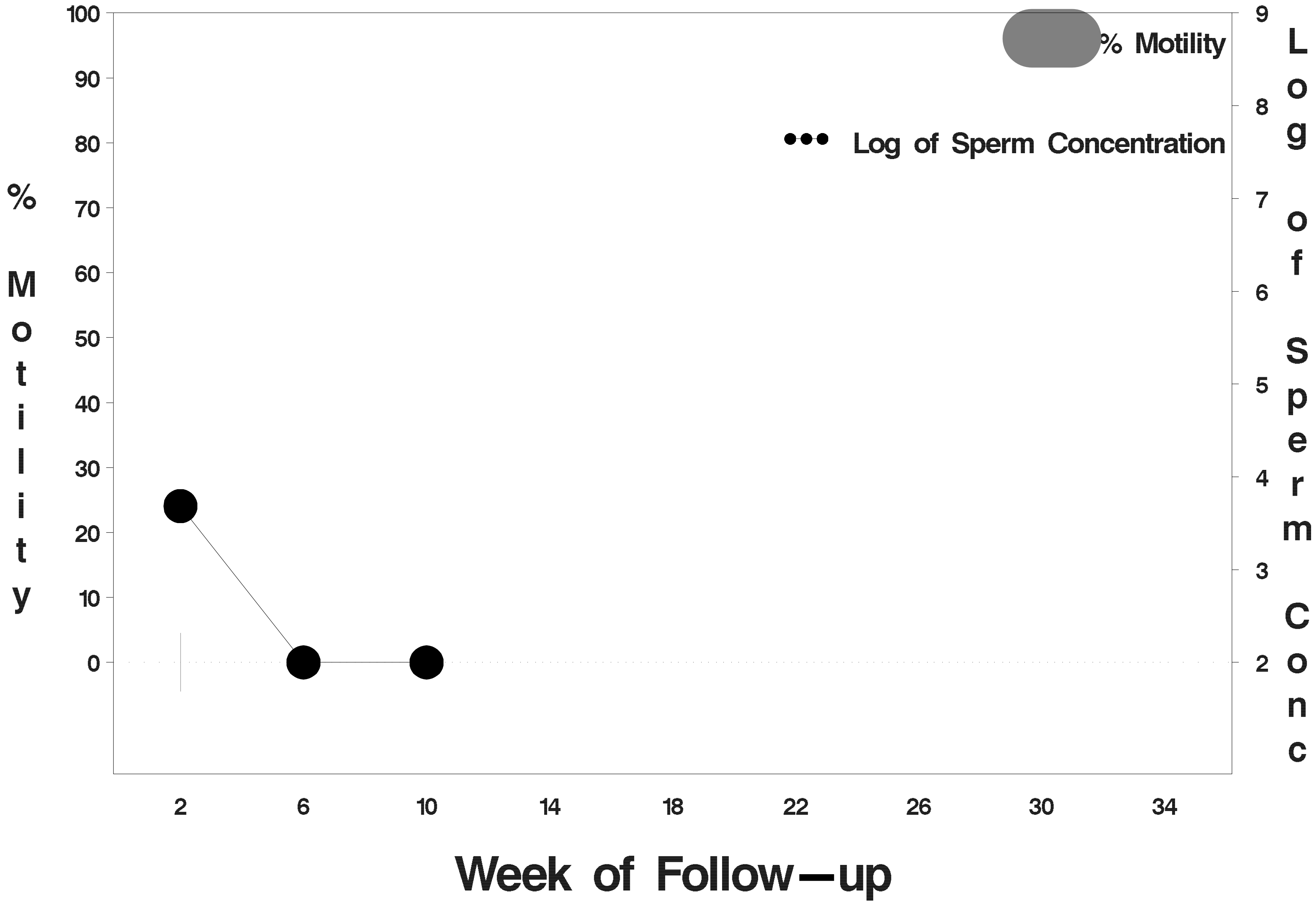

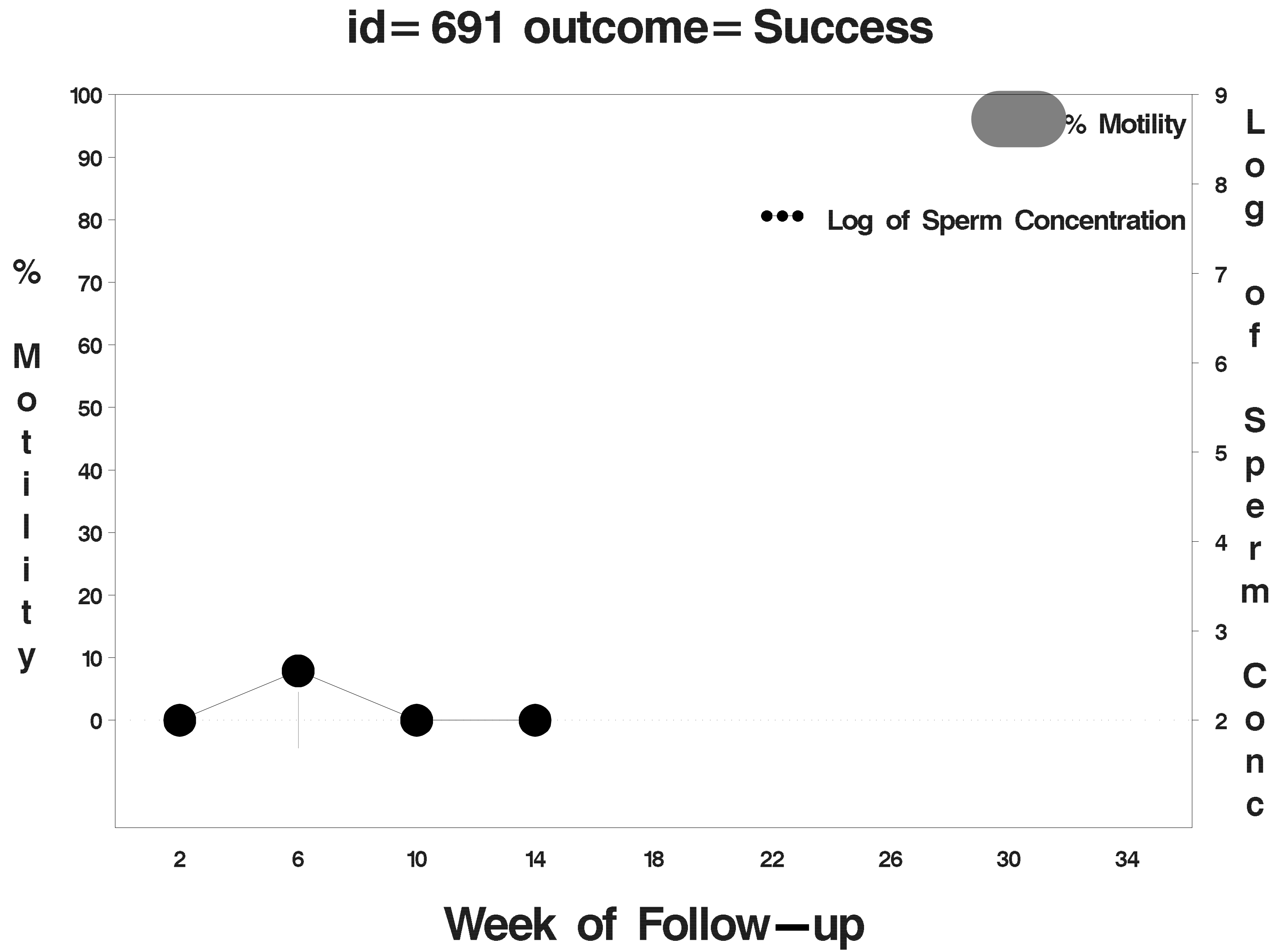
id= 691 outcome= Success
% Motility
Log of Sperm Concentration
100
90
80
70
60
50
40
30
20
10
0
9
8
7
6
5
4
3
2
2
6
10
14
18
22
26
30
34
% Motility
Log of Sperm Conc
Week of Follow—up

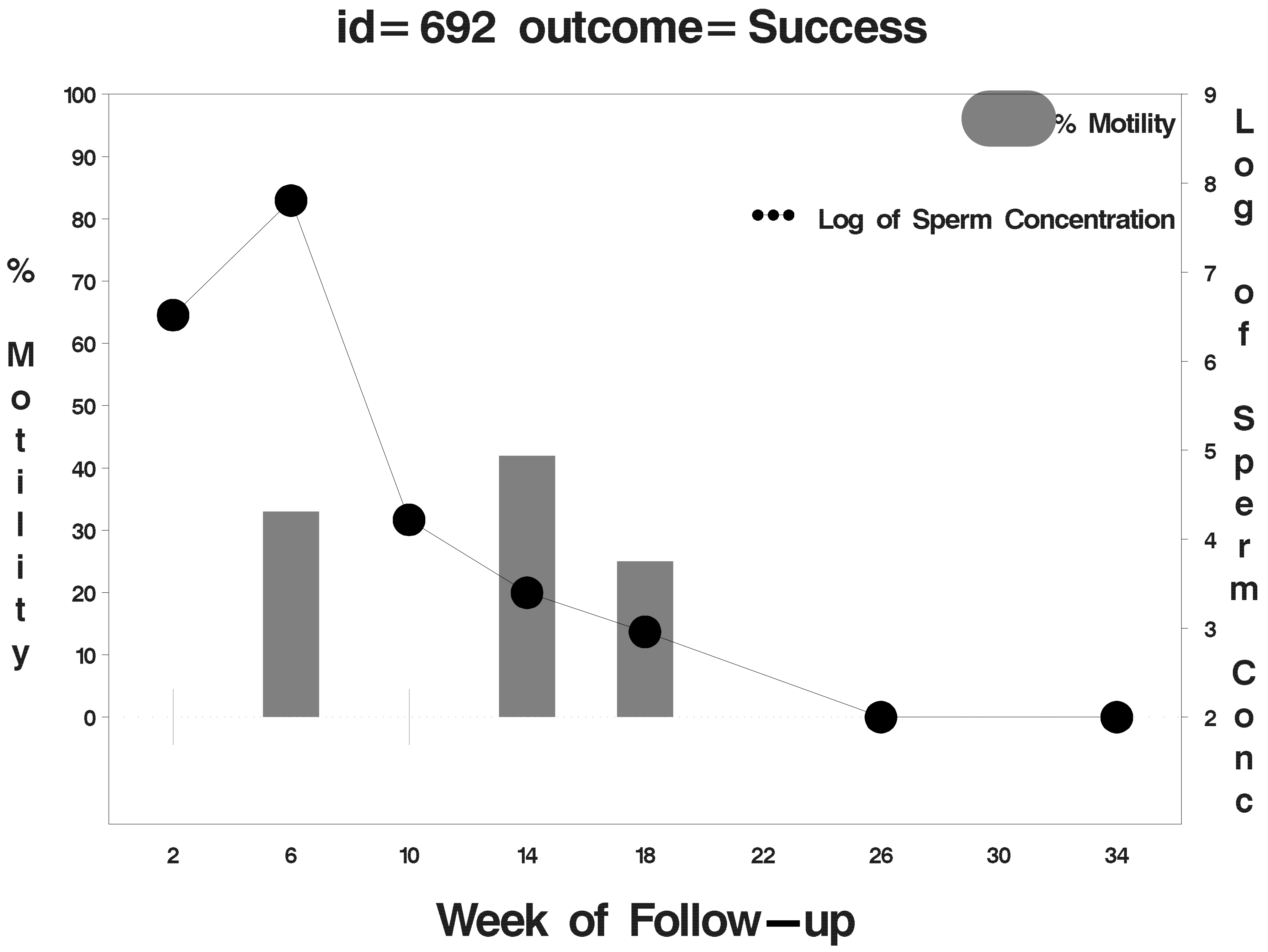

id= 692 outcome= Success
% Motility
Log of Sperm Concentration
100
90
80
70
60
50
40
30
20
10
0
9
8
7
6
5
4
3
2
2
6
10
14
18
22
26
30
34
Week of Follow−up
Log of Sperm Conc

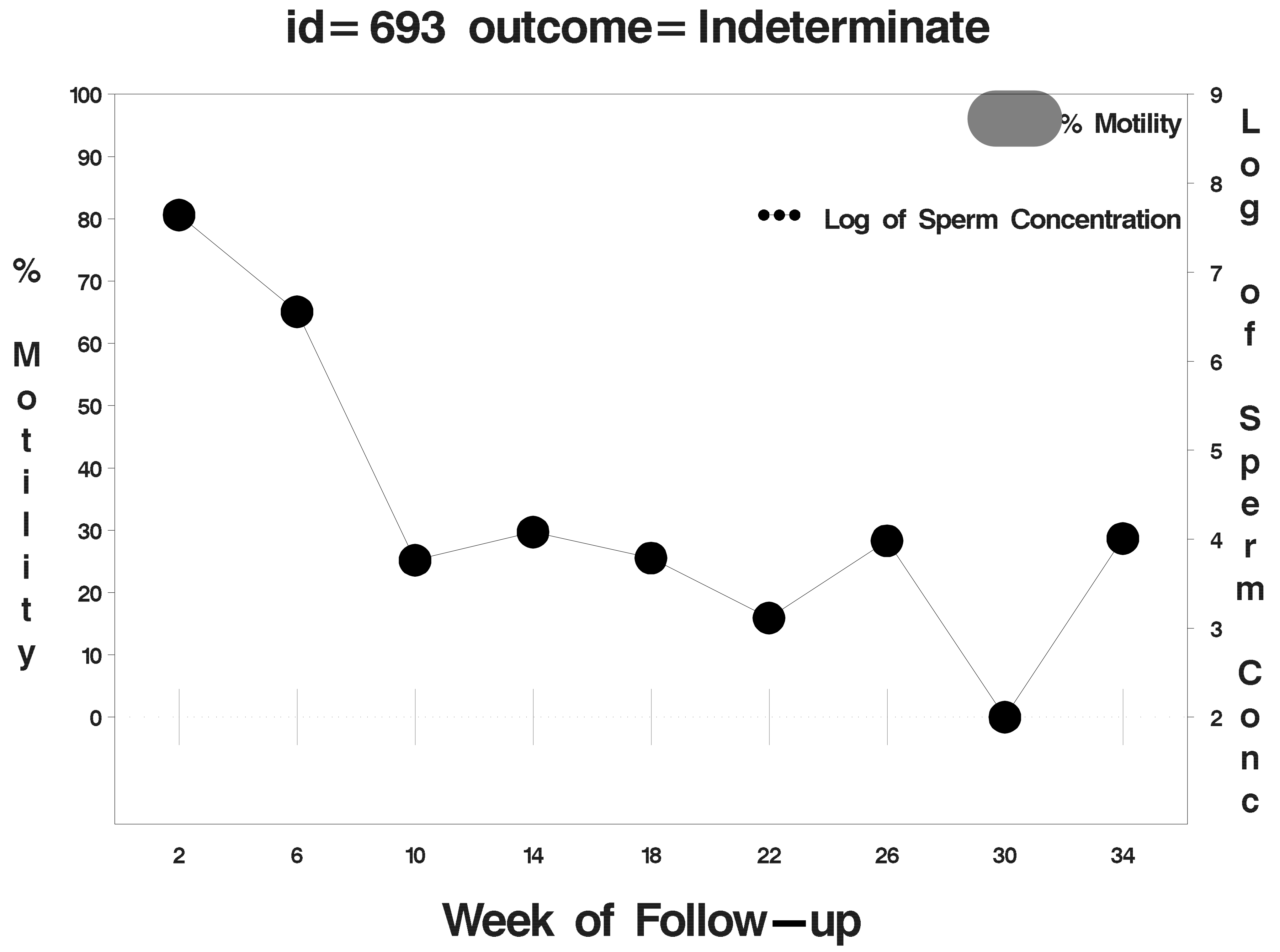
id=693 outcome=Indeterminate
% Motility
Log of Sperm Concentration
100
90
80
70
60
50
40
30
20
10
0
9
8
7
6
5
4
3
2
2
6
10
14
18
22
26
30
34
Week of Follow-up
Log of Sperm Conc

id= 694 outcome= Indeterminate

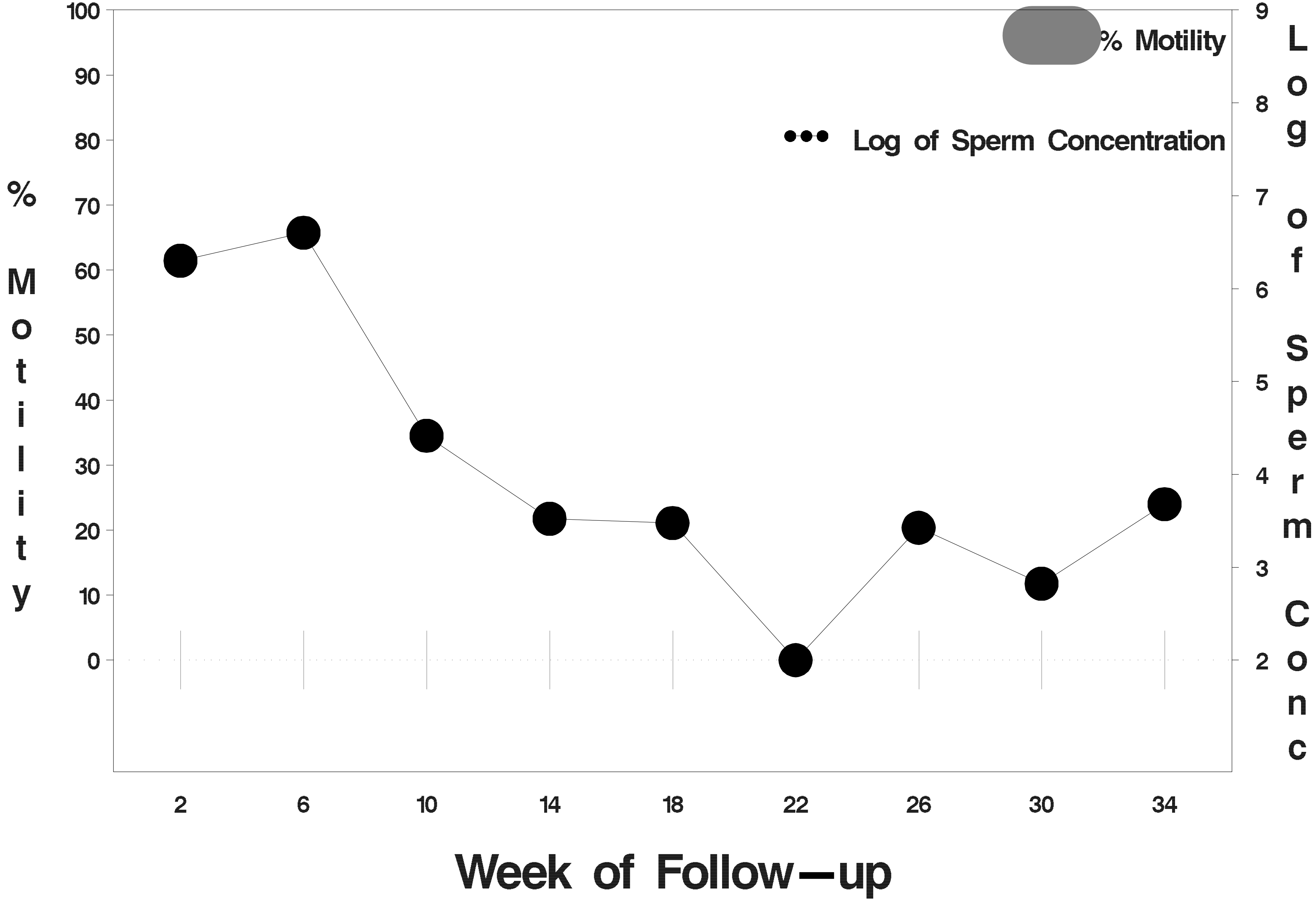

id= 695 outcome= Success

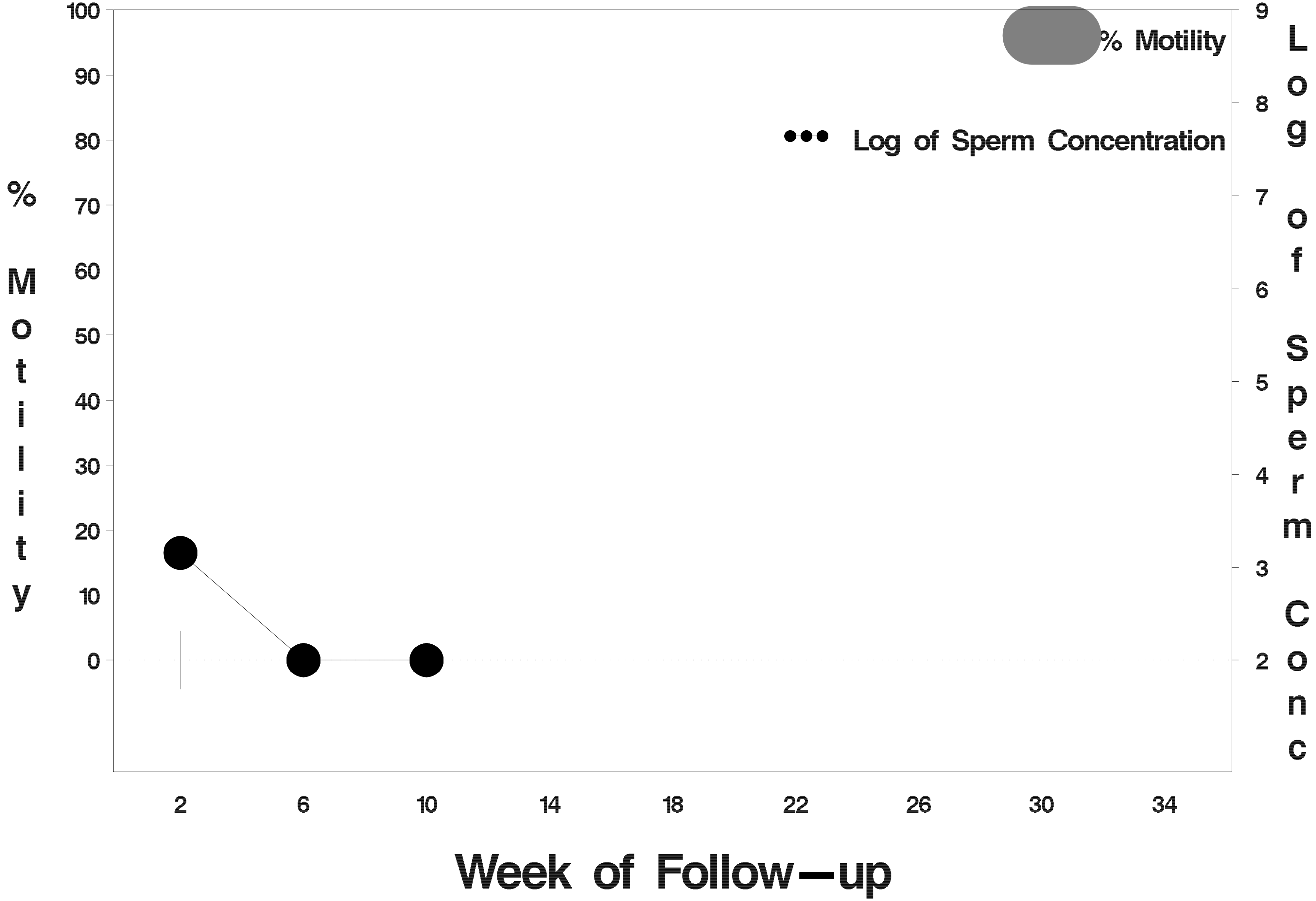

id= 696 outcome= Success

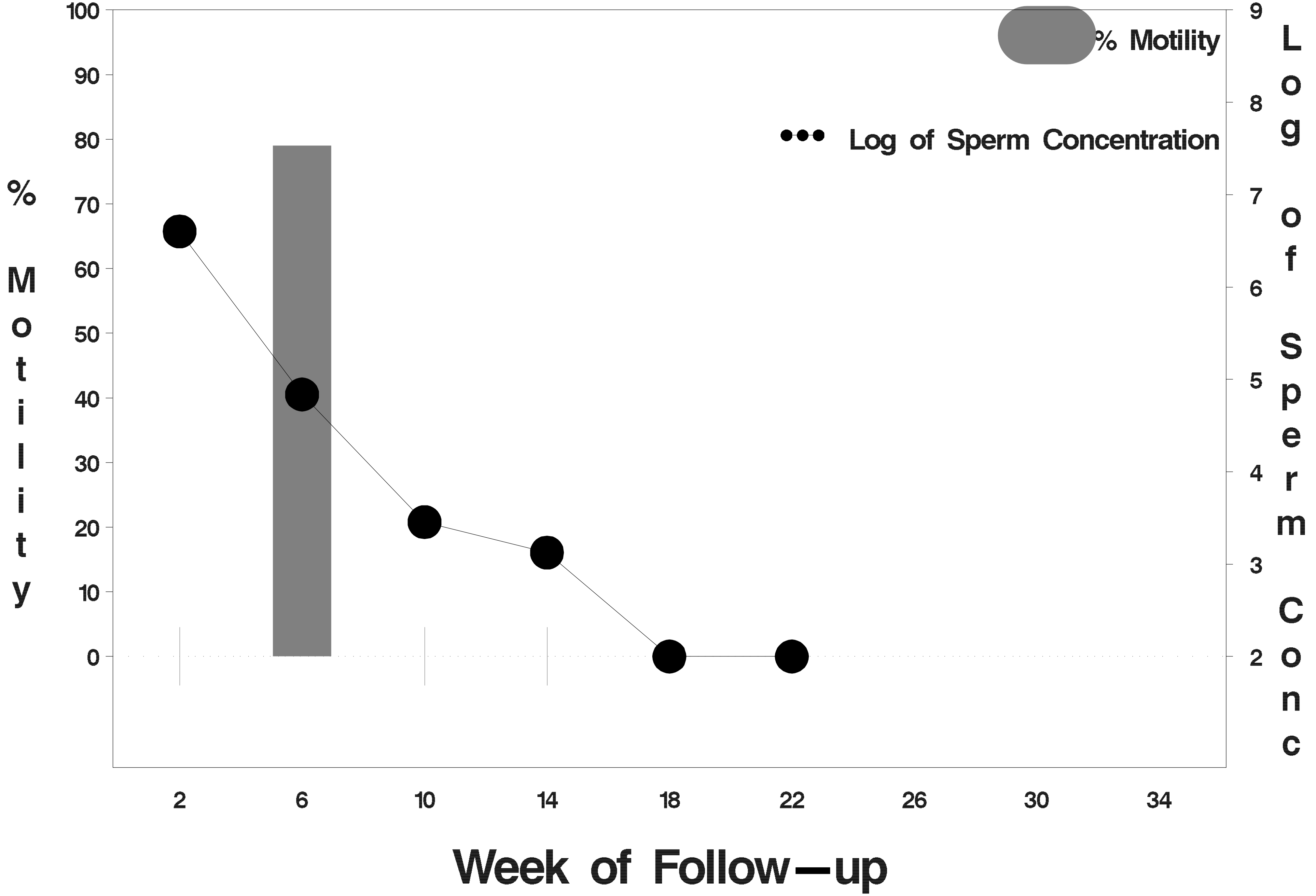

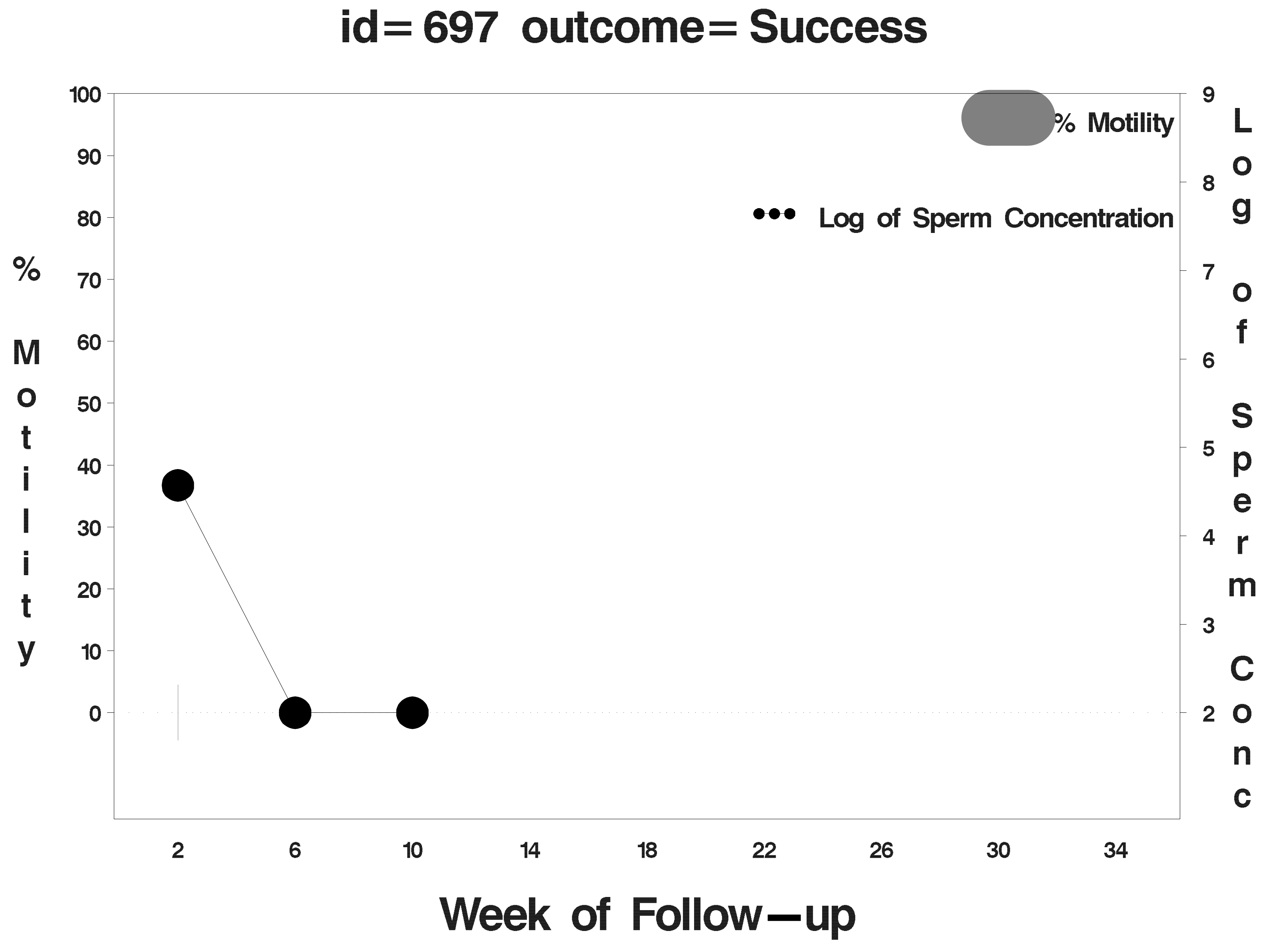

id= 697 outcome= Success
% Motility
Log of Sperm Concentration
% Motility
Log of Sperm Conc
100
90
80
70
60
50
40
30
20
10
0
9
8
7
6
5
4
3
2
2
6
10
14
18
22
26
30
34
Week of Follow—up

id= 698 outcome= Success

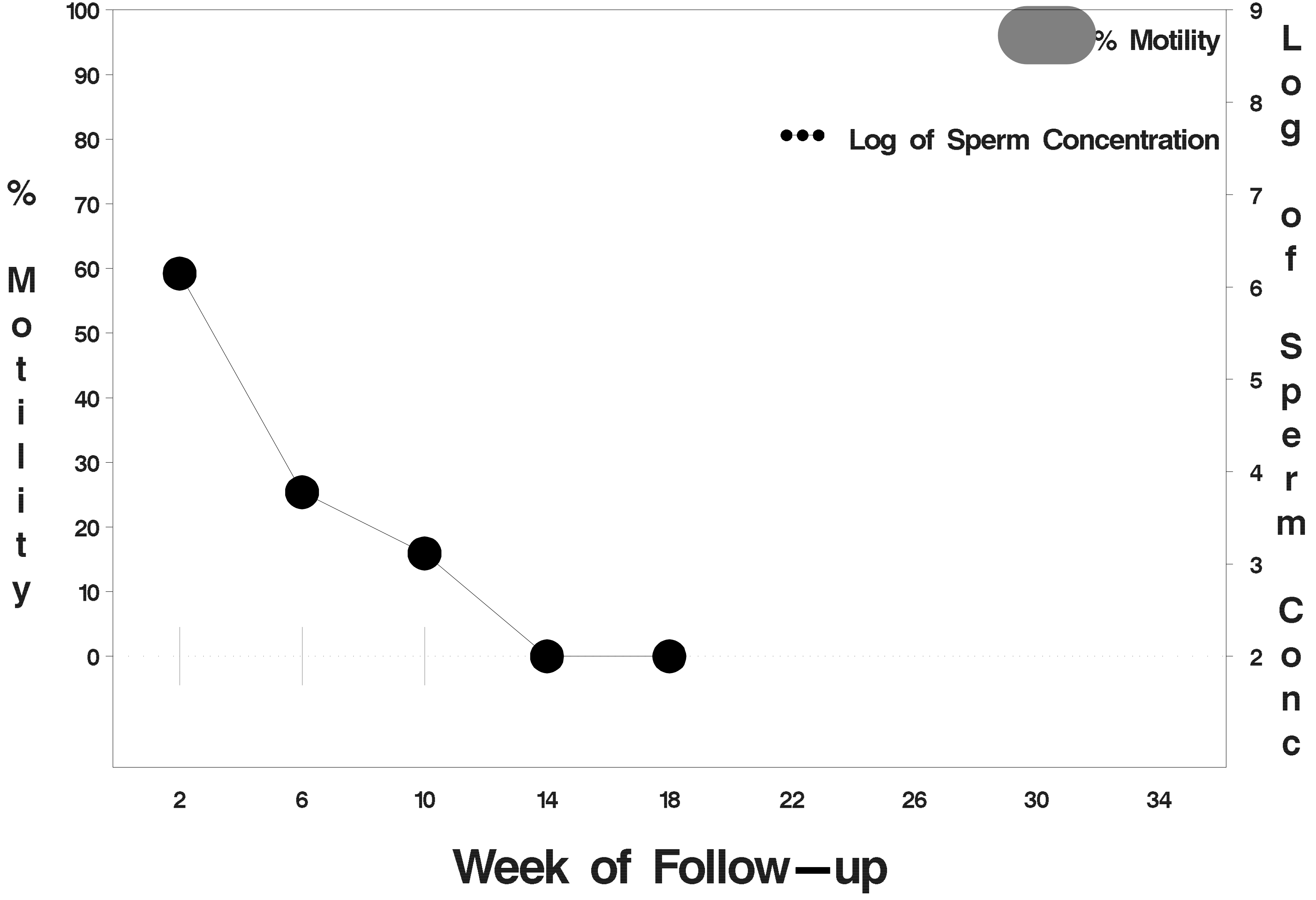

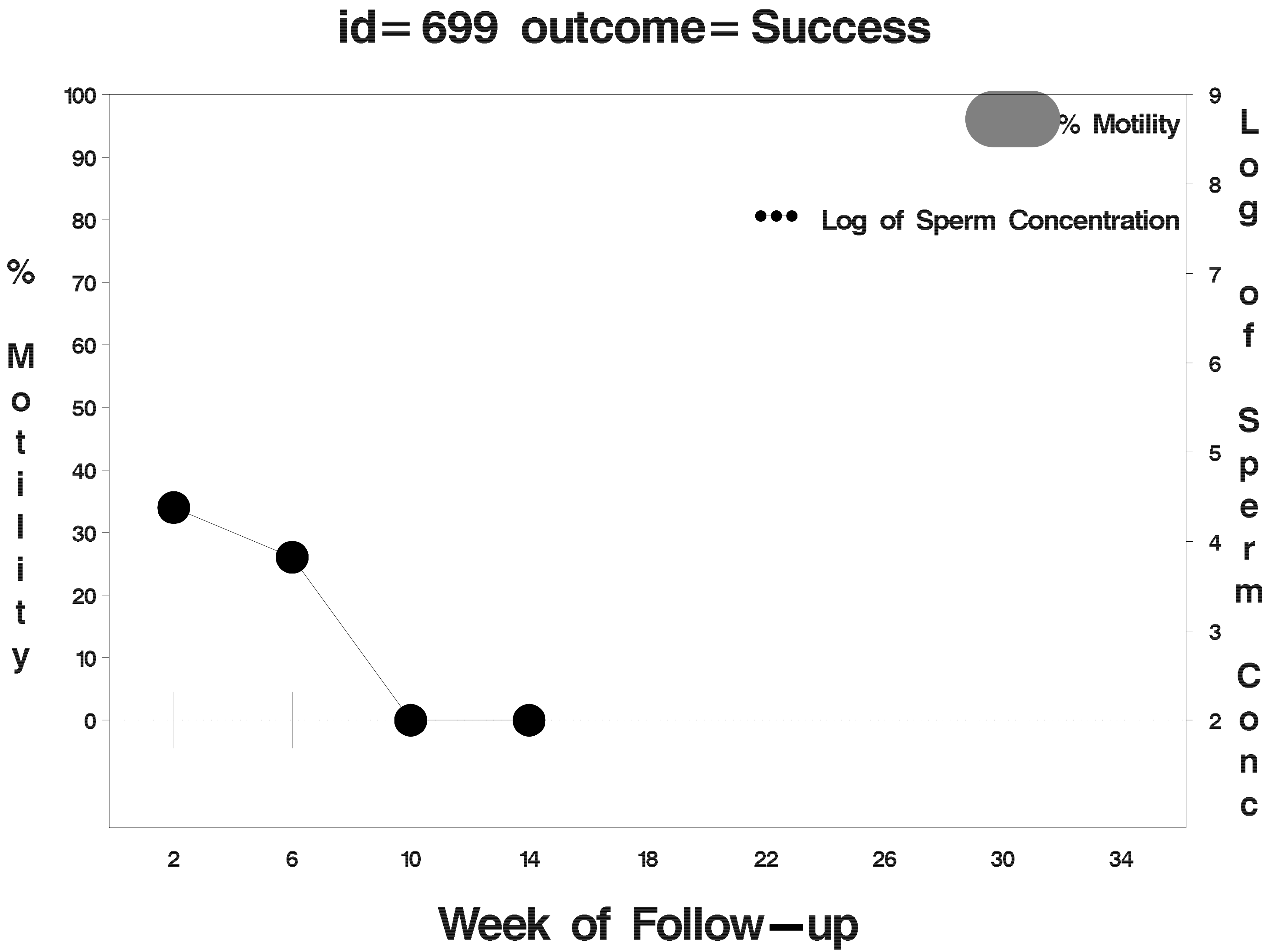
id= 699 outcome= Success
% Motility
Log of Sperm Concentration
100
90
80
70
60
50
40
30
20
10
0
9
8
7
6
5
4
3
2
2
6
10
14
18
22
26
30
34
Week of Follow−up
% Motility
Log of Sperm Conc

id= 700 outcome= Success

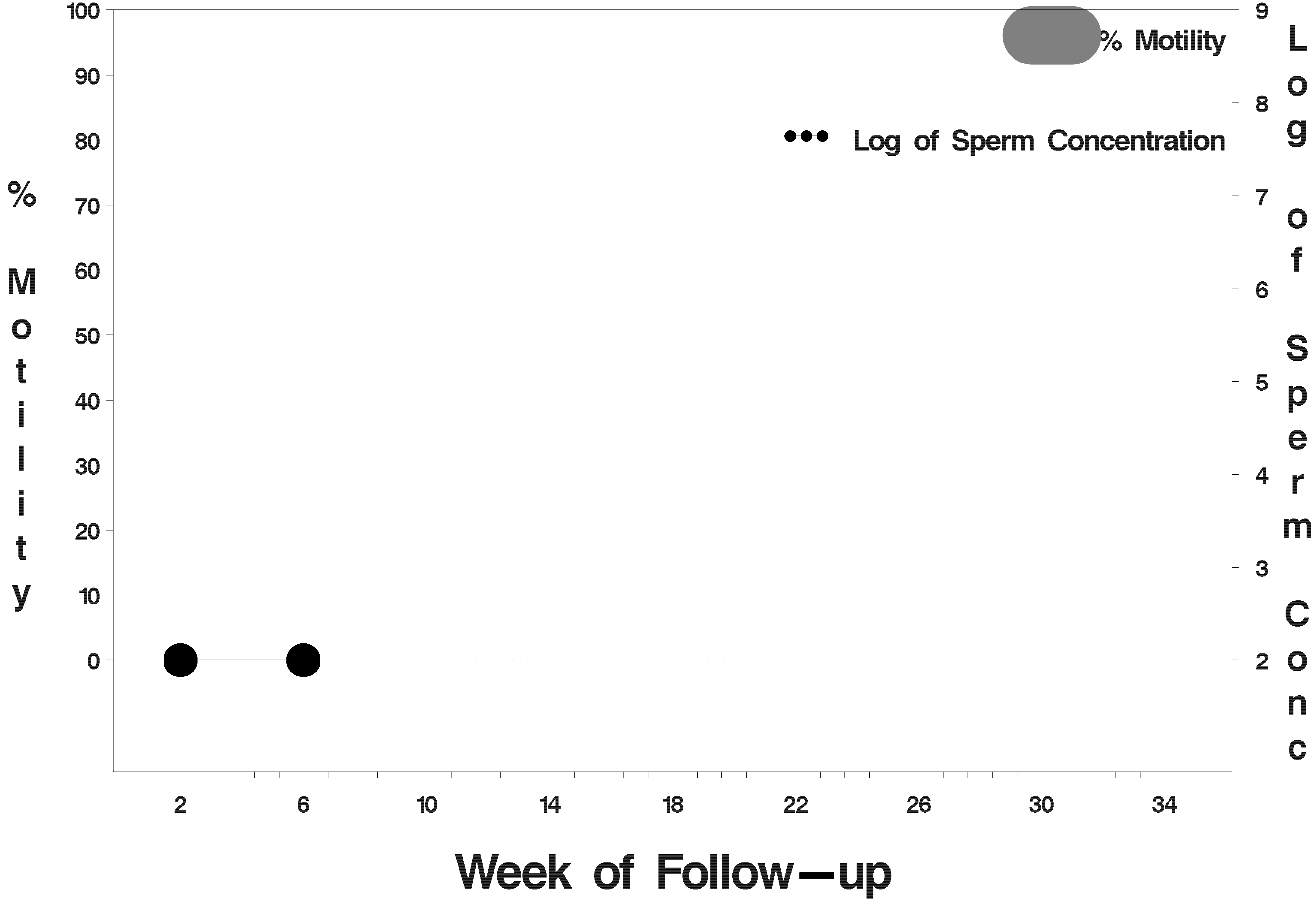

id= 701 outcome= Success

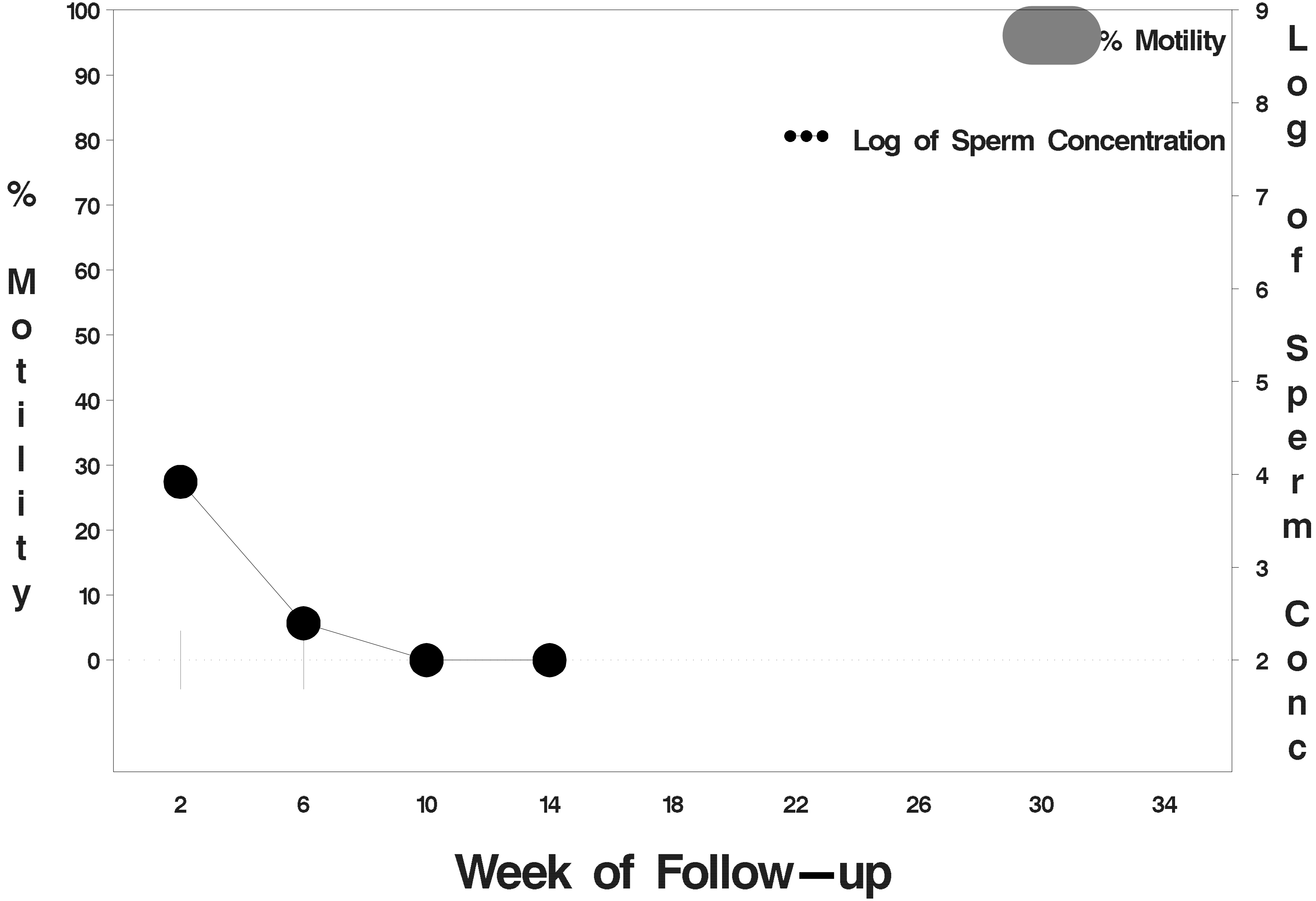

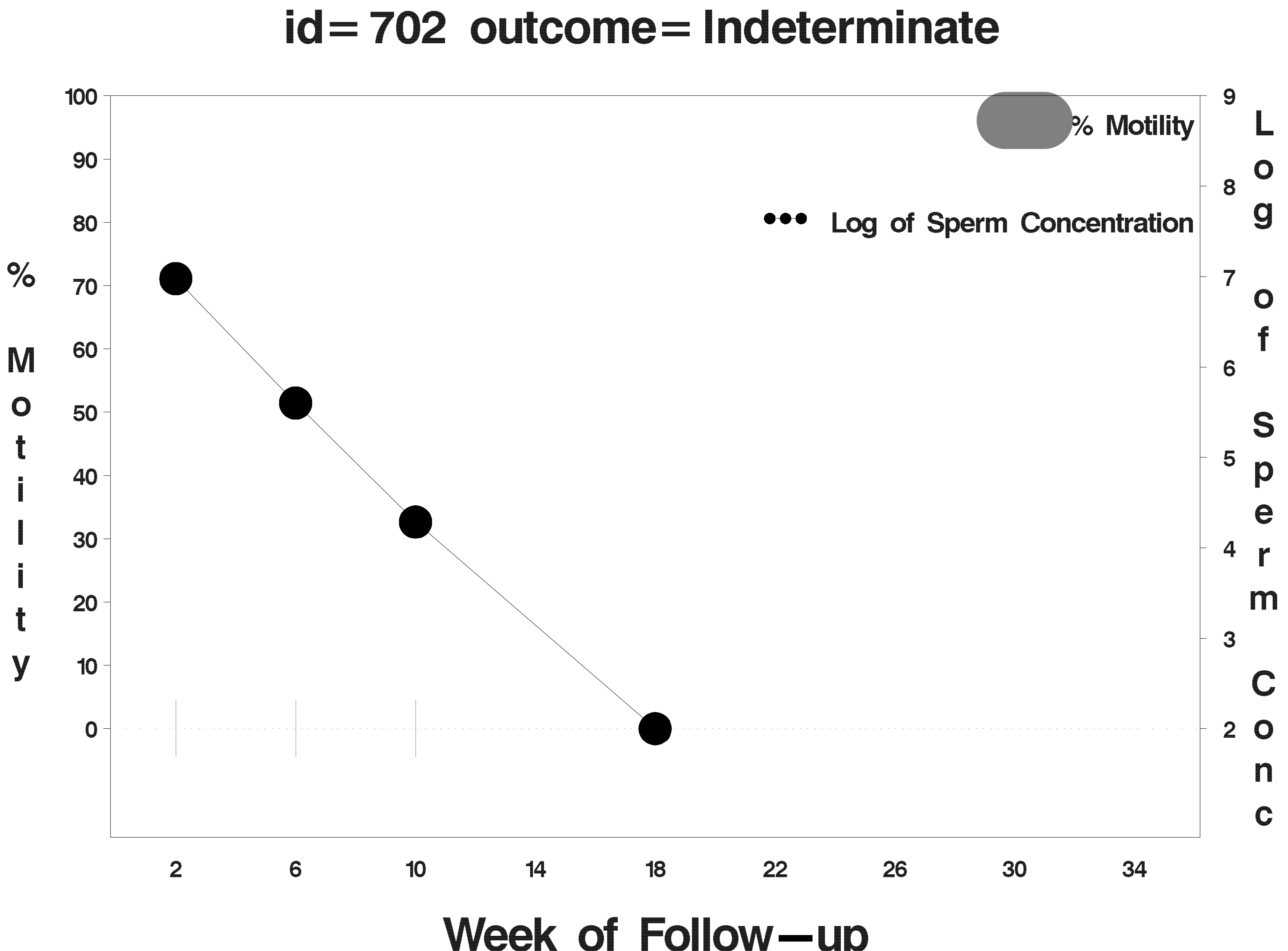

id= 702 outcome= Indeterminate
% Motility
Log of Sperm Concentration
% Motility
Log of Sperm Conc
100
90
80
70
60
50
40
30
20
10
0
9
8
7
6
5
4
3
2
2
6
10
14
18
22
26
30
34
Week of Follow−up

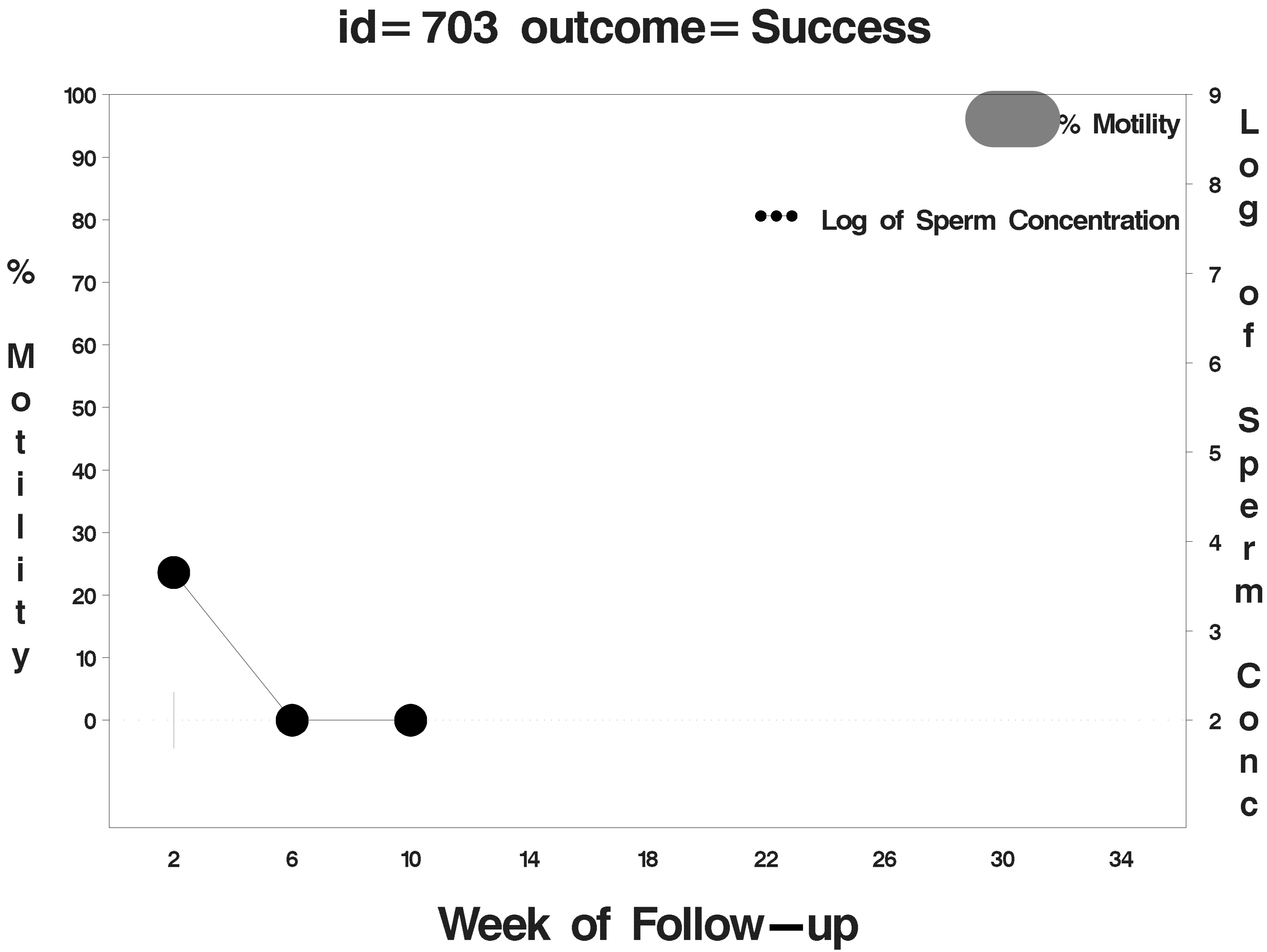

id= 703 outcome= Success
% Motility
Log of Sperm Concentration
% Motility
Log of Sperm Conc
Week of Follow-up
100
90
80
70
60
50
40
30
20
10
0
9
8
7
6
5
4
3
2
2
6
10
14
18
22
26
30
34

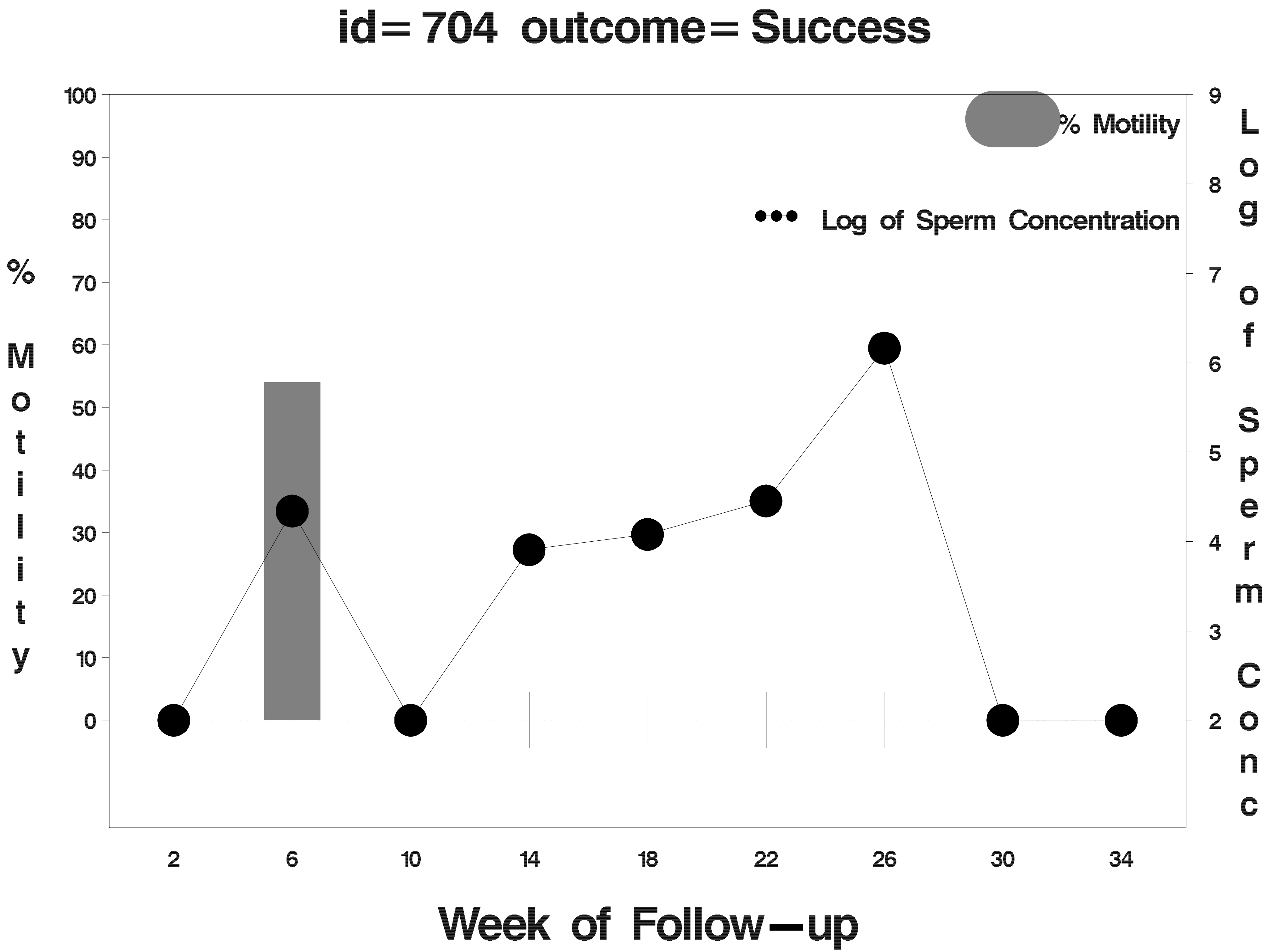
id= 704 outcome= Success
% Motility
Log of Sperm Concentration
% Motility
Log of Sperm Conc
Week of Follow−up
100
90
80
70
60
50
40
30
20
10
0
9
8
7
6
5
4
3
2
2
6
10
14
18
22
26
30
34

id= 705 outcome= Success

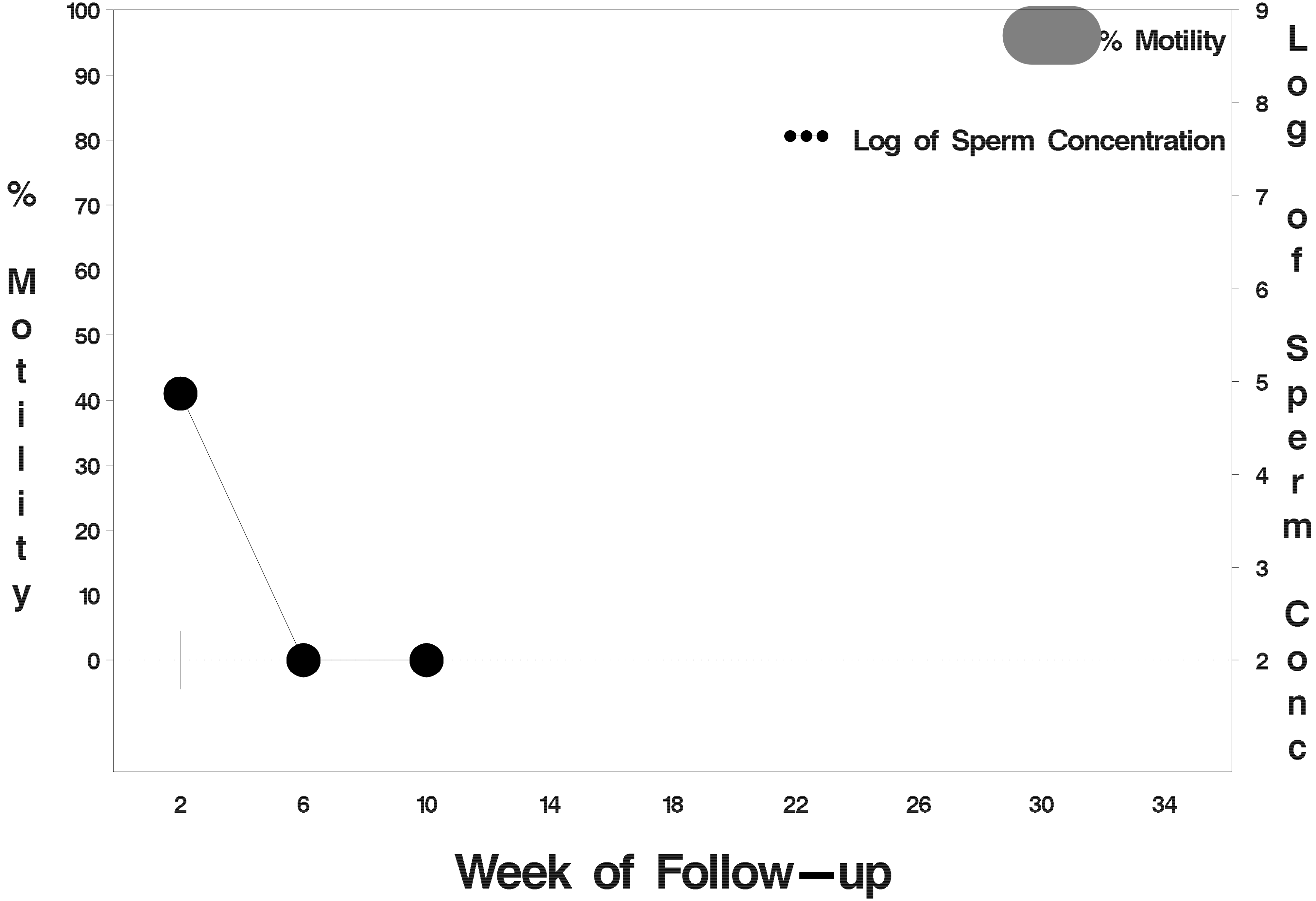

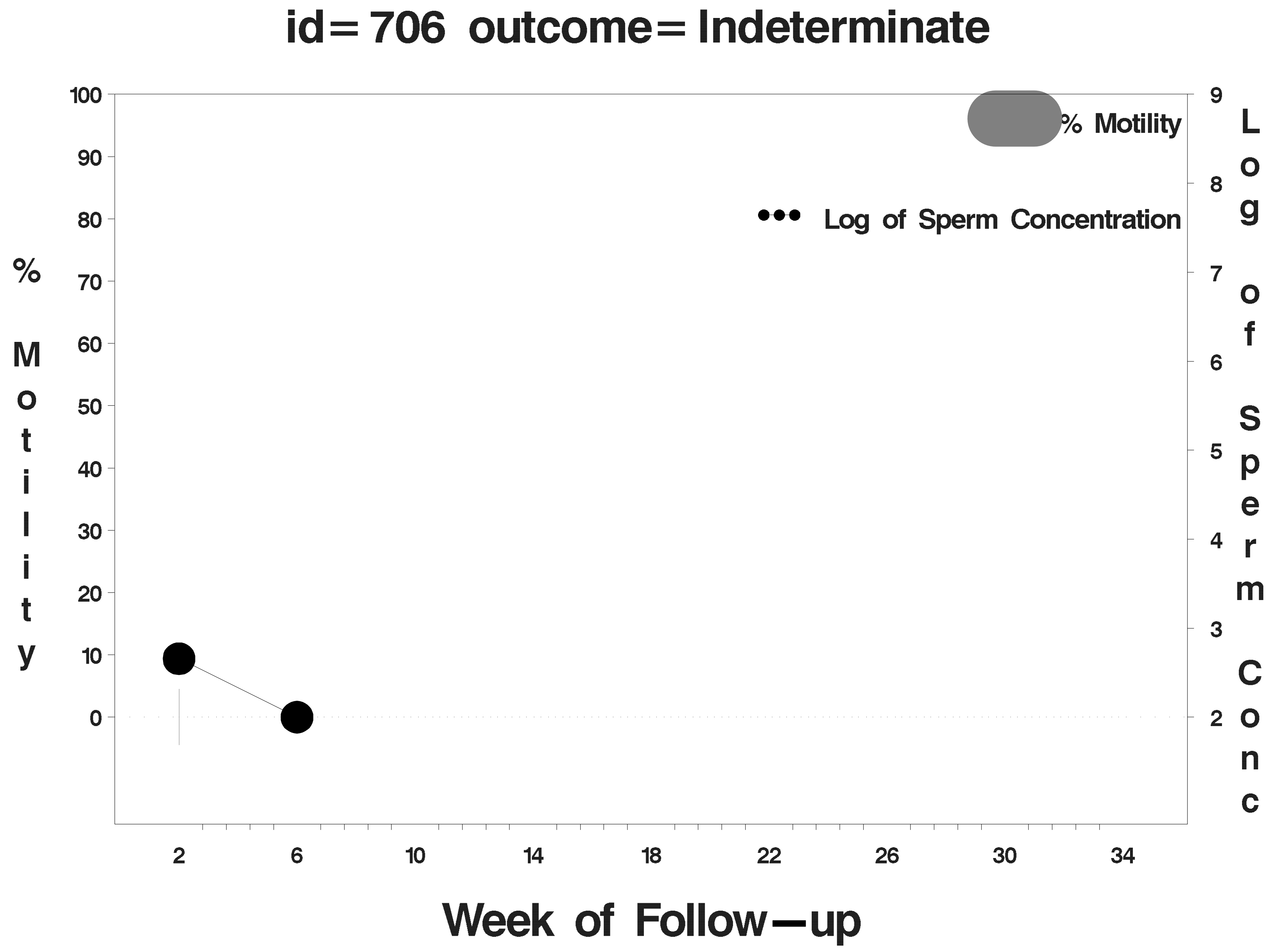
id= 706 outcome= Indeterminate
% Motility
Log of Sperm Concentration
100
90
80
70
60
50
40
30
20
10
0
9
8
7
6
5
4
3
2
2
6
10
14
18
22
26
30
34
Week of Follow—up
% Motility
Log of Sperm Conc

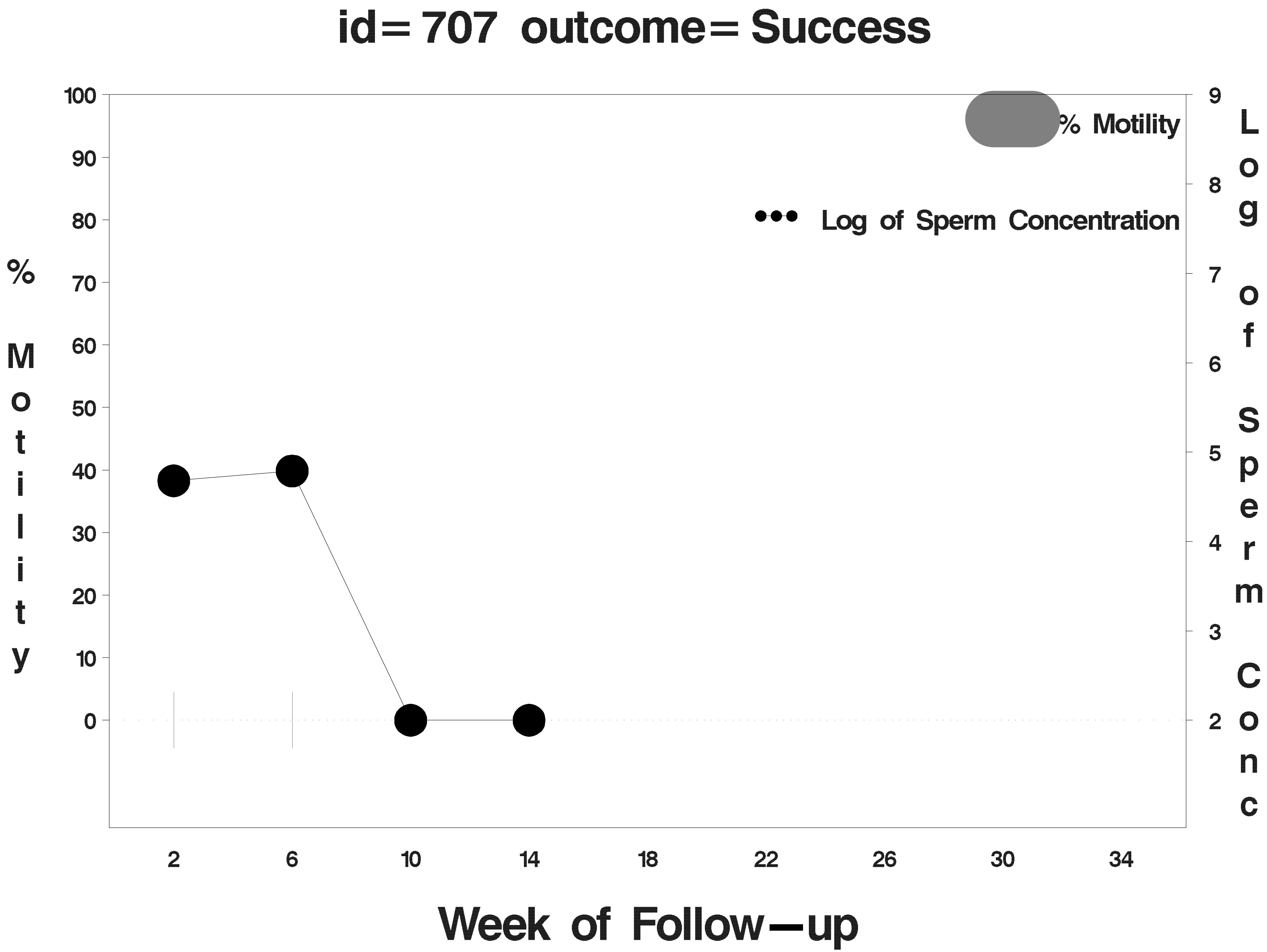
id= 707 outcome= Success
% Motility
Log of Sperm Concentration
100
90
80
70
60
50
40
30
20
10
0
9
8
7
6
5
4
3
2
2
6
10
14
18
22
26
30
34
Week of Follow−up
% Motility
Log of Sperm Conc

id= 708 outcome= Indeterminate

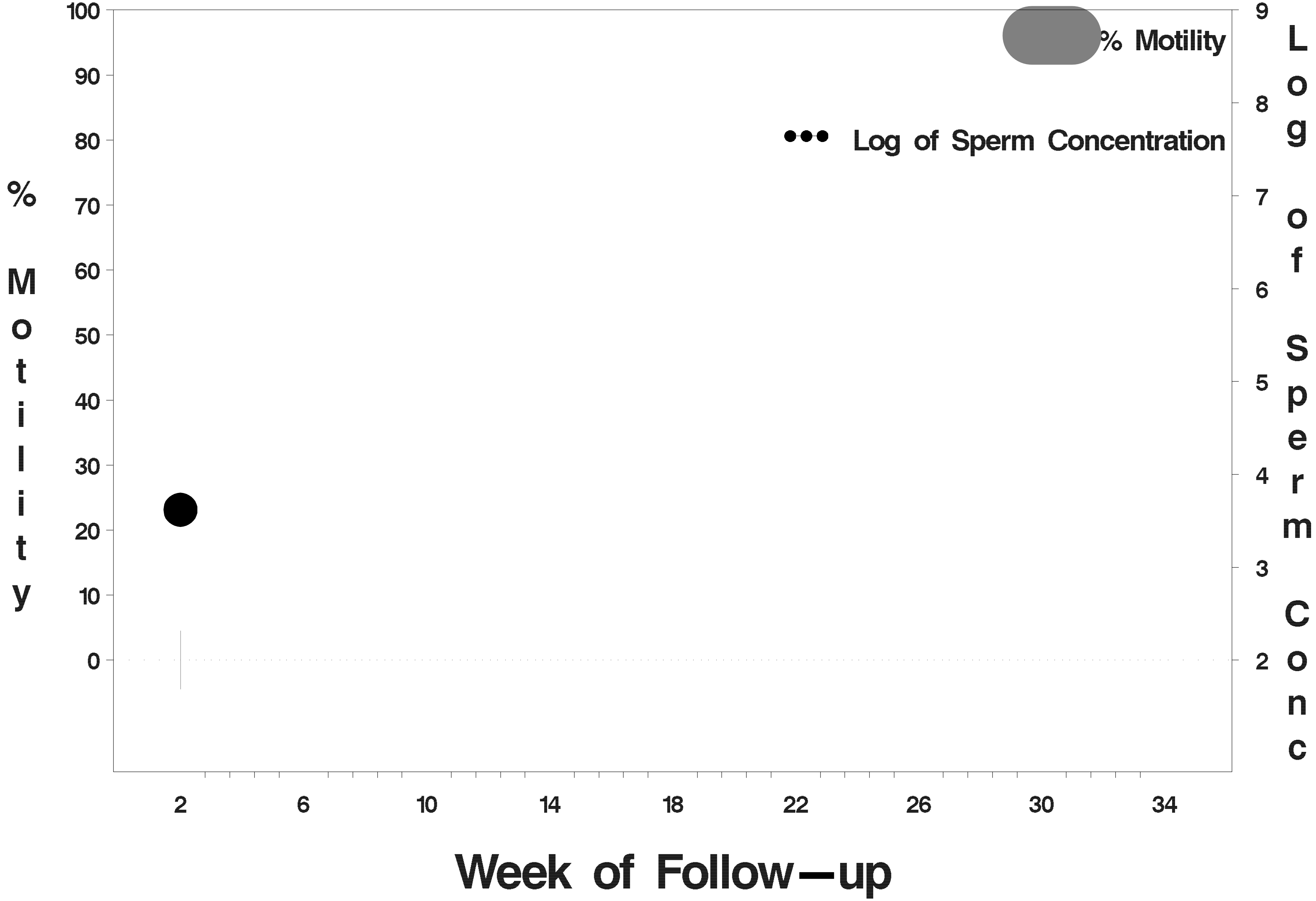

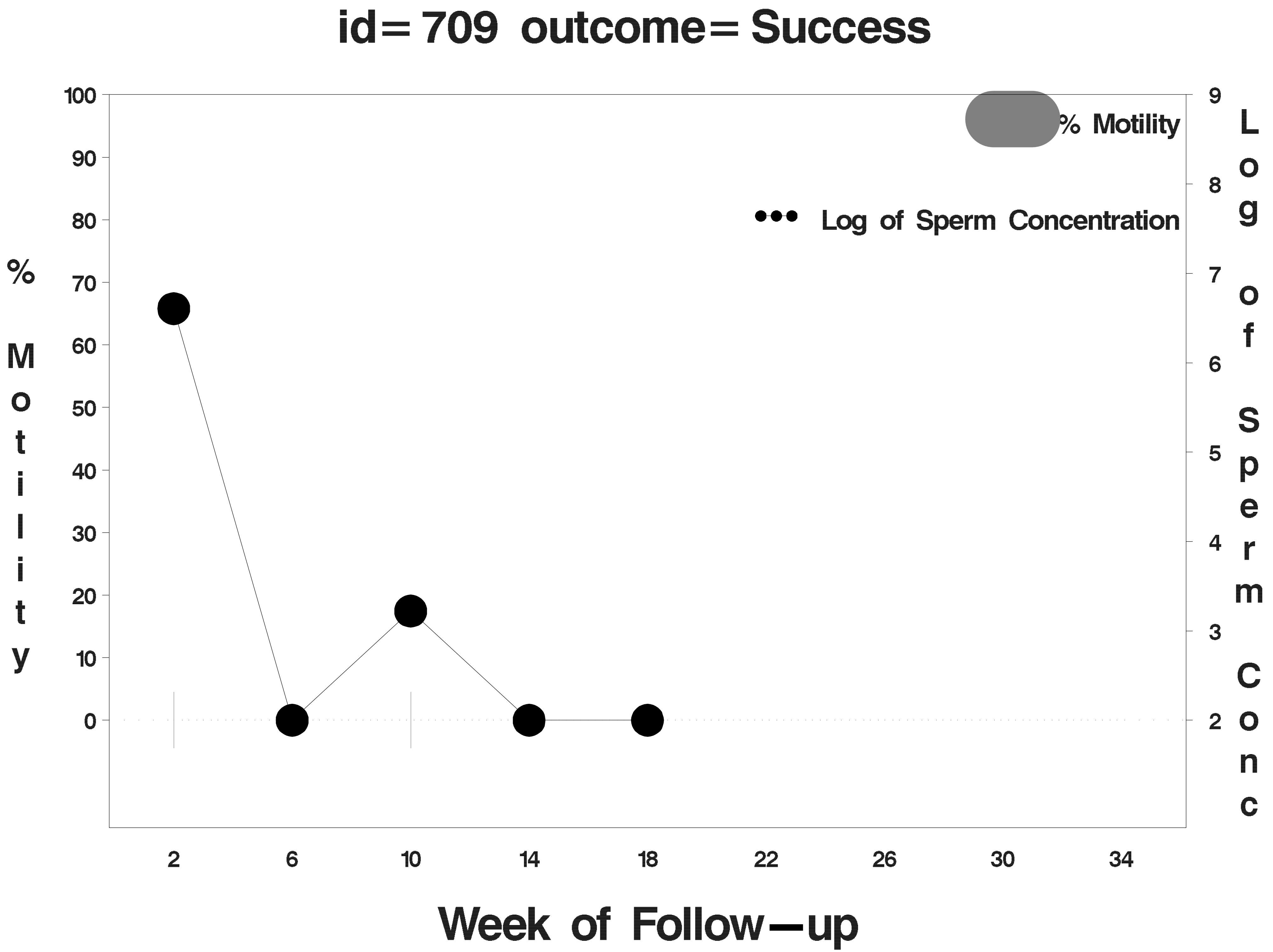
id= 709 outcome= Success
% Motility
Log of Sperm Concentration
% Motility
Log of Sperm Conc
Week of Follow—up
100
90
80
70
60
50
40
30
20
10
0
9
8
7
6
5
4
3
2
2
6
10
14
18
22
26
30
34

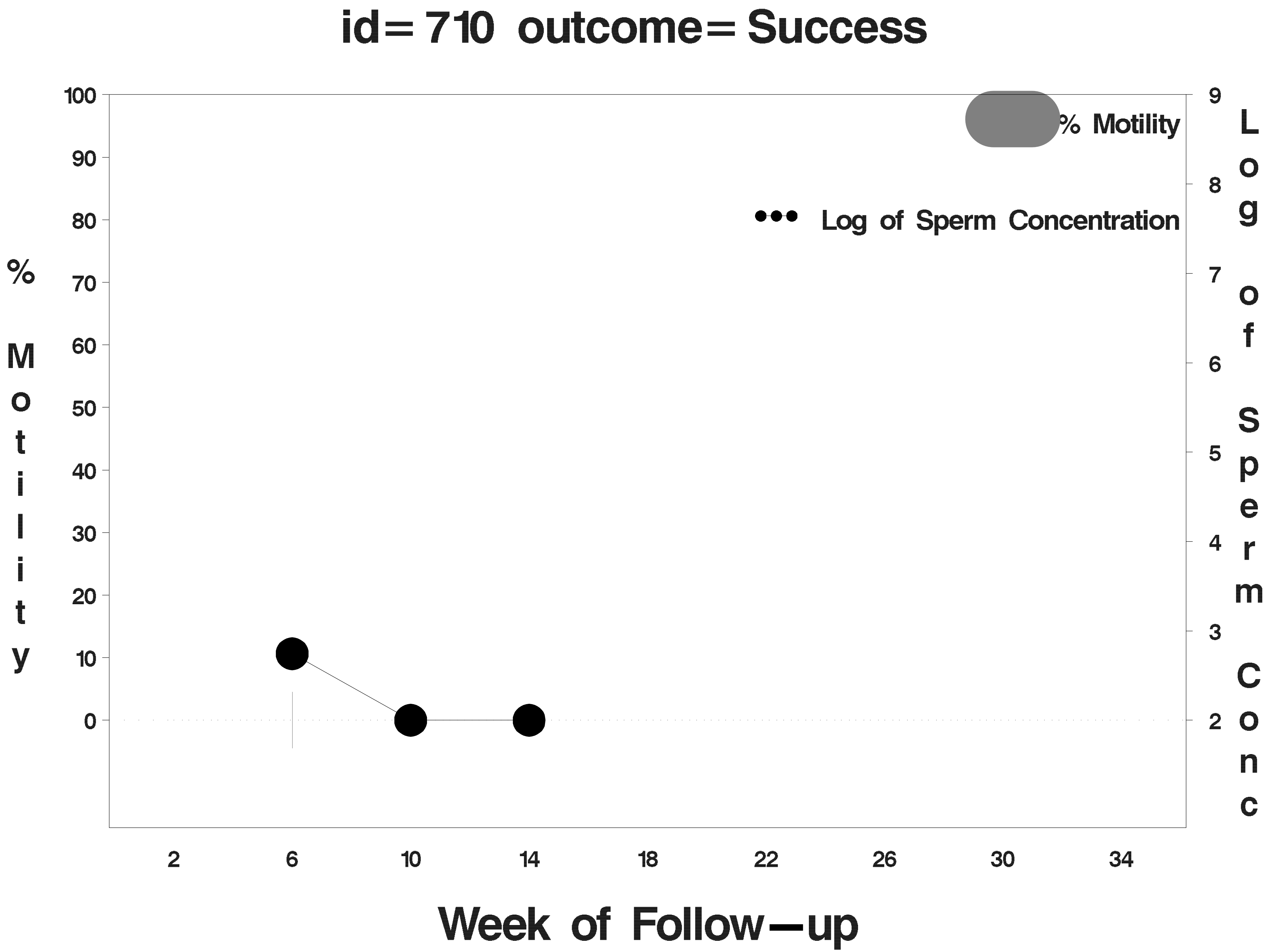
id= 710 outcome= Success
% Motility
Log of Sperm Concentration
100
90
80
70
60
50
40
30
20
10
0
9
8
7
6
5
4
3
2
2
6
10
14
18
22
26
30
34
Week of Follow—up
% Motility
Log of Sperm Conc

id=711 outcome=Success

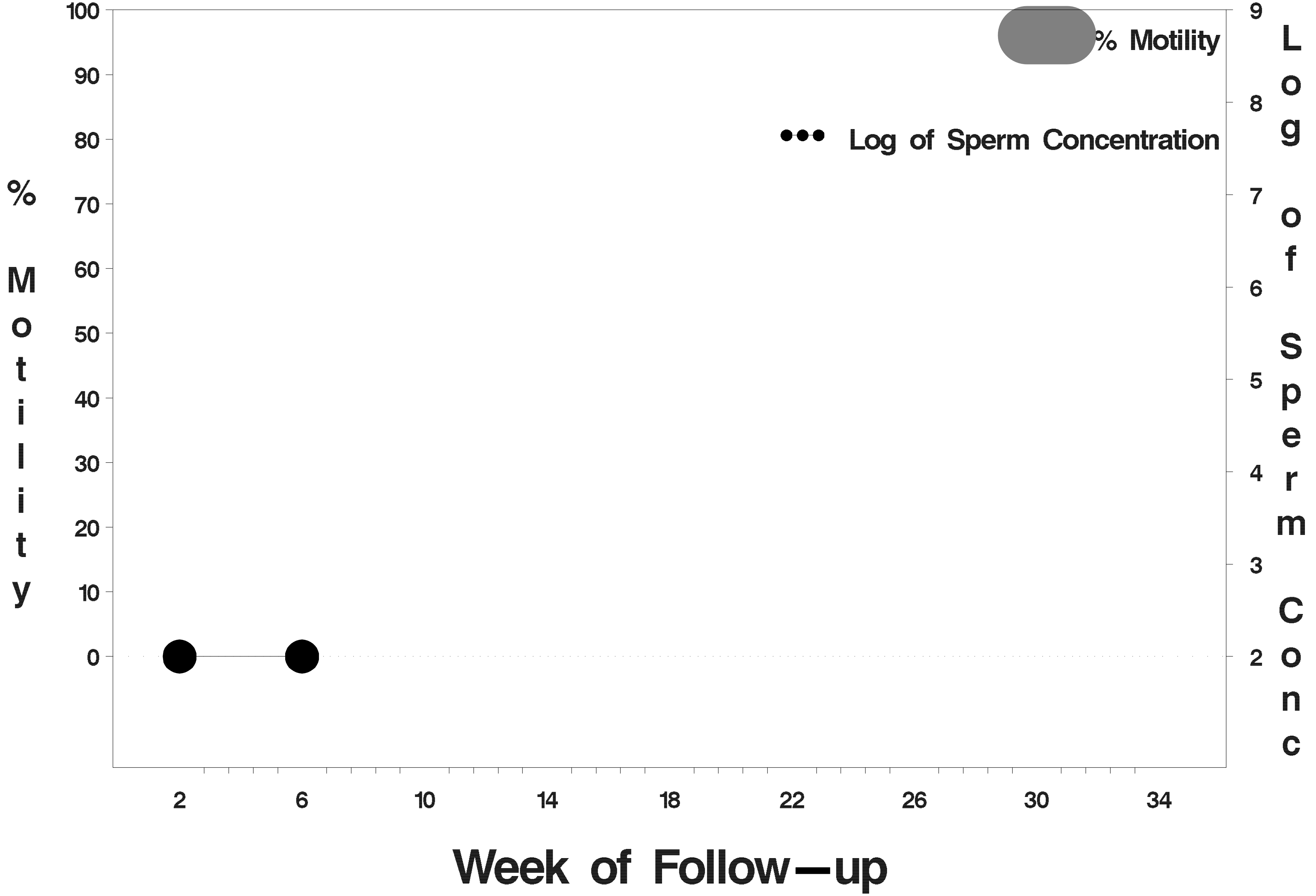

id= 712 outcome= Success

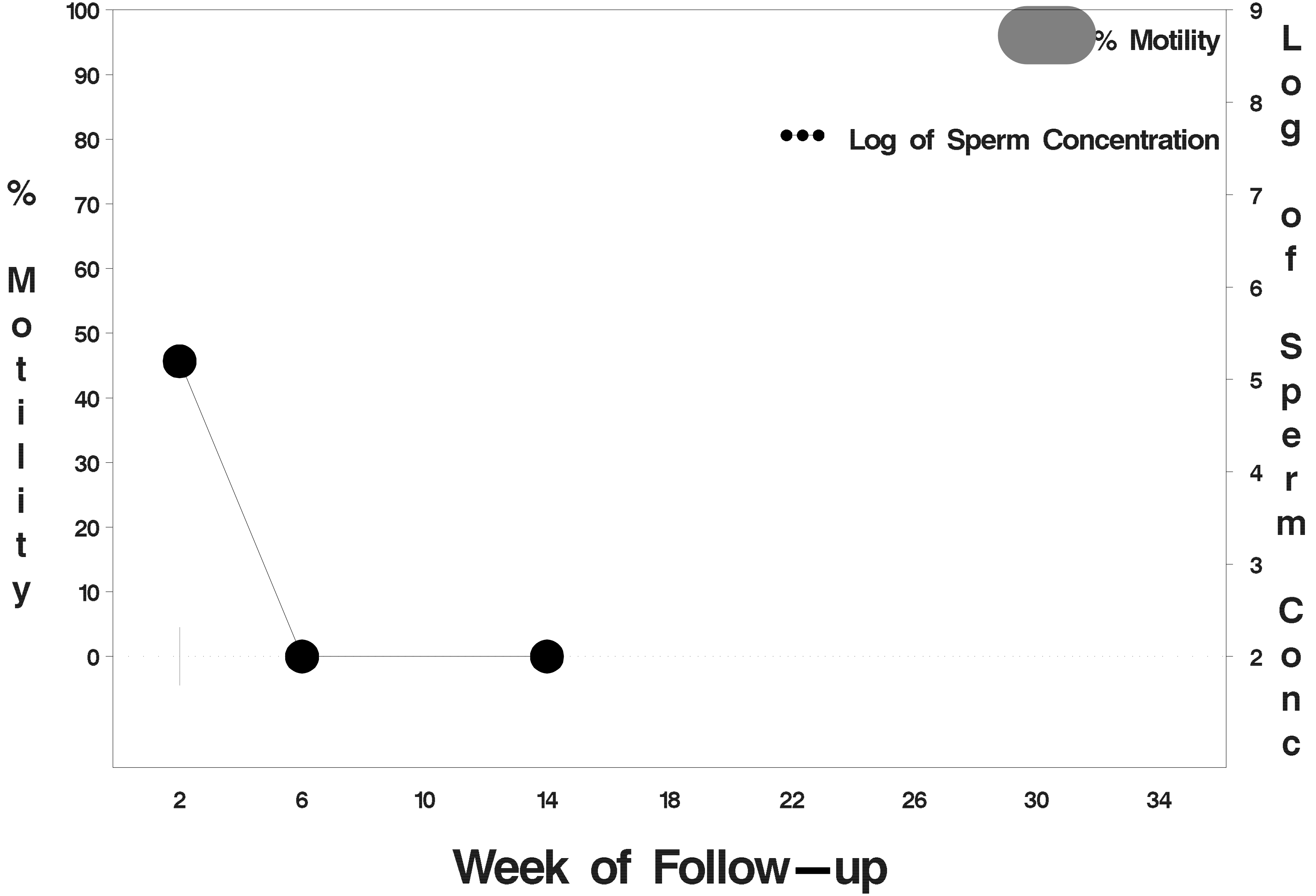

id= 713 outcome= Success

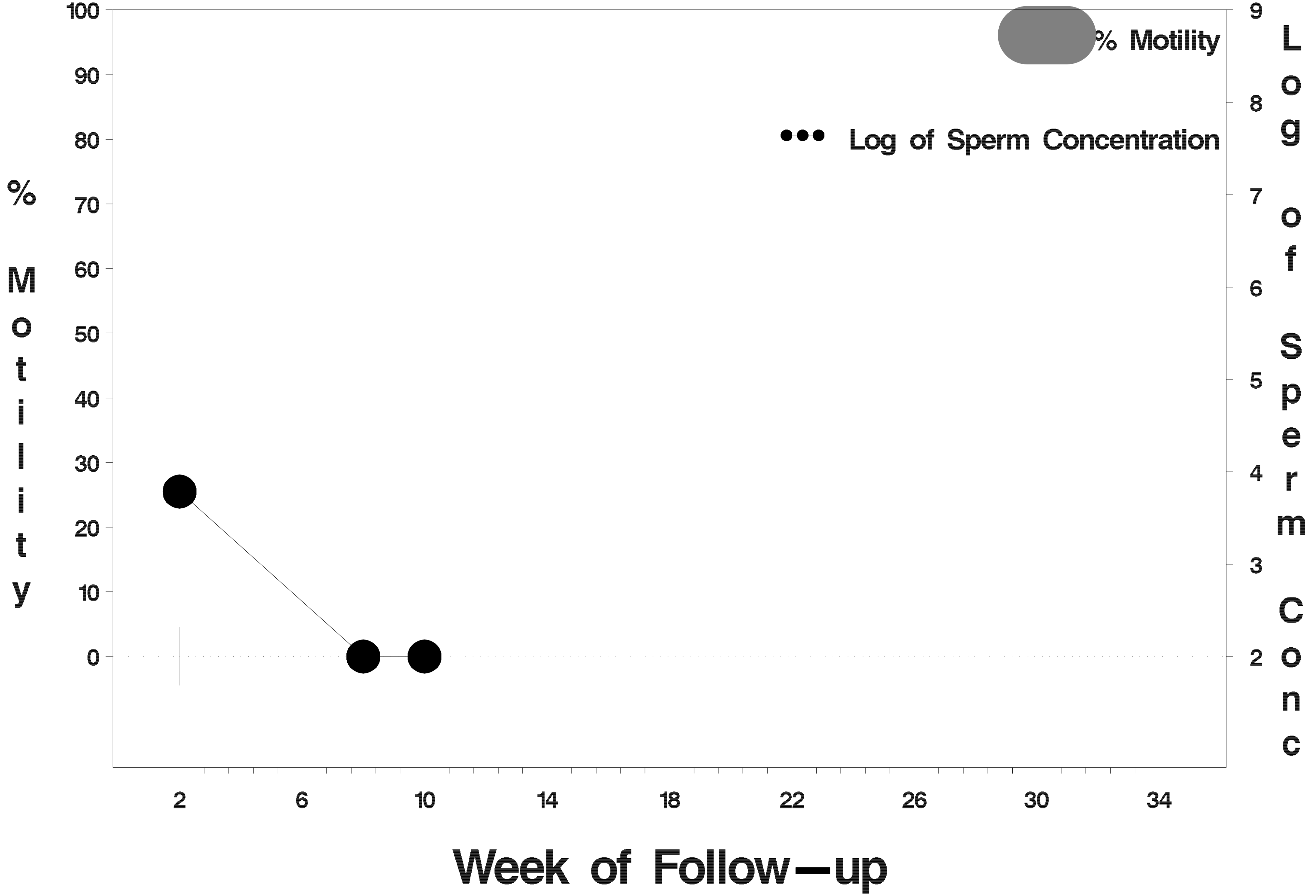

id= 714 outcome= Success

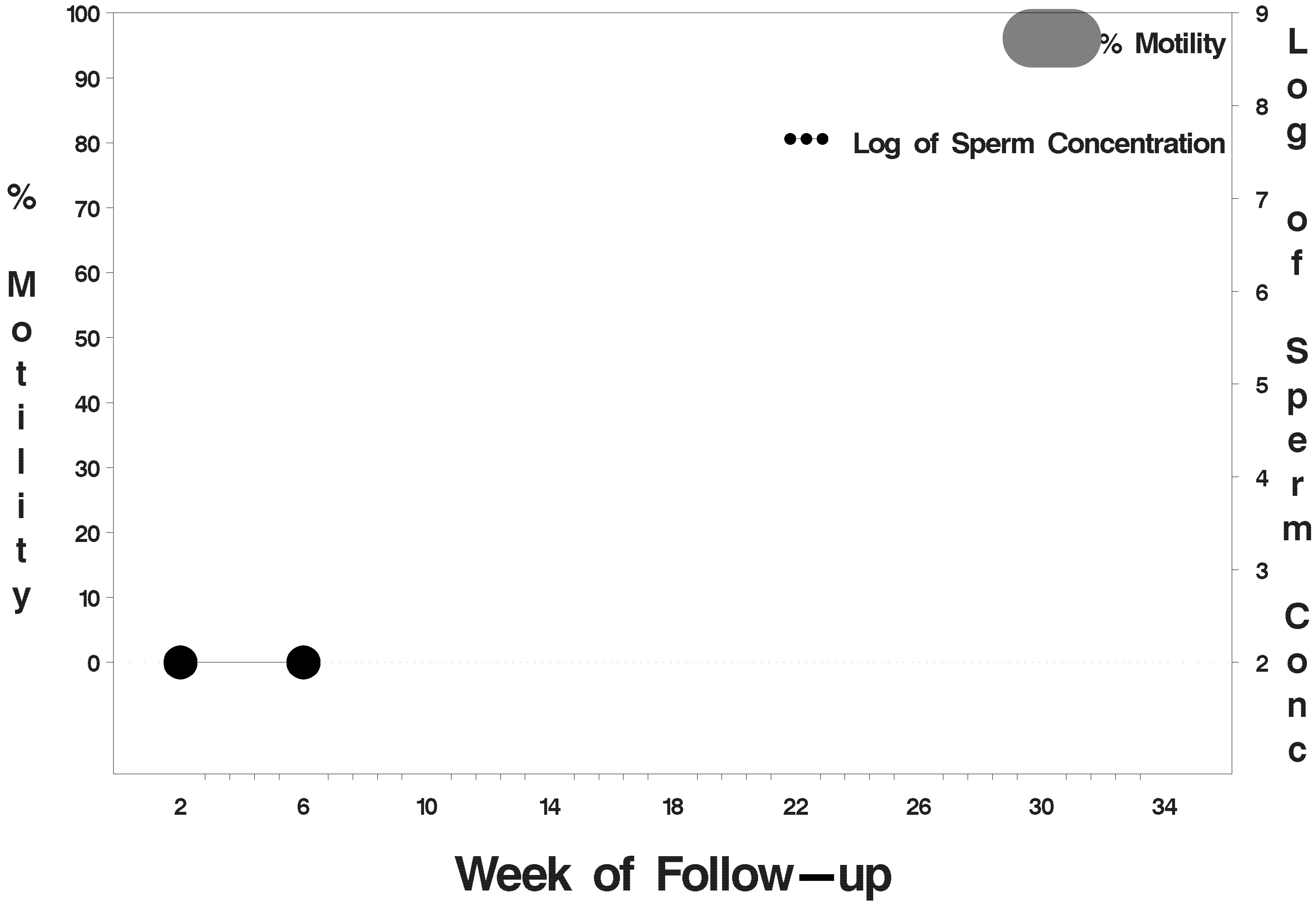

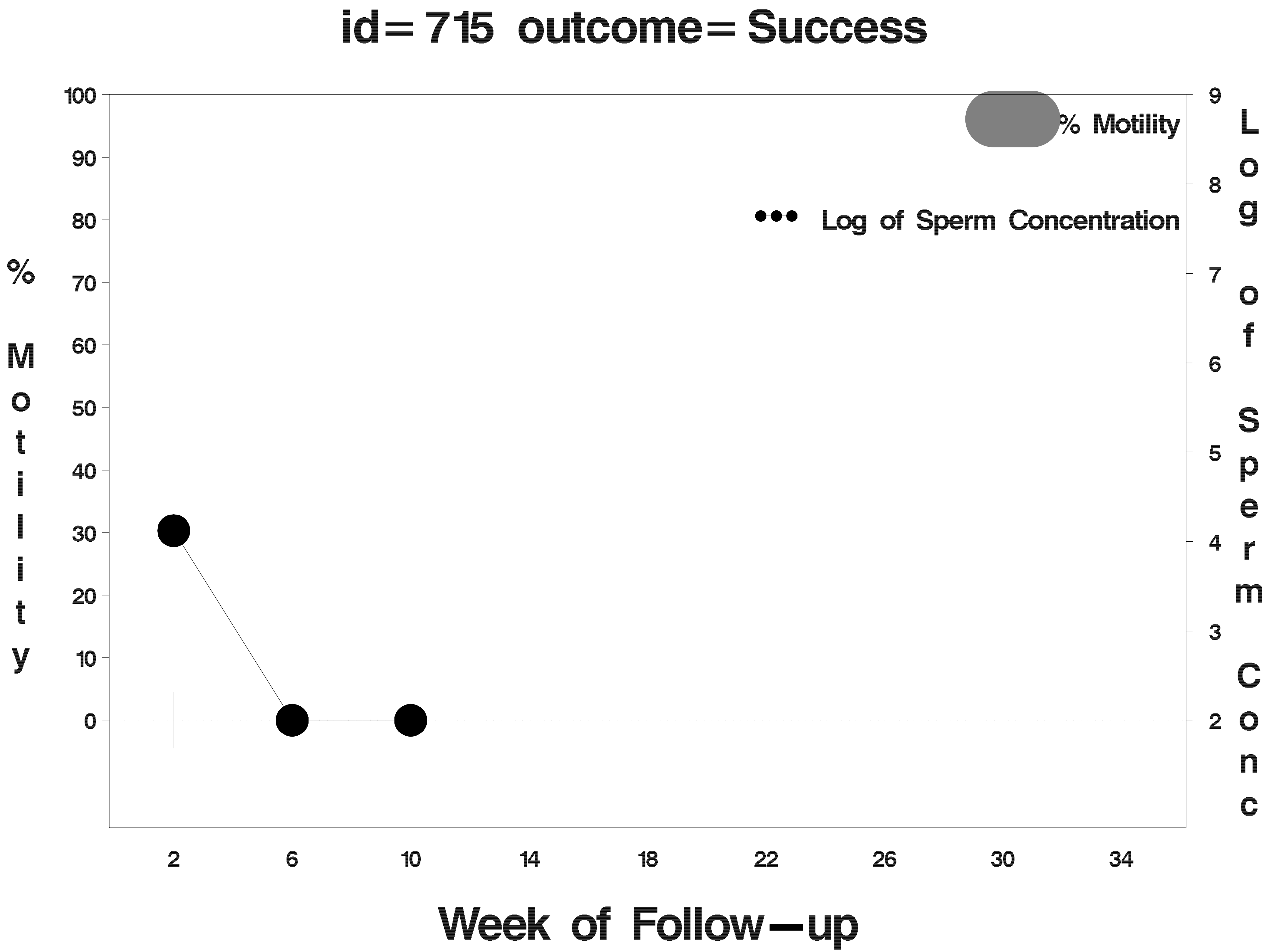
id= 715 outcome= Success
% Motility
Log of Sperm Concentration
100
90
80
70
60
50
40
30
20
10
0
9
8
7
6
5
4
3
2
% Motility
Log of Sperm Conc
2
6
10
14
18
22
26
30
34
Week of Follow−up

id= 716 outcome= Success

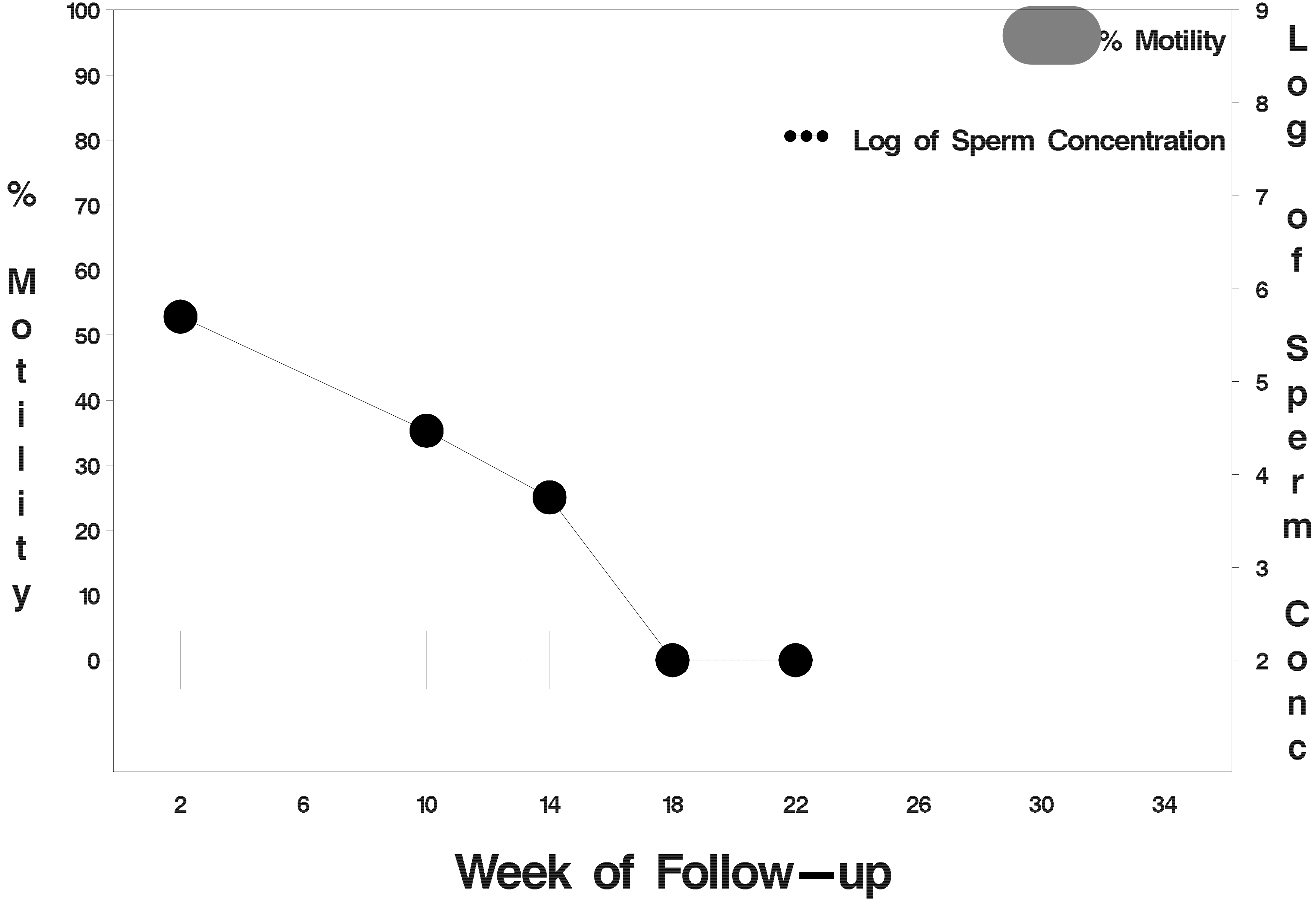

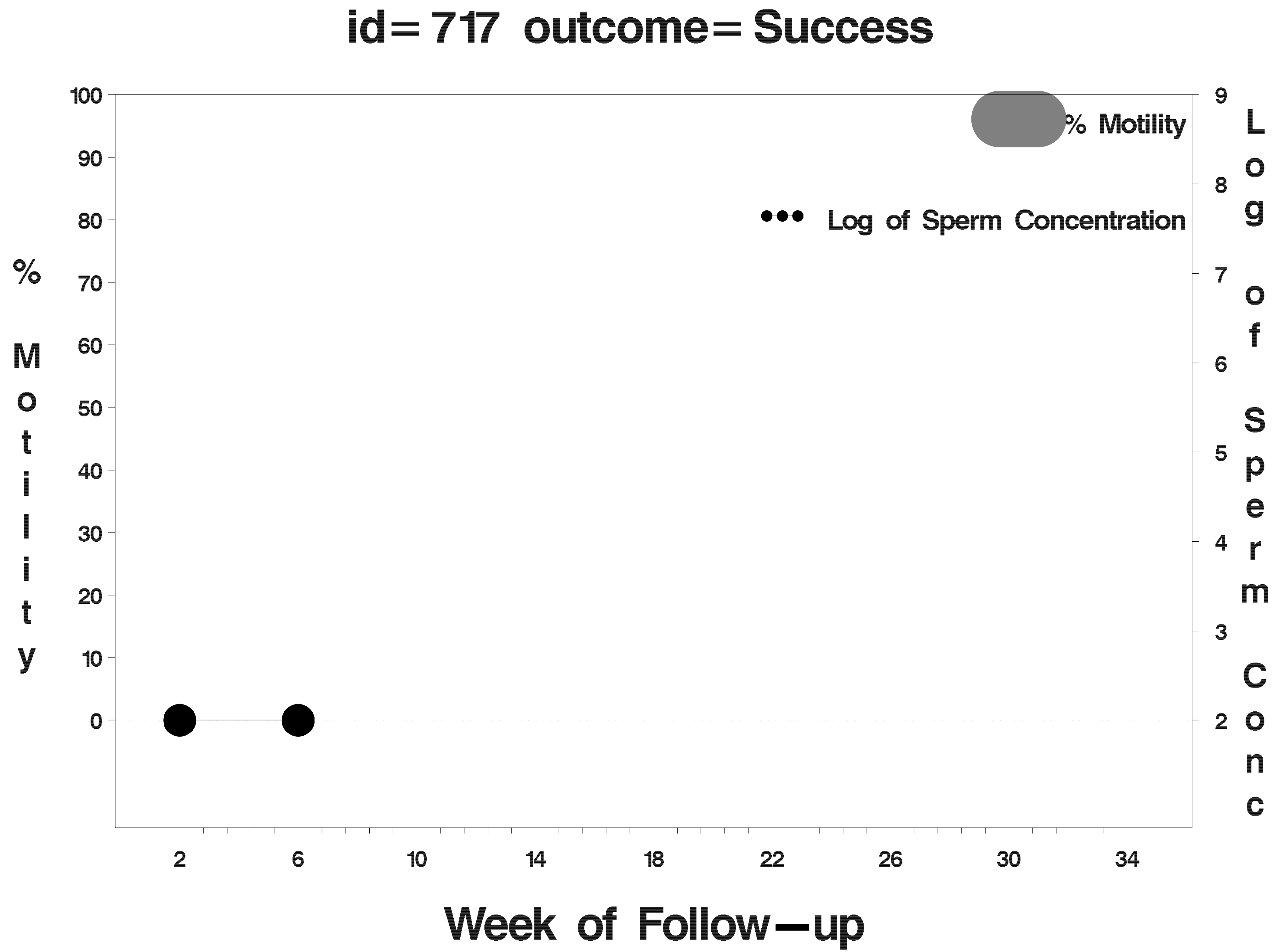
id= 717 outcome= Success
% Motility
Log of Sperm Concentration
100
90
80
70
60
50
40
30
20
10
0
9
8
7
6
5
4
3
2
2
6
10
14
18
22
26
30
34
Week of Follow—up
% Motility
Log of Sperm Conc

id= 718 outcome= Success

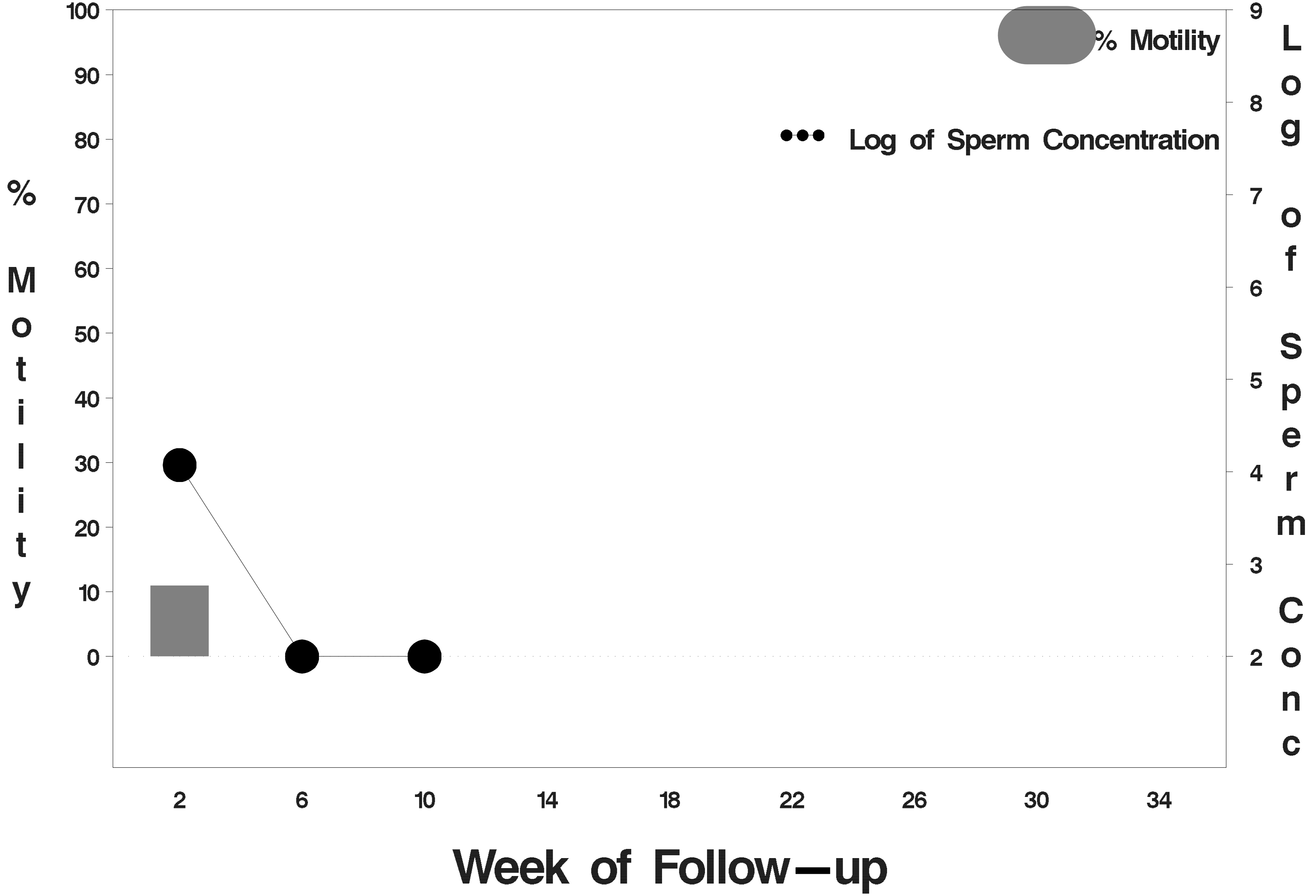

id= 719 outcome= Success

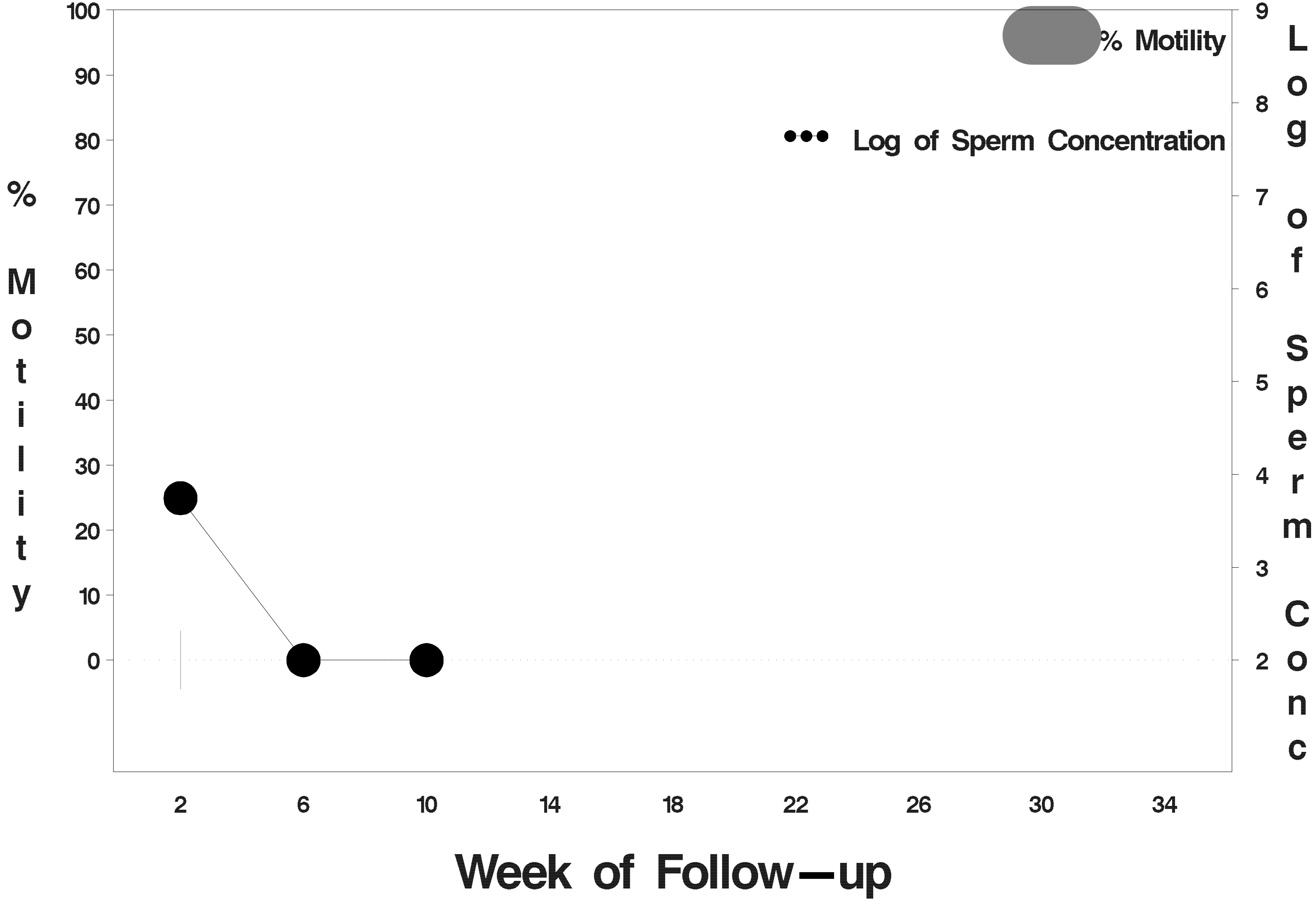

id= 720 outcome= Success

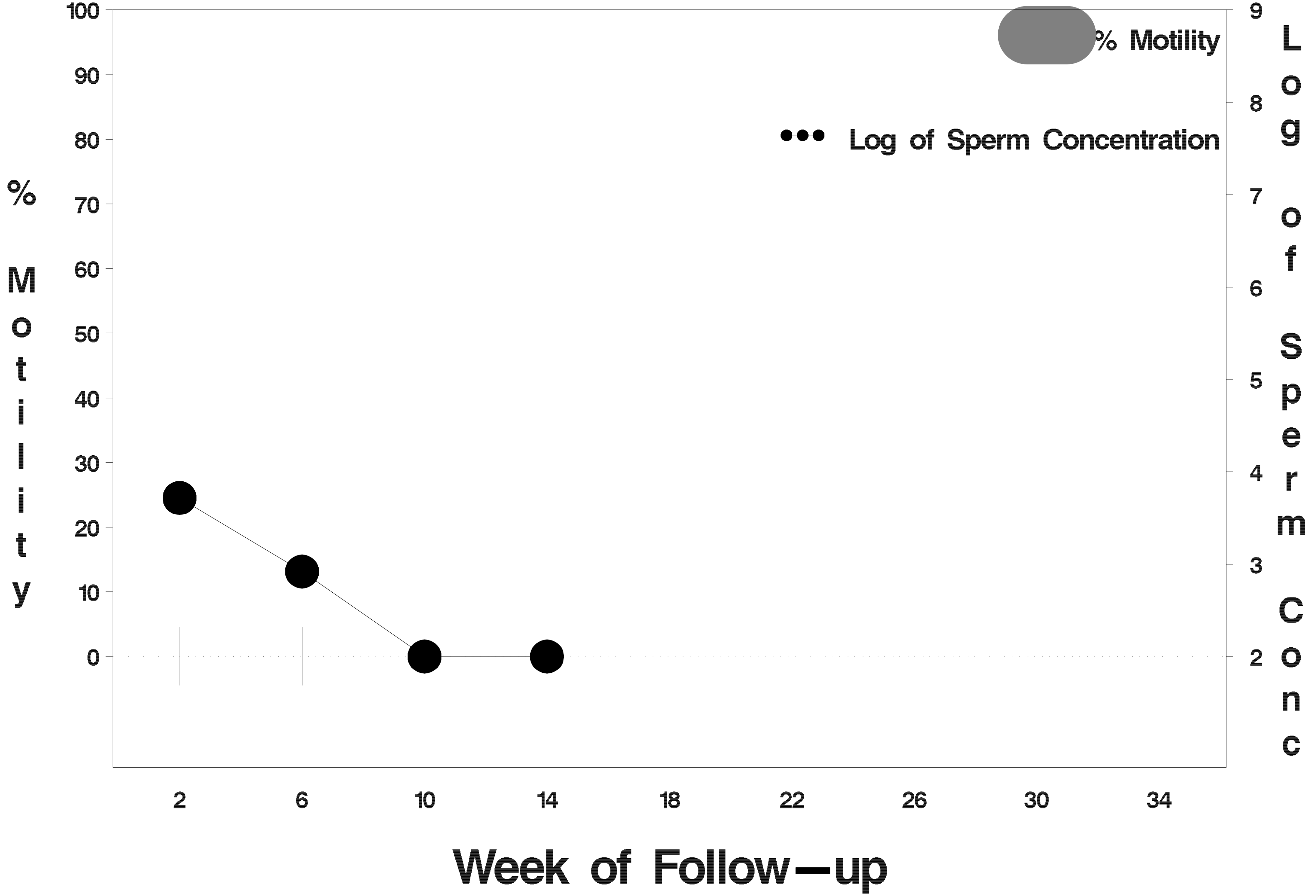

id= 721 outcome= Success

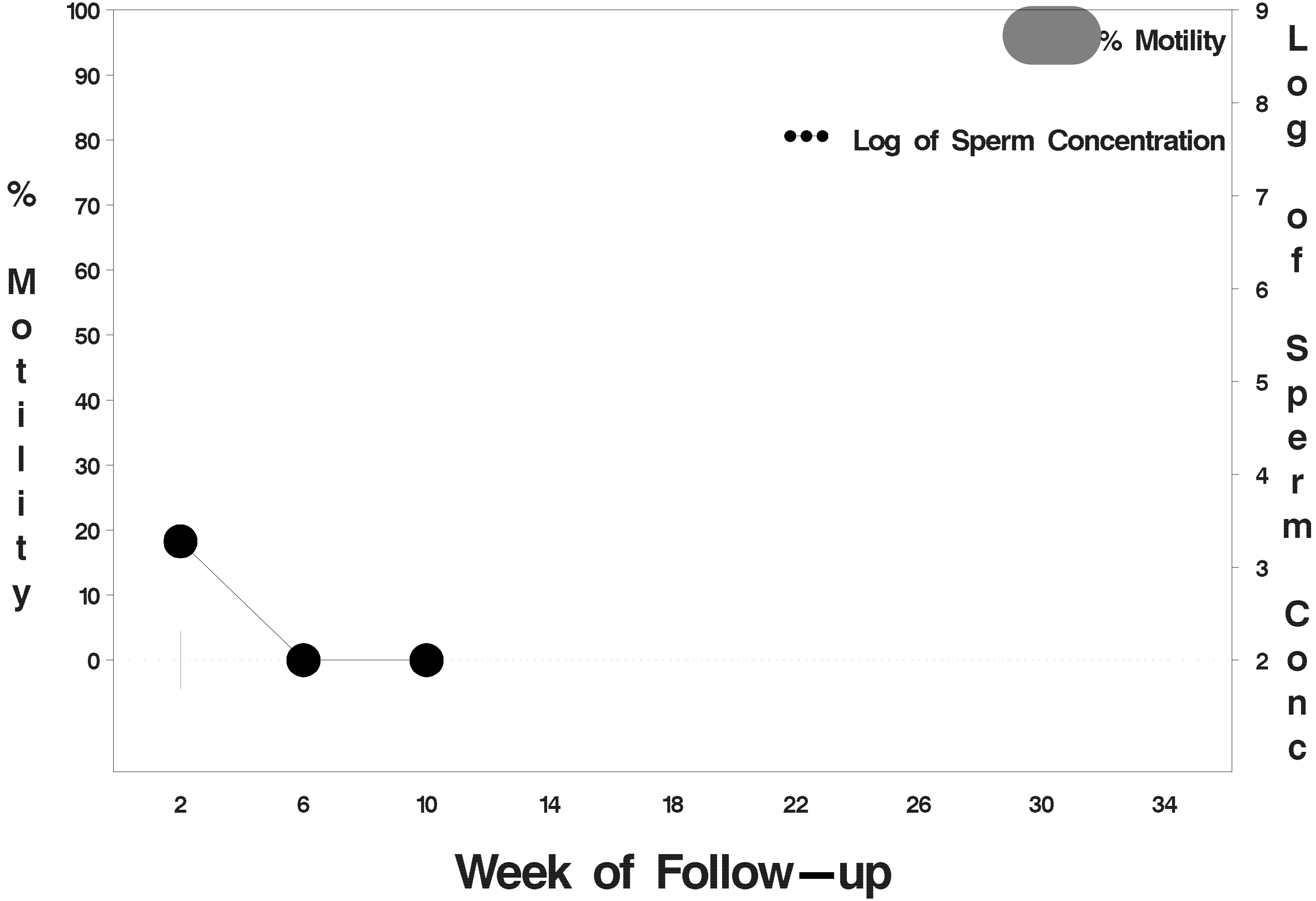

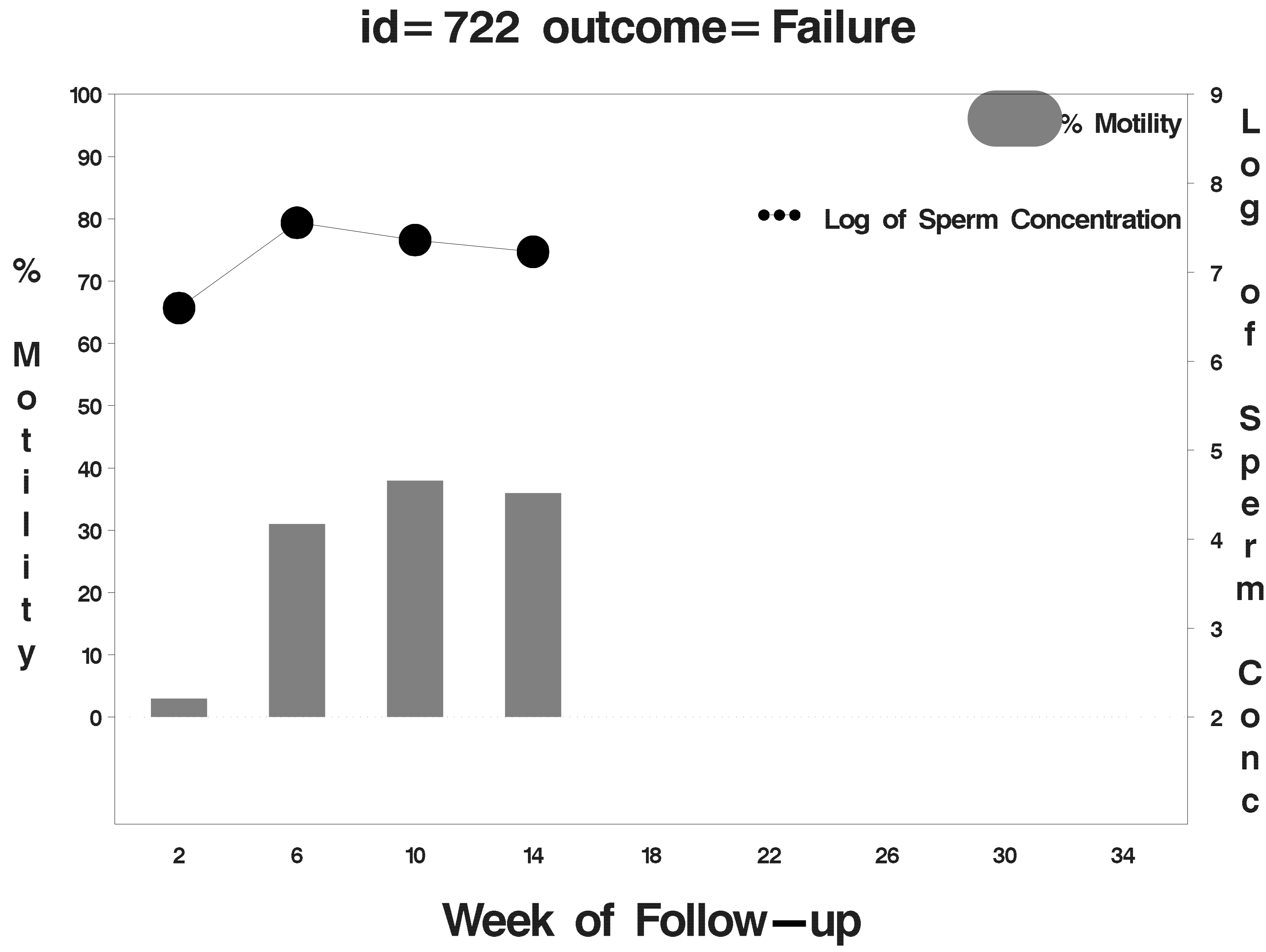

id= 722 outcome= Failure
% Motility
Log of Sperm Concentration
% Motility
Log of Sperm Conc
Week of Follow−up
100
90
80
70
60
50
40
30
20
10
0
9
8
7
6
5
4
3
2
2
6
10
14
18
22
26
30
34

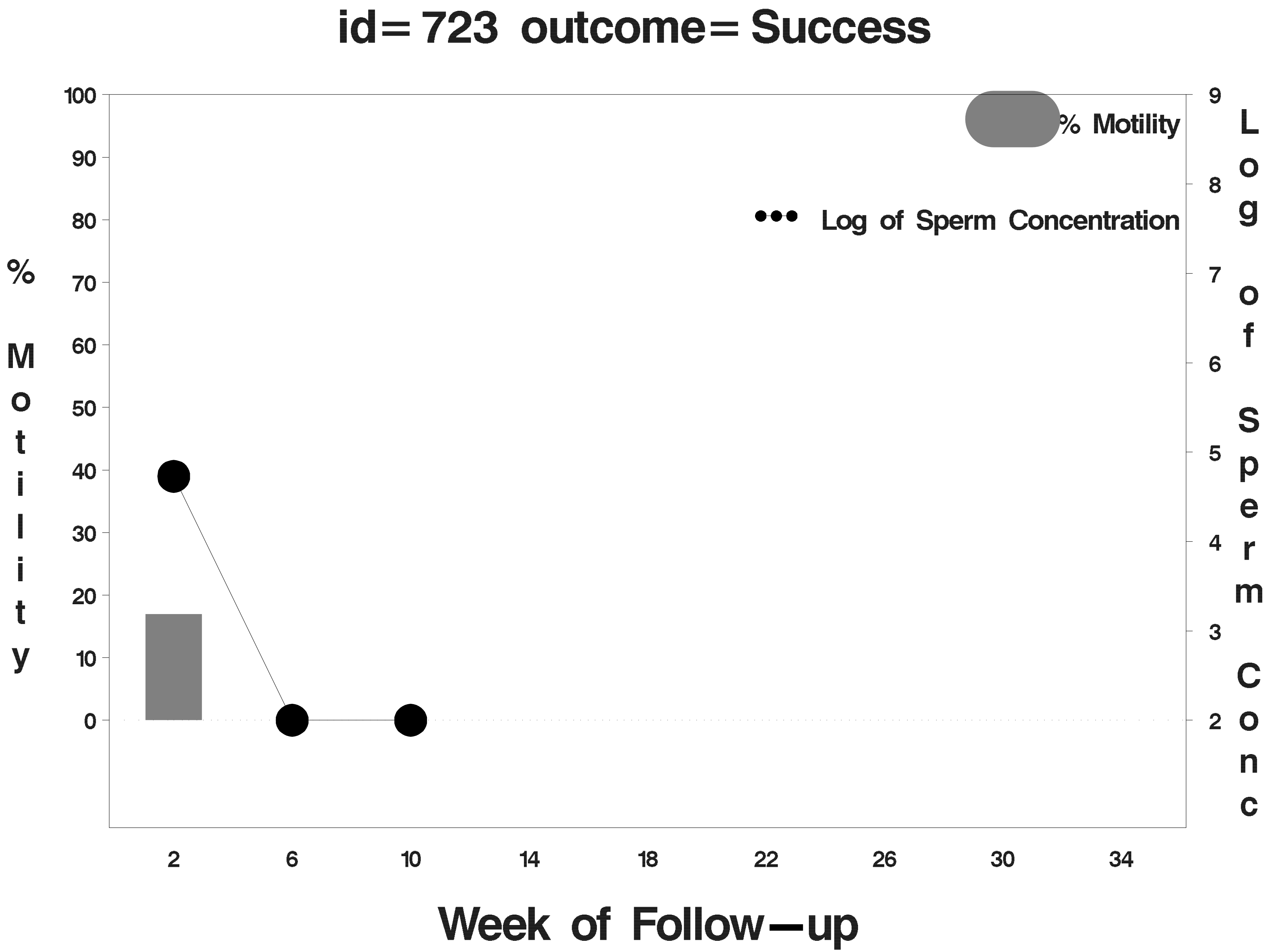
id= 723 outcome= Success
% Motility
Log of Sperm Concentration
100
90
80
70
60
50
40
30
20
10
0
9
8
7
6
5
4
3
2
2
6
10
14
18
22
26
30
34
Week of Follow—up
Log of Sperm Conc

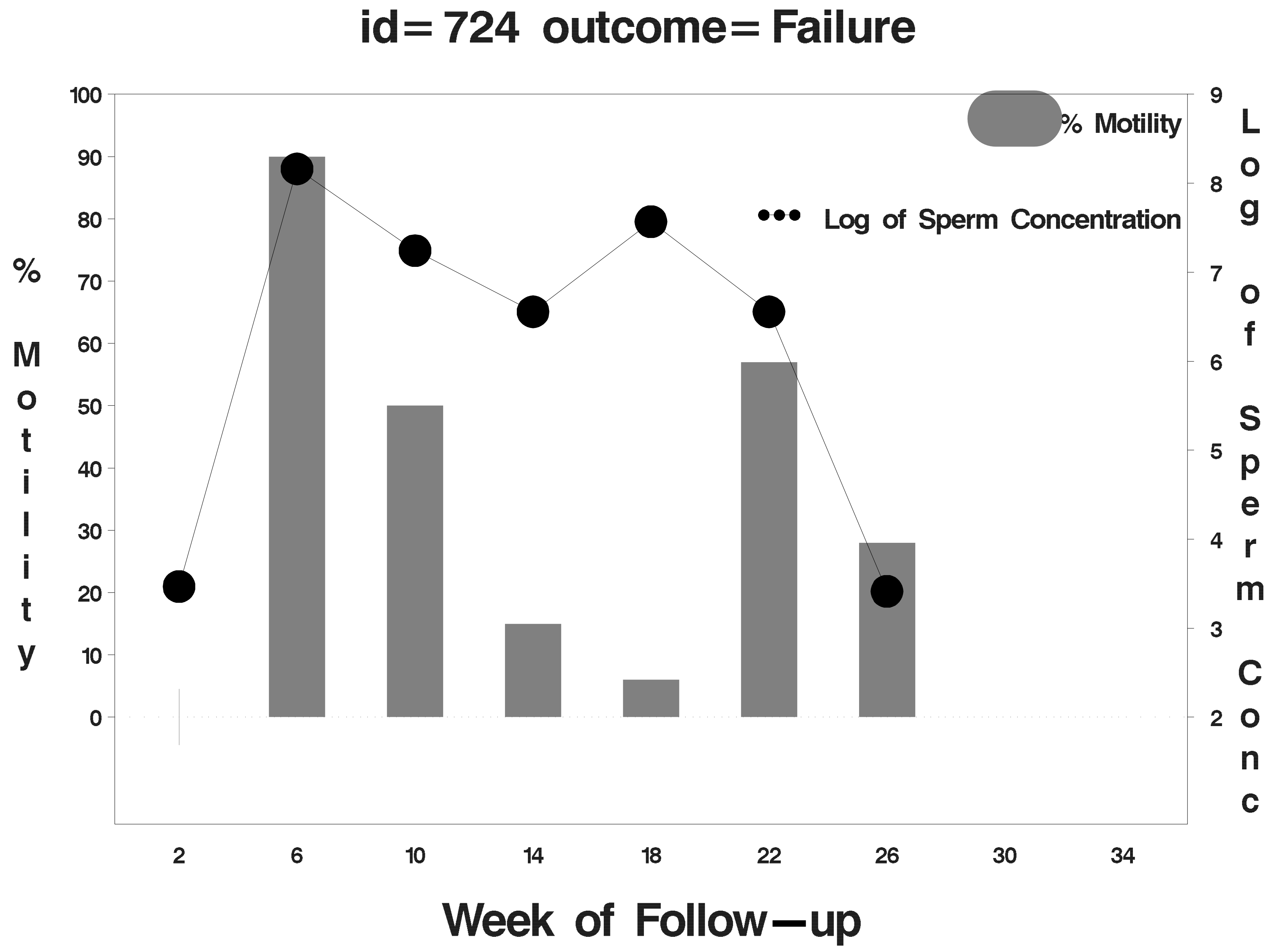

id= 724 outcome= Failure
% Motility
Log of Sperm Concentration
100
90
80
70
60
50
40
30
20
10
0
9
8
7
6
5
4
3
2
% Motility
Log of Sperm Conc
2
6
10
14
18
22
26
30
34
Week of Follow—up

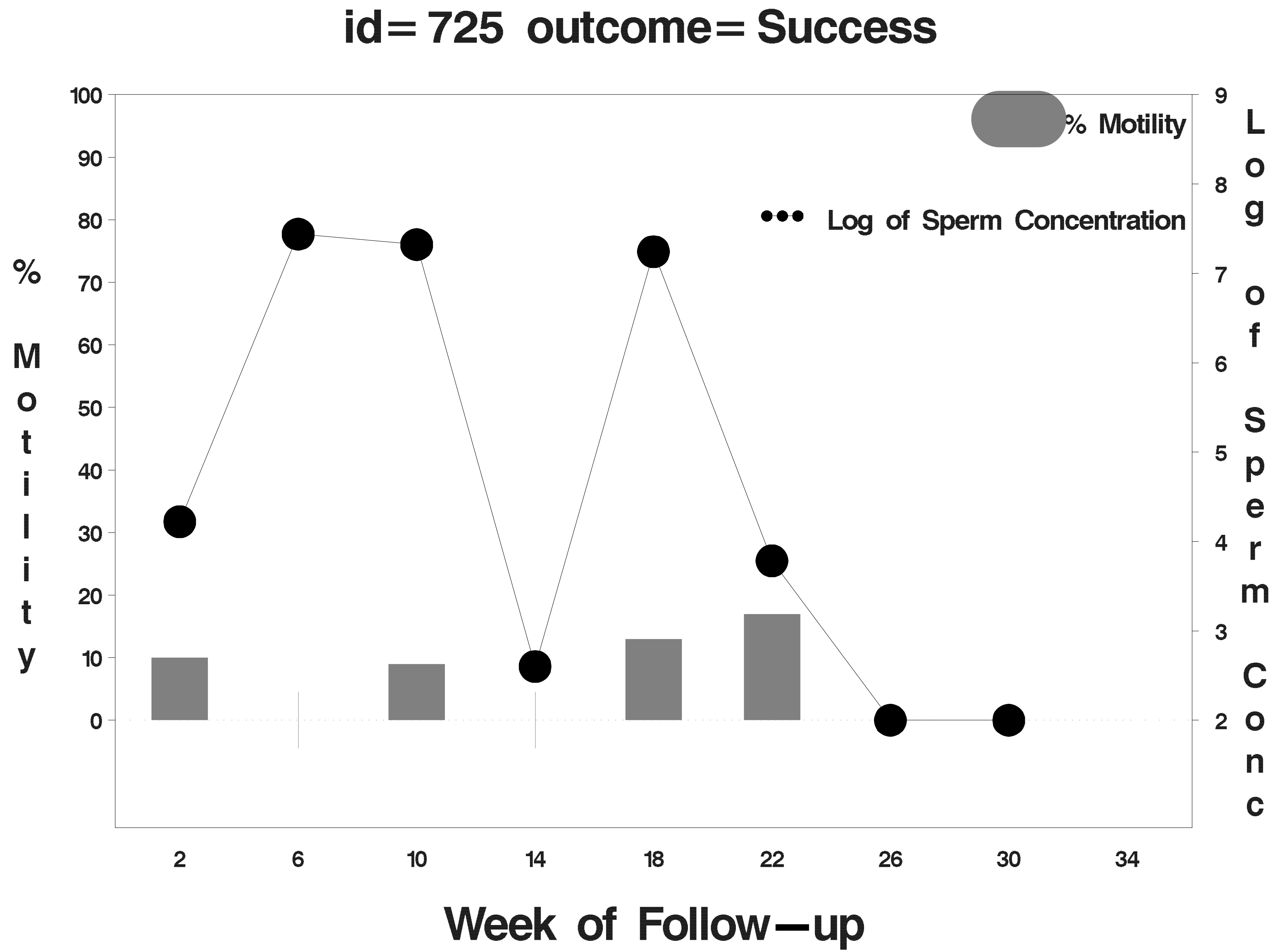

id= 725 outcome= Success
% Motility
Log of Sperm Concentration
100
90
80
70
60
50
40
30
20
10
0
9
8
7
6
5
4
3
2
2
6
10
14
18
22
26
30
34
Week of Follow−up
Log of Sperm Conc

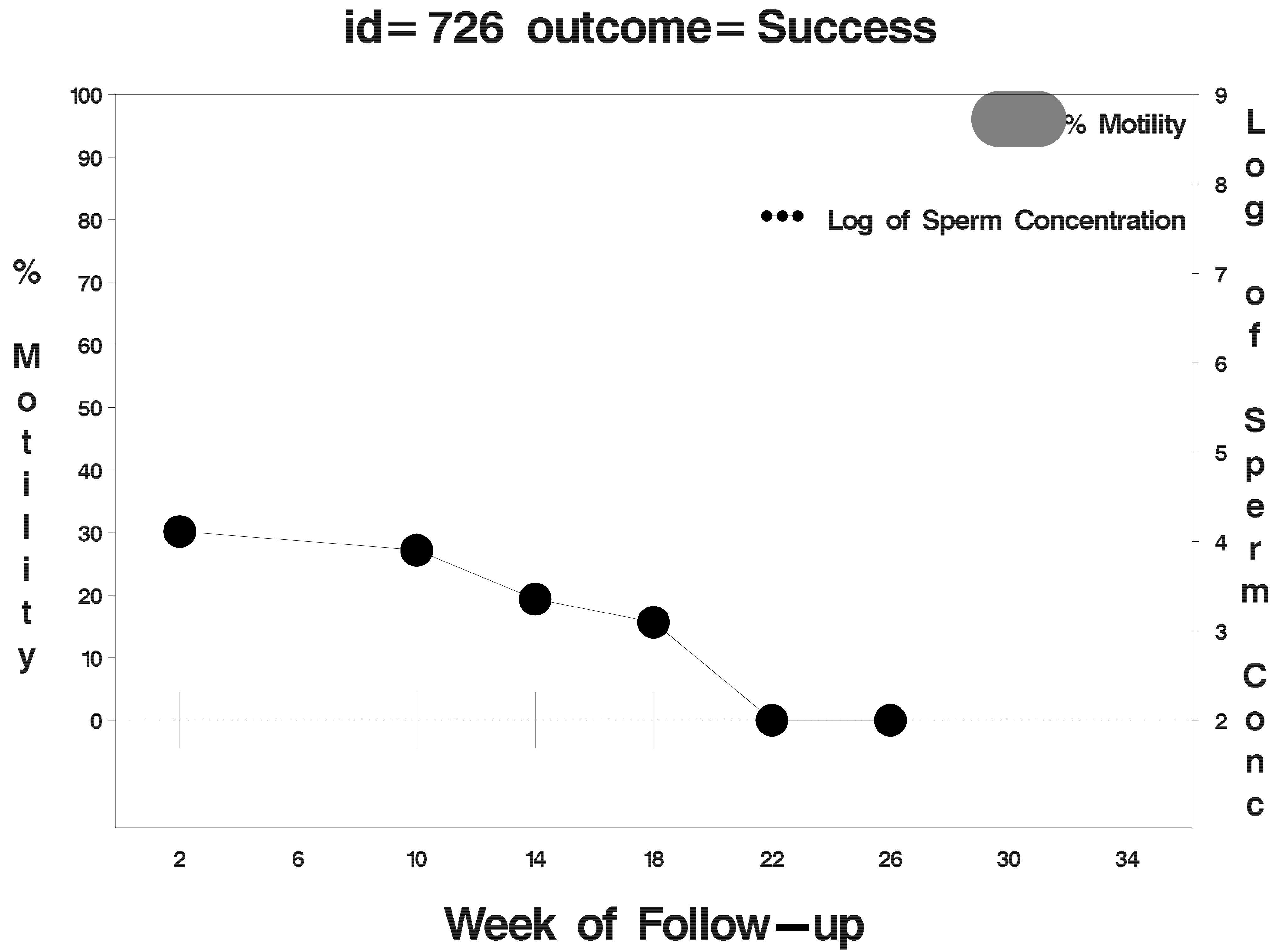
id= 726 outcome= Success
% Motility
Log of Sperm Concentration
100
90
80
70
60
50
40
30
20
10
0
9
8
7
6
5
4
3
2
2
6
10
14
18
22
26
30
34
Week of Follow—up
% Motility
Log of Sperm Conc

id= 727 outcome= Success

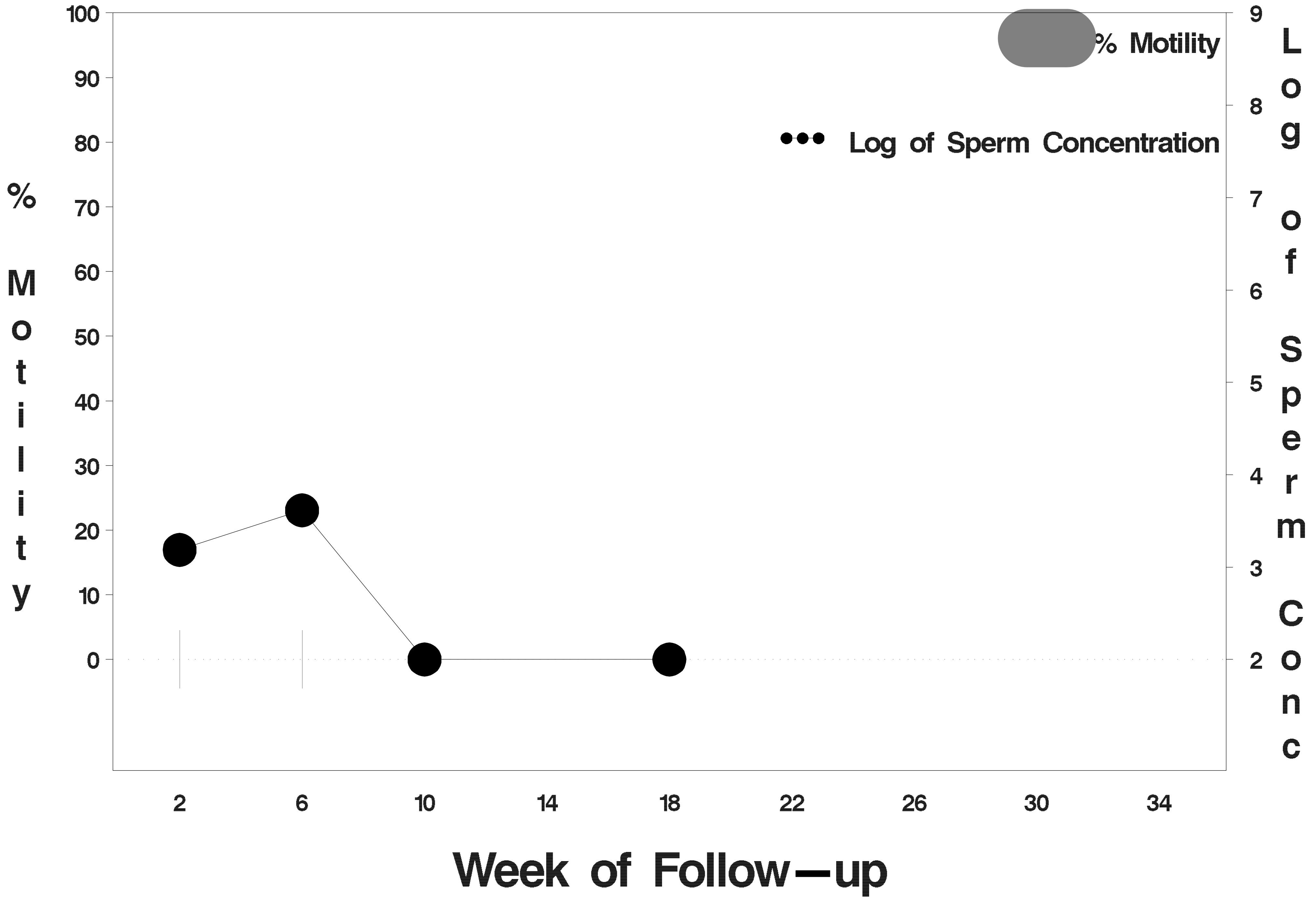

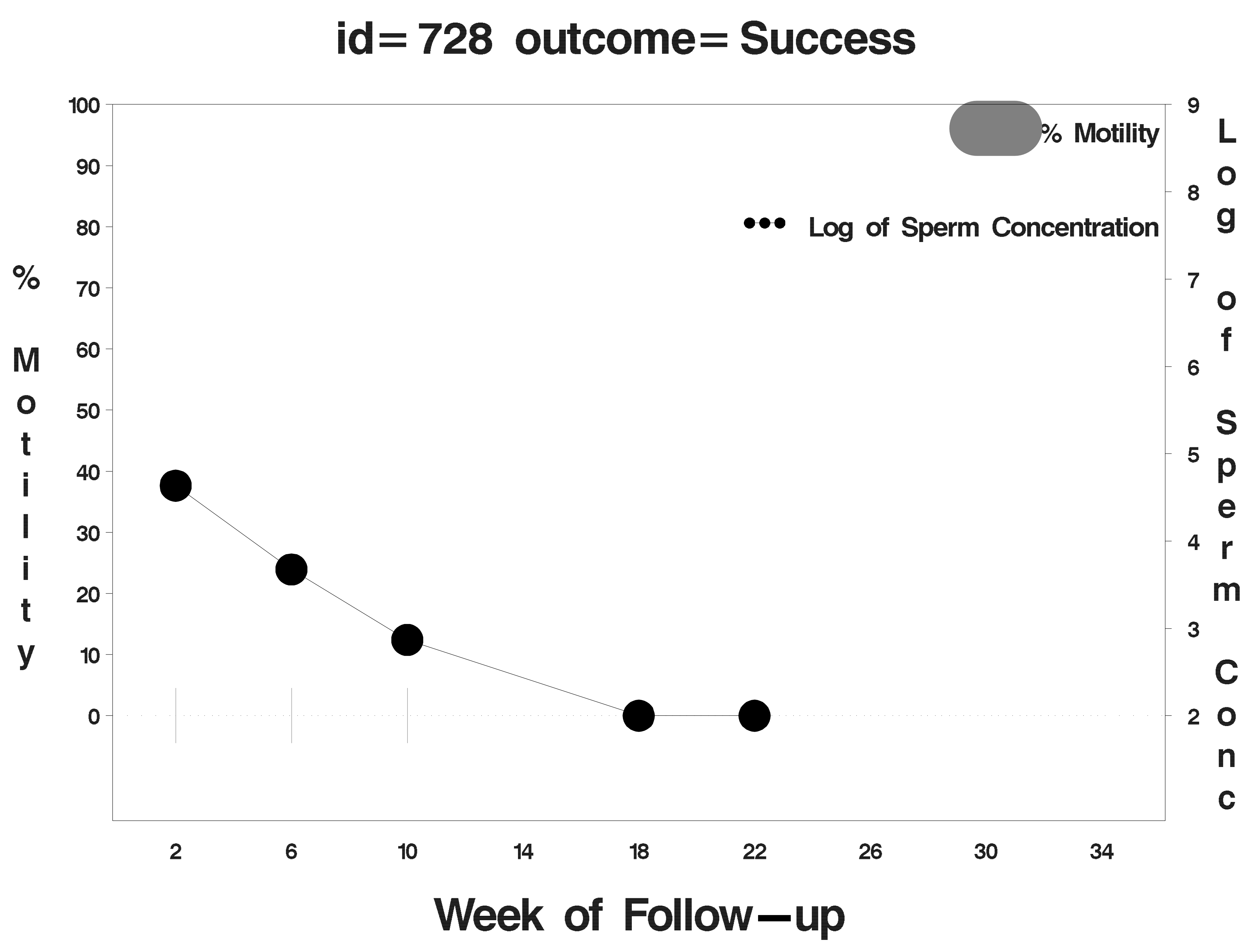

id= 728 outcome= Success
% Motility
Log of Sperm Concentration
% Motility
Log of Sperm Conc
Week of Follow−up
100
90
80
70
60
50
40
30
20
10
0
9
8
7
6
5
4
3
2
2
6
10
14
18
22
26
30
34

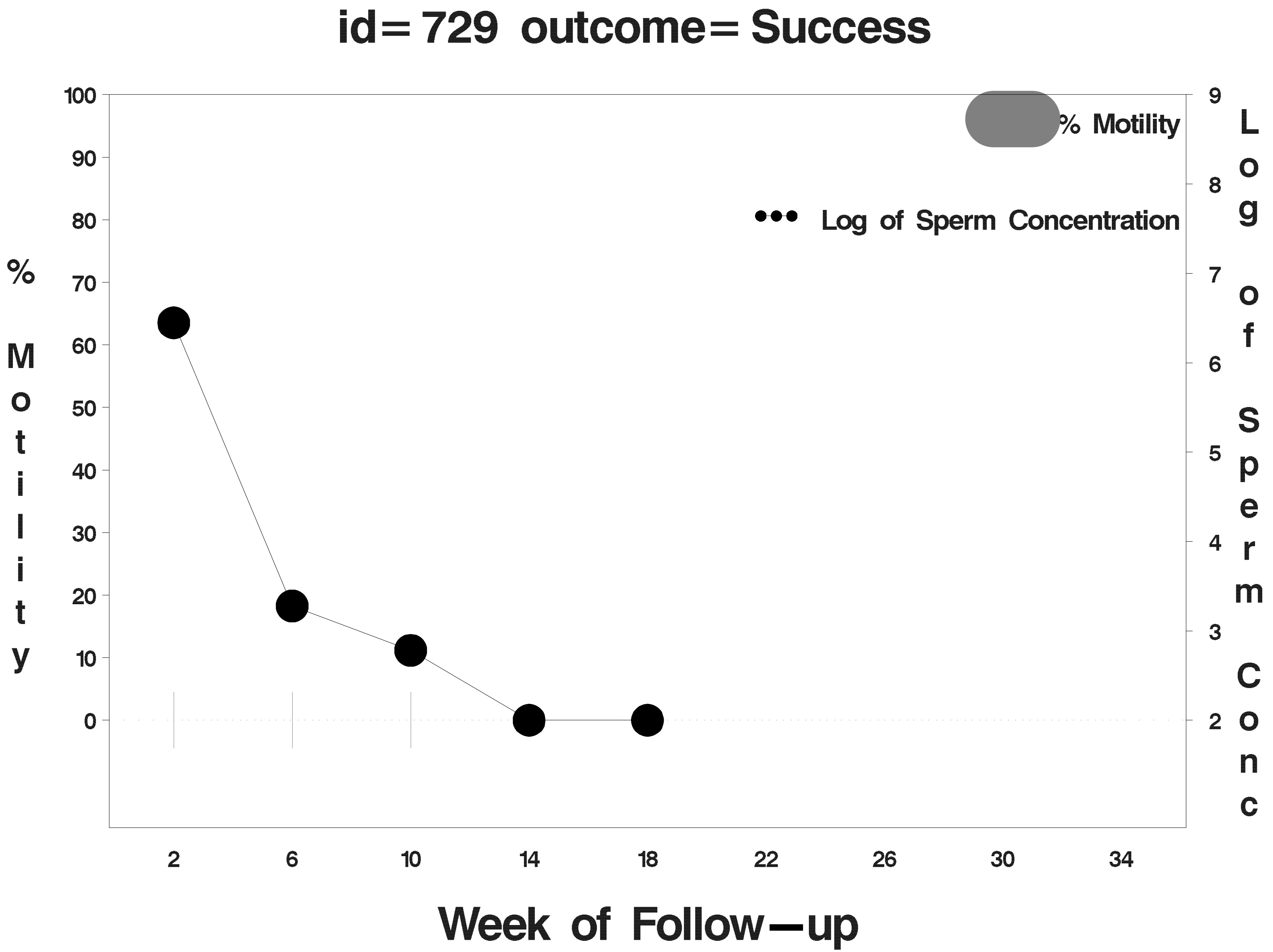
id= 729 outcome= Success
% Motility
Log of Sperm Concentration
100
90
80
70
60
50
40
30
20
10
0
9
8
7
6
5
4
3
2
2
6
10
14
18
22
26
30
34
% Motility
Log of Sperm Conc
Week of Follow-up

id= 730 outcome= Success

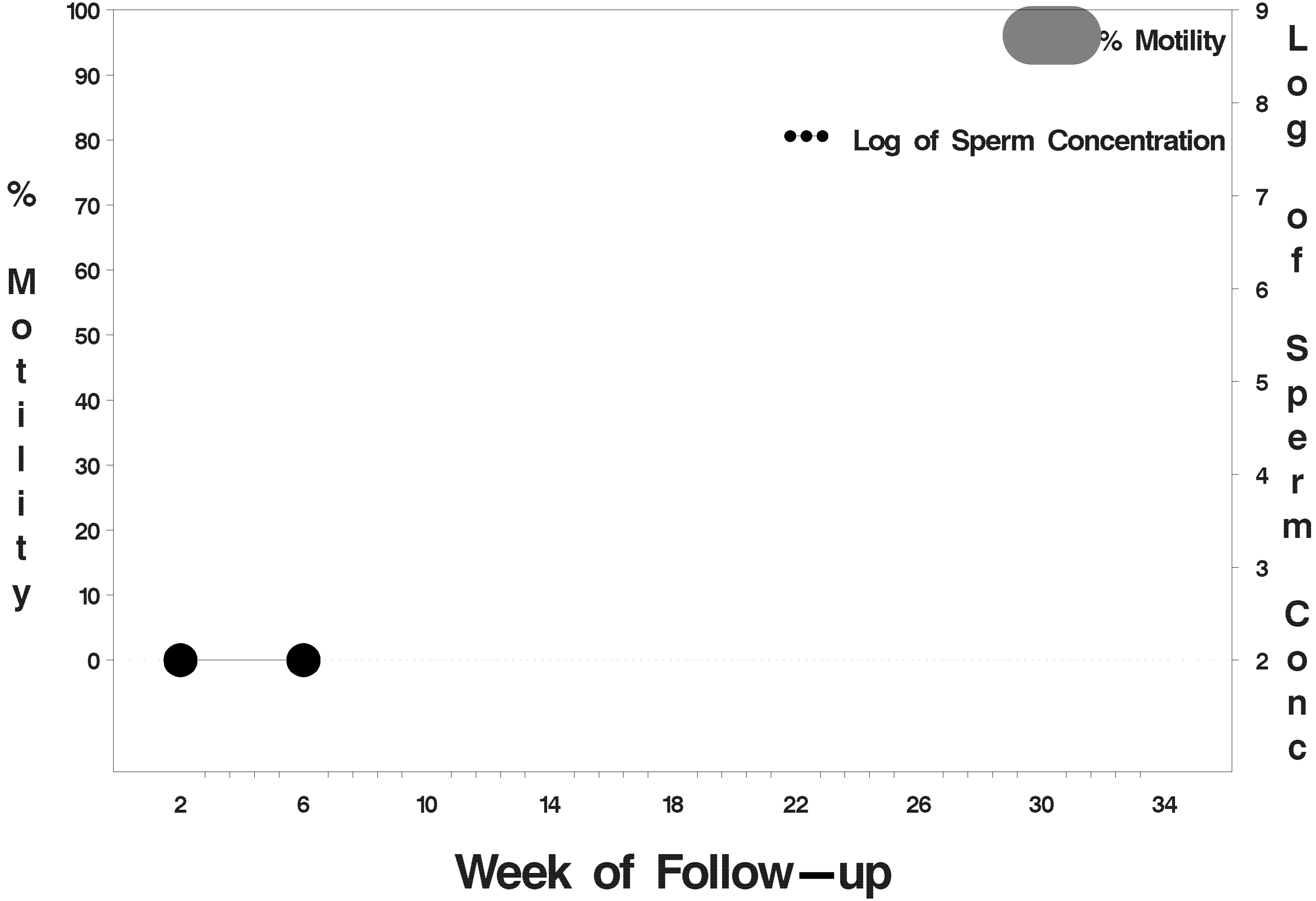

id= 731 outcome= Success

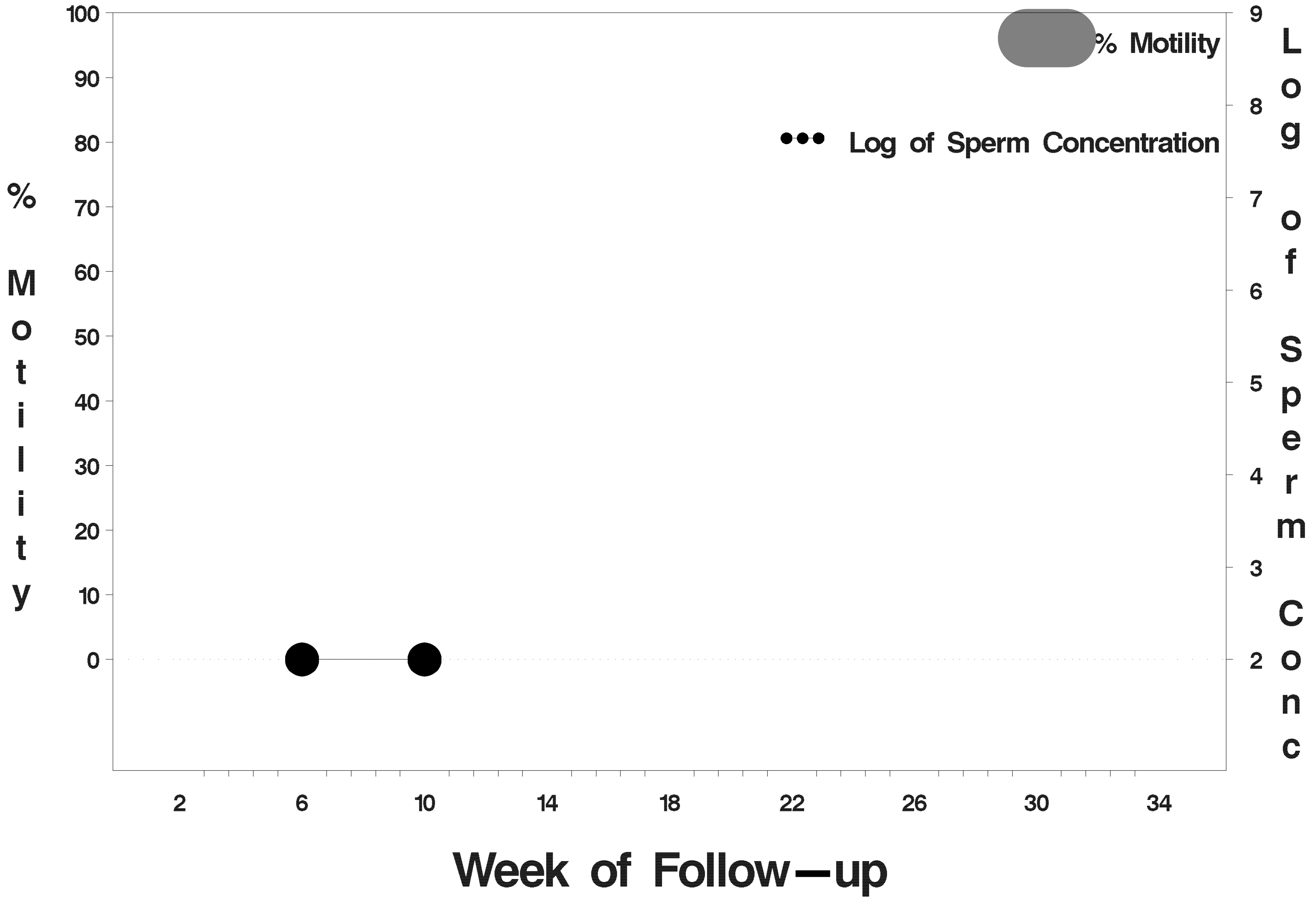

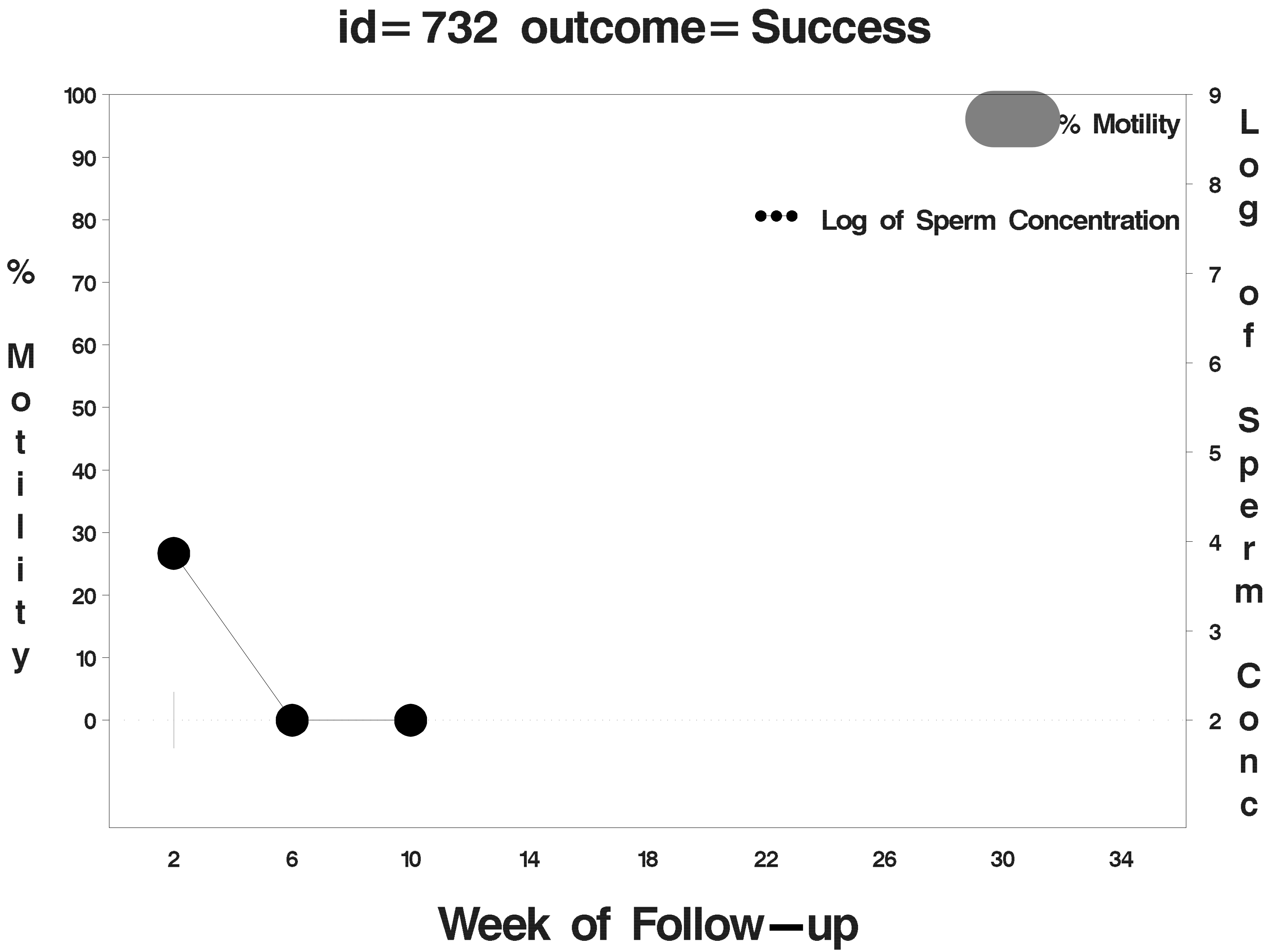

id= 732 outcome= Success
% Motility
Log of Sperm Concentration
100
90
80
70
60
50
40
30
20
10
0
9
8
7
6
5
4
3
2
Log of Sperm Conc
2
6
10
14
18
22
26
30
34
Week of Follow−up

id= 733 outcome= Success

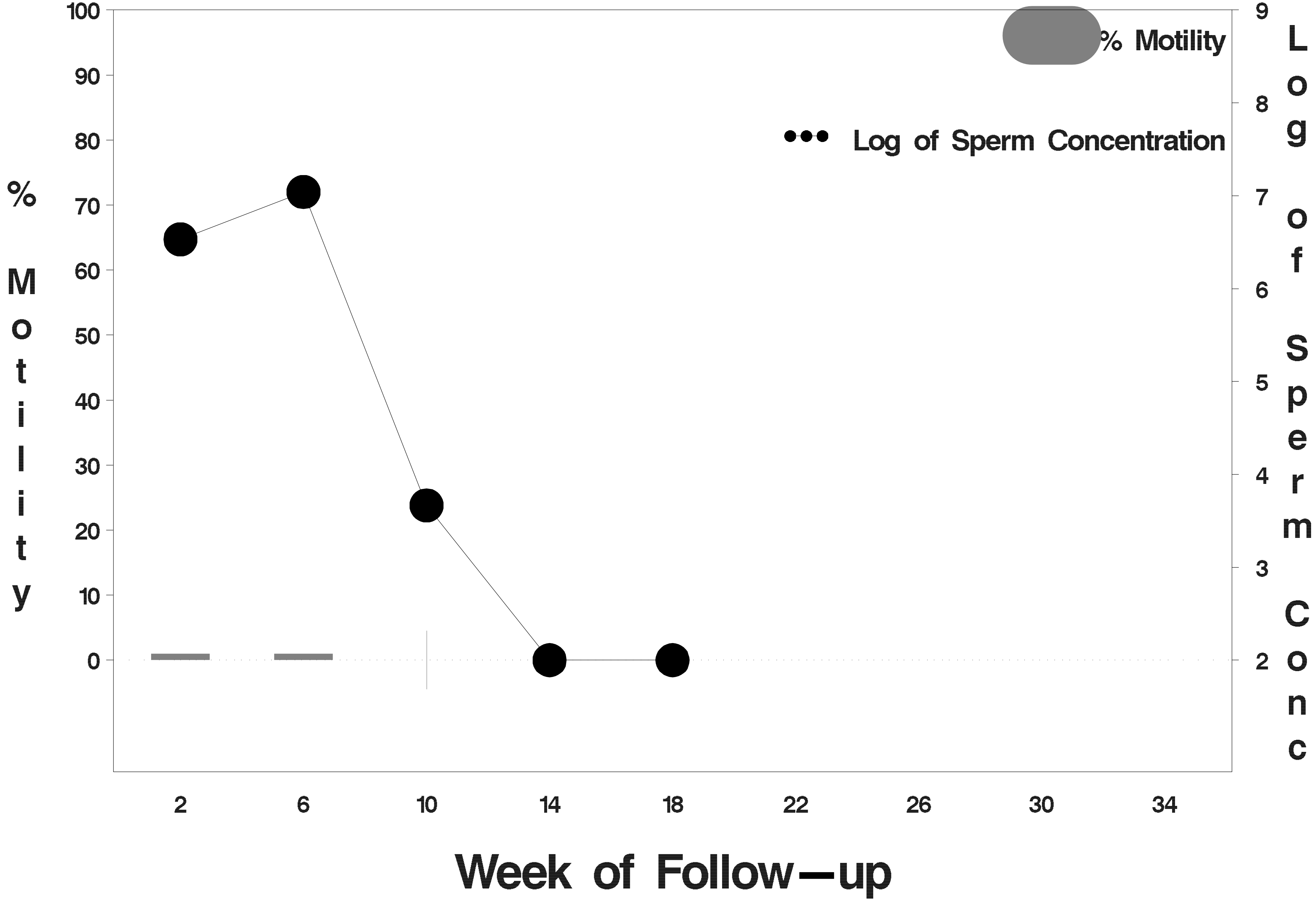

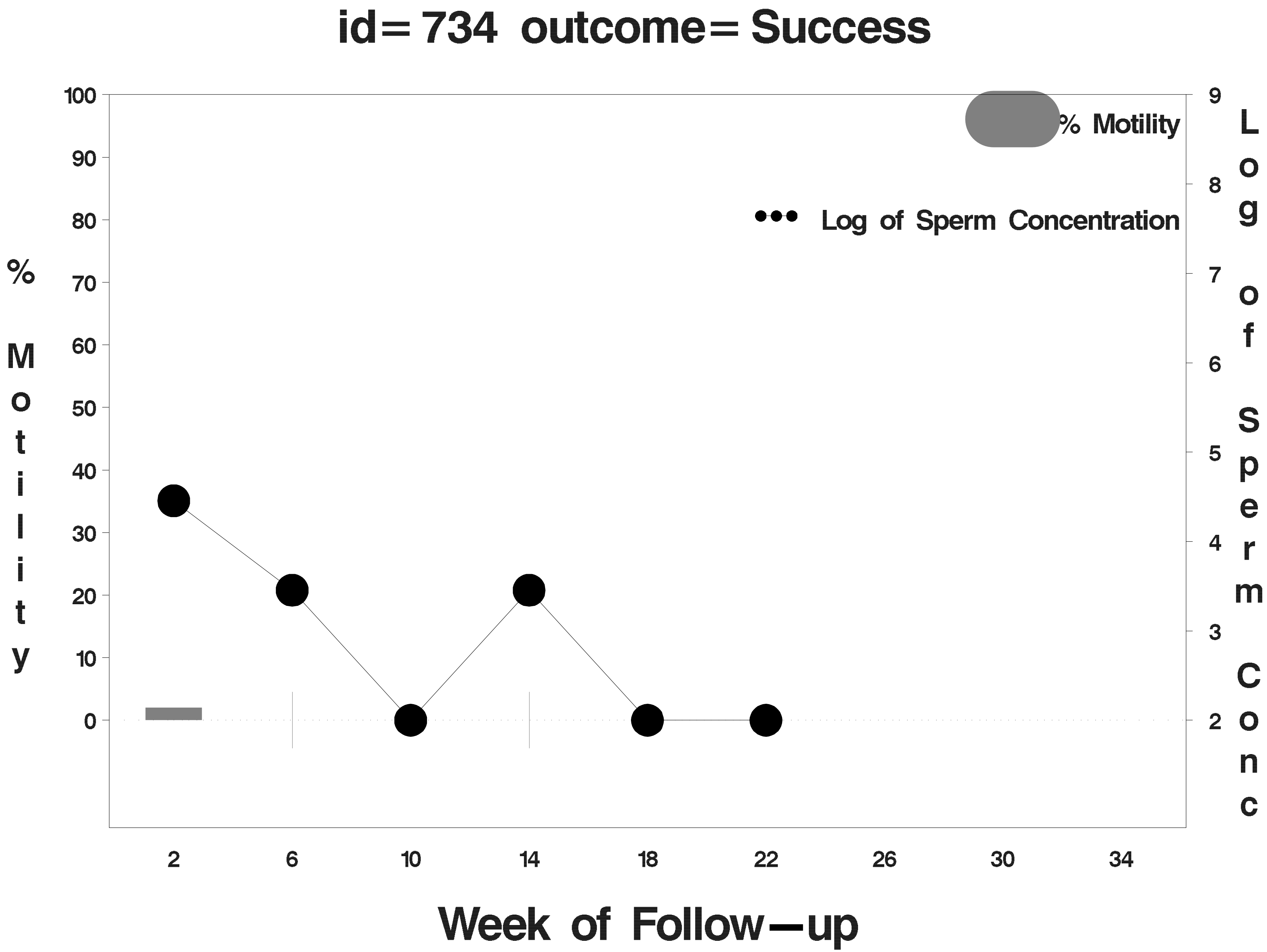

id= 734 outcome= Success
% Motility
Log of Sperm Concentration
100
90
80
70
60
50
40
30
20
10
0
9
8
7
6
5
4
3
2
% Motility
Log of Sperm Conc
2
6
10
14
18
22
26
30
34
Week of Follow-up

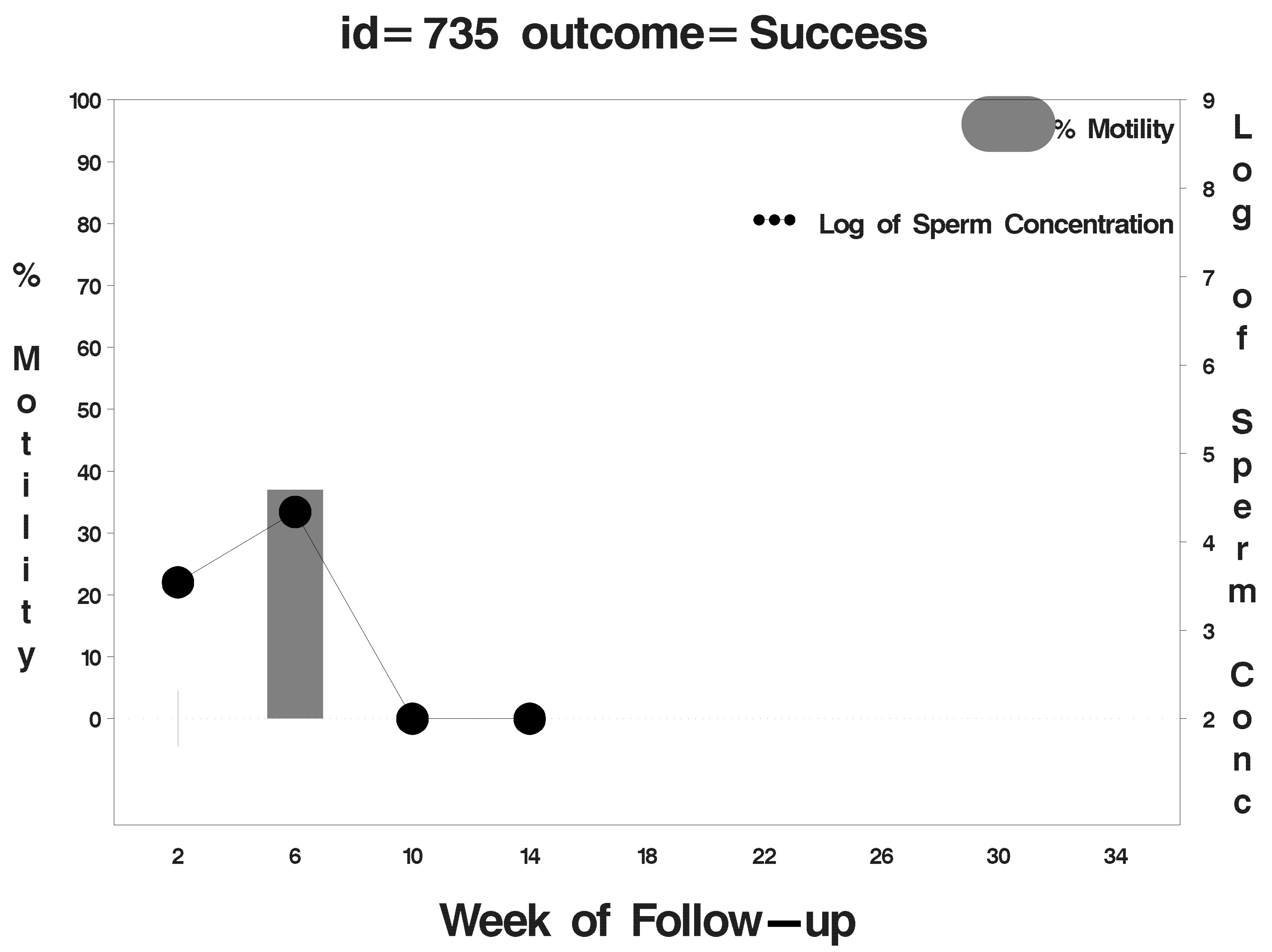
id= 735 outcome= Success
% Motility
Log of Sperm Concentration
100
90
80
70
60
50
40
30
20
10
0
9
8
7
6
5
4
3
2
% Motility
Log of Sperm Conc
2
6
10
14
18
22
26
30
34
Week of Follow—up

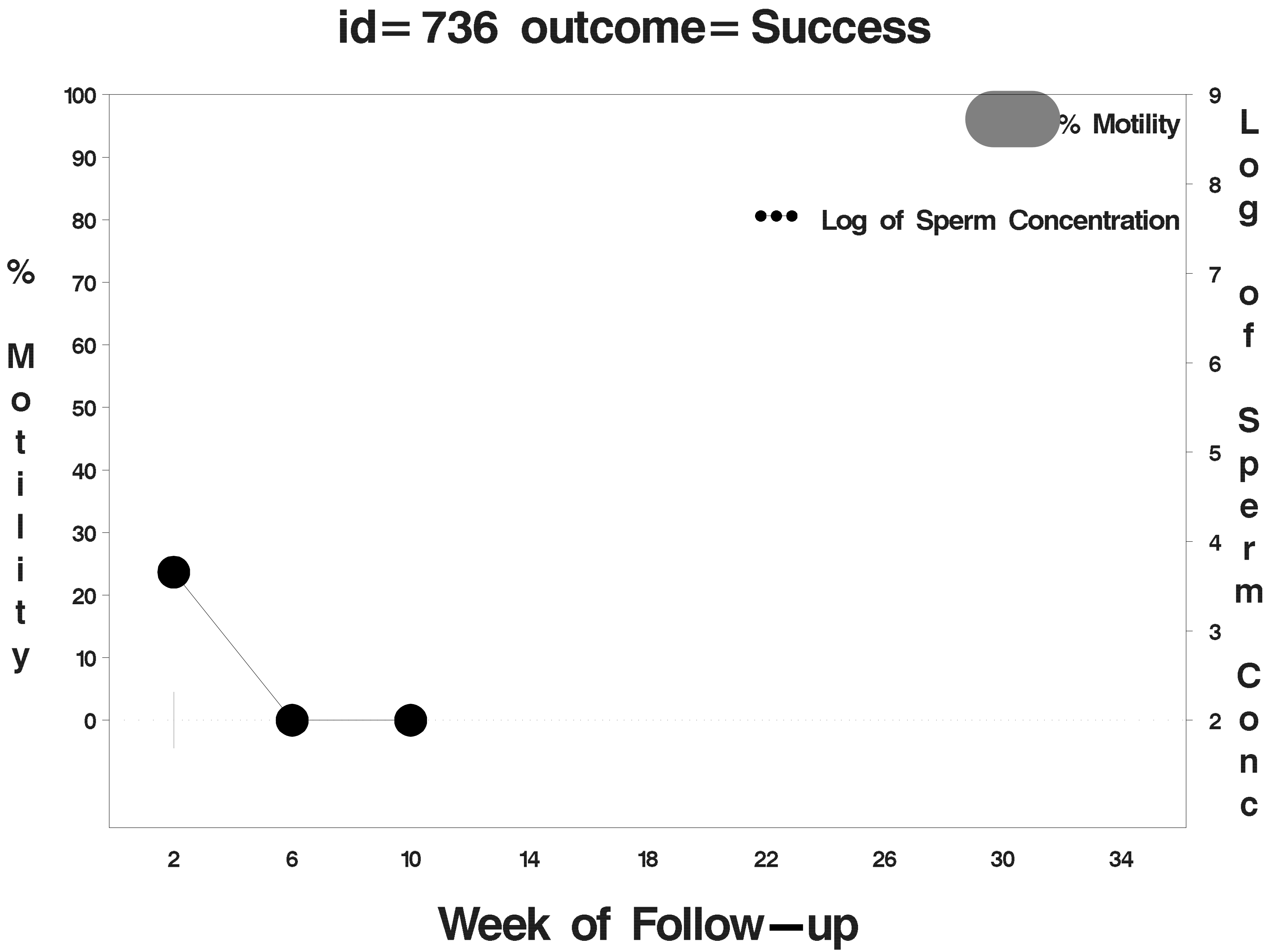
id= 736 outcome= Success
% Motility
Log of Sperm Concentration
100
90
80
70
60
50
40
30
20
10
0
9
8
7
6
5
4
3
2
2
6
10
14
18
22
26
30
34
Week of Follow—up
% Motility
Log of Sperm Conc

id= 737 outcome= Indeterminate

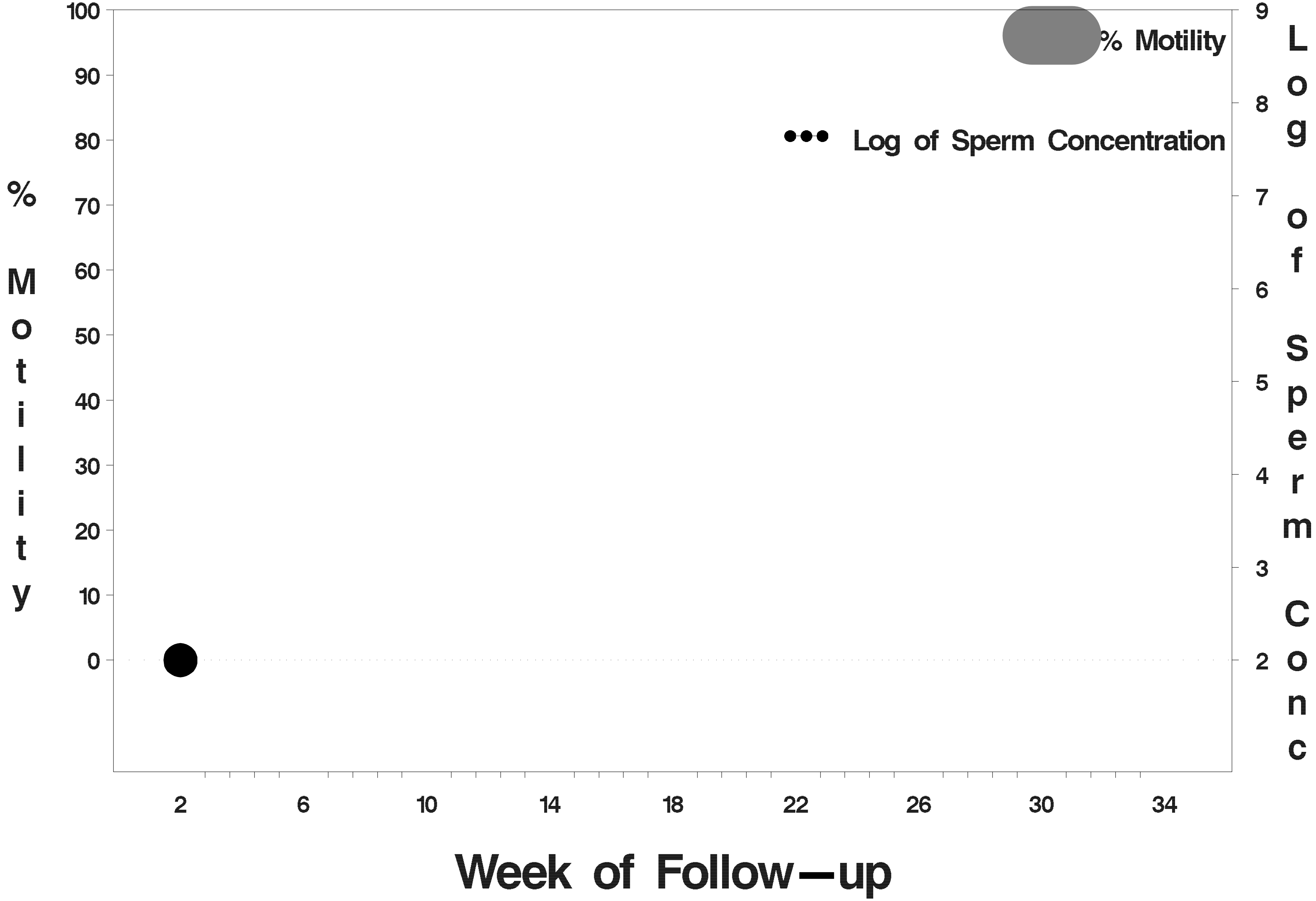

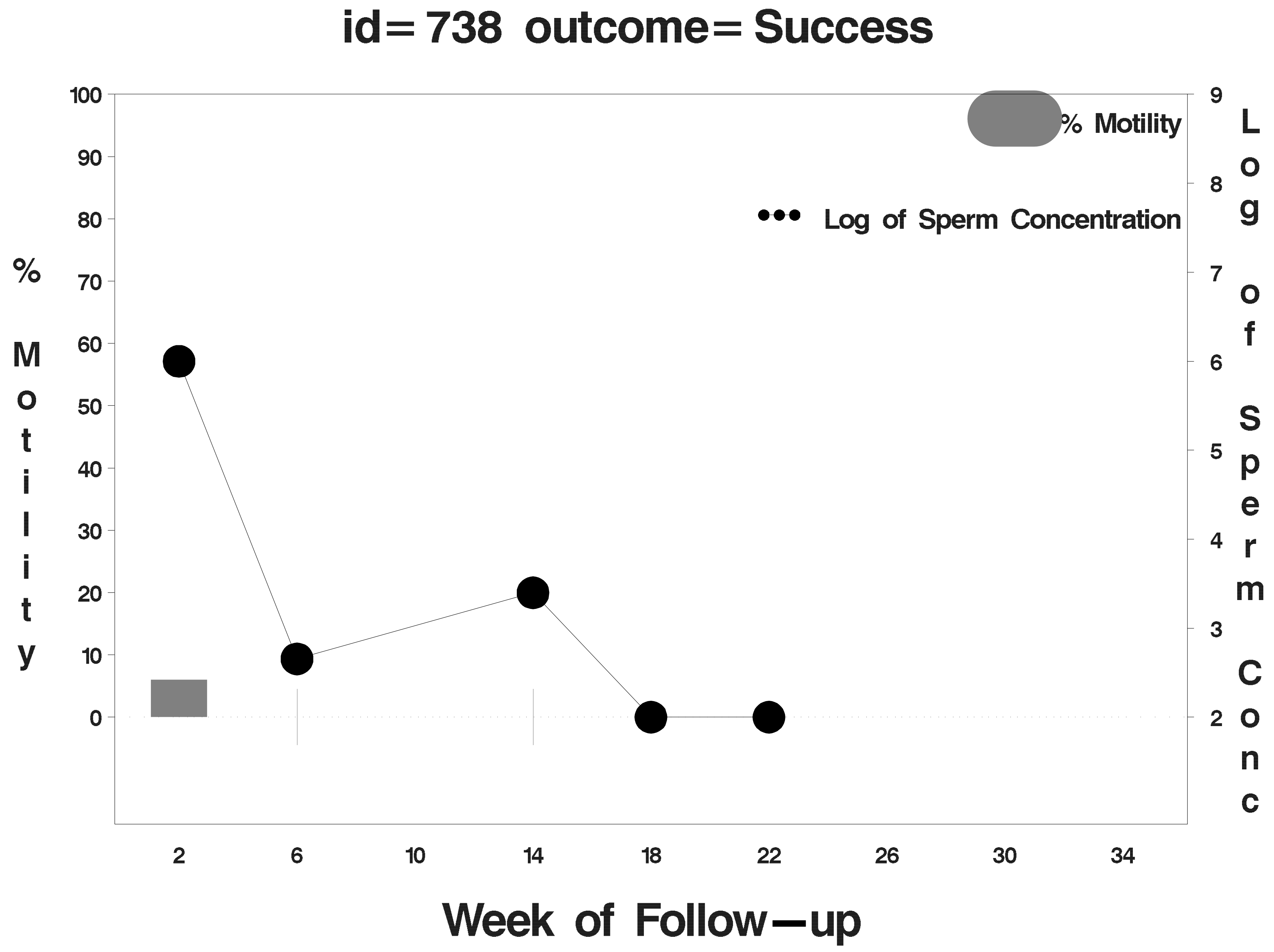
id= 738 outcome= Success
% Motility
Log of Sperm Concentration
100
90
80
70
60
50
40
30
20
10
0
9
8
7
6
5
4
3
2
2
6
10
14
18
22
26
30
34
% Motility
Log of Sperm Conc
Week of Follow−up

id= 739 outcome= Success

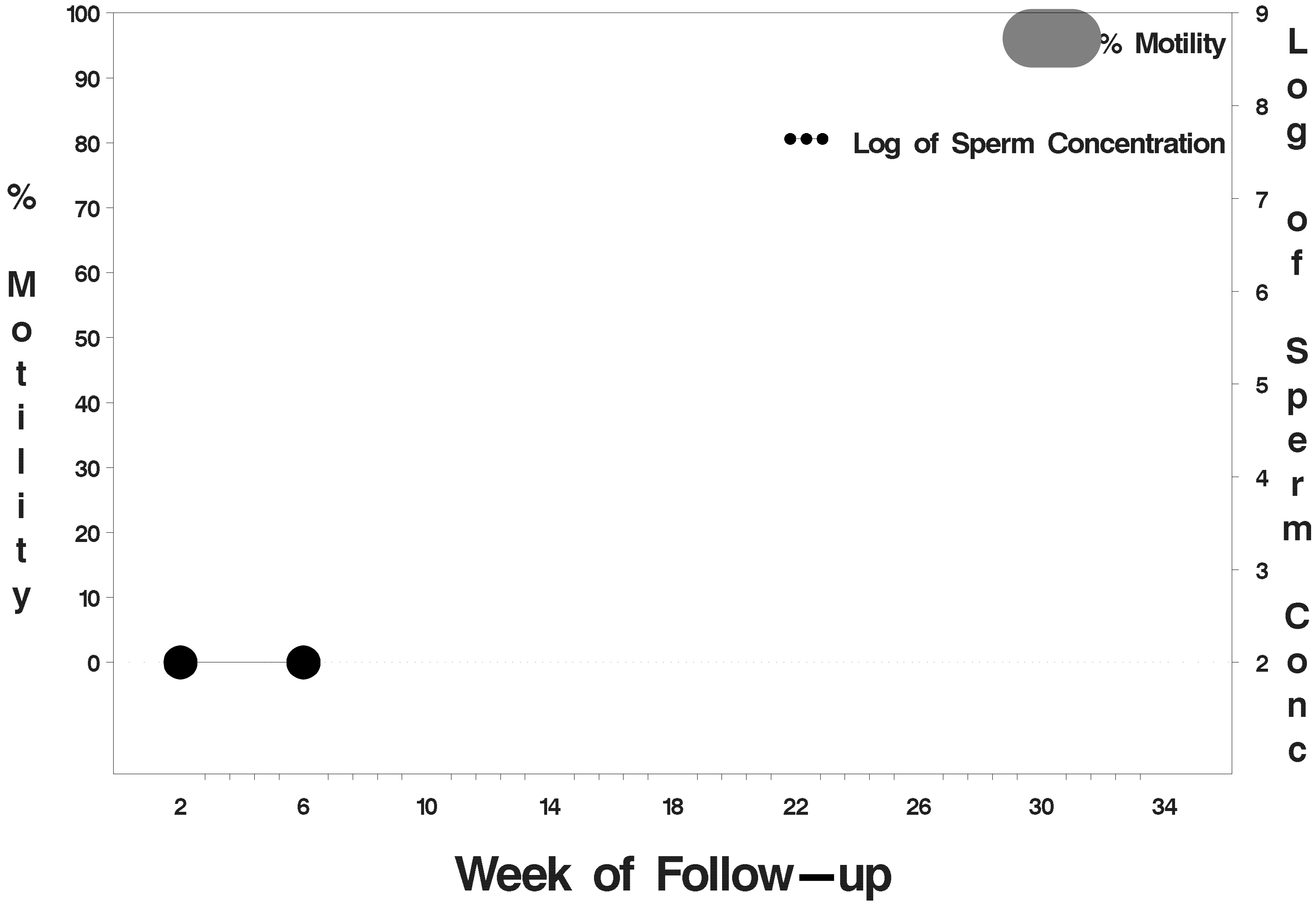

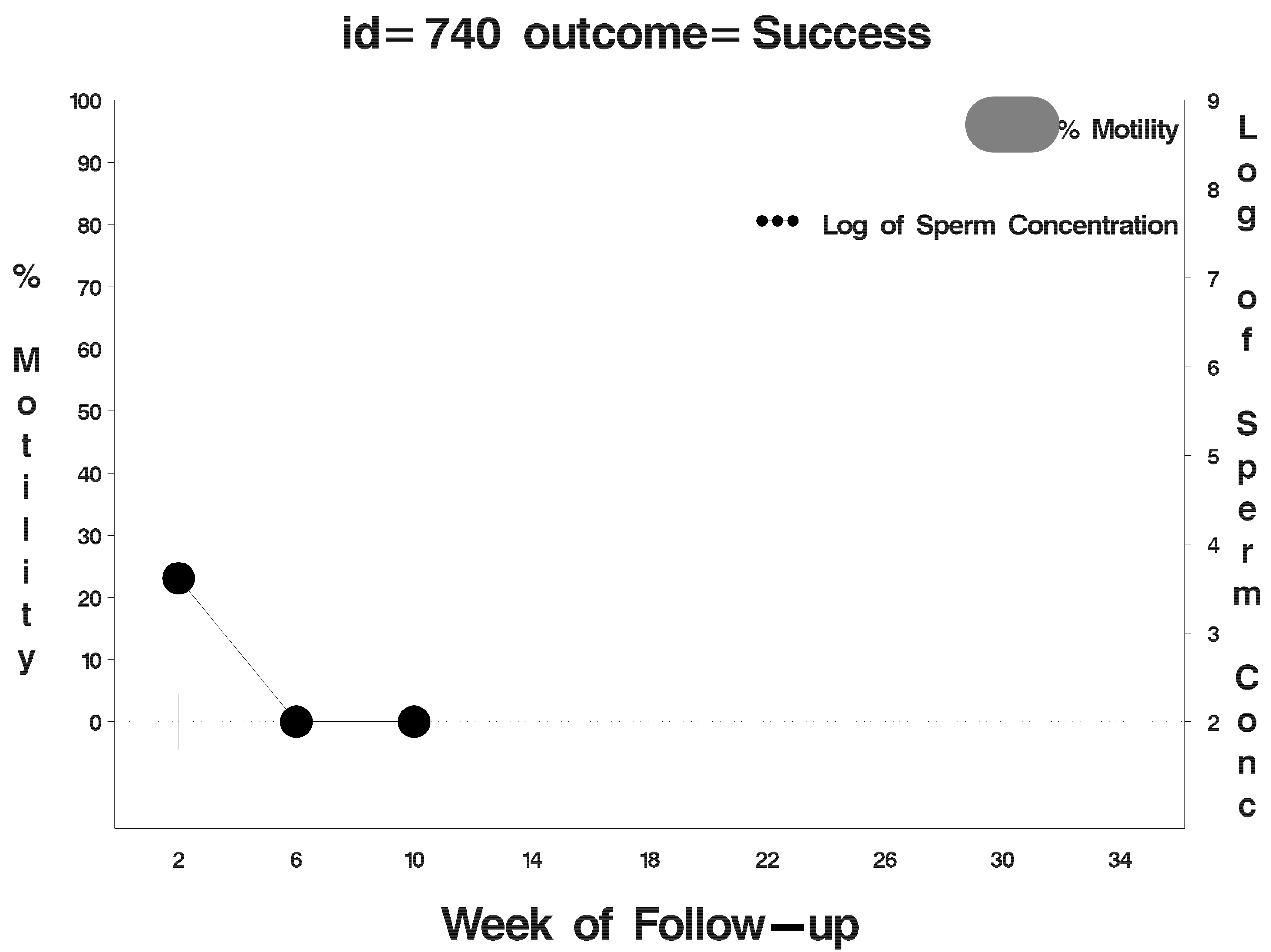

id= 740 outcome= Success
% Motility
Log of Sperm Concentration
100
90
80
70
60
50
40
30
20
10
0
9
8
7
6
5
4
3
2
2
6
10
14
18
22
26
30
34
Week of Follow−up
Log of Sperm Conc

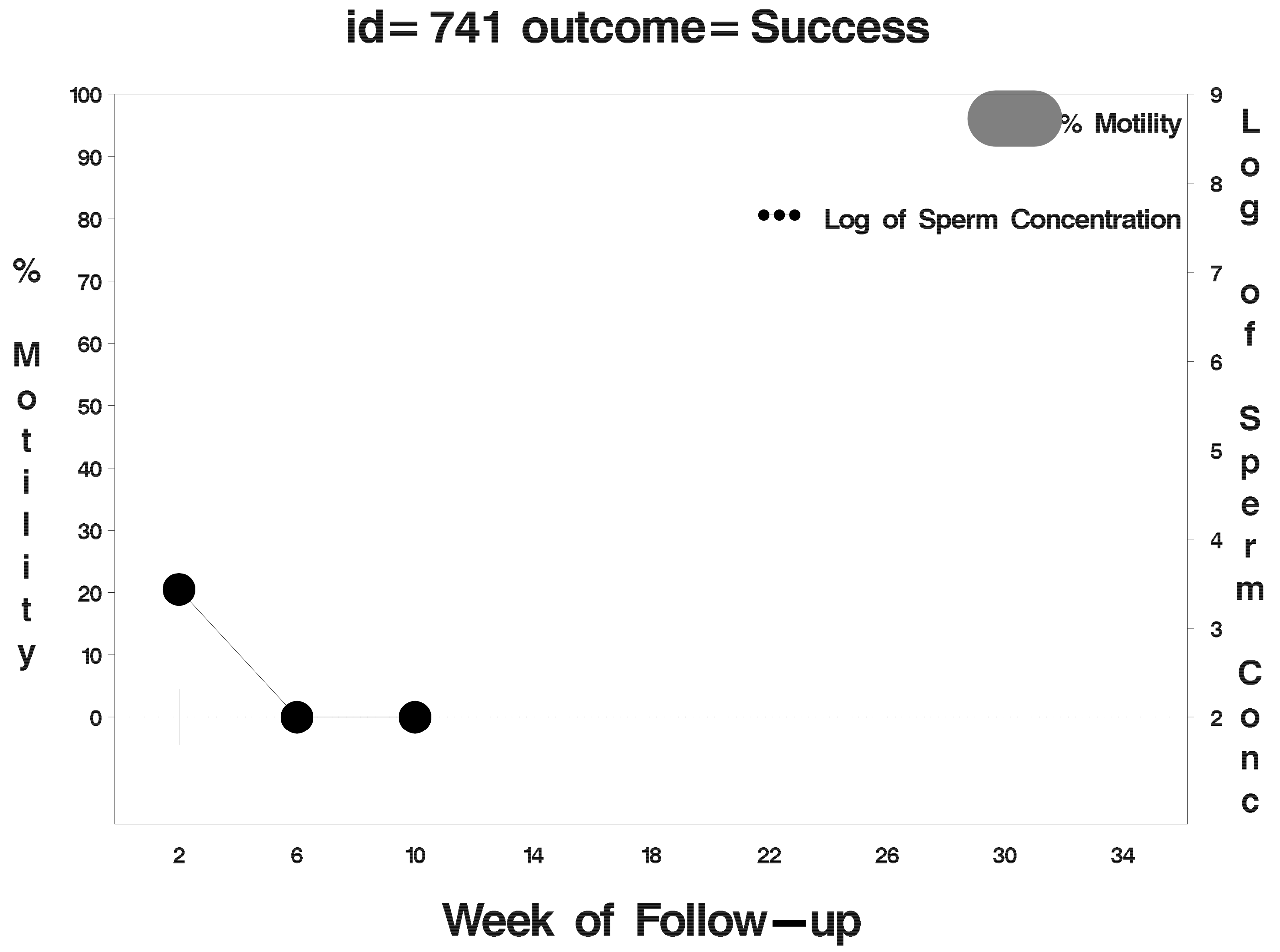
id= 741 outcome= Success
% Motility
Log of Sperm Concentration
100
90
80
70
60
50
40
30
20
10
0
9
8
7
6
5
4
3
2
2
6
10
14
18
22
26
30
34
Week of Follow−up
Log of Sperm Conc

id= 742 outcome= Success

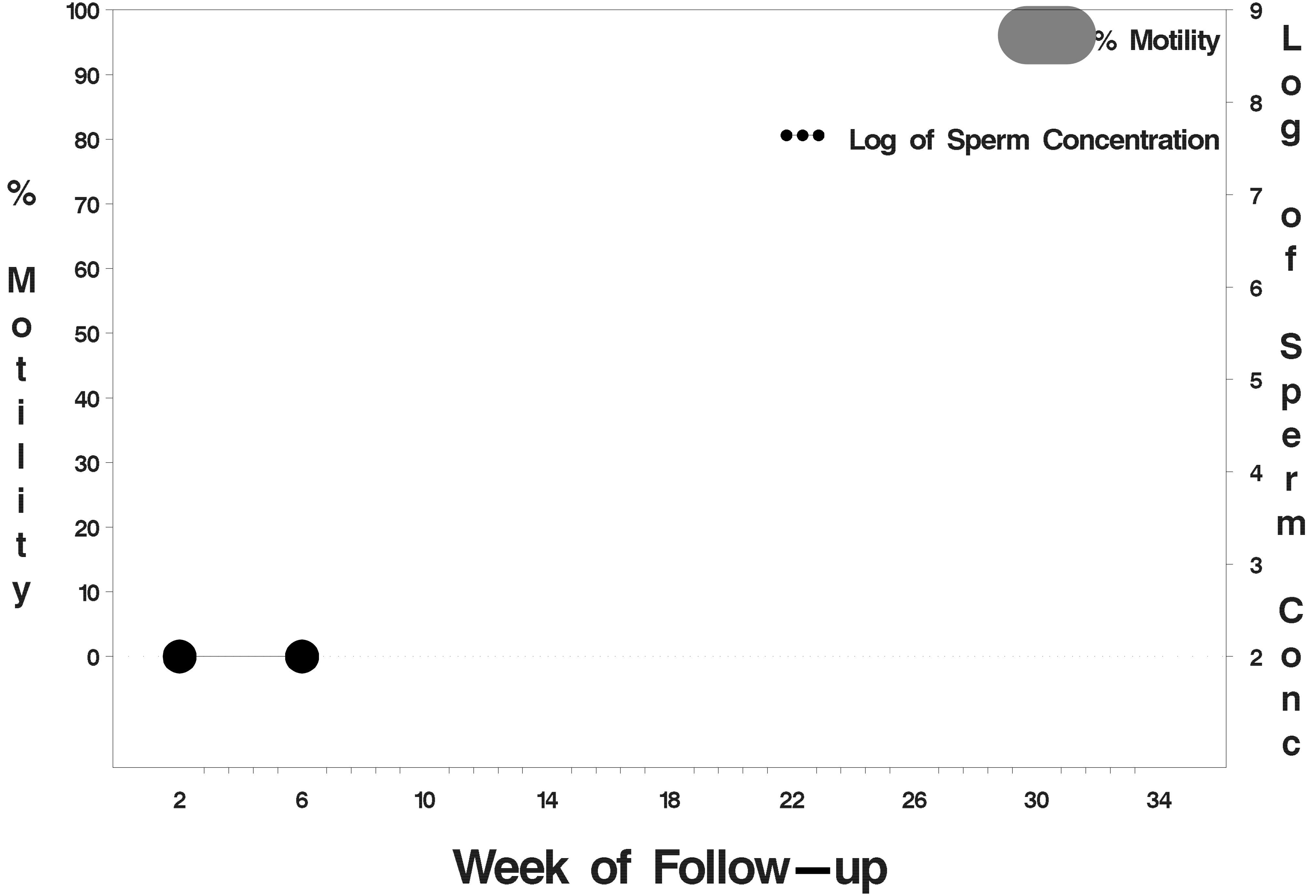

id= 743 outcome= Success

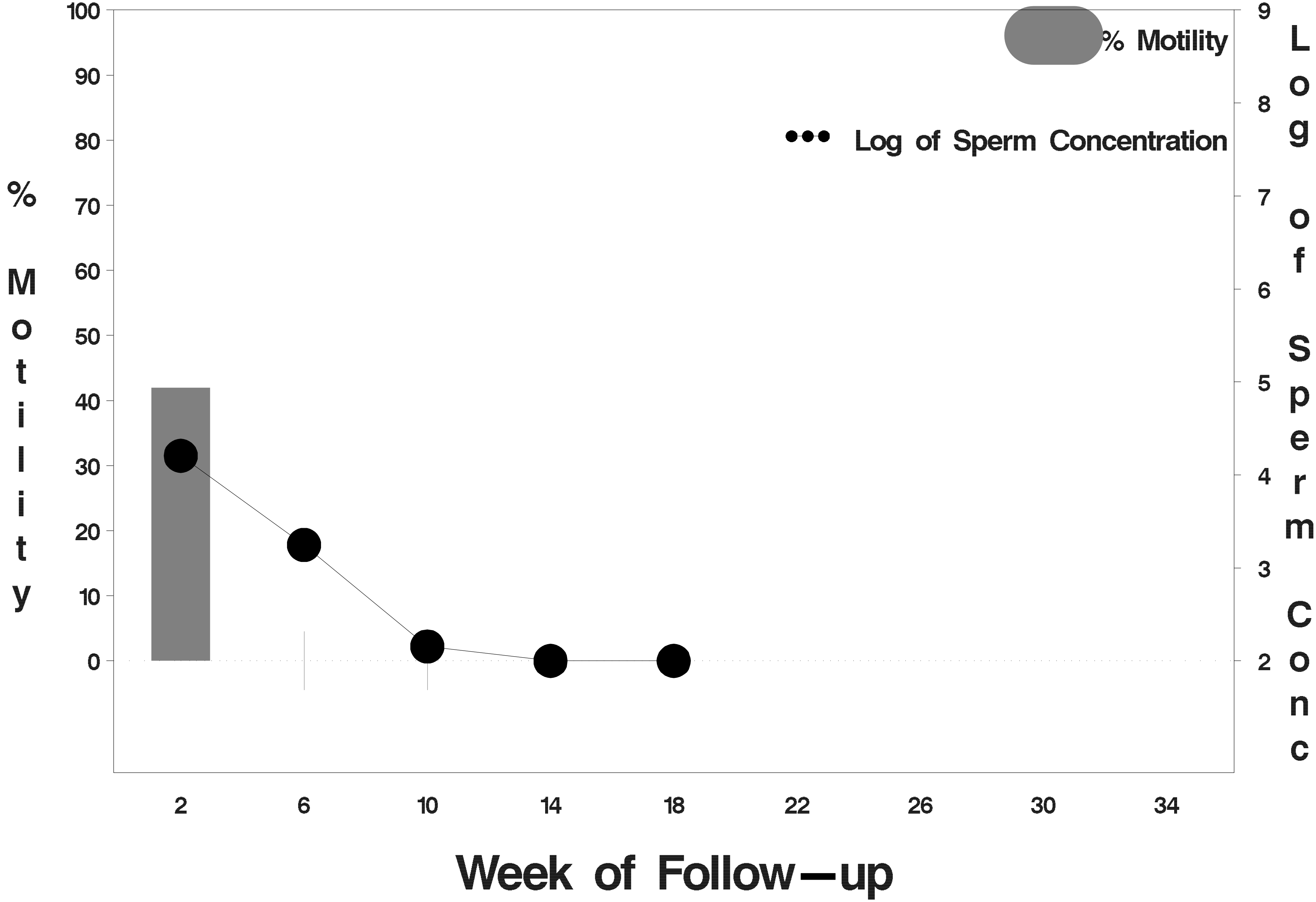

id= 744 outcome= Success

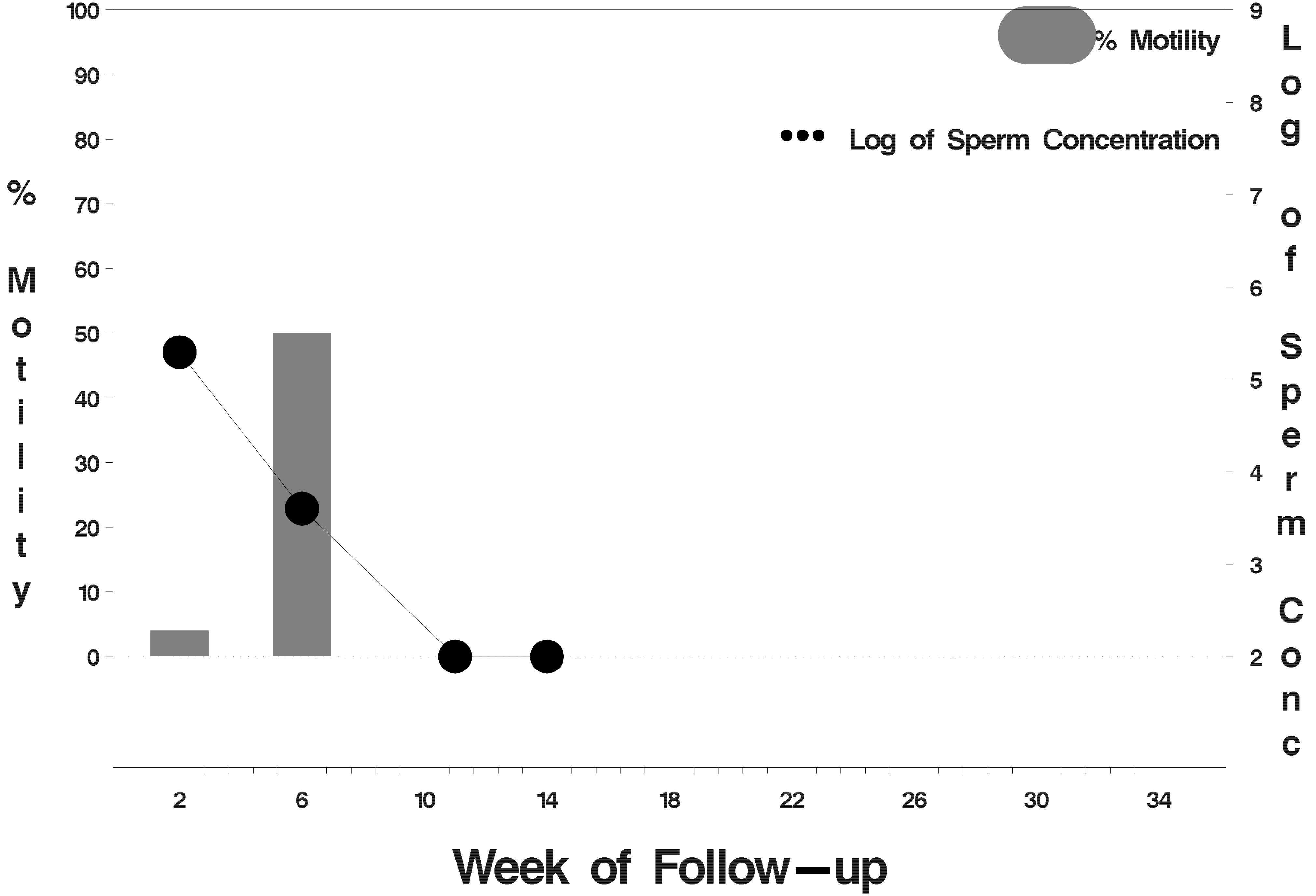

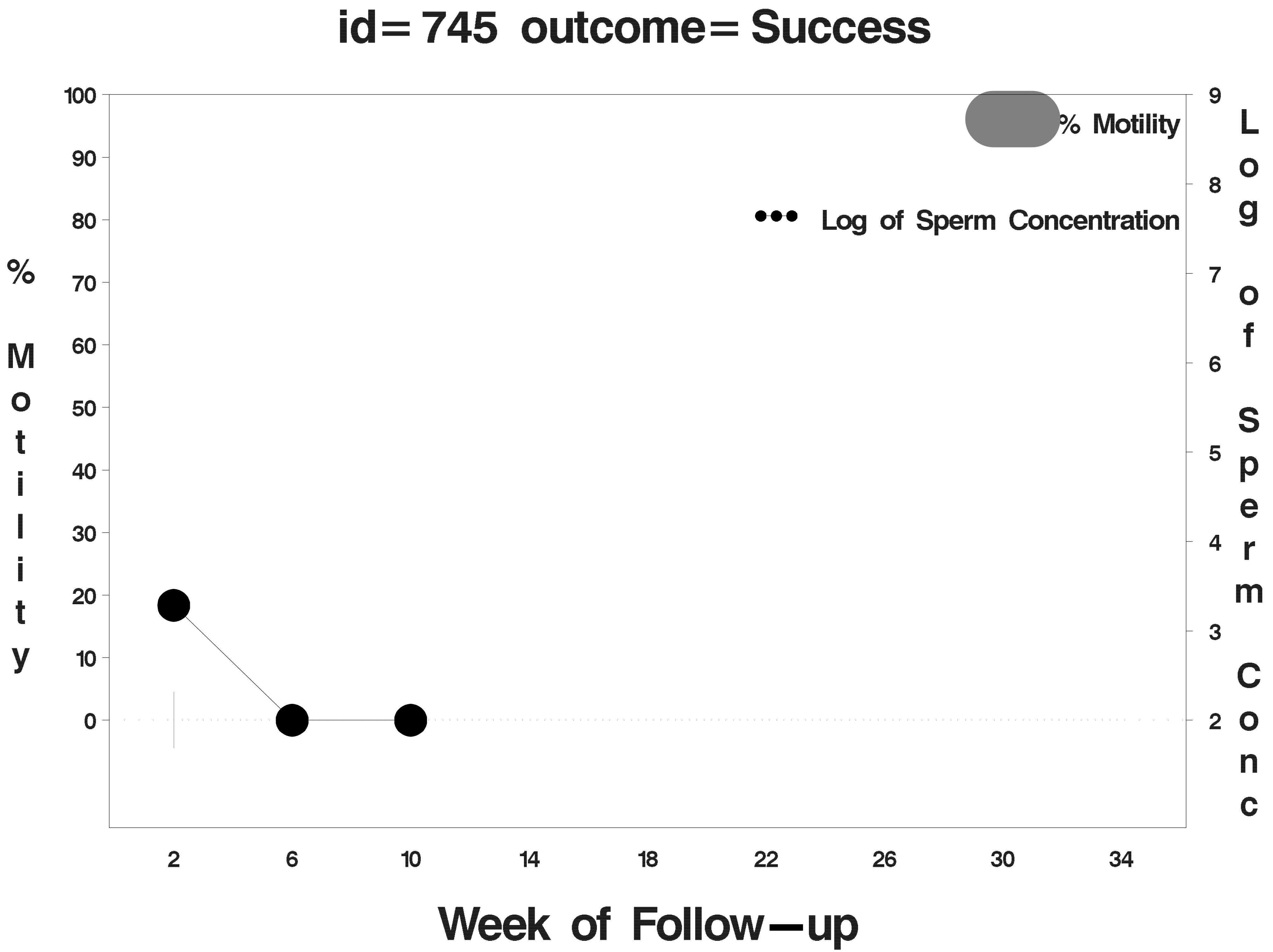
id= 745 outcome= Success
% Motility
Log of Sperm Concentration
100
90
80
70
60
50
40
30
20
10
0
9
8
7
6
5
4
3
2
2
6
10
14
18
22
26
30
34
Week of Follow−up
Log of Sperm Conc

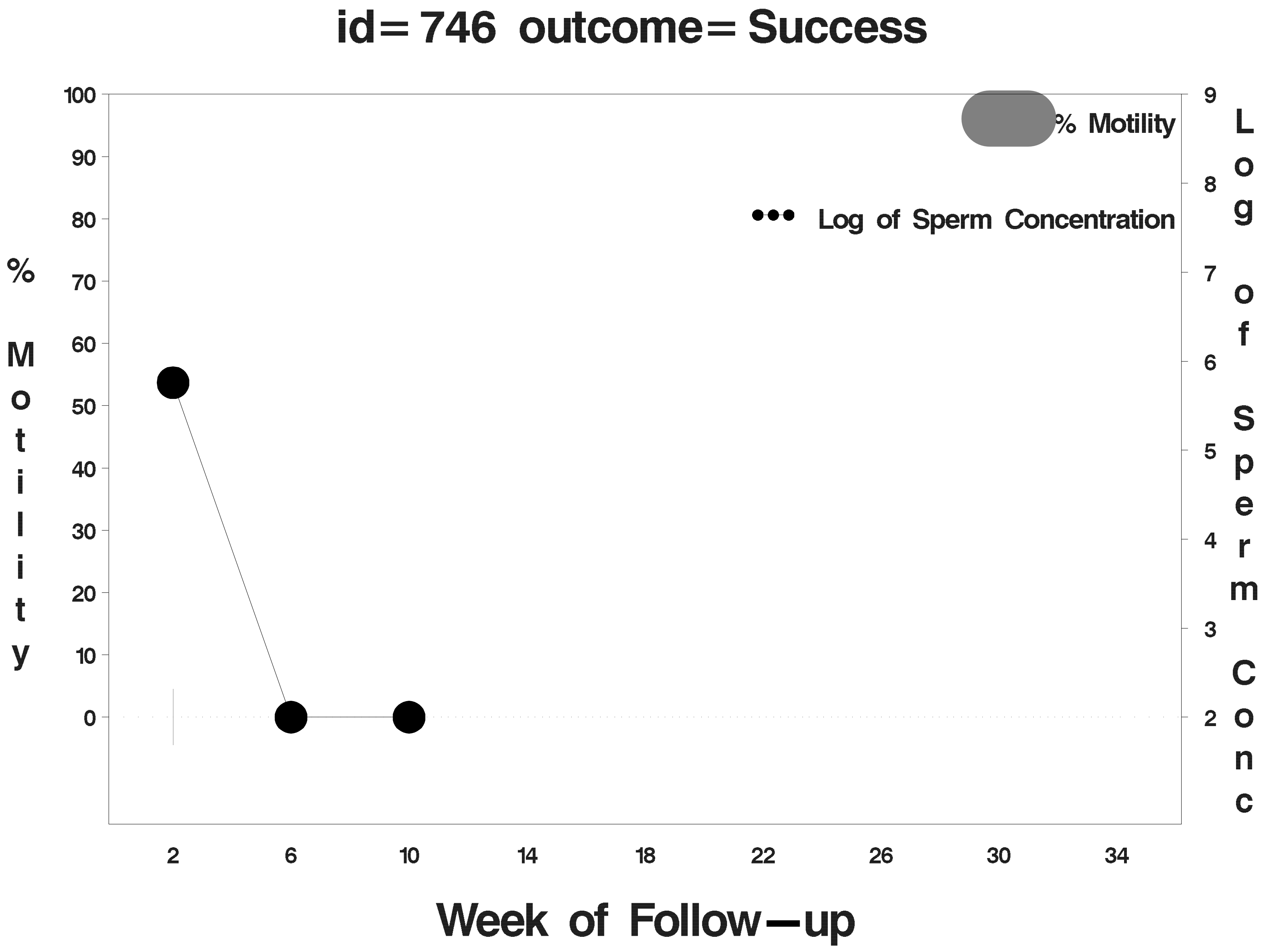

id= 746 outcome= Success
% Motility
Log of Sperm Concentration
% Motility
Log of Sperm Conc
Week of Follow-up
100
90
80
70
60
50
40
30
20
10
0
9
8
7
6
5
4
3
2
2
6
10
14
18
22
26
30
34

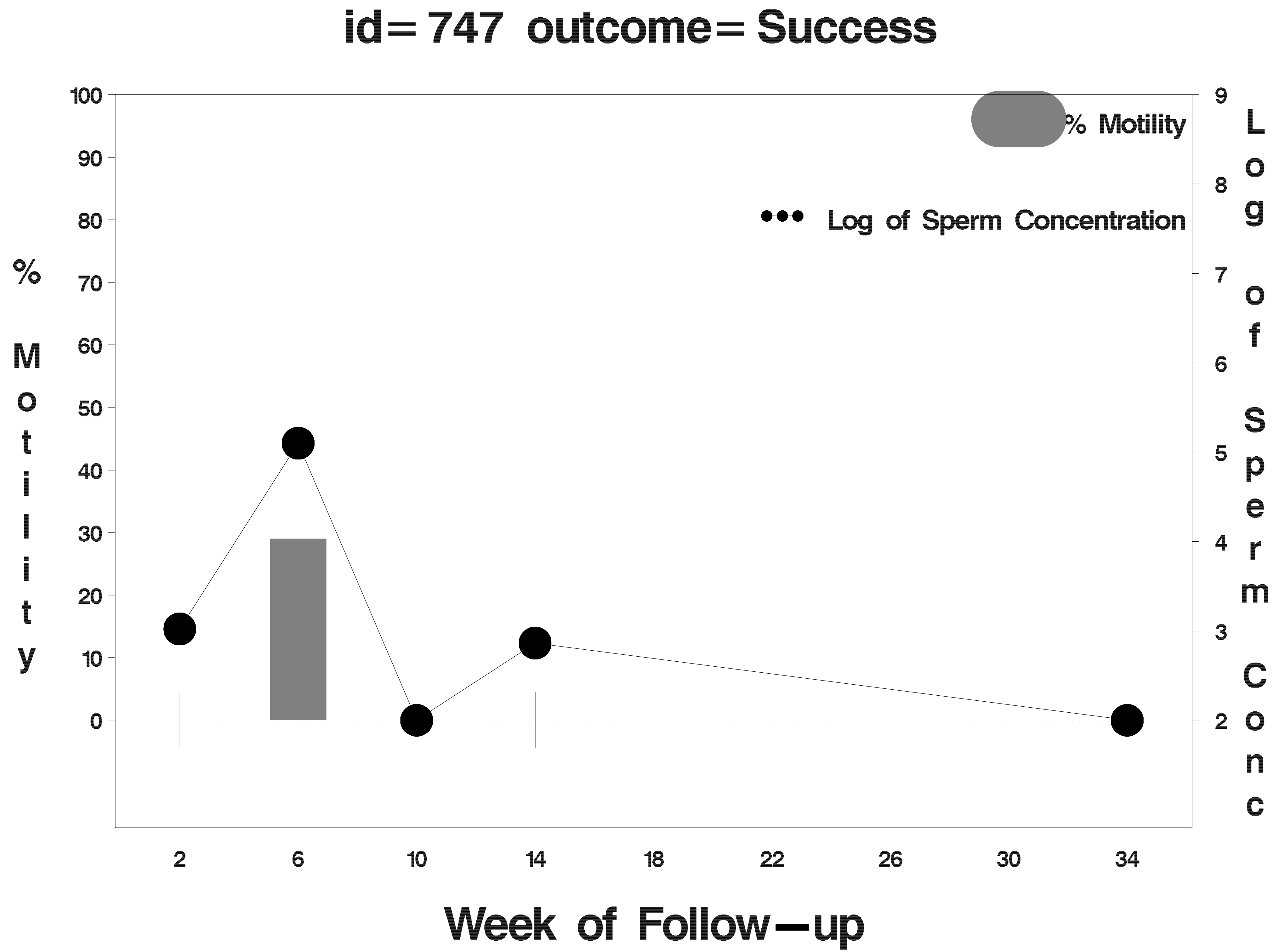
id= 747 outcome= Success
% Motility
Log of Sperm Concentration
100
90
80
70
60
50
40
30
20
10
0
9
8
7
6
5
4
3
2
2
6
10
14
18
22
26
30
34
Week of Follow—up
Log of Sperm Conc

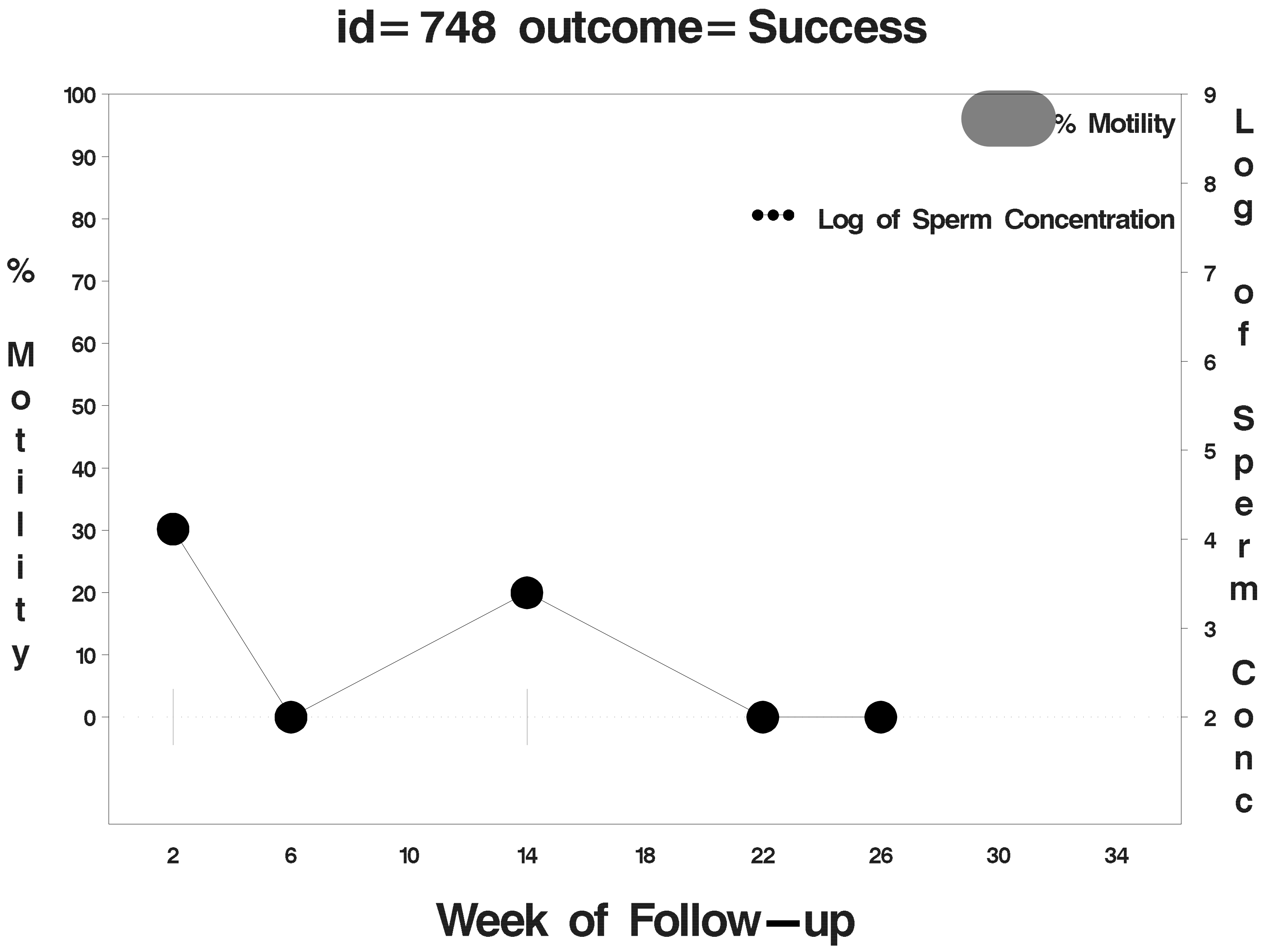
id= 748 outcome= Success
% Motility
Log of Sperm Concentration
% Motility
Log of Sperm Conc
Week of Follow-up
100
90
80
70
60
50
40
30
20
10
0
9
8
7
6
5
4
3
2
2
6
10
14
18
22
26
30
34

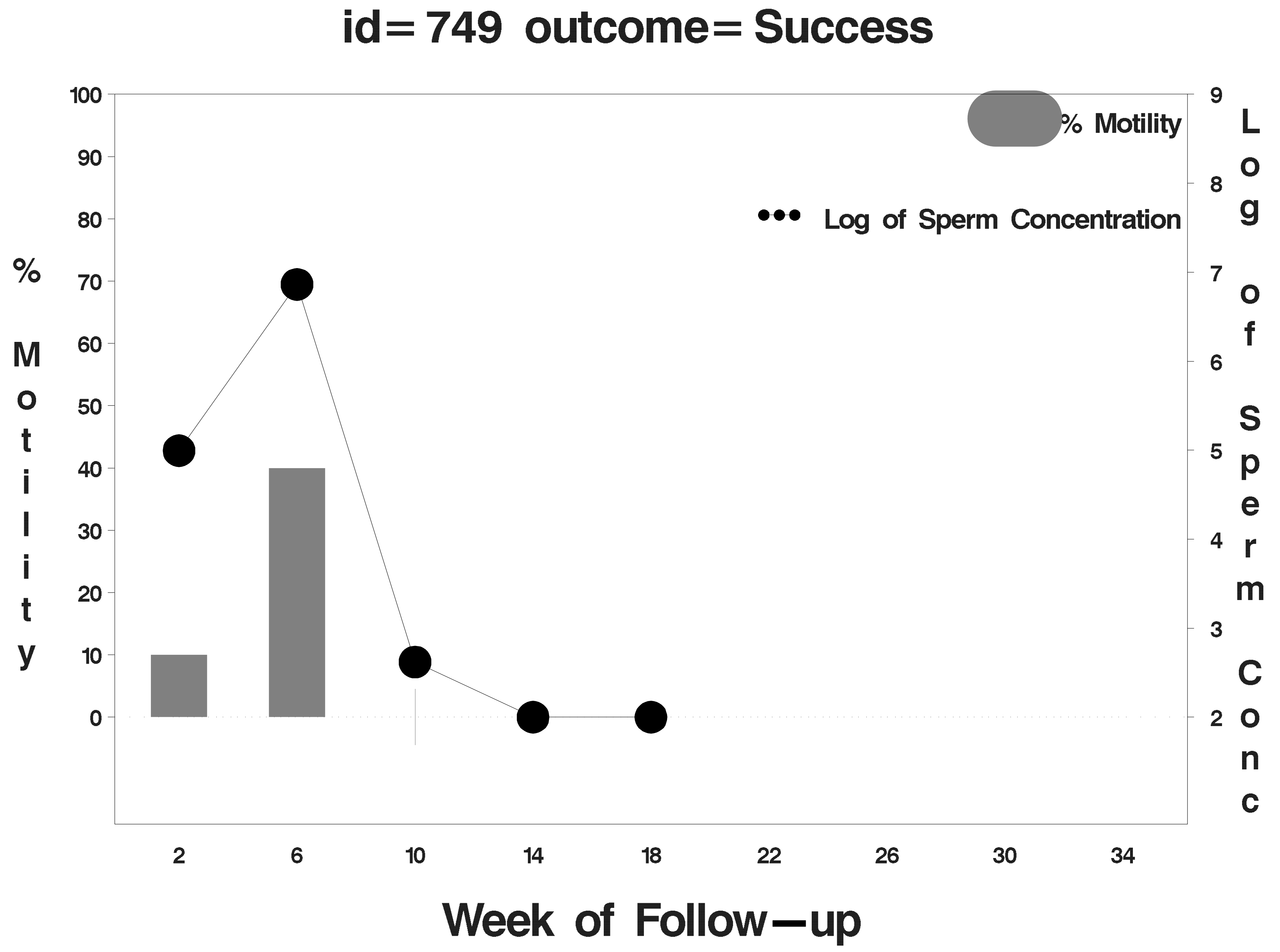

id= 749 outcome= Success
% Motility
Log of Sperm Concentration
100
90
80
70
60
50
40
30
20
10
0
9
8
7
6
5
4
3
2
2
6
10
14
18
22
26
30
34
Week of Follow—up
Log of Sperm Conc

id= 750 outcome= Success

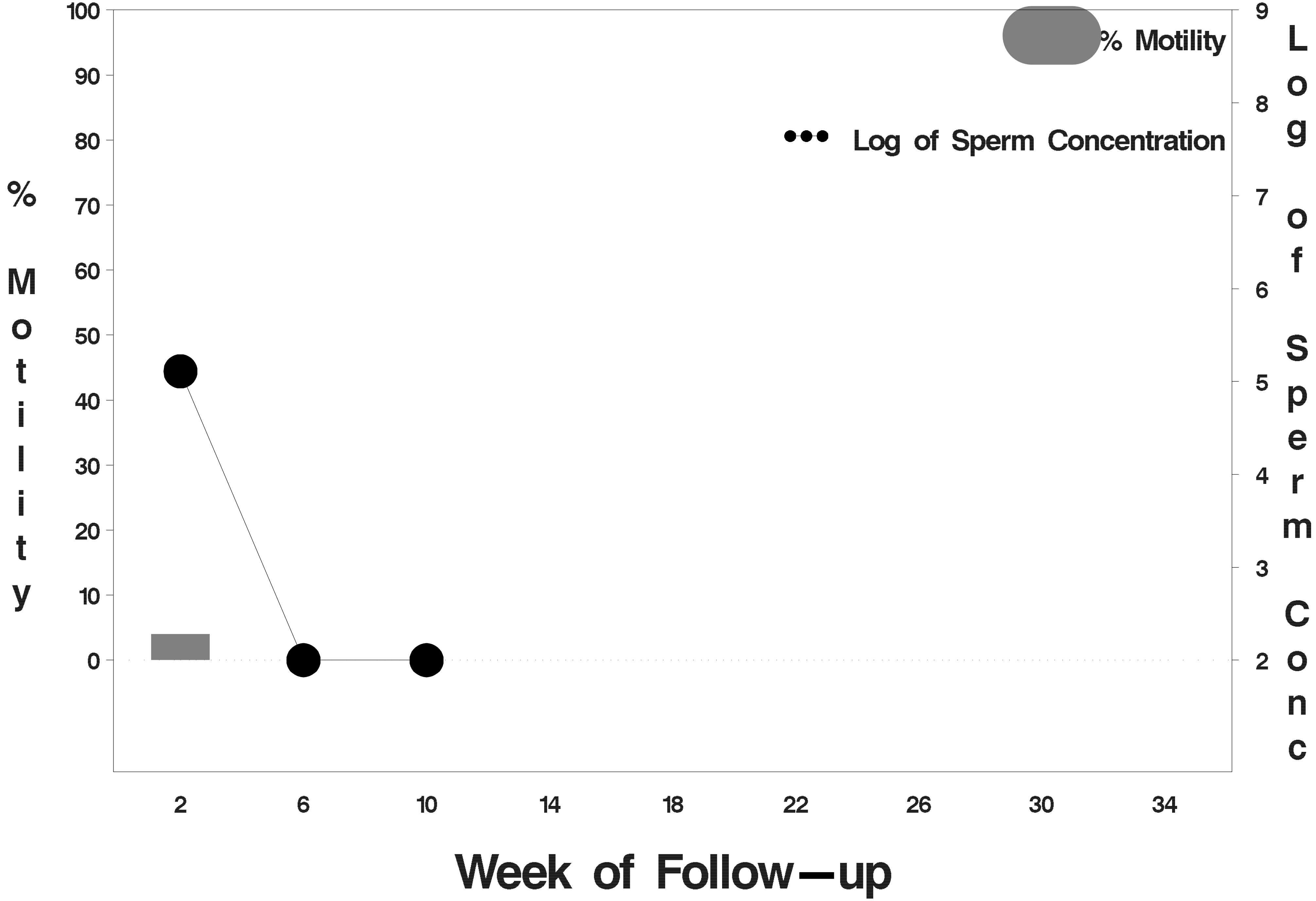

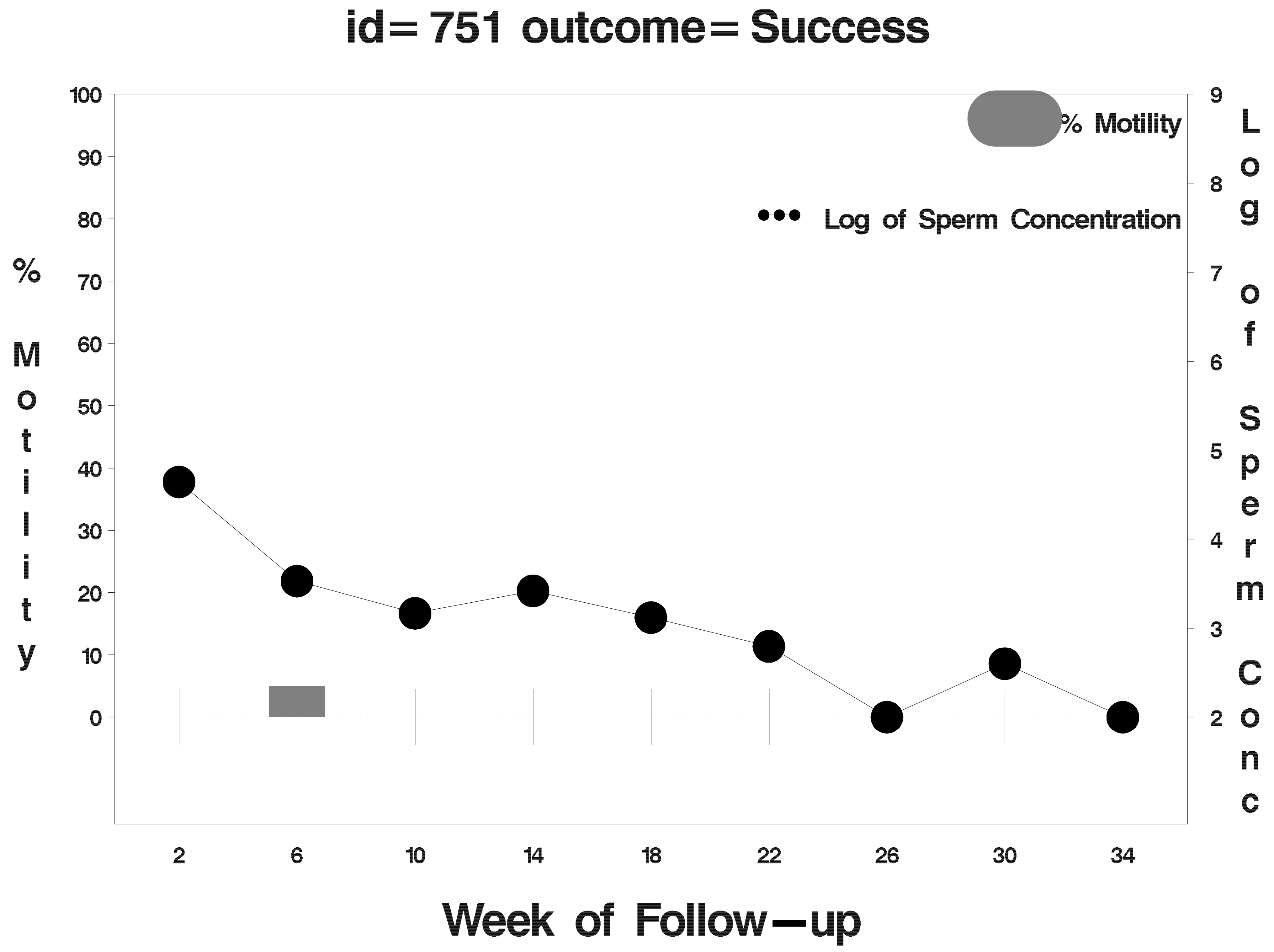
id= 751 outcome= Success
% Motility
Log of Sperm Concentration
% Motility
Log of Sperm Conc
Week of Follow−up
100
90
80
70
60
50
40
30
20
10
0
9
8
7
6
5
4
3
2
2
6
10
14
18
22
26
30
34

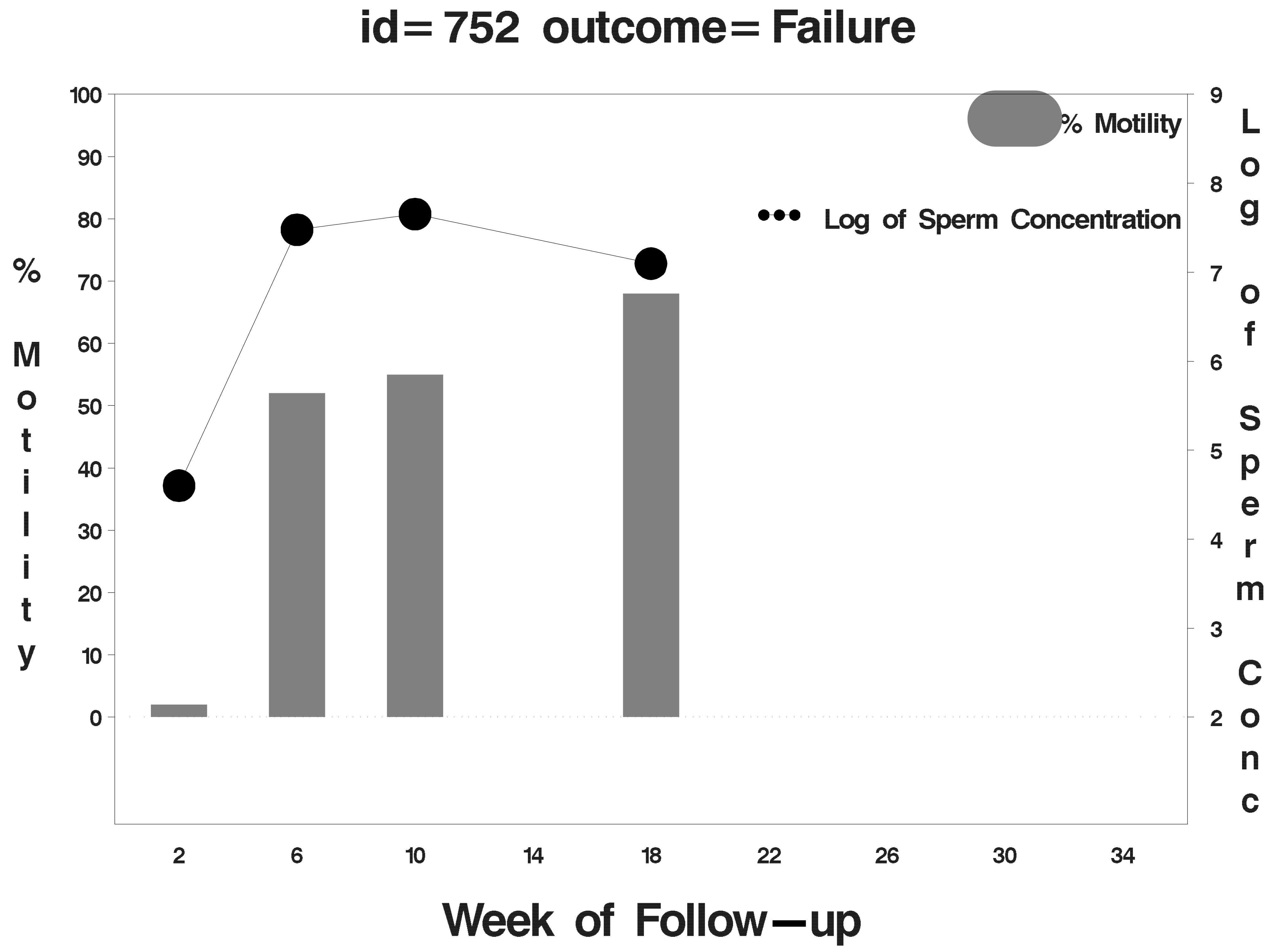
id= 752 outcome= Failure
% Motility
Log of Sperm Concentration
% Motility
Log of Sperm Conc
Week of Follow-up
100
90
80
70
60
50
40
30
20
10
0
9
8
7
6
5
4
3
2
2
6
10
14
18
22
26
30
34

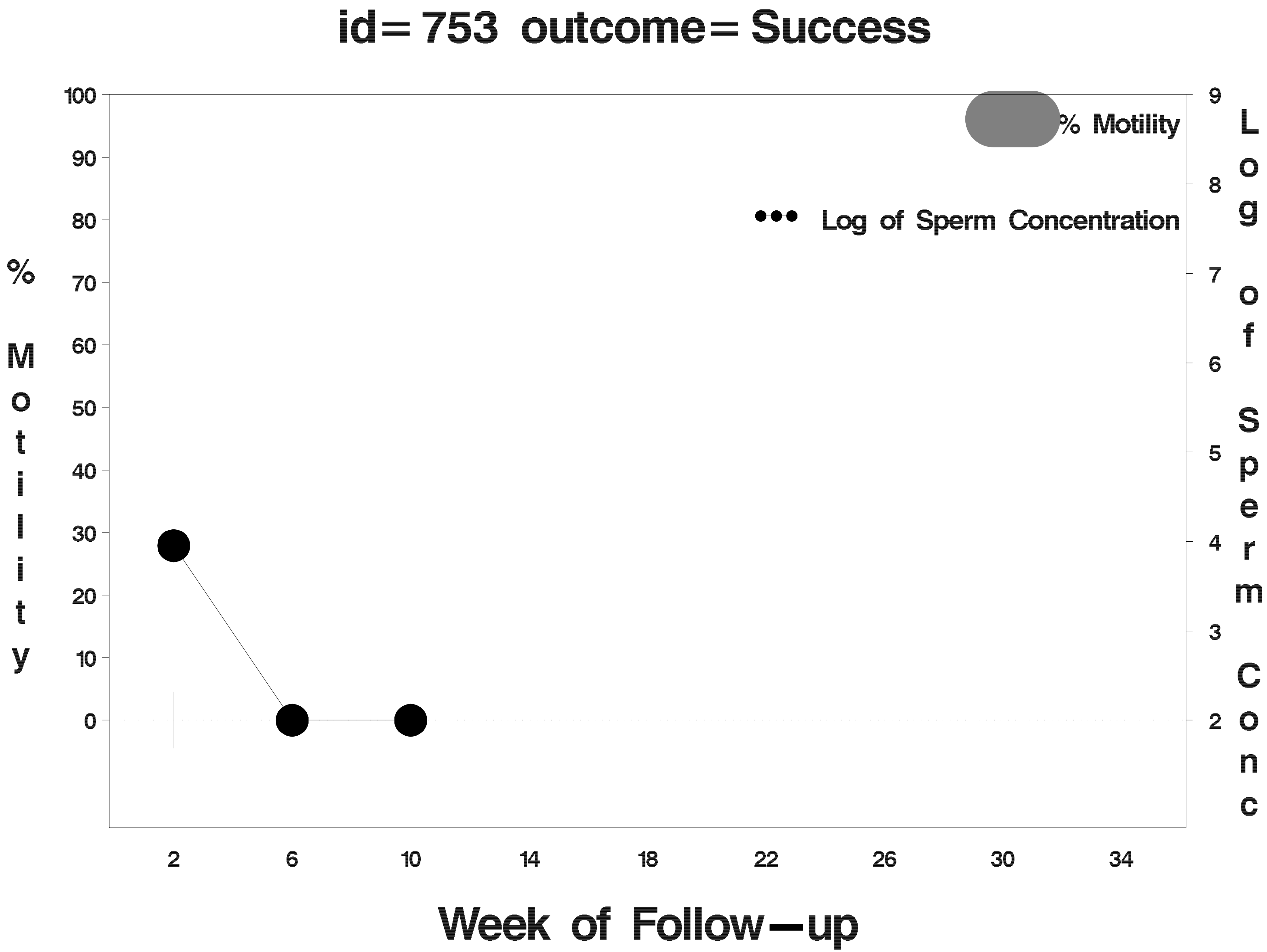
id= 753 outcome= Success
% Motility
Log of Sperm Concentration
100
90
80
70
60
50
40
30
20
10
0
9
8
7
6
5
4
3
2
2
6
10
14
18
22
26
30
34
Week of Follow-up
Log of Sperm Conc

id= 754 outcome= Success

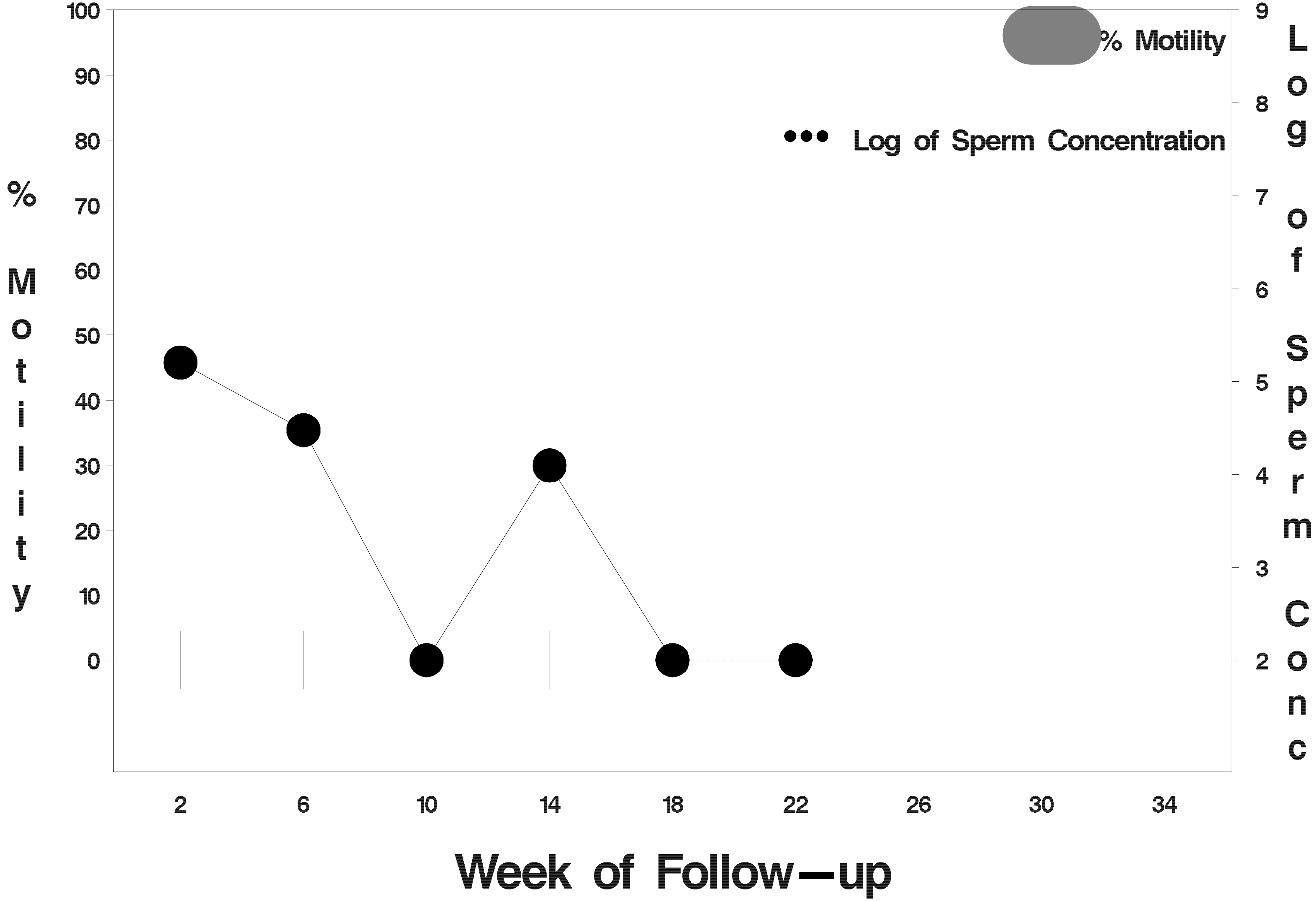

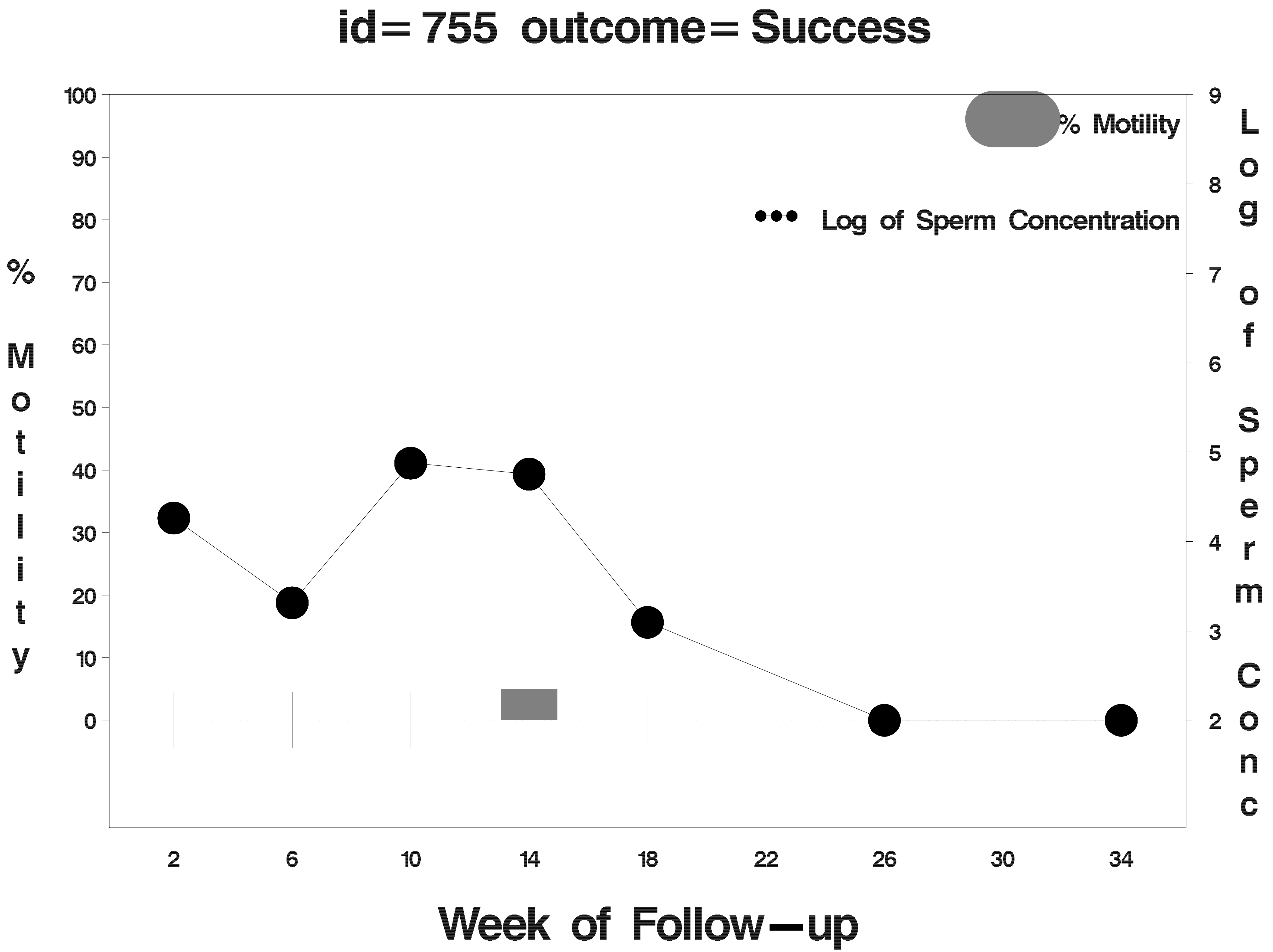

id= 755 outcome= Success
% Motility
Log of Sperm Concentration
100
90
80
70
60
50
40
30
20
10
0
9
8
7
6
5
4
3
2
2
6
10
14
18
22
26
30
34
Week of Follow−up
Log of Sperm Conc

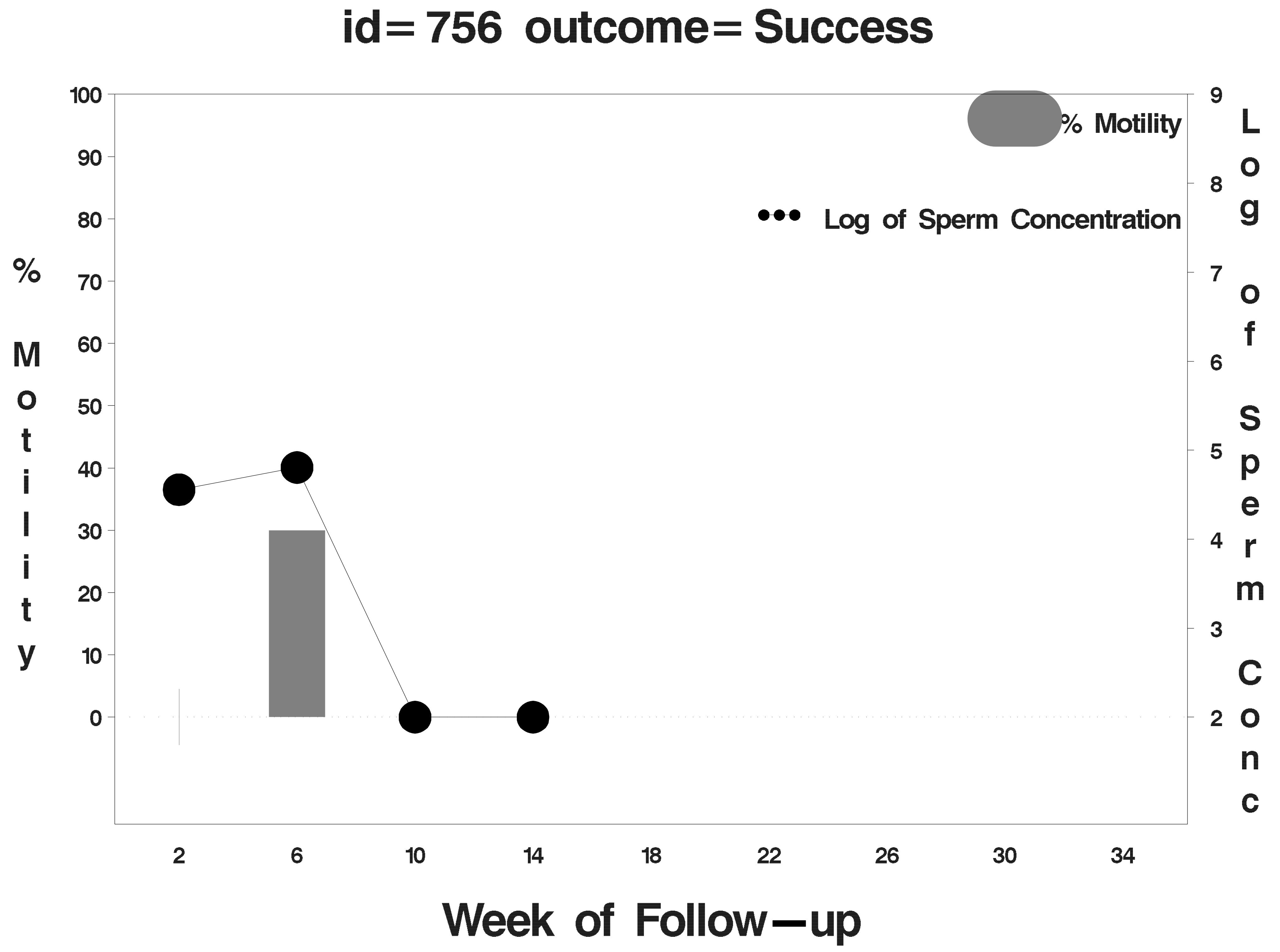

id= 756 outcome= Success
% Motility
Log of Sperm Concentration
100
90
80
70
60
50
40
30
20
10
0
9
8
7
6
5
4
3
2
2
6
10
14
18
22
26
30
34
Week of Follow−up
% Motility
Log of Sperm Conc

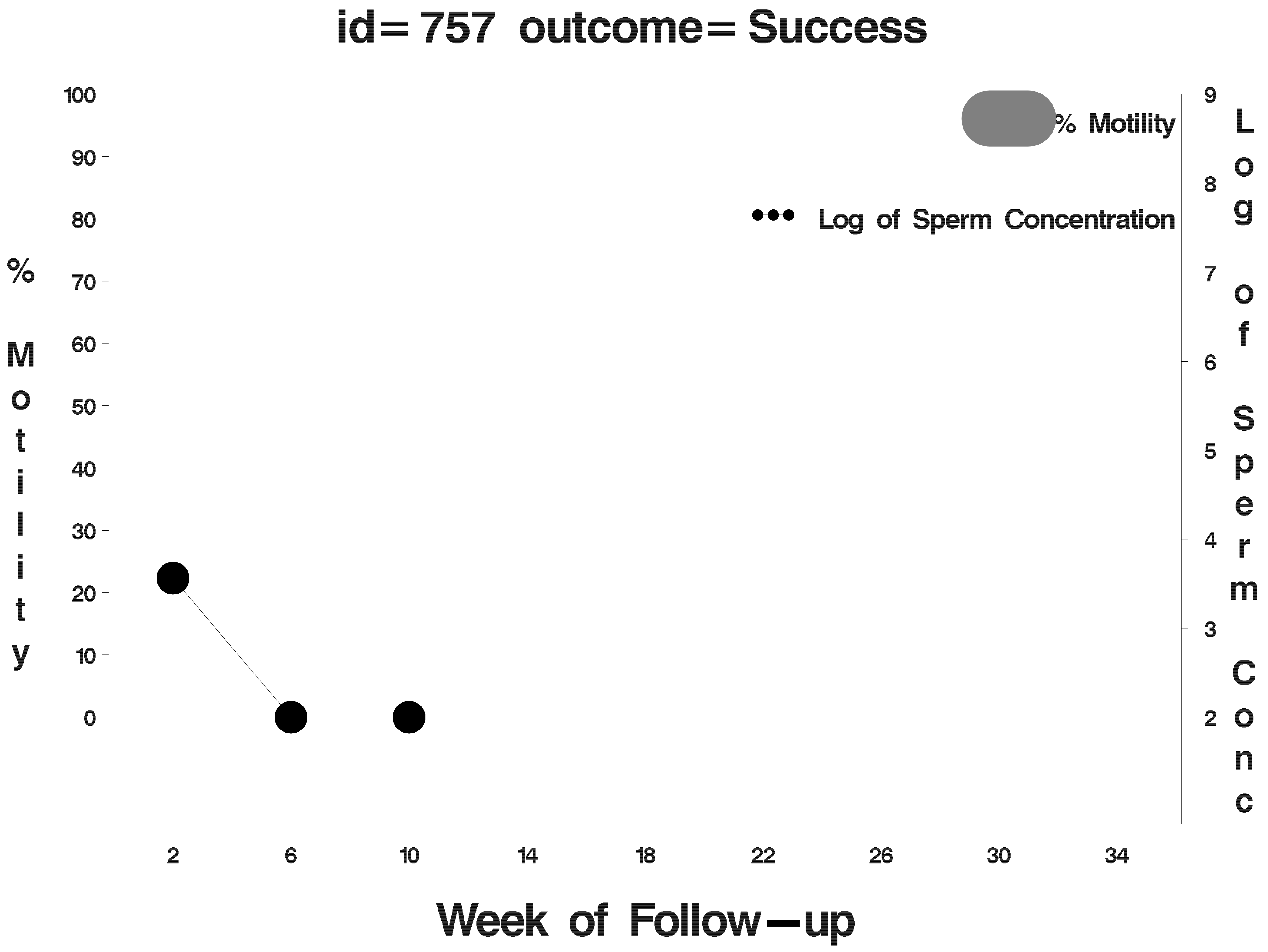

id= 757 outcome= Success
% Motility
Log of Sperm Concentration
100
90
80
70
60
50
40
30
20
10
0
9
8
7
6
5
4
3
2
2
6
10
14
18
22
26
30
34
Week of Follow−up
Log of Sperm Conc

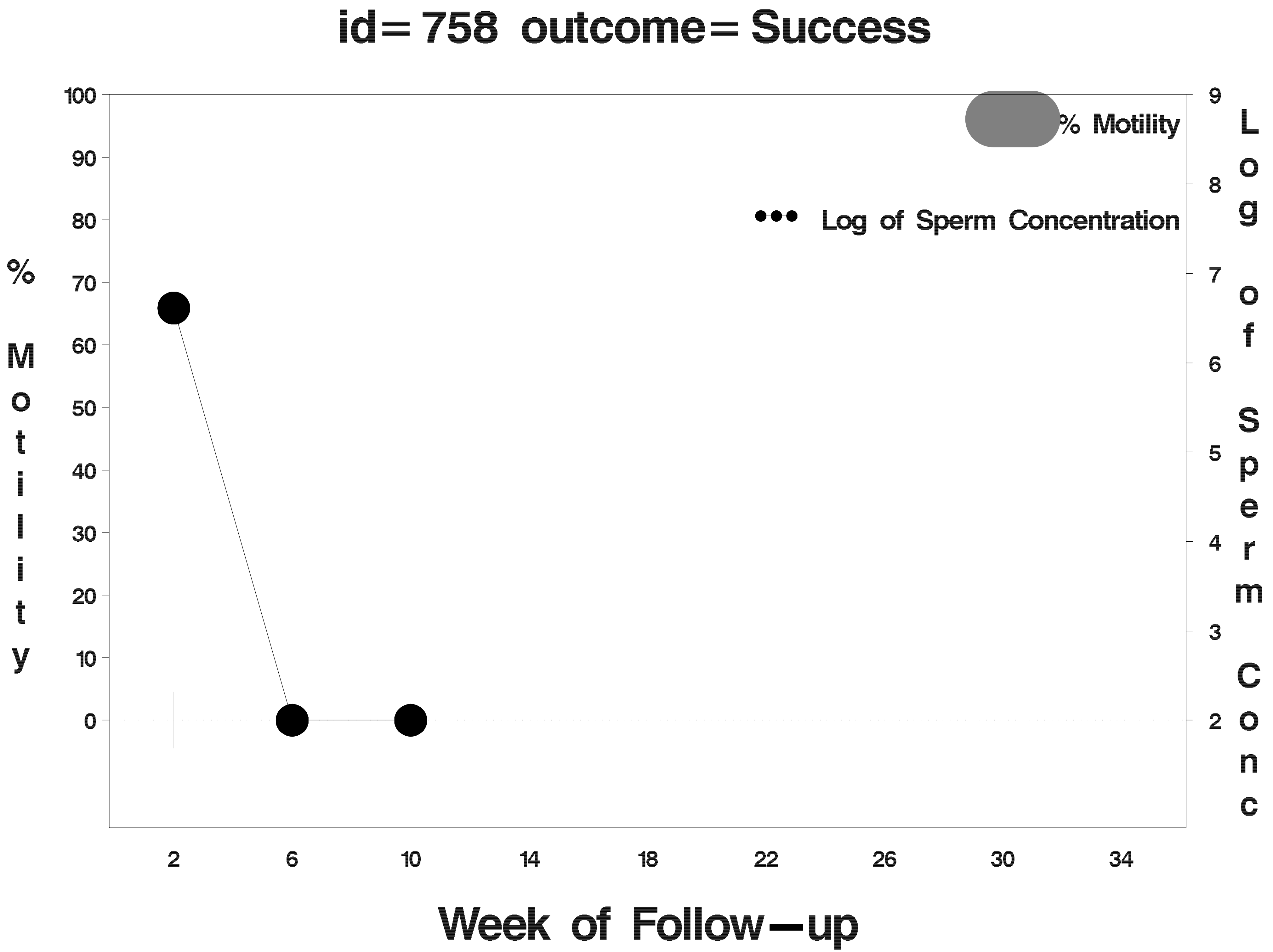

id= 758 outcome= Success
% Motility
Log of Sperm Concentration
100
90
80
70
60
50
40
30
20
10
0
9
8
7
6
5
4
3
2
2
6
10
14
18
22
26
30
34
Week of Follow—up
Log of Sperm Conc

id= 759 outcome= Indeterminate

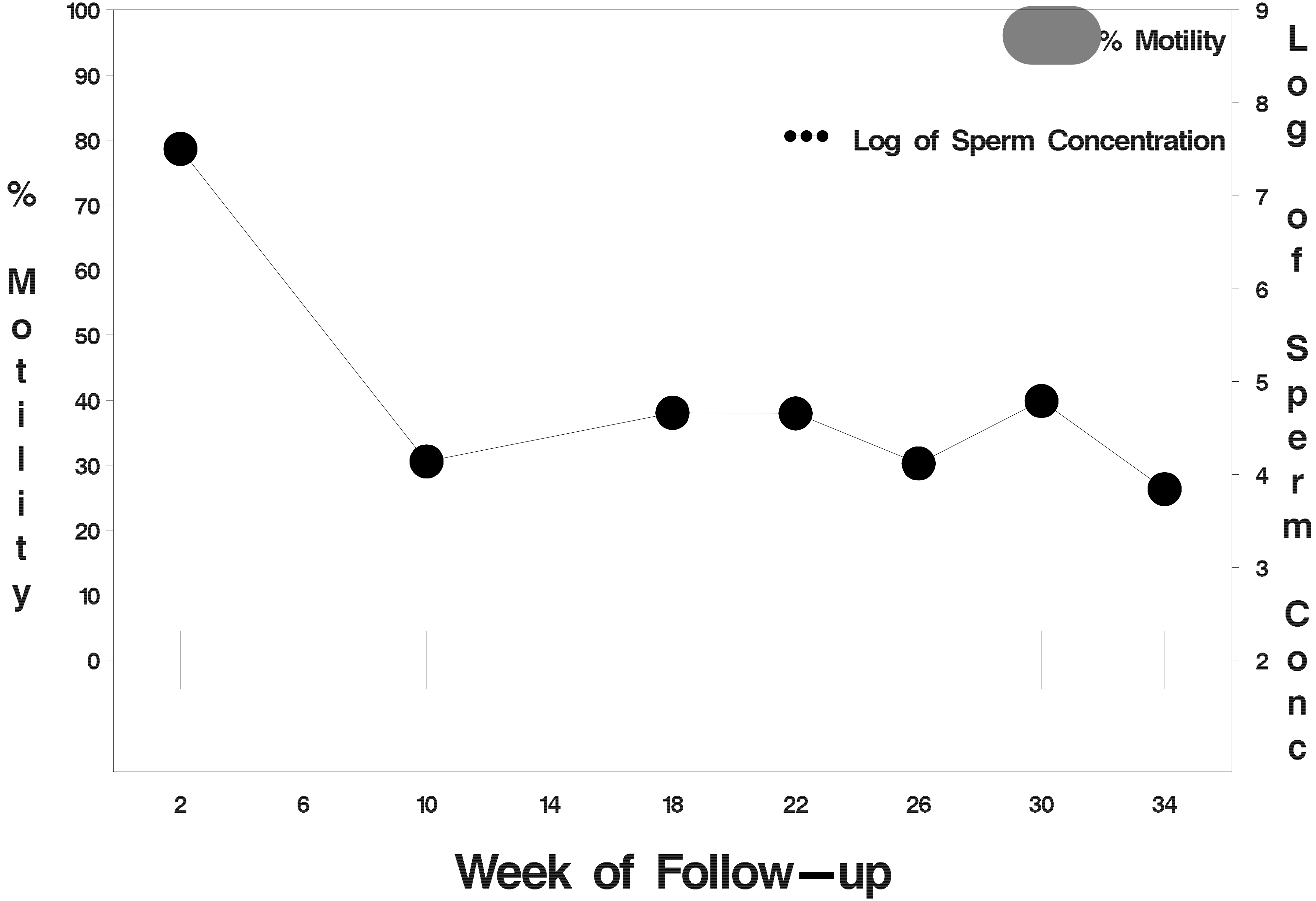

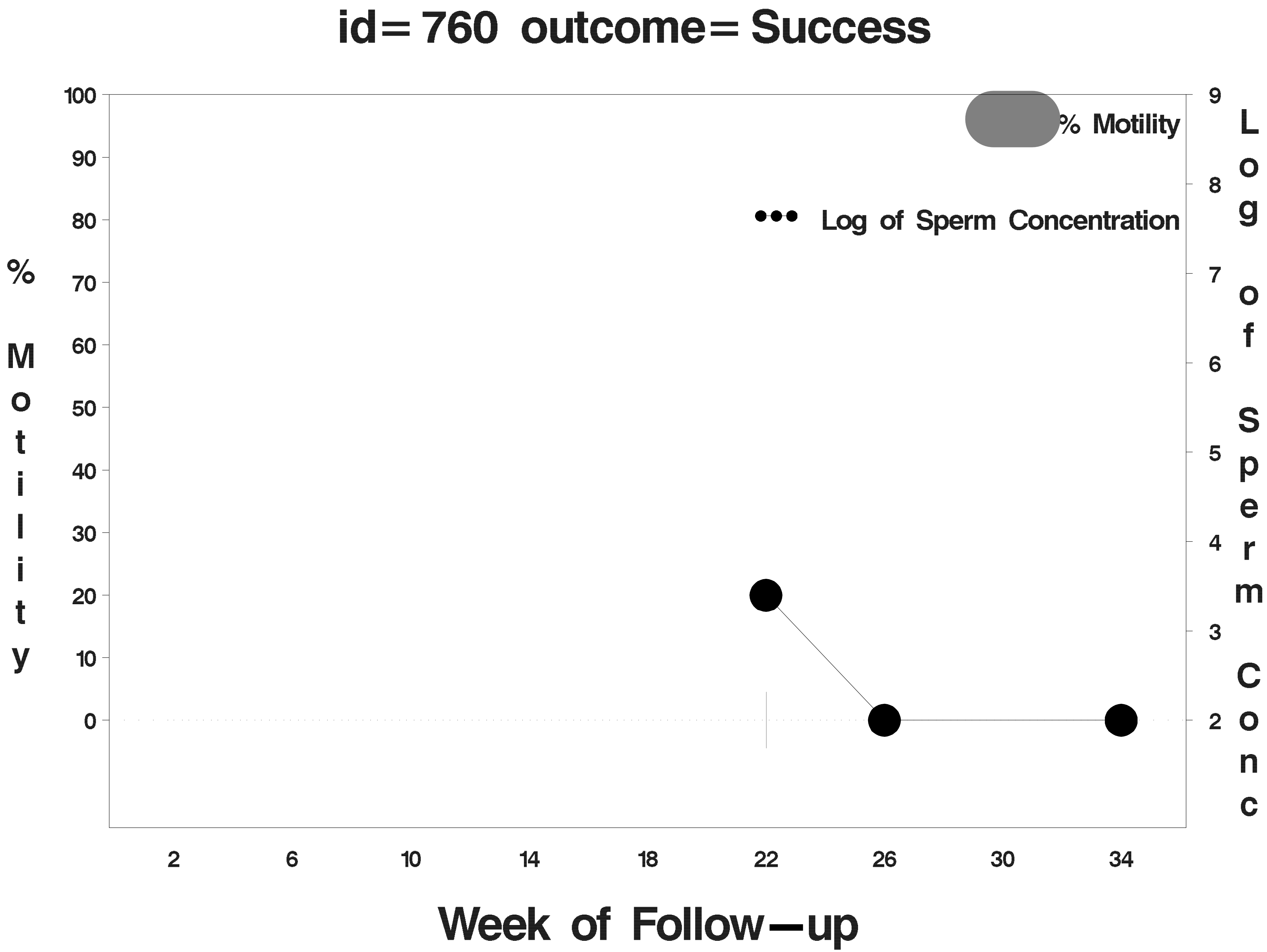
id= 760 outcome= Success
% Motility
Log of Sperm Concentration
100
90
80
70
60
50
40
30
20
10
0
9
8
7
6
5
4
3
2
2
6
10
14
18
22
26
30
34
Week of Follow−up
% Motility
Log of Sperm Conc

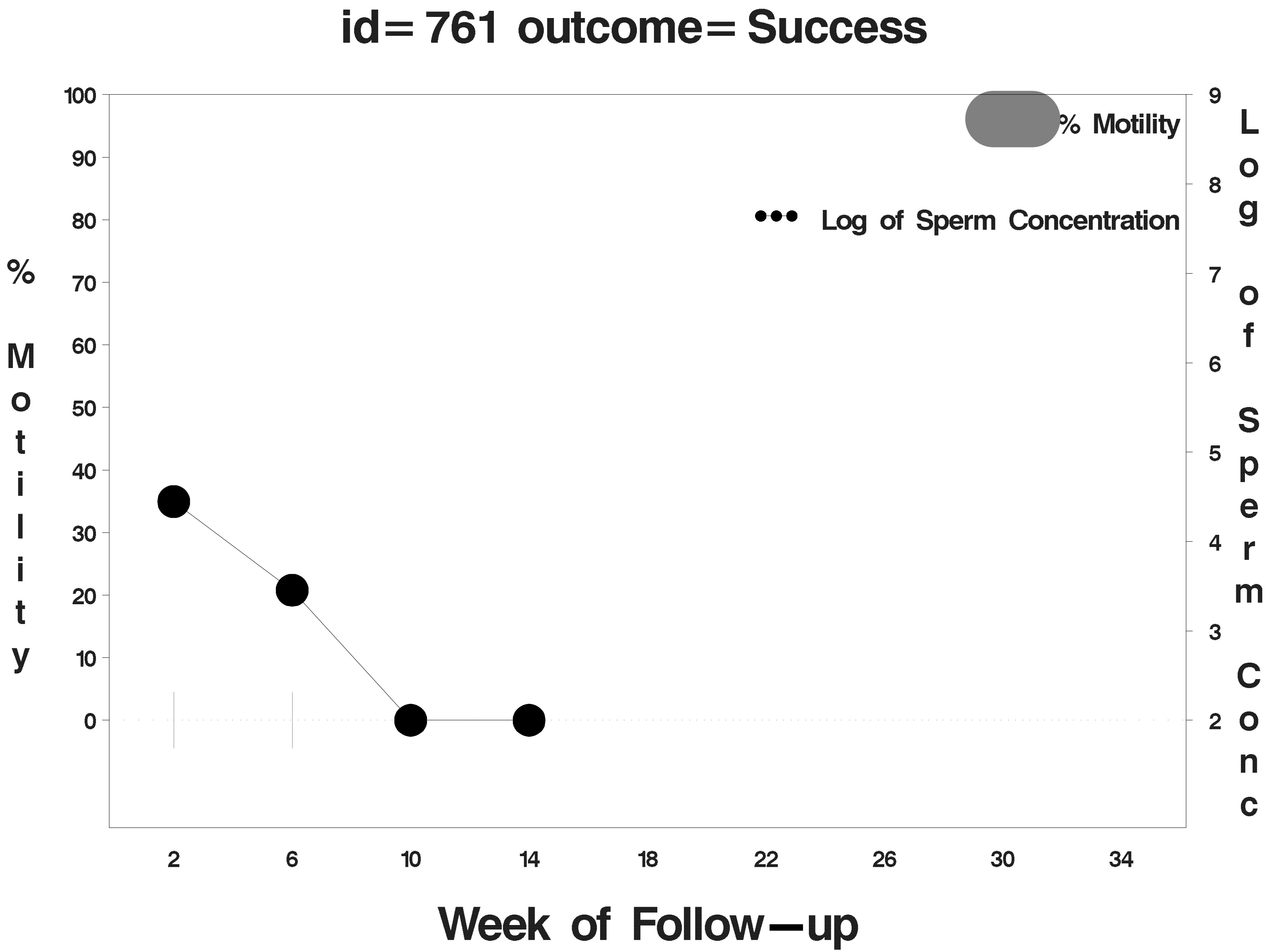
id= 761 outcome= Success
% Motility
Log of Sperm Concentration
100
90
80
70
60
50
40
30
20
10
0
9
8
7
6
5
4
3
2
2
6
10
14
18
22
26
30
34
Week of Follow−up
Log of Sperm Conc

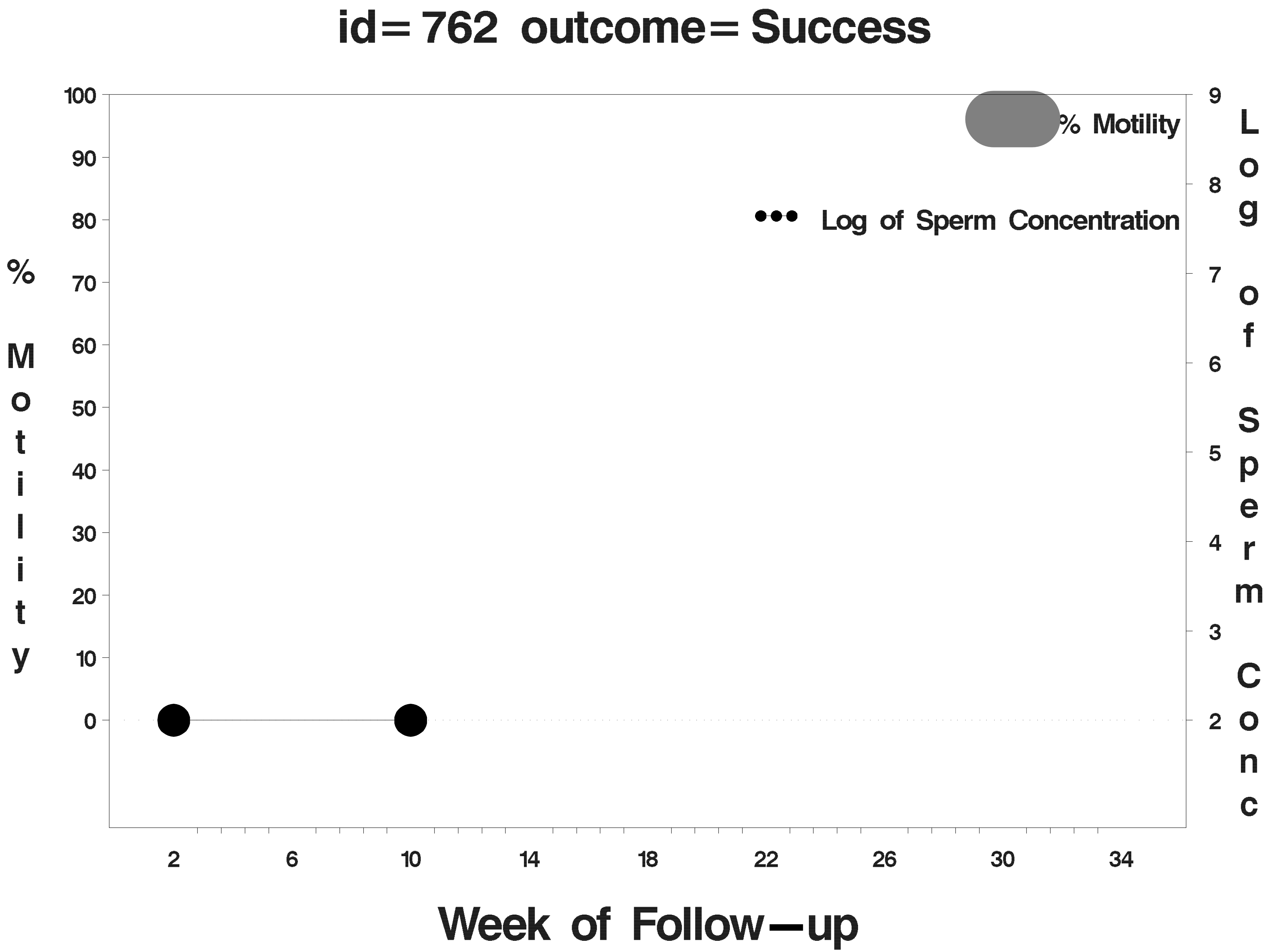
id= 762 outcome= Success
% Motility
Log of Sperm Concentration
100
90
80
70
60
50
40
30
20
10
0
9
8
7
6
5
4
3
2
2
6
10
14
18
22
26
30
34
Week of Follow—up
% Motility
Log of Sperm Conc

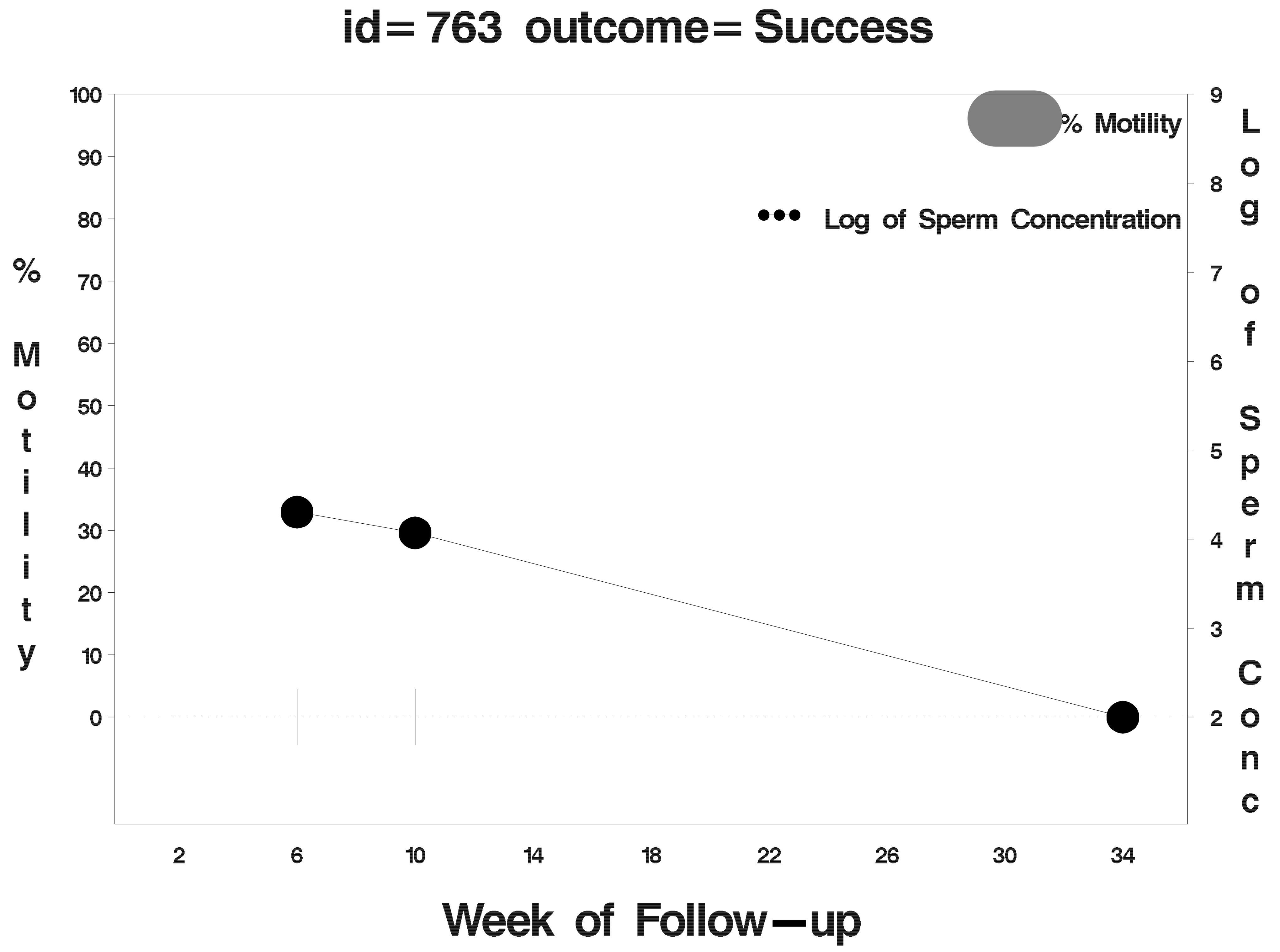

id= 763 outcome= Success
% Motility
Log of Sperm Concentration
% Motility
Log of Sperm Conc
Week of Follow−up
100
90
80
70
60
50
40
30
20
10
0
9
8
7
6
5
4
3
2
2
6
10
14
18
22
26
30
34

id= 764 outcome= Success

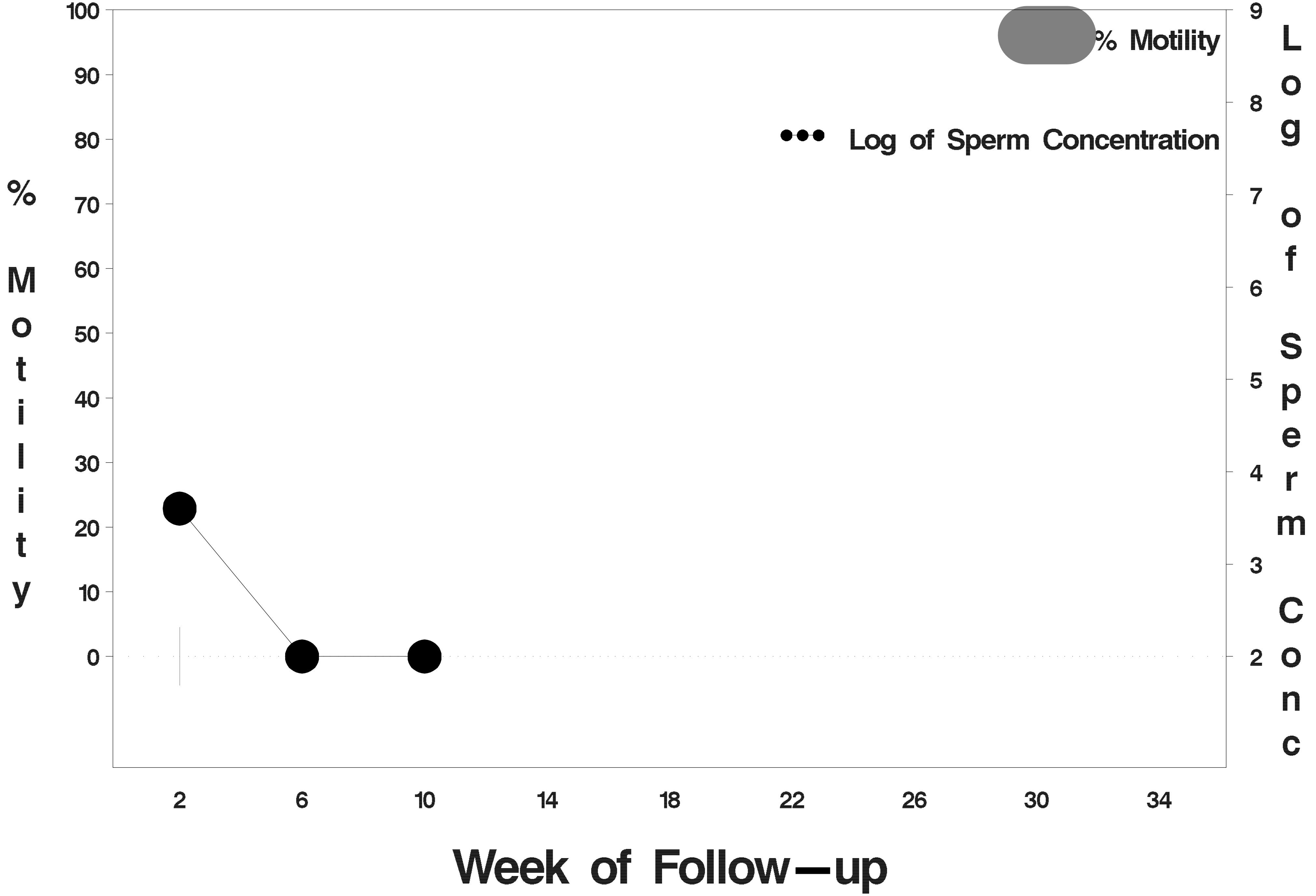

id= 765 outcome= Indeterminate

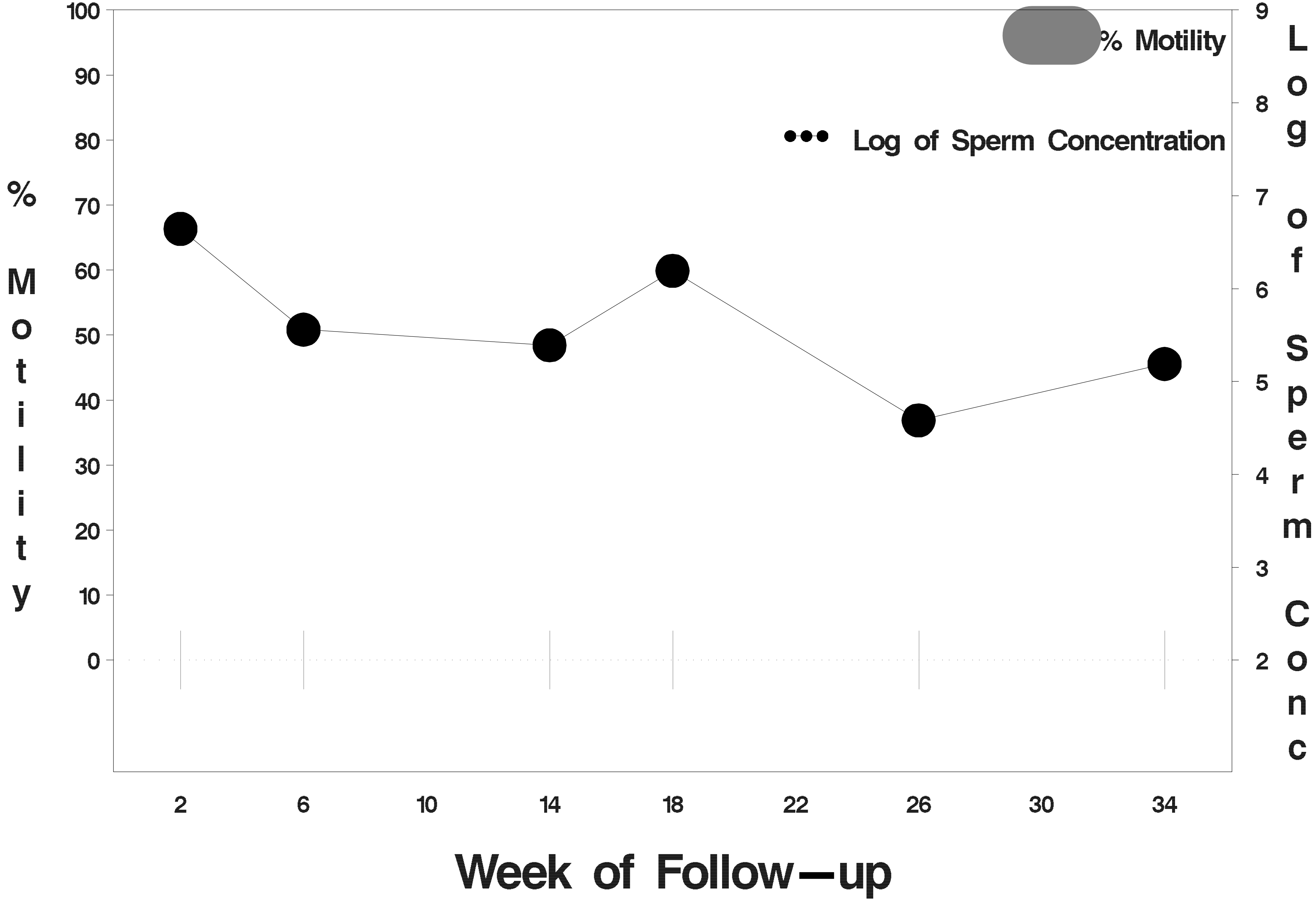

id= 766 outcome= Success

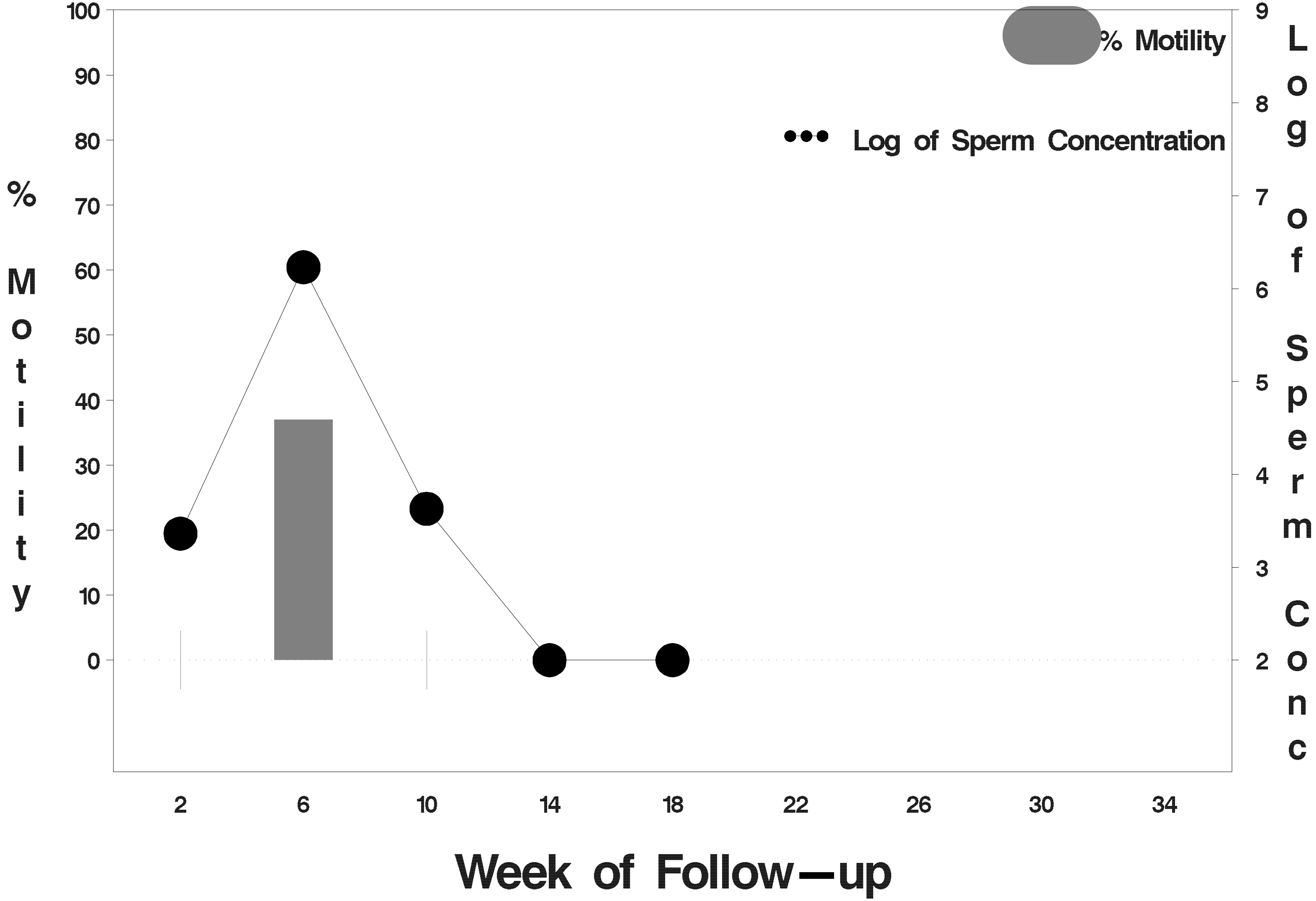

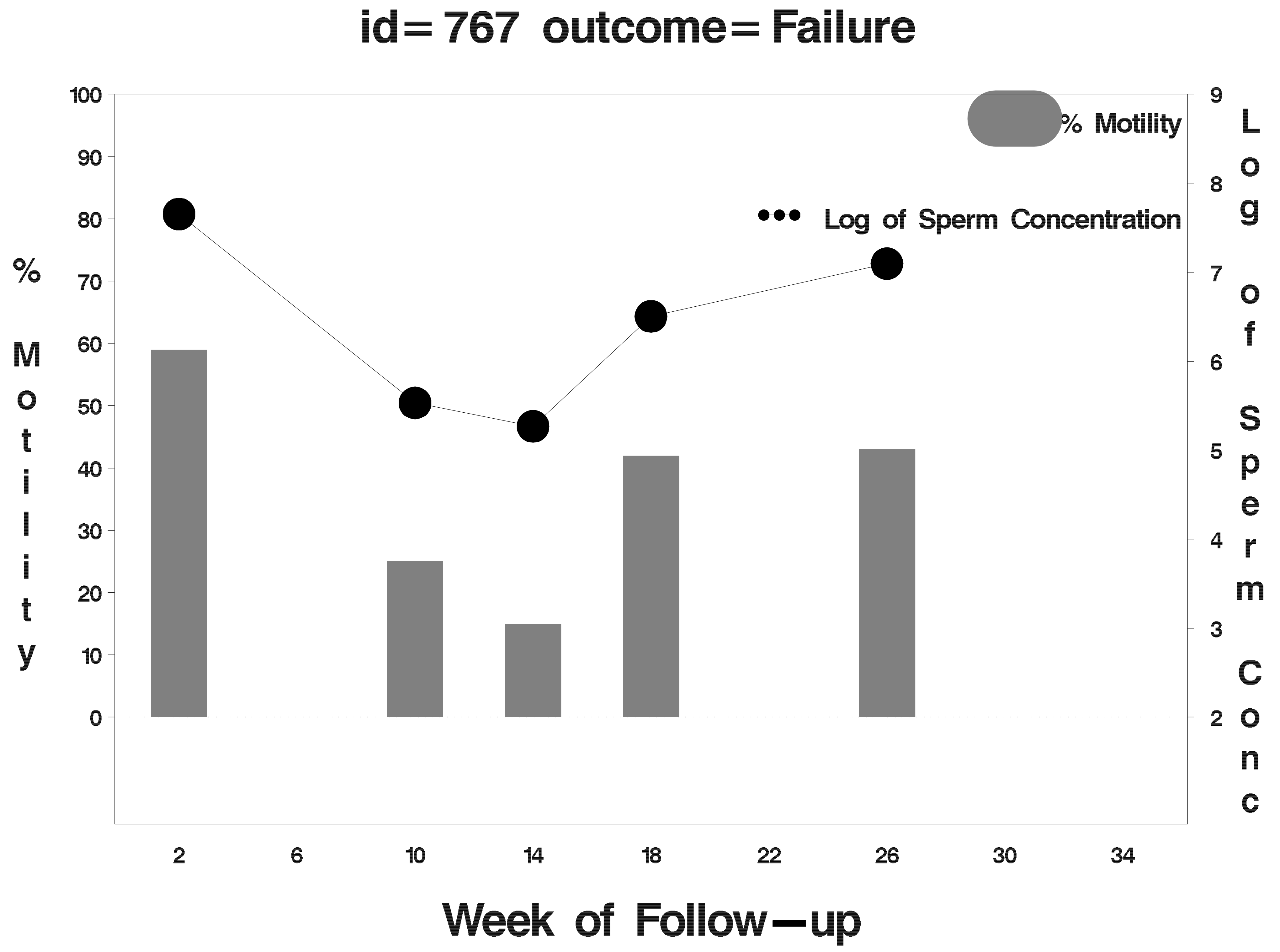

id= 767 outcome= Failure
% Motility
Log of Sperm Concentration
100
90
80
70
60
50
40
30
20
10
0
9
8
7
6
5
4
3
2
2
6
10
14
18
22
26
30
34
Week of Follow−up
Log of Sperm Conc

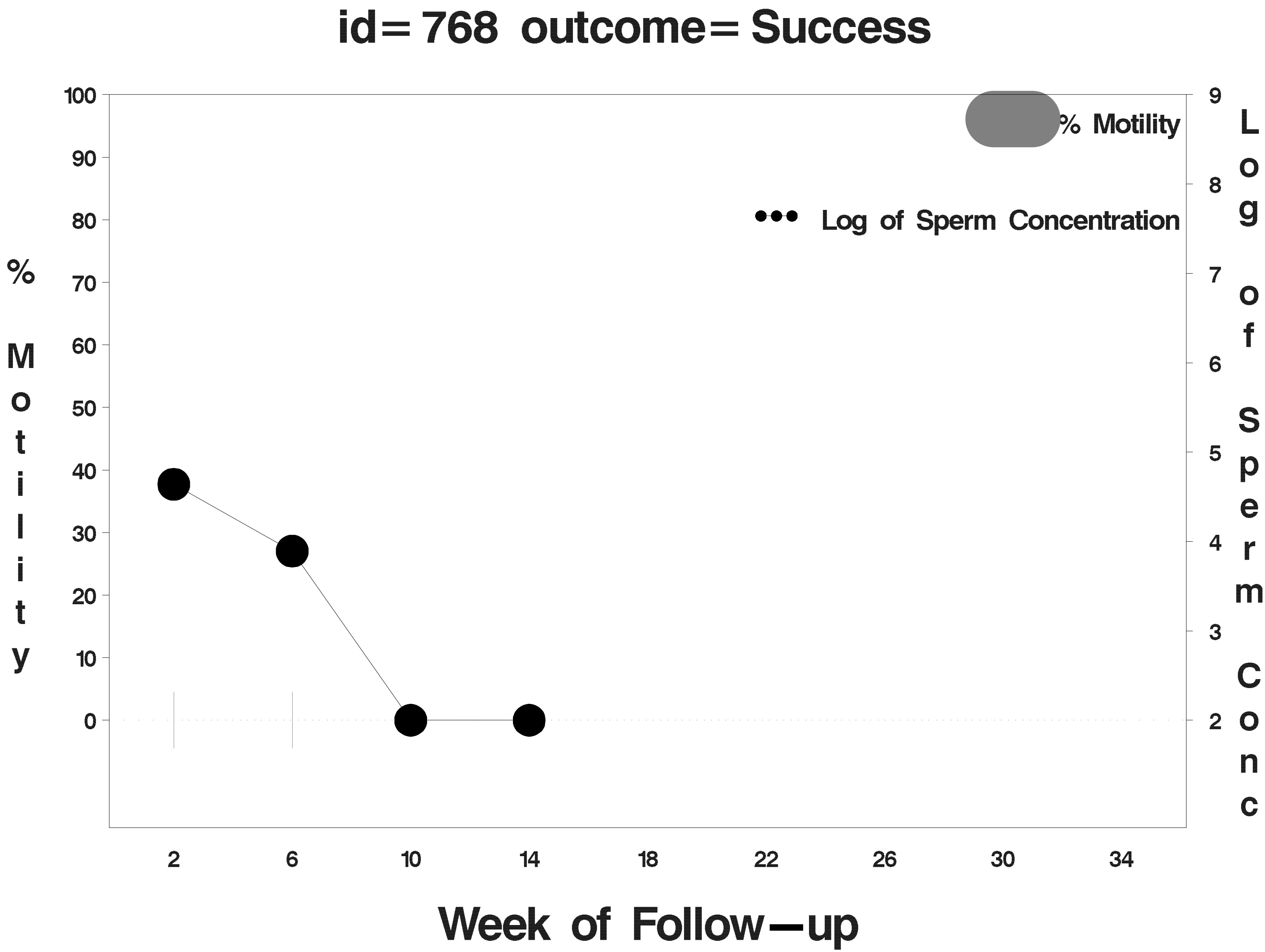

id= 768 outcome= Success
% Motility
Log of Sperm Concentration
% Motility
Log of Sperm Conc
Week of Follow−up
100
90
80
70
60
50
40
30
20
10
0
9
8
7
6
5
4
3
2
2
6
10
14
18
22
26
30
34

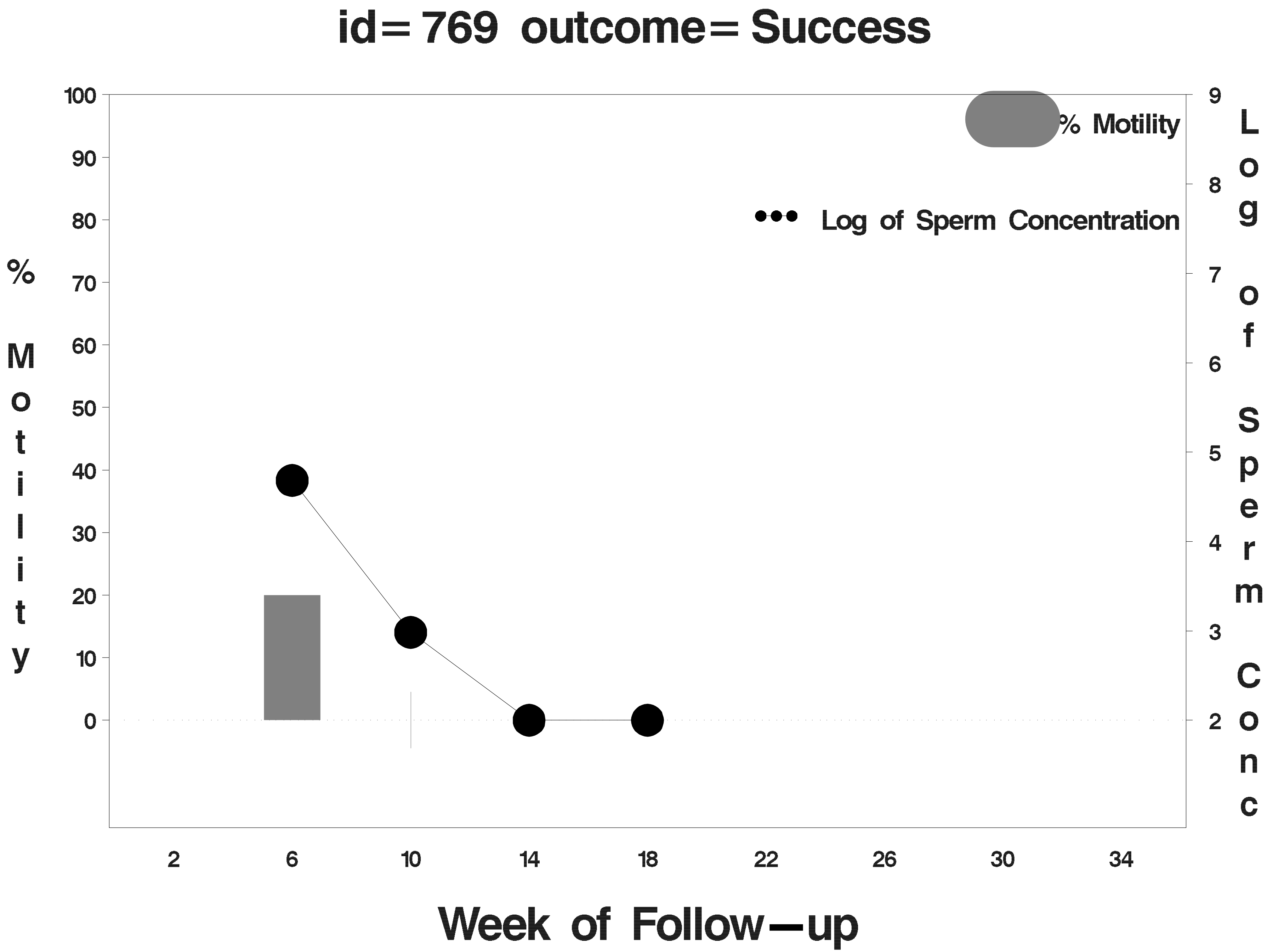
id= 769 outcome= Success
% Motility
Log of Sperm Concentration
100
90
80
70
60
50
40
30
20
10
0
9
8
7
6
5
4
3
2
2
6
10
14
18
22
26
30
34
Week of Follow—up
% Motility
Log of Sperm Conc

id= 770 outcome= Indeterminate

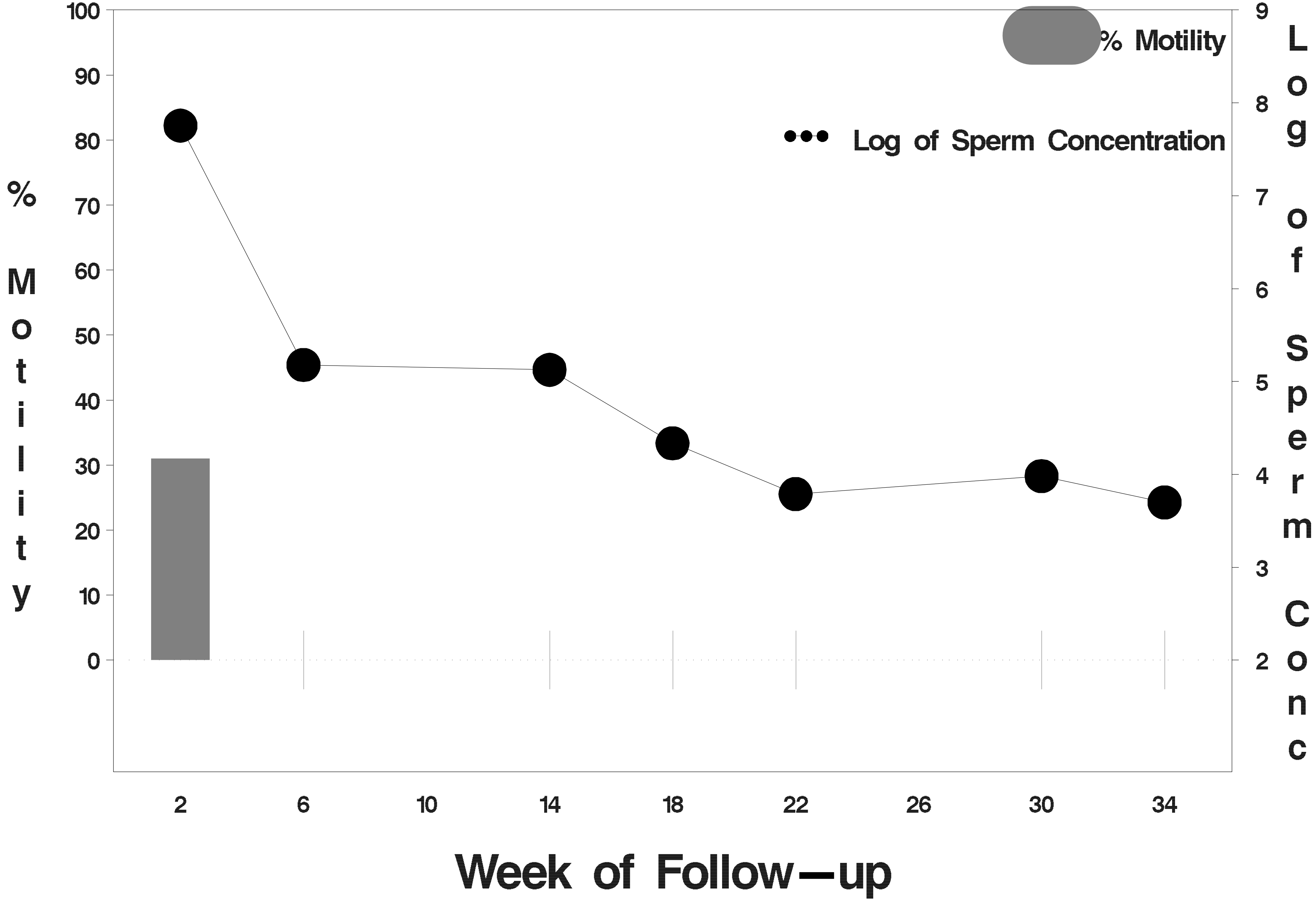

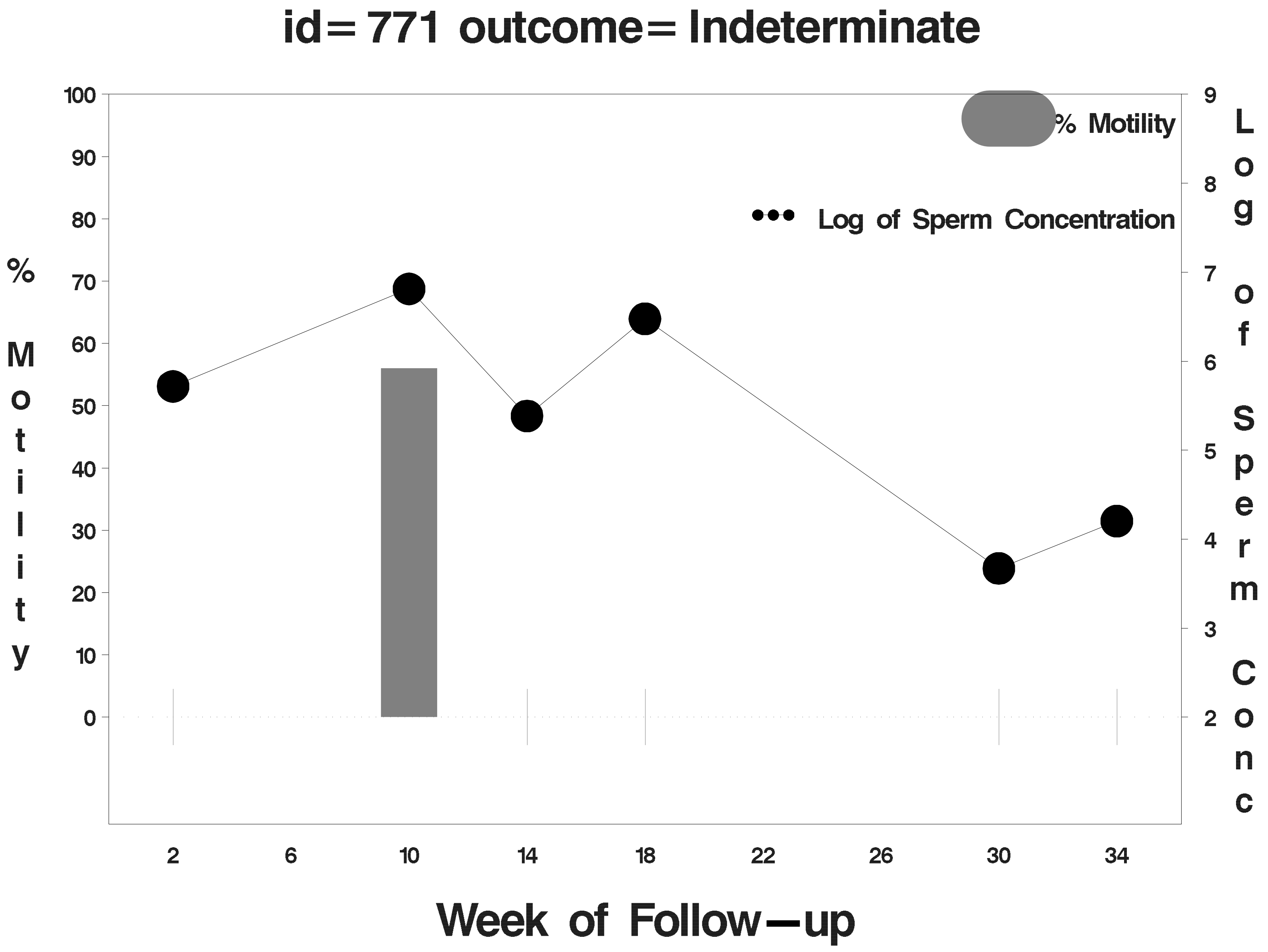
id= 771 outcome= Indeterminate
% Motility
Log of Sperm Concentration
% Motility
Log of Sperm Conc
Week of Follow−up
100
90
80
70
60
50
40
30
20
10
0
9
8
7
6
5
4
3
2
2
6
10
14
18
22
26
30
34

id= 772 outcome= Indeterminate

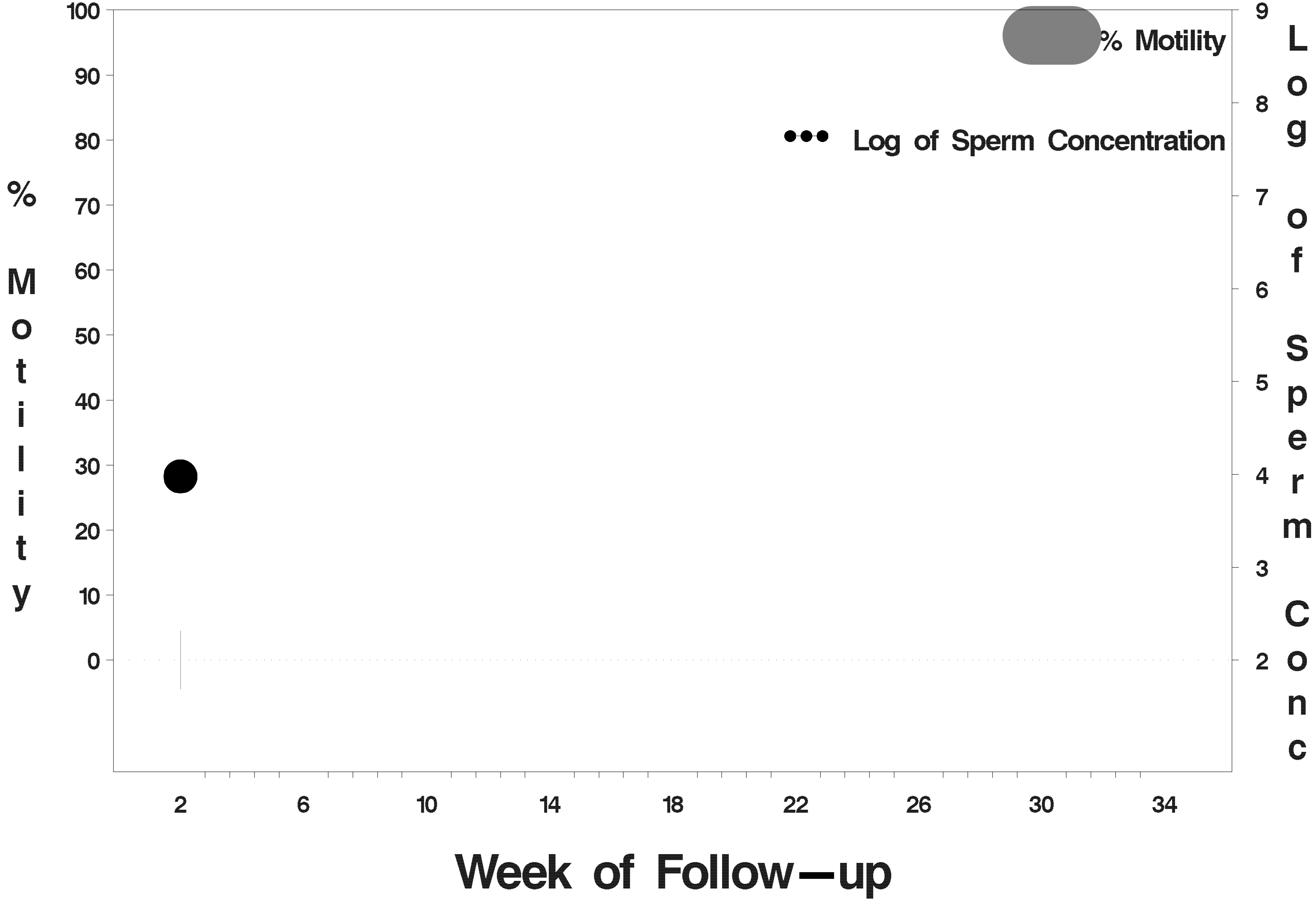

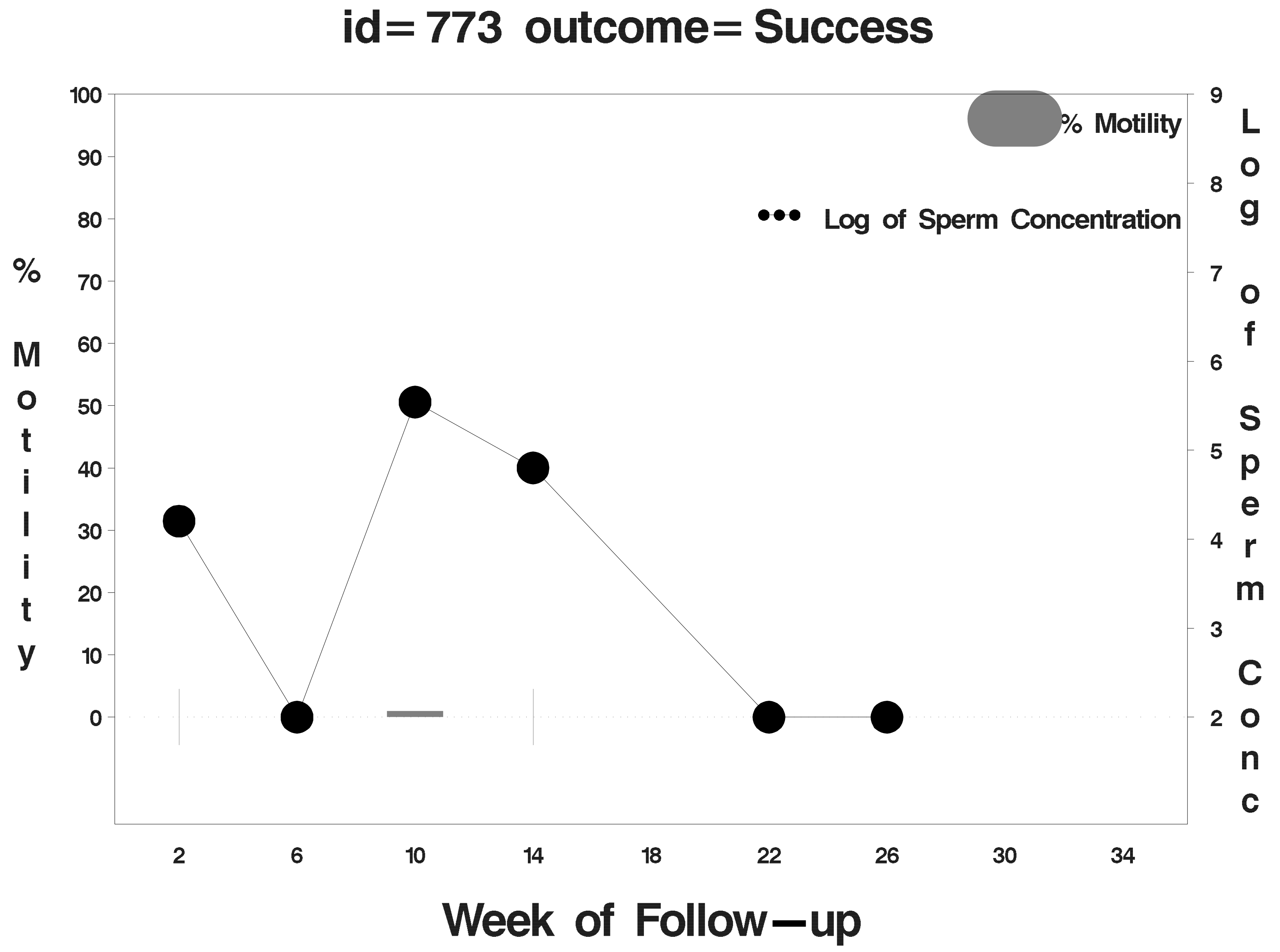

id= 773 outcome= Success
% Motility
Log of Sperm Concentration
100
90
80
70
60
50
40
30
20
10
0
9
8
7
6
5
4
3
2
% Motility
Log of Sperm Conc
2
6
10
14
18
22
26
30
34
Week of Follow—up

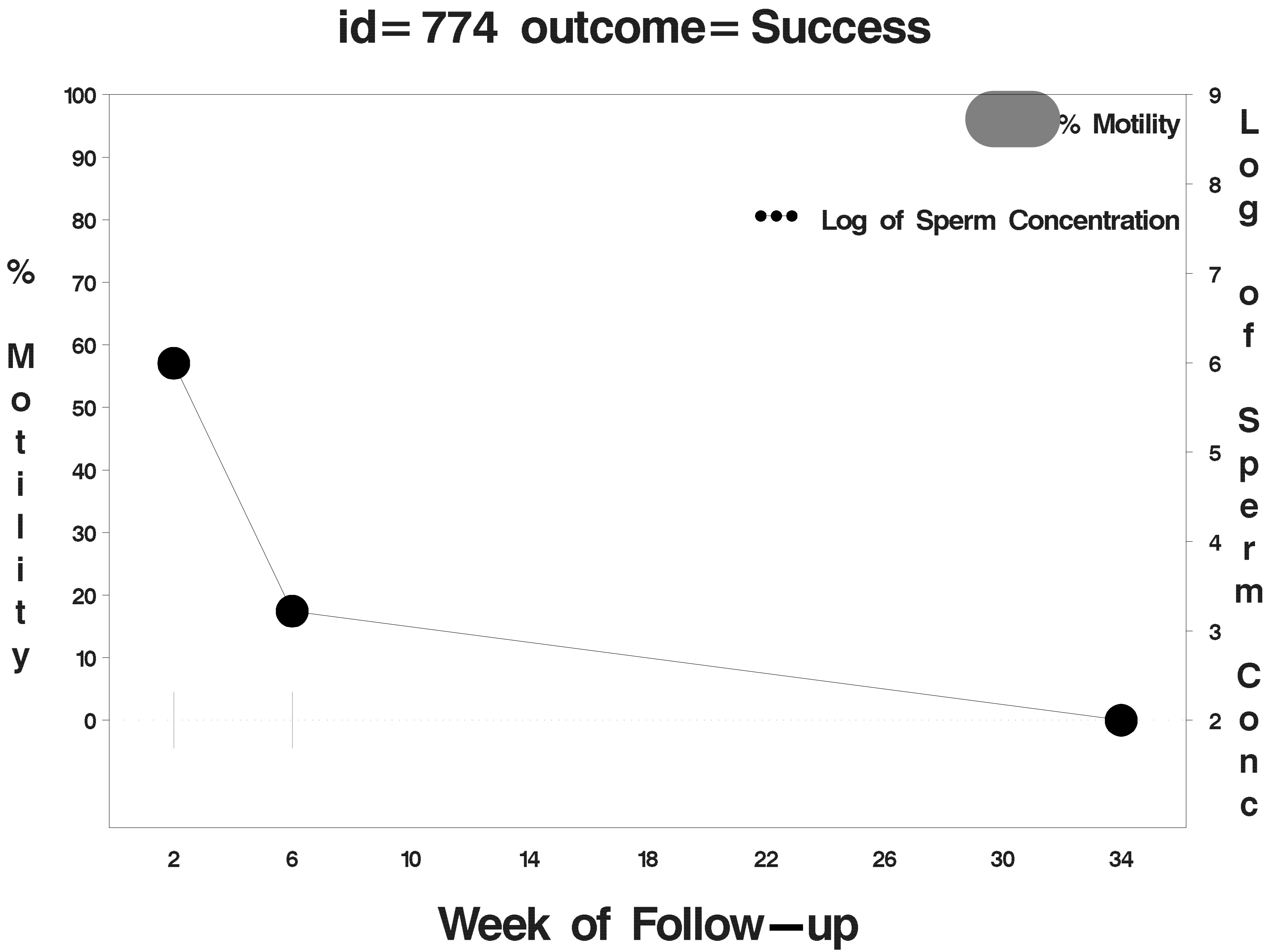

id= 774 outcome= Success
% Motility
Log of Sperm Concentration
100
90
80
70
60
50
40
30
20
10
0
9
8
7
6
5
4
3
2
2
6
10
14
18
22
26
30
34
Week of Follow—up
Log of Sperm Conc

id= 775 outcome= Success

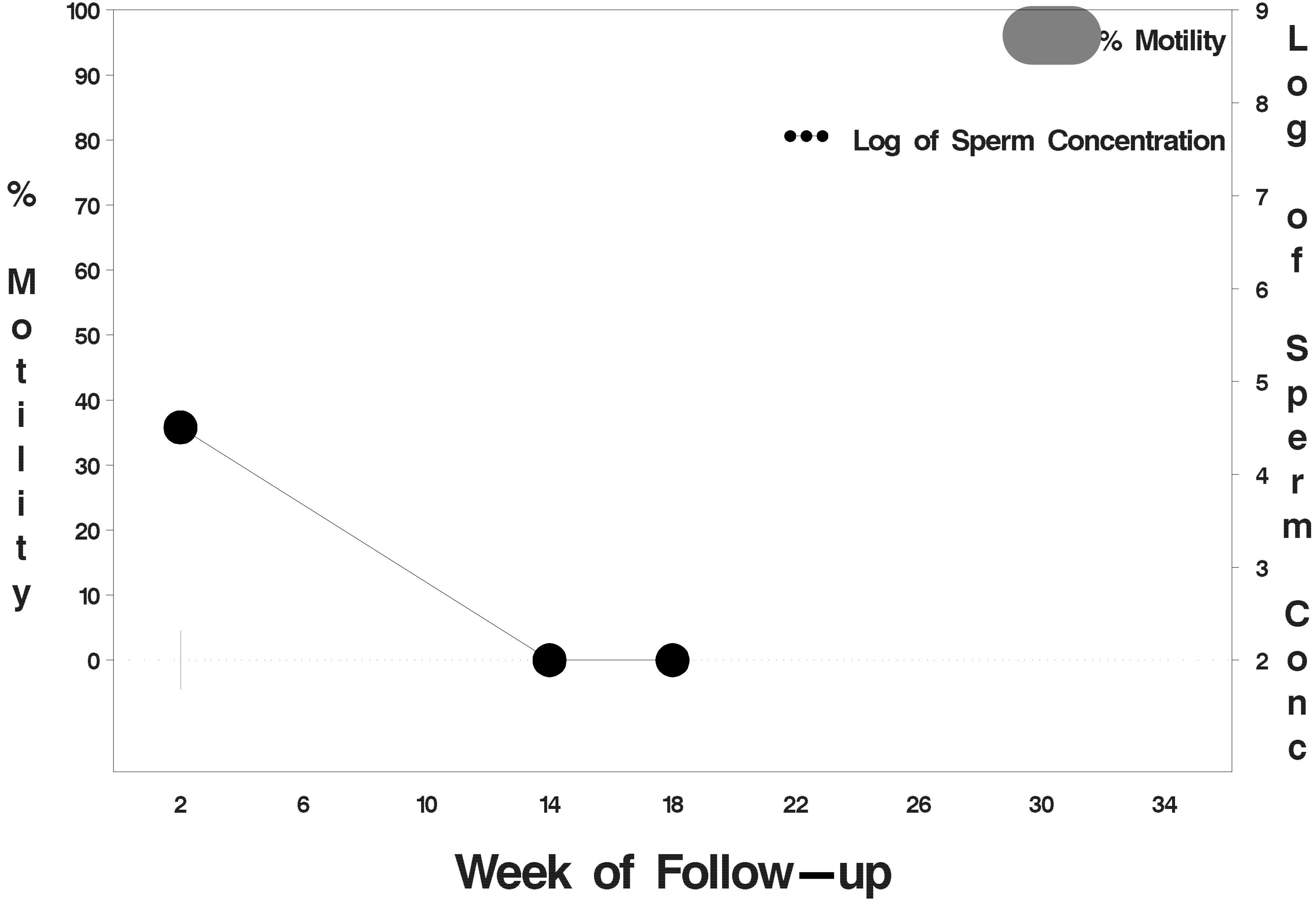

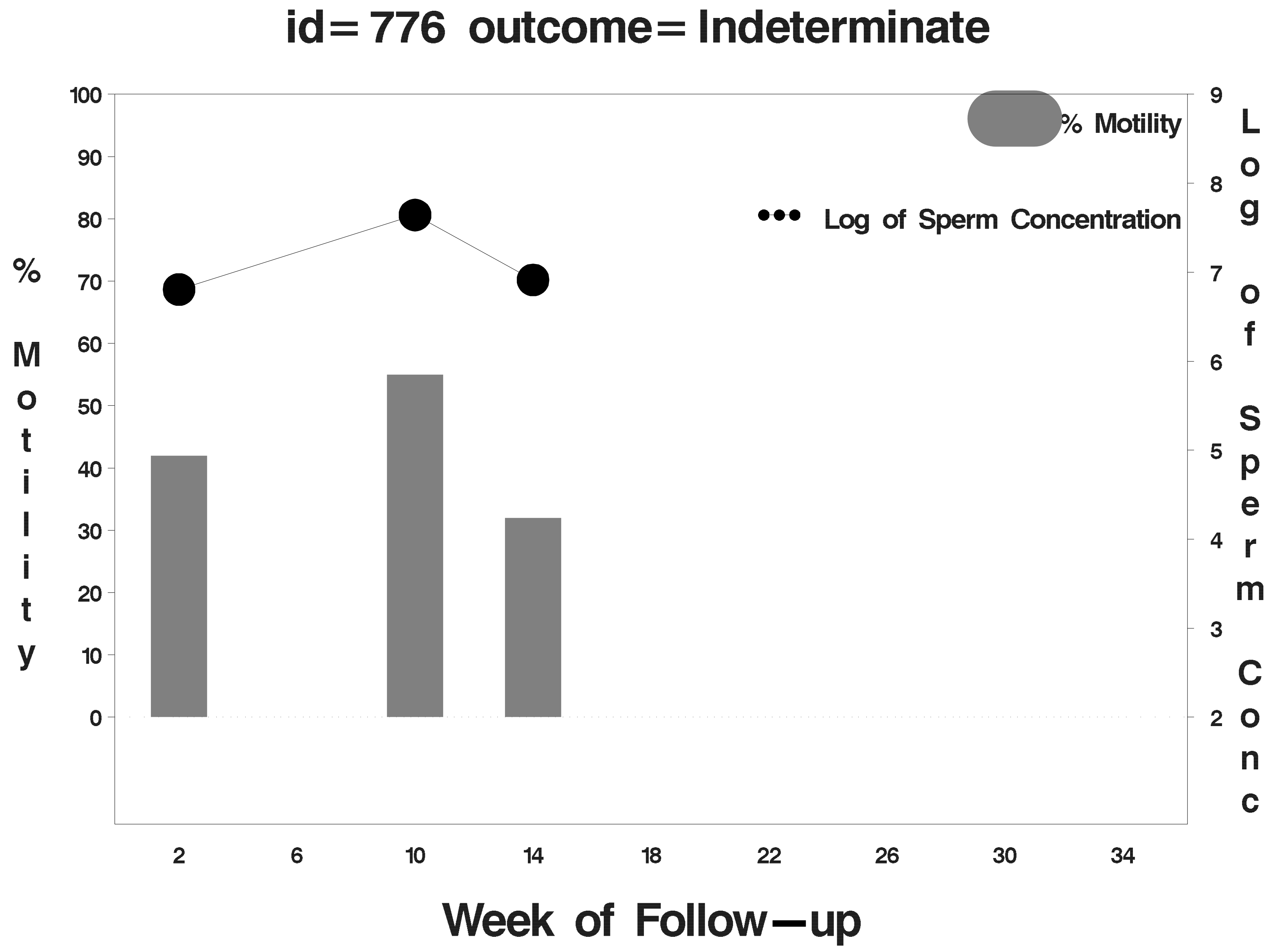

id= 776 outcome= Indeterminate
% Motility
Log of Sperm Concentration
100
90
80
70
60
50
40
30
20
10
0
9
8
7
6
5
4
3
2
2
6
10
14
18
22
26
30
34
Week of Follow−up
Log of Sperm Conc

id= 777 outcome= Success

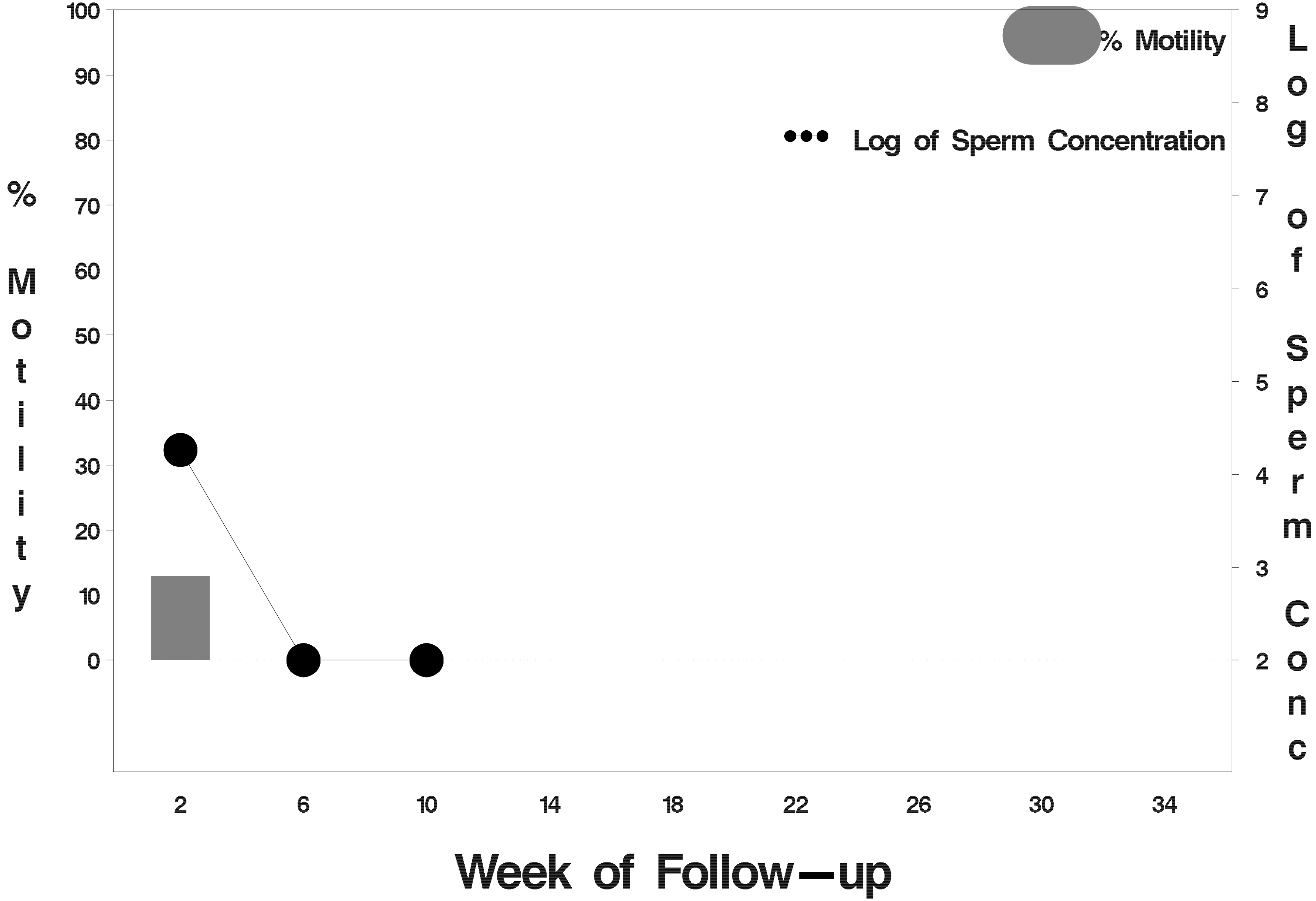

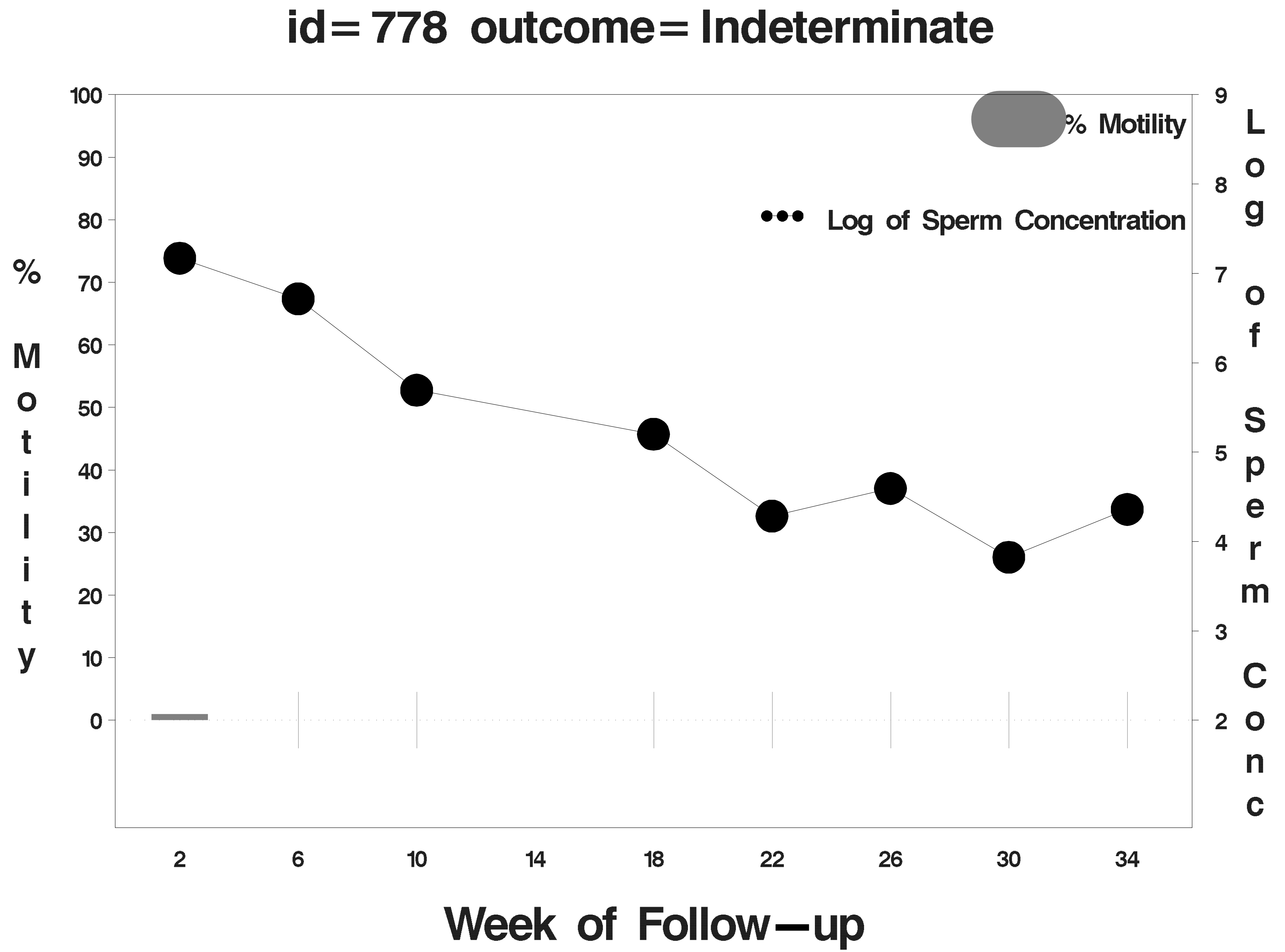

id= 778 outcome= Indeterminate
% Motility
Log of Sperm Concentration
% Motility
Log of Sperm Conc
Week of Follow−up
100
90
80
70
60
50
40
30
20
10
0
9
8
7
6
5
4
3
2
2
6
10
14
18
22
26
30
34

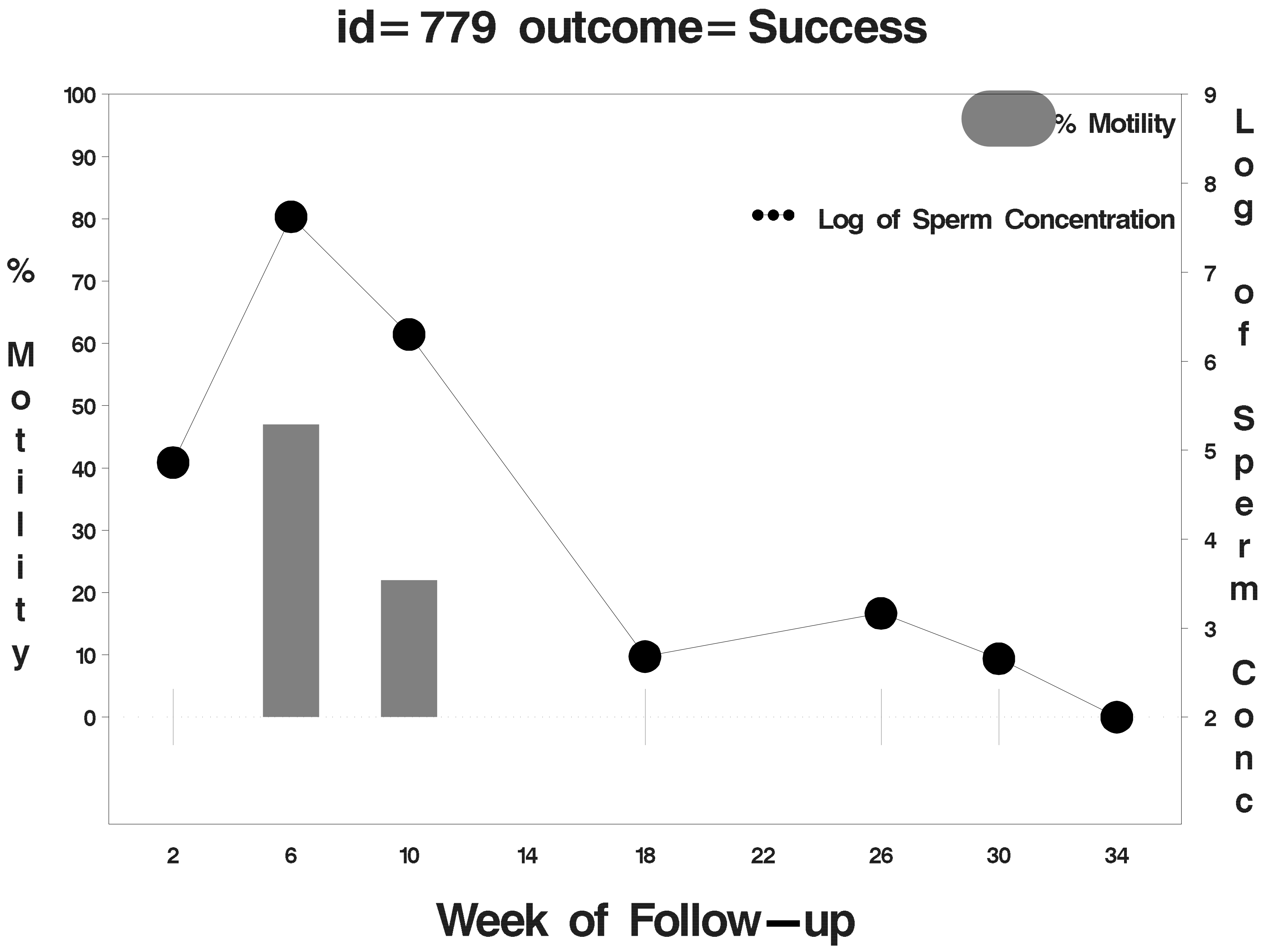

id= 779 outcome= Success
% Motility
Log of Sperm Concentration
% Motility
0
10
20
30
40
50
60
70
80
90
100
Log of Sperm Conc
2
3
4
5
6
7
8
9
2
6
10
14
18
22
26
30
34
Week of Follow−up

id= 780 outcome= Indeterminate

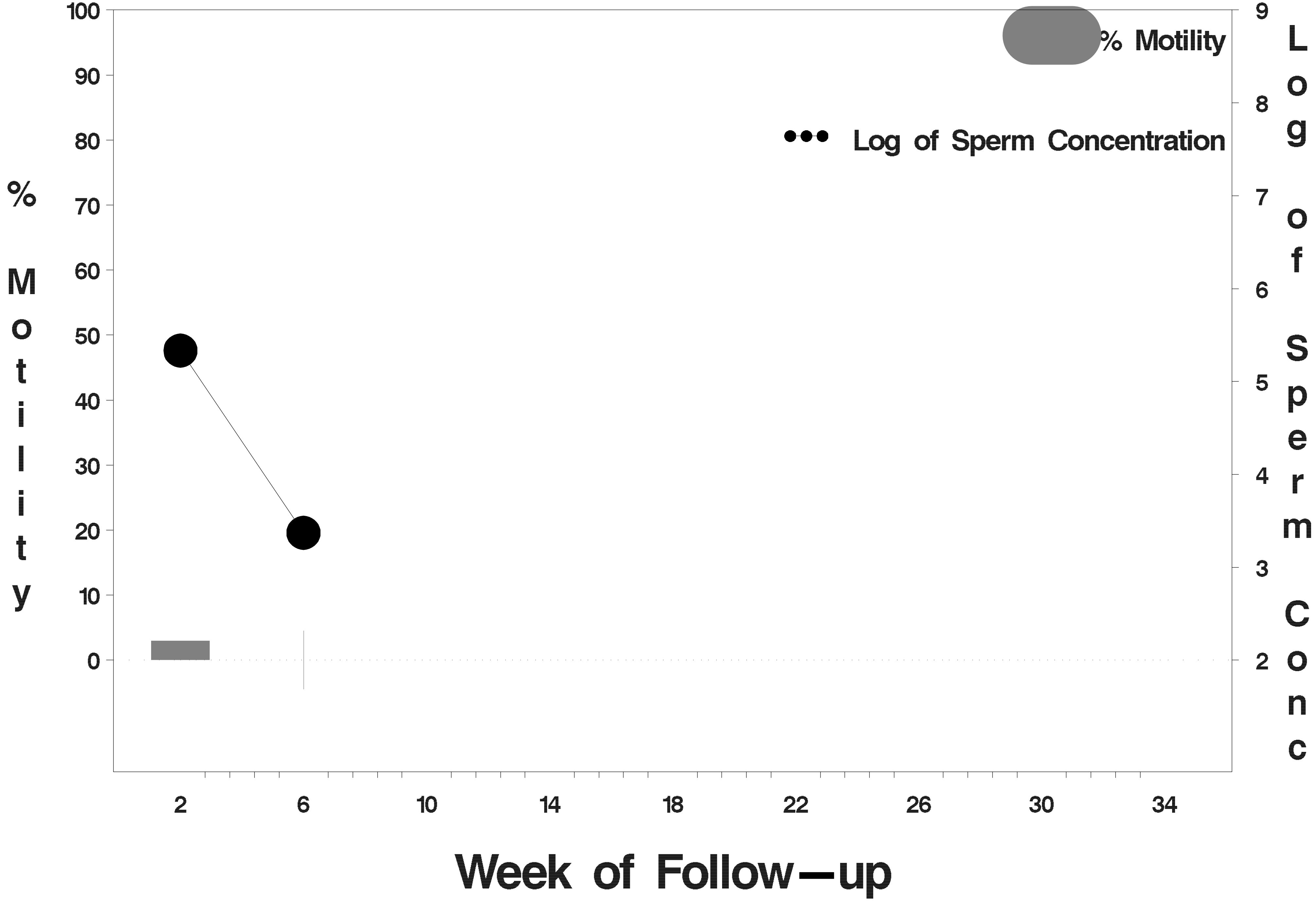

id= 781 outcome= Success

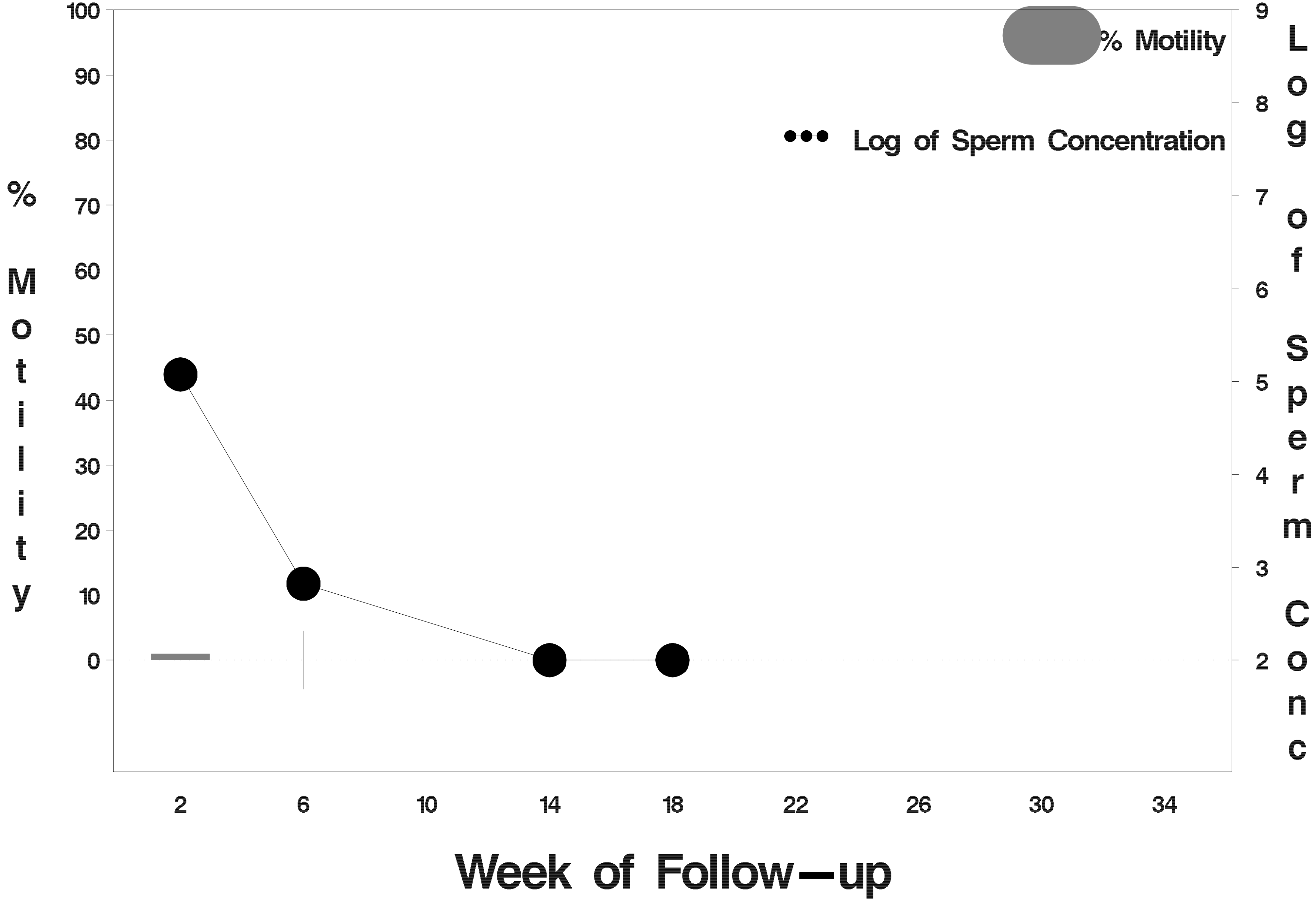

id= 782 outcome= Indeterminate

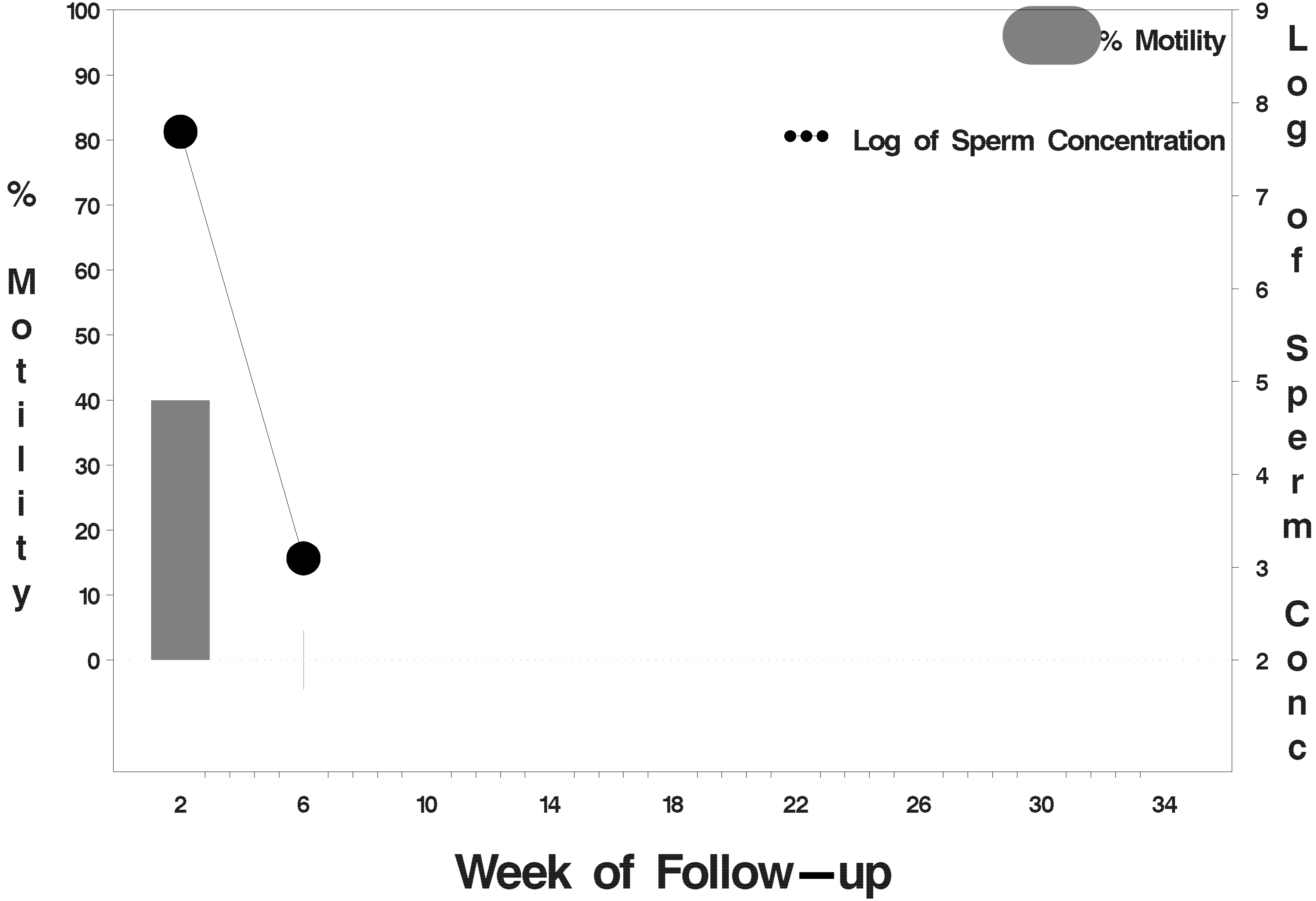

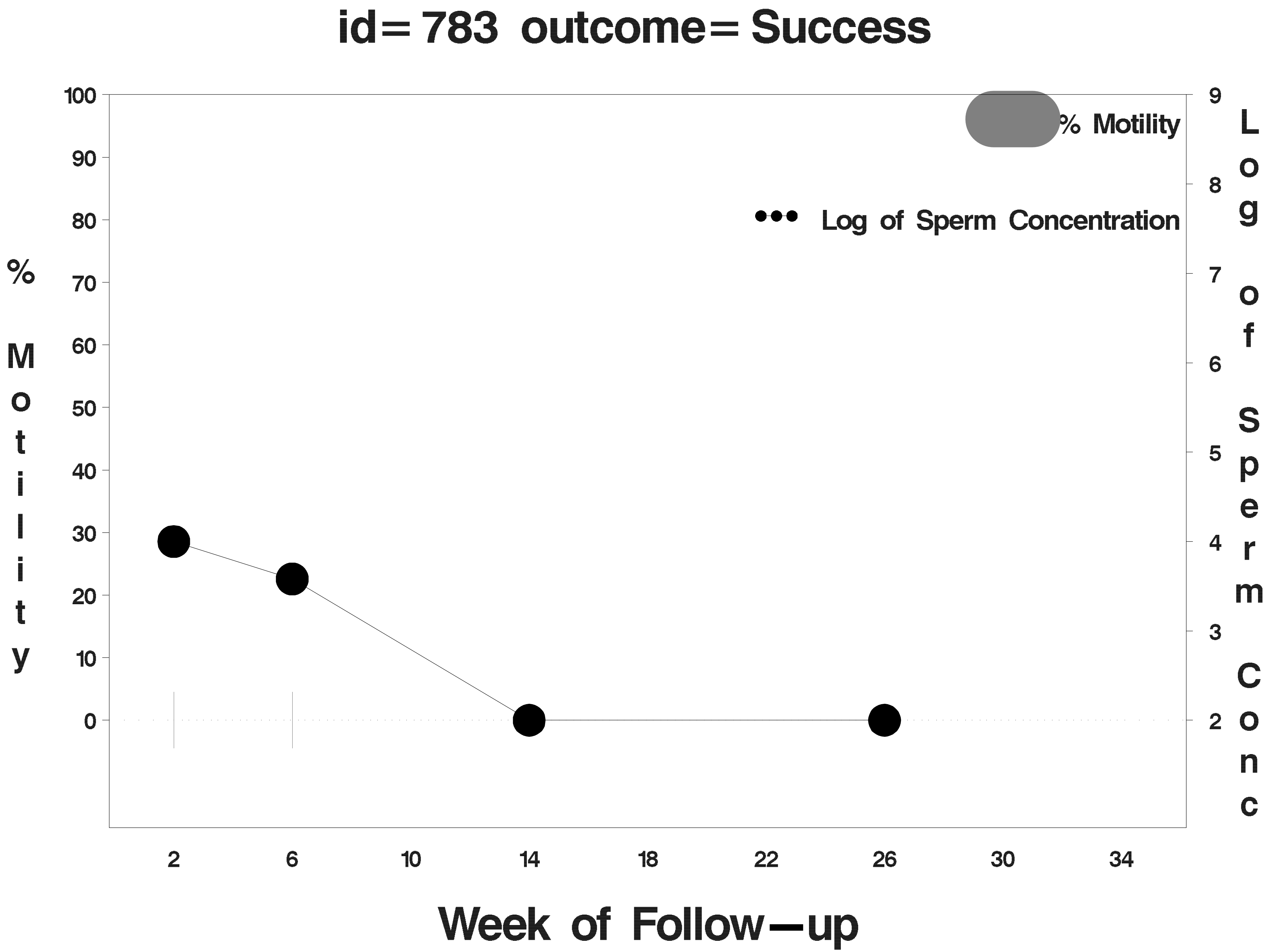

id= 783 outcome= Success
% Motility
Log of Sperm Concentration
% Motility
Log of Sperm Conc
Week of Follow—up
100
90
80
70
60
50
40
30
20
10
0
9
8
7
6
5
4
3
2
2
6
10
14
18
22
26
30
34

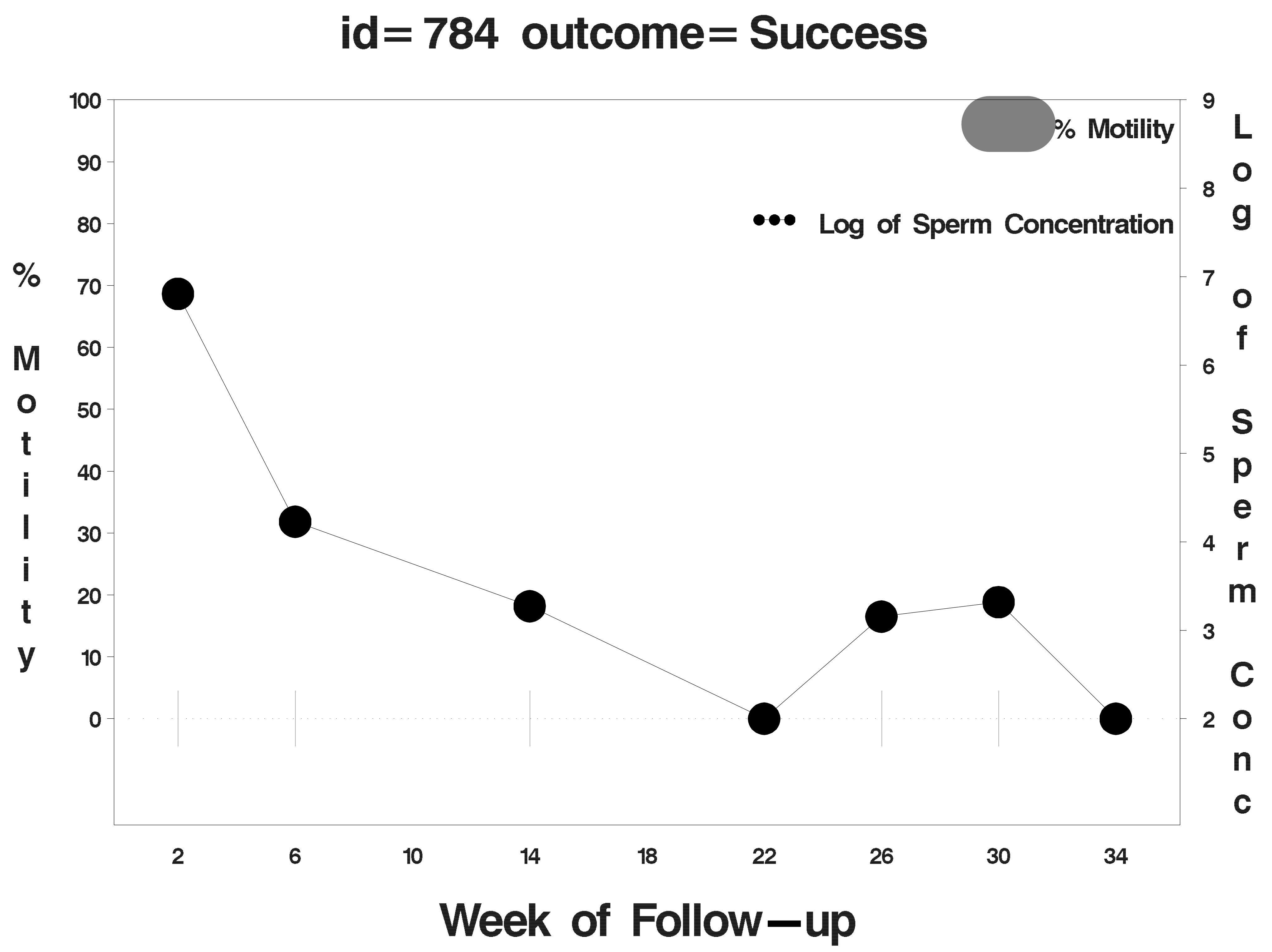

id= 784 outcome= Success
% Motility
Log of Sperm Concentration
% Motility
Log of Sperm Conc
Week of Follow—up
100
90
80
70
60
50
40
30
20
10
0
9
8
7
6
5
4
3
2
2
6
10
14
18
22
26
30
34

id= 785 outcome= Failure

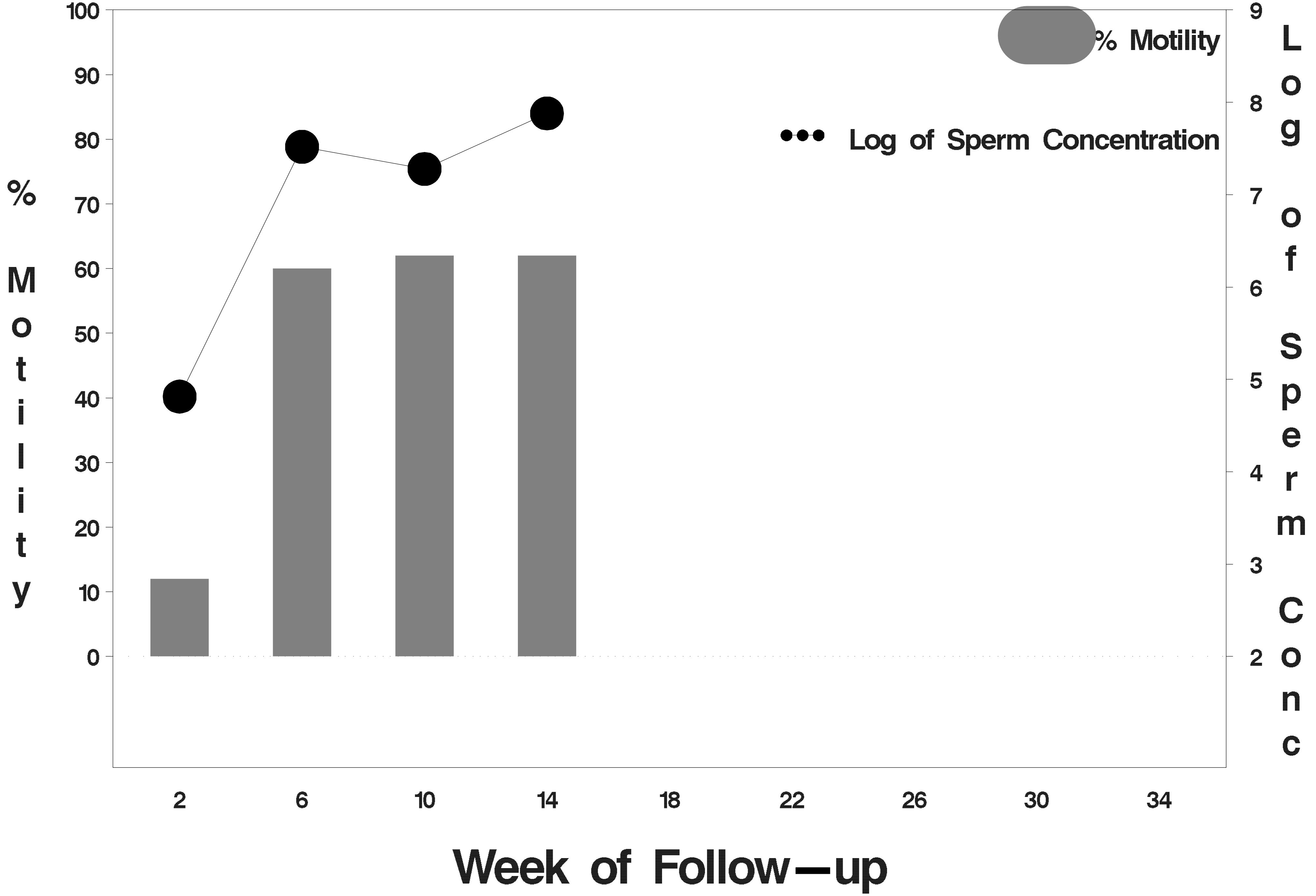

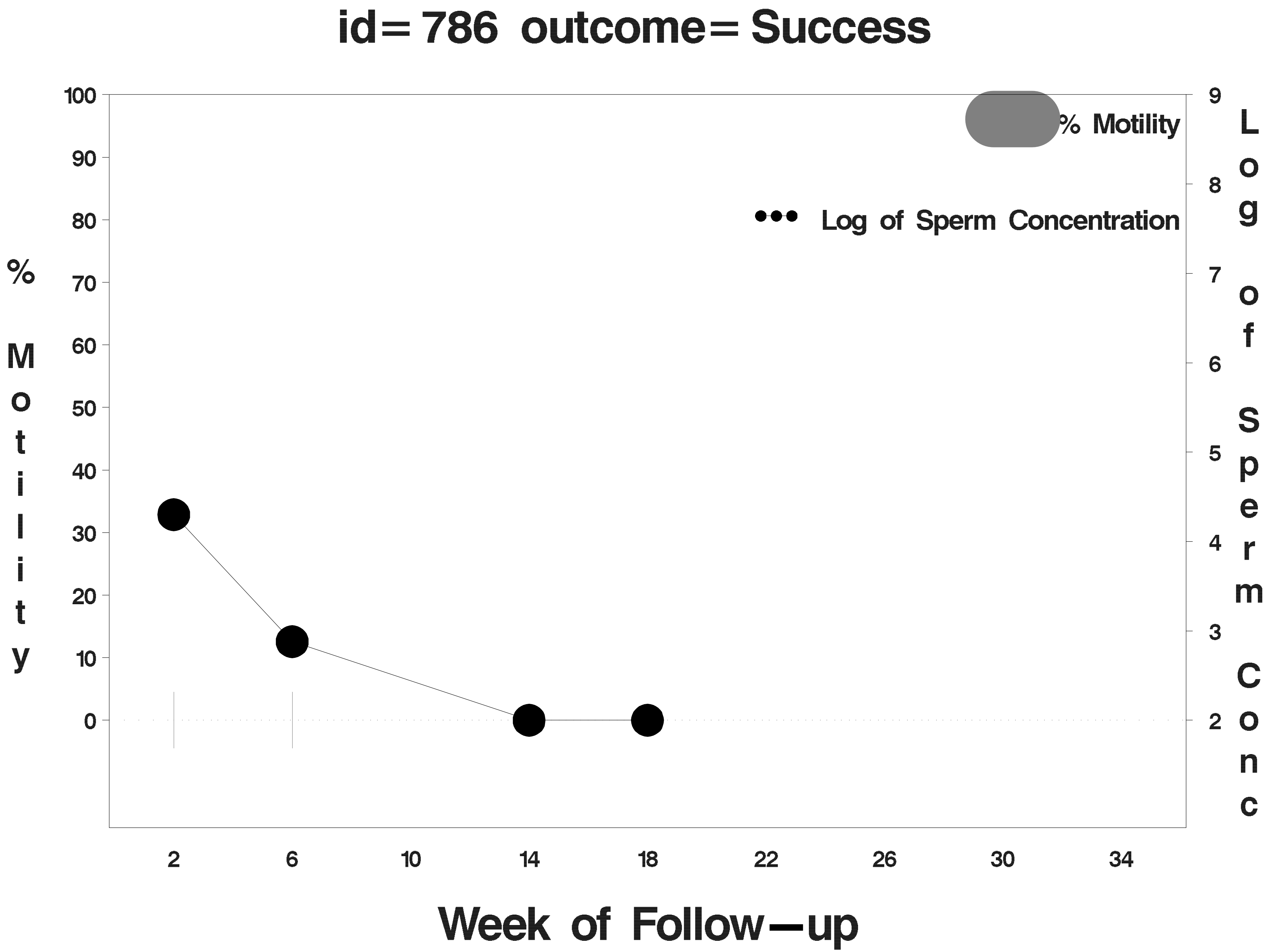
id= 786 outcome= Success
% Motility
Log of Sperm Concentration
100
90
80
70
60
50
40
30
20
10
0
9
8
7
6
5
4
3
2
2
6
10
14
18
22
26
30
34
Week of Follow−up
Log of Sperm Conc

id= 787 outcome= Indeterminate

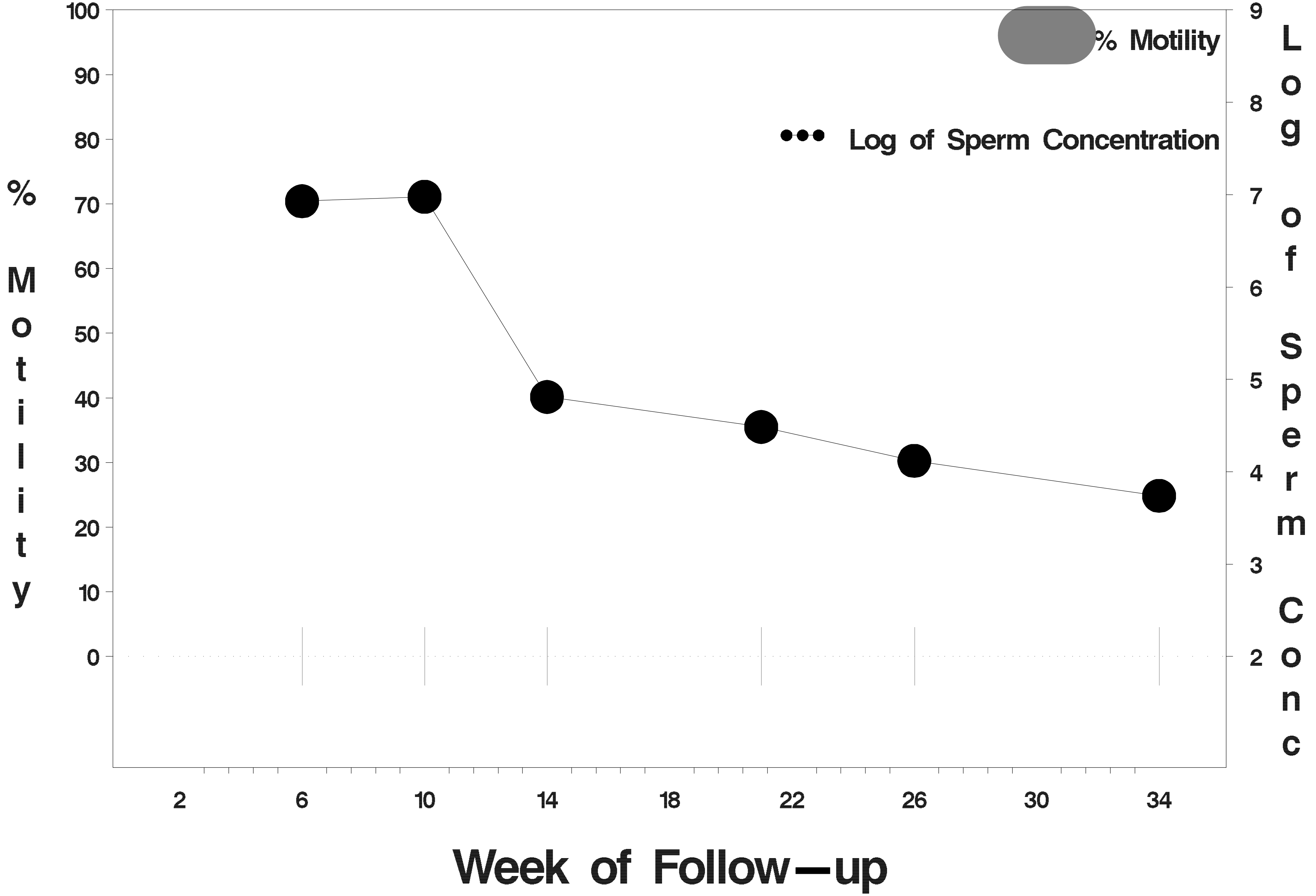

id= 788 outcome= Success

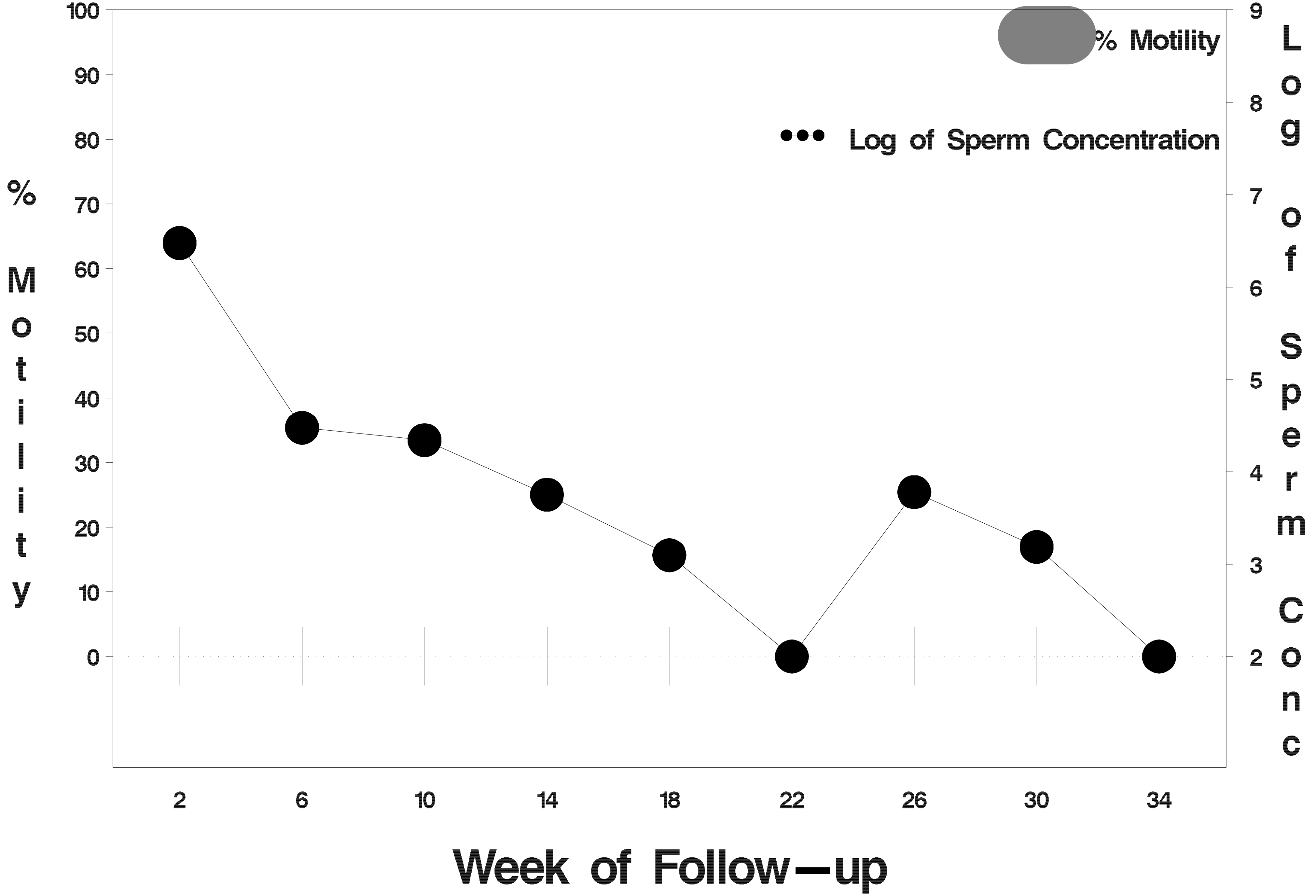

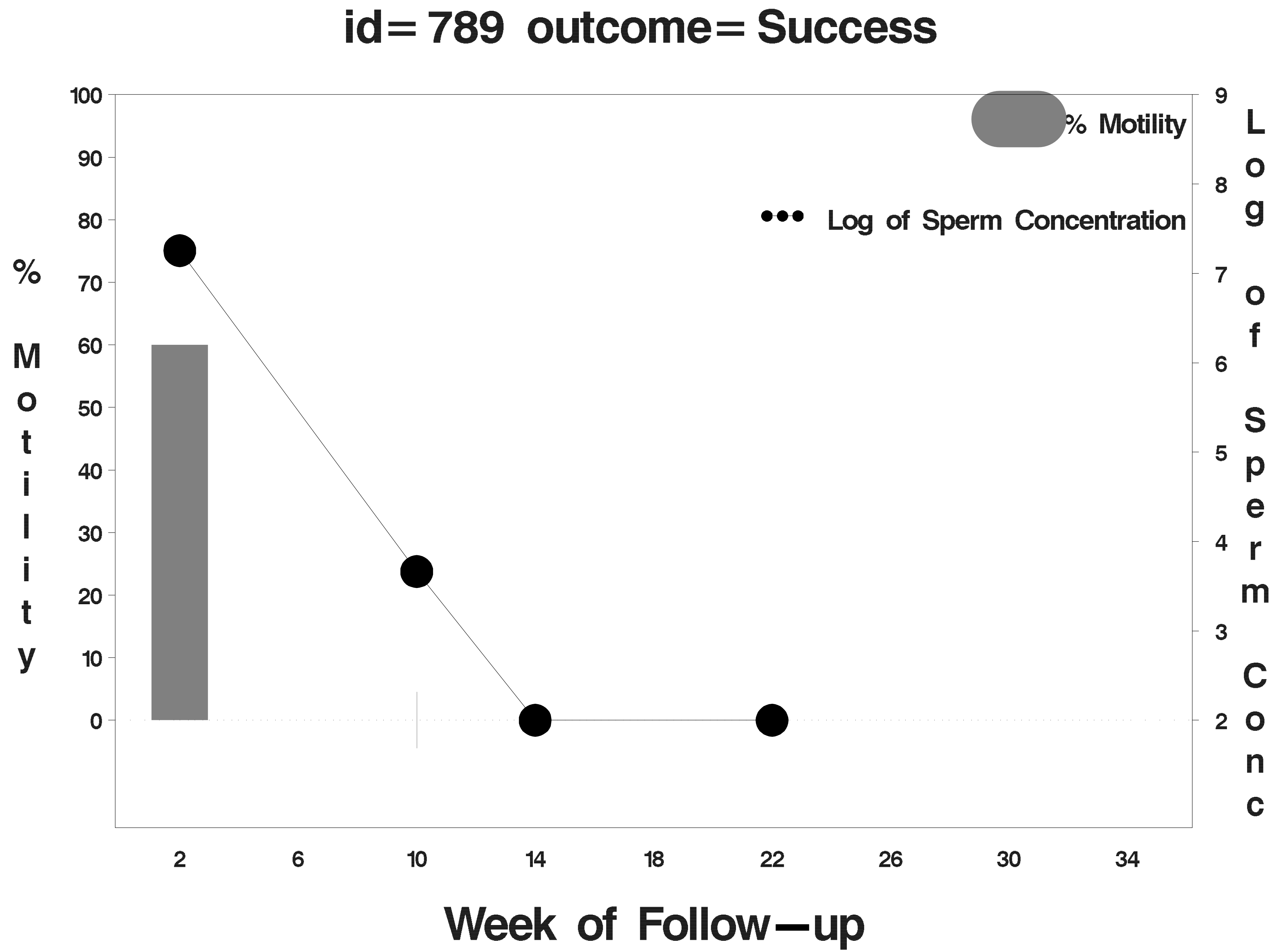

id= 789 outcome= Success
% Motility
Log of Sperm Concentration
100
90
80
70
60
50
40
30
20
10
0
9
8
7
6
5
4
3
2
2
6
10
14
18
22
26
30
34
Week of Follow−up
Log of Sperm Conc

id= 790 outcome= Success

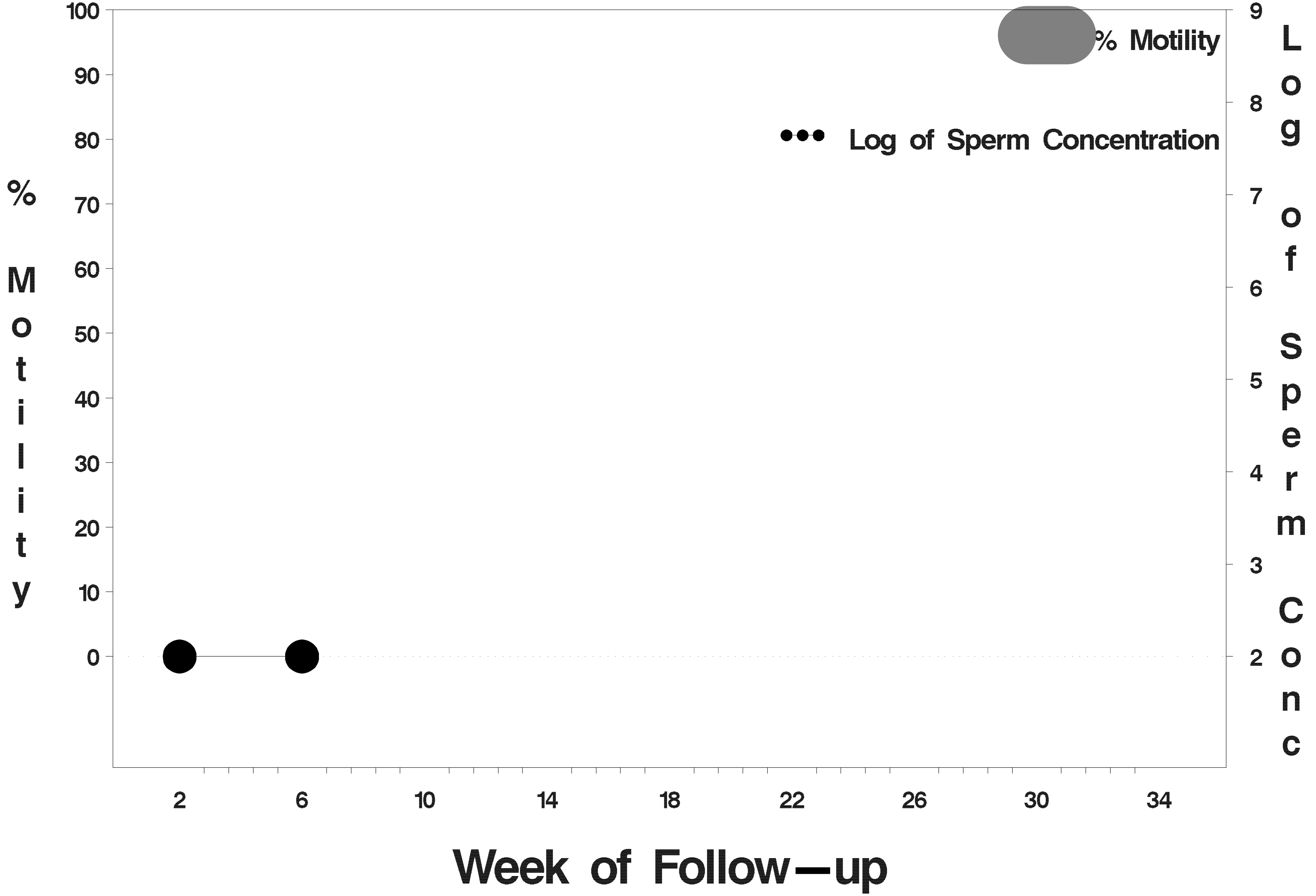

id= 791 outcome= Success

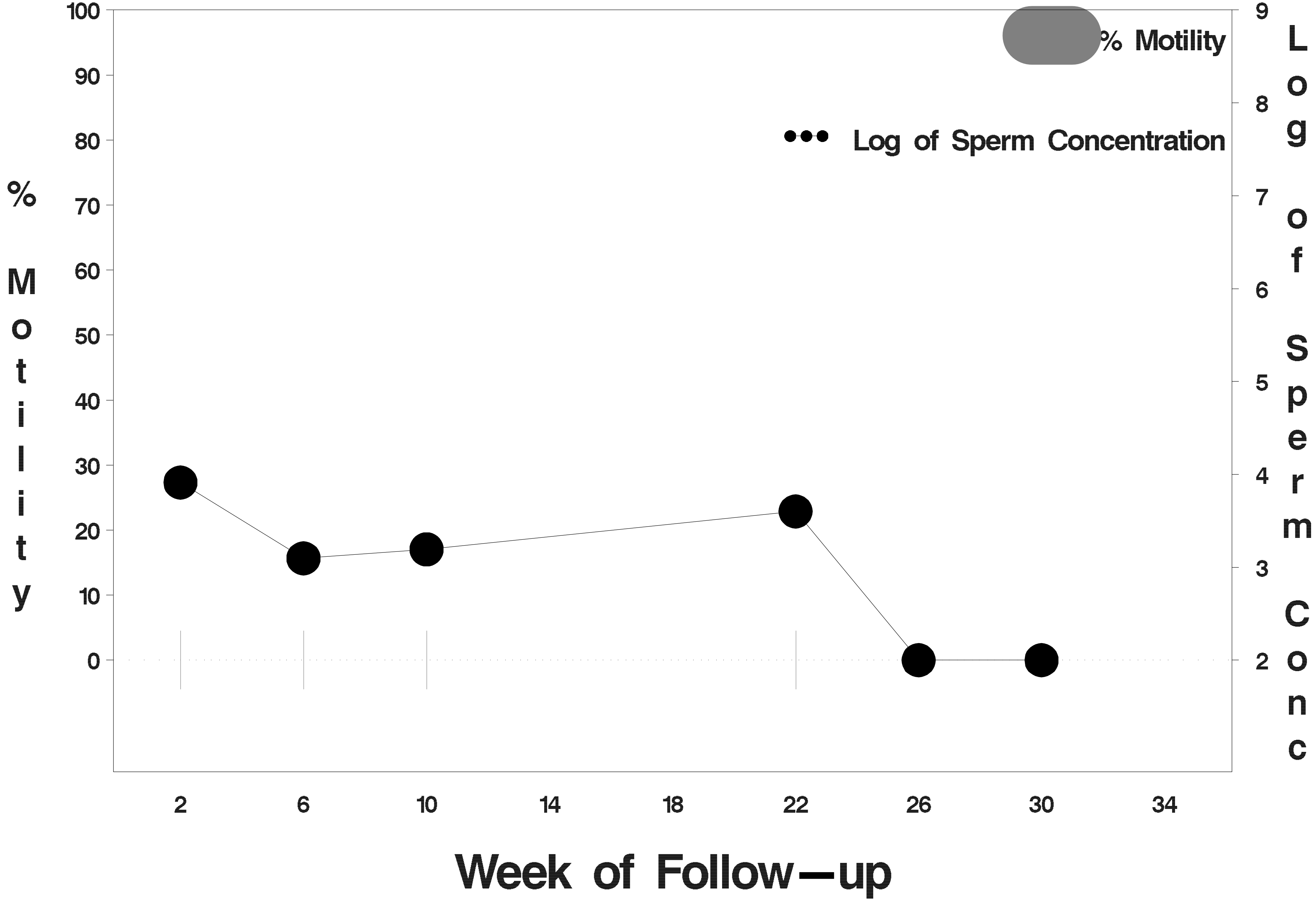

id= 792 outcome= Indeterminate

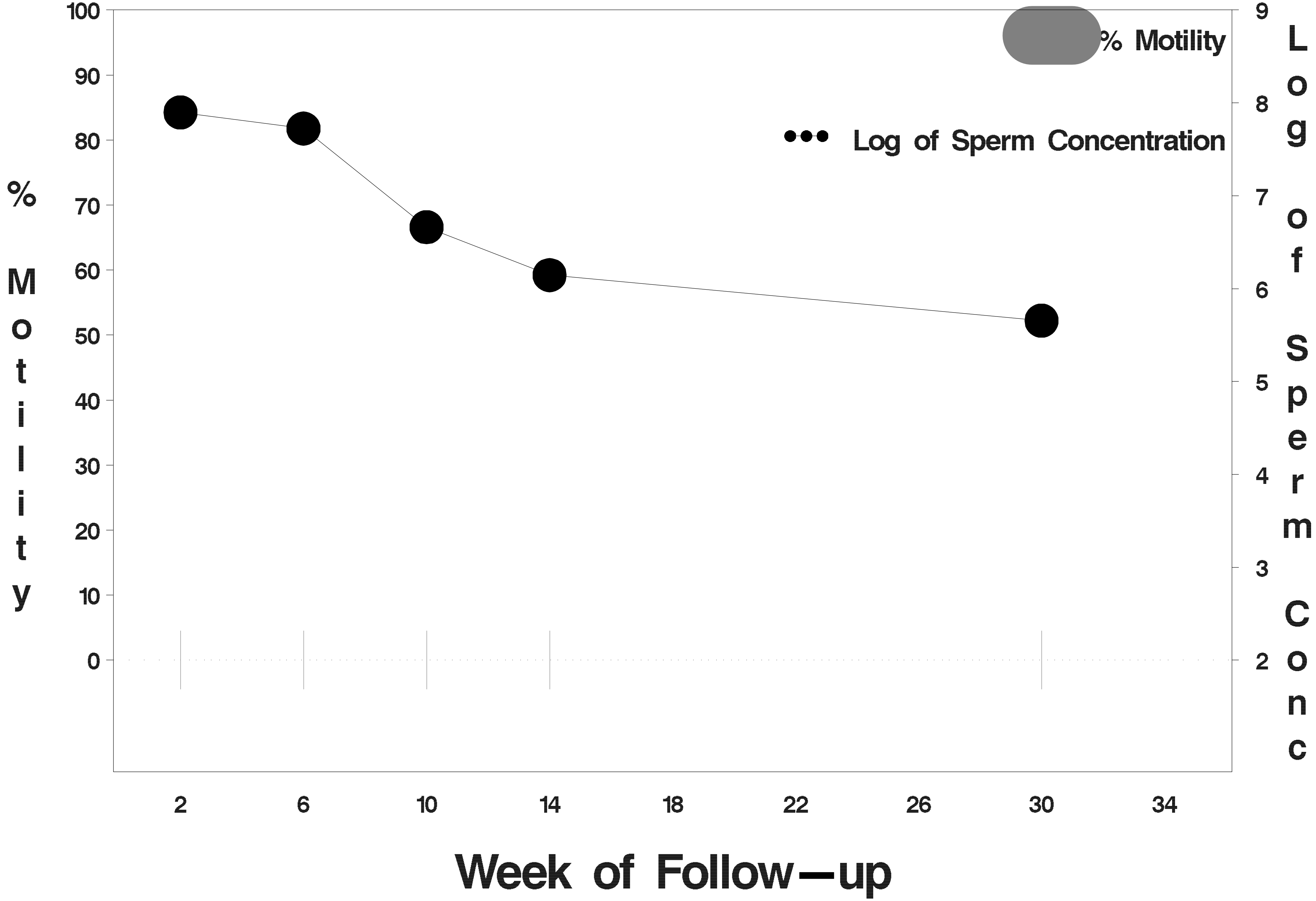

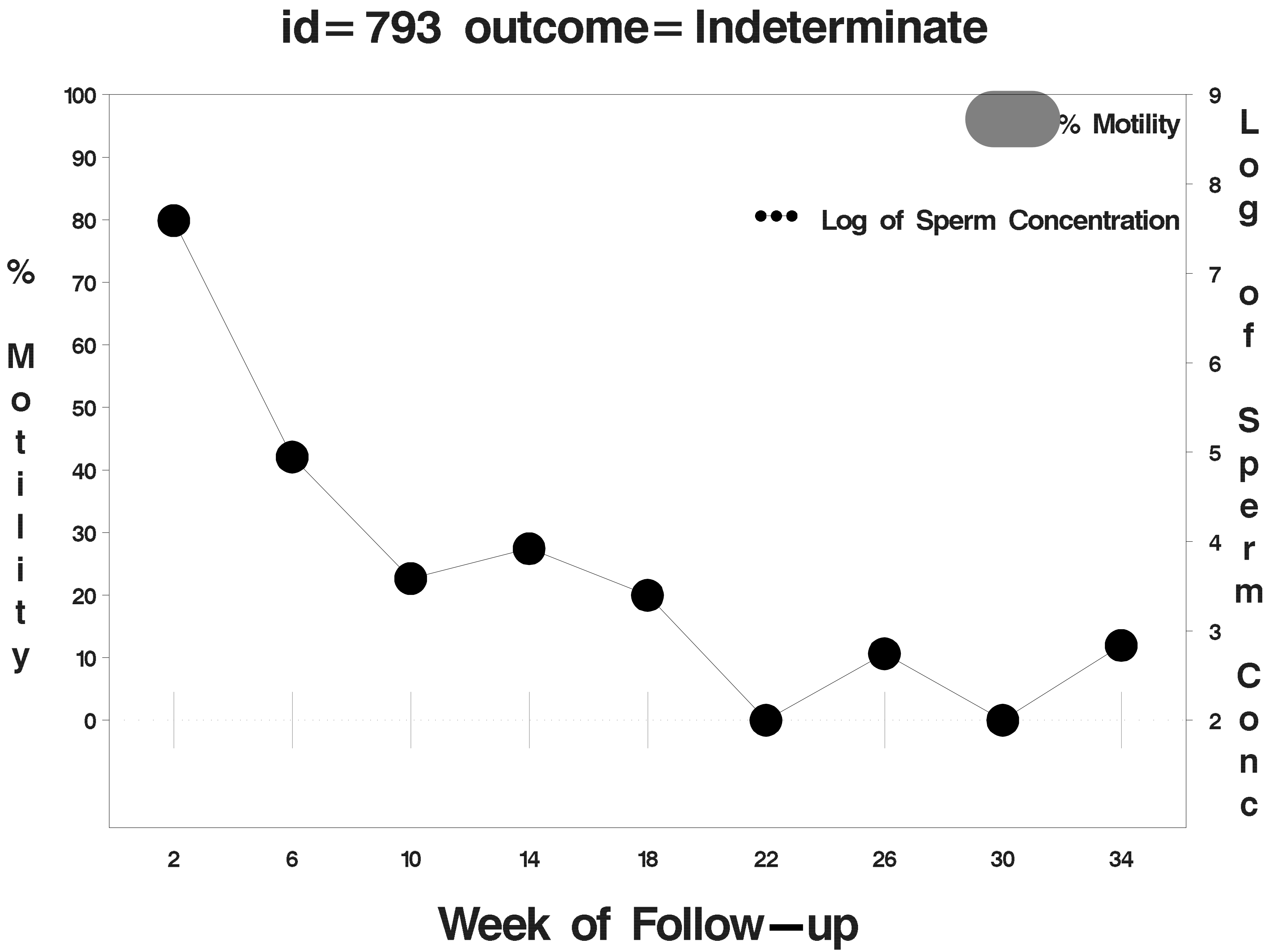

id= 793 outcome= Indeterminate
% Motility
Log of Sperm Concentration
% Motility
Log of Sperm Conc
Week of Follow—up
100
90
80
70
60
50
40
30
20
10
0
9
8
7
6
5
4
3
2
2
6
10
14
18
22
26
30
34

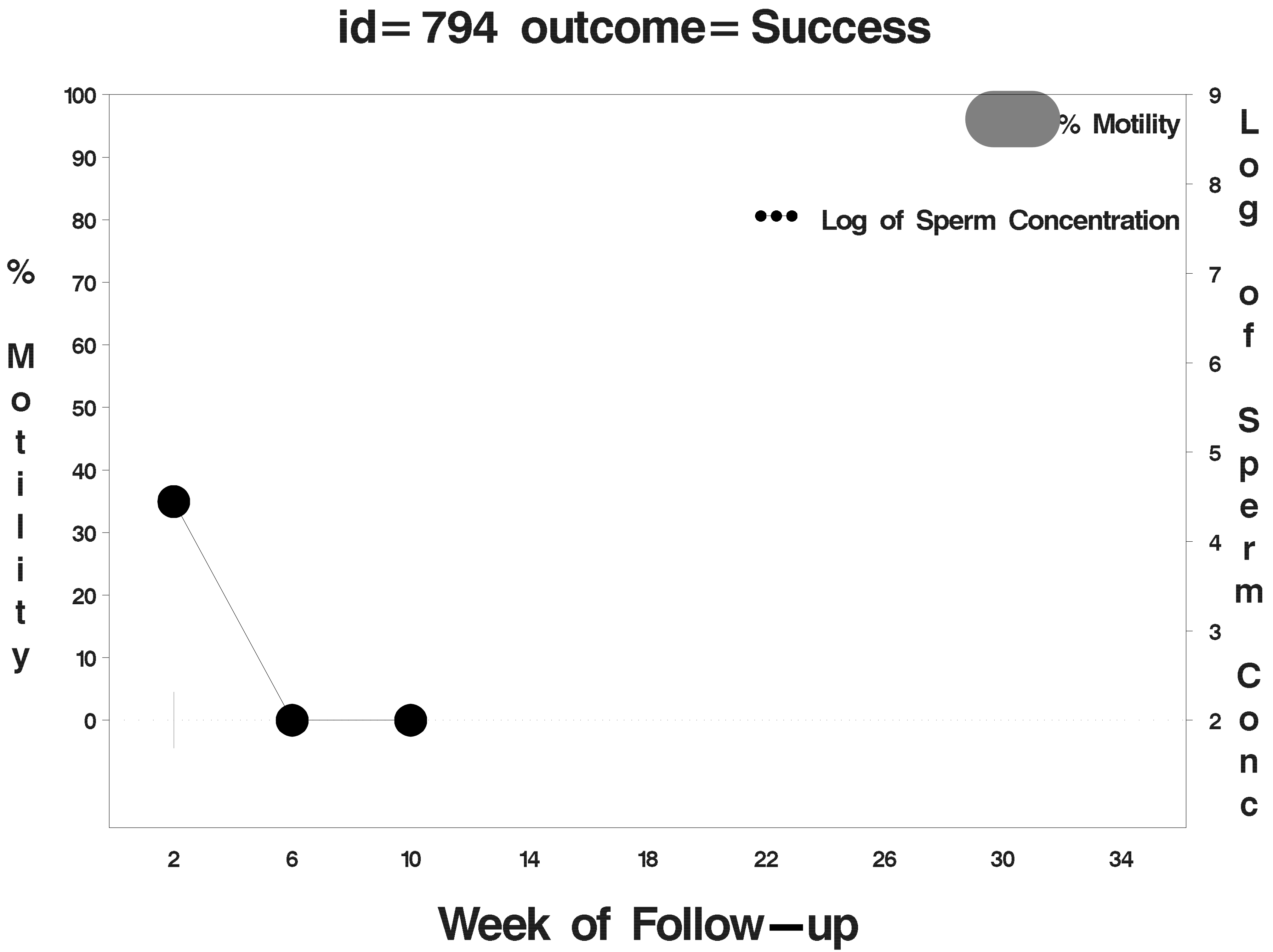

id= 794 outcome= Success
% Motility
Log of Sperm Concentration
100
90
80
70
60
50
40
30
20
10
0
9
8
7
6
5
4
3
2
2
6
10
14
18
22
26
30
34
Week of Follow−up
Log of Sperm Conc

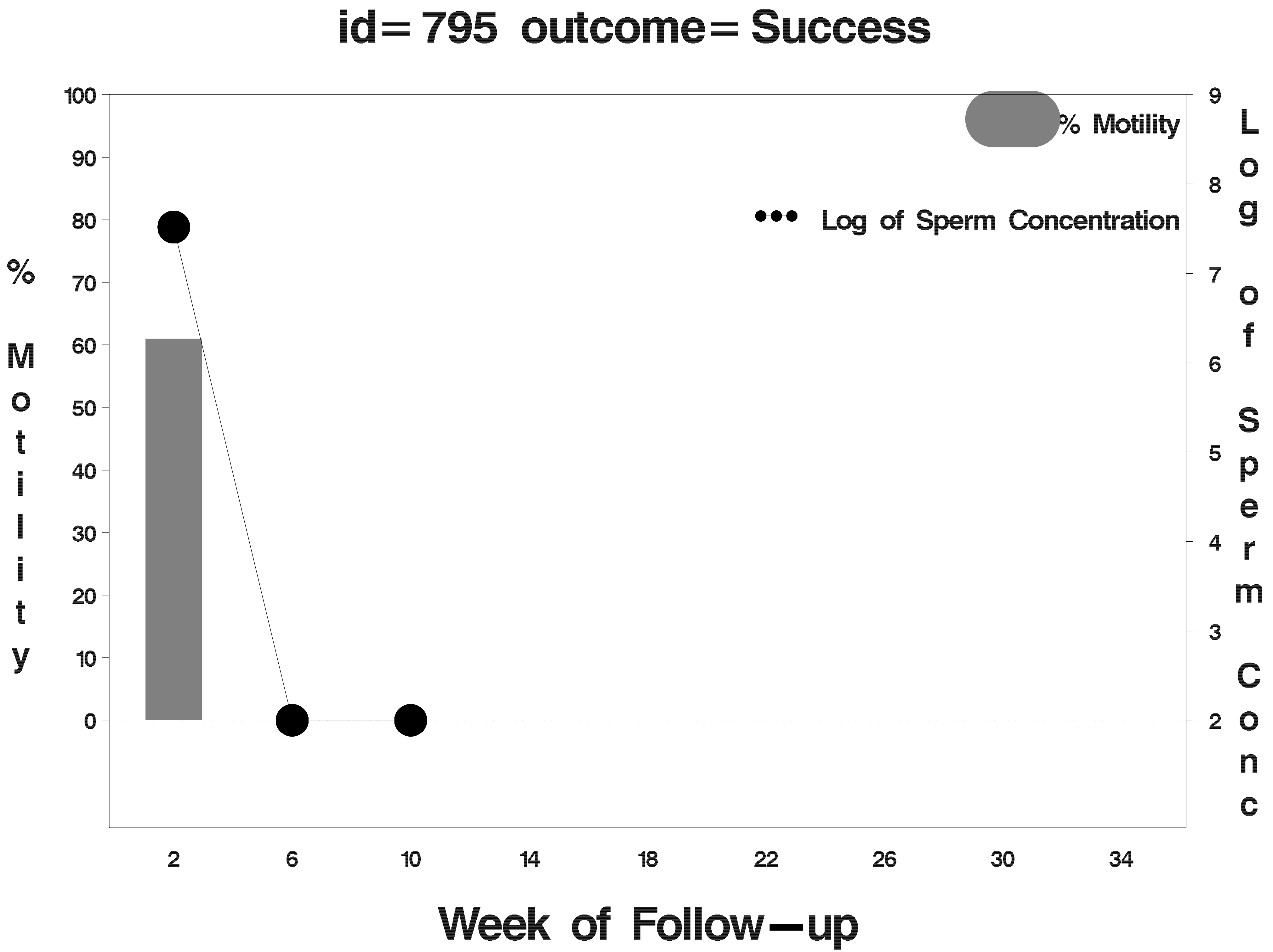

id= 795 outcome= Success
% Motility
Log of Sperm Concentration
% Motility
Log of Sperm Conc
Week of Follow−up
100
90
80
70
60
50
40
30
20
10
0
9
8
7
6
5
4
3
2
2
6
10
14
18
22
26
30
34

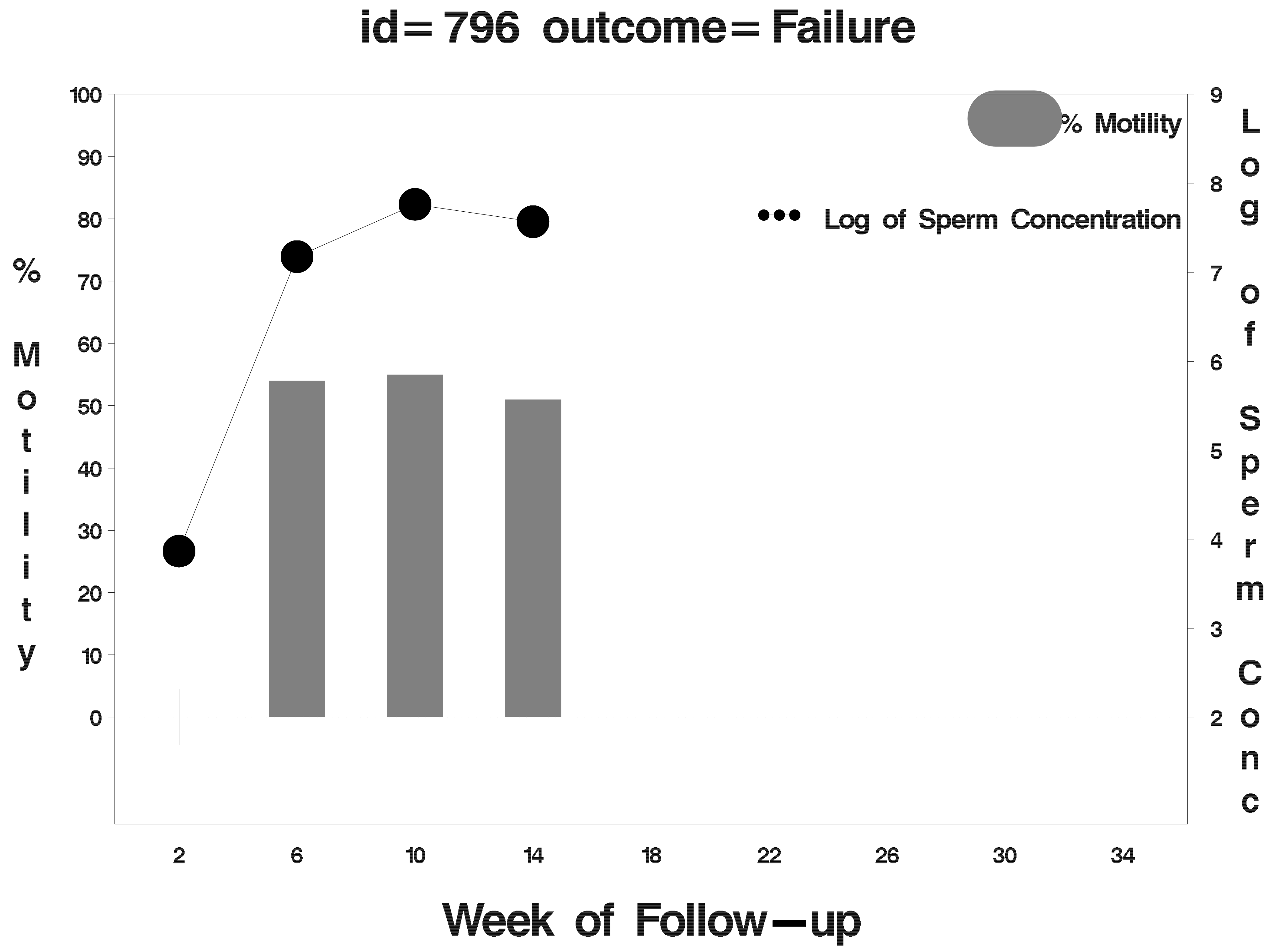
id= 796 outcome= Failure
% Motility
Log of Sperm Concentration
100
90
80
70
60
50
40
30
20
10
0
9
8
7
6
5
4
3
2
2
6
10
14
18
22
26
30
34
Week of Follow−up
% Motility
Log of Sperm Conc

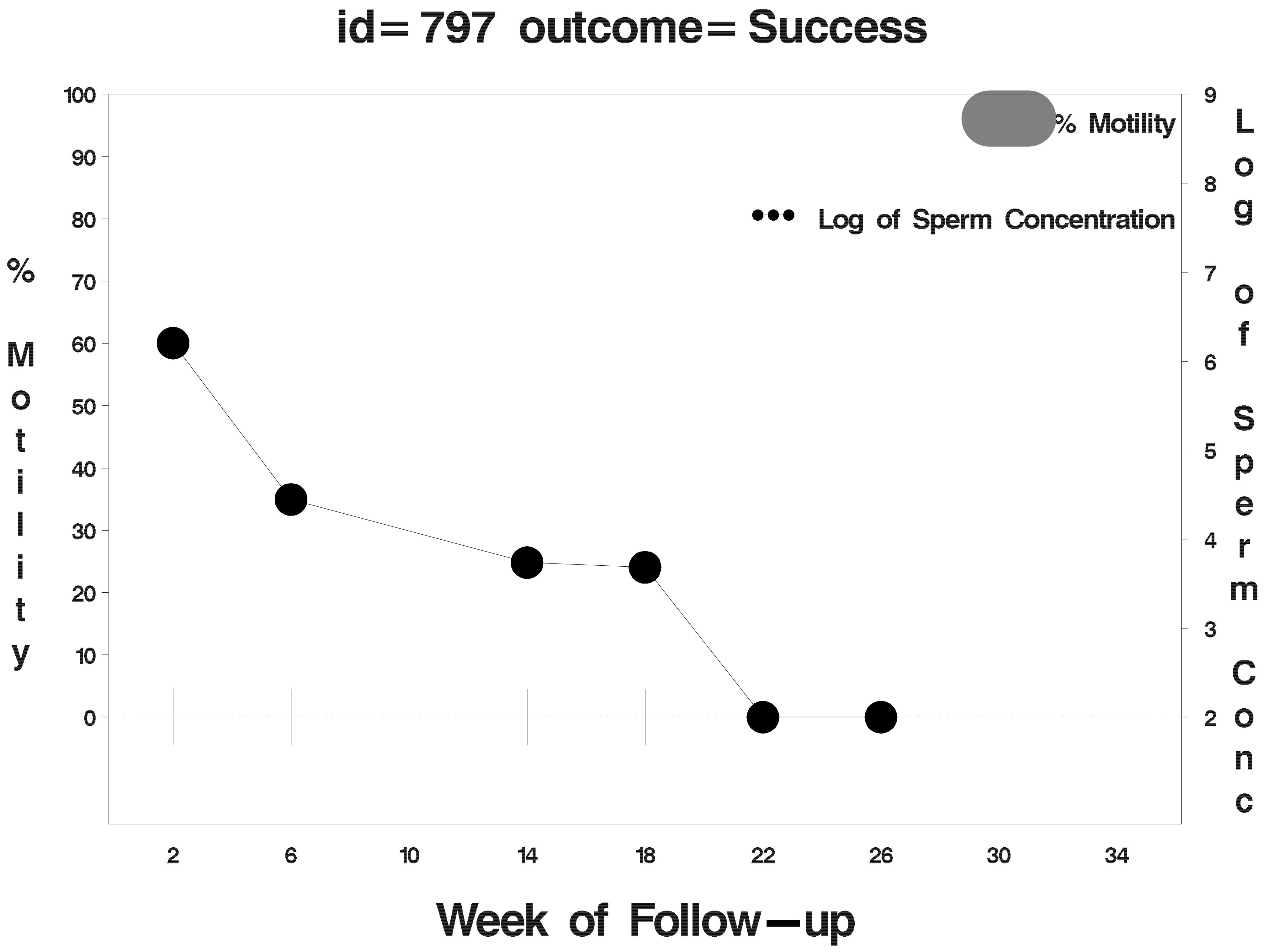

id= 797 outcome= Success
% Motility
Log of Sperm Concentration
100
90
80
70
60
50
40
30
20
10
0
9
8
7
6
5
4
3
2
2
6
10
14
18
22
26
30
34
Week of Follow—up
Log of Sperm Conc

id= 798 outcome= Success

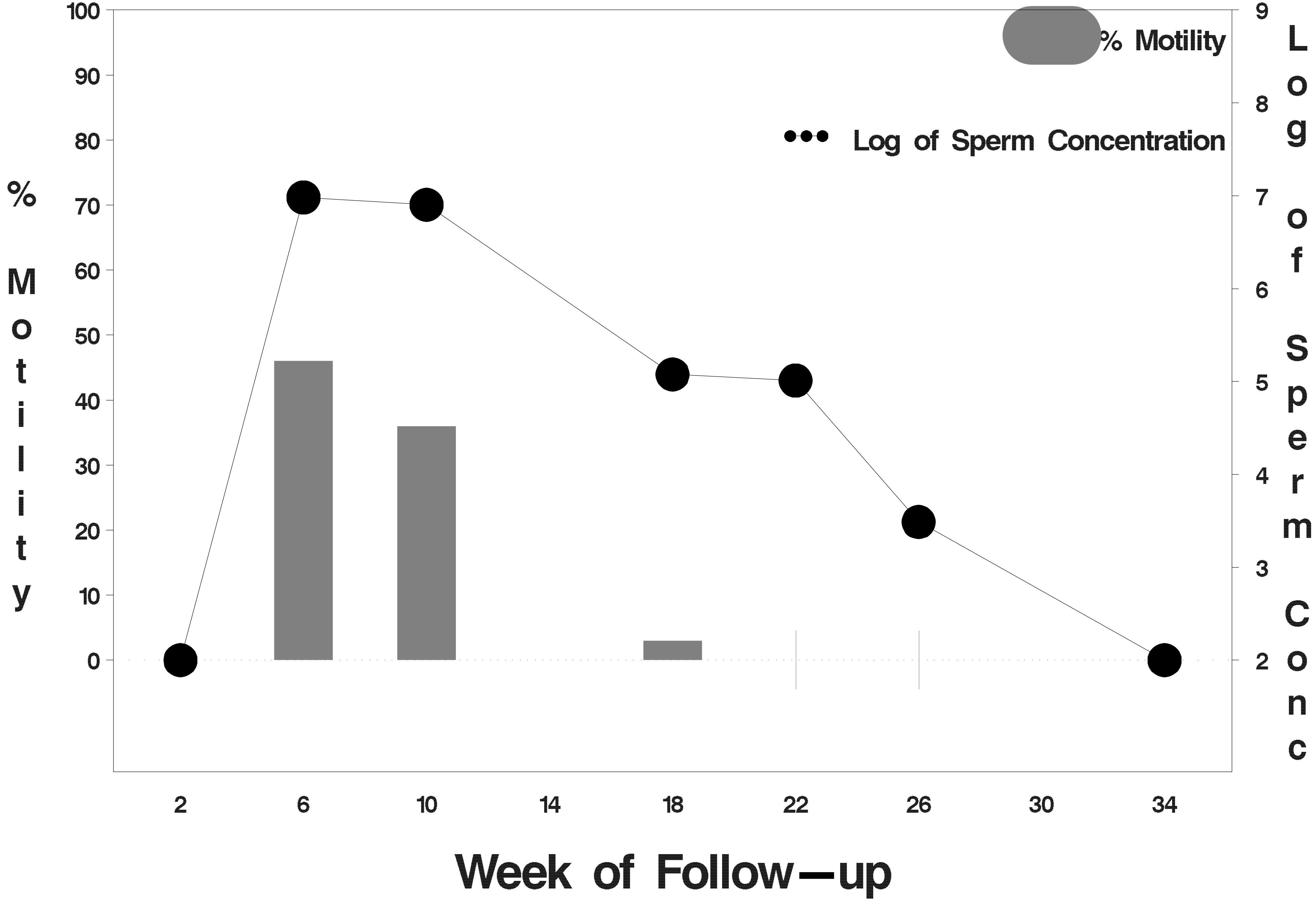

id= 799 outcome= Success

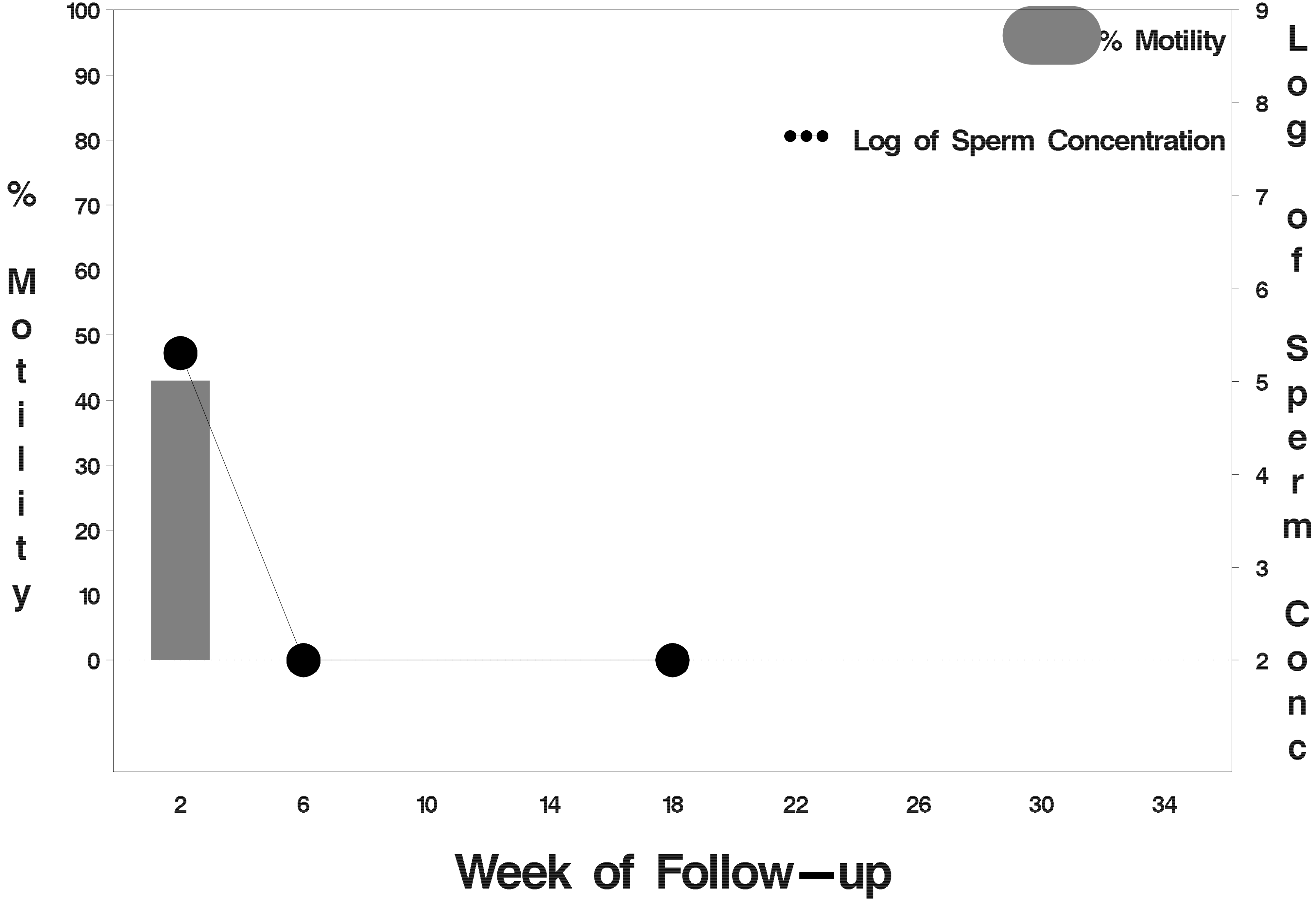

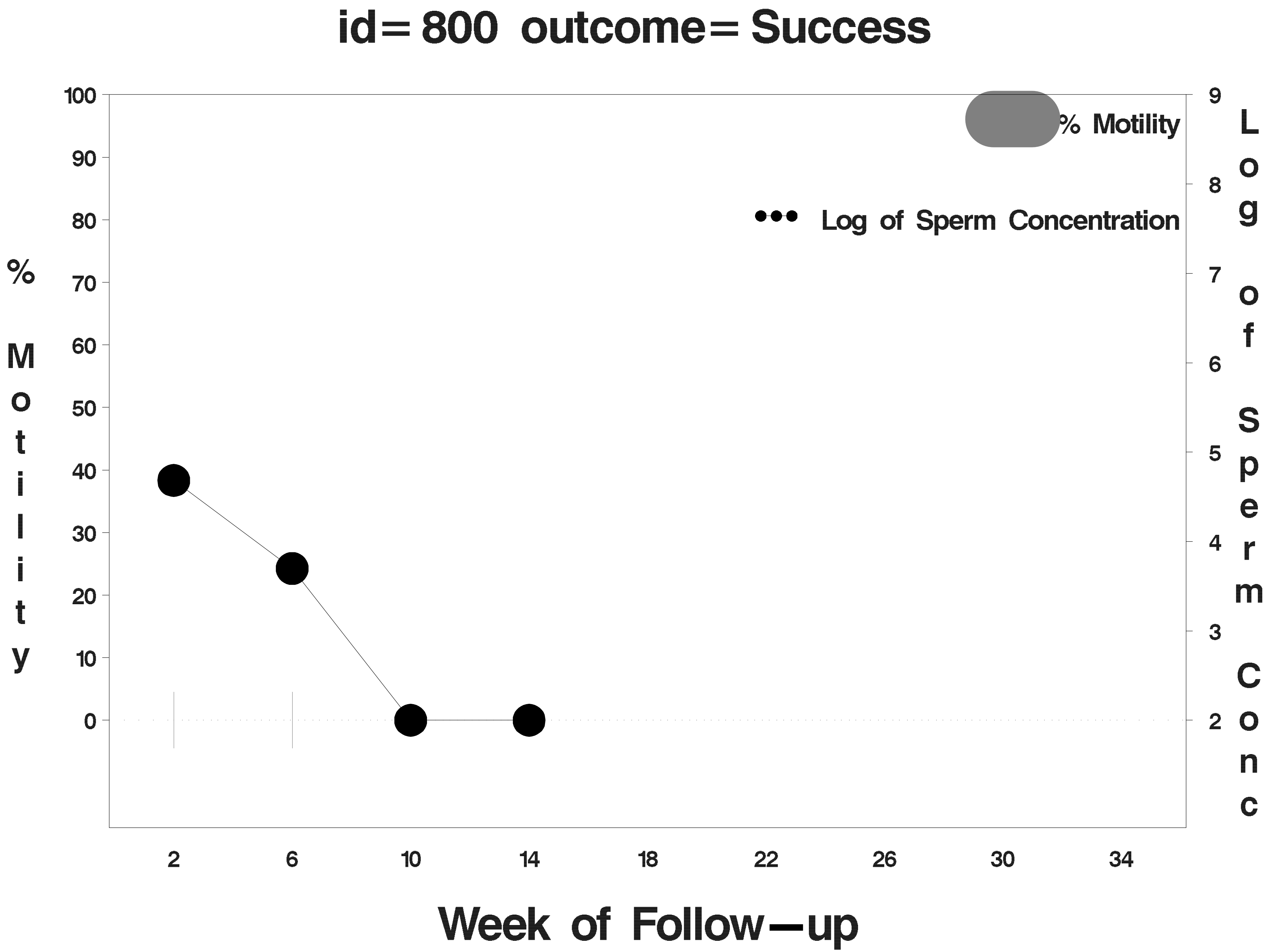

id= 800 outcome= Success
% Motility
Log of Sperm Concentration
% Motility
Log of Sperm Conc
Week of Follow-up
100
90
80
70
60
50
40
30
20
10
0
9
8
7
6
5
4
3
2
2
6
10
14
18
22
26
30
34

id= 801 outcome= Indeterminate

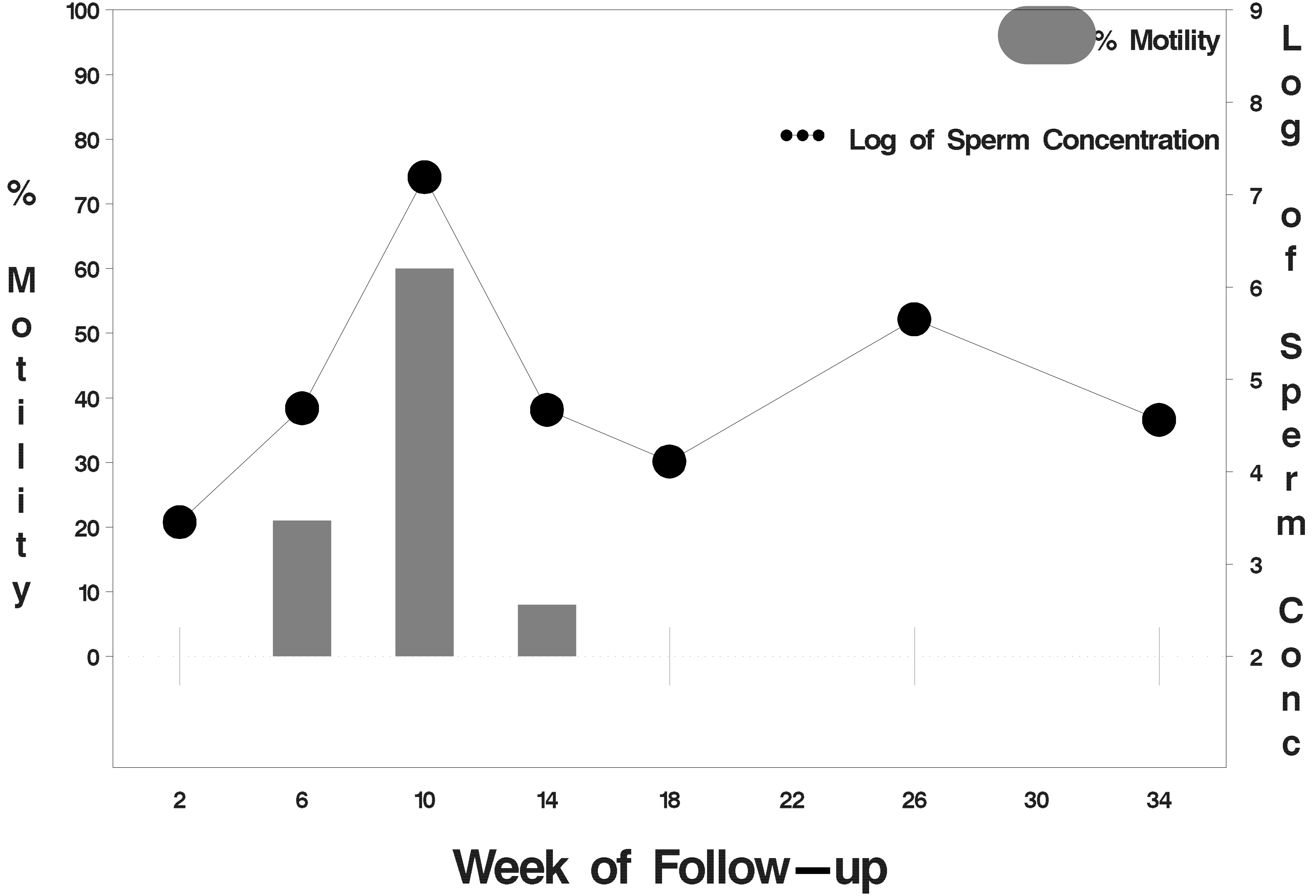

id= 802 outcome= Success

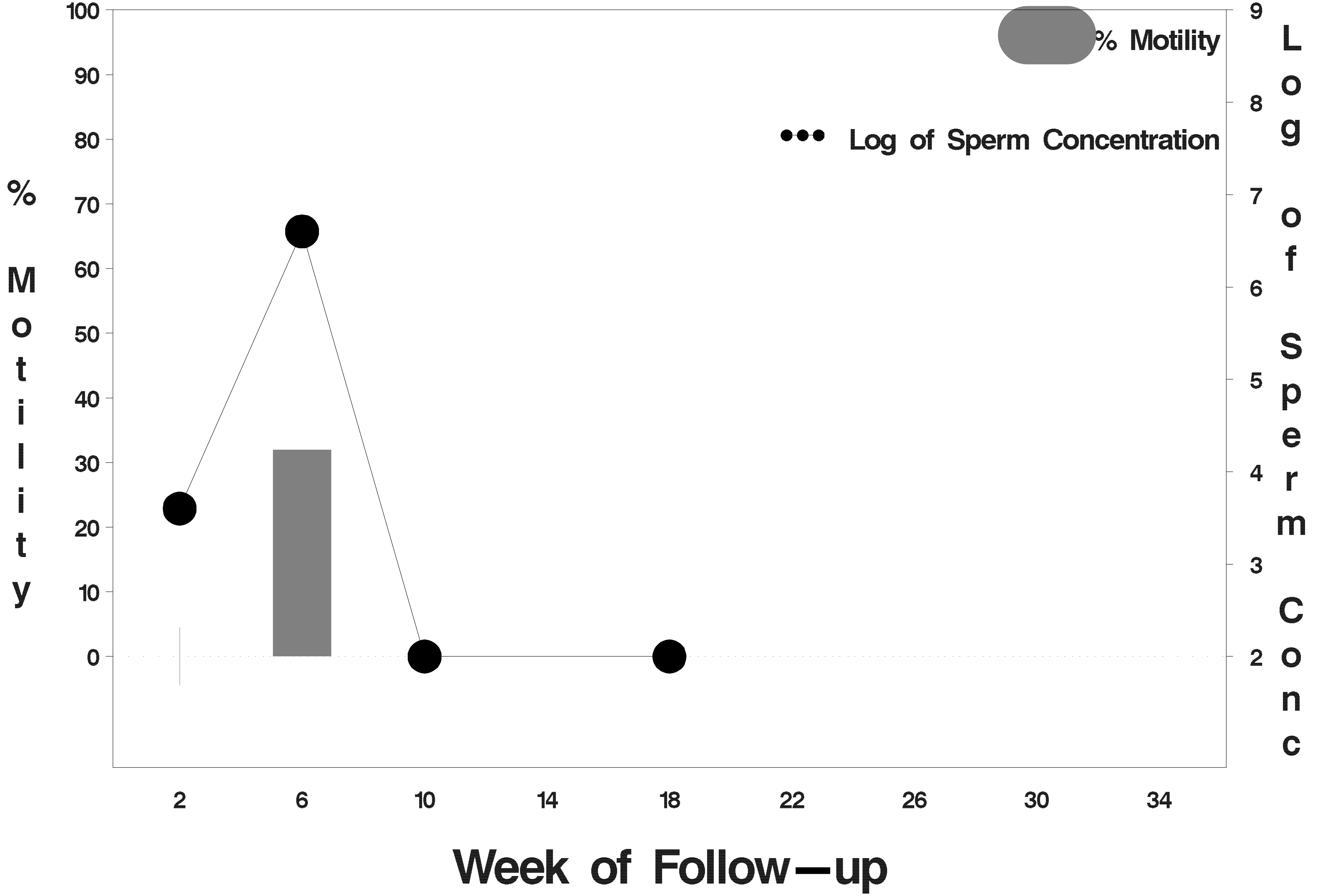

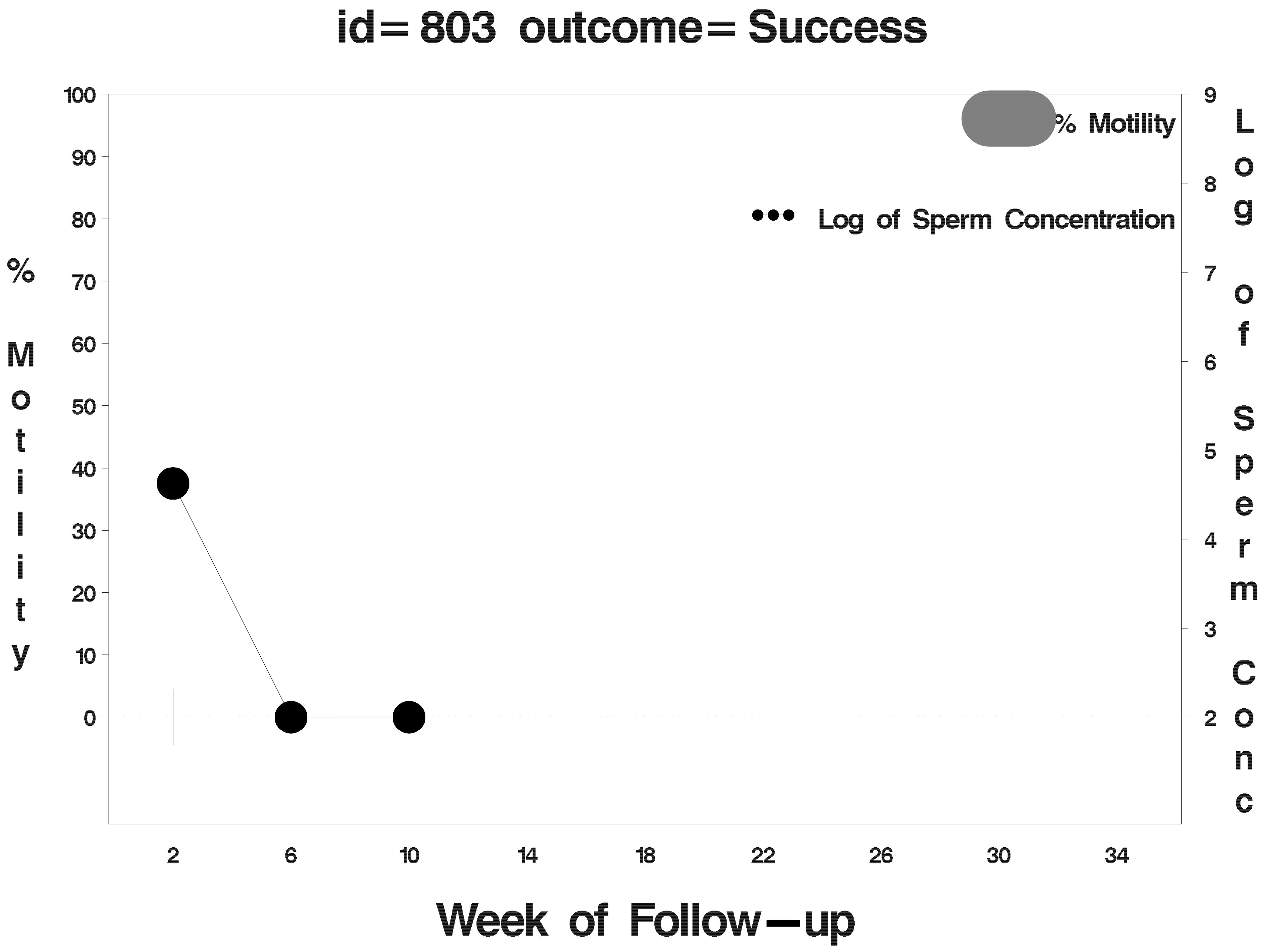
id= 803 outcome= Success
% Motility
Log of Sperm Concentration
100
90
80
70
60
50
40
30
20
10
0
9
8
7
6
5
4
3
2
2
6
10
14
18
22
26
30
34
% Motility
Log of Sperm Conc
Week of Follow-up

id= 804 outcome= Success

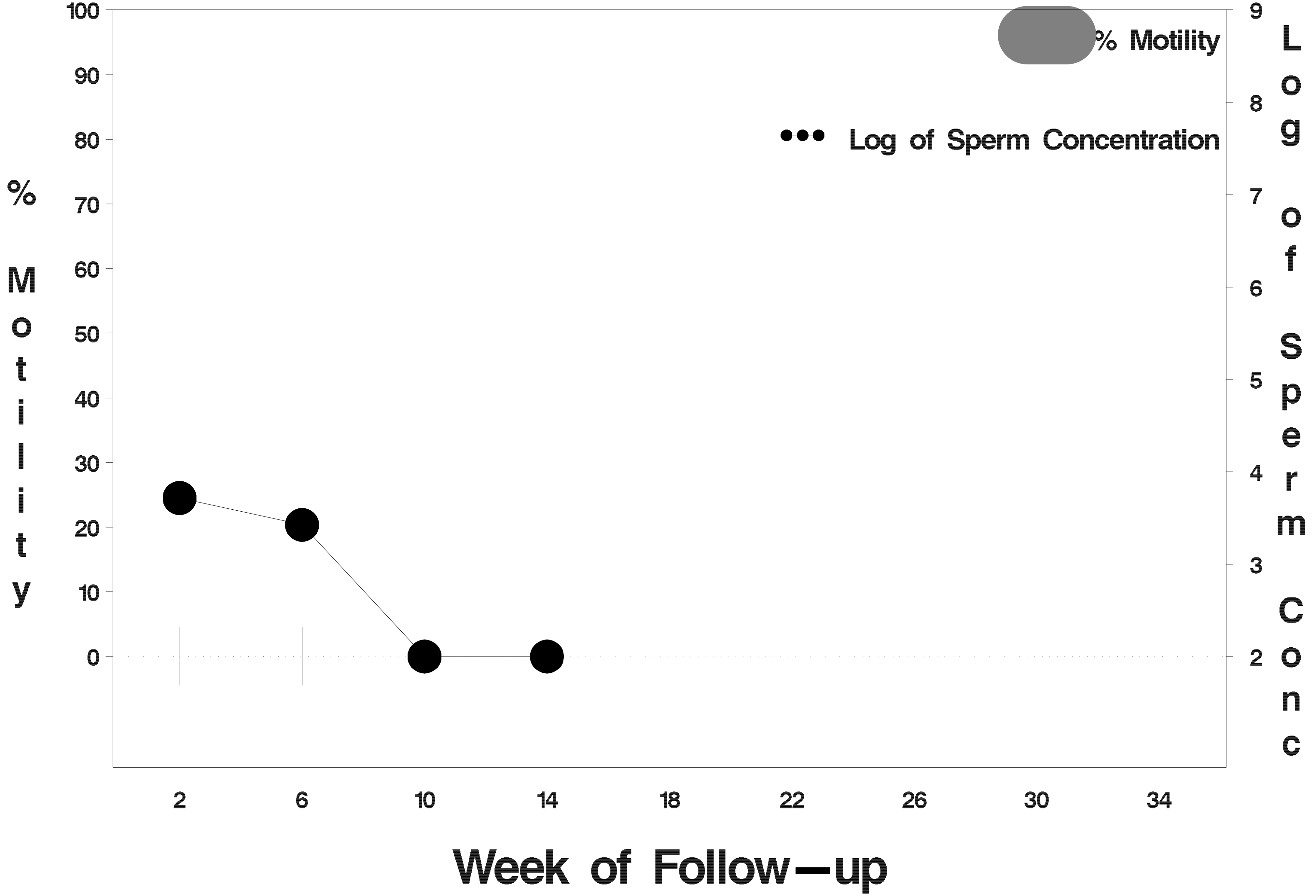

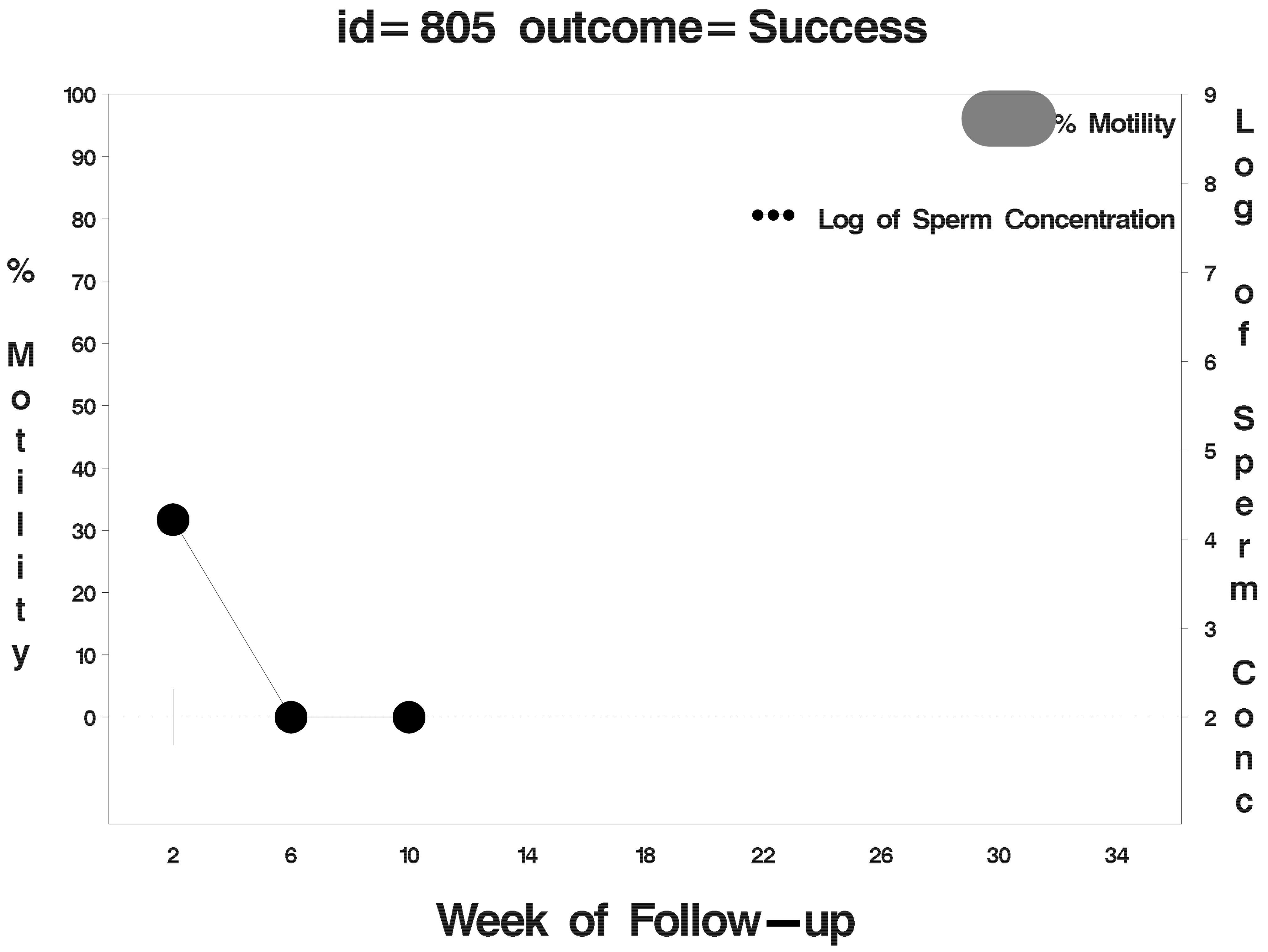

id= 805 outcome= Success
% Motility
Log of Sperm Concentration
100
90
80
70
60
50
40
30
20
10
0
9
8
7
6
5
4
3
2
2
6
10
14
18
22
26
30
34
Week of Follow−up
Log of Sperm Conc

id= 806 outcome= Success

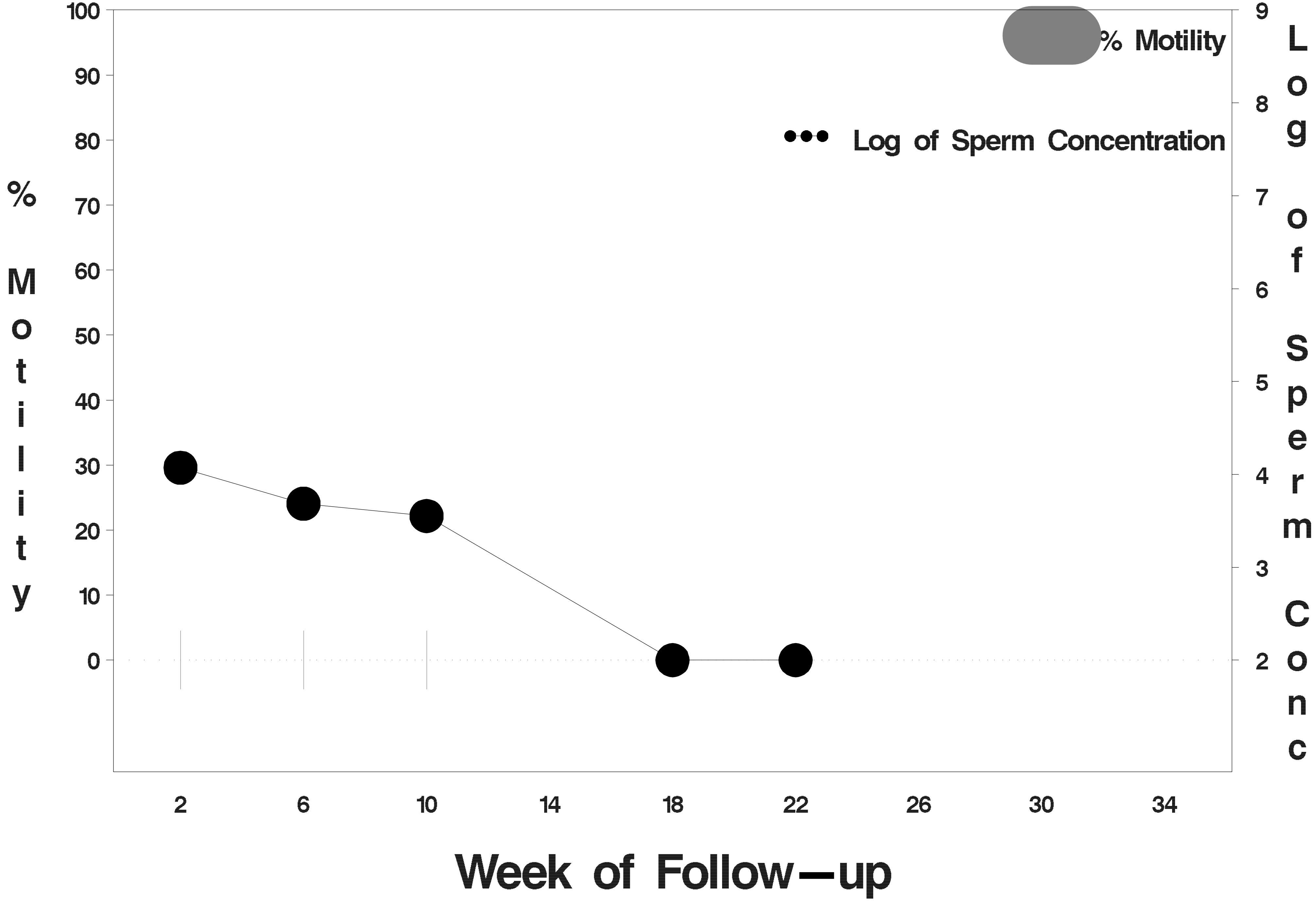

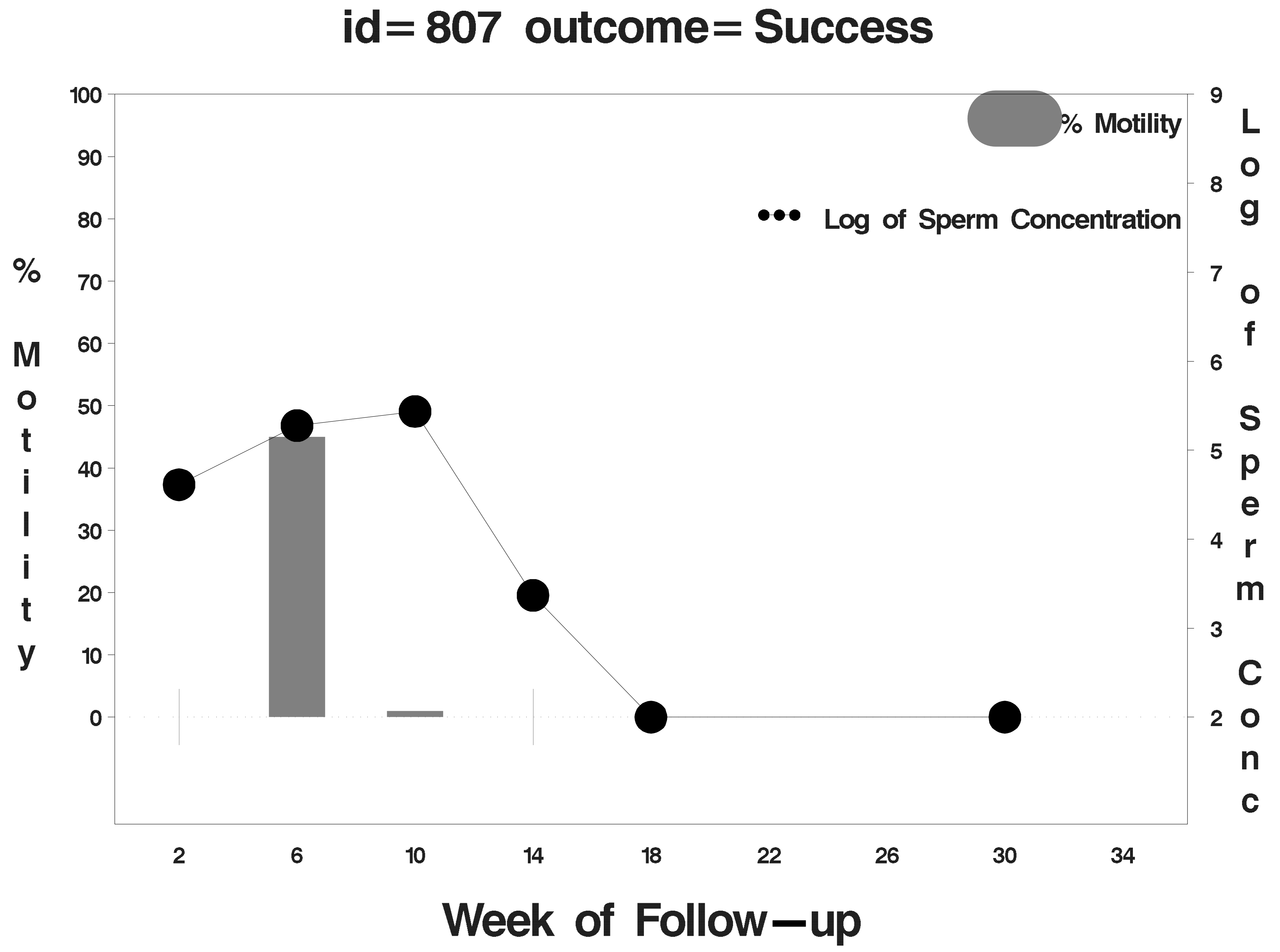
id= 807 outcome= Success
% Motility
Log of Sperm Concentration
% Motility
Log of Sperm Conc
100
90
80
70
60
50
40
30
20
10
0
9
8
7
6
5
4
3
2
2
6
10
14
18
22
26
30
34
Week of Follow—up

id= 808 outcome= Success

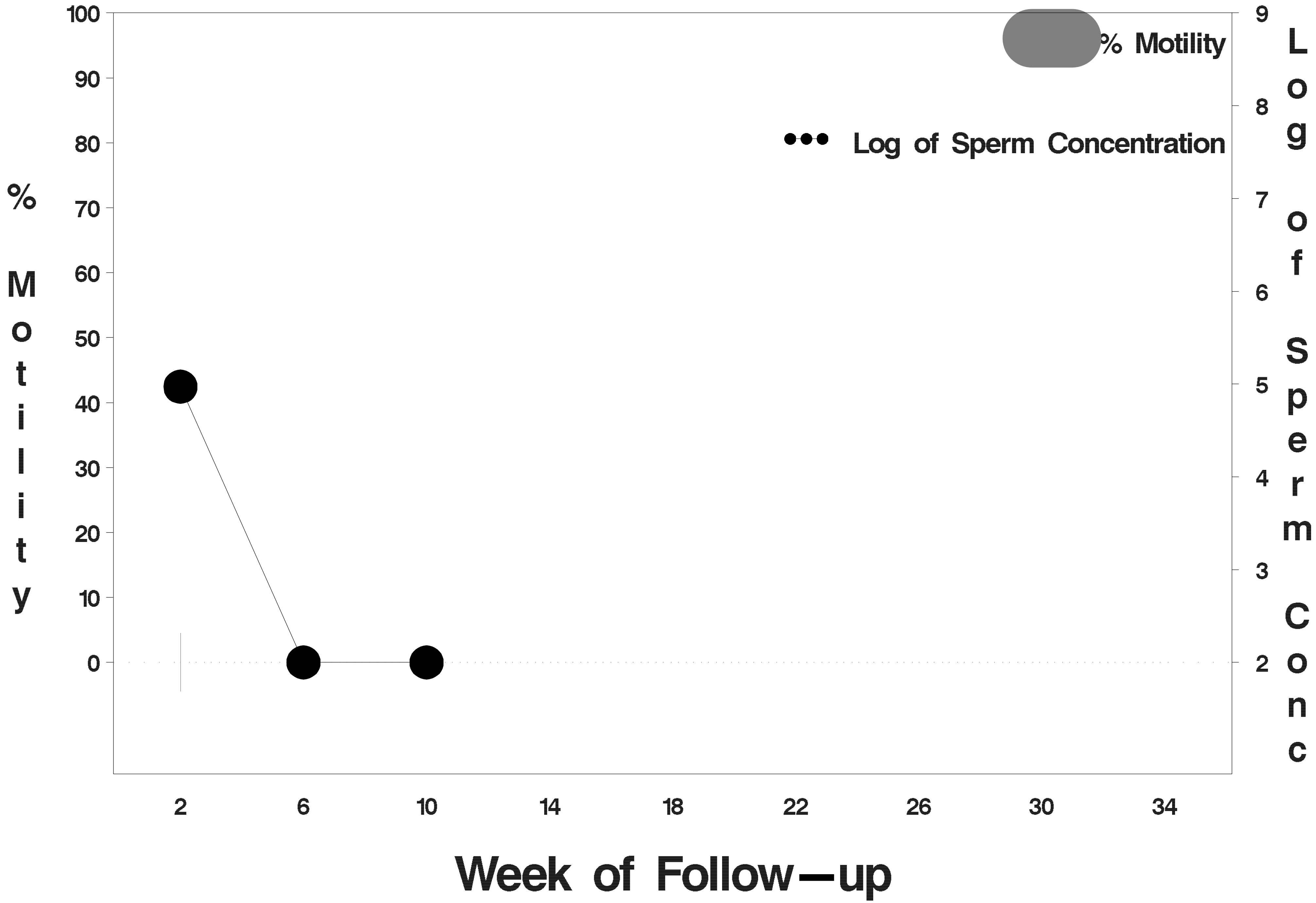

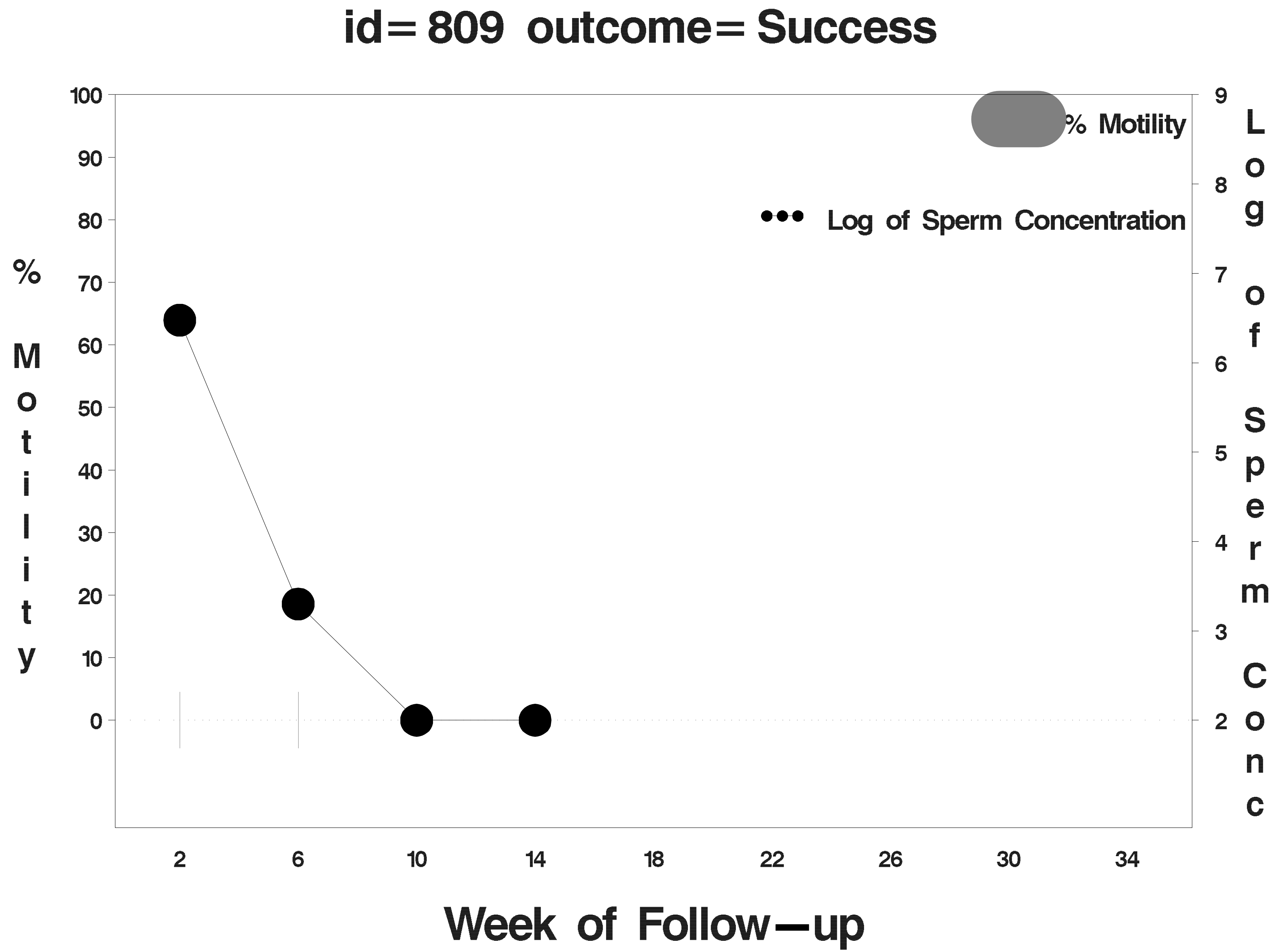
id= 809 outcome= Success
% Motility
Log of Sperm Concentration
% Motility
Log of Sperm Conc
Week of Follow-up
100
90
80
70
60
50
40
30
20
10
0
9
8
7
6
5
4
3
2
2
6
10
14
18
22
26
30
34

id= 810 outcome= Success

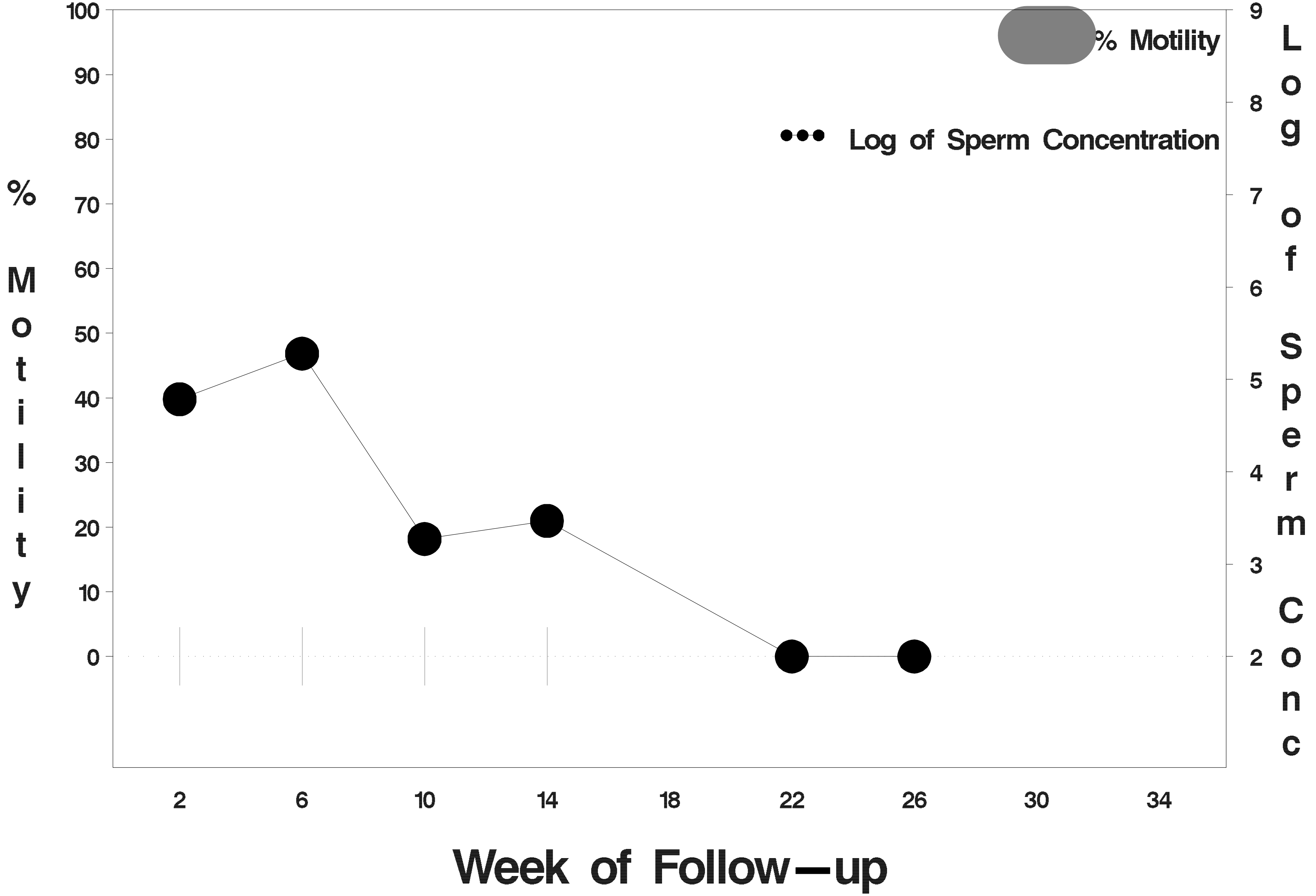

id= 811 outcome= Success

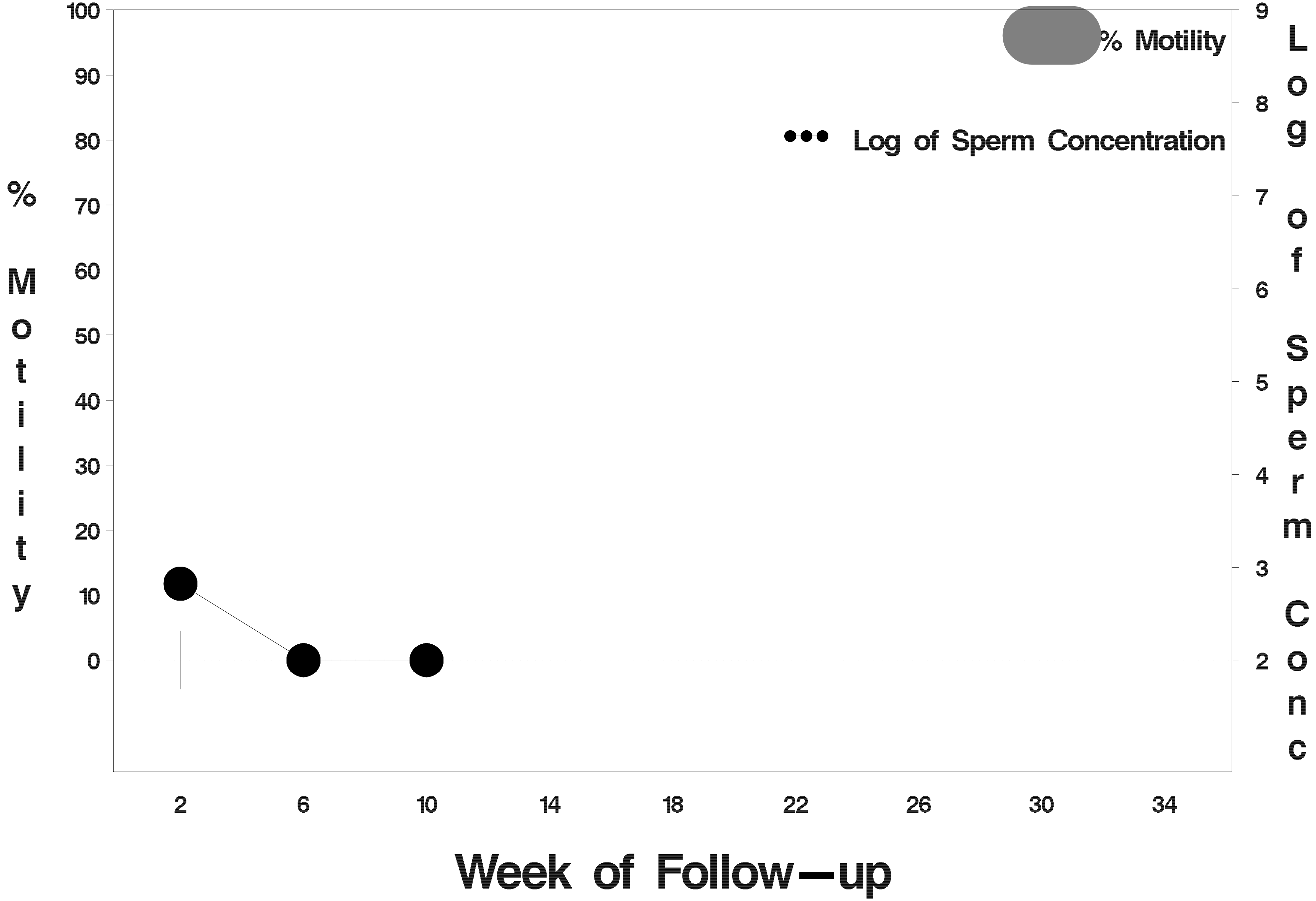

id= 812 outcome= Indeterminate

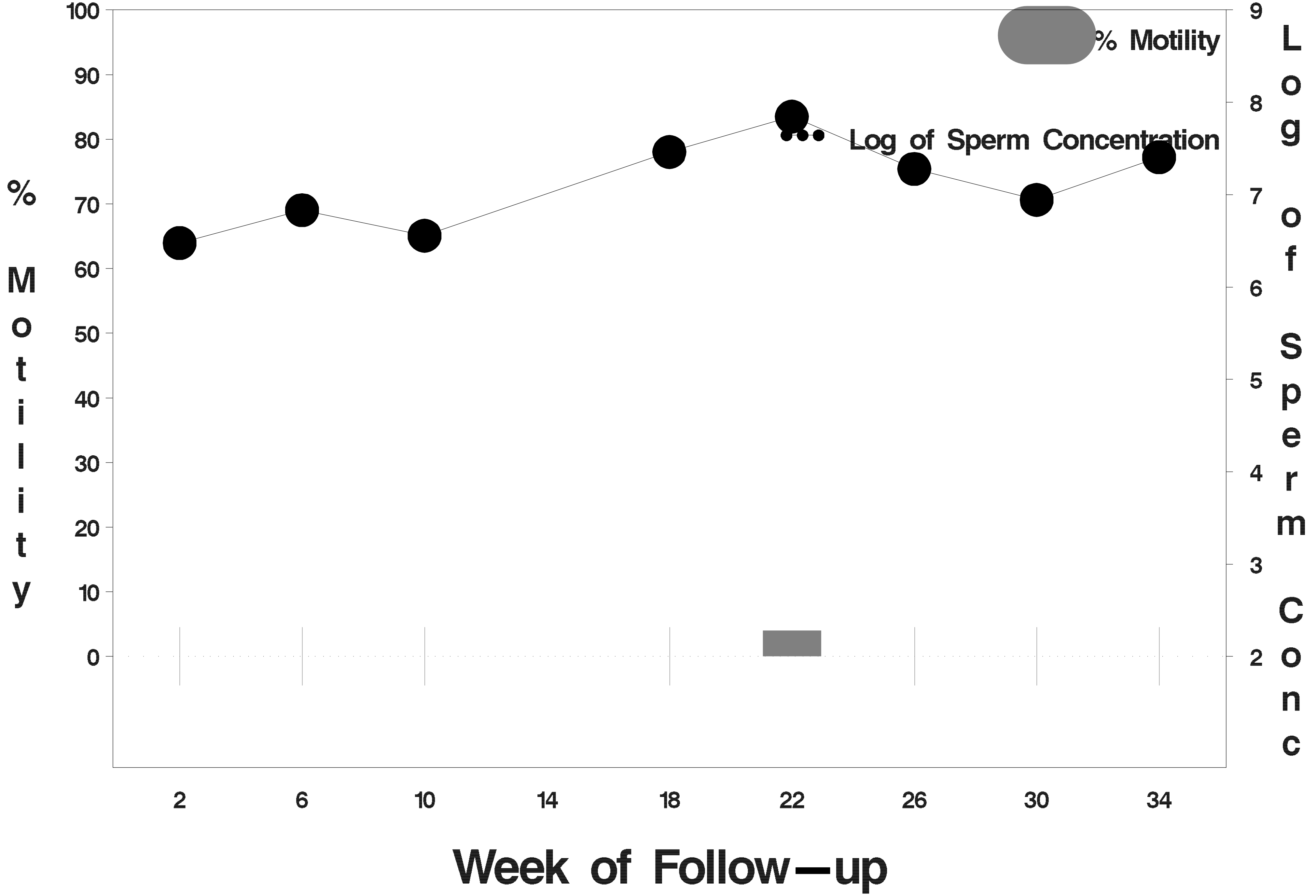

id= 813 outcome= Indeterminate

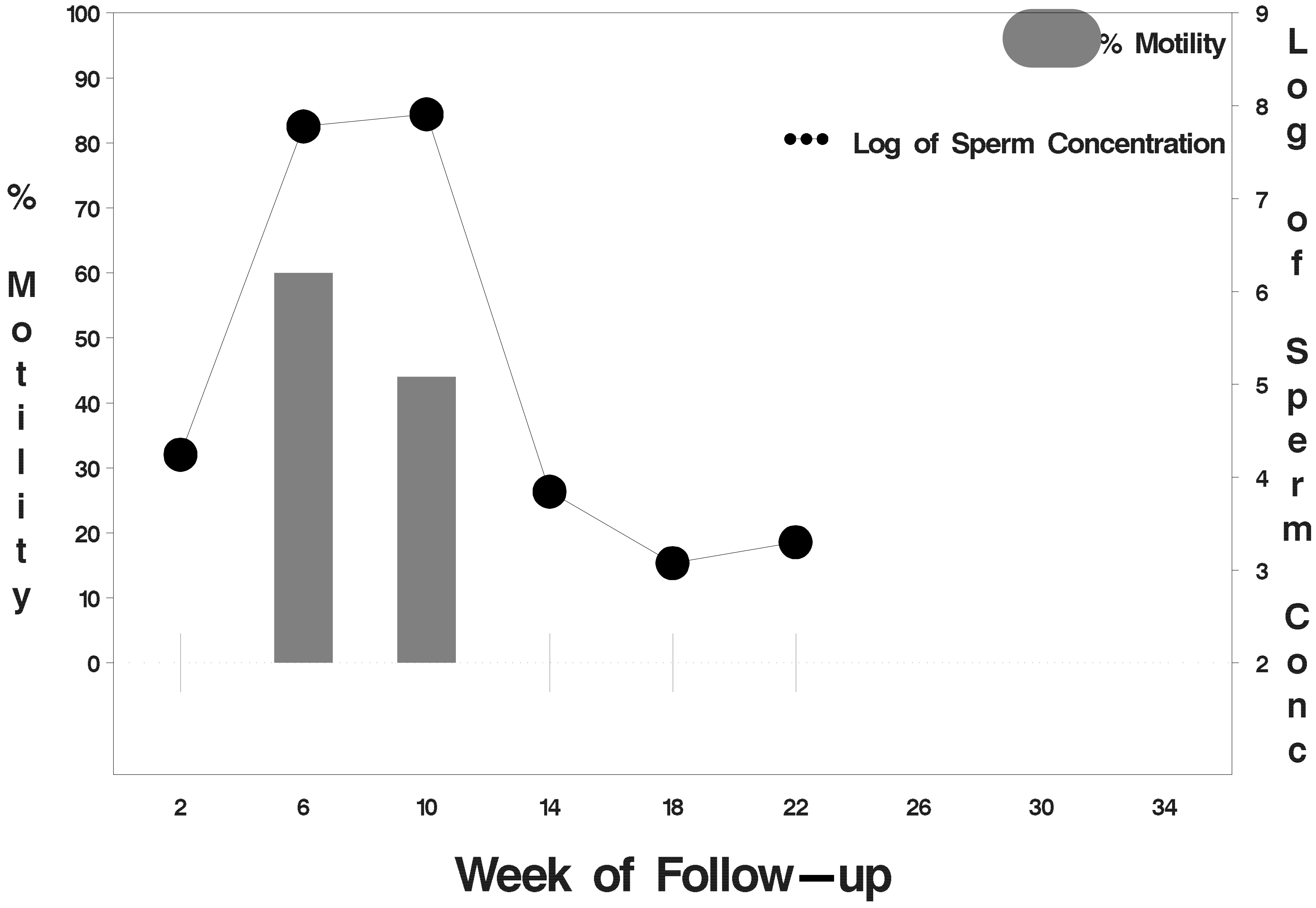

id= 814 outcome= Success

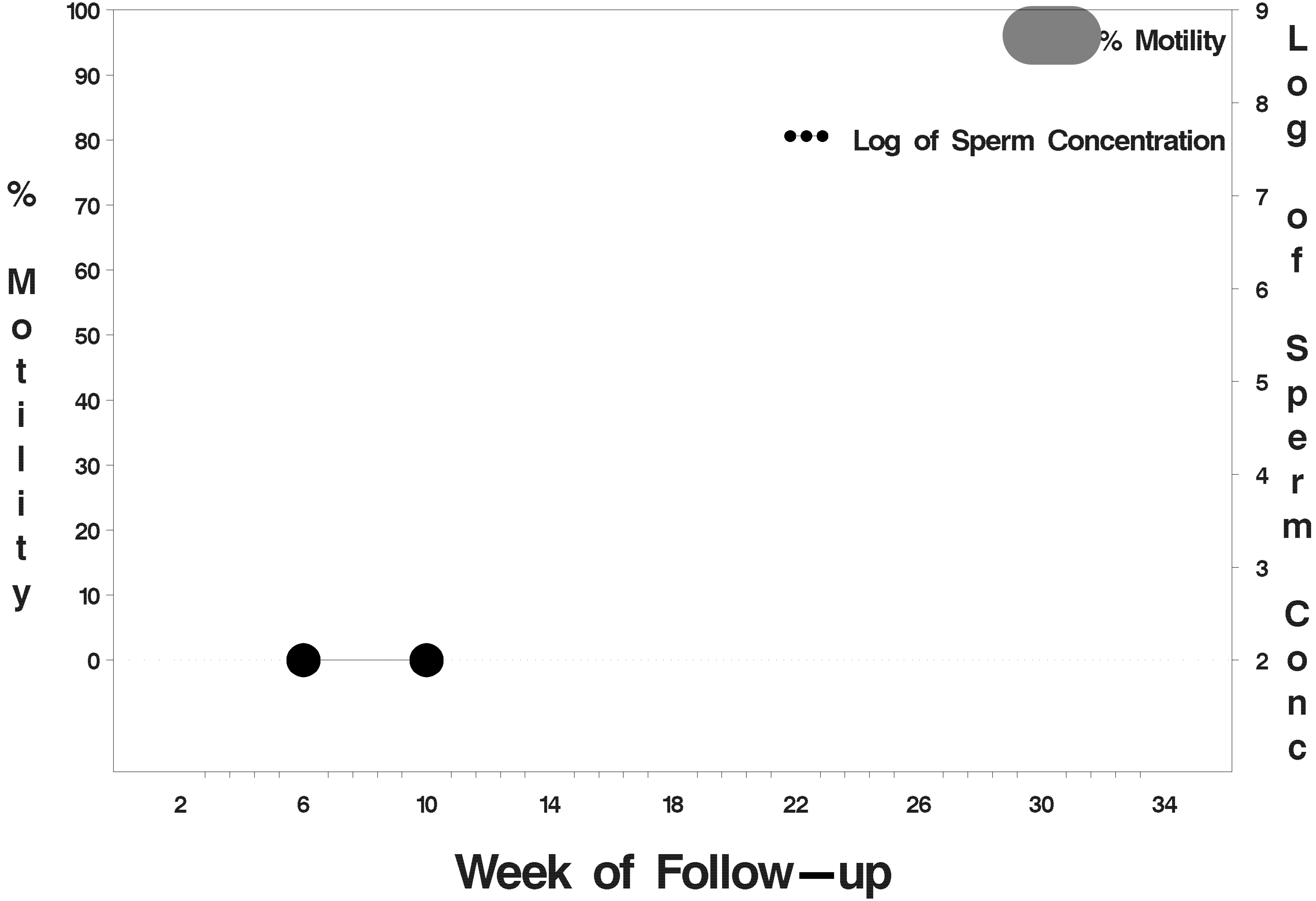

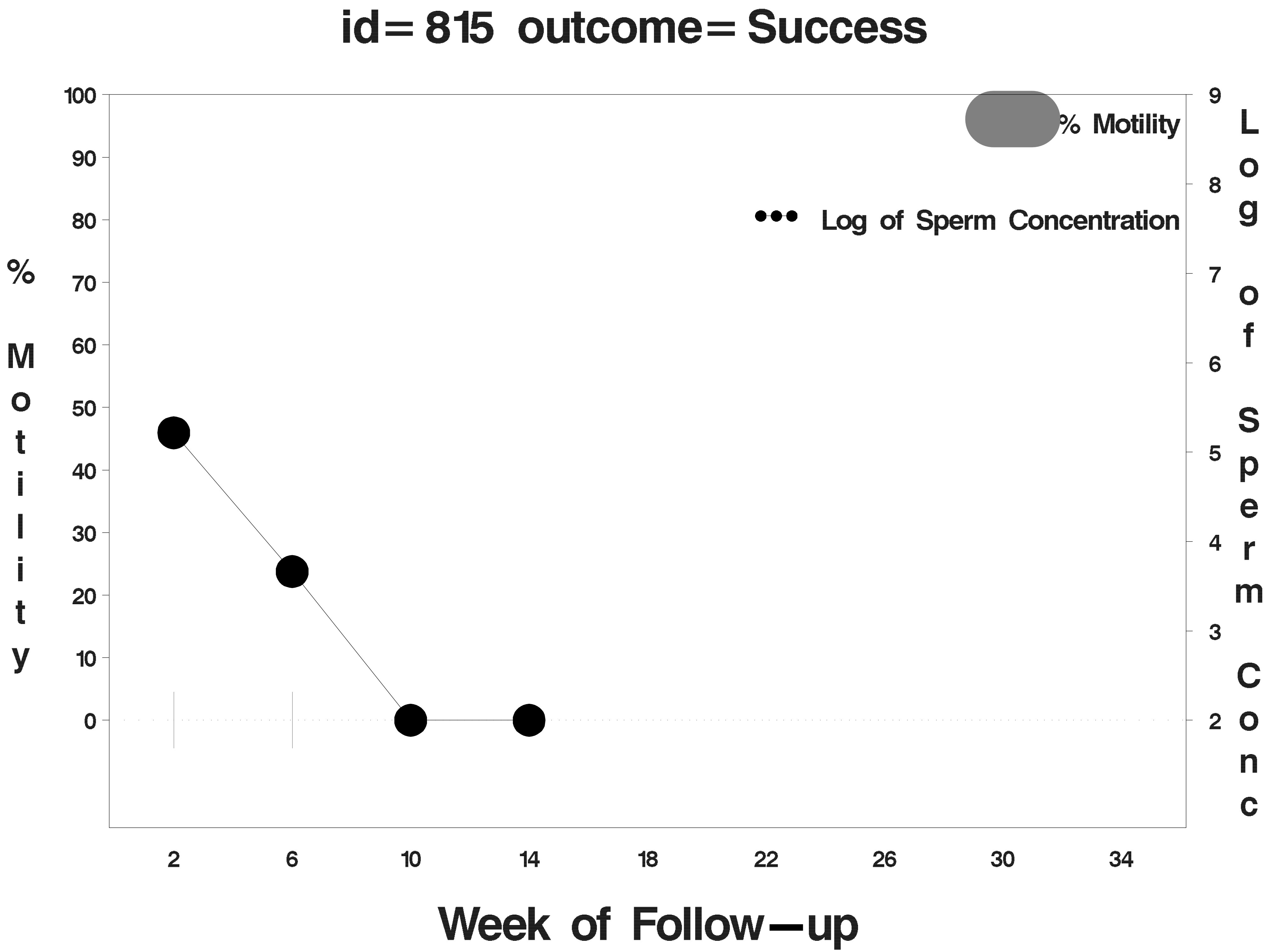
id= 815 outcome= Success
% Motility
Log of Sperm Concentration
% Motility
Log of Sperm Conc
Week of Follow-up
100
90
80
70
60
50
40
30
20
10
0
9
8
7
6
5
4
3
2
2
6
10
14
18
22
26
30
34

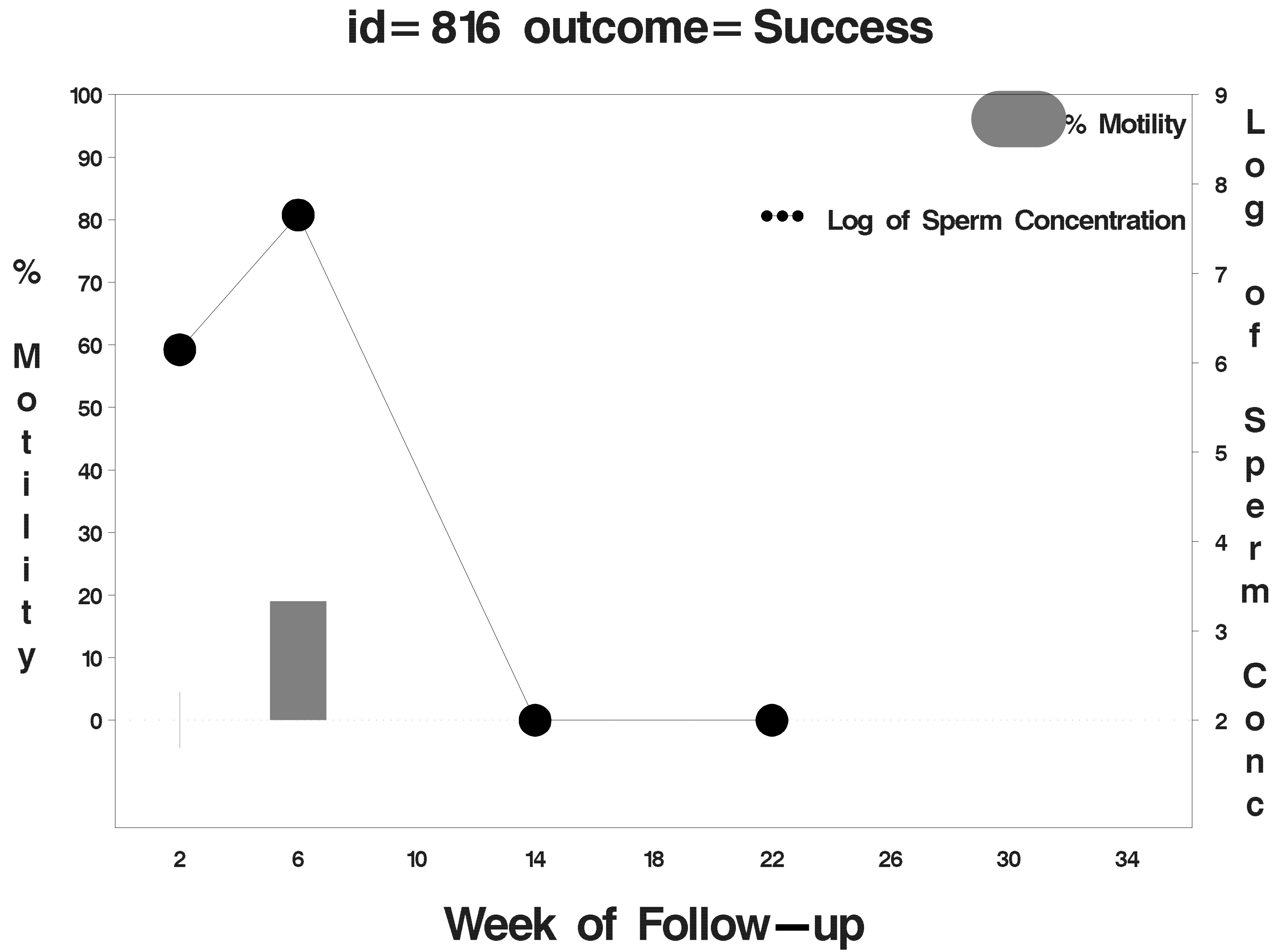

id= 816 outcome= Success
% Motility
Log of Sperm Concentration
100
90
80
70
60
50
40
30
20
10
0
9
8
7
6
5
4
3
2
2
6
10
14
18
22
26
30
34
Week of Follow−up
Log of Sperm Conc

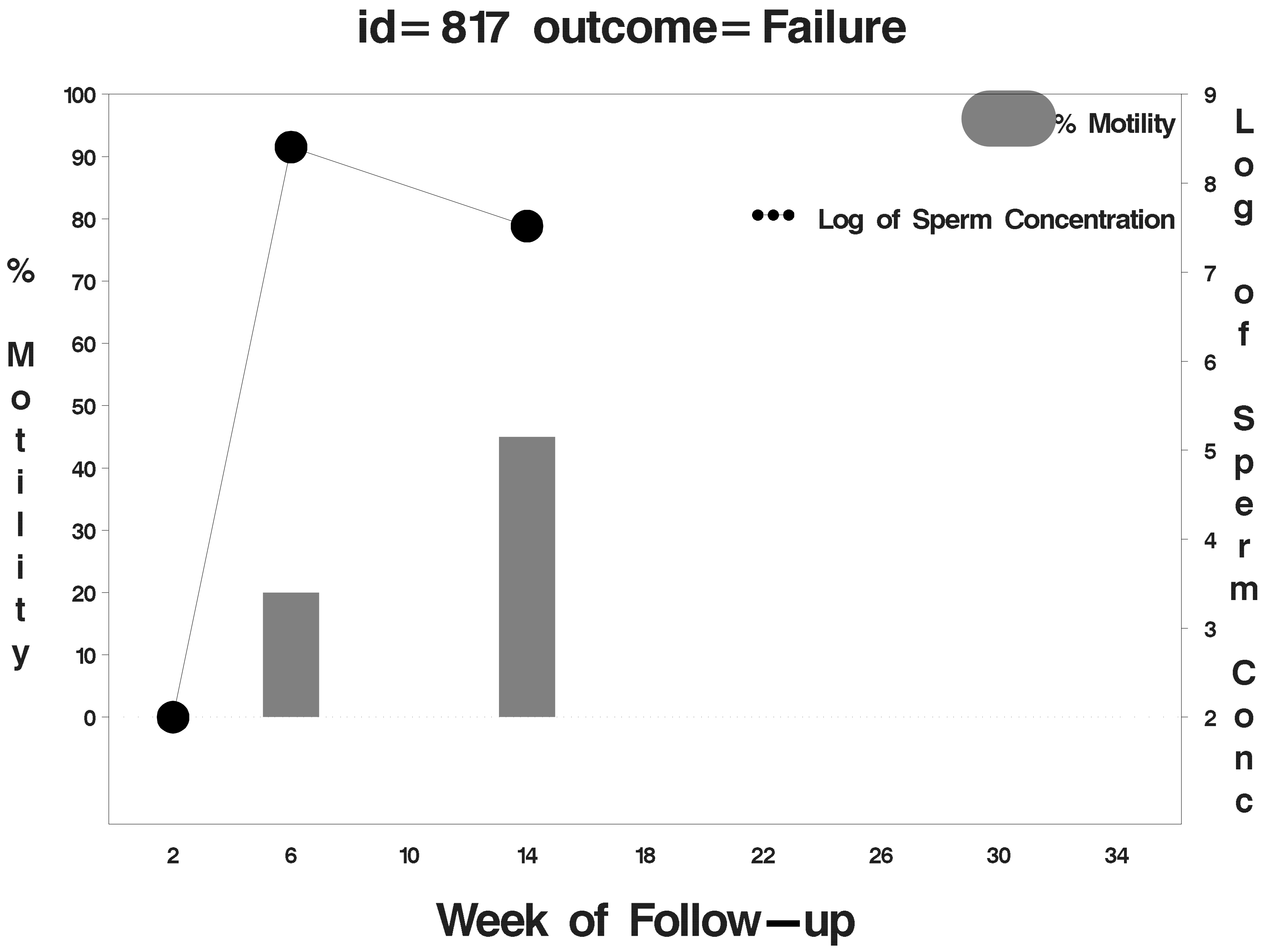

id= 817 outcome= Failure
% Motility
Log of Sperm Concentration
100
90
80
70
60
50
40
30
20
10
0
9
8
7
6
5
4
3
2
2
6
10
14
18
22
26
30
34
Week of Follow−up
Log of Sperm Conc

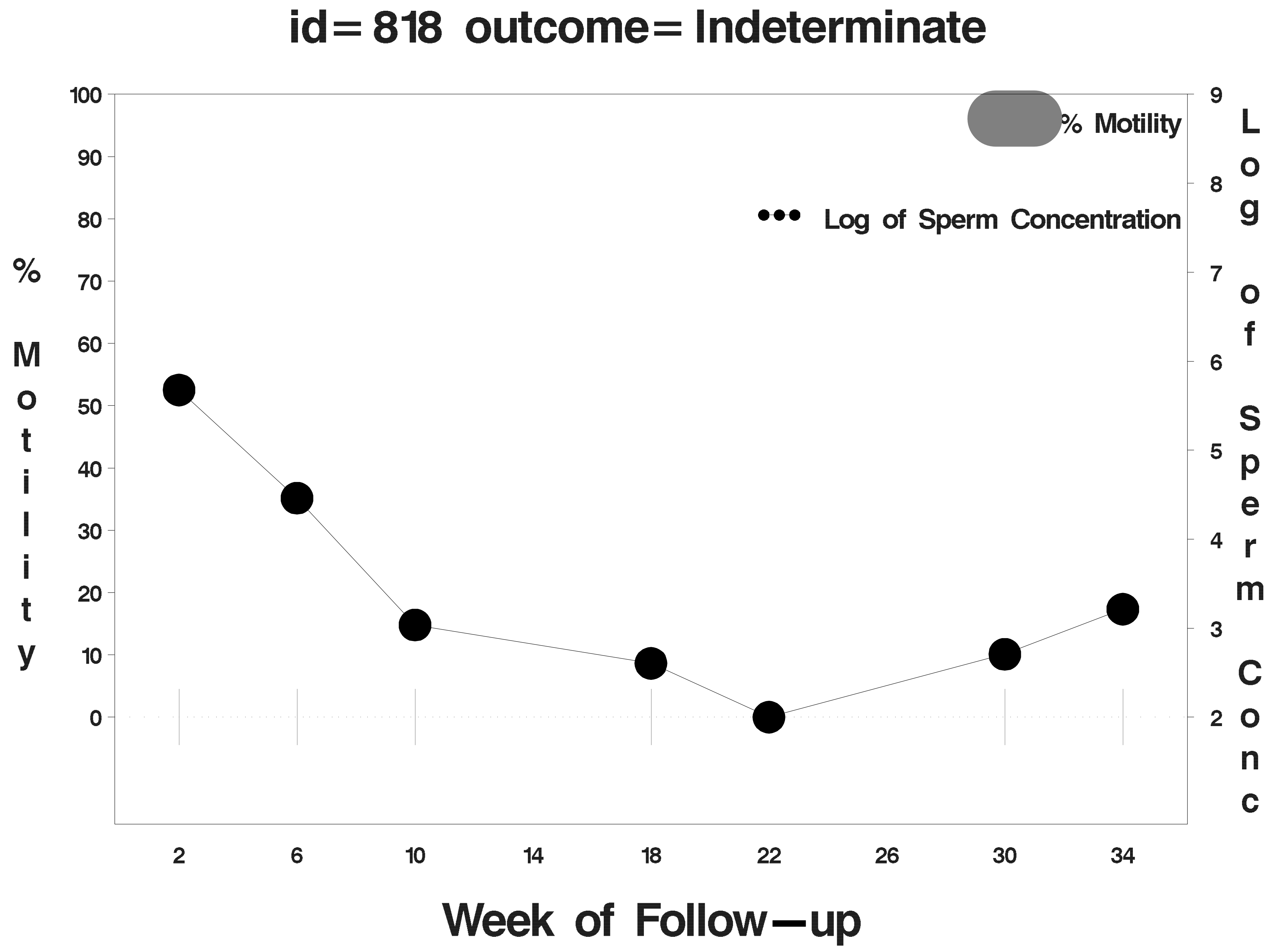

id= 818 outcome= Indeterminate
% Motility
Log of Sperm Concentration
% Motility
Log of Sperm Conc
Week of Follow—up
100
90
80
70
60
50
40
30
20
10
0
9
8
7
6
5
4
3
2
2
6
10
14
18
22
26
30
34

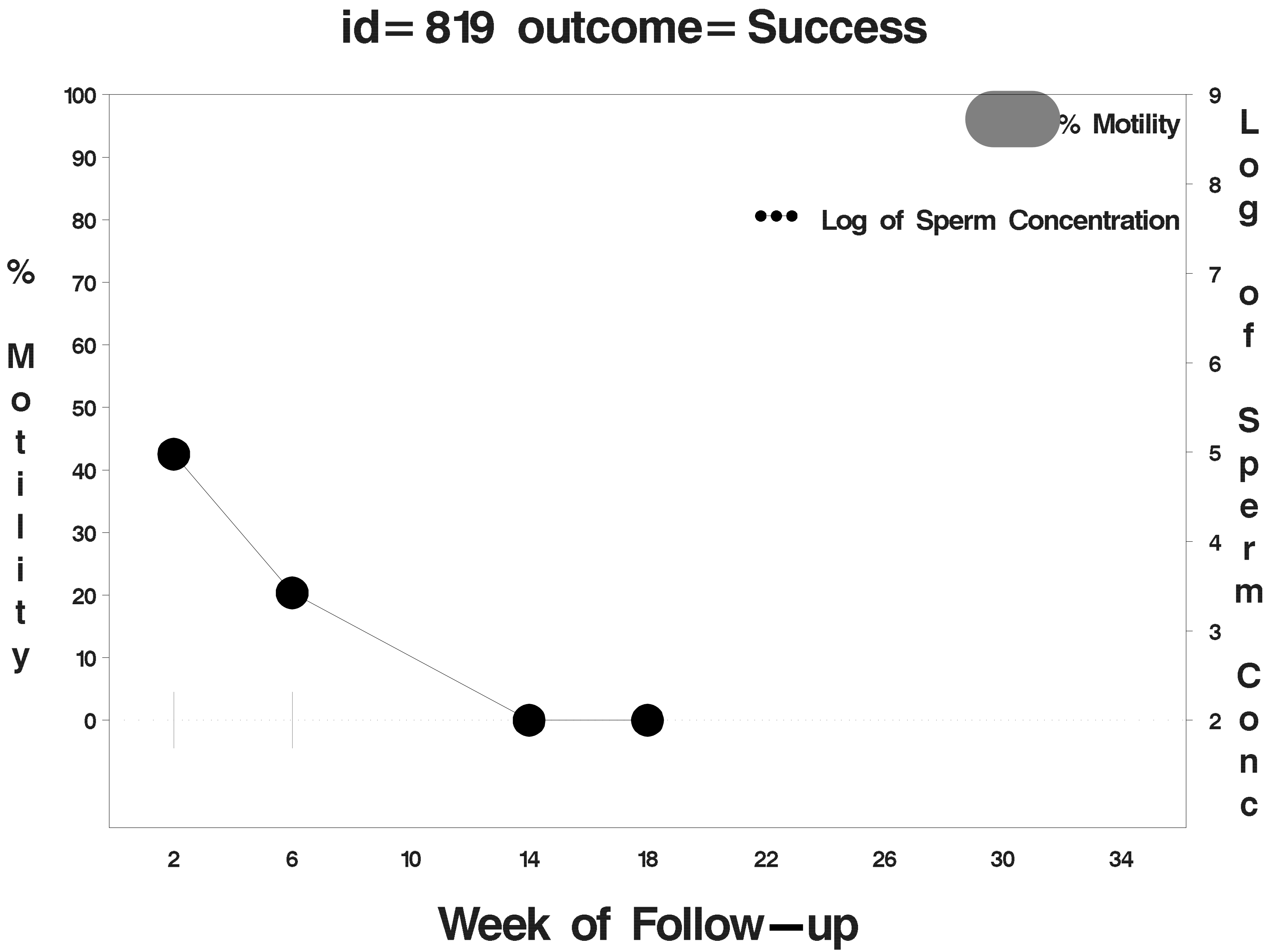

id= 819 outcome= Success
% Motility
Log of Sperm Concentration
% Motility
Log of Sperm Conc
Week of Follow-up
100
90
80
70
60
50
40
30
20
10
0
9
8
7
6
5
4
3
2
2
6
10
14
18
22
26
30
34

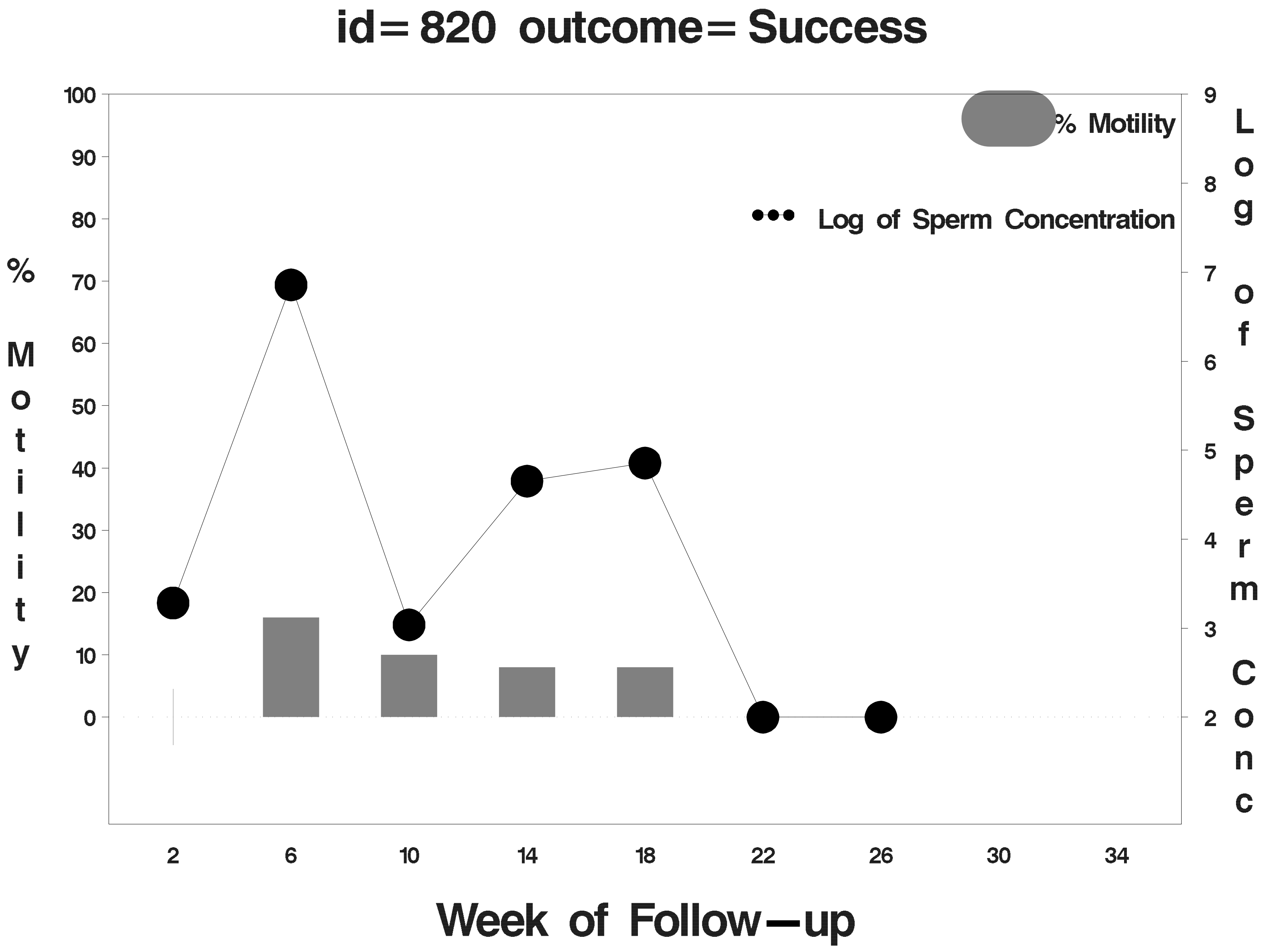

id= 820 outcome= Success
% Motility
Log of Sperm Concentration
% Motility
Log of Sperm Conc
Week of Follow−up
100
90
80
70
60
50
40
30
20
10
0
9
8
7
6
5
4
3
2
2
6
10
14
18
22
26
30
34

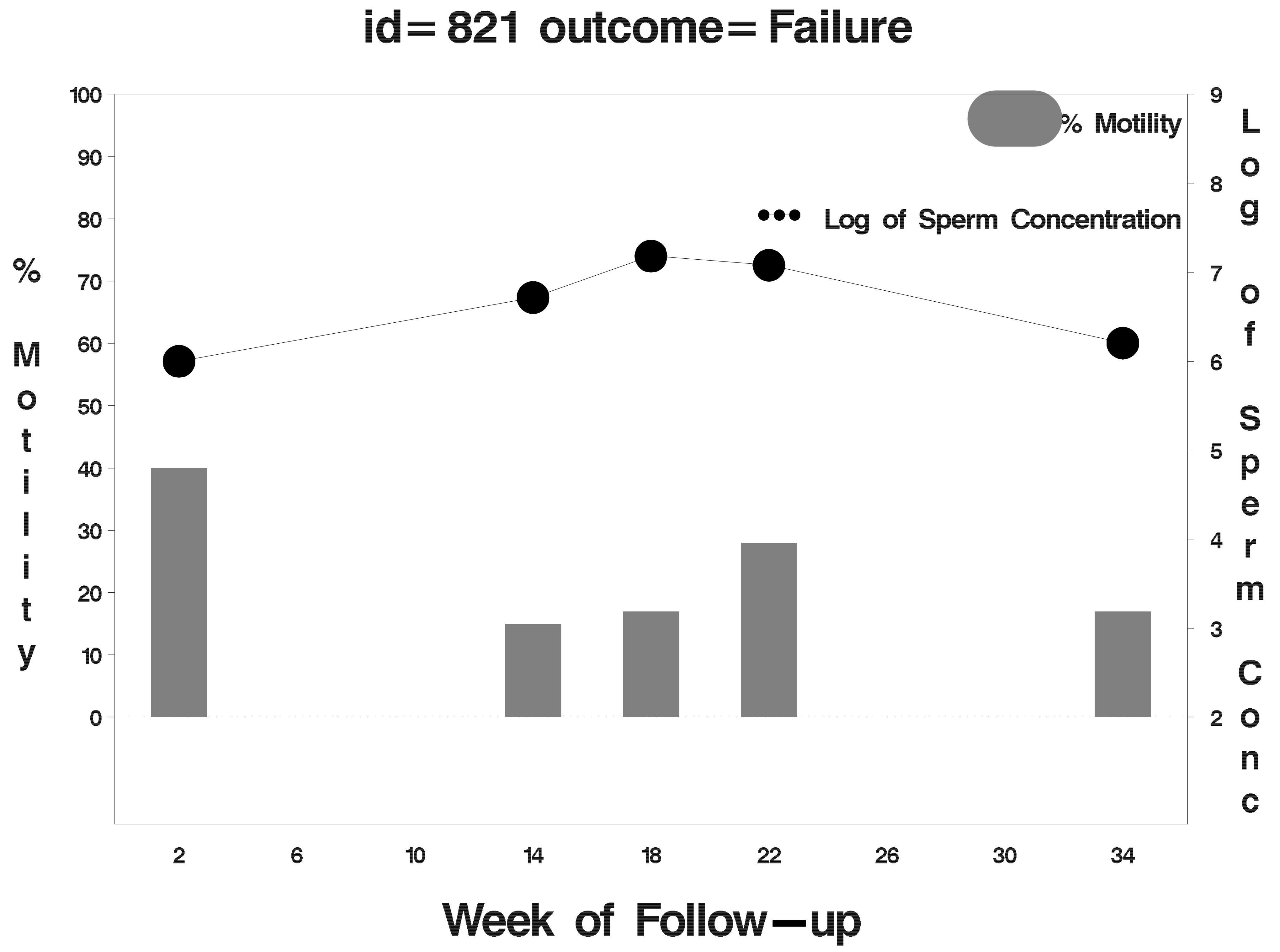

id= 821 outcome= Failure
% Motility
Log of Sperm Concentration
100
90
80
70
60
50
40
30
20
10
0
9
8
7
6
5
4
3
2
2
6
10
14
18
22
26
30
34
Week of Follow−up
Log of Sperm Conc

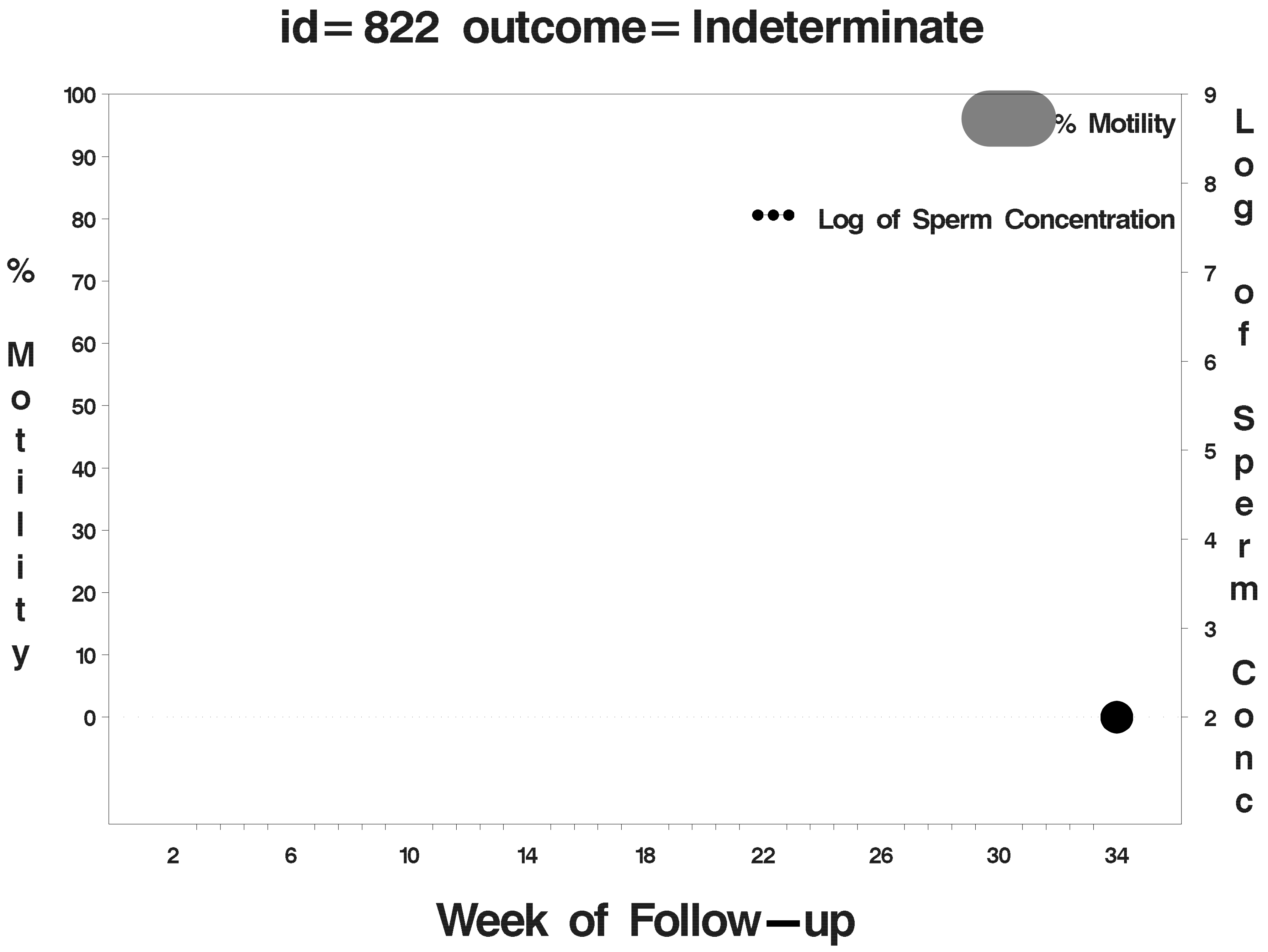

id= 822 outcome= Indeterminate
% Motility
Log of Sperm Concentration
100
90
80
70
60
50
40
30
20
10
0
9
8
7
6
5
4
3
2
2
6
10
14
18
22
26
30
34
Week of Follow-up
Log of Sperm Conc

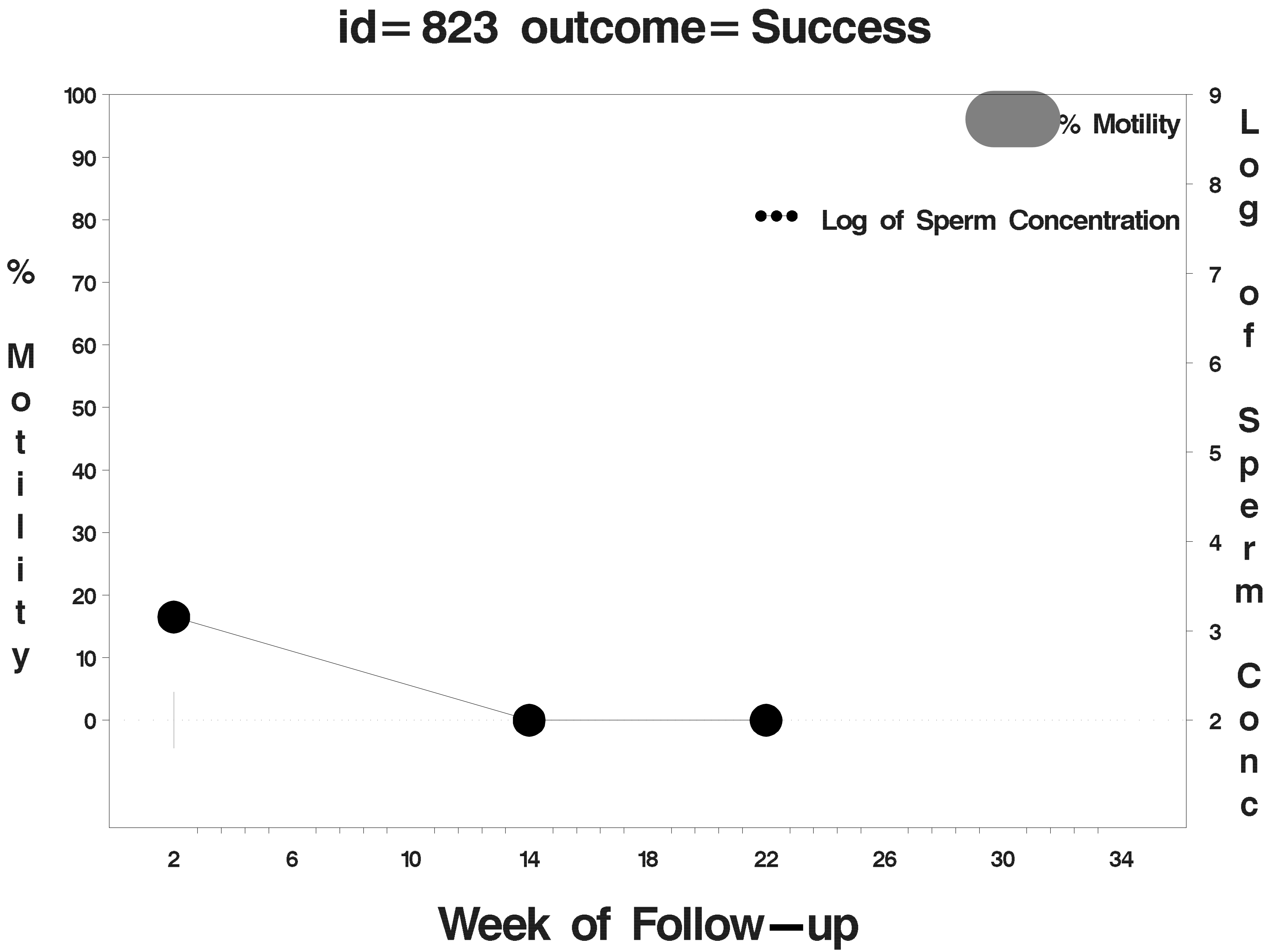
id= 823 outcome= Success
% Motility
Log of Sperm Concentration
% Motility
Log of Sperm Conc
Week of Follow—up
100
90
80
70
60
50
40
30
20
10
0
9
8
7
6
5
4
3
2
2
6
10
14
18
22
26
30
34

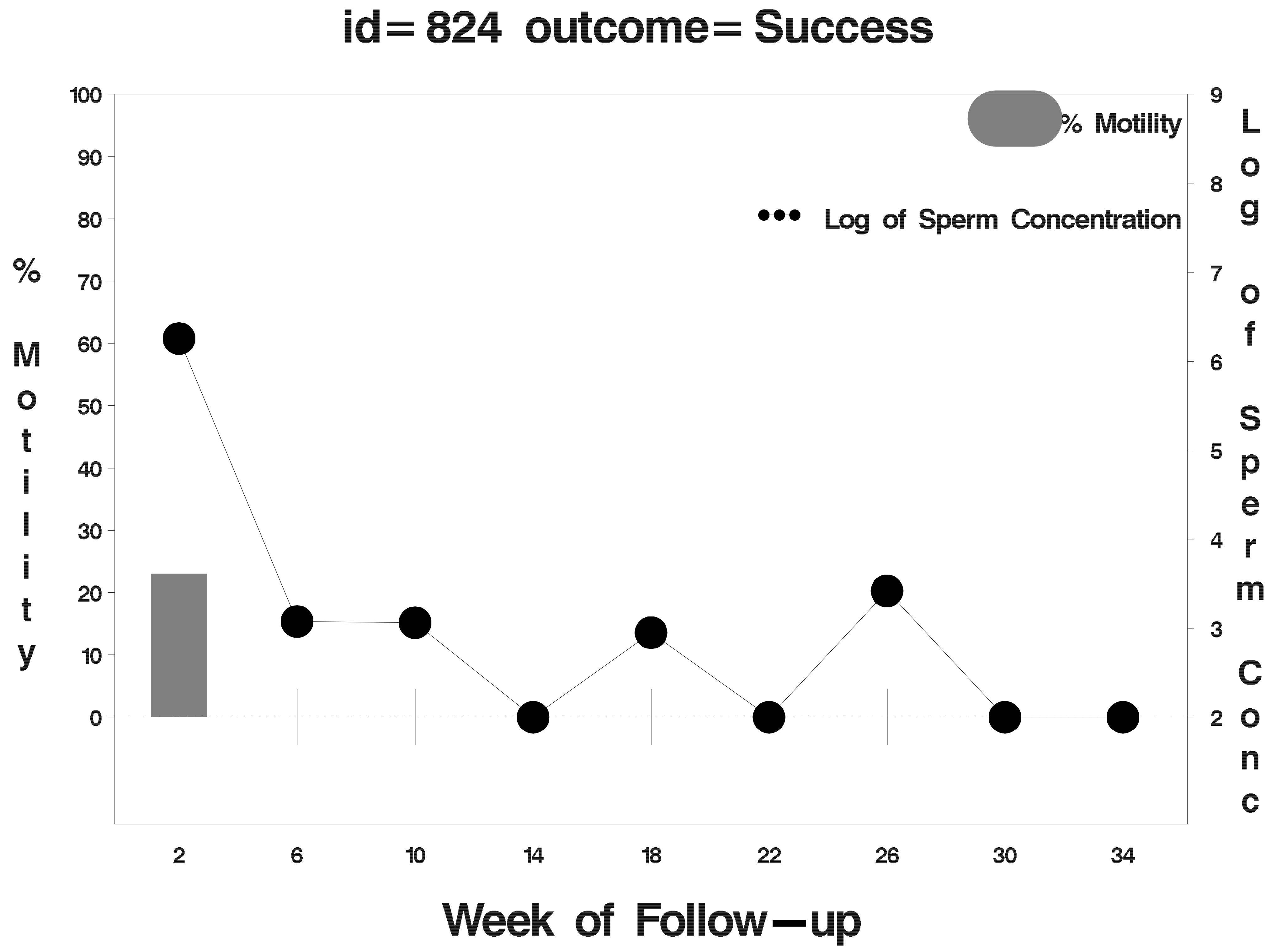
id= 824 outcome= Success
% Motility
Log of Sperm Concentration
100
90
80
70
60
50
40
30
20
10
0
9
8
7
6
5
4
3
2
2
6
10
14
18
22
26
30
34
Week of Follow—up
Log of Sperm Conc

id= 825 outcome= Success

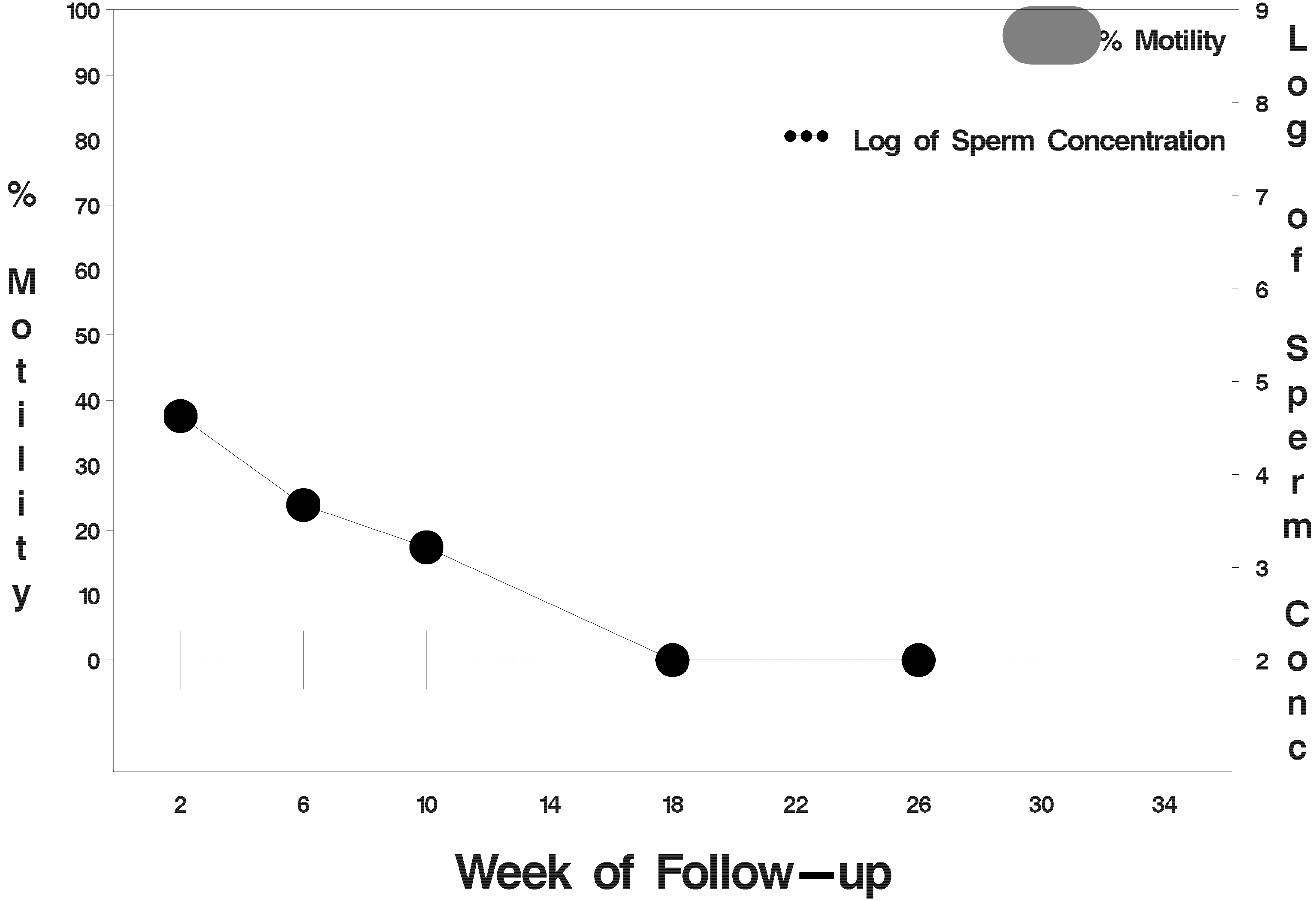

id= 826 outcome= Success

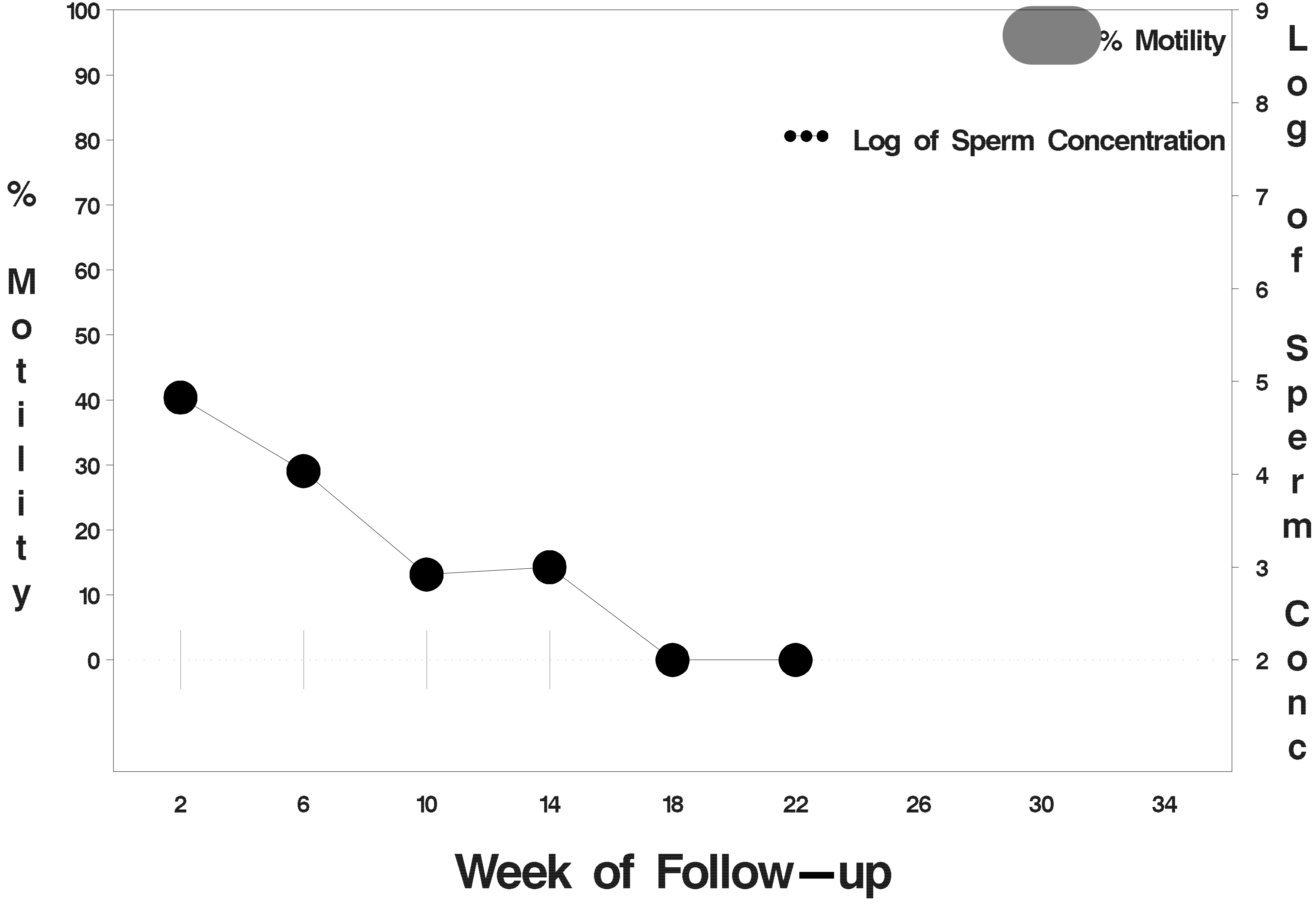

Supplement: Additional file 2 — Semen analysis charts from the Fascial Interposition trial. This file provides all semen analysis charts from the Fascial Interposition trial. [file 1471-2490-6-25-S2.pdf]
