# Supplementary material for: Frequency and patterns of early recanalization after vasectomy
Source: BMC Urol. 2006 Sep 19;6:25. doi: 10.1186/1471-2490-6-25 (PMC1586021; doi:10.1186/1471-2490-6-25)
Supplement: Additional file 3 — Semen analysis charts from the Cautery study. This file provides all semen analysis charts from the Cautery study. [file 1471-2490-6-25-S3.pdf]

Participant number= 101

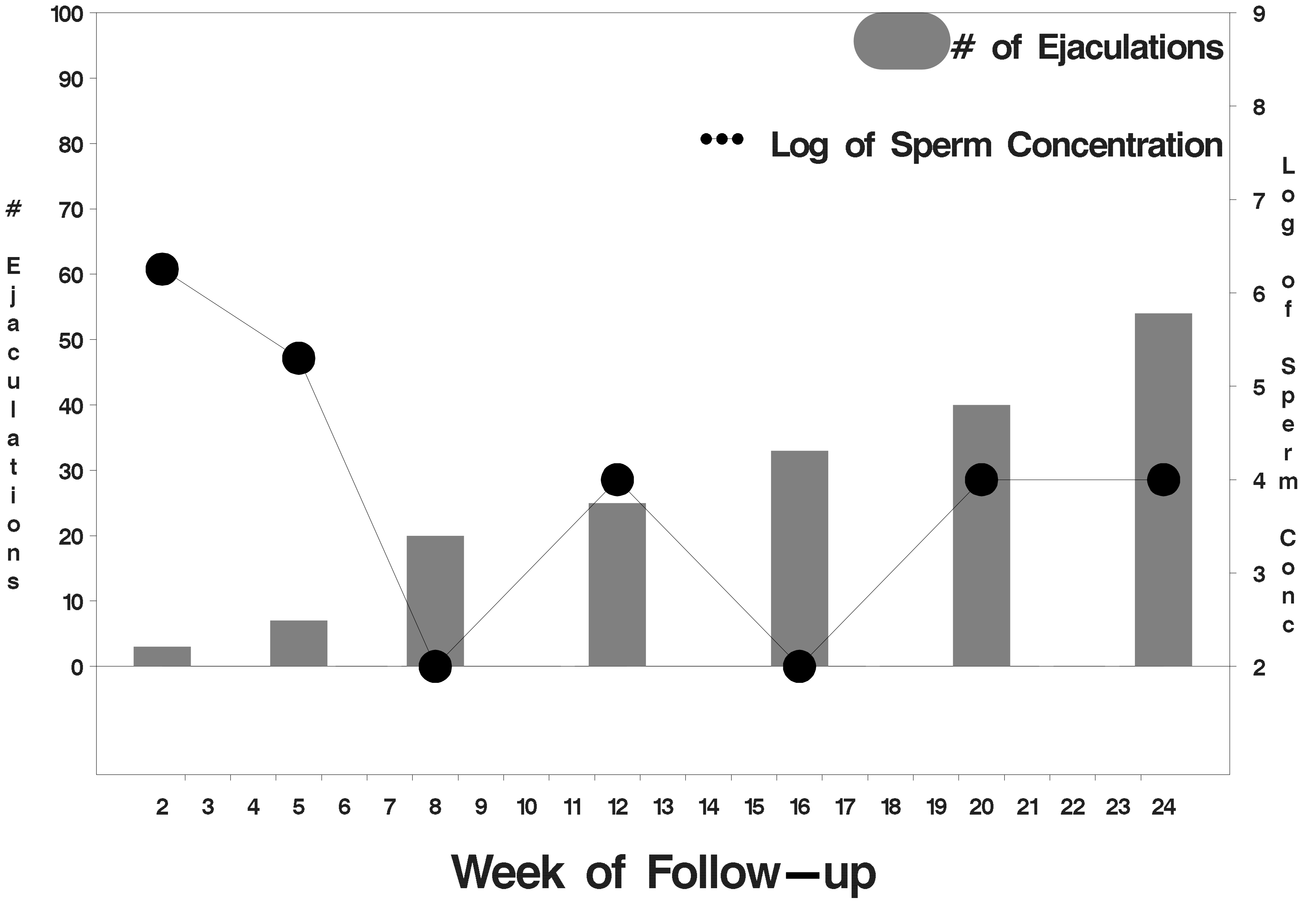

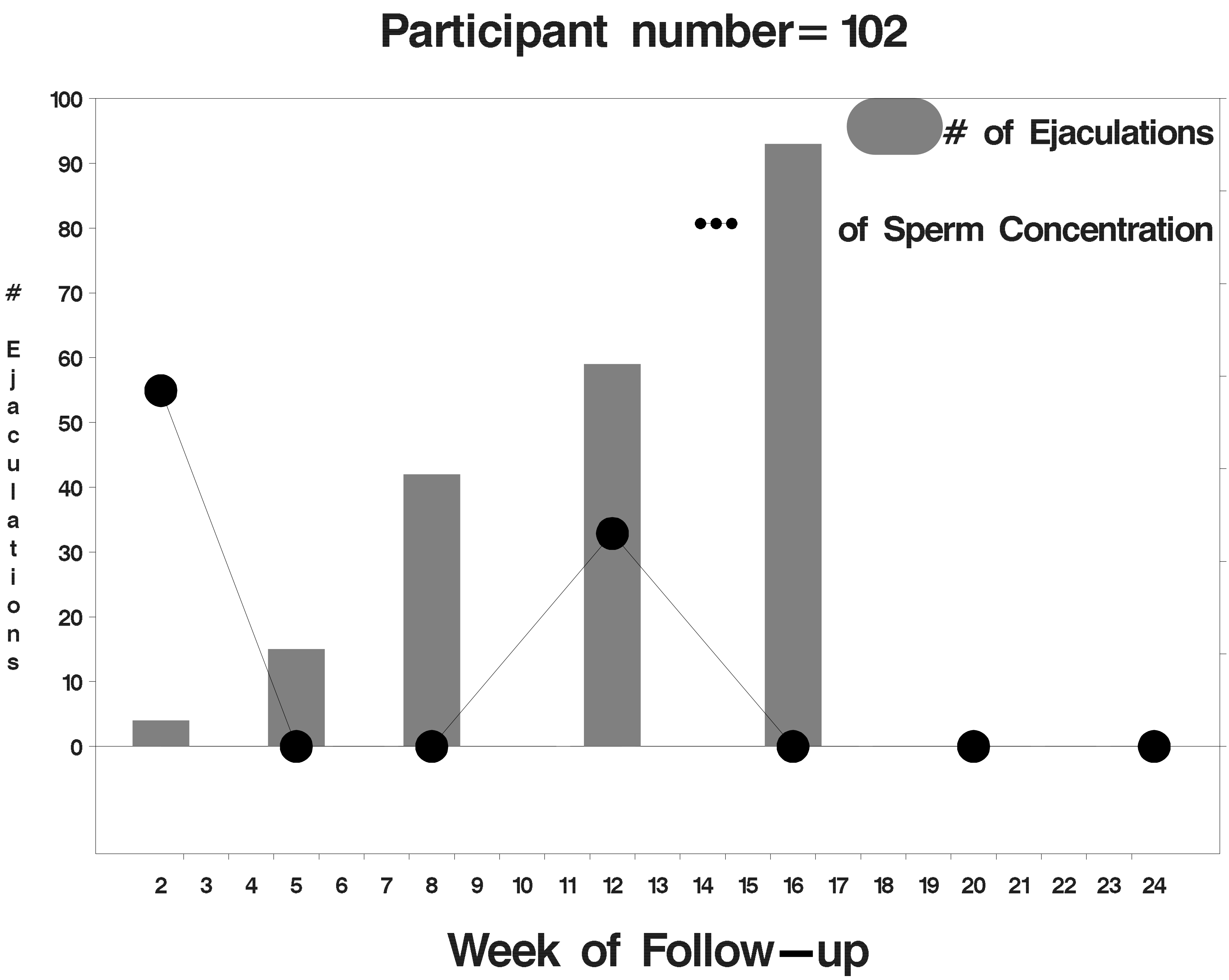
Participant number= 102
# of Ejaculations
of Sperm Concentration
Ejaculations
Log of Sperm Conc
100
90
80
70
60
50
40
30
20
10
0
9
8
7
6
5
4
3
2
2 3 4 5 6 7 8 9 10 11 12 13 14 15 16 17 18 19 20 21 22 23 24
Week of Follow-up

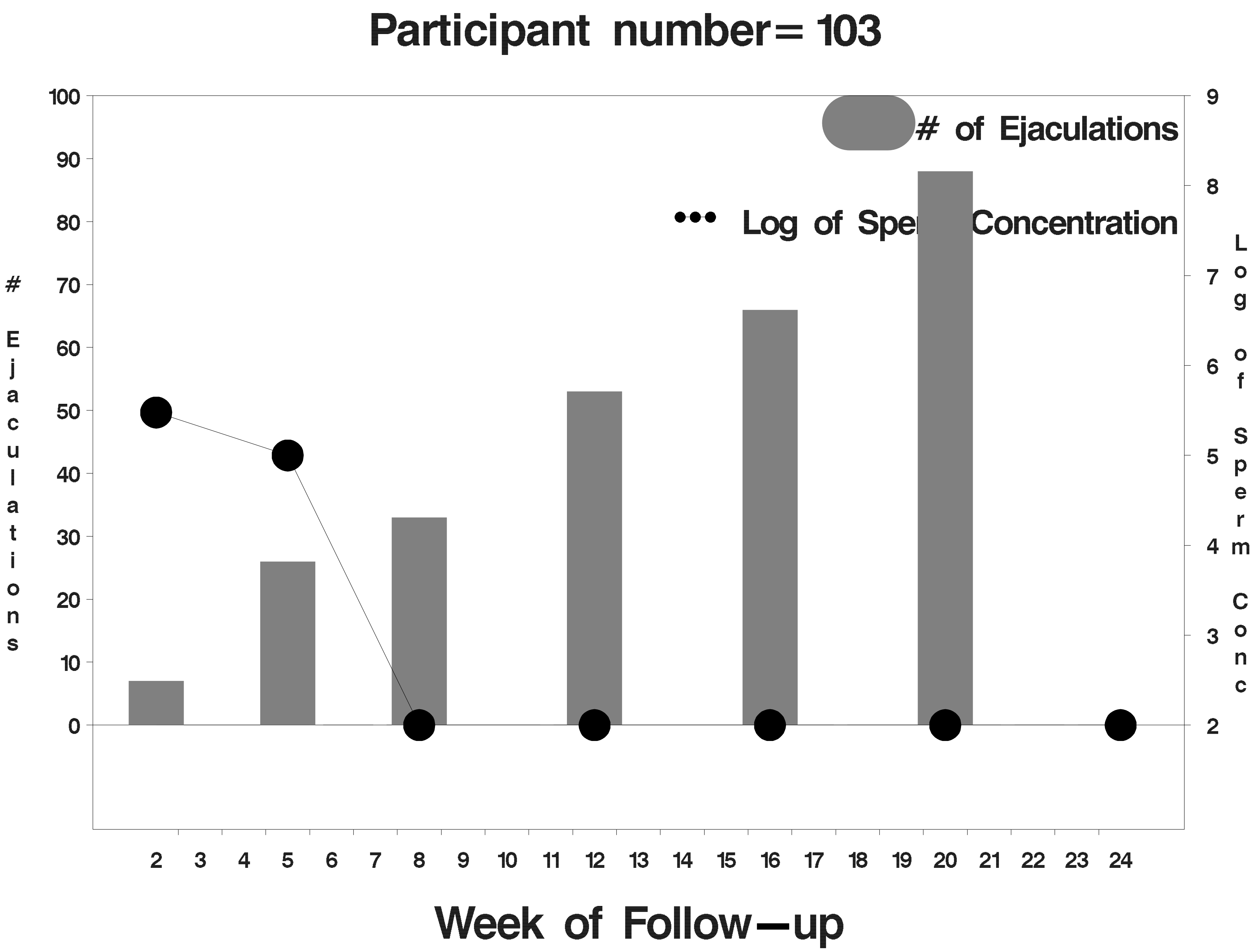

Participant number= 103
# of Ejaculations
Log of Sperm Concentration
Ejaculations
Log of Sperm Conc
100
90
80
70
60
50
40
30
20
10
0
9
8
7
6
5
4
3
2
2 3 4 5 6 7 8 9 10 11 12 13 14 15 16 17 18 19 20 21 22 23 24
Week of Follow−up

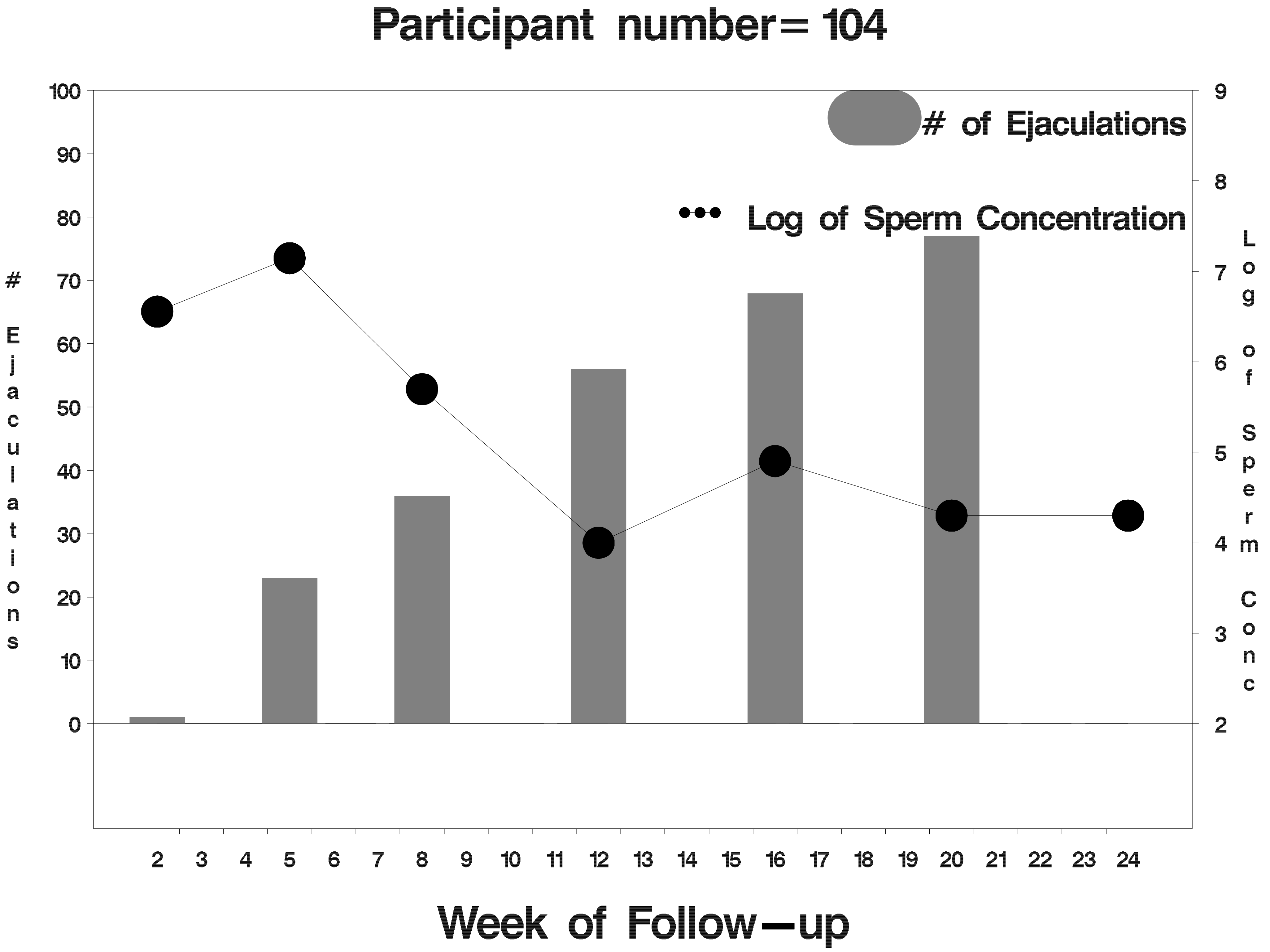

Participant number= 104
# of Ejaculations
Log of Sperm Concentration
Ejaculations
Log of Sperm Conc
0
10
20
30
40
50
60
70
80
90
100
2
3
4
5
6
7
8
9
2 3 4 5 6 7 8 9 10 11 12 13 14 15 16 17 18 19 20 21 22 23 24
Week of Follow-up

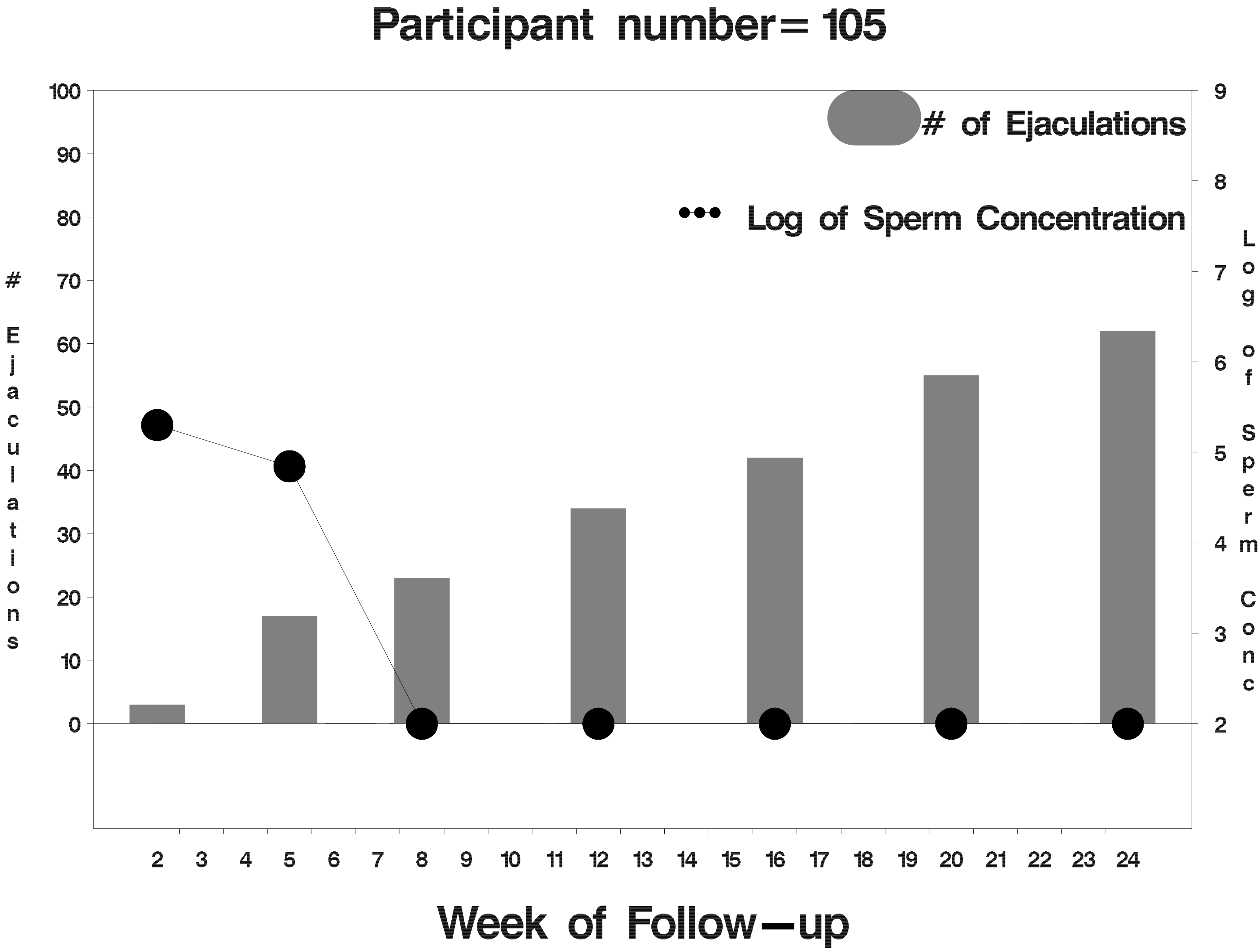

Participant number= 105
# of Ejaculations
Log of Sperm Concentration
Ejaculations
Log of Sperm Conc
100
90
80
70
60
50
40
30
20
10
0
9
8
7
6
5
4
3
2
2 3 4 5 6 7 8 9 10 11 12 13 14 15 16 17 18 19 20 21 22 23 24
Week of Follow-up

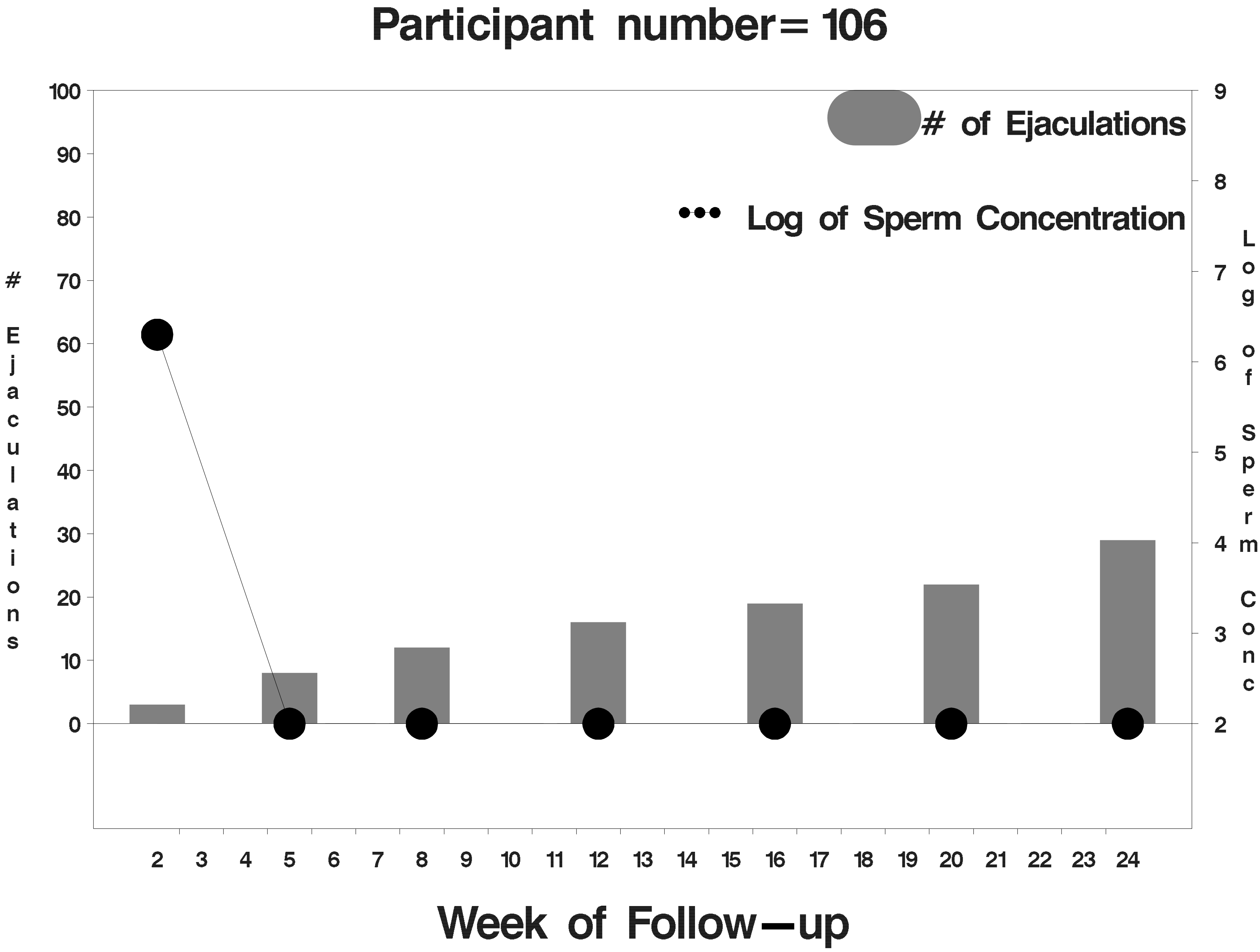
Participant number= 106
# of Ejaculations
Log of Sperm Concentration
Ejaculations
Log of Sperm Conc
100
90
80
70
60
50
40
30
20
10
0
9
8
7
6
5
4
3
2
2 3 4 5 6 7 8 9 10 11 12 13 14 15 16 17 18 19 20 21 22 23 24
Week of Follow-up

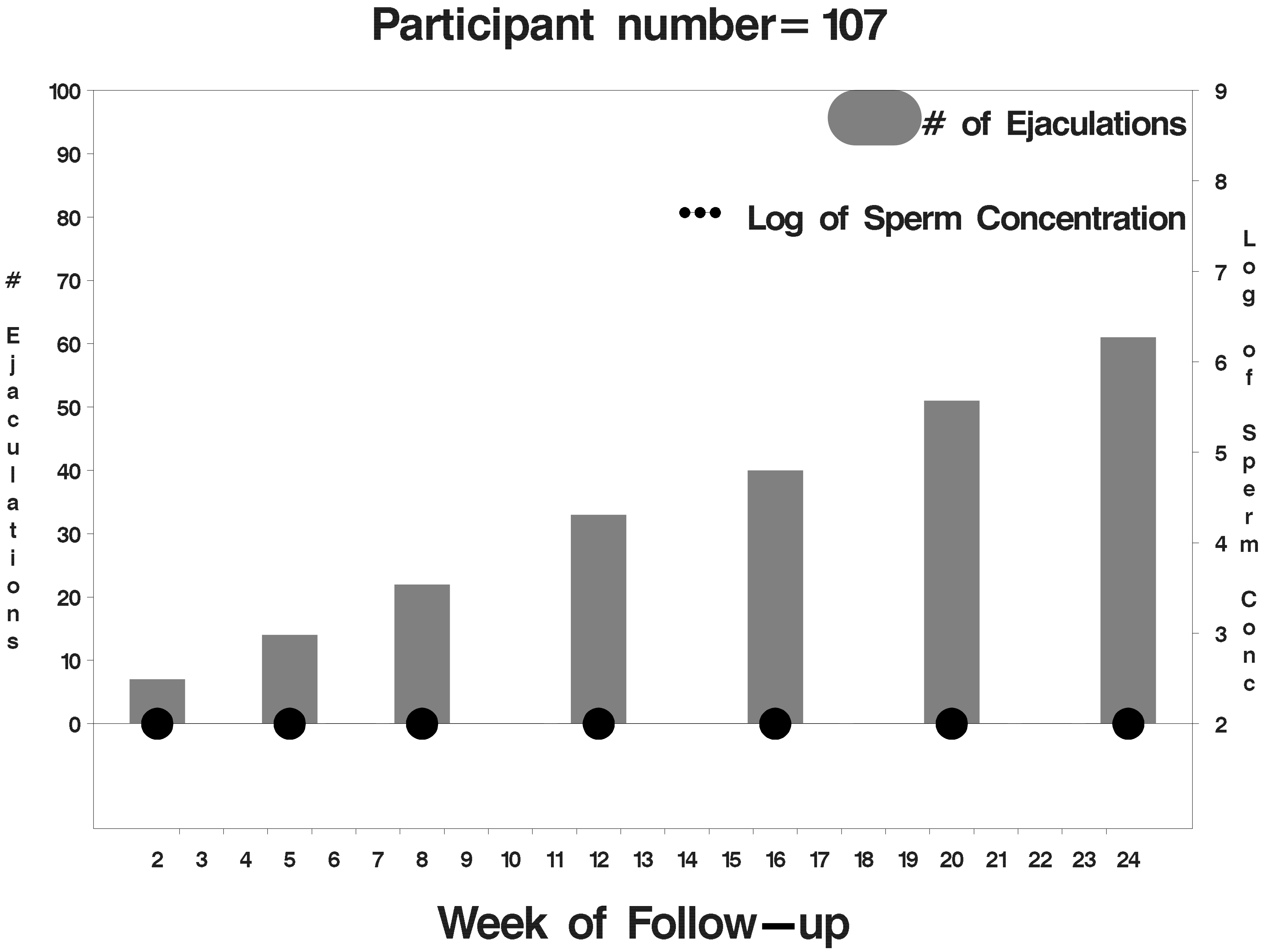

Participant number= 107
# of Ejaculations
Log of Sperm Concentration
Ejaculations
Log of Sperm Conc
100
90
80
70
60
50
40
30
20
10
0
9
8
7
6
5
4
3
2
2 3 4 5 6 7 8 9 10 11 12 13 14 15 16 17 18 19 20 21 22 23 24
Week of Follow-up

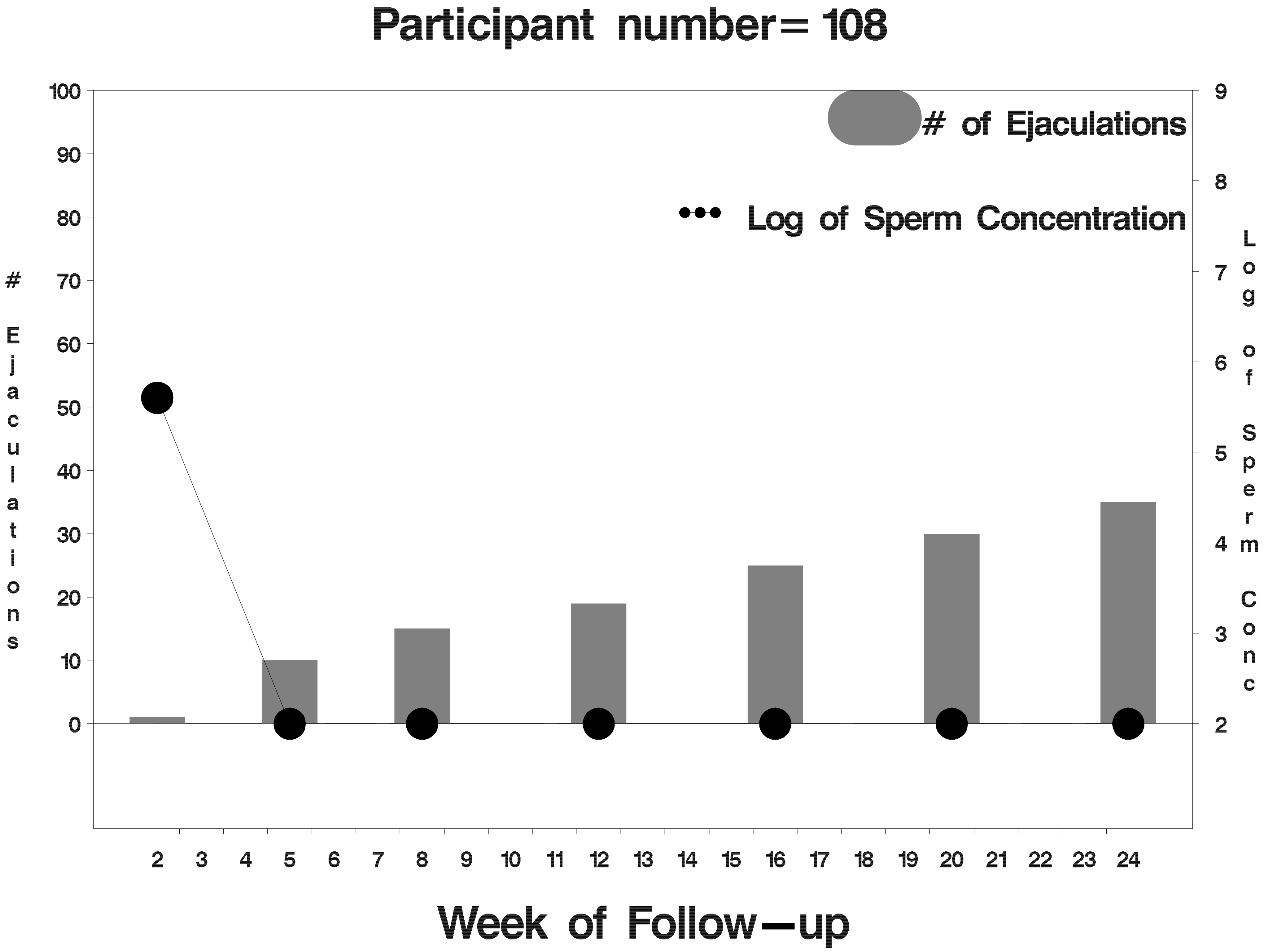

Participant number= 108
# of Ejaculations
Log of Sperm Concentration
Ejaculations
Log of Sperm Conc
100
90
80
70
60
50
40
30
20
10
0
9
8
7
6
5
4
3
2
2 3 4 5 6 7 8 9 10 11 12 13 14 15 16 17 18 19 20 21 22 23 24
Week of Follow-up

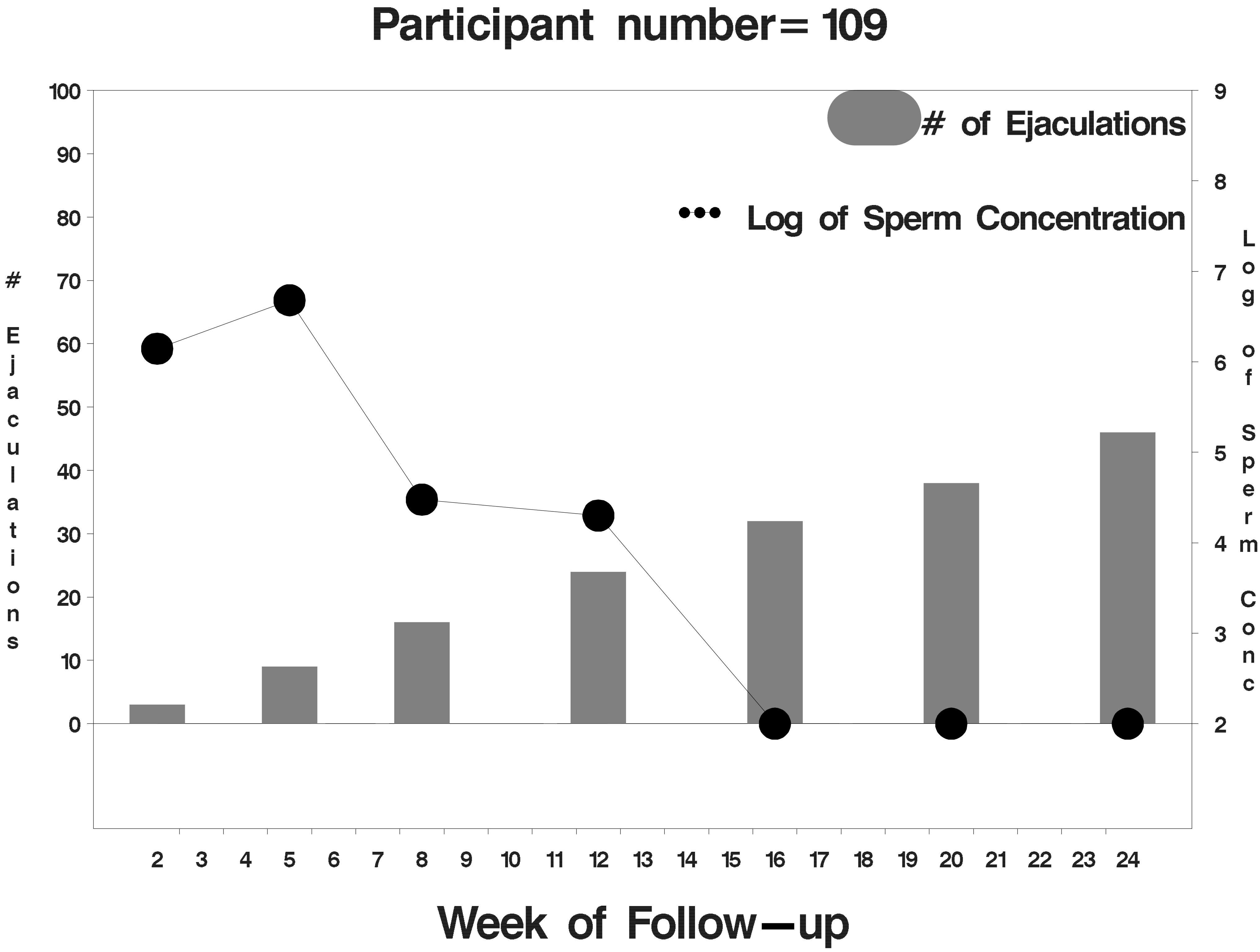

Participant number= 109
# of Ejaculations
Log of Sperm Concentration
Ejaculations
Log of Sperm Conc
100
90
80
70
60
50
40
30
20
10
0
9
8
7
6
5
4
3
2
2 3 4 5 6 7 8 9 10 11 12 13 14 15 16 17 18 19 20 21 22 23 24
Week of Follow−up

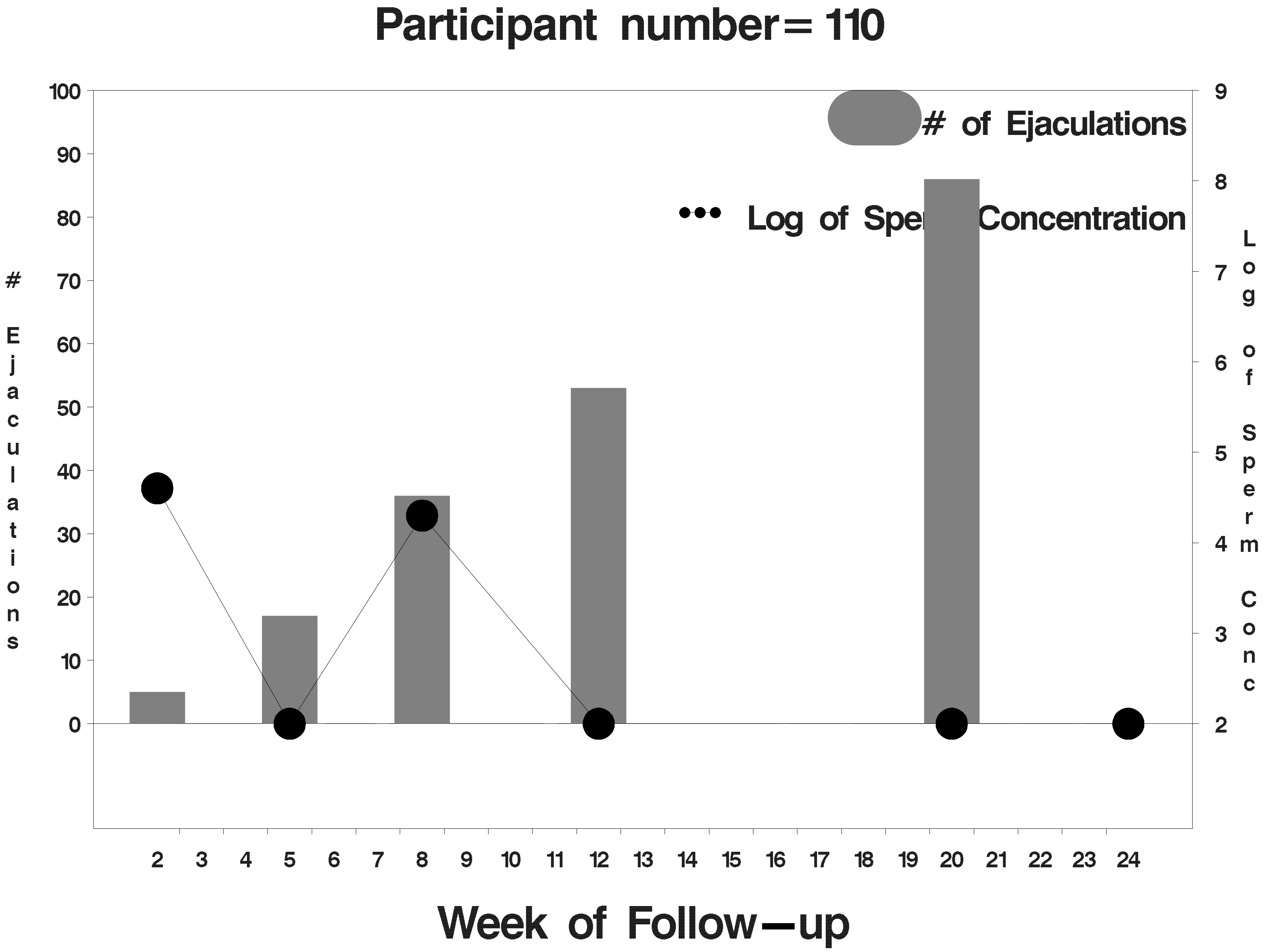

Participant number= 110
# of Ejaculations
Log of Sperm Concentration
Ejaculations
Log of Sperm Conc
100
90
80
70
60
50
40
30
20
10
0
9
8
7
6
5
4
3
2
2 3 4 5 6 7 8 9 10 11 12 13 14 15 16 17 18 19 20 21 22 23 24
Week of Follow−up

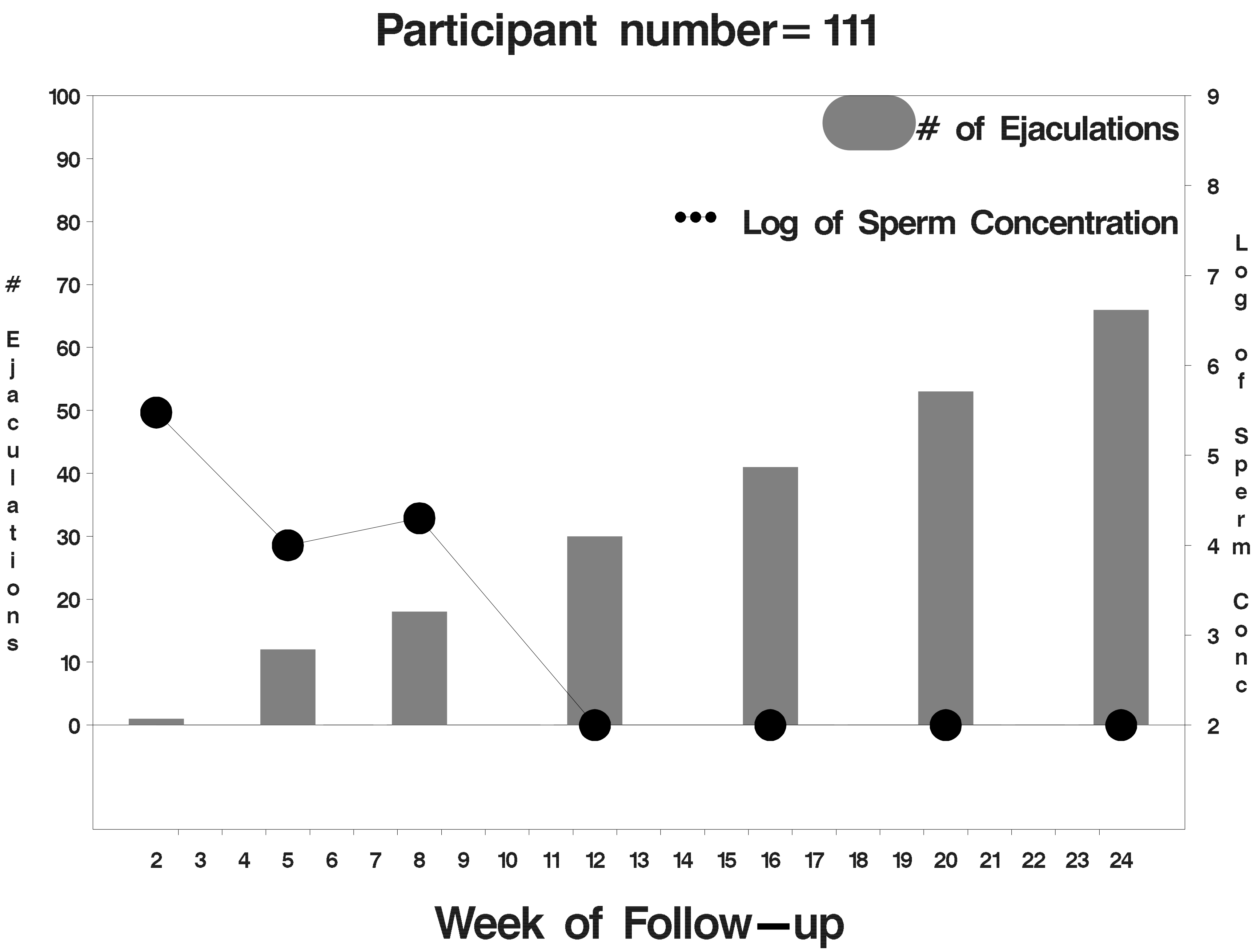

Participant number= 111
# of Ejaculations
Log of Sperm Concentration
Ejaculations
Log of Sperm Conc
100
90
80
70
60
50
40
30
20
10
0
9
8
7
6
5
4
3
2
2 3 4 5 6 7 8 9 10 11 12 13 14 15 16 17 18 19 20 21 22 23 24
Week of Follow-up

Participant number= 112

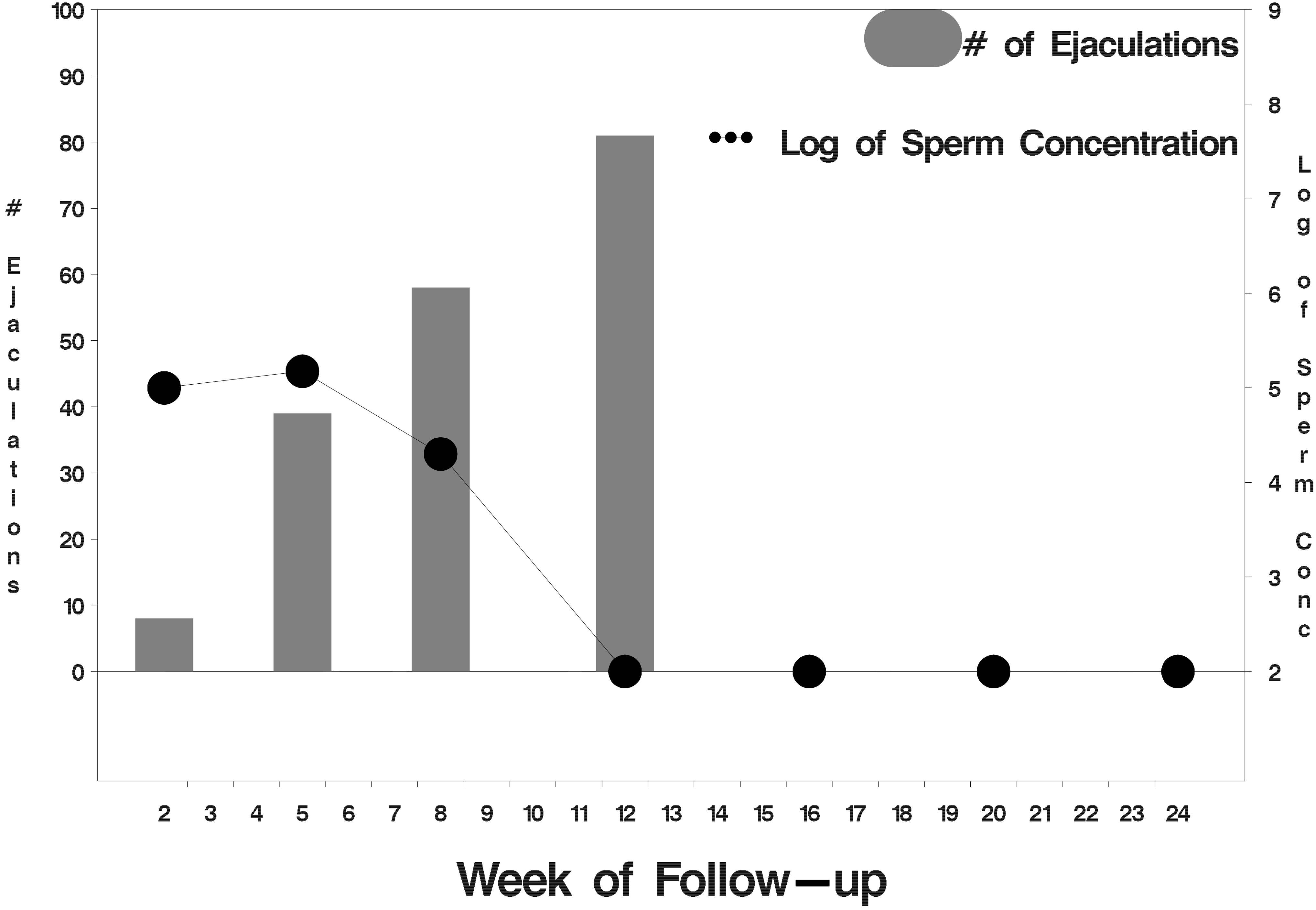

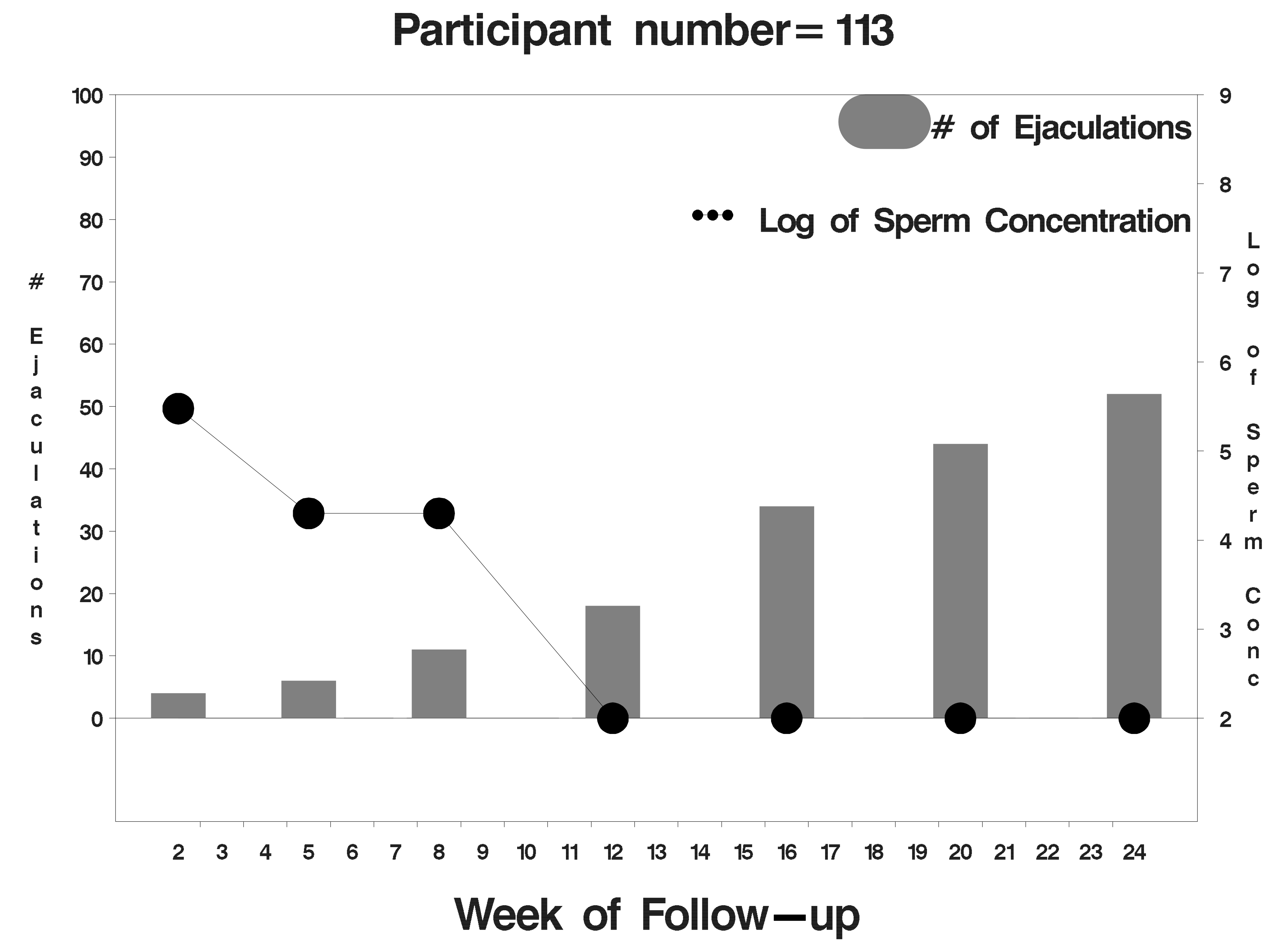

Participant number= 113
# of Ejaculations
Log of Sperm Concentration
Ejaculations
Log of Sperm Conc
100
90
80
70
60
50
40
30
20
10
0
9
8
7
6
5
4
3
2
2 3 4 5 6 7 8 9 10 11 12 13 14 15 16 17 18 19 20 21 22 23 24
Week of Follow-up

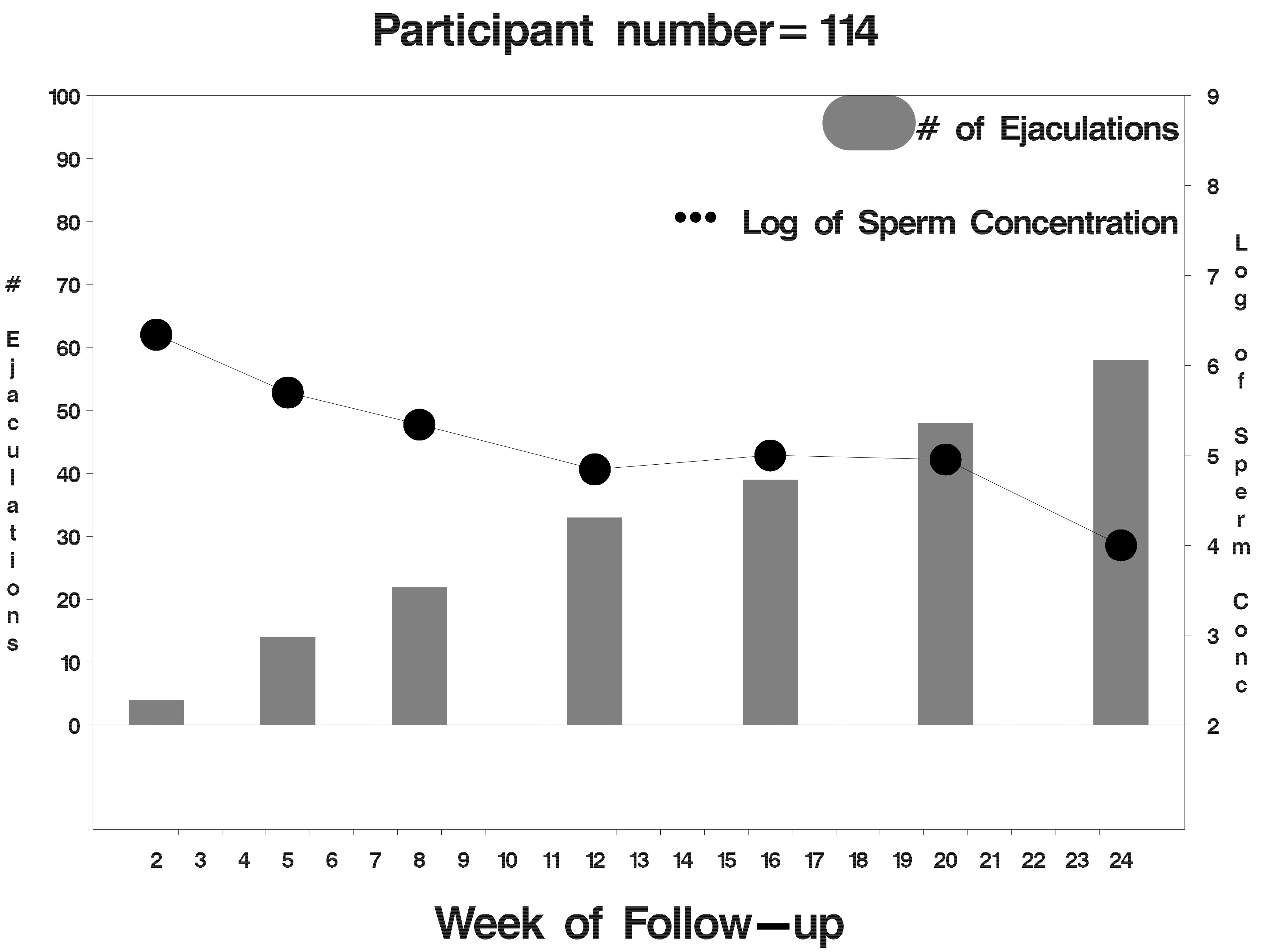

Participant number= 114
# of Ejaculations
Log of Sperm Concentration
Ejaculations
Log of Sperm Conc
100
90
80
70
60
50
40
30
20
10
0
9
8
7
6
5
4
3
2
2 3 4 5 6 7 8 9 10 11 12 13 14 15 16 17 18 19 20 21 22 23 24
Week of Follow-up

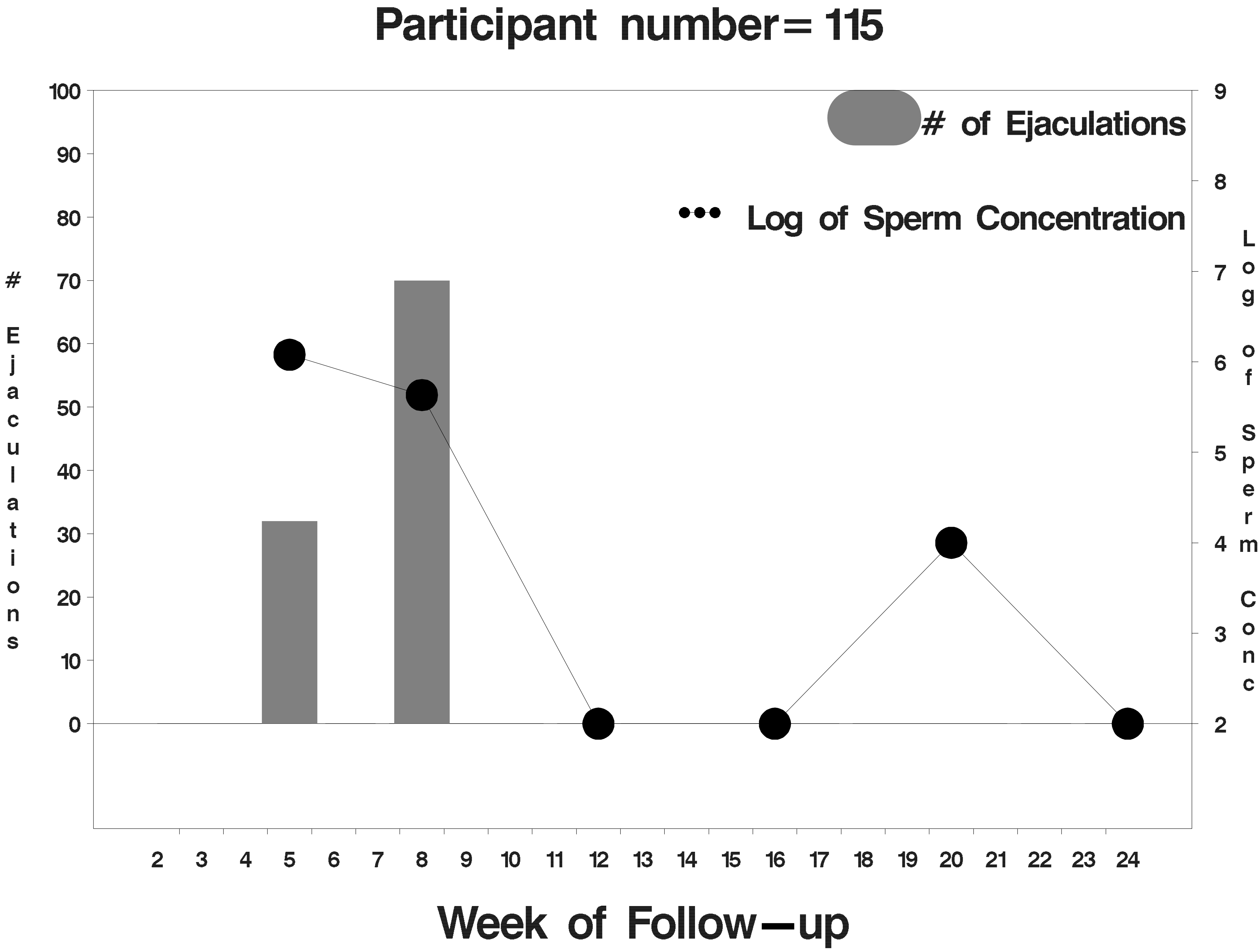

Participant number= 115
# of Ejaculations
Log of Sperm Concentration
Ejaculations
Log of Sperm Conc
100
90
80
70
60
50
40
30
20
10
0
9
8
7
6
5
4
3
2
2 3 4 5 6 7 8 9 10 11 12 13 14 15 16 17 18 19 20 21 22 23 24
Week of Follow-up

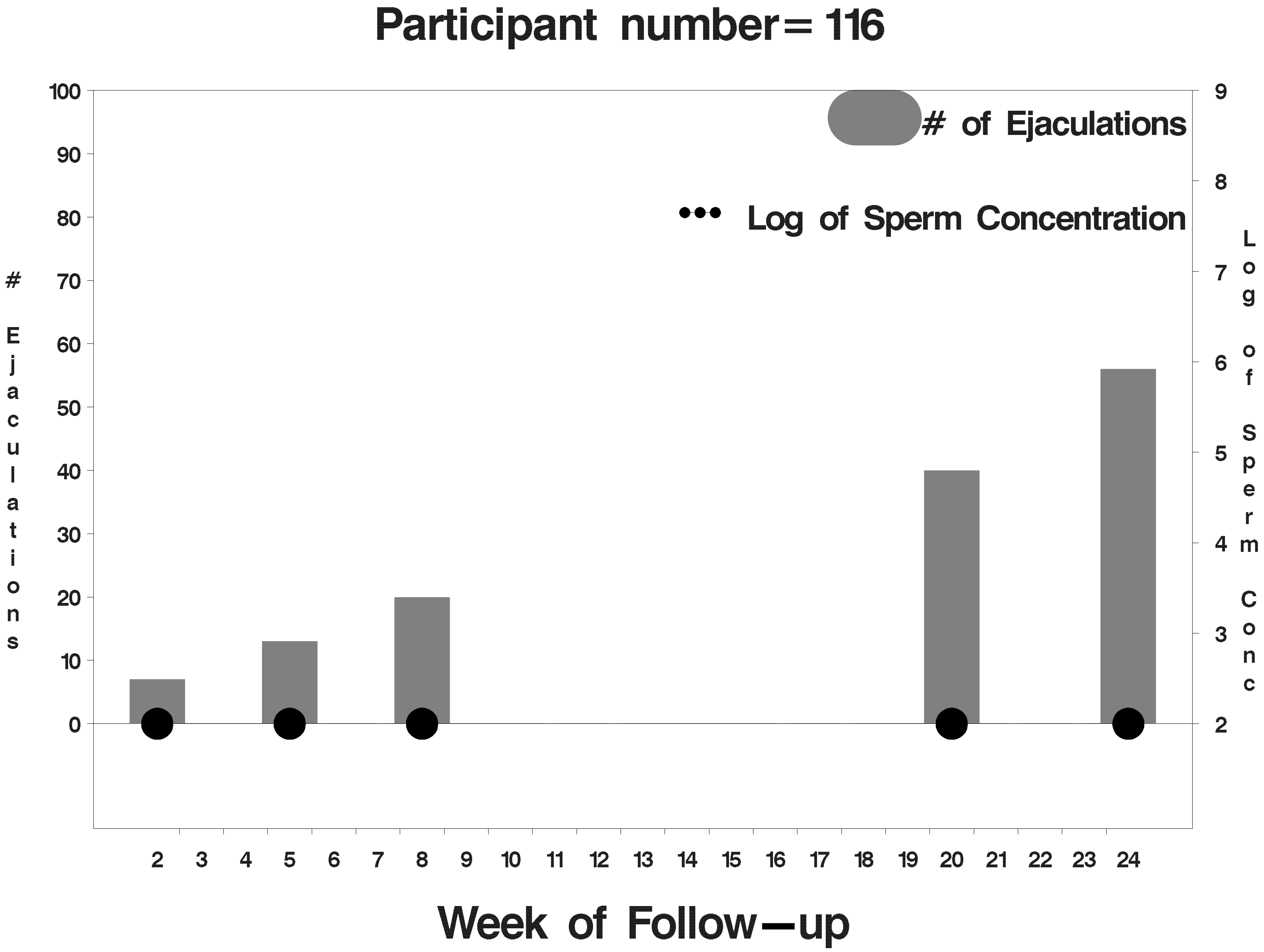

Participant number= 116
# of Ejaculations
Log of Sperm Concentration
Ejaculations
Log of Sperm Conc
100
90
80
70
60
50
40
30
20
10
0
9
8
7
6
5
4
3
2
2 3 4 5 6 7 8 9 10 11 12 13 14 15 16 17 18 19 20 21 22 23 24
Week of Follow-up

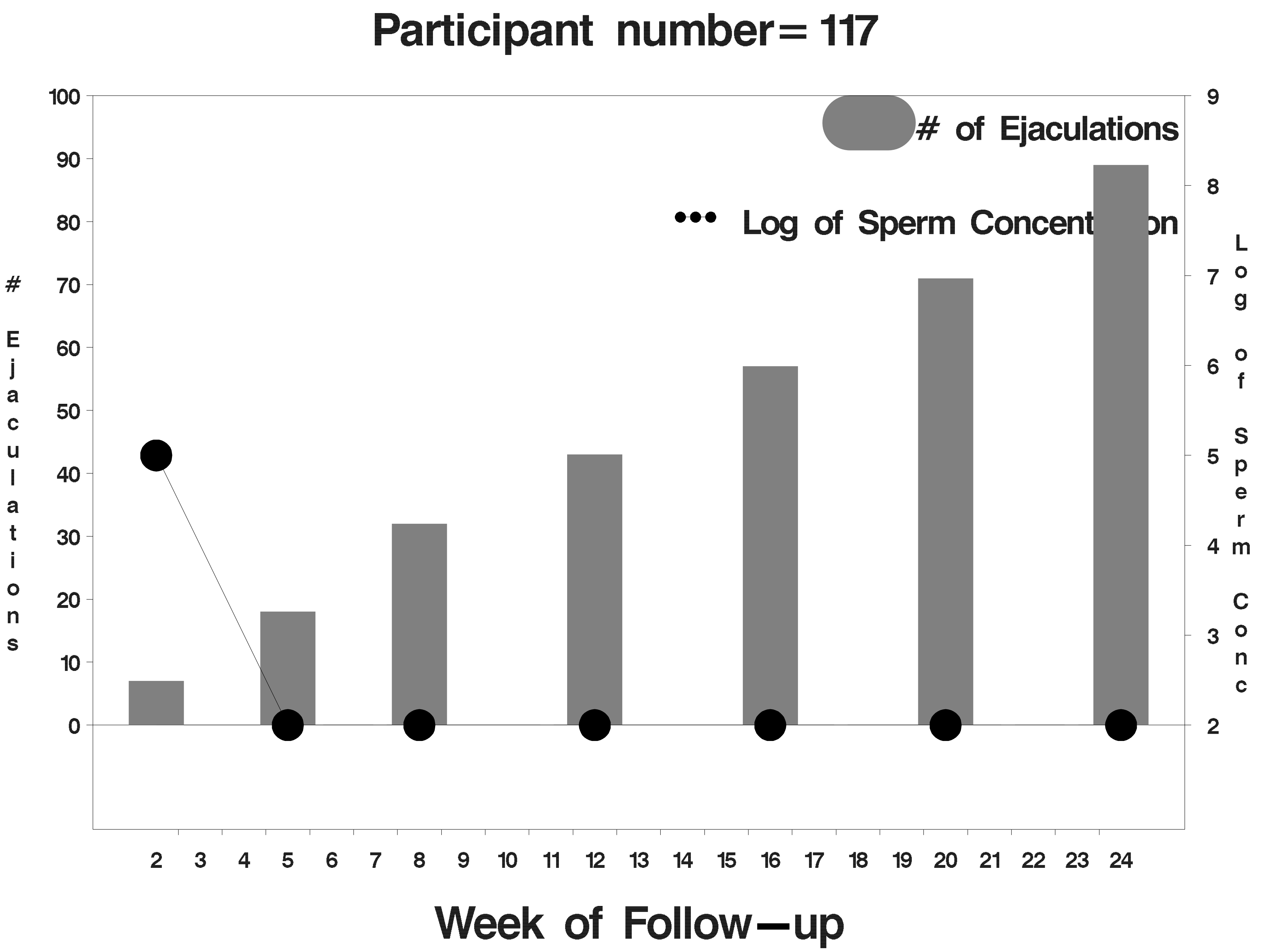

Participant number= 117
# of Ejaculations
Log of Sperm Concent[illegible]on
Ejaculations
Log of Sperm Conc
100
90
80
70
60
50
40
30
20
10
0
9
8
7
6
5
4
3
2
2 3 4 5 6 7 8 9 10 11 12 13 14 15 16 17 18 19 20 21 22 23 24
Week of Follow-up

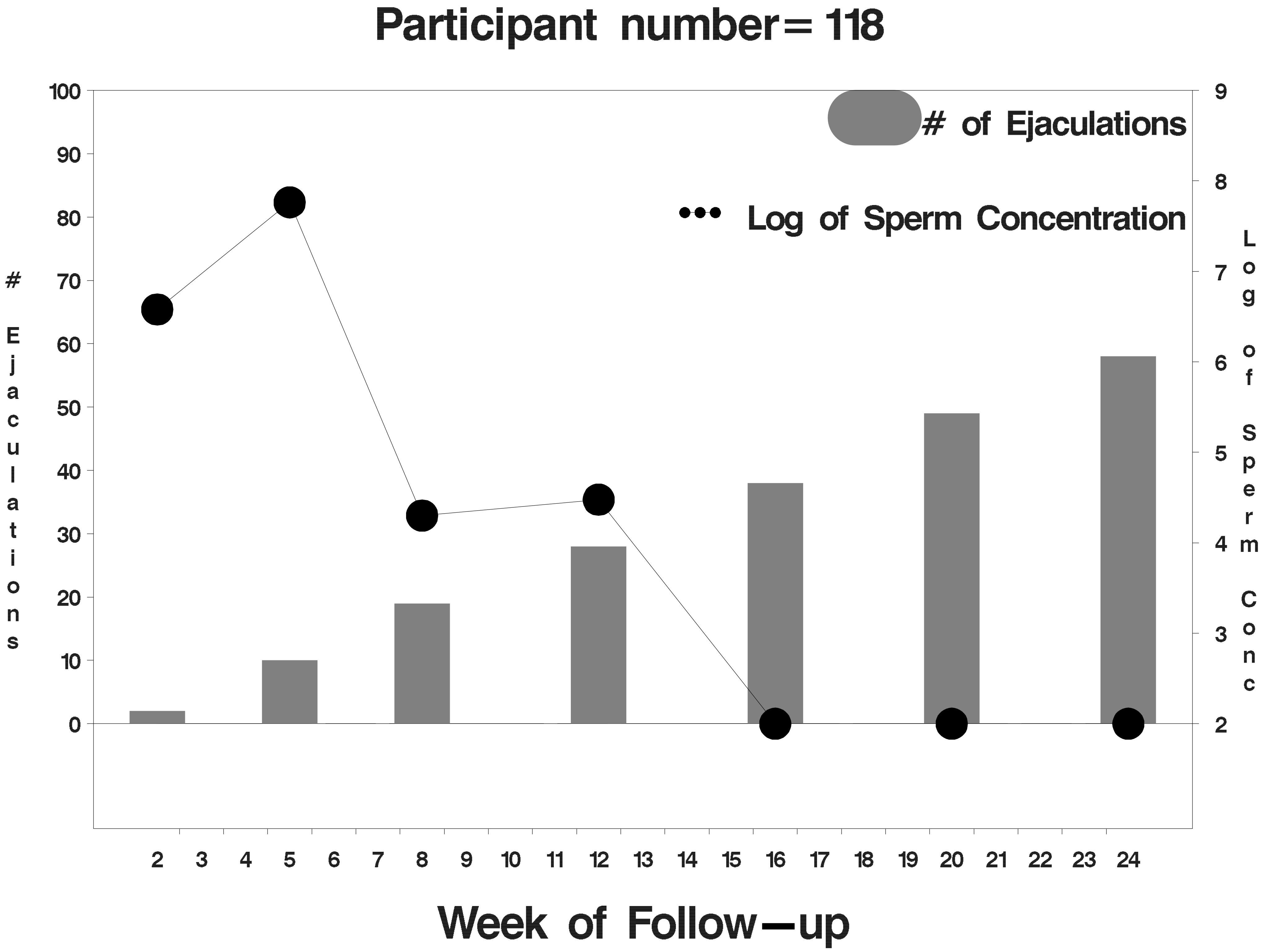

Participant number= 118
# of Ejaculations
Log of Sperm Concentration
Ejaculations
Log of Sperm Conc
100
90
80
70
60
50
40
30
20
10
0
9
8
7
6
5
4
3
2
2 3 4 5 6 7 8 9 10 11 12 13 14 15 16 17 18 19 20 21 22 23 24
Week of Follow-up

Participant number= 119

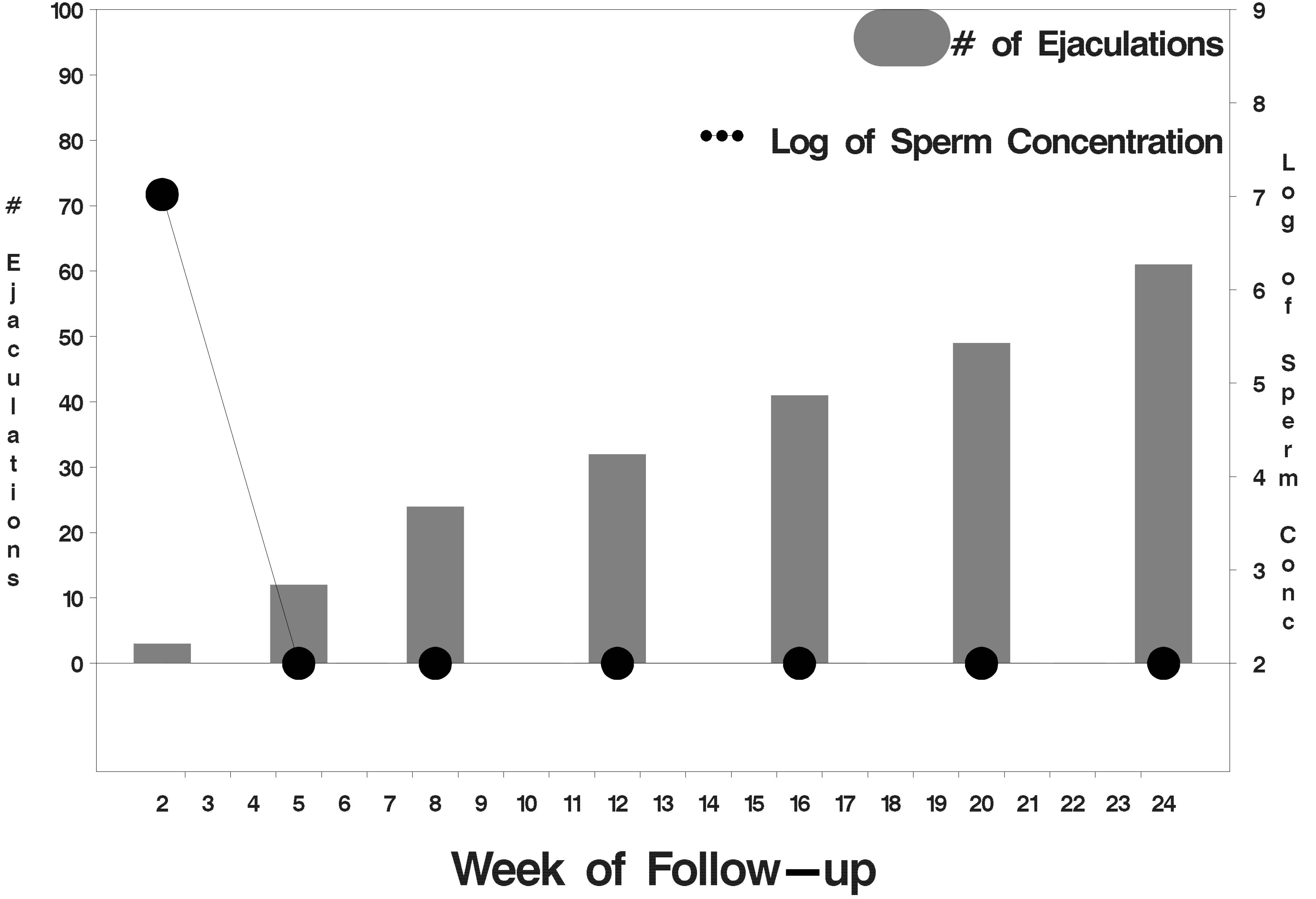

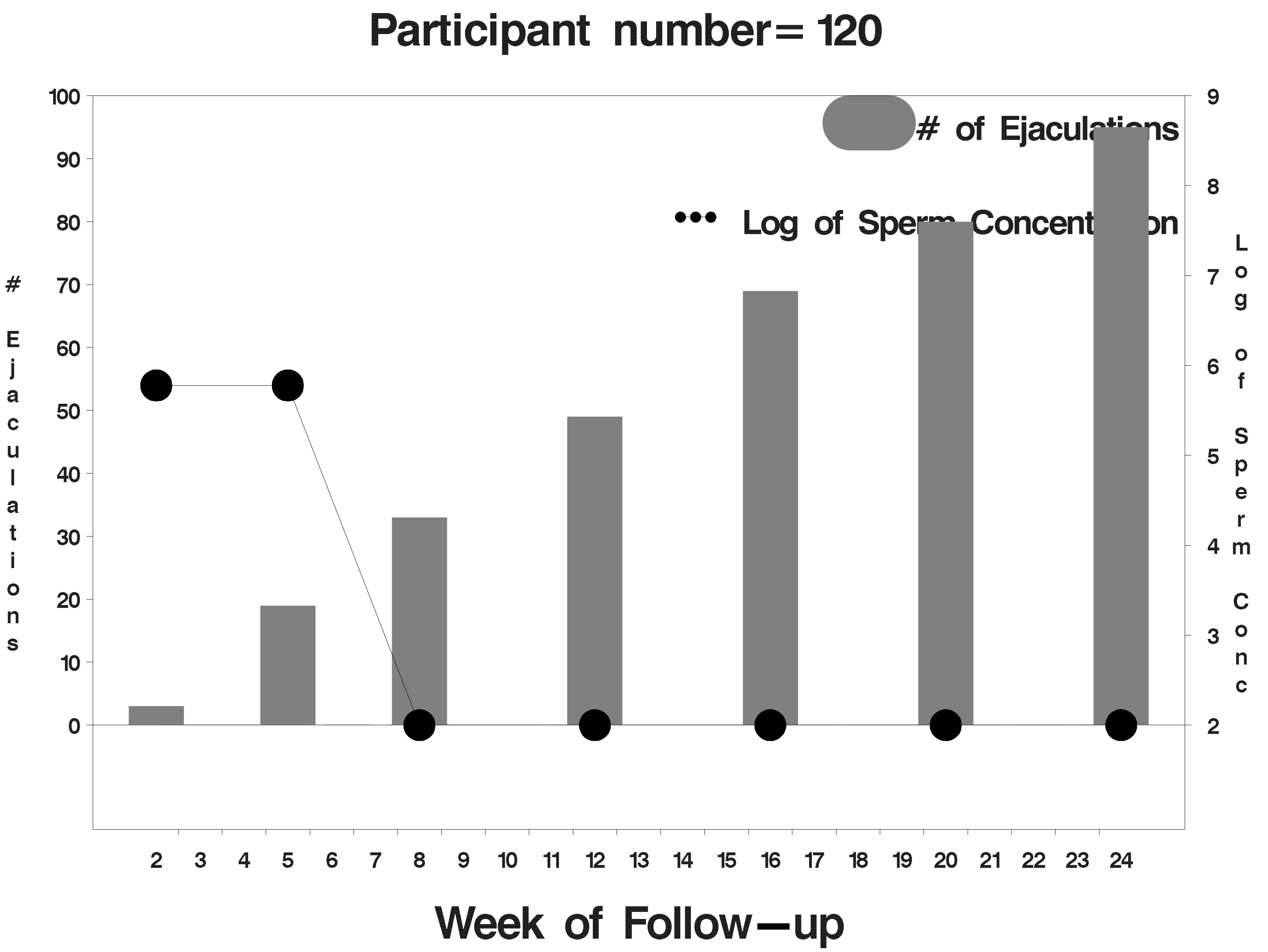

Participant number= 120
# of Ejaculations
Log of Sperm Concentration
Ejaculations
Log of Sperm Conc
100
90
80
70
60
50
40
30
20
10
0
9
8
7
6
5
4
3
2
2 3 4 5 6 7 8 9 10 11 12 13 14 15 16 17 18 19 20 21 22 23 24
Week of Follow-up

Participant number= 121

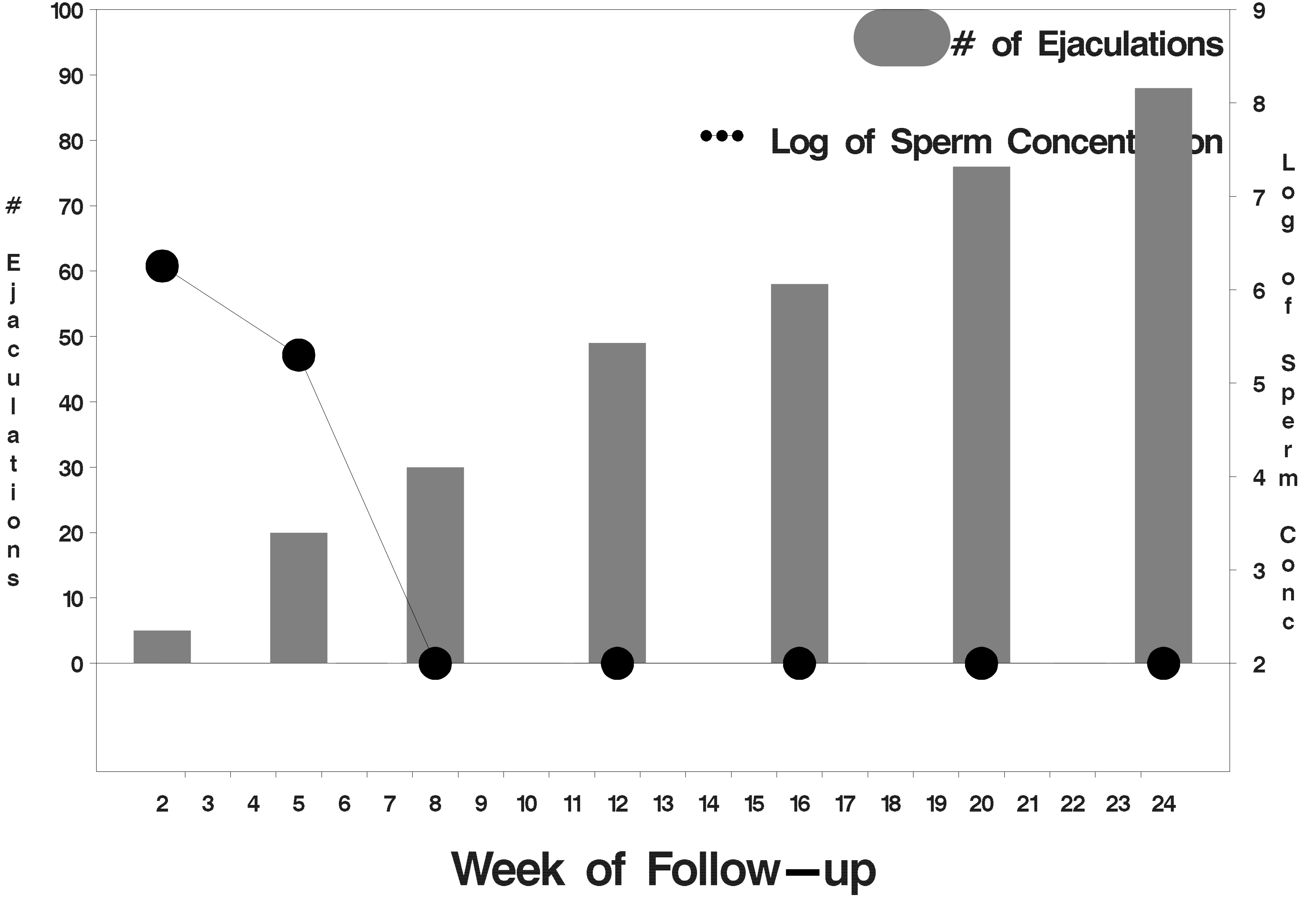

Participant number= 122

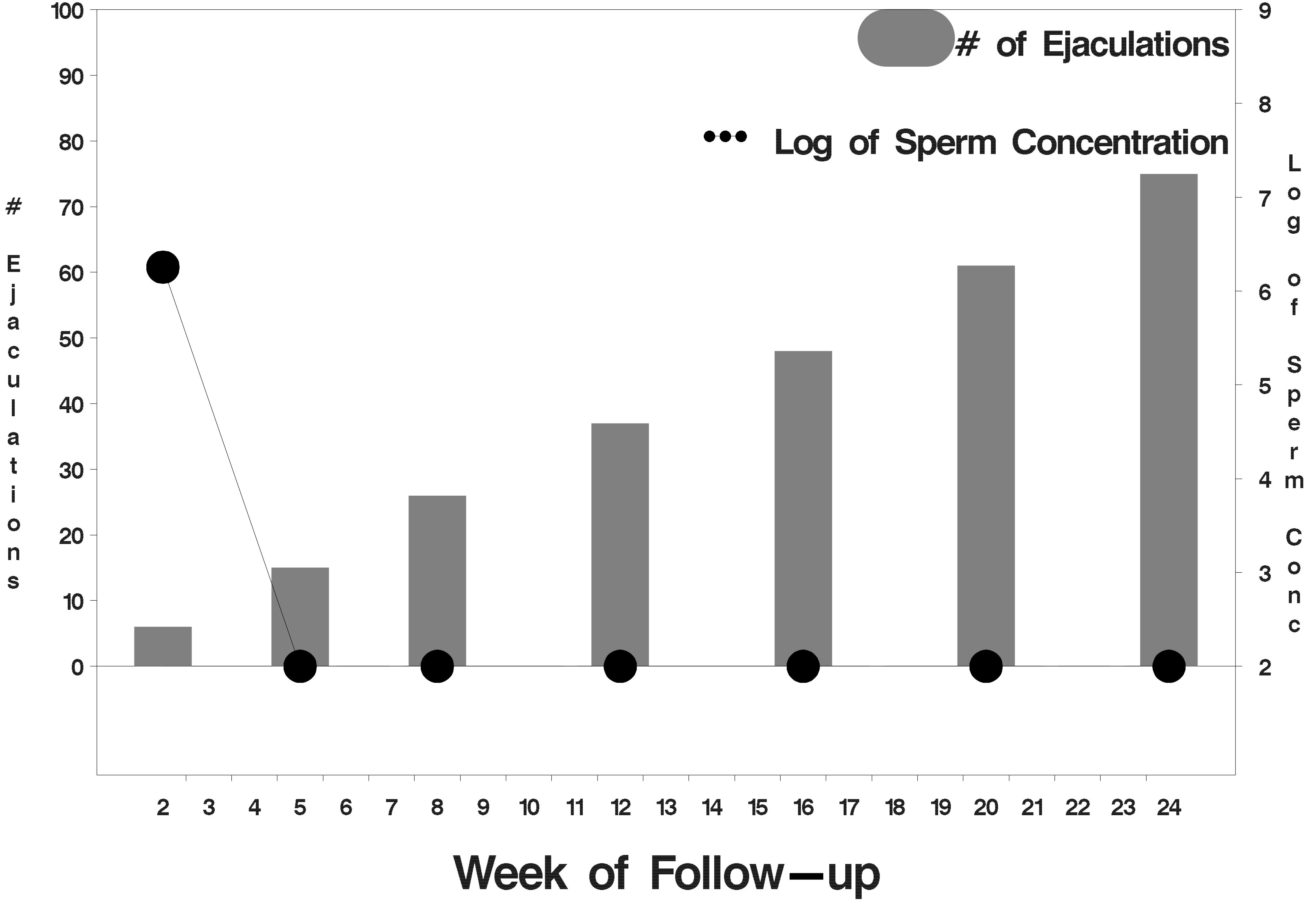

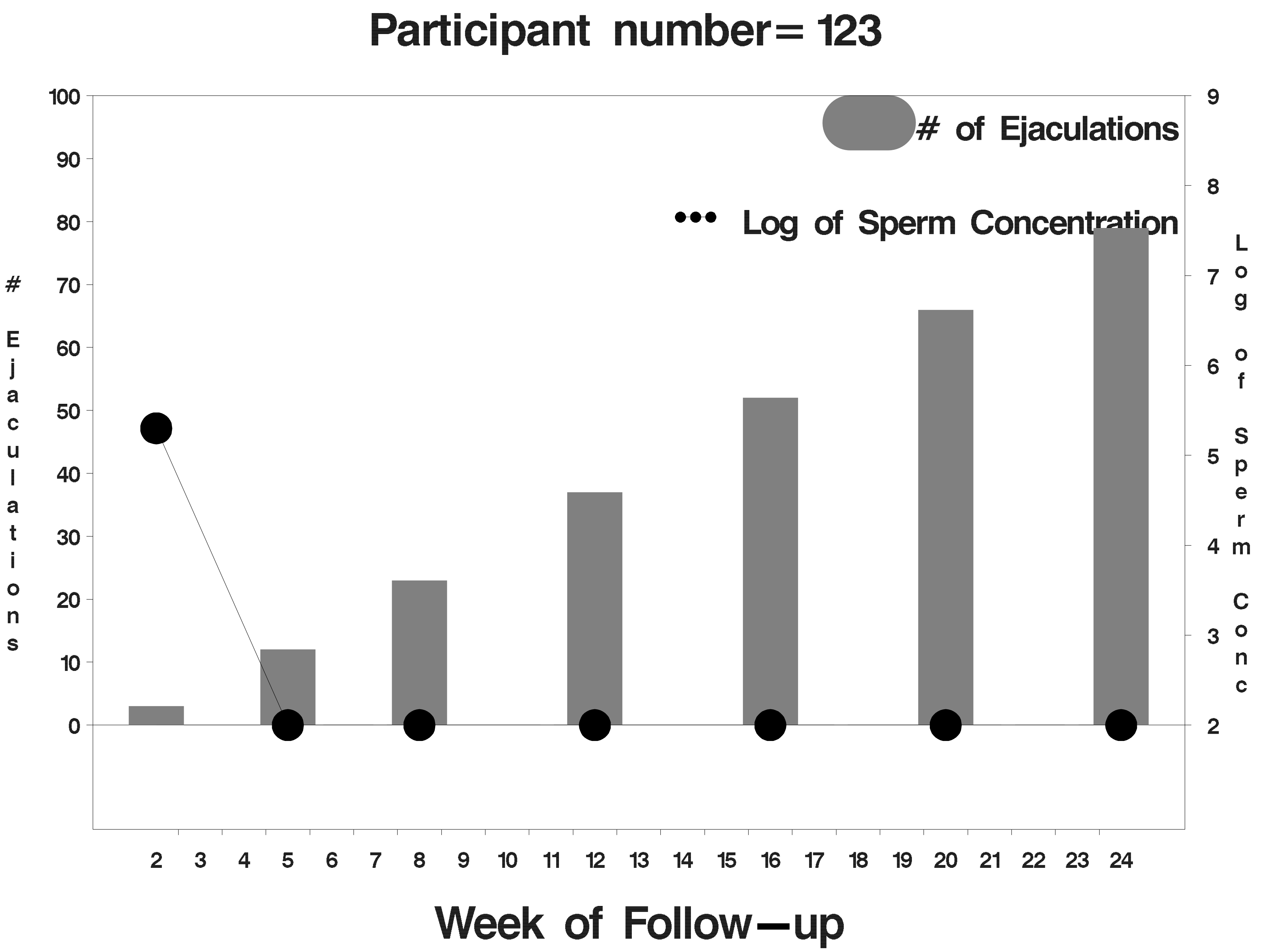

Participant number= 123
# of Ejaculations
Log of Sperm Concentration
Ejaculations
Log of Sperm Conc
100
90
80
70
60
50
40
30
20
10
0
9
8
7
6
5
4
3
2
2 3 4 5 6 7 8 9 10 11 12 13 14 15 16 17 18 19 20 21 22 23 24
Week of Follow-up

Participant number= 124

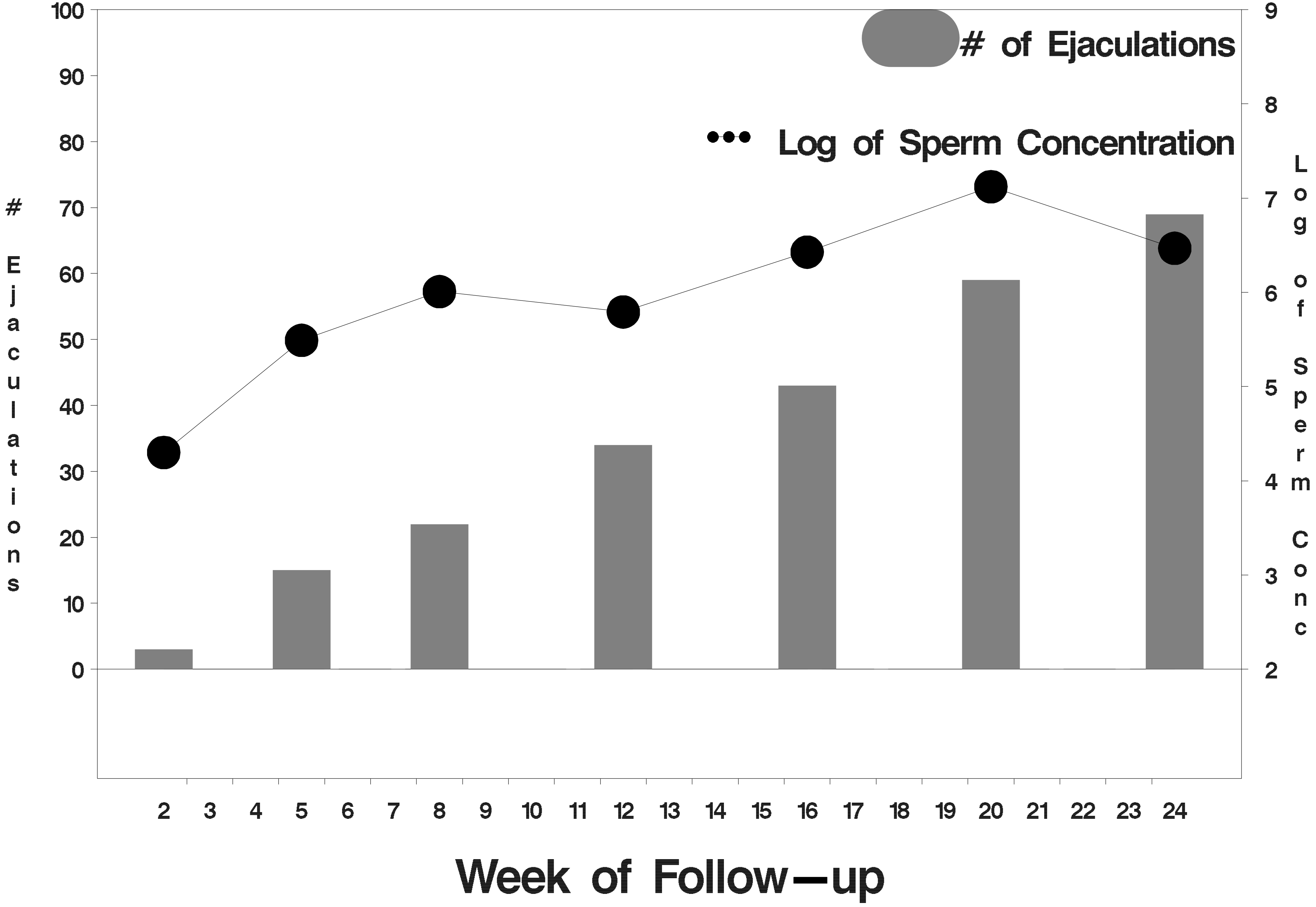

Participant number= 125

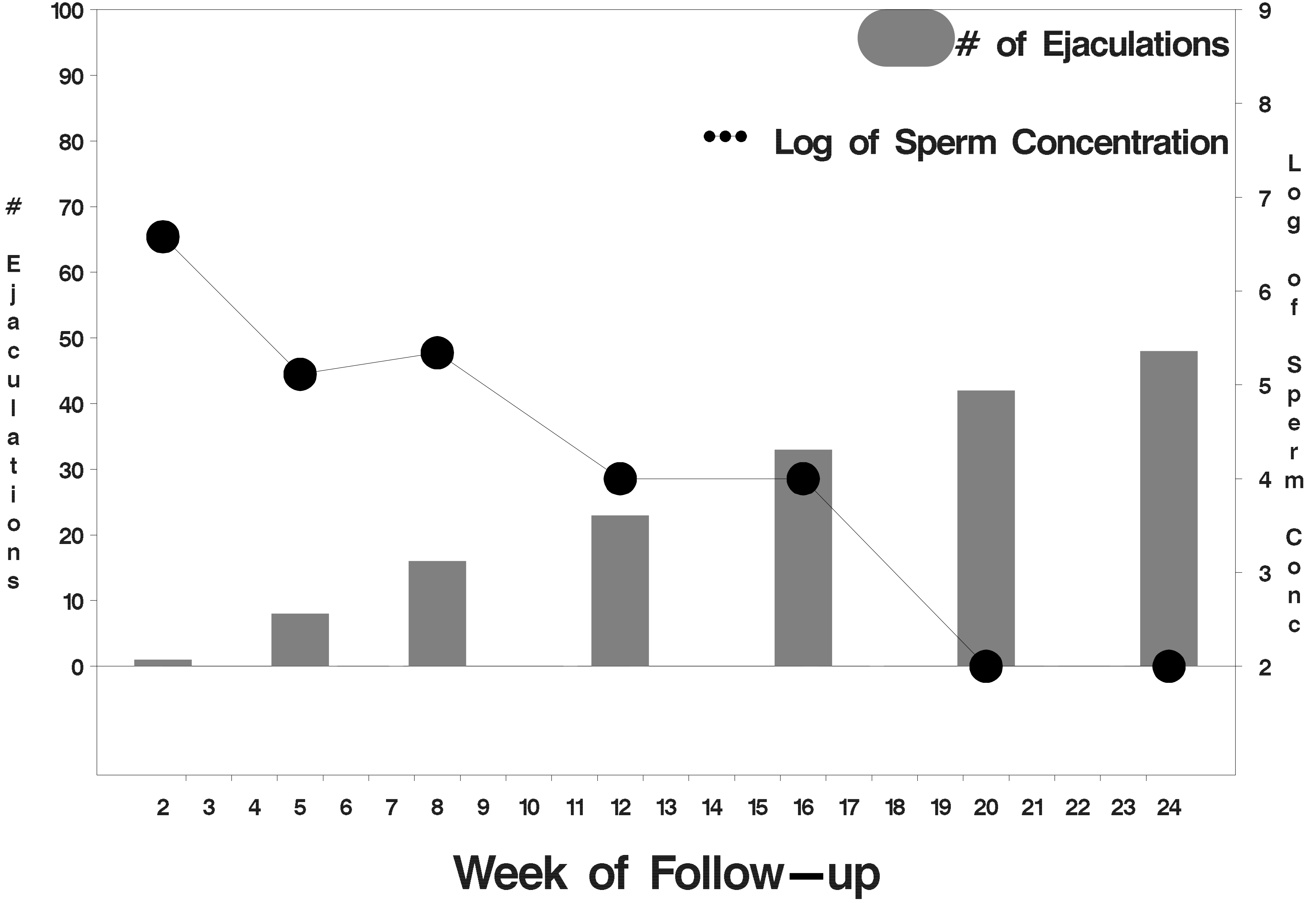

Participant number= 126

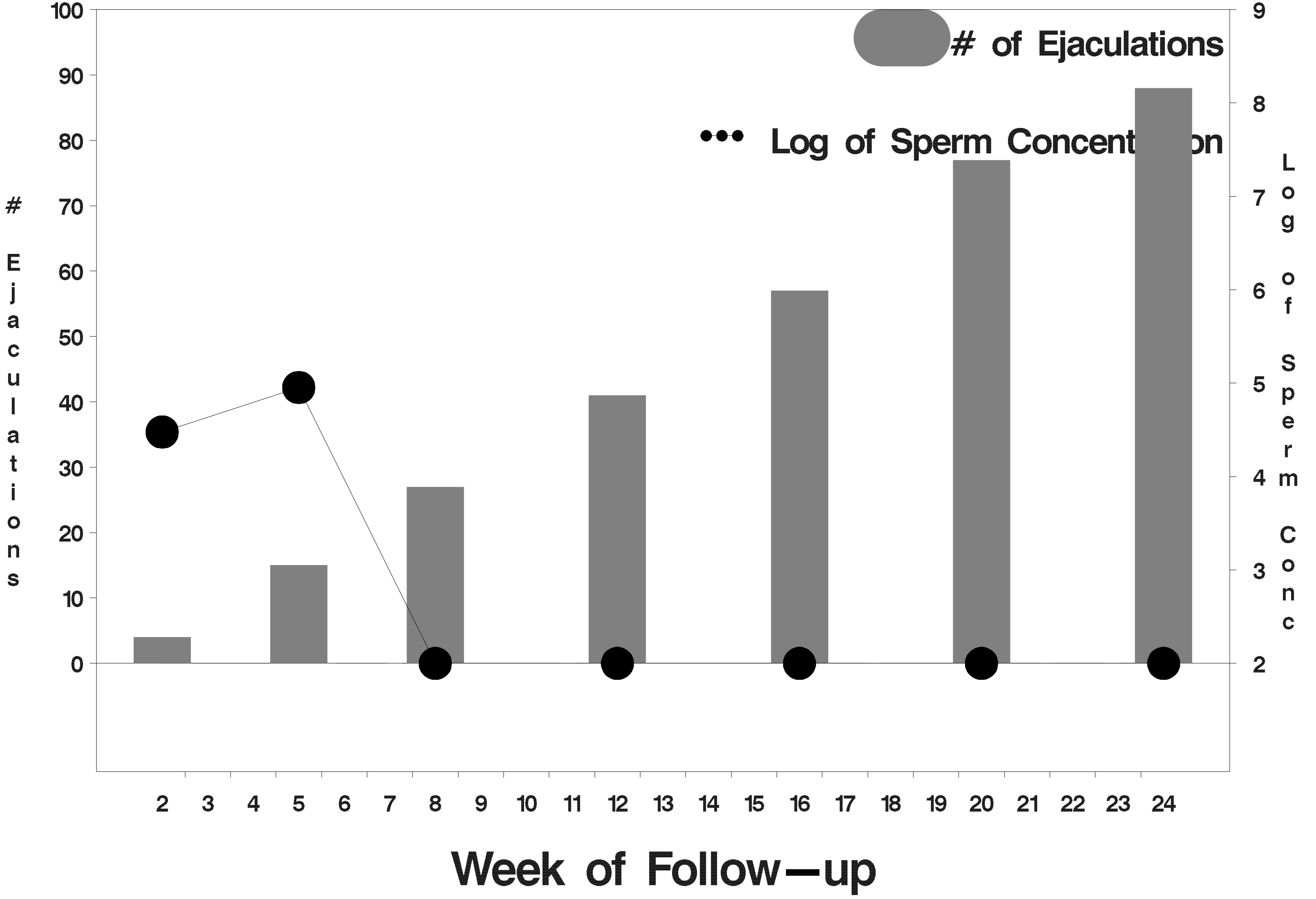

Participant number= 127

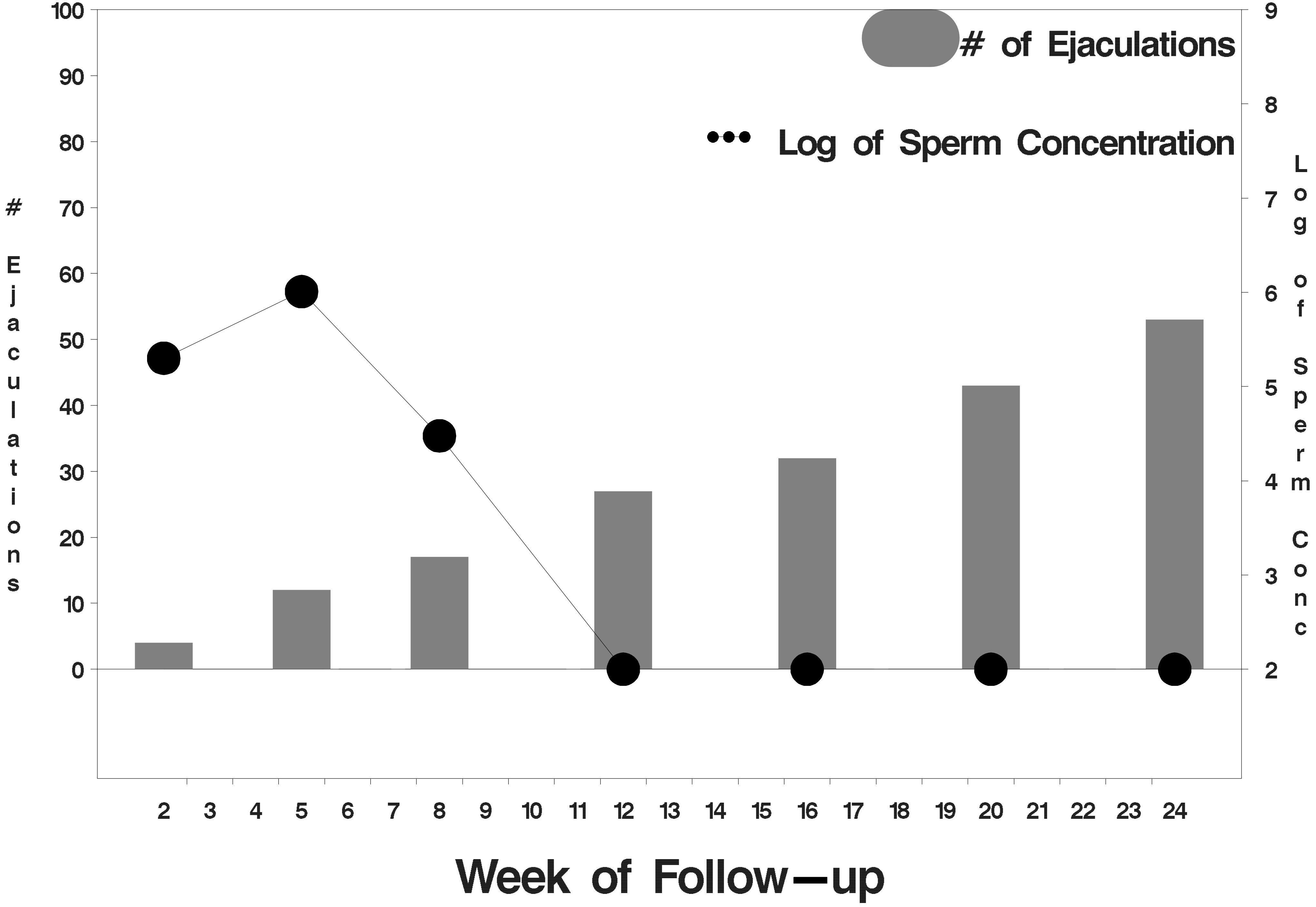

Participant number= 128

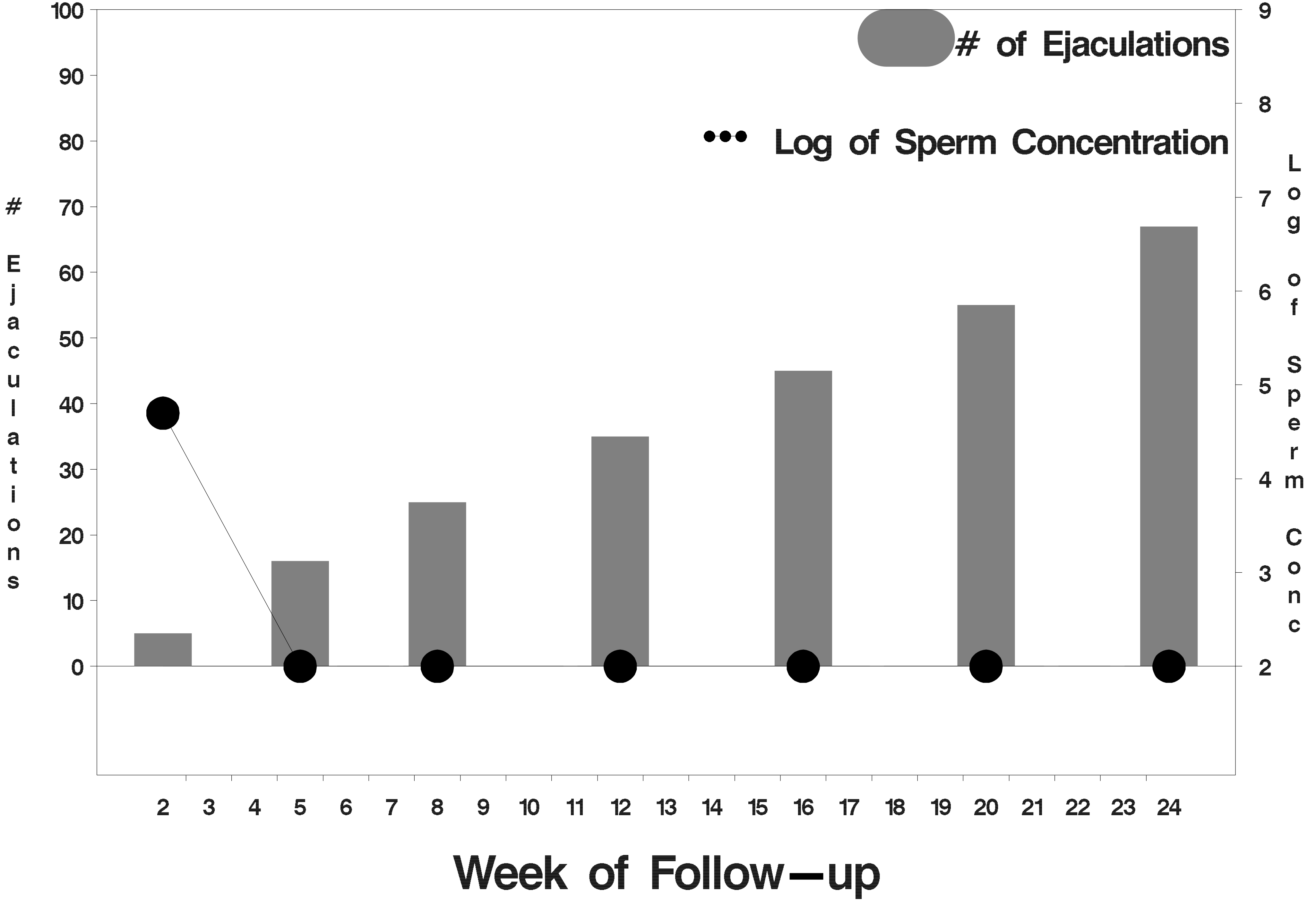

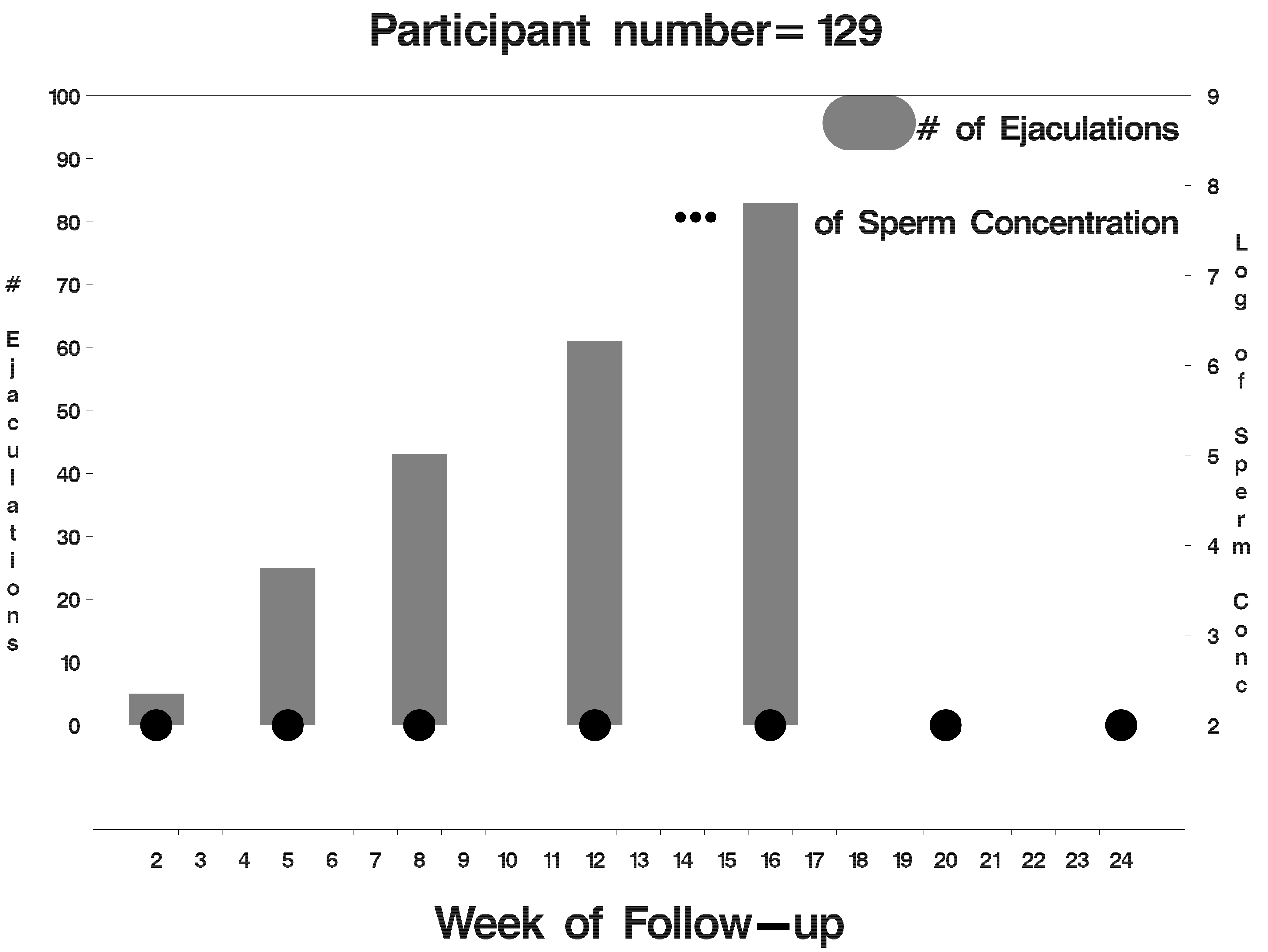
Participant number= 129
# of Ejaculations
of Sperm Concentration
Ejaculations
Log of Sperm Conc
100
90
80
70
60
50
40
30
20
10
0
9
8
7
6
5
4
3
2
2 3 4 5 6 7 8 9 10 11 12 13 14 15 16 17 18 19 20 21 22 23 24
Week of Follow-up

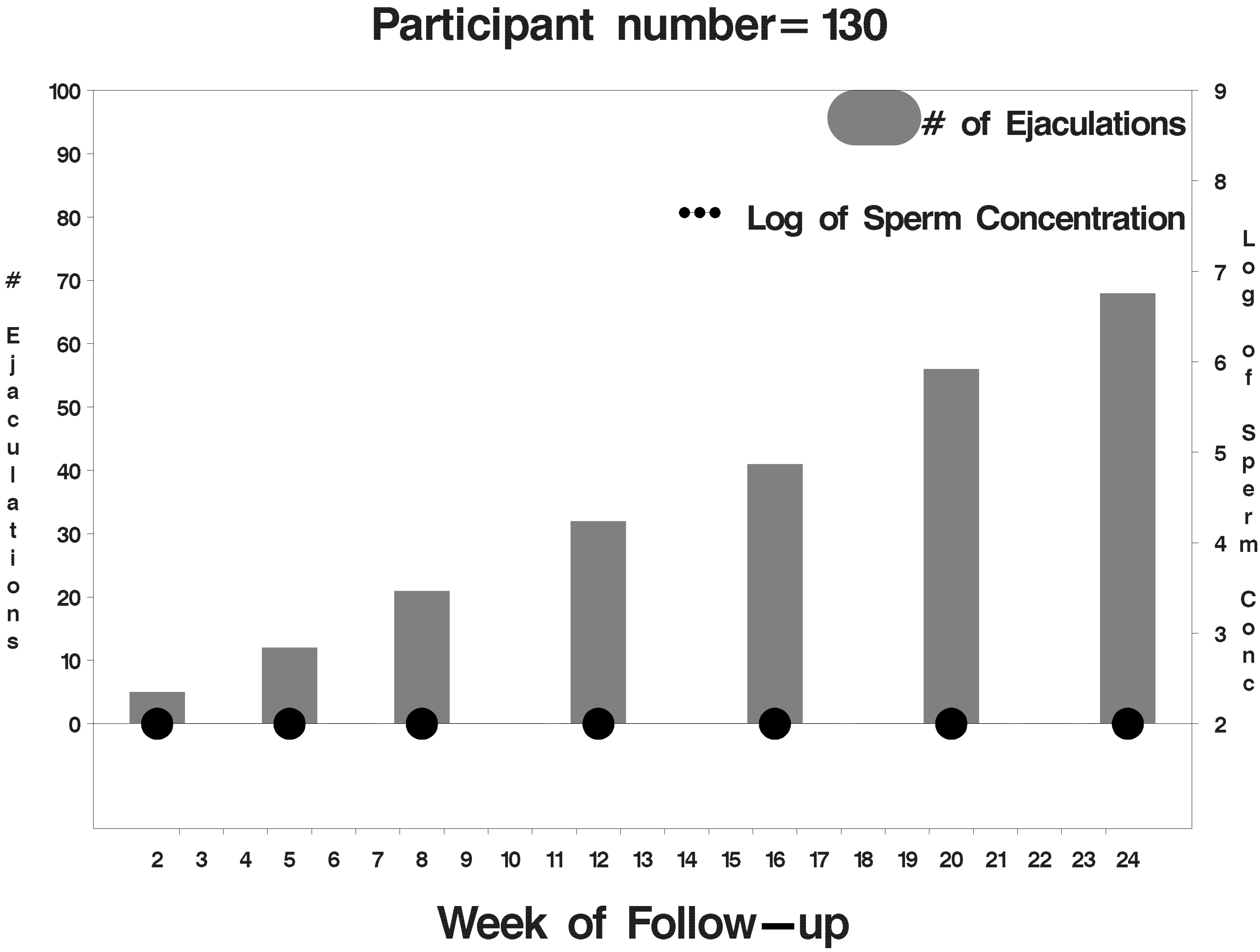

Participant number= 130
# of Ejaculations
Log of Sperm Concentration
Ejaculations
Log of Sperm Conc
100
90
80
70
60
50
40
30
20
10
0
9
8
7
6
5
4
3
2
2 3 4 5 6 7 8 9 10 11 12 13 14 15 16 17 18 19 20 21 22 23 24
Week of Follow-up

Participant number= 131

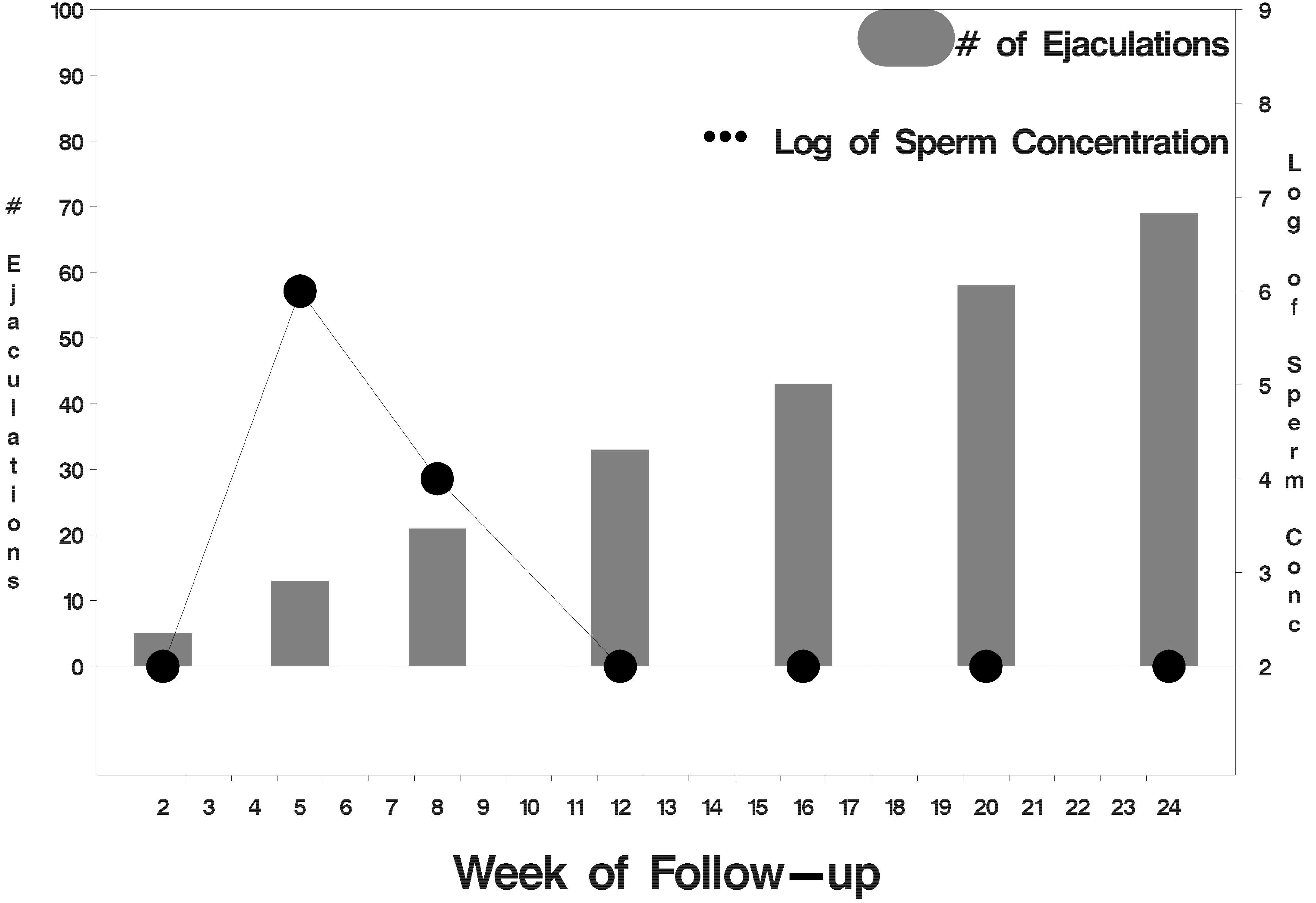

Participant number= 132

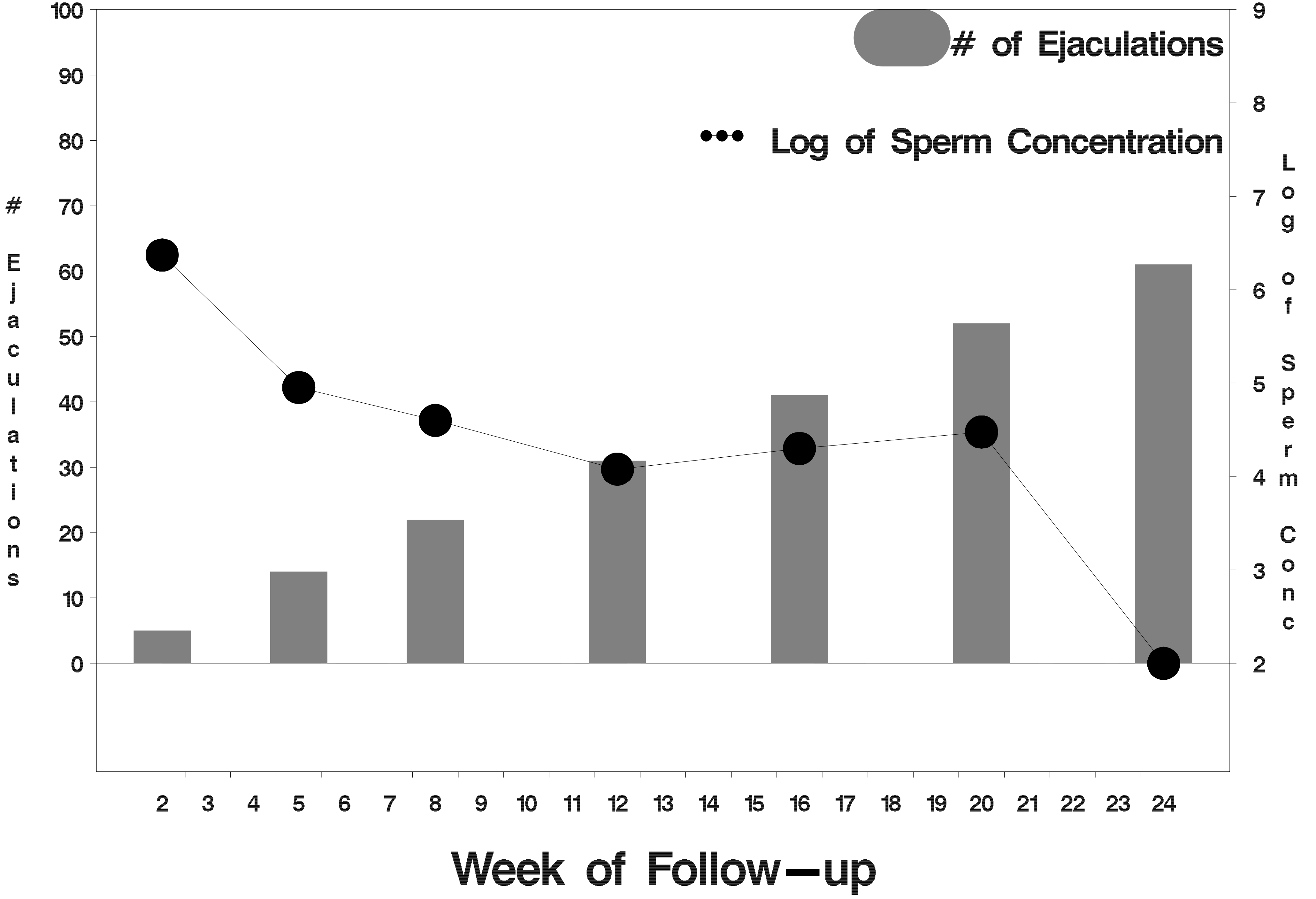

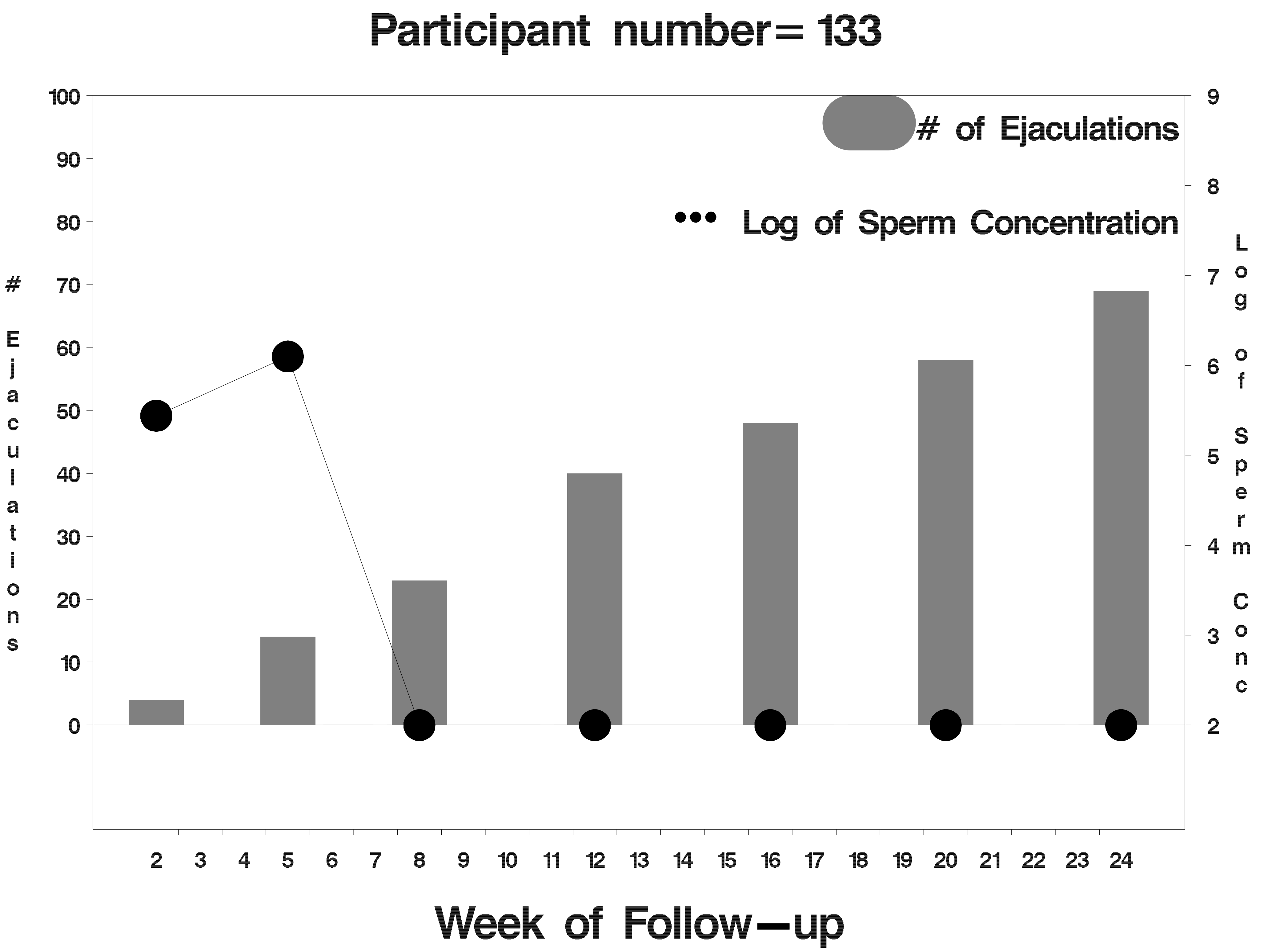

Participant number= 133
# of Ejaculations
Log of Sperm Concentration
Ejaculations
Log of Sperm Conc
0
10
20
30
40
50
60
70
80
90
100
2
3
4
5
6
7
8
9
2 3 4 5 6 7 8 9 10 11 12 13 14 15 16 17 18 19 20 21 22 23 24
Week of Follow—up

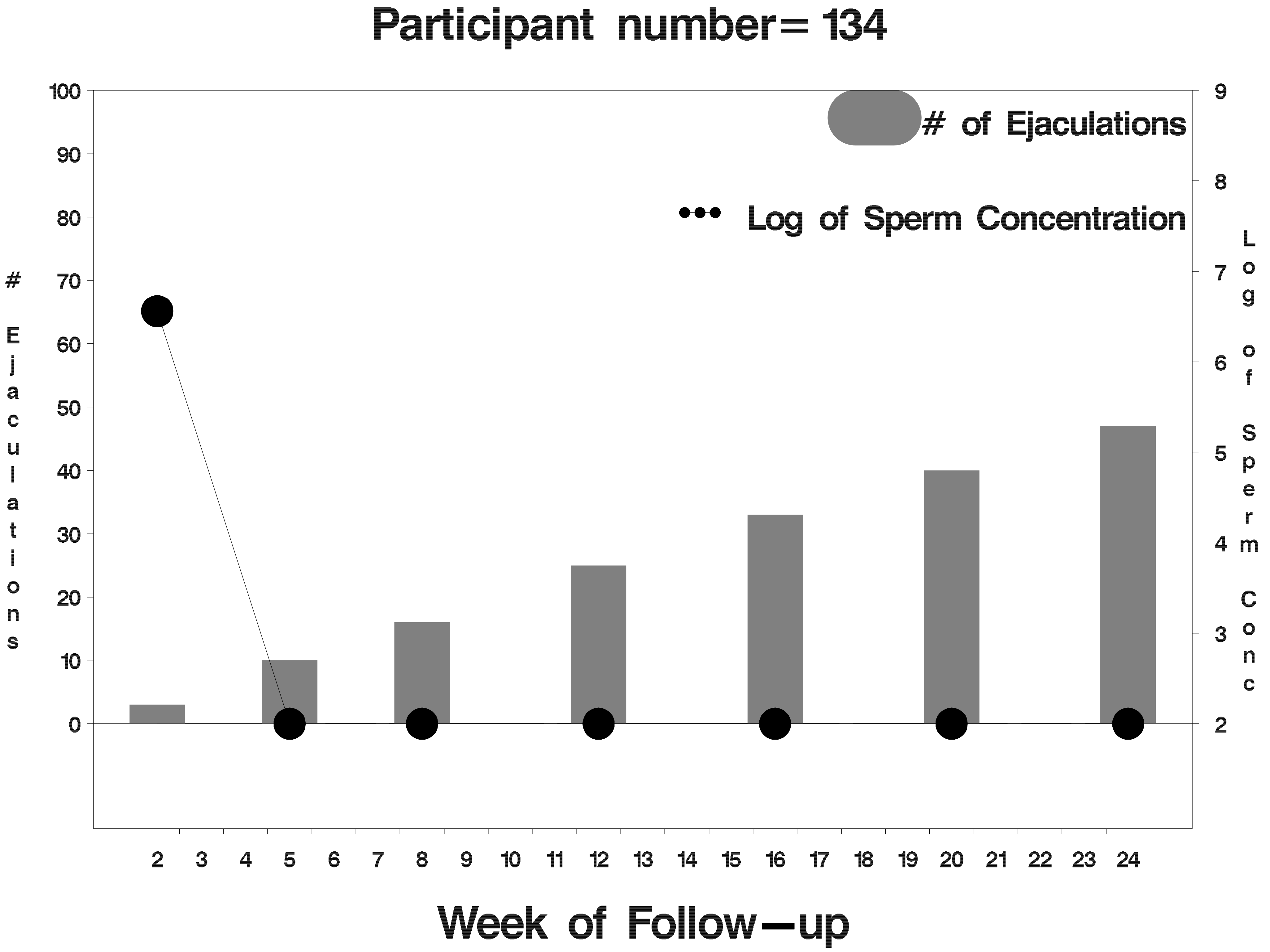

Participant number= 134
# of Ejaculations
Log of Sperm Concentration
Ejaculations
Log of Sperm Conc
100
90
80
70
60
50
40
30
20
10
0
9
8
7
6
5
4
3
2
2 3 4 5 6 7 8 9 10 11 12 13 14 15 16 17 18 19 20 21 22 23 24
Week of Follow-up

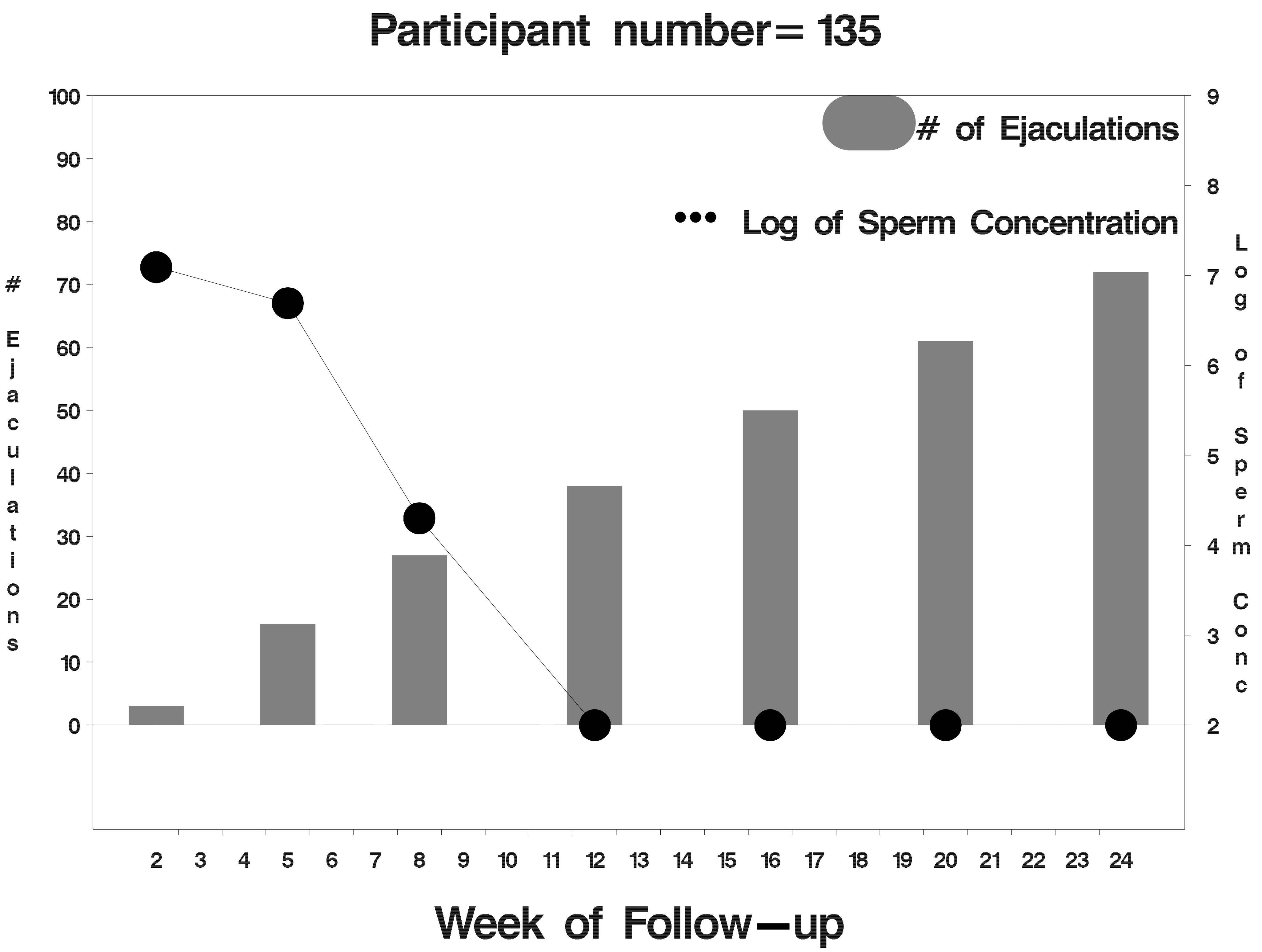
Participant number= 135
# of Ejaculations
Log of Sperm Concentration
Ejaculations
Log of Sperm Conc
Week of Follow-up
0
10
20
30
40
50
60
70
80
90
100
2
3
4
5
6
7
8
9
2
3
4
5
6
7
8
9
10
11
12
13
14
15
16
17
18
19
20
21
22
23
24

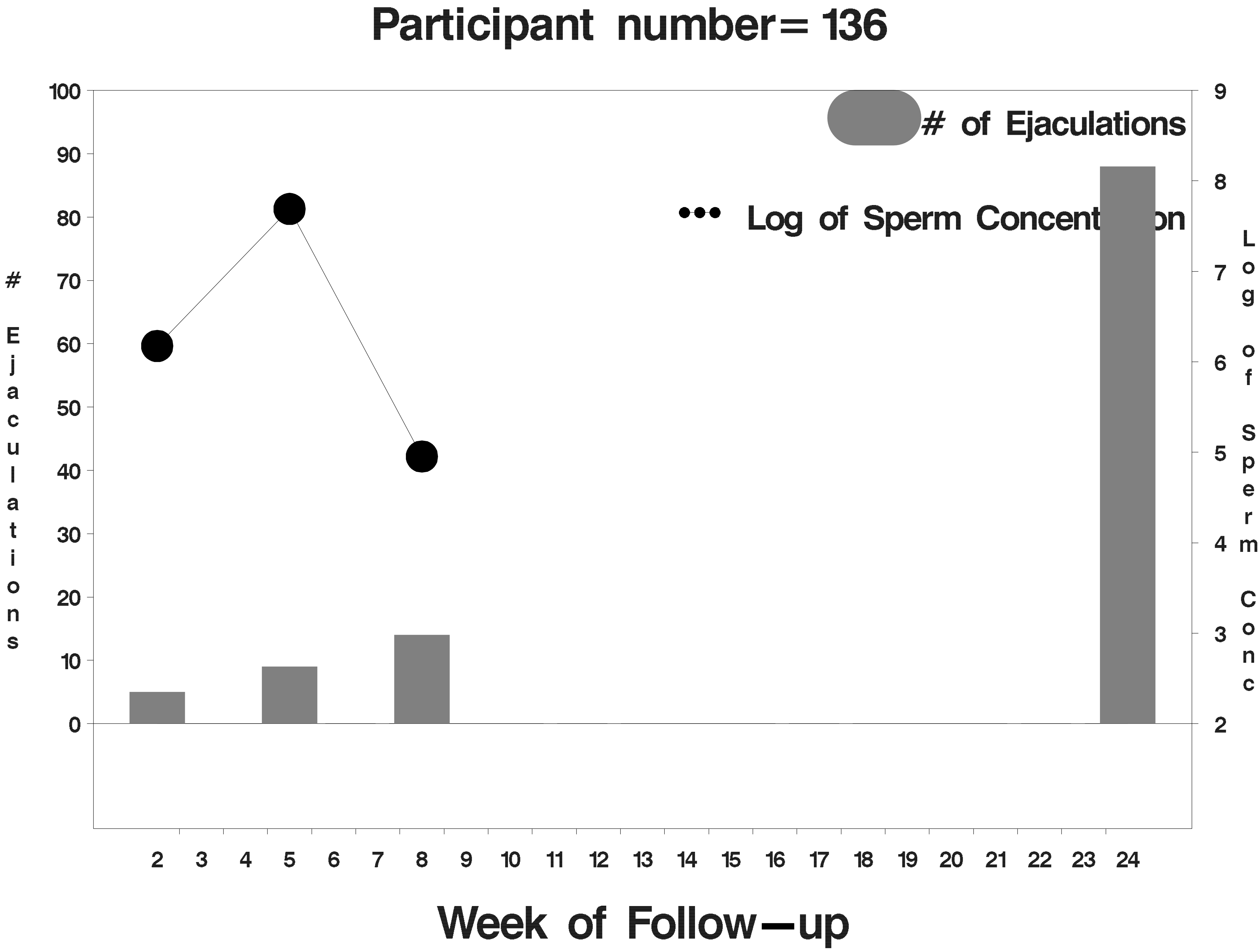

Participant number= 136
# of Ejaculations
Log of Sperm Concent on
Ejaculations
Log of Sperm Conc
100
90
80
70
60
50
40
30
20
10
0
9
8
7
6
5
4
3
2
2 3 4 5 6 7 8 9 10 11 12 13 14 15 16 17 18 19 20 21 22 23 24
Week of Follow—up

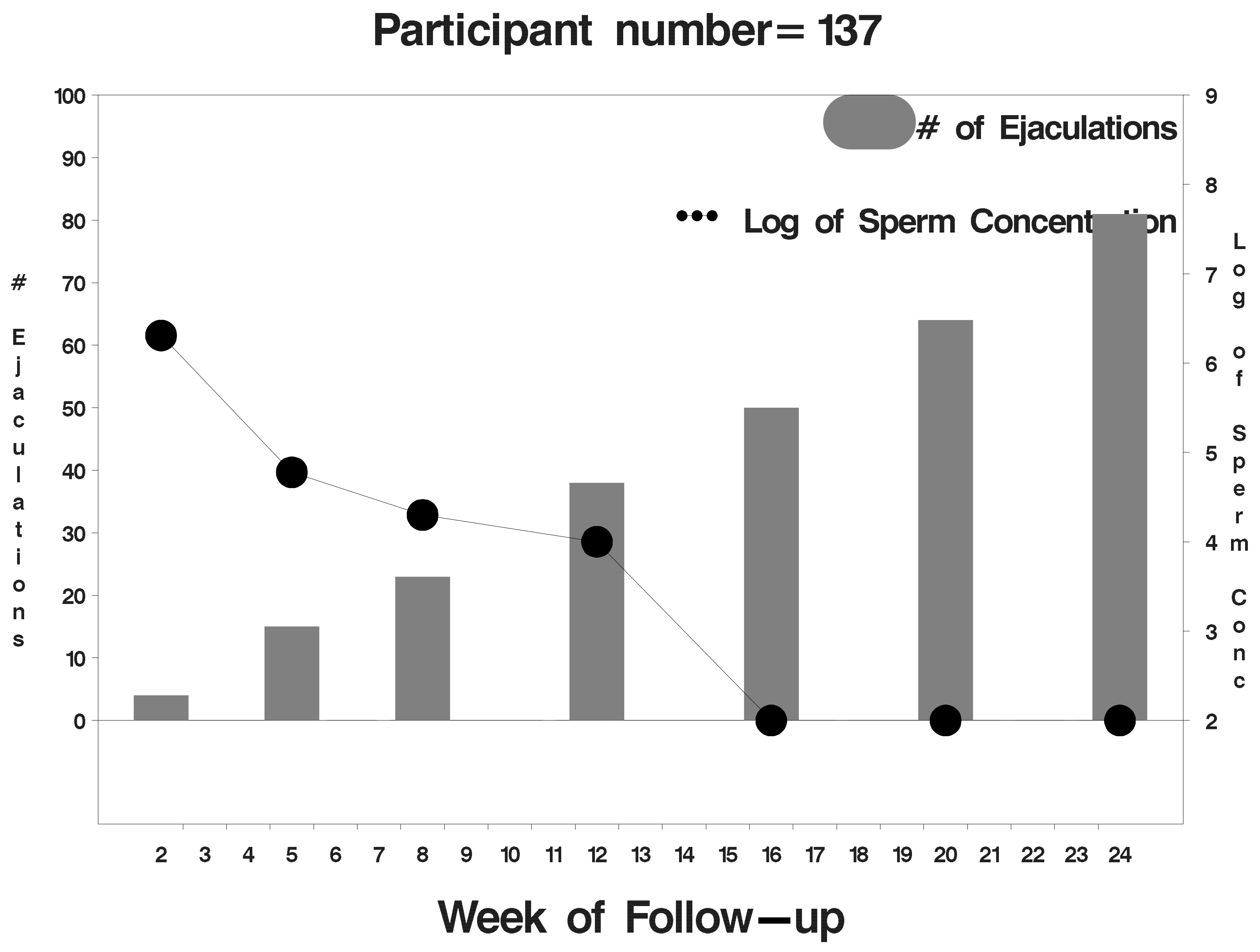

Participant number= 137
# of Ejaculations
Log of Sperm Concentration
Ejaculations
Log of Sperm Conc
0
10
20
30
40
50
60
70
80
90
100
2
3
4
5
6
7
8
9
2 3 4 5 6 7 8 9 10 11 12 13 14 15 16 17 18 19 20 21 22 23 24
Week of Follow-up

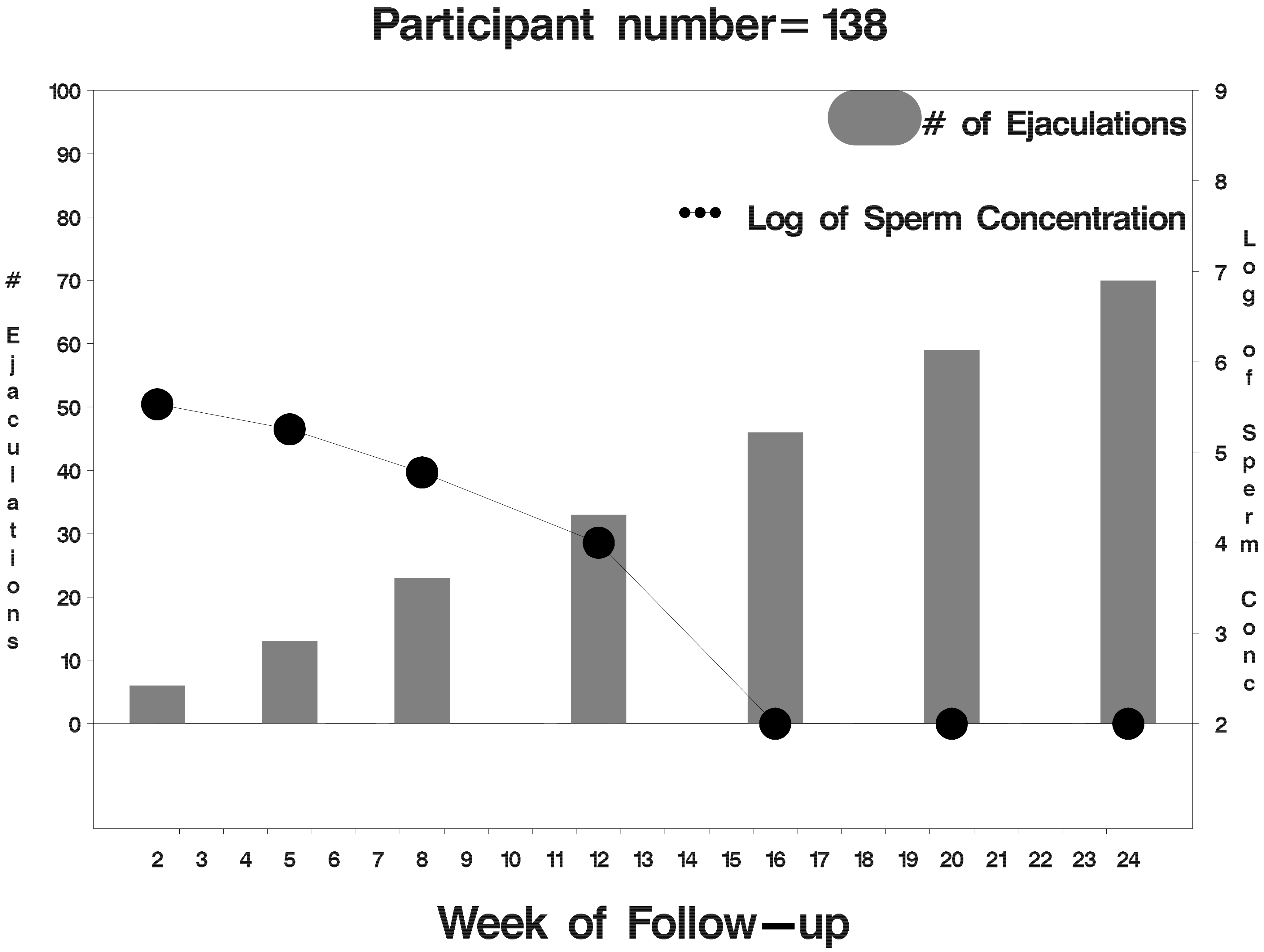

Participant number= 138
# of Ejaculations
Log of Sperm Concentration
Ejaculations
Log of Sperm Conc
100
90
80
70
60
50
40
30
20
10
0
9
8
7
6
5
4
3
2
2 3 4 5 6 7 8 9 10 11 12 13 14 15 16 17 18 19 20 21 22 23 24
Week of Follow−up

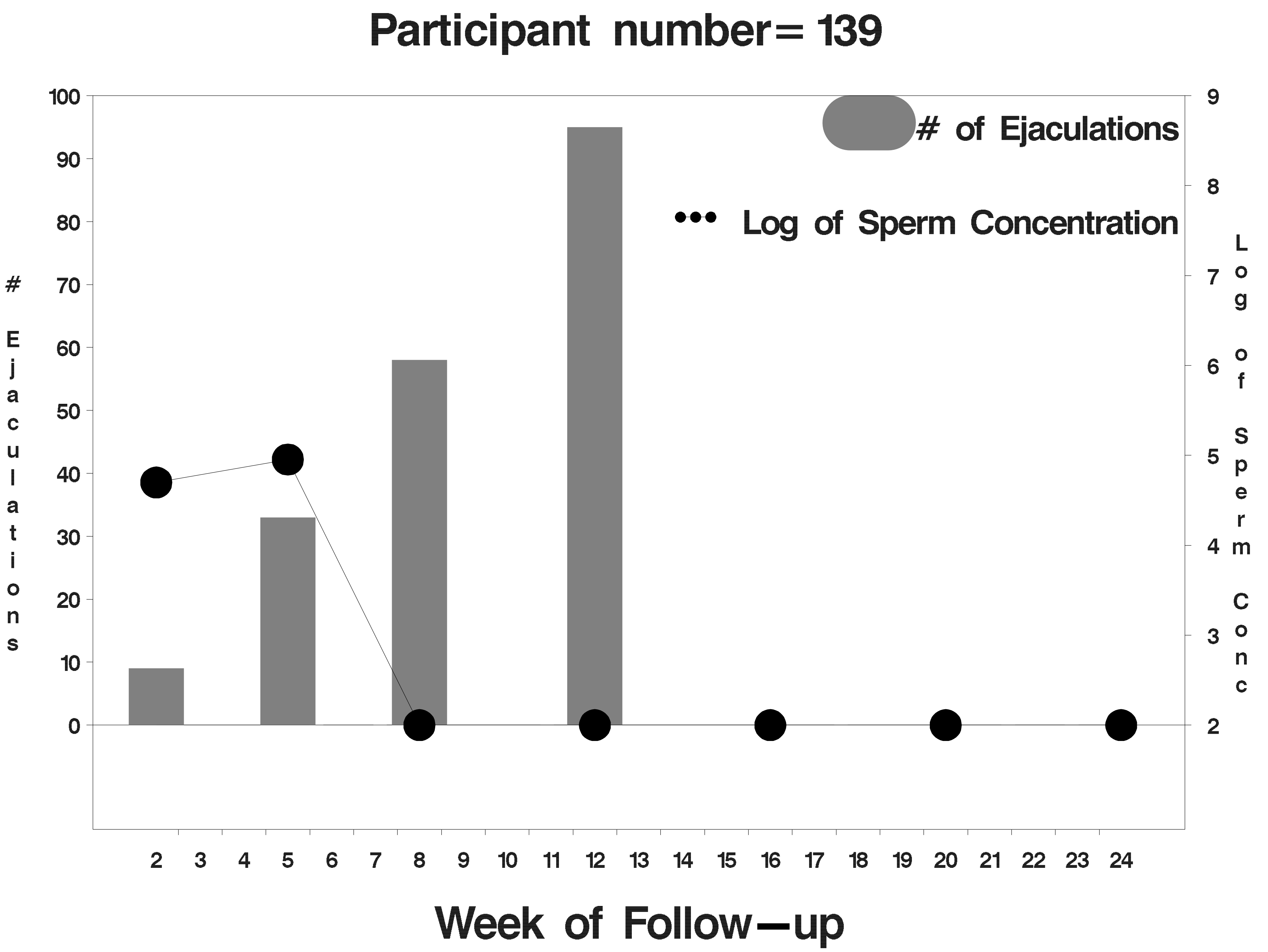

Participant number= 139
# of Ejaculations
Log of Sperm Concentration
Ejaculations
Log of Sperm Conc
100
90
80
70
60
50
40
30
20
10
0
9
8
7
6
5
4
3
2
2 3 4 5 6 7 8 9 10 11 12 13 14 15 16 17 18 19 20 21 22 23 24
Week of Follow-up

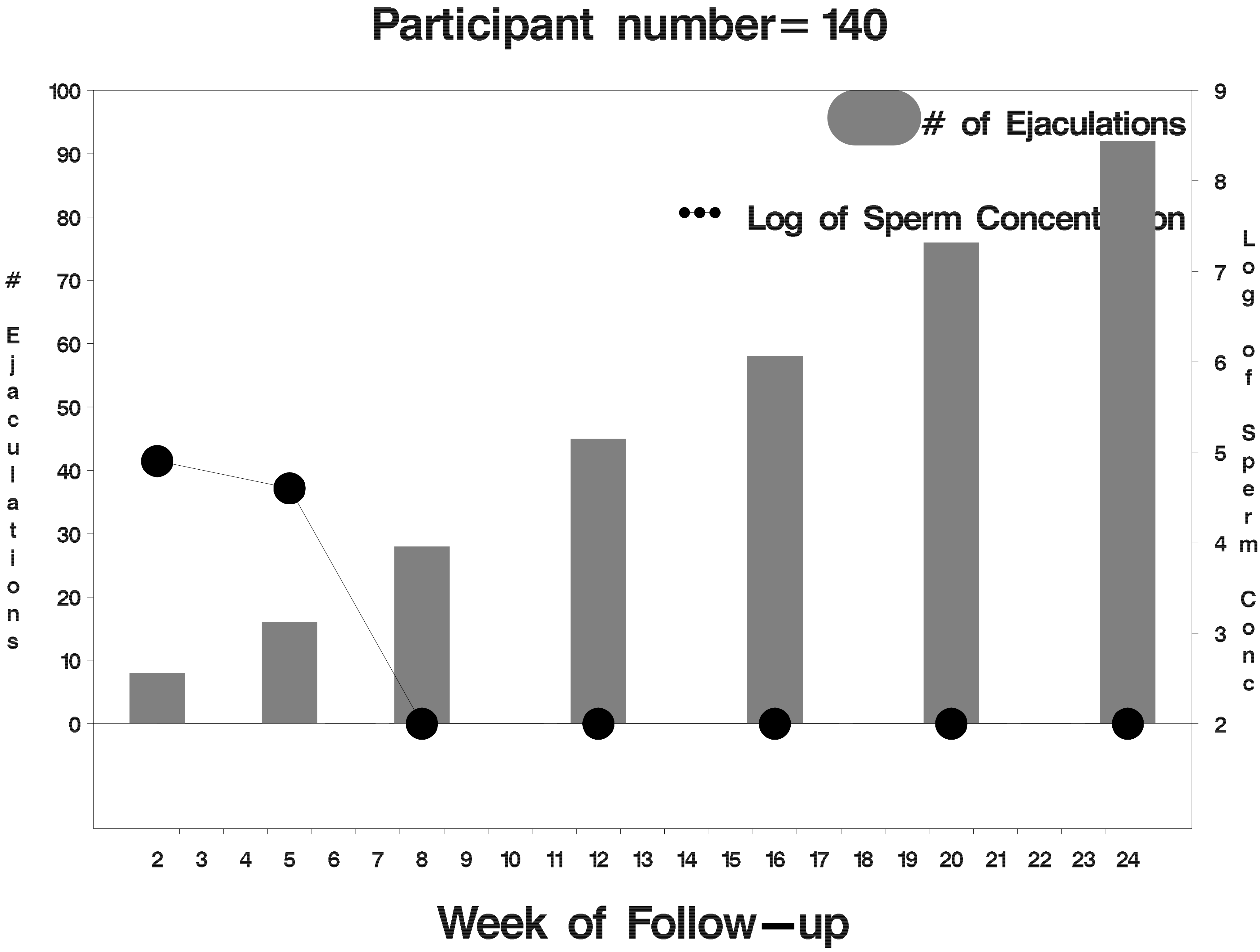

Participant number= 140
# of Ejaculations
Log of Sperm Concent on
Ejaculations
Log of Sperm Conc
100
90
80
70
60
50
40
30
20
10
0
9
8
7
6
5
4
3
2
2 3 4 5 6 7 8 9 10 11 12 13 14 15 16 17 18 19 20 21 22 23 24
Week of Follow—up

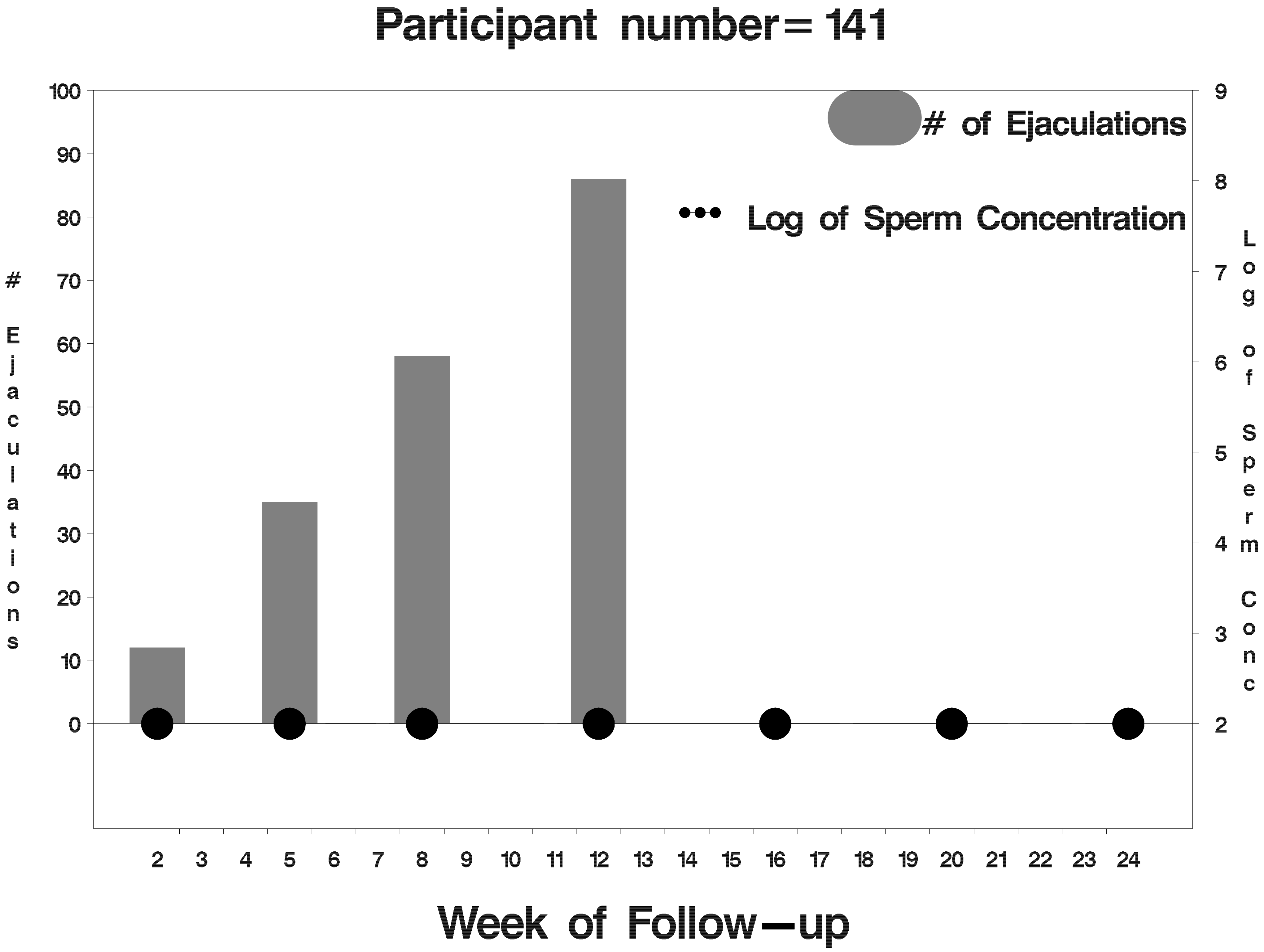

Participant number= 141
# of Ejaculations
Log of Sperm Concentration
Ejaculations
Log of Sperm Conc
100
90
80
70
60
50
40
30
20
10
0
9
8
7
6
5
4
3
2
2 3 4 5 6 7 8 9 10 11 12 13 14 15 16 17 18 19 20 21 22 23 24
Week of Follow-up

Participant number= 142

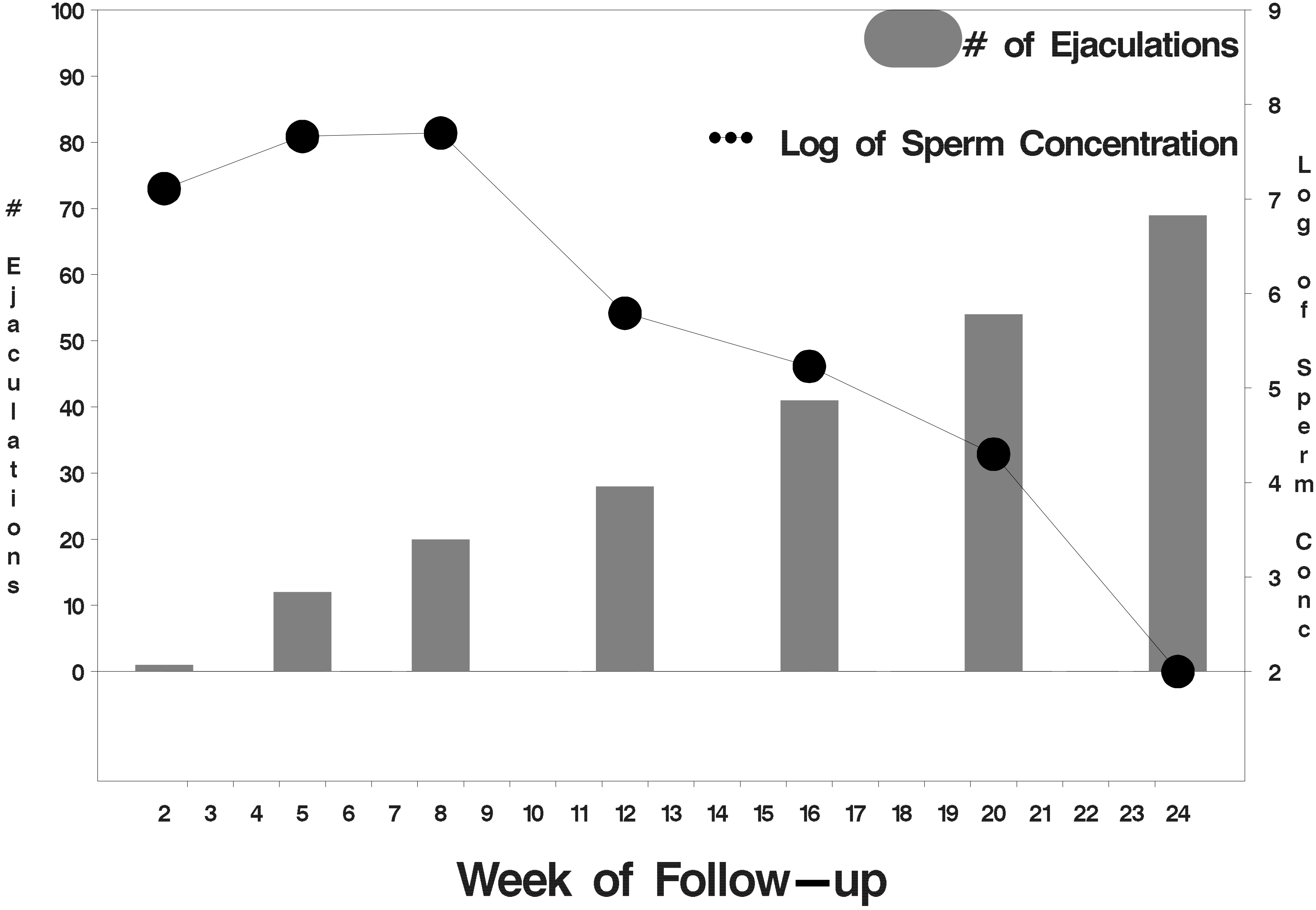

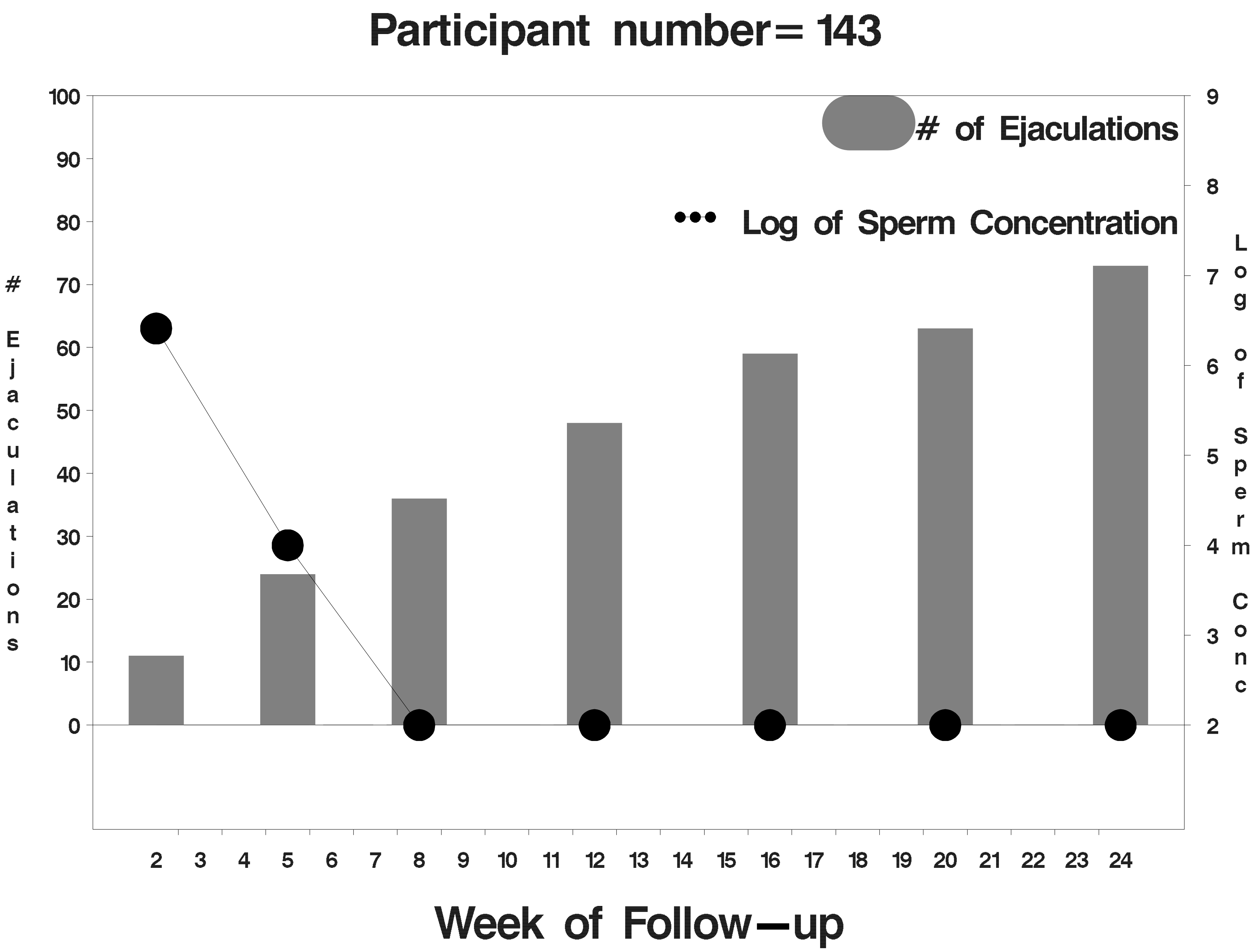
Participant number= 143
# of Ejaculations
Log of Sperm Concentration
Ejaculations
Log of Sperm Conc
100
90
80
70
60
50
40
30
20
10
0
9
8
7
6
5
4
3
2
2 3 4 5 6 7 8 9 10 11 12 13 14 15 16 17 18 19 20 21 22 23 24
Week of Follow-up

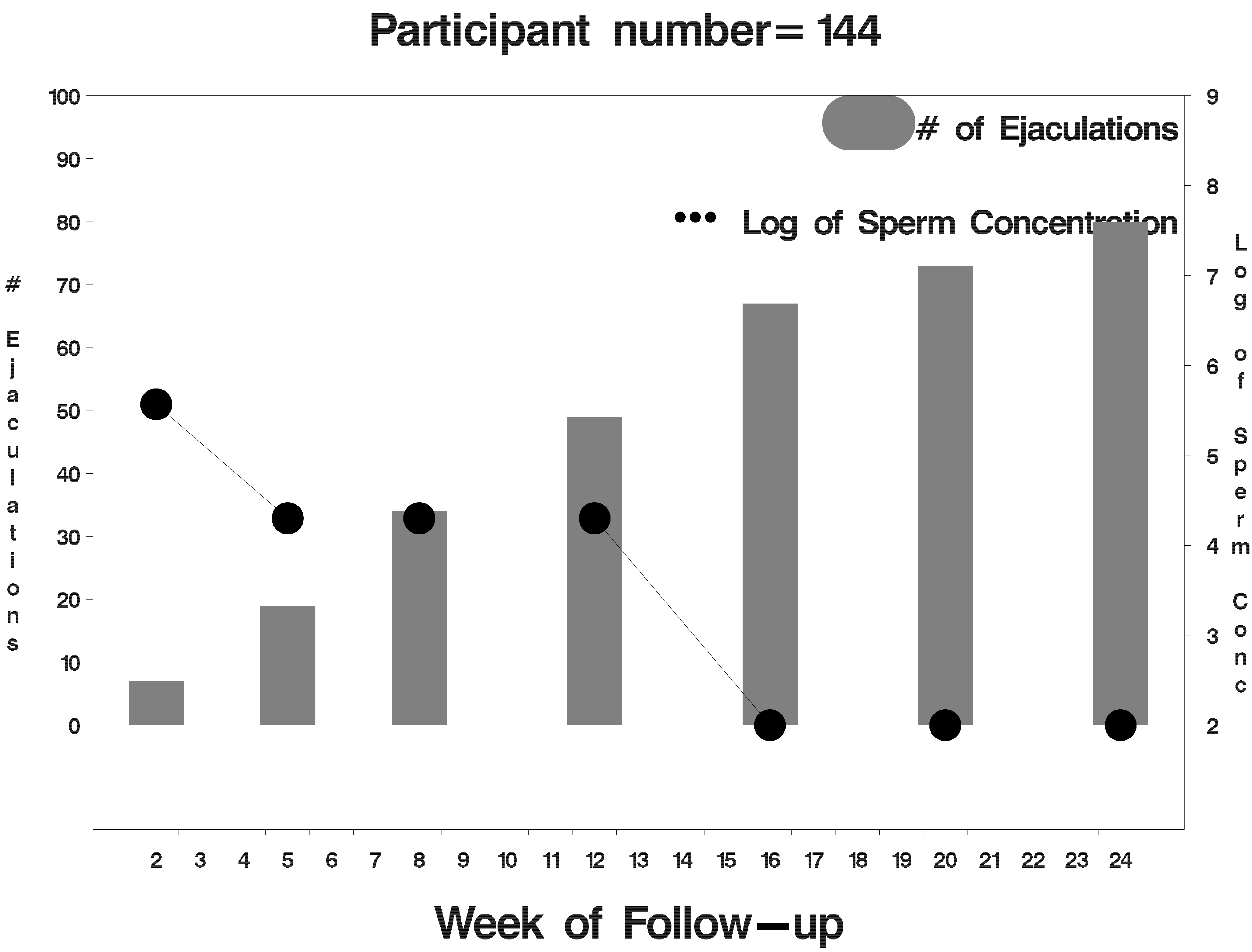

Participant number= 144
# of Ejaculations
Log of Sperm Concentration
Ejaculations
Log of Sperm Conc
100
90
80
70
60
50
40
30
20
10
0
9
8
7
6
5
4
3
2
2 3 4 5 6 7 8 9 10 11 12 13 14 15 16 17 18 19 20 21 22 23 24
Week of Follow-up

Participant number= 145

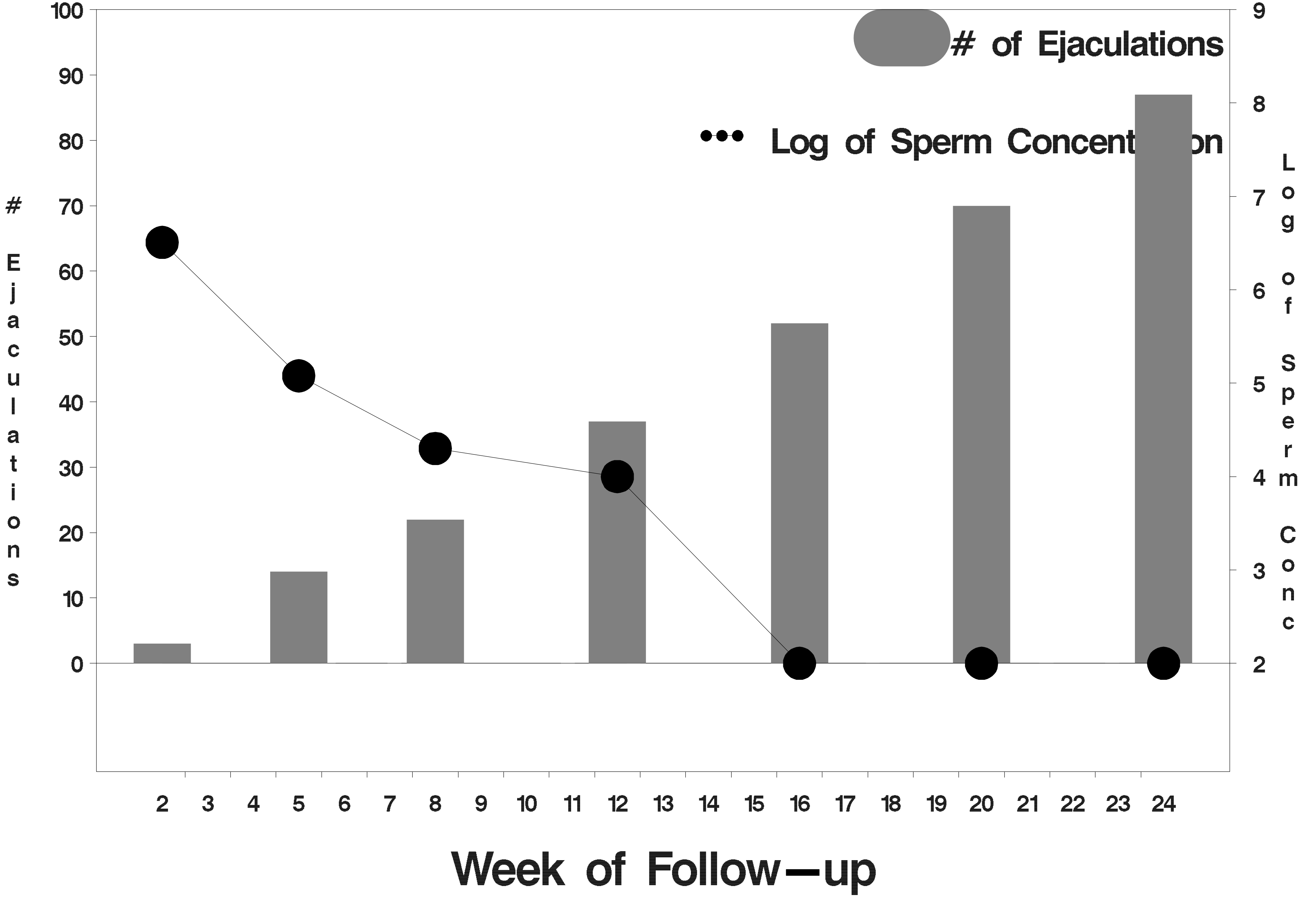

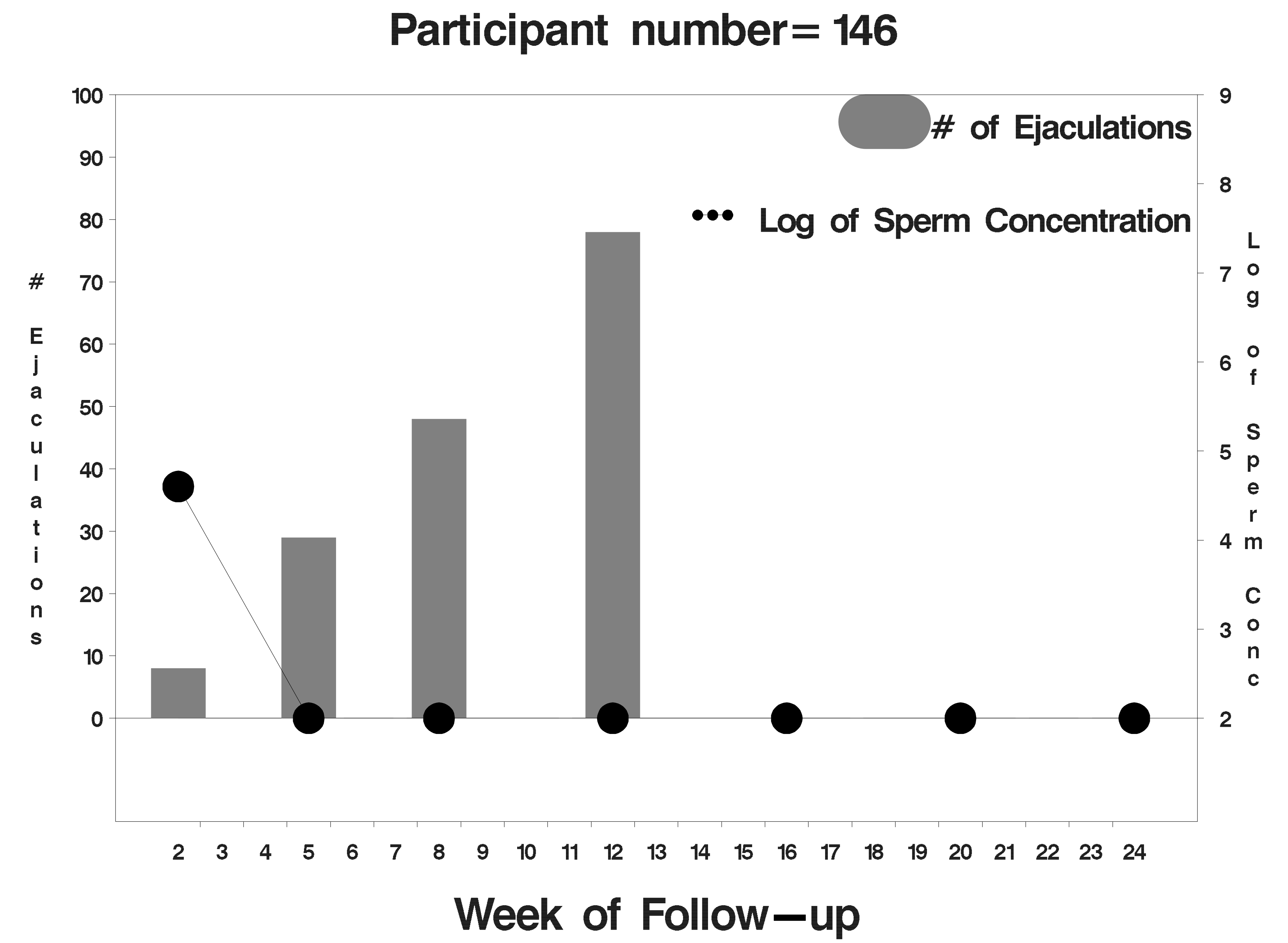

Participant number= 146
# of Ejaculations
Log of Sperm Concentration
Ejaculations
Log of Sperm Conc
100
90
80
70
60
50
40
30
20
10
0
9
8
7
6
5
4
3
2
2 3 4 5 6 7 8 9 10 11 12 13 14 15 16 17 18 19 20 21 22 23 24
Week of Follow-up

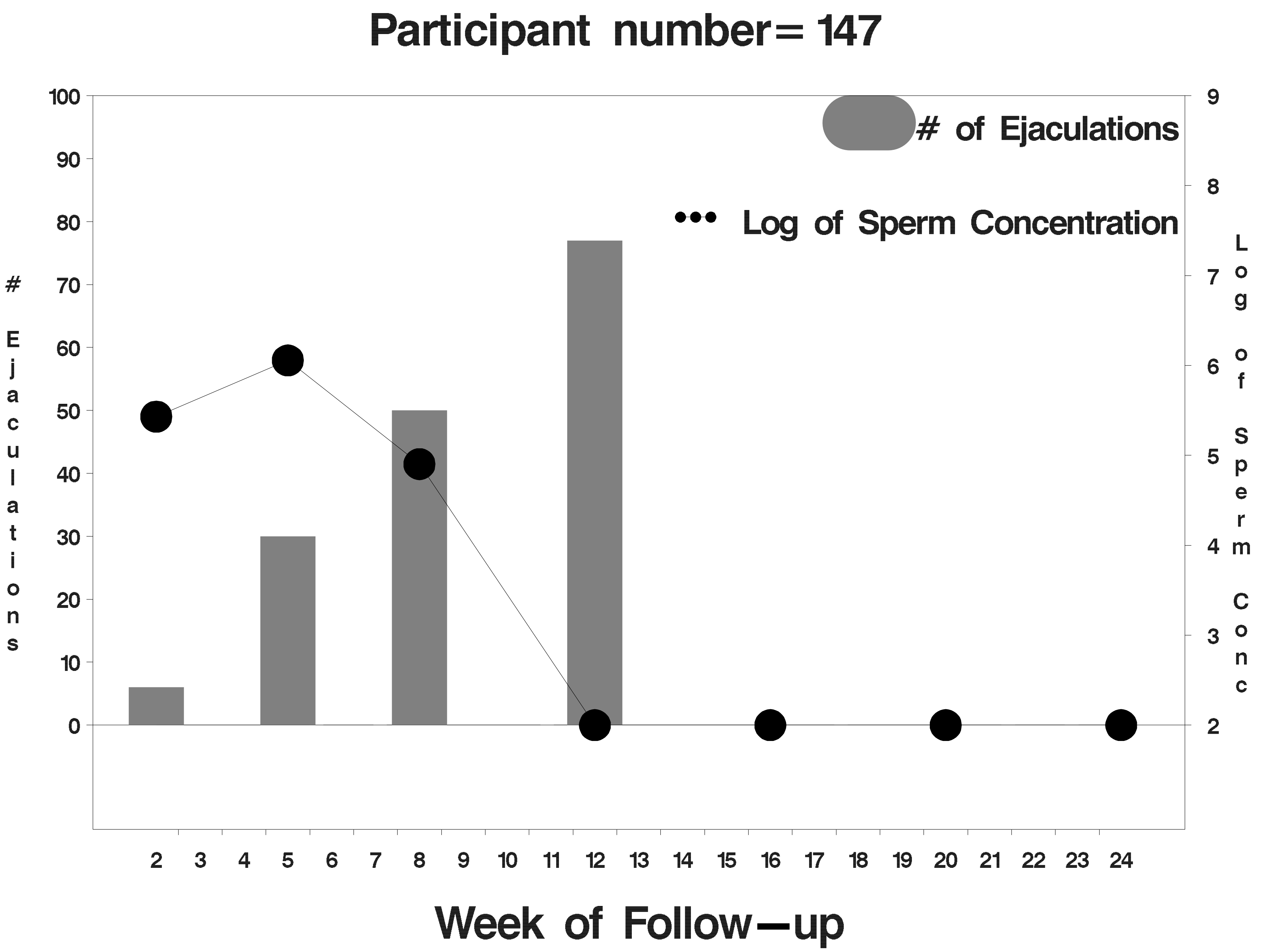

Participant number= 147
# of Ejaculations
Log of Sperm Concentration
Ejaculations
Log of Sperm Conc
100
90
80
70
60
50
40
30
20
10
0
9
8
7
6
5
4
3
2
2 3 4 5 6 7 8 9 10 11 12 13 14 15 16 17 18 19 20 21 22 23 24
Week of Follow-up

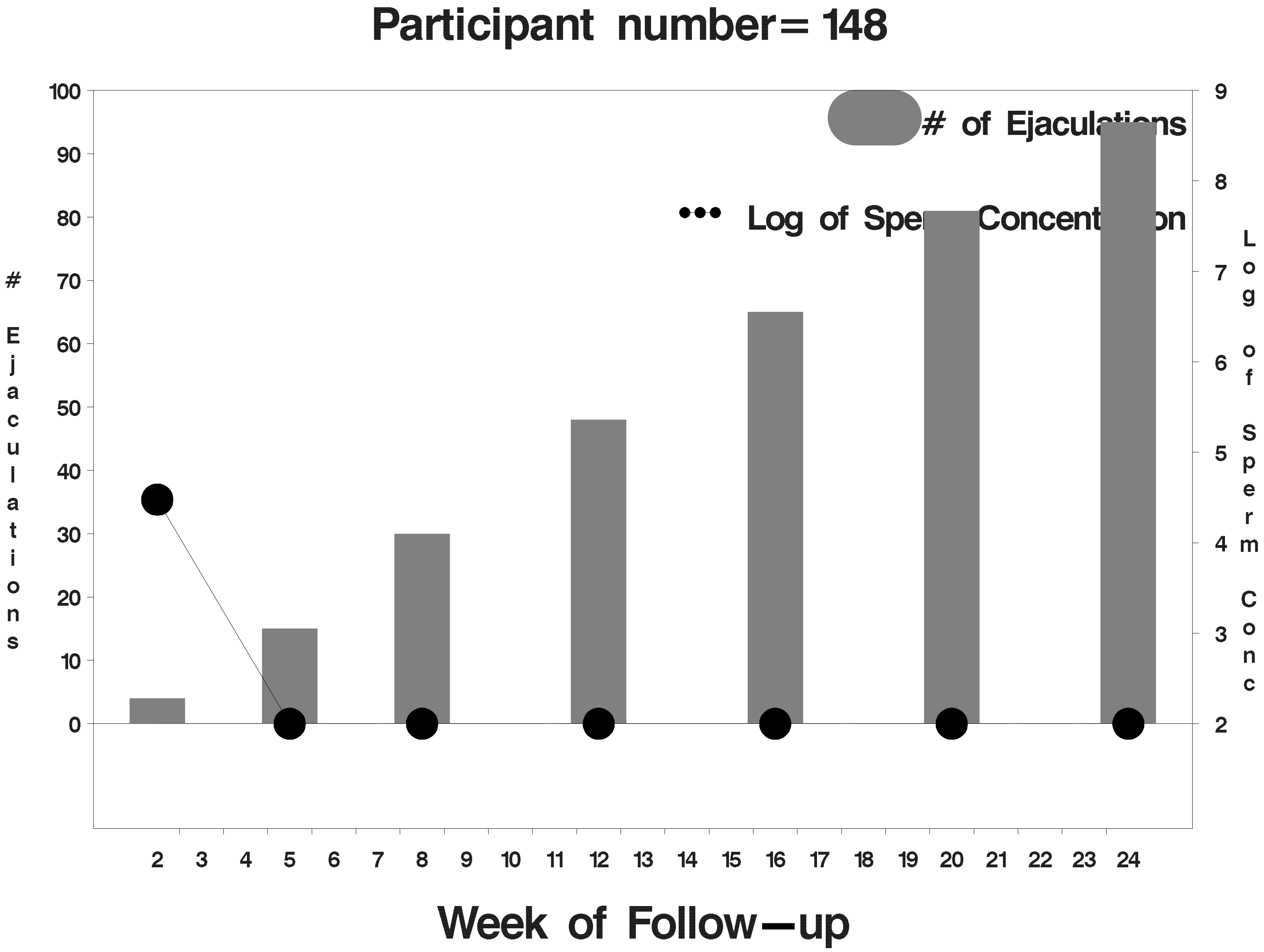

Participant number= 148
# of Ejaculations
Log of Sperm Concentration
Ejaculations
Log of Sperm Conc
Week of Follow-up
100
90
80
70
60
50
40
30
20
10
0
9
8
7
6
5
4
3
2
2 3 4 5 6 7 8 9 10 11 12 13 14 15 16 17 18 19 20 21 22 23 24

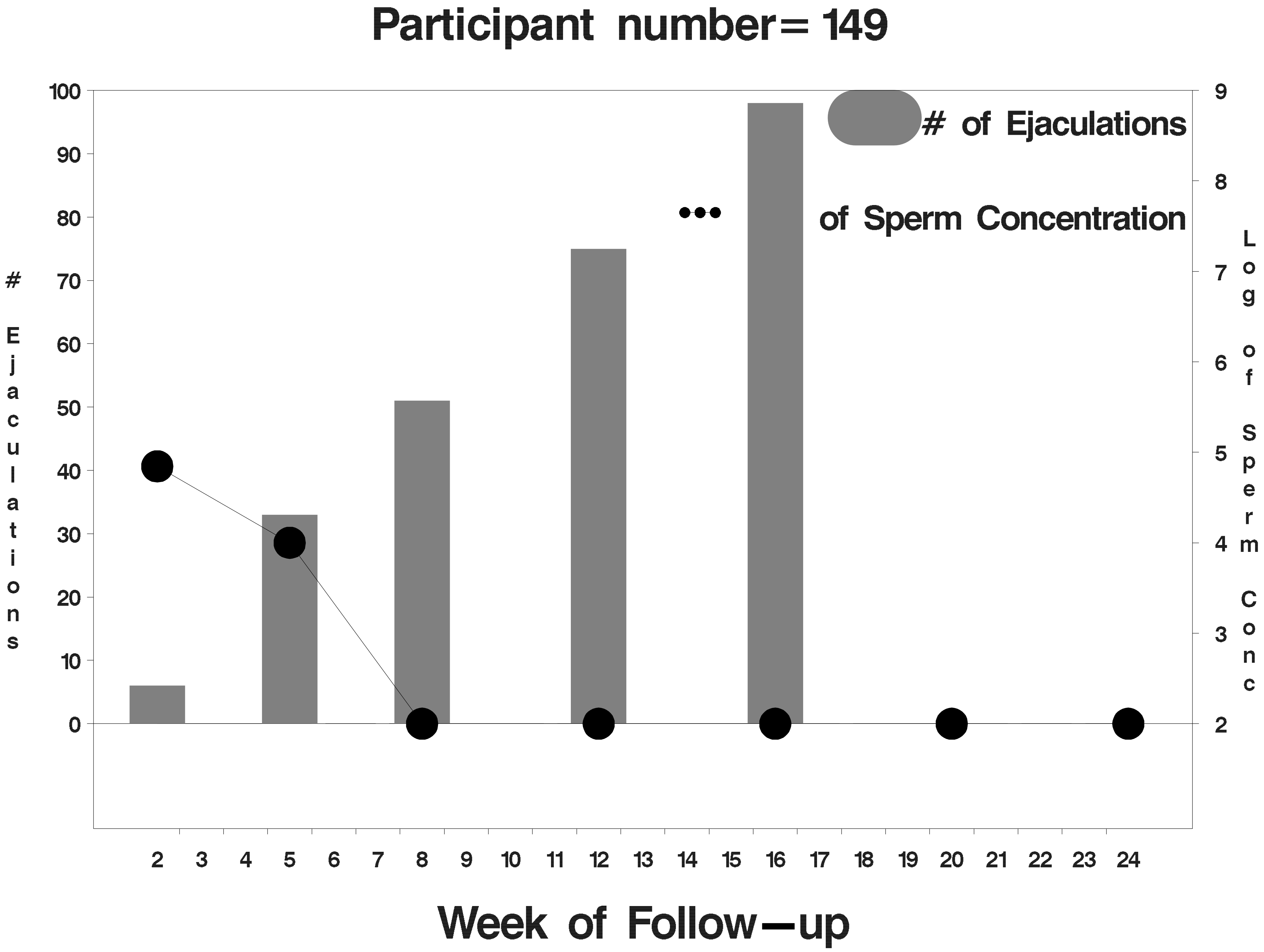
Participant number= 149
# of Ejaculations
of Sperm Concentration
Ejaculations
Log of Sperm Conc
100
90
80
70
60
50
40
30
20
10
0
9
8
7
6
5
4
3
2
2 3 4 5 6 7 8 9 10 11 12 13 14 15 16 17 18 19 20 21 22 23 24
Week of Follow-up

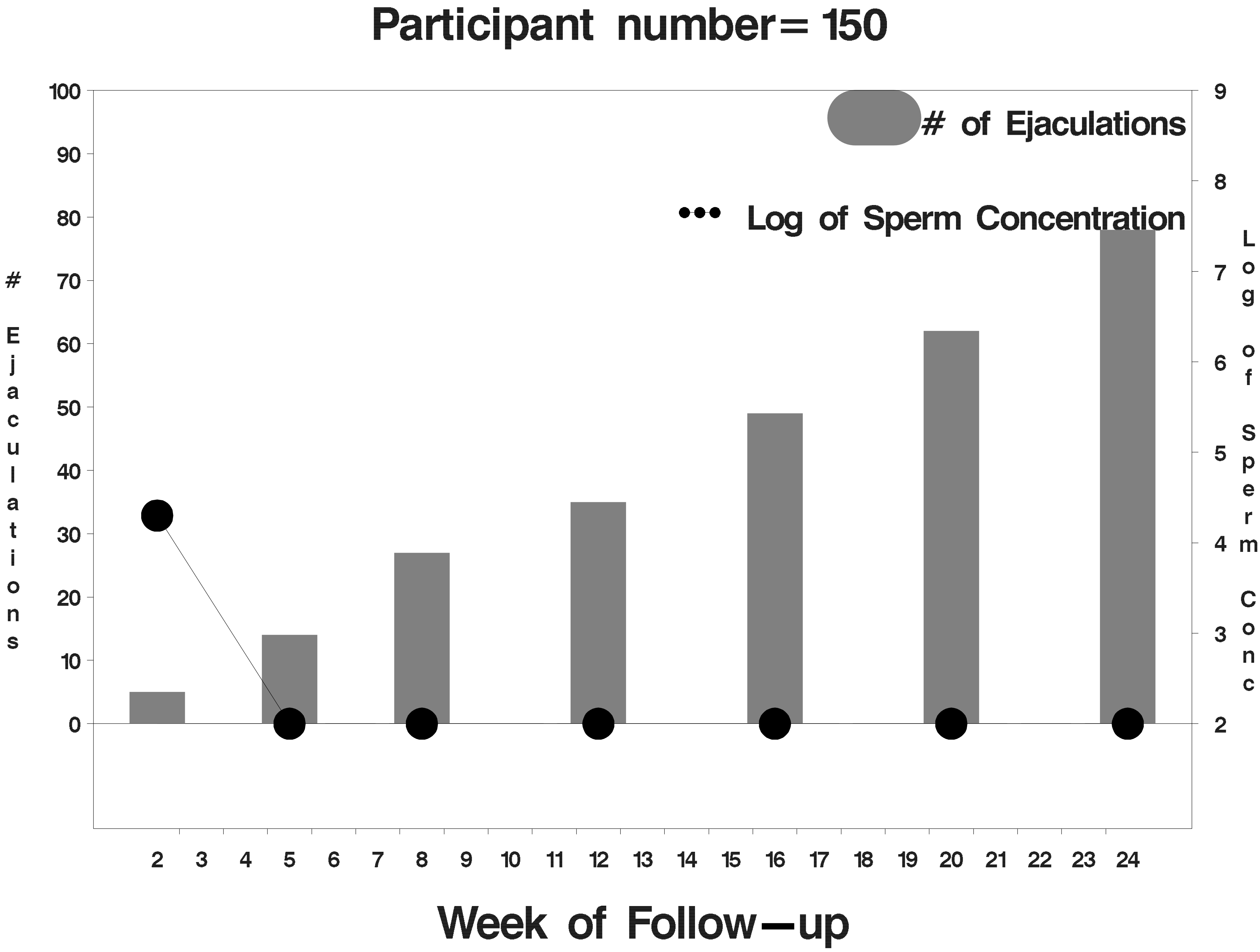

Participant number= 150
# of Ejaculations
Log of Sperm Concentration
Ejaculations
Log of Sperm Conc
100
90
80
70
60
50
40
30
20
10
0
9
8
7
6
5
4
3
2
2 3 4 5 6 7 8 9 10 11 12 13 14 15 16 17 18 19 20 21 22 23 24
Week of Follow-up

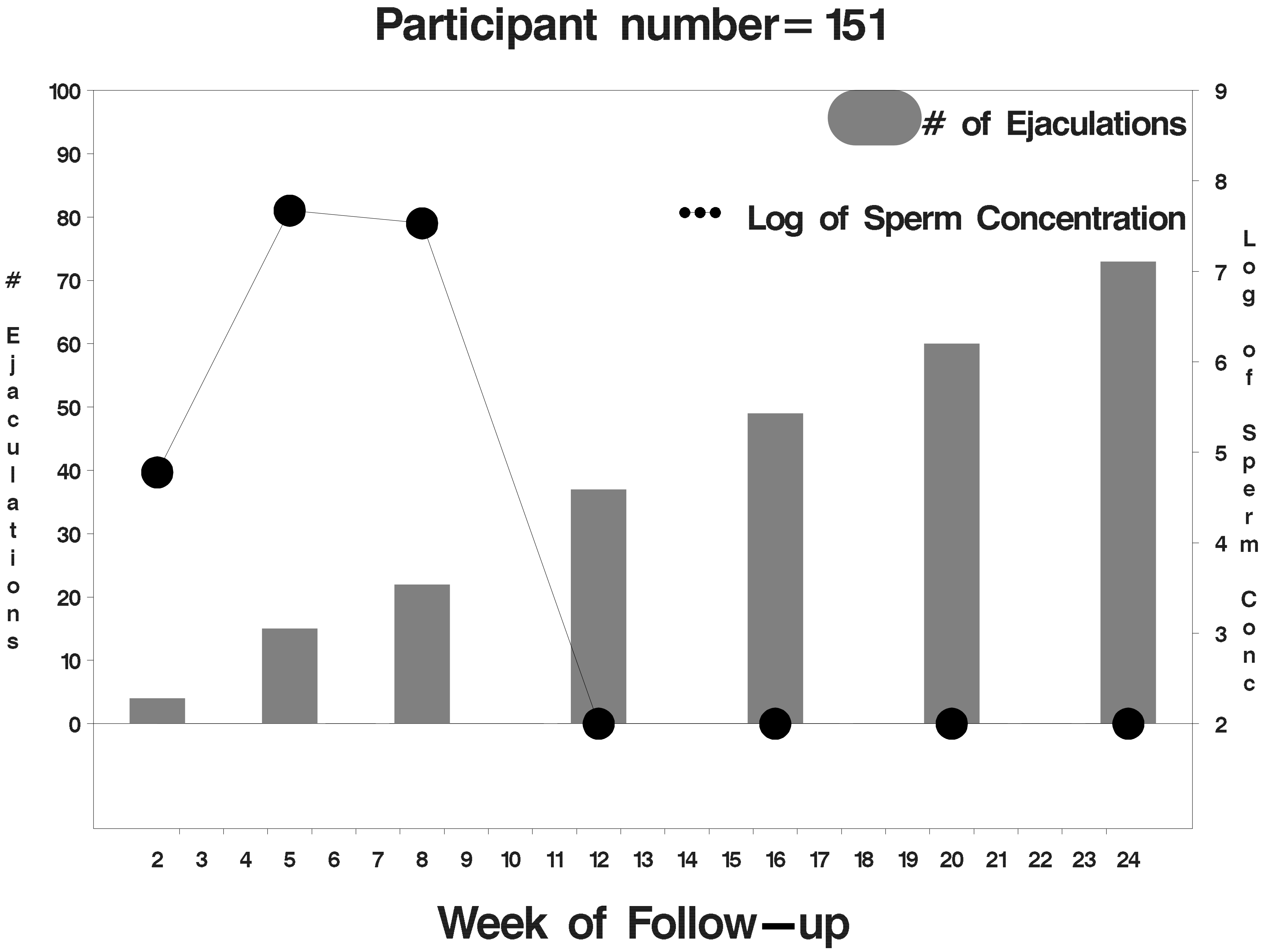

Participant number= 151
# of Ejaculations
Log of Sperm Concentration
Ejaculations
Log of Sperm Conc
100
90
80
70
60
50
40
30
20
10
0
9
8
7
6
5
4
3
2
2 3 4 5 6 7 8 9 10 11 12 13 14 15 16 17 18 19 20 21 22 23 24
Week of Follow-up

Participant number= 152

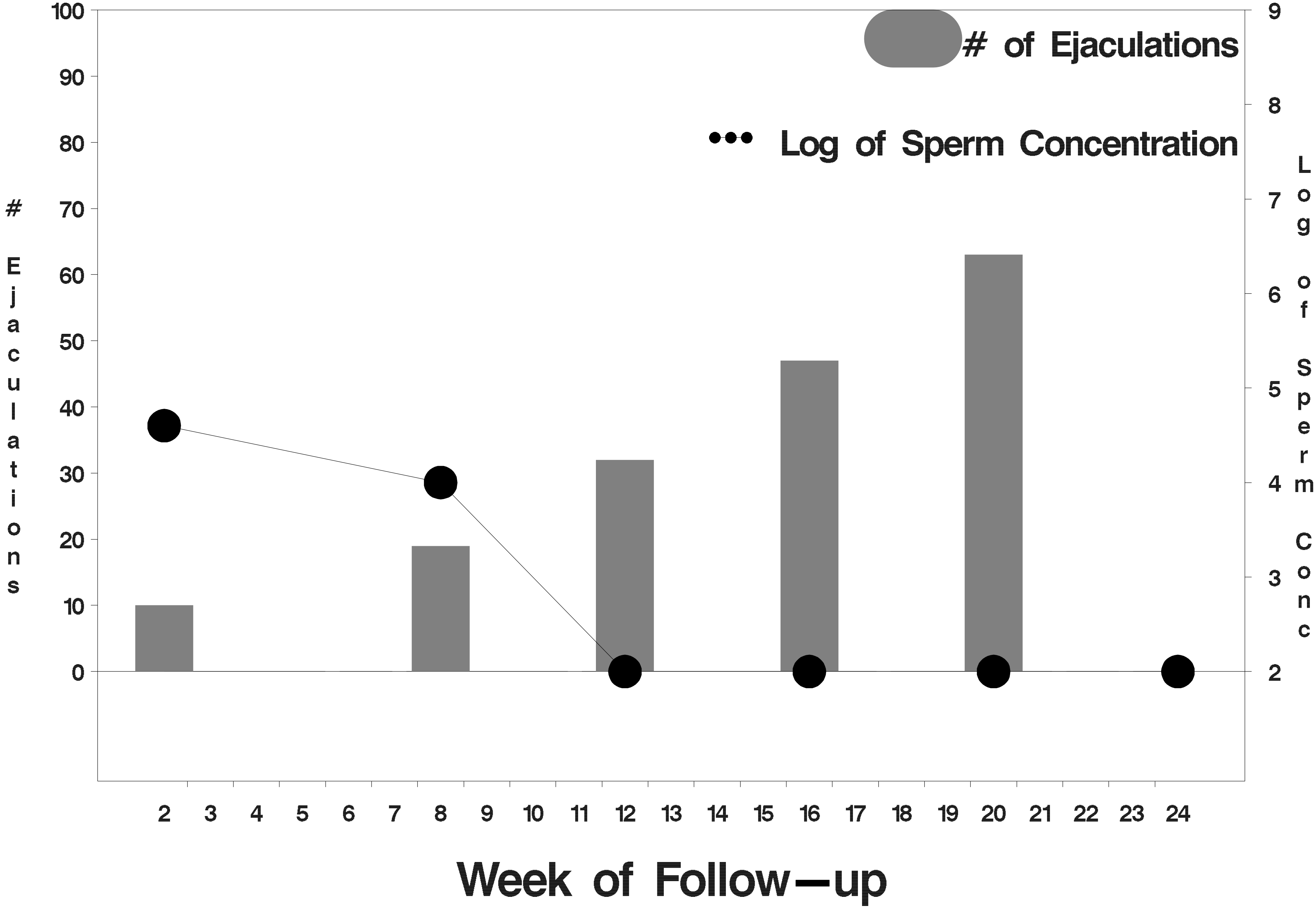

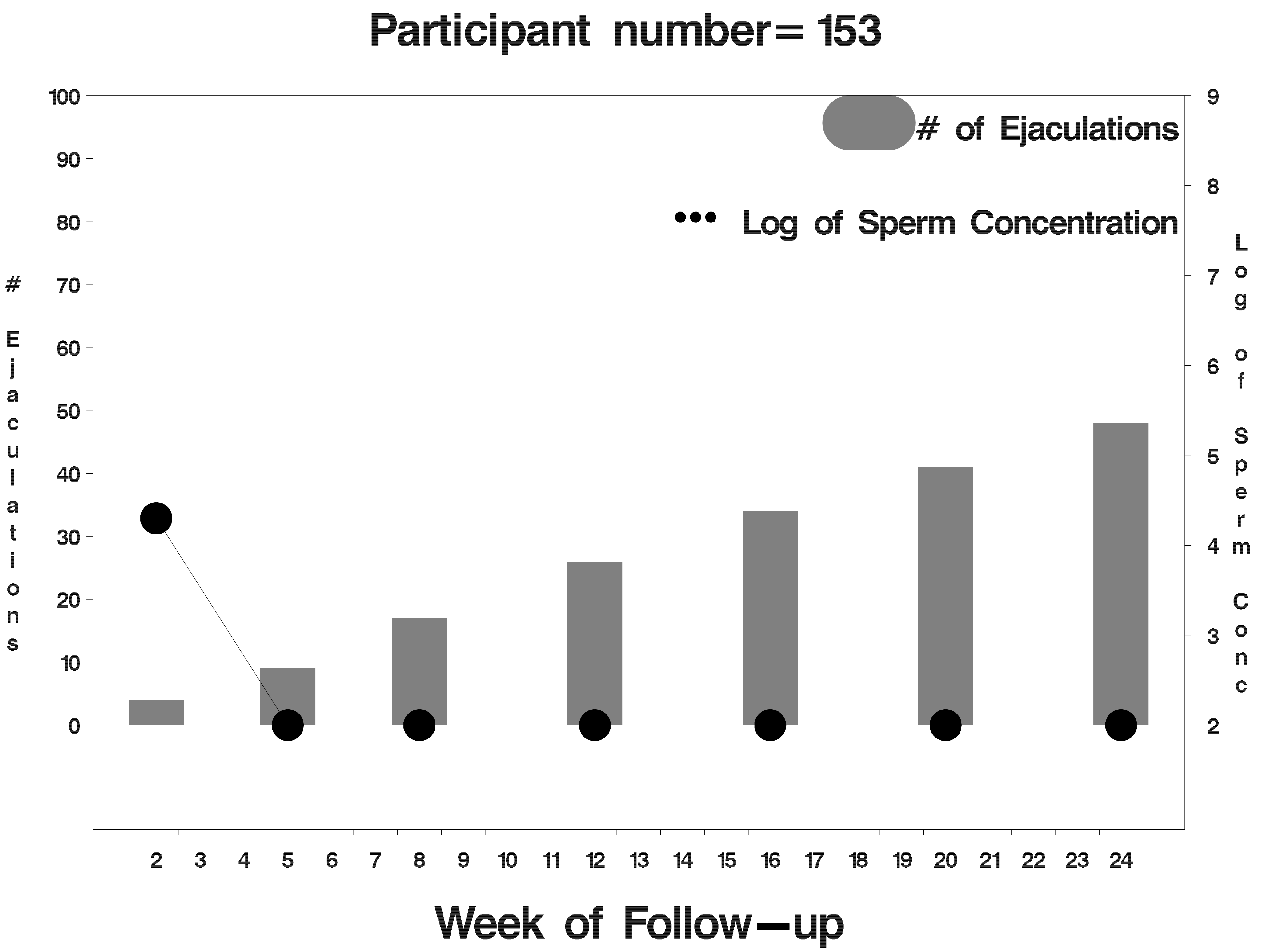

Participant number= 153
# of Ejaculations
Log of Sperm Concentration
Ejaculations
Log of Sperm Conc
100
90
80
70
60
50
40
30
20
10
0
9
8
7
6
5
4
3
2
2 3 4 5 6 7 8 9 10 11 12 13 14 15 16 17 18 19 20 21 22 23 24
Week of Follow-up

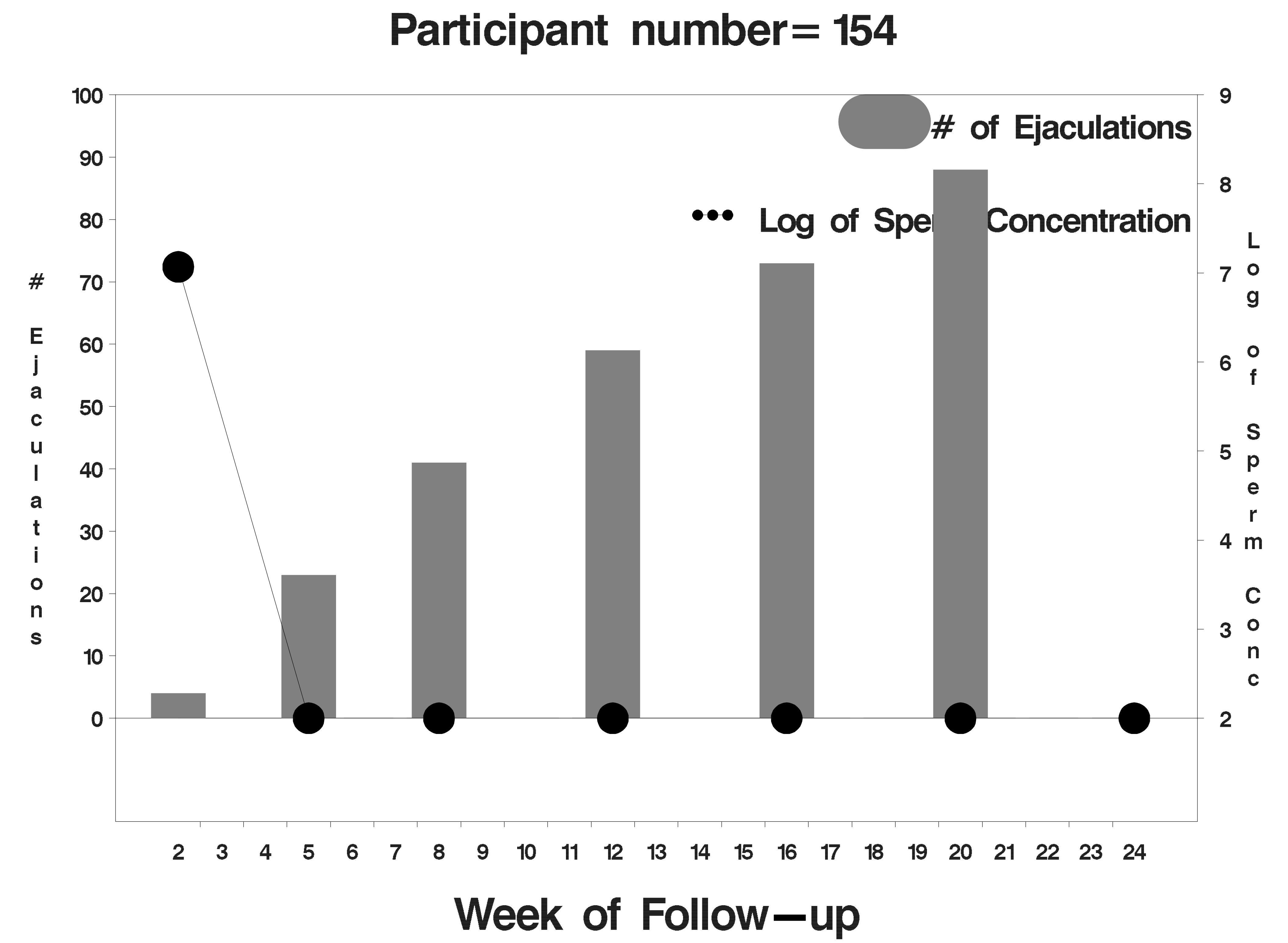

Participant number= 154
# of Ejaculations
Log of Sperm Concentration
Ejaculations
Log of Sperm Conc
100
90
80
70
60
50
40
30
20
10
0
9
8
7
6
5
4
3
2
2 3 4 5 6 7 8 9 10 11 12 13 14 15 16 17 18 19 20 21 22 23 24
Week of Follow−up

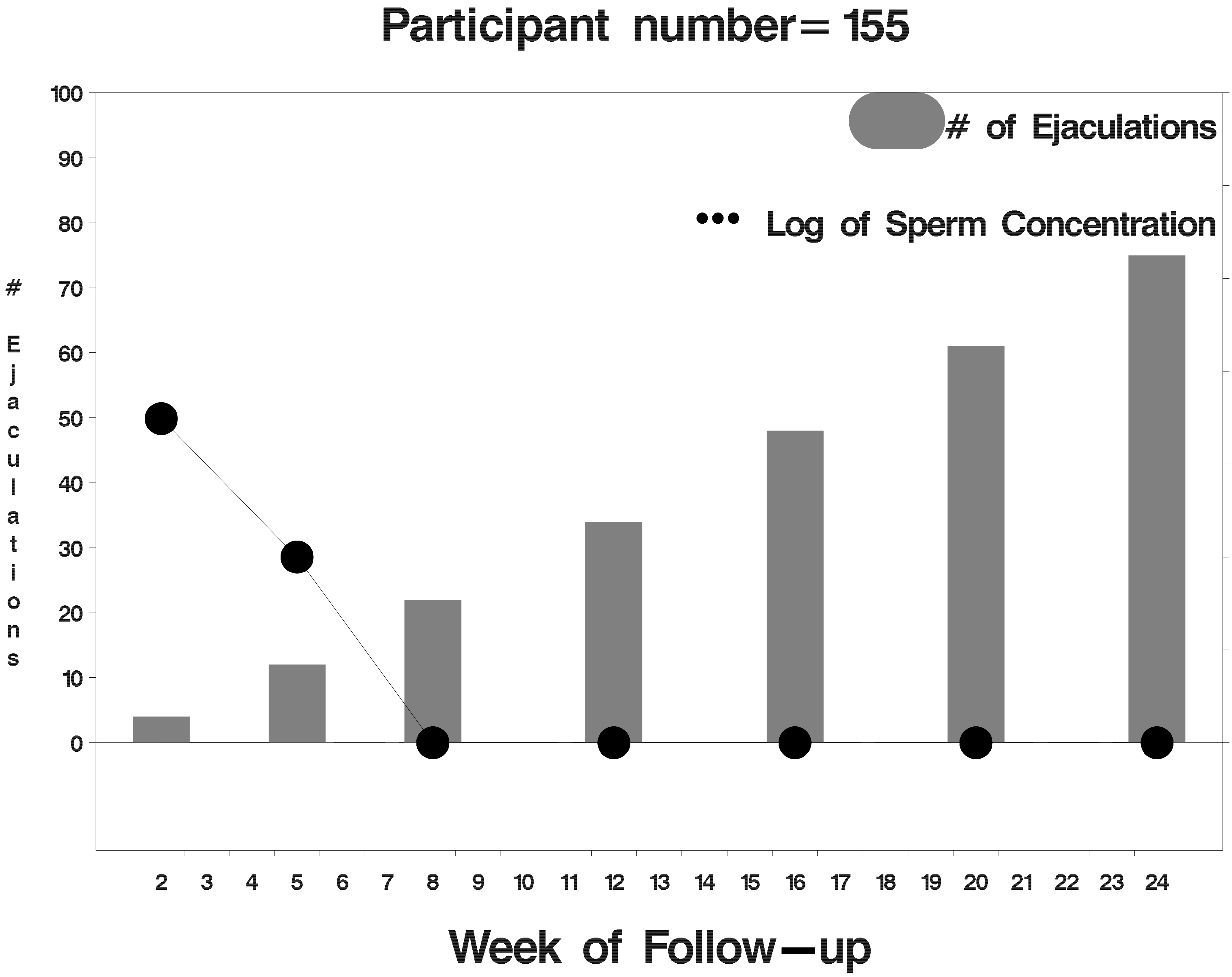
Participant number= 155
# of Ejaculations
Log of Sperm Concentration
Ejaculations
Log of Sperm Conc
100
90
80
70
60
50
40
30
20
10
0
9
8
7
6
5
4
3
2
2 3 4 5 6 7 8 9 10 11 12 13 14 15 16 17 18 19 20 21 22 23 24
Week of Follow-up

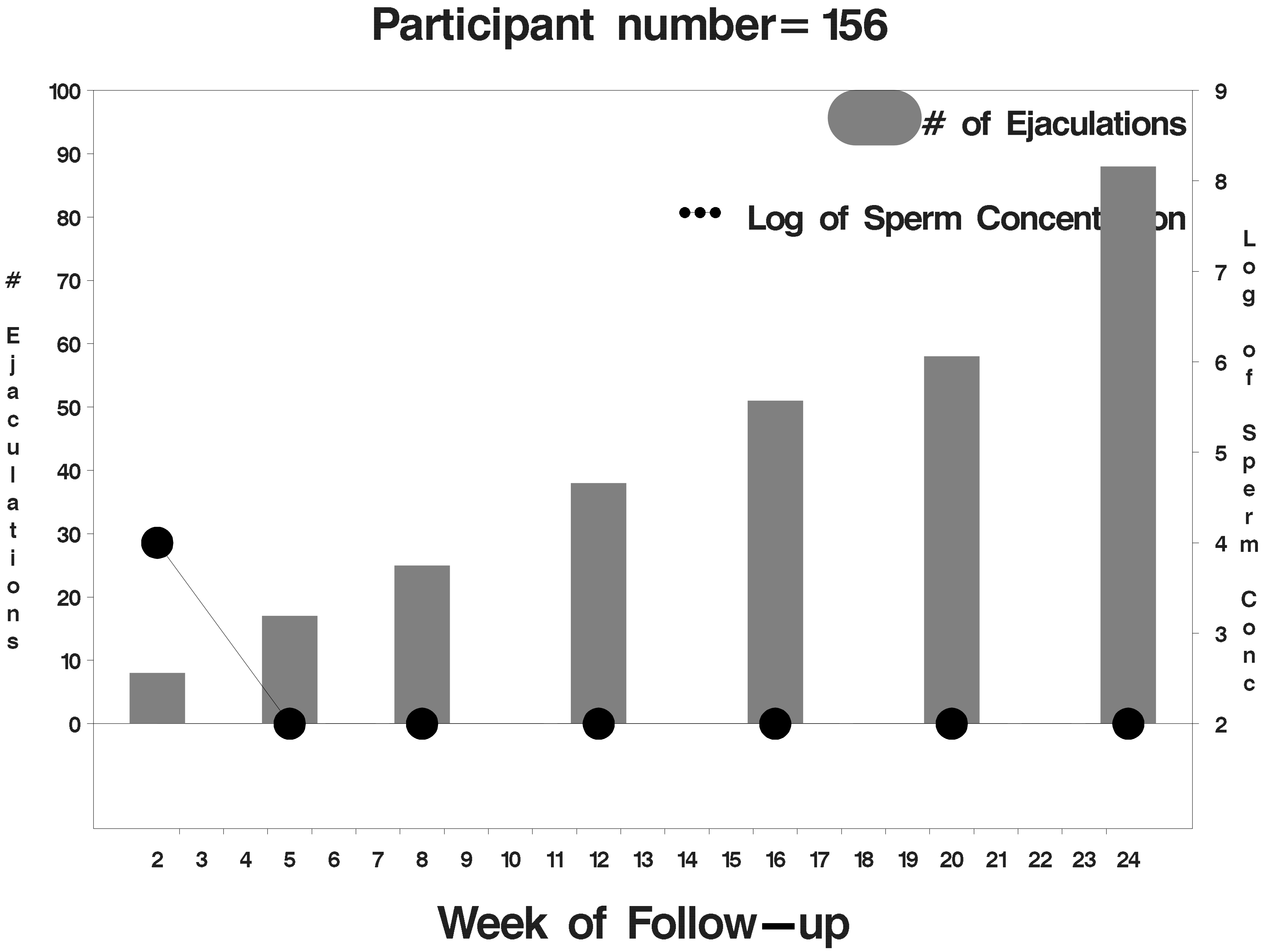

Participant number= 156
# of Ejaculations
Log of Sperm Concent[illegible]on
Ejaculations
Log of Sperm Conc
100
90
80
70
60
50
40
30
20
10
0
9
8
7
6
5
4
3
2
2 3 4 5 6 7 8 9 10 11 12 13 14 15 16 17 18 19 20 21 22 23 24
Week of Follow-up

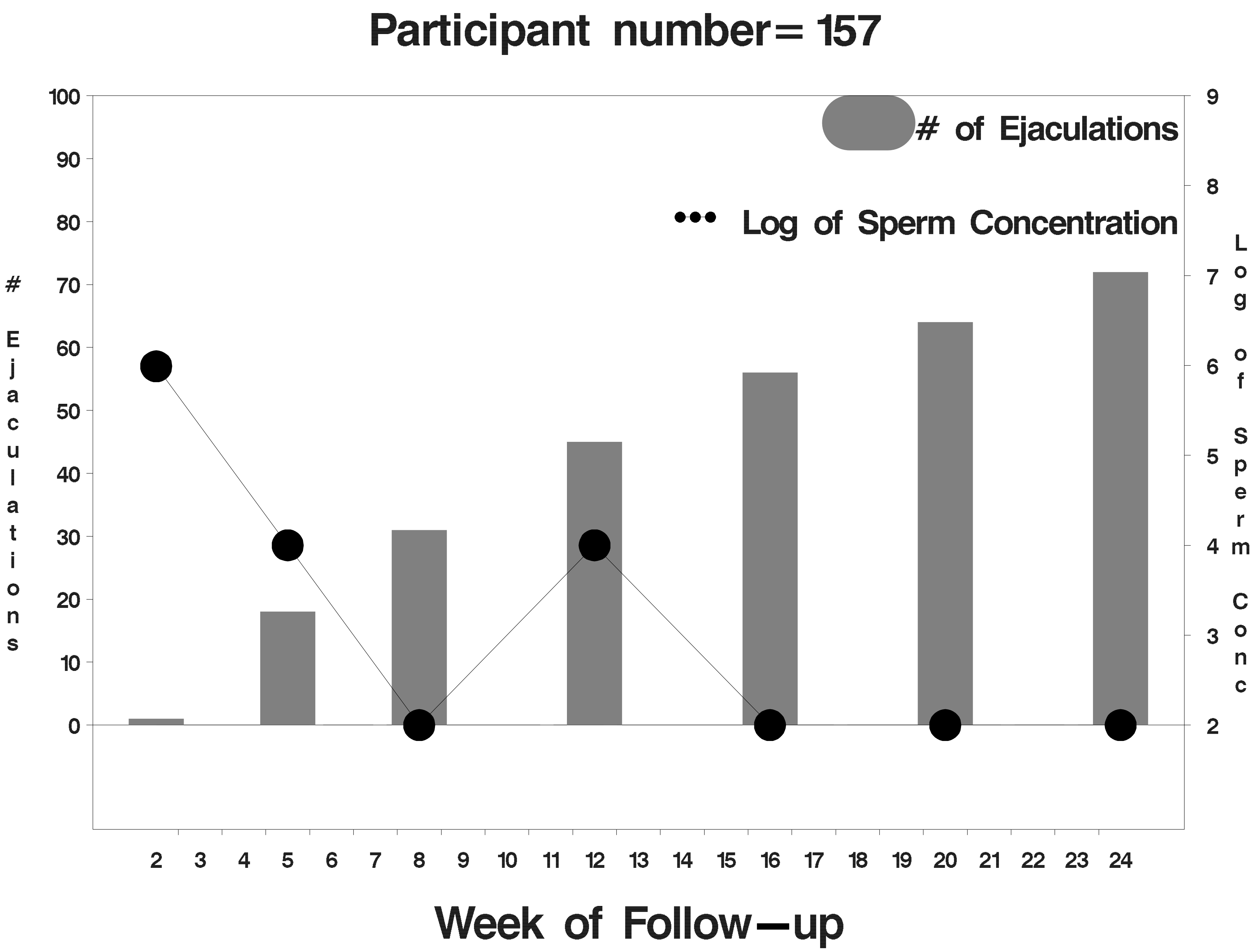
Participant number= 157
# of Ejaculations
Log of Sperm Concentration
Ejaculations
Log of Sperm Conc
Week of Follow-up
100
90
80
70
60
50
40
30
20
10
0
9
8
7
6
5
4
3
2
2 3 4 5 6 7 8 9 10 11 12 13 14 15 16 17 18 19 20 21 22 23 24

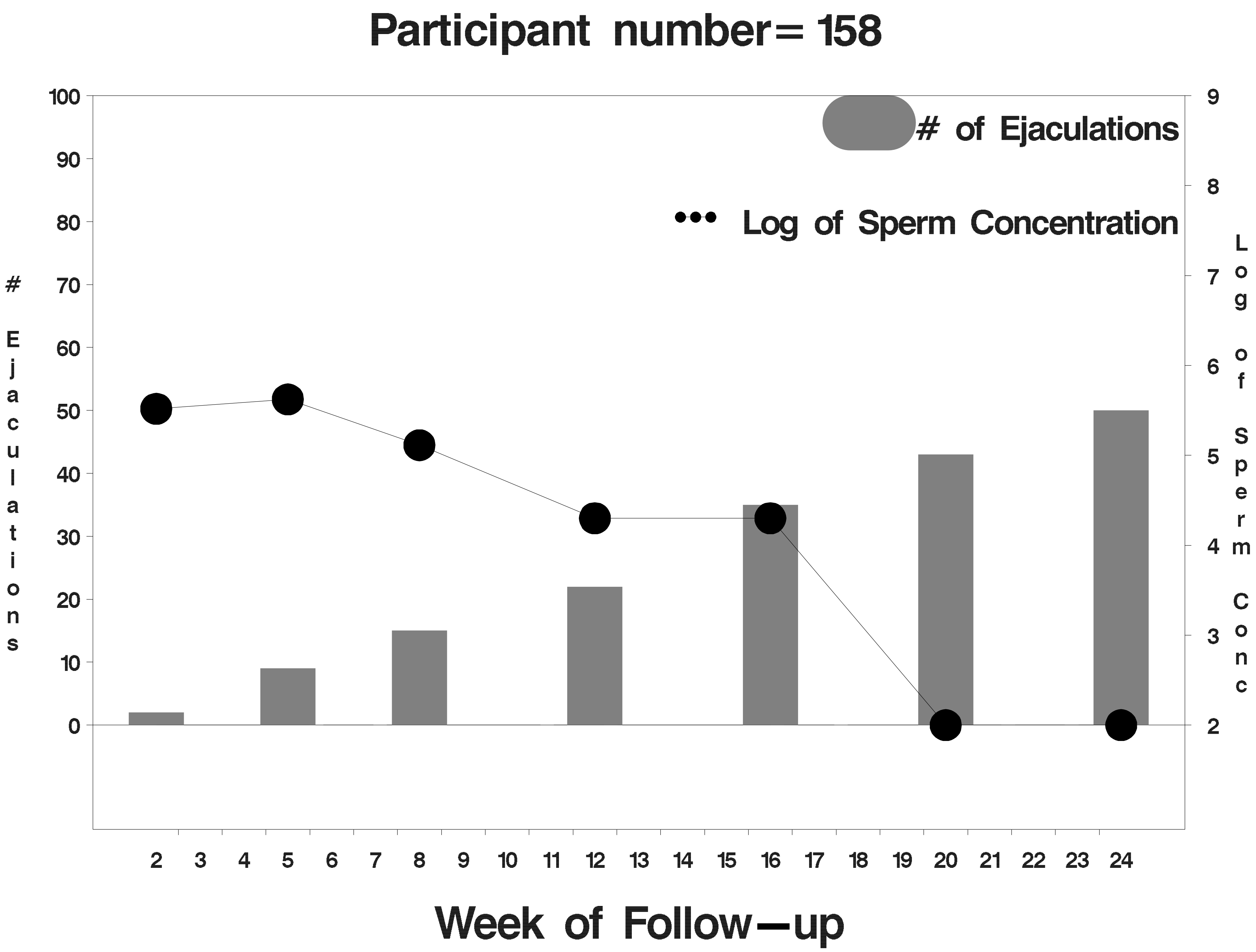
Participant number= 158
# of Ejaculations
Log of Sperm Concentration
Ejaculations
Log of Sperm Conc
Week of Follow-up
100
90
80
70
60
50
40
30
20
10
0
9
8
7
6
5
4
3
2
2 3 4 5 6 7 8 9 10 11 12 13 14 15 16 17 18 19 20 21 22 23 24

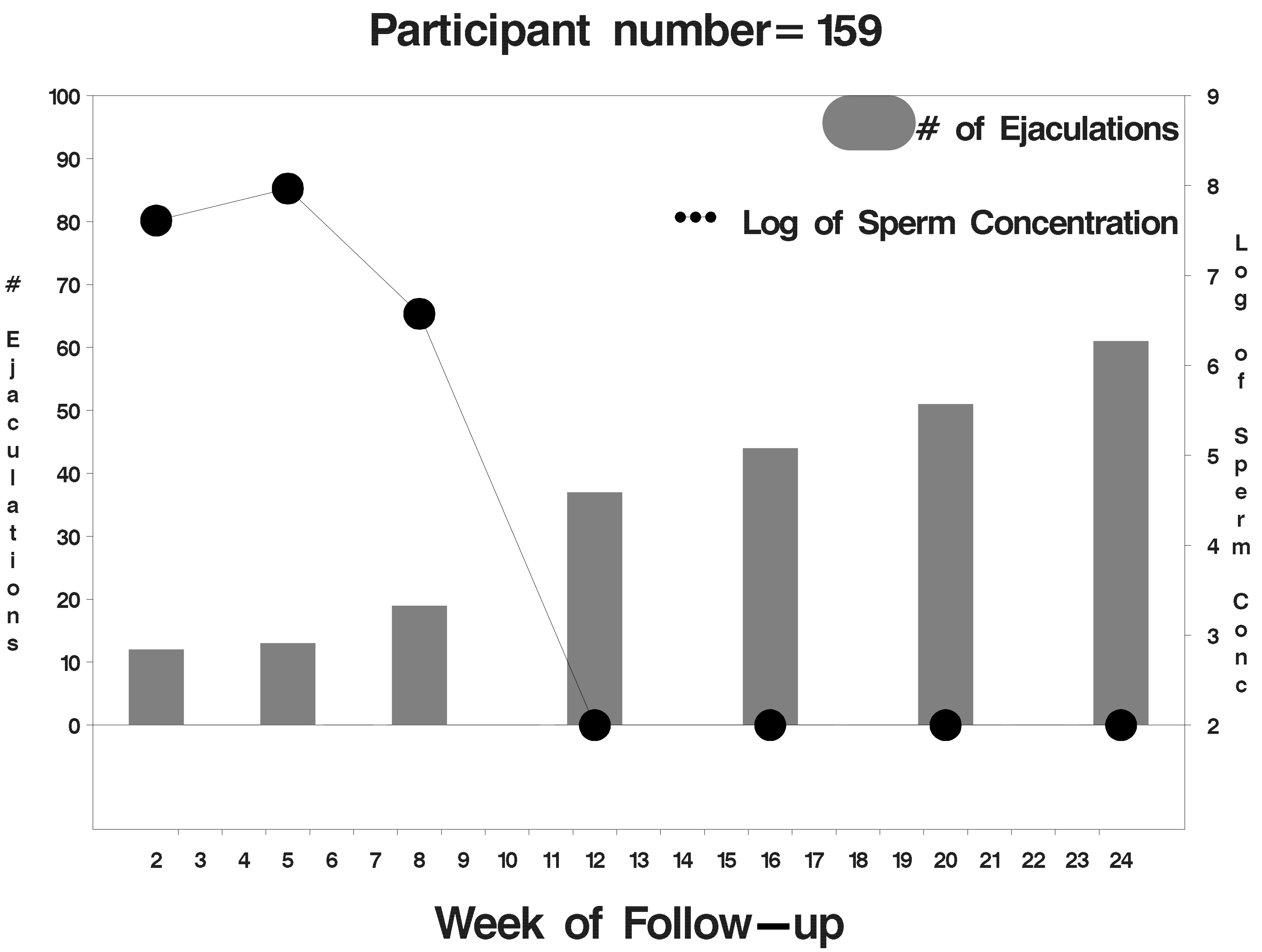

Participant number= 159
# of Ejaculations
Log of Sperm Concentration
Ejaculations
Log of Sperm Conc
100
90
80
70
60
50
40
30
20
10
0
9
8
7
6
5
4
3
2
2 3 4 5 6 7 8 9 10 11 12 13 14 15 16 17 18 19 20 21 22 23 24
Week of Follow-up

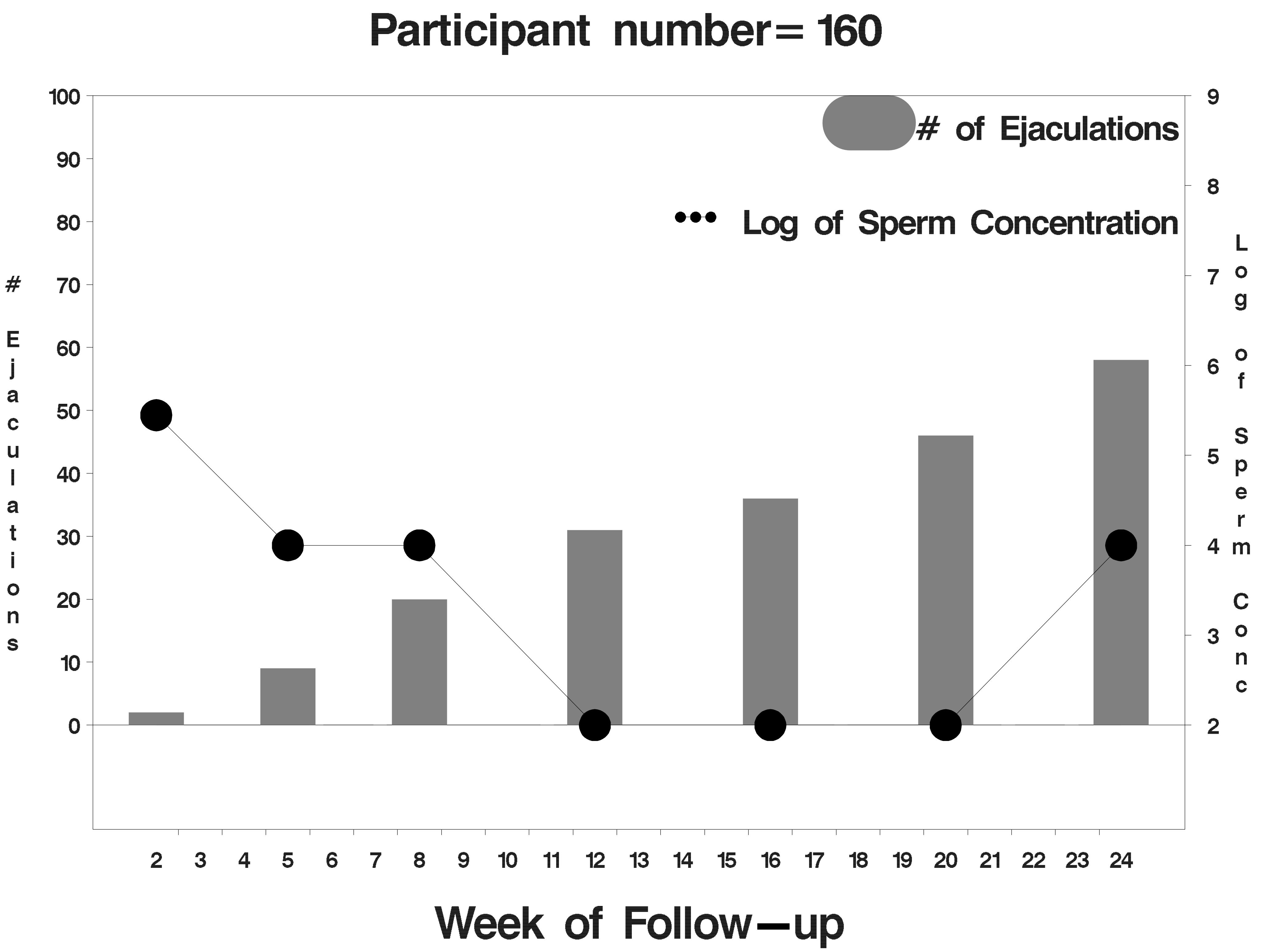

Participant number= 160
# of Ejaculations
Log of Sperm Concentration
Ejaculations
Log of Sperm Conc
100
90
80
70
60
50
40
30
20
10
0
9
8
7
6
5
4
3
2
2 3 4 5 6 7 8 9 10 11 12 13 14 15 16 17 18 19 20 21 22 23 24
Week of Follow-up

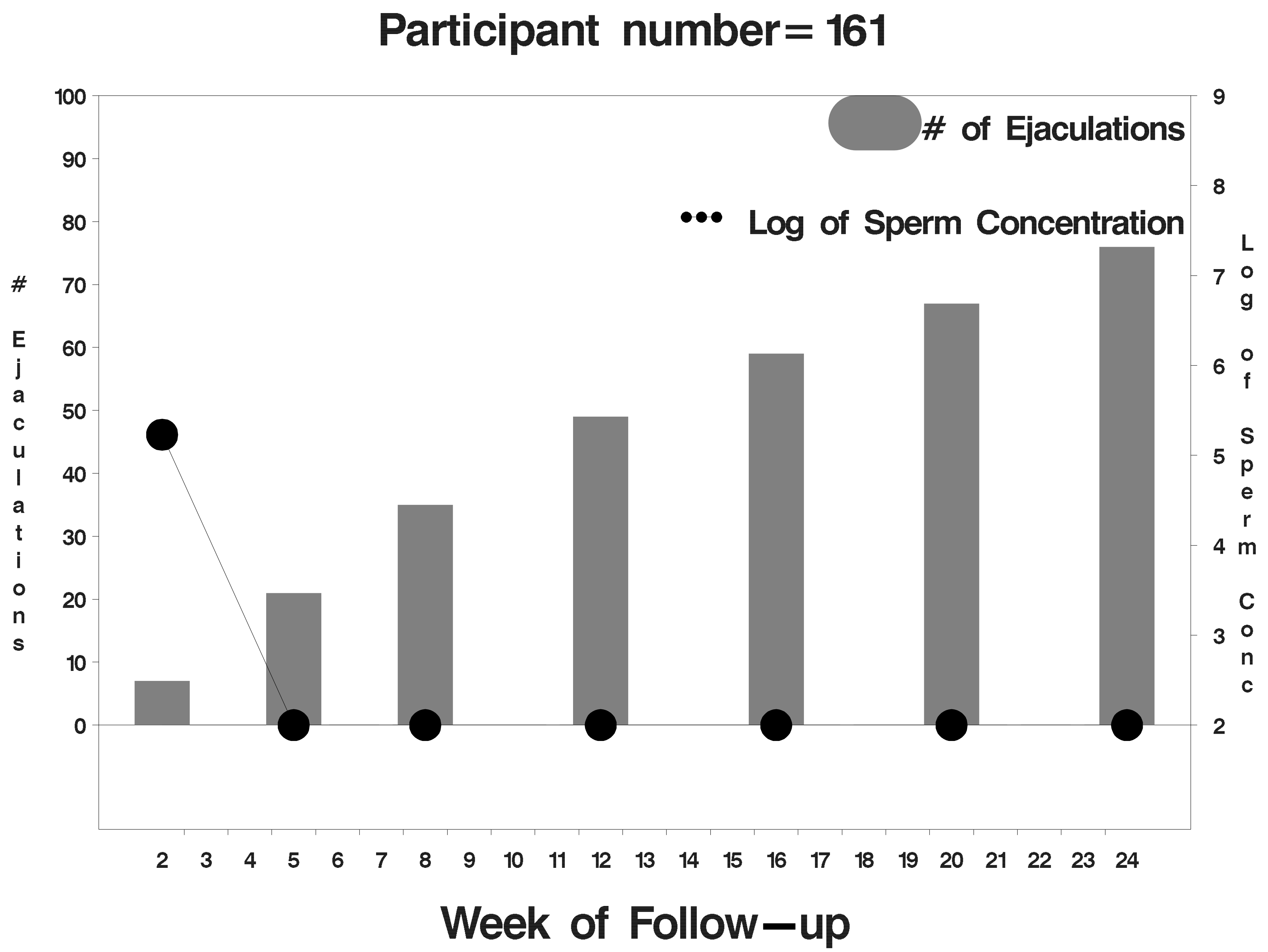

Participant number= 161
# of Ejaculations
Log of Sperm Concentration
Ejaculations
Log of Sperm Conc
100
90
80
70
60
50
40
30
20
10
0
9
8
7
6
5
4
3
2
2 3 4 5 6 7 8 9 10 11 12 13 14 15 16 17 18 19 20 21 22 23 24
Week of Follow-up

Participant number= 162

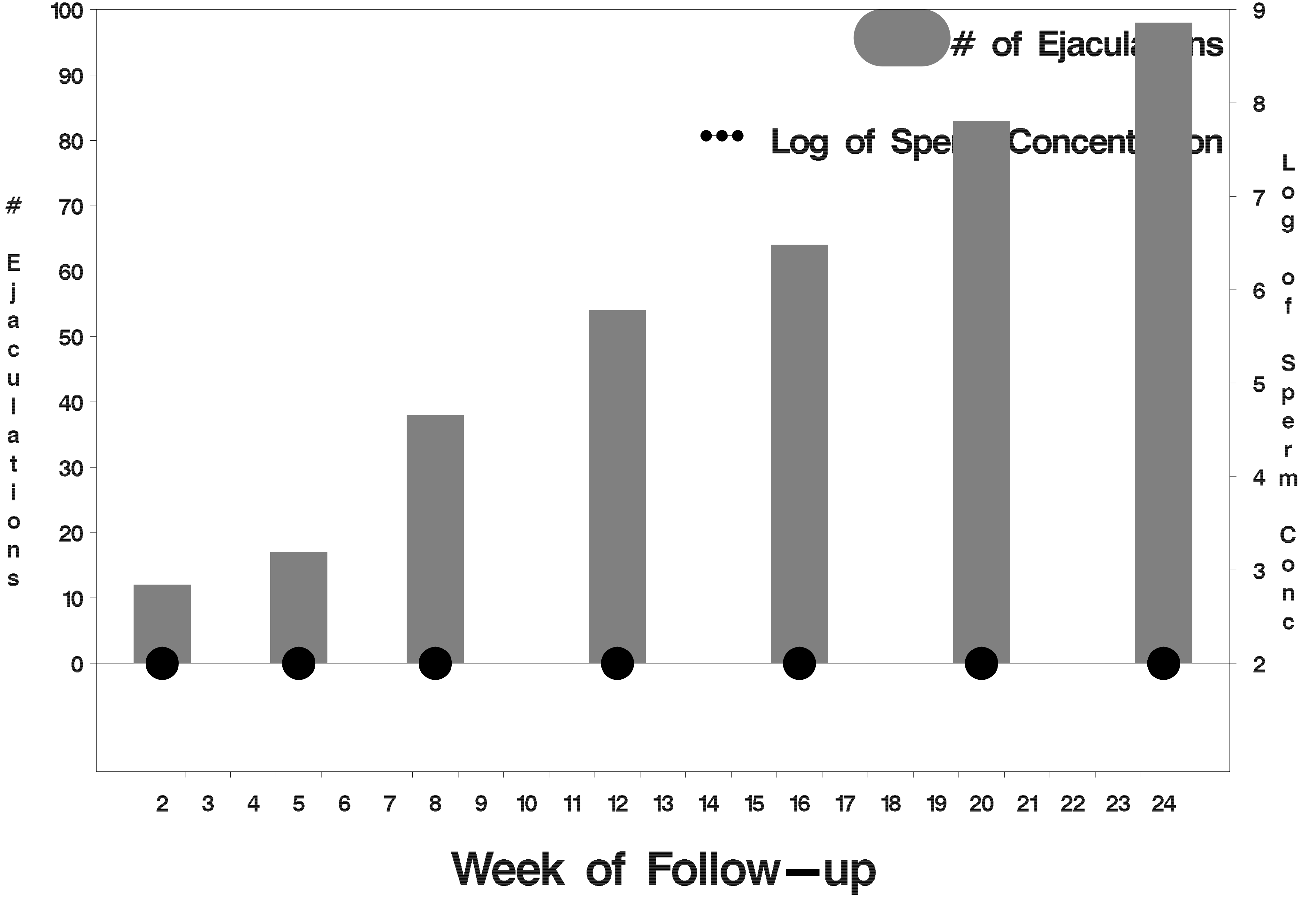

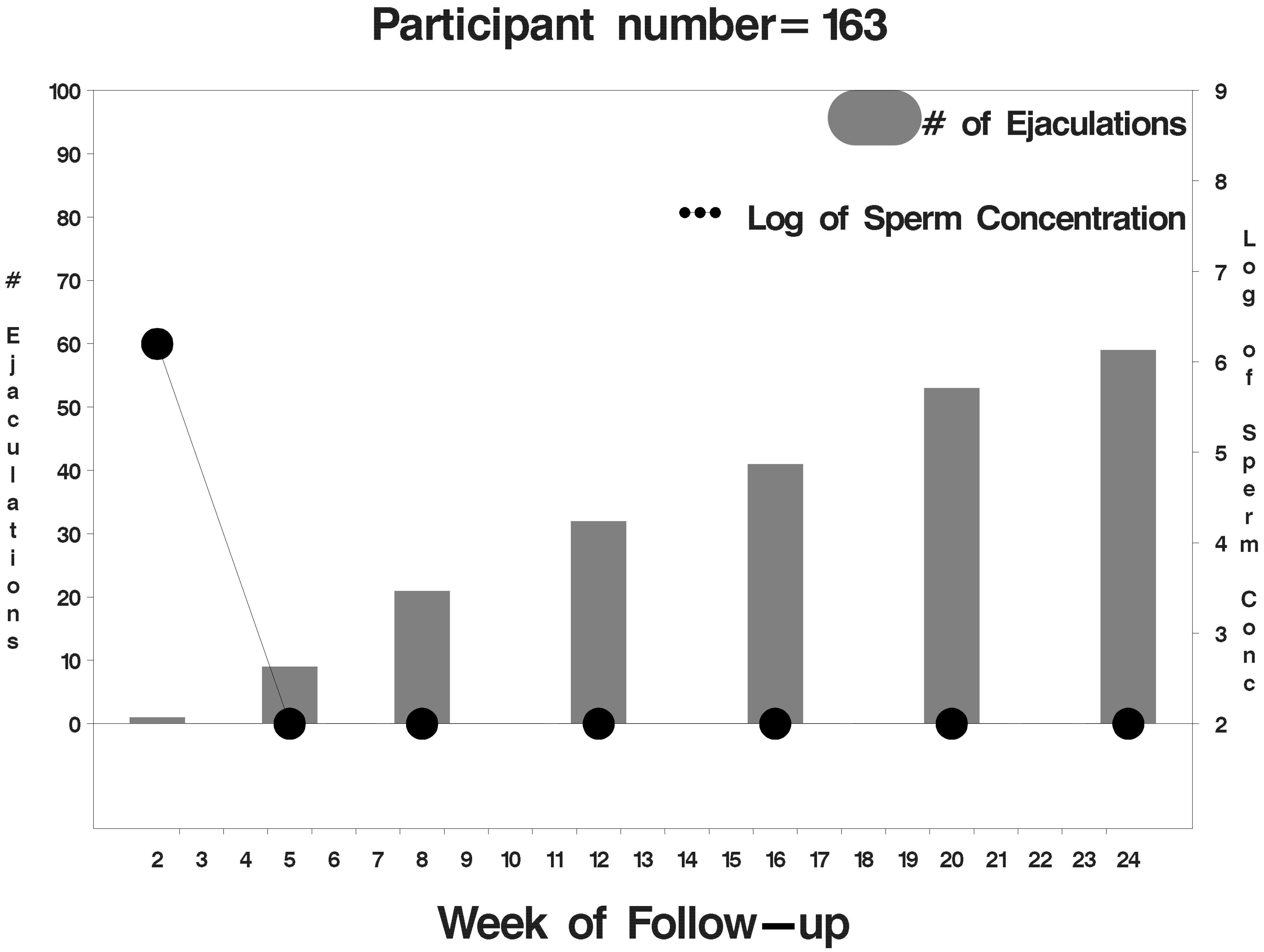

Participant number= 163
# of Ejaculations
Log of Sperm Concentration
Ejaculations
Log of Sperm Conc
100
90
80
70
60
50
40
30
20
10
0
9
8
7
6
5
4
3
2
2 3 4 5 6 7 8 9 10 11 12 13 14 15 16 17 18 19 20 21 22 23 24
Week of Follow—up

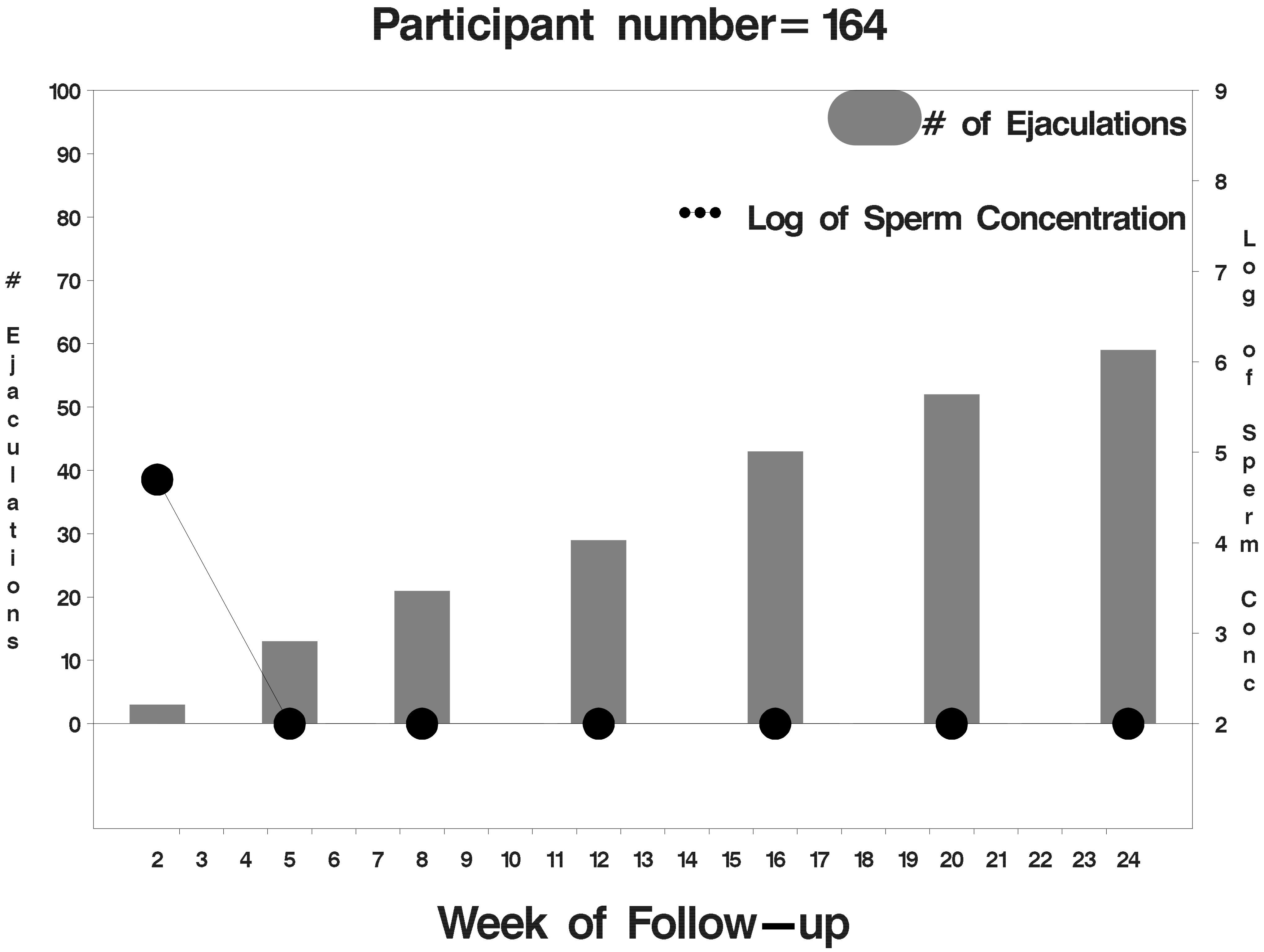

Participant number= 164
# of Ejaculations
Log of Sperm Concentration
Ejaculations
Log of Sperm Conc
100
90
80
70
60
50
40
30
20
10
0
9
8
7
6
5
4
3
2
2 3 4 5 6 7 8 9 10 11 12 13 14 15 16 17 18 19 20 21 22 23 24
Week of Follow-up

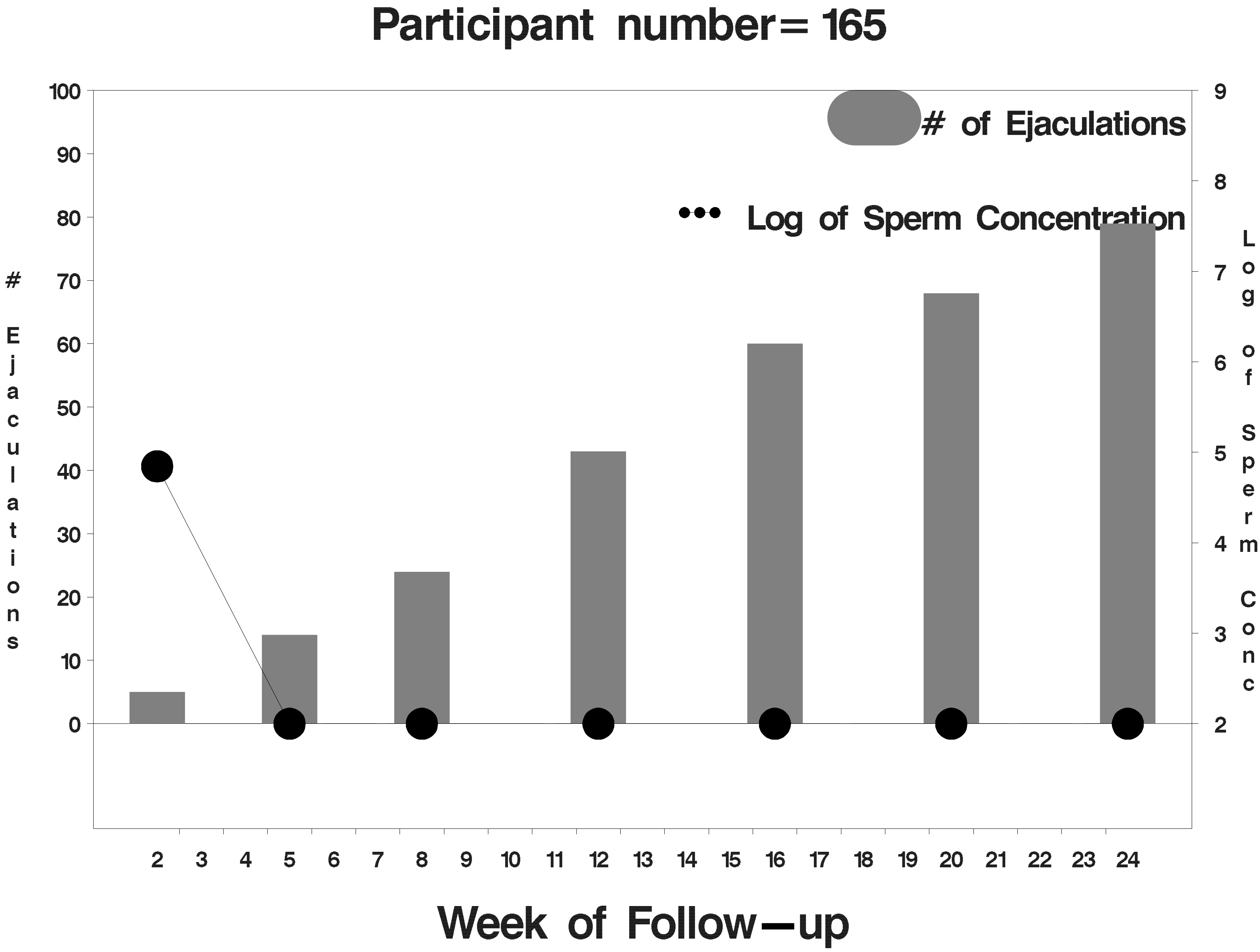

Participant number= 165
# of Ejaculations
Log of Sperm Concentration
Ejaculations
Log of Sperm Conc
100
90
80
70
60
50
40
30
20
10
0
9
8
7
6
5
4
3
2
2 3 4 5 6 7 8 9 10 11 12 13 14 15 16 17 18 19 20 21 22 23 24
Week of Follow-up

Participant number= 166

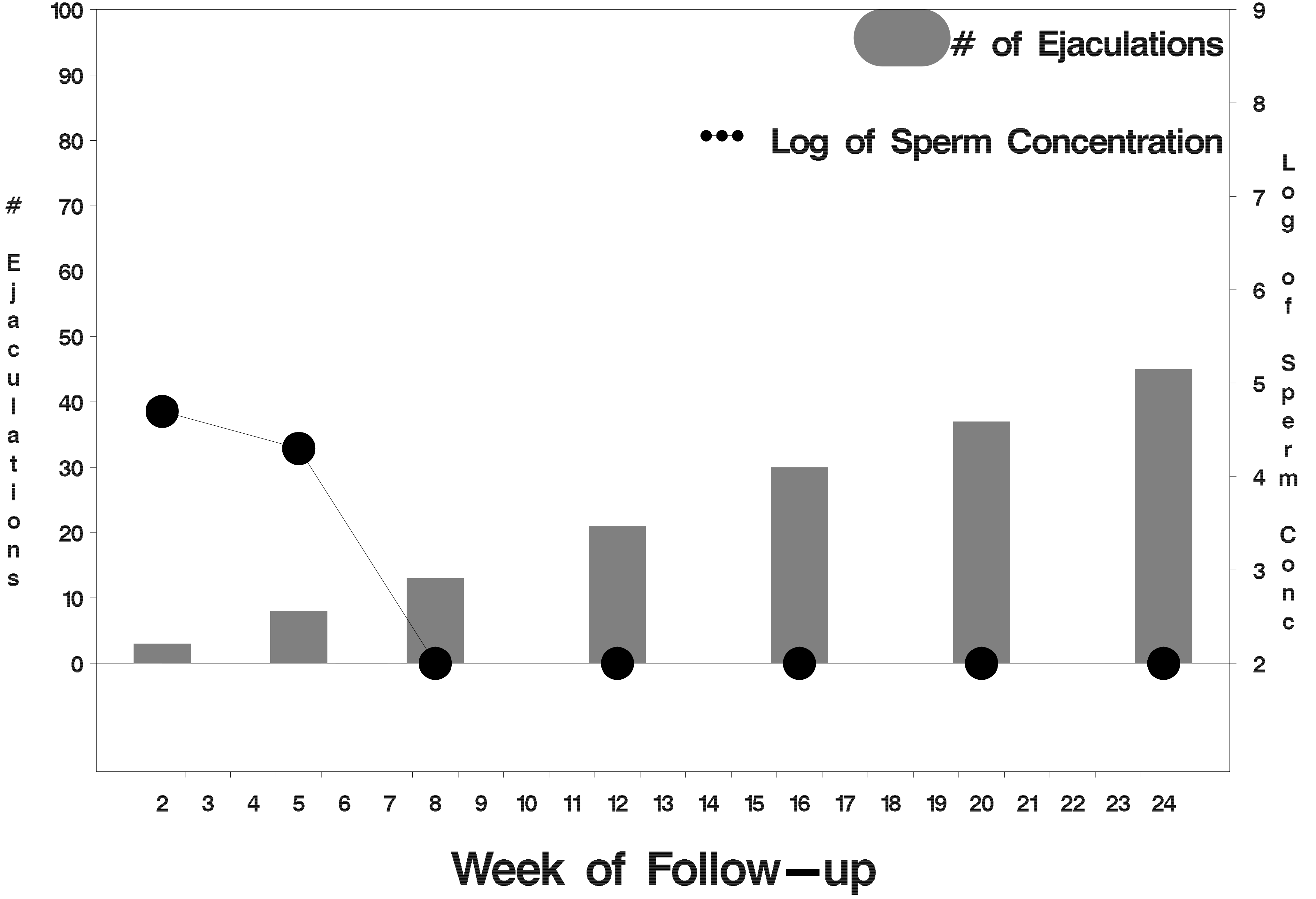

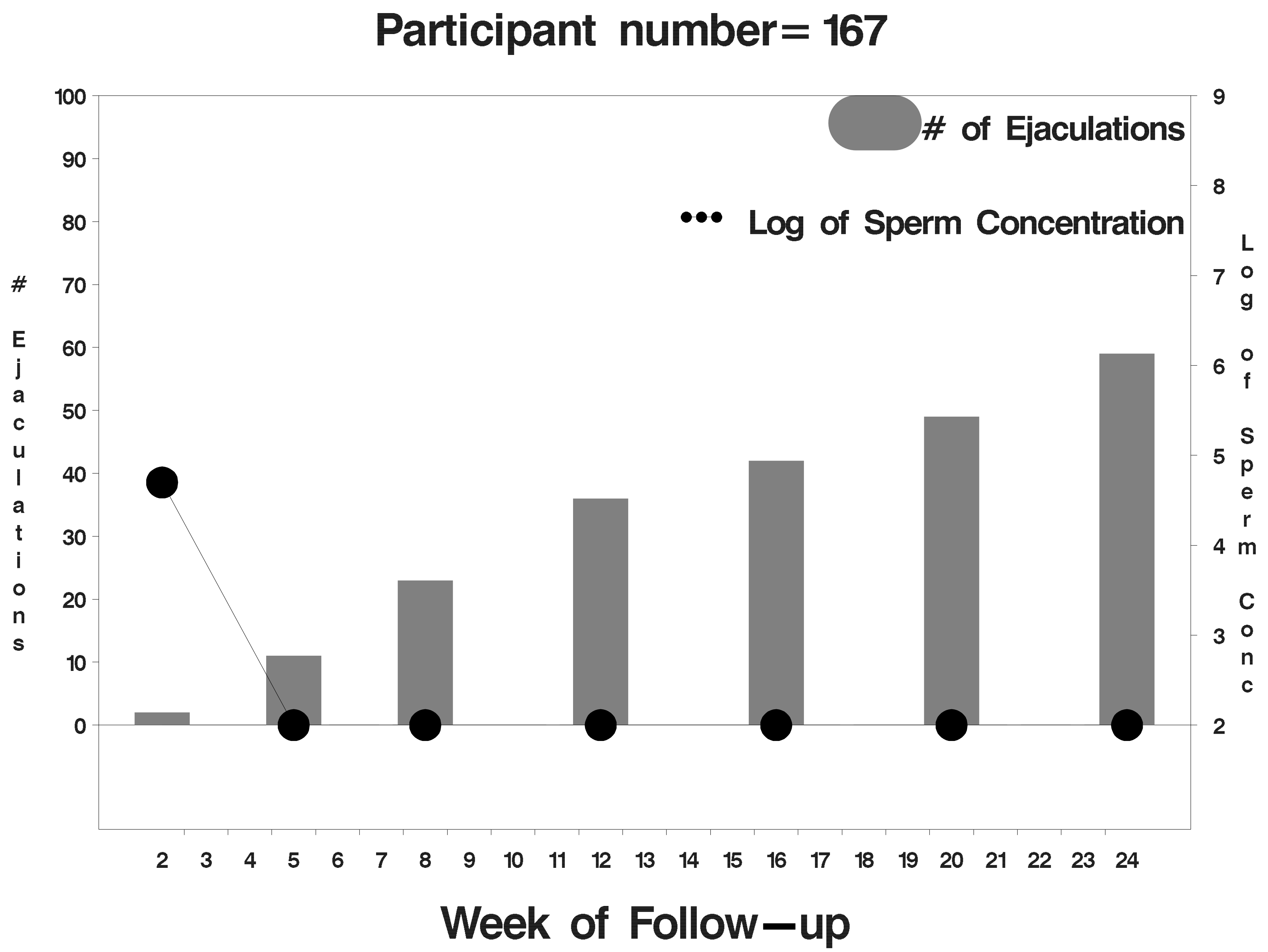

Participant number= 167
# of Ejaculations
Log of Sperm Concentration
Ejaculations
Log of Sperm Conc
100
90
80
70
60
50
40
30
20
10
0
9
8
7
6
5
4
3
2
2 3 4 5 6 7 8 9 10 11 12 13 14 15 16 17 18 19 20 21 22 23 24
Week of Follow-up

Participant number= 168

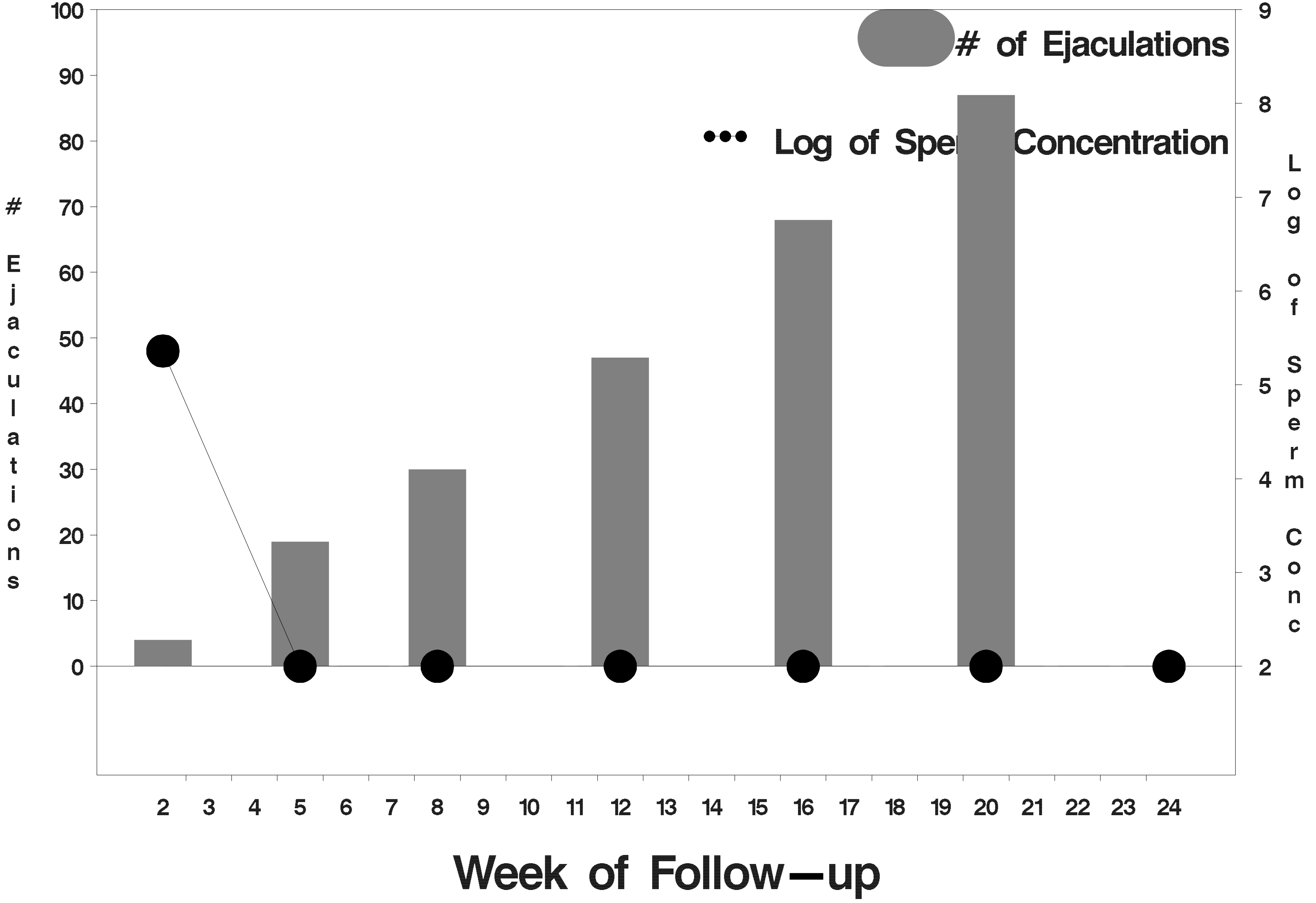

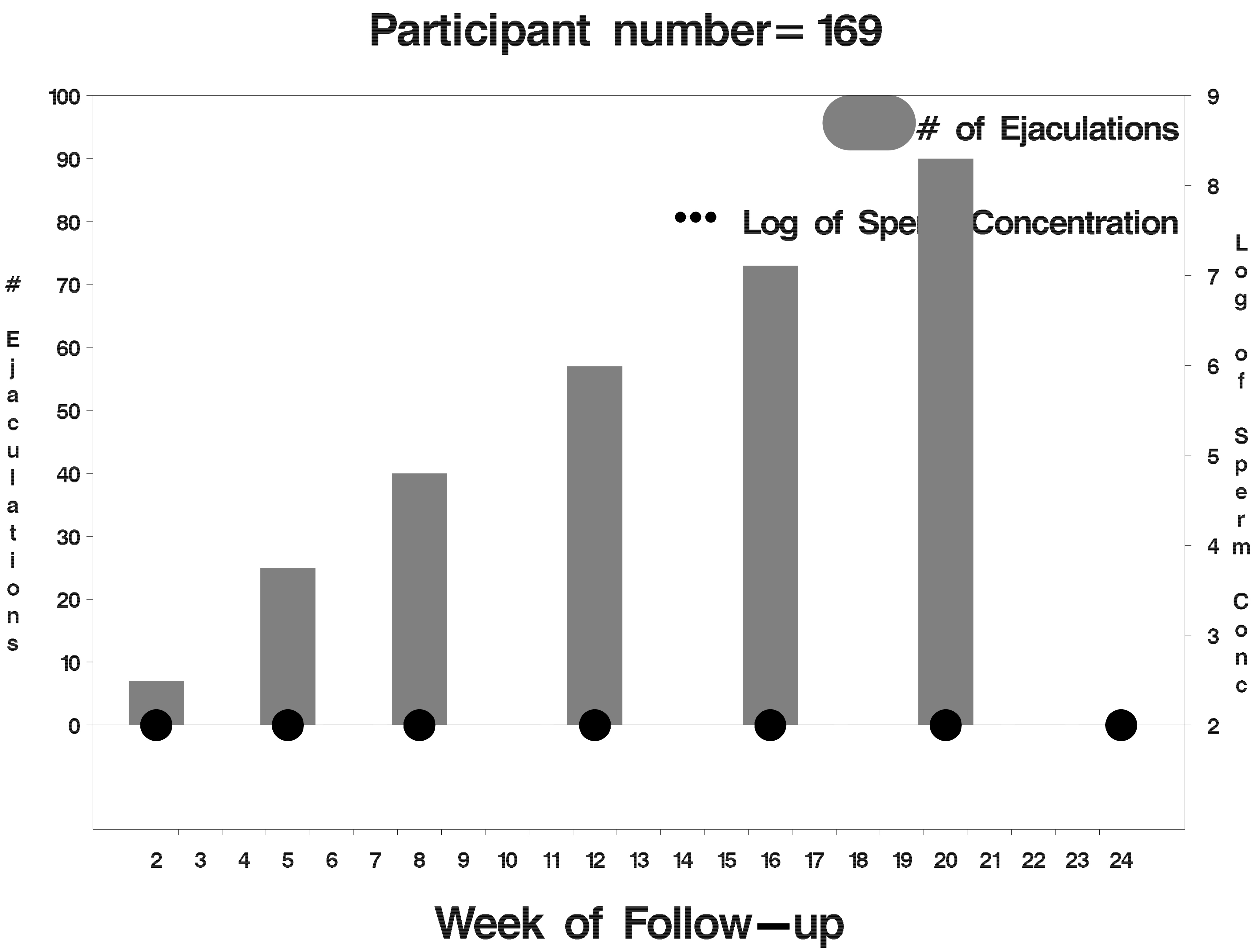

Participant number= 169
# of Ejaculations
Log of Sperm Concentration
Ejaculations
Log of Sperm Conc
100
90
80
70
60
50
40
30
20
10
0
9
8
7
6
5
4
3
2
2 3 4 5 6 7 8 9 10 11 12 13 14 15 16 17 18 19 20 21 22 23 24
Week of Follow-up

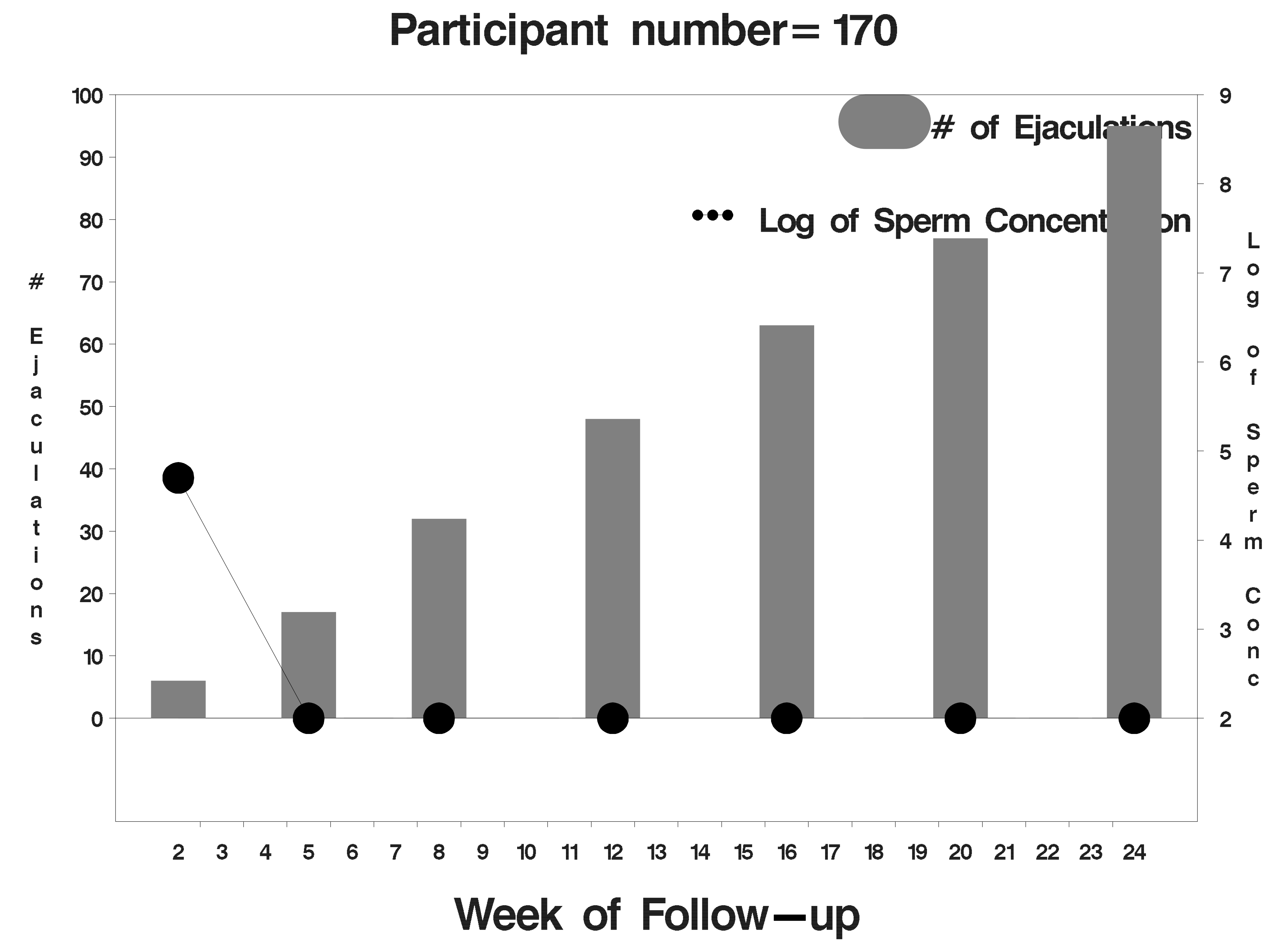

Participant number= 170
# of Ejaculations
Log of Sperm Concentration
Ejaculations
Log of Sperm Conc
100
90
80
70
60
50
40
30
20
10
0
9
8
7
6
5
4
3
2
2 3 4 5 6 7 8 9 10 11 12 13 14 15 16 17 18 19 20 21 22 23 24
Week of Follow-up

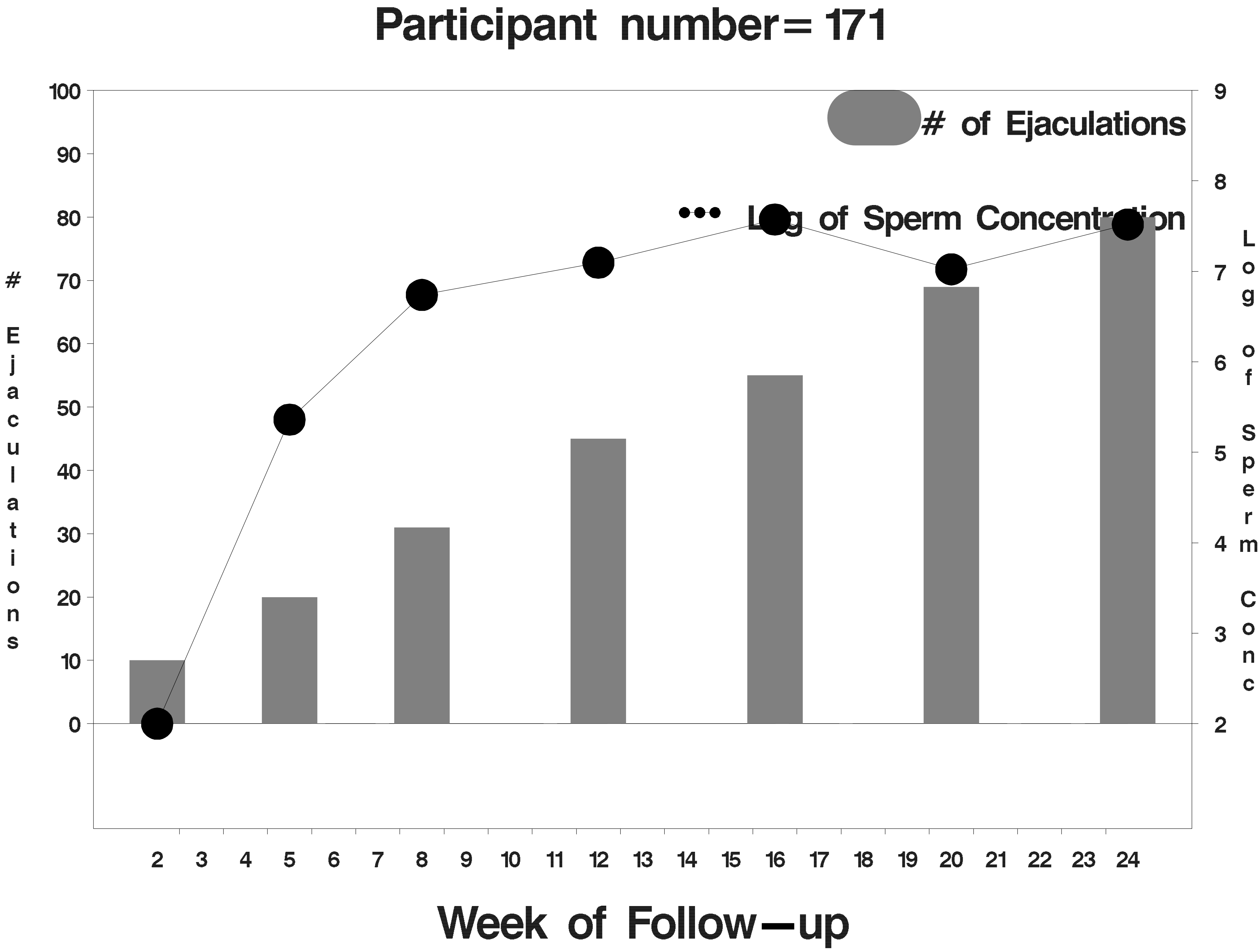

Participant number= 171
# of Ejaculations
Log of Sperm Concentration
Ejaculations
Log of Sperm Conc
100
90
80
70
60
50
40
30
20
10
0
9
8
7
6
5
4
3
2
2 3 4 5 6 7 8 9 10 11 12 13 14 15 16 17 18 19 20 21 22 23 24
Week of Follow-up

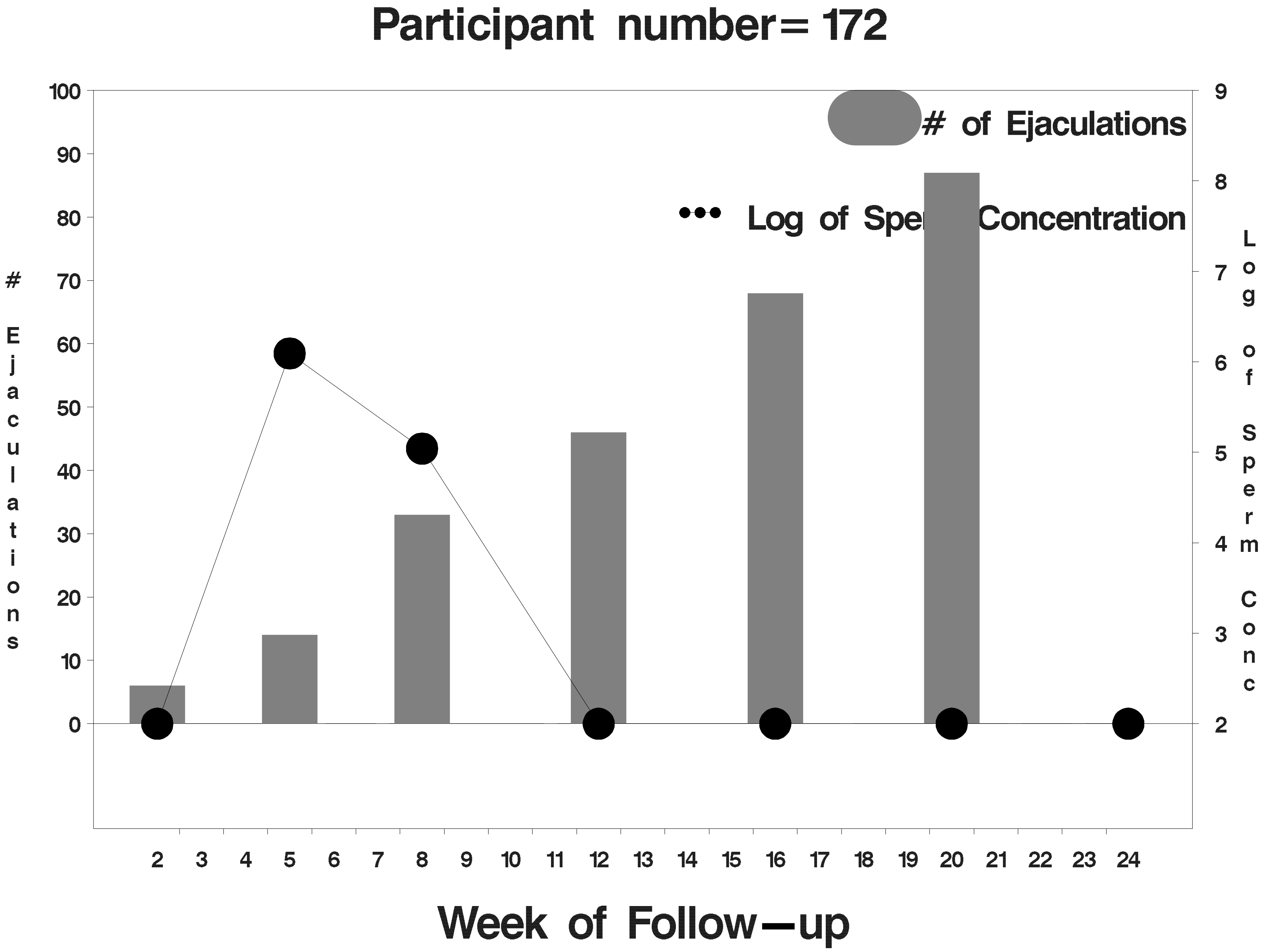

Participant number= 172
# of Ejaculations
Log of Sperm Concentration
Ejaculations
Log of Sperm Conc
100
90
80
70
60
50
40
30
20
10
0
9
8
7
6
5
4
3
2
2 3 4 5 6 7 8 9 10 11 12 13 14 15 16 17 18 19 20 21 22 23 24
Week of Follow-up

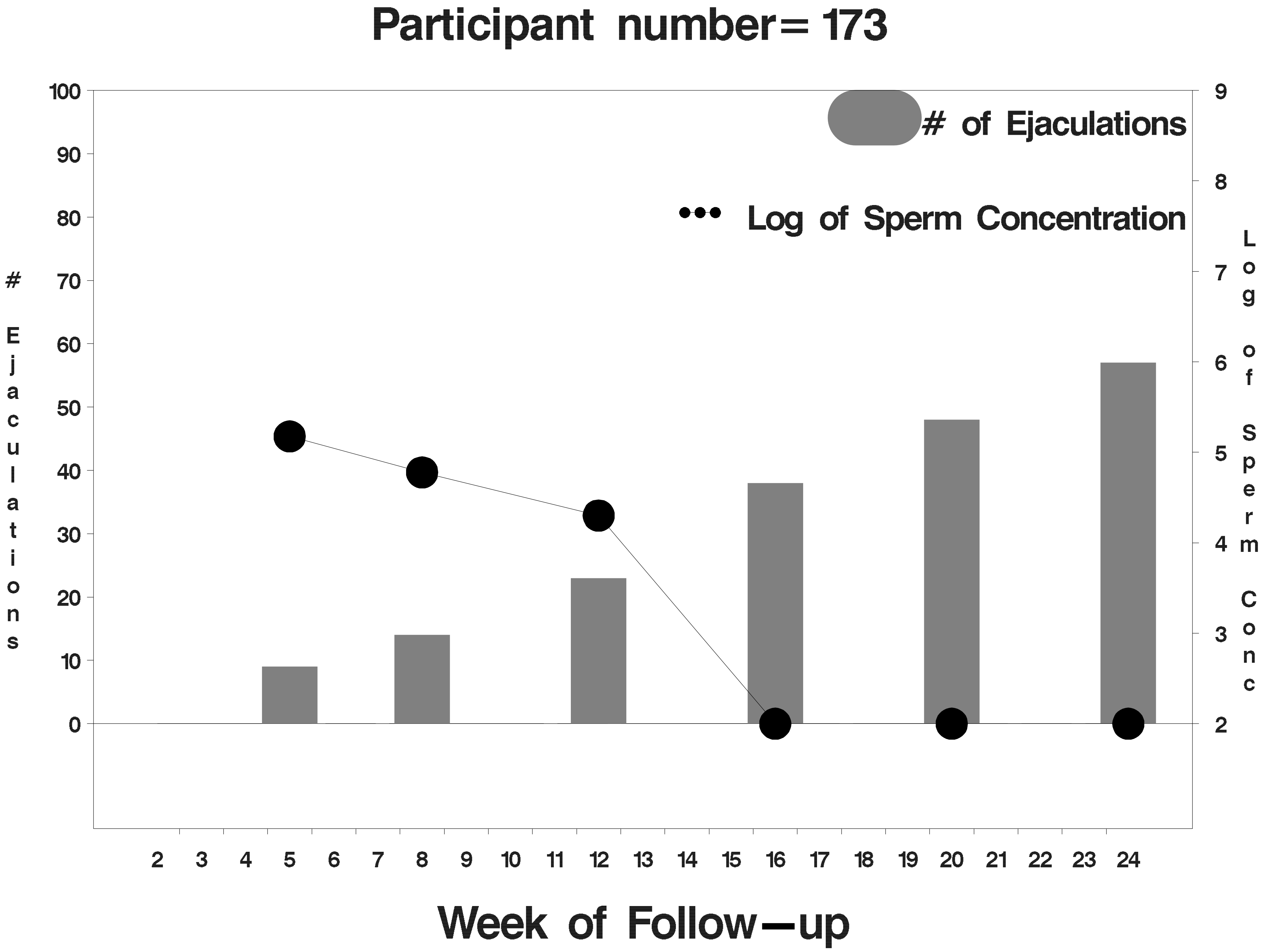
Participant number= 173
# of Ejaculations
Log of Sperm Concentration
Ejaculations
Log of Sperm Conc
100
90
80
70
60
50
40
30
20
10
0
9
8
7
6
5
4
3
2
2 3 4 5 6 7 8 9 10 11 12 13 14 15 16 17 18 19 20 21 22 23 24
Week of Follow-up

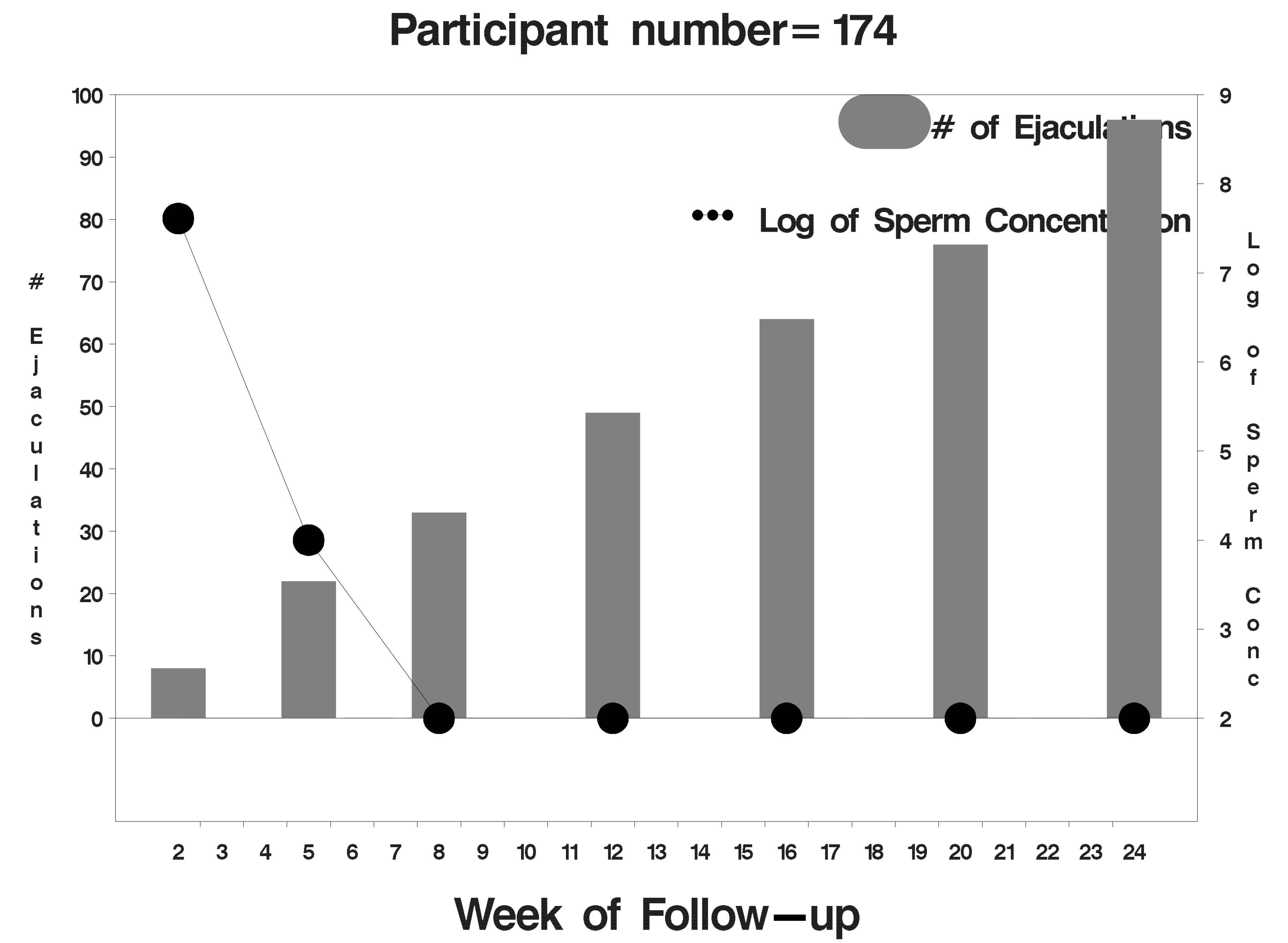

Participant number= 174
# of Ejaculations
Log of Sperm Concentration
Ejaculations
Log of Sperm Conc
100
90
80
70
60
50
40
30
20
10
0
9
8
7
6
5
4
3
2
2 3 4 5 6 7 8 9 10 11 12 13 14 15 16 17 18 19 20 21 22 23 24
Week of Follow-up

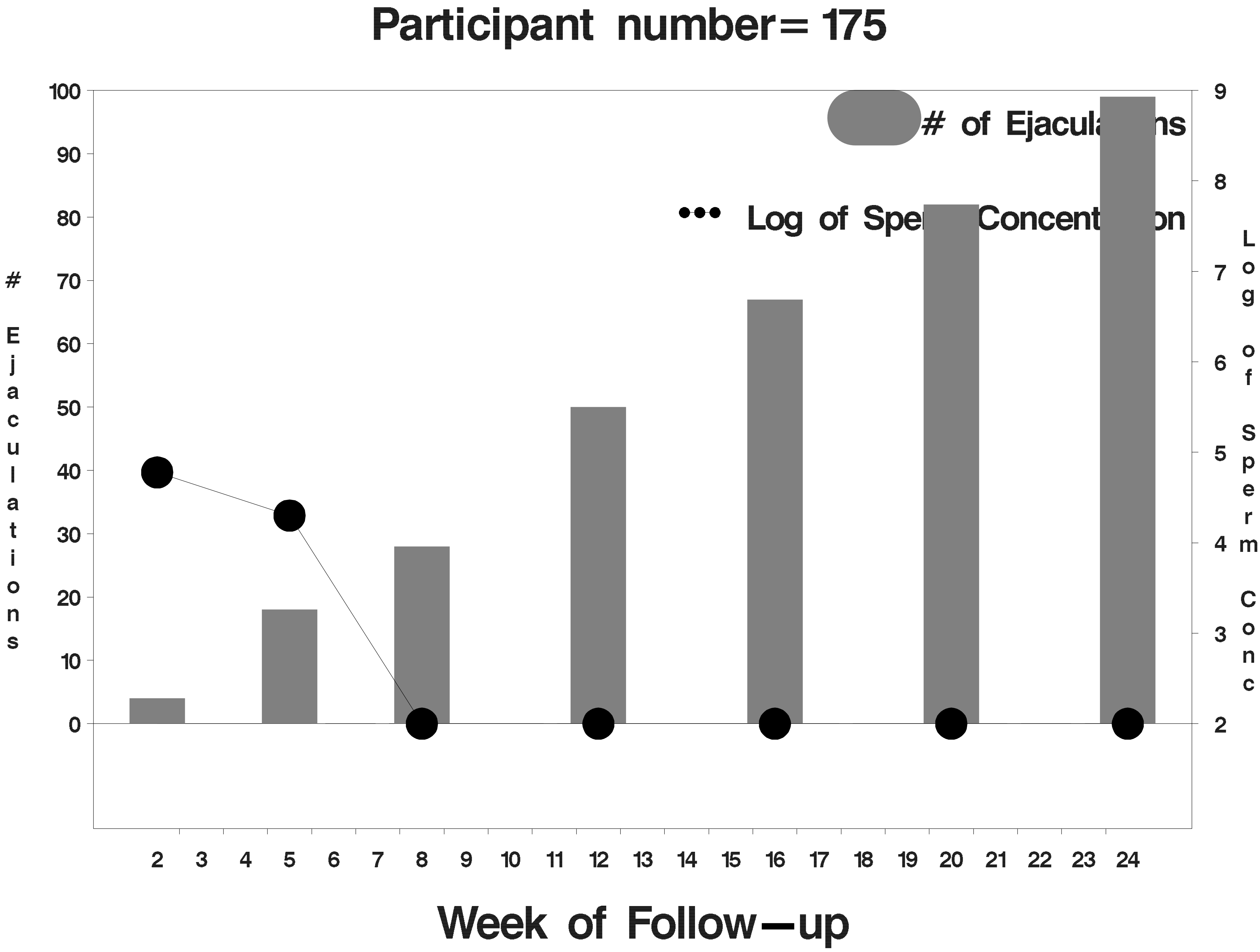
Participant number= 175
# of Ejaculations
Log of Sperm Concentration
Ejaculations
Log of Sperm Conc
100
90
80
70
60
50
40
30
20
10
0
9
8
7
6
5
4
3
2
2 3 4 5 6 7 8 9 10 11 12 13 14 15 16 17 18 19 20 21 22 23 24
Week of Follow-up

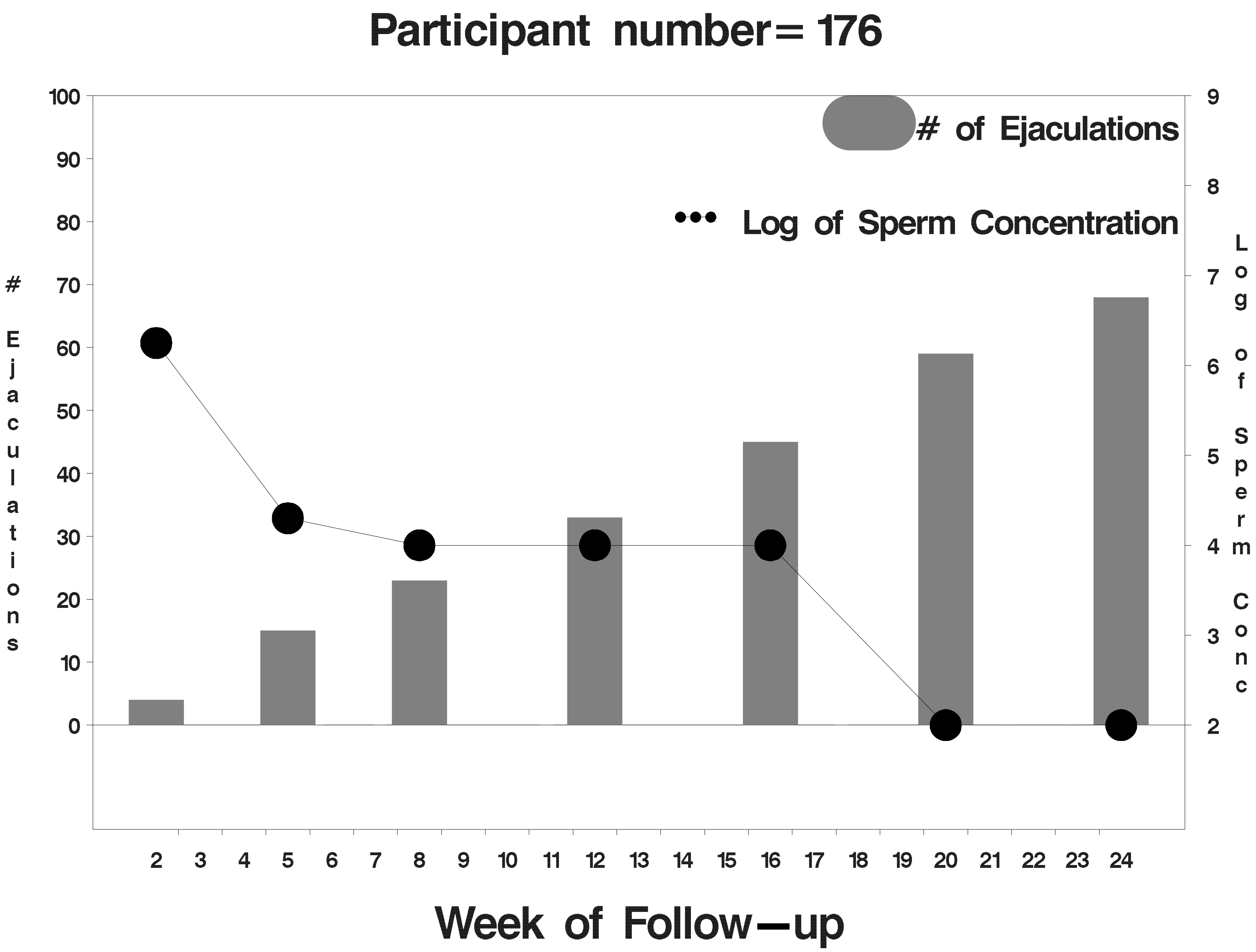

Participant number= 176
# of Ejaculations
Log of Sperm Concentration
Ejaculations
Log of Sperm Conc
0
10
20
30
40
50
60
70
80
90
100
2
3
4
5
6
7
8
9
2 3 4 5 6 7 8 9 10 11 12 13 14 15 16 17 18 19 20 21 22 23 24
Week of Follow-up

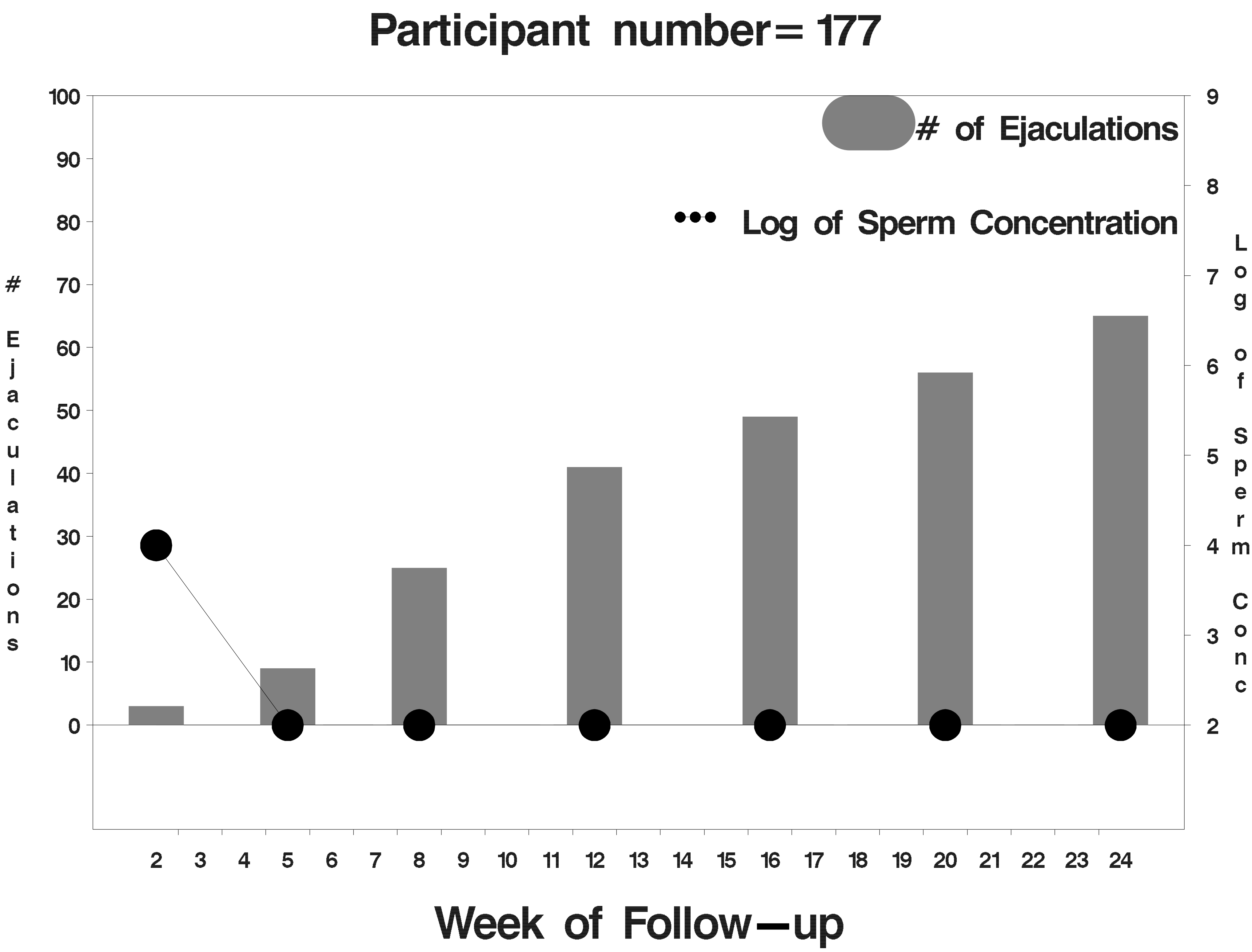

Participant number= 177
# of Ejaculations
Log of Sperm Concentration
Ejaculations
Log of Sperm Conc
100
90
80
70
60
50
40
30
20
10
0
9
8
7
6
5
4
3
2
2 3 4 5 6 7 8 9 10 11 12 13 14 15 16 17 18 19 20 21 22 23 24
Week of Follow-up

Participant number= 178

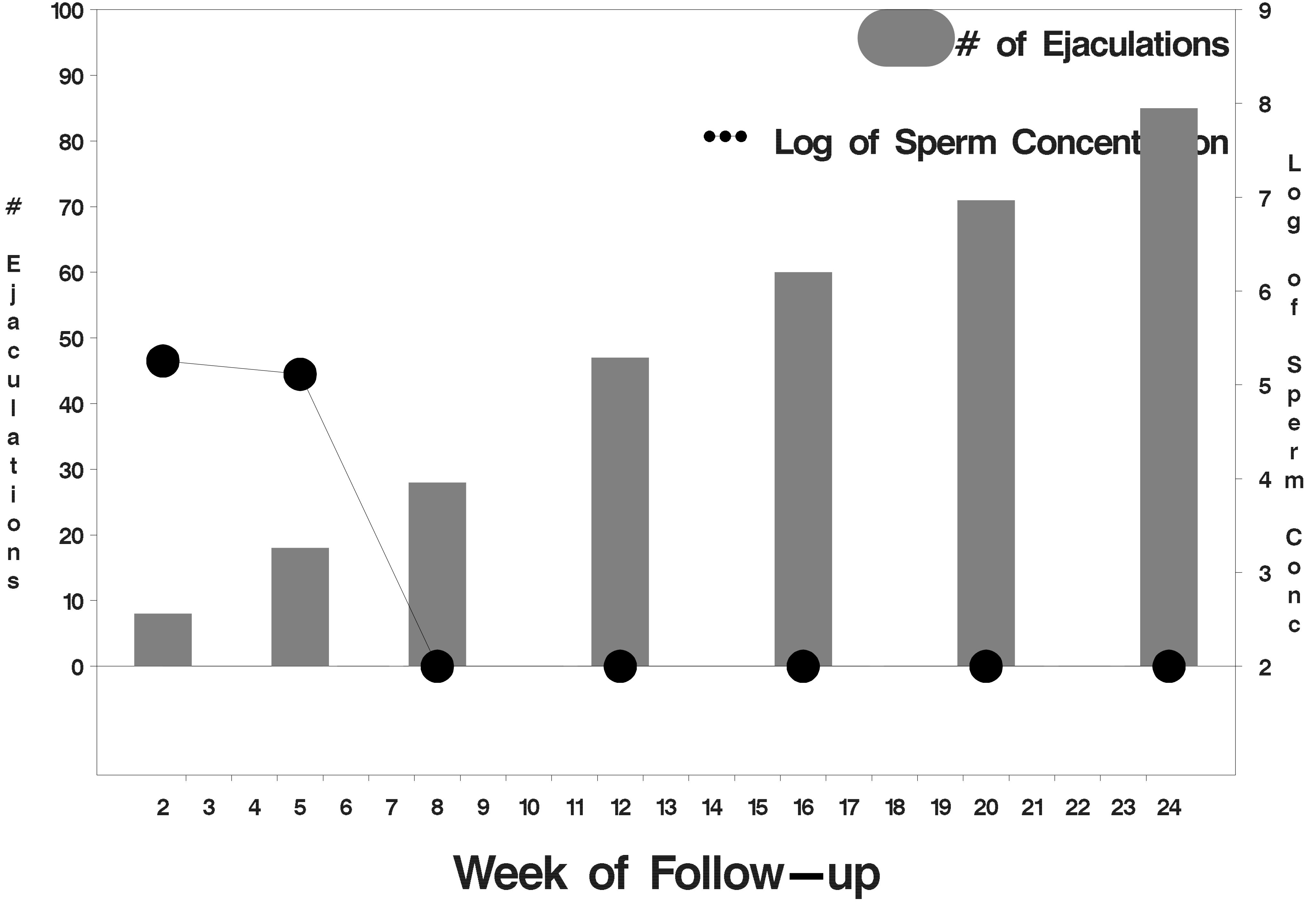

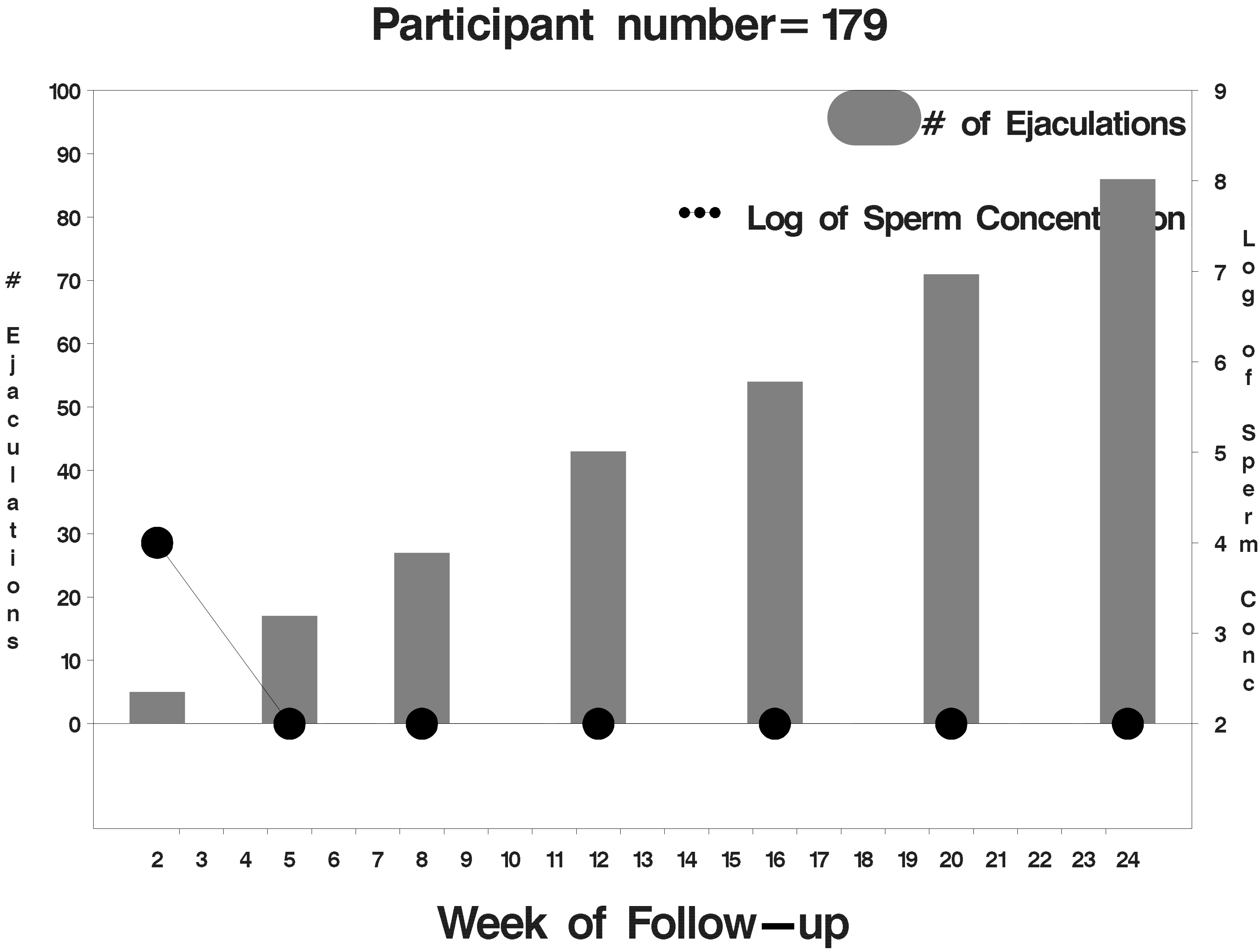

Participant number= 179
# of Ejaculations
Log of Sperm Concentration
Ejaculations
Log of Sperm Conc
100
90
80
70
60
50
40
30
20
10
0
9
8
7
6
5
4
3
2
2 3 4 5 6 7 8 9 10 11 12 13 14 15 16 17 18 19 20 21 22 23 24
Week of Follow-up

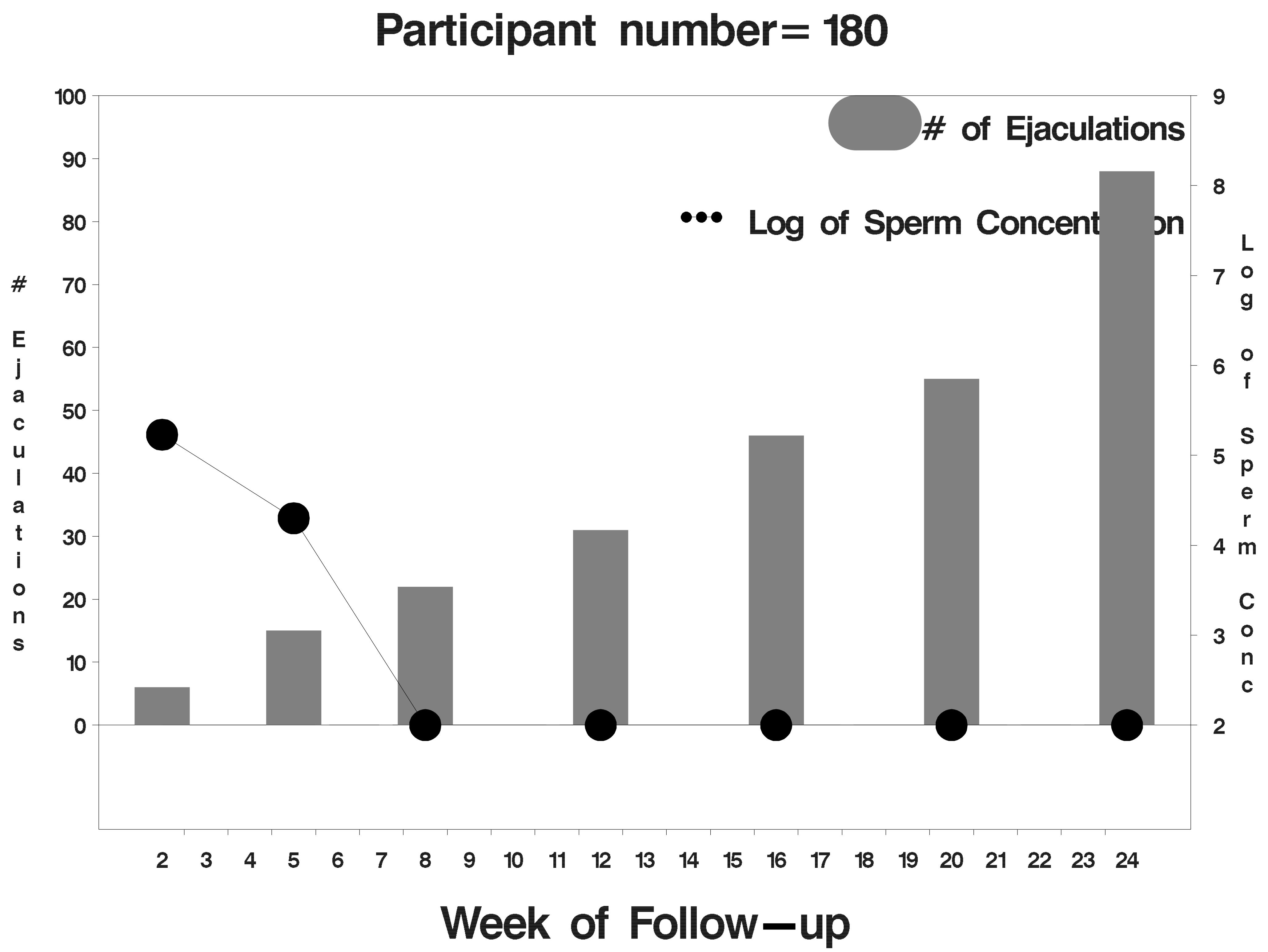

Participant number= 180
# of Ejaculations
Log of Sperm Concentration
Ejaculations
Log of Sperm Conc
100
90
80
70
60
50
40
30
20
10
0
9
8
7
6
5
4
3
2
2 3 4 5 6 7 8 9 10 11 12 13 14 15 16 17 18 19 20 21 22 23 24
Week of Follow—up

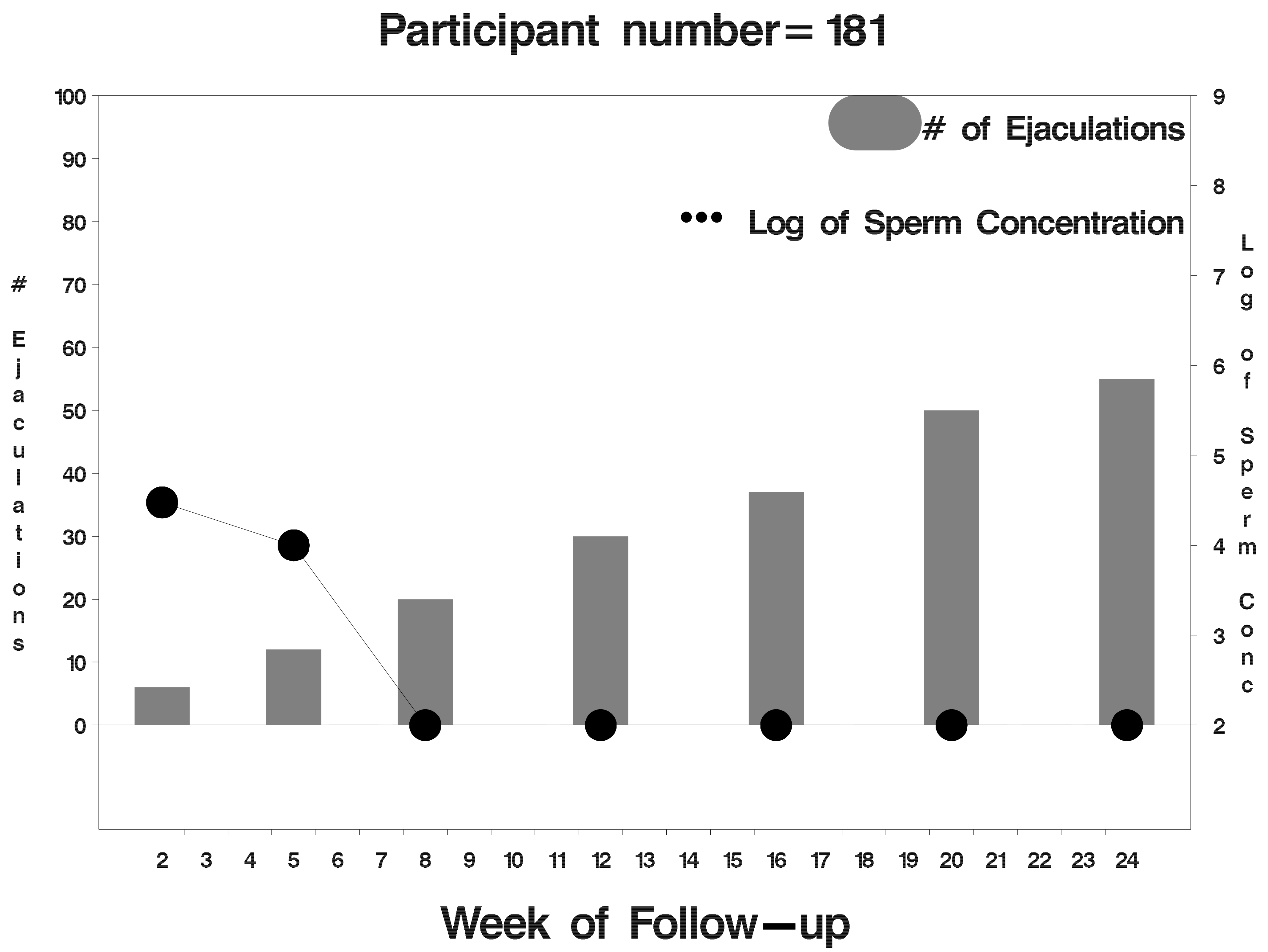
Participant number= 181
# of Ejaculations
Log of Sperm Concentration
Ejaculations
Log of Sperm Conc
100
90
80
70
60
50
40
30
20
10
0
9
8
7
6
5
4
3
2
2 3 4 5 6 7 8 9 10 11 12 13 14 15 16 17 18 19 20 21 22 23 24
Week of Follow-up

Participant number= 182

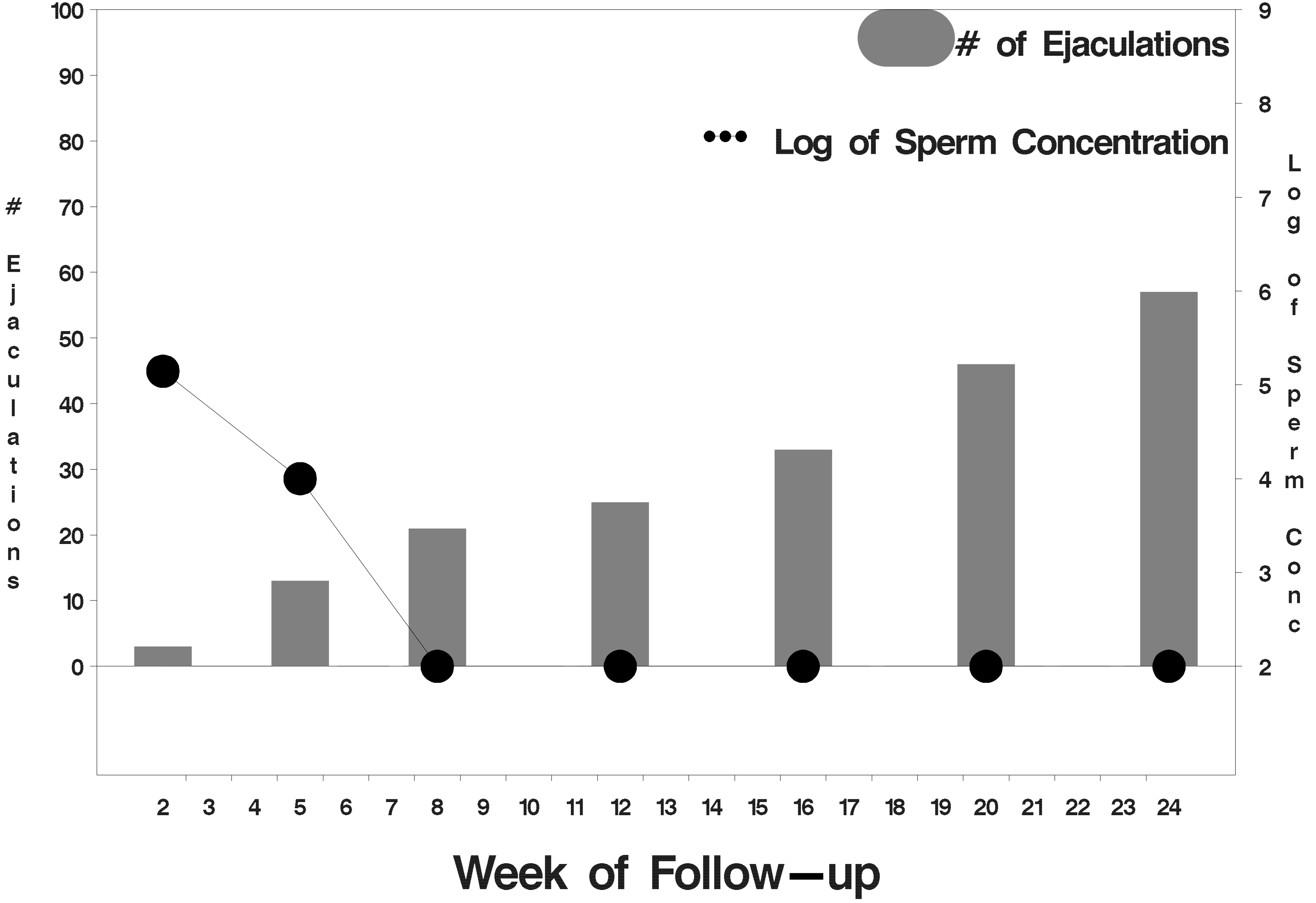

Participant number= 183

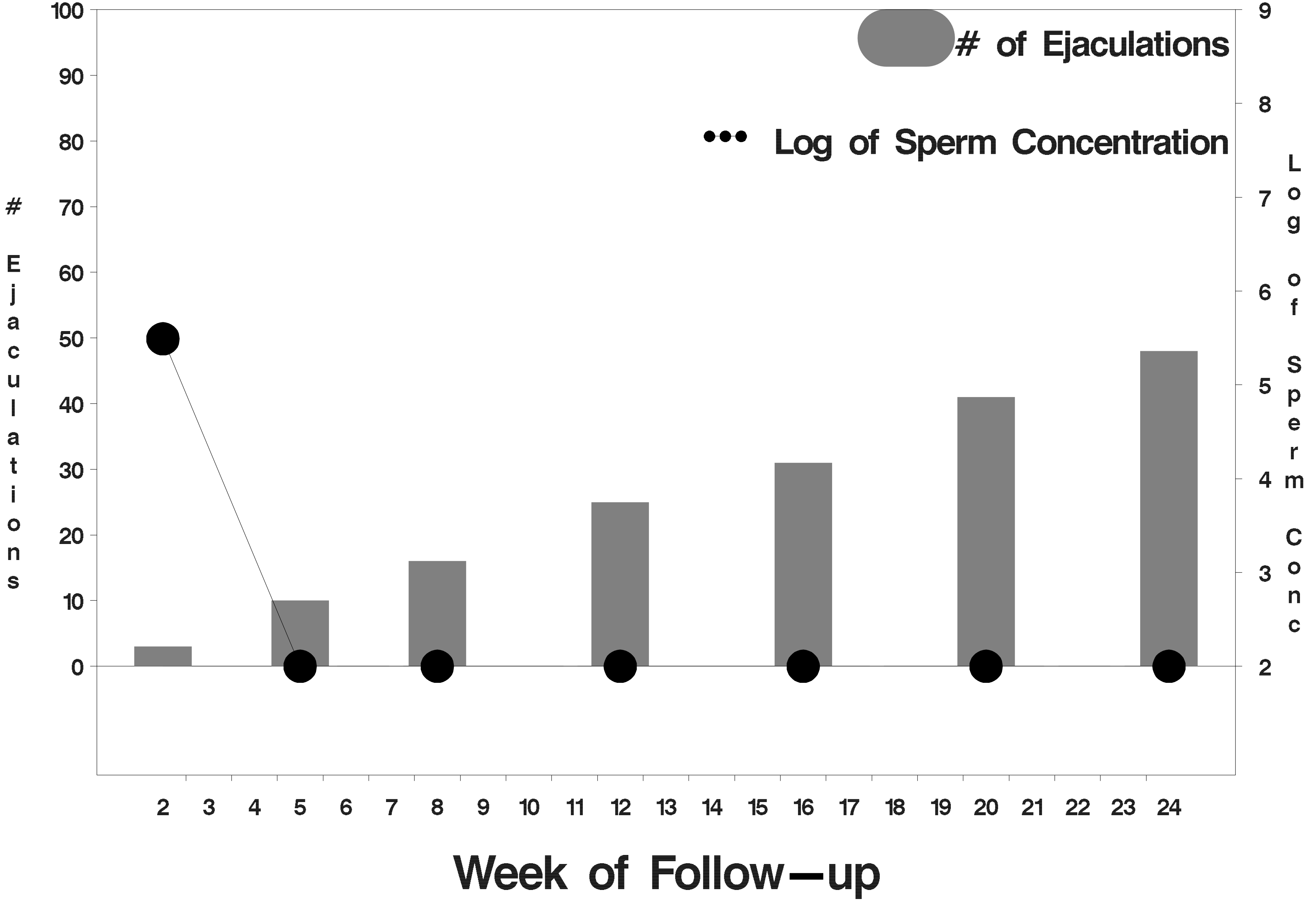

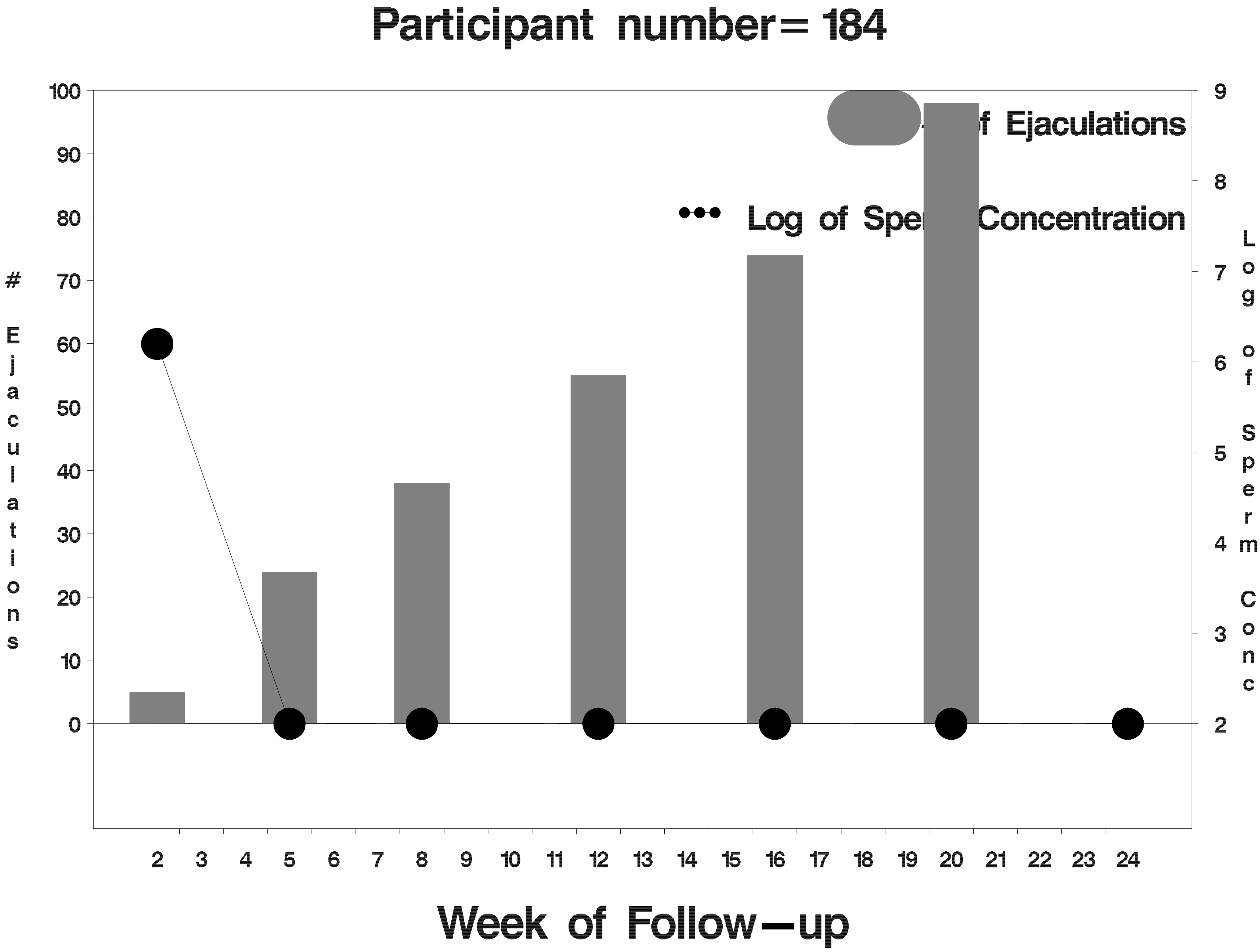

Participant number= 184
# of Ejaculations
Log of Sperm Concentration
Ejaculations
Log of Sperm Conc
Week of Follow-up
100
90
80
70
60
50
40
30
20
10
0
9
8
7
6
5
4
3
2
2 3 4 5 6 7 8 9 10 11 12 13 14 15 16 17 18 19 20 21 22 23 24

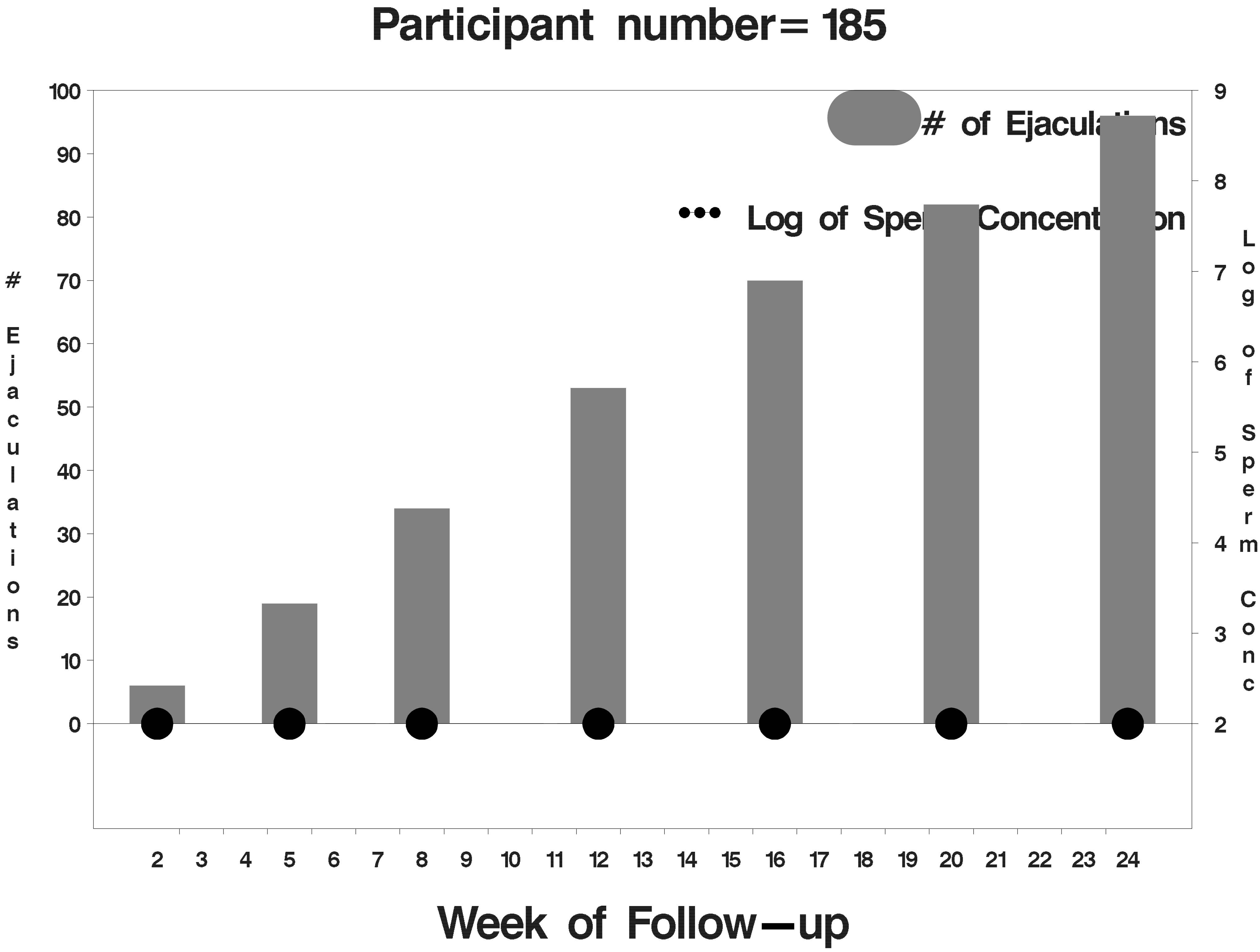

Participant number= 185
# of Ejaculations
Log of Sperm Concentration
Ejaculations
Log of Sperm Conc
100
90
80
70
60
50
40
30
20
10
0
9
8
7
6
5
4
3
2
2 3 4 5 6 7 8 9 10 11 12 13 14 15 16 17 18 19 20 21 22 23 24
Week of Follow—up

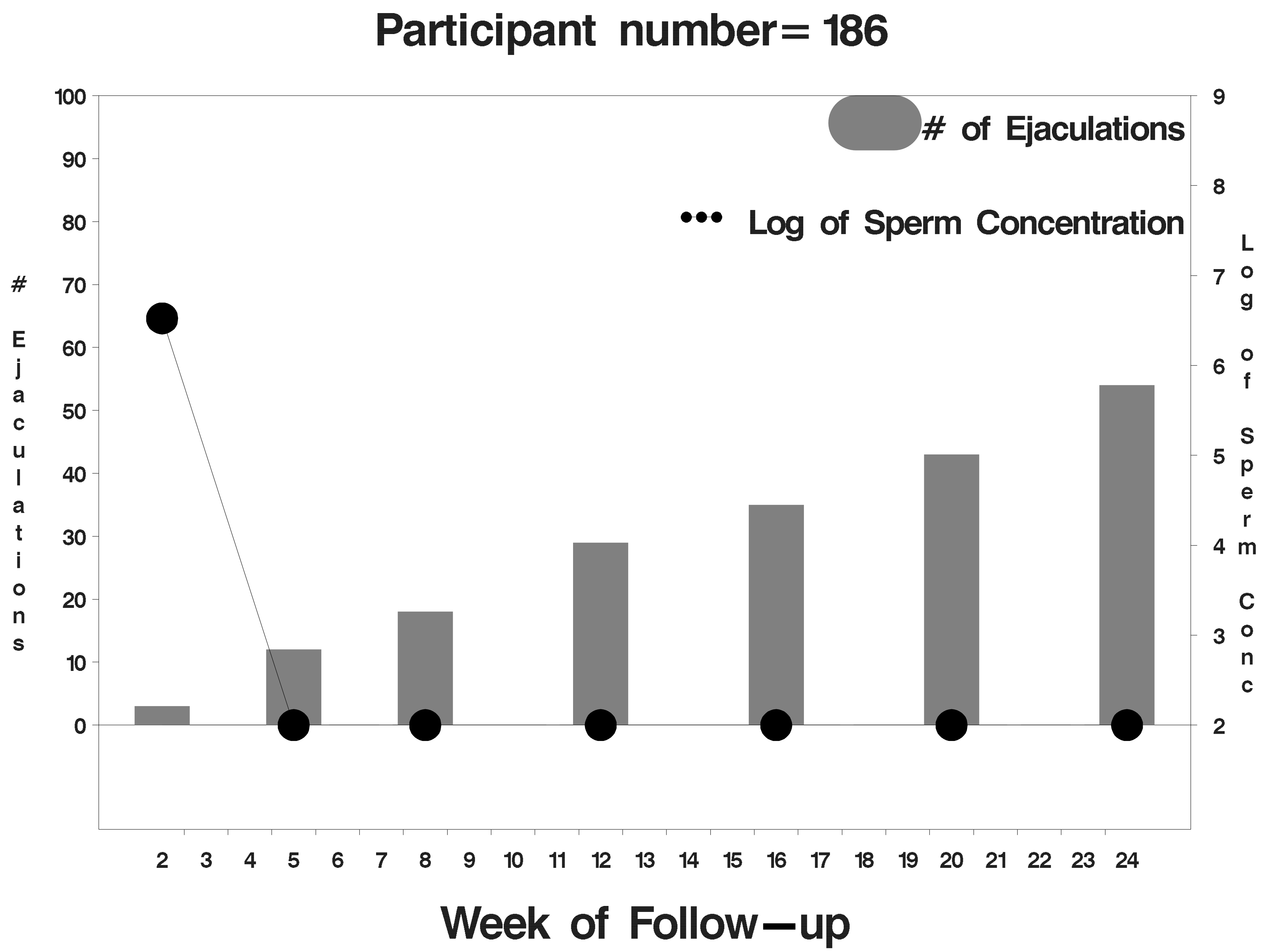

Participant number= 186
# of Ejaculations
Log of Sperm Concentration
Ejaculations
Log of Sperm Conc
100
90
80
70
60
50
40
30
20
10
0
9
8
7
6
5
4
3
2
2 3 4 5 6 7 8 9 10 11 12 13 14 15 16 17 18 19 20 21 22 23 24
Week of Follow-up

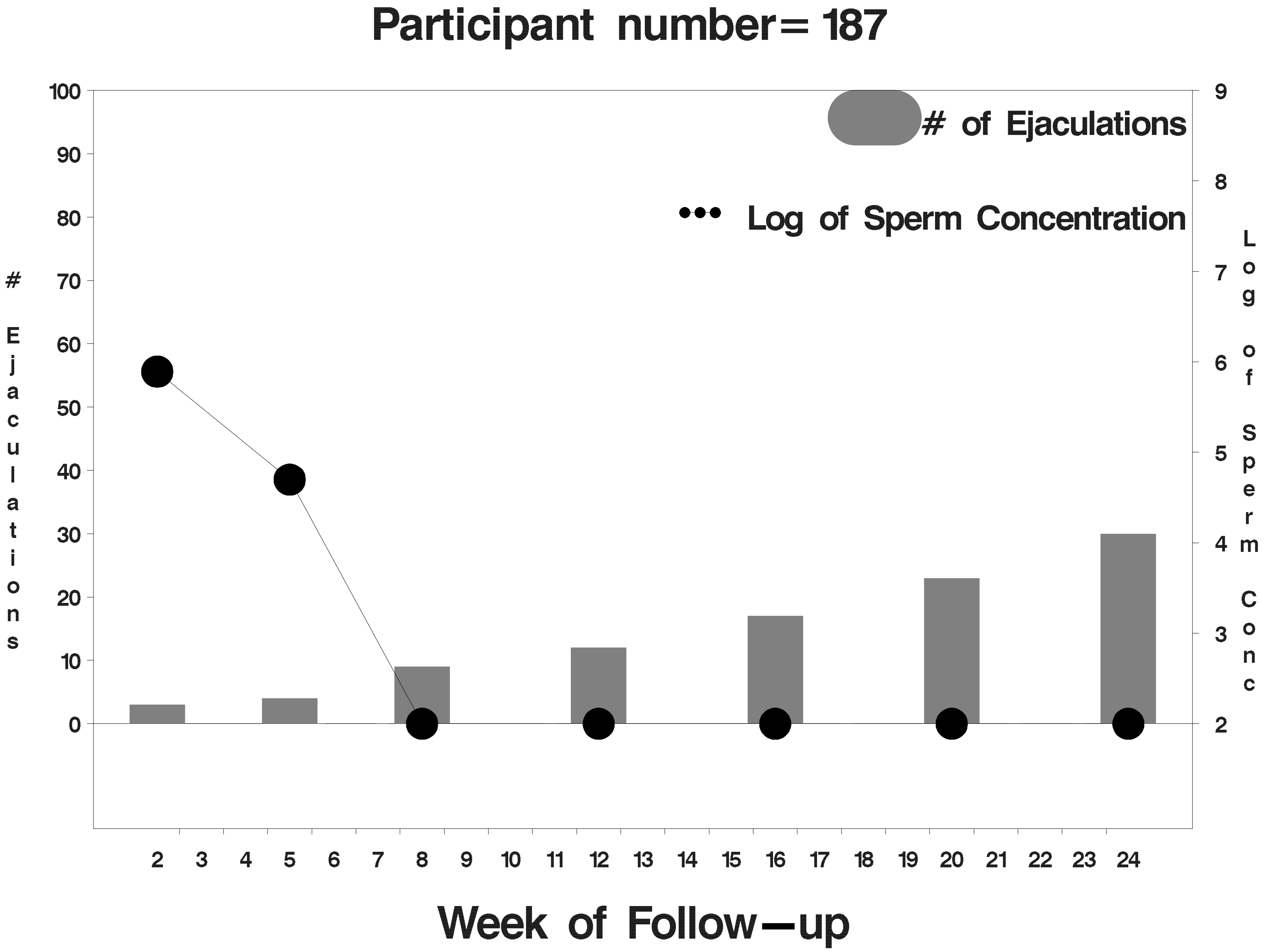

Participant number= 187
# of Ejaculations
Log of Sperm Concentration
Ejaculations
Log of Sperm Conc
100
90
80
70
60
50
40
30
20
10
0
9
8
7
6
5
4
3
2
2 3 4 5 6 7 8 9 10 11 12 13 14 15 16 17 18 19 20 21 22 23 24
Week of Follow-up

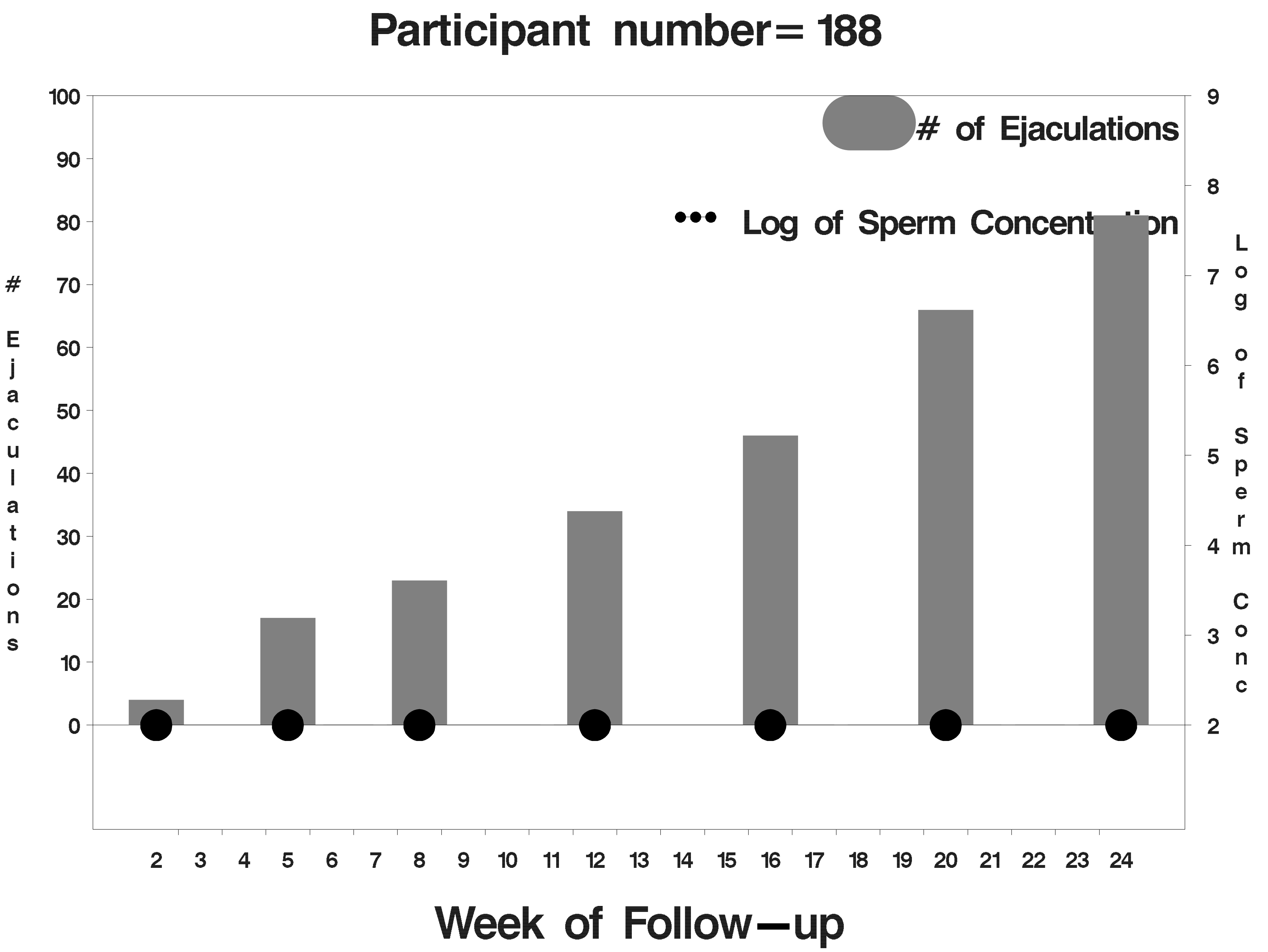
Participant number= 188
# of Ejaculations
Log of Sperm Concentration
Ejaculations
Log of Sperm Conc
Week of Follow-up
100
90
80
70
60
50
40
30
20
10
0
9
8
7
6
5
4
3
2
2 3 4 5 6 7 8 9 10 11 12 13 14 15 16 17 18 19 20 21 22 23 24

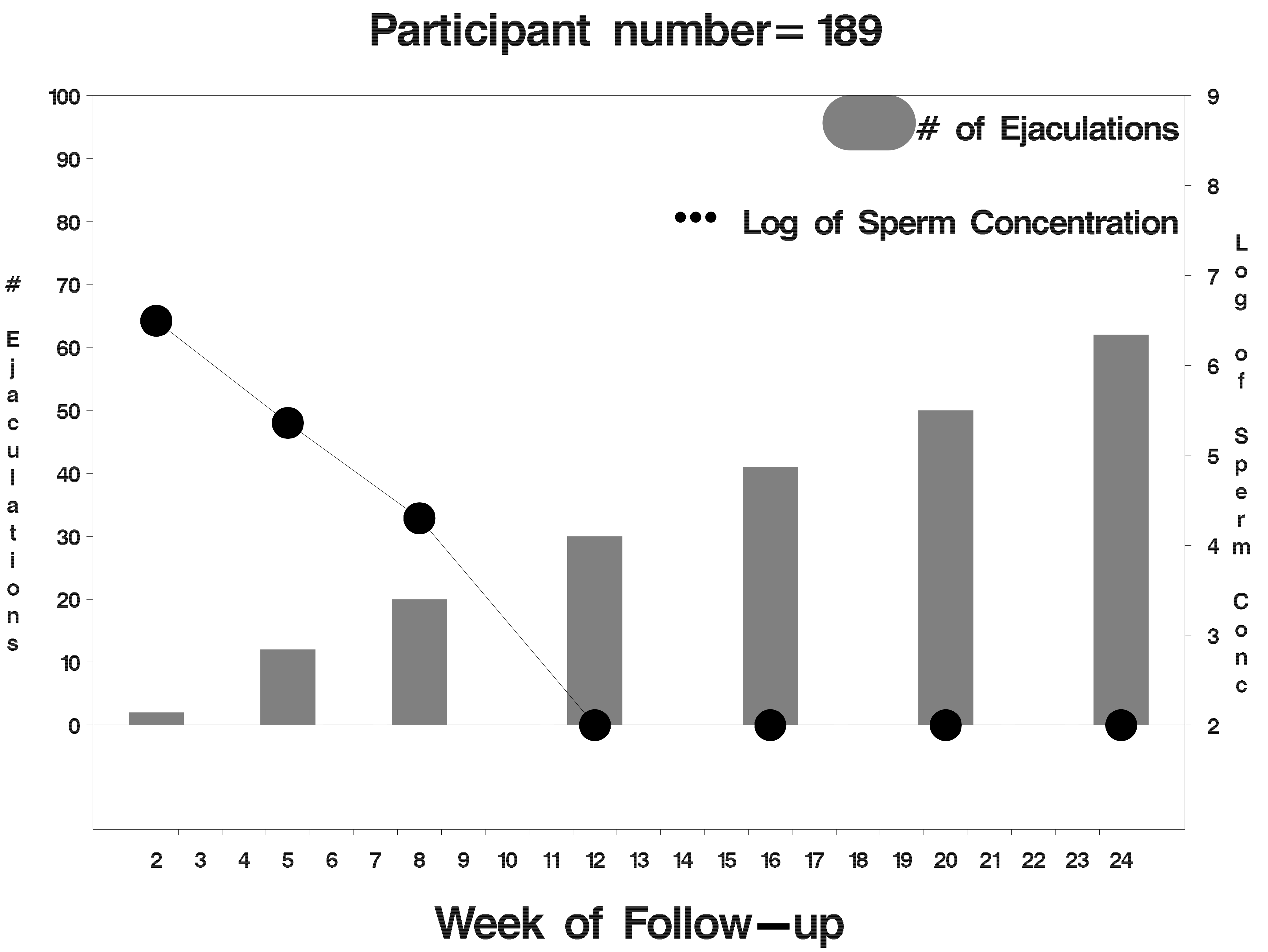

Participant number= 189
# of Ejaculations
Log of Sperm Concentration
Ejaculations
Log of Sperm Conc
100
90
80
70
60
50
40
30
20
10
0
9
8
7
6
5
4
3
2
2 3 4 5 6 7 8 9 10 11 12 13 14 15 16 17 18 19 20 21 22 23 24
Week of Follow—up

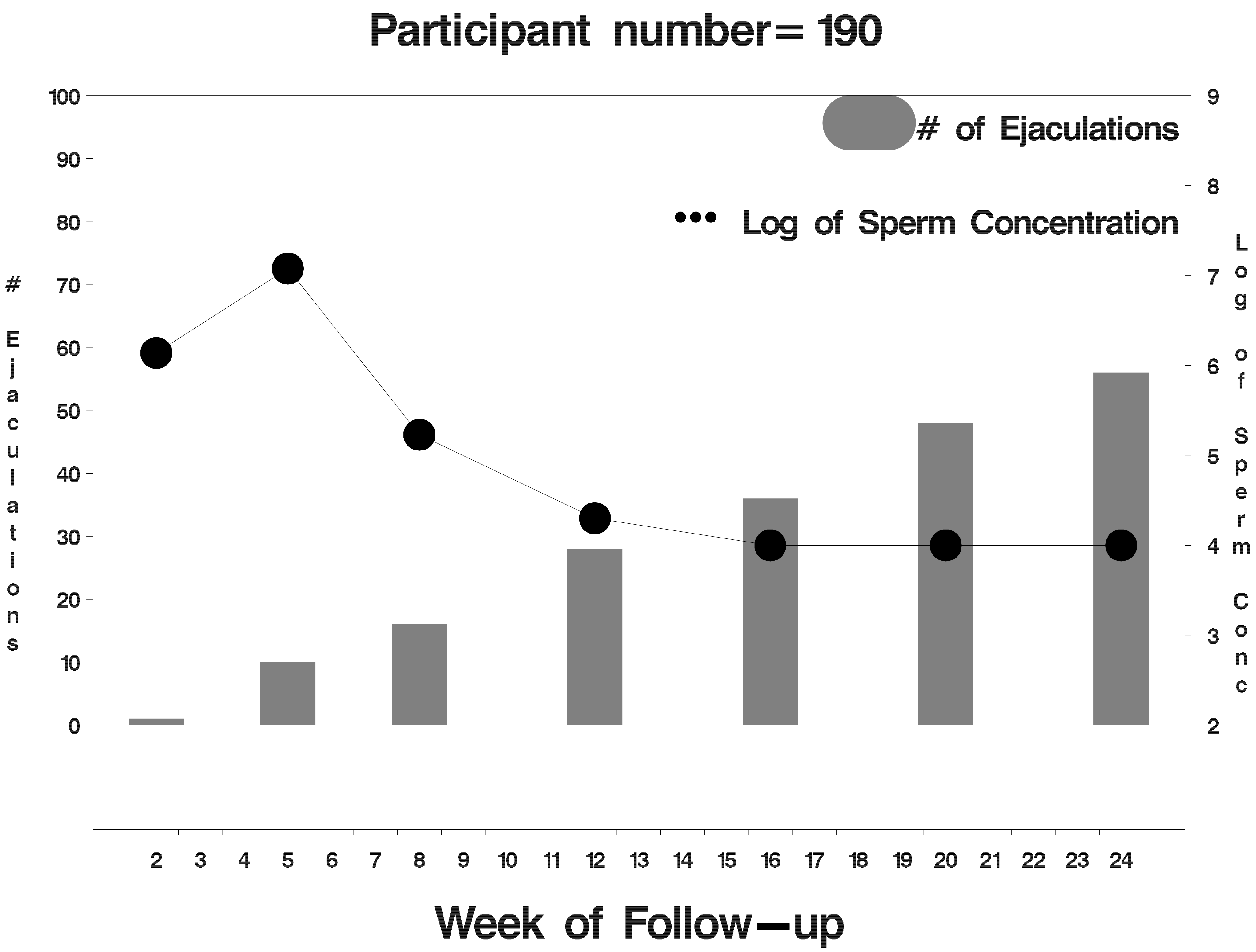

Participant number= 190
# of Ejaculations
Log of Sperm Concentration
Ejaculations
Log of Sperm Conc
100
90
80
70
60
50
40
30
20
10
0
9
8
7
6
5
4
3
2
2 3 4 5 6 7 8 9 10 11 12 13 14 15 16 17 18 19 20 21 22 23 24
Week of Follow−up

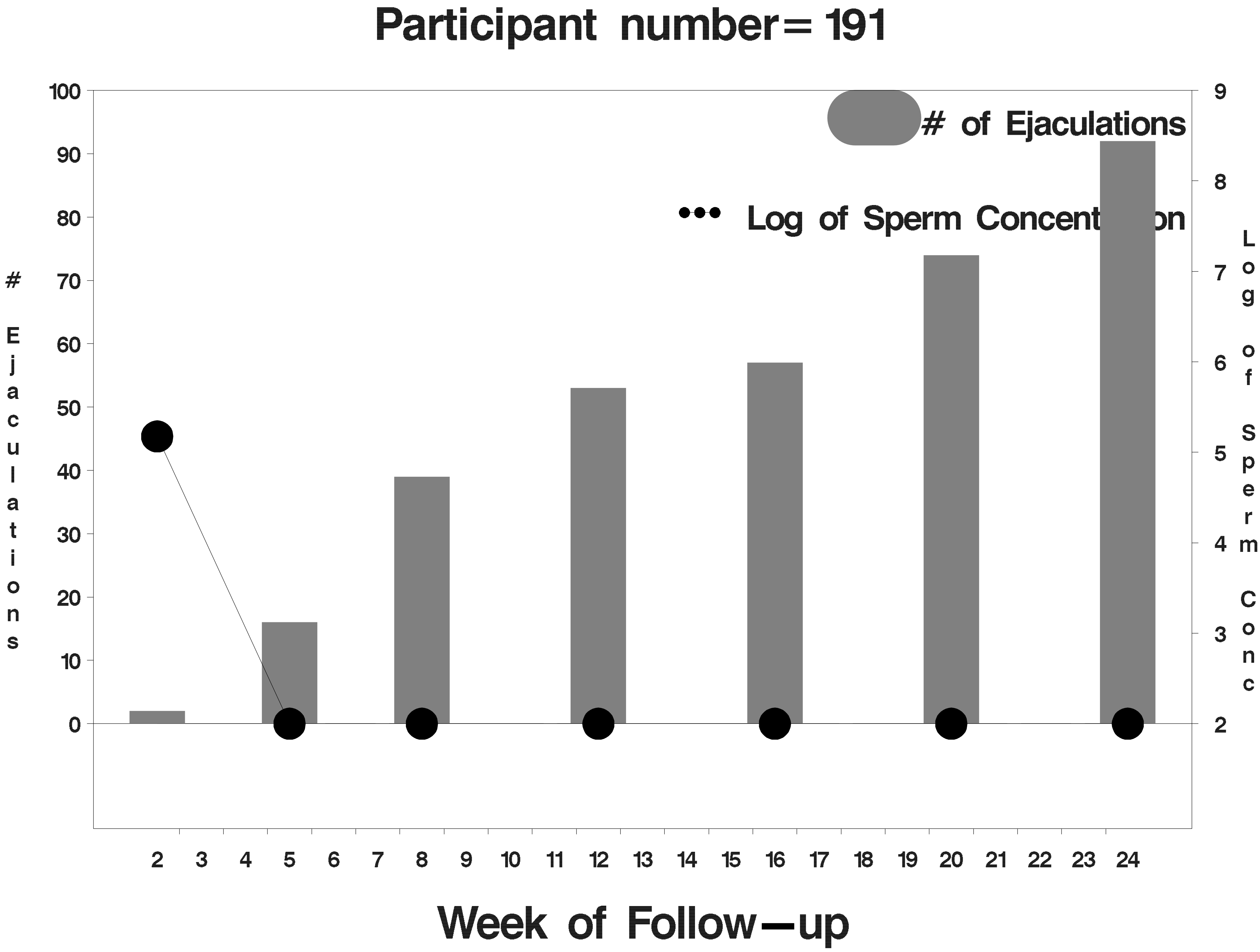

Participant number= 191
# of Ejaculations
Log of Sperm Concentration
Ejaculations
Log of Sperm Conc
100
90
80
70
60
50
40
30
20
10
0
9
8
7
6
5
4
3
2
2 3 4 5 6 7 8 9 10 11 12 13 14 15 16 17 18 19 20 21 22 23 24
Week of Follow-up

Participant number= 192

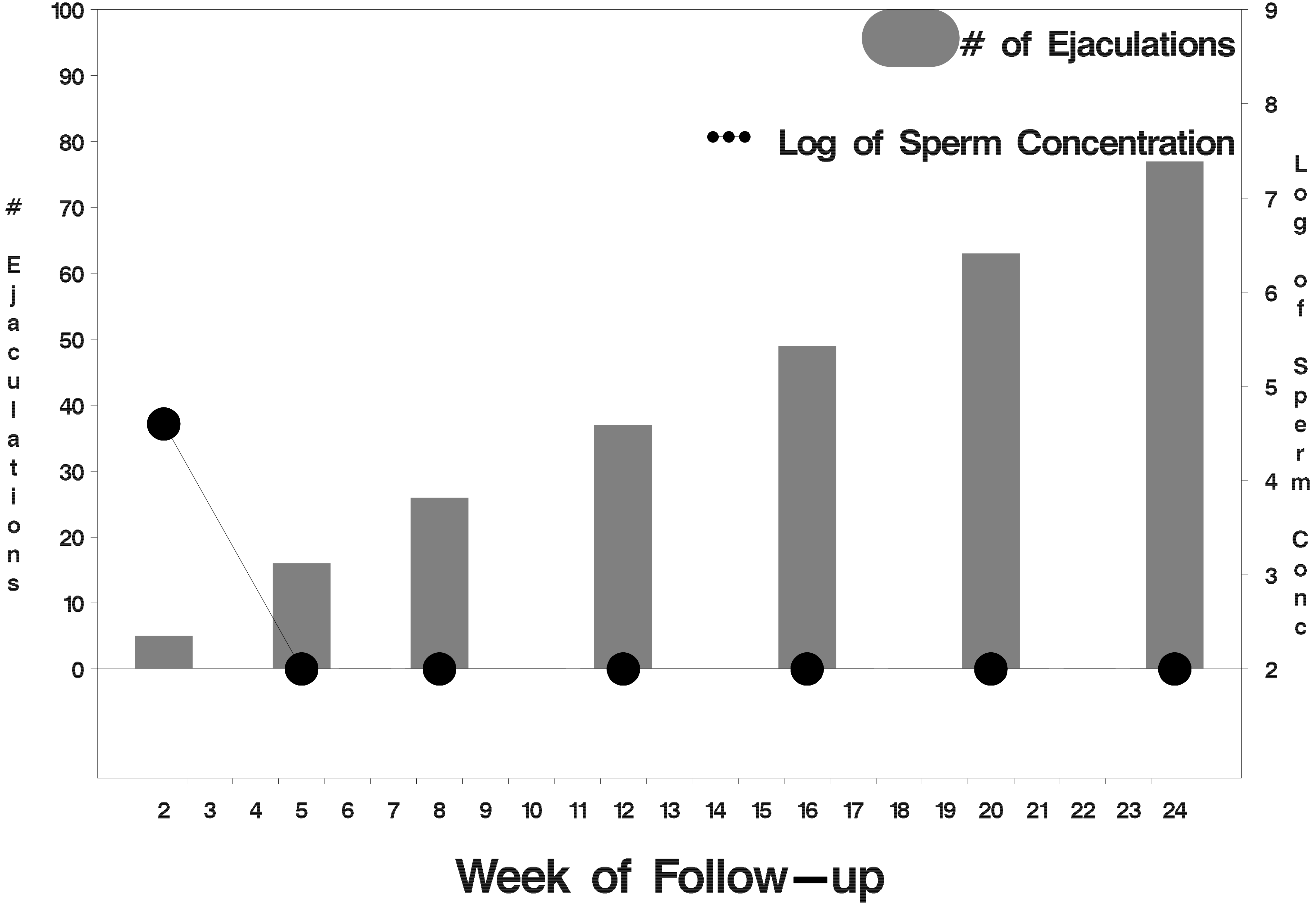

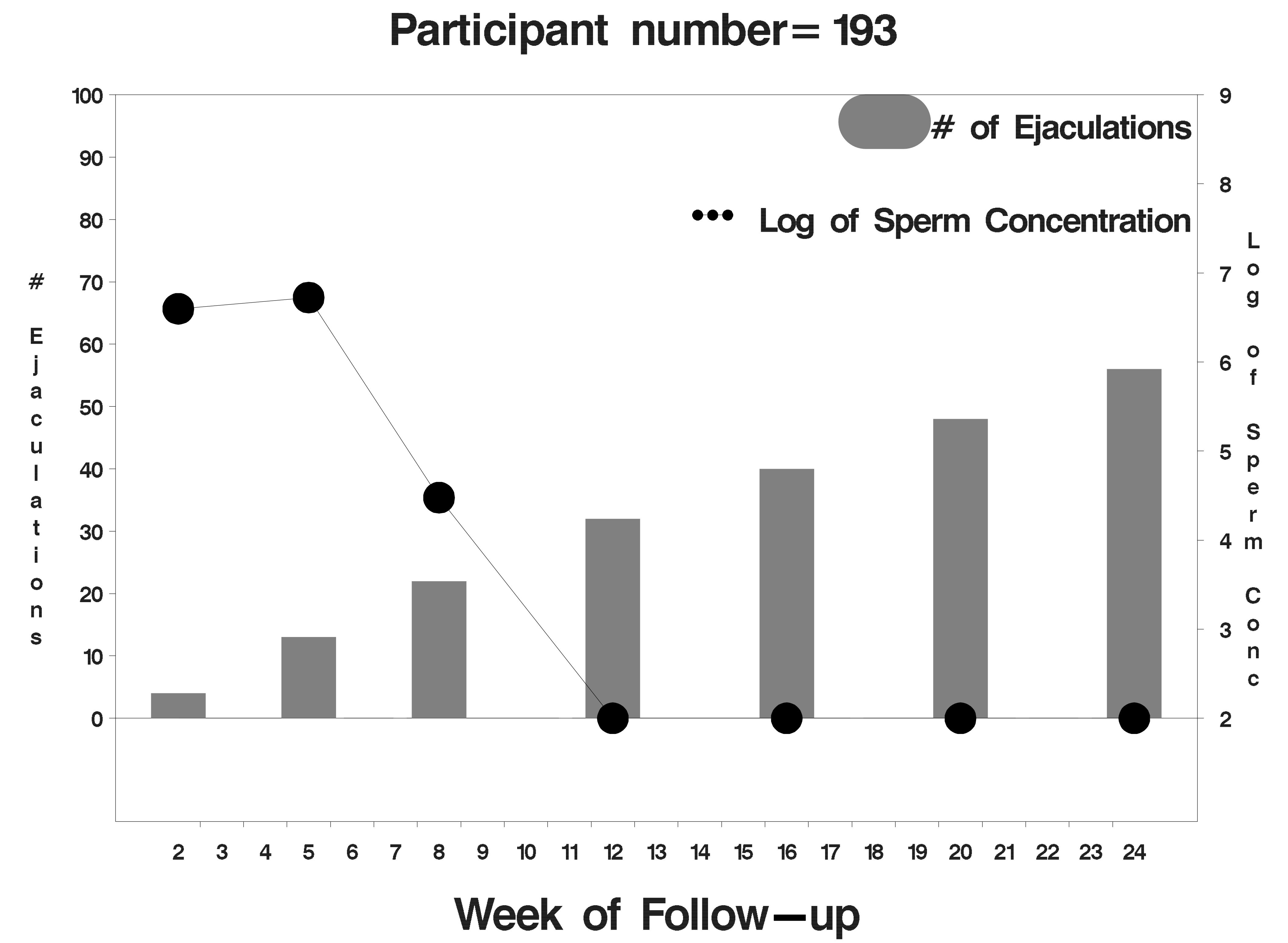

Participant number= 193
# of Ejaculations
Log of Sperm Concentration
Ejaculations
Log of Sperm Conc
100
90
80
70
60
50
40
30
20
10
0
9
8
7
6
5
4
3
2
2 3 4 5 6 7 8 9 10 11 12 13 14 15 16 17 18 19 20 21 22 23 24
Week of Follow-up

Participant number= 194

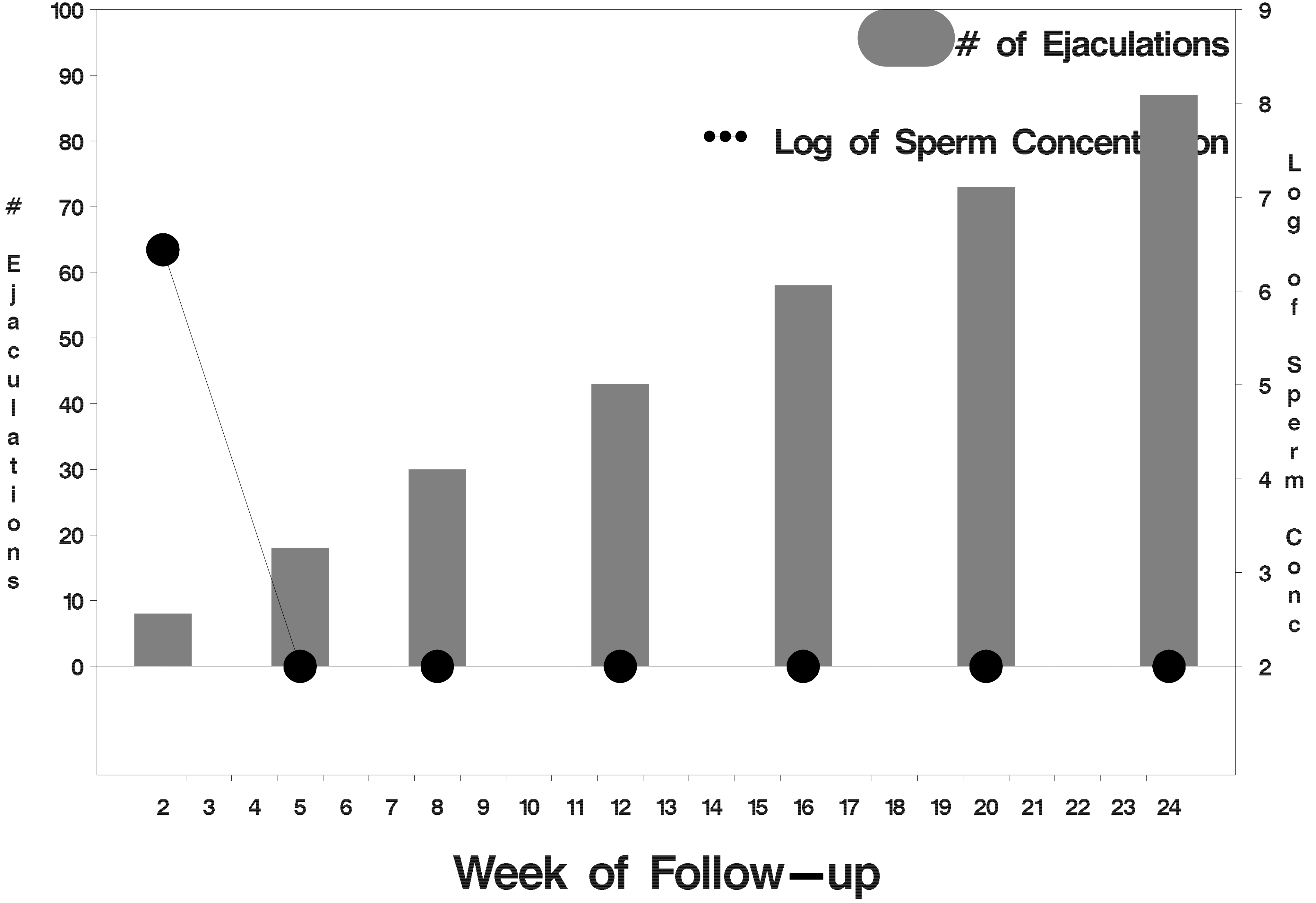

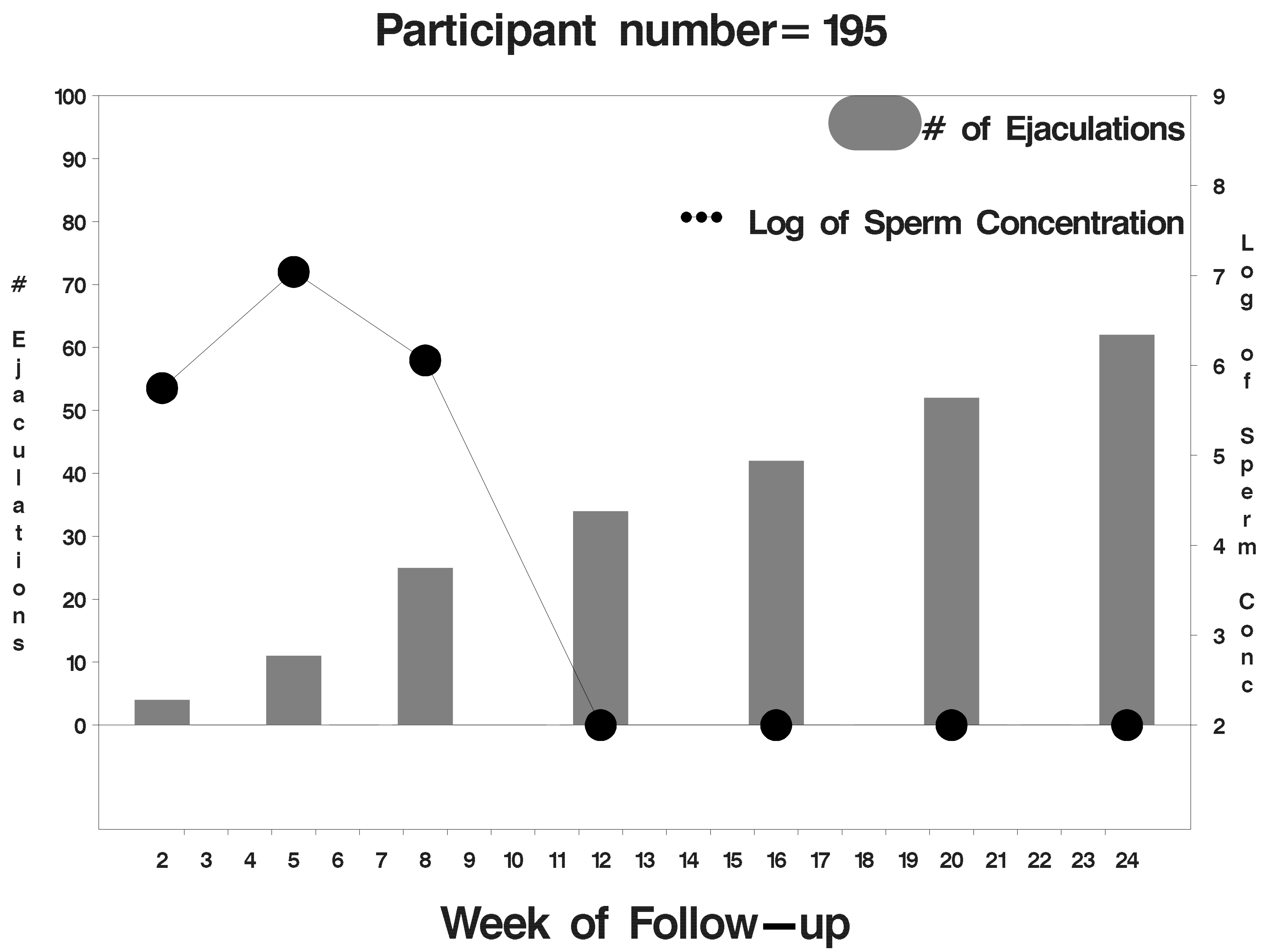

Participant number= 195
# of Ejaculations
Log of Sperm Concentration
Ejaculations
Log of Sperm Conc
100
90
80
70
60
50
40
30
20
10
0
9
8
7
6
5
4
3
2
2 3 4 5 6 7 8 9 10 11 12 13 14 15 16 17 18 19 20 21 22 23 24
Week of Follow−up

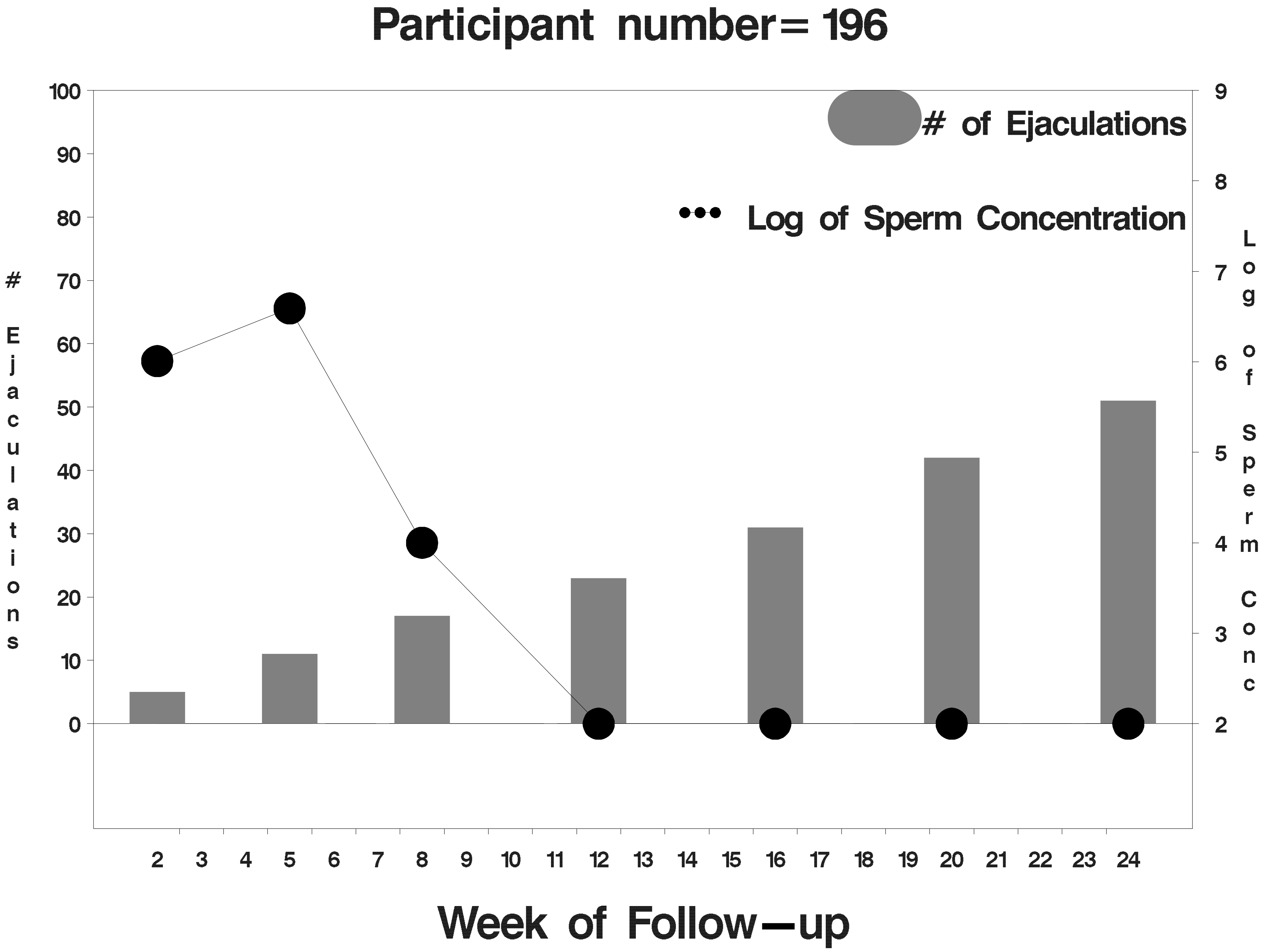
Participant number= 196
# of Ejaculations
Log of Sperm Concentration
Ejaculations
Log of Sperm Conc
100
90
80
70
60
50
40
30
20
10
0
9
8
7
6
5
4
3
2
2 3 4 5 6 7 8 9 10 11 12 13 14 15 16 17 18 19 20 21 22 23 24
Week of Follow-up

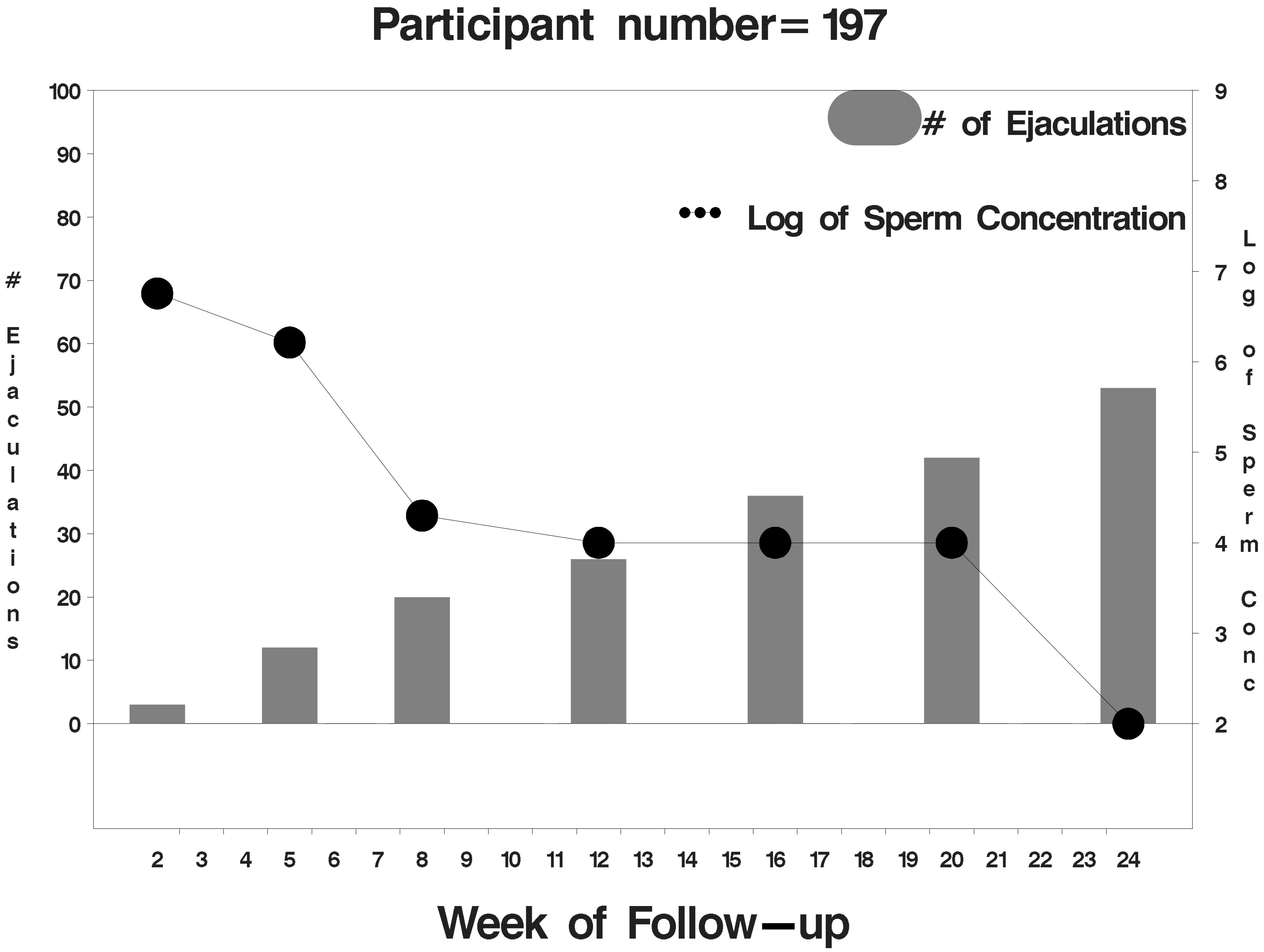

Participant number= 197
# of Ejaculations
Log of Sperm Concentration
Ejaculations
Log of Sperm Conc
100
90
80
70
60
50
40
30
20
10
0
9
8
7
6
5
4
3
2
2 3 4 5 6 7 8 9 10 11 12 13 14 15 16 17 18 19 20 21 22 23 24
Week of Follow-up

Participant number= 198

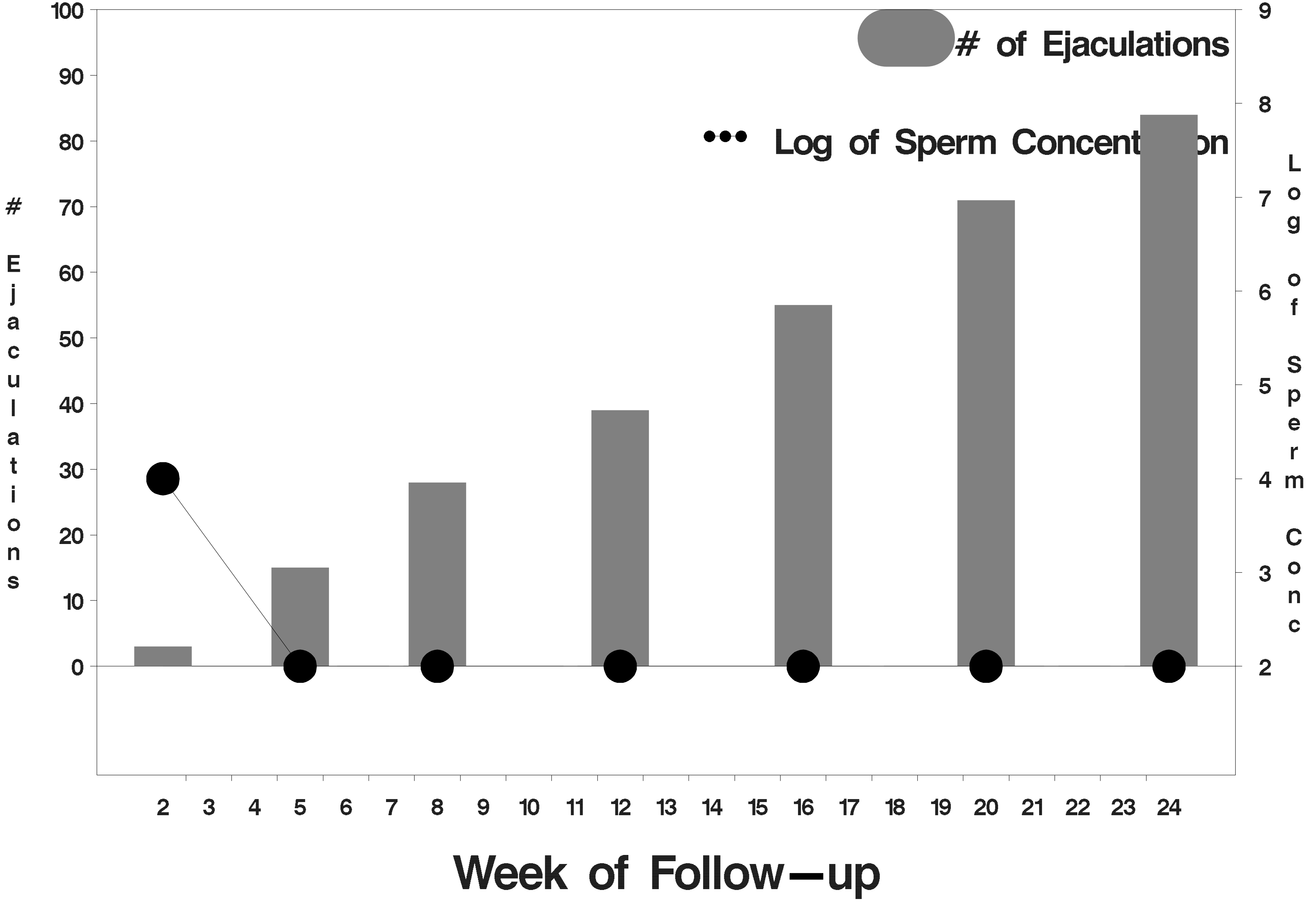

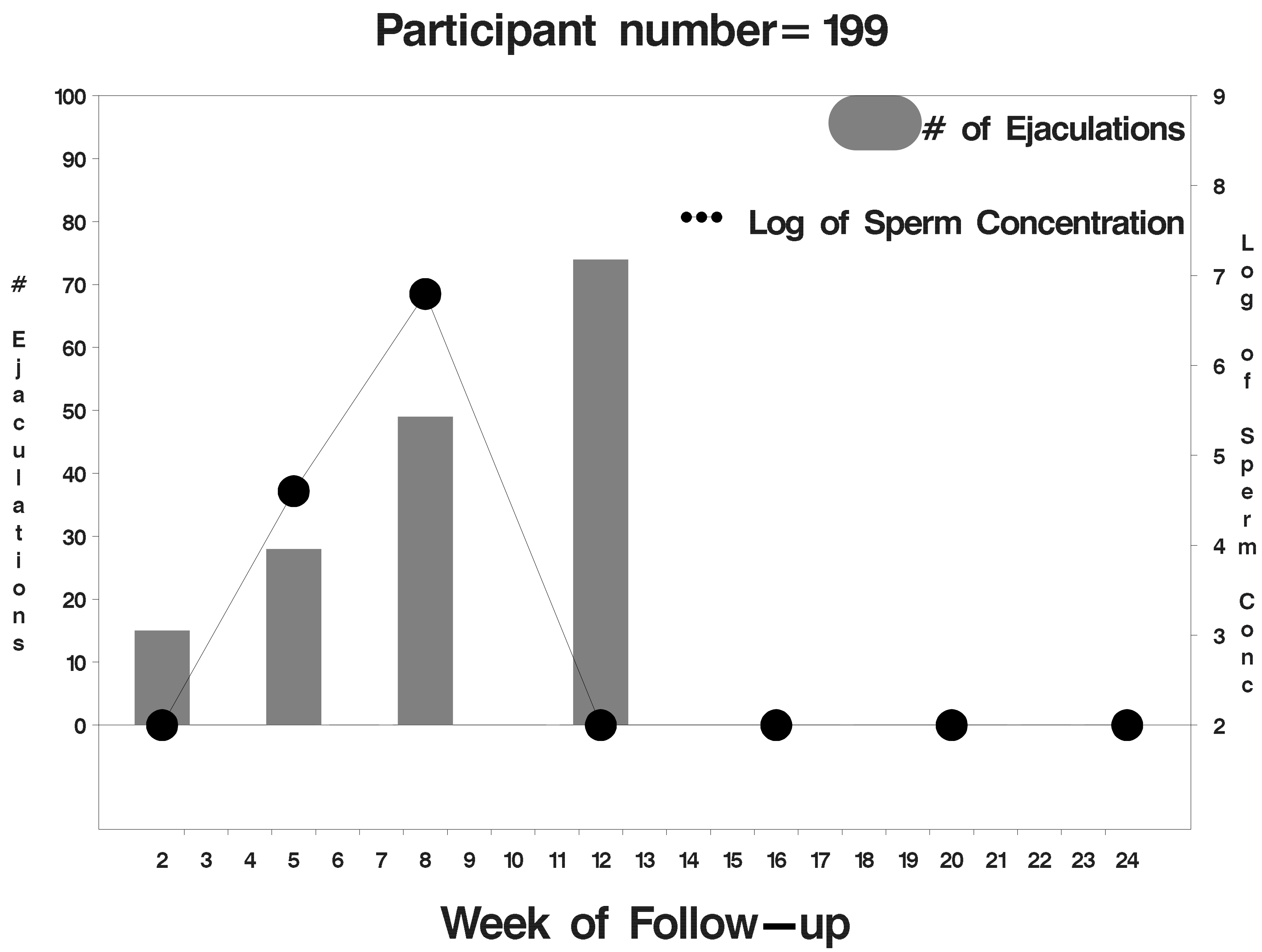

Participant number= 199
# of Ejaculations
Log of Sperm Concentration
Ejaculations
Log of Sperm Conc
100
90
80
70
60
50
40
30
20
10
0
9
8
7
6
5
4
3
2
2 3 4 5 6 7 8 9 10 11 12 13 14 15 16 17 18 19 20 21 22 23 24
Week of Follow-up

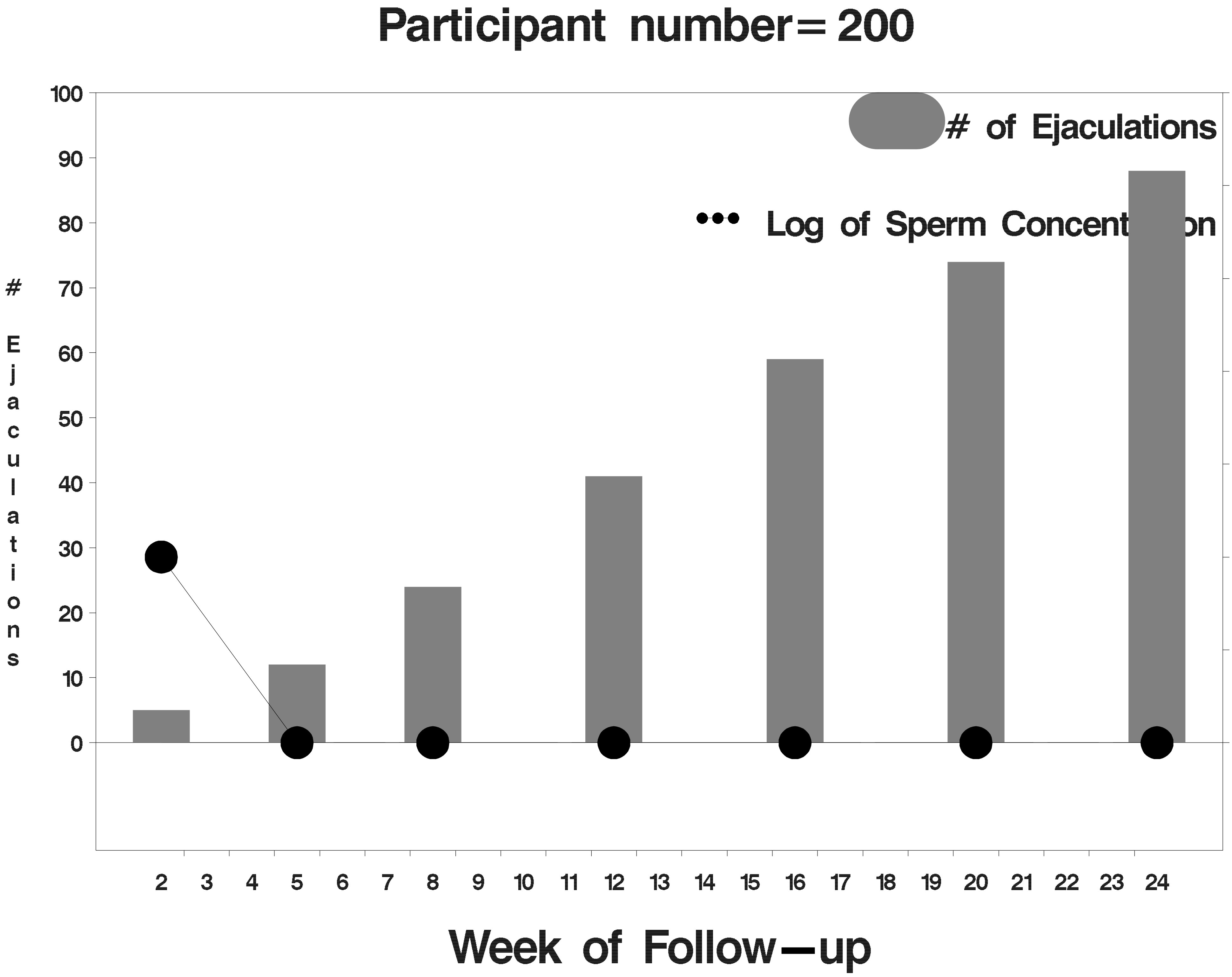

Participant number=200
# of Ejaculations
Log of Sperm Concent[illegible]on
Ejaculations
Log of Sperm Conc
100
90
80
70
60
50
40
30
20
10
0
9
8
7
6
5
4
3
2
2 3 4 5 6 7 8 9 10 11 12 13 14 15 16 17 18 19 20 21 22 23 24
Week of Follow-up

Participant number=301

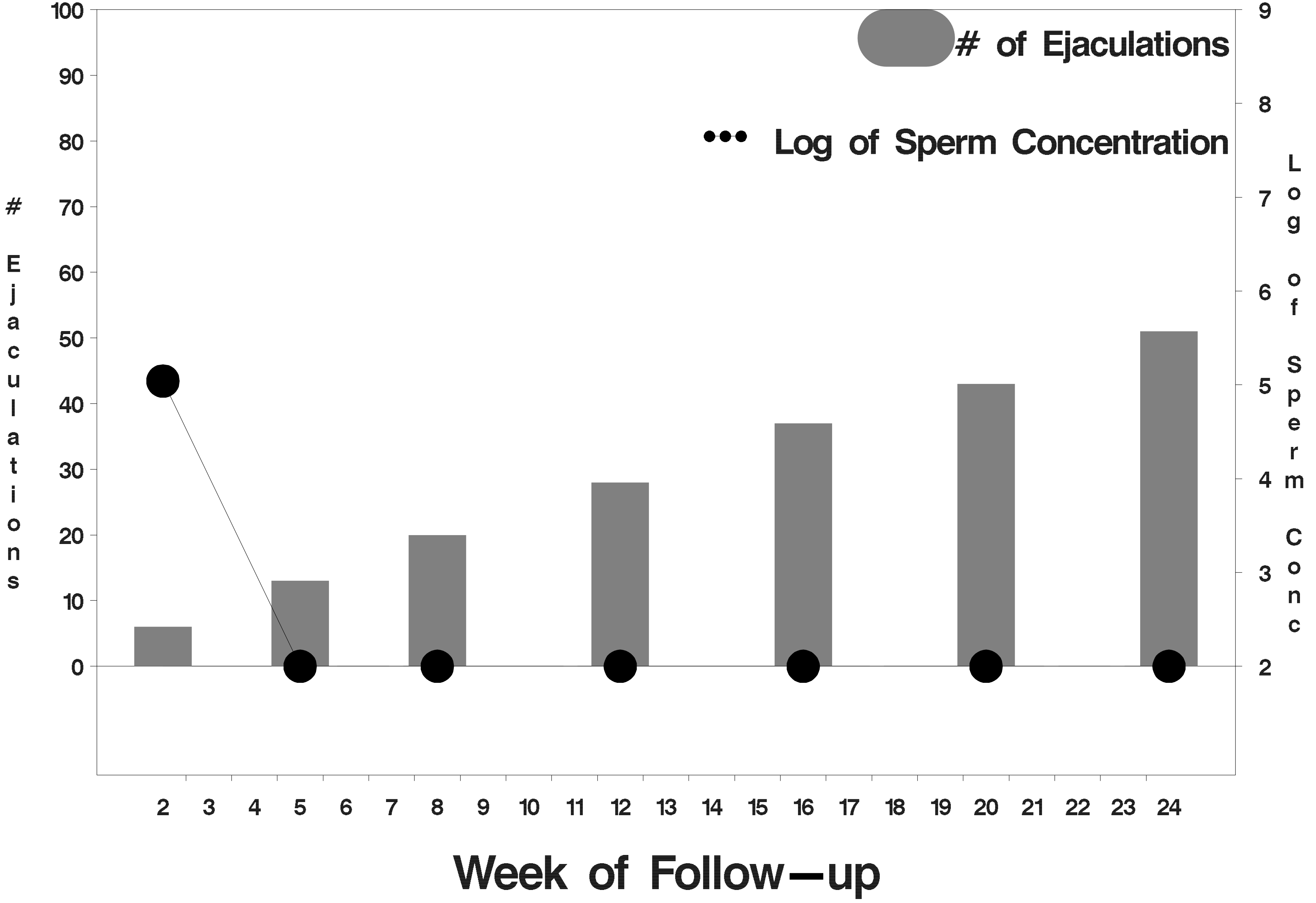

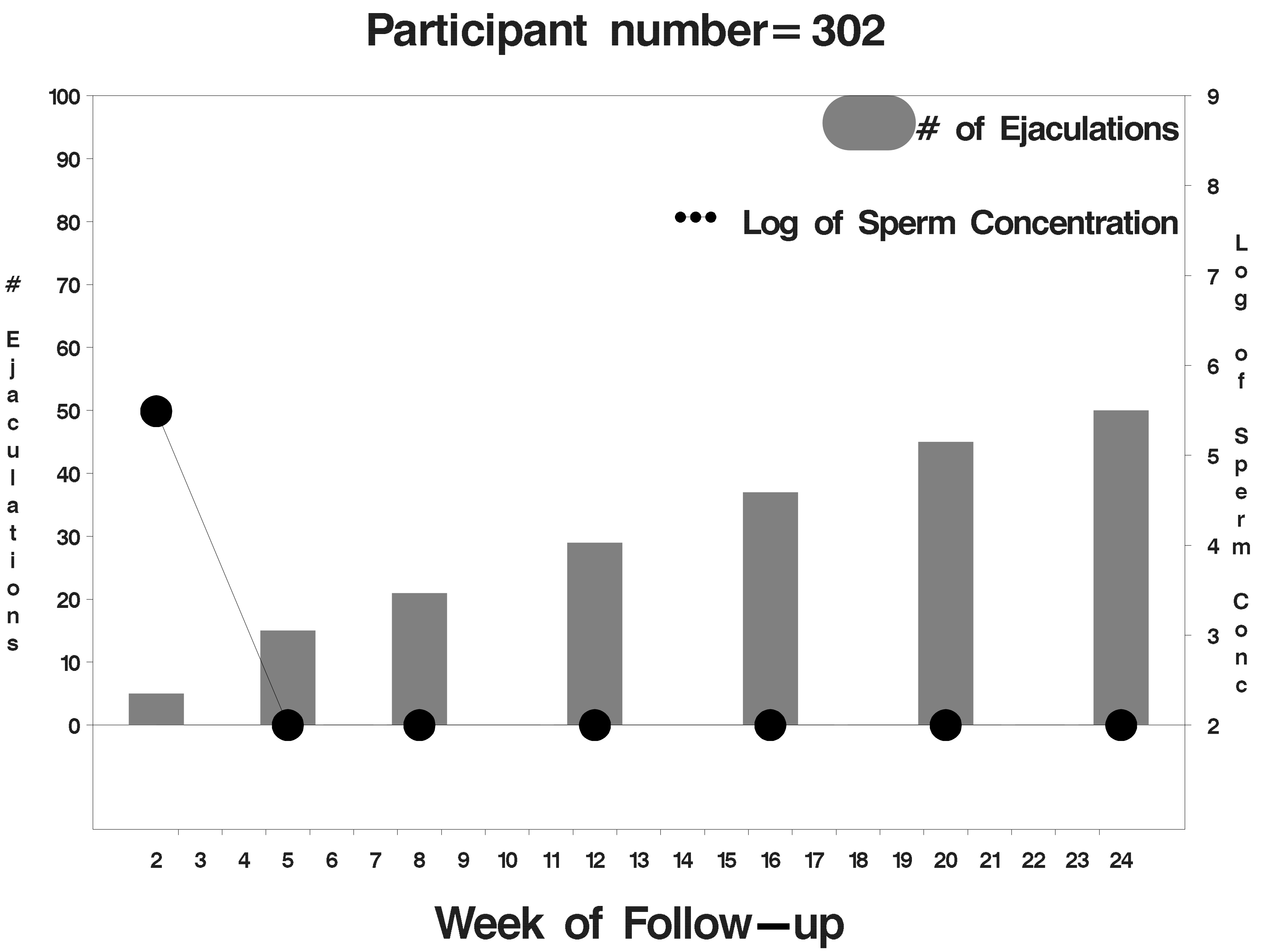

Participant number=302
# of Ejaculations
Log of Sperm Concentration
Ejaculations
Log of Sperm Conc
100
90
80
70
60
50
40
30
20
10
0
9
8
7
6
5
4
3
2
2 3 4 5 6 7 8 9 10 11 12 13 14 15 16 17 18 19 20 21 22 23 24
Week of Follow-up

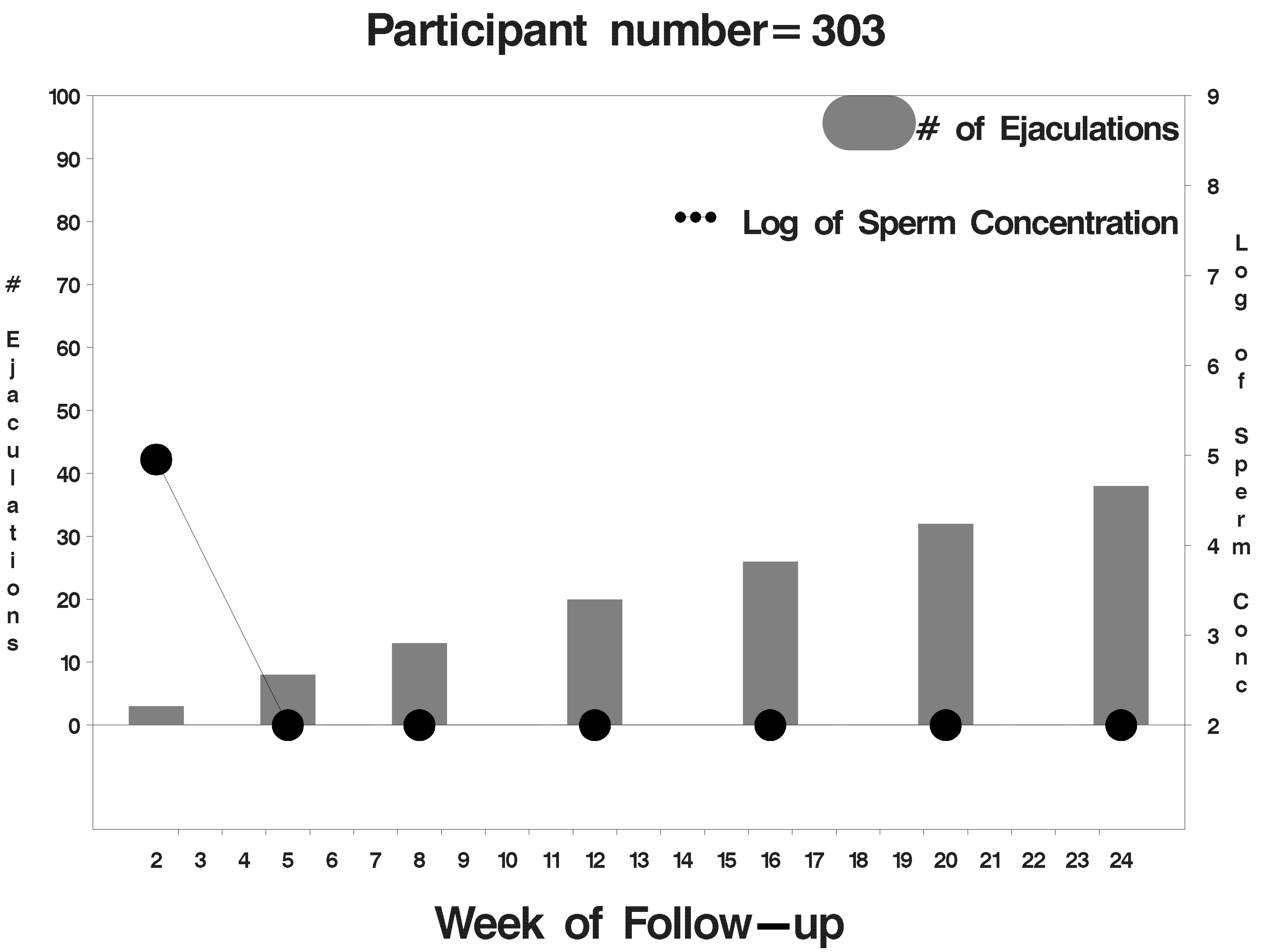
Participant number=303
# of Ejaculations
Log of Sperm Concentration
Ejaculations
Log of Sperm Conc
100
90
80
70
60
50
40
30
20
10
0
9
8
7
6
5
4
3
2
2 3 4 5 6 7 8 9 10 11 12 13 14 15 16 17 18 19 20 21 22 23 24
Week of Follow-up

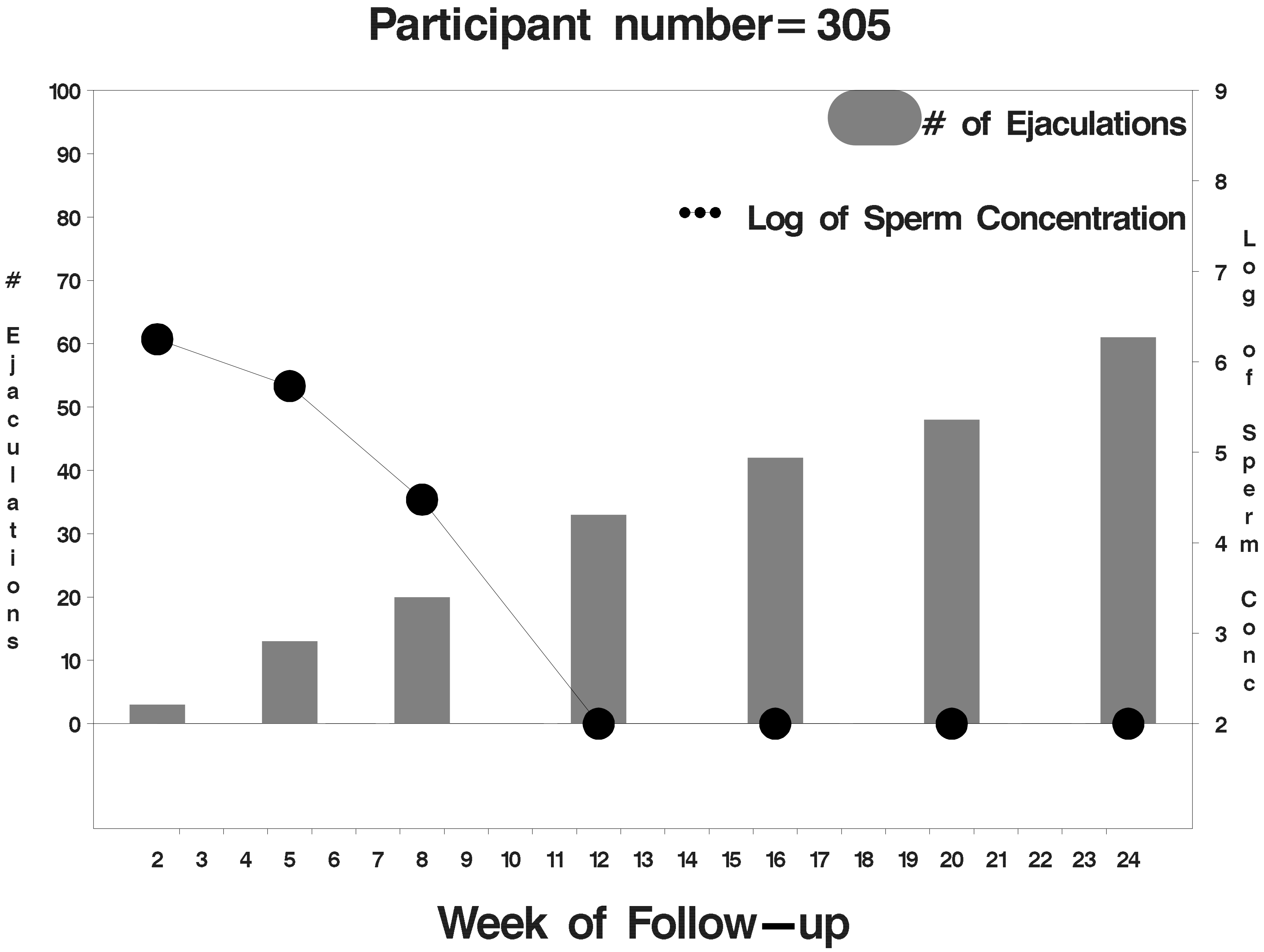

Participant number= 305
# of Ejaculations
Log of Sperm Concentration
Ejaculations
Log of Sperm Conc
100
90
80
70
60
50
40
30
20
10
0
9
8
7
6
5
4
3
2
2 3 4 5 6 7 8 9 10 11 12 13 14 15 16 17 18 19 20 21 22 23 24
Week of Follow—up

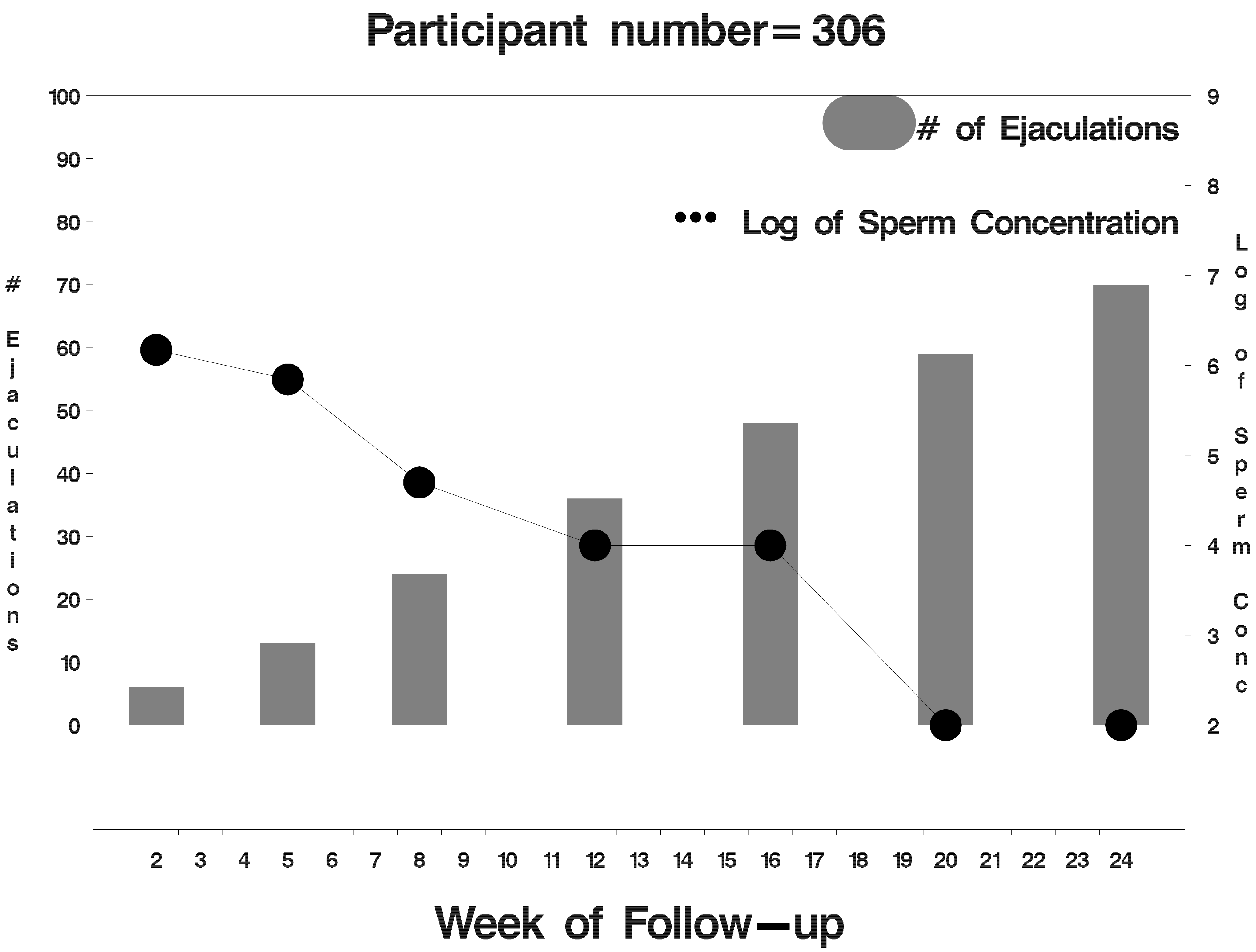
Participant number=306
# of Ejaculations
Log of Sperm Concentration
Ejaculations
Log of Sperm Conc
100
90
80
70
60
50
40
30
20
10
0
9
8
7
6
5
4
3
2
2 3 4 5 6 7 8 9 10 11 12 13 14 15 16 17 18 19 20 21 22 23 24
Week of Follow-up

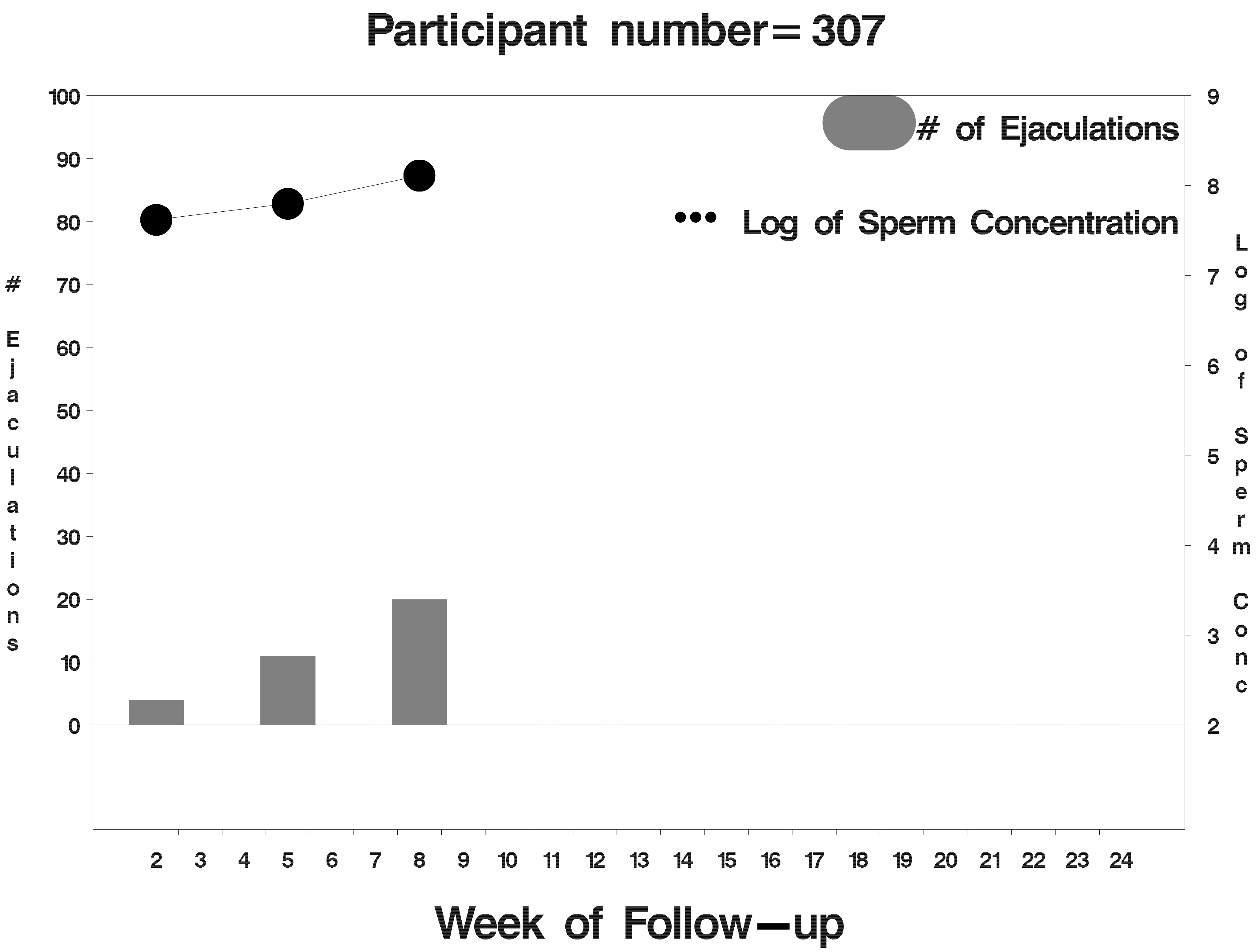
Participant number= 307
# of Ejaculations
Log of Sperm Concentration
Ejaculations
Log of Sperm Conc
100
90
80
70
60
50
40
30
20
10
0
9
8
7
6
5
4
3
2
2 3 4 5 6 7 8 9 10 11 12 13 14 15 16 17 18 19 20 21 22 23 24
Week of Follow-up

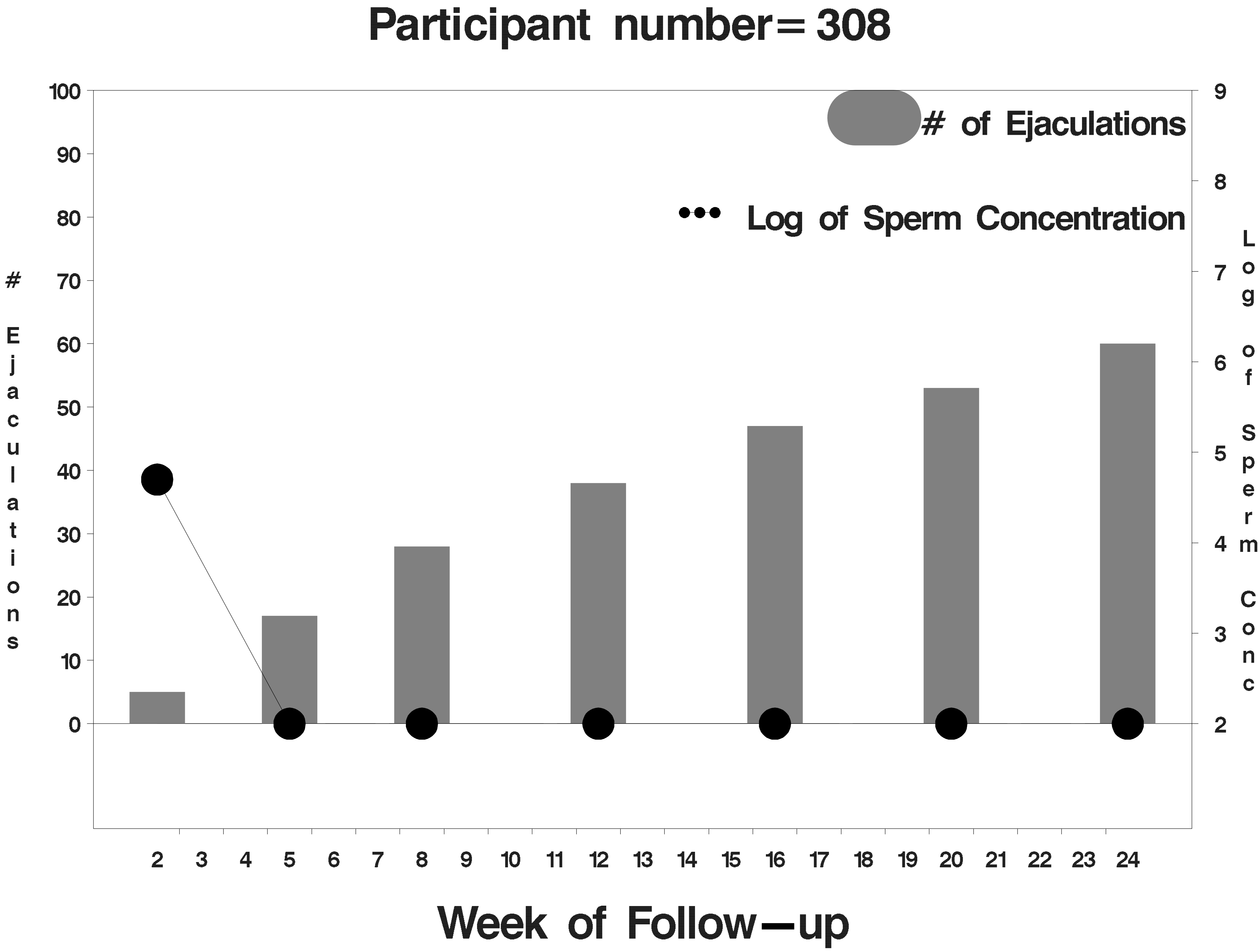
Participant number=308
# of Ejaculations
Log of Sperm Concentration
Ejaculations
Log of Sperm Conc
100
90
80
70
60
50
40
30
20
10
0
9
8
7
6
5
4
3
2
2 3 4 5 6 7 8 9 10 11 12 13 14 15 16 17 18 19 20 21 22 23 24
Week of Follow-up

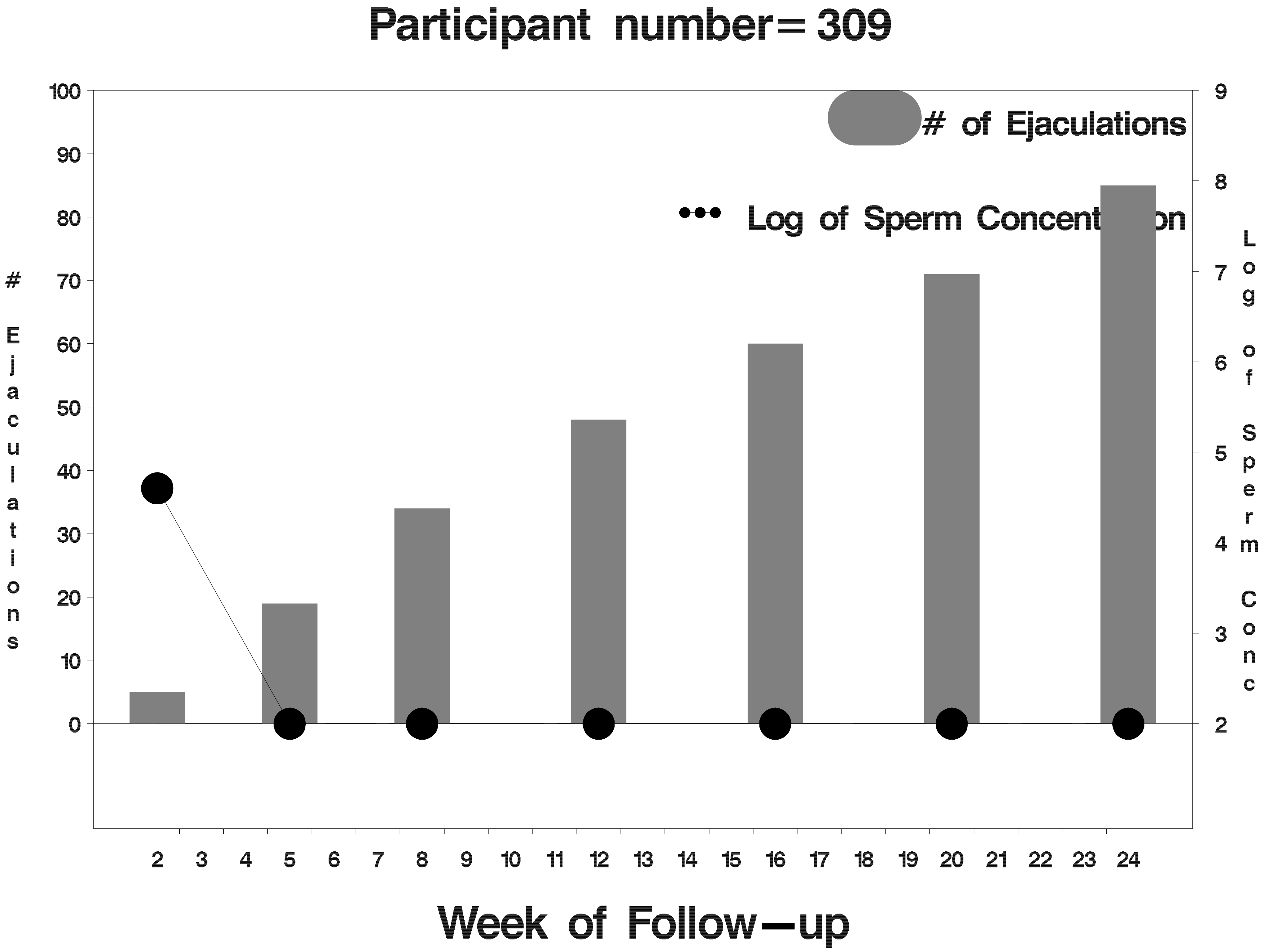
Participant number=309
# of Ejaculations
Log of Sperm Concentration
Ejaculations
Log of Sperm Conc
100
90
80
70
60
50
40
30
20
10
0
9
8
7
6
5
4
3
2
2 3 4 5 6 7 8 9 10 11 12 13 14 15 16 17 18 19 20 21 22 23 24
Week of Follow-up

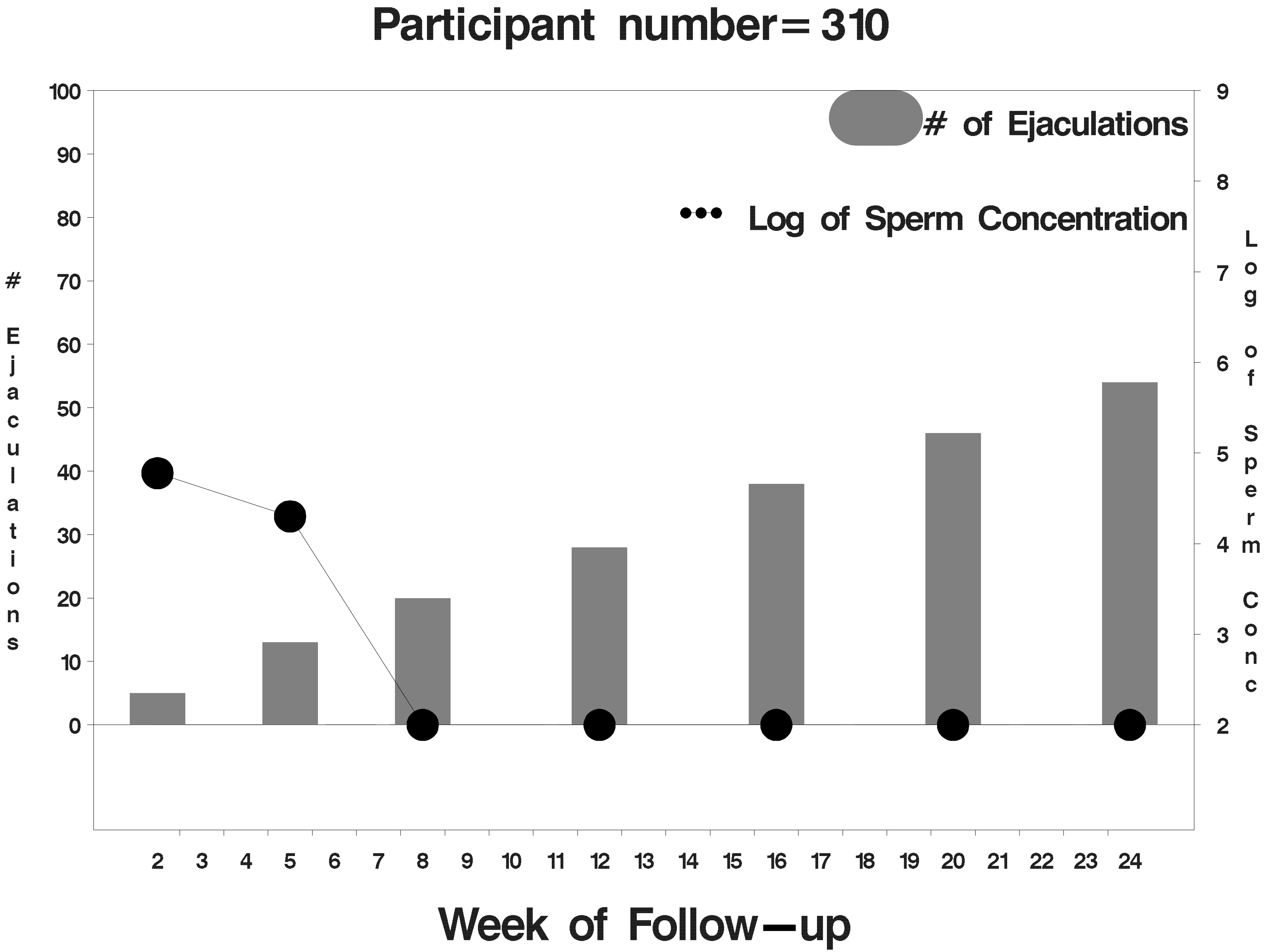

Participant number=310
# of Ejaculations
Log of Sperm Concentration
Ejaculations
Log of Sperm Conc
100
90
80
70
60
50
40
30
20
10
0
9
8
7
6
5
4
3
2
2 3 4 5 6 7 8 9 10 11 12 13 14 15 16 17 18 19 20 21 22 23 24
Week of Follow-up

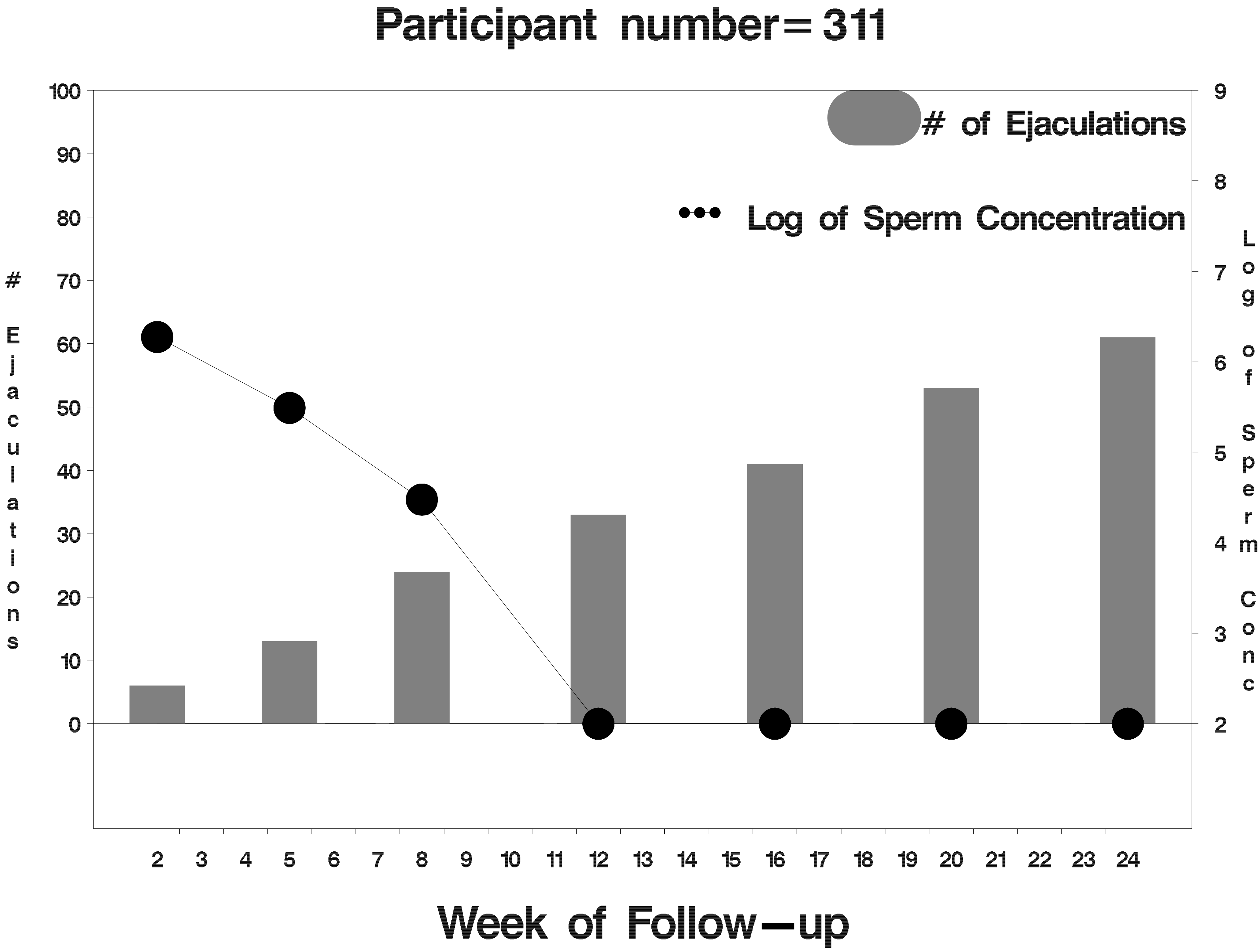

Participant number=311
100
90
80
70
60
50
40
30
20
10
0
Ejaculations
9
8
7
6
5
4
3
2
Log of Sperm Conc
# of Ejaculations
Log of Sperm Concentration
2 3 4 5 6 7 8 9 10 11 12 13 14 15 16 17 18 19 20 21 22 23 24
Week of Follow-up

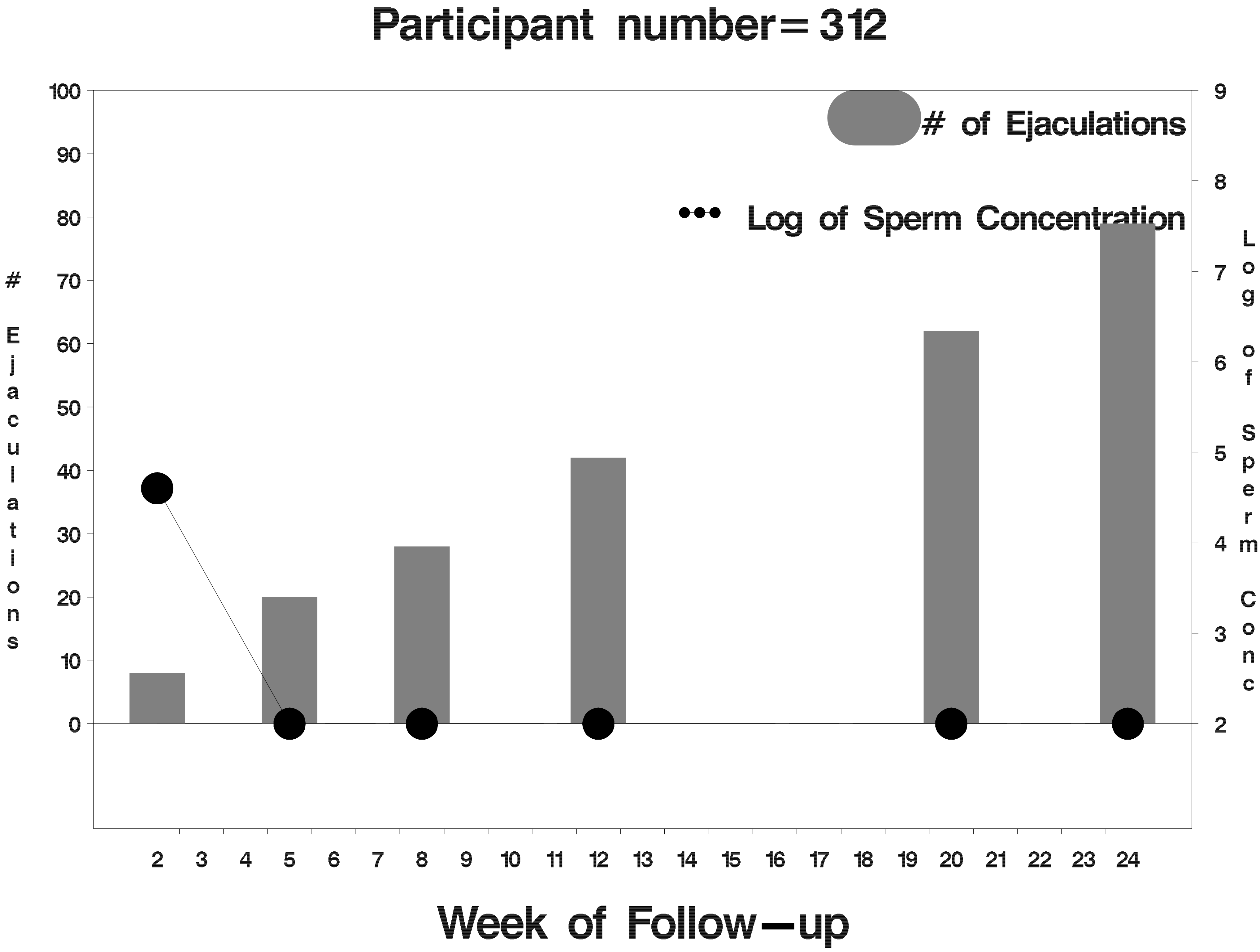

Participant number=312
# of Ejaculations
Log of Sperm Concentration
Ejaculations
Log of Sperm Conc
100
90
80
70
60
50
40
30
20
10
0
9
8
7
6
5
4
3
2
2 3 4 5 6 7 8 9 10 11 12 13 14 15 16 17 18 19 20 21 22 23 24
Week of Follow-up

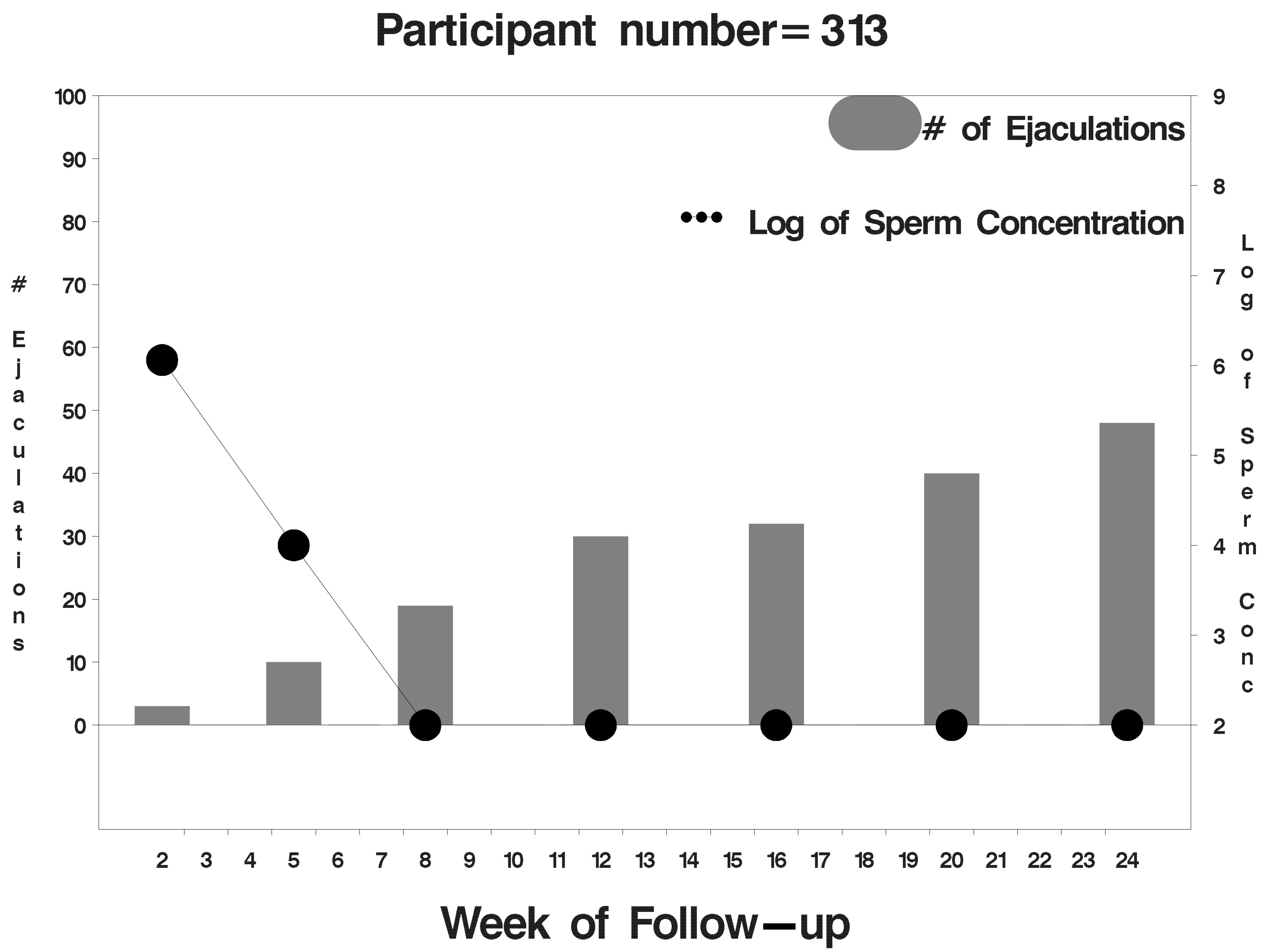

Participant number=313
# of Ejaculations
Log of Sperm Concentration
Ejaculations
Log of Sperm Conc
100
90
80
70
60
50
40
30
20
10
0
9
8
7
6
5
4
3
2
2 3 4 5 6 7 8 9 10 11 12 13 14 15 16 17 18 19 20 21 22 23 24
Week of Follow-up

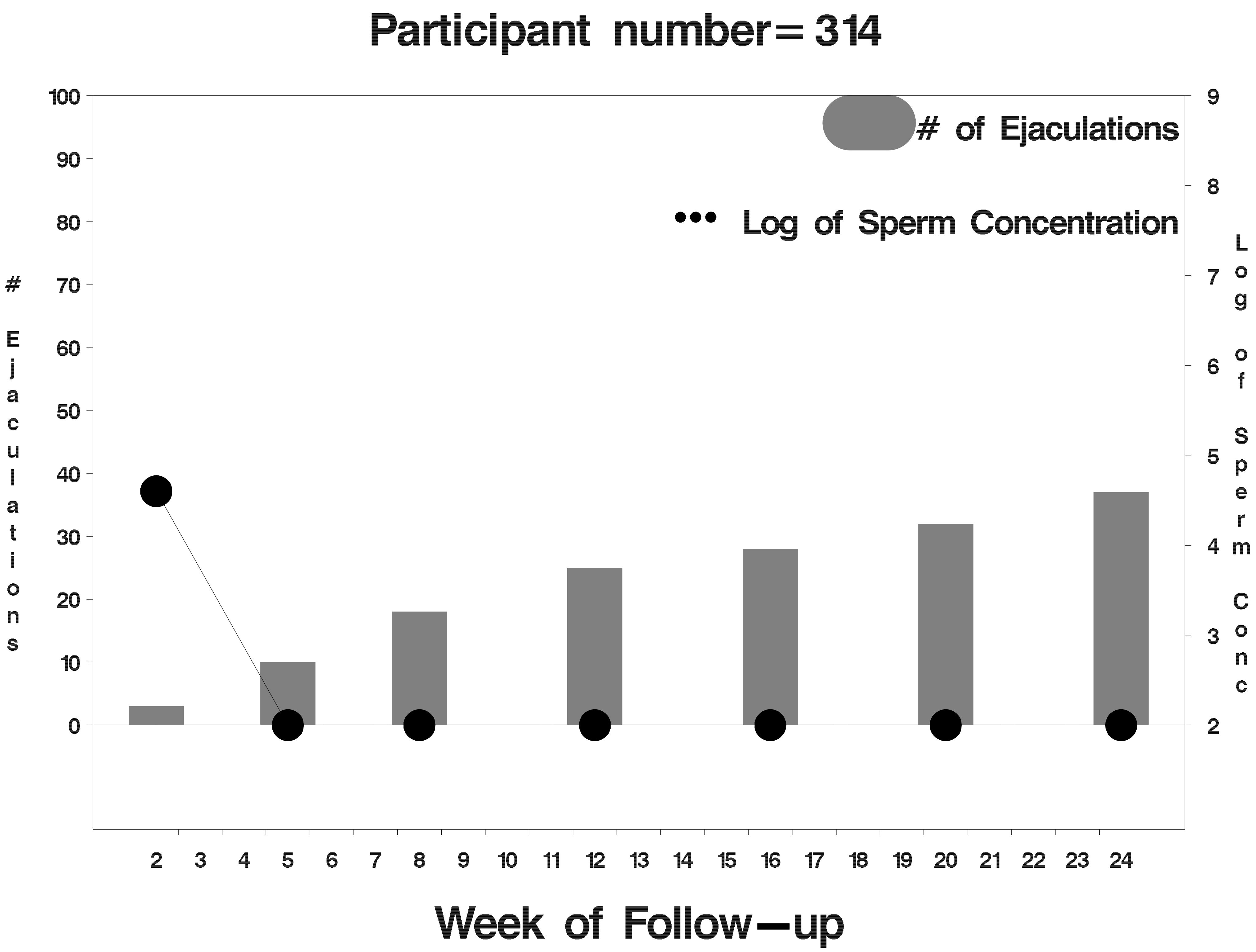
Participant number=314
# of Ejaculations
Log of Sperm Concentration
Ejaculations
Log of Sperm Conc
100
90
80
70
60
50
40
30
20
10
0
9
8
7
6
5
4
3
2
2 3 4 5 6 7 8 9 10 11 12 13 14 15 16 17 18 19 20 21 22 23 24
Week of Follow-up

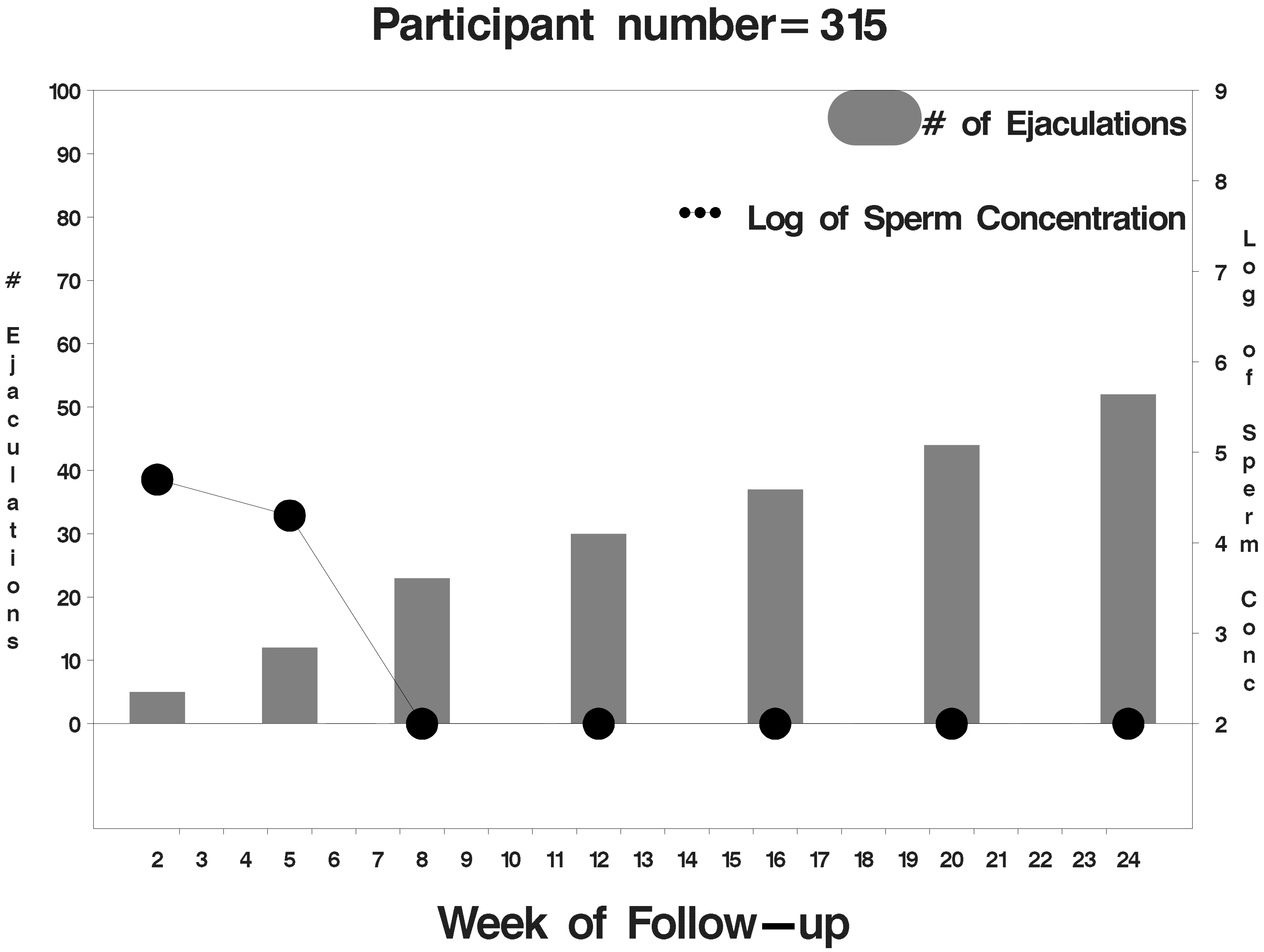
Participant number=315
# of Ejaculations
Log of Sperm Concentration
Ejaculations
Log of Sperm Conc
100
90
80
70
60
50
40
30
20
10
0
9
8
7
6
5
4
3
2
2 3 4 5 6 7 8 9 10 11 12 13 14 15 16 17 18 19 20 21 22 23 24
Week of Follow-up

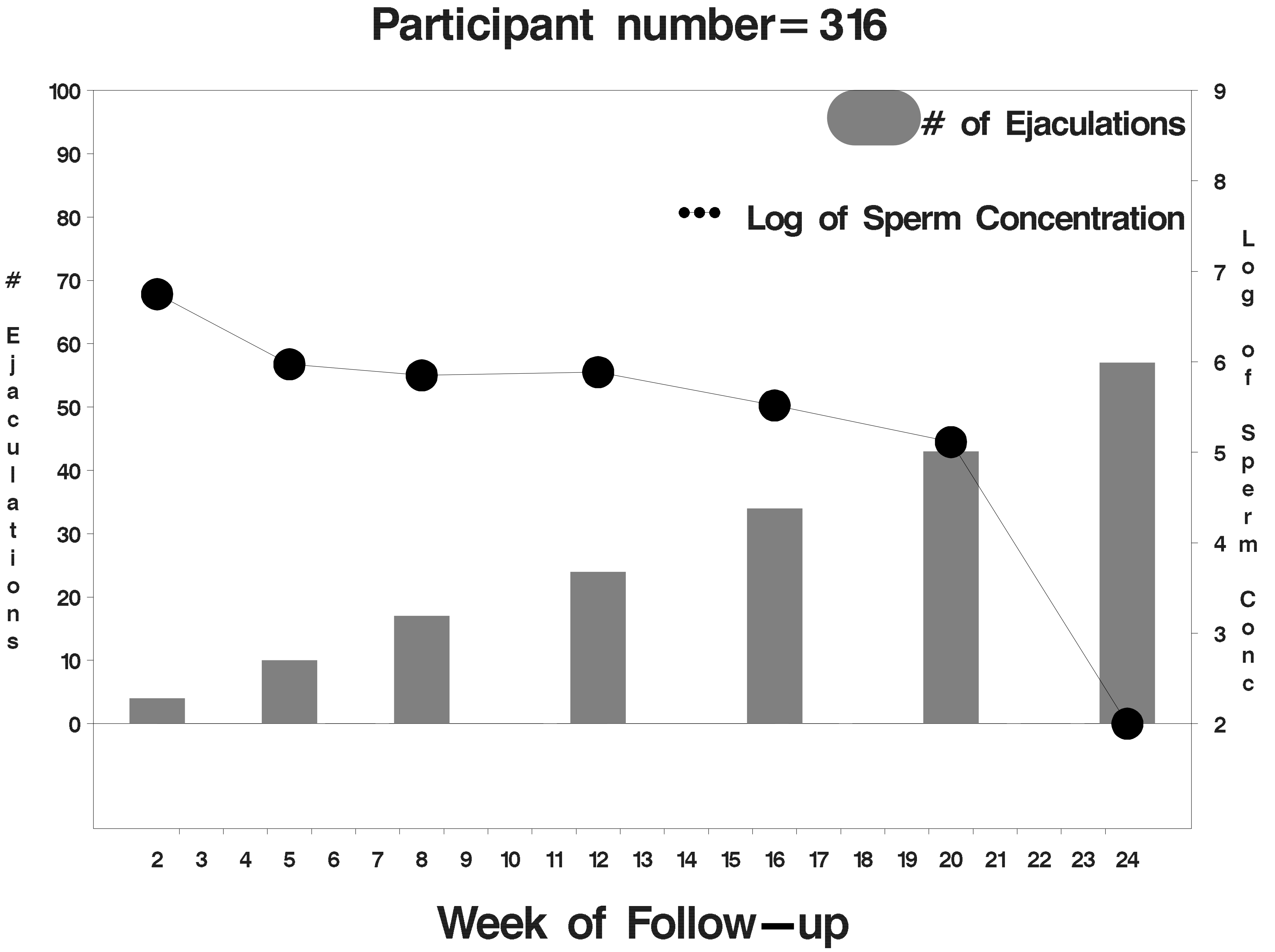

Participant number=316
# of Ejaculations
Log of Sperm Concentration
Ejaculations
Log of Sperm Conc
100
90
80
70
60
50
40
30
20
10
0
9
8
7
6
5
4
3
2
2 3 4 5 6 7 8 9 10 11 12 13 14 15 16 17 18 19 20 21 22 23 24
Week of Follow-up

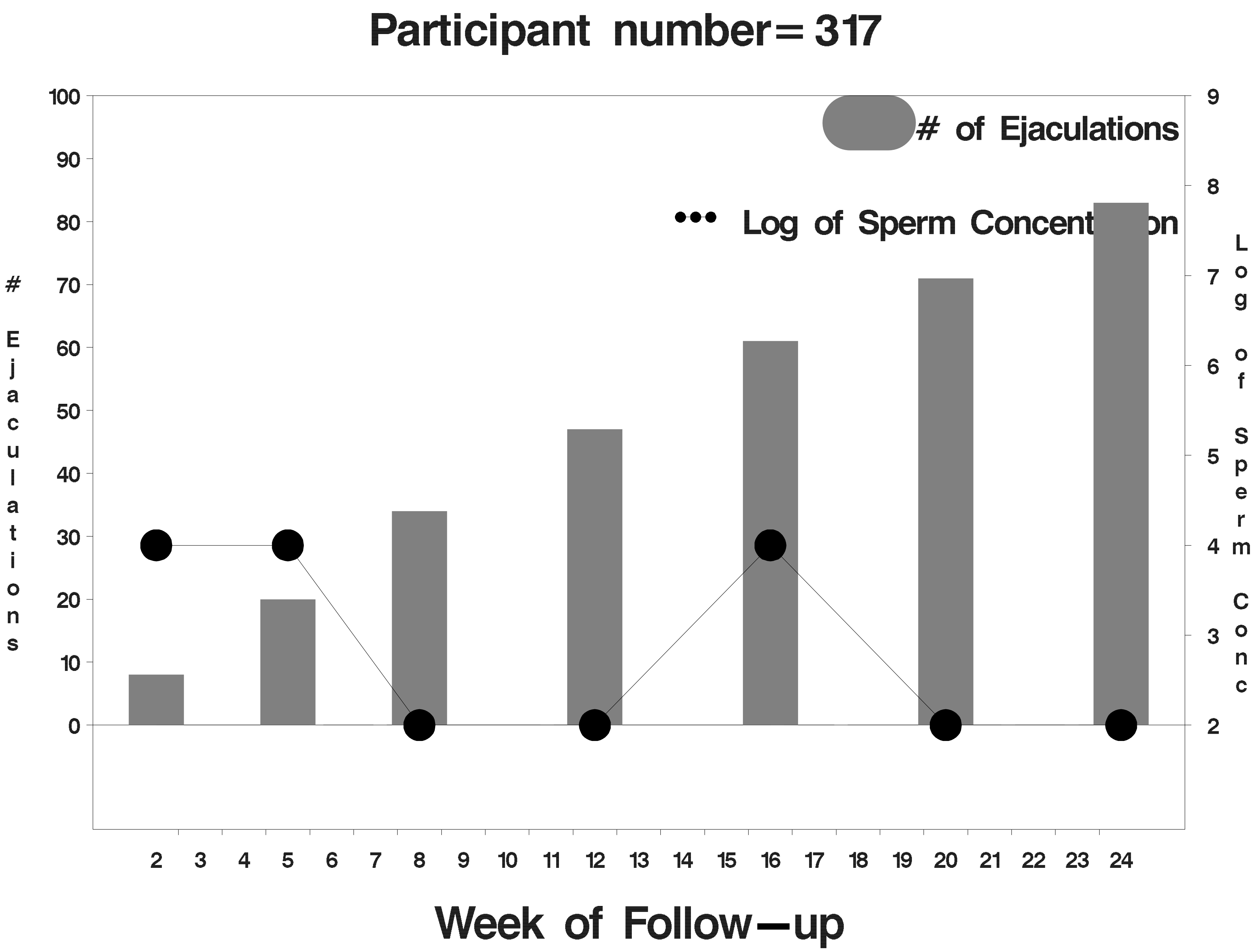

Participant number=317
# of Ejaculations
Log of Sperm Concent[illegible]on
Ejaculations
Log of Sperm Conc
100
90
80
70
60
50
40
30
20
10
0
9
8
7
6
5
4
3
2
2 3 4 5 6 7 8 9 10 11 12 13 14 15 16 17 18 19 20 21 22 23 24
Week of Follow-up

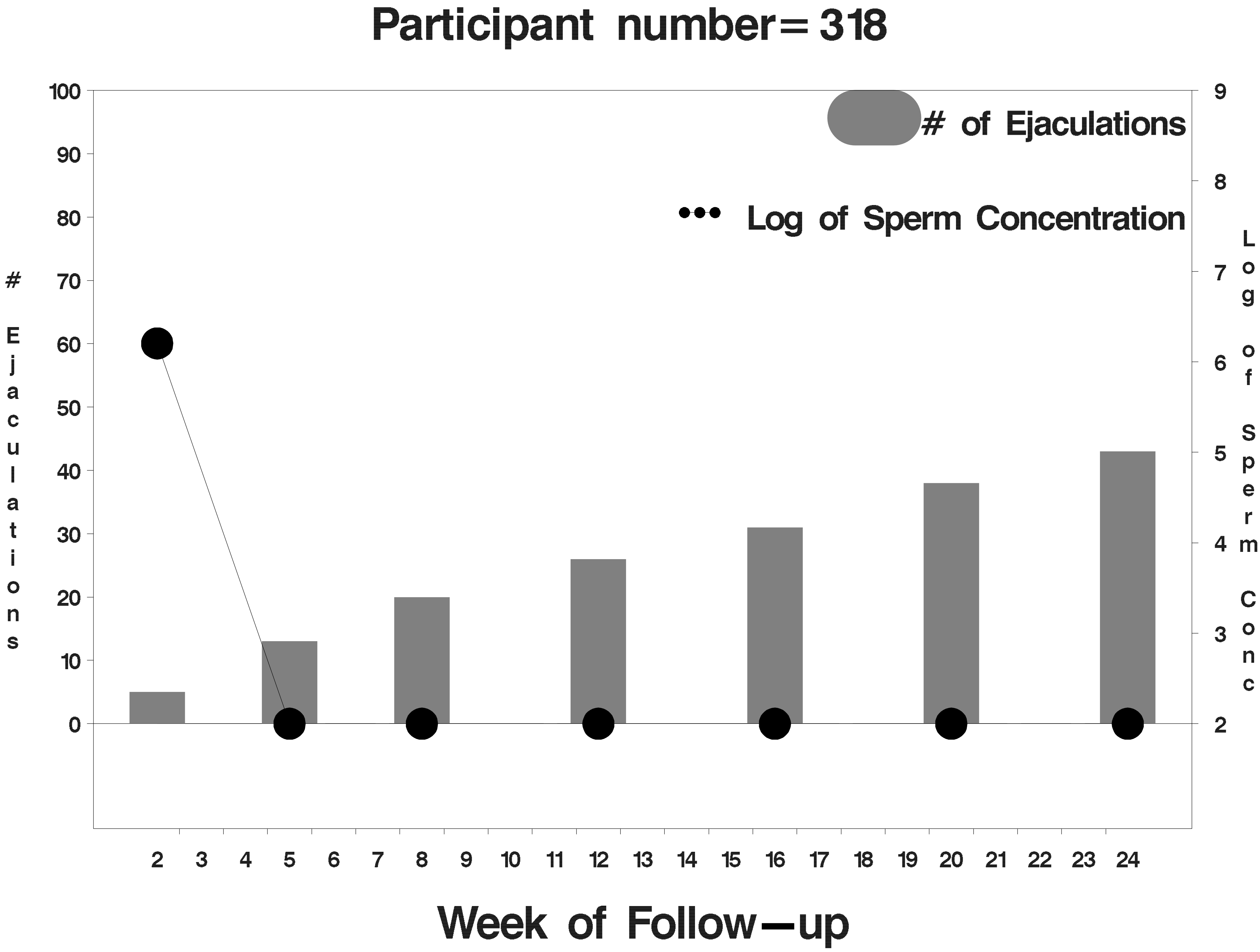

Participant number=318
# of Ejaculations
Log of Sperm Concentration
Ejaculations
Log of Sperm Conc
100
90
80
70
60
50
40
30
20
10
0
9
8
7
6
5
4
3
2
2 3 4 5 6 7 8 9 10 11 12 13 14 15 16 17 18 19 20 21 22 23 24
Week of Follow-up

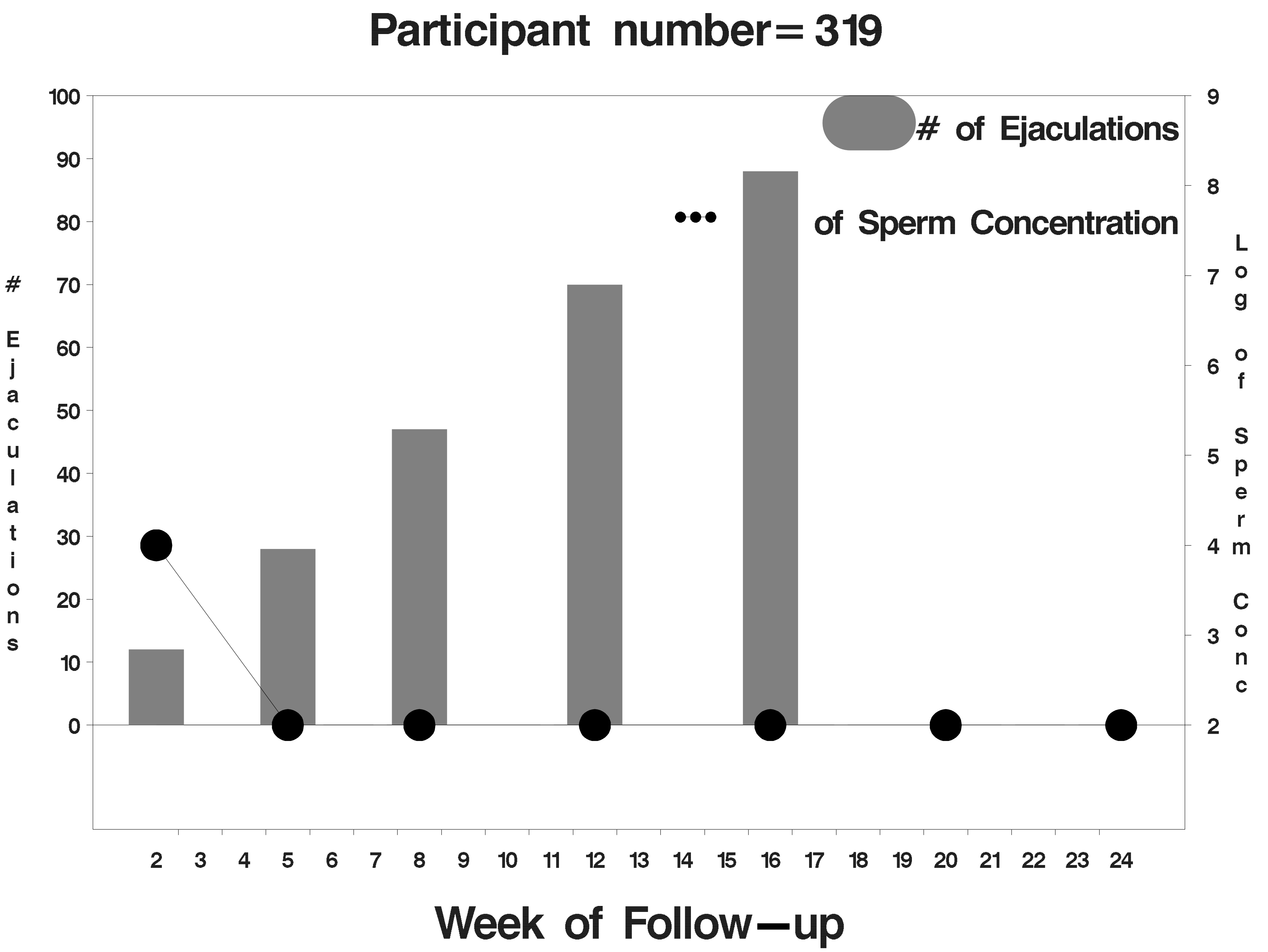

Participant number=319
# of Ejaculations
of Sperm Concentration
Ejaculations
Log of Sperm Conc
100
90
80
70
60
50
40
30
20
10
0
9
8
7
6
5
4
3
2
2 3 4 5 6 7 8 9 10 11 12 13 14 15 16 17 18 19 20 21 22 23 24
Week of Follow-up

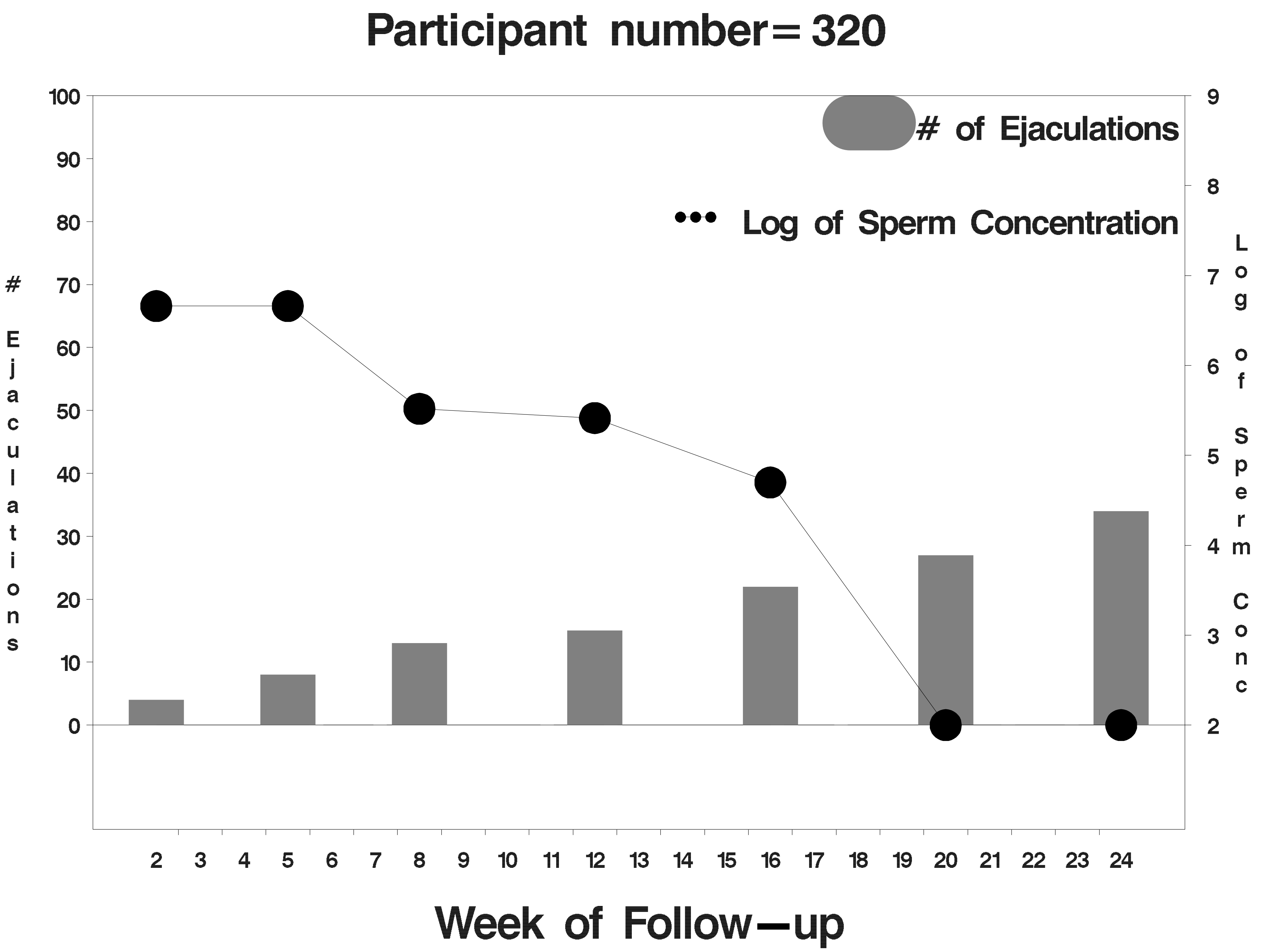

Participant number=320
# of Ejaculations
Log of Sperm Concentration
Ejaculations
Log of Sperm Conc
100
90
80
70
60
50
40
30
20
10
0
9
8
7
6
5
4
3
2
2 3 4 5 6 7 8 9 10 11 12 13 14 15 16 17 18 19 20 21 22 23 24
Week of Follow−up

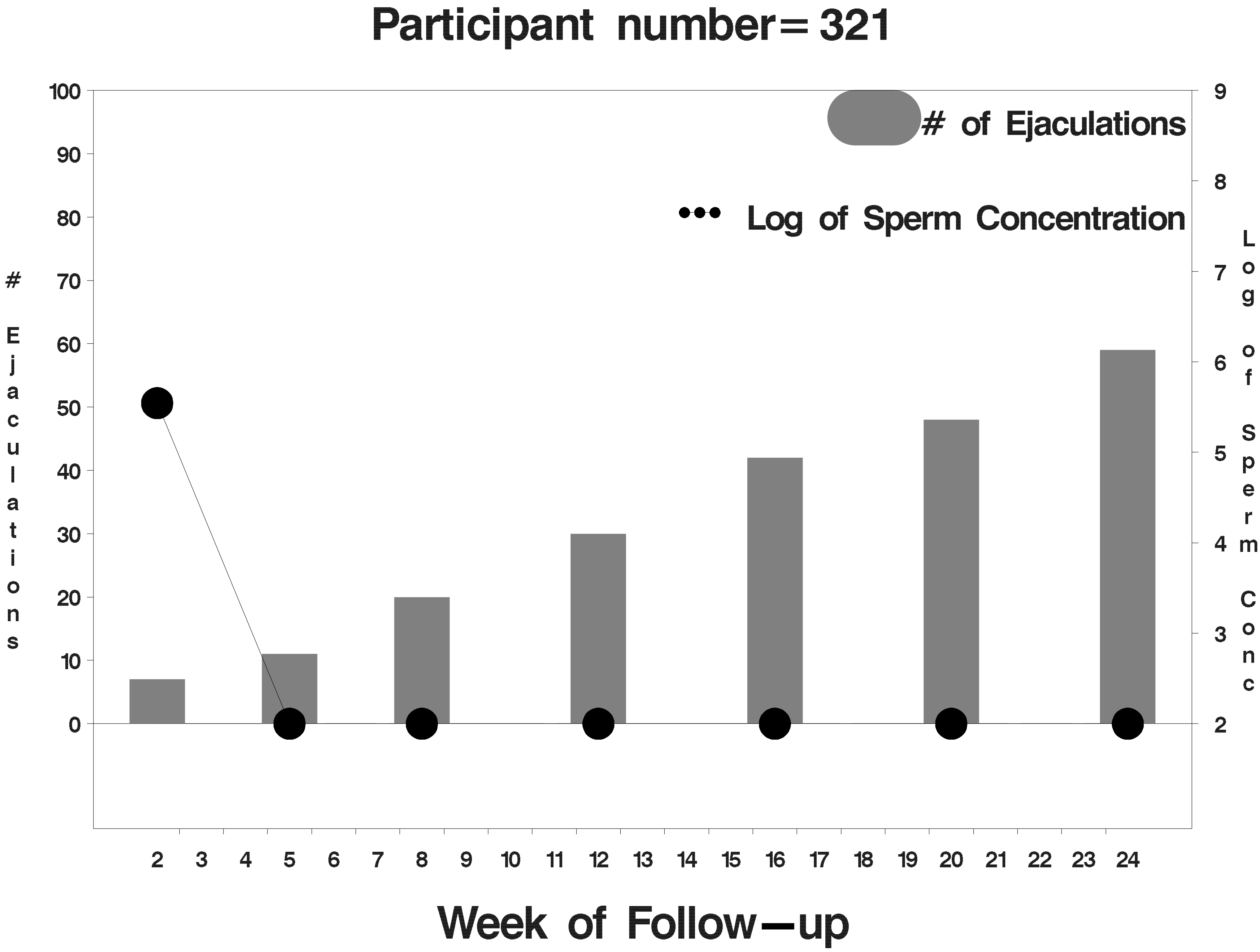

Participant number=321
# of Ejaculations
Log of Sperm Concentration
Ejaculations
Log of Sperm Conc
Week of Follow-up
100
90
80
70
60
50
40
30
20
10
0
9
8
7
6
5
4
3
2
2 3 4 5 6 7 8 9 10 11 12 13 14 15 16 17 18 19 20 21 22 23 24

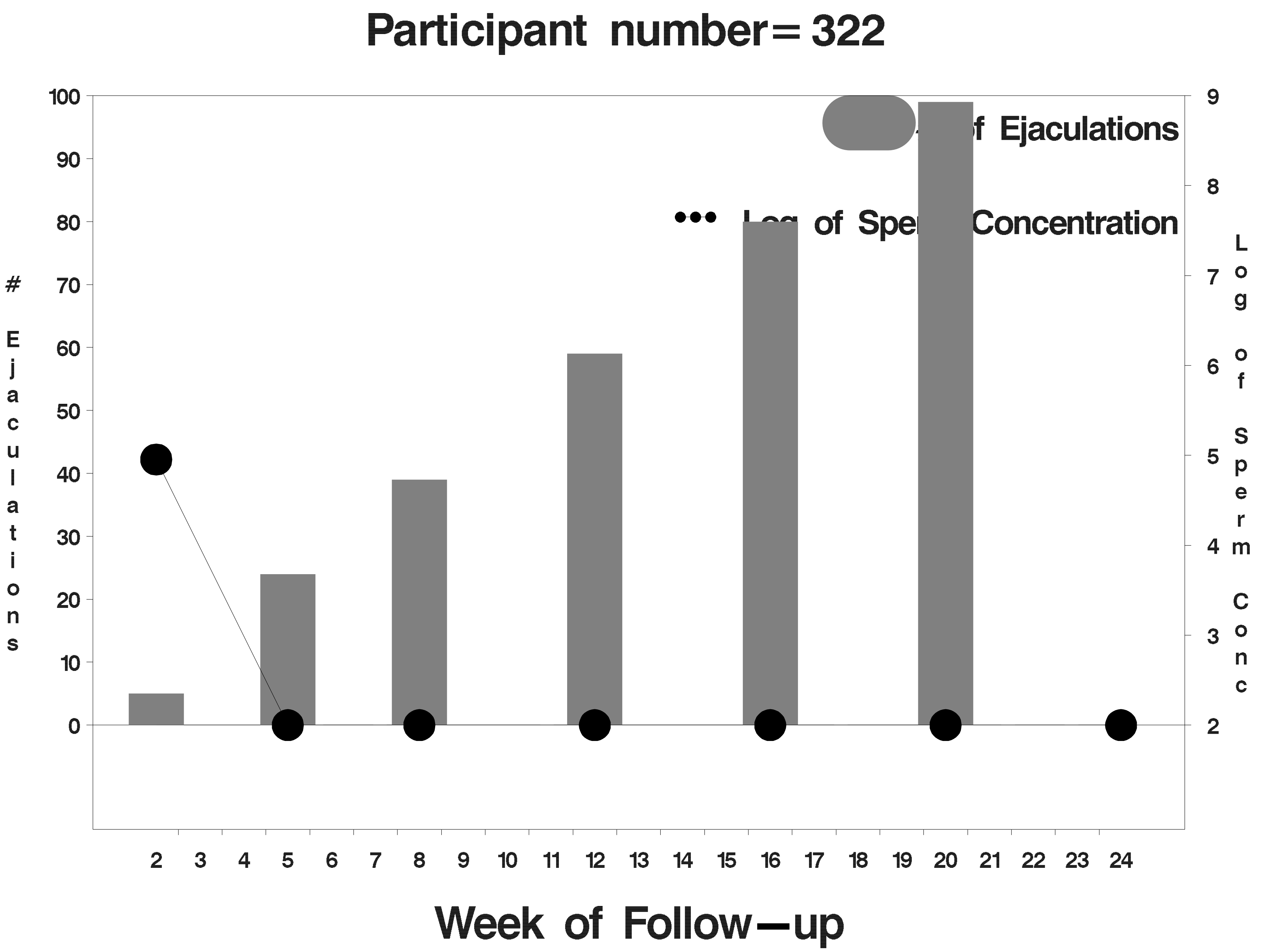
Participant number= 322
# of Ejaculations
Log of Sperm Concentration
Ejaculations
Log of Sperm Conc
100
90
80
70
60
50
40
30
20
10
0
9
8
7
6
5
4
3
2
2 3 4 5 6 7 8 9 10 11 12 13 14 15 16 17 18 19 20 21 22 23 24
Week of Follow-up

Participant number=323

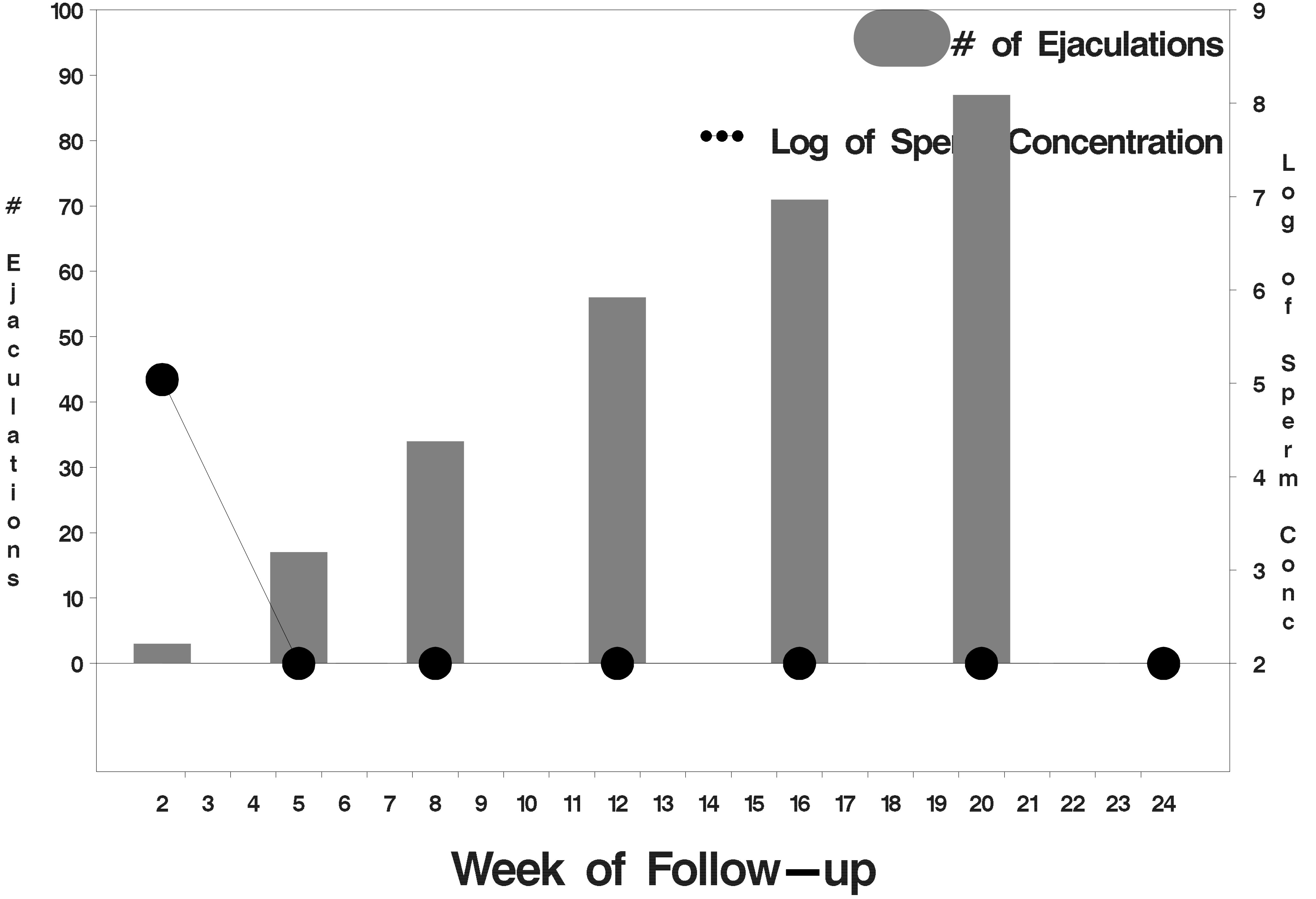

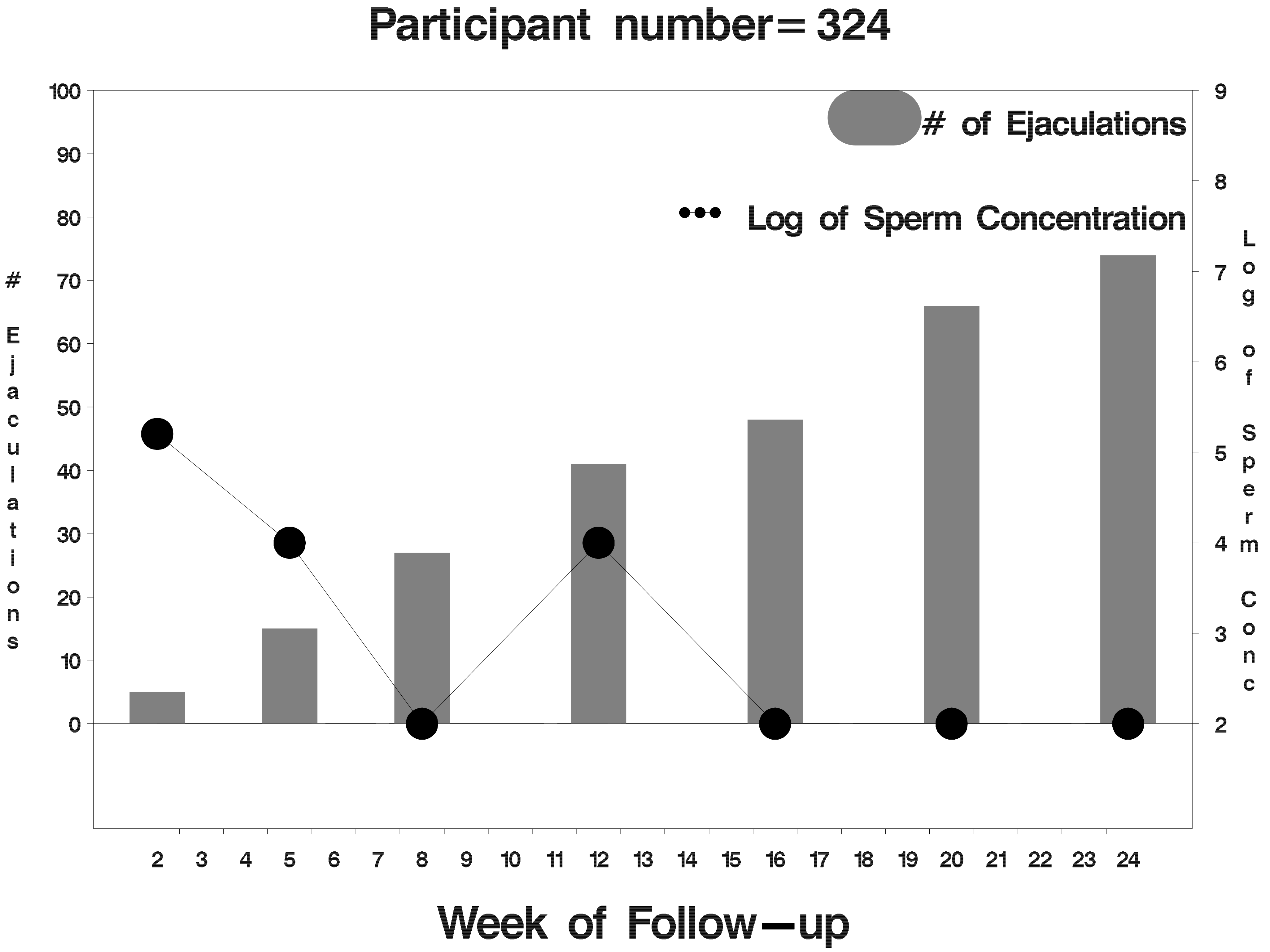
Participant number=324
# of Ejaculations
Log of Sperm Concentration
Ejaculations
Log of Sperm Conc
100
90
80
70
60
50
40
30
20
10
0
9
8
7
6
5
4
3
2
2 3 4 5 6 7 8 9 10 11 12 13 14 15 16 17 18 19 20 21 22 23 24
Week of Follow-up

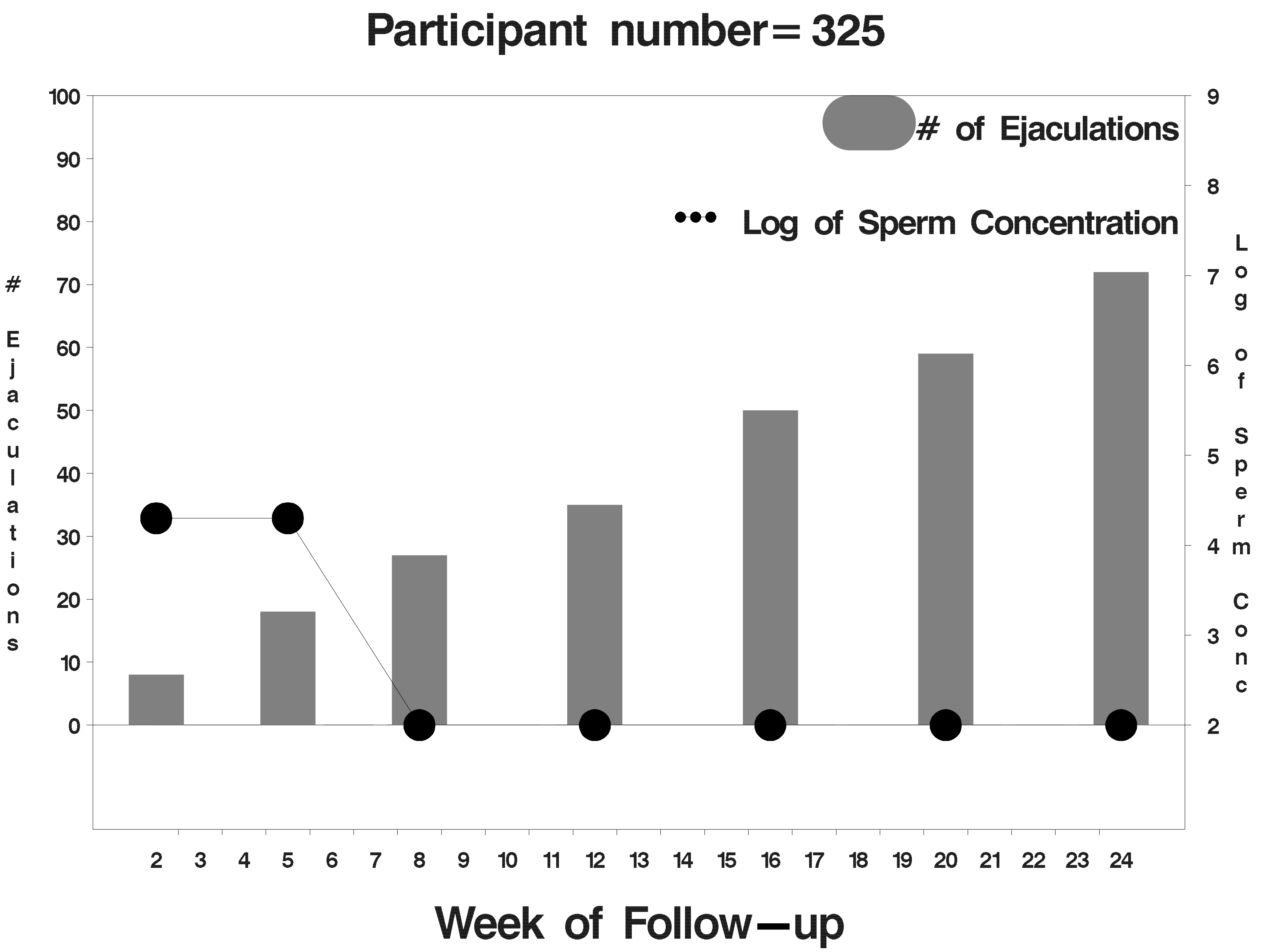

Participant number=325
# of Ejaculations
Log of Sperm Concentration
Ejaculations
Log of Sperm Conc
100
90
80
70
60
50
40
30
20
10
0
9
8
7
6
5
4
3
2
2 3 4 5 6 7 8 9 10 11 12 13 14 15 16 17 18 19 20 21 22 23 24
Week of Follow-up

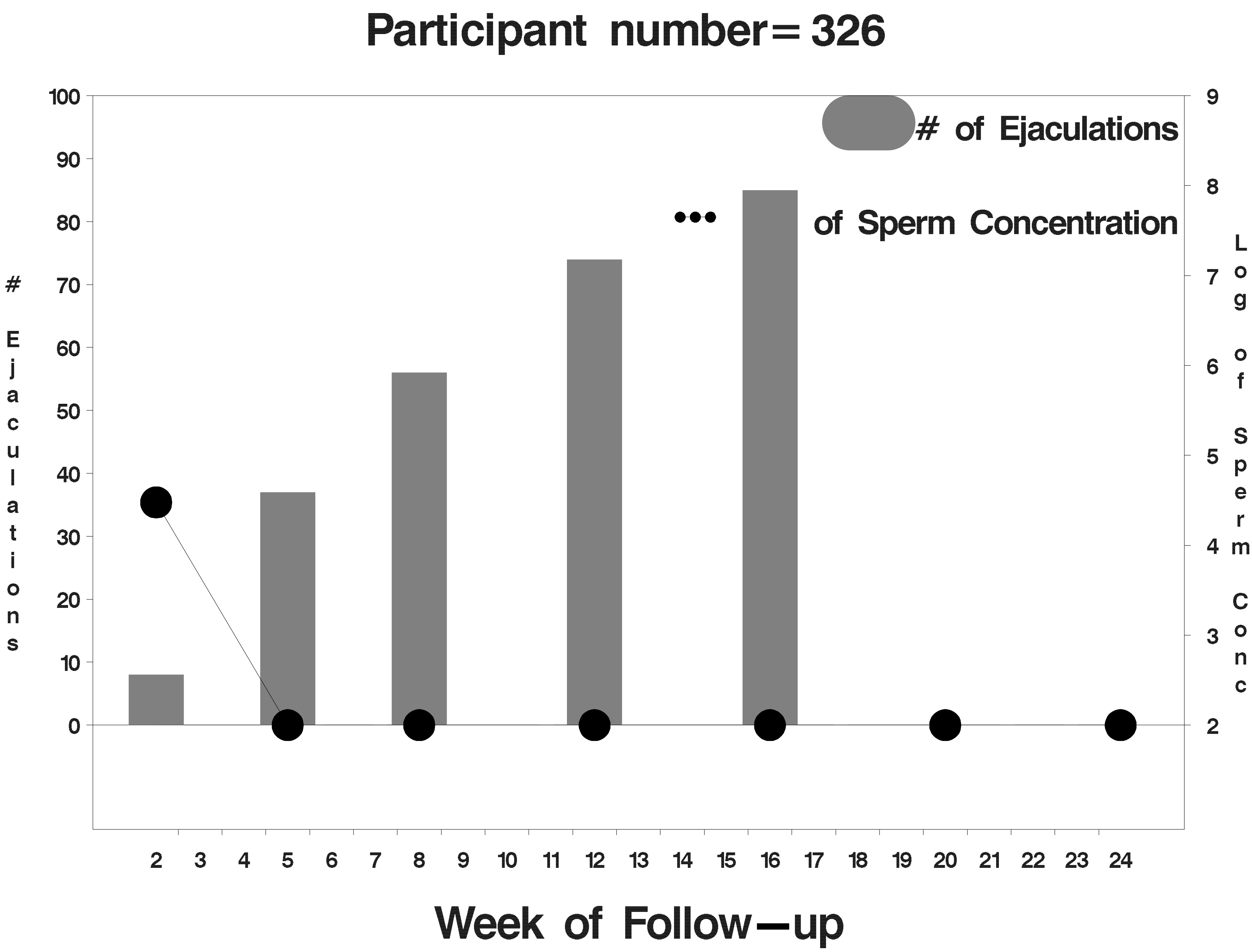

Participant number=326
100
90
80
70
60
50
40
30
20
10
0
Ejaculations
9
8
7
6
5
4
3
2
Log of Sperm Conc
# of Ejaculations
of Sperm Concentration
2 3 4 5 6 7 8 9 10 11 12 13 14 15 16 17 18 19 20 21 22 23 24
Week of Follow-up

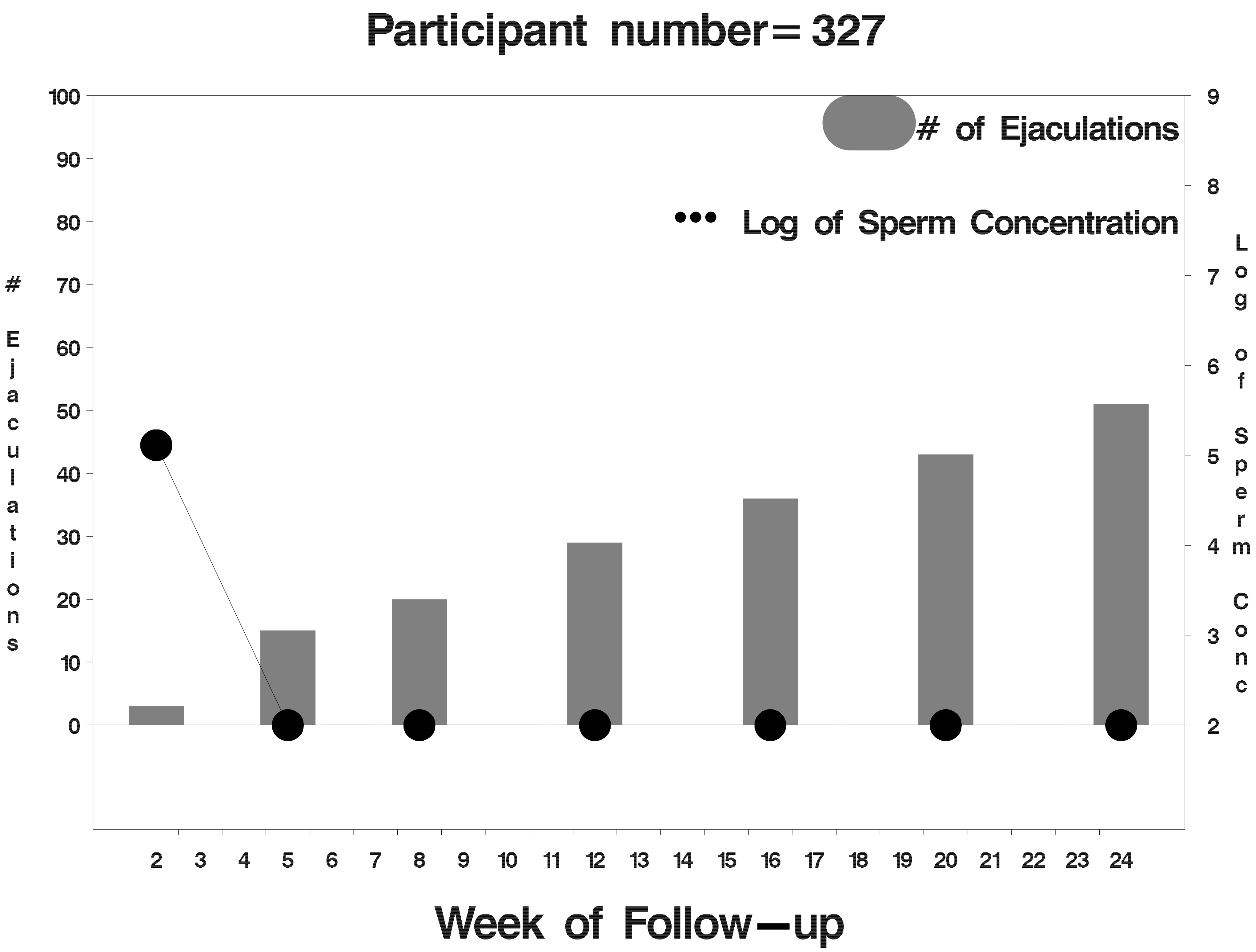

Participant number=327
# of Ejaculations
Log of Sperm Concentration
Ejaculations
Log of Sperm Conc
100
90
80
70
60
50
40
30
20
10
0
9
8
7
6
5
4
3
2
2 3 4 5 6 7 8 9 10 11 12 13 14 15 16 17 18 19 20 21 22 23 24
Week of Follow-up

Participant number= 328

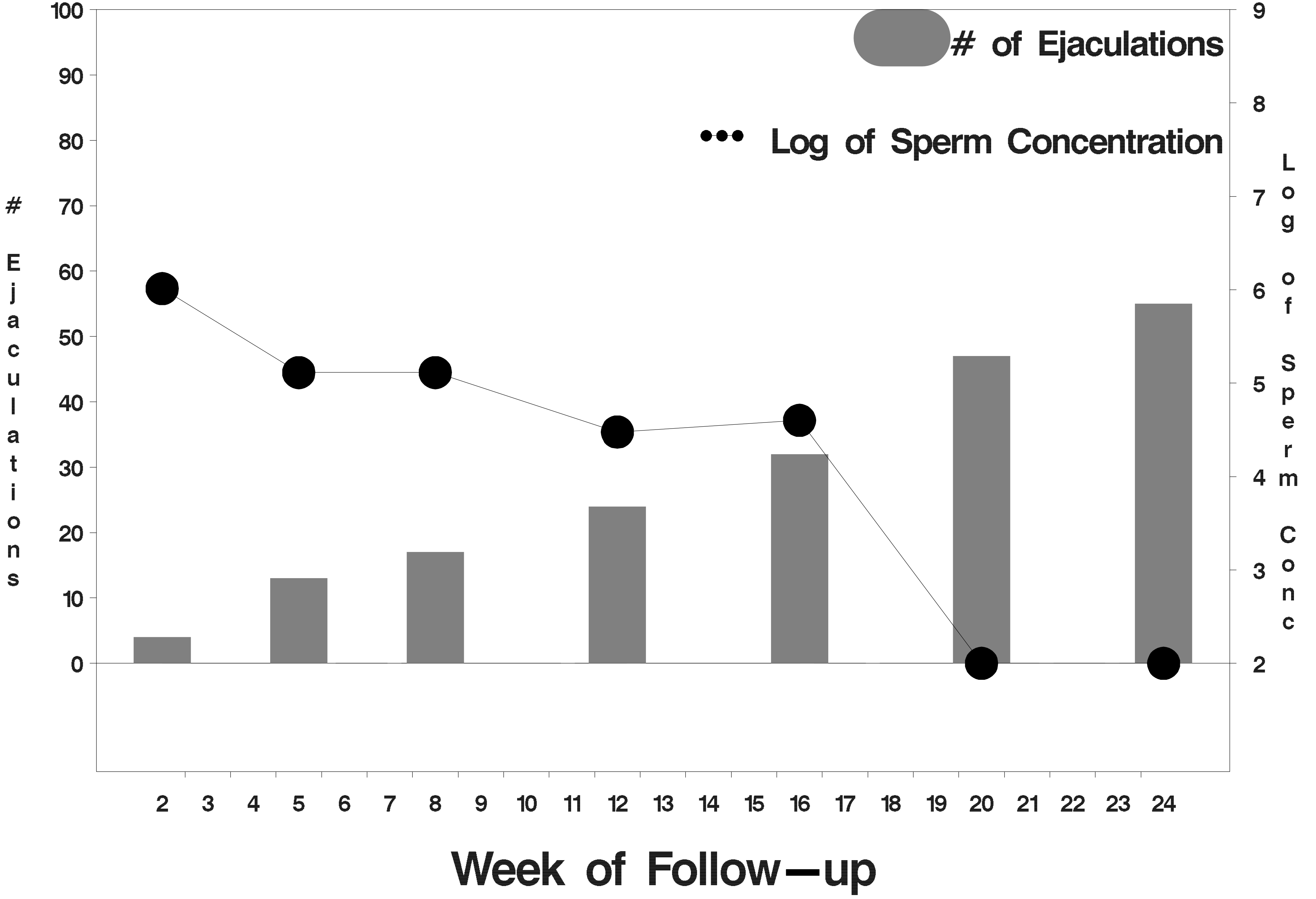

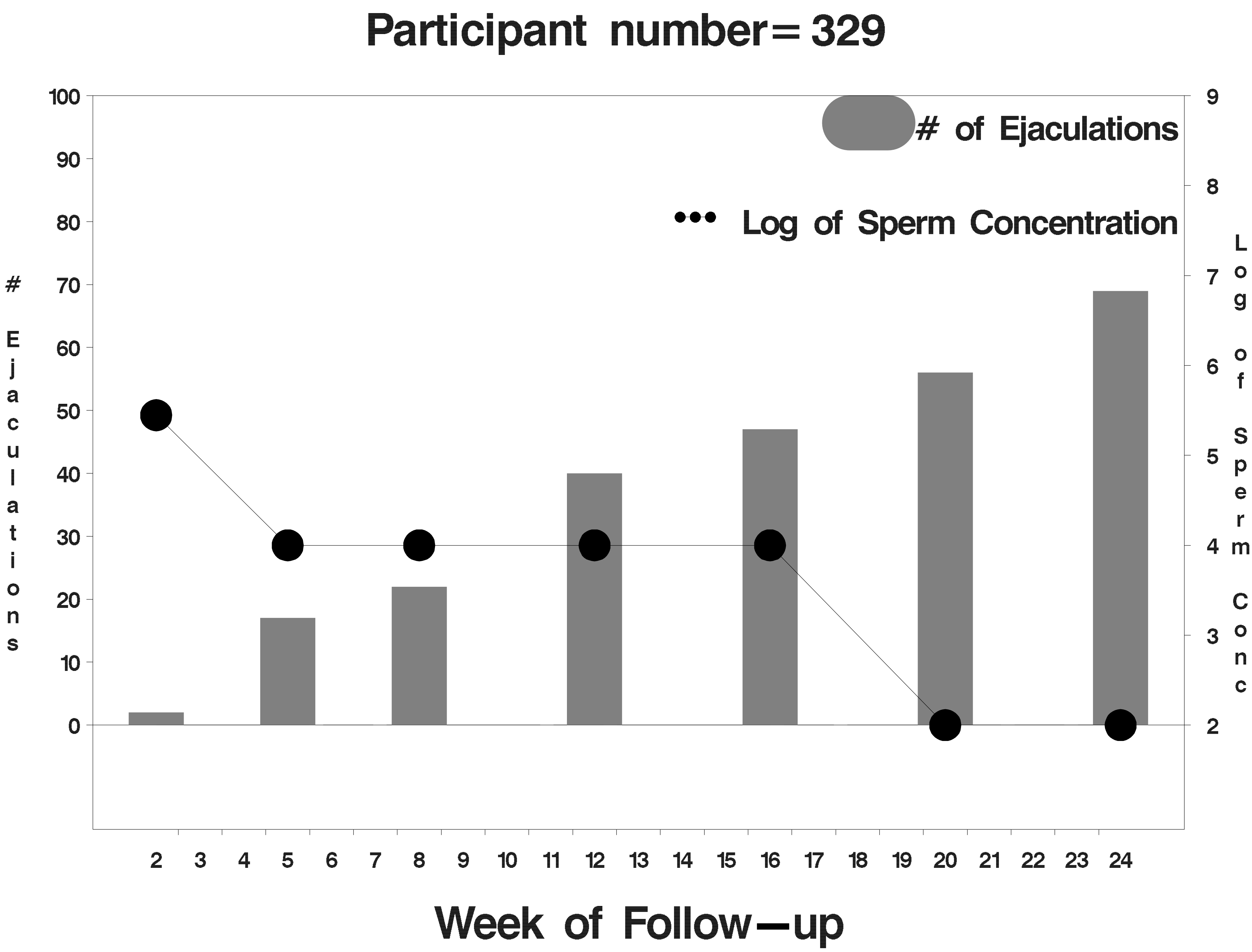

Participant number= 329
# of Ejaculations
Log of Sperm Concentration
Ejaculations
Log of Sperm Conc
100
90
80
70
60
50
40
30
20
10
0
9
8
7
6
5
4
3
2
2 3 4 5 6 7 8 9 10 11 12 13 14 15 16 17 18 19 20 21 22 23 24
Week of Follow-up

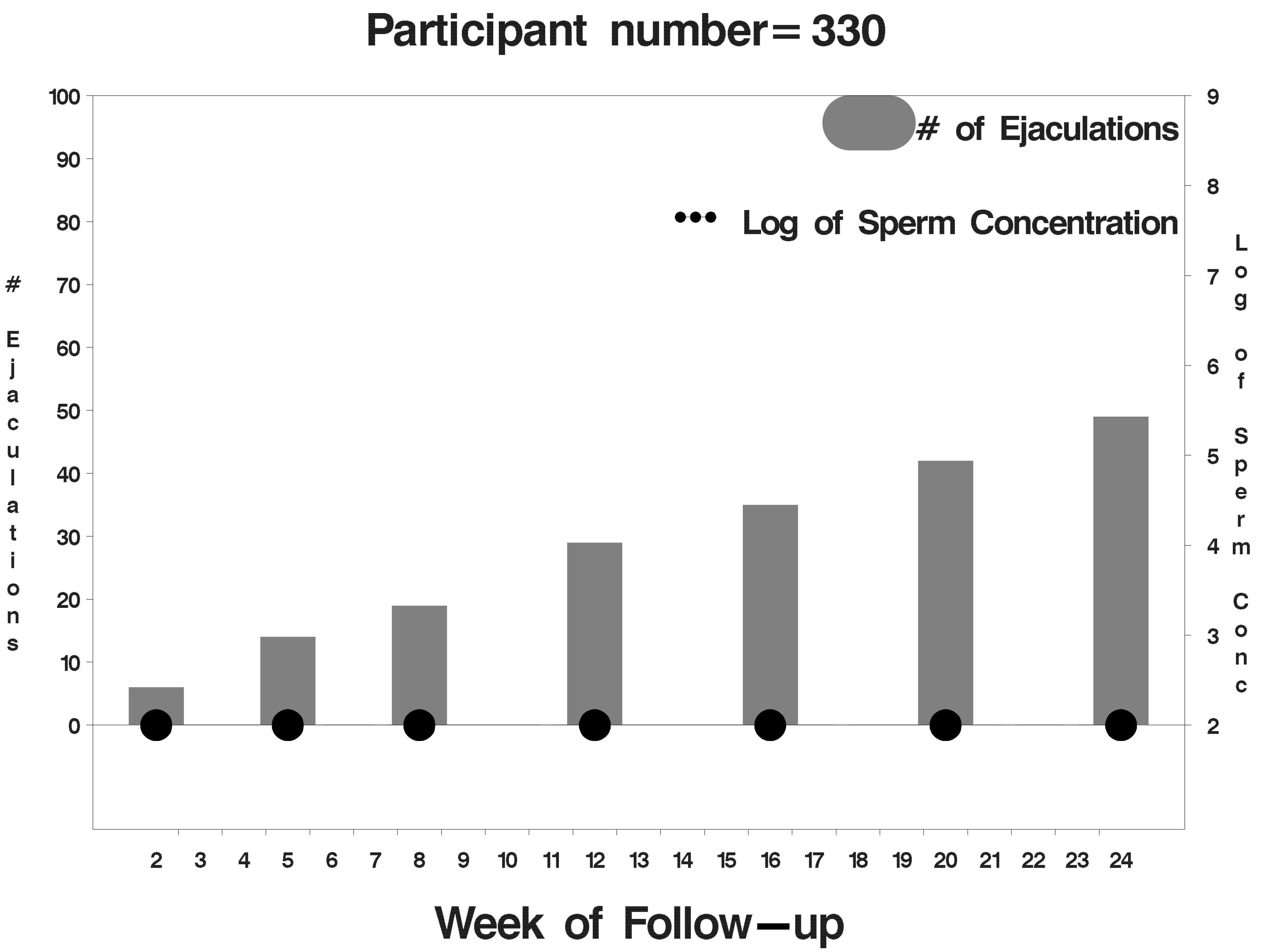
Participant number=330
# of Ejaculations
Log of Sperm Concentration
Ejaculations
Log of Sperm Conc
100
90
80
70
60
50
40
30
20
10
0
9
8
7
6
5
4
3
2
2 3 4 5 6 7 8 9 10 11 12 13 14 15 16 17 18 19 20 21 22 23 24
Week of Follow-up

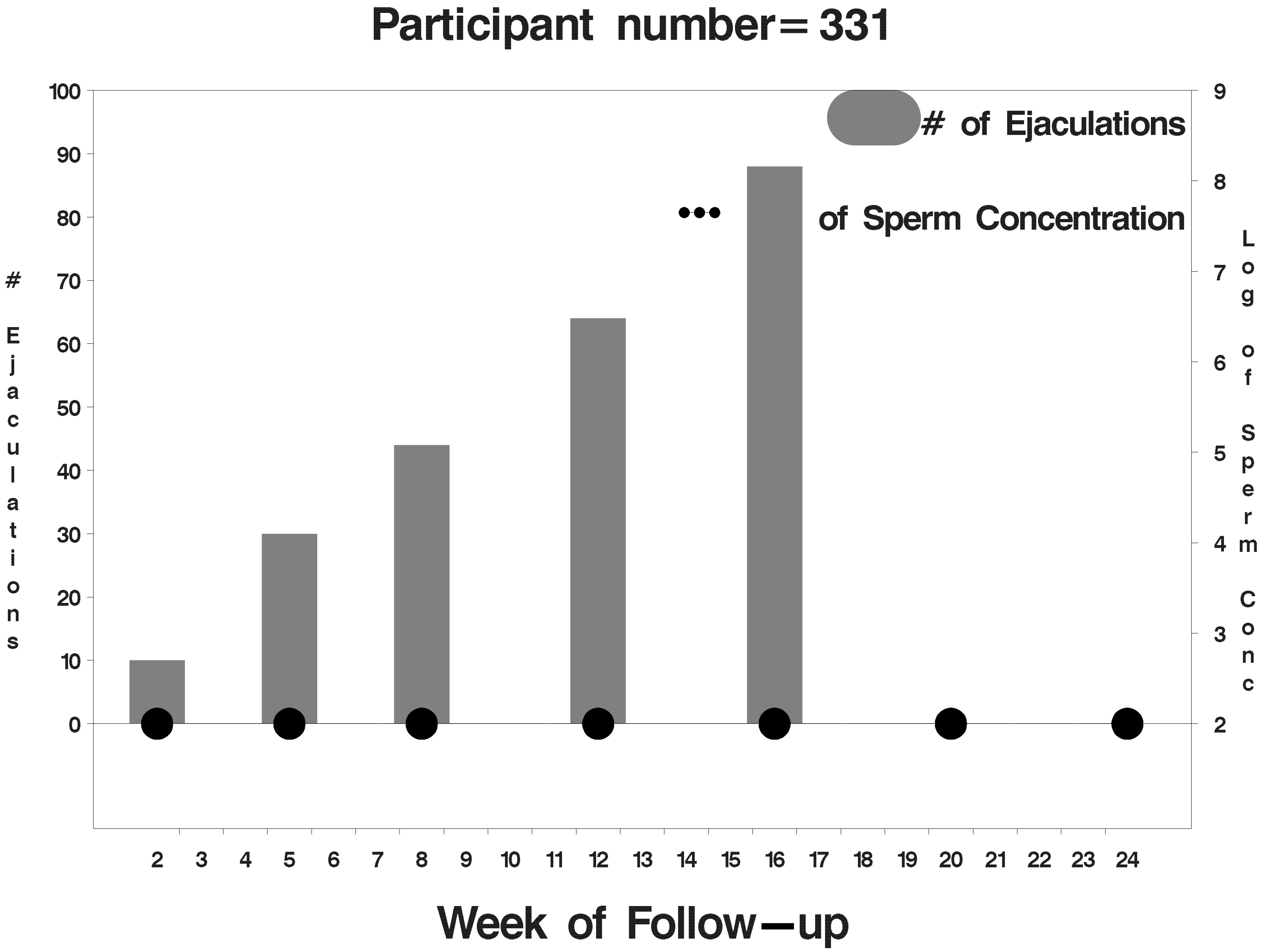
Participant number=331
# of Ejaculations
of Sperm Concentration
Ejaculations
Log of Sperm Conc
100
90
80
70
60
50
40
30
20
10
0
9
8
7
6
5
4
3
2
2 3 4 5 6 7 8 9 10 11 12 13 14 15 16 17 18 19 20 21 22 23 24
Week of Follow-up

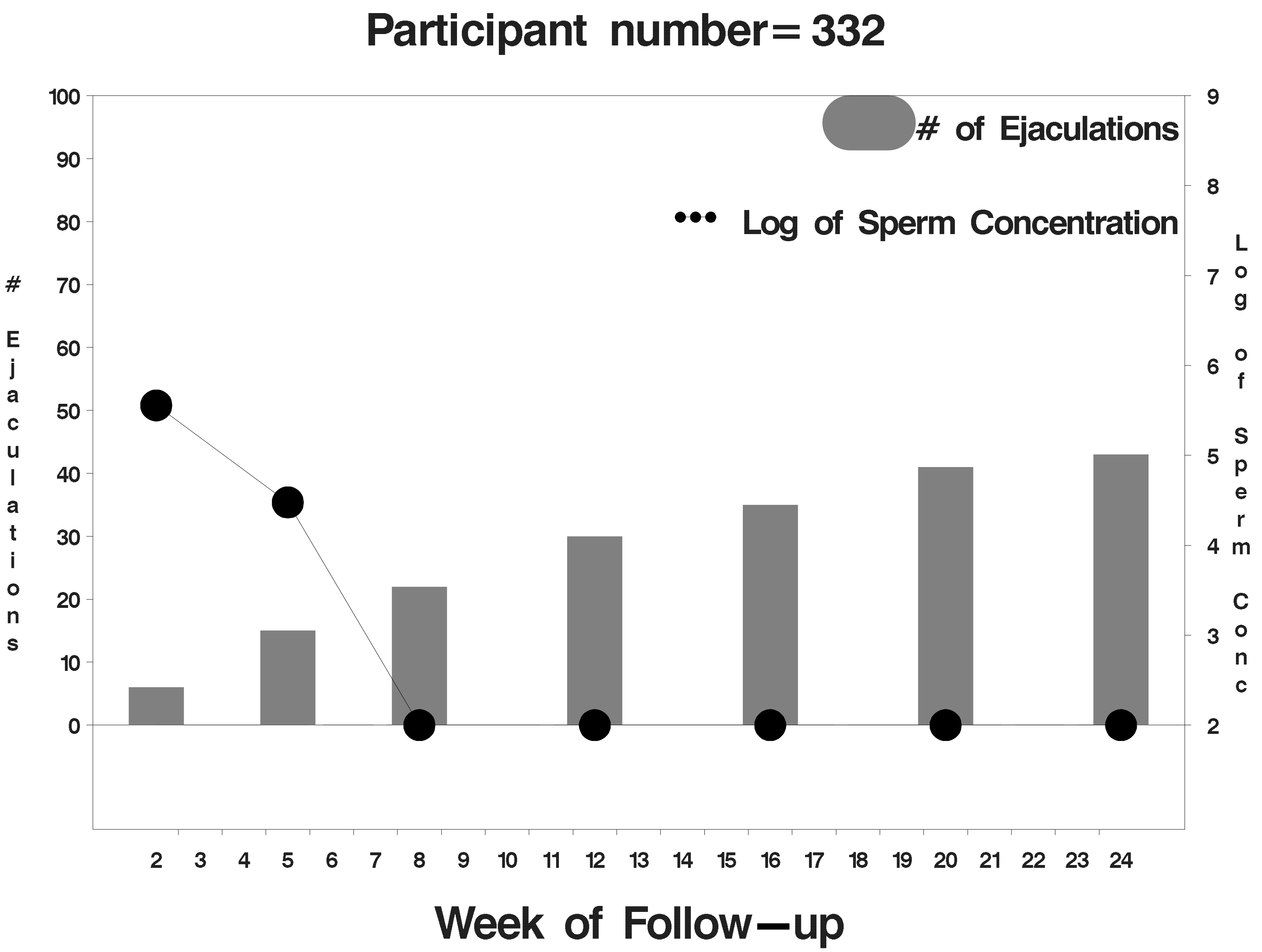
Participant number= 332
# of Ejaculations
Log of Sperm Concentration
Ejaculations
Log of Sperm Conc
0
10
20
30
40
50
60
70
80
90
100
2
3
4
5
6
7
8
9
2 3 4 5 6 7 8 9 10 11 12 13 14 15 16 17 18 19 20 21 22 23 24
Week of Follow-up

Participant number=333

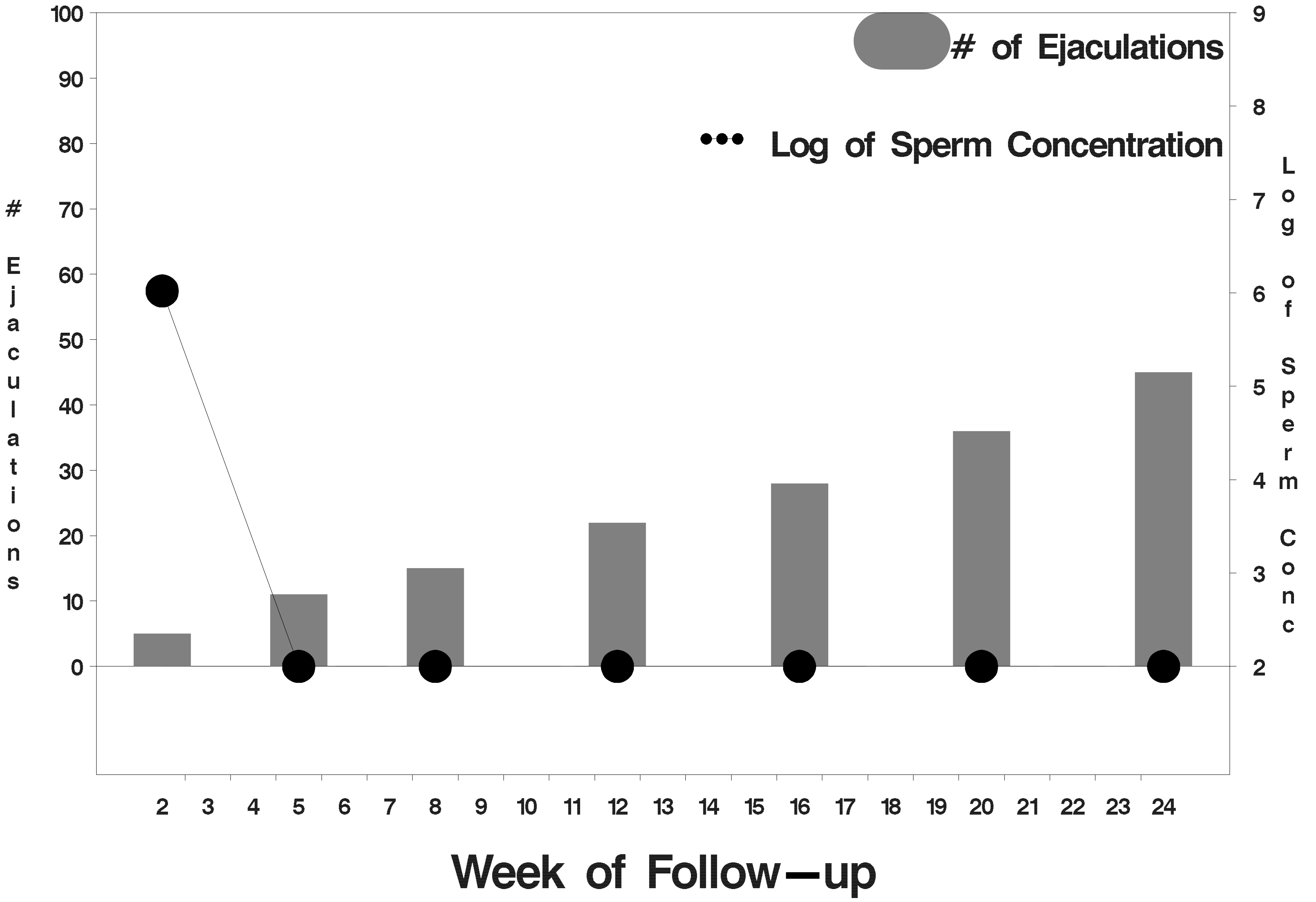

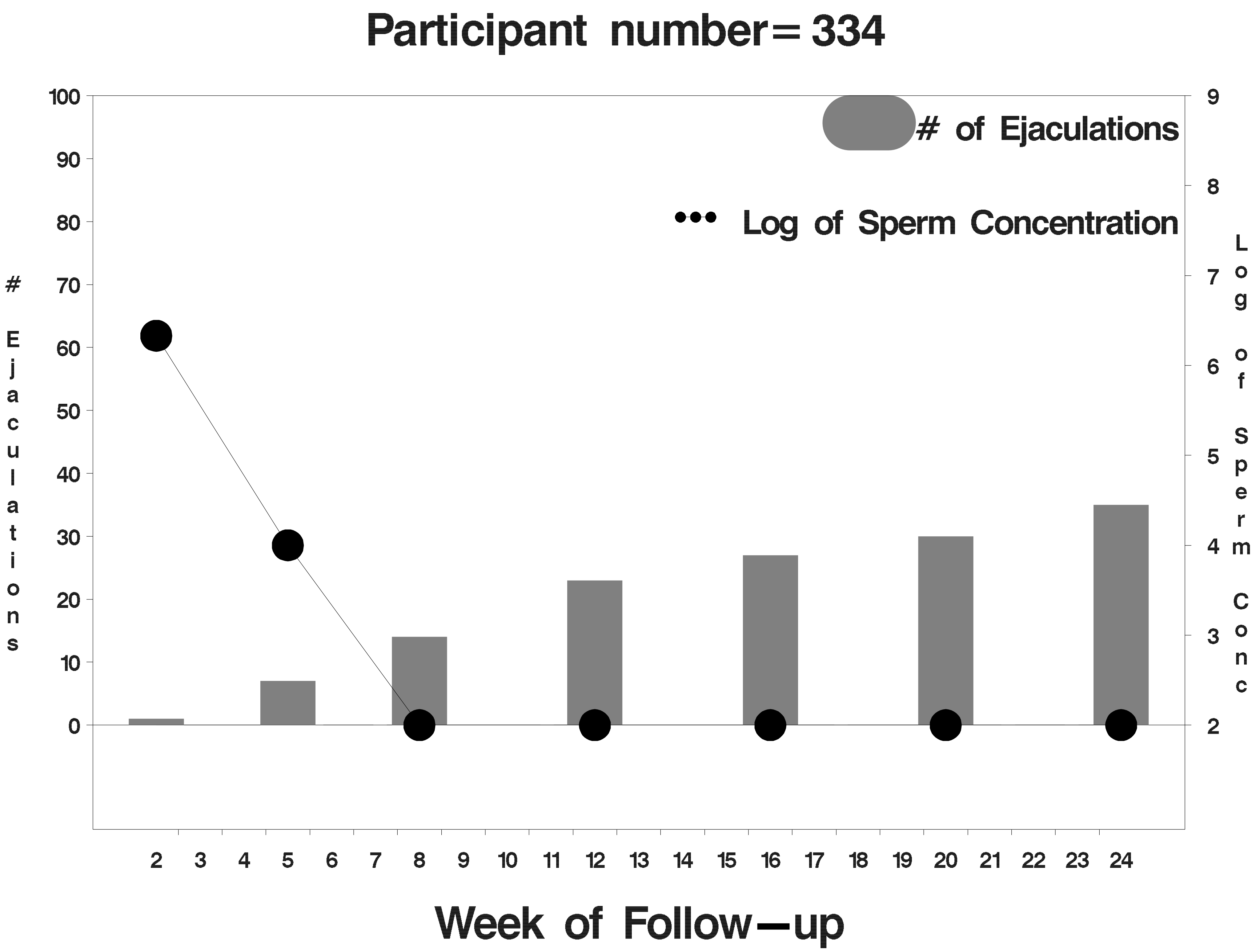

Participant number=334
# of Ejaculations
Log of Sperm Concentration
Ejaculations
Log of Sperm Conc
100
90
80
70
60
50
40
30
20
10
0
9
8
7
6
5
4
3
2
2 3 4 5 6 7 8 9 10 11 12 13 14 15 16 17 18 19 20 21 22 23 24
Week of Follow-up

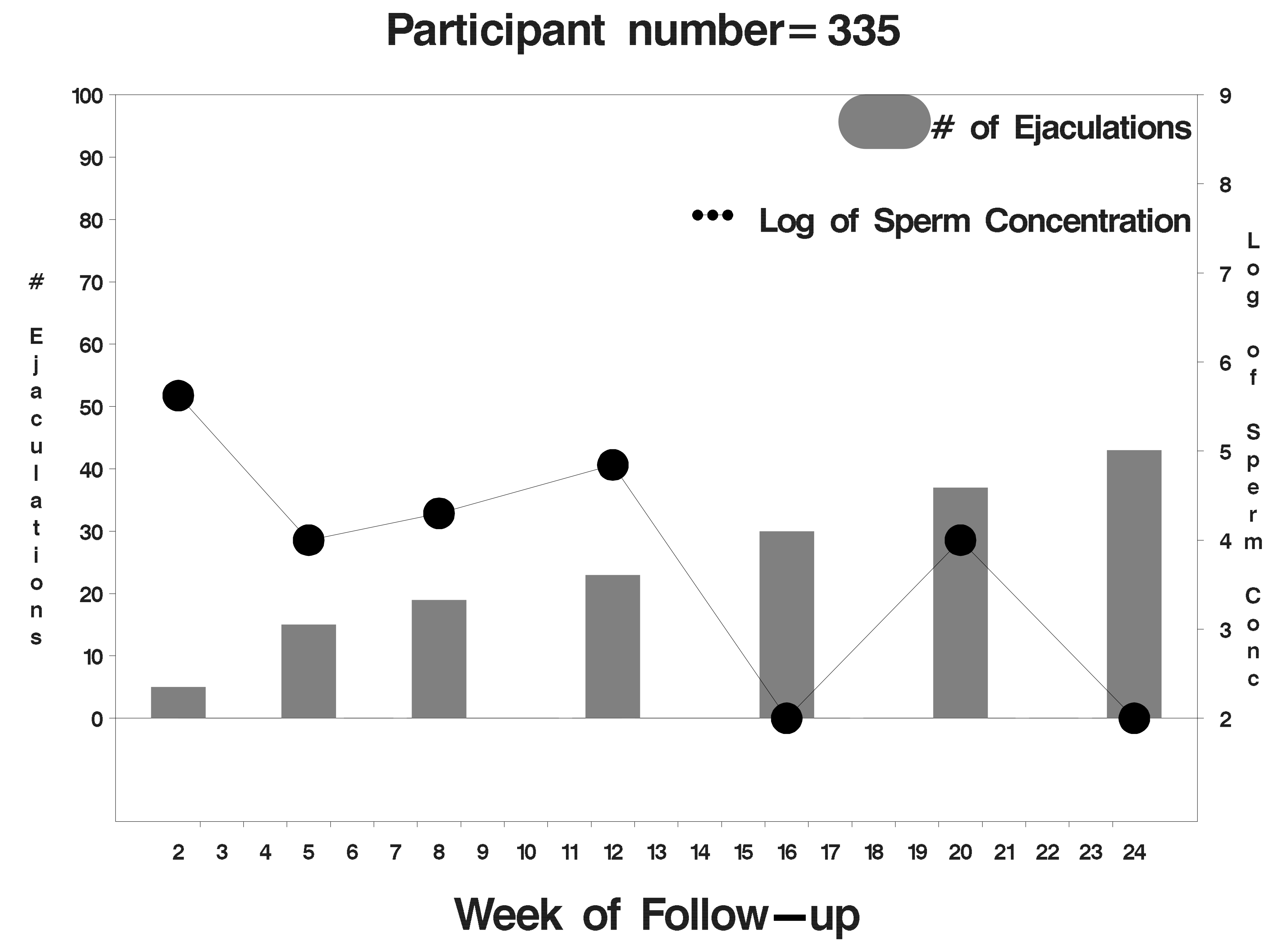

Participant number=335
# of Ejaculations
Log of Sperm Concentration
Ejaculations
Log of Sperm Conc
100
90
80
70
60
50
40
30
20
10
0
9
8
7
6
5
4
3
2
2 3 4 5 6 7 8 9 10 11 12 13 14 15 16 17 18 19 20 21 22 23 24
Week of Follow-up

Participant number=336

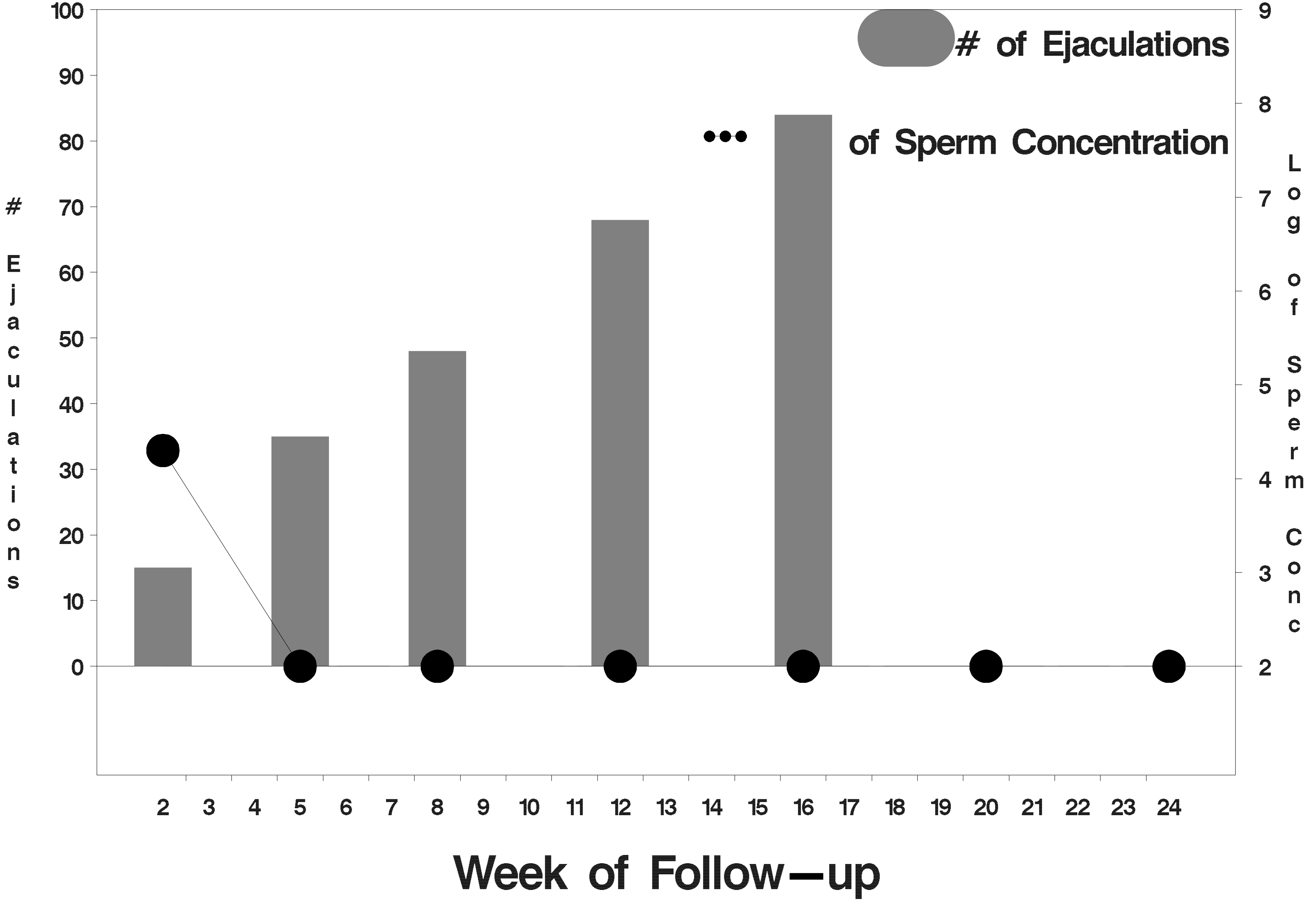

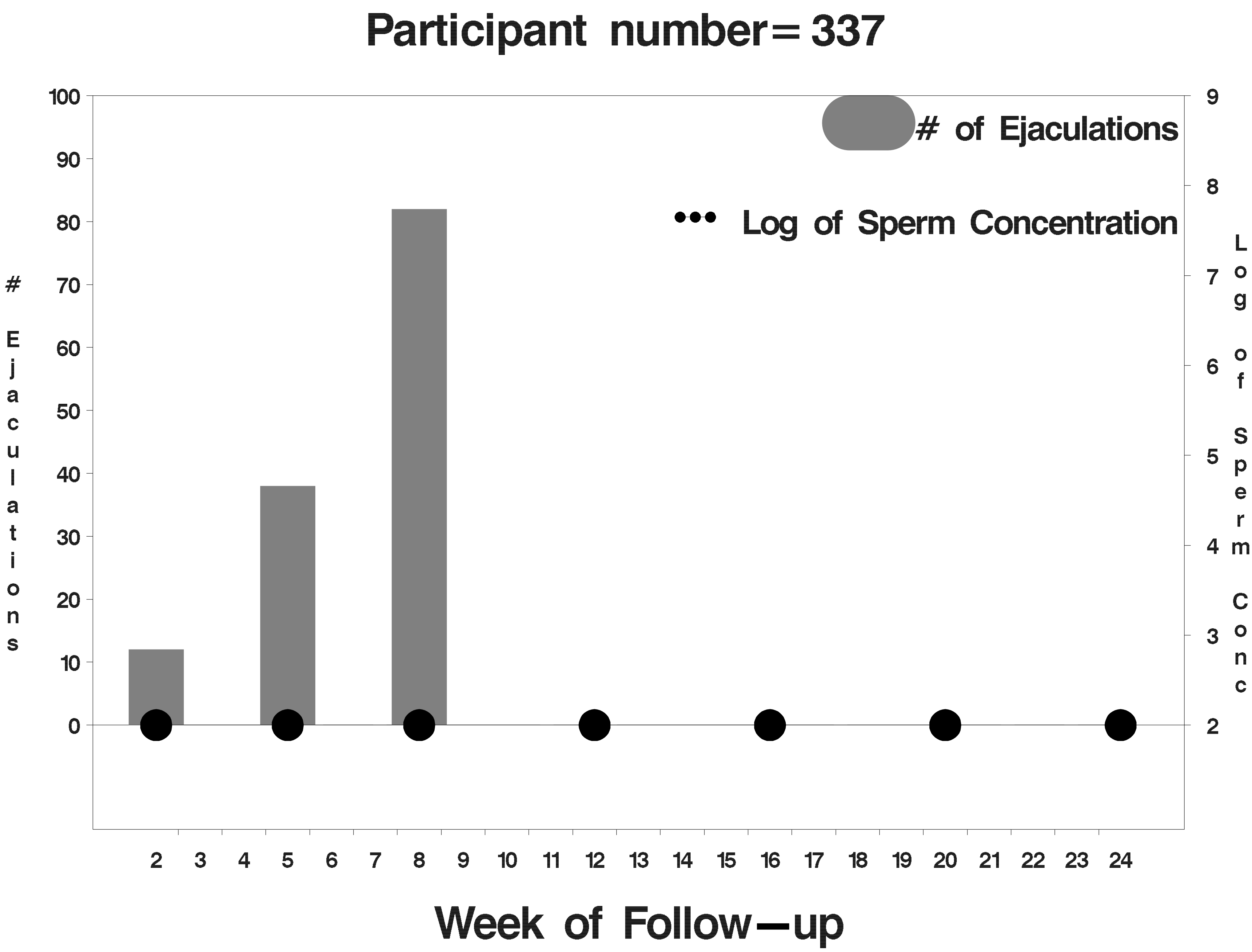

Participant number=337
# of Ejaculations
Log of Sperm Concentration
Ejaculations
Log of Sperm Conc
100
90
80
70
60
50
40
30
20
10
0
9
8
7
6
5
4
3
2
2 3 4 5 6 7 8 9 10 11 12 13 14 15 16 17 18 19 20 21 22 23 24
Week of Follow-up

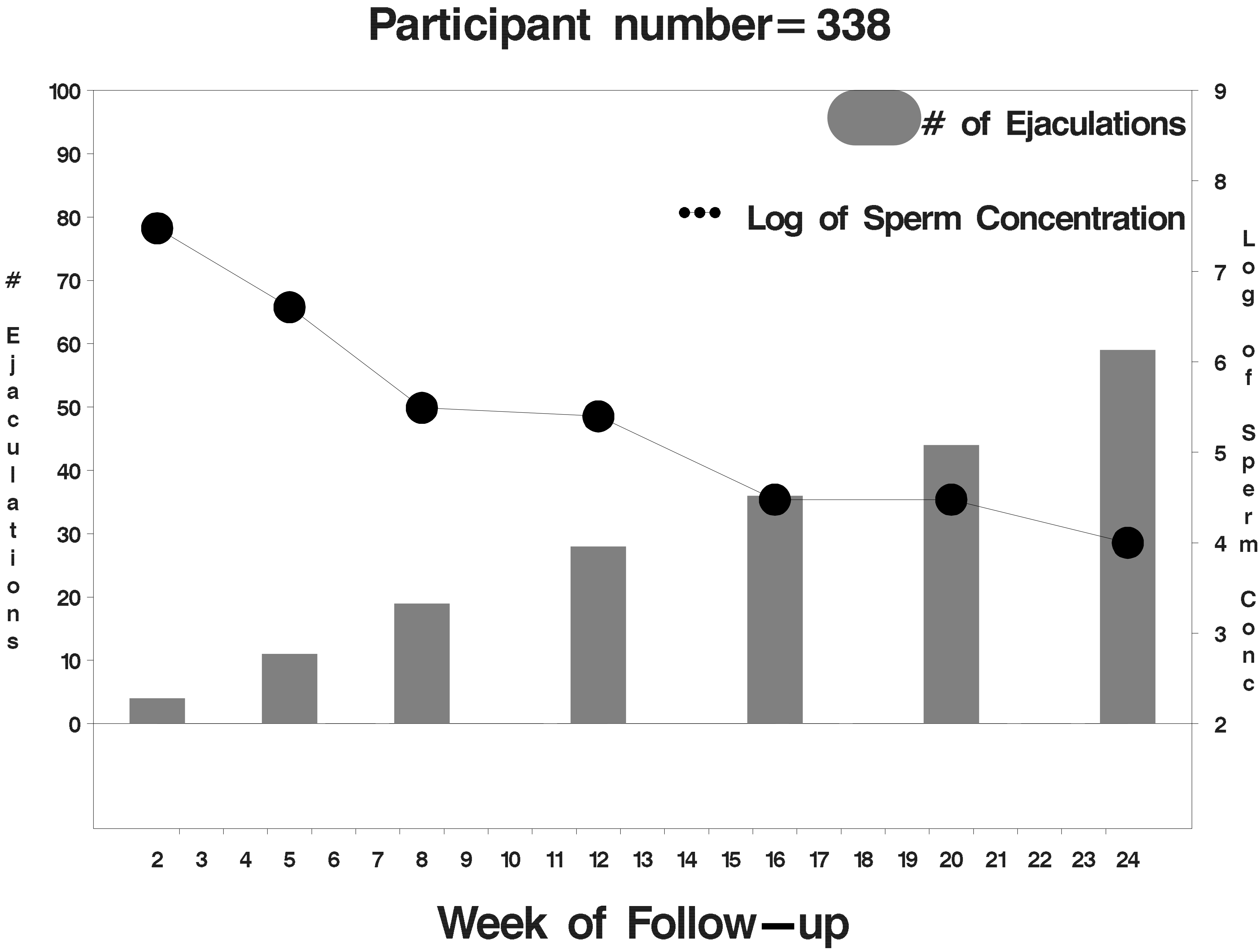

Participant number=338
# of Ejaculations
Log of Sperm Concentration
Ejaculations
Log of Sperm Conc
100
90
80
70
60
50
40
30
20
10
0
9
8
7
6
5
4
3
2
2 3 4 5 6 7 8 9 10 11 12 13 14 15 16 17 18 19 20 21 22 23 24
Week of Follow-up

Participant number=339

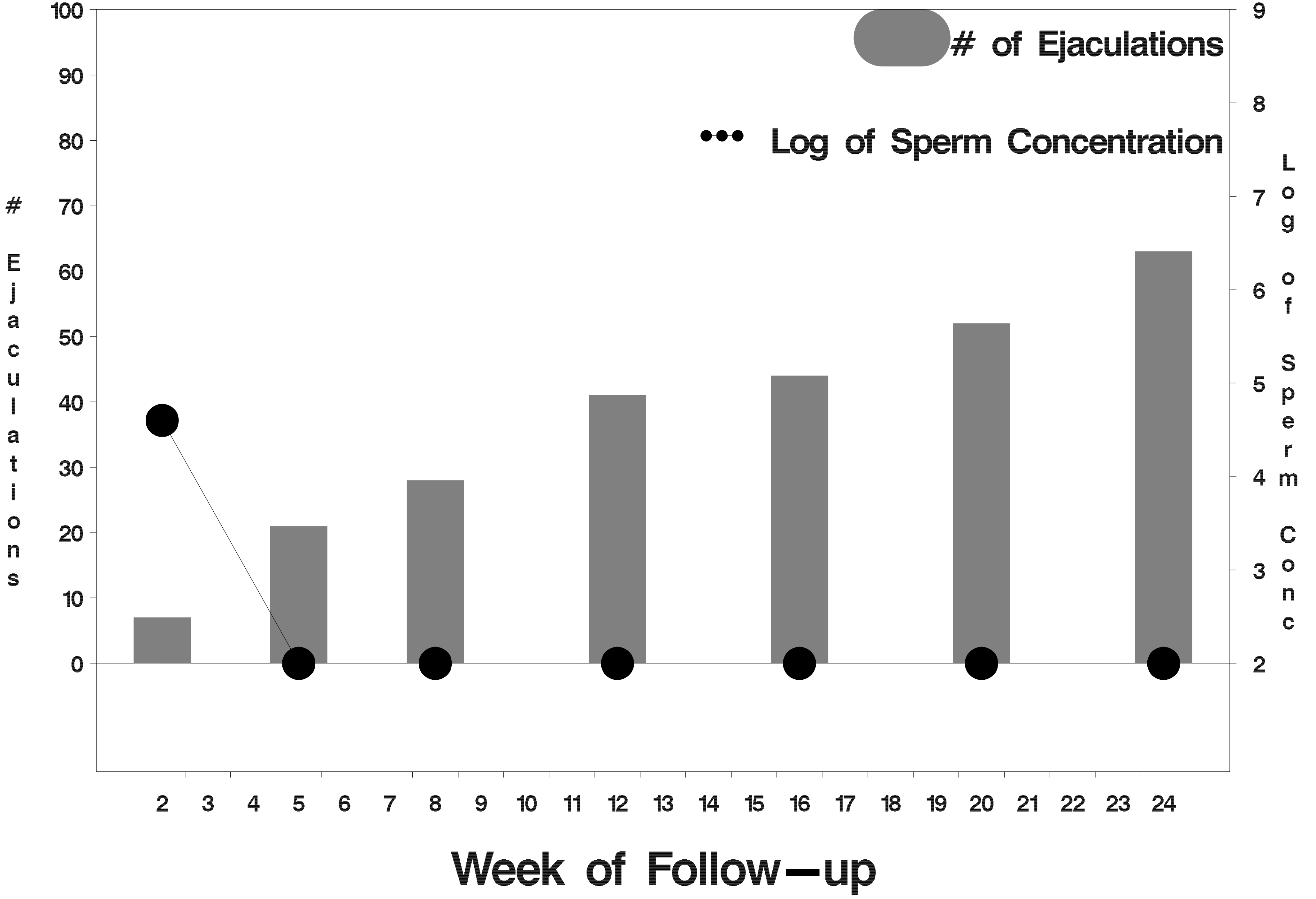

Participant number= 340

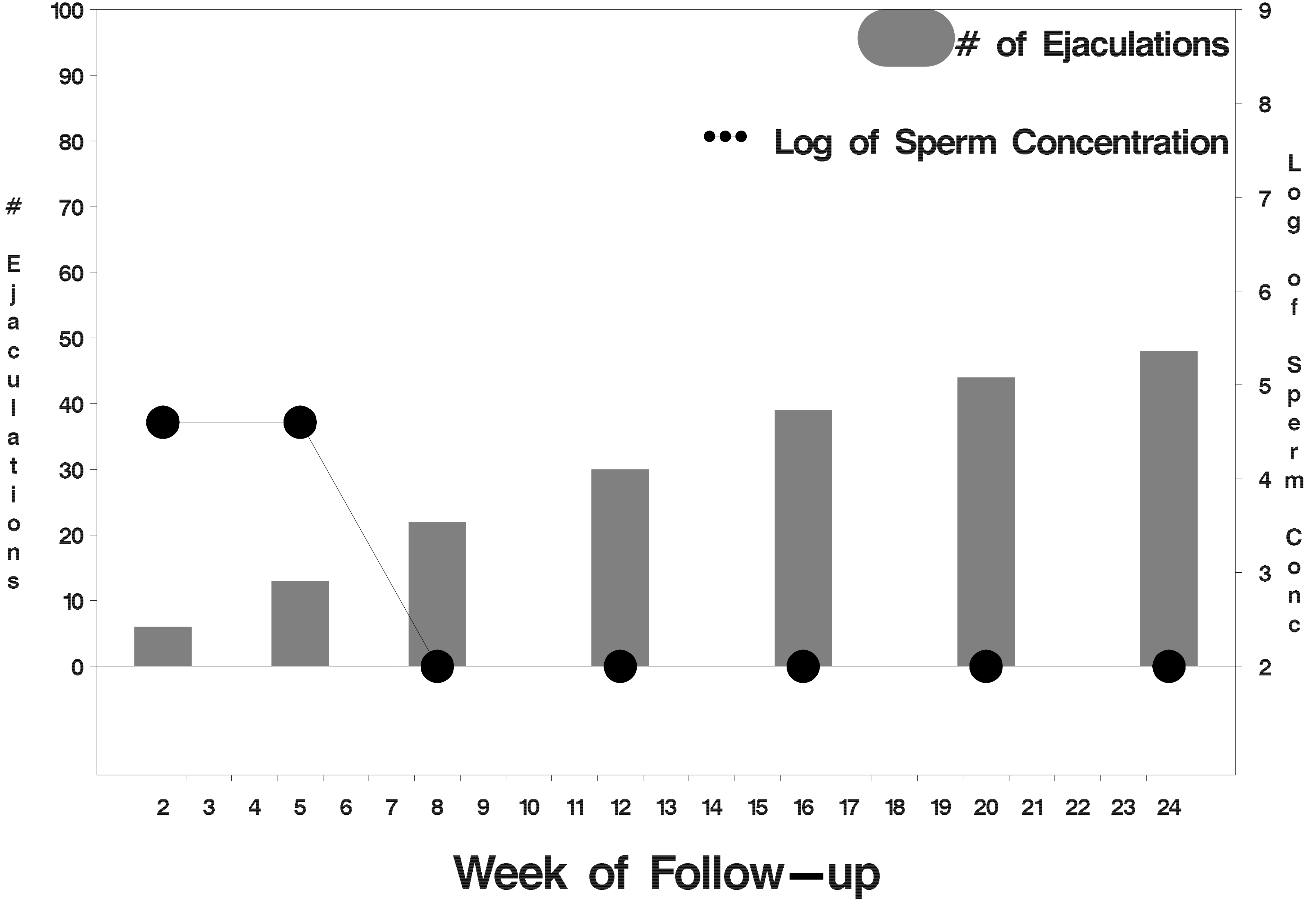

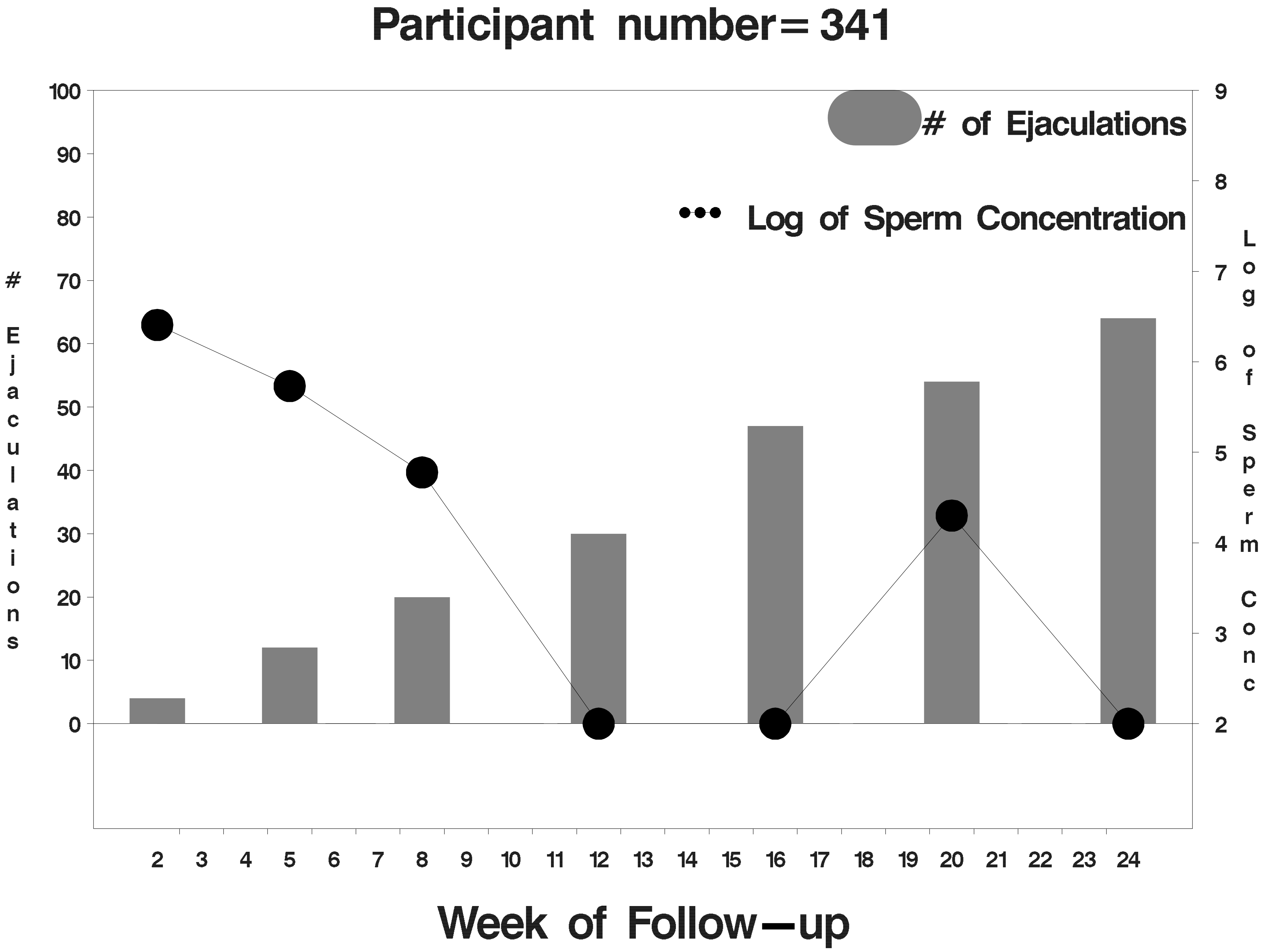

Participant number=341
# of Ejaculations
Log of Sperm Concentration
Ejaculations
Log of Sperm Conc
100
90
80
70
60
50
40
30
20
10
0
9
8
7
6
5
4
3
2
2 3 4 5 6 7 8 9 10 11 12 13 14 15 16 17 18 19 20 21 22 23 24
Week of Follow-up

Participant number= 342

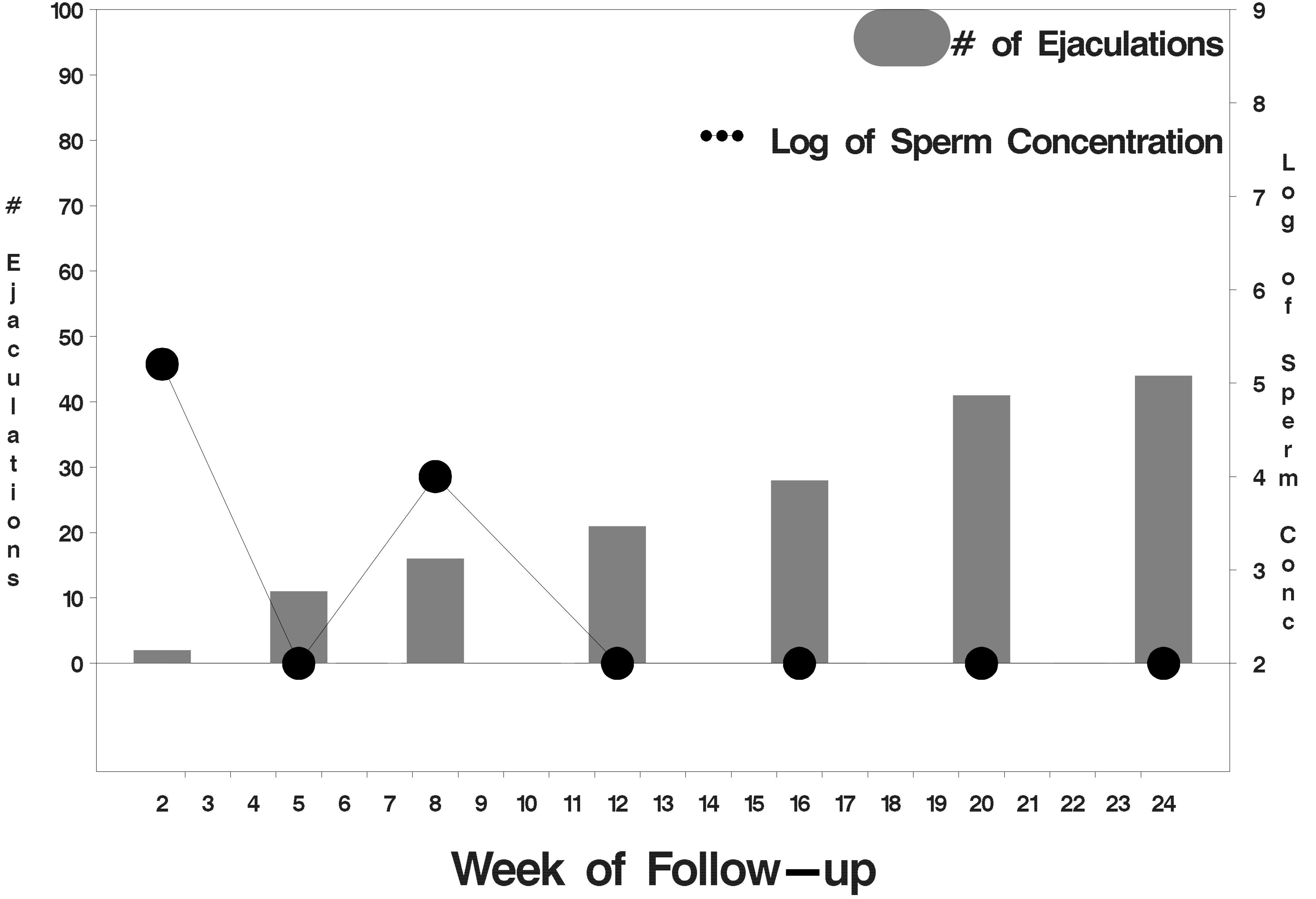

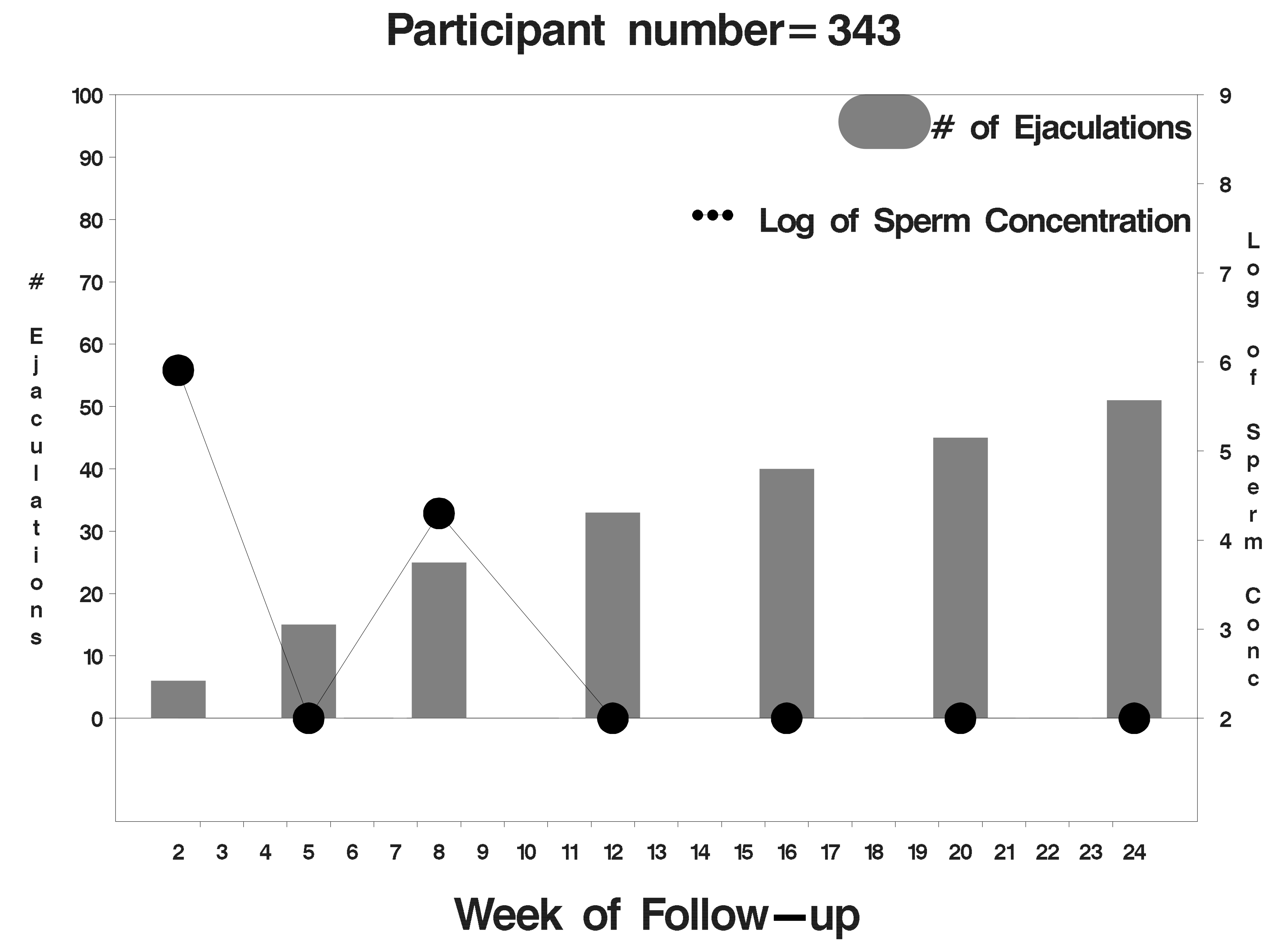

Participant number= 343
# of Ejaculations
Log of Sperm Concentration
Ejaculations
Log of Sperm Conc
100
90
80
70
60
50
40
30
20
10
0
9
8
7
6
5
4
3
2
2 3 4 5 6 7 8 9 10 11 12 13 14 15 16 17 18 19 20 21 22 23 24
Week of Follow-up

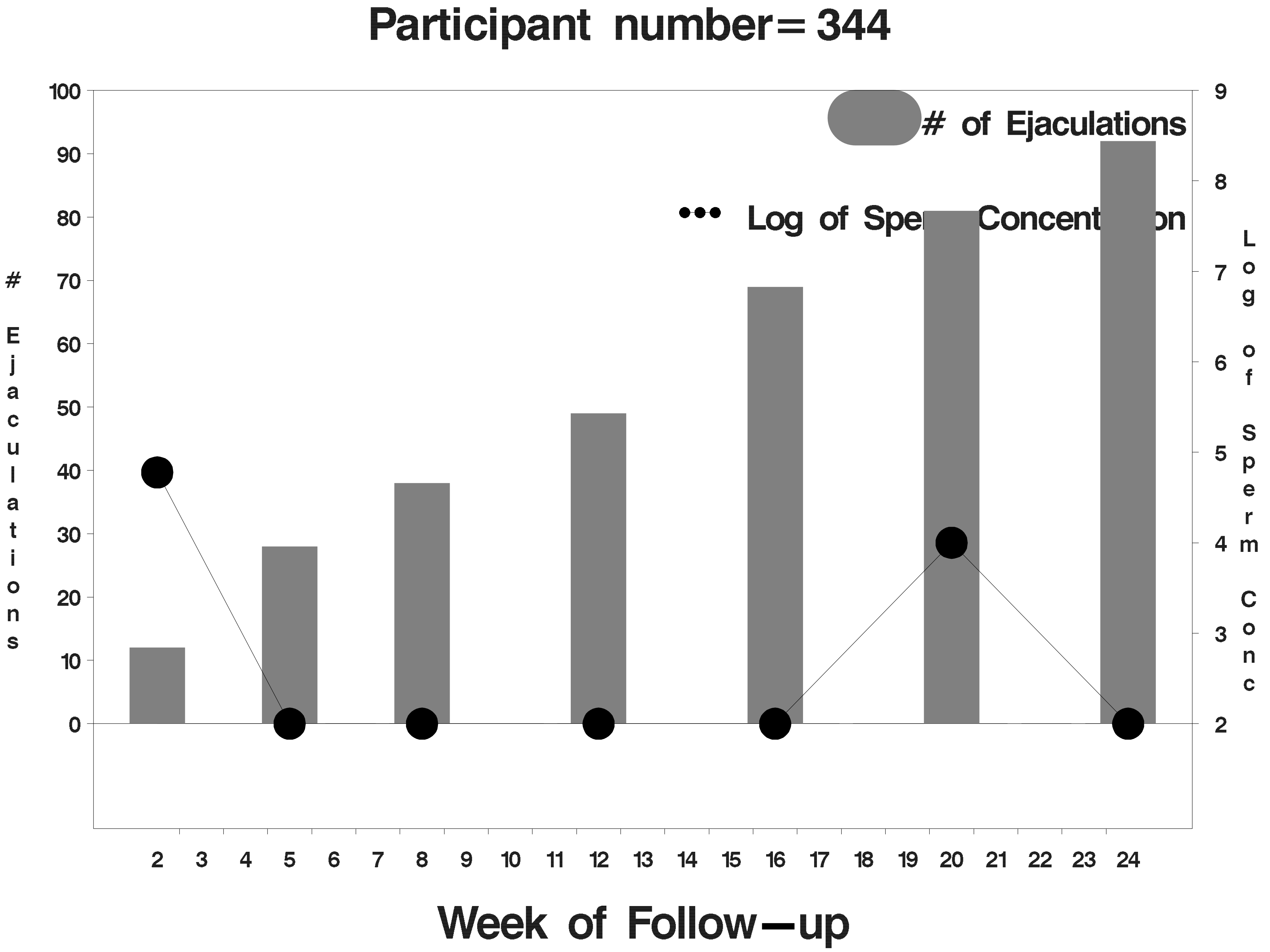

Participant number= 344
100
90
80
70
60
50
40
30
20
10
0
Ejaculations
of Ejaculations
Log of Sperm Concentration
9
8
7
6
5
4
3
2
Log of Sperm Conc
2 3 4 5 6 7 8 9 10 11 12 13 14 15 16 17 18 19 20 21 22 23 24
Week of Follow-up

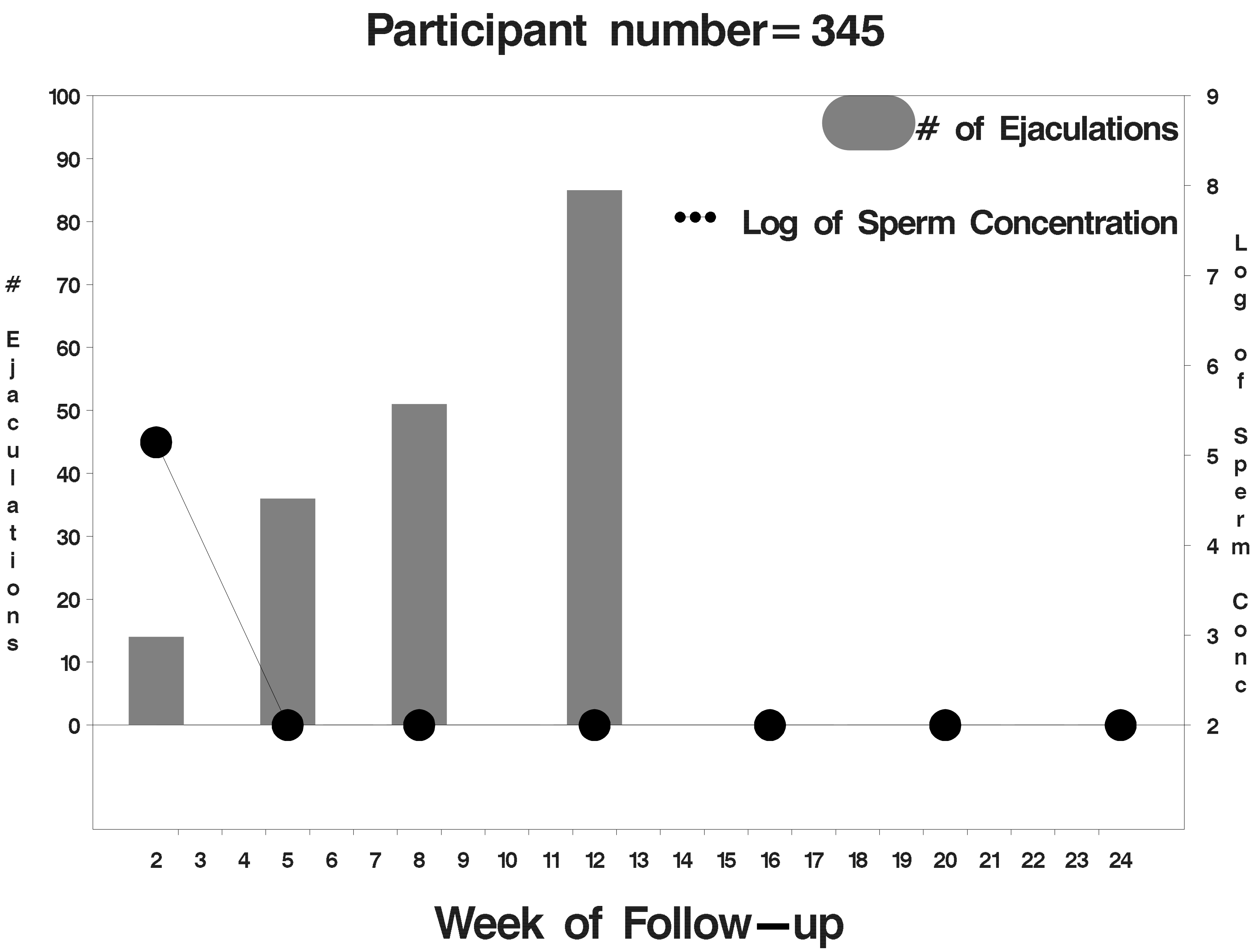

Participant number=345
# of Ejaculations
Log of Sperm Concentration
Ejaculations
Log of Sperm Conc
Week of Follow-up

Participant number=346

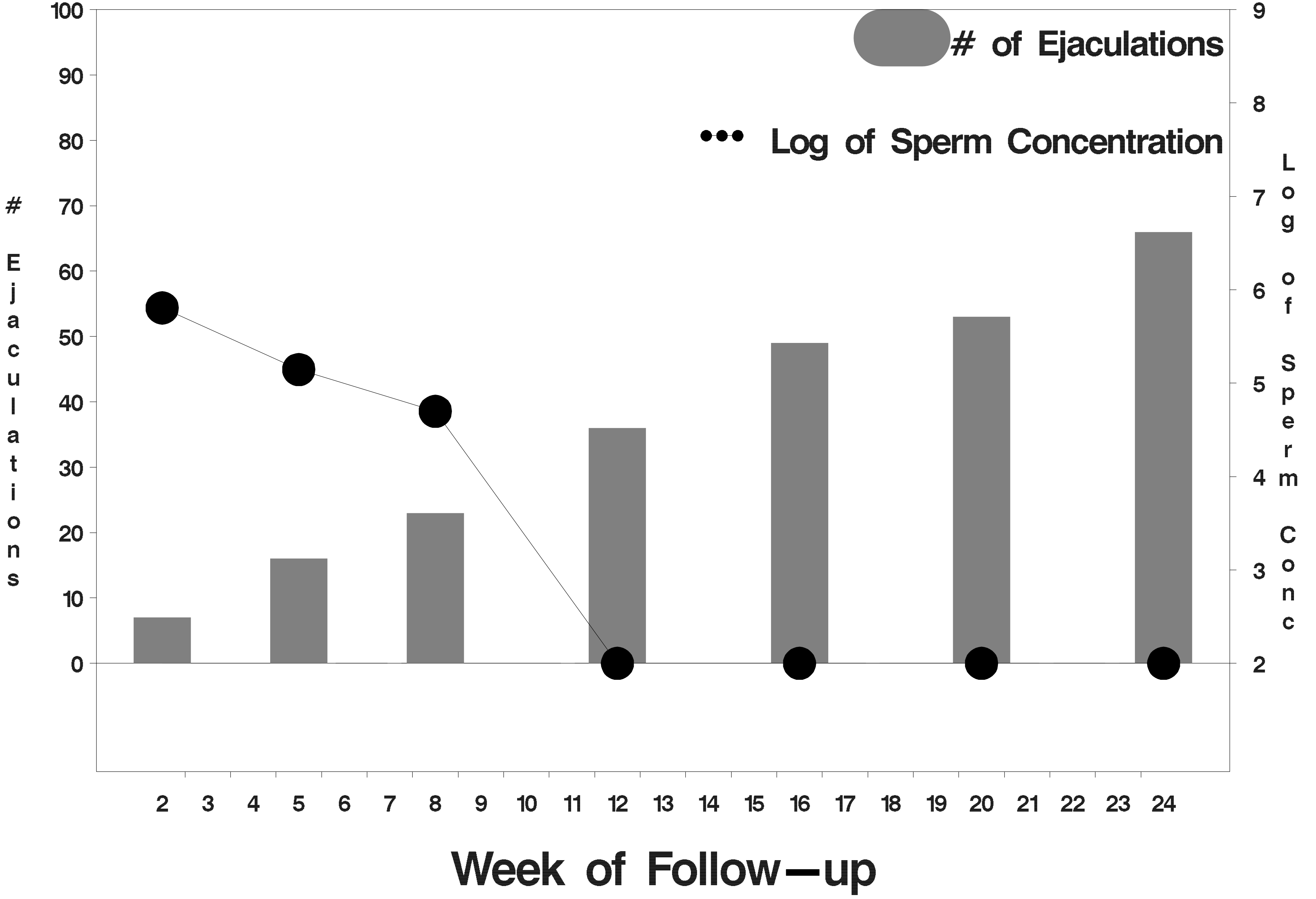

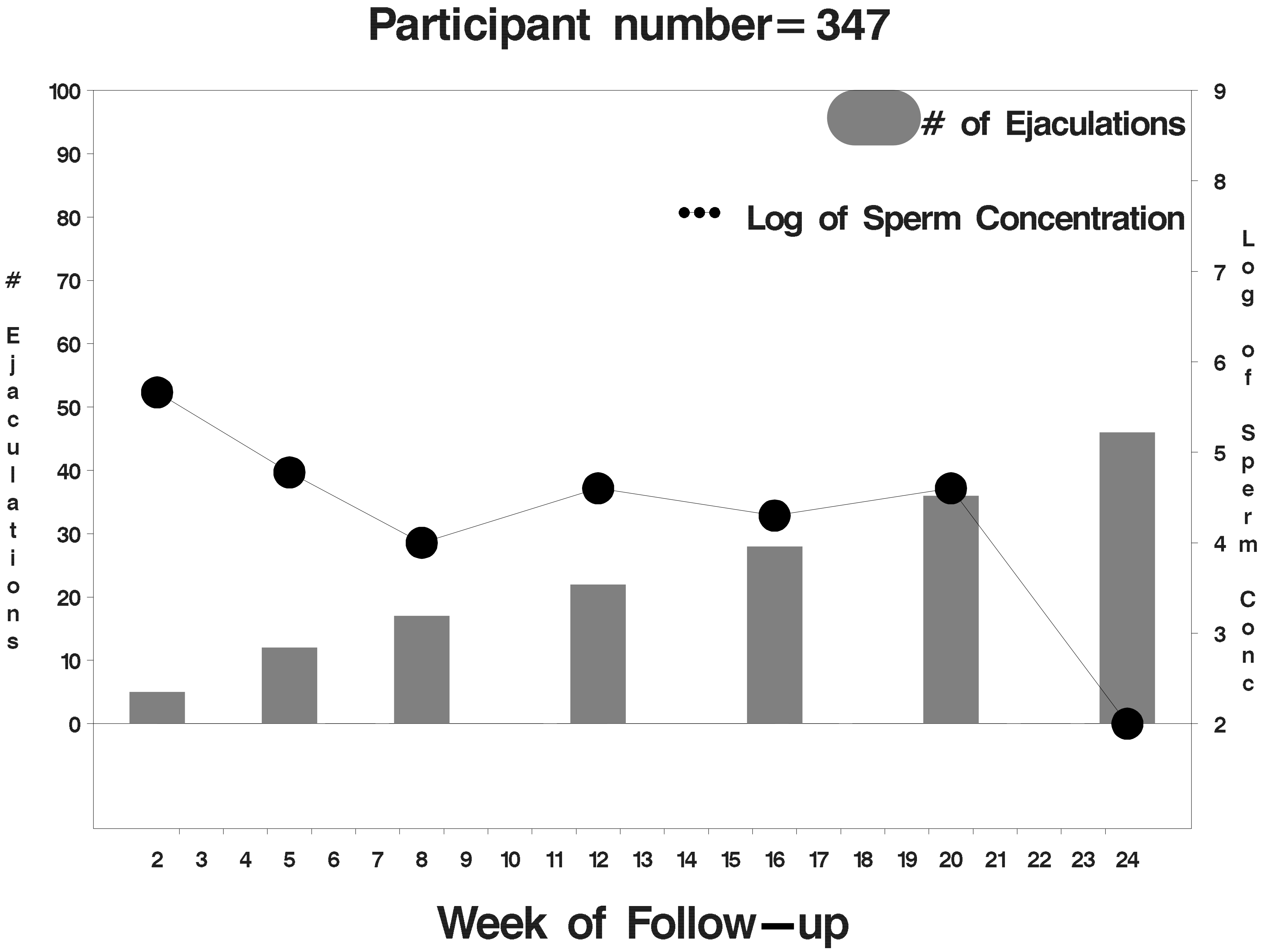

Participant number= 347
# of Ejaculations
Log of Sperm Concentration
Ejaculations
Log of Sperm Conc
100
90
80
70
60
50
40
30
20
10
0
9
8
7
6
5
4
3
2
2 3 4 5 6 7 8 9 10 11 12 13 14 15 16 17 18 19 20 21 22 23 24
Week of Follow-up

Participant number = 348

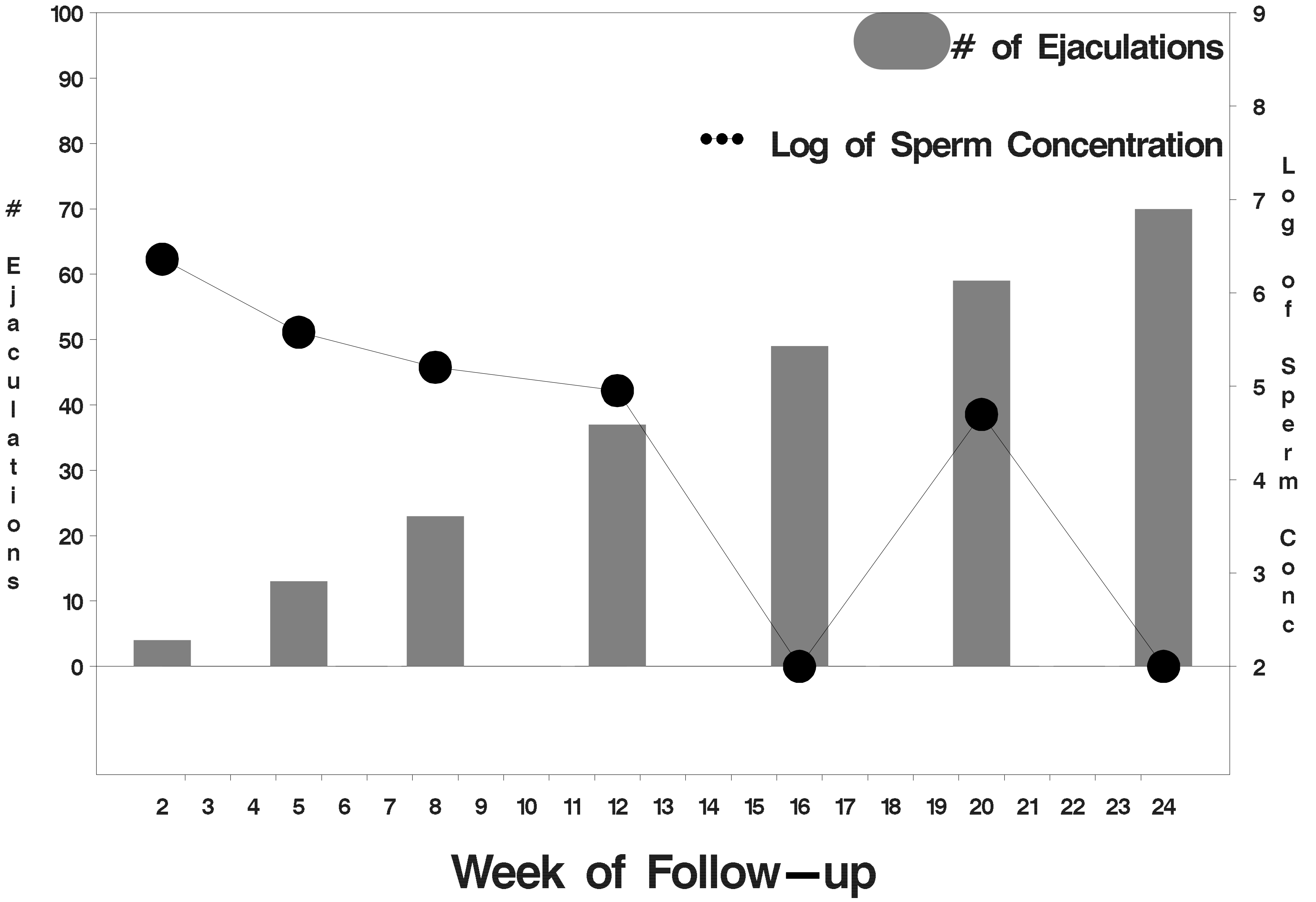

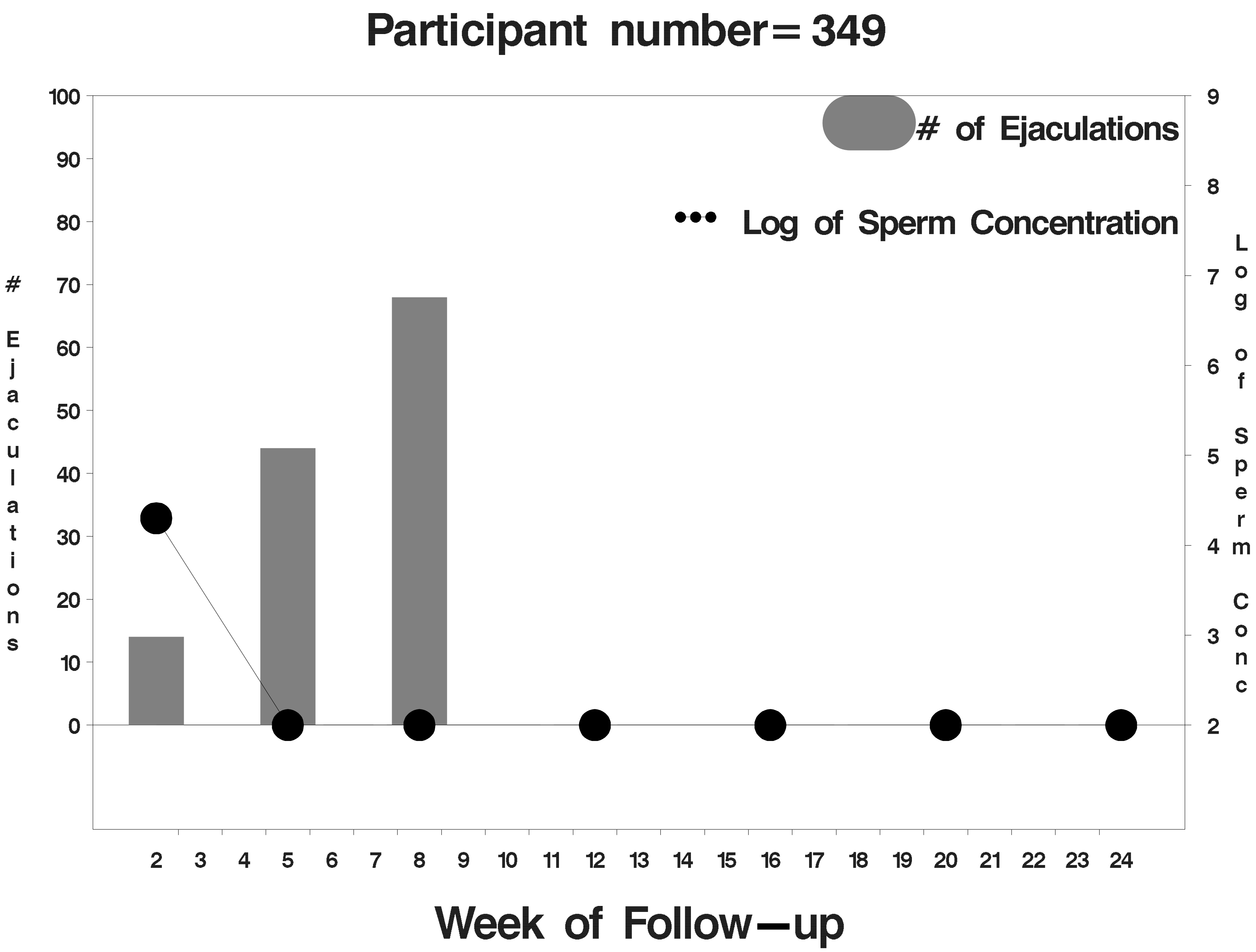
Participant number=349
# of Ejaculations
Log of Sperm Concentration
Ejaculations
Log of Sperm Conc
Week of Follow—up
100
90
80
70
60
50
40
30
20
10
0
9
8
7
6
5
4
3
2
2 3 4 5 6 7 8 9 10 11 12 13 14 15 16 17 18 19 20 21 22 23 24

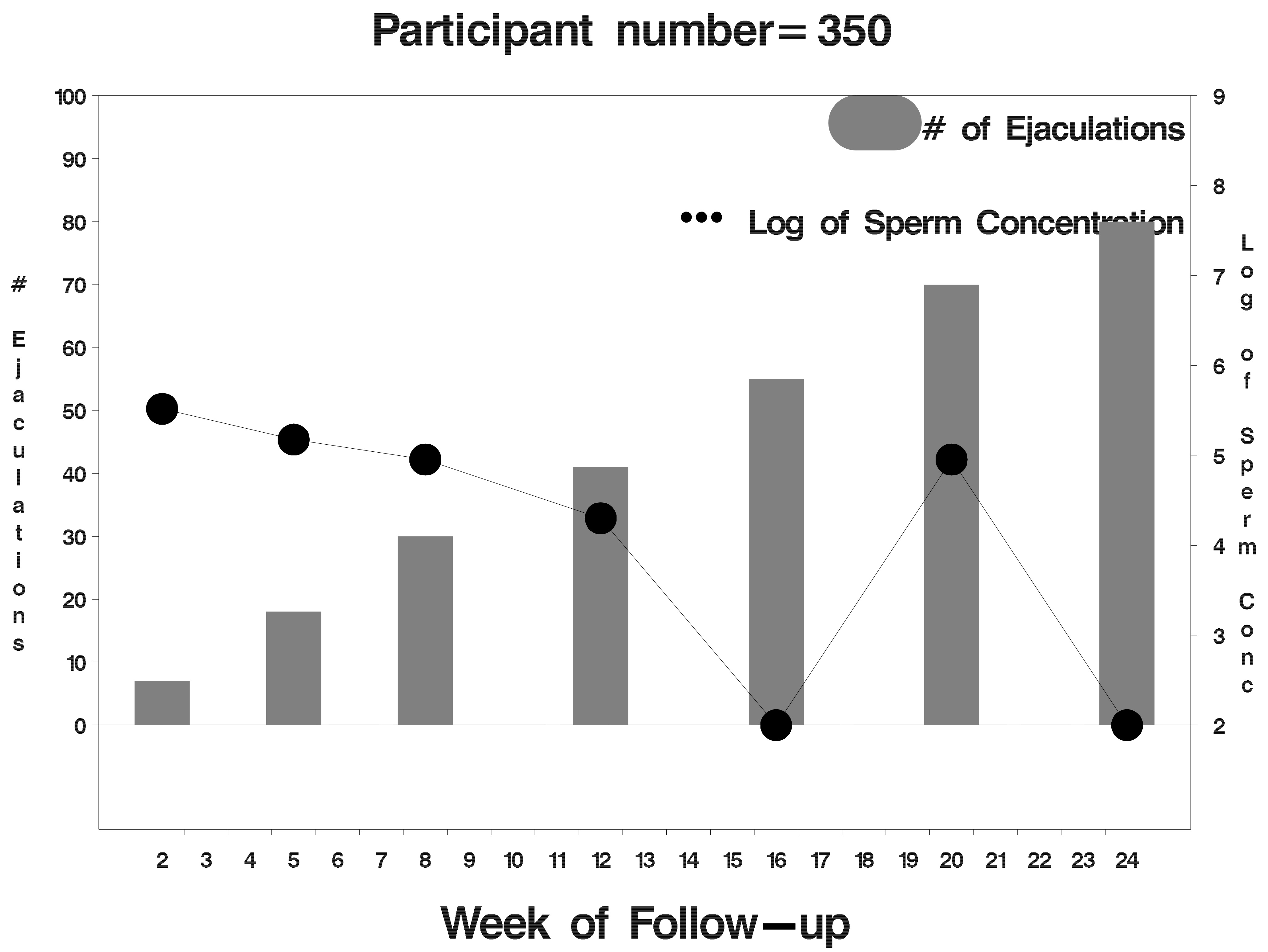

Participant number= 350
# of Ejaculations
Log of Sperm Concentration
Ejaculations
Log of Sperm Conc
100
90
80
70
60
50
40
30
20
10
0
9
8
7
6
5
4
3
2
2 3 4 5 6 7 8 9 10 11 12 13 14 15 16 17 18 19 20 21 22 23 24
Week of Follow-up

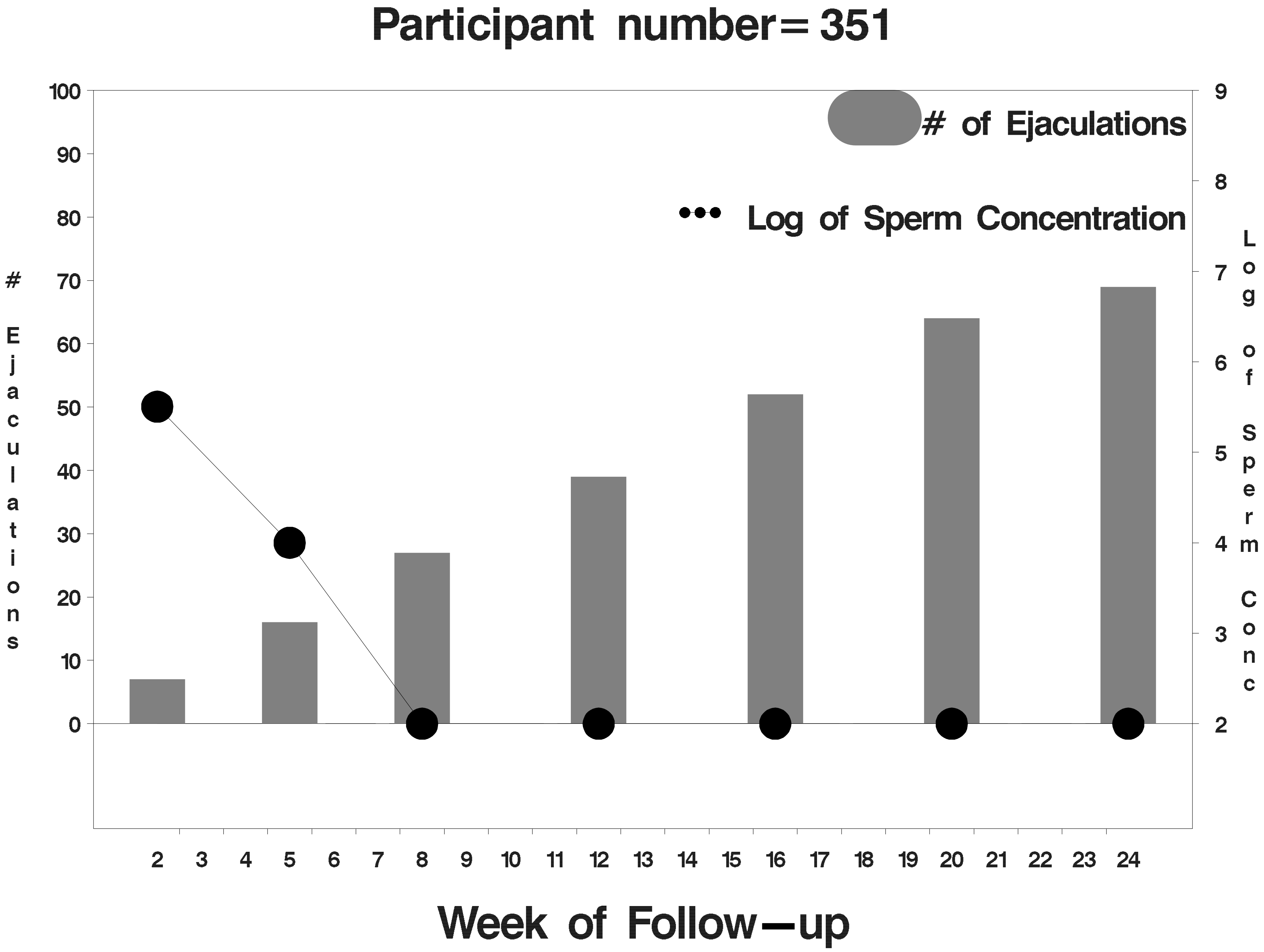

Participant number= 351
# of Ejaculations
Log of Sperm Concentration
Ejaculations
Log of Sperm Conc
100
90
80
70
60
50
40
30
20
10
0
9
8
7
6
5
4
3
2
2 3 4 5 6 7 8 9 10 11 12 13 14 15 16 17 18 19 20 21 22 23 24
Week of Follow-up

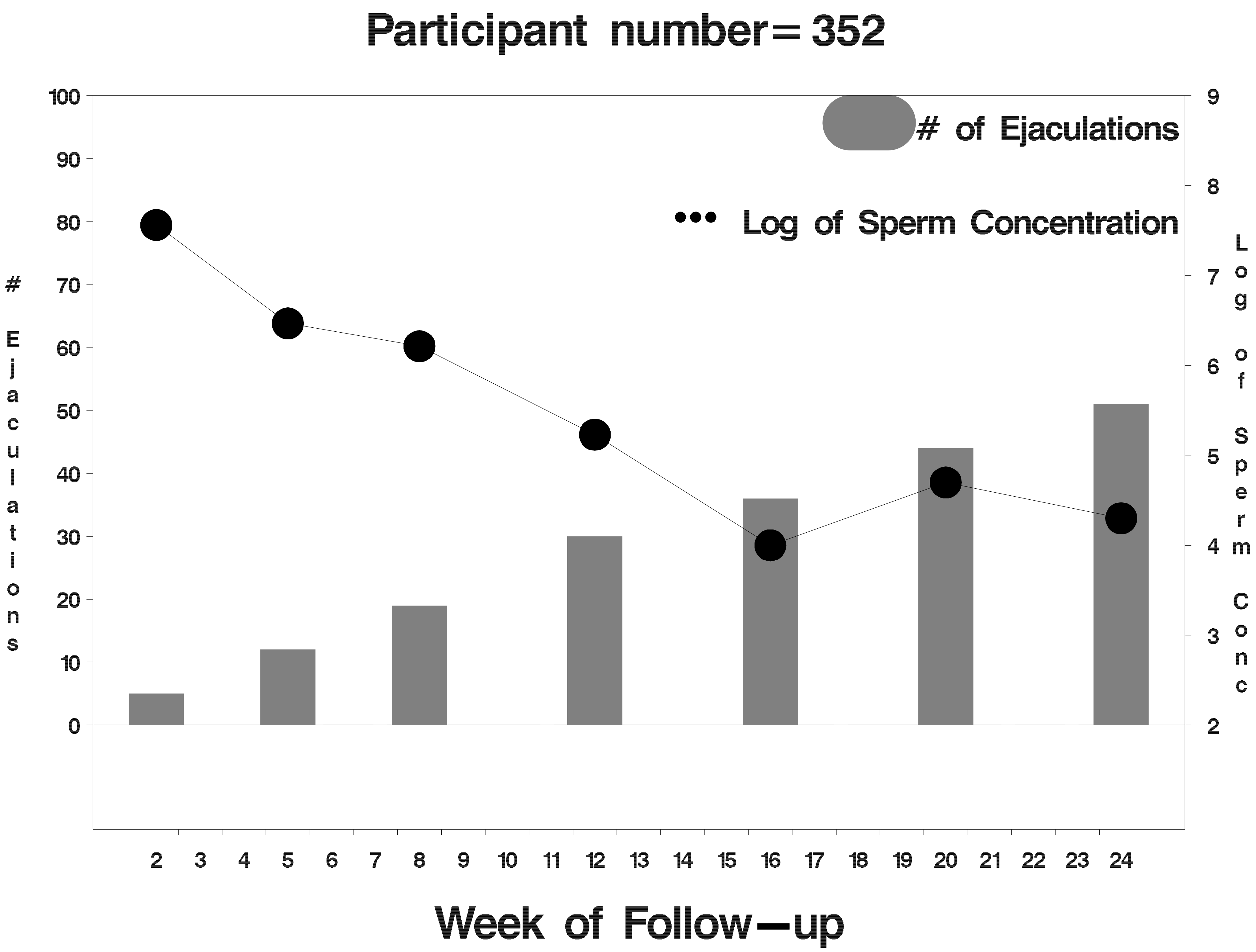

Participant number= 352
# of Ejaculations
Log of Sperm Concentration
Ejaculations
Log of Sperm Conc
100
90
80
70
60
50
40
30
20
10
0
9
8
7
6
5
4
3
2
2 3 4 5 6 7 8 9 10 11 12 13 14 15 16 17 18 19 20 21 22 23 24
Week of Follow-up

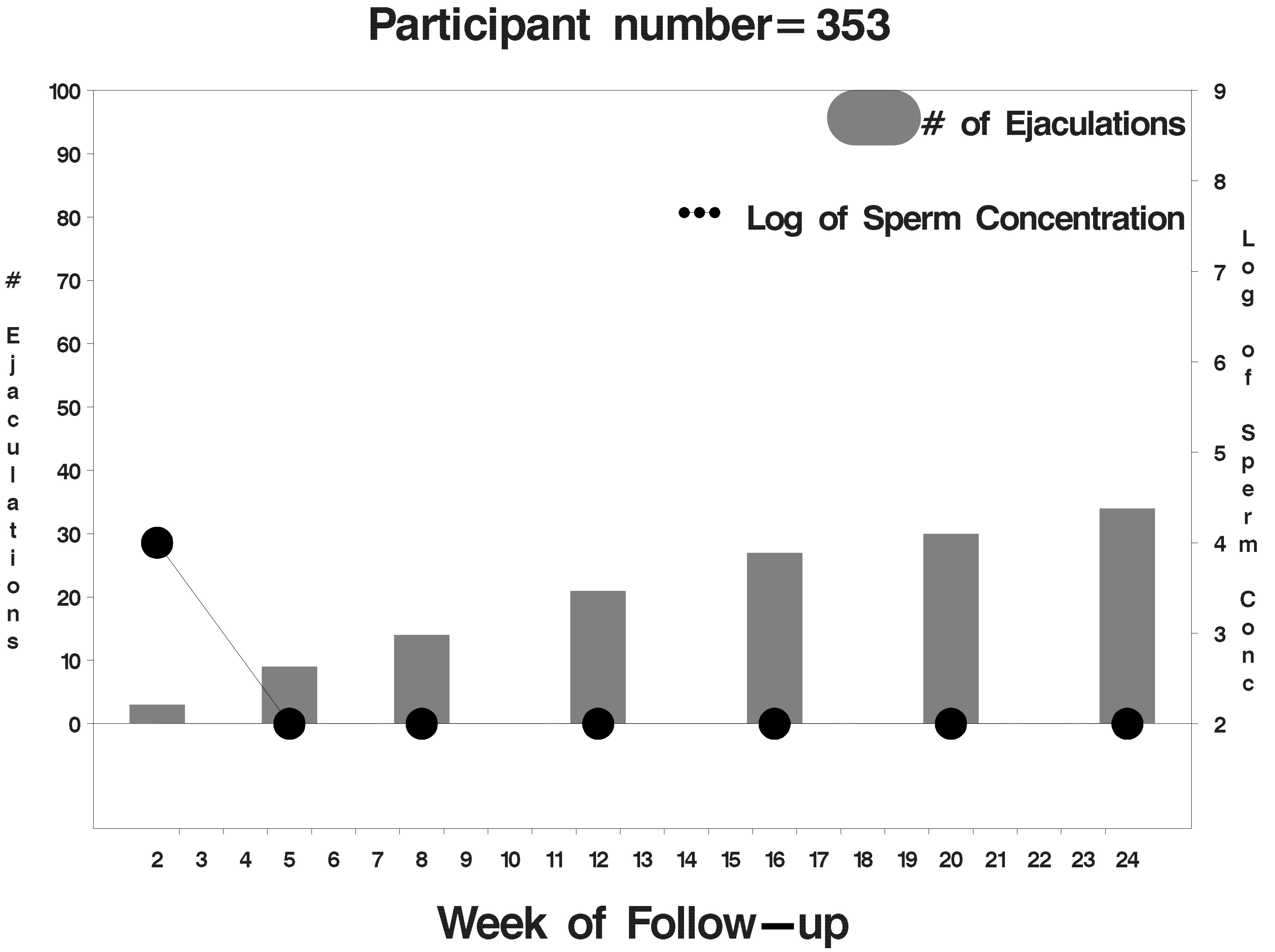
Participant number=353
# of Ejaculations
Log of Sperm Concentration
Ejaculations
Log of Sperm Conc
100
90
80
70
60
50
40
30
20
10
0
9
8
7
6
5
4
3
2
2 3 4 5 6 7 8 9 10 11 12 13 14 15 16 17 18 19 20 21 22 23 24
Week of Follow-up

Participant number=354

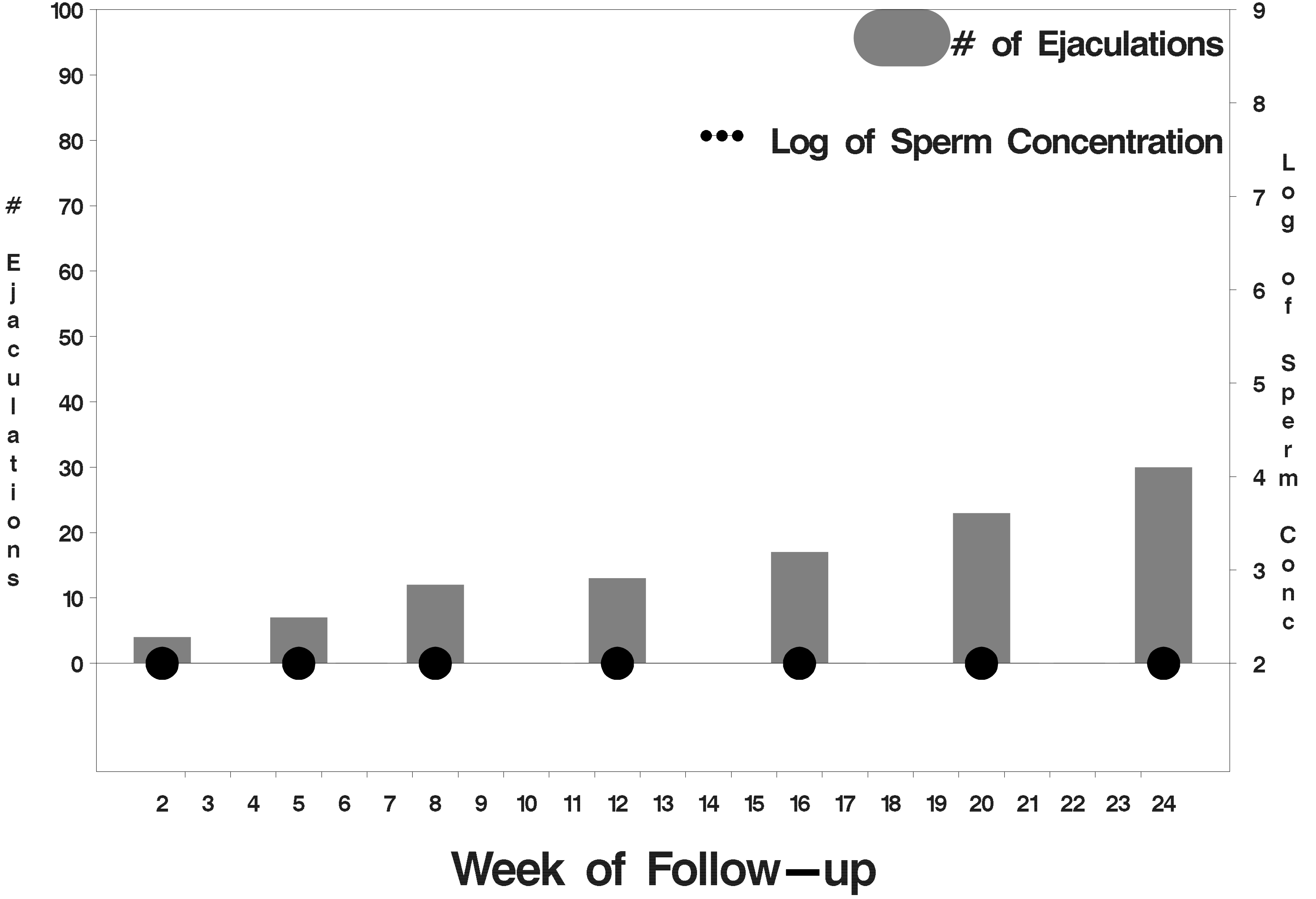

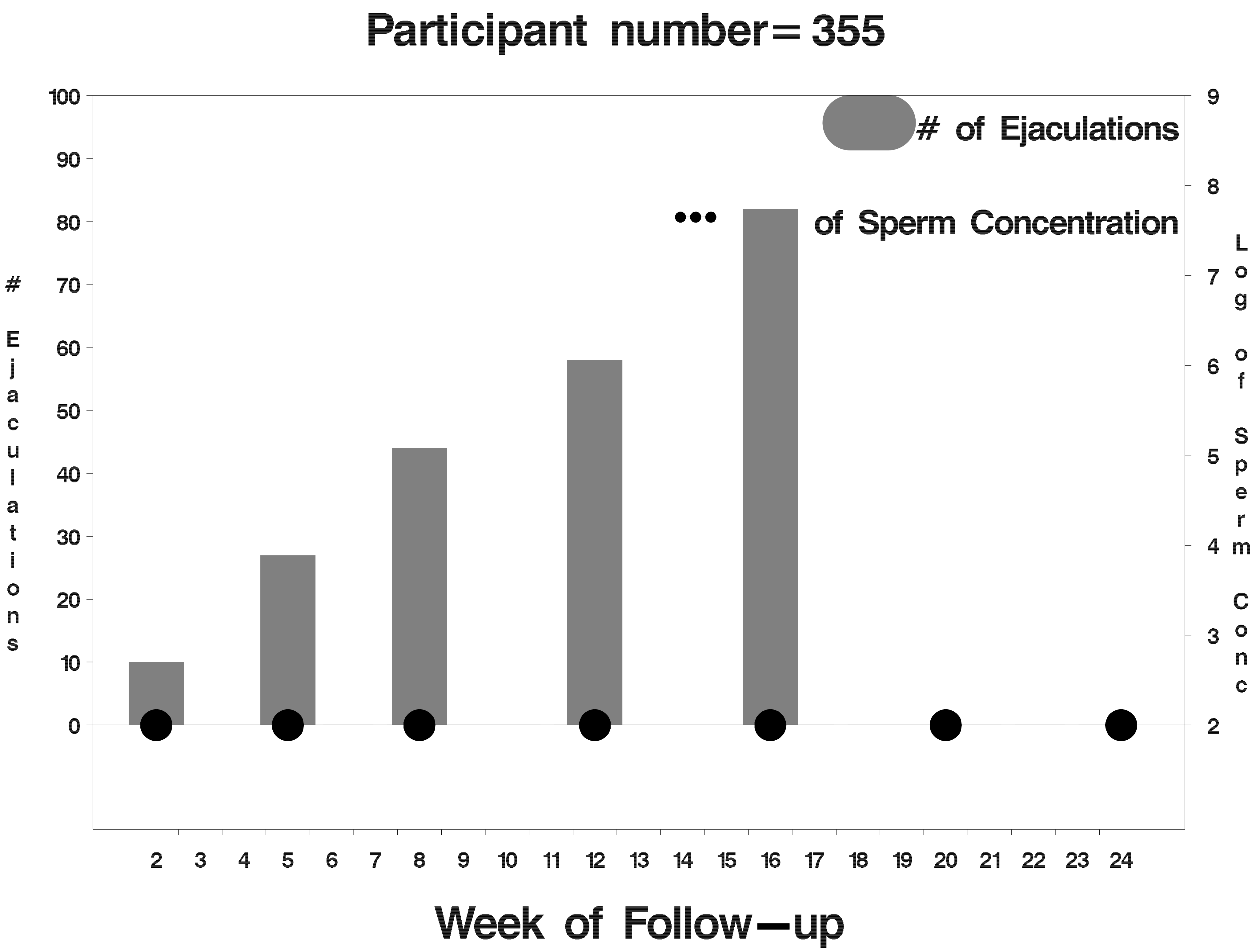

Participant number=355
# of Ejaculations
of Sperm Concentration
Ejaculations
Log of Sperm Conc
100
90
80
70
60
50
40
30
20
10
0
9
8
7
6
5
4
3
2
2 3 4 5 6 7 8 9 10 11 12 13 14 15 16 17 18 19 20 21 22 23 24
Week of Follow-up

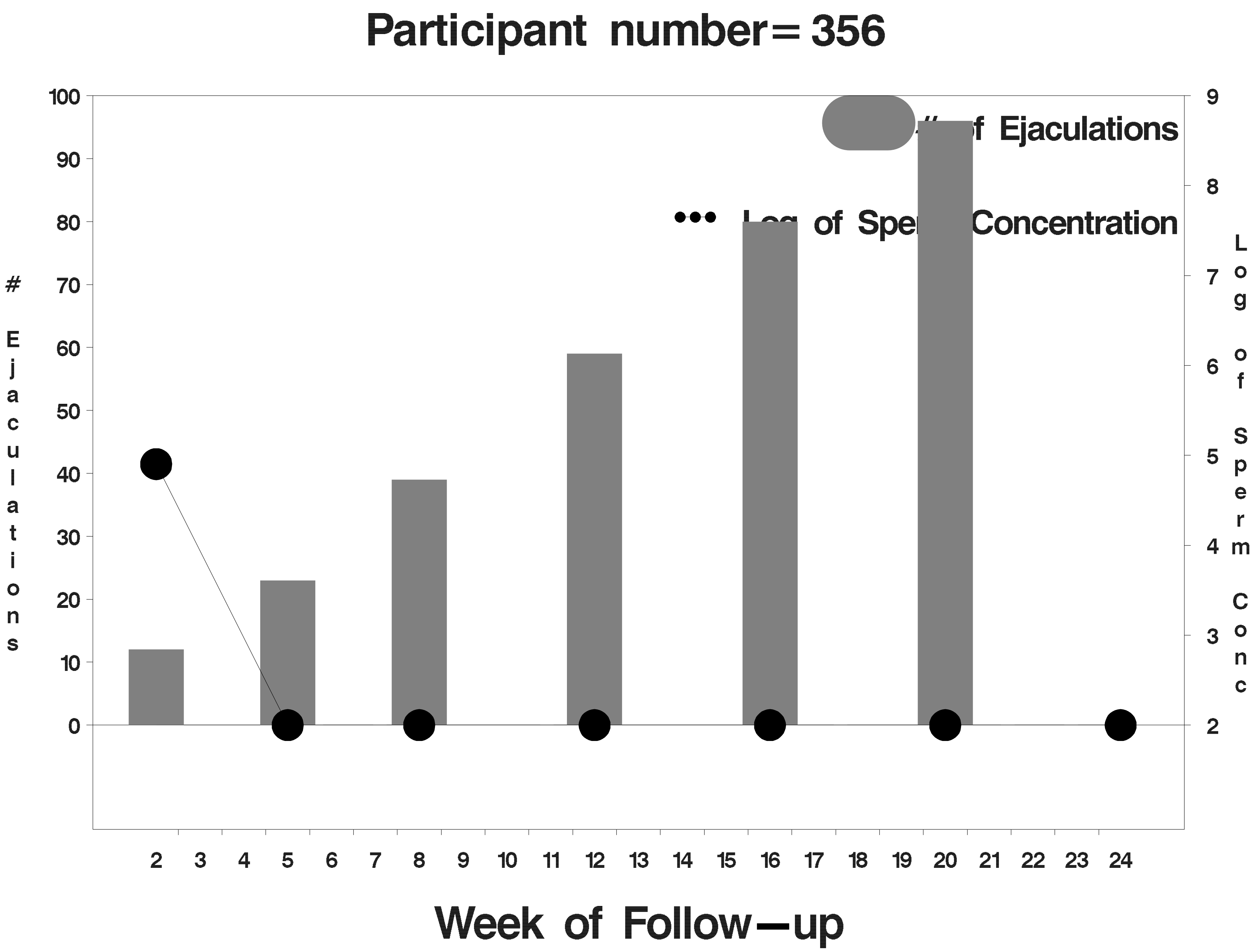

Participant number=356
# of Ejaculations
Log of Sperm Concentration
Ejaculations
Log of Sperm Conc
100
90
80
70
60
50
40
30
20
10
0
9
8
7
6
5
4
3
2
2 3 4 5 6 7 8 9 10 11 12 13 14 15 16 17 18 19 20 21 22 23 24
Week of Follow-up

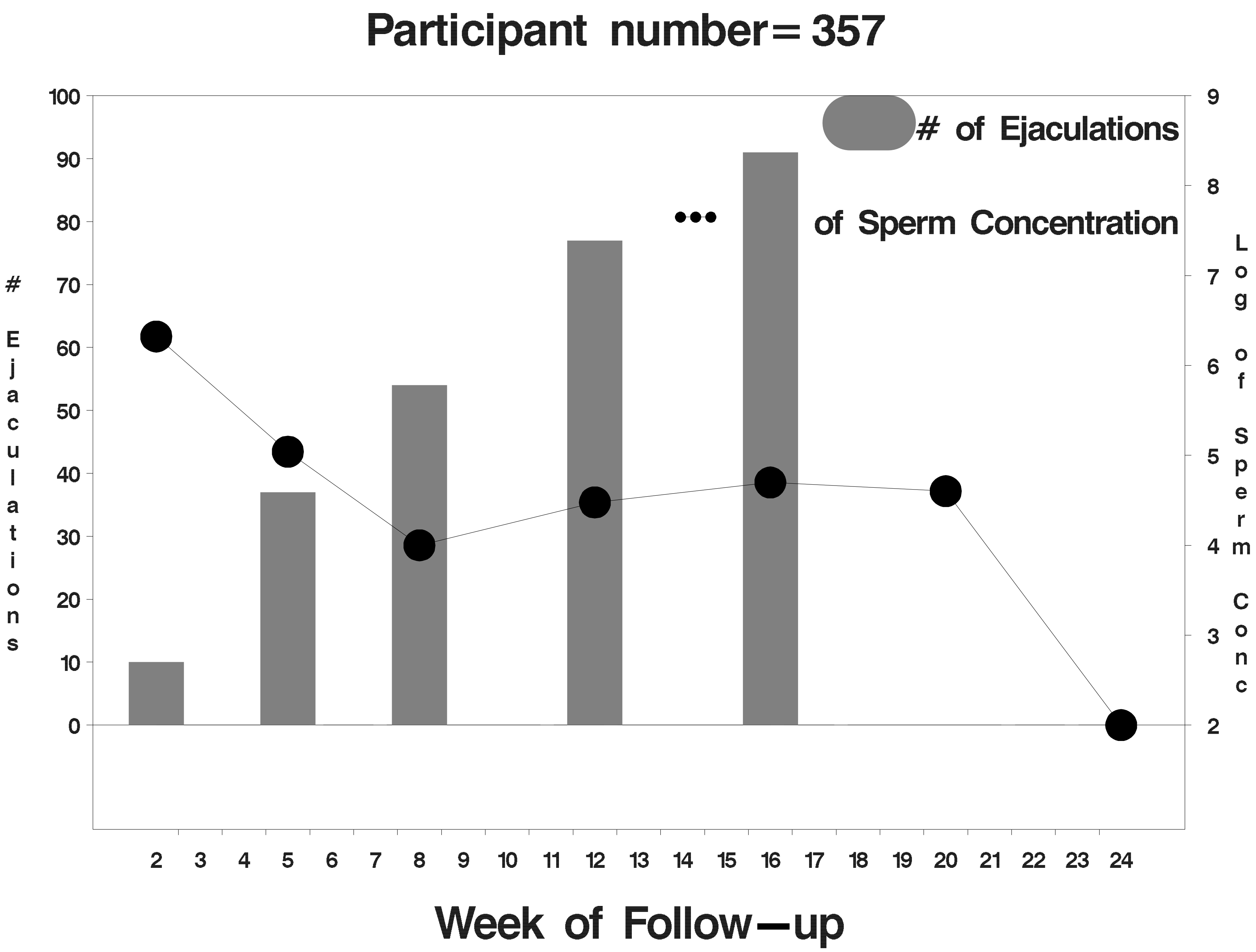

Participant number= 357
# of Ejaculations
of Sperm Concentration
Ejaculations
Log of Sperm Conc
100
90
80
70
60
50
40
30
20
10
0
9
8
7
6
5
4
3
2
2 3 4 5 6 7 8 9 10 11 12 13 14 15 16 17 18 19 20 21 22 23 24
Week of Follow-up

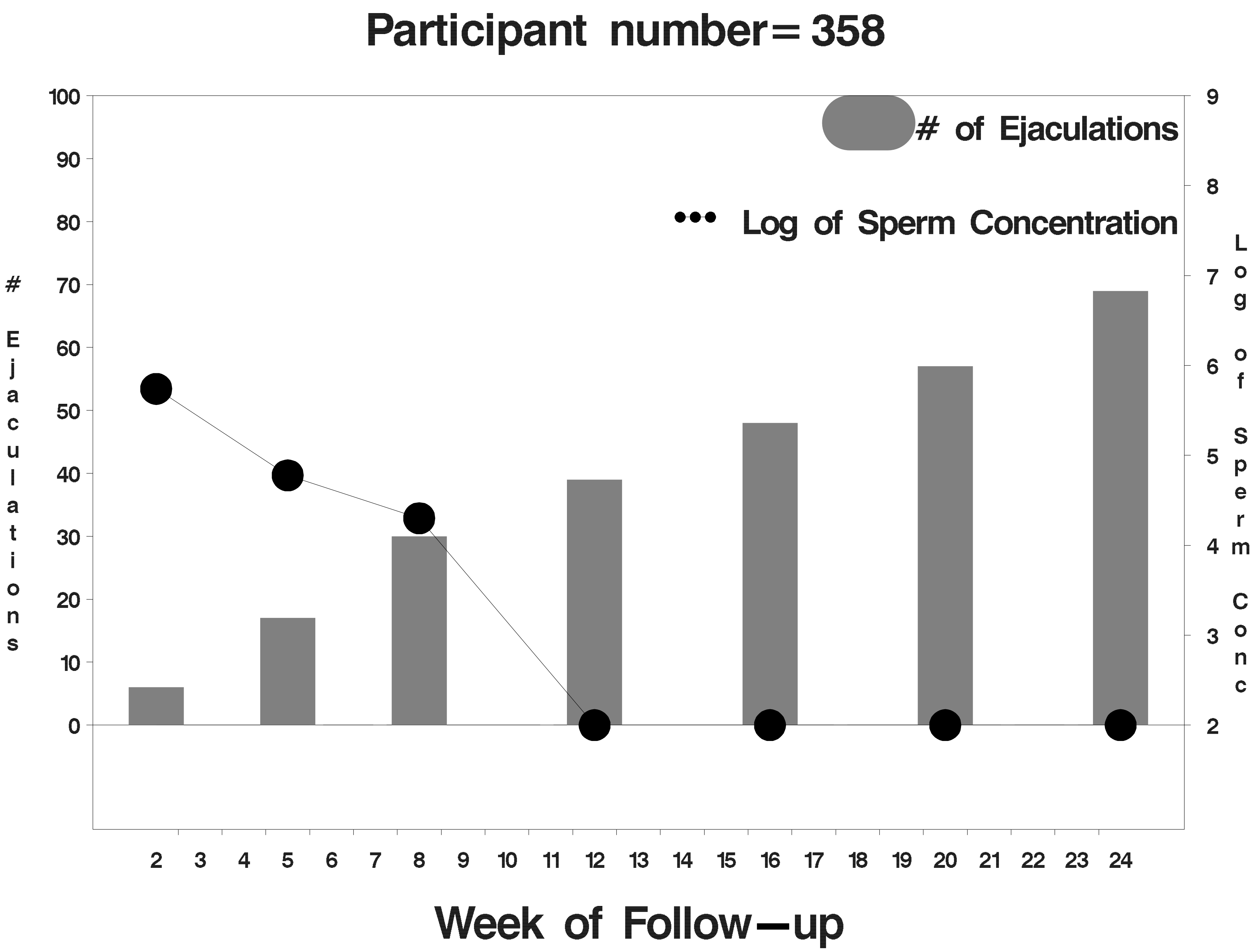

Participant number=358
# of Ejaculations
Log of Sperm Concentration
Ejaculations
Log of Sperm Conc
100
90
80
70
60
50
40
30
20
10
0
9
8
7
6
5
4
3
2
2 3 4 5 6 7 8 9 10 11 12 13 14 15 16 17 18 19 20 21 22 23 24
Week of Follow-up

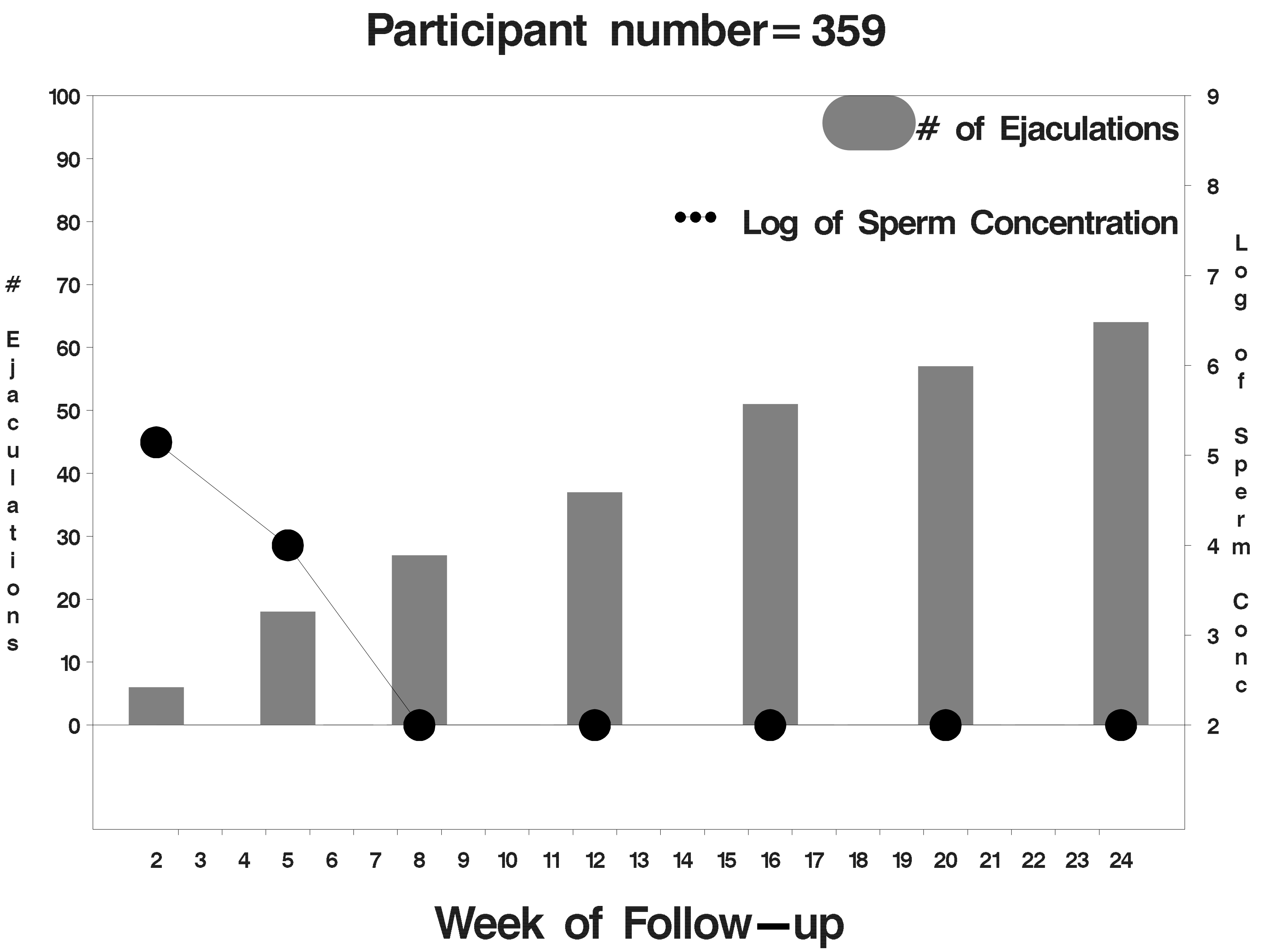
Participant number= 359
# of Ejaculations
Log of Sperm Concentration
Ejaculations
Log of Sperm Conc
100
90
80
70
60
50
40
30
20
10
0
9
8
7
6
5
4
3
2
2 3 4 5 6 7 8 9 10 11 12 13 14 15 16 17 18 19 20 21 22 23 24
Week of Follow-up

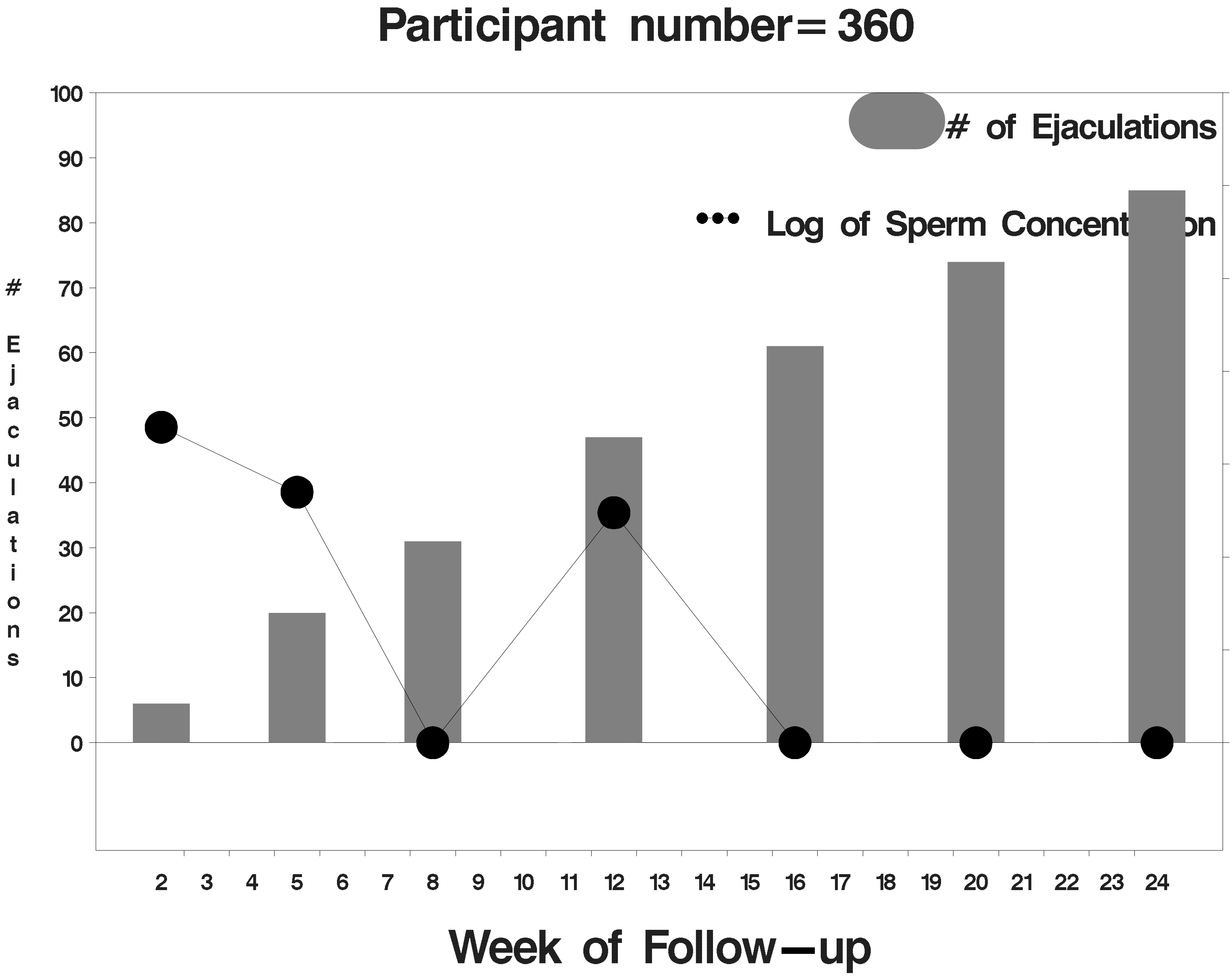

Participant number=360
# of Ejaculations
Log of Sperm Concentration
Ejaculations
Log of Sperm Conc
100
90
80
70
60
50
40
30
20
10
0
9
8
7
6
5
4
3
2
2 3 4 5 6 7 8 9 10 11 12 13 14 15 16 17 18 19 20 21 22 23 24
Week of Follow-up

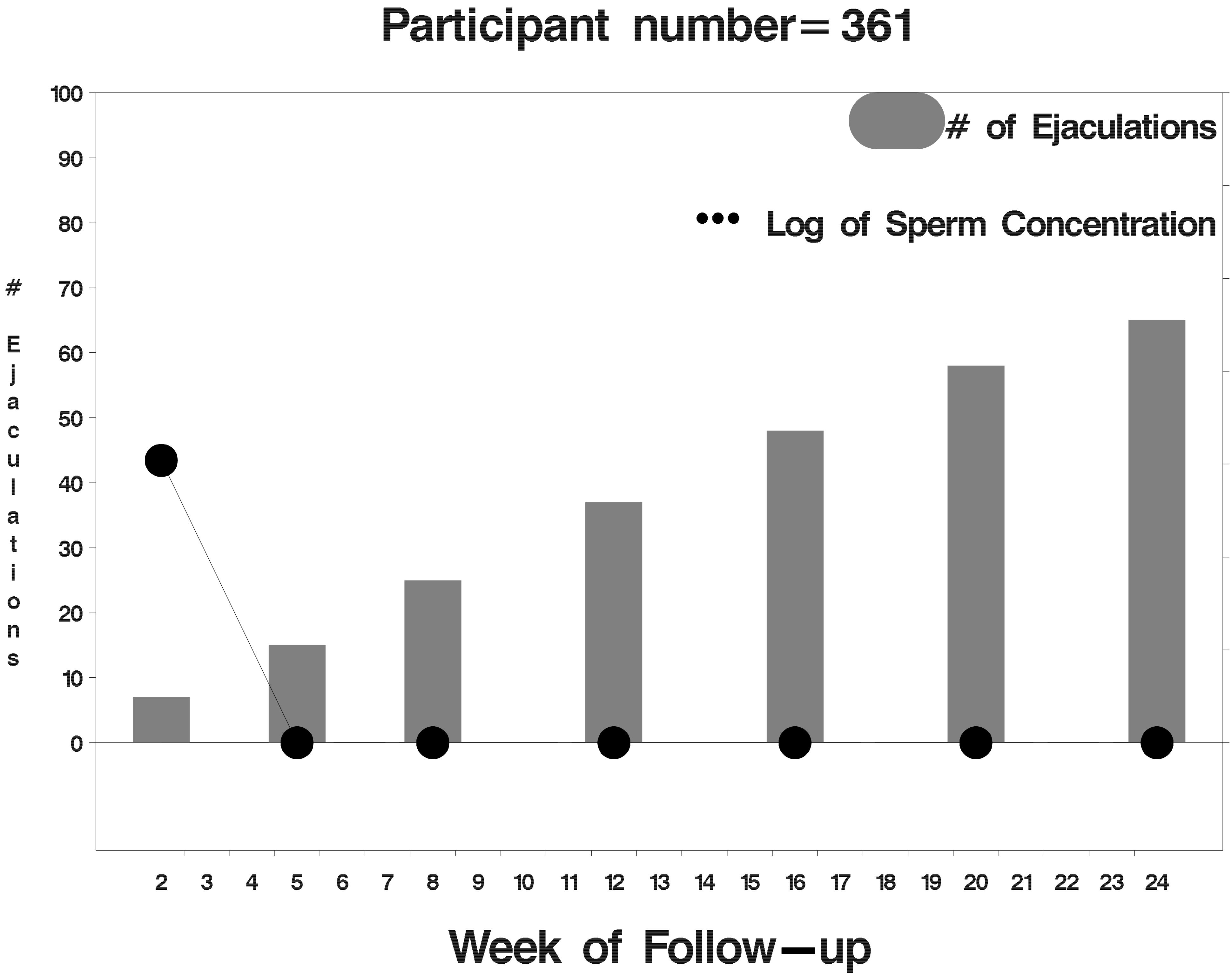

Participant number=361
# of Ejaculations
Log of Sperm Concentration
Ejaculations
Log of Sperm Conc
100
90
80
70
60
50
40
30
20
10
0
9
8
7
6
5
4
3
2
2 3 4 5 6 7 8 9 10 11 12 13 14 15 16 17 18 19 20 21 22 23 24
Week of Follow-up

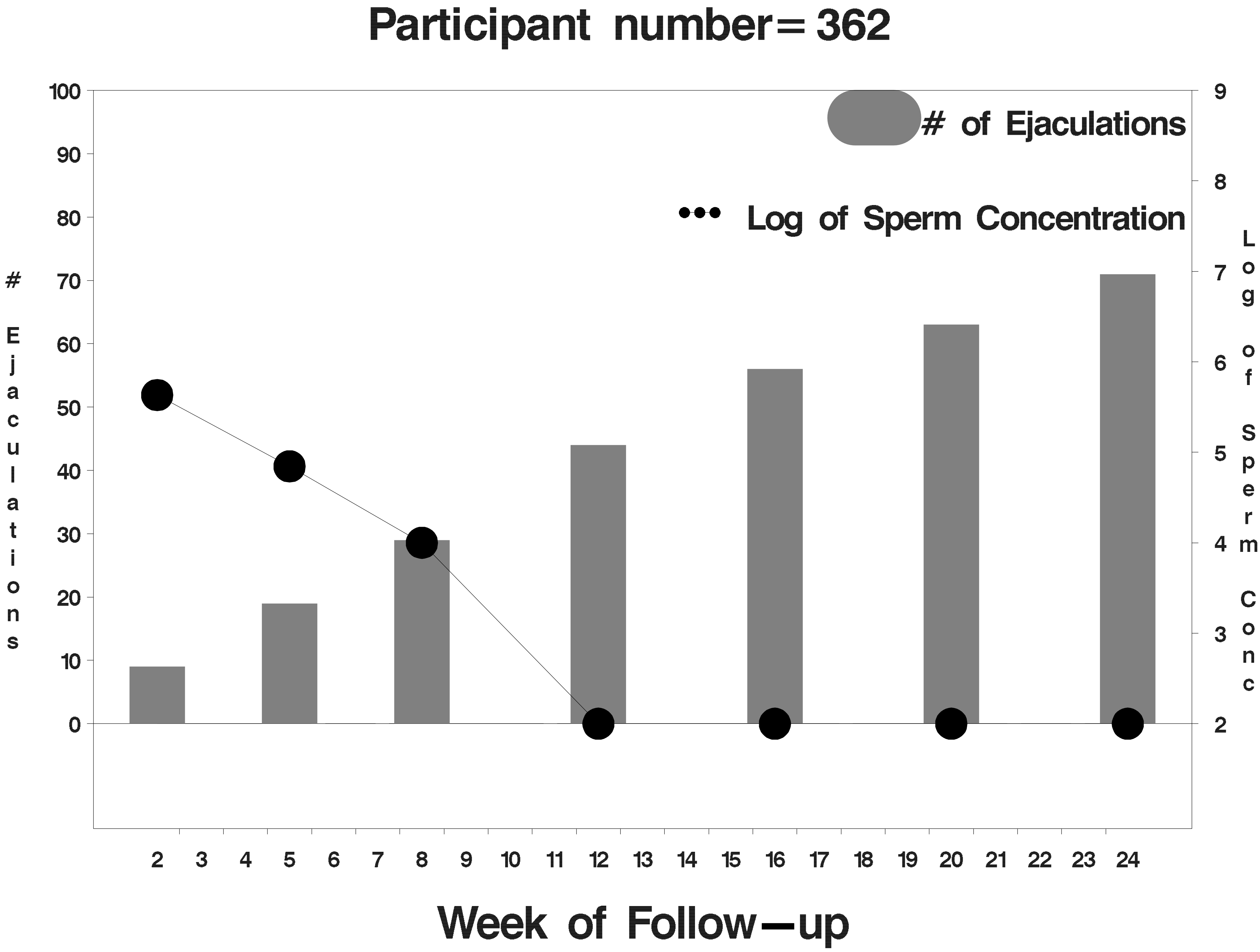

Participant number=362
# of Ejaculations
Log of Sperm Concentration
Ejaculations
Log of Sperm Conc
100
90
80
70
60
50
40
30
20
10
0
9
8
7
6
5
4
3
2
2 3 4 5 6 7 8 9 10 11 12 13 14 15 16 17 18 19 20 21 22 23 24
Week of Follow—up

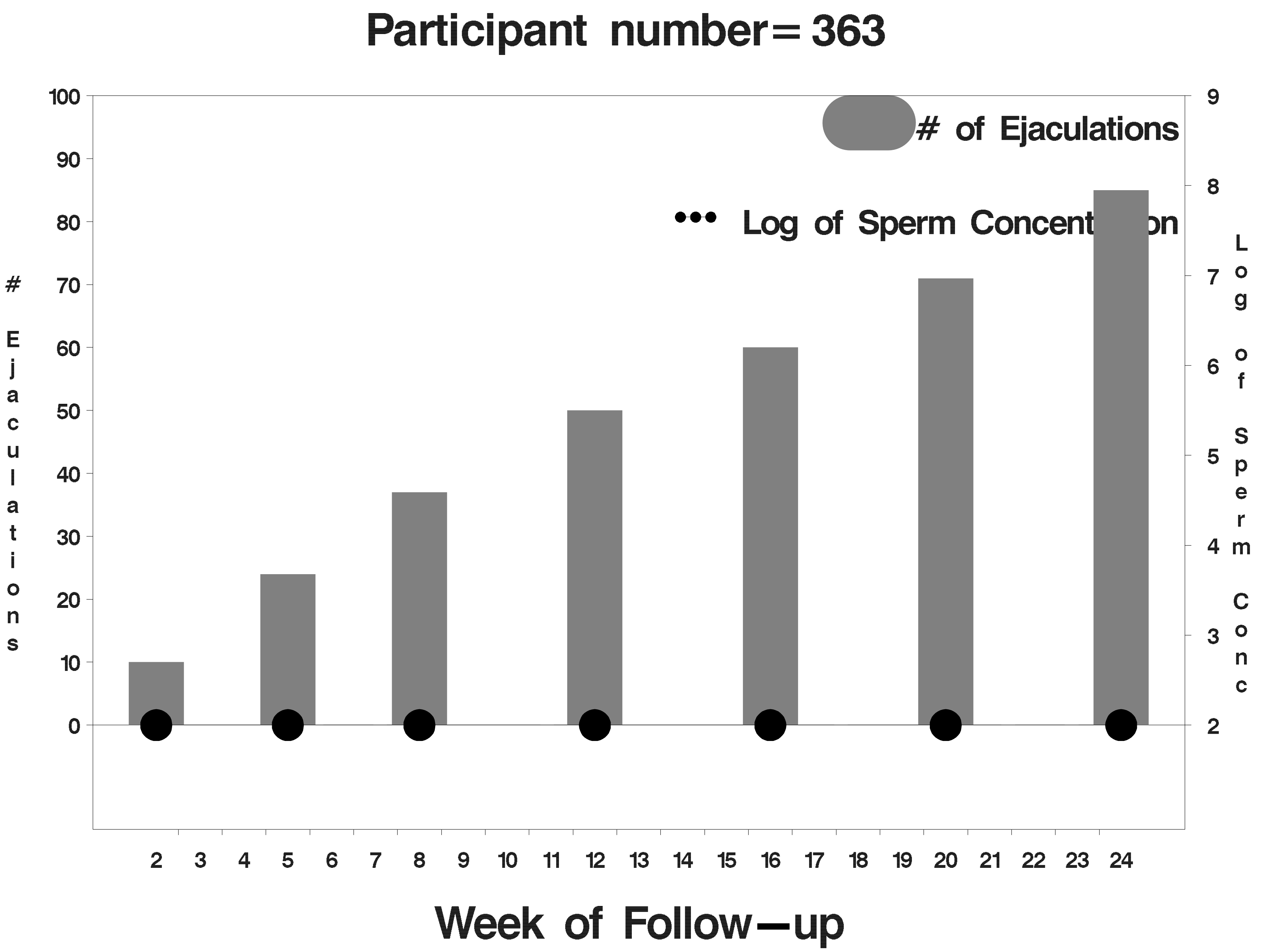
Participant number=363
# of Ejaculations
Log of Sperm Concentration
Ejaculations
Log of Sperm Conc
100
90
80
70
60
50
40
30
20
10
0
9
8
7
6
5
4
3
2
2 3 4 5 6 7 8 9 10 11 12 13 14 15 16 17 18 19 20 21 22 23 24
Week of Follow-up

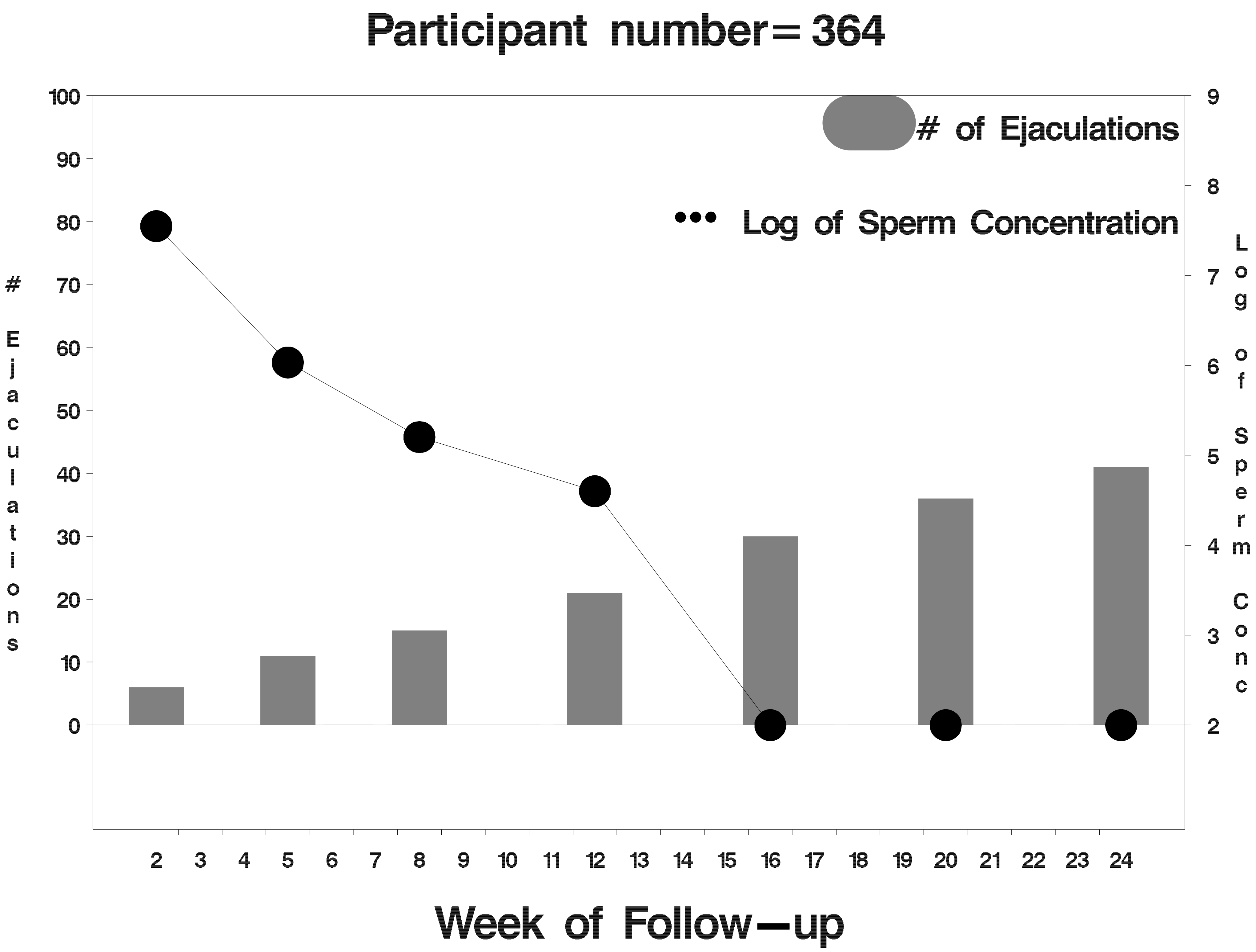

Participant number=364
# of Ejaculations
Log of Sperm Concentration
Ejaculations
Log of Sperm Conc
100
90
80
70
60
50
40
30
20
10
0
9
8
7
6
5
4
3
2
2 3 4 5 6 7 8 9 10 11 12 13 14 15 16 17 18 19 20 21 22 23 24
Week of Follow-up

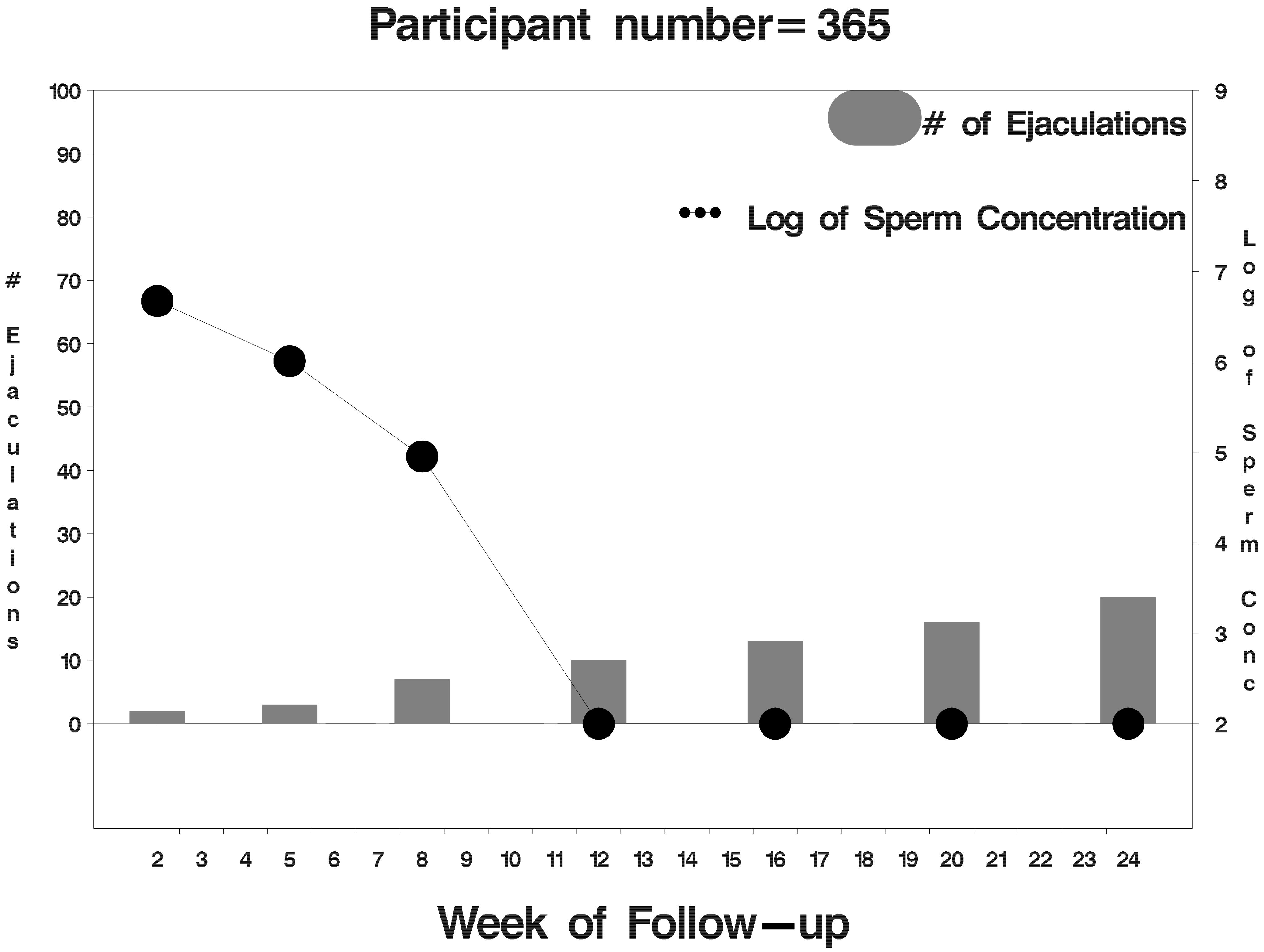

Participant number= 365
# of Ejaculations
Log of Sperm Concentration
Ejaculations
Log of Sperm Conc
100
90
80
70
60
50
40
30
20
10
0
9
8
7
6
5
4
3
2
2 3 4 5 6 7 8 9 10 11 12 13 14 15 16 17 18 19 20 21 22 23 24
Week of Follow−up

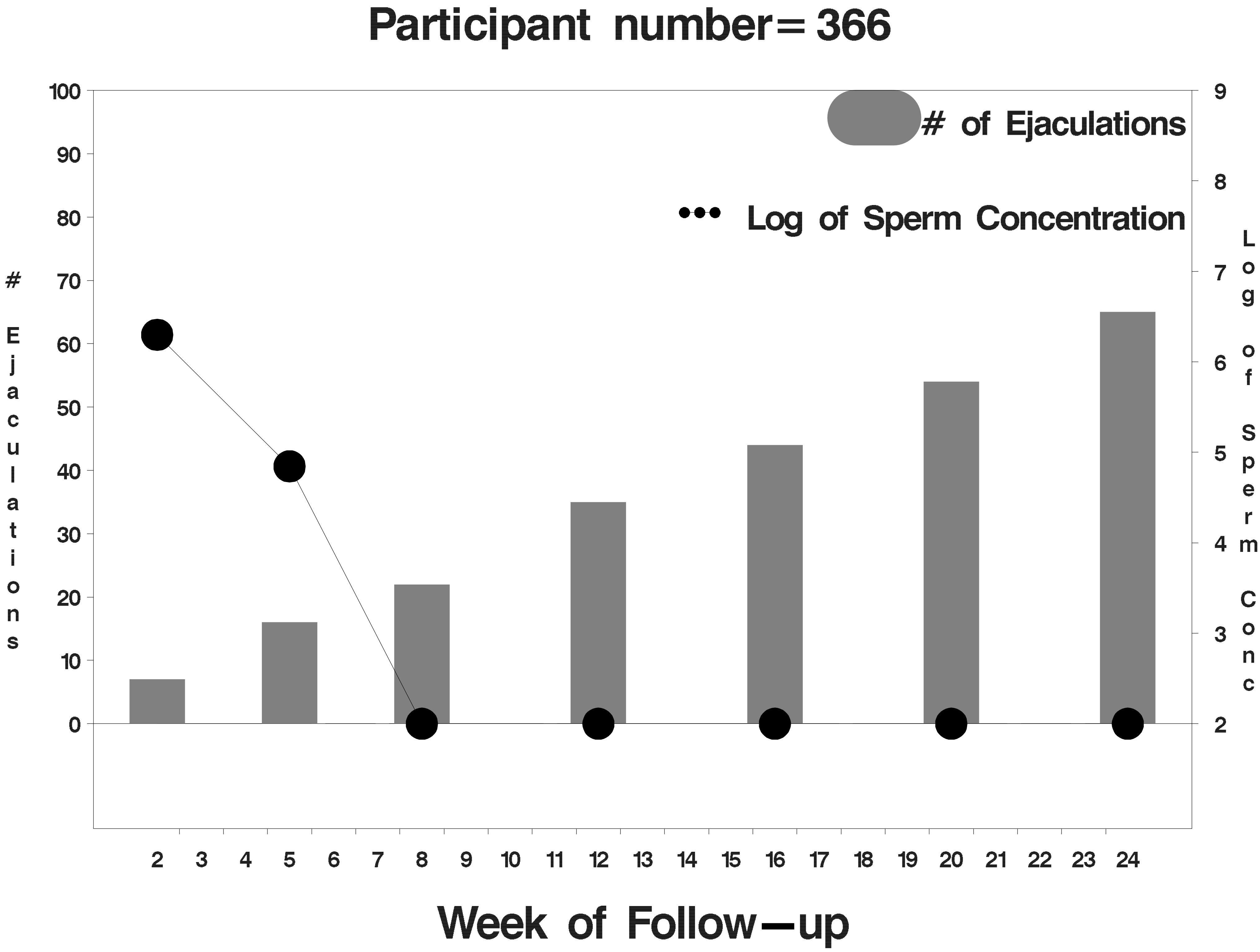

Participant number=366
# of Ejaculations
Log of Sperm Concentration
Ejaculations
Log of Sperm Conc
Week of Follow-up
0
10
20
30
40
50
60
70
80
90
100
2
3
4
5
6
7
8
9
2
3
4
5
6
7
8
9
10
11
12
13
14
15
16
17
18
19
20
21
22
23
24

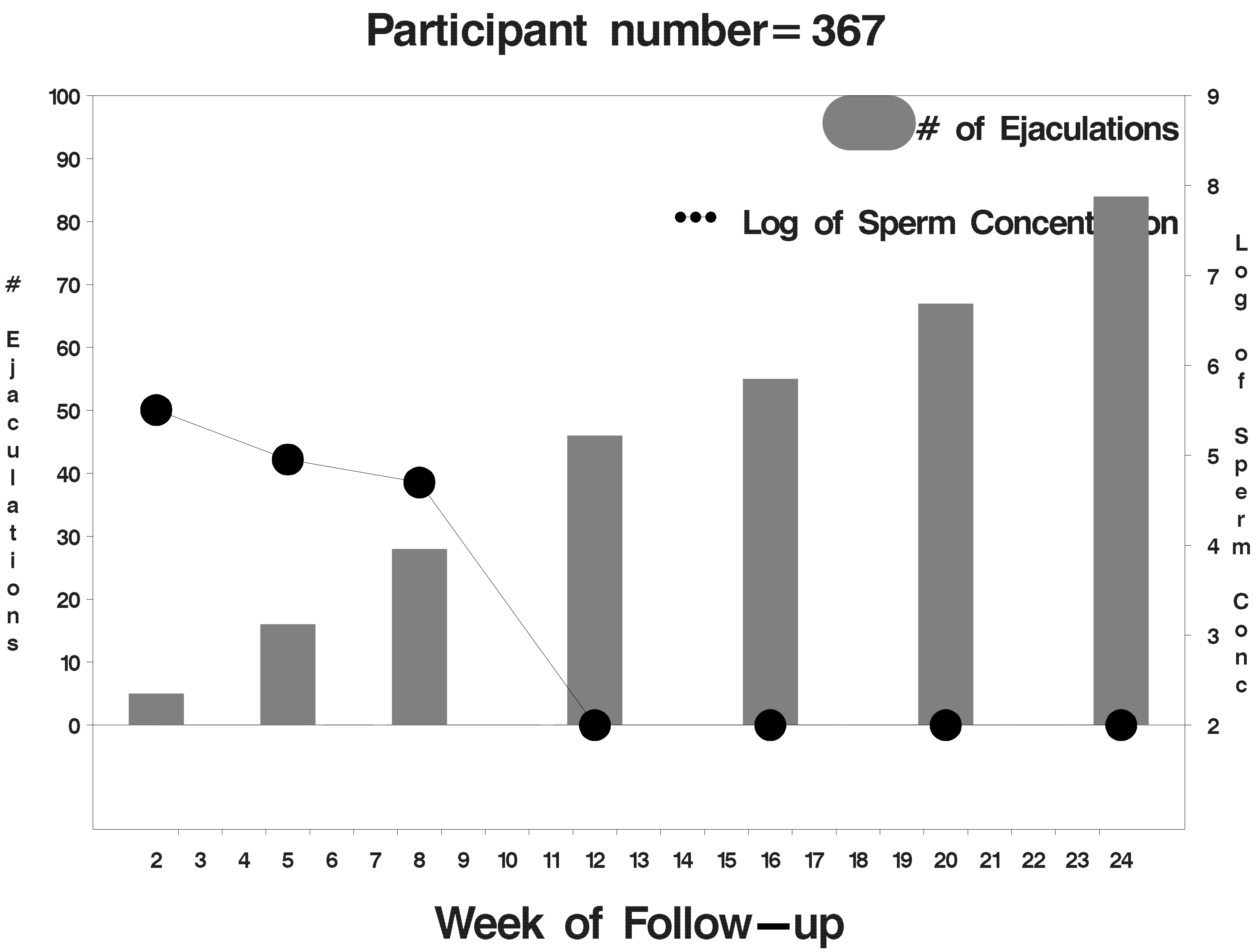

Participant number=367
# of Ejaculations
Log of Sperm Concentration
Ejaculations
Log of Sperm Conc
Week of Follow-up
100
90
80
70
60
50
40
30
20
10
0
9
8
7
6
5
4
3
2
2 3 4 5 6 7 8 9 10 11 12 13 14 15 16 17 18 19 20 21 22 23 24

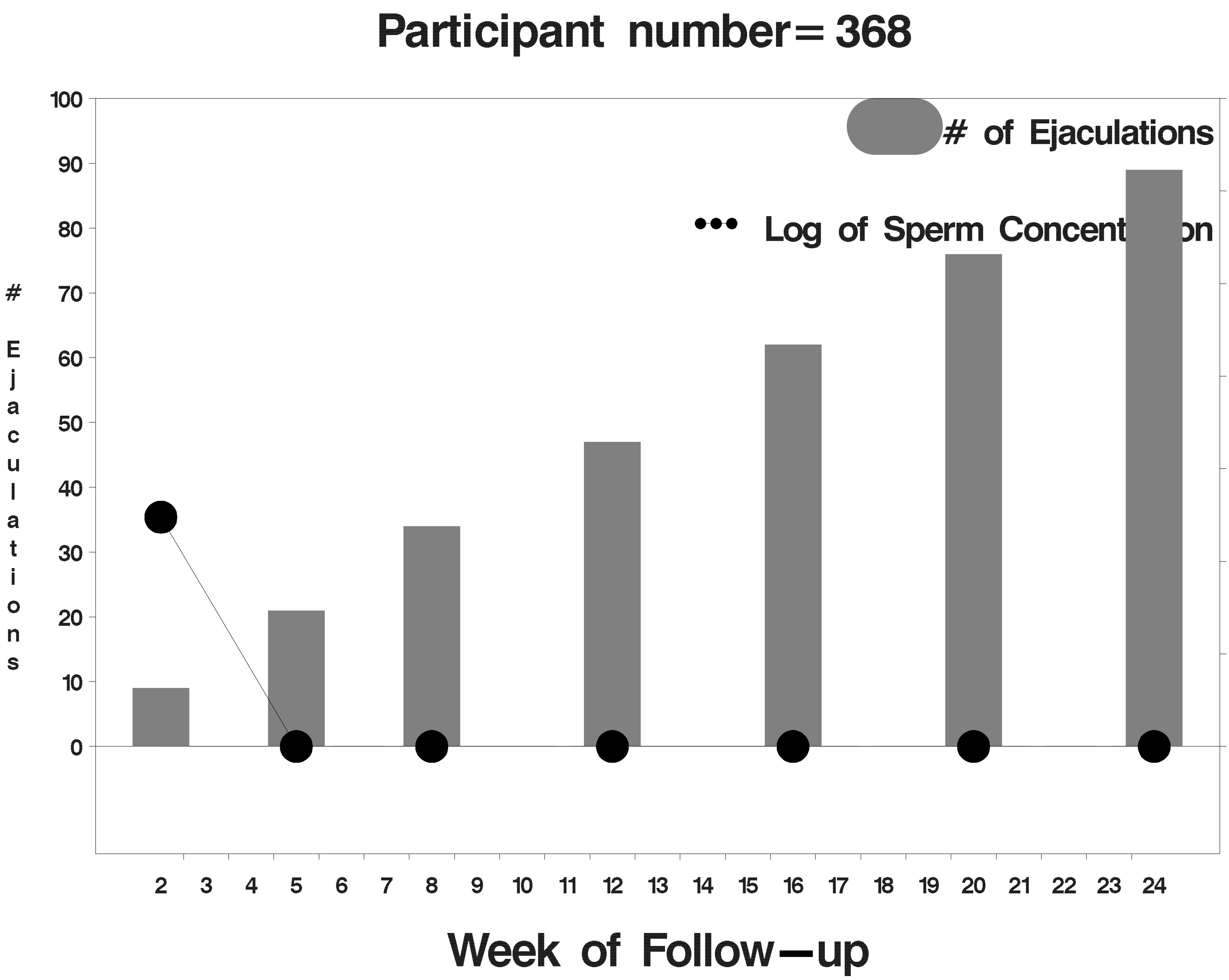
Participant number= 368
# of Ejaculations
Log of Sperm Concent[illegible]on
Ejaculations
Log of Sperm Conc
100
90
80
70
60
50
40
30
20
10
0
9
8
7
6
5
4
3
2
2 3 4 5 6 7 8 9 10 11 12 13 14 15 16 17 18 19 20 21 22 23 24
Week of Follow—up

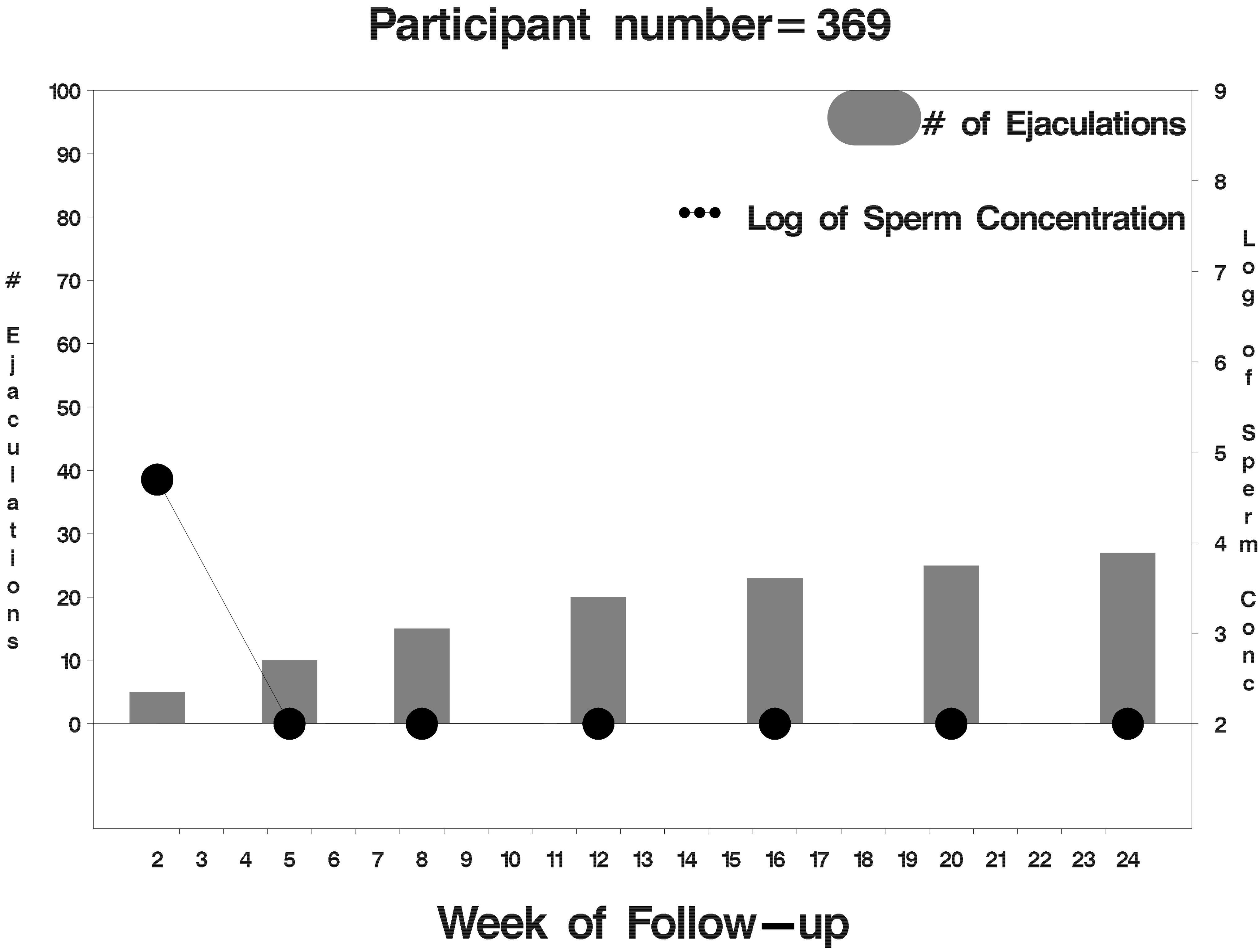

Participant number=369
# of Ejaculations
Log of Sperm Concentration
Ejaculations
Log of Sperm Conc
100
90
80
70
60
50
40
30
20
10
0
9
8
7
6
5
4
3
2
2 3 4 5 6 7 8 9 10 11 12 13 14 15 16 17 18 19 20 21 22 23 24
Week of Follow-up

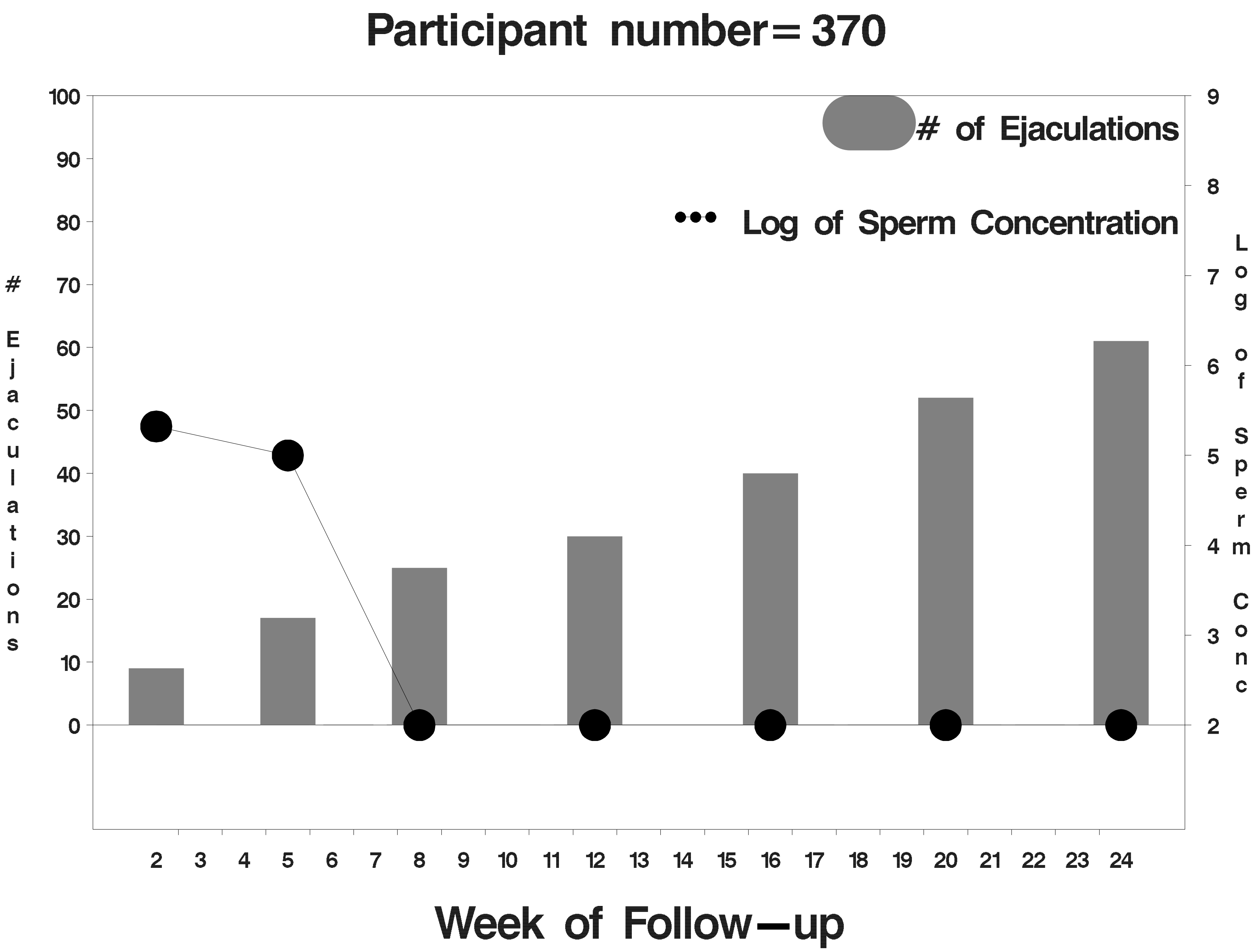

Participant number= 370
# of Ejaculations
Log of Sperm Concentration
Ejaculations
Log of Sperm Conc
100
90
80
70
60
50
40
30
20
10
0
9
8
7
6
5
4
3
2
2 3 4 5 6 7 8 9 10 11 12 13 14 15 16 17 18 19 20 21 22 23 24
Week of Follow-up

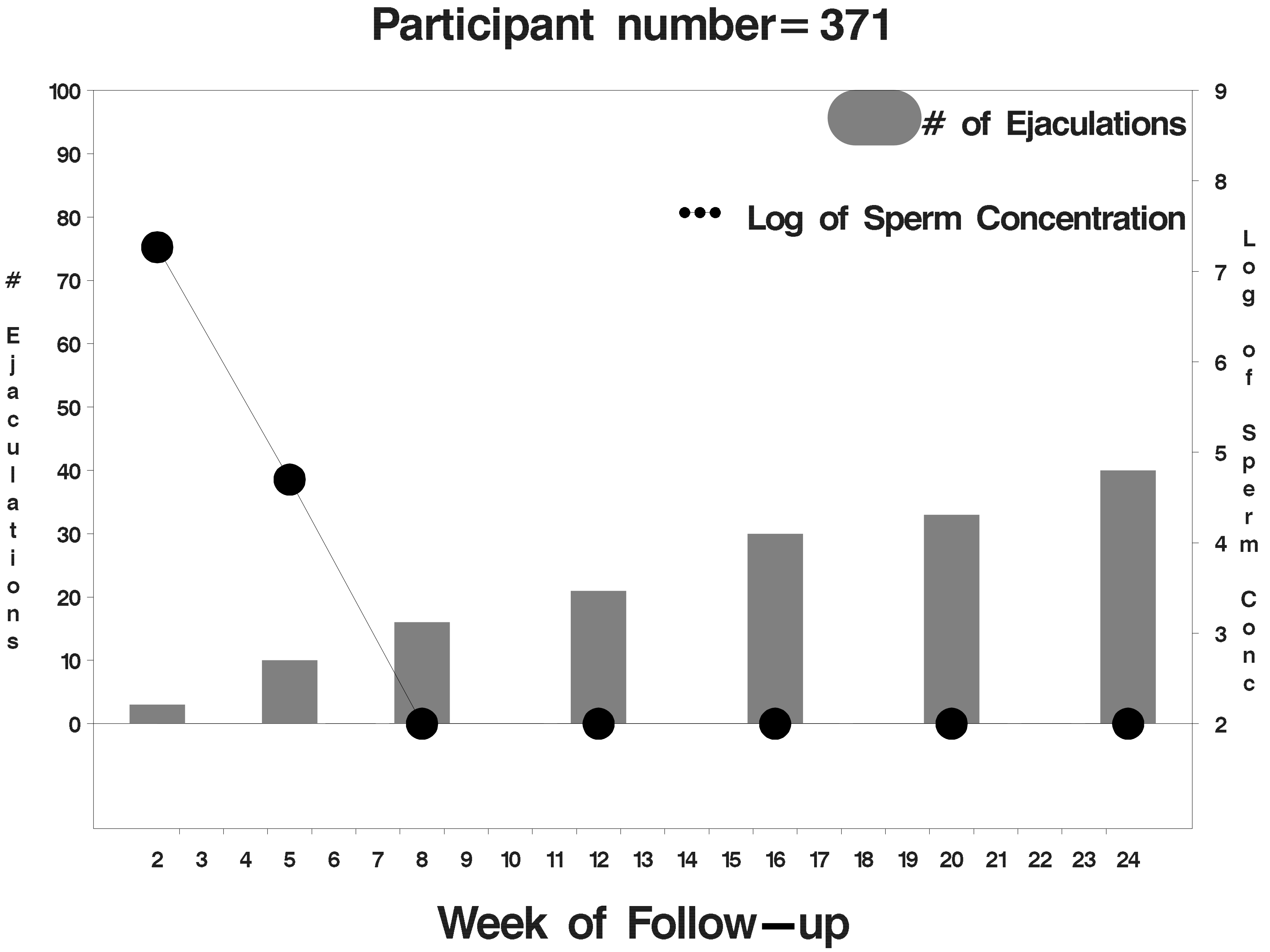

Participant number= 371
# of Ejaculations
Log of Sperm Concentration
Ejaculations
Log of Sperm Conc
100
90
80
70
60
50
40
30
20
10
0
9
8
7
6
5
4
3
2
2 3 4 5 6 7 8 9 10 11 12 13 14 15 16 17 18 19 20 21 22 23 24
Week of Follow-up

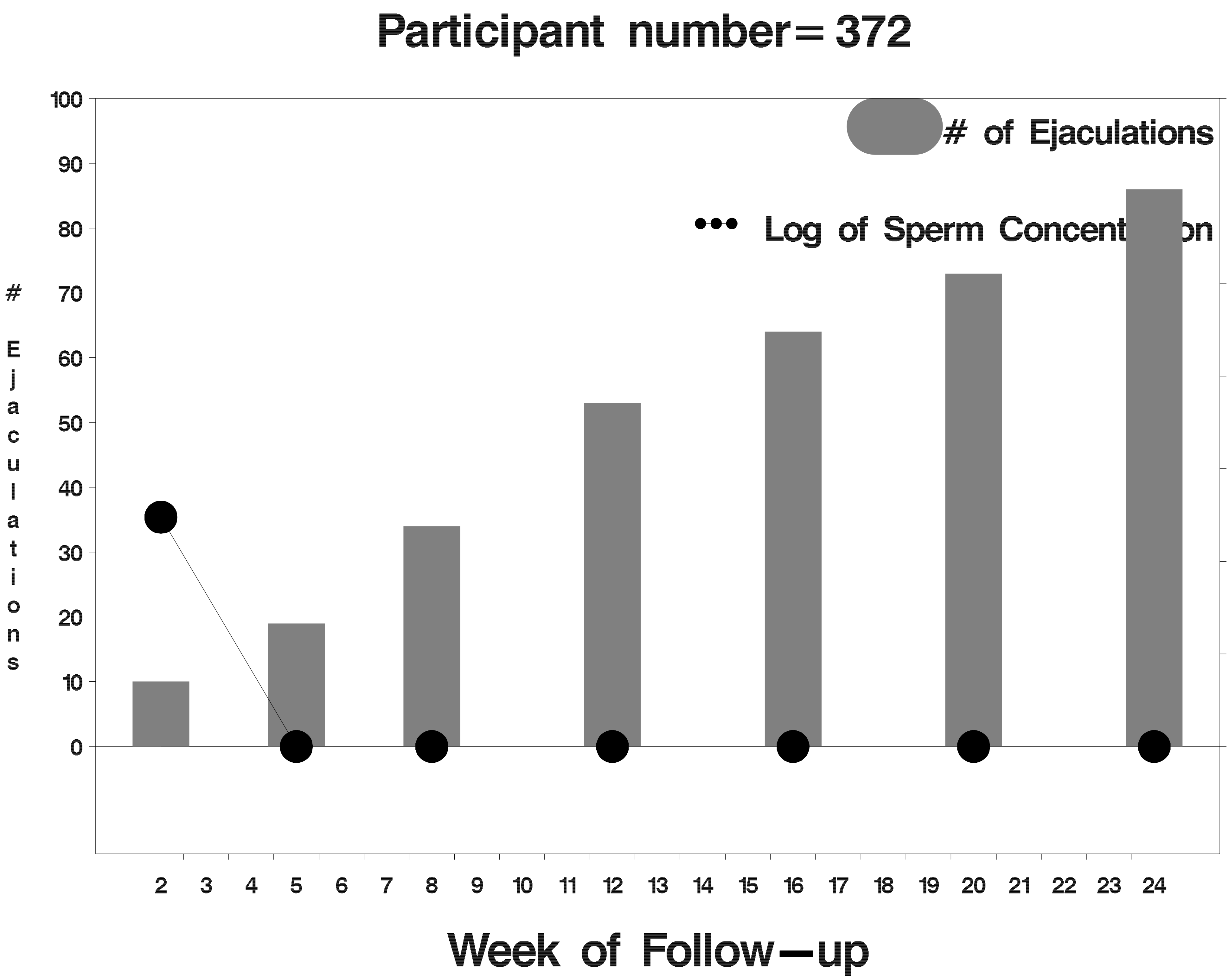

Participant number=372
# of Ejaculations
Log of Sperm Concent[illegible]on
Ejaculations
Log of Sperm Conc
0
10
20
30
40
50
60
70
80
90
100
2
3
4
5
6
7
8
9
2 3 4 5 6 7 8 9 10 11 12 13 14 15 16 17 18 19 20 21 22 23 24
Week of Follow−up

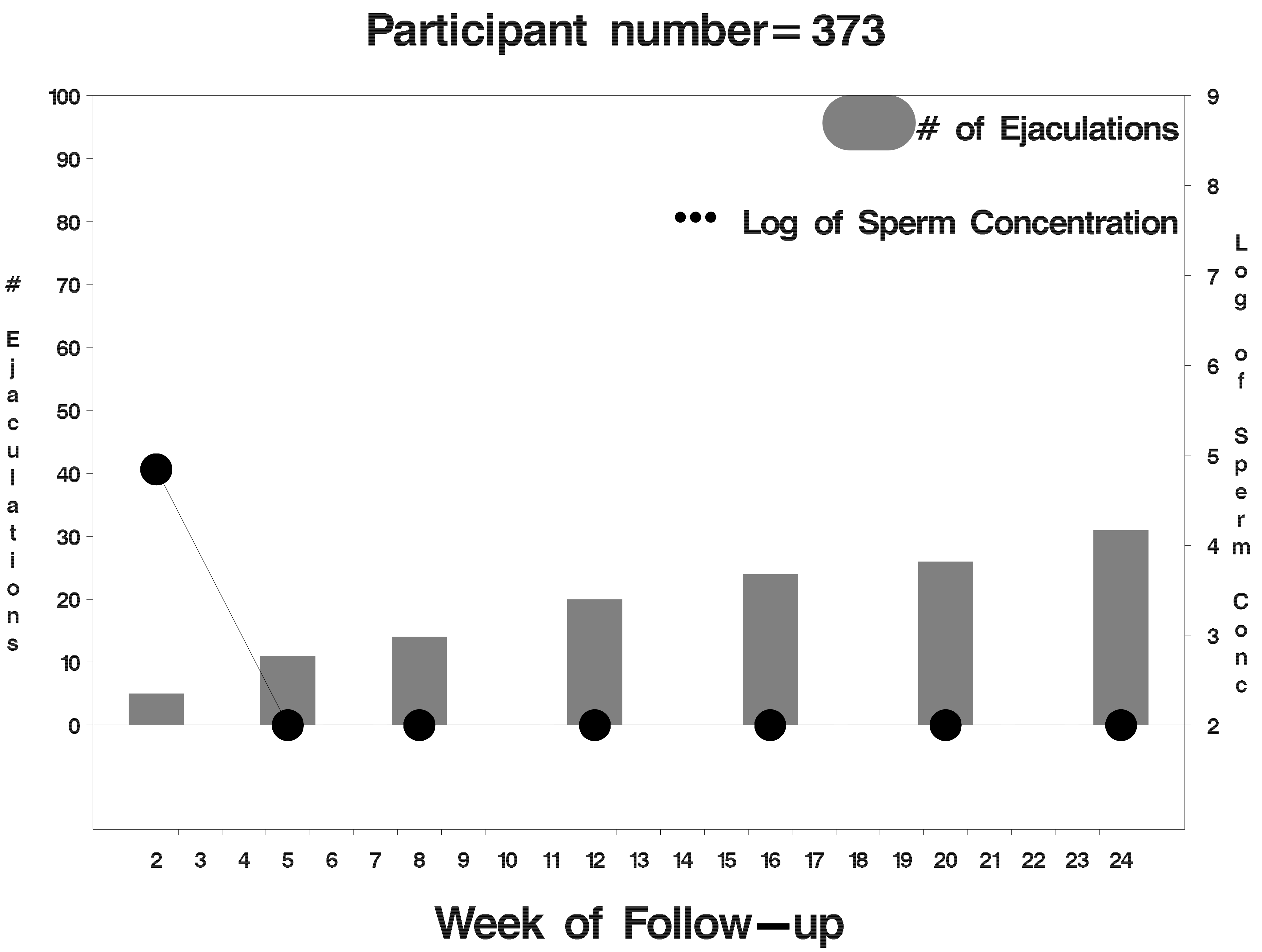

Participant number= 373
# of Ejaculations
Log of Sperm Concentration
Ejaculations
Log of Sperm Conc
100
90
80
70
60
50
40
30
20
10
0
9
8
7
6
5
4
3
2
2 3 4 5 6 7 8 9 10 11 12 13 14 15 16 17 18 19 20 21 22 23 24
Week of Follow-up

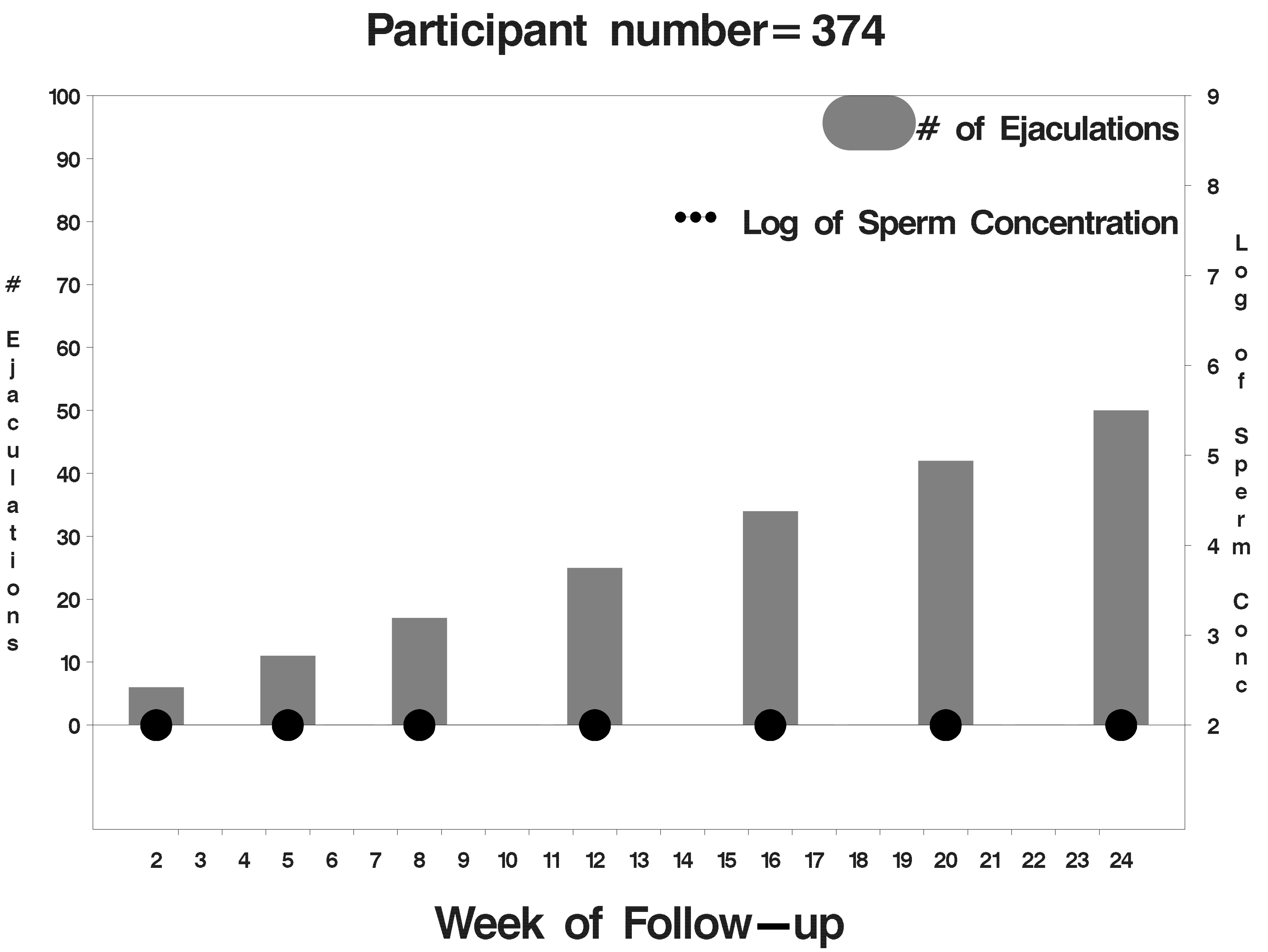

Participant number=374
# of Ejaculations
Log of Sperm Concentration
Ejaculations
Log of Sperm Conc
100
90
80
70
60
50
40
30
20
10
0
9
8
7
6
5
4
3
2
2 3 4 5 6 7 8 9 10 11 12 13 14 15 16 17 18 19 20 21 22 23 24
Week of Follow-up

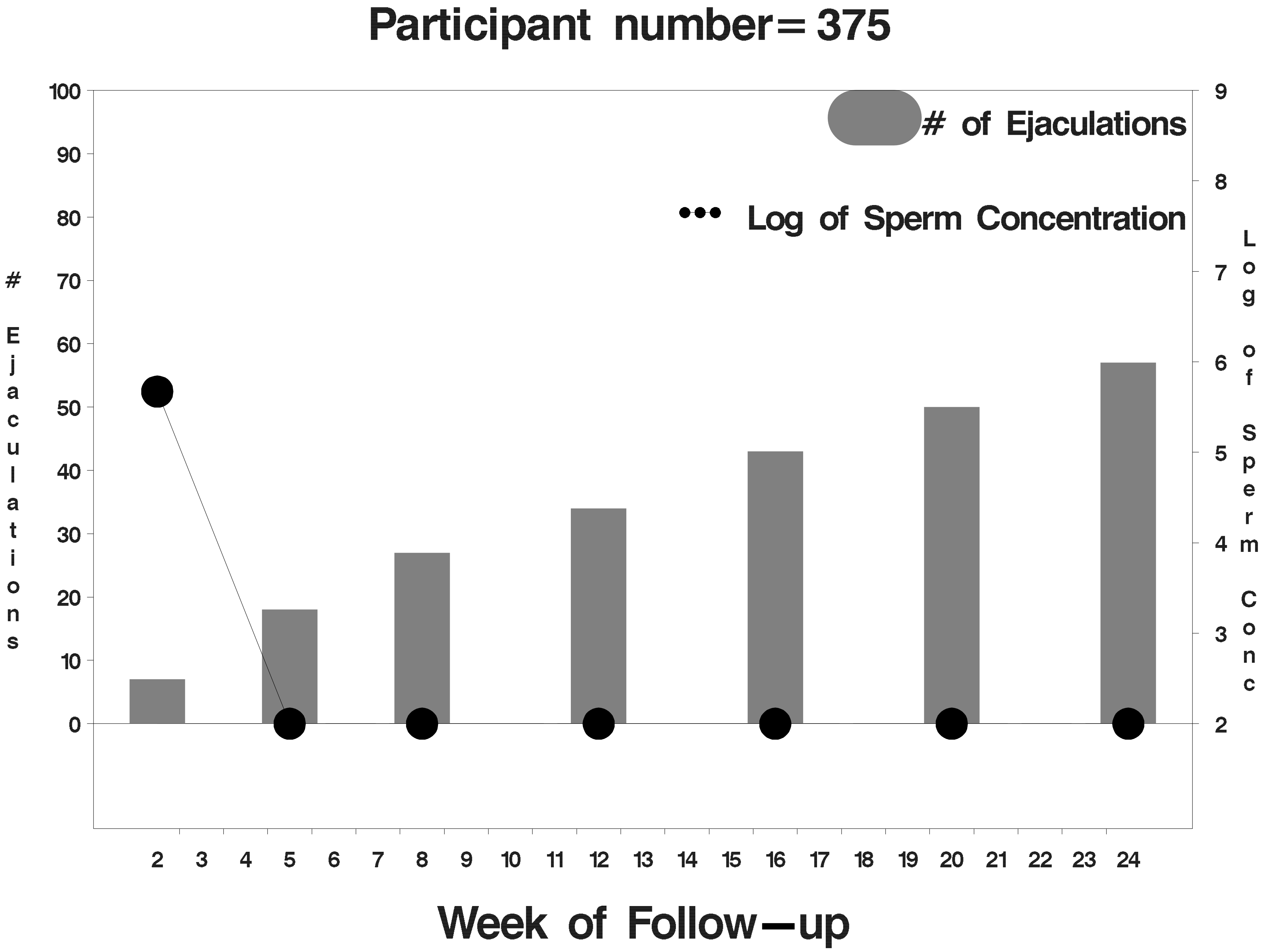

Participant number= 375
# of Ejaculations
Log of Sperm Concentration
Ejaculations
Log of Sperm Conc
100
90
80
70
60
50
40
30
20
10
0
9
8
7
6
5
4
3
2
2 3 4 5 6 7 8 9 10 11 12 13 14 15 16 17 18 19 20 21 22 23 24
Week of Follow-up

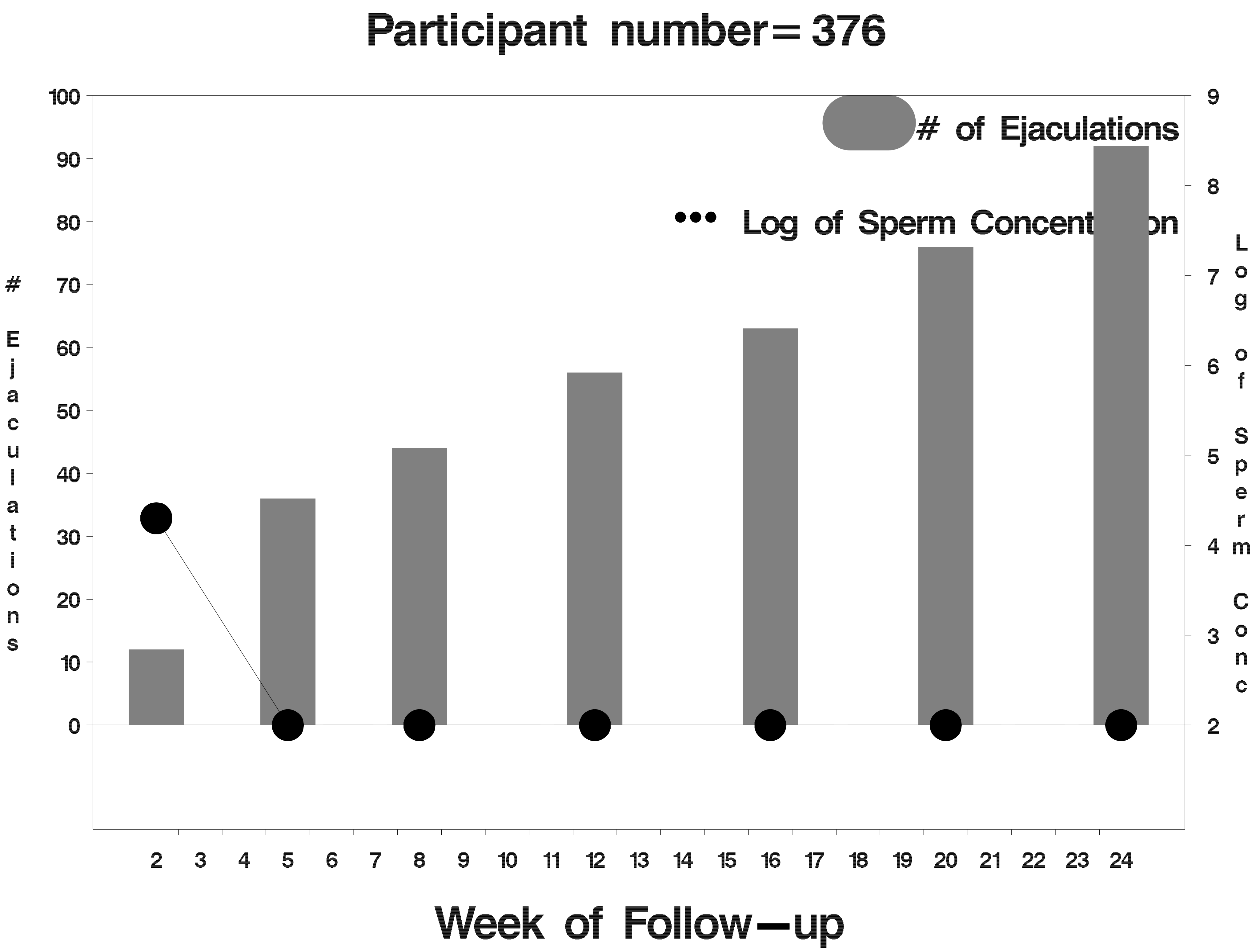
Participant number= 376
# of Ejaculations
Log of Sperm Concent[illegible]on
Ejaculations
Log of Sperm Conc
100
90
80
70
60
50
40
30
20
10
0
9
8
7
6
5
4
3
2
2 3 4 5 6 7 8 9 10 11 12 13 14 15 16 17 18 19 20 21 22 23 24
Week of Follow-up

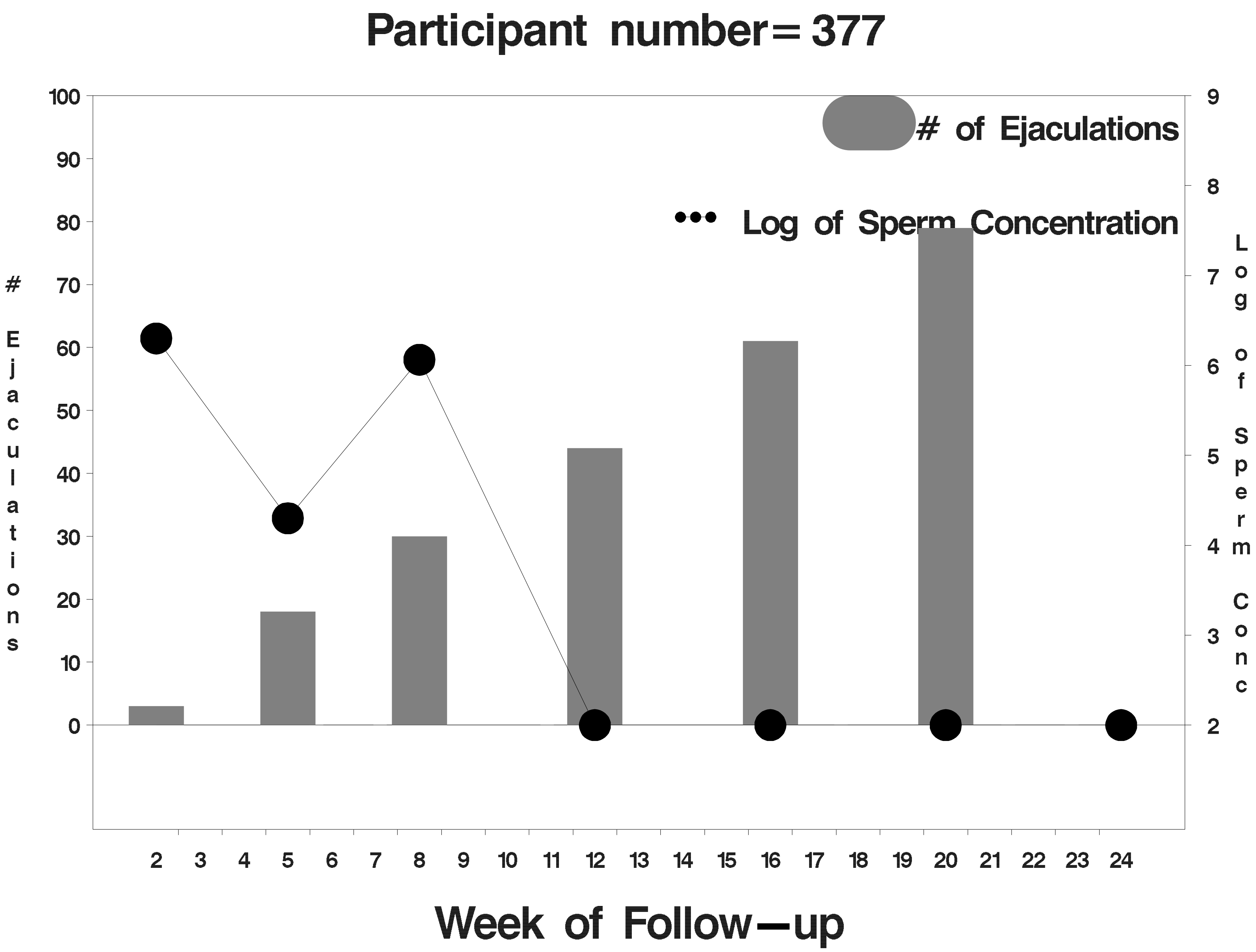

Participant number= 377
# of Ejaculations
Log of Sperm Concentration
Ejaculations
Log of Sperm Conc
100
90
80
70
60
50
40
30
20
10
0
9
8
7
6
5
4
3
2
2 3 4 5 6 7 8 9 10 11 12 13 14 15 16 17 18 19 20 21 22 23 24
Week of Follow-up

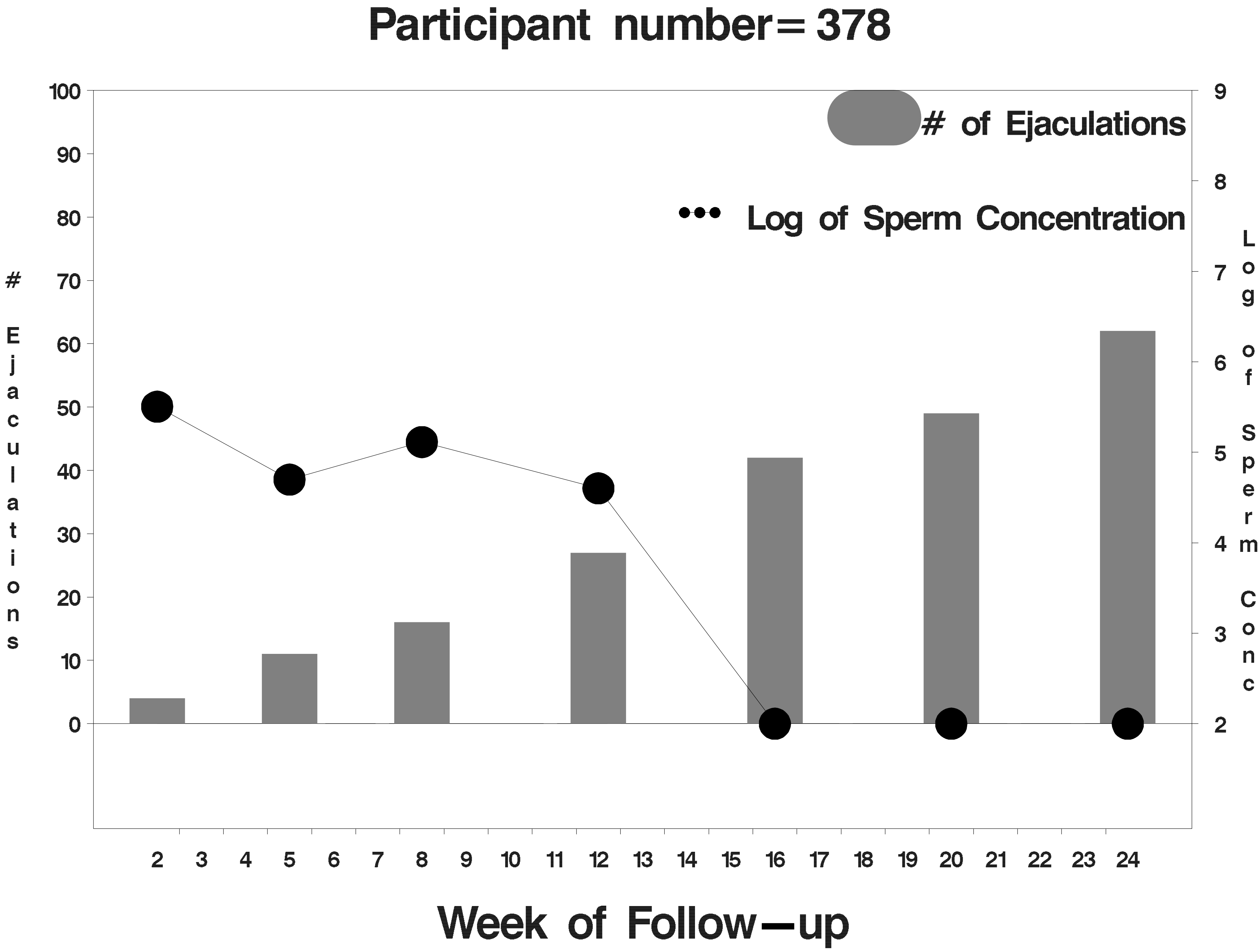

Participant number=378
# of Ejaculations
Log of Sperm Concentration
Ejaculations
Log of Sperm Conc
Week of Follow-up
100
90
80
70
60
50
40
30
20
10
0
9
8
7
6
5
4
3
2
2 3 4 5 6 7 8 9 10 11 12 13 14 15 16 17 18 19 20 21 22 23 24

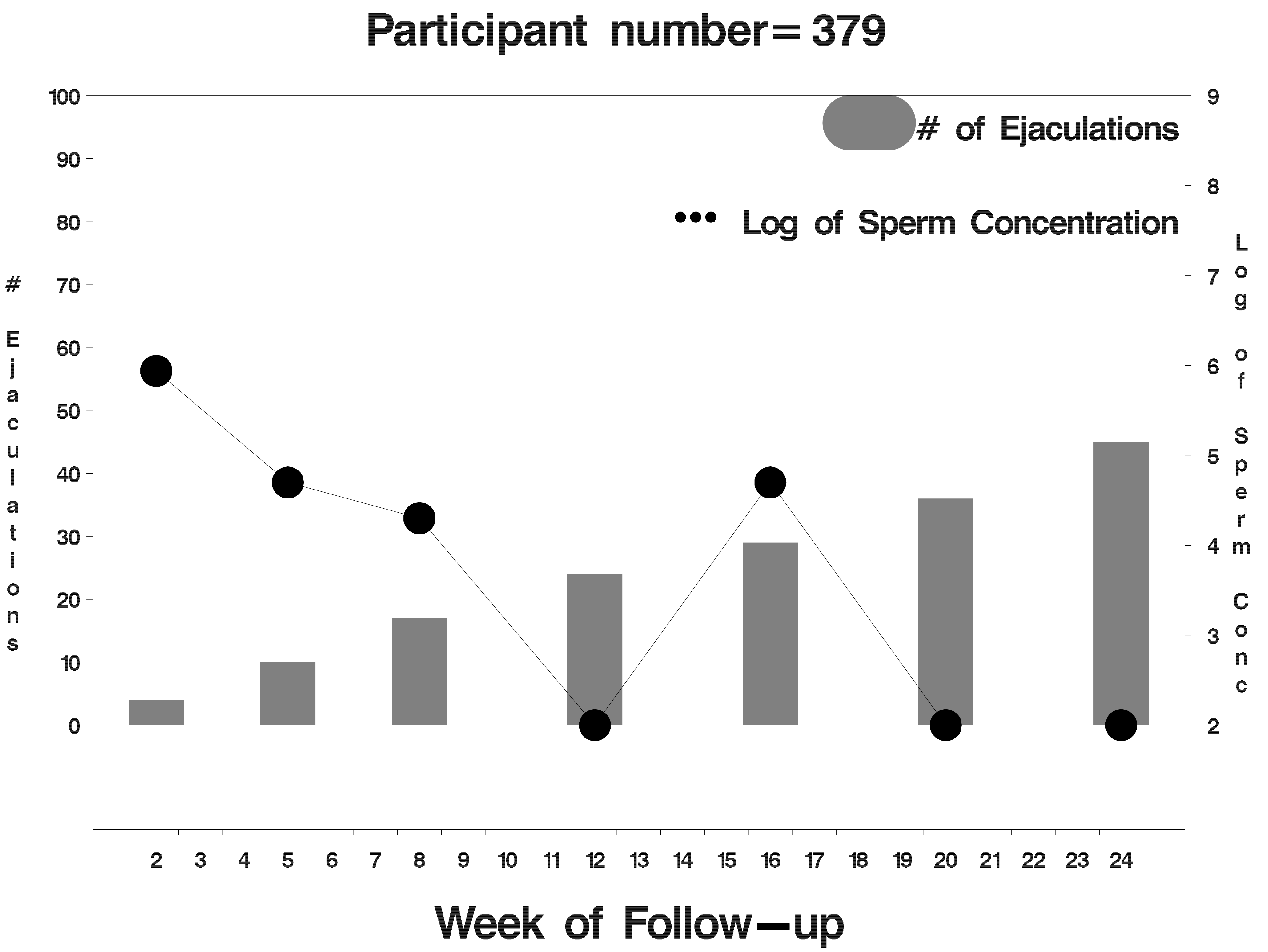

Participant number=379
100
90
80
70
60
50
40
30
20
10
0
Ejaculations
9
8
7
6
5
4
3
2
Log of Sperm Conc
# of Ejaculations
Log of Sperm Concentration
2 3 4 5 6 7 8 9 10 11 12 13 14 15 16 17 18 19 20 21 22 23 24
Week of Follow-up

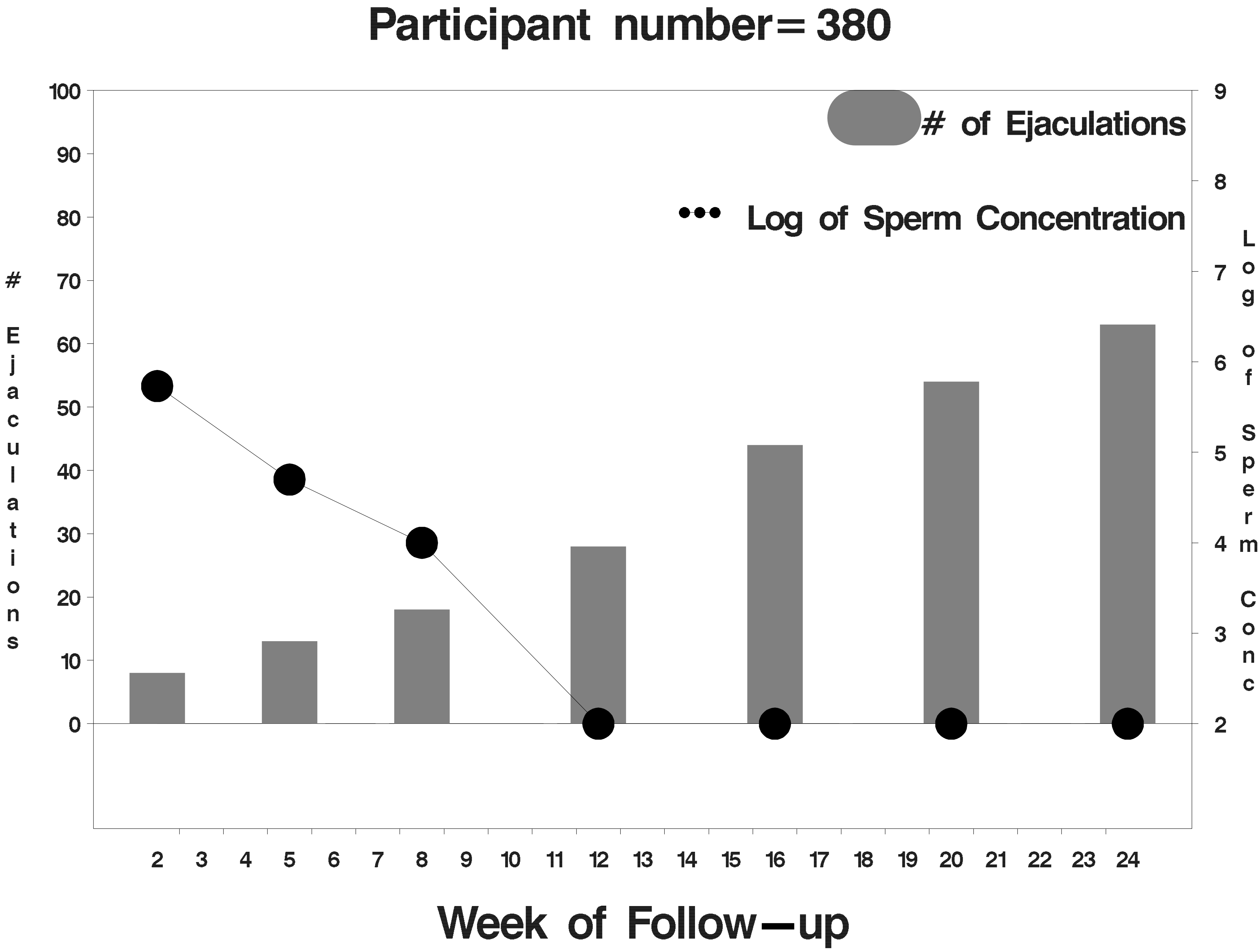

Participant number= 380
# of Ejaculations
Log of Sperm Concentration
Ejaculations
Log of Sperm Conc
100
90
80
70
60
50
40
30
20
10
0
9
8
7
6
5
4
3
2
2 3 4 5 6 7 8 9 10 11 12 13 14 15 16 17 18 19 20 21 22 23 24
Week of Follow-up

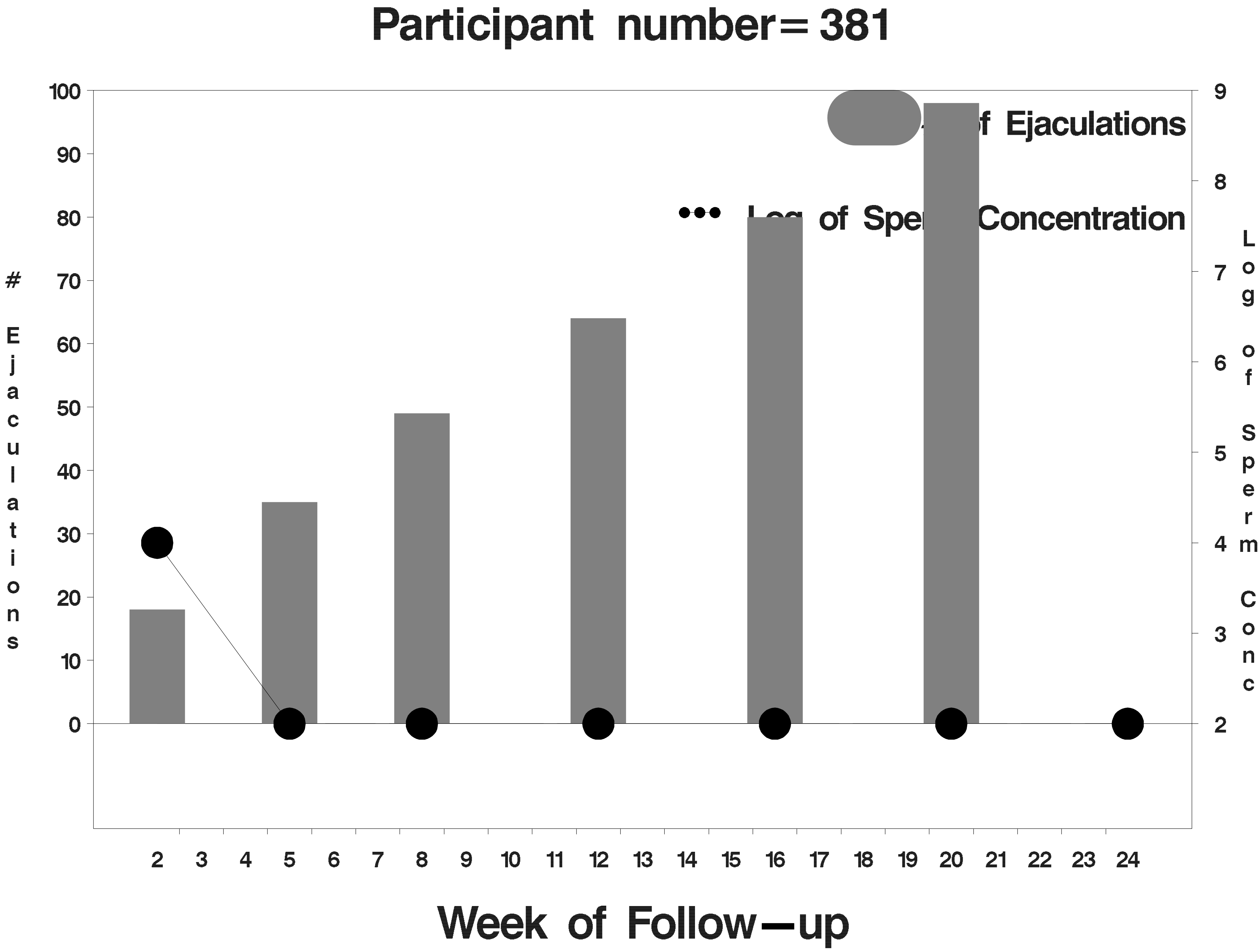

Participant number= 381
# of Ejaculations
Log of Sperm Concentration
Ejaculations
Log of Sperm Conc
100
90
80
70
60
50
40
30
20
10
0
9
8
7
6
5
4
3
2
2 3 4 5 6 7 8 9 10 11 12 13 14 15 16 17 18 19 20 21 22 23 24
Week of Follow-up

Participant number=382

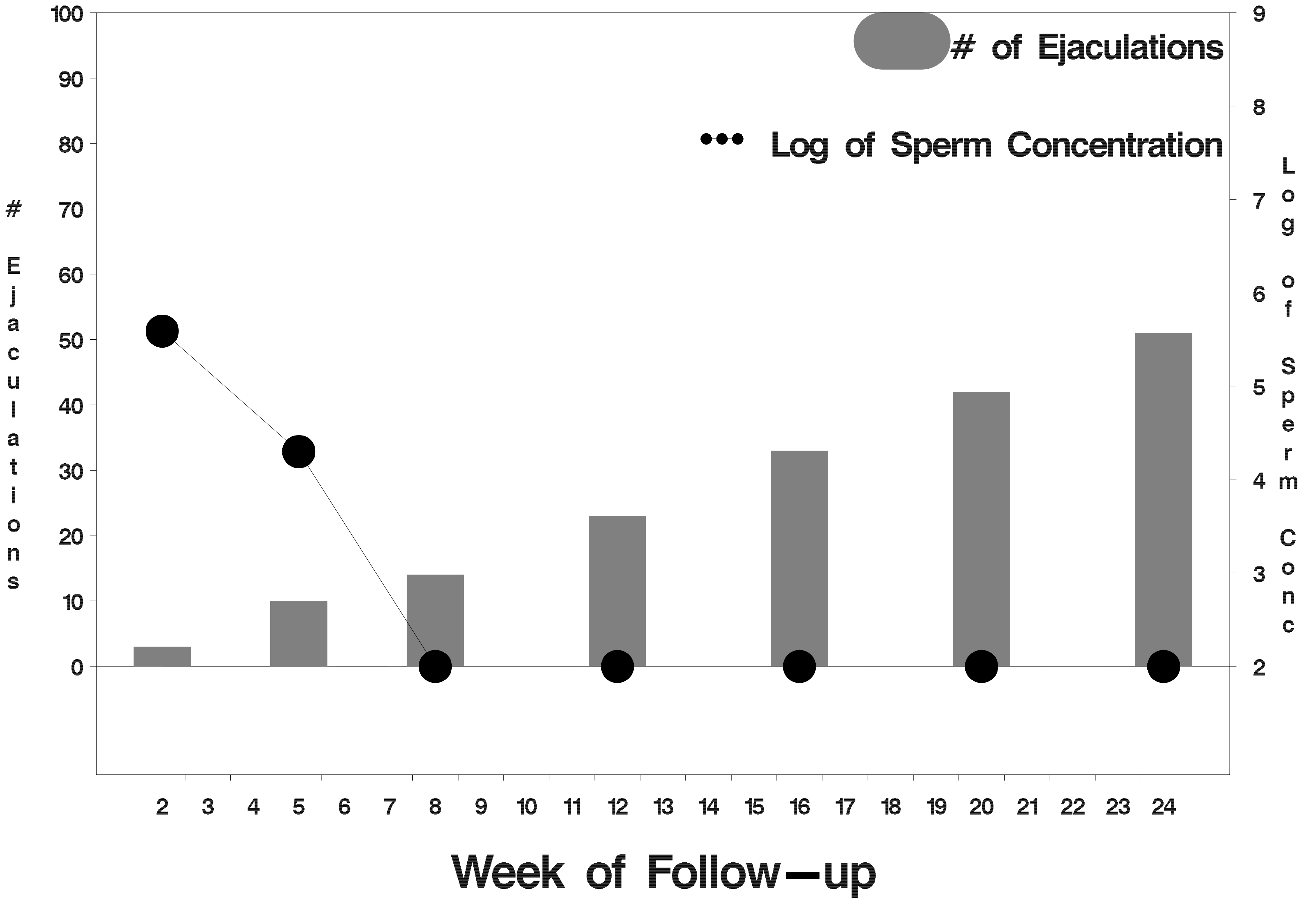

Participant number= 383

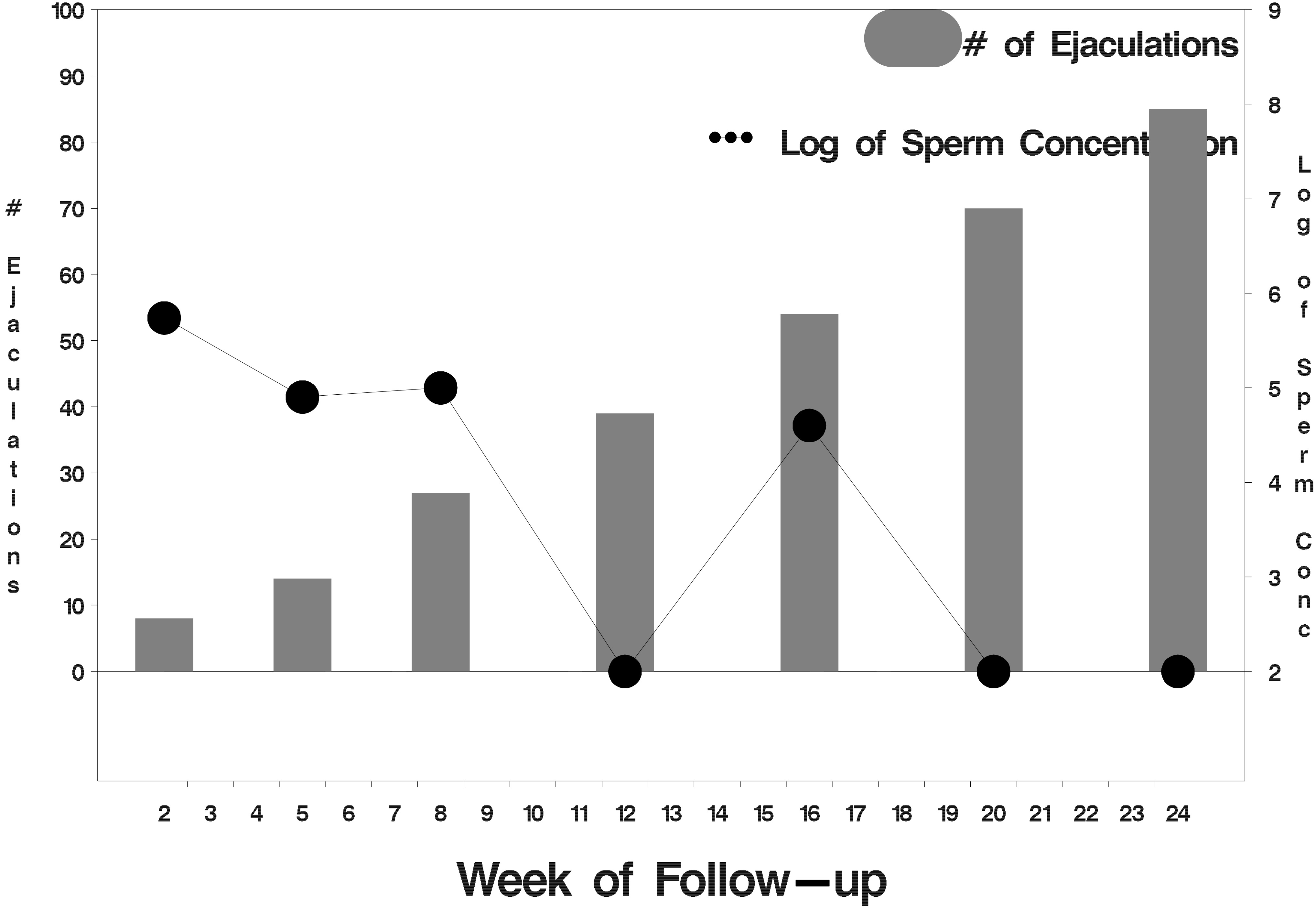

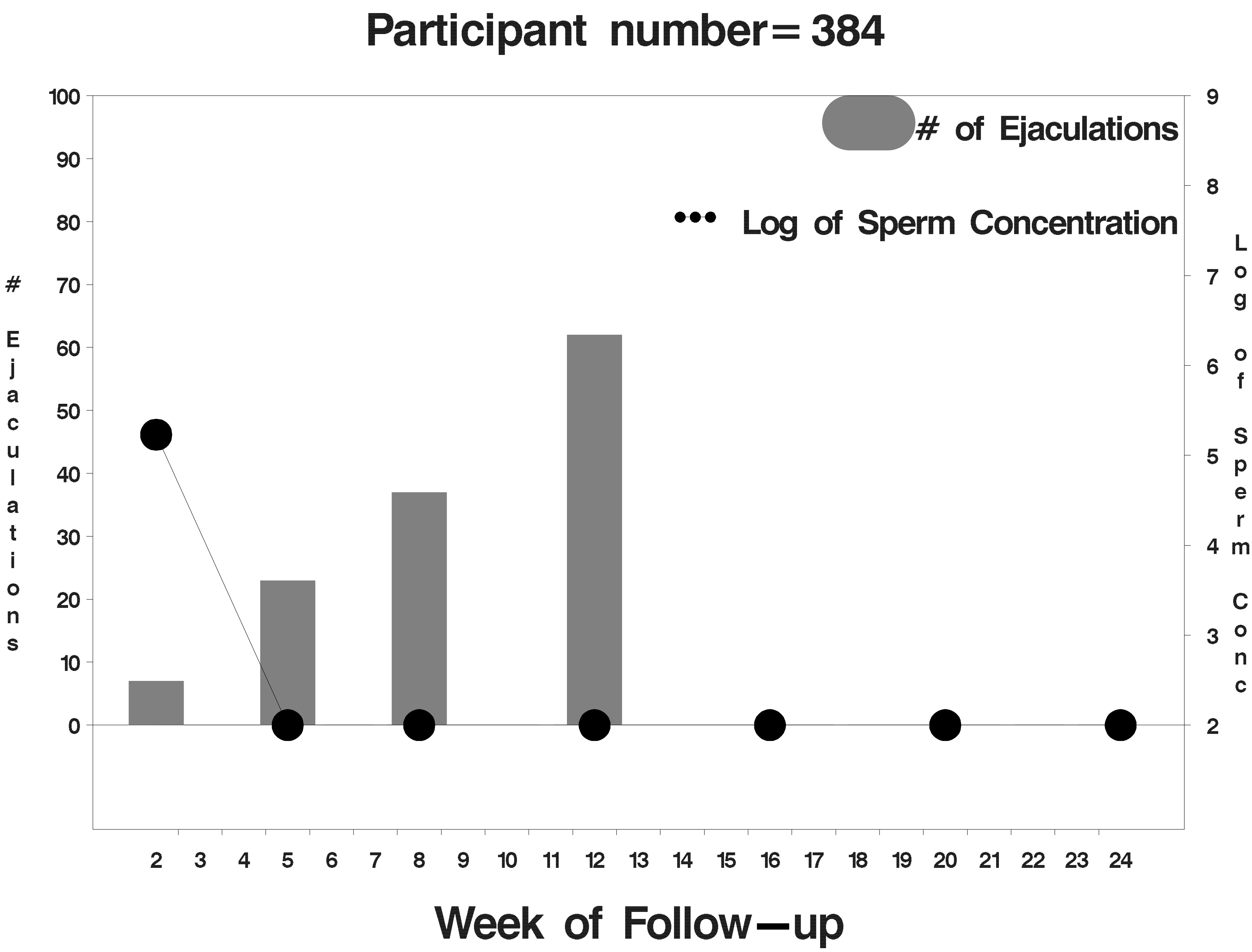

Participant number=384
# of Ejaculations
Log of Sperm Concentration
Ejaculations
Log of Sperm Conc
100
90
80
70
60
50
40
30
20
10
0
9
8
7
6
5
4
3
2
2 3 4 5 6 7 8 9 10 11 12 13 14 15 16 17 18 19 20 21 22 23 24
Week of Follow-up

Participant number= 385

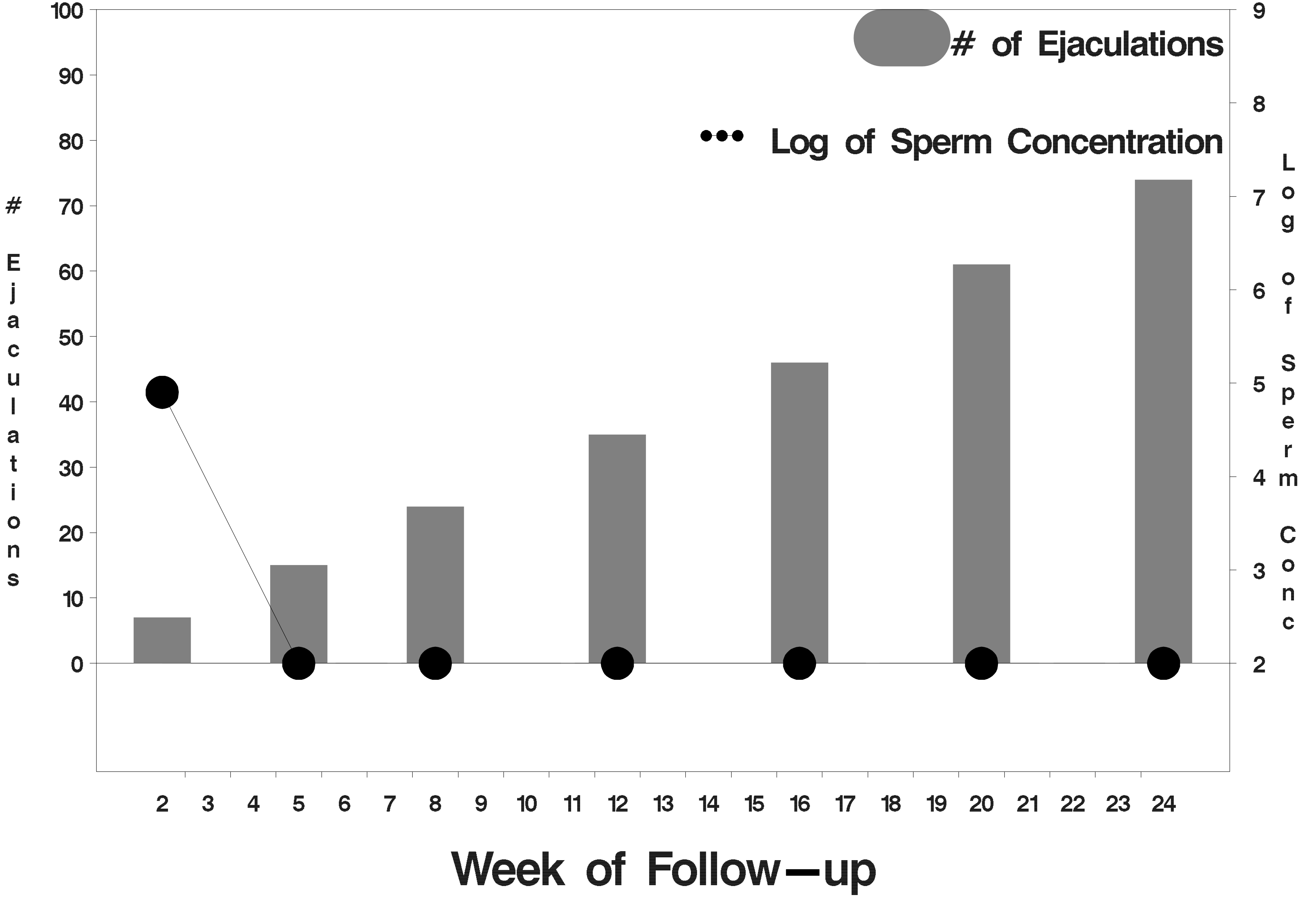

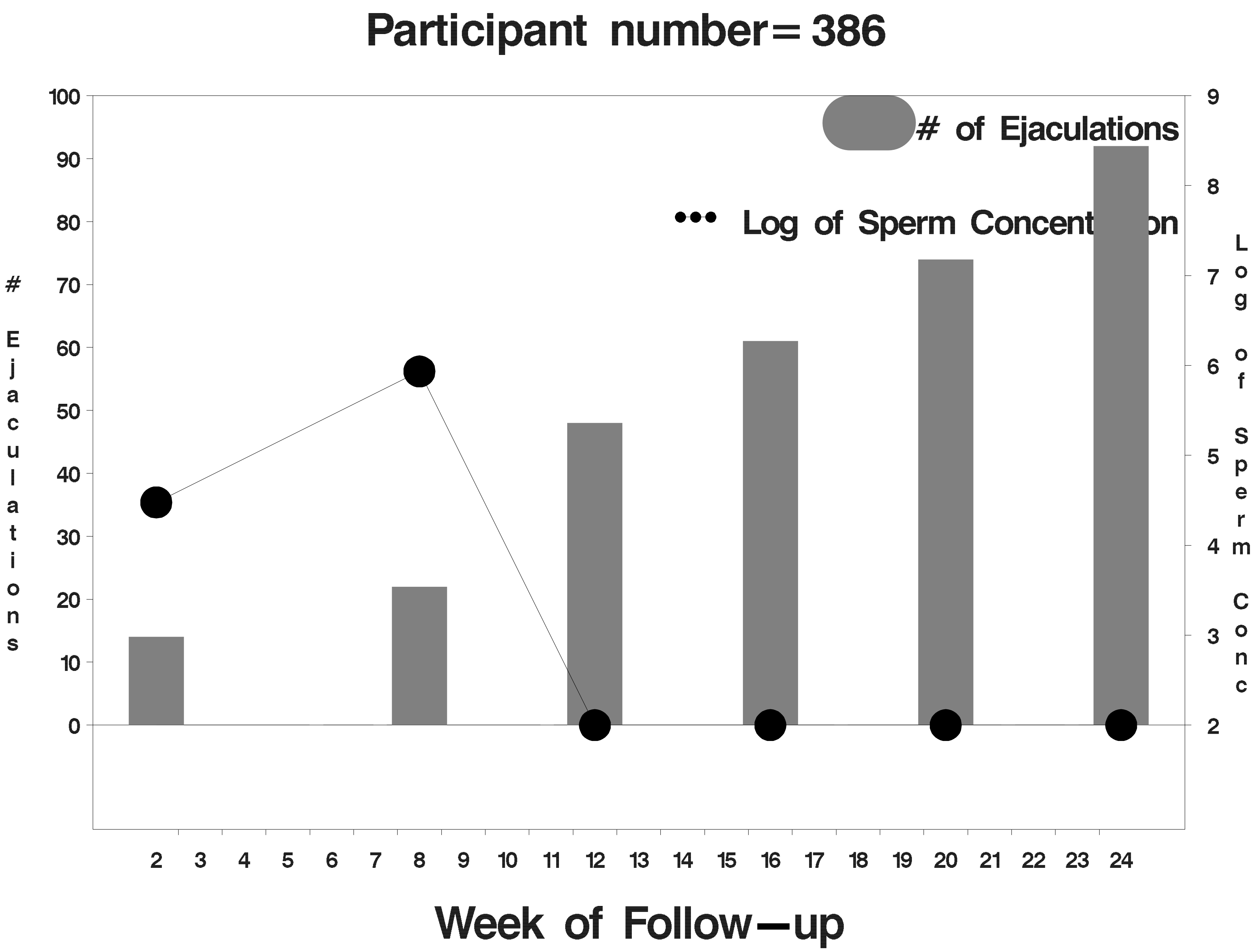

Participant number= 386
# of Ejaculations
Log of Sperm Concentration
Ejaculations
Log of Sperm Conc
0
10
20
30
40
50
60
70
80
90
100
2
3
4
5
6
7
8
9
2 3 4 5 6 7 8 9 10 11 12 13 14 15 16 17 18 19 20 21 22 23 24
Week of Follow-up

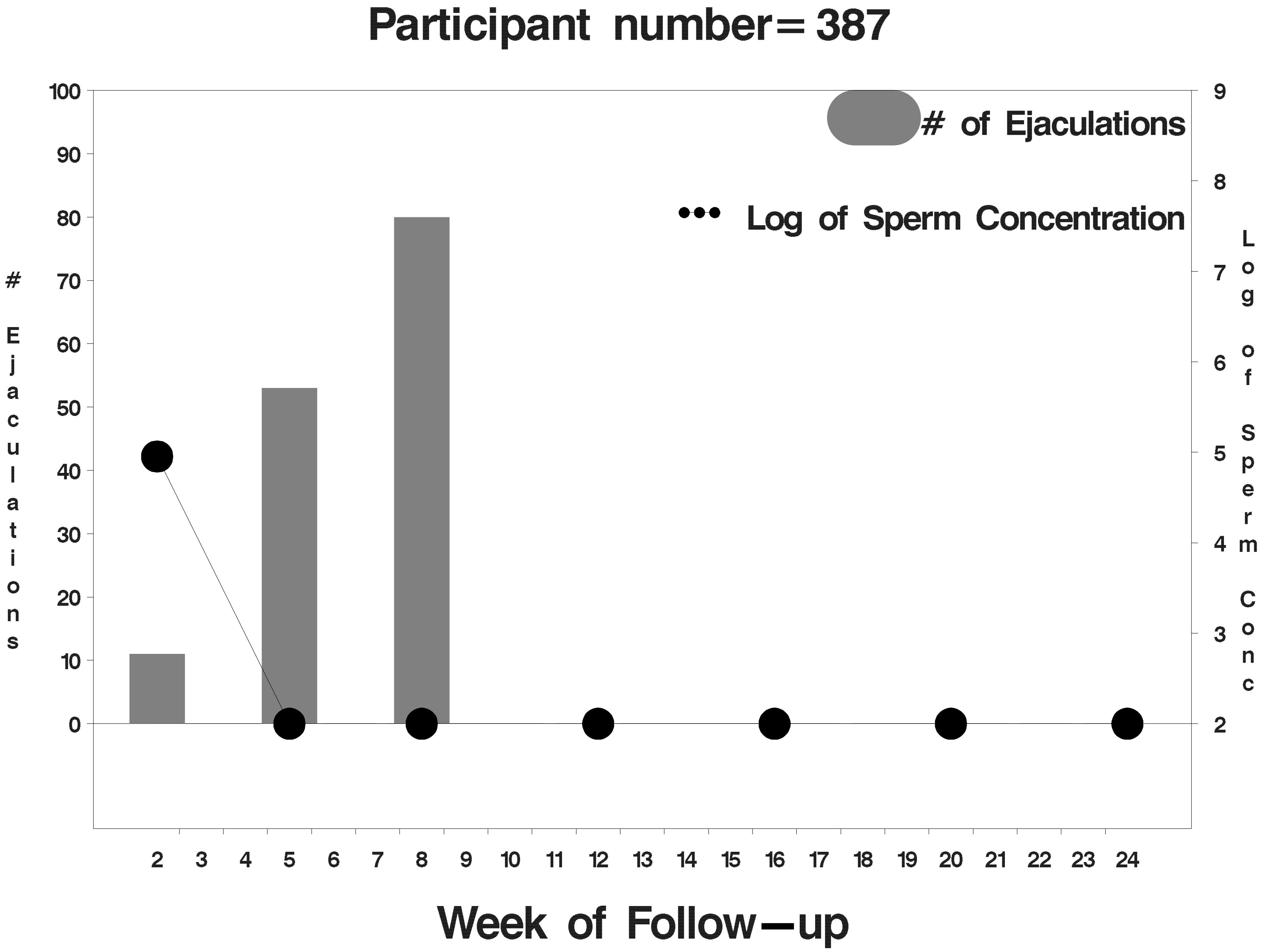

Participant number=387
# of Ejaculations
Log of Sperm Concentration
Ejaculations
Log of Sperm Conc
100
90
80
70
60
50
40
30
20
10
0
9
8
7
6
5
4
3
2
2 3 4 5 6 7 8 9 10 11 12 13 14 15 16 17 18 19 20 21 22 23 24
Week of Follow-up

Participant number= 388

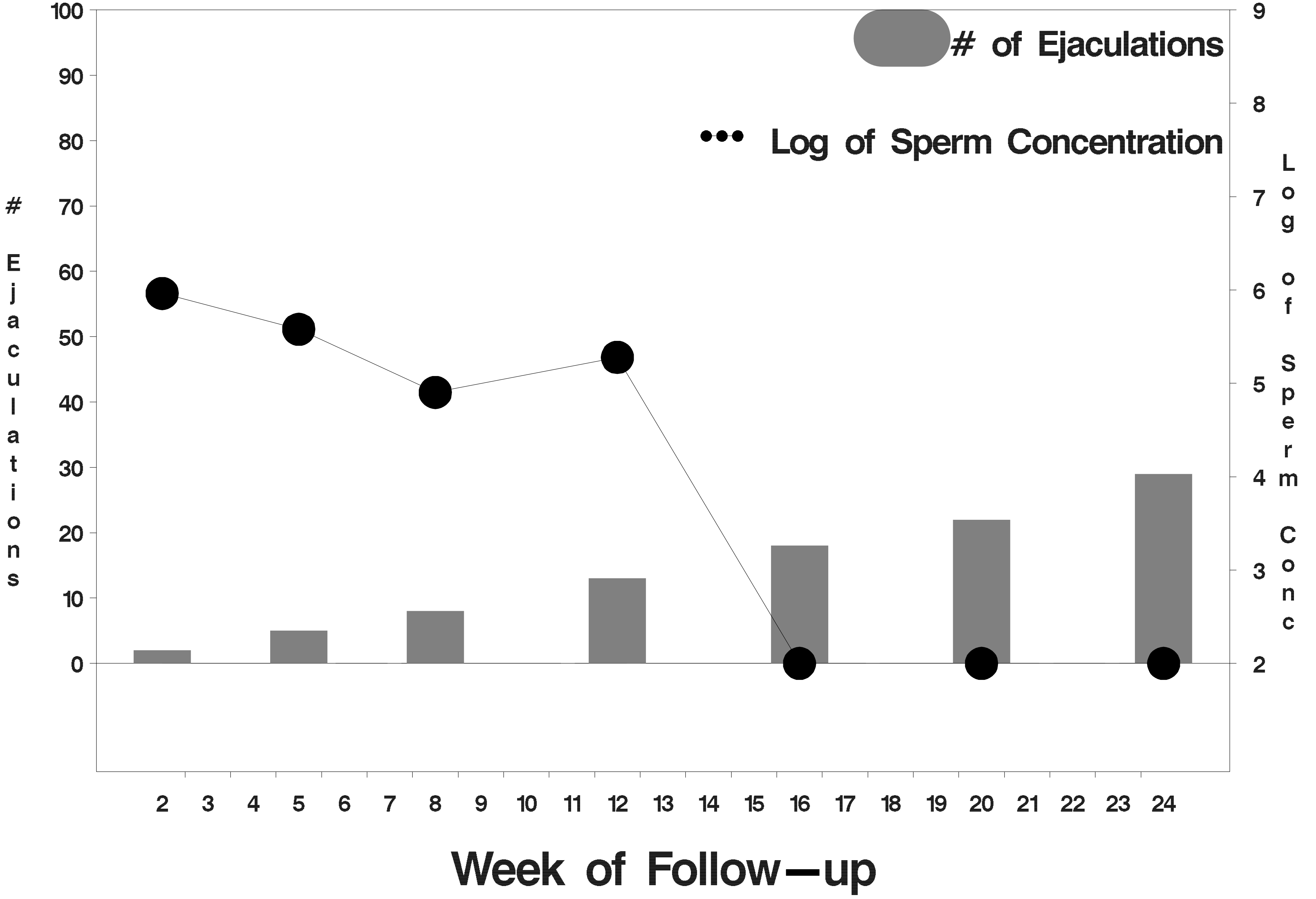

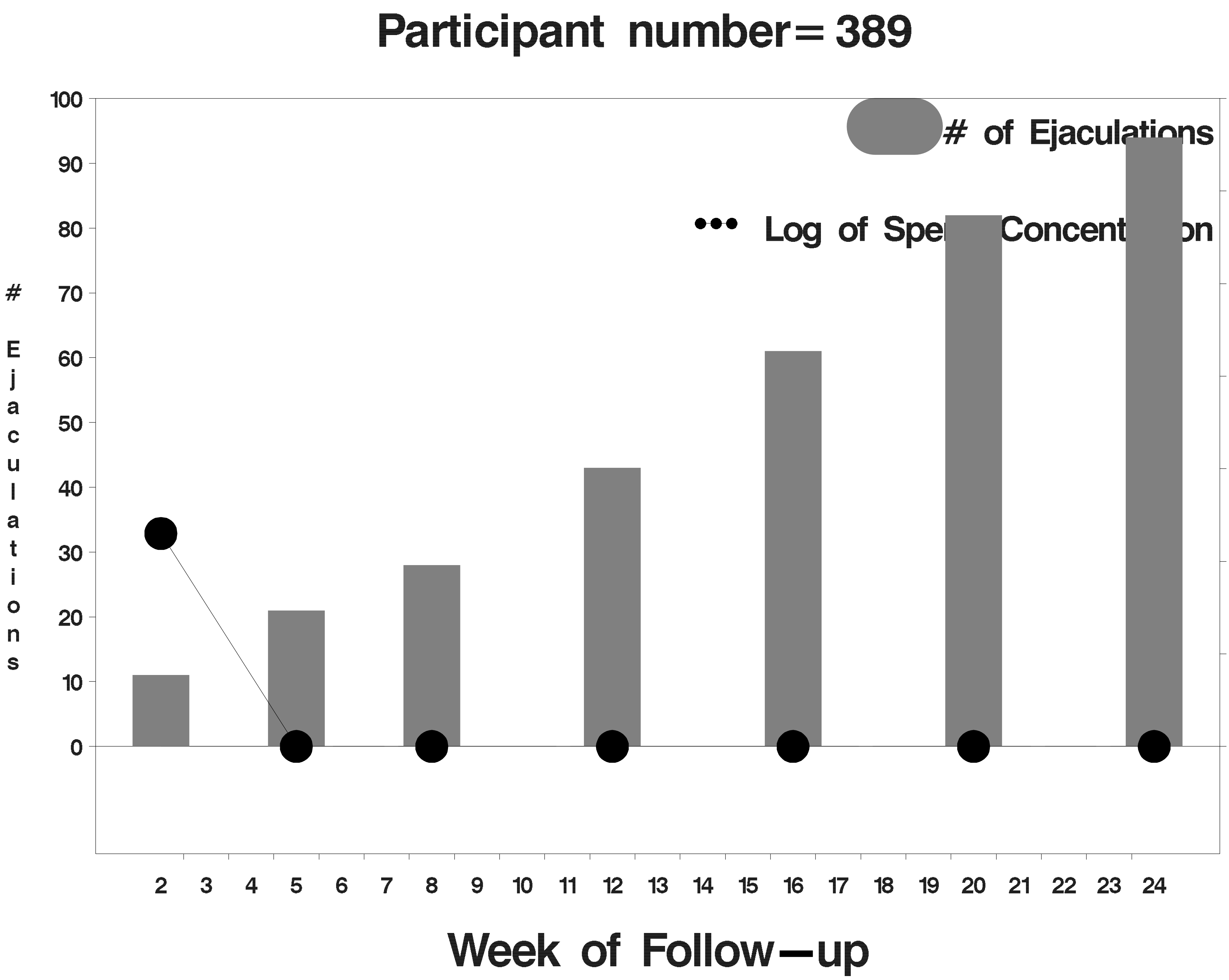

Participant number= 389
# of Ejaculations
Log of Sperm Concentration
Ejaculations
Log of Sperm Conc
Week of Follow-up
100
90
80
70
60
50
40
30
20
10
0
9
8
7
6
5
4
3
2
2 3 4 5 6 7 8 9 10 11 12 13 14 15 16 17 18 19 20 21 22 23 24

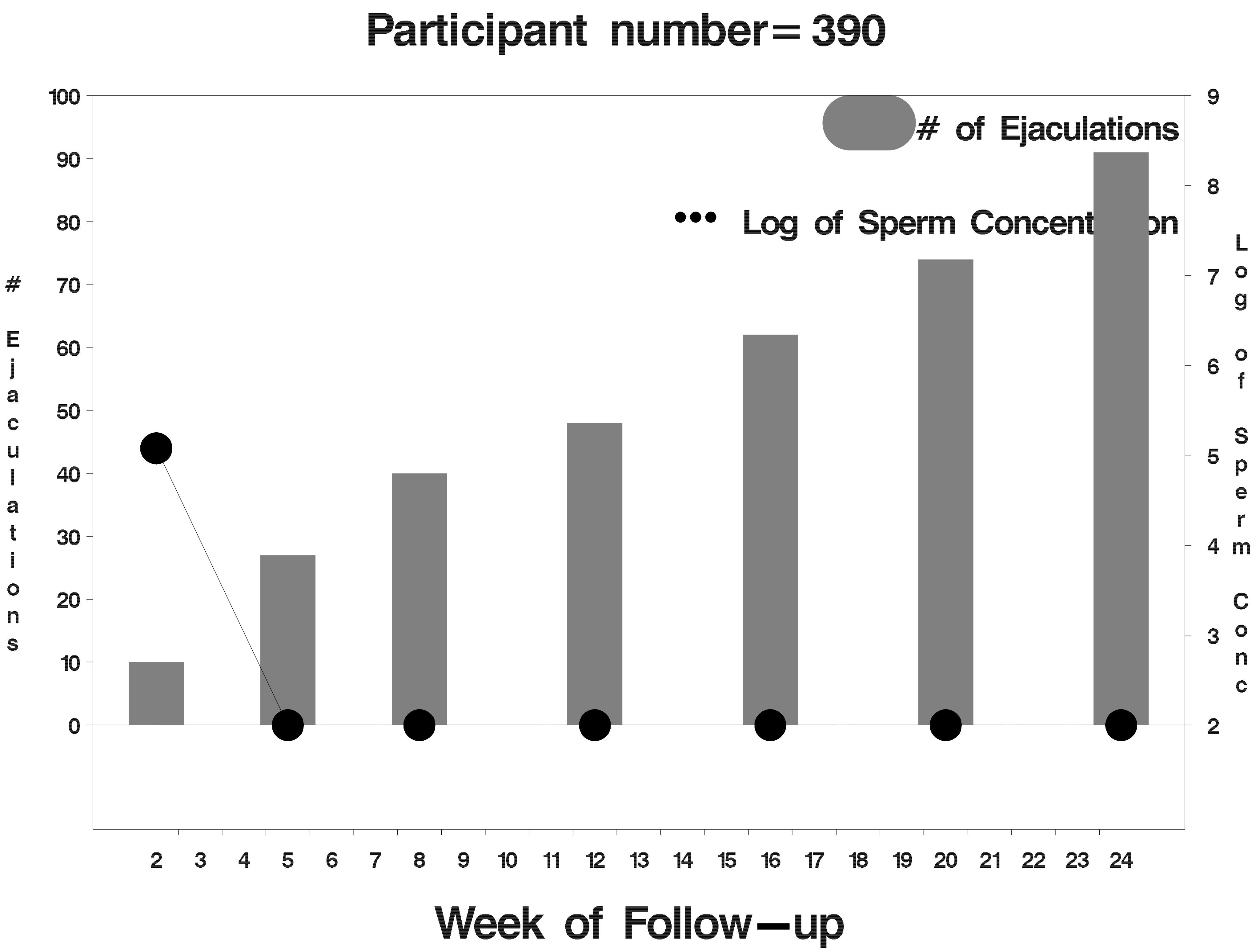

Participant number=390
# of Ejaculations
Log of Sperm Concent[illegible]on
Ejaculations
Log of Sperm Conc
100
90
80
70
60
50
40
30
20
10
0
9
8
7
6
5
4
3
2
2 3 4 5 6 7 8 9 10 11 12 13 14 15 16 17 18 19 20 21 22 23 24
Week of Follow-up

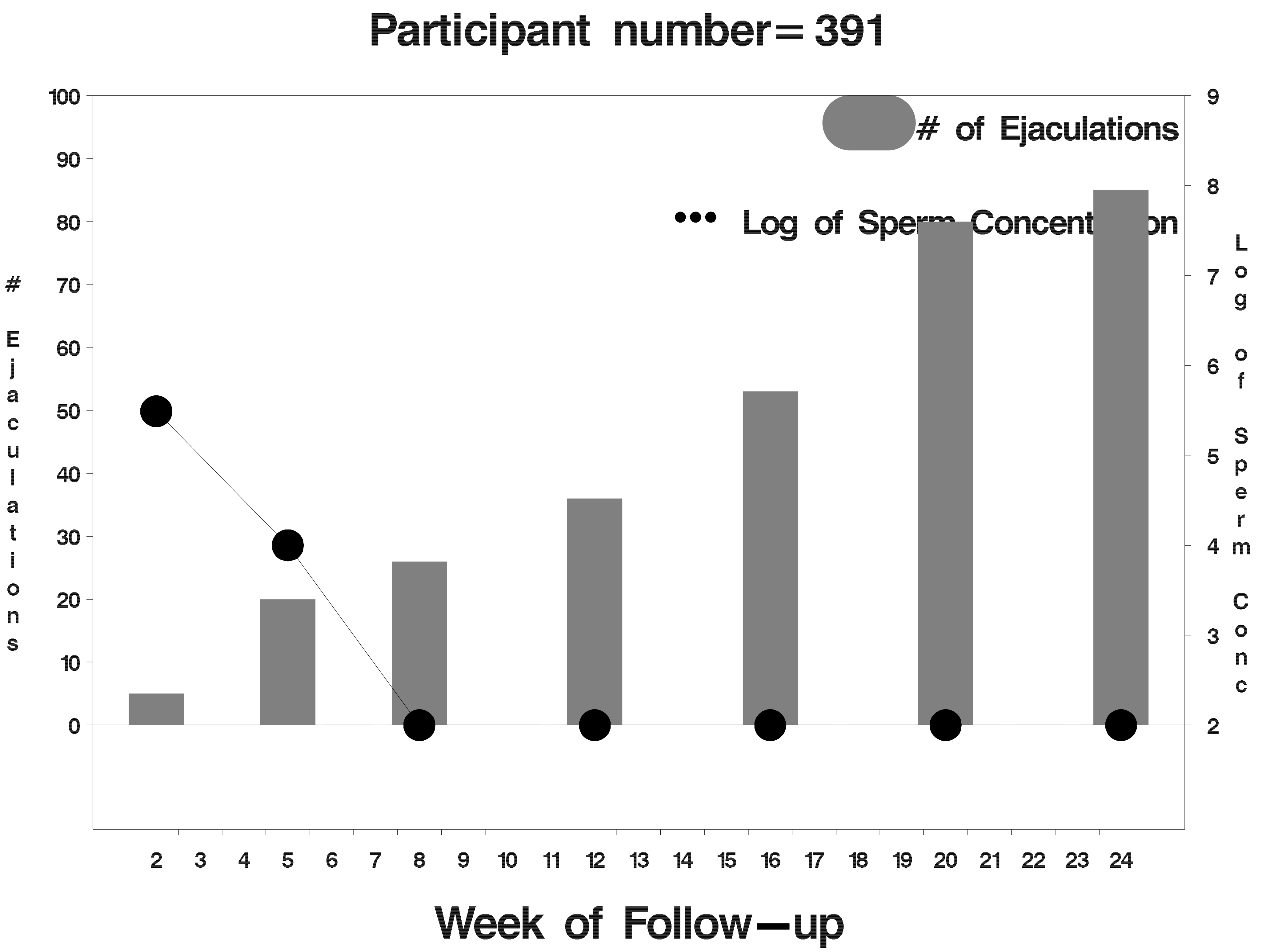

Participant number= 391
# of Ejaculations
Log of Sperm Concentration
Ejaculations
Log of Sperm Conc
100
90
80
70
60
50
40
30
20
10
0
9
8
7
6
5
4
3
2
2 3 4 5 6 7 8 9 10 11 12 13 14 15 16 17 18 19 20 21 22 23 24
Week of Follow-up

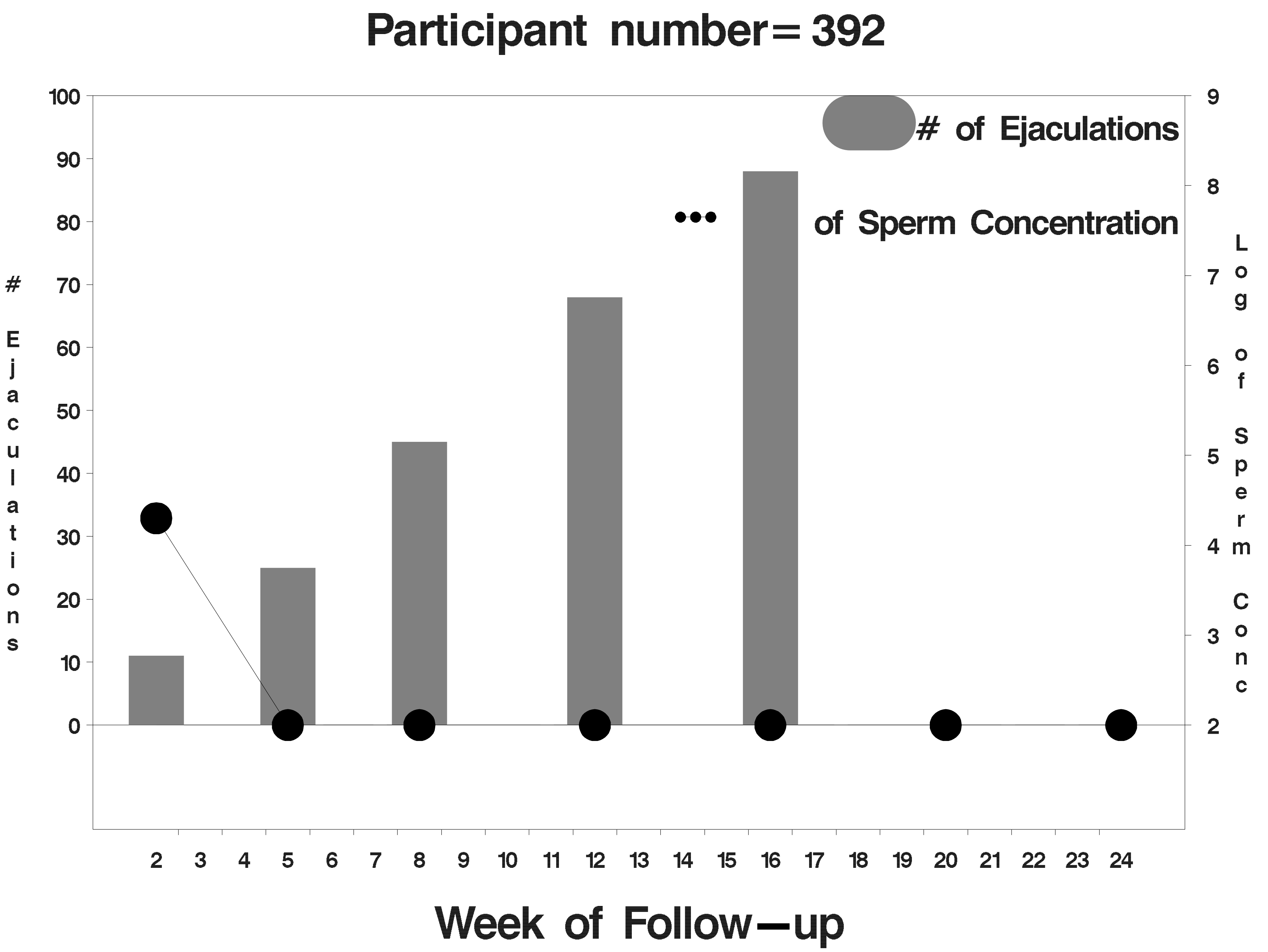

Participant number=392
# of Ejaculations
of Sperm Concentration
Ejaculations
Log of Sperm Conc
100
90
80
70
60
50
40
30
20
10
0
9
8
7
6
5
4
3
2
2 3 4 5 6 7 8 9 10 11 12 13 14 15 16 17 18 19 20 21 22 23 24
Week of Follow-up

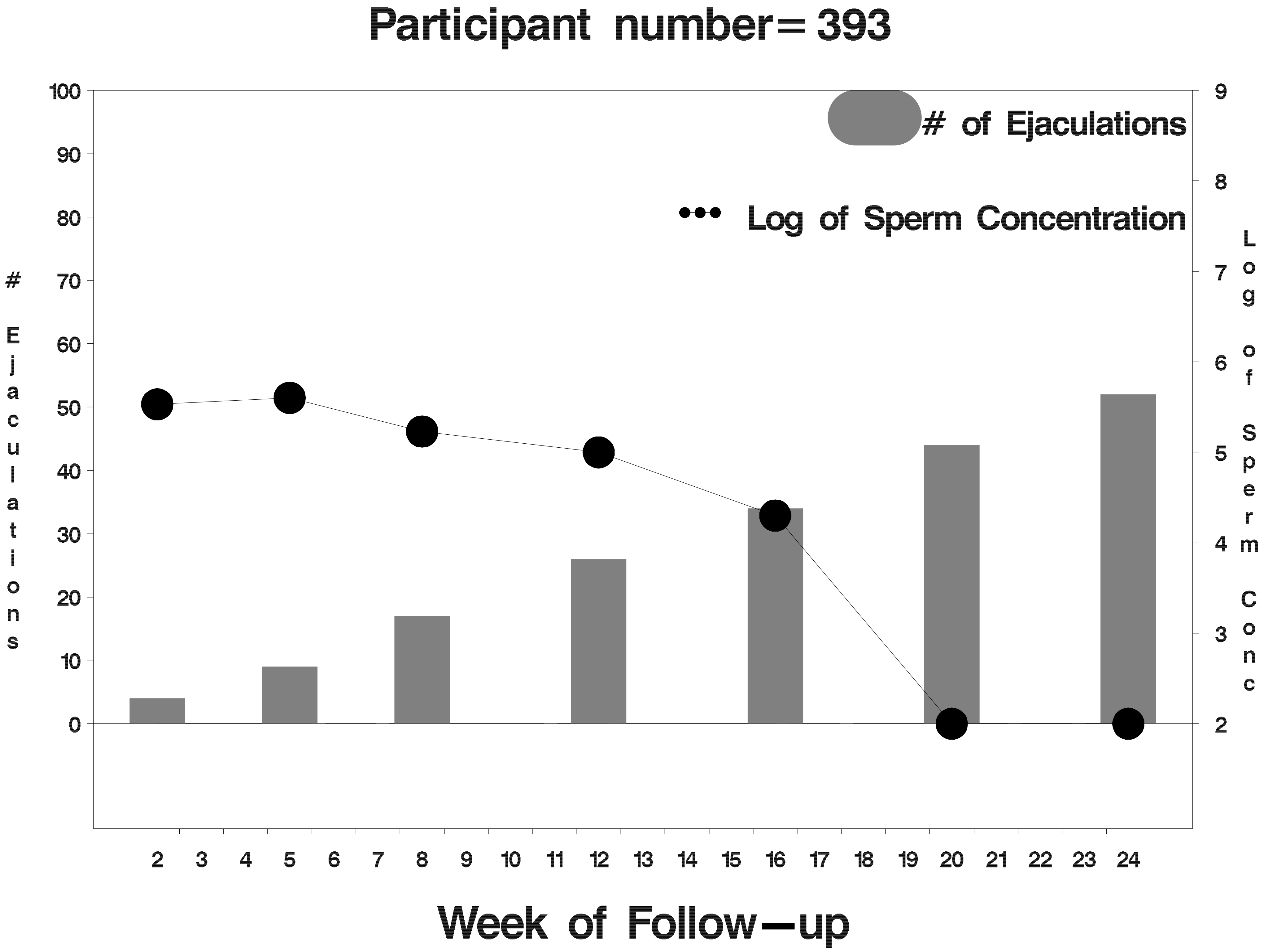

Participant number=393
# of Ejaculations
Log of Sperm Concentration
Ejaculations
Log of Sperm Conc
100
90
80
70
60
50
40
30
20
10
0
9
8
7
6
5
4
3
2
2 3 4 5 6 7 8 9 10 11 12 13 14 15 16 17 18 19 20 21 22 23 24
Week of Follow−up

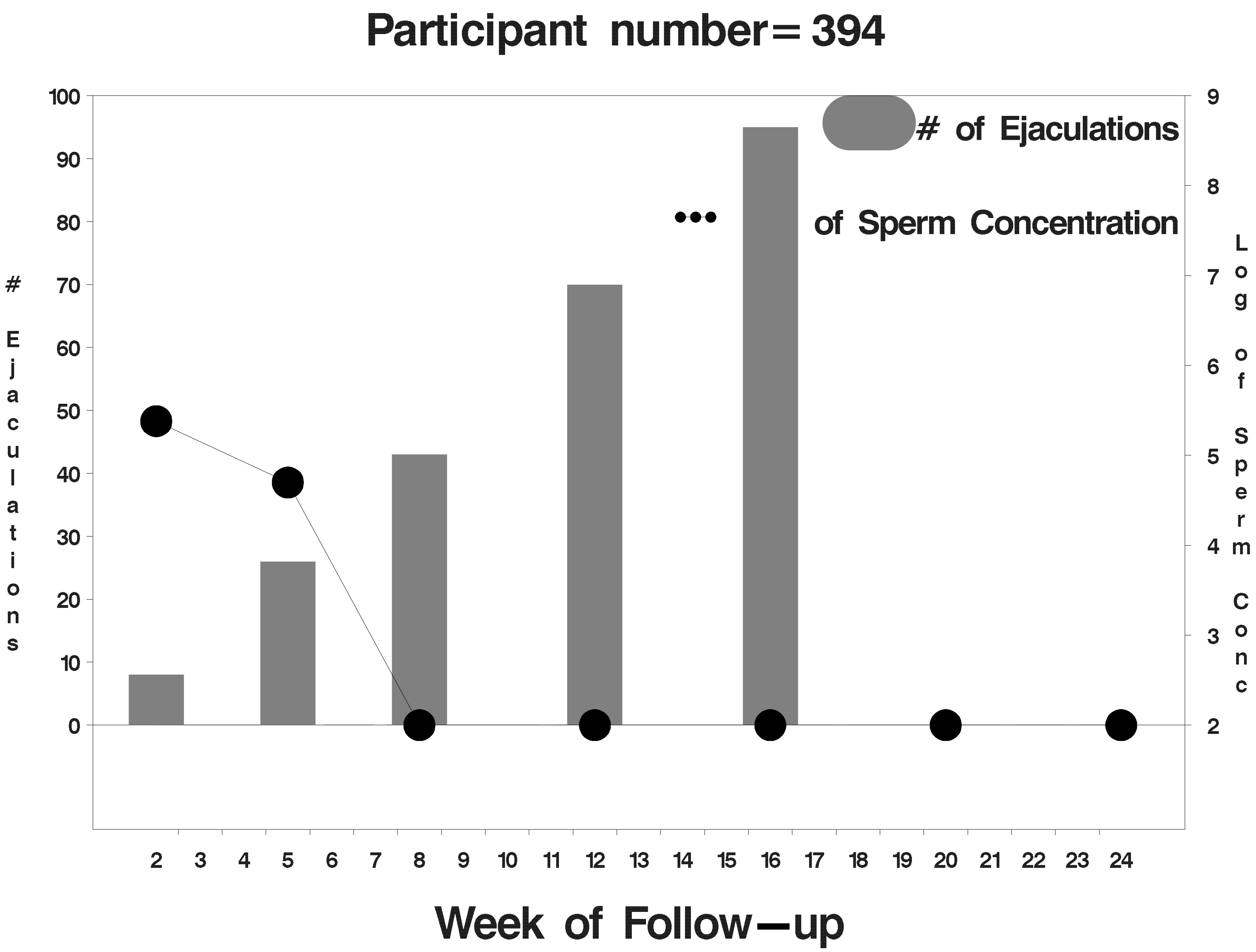

Participant number= 394
# of Ejaculations
of Sperm Concentration
Ejaculations
Log of Sperm Conc
100
90
80
70
60
50
40
30
20
10
0
9
8
7
6
5
4
3
2
2 3 4 5 6 7 8 9 10 11 12 13 14 15 16 17 18 19 20 21 22 23 24
Week of Follow-up

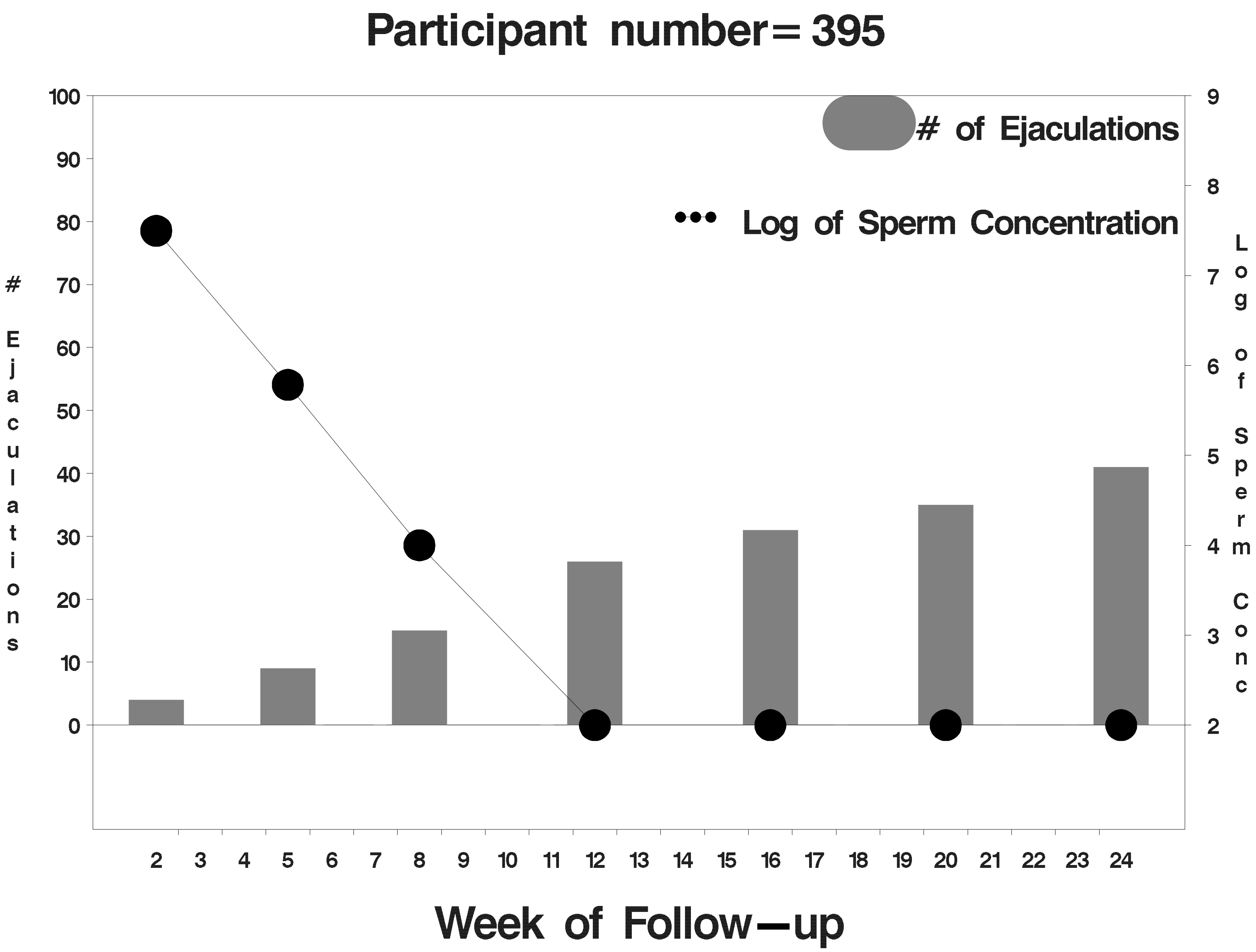
Participant number=395
# of Ejaculations
Log of Sperm Concentration
Ejaculations
Log of Sperm Conc
100
90
80
70
60
50
40
30
20
10
0
9
8
7
6
5
4
3
2
2 3 4 5 6 7 8 9 10 11 12 13 14 15 16 17 18 19 20 21 22 23 24
Week of Follow-up

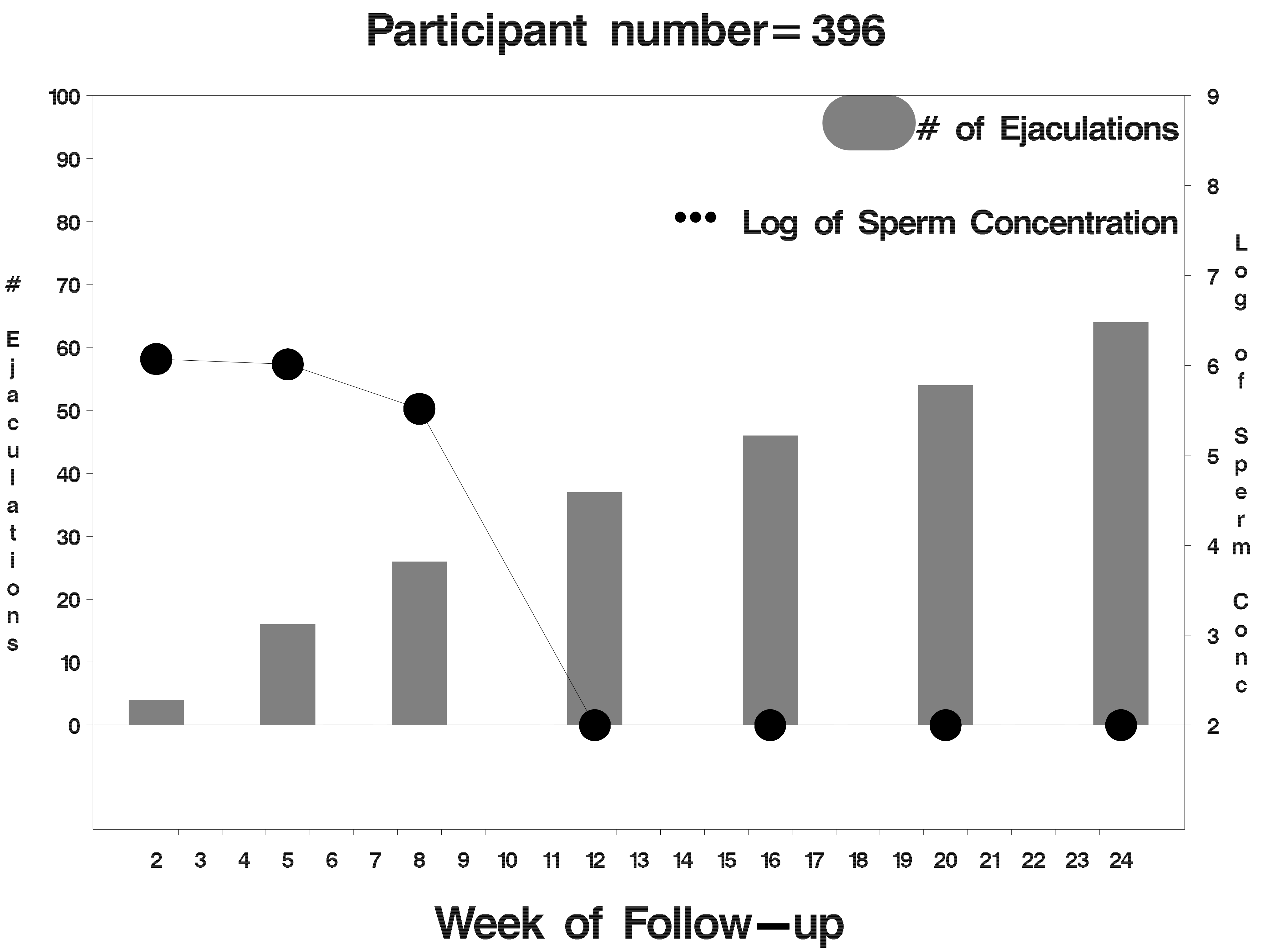
Participant number= 396
# of Ejaculations
Log of Sperm Concentration
Ejaculations
Log of Sperm Conc
Week of Follow—up
0
10
20
30
40
50
60
70
80
90
100
2
3
4
5
6
7
8
9
2 3 4 5 6 7 8 9 10 11 12 13 14 15 16 17 18 19 20 21 22 23 24

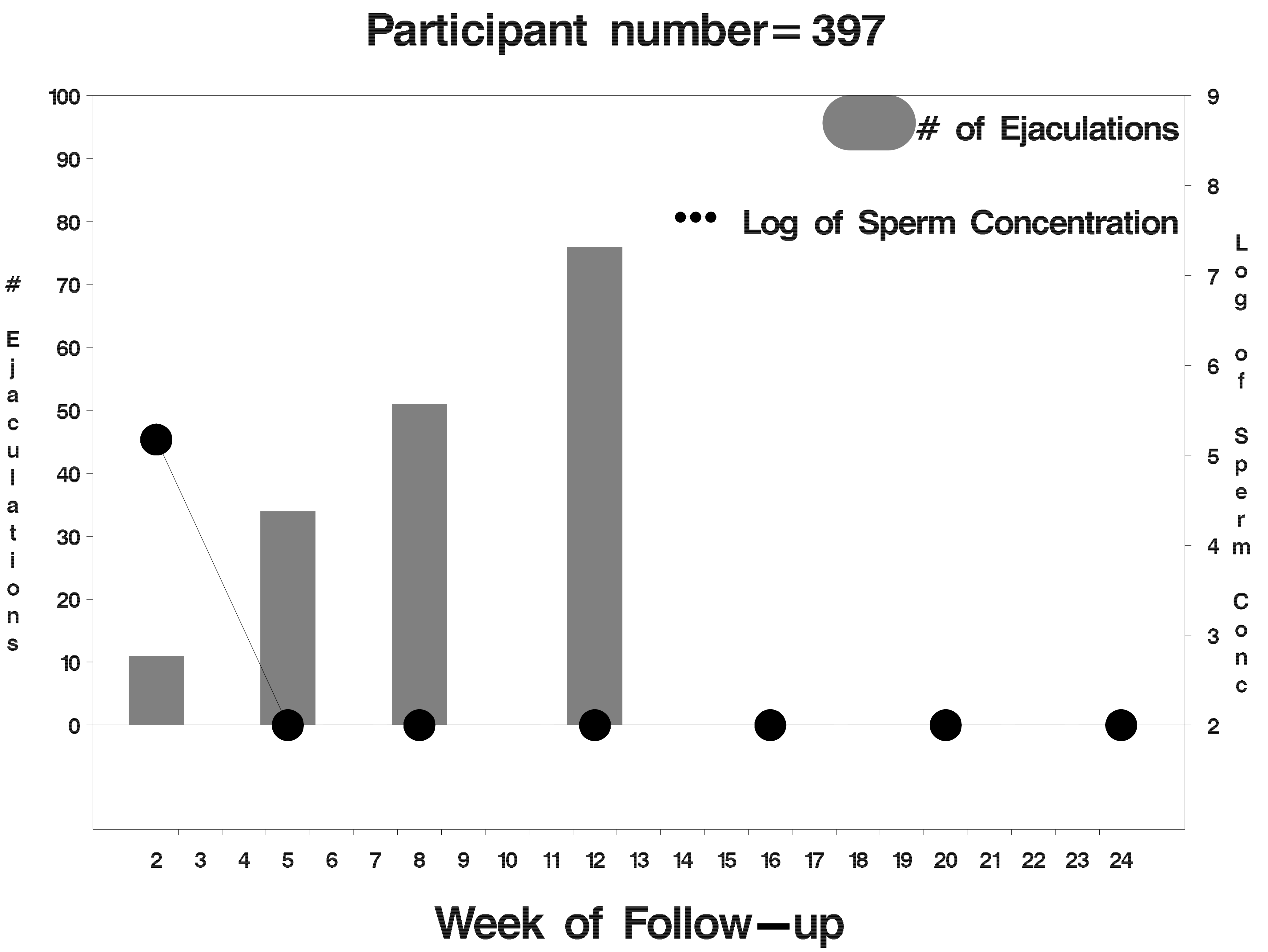

Participant number= 397
# of Ejaculations
Log of Sperm Concentration
Ejaculations
Log of Sperm Conc
100
90
80
70
60
50
40
30
20
10
0
9
8
7
6
5
4
3
2
2 3 4 5 6 7 8 9 10 11 12 13 14 15 16 17 18 19 20 21 22 23 24
Week of Follow—up

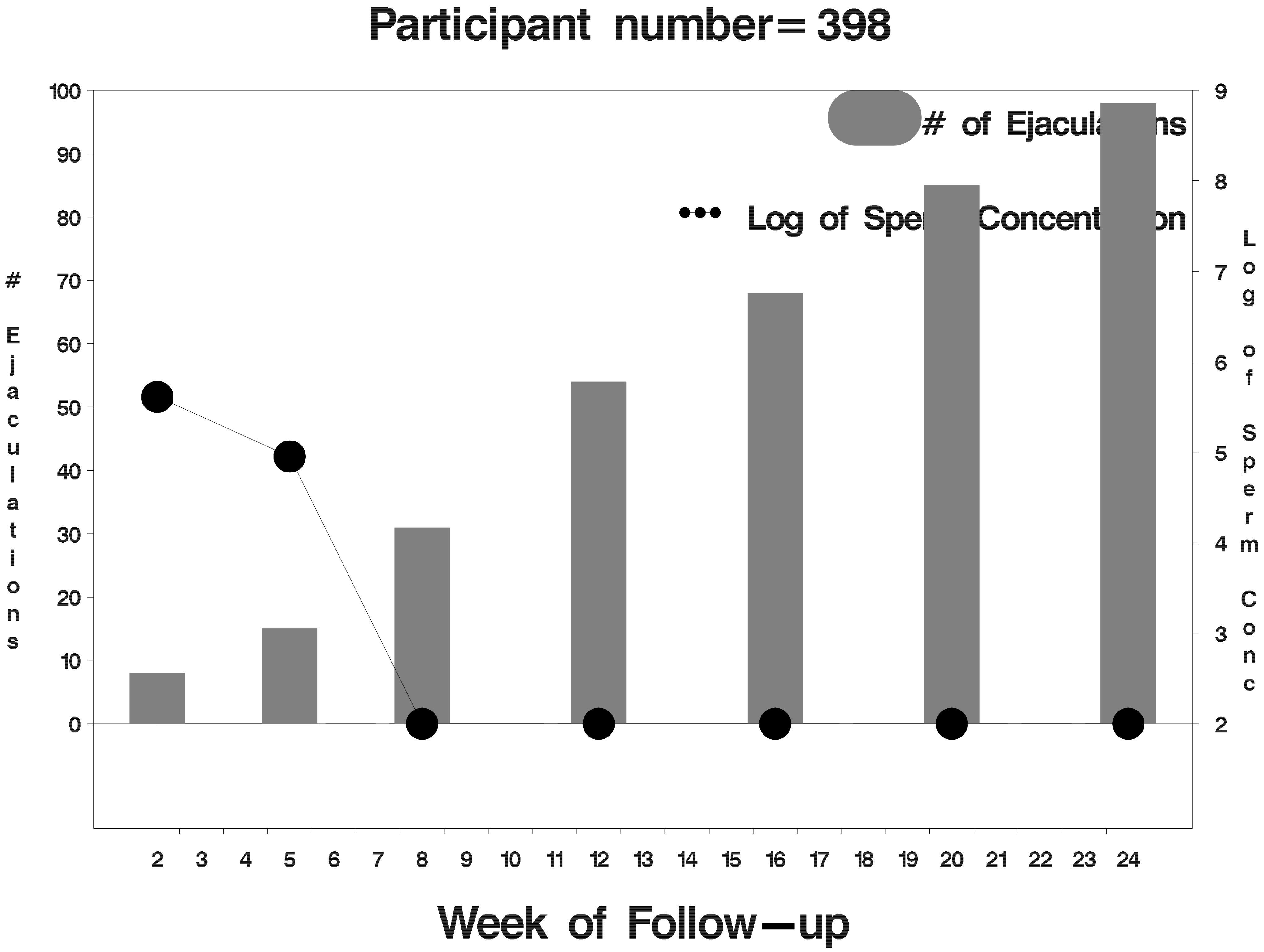
Participant number= 398
# of Ejaculations
Log of Sperm Concentration
Ejaculations
Log of Sperm Conc
Week of Follow-up
100
90
80
70
60
50
40
30
20
10
0
9
8
7
6
5
4
3
2
2 3 4 5 6 7 8 9 10 11 12 13 14 15 16 17 18 19 20 21 22 23 24

Participant number=399

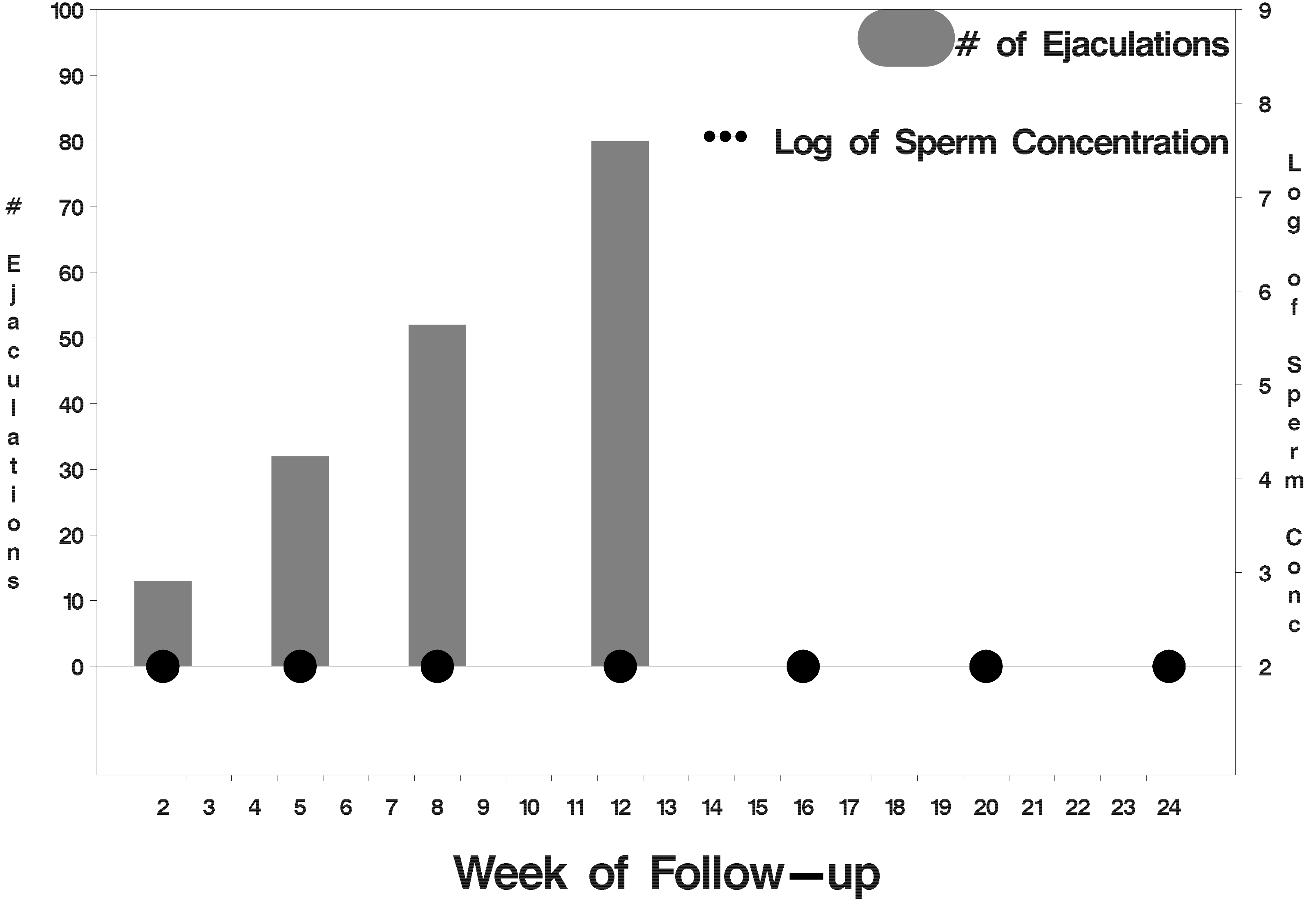

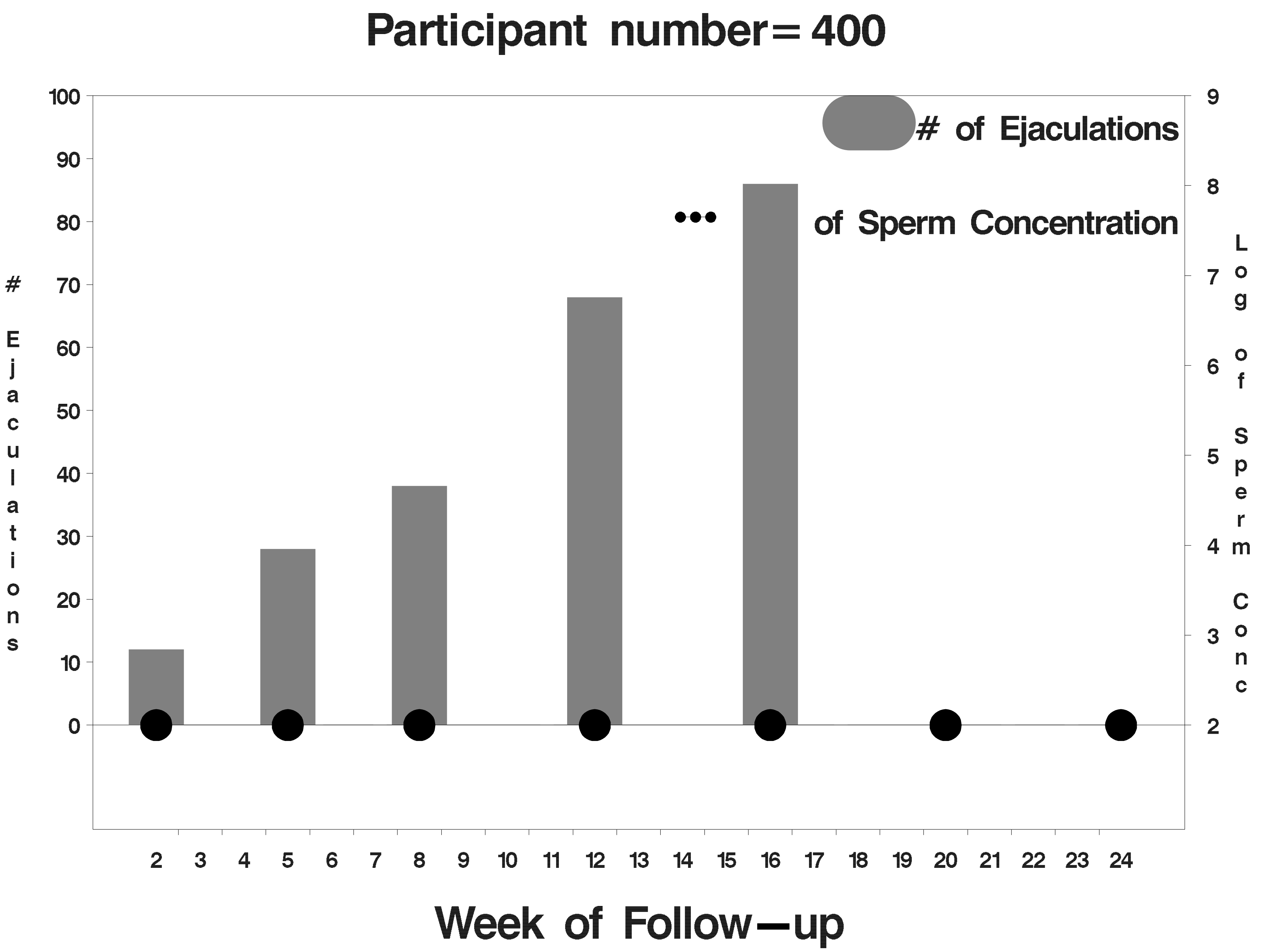

Participant number=400
# of Ejaculations
of Sperm Concentration
Ejaculations
Log of Sperm Conc
100
90
80
70
60
50
40
30
20
10
0
9
8
7
6
5
4
3
2
2 3 4 5 6 7 8 9 10 11 12 13 14 15 16 17 18 19 20 21 22 23 24
Week of Follow-up

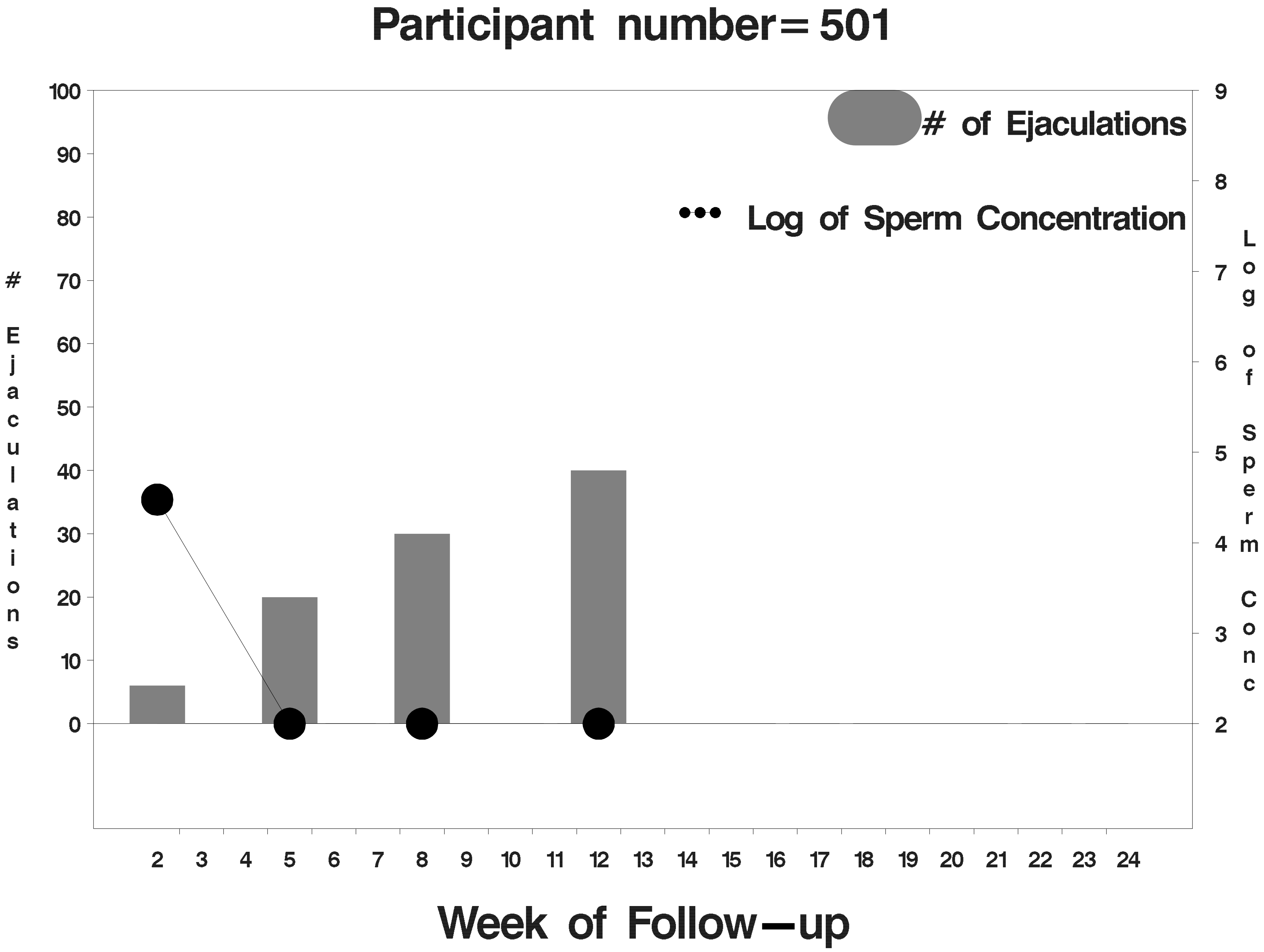
Participant number=501
# of Ejaculations
Log of Sperm Concentration
Ejaculations
Log of Sperm Conc
Week of Follow-up
0
10
20
30
40
50
60
70
80
90
100
2
3
4
5
6
7
8
9
2 3 4 5 6 7 8 9 10 11 12 13 14 15 16 17 18 19 20 21 22 23 24

Participant number= 502

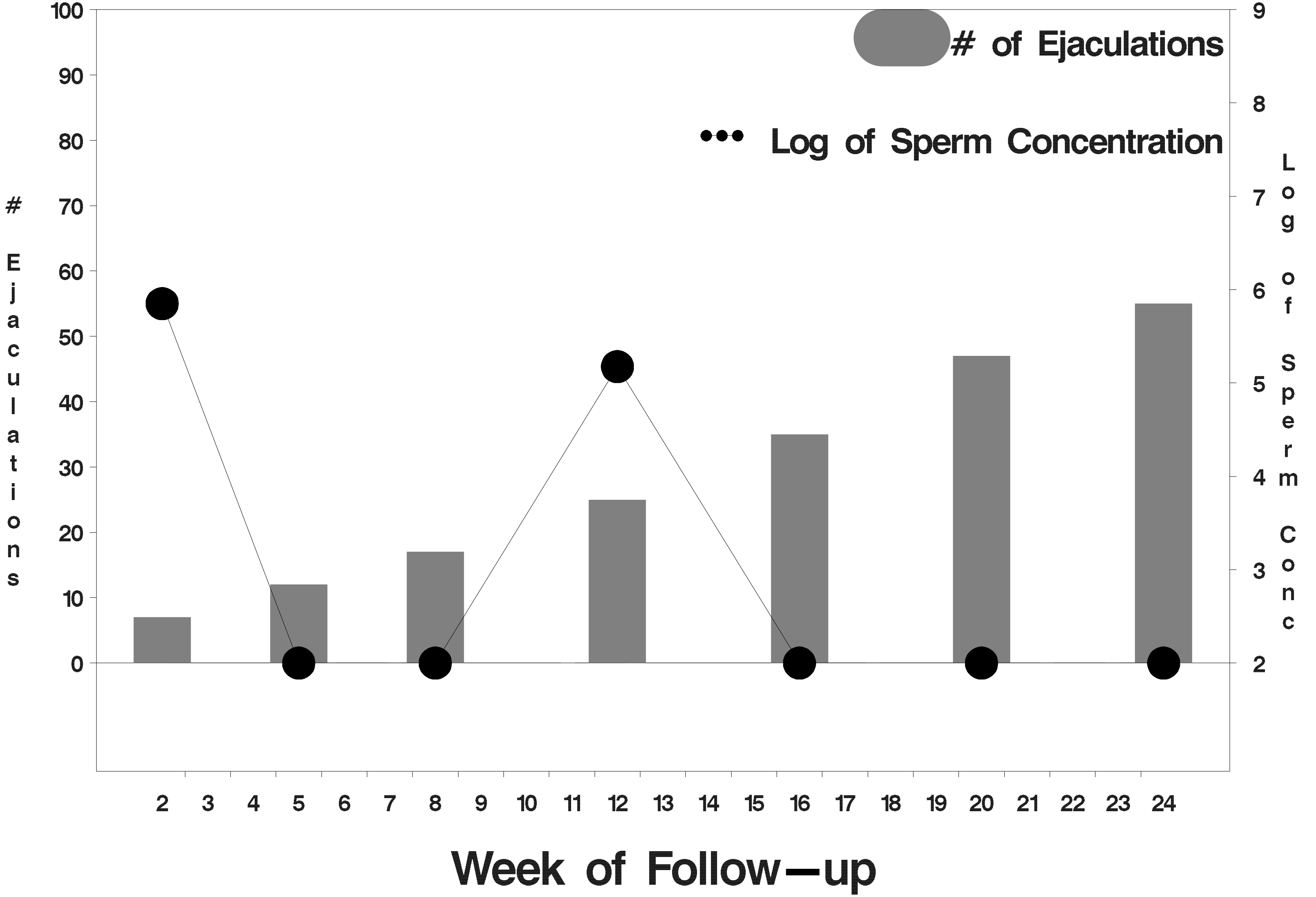

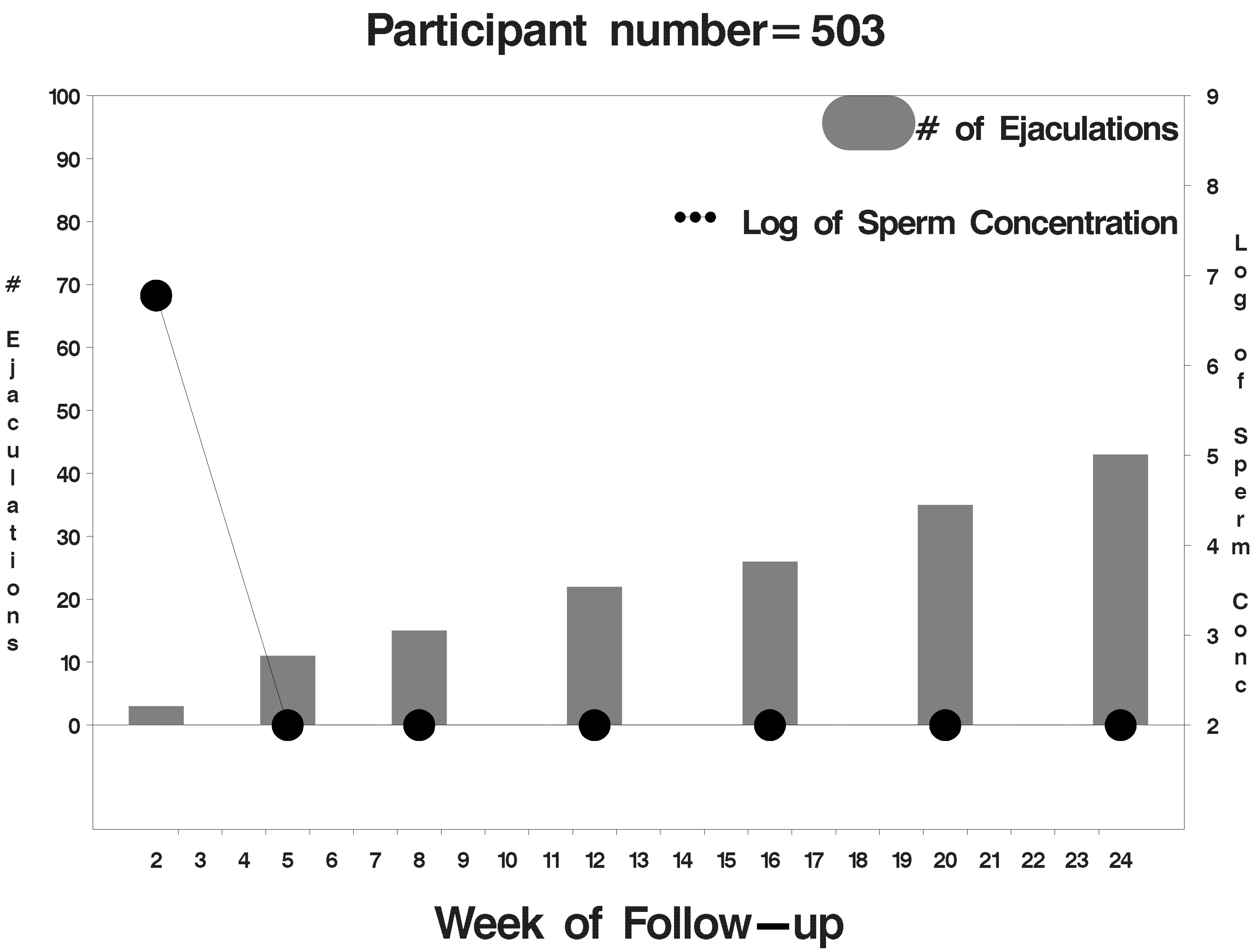

Participant number=503
# of Ejaculations
Log of Sperm Concentration
Ejaculations
Log of Sperm Conc
100
90
80
70
60
50
40
30
20
10
0
9
8
7
6
5
4
3
2
2 3 4 5 6 7 8 9 10 11 12 13 14 15 16 17 18 19 20 21 22 23 24
Week of Follow-up

Participant number= 504

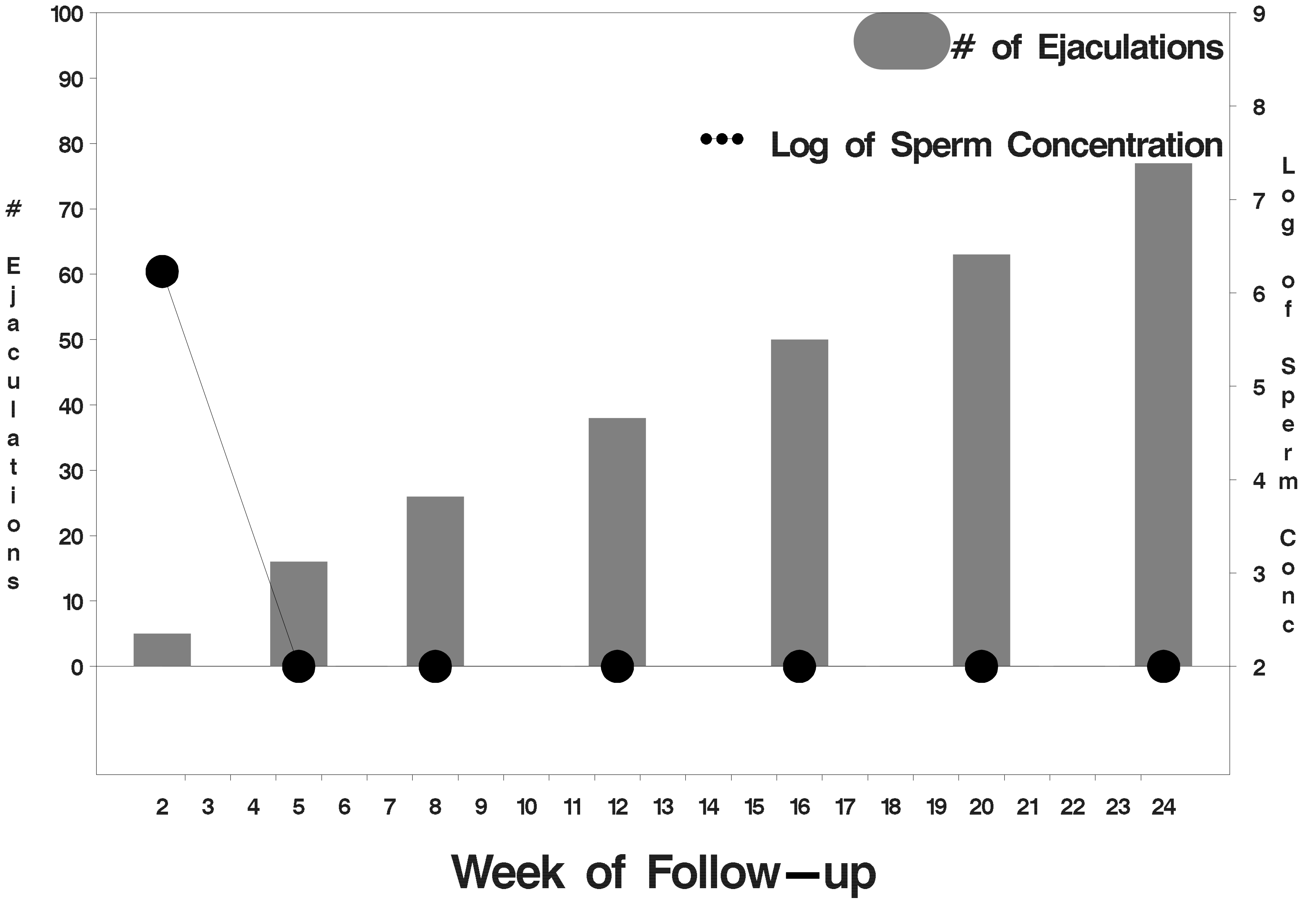

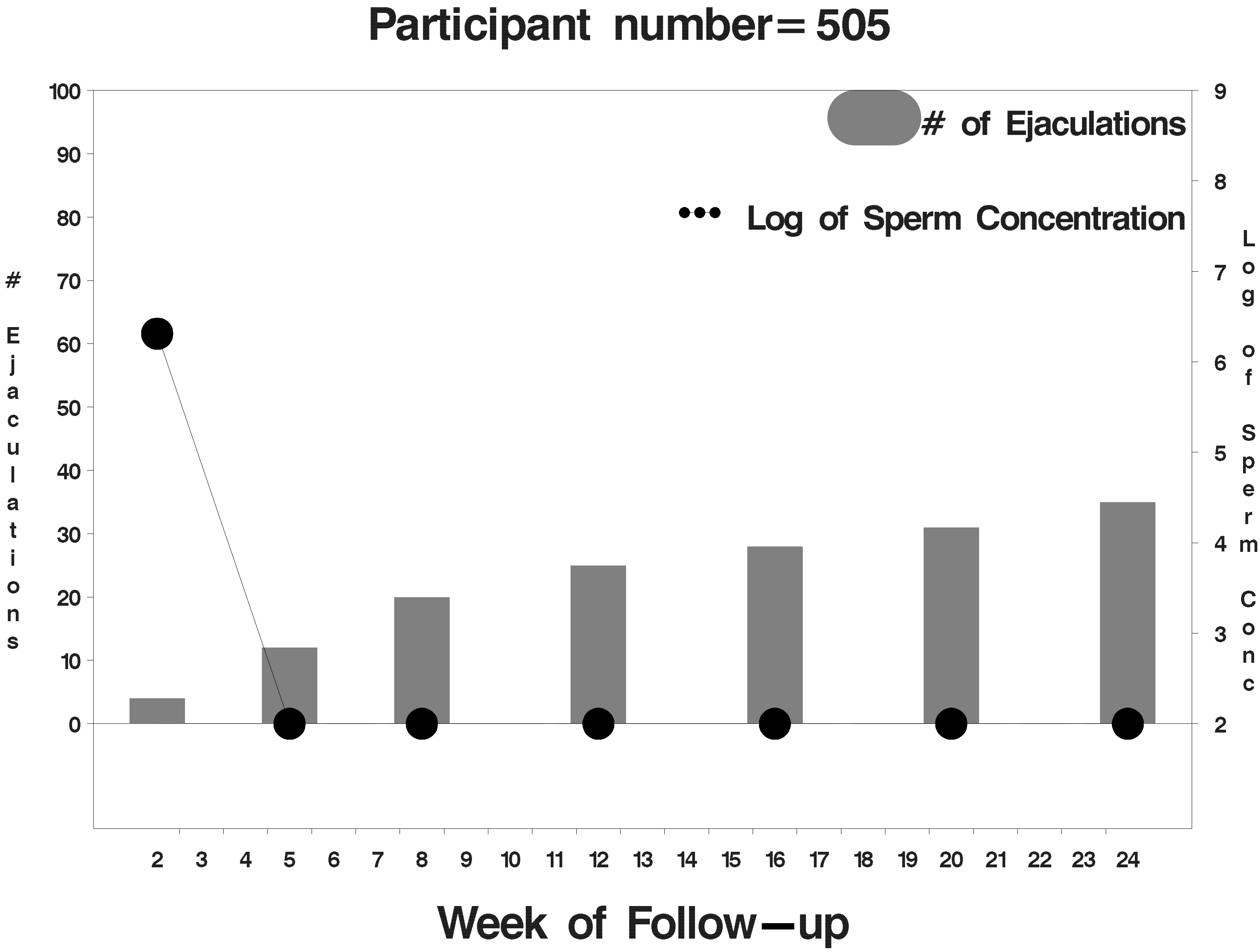

Participant number=505
# of Ejaculations
Log of Sperm Concentration
Ejaculations
Log of Sperm Conc
100
90
80
70
60
50
40
30
20
10
0
9
8
7
6
5
4
3
2
2 3 4 5 6 7 8 9 10 11 12 13 14 15 16 17 18 19 20 21 22 23 24
Week of Follow-up

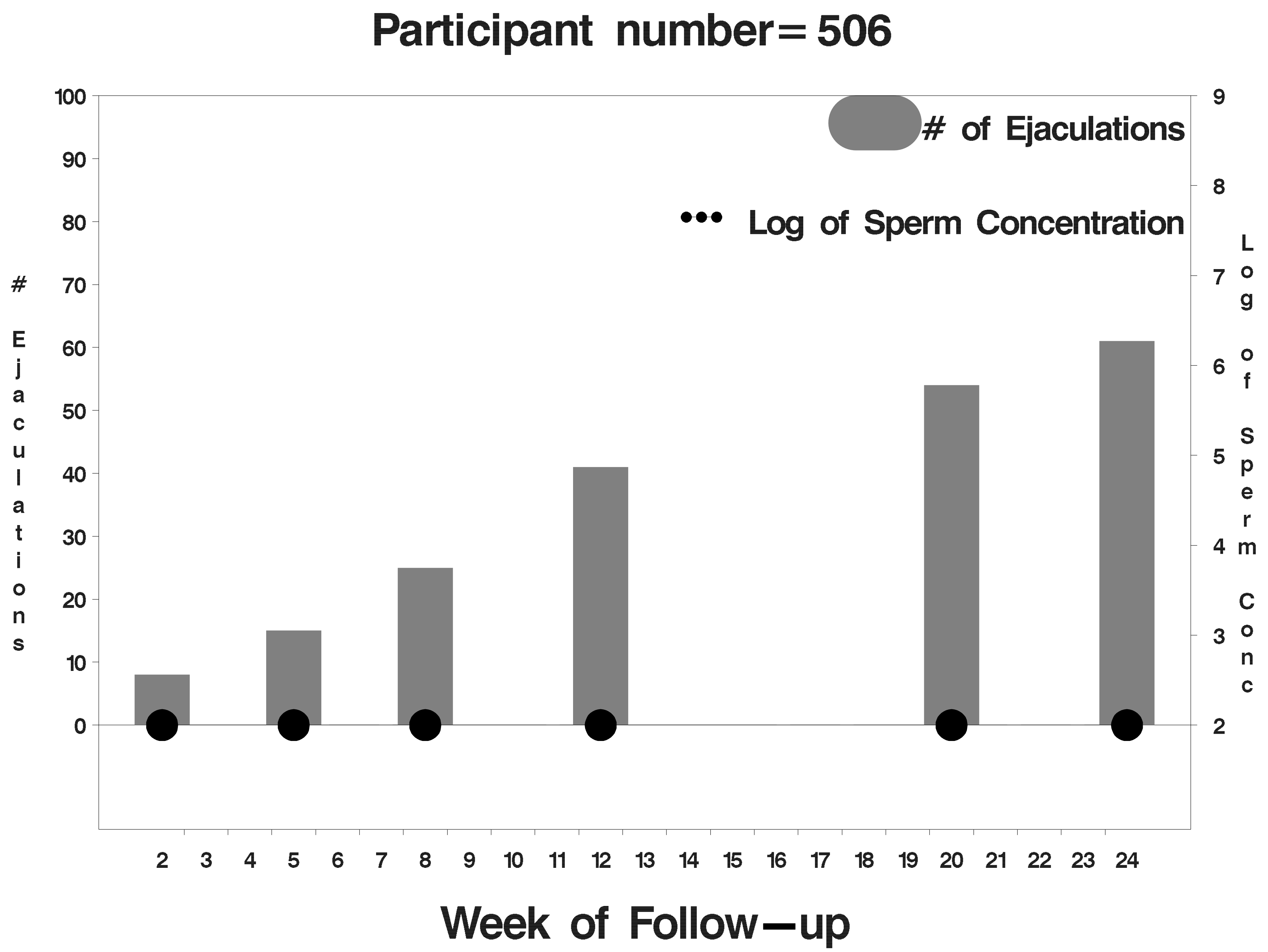

Participant number=506
# of Ejaculations
Log of Sperm Concentration
Ejaculations
Log of Sperm Conc
Week of Follow-up
100
90
80
70
60
50
40
30
20
10
0
9
8
7
6
5
4
3
2
2 3 4 5 6 7 8 9 10 11 12 13 14 15 16 17 18 19 20 21 22 23 24

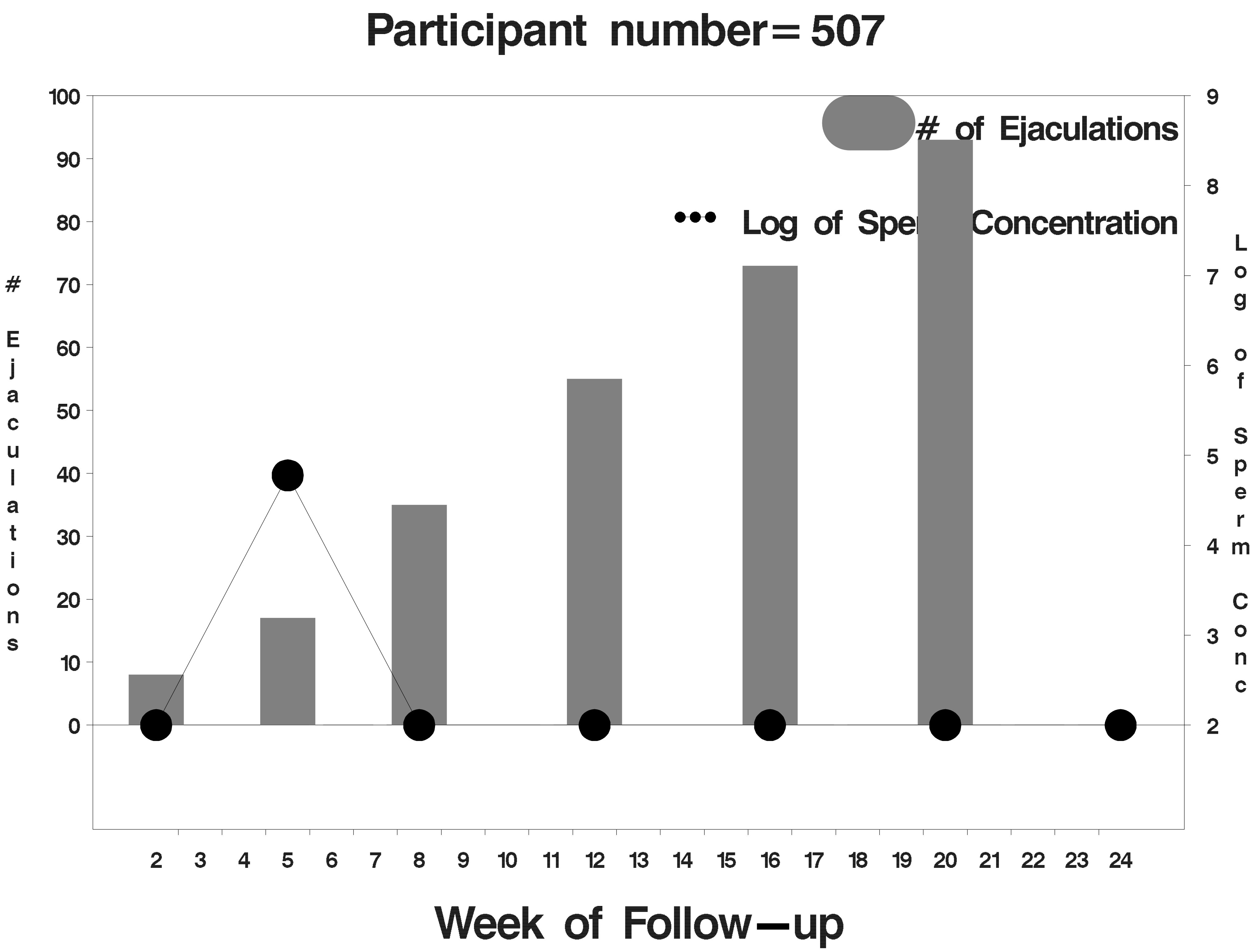
Participant number= 507
# of Ejaculations
Log of Sperm Concentration
Ejaculations
Log of Sperm Conc
100
90
80
70
60
50
40
30
20
10
0
9
8
7
6
5
4
3
2
2 3 4 5 6 7 8 9 10 11 12 13 14 15 16 17 18 19 20 21 22 23 24
Week of Follow-up

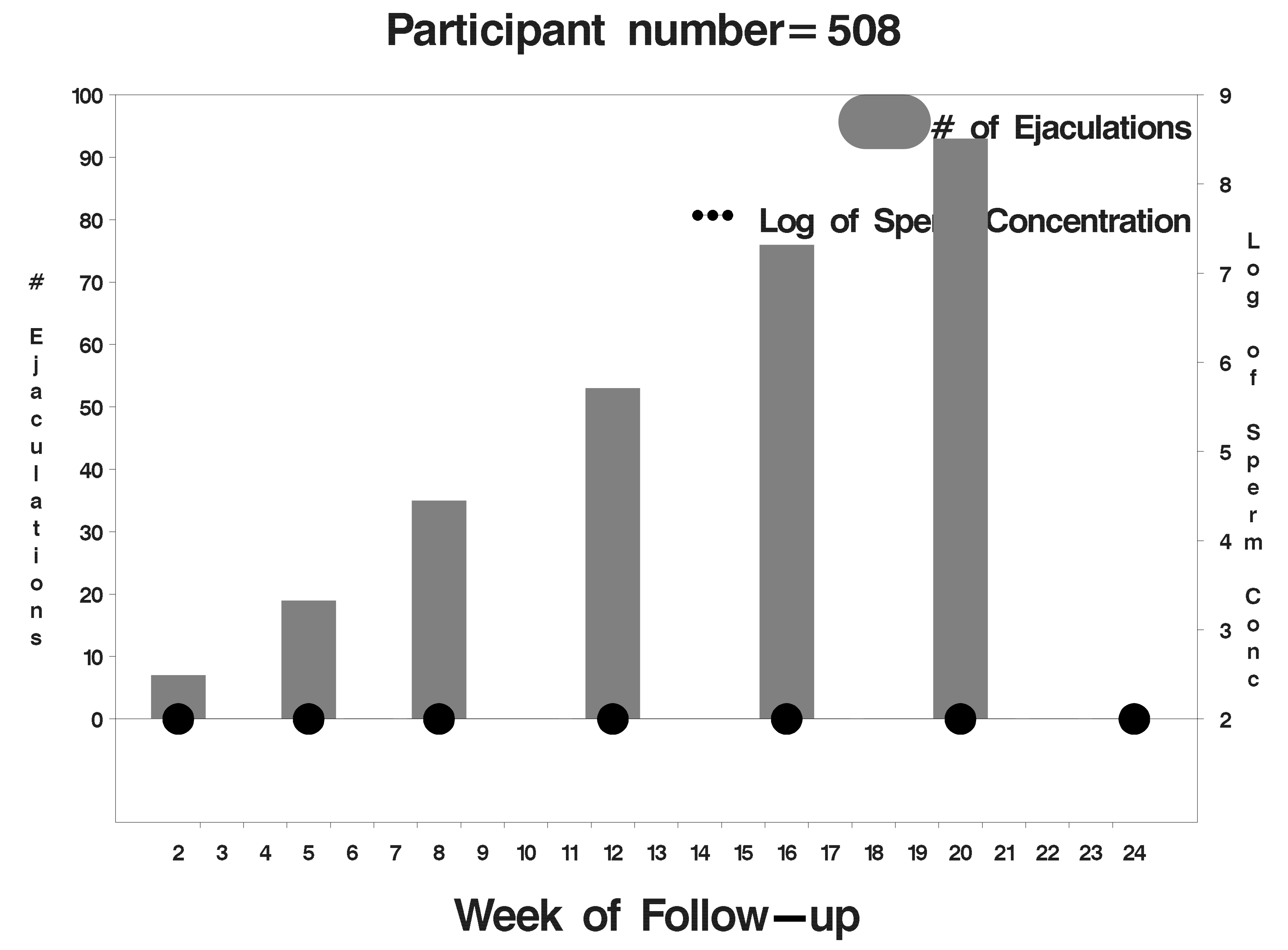

Participant number=508
# of Ejaculations
Log of Sperm Concentration
Ejaculations
Log of Sperm Conc
100
90
80
70
60
50
40
30
20
10
0
9
8
7
6
5
4
3
2
2 3 4 5 6 7 8 9 10 11 12 13 14 15 16 17 18 19 20 21 22 23 24
Week of Follow-up

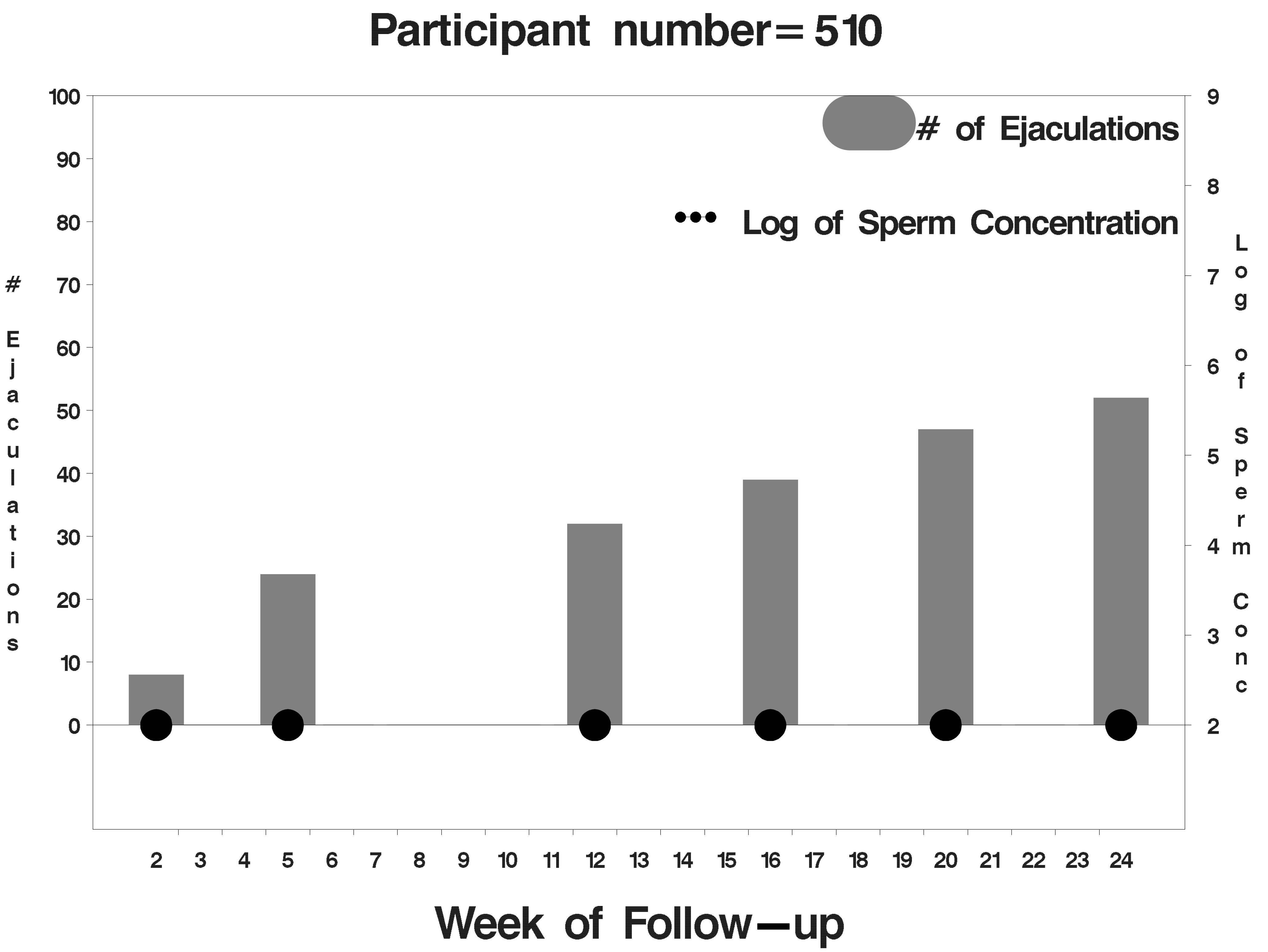

Participant number=510
# of Ejaculations
Log of Sperm Concentration
Ejaculations
Log of Sperm Conc
100
90
80
70
60
50
40
30
20
10
0
9
8
7
6
5
4
3
2
2 3 4 5 6 7 8 9 10 11 12 13 14 15 16 17 18 19 20 21 22 23 24
Week of Follow−up

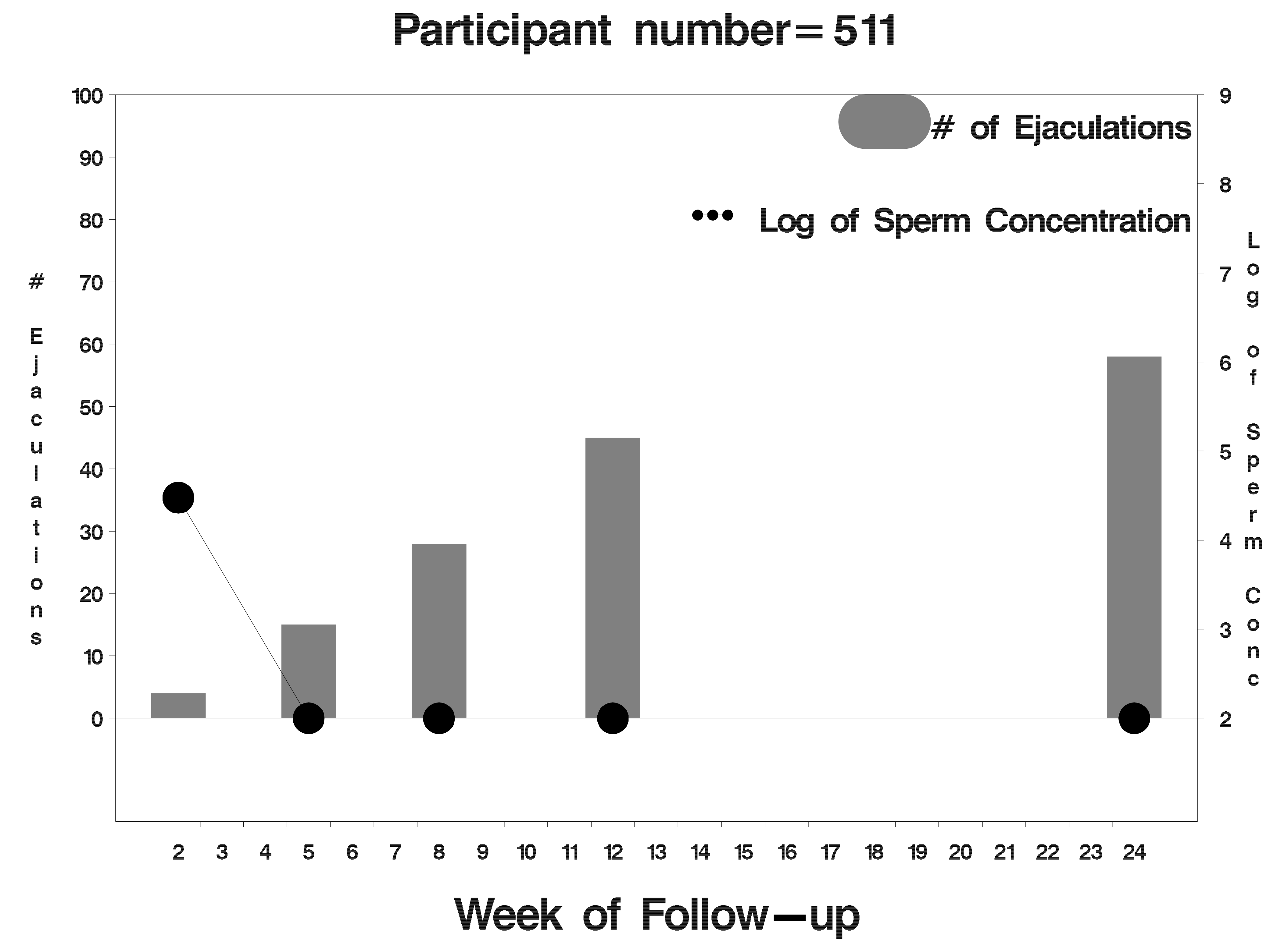

Participant number=511
# of Ejaculations
Log of Sperm Concentration
Ejaculations
Log of Sperm Conc
100
90
80
70
60
50
40
30
20
10
0
9
8
7
6
5
4
3
2
2 3 4 5 6 7 8 9 10 11 12 13 14 15 16 17 18 19 20 21 22 23 24
Week of Follow-up

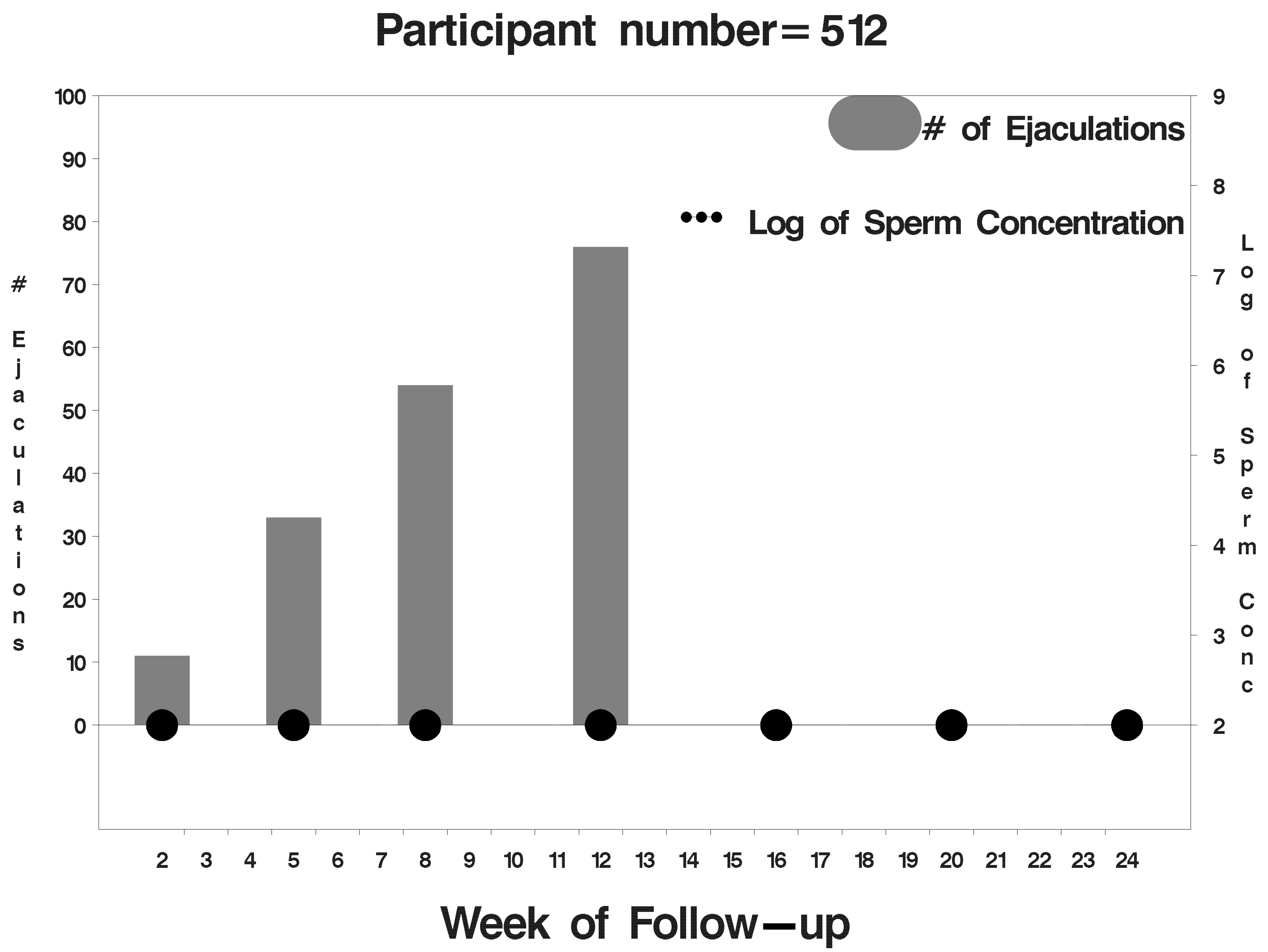

Participant number=512
# of Ejaculations
Log of Sperm Concentration
Ejaculations
Log of Sperm Conc
100
90
80
70
60
50
40
30
20
10
0
9
8
7
6
5
4
3
2
2 3 4 5 6 7 8 9 10 11 12 13 14 15 16 17 18 19 20 21 22 23 24
Week of Follow-up

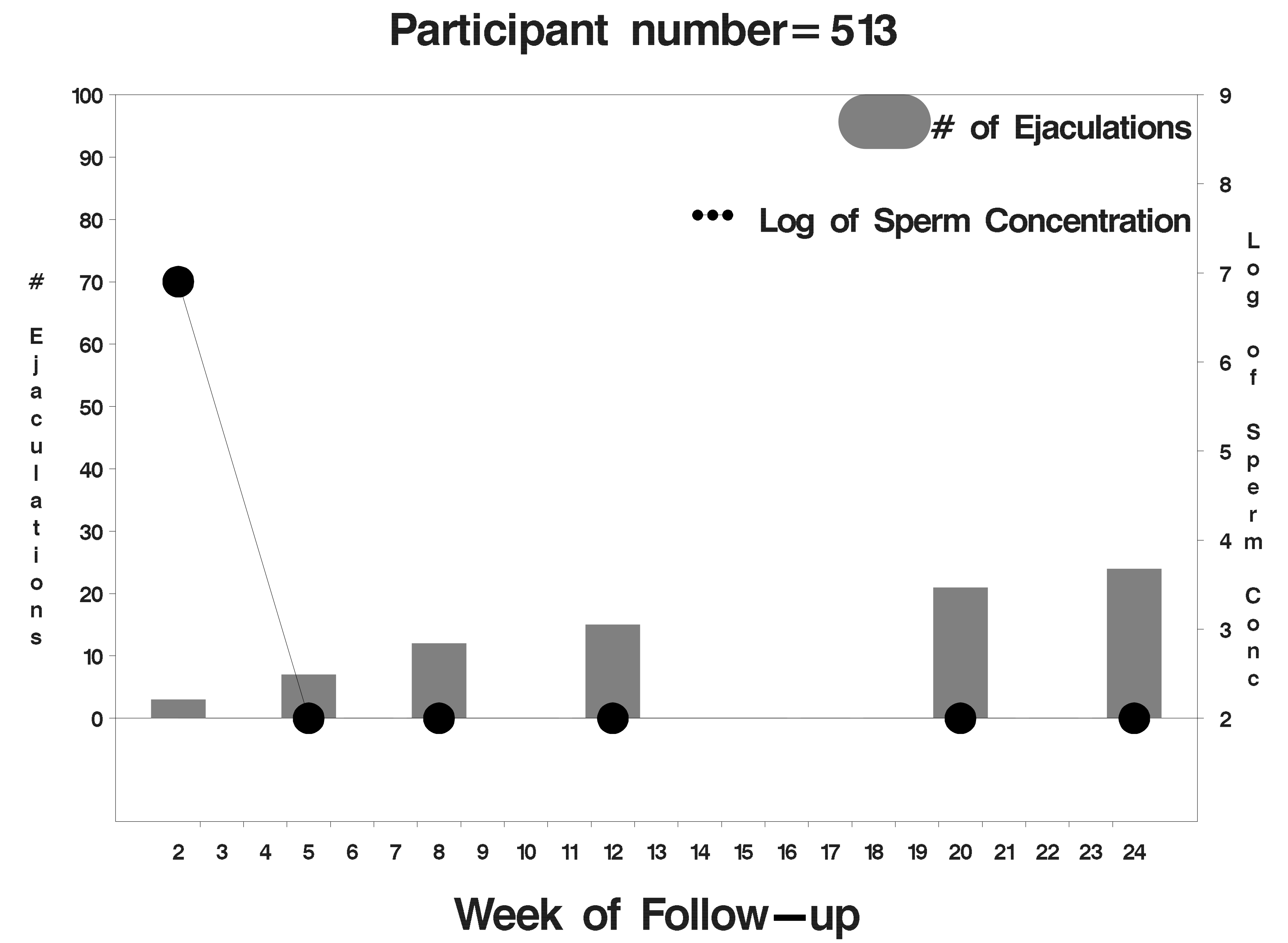

Participant number=513
# of Ejaculations
Log of Sperm Concentration
Ejaculations
Log of Sperm Conc
100
90
80
70
60
50
40
30
20
10
0
9
8
7
6
5
4
3
2
2 3 4 5 6 7 8 9 10 11 12 13 14 15 16 17 18 19 20 21 22 23 24
Week of Follow-up

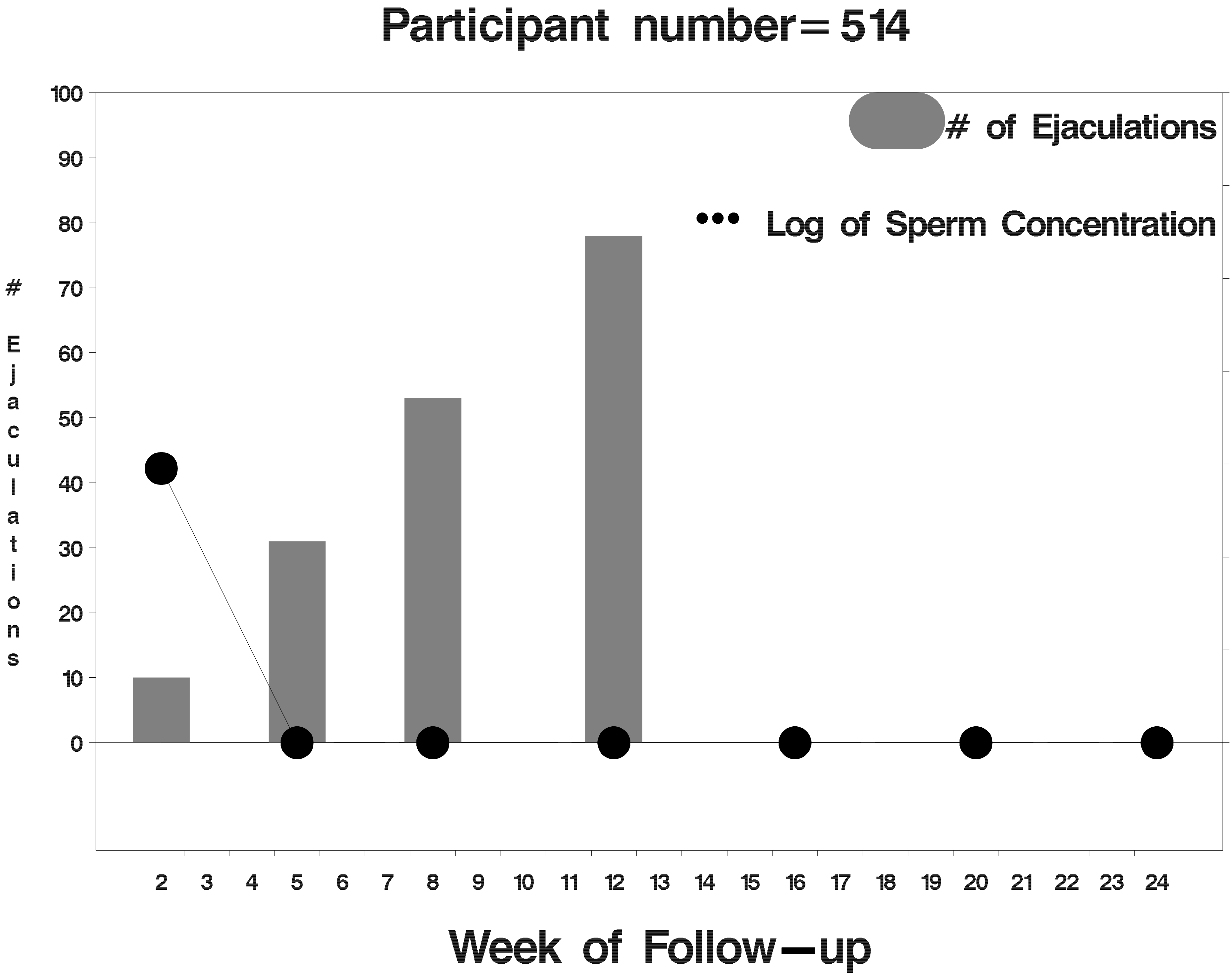
Participant number=514
# of Ejaculations
Log of Sperm Concentration
Ejaculations
Log of Sperm Conc
100
90
80
70
60
50
40
30
20
10
0
9
8
7
6
5
4
3
2
2 3 4 5 6 7 8 9 10 11 12 13 14 15 16 17 18 19 20 21 22 23 24
Week of Follow-up

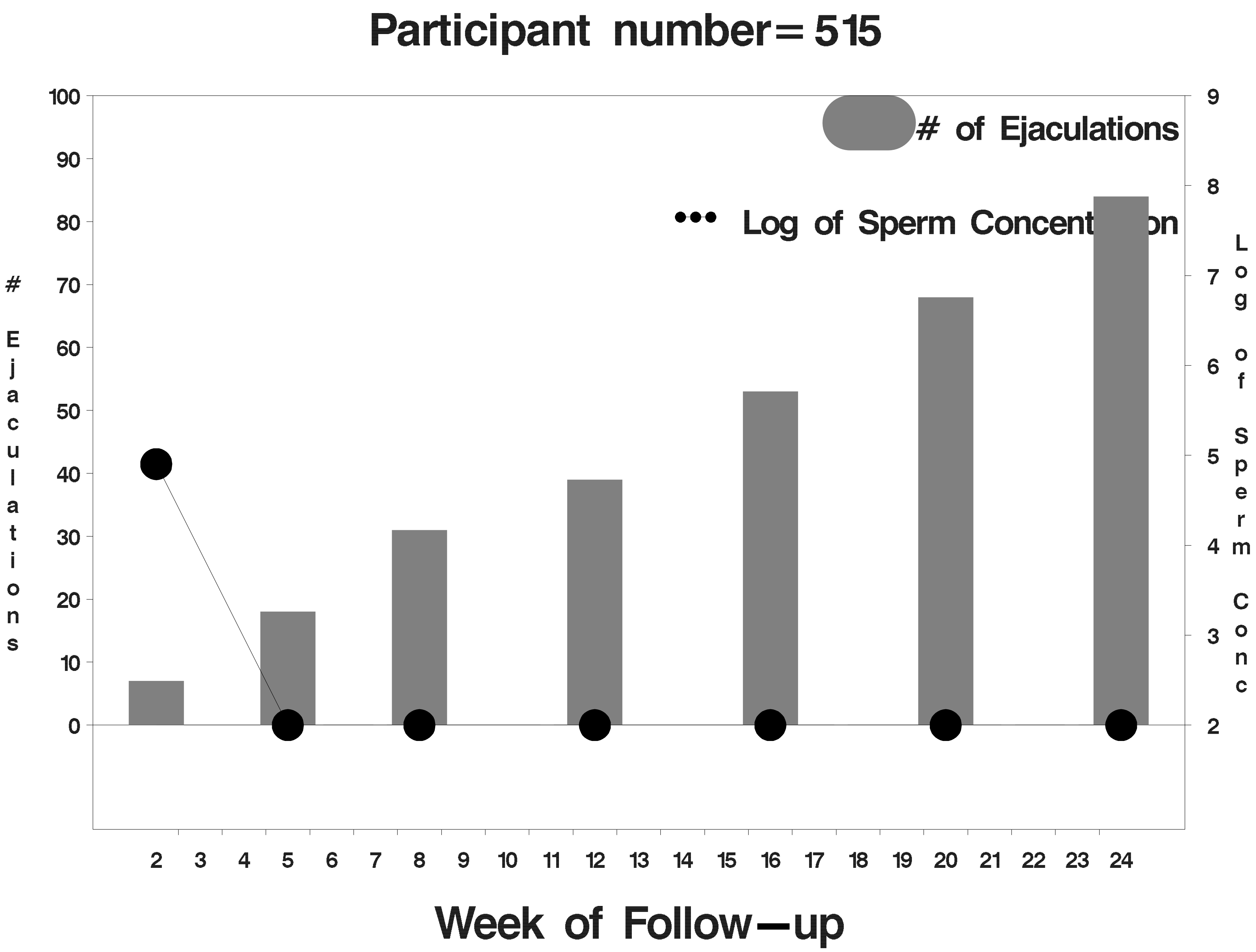

Participant number=515
# of Ejaculations
Log of Sperm Concent[illegible]on
Ejaculations
Log of Sperm Conc
100
90
80
70
60
50
40
30
20
10
0
9
8
7
6
5
4
3
2
2 3 4 5 6 7 8 9 10 11 12 13 14 15 16 17 18 19 20 21 22 23 24
Week of Follow-up

Participant number=516

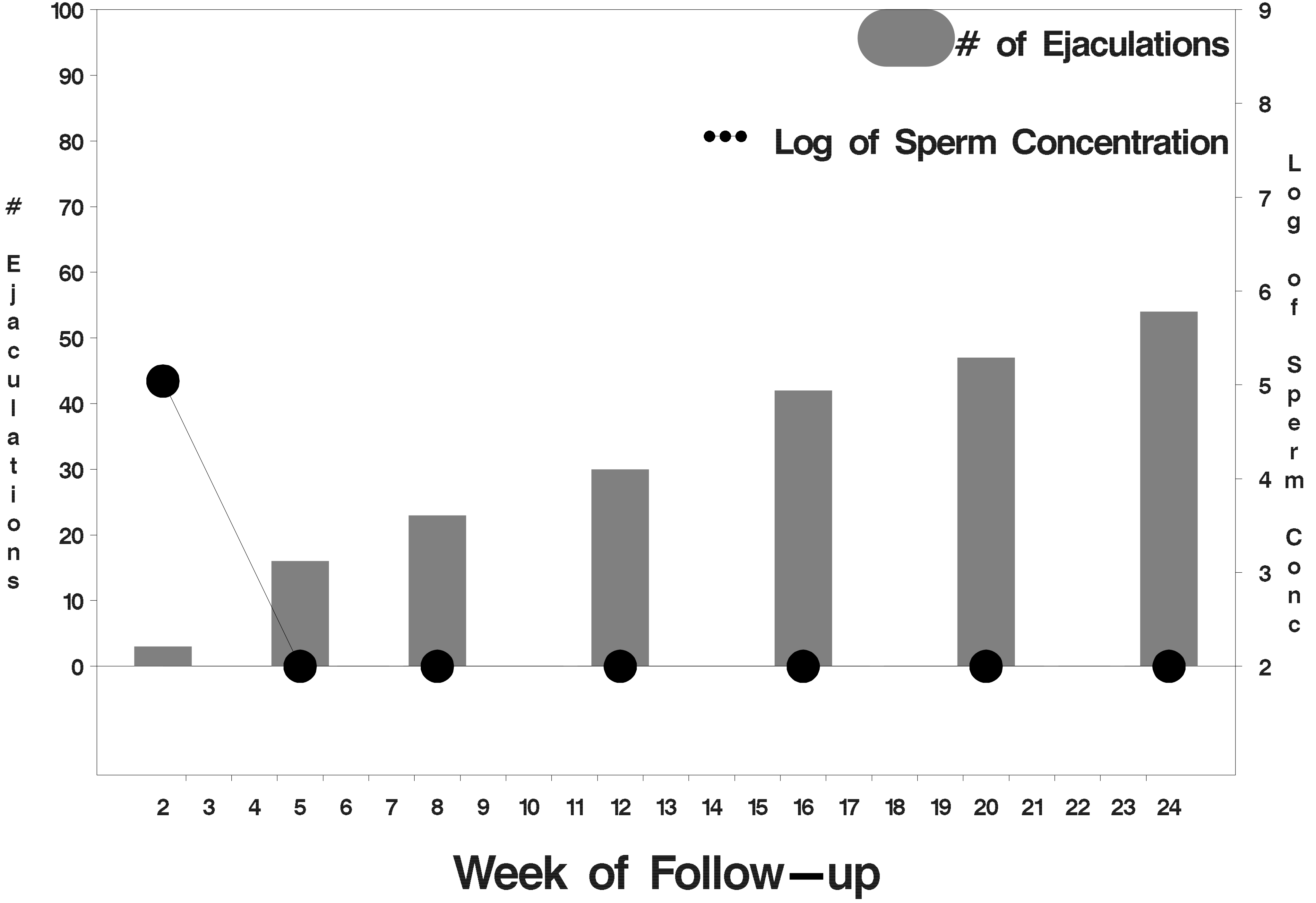

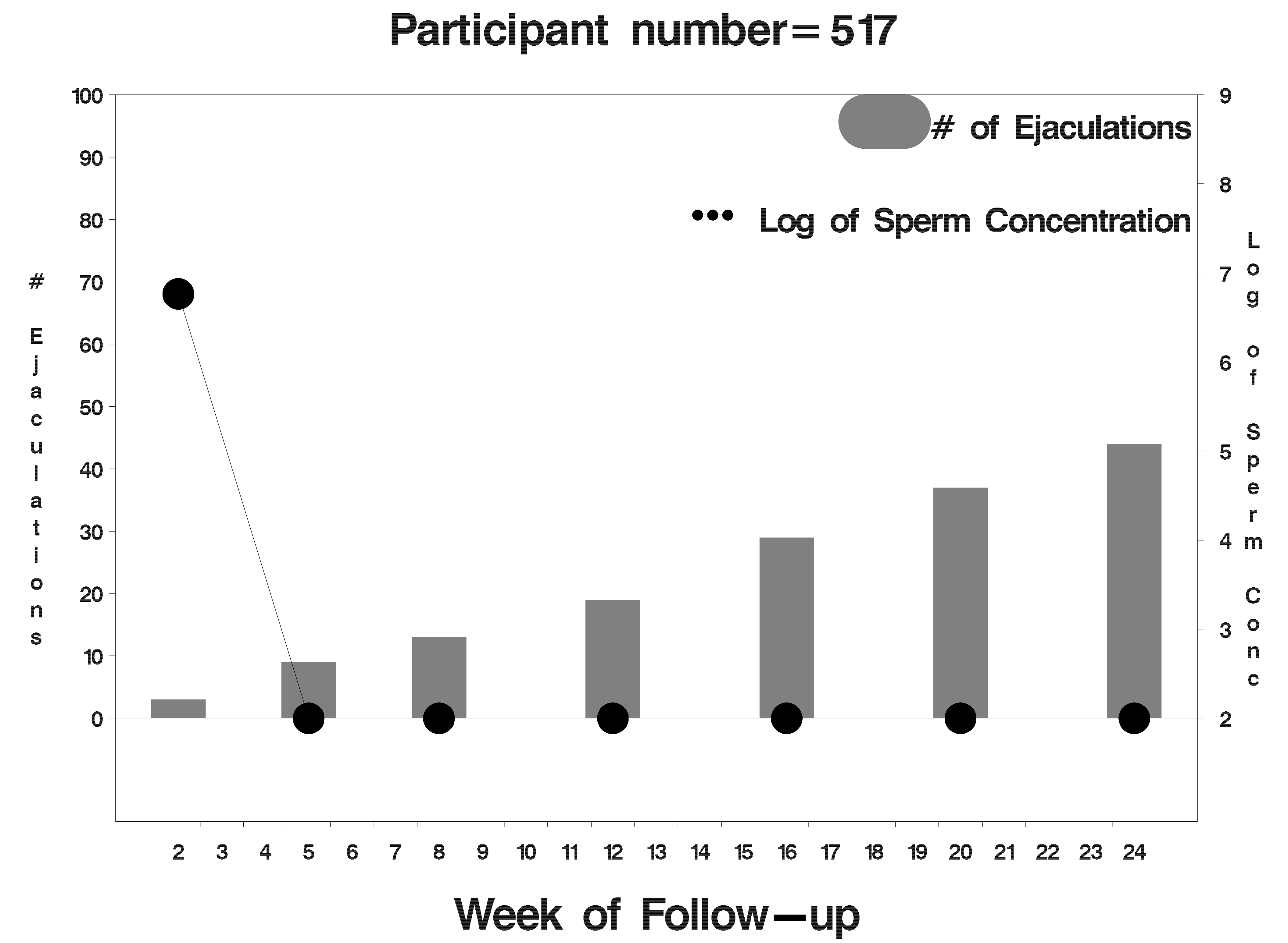

Participant number=517
# of Ejaculations
Log of Sperm Concentration
Ejaculations
Log of Sperm Conc
100
90
80
70
60
50
40
30
20
10
0
9
8
7
6
5
4
3
2
2 3 4 5 6 7 8 9 10 11 12 13 14 15 16 17 18 19 20 21 22 23 24
Week of Follow-up

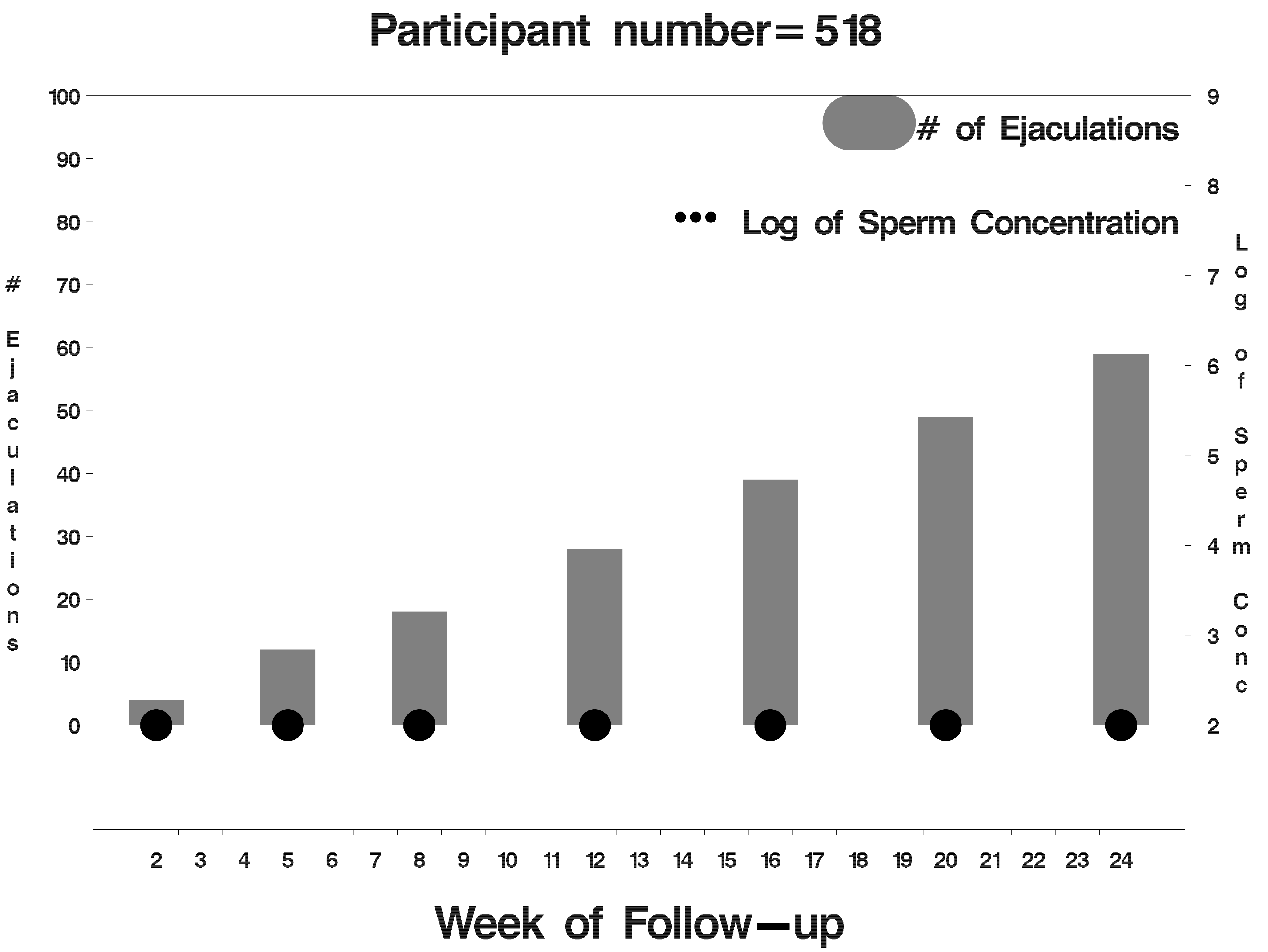
Participant number=518
# of Ejaculations
Log of Sperm Concentration
Ejaculations
Log of Sperm Conc
100
90
80
70
60
50
40
30
20
10
0
9
8
7
6
5
4
3
2
2 3 4 5 6 7 8 9 10 11 12 13 14 15 16 17 18 19 20 21 22 23 24
Week of Follow-up

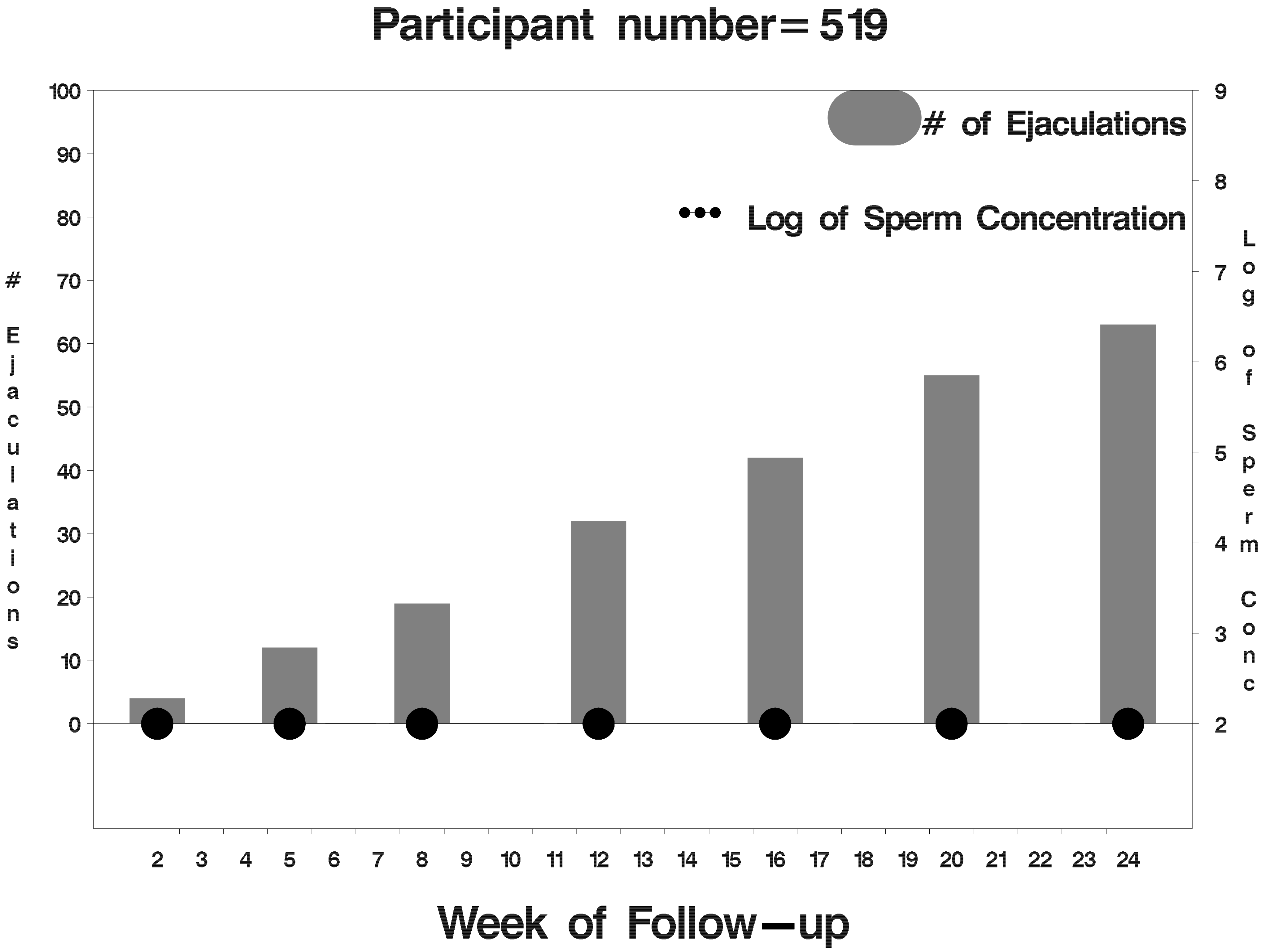

Participant number=519
# of Ejaculations
Log of Sperm Concentration
Ejaculations
Log of Sperm Conc
100
90
80
70
60
50
40
30
20
10
0
9
8
7
6
5
4
3
2
2 3 4 5 6 7 8 9 10 11 12 13 14 15 16 17 18 19 20 21 22 23 24
Week of Follow-up

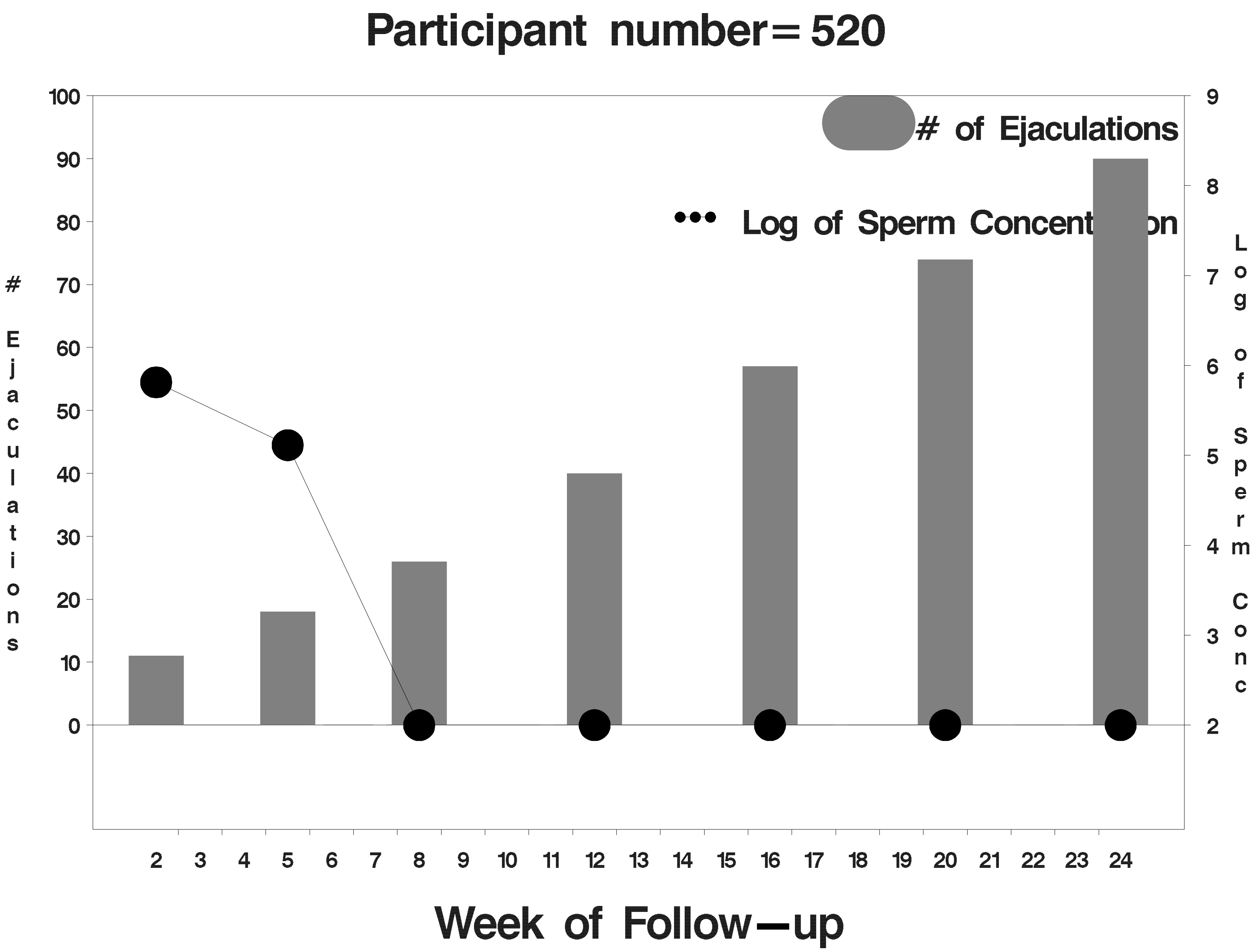

Participant number= 520
# of Ejaculations
Log of Sperm Concent[illegible]on
Ejaculations
Log of Sperm Conc
0
10
20
30
40
50
60
70
80
90
100
2
3
4
5
6
7
8
9
2 3 4 5 6 7 8 9 10 11 12 13 14 15 16 17 18 19 20 21 22 23 24
Week of Follow-up

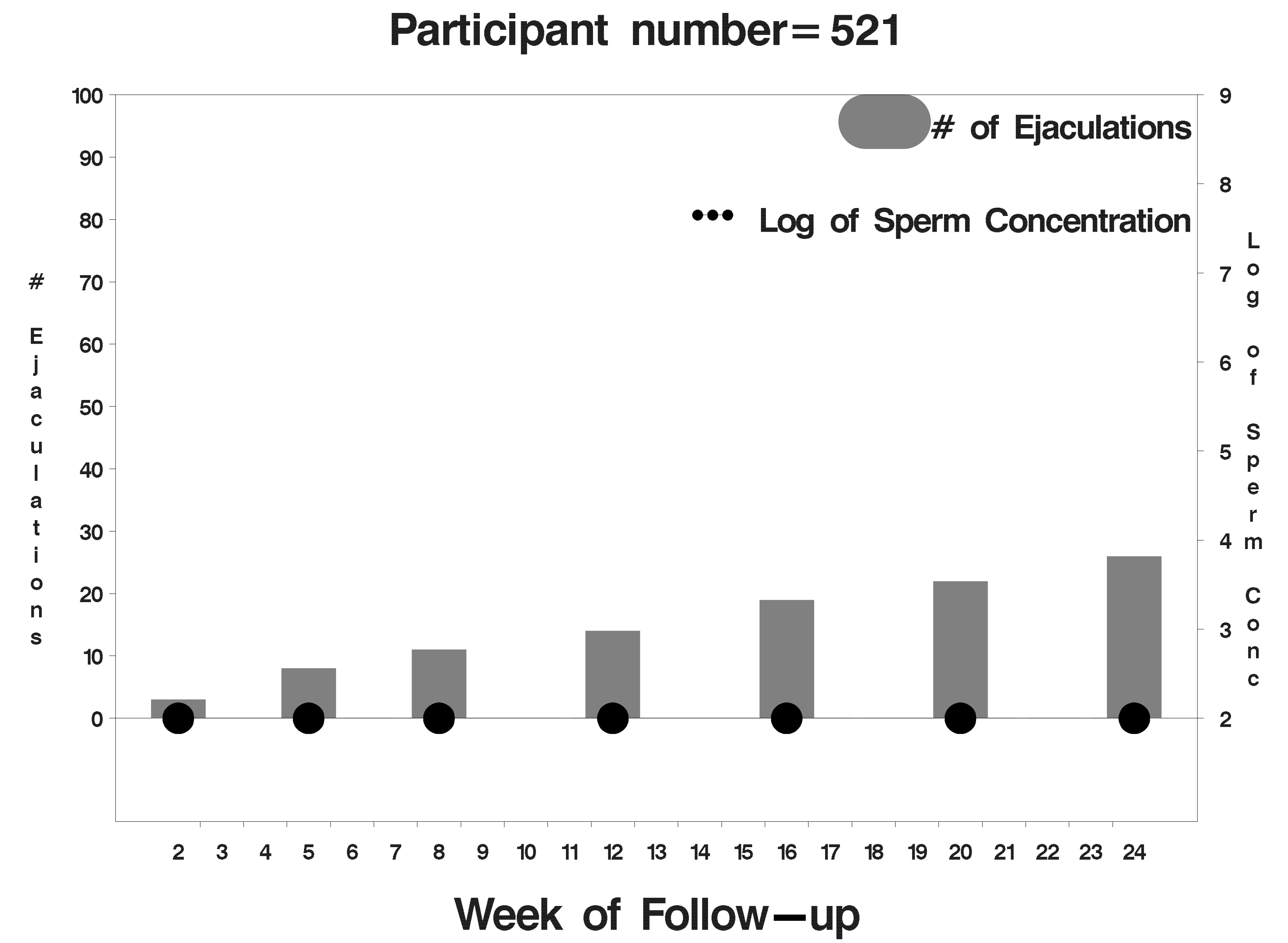

Participant number=521
# of Ejaculations
Log of Sperm Concentration
Ejaculations
Log of Sperm Conc
100
90
80
70
60
50
40
30
20
10
0
9
8
7
6
5
4
3
2
2 3 4 5 6 7 8 9 10 11 12 13 14 15 16 17 18 19 20 21 22 23 24
Week of Follow-up

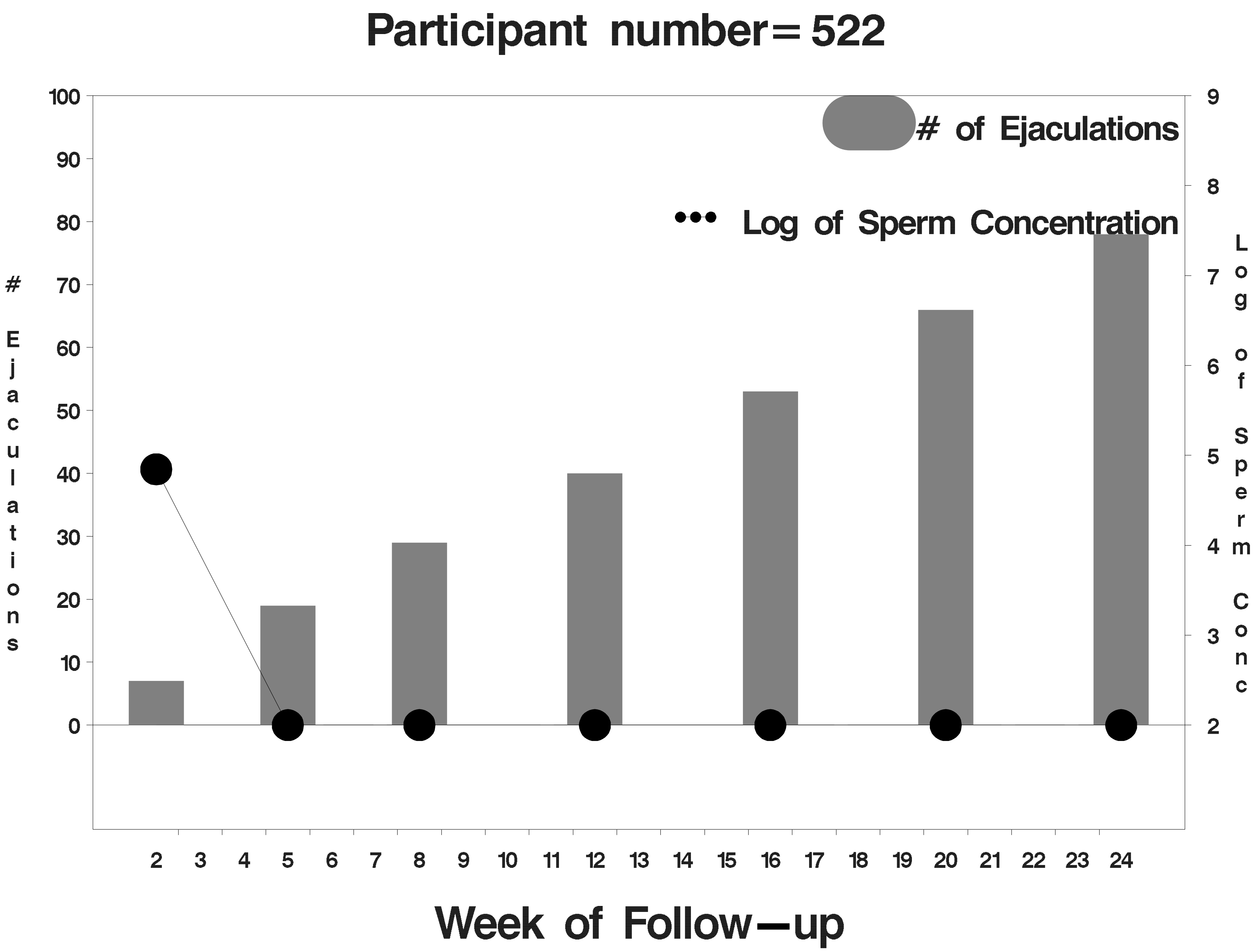

Participant number= 522
# of Ejaculations
Log of Sperm Concentration
Ejaculations
Log of Sperm Conc
100
90
80
70
60
50
40
30
20
10
0
9
8
7
6
5
4
3
2
2 3 4 5 6 7 8 9 10 11 12 13 14 15 16 17 18 19 20 21 22 23 24
Week of Follow-up

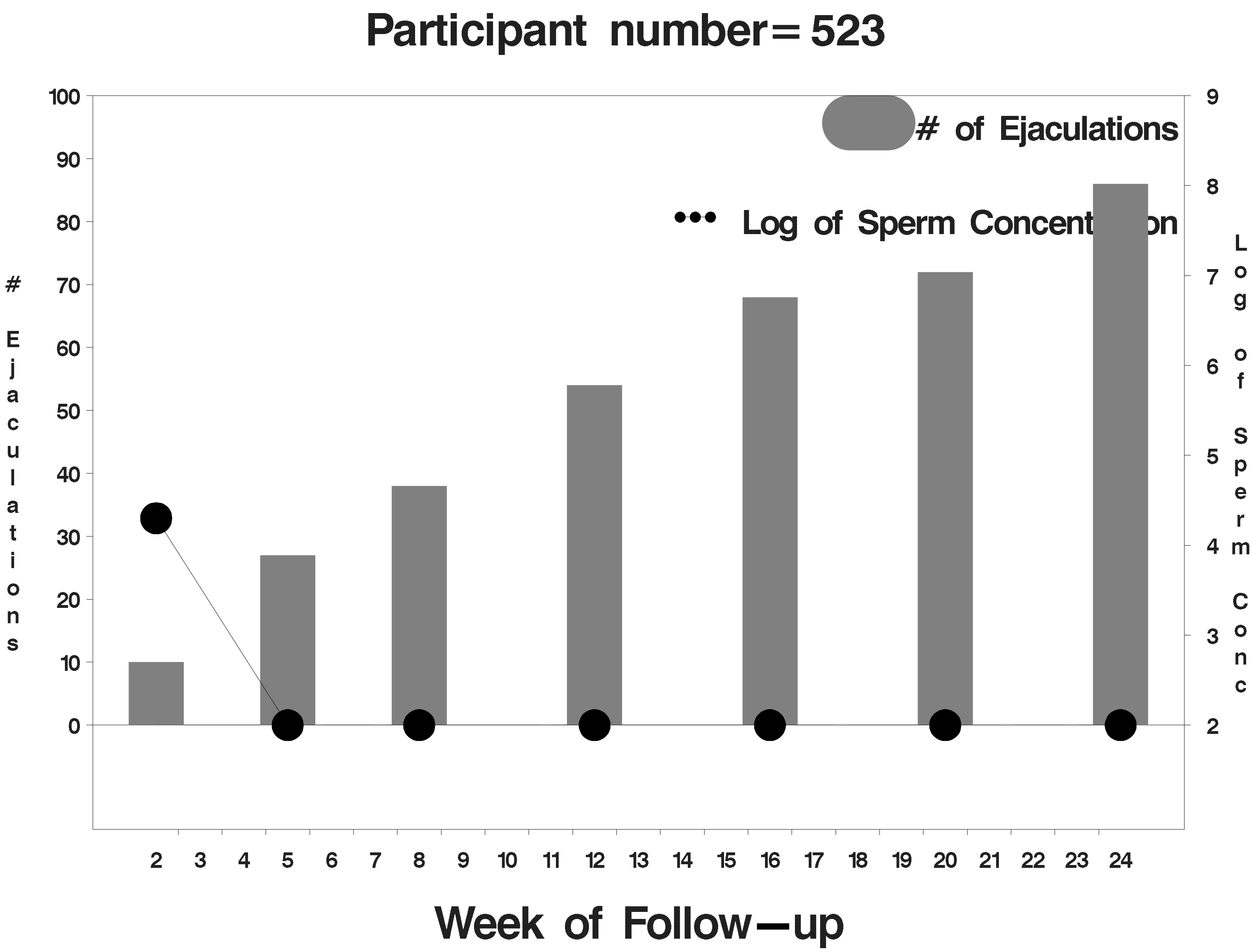

Participant number= 523
# of Ejaculations
Log of Sperm Concentration
Ejaculations
Log of Sperm Conc
Week of Follow-up
100
90
80
70
60
50
40
30
20
10
0
9
8
7
6
5
4
3
2
2 3 4 5 6 7 8 9 10 11 12 13 14 15 16 17 18 19 20 21 22 23 24

Participant number=524

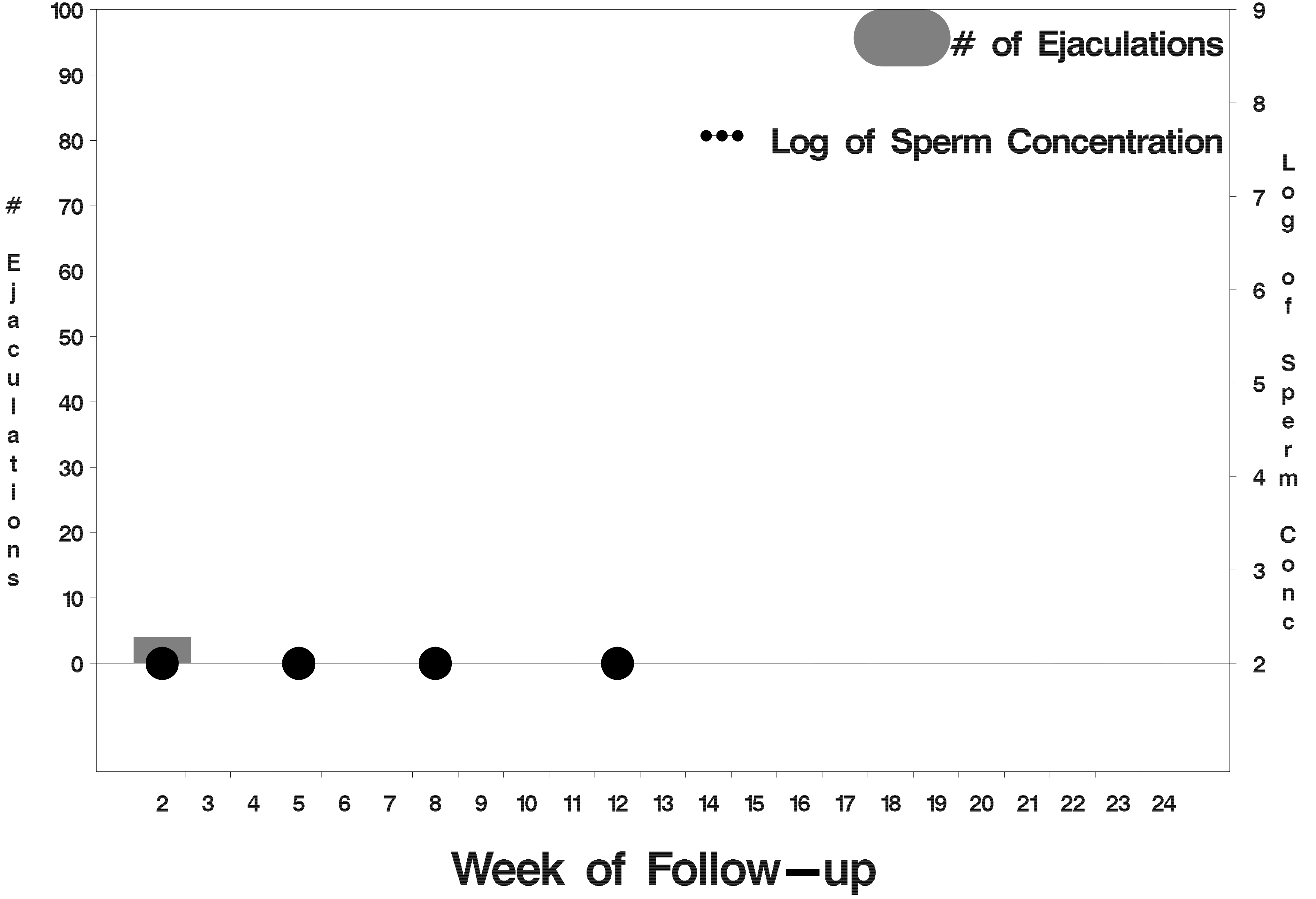

Participant number= 525

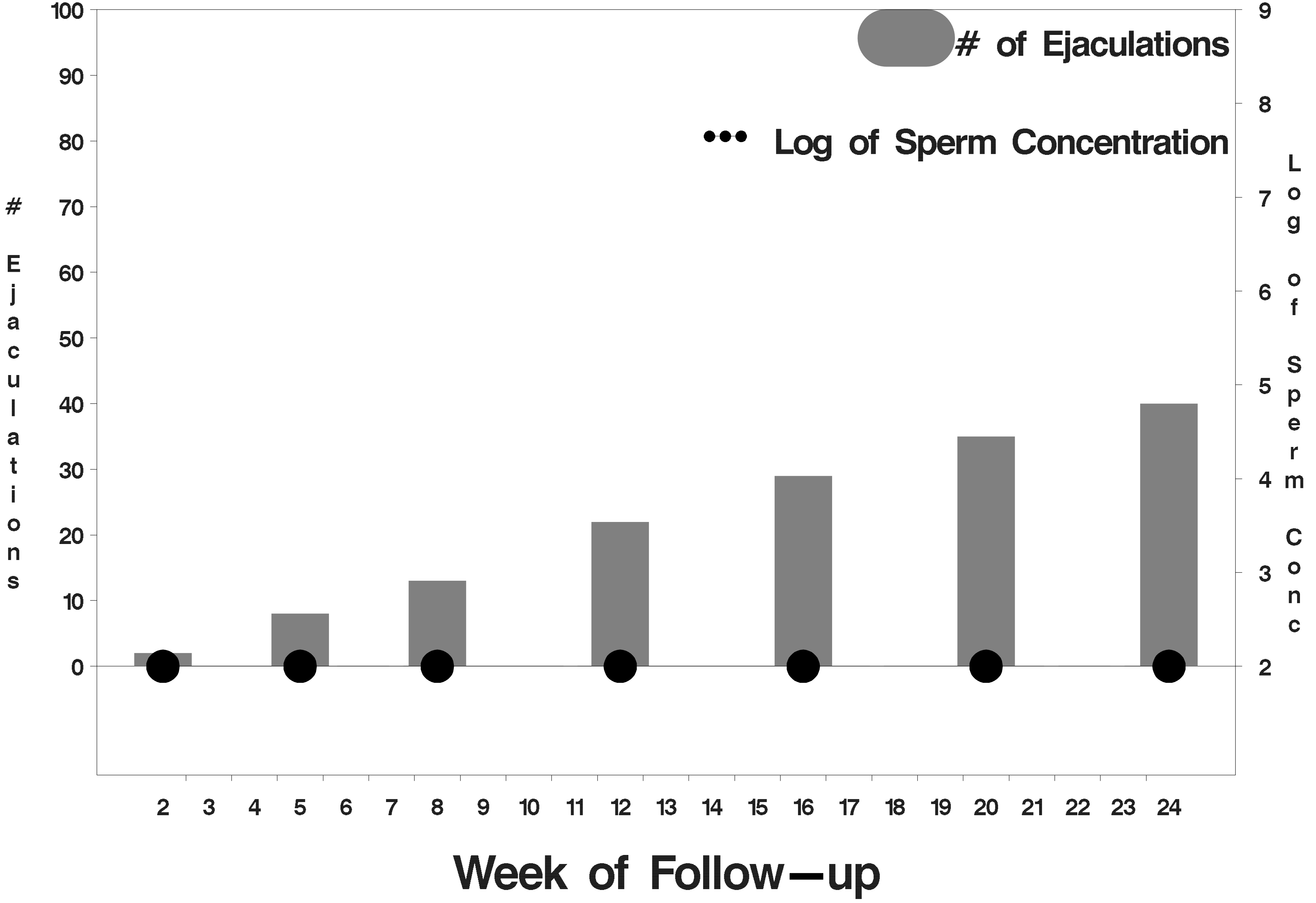

Participant number=526

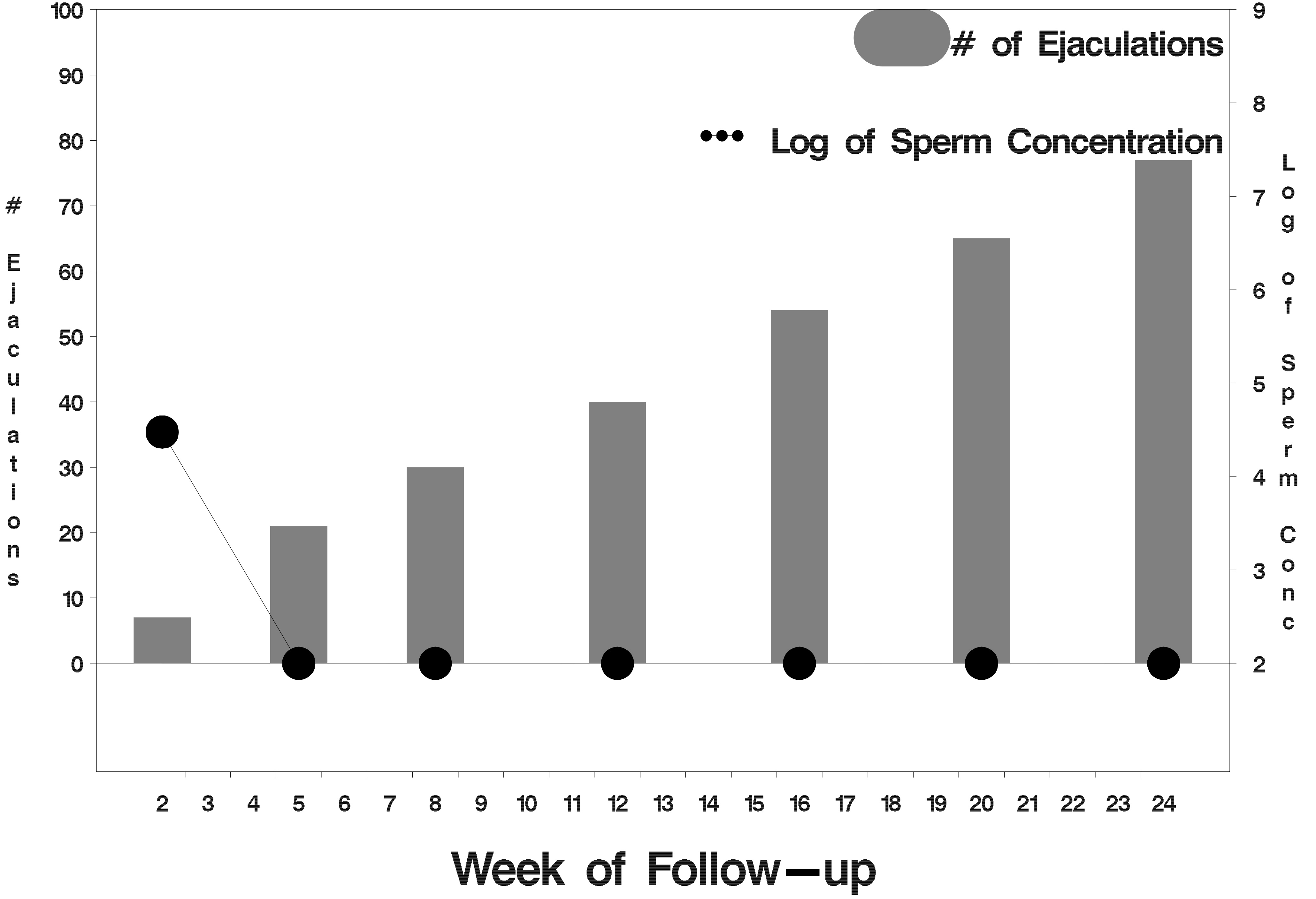

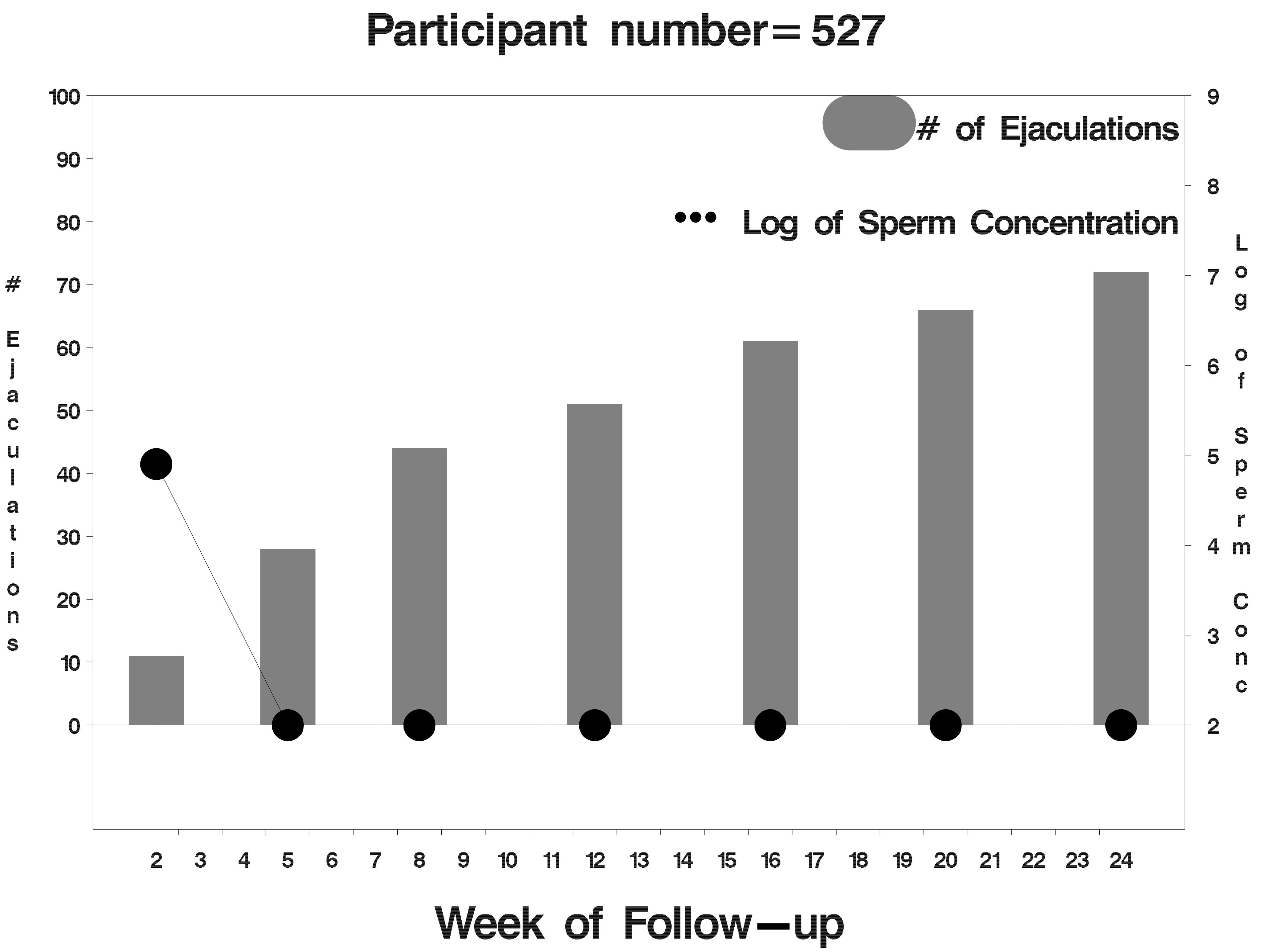

Participant number= 527
# of Ejaculations
Log of Sperm Concentration
Ejaculations
Log of Sperm Conc
100
90
80
70
60
50
40
30
20
10
0
9
8
7
6
5
4
3
2
2 3 4 5 6 7 8 9 10 11 12 13 14 15 16 17 18 19 20 21 22 23 24
Week of Follow—up

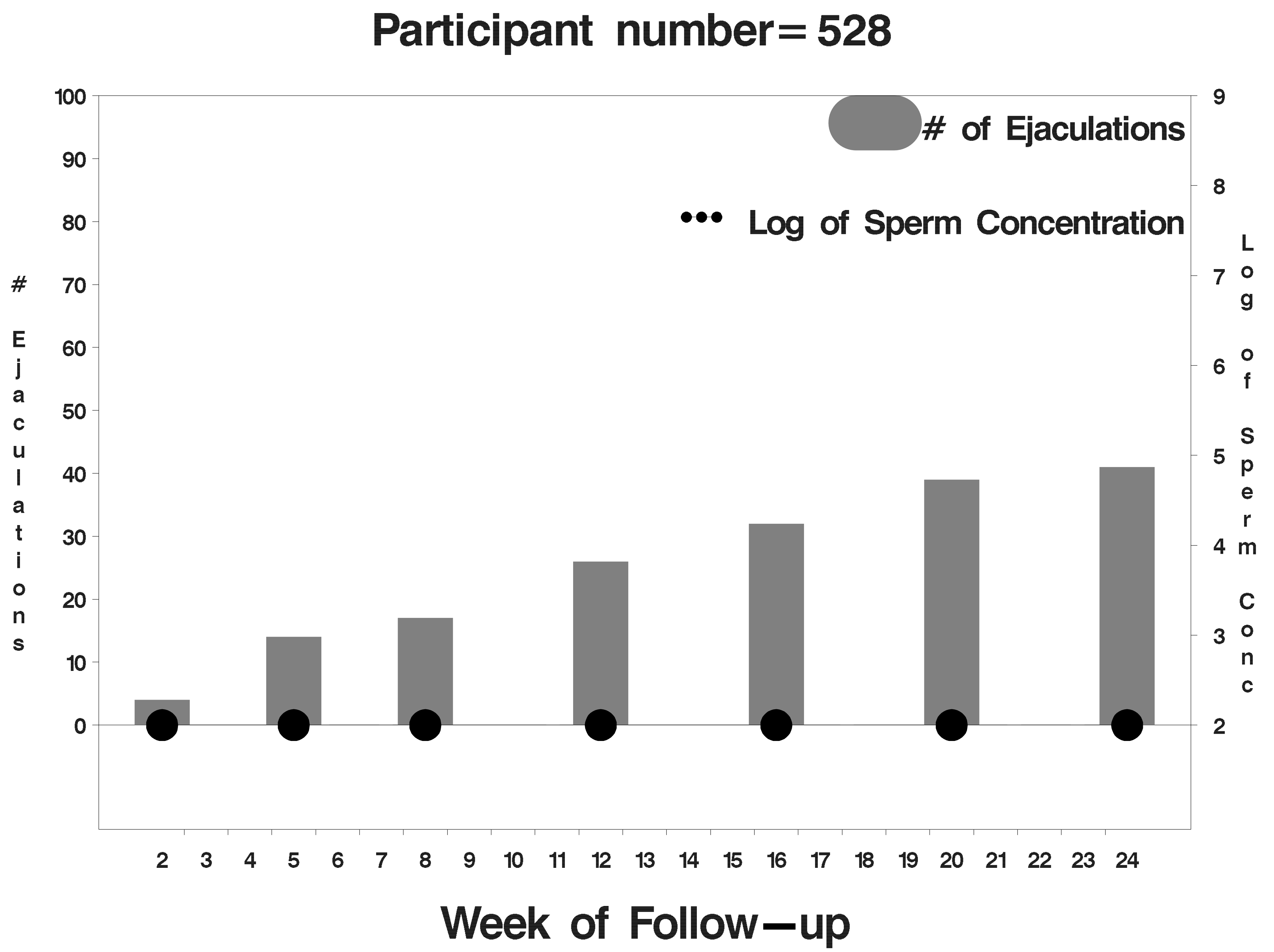

Participant number= 528
# of Ejaculations
Log of Sperm Concentration
Ejaculations
Log of Sperm Conc
100
90
80
70
60
50
40
30
20
10
0
9
8
7
6
5
4
3
2
2 3 4 5 6 7 8 9 10 11 12 13 14 15 16 17 18 19 20 21 22 23 24
Week of Follow-up

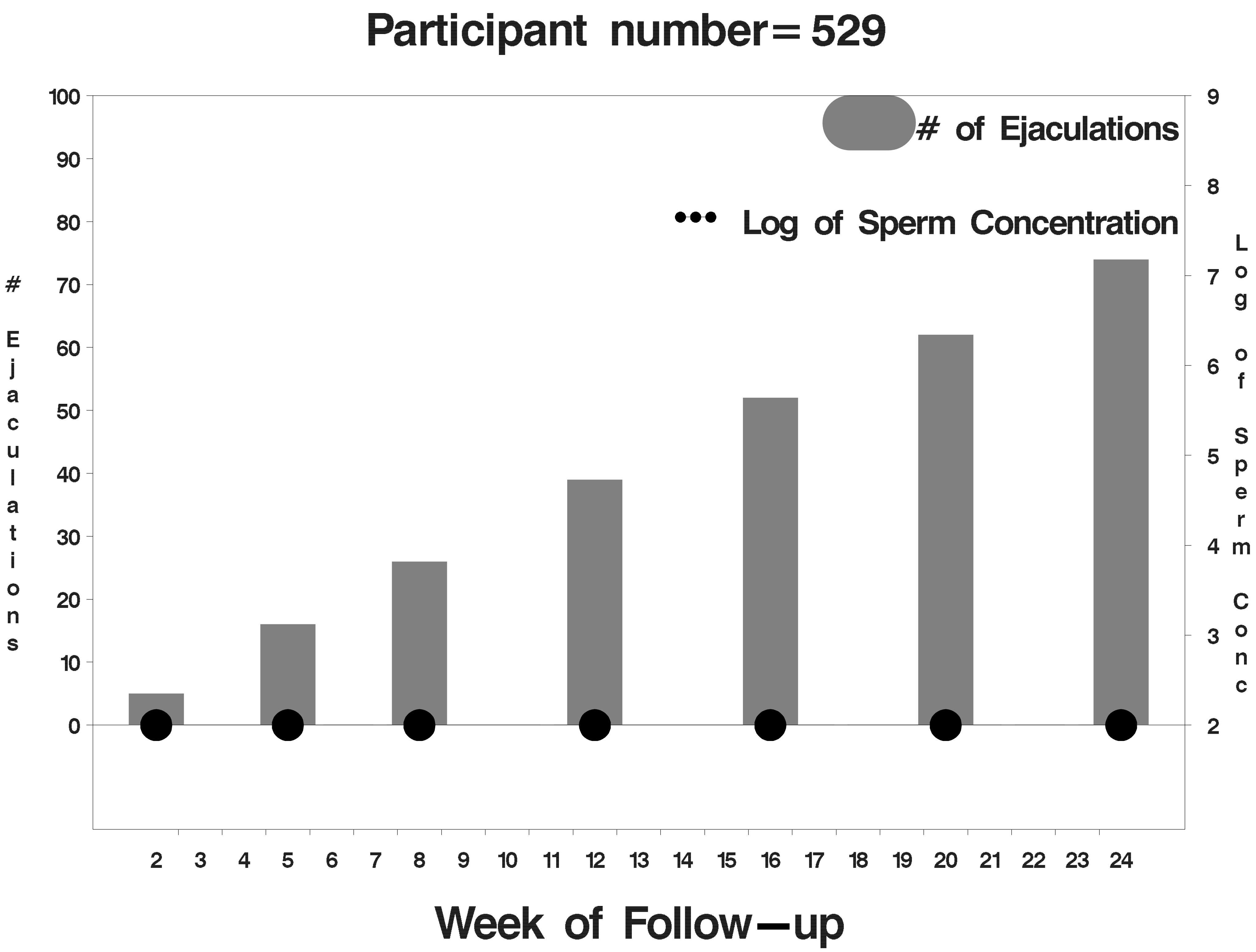
Participant number= 529
# of Ejaculations
Log of Sperm Concentration
Ejaculations
Log of Sperm Conc
100
90
80
70
60
50
40
30
20
10
0
9
8
7
6
5
4
3
2
2 3 4 5 6 7 8 9 10 11 12 13 14 15 16 17 18 19 20 21 22 23 24
Week of Follow—up

Participant number= 530

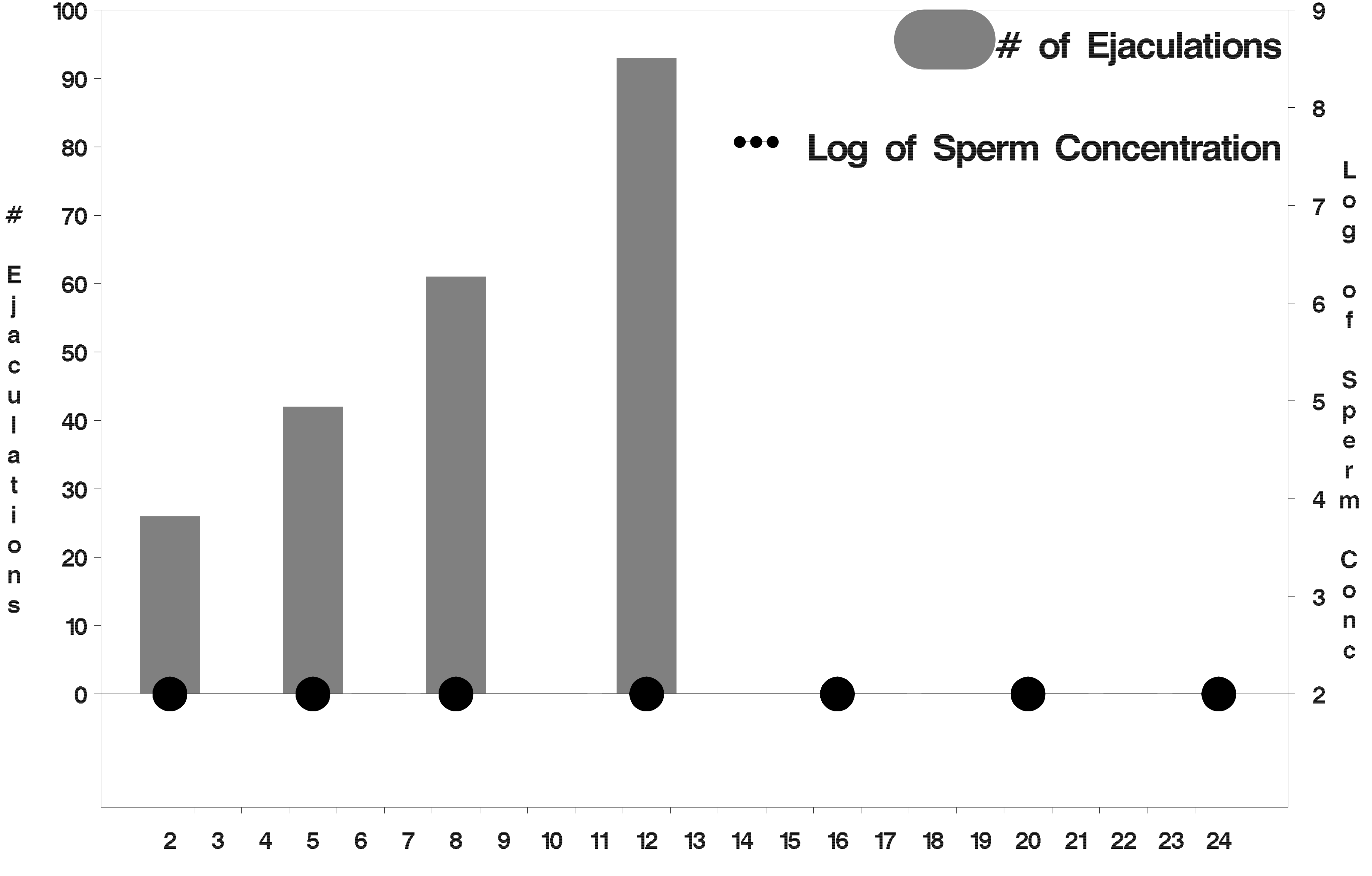

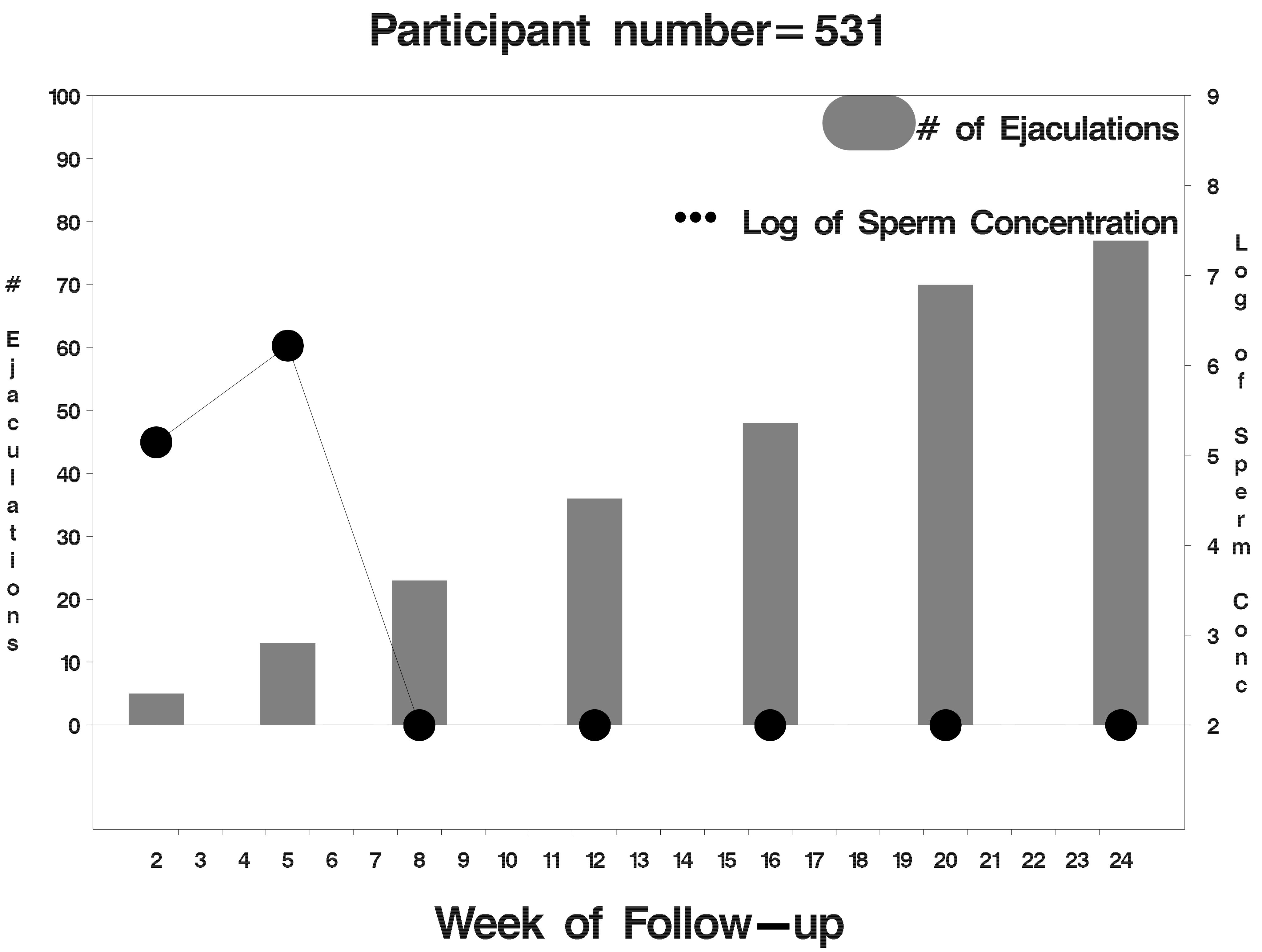

Participant number=531
# of Ejaculations
Log of Sperm Concentration
Ejaculations
Log of Sperm Conc
100
90
80
70
60
50
40
30
20
10
0
9
8
7
6
5
4
3
2
2 3 4 5 6 7 8 9 10 11 12 13 14 15 16 17 18 19 20 21 22 23 24
Week of Follow-up

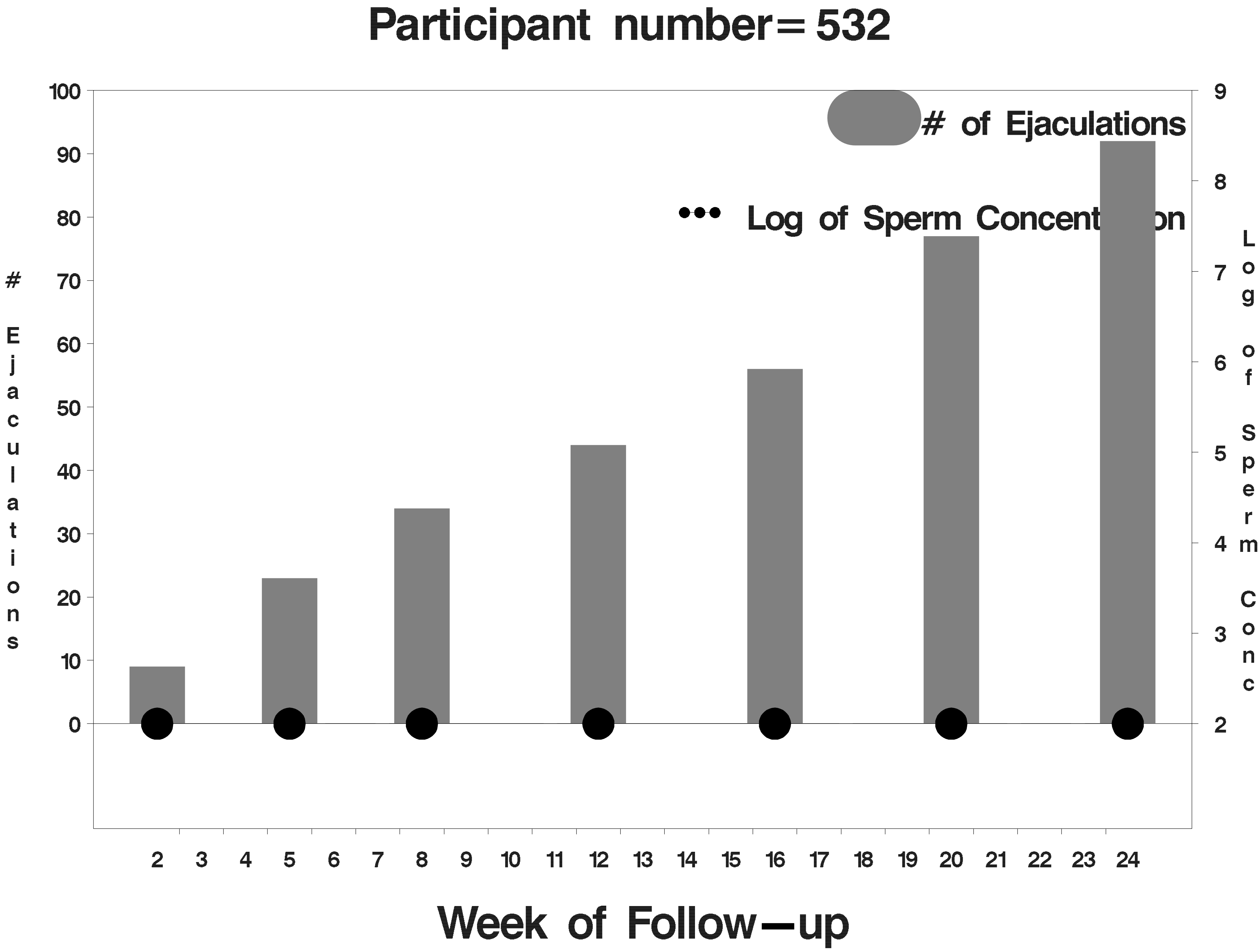
Participant number= 532
# of Ejaculations
Log of Sperm Concentration
Ejaculations
Log of Sperm Conc
100
90
80
70
60
50
40
30
20
10
0
9
8
7
6
5
4
3
2
2 3 4 5 6 7 8 9 10 11 12 13 14 15 16 17 18 19 20 21 22 23 24
Week of Follow-up

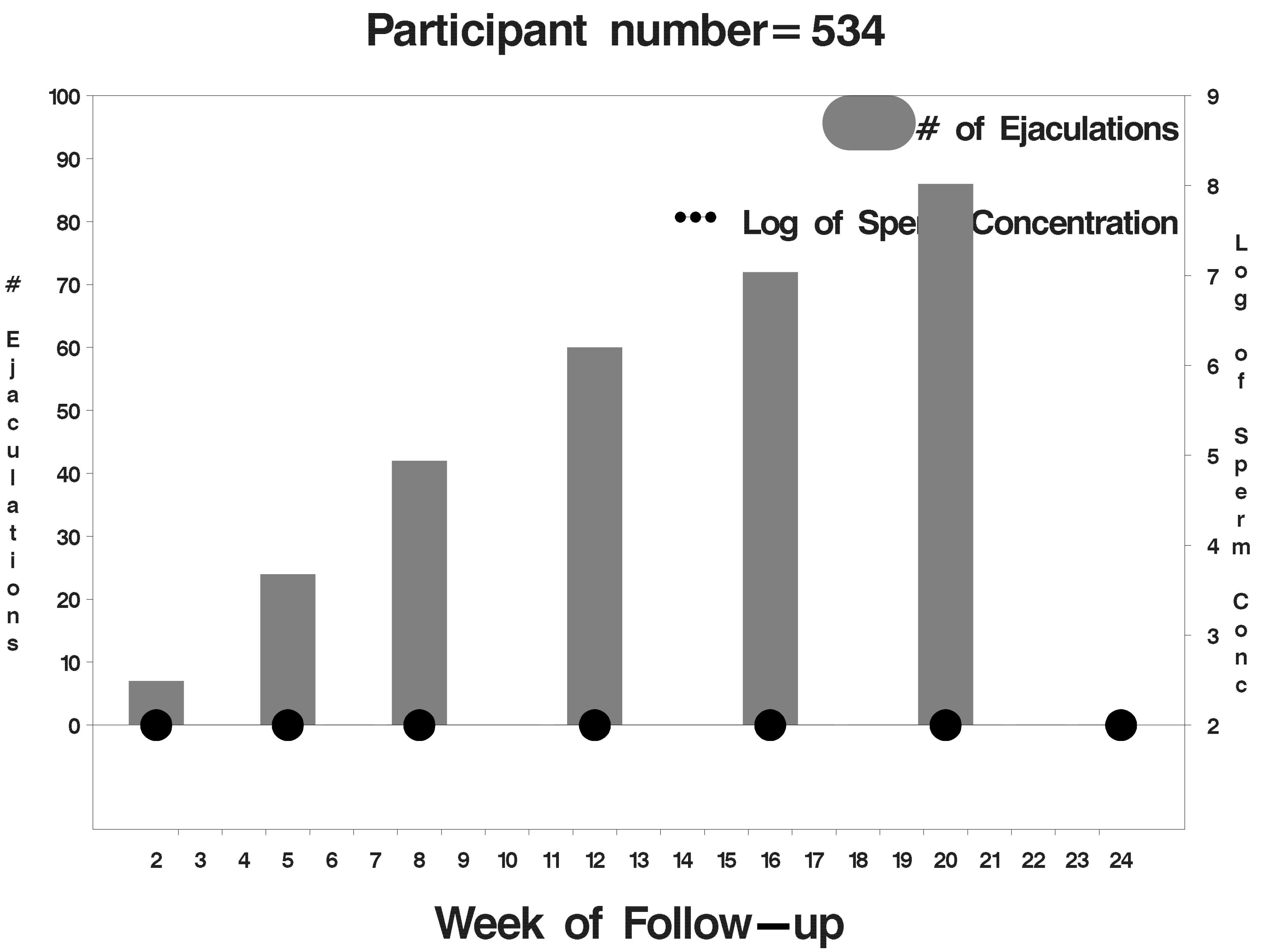

Participant number=534
# of Ejaculations
Log of Sperm Concentration
Ejaculations
Log of Sperm Conc
Week of Follow-up
100
90
80
70
60
50
40
30
20
10
0
9
8
7
6
5
4
3
2
2 3 4 5 6 7 8 9 10 11 12 13 14 15 16 17 18 19 20 21 22 23 24

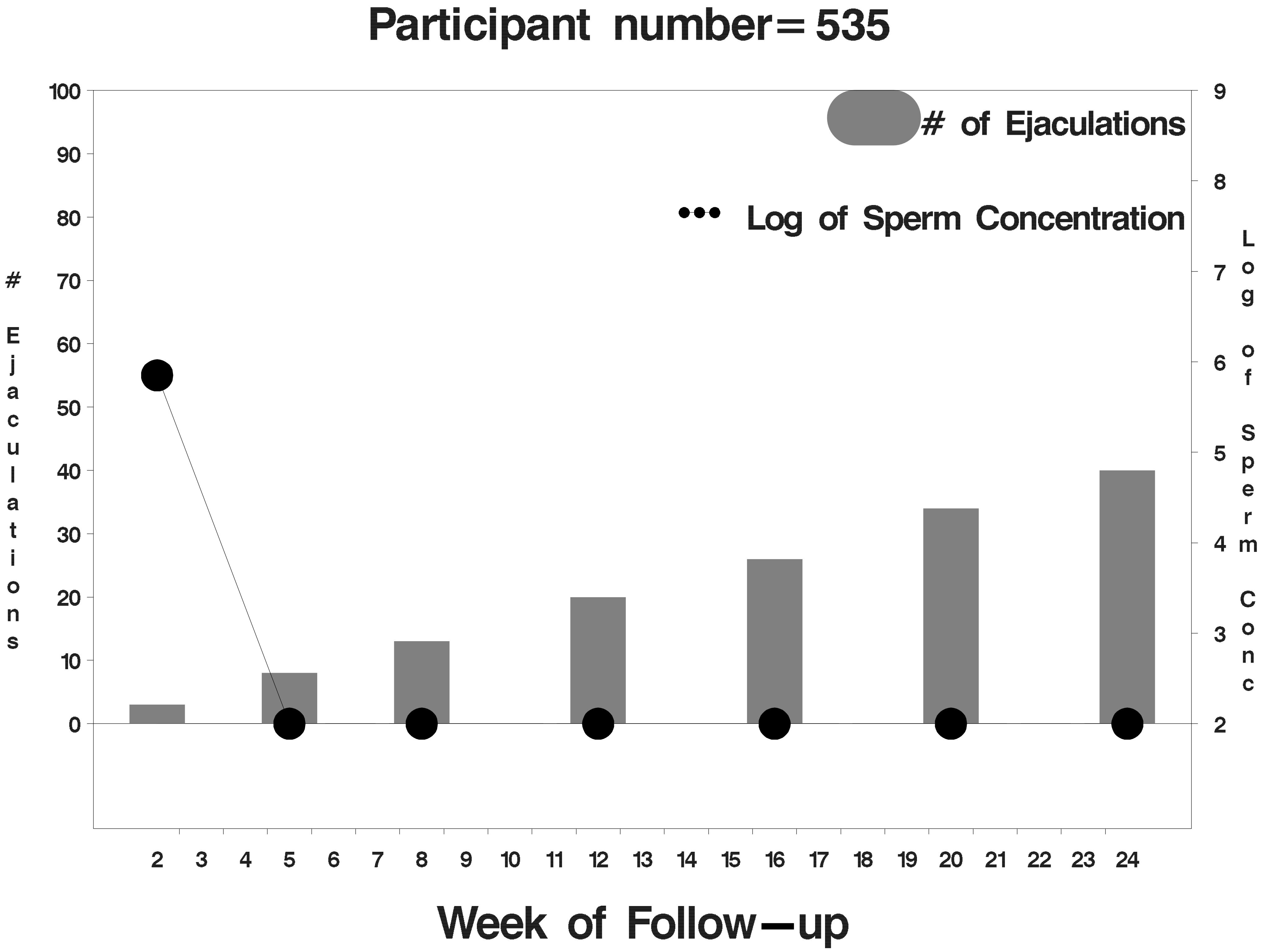

Participant number=535
# of Ejaculations
Log of Sperm Concentration
Ejaculations
Log of Sperm Conc
0
10
20
30
40
50
60
70
80
90
100
2
3
4
5
6
7
8
9
2 3 4 5 6 7 8 9 10 11 12 13 14 15 16 17 18 19 20 21 22 23 24
Week of Follow-up

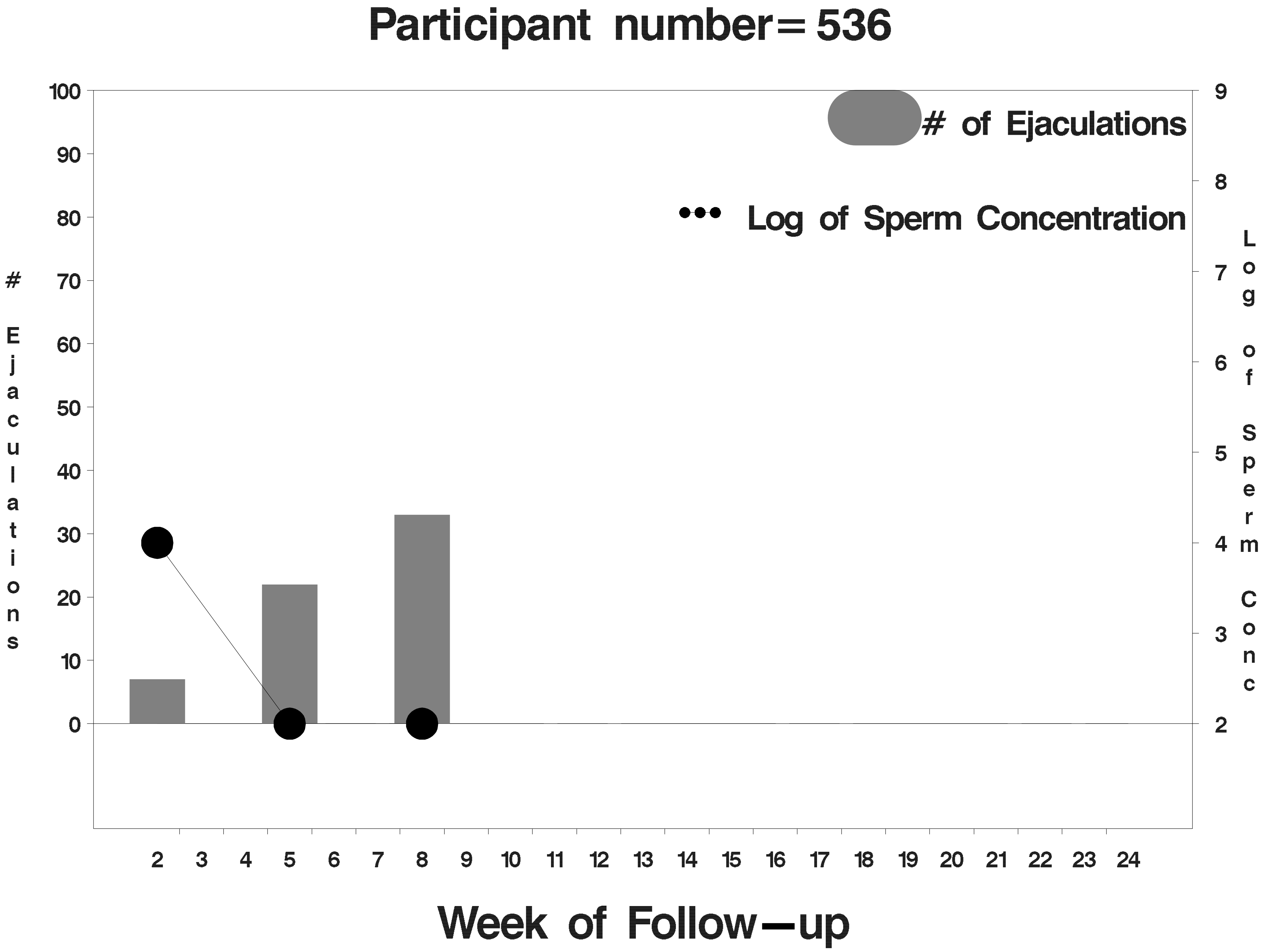
Participant number=536
# of Ejaculations
Log of Sperm Concentration
Ejaculations
Log of Sperm Conc
100
90
80
70
60
50
40
30
20
10
0
9
8
7
6
5
4
3
2
2 3 4 5 6 7 8 9 10 11 12 13 14 15 16 17 18 19 20 21 22 23 24
Week of Follow-up

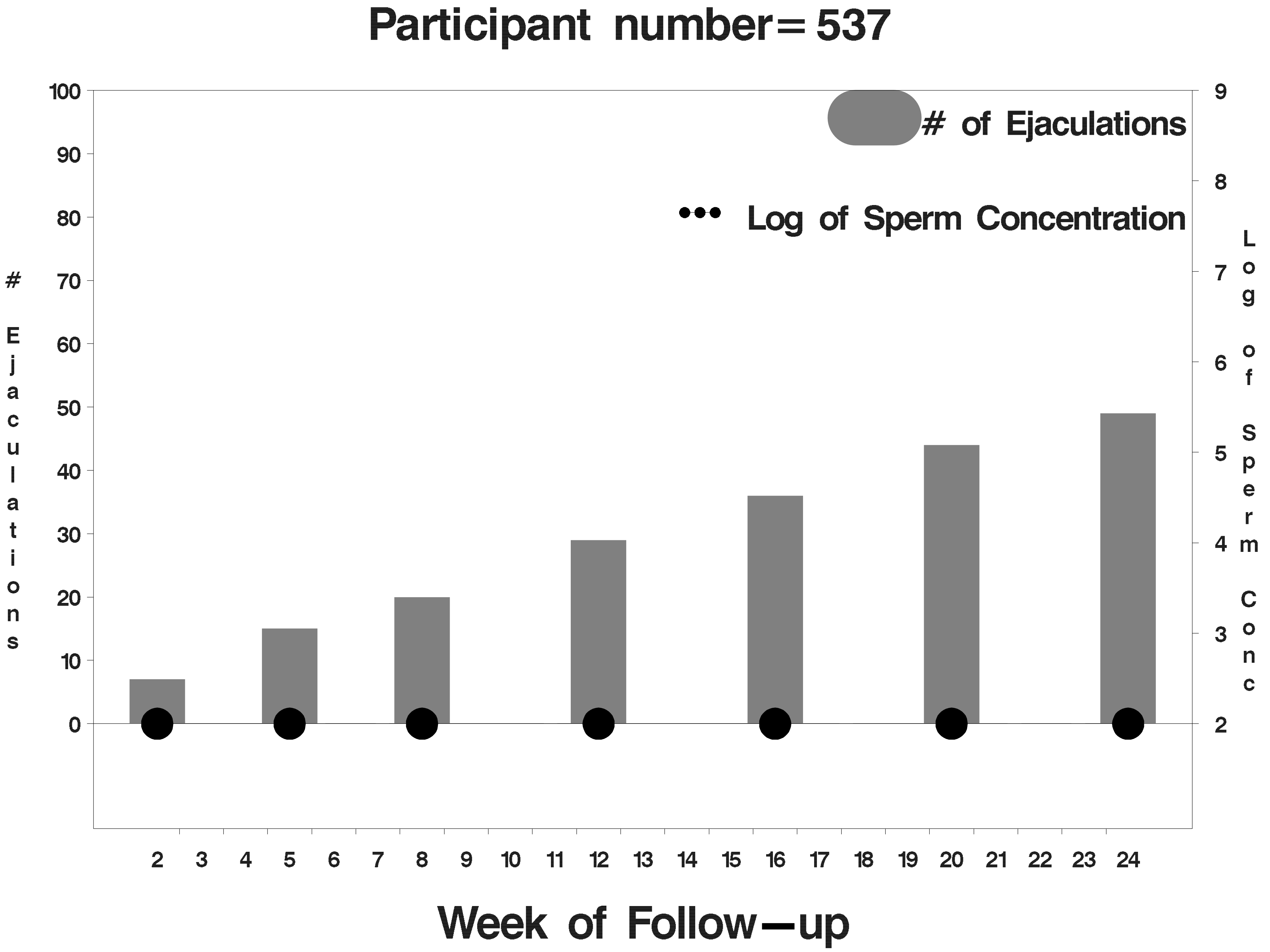

Participant number=537
# of Ejaculations
Log of Sperm Concentration
Ejaculations
Log of Sperm Conc
100
90
80
70
60
50
40
30
20
10
0
9
8
7
6
5
4
3
2
2 3 4 5 6 7 8 9 10 11 12 13 14 15 16 17 18 19 20 21 22 23 24
Week of Follow-up

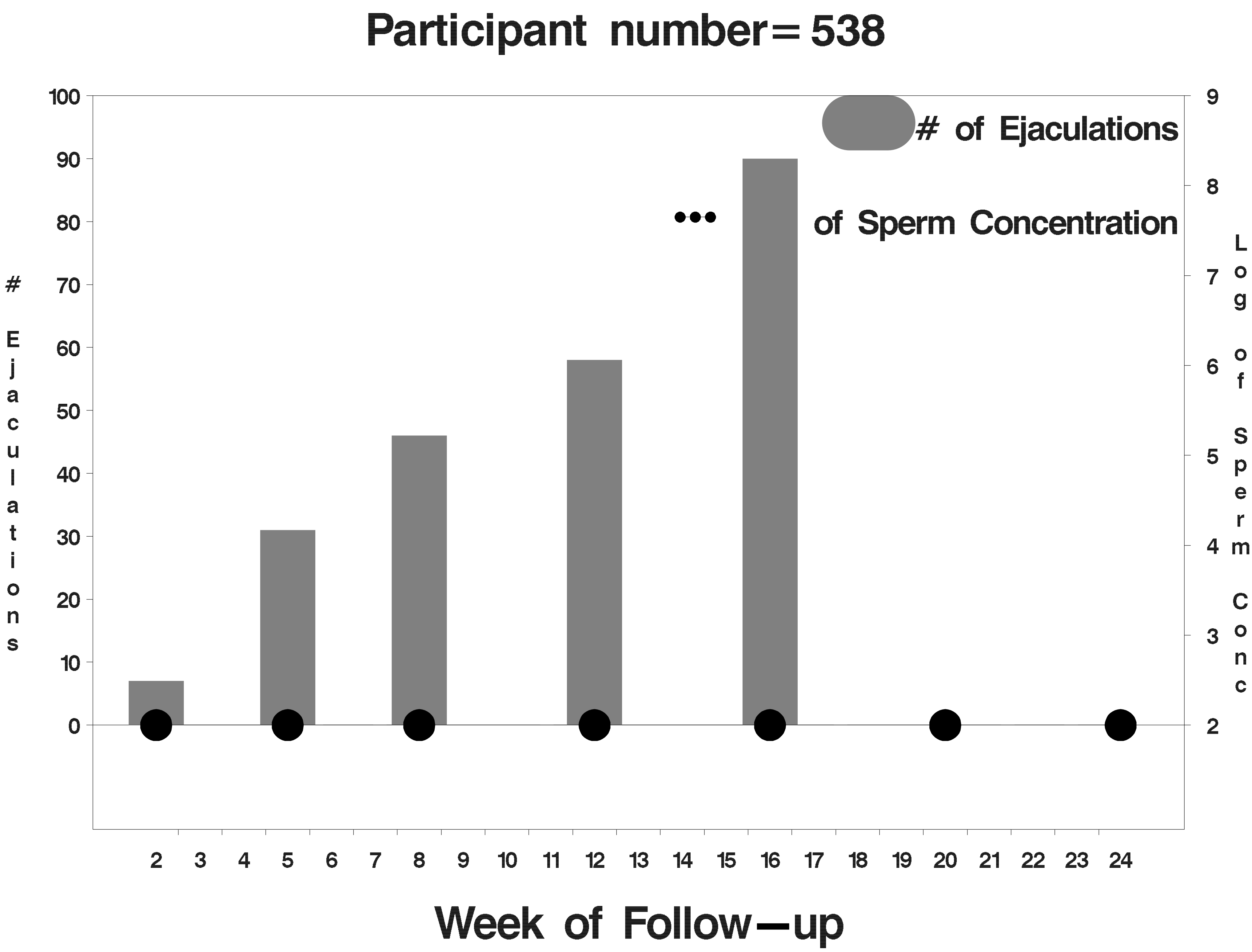

Participant number=538
# of Ejaculations
of Sperm Concentration
Ejaculations
Log of Sperm Conc
100
90
80
70
60
50
40
30
20
10
0
9
8
7
6
5
4
3
2
2 3 4 5 6 7 8 9 10 11 12 13 14 15 16 17 18 19 20 21 22 23 24
Week of Follow-up

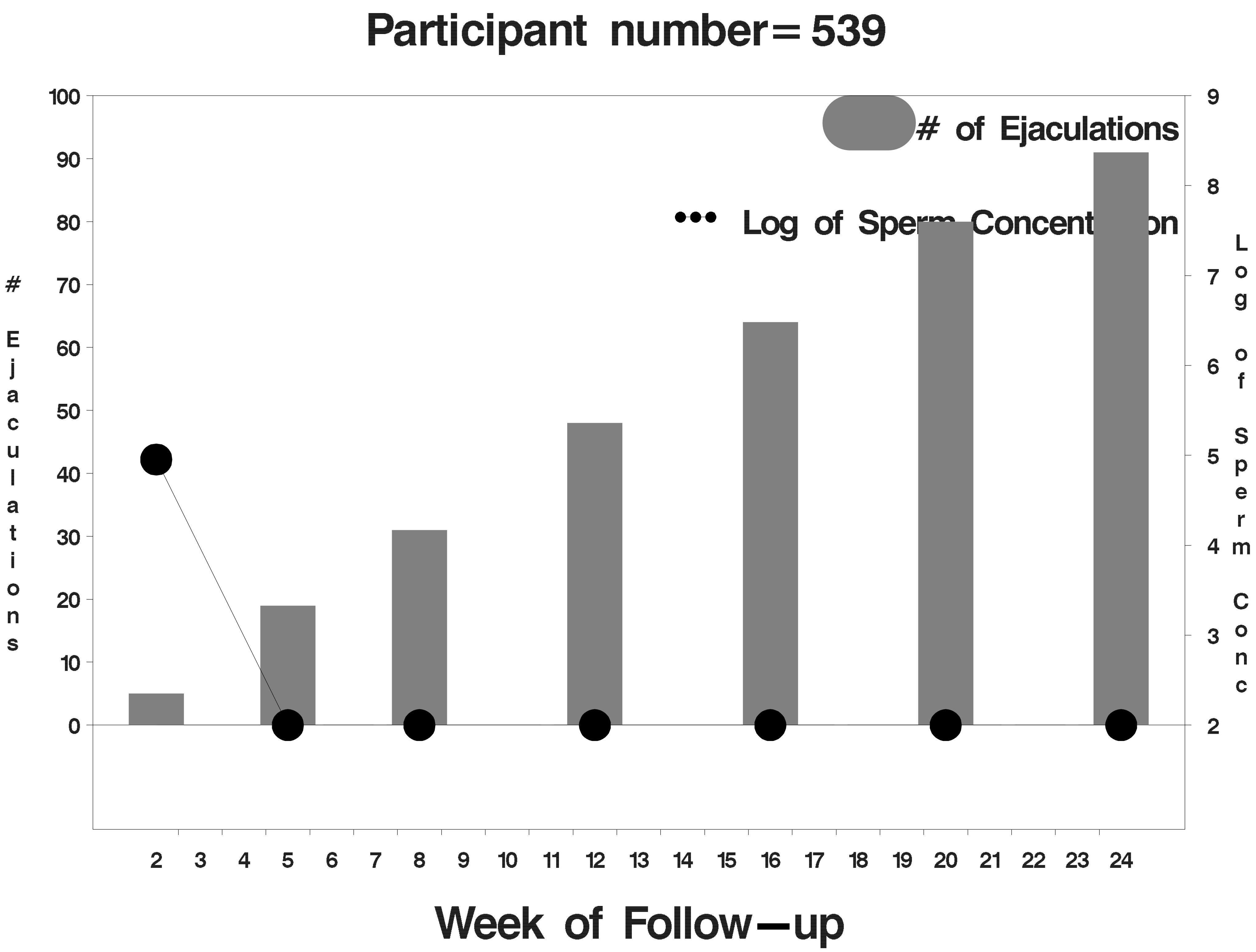

Participant number=539
# of Ejaculations
Log of Sperm Concentration
Ejaculations
Log of Sperm Conc
100
90
80
70
60
50
40
30
20
10
0
9
8
7
6
5
4
3
2
2 3 4 5 6 7 8 9 10 11 12 13 14 15 16 17 18 19 20 21 22 23 24
Week of Follow-up

Participant number=540

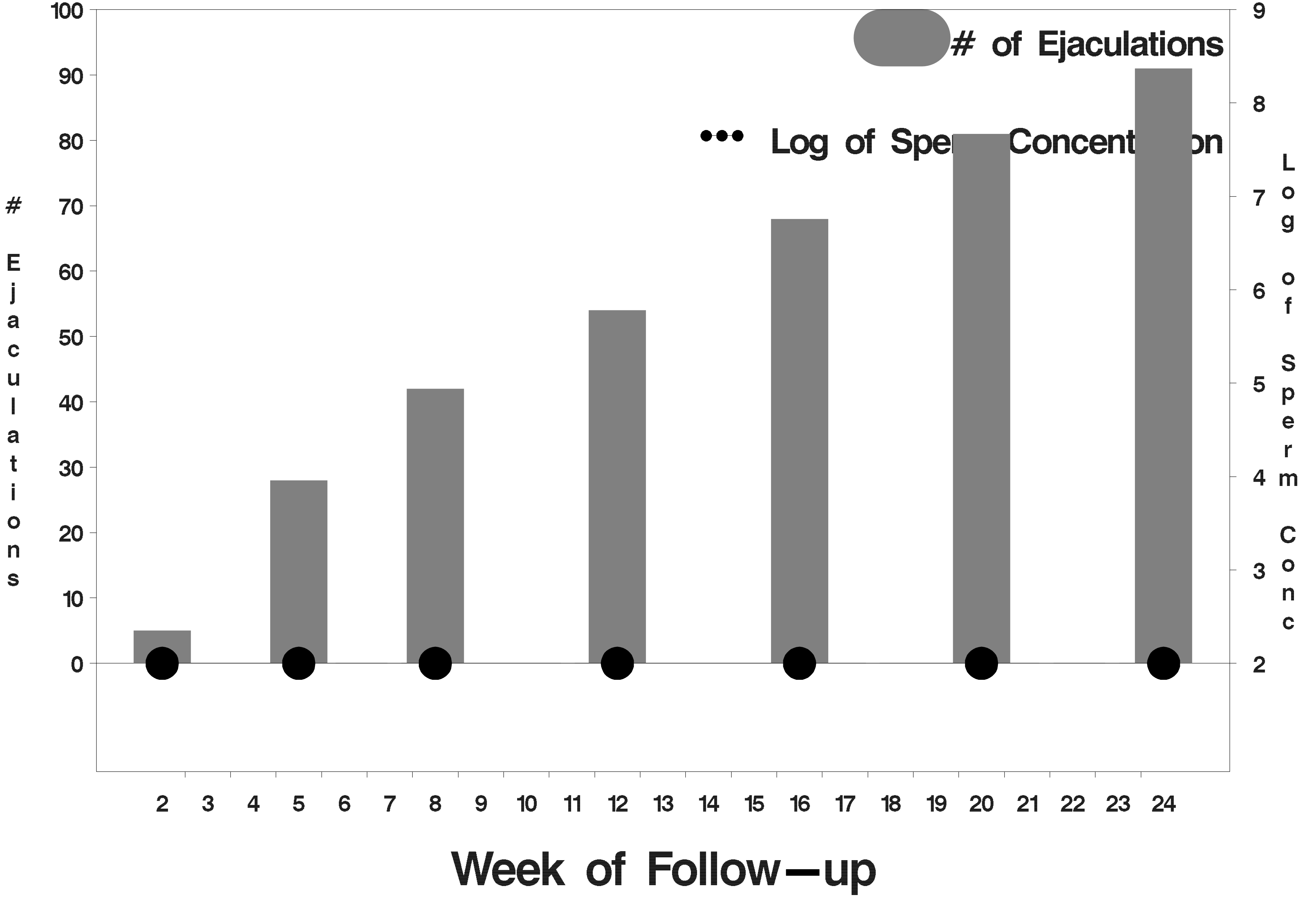

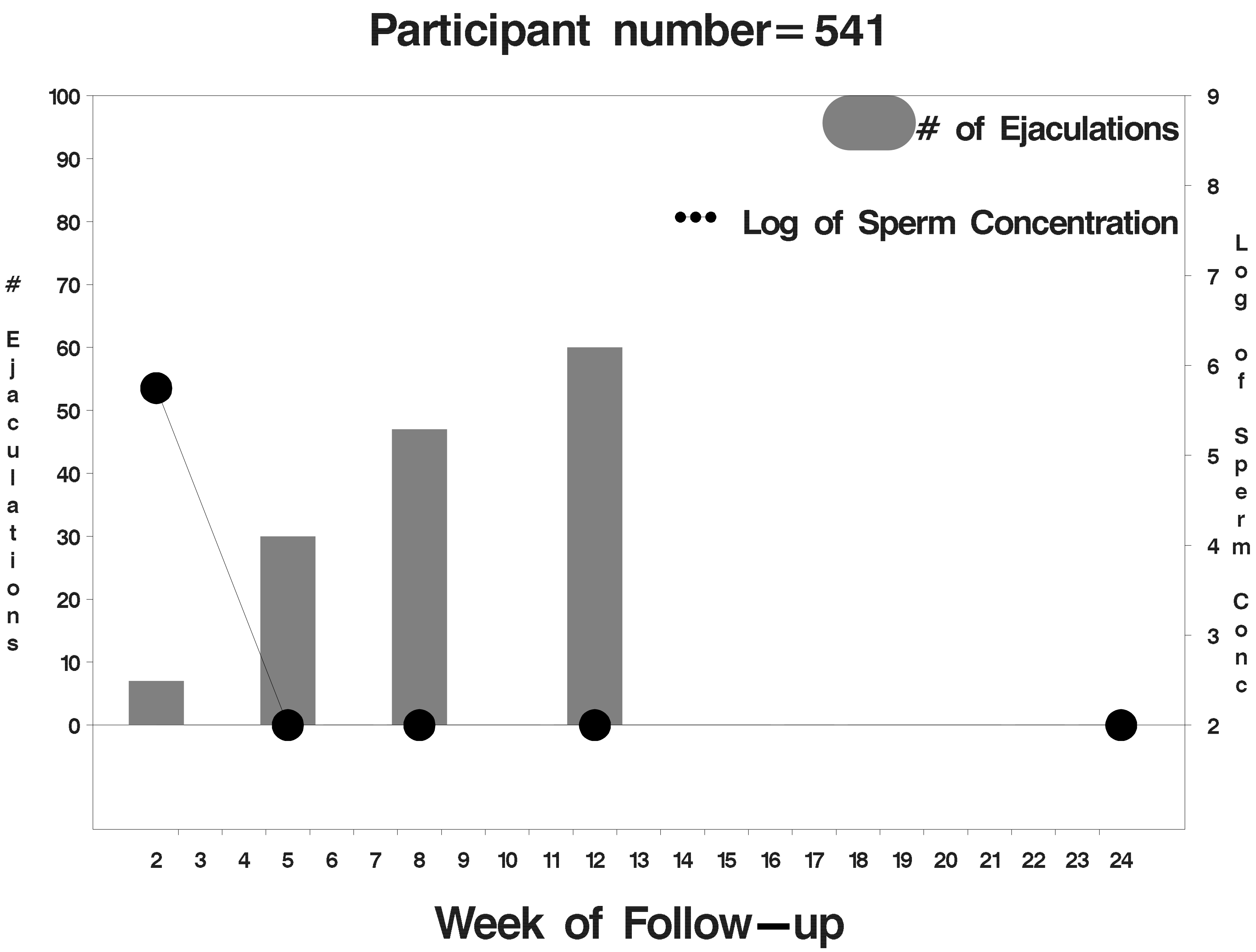
Participant number=541
# of Ejaculations
Log of Sperm Concentration
Ejaculations
Log of Sperm Conc
Week of Follow-up
100
90
80
70
60
50
40
30
20
10
0
9
8
7
6
5
4
3
2
2 3 4 5 6 7 8 9 10 11 12 13 14 15 16 17 18 19 20 21 22 23 24

Participant number= 545

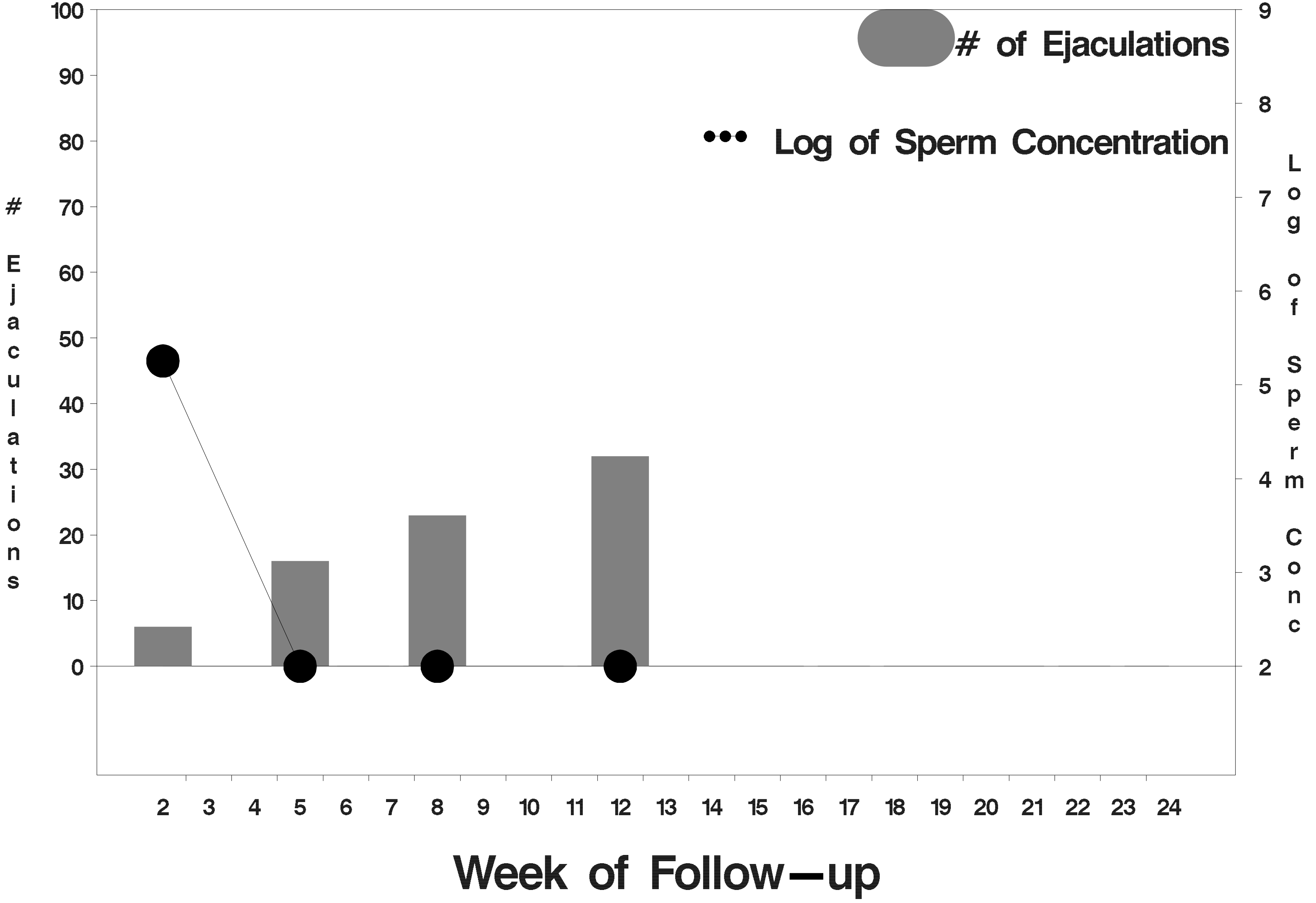

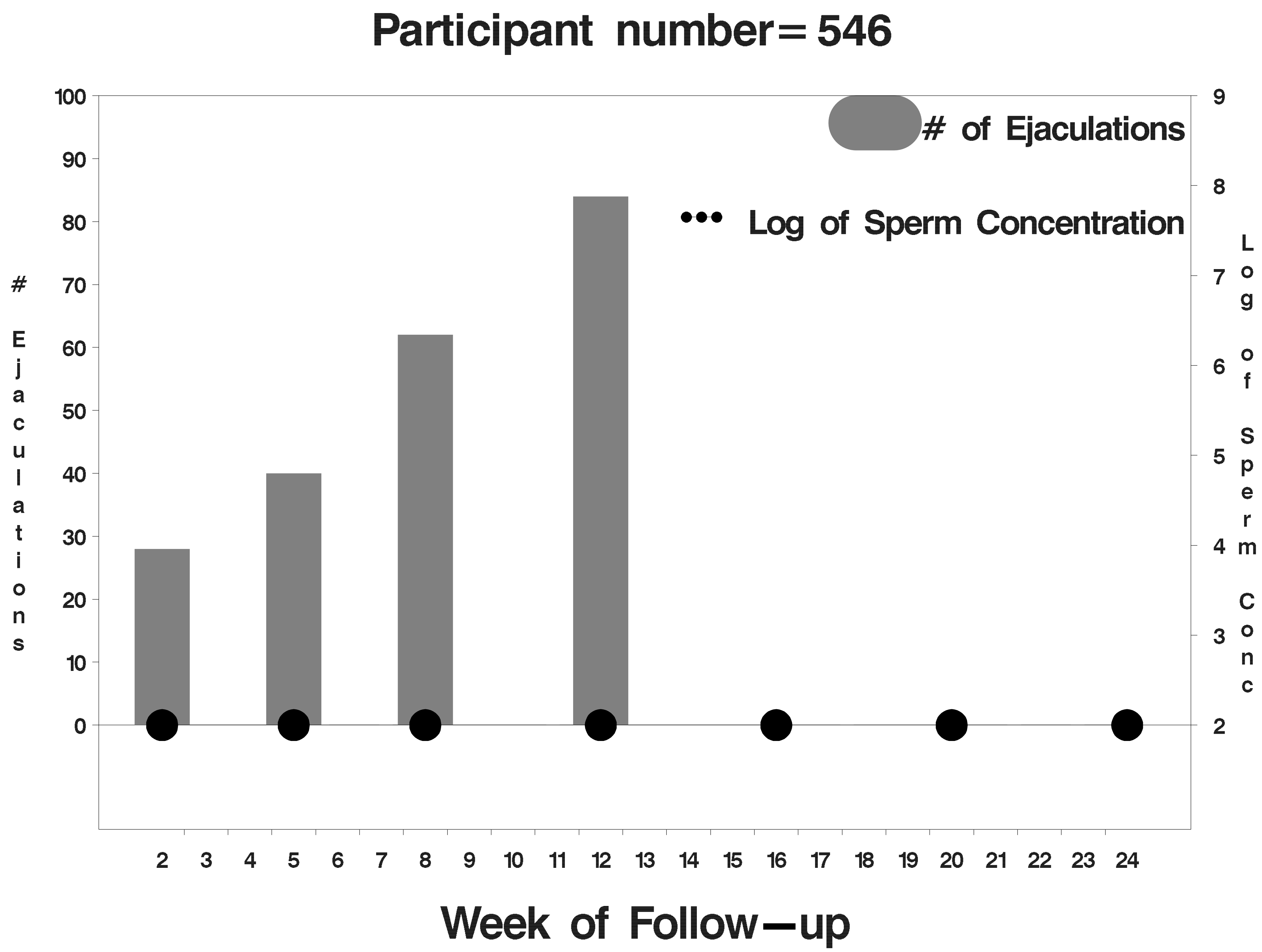

Participant number=546
# of Ejaculations
Log of Sperm Concentration
Ejaculations
Log of Sperm Conc
100
90
80
70
60
50
40
30
20
10
0
9
8
7
6
5
4
3
2
2 3 4 5 6 7 8 9 10 11 12 13 14 15 16 17 18 19 20 21 22 23 24
Week of Follow-up

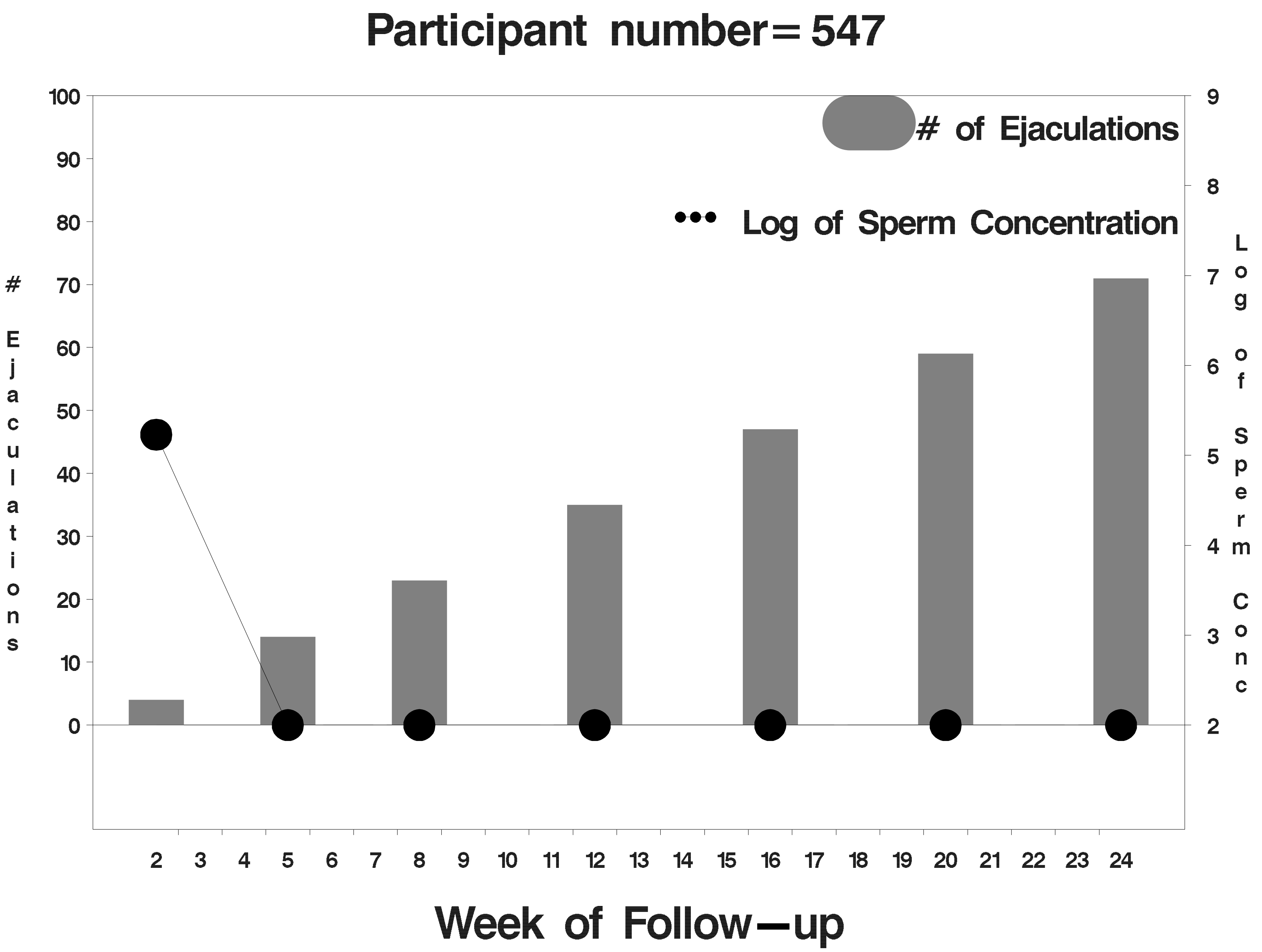

Participant number= 547
# of Ejaculations
Log of Sperm Concentration
Ejaculations
Log of Sperm Conc
100
90
80
70
60
50
40
30
20
10
0
9
8
7
6
5
4
3
2
2 3 4 5 6 7 8 9 10 11 12 13 14 15 16 17 18 19 20 21 22 23 24
Week of Follow-up

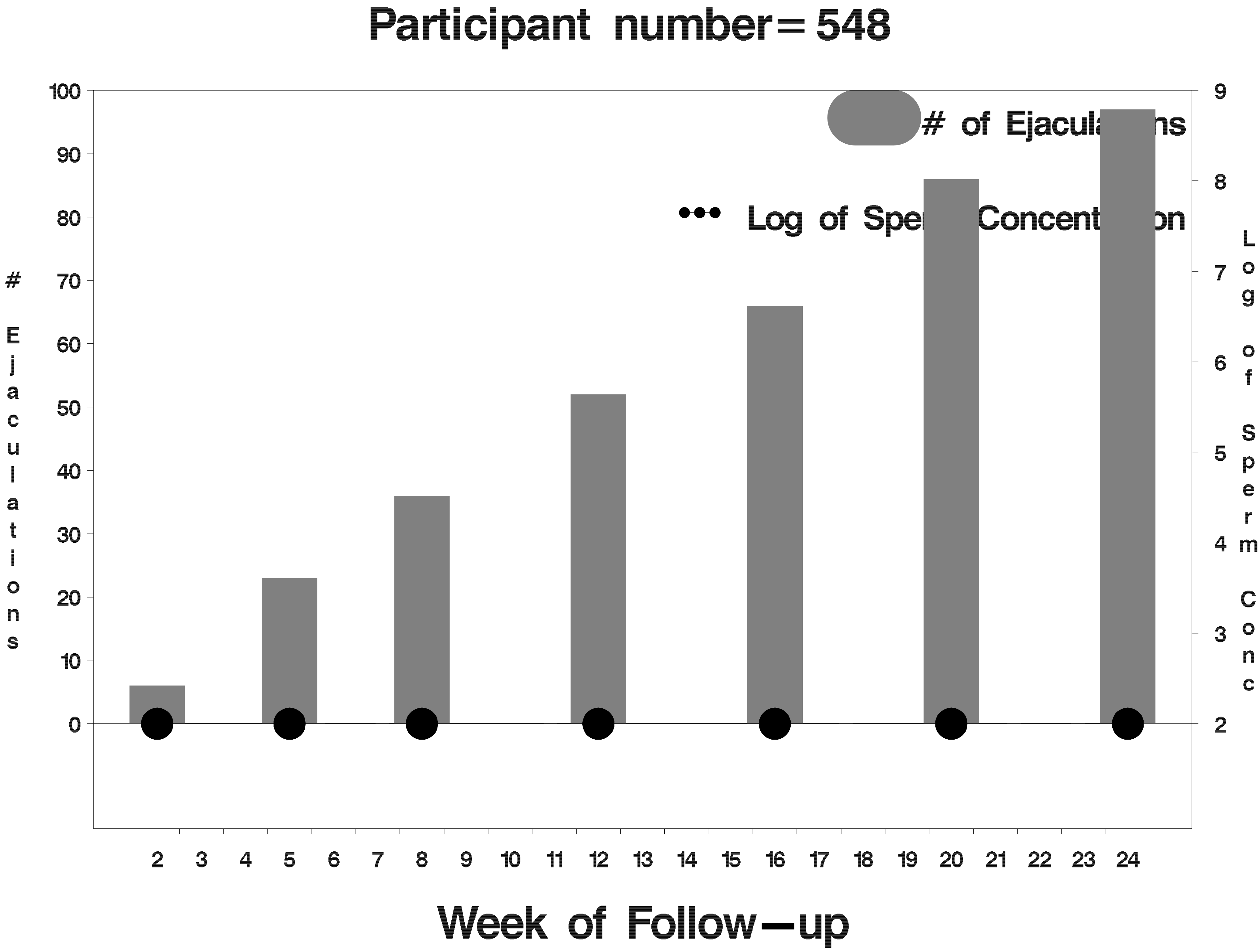

Participant number=548
# of Ejaculations
Log of Sperm Concentration
Ejaculations
Log of Sperm Conc
100
90
80
70
60
50
40
30
20
10
0
9
8
7
6
5
4
3
2
2 3 4 5 6 7 8 9 10 11 12 13 14 15 16 17 18 19 20 21 22 23 24
Week of Follow−up

Participant number= 549

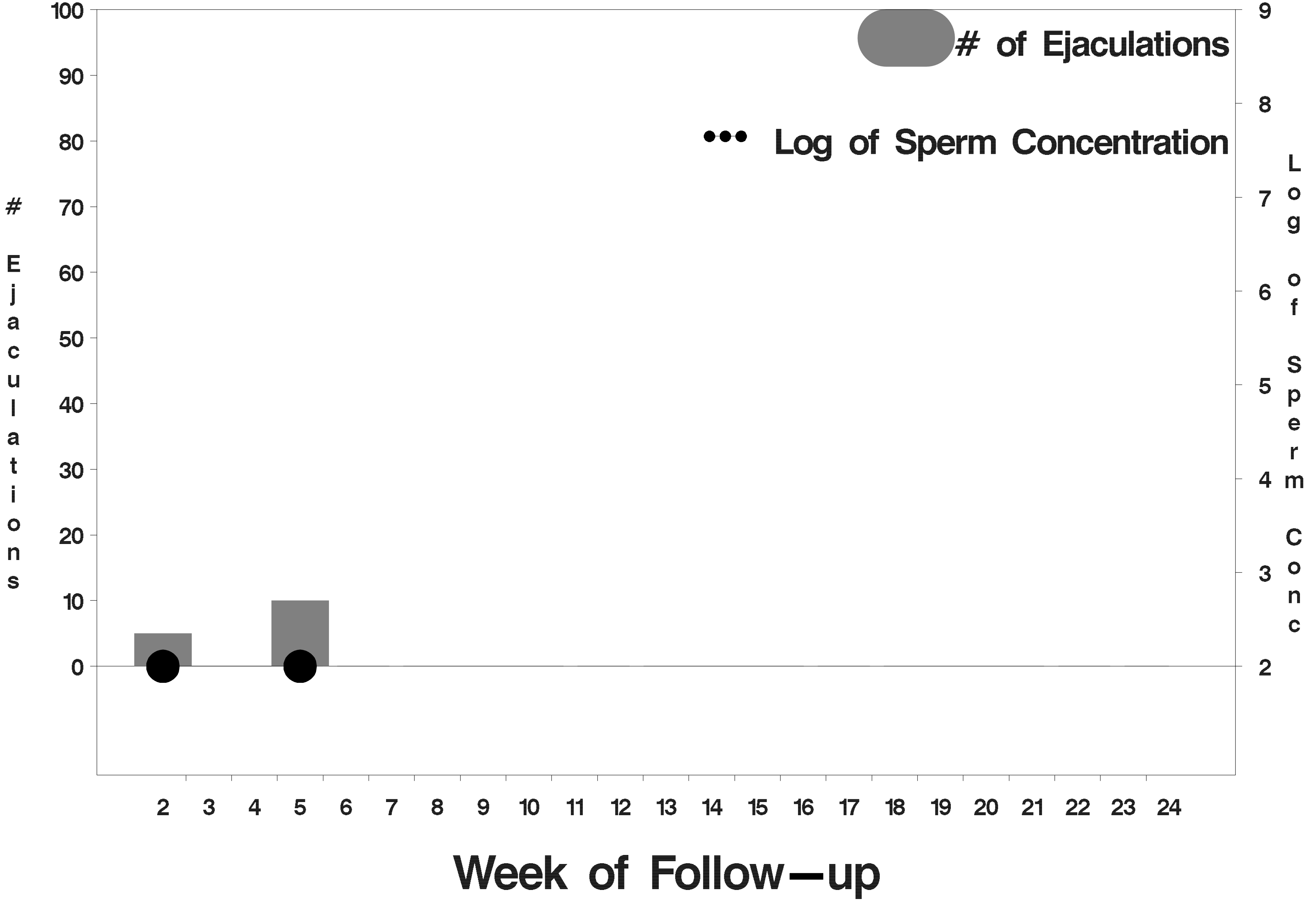

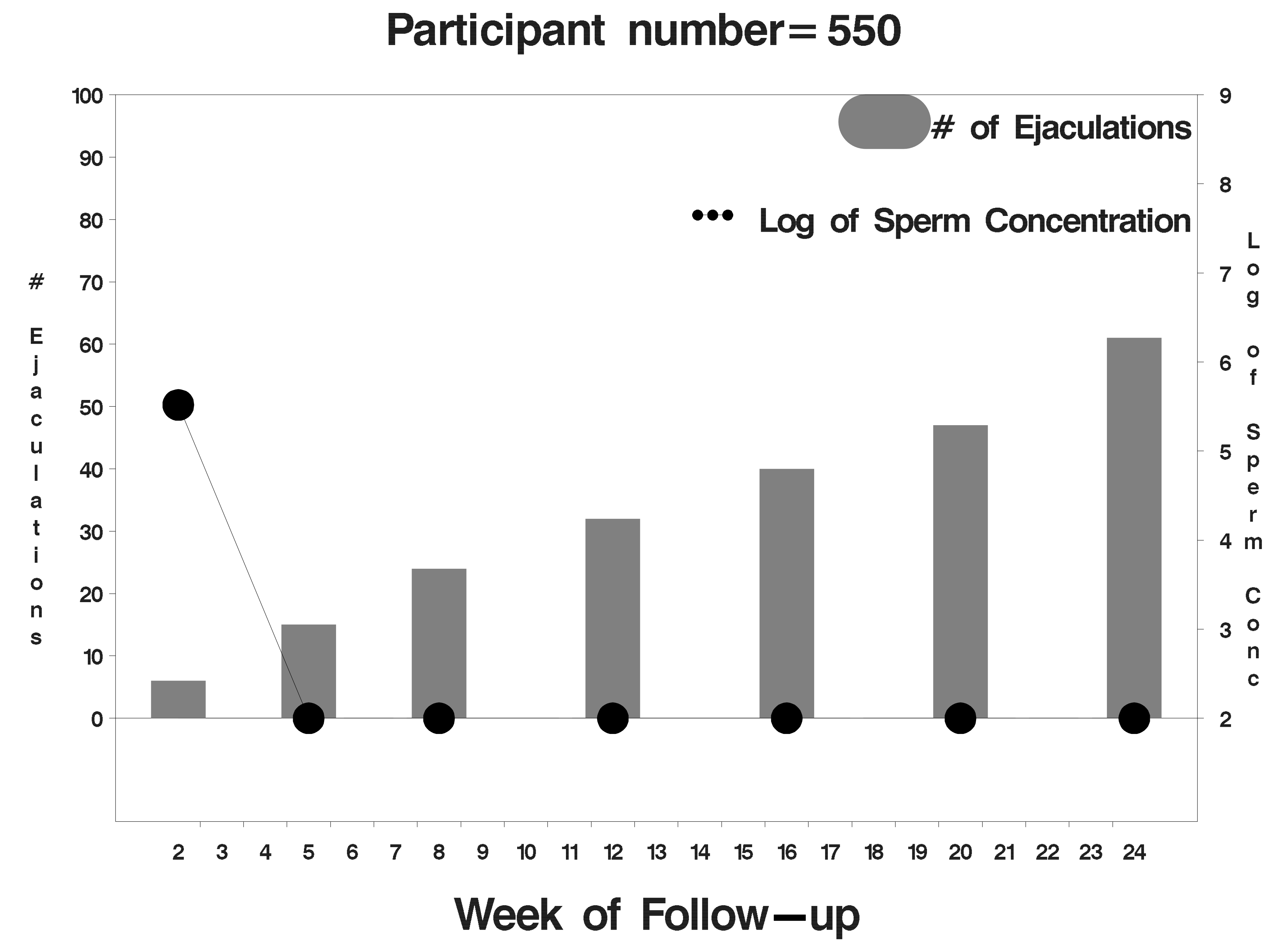
Participant number= 550
# of Ejaculations
Log of Sperm Concentration
Ejaculations
Log of Sperm Conc
100
90
80
70
60
50
40
30
20
10
0
9
8
7
6
5
4
3
2
2 3 4 5 6 7 8 9 10 11 12 13 14 15 16 17 18 19 20 21 22 23 24
Week of Follow-up

Participant number= 551

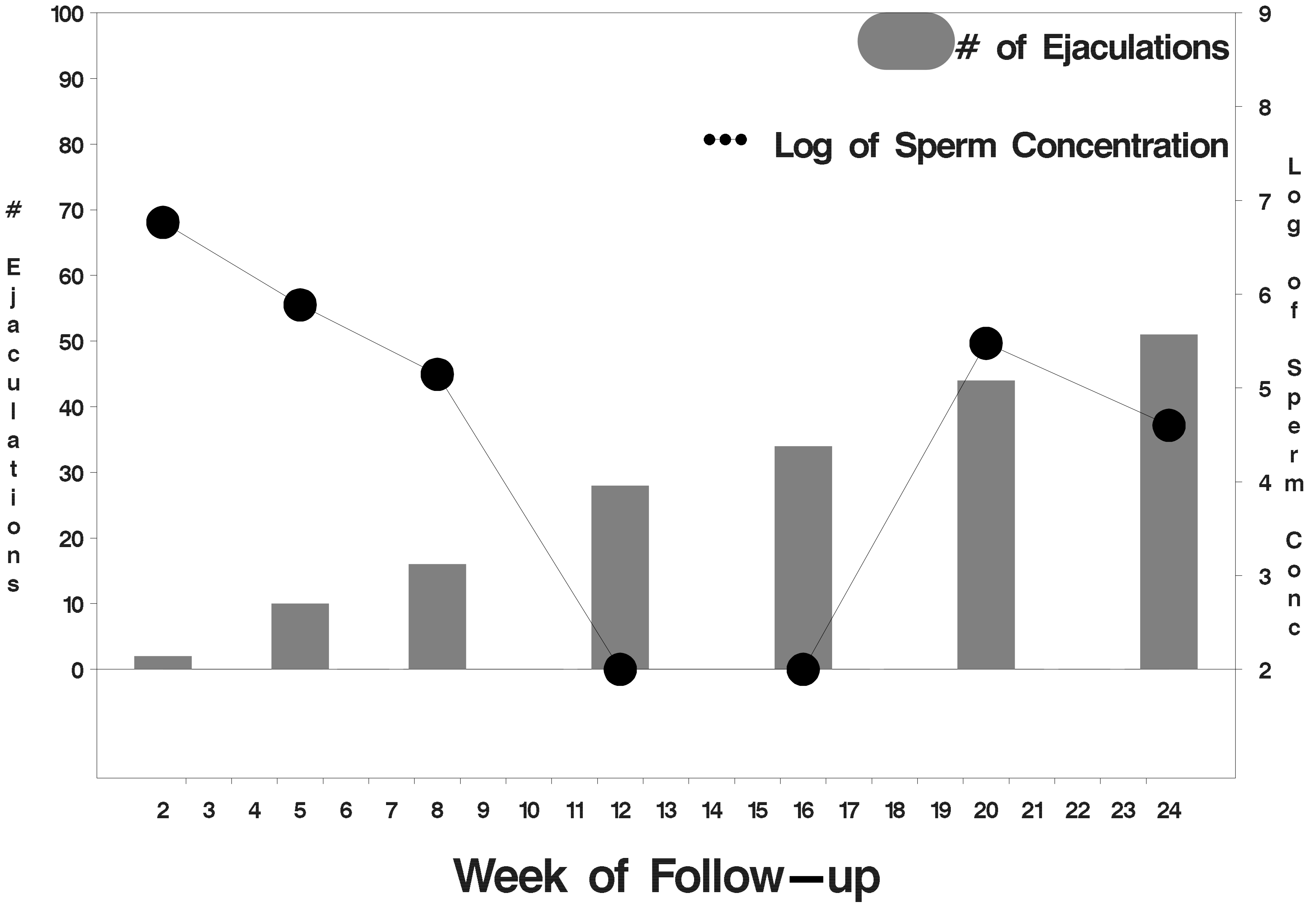

Participant number=552

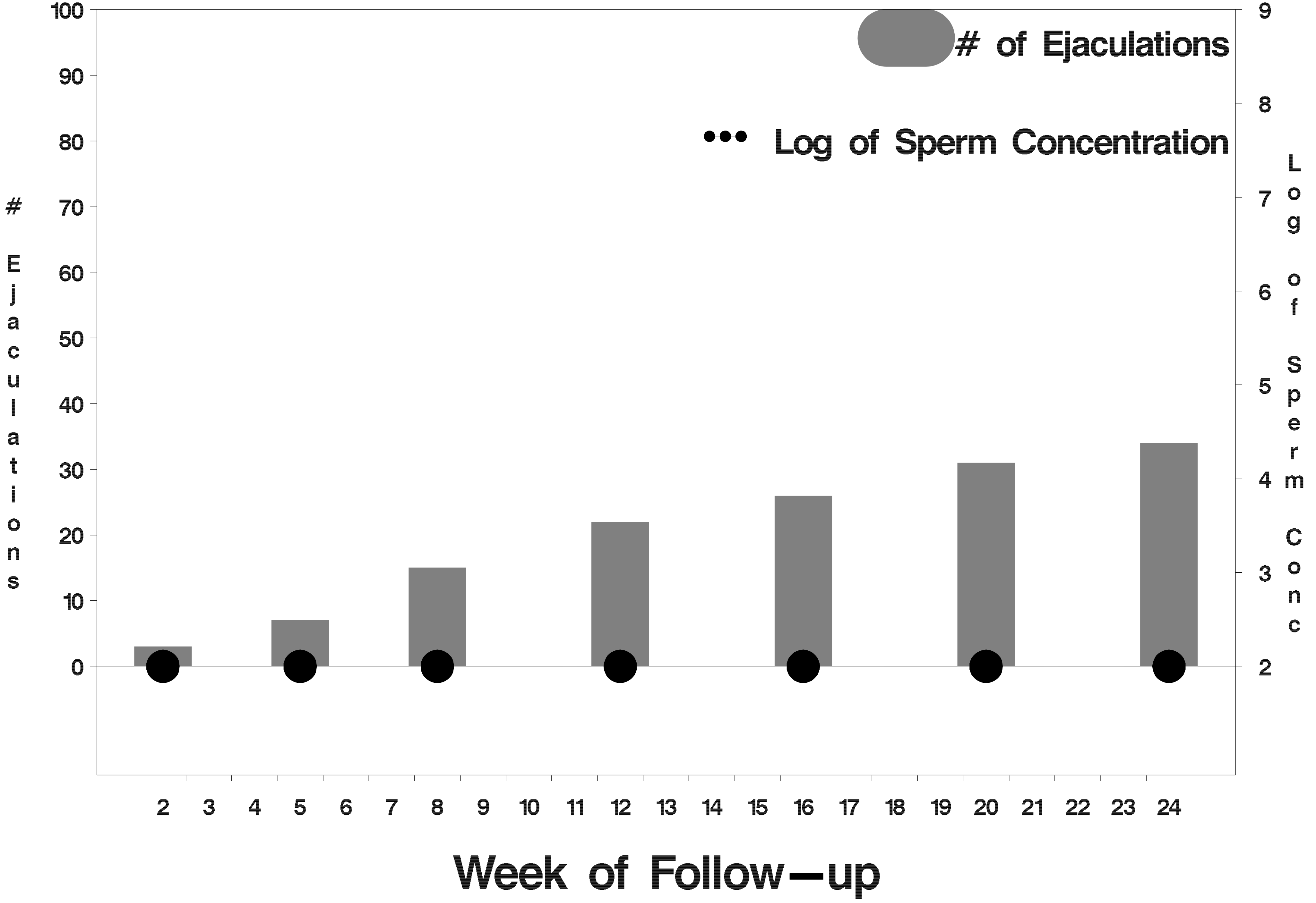

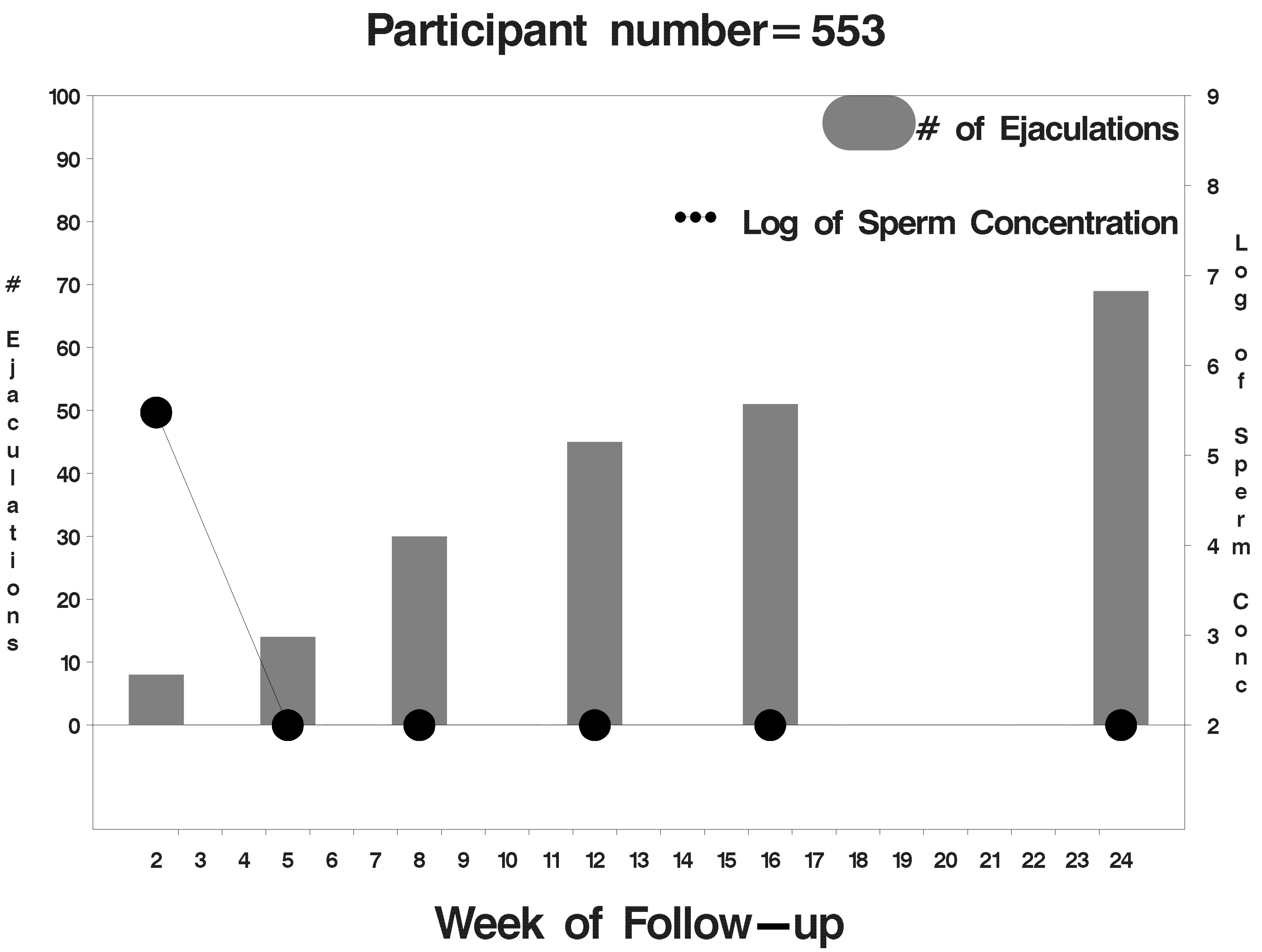

Participant number= 553
# of Ejaculations
Log of Sperm Concentration
Ejaculations
Log of Sperm Conc
100
90
80
70
60
50
40
30
20
10
0
9
8
7
6
5
4
3
2
2 3 4 5 6 7 8 9 10 11 12 13 14 15 16 17 18 19 20 21 22 23 24
Week of Follow−up

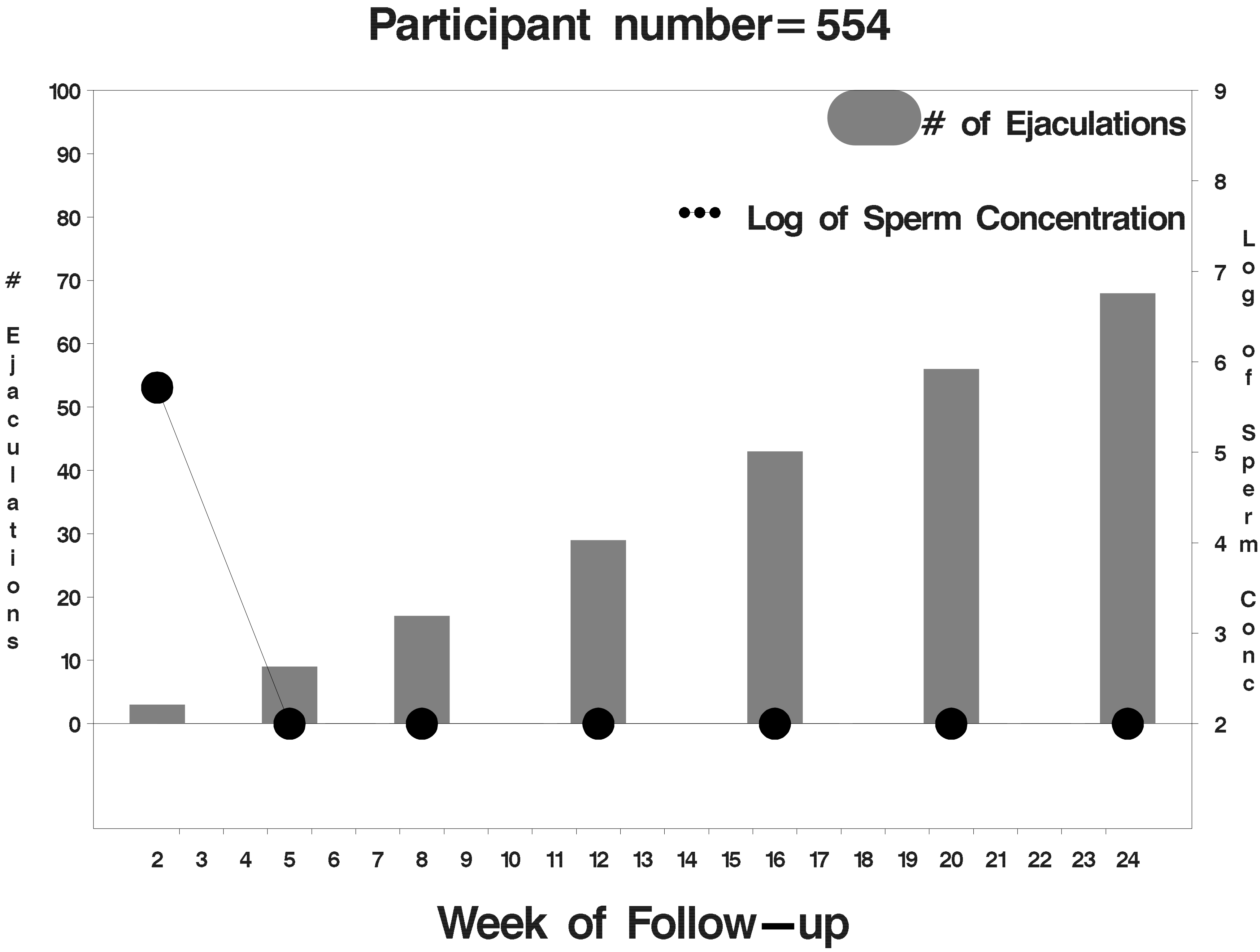

Participant number= 554
# of Ejaculations
Log of Sperm Concentration
Ejaculations
Log of Sperm Conc
0
10
20
30
40
50
60
70
80
90
100
2
3
4
5
6
7
8
9
2 3 4 5 6 7 8 9 10 11 12 13 14 15 16 17 18 19 20 21 22 23 24
Week of Follow-up

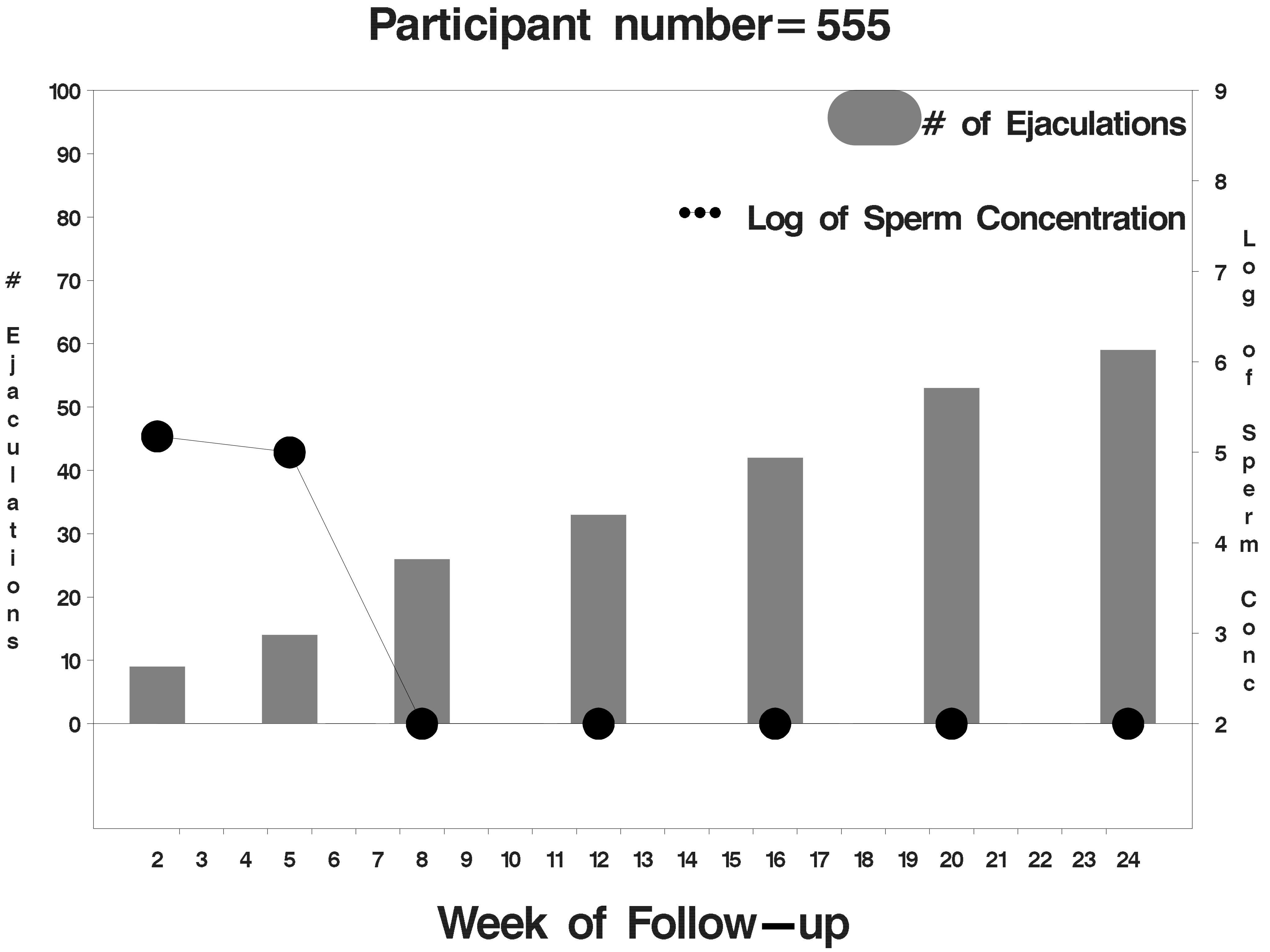

Participant number= 555
# of Ejaculations
Log of Sperm Concentration
Ejaculations
Log of Sperm Conc
100
90
80
70
60
50
40
30
20
10
0
9
8
7
6
5
4
3
2
2 3 4 5 6 7 8 9 10 11 12 13 14 15 16 17 18 19 20 21 22 23 24
Week of Follow-up

Participant number= 556

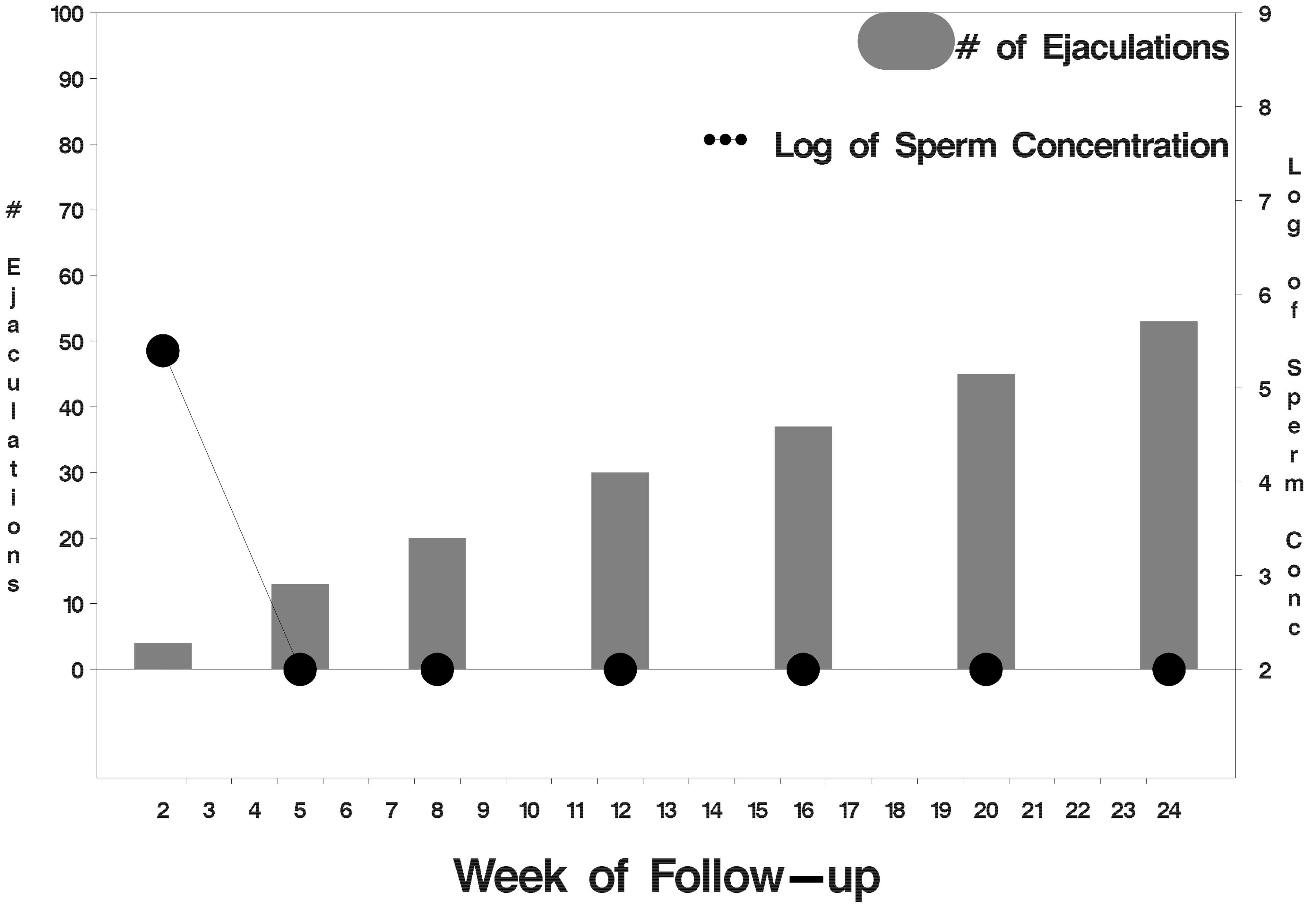

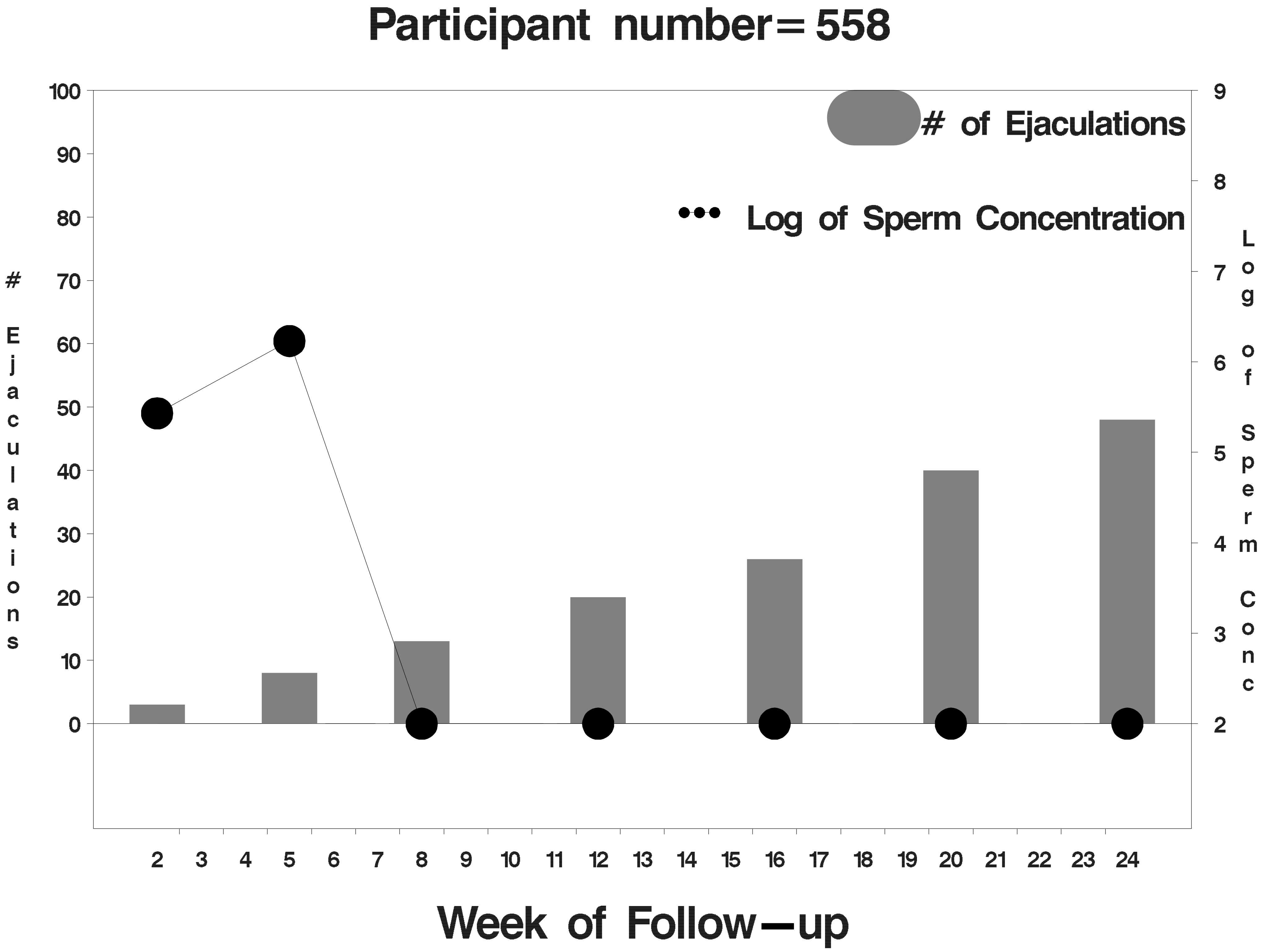
Participant number= 558
# of Ejaculations
Log of Sperm Concentration
Ejaculations
Log of Sperm Conc
100
90
80
70
60
50
40
30
20
10
0
9
8
7
6
5
4
3
2
2 3 4 5 6 7 8 9 10 11 12 13 14 15 16 17 18 19 20 21 22 23 24
Week of Follow-up

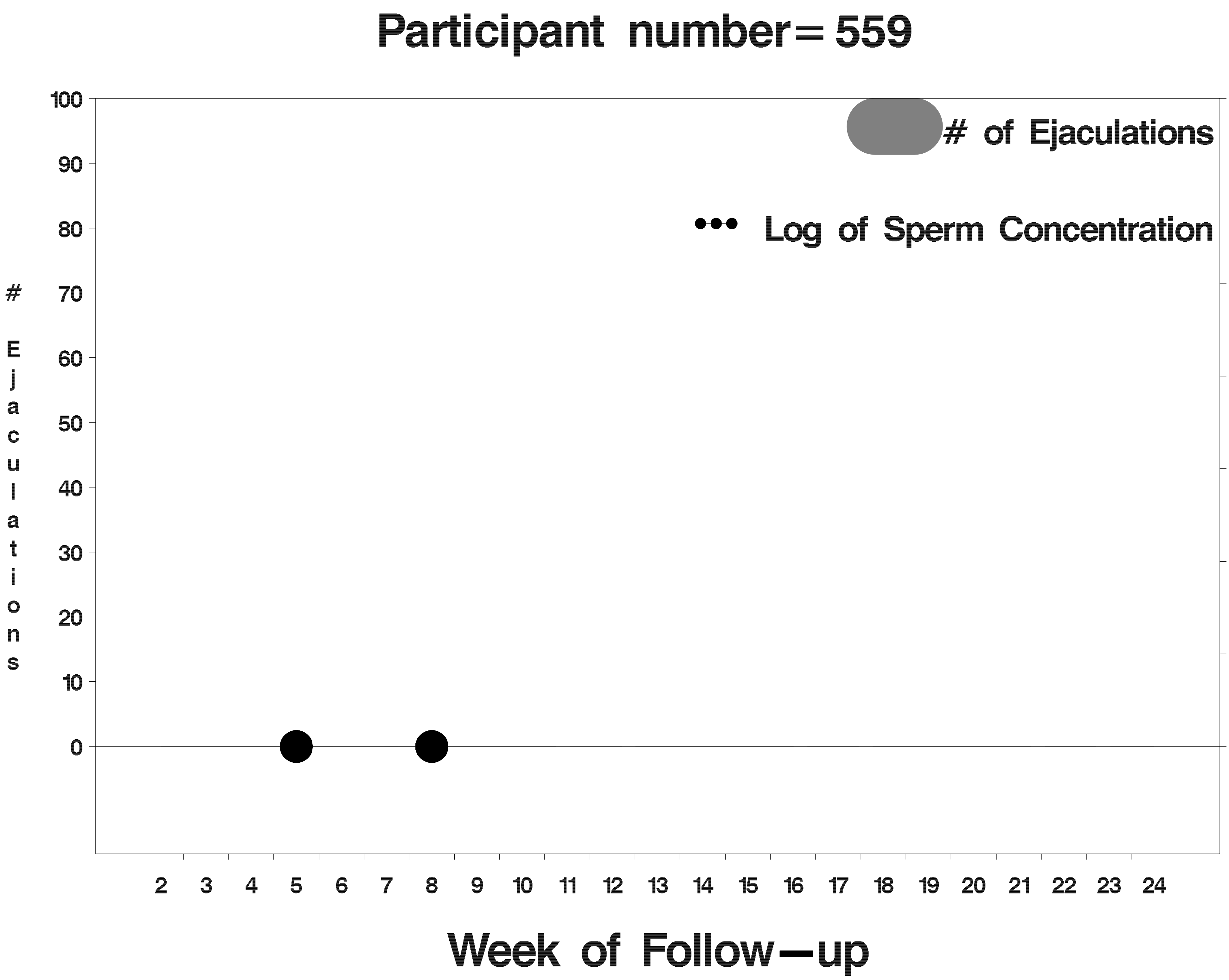
Participant number=559
# of Ejaculations
Log of Sperm Concentration
Ejaculations
Log of Sperm Conc
100
90
80
70
60
50
40
30
20
10
0
9
8
7
6
5
4
3
2
2 3 4 5 6 7 8 9 10 11 12 13 14 15 16 17 18 19 20 21 22 23 24
Week of Follow-up

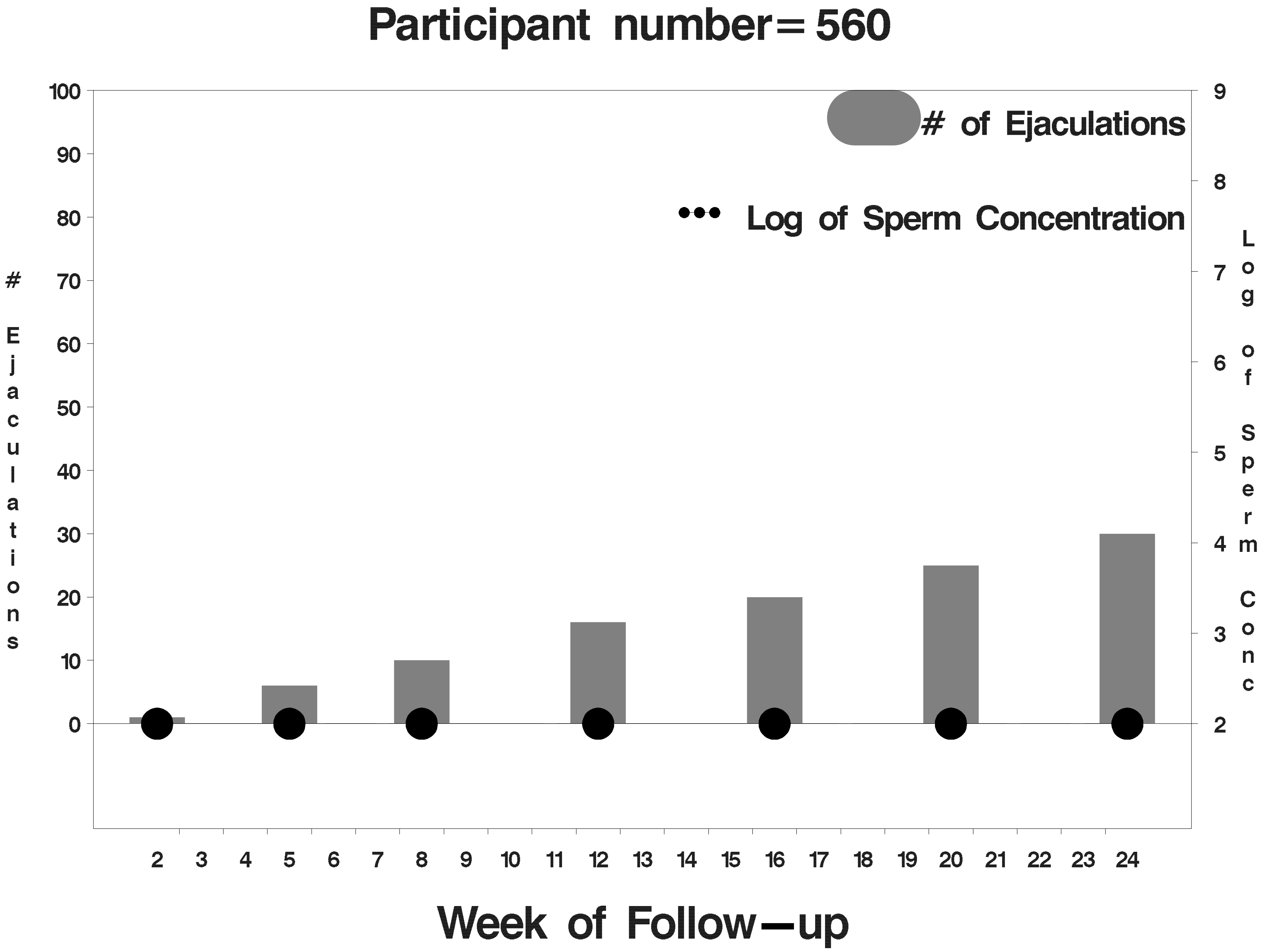

Participant number=560
# of Ejaculations
Log of Sperm Concentration
Ejaculations
Log of Sperm Conc
100
90
80
70
60
50
40
30
20
10
0
9
8
7
6
5
4
3
2
2 3 4 5 6 7 8 9 10 11 12 13 14 15 16 17 18 19 20 21 22 23 24
Week of Follow-up

Participant number=561

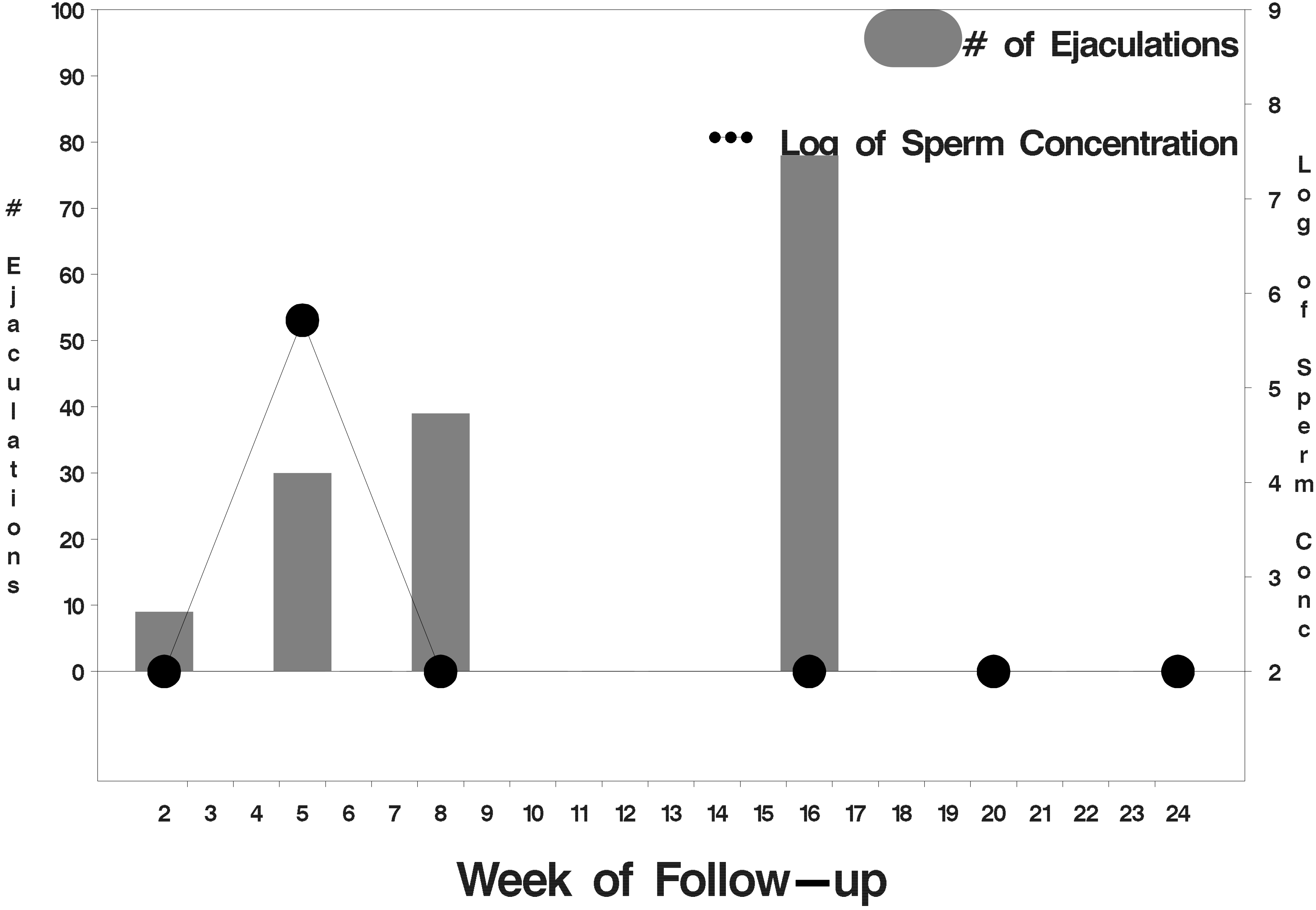

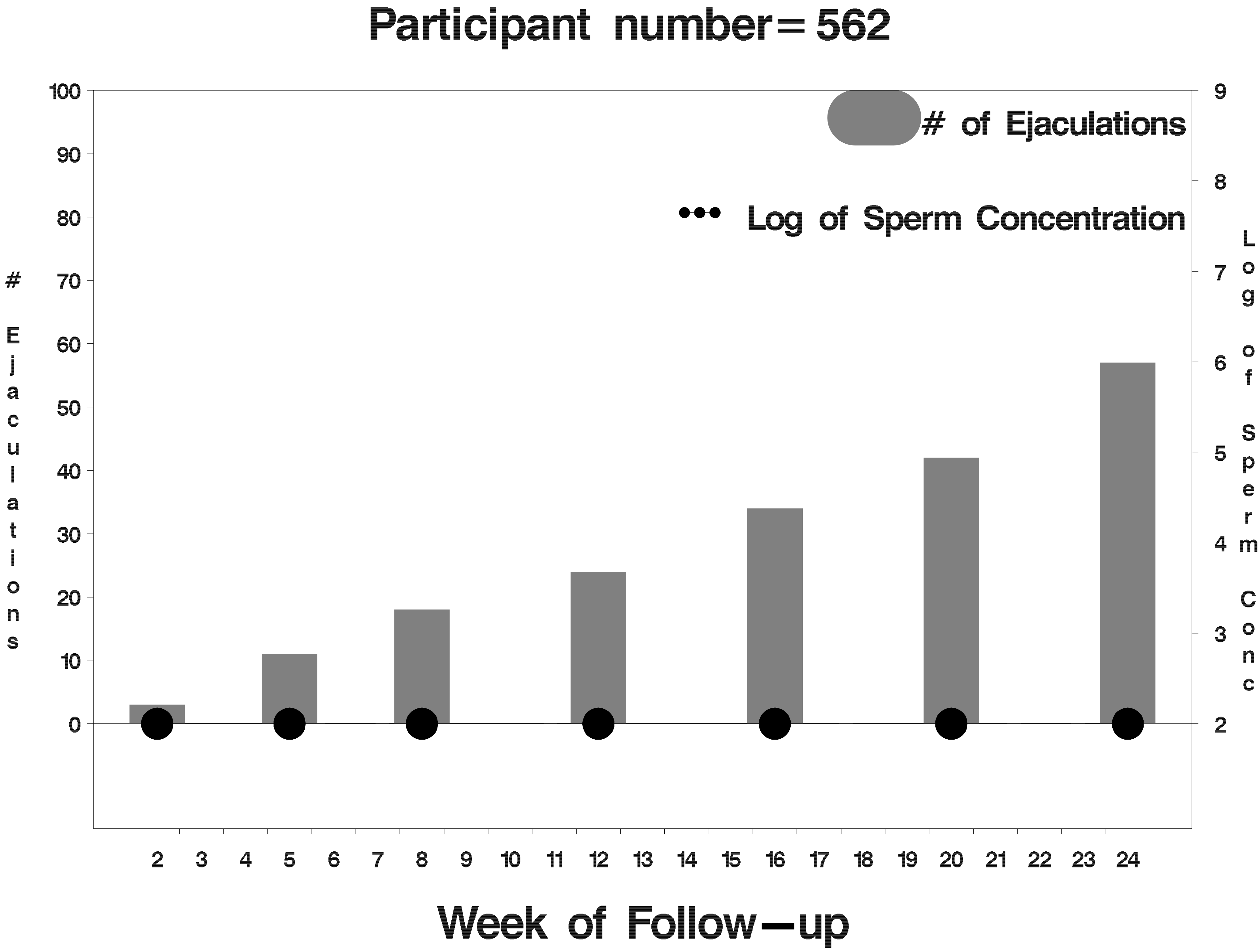

Participant number= 562
# of Ejaculations
Log of Sperm Concentration
Ejaculations
Log of Sperm Conc
100
90
80
70
60
50
40
30
20
10
0
9
8
7
6
5
4
3
2
2 3 4 5 6 7 8 9 10 11 12 13 14 15 16 17 18 19 20 21 22 23 24
Week of Follow—up

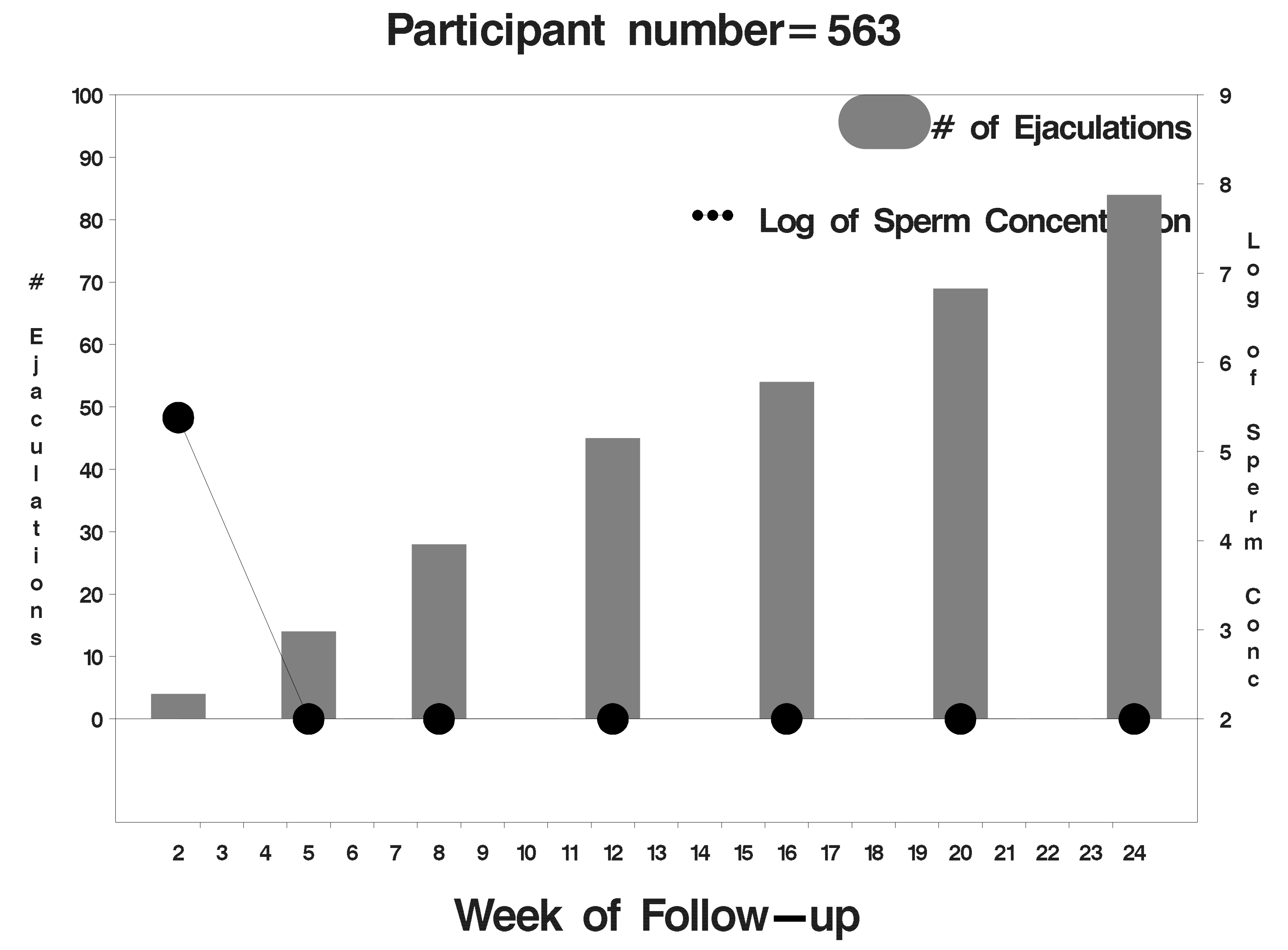

Participant number=563
# of Ejaculations
Log of Sperm Concentration
Ejaculations
Log of Sperm Conc
Week of Follow-up
0
10
20
30
40
50
60
70
80
90
100
2
3
4
5
6
7
8
9
2 3 4 5 6 7 8 9 10 11 12 13 14 15 16 17 18 19 20 21 22 23 24

Participant number=564

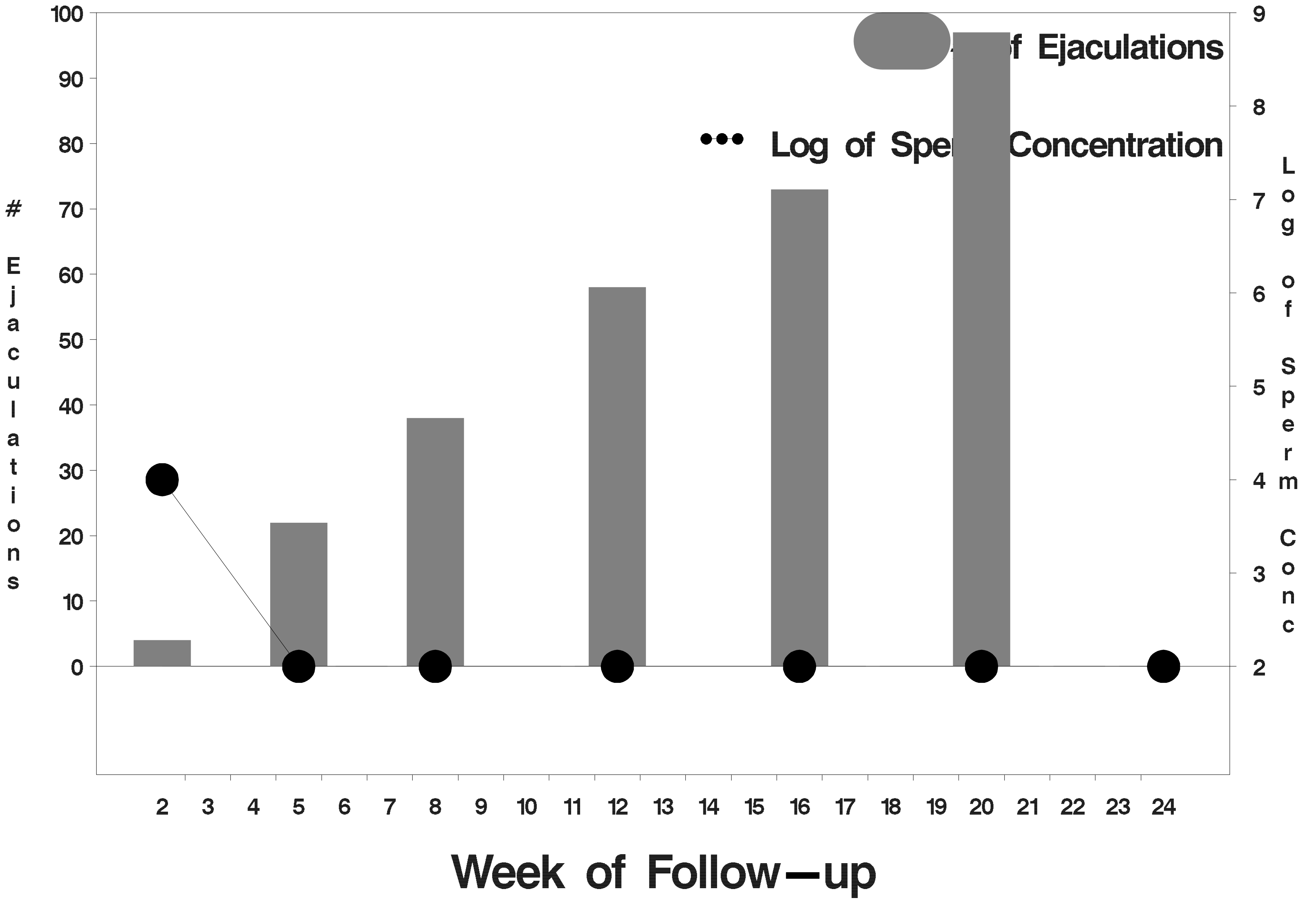

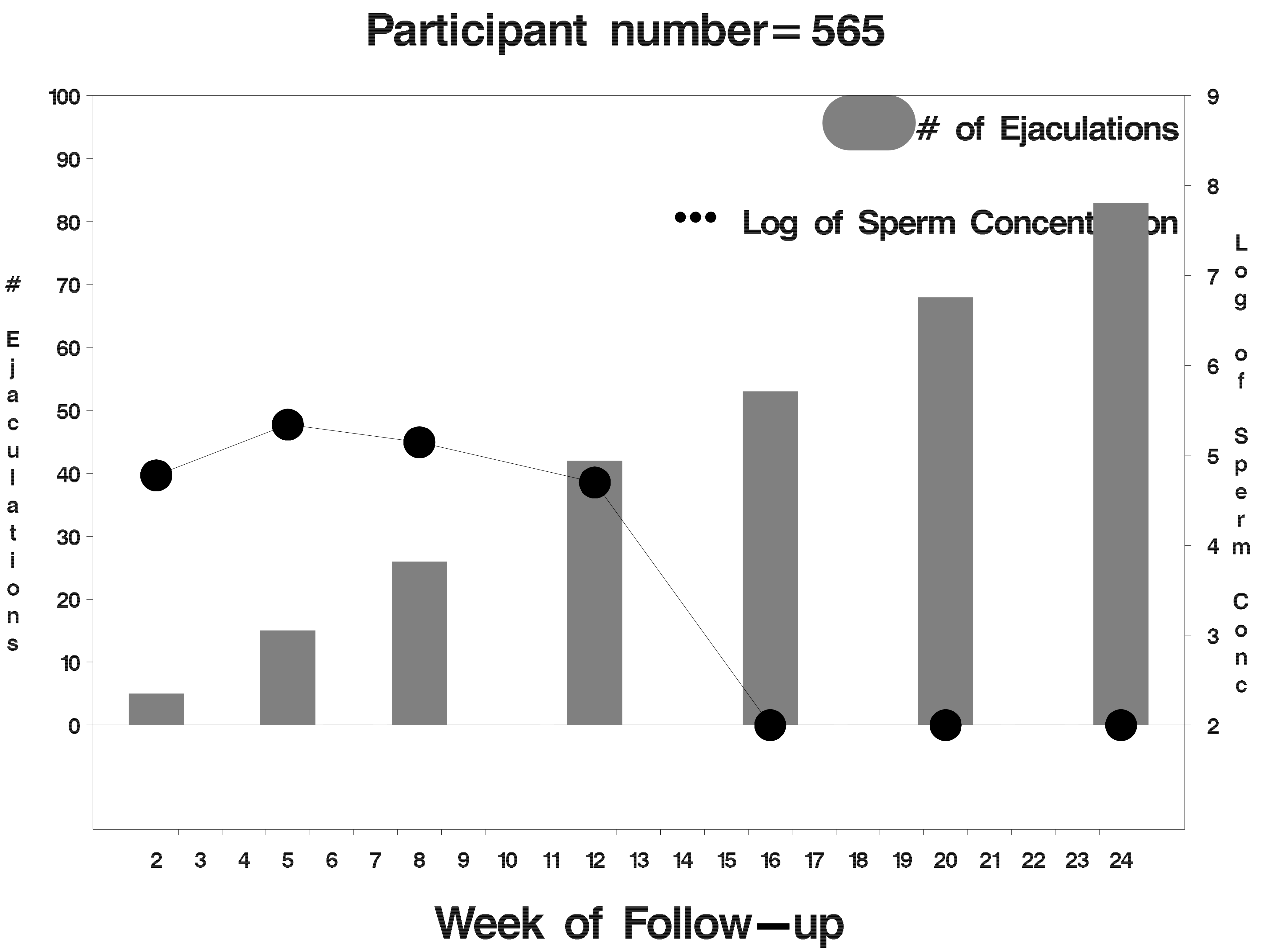

Participant number= 565
# of Ejaculations
Log of Sperm Concentration
Ejaculations
Log of Sperm Conc
100
90
80
70
60
50
40
30
20
10
0
9
8
7
6
5
4
3
2
2 3 4 5 6 7 8 9 10 11 12 13 14 15 16 17 18 19 20 21 22 23 24
Week of Follow-up

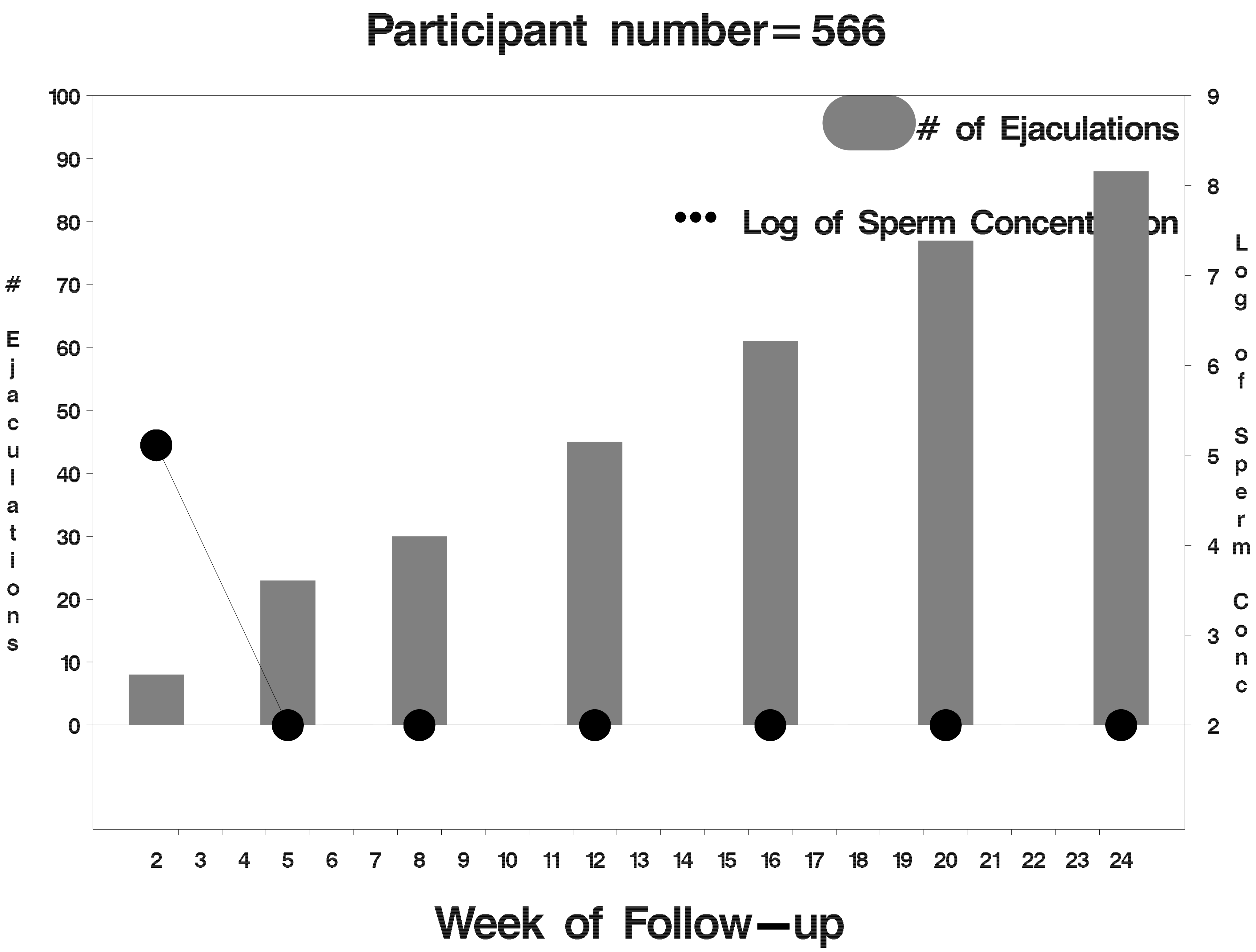

Participant number=566
# of Ejaculations
Log of Sperm Concent[illegible]on
Ejaculations
Log of Sperm Conc
100
90
80
70
60
50
40
30
20
10
0
9
8
7
6
5
4
3
2
2 3 4 5 6 7 8 9 10 11 12 13 14 15 16 17 18 19 20 21 22 23 24
Week of Follow-up

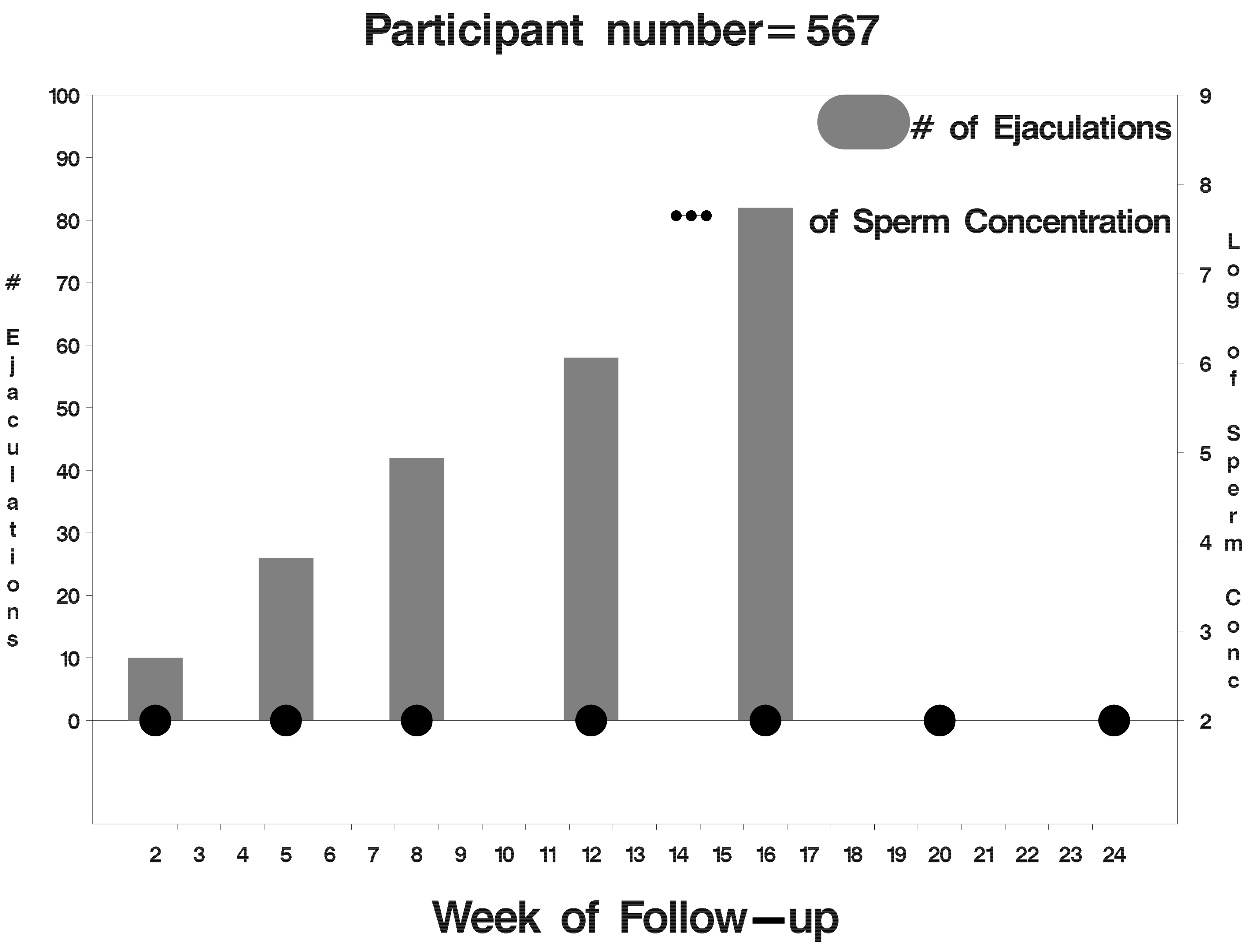

Participant number=567
# of Ejaculations
of Sperm Concentration
Ejaculations
Log of Sperm Conc
100
90
80
70
60
50
40
30
20
10
0
9
8
7
6
5
4
3
2
2 3 4 5 6 7 8 9 10 11 12 13 14 15 16 17 18 19 20 21 22 23 24
Week of Follow-up

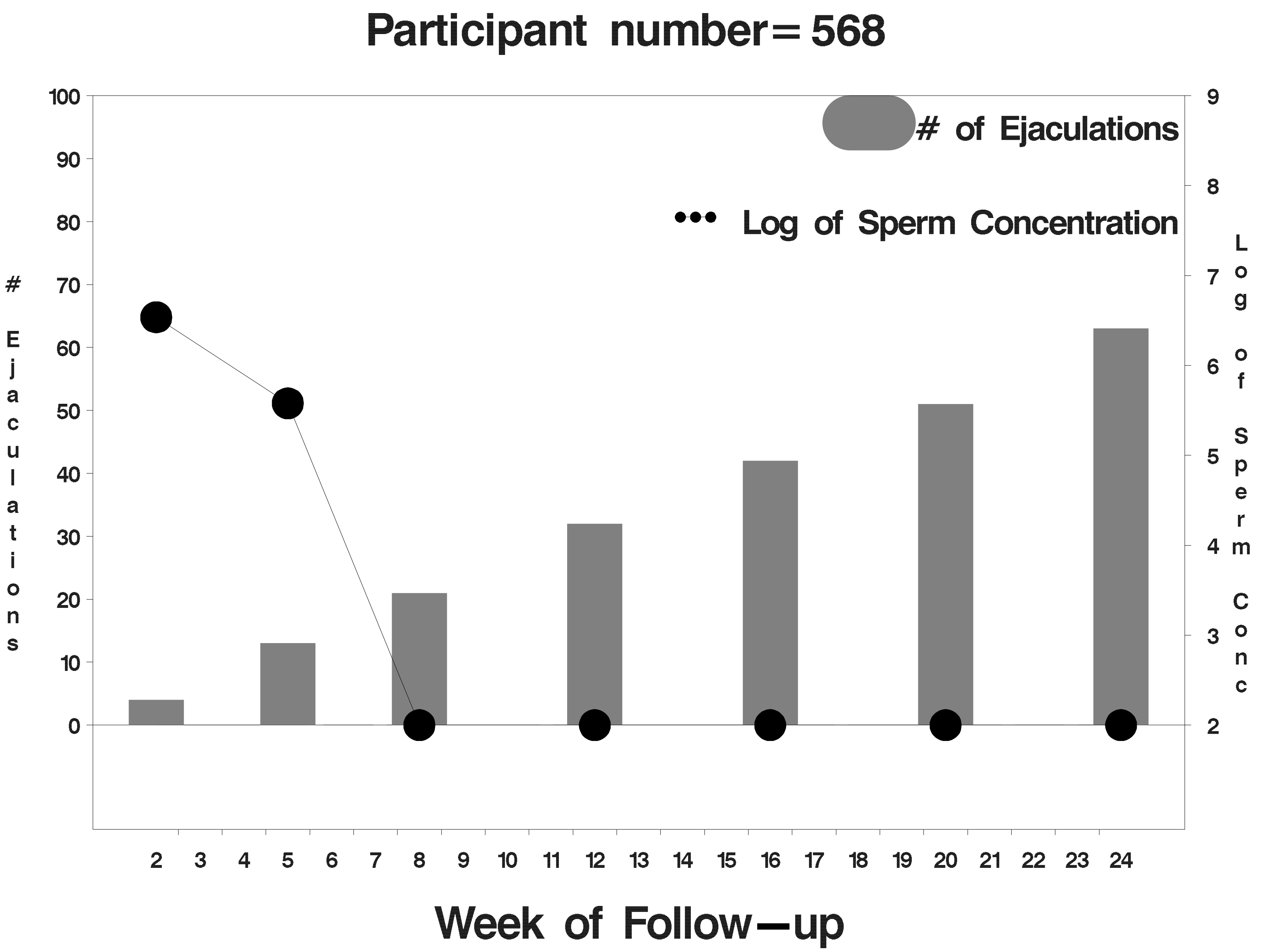

Participant number=568
# of Ejaculations
Log of Sperm Concentration
Ejaculations
Log of Sperm Conc
100
90
80
70
60
50
40
30
20
10
0
9
8
7
6
5
4
3
2
2 3 4 5 6 7 8 9 10 11 12 13 14 15 16 17 18 19 20 21 22 23 24
Week of Follow-up

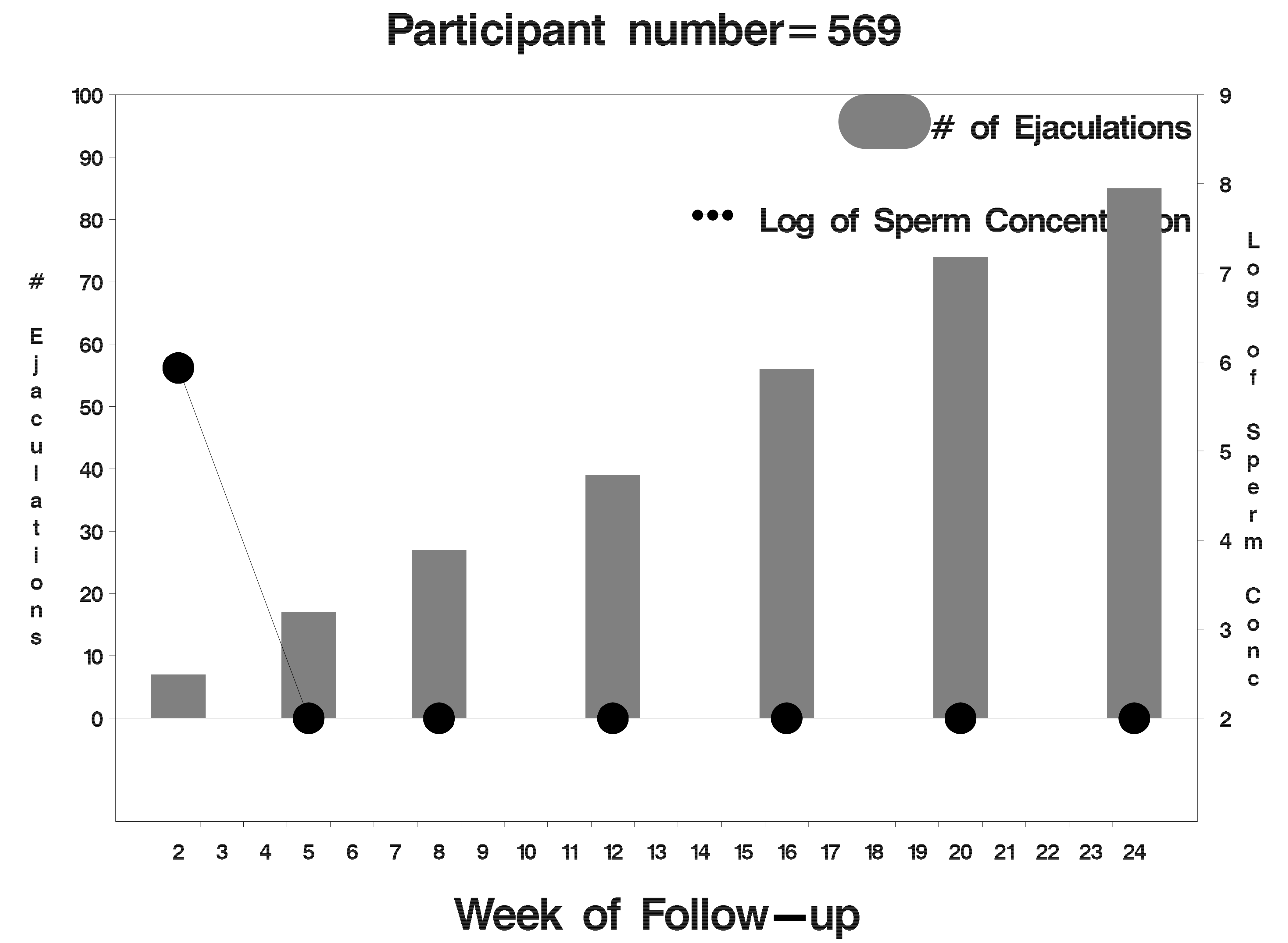

Participant number= 569
# of Ejaculations
Log of Sperm Concentration
Ejaculations
Log of Sperm Conc
100
90
80
70
60
50
40
30
20
10
0
9
8
7
6
5
4
3
2
2 3 4 5 6 7 8 9 10 11 12 13 14 15 16 17 18 19 20 21 22 23 24
Week of Follow-up

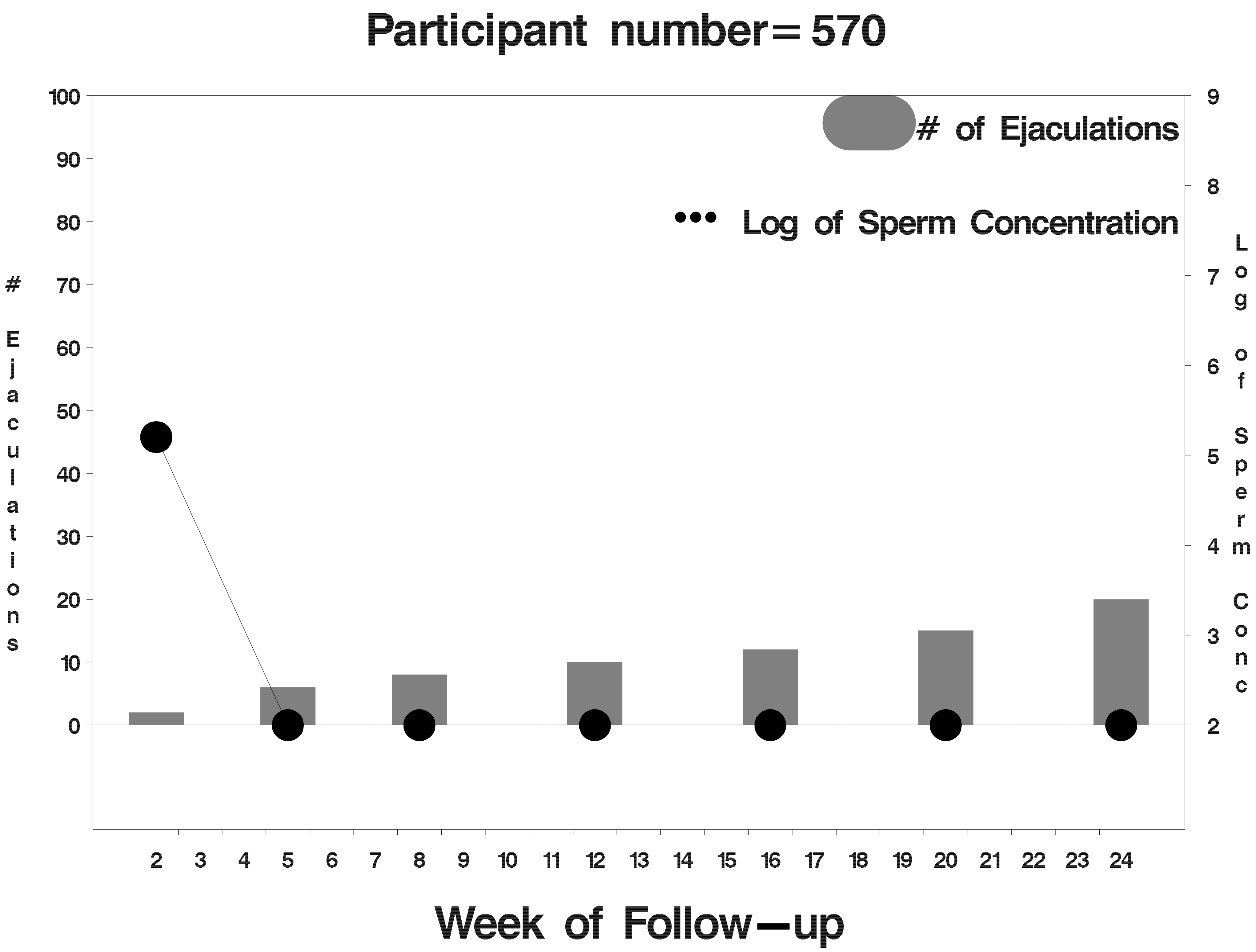

Participant number= 570
# of Ejaculations
Log of Sperm Concentration
Ejaculations
Log of Sperm Conc
100
90
80
70
60
50
40
30
20
10
0
9
8
7
6
5
4
3
2
2 3 4 5 6 7 8 9 10 11 12 13 14 15 16 17 18 19 20 21 22 23 24
Week of Follow—up

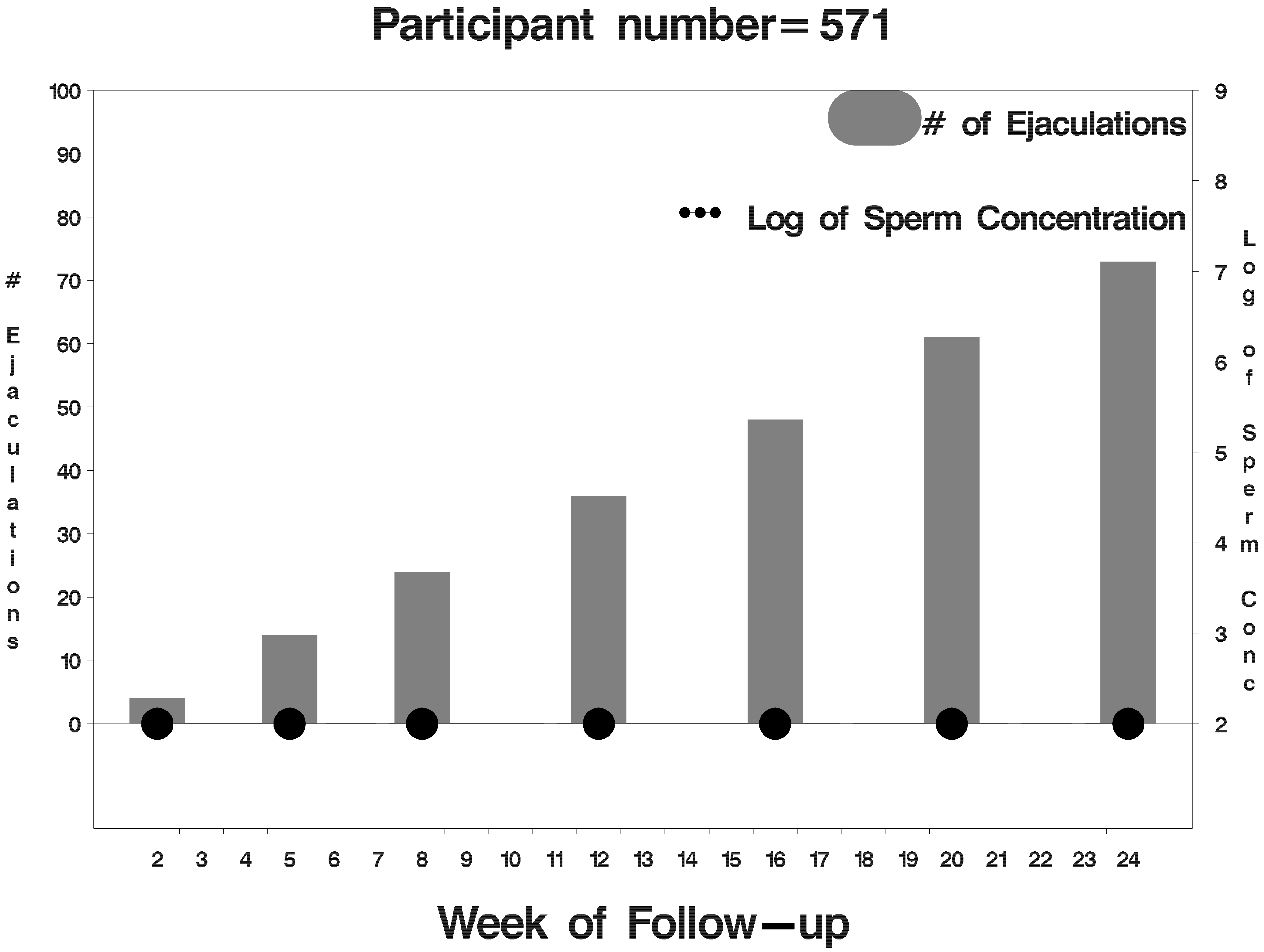

Participant number= 571
# of Ejaculations
Log of Sperm Concentration
Ejaculations
Log of Sperm Conc
100
90
80
70
60
50
40
30
20
10
0
9
8
7
6
5
4
3
2
2 3 4 5 6 7 8 9 10 11 12 13 14 15 16 17 18 19 20 21 22 23 24
Week of Follow-up

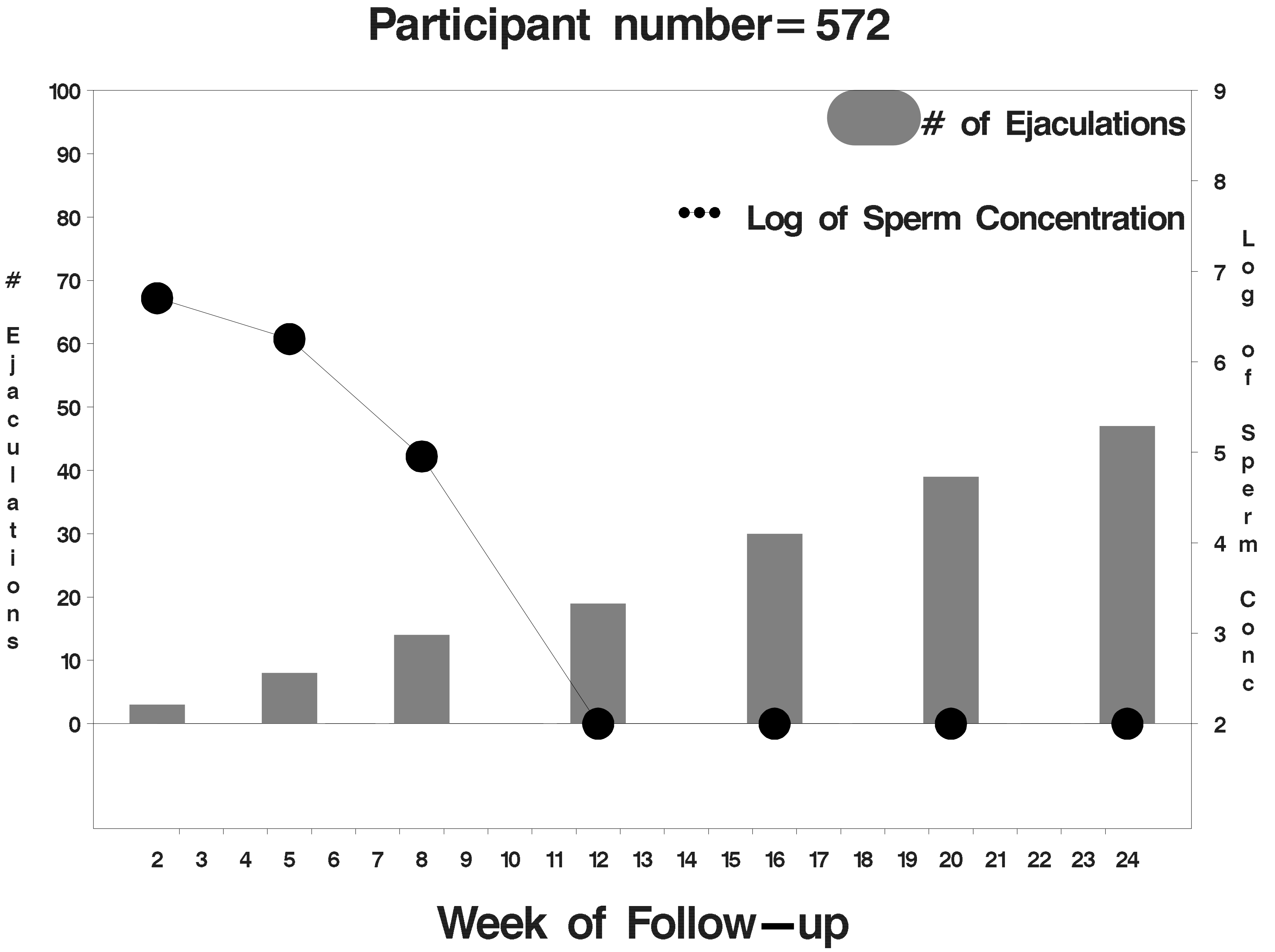

Participant number=572
# of Ejaculations
Log of Sperm Concentration
Ejaculations
Log of Sperm Conc
100
90
80
70
60
50
40
30
20
10
0
9
8
7
6
5
4
3
2
2 3 4 5 6 7 8 9 10 11 12 13 14 15 16 17 18 19 20 21 22 23 24
Week of Follow-up

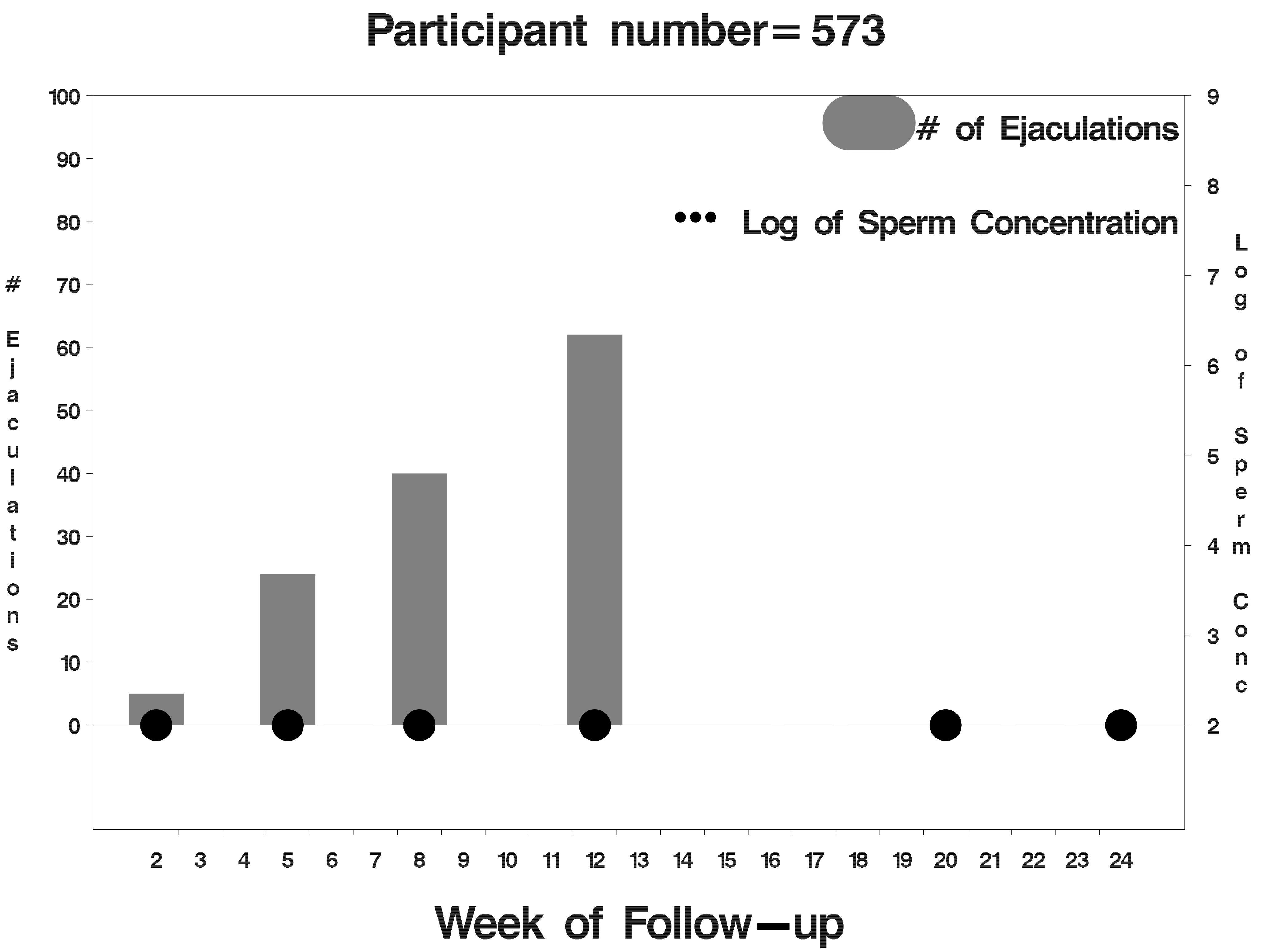

Participant number=573
# of Ejaculations
Log of Sperm Concentration
Ejaculations
Log of Sperm Conc
100
90
80
70
60
50
40
30
20
10
0
9
8
7
6
5
4
3
2
2 3 4 5 6 7 8 9 10 11 12 13 14 15 16 17 18 19 20 21 22 23 24
Week of Follow-up

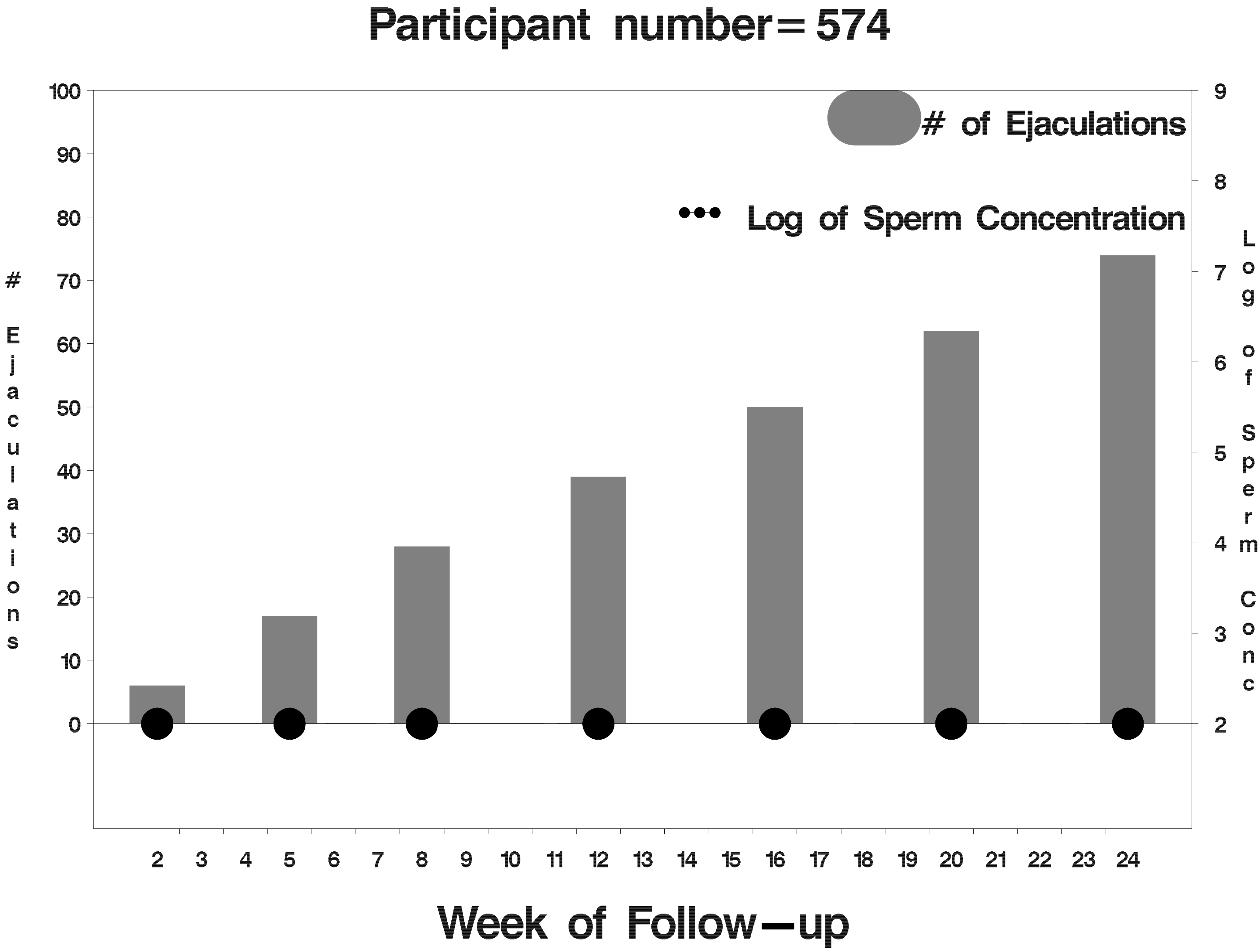

Participant number=574
# of Ejaculations
Log of Sperm Concentration
Ejaculations
Log of Sperm Conc
100
90
80
70
60
50
40
30
20
10
0
9
8
7
6
5
4
3
2
2 3 4 5 6 7 8 9 10 11 12 13 14 15 16 17 18 19 20 21 22 23 24
Week of Follow-up

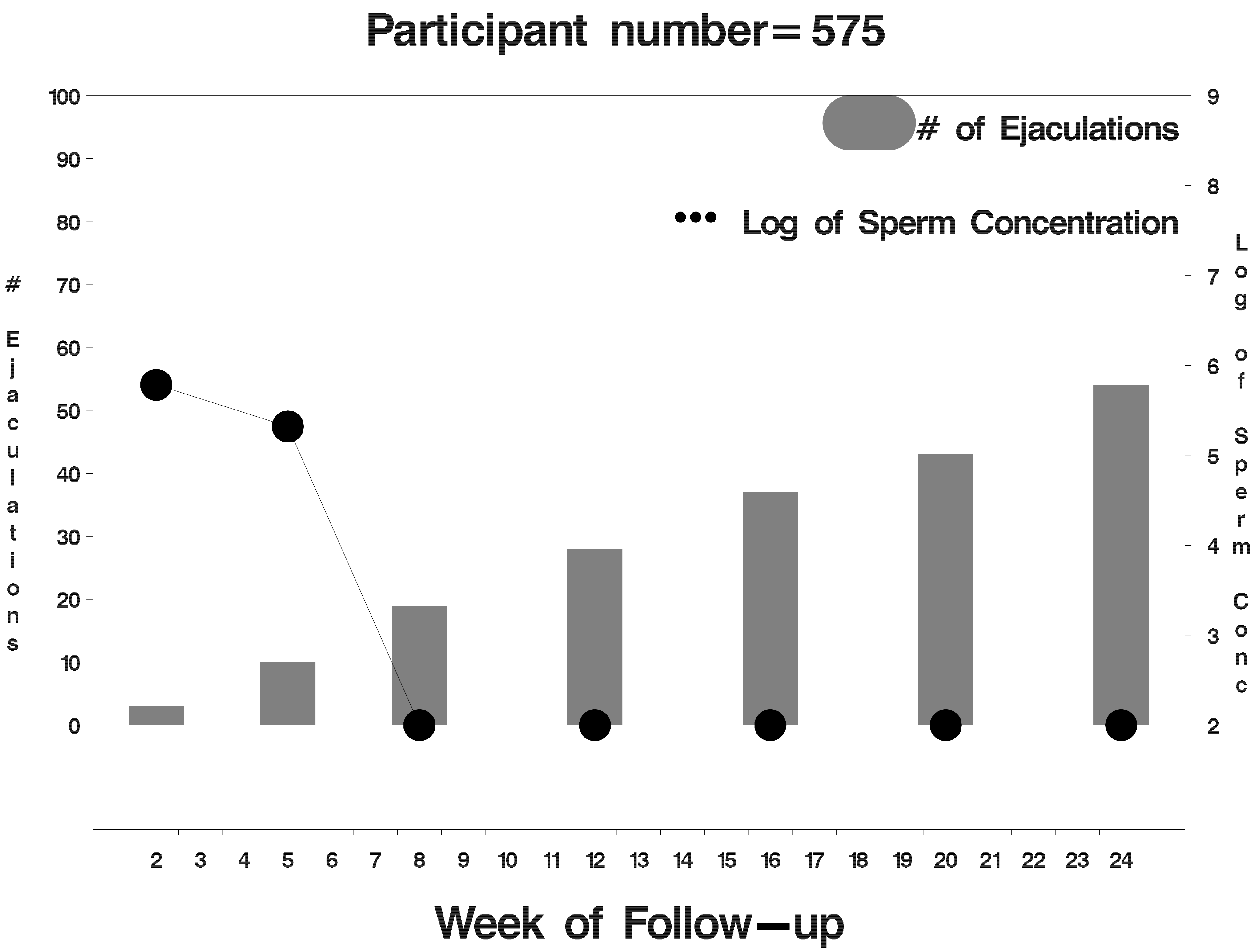

Participant number= 575
# of Ejaculations
Log of Sperm Concentration
Ejaculations
Log of Sperm Conc
100
90
80
70
60
50
40
30
20
10
0
9
8
7
6
5
4
3
2
2 3 4 5 6 7 8 9 10 11 12 13 14 15 16 17 18 19 20 21 22 23 24
Week of Follow-up

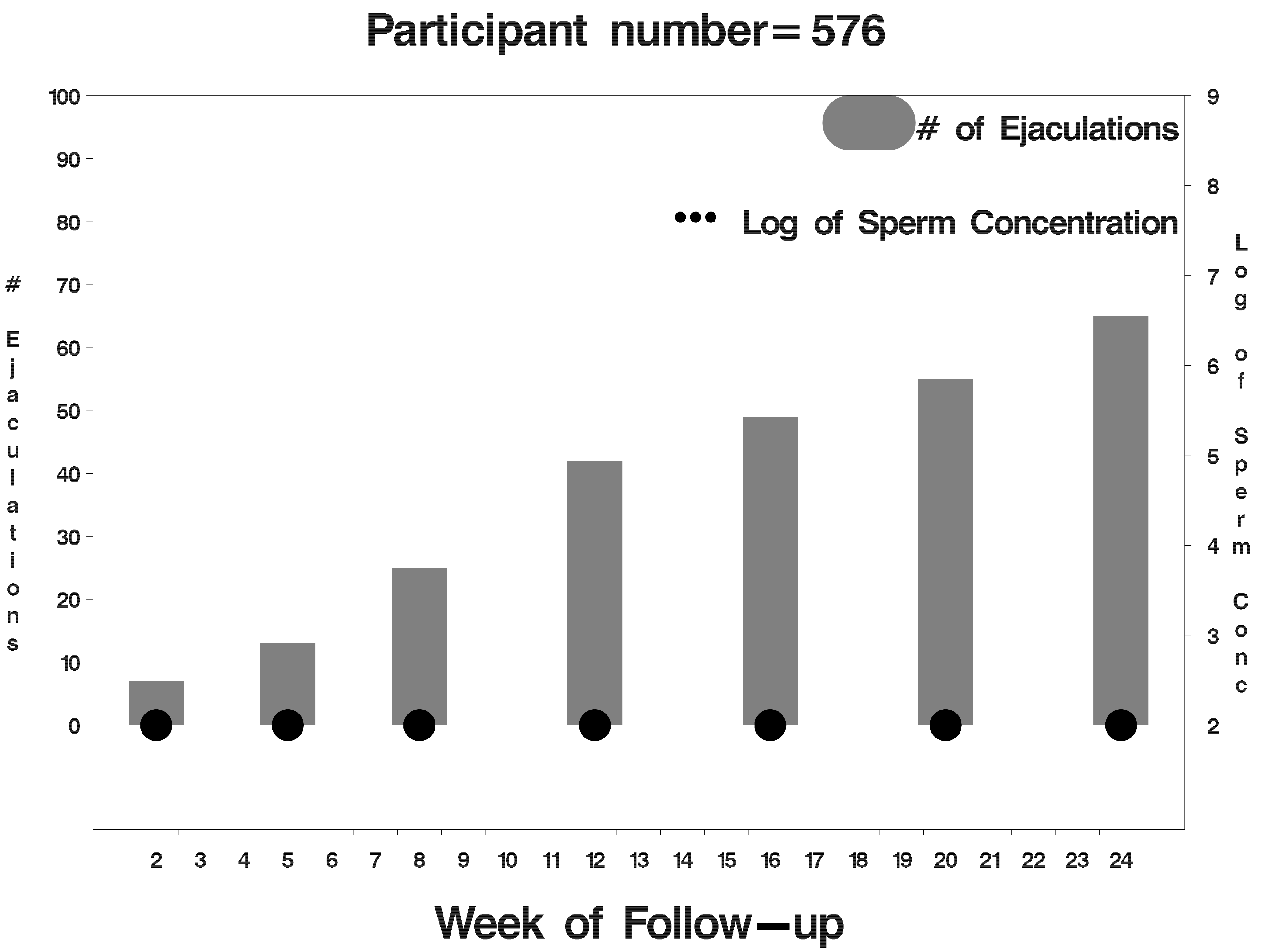

Participant number=576
# of Ejaculations
Log of Sperm Concentration
Ejaculations
Log of Sperm Conc
100
90
80
70
60
50
40
30
20
10
0
9
8
7
6
5
4
3
2
2 3 4 5 6 7 8 9 10 11 12 13 14 15 16 17 18 19 20 21 22 23 24
Week of Follow-up

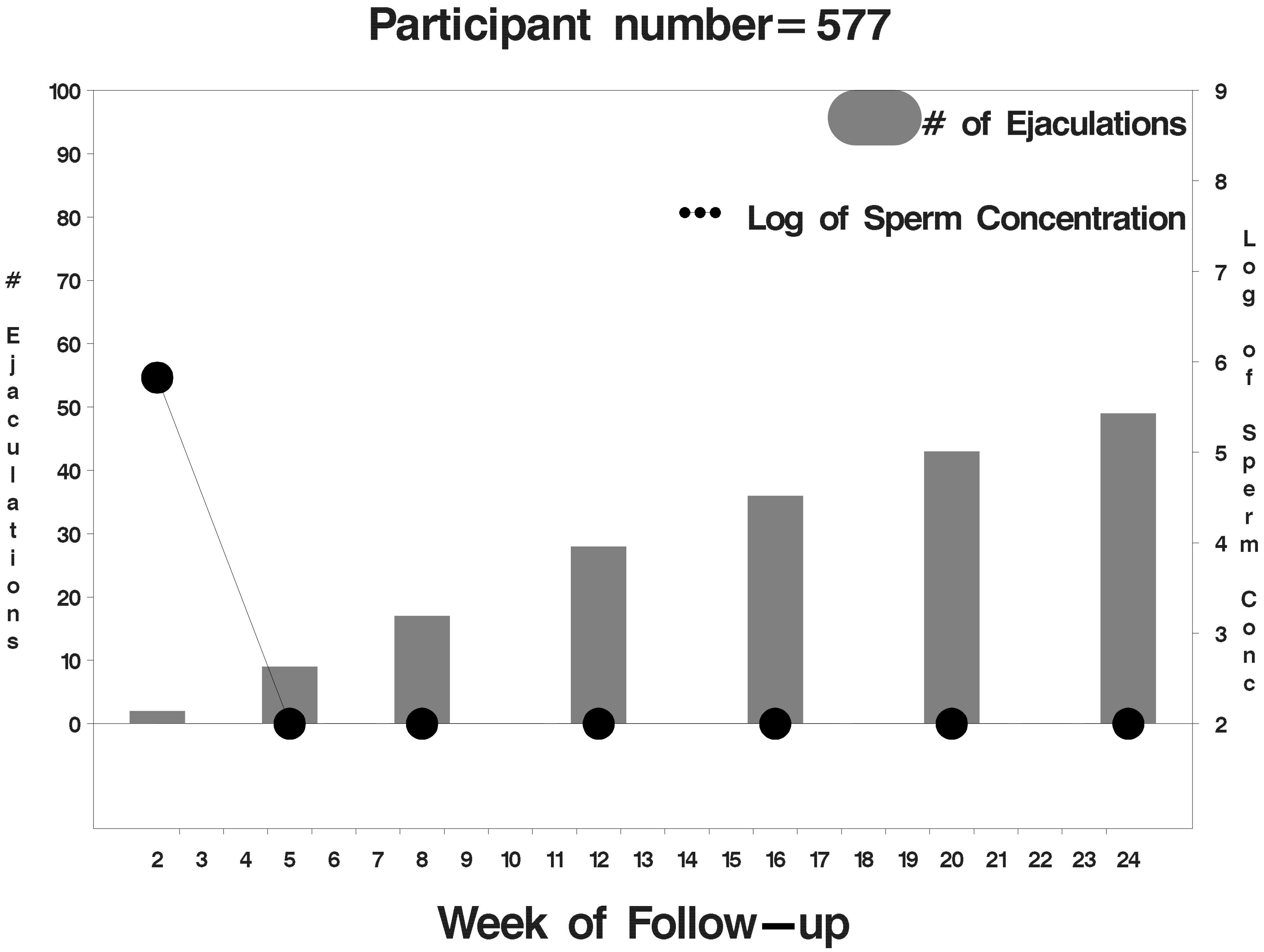

Participant number= 577
# of Ejaculations
Log of Sperm Concentration
Ejaculations
Log of Sperm Conc
0
10
20
30
40
50
60
70
80
90
100
2
3
4
5
6
7
8
9
2 3 4 5 6 7 8 9 10 11 12 13 14 15 16 17 18 19 20 21 22 23 24
Week of Follow-up

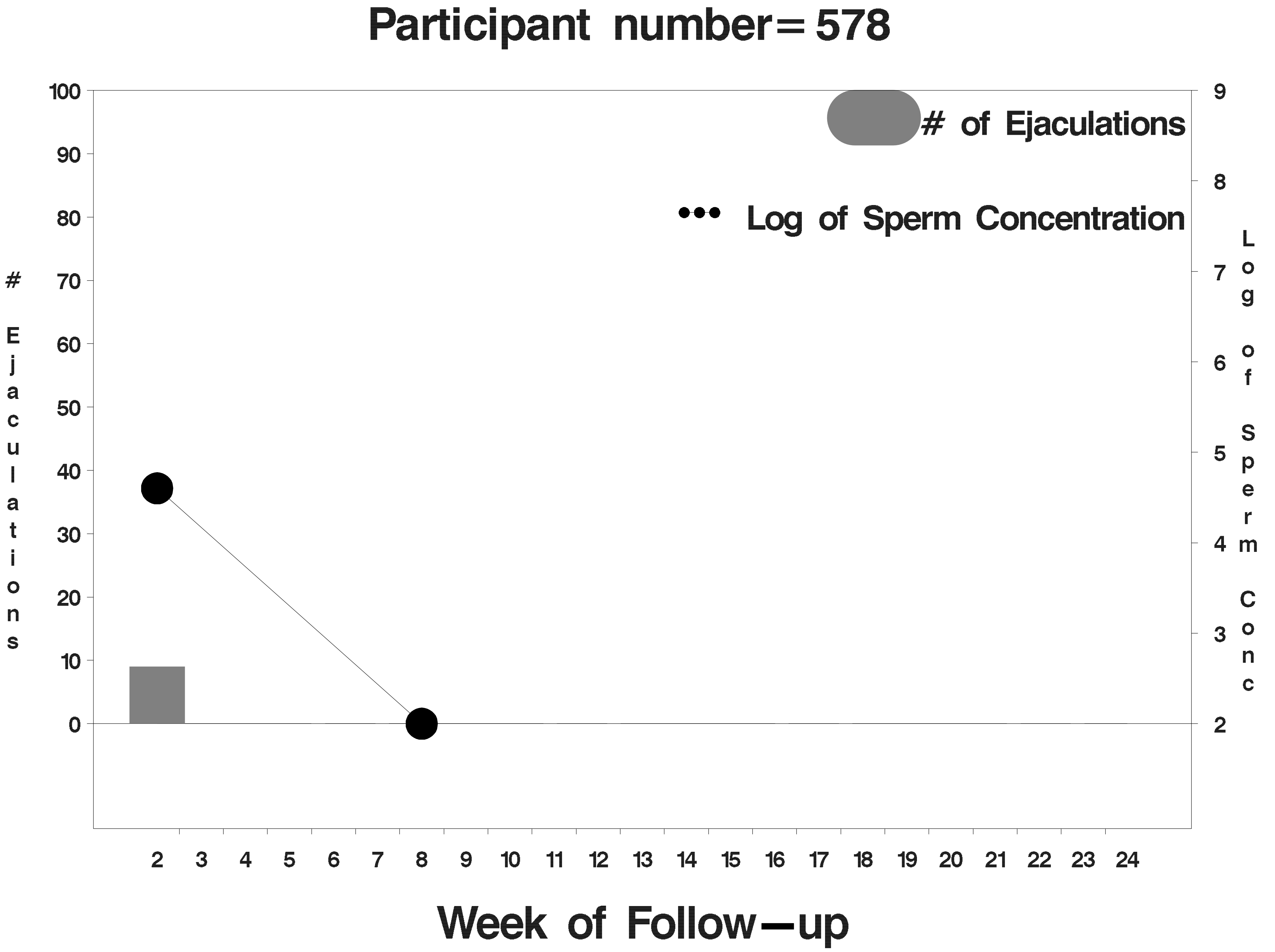

Participant number=578
# of Ejaculations
Log of Sperm Concentration
Ejaculations
Log of Sperm Conc
100
90
80
70
60
50
40
30
20
10
0
9
8
7
6
5
4
3
2
2 3 4 5 6 7 8 9 10 11 12 13 14 15 16 17 18 19 20 21 22 23 24
Week of Follow-up

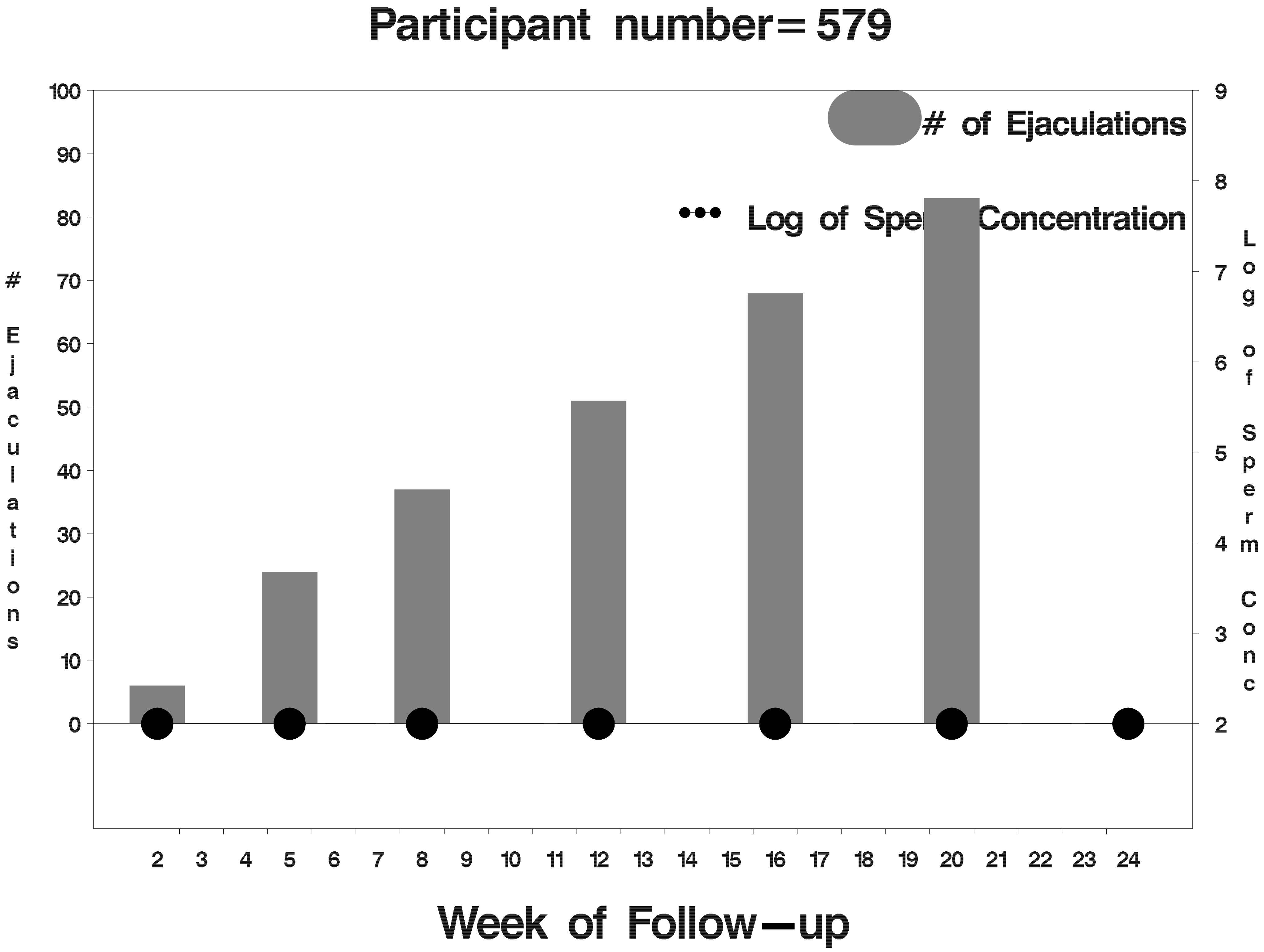
Participant number=579
# of Ejaculations
Log of Sperm Concentration
Ejaculations
Log of Sperm Conc
100
90
80
70
60
50
40
30
20
10
0
9
8
7
6
5
4
3
2
2 3 4 5 6 7 8 9 10 11 12 13 14 15 16 17 18 19 20 21 22 23 24
Week of Follow-up

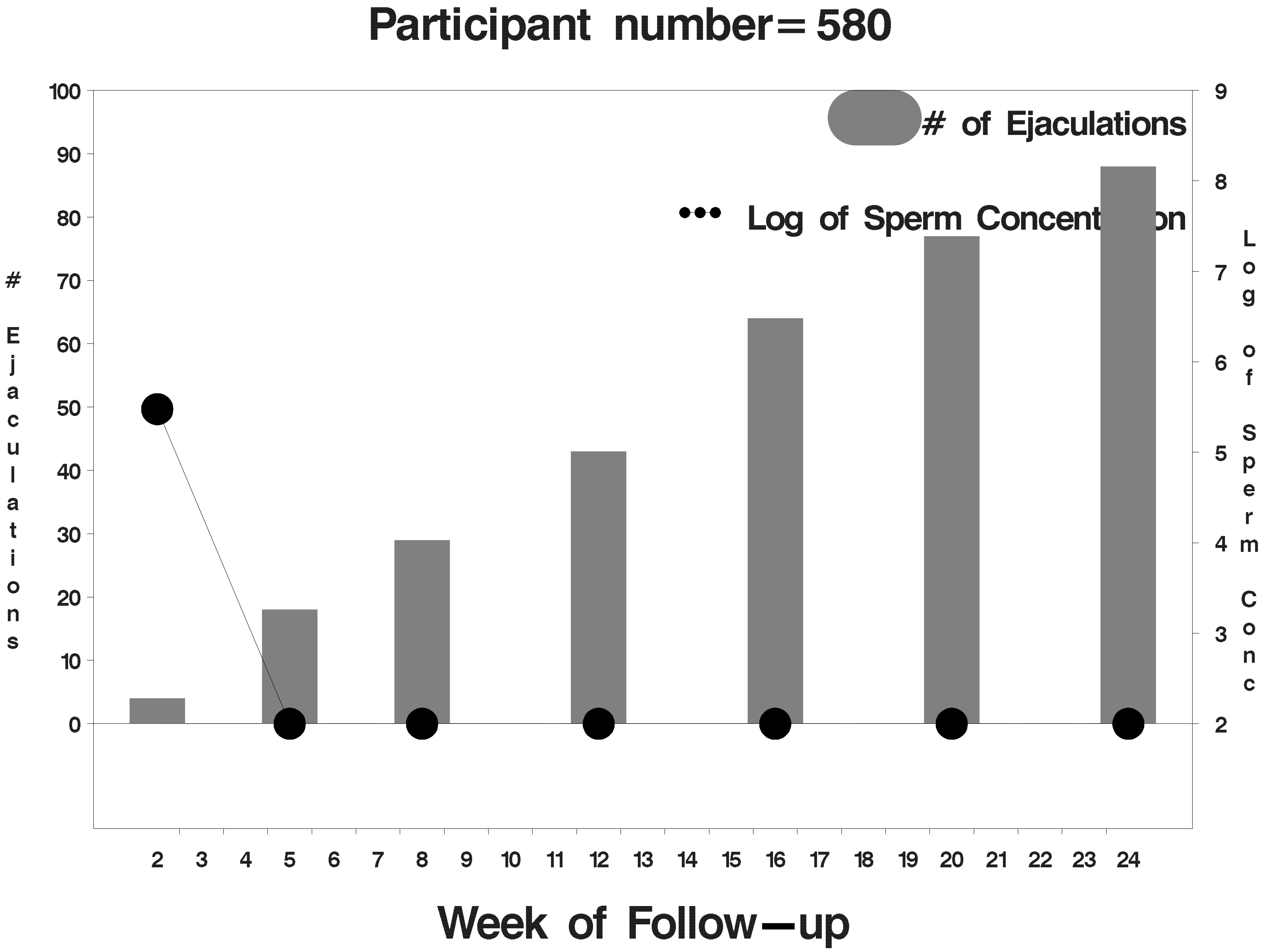

Participant number=580
# of Ejaculations
Log of Sperm Concentration
Ejaculations
Log of Sperm Conc
100
90
80
70
60
50
40
30
20
10
0
9
8
7
6
5
4
3
2
2 3 4 5 6 7 8 9 10 11 12 13 14 15 16 17 18 19 20 21 22 23 24
Week of Follow-up

Participant number=581

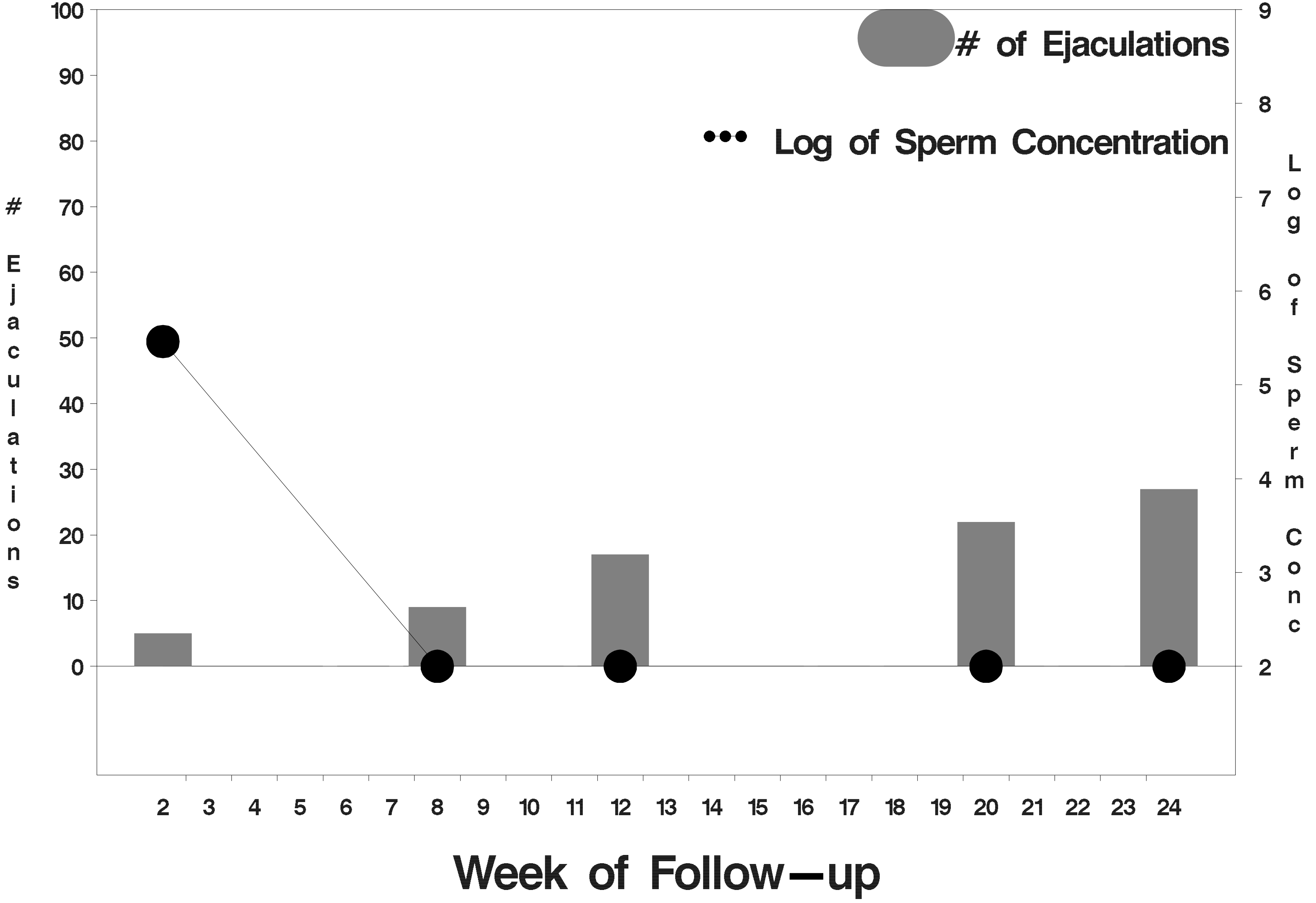

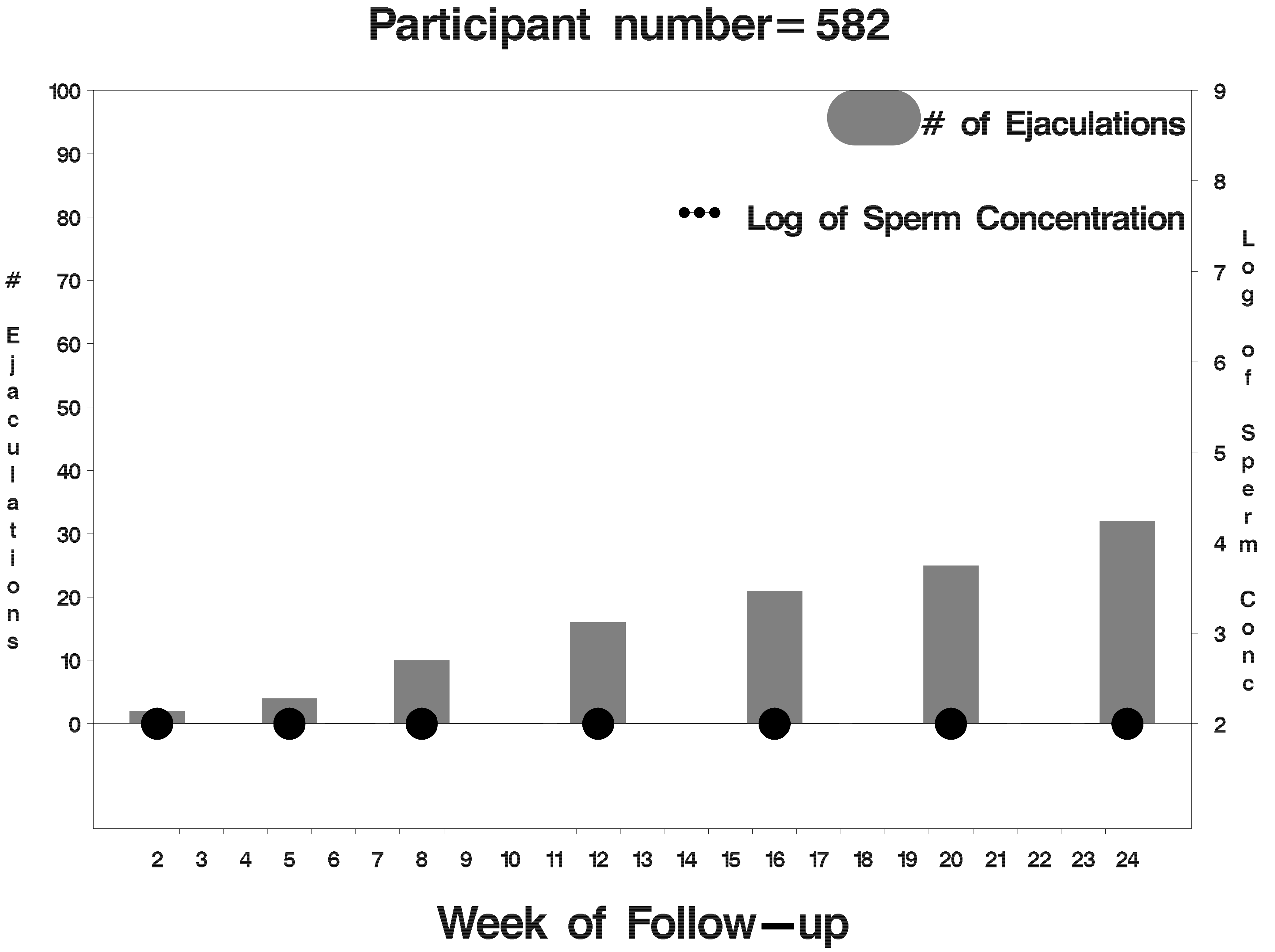

Participant number= 582
# of Ejaculations
Log of Sperm Concentration
Ejaculations
Log of Sperm Conc
100
90
80
70
60
50
40
30
20
10
0
9
8
7
6
5
4
3
2
2 3 4 5 6 7 8 9 10 11 12 13 14 15 16 17 18 19 20 21 22 23 24
Week of Follow-up

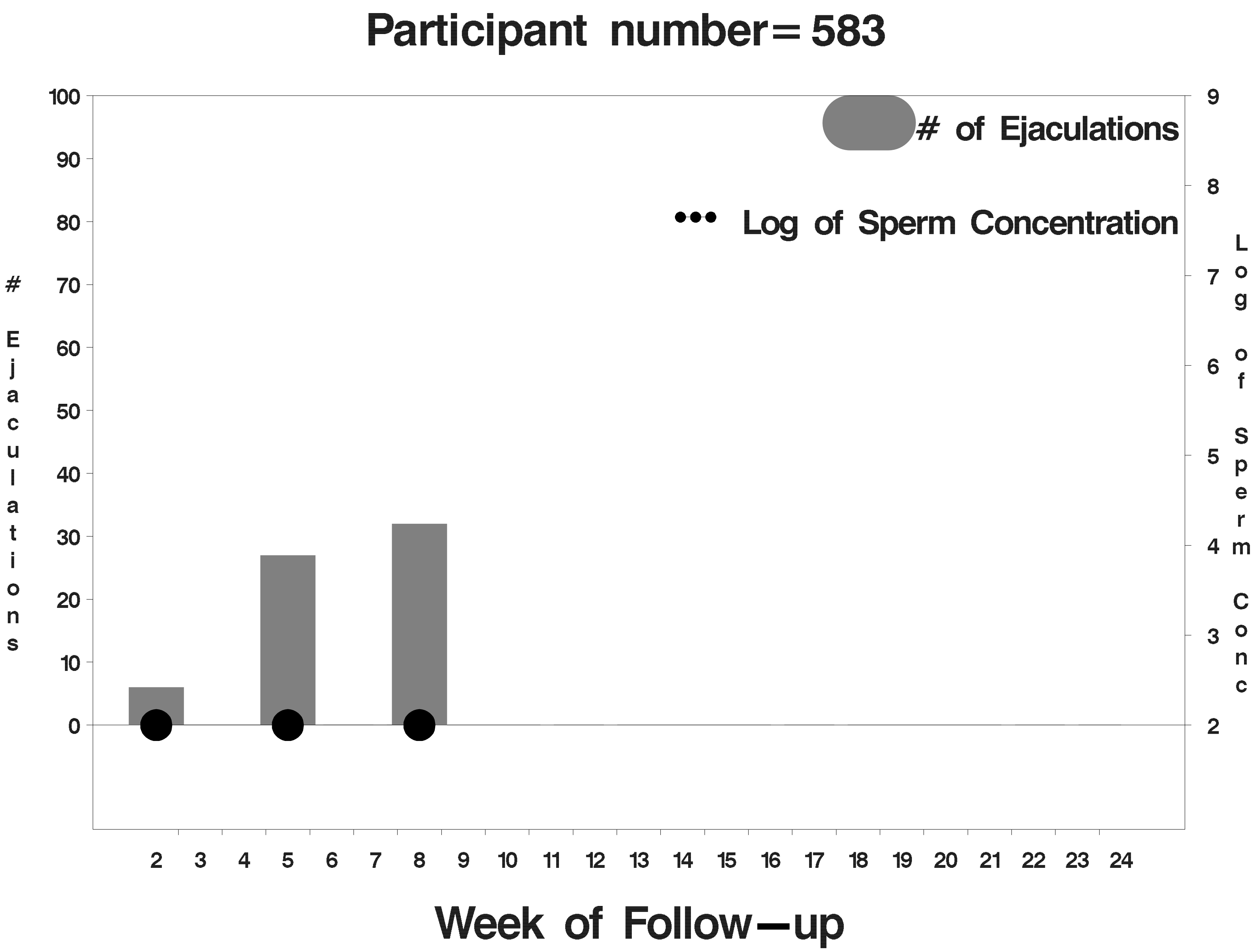

Participant number=583
# of Ejaculations
Log of Sperm Concentration
Ejaculations
Log of Sperm Conc
100
90
80
70
60
50
40
30
20
10
0
9
8
7
6
5
4
3
2
2 3 4 5 6 7 8 9 10 11 12 13 14 15 16 17 18 19 20 21 22 23 24
Week of Follow-up

Participant number=584

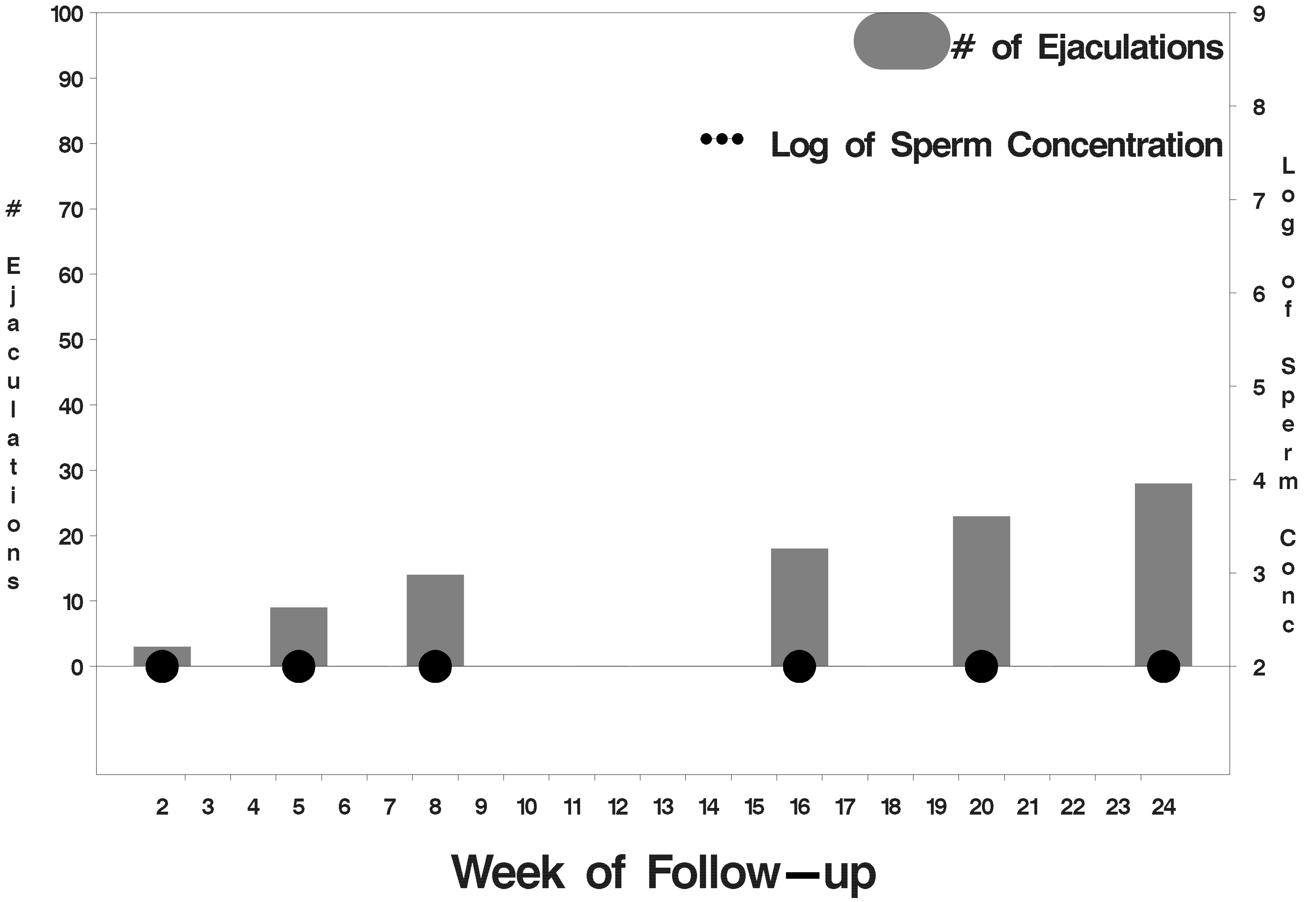

Participant number=585

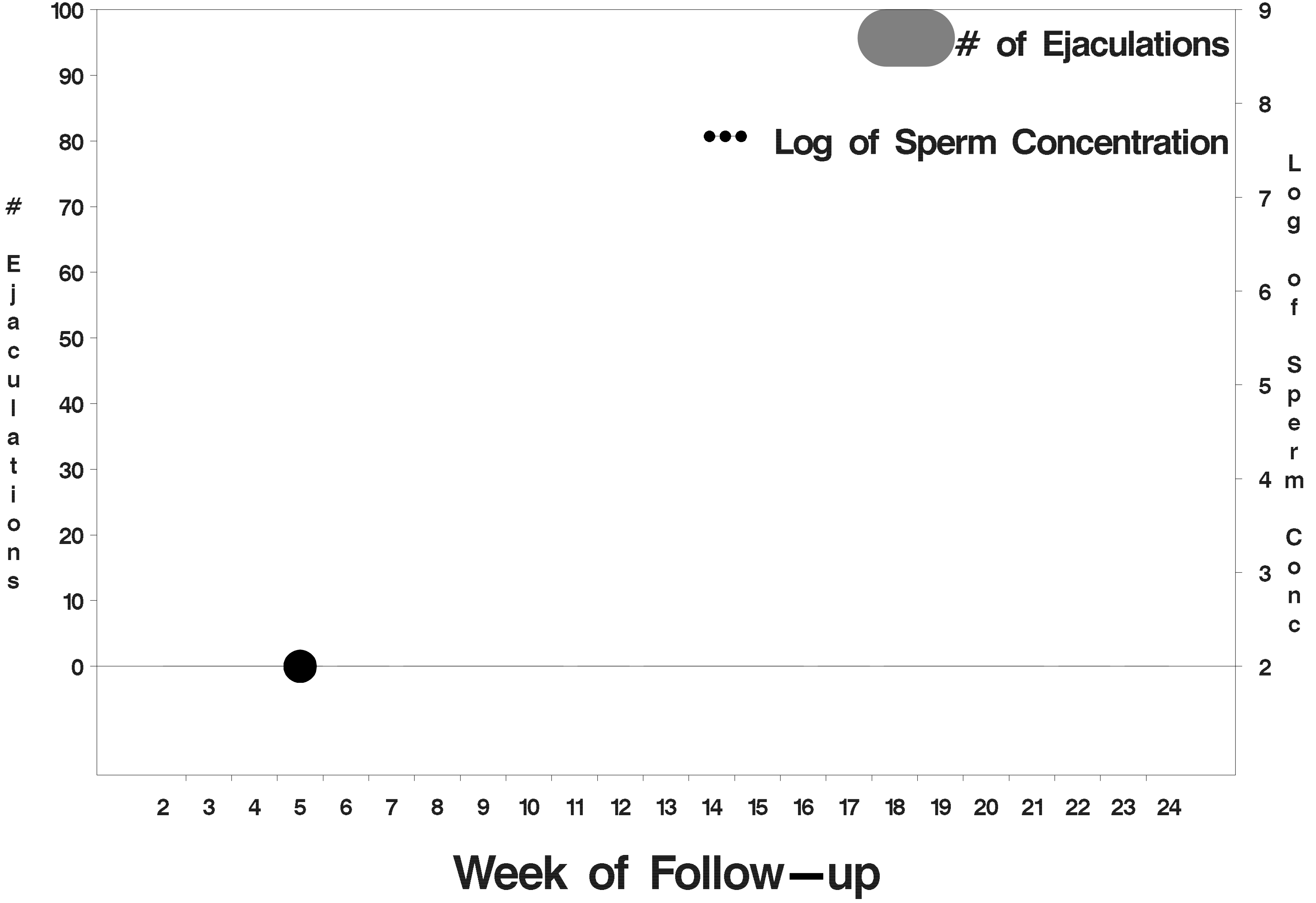

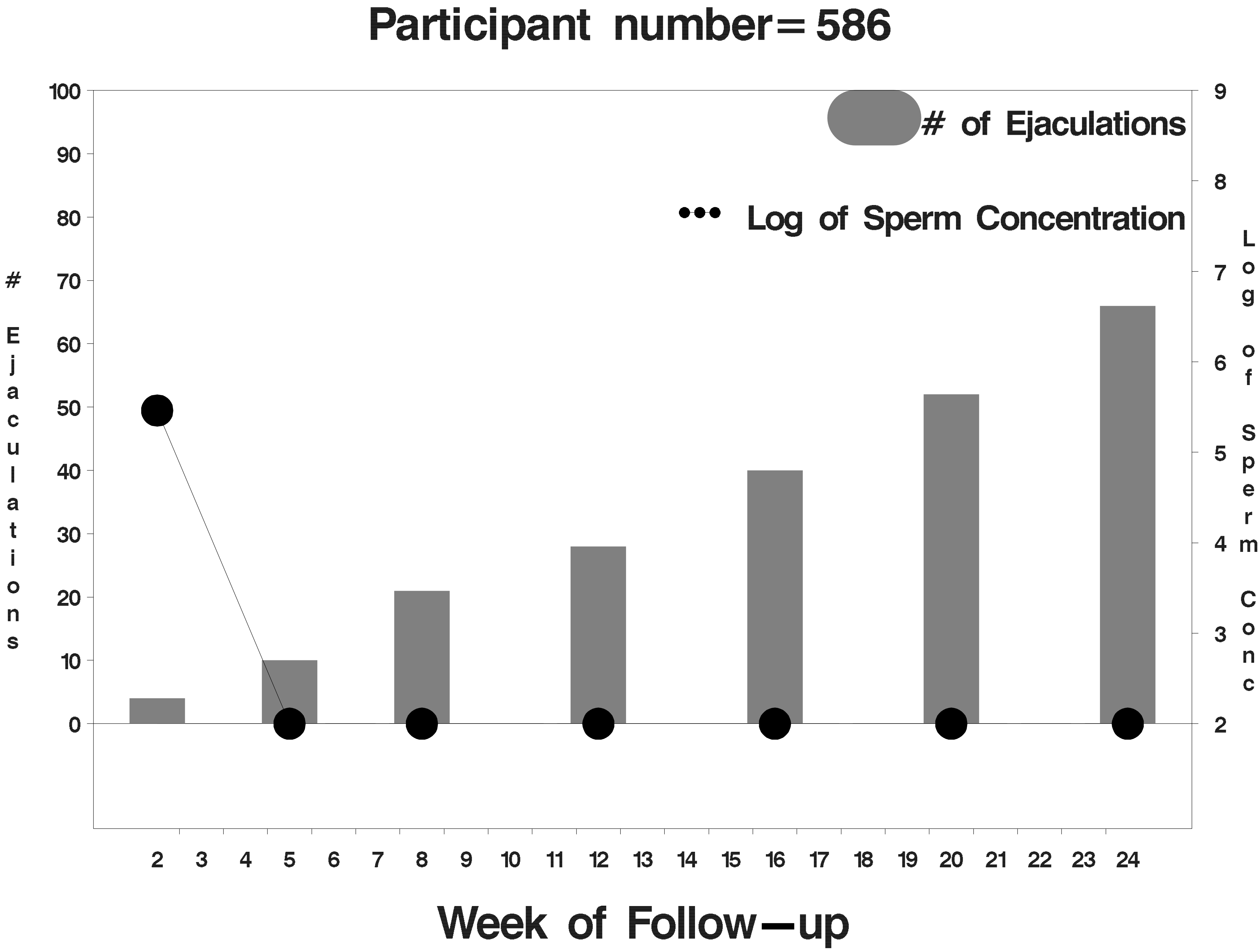

Participant number= 586
# of Ejaculations
Log of Sperm Concentration
Ejaculations
Log of Sperm Conc
100
90
80
70
60
50
40
30
20
10
0
9
8
7
6
5
4
3
2
2 3 4 5 6 7 8 9 10 11 12 13 14 15 16 17 18 19 20 21 22 23 24
Week of Follow−up

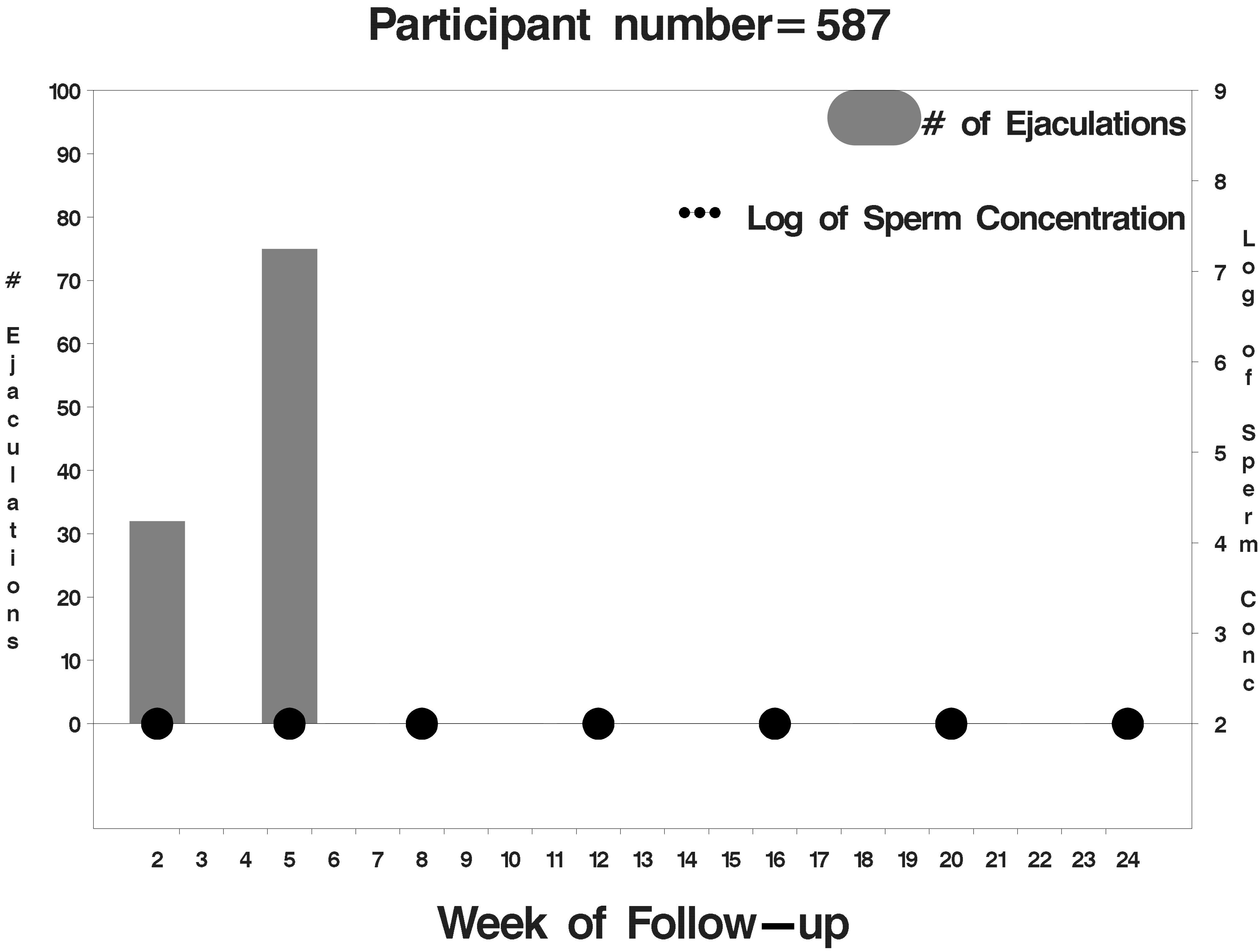

Participant number=587
# of Ejaculations
Log of Sperm Concentration
Ejaculations
Log of Sperm Conc
100
90
80
70
60
50
40
30
20
10
0
9
8
7
6
5
4
3
2
2 3 4 5 6 7 8 9 10 11 12 13 14 15 16 17 18 19 20 21 22 23 24
Week of Follow-up

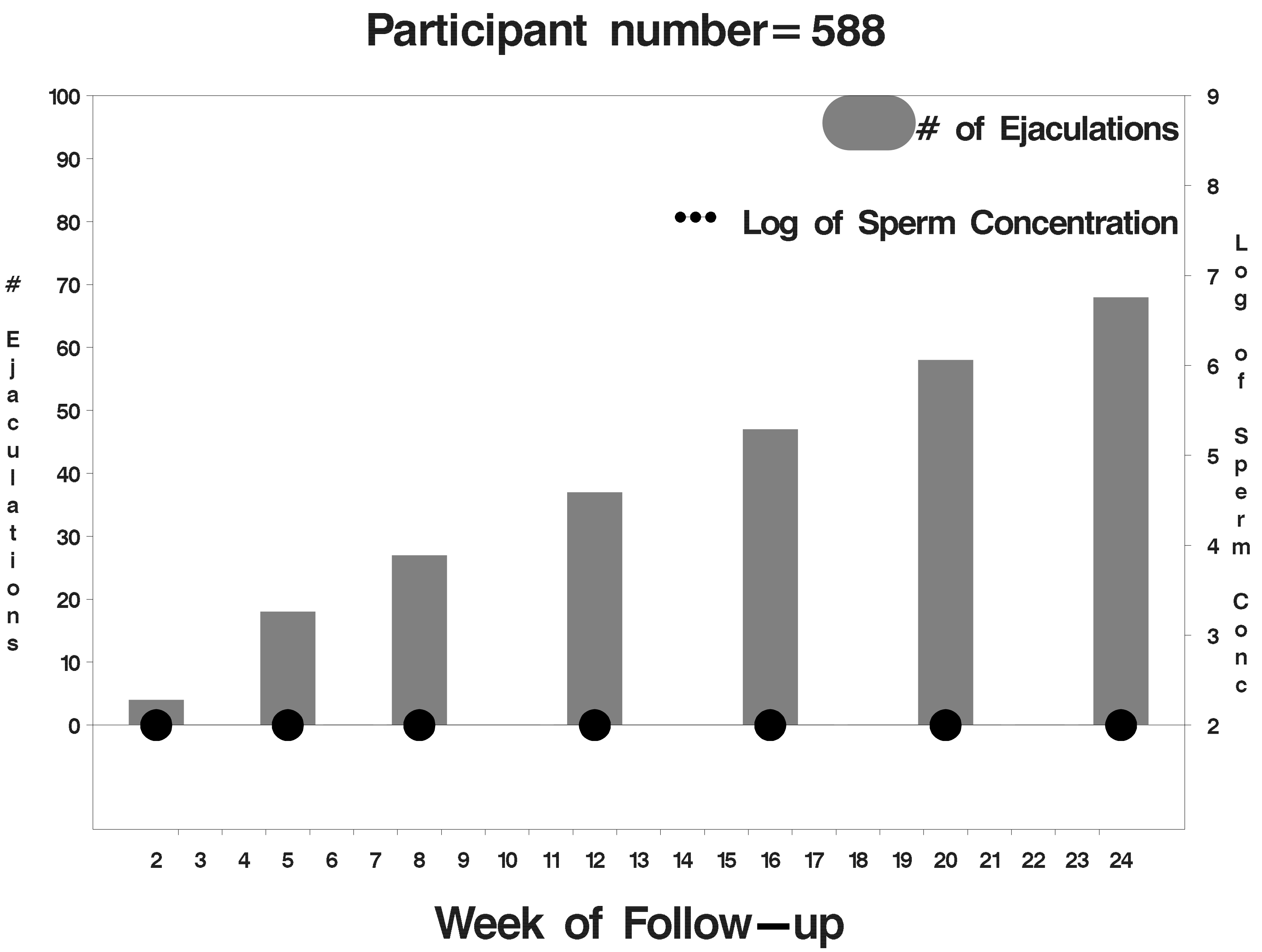
Participant number= 588
# of Ejaculations
Log of Sperm Concentration
Ejaculations
Log of Sperm Conc
100
90
80
70
60
50
40
30
20
10
0
9
8
7
6
5
4
3
2
2 3 4 5 6 7 8 9 10 11 12 13 14 15 16 17 18 19 20 21 22 23 24
Week of Follow-up

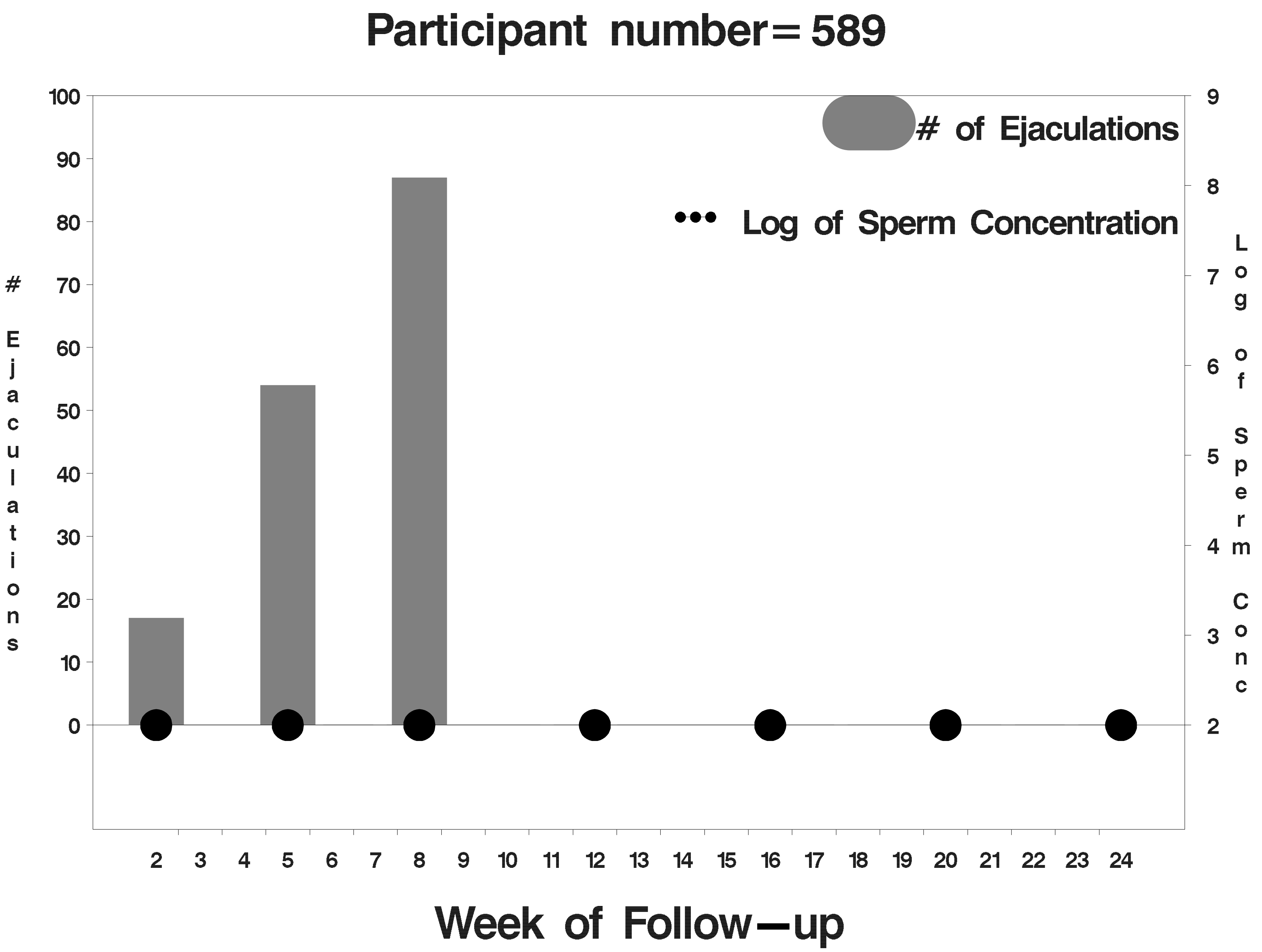

Participant number=589
# of Ejaculations
Log of Sperm Concentration
Ejaculations
Log of Sperm Conc
Week of Follow-up
0
10
20
30
40
50
60
70
80
90
100
2
3
4
5
6
7
8
9
2 3 4 5 6 7 8 9 10 11 12 13 14 15 16 17 18 19 20 21 22 23 24

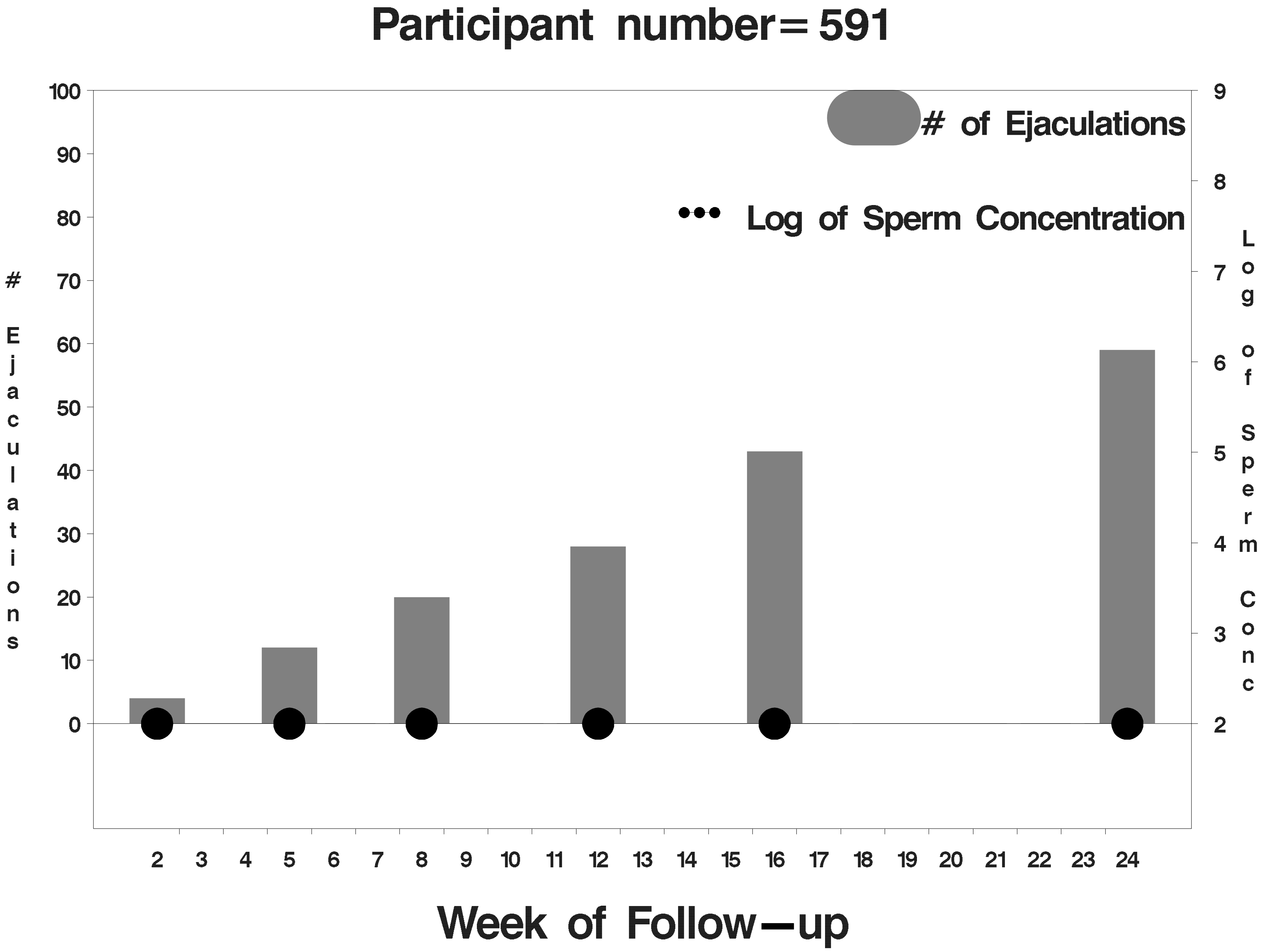

Participant number=591
# of Ejaculations
Log of Sperm Concentration
Ejaculations
Log of Sperm Conc
100
90
80
70
60
50
40
30
20
10
0
9
8
7
6
5
4
3
2
2 3 4 5 6 7 8 9 10 11 12 13 14 15 16 17 18 19 20 21 22 23 24
Week of Follow−up

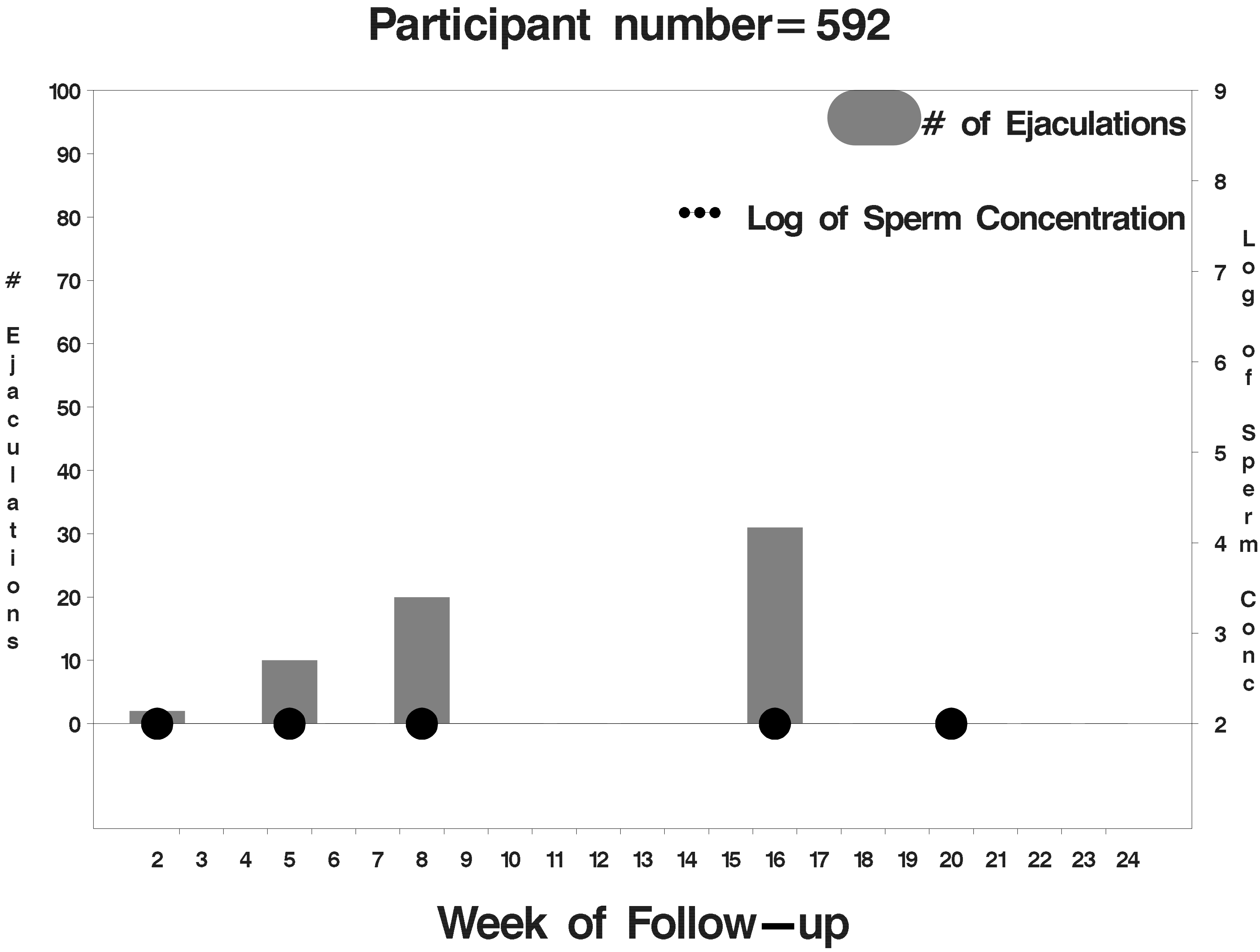
Participant number=592
# of Ejaculations
Log of Sperm Concentration
Ejaculations
Log of Sperm Conc
100
90
80
70
60
50
40
30
20
10
0
9
8
7
6
5
4
3
2
2 3 4 5 6 7 8 9 10 11 12 13 14 15 16 17 18 19 20 21 22 23 24
Week of Follow-up

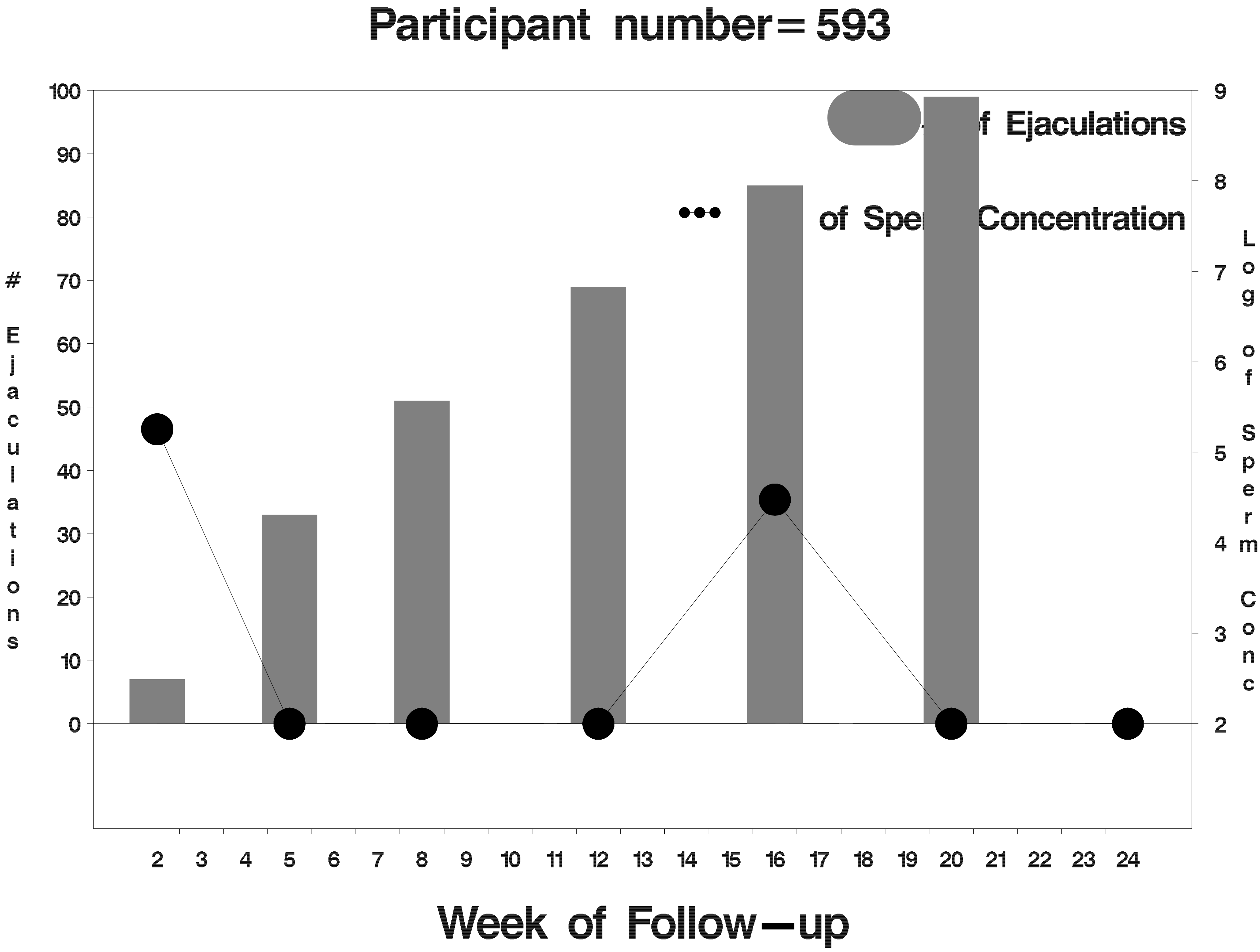
Participant number= 593
of Ejaculations
of Sperm Concentration
Ejaculations
Log of Sperm Conc
100
90
80
70
60
50
40
30
20
10
0
9
8
7
6
5
4
3
2
2 3 4 5 6 7 8 9 10 11 12 13 14 15 16 17 18 19 20 21 22 23 24
Week of Follow-up

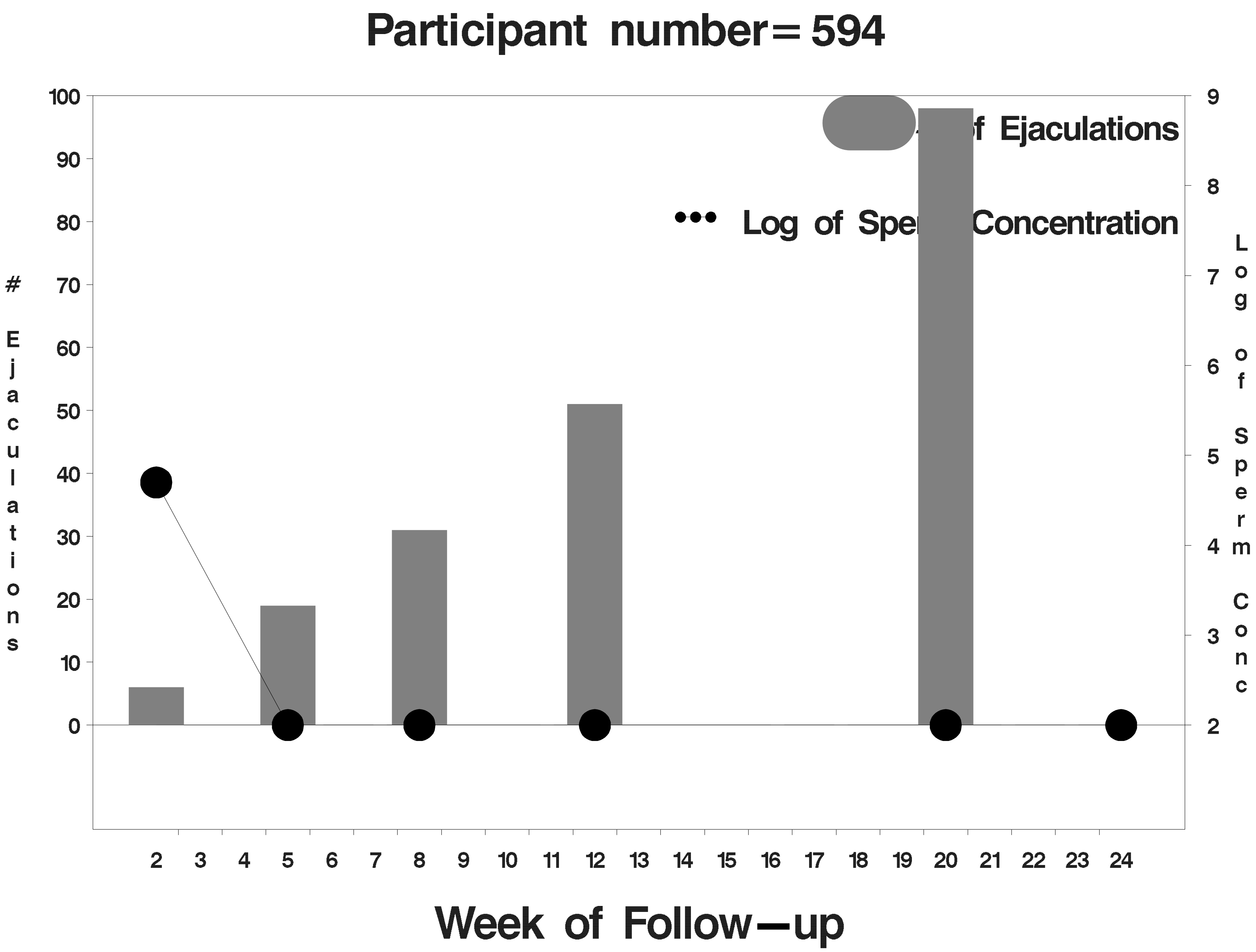

Participant number=594
Ejaculations
Log of Sperm Concentration
Log of Sperm Conc
100
90
80
70
60
50
40
30
20
10
0
9
8
7
6
5
4
3
2
2 3 4 5 6 7 8 9 10 11 12 13 14 15 16 17 18 19 20 21 22 23 24
Week of Follow-up

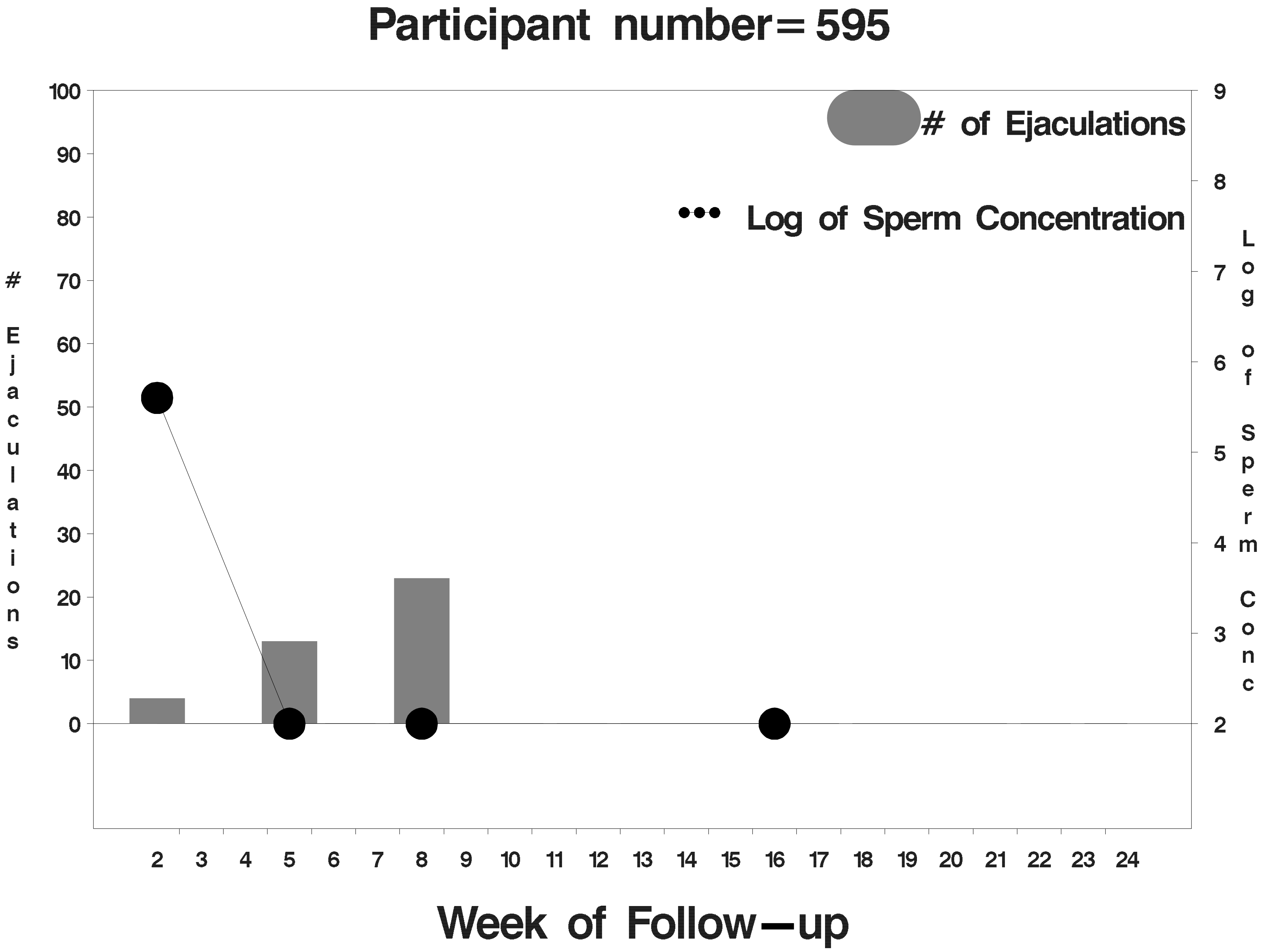

Participant number= 595
# of Ejaculations
Log of Sperm Concentration
Ejaculations
Log of Sperm Conc
100
90
80
70
60
50
40
30
20
10
0
9
8
7
6
5
4
3
2
2 3 4 5 6 7 8 9 10 11 12 13 14 15 16 17 18 19 20 21 22 23 24
Week of Follow-up

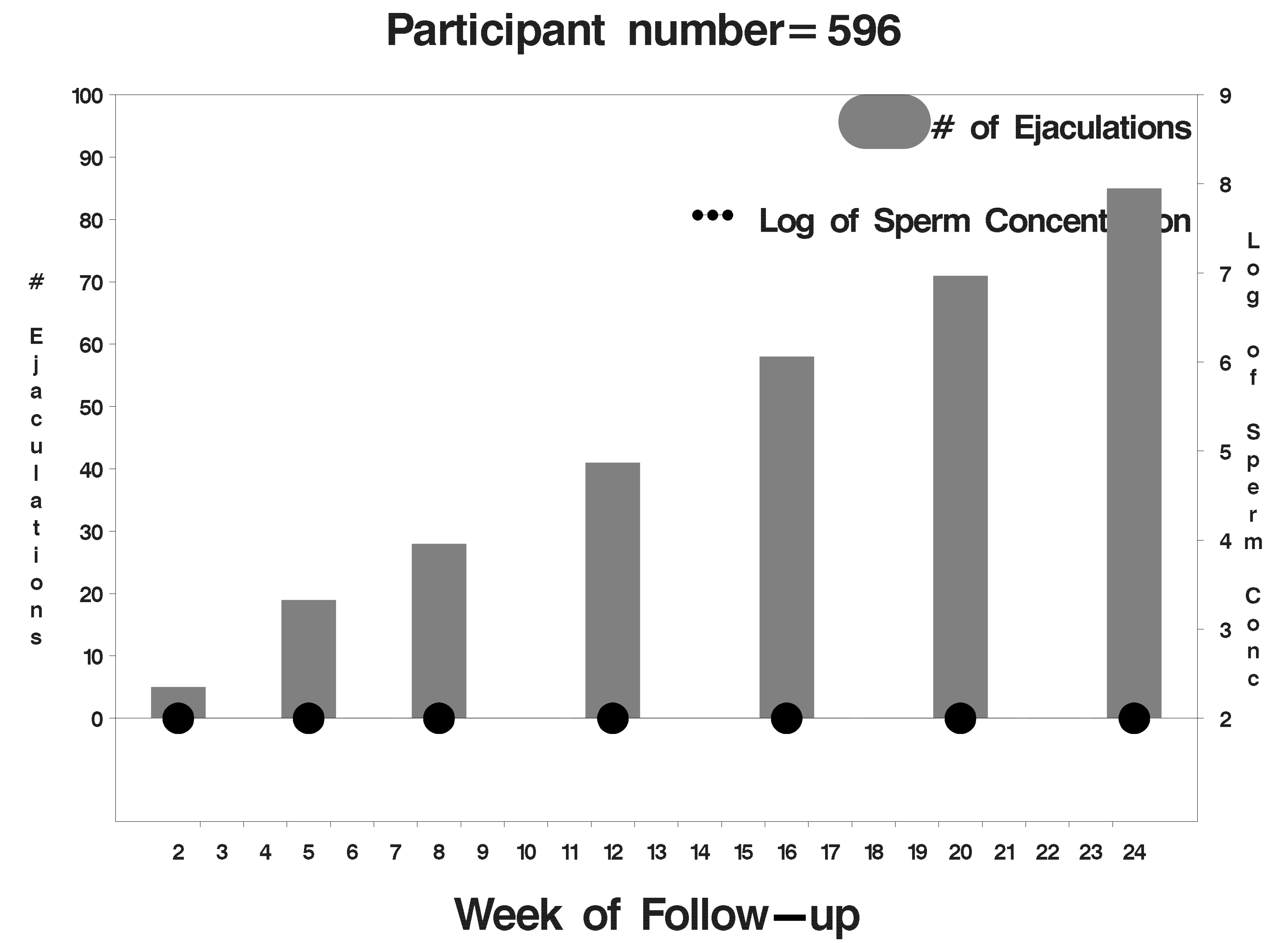

Participant number=596
# of Ejaculations
Log of Sperm Concentration
Ejaculations
Log of Sperm Conc
100
90
80
70
60
50
40
30
20
10
0
9
8
7
6
5
4
3
2
2 3 4 5 6 7 8 9 10 11 12 13 14 15 16 17 18 19 20 21 22 23 24
Week of Follow-up

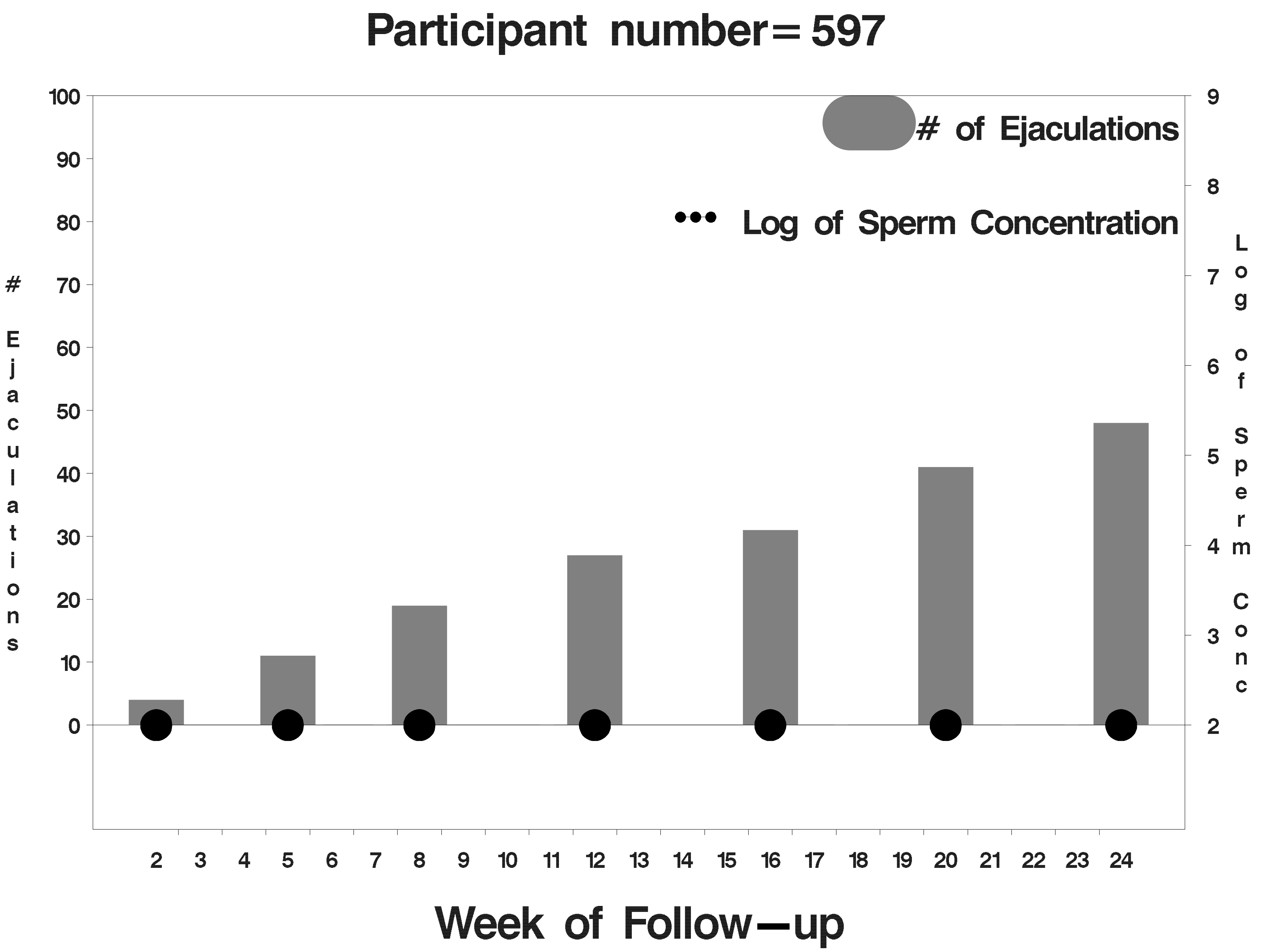

Participant number=597
# of Ejaculations
Log of Sperm Concentration
Ejaculations
Log of Sperm Conc
100
90
80
70
60
50
40
30
20
10
0
9
8
7
6
5
4
3
2
2 3 4 5 6 7 8 9 10 11 12 13 14 15 16 17 18 19 20 21 22 23 24
Week of Follow—up

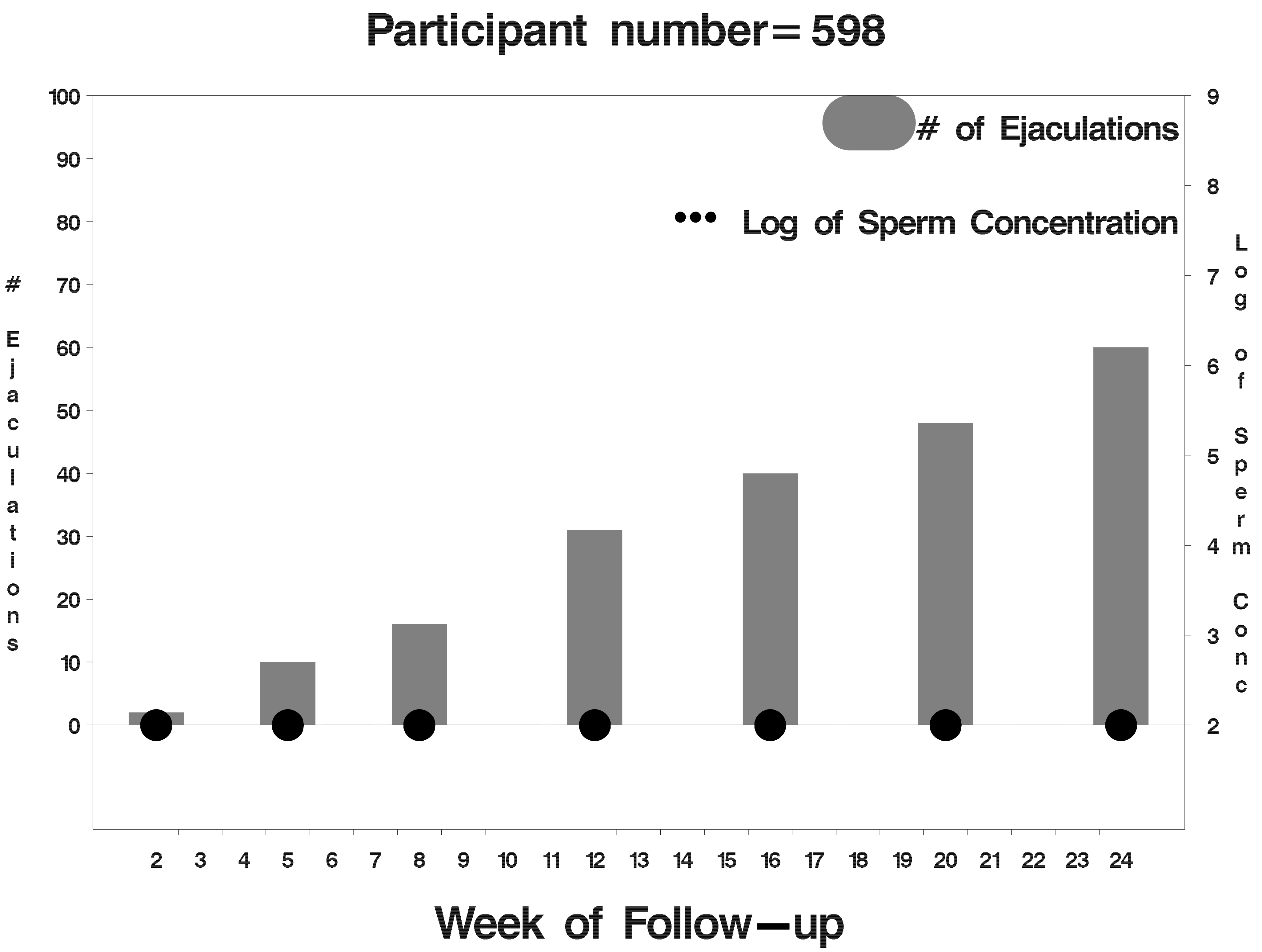

Participant number= 598
# of Ejaculations
Log of Sperm Concentration
Ejaculations
Log of Sperm Conc
100
90
80
70
60
50
40
30
20
10
0
9
8
7
6
5
4
3
2
2 3 4 5 6 7 8 9 10 11 12 13 14 15 16 17 18 19 20 21 22 23 24
Week of Follow-up

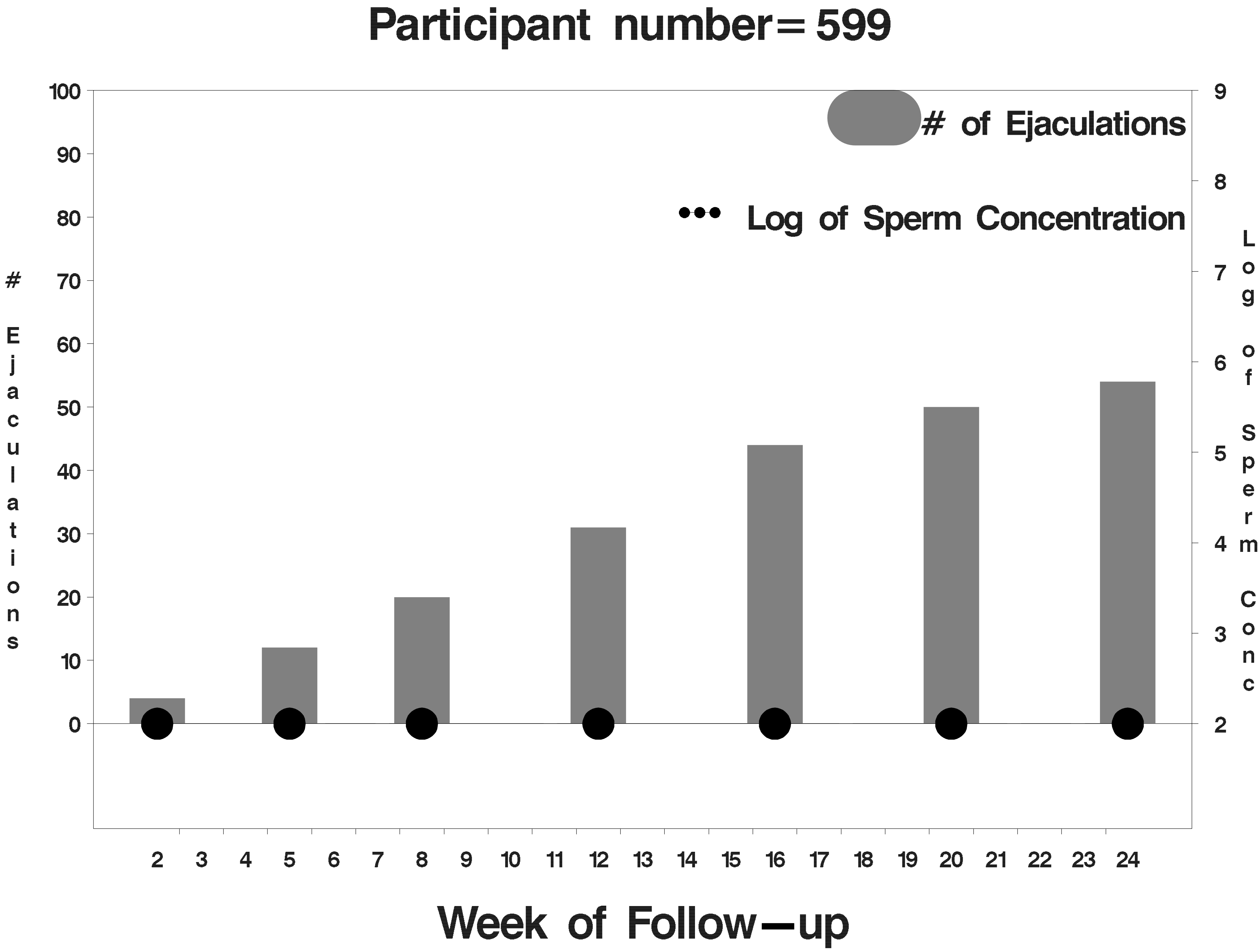

Participant number=599
# of Ejaculations
Log of Sperm Concentration
Ejaculations
Log of Sperm Conc
100
90
80
70
60
50
40
30
20
10
0
9
8
7
6
5
4
3
2
2 3 4 5 6 7 8 9 10 11 12 13 14 15 16 17 18 19 20 21 22 23 24
Week of Follow−up

Participant number= 600

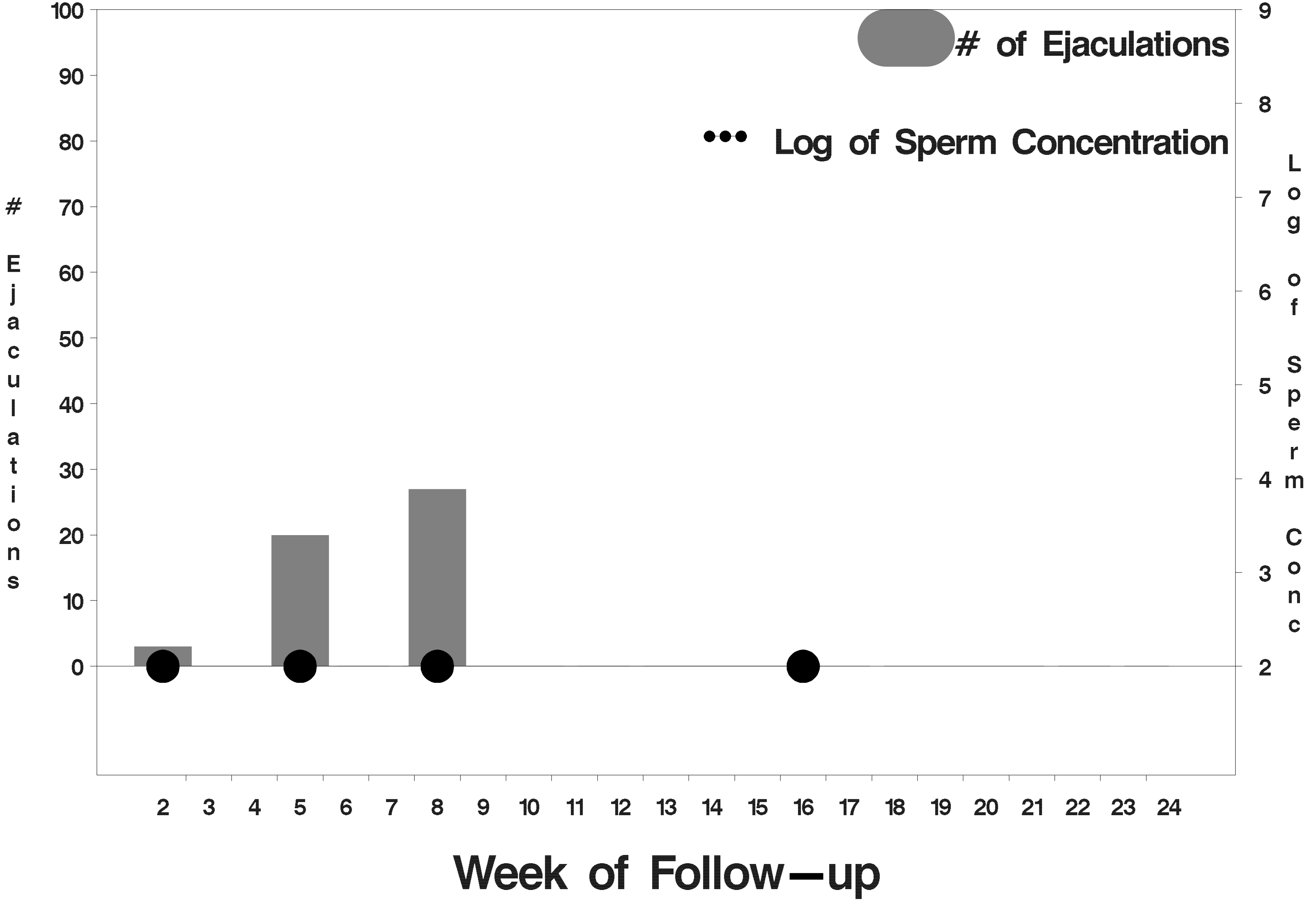

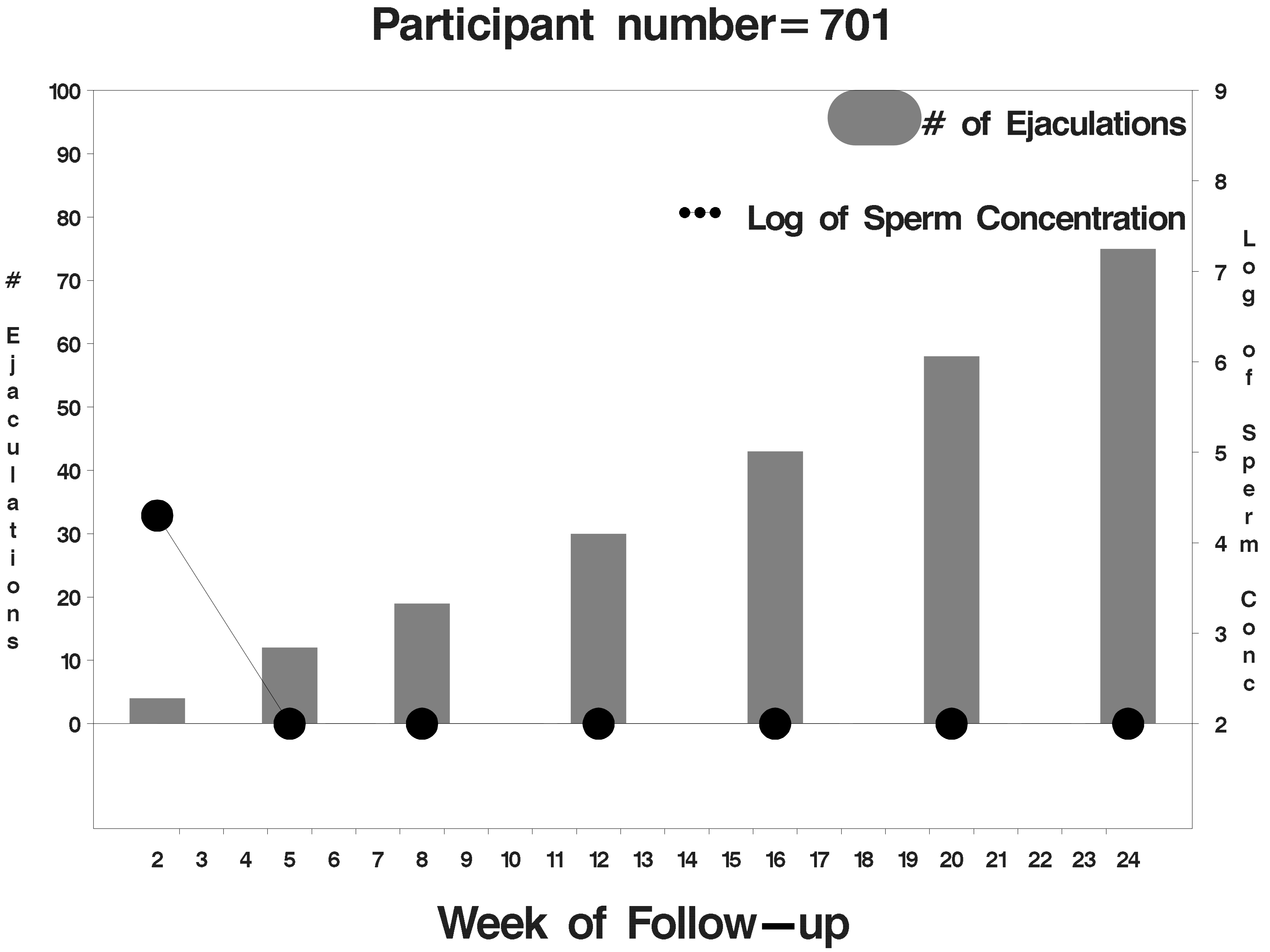
Participant number= 701
# of Ejaculations
Log of Sperm Concentration
Ejaculations
Log of Sperm Conc
0
10
20
30
40
50
60
70
80
90
100
2
3
4
5
6
7
8
9
2
3
4
5
6
7
8
9
10
11
12
13
14
15
16
17
18
19
20
21
22
23
24
Week of Follow-up

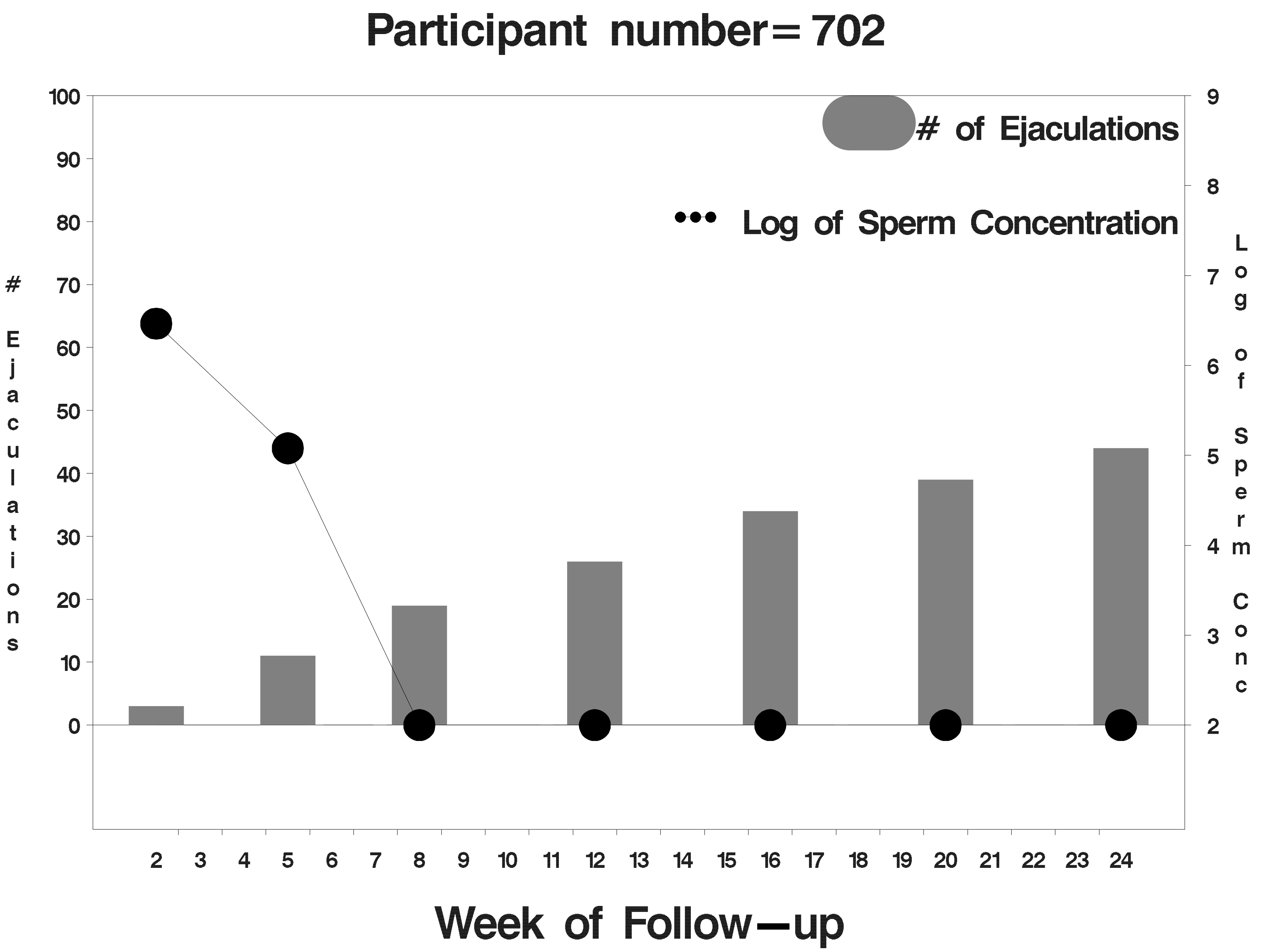

Participant number= 702
# of Ejaculations
Log of Sperm Concentration
Ejaculations
Log of Sperm Conc
100
90
80
70
60
50
40
30
20
10
0
9
8
7
6
5
4
3
2
2 3 4 5 6 7 8 9 10 11 12 13 14 15 16 17 18 19 20 21 22 23 24
Week of Follow-up

Participant number= 703

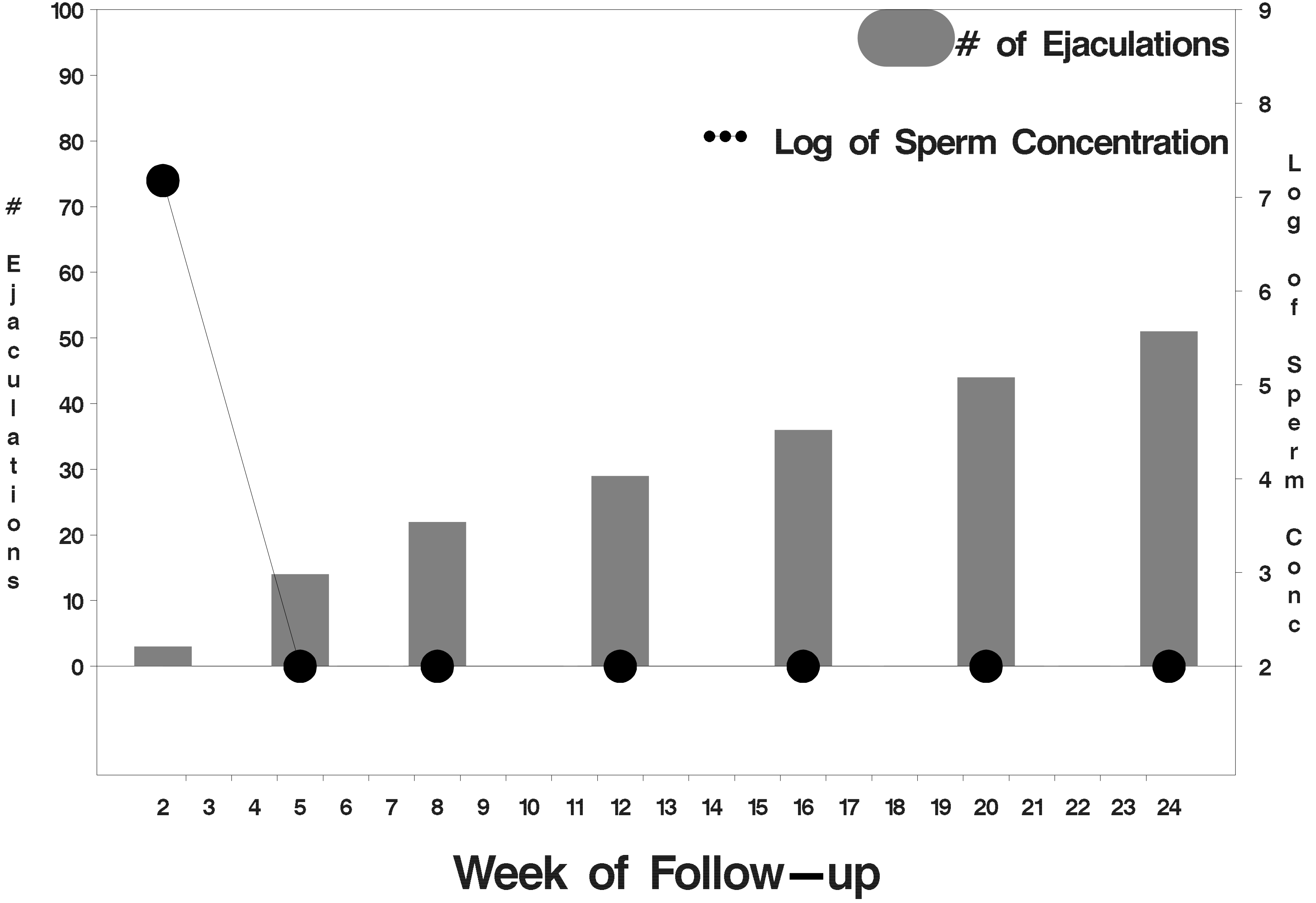

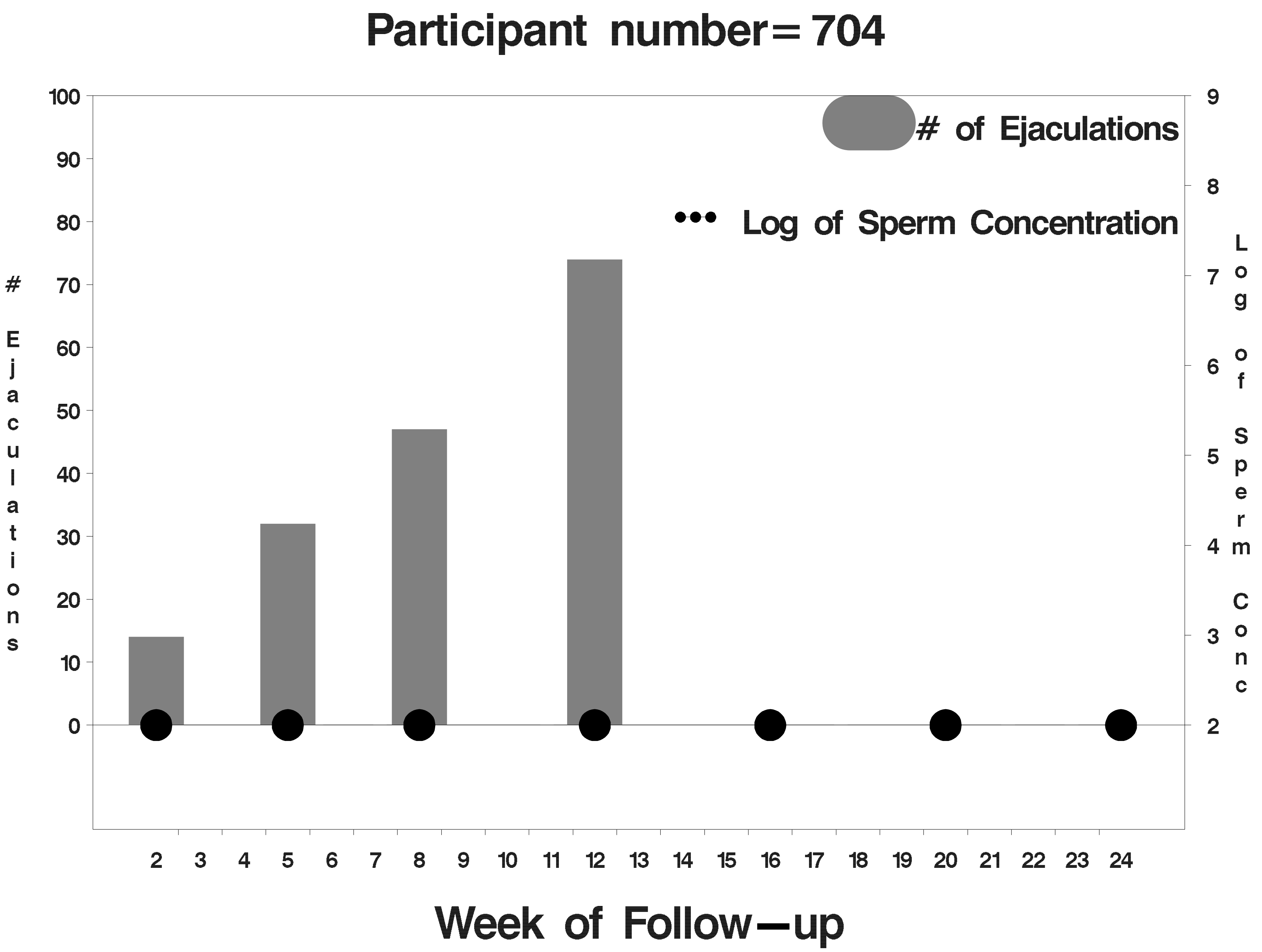
Participant number=704
# of Ejaculations
Log of Sperm Concentration
Ejaculations
Log of Sperm Conc
Week of Follow-up
100
90
80
70
60
50
40
30
20
10
0
9
8
7
6
5
4
3
2
2 3 4 5 6 7 8 9 10 11 12 13 14 15 16 17 18 19 20 21 22 23 24

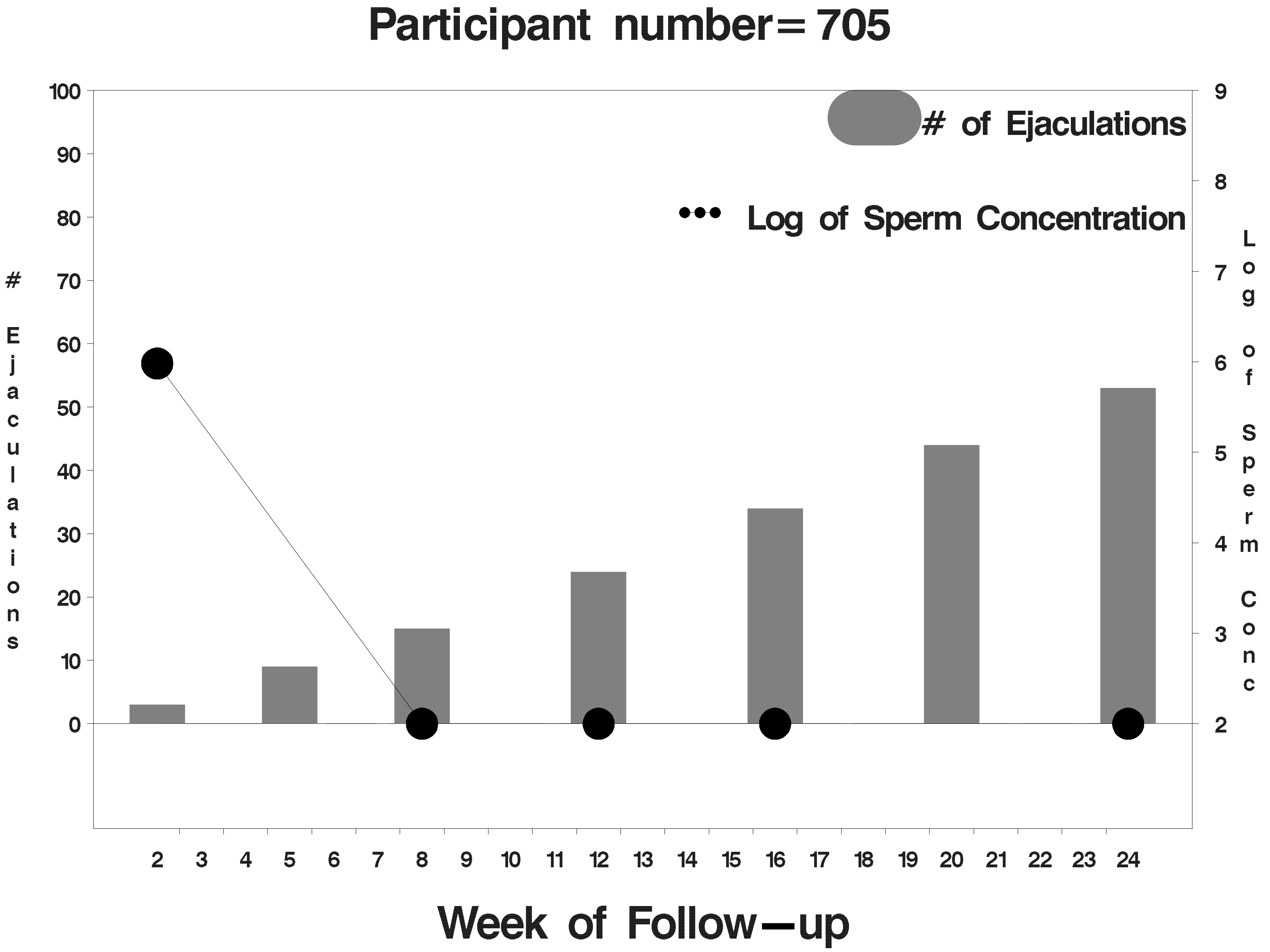
Participant number= 705
# of Ejaculations
Log of Sperm Concentration
Ejaculations
Log of Sperm Conc
100
90
80
70
60
50
40
30
20
10
0
9
8
7
6
5
4
3
2
2 3 4 5 6 7 8 9 10 11 12 13 14 15 16 17 18 19 20 21 22 23 24
Week of Follow-up

Participant number= 706

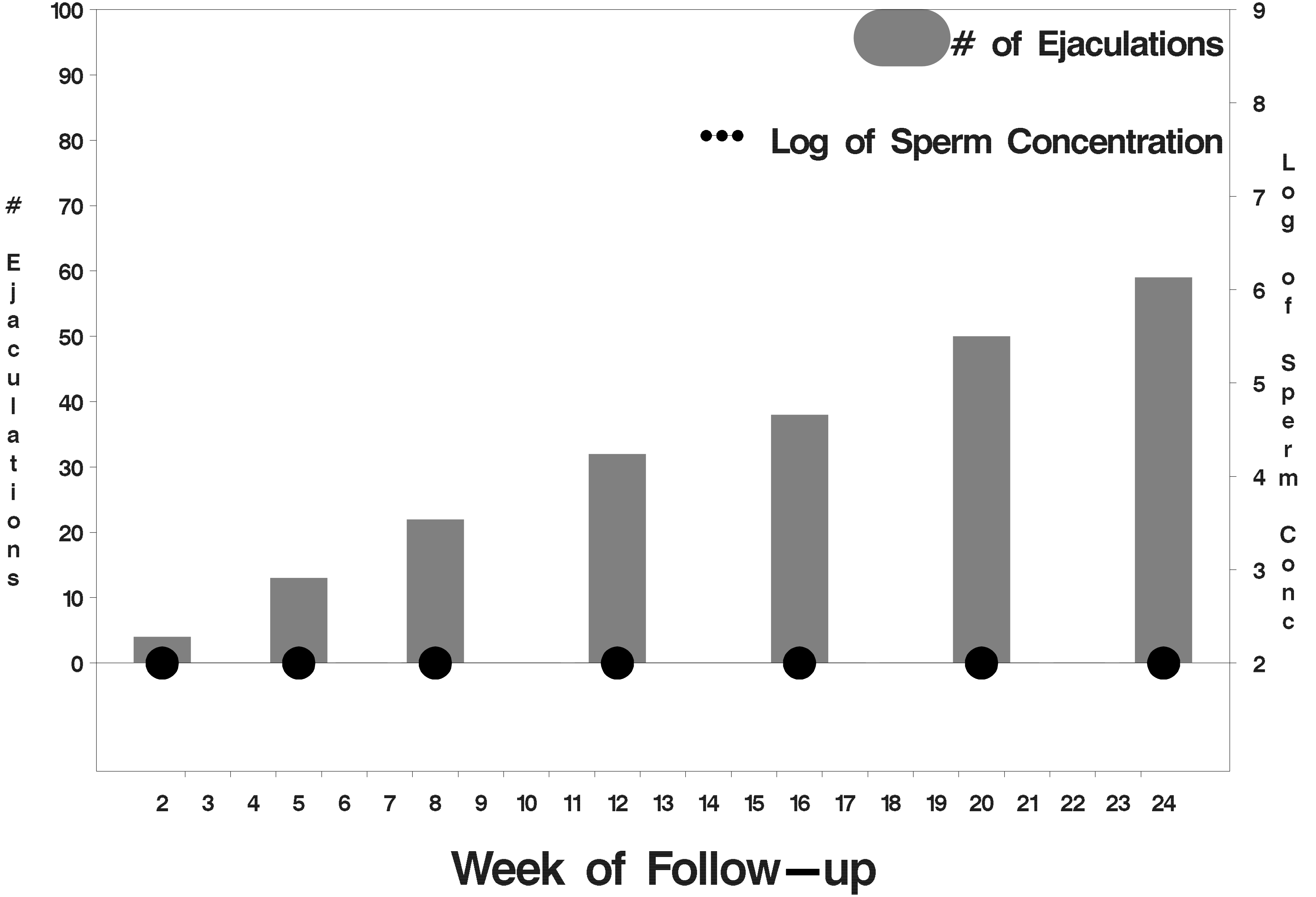

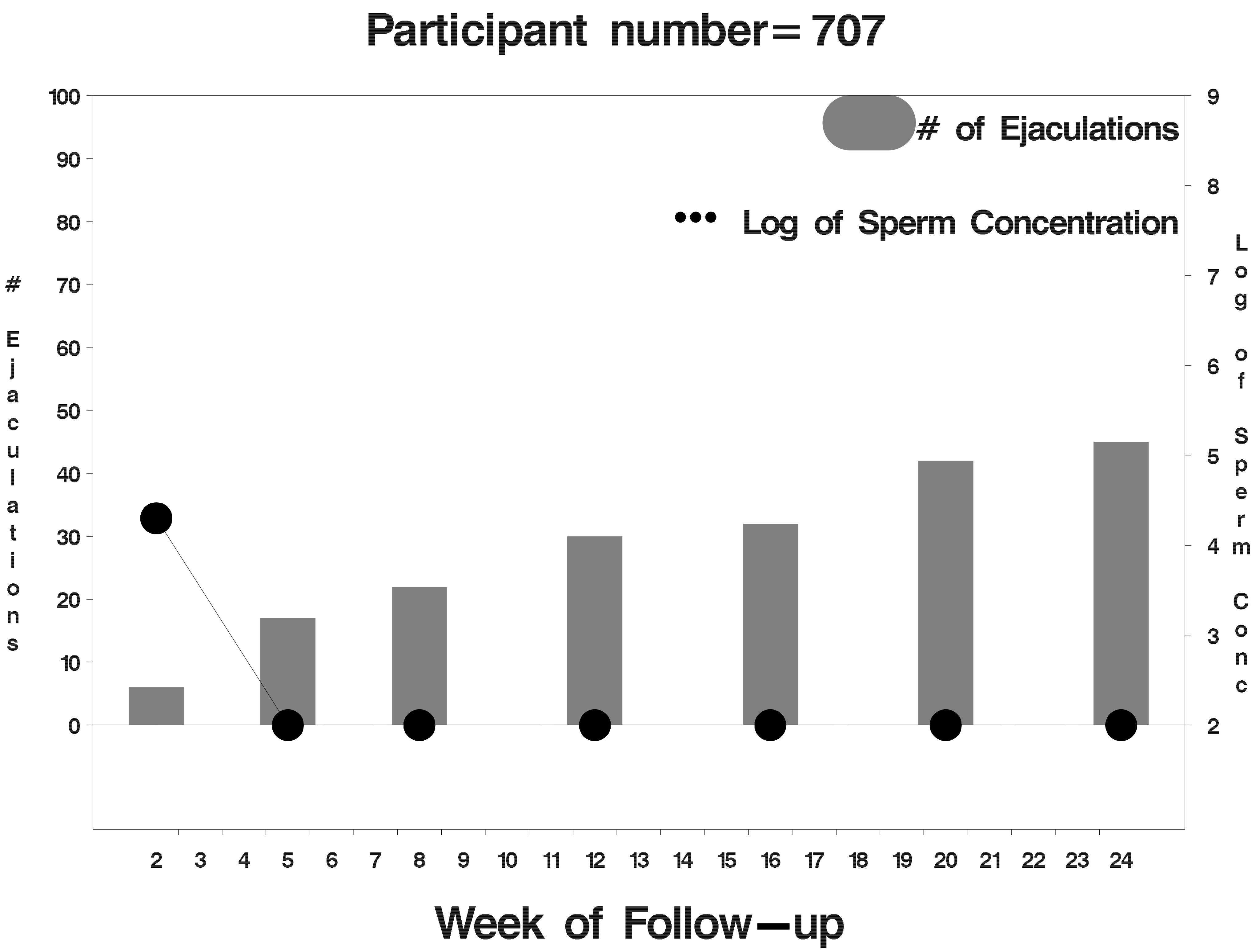

Participant number=707
# of Ejaculations
Log of Sperm Concentration
Ejaculations
Log of Sperm Conc
100
90
80
70
60
50
40
30
20
10
0
9
8
7
6
5
4
3
2
2 3 4 5 6 7 8 9 10 11 12 13 14 15 16 17 18 19 20 21 22 23 24
Week of Follow-up

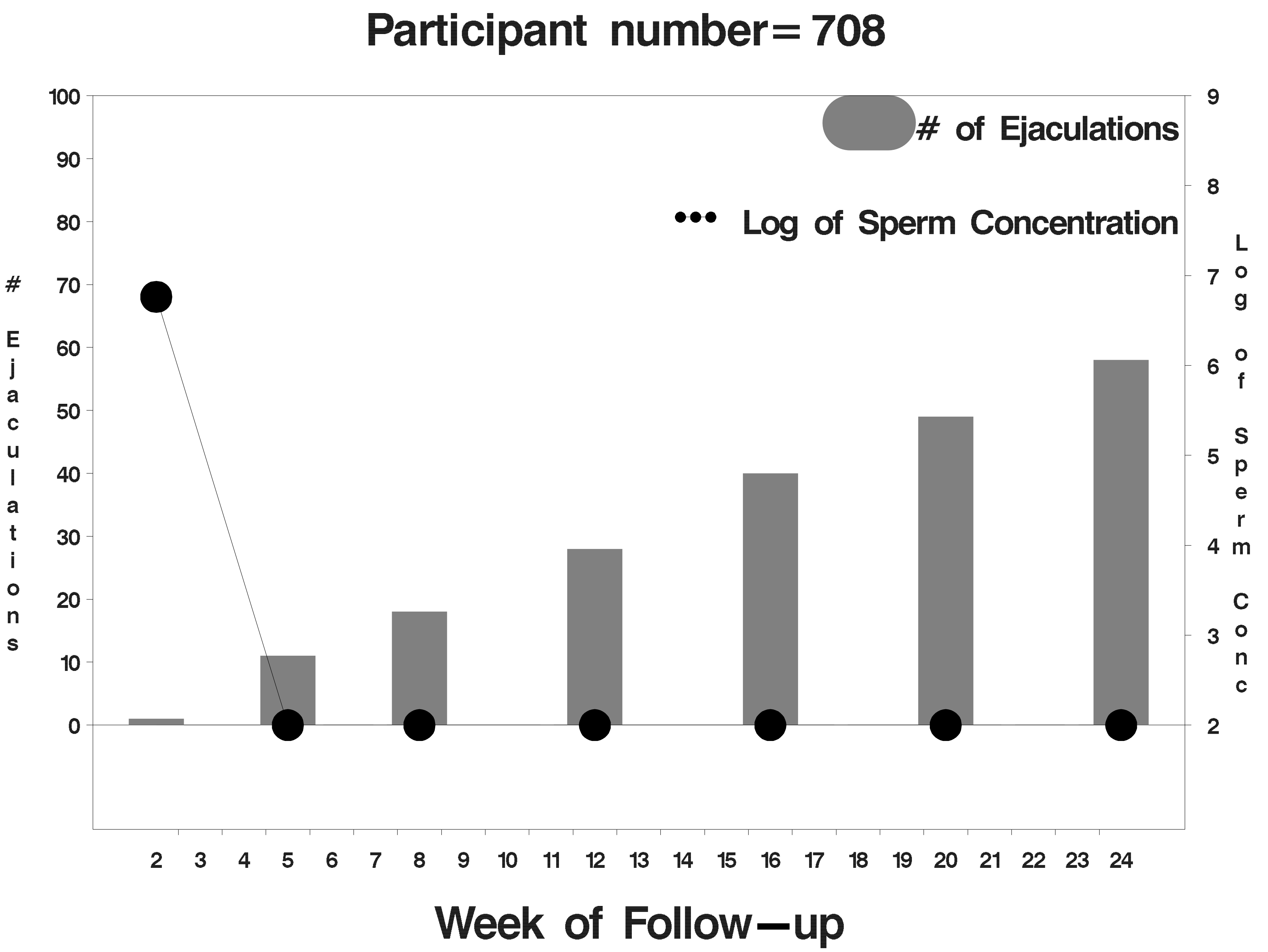

Participant number= 708
# of Ejaculations
Log of Sperm Concentration
Ejaculations
Log of Sperm Conc
100
90
80
70
60
50
40
30
20
10
0
9
8
7
6
5
4
3
2
2 3 4 5 6 7 8 9 10 11 12 13 14 15 16 17 18 19 20 21 22 23 24
Week of Follow-up

Participant number= 709

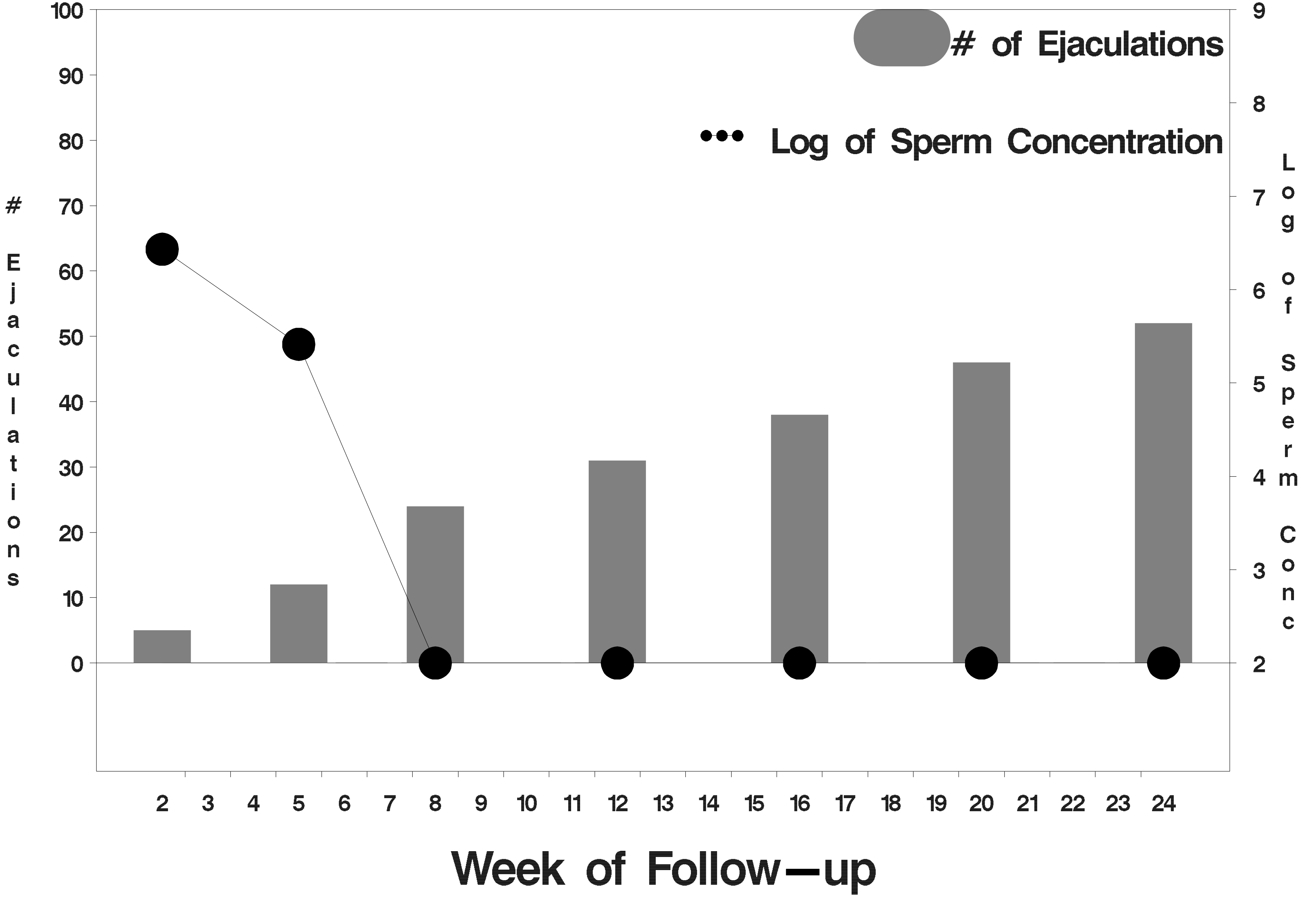

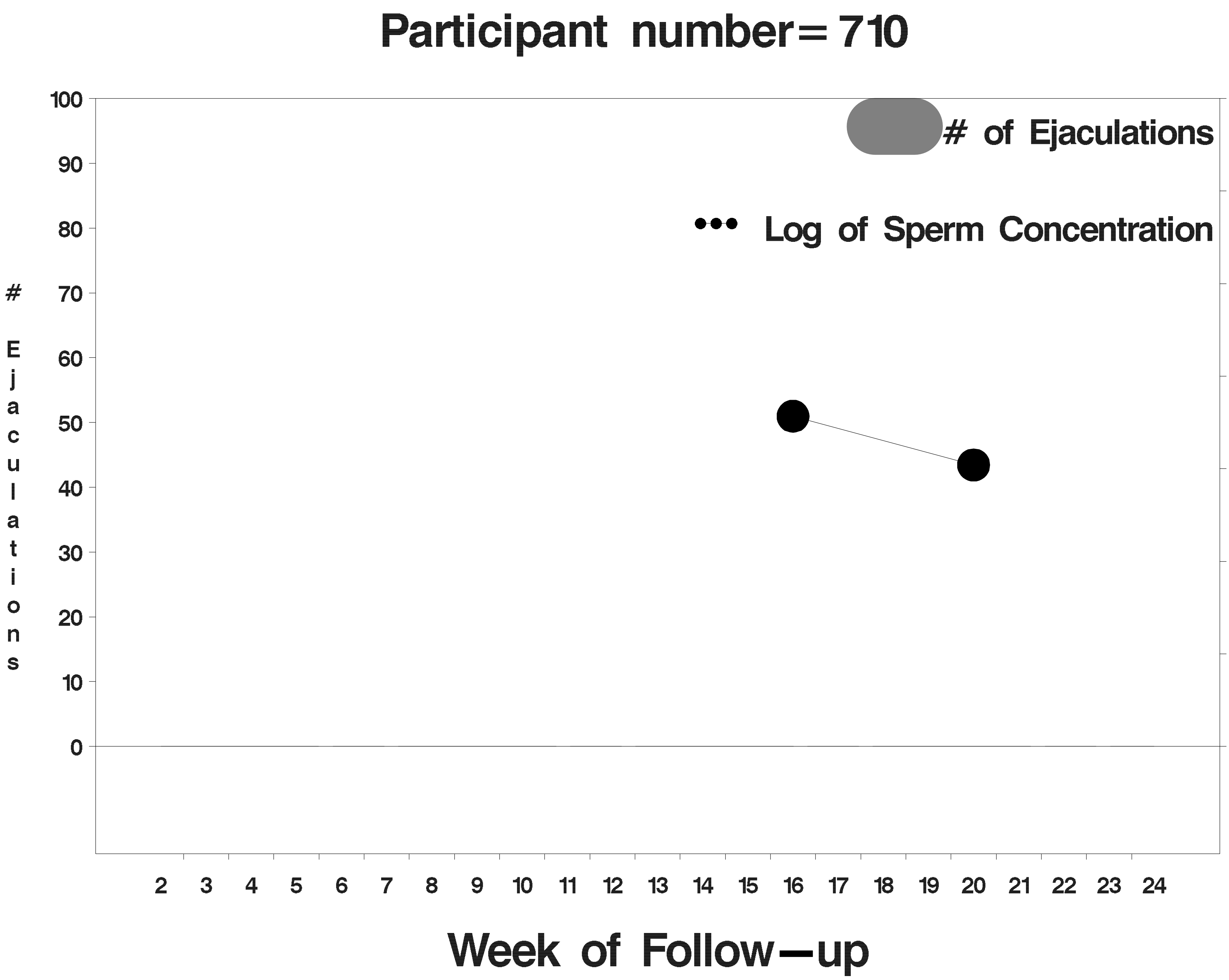

Participant number=710
# of Ejaculations
Log of Sperm Concentration
Ejaculations
Log of Sperm Conc
100
90
80
70
60
50
40
30
20
10
0
9
8
7
6
5
4
3
2
2 3 4 5 6 7 8 9 10 11 12 13 14 15 16 17 18 19 20 21 22 23 24
Week of Follow-up

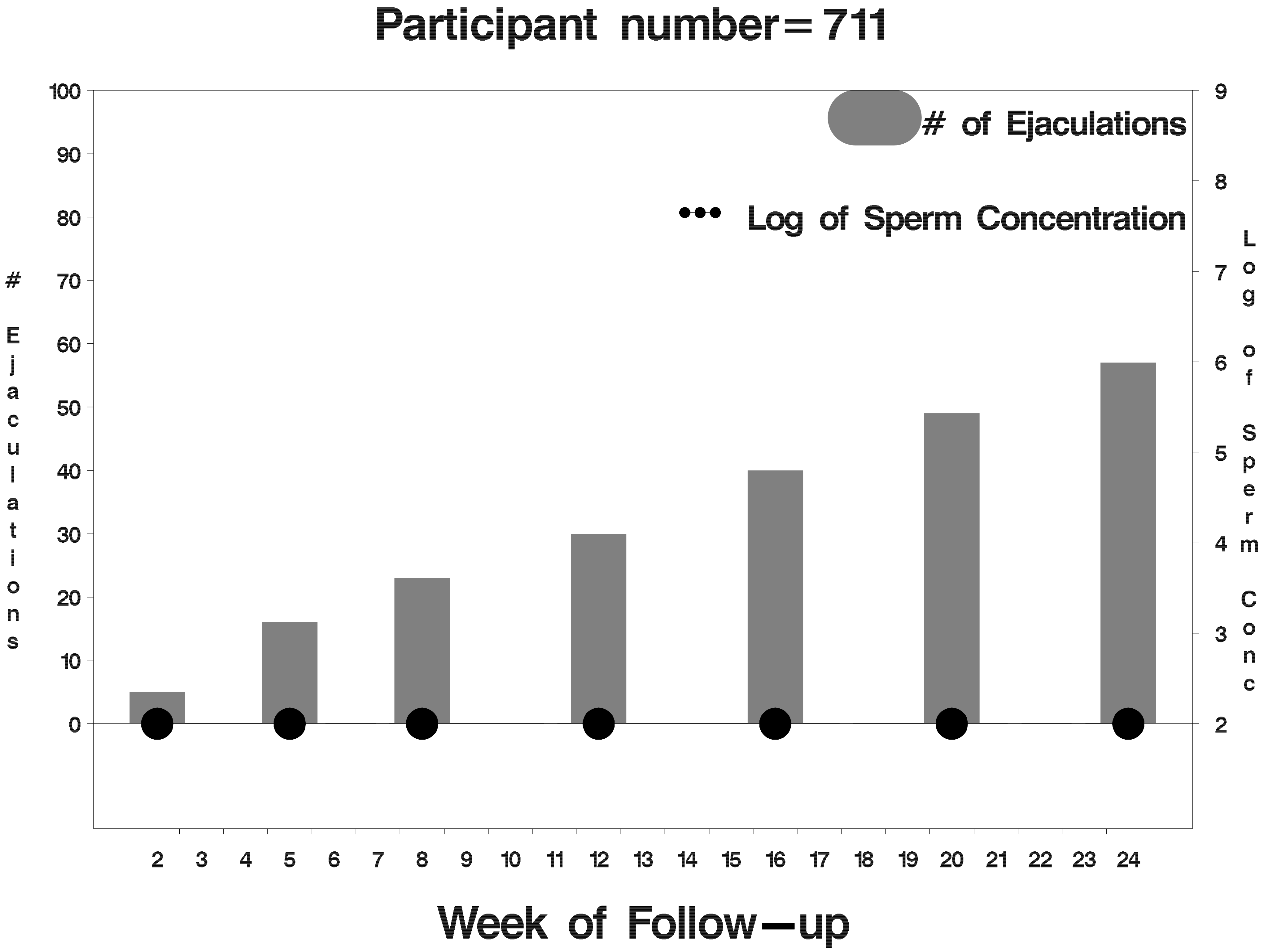

Participant number=711
# of Ejaculations
Log of Sperm Concentration
Ejaculations
Log of Sperm Conc
100
90
80
70
60
50
40
30
20
10
0
9
8
7
6
5
4
3
2
2 3 4 5 6 7 8 9 10 11 12 13 14 15 16 17 18 19 20 21 22 23 24
Week of Follow-up

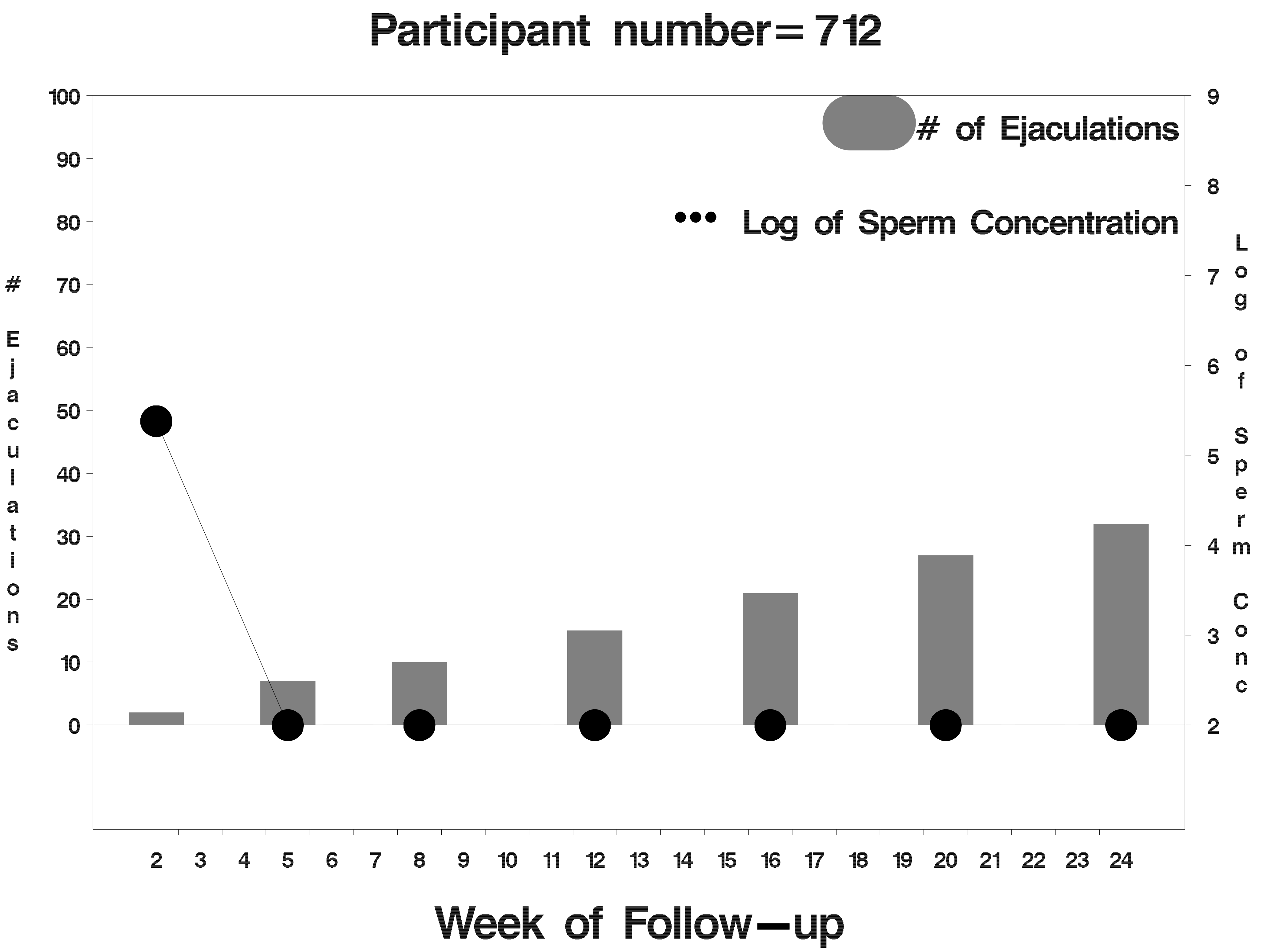
Participant number=712
# of Ejaculations
Log of Sperm Concentration
Ejaculations
Log of Sperm Conc
100
90
80
70
60
50
40
30
20
10
0
9
8
7
6
5
4
3
2
2 3 4 5 6 7 8 9 10 11 12 13 14 15 16 17 18 19 20 21 22 23 24
Week of Follow-up

Participant number=713

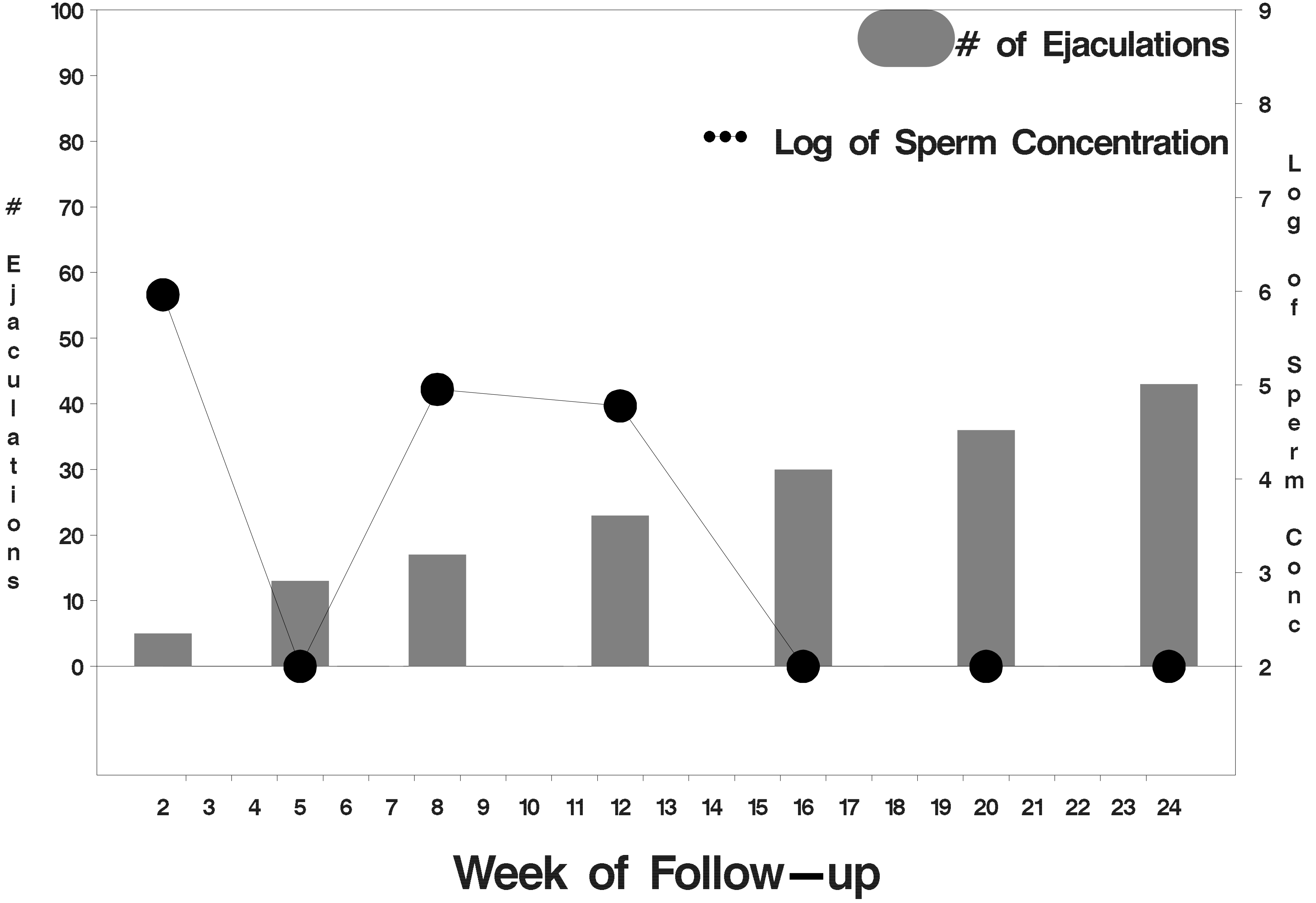

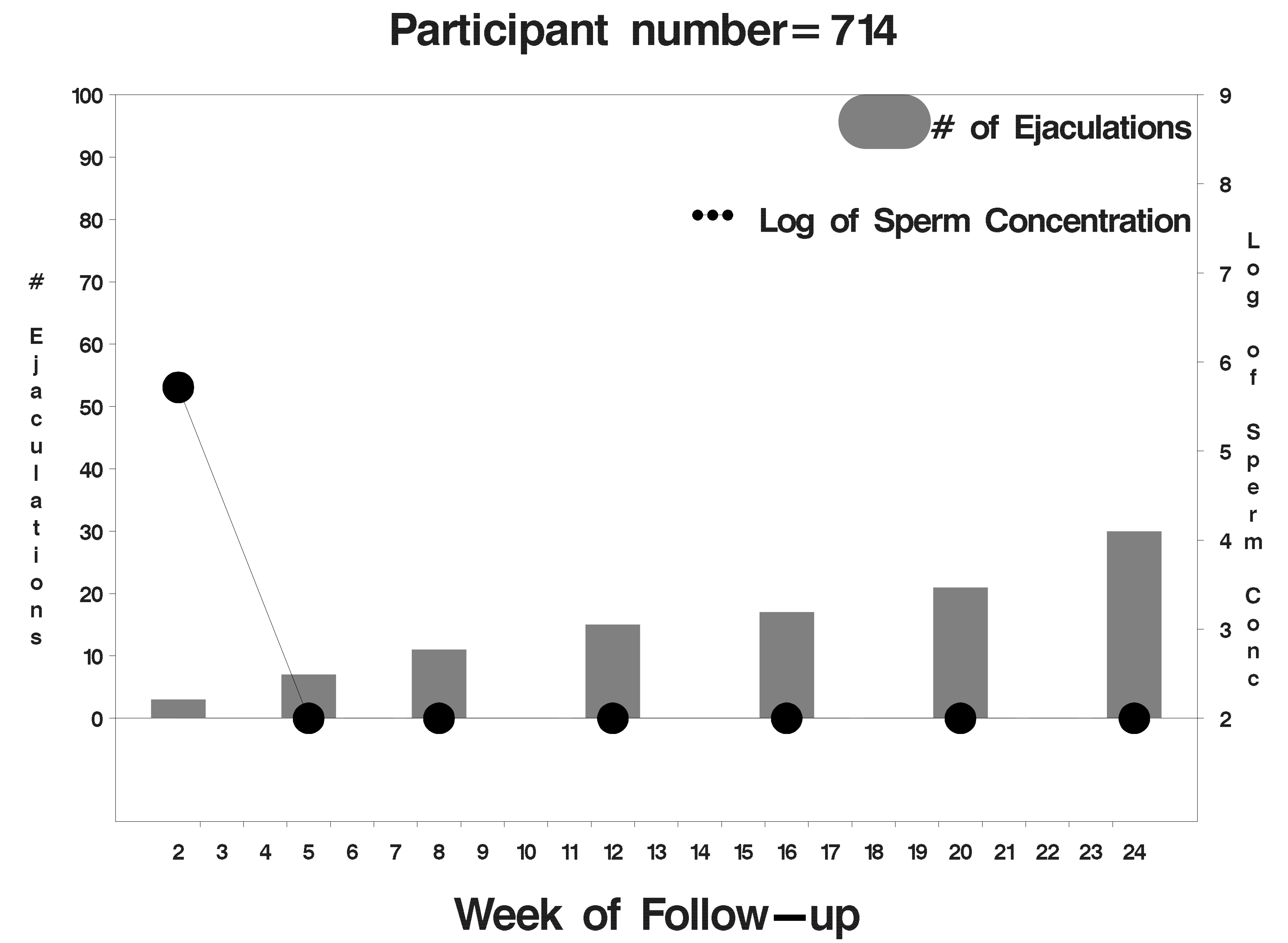

Participant number=714
# of Ejaculations
Log of Sperm Concentration
Ejaculations
Log of Sperm Conc
100
90
80
70
60
50
40
30
20
10
0
9
8
7
6
5
4
3
2
2 3 4 5 6 7 8 9 10 11 12 13 14 15 16 17 18 19 20 21 22 23 24
Week of Follow-up

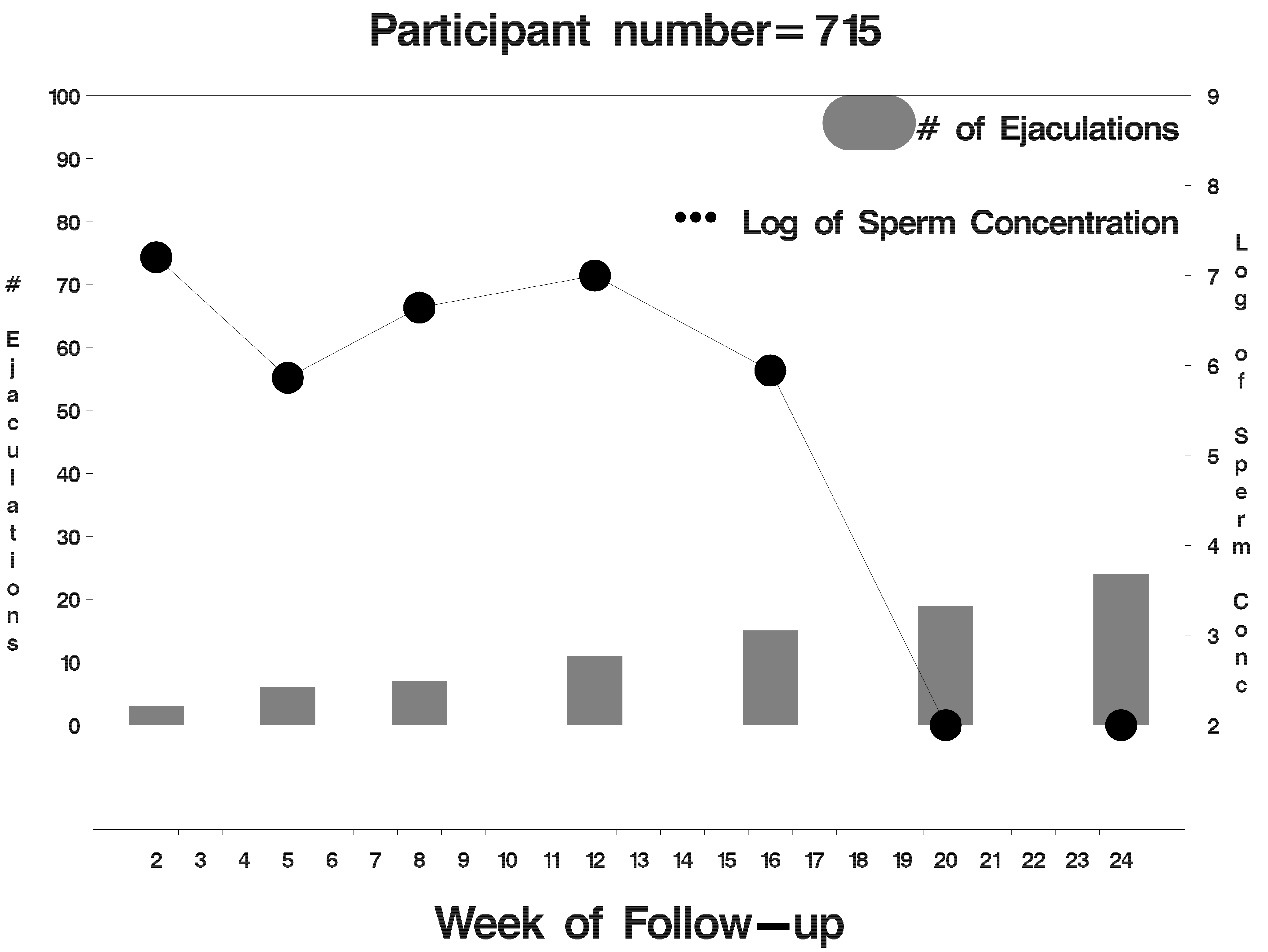

Participant number=715
# of Ejaculations
Log of Sperm Concentration
Ejaculations
Log of Sperm Conc
100
90
80
70
60
50
40
30
20
10
0
9
8
7
6
5
4
3
2
2 3 4 5 6 7 8 9 10 11 12 13 14 15 16 17 18 19 20 21 22 23 24
Week of Follow-up

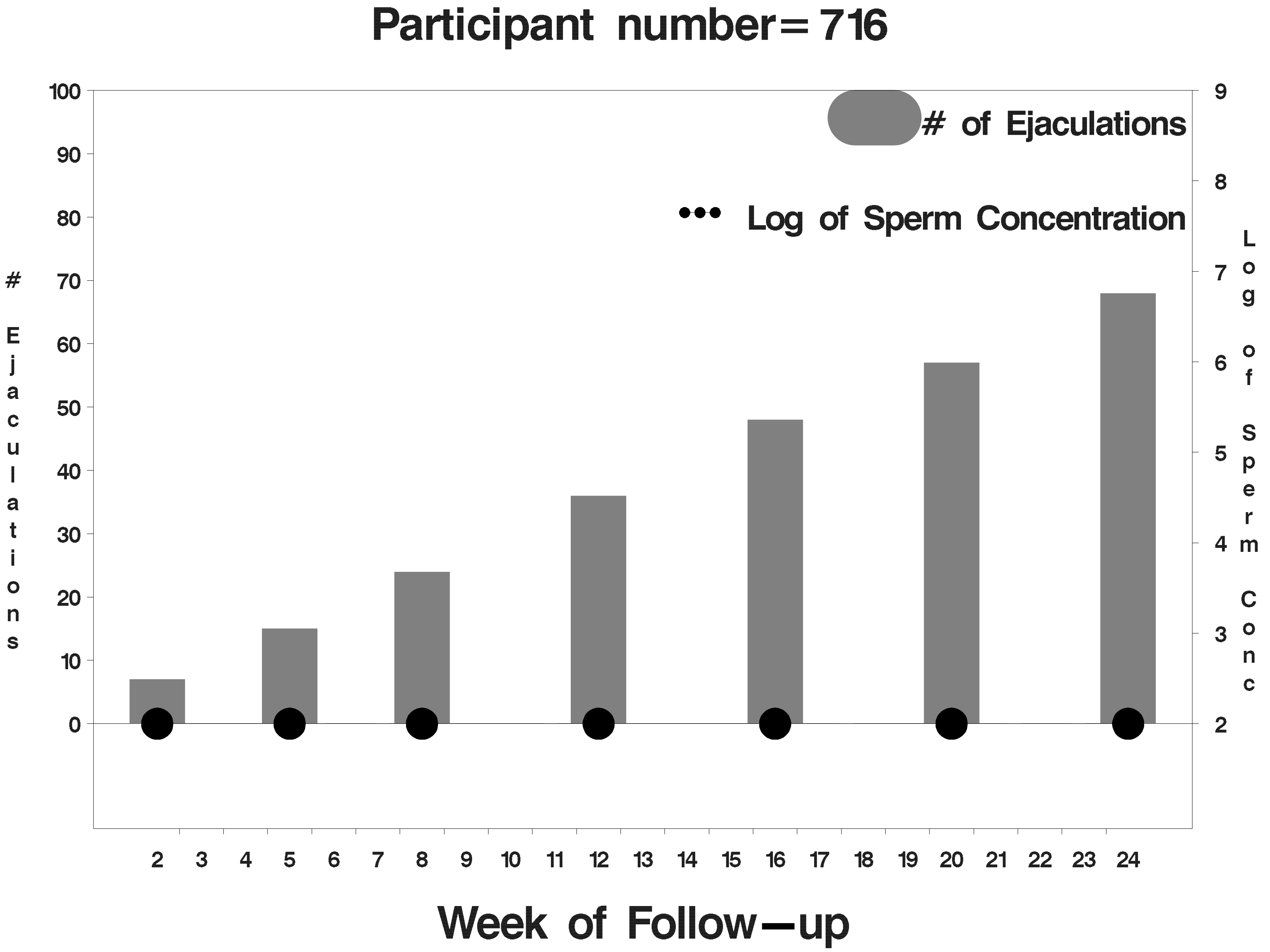

Participant number=716
# of Ejaculations
Log of Sperm Concentration
Ejaculations
Log of Sperm Conc
100
90
80
70
60
50
40
30
20
10
0
9
8
7
6
5
4
3
2
2 3 4 5 6 7 8 9 10 11 12 13 14 15 16 17 18 19 20 21 22 23 24
Week of Follow−up

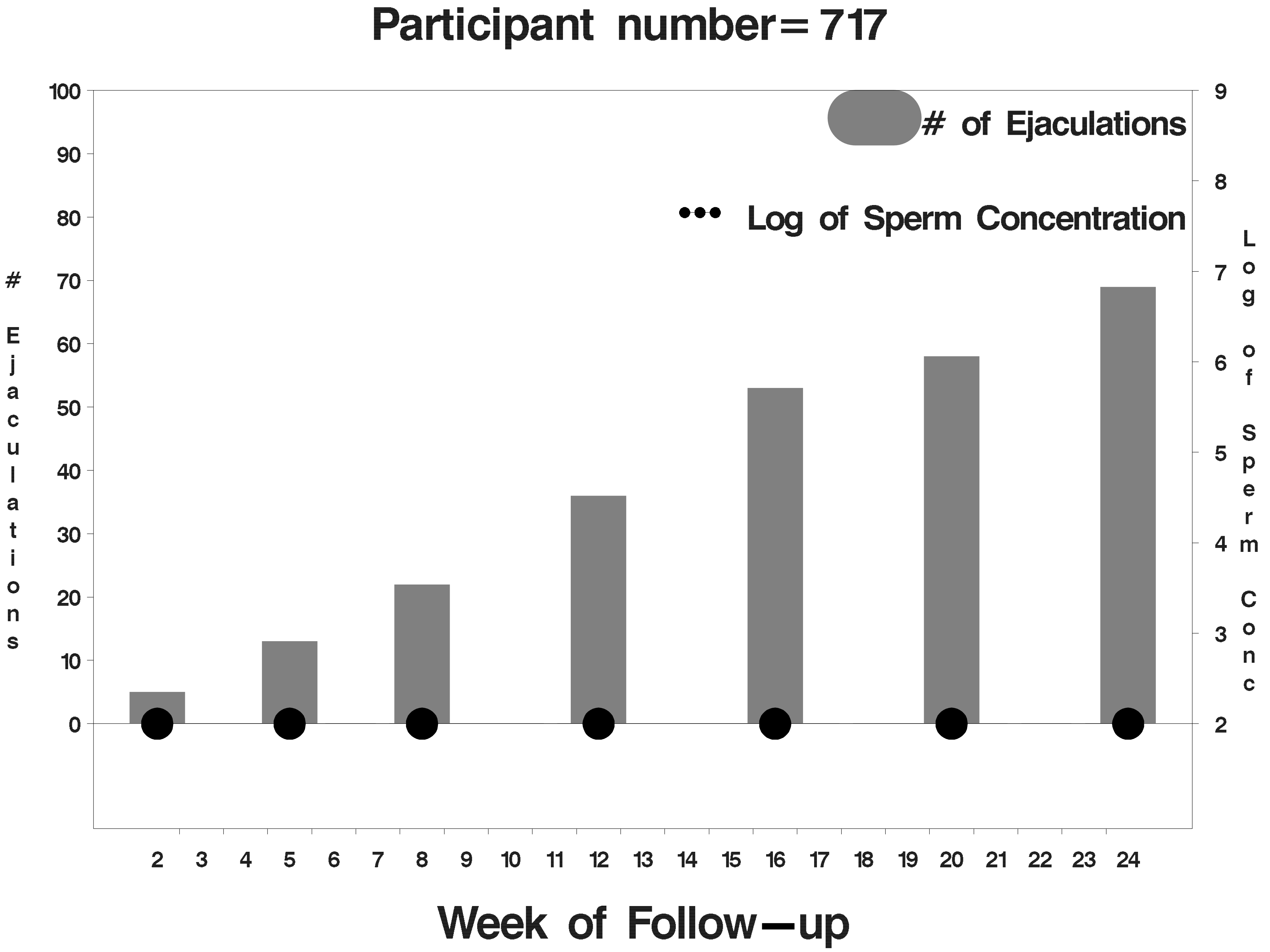

Participant number= 717
# of Ejaculations
Log of Sperm Concentration
Ejaculations
Log of Sperm Conc
100
90
80
70
60
50
40
30
20
10
0
9
8
7
6
5
4
3
2
2 3 4 5 6 7 8 9 10 11 12 13 14 15 16 17 18 19 20 21 22 23 24
Week of Follow-up

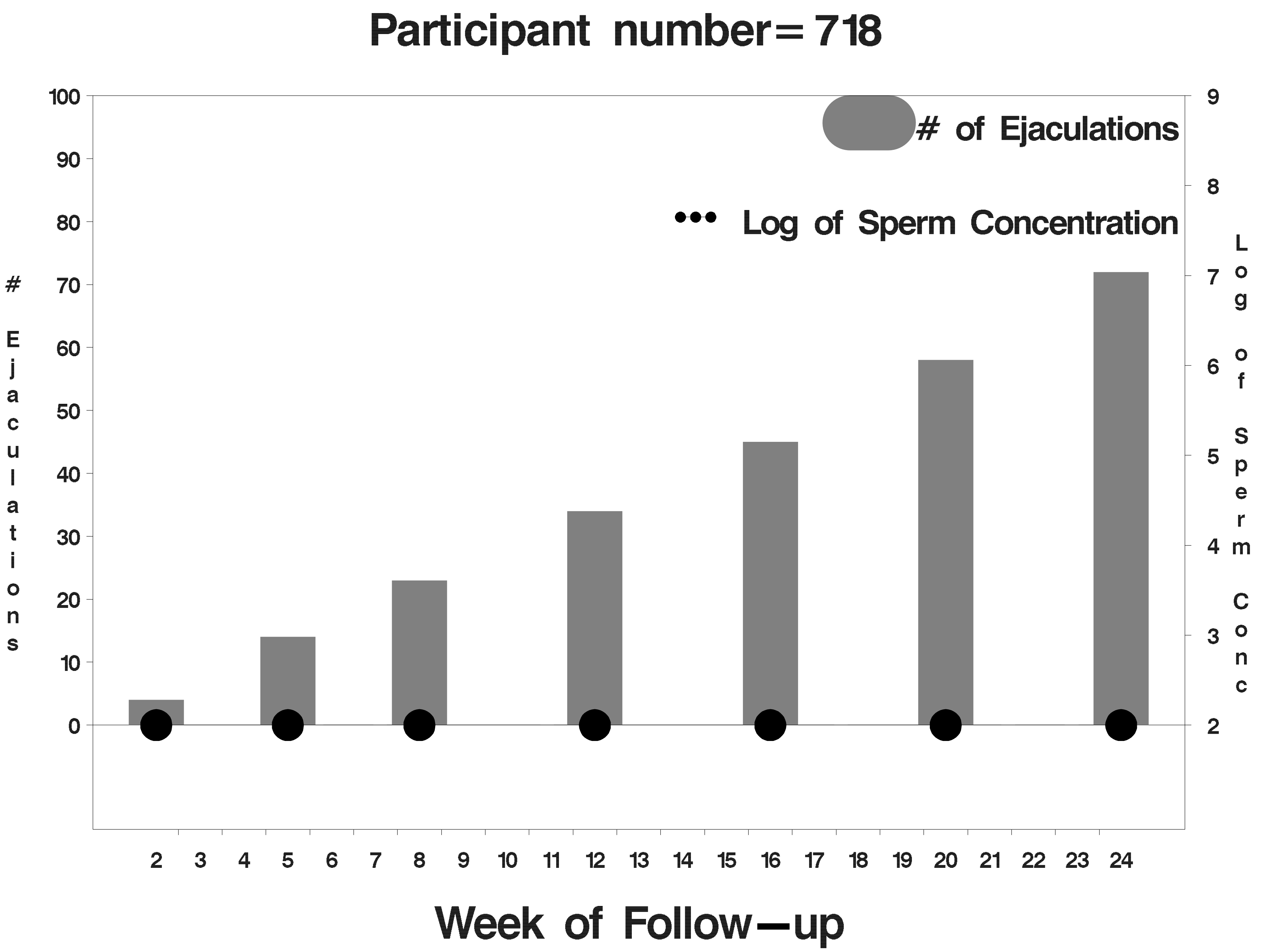

Participant number=718
# of Ejaculations
Log of Sperm Concentration
Ejaculations
Log of Sperm Conc
100
90
80
70
60
50
40
30
20
10
0
9
8
7
6
5
4
3
2
2 3 4 5 6 7 8 9 10 11 12 13 14 15 16 17 18 19 20 21 22 23 24
Week of Follow-up

Participant number=719

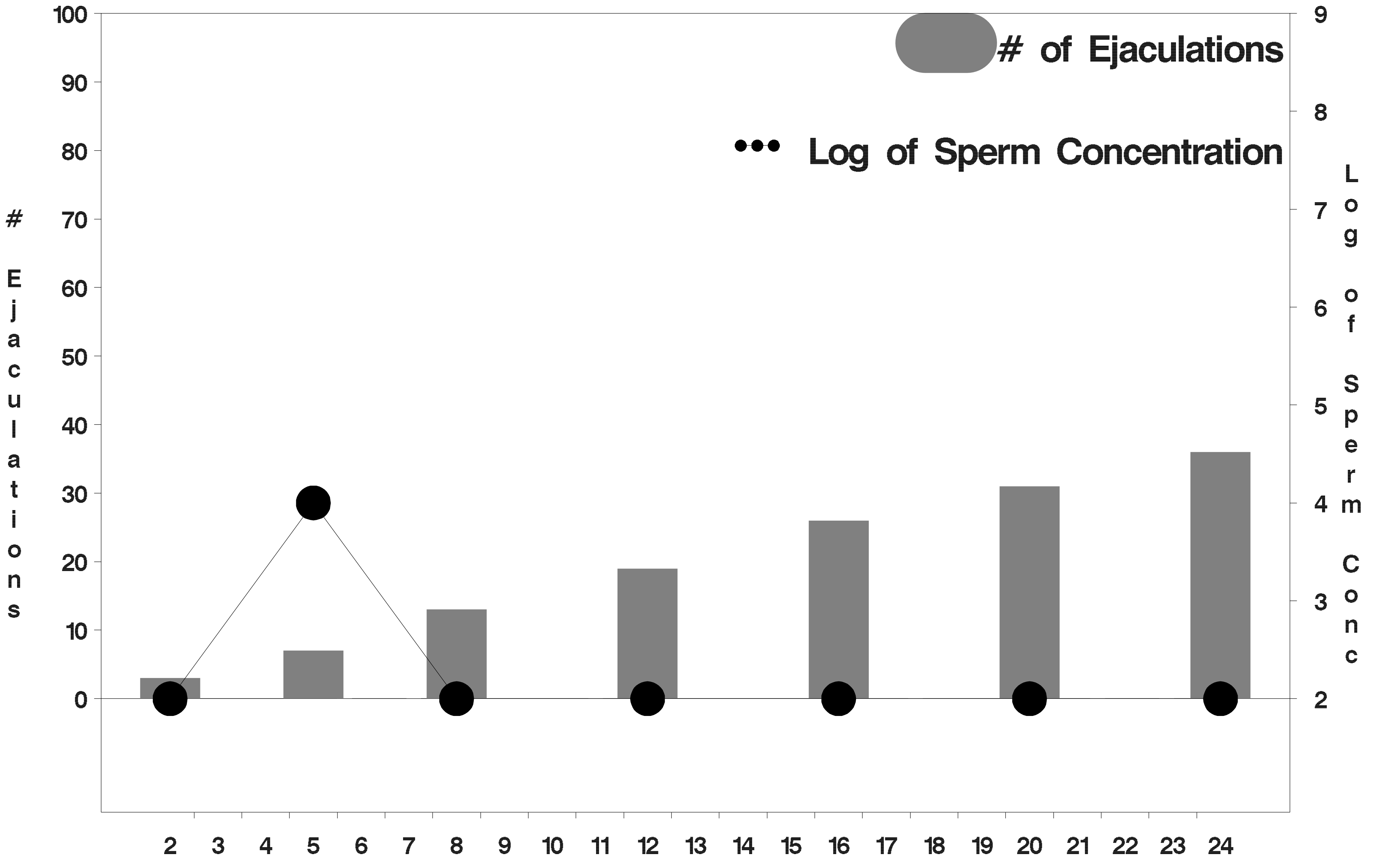

Week of Follow−up

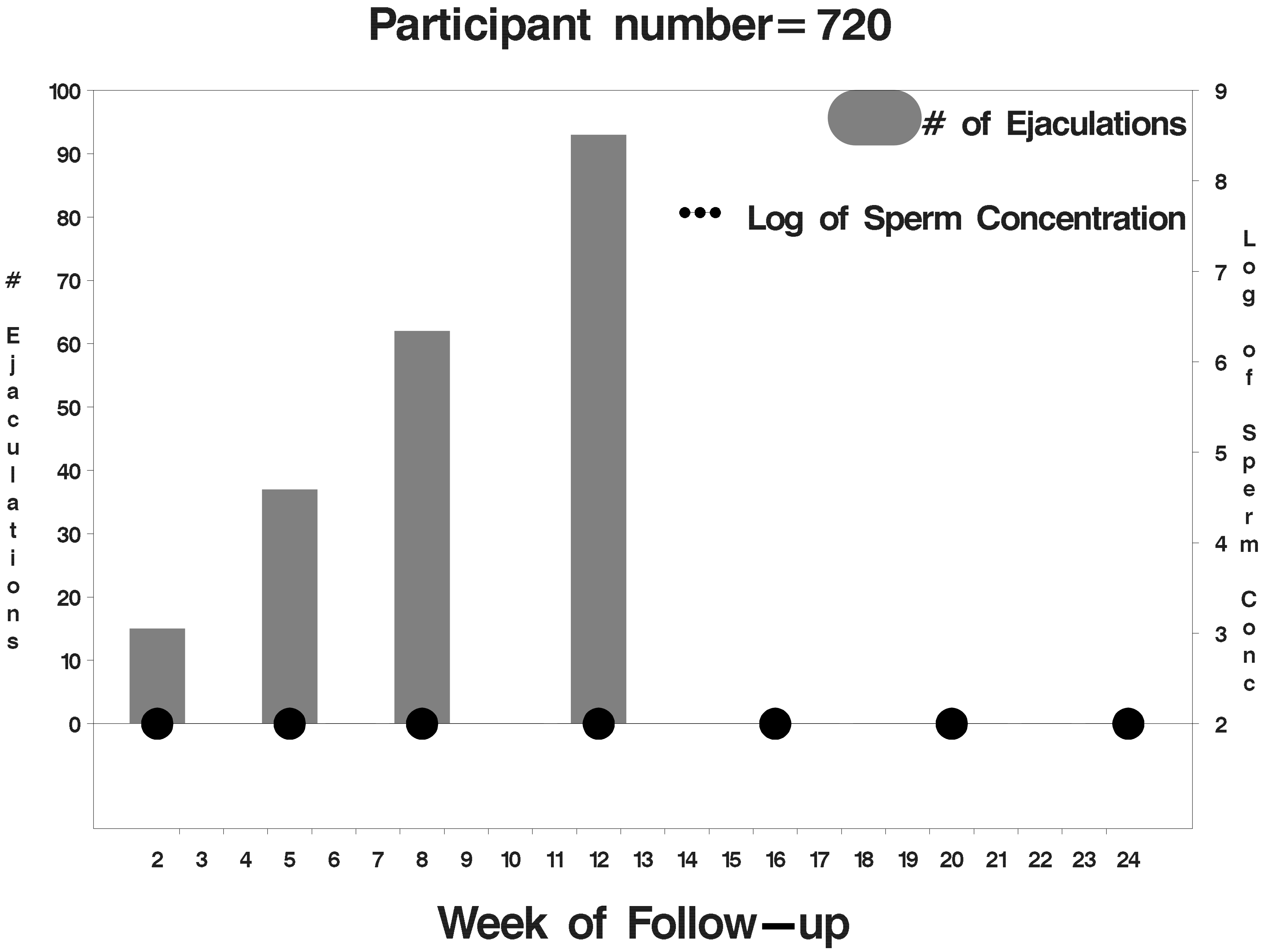

Participant number= 720
# of Ejaculations
Log of Sperm Concentration
Ejaculations
Log of Sperm Conc
100
90
80
70
60
50
40
30
20
10
0
9
8
7
6
5
4
3
2
2 3 4 5 6 7 8 9 10 11 12 13 14 15 16 17 18 19 20 21 22 23 24
Week of Follow-up

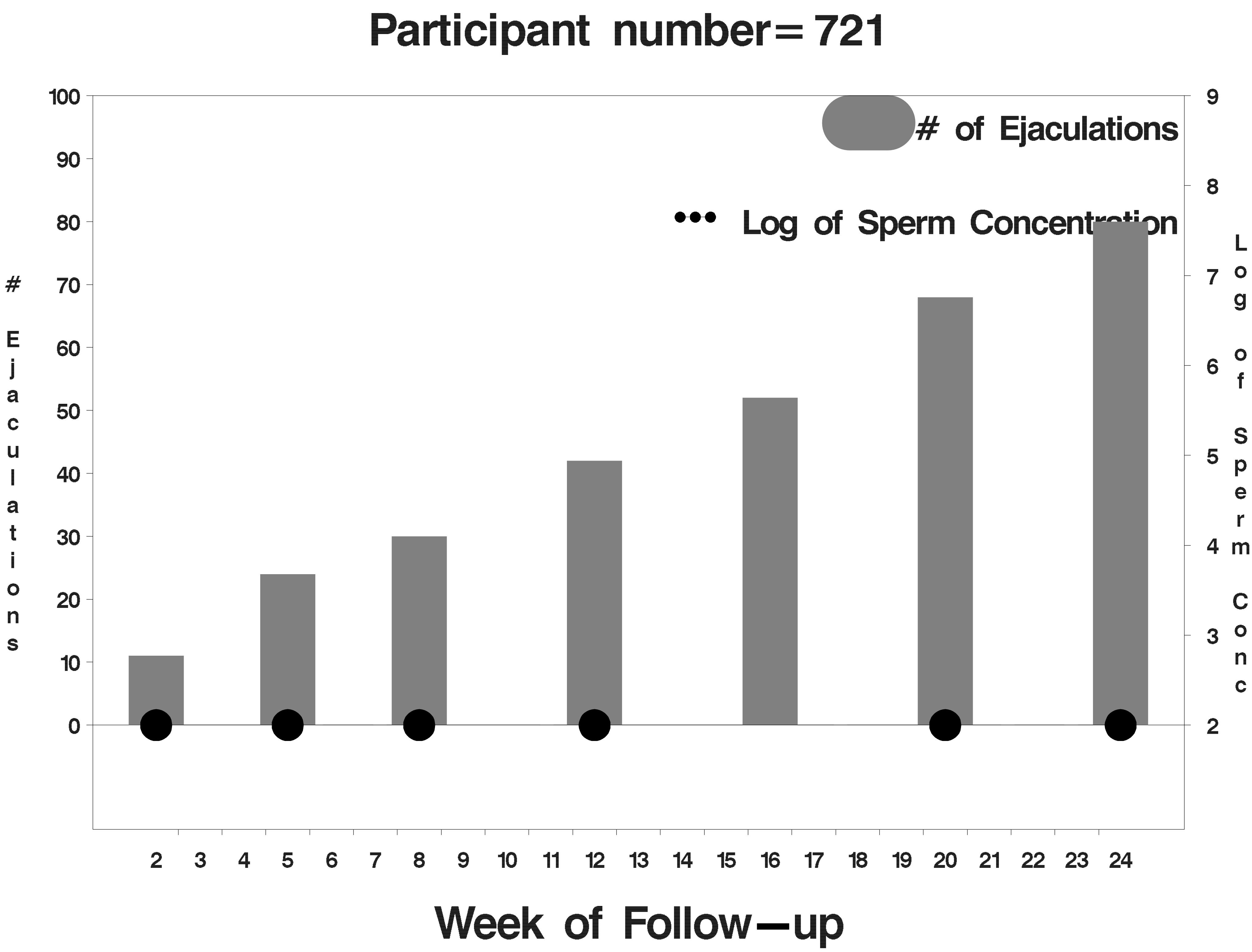

Participant number=721
# of Ejaculations
Log of Sperm Concentration
Ejaculations
Log of Sperm Conc
Week of Follow-up
100
90
80
70
60
50
40
30
20
10
0
9
8
7
6
5
4
3
2
2 3 4 5 6 7 8 9 10 11 12 13 14 15 16 17 18 19 20 21 22 23 24

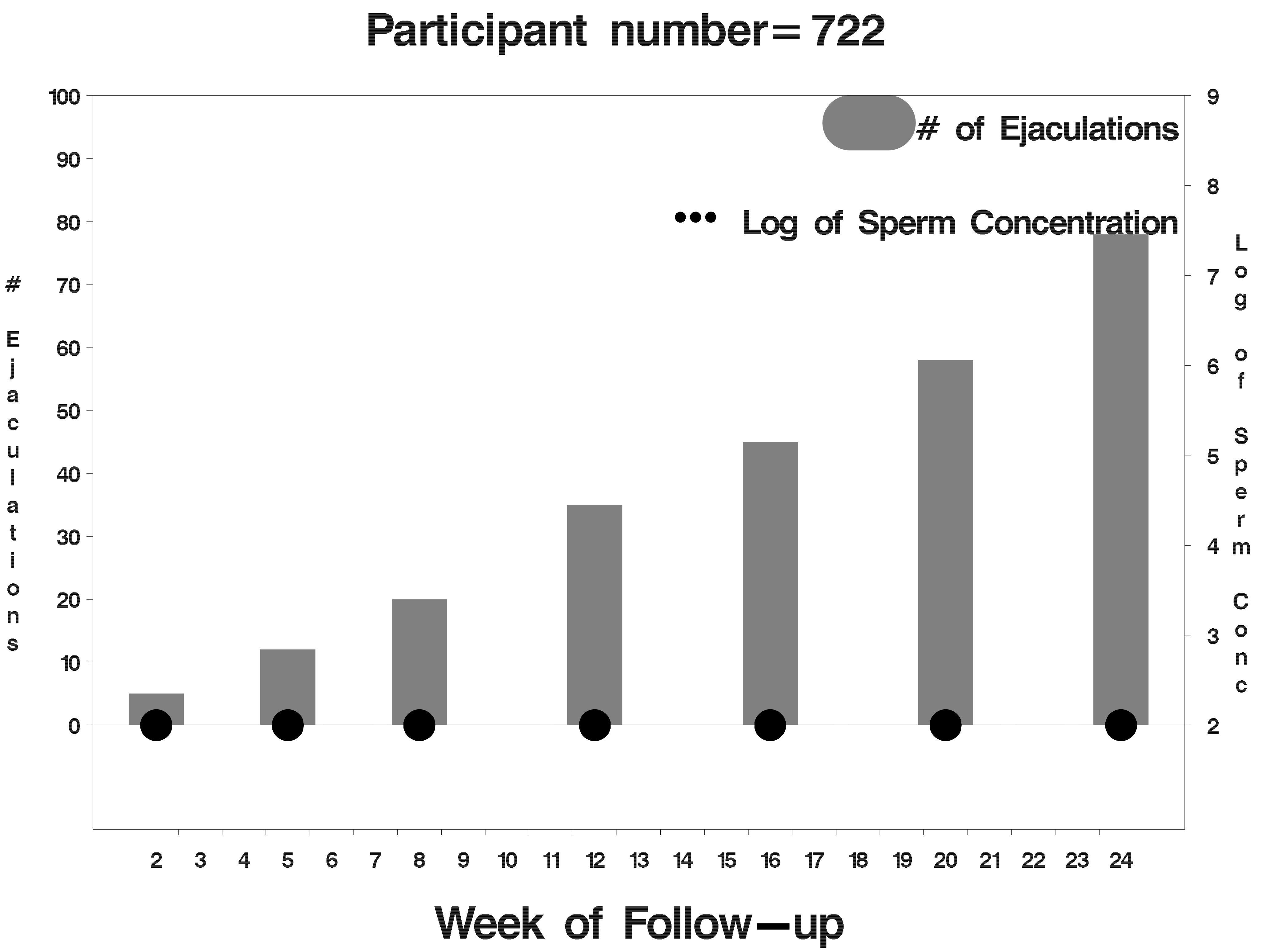

Participant number= 722
# of Ejaculations
Log of Sperm Concentration
Ejaculations
Log of Sperm Conc
100
90
80
70
60
50
40
30
20
10
0
9
8
7
6
5
4
3
2
2 3 4 5 6 7 8 9 10 11 12 13 14 15 16 17 18 19 20 21 22 23 24
Week of Follow-up

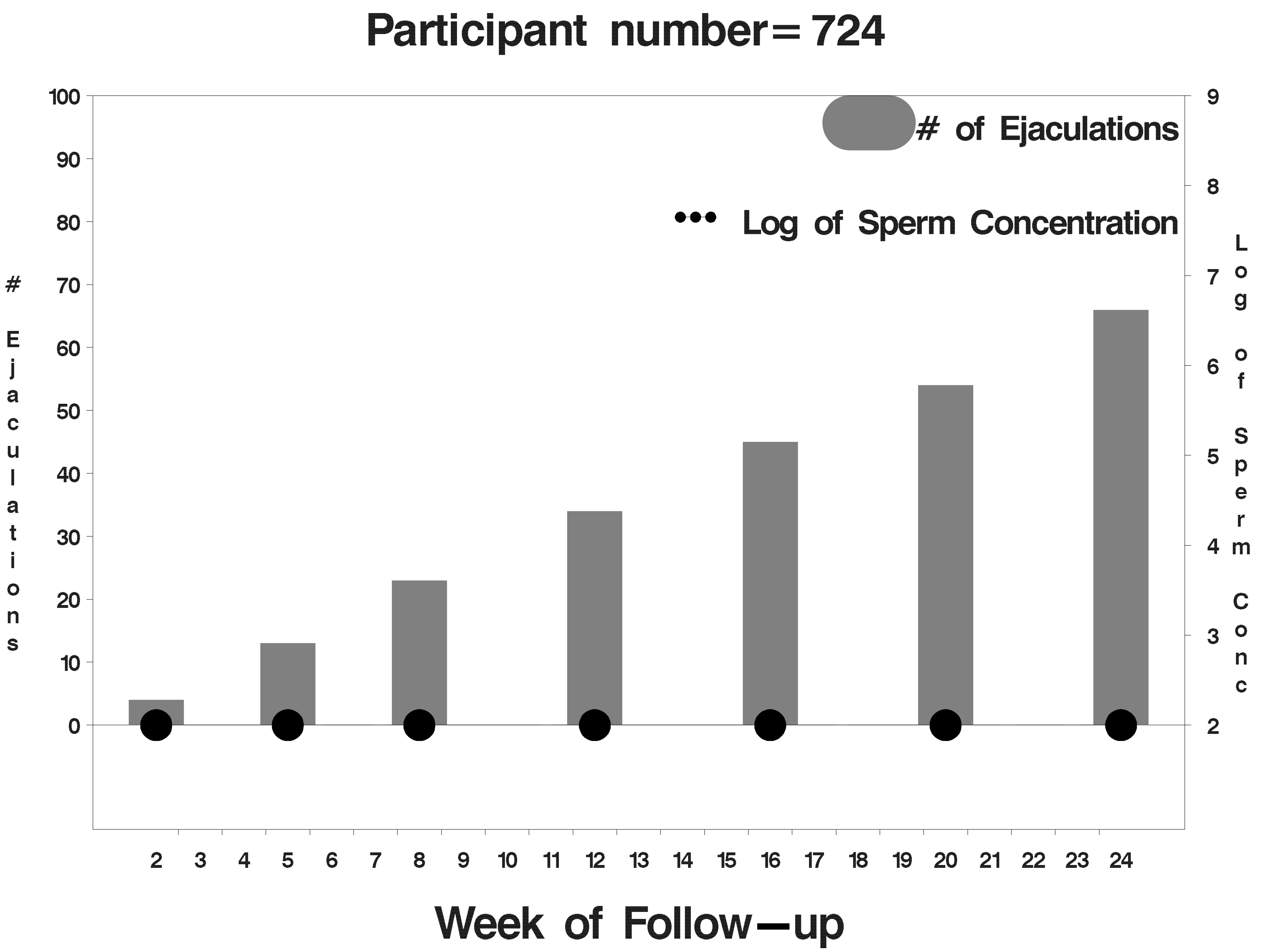

Participant number= 724
# of Ejaculations
Log of Sperm Concentration
Ejaculations
Log of Sperm Conc
100
90
80
70
60
50
40
30
20
10
0
9
8
7
6
5
4
3
2
2 3 4 5 6 7 8 9 10 11 12 13 14 15 16 17 18 19 20 21 22 23 24
Week of Follow−up

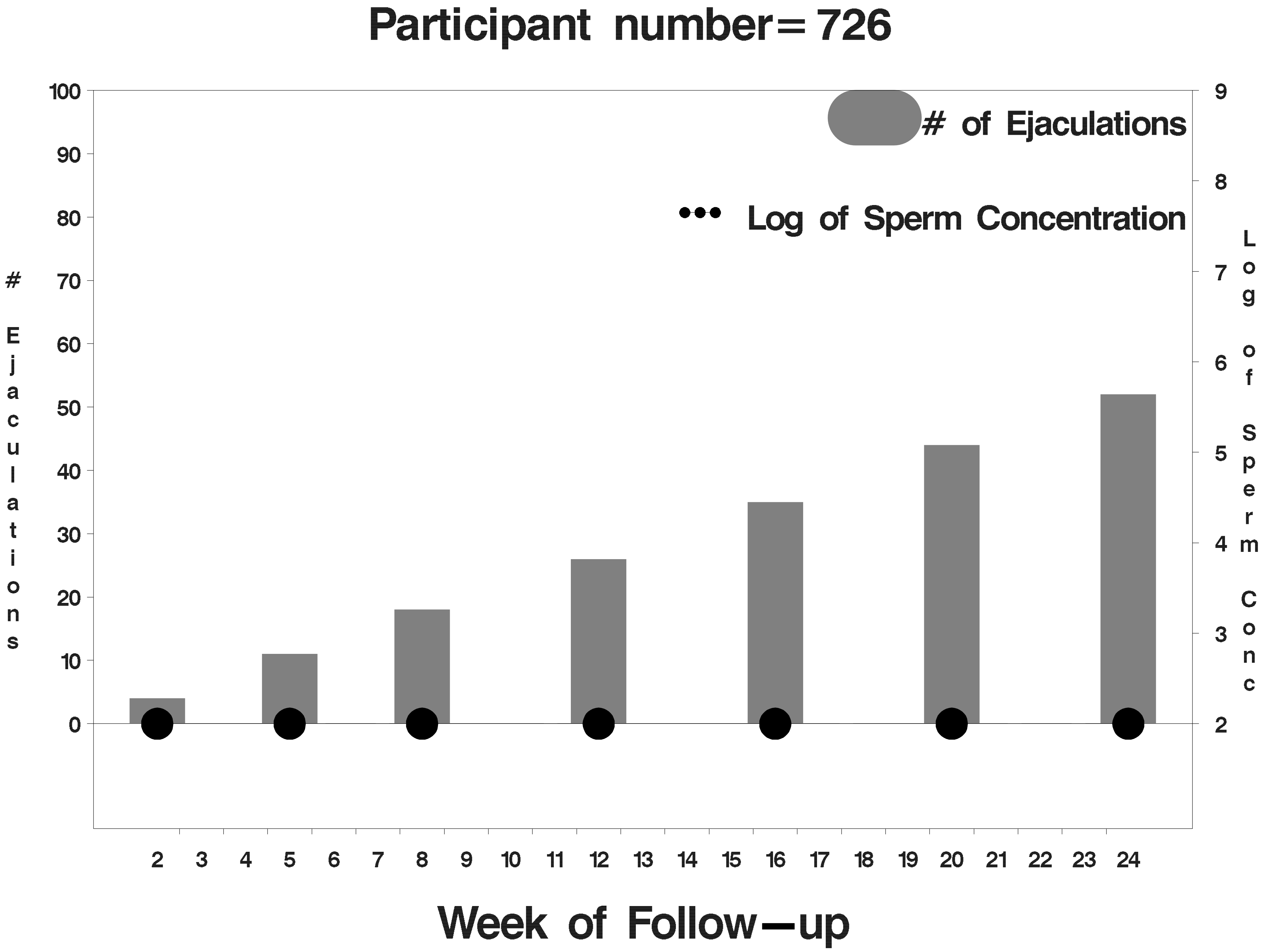

Participant number= 726
# of Ejaculations
Log of Sperm Concentration
Ejaculations
Log of Sperm Conc
100
90
80
70
60
50
40
30
20
10
0
9
8
7
6
5
4
3
2
2 3 4 5 6 7 8 9 10 11 12 13 14 15 16 17 18 19 20 21 22 23 24
Week of Follow—up

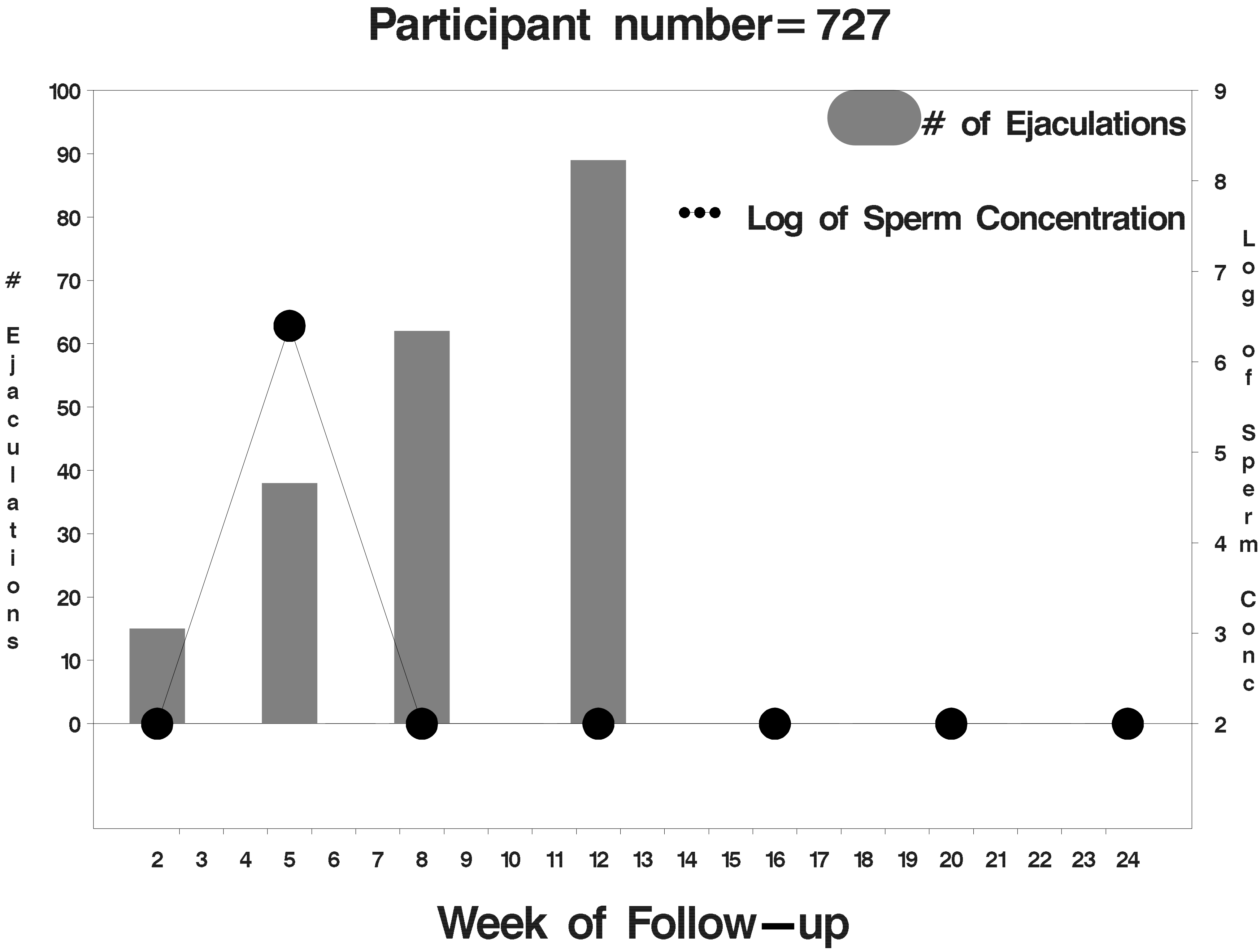

Participant number= 727
# of Ejaculations
Log of Sperm Concentration
Ejaculations
Log of Sperm Conc
100
90
80
70
60
50
40
30
20
10
0
9
8
7
6
5
4
3
2
2 3 4 5 6 7 8 9 10 11 12 13 14 15 16 17 18 19 20 21 22 23 24
Week of Follow-up

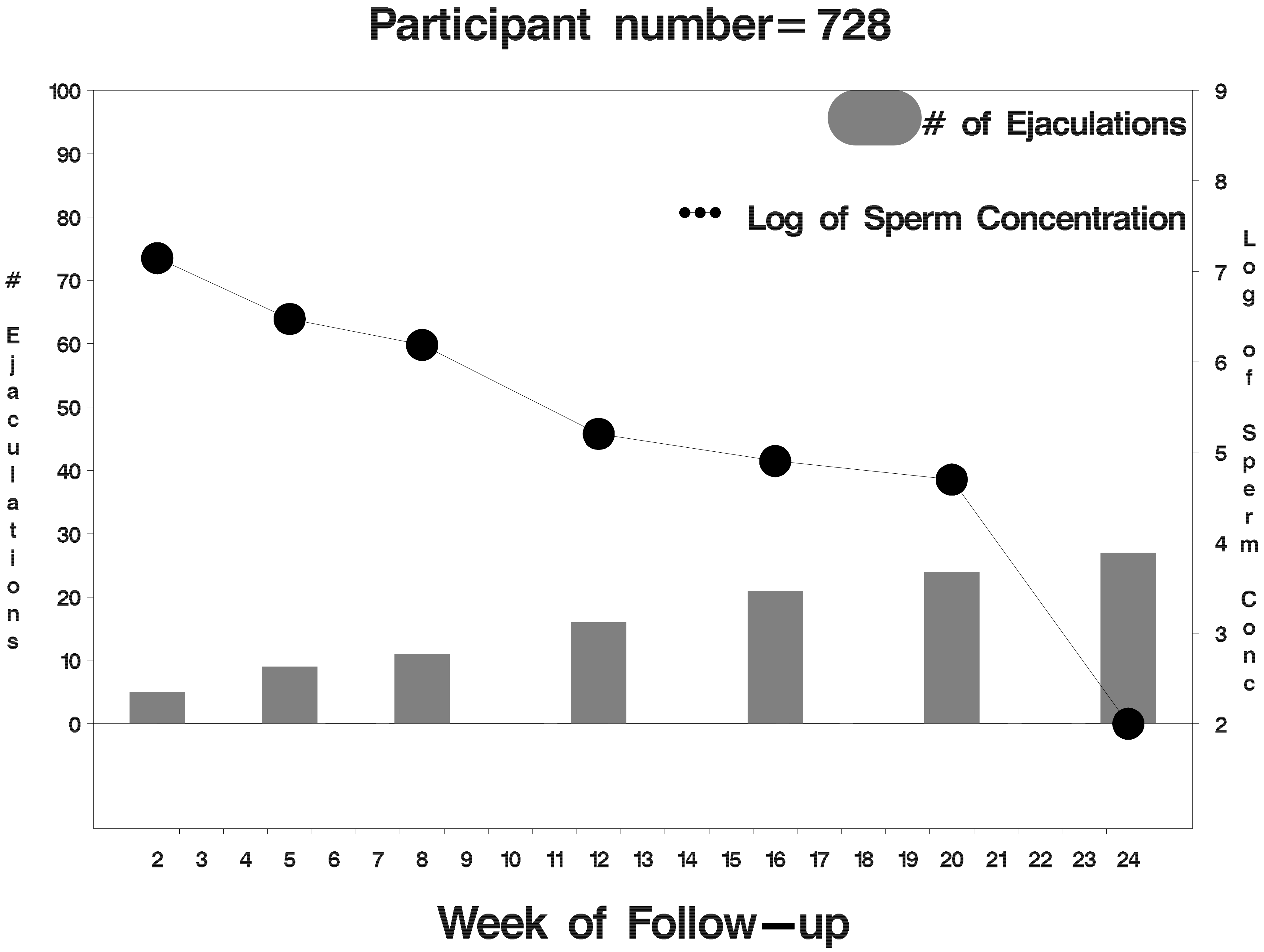

Participant number= 728
# of Ejaculations
Log of Sperm Concentration
Ejaculations
Log of Sperm Conc
100
90
80
70
60
50
40
30
20
10
0
9
8
7
6
5
4
3
2
2 3 4 5 6 7 8 9 10 11 12 13 14 15 16 17 18 19 20 21 22 23 24
Week of Follow-up

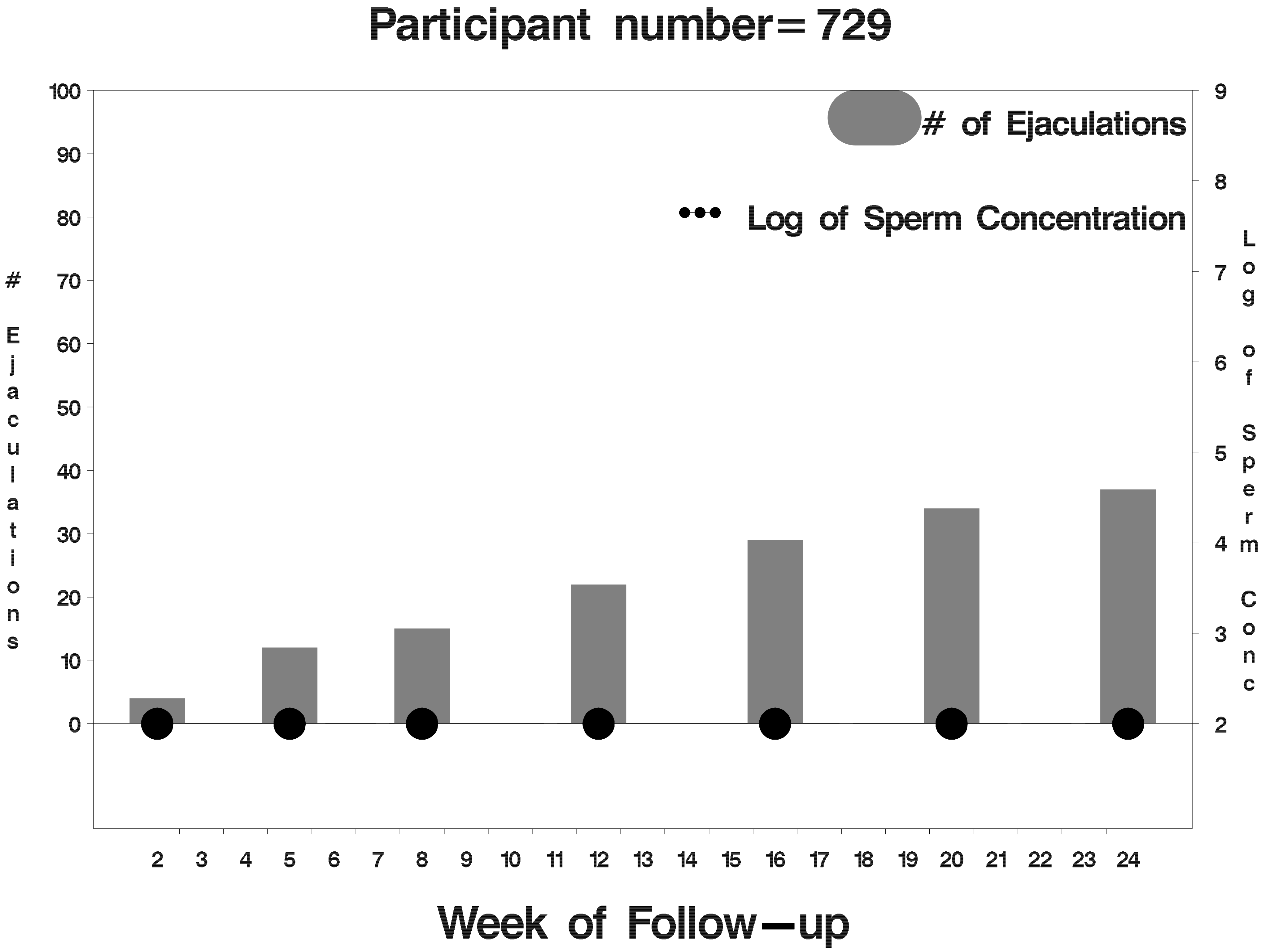

Participant number= 729
# of Ejaculations
Log of Sperm Concentration
Ejaculations
Log of Sperm Conc
100
90
80
70
60
50
40
30
20
10
0
9
8
7
6
5
4
3
2
2 3 4 5 6 7 8 9 10 11 12 13 14 15 16 17 18 19 20 21 22 23 24
Week of Follow-up

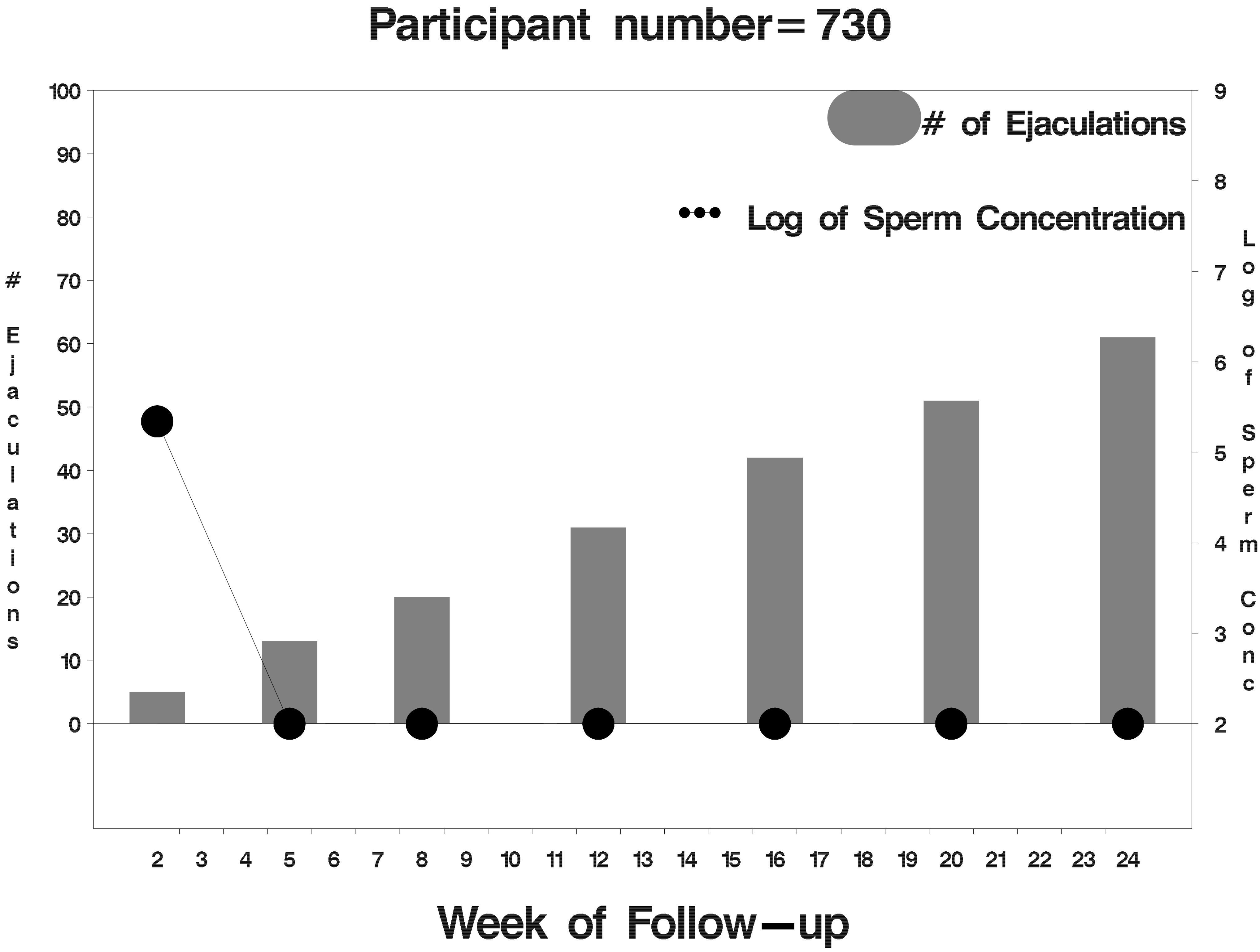

Participant number= 730
# of Ejaculations
Log of Sperm Concentration
Ejaculations
Log of Sperm Conc
100
90
80
70
60
50
40
30
20
10
0
9
8
7
6
5
4
3
2
2 3 4 5 6 7 8 9 10 11 12 13 14 15 16 17 18 19 20 21 22 23 24
Week of Follow-up

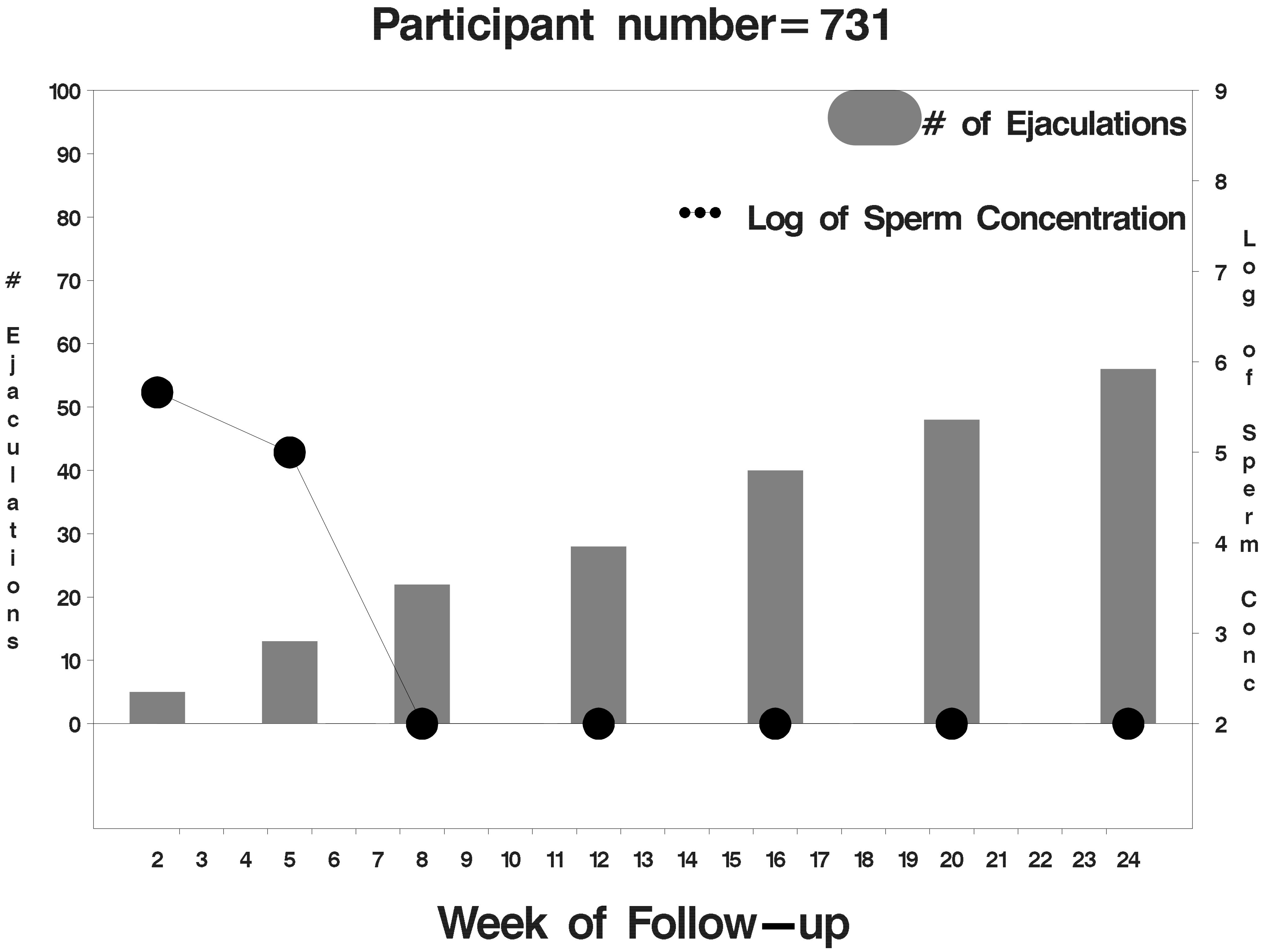

Participant number= 731
# of Ejaculations
Log of Sperm Concentration
Ejaculations
Log of Sperm Conc
0
10
20
30
40
50
60
70
80
90
100
2
3
4
5
6
7
8
9
2 3 4 5 6 7 8 9 10 11 12 13 14 15 16 17 18 19 20 21 22 23 24
Week of Follow-up

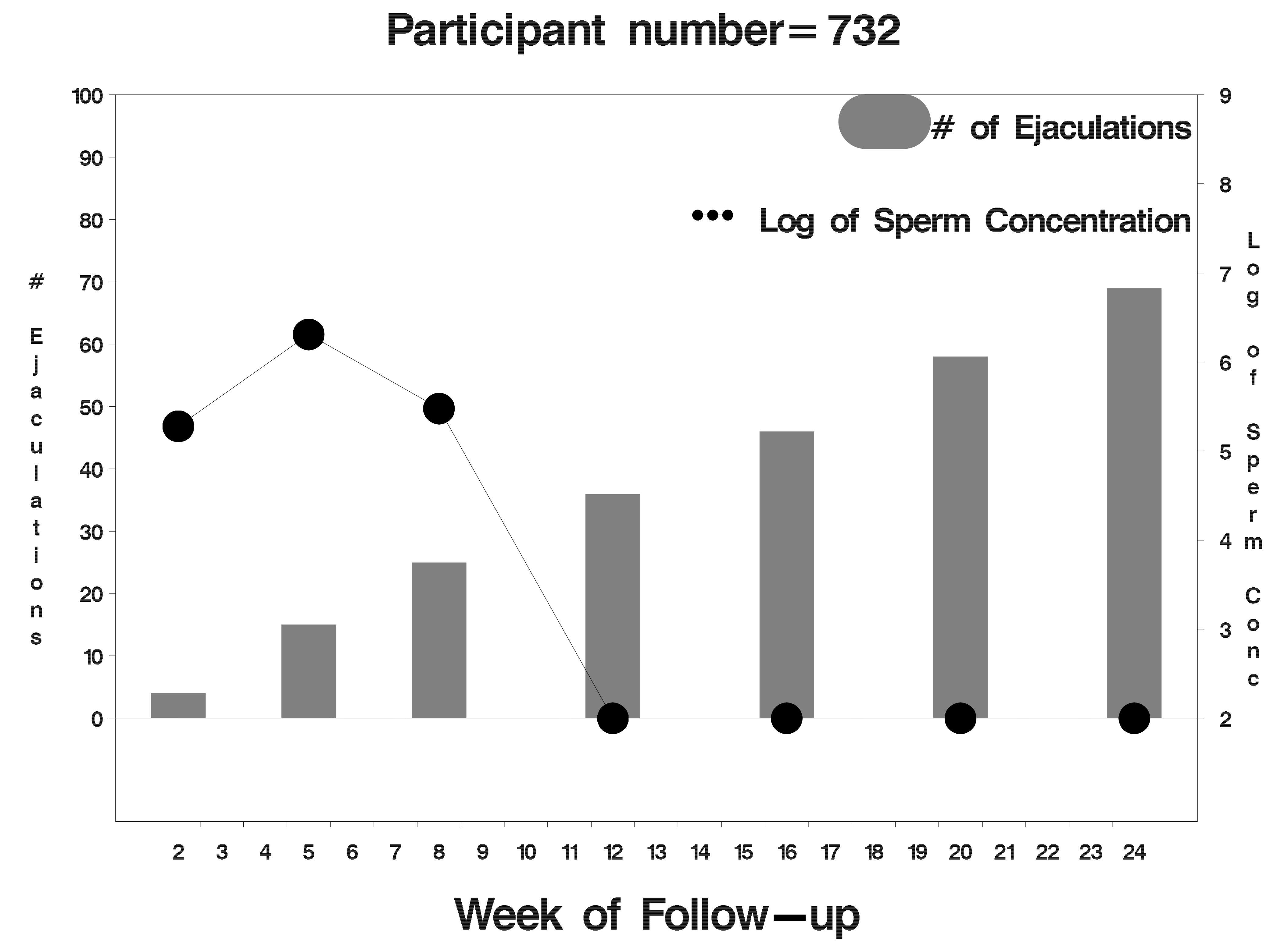
Participant number= 732
# of Ejaculations
Log of Sperm Concentration
Ejaculations
Log of Sperm Conc
100
90
80
70
60
50
40
30
20
10
0
9
8
7
6
5
4
3
2
2 3 4 5 6 7 8 9 10 11 12 13 14 15 16 17 18 19 20 21 22 23 24
Week of Follow-up

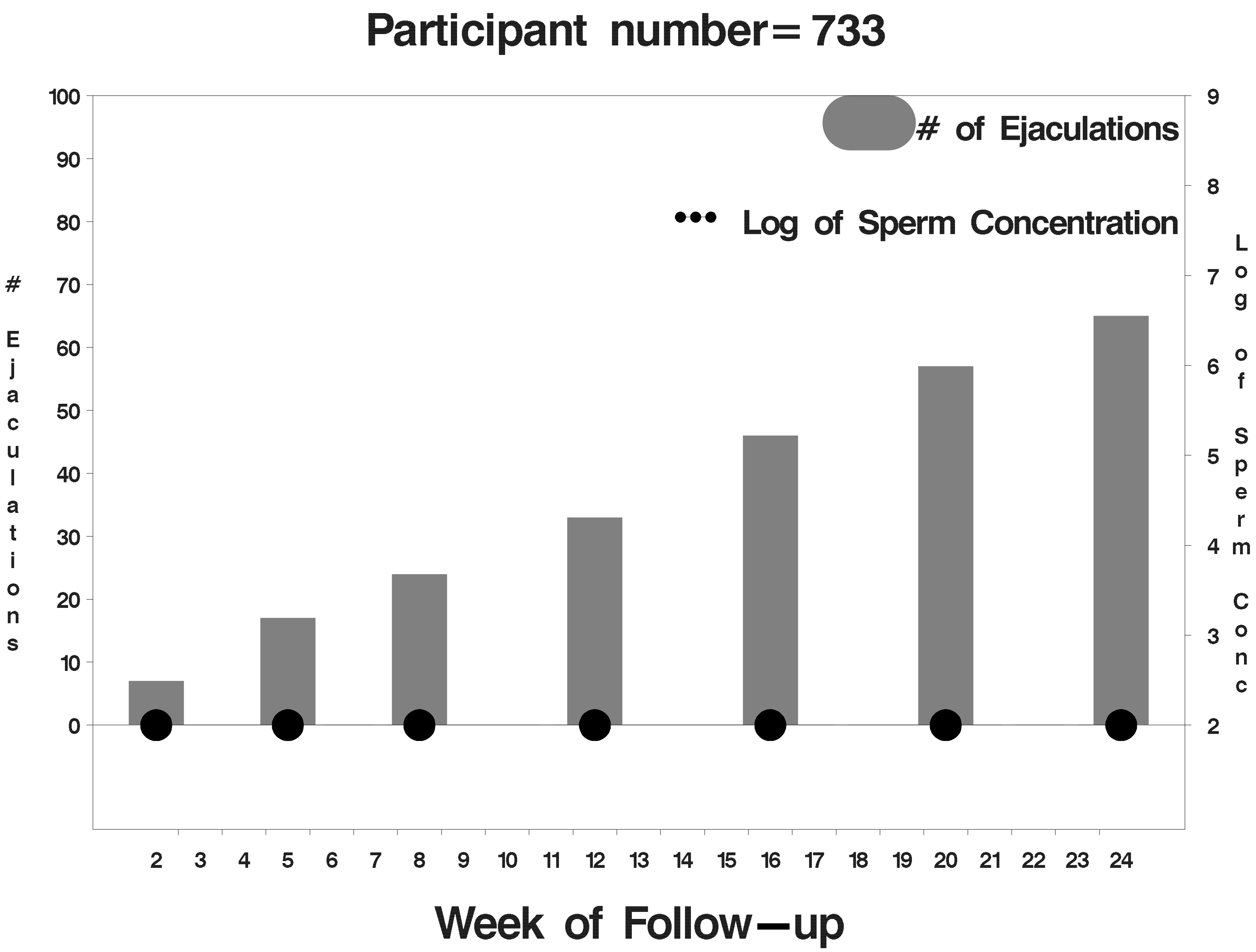
Participant number=733
# of Ejaculations
Log of Sperm Concentration
Ejaculations
Log of Sperm Conc
Week of Follow-up
0
10
20
30
40
50
60
70
80
90
100
2
3
4
5
6
7
8
9
2 3 4 5 6 7 8 9 10 11 12 13 14 15 16 17 18 19 20 21 22 23 24

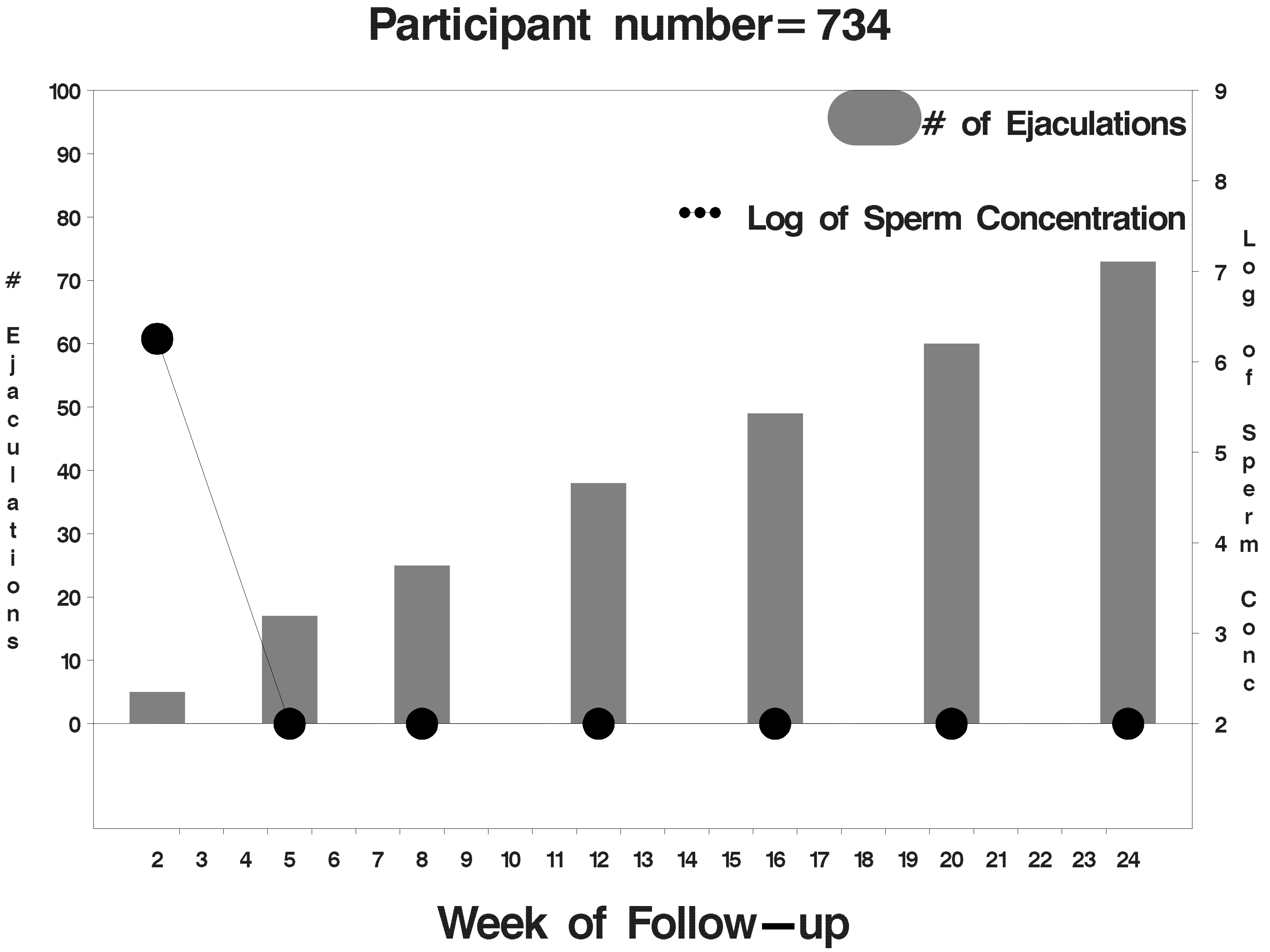

Participant number= 734
# of Ejaculations
Log of Sperm Concentration
Ejaculations
Log of Sperm Conc
100
90
80
70
60
50
40
30
20
10
0
9
8
7
6
5
4
3
2
2 3 4 5 6 7 8 9 10 11 12 13 14 15 16 17 18 19 20 21 22 23 24
Week of Follow-up

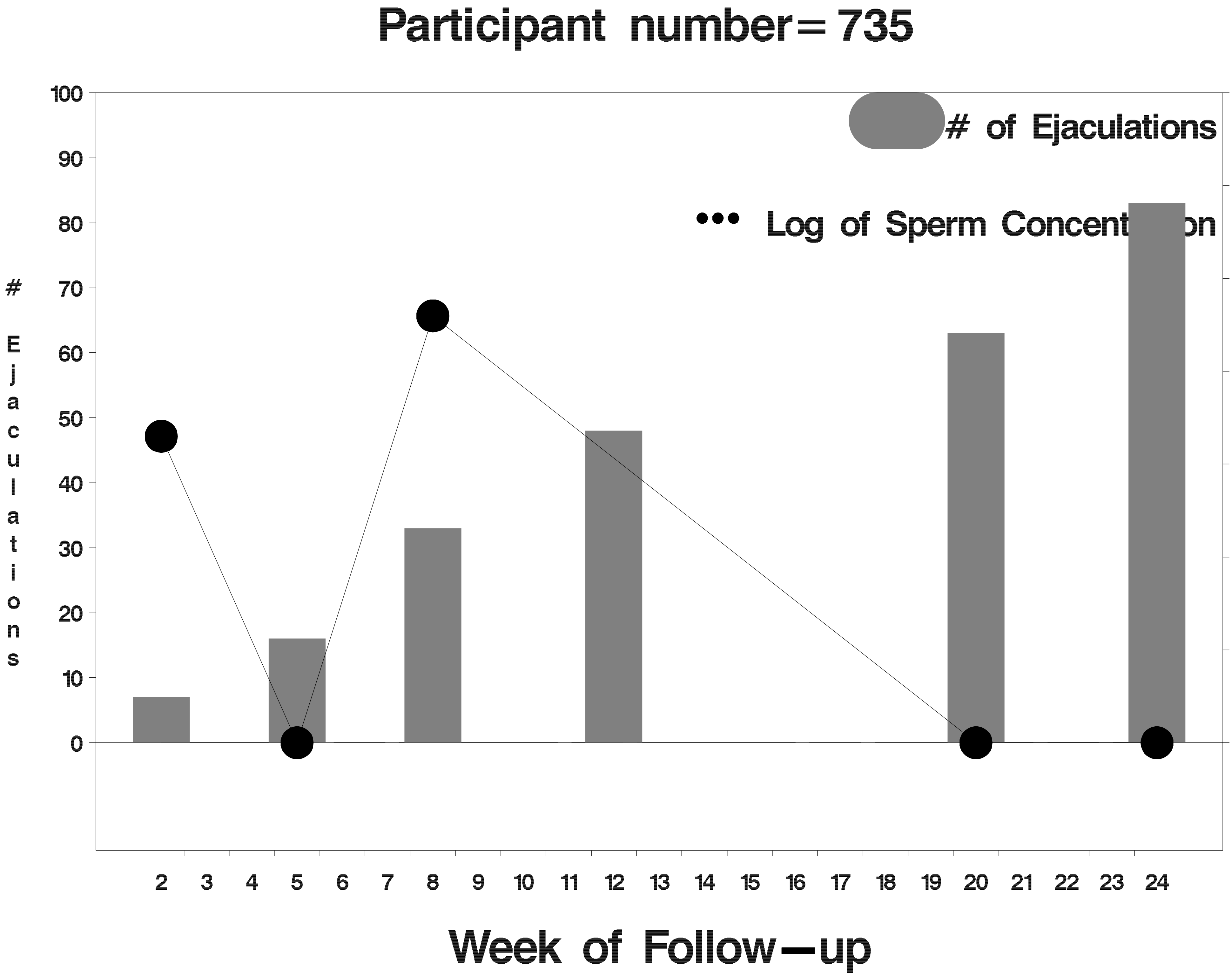

Participant number=735
# of Ejaculations
Log of Sperm Concent[illegible]on
Ejaculations
Log of Sperm Conc
100
90
80
70
60
50
40
30
20
10
0
9
8
7
6
5
4
3
2
2 3 4 5 6 7 8 9 10 11 12 13 14 15 16 17 18 19 20 21 22 23 24
Week of Follow-up

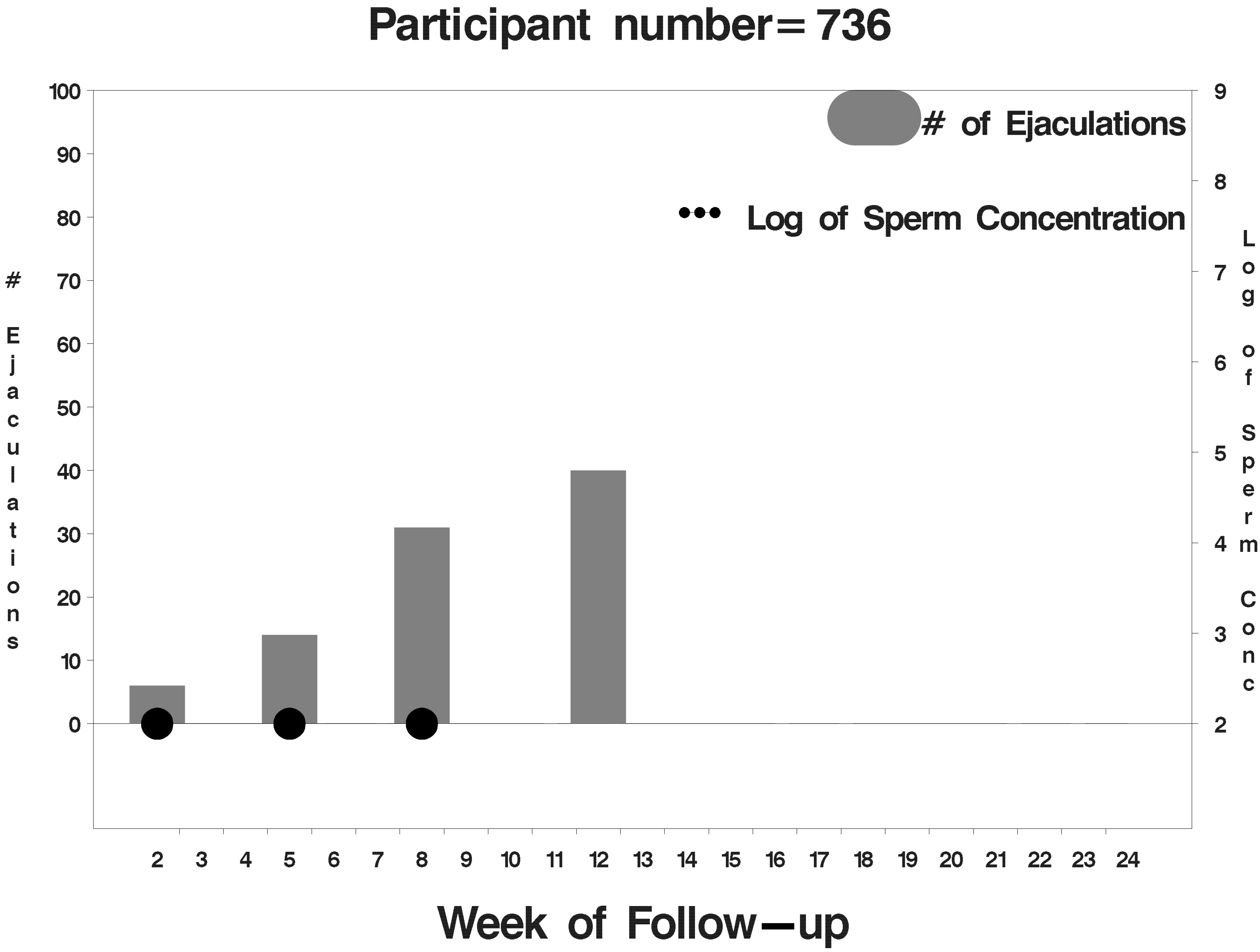

Participant number=736
# of Ejaculations
Log of Sperm Concentration
Ejaculations
Log of Sperm Conc
100
90
80
70
60
50
40
30
20
10
0
9
8
7
6
5
4
3
2
2 3 4 5 6 7 8 9 10 11 12 13 14 15 16 17 18 19 20 21 22 23 24
Week of Follow-up

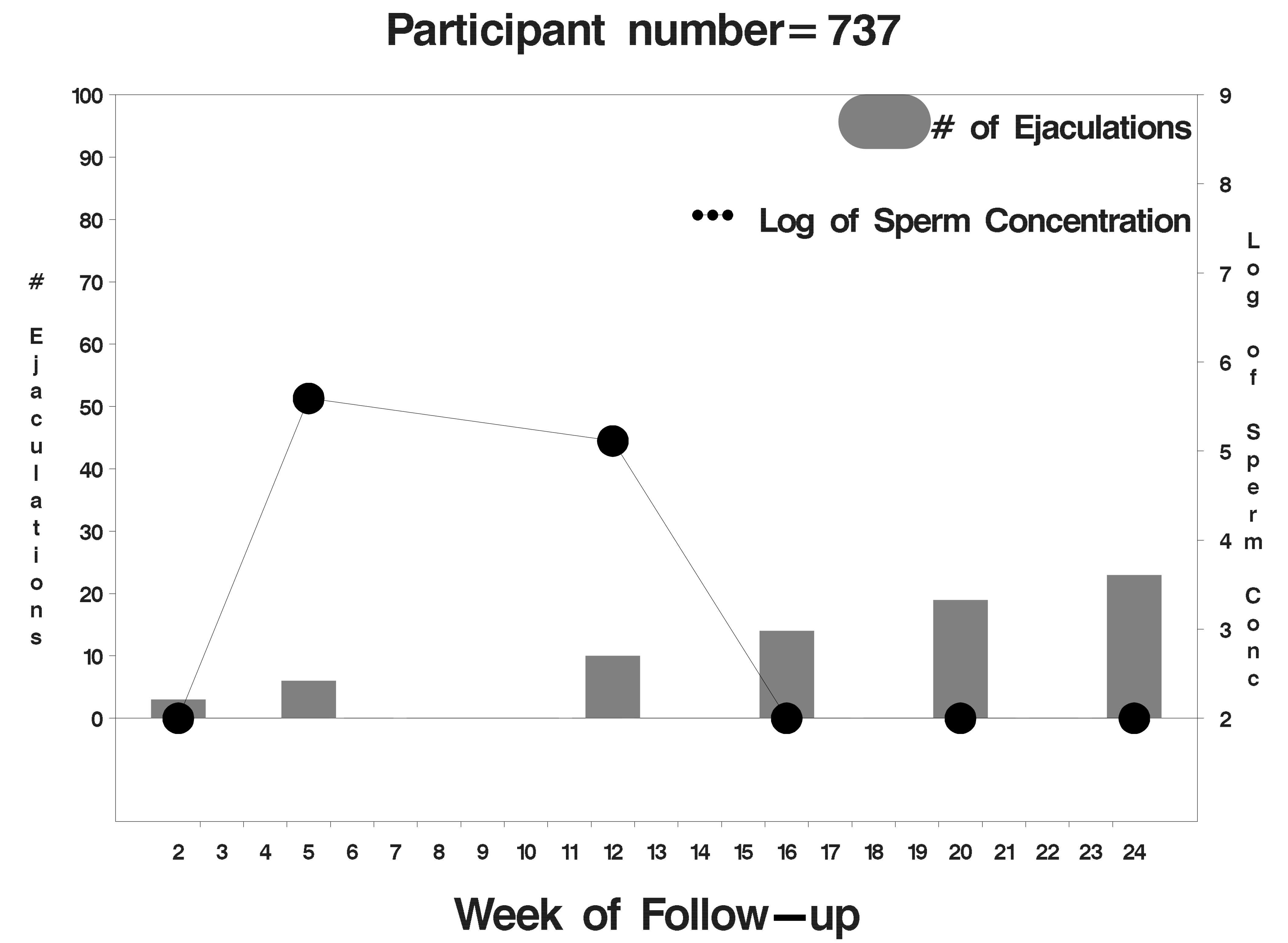

Participant number= 737
# of Ejaculations
Log of Sperm Concentration
Ejaculations
Log of Sperm Conc
100
90
80
70
60
50
40
30
20
10
0
9
8
7
6
5
4
3
2
2 3 4 5 6 7 8 9 10 11 12 13 14 15 16 17 18 19 20 21 22 23 24
Week of Follow-up

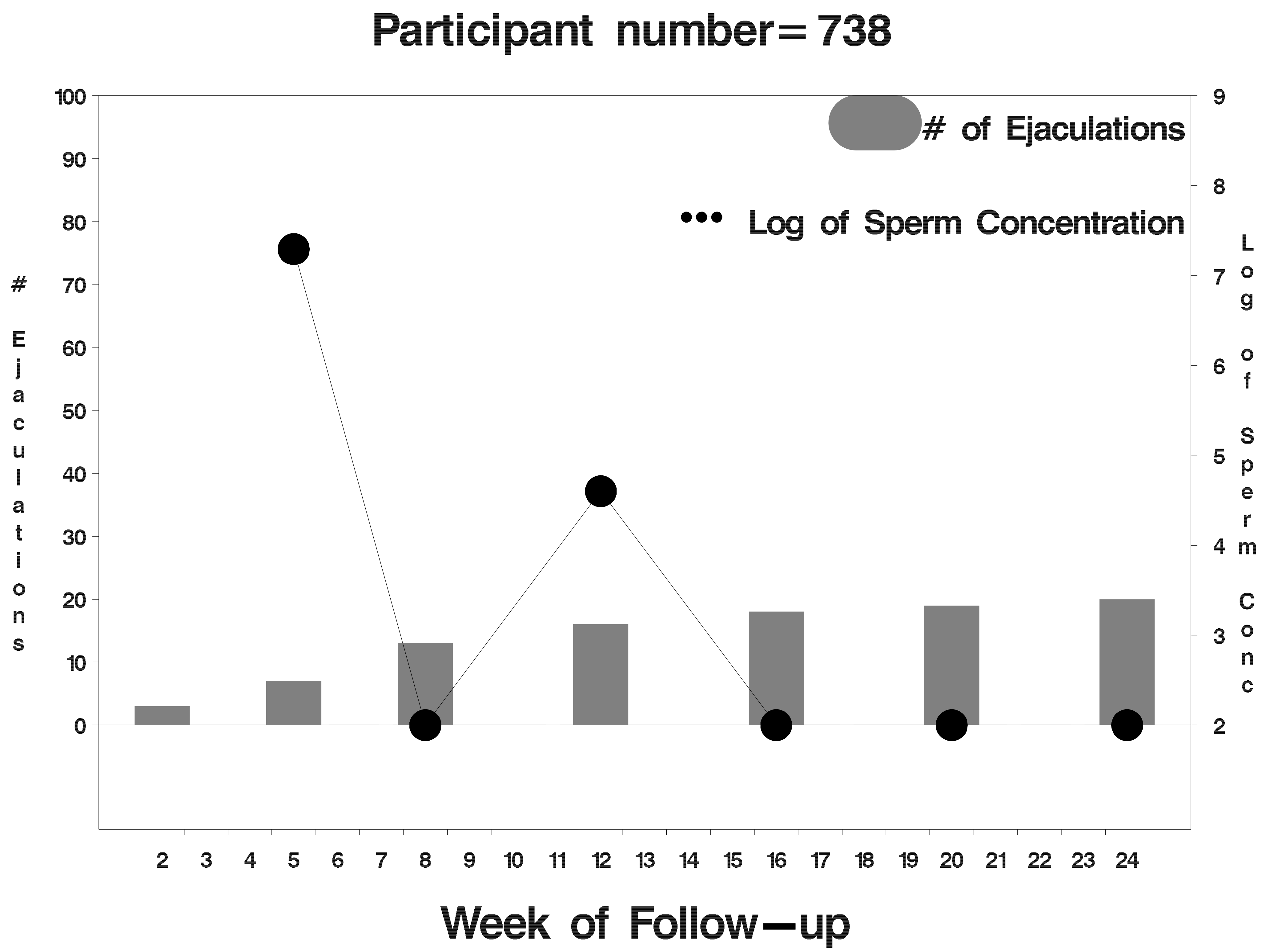

Participant number= 738
# of Ejaculations
Log of Sperm Concentration
Ejaculations
Log of Sperm Conc
100
90
80
70
60
50
40
30
20
10
0
9
8
7
6
5
4
3
2
2 3 4 5 6 7 8 9 10 11 12 13 14 15 16 17 18 19 20 21 22 23 24
Week of Follow-up

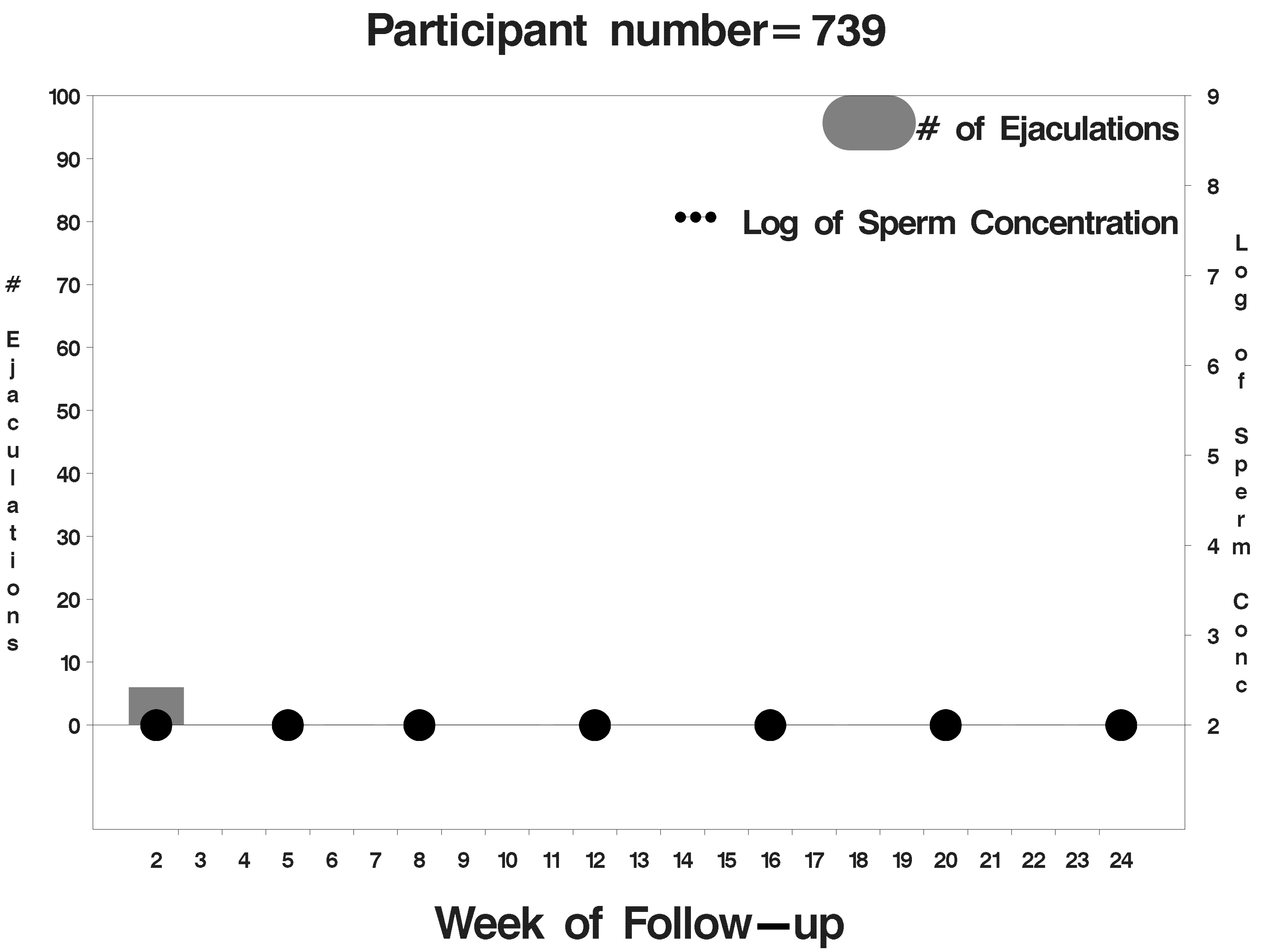

Participant number= 739
# of Ejaculations
Log of Sperm Concentration
Ejaculations
Log of Sperm Conc
100
90
80
70
60
50
40
30
20
10
0
9
8
7
6
5
4
3
2
2 3 4 5 6 7 8 9 10 11 12 13 14 15 16 17 18 19 20 21 22 23 24
Week of Follow—up

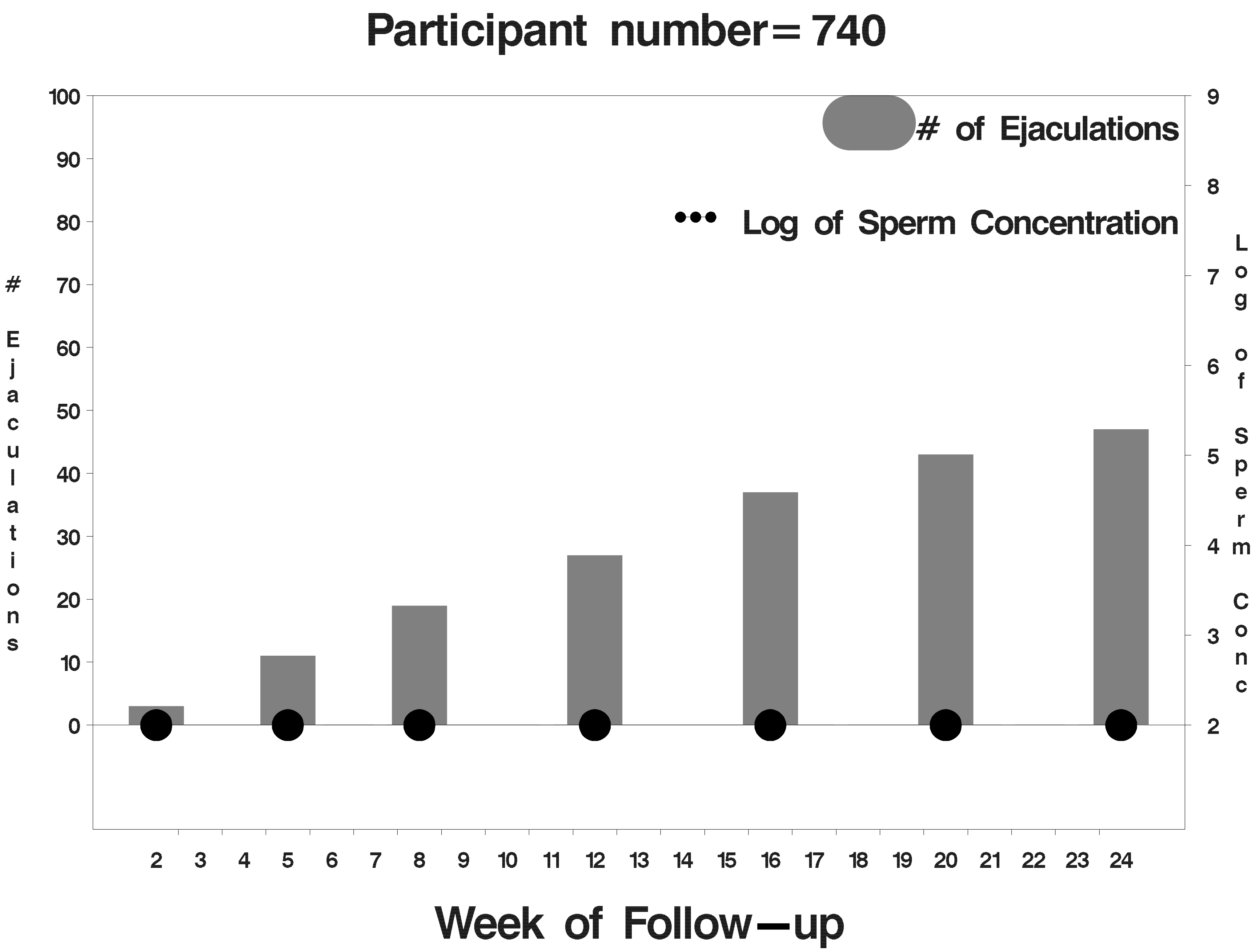
Participant number= 740
# of Ejaculations
Log of Sperm Concentration
Ejaculations
Log of Sperm Conc
100
90
80
70
60
50
40
30
20
10
0
9
8
7
6
5
4
3
2
2 3 4 5 6 7 8 9 10 11 12 13 14 15 16 17 18 19 20 21 22 23 24
Week of Follow-up

Participant number=741

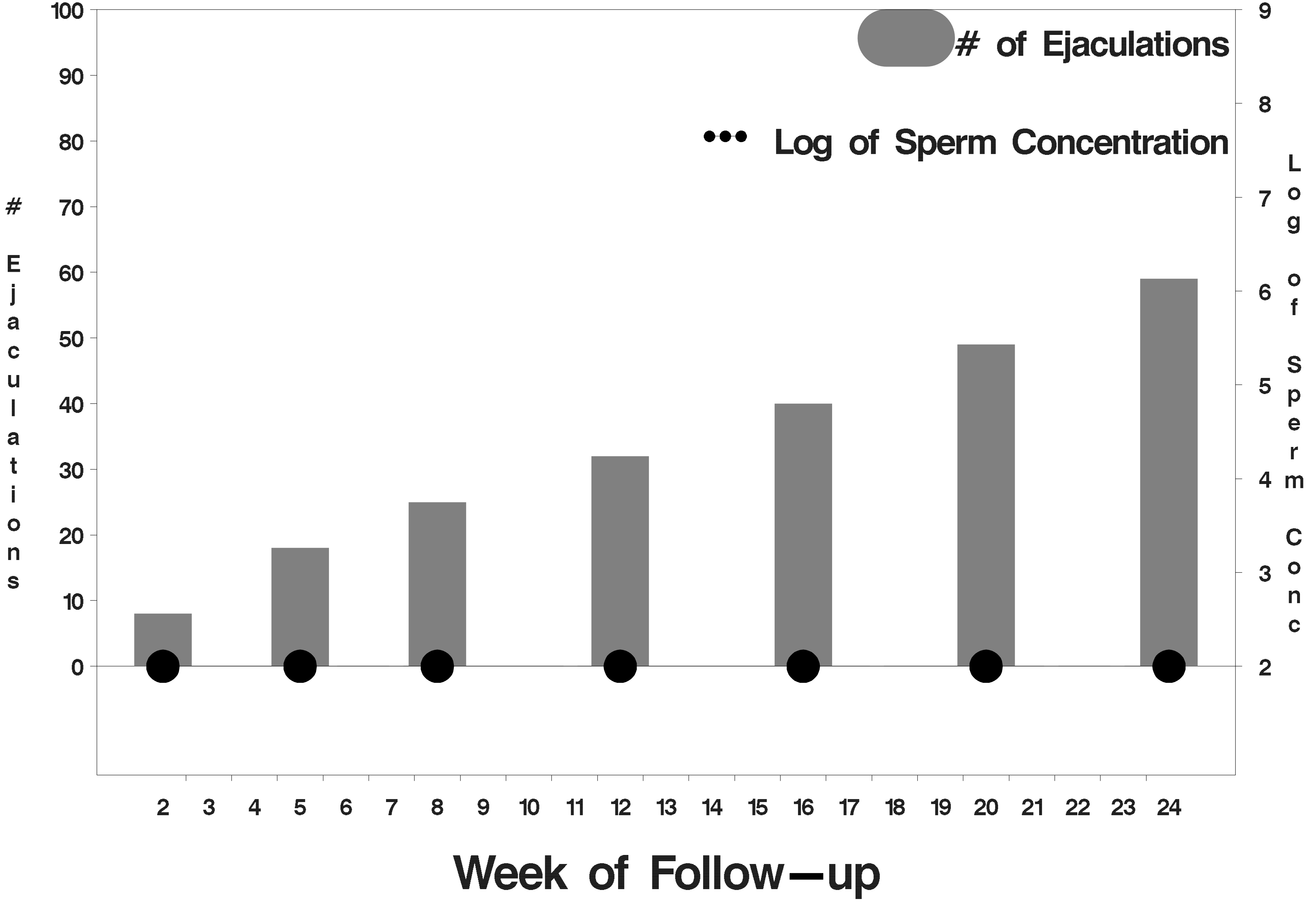

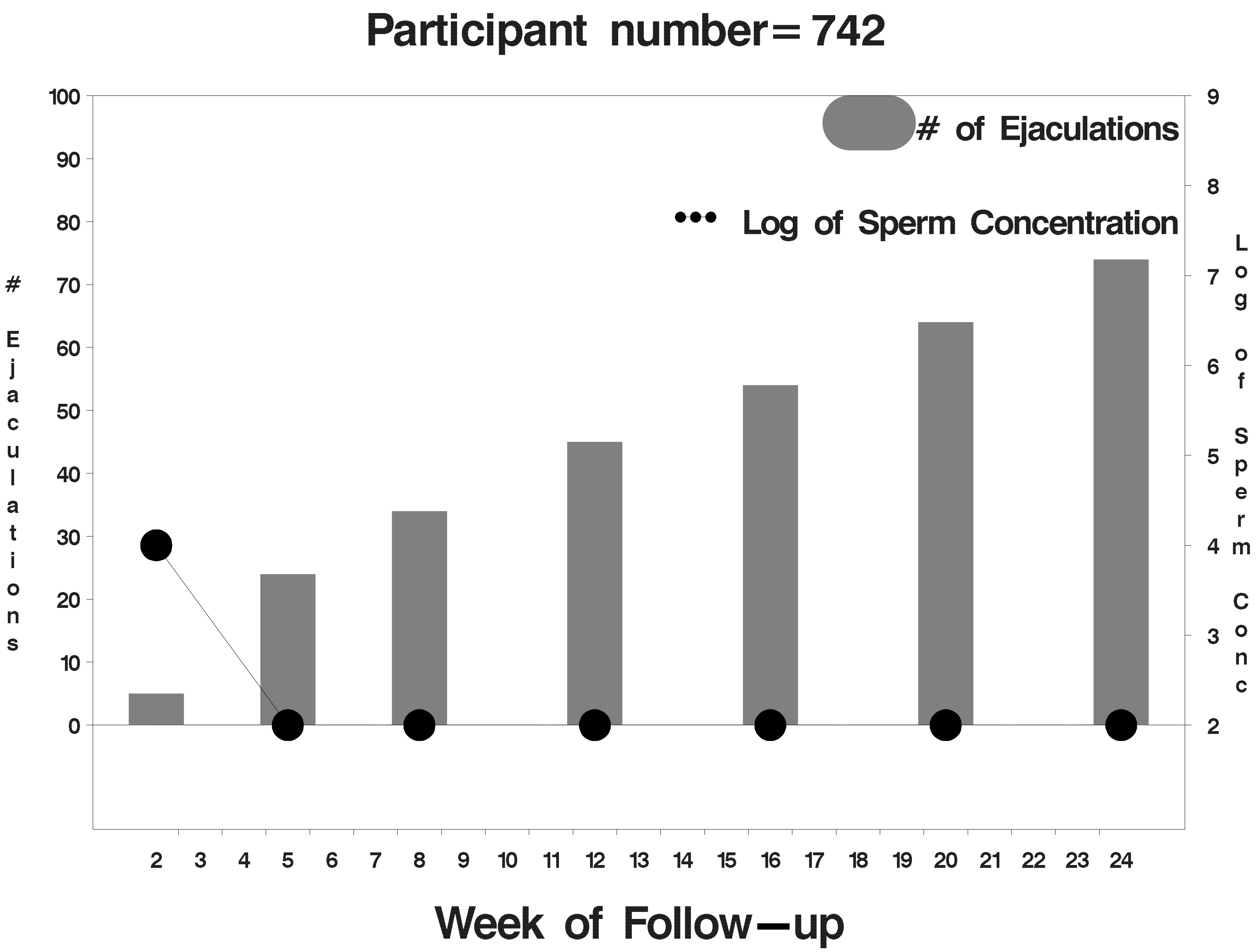

Participant number=742
# of Ejaculations
Log of Sperm Concentration
Ejaculations
Log of Sperm Conc
100
90
80
70
60
50
40
30
20
10
0
9
8
7
6
5
4
3
2
2 3 4 5 6 7 8 9 10 11 12 13 14 15 16 17 18 19 20 21 22 23 24
Week of Follow—up

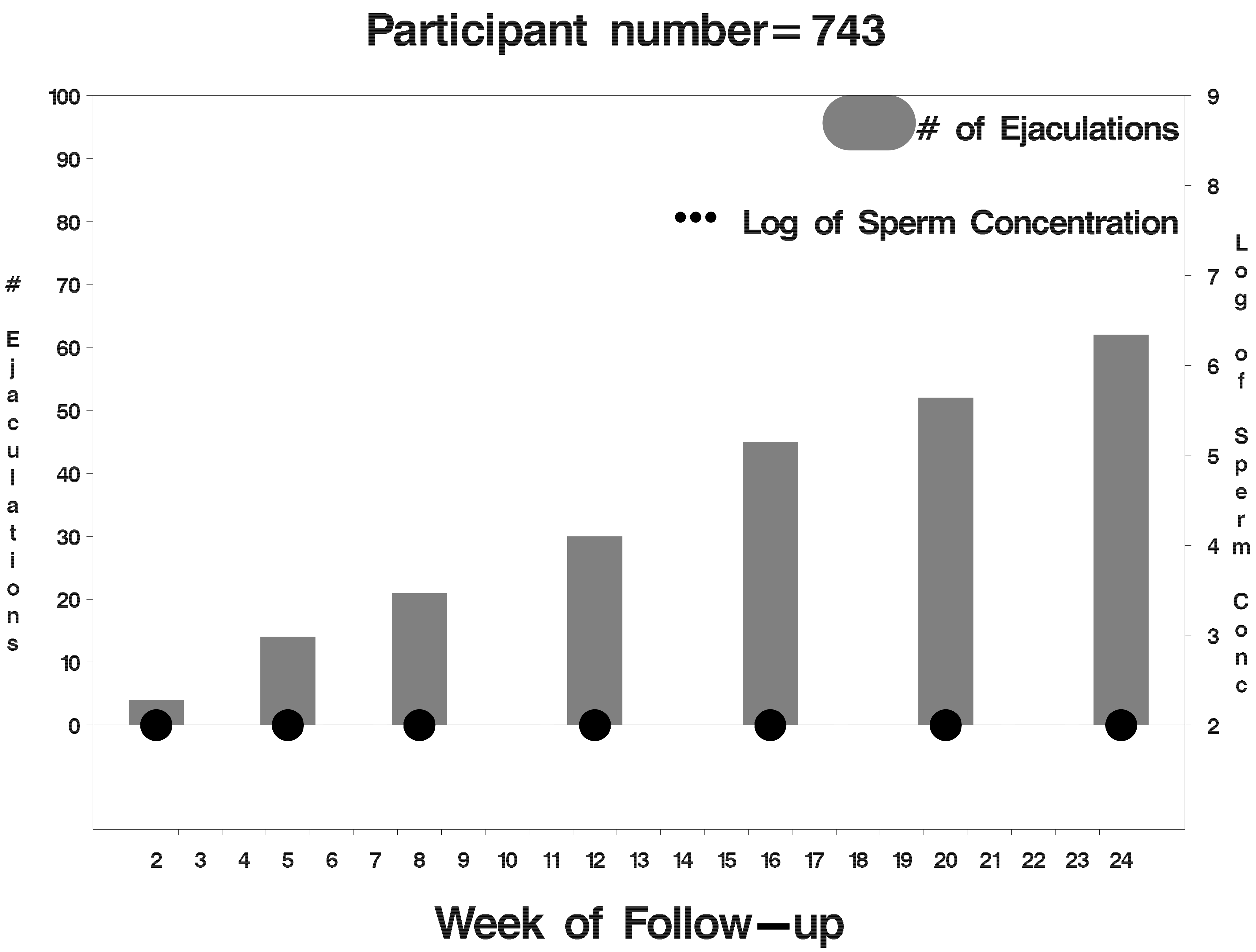

Participant number= 743
# of Ejaculations
Log of Sperm Concentration
Ejaculations
Log of Sperm Conc
100
90
80
70
60
50
40
30
20
10
0
9
8
7
6
5
4
3
2
2 3 4 5 6 7 8 9 10 11 12 13 14 15 16 17 18 19 20 21 22 23 24
Week of Follow-up

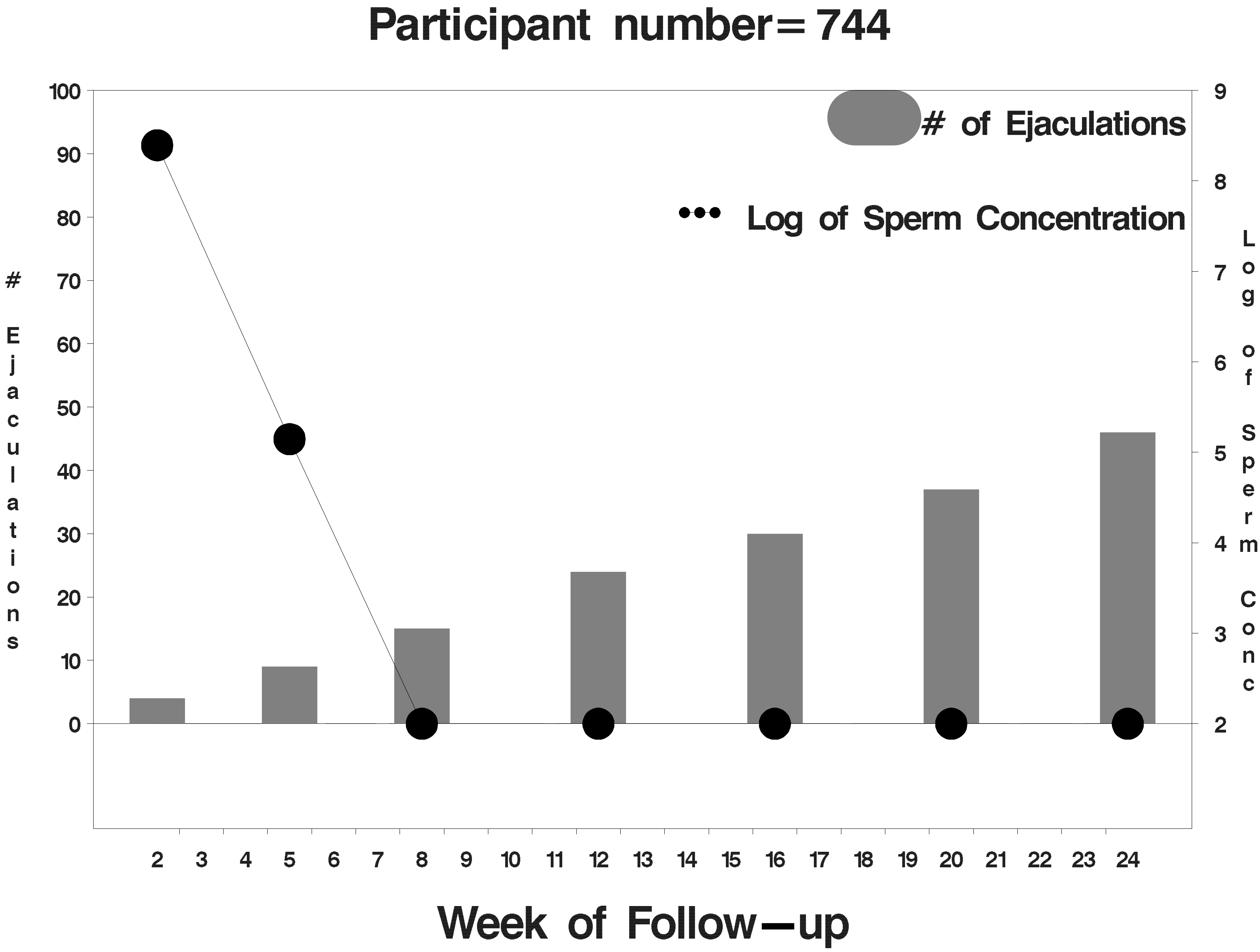
Participant number= 744
# of Ejaculations
Log of Sperm Concentration
Ejaculations
Log of Sperm Conc
100
90
80
70
60
50
40
30
20
10
0
9
8
7
6
5
4
3
2
2 3 4 5 6 7 8 9 10 11 12 13 14 15 16 17 18 19 20 21 22 23 24
Week of Follow-up

Participant number= 745

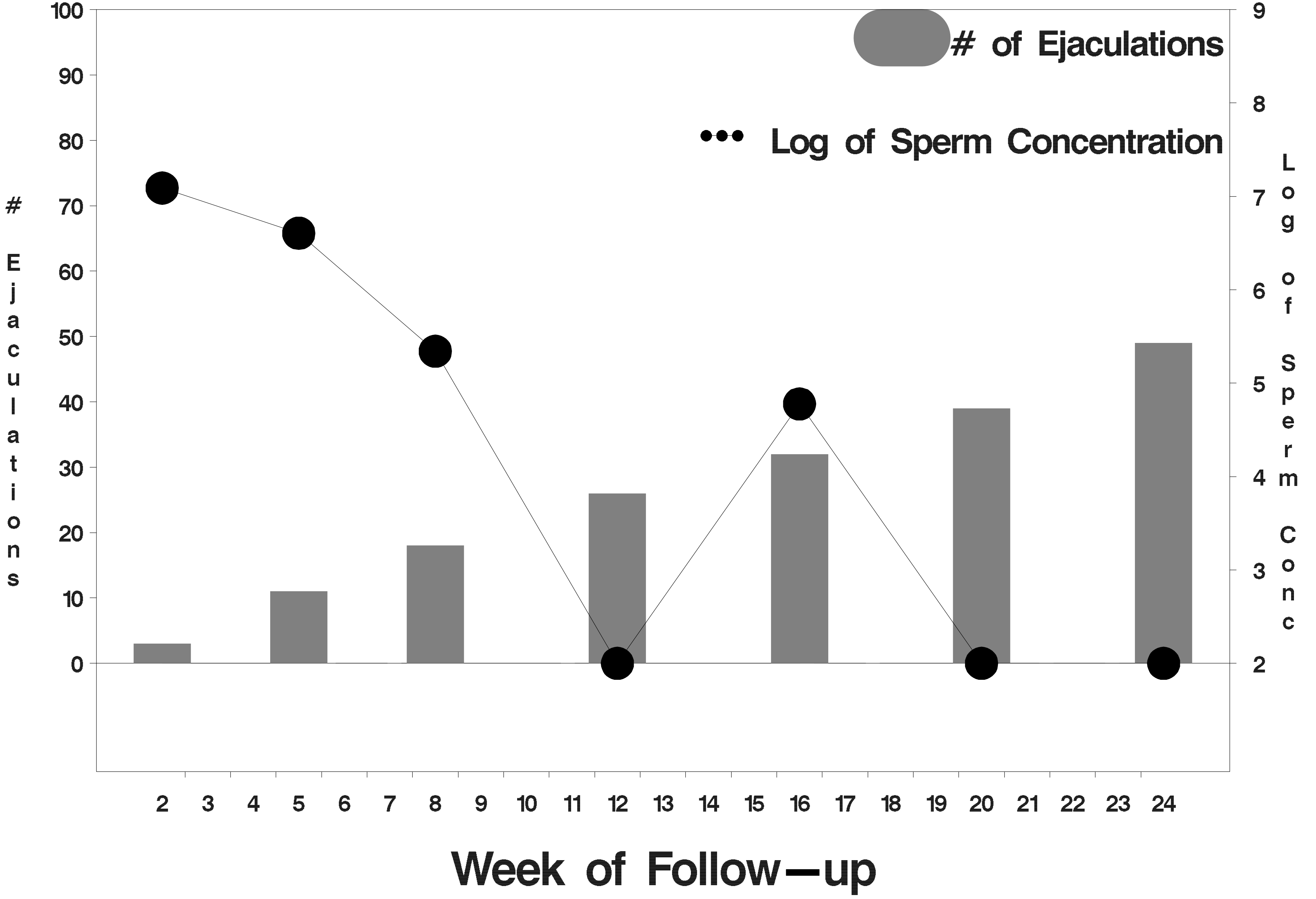

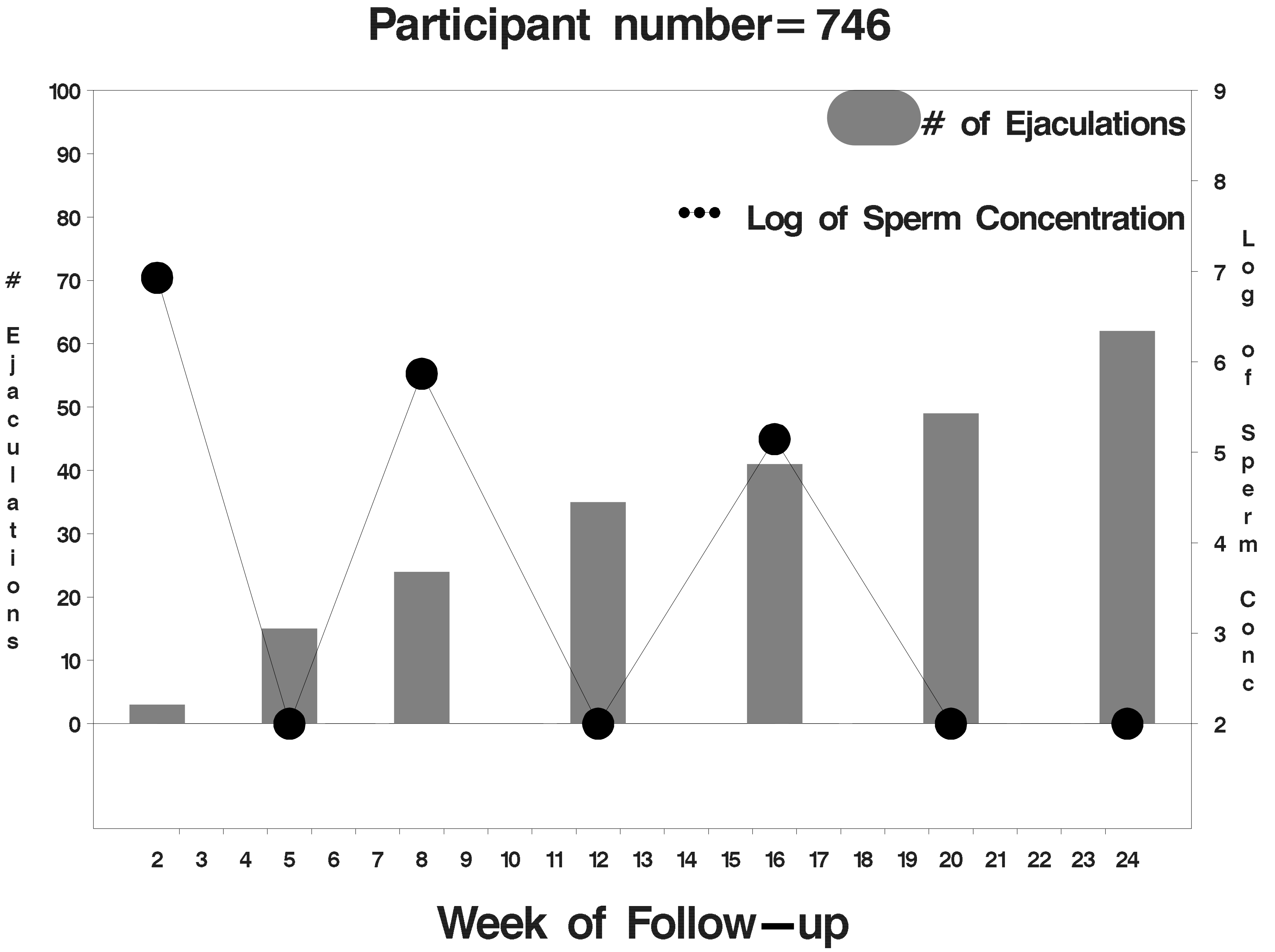

Participant number= 746
# of Ejaculations
Log of Sperm Concentration
Ejaculations
Log of Sperm Conc
100
90
80
70
60
50
40
30
20
10
0
9
8
7
6
5
4
3
2
2 3 4 5 6 7 8 9 10 11 12 13 14 15 16 17 18 19 20 21 22 23 24
Week of Follow-up

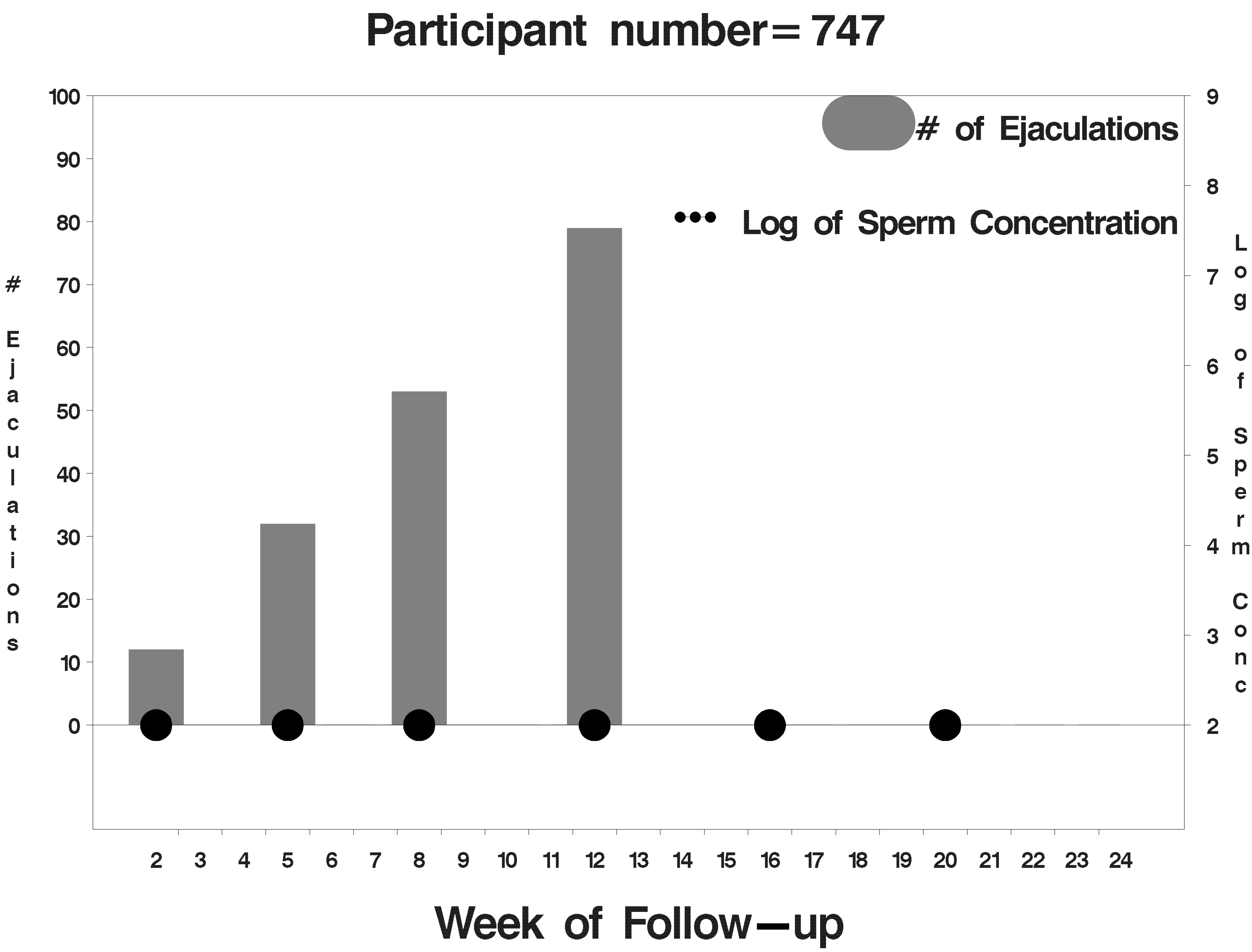

Participant number= 747
# of Ejaculations
Log of Sperm Concentration
Ejaculations
Log of Sperm Conc
100
90
80
70
60
50
40
30
20
10
0
9
8
7
6
5
4
3
2
2 3 4 5 6 7 8 9 10 11 12 13 14 15 16 17 18 19 20 21 22 23 24
Week of Follow-up

Participant number= 748

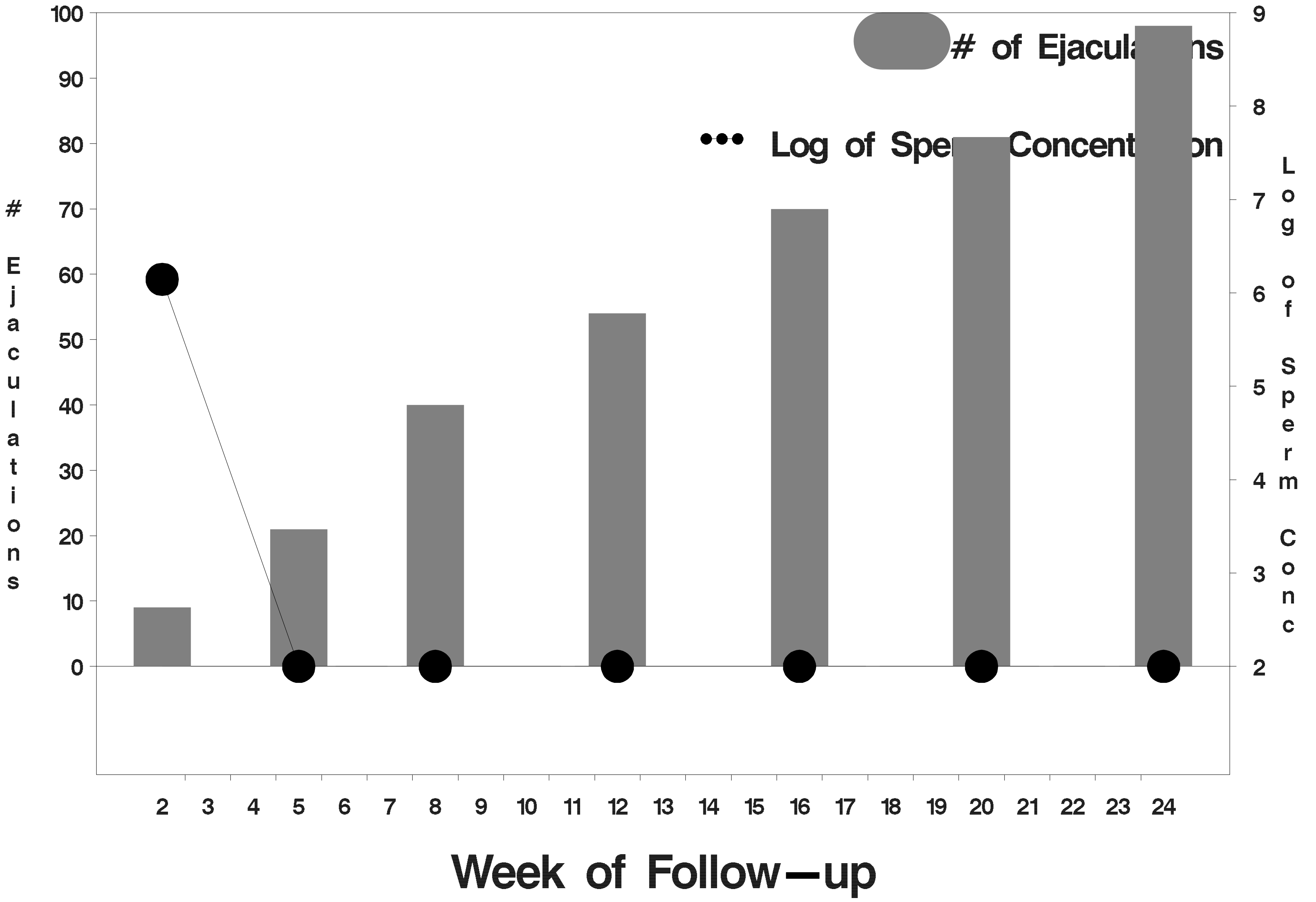

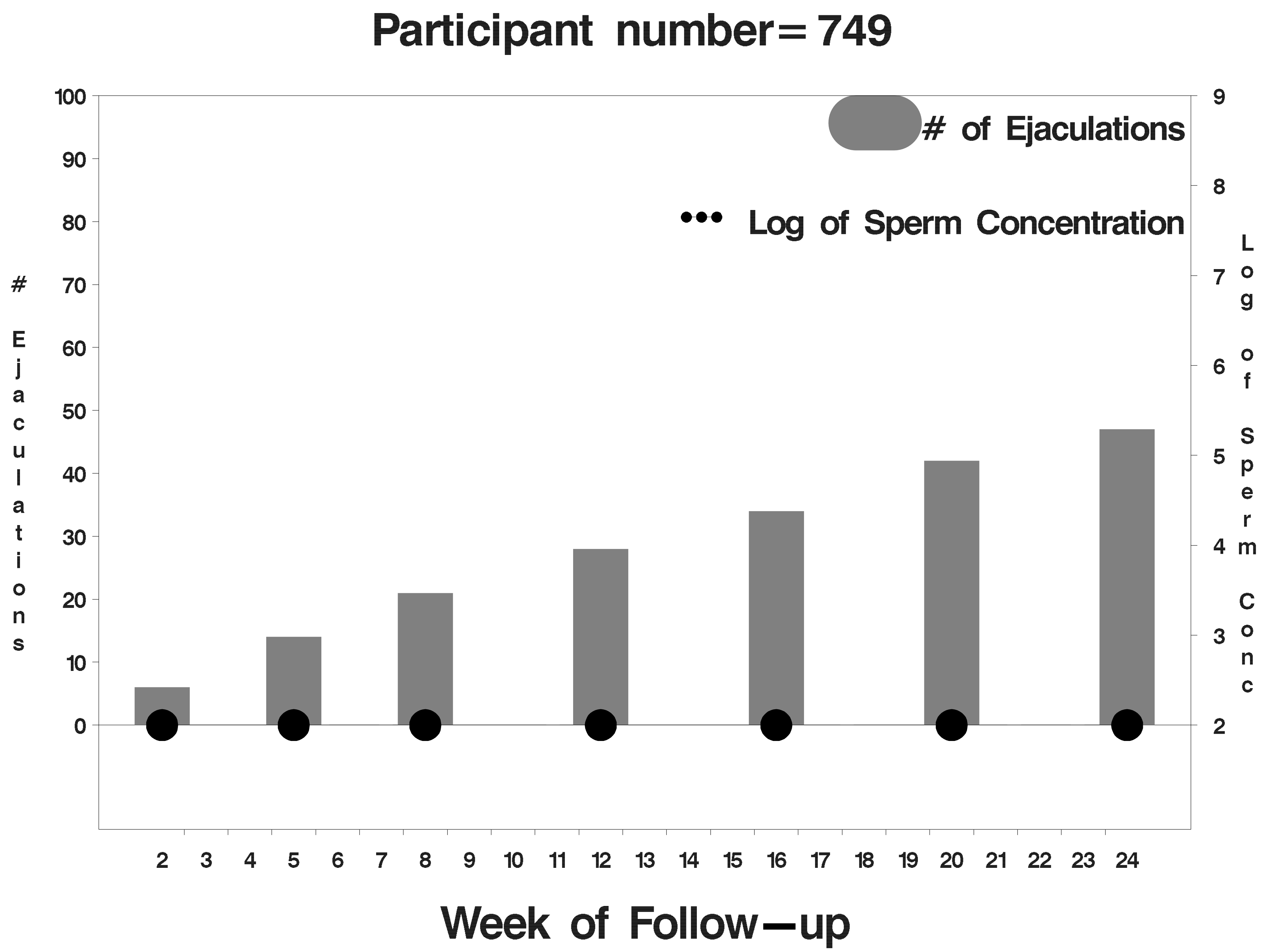

Participant number= 749
# of Ejaculations
Log of Sperm Concentration
Ejaculations
Log of Sperm Conc
100
90
80
70
60
50
40
30
20
10
0
9
8
7
6
5
4
3
2
2 3 4 5 6 7 8 9 10 11 12 13 14 15 16 17 18 19 20 21 22 23 24
Week of Follow-up

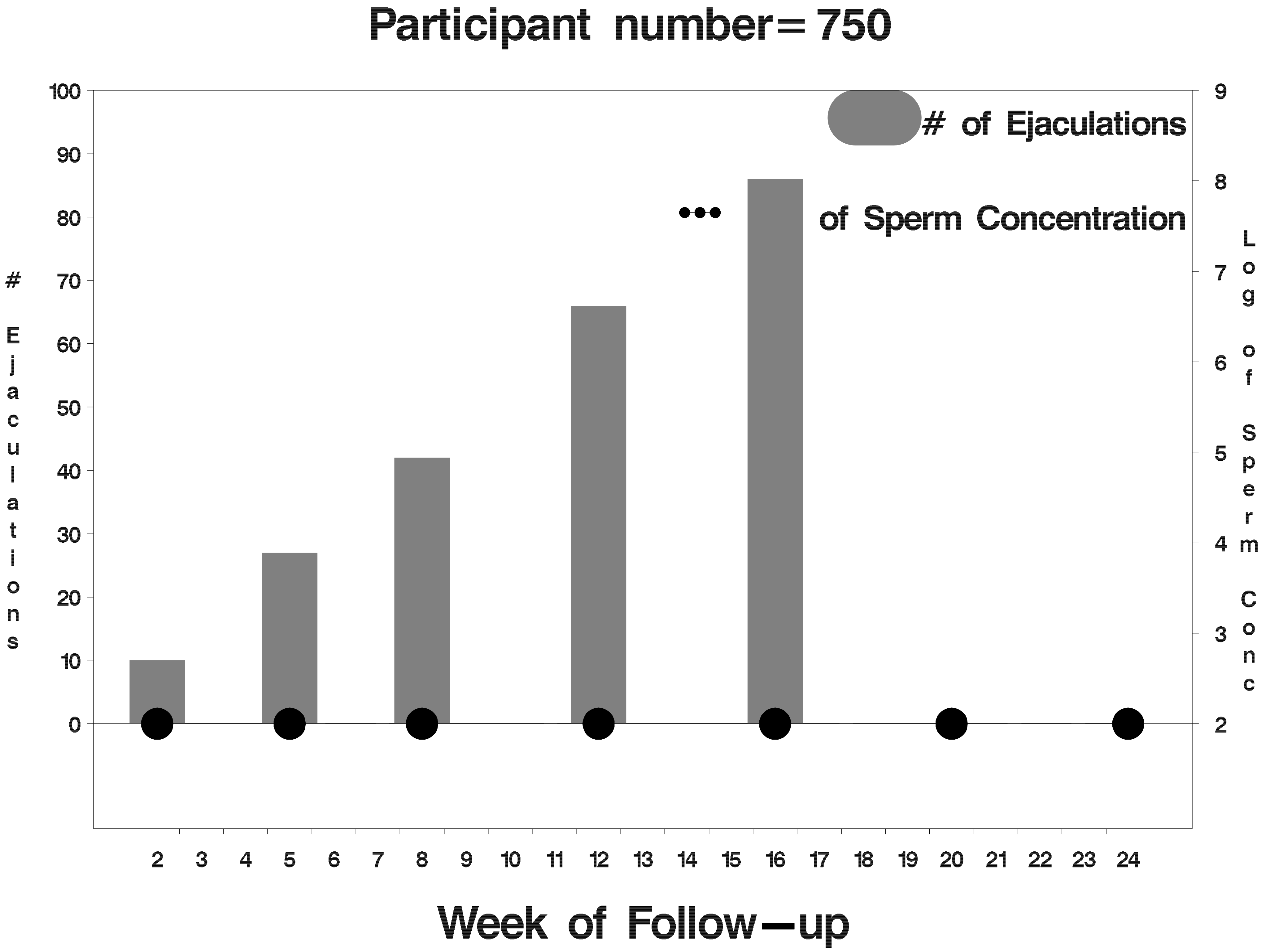

Participant number= 750
# of Ejaculations
of Sperm Concentration
Ejaculations
Log of Sperm Conc
100
90
80
70
60
50
40
30
20
10
0
9
8
7
6
5
4
3
2
2 3 4 5 6 7 8 9 10 11 12 13 14 15 16 17 18 19 20 21 22 23 24
Week of Follow-up

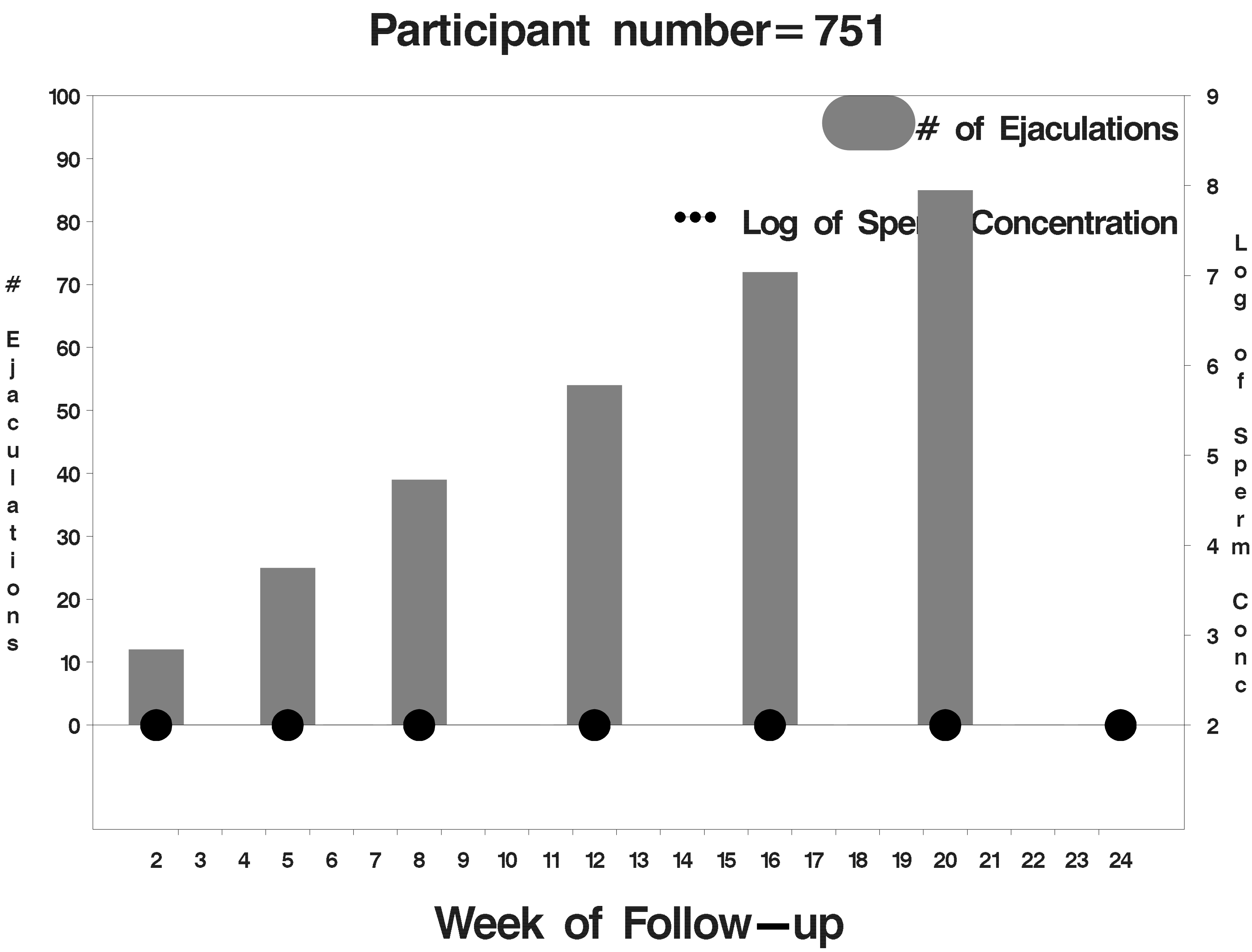

Participant number=751
# of Ejaculations
Log of Sperm Concentration
Ejaculations
Log of Sperm Conc
Week of Follow-up
100
90
80
70
60
50
40
30
20
10
0
9
8
7
6
5
4
3
2
2 3 4 5 6 7 8 9 10 11 12 13 14 15 16 17 18 19 20 21 22 23 24

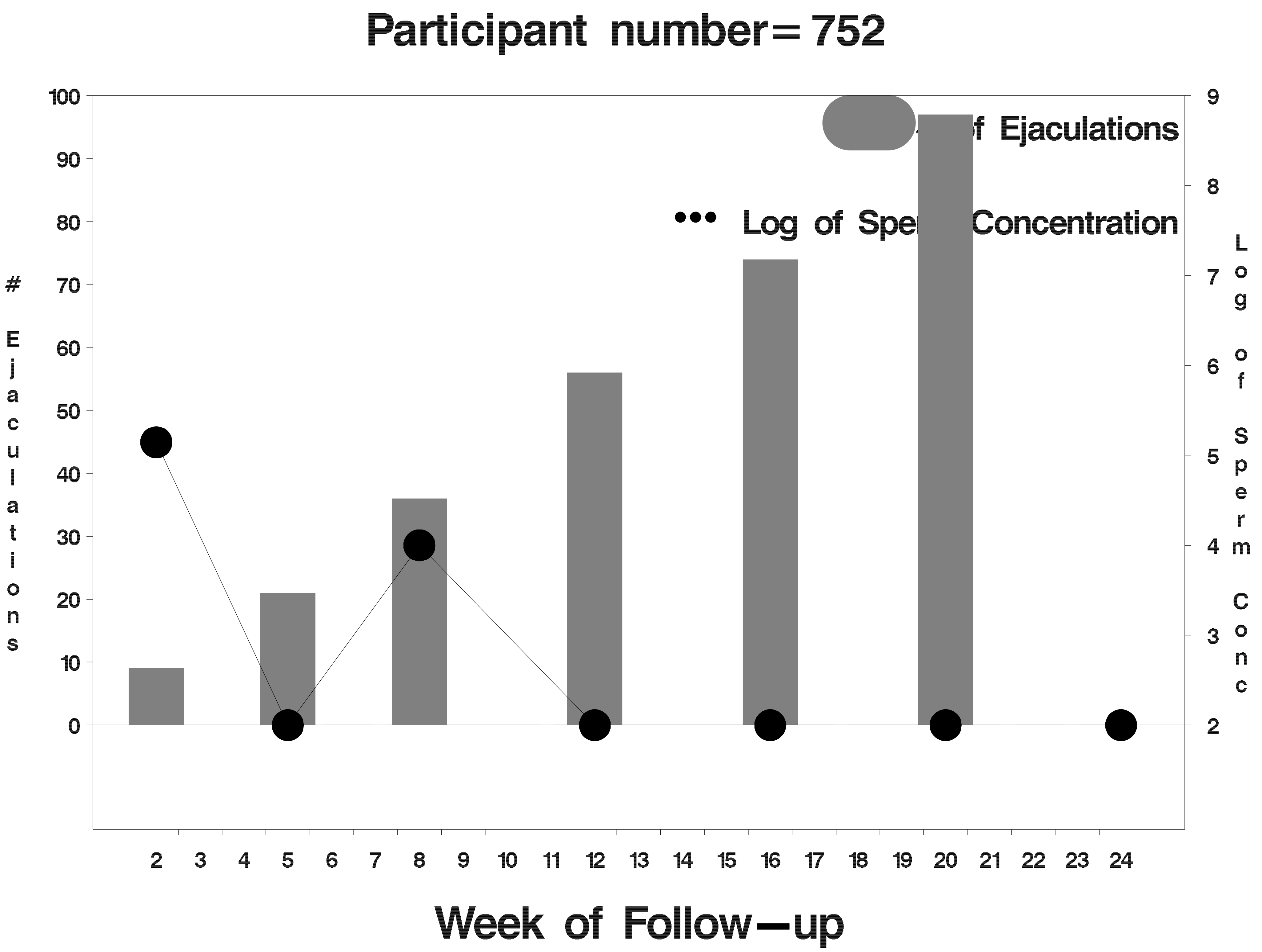

Participant number= 752
100
90
80
70
60
50
40
30
20
10
0
Ejaculations
9
8
7
6
5
4
3
2
Log of Sperm Conc
# of Ejaculations
Log of Sperm Concentration
2 3 4 5 6 7 8 9 10 11 12 13 14 15 16 17 18 19 20 21 22 23 24
Week of Follow-up

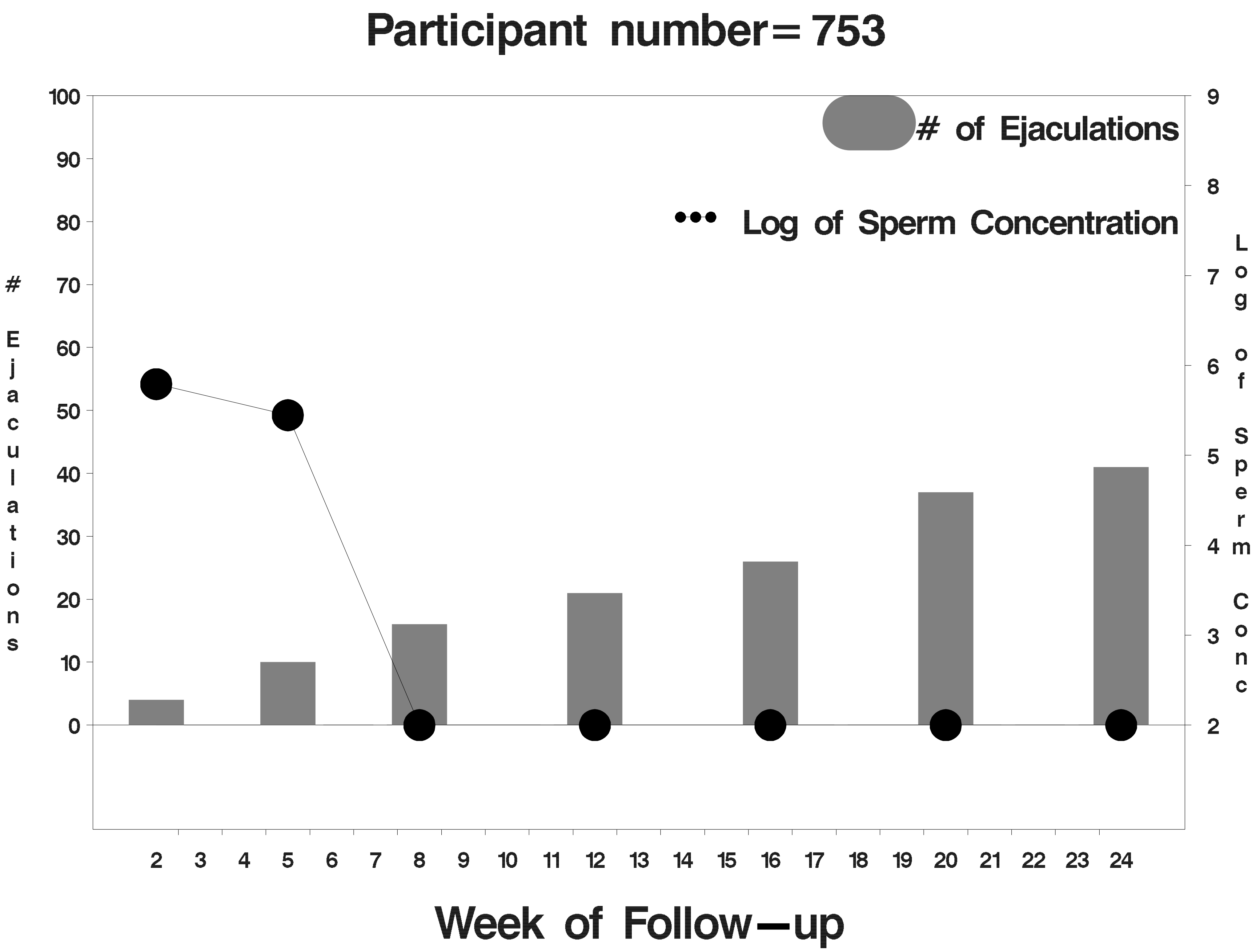

Participant number= 753
# of Ejaculations
Log of Sperm Concentration
Ejaculations
Log of Sperm Conc
100
90
80
70
60
50
40
30
20
10
0
9
8
7
6
5
4
3
2
2 3 4 5 6 7 8 9 10 11 12 13 14 15 16 17 18 19 20 21 22 23 24
Week of Follow-up

Participant number= 754

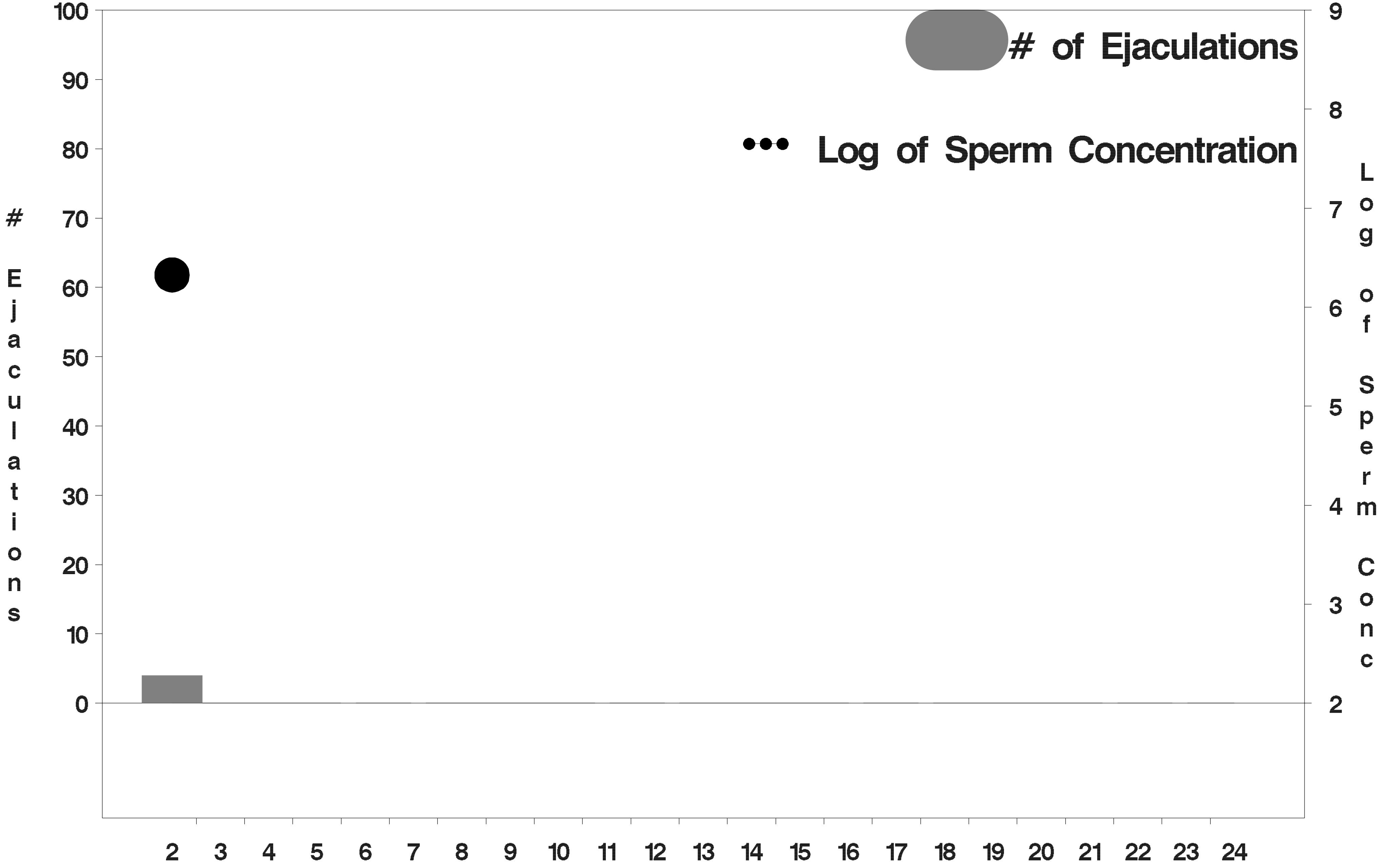

Week of Follow—up

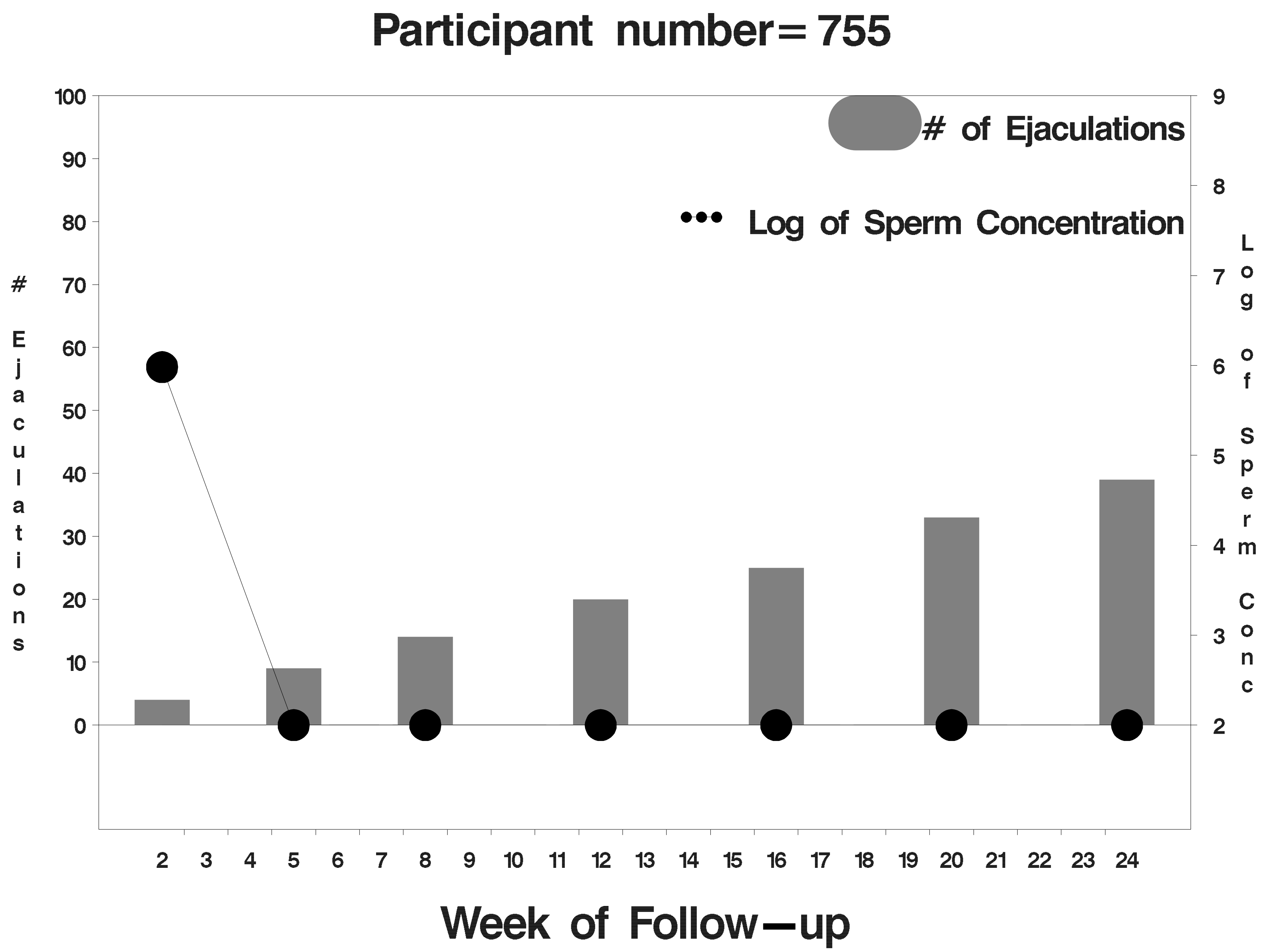

Participant number= 755
# of Ejaculations
Log of Sperm Concentration
Ejaculations
Log of Sperm Conc
100
90
80
70
60
50
40
30
20
10
0
9
8
7
6
5
4
3
2
2 3 4 5 6 7 8 9 10 11 12 13 14 15 16 17 18 19 20 21 22 23 24
Week of Follow—up

Participant number= 756

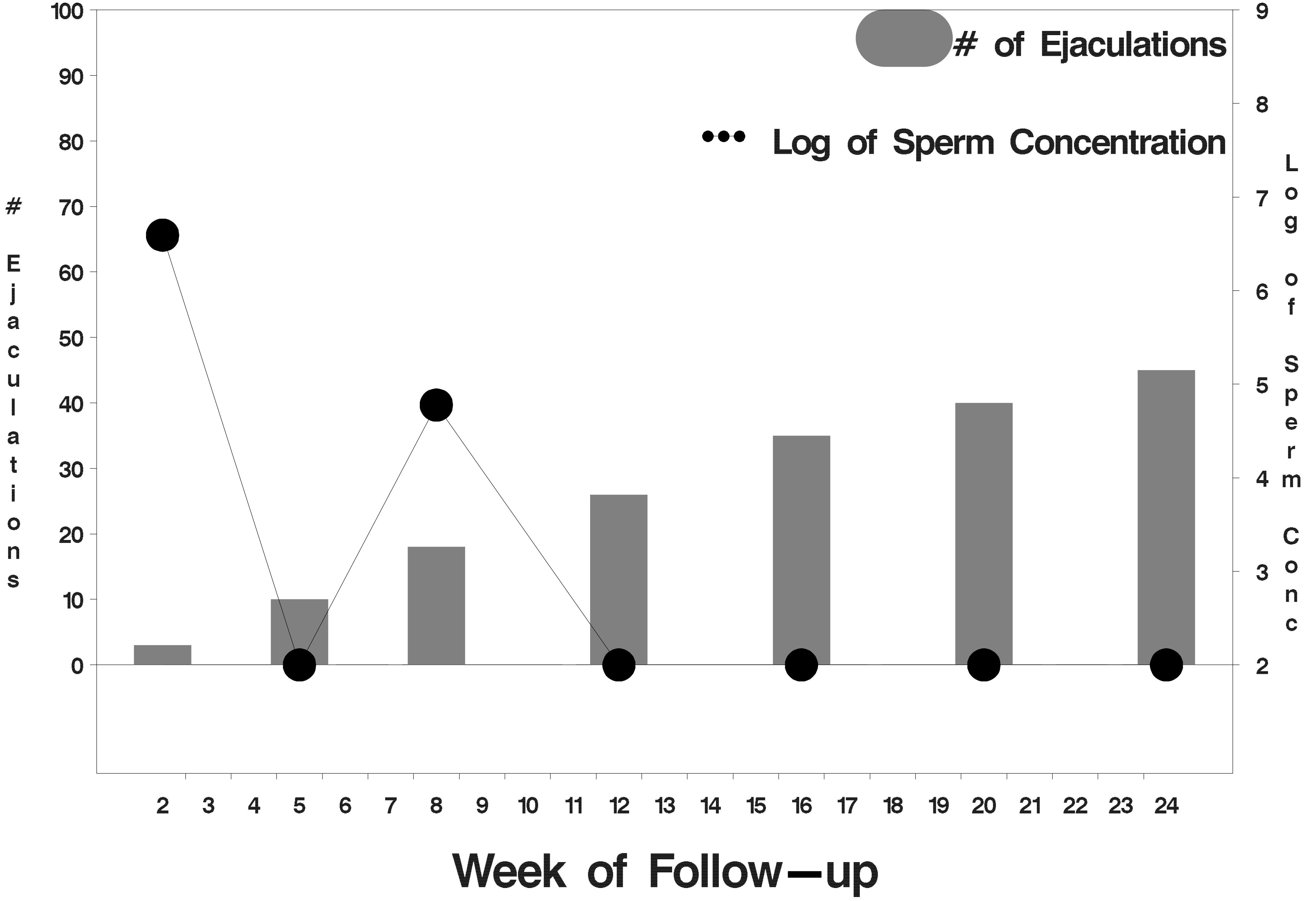

Participant number=757

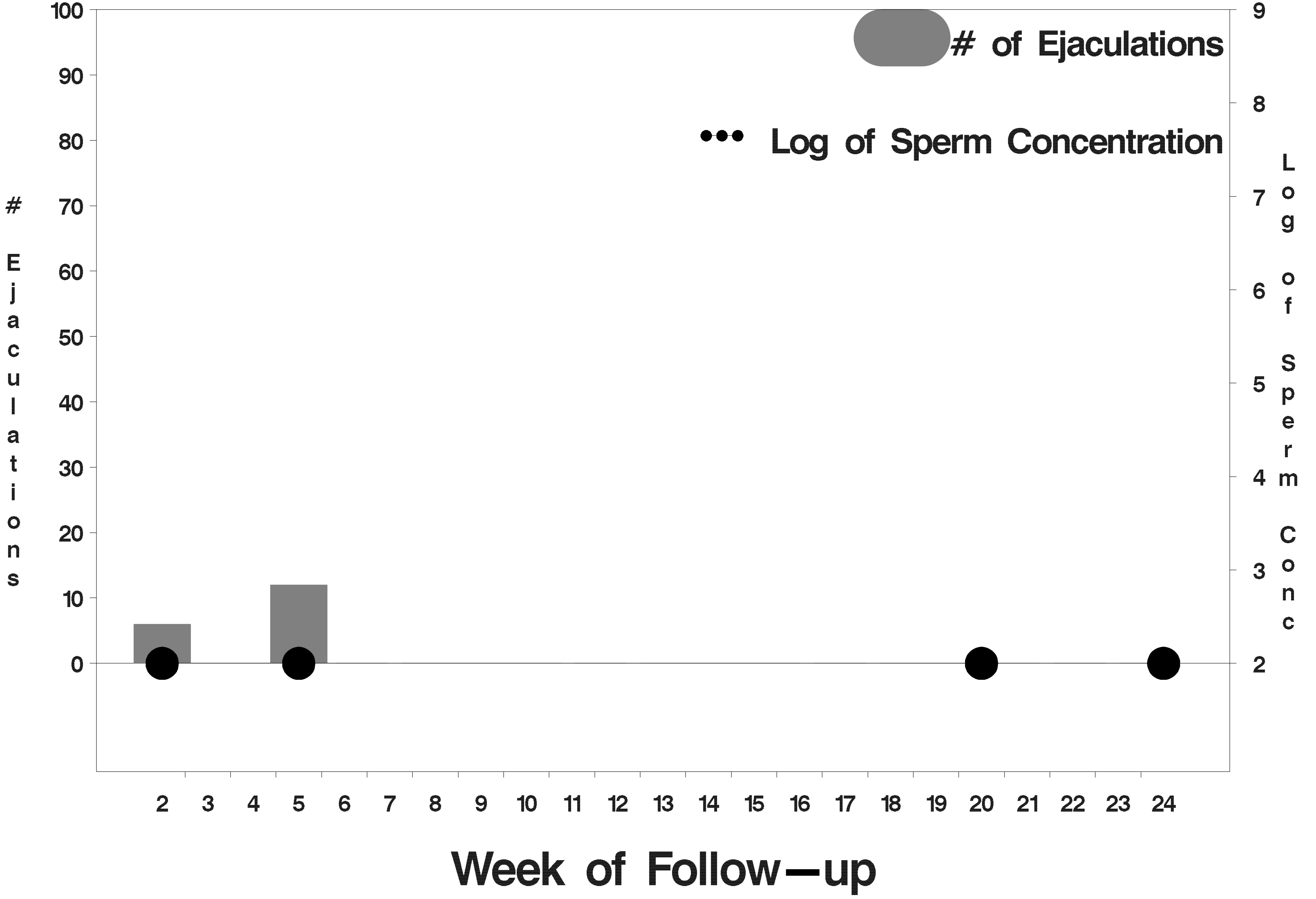

Participant number= 758

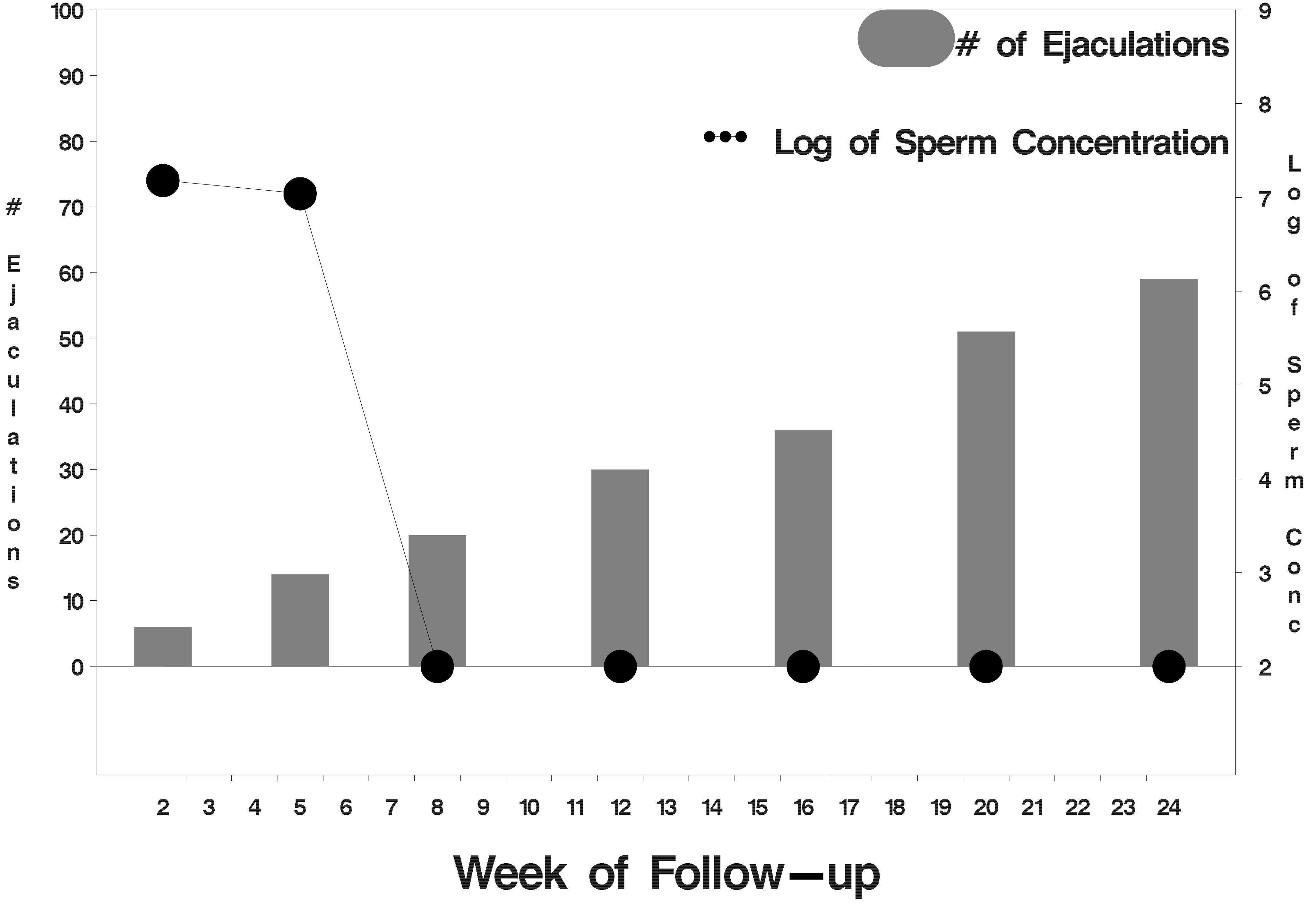

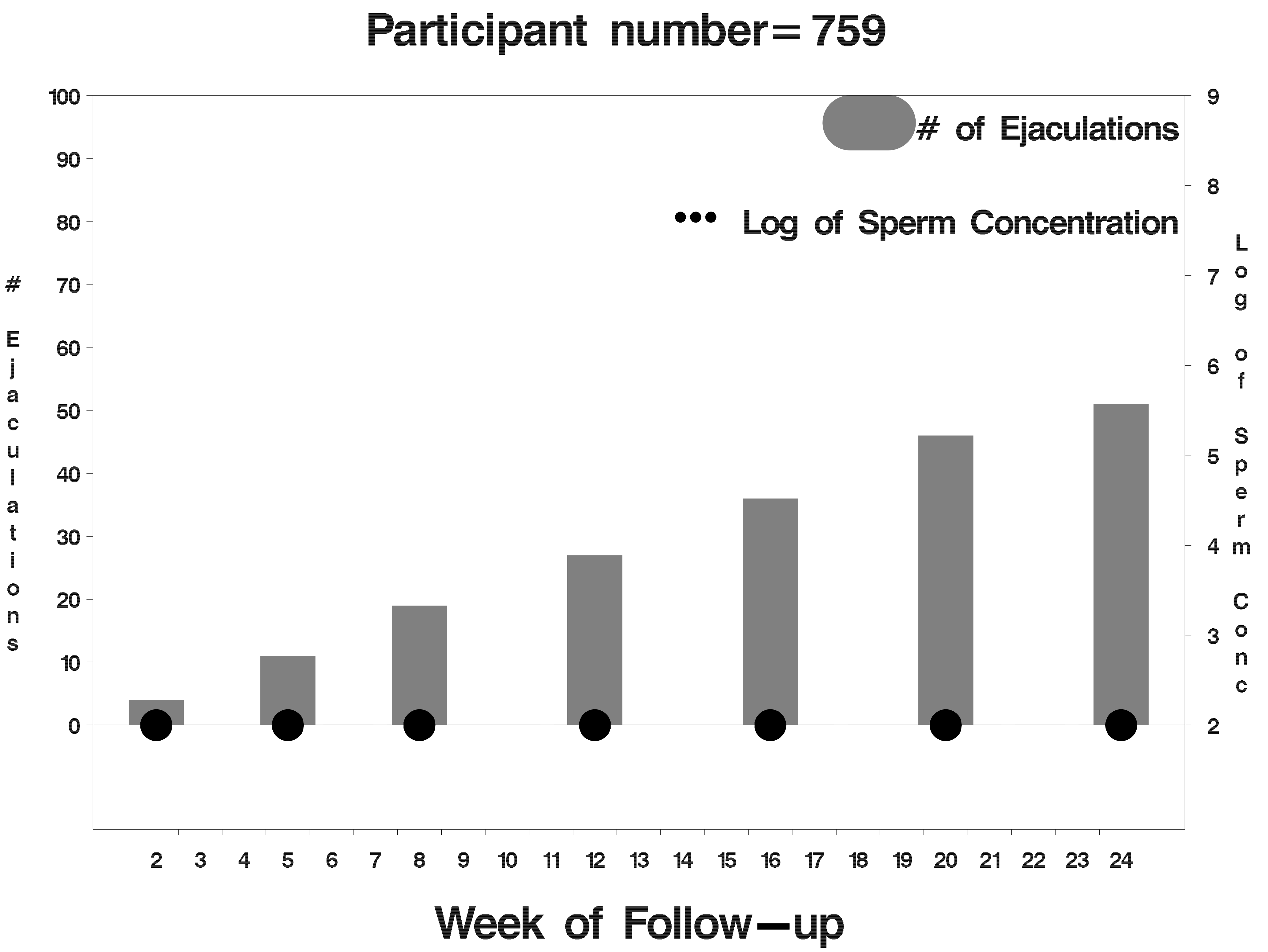

Participant number= 759
# of Ejaculations
Log of Sperm Concentration
Ejaculations
Log of Sperm Conc
100
90
80
70
60
50
40
30
20
10
0
9
8
7
6
5
4
3
2
2 3 4 5 6 7 8 9 10 11 12 13 14 15 16 17 18 19 20 21 22 23 24
Week of Follow—up

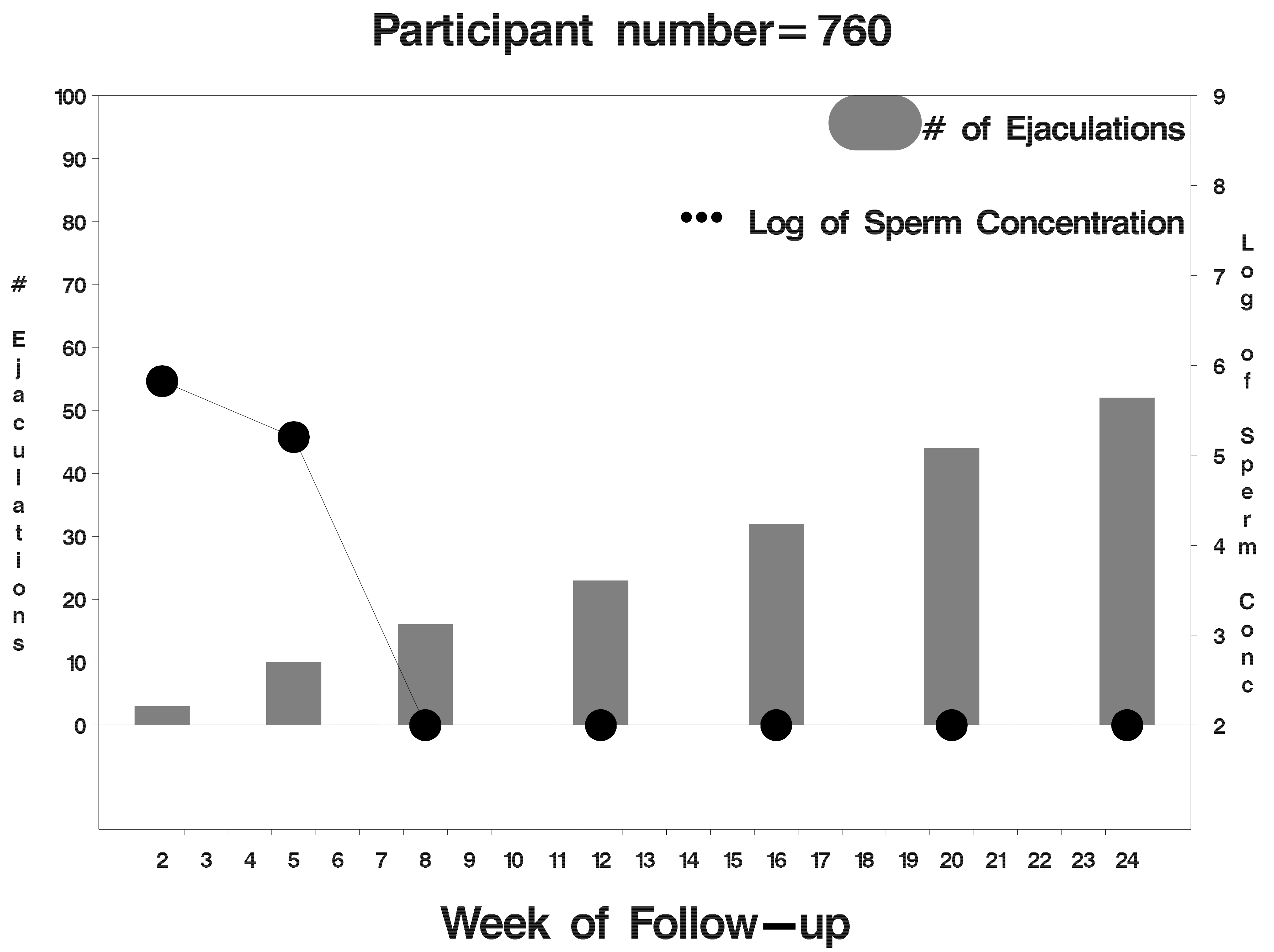

Participant number= 760
# of Ejaculations
Log of Sperm Concentration
Ejaculations
Log of Sperm Conc
100
90
80
70
60
50
40
30
20
10
0
9
8
7
6
5
4
3
2
2 3 4 5 6 7 8 9 10 11 12 13 14 15 16 17 18 19 20 21 22 23 24
Week of Follow-up

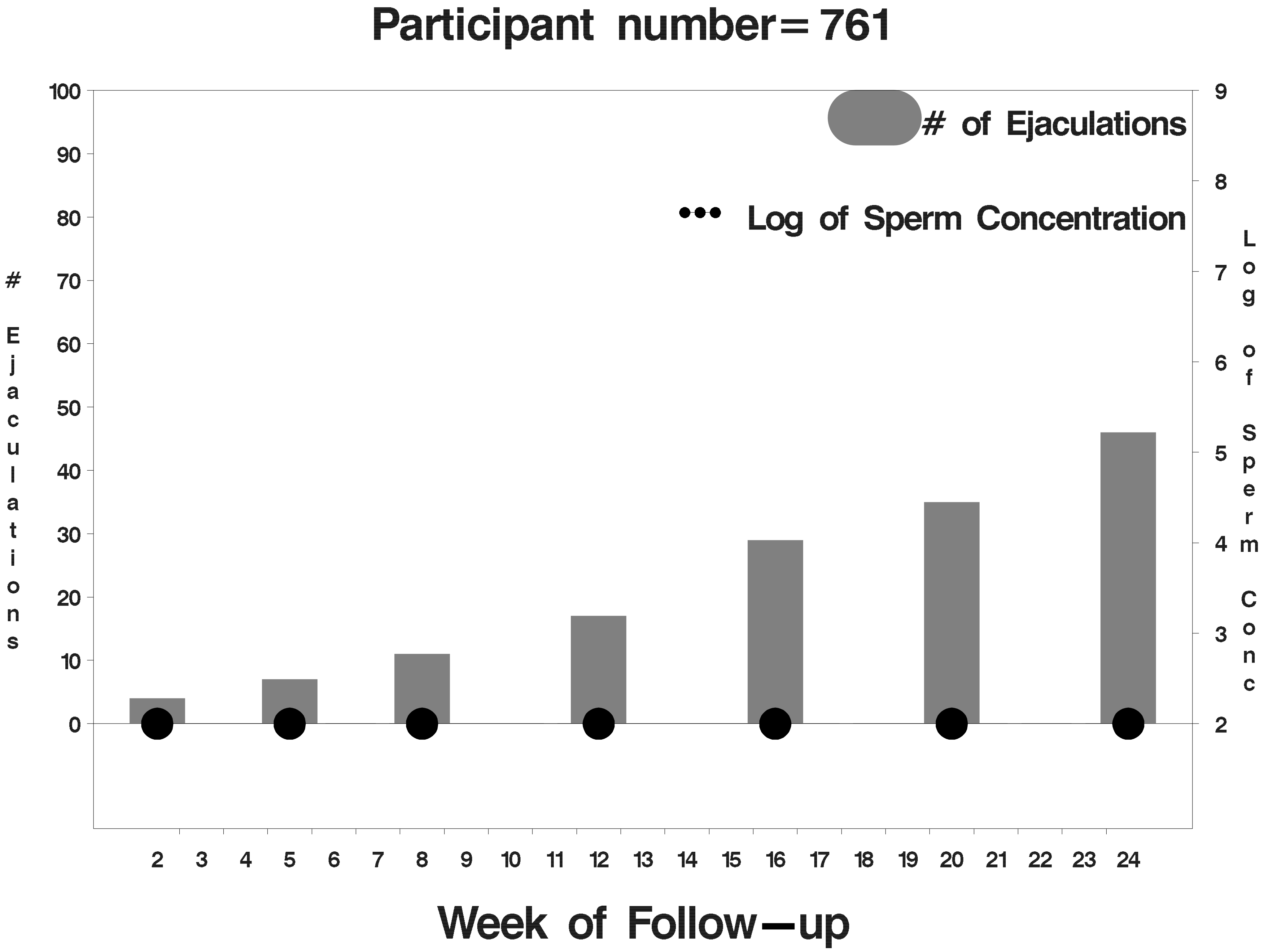
Participant number=761
# of Ejaculations
Log of Sperm Concentration
Ejaculations
Log of Sperm Conc
100
90
80
70
60
50
40
30
20
10
0
9
8
7
6
5
4
3
2
2 3 4 5 6 7 8 9 10 11 12 13 14 15 16 17 18 19 20 21 22 23 24
Week of Follow-up

Participant number= 762

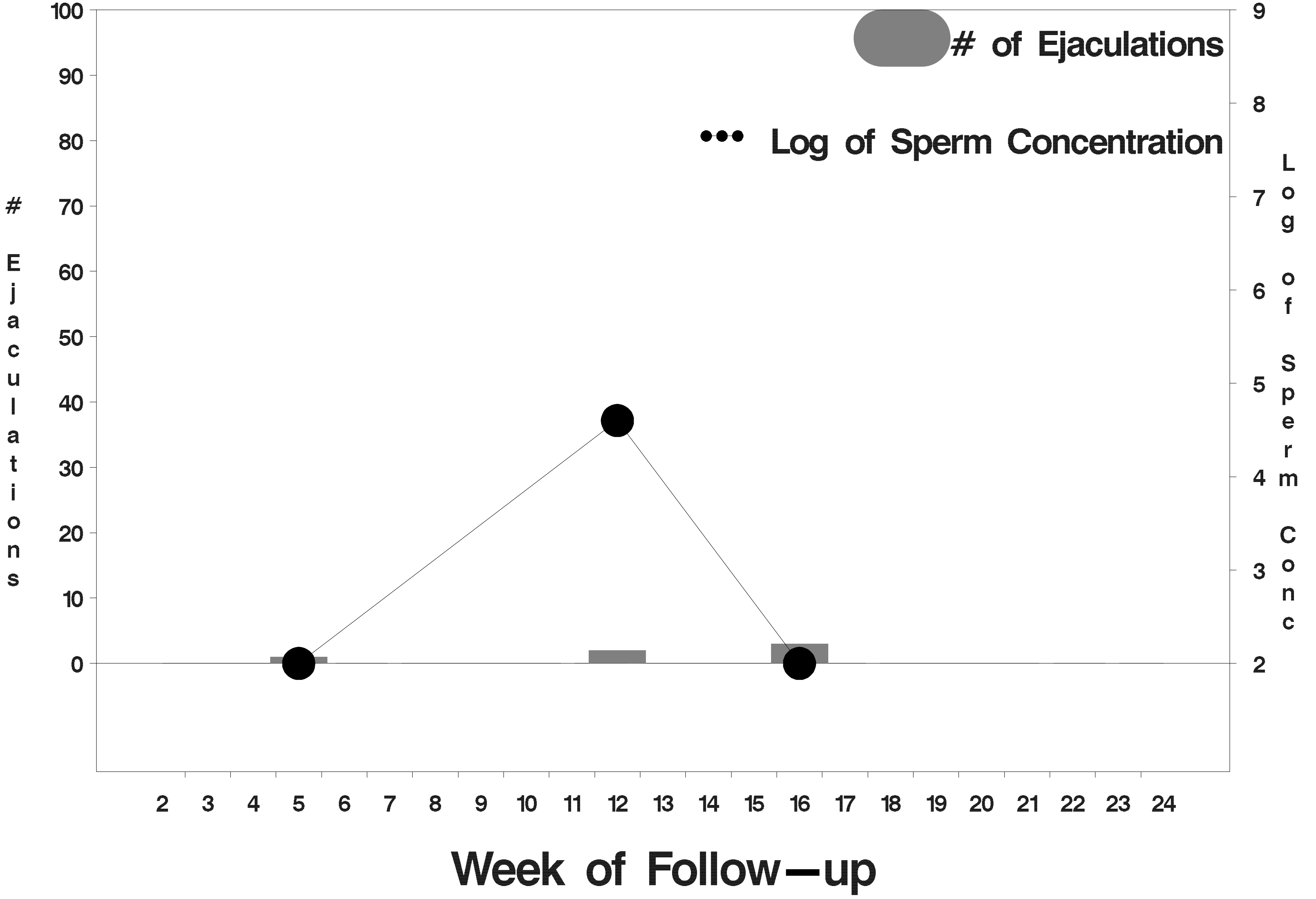

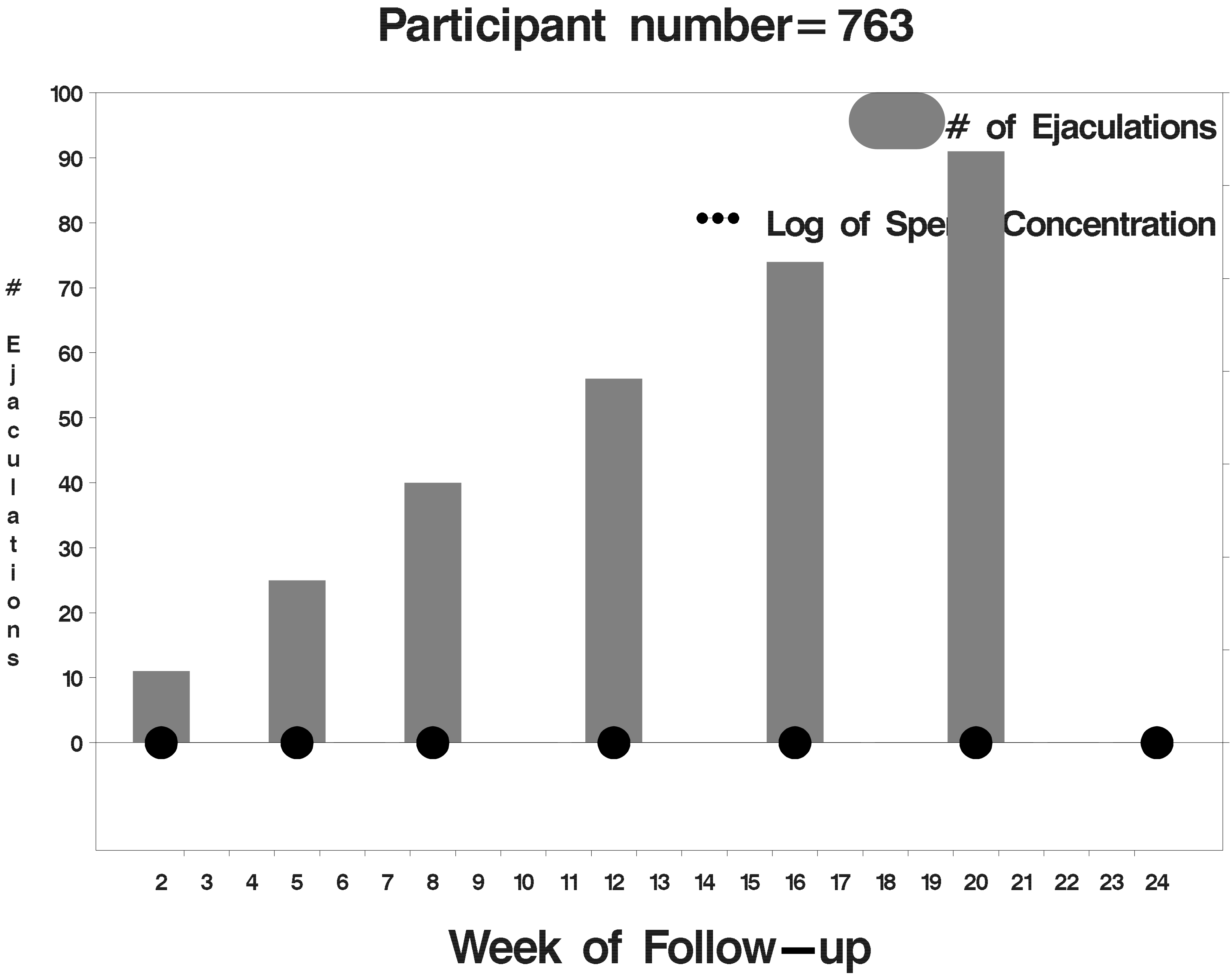
Participant number=763
# of Ejaculations
Log of Sperm Concentration
Ejaculations
Log of Sperm Conc
100
90
80
70
60
50
40
30
20
10
0
9
8
7
6
5
4
3
2
2 3 4 5 6 7 8 9 10 11 12 13 14 15 16 17 18 19 20 21 22 23 24
Week of Follow-up

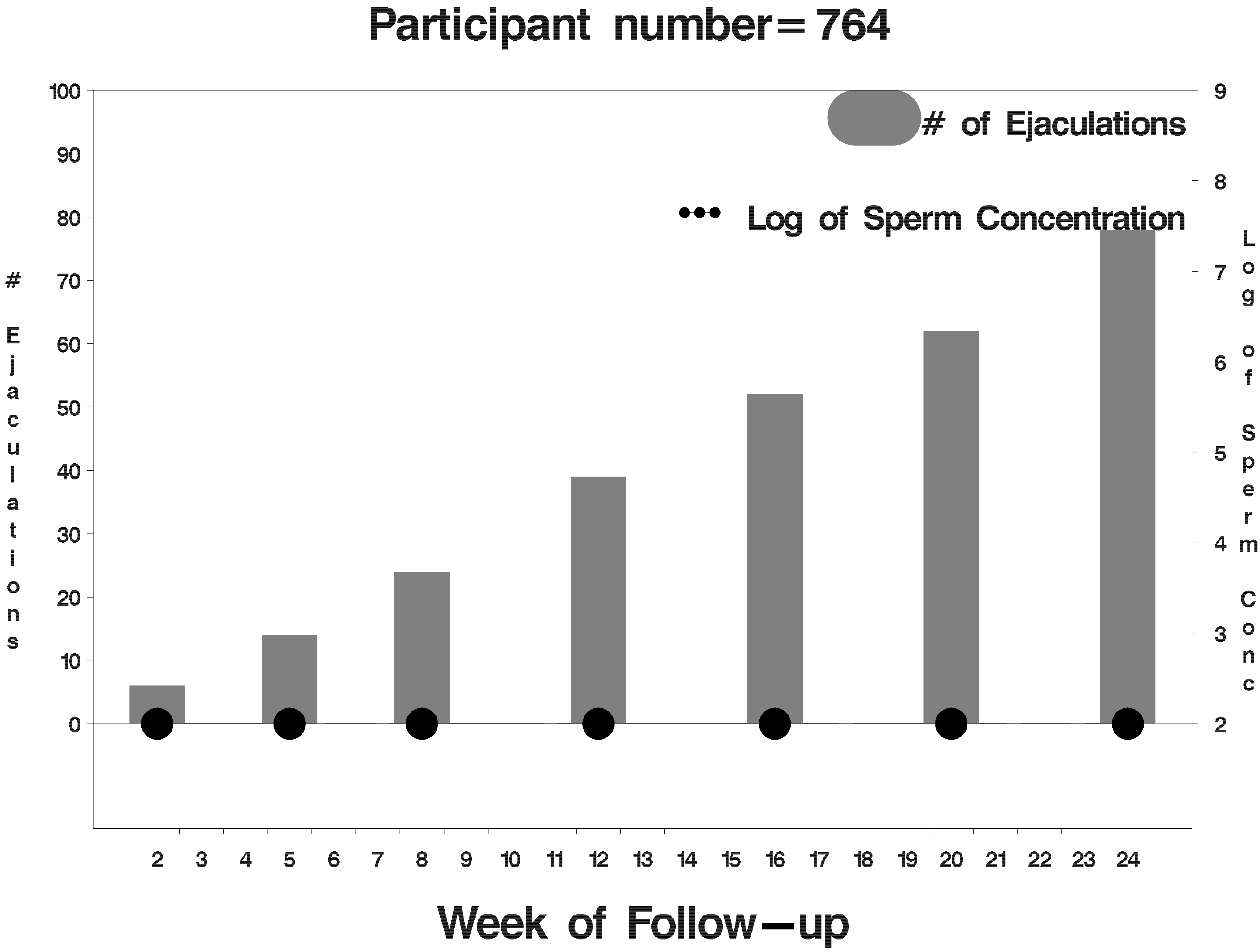

Participant number=764
# of Ejaculations
Log of Sperm Concentration
Ejaculations
Log of Sperm Conc
Week of Follow-up
0
10
20
30
40
50
60
70
80
90
100
2
3
4
5
6
7
8
9
2 3 4 5 6 7 8 9 10 11 12 13 14 15 16 17 18 19 20 21 22 23 24

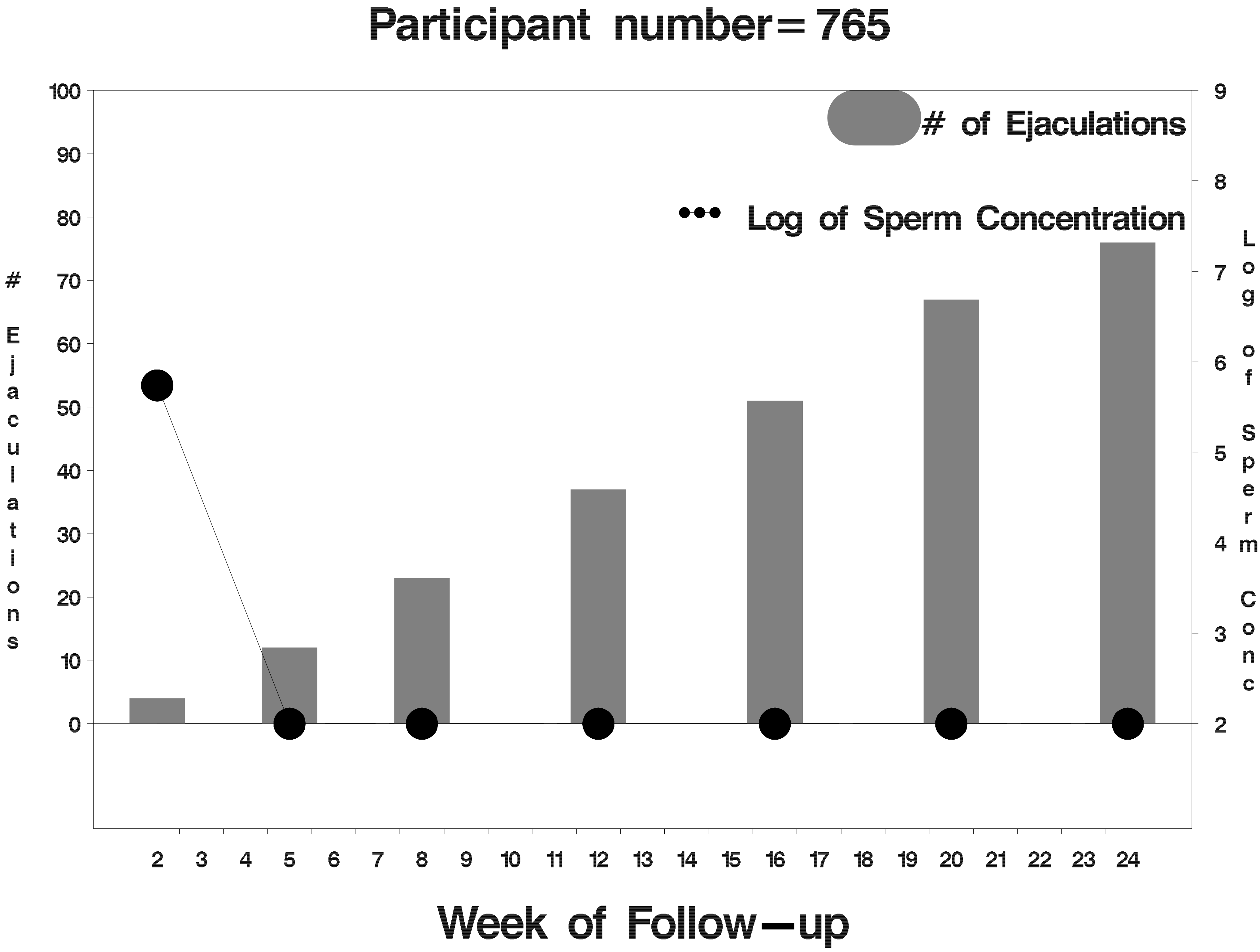

Participant number=765
# of Ejaculations
Log of Sperm Concentration
Ejaculations
Log of Sperm Conc
100
90
80
70
60
50
40
30
20
10
0
9
8
7
6
5
4
3
2
2 3 4 5 6 7 8 9 10 11 12 13 14 15 16 17 18 19 20 21 22 23 24
Week of Follow-up

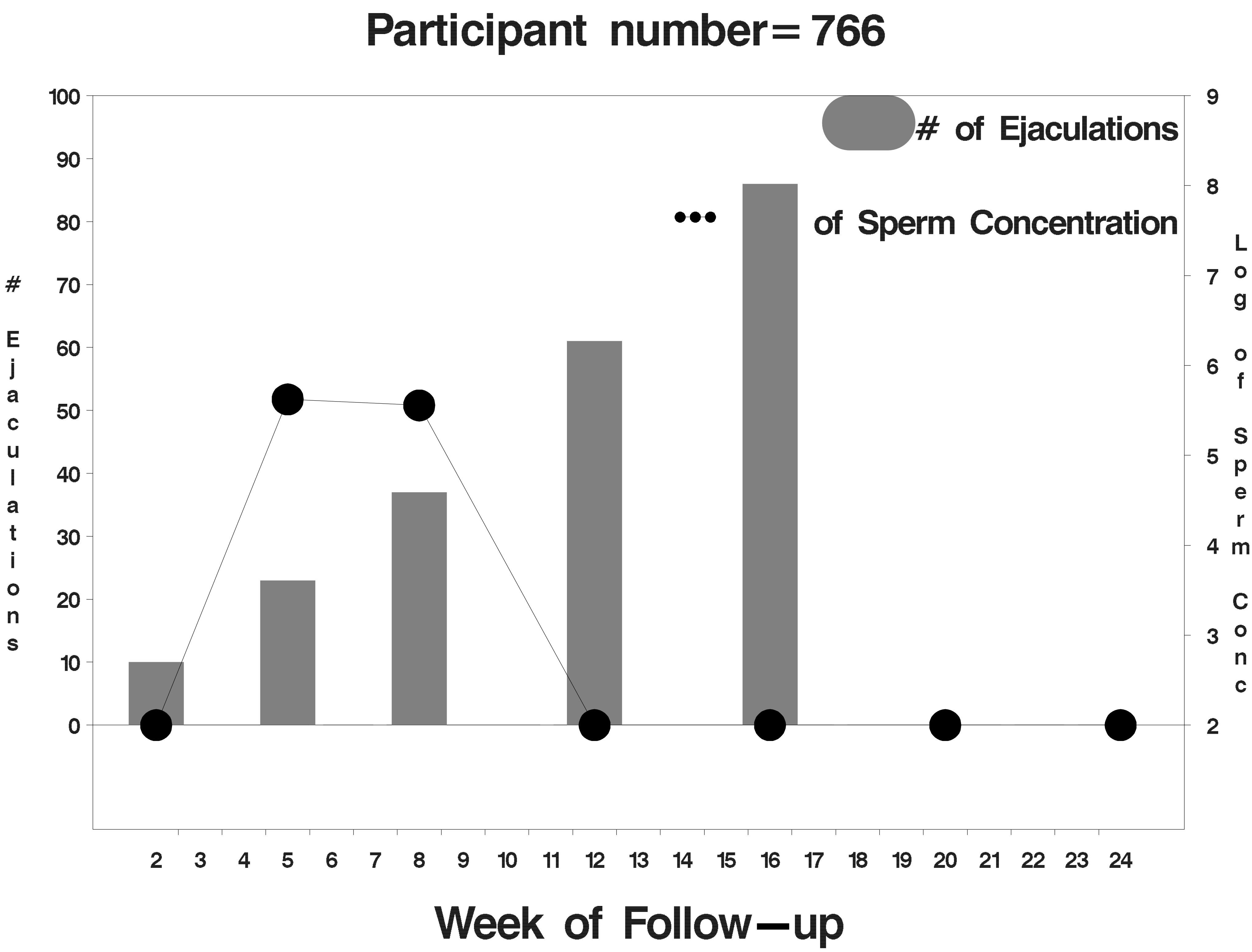

Participant number=766
# of Ejaculations
of Sperm Concentration
Ejaculations
Log of Sperm Conc
100
90
80
70
60
50
40
30
20
10
0
9
8
7
6
5
4
3
2
2 3 4 5 6 7 8 9 10 11 12 13 14 15 16 17 18 19 20 21 22 23 24
Week of Follow-up

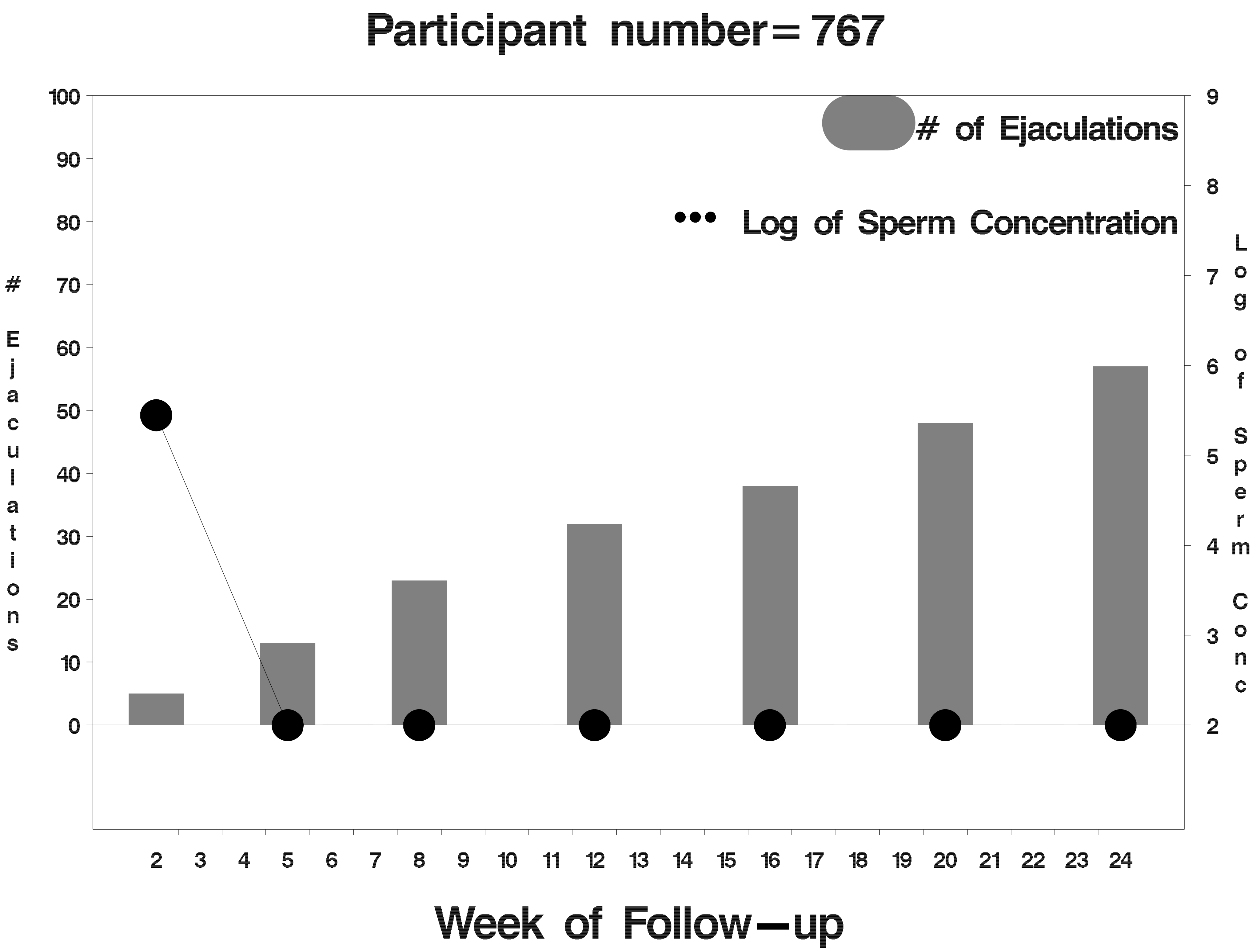

Participant number= 767
# of Ejaculations
Log of Sperm Concentration
Ejaculations
Log of Sperm Conc
100
90
80
70
60
50
40
30
20
10
0
9
8
7
6
5
4
3
2
2 3 4 5 6 7 8 9 10 11 12 13 14 15 16 17 18 19 20 21 22 23 24
Week of Follow-up

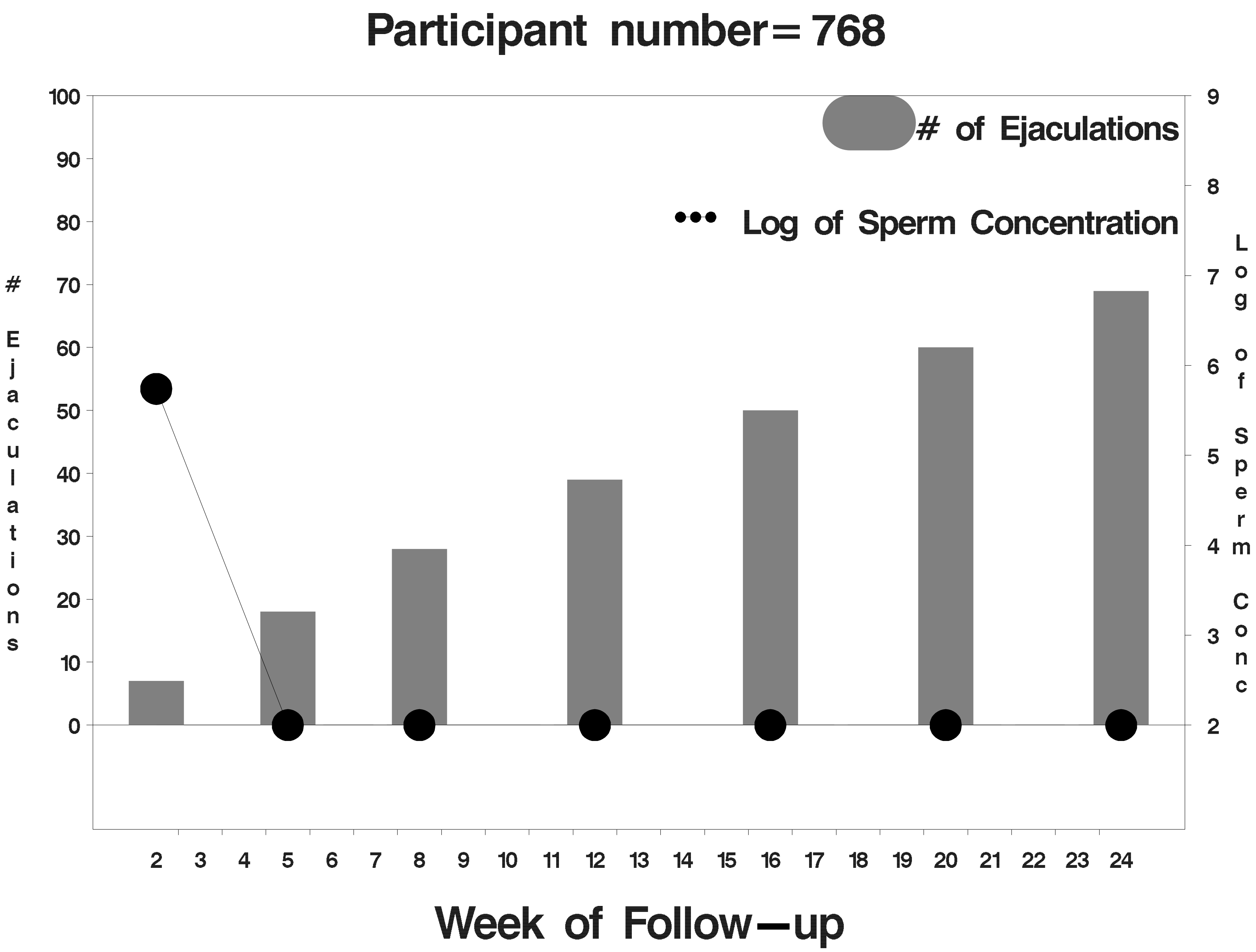

Participant number= 768
# of Ejaculations
Log of Sperm Concentration
Ejaculations
Log of Sperm Conc
100
90
80
70
60
50
40
30
20
10
0
9
8
7
6
5
4
3
2
2 3 4 5 6 7 8 9 10 11 12 13 14 15 16 17 18 19 20 21 22 23 24
Week of Follow-up

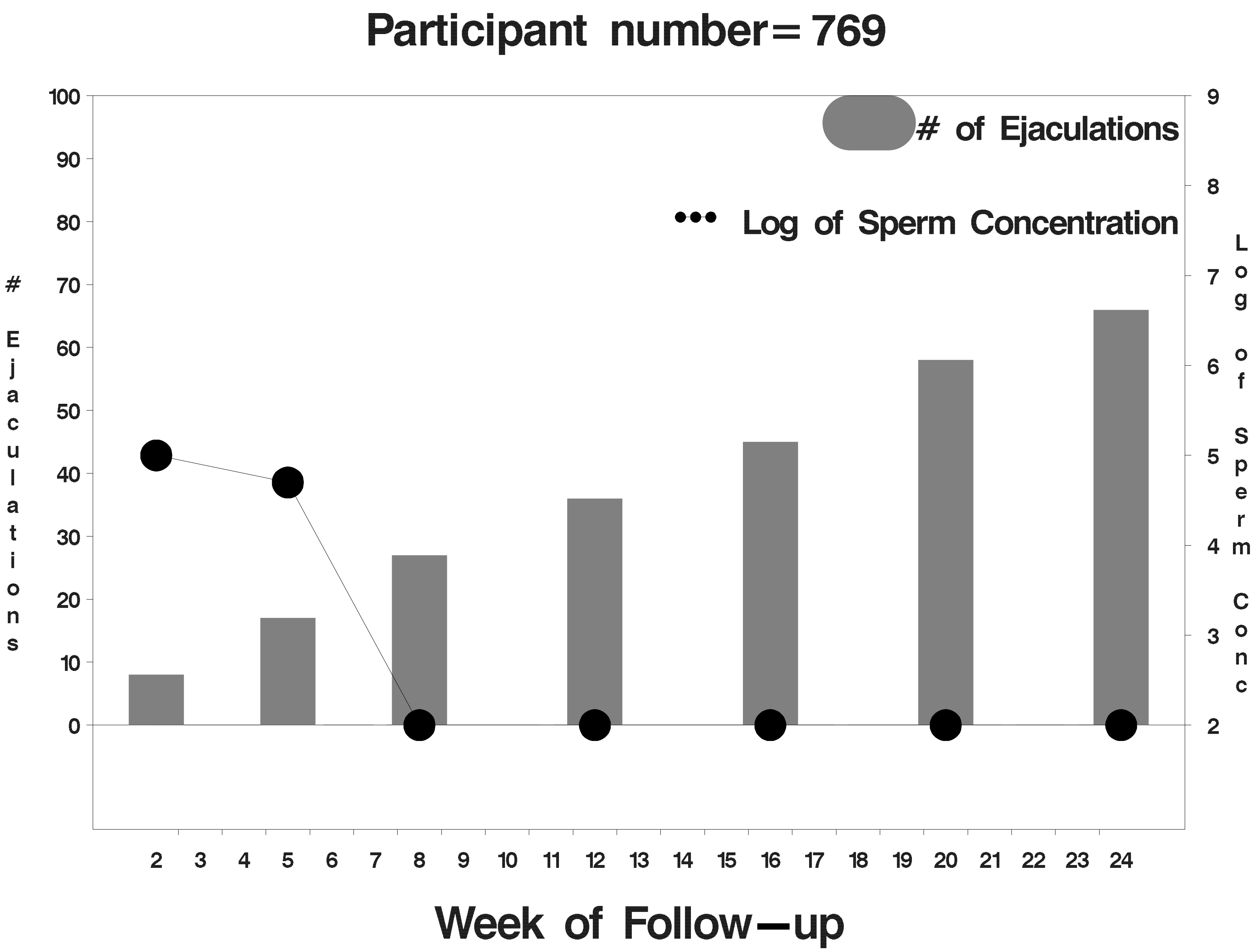

Participant number=769
# of Ejaculations
Log of Sperm Concentration
Ejaculations
Log of Sperm Conc
Week of Follow-up
100
90
80
70
60
50
40
30
20
10
0
9
8
7
6
5
4
3
2
2 3 4 5 6 7 8 9 10 11 12 13 14 15 16 17 18 19 20 21 22 23 24

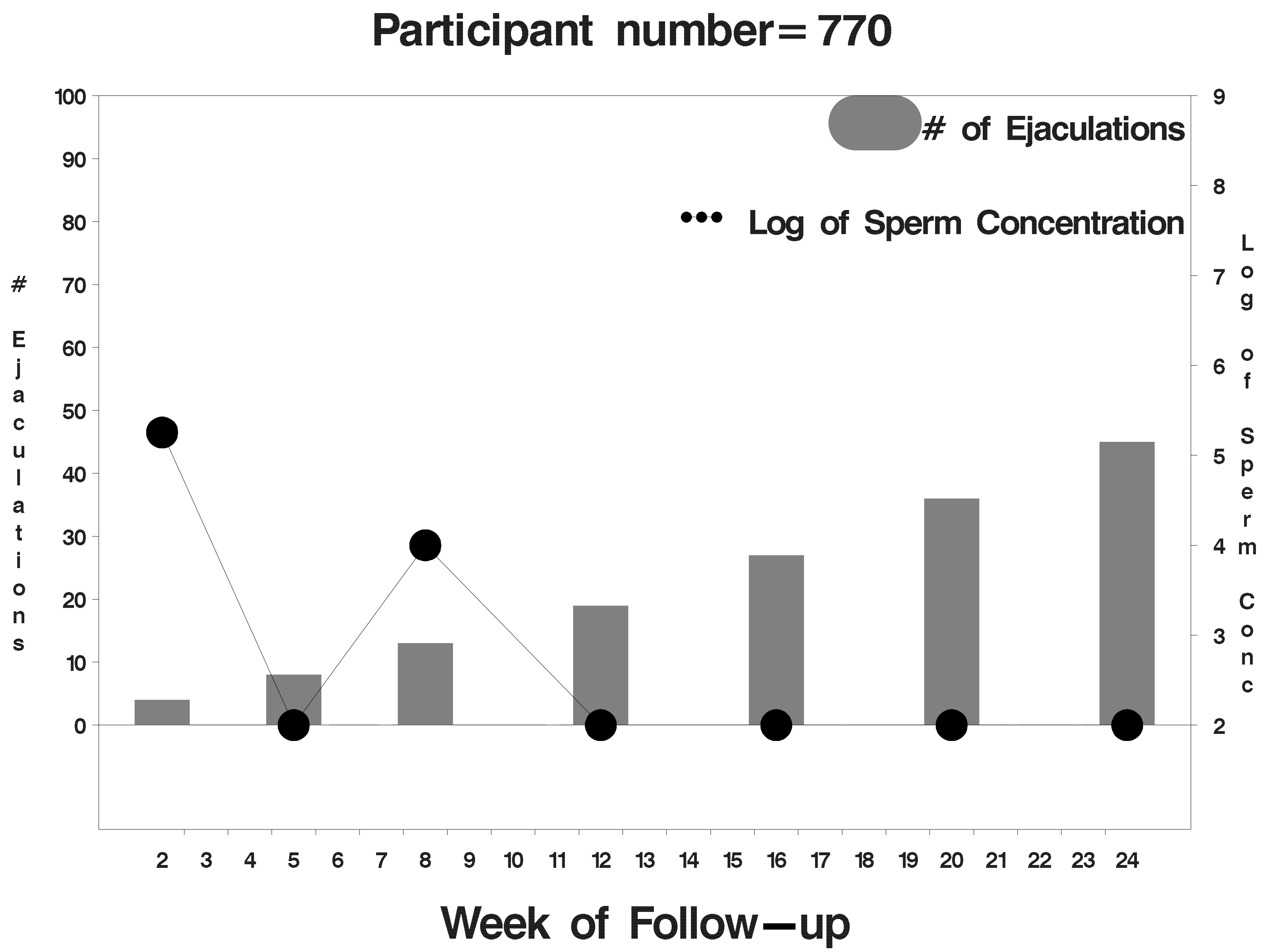

Participant number= 770
# of Ejaculations
Log of Sperm Concentration
Ejaculations
Log of Sperm Conc
100
90
80
70
60
50
40
30
20
10
0
9
8
7
6
5
4
3
2
2 3 4 5 6 7 8 9 10 11 12 13 14 15 16 17 18 19 20 21 22 23 24
Week of Follow-up

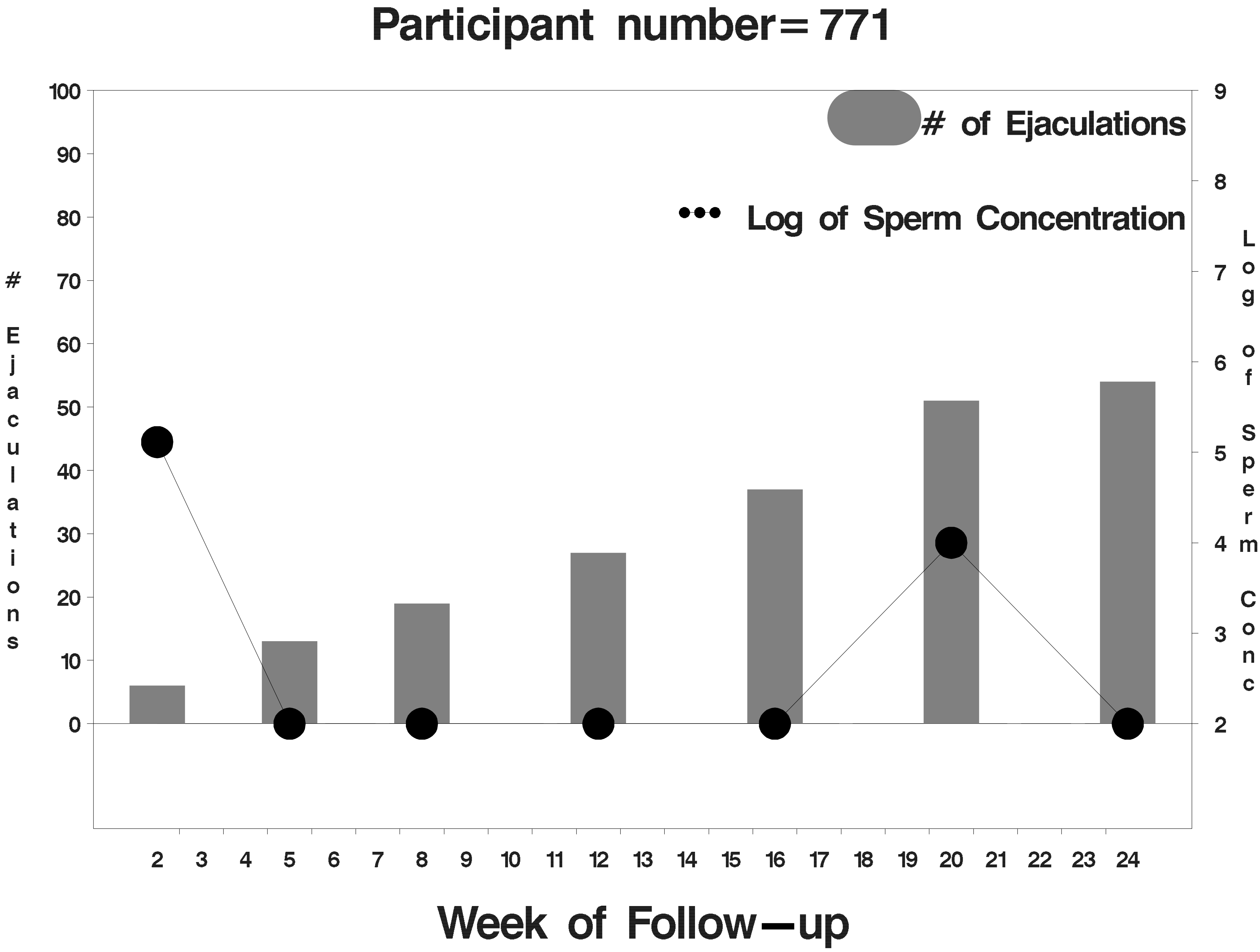
Participant number= 771
# of Ejaculations
Log of Sperm Concentration
Ejaculations
Log of Sperm Conc
100
90
80
70
60
50
40
30
20
10
0
9
8
7
6
5
4
3
2
2 3 4 5 6 7 8 9 10 11 12 13 14 15 16 17 18 19 20 21 22 23 24
Week of Follow-up

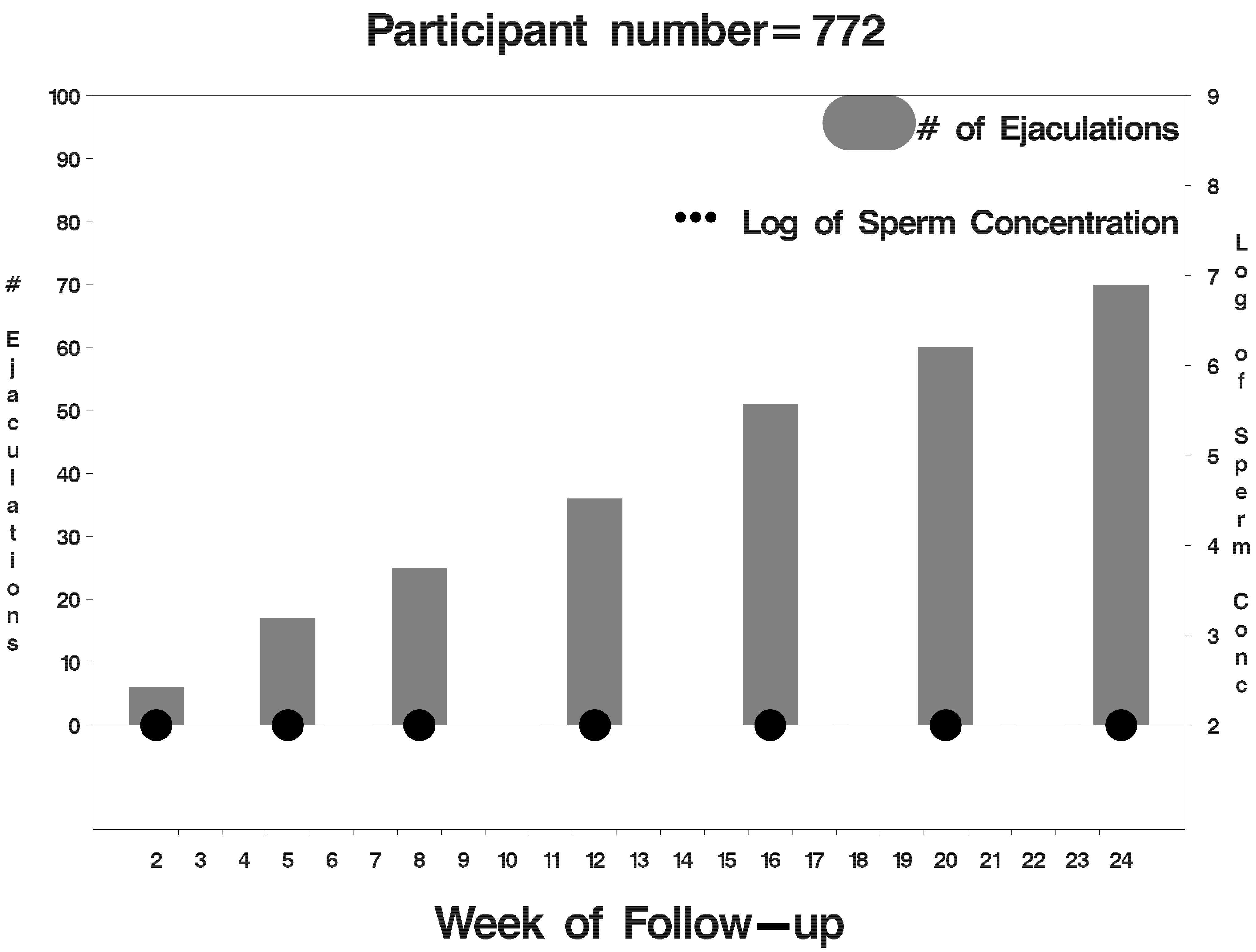

Participant number=772
# of Ejaculations
Log of Sperm Concentration
Ejaculations
Log of Sperm Conc
100
90
80
70
60
50
40
30
20
10
0
9
8
7
6
5
4
3
2
2 3 4 5 6 7 8 9 10 11 12 13 14 15 16 17 18 19 20 21 22 23 24
Week of Follow-up

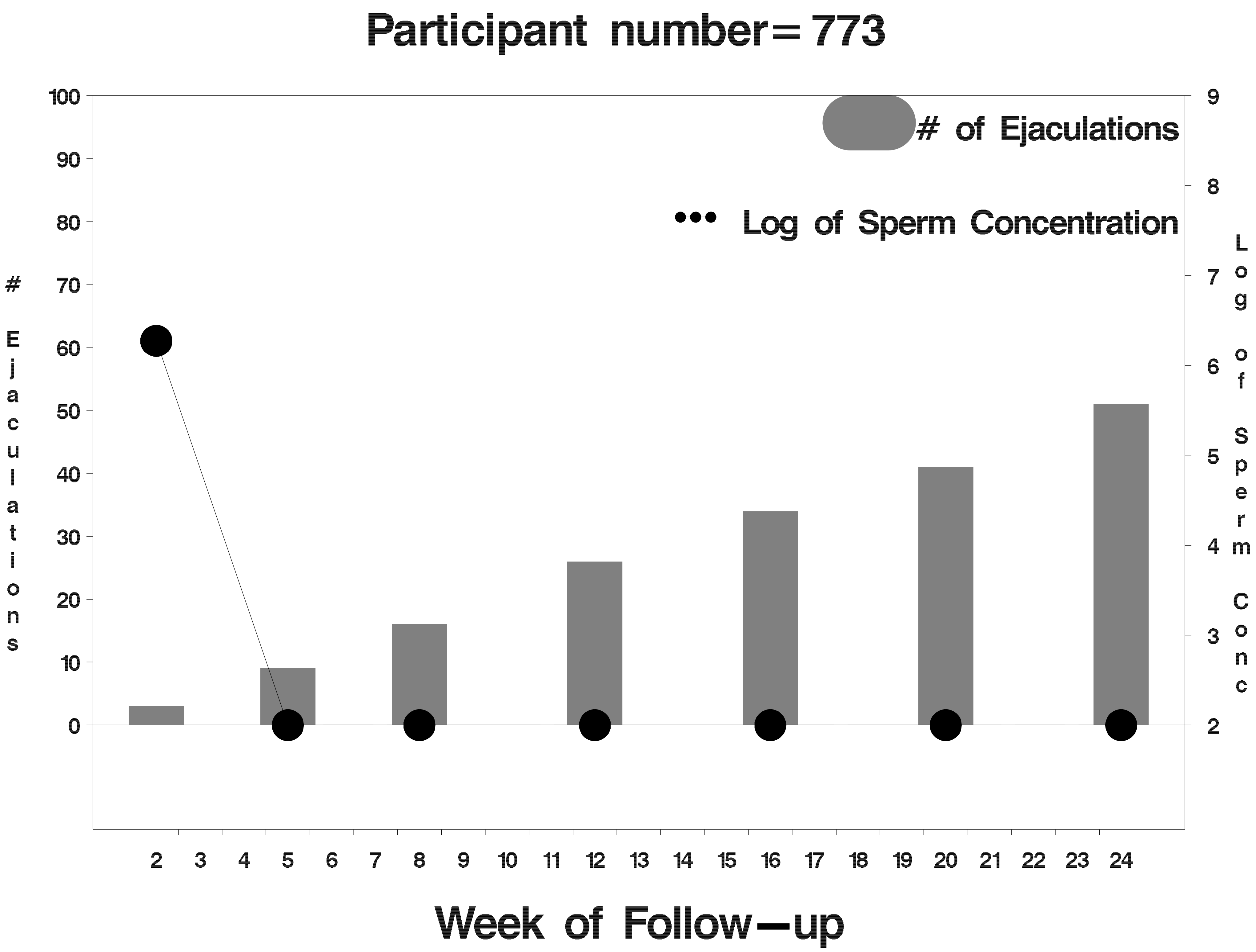

Participant number=773
# of Ejaculations
Log of Sperm Concentration
Ejaculations
Log of Sperm Conc
100
90
80
70
60
50
40
30
20
10
0
9
8
7
6
5
4
3
2
2 3 4 5 6 7 8 9 10 11 12 13 14 15 16 17 18 19 20 21 22 23 24
Week of Follow-up

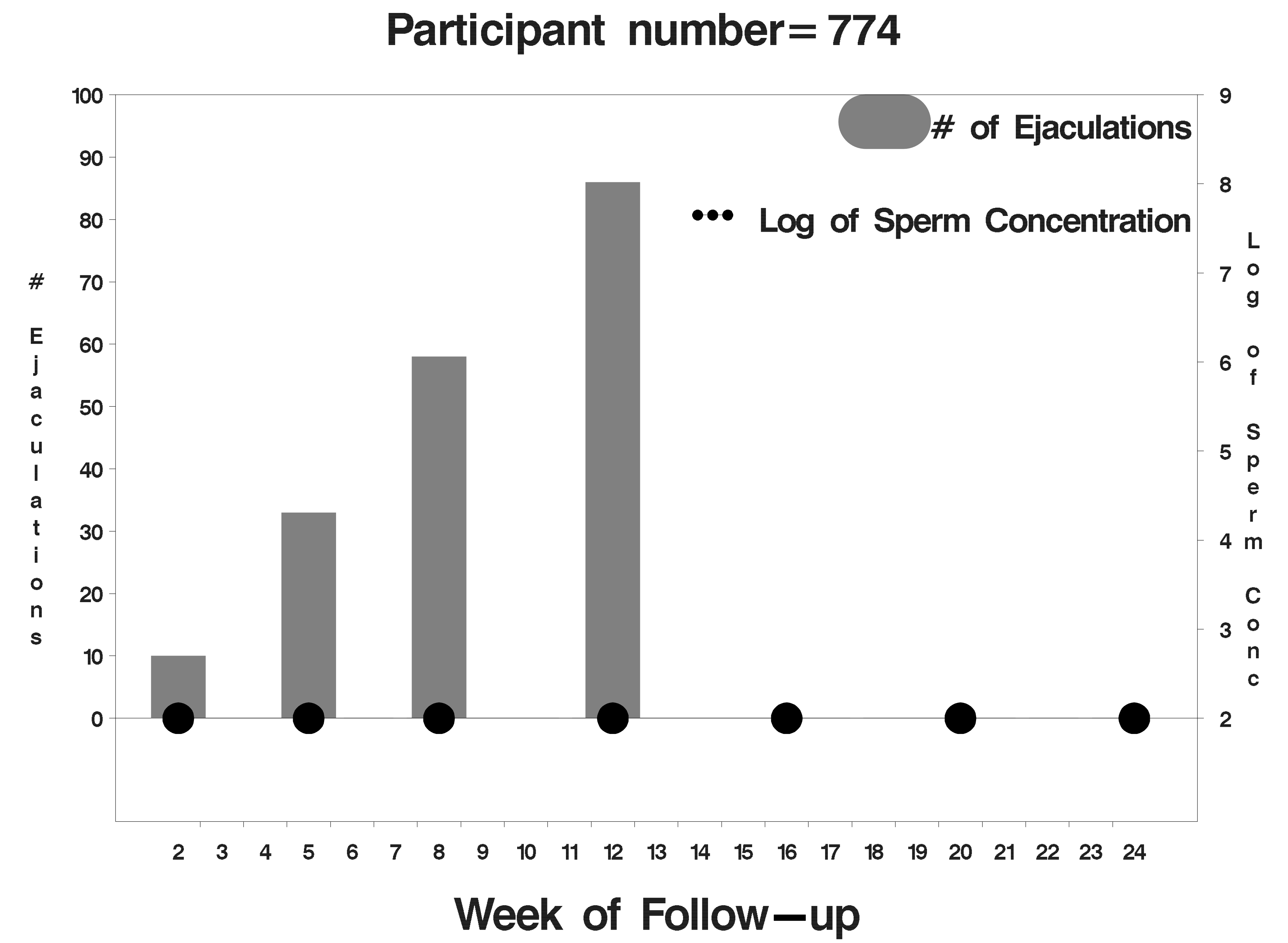

Participant number= 774
# of Ejaculations
Log of Sperm Concentration
Ejaculations
Log of Sperm Conc
100
90
80
70
60
50
40
30
20
10
0
9
8
7
6
5
4
3
2
2 3 4 5 6 7 8 9 10 11 12 13 14 15 16 17 18 19 20 21 22 23 24
Week of Follow-up

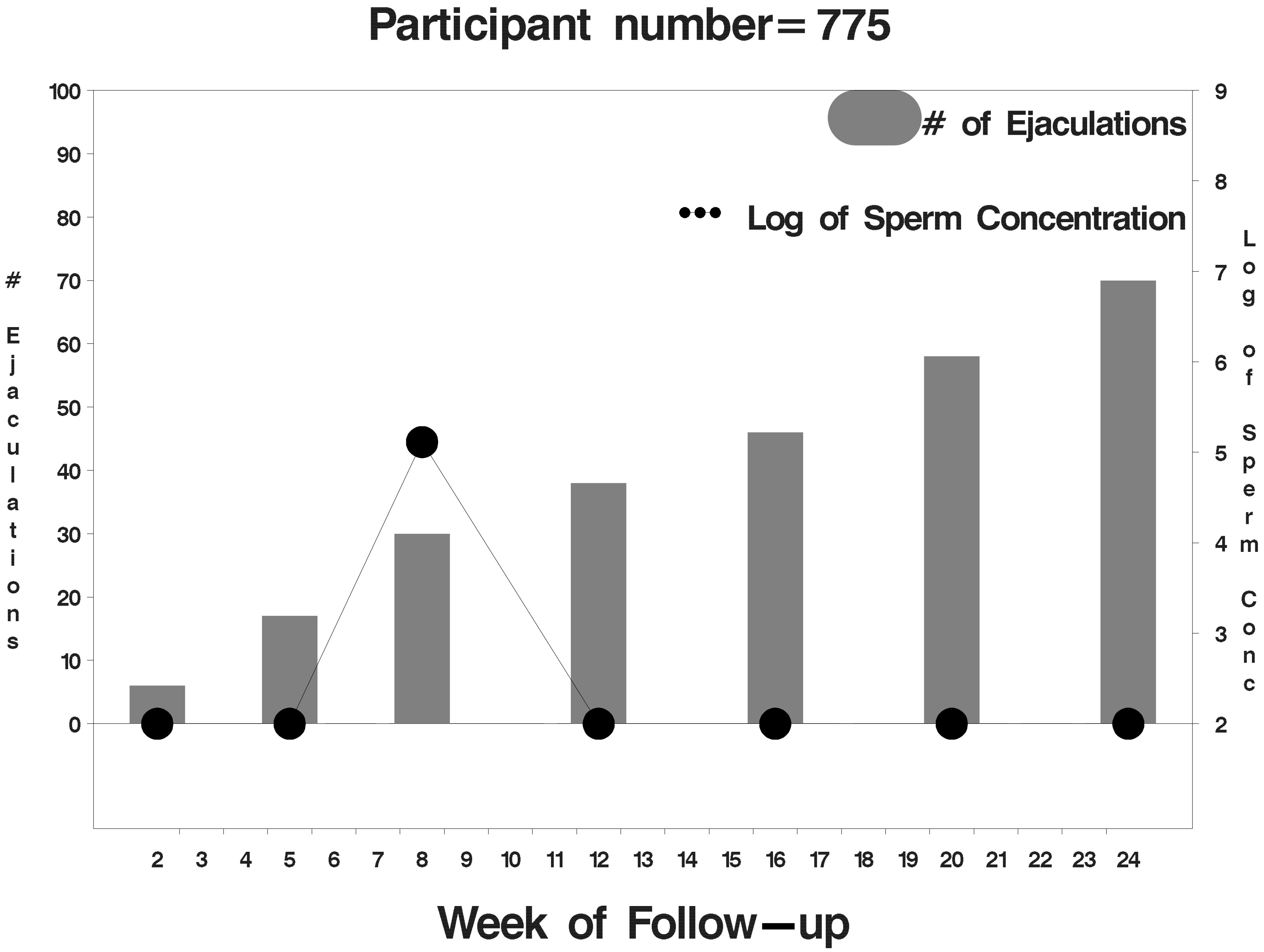

Participant number= 775
# of Ejaculations
Log of Sperm Concentration
Ejaculations
Log of Sperm Conc
100
90
80
70
60
50
40
30
20
10
0
9
8
7
6
5
4
3
2
2 3 4 5 6 7 8 9 10 11 12 13 14 15 16 17 18 19 20 21 22 23 24
Week of Follow-up

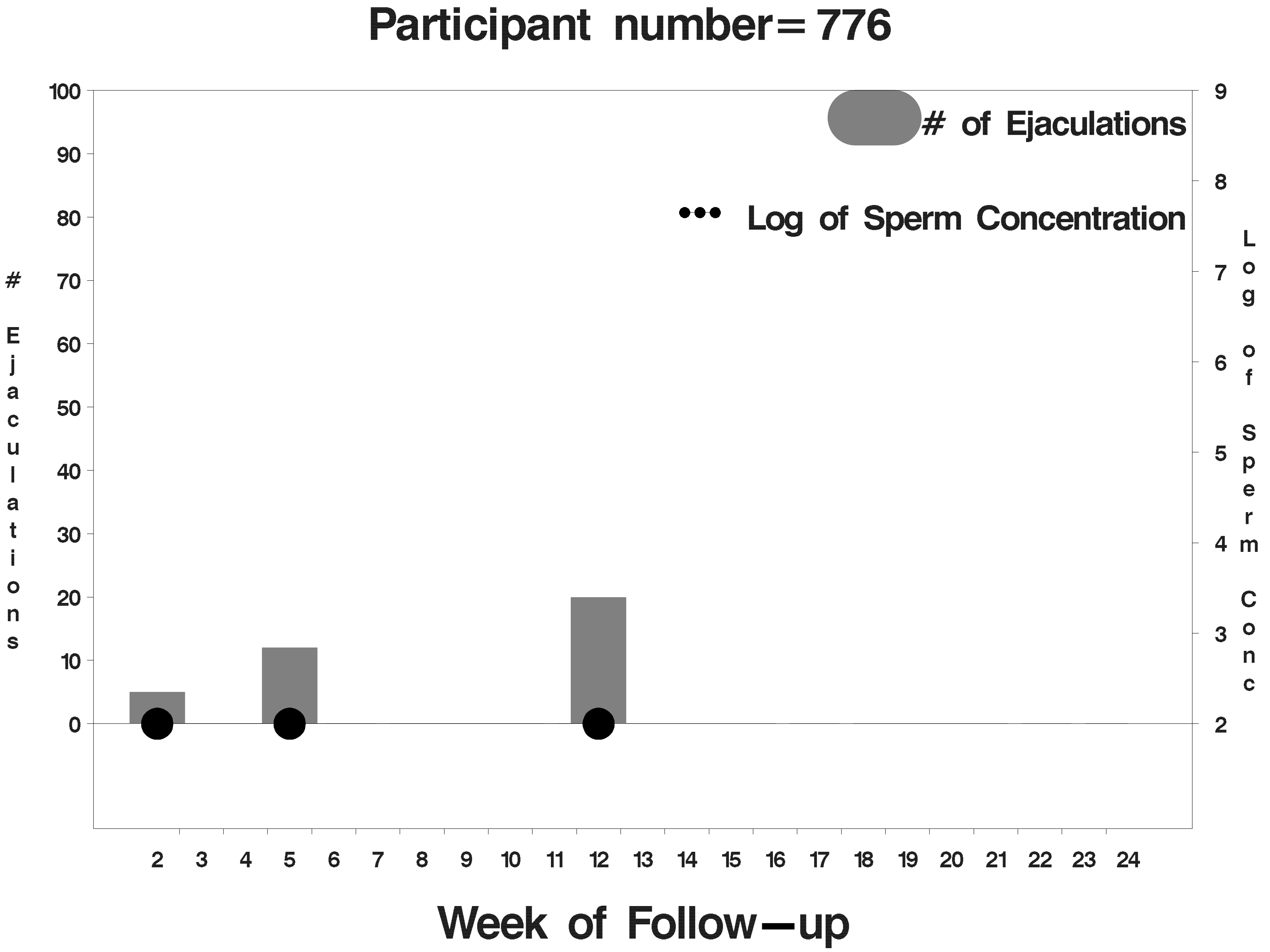

Participant number=776
# of Ejaculations
Log of Sperm Concentration
Ejaculations
Log of Sperm Conc
100
90
80
70
60
50
40
30
20
10
0
9
8
7
6
5
4
3
2
2 3 4 5 6 7 8 9 10 11 12 13 14 15 16 17 18 19 20 21 22 23 24
Week of Follow−up

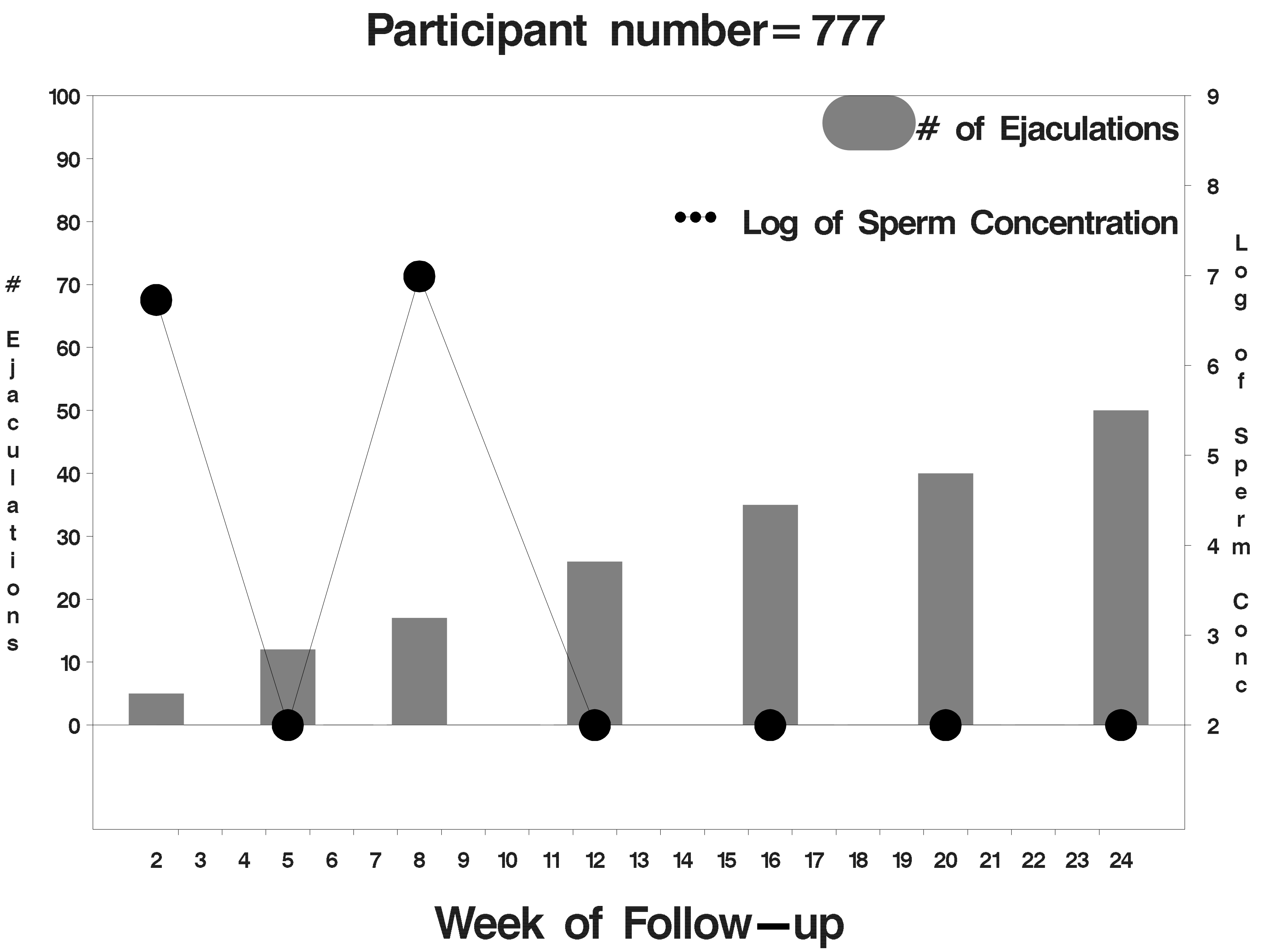

Participant number=777
# of Ejaculations
Log of Sperm Concentration
Ejaculations
Log of Sperm Conc
100
90
80
70
60
50
40
30
20
10
0
9
8
7
6
5
4
3
2
2 3 4 5 6 7 8 9 10 11 12 13 14 15 16 17 18 19 20 21 22 23 24
Week of Follow−up

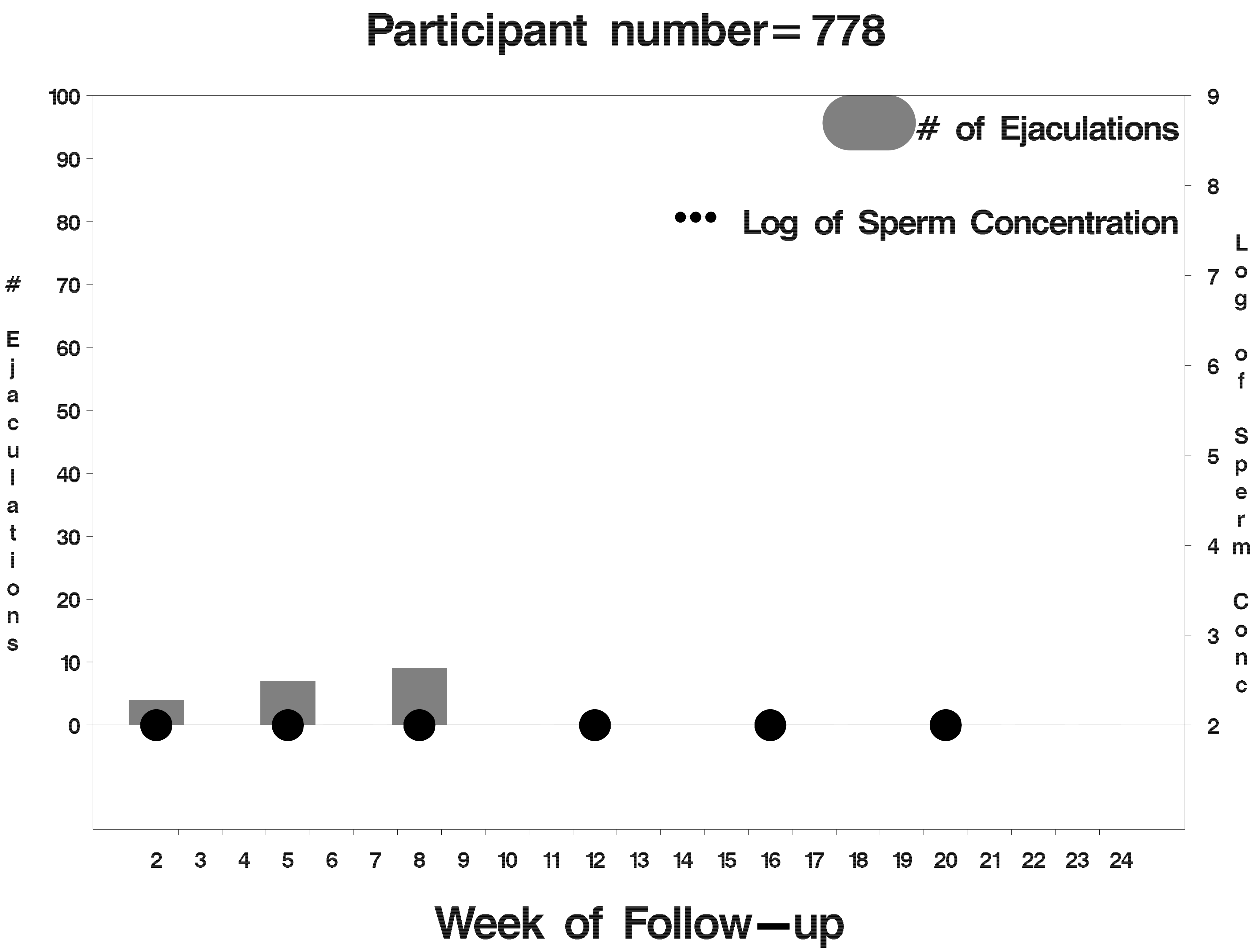

Participant number=778
# of Ejaculations
Log of Sperm Concentration
Ejaculations
Log of Sperm Conc
100
90
80
70
60
50
40
30
20
10
0
9
8
7
6
5
4
3
2
2 3 4 5 6 7 8 9 10 11 12 13 14 15 16 17 18 19 20 21 22 23 24
Week of Follow-up

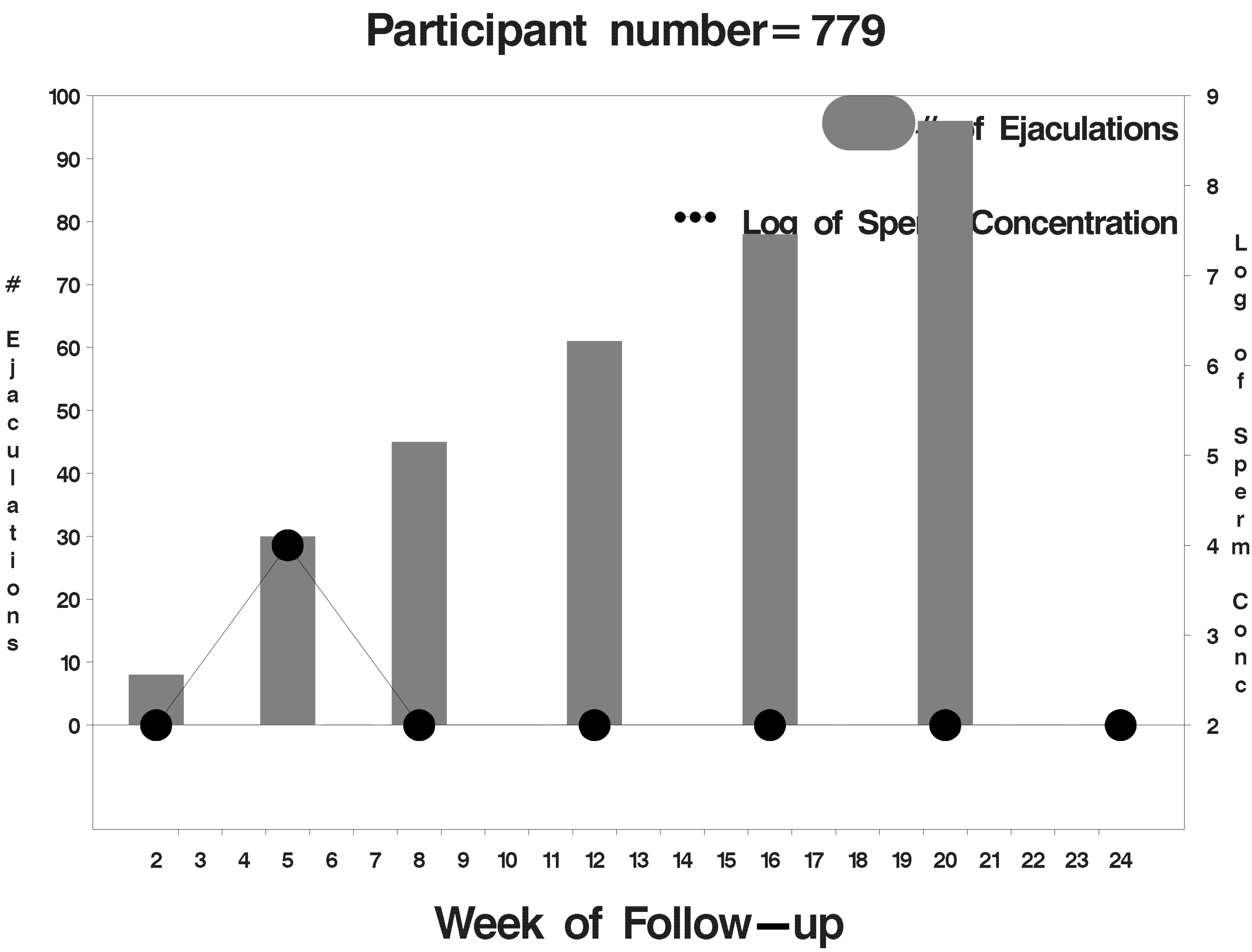

Participant number= 779
# of Ejaculations
Log of Sperm Concentration
Ejaculations
Log of Sperm Conc
100
90
80
70
60
50
40
30
20
10
0
9
8
7
6
5
4
3
2
2 3 4 5 6 7 8 9 10 11 12 13 14 15 16 17 18 19 20 21 22 23 24
Week of Follow-up

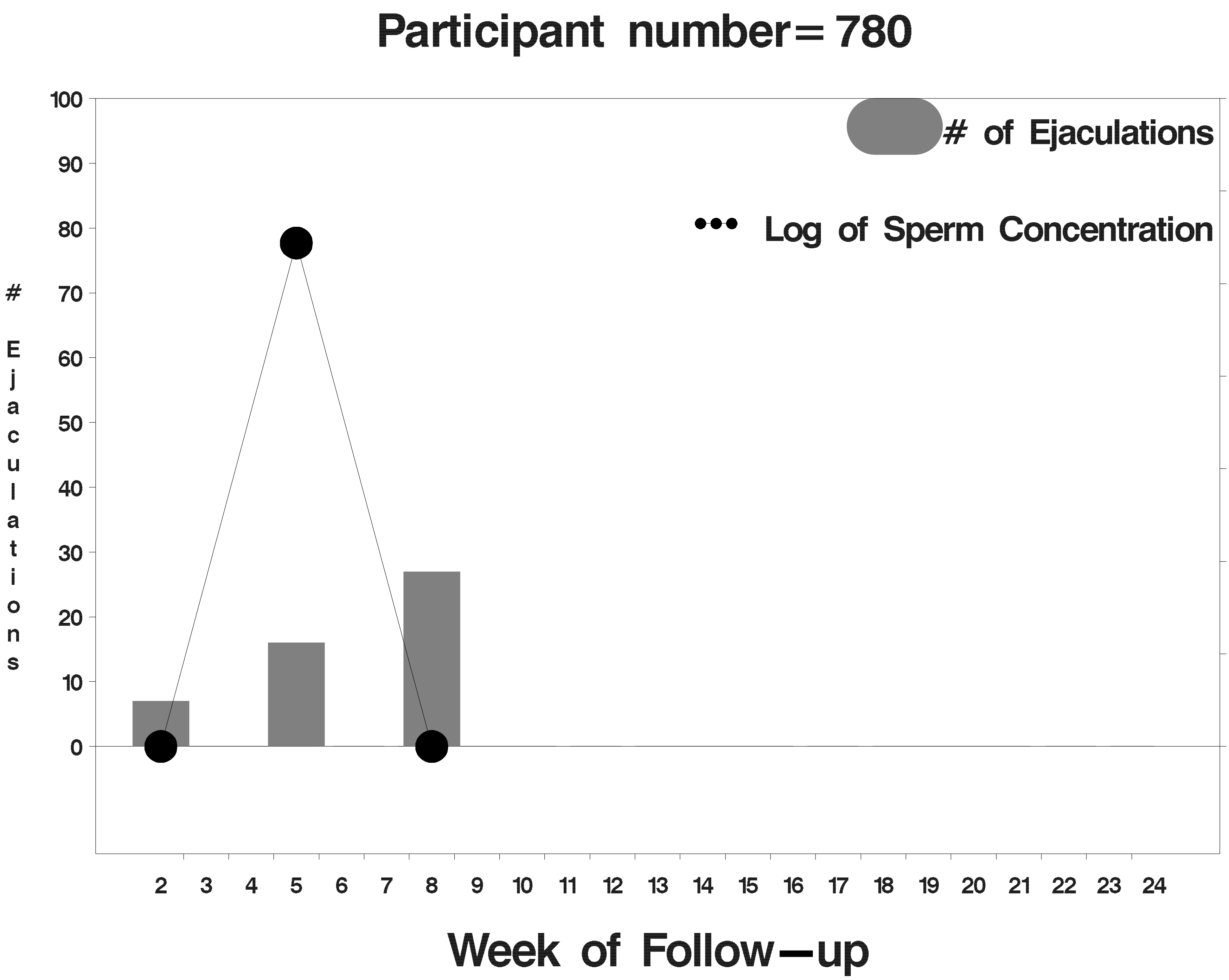

Participant number= 780
# of Ejaculations
Log of Sperm Concentration
Ejaculations
Log of Sperm Conc
Week of Follow-up
0
10
20
30
40
50
60
70
80
90
100
2
3
4
5
6
7
8
9
2 3 4 5 6 7 8 9 10 11 12 13 14 15 16 17 18 19 20 21 22 23 24

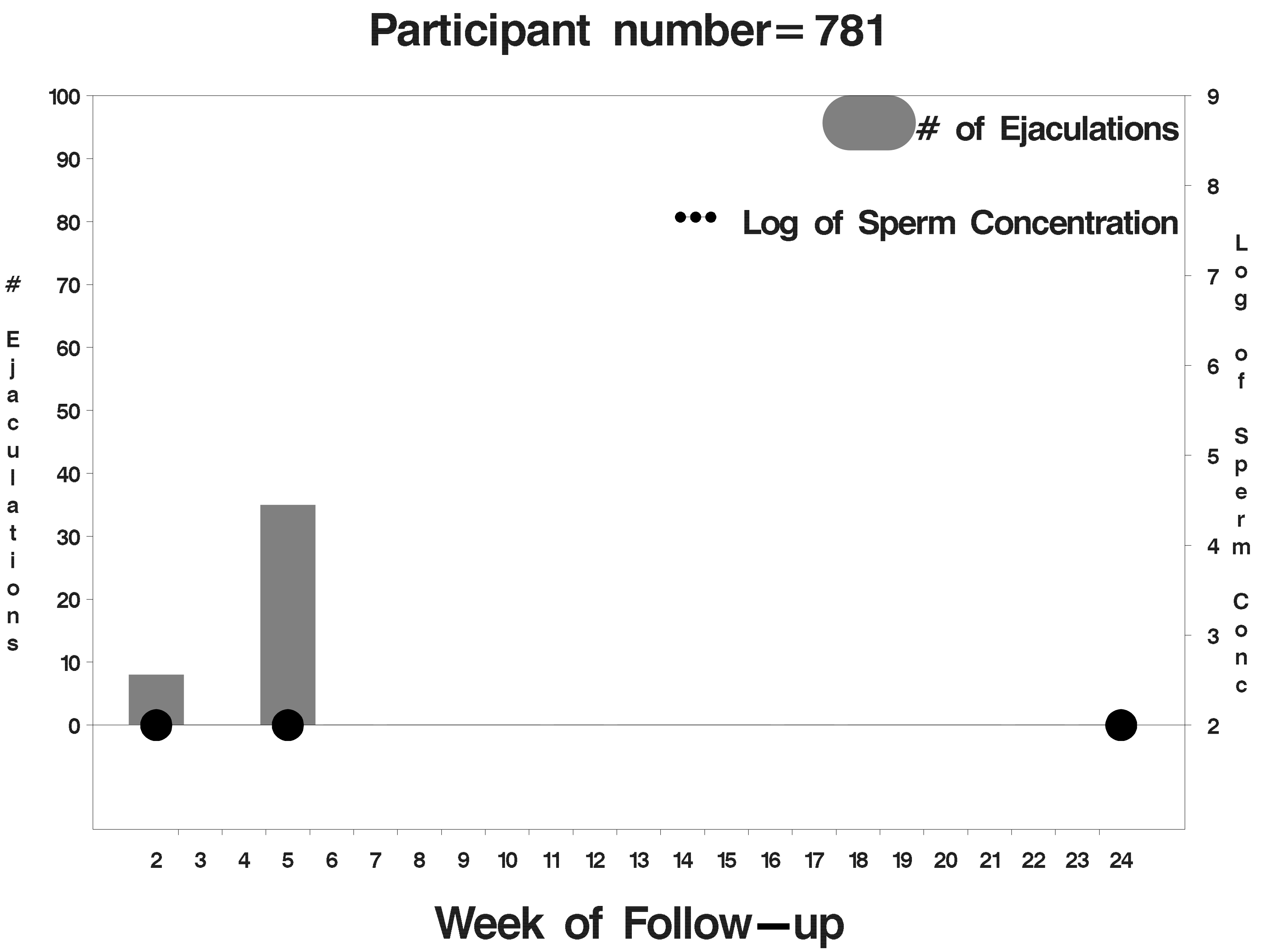

Participant number= 781
# of Ejaculations
Log of Sperm Concentration
Ejaculations
Log of Sperm Conc
100
90
80
70
60
50
40
30
20
10
0
9
8
7
6
5
4
3
2
2 3 4 5 6 7 8 9 10 11 12 13 14 15 16 17 18 19 20 21 22 23 24
Week of Follow-up

Participant number= 782

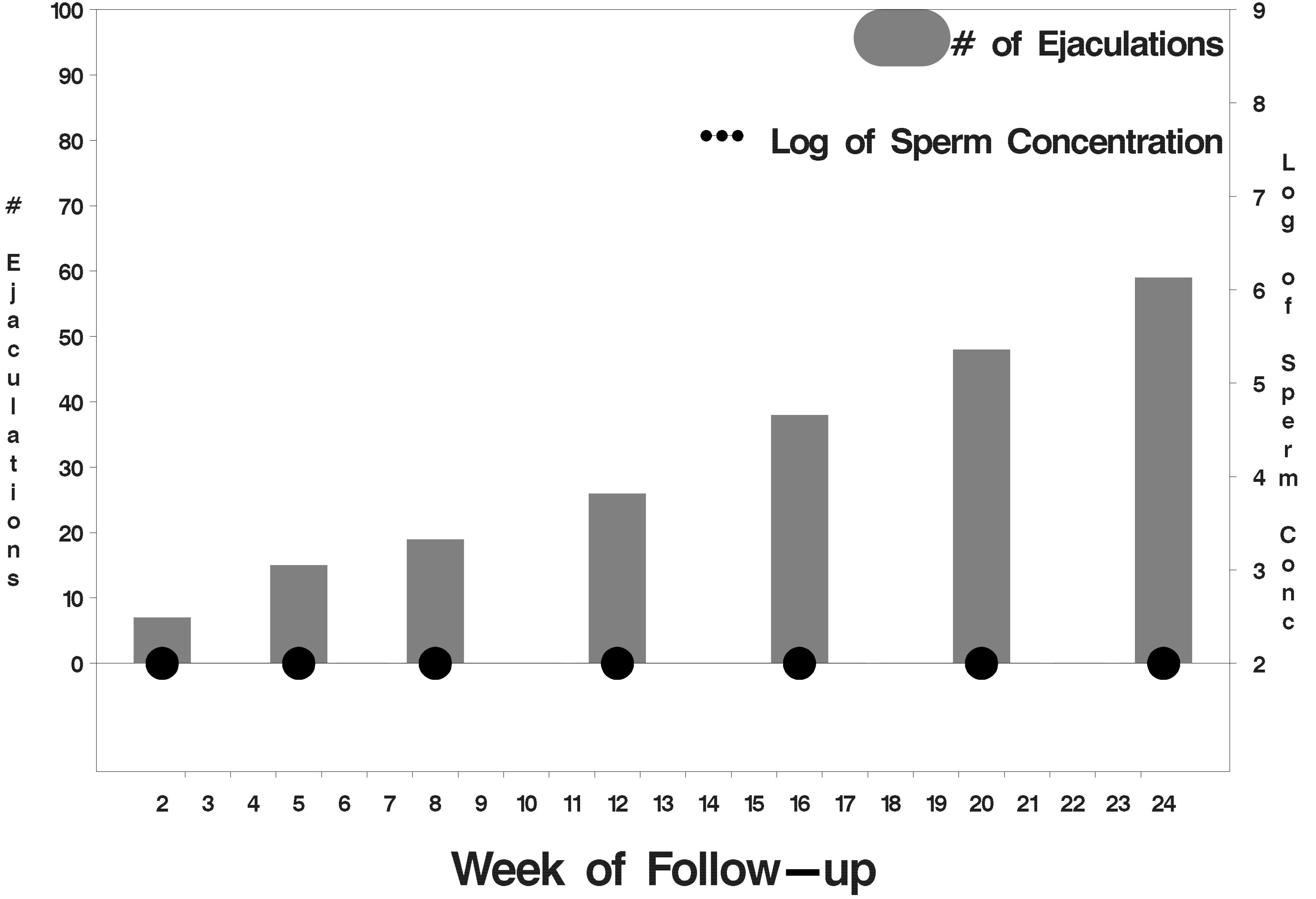

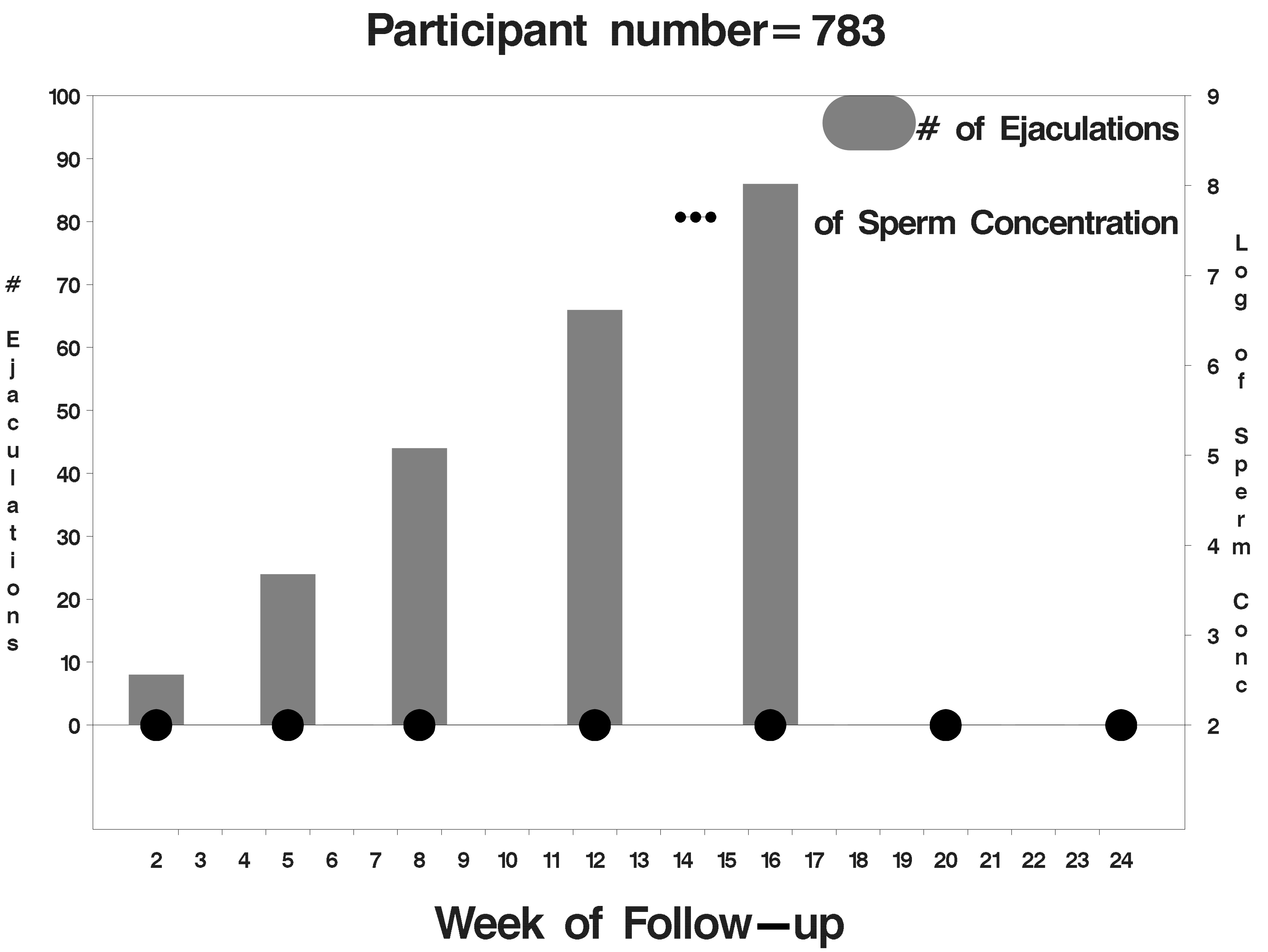
Participant number= 783
# of Ejaculations
of Sperm Concentration
Ejaculations
Log of Sperm Conc
100
90
80
70
60
50
40
30
20
10
0
9
8
7
6
5
4
3
2
2 3 4 5 6 7 8 9 10 11 12 13 14 15 16 17 18 19 20 21 22 23 24
Week of Follow-up

Participant number= 784

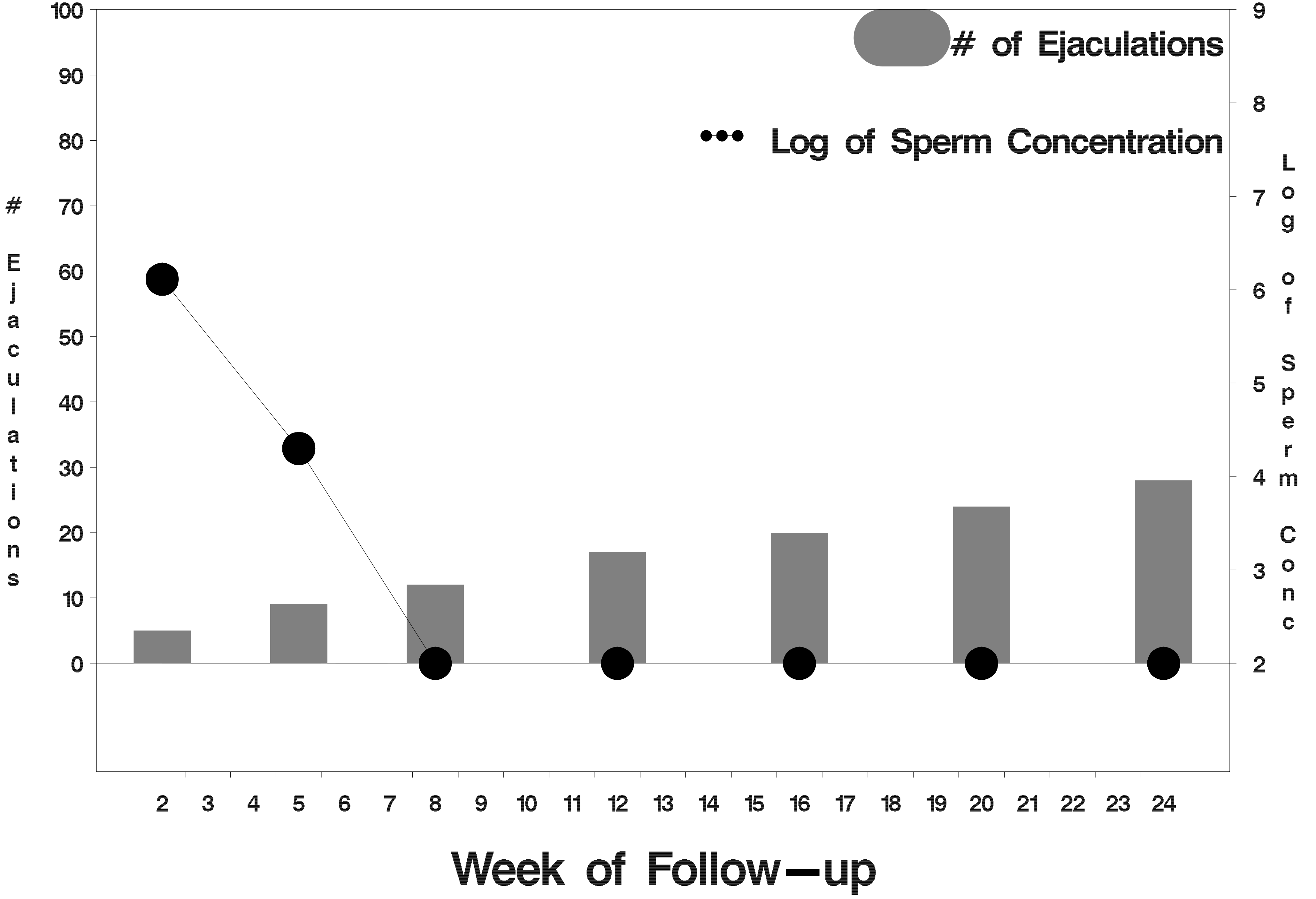

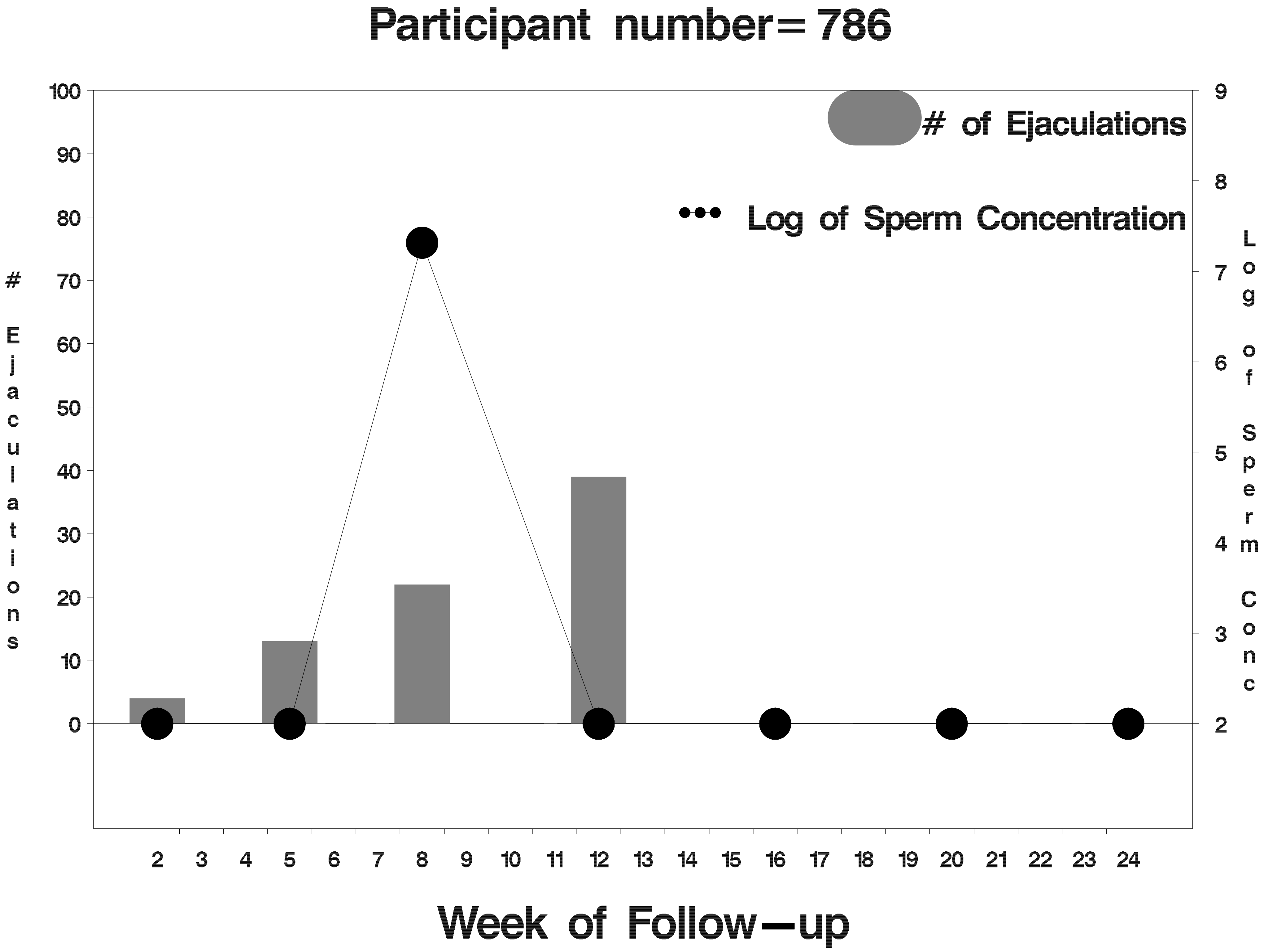

Participant number= 786
# of Ejaculations
Log of Sperm Concentration
Ejaculations
Log of Sperm Conc
100
90
80
70
60
50
40
30
20
10
0
9
8
7
6
5
4
3
2
2 3 4 5 6 7 8 9 10 11 12 13 14 15 16 17 18 19 20 21 22 23 24
Week of Follow-up

Participant number= 787

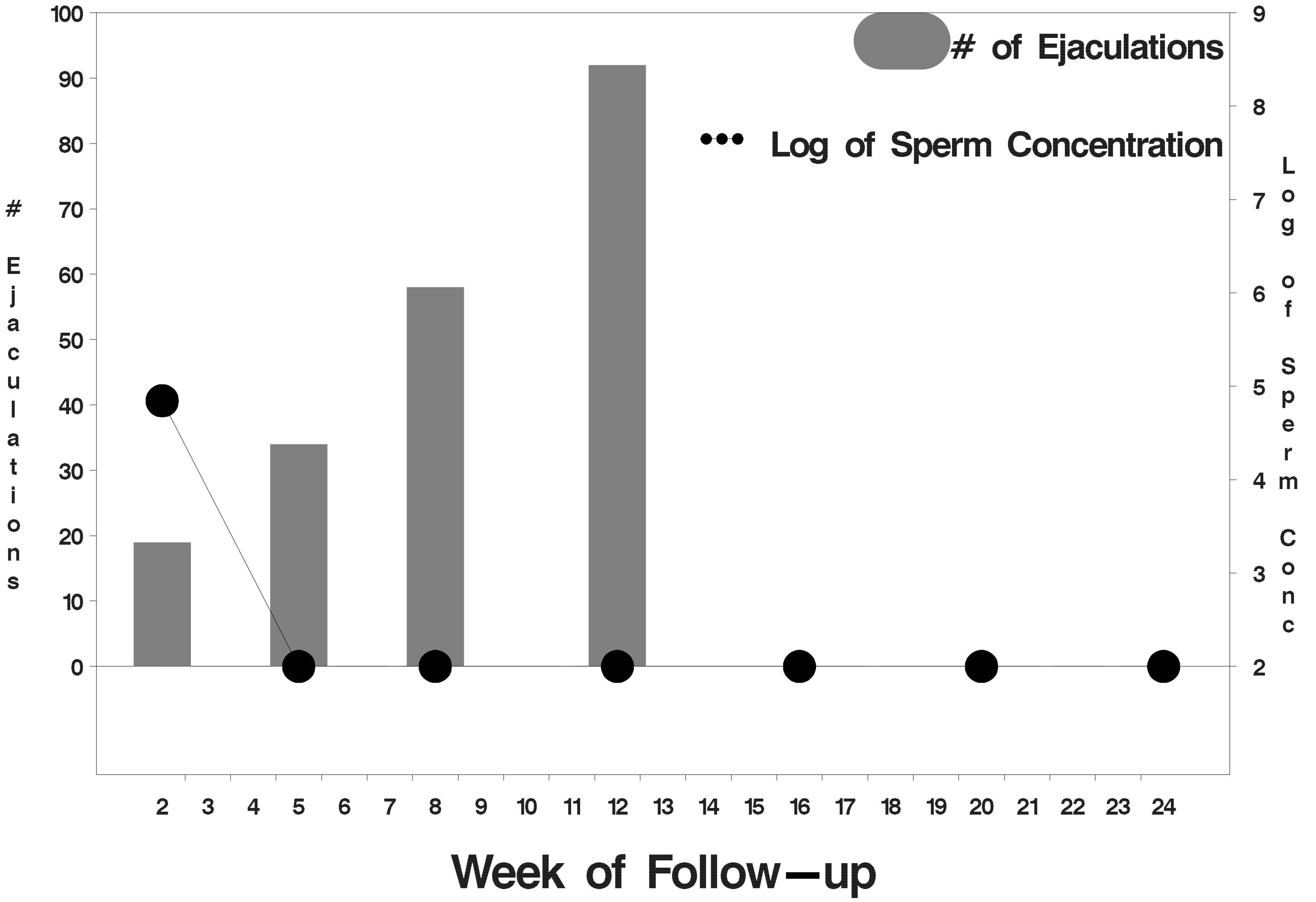

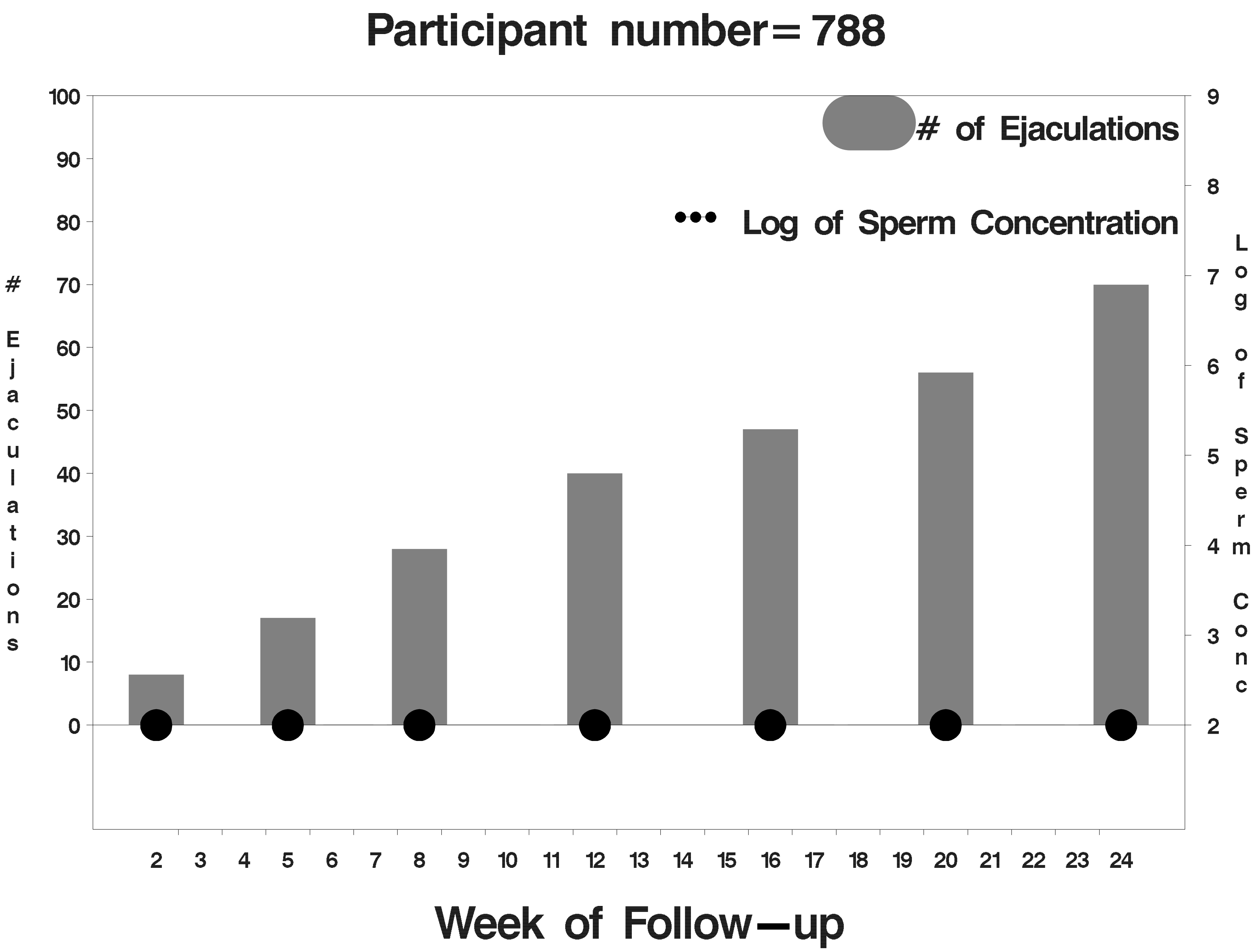

Participant number= 788
# of Ejaculations
Log of Sperm Concentration
Ejaculations
Log of Sperm Conc
100
90
80
70
60
50
40
30
20
10
0
9
8
7
6
5
4
3
2
2 3 4 5 6 7 8 9 10 11 12 13 14 15 16 17 18 19 20 21 22 23 24
Week of Follow-up

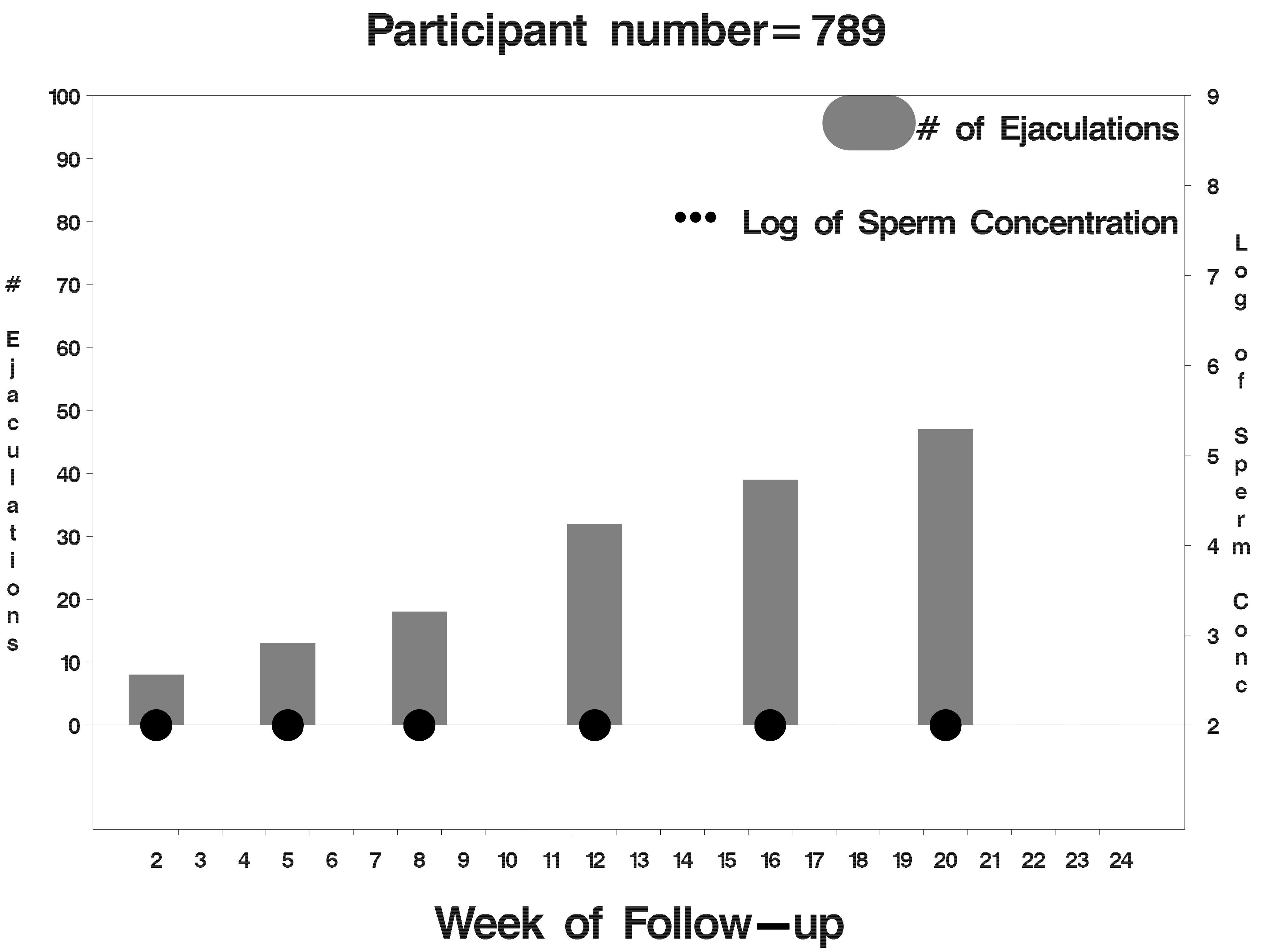

Participant number= 789
# of Ejaculations
Log of Sperm Concentration
Ejaculations
Log of Sperm Conc
100
90
80
70
60
50
40
30
20
10
0
9
8
7
6
5
4
3
2
2 3 4 5 6 7 8 9 10 11 12 13 14 15 16 17 18 19 20 21 22 23 24
Week of Follow-up

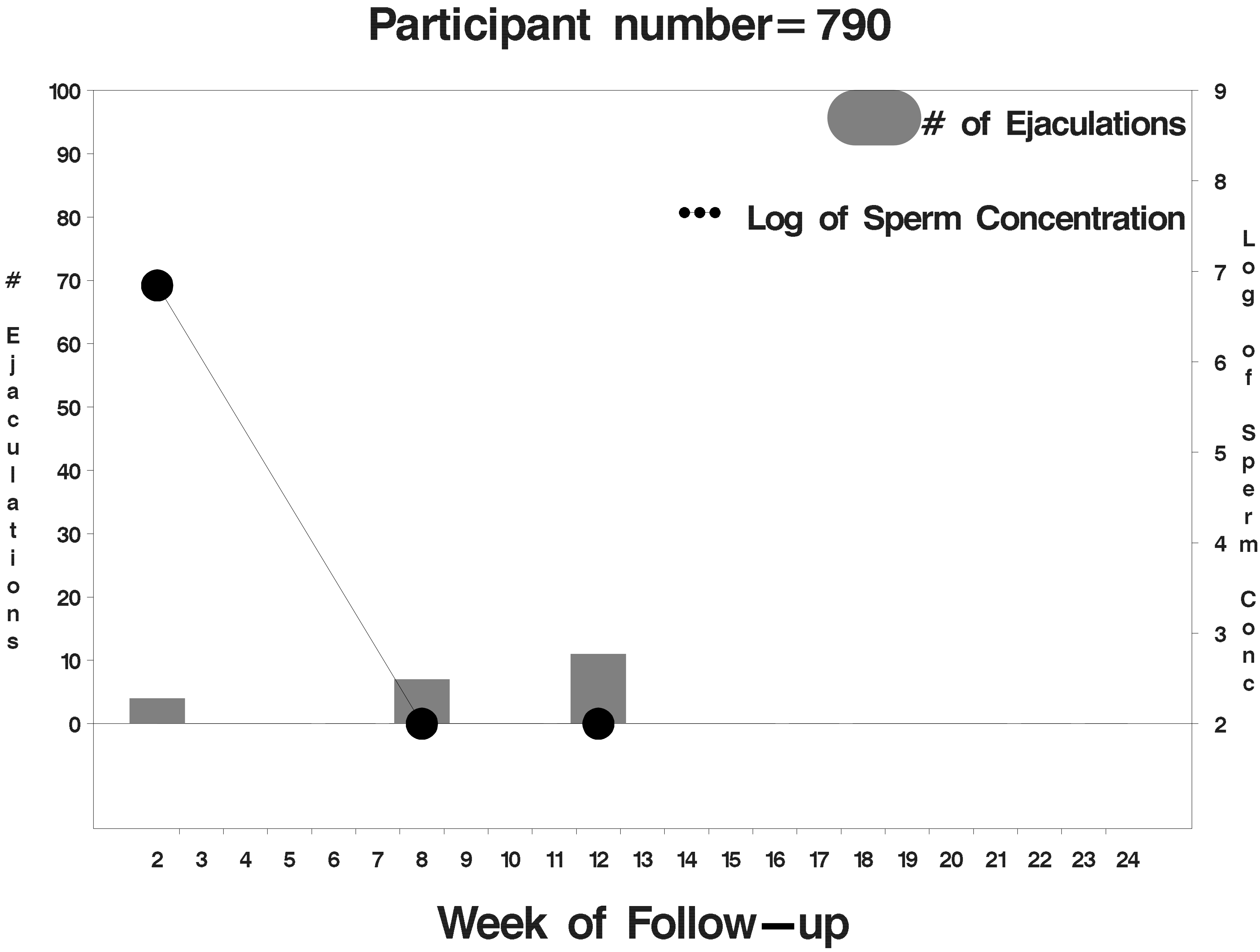

Participant number= 790
# of Ejaculations
Log of Sperm Concentration
Ejaculations
Log of Sperm Conc
0
10
20
30
40
50
60
70
80
90
100
2
3
4
5
6
7
8
9
2 3 4 5 6 7 8 9 10 11 12 13 14 15 16 17 18 19 20 21 22 23 24
Week of Follow-up

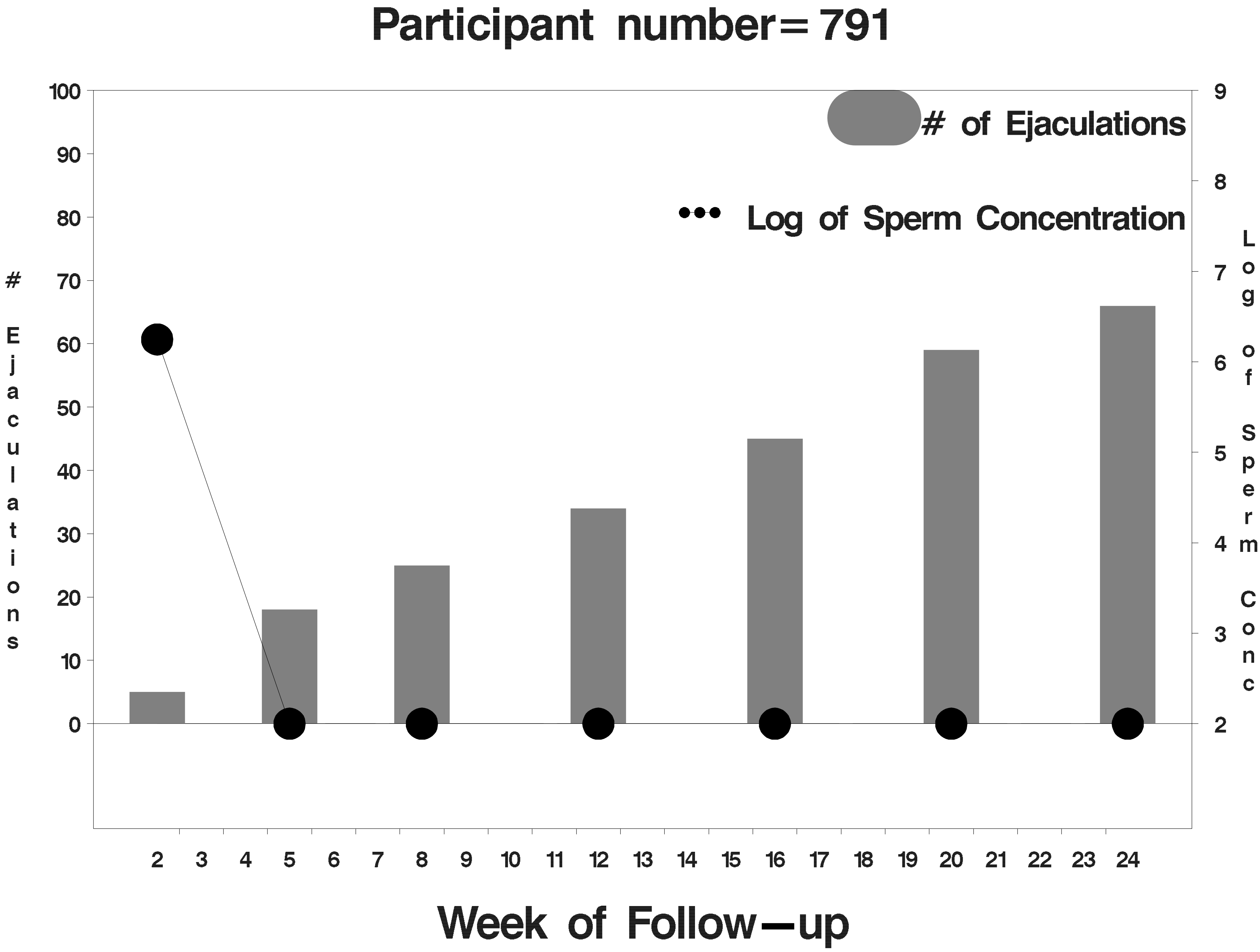

Participant number= 791
# of Ejaculations
Log of Sperm Concentration
Ejaculations
Log of Sperm Conc
100
90
80
70
60
50
40
30
20
10
0
9
8
7
6
5
4
3
2
2 3 4 5 6 7 8 9 10 11 12 13 14 15 16 17 18 19 20 21 22 23 24
Week of Follow−up

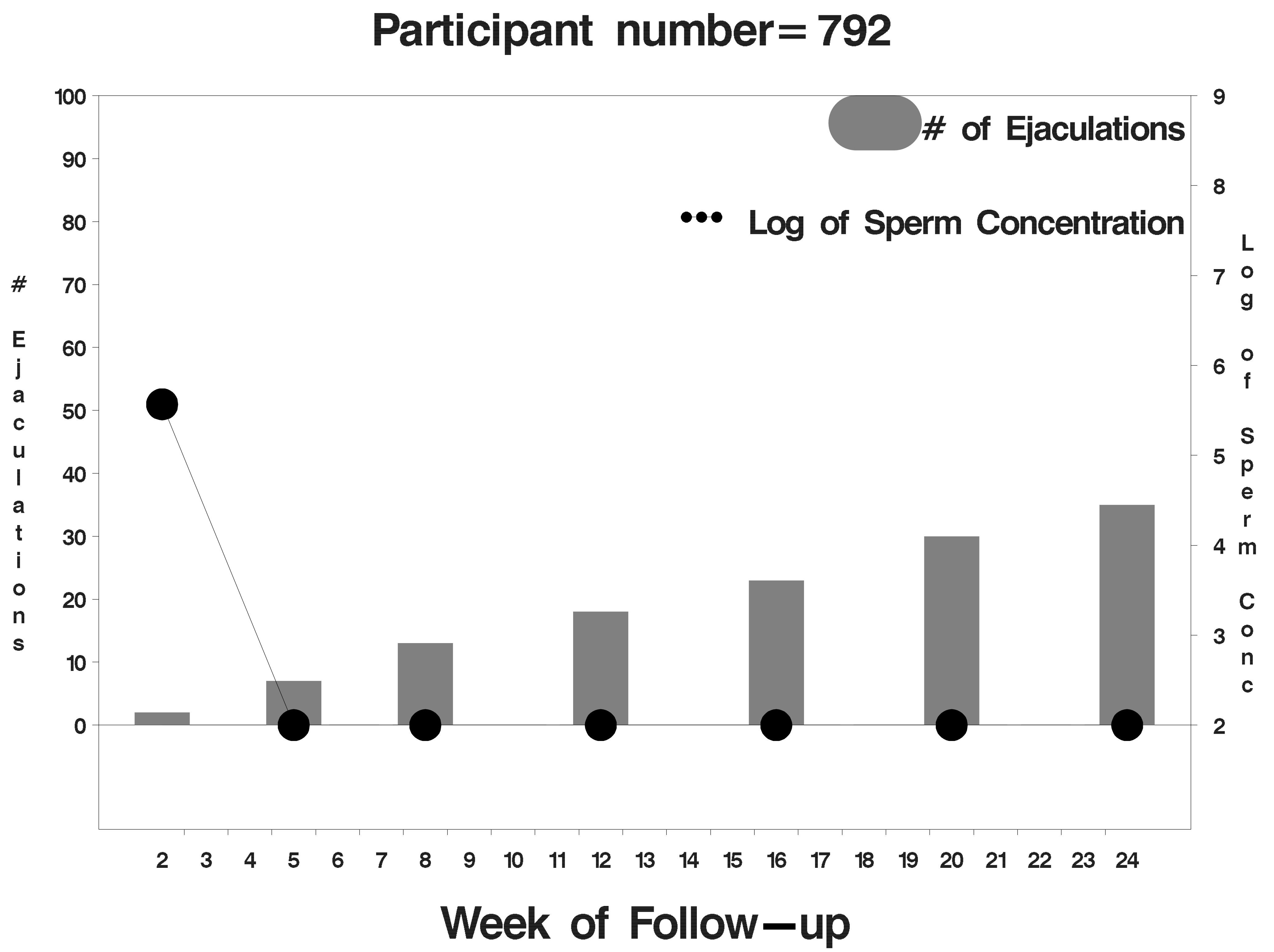

Participant number=792
# of Ejaculations
Log of Sperm Concentration
Ejaculations
Log of Sperm Conc
100
90
80
70
60
50
40
30
20
10
0
9
8
7
6
5
4
3
2
2 3 4 5 6 7 8 9 10 11 12 13 14 15 16 17 18 19 20 21 22 23 24
Week of Follow-up

Participant number= 793

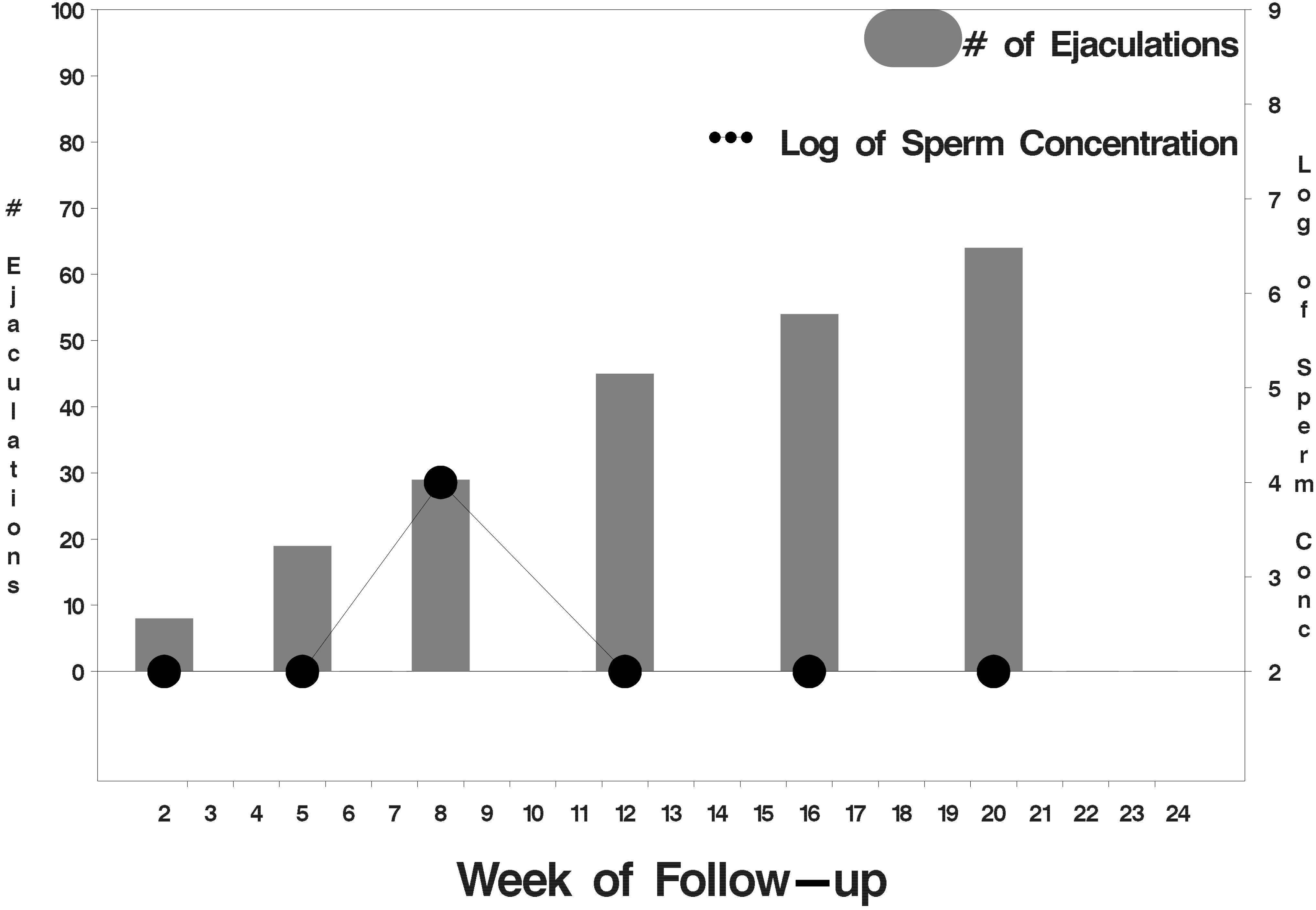

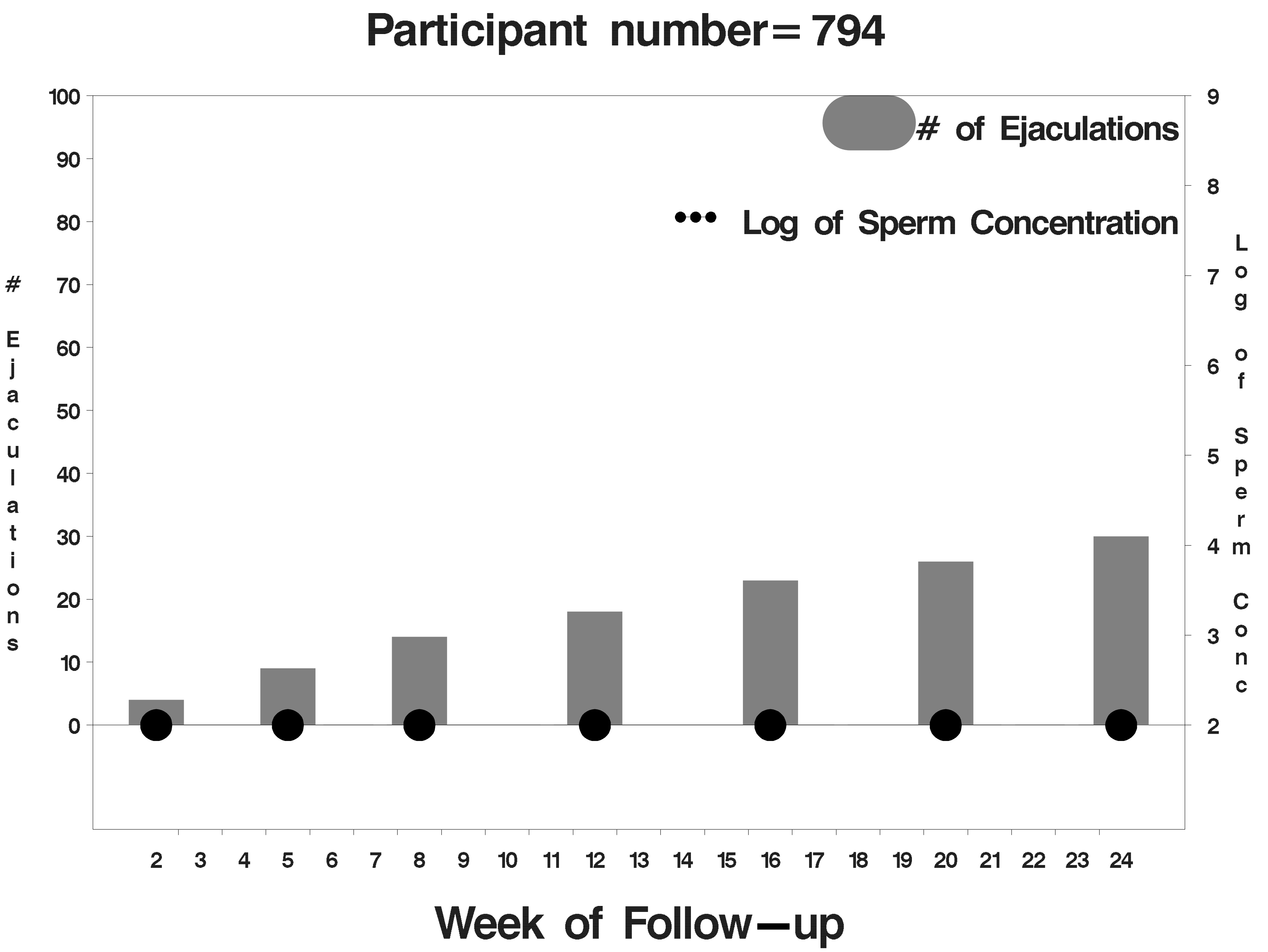

Participant number=794
# of Ejaculations
Log of Sperm Concentration
Ejaculations
Log of Sperm Conc
100
90
80
70
60
50
40
30
20
10
0
9
8
7
6
5
4
3
2
2 3 4 5 6 7 8 9 10 11 12 13 14 15 16 17 18 19 20 21 22 23 24
Week of Follow-up

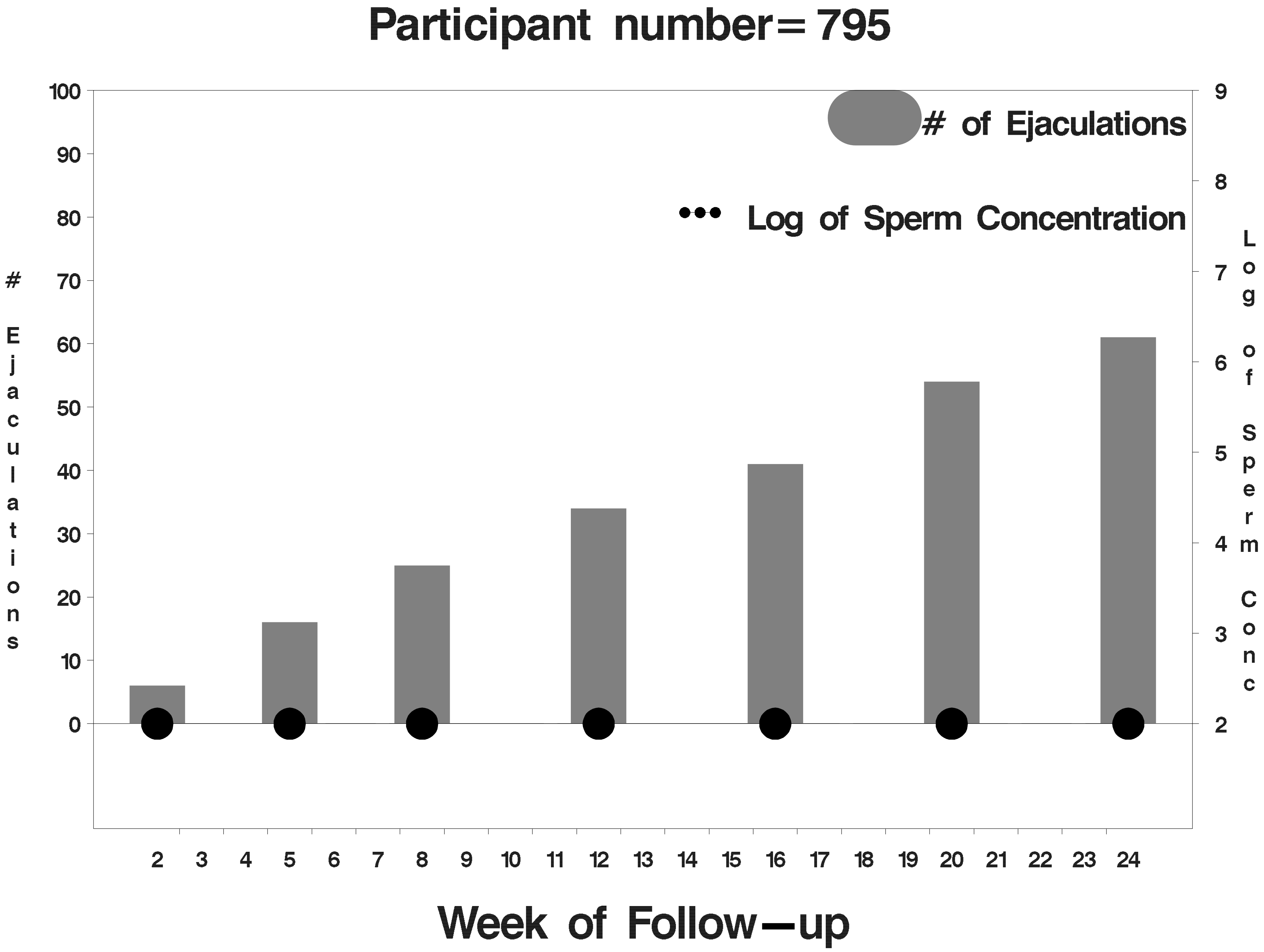

Participant number= 795
# of Ejaculations
Log of Sperm Concentration
Ejaculations
Log of Sperm Conc
100
90
80
70
60
50
40
30
20
10
0
9
8
7
6
5
4
3
2
2 3 4 5 6 7 8 9 10 11 12 13 14 15 16 17 18 19 20 21 22 23 24
Week of Follow-up

Participant number=796

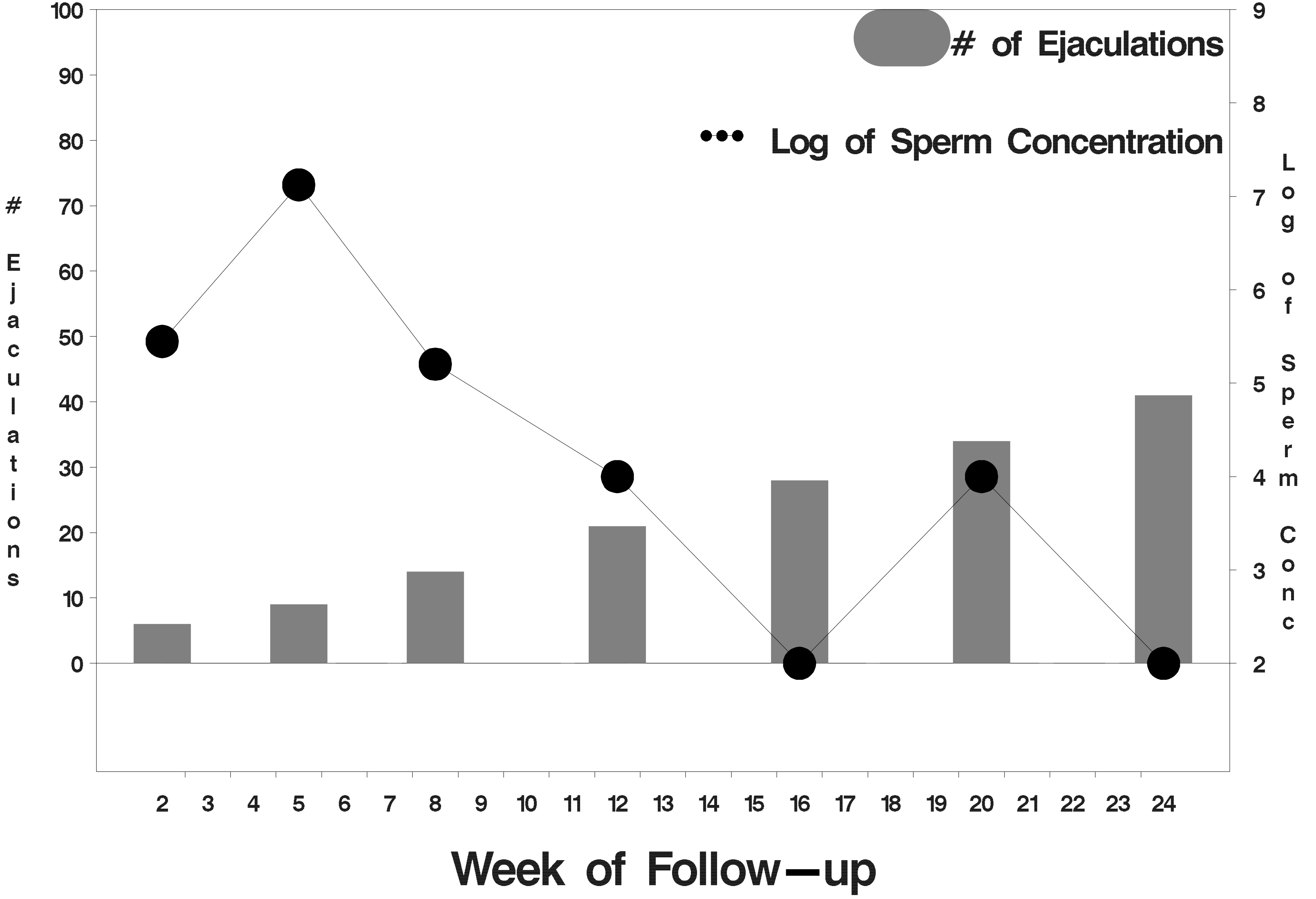

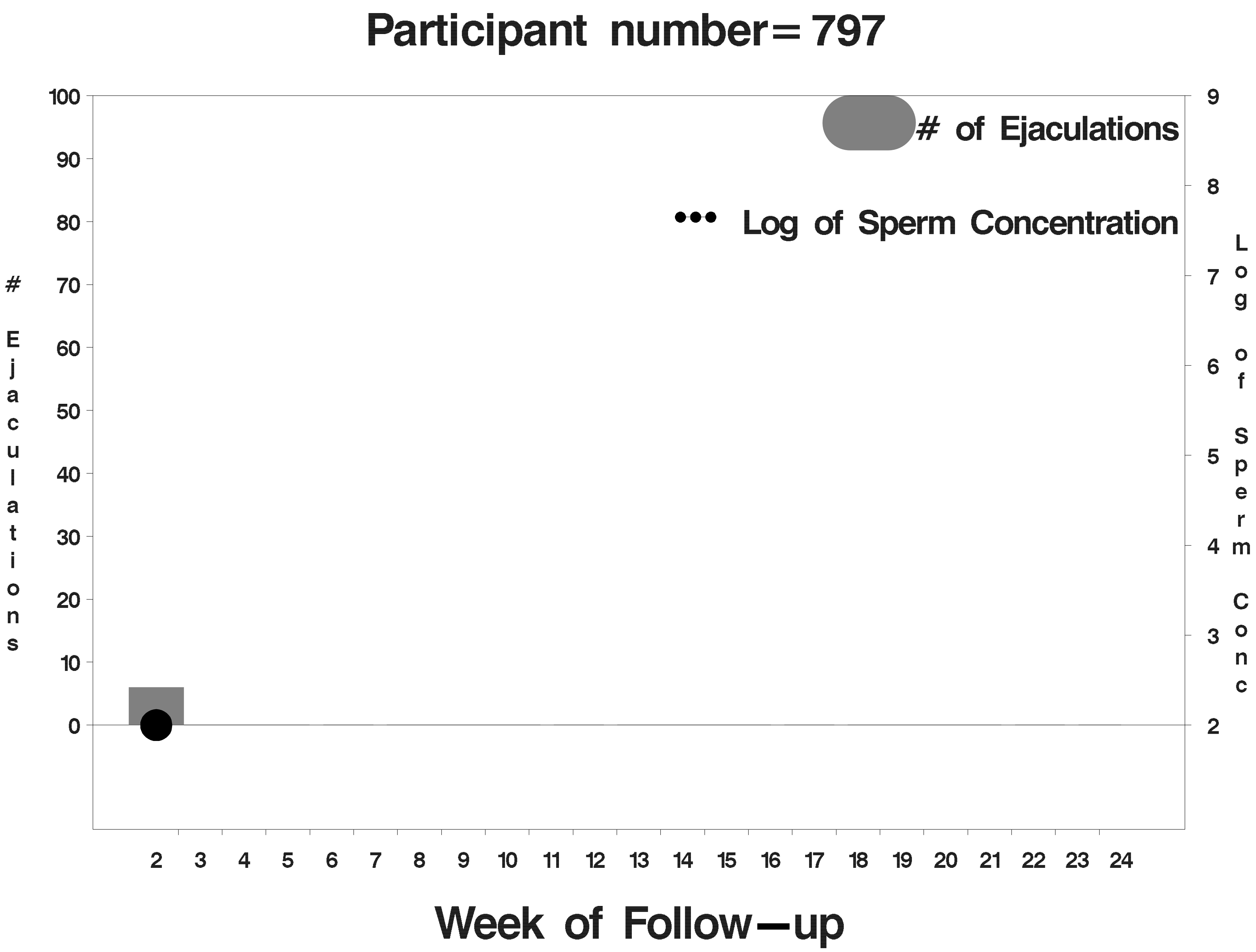

Participant number= 797
# of Ejaculations
Log of Sperm Concentration
Ejaculations
Log of Sperm Conc
100
90
80
70
60
50
40
30
20
10
0
9
8
7
6
5
4
3
2
2 3 4 5 6 7 8 9 10 11 12 13 14 15 16 17 18 19 20 21 22 23 24
Week of Follow−up

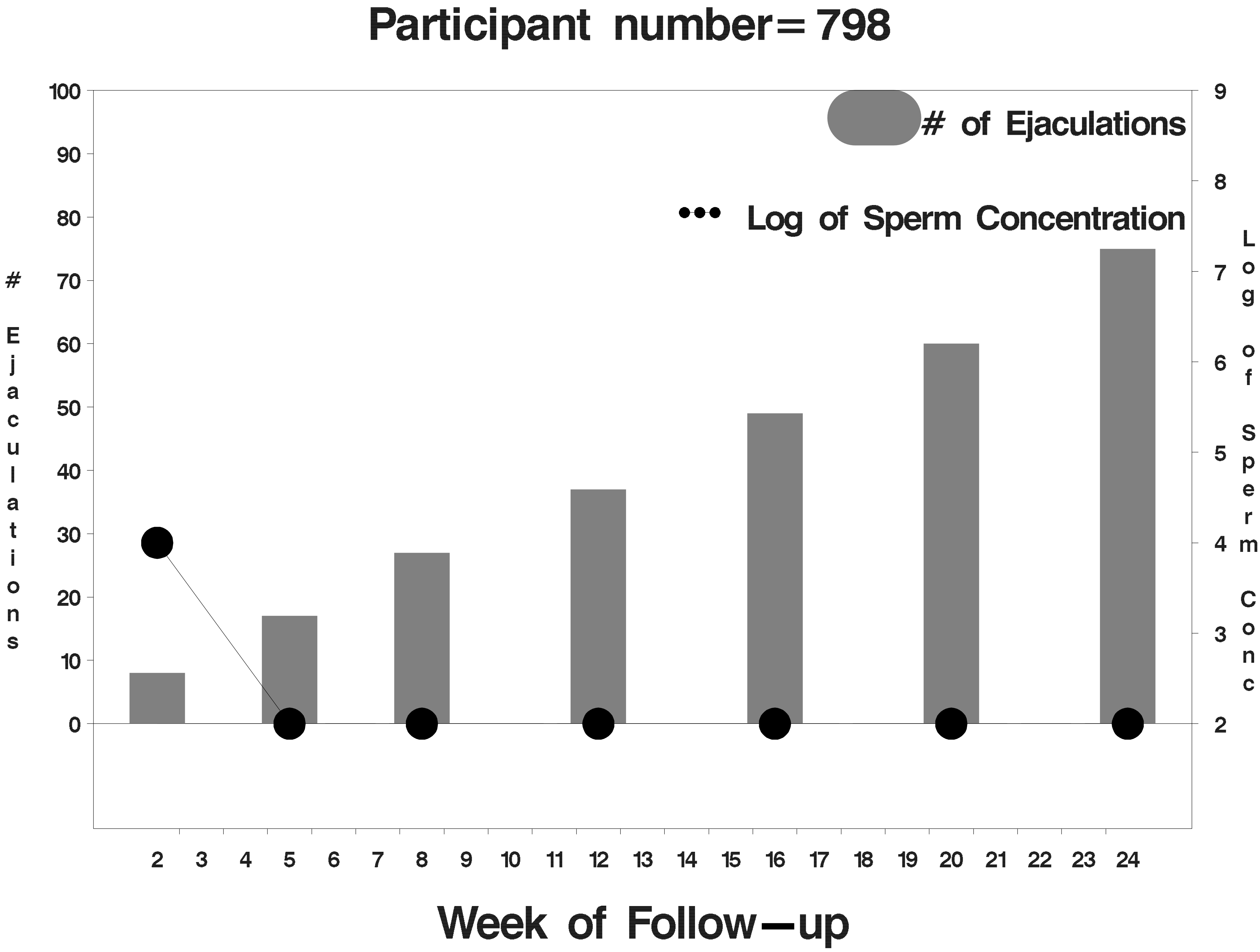

Participant number= 798
# of Ejaculations
Log of Sperm Concentration
Ejaculations
Log of Sperm Conc
100
90
80
70
60
50
40
30
20
10
0
9
8
7
6
5
4
3
2
2 3 4 5 6 7 8 9 10 11 12 13 14 15 16 17 18 19 20 21 22 23 24
Week of Follow-up

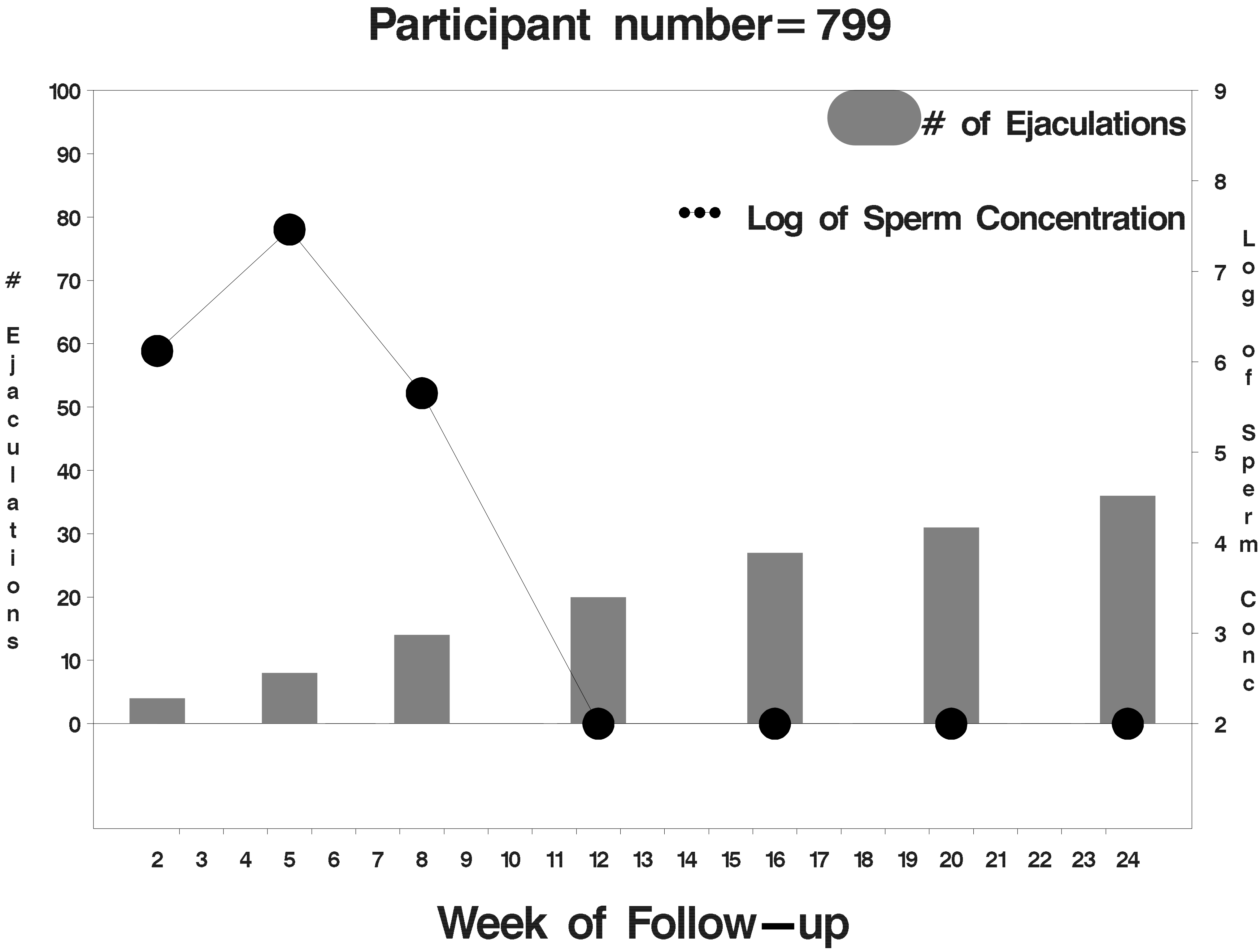

Participant number= 799
# of Ejaculations
Log of Sperm Concentration
Ejaculations
Log of Sperm Conc
100
90
80
70
60
50
40
30
20
10
0
9
8
7
6
5
4
3
2
2 3 4 5 6 7 8 9 10 11 12 13 14 15 16 17 18 19 20 21 22 23 24
Week of Follow-up

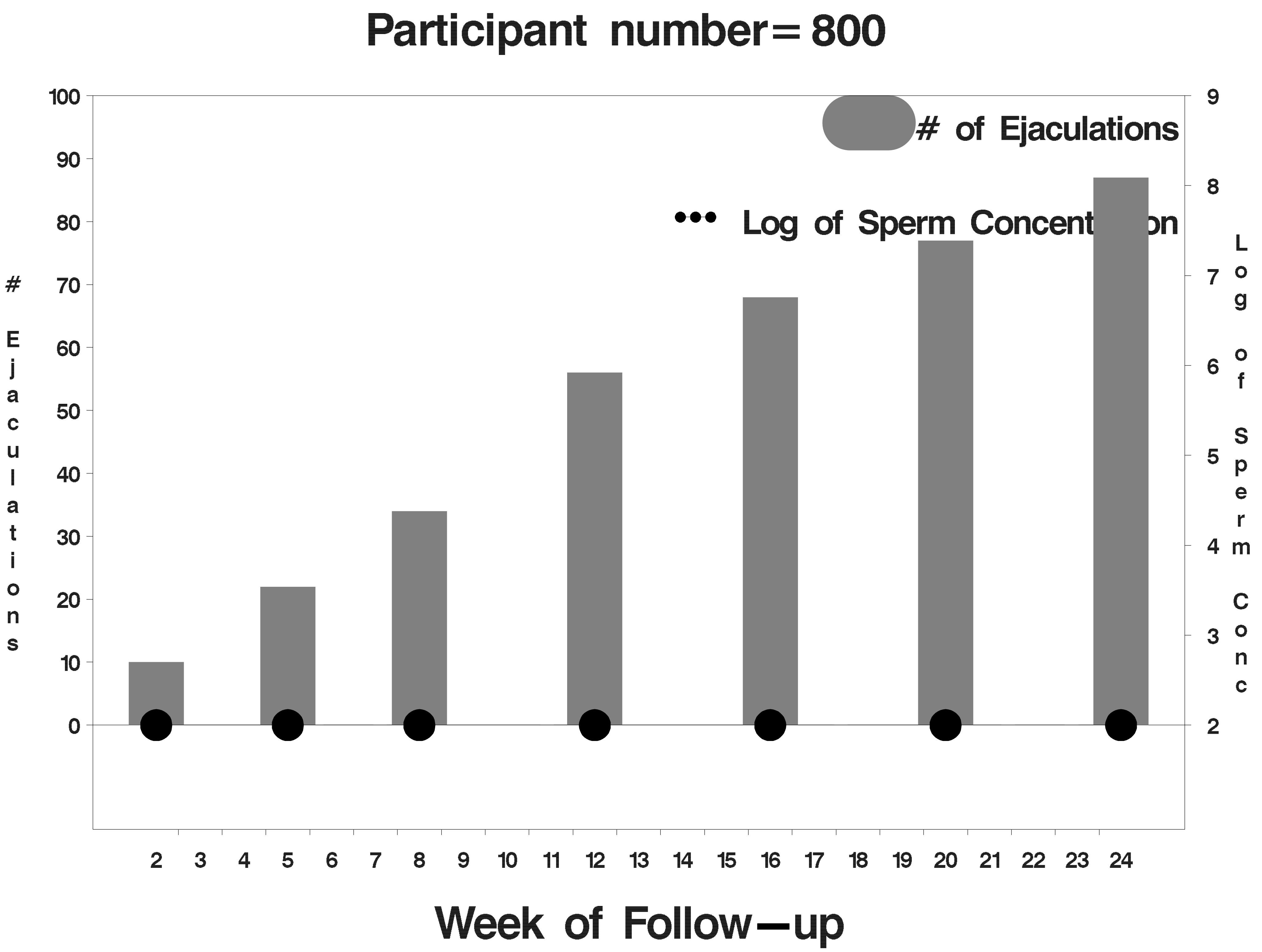
Participant number=800
# of Ejaculations
Log of Sperm Concent[illegible]on
Ejaculations
Log of Sperm Conc
100
90
80
70
60
50
40
30
20
10
0
9
8
7
6
5
4
3
2
2 3 4 5 6 7 8 9 10 11 12 13 14 15 16 17 18 19 20 21 22 23 24
Week of Follow-up
